# Molecular evolution of a widely-adopted taxonomic marker (COI) across the animal tree of life

**DOI:** 10.1038/srep35275

**Published:** 2016-10-13

**Authors:** Mikko Pentinsaari, Heli Salmela, Marko Mutanen, Tomas Roslin

**Affiliations:** 1 Department of Genetics and Physiology, University of Oulu, P.O.Box 3000 (Pentti Kaiteran katu 1), FI-90014, Finland; 2 Department of Biosciences, Centre of Excellence in Biological Interactions, University of Helsinki, Viikinkaari 1, FI-00014, Finland; 3 Spatial Foodweb Ecology Group, Department of Agricultural Sciences, University of Helsinki, Latokartanonkaari 5, FI-00014, Finland; 4 Department of Ecology, Swedish University of Agricultural Sciences, Box 7044, 750 07 Uppsala, Sweden

## Abstract

DNA barcodes are widely used for identification and discovery of species. While such use draws on information at the DNA level, the current amassment of ca. 4.7 million COI barcodes also offers a unique resource for exploring functional constraints on DNA evolution. Here, we explore amino acid variation in a crosscut of the entire animal kingdom. Patterns of DNA variation were linked to functional constraints at the level of the amino acid sequence in functionally important parts of the enzyme. Six amino acid sites show variation with possible effects on enzyme function. Overall, patterns of amino acid variation suggest convergent or parallel evolution at the protein level connected to the transition into a parasitic life style. Denser sampling of two diverse insect taxa revealed that the beetles (Coleoptera) show more amino acid variation than the butterflies and moths (Lepidoptera), indicating fundamental difference in patterns of molecular evolution in COI. Several amino acid sites were found to be under notably strong purifying selection in Lepidoptera as compared to Coleoptera. Overall, these findings demonstrate the utility of the global DNA barcode library to extend far beyond identification and taxonomy, and will hopefully be followed by a multitude of work.

In 2003, a standardized 658 bp fragment of the mitochondrial cytochrome C oxidase subunit I gene (cox1 or COI) was proposed as a universal marker for species identification – to be used as a “DNA barcode” tagging any taxon in the animal kingdom^1^. Following this seminal idea, the number of partial COI gene sequences available in public data repositories has skyrocketed. As of March 2016, about 4.7 million COI barcode sequences are stored in the Barcode of Life Datasystems database (BOLD, http://www.boldsystems.org/), and more than 3000 papers have been published on the application of COI barcodes to identification and discovery of animal species. Due to these efforts, COI is now by far the most extensively sequenced gene region of the animal kingdom. Importantly, most DNA barcoding studies published to date treat this gene region as a mere identification tag – in exact accordance with the concept of a conveniently readable “barcode”. Yet, the barcode fragment is located at the core of energy production within cells: COI is one of the building blocks of the cytochrome C oxidase protein (COX).

The COX protein is a dimer composed of two identical parts. These, in turn, consist of several amino acid chains (11 nuclear-encoded and three mitochondrial-encoded in mammals) as well as several metallic ligands: two iron atoms bound in heme groups, three coppers, one zinc and one magnesium^2^,^3^. COX is the last enzyme in the electron transport chain, reducing oxygen and pumping protons across the inner mitochondrial membrane. Thus, changes in the amino acid sequence that modify the protein structure may affect energy metabolism. Changes close to the enzymatically active sites or contact regions of amino acid chains are particularly likely to mediate such changes^4^.

Mitochondrial protein-coding genes are predominantly under purifying selection^5^. Amino acid substitutions are rare especially in the cytochrome oxidase genes^6^,^7^. The selective constraints on the amino acid sequence are reflected at the DNA sequence level: the DNA barcode sequence cannot vary freely and its evolution is far from neutral^5^,^8^. Evidence of positive selection on mtDNA has also been found^5^,^9^,^10^. At the same time, patterns of COI evolution may vary between taxa: In previous studies, we have detected distinctly higher DNA barcode divergences between species of Coleoptera than species of Lepidoptera^11^,^12^. Remarkable redesign of mitochondrial proteins has been observed in snakes, probably due to adaptation to a lifestyle where the metabolic rate can vary suddenly and dramatically^7^. Likewise, some endoparasitic taxa are characterized by unusually high rates of amino acid substitution in mitochondrial genes, potentially related to adaptation into living in anoxic environments^13^. Thus, patterns of DNA variation within the barcode region may reflect both constraints and opportunities at the protein level. Overall, these two levels of information should thus be explicitly related to understand the utility of DNA barcodes for taxon-specific purposes of species identification and delimitation, and for deriving functional insights from the large data set amassed to date.

In this paper, we draw on the massive number of COI sequences generated by the DNA barcoding initiative to examine amino acid variation in the DNA barcode region across the Metazoa. We do so at two hierarchical scales using three datasets: 1) across the full kingdom, and focusing on two megadiverse animal taxa, 2) Coleoptera (beetles) and 3) Lepidoptera (butterflies and moths). With approximately 390,000 and 160,000 described species, respectively, these two insect groups include approximately 35% of all described animal species^14^,^15^,^16^. We measure variation at each amino acid site, and map the conserved and variable sites onto a three-dimensional model of the COI protein structure. We project the observed variation on the animal tree of life. Finally, we study patterns of evolution in the DNA barcode sequence in Coleoptera and Lepidoptera to gain insight into the possible reasons for the observed differences in their DNA barcode variation.

## Results

### Nucleotide and amino acid statistics

**Figure 1:**
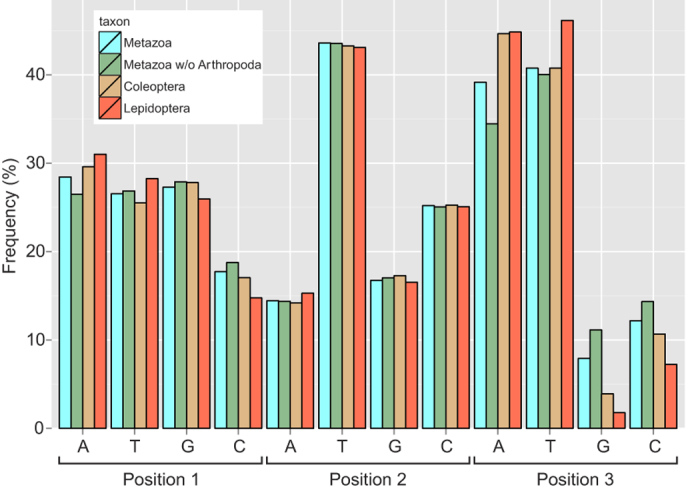
Nucleotide frequencies at each codon position. Due to high representation of Arthropoda in the Metazoa dataset, the values with Arthropoda excluded from the data are also provided.

**Figure 2:**
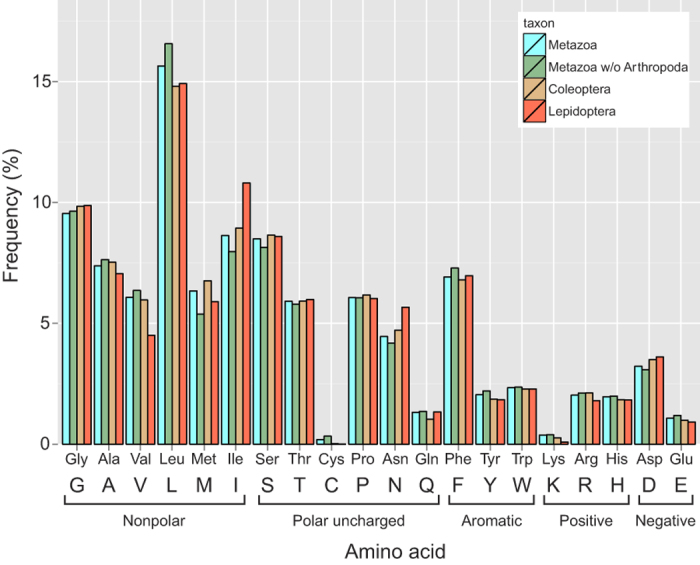
Average amino acid frequencies in barcode sequences of Coleoptera, Lepidoptera and Metazoa (also presented with the densely sampled Arthropoda excluded). Both the three-letter abbreviations and the single-letter standard codes for the amino acids are provided. The amino acids are grouped by their biochemical properties as explained in Methods. More than half of the amino acids in the protein fragment coded by the DNA barcode sequence are nonpolar.

The DNA sequences analyzed proved AT-biased (mean AT content >60% in all datasets), as is usually the case with animal mitochondrial DNA. The nucleotide composition of the three datasets (with and without the densely sampled Arthropoda in case of the Metazoa dataset) is shown in Fig. 1. The AT bias is slightly higher in Lepidoptera than in the other datasets. As expected for a membrane-embedded protein, the fragment coded by the DNA barcode sequence consists largely of nonpolar amino acids (ca. 53% in all three datasets; Fig. 2). The overall amino acid composition is mostly similar between Coleoptera and Lepidoptera, and both groups closely resemble the metazoan mean.

### Amino acid variation across Metazoa

**Figure 3:**
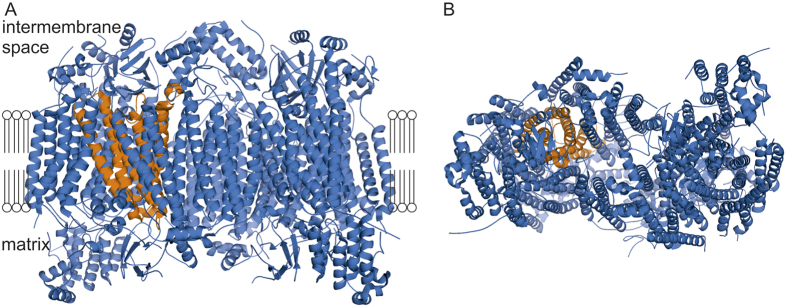
Location of the protein fragment coded by the DNA barcoding region of the COI gene. Most of this fragment is located in the transmembrane part of the cytochrome oxidase (COX) protein. For reference, the complete COX molecule (reconstructed from cattle, *Bos taurus*) is shown in blue, with the barcode sequence indicated in orange. Note that the complete COX molecule is built of two identical sets of subunits; for clarity, we have here highlighted only the barcode area at the left side of the protein from two different angles, showing (**A**) a side view of the transmembrane surface and (**B**) a view from the mitochondrial matrix. See Methods for details on how the structural model was inferred.

**Figure 4:**
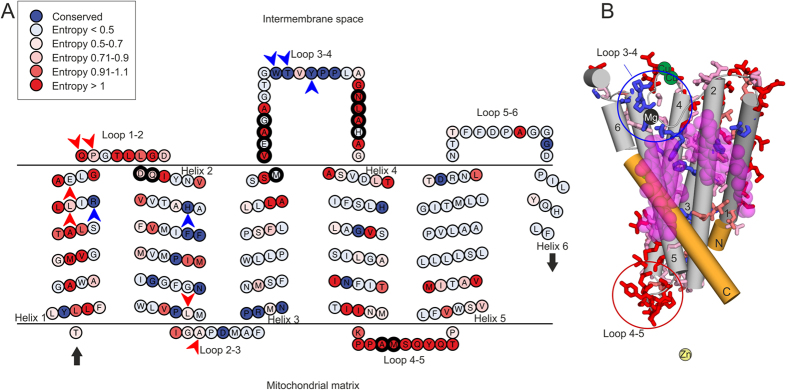
Amino acid variation in the CO1 barcode sequence across the Metazoa. Shown for reference is the amino acid sequence of cattle, *Bos taurus*. In (**A**), we map variation onto a schematic drawing of transmembrane helices versus connecting loops: Completely conserved amino acid residues are identified in blue, whereas variable amino acid residues are labeled from light pink to bright red according to their entropy values. The black arrows mark the N- and the C-termini. Because the primer binding sites of the COI barcode sequence are located within helices, Helix 1 lacks its first six amino acids and the last helix is shortened by 27 amino acids. The 13 amino acid residues missing in Dicyemida (encircled; cf. Fig. 5) mostly correspond to the highly variable amino acid residues in or close to the loop structures. The variable amino acids located at <5 Å distance from the heme ligands (positions 20, 24, 27, 28, 69 and 73) are indicated with red arrowheads. Blue arrowheads indicate the conserved amino acids situated at <5 Å from the protein ligands. In (**B**) we map conserved and variable amino acids onto a three-dimensional model of the protein structure. Helices are shown as grey columns and labeled with numbers 1–6. The parts of helices 1 and 6 not covered by the barcode sequence are shown in orange. Loop 3–4 (containing many conserved residues), and loop 4–5 (containing extensive variation in residues) are highlighted by circles. The two heme groups and the metal ion ligands are shown as transparent spheres. Color coding for ligands: heme groups = magenta, copper = green, zinc = yellow and magnesium = black.

The animal DNA barcode fragment was found to cover 219 amino acids in the enzymatically active part of COX, i.e. around the very site where the electron transfer from Cu to heme occurs (Fig. 3). The secondary structure of the barcode region consists of six α-helices (hereafter called Helix 1, Helix 2 etc., beginning from the N-terminus) connected by five loops (hereafter called Loop 1–2, Loop 2–3 etc.). These loops encompass a total of 60 amino acids (Fig. 4). In evidence of the functional constraints affecting variation in amino acid sequences, we found 23 of the 219 amino acids to be completely conserved across the Metazoa. The reasons were revealed by our examination of the 3D protein structure: Most of the conserved amino acid residues (16/23) are situated in the helices that penetrate the inner mitochondrial membrane (Fig. 4). However, one of the loops of the protein was also characterized by conserved amino acids. Unlike the other loops, this loop is pointing towards the heme group at the active site of the protein (cf. Fig. 4A,B: Loop 3–4). Five out of the 23 conserved residues were indeed strategically located at atomic interaction distances (<5 Å) from the protein ligands, and thus likely to directly affect electron transfer properties (Fig. 4A). The difference between the loops and helices in the number of conserved amino acids is not statistically significant (G = 0.11359, df = 1, p = 0.74), likely due to the conserved stretch in Loop 3–4.

**Figure 5:**
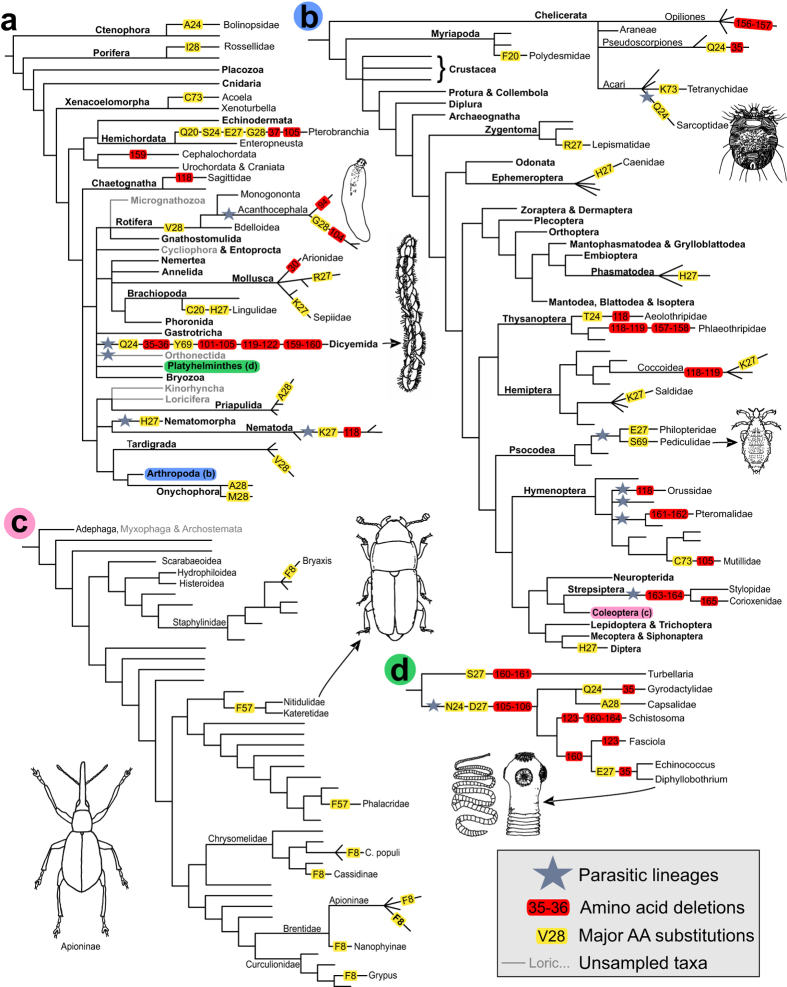
Amino acid substitutions and deletions superimposed on previously published comprehensive phylogenetic trees of the animal kingdom. The trees are based on references^42^,^43^,^44^,^45^ (**A**–**D**). Tree (**A**) represents the entire kingdom, whereas trees **B**–**D** represent blowups of individual branches as identified by color codes: (**B**) the Arthropoda, (**C**) beetles (Coleoptera) and (**D**) flatworms (Platyhelminthes). Fully or predominantly parasitic lineages are indicated by blue stars. Yellow labels on the branches represent amino acid substitutions with potential effects on enzyme function, showing the affected amino acid position and the residue observed in the branch. Red labels indicate deletions, with each deleted stretch in each branch presented separately. Importantly, the vast majority of deletions observed in the Metazoa dataset were found among parasites representing several different phyla, mainly Platyhelminthes, Dicyemida and Arthropoda. Unsampled major taxa are shown with gray branches and/or taxon labels. The bold print in the taxon labels represents phyla in tree (**A**) and subphyla and insect orders in tree (**B**).

Across the Metazoan DNA barcode dataset, 99 amino acids showed high variability (entropy >0.5, see Materials and Methods). Most of the observed variation occurred at sites far from the active site of the protein and was thus unlikely to affect its functioning (Fig. 4A,B: Helix 1; Loops 1–2, 3–4 and 4–5,). The most variable amino acids (entropy >1.1) were found significantly more often in loops than in helices (G = 14.689, df = 1, p = 0.0001). Yet, six variable sites (corresponding to amino acids 20, 24, 27, 28, 69 and 73 of the barcoding region; Fig. 4A) occur at atomic interaction distances (<5 Å) from the heme ligands. At these sites, major amino acid transitions from one biochemical group to another have been indicated in the phylogenies in Fig. 5. Strikingly many of them occur in parasitic lineages representing several different phyla (Fig. 5). At position 20, only three taxa (two marine filter-feeders and one detritivore) have an amino acid that is not nonpolar. Most taxa have a negatively charged glutamate at pos. 24, but in some cases this has been substituted with either a nonpolar (alanine in a ctenophore, glycine in a stick insect, Phasmatidae) or an uncharged polar amino acid (12 taxa, 9 of which are parasites). The change to uncharged polar amino acid has occurred in three unrelated parasite lineages: Dicyemida, parasitic flatworms (glutamate in free-living trematode flatworms) and sarcoptid mites. Position 27 shows an extremely wide scale of variation as all amino acid groups are represented. Most dipterans have a positively charged histidine at this site, but otherwise the positively and negatively charged amino acids seem to be occurring notably often in parasitic lineages (11 of 23 non-dipteran taxa with charged amino acids are parasites). The majority of metazoans have proline or one of the other polar amino acids at pos. 28. It has been replaced by a nonpolar AA in many different lineages without obvious connections to any particular lifestyle. At position 69, all studied animals hold nonpolar amino acids except for two parasite taxa (serine in human louse, *Pediculus humanus*, and tyrosine in Dicyemida). Variation at position 73 shows no obvious connections to habitat or life history.

Beyond the patterns in variable and conserved sites examined above, sequence deletions can give valuable information about the protein areas necessary (and potentially unnecessary) for enzymatic function. Again, the vast majority of deletions observed in the Metazoa dataset were found among parasites, and again they were spread among several different phyla (Fig. 5). For example, parasitic flatworm lineages show multiple deletions which are not found in free-living flatworms (Fig. 5D). The observed difference in the occurrence of deletions between parasites and non-parasites is also statistically significant (G = 47.012, df = 1, p = 7 × 10^−12^). The deletions are largely concentrated on both sides of the conserved amino acid stretch in Loop 3–4. Multiple deletions were also observed in Loop 4–5. The Dicyemida (small endoparasites of squids and octopuses) have exceptionally many (13) deletions in the barcode sequence (Figs 4A and 5A). Single deletions are found scattered across the metazoan barcode dataset, but multiple deletions in non-parasites are rare in our data. In addition to parasites, the Thysanoptera and the plant-sucking scale insects (Hemiptera: Coccoidea) also had multiple amino acid deletions.

### Coleoptera vs. Lepidoptera

**Figure 6:**
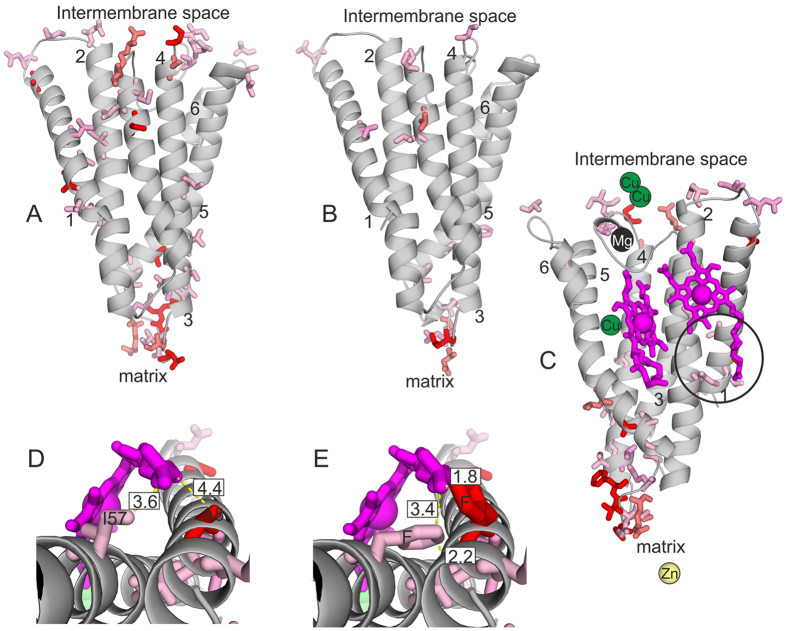
Homology models and amino acid variation of the barcode sequence in Coleoptera (**A**; Elateridae: *Agrypnis murinus*) and Lepidoptera (**B**; Geometridae: *Abraxas grossulariata*). The numbers refer to the α-helices as numbered in Fig. 4, with variable amino acids highlighted as sticks. The color reflects the level of variation as in Fig. 4. The lower end of the models points to the mitochondrial matrix, and the upper end to the intermembrane space. In (**C**) we offer a view of the *A*. *murinus* barcode protein from the side opposite to that of (**A**) with ligands added. Here, heme groups are shown in magenta, copper in green, zinc in yellow and magnesium in black. Note that most variable amino acid sites occur in locations not facing the ligands. The encircled area is shown in detail in (**D**,**E**). In (**D**) we show distances between variable amino acid sites and cytochrome oxidase ligands in Coleoptera. Two variable sites occur at atomic interaction distance from a heme group: In the sequence of *Agrypnus murinus*, sites 8 and 57 occur at distances of 3.6 Å and 4.4 Å from the heme, respectively. In (**E**) we highlight specific mutations occurring among 103 beetle species: here, one of the two sites (8 or 57) has mutated into a bulky phenylalanine. When site 8 is mutated, the heme group (located at a distance of only 1.8 Å) is left with little space and is likely pushed away from the phenylalanine. When site 57 is mutated, the phenylalanine side chain likely restricts the nearby helix (located at a distance of only 2.2 Å).

Turning from patterns across the Metazoa to patterns within the highly-resolved Coleoptera and Lepidoptera, the amino acid sequence proved much less variable in the latter than the former (Fig. 6A,B; for details, see Supplementary Text S1). The 14 variable sites in Lepidoptera overlapped with the 39 variable sites in Coleoptera, except for one lepidopteran site (93) which was non-variable (<0.5 entropy) in Coleoptera (see Supplementary Text S1). Half of the variable amino acids, including most of the variation in charge, were found in the loops. The difference in the number of variable amino acids between loops and helices was significant in Coleoptera (G = 5.729, df = 1, p = 0.02) and notable also in Lepidoptera (G = 3.2905, df = 1, p = 0.07). Loop 4–5 emerged as a hotspot of variability: 7 variable amino acids in Coleoptera and 5 in Lepidoptera were located in this loop, which consists of 10 amino acids and points to the mitochondrial matrix and may thus be functionally redundant.

When subsets of the orders with similar life histories and evolutionary age (see Methods) were compared, the number of variable amino acids (entropy >0.5 and within-group variation only excluded) was still clearly higher in Coleoptera (weevils, the Curculionidae + Apionidae clade) compared to the Lepidoptera subset (37 vs 12 amino acid positions with entropy >0.5). The observed lower count of completely conserved amino acids in the Lepidoptera subset (83, versus 111 in the weevil subset) is apparently due to sampling: more than 10× the number of weevil beetle sequences were sampled from Lepidoptera, and even a single sequence deviating from the consensus causes the AA position to lose its “completely conserved” status. If the criterion of “conserved” is changed from 100% to 99.5% consensus, the conserved AA count is notably higher in the lepidopteran subsample than in weevils (160 vs. 125), in line with the numbers of variable amino acids.

Most variable amino acid sites occurred far from the protein ligands (Fig. 6C). However, in Coleoptera we detected two variable sites within 5 Å from the heme ligand (Fig. 6D), with no corresponding variation in Lepidoptera. At these two sites in Coleoptera, the bulky phenylalanine observed in some lineages may steal space from the heme or push away the nearby helix which aligns the heme group (Fig. 6E). As a likely consequence, only one of the two sites (never both) had a phenylalanine in any one beetle species (Fig. 5C). The change to phenylalanine at position 8 has occurred at least seven times independently among the beetles, and all occurrences except for a single staphylinid species are found in the herbivorous weevils and leaf beetles (Fig. 5C). At pos. 57, the change has occurred in two beetle clades (Phalacridae, and Nitidulidae + Kateretidae), both of which are thought to be ancestrally fungivorous^17^ although modern representatives show a variety of other diets as well^18^,^19^.

**Figure 7:**
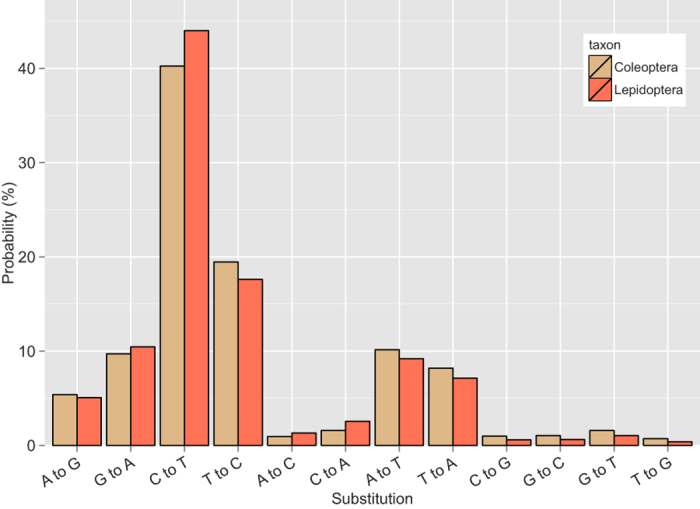
Maximum likelihood estimates for substitution probabilities in Coleoptera and Lepidoptera. The substitution pattern and rates were estimated under the GTR + G + I model. All three codon positions were included in the estimation. A discrete Gamma distribution was used to model evolutionary rate differences among sites (5 categories, gamma parameter = 0.5027). The rate variation model allowed for some sites to be evolutionarily invariable (24.5% sites). For simplicity, the sum of rate values in both datasets is made equal to 100.

The estimated nucleotide substitution matrices for Coleoptera and Lepidoptera show similar substitution probabilities, transitions not surprisingly being more common than transversions (Fig. 7). Both Coleoptera and Lepidoptera have a notable bias towards C to T and G to A transitions versus T to C and A to G (Fig. 7), as expected if the main cause of mutations is (oxidative) damage to DNA^20^. This bias is more pronounced in Lepidoptera than Coleoptera.

**Figure 8:**
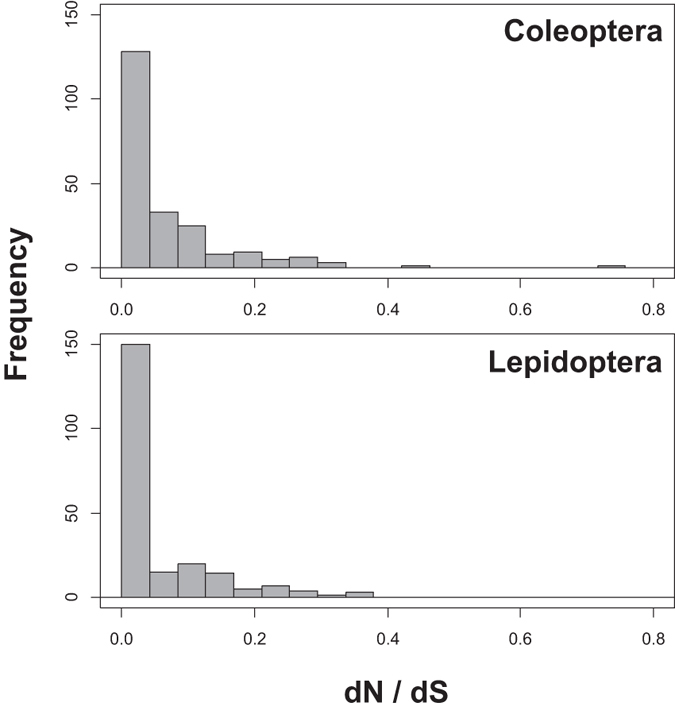
Codon-wise purifying selection in Coleoptera and Lepidoptera, measured as dN/dS. The sample size was 3208/1764 sequences/species in Coleoptera and 4628/2547 sequences/species in Lepidoptera.

The barcode sequence is predominantly under purifying selection in both Lepidoptera and Coleoptera, as expected. The distribution of dN/dS for the full Coleoptera and Lepidoptera datasets is shown in Fig. 8. The median value for dN/dS was 0.0131 in Coleoptera and 0.0072 in Lepidoptera. Although there seems to be some difference in the intensity of selection between the taxa based on the dN/dS values, with some sites in Coleoptera showing more relaxed selection, this difference is not statistically significant (Wilcoxon test; W = 26028, p = 0.12).The same pattern can be seen in the herbivore subsets (W = 25278, p = 0.32, with several sites apparently under relaxed selection in weevil beetles). The median values for dN/dS was 0.0164 in the weevils and 0.0045 in the ditrysian Lepidoptera subset.

## Discussion

Animal DNA barcodes are continuously generated for species identification and taxonomic purposes. What was originally proposed as a bold vision^1^ has developed into a common initiative, with biologists across the globe contributing both samples and species identifications to a global infrastructure. In this paper, we demonstrate that the database generated over the past decade shows potential for much more than it was originally constructed for. Given that it features more than four million sequences of the same gene region from all major (and most minor) animal lineages, it offers an unparalleled resource for examining patterns in and constraints on the evolution of a core metabolic protein. By screening national and global DNA barcode databases, by translating the patterns of DNA variation to variation at the level of amino acids and protein structure, and by mapping the variation uncovered onto the metazoan phylogeny, we make use of the depth of the data stemming from the barcoding initiative. What we find are strong functional constraints, and suggestions of convergent or parallel evolution among taxa sharing a similar, endoparasitic life style.

The choice of the COI Folmer region for species identification was originally based on its patterns of variation at the DNA level, and the relative ease of retrieving the sequence. The region was shown to be sufficiently conserved within species, yet sufficiently variable between species to enable reliable identification of each taxon^1^. Universal Folmer primers also allowed its PCR amplification from most animal phyla^1^,^21^.

Despite extensive variation at the DNA level, we have here observed signs of strong constraints on function, as determined by the amino acid sequence and resultant protein structure. By mapping the animal DNA barcode fragment onto extant models of the cytochrome oxidase protein^2^,^22^,^23^, we found this region to be located in the enzymatically active part of COI, i.e. around the site where the electron transfer from Cu to heme occurs. Being located at the core of cell respiration, the barcode region cannot vary freely, and mutations affecting protein function will likely most often be lethal. The highest level of variation was observed in regions deemed to be functionally redundant (like the loop structures of the protein) and the highest conservatism close to the active site, where functional constraints likely restrict variation in the amino acid sequence. Thus, the level of variation in DNA sequence so convenient from a taxonomic perspective will directly reflect the information encoded in these sequences. A similar pattern of variation has been detected in ribosomal RNA sequences, where the stem regions formed of paired nucleotides show less variation than the loops consisting of unpaired nucleotides^24^.

DNA variation in different parts of the COI barcoding region should be related to the functional role of these sections in the protein. Of the most variable amino acids (entropy >1.1) that we detected in Metazoa, approximately half were located in the loop structures of the protein. This finding agrees with previous knowledge of amino acid variation occurring with higher frequency in loop structures than in the rigid α-helices^25^. In the case of COI, the loops are mostly extra-membranous. This may allow more variation in amino acid charge and size than in membrane-embedded helices, which are limited by their lipophilic and crowded environment. However, in other transmembrane proteins, the structure of extra-membranous loops has been found to affect protein stability and membrane dynamics^26^. Whether or not variation in the COI loop sequences has functional consequences will thus call for further scrutiny. The extensive variation and multiple deletions observed in the Metazoa dataset also caused difficulties in aligning the most variable parts of the barcode sequences. Despite careful refinement of the alignment, it is likely that some alignment errors remain in the (mainly endoparasitic) taxa showing deletions of two or more amino acids at one or more points in the barcode sequence, such as the Dicyemida. However, these errors should have no significant effect on our results, as they are concentrated in the most variable regions of the protein, and not close to the conserved amino acids or those showing potentially functionally relevant variation.

The vast majority of the variable amino acids were located relatively far from the COX ligands. This is not surprising, considering the crucial role of COX and its ligands in the respiratory chain. However, some of the variable sites within the barcode sequence may directly affect enzymatic activity – by being located at atomic interaction distance from the heme groups, the center of COX enzymatic activity^2^,^22^,^23^. Six such variable sites were identified in the Metazoa dataset, and two in Coleoptera. At these sites, major shifts between amino acids of different biochemical groups were often found among metazoan parasites. As we have not inferred ancestral amino acid sequences due to the relatively sparse sampling of the Metazoa dataset, we cannot distinguish between convergent and parallel amino acid substitutions (independent changes to the same descendant amino acid from different ancestral amino acids in different lineages, versus independent transitions from the same ancestral amino acid to the same descendant amino acid, respectively). In addition, further development in statistical analysis of categorical data in a phylogenetic context is required before statistical support can be inferred for the effect of transitions to parasitism on the amino acid changes observed in the barcode region^27^.

Long branches in phylogenetic trees and exceptionally high rates of amino acid substitution are often associated with parasitic lifestyles, but an accelerated substitution rate has also been found in many non-parasitic lineages^13^,^28^,^29^. Long branches may also appear in phylogenies due to incomplete sampling or unobserved extinctions. Endoparasites often face hypoxic or anoxic conditions during their life cycles, but the same is true for e.g. nematodes living in decaying organic material. Thus, high substitution rates in mitochondria, and the amino acid transitions in many parasites with potential functional relevance we observe here, may be related to adaptation to hypoxic conditions.

The G-test that we used to evaluate the difference in occurrence of deletions between parasites and non-parasites admittedly comes with an elevated risk of a type I error as it does not account for the effect of phylogeny. Nonetheless, it seems obvious that transition to a parasitic lifestyle has often led to a reduction in barcode sequence length. Parasite genomes, both nuclear and mitochondrial, can be significantly reduced as some functions are taken over by the host^30^. The length of the mitochondrial genome is known to be associated with the thermal environment inside the host in parasitic nematodes: A shorter genome and thus faster replication rate is apparently selected for in parasites of endotherms^31^. There is no obvious association between the host type and extent of deletions in our Metazoa barcode dataset. However, the DNA barcode region is only a short fragment of the complete mitochondrial genome, and most of the length variation is expected to occur in non-coding regions^31^.

When focusing on two densely sampled insect orders, beetles (Coleoptera) and butterflies & moths (Lepidoptera), we found much more amino acid variation in the former than the latter. This pattern conforms with the presumed age of the orders: Coleoptera is an older group than Lepidoptera, and many of the major beetle lineages had already appeared by the time of the great radiation of Lepidoptera with the rise of the angiosperm plants^17^,^32^,^33^. Beetles have therefore had more time to accumulate amino acid differences between lineages. However, when we compared subsets of these taxa sharing a similar evolutionary age and history, beetles still showed considerably more amino acid variation than butterflies and moths. This indicates a fundamental difference in patterns and/or rates of molecular evolution in COI between the two taxa, and is in line with previous observations of higher DNA barcode divergence in Coleoptera than Lepidoptera^11^. Although the average intensity of purifying selection does not seem to differ between the taxa, several sites in Lepidoptera do show a very high intensity of selection compared to Coleoptera.

Increase in weight-specific metabolic rate (SMR) increases the production of highly reactive oxygen radicals. Presumably as a result of this, the rate of mutation and DNA damage also shows a positive correlation with SMR^20^. At least in mammals, differences in SMR seem to largely explain differences in AT content between lineages^20^. The metabolic rate can also affect selective constraints on mitochondrial genes. For example, salamanders experience weaker purifying selection on mitochondrial protein-coding genes than frogs, a pattern probably explained by the higher metabolic rate in frogs^34^. A similar pattern has been observed in the mitochondrial genomes of flightless vs. flight-capable birds, and slow vs. fast-moving mammals^35^. We speculate that these metabolism-related factors may be the cause to the observed differences in patterns of variation, substitution and selection between Coleoptera and Lepidoptera, but further studies are needed to confirm this. In actively flying insect species (like most Lepidoptera), even the resting metabolic rates are generally higher than in non-flying species^36^. This is possibly due to selection for higher active metabolic rate which also results in an increase of the resting metabolic rate. The higher relative rate of CT/GA transitions in Lepidoptera may be caused by more oxidative damage to DNA, and higher variation at both the nucleotide and the amino acid level in Coleoptera may be due to weaker purifying selection in beetles, many of which are not as ready and active fliers as butterflies and moths generally are.

Taken together, COI DNA barcodes can provide insights into molecular evolution and protein function in animals at different taxonomic scales. Our findings illustrate how patterns at the level of DNA variation should explicitly be related to what this DNA does – i.e. encodes protein structure. Yet, these two levels of information are frequently disconnected in analyses targeting one or the other. As a particularly promising avenue for further exploitation of the sequence data generated by the global DNA barcoding initiative, we encourage combinations of phylogenetic and biochemical research on the COX enzyme. Several commercial kits for measuring COX activity in animal tissue samples are readily available. Such measurements could be used to directly test if the amino acid changes we observed close to the active site of the enzyme in the Metazoa-wide sample and in Coleoptera truly have consequences for metabolism. We anticipate that the dredging of the unique data base on COI sequences amassed by the global biologist community will yield interesting insights into evolution. By taking the first step in this paper, we hope to have stimulated such a development.

## Methods

### Sampling

To cover the diversity of Metazoa, we searched the publicly available sequence data in the BOLD database (http://boldsystems.org/) for full-length, high-quality COI barcodes from all Metazoan phyla, selecting at least one representative from each major lineage within each phylum whenever publicly available. Within the megadiverse Arthropoda and particularly insects, our sampling was denser, covering all insect orders and the biggest families within orders, again provided that high quality data were publicly available. This sampling strategy resulted in a set of 292 sequences representing 26 of the 32 known animal phyla. The complete list and taxonomic classification of these sequences, including the process ID numbers with which they can be accessed in BOLD and GenBank accessions, is provided in Supplementary Table S1.

To add resolution within megadiverse taxa, we sampled extensively within the Coleoptera (beetles) and Lepidoptera (butterflies and moths). Our datasets of Coleoptera and Lepidoptera are based on the sequence libraries assembled as a part of the Finnish Barcode of Life project (http://finbol.org/). The Coleoptera dataset is a subset of the previously published North European beetle data^11^. The Lepidoptera dataset consists mostly of previously unpublished records. The material for tissue sampling for both datasets has been collected mainly from Finland and to a smaller extent from other Nordic and Baltic countries. Only full-length sequences with less than 1% ambiguous bases were selected for analysis, and after filtering for quality, the sequences were collapsed into haplotypes in ALTER^37^. The final datasets encompass 3208/1764 and 4628/2547 sequences/species for Coleoptera and Lepidoptera, respectively.

All insect sampling for DNA barcoding was made in accordance with the laws of the countries where the samples were collected. A sampling permit for beetles covering all government-owned protected areas in Finland was issued to the Finnish Expert Group on Coleoptera including MP by Metsähallitus (Finnish Forest and Park Service, permit number 2322/662/2012). The Centre for Economic Development, Transport and the Environment in Lapland permitted sampling of *Pytho kolwensis* Sahlberg, 1833, a species protected by law in the European Union (permit number LAPELY/275/07.01/2012). Sampling non-protected insect species outside national parks and other protected areas does not require special permits in the Nordic countries.

### Alignment and translation

The DNA barcodes were downloaded from BOLD as DNA sequences, and aligned and translated into amino acids in MEGA v. 6.06^38^. The sequences were first algorithmically aligned with ClustalW^39^ using the default options, and the resulting alignment was manually refined before translation. The Metazoa dataset was translated in several batches using taxon-specific mitochondrial translation tables (reported in Table S1). The Coleoptera and Lepidoptera datasets were translated using the invertebrate mitochondrial code (translation table 5 in GenBank).

### Detection and visualization of variable amino acid sites

To measure variation at each amino acid position in our three selected barcode sequence datasets (Metazoa, Coleoptera and Lepidoptera), we calculated entropy (uncertainty; H(x)) values for all positions in BioEdit^40^. Zero variation results in an entropy value of 0, and increasing variability is reflected by increasing entropy. Based on the resultant values, we further divided the variable amino acids into four (arbitrary) categories according to increasing entropy: H(x) 0.5–0.7, 0.71–0.9, 0.91–1.1 and >1.1. Amino acid positions with entropy below 0.5 were considered non-variable, and residues that showed no variation at all were defined as conserved. To characterize the chemical properties of amino acids at each site, we divided them into standard groups: nonpolar aliphatic (G, A, V, L, M, I); polar uncharged (S, T, C, P, N, Q); aromatic (F, Y, W); positively charged (K, R, H); and negatively charged (D, E). When the amino acids at a given position showed variation only among amino acids within such groups, we considered the site non-variable and, regardless of the entropy value, treated it as equal to those sites with entropy <0.5.

To visualize the topology of the barcode protein, we used the TOPO2 software^41^. Cattle (*Bos taurus*) was used as a reference for structural modeling of all other sequences, as its cytochrome oxidase protein structure is particularly well-studied at a fine resolution^2^,^22^,^23^. Homology models representing the COI barcode region were built based on the bovine protein X-ray structure (Protein Data Bank ID: 1V54) using the MODELER software in the Discovery Studio 4.0 Modeling Environment (Accelrys Software Inc., San Diego 2013). The model optimization level was set to ‘high’, and loop refinement was included. The model quality was assessed with the 3D-profile option in the software, which compares the compatibility of the 3D structure and the sequence. Ten models per protein were built and the lowest energy model was selected for visualization and distance measurements between amino acids and other enzyme components with PyMOL Molecular Graphics System 1.7 (Schrödinger, LLC). Particular attention was paid to amino acid substitutions from one chemical group to another close to the enzyme ligands, as these changes can potentially affect enzyme function.

### Mapping amino acid changes on phylogenies

To explore how many times the major amino acid changes observed here have appeared during evolution, we mapped amino acid substitutions with potential impact on protein function onto recently published, comprehensive phylogenies. For phylum-level relationships, we used the phylogeny compiled from multiple recent studies by Dunn *et al*.^42^. For relationships within the Arthropoda, we used the phylogenomic tree published by Misof *et al*.^43^ For Coleoptera, we relied on the recent comprehensive molecular phylogenies by Hunt *et al*.^17^ and McKenna *et al*.^44^, whereas the phylogeny of flatworms was based on the work of Park *et al*.^45^.

### Statistical analysis of amino acid variation

Tests of correlated evolution on categorical data, such as amino acids or groups of amino acids, are problematic as none of the currently available phylogeny-aware methods fully eliminate pseudoreplication^27^. We used the G-test of independence to assess the statistical significance of the association between parasitic lifestyle and deletions in the barcode sequence. The G-test does not take phylogeny into account, which results in an elevated risk of type I error. This must be taken into account when interpreting the results. The G-test was also used in testing if the occurrence of variable amino acids differs in loops vs. helices of the protein. No suitable statistical tests for correlated evolution of categorical characters with more than two possible values (such as the amino acid groups studied in this paper) are available^27^, so we must settle for a descriptive examination of the amino acid variation in different lineages.

### Selection, substitution patterns and amino acid variation in Coleoptera and Lepidoptera

To account for the old evolutionary age of many Coleoptera lineages and the resulting possible bias in comparing amino acid variation between the full Coleoptera and Lepidoptera datasets, we compared the amino acid variation in phylogenetically restricted subsets of these taxa in addition to the analyses on full datasets. For this purpose, we excluded the basal lineages of Lepidoptera and concentrated on the derived phytophagous ditrysian families (the sister group of Tineidae in ref. 33; “non-tineoid Ditrysia” in ref. 28). We contrasted this clade with the likewise phytophagous Curculionidae + Brentidae clade of weevil beetles, which is estimated to be approximately equally old (ca. 150 million years) and to have experienced its major radiation at the same time in connection with the diversification of the angiosperm plants^46^. These subsets included 376/206 and 4285/2363 sequences/species for Coleoptera and Lepidoptera, respectively.

We estimated the nucleotide substitution patterns for Coleoptera and Lepidoptera using the Estimate Substitution Matrix feature in MEGA v. 6.06. We adopted the GTR + G + I model of nucleotide substitution (with 5 distinct gamma categories), as it showed the best fit to all datasets based on ML model tests performed in MEGA. The strength and nature of selection acting on the barcode sequence in Coleoptera and Lepidoptera was estimated using the HyPhy package^47^ implemented in MEGA 6.06. The codon-wise dN/dS values were used in comparing the two taxa. The selection analysis was performed both on the full datasets of Coleoptera and Lepidoptera and on the phytophagous subsets.

### Availability of Data

The Metazoa dataset was compiled from publicly available data in the BOLD database (http://www.boldsystems.org/), and the analyzed sequences can be accessed in BOLD with the process ID codes provided in Table S1. The Coleoptera and Lepidoptera sequences are publicly available as BOLD datasets (doi: 10.5883/DS-FCPROT and 10.5883/DS-FILEPRO, respectively) along with the original sequencing trace files, specimen metadata and photographs. The sequences are also available in GenBank (accession numbers provided in the BOLD datasets).

## Additional Information

**How to cite this article**: Pentinsaari, M. *et al*. Molecular evolution of a widely-adopted taxonomic marker (COI) across the animal tree of life. *Sci. Rep.* **6**, 35275; doi: 10.1038/srep35275 (2016).

## Supplementary Material

Supplementary Information
