# Supplementary material for: Molecular evolution of a widely-adopted taxonomic marker (COI) across the animal tree of life
Source: Sci Rep. 2016 Oct 13;6:35275. doi: 10.1038/srep35275 (PMC5062346; doi:10.1038/srep35275)
Supplement: Supplementary Information [file srep35275-s1.pdf]

Supplementary information for:

Molecular evolution of a widely-adopted taxonomic marker (COI) across the animal tree of life

Mikko Pentinsaari*
Department of Genetics and Physiology, University of Oulu, P.O.Box 3000 (Pentti Kaiteran katu 1), FI-90014 University of Oulu, Finland. E-mail: mikko.pentinsaari@oulu.fi

Heli Salmela
Department of Biosciences, University of Helsinki, Viikinkaari 1, FI-00014 University of Helsinki, Finland. E-mail: heli.havukainen@helsinki.fi

Marko Mutanen
Department of Genetics and Physiology, University of Oulu, P.O.Box 3000 (Pentti Kaiteran katu 1), FI-90014 University of Oulu, Finland. E-mail: marko.mutanen@oulu.fi

Tomas Roslin
1) Spatial Foodweb Ecology Group, Department of Agricultural Sciences, University of Helsinki, Latokartanonkaari 5, FI-00014 University of Helsinki, Finland
2) Department of Ecology, Swedish University of Agricultural Sciences, Box 7044, 750 07 Uppsala, Sweden
E-mail: tomas.roslin@helsinki.fi

* CORRESPONDING AUTHOR

Supplementary Text S1: Variable amino acids in Coleoptera and Lepidoptera

The variable amino acids in Coleoptera and Lepidoptera are shown schematically in relation to the secondary structure of the modelled barcode protein structure. The amino acid sequence is that of *Agrypnus murinus* (Linnaeus) (Coleoptera: Elateridae). In the secondary structure, H stands for helix and dash (-) for loop structures. The variable amino acids (entropy >0.5) are indicated with an asterisk (*) for both Coleoptera and Lepidoptera. Non-variable amino acids (entropy <0.5) are represented by dots (.).

```
                             Helix 1
Secondary structure        HHHHHHHHHHHHHHHHHHHHHHHHHH--------H
Sequence                   TLYFLFGAWAGMLGTSLSLLIRAELGNPGSLIGND 35
Coleoptera                 ....*..*.*..*.........*...*..*...*.
Lepidoptera                ............*.............*........
                            Helix 2
Secondary structure        HHHHHHHHHHHHHHHHHHHHHHHHHHHHHHHHHHH
Sequence                   QIYNVVVTAHAFIMIFFMVMPIMIGGFGNWLVPLM 70
Coleoptera                 ............*........*.............
Lepidoptera                ............................ .....
                                     Helix 3
Secondary structure        --------HHHHHHHHHHHHHHHHHHHHHHH----
Sequence                   LGAPDMAFPRMNNMSFWLLPPSLSLLLMSSIVENG 105
Coleoptera                 .......................*..**..*..*.
Lepidoptera                ......................**......*....
                                                Helix 4
Secondary structure        -------------------HHHHHHHHHHHHHHHH
Sequence                   AGTGWTVYPPLSANIAHSGSSVDLAIFSLHLAGIS 140
Coleoptera                 ............***..*.*.............*.
Lepidoptera                .................*.................
                                                       Helix5
Secondary structure        HHHHHHHHHHHHHHH-----------HHHHHHHHH
Sequence                   SILGAVNFISTVINMRSTGITFDRMPLFVWAVAIT 175
Coleoptera                 .....*...*.**...******.**...*.*.*..
Lepidoptera                ................*.****......*...*..

Secondary structure        HHHHHHHHHHHHHHHHHHHHHHHH-----------
Sequence                   ALLLLLSLPVLAGAITMLLTDRNLNTSFFDPAGGG 210
Coleoptera                 .*.....................*.......*...
Lepidoptera                .*.................................
                              Helix6
Secondary structure        -HHHHHHHH
Sequence                   DPILYQHLF 219
Coleoptera                 .........
Lepidoptera                .........
```

Supplementary Table S1. BOLD process ID numbers, GenBank accessions, translation matrices and BOLD taxonomic classification of the analyzed Metazoan sequences.

BOLD process ID	GenBank Accession	Translation matrix	Phylum	Class	Order	Family	Genus	Species
GBSP8040-13	NC_019808	Invertebrate Mitochondrial	Acanthocephala	Archiacanthocephala	Oligacanthorhynchida	Oligacanthorhynchidae	Macracanthorhynchus	Macracanthorhynchus hirudinaceus
GBSP8064-13	FR856885	Invertebrate Mitochondrial	Acanthocephala	Eoacanthocephala	Neoechinorhynchida	Tenuisentidae		
GBSP8066-13	FR856883	Invertebrate Mitochondrial	Acanthocephala	Palaeacanthocephala	Echinorhynchida	Echinorhynchidae		
ANCN089-09	KM612104	Invertebrate Mitochondrial	Annelida	Clitellata	Arhynchobdellida	Erpobdellidae		
GBMIN1558-12	JN104648	Invertebrate Mitochondrial	Annelida	Clitellata	Arhynchobdellida	Hirudinidae	Hirudo	Hirudo orientalis
GENHP1048-12		Invertebrate Mitochondrial	Annelida	Clitellata	Haplotaxida	Enchytraeidae		
ANCN077-09	KM612089	Invertebrate Mitochondrial	Annelida	Clitellata	Haplotaxida	Lumbricidae	Eiseniella	Eiseniella tetraedra
ANNCA025-10	KM612071	Invertebrate Mitochondrial	Annelida	Clitellata	Haplotaxida	Lumbricidae	Dendrodrilus	Dendrodrilus rubidus
ANCN118-10	HQ920532	Invertebrate Mitochondrial	Annelida	Clitellata	Haplotaxida	Tubificidae		
ANNCA064-10	KM612030	Invertebrate Mitochondrial	Annelida	Clitellata	Rhynchobdellida	Glossiphoniidae		
GBAN4626-13	JX503029	Invertebrate Mitochondrial	Annelida	Polychaeta	Phyllodocida	Nereididae	Pseudonereis	Pseudonereis variegata
CMBIA433-11		Invertebrate Mitochondrial	Annelida	Polychaeta	Spionida	Spionidae	Spiophanes	Spiophanes kimballi
LABBI062-09	HQ023927	Invertebrate Mitochondrial	Annelida	Polychaeta	Terebellida	Terebellidae	Thelepus	Thelepus sp. CMC01
GBADC044-10	HQ956705	Invertebrate Mitochondrial	Arthropoda	Arachnida	Araneae	Dictynidae	Dictyna	Dictyna major
SPICA1778-11	JF885356	Invertebrate Mitochondrial	Arthropoda	Arachnida	Araneae	Hahniidae	Hahnia	Hahnia ononidum
SPICA1611-10	JF885193	Invertebrate Mitochondrial	Arthropoda	Arachnida	Araneae	Lycosidae	Arctosa	Arctosa insignita
SPICA1677-10	JF885256	Invertebrate Mitochondrial	Arthropoda	Arachnida	Araneae	Tetragnathidae	Tetragnatha	Tetragnatha dearmata
GBA16272-14	KJ748528	Invertebrate Mitochondrial	Arthropoda	Arachnida	Astigmata	Sarcoptidae	Sarcoptes	Sarcoptes scabiei
GBA17391-14	FN394341	Invertebrate Mitochondrial	Arthropoda	Arachnida	Ixodida	Argasidae	Argas	Argas persicus
GBCH12423-13	KC488312	Invertebrate Mitochondrial	Arthropoda	Arachnida	Ixodida	Ixodidae	Ixodes	Ixodes scapularis
CNPCR148-13	KM832079	Invertebrate Mitochondrial	Arthropoda	Arachnida	Mesostigmata	Ascidae		
MIONT623-09	HM379389	Invertebrate Mitochondrial	Arthropoda	Arachnida	Mesostigmata	Parasitidae		
UAMIC1807-14		Invertebrate Mitochondrial	Arthropoda	Arachnida	Opiliones	Ceratolasmatidae	Hesperonemastoma	Hesperonemastoma modestum
OPILI311-11	KM837281	Invertebrate Mitochondrial	Arthropoda	Arachnida	Opiliones	Phalangiidae	Phalangium	Phalangium opilio
UAMIC1778-14		Invertebrate Mitochondrial	Arthropoda	Arachnida	Opiliones	Sabaconidae	Sabacon	
OPILI385-11	KM824955	Invertebrate Mitochondrial	Arthropoda	Arachnida	Opiliones	Sclerosomatidae	Nelima	Nelima paessleri
GENHP1398-12		Invertebrate Mitochondrial	Arthropoda	Arachnida	Pseudoscorpiones	Chthoniidae	Chthonius	Chthonius ischnocheles
UAMIC1763-14		Invertebrate Mitochondrial	Arthropoda	Arachnida	Pseudoscorpiones	Neobisiidae	Halobisium	Halobisium occidentale
SSWLA032-13	KM824002	Invertebrate Mitochondrial	Arthropoda	Arachnida	Sarcoptiformes	Ceratozetidae		
CNBPB528-12	KM830640	Invertebrate Mitochondrial	Arthropoda	Arachnida	Sarcoptiformes	Oripodidae		
CHACA811-09	JX835945	Invertebrate Mitochondrial	Arthropoda	Arachnida	Sarcoptiformes	Trhypochthoniidae	Trhypochthonius	Trhypochthonius tectorum
GBCH6916-13	JF700145	Invertebrate Mitochondrial	Arthropoda	Arachnida	Scorpiones	Buthidae	Mesobuthus	Mesobuthus martensii
GBCH0452-06	AY156575	Invertebrate Mitochondrial	Arthropoda	Arachnida	Scorpiones	Scorpionidae	Heterometrus	Heterometrus swammerdami
SSBAB1929-12	KM833288	Invertebrate Mitochondrial	Arthropoda	Arachnida	Trombidiformes	Rhagidiidae		
CNGIB587-12	KM827486	Invertebrate Mitochondrial	Arthropoda	Arachnida	Trombidiformes	Tetranychidae		
LFWM005-10	JN034799	Invertebrate Mitochondrial	Arthropoda	Arachnida	Trombidiformes	Unionicolidae	Unionicola	Unionicola crassipes
GBCB0206-06	DQ426848	Invertebrate Mitochondrial	Arthropoda	Branchiopoda	Anostraca	Artemiidae	Artemia	Artemia salina
ZPII1536-11	KC616942	Invertebrate Mitochondrial	Arthropoda	Branchiopoda	Diplostraca	Daphniidae	Daphnia	Daphnia laevis
SABRA124-08	JN233969	Invertebrate Mitochondrial	Arthropoda	Branchiopoda	Diplostraca	Sididae	Sida	Sida crystallina BER1
BBMYR073-10	KM611678	Invertebrate Mitochondrial	Arthropoda	Chilopoda	Geophilomorpha			
COLNO228-09		Invertebrate Mitochondrial	Arthropoda	Chilopoda	Lithobiomorpha	Lithobiidae	Lithobius	Lithobius forficatus
GACCH080-13	JF700178	Invertebrate Mitochondrial	Arthropoda	Chilopoda	Scolopendromorpha	Scolopendridae	Scolopendra	Scolopendra subspinipes
MHCLB433-09	GU657253	Invertebrate Mitochondrial	Arthropoda	Collembola	Entomobryomorpha	Isotomidae	Isotoma	Isotoma riparia
MHCLM550-08	KF642224	Invertebrate Mitochondrial	Arthropoda	Collembola	Poduromorpha	Hypogastruridae	Hypogastrura	Hypogastrura arctandria
MHCLM689-08	KF642150	Invertebrate Mitochondrial	Arthropoda	Collembola	Symphypleona	Bourletiellidae	Heterosminthurus	Heterosminthurus sp2
SSROA1447-14		Invertebrate Mitochondrial	Arthropoda	Diplopoda	Julida	Julidae	Julus	Julus scandinavius
COLNO227-09		Invertebrate Mitochondrial	Arthropoda	Diplopoda	Polydesmida	Polydesmidae	Polydesmus	Polydesmus angustus
MYFAB206-10	HQ966144	Invertebrate Mitochondrial	Arthropoda	Diplopoda	Polyxenida	Polyxenidae	Polyxenus	Polyxenus lagurus

RBNII219-13		Invertebrate Mitochondrial	Arthropoda	Diplopoda	Polyzoniida	Polyzoniidae		
GBA8902-12	AB608779	Invertebrate Mitochondrial	Arthropoda	Diplopoda	Spirobolida	Spirobolidae	Paraspirobolus	Paraspirobolus lucifugus
GBA11456-13	JQ846461	Invertebrate Mitochondrial	Arthropoda	Entognatha	Protura	Acerentomidae	Yamatentomon	Yamatentomon guoi
PROAT068-12		Invertebrate Mitochondrial	Arthropoda	Entognatha	Protura	Eosentomidae	Eosentomon	Eosentomon cetium
SIOCA201-10	JN309503	Invertebrate Mitochondrial	Arthropoda	Insecta	Archaeognatha	Machilidae	Pedetontus	Pedetontus submutans
GBMH1509-06	AY793551	Invertebrate Mitochondrial	Arthropoda	Insecta	Archaeognatha	Meinertellidae	Nesomachilis	Nesomachilis australica
BMBLA079-10	JN297486	Invertebrate Mitochondrial	Arthropoda	Insecta	Blattodea	Blaberidae	Geoscapheus	Geoscapheus dilatatus
GBA8745-12	JQ350728	Invertebrate Mitochondrial	Arthropoda	Insecta	Blattodea	Blattellidae	Blattella	Blattella germanica
INRMA361-12		Invertebrate Mitochondrial	Arthropoda	Insecta	Blattodea	Blattidae	Periplaneta	Periplaneta australasiae
GMGSG370-12		Invertebrate Mitochondrial	Arthropoda	Insecta	Blattodea	Cryptocercidae	Cryptocercus	
COLFD501-12	KJ965833	Invertebrate Mitochondrial	Arthropoda	Insecta	Coleoptera	Carabidae	Carabus	Carabus cancellatus
COLFE1084-13	KJ964698	Invertebrate Mitochondrial	Arthropoda	Insecta	Coleoptera	Cryptophagidae	Atomaria	Atomaria wollastoni
COLFB441-12	KJ966199	Invertebrate Mitochondrial	Arthropoda	Insecta	Coleoptera	Curculionidae	Anthonomus	Anthonomus rubi
COLFF204-13	KJ963738	Invertebrate Mitochondrial	Arthropoda	Insecta	Coleoptera	Elateridae	Agrypnus	Agrypnus murinus
COLFE846-13	KJ963185	Invertebrate Mitochondrial	Arthropoda	Insecta	Coleoptera	Staphylinidae	Atheta	Atheta volans
SIOCA269-10	KM536227	Invertebrate Mitochondrial	Arthropoda	Insecta	Dermaptera	Forficulidae	Forficula	Forficula auricularia-A
GBA8702-12	JN241998	Invertebrate Mitochondrial	Arthropoda	Insecta	Dermaptera	Labiduridae	Labidura	Labidura riparia
GBA0018-06	DQ529237	Invertebrate Mitochondrial	Arthropoda	Insecta	Diplura	Campodeidae	Campodea	Campodea lubbocki
GBA11473-13	JQ796635	Invertebrate Mitochondrial	Arthropoda	Insecta	Diplura	Parajapygidae	Parajapyx	Parajapyx emeryanus
TTDFW099-08	KM571291	Invertebrate Mitochondrial	Arthropoda	Insecta	Diptera	Acroceridae		
DIPNO102-09	HM909572	Invertebrate Mitochondrial	Arthropoda	Insecta	Diptera	Anisopodidae		
ASRMA103-10	JN289753	Invertebrate Mitochondrial	Arthropoda	Insecta	Diptera	Asilidae	Laphria	Laphria postica
BBDCM702-10	JF867395	Invertebrate Mitochondrial	Arthropoda	Insecta	Diptera	Bibionidae	Bibio	
TTDBW501-09	KM570561	Invertebrate Mitochondrial	Arthropoda	Insecta	Diptera	Chaoboridae	Chaoborus	
CDINV096-07	HM137906	Invertebrate Mitochondrial	Arthropoda	Insecta	Diptera	Chironomidae	Chironomus	Chironomus riparius
GBMIN13613-13	HF562807	Invertebrate Mitochondrial	Arthropoda	Insecta	Diptera	Culicidae	Culex	Culex pipiens
CNSLB427-12	KP048218	Invertebrate Mitochondrial	Arthropoda	Insecta	Diptera	Empididae	Empis	Empis sp. 7
GBMIN18768-13	JQ246707	Invertebrate Mitochondrial	Arthropoda	Insecta	Diptera	Hippoboscidae	Ornithoctona	Ornithoctona erythrocephala
CNEIB1014-12		Invertebrate Mitochondrial	Arthropoda	Insecta	Diptera	Lonchopteridae	Lonchoptera	
TTDFW319-08	KM570270	Invertebrate Mitochondrial	Arthropoda	Insecta	Diptera	Muscidae	Musca	Musca domestica
NODIP096-13	KF147195	Invertebrate Mitochondrial	Arthropoda	Insecta	Diptera	Oestridae	Hypoderma	Hypoderma tarandi
PSYFI053-11	JQ349597	Invertebrate Mitochondrial	Arthropoda	Insecta	Diptera	Psychodidae	Psychoda	Psychoda lativentris
TTDFW481-08	KM570254	Invertebrate Mitochondrial	Arthropoda	Insecta	Diptera	Scathophagidae	Scathophaga	Scathophaga stercoraria
CNGLF1290-13		Invertebrate Mitochondrial	Arthropoda	Insecta	Diptera	Sciomyzidae		
CFWIG566-10	JF870372	Invertebrate Mitochondrial	Arthropoda	Insecta	Diptera	Simuliidae	Simulium	
SSBAB1396-12	KM933989	Invertebrate Mitochondrial	Arthropoda	Insecta	Diptera	Sphaeroceridae		
SSBAF2762-13	KM926940	Invertebrate Mitochondrial	Arthropoda	Insecta	Diptera	Stratiomyidae		
TTDFW423-08	KM570738	Invertebrate Mitochondrial	Arthropoda	Insecta	Diptera	Syrphidae	Syrphus	Syrphus vitripennis
GBMIN18091-13	KC136014	Invertebrate Mitochondrial	Arthropoda	Insecta	Diptera	Tabanidae	Tabanus	Tabanus taiwanus
UAMIC1122-13		Invertebrate Mitochondrial	Arthropoda	Insecta	Diptera	Tipulidae	Tipula	Tipula aleutica
CNRMG032-12	KP041176	Invertebrate Mitochondrial	Arthropoda	Insecta	Diptera	Ulidiidae		
CNPCK058-13		Invertebrate Mitochondrial	Arthropoda	Insecta	Diptera	Xylophagidae		
CFWIE183-10	JN297628	Invertebrate Mitochondrial	Arthropoda	Insecta	Ephemeroptera	Baetidae	Baetis	Baetis tricaudatus
FAMAY081-07	JQ663178	Invertebrate Mitochondrial	Arthropoda	Insecta	Ephemeroptera	Caenidae	Caenis	Caenis eglinensis
EPHNB752-09	GU682091	Invertebrate Mitochondrial	Arthropoda	Insecta	Ephemeroptera	Ephemeridae	Ephemera	Ephemera varia
LJMAY262-11	JQ661982	Invertebrate Mitochondrial	Arthropoda	Insecta	Ephemeroptera	Leptophlebiidae	Paraleptophlebia	Paraleptophlebia memorialis
SSJAA1402-13	KM530619	Invertebrate Mitochondrial	Arthropoda	Insecta	Grylloblattodea	Grylloblattidae	Grylloblatta	
MAWFL243-13	KC626996	Invertebrate Mitochondrial	Arthropoda	Insecta	Hemiptera	Aleyrodidae	Bemisia	Bemisia tabaci
TTHFW053-08		Invertebrate Mitochondrial	Arthropoda	Insecta	Hemiptera	Cercopidae		
NCCD155-11		Invertebrate Mitochondrial	Arthropoda	Insecta	Hemiptera	Cicadellidae	Empoasca	

ANICY856-11		Invertebrate Mitochondrial	Arthropoda	Insecta	Hemiptera	Cicadidae	Yoyetta	Yoyetta sp. 513 nr. abdominalis
UAMIC640-13		Invertebrate Mitochondrial	Arthropoda	Insecta	Hemiptera	Cimicidae	Cimex	Cimex lectularius
BBPEC014-09	GU692449	Invertebrate Mitochondrial	Arthropoda	Insecta	Hemiptera	Cixiidae	Cixius	Cixius nervosus
LHASA725-07		Invertebrate Mitochondrial	Arthropoda	Insecta	Hemiptera	Coccidae		
UDCC044-13		Invertebrate Mitochondrial	Arthropoda	Insecta	Hemiptera	Delphacidae	Parkana	Parkana alata
ASMEL102-11	KF461381	Invertebrate Mitochondrial	Arthropoda	Insecta	Hemiptera	Diaspididae		
NCCD1136-11		Invertebrate Mitochondrial	Arthropoda	Insecta	Hemiptera	Enicocephalidae	Systelloderes	Systelloderes biceps
SIHET1180-13		Invertebrate Mitochondrial	Arthropoda	Insecta	Hemiptera	Fulgoridae	Neolieftinckana	Neolieftinckana dorsirubra
CNCHB920-11		Invertebrate Mitochondrial	Arthropoda	Insecta	Hemiptera	Gerridae	Gerris	Gerris buenoi
GBMHH1383-13	GQ884145	Invertebrate Mitochondrial	Arthropoda	Insecta	Hemiptera	Peloridiidae	Hackeriella	Hackeriella veitchi
USHEM038-10	HQ985126	Invertebrate Mitochondrial	Arthropoda	Insecta	Hemiptera	Pentatomidae	Acrosternum	Acrosternum hilare
SSBAB1692-12		Invertebrate Mitochondrial	Arthropoda	Insecta	Hemiptera	Pseudococcidae	Puto	
BBHCM075-10		Invertebrate Mitochondrial	Arthropoda	Insecta	Hemiptera	Psyllidae		
EUBUG1200-12	KM022393	Invertebrate Mitochondrial	Arthropoda	Insecta	Hemiptera	Saldidae	Saldula	Saldula saltatoria
GBMIN31766-13	HQ264042	Invertebrate Mitochondrial	Arthropoda	Insecta	Hymenoptera	Braconidae	Meteorus	Meteorus pendulus
TTHYB370-09		Invertebrate Mitochondrial	Arthropoda	Insecta	Hymenoptera	Cephidae	Cephus	Cephus cinctus
TTHYW547-08		Invertebrate Mitochondrial	Arthropoda	Insecta	Hymenoptera	Chrysididae	Chrysis	Chrysis impressa
BBFOB261-10	JN292029	Invertebrate Mitochondrial	Arthropoda	Insecta	Hymenoptera	Formicidae	Formica	Formica glacialis
BBHEC575-09	HM414254	Invertebrate Mitochondrial	Arthropoda	Insecta	Hymenoptera	Halictidae	Halictus	Halictus rubicundus
BBHYL803-10	JN294109	Invertebrate Mitochondrial	Arthropoda	Insecta	Hymenoptera	Ichneumonidae	Ichneumon	
CYTC3565-12	NC_014485	Invertebrate Mitochondrial	Arthropoda	Insecta	Hymenoptera	Mutillidae	Radoszkowskius	Radoszkowskius oculata
GBAH2225-06	EF032284	Invertebrate Mitochondrial	Arthropoda	Insecta	Hymenoptera	Orussidae	Orussus	Orussus minutus
SSBAF7386-13	KM558287	Invertebrate Mitochondrial	Arthropoda	Insecta	Hymenoptera	Pteromalidae	Pteromalus	Pteromalus phycidis
GBMIN8495-12	JQ619794	Invertebrate Mitochondrial	Arthropoda	Insecta	Hymenoptera	Siricidae	Sirex	Sirex nigricornis
BBHYL869-10	JN294155	Invertebrate Mitochondrial	Arthropoda	Insecta	Hymenoptera	Tenthredinidae	Tenthredo	Tenthredo stricklandi
TTHYW345-08	KM560699	Invertebrate Mitochondrial	Arthropoda	Insecta	Hymenoptera	Vespidae	Vespula	Vespula alascensis
GBAH2184-06	EF032210	Invertebrate Mitochondrial	Arthropoda	Insecta	Hymenoptera	Xyelidae	Xyela	Xyela sp. Sus1
GBMH6241-09	EU253842	Invertebrate Mitochondrial	Arthropoda	Insecta	Isoptera	Kalotermitidae	Kalotermes	Kalotermes flavicollis
TDWGB463-10	HQ978941	Invertebrate Mitochondrial	Arthropoda	Insecta	Isoptera	Rhinotermitidae	Reticulitermes	
GAISO145-13	KF430109	Invertebrate Mitochondrial	Arthropoda	Insecta	Isoptera	Termitidae	Aparatermes	Aparatermes cingulatus
GAISO059-13	KC136611	Invertebrate Mitochondrial	Arthropoda	Insecta	Isoptera	Termopsidae	Zootermopsis	Zootermopsis angusticollis
LEFIG341-10	HM876018	Invertebrate Mitochondrial	Arthropoda	Insecta	Lepidoptera	Micropterigidae	Micropterix	Micropterix aruncella
LEFIF304-10	HM874991	Invertebrate Mitochondrial	Arthropoda	Insecta	Lepidoptera	Noctuidae	Noctua	Noctua comes
LEFIC010-10		Invertebrate Mitochondrial	Arthropoda	Insecta	Lepidoptera	Tineidae	Niditinea	Niditinea fuscella
LEFIF835-10	HM875518	Invertebrate Mitochondrial	Arthropoda	Insecta	Lepidoptera	Tortricidae	Acleris	Acleris variegana
LEFIL258-10		Invertebrate Mitochondrial	Arthropoda	Insecta	Lepidoptera	Yponomeutidae	Yponomeuta	Yponomeuta cagnagella
JSMTW018-11	KM529415	Invertebrate Mitochondrial	Arthropoda	Insecta	Mantodea	Mantidae	Mantis	Mantis religiosa
TTSOW540-11	KM529089	Invertebrate Mitochondrial	Arthropoda	Insecta	Mantodea	Mantidae		
GBMH1667-06	DQ241798	Invertebrate Mitochondrial	Arthropoda	Insecta	Mantophasmatodea	Mantophasmatidae	Sclerophasma	Sclerophasma paresisense
JSMW004-11	KM535499	Invertebrate Mitochondrial	Arthropoda	Insecta	Mecoptera	Bittacidae	Bittacus	Bittacus strigosus
UAMIC852-13		Invertebrate Mitochondrial	Arthropoda	Insecta	Mecoptera	Boreidae	Boreus	Boreus borealis
WEAI089-11		Invertebrate Mitochondrial	Arthropoda	Insecta	Mecoptera	Panorpidae	Panorpa	Panorpa subfurcata
WEAI081-11		Invertebrate Mitochondrial	Arthropoda	Insecta	Megaloptera	Sialidae	Sialis	Sialis BIO6
HETFI064-11	JX438308	Invertebrate Mitochondrial	Arthropoda	Insecta	Neuroptera	Hemerobiidae	Hemerobius	Hemerobius perelegans
SISAF329-12		Invertebrate Mitochondrial	Arthropoda	Insecta	Neuroptera	Myrmeleontidae		
HETFI077-11	JX438303	Invertebrate Mitochondrial	Arthropoda	Insecta	Neuroptera	Sisyridae	Sisyra	Sisyra nigra
ECODB311-09	GU713015	Invertebrate Mitochondrial	Arthropoda	Insecta	Odonata	Aeshnidae	Basiaeschna	Basiaeschna janata
ECODN159-09	JN419409	Invertebrate Mitochondrial	Arthropoda	Insecta	Odonata	Calopterygidae	Calopteryx	
ODSO315-08	KM532506	Invertebrate Mitochondrial	Arthropoda	Insecta	Odonata	Lestidae	Lestes	Lestes dryas
ODRMA115-10	JF839318	Invertebrate Mitochondrial	Arthropoda	Insecta	Odonata	Libellulidae	Sympetrum	Sympetrum danae

GBA7732-12	GU122415	Invertebrate Mitochondrial	Arthropoda	Insecta	Orthoptera	Acrididae	Locusta	Locusta migratoria
INRMA006-12		Invertebrate Mitochondrial	Arthropoda	Insecta	Orthoptera	Tetrigidae	Tetrix	Tetrix brunnerii
GBMIN15439-13	HQ609201	Invertebrate Mitochondrial	Arthropoda	Insecta	Orthoptera	Tettigoniidae	Conanalus	Conanalus pieli
TTSOW546-11	KM531234	Invertebrate Mitochondrial	Arthropoda	Insecta	Phasmatodea	Heteronemiidae	Diapheromera	Diapheromera femorata
GBMH8642-13	AB477461	Invertebrate Mitochondrial	Arthropoda	Insecta	Phasmatodea	Phasmatidae	Phyllium	Phyllium giganteum
GBMH8260-13	JQ339221	Invertebrate Mitochondrial	Arthropoda	Insecta	Phasmatodea		Timema	Timema cristinae
MAPTH590-13	KJ840111	Invertebrate Mitochondrial	Arthropoda	Insecta	Phthiraptera	Pediculidae	Pediculus	Pediculus humanus_D
GBMHP027-13	JX855267	Invertebrate Mitochondrial	Arthropoda	Insecta	Phthiraptera	Philopteridae	Brueelia	Brueelia interposita
GMNCA119-12		Invertebrate Mitochondrial	Arthropoda	Insecta	Plecoptera	Leuctridae		
PLCHU220-08	GU115803	Invertebrate Mitochondrial	Arthropoda	Insecta	Plecoptera	Nemouridae	Nemoura	Nemoura arctica
CFWIB247-10	HQ939318	Invertebrate Mitochondrial	Arthropoda	Insecta	Plecoptera	Perlidae	Calineuria	Calineuria californica
GMNCG451-12		Invertebrate Mitochondrial	Arthropoda	Insecta	Psocoptera	Lachesillidae		
TDWGB368-10	HQ978905	Invertebrate Mitochondrial	Arthropoda	Insecta	Psocoptera			
INRMA371-12		Invertebrate Mitochondrial	Arthropoda	Insecta	Raphidioptera	Raphidiidae	Agulla	Agulla adnixa
SIOCA226-10	JN309510	Invertebrate Mitochondrial	Arthropoda	Insecta	Siphonaptera	Ceratophyllidae	Megabothris	Megabothris quirini
HSYE170-11		Invertebrate Mitochondrial	Arthropoda	Insecta	Siphonaptera	Hystrichopsyllidae	Dinopsyllus	Dinopsyllus lypusus
HSYE497-13		Invertebrate Mitochondrial	Arthropoda	Insecta	Siphonaptera	Pulicidae	Xenopsylla	Xenopsylla cheopis
MAMTL298-12		Invertebrate Mitochondrial	Arthropoda	Insecta	Strepsiptera	Corioxenidae		
GBMH5443-09	AM286745	Invertebrate Mitochondrial	Arthropoda	Insecta	Strepsiptera	Stylopidae	Xenos	Xenos vesparum
CNWLB199-12	KM529474	Invertebrate Mitochondrial	Arthropoda	Insecta	Thysanoptera	Aeolothripidae	Aeolothrips	
BBTHY012-09	KM530889	Invertebrate Mitochondrial	Arthropoda	Insecta	Thysanoptera	Phlaeothripidae		
CYTC4417-12	AY191994	Invertebrate Mitochondrial	Arthropoda	Insecta	Thysanura	Lepidotrichidae	Tricholepidion	Tricholepidion gertschi
GBMH1943-06	NC_006080	Invertebrate Mitochondrial	Arthropoda	Insecta	Thysanura	Lepismatidae	Thermobia	Thermobia domestica
NBTRI303-08	JN658546	Invertebrate Mitochondrial	Arthropoda	Insecta	Trichoptera	Hydropsychidae	Hydropsyche	Hydropsyche sparna
SMCAD168-07	HM101883	Invertebrate Mitochondrial	Arthropoda	Insecta	Trichoptera	Hydroptilidae	Hydroptila	Hydroptila grandiosa
BBEPT290-11	KM535206	Invertebrate Mitochondrial	Arthropoda	Insecta	Trichoptera	Phryganeidae	Phryganea	Phryganea cinerea
TFLAN070-11		Invertebrate Mitochondrial	Arthropoda	Insecta	Trichoptera	Rhyacophilidae	Rhyacophila	Rhyacophila fasciata
FCGA082-05	GQ341860	Invertebrate Mitochondrial	Arthropoda	Malacostraca	Amphipoda	Gammaridae	Gammarus	Gammarus tigrinus
GBCMD10249-13	HM463571	Invertebrate Mitochondrial	Arthropoda	Malacostraca	Decapoda	Paguridae		Anomura sp. LPdivOTU161
WW131-07	FJ581922	Invertebrate Mitochondrial	Arthropoda	Malacostraca	Euphausiacea	Euphausiidae	Thysanoessa	Thysanoessa raschii
GBCMI2426-13	JQ921353	Invertebrate Mitochondrial	Arthropoda	Malacostraca	Isopoda	Asellidae	Proasellus	
ACM032-06		Invertebrate Mitochondrial	Arthropoda	Malacostraca	Mysida	Mysidae	Gastrosaccus	Gastrosaccus bispinosus
GBA14335-13	KC616794	Invertebrate Mitochondrial	Arthropoda	Maxillopoda	Calanoida			
GBA11524-13	JX502999	Invertebrate Mitochondrial	Arthropoda	Maxillopoda	Scalpelliformes	Pollicipedidae	Capitulum	Capitulum mitella
DQCS106-10	KM611719	Invertebrate Mitochondrial	Arthropoda	Maxillopoda	Sessilia	Balanidae	Balanus	
SLOBC172-07	FJ411793	Invertebrate Mitochondrial	Arthropoda	Maxillopoda	Siphonostomatoida	Caligidae	Lepeophtheirus	Lepeophtheirus salmonis
SBBM082-13		Invertebrate Mitochondrial	Arthropoda	Maxillopoda	Siphonostomatoida	Eudactylinidae	Nemesis	
GBA10398-13	HQ588747	Invertebrate Mitochondrial	Arthropoda	Merostomata	Xiphosurida	Limulidae	Limulus	Limulus polyphemus
GBFCG353-14	GU073327	Invertebrate Mitochondrial	Arthropoda	Ostracoda	Halocyprida	Halocyprididae	Conchoecia	Conchoecia hyalophyllum
GBA10022-13	AB519692	Invertebrate Mitochondrial	Arthropoda	Ostracoda	Podocopida	Loxoconchidae		
GENHP137-11		Invertebrate Mitochondrial	Arthropoda	Pauropoda				
GBA0034-06	NC_008453	Invertebrate Mitochondrial	Arthropoda	Symphyla		Scutigerellidae	Scutigerella	Scutigerella causeyae
GBSP0058-06	AB178773	Invertebrate Mitochondrial	Brachiopoda	Lingulata	Lingulida	Lingulidae	Lingula	Lingula anatina
GBSP1323-06	NC_002322	Invertebrate Mitochondrial	Brachiopoda	Rhynchonellata	Terebratulida	Laqueidae	Laqueus	Laqueus rubellus
GBSP10165-13	EU560978	Invertebrate Mitochondrial	Bryozoa	Gymnolaemata	Cheilostomatida	Calloporidae	Cauloramphus	Cauloramphus multispinosus
GBMAA480-14	KF805632	Invertebrate Mitochondrial	Bryozoa	Phylactolaemata	Plumatellida	Plumatellidae	Plumatella	Plumatella fungosa
GBMAA1543-14	KF977334	Invertebrate Mitochondrial	Chaetognatha	Sagittoidea	Aphragmophora	Sagittidae	Zonosagitta	Zonosagitta nagae
GBSP1644-10	GQ368390	Invertebrate Mitochondrial	Chaetognatha	Sagittoidea	Phragmophora	Eukrohniidae	Eukrohnia	Eukrohnia hamata
GBGCA4747-13	NC_021757	Vertebrate Mitochondrial	Chordata	Actinopterygii	Acipenseriformes	Acipenseridae	Acipenser	Acipenser schrenckii
BNAF367-09	HQ557132	Vertebrate Mitochondrial	Chordata	Actinopterygii	Perciformes	Percidae	Perca	Perca flavescens

GBAP1791-13	AB511303	Vertebrate Mitochondrial	Chordata	Amphibia	Anura	Ranidae	Rana	Rana catesbeiana
GBIR3074-12	JN850729	Vertebrate Mitochondrial	Chordata	Aves	Anseriformes	Anatidae	Anas	Anas platyrhynchos
GBIR3040-12	JN850695	Vertebrate Mitochondrial	Chordata	Aves	Passeriformes	Passeridae	Passer	Passer domesticus
GBIR0247-06	NC_002785	Vertebrate Mitochondrial	Chordata	Aves	Struthioniformes	Struthionidae	Struthio	Struthio camelus
FMV444-09	JQ354155	Vertebrate Mitochondrial	Chordata	Cephalaspidomorphi	Petromyzontiformes	Petromyzontidae	Lampetra	Lampetra ayresii
GBGC11410-13	KC526959	Vertebrate Mitochondrial	Chordata	Elasmobranchii	Rajiformes	Dasyatidae	Dasyatis	Dasyatis akajei
GBGC9617-09	AB478571	Vertebrate Mitochondrial	Chordata	Leptocardii		Branchiostomidae	Branchiostoma	Branchiostoma lanceolatum
GBMA4658-13	JF700141	Vertebrate Mitochondrial	Chordata	Mammalia	Artiodactyla	Bovidae	Bos	Bos taurus
GBMA0669-06	NC_008133	Vertebrate Mitochondrial	Chordata	Mammalia	Diprotodontia	Phascolarctidae	Phascolarctos	Phascolarctos cinereus
GBMA0517-06	NC_000891	Vertebrate Mitochondrial	Chordata	Mammalia	Monotremata	Ornithorhynchidae	Ornithorhynchus	Ornithorhynchus anatinus
GBHS0859-06	AY275535	Vertebrate Mitochondrial	Chordata	Mammalia	Primates	Hominidae	Homo	Homo sapiens
CYTC4142-12	AF534390	Vertebrate Mitochondrial	Chordata	Reptilia	Sphenodontia	Sphenodontidae	Sphenodon	Sphenodon punctatus
GBGC12668-13	NC_021766	Vertebrate Mitochondrial	Chordata	Reptilia	Squamata	Lacertidae	Lacerta	Lacerta agilis
EANAH439-12		Vertebrate Mitochondrial	Chordata	Reptilia	Squamata	Viperidae	Vipera	Vipera russelli russelli
LOBO017-12	KF369192	Mold, Protozoan, and Coelenterate Mitochondrial Code	Cnidaria	Anthozoa	Pennatulacea	Veretillidae	Veretillum	Veretillum cynomorium
GBCI1767-13	JN700978	Mold, Protozoan, and Coelenterate Mitochondrial Code	Cnidaria	Cubozoa	Cubomedusae	Carybdeidae	Carybdea	Carybdea xaymacana
RBNII103-13		Mold, Protozoan, and Coelenterate Mitochondrial Code	Cnidaria	Hydrozoa	Anthoathecata	Hydridae	Hydra	Hydra oligactis
GBCI1907-13	KC533760	Mold, Protozoan, and Coelenterate Mitochondrial Code	Cnidaria	Hydrozoa	Leptothecata	Campanulariidae	Clytia	
GBCI1668-13	JN215546	Mold, Protozoan, and Coelenterate Mitochondrial Code	Cnidaria	Scyphozoa	Rhizostomeae	Magistiidae	Mastigias	
GBCI1763-13	JN700976	Mold, Protozoan, and Coelenterate Mitochondrial Code	Cnidaria	Staurozoa	Stauromedusae	Depastridae	Craterolophus	Craterolophus convolvulus
GBCL19693-14	NC_023246	Mold, Protozoan, and Coelenterate Mitochondrial Code	Ctenophora	Tentaculata	Cydippida	Euchloridae	Cheirotonus	Cheirotonus jansoni
CYTC5087-12	JF760210	Mold, Protozoan, and Coelenterate Mitochondrial Code	Ctenophora	Tentaculata	Lobata	Bolinopsidae	Mnemiopsis	Mnemiopsis leidyi
EQCS213-11		Echinoderm and Flatworm Mitochondrial Code	Echinodermata	Asteroidea	Forcipulatida	Pycnopodiidae	Pycnopodia	Pycnopodia helianthoides
WSEC022-09	GU672429	Echinoderm and Flatworm Mitochondrial Code	Echinodermata	Asteroidea	Velatida	Pterasteridae	Pteraster	Pteraster militaris
CHEC034-11		Echinoderm and Flatworm Mitochondrial Code	Echinodermata	Crinoidea	Comatulida	Antedonidae	Florometra	Florometra serratissima
NZECA506-10		Echinoderm and Flatworm Mitochondrial Code	Echinodermata	Echinoidea	Echinoida	Echinidae	Sterechinus	Sterechinus dentifer
NZEC042-08		Echinoderm and Flatworm Mitochondrial Code	Echinodermata	Echinoidea	Echinothuroida	Echinothuridae	Tromikosoma	
NZEC166-08		Echinoderm and Flatworm Mitochondrial Code	Echinodermata	Holothuroidea	Dendrochirotida	Cucumariidae	Heterocucumis	Heterocucumis denticulata
EQCS185-11		Echinoderm and Flatworm Mitochondrial Code	Echinodermata	Holothuroidea	Dendrochirotida	Cucumariidae	Cucumaria	Cucumaria miniata
EQCS013-08	HM473910	Echinoderm and Flatworm Mitochondrial Code	Echinodermata	Ophiuroidea	Euryalida	Gorgonocephalidae	Gorgonocephalus	Gorgonocephalus eucnemis
ECHOZ360-10	HQ946179	Echinoderm and Flatworm Mitochondrial Code	Echinodermata	Ophiuroidea	Ophiurida	Ophiolepididae	Ophiomusium	Ophiomusium australe
GBMAA1272-14	JQ614997	Invertebrate Mitochondrial	Entoprocta		Loxosomatida	Loxosomatidae	Loxosomella	Loxosomella sp. JM2012
GBSP2865-10	FJ196081	Invertebrate Mitochondrial	Entoprocta			Pedicellinidae	Pedicellina	Pedicellina cernua
GBSP1196-06	DQ079965	Invertebrate Mitochondrial	Gnathostomulida		Bursovaginoidea	Austrognathiidae	Austrognathia	Austrognathia sp. MVS-2005
GBSP1197-06	DQ079966	Invertebrate Mitochondrial	Gnathostomulida		Bursovaginoidea	Gnathostomulidae	Gnathostomula	Gnathostomula axi
CYTC4099-12	AF051097	Invertebrate Mitochondrial	Hemichordata	Enteropneusta		Ptychoderidae	Balanoglossus	Balanoglossus carnosus
CYTC4675-12	FN908482	Pterobranchia Mitochondrial	Hemichordata	Pterobranchia	Rhabdopleurida	Rhabdopleuridae	Rhabdopleura	Rhabdopleura compacta
GBMBV1076-13	JF301762	Invertebrate Mitochondrial	Mollusca	Bivalvia	Euheterodonta_incertae_sedis	Pharidae	Ensis	Ensis macha
GBMBM1111-13	KC509712	Invertebrate Mitochondrial	Mollusca	Bivalvia	Mytiloida	Mytilidae	Limnoperna	Limnoperna securis
LOBO004-12	KF369158	Invertebrate Mitochondrial	Mollusca	Bivalvia	Nuculida	Nuculidae	Nucula	Nucula sulcata
NZCOC061-10	JN201138	Invertebrate Mitochondrial	Mollusca	Bivalvia	Veneroida	Veneridae	Austrovenus	Austrovenus stutchburyi
GBCPH1394-13	AB749277	Invertebrate Mitochondrial	Mollusca	Cephalopoda	Oegopsida	Gonatidae	Berryteuthis	Berryteuthis anonychus
GBMIN2572-12	HQ846088	Invertebrate Mitochondrial	Mollusca	Cephalopoda	Sepiida	Sepiidae	Sepia	Sepia esculenta
JUT206-12		Invertebrate Mitochondrial	Mollusca	Gastropoda	Littorinimorpha	Bithyniidae	Wattebledia	Wattebledia crosseana
FRANZ301-08	EU820640	Invertebrate Mitochondrial	Mollusca	Gastropoda	Neogastropoda	Turridae	Gemmula	Gemmula diomedea
CONO1265-12		Invertebrate Mitochondrial	Mollusca	Gastropoda	Sorbeoconcha	Terebridae	Terebra	Terebra larvaeformis
JSBIC067-14		Invertebrate Mitochondrial	Mollusca	Gastropoda	Stylommatophora	Arionidae	Arion	Arion subfuscus
GBMLG12884-13	JX183599	Invertebrate Mitochondrial	Mollusca	Gastropoda	Thecosomata	Cavoliniidae	Diacavolinia	Diacavolinia longirostris
GBMLG10186-13	FR693840	Invertebrate Mitochondrial	Mollusca	Gastropoda		Turbinidae	Lunella	Lunella jungi
GBML1568-13	JQ950256	Invertebrate Mitochondrial	Mollusca	Polyplacophora	Lepidopleurida	Nierstraszellidae	Nierstraszella	Nierstraszella lineata

GBML1581-13	JQ950243	Invertebrate Mitochondrial	Mollusca	Polyplacophora	Neoloricata	Lepidopleuridae	Leptochiton	Leptochiton boucheti
NEMNO042-10		Invertebrate Mitochondrial	Nematoda	Adenophorea	Chromadorida	Plectidae	Plectus	
NEMMX084-11	KC130730	Invertebrate Mitochondrial	Nematoda	Enoplea	Ascaridida	Cosmocercidae	Aplectana	
RFNPM078-07	EU646605	Invertebrate Mitochondrial	Nematoda	Secernentea	Spirurida	Dracunculidae	Dracunculus	Dracunculus lutrae
GBSP9455-13	JN881995	Invertebrate Mitochondrial	Nematomorpha	Gordioida	Gordioidea	Chordodidae	Neochordodes	Neochordodes occidentalis
GBSP9454-13	JN881996	Invertebrate Mitochondrial	Nematomorpha	Gordioida	Gordioidea	Chordodidae	Neochordodes	Neochordodes occidentalis
CMBIA368-11		Invertebrate Mitochondrial	Nemertea	Anopla	Paleonemertea	Tubulanidae	Tubulanus	Tubulanus polymorphus
GBSP8730-13	JX220620	Invertebrate Mitochondrial	Nemertea	Enopla	Bdellonemertea	Malacobdellidae	Malacobdella	Malacobdella arrokeana
GBMAA1418-14	KC754642	Invertebrate Mitochondrial	Onychophora	Onychophorida	Euonychophora	Peripatidae	Principapillatus	Principapillatus hitoyensis
GBMAA1395-14	KC754646	Invertebrate Mitochondrial	Onychophora	Onychophorida	Euonychophora	Peripatidae	Peripatus	Peripatus dominicae
GBMAA937-14	KC754647	Invertebrate Mitochondrial	Onychophora	Onychophorida	Euonychophora	Peripatopsidae	Aethrikos	Aethrikos setosa
GBMAA1312-14	KC754670	Invertebrate Mitochondrial	Onychophora	Onychophorida	Euonychophora	Peripatopsidae	Ooperipatellus	Ooperipatellus sp. DNA103742
CMBIA537-12		Invertebrate Mitochondrial	Phoronida			Phoronidae	Phoronis	Phoronis sp.
CMBIA320-11		Invertebrate Mitochondrial	Phoronida				Phoronopsis	Phoronopsis sp.
GBSP3330-11	DQ889456	Mold, Protozoan, and Coelenterate Mitochondrial Code	Placozoa				Unclassified_Placozoa	Unclassified_placozoa sp. BZ10101
CYTC5765-12	DQ889458	Mold, Protozoan, and Coelenterate Mitochondrial Code	Placozoa				Unclassified_Placozoa	Unclassified_Placozoa sp.
GBPL1207-09	AB477010	Echinoderm and Flatworm Mitochondrial Code	Platyhelminthes	Cestoda	Cyclophyllidea	Taeniidae	Echinococcus	Echinococcus multilocularis
GBPL1189-09	FM209181	Echinoderm and Flatworm Mitochondrial Code	Platyhelminthes	Cestoda	Pseudophyllidea	Diphyllobothriidae	Diphyllobothrium	Diphyllobothrium latum
GBPL3945-14	GU252718	Echinoderm and Flatworm Mitochondrial Code	Platyhelminthes	Monogenea	Monopisthocotylea	Gyrodactylidae	Macrogyrodactylus	Macrogyrodactylus clarii
GBPL4130-14	JQ038228	Echinoderm and Flatworm Mitochondrial Code	Platyhelminthes	Monogenea		Capsalidae	Neobenedenia	Neobenedenia melleni
GBSP5059-13		Echinoderm and Flatworm Mitochondrial Code	Platyhelminthes	Trematoda	Echinostomida	Fasciolidae	Fasciola	Fasciola hepatica
CYTC4903-12	HE601612	Echinoderm and Flatworm Mitochondrial Code	Platyhelminthes	Trematoda	Strigeidida	Schistosomatidae	Schistosoma	Schistosoma mansoni
GBSP4342-12	FR832385	Echinoderm and Flatworm Mitochondrial Code	Platyhelminthes	Turbellaria	Acoela	Childiidae	Philactinoposthia	Philactinoposthia brevis
GBSP4339-12	FR832382	Echinoderm and Flatworm Mitochondrial Code	Platyhelminthes	Turbellaria	Acoela	Isodiametridae	Pharyngia	Pharyngia furva
CYTC5662-12	AB618487	Echinoderm and Flatworm Mitochondrial Code	Platyhelminthes	Turbellaria	Tricladida	Dugesiidae	Dugesia	Dugesia japonica
GBSP4653-12	HM592735	Mold, Protozoan, and Coelenterate Mitochondrial Code	Porifera	Demospongiae	Astrophorida	Ancorinidae	Stelletta	Stelletta tuberosa
GBSP8758-13	HE611623	Mold, Protozoan, and Coelenterate Mitochondrial Code	Porifera	Demospongiae	Poecilosclerida	Iotrochotidae	Iotrochota	Iotrochota coccinea
CYTC3322-12	NC_010769	Mold, Protozoan, and Coelenterate Mitochondrial Code	Porifera	Hexactinellida	Hexactinosida	Aphrocallistidae	Aphrocallistes	Aphrocallistes vastus
GBSP7503-13	FR819684	Mold, Protozoan, and Coelenterate Mitochondrial Code	Porifera	Hexactinellida	Lyssacinosida	Rossellidae	Caulophacus	Caulophacus arcticus
GBSP1301-06	DQ463747	Invertebrate Mitochondrial	Priapulida	Priapulimorpha	Priapulimorphida	Priapulidae	Priapulus	Priapulus caudatus
GBSP8004-13	FN689349	Invertebrate Mitochondrial	Priapulida	Priapulimorpha	Priapulimorphida	Priapulidae	Halicryptus	Halicryptus spinulosus
GBMAA187-14	KF208357	Invertebrate Mitochondrial	Rhombozoa	Rhombozoa_incertae_sedis	Rhombozoa_order_insertae_sedis	Dicyemidae	Dicyemennea	Dicyemennea floscephalum
GBMAA207-14	KF208353	Invertebrate Mitochondrial	Rhombozoa	Rhombozoa_incertae_sedis	Rhombozoa_order_insertae_sedis	Dicyemidae	Dicyema	Dicyema papuceum
GBSP8586-13	JX183999	Invertebrate Mitochondrial	Rotifera	Bdelloidea	Philodinida	Philodinidae	Macrotrachela	Macrotrachela quadricornifera
GBMAA008-14	EF524548	Invertebrate Mitochondrial	Rotifera	Monogononta	Ploima	Brachionidae	Brachionus	Brachionus plicatilis
GBMAA950-14	GU230171	Invertebrate Mitochondrial	Sipuncula	Phascolosomatidea	Aspidosiphonida	Aspidosiphonidae	Aspidosiphon	Aspidosiphon laevis
GBAN4980-13	JQ904316	Invertebrate Mitochondrial	Sipuncula	Phascolosomatidea	Phascolosomatida	Phascolosomatidae	Phascolosoma	Phascolosoma agassizii
GBAN4942-13	JQ904354	Invertebrate Mitochondrial	Sipuncula	Sipunculidea	Golfingiida	Golfingiidae	Thysanocardia	Thysanocardia nigra
GBAN4923-13	JQ904373	Invertebrate Mitochondrial	Sipuncula	Sipunculidea	Golfingiida	Themistidae	Themiste	Themiste pyroides
GBSP7732-13	FJ435797	Invertebrate Mitochondrial	Tardigrada	Eutardigrada	Parachaela	Hypsibiidae	Isohypsibius	Isohypsibius sp. Tar195
GBSP8061-13	JX865305	Invertebrate Mitochondrial	Tardigrada	Eutardigrada	Parachaela	Hypsibiidae	Acutuncus	Acutuncus antarcticus
GBSP7727-13	FJ435802	Invertebrate Mitochondrial	Tardigrada	Eutardigrada	Parachaela	Macrobiotidae	Minibiotus	Minibiotus furcatus
GBSP9217-13	JQ689525	Invertebrate Mitochondrial	Tardigrada	Heterotardigrada	Echiniscoidea	Echiniscoididae	Echiniscoides	
GBSP10183-13	AM296016	Invertebrate Mitochondrial	Xenoturbellida				Xenoturbella	Xenoturbella bocki

Amino acid alignment of the analyzed Metazoa DNA barcodes (Dataset 1, FASTA format)

```
>GBSP8040-13|NC_019808|NC_019808|Macracanthorhynchus_hirudinaceus
IMYFILSVWSGLMGMVISVVMRLELGSVGSYLMDDHLYNSLVSSHAVLMIFFLVMPIMMGGFGNWLIPVMMGIGDMAFPRLNNFSFWLLPTSLMFLMVSMFVG-
GAGTGWTIYPPLSSGEFSGGVSVDFMVLSLHVAGLSSILASVNMISTVWCCKANNIGFEKLTLFTWSMMVTSGLIILTVPVLAAALTMLLLDRNMMTTFFDPAGGGSAVMYQHL
>GBSP8064-13|FR856885|FR856885|Tenuisentidae
MLYFLISVWGGLLGFSLSGLIRLELSVGGCWLGSEALYNMIVTSHAILMVFFLVMPLFMGGLGNLLTPLMMGVSDMAFPRLNNISVLMVYLSLGLYMFSMLKE-
GLSPGWTFYPPLSSSVFSPDVSVDISIFSLHLLGVSSILNSINILSTVYMLSSNSMSLEMTPLFVWSLSVTSVLVVITIPVLAAALAMLLLDRSLNSSFFDPAGAGGLVLYQHL
>GBSP8066-13|FR856883|FR856883|Echinorhynchidae
LMYFLVSIWGGLVGFSLSLLIRLELGSGGQWMGDEHLYNVVVTAHAIMMVFFLVMPMFMGGFGNWLMPVMLGLSDMVLPRLNNLSFLILPFSL-
LLMASSLMLKGGGAGWTMYPPLMLSDYSSGVSVDMMILSLHLAGLSSILGSINVMVTGVVGSKIAGSVEQLPLLIWALLVTAGLVLLTVPVLAAALTMLLLDRNFSSSFFDPTGGGSPLLYQHL
>ANCN089-09|KM612104|08BBANN-089|Erpobdellidae
TLYFILGAWSAMAGTGMSVLIRIELAQPGTFLGNDQIYNTIVTAHGLVMIFFMVMPILIGGFGNWLIPLMIGAPDMAFPRLNNLSFWLLPPSMIMLVFSAFVENGVGTGWTVYPPLAYNIAHSGPSVDMAIFSLHLAGASSILGSLNFISTVANMRWK
GMTLDRIPLFIWSVVITTVLLLLSLPVLAAAITMLLTDRNLNTSFFDPAGGGDPILFQHL
>GBMIN1558-12|JN104648|JN104648|Hirudo_orientalis
TLYLILGSWSAMLGSSMSSIIRIELAQPGKFLGDDQLYNSLVTAHGLVMIFFMVMPILIGGFGNWLLPLMVGAIDMSFPRMNNFSFWLLPPSMIMLLSSSMIENGVGTGWTLYPPLADNISHSGPSVDMAIFSLHMAGASSILGSLNFISTIINMRISGM
SSERVPLFVWSVVITTILLLLSLPVLAAAITMLLTDRNLNTTFFDPIGGGDPILFQHL
>GENHP1048-12|16711-G11|Enchytraeidae
TLYFILGVWAGMVGAGMSLLIRIELSQPGAFLGSDQLYNTIVTAHAFVMIFFLVMPVFIGGFGNWLLPLMLGAPDMAFPRLNNMSFWLLPPSLILLVSSAAVEKGAGTGWTVYPPLASNLAHAGPSVDLAIFSLHLAGASSILGAINFITTVINMRWSGL
RLERIPLFVWAVLITVVLLLLSLPVLAGAITMLLTDRNLNTSFFDPAGGGDPILYQHL
>ANCN077-09|KM612089|08BBANN-077|Eiseniella_tetraedra
TLYFILGIWAGMVGAGMSLLIRIELSQPGAFLGSDQLYNTIVTAHAFVMIFFLVMPVFIGGFGNWLLPLMLGAPDMAFPRLNNMSFWLLPPSLILLVSSAAVEKGAGTGWTVYPPLASNLAHAGPSVDLAIFSLHLAGASSILGAINFITTVINMRWSGL
RLERIPLFVWAVLITVILLLLSLPVLAGAITMLLTDRNLNTSFFDPAGGGDPILYQHL
>ANNCA025-10|KM612071|BIOUG00690-E04|Dendrodrilus_rubidus
TLYFILGVWAGMVGAGMSLLIRIELSQPGAFLGSDQLYNTIVTAHAFVMIFFLVMPVFIGGFGNWLLPLMLGAPDMAFPRLNNMSFWLLPPALILLVSSAAVEKGAGTGWTVYPPLSSNIAHAGPSVDLAIFSLHLAGASSILGAINFITTVINMRWSGL
RLERIPLFVWAVLITVVLLLLSLPVLAGAITMLLTDRNLNTSFFDPAGGGDPILYQHL
>ANCN118-10|HQ920532|08BBANN-118|Tubificidae
TLYMVFGLWAGMVGTGTSLLIRFELAQPGSFLGSDQLYNTLVTAHGFLMIFFMVMPIFIGGFGNWLVPLMLGAPDMAFPRLNNLSFWLMPPSLILLVSSAAVEKGAGTGWTVYPPLASNLAHSGPSVDLAIFSLHLAGAASILGAINFITTMINMRWK
GMRLERIPLFVWSVIITVILLLLTLPVLAGAITMLLTDRNLNTSFFDPAGGGDPVLYQHL
>ANNCA064-10|KM612030|BIOUG00690-H07|Glossiphoniidae
TLYFMFGAWSAMVGTAMSIIIRIELAQPGSFLANDQLYNCIITAHGLIMIFFMVMPILIGGFGNWLIPLMIGAPDMAFPRLNNLSFWLLPPSMILLVTSALVEKGAGTGWTVYPPLAANLAHSGPSVDLAIFSLHLAGASSILGALNFITTVMNMRWQG
MRVERMPLFVWAVFITVILLLLSLPVLAAAITMLLTDRNLNTAFFDPMGGGDPVLYQHL
>GBAN4626-13|JX503029|JX503029|Pseudonereis_variegata
TLYFIFGMWSGLLGTSMSLLIRAELGQPGSLLGSDQLYNTIVTAHAFLMIFFLVMPVMIGGFGNWLVPLMLGAPDMAFPRLNNMSFWLLPPSLTLLLSSAAVEKGVGTGWTVYPPLASNIAHAGPSVDLAIFSLHLAGVSSIMGALNFITTVINMRSKG
LRLERVPLFVWSVVITAILLLLSLPVLAGAITMLLTDRNLNTAFFDPAGGGDPILYQHL
>CMBIA433-11|MBI-SCCWRP-00566|Spiophanes_kimballi
TLYFILGIWSGLLGTSMSLLIRAELGQPGSLLGSDQLYNTIVTAHAFLMIFFLVMPVFIGGFGNWLLPLMLGAPDMAFPRLNNMSFWLLPPSLTLLVSSAAVESGVGTGWTVYPPLAGNLAHAGPSVDLAIFSLHLAGVSSILGALNFITTIINMRSSGMRF
ERVPLFIWSVKITAVLLLLSLPVLAGGITMLLTDRNLNTSFFDPAGGGDPILYQHL
>LABBI062-09|HQ023927|TBLABR-062|Thelepus_sp._CMC01
TLYFIFGIWGGLLGTSMSLLIRIELGQPGAFLGSDQLYNTVVTAHGLLMIFFLVMPILIGGFGNWLIPLMLGAPDMAFPRMNNMSFWLLPPALLLLLSSAAVEKGAGTGWTVYPPLSSNLAHAGPSVDLAIFSLHLAGISSILGAINFITTVANMRWKGLR
LERIPLFVWAVNITVILLLLSLPVLAGAITMLLTDRNVNTSFFDPAGGGDPILYQHL
>GBADC044-10|HQ956705|10PROBE-21164|Dictyna_major
TLYLLFGAWAAMVGTAMSVLIRIELGQPGSFLGNDQIYNVMVTAHAFVMIFFMVMPILIGGFGNWLVPLMLGAPDMAFPRMNNLSFWLLPPSLLLLFISSMVEMGVGAGWTIYPPLASLVGHSGSSVDFAIFSLHLAGASSIMGAINFISTIINMRME
GMTMEKVPLFVWSVLITAVLLLLSLPVLAGAITMLLTDRNFNTSFFDPAGGGDPILFQHL
>SPICA1778-11|JF885356|BIOUG00520-G05|Hahnia_ononidum
TLYLIFGSWSAMVGTAMSVLIRIELGQPGSFIGDDHLYNVIVTAHAFVMIFFMVMPIMIGGFGNWLVPLMLGAPDMAFPRMNNLSFWLLPPSLVLLFLSSMVEMGVGAGWTVYPPLASITGHAGSSVDFAIFSLHLAGASSIMGAINFITTIMNMRS
YGMTMEKVPLFVWSVLITAILLLLSLPVLAGAITMLLTDRNFNTSFFDPAGGGDPILFQHL
```

>SPICA1611-10|JF885193|BIOUG00519-A05|Arctosa_insignita
TLYLMFGLWSAMAGTAMSVLIRLELGHSGSLLGDDHLYNVVVTAHAFVMIFFMVMPILIGGFGNWLIPLMLGAPDMSFPRMNNLSFWLLPPSLFLLFMSSMVEMGVGAGWTVYPPLASSVGHMGSSMDFAIFSLHLAGASSIMGAVNFISTIINM
RLVGMSMEKVPLFVWSVLITAVLLLLSLPVLAGAITMLLTDRNFNTSFFDPAGGGDPILFQHL
>SPICA1677-10|JF885256|BIOUG00519-F11|Tetragnatha_dearmata
SLYFLFGVWSAMVGTAMSVLIRIELGQSGSFLGDDQLYNVIVTAHAFVMIFFMVMPILIGGFGNWLVPLMLGAPDMAFPRMNNLSFWLLPPSLFMLFISSMVDMGVGAGWTVYPPLASLEGHSGSSVDFAIFSLHLAGVSSIMGAINFISTIINMRM
SGVSMEKVPLFVWSVLITAVLLLLSLPVLAGAITMLLTDRNFNTSFFDPSGGGDPVLFQHL
>GBA16272-14|KJ748528|KJ748528|Sarcoptes_scabiei
TLYFIFGMWSGFLGAGFSMLIRYQLSQPMGISMNSMFYNSVVTAHAFIMIFFMVMPIMMGGFGNLLIPLMLGSADMAYPRLNNMSFWLLPPSLTLLLISLLCGTGSGTGWTIYPPLSSITYHSNMSVDFTIVSLHIAGISSILSSINFIVTIYNMKMKGM
SWSNLTLFAWSVLLTSFLLVFSLPVLAAALTMLLTDRNLSTSFFDPIGGGDPILYQHL
>GBA17391-14|FN394341|FN394341|Argas_persicus
TMYLIFGAWSMMIGMSLSVLIRAELGQPGSMIGDDQIYNVIVTAHAFIMIFFMVMPIMIGGFGNWLVPIMLGVPDMAFPRMNNMSFWLLPPSLLLLISSSLVESGAGTGWTVYPPLASNISHSGMSVDLAIFSLHMAGISSILGAINFISTIMNMRSD
GMILERIPLFLWSVMITAILLLLSLPVLAGAITMLLTDRNFNTSFFDPAGGGDPILYQHL
>GBCH12423-13|KC488312|KC488312|Ixodes_scapularis
TMYLIFGSWATMVGMSMSILIRTELGQPGSLIGNDQIYNVIVTAHAFIMIFFMVMPVMIGGFGNWLIPLMLGAPDMAFPRMNNLSFWLLPPSLFLLINSSLVESGAGTGWTVYPPLSSNISHSGASVDMAIFSLHLAGISSILGAINFITTIINMRSPGM
SLERMPLFVWSVFITAILLLLSLPVLAGAITMLLTDRNFNTSFFDPSGGGDPILYQHL
>CNPCR148-13|KM832079|BIOUG07637-B06|Ascidae
TLYFLFASWAGMAGSSMSLLIRCELSQSGSFIGDDCIYNVIVTAHAFVMIFFMVMPAMIGGFGNWLVPLMIGSPDMAFPRMNNMSFWLLPPSFIMLIFSGFVESGAGTGWTVYPPLSSSIFHSGSSVDMVIFSLHLAGASSILGAINFISTILNMRPKS
MSFERMPLFVWSVFITAILLLLSLPVLAGAITMLLTDRNFNTSFFDPSGGGDPVLYQHL
>MIONT623-09|HM379389|09DPMIT-0204|Parasitidae
TMYLIFAAWAGMLGTALSMIIRAELGQPGSLIGDDQIYNVVVTAHAFIMIFFMVMPALIGGFGNWLVPLMVSAPDMAFPRMNNMSFWLLPPSLLLLSSSMVESGAGTGWTVYPPLAGNMSHSGGAVDLAIFSLHLAGISSILGSINFITTIMNMRP
KEMTMERMPLFVWSVFITTILLLLSLPVLAGAITMLLTDRNFNTSFFDPSGGGDPILYQHL
>UAMIC1807-14|UAM:Ento:232200|Hesperonemastoma_modestum
TLYLVFGMWSAMLGTSLSMLIRVELGQVGSILGDDQLYNVVVTAHAFVMIFFMVMPLMIGGFGNWLVPLMIGAPDMAFPRLNNMSFWLLPPSLFLLLSSSMVESGAGTGWTVYPPLSSVMSHPGSSVDLTIFSLHLAGVSSILGAINFITTVINM--
KKMPFEQLPLFVWSVFITAILLLLSLPVLAGAITMLLVDRNFNTSFFDPSGGGDPILYQHL
>OPILI311-11|KM837281|BIOUG00881-A03|Phalangium_opilio
TMYMIFGMWAAMIGTALSMLIRAELGQPGSMMNDDQIYNVIVTAHAFVMIFFMVMPIMIGGFGNWLVPLMLGAPDMAFPRLNNMSFWLLPPSFLLLLSSTMVENGAGTGWTVYPPLSSNIAHTGASVDLTIFSLHLAGVSSILGAINFITTIINMR
TQGMIMERMPLFVWSVKITAILLLLSLPVLAGAITMLLTDRNFNTSFFDPAGGGDPILYQHL
>UAMIC1778-14|UAM:Ento:148825|Sabacon
TLYLMLGMWSAMMGTALSVIIRSELGQPGSILNDDQIYNVVVTAHAFLMIFFMVMPIMIGGFGNWLVPLMLGAPDMAFPRLNNMSFWLLPPSLLLLTLSSLMESGAGTGWTVYPPLSTNLAHPGASVDLTIFSLHLAGVSSILGAINFITTIMNMRTK
GMLMEQMPLFVWSVLITAILLLLSLPVLAGAITMLLMDRNFNTSFFDPAGGGDPILYQHL
>OPILI385-11|KM824955|BIOUG00881-G05|Nelima_paessleri
TMYMIFGFWAAMAGSALSVLIRTELGQPGSLMNDDQVYNVIVTSHAFVMIFFMVMPIMMGSFGNWLVPLMLGAPDMAFPRLNNMSFWLLPPSFLLLLSSTMVESGAGTGWTVYPPLSSNTAHSGPSVDLTIFSLHLAGISSILGAVNFITTIINMRT
KGVIYERLPLFIWSIKITAILLLLSLPVLAGAITMLLTDRNFNTSFFDPSGGGDPILYQHL
>GENHP1398-12|16707-H08|Chthonius_ischnocheles
TMYLILGVWSGCLGLSLSMIIRSELSQAGGIIGSDQIFNVMVTAHAFIMIFFMVMPMMIGGFGNWLVPMMIGAPDMAFPRMNNMSFWLLPPSLLLLMLSSLIEMGCGTGWTMYPPLANNFSHNGSAVDLAIFSLHLAGVSSILGAVNFISTILNMR
SNNFSLSKTPLFVWSILITTVLLLLALPVLAGAITMLLTDRNFNTSFFDPVGGGDPILFQHL
>UAMIC1763-14|UAM:Ento:217540|Halobisium_occidentale
TLYLIFGVWSGIMGMSLSMIIRLQLIIPGMLISD-
HNYNVVVTTHAFIMIFFMIMPMMIGGFGNWLVPLMIGSPDMAFPRMNNLSFWLLPPSLSLLILSSTMEMGAGTGWTIYPPLASNLFHTGKSVDLLIFSLHLAGISSILGAVNFITTILNMRMPSMKLNSMPLFVWSILFTTILLLLAIPVLAGAITMLLTD
RNFNTAFFVPAGGGDPILFQHL
>SSWLA032-13|KM824002|BIOUG04238-G12|Ceratozetidae
TLYFIFGAWAGLLGSALSGLIRLELGQPGSLMENDQIYNTIVTAHAFVMIFFMVMPVMIGGFGNWLVPMMIGAQDMAFPRMNNMSFWLLPPSLALLSSSAFAGMGAGTGWTVYPPLSGNLFHSGISVDLAIFSLHLAGASSILGAINFITTIMNMRS
SSMTLDTIPLFVWSVLITAVLLLLSLPVLAGAITMLLTDRNFNTTFFDPAGGGDPILYQHL
>CNBPB528-12|KM830640|BIOUG04308-A03|Oripodidae
TLYFIFGAWSGILGSSLSSAIRLELSQSGTFLGNDQIYNVIVTAHAFVMIFFMVMPIMIGGFGNWLVPLMIGAQDMAFPRMNNMSFWLLPPSLSLLSISSLAGLGAGTGWTVYPPLAGNLFHHGFSVDLAIFSLHLAGASSILGAINFISTIFNMRSDSTM
MESLPLFVWSVLITAILLLLSLPVLAGALTMLLTDRNFNTSFFDPSGGGDPILYQHL
>CHACA811-09|JX835945|08DPMIT-0811|Trhypochthonius_tectorum

TLYFIFGTWAGLMGSSLSALIRLELGQPGSLLENDQIYNTIVTAHAFVMIFFMVMPVMIGGFGNWLVPMMIGAQDMAFPRMNNMSFWLLPPSLMLLSASAFAGMGVGTGWTVYPPLAGNLFHSGISVDLAIFSLHLAGASSILGAINFITTIMNMRS
SSLSLDSIPLFVWSVLITAVLLLLALPVLAGAITMLLTDRNFNTSFFDPTGGGDPILYQHL
>GBCH6916-13|JF700145|JF700145|Mesobuthus_martensii
TMYLVFGVWASMVGTALSLLIRGEIGMPGALMGDDQVYNVVVTAHAFVMIFFMVMPIMIGGFGNWLIPLMVGAPDMAFPRMNNMSFWLLPPAFFLLLSSAMLESGAGTGWTVYPPLSSSLAHMGGSVDLTIFSLHLAGVSSILGAINFMTTIIN
MRSMGMTLDKVPLFVWSVFVTAVLLLLSLPVLAGAITMLLTDRNFNTSFFDPAGGGDPILYQHL
>GBCH0452-06|AY156575|AY156575|Heterometrus_swammerdami
TMYLILGGWASMVGTALSMMIRIETGGPGSFIGDDQIYNVVVTAHAFVMIFFMVMPIMIGGFGNWLVPLMLGAPDMAFPRLNNMSFWLLPPAFFLLLGSAALESGAGTGWTVYPPLSSSVFHSGGSVDMTIFSLHLAGASSILGAINFITTIVNMRS
DGMVLDRIPLFVWSVLVTAILLLLSLPVLAGAITMLLTDRNFNTSFFDPAGGGDPILYQHL
>SSBAB1929-12|KM833288|BIOUG03892-F04|Rhagidiidae
TMYFAFGLWSALLGASMSILIRMELGQPGSMMQNDQIYNTIVTAHAFVMIFFMVMPIMIGGFGNWLVPMMIGAPDMAFPRMNNMSFWLLPPSLSLLLMSAFTSAGVGTGWTVYPPLSSNIAHPGGAVDLGIFSLHLAGISSILGAINFMTTIINM
RSPAMSFERIPLFVWSVFITALLLLLSLPVLAGAITMLLTDRNFNTSFFDPAGGGDPILYQHL
>CNGIB587-12|KM827486|BIOUG04183-E06|Tetranychidae
TMYFMFSLFSGLIGASMSIIIRMELMQPGSLIQNDFLYNSLVTSHAMIMIFFMVMPAMIGGFGNWLIPIMMNKLDMCYPRLNNMSFWLLMPSIFLMLASSTNNILNGVGWTMYPPLTSFMFNTSSSIEMMIFSLHIAGVSSIMSAVNFITTILNLKNI
NLSINTLPLFSISILITTFLLLLALPVLAGAITMIITDRNFNTSFFDPSGGGDPILYQHL
>LFWM005-10|JN034799|NHRS-ACAR_000000005|Unionicola_crassipes
TLYFAFGSWSGMLGASLSAIIRLELGQPGSLVGNDQIYNTIVTAHAFIMIFFMVMPMLIGGFGNWLIPLMISAPDMAFPRMNNMSFWLLPPALILLCTGSLTNMGAGTGWTVYPPLSGNIAHGGVSVDLSIFSLHLAGISSILGAINFMATILNMKPKF
MKMEQMPLFVWSIFITVILLLLSLPVLAGAITMLLTDRNFNTSFFDPAGGGDPILYQHL
>GBCB0206-06|DQ426848|DQ426848|Artemia_salina
TLYFIFGAWAGMVGTSLSMLIRAELGQPGSLIGDEQVYNLIVTAHAFVMIFFMVMPILIGGFGNWLVPIMLGAPDMAFPRLNNLSFWMLPPSLTLLLASSMVESGAGTGWTVYPPLSSAIAHAGPSVDLAIFSLHLAGISSILGAVNFITTIVNMRPQS
MSVDRMPLLVWAVGITAVLLLLSLPVLAGAITMLLTDRNLNTSFFDPAGGGDPILYQHL
>ZPII1536-11|KC616942|HE-360|Daphnia_laevis
TLYFIFGIWSGMVGTALSLLIRAELGQSGSLIGDDQIYNVIVTAHAFVMIFFMVMPIMIGGFGNWLVPLMLGAPDMAFPRLNNLSFWLLPPALTLLLVGGAVESGAGTGWTVYPPLSAGIAHAGASVDLSIFSLHLAGISSILGAVNFITTIINMRSLGMT
LDRIPLFVWAVGITALLLLLSLPVLAGAITMLLTDRNLNTSFFDPAGGGDPILYQHL
>SABRA124-08|JN233969|08PROBE-2075|Sida_crystallina_BER1
SLYFMFGIWAGMVGTALSLLIRAELGQSGSLIGDDQIYNVIVTAHAFVMIFFMVMPILIGGFGNWLVPLMLGAPDMAFPRLNNLSFWLLPPALTLLLVGSGVESGAGTGWTVYPPLSSTIAHAGAAVDLSIFSLHLAGISSILGAVNFITTIINMRSQGLTL
DRIPLFVWAVGITALLLLLSLPVLAGAITMLLTDRNLNTSFFDPAGGGDPILYQHL
>BBMYR073-10|KM611678|10BBMYR-091|Geophilomorpha
TMYLIFGTWAAMAGTALSLIVRLELSQPGTLIGDDQIYNVIVTAHAFVMIFFMVMPIMMGGFGNWMLPLMLGAPDMAFPRMNNLSFWLLPPSLTLLMASMAVESGAGTGWTVYPPLAANISHSGPSVDMTIFALHLAGVSSILGSINFITTIINMRS
TGMVLERIPLFVWSVKITAVLLLLSLPVLAGAITMLLTDRNLNTSFFDPVGGGDPILYQHL
>COLNO228-09|MYRNO-0024|Lithobius_forficatus
TMYFVLGIWSAMIGTGLSLLIRLELSQPGSLIGDDQIYNVIVTAHAFIMIFFMVMPIMIGGFGNWLVPLMLGAPDMAFPRMNNMSFWLLPPSLTLLLCSAAVESGAGTGWTVYPPLSSNISHSGASVDMTIFSLHLAGASSILGAINFISTIINMRTSGM
SFERVPLFVWSVKITVILLLLSLPVLAGAITMLLTDRNLNTSFFDPTGGGDPILYQHL
>GACCH080-13|JF700178|JF700178|Scolopendra_subspinipes
TMYFIFGAWASMIGTALSLIIRLELSQPGSLIGDDQTYNVMVTAHAFIMIFFMVMPIMIGGFGNWLVPLMLGAPDMAFPRMNNLSFWLLPPSIMLLLTSALIENGAGTGWTVYPPLASNITHAGPSVDMTIFSLHLAGASSILGSINFITTIMNMRSSG
MLMERTPLFVWSVLITTILLLLSLPVLAGAITMLLTDRNFNTSFFDPAGGGDPILYQHL
>MHCLB433-09|GU657253|CHU06-COL-1078|Isotoma_riparia
TMYLIFGVWSAMVGTAFSVLIRLELGQPGSFIGDDQIYNVMVTAHAFIMIFFMVMPIMIGGFGNWLVPLMIGAPDMAFPRMNNMSFWLLPPSLTLLLAGGLVESGAGTGWTVYPPLSSGIAHAGASVDLSIFSLHLAGASSILGAVNFITTIINMRST
GMSWDRTPLFVWSVFLTAILLLLSLPVLAGAITMLLTDRNLNTSFFDPAGGGDPILYQHL
>MHCLM550-08|KF642224|CHU06-COL-0362|Hypogastrura_arctandria
TMYLIFGAWSALVGTAFSILIRMELGQPGAFIGDDQTYNVMVTAHAFIMIFFMVMPIMIGGFGNWLIPLMIGAPDMAFPRMNNMSFWLLPPSLTLLLAGGLVESGAGTGWTVYPPLASGLAHAGGSVDLSIFSLHLAGASSILGAVNFITTIINMRAP
GMSWDQTPLFVWSVFLTAILLLLSLPVLAGAITMLLTDRNFNTSFFDPAGGGDPILYQHL
>MHCLM689-08|KF642150|CHU06-COL-0501|Heterosminthurus_sp2
TMYLIFGFWSAMVGTAFSMLIRLELGQPGSFIGDDQIYNVMVTAHAFVMIFFMVMPIMIGGFGNWLVPLMIGAPDMAFPRMNNMSFWLLPPSLTLLLTGGMVESGAGTGWTVYPPLSTNISHSGGSVDLSIFSLHLAGVSSILGAINFITTIINMRSP
GMSWDQTPLFVWSVFLTAILLLLSLPVLAGAITMLLTDRNLNTTFFDPAGGGDPILYQHL
>SSROA1447-14|BIOUG12147-H02|Julus_scandinavius
TLYLIFGAWAAMAGTSLSMLIRMELSYPGSLIGDDQIYNVIVTAHAFVMIFFMVMPIMIGGFGNWLVPLMLGAPDMAFPRLNNMSFWLLPPSLFLLLMSSAVEKGAGTGWTVYPPLAGNISHSGPSVDMAIFSLHLAGASSILGAINFITTIINMRTIG
MGFEHLPLFVWAVKITAILLLLSLPVLAGAITMLLTDRNFNTSFFDPSGGGDPILYQHL

>COLNO227-09|MYRNO-0023|Polydesmus_angustus
TLYLILGAWAGLSGSAMSGFIRLELGVPGSFMGDDHIFNVVVTAHAFVMIFFMVMPIMIGGFGNWLVPIMIGAPDMAFPRLNNLSFWLLPPSLLLLLASSMVEIGVGTGWTVYPPLAGGLFHSGGAVDLAIFSLHLAGASSILGAINFISTVINMRSCG
MIYERLPLFVWSVIVTVVLLLLSLPVLAGAITMLLSDRNFNSSFFDPAGGGDPILYQHL
>MYFAB206-10|HQ966144|BC_ZSM_MYR_00206|Polyxenus_lagurus
TIYLLFGAWAGLIGTALSMLIRIELGSVGSLIGDDQIYNTVVTAHAFVMIFFMVMPIMIGGFGNWLVPLMLGAPDMAFPRMNNMSFWLLPPAFMFLLGSSMVGQGVGTGWTVYPPLAGVIAHGGGSVDLAIFSLHLAGVSSILGAINFITTILNMRSP
GMSFEKIPLFVWSVGITAVLLLLSLPVLAGAITMLLCDRNFNCSFFDPAGGGDPILYQHL
>RBNII219-13|BIOUG08056-C02|Polyzoniidae
TLYFIFGIWAGMTGTAMSIIIRSELGLPGSMINNDQIYNTTVTSHALIMIFFMVMPVMMGGFGNWLVPLMLGAPDMAFPRLNNMSFWLIPPAFFLLLSSTLVDRGVGTGWTAYPPLSASLGHPGMSMDLAIVALHLAGASSILGSTNFITTIMNMRT
MKMELDRMPLFVWSVMLTAILLLLSLPVLAGAITMLLTDRNFNTSFFDPSGGGDPILYQHL
>GBA8902-12|AB608779|AB608779|Paraspirobolus_lucifugus
TMYLIFGAWAAMVGTALSMLIRLELGQPGSLIGDDQIYNVIVTAHAFVMIFFMVMPIMIGGFGNWLVPLMLGAPDMAFPRLNNMSFWLLPPALFLLVGSSAVESGAGTGWTVYPPLAANLAHAGPSVDMAIFSLHLAGASSILGAINFITTIINMRP
YGMLLENMPLFVWAVKITAILLLLSLPVLAGAITMLLTDRNFNTSFFDPAGGGDPILYQHL
>GBA11456-13|JQ846461|JQ846461|Yamatentomon_guoi
SLYFIFGVWAGMIGLSLSVLIRTELSTPGDLIGDDQIFNVIVTAHAFIMIFFMVMPILIGGFGNWLIPLMLSAPDMAFPRMNNLSFWFLPPSVFFLIMSSMAESGVGTGWTAYPPLSDNLSHSGVSVDMAIFSLHLAGVSSILGAVNFITTIINMRSISLKM
SKISLFSWSVLITALLLLLSLPVLAAAITMLLTDRNLNSSFFNPSGGGDPILFQHL
>PROAT068-12|HP149|Eosentomon_cetium
SLYFLFGAWSAMLGTSLSLLIRAELSSCGQLIGNDQLYNVIVTAHAFIMIFFMVMPILIGGFGNWLVPLMLGSPDMAFPRMNNLSFWLLPPSLLLLVLSSIVEMGVGTGWTVYPPLSSNISHLGASVDLGIFSLHLAGASSILGAINFITTIVNTGSMKFKM
EKITLFTWSVLLTAILLLVSLPVLAGAITMLLTDRNINTSFFDPLGGGDPILFQHL
>SIOCA201-10|JN309503|10BBSIO-0201|Pedetontus_submutans
TLYLIFGVWAGLVGTSLSILIRAELGQPGSLIGDDQIYNVIVTAHAFIMIFFMVMPIMIGGFGNWLVPLMLGAPDMAFPRLNNMSFWLLPPSLLLLLSGSIVENGAGTGWTVYPPLSSGMAHAGGSVDLSIFSLHLAGASSILGAANFITTVINMRPSGM
TLDRMPLFVWSVLITAILLLLSLPVLAGAITMLLTDRNLNTSFFDPAGGGDPILYQHL
>GBMH1509-06|AY793551|AY793551|Nesomachilis_australica
TLYLIFGVWAGLIGTSLSILIRAELGQPGSLIGDDQIYNVIVTAHAFIMIFFMVMPIMIGGFGNWLVPLMLGAPDMAFPRLNNMSFWLLPPALLLLLTGSVVENGAGTGWTVYPPLSANMAHGGGSVDLSIFSLHLAGVSSILGAANFITTVINMRAPG
MTFDRIPLFVWSVLITAVLLLLSLPVLAGAITMLLTDRNLNTSFFDPAGGGDPILYQHL
>BMBLA079-10|JN297486|ANIC_Database_No.9_000218|Geoscapheus_dilatatus
TLYFIFGAWAGMVGTSLSMLIRAELNQPGSLIGDDQIYNVIVTAHAFIMIFFMVMPILIGGFGNWLVPLMLGAPDMAFPRMNNMSFWLLPPSLTLLLASSMVESGAGTGWTVYPPLASSIAHAGASVDLAIFSLHLAGVSSILGAVNFISTIINMKPISM
KPERIPLFVWAVGITALLLLLSLPVLAGAITMLLTDRNLNTSFFDPAGGGDPILYQHL
>GBA8745-12|JQ350728|JQ350728|Blattella_germanica
TLYFIFGAWSGMVGMSLSMLIRAELNQPGSLIGDDQIYNVIVTAHAFVMIFFMVMPILIGGFGNWLVPLMLGAPDMAFPRMNNMSFWFLPPSLSLLLASSLVESGAGTGWTLYPPLASGIAHAGASVDLAIFSLHLAGVSSILGAVNFISTIINMKPIN
MSPERIPLFVWSVGITALLLLLSLPVLAGAITMLLTDRNLNTSFFDPAGGGDPILYQHL
>INRMA361-12|BIOUG02794-A06|Periplaneta_australasiae
TLYFIFGAWSGMVGTSLSMLIRAELGQPGSLIGDDQIYNVIVTAHAFIMIFFMVMPIMIGGFGNWLVPLMLGAPDMAFPRMNNMSFWLLPPSLTLLLASSMVESGAGTGWTVYPPLASGIAHAGASVDLAIFSLHLAGVSSILGAVNFISTTINMKPIN
MKPERIPLFVWSVAITALLLLLSLPVLAGAITMLLTDRNLNTSFFDPAGGGDPILYQHL
>GMGSG370-12|BIOUG02950-H11|Cryptocercus
TLYFIFGAWSGMMGTSLSMLIRTELGQPGFLIGDDQIYNVIVTAHAFVMIFFMVMPILIGGFGNWLVPLMLGAPDMAFPRMNNMSFWLLPPSLSLLLMSSMIESGAGTGWTVYPPLASSIAHTGASVDLAIFSLHLAGASSILGAVNFISTTINMKPN
NMKPERIPLFVWSVGITALLLLLSLPVLAGAITMLLTDRNLNTSFFDPAGGGDPILYQHL
>COLFD501-12|KJ965833|ZMUO.004301|Carabus_cancellatus
TLYFIFGAWSGMVGTSLSMLIRAELGNPGSLIGDDQIYNVIVTAHAFVMIFFMVMPIMIGGFGNWLVPLMLGAPDMAFPRMNNMSFWLLPPSLTLLLMSSMVEKGAGTGWTVYPPLSSGIAHSGASVDLAIFSLHLAGISSILGAVNFITTIINMRSV
GMTFDRMPLFVWSVGITALLLLLSLPVLAGAITMLLTDRNLNTSFFDPAGGGDPILYQHL
>COLFE1084-13|KJ964698|ZMUO.006879|Atomaria_wollastoni
TLYFIFGAWAGMVGTSLSLLIRAELGTPGSLIGDDQIYNVIVTAHAFIMIFFMVMPMMIGGFGNWLVPLMLGAPDMAFPRLNNMSFWLLPPSLMFLLMSSLVEKGAGTGWTVYPPLSSNVAHAGASVDLAIFSLHMAGISSILGAVNFITTVINMRPK
GMKFDRLPLFVWAVKITAILLLLSLPVLAGAITMLLTDRNMNTSFFDPAGGGDPILYQHL
>COLFB441-12|KJ966199|ZMUO.001866|Anthonomus_rubi
TLYFIFGAWSGAVGTSLSMLIRTELGNPGSLIGDDQIYNTIVTAHAFIMIFFMVMPIMIGGFGNWLIPLMLAAPDMAFPRLNNMSFWLLPPSLTLLIMSSIISKGAGTGWTVYPPLSSNLAHEGASVDFAIFSLHMAGISSILGAMNFISTILNMKPMKM
KFEQMPLFIWAVKITAILLLISLPVLAGAITMLLTDRNINTSFFDPAGGGDPILYQHL
>COLFF204-13|KJ963738|ZMUO.005809|Agrypnus_murinus

TLYFLFGAWAGMLGTSLSLLIRAELGNPGSLIGNDQIYNVVVTAHAFIMIFFMVMPIMIGGFGNWLVPLMLGAPDMAFPRMNNMSFWLLPPSLSLLLMSSIVENGAGTGWTVYPPLSANIAHSGSSVDLAIFSLHLAGISSILGAVNFISTVINMRSTGI
TFDRMPLFVWAVAITALLLLSLPVLAGAITMLLTDRNLNTSFFDPAGGGDPILYQHL
>COLFE846-13|KJ963185|ZMUO.005691|Atheta_volans
TLYFIFGAWAGMVGTSLSLLIRAELGNPGSLIGDDQIYNVIVTAHAFIMIFFMVMPIVIGGFGNWLVPLMLGAPDMAFPRMNNMSFWLLPPSLTLLLMSSMVESGAGTGWTVYPPLSSNIAHGGSSVDLAIFSLHLAGISSILGAVNFISTVINMRSTGI
SFDRMPLFVWSVAITALLLLSLPVLAGAITMLLTDRNLNTSFFDPAGGGDPILYQHL
>SIOCA269-10|KM536227|BIOUG00571-G01|Forficula_auricularia-A
TLYFVFGAWSGMVGTSLSLLIRAELGQPGALIGDDQIYNVIVTAHAFVMIFFMVMPIMIGGFGNWLVPLMLSAPDMAFPRMNNMSFWLLPPSLMLLLSGSMVDSGAGTGWTVYPPLSGAIAHAGASVDLSIFSLHLAGISSILGAINFITTVINMRPS
GLKLERMPLFVWSVAITALLLLSLPVLAGAITMLLTDRNLNTSFFDPAGGGDPILYQHL
>GBA8702-12|JN241998|JN241998|Labidura_riparia
TLYFLFGAWSGMVGTSLSLLVRAELGSPGSLIGDDQIYNVIVTAHAFVMIFFMVMPIMIGGFGNWLVPLMLSAPDMAFPRMNNMSFWLLPPSLMLLMSSSMVDSGAGTGWTVYPPLSAAIAHAGASVDLTIFSLHLAGVSSILGAINFITTVINMRP
VGLSLERMPLFVWSVAITALLFLLSLPVLAGAITMLLTDRNLNTFFFDLCVGGVSVLFLHL
>GBA0018-06|DQ529237|DQ529237|Campodea_lubbocki
TLYLIFGAWAALVGTALSLLIRAELGQTGSLIGDYQIYNVIVTAHAFIMIFFVVMPIMIGGFGNWLVPLMLGAPDMAFPRMNNMSFWLLPPSLTLLLSSKMVESGAGTGWTVYPPLSSILAHAGASVDLTIFSLHLAGISSILGAINFITTVINMQSTGMT
MERTPLFVWSVLITAILLLLSLHVLAGAITMLLTDRNLNTSLFDPAGGGDPILYPHL
>GBA11473-13|JQ796635|JQ796635|Parajapyx_emeryanus
TMYLILGAWSAMLGTALSLLIRAELGQPGSMIGDDQIYNVIVTAHAFIMIFFMVMPIMIGGFGNWLVPLMLGAPDMAFPRLNNMSFWLLPPSLTLLLAGSAVENGAGTGWTVYPPLASNVAHAGGSVDLTIFSLHLAGASSILGAVNFITTVINMRTN
GMTMERMPLFVWAVFITAILLLLSLPVLAGAITMLLTDRNLNTSFFDPAGGGDPILYQHL
>TTDFW099-08|KM571291|08BBDIP-0159|Acroceridae
ILYFIFGMWSGMIGTSLSVLIRTELSHPGSLLENQQIYNSIVTTHAFIMIFFMVMPIMIGGFGNWLIPLMLGAPDMAFPRMNNMSFWLLPPSLTLMLTSNMADLGIGTGWTVYPPLSSNLFHNSPSVDLAIFSLHLAGISSILGAINFITTIINMHVPNMT
FDRMPLFVWSVFITAILLLLSLPVLAGAITMLLSDRNFNTTFFDPAGGGDPILYQHL
>DIPNO102-09|HM909572|DIPNO-0113|Anisopodidae
TLYFIFGAWASMVGTSLSMLIRAELGHPGALIGDDQIYNVIVTAHAFVMIFFMVMPIMIGGFGNWLVPLMLGAPDMAFPRMNNMSFWMLPPSLTLLLASSMVENGAGTGWTVYPPLSSGIAHAGASVDLAIFSLHLAGISSILGAVNFITTVINMRS
SGITFDRMPLFVWSVVITAILLLLSLPVLAGAITMLLTDRNLNTSFFDPAGGGDPILYQHL
>ASRMA103-10|JN289753|10-SKAS-103|Laphria_postica
TLYFIFGAWAGMVGTSLSILIRAELGHPGSLIGDDQIYNVIVTAHAFIMIFFMVMPIMIGGFGNWLVPLMLGAPDMAFPRMNNMSFWLLPPSLTLLLASSMVDNGAGTGWTVYPPLSAGIAHGGASVDLAIFSLHLAGVSSILGAVNFITTVINMRST
GITFDRMPLFVWSVVITAILLLLSLPVLAGAITMLLTDRNLNTSFFDPAGGGDPILYQHL
>BBDCM702-10|JF867395|10BBCDIP-1417|Bibio
TLYFIFGAWAGMLGTSLSMLIRAELGHPGSLLGNDQIYNVIVTAHAFIMIFFMVMPIMIGGFGNWLVPLMLGAPDMAFPRMNNMSFWMLPPSLTLLLMSSMVENGAGTGWTVYPPLSSTLSHSGGSVDLAIFSLHLAGISSILGAVNFITTVINMRAI
GISFDRMPLFVWSVVITAILLLLSLPVLAGAITMLLTDRNLNTSFFDPMGGGDPILYQHL
>TTDBW501-09|KM570561|08BBDIP-0676|Chaoborus
TLYFIFGAWAGMIGTSLSLLIRAELGHAGSLIGNDQIYNVIVTAHAFIMIFFMVMPIMIGGFGNWLVPLMLGAPDMAFPRMNNMSFWLLPPSLTLLLSSSMVEVGAGTGWTVYPPLSSNIAHAGASVDLAIFSLHLAGISSILGAINFITTTINMRAYGIS
FDRMPLFVWSVFITAILLLLSLPVLAGAITMLLTDRNLNTSFFDPAGGGDPILYQHL
>CDINV096-07|HM137906|Chi2woS_F02|Chironomus_riparius
TLYIIFGAWSGMVGTSLSMLIRAELGRPGTFIGDDQIYNVVVTAHAFIMIFFMVMPILIGGFGNWLVPLMLGAPDMAFPRMNNMSFWLLPPSLTLLLSSSFVENGAGTGWTVYPPLSSAIAHSGASVDLAIFSLHLAGISSILGSVNFITTVINMRANGIT
LDRMPLFVWSVVITTVLLLLSLPVLAGAITMLLTDRNLNTSFFDPAGGGDPILYQHL
>GBMIN13613-13|HF562807|HF562807|Culex_pipiens
TLYFIFGAWAGMVGTSLSLLIRAELSQPGVFIGNDQIYNVIVTAHAFIMIFFMVMPIMIGGFGNWLVPLMLGAPDMAFPRMNNMSFWMLPPSLTLLLSSSLVENGAGTGWTVYPPLSSGTAHAGASVDLAIFSLHLAGISSILGAVNFITTVINMRSSGI
TLDRMPLFVWSVVITAVLLLLSLPVLAGAITMLLTDRNLNTSFFDPIGGGDPILYQHL
>CNSLB427-12|KP048218|BIOUG02740-A06|Empis_sp._7
TLYFIFGAWAGMVGTSLSILIRMELGHPGALIGDDQIYNVIVTAHAFVMIFFMVMPIMIGGFGNWLVPLMLGAPDMAFPRMNNMSFWMLPPSLTLLLSSSMVENGAGTGWTVYPPLSSGIAHGGASVDLAIFSLHLAGISSILGAVNFITTVINMRST
GITFDRMPLFVWSVVITAILLLLSLPVLAGAITMLLTDRNLNTSFFDPAGGGDPILYQHL
>GBMIN18768-13|JQ246707|JQ246707|Ornithoctona_erythrocephala
TLYFIFGAWSGMIGTSLSMLIRAELGHPGALIGDDQIYNVIVTAHAFIMIFFMVMPTVIGGFGNWLVPLMLGAPDMAFPRMNNMSFWLLPPALTLIIMSSLTENGAGTGWTVYPPLSSNIAHMGASVDLAIFSLHLAGISSILGAINFITTVINMRSTGIT
FDRMPLFVWSVMITAFLLLLSLPVLAGAITMLLTDRNINTSFFDPAGGGDPILYQHL
>CNEIB1014-12|BIOUG03190-F08|Lonchoptera
TLYFIFGAWAGMVGTSLSILIRAELGNPGALIGDDQIYNVIVTAHAFVMIFFMVMPIMIGGFGNWLVPLMLGAPDMAFPRMNNMSFWMLPPSLTLLLASSMVENGAGTGWTVYPPLSSGIAHGGASVDLAIFSLHLAGISSILGAVNFITTVINMRSS
GMSFDRMPLFVWSVVITALLLLLSLPVLAGAITMLLTDRNLNTSFFDPAGGGDPILYQHL

>TTDFW319-08|KM570270|08BBDIP-0878|Musca_domestica
TLYFIFGAWSGMVGTSLSILIRAELGHPGALIGDDQIYNVIVTAHAFIMIFFMVMPIMIGGFGNWLVPLMLGAPDMAFPRMNNMSFWLLPPALTLLLVSSMVEKGAGTGWTVYPPLSSIIAHGGASVDLAIFSLHLAGISSILGAVNFITTVINMRSTGIT
FDRMPLFVWSVVITALLLLLSLPVLAGAITMLLTDRNLNTSFFDPAGGGDPILYQHL
>NODIP096-13|KF147195|NHMO_DIP00004|Hypoderma_tarandi
TLYFIFGAWSGMIGTSLSILIRAELGHPGALIGDDQIYNVIVTAHAFIMIFFMVMPIMIGGFGNWLVPLMLGAPDMAFPRMNNMSFWLLPPSLTLLLLSSMVENGAGTGWTVYPPLSSNMAHGGPSVDLAIFSLHLAGISSILGAVNFITTIINMRSTGIS
LDRMPLFVWSVGITALLLLLSLPVLAGAITMLLTDRNLNTSFFDPAGGGDPILYQHL
>PSYFI053-11|JQ349597|FinPsyc53|Psychoda_lativentris
TLYFIFGSWASMVGTSLSMIIRAELGHPGSLIGNDQIYNTIVTAHAFVMIFFMVMPIMIGGFGNWLVPLMLGAPDMAFPRMNNMSFWLLPPSLLLLLNSSMVDTGAGTGWTVYPPLSSLISHGGPSVDLAIFSLHLAGISSILGAVNFITTIINMRSIGIT
FERMPLFVWSVLITAVLLLLSLPVLAGAITMLLTDRNLNTSFFDPAGGGDPILYQHL
>TTDFW481-08|KM570254|08BBDIP-1112|Scathophaga_stercoraria
TLYFIFGAWSGMIGTSLSILIRAELGHPGALIGDDQIYNVIVTAHAFIMIFFMVMPIMIGGFGNWLVPLMLGAPDMAFPRMNNMSFWLLPPALTLLLVSSMVENGAGTGWTVYPPLSSNIAHGGASVDLAIFSLHLAGISSILGAVNFITTVINMRSTGI
TFDRMPLFVWSVVITALLLLLSLPVLAGAITMLLTDRNLNTSFFDPAGGGDPILYQHL
>CNGLF1290-13|BIOUG05987-H03|Sciomyzidae
TLYFIFGAWAGMVGTSLSILIRAELGHPGALIGDDQIYNVIVTAHAFVMIFFMVMPIMIGGFGNWLVPLMLGAPDMAFPRMNNMSFWLLPPSLSLLLVSSMVENGAGTGWTVYPPLSSVIAHGGASVDLAIFSLHLAGVSSILGAVNFITTVINMRST
GISFDRMPLFVWSVAITALLLLLSLPVLAGAITMLLTDRNLNTSFFDPAGGGDPILYQHL
>CFWIG566-10|JF870372|10-SCCWRP-5601|Simulium
TLYFIFGAWAGMVGTSLSMLIRAELGHPGSLIGDDQIYNVIVTAHAFVMIFFMVMPIMIGGFGNWLVPLMLGAPDMAFPRMNNMSFWMLPPSLTLLLASSMVEAGAGTGWTVYPPLSSGIAHAGASVDLAIFSLHLAGISSILGAVNFITTIINMRSN
GITFDRMPLFVWSVVITAILLLLSLPVLAGAITMLLTDRNLNTSFFDPAGGGDPILYQHL
>SSBAB1396-12|KM933989|BIOUG03861-G06|Sphaeroceridae
TLYFMFGAWAGMVGTSLSILIRAELGHPGALIGDDQIYNVIVTAHAFVMIFFMVMPIMIGGFGNWLVPLMLGAPDMAFPRMNNMSFWLLPPSLTLLLVSSMVENGAGTGWTVYPPLSSGIAHGGASVDLAIFSLHLAGISSILGAVNFITTVINMRST
GITFDRMPLFVWSVVITALLLLLSLPVLAGAITMLLTDRNLNTSFFDPAGGGDPILYQHL
>SSBAF2762-13|KM926940|BIOUG06556-C02|Stratiomyidae
TLYFIFGAWAGMVGTSLSMLVRAELGHPGALIGDDQIYNVIVTAHAFVMIFFMVMPIMIGGFGNWLVPLMLGAPDMAFPRMNNMSFWLLPPSLTLLLASSMVDVGAGTGWTVYPPLAANIAHGGASVDLAIFSLHLAGISSILGAVNFITTVINMR
STGITFDRMPLFVWSVVITAVLLLLSLPVLAGAITMLLTDRNLNTSFFDPAGGGDPILYQHL
>TTDFW423-08|KM570738|08BBDIP-1027|Syrphus_vitripennis
TLYFIFGTWAGMVGTSLSVLIRAELGHPGALIGDDQIYNVIVTAHAFVMIFFMVMPIMIGGFGNWLVPLMLGAPDMAFPRMNNMSFWLLPPSLTLLLVSSMVENGAGTGWTVYPPLSASIAHGGASVDLAIFSLHLAGMSSILGAVNFITTVINMRS
SGLTYDRMPLFVWSVVITALLLLLSLPVLAGAITMLLTDRNLNTSFFDPAGGGDPILYQHL
>GBMIN18091-13|KC136014|KC136014|Tabanus_taiwanus
TLYFIFGAWAGMIGTSLSILIRAELGHPGSLIGDDQIYNVIVTAHAFVMIFFMVMPIMIGGFGNWLVPLMLGAPDMAFPRMNNMSFWLLPPSLTLLLASSMVENGAGTGWTVYPPLSAAIAHGGGSVDLAIFSLHLAGISSILGAVNFITTVINMRSTGI
TFDRMPLFVWAVVITAILLLLSLPVLAGAITMLLTDRNLNTSFFDPAGGGDPILYQHL
>UAMIC1122-13|UAM:Ento:151867|Tipula_aleutica
TLYFIFGAWAGMVGTSLSILIRAELGHPGALIGDDQIYNVIVTAHAFIMIFFMVMPIMIGGFGNWLVPLMLGAPDMAFPRMNNMSFWMLPPSITLLLASSMVENGAGTGWTVYPPLSSGIAHTGASVDLAIFSLHLAGISSILGAVNFITTVINMRSSGI
TLDRMPLFVWSVVITAILLLLSLPVLAGAITMLLTDRNLNTSFFDPAGGGDPILYQHL
>CNRMG032-12|KP041176|BIOUG03414-G02|Ulidiidae
TLYFIFGAWAGMVGTSLSILIRAELGHPGALIGDDQIYNVIVTAHAFVMIFFMVMPIMIGGFGNWLVPLMLGAPDMAFPRMNNMSFWLLPPSLTLLLVSSMVENGAGTGWTVYPPLSSVIAHGGASVDLAIFSLHLAGVSSILGAVNFITTVINMRST
GITFDRMPLFVWSVVITALLLLLSLPVLAGAITMLLTDRNLNTSFFDPAGGGDPILYQHL
>CNPCK058-13|BIOUG07588-C06|Xylophagidae
TLYFIFGAWAGMVGTSLSMMIRAELGHPGSLIGDDQIYNVIVTAHAFIMIFFMVMPIMIGGFGNWLVPLMLGAPDMAFPRMNNMSFWMLPPSLTLLLASSVVENGAGTGWTVYPPLSASIAHGGASVDLAIFSLHLAGISSILGAVNFITTVINMRST
GISFDRMPLFVWSVVITAILLLLSLPVLAGAITMLLTDRNLNTSFFDPAGGGDPILYQHL
>CFWIE183-10|JN297628|10-SCCWRP-3318|Baetis_tricaudatus
TLYFIFGAWAGMVGTSLSLLIRAELGNPGSLIGDDQIYNVIVTAHAFIMIFFMVMPIMIGGFGNWLVPLMLGAPDMAFPRMNNMSFWLLPPSLTLLISSSIVDVGAGTGWTVYPPLAANIAHGGSSVDFAIFSLHLAGVSSILGAVNFITTVVNMRSPG
MTLDRMPLFVWSVVITAVLLLLSLPVLAGAITMLLTDRNLNTSFFDPAGGGDPILYQHL
>FAMAY081-07|JQ663178|FAMUBOLD0081|Caenis_eglinensis
TLYFIFGVWSGMVGTSLSLVIRAELGHPGSLIGDDQIYNVIVTAHAFIMIFFMVMPIMIGGFGNWLVPLMLGAPDMAFPRMNNMSFWLLPPALTLLLTSSLVEAGAGTGWTVYPPLAAGIAHAGASVDLAIFSLHLAGISSILGAVNFITTTINMRSSG
MTMDRIPLFVWSVVITAVLLLLSLPVLAGAITMLLTDRNLNTSFFDPAGGGDPILYQHL
>EPHNB752-09|GU682091|09NBMAY-0752|Ephemera_varia

TLYFIFGAWSGMIGTSLSLLIRAELGQPGSLIGDDQIYNVIVTAHAFIMIFFMVMPIMIGGFGNWLVPLMLGAPDMAFPRMNNMSFWLLPPALTLLLASSMVESGAGTGWTVYPPLAAGIAHAGASVDLAIFSLHLAGISSILGAVNFITTTINMRTSG
MTMDRIPLFVWSVVITAVLLLLSLPVLAGAITMLLTDRNLNTSFFDPAGGGDPILYQHL
>LJMAY262-11|JQ661982|BIOUG00640-B04|Paraleptophlebia_memorialis
TLYFIFGAWSGMVGTSLSLLIRAELGQPGSLIGDDQIYNVIVTAHAFIMIFFMVMPIMIGGFGNWLVPLMLGAPDMAFPRMNNMSFWLLPPALTLLLASSMVESGAGTGWTVYPPLSAGIAHAGASVDLAIFSLHLAGVSSILGAVNFITTTINMRTSG
MTLDRIPLFVWSVVITAVLLLLSLPVLAGAITMLLTDRNLNTSFFDPAGGGDPILYQHL
>SSJAA1402-13|KM530619|BIOUG04768-H11|Grylloblatta
TLYFLFGAWSGMVGTSLSMLIRAELGQPGALIGDDQIYNVIVTAHAFVMIFFMVMPIMIGGFGNWLVPLMLGAPDMAFPRMNNMSFWLLPPSLTLLLTSSMVESGAGTGWTVYPPLSAGIAHAGASVDMAIFSLHLAGASSILGAVNFISTVINMR
STGITLERMPLFVWSVMITAVLLLLSLPVLAGAITMLLTDRNLNTSFFDPAGGGDPILYQHL
>MAWFL243-13|KC626996|NIBGE_WFL-00243|Bemisia_tabaci
VLYFIFGVWSGLIGTSFSMIIRSELMNIGSFLSNEHLYNVVVTSHAFIMIFFMTMPLVIGGFGNWLVPLMIGAPDMAFPRMNNLSFWLLVPSLIFMLASMLVSAGAGTGWTVYPPLSLGLTHSGLSVDLLIFSLHIAGVSSILGSVNFIVTIFNMRVLGMN
FEYLSLFVWSVLITVFLLLISLPVLAGAITMLLMDRNFNSSFYDPLGGGDPILYQHL
>TTHFW053-08|HEMI_0075.02|Cercopidae
TLYFLFGVWSGMIGTTLSLLIRVELGQPGSFIGDDQIYNVIVTSHAFIMIFFMVMPIMIGGFGNWLVPLMIGAPDMAFPRMNNMSFWMLPPSLTLLLSSSMVDNGVGTGWTVYPPLSSGMAHSGSCVDLAIFSLHLAGISSILGAVNFITTIFNMRCIG
MNMDRTPLFVWSVLITAILLLLSLPVLAGAITMLLTDRNLNTSFFDPAGGGDPILYQHL
>NCCD155-11|BIOUG01663-B10|Empoasca
TMYFIFGIWSGMVGMMLSMLIRVELAQPGAFFNNDQMYNVIVTSHAFIMIFFMVMPIMIGGFGNWLLPLMIGAPDMAFPRLNNMSFWLLIPSLFLLTLSSFVEVGAGTGWTVYPPLSSNIAHSGSSVDLAIFSLHLAGISSILGAVNFITTVFNMRCV
GMTFDRIPLFVWSVLITAVLLLLSLPVLAGAITMLLTDRNLNTSFFDPSGGGDPILYQHL
>ANICY856-11|11ANIC-16856|Yoyetta_sp._513_nr._abdominalis
TLYFIFGIWSGMIGTTLSVLIRVELGTPGSYIGDDQIYNVIVTAHAFIMIFFMVMPIMIGGFGNWLVPLMIGAPDMAFPRMNNMSFWLLPPSLTLLLVGSMVDSGAGTGWTVYPPLSSGIAHSGSCVDLTIFSLHLAGVSSILGAVNFISTIFNMRSVGI
WLDRMPLFVWAVLITAFLLLLSLPVLAGAITMLLTDRNLNTSFFDPAGGGDPILYQHL
>UAMIC640-13|UAM:Ento:230093|Cimex_lectularius
TLYFLFGMWAGMLGTSMSWIIRIELSQPGSFIGDDQIYNVIVTAHAFVMIFFMVMPIMIGGFGNWLVPLMIGAPDMAFPRLNNMSFWLLPPSLLLLLVSSTSSTGVGTGWTVYPPLSGNIAHMGYSVDFAIFSLHLAGMSSILGAINFISTILNMRPAG
MTLERTPLFVWSVGITAMLLLLSLPVLAGAITMLLTDRNFNTSFFDPVGGGDPVLYQHL
>BBPEC014-09|GU692449|09BBEHE-014|Cixius_nervosus
TLYFIFGLWSGLVGTMSSIIIRIELTQPGSIINNDQLYNVIVTSHAFIMIFFMVMPIMIGGFGNWLVPMMIGAPDMAFPRMNNMSFWLLPPSLLMLFSSSITGTGSGTGWTVYPPLSSFMSHSGPSVDMTIFSLHMAGISSILGAINFISTIMNMRSKEM
TMEKMPLFCWSVLITAILLLVSLPVLAGAITMLITDRNFNTSFFDPTGGGDPILYQHL
>UDCC044-13|CCDB-21319-G12|Parkana_alata
TLYFILGIWSGLLGTMSSILIRSELTQPGSLIKNDQIYNVLVTSHAFIMIFFMVMPILIGGFGNWLVPLMIGAPDMAFPRMNNMSFWLLPPSLTLLISSSIAGSGSGTGWTVYPPLSSITSHSGPSVDLTIFSLHIAGISSIMGAINFISTIINMRSKNIILEKMPL
FCWSVLITAFLLLLSLPVLAGAITMLLTDRNINTSFFDPTGGGDPILYQHL
>LHASA725-07|HUM-07-0006|Coccidae
IMYLMLGMWSGMMGMSMSMIMRIELMNMNNNFNNNLIYYMMITIHAFLMIFFMTMPIIIGSFSNWLIPLMMNSSDLMYPRLNNLSFWLLFPSLMMMVMSMFINNKIYTGWTLYPPLS---
IQNNTSINMIIFSIHMNGMSSILSSMNFMISMTNMNSNKQLMNNLSLYCWSIMITSMLLILSVPVLASGITMIILDHNLNTMFFNPMGNGNPIIFQHL
>ASMEL102-11|KF461381|CASENT0426459-D14|Diaspididae
LLYLILGIWSGMMGMSMSMIIRTELININNNFNNQSFYYSTITIHALMMIFFMTMPIIIGSMSNWLIPLMIMSLDLIYPRMNNLSFWFLLPSLSLMIINMFINNNINTGWTLYPPLI---
NQNNTSINFIIFSLHINGISSILSSMNFILTMIYLKSFNLNLINLNLFCWAIMITSTLLILSLPVLASGITMTLTDTNFKTMFFNPSGNGNPIMFQHL
>SSBAB1692-12|BIOUG02381-F07|Puto
FMYLMMGMWSSMLGMSISLIMRIEMMKMYINMNSSMIYNMMITIHAFIMIFFMTMPIIIGSFSNWLIPMMISSPDMILPRMNNFSLWLLFPSLMMMIMSMLFNNNINTGWTLYPPLS---
LQNNLSLNLIIFSLHLNGLSSMLSSMNFILSIYLMNFNLMLSNNLNLYSWSIIITSLLLILSIPVLSSAITMIIMDHNFNTMFFNPIGNGDPILYQHM
>NCCD1136-11|BIOUG01799-E05|Systelloderes_biceps
TLYFIFGIWAGILGMVLSWIIRIELGQPGSFINNDQIYNVIVTAHAFVMIFFMVMPIMIGGFGNWLIPLMIGAPDMAFPRMNNLSFWLLPPSITLLIISSIVENGSGTGWTVYPPLSNNISHNGPSVDLTIFSLHLAGISSILGAINFISTIMNMRTSNMKFN
KISLFTWSVFITAILLLLSLPVLAGAITMLLTDRNLNTSFFDPAGGGDPILYQHL
>SIHET1180-13|CCDB-21319-D04|Neolieftinckana_dorsirubra
TLYFIFGLWSSMLGTMSSLIIRMELLQPGSMIKNDQIYNTIVTSHAFIMIFFMVMPIMIGGFGNWLVPLMIGAPDMAFPRMNNMSFWLLPPSLILLISSSIVGDGSGTGWTVYPPLSAQLAHSGPSVDLTIFSLHIAGISSIMGAINFISTILNMRPKGMTL
EKMPLFCWSVLITAVLLLVSLPVLAGAITMLLTDRNFNTSFFDPTGGGDPILYQHL
>CNCHB920-11|CNC*HEM302839|Gerris_buenoi
TLYFMLGMWSGMIGMSMSWIIRIELGQPGSFIGNDQIYNVIVTAHAFIMIFFMVMPIMIGGFGNWLVPLMIGAPDMAFPRMNNMSFWLLPPSLTLLLVGSLVDAGAGTGWTVYPPLSSNIAHNGASVDLTIFSLHLAGVSSILGAVNFISTIINMRTT
GMTSEKIPLFVWSVGITALLLLLSLPVLAGAITMLLTDRNINTSFFDPAGGGDPVLYQHL

>GBMHH1383-13|GQ884145|GQ884145|Hackeriella_veitchi
TLYLILGMWSSMLGTSLSMIIRTELGQLNSFLSNDQIYNVMVTAHALVMIFFMVMPILVGGFGNWLVPMMIGAPDMAFPRLNNMSFWLLPPSLVLLISSSFIGSGAGTGWTVYPPLSSYTFHSSSAVDLTIFSLHLAGISSILGALNLITTILNMRSIGMS
MEKTPLFVWSILITSLLLLSLPVLAGAITMLLTDRNFNTSFFDPSGGGDPILYQHL
>USHEM038-10|HQ985126|10BBHEM-0038|Acrosternum_hilare
TLYFLFGMWAGMVGSAMSLIIRIELGQPGSFIGDDQIYNVVVTAHAFVMIFFMVMPIMIGGFGNWLVPLMIGAPDMAFPRMNNMSFWLLPPSLTLLMVSSLAESGAGTGWTVYPPLSSNLSHSGASVDLAIFSLHLAGVSSILGAVNFISTIINMRP
MGMTPERIPLFVWSVGITALLLLLSLPVLAGAITMLLTDRNFNTSFFDPSGGGDPILYQHL
>BBHCM075-10|10BBCHEM-1075|Psyllidae
TLYFIFGVWSGLLGLSLSMIIRLELSQSSPVLMNDQIYNTIVTSHAFIMIFFMTMPIIIGGFGNWLVPLMIGAPDMAFPRLNNLSFWLLIPSLYLLLMSSLIDQGVGTGWTVYPPLSNSMFHSGYSVDTAIFSLHLAGISSILGAINFITTIINMRSNLHTMEKL
PLFVWSVLITAFLLLLALPVLAGAITMLLTDRNMNTTFFDPAGGGDPILYQHL
>EUBUG1200-12|KM022393|EUBUG_1198_f_Saldsalt7|Saldula_saltatoria
TLYFMLGMWSGMVGTAMSWIIRIELGKPGSFIGDDQIYNVIVTAHAFIMIFFMVMPIMIGGFGNWLVPLMIGAPDMAFPRLNNMSFWLLPPSLTLLLKSSIVDSGAGTGWTVYPPLSSNVAHSGASVDLAIFSLHLAGASSILGAINFISTIINMRCTG
MTPERIPLFVWSVGITALLLLSLPVLAGAITMLLTDRNLNTSFFDPSGGGDPILYQHL
>GBMIN31766-13|HQ264042|HQ264042|Meteorus_pendulus
LLYFMFGMWSGMLGLSMSIIIRMELSSVGSLLGDDQIYNSIVTAHAFVMIFFMVMPIMIGGFGNWLIPMMLGAPDMAFPRMNNMSFWLLIPSLFLLLLSSVVNIGAGTGWTVYPPLSLNVSHGGVSVDLAIYSLHLAGASSIMGAVNFITTIVNMQL
MGLKFDMISLLIWSVLITAILLLLSLPVLAGAITMLLTDRNLNTSFFDPSGGGDPILYQHL
>TTHYB370-09|08BBHYM-0794|Cephus_cinctus
ILYFIFGIWAGMLGTSLSLIIRAELGTPGPLINNDQIYNSIVTAHAFIMIFFMIMPIMIGGFGNWLIPLMLGAPDMAFPRMNNMSFWLLPPSLTLLLISSVTNQGVGTGWTVYPPLSNNLYHSGPSVDLAIFSLHLAGMSSIMGAINFISTIINMRPLGLNLD
QMSLFGWAVGITAILLLLSLPVLAGAITMLLTDRNLNTTFFDPAGGGDPILYQHL
>TTHYW547-08|08BBHYM-0514|Chrysis_impressa
MLYFLFGMWSGMVGTALSMMIRMELGIPGSLIKNDQIYNVLITSHAFVMIFFMVMPFMIGGFGNWLVPLMLMSPDMAYPRMNNMSFWLLPPSMILMIFSSLVGSGVGTGWTVYPPLSSLVGHTGMSVDLSIFSLHIAGISSIMGAINFIVTILNMH
LKSLKLDQIYLLVWSIFITAILLLLSLPVLAGAITMLLTDRNLNTSFFDPAGGGDPILYQHL
>BBFOB261-10|JN292029|10BBCFO-0261|Formica_glacialis
ILYFLFAIWAGMIGSSMSMIIRLELGSSNSLINNDQIYNSLVTNHAFIMIFFMVMPFMIGGFGNFLIPLMLGSPDMAYPRMNNMSFWLLPPSITLLLLSNFINDGTGTGWTIYPPLSSNIFHNGPSVDLTIFSLHIAGMSSILGAINFISTILNMHHKNFSIDK
IPLLVWSILITAILLLLSLPVLAGAITMLLTDRNLNTSFFDPSGGGDPILYQHL
>BBHEC575-09|HM414254|09BBEHY-0606|Halictus_rubicundus
MLYFIFAMWSGMIGASLSMIIRMELSTPGSWINNDQIYNTIVTSHAFIMIFFMVMPFMIGGFGNWLVPLMIGAPDMAFPRMNNMSFWLLIPSLFMLMMSSTLSTGSGTGWTIYPPLSSIMYHSSFSVDFTIFSLHIAGISSIMGAINFIVSIMLMKNIS
LNMNQIPLFPWSVKITAILLLLSLPVLAGAITMLLTDRNLNTSFFDPSGGGDPILYQHL
>BBHYL803-10|JN294109|10BBCHY-3757|Ichneumon
ILYFMFGMWSGMIGSSMSLIIRMELGNPGYLINNDQIYNSIVTAHAFIMIFFMVMPIMIGGFGNWLIPLMLGAPDMAFPRMNNMSFWLLPPSILILISGSLTNQGAGTGWTVYPPLSLNLNHEGLSIDLSIFSLHMAGTSSIMGAINFITTILNMYPINM
KFEQLTLFTWSILITTILLLLAVPVLAGAITMLLTDRNLNTSFFDPSGGGDPILYQHL
>CYTC3565-12|NC_014485|NC_014485|Radoszkowskius_oculata
MLYFMFSTWSGLLGSSMSMIIRMELSSCGSMIMNDQIYNSMVTAHALVMIFFMVMPFMMGGFANTLIPLMLGCPDMAFPRMNNISFWILPPSLFMLLSTMLLEG-
VGTGWTMYPPLSSTMFHSDESIDLSIFSLHIAGASSIMGSINFITTMLNSLTEKIKMDQMPLFLWSVTLTGILLIIALPVLAGGITMLLFDRNMNTSFFDPMGGGDPVLFQHL
>GBAH2225-06|EF032284|EF032284|Orussus_minutus
VLYFIFGMWAGMVGSSLSMIIRMELGTPGPLIGNDQIYNSIVTSHAFVMIFFMVMPILIGGFGNWLIPLMLGAPDMAFPRMNNMSFWLLPPALIILSSSMFTSKGVGTGWTVYPPLS-
SSGHVGLAVDLSIFSLHIAGMSSIMGAINFISTIINMRNKNISMDKISLLSWSTNITAILLLLSLPVLAGAITMLLTDRNFNTSFFDPAGGGDPILYQHL
>SSBAF7386-13|KM558287|BIOUG06917-B10|Pteromalus_phycidis
ILYFIFGMWAGVMGLSMSMIIRLELGNPGSLIGNDQIYNSIVTTHAFTMIFFFVMPVMMGGFGNYLVPMILGAPDMAFPRMNNMSFWLLPPSLMLLISSMFIGSGTGTGWTVYPPLSSNLSHSGPSVDLSIFSLHIAGISSIMGSINFITTVLNMKIYKI-
-ENIPLLAWSMLLTAILLLLSLPVLAGAITMLLFDRNLNTSFFDPAGGGDPILYQHL
>GBMIN8495-12|JQ619794|JQ619794|Sirex_nigricornis
TLYFIFGSWAGIIGSSMSIIIRTELMSPKPFIYNDHLFNSIITGHGLIMIFFMIMPIMMGGFGNWLIPLILNTPDMAFPRMNNFSFWLLPPALLLLLLSMMNGLGPGTGWTLYPPLSSFPSHSNMSVDFGILSMHLAGISSILGSINFISTFMNMHCSSLNM
EHLPLFTWSINVTAILLVISLPVLAGAITMLLTDRNFNTTFFDPAGGGDPILFQHL
>BBHYL869-10|JN294155|10BBCHY-3823|Tenthredo_stricklandi
TLYFIFGFWSGMLGLSFSMLIRTELGMPGSMIGDDQIYNVIVTSHAFLMIFFMVMPIMIGGFGNWLIPLMLGAPDMAFPRLNNMSFWFLPPSITLLLSSSMVNSGSGTGWTVYPPLSSNMSHAGASVDLTIFSLHLAGISSILGAINFISTMINMRVKG
MNFERMPLFIWAVSLTALLLLLSLPVLAGAITMLLTDRNLNTSFFDPSGGGDPILYQHL
>TTHYW345-08|KM560699|08BBHYM-0312|Vespula_alascensis

TLYFIFALWAGTLGASMSMIIRLELSSPGALINNDQIYNTIITAHAFIMIFFMVMPFLVGGFGNWLIPLMLGVPDMAFPRMNNMSFWLLPPSLFLLILSNFIGTGVGTGWTLYPPLSSIVGHDSPSVDLGIFSIHIAGISSIMGSINFIVTILNMHTKTHSLNF
LPLFTWSILITAILLLLSLPVLAGAITMLLTDRNLNTSFFDPAGGGDPILYQHL
>GBAH2184-06|EF032210|EF032210|Xyela_sp._Sus1
TLYFIFGAWSGMVGTSLSMLIRAELGTPGTLIGNDQIYNVIVTAHAFVMIFFMVMPIMIGGFGNWLVPLMLGAPDMAFPRLNNMSFWLLPPSLTLLLASSIVDTGAGTGWTVYPPLSSGMAHAGASVDLAIFSLHLAGISSILGAINFITTIINMRTTGM
NFDRIPLFVWAVGITALLLLLSLPVLAGAITMLLTDRNLNTSFFDPAGGGDPILYQHL
>GBMH6241-09|EU253842|EU253842|Kalotermes_flavicollis
TLYFIFGAWSGMVGTSLSMLIRAELGQPGSLIGDDQIYNVIVTAHAFVMIFFMVMPIMIGGFGNWLVPLMLGAPDMAFPRMNNMSFWLLPPSLTLLLMSSMVESGAGTGWTVYPPLASGIAHAGASVDLAIFSLHLAGVSSILGAVNFISTTINMKP
NNMKPERIPLFVWAVGITALLLLLSLPVLAGAITMLLTDRNLNTSFFDPAGGGDPILYQHL
>TDWGB463-10|HQ978941|TDWG-0599|Reticulitermes
TLYFVFGAWSGMVGTSLSMLIRTELGQPGSLIGDDQIYNVIVTAHAFVMIFFMVMPIMIGGFGNWLVPLMLGAPDMAFPRMNNMSFWLLPPSLTLLLTSSTVESGAGTGWTVYPPLASGIAHAGASVDLAIFSLHLAGVSSILGAVNFISTTINMKPK
NMKPERIPLFVWSVAITALLLLLSLPVLAGAITMLLTDRNLNTSFFDPAGGGDPILYQHL
>GAISO145-13|KF430109|KF430109|Aparatermes_cingulatus
TLYFVFGAWSGMVGTSLSMLIRTELGQPGSLIGDDQIYNVIVTAHAFVMIFFMVMPIMIGGFGNWLVPLMLGAPDMAFPRMNNMSFWLLPPSLTLLLASSTVESGVGTGWTVYPPLASGIAHAGASVDLAIFSLHLAGVSSILGAVNFITTTINMKPK
SMKPERIPLFVWSIGITALLLLLSLPVLAGAITMLLTDRNLNTSFFDPAGGGDPILYQHL
>GAISO059-13|KC136611|KC136611|Zootermopsis_angusticollis
TLYFIFGAWSGMLGTSLSMLIRAELGQPGSLIGDDQIYNVIVTAHAFIMIFFMVMPILIGGFGNWLVPLMLGAPDMAFPRMNNMSFWLLPPSLTLLLTSSMVESGAGTGWTVYPPLASGMAHAGASVDLTIFSLHLAGVSSILGAVNFISTAINMKPSS
MKSEQMPLFVWAVIITAILLLLSLPVLAGAITMLLTDRNLNTSFFDPAGGGDPILYQHL
>LEFIG341-10|HM876018|MM14326|Micropterix_aruncella
ILYFLFGMWNGLIGMMFSMLIRIELSIPNYFLNNDQIFNLIITSHAFIMIFFMVMPILIGGFGNWLVPLMLGSPDMAFPRMNNLSFWLLPPSINLLLLSSFMELGTGTGWTMYPPLSSSIYHSGISVDLTIFSLHLAGISSILGAINFISTIMNMKIYNLNFNQI
PLFVWSVKITALLLLLSLPVLAGAITMLLTDRNLNTSFFDPAGGGDPILFQHL
>LEFIF304-10|HM874991|MM11092|Noctua_comes
TLYFIFGIWAGMVGTSLSLLIRAELGNPGSLIGDDQIYNTIVTAHAFIMIFFMVMPIMIGGFGNWLVPLMLGAPDMAFPRMNNMSFWLLPPSLTLLISSSIVENGAGTGWTVYPPLSSNIAHGGSSVDLAIFSLHLAGISSILGAINFITTIINMRLNSLSFD
QMPLFIWAVGITAFLLLLSLPVLAGAITMLLTDRNLNTSFFDPAGGGDPILYQHL
>LEFIC010-10|HM871880|MM03213|Niditinea_fuscella
TLYFIFGIWAGMLGTSLSLLIRAELGNPGLLIGDDQIYNTIVTAHAFIMIFFMVMPIMIGGFGNWLIPLMLGAPDMAFPRLNNMSFWLLPPSLLLLTSSSLVENGAGTGWTVYPPLSSNIAHGGSSVDLAIFSLHLAGISSILGAINFITTMINMRPMKMSL
DQMPLFAWAVLITAVLLLLSLPVLAGAITMLLTDRNLNTSFFDPAGGGDPILYQHL
>LEFIF835-10|HM875518|MM13178|Acleris_variegana
TLYFIFGIWAGMVGTSLSLLIRAELGNPGSLIGDDQIYNTIVTAHAFIMIFFMVMPIMIGGFGNWLVPLMLGAPDMAFPRMNNMSFWLLPPSIMLLISSSIVENGAGTGWTVYPPLSSNIAHSGSSVDLAIFSLHLAGISSILGAVNFITTIINMRPNNMSL
DQMPLFVWAVGITALLLLLSLPVLAGAITMLLTDRNLNTSFFDPAGGGDPILYQHL
>LEFIL258-10|MM19258|Yponomeuta_cagnagella
TLYFIFGIWSGMVGTSLSLLIRAELGNPGSLIGDDQIYNTIVTAHAFIMIFFMVMPIMIGGFGNWLVPLMLGAPDMAFPRMNNMSFWLLPPSLTLLISSSIVENGAGTGWTVYPPLSSNIAHSGSSVDLAIFSLHLAGISSILGAINFITTIINMKSNGMSFD
QMPLFVWAVGITALLLLLSLPVLAGAITMLLTDRNLNTSFFDPAGGGDPILYQHL
>JSMTW018-11|KM529415|BIOUG00871-C07|Mantis_religiosa
TLYFIFGAWAGMLGTSLSILIRTELGQPGSLIGDDQIYNVIVTAHAFIMIFFMVMPIMIGGFGNWLVPLMLGAPDMAFPRMNNMSFWLLPPSILLLLISSTVESGAGTGWTVYPPLSASIAHAGPAVDLTIFSLHLAGMSSIMGAVNFITTMINMKPIYM
NQTQVPLFVWSVGITALLLLLSLPVLAGAITMLLTDRNLNTSFFDPAGGGDPILYQHL
>TTSOW540-11|KM529089|BIOUG00863-A08|Mantidae
TLYFIFGAWAGMLGTSLSILIRTELGQPGSLIGDDQIYNVIVTAHAFIMIFFMVMPIMIGGFGNWLVPLMLGAPDMAFPRMNNMSFWLLPPSILLLLISSMVESGAGTGWTVYPPLSASIAHAGPAVDLTIFSLHLAGMSSIMGAVNFITTMINMKPIY
MNQTQVPLFVWSVGITALLLLLSLPVLAGAITMLLTDRNLNTSFFDPAGGGDPILYQHL
>GBMH1667-06|DQ241798|DQ241798|Sclerophasma_paresisense
TLYFIFGAWSGMIGTSLSMLIRTELGQPGSLIGDDQIYNVVVTAHAFIMIFFMVMPILIGGFGNWLVPLMLGAPDMAFPRMNNMSFWLLPPSLILLLSSGLVESGAGTGWTVYPPLASGIAHAGSSVDMAIFSLHLAGVSSILGAVNFISTVINMRSMG
MTLERTPLFVWAVLITAILLLLSLPVLAGAITMLLTDRNLNTSFFDPAGGGDPILYQHL
>JSMW004-11|KM535499|BIOUG00871-F11|Bittacus_strigosus
TLYFIFGAWSGMVGTSLSLLIRAELGQPGALIGDDQIYNVIVTAHAFIMIFFMVMPILIGGFGNWLVPLMLGAPDMAFPRMNNMSFWLLPPSLTLLLMSSLVENGAGTGWTVYPPLSSTIAHTGASVDLAIFSLHLAGISSILGAVNFITTVINMRMSG
MTLDRMPLFVWSVAITALLLLLSLPVLAGAITMLLTDRNLNTSFFDPAGGGDPILYQHL
>UAMIC852-13|UAM:Ento:235218|Boreus_borealis
TLYFIFGTWSGMVGTSLSILIRTELSQPGSLIESDQIYNVIVTAHAFVMIFFMVMPILIGGFGNWLVPLMLGAPDMAFPRMNNMSFWLLPPSLSLLLMSSIVENGAGTGWTVYPPLSSSIAHAGASVDLAIFSLHLAGISSILGAVNFITTVINMRPKMMT
LDRIPLFVWSVVITALLLLLSLPVLAGAITMLLTDRNLNTSFFDPAGGGDPILYQHL

>WEAI089-11|BIOUG00638-H04|Panorpa_subfurcata
TLYFIFGAWSGMVGTSLSLLIRAELGQPGALIGDDQIFNVIVTAHAFVMIFFMVMPILIGGFGNWLVPLMLGAPDMAFPRMNNMSFWLLPPSLTLLLTSSLVENGAGTGWTVYPPLSSTIAHAGASVDLAIFSLHLAGVSSILGAVNFITTVINMRSTGM
TLDRMPLFVWSVAITALLLLSLPVLAGAITMLLTDRNLNTSFFDPAGGGDPILYQHL
>WEAI081-11|BIOUG00638-G08|Sialis_BIO6
TLYFIFGAWSGMIGTSLSLLIRAELGNPGSLIGDDQIYNVIVTAHAFVMIFFMVMPILIGGFGNWLVPLMLGAPDMAFPRMNNMSFWLLPPSLTLLLSSSIVENGAGTGWTVYPPLSSAIAHAGASVDLAIFSLHLAGVSSILGAVNFITTVINMRASGM
TLDRMPLFVWSVVITAILLLLSLPVLAGAITMLLTDRNLNTSFFDPAGGGDPILYQHL
>HETFI064-11|JX438308|FinHet64|Hemerobius_perelegans
TLYFIFGIWSGLVGTSLSLLIRAELGQPGSLIGDDQIYNVIVTAHAFIMIFFMVMPIVIGGFGNWLVPLMLAAPDMAFPRMNNMSFWMLPPSLMLLLASSMVENGAGTGWTVYPPLSANIAHAGASVDLAIFSLHLAGVSSILGAVNFITTVINMRLNY
MTLDRMPLFVWSVVITALLLLLSLPVLAGAITMLLTDRNLNTSFFDPAGGGDPILYQHL
>SISAF329-12|BIOUG02800-G02|Myrmeleontidae
TLYFLFGIWAGLVGTSLSLLIRAELGQPGSLIGDDQIYNVIVTAHAFIMIFFMVMPIVIGGFGNWLVPLMLAAPDMAFPRMNNMSFWLLPPSLTLLLASSIVESGAGTGWTVYPPLSAGIAHAGASVDLAIFSLHLAGVSSILGAVNFITTVINMRLSYMTL
DRMPLFVWSVVITAILLLLSLPVLAGAITMLLTDRNLNTSFFDPAGGGDPILYQHL
>HETFI077-11|JX438303|FinHet77|Sisyra_nigra
TLYFIFGVWSGLIGTSLSVLIRAELGQPGSLIGDDQIYNVIVTAHAFIMIFFMVMPIVIGGFGNWLVPLMLGAPDMAFPRMNNMSFWLLPPSLTLLLASSMVESGAGTGWTVYPPLASNIAHAGASVDLAIFSLHLAGISSILGAVNFITTIINMRSYGMT
LDRMPLFIWSVAITALLLLLSLPVLAGAITMLLTDRNLNTSFFDPAGGGDPILYQHL
>ECODB311-09|GU713015|UNB-002-N01-IS|Basiaeschna_janata
TLYFLFGAWSGMVGTALSVLIRIELGQPGSLIGDDQIYNVIVTAHAFVMIFFMVMPIMIGGFGNWLVPLMLGAPDMAFPRLNNMSFWLLPPSLTLLLAGSMVESGAGTGWTVYPPLAGAIAHAGASVDLTIFSLHLAGVSSILGAINFITTTINMKSPG
MKMDQMPLFVWAVVITAVLLLLSLPVLAGAITMLLTDRNINTSFFDPAGGGDPILYQHL
>ECODN159-09|JN419409|UNB-010-N15-IE|Calopteryx
TLYLLFGAWAGMVGTALSMLIRVELGQPGSLIGDDQIYNVVVTAHAFVMIFFMVMPIMIGGFGNWLVPLMLGAPDMAFPRLNNMSFWLLPPALTLLLASSLVESGAGTGWTVYPPLAGVIGHAGGSVDLTIFSLHLAGVSSILGAINFITTTINMKAP
GMKLDQMPLLVWAVVITAVLLLLSLPVLAGAITMLLTDRNMNTSFFDPAGGGDPILYQHL
>ODSO315-08|KM532506|08OMSOD-0108|Lestes_dryas
TLYLMFGAWAGMVGTALSMLIRVELGQPGSLIGDDQIYNVIVTAHAFVMIFFMVMPIMIGGFGNWLVPLMLGAPDMAFPRLNNMSFWLLPPSLTLLLASSLVESGAGTGWTVYPPLAGAIAHAGASVDLTIFSLHLAGVSSILGAINFITTTINMKSP
GMKLDQMPLFVWAVVITAILLLLSLPVLAGAITMLLTDRNINTSFFDPAGGGDPILYQHL
>ODRMA115-10|JF839318|10-SKOD-115|Sympetrum_danae
TLYLIFGAWAGMIGTALSVLIRIELGQPGSLIGDDQIYNVIVTAHAFVMIFFMVMPIMIGGFGNWLVPLMLGAPDMAFPRLNNMSFWLLPPSFTLLLASSMVESGAGTGWTVYPPLAGAIAHAGASVDLTIFSLHLAGVSSILGAINFITTVINMKSPGM
KLDQMPLFVWAVVITAVLLLLSLPVLAGAITMLLTDRNINTSFFDPAGGGDPILYQHL
>GBA7732-12|GU122415|GU122415|Locusta_migratoria
TLYFMFGAWAGMVGTSVSMIIRAELGQPGTMINDDQVYNVIITAHAFVMIFFMVMPIMIGGFGNWLVPLMIGAPDMAFPRMNNMSFWLLPPSLTLLLMSSVVDNGAGTGWTVYPPLASVIAHSGASVDLAIFSLHLAGVSSILGAINFITTAINMR
SNNMTLDQTPLFVWSVAITALLLLLSLPVLAGAITMLLTDRNLNTSFFDPAGGGDPILYQHL
>INRMA006-12|BIOUG02790-A06|Tetrix_brunnerii
TLYFIFGAWAGLVGTALSMIIRMELSTPGHLINDDQIYNVVVTAHAFIMIFFMVMPIMIGGFGNWLVPLMIGAPDMAFPRMNNMSFWLLPPSLILLISSSIVDTGVGTGWTVYPPLAGPIAHSGAAVDLAIFSLHLAGVSSILGAINFITTTINMKAPEM
NMDQLPLFVWSVMITAILLLLSLPVLAGAITMLLTDRNLNTSFFDPAGGGDPILYQHL
>GBMIN15439-13|HQ609201|HQ609201|Conanalus_pieli
TLYFIFGAWAGMVGTSLSLLIRAELGQPGYLIGDDQIYNVIVTAHAFVMIFFMVMPIMIGGFGNWLVPLMLGAPDMAFPRMNNMSFWLLPPSLTLLLASSLVENGAGTGWTVYPPLSGGISHAGASVDLAIFSLHLAGISSILGAVNFITTTINMRTPG
MALDQTPLFVWAVAITALLLLLSLPVLAGAITMLLTDRNLNTSFFDPAGGGDPILYQHL
>TTSOW546-11|KM531234|BIOUG00863-B02|Diapheromera_femorata
TLYFLLGMWSGMIGLSMSMLIRMELGMPGSIIGNDQIYNTIVTAHAFVMIFFMVMPIMIGGFGNWLLPIMIGAPDMAFPRMNNMSFWLLPPSLTLLLLSSMIDSGVGTGWTLYPPLSSLVGHSGMSVDFSIFSLHMAGISSILGAVNFISTTINMKSP
GMSWEQVPLFVWSVIITAVLLLLSLPVLAGAITMLLTDRNMNTSFFDPSGGGDPILYQHL
>GBMH8642-13|AB477461|AB477461|Phyllium_giganteum
TLYFLLGMWSGMIGMSMSMIIRMGLGMPGSLVVNNNTYNTIVTAHAFVMIFFMIMPIMIGGFGNWLLPMMIGAPDMAFPRMNNMSFWLLPPSLMLLIAGSMIDFGVGTGWTLYPPLASLTGHNSMSVDLTIFSLHMAGMSSILGAVNFISTTIN
MTSPGTKMNQIPLFVWSVVITAILLLLSLPVLAGAITMLLTDRNMNTSFFDPSGGGDPILYQHL
>GBMH8260-13|JQ339221|JQ339221|Timema_cristinae
TLYFIFGAWSGMVGTSLSMLIRTELGHPGTLIGDDQTYNVIVTAHAFIMIFFMVMPILIGGFGNWLVPLMLGAPDMAFPRMNNMSFWLLPPSLVMLLTSSLIESGAGTGWTVYPPLSSIIAHAGGSVDMAIFSLHLAGISSILGAVNFISTVINMRPIGM
TFDRIPLFVWSVLITAILLLLSLPVLAGAITMLLTDRNLNTSFFDPAGGGDPILYQHL
>MAPTH590-13|KJ840111|NIBGE_PTH-00590|Pediculus_humanus_D

FLYLCSGVWFGLLGLSLSLMIRLELSSTGLLLSDSHLYNVFVTSHAFVMIFFMVMPVMMGGFANWLVPSMLGSPDMAFPRMNNMSYWLLTPSGILLISSSFVQGGVGTGWTVYPPLSSLEGQPSVSVDLAILSLHLAGVSSILGSVNFISTIFNMWPQY
FGLVRLPLFCWSVLVTAFLLLLSLPVLAGAITMLLMDRNFNCSFFDPLGGGDPVLYQHL
>GBMHP027-13|JX855267|JX855267|Brueelia_interposita
ILYLLFGLWSGLLGFSMSVIIRFELGEVGSYLGDGHLYNVLVTSHAFLMIFFMVMPIMIGGFANWLVPMMLGAPDMAFPRMNNMSFWLLPPSLVLLLISAYLDVGVGTGWTVYPPLS-GLGQPGSSVDYAIFSLHLAGLSSIMGAINFISTIKNMWVSS-
KWDSLSLFSWSVLITAVLLLLSLPVLAGAITMLLMDRNLNSSFFDPSGGGDPILYQHL
>GMNCA119-12|BIOUG03086-F06|Leuctridae
TLYFIFGAWAGMVGTSLSLLIRAELGQPGSLIGDDQIYNVIVTAHAFVMIFFMVMPIMIGGFGNWLVPLMLGAPDMAFPRMNNMSFWLLPPSLTLLLASSMVENGAGTGWTVYPPLAAGIAHAGASVDLAIFSLHLAGVSSILGAVNFITTVINMRS
NGMSLDRMPLFVWAVVITALLLLLSLPVLAGAITMLLTDRNLNTSFFDPAGGGDPILYQHL
>PLCHU220-08|GU115803|07PROBE-02704|Nemoura_arctica
TLYFIFGAWSGMVGTSLSLLIRAELGQPGSLIGDDQIYNVIVTAHAFVMIFFMVMPIMIGGFGNWLVPLMLGAPDMAFPRMNNMSFWLLPPSLTLLLASSLVENGAGTGWTVYPPLSAGIAHAGSSVDMAIFSLHLAGVSSILGAVNFITTVINMRSS
GMTLDRMPLFVWAVAITALLLLLSLPVLAGAITMLLTDRNLNTSFFDPAGGGDPILYQHL
>CFWIB247-10|HQ939318|10-SCCWRP-1197|Calineuria_californica
TLYFIFGAWSGMVGTSLSLLIRAELGQPGSLIGDDQIYNVIVTAHAFVMIFFMVMPIMIGGFGNWLVPLMLGAPDMAFPRMNNMSFWLLPPSLTLLLASSLVENGAGTGWTVYPPLSAGIAHAGASVDMAIFSLHLAGVSSILGAVNFITTVINMRSA
GMSFDRMPLFVWSVAITALLLLLSLPVLAGAITMLLTDRNLNTSFFDPAGGGDPILYQHL
>GMNCG451-12|BIOUG03280-A09|Lachesillidae
TLYFLFGIWAGMVGTSLSVLIRLELGQPGLFLEDDQTYNVMVTAHAFIMIFFMVMPIMIGGFGNWLVPLMLGAPDMAFPRMNNMSFWLLPPSLTLLLSSSLVNTGAGTGWTVYPPLSSSLAHPGASVDLAIFSLHLAGVSSILGAVNFITTIINMRPSSI
SLERIPLFVWSVLITAVLLLLSLPVLAGAITMLLTDRNLNTSFFDPAGGGDPILYQHL
>TDWGB368-10|HQ978905|TDWG-0504|Psocoptera
TLYFLFGIWAGMVGTSLSVLIRLELGQPGLFLEDDQIYNVIVTAHAFIMIFFMIMPIMIGGFGNWLVPLMLNAPDMAFPRMNNMSFWMLPPSLSLLLSSSMVNTGAGTGWTVYPPLSSTIAHPGASVDMAIFSLHLAGISSILGAVNFITTIINMRSQG
MTFERMPLFVWSVFITAILLLLSLPVLAGAITMLLTDRNINTSFFDPAGGGDPILYQHL
>INRMA371-12|BIOUG02794-B04|Agulla_adnixa
TLYFIFGAWSGMIGTSLSLLIRSELGSPGSLIGDDQIYNVIVTAHAFIMIFFMVMPIMIGGFGNWLVPLMLGAPDMAFPRLNNMSFWLLPPSLMLLLSGSMSESGAGTGWTVYPPLSSNIAHAGASVDLTIFSLHLAGVSSILGAINFITTTLNMRSNGLT
LERMPLFVWSVAITALLLLLSLPVLAGAITMLLTDRNLNTSFFDPAGGGDPILYQHL
>SIOCA226-10|JN309510|BIOUG00571-A11|Megabothris_quirini
TLYFIFGAWSGMIGTSLSILIRTELGQPGSLIGDDQIFNVIVTAHAFIMIFFMVMPILIGGFGNWLIPLMLGAPDMAFPRMNNMSFWLLPPSLILLLSSSMVESGAGTGWTVYPPLSSTIAHSGASVDLTIFSLHMAGISSILGAINFITTCINMRPVGMNL
DRMPLFVWSVFITAFLLLSLPVLAGAITMLLTDRNLNTSFFDPAGGGDPILYQHL
>HSYE170-11|USNM_ENT_00762345|Dinopsyllus_lypusus
TLYFIFGAWSGMIGTSLSILIRTELGQPGSLIGDDQIFNVIVTAHAFVMIFFMVMPILIGGFGNWLVPLMLGAPDMAFPRMNNMSFWLLPPSLILLLSSSMVESGAGTGWTVYPPLSSSIAHSGPSVDLTIFSLHLAGISSILGAINFITTCINMRPMGMSL
DRMPLFVWSVLITAFLLLSLPVLAGAITMLLTDRNLNTSFFDPAGGGDPILYQHL
>HSYE497-13|USNM_ENT_00867199|Xenopsylla_cheopis
TMYFIFGMWSGMVGTAMSMIIRTELSQPGSMIKDDQIFNVIVTAHAFIMIFFMVMPILIGGFGNWLIPLMLGAPDMAFPRMNNMSFWLLPPSLNLLLLGALVESGAGTGWTVYPPLSSNIAHSGSSIDLSIFSLHLAGISSILGAINFISTCLNMRPINM
SLDQMPLFVWSVLITAVLLLLSLPVLAGAITMLLTDRNFNTSFFDPSGGGDPILYQHL
>MAMTL298-12|BIOUG02547-E05|Corioxenidae
TMYMIFSMITGIMGMNFSLLTRLELMLSKTFLMDGNNFNLIITAHAIIMIFFLIMPITMGGFGNWLIPMMIGSPDMAFPRLNNFSFWLLIPSFLMFSLSMMTQLSMGAGWTFYPPLTLTYTFSDYAIDMMIFSLHLAGMSSILSSINFTTTILMMNNFN
SKT---PLFVWSILITAILLILTLPVLAGAITMLLFDRNFNTSFFDPLGGGDPVLFQHL
>GBMH5443-09|AM286745|AM286745|Xenos_vesparum
TLYFIFSAWAGMVGLSTSMVIRYELSNMSSMYFDTNSYNIMVTAHAIVMIFFLIMPVTMGGFGNWLIPLMITAPDMAFPRLNNFSFWLLIPAFILFMKSMLMEIGVGSGWTLYPPLTLNESHSDYSMDLTIFSLHLAGVSSIMSSINFITTSVNMSSLSL
MS--LPLFVWSIIFTSILLIISLPILAGAITMLLFDRNFNTSFFDPLGGGDPILFQHL
>CNWLB199-12|KM529474|BIOUG03259-A11|Aeolothrips
ILYFLFGAWAGMLGLSLSMLIRITLRNMKVLIEDDQFYNSVVTAHAFVMIFFTVMPILIGGFGNWLVPLMLGAPDMAFPRLNNMSFWLLPPSLTLLMAGLVVGEGAGTGWTVYPPLS-
TFYHSSISVDLTIFSLHLAGISSILGAINFITTIIHMHSKNLSTEFISLFAWSVLITAILLLLSLPVLAGAITMLLTDRNLNTTFFDPSGGGDPILYQHL
>BBTHY012-09|KM530889|08BBTHY-012|Phlaeothripidae
MLYFILGFWSGICGLFMSLLIRLELMQPGNNFLSGQAYNVMVTNHAFVMIFFTVMPIMMGGFGNWLVPIMLGSPDMCYPRMNNMSFWLLPPSLFLLLFSMILEEGSGTGWTIYPPLS--SLSSGICVDMTIISLHIAGVSSLLGSINFLSTVFNMS--
NMKMEYFSLFVWSIVVTSLLLLLSLPVLAGALTMLLVDRNLNTSYFDPSGGGDPTLYQHL
>CYTC4417-12|AY191994|AY191994|Tricholepidion_gertschi
TLYFIFGGWAGMVGTSLSILIRTELGQPGSLIGDDQIYNVIVTAHAFIMIFFMVMPIMIGGFGNWLVPLMLGAPDMAFPRMNNMSFWLLPPALTLLLMGSIVENGAGTGWTVYPPLAGSIAHAGASVDLSIFSLHLAGVSSILGAVNFITTVINMRSPG
MSMDRIPLFVWSVVITAILLLLSLPVLAGAITMLLTDRNLNTSFFDPAGGGDPILYQHL

>GBMH1943-06|NC_006080|NC_006080|Thermobia_domestica
TMYLIFGAWAGMVGTSLSLLIRAELGRPGSLIGDDQIYNVIVTAHAFIMIFFMVMPIMIGGFGNWLVPLMLGAPDMAFPRLNNMSFWLLPPSLTLLLTGSIVESGAGTGWTVYPPLSSAIAHAGASVDLSIFSLHLAGVSSILGAVNFITTVINMRTPGM
TLERMPLFVWSVVITAVLLLLSLPVLAGAITMLLTDRNLNTSFFDPSGGGDPILYQHL
>NBTRI303-08|JN658546|08NBEPT-1257|Hydropsyche_sparna
TLYFMFGIWSGLVGSSLSFIIRMELSTPGSFIGNDQIYNVIVTSHAFIMIFFMVMPIMIGGFGNWLVPLMLGSPDMAFPRMNNLSFWFLPPSLIFLLLSSMTNSGAGTGWTVYPPLSSNLSHAGSSVDLTIFSLHMAGISSILGAINFISTIMNMKFKNLNF
EMIPLFVWSILITAVLLLLSLPVLAGAITMLLTDRNLNTSFFDPAGGGDPILYQHL
>SMCAD168-07|HM101883|07GSM-0168|Hydroptila_grandiosa
MLYFILGIWSGMVGTSLSMLIRLELSSPGFLIGNDQIYNVIVTAHAFIMIFFMVMPIMVGGFGNWLVPLMLGIPDMAFPRMNNMSFWLLPPALLLLLLSSMINNGAGTGWTVYPPLASNIAHSGSSVDLTIFSLHLAGISSILGAINFISTIINMKPINMKF
DQMSLFVWSILITAILLLLSLPVLAGAITMLLTDRNINTSFFDPAGGGDPILYQHL
>BBEPT290-11|KM535206|BIOUG00838-A07|Phryganea_cinerea
TIYFIFGIWAGMVGTSLSMIIRTELGTTESLIKNDQIYNVLVTAHAFIMIFFMVMPIMIGGFGNWLVPLMIGAPDMAFPRMNNMSFWLLPPSLNFLLISSIVENGTGTGWTVYPPLSSNIAHAGSSVDISIFSLHLAGISSILGAINFISTTINMRSNLITLDRL
PLFVWSVAITALLLLLSLPVLAGAITMLLTDRNLNTSFFDPSGGGDPILYQHL
>TFLAN070-11|UA-SG-TRICH-A01|Rhyacophila_fasciata
TLYFIFGIWAGMVGSSLSLIIRTELGMPGSLIGNDQIYNVIVTAHAFVMIFFMVMPIMIGGFGNWLVPLMLGAPDMAFPRMNNMSFWLLPPSLTLLTMSMIVENGAGTGWTVYPPLSSNIGHNGSSVDLTIFSLHLAGVSSIMGAINFITTVINMRSK
GMSMDQMPLFVWSVAITALLLLLSLPVLAGAITMLLTDRNLNTSFFDPAGGGDPILYQHL
>FCGA082-05|GQ341860|FC-TIG85|Gammarus_tigrinus
TLYFILGAWASALGTSLSVIIRTELSAPGNLIGDDQLYNVMVTAHAFVMIFFMVMPIMIGGFGNWLVPLMLGSPDMAFPRMNNMSFWLLPPSLTLLLMSGMVESGVGTGWTVYPPLAATVAHSGGAVDLAIFSLHLAGASSILGAINFMSTVINMRS
PRMSFDQMPLFVWSVFITAILLLLSLPVLAGAITMLLTDRNLNTSFFDPSGGGDPILYQHL
>GBCMD10249-13|HM463571|HM463571|Anomura_sp._LPdivOTU161
TLYFIFGAWAGMVGTSLSLIIRAELGQPGSLIGDDQIYNVIVTAHAFVMIFFMVMPIMIGGFGNWLVPLMLGAPDMAFPRMNNMSFWLLPPSLTLLLMSGMVESGVGTGWTVYPPLAAGIAHAGASVDMGIFSLHLAGVSSILGAINFMTTVINMR
PKGMTMDRMPLFVWSVFITAILLLLSLPVLAGAITMLLTDRNLNTSFFDPAGGGDPILYQHL
>WW131-07|FJ581922|TR02GS0105|Thysanoessa_raschii
TLYFIFGAWAGMVGTSLSLIIRAELGQPGSLIGDDQIYNVVVTAHAFVMIFFMVMPIMIGGFGNWLVPLMLGAPDMAFPRMNNMSFWLLPPSLTLLLGSGLVESGVGTGWTVYPPLSAGIAHAGASVDMGIFSLHIAGASSILGAVNFITTVINMRSA
GMTMDRIPLFVWSVFITAILLLLSLPVLAGAITMLLTDRNLNTSFFDPAGGGDPILYQHL
>GBCMI2426-13|JQ921353|JQ921353|Proasellus
TLYFIFGAWSGSVGTALSVLIRTELGQPGSFIGSDQIYNVIVTAHAFVMIFFLVMPVMIGGFGNWLIPLMLGAPDMAFPRMNNMSFWLLPPSLTLLLSSGLIEGGVGTGWTVYPPLAASIAHSGPAVDLGIFSLHLAGASSILGSVNFISTVINMRSEGMS
FDKIPLFVWSVFITTILLLLSLPVLAGAITMLLTDRNLNTSFFDPSGGGDPILYQHL
>ACM032-06|2006-ACM-0032|Gastrosaccus_bispinosus
TLYFMFGAWAGMVGSSLSVLVRLELGQPGSLIGDDQIYNVIVTAHAFVMIFFMVMPIMIGGFGNWLVPLMLGAPDMAFPRMNNMSFWLLPPSLTLLLSSGLVESGVGTGWTVYPPLASGIAHAGASVDLGIFSLHLAGASSILGAVNFISTVINMRS
PGMTWDRLPLFVWAVFITAVLLLLSLPVLAGAITMLLTDRNLNTTFFDPTGGGDPILYQHL
>GBA14335-13|KC616794|KC616794|Calanoida
TLYLIAGAWSGMVGTGLSMIIRMELGQAGSLIGDDQVYNVVVTAHAFIMIFFMVMPILIGGFGNWLVPLMLGASDMAFPRMNNMSFWFLIPALVMLLSSSLVESGAGTGWTVYPPLSSNIAHAGSSVDFAIFSLHLAGVSSILGAVNFISTLGNLRAF
GMILDRMPLFAWAVLITAVLLLLSLPVLAGAITMLLTDRNLNSSFYDAGGGGDPILYQHL
>GBA11524-13|JX502999|JX502999|Capitulum_mitella
TLYLIFGAWSAMVGTALSMLIRAELGQPGSLIGDDQVYNVIVTAHAFIMIFFMVMPIMIGGFGNWLLPLMLGAPDMAFPRLNNMSFWLLPPALMLLISGSLVEAGAGTGWTVYPPLASNIAHSGASVDLTIFSLHLAGASSILGAINFMSTVINMRAE
TLTFDRLPLFVWSVFVTVILLLLSLPVLAGAITMLLTDRNLNTSFFDPTGGGDPILYQHL
>DQCS106-10|KM611719|BCC2010-005|Balanus
TLYLIFGAWSAMVGTALSMLIRAELGQPGSLIGDDQIYNVIVTAHAFIMIFFMVMPIMIGGFGNWLLPLMLGAPDMAFPRLNNMSFWLLPPALMLLISGSLVEAGAGTGWTVYPPLSSNIAHSGASVDLSIFSLHLAGASSILGAINFMSTVINMRAET
LTFDRLPLFVWSVFITVILLLLSLPVLAGAITMLLTDRNLNTSFFDPTGGGDPILYQHL
>SLOBC172-07|FJ411793|2007-BCSL-076|Lepeophtheirus_salmonis
TLYLLSGFWSGLVGLAMSIIIRLELSQPGAYLGDSQVYNVIVTAHAFIMIFFMVMPVLIGGFGNWLVPLMLGAPDMAFPRLNNMSFWFLMPSLSLLLMSALVESGAGTGWTVYPPLSSGVFHSGASVDFAIFSLHLAGVSSLLGAVNFISTITNLRCLGLL
VGQMPMFPWSVLITAVLLLLSLPVLAGAITMLLTDRNLNTTFFDPSGGGDPILYQHL
>SBBM082-13|5_NSB_8|Nemesis
TLYLISGMWSGFVGLAMSLIIRMELAQPGSLIHNDQTYNVMVTAHAFIMIFFMVMPVMIGGFGNWLVPLMIGAPDMAFPRLNNLSFWFLMPSLTLLLLSSMVETGAGTGWTVYPPLSLAQSHSGPSVDIVIFSLHLAGISSILGAINFITTTMNTRSIG
FMLDHVPMFPWSVLITAILLLLSLPILAGAITMLLTDRNLNTSFYDVAGGGDPILYQHL
>GBA10398-13|HQ588747|HQ588747|Limulus_polyphemus

TMYLIFGIWAAMVGTALSILIRAELGQPGSLIGDDQIYNVIVTAHAFVMIFFMVMPVMIGGFGNWLIPLMLGAPDMAFPRLNNMSFWLLPPSFLLLLSSAAVESGAGTGWTVYPPLASGMAHAGASVDLTIFSLHLAGVSSILGAINFITTIINMRTSG
MVLERMPLFVWSVKITAILLLLSLPVLAGAITMLLTDRNFNTSFFDPAGGGDPVLYQHL
>GBFCG353-14|GU073327|GU073327|Conchoecia_hyalophyllum
TLYFIFGAWSAMLGTSTSVIMRLELSQGGTFLNNGHIYNVVVTAHAFLMIFFAVMPMLMGGFGNWLMPLTLGAPDMAFPRLNNLSFWLLPPSLFLLLSSSLTNQGVGTGWTVYPPLSSLVGQNDPSVDLAIISLHLAGFSSIIASLNMMTTVLNIRLP
WVTLEMTPLLPWALLITALLLILALPVLAACITMLLTDRNLNTSFFDPAGGGDLILFQHL
>GBA10022-13|AB519692|AB519692|Loxoconchidae
TLYFIFGAWSGMLGAALSSLIRMELIQPGALIGDDQIYNTLVTAHAFVMIFFMVMPVLIGGFGNWLVPLMLGAPDMAFPRMNNMSFWLLPPSLSFLTLGALTGAGAGTGWTVYPPLSSASYHSASSVDLTIFSLHLAGISSILGAINFITTVLNMRPYSM
KLSGVPLFVWSVIITAVLLLLSLPVLAGAITMLLTDRNFNTTFFDPAGGGDPILYQHL
>GENHP137-11|11237-A10|Pauropoda
TLYFIFGMWAALIGTSFSMIIRLELSCPGSLIGDDQIYNSVVTSHALVMIFFMVMPVMIGGFGNWLIPLMNLAPDMSFPRLNNMSFWLLPPSLLLLLFSGLLGSGSGTGWTMYPPLSLVTGHNTISVDFSIFSLHVAGISSILGAINFISTIINMKIKNFLMN
FNTLFSWSVFLTATLLLLSLPVLAGAITMLLFDRNLNTSFFDPSGGGDPILFQHL
>GBA0034-06|NC_008453|NC_008453|Scutigerella_causeyae
TMYFIFGVWSAMLGTALSLIIRAELGQPGSMIGDDQTYNVIVTAHAFIMIFFMVMPIMMGGFGNWLVPIMIGAPDMAFPRMNNMSFWLLPPSLSLLLASAAVESGAGTGWTVYPPLASETAHAGASVDLAIFSLHLAGASSILGSANFITTIINMRSP
GMSFERMPLFVWSVLLTAILLLLSLPVLAGAITMLLTDRNYNTSFFNPVGGGDPILYQHL
>GBSP0058-06|AB178773|AB178773|Lingula_anatina
TIYLYMGLWSGVFGLSLSHCMRIELSHPGEWLQVGYMYHSIMTMHAFMMIFFFVMPTSIGGLGNWFIPLMIKIKDLSMPRLNNLSVWLALGSLFLMCMAFLSSGGLGCGWTMYPPLSNSEFMDGLPIDLAVFSLHMAGMSSIAGSINFLVTIFNMR
MGALFFMSLPMLIWTLFGTSILLVTSVPVLAAGLTLLLLDRHFSTSFYYPEGGGDPILWQHL
>GBSP1323-06|NC_002322|NC_002322|Laqueus_rubellus
TLYFIFGVWSGLVGLALSLLVRAELGQPGSMLGNDQLYNVIVTAHALVMIFFLVMPVMIGGFGNWLIPLMIGAPDMAYPRMNNMSFWLLPPALLLLLGSAAMGAGAGTGWTVYPPLSSVGAHGGPAVDLAIFSLHLAGASSILGAINFIGSVMNMK
PGGFKMENVPLIVWSVLNTVGLLLLSLPVMAGAITMLLLDRNFNTSFFDPAGGGDPVLFQHL
>GBSP10165-13|EU560978|EU560978|Cauloramphus_multispinosus
TLYFLFGLWAGMMGSGLSAMIRMELSQPGSFLGNDHLYNVIVTAHAFVMIFFMVMPVMIGGFGNWLVPLMLGLPDMAFPRLNNMSFWFLPPALMMLLVSMLVESGAGTGWTVYPPLSSNLSHSGASVDLVIFSLHLAGISSILGAVNFITTVLNM
RSGGMKMMNISLLVWSIFITAILLLLSLPVLAGAITMLLTDRNLNTSFFDPAGGGDPLLMQHL
>GBMAA480-14|KF805632|KF805632|Plumatella_fungosa
TLYFLFGMWSGLVGSSLSFLIRAELSQPGALFGDDQLYNVIVTAHAFIMIFFLVMPVMLGGFGNWLVPLMLGAPDMAFPRLNNMSFWLLPPSLLLLLSSALVESGAGTGWTVYPPLSSNVSHAGGSVDLAIFSLHLAGVSSILGALNFITTIVNMRWGG
MTFERVPLFAWSILITAILLLLSLPVLAGAITMLLTDRNFNTSFFDPAGGGDPILFQHL
>GBMAA1543-14|KF977334|KF977334|Zonosagitta_nagae
TLYFVFGVWSAFLGTALSALIRLELGSPGSLLGDDQLYNVIVTAHAFIMIFFFVMPTMMGGFGNWLVPLMLNAPDMAFPRMNNMSFWLLPPALTLLLMSGMVESGVGTGWTVYPPLS-
TLGHPGSAVDLGIFSLHIAGVSSILGSANFITTISNMRSEGMTFEIMPLFVWSVLLTAILLLLSLPVLAGAITMLLTDRNFNTSFFDPAGGGDPILYQHL
>GBSP1644-10|GQ368390|GQ368390|Eukrohnia_hamata
TLYFLLGVWSAFVGTGLSA?IRLELGCPGSLLGDDQLYNVIVTAHAFIMIFFFVMPIMIGGFGNWLCPLMLNSPDMAFPRMNNMSFWMLPPALIFLLTSGFVESGVGTGWTVYPPLSSISGHSGGAVDLGIFSLHLAGVSSILGAANFITTILNMRSEGM
TMELISLFVWSVLLTAILLLLSLPVLAGAITMLLTDRNFNTSFFDPAGGGDPILFQHL
>GBGCA4747-13|NC_021757|NC_021757|Acipenser_schrenckii
TLYLVFGAWAGMVGTALSLLIRAELSQPGALLGDDQIYNVIVTAHAFVMIFFMVMPIMIGGFGNWLVPLMIGAPDMAFPRMNNMSFWLLPPSFLLLLASSGVEAGAGTGWTVYPPLAGNLAHAGASVDLTIFSLHLAGVSSILGAINFITTIINMKPP
AVSQYQTPLFVWSVLITAVLLLLSLPVLAAGITMLLTDRNLNTTFFDPAGGGDPILYQHL
>BNAF367-09|HQ557132|PEFL-NY-HUR2-1|Perca_flavescens
TLYLVFGAWAGMVGTALSLLIRAELSQPGALLGDDQIYNVIVTAHAFVMIFFMVMPIMIGGFGNWLIPLMIGAPDMAFPRMNNMSFWLLPPSFLLLLASSGVEAGAGTGWTVYPPLAGNLAHAGASVDLTIFSLHLAGVSSILGAINFITTIINMKPPAI
SQYQTPLFVWAVLITAVLLLLSLPVLAAGITMLLTDRNLNTTFFDPAGGGDPILYQHL
>GBAP1791-13|AB511303|AB511303|Rana_catesbeiana
TLYLVFGAWAGMVGTALSLLIRAELSQPGTLLGDDQIYNVIVTAHAFVMIFFMVMPILIGGFGNWLVPLMIGAPDMAFPRMNNMSFWLLPPSFFLLLASSTVEAGAGTGWTVYPPLAGNLAHAGPSVDLAIFSLHLAGVSSILGAINFITTIINMKPSST
TQYQTPLFVWSVLITAVLLLLSLPVLAAGITMLLTDRNLNTTFFDPAGGGDPVLYQHL
>GBIR3074-12|JN850729|JN850729|Anas_platyrhynchos
TLYLIFGAWAGMIGTALSLLIRAELGQPGTLLGDDQIYNVIVTAHAFVMIFFMVMPIMIGGFGNWLVPLMIGAPDMAFPRMNNMSFWLLPPSFLLLLASSTVEAGAGTGWTVYPPLAGNLAHAGASVDLAIFSLHLAGVSSILGAINFITTAINMKPPA
LSQYQTPLFVWSVLITAILLLLSLPVLAAGITMLLTDRNLNTTFFDPAGGGDPILYQHL
>GBIR3040-12|JN850695|JN850695|Passer_domesticus
TLYLIFGAWAGMVGTALSLLIRAELGQPGALLGDDQVYNVVVTAHAFVMIFFMVMPIMIGGFGNWLVPLMIGAPDMAFPRMNNMSFWLLPPSFLLLLASSTVEAGAGTGWTVYPPLAGNLAHAGASVDLAIFSLHLAGISSILGAINFITTAINMKPP
ALSQYQTPLFVWSVLITAVLLLLSLPVLAAGITMLLTDRNLNTTFFDPAGGGDPVLYQHL

>GBIR0247-06|NC_002785|NC_002785|Struthio_camelus
TLYLIFGAWAGMVGTALSLLIRAELGQPGTLLGDDQIYNVIVTAHAFVMIFFMVMPVMIGGFGNWLVPLMIGAPDMAFPRMNNMSFWLLPPSFLLLLASSTVEAGAGTGWTVYPPLAGNLAHAGASVDLAIFSLHLAGVSSILGAINFITTAINMKPP
ALSQYQTPLFVWSVLITAILLLLSLPVLAAGITMLLTDRNLNTTFFDPAGGGDPVLYQHL
>FMV444-09|JQ354155|UW118537|Lampetra_ayresii
TLYLIFGAWAGMVGTALSILIRAELSQPGTLLGDDQIFNVIVTAHAFVMIFFMVMPIMIGGFGNWLVPLMLSAPDMAFPRMNNMSFWLLPPSLLLLLASAGVEAGAGTGWTVYPPLAGNLAHTGASVDLTIFSLHLAGISSILGAVNFITTIFNMKPPT
MTQYQTPLFVWSVLITAVLLLLSLPVLAAAITMLLTDRNLNTSFFDPAGGGDPILYQHL
>GBGC11410-13|KC526959|KC526959|Dasyatis_akajei
TLYLIFGAWAGMVGTGLSLLIRTELSQPGALLGDDQIYNVIVTAHAFVMIFFMVMPIMIGGFGNWLVPLMIGAPDMAFPRLNNMSFWLLPPSFLLLLASAGVEAGAGTGWTVYPPLAGNLAHAGASVDLAIFSLHLAGISSILASINFITTIINMKPPAIS
QYQTPLFVWSILITTVLLLLSLPVLAAGITMLLTDRNLNTTFFDPAGGGDPILYQHL
>GBGC9617-09|AB478571|AB478571|Branchiostoma_lanceolatum
TLYFIFGAWAAMVGTAMSLLIRAELSQPGALLGDDHLYNVIVTAHAFVMIFFMVMPIMIGGFGNWLVPMMIGAPDMAFPRMNNMSFWMLPPSFSLLLASSAVEAGVGTGWTVYPPLSSNIAHAGASVDLAIFSLHLAGVSSILGAINFITTIHNMR
AS-IEWNRVPLFVWSIWVTAYLLLLSLPVLAGAITMLLTDRNINTTFFDPSGGGDPILYEHL
>GBMA4658-13|JF700141|JF700141|Bos_taurus
TLYLLFGAWAGMVGTALSLLIRAELGQPGTLLGDDQIYNVVVTAHAFVMIFFMVMPIMIGGFGNWLVPLMIGAPDMAFPRMNNMSFWLLPPSFLLLLASSMVEAGAGTGWTVYPPLAGNLAHAGASVDLTIFSLHLAGVSSILGAINFITTIINMKPP
AMSQYQTPLFVWSVMITAVLLLLSLPVLAAGITMLLTDRNLNTTFFDPAGGGDPILYQHL
>GBMA0669-06|NC_008133|NC_008133|Phascolarctos_cinereus
TMYMLFGAWAGMVGTALSLLIRMELGQPGALIEDDQIYNVIVTAHAFVMIFFMVMPIMIGGFGNWLVPLMIGAPDMAFPRMNNMSFWLLPPSFLLLLASSTVEAGAGTGWTVYPPLASNLAHAGASVDLAIFSLHLAGISSILGAINFITTIINMKPP
ALSQYQIPLFVWSVMITAVLLLLSLPILAAGITMLLTDRNLNTTFFDPTGGGDPILYQHL
>GBMA0517-06|NC_000891|NC_000891|Ornithorhynchus_anatinus
TLYLLFGAWAGMAGTALSILIRSELGQPGSLLGDDQIYNVIVTAHAFVMIFFMVMPIMIGGFGNWLVPLMIGAPDMAFPRMNNMSFWLLPPSFLLLLVSSTVEAGAGTGWTVYPPLAGNLAHAGASVDLAIFSLHLAGVSSILGAINFITTIINMKPPA
MSQYQTPLFVWSVLITAVLLLLSLPVLAAGITMLLTDRNLNTTFFDPAGGGDPILYQHL
>GBHS0859-06|AY275535|AY275535|Homo_sapiens
TLYLLFGAWAGVLGTALSLLIRAELGQPGNLLGNDHIYNVIVTAHAFVMIFFMVMPIMIGGFGNWLVPLMIGAPDMAFPRMNNMSFWLLPPSLLLLLASAMVEAGAGTGWTVYPPLAGNYSHPGASVDLTIFSLHLAGVSSILGAINFITTIINMKPPA
MTQYQTPLFVWSVLITAVLLLLSLPVLAAGITMLLTDRNLNTTFFDPAGGGDPILYQHL
>CYTC4142-12|AF534390|AF534390|Sphenodon_punctatus
TLYLLFGAWAGMVGTSLSLLIRGELSYPGTLMGNDQIYNVIVTAHAFTMIFFMVMPVMIGGFGNWLIPLMIGAPDMAFPRMNNMSFWLLPPSFLLLLTSAWTETGAGTGWTVYPPLAGNLAHAGPSVDLTIFSLHLAGVSSILGAINFITTIINMKPP
NQSQYQMPLFIWSVLVTAVLLLLSLPVLAAGITMLLTDRNLNTSFFDPSGGGDPILYQHL
>GBGC12668-13|NC_021766|NC_021766|Lacerta_agilis
TLYLLFGAWAGMVGTALSLIIRTELSQPGTLLGDDQVYNVVVTAHAFVMIFFLVMPVMIGGFGNWLVPLMIGAPDMAFPRMNNMSFWLLPPSLLLLLSSSAIEAGAGTGWTVYPPLAGNLAHAGASVDLTIFSLHLAGISSILGAINFITTCINMKPPN
MSQYQTPLFVWSVLITAVLLLLSLPVLAAGITMLLTDRNLNTSFFDPAGGGDPILYQPL
>EANAH439-12|BIOUG-PW021|Vipera_russelli_russelli
TLYLIFGAWSGLVGACLSILMRMELTQPGSLFGSDQIFNVLVTAHAFIMIFFMVMPIMIGGFGNWLIPLMIGTPDMAFPRMNNMSFWLLPPALLLLLSSSYMEAGAGTGWTVYPPLSGNLVHSGPSVDLAIFSLHLAGASSILGAINFITTCINMKPKS
MPMFNFPLFVWSVMITAIMLLLALPVLAAAITMLLTDRNLNTSFFDPCGGGDPVLFQHL
>LOBO017-12|KF369192|LMBSAP37-001|Veretillum_cynomorium
TLYLIFGAFSGMAGTASSMLIRLELTAPGSMLGDDHLYNVIVTAHALLMIFFMVMPILIGGFGNWFVPIMIGAPDMAFPRLNNISFWLLPPSLILLTGSMFVEQGAGTGWTVYPPLSSIQAHSGGAVDMAIFSLHLAGVSSILSSINFITTIINMRVPGMS
MHRLPLFVWSVLITTILLLLTLPVLAGAITMLLTDRNFNTTFFDPAGGGDPILFQHL
>GBCI1767-13|JN700978|JN700978|Carybdea_xaymacana
TLYLIFGAFSGMVGTAFSMLIRLELSAPGSMLGDDQLYNVIVTAHAFVMIFFLVMPVMIGGFGNWLVPLYIGAPDMAFPRLNNISFWLLPPSLFLLLASSLVEQGAGTGWTVYPPLSAIQSHSGGAVDLAIFSLHLAGASSILGAMNFITTIFNMRAPGLT
LDKMPLFVWSVLVTAFLLLLSLPVLAGAITMLLTDRNFNTTFLDPAGGGDPILFQHL
>RBNII103-13|ON13-C4215-0001|Hydra_oligactis
TLYIVFGAFSGMIGTALSMLIRIELSAPGRIIGDDHLYNVIVTAHAFVMIFFLVMPVLIGGYGNWFVPIYIGAPDMAFPRLNNLSFWLLPPALILLLTSSLVEQGAGTGWTVYPPLSGPLAHSGGSVDLAIFSLHCAGFSSIAGAINFITTVFNMRTPGLTFDK
LPLFVWSVLITAFLLLLSLPVLAGAITMLLTDRNFNTTFFDPAGGGDPVLYQHL
>GBCI1907-13|KC533760|KC533760|Clytia
TLYIVFGAFSGMVGTALSMLIRLELAGPGAMFGDDHLYNVIVTAHAFVMIFFLVMPVLIGGFGNWLVPLYIGAPDMAFPRLNNLSFWLLPPALLLLLGSSLVEQGAGTGWTVYPPLAGPQTHSGGSVDMAIFSLHCAGASSIMGAINFITTIFNMRAPG
LTLDKLPLFVWSVLITAFLLLLSLPVLAGAITMLLTDRNFNTTFFDPAGGGDPVLYQHL
>GBCI1668-13|JN215546|JN215546|Mastigias

TLYLLFGIFSGVLGAGFSMIIRLELSGPGSMLGDDHLYNVAVTAHGLIMIFFFVMPVLLGGFGNWFVPLYVGAPDMAFPRLNNISFWLLPPALMLLLGSSLVEQGVGTGWTVYPPLSAIQAHSGGSVDMAIFSLHLGGVSSILASINFITTILNMRAPGM
TMDKMPLFVWAILITAVLLILSLPVFAGAITMLLTDRNFNTSFFDPAGGGDPILYQHL
>GBCI1763-13|JN700976|JN700976|Craterolophus_convolvulus
TLYLIFGAFSGMVGTAFSMLIRLELSSPGSMLGDDQLYNVLVTAHAFVMIFFLVMPVMIGGFGNWLVPLYIGAPDMAFPRLNNISFWLLPPALILLLGSSLIEQGVGTGWTVYPPLSGIQAHSGGAVDMGIFSLHLAGASSILGAMNFITTIFNMRAPGL
SWDKLPLFVWSVLITAFLLLLSLPVLAGAITMLLTDRNFNTSFFDPAGGGDPILFQHL
>GBCL19693-14|NC_023246|NC_023246|Cheirotonus_jansoni
TLYFLFGSWAGMVGTSLSLLIRAELGNPGSLIGDDQIYNVIVTAHAFIMIFFIVMPIIIGGFGNWLVPLILGAPDMAFPRINNIRFWLLPPSLTLLLIRRLVERGAGTGWTVYPPLSANIAHSGASVDLAIFSLHLAGVSSILGAVNFITTVINIRSVGITLDRMPL
FVWSVVLTAILLLLSLPVLAGAITMLLTDRNINTSFFDPAGGGDPILYQHL
>CYTC5087-12|JF760210|JF760210|Mnemiopsis_leidyi
SLYFFFSIIMGFCAFFYSFVMRLALVWPFAFIESGIIYLYYVTLHAVYMIFFFVMPFSIGGLSNLLIPLCFHLADMCLPRINNLSFWLLFASFIISLLSSFHYYGPSSGWTLYPPYSSYPASAYLSTDLIIFSLHLAGASSILSSINFIVTVFILPINSFSFFQYPLFIVAQIT
VSFLLLISLPVLAAAITMLLFDRNFNTSFFSNYLGGDALLYQHL
>EQCS213-11|10BIOBC-EC018|Pycnopodia_helianthoides
TLYLIFGAWAGMIGTAMSVIIRTELAQPGSLLQDDQIYNVVVTAHALVMIFFMVMPIMIGGFGNWLIPLMIGAPDMAFPRMNNMSFWLIPPSFLLLLASAGVESGAGTGWTIYPPLSSGLAHAGGSVDLAIFSLHLAGASSILASINFITTIINMRTPGM
SFDRLPLFVWSVFVTAFLLLLSLPVLAGAITMLLTDRNINTTFFDPAGGGDPILFQHL
>WSEC022-09|GU672429|WSECH0022|Pteraster_militaris
TLYLIFGAWAGMIGTAMSVIIRIELTQPGSLLQDDQIYNVIVTAHALIMIFFMVMPIMIGGFGNWLIPLMIGAPDMAFPRMNNMSFWLIPPSFLLLLASASVESGAGTGWTIYPPLSSNIAHAGGSVDLAIFSLHLAGASSILASINFITTIINMRTPGISFD
RLPLFIWSVFITAFLLLLSLPVLAGAITMLLTDRNINTTFFDPTGGGDPILFQHL
>CHEC034-11|10CHEC-034|Florometra_serratissima
TLYFLFGAWAGMVGTALSIIIRTELSQPGSFLGDDQIYNVIVTSHALIMIFFMVMPIMIGGFGNWLIPLMIGAPDLAFPRVNNMSFWLLPPSFLLLLASAGVESGAGTGWTIYPPLSSGLAHSGGSVDLAIFSLHIAGASSIVASINFITTVINMRSPGVTFD
RLPLFVWSAFITAFLLLLSLPVLAGAITMLLTDRNINTTFFDPAGGGDPILFQHL
>NZECA506-10|66508|Sterechinus_dentifer
TLYLIFGAWAGMVGTAMSVIIRAELAQPGSLLNDDQIYNVIVTAHALVMIFFMVMPIMIGGFGNWLIPLMIGAPDMAFPRMNNMSFWLIPPSFILLLASAGVESGAGTGWTIYPPLSSNIAHAGGSVDLAIFSLHLAGASSILASINFITTIINMRTPGM
SFDRLPLFVWSVFVTAFLLLLSLPVLAGAITMLLTDRNINTTFFDPAGGGDPILFQHL
>NZEC042-08|45092.2|Tromikosoma
TLYLIFGAWAGMVGTAMSIIIRAELAQPGSLLQDDQIYNVIVTAHALIMIFFMVMPIMIGGFGNWLIPLMIGAPDMAFPRMNNMSFWLLPPSFILLLASAGVESGAGTGWTIYPPLSSNIAHAGSSVDLAIFSLHLAGASSILASINFITTIINMRTPGVSF
DRLPLFVWSILITTFLLLLSLPILAGAITMLLTDRNINTTFFDPAGGGDPILFQHL
>NZEC166-08|42174|Heterocucumis_denticulata
TLYLIFGAWAGMVGTAMSVIIRSELAQPGSLLNDDQIYNVIVTAHALIMIFFMVMPIMIGGFGNWLIPLMIGAPDMAFPRMNNMSFWLIPPSFILLLASAGVESGAGTGWTIYPPLSSNMAHAGGSVDLAIFSLHLAGASSILASINFITTIINMRTPGIT
FDRLPLFVWSVFITAFLLLLSLPVLAGAITMLLTDRNINTTFFDPAGGGDPILFQHL
>EQCS185-11|10BIOBC-EC001|Cucumaria_miniata
TLYLLFGAWAGMIGTAMSVIIRTELAQPGSLLNDDQIYNTIVTAHALVMIFFMVMPIMIGGFGNWLIPLMIGAPDMAFPRMNNMSFWLIPPSFILLLASAGVENGAGTGWTIYPPLSSNIAHAGGSVDLAIFSLHLAGASSILASINFITTIINMRSPGITF
DRLPLFVWSVFITAFLLLLSLPVLAGAITMLLTDRNINTSFFDPAGGGDPILFQHL
>EQCS013-08|HM473910|HLC-24077|Gorgonocephalus_eucnemis
TLYLIFGAWAGTVGTAMSNIIRVELSQPGSLIQNDQTYNVMVTSHALIMIFFMVMPIMIGGFGNWLVPLMIGAPDMAFPRMNNMSFWLIPPSFLLLLASAGNESGVGTGWTLYPPLSGPTAHSGGCVDLAIFSIHLAGASSIMASINFISTIFNMRAP
GMSLDRTPLFVWSILITTFLLLLSLPVLAGAITMLLTDRNINTTFFDPTGGGDPILFQHL
>ECHOZ360-10|HQ946179|TOH_0930|Ophiomusium_australe
TLYLIFGTWAGTVGTAMSNIIRVELSQPGSLIQDDQTYNVMVTAHAFIMIFFMVMPIMIGGFGNWLIPLMIGAPDMAFPRMNNMSFWLIPPSFLLLTASAGNEGGVGTGWTIYPPLSGPVAHGGGCVDLAIFSLHLAGASSIMASINFISTIINMRAP
GMHLDRAPLFIWSIFITTFLLLLSLPVLAGAITMLLTDRNINTSFFDPTGGGDPILFQHL
>GBMAA1272-14|JQ614997|JQ614997|Loxosomella_sp._JM2012
TLYFIFGSWAGMVGTSLSILIRLELGQPGALLGDDQLYNVIVTAHAFVMIFFLVMPVLIGGFGNWLLPLMLGAPDMAFPRMNNMSFWLLPPSLTLLLSSSLVEMGAGTGWTVYPPLAGNIAHSGGSVDLAIFSLHLAGISSILGAINFITTTINMRWYGY
QFEHVPLFVWSVKLTAILLLLSLPVLAGGITMLLTDRNFNTSFFDPCGGGDPILYQHL
>GBSP2865-10|FJ196081|FJ196081|Pedicellina_cernua
TLYFLFGIWSGMVGTALSMLIRMELGQPGSLLGDDQLYNVVVTAHAFVMIFFLVMPVMIGGFGNWLVPLMLGAPDMAFPRMNNMSFWLLPPALTLLLSSSLVEMGAGTGWTVYPPLSSNLAHSGGSVDLAIFSLHLAGVSSILGAINFITTIINMRW
HGYQFERLPLFVWSVKITAVLLLLSLPVLAGGITMLLTDRNFNTSFFDPCGGGDPILYQHL
>GBSP1196-06|DQ079965|DQ079965|Austrognathia_sp._MVS-2005
MNYLWYGLTTGFVAAGLSMIIRLELGTTGSVLMNDHTYNVVITAHGLLMLFFVVTPVLMGSYGNYFVPMMLGAPDMAFPRMNNLSFWMAIPAFLLLISSSMMEGGAGTGWTVYPPLSSIEYHTSISVDLAIFSLHFSGMSLILGSINFIATITNMRTSS
LFLMRINLYSWSILITAILLITALPVFAGAITMLLVDRNFNCSFFDPMGGGNIILYQHL

>GBSP1197-06|DQ079966|DQ079966|Gnathostomula_axi MNYLWYGL
TTGFVAAGLSMIIRIELGTTGSILMNDQTYNVVITAHGLLMLFFVVTPVLMGSYGNFFIPIMLSSPDMAFPRMNNLSFWMAIPAFMLLISSSMMEGGAGTGWTVYPPLSSIEFHSSVAVDLAIFSLHFSGVSLILGSVNFIATITNMRTSALFLMRINLYT
WSIFITSVLLIISLPVFAGAITMLLVDRNFNCSFFDPMGGGNLILYQHL
>CYTC4099-12|AF051097|AF051097|Balanoglossus_carnosus
TLYFLLGAWAGMIGTGLSILIRAELAQPGPLLGDDQIYNVIVTAHAFVMIFFMVMPIMMGGFGNWLLPLMLGAPDMAFPRLNNMSFWLLPPSFLLLLSSAGVESGVGTGWTVYPPLAGNMAHAGGSVDLAIFSLHLAGISSILGAINFMTTVMNMR
APGVRFDRLPLFVWSVFITVILLLLSLPVLAGAITMLLTDRNLNTSFFDPAGGGDPILYQHL
>CYTC4675-12|FN908482|FN908482|Rhabdopleura_compacta
SLYFVFGFISAISGGGLSIQMRRSLSEGGLSITSDH-YNSMVTAHGLVMIFFFIMPVMIGGFGNWLIPLMIGSADMAFPRLNNLSFWLLPPAYFMLWVGLFSGK-
VGIGWTIYPPLSGGNFSSGWFGDFLLISLHIAGAGSILGGINFITTMSQLRVSGMSFNKLPVFCWALLVASVLLVVAMPVLAGAISMLLADRHFGGSFFDPLGGGDPILWQHL
>GBMBV1076-13|JF301762|JF301762|Ensis_macha
TLYMILAFWSGLVGTSLSLLIRLELARPGGFMGDEHLYNVIVTAHAFIMIFFLVMPMMVGGFGNWLLPLMLTSPDMSFPRLNNLSFWLLPCALFFLLWSGFVGSGIGAGWTIYPPLSGNLAHSSPSMDYGIFSMHLAGASSILGAANFVTTMINMRPG
VMELKRVSLFVWSVAITAFLLVLAMPVLAGALTMLLTDRHFNTSFFDPSGGGDPILFVHL
>GBMBM1111-13|KC509712|KC509712|Limnoperna_securis
TLYILLGMWSAMVGISLSMIIRIELGRPGSFLGDDQLYNTIVTAHALIMIFFMVMPLMVGGFGNWLLPLMMGSMDMIFPRLNNLSFWFMPASLYMLLSSMFIENGSGTGWTLYPPLSSYNGHSGPAVDMSLFALHLAGASSIGGSINFLTSIKNLPVE
GMRGERMVLFIWSMTVTAVLLVVSLPVLAGGITMLIFDRHFNTSFYDPSGGGDPVLYQHL
>LOBO004-12|KF369158|LMBLP11-001|Nucula_sulcata
TMYILFAIWSGLVGTSLSLLIRAELGQPGSLLGDDQLYNVIVTAHAFIMIFFMVMPMMIGGFGNWLIPLMLGGPDMAFPRLNNMSFWLLPPSLILLLSSAAVESGAGTGWTVYPPLSSNMAHSGPSVDLAIFSLHLAGASSILASVNFITTVINMRING
MPLERVPLFVWSVKITAILLLLSLPVLAGAITMLLTDRNFNTSFFDPAGGGDPVLFQHL
>NZCOC061-10|JN201138|NZCOC061|Austrovenus_stutchburyi
TLYFIFSIWAGLMGTAFSVIIRMELAMPGKMLDDGQLYNVIVTAHGLVMIFFLVMPMMIGGFGNWLVPLMLTMPDMAFPRMNNLSFWLLPVSMMLLLSSAYVEGGAGTGWTIYPPLSSSLSHYGSSMDYVIFSLHVGGVSSILASINFVTTSFLMRP
GVISLLRTSMFVWCVGVTGFLLIVAMPVLAAALTMLLTDRNFNTSFFDPVGLGDPVLFIHL
>GBCPH1394-13|AB749277|AB749277|Berryteuthis_anonychus
TLYFIFGIWAGLLGTSLSLMIRTELGQPGSLLNDDQLYNVVVTAHGFIMIFFLVMPIMIGGFGNWLVPLMLGAPDMAFPRMNNMSFWLLPPSLTLLLASSAVESGAGTGWTVYPPLSSNLSHAGPSVDLAIFSLHLAGVSSILGAINFITTILNMRWEGL
QMERLPLFAWSVFITAILLLLSLPVLAGAITMLLTDRNFNTTFFDPSGGGDPILYQHL
>GBMIN2572-12|HQ846088|HQ846088|Sepia_esculenta
TLYFIFGIWSGLLGTSLSLMIRSELGKPGTLLNDDQLYNVVVTAHGFIMIFFLVMPIMIGGFGNWLVPLMLGAPDMAFPRMNNMSFWLLPPSLTLLLSSSAVESGAGTGWTVYPPLSSNLSHAGPSVDLAIFSLHLAGVSSILGAINFITTILNMRWEGLQ
MERLPLFAWSVFITAILLLLSLPVLAGAITMLLTDRNFNTTFFDPSGGGDPILYQHL
>JUT206-12|KK108_003|Wattebledia_crosseana
TLYILFGMWSGLVGTALSLLIRAELGQPGTLLGDDQLYNVIVTAHAFVMIFFLVMPMMIGGFGNWLVPLMLGAPDMAFPRLNNMSFWLLPPALLLLLSSAAVEGGVGTGWTVYPPLAGNLAHAGGSVDLAIFSLHLAGVSSILGAVNFITTIINMRWR
GMQFERLPLFVWSVKITAVLLLLSLPVLAGAITMLLTDRNFNTSFFDPAGGGDPILYQHL
>FRANZ301-08|EU820640|MNHN-IM-2007-40844|Gemmula_diomedea
TLYILFGMWSGLVGTALSLLIRAELGQPGALLGDDQLYNVIVTAHAFVMIFFLVMPMMIGGFGNWLVPLMLGAPDMAFPRLNNMSFWLLPPSLLLLLSSAAVESGAGTGWTVYPPLAGNLAHAGGSVDLAIFSLHLAGASSILGAVNFITTIINMRWK
GMQFERLSLFVWSVKITAILLLLSLPVLAGAITMLLTDRNLNTAFFDPAGGGDPILYQHL
>CONO1265-12|MNHN-IM-2007-42083|Terebra_larvaeformis
TLYILFGMWSGLVGTALSLLIRAELGQPGALLGDDQLYNVIVTAHAFVMIFFLVMPMMIGGFGNWLVPLMLGAPDMAFPRLNNMSFWLLPPALLLLLSSSAVESGVGTGWTVYPPLASNLAHAGGSVDLAIFSLHLAGASSILGAVNFITTIINMRWQ
GMQLERMPLFVWSVKITAVLLLLSLPVLAGAITMLLTDRNLNTAFFDPAGGGDPILYQHL
>JSBIC067-14|BIOUG12146-C04|Arion_subfuscus
TLYLIFGIWSGLVGTGMSLLIRLELGTTG-
ILTDDQFFNVLITAHAFVMIFFMVMPLMIGGFGNWMVPLLIGAPDMSFPRMNNMSFWLLPPSLLLLISSSMVEGGAGTGWTVYPPLSGSMAHSGAAVDLAIFSLHLAGMSSILGAINFISTIFNMRPKALTLERMSLFVWSILITVFLLLLSLPVLAGAIT
MLLTDRNFNTSFFDPAGGGDPILYQHL
>GBMLG12884-13|JX183599|JX183599|Diacavolinia_longirostris
TLYLIFGVWCGMVGTGLSLLIRYELSMPGTLLMDGHLYNVIVTAHAFVMIFFLVMPVLIGGFGNWMLPLLIGAPDMSFPRMNNLSFWLLPPSFIFLLSSSMTESGVGTGWTVYPPLSGPIAHAGAGVDLAIFSLHVAGISSILGAINFMTTVINMRAPGI
TWERLNLFVWSVFLTTILLLLSLPVLAGAITMLLTDRNFNTSFFDPSGGGDPILFQHL
>GBMLG10186-13|FR693840|FR693840|Lunella_jungi
TLYLILGIWSGLVGTALSLLIRAELGQPGALLGDDQLYNVIVTAHAFVMIFFLVMPLMIGGFGNWLIPLMLGAPDMAFPRLNNMSFWLLPPSLTLLLTSAAVESGAGTGWTVYPPLAGNLAHAGASVDLAIFSLHLAGISSILGAVNFITTVINMRWQG
MKFERLPLFVWSVKITAILLLLSLPVLAGAITMLLTDRNFNTSFFDPAGGGDPILYQHL
>GBML1568-13|JQ950256|JQ950256|Nierstraszella_lineata

TLYIILGIWAGLIGTALSLLIRAELGQPGTLLGNDQLYNVIVTAHAFVMIFFLVMPMMIGGFGNWLIPLMLGAPDMAFPRLNNMSFWLLPPALLLLMSAAVESGVGTGWTVYPPLASNVAHAGGSVDLAIFSLHLAGVSSILASVNFISTIGNMRSKG
MQLERVPLFIWSVKITAILLLLSLPVLAGAITMLLTDRNFNTTFFDPAGGGDPILYQHL
>GBML1581-13|JQ950243|JQ950243|Leptochiton_boucheti
TLYIIFGIWSGLVGTALSLLIRAELGQPGALLGDDQLYNVIVTAHAFVMIFFLVMPMMIGGFGNWLVPLMLGAPDMAFPRLNNMSFWLLPPALILLLTSGAVESGVGTGWTVYPPLASNIAHAGGSVDLAIFSLHLAGASSILAAVNFITTIINMRWRG
MQLERVPLFVWSVKITAILLLLSLPVLAGAITMLLTDRNFNTAFFDPAGGGDPILYQHL
>NEMNO042-10|CV-ECOSOLS-42|Plectus
TFYLLFSLWSGLMGGSFSVLLRFELATPGSFYNNAQLYNSILTAHAFIMIFFMVMPGLVGGFGNWMLPLMLCAPDMSYPRLNNLSFWLLPSSILLMVSSAMTDSGAGTSWTVYPPLSSTLGHPGSAVDLAIFSLHLAGVSSILGSINFMVTTSNLRNVSI
SINQLSLFIWTTIVTTFLLVLSLPVLAGAITMVLTDRNISTAFFDPSAGGNVVLYQHL
>NEMMX084-11|KC130730|MXHEL442|Aplectana
TLYFLFGLWSGMVGTGLSLIIRLELAKPGVFLGDGQLYNSVITAHAILMIFFMVMPTMIGGFGNWMLPLMLGAPDMSFPRLNNLSFWLLPTAMILILTACFVDKGCGTSWTVYPPLS-
TLGHPGSSVDLAIFSLHCAGVSSILGGINFMTTTKNLRSSSISLEHMSLFVWTVFVTVFLLILSLPVLAGAITMLLTDRNLNTSFFDPSAGGNPLIYQHL
>RFNPM078-07|EU646605|OTT2105-1|Dracunculus_lutrae
SLYLIFGFWSGMVGAGLSILIRAELCKPGFLLGSGQLYNAVITSHAIMMIFFMVMPSLIGGFGNWMVPLMLGAPDMSFPRLNNVSYWLMPVSLVLILSACLVDSSCGTSWTVYPPLS-
TSGHPGNSVDLAILSLHCSGISSILGGINFMSTVKNMRSASISLEHLALFVWTVFVTVFLLILTLPVLAGAITMLLMDRSFNTSFFDPSSGGNPLTYQHL
>GBSP9455-13|JN881995|JN881995|Neochordodes_occidentalis
MLYFIFGFWSAFLGTSLSMIIRTELSHSGSILMSGSSYNTVITAHALVMIFFAVMPILVGGFGNWLIPMMVSAPDMAFPRMNNLSFWILPPSLFLLLTSSLTESGCATGWTLYPPLSSFLGHPSPSTDLAILSLHLAGVSSLLGAINFITTIMVMRPKGLAIEK
TSLFIWASLITAILLLISLPVLAGALTMLILDRNINTSFFDPIGGGDPILFQHL
>GBSP9454-13|JN881996|JN881996|Neochordodes_occidentalis
MLYFIFGFWSAFLGTSLSMIIRTELSHSGSILMSGSSYNTVITAHALVMIFFAVMPILVGGFGNWLIPMMVSAPDMAFPRMNNLSFWILPPSLFLLLTSSLTESGCATGWTLYPPLSSFLGHPSPSTDLAILSLHLAGVSSLLGAINFITTIMVMRPKGLAIEK
TSLFIWASLITAILLLISLPVLAGALTMLILDRNINTSFFDPIGGGDPILFQHL
>CMBIA368-11|MBI-SCCWRP-00368|Tubulanus_polymorphus
TLYFIFGVWSGLVGTALSMLIRAELGQPGALLGDDHLYNVIVTAHAFVMIFFLVMPVMIGGFGNWLVPLMLGAPDMAFPRMNNMSFWLLPPSLALLLFSGAVESGVGTGWTVYPPLASNLAHSGGSVDLAIFSLHLAGVSSILGAINFITTIINMRWF
GLQFERLPLFVWSVKITAILLLLSLPVLAGAITMLLTDRNFNTSFFDPAGGGDPILYQHL
>GBSP8730-13|JX220620|JX220620|Malacobdella_arrokeana
SLYFIFGVWSGLVGTALSMLIRAELGQPGALLGDDQLYNVIVTAHAFVMIFFLVMPVMIGGFGNWLVPLMLGAPDMAFPRMNNMSFWLLPPALILLLCSGAVESGVGTGWTVYPPLSGNISHSGGSVDLAIFSLHLAGVSSILGAINFITTIMNMRWY
GMQLERLPLFVWSVKITAILLLLSLPVLAGAITMLLTDRNFNTSFFDPAGGGDPVLYQHL
>GBMAA1418-14|KC754642|KC754642|Principapillatus_hitoyensis
TMYFIFGVWSAMVGTSLSLLIRTELMVMGNLLGDDQLFNVIVTAHAFVMIFFMVMPIMIGGFGNWLVPLMLGAPDMAFPRLNNLSFWLLPPSFFLLLSSSMVESGAGTGWTVYPPLSSNLTHSGGSVDLTIFSLHLAGVSSILGALNFITTVINMRTF
GMVFERVPLFVWSVKITAILLLLSLPVLAGAITMLLTDRNLNTSFFDPAGGGDPILYQHL
>GBMAA1395-14|KC754646|KC754646|Peripatus_dominicae
TMYFVFGVWSAMVGTSLSLLIRTELMVMGNLLGDDQLFNVIVTAHAFVMIFFMVMPMMIGGFGNWLIPLMLGAPDMAFPRLNNLSFWLLPPSFFLLLSSSMVESGAGTGWTVYPPLSSNLTHSGGSVDLTIFSLHLAGVSSILGALNFITTVVNMRT
FGMIFERVPLFVWSVKITAVLLLLSLPVLAGAITMLLTDRNLNTSFFDPAGGGDPILYQHL
>GBMAA937-14|KC754647|KC754647|Aethrikos_setosa
TMYFIFGGWAAMVGTSLSLLIRVELSQAGNLMGDDQLYNVVVTAHAFVMIFFMVMPIMIGGFGNWLVPLMLGSPDMAFPRLNNMSFWLLPPSFLLLIASSMVESGAGTGWTVYPPLSSNMSHSGASVDLTIFSLHLAGVSSILGALNFITTVINMRT
YGMVMERVPLFVWSVKITAILLLLSLPVLAGAITMLLTDRNLNTSFFDPAGGGDPILYQHL
>GBMAA1312-14|KC754670|KC754670|Ooperipatellus_sp._DNA103742
TMYFIFGAWAAMVGTSLSLLIRVELSQAGNLMGDDQLYNVVVTAHAFVMIFFMVMPIMIGGFGNWLVPLMLGAPDMAFPRLNNMSFWLLPPSFLLLIGSSLVESGAGTGWTVYPPLSSNLSHSGASVDLTIFSLHLAGVSSILGALNFITTVINMRTY
GMVMERVSLFVWSVKITAILLLLSLPVLAGAITMLLSDRNLNTSFFDPAGGGDPILYQHL
>CMBIA537-12|MBI-SCCWRP-00473|Phoronis_sp.
TLYLIFGVWAGLVGTGLSALIRLELGQPGTLLGDDQLYNVIVTAHAFIMIFFMVMPVMMGGFGNWLVPLMIGAPDMAFPRLNNMSFWLLPPSLTLLLSSAAVESGVGTGWTVYPPLSGNIAHSGGSVDLAIFSLHLAGVSSILGSVNFITTIINMRWEG
YQLERVPLFVWSVKFTAILLVLSLPVLAGAITMLLTDRNFNTSFFDPAGGGDPILYQHL
>CMBIA320-11|MBI-SCCWRP-00320|Phoronopsis_sp.
TLYLIFGVWAGLVGTGLSALIRLELGQPGTLLGDDQLYNVIVTAHAFIMIFFMVMPVMMGGFGNWLVPLMIGAPDMAFPRLNNMSFWLLPPSLTLLLSSAAVESGVGTGWTVYPPLAGNIAHSGGSVDLAIFSLHLAGISSILGSINFITTIVNMRWEG
YQLERVPLFVWSVKFTAILLVLSLPVLAGAITMLLTDRNFNTSFFDPAGGGDPILYQHL
>GBSP3330-11|DQ889456|DQ889456|Unclassified_placozoa_sp._BZ10101
SLYLVFGALSGAIGTAFSMLIRLELSSPGSMLGDDHLYNVIVTAHAFVMIFFLVMPTMIGGFGNWFVPLMIGAPDMAFPRLNNISFWLLPPALFLLLGSSLVEQGAGTGWTVYPPLASIQAHSGGSVDMAIFSLHLAGLSSILGAMNFITTVLNMRTPG
MTMSRIPLFVWSVFITAILLLLSLPVLAGAITMLLTDRNFNTTFFDPAGGGDPILYQHL

>CYTC5765-12|DQ889458|DQ889458|Unclassified_Placozoa_sp.
SLYLVFGALSGAIGTAFSMLIRLELSSPGSMLGDDHLYNVIVTAHAFVMIFFLVMPTMIGGFGNWFVPLMIGAPDMAFPRLNNISFWLLPPALFLLLGSSLVEQGAGTGWTVYPPLASIQAHSGGSVDMAIFSLHLAGLSSILGAMNFITTVMNMRTPG
MTMSRIPLFVWSVLITAILLLLSLPVLAGAITMLLTDRYFNTTFFDPAGGGDPILYQHL
>GBPL3945-14|GU252718|GU252718|Macrogyrodactylus_clarii
VIYSVIGTWAGFVGLGLSLLIRIQVSDPYNLIPF-EVYNYVITSHGIIMIFFFLMPVLIGGFGNYLIPLLMGLHDLNLPRLNALSAWLLVPSSVLVFSSLWLGS--
GTGWTFYPPLSSVAYSSNVGVDLLMFSLHLAGISSIFSSLNFICTIISAWGVSILVSDTPIVVWSFLFTSILLILSLPVLAAGITMLLFDRNFNSSFFDPVGGGDPVLFQHL
>GBPL4130-14|JQ038228|JQ038228|Neobenedenia_melleni
MIYSLIGVWAGFIGLGLSILIRTNMGDAYNNILPTEVYNQVITSHGIIMIFFFLMPVLIGGFGNYLIPLLLNLPDLNLPRLNALSAWLLLPSVICLITSLFKGS--
GVGWTFYPPLSGGIFSSGSGTDYLMFSLHLAGISSILSSINFICTINSAIYFNINHEYISVVIWSYLFTSILLLLSLPVLAAGITMLLFDRNFSSSFFDPMGGGDPVLFQHM
>GBSP5059-13|M93388|Fasciola_hepatica
LIYMVIGLWGGFFGLSLSVLVRLNYLDPYFNLVSPEVYNYVVTGHGIIMIFFFLMPVLIGGFGNYLLPLLLGIPDLNLPRLNALSAWLLLPACVCLSFGLIGGV--GVGWTFYPPLSSLDYS-SWGVDFLMFSLHLAGVSSLLGSINFICTILEVMLDE-
GTGRHSILVWAYLFTSVLLLLSLPVLAAAITMLLFDRNFGSAFFDPMGGGDPVLFQHL
>CYTC4903-12|HE601612|HE601612|Schistosoma_mansoni
LLYFVFGLWGGFIGLGLSLLIRLNLCDPYYNLVSVDVYNYLVTNHGVAMIFFFLMPILIGGFGNYFLPIFLCLDDLLLPRLNSLSLWLMVPSIFYMELSLYYGC--GIGWTLYPPLSIWEGS-GFGVDYLMFSLHLAGVSSLIGSVNFISTIFSRLSFN-----
CSIIVWAYLFTSVLLLLSLPVLASGITMLLFDRNFGTAFFEPSGGGDPILFQHL
>GBPL1207-09|AB477010|AB477010|Echinococcus_multilocularis
VIYSLLGIWSGFVGLSFSLLIRVNFLEPYNVIPL-DCYNFLVTNHGIIMIFFFLMPILIGGFGNYLLPLLGGLSDLNLPRLNALSAWLLIPSLVLLLISMCLGA--GVGWTFYPPLSSSYFSSSSGVDFLMFSLHLAGVSSVFSSINFICTLYSVFMTN-
VFSRTSIVLWSYLFTSILLLVTLPVLAAAITMLLFDRNFCSAFFDPLGGGDPILFQHM
>GBPL1189-09|FM209181|FM209181|Diphyllobothrium_latum
MIYTLIGVWSGFVGLSLSVMIRINFVEPYNVISS-DCYNFLITNHGIIMIFFFLMPVLIGGFGNYLIPLLSGLPDLNLPRLNALSAWLLFPSILFLVLSMCFGA--GIGWTFYPPLSSSLFSDSKGVDFLMFSLHLAGLSSVLGSINFICTLYTAFVDN-
FISRSSILLWSYLFTSILLLLTIPVLAAAITMLLFDRNFGSAFFDPLGGGDPILFQHM
>GBSP4342-12|FR832385|FR832385|Philactinoposthia_brevis
TLYFLFGIFSGIIGTLISLLIRTELRSPNTNLLNENIYNVLITAHGLIIIFFFVMPILMGGFGNWLLPLMLGCADIAFPRLNNLSFWFLPPSFFLLILSSLSELGVGAGWTIYPPISDLIGQPGLSVDLAIFSLHIAGASSIGGAINFLCTIINLRNPSMSWDNLPLFVW
AIFFTAILLVISLPVFAGGITILLTDRNFNTAFFDPSGGGDPILFVHL
>GBSP4339-12|FR832382|FR832382|Pharyngia_furva
TLYFFLGIGSGIIGALISLIIRTELRYPGDGITNEICYNVLITAHGLIIIFFFVIPVLMGGFGNWLLPLILGCADIAFPRLNNFSFWLLPPSFSLLLISSIVEIGVGSGWTIYPPISDLVGQPGISVDLAIFSLHIAGASSIGGSINFLCTIINLRNPSISWENIPLFIWTVFFT
AILLVLSLPVLAGGITILLTDRNFNTSFFDPGGGGDPVLFVHL
>CYTC5662-12|AB618487|AB618487|Dugesia_japonica
TLYFIFGIFMGLFGGSLSLVLRLELASPGSLLGSLSLYYGIMTAHGLVMIFFFVMPILIGGFGNWLIPLMLGTVDMAFPRANNLSFWLLIPSVTFLFFSVFCFGGVIAGWTIYPPLSSIKYSTGVGLDFAILSLHVAGASSILGSINFIVTIYGMSNCN--
LVRLPLYLWSLFITSWLLLLSLPVLASALTMLITDRNFNTSFFDPSGGGDPVLFQHM
>GBSP4653-12|HM592735|HM592735|Stelletta_tuberosa
TLYLLFGAFSGMIGTGLSFLIRLELSAPGSMLGDDHLYNVIITAHGLIMIFFLVMPIMIGGFGNWFVPLYIGAPDMAFPRLNNISFWVLPPSLVLLLGSAFVEQGVGAGWTIYPPLSSVQAHSGGSVDAAIFSLHLAGISSILGSMNFITTIFNMRAPGVTM
DRLPLFVWSILVTTYLLILSLPVLAGAITMLLTDRNFNTTFFDPAGGGDPILFQHL
>GBSP8758-13|HE611623|HE611623|Iotrochota_coccinea
TLYLLFGAFAGMIGTAFSMLIRLELSAPGSMLGDDHLYNVIVTAHAFVMIFFLVMPVMIGGFGNWFVPLYIGAPDMAFPRLNNISFWLLPPALSLLLASVFVEQGAGTGWTVYPPLSGIQAHSGGSVDLVIFSLHLAGISSILGAMNFITTIINMRAPGIT
MDRMPLFVWSILVTAVLLLLSLPVLAGAITMLLTDRNFNTAFFDPAGGGDPILYQHL
>CYTC3322-12|NC_010769|NC_010769|Aphrocallistes_vastus
TLYLIFGAFAAFAGTSLRTLIRLELSQTGSLLEDDHIYNVVVTAHALIMIFFFVMPVLIGGFGNWFLPLCIGAPDMAFPRLNNIRFWLLPPALLLLLCSSLVEEGVGTGWTLYPPLSRIQSHSRGGVDLAIFSLHLAGISSILRSINFLTTIINMRTGGITIYRTPLFI
WAIFFTAFLLVLALPVLAGGITMLLTDRNFNTTFFDPAGGGDPILFQHL
>GBSP7503-13|FR819684|FR819684|Caulophacus_arcticus
TLYLIFGIFAAFIGTSLRILIRLELSQIGTLLENDHTYNVIVTAHALIMIFFFIMPVLIGGFGNWFLPLCIGAPDMAFPRLNNIRFWLLPPSLFLLLSSSFIENGVGTGWTLYPPLSNIQAHSGRGVDLVIFRLHLAGISSILSSINFLTTIINMRTRGITAYRIPLFIWAI
FFTAFLLVLALPVLAGGITMLLTDRNFNTTFFDPAGGGDPILFQHL
>GBSP1301-06|DQ463747|DQ463747|Priapulus_caudatus
TMYLIFGFWSAMVGSGLSLIIRAELGQAGSLLGDDHLYNVIVTAHAFVMIFFMVMPILIGGFGNWMLPLMLGAPDMAFPRLNNLSFWLLPPSLTLLLASSLVEAGAGTGWTVYPPLASNIAHAGGSVDLAIFSLHLAGASSILGAVNFITTVINMRTEG
MVFERIPLFVWAVKITVVLLLLSLPVLAGAITMLLTDRNLNTSFFDPAGGGDPILYQHL
>GBSP8004-13|FN689349|FN689349|Halicryptus_spinulosus

TMYLIFGIWSSMVGSGLSLLIRAELGQPGSLFADDHLYNVLVTAHAFVMIFFMVMPILIGGFGNWLLPLMLGAPDMAFPRLNNLSFWLLPPSLTLLLASSLVEAGVGTGWTVYPPLSSNIAHAGGSVDLAIFSLHLAGASSILGAINFITTVVNMRTEGM
VFERIPLFVWAVKITVVLLLLSLPVLAGAITMLLTDRNLNTSFFDPAGGGDPILYQHL
>GBMAA187-14|KF208357|KF208357|Dicyemennea_floscephalum
MLYLFLGVFSGVYGSSMSLLFRLQLSYPVLLTSD--LYNSFITLHGLIMVFFAAMPTAIGFYSNMLIPYHCGLPDLVFPRLNALSFWIMPMSLFMLTSSM-----ASTGWTLYPPLST----LSANIEYTVFSLHLAGLSSILSSCNFLVSMLSTRSY--
AWYSLSLFCWSNVFVIALLLLSLPVLAVAITLILLDLHLSTSFYDSSLGGDPLLYQHL
>GBMAA207-14|KF208353|KF208353|Dicyema_papuceum
IYYLLVGVFSAVLGSSMSFLFRLQLSYPILLTSD--LYNSLVTLHGLIMVFFAAMPTAIGFFANLLIPYHCNLPDLLFPRMNALSFWLLPGSLCMLVYSM-----ACAGWTLYPPLST----LSMGVEHTIFSLHIAGISSLISSCNFMVTMMSTRSQ--
TWYSLSLFCWSILLVSFLLVLSLPVLAVGITLILTDKHLSTCFYDSSIGGDPVLYQHL
>GBSP8586-13|JX183999|JX183999|Macrotrachela_quadricornifera
TLYFIIGVWSGFIGASLSLIIRTELGMVGSIIMDEQIFNSVVTAHAFLMIFFFVMPVAVGGFGNWLLPLMMNVMDMAFPRLNNLSFWLVPVALLFMTLSLLVGLGPGTGWTVYPPLSNSIYHFGGSVDFAIFSLHVAGVSSILGGINFITTCLKGKISVMSF
EFLTLFVWAMIVTSFLLVLSLPVLAGGITMLLLDRNFGASFFDPSGGGNPILYQHL
>GBMAA008-14|EF524548|EF524548|Brachionus_plicatilis
TLYFIFGIWAGLIGLSMSFLIRLELGVVGSYLGDEHLYNVLVTAHAFVMIFFMVMPVSMGGFGNWLIPLMLGVADMAFPRMNNLSFWLLVPAFMFLLLSSAIDAGAGTGWTVYPPLSDSTYHAGVSVDLAIFSLHLSGVSSILGSINFLTTIICSRTKSVSL
DRLPLMLWAIAVTAVLLITSLPVLAGAITMLLTDRNFNTSFFDPAGGGNPVLYQHL
>GBMAA950-14|GU230171|GU230171|Aspidosiphon_laevis
TLYFMLGIWAGLMGTSMSLLIRAELGQPGSLLGSDQLYNVIVTAHAFLMIFFLVMPVLIGGFGNWLIPLMIGAPDMAFPRLNNLSFWLLPPALALLLFSSAVEKGAGTGWTVYPPLSGALAHAGPSVDFAIFSLHLAGVSSILGALNFITTVINMRVGQM
TWERLPLFIWAAFITVVLLLLALPVLAGALTMLLTDRNLNTSFFDPSGGGDPVLFSHL
>GBAN4980-13|JQ904316|JQ904316|Phascolosoma_agassizii
TLYFILGIWSGLMGTSMSLLIRAELGQPGSLLGSDQLYNVIVTAHAFLMIFFLVMPVLIGGFGNWLIPLMIGAPDMAFPRLNNLSFWLLPPALCLLLASSAVEKGVGTGWTVYPPLSGALAHAGASVDLAIFSLHLAGVSSILGALNFISTVTNMRPSMFS
WERVPLFVWAAFITVILLLLALPVLAGAITMLLTDRNLNTAFFDPGGGGDPILFSHL
>GBAN4942-13|JQ904354|JQ904354|Thysanocardia_nigra
TMYFILGVWSGLLGTSMSLLIRAELGQPGSLLGSDHLFNVLVTAHAFLMIFFLVMPILIGGFGNWLLPMMLGAPDMAFPRLNNMSFWLLPPALTLLVASAAVEKGAGTGWTVYPPLANAIAHAGPSVDLAIFSLHLAGVSSILASLNFITTIYIMRGKGY
WMFRVPLFVWAVMLTTILLVLALPVLAGAITMLLTDRNLNTCFFDPSGGGDPILFSHL
>GBAN4923-13|JQ904373|JQ904373|Themiste_pyroides
TLYFIFGVWAGLLGTSMSLLIRAELGQPGSLLGSDHLYNVIVTAHAFLMIFFLVMPVLIGGFGNWLLPLMLGAPDMAFPRLNNMSFWLLPPALILLVGSAAVEKGAGTGWTVYPPLSNAIAHAGPSVDLAIFSLHLAGVSSILASLNFITTVYNMRGKGF
WMFRVPLFVWAVMLTTILLLLALPVLAGAITMLLTDRNLNTCFFDPTGGGDPILFSHL
>GBSP7732-13|FJ435797|FJ435797|Isohypsibius_sp._Tar195
TLYFMFGLWAATIGTSLSFIIRMELSQPGNFLGDEQIYNVTVTAHAFVMIFFFVMPILIGGFGNWLVPLMIGAPDMAFPRMNNLSFWLLPPSFLLIISSTVAEQGAGTGWTVY?PLSHYFAHSGPAVDLAIFSLHMAGISSILGAINFISTIINMRSPTMSM
DQLPLFVWSVFLTAILLLLSLPVLAGAITMLLLDRNFNTSFFDPAGGGDPILYHHL
>GBSP8061-13|JX865305|JX865305|Acutuncus_antarcticus
TLYFIFGVWAATVGTSLSMIIRSELSQPGSLFSDEQLYNVTVTSHAFVMIFFFVMPILIGGFGNWLVPLMISAPDMAFPRMNNLSFWLLPPSFMLITMSSMAEQGAGTGWTVYPPLAHYFAHSGPAVDLTIFSLHVAGASSILGAVNFISTIMNMRAPSI
SLEQMPLFVWSVLLTAILLLLALPVLAGAITMLLLDRNFNTSFFDPAGGGDPILYQHL
>GBSP7727-13|FJ435802|FJ435802|Minibiotus_furcatus
TLYFIFGLWAATVGTSLSFIIRSELSQPGQLFCDEQLYNVTVTSHAFVMIFFFVMPILIGGFGNWLVPLMIGAPDMAFPRMNNLSFWLLPPSFTLISASTMAEQGAGTGWTVYPPLSNYFAHSGPSVDLTIFSLHIAGVSSILGAINFISTIINMRAPHLSLE
QMPLFVWSVLITAILLLLALPVLAGGITMLLLDRNFNTSFFDPAGGGDPILYQHL
>GBSP9217-13|JQ689525|JQ689525|Echiniscoides
TIYFIFGVWSGLIGSSMSFLIRMELSTVTQFMGDEQTYNVLVTAHALLMIFFMVMPIMIGGFGNWLVPMMIGAPDMAFPRMNNLSFWLLIPSLMLIVLSSFIGVGAGTGWTIYPPLSSLLGHANKSVDLAIFSLHLAGASSILGAINFICTIMNMRPISM
KMEVVPLFVWSVFITAILLLLSLPVLAGAITMLILDRNFNTSFFDPAGGGDPILFQHL
>GBSP10183-13|AM296016|AM296016|Xenoturbella_bocki
TLYIFFGIWAAMVGTGLSMIIRLELTQPGALLGDDQIYNVVVTAHALVMIFFMAMPIMIGGFGNWLLPLMIGAPDMAFPRLNNMSFWLLPPSFLLLIASA?VES?VGTGWTLYPPLSNNLAHAGGSVDLAIFSLHLAGASSILGAANFITTSINMRAPGI
TLDRLPLFVWSVIITAILLLLSLPVLAGGITMLLTDRNLNTSFFDPAGGGDPVLFQHL

Amino acid alignment of the analyzed Coleoptera DNA barcodes (Dataset 2, FASTA format)

>COLFF204-13|KJ963738|ZMUO.005809|Agrypnus_murinus
TLYFLFGAWAGMLGTSLSLLIRAELGNPGSLIGNDQIYNVVVTAHAFIMIFFMVMPIMIGGFGNWLVPLMLGAPDMAFPRMNNMSFWLLPPSLSLLLMSSIVENGAGTGWTVYPPLSANIAHSGSSVDLAIFSLHLAGISSILGAVNFISTVINMRSTGI
TFDRMPLFVWAVAITALLLLLSLPVLAGAITMLLTDRNLNTSFFDPAGGGDPILYQHL
>COLFF192-13|KJ963078|ZMUO.005797|Cardiophorus_ruficollis
TLYFLFGAWSGMLGTSLSLLIRAELGSPGSLIGNDQIYNVIVTAHAFIMIFFMVMPIMIGGFGNWLMPLMLGAPDMAFPRMNNMSFWFLPPSLSLLLMSSIVENGAGTGWTVYPPLSSNIAHSGSSVDLAIFSLHLAGISSILGAVNFISTVINMRSPGM
TFERMPLFVWAVVITALLLLLSLPVLAGAITMLLTDRNLNTSFFDPAGGGDPILYQHL
>COLFG119-13|KJ961842|ZMUO.007814|Ptenidium_punctatum
ILYFMFGSWAGMVGTSLSLLIRSELSIPGSLIGDDQIYNVIVTAHAFIMIFFMVMPILIGGFGNCLVPLMLGAPDMAFPRMNNMSFWLLPPSLMLLLMSSMVESGAGTGWTVYPPLASNIAHSGASVDLAIFSLHLAGISSILGAVNFITTVINMRTPGM
SFDQMPLFVWAVAITALLLLLSLPVLAGAITMLLTDRNLNTSFFDPSGVGDPILYQHL
>COLFG133-13|KJ962041|ZMUO.007828|Ptenidium_formicetorum
ILYFMFGSWAGMIGTSLSLLIRSELSIPGSLIGDDQIYNVIVTAHAFIMIFFMVMPILIGGFGNCLVPLMLGAPDMAFPRMNNMSFWLLPPSLMLLLMSSMVESGAGTGWTVYPPLASNIAHSGASVDLAIFSLHLAGISSILGAVNFITTVINMRTPGMS
FDQMPLFVWSVAITALLLLLSLPVLAGAITMLLTDRNLNTSFFDPSGGGHPILYQHL
>COLFG123-13|KJ967041|ZMUO.007818|Aspidiphorus_orbiculatus
TLYFIFGIWSGMIGTSLSMLIRMELSMPGALIGDDQIYNVIVTAHAFIMIFFMVMPIMIGGFGNWLIPLMMGAPDMAFPRMNNMSFWLLPPSLSLLLMSSIVEKGTGTGWTVYPPLSSNISHNGASVDLSIFSLHLAGMSSIMGAINFISTILNMRPSN
MSLDQMPLFIWAVFITAFLLALSLPVLAGAITMLLTDRNLNTSFFDPSGGGDPILYQHL
>COLFE1240-13|KJ963053|ZMUO.007035|Gonioctena_arctica
TLYFIFGIWAGMVGTSLSILIRAELGNPGTLIGNDQIYNVIVTAHAFIMIFFMVMPIMIGGFGNWLVPLMIGAPDMAFPRMNNMSFWLLPPSLFFLIMSSVVESGAGTGWTVYPPLSANIAHSGSSVDLAIFSLHLAGISSILGAINFITTIINMRPMGMT
MDRMPLFVWAVLITAILLLLSLPVLAGAITMLLTDRNLNTSFFDPAGGGDPILYQHL
>COLFF280-13|KJ966561|ZMUO.005885|Selatosomus_aeneus
TLYFLFGAWAGMLGTSLSLLIRAELGNPGSLIGNDQIYNVIVTAHAFIMIFFMVMPIMIGGFGNWLVPLMLGAPDMAFPRMNNMSFWFLPPSLSLLLMSSIVENGAGTGWTVYPPLSANIAHSGSSVDLAIFSLHLAGISSILGAVNFISTVINMRSTGIT
FDRMPLFVWAVAITALLLLLSLPVLAGAITMLLTDRNLNTSFFDPAGGGDPILYQHL
>COLFG134-13|KJ962489|ZMUO.007829|Ptenidium_formicetorum
ILYFMFGSWAGMIGTSLSLLIRSELSIPGSLIGDDQIYNVIVTAHAFIMIFFMVMPILIGGFGNCLVPLMLGAPDMAFPRMNNMSFWLLPPSLMLLLMSSMVESGAGTGWTVYPPLASNIAHSGASVDLAIFSLHLAGISSILGAVNFITTVINMRTPGMS
FDQMPLFVWSVAITALLLLLSLPVLAGAITMLLTDRNLNTSFFDPSGGGDPILYQHL
>COLFG179-13|KJ967166|ZMUO.007874|Corticaria_elongata
SLYFLFGMWSGMVGTSLSLLIRLELGNPGSLIGDDQIYNVIVTAHAFIMIFFMVMPIMIGGFGNWLVPLMLGAPDMAFPRLNNMSFWLLPPSLSLLIMSSIVESGAGTGWTVYPPLSSNIAHGGSSVDLAIFSLHLAGISSILGAVNFITTVINMRPTGMS
LDQMPLFVWSVVITAILLLLSLPVLAGAITMLLTDRNLNTSFFDPAGGGDPILYQHL
>COLFG130-13|KJ967075|ZMUO.007825|Abraeus_perpusillus
TLYFIFGAWSGMVGTSLSLLIRTELGNPGSLIGNDQIYNVIVTAHAFIMIFFMVMPIMIGGFGNWLVPLMLGAPDMAFPRMNNMSFWLLPPSLTLLLMSSVVESGAGTGWTVYPPLASNIAHGGASVDLAIFSLHLAGISSILGAVNFITTVINMRSPG
MLFDQMPLFVWSVVITALLLLLSLPVLAGAITMLLTDRNLNTTFFDPAGGGDPILYQHL
>COLFC295-12|KJ962573|ZMUO.002480|Stenus_bifoveolatus
SLYFIFGAWSGMLGTSLSLLIRAELGNPGALIGDDQIYNVIVTAHAFIMIFFMVMPIMIGGFGNWLVPLMLGAPDMAFPRMNNMSFWLLPPSLSLLLTSSIVESGAGTGWTVYPPLSSNIAHGGASVDLAIFSLHLAGISSILGAINFITTILNMRTMKMQ
LDCLPLFVWSVGITALLLLLSLPVLAGAITMLLTDRNLNTSFFDPAGGGDPILYQHL
>COLFG104-13|KJ965960|ZMUO.007799|Corticarina_latipennis
SLYFLFGMWSGMVGTSLSLLIRLELGNPGSLIGDDQIYNVIVTAHAFIMIFFMVMPIMIGGFGNWLVPLMLGAPDMAFPRLNNMSFWLLPPSLSLLIMSSIVESGAGTGWTVYPPLSSNIAHGGSSVDLAIFSLHLAGISSILGAVNFITTVINMRPTGM
MLDQMPLFVWSVVITAILLLLSLPVLAGAITMLLTDRNLNTSFFDPAGGGDPILYQHL
>COLFF262-13|KJ963733|ZMUO.005867|Athous_haemorrhoidalis
TLYFLFGAWAGMLGTSLSLLIRAELGNPGSLIGNDQIYNVIVTAHAFIMIFFMVMPIMIGGFGNWLVPLMLGAPDMAFPRMNNMSFWFLPPSLSLLLMSSIVENGAGTGWTVYPPLSANIAHSGSSVDLAIFSLHLAGISSILGAVNFISTVINMRSTGIT
FDRMPLFVWAVVITALLLLLSLPVLAGAITMLLTDRNLNTSFFDPAGGGDPILYQHL
>COLFG110-13|KJ961998|ZMUO.007805|Corticaria_elongata
SLYFLFGMWSGMVGTSLSLLIRLELGNPGSLIGDDQIYNVIVTAHAFIMIFFMVMPIMIGGFGNWLVPLMLGAPDMAFPRLNNMSFWLLPPSLSLLIMSSIVESGAGTGWTVYPPLSSNIAHGGSSVDLAIFSLHLAGISSILGAVNFITTVINMRPAGM
SLDQMPLFVWSVVITAILLLLSLPVLAGAITMLLTDRNLNTSFFDPAGGGDPILYQHL
>COLFG111-13|KJ962546|ZMUO.007806|Trimium_brevicorne
TLYMILGLFAGMMGTSLSILIRMELSNPGSLIENDQIYNVIVTMHAFIMIFFMVMPIMIGGFSNWLIPLMLGAPDMAFPRLNNMSFWLLPPSLILLFTSSMVENGTGTGWTIYPPLSSNITHSSSSVDLTIFSLHLAGISSILGAMNFISTMLNMRSMNFK
FDQMTLFIWSVKITAFLLLLSLPVLAGAITMLLTDRNLNTSFFDPMGGGDPVLFQHL

>COLFB374-12|KJ964457|ZMUO.001704|Nedyus_quadrimaculatus
TLYFIFGAWAAMAGTSLSMLIRTELGNPGSLIGDDQIYNSIVTAHAFIMIFFMVMPILIGGFGNWLVPLMLGAPDMAFPRLNNMSFWLLPPALSLLLMSSIVNKGAGTGWTVYPPLSTNTAHEGMSVDLAIFSLHLAGLSSILGAINFISTVMNMRPQG
MSPEFTPLFVWAVKITAILLLLSLPVLAGAITMLLTDRNINTSFFDPSGGGDPI?YQHL
>COLFE126-12|KJ963480|ZMUO.005161|Oedemera_subrobusta
TLYL?FGAWAGMVGTSLSLLIRAELGNPGSLISDDQIYNVIVTAHAFIMIFFMVMPIMIGGFGNWLVPLMLGAPDMAFPRMNNMSFWLLPPSLTLLIMSSMVESGVGTGWTVYPPLSSNIAHSGSSVDLAIFSLHLAGVSSILGAVNFITTVINMRPVG
MTLDRMPLFVWAVVITAILLLLSLPVLAGAITMLLTDRNLNTSFFDPAGGGDPILYQHL
>COLFD442-12|KJ963837|ZMUO.003862|Adrastus_pallens
TLYFLFGAWAGMLGTSLSLLIRAELGNPGSLIGNDQIYNVIVTAHAFIMIFFMVMPIMIGGFGNWLVPLMLGAPDMAFPRMNNMSFWFLPPSLSLLLMSSIVENGAGTGWTVYPPLSSNIAHSGSSVDLAIFSLHLAGISSILGAVNFISTVINMRSVGIT
FDRMPLFVWAVAITALLLLLSLPVLAGAITMLLTDRNLNTSFFDPAGGGDPILYQHL
>COLFF216-13|KJ963679|ZMUO.005821|Lampyris_noctiluca
TLYFIFGAWAGMLGTSFSLLIRAELGSAGTLIGNDHIFNVIVTSHAFIMIFFMVMPIMIGGFGNWLVPLMLGAPDMAFPRMNNMSFWLLPPSLSLLIMSSLIESGAGTGWTVYPPLSANIAHSGPSVDLAIFSLHLAGVSSILGAVNFISTIINMRPNSM
MFDQMPLFVWAVLITAILLLLSLPVLAGAITMLLTDRNLNTSFFDPAGGGDPILYQHL
>COLFC804-12|KJ966916|ZMUO.003369|Hypera_suspiciosa
TLYFIFGTWAGTVGTSLSILIRTELGNPGSLIGNDQIYNTIVTAHAFIMIFFMVMPIMIGGFGNWLVPLMLGAPDMAFPRLNNMSFWLLPPSLSLLLMSSMVDSGAGTGWTVYPPLSSNIAHEGSSVDLAIFSLHMAGVSSILGAINFISTVLNMRPSGM
SLDKMALFIWAVKITAILLLLSLPVLAGAITMLLTDRNINTSFFDPAGGGDPILYQHL
>COLFF563-13|KJ962118|ZMUO.006643|Bitoma_crenata
TLYFIFGAWSGMVGTSLSLLIRAELGNPGSLIGDDQIYNVIVTAHAFIMIFFMVMPIMIGGFGNWLVPLMLGAPDMAFPRMNNMSFWLLPPSLTLLLMSSIVENGAGTGWTVYPPLSSNIAHGGSSVDLAIFSLHLAGISSILGAVNFITTVINMRPSGM
SFDRMPLFAWAVVITAVLLLLSLPVLAGAITMLLTDRNLNTSFFDPAGGGDPILYQHL
>COLFE520-13|KJ962531|ZMUO.005365|Hylastes_cunicularius
TLYFIFGAWAGMVGTSLSMLIRSELGTPGSLIGDDQLYNTLVTSHAFIMIFFMVMPIMIGGFGNWLIPLMLGAPDMAFPRLNNMSFWFLPPSLILLLMSSIIDKGAGTGWTVYPPLASNISHEGSSVDLAIFSLHMAGISSILGAMNFISTIINMYPSGMK
PDSLTLFTWSVKITAILLLLSLPVLAGAITMLLTDRNINTTFFDPSGGGDPILYQHL
>LEFIJ1817-13|KJ963020|ZMUO.004572|Psylliodes_napi
TLYFIFGIWSGMIGTSLSILIRAELGSPGSLIGNDQIYNVIVTAHAFIMIFFMVMPIMIGGFGNWLVPLMIGAPDMAFPRMNNMSFWLLPPSLLLLLMSSMVESGAGTGWTVYPPLSSNIAHGGSSVDLAIFSLHLAGISSILGAINFITTVINMRPEGMTL
DRMPLFVWAVVITAILLLLSLPVLAGAITMLLTDRNLNTSFFDPIGGGDPILYQHL
>COLFE1537-13|KJ965662|ZMUO.007427|Tetrops_starkii
ILYFIFGAWASMIGTSLSLLIRSELGNPGSLIGNDQIYNTIVTAHAFIMIFFMVMPIMIGGFGNWLIPLMLGAPDMAFPRMNNMSFWLLPPSLILLIMSSIVENGAGTGWTVYPPLASNIAHSGASVDLAIFSLHLAGISSILGAVNFITTVINMRPKEMSLD
QLSLFIWAVKITAILLLLSLPVLAGAITMLLTDRNLNTSFFDPASGGDPILYQHL
>COLFD182-12|KJ967395|ZMUO.004077|Rugilus_rufipes
TLYFIFGAWAGMVGTSLSLLIRAELGAPGSLIGDDQIYNVIVTAHAFVMIFFMVMPIVIGGFGNWLVPLMLGAPDMAFPRMNNMSFWLLPPSLSLLLMSSMVESGAGTGWTVYPPLSSNIAHGGASVDLAIFSLHLAGISSILGAVNFITTIINMRSKG
MTYERMPLFVWSVGITALLLLLSLPVLAGAITMLLTDRNLNTSFFDPAGGGDPILYQHL
>COLFE040-12|KJ965957|ZMUO.005265|Amara_aulica
TLYFIFGAWSGMVGTSLSMLIRAELGNPGALIGDDQIYNVIVTAHAFIMIFFMVMPIMIGGFGNWLVPLMLGAPDMAFPRMNNMSFWMLPPSLILLLVSSMVESGVGTGWTVYPPLSSGIAHAGASVDLAIFSLHLAGISSILGAVNFITTIINMRTTG
MTFDRMPLFVWSVGITALLLLLSLPVLAGAITMLLTDRNLNTSFFDPAGGGDPILYQHL
>COLFE1556-13|KJ963872|ZMUO.007446|Eutrichapion_viciae
TLYFIFGLWSGMVGTSLSMLIRIELGGPGSLIGDDQIYNVIVTAHAFIMIFFMVMPIMIGGFGNWLVPLMLGAPDMAFPRMNNMSFWLLPPSLTLLLMSSIVEKGAGTGWTVYPPLASNIAHGGASVDLAIFSLHLAGISSILGAVNFISTVINMYPNGL
SLDQLSLFTWAVKITAILLLLSLPVLAGAITMLLTDRNINTSFFDPAGGGDPILYQHL
>COLFF388-13|KJ963578|ZMUO.005906|Cis_comptus
ILYFIFGAWSGMVGTSMSMMIRSELGTPGSLIGNDQIYNVIVTAHAFVMIFFMVMPIMIGGFGNWLVPLMLGAPDMAFPRMNNMSFWLLPPSLSLLLMSSIVENGAGTGWTVYPPLSSNVAHSGSSVDLAIFSLHLAGISSILGAVNFISTVINMRPS
GMTPDRMPLFVWAVVITAVLLLLSLPVLAGAITMLLTDRNFNTSFFDPAGGGDPILYQHL
>COLFE660-13|KJ965572|ZMUO.005505|Dromius_agilis
TLYFIFGAWAGMVGTSLSMLIRAELGNPGALIGDDQIYNVIVTAHAFIMIFFMVMPIMIGGFGNWLVPLMLGAPDMAFPRMNNMSFWLLPPSLSLLLMSSMVESGAGTGWTVYPPLSSGIAHAGASVDLAIFSLHLAGVSSILGAVNFITTIINMRSIG
MTFDRMPLFVWSVGITALLLLLSLPVLAGAITMLLTDRNLNTSFFDPAGGGDPILYQHL
>COLFE1198-13|KJ965691|ZMUO.006993|Tetartopeus_terminatus
TLYFIFGAWAGMVGTSLSLLIRTELANPGSLIGDDQIYNVIVTAHAFVMIFFMVMPIVIGGFGNWLVPLMLGAPDMAFPRMNNMSFWLLPPSLSLLLASSMVESGAGTGWTVYPPLSSNIAHGGASVDLAIFSLHLAGISSILGAVNFITTIINMRSPGM
LYERMPLFVWSVGITALLLLLSLPVLAGAITMLLTDRNLNTSFFDPAGGGDPILYQHL
>COLFB415-12|KJ962021|ZMUO.001840|Mycetochara_axillaris

TLYFIFGAWSGMVGTSLSLLIRAELGNPGSLIGDDQIYNVIVTAHAFIMIFFMVMPIMIGGFGNWLVPIMLGAPDMAFPRMNNMSFWLLPPSLTLLLMSSMVESGAGTGWTVYPPLSSNIAHGGASVDLSIFSLHLAGISSILGAVNFITTVINMKPQG
MTFDRMPLFVWAVVITAVLLLLSLPVLAGAITMLLTDRNINTSFFDPAGGGDPILYQHL
>COLFA651-12|KJ966754|ZMUO.000786|Tachinus_lignorum
TLYFIFGAWAGMVGTSLSLLIRAELGNPGTLIGDDQIYNVIVTAHAFIMIFFMVMPIVIGGFGNWLVPLMLGAPDMAFPRMNNMSFWLLPPSLSLLLMSSMVESGAGTGWTVYPPLSSNIAHGGSSVDLAIFSLHLAGISSILGAVNFITTVINMRSIGM
TFDRMPLFVWSVAITALLLLLSLPVLAGAITMLLTDRNLNTTFFDPAGGGDPILYQHL
>COLFF604-13|KJ966690|ZMUO.006494|Cleopus_pulchellus
TLYFIFGMWSGTIGTSLSMLIRTELGNPGSLIGDDQIYNTIVTAHAFIMIFFMVMPIMIGGFGNWLVPLMLGAPDMAFPRLNNMSFWLLPPSITLLLMSSIVEKGAGTGWTVYPPLSNNIAHEGASVDSAIFSLHMAGVSSILGAINFISTVSNMRIKGM
NYDRTPLFVWSVNITAFLLLLSLPVLAGGITMLLTDRNVNTSFFDPSGGGDPILYQHL
>COLFD599-12|KJ963714|ZMUO.004399|Oodes_helopioides
TLYFIFGAWSGMVGTSLSMLIRAELGNPGALIGDDQIYNVIVTAHAFIMIFFMVMPIMIGGFGNWLVPLMLGAPDMAFPRMNNMSFWLLPPSLSLLLMSSMVESGAGTGWTVYPPLSSEIAHSGASVDLAIFSLHLAGVSSILGAVNFITTIINMRSVG
MTFDRMPLFVWSVGITALLLLLSLPVLAGAITMLLTDRNLNTSFFDPAGGGDPILYQHL
>COLFE1428-13|KJ966729|ZMUO.007223|Aleochara_bipustulata
TLYFIFGAWAGMVGTSLSLLIRAELGNPGSLIGDDQIYNVIVTAHAFIMIFFMVMPIVIGGFGNWLVPLMLGAPDMAFPRMNNMSFWLLPPSLTLLLMSSMVESGAGTGWTVYPPLSSNIAHGGSSVDLAIFSLHLAGISSILGAVNFISTVINMRSMG
MTFDKMPLFVWSVAITALLLLLSLPVLAGAITMLLTDRNLNTSFFDPAGGGDPILYQHL
>COLFC323-12|KJ961716|ZMUO.002508|Atheta_spatuloides
TLYFIFGAWAGMVGTSLSLLIRAELGNPGSLIGDDQIYNVIVTAHAFIMIFFMVMPIMIGGFGNWLIPLMLGAPDMAFPRMNNMSFWLLPPSLSLLLMSSMVESGAGTGWTVYPPLSSNIAHGGASVDLAIFSLHLAGISSILGAVNFISTVINMRSTGI
TFDRMPLFVWAVVITALLLLLSLPVLAGAITMLLTDRNLNTSFFDPAGGGDPILYQHL
>COLFB144-12|KJ964461|ZMUO.001474|Byrrhus_fasciatus
TLYFIFGAWAGIVGTSLSMLIRTELGNPGSLIGDDQIYNVIVTAHAFIMIFFMVMPIMIGGFGNWLVPLMLGAPDMAFPRMNNMSFWLLPPSLSLLLMSSVVESGAGTGWTVYPPLSANIAHSGSSVDLAIFSLHLAGISSILGAVNFISTVINMRSPGM
SFDQMSLFSWSVVITALLLLLSLPVLAGAITMLLTDRNVNTSFFDPAGGGDPILYQHL
>COLFF625-13|KJ963892|ZMUO.006515|Crepidodera_aurata
TLYFIFGIWSGMVGTSLSMLIRTELGNPGSLIGNDQIYNVIVTAHAFIMIFFMVMPIMIGGFGNWLVPLMIGAPDMAFPRMNNMSFWLLPPSLFLLLMSSLVESGAGTGWTVYPPLSSNIAHGGSSVDLAIFSLHLAGISSILGAINFITTIINMRPKGMS
LDRMPLFVWAVAITAILLLLSLPVLAGAITMLLTDRNLNTSFFDPIGGGDPILYQHL
>COLFD360-12|KJ966299|ZMUO.004255|Protapion_fulvipes
TLYFIFGLWSGMIGTSLSMLIRIELGNPGSLIGNDQIYNVIVTAHAFIMIFFMVMPVMIGGFGNWLVPLMLGAPDMAFPRMNNMSFWLLPPSLTLLLMSSIVEKGAGTGWTVYPPLAANIAHSGASVDLAIFSLHLAGISSILGAVNFISTIMNMRPTG
MSLDQLSLFTWAVKITAILLLLSLPVLAGAITMLLTDRNINTSFFDPAGGGDPILYQHL
>COLFC208-12|KJ966307|ZMUO.002393|Atheta_corvina
TLYFIFGAWAGMVGTSLSLLIRAELGNPGSLIGDDQIYNVIVTAHAFIMIFFMVMPIVIGGFGNWLVPLMLGAPDMAFPRMNNMSFWLLPPSLTLLLMSSMVESGAGTGWTVYPPLSSNIAHGGSSVDLAIFSLHLAGISSILGAVNFISTVINMRSTGI
TFDRMPLFVWSVVITALLLLLSLPVLAGAITMLLTDRNLNTSFFDPAGGGDPILYQHL
>COLFB243-12|KJ966899|ZMUO.001573|Laccobius_decorus
TLYFIFGAWAGMVGTSLSILIRAELGNPGTLIGDDQIYNVIVTAHAFIMIFFMVMPIMIGGFGNWLVPLMLGAPDMAFPRMNNMSFWLLPPSLTLLLMSSMVESGAGTGWTVYPPLSSNIAHGGASVDLAIFSLHLAGISSILGAVNFITTVINMRSNN
MTYDRLPLFVWSVAITALLLLLSLPVLAGAITMLLTDRNLNTSFFDPAGGGDPILYQHL
>COLFD651-12|KJ962702|ZMUO.004451|Psammoecus_bipunctatus
TLYFIFGAWSGMIGTSLSMLIRTELGTPGSLIGDDQIYNVIVTAHAFIMIFFMVMPIMIGGFGNWLVPLMLGAPDMAFPRMNNMSFWLLPPSLSLLLMSSIVESGAGTGWTVYPPLSSNIAHSGSSVDLAIFSLHLAGISSILGAANFITTVINMRPFGM
NLDQMPLFVWAVVITAILLLLSLPVLAGAITMLLTDRNINTSFFDPAGGGDPILYQHL
>COLFC209-12|KJ965101|ZMUO.002394|Cercyon_borealis
TLYFIFGAWAGMVGTSLSILIRAELGNPGTLIGDDQIYNVIVTAHAFIMIFFMVMPIMIGGFGNWLVPLMLGAPDMAFPRMNNMSFWLLPPSLTLLLMSSMVESGAGTGWTVYPPLSSNIAHGGSSVDLAIFSLHLAGISSILGAVNFITTVINMRSPNL
TYDRLPLFVWSVAITALLLLLSLPVLAGAITMLLTDRNLNTSFFDPAGGGDPILYQHL
>COLFD090-12|KJ963105|ZMUO.003985|Metoecus_paradoxus
TLYFLFGAWAGMVGTSLSLLVRIELGNSGSLIGNDQIYNVIVTAHAFVMIFFMVMPIMIGGFGNWLVPLMLGAPDMAFPRMNNMSFWLLPPSLSLLMMSSMVESGAGTGWTVYPPLSSNIAHGGASVDLAIFSLHLAGISSILGAVNFITTVINMRP
EGMSFDRMPLFVWAVVITAVLLLLSLPVLAGAITMLLTDRNLNTSFFDPAGGGDPILYQHL
>COLFC272-12|KJ966498|ZMUO.002457|Meotica_pallens
SLYFIFGSWAGMIGTSLSLLIRAELGNPGSLIGDDQIYNVIVTAHAFIMIFFMVMPIMIGGFGNWLVPLMLGAPDMAFPRMNNMSFWLLPPSLTLLLMSSMVESGAGTGWTVYPPLSSNIAHSGSSVDLAIFSLHLAGISSILGAVNFLSTIINMRSVGM
TFDRMPLFVWSVGITALLLLLSLPVLAGAITMLLTDRNLNTSFFDPAGGGDPILYQHL
>COLFC507-12|KJ965358|ZMUO.003072|Olisthaerus_megacephalus
TLYFIFGAWSGMVGTSLSLLIRAELGNPGSLIGDDQIYNVIVTAHAFVMIFFMVMPIVIGGFGNWLVPLMLGAPDMAFPRMNNMSFWLLPPSLSLLLMSSMVESGAGTGWTVYPPLSSNIAHGGASVDLAIFSLHLAGISSILGAVNFITTVINMRSTG
MTFDRMPLFVWSVAITALLLLLSLPVLAGAITMLLTDRNLNTSFFDPAGGGDPILYQHL

>COLFB376-12|KJ964377|ZMUO.001706|Corticaria_impressa
SLYFLFGMWSGMVGTSLSLLIRLELGNPGSLIGDDQIYNVIVTAHAFIMIFFMVMPIMIGGFGNWLVPLMLGAPDMAFPRLNNMSFWLLPPSLSLLIMSSIVESGAGTGWTVYPPLSSNIAHGGSSVDLAIFSLHLAGISSILGAVNFITTVINMRPAGM
NLDQMPLFVWSVVITAILLLLSLPVLAGAITMLLTDRNLNTSFFDPAGGGDPILYQHL
>COLFF084-13|KJ963990|ZMUO.005784|Brychius_elevatus
TLYFIFGAWAGMVGTSLSMLIRAELGTPGSLIGDDQIYNVIVTAHAFIMIFFMVMPIMIGGFGNWLVPLMLGAPDMAFPRMNNMSFWLLPPSLSLLLMSSMVESGAGTGWTVYPPLSAGIAHSGASVDLAIFSLHLAGISSILGAVNFITTIINMRSM
GMSFDRMPLFVWSVGITALLLLLSLPVLAGAITMLLTDRNLNTSFFDPAGGGDPILYQHL
>LEFIJ1987-13|KJ962906|ZMUO.003721|Saperda_scalaris
TLYFIFGAWAGMVGTSLSLLIRSELGTPGSLIGDDQIYNVIVTAHAFIMIFFMVMPIMIGGFGNWLVPLMLGAPDMAFPRMNNMSFWLLPPALSLLIMSSIVDKGAGTGWTVYPPLAANVAHNGSSVDLAIFSLHLAGISSILGAVNFITTVINMRPKG
MTLDRMPLFVWAVKITAILLLLSLPVLAGAITMLLTDRNLNTSFFDPAGGGDPILYQHL
>COLFD786-12|KJ966142|ZMUO.004776|Brachypterus_glaber
TLYFIFGAWSGMVGTSLSLLIRSELGNPGSLIGDDQIYNVIVTAHAFVMIFFMVMPFMIGGFGNWLVPLMLGAPDMAFPRMNNMSFWLLPPSLTLLLMSSIVESGAGTGWTVYPPLSSNIAHGGSSVDLAIFSLHLAGISSILGAVNFITTVINMRPTG
MNFDRMPLFVWAVAITALLLLLSLPVLAGAITMLLTDRNLNTSFFDPAGGGDPILYQHL
>COLFE1161-13|KJ966134|ZMUO.006956|Bembidion_ruficolle
TLYFIFGAWSGMVGTSLSMLIRAELGNPGSLIGDDQIYNVIVTAHAFVMIFFMVMPILIGGFGNWLVPLMLGAPDMAFPRMNNMSFWLLPPSLTLLLMSSMVESGAGTGWTVYPPLSSSIAHSGASVDLAIFSLHLAGVSSILGAVNFITTIINMRSVG
MTFDRMPLFVWSVGITALLLLLSLPVLAGAITMLLTDRNLNTSFFDPAGGGDPILYQHL
>COLFB151-12|KJ963884|ZMUO.001481|Broscus_cephalotes
TLYFIFGTWSGMVGTSLSMMIRAELGNPGSLIGDDQIYNVIVTAHAFVMIFFMVMPIMIGGFGNWLVPLMLGAPDMAFPRMNNMSFWLLPPSLTLLLMSSMVESGAGTGWTVYPPLSSSIAHSGASVDLAIFSLHLAGISSILGAVNFISTIINMRSV
GIKFDRMPLFVWSVGITALLLLLSLPVLAGAITMLLTDRNLNSSFFDPAGGGDPILYQHL
>COLFF744-13|KJ962488|ZMUO.006444|Anaspis_flava
TLYFIFGAWSGMVGTSLSLLIRSELGTPGSLIGDDQIYNVIVTAHAFIMIFFMVMPILIGGFGNWLVPLMLGAPDMAFPRMNNMSFWLLPPSLTLLIMSSVVENGAGTGWTVYPPLASNIAHSGSSVDLAIFSLHLAGVSSILGAVNFITTVINMRPQGM
TLDRMPLFVWAVVITAILLLLSLPVLAGAITMLLTDRNLNTSFFDPAGGGDPILYQHL
>COLFA311-12|KJ965357|ZMUO.000446|Cercyon_nigriceps
TLYFIFGAWAGMVGTSLSILIRAELGNPGTLIGDDQIYNVIVTAHAFIMIFFMVMPIMIGGFGNWLVPLMLGAPDMAFPRMNNMSFWLLPPSLTLLLMSSMVESGAGTGWTVYPPLSSNIAHGGSSVDLAIFSLHLAGISSILGAVNFITTVINMRSPNL
TYDRLPLFVWSVAITALLLLLSLPVLAGAITMLLTDRNLNTSFFDPAGGGDPILYQHL
>COLFC072-12|KJ965177|ZMUO.002067|Bledius_talpa
TLYFIFGAWAGMVGTSLSMLIRTELGTPGSLIGNDQIYNVIVTAHAFVMIFFMVMPIVIGGFGNWLIPLMLGAPDMAFPRMNNMSFWLLPPSLTLLLMSSMVESGAGTGWTVYPPLSSNIAHSGSSVDLAIFSLHLAGISSILGAVNFISTIINMRSIGM
TFDRMPLFIWSVKITAILLLLSLPVLAGAITMLLTDRNLNTSFFDPAGGGDPILYQHL
>COLFE1529-13|KJ967163|ZMUO.007419|Ceratomegilla_notata
TLYFLFGMWAGMIGTSLSILIRLELGTTGSLIGNDQIYNVIVTAHAFIMIFFMVMPIMIGGFGNWLVPLMIGAPDMAFPRLNNMSFWLLPPALILLLFSSLVEMGAGTGWTVYPPLSSNLAHNGPSVDLVIFSLHLAGISSILGAVNFISTIMNMRPYGM
KLDKTPLFVWSVLITAILLLLSLPVLAGAITMLLTDRNLNTSFFDPTGGGDPILYQHL
>COLFA416-12|KJ967151|ZMUO.000646|Cercyon_lateralis
TLYFIFGAWAGMVGTSLSILIRAELGNPGTLIGDDQIYNVIVTAHAFIMIFFMVMPIMIGGFGNWLVPLMLGAPDMAFPRMNNMSFWLLPPSLTLLLMSSMVESGAGTGWTVYPPLSSNIAHGGSSVDLAIFSLHLAGISSILGAVNFITTVINMRSPSL
TYDRLPLFVWSVAITALLLLLSLPVLAGAITMLLTDRNLNTSFFDPAGGGDPILYQHL
>COLFE1551-13|KJ963954|ZMUO.007441|Meligethes_difficilis
TLYFIFGAWSGMVGTSLSMLIRTELGNPGSLIGNDQIYNVIVTAHAFVMIFFMVMPFMIGGFGNWLVPLMLGAPDMAFPRMNNMSFWLLPPSLSLLLTSSIVESGAGTGWTVYPPLSSNIAHGGASVDLAIFSLHLAGISSILGAVNFITTVMNMRPS
GMTFDRMPLFVWAVVITAILLLLSLPVLAGAITMLLTDRNLNTTFFDPSGGGDPILYQHL
>COLFA516-12|KJ963547|ZMUO.000556|Microcara_testacea
TLYFIFGSWAGMVGTSLSLLIRAELGTPGSLIGDDQIYNVIVTAHAFIMIFFMVMPIMIGGFGNWLVPLMLGAPDMAFPRMNNMSFWLLPPSLTLLLMSSMVENGAGTGWTVYPPLSAGMAHSGASVDLAIFSLHLAGISSILGAVNFISTVINMRSS
GMTFDRMPLFVWSVAITALLLLLSLPVLAGAITMLLTDRNLNTSFFDPAGGGDPILYQHL
>COLFF240-13|KJ965038|ZMUO.005845|Malthinus_facialis
TLYFLFGAWSGMLGTSLSLLIRAELGNPGSLIGNDQIYNVIVTAHAFIMIFFMVMPIMIGGFGNWLVPLMLGAPDMAFPRMNNMSFWFLPPSLSLLLMSSIVENGAGTGWTVYPPLSANIAHSGSSVDLAIFSLHMAGISSILGAVNFISTVINMRSTG
MSFDRMPLFVWAVAITALLLLLSLPVLAGAITMLLTDRNLNTSFFDPAGGGDPILYQHL
>COLFA611-12|KJ964503|ZMUO.000746|Hydrobius_fuscipes
TLYFIFGAWAGMVGTSLSILIRAELGNPGTLIGDDQIYNVIVTAHAFIMIFFMVMPIMIGGFGNWLVPLMLGAPDMAFPRMNNMSFWLLPPSLTLLLMSSMVENGAGTGWTVYPPLSSNIAHGGASVDLAIFSLHLAGISSILGAVNFITTVINMRSPN
LTYDRLPLFVWSVAITALLLLLSLPVLAGAITMLLTDRNLNTSFFDPAGGGDPILYQHL
>COLFF770-13|KJ964923|ZMUO.006090|Dyschirius_thoracicus

TLYFIFGIWSGMVGTSLSILIRTELGNPGSLIGDDQIYNVIVTAHAFIMIFFMVMPIMIGGFGNWLVPLMLGAPDMAFPRMNNMSFWLLPPSLTLLLMSSMVESGAGTGWTVYPPLSSGIAHSGASVDLAIFSLHLAGISSILGAVNFITTIINMRSTGMT
FERMPLFVWSVGITALLLLLSLPVLAGAITMLLTDRNLNTSFFDPAGGGDPILYQHL
>COLFD362-12|KJ966413|ZMUO.004257|Amara_gebleri
TLYFIFGAWSGMLGTSLSMLIRAELGNPGALIGDDQIYNVIVTAHAFIMIFFMVMPIMIGGFGNWLVPLMLGAPDMAFPRMNNMSFWMLPPSLTFLLASSMVESGVGTGWTVYPPLSSGIAHAGASVDLAIFSLHLAGISSILGAVNFITTIINMRTTG
MTFDRMPLFVWSVGITALLLLLSLPVLAGAITMLLTDRNLNTSFFDPAGGGDPILYQHL
>COLFE1186-13|KJ964343|ZMUO.006981|Agonum_consimile
TLYFIFGAWAGMVGTSLSMLIRAELGNPGALIGDDQIYNVIVTAHAFIMIFFMVMPIMIGGFGNWLVPLMLGAPDMAFPRMNNMSFWLLPPSLTLLLMSSLVESGAGTGWTIYPPLSSGIAHAGASVDLAIFSLHLAGISSILGAVNFITTIINMRSVG
MTFDRMPLFVWSVGITALLLLLSLPVLAGAITMLLTDRNLNTSFFDPAGGGDPILYQHL
>COLFB052-12|KJ961918|ZMUO.001382|Cercyon_marinus
TLYFIFGAWAGMVGTSLSILIRAELGNPGTLIGDDQIYNVIVTAHAFIMIFFMVMPIMIGGFGNWLVPLMLGAPDMAFPRMNNMSFWLLPPSLTLLLMSSMVESGAGTGWTVYPPLSSNIAHGGASVDLAIFSLHLAGISSILGAVNFITTVINMRSPN
LTYDRLPLFVWSVAITALLLLLSLPVLAGAITMLLTDRNLNTSFFDPAGGGDPILYQHL
>COLFD507-12|KJ961989|ZMUO.004307|Acupalpus_flavicollis
TLYFIFGVWAGMVGTSLSMLIRAELGTPGALIGDDQIYNVIVTAHAFIMIFFMVMPIMIGGFGNWLVPLMLGAPDMAFPRMNNMSFWLLPPSLTLLLMSSLVENGAGTGWTVYPPLSSGIAHSGASVDLTIFSLHLAGVSSILGAVNFITTIINMRSVG
MTFERMPLFVWSVGITALLLLLSLPVLAGAITMLLTDRNLNTSFFDPAGGGDPILYQHL
>COLFC051-12|KJ962453|ZMUO.002046|Bembidion_hastii
TLYFIFGAWSGMVGTSLSMLIRAELGNPGSLIGDDQIYNVIVTAHAFVMIFFMVMPILIGGFGNWLVPLMLGAPDMAFPRMNNMSFWLLPPSLTLLLMSSMVESGAGTGWTVYPPLSSSIAHSGASVDLAIFSLHLAGVSSILGAVNFITTIINMRSVG
MSFDRMPLFVWSVGITALLLLLSLPVLAGAITMLLTDRNLNTTFFDPAGGGDPILYQHL
>COLFB205-12|KJ963856|ZMUO.001535|Hydroporus_pubescens
TLYFLFGAWSGMVGTSLSMLIRAELGNPGSLIGDDQIYNVIVTAHAFIMIFFMVMPIMIGGFGNWLVPLMLGAPDMAFPRMNNMSFWLLPPSLTLLLMSSMVENGAGTGWTVYPPLSSGIAHSGASVDLAIFSLHLAGVSSILGAVNFITTIINMRSI
GMTFDRMPLFVWSVGITALLLLLSLPVLAGAITMLLTDRNLNTSFFDPAGGGDPILYQHL
>COLFC854-12|KJ962295|ZMUO.003419|Bembidion_doris
TLYFIFGAWSGMVGTSLSMLIRAELGNPGSLIGDDQIYNVIVTAHAFVMIFFMVMPILIGGFGNWLVPLMLGAPDMAFPRMNNMSFWLLPPSLTLLLMSSMVENGAGTGWTVYPPLSSSIAHSGASVDLAIFSLHLAGVSSILGAVNFITTIINMRSM
GMTFDRMPLFVWSVGITALLLLLSLPVLAGAITMLLTDRNLNTSFFDPAGGGDPILYQHL
>COLFD821-12|KJ965978|ZMUO.004811|Longitarsus_rubiginosus
TLYFIFGIWAGMIGTSLSMLIRTELGNPGSLIGNDQIYNVIVTAHAFIMIFFMVMPIMIGGFGNWLVPLMIGAPDMAFPRMNNMSFWLLPPSLFLLVMSSMVENGAGTGWTVYPPLSSNIAHSGSSIDLAIFSLHLAGISSILGAINFITTVINMRPVGM
TLDRMPLFIWAVTITAILLLLSLPVLAGAITMLLTDRNLNTTFFDPAGGGDPILYQHL
>COLFA570-12|KJ965870|ZMUO.000705|Aromia_moschata
TLYFIFGAWSGMVGTSLSMLIRSELGNPGSLIGDDQIYNVIVTAHAFIMIFFMVMPIMIGGFGNWLVPLMLGAPDMAFPRMNNMSFWLLPPSLTFLILSSIVESGAGTGWTVYPPLSSNIAHSGSSVDLAIFSLHLAGISSILGAVNFISTVINMRPSGMS
LDRMPLFVWAVVITAILLLLSLPVLAGAITMLLTDRNINTSFFDPAGGGDPILYQHL
>COLFD054-12|KJ962020|ZMUO.003949|Quedius_fuliginosus
TLYFIFGAWAGMVGTSLSLLIRAELGNPGSLIGDDQIYNVIVTAHAFIMIFFMVMPIVIGGFGNWLVPLMLGAPDMAFPRMNNMSFWLLPPSLSLLLMSSMVESGAGTGWTVYPPLSSNIAHGGASVDLAIFSLHLAGISSILGAVNFITTVINMRSIG
MTFDRMPLFVWSVAITALLLLLSLPVLAGAITMLLTDRNLNTSFFDPAGGGDPILYQHL
>COLFF561-13|KJ963488|ZMUO.006641|Tachyta_nana
TLYFIFGAWSGMVGTSLSMLIRAELGNPGSLIGDDQIYNVIVTAHAFIMIFFMVMPILIGGFGNWLVPLMLGAPDMAFPRMNNMSFWLLPPSLTLLLMSSMVESGAGTGWTVYPPLSSVIAHSGASVDLAIFSLHLAGISSILGAVNFITTIINMRSIGM
TFDRMPLFVWSVGITALLLLLSLPVLAGAITMLLTDRNLNTSFFDPAGGGDPILYQHL
>COLFB284-12|KJ964419|ZMUO.001614|Ampedus_balteatus
TLYFIFGAWAGMLGTSLSLLIRAELGNPGSLIGNDQIYNVIVTAHAFIMIFFMVMPIMIGGFGNWLVPLMLGAPDMAFPRMNNMSFWLLPPSLSLLLMSSIVENGAGTGWTVYPPLSSNIAHSGSSVDLAIFSLHLAGISSILGAVNFISTVINMRSTGIT
FDRMPLFVWAVAITALLLLLSLPVLAGAITMLLTDRNLNTSFFDPAGGGDPILYQHL
>COLFD674-12|KJ962498|ZMUO.004664|Anoplodera_sanguinolenta
TLYFIFGAWASMVGTSLSLLIRSELGNPGSLIGNDQIYNVIVTAHAFVMIFFMVMPIMIGGFGNWLVPLMLGAPDMAFPRMNNMSFWLLPPSLTLLIMSSIVESGAGTGWTVYPPLSSNIAHSGSSVDLAIFSLHLAGISSILGAVNFITTVINMRPAGLK
PEQMPLFVWAVVITAVLLLLSLPVLAGAITMLLTDRNLNTSFFDPAGGGDPILYQHL
>COLFE1328-13|KJ964528|ZMUO.007123|Dyschirius_angustatus
TLYFIFGIWSGMVGTSLSILIRTELGNPGSLIGDDQIYNVIVTAHAFIMIFFMVMPIMIGGFGNWLVPLMLGAPDMAFPRMNNMSFWLLPPSLTLLLMSSMVENGAGTGWTVYPPLSSSIAHSGASVDLAIFSLHLAGISSILGAVNFITTIINMRSTGMT
FERMPLFVWSVGITALLLLLSLPVLAGAITMLLTDRNLNTSFFDPAGGGDPILYQHL
>COLFA204-10|HM909123|MP00051|Agrilus_viridis
TLYFIFGVWSGMVGTALSLLIRAELGNPGALIGNDQIYNVIVTAHAFIMIFFMVMPIMMGGFGNWLVPLMLGAPDMAFPRMNNMSFWLLPPSLTLLLMSSMVESGAGTGWTVYPPLAANIAHSGGSVDLAIFSLHLAGISSILGAINFITTVINMRAV
GMTMDRVPLLVWSIAITALLLLLSLPVLAGAITMLLTDRNLNTSFFDPAGGGDPILYQHL

>COLFF160-13|KJ965422|ZMUO.003794|Tychius_breviusculus
TLYFIFGSWSGMVGTSLSMLIRTELGNPGSLIGDDQIYNSIVTAHAFIMIFFMVMPIMIGGFGNWLVPLMLGAPDMAFPRLNNMSFWLLPPSISLLLMSSIVNKGAGTGWTVYPPLSSNMAHEGASVDLAIFSLHMAGASSILGAINFISTAMNMRPF
GMNSERVTLFTWAVQITAILLLLSLPVLAGAITMLLTDRNVNTTFFDPAGGGDPILYQHL
>COLFB468-12|KJ962536|ZMUO.001893|Rhagonycha_lignosa
TLYFIFGAWSGSLGLALSLLIRAELGTPGTLIGNDQIYNVIVTAHAFIMIFFMVMPIMIGGFGNWLVPLMLGAPDMAFPRMNNMSFWFLPPSLMFLLMSSMVESGAGTGWTVYPPLSANIAHSGPSVDLAIFSLHMAGISSILGAVNFISTILNMKPPS
MKFDQMPLFVWSVGITALLLLLSLPVLAGAITMLLSDRNLNTSFFDPMGGGDPILYQHL
>COLFE812-13|KJ963648|ZMUO.005657|Tachyporus_pallidus
TLYFIFGAWSGMVGTSLSLLIRAELGNPGSLIGDDQIYNVIVTAHAFIMIFFMVMPIVIGGFGNWLVPLMLGAPDMAFPRMNNMSFWLLPPSLTLLLMSSMVESGAGTGWTVYPPLSANIAHSGSSVDLAIFSLHLAGISSILGAVNFITTVINMRASEM
NFDQMPLFIWSVAITALLLLLSLPVLAGAITMLLTDRNLNTSFFDPAGGGDPILYQHL
>COLFD326-12|KJ966626|ZMUO.004221|Anthicus_ater
TLYLIFGAWAGMVGTSLSLLIRSELGNPGTLIGNDQIYNVIVTAHAFIMIFFMVMPIVIGGFGNWLVPLMLGAPDMAFPRMNNMSFWLLPPSLSLLIMSSIVESGSGTGWTVYPPLSANIAHSGSSVDLAIFSLHLAGISSILGAVNFITTVINMRPTGMS
LDRMPLFVWAVVITAVLLLLSLPVLAGAITMLLTDRNLNTSFFDPAGGGDPILYQHL
>COLFE649-13|KJ961834|ZMUO.005494|Plateumaris_discolor
TLYFIFGAWSGMMGTSLSMLIRTELMNPGSLIGNDQIYNVIVTAHAFIMIFFMVMPIMIGGFGNWLIPLMLGAPDMAFPRMNNMSFWLLPPSLTFLIMSSIVENGAGTGWTVYPPLSSNIAHSGASVDLAIFSLHLAGVSSILGAVNFITTIINMRPMG
MKMDKVPLFAWAVMITAILLLLSLPVLAGAITMLLTDRNLNTSFFDPAGGGDPVLYQHL
>COLFE1525-13|KJ963471|ZMUO.007415|Cantharis_pellucida
TLYFIFGAWSGSLGLALSLLIRAELGTPGTLIGNDQIYNVIVTAHAFIMIFFMVMPIMIGGFGNWLVPLMLGAPDMAFPRMNNMSFWFLPPSLMFLLMSSMVESGAGTGWTVYPPLSANIAHSGPSVDLAIFSLHMAGISSILGAVNFISTIMNMKPPS
MKFDQMPLFVWSVGITALLLLLSLPVLAGAITMLLSDRNLNTSFFDPMGGGDPILYQHL
>COLFC182-12|KJ966646|ZMUO.002367|Corticeus_fraxini
TLYFIFGAWSGMVGTSLSLLIQAELGNPGSLIGDDQIYNVIVTAHAFIMIFFMVMPIMMGGFGNWLVPLMLGAPDMAFPWMNNMSFWLLPPSLTLLLMSSVVESGAGTGWTVYPPLSSNIAHGGASVDLAIFSLHLAGISSILGAVNFITTVINMRP
QGMTFDRMPLFVWAVVLTAVLLLLSLPVLAGAITMLLTDRNLNTSFFDPAGGGDPILYQHL
>COLFF895-13|KJ967388|ZMUO.006690|Agonum_emarginatum
TLYFIFGAWSGMVGTSLSMLIRAELGNPGALIGDDQIYNVIVTAHAFIMIFFMVMPIMIGGFGNWLVPLMLGAPDMAFPRMNNMSFWLLPPSLTLLLMSSMVESGAGTGWTVYPPLSSGIAHAGASVDLAIFSLHLAGVSSILGAVNFITTIINMRSV
GMTFDRMPLFVWSVGITALLLLLSLPVLAGAITMLLTDRNLNTSFFDPAGGGDPILYQHL
>COLFF568-13|KJ964668|ZMUO.006648|Smaragdina_salicina
TLYFIFGAWSGMVGTSLSLLIRVELGNPGTLIGNDQIYNTIVTAHAFIMIFFMVMPIMIGGFGNWLVPLMLGAPDMAFPRMNNMSFWLLPPSLTLLLMSSIVENGAGTGWTVYPPLSANLAHSGASVDLAIFSLHLAGVSSIMGAINFISTVINMRPQG
MLLDRTPLFVWAVVITAILLLLSLPVLAGAITMLLTDRNLNTSFFDPAGGGDPILYQHL
>COLFD510-12|KJ964833|ZMUO.004310|Epuraea_unicolor
TLYFIFGAWSGMVGTSLSILIRTELGSPGSLIGNDQIYNVIVTAHAFIMIFFMVMPFMIGGFGNWLVPLMLGAPDMAFPRMNNMSFWLLPPSLSLLLMSSIVESGAGTGWTVYPPLSSNIAHGGSSVDLAIFSLHLAGISSILGAVNFITTIINMRPVGMT
LDRMPLFVWSVMITAVLLLLSLPVLAGAITMLLTDRNLNTTFFDPSGGGDPILYQHL
>COLFB458-12|KJ962224|ZMUO.001883|Anthicus_antherinus
TLYLIFGAWAGMVGTSLSLLIRSELGNPGTLIGNDQIYNVIVTAHAFIMIFFMVMPIVIGGFGNWLVPLMLGAPDMAFPRMNNMSFWLLPPSLSLLIMSSIVESGSGTGWTVYPPLSANIAHSGSSVDLAIFSLHLAGISSILGAVNFITTVINMRPAGMT
LDRMPLFVWAVVITAVLLLLSLPVLAGAITMLLTDRNLNTSFFDPAGGGDPILYQHL
>COLFE056-12|KJ967332|ZMUO.005281|Anthicus_flavipes
TLYLIFGAWAGMVGTSLSMLIRSELGNPGTLIGNDQIYNVIVTAHAFIMIFFMVMPIVIGGFGNWLVPLMLGAPDMAFPRMNNMSFWLLPPSLSLLIMSSIVESGAGTGWTVYPPLSANIAHSGSSVDLAIFSLHLAGISSILGAVNFITTVINMRPVGM
TLDRMPLFVWAVVITAVLLLLSLPVLAGAITMLLTDRNLNTSFFDPAGGGDPILYQHL
>COLFG115-13|KJ966112|ZMUO.007810|Phloeostiba_plana
TLYFIFGAWAGMVGTSLSLLIRAELGNPGSLIGDDQIYNVIVTAHAFVMIFFMVMPIVIGGFGNWLVPLMLGAPDMAFPRMNNMSFWLLPPSLTLLLMSSMVESGAGTGWTVYPPLSSNIAHGGSSVDLAIFSLHLAGISSILGAVNFITTVINMRAM
GMTFDRMPLFVWSVVITALLLLLSLPVLAGAITMLLTDRNLNTSFFDPAGGGDPILYQHL
>COLFE1110-13|KJ963341|ZMUO.006905|Agabus_lapponicus
TLYFIFGAWAGMVGTSLSMLIRAELGNPGSLIGDDQIYNVIVTAHAFVMIFFMVMPIMIGGFGNWLIPLMLGAPDMAFPRMNNMSFWLLPPSLTLLLMSSMVESGAGTGWTVYPPLSSGIAHGGASVDLAIFSLHLAGISSILGAVNFITTIINMRSVG
MTFDRMPLFVWSVGITALLLLLSLPVLAGAITMLLTDRNLNTSFFDPAGGGDPILYQHL
>COLFF200-13|KJ965924|ZMUO.005805|Polydrusus_cervinus
TLYFIFGAWSGMVGTSLSMLIRTELGNPGSLIGDDQIYNVIVTAHAFIMIFFMVMPIMIGGFGNWLVPLMLGAPDMAFPRLNNMSFWLLPPSLTLLLMSSIVDKGAGTGWTVYPPLSTNIAHEGSSVDLAIFSLHMAGVSSILGAINFISTIINMRPTGM
SYDRMPLFVWAVKITAILLLLSLPVLAGAITMLLTDRNINTSFFDPAGGGDPILYQHL
>COLFB539-12|KJ963278|ZMUO.001014|Aloconota_gregaria

TLYFIFGAWAGMVGTSLSLLIRAELGNPGSLIGDDQIYNVIVTAHAFIMIFFMVMPIVIGGFGNWLIPLMLGAPDMAFPRMNNMSFWLLPPSLSLLLMSSMVESGAGTGWTVYPPLSSNIAHGGSSVDLAIFSLHLAGISSILGAVNFISTIINMRSSGISF
DRMPLFVWSVAITALLLLLSLPVLAGAITMLLTDRNLNTSFFDPAGGGDPILYQHL
>COLFD368-12|KJ962399|ZMUO.004263|Aleochara_brevipennis
TLYFIFGAWSGMVGTSLSLLIRAELGNPGSLIGDDQIYNVIVTAHAFIMIFFMVMPIMIGGFGNWLVPLMLGAPDMAFPRMNNMSFWLLPPSLTLLLMSSMVESGAGTGWTVYPPLSSNIAHGGASVDLAIFSLHLAGISSILGAVNFISTVINMRSMG
MTFDKMPLFVWAVAITALLLLLSLPVLAGAITMLLTDRNLNTSFFDPAGGGDPILYQHL
>COLFF025-13|KJ964121|ZMUO.005725|Nedyus_quadrimaculatus
TLYFIFGAWAAMAGTSLSMLIRTELGNPGSLIGDDQIYNSIVTAHAFIMIFFMVMPILIGGFGNWLVPLMLGAPDMAFPRLNNMSFWLLPPALSLLLMSSIVNKGAGTGWTVYPPLSTNTAHEGMSVDLAIFSLHLAGLSSILGAINFISTVMNMRPQG
MSPEFTPLFVWAVKITAILLLLSLPVLAGAITMLLTDRNINTSFFDPSGGGDPILYQHL
>COLFA379-12|KJ963650|ZMUO.000609|Nicrophorus_investigator
TLYFIFGAWAGMVGMSLSMLIRVELSTPGTLLGDDQMYNVIVTAHAFIMIFFMVMPIVIGGFGNWLVPLMLGAPDMAFPRLNNMSFWLLPPSLSLLLISSMVESGAGTGWTVYPPLSANIAHSGSSVDLAIFSLHLAGISSILGAVNFITTVINMRSPG
MTFDRMPLFVWSVVITALLLLLSLPVLAGAITMLLTDRNLNTSFFDPAGGGDPILYQHL
>COLFE075-12|KJ962521|ZMUO.005300|Hemicrepidius_niger
TLYFLFGAWSGMLGTSLSLLIRAELGNPGSLIGNDQIYNVIVTAHAFIMIFFMVMPIMIGGFGNWLVPLMLGAPDMAFPRMNNMSFWFLPPSLSLLLMSSIVENGAGTGWTVYPPLSANIAHSGSSVDLAIFSLHLAGISSILGAVNFISTVINMRSTGIT
FDRMPLFVWAVAITALLLLLSLPVLAGAITMLLTDRNLNTSFFDPAGGGDPILYQHL
>COLFD228-12|KJ966928|ZMUO.004123|Corticaria_foveola
SLYFLFGMWAGMVGTSLSLLIRLELGNPGSLIGDDQIYNVIVTAHAFVMIFFMVMPIMIGGFGNWLVPLMLGAPDMAFPRLNNMSFWLLPPSLSLLIMSSIVESGAGTGWTVYPPLSSNIAHGGSSVDLAIFSLHLAGISSILGAVNFITTVINMRPTGM
NLDQMPLFVWSVVITAILLLLSLPVLAGAITMLLTDRNLNTSFFDPAGGGDPILYQHL
>COLFD117-12|KJ965440|ZMUO.004012|Corticaria_lapponica
SLYFLFGMWAGMVGTSLSLLIRLELGNPGSLIGDDQIYNVIVTAHAFVMIFFMVMPIMIGGFGNWLVPLMLGAPDMAFPRLNNMSFWLLPPSLSLLIMSSIVEKGAGTGWTVYPPLSSNIAHGGSSVDLAIFSLHLAGISSILGAVNFITTVINMRPTGM
NLDQMPLFVWSVVITAILLLLSLPVLAGAITMLLTDRNLNTSFFDPAGGGDPILYQHL
>COLFF210-13|KJ966249|ZMUO.005815|Agriotes_sputator
TLYFLFGAWAGMLGTSLSLLIRAELGNPGSLIGNDQIYNVIVTAHAFIMIFFMVMPIMIGGFGNWLVPLMLGAPDMAFPRMNNMSFWFLPPSLSLLLMSSIVENGAGTGWTVYPPLSSNIAHSGSSVDLAIFSLHLAGISSILGAVNFISTVINMRSTGIT
FDRMPLFVWAVAITALLLLLSLPVLAGAITMLLTDRNLNTSFFDPAGGGDPILYQHL
>COLFE022-12|KJ964700|ZMUO.005247|Larinus_sturnus
TLYFIFGAWSGMVGTSLSMLIRTELGNPGSLIGDDQIYNTIVTAHAFIMIFFMVMPIMIGGFGNWLVPLMLGAPDMAFPRLNNMSFWLLPPSLTLLLMSSIVDKGAGTGWTVYPPLSTNIAHEGASVDLAIFSLHMAGISSILGAINFISTVLNMRPMG
MKPDQTTLFTWAVEITAILLLLSLPVLAGAITMLLTDRNINTSFFDPAGGGDPILYQHL
>COLFC837-12|KJ964491|ZMUO.003402|Neocrepidodera_nigritula
TLYFIFGLWSGMVGASLSMLIRTELGSPGSLIGNDQIYNVIVTAHAFIMIFFMVMPIMIGGFGNWLVPLMIGAPDMAFPRMNNMSFWLLPPSLFLLLMSSMVESGAGTGWTVYPPLSSNIAHGGSSVDLAIFSLHLAGISSILGAINFITTVINMRPMG
MTLDRMPLFVWAVVITAVLLLLSLPVLAGAITMLLTDRNLNTSFFDPSGGGDPILYQHL
>COLFF731-13|KJ965437|ZMUO.006431|Isomira_murina
TLYFIFGAWSGMVGTSLSLLIRAELGNPGSLIGDDQIYNVIVTAHAFVMIFFMVMPIMIGGFGNWLVPLMLGAPDMAFPRMNNMSFWLLPPSLSLLLMSSLVENGAGTGWTVYPPLSANIAHSGASVDLAIFSLHLAGISSILGAVNFITTVINMRPQG
MTFDRMPLFVWSVVITAVLLLLSLPVLAGAITMLLTDRNINTSFFDPAGGGDPILYQHL
>COLFB867-12|KJ964846|ZMUO.001912|Aromia_moschata
TLYFIFGAWSGMVGTSLSMLIRSELGNPGSLIGDDQIYNVIVTAHAFIMIFFMVMPIMIGGFGNWLVPLMLGAPDMAFPRMNNMSFWLLPPSLTFLILSSIVESGAGTGWTVYPPLSSNIAHSGSSVDLAIFSLHLAGISSILGAVNFISTVINMRPSGMS
PDRMPLFVWAVVITAILLLLSLPVLAGAITMLLTDRNINTSFFDPAGGGDPILYQHL
>LEFIJ1809-13|KJ967322|ZMUO.004564|Ceutorhynchus_contractus
TLYFIFGSWAGMAGTSLSMLIRTELGNPGSLIGNDQIYNSIVTAHAFIMIFFMVMPILIGGFGNWLVPLMLGAPDMAFPRLNNMSFWLLPPSLSLLLMSSIVNKGAGTGWTVYPPLSGNVAHEGMSVDLAIFSLHMAGISSILGAINFISTVMNMQPK
GMTPELMPLFVWAVEITAILLLLSLPVLAGAITMLLTDRNINTSFFDPSGGGDPILYQHL
>COLFF083-13|KJ962594|ZMUO.005783|Bembidion_prasinum
TLYFIFGAWSGMVGTSLSMLIRAELGNPGSLIGDDQIYNVIVTAHAFVMIFFMVMPILIGGFGNWLVPLMLGAPDMAFPRMNNMSFWLLPPSLSLLLMSSMVESGAGTGWTVYPPLSSSIAHSGASVDLAIFSLHLAGVSSILGAVNFITTIVNMRSVG
MSFDRMPLFVWSVGITALLLLLSLPVLAGAITMLLTDRNLNTTFFDPAGGGDPILYQHL
>COLFA025-10|HM909052|MP00163|Zorochros_minimus
TLYFLFGAWAGMLGTSLSLLIRAELGNPGSLIGNDQIYNVIVTAHAFIMIFFMVMPIMIGGFGNWLVPLMLGAPDMAFPRMNNMSFWFLPPSLSLLLMSSIVENGAGTGWTVYPPLSANIAHSGSSVDLAIFSLHLAGISSILGAVNFISTVINMRSTGIT
FDRMPLFVWAVAITALLLLLSLPVLAGAITMLLTDRNLNTSFFDPAGGGDPILYQHL
>COLFE477-13|KJ961796|ZMUO.005322|Sibinia_viscariae
TLYFIFGSWSGMIGTSMSLLIRAELGNPGKLIGNDQIYNTIVTAHAFIMIFFMVMPIMIGGFGNWLVPLMLGAPDMAFPRLNNMSFWLLPPSLFLLIMSSMINKGVGTGWTVYPPLSSNKAHEGISVDLAIFSLHLAGMSSILGAINFISTVMNMQPTG
MKPEQMSLFTWAVQITAILLLLSLPVLAGAITMLLTDRNVNTSFFDPAGGGDPILYQHL

>COLFF703-13|KJ962780|ZMUO.006403|Nedyus_quadrimaculatus
TLYFIFGAWAAMAGTSLSMLIRTELGNPGKLIGDDQIYNSIVTAHAFIMIFFMVMPILIGGFGNWLVPLMLGAPDMAFPRLNNMSFWLLPPALSLLLMSSIVNKGAGTGWTVYPPLSTNTAHEGMSVDLAIFSLHLAGLSSILGAINFISTVMNMRPQG
MNPEFTPLFVWAVKITAILLLLSLPVLAGAITMLLTDRNINTSFFDPSGGGDPILYQHL
>COLFE628-13|KJ964870|ZMUO.005473|Dicerca_moesta
TLYFIFGAWSGMVGTGLSLLIRAELGNPGALIGDDQIYNVIVTAHAFIMIFFMVMPIMMGGFGNWLVPLMLGAPDMAFPRMNNMSFWLLPPSLTLLLMSSIVENGAGTGWTVYPPLAANVAHSGASVDLAIFSLHLAGISSILGAVNFITTVINMRST
GMSFDRMPLFVWSVAITALLLLLSLPVLAGAITMLLTDRNLNTSFFDPAGGGDPILYQHL
>COLFB010-12|KJ965573|ZMUO.001340|Dermestes_lardarius
TLYFIFGAWAGMVGTSLSMLIRTELGMPGSLIGNDQIFNVIVTAHAFIMIFFMVMPIMIGGFGNWLVPLMLGAPDMAFPRMNNMSFWLLPPSLSLLLMSSMVESGAGTGWTVYPPLSANIAHSGASVDLAIFSLHLAGISSILGAVNFITTVINMRSK
GMTPDRMPLFVWSVAITALLLLLSLPVLAGAITMLLTDRNLNTSFFDPAGGGDPILYQHL
>COLFC201-12|KJ965020|ZMUO.002386|Atheta_vaga
TLYFIFGAWAGMVGTSLSLLIRAELGNPGSLIGDDQIYNVIVTAHAFVMIFFMVMPIVIGGFGNWLIPLMLGAPDMAFPRMNNMSFWLLPPSLTLLLMSSMVESGAGTGWTVYPPLSSNIAHGGSSVDLAIFSLHLAGISSILGAVNFISTVINMRSTGI
TFDRMPLFVWSVAITALLLLLSLPVLAGAITMLLTDRNLNTSFFDPAGGGDPILYQHL
>COLFC152-12|KJ961890|ZMUO.002337|Quedius_nitipennis
TLYFIFGAWAGMVGTSLSLLIRAELGNPGTLIGDDQIYNVIVTAHAFIMIFFMVMPIVIGGFGNWLVPLMLGAPDMAFPRMNNMSFWLLPPSLSLLLMSSMVESGAGTGWTVYPPLSSNIAHGGASVDLAIFSLHLAGISSILGAVNFITTVINMRSIG
MSFDRMPLFVWSVAITALLLLLSLPVLAGAITMLLTDRNLNTSFFDPAGGGDPILYQHL
>COLFB840-12|KJ962703|ZMUO.001315|Atheta_aeneipennis
TLYFIFGAWAGMVGTSLSLLIRAELGNPGSLIGDDQIYNVIVTAHAFIMIFFMVMPIVIGGFGNWLVPLMLGAPDMAFPRMNNMSFWLLPPSLTLLLMSSMVESGAGTGWTVYPPLSSNIAHGGSSVDLAIFSLHLAGISSILGAVNFISTVINMRSTGI
SFDRMPLFVWSVAITALLLLLSLPVLAGAITMLLTDRNLNTSFFDPAGGGDPILYQHL
>COLFE831-13|KJ962830|ZMUO.005676|Oulema_melanopus
TLYFIFGTWSGMVGTSLSMMIRTELGNPGSFIGNDQIYNVIVTAHAFIMIFFMVMPIMIGGFGNWLVPLMLGAPDMAFPRMNNMSFWLLPPSISLLLMSSIVENGAGTGWTVYPPLSANISHNGASVDLAIFSLHLAGISSILGAVNFISTVSNMRPE
GMTLDRMSLFVWAVLITAILLLLSLPVLAGAITMLLTDRNLNTSFFDPSSGGDPILYQHL
>COLFE1347-13|KJ964370|ZMUO.007142|Oedemera_lurida
TLYLIFGAWAGMVGTSLSLLIRAELGNPGSLIGDDQIYNVIVTAHAFIMIFFMVMPIVIGGFGNWLVPLMLGAPDMAFPRMNNMSFWLLPPSLTLLIMSSMVESGTGTGWTVYPPLSSNIAHSGSSVDLAIFSLHLAGVSSILGAVNFITTVINMRPVG
MTLDRMPLFVWAVVITAILLLLSLPVLAGAITMLLTDRNLNTSFFDPAGGGDPILYQHL
>COLFC646-12|KJ964477|ZMUO.003211|Hydroporus_geniculatus
TLYFLFGAWSGMVGTSLSMLIRAELGNPGSLIGDDQIYNVIVTAHAFIMIFFMVMPIMIGGFGNWLVPLMLGAPDMAFPRMNNMSFWLLPPSLTLLLMSSMVENGAGTGWTVYPPLSSGIAHSGASVDLAIFSLHLAGVSSILGAVNFITTIINMRSI
GMTFDRMPLFVWSVGITALLLLLSLPVLAGAITMLLTDRNLNTSFFDPAGGGDPILYQHL
>COLFA096-10|JN297959|MP00336|Ptenidium_pusillum
ILYFMFGSWAGMVGTSLSMLIRSELSIPGSLIGDDQIYNVIVTAHAFVMIFFMVMPILIGGFGNCLVPLMLGAPDMAFPRMNNMSFWLLPPSLMLLLMSSMVESGAGTGWTVYPPLASNIAHGGASVDLAIFSLHLAGISSILGAVNFITTVINMRTPG
MTFDQMPLFVWAVVITALLLLLSLPVLAGAITMLLTDRNLNTSFFDPSGGGDPILYQHL
>COLFE023-12|KJ966093|ZMUO.005248|Nicrophorus_vespilloides
TLYFIFGAWAGMVGMSLSMLIRVELSTPGTLLGDDQMYNVIVTAHAFIMIFFMVMPIVIGGFGNWLVPLMLGAPDMAFPRLNNMSFWLLPPSLSLLLISSMVESGAGTGWTVYPPLSANIAHSGSSVDLAIFSLHLAGISSILGAVNFITTVINMRSPG
MTFDRMPLFVWSVVITALLLLLSLPVLAGAITMLLTDRNLNTSFFDPAGGGDPILYQHL
>COLFF779-13|KJ964283|ZMUO.006099|Calathus_micropterus
TLYFIFGAWAGMVGTSLSMLIRAELGNPGALIGDDQIYNVIVTAHAFVMIFFMVMPIMIGGFGNWLVPLMLGAPDMAFPRMNNMSFWLLPPALSLLLMSSMVESGAGTGWTVYPPLSSGIAHSGASVDLAIFSLHLAGVSSILGAVNFITTIINMRSV
GMTFDRMPLFVWSVGITALLLLLSLPVLAGAITMLLTDRNLNTSFFDPAGGGDPILYQHL
>COLFE1445-13|KJ962526|ZMUO.007240|Anaulacaspis_nigra
TLYFIFGAWAGMVGTSLSLLIRAELGNPGSLIGDDQIYNVIVTAHAFIMIFFMVMPIMIGGFGNWLVPLMLGAPDMAFPRKNNMSFWLLPPSLTLLLMSSMVESGAGTGWTVYPPLSSNIAHAGATVDLAIFSLHLAGISSILGTVNFISTVINMRSIGIS
FDRMPLFVWAVAITALLLLLSLPVLAGAITMLLTDRNLNTSFFDPAGGGDPILYQHL
>LEFIJ1825-13|KJ965333|ZMUO.004580|Ceutorhynchus_ignitus
TLYFIFGSWAGMAGTSLSMLIRTELGNPGSLIGNDQIYNSIVTAHAFIMIFFMVMPILIGGFGNWLVPLMLGAPDMAFPRLNNMSFWLLPPSLSLLLMSSIVNKGAGTGWTVYPPLSSNVAHEGMSVDLAIFSLHMAGISSILGAINFISTVMNMQPK
GMTPELMPLFVWAVEITAILLLLSLPVLAGAITMLLTDRNINTSFFDPSGGGDPILYQHL
>COLFD705-12|KJ963885|ZMUO.004695|Philonthus_quisquiliarius
TLYFIFGSWAGMVGTSLSLLIRAELGNPGSLIGDDQIYNVIVTAHAFIMIFFMVMPIVIGGFGNWLVPLMLGAPDMAFPRMNNMSFWLLPPSLTLLLMSSMVESGAGTGWTVYPPLSSNIAHGGASVDLAIFSLHLAGISSILGAVNFITTVINMRSSG
MSFDRMPLFVWSVAITALLLLLSLPVLAGAITMLLTDRNLNTTFFDPAGGGDPILYQHL
>COLFE1190-13|KJ967020|ZMUO.006985|Anisotoma_glabra

TLYFIFGAWSGMVGTSLSILIRAELGTPGSLIGDDQIYNVIVTAHAFVMIFFMVMPIVIGGFGNWLVPLMLGAPDMAFPRMNNMSFWLLPPSLTLLLMSSMVENGAGTGWTVYPPLSANISHSGSSVDLAIFSLHLAGISSILGAVNFITTVINMRSIGM
TFDKMPLFVWSVAITALLLLLSLPVLAGAITMLLTDRNLNTSFFDPAGGGDPILYQHL
>COLFB865-12|KJ964213|ZMUO.001910|Anoplodera_reyi
TLYFIFGAWASMVGTSLSLLIRSELGNPGSLIGDDQIYNVIVTAHAFVMIFFMVMPIMIGGFGNWLVPLMLGAPDMAFPRMNNMSFWLLPPSLTLLIMSSIVESGAGTGWTVYPPLSSNIAHGGSSVDLAIFSLHLAGISSILGAVNFITTVINMRPVGM
SPERMPLFVWAVVITAVLLLLSLPVLAGAITMLLTDRNLNTSFFDPAGGGDPILYQHL
>COLFC617-12|KJ966583|ZMUO.003182|Gyrinus_minutus
TLYFIFGAWSGMVGTSLSMLIRAELGNPGSLIGDDQVYNVIVTAHAFIMIFFMVMPIMIGGFGNWLVPLMLGAPDMAFPRMNNMSFWLLPPSLTLLLMSSMVENGAGTGWTVYPPLSSNIAHGGASVDLAIFSLHLAGISSILGAVNFITTIINMRSI
GMTLDRMPLFVWSVGITALLLLLSLPVLAGAITMLLTDRNLNTSFFDPAGGGDPILYQHL
>COLFD074-12|KJ962215|ZMUO.003969|Ampedus_nigrinus
TLYFIFGAWAGMLGTSLSLLIRAELGNPGSLIGNDQIYNVIVTAHAFIMIFFMVMPIMIGGFGNWLVPLMLGAPDMAFPRMNNMSFWLLPPSLSLLLMSSIVENGAGTGWTVYPPLSSNIAHSGSSVDLAIFSLHLAGISSILGAVNFISTVINMRSTGIS
FDRMPLFVWAVAITALLLLLSLPVLAGAITMLLTDRNLNTSFFDPAGGGDPILYQHL
>COLFC587-12|KJ966933|ZMUO.003152|Athous_subfuscus
TLYFLFGAWAGMLGTSLSLLIRAELGNPGSLIGNDQIYNVIVTAHAFIMIFFMVMPIMIGGFGNWLVPLMLGAPDMAFPRMNNMSFWFLPPSLSLLLMSSIVENGAGTGWTVYPPLSANIAHSGSSVDLAIFSLHLAGISSILGAVNFISTVINMRSTGIT
FDRMPLFVWAVAITALLLLLSLPVLAGAITMLLTDRNLNTSFFDPAGGGDPILYQHL
>COLFD690-12|KJ963397|ZMUO.004680|Anthonomus_pinivorax
TLYFIFGAWSGTVGTSLSMLIRTELGNPGSLIGDDQIYNTIVTAHAFIMIFFMVMPIMIGGFGNWLVPLMLAAPDMAFPRLNNMSFWLLPPSLTLLLMSSIVSKGAGTGWTVYPPLSSNISHEGASVDFAIFSLHMAGISSILGAMNFISTVLNMKPKG
MKLEQMALFIWAVKITAILLLISLPVLAGAITMLLTDRNINTSFFDPAGGGDPILYQHL
>COLFA420-12|KJ963286|ZMUO.000650|Boreophilia_eremita
TLYFIFGAWAGMIGTSLSLLIRAELGNPGSLIGDDQIYNVIVTAHAFIMIFFMVMPIVIGGFGNWLVPLMLGAPDMAFPRMNNMSFWLLPPSLTLLLISSMVESGAGTGWTVYPPLSSNIAHGGSSVDLAIFSLHLAGISSILGAVNFISTVINMRSTGVSF
DRMPLFVWSVAITALLLLLSLPVLAGAITMLLTDRNLNTSFFDPAGGGDPILYQHL
>COLFE1314-13|KJ965368|ZMUO.007109|Stenus_cicindeloides
TLYFIFGAWSGMIGTSLSLLIRAELGNPGSLIGDDQIYNVIVTAHAFIMIFFMVMPAMIGGFGNWLVPLMLGAPDMAFPRMNNMSFWLLPPSLSLLLMSSIVESGAGTGWTVYPPLSSNIAHGGSSVDLAIFSLHLAGISSILGAINFITTILNMRAMKIQ
LDCMPLFVWSVGITALLLLLSLPVLAGAITMLLTDRNLNTSFFDPAGGGDPILYQHL
>COLFE1518-13|KJ967197|ZMUO.007313|Anomala_dubia
TLYFLFGSWAGMVGTSLSLLIRAELGNPGSLIGDDQIYNVIVTAHAFIMIFFMVMPIMIGGFGNWLVPLMLGAPDMAFPRMNNMSFWLLPPSLTLLLMSSLVENGAGTGWTVYPPLSANIAHSGASVDLAIFSLHLAGISSILGAVNFITTVINMRSTG
MTFDRMPLFVWSVVLTALLLLLSLPVLAGAITMLLTDRNINTSFFDPAGGGDPILYQHL
>COLFF263-13|KJ962254|ZMUO.005868|Rhinocyllus_conicus
TLYFIFGAWSGMVGTSLSMLIRTELGNPGSLIGDDQIYNTIVTAHAFIMIFFMVMPIMIGGFGNWLVPLMLGAPDMAFPRLNNMSFWLLPPSLTLLLMSSIIDKGMGTGWTVYPPLSSNIAHEGTTVDLAIFSLHMAGVSSILGAINFISTVLNMQPMG
IKLDQTTLFIWAVEITAILLLLSLPVLAGAITMLLTDRNINTSFFDPAGGGDPILYQHL
>LEFIJ1853-13|KJ964538|ZMUO.004608|Rhinoncus_castor
TLYFIFGSWAGTVGTSLSMLIRTELGTPGSLIGNDQIYNSIVTAHAFIMIFFMVMPILIGGFGNWLVPLMLGAPDMAFPRLNNMSFWLLPPSIMLLLMSSIVNKGAGTGWTVYPPLSSNVTHEGASVDLAIFSLHMAGISSILGAINFISTIMNMRPQGM
SYDKMPLFSWAVLITAILLLLSLPVLAGAITMLLTDRNINTSFFDPAGGGDPILYQHL
>COLFB477-12|KJ963256|ZMUO.000952|Cyphon_variabilis
TLYFIFGSWSGMVGTSLSLLIRAELGTPGSLIGDDQIYNVIVTAHAFIMIFFMVMPIMIGGFGNWLVPLMLGAPDMAFPRMNNMSFWLLPPSLTLLLMSSMVENGAGTGWTVYPPLSAGVAHSGASVDLAIFSLHLAGISSILGAVNFISTVINMRSVG
MTFDRMPLFVWAVAITALLLLLSLPVLAGAITMLLTDRNLNTSFFDPAGGGDPILYQHL
>COLFE888-13|KJ963019|ZMUO.006208|Pissodes_harcyniae
TLYFIFGAWSGMIGTSMSIIIRTELGTPSNMIGDDQIYNTIVTAHAFIMIFFMVMPIMIGGFGNWLIPLMLGAPDMAFPRLNNMSFWLLPPSIMLLLMSSVADKGAGTGWTVYPPLSTNIAHEGPSVDLAIFSLHLAGISSILGAVNFISTIINMRPMGM
NPDQMSLFIWAVKITAILLLLSLPVLAGAITMLLTDRNINTSFFDPAGGGGPILYQHL
>COLFA011-10|HM909041|MP00141|Atomaria_rubella
TLYFIFGAWSGMVGTSLSMLIRTELGNPGSLIGDDQIYNVIVTAHAFIMIFFMVMPMMIGGFGNWLVPLMLGAPDMAFPRLNNMSFWLLPPSLMFLLMSSIVEKGAGTGWTVYPPLSSNVAHAGSSVDLAIFSLHLAGISSILGSVNFITTVINMRPK
GMNFDRLPLFVWAVKITTILLLLSLPVLAGAITMLLTDRNINTSFFDPAGGGDPILYQHL
>COLFE1079-13|KJ965658|ZMUO.006874|Tachyerges_salicis
TLYFMLGAWSGMVGTSLSMLIRTELGTPGKLIGDDQIYNTIVTAHAFVMIFFMVMPIMIGGFGNWLIPLMLGAPDMAFPRLNNMSFWLLPPSLAFLLFSSIIDKGVGTGWTVYPPLSTNISHEGASVDMAIFSLHMAGVSSILGAMNFISTAMNMFP
KGVIKDQMSLFIWAVKVTAILLLISLPVLAGAITMLLTDRNVNTSFFDPAGGGDPILYQHL
>COLFB131-12|KJ961779|ZMUO.001461|Leptusa_fumida
TLYFIFGAWAGMVGTSLSLLIRAELGNPGSLIGDDQIYNVIVTAHAFIMIFFMVMPIMIGGFGNWLIPLMLGAPDMAFPRMNNMSFWLLPPSLTFLLMSSMVESGAGTGWTVYPPLSSNIAHGGSSVDLAIFSLHLAGISSILGAVNFISTIINMRSPGM
TFDRMPLFVWSVAITALLLLLSLPVLAGAITMLLTDRNLNTSFFDPAGGGDPILYQHL

>COLFF296-13|KJ964484|ZMUO.005996|Rhantus_suturellus
TLYFIFGAWAGMVGTSLSMLIRAELGNPGSLIGDDQIYNVIVTAHAFVMIFFMVMPIMIGGFGNWLVPLMLGAPDMAFPRMNNMSFWLLPPSLTLLLMSSMVESGAGTGWTVYPPLSSGIAHGGASVDLAIFSLHLAGISSILGAVNFITTIINMRSV
GMTFDRMPLFVWSVGITALLLLLSLPVLAGAITMLLTDRNLNTSFFDPAGGGDPILYQHL
>COLFA529-12|KJ965077|ZMUO.000569|Leptura_melanura
TLYFIFGAWAAMVGTSLSLLIRSELGNPGSLIGDDQIYNVIVTAHAFVMIFFMVMPIMIGGFGNWLVPLMLGAPDMAFPRMNNMSFWLLPPSLTLLIMSSIVETGAGTGWTVYPPLSSNIAHSGSSVDLAIFSLHLAGISSILGAVNFITTVINMHPVGM
TPDRMPLFVWAVVITAVLLLLSLPVLAGAITMLLTDRNLNTSFFDPAGGGDPILYQHL
>COLFB299-12|KJ962468|ZMUO.001629|Atheta_hygrotopora
TLYFIFGAWAGMVGTSLSLLIRAELGNPGSLIGDDQIYNVIVTAHAFIMIFFMVMPIVIGGFGNWLVPLMLGAPDMAFPRMNNMSFWLLPPSLTLLLMSSMVESGAGTGWTVYPPLSSNIAHGGSSVDLAIFSLHLAGISSILGAVNFISTVINMRSAGI
SFDRMPLFVWSVVITALLLLLSLPVLAGAITMLLTDRNLNTSFFDPAGGGDPILYQHL
>COLFB055-12|KJ964784|ZMUO.001385|Ilybius_subtilis
TLYFIFGAWAGMVGTSLSMLIRAELGNPGSLIGDDQIYNVIVTAHAFVMIFFMVMPIMIGGFGNWLVPLMLGAPDMAFPRMNNMSFWLLPPSLSLLLMSSMVESGAGTGWTVYPPLSSGIAHSGASVDLAIFSLHLAGISSILGAVNFITTIINMRSV
GMTFDRMPLFVWSVGITALLLLLSLPVLAGAITMLLTDRNLNTSFFDPAGGGDPILYQHL
>COLFD839-12|KJ964137|ZMUO.004829|Acilius_sulcatus
TLYFIFGAWSGMVGTSLSMLIRAELGNPGSLIGDDQIYNVIVTAHAFIMIFFMVMPIMIGGFGNWLVPLMLGAPDMAFPRMNNMSFWLLPPSLTLLLMSSMVENGAGTGWTVYPPLSAGIAHGGASVDLAIFSLHLAGISSILGAVNFITTIINMRSV
GMTFDRMPLFVWSVGITALLLLLSLPVLAGAITMLLTDRNLNTSFFDPAGGGDPILYQHL
>COLFB491-12|KJ965249|ZMUO.000966|Aphodius_rufus
TLYFLFGSWAGMVGTSLSLLIRAELGNPGSLIGDDQIYNVIVTAHAFVMIFFMVMPILIGGFGNWLVPLMLGAPDMAFPRMNNMSFWLLPPSLTLLLMSSMVESGAGTGWTVYPPLSSNIAHGGASVDLAIFSLHLAGISSILGAVNFITTVINMRSM
GMTFDRMPLFVWSVAITALLLLLSLPVLAGAITMLLTDRNLNTSFFDPAGGGDPILYQHL
>COLFE234-12|KJ966461|ZMUO.005079|Anthocomus_rufus
TLYFIFGAWSGMVGLSLSLLIRSELSIPGTLIGNDQIYNVIVTAHAFIMIFFMVMPILIGGFGNWLVPLMLGAPDMAFPRMNNMSFWLLPPSLTLLLLSSMVENGAGTGWTVYPPLSANIAHSGSSVDLAIFSLHLAGISSILGAVNFITTVINMRPQGMT
LDRTPLFVWAVVITALLLLLSLPVLAGAITMLLTDRNLNTSFFDPAGGGDPILYQHL
>COLFD759-12|KJ966993|ZMUO.004749|Amara_ovata
TLYFIFGAWSGMVGTSLSMLIRAELGNPGALIGDDQIYNVIVTAHAFVMIFFMVMPIMIGGFGNWLVPLMLGAPDMAFPRMNNMSFWLLPPSLSLLLMSSMVESGAGTGWTVYPPLSSGIAHAGASVDLAIFSLHLAGISSILGAVNFITTIINMRSV
GMTFDRMPLFVWSVGITALLLLLSLPVLAGAITMLLTDRNLNTSFFDPAGGGDPILYQHL
>COLFA326-12|KJ962406|ZMUO.000461|Aphodius_borealis
TLYFLFGSWAGMVGTSLSLLIRAELGNPGTLIGDDQIYNVIVTAHAFVMIFFMVMPILIGGFGNWLVPLMLGAPDMAFPRMNNMSFWLLPPSLTLLLMSSMVESGAGTGWTVYPPLSSNIAHGGASVDLAIFSLHLAGISSILGAVNFITTVINMRSPG
LTFDRMPLFVWSVAITALLLLLSLPVLAGAITMLLTDRNLNTSFFDPAGGGDPILYQHL
>COLFA202-10|HM909121|MP00049|Agrilus_viridis
TLYFIFGVWSGMVGTALSLLIRAELGNPGALIGNDQIYNVIVTAHAFIMIFFMVMPIMMGGFGNWLVPLMLGAPDMAFPRMNNMSFWLLPPSLTLLLMSSMVESGAGTGWTVYPPLAANIAHSGGSVDLAIFSLHLAGISSILGAINFITTVINMRAV
GMTMDRVPLLVWSIAITALLLLLSLPVLAGAITMLLTDRNLNTSFFDPAGGGDPILYQHL
>COLFB160-12|KJ965519|ZMUO.001490|Hydrochus_brevis
TLYFIFGAWSGMVGTSLSLLIRTELGNPGSLIGDDQIYNVIVTAHAFIMIFFMVMPIMIGGFGNWLVPLMLGAPDMAFPRMNNMSFWLLPPSLTLLLMSSMVESGAGTGWTVYPPLSSNIAHGGASVDLAIFSLHLAGISSILGAVNFITTIINMRSNN
MTYDRLPLFVWSVGITALLLLLSLPVLAGAITMLLTDRNLNTSFFDPIGGGDPILYQHL
>COLFG106-13|KJ962649|ZMUO.007801|Cartodere_nodifer
SLYFLFGMWSGMVGTSLSLLIRLELGNPGSLIGDDQIYNVIVTAHAFIMIFFMVMPLMMGGFGNWLVPLMLGAPDMAFPRLNNMSFWLLPPSLSLLIMSSVVESGVGTGWTVYPPLSSNIAHGGSSVDLAIFSLHLAGISSILGAVNFITTVMNMRPV
GMTLEQTPLFVWSVLLTAILLLLSLPVLAGAITMLLTDRNLNTSFFDPAGGGDPILYQHL
>COLFF408-13|KJ964609|ZMUO.005926|Prosternon_tessellatum
TLYFLFGAWSGMLGTSLSLLIRAELGNPGSLIGNDQIYNVIVTAHAFIMIFFMVMPIMIGGFGNWLVPLMLGAPDMAFPRMNNMSFWFLPPSLSLLLMSSIVENGAGTGWTVYPPLSANIAHSGSSVDLAIFSLHLAGISSILGAVNFISTVINMRTTGIT
FDRMPLFVWAVAITALLLLLSLPVLAGAITMLLTDRNLNTSFFDPAGGGDPILYQHL
>COLFD489-12|KJ966975|ZMUO.004289|Cryptocephalus_labiatus
TLYFLFGAWAGMIGTSLSLLIRIELGNPGSLIGNDQIYNTIVTAHAFIMIFFMVMPIMIGGFGNWLVPLMLGAPDMAFPRMNNMSFWLLPPSLTLLLMSSIVENGAGTGWTVYPPLSTTIAHAGASVDLAIFSLHLAGISSIMGAINFISTVINMRPQGM
FMDRTPLFVWAVLITAILLLLSLPVLAGAITMLLTDRNLNTSFFDPAGGGDPILYQHL
>COLFD352-12|KJ966580|ZMUO.004247|Altica_brevicollis
TLYFLFGIWAGMIGTSMSLLIRTELGSPGSLIGNDQIYNVIVTAHAFVMIFFMVMPMMIGGFGNWLVPLMIGAPDMAFPRMNNMSFWLLPPSIFLLLMSSFTESGAGTGWTVYPPLSSNLAHNGPSVDLAIFSLHLAGISSILGAINFITTMVNMRPQ
GMSMDQMPLFVWAVFITAILLLLSLPVLAGAITMLLTDRNLNTSFFEPAGGGDPILYQHL
>COLFF085-13|KJ961802|ZMUO.005785|Brychius_elevatus

TLYFIFGAWAGMVGTSLSMLIRAELGTPGSLIGDDQIYNVIVTAHAFIMIFFMVMPIMIGGFGNWLVPLMLGAPDMAFPRMNNMSFWLLPPSLSLLLMSSMVESGAGTGWTVYPPLSAGIAHSGASVDLAIFSLHLAGISSILGAVNFITTIINMRSM
GMSFDRMPLFVWSVGITALLLLLSLPVLAGAITMLLTDRNLNTSFFDPAGGGDPILYQHL
>COLFA077-10|HM909094|MP00281|Enochrus_testaceus
TLYFIFGAWAGMVGTSLSILIRAELGNPGSLIGDDQIYNVIVTAHAFIMIFFMVMPIMIGGFGNWLVPLMLGAPDMAFPRMNNMSFWLLPPSLTLLLMSSMVESGAGTGWTVYPPLSSNIAHGGASVDLAIFSLHLAGISSILGAVNFITTVINMRSPS
MTYDRLPLFVWSVAITALLLLLSLPVLAGAITMLLTDRNLNTSFFDPAGGGDPILYQHL
>COLFE183-12|KJ962285|ZMUO.005218|Rhamphus_pulicarius
TLYFLFGGWSGMVGTSLSMLIRTELGNPGKLIGDDQIYNTIVTAHAFIMIFFMVMPIMIGGFGNWLVPLMLGAPDMAFPRLNNMSFWLLPPSLTFLLLSSVMDKGAGTGWTVYPPLSANIAHEGSSVDLAIFSLHMAGVSSILGAMNFISTIINMKPK
NMSMDQMSLFVWSVKITAILLLLSLPVLAGAITMLLTDRNINTSFFDPAGGGDPILYQHL
>COLFE448-12|KJ964612|ZMUO.005008|Taeniapion_urticarium
TLYFIFGVWSGLIGTSLSMLIRIELGNPGSLIGDDQIYNVIITAHAFIMIFFMVMPIMIGGFGNWLIPLMLGAPDMAFPRMNNMSFWLLPPSLSLLLMSSIVEKGAGTGWTVYPPLASNIAHSGASVDLAIFSLHLAGISSILGAVNFISTMMNMQPMGL
SLEQLSLFTWAVKITAILLLLSLPVLAGAITMLLTDRNTNTSFFDPAGGGDPILYQHL
>COLFA705-12|KJ966341|ZMUO.000840|Cilea_silphoides
TLYFIFGIWAGMIGTSLSLLIRAELGNPGSLIGDDQIYNVIVTAHAFIMIFFMVMPIMIGGFGNWLVPLMLGAPDMAFPRMNNMSFWLLPPSITLLLMSSMVESGAGTGWTVYPPLSSNIAHGGSSVDLAIFSLHLAGISSILGAINFITTVINMRSPGMT
FERMPLFVWAVVITALLLLLSLPVLAGAITMLLTDRNLNTSFFDPAGGGDPILYQHL
>COLFE195-12|KJ963481|ZMUO.005040|Amara_equestris
TLYFIFGTWSGMVGTSLSMLIRAELGNPGALIGDDQIYNVIVTAHAFVMIFFMVMPIMIGGFGNWLVPLMLGAPDMAFPRMNNMSFWLLPPSLTLLLMSSMVESGAGTGWTVYPPLSSSIAHAGASVDLAIFSLHLAGISSILGAVNFITTIINMRSPG
MMFDRMPLFVWSVGITALLLLLSLPVLAGAITMLLTDRNLNTSFFDPSGGGDPILYQHL
>COLFE210-12|KJ966889|ZMUO.005055|Calathus_fuscipes
TLYFIFGAWAGMVGTSLSMLIRAELGNPGALIGDDQVYNVIVTAHAFVMIFFMVMPIMIGGFGNWLVPLMLGAPDMAFPRMNNMSFWLLPPSLTLLLMSSMVESGAGTGWTVYPPLSSGIAHSGASVDLAIFSLHLAGISSILGAVNFITTIINMRSV
GMTFDRMPLFVWSVGITALLLLLSLPVLAGAITMLLTDRNLNTSFFDPAGGGDPILYQHL
>COLFC224-12|KJ967329|ZMUO.002409|Stenus_palustris
TLYFILGAWAGLTGTSLSLLIRTELGSPGSFIGDDQIYNVIVTAHAFIMIFFMVMPIMIGGFGNWLIPLMLGAPDMAFPRMNNMSFWLLPPSLSLLLMSSIVESGAGTGWTVYPPLSSNIAHSGASVDLTIFSLHLAGISSILGAINFITTIFNMRAMKLQLD
CLPLFIWSVNVTTFLLLLSLPVLAGAITMLLTDRNVNTSFFDPGGGGDPILYQHL
>COLFC010-12|KJ966288|ZMUO.002005|Dyschirius_tristis
TLYFIFGIWSGMVGTSLSILIRAELGNPGSLIGDDQIYNVIVTAHAFIMIFFMVMPIMIGGFGNWLVPLMLGAPDMAFPRMNNMSFWLLPPSLSLLLMSSMVEKGAGTGWTVYPPLSSSIAHSGASVDLAIFSLHLAGISSILGAVNFITTIINMRSTGMT
FERMPLFVWSVGITALLLLLSLPVLAGAITMLLTDRNLNTSFFDPAGGGDPILYQHL
>COLFF281-13|KJ962734|ZMUO.005886|Callidium_violaceum
TLYFLFGAWASMVGTSLSMLIRTELGNPGSLIGDDQIYNVIVTAHAFVMIFFMVMPIMIGGFGNWLVPLMIGAPDMAFPRMNNLSFWLLPPALILLILSSIVENGAGTGWTVYPPLSANVAHSGSSVDLAIFSLHLAGISSILGAVNFISTVINMRPMG
MTPERMPLFVWAVVITAILLLLSLPVLAGAITMLLTDRNINTSFFDPAGGGDPILYQHL
>COLFE1380-13|KJ963219|ZMUO.007175|Adalia_bipunctata
TLYFLFGLWAGMVGTSLSIIIRLELGTTNSLIGNDQIYNVIVTAHAFIMIFFMVMPIMIGGFGNWLVPLMIGAPDMAFPRLNNMSFWLLPPALTLLISSSVIEMGAGTGWTVYPPLSSNMAHNGPSVDLVIFSLHLAGISSILGAVNFISTIMNMRPNGM
NLDKTPLFVWSVLITAILLLLSLPVLAGAITMLLTDRNINTSFFDPTGGGDPVLYQHL
>COLFA272-12|KJ965410|ZMUO.000407|Agriotes_lineatus
TLYFLFGAWAGMLGTSLSLLIRAELGNPGSLIGNDQIYNVIVTAHAFIMIFFMVMPIMIGGFGNWLVPLMLGAPDMAFPRMNNMSFWFLPPSLSLLLMSSIVENGAGTGWTVYPPLSSNIAHSGSSVDLAIFSLHLAGISSILGAVNFISTVINMRSTGIT
FDRMPLFVWAVAITALLLLLSLPVLAGAITMLLTDRNLNTSFFDPAGGGDPILYQHL
>COLFE591-13|KJ962185|ZMUO.005436|Poecilus_versicolor
TLYFIFGAWSGMVGTSLSMLIRAELGNPGSLIGDDQIYNVIVTAHAFVMIFFMVMPIMIGGFGNWLVPLMLGAPDMAFPRMNNMSFWLLPPSLTLLLMSSMVESGAGTGWTVYPPLSSGIAHAGASVDLAIFSLHLAGVSSILGAVNFITTIINMRSI
GMTFDRMPLFVWSVGITALLLLLSLPVLAGAITMLLTDRNLNTSFFDPAGGGDPILYQHL
>COLFD683-12|KJ961763|ZMUO.004673|Psylliodes_marcidus
TLYFIFGIWSGMIGTSLSILIRAELGSPGSLIGNDQIYNVIVTAHAFIMIFFMVMPIMIGGFGNWLVPLMIGAPDMAFPRMNNMSFWLLPPSLLLLMSSMVESGAGTGWTVYPPLSSNIAHGGSSVDLAIFSLHLAGISSILGAINFITTVINMRPEGMTL
DRMPLFVWAVVITAILLLLSLPVLAGAITMLLTDRNLNTSFFDPIGGGDPILYQHL
>COLFD693-12|KJ962775|ZMUO.004683|Paradromius_linearis
TLYFIFGAWAGMVGTSLSMLIRAELGNPGALIGDDQIYNVIVTAHAFIMIFFMVMPIMIGGFGNWLVPLMLGAPDMAFPRMNNMSFWLLPPSLTLLLMSSMVESGAGTGWTVYPPLSSGIAHAGASVDLAIFSLHLAGISSILGAVNFITTIINMRLIG
MTFDRMPLFVWSVGITALLLLLSLPVLAGAITMLLTDRNLNTSFFDPAGGGDPILYQHL
>COLFF171-13|KJ967478|ZMUO.005895|Aphodius_depressus
TLYFLFGSWAGMVGTSLSLLIRAELGNPGTLIGDDQIYNVIVTAHAFIMIFFMVMPILIGGFGNWLVPLMLGAPDMAFPRMNNMSFWLLPPSLTLLLMSSMVESGAGTGWTVYPPLSSNIAHGGASVDLAIFSLHLAGISSILGAVNFITTVINMRSPG
MTFDRMPLFVWSVAITALLLLLSLPVLAGAITMLLTDRNLNTSFFDPAGGGDPILYQHL

>COLFA524-12|KJ965747|ZMUO.000564|Helophorus_brevipalpis
TLYFIFGAWAGMVGTSLSILIRAELGNPGTLIGDDQIYNVIVTAHAFIMIFFMVMPIMIGGFGNWLVPLMLGAPDMAFPRMNNMSFWLLPPSLTLLLMSSMVESGAGTGWTVYPPLSSNIAHSGASVDLAIFSLHLAGISSILGAVNFITTVINMRSISM
TYDRLPLFVWSVAITALLLLLSLPVLAGAITMLLTDRNLNTSFFDPAGGGDPILYQHL
>COLFE588-13|KJ963831|ZMUO.005433|Platystomos_albinus
TLYFIFGAWAGMMGTSLSILIRTELGTPSSLIGDDQIYNVIVTAHAFVMIFFMVMPTMIGGFGNWLVPLMLGAPDMAFPRMNNMSFWLLPPSLILLTMSSIVEKGAGTGWTVYPPLASNIAHSGASVDLAIFSLHLAGVSSILGAVNFITTIINMRPEG
MTPDRLPLFVWAVGITALLLLLSLPVLAGAITMLLTDRNLNTTFFDPAGGGDPILYQHL
>COLFE331-12|KJ966490|ZMUO.004891|Hypera_arator
TLYFIFGIWAGAVGTSLSILIRTELGNPGSLIGNDQIYNTIVTAHAFIMIFFMVMPIMIGGFGNWLVPLMLGAPDMAFPRLNNMSFWLLPPSLSLLLMSSMVDSGAGTGWTVYPPLSSNIAHEGSSVDLAIFSLHMAGISSILGAINFISTVLNMRPKGLSL
DKMTLFIWAVKITAILLLVSLPVLAGAITMLLTDRNINTSFFDPAGGGDPILYQHL
>COLFA581-12|KJ966945|ZMUO.000716|Protaetia_marmorata
TLYFLFGSWAGMVGTSLSLLIRAELGNPGSLIGDDQIYNVIVTAHAFIMIFFMVMPVMIGGFGNWLVPLMLGAPDMAFPRMNNMSFWLLPPSLTLLLMSSMVESGAGTGWTVYPPLSSNIAHSGASVDLAIFSLHLAGISSILGAVNFITTVINMRSTG
MTFDRMPLFVWSVVLTALLLLLSLPVLAGAITMLLTDRNINTSFFDPAGGGDPILYQHL
>COLFD214-12|KJ961850|ZMUO.004109|Deliphrum_tectum
TLYFIFGAWAGMVGTSLSILIRAELGNPGTLIGDDQIYNVIVTAHAFVMIFFMVMPIVIGGFGNWLVPLMLGAPDMAFPRMNNMSFWLLPPSLTLLLMSSMVESGAGTGWTVYPPLSSNIAHGGSSVDLAIFSLHLAGISSILGAVNFITTVINMRATG
MTFDRMPLFVWSVAITALLLLLSLPVLAGAITMLLTDRNLNTSFFDPAGGGDPILYQHL
>COLFB147-12|KJ967137|ZMUO.001477|Quedius_boops
TLYFIFGAWAGMVGTSLSLLIRAELGNPGSLIGDDQIYNVIVTAHAFIMIFFMVMPTLIGGFGNWLVPLMLGAPDMAFPRMNNMSFWLLPPSLSLLLMSSMVESGAGTGWTVYPPLSSNIAHSGASVDLAIFSLHLAGISSILGAVNFITTVINMRSIGM
TFDRMPLFVWSVAITALLLLLSLPVLAGAITMLLTDRNLNTTFFDPAGGGDPILYQHL
>COLFF419-13|KJ967073|ZMUO.005937|Cassida_rubiginosa
TLYFIFGFWSGMVGTSLSILIRAELGNPGTLIGNDQIYNSIVTAHAFIMIFFMVMPIMIGGFGNWLVPLMLGAPDMAFPRLNNMSFWLLPPSITFLIMSSVIESGAGTGWTVYPPLSSNIAHSGASVDMAIFSLHLAGISSILGAINFISTIMNMQPSGMS
LDKMPLFVWAVIITAILLLLSLPVLAGAITMLLTDRNINTSFFDPAGGGDPILYQHL
>COLFE1049-13|KJ966280|ZMUO.006844|Agabus_affinis
TLYFIFGAWAGMVGTSLSMLIRAELGNPGSLIGDDQIYNVIVTAHAFVMIFFMVMPIMIGGFGNWLVPLMLGAPDMAFPRMNNMSFWLLPPSLTLLLMSSMVEKGAGTGWTVYPPLSSGIAHSGASVDLAIFSLHLAGISSILGAVNFITTIINMRSM
GMTFDRMPLFVWSVGITALLLLLSLPVLAGAITMLLTDRNLNTSFFDPAGGGDPILYQHL
>COLFB912-12|KJ962530|ZMUO.001957|Phaenops_cyanea
TLYFIFGAWSGMVGTALSLLIRAELGNPGALIGDDQIYNTIVTAHAFIMIFFMVMPIMMGGFGNWLVPLMLGAPDMAFPRMNNMSFWLLPPSLTLLLMSSIVENGAGTGWTVYPPLAANTAHSGASVDLAIFSLHLAGISSILGAVNFITTVINMRSV
GMTFDQMPLFVWSVAITALLLLLSLPVLAGAITMLLTDRNLNTSFFDPAGGGDPILYQHL
>COLFF971-13|KJ965284|ZMUO.007336|Aphodius_contaminatus
TLYFLFGSWAGMVGTSLSLLIRAELGNPGSLIGDDQIYNVIVTAHAFVMIFFMVMPILIGGFGNWLVPLMLGAPDMAFPRMNNMSFWLLPPSLTLLLMSSMVESGAGTGWTVYPPLSSNIAHGGASVDLAIFSLHLAGISSILGAVNFITTVINMRSPG
MTFDRMPLFVWSVAITALLLLLSLPVLAGAITMLLTDRNLNTSFFDPAGGGDPILYQHL
>COLFD068-12|KJ963165|ZMUO.003963|Selatosomus_impressus
TLYFLFGAWAGMLGTSLSLLIRAELGNPGSLIGNDQIYNVIVTAHAFIMIFFMVMPIMIGGFGNWLVPLMLGAPDMAFPRMNNMSFWFLPPSLSLLLMSSIVENGAGTGWTVYPPLSANIAHSGSSVDLAIFSLHLAGISSILGAVNFISTVINMRSTGIT
FDRMPLFVWAVAITALLLLLSLPVLAGAITMLLTDRNLNTSFFDPAGGGDPILYQHL
>COLFC425-12|KJ964567|ZMUO.002990|Negastrius_sp.
TLYFLFGAWAGMLGTSLSLLIRAELGNPGSLIGNDQIYNVIVTAHAFIMIFFMVMPIMIGGFGNWLVPLMLGAPDMAFPRMNNMSFWFLPPSLSLLLMSSIVENGAGTGWTVYPPLSANIAHSGSSVDLAIFSLHLAGISSILGAVNFISTVINMRSTGIT
FDRMPLFVWAVVITALLLLLSLPVLAGAITMLLTDRNLNTSFFDPAGGGDPILYQHL
>COLFB564-12|KJ965618|ZMUO.001039|Pterostichus_sp.
TLYFIFGAWAGMVGTSLSMLIRAELGNPGSLIGDDQIYNVIVTAHAFIMIFFMVMPIMIGGFGNWLVPLMLGAPDMAFPRMNNMSFWLLPPSLTLLLMSSMVESGAGTGWTVYPPLSSGIAHAGASVDLAIFSLHLAGVSSILGAVNFITTIINMRSV
GMTFDRMPLFVWSVGITALLLLLSLPVLAGAITMLLTDRNLNTSFFDPAGGGDPILYQHL
>COLFF404-13|KJ965383|ZMUO.005922|Zeugophora_subspinosa
TLYFIFGVWAGMVGTSLSLLIRSELGTPGSLIGDDQIYNVIVTAHAFVMIFFMVMPIMIGGFGNWLVPLMLGAPDMAFPRMNNMSFWFLPPSLILLLMSSIVESGAGTGWTVYPPLSANIAHSGSSVDLAIFSLHMAGISSILGAVNFITTVINMRPMG
LNLDKMPLFVWAVVITAILLLLSLPVLAGAITMLLTDRNINTSFFDPSGGGDPILYQHL
>COLFC629-12|KJ962618|ZMUO.003194|Hygrotus_marklini
TLYFLFGAWSGMVGTSMSMLIRAELGNPGSLIGDDQIYNVIVTAHAFIMIFFMVMPIMIGGFGNWLVPLMLGAPDMAFPRMNNMSFWMLPPSLTLLLMSSMVESGAGTGWTVYPPLSAGIAHGGASVDLAIFSLHLAGISSILGAVNFITTIINMRS
VGMTFDRMPLFVWSVGITALLLLLSLPVLAGAITMLLTDRNLNTSFFDPAGGGDPILYQHL
>COLFC014-12|KJ966158|ZMUO.002009|Patrobus_septentrionis

TLYFIFGAWSGMVGTSLSMLIRAELGNPGSLIGDDQIYNVIVTAHAFVMIFFMVMPIMIGGFGNWLVPLMLGAPDMAFPRMNNMSFWLLPPSLTLLLMSSMVESGAGTGWTVYPPLSSGIAHSGASVDLAIFSLHLAGISSILGAVNFITTIINMRSVG
MSFDRMPLFVWSVGITALLLLLSLPVLAGAITMLLTDRNLNTSFFDPAGGGDPILYQHL
>COLFA612-12|KJ962223|ZMUO.000747|Ceratomegilla_notata
TLYFLFGMWAGMIGTSLSILIRLELGTTGSLIGNDQIYNVIVTAHAFIMIFFMVMPIMIGGFGNWLVPLMIGAPDMAFPRLNNMSFWLLPPALILLLFSSLVEMGAGTGWTVYPPLSSNLAHNGPSVDLVIFSLHLAGISSILGAVNFISTIMNMRPYGM
KLDKTPLFVWSVLITAILLLLSLPVLAGAITMLLTDRNLNTSFFDPTGGGDPILYQHL
>COLFE1046-13|KJ963555|ZMUO.006841|Agabus_thomsoni
TLYFIFGAWAGMVGTSLSMLIRAELGNPGSLIGDDQIYNVIVTAHAFVMIFFMVMPIMIGGFGNWLIPLMLGAPDMAFPRMNNMSFWLLPPSLTLLLMSSMVESGAGTGWTVYPPLSSGIAHGGASVDLAIFSLHLAGISSILGAVNFITTIINMRSVG
MTFDRMPLFVWSVGITALLLLLSLPVLAGAITMLLTDRNLNTSFFDPAGGGDPILYQHL
>COLFE1421-13|KJ966834|ZMUO.007216|Lithocharis_nigriceps
TLYFIFGAWAGMVGTSLSLLIRAELGVPGSLIGDDQIYNVIVTAHAFVMIFFMVMPIVIGGFGNWLVPLMLGAPDMAFPRMNNMSFWLLPPSLSLLLMSSMVESGAGTGWTVYPPLSSNIAHGGASVDLAIFSLHLAGISSILGAVNFITTVINMRSSG
MTYERMPLFIWSVAITALLLLLSLPVLAGAITMLLTDRNLNTSFFDPAGGGDPILYQHL
>COLFD253-12|KJ963871|ZMUO.004148|Rhantus_frontalis
TLYFIFGAWAGMVGTSLSMLIRAELGNPGSLIGDDQIYNVIVTAHAFVMIFFMVMPIMIGGFGNWLVPLMLGAPDMAFPRMNNMSFWLLPPSLTLLLMSSMVESGAGTGWTVYPPLSSGIAHGGASVDLAIFSLHLAGISSILGAVNFITTIINMRSIG
MTFDRMPLFVWSVGITALLLLLSLPVLAGAITMLLTDRNLNTSFFDPAGGGDPILYQHL
>COLFE1398-13|KJ966818|ZMUO.007193|Ceutorhynchus_hampei
TLYFIFGSWAGMVGTSLSMLIRTELGNPGSLIGNDQIYNSTVTAHAFIMIFFMVMPILIGGFGNWLVPLMLGAPDMAFPRLNNMSFWLLPPSLSLLLMSSIVNKGAGTGWTVYPPLSGNVAHEGMSVDLAIFSLHMAGISSILGAINFISTVMNMQP
MGMVPELMPLFVWAVQITAILLLISLPVLAGAITMLLTDRNINTSFFDPSGGGDPILYQHL
>COLFF730-13|KJ962998|ZMUO.006430|Drilus_concolor
TLYFLFGAWAGMLGTSLSLMIRTELGNPGSLIGNDQTYNVIVTAHAFVMIFFMVMPIMIGGFGNWLVPLMIGAPDMAFPRMNNMSFWLLPPSLSLLLMSGMVESGAGTGWTVYPPLSSNIAHSGPSVDLTIFSLHLAGISSILGAVNFISTVINMRSP
GITLDRLPLFVWAVVITAVLLLLSLPVLAGAITMLLTDRNLNTSFFDPAGGGDPILYQHL
>COLFD889-12|KJ966205|ZMUO.004499|Galerucella_sagittariae
TLYFIFGVWAGMVGTSLSILVRVELGNPGSLIGNDQIYNVIVTAHAFIMIFFMVMPIMIGGFGNWLVPLMIGAPDMAFPRMNNMSFWLLPPSLFLLIMSSIVESGAGTGWTVYPPLSSNIAHGGSSVDLAIFSLHLAGISSILGAINFITTIINMRPKGMT
LDRMPLFVWAVMITAILLLLSLPVLAGAITMLLTDRNLNTSFFDPAGGGDPILYQHL
>COLFC597-12|KJ966966|ZMUO.003162|Brachypterolus_linariae
TLYFIFGAWSGMIGTSLSLLIRSELGNPGSLIGDDQIYNVIVTAHAFVMIFFMVMPFMIGGFGNWLVPLMLGAPDMAFPRMNNMSFWLLPPSLFLLLMSSIVESGAGTGWTVYPPLSSNIAHGGSSVDLAIFSLHLAGISSILGAVNFITTVINMRPSGM
TFDRMPLFIWAVVITALLLLLSLPVLAGAITMLLTDRNLNTSFFDPAGGGDPILYQHL
>COLFD187-12|KJ967022|ZMUO.004082|Ischnosoma_splendidum
TLYFIFGAWAGMVGTSLSLLIRAELGNPGSLIGDDQIYNVIVTAHAFVMIFFMVMPIVIGGFGNWLVPLMLGAPDMAFPRMNNMSFWLLPPSLSLLLMSSLVESGAGTGWTVYPPLSSNIAHGGASVDLAIFSLHLAGISSILGAVNFITTVINMRSTG
MTFDRMPLFVWSVVITALLLLLSLPVLAGAITMLLTDRNLNTSFFDPAGGGDPILYQHL
>COLFF449-13|KJ962228|ZMUO.005967|Myllaena_intermedia
TLYFIFGAWAGMIGSSLSLLIRAELGNPGSLIGDDQIYNVIVTAHAFIMIFFMVMPIVIGGFGNWLVPLMLGAPDMAFPRMNNMSFWLLPPSLSLLLMSSMVESGAGTGWTVYPPLSANIAHSGPSVDLAIFSLHLAGISSILGAVNFISTIINMRSTGMS
FDRMPLFVWSVGITALLLLLSLPVLAGAITMLLTDRNLNTSFFDPAGGGDPILYQHL
>COLFA517-12|KJ965113|ZMUO.000557|Dyschirius_politus
TLYFIFGIWSGMVGTSLSILIRAELGNPGSLIGDDQIYNVIVTAHAFIMIFFMVMPIMIGGFGNWLVPLMLGAPDMAFPRMNNMSFWMLPPSLSLLLMSSMVEKGAGTGWTVYPPLSSSIAHSGASVDLAIFSLHLAGISSILGAVNFITTIINMRSTGM
TFEKMPLFVWSVGITALLLLLSLPVLAGAITMLLTDRNLNTSFFDPAGGGDPILYQHL
>COLFA291-12|KJ964596|ZMUO.000426|Leptacinus_intermedius
TLYFIFGAWAGMVGTSLSLLIRAELGNPGSLIGDDQIYNVIVTAHAFIMIFFMVMPIVIGGFGNWLVPLMLGAPDMAFPRMNNMSFWLLPPSLTLLLTSSLVESGAGTGWTVYPPLSSNIAHGGASVDLAIFSLHLAGISSILGAVNFITTVLNMRSFGM
TFDRMPLFVWSVAITALLLLLSLPVLAGAITMLLTDRNLNTSFFDPMGGGDPILYQHL
>COLFE1595-13|KJ964660|ZMUO.007485|Anacaena_globulus
TLYFIFGAWAGMVGTSLSILIRAELGNPGSLIGDDQIYNVIVTAHAFIMIFFMVMPIMIGGFGNWLVPLMLGAPDMAFPRMNNMSFWLLPPSLTLLLMSSMVESGAGTGWTVYPPLSSNIAHSGASVDLAIFSLHLAGISSILGAVNFITTVINMRSSM
MTYDRLPLFVWSVAITALLLLLSLPVLAGAITMLLTDRNLNTSFFDPAGGGDPILYQHL
>COLFA014-10|HM909043|MP00145|Morychus_aeneus
TLYFIFGAWAGMLGTSLSMLIRSELGNPGSLIGDDQIYNVIVTAHAFIMIFFMVMPIMIGGFGNWLVPLTLGAPDMAFPRMNNMSFWLLPPSLSLLLMSSIVESGVGTGWTVYPPLSANIAHSGSSVDLAIFSLHLAGISSILGAVNFISTVINMRASGM
TFDQMPLFSWSVVITAVLLLLSLPVLAGAITMLLTDRNLNTSFFDPAGGGDPILYQHL
>COLFA494-12|KJ963040|ZMUO.000534|Xantholinus_tricolor
TLYFIFGSWAGMVGTSLSLLIRAELGNPGTLIGDDQIYNVIVTAHAFVMIFFMVMPIVIGGFGNWLVPLMLGAPDMAFPRMNNMSFWLLPPSLTLLLMSSMVESGAGTGWTVYPPLSSNIAHSGASVDLAIFSLHLAGISSILGAVNFITTIINMRATG
MSFDRMPLFVWSVAITALLLLLSLPVLAGAITMLLTDRNLNTSFFDPTGGGDPILYQHL

>COLFF920-13|KJ966852|ZMUO.006715|Cyphon_coarctatus
TLYFIFGSWSGMVGTSLSLLIRAELGTPGSLIGDDQIYNVIVTAHAFIMIFFMVMPIMIGGFGNWLVPLMLGAPDMAFPRMNNMSFWLLPPSLTLLLMSSMVENGAGTGWTVYPPLSAGVAHSGASVDLAIFSLHLAGISSILGAVNFISTVINMRSVG
MTFDRMPLFVWAVAITALLLLLSLPVLAGAITMLLTDRNLNTSFFDPAGGGDPILYQHL
>COLFF882-13|KJ962281|ZMUO.006677|Acrotona_parvula
TLYFIFGAWAGMVGTSLSLLIRAELGNPGSLIGDDQIYNVIVTAHAFIMIFFMVMPIVIGGFGNWLVPLMLGAPDMAFPRMNNMSFWLLPPSLTLLLMSSLVESGAGTGWTVYPPLSSNIAHGGSSVDLAIFSLHLAGISSILGAVNFISTVINMRSTGIT
FDRMPLFVWSVVITALLLLLSLPVLAGAITMLLTDRNLNTSFFDPAGGGDPILYQHL
>COLFE595-13|KJ962125|ZMUO.005440|Nudobius_lentus
TLYFIFGAWAGMVGTSLSLLIRAELGNPGSLIGDDQIYNVIVTAHAFVMIFFMVMPIVIGGFGNWLVPLMLGAPDMAFPRMNNMSFWLLPPSLTLLLMSSMVENGAGTGWTVYPPLSSNIAHSGASVDLAIFSLHLAGISSILGAVNFITTIINMRSYG
MTFDRMPLFVWSVAITALLLLLSLPVLAGAITMLLTDRNLNTSFFDPTGGGDPILYQHL
>LEFIJ1868-13|KJ966467|ZMUO.004623|Ceutorhynchus_inaffectatus
TLYFIFGSWAGMAGTSLSMLIRTELGNPGSLIGNDQIYNSIVTAHAFIMIFFMVMPILIGGFGNWLVPLMLGAPDMAFPRLNNMSFWLLPPSLSLLLMSSIVNKGVGTGWTVYPPLSGNVAHEGMSVDLAIFSLHMAGISSILGAINFISTVLNMQPKG
MTPELMPLFVWAVEITAILLLISLPVLAGAITMLLTDRNINTSFFDPSGGGDPILYQHL
>COLFB366-12|KJ964481|ZMUO.001696|Devia_prospera
TLYFIFGAWAGMIGTSLSLLIRAELGNPGSLIGDDQIYNVIVTAHAFVMIFFMVMPIVIGGFGNWLVPIMLGAPDMAFPRMNNMSFWLLPPSLSLLLMSSLVESGAGTGWTVYPPLSSNIAHGGSSVDLAIFSLHLAGISSILGAVNFISTIINMRTTGMY
FDRMPLFVWSVAITALLLLLSLPVLAGAITMLLTDRNLNTSFFDPAGGGDPILYQHL
>COLFD481-12|KJ967240|ZMUO.004281|Hypera_suspiciosa
TLYFIFGTWAGTVGTSLSILIRTELGNPGSLIGNDQIYNTIVTAHAFIMIFFMVMPIMIGGFGNWLVPLMLGAPDMAFPRLNNMSFWLLPPSLSLLLMSSMVDSGAGTGWTVYPPLSSNIAHEGSSVDLAIFSLHMAGVSSILGAINFISTVLNMRPSGM
SLDKMALFIWAVKITAILLLLSLPVLAGAITMLLTDRNINTSFFDPAGGGDPILYQHL
>COLFE849-13|KJ962764|ZMUO.005694|Atomaria_rubella
TLYFIFGTWSGMVGTSLSMLIRTELGTPGSLIGDDQIYNVIVTAHAFIMIFFMVMPMMIGGFGNWLVPLMLGAPDMAFPRLNNMSFWLLPPSLMFLLMSSIVEKGAGTGWTVYPPLSSNVAHAGSSVDLAIFSLHLAGISSILGSVNFITTVINMRPKG
MNFDRLPLFVWAVKITTILLLLSLPVLAGAITMLLTDRNINTSFFDPAGGGDPILYQHL
>COLFE016-12|KJ963632|ZMUO.005241|Denticollis_linearis
TLYFLFGAWAGMLGTSLSLLIRAELGNPGSLIGNDQIYNVIVTAHAFIMIFFMVMPIMIGGFGNWLVPLMLGAPDMAFPRMNNMSFWFLPPSLSLLLMSSIVENGAGTGWTVYPPLSANIAHSGSSVDLAIFSLHLAGISSILGAVNFISTVINMRSTGIT
FDRMPLFVWAVVITALLLLLSLPVLAGAITMLLTDRNLNTSFFDPAGGGDPILYQHL
>COLFE596-13|KJ962405|ZMUO.005441|Lochmaea_caprea
TLYFIFGIWAGMVGTSLSILVRAELGSPGTLIGNDQIYNVIVTAHAFIMIFFMVMPIMIGGFGNWLVPLMIGAPDMAFPRMNNMSFWLLPPSLFLLIMSSIVESGAGTGWTVYPPLSSNIAHGGSSVDLAIFSLHLAGISSILGAINFITTIINMRPKGMTL
DRIPLFVWAVMITAILLLLSLPVLAGAITMLLTDRNLNTSFFDPAGGGDPILYQHL
>COLFA288-12|KJ967203|ZMUO.000423|Stenus_clavicornis
TLYFIFGSWAGMVGTSLSMLIRSELGSPGSLIGDDQIYNVIVTAHAFIMIFFMVMPIMIGGFGNWLVPLMLGAPDMAFPRMNNMSFWLLPPSLSLLLMSSIVESGAGTGWTVYPPLSSNIAHSGASVDLAIFSLHLAGISSILGAINFITTIINMRTMKM
QLDCLPLFVWSVSITALLLLLSLPVLAGAITMLLTDRNLNTSFFDPAGGGDPILYQHL
>COLFE1159-13|KJ964593|ZMUO.006954|Cantharis_quadripunctata
TLYFIFGAWSGSLGLALSLLIRAELGTPGTLIGNDQIYNVIVTAHAFIMIFFMVMPIMIGGFGNWLVPLMLGAPDMAFPRMNNMSFWFLPPSLMFLLMSSMVESGAGTGWTVYPPLSANIAHSGPSVDLAIFSLHMAGISSILGAVNFISTIMNMKPPS
MKFDQMPLFVWSVGITALLLLLSLPVLAGAITMLLSDRNLNTSFFDPMGGGDPILYQHL
>COLFC618-12|KJ965638|ZMUO.003183|Stictotarsus_griseostriatus
TLYFLFGAWSGMVGTSLSMLIRAELGNPGSLIGDDQIYNVIVTAHAFIMIFFMVMPIMIGGFGNWLVPLMLGAPDMAFPRMNNMSFWLLPPSLTLLLMSSMVENGAGTGWTVYPPLSAGIAHGGASVDLAIFSLHLAGISSILGAVNFITTIINMRSV
GMTFDRMPLFVWSVGITALLLLLSLPVLAGAITMLLTDRNLNTSFFDPAGGGDPILYQHL
>COLFF241-13|KJ962452|ZMUO.005846|Archarius_pyrrhoceras
TLYFIFGAWSGMVGTSLSMLIRTELGNPGSLIGDDQIYNTIVTAHAFIMIFFMVMPTLIGGFGNWLIPLMLGAPDMAFPRLNNMSFWLLPPSLFLLLLSSIADKGAGTGWTVYPPLSTNIAHEGSSVDLAIFSLHMAGISSNLGAMNFISTIMNMRPTSM
KMDQMSLFIWAVKITAILLLLSLPVLAGAITMLLTDRNINTSFFDPAGGGDPILYQHL
>COLFF250-13|KJ967127|ZMUO.005855|Rhamphus_pulicarius
TLYFLFGGWSGMVGTSLSMLIRTELGNPGKLIGDDQIYNTIVTAHAFIMIFFMVMPIMIGGFGNWLVPLMLGAPDMAFPRLNNMSFWLLPPSLTFLLLSSVMDKGAGTGWTVYPPLSANIAHEGSSVDLAIFSLHMAGVSSILGAMNFISTIINMKPK
NMSMDQMSLFVWSVKITAILLLLSLPVLAGAITMLLTDRNINTSFFDPAGGGDPILYQHL
>COLFD427-12|KJ964634|ZMUO.003847|Chrysolina_fastuosa
TLYFIFGTWAGMVGTSLSILIRAELGNPGTLIGNDQIYNVIVTAHAFIMIFFMVMPIMIGGFGNWLVPLMLGAPDMAFPRMNNMSFWLLPPSLIFLLMSSIVENGVGTGWTVYPPLSANVAHSGPSVDLAIFSLHLAGISSILGAINFITTVINMRPTGM
KLEQMPLFSWAVLITAILLLLSLPVLAGAITMLLTDRNLNTSFFDPASGGDPILYQHL
>COLFC783-12|KJ966772|ZMUO.003348|Bembidion_saxatile

TLYFIFGAWSGMVGTSLSMLIRAELGNPGSLIGDDQIYNVIVTAHAFVMIFFMVMPILIGGFGNWLVPLMLGAPDMAFPRMNNMSFWLLPPSLSLLLMSSIVESGAGTGWTVYPPLSSSIAHSGASVDLAIFSLHLAGVSSILGAVNFITTIINMRSIGM
SFDRMPLFVWSVGITALLLLLSLPVLAGAITMLLTDRNLNTSFFDPAGGGDPILYQHL
>COLFA155-10|HQ559257|MP00417|Platydracus_fulvipes
TLYFIFGAWSGMVGTSLSLLIRAELGNPGTLIGDDQIYNVIVTAHAFVMIFFMVMPIVIGGFGNWLVPLMLGAPDMAFPRMNNMSFWLLPPSLTLLLMSSMAESGAGTGWTVYPPLSANVAHSGTSVDLAIFSLHLAGISSILGAVNFITTVINMRSTG
MSFDRMPLFVWSVAITALLLLLSLPVLAGAITMLLTDRNLNTSFFDPAGGGDPILYQHL
>COLFF520-13|KJ967146|ZMUO.006600|Bledius_erraticus
TLYFIFGAWASMVGTSLSMLIRAELGTPGSLIGDDQIYNVIVTAHAFVMIFFMVMPVVIGGFGNWLVPLMLGAPDMAFPRMNNMSFWLLPPSLTLLLMSSMVESGAGTGWTVYPPLSSNIAHSGSSVDLAIFSLHLAGISSILGAVNFISTVINMRSIG
MTFDRMPLFVWSVKITAILLLLSLPVLAGAITMLLTDRNLNTSFFDPSGGGDPILYQHL
>COLFA466-12|KJ966080|ZMUO.000506|Pityophagus_ferrugineus
TLYFIFGAWSGMIGTSLSLLIRSELGNPGSLIGDDQIYNVIVTAHAFIMIFFMVMPFMIGGFGNWLVPLMLGAPDMAFPRLNNMSFWLLPPSISLLLMSSIVESGAGTGWTVYPPLSSNIAHGGSSVDLAIFSLHLAGISSILGAVNFITTVINMRPSGMSF
DRMPLFVWAVTITAILLLLSLPVLAGAITMLLTDRNLNTSFFDPAGGGDPILYQHL
>COLFF645-13|KJ964543|ZMUO.006535|Limnobaris_t-album
TLYFIFGAWAGTIGTSLSMLIRSELGNPGSLIGDDQIYNTIVTAHAFIMIFFMVMPIMIGGFGNWLVPLMLGAPDMAFPRLNNMSFWLLPPSLTLLLMSSIVDKGVGTGWTVYPPLSSNNAHEGASVDLGIFSLHMAGISSILGAMNFISTAMNMRPT
GLKSDQMSLFIWAVKITAILLLLSLPVLAGAITMLLTDRNINTSFFDPIGGGDPILYQHL
>COLFE396-12|KJ966873|ZMUO.004956|Cantharis_figurata
TLYFIFGAWSGSLGLALSLLIRAELGTPGTLIGNDQIYNVIVTAHAFIMIFFMVMPIMIGGFGNWLVPLMLGAPDMAFPRMNNMSFWFLPPSLMFLLMSSMVESGAGTGWTVYPPLSANIAHSGPSVDLAIFSLHMAGISSILGAVNFISTIMNMKPPS
MKFDQMPLFVWSVGITALLLLLSLPVLAGAITMLLSDRNLNTSFFDPMGGGDPILYQHL
>COLFB710-12|KJ964364|ZMUO.001185|Aphodius_conspurcatus
TLYFLFGSWAGMVGTSLSLLIRAELGNPGSLIGDDQIYNVIVTAHAFVMIFFMVMPILIGGFGNWLVPLMLGAPDMAFPRMNNMSFWLLPPSLTLLLMSSMVESGAGTGWTVYPPLSSNIAHGGASVDLAIFSLHLAGISSILGAVNFITTVINMRAPG
LTFDQMPLFVWSVAITALLLLLSLPVLAGAITMLLTDRNLNTSFFDPAGGGDPILYQHL
>COLFA304-12|KJ967429|ZMUO.000439|Bagous_longitarsis
TLYFIFGAWSGMLGTSLSMLIRLELGSPGSLIGNDQIYNTIVTAHAFIMIFFMVMPIMIGGFGNWLVPLMLGAPDMAFPRLNNMSFWLLPPSIILLILSSIVDSGAGTGWTVYPPLSSNIAHEGSSVDLAIFSLHMAGVSSILGAVNFISTVLNMRPMGML
PEQMSLFTWAVEITAILLLLSLPVLAGAITMLLTDRNINTSFFDPAGGGDPILYQHL
>COLFD194-12|KJ965215|ZMUO.004089|Stenus_melanarius
TLYFIFGAWAGMVGTSLSLLIRAELGNPGSLIGDDQIYNVIVTAHAFVMIFFMVMPIMIGGFGNWLVPLMLGAPDMAFPRMNNMSFWLLPPSLSLLLMSSIVENGAGTGWTVYPPLSSNIAHGGASVDLAIFSLHLAGISSILGAINFITTIFNMRTMKL
QLDCLPLFVWSVGITALLLLLSLPVLAGAITMLLTDRNLNTSFFDPAGGGDPILYQHL
>COLFE1153-13|KJ964136|ZMUO.006948|Quedius_boops
TLYFIFGAWAGMVGTSLSLLIRAELGNPGSLIGDDQIYNVIVTAHAFIMIFFMVMPTLIGGFGNWLVPLMLGAPDMAFPRMNNMSFWLLPPSLSLLLMSSMVESGAGTGWTVYPPLSSNIAHSGASVDLAIFSLHLAGISSILGAVNFITTVINMRSIGM
TFDRMPLFVWSVAITALLLLLSLPVLAGAITMLLTDRNLNTTFFDPAGGGDPILYQHL
>COLFD036-12|KJ967410|ZMUO.003931|Anaspis_arctica
TLYFIFGAWSGMVGTSLSLLIRSELGTPGSLIGDDQIYNVIVTAHAFIMIFFMVMPILIGGFGNWLVPLMLGAPDMAFPRMNNMSFWLLPPSLTLLIMSSVVENGAGTGWTVYPPLAANIAHSGSSVDLAIFSLHLAGVSSILGAVNFITTVINMRPQG
MTLDRMPLFVWAVVITAILLLLSLPVLAGAITMLLTDRNLNTSFFDPAGGGDPILYQHL
>COLFB254-12|KJ964406|ZMUO.001584|Gabrius_trossulus
TLYFIFGSWAGMVGTSLSLLIRAELGNPGSLIGDDQIYNVIVTAHAFIMIFFMVMPIVIGGFGNWLVPLMLGAPDMAFPRMNNMSFWLLPPSITLLLMSSMVESGAGTGWTVYPPLSSNIAHGGASVDLAIFSLHLAGISSILGAVNFITTVINMRSFG
MSFDRMPLFVWSVAITALLLLLSLPVLAGAITMLLTDRNLNTSFFDPAGGGDPILYQHL
>COLFF233-13|KJ963731|ZMUO.005838|Plagiosterna_aenea
TLYFIFGIWAGMVGTSLSMLIRTELGNPGSLIGNDQIYNVIVTAHAFIMIFFMVMPIMIGGFGNWLVPLMIGAPDMAFPRLNNMSFWLLPPSLFLLLMSSVVESGAGTGWTVYPPLSANITHSGSSVDLAIFSLHLAGISSILGAINFITTVINMRPEGMN
FEQTPLFVWAVLITAILLLLSLPVLAGAITMLLTDRNLNTSFFDPAGGGDPILYQHL
>COLFC174-12|KJ962575|ZMUO.002359|Cercyon_sternalis
TLYFIFGAWAGMVGTSLSILIRAELGNPGTLIGDDQIYNVIVTAHAFIMIFFMVMPIMIGGFGNWLVPLMLGAPDMAFPRMNNMSFWLLPPSLSLLLMSSMVESGAGTGWTVYPPLSSNIAHGGSSVDLAIFSLHLAGISSILGAVNFITTVINMRSPSL
TYDRLPLFVWSVAITALLLLLSLPVLAGAITMLLTDRNLNTSFFDPAGGGDPILYQHL
>COLFE1437-13|KJ962231|ZMUO.007232|Hypera_plantaginis
TLYFIFGTWAGTVGTSLSILIRTELGNPGSLIGNDQIYNTIVTAHAFIMIFFMVMPIMIGGFGNWLVPLMLGAPDMAFPRLNNMSFWLLPPSLSLLLMSSMVDSGAGTGWTVYPPLSSNIAHEGSSVDLAIFSLHMAGVSSILGAINFISTVLNMRPSGM
SLDKMALFIWAVKITAILLLLSLPVLAGAITMLLTDRNINTSFFDPAGGGDPILYQHL
>COLFC203-12|KJ966725|ZMUO.002388|Atheta_strandiella
TLYFIFGTWAGMVGTSLSLLIRAELGNPGSLIGDDQIYNVIVTAHAFVMIFFMVMPVVIGGFGNWLVPLMLGAPDMAFPRMNNMSFWLLPPSLTLLLMSSMVESGAGTGWTVYPPLSSNIAHGGSSVDLAIFSLHLAGISSILGAVNFISTVINMRSTG
ISFDRMPLFVWSVAITALLLLLSLPVLAGAITMLLTDRNLNTSFFDPAGGGDPILYQHL

>COLFC850-12|KJ961761|ZMUO.003415|Quedius_nitipennis
TLYFIFGAWAGMVGTSLSLLIRAELGNPGTLIGDDQIYNVIVTAHAFIMIFFMVMPIVIGGFGNWLVPLMLGAPDMAFPRMNNMSFWLLPPSLSLLLMSSMVESGAGTGWTVYPPLSSNIAHGGASVDLAIFSLHLAGISSILGAVNFITTVINMRSIG
MSFDRMPLFVWSVAITALLLLLSLPVLAGAITMLLTDRNLNTSFFDPAGGGDPILYQHL
>COLFC397-12|KJ963081|ZMUO.002962|Quedius_fellmani
TLYFIFGAWAGMVGTSLSLLIRAELGNPGTLIGDDQIYNVIVTAHAFIMIFFMVMPIVIGGFGNWLVPLMLGAPDMAFPRMNNMSFWLLPPSLSLLLMSSMVESGAGTGWTVYPPLSSNIAHGGASVDLAIFSLHLAGISSILGAVNFITTVINMRSIG
MSFDRMPLFIWSVAITALLLLLSLPVLAGAITMLLTDRNLNTSFFDPAGGGDPILYQHL
>COLFA449-12|KJ963625|ZMUO.000489|Acilius_canaliculatus
TLYFIFGAWSGMVGTSLSMLIRAELGNPGSLIGDDQIYNVIVTAHAFIMIFFMVMPIMIGGFGNWLVPLMLGAPDMAFPRMNNMSFWLLPPSLTLLLMSSMVENGAGTGWTVYPPLSAGIAHGGASVDLAIFSLHLAGISSILGAVNFITTIINMRSIG
MTFDRMPLFVWSVGITALLLLLSLPVLAGAITMLLTDRNLNTSFFDPAGGGDPILYQHL
>COLFF159-13|KJ966960|ZMUO.003793|Stenopterapion_meliloti
TLYFIFGLWAGMVGTSLSMLIRIELGTPGSLIGNDQIYNVIVTAHAFIMIFFMVMPIMIGGFGNWLVPLMLGAPDMAFPRMNNMSFWLLPPSLTLLLMSSIVEKGAGTGWTVYPPLASNIAHGGASVDLAIFSLHLAGISSILGAVNFISTIINMHPNGLS
FDQMSLFTWAVKITAILLLLSLPVLAGAITMLLTDRNINTSFFDPAGGGDPILYQHL
>COLFE1431-13|KJ963880|ZMUO.007226|Aleochara_inconspicua
TLYFIFGAWSGMVGTSLSLLIRAELGNPGSLIGDDQIYNVIVTAHAFVMIFFMVMPIVIGGFGNWLVPLMLGAPDMAFPRMNNMSFWLLPPSLTLLLMSSMVESGAGTGWTVYPPLSSNIAHGGSSVDLAIFSLHLAGISSILGAVNFISTVINMRTMG
MSFDKMPLFVWSVAITALLLLLSLPVLAGAITMLLTDRNLNTSFFDPAGGGDPILYQHL
>COLFA160-10|HQ559261|MP00422|Silpha_tristis
TLYFIFGAWAGIVGMSLSILIRMELSTPSSLLGDDQMYNVIVTAHAFIMIFFMVMPIVIGGFGNWLIPLMLGAPDMAFPRMNNMSFWLLPPSLSLLLVSSMVESGAGTGWTVYPPLSSNIAHGGSSVDLAIFSLHLAGISSILGAVNFITTIINMRSSGMT
FDRMPLFVWSVGITALLLLLSLPVLAGAITMLLTDRNLNTSFFDPAGGGDPILYQHL
>COLFC132-12|KJ961781|ZMUO.002317|Trichophya_pilicornis
TLYFIFGAWAGMVGTSLSLLIRAELGNPGSLIGDDQIYNVIVTAHAFVMIFFMVMPIVIGGFGNWLVPLMLGAPDMAFPRMNNMSFWLLPPSLTLLLMSSMVESGAGTGWTVYPPLSSNIAHGGASVDLAIFSLHLAGISSILGAVNFITTVINMRSIG
MTFDRMPLFVWSVAITALLLLLSLPVLAGAITMLLTDRNLNTSFFDPAGGGDPILYQHL
>COLFB687-12|KJ964218|ZMUO.001162|Atheta_laticollis
TLYFIFGAWAGMVGTSLSLLIRAELGNPGSLIGDDQIYNVIVTAHAFIMIFFMVMPIVIGGFGNWLVPLMLGAPDMAFPRMNNMSFWLLPPSLTLLLMSSMVESGAGTGWTVYPPLSSNIAHGGSSVDLAIFSLHLAGISSILGAVNFISTVINMRSVGI
TFDRMPLFVWSVAITALLLLLSLPVLAGAITMLLTDRNLNTSFFDPAGGGDPILYQHL
>COLFC704-12|KJ965721|ZMUO.003269|Ocypus_fuscatus
TLYFIFGVWSSMVGTSLSLLIRAELGNPGSLIGDDQIYNVIVTAHAFVMIFFMVMPIVIGGFGNWLVPLMLGAPDMAFPRMNNMSFWLLPPSLTLLLTSSMAESGAGTGWTVYPPLSANMAHSGTSVDLAIFSLHLAGISSILGAVNFITTIMNMRAT
GITFDRMPLFVWSVAITALLLLLSLPVLAGAITMLLTDRNINTSFFDPAGGGDPILYQHL
>COLFC605-12|KJ963238|ZMUO.003170|Crepidodera_fulvicornis
TLYFIFGIWSGMVGTSLSILIRTELGSPGSLIGNDQIYNVIVTAHAFIMIFFMVMPIMIGGFGNWLIPLMIGAPDMAFPRMNNMSFWLLPPSLFLLLMSSLVESGAGTGWTVYPPLSSNIAHGGSSVDLAIFSLHLAGISSILGAINFITTIINMRPKGMNLD
RMPLFVWAVAITAILLLLSLPVLAGAITMLLTDRNMNTSFFDPIGGGDPILYQHL
>COLFC077-12|KJ965008|ZMUO.002072|Hygrotus_inaequalis
TLYFLFGAWAGMVGTSLSMLIRAELGNPGSLIGDDQIYNVIVTAHAFIMIFFMVMPIMIGGFGNWLVPLMLGAPDMAFPRMNNMSFWMLPPSLTLLLMSSMVESGAGTGWTVYPPLSAGIAHGGASVDLAIFSLHLAGISSILGAVNFITTIINMRS
VGMTFDRMALFVWSVGITALLLLLSLPVLAGAITMLLTDRNLNTSFFDPAGGGDPILYQHL
>COLFF980-13|KJ967066|ZMUO.007345|Agonum_marginatum
TLYFIFGAWAGMVGTSLSMLIRAELGNPGALIGDDQIYNVIVTAHAFIMIFFMVMPIMIGGFGNWLVPLMLGAPDMAFPRMNNMSFWLLPPSLTLLLMSSLVESGAGTGWTVYPPLSSGIAHAGASVDLAIFSLHLAGVSSILGAVNFITTIINMRSVG
MTFDRMPLFVWSVGITALLLLLSLPVLAGAITMLLTDRNLNTSFFDPAGGGDPILYQHL
>COLFB392-12|KJ963110|ZMUO.001817|Rugilus_orbiculatus
TLYFIFGAWAGMVGTSLSLLIRAELSAPGSLIGDDQIYNVIVTAHAFVMIFFMVMPIVIGGFGNWLVPLMLGAPDMAFPRMNNMSFWLLPPSLSLLLMSSMVESGAGTGWTVYPPLSSNIAHGGASVDLAIFSLHLAGISSILGAVNFITTIINMRSKG
MTYERMPLFVWSVGITALLLLLSLPVLAGAITMLLTDRNLNTSFFDPAGGGDPILYQHL
>COLFD047-12|KJ962540|ZMUO.003942|Sphindus_dubius
TLYFIFGIWSGMVGTSLSMLIRAELSTPNALIGDDQIYNVIVTAHAFIMIFFMVMPIMIGGFGNWLIPLMMGAPDMAFPRMNNMSFWLLPPSLSLLLMSSMVEKGTGTGWTVYPPLSSNVSHNGASVDLSIFSLHMAGVSSILGAINFISTIVNMRPM
NMKMDQLPLFIWAVLITAFLLVLSLPVLAGAITMLLTDRNINTSFFDPSGGGDPILYQHL
>COLFE1442-13|KJ965166|ZMUO.007237|Ischnopterapion_loti
TLYFIFGLWSGMAGTSLSMLIRIELGTPGSLIGNDQIYNVIVTAHAFIMIFFMVMPIMIGGFGNWLVPLMLGAPDMAFPRMNNMSFWLLPPSLTFLLMSSIVEKGAGTGWTVYPPLASNIAHGGASVDLAIFSLHLAGISSILGAVNFISTIINMHPNELS
FDQLSLFTWAVKITAILLLLSLPVLAGAITMLLTDRNINTSFFDPAGGGDPILYQHL
>COLFE239-12|KJ965198|ZMUO.005084|Tasgius_melanarius

TLYFIFGTWSGMVGTSLSLLIRAELGNPGSLIGNDQIYNVIVTAHAFVMIFFMVMPIVIGGFGNWLVPLMLGAPDMAFPRMNNMSFWLLPPSLNLLLTSSMAESGAGTGWTVYPPLSANVAHSGTSVDLAIFSLHLAGISSILGAVNFITTVLNMRATG
MTFDRMPLFVWSVTITALLLLSLPVLAGAITMLLTDRNLNTSFFDPSGGGDPILYQHL
>COLFC032-12|KJ961863|ZMUO.002027|Aleochara_lanuginosa
TLYFIFGAWAGMVGTSLSLLIRAELGNPGSLIGDDQIYNVIVTAHAFIMIFFMVMPIVIGGFGNWLVPLMLGAPDMAFPRMNNMSFWLLPPSLTLLLMSSMVESGAGTGWTVYPPLSSNIAHGGSSVDLAIFSLHLAGISSILGAVNFISTVINMRSSG
MTFDKMPLFVWSVAITALLLLSLPVLAGAITMLLTDRNLNTSFFDPAGGGDPILYQHL
>COLFC847-12|KJ966276|ZMUO.003412|Brachonyx_pineti
TLYFIFGMWAGSIGTSLSVLIRAELGTPGNLIGDDQIYNTIVTAHAFVMIFFMVMPIMIGGFGNWLIPLMLAAPDMAFPRLNNLSFWLLPPSLTLLIMSSIIGKGAGTGWTVYPPLSANLAHEGPSVDFAIFSLHMAGISSILGAMNFISTILNMKPMKMK
FDQMPLFIWAVKITAILLLISLPVLAGAITMLLTDRNINTSFFDPAGGGDPILYQHL
>COLFA264-12|KJ964285|ZMUO.000399|Migneauxia_lederi
SLYFLFGMWSGMVGTSLSLLIRLELGNPGSLIGDDQIYNVIVTAHAFIMIFFMVMPIMIGGFGNWLVPLMLGAPDMAFPRLNNMSFWLLPPSLSLLIMSSIVESGAGTGWTVYPPLSSNIAHGGSSVDLAIFSLHLAGISSILGAVNFITTVINMRPKGMS
LDRMPLFVWSVVITAVLLLLSLPVLAGAITMLLTDRNLNTSFFDPAGGGDPILYQHL
>COLFE1371-13|KJ964463|ZMUO.007166|Omphalapion_hookerorum
TLYFIFGFWSGMIGTSLSMLIRVELGNPGSLIGDDQIYNVIVTAHAFIMIFFMVMPIMIGGFGNWLIPLMLGAPDMAFPRLNNMSFWLLPPSMAFLLLSSIIEKGVGTGWTVYPPLASNIAHSGASVDLAIFSLHLAGISSILGAVNFISTILNMRPSGLSLD
QMSLFSWAVKVTATLLLLSLPVLAGAITMLLTDRNINTSFFDPAGGGDPILYQHL
>COLFD014-12|KJ962481|ZMUO.003909|Lebia_cruxminor
TLYFIFGAWAGMVGTSLSMLIRAELGNPGALIGDDQIYNVIVTAHAFIMIFFMVMPIMIGGFGNWLVPLMLGAPDMAFPRMNNMSFWLLPPSLTLLLMSSLVESGAGTGWTVYPPLSSGIAHAGASVDLAIFSLHLAGISSILGAVNFITTIINMRSVG
MTFDRMPLFVWSVGITALLLLSLPVLAGAITMLLTDRNLNTSFFDPAGGGDPILYQHL
>COLFB602-12|KJ964623|ZMUO.001077|Amara_ingenua
TLYFIFGAWSGMVGTSLSMLIRAELGNPGALIGDDQIYNVIVTAHAFVMIFFMVMPIMIGGFGNWLVPLMLGAPDMAFPRMNNMSFWLLPPSLTLLLMSSMVESGAGTGWTVYPPLSSGIAHAGASVDLAIFSLHLAGISSILGAVNFITTIINMRSV
GMTFDRMPLFVWSVGITALLLLSLPVLAGAITMLLTDRNLNTSFFDPAGGGDPILYQHL
>COLFB434-12|KJ966325|ZMUO.001859|Phyllotreta_undulata
TLYFIFGIWSGMLGMSMSMLIRVELAAPGSLIGNDQIYNVIVTAHAFIMIFFMVMPIMIGGFGNWLIPLMIGAPDMAFPRMNNMSFWLLPPSLFLLIMSSIIENGAGTGWTVYPPLSSNISHAGASVDLTIFSLHLAGISSILGAINFITTVINMRPKGMT
LDRLPLFVWAVLITAILLLLSLPVLAGAITMLLTDRNLNTSFFDPMGGGDPILYQHL
>COLFG052-13|KJ961784|ZMUO.007557|Agrilus_sulcicollis
TLYFIFGAWSGMVGTALSLLVRAELGNPGALIGNDQIYNVIVTAHAFIMIFFMVMPIMMGGFGNWLVPLMLGAPDMAFPRMNNMSFWLLPPSLTLLLMSSMVESGAGTGWTVYPPLAANIAHSGASVDLAIFSLHLAGISSILGAINFITTVINMRA
SGMTLDRMPLLVWSIAITALLLLLSLPVLAGAITMLLTDRNLNTSFFDPAGGGDPILYQHL
>COLFE1026-13|KJ963402|ZMUO.006821|Amara_brunnea
TLYFIFGTWSGMVGTSLSMLIRAELGNPGALIGDDQIYNVIVTAHAFVMIFFMVMPIMIGGFGNWLVPLMLGAPDMAFPRMNNMSFWLLPPSLSLLLMSSLVESGAGTGWTVYPPLSSNIAHAGASVDLAIFSLHLAGISSILGAVNFITTIINMRSIG
MTFDRMPLFVWSVGITALLLLSLPVLAGAITMLLTDRNLNTSFFDPAGGGDPILYQHL
>COLFF433-13|KJ965174|ZMUO.005951|Agonum_viduum
TLYFIFGAWSGMVGTSLSMLIRAELGNPGALIGDDQIYNVIVTAHAFIMIFFMVMPIMIGGFGNWLVPLMLGAPDMAFPRMNNMSFWLLPPSLTLLLMSSMVESGAGTGWTVYPPLSSGIAHAGASVDLAIFSLHLAGVSSILGAVNFITTIINMRSV
GMTFDRMPLFVWSVGITALLLLSLPVLAGAITMLLTDRNLNTSFFDPAGGGDPILYQHL
>COLFB622-12|KJ962462|ZMUO.001097|Agabus_uliginosus
TLYFIFGAWAGMVGTSLSMLIRAELGNPGSLIGDDQIYNVIVTAHAFVMIFFMVMPIMIGGFGNWLVPLMLGAPDMAFPRMNNMSFWLLPPSLTLLLMSSMVEKGAGTGWTVYPPLSSGIAHGGASVDLAIFSLHLAGISSILGAVNFITTIINMRSV
GMTFDRMPLFVWSVGITALLLLSLPVLAGAITMLLTDRNLNTSFFDPAGGGDPILYQHL
>COLFA707-12|KJ966835|ZMUO.000842|Nehemitropia_lividipennis
TLYFIFGTWAGMVGTSLSLLIRAELGNPGSLIGDDQIYNVIVTAHAFVMIFFMVMPIVIGGFGNWLVPLMLGAPDMAFPRMNNMSFWLLPPSLTLLLMSSMVESGAGTGWTVYPPLSANIAHGGSSVDLAIFSLHLAGISSILGAVNFISTIINMRAPG
MSFDRMPLFVWSVAITALLLLSLPVLAGAITMLLTDRNINTSFFDPAGGGDPILYQHL
>COLFD675-12|KJ963360|ZMUO.004665|Anoplodera_sanguinolenta
TLYFIFGAWASMVGTSLSLLIRSELGNPGSLIGNDQIYNVIVTAHAFVMIFFMVMPIMIGGFGNWLVPLMLGAPDMAFPRMNNMSFWLLPPSLTLLIMSSIVESGAGTGWTVYPPLSSNIAHSGSSVDLAIFSLHLAGISSILGAVNFITTVINMRPAGLK
PEQMPLFVWAVVITAVLLLLSLPVLAGAITMLLTDRNLNTSFFDPAGGGDPILYQHL
>COLFA568-12|KJ963735|ZMUO.000703|Phymatodes_testaceus
TLYFIFGAWAGMVGTSLSMLIRSELGNPGSLIGDDQIYNVIVTAHAFIMIFFMVMPVMIGGFGNWLVPLMLGAPDMAFPRMNNMSFWLLPPSLTLLIMSSIVENGAGTGWTVYPPLSANVAHSGSSVDLAIFSLHLAGVSSILGAVNFISTVINMKPA
GMTPEQMPLFVWAVLITAILLLLSLPVLAGAITMLLTDRNLNTSFFDPAGGGDPILYQHL
>COLFE1109-13|KJ962108|ZMUO.006904|Hydroporus_morio
TLYFLFGAWSGMVGTSLSMLIRAELGNPGSLIGDDQIYNVIVTAHAFIMIFFMVMPIMIGGFGNWLVPLMLGAPDMAFPRMNNMSFWLLPPSLSLLLMSSMVENGAGTGWTVYPPLSSGIAHSGASVDLAIFSLHLAGVSSILGAVNFITTIINMRSIG
MTFDRMPLFVWSVGITALLLLSLPVLAGAITMLLTDRNLNTSFFDPAGGGDPILYQHL

>COLFA511-12|KJ964773|ZMUO.000551|Dasytes_plumbeus
TLYFIFGAWSGMVGMSLSLLIRSELNNPGTLIGNDQIYNVIVTAHAFIMIFFMVMPILIGGFGNWLVPLMLGAPDMAFPRMNNMSFWLLPPSLTLLLMSSMVEQGAGTGWTVYPPLSANIAHGGASVDLAIFSLHLAGISSILGAVNFITTVINMRPIG
MTLDRTPLFVWAVAITALLLLLSLPVLAGAITMLLTDRNLNTSFFDPAGGGDPILYQHL
>COLFF620-13|KJ963282|ZMUO.006510|Cardiophorus_ruficollis
TLYFLFGAWSGMLGTSLSLLIRAELGSPGSLIGNDQIYNVIVTAHAFIMIFFMVMPIMIGGFGNWLMPLMLGAPDMAFPRMNNMSFWFLPPSLSLLLMSSIVENGAGTGWTVYPPLSSNIAHSGSSVDLAIFSLHLAGISSILGAVNFISTVINMRSPGM
TFERMPLFVWAVVITALLLLLSLPVLAGAITMLLTDRNLNTSFFDPAGGGDPILYQHL
>COLFF614-13|KJ965426|ZMUO.006504|Cicindela_hybrida
TLYFIFGAWSGMVGTSLSMLIRAELGSPGSLIGDDQIYNVIVTAHAFVMIFFMVMPIMIGGFGNWLVPLMLGAPDMAFPRMNNMSFWLLPPSLTLLLMSSMVGDGAGTGWTVYPPLSAGIAHAGASVDLAIFSLHLAGVSSILGAVNFITTIINMRS
VGMTFDRMPLFVWSVGITALLLLLSLPVLAGAITMLLTDRNLNTSFFDPAGGGDPILYQHL
>COLFC157-12|KJ965965|ZMUO.002342|Stenus_flavipes
TLYFIFGAWAGMVGTSLSLLIRAELGTPSSLIGDDQIYNVIVTAHAFIMIFFMVMPIMIGGFGNWLVPLMMGAPDMAFPRMNNMSFWLLPPSLSLLLMSSIVESGAGTGWTVYPPLSSNIAHSGASVDLAIFSLHLAGISSILGAINFITTIINMRTMKM
NFDCLPLFVWSVSITALLLLLSLPVLAGAITMLLTDRNLNTSFFDPAGGGDPVLYQHL
>COLFF769-13|KJ965279|ZMUO.006089|Meligethes_lugubris
TLYFIFGAWSGMVGTSLSMLIRTELGNPGSLIGNDQIYNVIVTAHAFVMIFFMVMPFMIGGFGNWLVPLMLGAPDMAFPRMNNMSFWLLPPSLSLLLMSSIIESGAGTGWTVYPPLSSNIAHGGASVDLAIFSLHLAGISSILGAVNFITTVINMRPSG
MTFDRMPLFVWAVAITALLLLLSLPVLAGAITMLLTDRNLNTTFFDPSGGGDPILYQHL
>COLFB369-12|KJ962729|ZMUO.001699|Stenus_palustris
TLYFILGAWAGLTGTSLSLLIRTELGSPGSFIGDDQIYNVIVTAHAFIMIFFMVMPIMIGGFGNWLIPLMLGAPDMAFPRMNNMSFWLLPPSLSLLLMSSIVESGAGTGWTVYPPLSSNIAHSGASVDLTIFSLHLAGISSILGAINFITTIFNMRTMKLQLD
CLPLFIWSVNVTTFLLLLSLPVLAGAITMLLTDRNVNTSFFDPGGGGDPILYQHL
>COLFD067-12|KJ961831|ZMUO.003962|Melanotus_castanipes
TLYFIFGAWAGMLGTSLSLLIRAELGNPGSLIGNDQIYNVIVTAHAFIMIFFMVMPIMIGGFGNWLVPLMLGAPDMAFPRMNNMSFWLLPPSLSLLLMSSIVENGAGTGWTVYPPLSSNIAHSGSSVDLAIFSLHLAGISSILGAVNFISTVINMRSTGM
TFDRMPLFVWAVALTALLLLLSLPVLAGAITMLLTDRNLNTSFFDPAGGGDPILYQHL
>COLFD091-12|KJ966195|ZMUO.003986|Philopedon_plagiatum
TLYFIFGAWSGMVGTSLSMLIRTELGNPGSLIGDDQIYNVIVTAHAFIMIFFMVMPMMIGGFGNWLVPLMLGAPDMAFPRLNNMSFWLLPPSLSLLLMSSIVDKGAGTGWTVYPPLSANIAHEGSSVDLAIFSLHMAGVSSILGAINFISTVINMRPQ
GMSPERISLFVWSVKITAVLLLLSLPVLAGAITMLLTDRNINTSFFDPAGGGDPILYQHL
>COLFE221-12|KJ966672|ZMUO.005066|Agonum_marginatum
TLYFIFGAWAGMVGTSLSMLIRAELGNPGALIGDDQIYNVIVTAHAFIMIFFMVMPIMIGGFGNWLVPLMLGAPDMAFPRMNNMSFWLLPPSLTLLLMSSLVESGAGTGWTVYPPLSSGIAHAGASVDLAIFSLHLAGVSSILGAVNFITTIINMRSVG
MTFDRMPLFVWSVGITALLLLLSLPVLAGAITMLLTDRNLNTSFFDPAGGGDPILYQHL
>COLFD482-12|KJ965570|ZMUO.004282|Hypera_fornicata
TLYFIFGTWAGTVGTSLSILIRTELGNPGSLIGNDQIYNTIVTAHAFIMIFFMVMPIMIGGFGNWLVPLMLGAPDMAFPRLNNMSFWLLPPSLSLLLMSSTVDKGAGTGWTVYPPLSSNIAHEGSSVDLAIFSLHMAGVSSILGAINFISTVLNMRPSGM
SLDKMALFIWAVKITAILLLLSLPVLAGAITMLLTDRNINTSFFDPAGGGDPILYQHL
>COLFF749-13|KJ966381|ZMUO.006449|Stenopterapion_meliloti
TLYFIFGLWAGMVGTSLSMLIRIELGTPGSLIGNDQIYNVIVTAHAFIMIFFMVMPIMIGGFGNWLVPLMLGAPDMAFPRMNNMSFWLLPPSLTLLLMSSIVEKGAGTGWTVYPPLASNIAHGGASVDLAIFSLHLAGISSILGAVNFISTIINMHPNGLS
FDQMSLFTWAVKITAILLLLSLPVLAGAITMLLTDRNINTSFFDPAGGGDPILYQHL
>COLFC147-12|KJ963514|ZMUO.002332|Thinonoma_atra
TLYFIFGAWAGMVGTSLSLLIRAELGNPGSLIGDDQIYNVIVTAHAFVMIFFMVMPVVIGGFGNWLVPLMLGAPDMAFPRMNNMSFWLLPPSLTLLLMSSMVESGAGTGWTVYPPLSSNIAHGGSSVDLAIFSLHLAGISSILGAVNFISTVINMRSTG
ITFDRMPLFVWSVAITALLLLLSLPVLAGAITMLLTDRNLNTSFFDPAGGGDPILYQHL
>COLFD538-12|KJ963283|ZMUO.004338|Altica_chamaenerii
TLYFLFGIWAGMIGTSMSLLIRTELGSPGSLIGNDQIYNVIVTAHAFVMIFFMVMPMMIGGFGNWLVPLMIGAPDMAFPRMNNMSFWLLPPSIFLLLMSSFTESGAGTGWTVYPPLSSNLAHNGPSVDLAIFSLHLAGISSILGAINFITTMVNMRPQ
GMSMDQMPLFVWAVFITAILLLLSLPVLAGAITMLLTDRNLNTSFFEPAGGGDPILYQHL
>COLFG139-13|KJ964707|ZMUO.007834|Baeocrara_variolosa
TLYFIFGAWAGMVGTSLSILIRTELGTPGSLIGDDQIYNVIVTAHAFVMIFFMVMPILIGGFGNWLVPLMLGAPDMAFPRMNNMSFWLLPPSLTLLLMSSMVESGAGTGWTVYPPLASNIAHGGSSVDLAIFSLHLAGISSILGAVNFITTIINMRTPNM
KFDQMPLFVWAVGITALLLLLSLPVLAGAITMLLTDRNLNTSFFDPAGGGDPILYQHL
>COLFE1105-13|KJ961858|ZMUO.006900|Hydroporus_memnonius
TLYFLFGAWSGMVGTSLSMLIRAELGNPGSLIGDDQIYNVIVTAHAFIMIFFMVMPIMIGGFGNWLVPLMLGAPDMAFPRMNNMSFWLLPPSLTLLLMSSMVENGAGTGWTVYPPLSSGIAHSGASVDLAIFSLHLAGVSSILGAVNFITTIINMRSI
GMTFDRMPLFVWSVGITALLLLLSLPVLAGAITMLLTDRNLNTSFFDPAGGGDPILYQHL
>COLFB465-12|KJ966394|ZMUO.001890|Carpelimus_bilineatus

TLYFIFGAWSGMVGTSLSMLIRIELGTPGSLIGDDQIYNVIVTAHAFIMIFFMVMPIVIGGFGNWLVPLMLGAPDMAFPRMNNMSFWLLPPSLTLLLFSSMVESGAGTGWTVYPPLSSNIAHSGSSVDLAIFSLHLAGISSILGAVNFISTIINMRSIGMSF
DRMPLFVWSVNITAILLLLSLPVLAGAITMLLTDRNLNTSFFDPAGGGDPILYQHL
>COLFE474-12|KJ963475|ZMUO.005034|Aplotarsus_incanus
TLYFLFGAWAGMLGTSLSLLIRAELGNPGSLIGNDQIYNVIVTAHAFIMIFFMVMPIMIGGFGNWLVPLMLGAPDMAFPRMNNMSFWFLPPSLSLLLMSSIVENGAGTGWTVYPPLSANIAHSGSSVDLAIFSLHLAGISSILGAVNFISTVINMRSTGIT
FDRMPLFVWAVAITALLLLLSLPVLAGAITMLLTDRNLNTSFFDPAGGGDPILYQHL
>COLFA079-10|HM909096|MP00283|Hygrotus_inaequalis
TLYFLFGAWAGMVGTSLSMLIRAELGNPGSLIGDDQIYNVIVTAHAFIMIFFMVMPIMIGGFGNWLVPLMLGAPDMAFPRMNNMSFWMLPPSLTLLLMSSMVESGAGTGWTVYPPLSAGIAHGGASVDLAIFSLHLAGISSILGAVNFITTIINMRS
VGMTFDRMALFVWSVGITALLLLLSLPVLAGAITMLLTDRNLNTSFFDPAGGGDPILYQHL
>COLFD496-12|KJ965772|ZMUO.004296|Agabus_thomsoni
TLYFIFGAWAGMVGTSLSMLIRAELGNPGSLIGDDQIYNVIVTAHAFVMIFFMVMPIMIGGFGNWLIPLMLGAPDMAFPRMNNMSFWLLPPSLTLLLMSSMVESGAGTGWTVYPPLSSGIAHGGASVDLAIFSLHLAGISSILGAVNFITTIINMRSVG
MTFDRMPLFVWSVGITALLLLLSLPVLAGAITMLLTDRNLNTSFFDPAGGGDPILYQHL
>COLFE1073-13|KJ966474|ZMUO.006868|Nebrioporus_depressus
TLYFLFGAWSGMVGTSLSMLIRAELGNPGSLIGDDQIYNVIVTAHAFIMIFFMVMPIMIGGFGNWLVPLMLGAPDMAFPRMNNMSFWMLPPSLSLLLMSSMVENGAGTGWTVYPPLSAGLAHGGASVDLAIFSLHLAGISSILGAVNFITTIINMRS
VGMTFDRMPLFVWSVGITALLLLLSLPVLAGAITMLLTDRNLNTSFFDPAGGGDPILYQHL
>COLFC454-12|KJ962547|ZMUO.003019|Podistra_schoenherri
TLYFIFGAWSGSLGLALSLLIRAELGTPGTLIGNDQIYNVIVTAHAFIMIFFMVMPIMIGGFGNWLVPLMLGAPDMAFPRMNNMSFWFLPPSLMFLLMSSMVESGAGTGWTVYPPLSANIAHSGPSVDLAIFSLHMAGISSILGAVNFISTIMNMKPPS
MKFDQMPLFVWSVGITALLLLLSLPVLAGAITMLLSDRNLNTSFFDPMGGGDPILYQHL
>COLFB916-12|KJ962832|ZMUO.001961|Acidota_crenata
TLYFIFGAWAGMVGTSLSILIRAELGNPGTLIGDDQIYNVIVTAHAFVMIFFMVMPIVIGGFGNWLVPLMLGAPDMAFPRMNNMSFWLLPPSLSLLLMSSMVESGAGTGWTVYPPLSSNIAHGGASVDLAIFSLHLAGISSILGAVNFITTVINMRATG
MTFDRMPLFVWSVAITALLLLLSLPVLAGAITMLLTDRNLNTSFFDPAGGGDPILYQHL
>COLFA091-10|HM909107|MP00320|Helophorus_glacialis
TLYFIFGAWAGMVGTSLSILIRAELGNPGTLIGDDQIYNVIVTAHAFIMIFFMVMPIMIGGFGNWLVPLMLGAPDMAFPRMNNMSFWLLPPSLTLLLMSSMVESGAGTGWTVYPPLSSNIAHSGASVDLAIFSLHLAGISSILGAVNFITTVINMRSVS
MTYDRLPLFVWSVAITALLLLLSLPVLAGAITMLLTDRNLNTSFFDPAGGGDPILYQHL
>COLFB498-12|KJ962638|ZMUO.000973|Aploderus_caelatus
TLYFIFGAWSGMIGTSLSMLIRAELGAPGSLIGDDQIYNVVVTAHAFIMIFFMVMPIVIGGFGNWLVPLMLGAPDMAFPRMNNMSFWLLPPSLTLLLFSSMVESGAGTGWTVYPPLSSNIAHSGSSVDLAIFSLHLAGISSILGAVNFISTIINMRSVGM
SFDRMPLFIWSVNITAILLLLSLPVLAGAITMLLTDRNLNTSFFDPAGGGDPILYQHL
>COLFD934-12|KJ965068|ZMUO.004544|Labidostomis_tridentata
TLYFIFGAWSGMVGTSLSLLIRVELGNPGTLIGNDQIYNTIVTAHAFIMIFFMVMPIMIGGFGNWLVPLMLGAPDMAFPRMNNMSFWLLPPSLTLLLMSSIVENGAGTGWTVYPPLSANIAHSGSSVDLAIFSLHLAGVSSIMGAINFISTVINMRPQG
MLLDRTPLFVWAVVITAVLLLLSLPVLAGAITMLLTDRNLNTSFFDPAGGGDPILYQHL
>COLFB844-12|KJ963366|ZMUO.001319|Atheta_cinnamoptera
TLYFIFGAWAGMVGTSLSLLIRAELGNPGSLIGDDQIYNVIVTAHAFIMIFFMVMPIVIGGFGNWLVPLMLGAPDMAFPRMNNMSFWLLPPSLTLLLMSSMVESGAGTGWTVYPPLSSNIAHGGSSVDLAIFSLHLAGISSILGAVNFISTVINMRSTGI
SFDRMPLFVWSVAITALLLLLSLPVLAGAITMLLTDRNLNTSFFDPAGGGDPILYQHL
>COLFD288-12|KJ964672|ZMUO.004183|Oreodytes_septentrionalis
TLYFLFGAWSGMVGTSLSMLIRAELGNPGSLIGDDQIYNVIVTAHAFIMIFFMVMPIMIGGFGNWLVPLMLGAPDMAFPRMNNMSFWLLPPSLTLLLMSSMVESGAGTGWTVYPPLSAGIAHGGASVDLAIFSLHLAGISSILGAVNFITTIINMRSIG
MTFDRMPLFVWSVGITALLLLLSLPVLAGAITMLLTDRNLNTSFFDPAGGGDPILYQHL
>COLFD783-12|KJ963066|ZMUO.004773|Meligethes_carinulatus
TLYFIFGAWSGMVGTSLSMLIRTELGNPGSLIGNDQIYNVIVTAHAFVMIFFMVMPFMIGGFGNWLVPLMLGAPDMAFPRMNNMSFWLLPPSLSLLLMSSIVESGAGTGWTVYPPLSSNIAHGGASVDLAIFSLHLAGISSILGAVNFITTVINMRPSG
MNFDQMPLFVWSVVITAFLLLLSLPVLAGAITMLLTDRNLNTTFFDPSGGGDPILYQHL
>COLFE541-13|KJ964741|ZMUO.005386|Trachyphloeus_bifoveolatus
TLYFIFGSWSGMVGTSLSILIRTELGNPGSLIGDDQIYNMIVTAHAFIMIFFMVMPILIGGFGNWLIPLMLGAPDMAFPRLNNMSFWLLPPSLSLLLMSSIVDKGVGTGWTIYPPLSANIAHEGSSVDLAIFSLHMAGISSILGAINFISTIINMRPKGMSID
RMPLFIWSIKITAILLLLSLPVLAGAITMLLTDRNINTSFFDPAGGGDPILYQHL
>COLFA090-10|HM909106|MP00316|Xyleborus_cryptographus
TLYFIFGTWAGMVGTSLSILIRTELGTPGSLIMDDQIFNTIITAHAFIMIFFMVMPILIGGFGNWLVPLMLGAPDMAFPRLNNMSFWLLPPSLSLLLMSSIIDKGAGTGWTVYPPLAANIAHEGASIDLAIFSLHMSGASSILGAINFISTIINMHPMGMKP
EQLSLFTWAVKITAILLLLSLPVLAGGITMLLTDRNINTSFFDPAGGGDPILYQHL
>COLFD647-12|KJ965612|ZMUO.004447|Chaetocnema_aridula
TLYFIFGIWSGMVGTSMSILIRAELGSPGSLIGNDQIYNVIVTAHAFVMIFFMVMPIMIGGFGNWLVPLMIGAPDMAFPRMNNMSFWLLPPSLFLLLMSSMVESGAGTGWTVYPPLSSNIAHGGSSVDLAIFSLHLAGISSILGAINFITTIINMRPQGM
SFDQMPLFVWAVLITAILLLLSLPVLAGAITMLLTDRNLNTSFFDPMGGGDPILYQHL

>COLFD153-12|KJ965305|ZMUO.004048|Ilybius_chalconatus
TLYFIFGAWAGMVGTSLSMLIRAELGNPGSLIGDDQIYNVIVTAHAFVMIFFMVMPIMIGGFGNWLVPLMLGAPDMAFPRMNNMSFWLLPPSLSLLLMSSMVESGAGTGWTVYPPLSSGIAHSGASVDLAIFSLHLAGISSILGAVNFITTIINMRSV
GMTFDRMPLFVWSVGITALLLLLSLPVLAGAITMLLTDRNLNTSFFDPAGGGDPILYQHL
>COLFF957-13|KJ965091|ZMUO.007322|Stenus_bimaculatus
TLYFIFGSWSGMVGTSLSMLIRSELGNPGSLIGDDQIYNVIVTAHAFIMIFFMVMPIMIGGFGNWLIPLMLGAPDMAFPRMNNMSFWLLPPSLSLLLTSSIVESGAGTGWTVYPPLSSNIAHSGASVDLAIFSLHLAGISSILGAINFITTIFNMRSMKLQL
DCLSLFIWSVKITAFLLLLSLPVLAGAITMLLTDRNLNTSFFDPAGGGDPILYQHL
>COLFA541-12|KJ963379|ZMUO.000676|Aromia_moschata
TLYFIFGAWSGMVGTSLSMLIRSELGNPGSLIGDDQIYNVIVTAHAFIMIFFMVMPIMIGGFGNWLVPLMLGAPDMAFPRMNNMSFWLLPPSLTFLILSSIVESGAGTGWTVYPPLSSNIAHSGSSVDLAIFSLHLAGISSILGAVNFISTVINMRPSGMS
PDRMPLFVWAVVITAILLLLSLPVLAGAITMLLTDRNINTSFFDPAGGGDPILYQHL
>COLFB146-12|KJ966160|ZMUO.001476|Geodromicus_plagiatus
TLYFIFGAWAGMVGTSLSILIRSELGNPGSLIGDDQIYNVIVTAHAFVMIFFMVMPVVIGGFGNWLVPLMLGAPDMAFPRMNNMSFWLLPPSLTLLLMSSMVESGAGTGWTVYPPLSANIAHSGASVDLAIFSLHLAGISSILGAVNFITTVINMRSTG
ISFDRMPLFVWSVAITALLLLSLPVLAGAITMLLTDRNLNTSFFDPAGGGDPILYQHL
>COLFB474-12|KJ962194|ZMUO.001899|Trixagus_dermestoides
TLYFIFGSWAGMIGTAMSMIIRMELGTPGSLIGNDQIYNVIVTSHAFIMIFFMVMPIMIGGFGNWLVPLMIGAPDMAFPRLNNMSFWLLPPSMSLILTSNMTENGAGTGWTIYPPLSSNLAHSGPAVDFTIFSLHLAGMSSILGAINFITTSINMRSPLI
KPERLTLFAWSVSITALLLLLSLPVLAGAITMLLTDRNLNTSFFDPAGGGDPILYQHL
>COLFD133-12|KJ963183|ZMUO.004028|Gyrinus_opacus
TLYFIFGAWSGMVGTSLSMLIRAELGNPGSLIGDDQIYNVIVTAHAFIMIFFMVMPIMIGGFGNWLVPLMLGAPDMAFPRMNNMSFWLLPPSLTLLLMSSMVENGAGTGWTVYPPLSSNIAHGGASVDLAIFSLHLAGISSILGAVNFITTIINMRSV
GMTLDRMPLFVWSVGITALLLLLSLPVLAGAITMLLTDRNLNTSFFDPAGGGDPILYQHL
>COLFC100-12|KJ964447|ZMUO.002285|Bradycellus_caucasicus
TLYFIFGAWSGMVGTSLSMLIRAELGTPGALIGDDQIYNVIVTAHAFIMIFFMVMPIMIGGFGNWLVPLMLGAPDMAFPRMNNMSFWLLPPSLSLLLMSSMVEKGAGTGWTVYPPLSSGIAHSGASVDLAIFSLHLAGVSSILGAVNFITTIINMRSVG
MTFDRMPLFVWSVGITALLLLLSLPVLAGAITMLLTDRNLNTSFFDPAGGGDPILYQHL
>COLFD057-12|KJ963949|ZMUO.003952|Anaspis_thoracica
TLYFIFGAWSGMVGTSLSLLIRSELGTPGSLIGDDQIYNVIVTAHAFIMIFFMVMPILIGGFGNWLVPLMLGAPDMAFPRMNNMSFWLLPPSLTLLIMSSVVENGAGTGWTVYPPLAANIAHSGSSVDLAIFSLHLAGVSSILGAVNFITTVINMRPQG
MTLDRMPLFVWAVVITAILLLLSLPVLAGAITMLLTDRNLNTSFFDPAGGGDPILYQHL
>COLFE275-12|KJ962522|ZMUO.005120|Pterostichus_crenatus
TLYFIFGAWSGMVGTSLSMLIRAELGNPGSLIGDDQIYNVIVTAHAFVMIFFMVMPIMIGGFGNWLVPLMLGAPDMAFPRMNNMSFWLLPPSLTLLLMSSMVESGAGTGWTVYPPLSSGIAHAGASVDLAIFSLHLAGISSILGAVNFITTILNMRST
GMTFDRMPLFVWSVGITALLLLLSLPVLAGAITMLLTDRNLNTSFFDPAGGGDPILYQHL
>COLFE646-13|KJ963132|ZMUO.005491|Cicindela_sylvatica
TLYFIFGAWSGMVGTSLSMLIRAELGSPGSLIGDDQIYNVIVTAHAFVMIFFMVMPIMIGGFGNWLVPLMLGAPDMAFPRMNNMSFWLLPPSLTLLLMSSMVGDGAGTGWTVYPPLSAGIAHAGASVDLAIFSLHLAGVSSILGAVNFITTIINMRS
VGMTFDRMPLFVWSVGITALLLLLSLPVLAGAITMLLTDRNLNTSFFDPAGGGDPILYQHL
>COLFD025-12|KJ965298|ZMUO.003920|Haliplus_ruficollis
TLYFIFGAWAGMVGTSLSMLIRAELGTPGSLIGDDQIYNVIVTAHAFIMIFFMVMPIMIGGFGNWLVPLMLGAPDMAFPRMNNMSFWLLPPSLSLLLMSSMVENGAGTGWTVYPPLSAGIAHSGASVDLAIFSLHLAGISSILGAVNFITTIINMRSIG
MTFDRMPLFVWSVGITALLLLLSLPVLAGAITMLLTDRNLNTSFFDPAGGGDPILYQHL
>COLFE1300-13|KJ963644|ZMUO.007095|Tetartopeus_quadratus
TLYFIFGAWAGMVGTSLSLLIRTELANPGSLIGDDQIYNVIVTAHAFVMIFFMVMPIVIGGFGNWLVPLMLGAPDMAFPRMNNMSFWLLPPSLSLLLASSMVESGAGTGWTVYPPLSSNIAHGGASVDLAIFSLHLAGISSILGAVNFITTIINMRSPGM
LYERMPLFVWSVGITALLLLLSLPVLAGAITMLLTDRNLNTSFFDPAGGGDPILYQHL
>COLFG076-13|KJ963848|ZMUO.007581|Trachodes_hispidus
TLYFIFGSWSGMVGTSLSMMIRTELGTPGSVIGNDQIYNTIVTAHAFIMIFFMVMPIMIGGFGNWLVPLMLGAPDMAFPRLNNMSFWLLPPSLTLLLMSSIIDKGAGTGWTVYPPLSTNIAHEGMSVDLAIFSLHLAGISSILGAMNFISTVINMHPTG
MKLDQLPLFVWSVKITAILLLLSLPVLAGAITMLLTDRNINTTFFDPAGGGDPILYQHL
>COLFC652-12|KJ967351|ZMUO.003217|Gonioctena_linnaeana
TLYFIFGIWAGMVGTSLSILIRAELGNPGTLIGNDQIYNVIVTAHAFIMIFFMVMPIMIGGFGNWLVPLMIGAPDMAFPRMNNMSFWLLPPSLFFLIMSSIVESGAGTGWTVYPPLSANIAHSGSSVDLAIFSLHLAGISSILGAINFITTIINMRPMGMS
MDRMPLFVWAVLITAILLLLSLPVLAGAITMLLTDRNLNTSFFDPAGGGDPILYQHL
>COLFF297-13|KJ962840|ZMUO.005997|Diacanthous_undulatus
TLYFPIGAWAGMLGTSLSLLIRAELGNPGSLIGNDQIYNVIVTAHAFIMIFFMVMPIMIGGFGNWLVPLMLGAPDMAFPRMNNMSFWFLPPSLSLLLMSSIVENGAGTGWTVYPPLSANIAHSGSSVDLAIFSLHLAGISSILGAVNFISTVINMRSTGIT
FDRMPLFVWAVAITALLLLLSLPVLAGAITMLLTDRNLNTSFFDPAGGGDPILYQHL
>COLFC672-12|KJ962012|ZMUO.003237|Eusphalerum_minutum

TLYFIFGAWAGMVGTSLSMLIRAELGNPGSLIGDDQIYNVIVTAHAFIMIFFMVMPIVIGGFGNWLVPLMLGAPDMAFPRMNNMSFWLLPPSLTLLLMSSMVESGAGTGWTVYPPLSSNIAHGGASVDLAIFSLHLAGISSILGAVNFITTVINMRSTG
MTFDRMPLFIWSVVITALLLLLSLPVLAGAITMLLTDRNLNTSFFDPAGGGDPILYQHL
>COLFC576-12|KJ966265|ZMUO.003141|Berosus_luridus
TLYFIFGAWAGMVGTSLSLLIRAELGNPGTLIGDDQIYNVIVTAHAFIMIFFMVMPIMIGGFGNWLVPLMLGAPDMAFPRMNNMSFWLLPPSLTLLLSSMVESGAGTGWTVYPPLSSNIAHGGASVDLAIFSLHLAGISSILGAVNFITTVINMRAPN
MTYDRMPLFVWSVAITALLLLLSLPVLAGAITMLLTDRNLNTSFFDPAGGGDPILYQHL
>COLFF027-13|KJ964996|ZMUO.005727|Tachyporus_scitulus
TLYFIFGAWSGMVGTSLSLLIRAELGNPGSLIGDDQIYNVIVTAHAFIMIFFMVMPIVIGGFGNWLVPLMLGAPDMAFPRMNNMSFWLLPPSLTLLLMSSMVESGAGTGWTVYPPLSANIAHSGPSVDLAIFSLHLAGISSILGAVNFITTVINMRASG
MYFDRMPLFIWSVAITALLLLLSLPVLAGAITMLLTDRNLNTSFFDPAGGGDPILYQHL
>COLFF857-13|KJ961958|ZMUO.006652|Aphodius_paykulli
TLYFLFGSWAGMVGTSLSLLIRAELGNPGSLIGDDQIYNVIVTAHAFVMIFFMVMPILIGGFGNWLVPLMLGAPDMAFPRMNNMSFWLLPPSLTLLLMSSMVESGAGTGWTVYPPLSSNIAHGGASVDLAIFSLHLAGISSILGAVNFITTVINMRSPG
LTFDRMPLFVWSVAITALLLLLSLPVLAGAITMLLTDRNLNTSFFDPAGGGDPILYQHL
>COLFD504-12|KJ965145|ZMUO.004304|Pissodes_piniphilus
TLYFIFGTWSGMIGTSLSIIIRTELGTPSSMIGDDQIYNTIVTAHAFIMIFFMVMPIMIGGFGNWLIPLMLGAPDMAFPRLNNMSFWLLPPSIMLLLMSSITDKGAGTGWTVYPPLSTNIAHEGPSVDLAIFSLHMAGISSILGAVNFISTIINMRPIGMNP
DQMSLFIWAVKITAILLLLSLPVLAGAITMLLTDRNINTSFFDPAGGGDPILYQHL
>COLFF757-13|KJ964339|ZMUO.006457|Meligethes_ruficornis
TLYFIFGTWSGMIGTSLSMLIRTELGNPGSLIGNDQIYNVIVTAHAFVMIFFMVMPFMIGGFGNWLVPLMLGAPDMAFPRMNNMSFWLLPPSLSLLLMSSIVESGAGTGWTVYPPLSSNIAHSGASVDLAIFSLHLAGISSILGAVNFITTVINMRPVG
MTFDRMPLFVWAVAITALLLLLSLPVLAGAITMLLTDRNLNTTFFDPSGGGDPILYQHL
>COLFG044-13|KJ967420|ZMUO.007549|Monochamus_urussovii
TLYFIFGAWSGMVGTSLSLLIRSELGMPGSLIGNDQIYNVIVTAHAFIMIFFMVMPIMIGGFGNWLVPLMLGAPDMAFPRMNNMSFWLLPPSLTLLIMSSIVENGAGTGWTVYPPLAANVAHSGSSVDLAIFSLHLAGVSSILGAVNFITTVINMRPSG
MNLDRLPLFVWAVKITAILLLLSLPVLAGAITMLLTDRNLNTSFFDPAGGGDPILYQHL
>COLFF808-13|KJ965229|ZMUO.006128|Elmis_aenea
TLYFILGSWSGMLGMALSLLIRAELGTPGSLIGDDQIYNVIVTAHAFVMIFFMVMPIMIGGFGNWLVPLMLGAPDMAFPRMNNMSFWLLPPSLSLLLMSSIVESGVGTGWTVYPPLSANIAHSGSSVDLAIFSLHLAGISSILGAVNFITTVINMRSPG
MTFDRMPLFVWSVAITALLLLLSLPVLAGAITMLLTDRNLNTSFFDPAGGGDPILYQHL
>COLFF354-13|KJ963645|ZMUO.006054|Agonum_consimile
TLYFIFGAWAGMVGTSLSMLIRAELGNPGALIGDDQIYNVIVTAHAFIMIFFMVMPIMIGGFGNWLVPLMLGAPDMAFPRMNNMSFWLLPPSLTLLLMSSLVESGAGTGWTVYPPLSSGIAHAGASVDLAIFSLHLAGISSILGAVNFITTIINMRSVG
MTFDRMPLFVWSVGITALLLLLSLPVLAGAITMLLTDRNLNTSFFDPAGGGDPILYQHL
>COLFE523-13|KJ962690|ZMUO.005368|Anisotoma_humeralis
TLYFIFGAWSGMVGTSLSILIRAELGTPGSLIGDDQIYNVIVTAHAFVMIFFMVMPIMIGGFGNWLVPLMLGAPDMAFPRMNNMSFWLLPPSLTLLLMSSMVENGAGTGWTVYPPLSANISHSGSSVDLAIFSLHLAGISSILGAVNFITTVINMRSIG
MTFDKMPLFVWSVVITALLLLLSLPVLAGAITMLLTDRNLNTSFFDPAGGGDPILYQHL
>COLFE870-13|KJ966086|ZMUO.006190|Ampedus_suecicus
TLYFIFGAWAGMLGTSLSLLIRAELGNPGSLIGNDQIYNVIVTAHAFIMIFFMVMPIMIGGFGNWLVPLMLGAPDMAFPRMNNMSFWLLPPSLSLLLMSSIVENGAGTGWTVYPPLSSNIAHSGSSVDLAIFSLHLAGISSILGAVNFISTVINMRSTGIT
FDRMPLFVWAVAITALLLLLSLPVLAGAITMLLTDRNLNTSFFDPAGGGDPILYQHL
>COLFC503-12|KJ965054|ZMUO.003068|Cryptophagus_dorsalis
TLYFIFGAWASMVGTSLSMLIRSELGMPGSLIGDDQIYNVIVTAHAFVMIFFMVMPIMIGGFGNWLIPLMLGAPDMAFPRMNNMSFWLLPPSLSLLLMSSITEKGVGTGWTVYPPLSSNIAHGGSSVDLAIFSLHLAGISSILGAVNFISTIMNMHPKG
MTLDRMPLFVWAVMITAILLLLSLPVLAGAITMLLTDRNLNTSFFDPAGGGDPILYQHL
>COLFB035-12|KJ964200|ZMUO.001365|Philonthus_subvirescens
TLYFIFGSWAGMVGTSLSLLIRAELGNPGTLIGDDQIYNVIVTAHAFIMIFFMVMPIVIGGFGNWLVPLMLGAPDMAFPRMNNMSFWLLPPSLTLLLMSSLVESGAGTGWTVYPPLSSNMAHGGASVDLAIFSLHLAGISSILGAVNFITTVINMRSTG
MNFDRMPLFVWSVVITALLLLLSLPVLAGAITMLLTDRNINTTFFDPAGGGDPILYQHL
>COLFE333-12|KJ963715|ZMUO.004893|Triplax_rufipes
TLYFIFGMWAGMVGTALSVLIRTELGNPGSLIGNDQIYNVIVTAHAFIMIFFMVMPIMMGGFGNWLVPLMLGAPDMAFPRLNNMSFWLLPPSLSLLIMSSIVETGAGTGWTVYPPLSSNIAHSGASVDLAIFSLHLAGISSILGAMNFITTMMNMRP
SGMTMDQMPLFVWAVFITSILLLLSLPVLAGGITMLLTDRNINTSFFDPAGGGDPILYQHL
>COLFD583-12|KJ964496|ZMUO.004383|Demetrias_imperialis
TLYFIFGAWAGMVGTSLSMLIRAELGNPGALIGDDQIYNVIVTAHAFIMIFFMVMPIMIGGFGNWLVPLMLGAPDMAFPRMNNMSFWLLPPSLSLLMMSSMVESGAGTGWTVYPPLSSNIAHAGASVDLAIFSLHLAGVSSILGAVNFITTIINMRSI
GMTFDRMPLFVWSVGITALLLLLSLPVLAGAITMLLTDRNLNTSFFDPAGGGDPILYQHL
>COLFF521-13|KJ964210|ZMUO.006601|Hydroporus_palustris
TLYFLFGAWSGMVGTSLSMLIRAELGNPGSLIGDDQIYNVIVTAHAFIMIFFMVMPIMIGGFGNWLVPLMLGAPDMAFPRMNNMSFWLLPPSLTLLLMSSMVENGAGTGWTVYPPLSSGIAHSGASVDLAIFSLHLAGVSSILGAVNFITTIINMRSI
GMTFDRMPLFVWSVGITALLLLLSLPVLAGAITMLLTDRNLNTSFFDPAGGGDPILYQHL

>COLFF827-13|KJ961903|ZMUO.006147|Amara_aenea
TLYFIFGAWSGMVGTSLSMLIRAELGNPGALIGDDQIYNVIVTAHAFVMIFFMVMPIMIGGFGNWLVPLMLGAPDMAFPRMNNMSFWLLPPSLTLLLMSSMVESGAGTGWTVYPPLSSGIAHAGASVDLAIFSLHLAGISSILGAVNFITTIINMRSV
GMTFDRMPLFVWSVGITALLLLLSLPVLAGAITMLLTDRNLNTSFFDPAGGGDPILYQHL
>COLFD501-12|KJ965833|ZMUO.004301|Carabus_cancellatus
TLYFIFGAWSGMVGTSLSMLIRAELGNPGSLIGDDQIYNVIVTAHAFVMIFFMVMPIMIGGFGNWLVPLMLGAPDMAFPRMNNMSFWLLPPSLTLLLMSSMVEKGAGTGWTVYPPLSSGIAHSGASVDLAIFSLHLAGISSILGAVNFITTIINMRSV
GMTFDRMPLFVWSVGITALLLLLSLPVLAGAITMLLTDRNLNTSFFDPAGGGDPILYQHL
>COLFE846-13|KJ963185|ZMUO.005691|Atheta_volans
TLYFIFGAWAGMVGTSLSLLIRAELGNPGSLIGDDQIYNVIVTAHAFIMIFFMVMPIVIGGFGNWLVPLMLGAPDMAFPRMNNMSFWLLPPSLTLLLMSSMVESGAGTGWTVYPPLSSNIAHGGSSVDLAIFSLHLAGISSILGAVNFISTVINMRSTGI
SFDRMPLFVWSVAITALLLLLSLPVLAGAITMLLTDRNLNTSFFDPAGGGDPILYQHL
>COLFE1084-13|KJ964698|ZMUO.006879|Atomaria_wollastoni
TLYFIFGAWAGMVGTSLSLLIRAELGTPGSLIGDDQIYNVIVTAHAFIMIFFMVMPMMIGGFGNWLVPLMLGAPDMAFPRLNNMSFWLLPPSLMFLLMSSLVEKGAGTGWTVYPPLSSNVAHAGASVDLAIFSLHMAGISSILGAVNFITTVINMRPK
GMKFDRLPLFVWAVKITAILLLLSLPVLAGAITMLLTDRNMNTSFFDPAGGGDPILYQHL
>COLFD497-12|KJ965763|ZMUO.004297|Agabus_bipustulatus
TLYFIFGAWAGMVGTSLSMLIRAELGNPGSLIGDDQIYNVIVTAHAFVMIFFMVMPIMIGGFGNWLVPLMLGAPDMAFPRMNNMSFWLLPPSLTLLLMSSMVESGAGTGWTVYPPLSSGIAHGGASVDLAIFSLHLAGISSILGAVNFITTIINMRSV
GMTFDRMPLFVWSVGITALLLLLSLPVLAGAITMLLTDRNLNTSFFDPAGGGDPILYQHL
>COLFE090-12|KJ965674|ZMUO.005315|Lagria_hirta
ILYFIFGSWAGMVGTSLSLLIRVELSNPGSFIGDDQVYNVVVTAHAFIMIFFMVMPIMIGGFGNWLVPLMLGAPDMAFPRMNNMSFWLLPPSLSLLLMSSLVESGAGTGWTVYPPLSSNLAHSGSSVDLAIFSLHLAGISSILGAVNFITTVINMRPLG
MKFDRLPLFVWSVMITAILLLLSLPVLAGAITMLLTDRNINTSFFDPSGGGDPILYQHL
>COLFE242-12|KJ965967|ZMUO.005087|Bembidion_tetracolum
TLYFIFGAWSGMVGTSLSMLIRAELGNPGSLIGDDQIYNVIVTAHAFVMIFFMVMPILIGGFGNWLVPLMLGAPDMAFPRMNNMSFWLLPPSLTLLLMSSMVESGAGTGWTVYPPLSSSIAHSGASVDLAIFSLHLAGVSSILGAVNFITTIINMRSVG
MSFDRMPLFVWSVGITALLLLLSLPVLAGAITMLLTDRNLNTSFFDPAGGGDPILYQHL
>COLFE1336-13|KJ961910|ZMUO.007131|Bledius_opacus
TLYFIFGAWAGMVGTSLSMLIRAELSTPGSLIGDDQIYNVIVTAHAFVMIFFMVMPIVIGGFGNWLVPLMLGAPDMAFPRMNNMSFWLLPPSLTLLLMSSLVESGVGTGWTVYPPLSSNIAHSGSSVDLAIFSLHLAGISSILGAVNFISTIINMRSIGITF
DRMPLFVWSVKITAILLLLSLPVLAGAITMLLTDRNLNTSFFDPAGGGDPILYQHL
>COLFE054-12|KJ964652|ZMUO.005279|Dasytes_obscurus
TLYFIFGAWSGMVGMSLSLLIRSELNNPGTLIGNDQIYNVIVTAHAFIMIFFMVMPILIGGFGNWLVPLMLGAPDMAFPRMNNMSFWLLPPSLTLLLMSSMVEQGAGTGWTVYPPLSANIAHSGASVDLAIFSLHLAGISSILGAVNFITTVINMRPVG
MTLDRTPLFVWAVAITALLLLLSLPVLAGAITMLLTDRNLNTSFFDPAGGGDPILYQHL
>COLFA712-12|KJ966790|ZMUO.000847|Gyrohypnus_angustatus
TLYFIFGAWSGMVGTSLSLLIRAELGNPGTLIGDDQIYNVIVTAHAFIMIFFMVMPIVIGGFGNWLVPLMLGAPDMAFPRMNNMSFWLLPPSLTLLLSSSLVESGAGTGWTVYPPLSSNIAHSGSSVDLAIFSLHLAGVSSILGAVNFITTVINMRSSGMS
FDRMPLFVWSVAITALLLLLSLPVLAGAITMLLTDRNLNTSFFDPTGGGDPILYQHL
>COLFF069-13|KJ961853|ZMUO.005769|Platambus_maculatus
TLYFIFGAWAGMVGTSLSMLIRAELGNPGSLIGDDQIYNVIVTAHAFVMIFFMVMPIMIGGFGNWLVPLMLGAPDMAFPRMNNMSFWLLPPSLSLLLMSSLVENGAGTGWTVYPPLSSGIAHGGASVDLAIFSLHLAGISSILGAVNFITTIINMRSVG
MTFDRMPLFVWSVGITALLLLLSLPVLAGAITMLLTDRNLNTSFFDPAGGGDPILYQHL
>COLFD042-12|KJ962759|ZMUO.003937|Amara_bifrons
TLYFIFGAWSGMVGTSLSMLIRAELGNPGALIGDDQIYNVIVTAHAFVMIFFMVMPIMIGGFGNWLVPLMLGAPDMAFPRMNNMSFWLLPPSLTLLLMSSLVESGAGTGWTVYPPLSSNIAHAGASVDLAIFSLHLAGISSILGAVNFITTIINMRSVG
MTFDRMPLFVWSVGITALLLLLSLPVLAGAITMLLTDRNLNTSFFDPAGGGDPILYQHL
>COLFB831-12|KJ962166|ZMUO.001306|Omalium_strigicolle
TLYFIFGAWAGMVGTSLSLLIRAELGNPGSLIGDDQIYNVIVTAHAFIMIFFMVMPIVIGGFGNWLVPLMLGAPDMAFPRMNNMSFWLLPPSLTLLLMSSMVESGAGTGWTVYPPLSSNIAHSGSSVDLAIFSLHLAGISSILGAVNFITTVINMRAMG
MTFDRMPLFVWSVVITALLLLLSLPVLAGAITMLLTDRNLNTSFFDPAGGGDPILYQHL
>COLFF492-13|KJ962094|ZMUO.006572|Typhaea_haagi
TLYFIFGAWSGMVGTSLSLLIRSELGNPGSLIGDDQIYNVIVTAHAFIMIFFMVMPIMMGGFGNWLVPLMLGAPDMAFPRMNNMSFWLLPPSLTLLIMSSVVENGAGTGWTVYPPLSSNIAHSGSSVDLAIFSLHLAGISSILGAVNFITTIINMRPMG
MSFDRMPLFVWAVAITALLLLLSLPVLAGAITMLLTDRNLNTSFFDPAGGGDPILYQHL
>COLFC466-12|KJ966497|ZMUO.003031|Liotrichus_affinis
TLYFLFGAWAGMLGTSLSLLIRAELGNPGSLIGNDQIYNVIVTAHAFIMIFFMVMPIMIGGFGNWLVPLMLGAPDMAFPRMNNMSFWFLPPSLSLLLMSSIVENGAGTGWTVYPPLSANIAHSGSSVDLAIFSLHLAGISSILGAVNFISTVINMRSTGIT
FDRMPLFVWAVAITALLLLLSLPVLAGAITMLLTDRNLNTSFFDPAGGGDPILYQHL
>COLFE1188-13|KJ967186|ZMUO.006983|Philonthus_nigrita

TLYFIFGSWAGMVGTSLSLLIRAELGNPGTLIGDDQIYNVIVTAHAFIMIFFMVMPIVIGGFGNWLIPLMLGAPDMAFPRMNNMSFWLLPPSLTLLLMSSMVESGAGTGWTVYPPLSSNMAHGGASVDLAIFSLHLAGISSILGAVNFISTVINMRSTG
MTFDRMPLFIWSVAITALLLLLSLPVLAGAITMLLTDRNLNTTFFDPAGGGDPILYQHL
>COLFD649-12|KJ965238|ZMUO.004449|Dicheirotrichus_placidus
TLYFIFGAWSGMVGTSLSMLIRAELGTPGALIGDDQIYNVIVTAHAFVMIFFMVMPIMIGGFGNWLVPLMLGAPDMAFPRMNNMSFWLLPPSLTLLLMSSMVEKGAGTGWTVYPPLSSGIAHGGASVDLAIFSLHLAGISSILGAVNFITTIINMRSV
GMTFDRMPLFVWSVGITALLLLLSLPVLAGAITMLLTDRNLNTSFFDPAGGGDPILYQHL
>COLFF089-13|KJ966902|ZMUO.005789|Stenus_bohemicus
TLYFIFGVWAGMVGTSLSLLIRTELGNPGSLIGDDQIYNVIVTAHAFIMIFFMVMPVMIGGFGNWLIPLMLGAPDMAFPRMNNMSFWLLPPSLSLLLMSSIVESGAGTGWTVYPPLSSNIAHSGASVDLAIFSLHLAGISSILGAINFITTIFNMRTMKIQ
LDCLPLFIWSVGITALLLLLSLPVLAGAITMLLTDRNINTSFFDPAGGGDPILYQHL
>COLFE620-13|KJ962148|ZMUO.005465|Arpedium_quadrum
TLYFIFGAWAGMVGTSLSILIRAELGNPGTLIGDDQIYNVIVTAHAFVMIFFMVMPIVIGGFGNWLVPLMLGAPDMAFPRMNNMSFWLLPPSLTLLLMSSMVESGAGTGWTVYPPLSSNIAHGGSSVDLAIFSLHLAGISSILGAVNFITTVINMRATG
MTFDRMPLFVWSVAITALLLLLSLPVLAGAITMLLTDRNLNTSFFDPAGGGDPILYQHL
>COLFB765-12|KJ964536|ZMUO.001240|Coccinella_undecimpunctata
TLYFLFGMWAGMIGTSLSILIRLELGTTNSLIGNDQIYNVIVTAHAFIMIFFMVMPIMIGGFGNWLVPLMIGAPDMAFPRLNNMSFWLLPPALTLLIISSLVEMGAGTGWTVYPPLSSNLAHNGPSVDLVIFSLHLAGISSILGAVNFISTIMNMRPFGM
NLDKTPLFVWSVLITAILLLLSLPVLAGAITMLLTDRNINTSFFDPTGGGDPILYQHL
>COLFC543-12|KJ966354|ZMUO.003108|Thanatophilus_lapponicus
TLYFIFGAWAGMVGMSLSILIRAELSTPGTLLGDDQMYNVIVTAHAFIMIFFMVMPIVIGGFGNWLVPLMLGAPDMAFPRMNNMSFWLLPPSLSLLLVSSMVESGAGTGWTVYPPLSSNIAHGGSSVDLAIFSLHLAGISSILGAVNFITTIINMRSSG
MTFDRMPLFVWSVAITALLLLLSLPVLAGAITMLLTDRNINTSFFDPAGGGDPILYQHL
>COLFB196-12|KJ964095|ZMUO.001526|Stenus_nitens
TLYFIFGAWAGMVGTSLSLLIRAELGNPGSLIGDDQIYNVIVTAHAFVMIFFMVMPIMIGGFGNWLVPLMLGAPDMAFPRMNNMSFWLLPPSLSLLLMSSIVENGAGTGWTVYPPLSSNIAHSGASVDLAIFSLHLAGISSILGAINFITTIFNMRTMKL
QLDCLPLFVWSVAITALLLLLSLPVLAGAITMLLTDRNLNTSFFDPAGGGDPILYQHL
>COLFE1052-13|KJ962350|ZMUO.006847|Agabus_sturmii
TLYFIFGAWAGMVGTSLSMLIRAELGNPGSLIGDDQIYNVIVTAHAFVMIFFMVMPIMIGGFGNWLVPLMLGAPDMAFPRMNNMSFWLLPPSLTLLLMSSMVEKGAGTGWTVYPPLSSGIAHGGASVDLAIFSLHLAGISSILGAVNFITTIINMRSV
GMTFDRMPLFVWSVGITALLLLLSLPVLAGAITMLLTDRNLNTSFFDPAGGGDPILYQHL
>COLFD434-12|KJ961956|ZMUO.003854|Sciaphilus_asperatus
TLYFIFGSWSGMIGTSLSMLIRTELGNPGSLIGDDQIYNVIVTAHAFIMIFFMVMPIMIGGFGNWLVPLMLGAPDMAFPRLNNMSFWLLPPSLSLLLMSSIVDKGAGTGWTVYPPLSANIAHEGSSVDLAIFSLHMAGVSSILGTINFISTVINMRPLGM
TPERMPLFVWAVKITAILLLLSLPVLAGAITMLLTDRNVNTSFFDPAGGGDPILYQHL
>COLFF616-13|KJ966428|ZMUO.006506|Poecilus_cupreus
TLYFIFGAWSGMVGTSLSMLIRAELGNPGSLIGDDQIYNVIVTAHAFVMIFFMVMPIMIGGFGNWLVPLMLGAPDMAFPRMNNMSFWLLPPSLTLLLMSSMVESGAGTGWTVYPPLSSGIAHAGASVDLAIFSLHLAGVSSILGAVNFITTIINMRSI
GMTFDRMPLFVWSVGITALLLLLSLPVLAGAITMLLTDRNLNTSFFDPAGGGDPILYQHL
>COLFD622-12|KJ966252|ZMUO.004422|Paranchus_albipes
TLYFIFGAWSGMVGTSLSMLIRAELGNPGALIGDDQIYNVIVTAHAFIMIFFMVMPIMIGGFGNWLVPLMLGAPDMAFPRMNNMSFWLLPPSLTLLLMSSMVESGAGTGWTVYPPLSSGIAHAGASVDLAIFSLHLAGVSSILGAVNFITTIINMRSM
GMTFDRMPLFVWSVGITALLLLLSLPVLAGAITMLLTDRNLNTSFFDPAGGGDPILYQHL
>COLFE1321-13|KJ965810|ZMUO.007116|Asaphidion_pallipes
TLYFIFGAWAGMVGTSLSMLIRAELGNPGSLIGDDQIYNVIVTAHAFVMIFFMVMPILIGGFGNWLVPLMLGAPDMAFPRMNNMSFWLLPPSLTLLLMSSMVESGAGTGWTVYPPLSSSIAHSGASVDLAIFSLHLAGVSSILGAVNFITTIINMRSIG
MTFDRMPLFVWSVGITALLLLLSLPVLAGAITMLLTDRNINTSFFDPAGGGDPILYQHL
>COLFF794-13|KJ962715|ZMUO.006114|Eucnecosum_brachypterum
TLYFIFGAWAGMVGTSLSILIRAELGNPGTLIGDDQIYNVIVTAHAFVMIFFMVMPIVIGGFGNWLVPLMLGAPDMAFPRMNNMSFWLLPPSLTLLLMSSMVESGAGTGWTVYPPLSSNIAHGGSSVDLAIFSLHLAGISSILGAVNFITTVINMRTTG
MTFDRMPLFVWSVAITALLLLLSLPVLAGAITMLLTDRNLNTSFFDPAGGGDPILYQHL
>COLFF100-13|KJ962817|ZMUO.003734|Rhacopus_sahlbergi
TLYFIFGVWSGMIGTSLSLLIRLELGMPGTLIGNDQLFNVIVTAHAFIMIFFMVMPVMMGGFGNWLIPLMLSSPDMAFPRMNNMSFWLLPPALSLLLLSSMVESGAGTGWTVYPPLASNIAHSGSSVDLAIFSLHLAGISSILGAINFISTIFNMRPLMM
TLDKMPLFVWSVLLTAVLLLLSLPVLAGAITMLLTDRNLNTSFFEPSGGGDPILYQHL
>LEFIJ1852-13|KJ966668|ZMUO.004607|Rhinoncus_castor
TLYFIFGSWAGTVGTSLSMLIRTELGTPGSLIGNDQIYNSIVTAHAFIMIFFMVMPILIGGFGNWLVPLMLGAPDMAFPRLNNMSFWLLPPSIMLLLMSSIVNKGAGTGWTVYPPLSSNITHEGASVDLAIFSLHMAGISSILGAINFISTIMNMRPQGM
SYDKMPLFSWAVLITAILLLLSLPVLAGAITMLLTDRNINTSFFDPAGGGDPILYQHL
>COLFF284-13|KJ964848|ZMUO.005889|Hypera_arator
TLYFIFGTWAGTVGTSLSILIRTELGNPGSLIGNDQIYNTIVTAHAFIMIFFMVMPIMIGGFGNWLVPLMLGAPDMAFPRLNNMSFWLLPPSLSLLLMSSMIDKGAGTGWTVYPPLSSNIAHEGSSVDLAIFSLHMAGVSSILGAINFISTVLNMRPSGM
SLDKMALFIWAVKITAILLLLSLPVLAGAITMLLTDRNINTSFFDPAGGGDPILYQHL

>COLFB451-12|KJ963768|ZMUO.001876|Bembidion_bruxellense
TLYFIFGAWSGMVGTSLSMLIRAELGNPGSLIGDDQIYNVIVTAHAFVMIFFMVMPILIGGFGNWLVPLMLGAPDMAFPRMNNMSFWLLPPSLTLLLMSSMVESGAGTGWTVYPPLSSSIAHSGASVDLAIFSLHLAGVSSILGAVNFITTIINMRSIG
MSFDRMPLFVWSVGITALLLLLSLPVLAGAITMLLTDRNLNTSFFDPAGGGDPILYQHL
>COLFG160-13|KJ966882|ZMUO.007855|Gyrophaena_boleti
TLYFIFGAWSGMVGTSLSLLIRAELGNPGSLIGDDQIYNVIVTAHAFVMIFFMVMPIVIGGFGNWLIPLMLGAPDMAFPRMNNMSFWLLPPSLSLLLMSSMVESGAGTGWTVYPPLSSNIAHGGASVDLAIFSLHLAGISSILGAVNFISTIINMRSIGM
TFDRMPLFIWSVAITALLLLLSLPVLAGAITMLLTDRNLNTSFFDPAGGGDPILYQHL
>COLFE1169-13|KJ965287|ZMUO.006964|Phaedon_cochleariae
MLYFIFGIWAGMVGTSLSIMIRSELGNPGTLIGNDQIYNVIVTAHAFVMIFFMVMPLMIGGFGNWLVPLMIGAPDMAFPRMNNMSFWLLPPSLLLLLMSSVVENGAGTGWTVYPPLSANIAHSGSSVDLAIFSLHLAGISSILGAINFITTVINMRPEG
MTLEQIPLFVWAVLITAILLLLSLPVLAGAITMLLTDRNLNTSFFDPAGGGDPILYQHL
>COLFG099-13|KJ963522|ZMUO.007794|Gyrophaena_affinis
TLYFIFGAWSGMVGTSLSLLIRAELGNPGSLIGDDQIYNVIVTAHAFIMIFFMVMPVVIGGFGNWLVPLMLGAPDMAFPRMNNMSFWLLPPSLSLLLMSSMVESGAGTGWTVYPPLSSNIAHGGASVDLAIFSLHLAGISSILGAVNFISTIINMRALG
MSFDRMPLFVWSVAITALLLLLSLPVLAGAITMLLTDRNLNTSFFDPAGGGDPILYQHL
>COLFC669-12|KJ965248|ZMUO.003234|Ilybius_sp.
TLYFIFGAWAGMVGTSLSMLIRAELGNPGSLIGDDQIYNVIVTAHAFVMIFFMVMPIMIGGFGNWLVPLMLGAPDMAFPRMNNMSFWLLPPSLSLLLMSSMVESGAGTGWTVYPPLSAGIAHSGASVDLAIFSLHLAGISSILGAVNFITTIINMRSV
GMTFDRMPLFVWSVGITALLLLLSLPVLAGAITMLLTDRNLNTSFFDPAGGGDPILYQHL
>COLFD476-12|KJ963394|ZMUO.004276|Cryptocephalus_bipunctatus
TLYFLFGAWAGMIGTSLSLLIRIELGNPGSLIGNDQIYNTIVTAHAFIMIFFMVMPIMIGGFGNWLVPLMLGAPDMAFPRMNNMSFWLLPPSLMLLLMSSIVENGAGTGWTVYPPLSATIAHTGPSVDLAIFSLHLAGASSIMGAINFISTVINMRPQG
MMMDRTPLFVWAVLITAILLLLSLPVLAGAITMLLTDRNINTSFFDPAGGGDPILYQHL
>COLFB029-12|KJ965697|ZMUO.001359|Amara_fulva
TLYFIFGAWSGMVGTSLSMLIRTELGNPGALIGDDQIYNVIVTAHAFVMIFFMVMPIMIGGFGNWLVPLMLGAPDMAFPRMNNMSFWMLPPSLTLLLMSSMVESGAGTGWTVYPPLSSGIAHAGASVDLAIFSLHLAGISSILGAVNFITTIINMRSV
GMTFDRMPLFVWSVGITALLLLLSLPVLAGAITMLLTDRNLNTSFFDPAGGGDPILYQHL
>COLFF511-13|KJ967393|ZMUO.006591|Bembidion_litorale
TLYFIFGAWAGMVGTSLSMLIRAELGNPGSLIGDDQIYNVIVTAHAFVMIFFMVMPILIGGFGNWLVPLMLGAPDMAFPRMNNMSFWLLPPSLTLLLMSSMVESGAGTGWTVYPPLSSSIAHSGASVDLAIFSLHLAGVSSILGAVNFITTIINMRSM
GMTFDRMPLFVWSVGITALLLLLSLPVLAGAITMLLTDRNLNTSFFDPAGGGDPILYQHL
>COLFD737-12|KJ963272|ZMUO.004727|Acanthocinus_aedilis
TLYFIFGAWSGMVGTSLSMLIRSELGNPGTLIGNDQIYNTIVTAHAFIMIFFMVMPIMIGGFGNWLIPLMLGAPDMAFPRMNNMSFWLLPPSLTLLIMSSIVENGAGTGWTVYPPLSSNIAHSGSSVDLAIFSLHLAGISSILGAVNFITTIINMRPMGM
TFDRMPLFVWAVKITAILLLLSLPVLAGAITMLLTDRNINTSFFDPAGGGDPILYQHL
>COLFE304-12|KJ966533|ZMUO.004864|Anthrenus_museorum
TLYFIFGAWAGMVGTSLSILIRTELGNPGSLIGDDQTFNVIVTAHAFIMIFFMVMPMMIGGFGNWLVPLMLGAPDMAFPRMNNMSFWLLPPSLSLLLLSSMVESGAGTGWTVYPPLSSNIAHGGASVDLAIFSLHLAGISSILGAVNFITTAINMRAA
GMTPERMPLFVWSVAITAILLLLSLPVLAGAITMLLTDRNLNTSFFDPAGGGDPILYQHL
>COLFC713-12|KJ966743|ZMUO.003278|Platycerus_caprea
TLYFLLGSWSGMVGTSLSMLIRAELGNPGSLIGDDQIYNVIVTAHAFVMIFFMVMPILIGGFGNWLVPLMLGAPDMAFPRMNNMSFWLLPPSLTLLLLSSMVEKGAGTGWTVYPPLSSNIAHGGASVDLAIFSLHLAGISSILGAVNFITTVINMRSIG
MTFDRMPLFVWSVVLTAILLLLSLPVLAGAITMLLTDRNINTSFFDPAGGGDPILYQHL
>COLFE1393-13|KJ963161|ZMUO.007188|Leiodes_ferruginea
TLYFIFGAWAGMVGTSLSILIRTELGNPGSLIGDDQIYNVIVTAHAFIMIFFMVMPIVIGGFGNWLVPLMLGAPDMAFPRMNNMSFWLLPPSLTLLLMSSVVENGAGTGWTVYPPLSSNIAHSGSSVDLAIFSLHLAGISSILGAVNFITTVINMRPVGM
SFDKMPLFVWSVAITALLLLLSLPVLAGAITMLLTDRNLNTSFFDPAGGGDPILYQHL
>COLFB129-12|KJ967360|ZMUO.001459|Harpalus_affinis
TLYFIFGAWAGMVGTSLSMLIRAELGTPGALIGDDQIYNVIVTAHAFIMIFFMVMPIMIGGFGNWLVPLMLGAPDMAFPRMNNMSFWLLPPSLTLLLMSSMVESGAGTGWTVYPPLSSGIAHSGASVDLAIFSLHLAGVSSILGAVNFITTIINMRSV
GMTFDRMPLFVWSVGITALLLLLSLPVLAGAITMLLTDRNLNTSFFDPAGGGDPILYQHL
>COLFB911-12|KJ964067|ZMUO.001956|Poecilonota_variolosa
TLYFIFGAWSGMVGTALSLLIRAELGNPGALIGDDQIYNVIVTAHAFIMIFFMVMPIMMGGFGNWLVPLMLGAPDMAFPRMNNMSFWLLPPSLTLLLMSSIIENGAGTGWTVYPPLAANVAHSGASVDLAIFSLHLAGISSILGAVNFITTVINMRST
GMTFDRMPLFVWSVAITALLLLLSLPVLAGAITMLLTDRNLNTSFFDPAGGGDPILYQHL
>COLFF569-13|KJ967466|ZMUO.006649|Opatrum_riparium
TLYFIFGAWSGMVGTSLSILIRAELGNPGSLIGDDQIYNVIVTAHAFIMIFFMVMPIMIGGFGNWLVPLMLGAPDMAFPRMNNMSFWLLPPSLTLLLMSSIVENGAGTGWTVYPPLSSNIAHGGSSVDLAIFSLHLAGISSILGAVNFITTVINMRPQG
MTFDRMPLFVWAVVITAVLLLLSLPVLAGAITMLLTDRNINTSFFDPAGGGDPILYQHL
>COLFC376-12|KJ964603|ZMUO.002561|Aleochara_curtula

TLYFIFGAWAGMVGTSLSLLIRAELGNPGSLIGDDQIYNVIVTAHAFIMIFFMVMPIVIGGFGNWLVPLMLGAPDMAFPRMNNMSFWLLPPSLTLLLMSSMVESGAGTGWTVYPPLSSNIAHGGSSVDLAIFSLHLAGISSILGAVNFISTVINMRSMG
MSFDKMPLFVWSVVITALLLLLSLPVLAGAITMLLTDRNLNTSFFDPAGGGDPILYQHL
>COLFC048-12|KJ966061|ZMUO.002043|Autalia_puncticollis
TLYFIFGAWSGMVGTSLSLLIRAELGNPGSLIGDDQIYNVIVTAHAFIMIFFMVMPIMIGGFGNWLVPLMLGAPDMAFPRMNNMSFWLLPPSLTLLLMSSMVESGAGTGWTVYPPLSSNIAHGGSSVDLAIFSLHLAGISSILGAVNFISTIINMRSMGI
TFDRMPLFVWSVGITALLLLLSLPVLAGAITMLLTDRNLNTSFFDPAGGGDPILYQHL
>COLFF173-13|KJ964697|ZMUO.005897|Donacia_obscura
TLYFIFGAWSGMMGTSLSMLIRSELSNPGSLIGNDQIYNVIVTAHAFIMIFFMVMPIMIGGFGNWLVPLMLGAPDMAFPRMNNMSFWLLPPSLTFLTMSSITESGAGTGWTVYPPLSNNLAHSGSSVDLAIFSLHLAGISSILGAVNFISTIINMRPAG
MTMEKMSLFSWAVMITAILLLVSLPVLAGAITMLLTDRNINTSFFDPAGGGDPILYQHL
>COLFE062-12|KJ963419|ZMUO.005287|Syneta_betulae
TLYFIFGTWSGMVGTSLSMLIRAELGNPGTLIGNDQIYNVIVTAHAFIMIFFMVMPIMIGGFGNWLVPLMLGAPDMAFPRMNNMSFWLLPPSLSLLLMSSIVENGAGTGWTVYPPLSANIAHSGSSVDLAIFSLHLAGISSILGAVNFITTVINMRPEK
MTLDRMSLFIWAVTITAILLLLSLPVLAGAITMLLTDRNLNTSFFDPAGGGDPILYQHL
>COLFA226-10|HM909141|MP00092|Agrilus_viridis
TLYFIFGAWSGMVGTALSLLIRAELGNPGALIGNDQIYNVIVTAHAFIMIFFMVMPIMMGGFGNWLVPLMLGAPDMAFPRMNNMSFWLLPPSLTLLLMSSMVESGAGTGWTVYPPLAANIAHSGGSVDLAIFSLHLAGISSILGAINFITTVINMRAV
GMTMDRVPLLVWSIAITALLLLLSLPVLAGAITMLLTDRNLNTSFFDPAGGGDPILYQHL
>COLFF001-13|KJ964665|ZMUO.005701|Carabus_hortensis
TLYFIFGAWSGMVGTSLSMLIRAELGNPGSLIGDDQIYNVIVTAHAFVMIFFMVMPIMIGGFGNWLVPLMLGAPDMAFPRMNNMSFWLLPPSLTLLLMSSMVEKGAGTGWTVYPPLSSGIAHSGASVDLAIFSLHLAGISSILGAVNFITTIINMRSV
GMTFDRMPLFVWSVGITALLLLLSLPVLAGAITMLLTDRNLNTSFFDPAGGGDPILYQHL
>COLFB775-12|KJ963979|ZMUO.001250|Aleochara_moerens
TLYFIFGAWAGMVGTSLSLLIRAELGNPGSLIGDDQIYNVIVTAHAFIMIFFMVMPIVIGGFGNWLVPLMLGAPDMAFPRMNNMSFWLLPPSLTLLLMSSMVESGVGTGWTVYPPLSANIAHSGSSVDLAIFSLHLAGISSILGAVNFISTIINMRSSGM
SFDKMPLFVWSVAITALLLLLSLPVLAGAITMLLTDRNLNTSFFDPAGGGDPILYQHL
>COLFE1060-13|KJ962518|ZMUO.006855|Hydroporus_tristis
TLYFLFGAWSGMVGTSLSMLIRAELGNPGSLIGDDQIYNVIVTAHAFIMIFFMVMPIMIGGFGNWLVPLMLGAPDMAFPRMNNMSFWLLPPSLTLLLMSSMVENGAGTGWTVYPPLSSGIAHSGASVDLAIFSLHLAGVSSILGAVNFITTIINMRSI
GMTFDRMPLFVWSVGITALLLLLSLPVLAGAITMLLTDRNLNTSFFDPAGGGDPILYQHL
>COLFD906-12|KJ964253|ZMUO.004516|Halobrecta_puncticeps
TLYFIFGAWAGMVGTSLSLLIRAELGNPGSLIGDDQIYNVIVTAHAFIMIFFMVMPIVIGGFGNWLVPLMLGAPDMAFPRMNNMSFWLLPPSLTLLLMSSMVESGAGTGWTVYPPLSSNIAHGGSSVDLAIFSLHLAGISSILGAVNFISTVINMRSPG
MSFDRMPLFVWSVAITALLLLLSLPVLAGAITMLLTDRNLNTSFFDPAGGGDPILYQHL
>COLFD927-12|KJ965366|ZMUO.004537|Pachyta_quadrimaculata
TLYFIFGAWAGMVGTSLSLLIRSELGNPGSLIGDDQIYNVIVTAHAFIMIFFMVMPIMIGGFGNWLVPLMLGAPDMAFPRMNNMSFWLLPPSLTLLIMSSIVESGAGTGWTVYPPLSSNIAHGGSSVDLAIFSLHLAGISSILGAVNFITTVINMRPVGM
TLDRMPLFVWAVVITAILLLLSLPVLAGAITMLLTDRNLNTSFFDPAGGGDPILYQHL
>COLFA562-12|KJ961983|ZMUO.000697|Gaurotes_virginea
MLYFIFGAWAGMVGTSLSLLIRSELGNPGSLIGNDQIYNVIVTAHAFIMIFFMVMPIMIGGFGNWLVPLMLGAPDMAFPRLNNMSFWLLPPSLTLLIMSSVVESGAGTGWTVYPPLSSNIAHGGSSVDLAIFSLHLAGISSILGAINFITTVINMRPKGM
TFDRMPLFVWAVVITAILLLLSLPVLAGAITMLLTDRNLNTSFFDPAGGGDPILYQHL
>COLFB545-12|KJ962810|ZMUO.001020|Syntomus_truncatellus
TLYFIFGMWAGMVGTSLSILIRAELGNPGALIGDDQIYNVIVTAHAFIMIFFMVMPIMIGGFGNWLVPLMLGAPDMAFPRMNNMSFWLLPPSLTLLLMSSMVESGAGTGWTVYPPLSSGIAHAGASVDLAIFSLHLAGISSILGAVNFITTIINMRSVGI
TFDRMPLFVWSVGITALLLLLSLPVLAGAITMLLTDRNLNTSFFDPAGGGDPILYQHL
>COLFF806-13|KJ964224|ZMUO.006126|Notaris_acridulus
TLYFIFGAWSGMVGTSLSMLIRMELGNPGSLIGDDQIYNVIVTAHAFIMIFFMVMPIMIGGFGNWLVPLMLGAPDMAFPRLNNLSFWLLPPSLIILLSSSLIEKGAGTGWTVYPPLSSNIAHSGPSVDLAIFSLHMAGISSILGAINFISTVINMRPVGMSP
DRMTLFVWAVEITAILLLLSLPVLAGAITMLLTDRNINTSFFDPAGGGDPILYQHL
>COLFC563-12|KJ964293|ZMUO.003128|Hylobius_abietis
TLYFIFGTWSGMVGTSLSMLIRTELGNPGSLIGDDQIYNTIVTAHAFIMIFFMVMPIMIGGFGNWLVPLMLGAPDMAFPRLNNMSFWLLPPSLTLLLMSSIVDKGAGTGWTVYPPLSANIAHEGASVDFAIFSLHMAGISSILGAINFISTAINMRSSGM
KSDQMSLFIWAVKITAILLLLSLPVLAGAITMLLTDRNINTSFFDPAGGGDPILYQHL
>COLFC664-12|KJ963920|ZMUO.003229|Cerylon_histeroides
TLYFMFGMWSGMVGTSMSMMIRLELGNPGSLIGDDQIYNVIVTAHAFVMIFFMVMPIMIGGFGNWLVPLMLGAPDMAFPRMNNMSFWLLPPSLTLLIMSSIVEKGAGTGWTVYPPLSANLTHSGSSVDLAIFSLHLAGISSILGAVNFITTVINMR
PSGMTWDRLPLFVWSVIITAVLLLLSLPVLAGAITMLLTDRNLNTSFFDPAGGGDPILYQHL
>COLFE430-12|KJ964341|ZMUO.004990|Plagiodera_versicolora
TLYFIFGIWAGMVGTSLSILIRAELGNPGSLIGNDQIYNVIVTAHAFIMIFFMVMPIMIGGFGNWLVPLMIGAPDMAFPRLNNMSFWLLPPSLFLLLMSSVVENGAGTGWTVYPPLSANIAHSGSSVDLAIFSLHLAGISSILGAINFITTIINMRPHGMNF
EQTPLFVWAVLITAILLLLSLPVLAGAITMLLTDRNLNTSFFDPAGGGDPILYQHL

>COLFE1043-13|KJ966353|ZMUO.006838|Proteinus_brachypterus
TLYFIFGAWAGMVGTSLSILIRAELGNPGTLIGDDQIYNVIVTAHAFIMIFFMVMPIVIGGFGNWLVPLMLGAPDMAFPRMNNMSFWLLPPSITLLLMSSMVENGAGTGWTVYPPLSSNISHSGSSVDLAIFSLHLAGISSILGAVNFITTVINMRSPGM
TFDRMPLFVWSVAITALLLLSLPVLAGAITMLLTDRNINTTFFDPAGGGDPILYQHL
>COLFB809-12|KJ966384|ZMUO.001284|Eucnecosum_brachypterum
TLYFIFGAWAGMVGTSLSILIRAELGNPGTLIGDDQIYNVIVTAHAFVMIFFMVMPIVIGGFGNWLVPLMLGAPDMAFPRMNNMSFWLLPPSLTLLLMSSMVESGAGTGWTVYPPLSSNIAHGGSSVDLAIFSLHLAGISSILGAVNFITTVINMRTTG
MTFDRMPLFVWSVAITALLLLSLPVLAGAITMLLTDRNLNTSFFDPAGGGDPILYQHL
>COLFF709-13|KJ963787|ZMUO.006409|Dorcatoma_chrysomelina
TLYFIFGAWSGMIGTSMSILIRSELGNPGALIGNDQIYNVIVTAHAFVMIFFMVMPIMIGGFGNWLVPLMLGAPDMAFPRMNNMSFWLLPPSISLLLLSSAVNNGAGTGWTVYPPLSSNIAHSGASVDLAIFSLHLAGISSILGAVNFISTVINMRANG
MSFDKMPLFIWSVAITALLLLSLPVLAGAITMLLTDRNLNTSFFDPAGGGDPILYQHL
>COLFA564-12|KJ967363|ZMUO.000699|Pseudovadonia_livida
TLYFIFGAWASMVGTSLSLLIRSELGNPGSLIGDDQIYNVIVTAHAFVMIFFMVMPIMIGGFGNWLVPLMLGAPDMAFPRMNNMSFWLLPPSLTLLIMSSVVENGAGTGWTVYPPLSANIAHSGSSVDLAIFSLHLAGISSILGAVNFITTVINMRPKG
MNLDQMPLFVWAVVITAILLLSLPVLAGAITMLLTDRNLNTSFFDPAGGGDPILYQHL
>COLFF624-13|KJ964537|ZMUO.006514|Chaetocnema_hortensis
TLYFIFGIWSGMVGTSMSILIRAELGSPGSLIGNDQIYNVIVTAHAFVMIFFMVMPIMIGGFGNWLVPLMIGAPDMAFPRMNNMSFWLLPPSLFLLLMSSLVESGAGTGWTVYPPLSSNIAHGGSSVDLAIFSLHLAGVSSILGAINFITTIINMRPQGM
SFDQMPLFVWAVLITAILLLSLPVLAGAITMLLTDRNLNTSFFDPIGGGDPILYQHL
>COLFC314-12|KJ962015|ZMUO.002499|Boreaphilus_henningianus
TLYFIFGAWAGMVGTSLSILIRAELGNPGSLIGDDQIYNVIVTAHAFVMIFFMVMPIVIGGFGNWLVPLMLGAPDMAFPRMNNMSFWLLPPSLNLLLMSSMVESGAGTGWTVYPPLSSNIAHGGSSVDLAIFSLHLAGVSSILGAVNFITTVINMRSTG
MSFDRMPLFVWSVAITALLLLSLPVLAGAITMLLTDRNLNTSFFDPAGGGDPILYQHL
>COLFB176-12|KJ963521|ZMUO.001506|Anisotoma_axillaris
TLYFIFGAWSGMVGTSLSILIRAELGTPGSLIGDDQIYNVIVTAHAFVMIFFMVMPIMIGGFGNWLVPLMLGAPDMAFPRMNNMSFWLLPPSLTLLLMSSMVENGAGTGWTVYPPLSANISHSGSSVDLAIFSLHLAGISSILGAVNFITTVINMRSIG
MTFDKMPLFVWSVVITALLLLSLPVLAGAITMLLTDRNLNTSFFDPAGGGDPILYQHL
>COLFD168-12|KJ967401|ZMUO.004063|Omosita_colon
TLYFIFGTWSGMIGTSLSILIRTELGNPGSLIGNDQIYNVIVTAHAFIMIFFMVMPFMIGGFGNWLVPLMLGAPDMAFPRMNNMSFWLLPPSLSLLLMSSIVESGAGTGWTVYPPLSSNIAHGGSSVDLAIFSLHLAGISSILGAVNFITTVINMRPTGMS
FDRMPLFVWAVVITALLLLSLPVLAGAITMLLTDRNINTTFFDPSGGGDPILYQHL
>COLFA447-12|KJ967045|ZMUO.000487|Chrysomela_populi
TLYFIFGFWAGMTGTSLSILIRAELGNPGTLIGNDQIYNVIVTAHAFIMIFFMVMPIMIGGFGNWLVPLMIGAPDMAFPRLNNMSFWLLPPSLFLLLMSSVVENGAGTGWTVYPPLSANIAHSGSSVDLAIFSLHLAGISSILGAMNFITTVINMRPEGM
NFEQTPLFVWAVLITAVLLLSLPVLAGAITMLLTDRNLNTSFFDPAGGGDPILYQHL
>COLFA286-12|KJ967344|ZMUO.000421|Amischa_analis
TLYFIFGAWAGMVGTSLSLLIRAELGNPGSLIGDDQIYNVIVTAHAFIMIFFMVMPIVIGGFGNWLVPLMLGAPDMAFPRMNNMSFWLLPPSLTLLLMSSMVESGAGTGWTVYPPLSSNIAHGGSSVDLAIFSLHLAGISSILGAVNFISTVINMRSTGI
SFDRMPLFVWSVAITALLLLSLPVLAGAITMLLTDRNLNTSFFDPAGGGDPILYQHL
>COLFE434-12|KJ967352|ZMUO.004994|Ochthephilum_collare
TLYFIFGAWAGMVGTSLSLLIRSELANPGSLIGDDQIYNVIVTAHAFIMIFFMVMPVMIGGFGNWLVPLMLGAPDMAFPRMNNMSFWLLPPALTLLLMSSMVESGAGTGWTVYPPLSSNSFHNGSSVDLAIFSLHLAGISSILGAINFITTAINMRTTG
MNYERMPLFIWSVAITALLLLSLPVLAGAITMLLTDRNLNTSFFDPAGGGDPILYQHL
>COLFC711-12|KJ963610|ZMUO.003276|Aphthona_erichsoni
TMYFIFGVWSGMVGTSLSVLIRTELGNPGSLIGNDQIYNVIVTAHAFIMIFFMVMPIMIGGFGNWLVPLMIGAPDMAFPRMNNMSFWLLPPSLFFLIMSSMIENGAGTGWTVYPPLSANIAHSGSSVDLAIFSLHLAGISSILGAINFITTVINMRPKG
MNLDKMPLFVWAVTITAILLLSLPVLAGAITMLLTDRNLNTSFFDPAGGGDPILYQHL
>COLFE071-12|KJ963452|ZMUO.005296|Tetropium_castaneum
TLYFIFGAWAGMVGTSLSILIRSELGNPGSLIGNDQIYNVIVTAHAFIMIFFMVMPIMIGGFGNWLVPLMLGAPDMSFPRLNNLSFWFLPPSLILLIMGMIVEKGAGTGWTVYPPLSANIAHSGSSVDLTIFSLHLAGISSILSAINFITTIMNMRPKGMTL
DQMPLFVWAVMITTILLLISLPVLAGAITMLLTDRNINTSFFDPAGGGDPILYQHL
>COLFC144-12|KJ962102|ZMUO.002329|Oxypoda_brevicornis
TLYFIFGAWAGMVGTSLSLLIRAELGNPGSLIGDDQIYNVIVTAHAFVMIFFMVMPIVIGGFGNWLVPLMLGAPDMAFPRMNNMSFWLLPPSLTLLLMSSMVESGAGTGWTVYPPLSSNIAHGGSSVDLAIFSLHLAGISSILGAVNFISTIINMRTLG
MTFDRMPLFVWSVAITALLLLSLPVLAGAITMLLTDRNLNTSFFDPAGGGDPILYQHL
>COLFC521-12|KJ963162|ZMUO.003086|Pityogenes_bidentatus
TLYFIFGAWSGMVGTSLSMLIRTELGTPGSLIGDDQIFNTIVTAHAFIMIFFMVMPILIGGFGNWLVPLMLGAPDMAFPRLNNMSFWLLPPSLTFLIMSSIIDKGAGTGWTVYPPLSSNIAHEGASVDLAIFSLHMSGVSSILGAINFISTIINMHPKGVAP
EQLSLFTWAVKITAILLLLSLPVLAGAITMLLTDRNVNTSFFDPAGGGDPILYQHL
>COLFD464-12|KJ966014|ZMUO.003884|Phyllotreta_nigripes

TLYFIFGIWSGMVGLSMSMLIRIELATPGSLIGNDQIYNVIVTAHAFIMIFFMVMPIMIGGFGNWLVPLMIGAPDMAFPRMNNMSFWLLPPSLFLLIMSSIVENGAGTGWTVYPPLSANTSHAGSSVDLTIFSLHLAGISSILGAINFITTVINMRPKGMKFDRLPLFVWAVLITAILLLLSLPVLAGAITMLLTDRNLNTSFFDPMGGGDPILYQHL
>COLFB905-12|KJ963967|ZMUO.001950|Monochamus_urussovii
TLYFIFGAWSGMVGTSLSLLIRSELGMPGSLIGNDQIYNVIVTAHAFIMIFFMVMPIMIGGFGNWLVPLMLGAPDMAFPRMNNMSFWLLPPSLTLLIMSSIVENGAGTGWTVYPPLAANVAHSGSSVDLAIFSLHLAGVSSILGAVNFITTVINMRPSGMNLDRLPLFVWAVKITAILLLLSLPVLAGAITMLLTDRNLNTSFFDPAGGGDPILYQHL
>COLFB719-12|KJ966526|ZMUO.001194|Atheta_setigera
TLYFIFGAWSGMVGTSLSLLIRAELGNPGSLIGDDQIYNVIVTAHAFIMIFFMVMPVVIGGFGNWLVPLMLGAPDMAFPRMNNMSFWLLPPSLTLLLMSSMVESGAGTGWTVYPPLSSNIAHGGSSVDLAIFSLHLAGISSILGAVNFISTVINMRSTGISFDRMPLFVWSVAITALLLLLSLPVLAGAITMLLTDRNLNTSFFDPAGGGDPILYQHL
>COLFE951-13|KJ966176|ZMUO.006746|Aphodius_subterraneus
TLYFLFGSWAGMVGTSLSLLIRAELGNPGTLIGDDQIYNVIVTAHAFVMIFFMVMPILIGGFGNWLVPLMLGAPDMAFPRMNNMSFWLLPPSLTLLLMSSMVESGAGTGWTVYPPLSSNIAHGGASVDLAIFSLHLAGISSILGAVNFITTVINMRSPGMTFDRMPLFVWSVAITALLLLLSLPVLAGAITMLLTDRNLNTSFFDPAGGGDPILYQHL
>LEFIJ1835-13|KJ965047|ZMUO.004590|Psylliodes_affinis
TLYFIFGIWSGMIGTSLSMLIRTELGAPGSLIGNDQIYNVIVTAHAFIMIFFMVMPTMIGGFGNWLIPLMIGAPDMAFPRMNNMSFWLLPPSLTLLLMSSMVENGAGTGWTVYPPLSSNIAHGGSSVDLAIFSLHLAGISSILGAINFITTVINMRPKGMTLDRMPLFVWAVVITAILLLLSLPVLAGAITMLLTDRNLNTSFFDPIGGGDPILYQHL
>COLFA403-12|KJ967183|ZMUO.000633|Leiodes_silesiaca
TLYFIFGAWAGMVGTSLSILIRAELGNPGSLIGDDQIYNVIVTAHAFVMIFFMVMPIVIGGFGNWLIPLMLGAPDMAFPRMNNMSFWLLPPSLTLLLMSSVVENGAGTGWTVYPPLSSNIAHSGSSVDLAIFSLHLAGISSILGAVNFITTVINMRSAGMTFDKMPLFVWSVAITALLLLLSLPVLAGAITMLLTDRNLNTSFFDPAGGGDPILYQHL
>COLFB698-12|KJ966560|ZMUO.001173|Otiorhynchus_raucus
TLYFIFGAWSGMVGTSLSMLIRTELGNPGSLIGNDQIYNVIVTAHAFIMIFFMVMPMMIGGFGNWLVPLMLGAPDMAFPRLNNMSFWLLPPSLILLLMSSIIDKGAGTGWTVYPPLSSNIAHEGASVDLAIFSLHMAGVSSILGAVNFISTVANMRPNGMTPDRISLFIWAVKITAILLLLSLPVLAGAITMLLTDRNINTSFFDPAGGGDPILYQHL
>COLFE600-13|KJ967005|ZMUO.005445|Oulema_erichsonii
TLYFIFGTWSGMVGTSLSMMIRTELGNPGSFIGNDQIYNVIVTAHAFIMIFFMVMPIMIGGFGNWLVPLMLGAPDMAFPRMNNMSFWLLPPSISLLLMSSIVENGAGTGWTVYPPLSANISHNGASVDLAIFSLHLAGISSILGAVNFISTVSNMRPEGMTLDRMSLFVWAVLITAILLLLSLPVLAGAITMLLTDRNLNTSFFDPSSGGDPILYQHL
>COLFC094-12|KJ965494|ZMUO.002089|Phyllodrepa_melanocephala
TLYFIFGAWAGMVGTSLSILIRAELGNPGSLIGDDQIYNVIVTAHAFVMIFFMVMPIVIGGFGNWLVPLMLGAPDMAFPRMNNMSFWLLPPSLTLLLMSSMVESGAGTGWTVYPPLSSNIAHGGSSVDLAIFSLHLAGISSILGAVNFITTVINMRSMGMTFDRMPLFVWSVAITALLLLLSLPVLAGAITMLLTDRNLNTSFFDPAGGGDPILYQHL
>COLFE1387-13|KJ967251|ZMUO.007182|Rhinoncus_inconspectus
TLYFIFGSWAGTVGTSLSMLIRTELGTPGSLIGNDQIYNSIVTAHAFIMIFFMVMPILIGGFGNWLVPLMLGAPDMAFPRLNNMSFWLLPPSIMLLLMSSIVNKGAGTGWTVYPPLSSNITHEGASVDLAIFSLHMAGISSILGAINFISTIMNMRPQGMTYDKMPLFSWAVLITAILLLLSLPVLAGAITMLLTDRNVNTSFFDPAGGGDPILYQHL
>COLFB072-12|KJ961807|ZMUO.001402|Stenus_strandi
TLYFIFGAWAGMVGTSLSLLIRAELGNPGSLIGDDQIYNVIVTAHAFVMIFFMVMPVMIGGFGNWLVPLMLGAPDMAFPRMNNMSFWLLPPSLSLLLMSSIVENGAGTGWTVYPPLSSNIAHSGASVDLAIFSLHLAGISSILGAINFITTIFNMRTMKLHLDCLPLFVWSVGITALLLLLSLPVLAGAITMLLTDRNLNTSFFDPAGGGDPILYQHL
>COLFA316-12|KJ965189|ZMUO.000451|Tachyporus_scitulus
TLYFIFGAWSGMVGTSLSLLIRAELGNPGSLIGDDQIYNVIVTAHAFIMIFFMVMPIVIGGFGNWLVPLMLGAPDMAFPRMNNMSFWLLPPSLTLLLMSSMVESGAGTGWTVYPPLSANIAHSGPSVDLAIFSLHLAGISSILGAVNFITTVINMRASGMYFDRMPLFIWSVAITALLLLLSLPVLAGAITMLLTDRNLNTSFFDPAGGGDPILYQHL
>COLFF1001-13|KJ965798|ZMUO.007366|Sirocalodes_depressicollis
TLYFIFGSWAGMAGTSLSMLIRTELGNPGSLIGDDQIYNSIVTAHAFIMIFFMVMPILIGGFGNWLVPLMLGAPDMAFPRLNNMSFWLLPPSLALLLMSSIVNKGAGTGWTVYPPLSMNVAHEGMSVDLAIFSLHMAGISSILGAINFISTIMNMRPTGMTAEYMPLFVWAVKITAILLLLSLPVLAGAITMLLTDRNINTSFFDPAGGGDPILYQHL
>COLFD608-12|KJ965070|ZMUO.004408|Badister_bullatus
TLYFIFGAWAGMVGTSLSMLIRAELGNPGSLIGDDQIYNVIVTAHAFVMIFFMVMPIMIGGFGNWLVPLMLGAPDMAFPRMNNMSFWLLPPSLTLLLMSSLVESGAGTGWTVYPPLSSSIAHSGASVDLAIFSLHLAGISSILGAVNFITTIINMRSTGITFDRMPLFVWSVGITAILLLLSLPVLAGAITMLLTDRNLNTSFFDPAGGGDPILYQHL
>COLFD113-12|KJ963287|ZMUO.004008|Stephostethus_pandellei
SLYFLFGMWSGMVGTSLSLLIRLELGNPGSLIGDDQIYNVIVTAHAFIMIFFMVMPVMMGGFGNWLVPLMLGAPDMAFPRLNNMSFWLLPPSLSLLIMSSIVESGVGTGWTVYPPLSSNIAHGGSSVDLAIFSLHLAGISSILGAVNFITTMINMRPTGMKLELMSLFAWSVLLTAILLLLSLPVLAGAITMLLTDRNLNTSFFDPAGGGDPILYQHL
>COLFG108-13|KJ963754|ZMUO.007803|Smicrus_filicornis
TLYFMFGAWAGMVGTSLSILIRTELGTPGSLIGDDQIYNVIVTAHAFVMIFFMVMPILIGGFGNWLVPLMLGAPDMAFPRMNNMSFWFLPPSLTLLLMSSMVESGAGTGWTVYPPLASNIAHGGASVDLAIFSLHLAGISSILGAVNFITTIINMRAPQMNFDQMPLFVWAVGITALLLLLSLPVLAGAITMLLTDRNLNTSFFDPAGGGDPILYQHL

>COLFE1511-13|KJ964681|ZMUO.007306|Phyllotreta_nigripes
TLYFIFGIWSGMVGLSMSMLIRIELATPGSLIGNDQIYNVIVTAHAFIMIFFMVMPIMIGGFGNWLVPLMIGAPDMAFPRMNNMSFWLLPPSLFLLIMSSIVENGAGTGWTVYPPLSANTSHAGSSVDLTIFSLHLAGISSILGAINFITTVINMRPKGMK
FDRLPLFVWAVLITAILLLLSLPVLAGAITMLLTDRNLNTSFFDPMGGGDPILYQHL
>COLFB074-12|KJ965935|ZMUO.001404|Dyschirius_nigricornis
TLYFIFGIWSGMVGTSLSIMIRAELGNPGSLIGDDQIYNVIVTAHAFIMIFFMVMPIMIGGFGNWLVPLMLGAPDMAFPRMNNMSFWLLPPSLSLLMSSMVEKGAGTGWTVYPPLSSSIAHSGASVDLAIFSLHLAGVSSILGAVNFITTIINMRSTG
MTFERMPLFVWSVGITALLLLLSLPVLAGAITMLLTDRNLNTSFFDPAGGGDPILYQHL
>COLFF854-13|KJ965913|ZMUO.006174|Phyllobius_argentatus
TLYFIFGAWSGMVGTSLSILIRIELGNPGSLIGDDQIYNVIVTAHAFIMIFFMVMPMMIGGFGNWLVPLMLGAPDMAFPRLNNMSFWLLPPSLSLLMSSIVDKGAGTGWTVYPPLSANIAHEGSSVDLAIFSLHMAGVSSILGAINFISTVINMRPSGL
SPDRMSLFIWAVKITAVLLLSLPVLAGAITMLLTDRNMNTSFFDPAGGGDPILYQHL
>COLFD327-12|KJ963995|ZMUO.004222|Anthicus_flavipes
TLYLIFGAWASMVGTSLSLLIRSELGNPGTLIGNDQIYNVIVTAHAFIMIFFMVMPIVIGGFGNWLVPLMLGAPDMAFPRMNNMSFWLLPPSLSLLIMSSIVESGAGTGWTVYPPLSANIAHSGSSVDLAIFSLHLAGISSILGAVNFITTVINMRPVGMT
LDRMPLFVWAVVITAVLLLSLPVLAGAITMLLTDRNLNTSFFDPAGGGDPILYQHL
>COLFE314-12|KJ965677|ZMUO.004874|Cionus_hortulanus
TLYFIFGMWSGTVGTSLSMLIRTELGNPGSLIGDDQIYNTIVTAHAFIMIFFMVMPIMIGGFGNWLVPLMLGAPDMAFPRLNNMSFWLLPPSITLLLMSSIVEKGAGTGWTVYPPLSNNVTHEGASVDLAIFSLHMAGISSILGAINFISTVSNMRTKG
MDYDRTPLFVWSVNITAFLLLSLPVLAGAITMLLTDRNINTSFFDPSGGGDPILYQHL
>COLFB426-12|KJ964270|ZMUO.001851|Aloconota_gregaria
TLYFIFGAWAGMVGTSLSLLIRAELGNPGSLIGDDQIYNVIVTAHAFIMIFFMVMPIVIGGFGNWLIPLMLGAPDMAFPRMNNMSFWLLPPSLSLLMSSMVESGAGTGWTVYPPLSSNIAHGGSSVDLAIFSLHLAGISSILGAVNFISTIINMRSSGISF
DRMPLFVWSVAITALLLLLSLPVLAGAITMLLTDRNLNTSFFDPAGGGDPILYQHL
>COLFA327-12|KJ962028|ZMUO.000462|Notiophilus_aquaticus
TLYFIFGAWSGMVGTSLSMLIRAELGNPGSLIGDDQIYNVIVTAHAFVMIFFMVMPIMIGGFGNWLVPLMLGAPDMAFPRMNNMSFWLLPPSLTLLLTSSMVESGAGTGWTVYPPLSSGIAHSGASVDLAIFSLHLAGVSSILGAVNFITTIINMRSVG
MTFDRMPLFVWSVGITALLLLLSLPVLAGAITMLLTDRNLNTSFFDPAGGGDPILYQHL
>COLFD281-12|KJ962117|ZMUO.004176|Stagetus_borealis
TLYFIFGAWSGMVGTSLSMLIRSELGNPGALIGNDQIYNVIVTAHAFIMIFFMVMPIMIGGFGNWLVPLMLGAPDMAFPRMNNMSFWLLPPSLSLLMSSAVNSGAGTGWTVYPPLSSNIAHSGASVDLAIFSLHLAGISSILGAVNFITTVINMRPM
QMTFDRMPLFVWSVAITALLLLLSLPVLAGAITMLLTDRNLNTSFFDPAGGGDPILYQHL
>COLFB645-12|KJ965922|ZMUO.001120|Ocyusa_maura
TLYFIFGAWSGMVGTSLSLLIRAELGNPGSLIGDDQIYNVIVTAHAFVMIFFMVMPIVIGGFGNWLIPLMLGAPDMAFPRMNNMSFWLLPPSLSLLMSSMVESGAGTGWTVYPPLSSNIAHGGSSVDLAIFSLHLAGISSILGAVNFISTIINMRTSGM
SFDRMPLFVWSVAITALLLLSLPVLAGAITMLLTDRNLNTSFFDPAGGGDPILYQHL
>COLFC613-12|KJ961945|ZMUO.003178|Orthocis_alni
TLYFIFGIWSGMVGTSLSMLIRSELGMPGSLIGNDQIYNVIVTAHAFIMIFFMVMPILIGGFGNWLVPLMLGAPDMAFPRMNNMSFWLLPPSLSLLLLSSIVENGAGTGWTVYPPLSSNIAHSGASVDMAIFSLHLAGISSILGAVNFISTIINMRPKGM
NPDQMPLFVWAVVITAILLLLSLPVLAGAITMLLTDRNFNTSFFDPAGGGDPILYQHL
>COLFC189-12|KJ966831|ZMUO.002374|Orithales_serraticornis
TLYFLFGAWAGMLGTSLSLLIRAELGNPGSLIGNDQIYNVIVTAHAFIMIFFMVMPIMIGGFGNWLVPLMLGAPDMAFPRMNNMSFWFLPPSLSLLLMSSIVENGAGTGWTVYPPLSANIAHSGSSVDLAIFSLHLAGISSILGAVNFISTVINMRSTGIT
FDRMPLFVWAVAITALLLLLSLPVLAGAITMLLTDRNLNTSFFDPAGGGDPILYQHL
>COLFA486-12|KJ963552|ZMUO.000526|Rhizophagus_dispar
TLYFIFGAWAGMVGTSLSLLIRAELGTPGQLIGDDQIYNVIVTAHAFIMIFFMVMPILIGGFGNWLVPLMLGAPDMAFPRMNNMSFWLLPPSLTLLIMSSIVENGAGTGWTVYPPLSSNIAHSGSSVDLAIFSLHLAGISSILGAVNFITTVINMRPSGMT
LDRTPLFVWAVIITATLLLLSLPVLAGAITMLLTDRNLNTSFFDPAGGGDPILYQHL
>COLFE1088-13|KJ963351|ZMUO.006883|Magdalis_carbonaria
TLYLIFGAWSGMTGTSLSMLIRVELGNPGSLIGNDQIYNTIVTAHAFIMIFFMVMPIMIGSFGNWLIPLMLGAPDMAFPRLNNMSFWLLPPSLTLLLMSSIIDKGAGTGWTVYPPLSSNIAHEGSSIDLAIFSLHLAGISSILGALNFISTIINMRPTNMKLD
QMSLFIWAVKITAILLLLSLPVLAGAITMLLTDRNINTSFFDPAGGGDPILYQHL
>COLFF202-13|KJ963218|ZMUO.005807|Rhynchaenus_rusci
TLYFMFGAWSGMVGTSLSMLIRTELGTPGKLIGDDQIYNTIVTAHAFIMIFFMVMPILIGGFGNWLIPLMLGAPDMAFPRLNNMSFWLLPPSLTLLLTSSIMDKGAGTGWTVYPPLSGNVSHEGASVDLAIFSLHMAGISSILGAINFISTVANMRASG
MNTDQMSLFVWAVKITAILLLLSLPVLAGAITMLLTDRNINTSFFDPAGGGDPILYQHL
>COLFE1345-13|KJ965688|ZMUO.007140|Aphodius_distinctus
TLYFLFGSWAGMVGTSLSLLIRAELGNPGTLIGDDQIYNVIVTAHAFVMIFFMVMPILIGGFGNWLVPLMLGAPDMAFPRMNNMSFWLLPPSLTLLLMSSMVESGAGTGWTVYPPLSSNIAHGGASVDLAIFSLHLAGISSILGAVNFITTVINMRSPG
MTFDRMPLFVWSVAITALLLLLSLPVLAGAITMLLTDRNLNTSFFDPAGGGDPILYQHL
>COLFF222-13|KJ964005|ZMUO.005827|Othius_angustus

TLYFIFGAWAGMVGTSLSLLIRAELGNPGSLIGDDQIYNVIVTAHAFIMIFFMVMPIMIGGFGNWLVPLMLGAPDMAFPRMNNMSFWLLPPSLSLLLMSSMIESGAGTGWTVYPPLSSNIAHGGASVDLAIFSLHLAGISSILGAVNFITTVINMRSIG
MTFDRMPLFVWSVAITALLLLLSLPVLAGAITMLLTDRNLNTSFFDPAGGGDPILYQHL
>COLFB116-12|KJ963461|ZMUO.001446|Anthaxia_quadripunctata
TLYFIFGAWSGMVGTALSLLIRAELGNPGTLIGNDQIYNVIVTAHAFVMIFFMVMPIMMGGFGNWLVPLMLGAPDMAFPRMNNMSFWLLPPSLTLLLMSSIVESGAGTGWTVYPPLASNIAHSGASVDLAIFSLHLAGISSILGAINFITTVINMRSVG
MSFDRMPLFVWSVAITAVLLLLSLPVLAGAITMLLTDRNLNTSFFDPAGGGDPILYQHL
>COLFA515-12|KJ964971|ZMUO.000555|Saperda_carcharias
TLYFLFGAWASMVGTSLSLLIRSELGTPGSLIGDDQIYNVIVTAHAFIMIFFMVMPIMIGGFGNWLVPLMLGAPDMAFPRMNNMSFWLLPPSLSLLIMSSIVDNGAGTGWTVYPPLAANIAHSGSSVDLAIFSLHLAGISSILGAVNFITTVINMRPQG
MSMDRMPLFVWAVKITAILLLLSLPVLAGAITMLLTDRNLNTSFFDPAGGGDPILYQHL
>COLFE1474-13|KJ963171|ZMUO.007269|Gonioctena_intermedia
TLYFIFGIWAGMVGTSLSILIRAELGNPGTLIGNDQIYNVIVTAHAFIMIFFMVMPIMIGGFGNWLVPLMLGAPDMAFPRMNNMSFWLLPPSLFFLIMSSIVESGAGTGWTVYPPLSANIAHSGSSVDLAIFSLHLAGISSILGAINFITTIINMRPMGMT
MDQMPLFAWAVLITTILLLLSLPVLAGAITMLLTDRNLNTSFFDPAGGGDPILYQHL
>COLFC740-12|KJ962742|ZMUO.003305|Hylesinus_crenatus
TLYFILGAWSGMVGTSLSMIIRTELGTPGSLIGNDQIYNSIVTAHAFIMIFFMVMPIMIGGFGNWLVPLMLGAPDMAFPRLNNMSFWLLPPSLLFLLMSSIIDKGAGTGWTVYPPLANNIAHEGASVDLAIFSLHMAGVSSILGALNFISTIINMHPMG
MKLDRLTLFTWAVKITAILLLLSLPVLAGAITMLLTDRNINTSFFDPAGGGDPILYQHL
>COLFB489-12|KJ964708|ZMUO.000964|Anotylus_rugosus
TLYFIFGAWSGMVGTSLSMLIRAELGAPGSLIGDDQIYNVVVTAHAFIMIFFMVMPIVIGGFGNWLVPLMLGAPDMAFPRMNNMSFWLLPPSLTLLLFSSIVESGAGTGWTVYPPLSSNIAHSGSSVDLAIFSLHLAGISSILGAVNFISTIINMRSTGMS
FDRMPLFVWSVNITAILLLLSLPVLAGAITMLLTDRNLNTSFFDPAGGGDPILYQHL
>COLFB683-12|KJ965415|ZMUO.001158|Acrotona_obfuscata
TLYFVFGAWAGMVGTSLSLLIRAELGNPGSLIGDDQIYNVIVTAHAFVMIFFMVMPIVIGGFGNWLIPLMLGAPDMAFPRMNNMSFWLLPPSLTLLLMSSMVESGAGTGWTVYPPLSSNIAHGGASVDLAIFSLHLAGISSILGAVNFISTVINMRATG
ISFDRMPLFVWSVAITALLLLLSLPVLAGAITMLLTDRNLNTSFFDPAGGGDPILYQHL
>COLFE078-12|KJ963136|ZMUO.005303|Chrysanthia_geniculata
TLYLIFGAWAGMVGTSLSLLIRAELGNPGSLIGDDQIYNVIVTAHAFIMIFFMVMPIVIGGFGNWLVPLMLGAPDMAFPRMNNMSFWLLPPSLTLLIMSSIVENGAGTGWTVYPPLSSNIAHSGSSVDLAIFSLHLAGISSILGAVNFITTVINMRPVGM
TLDRMPLFVWAVVITAVLLLLSLPVLAGAITMLLTDRNLNTSFFDPAGGGDPILYQHL
>COLFA431-12|KJ966551|ZMUO.000661|Hylastes_cunicularius
TLYFIFGAWAGMVGTSLSMLIRSELGTPGSLIGDDQLYNTLVTSHAFIMIFFMVMPIMIGGFGNWLIPLMLGAPDMAFPRLNNMSFWFLPPSLILLLMSSIIDKGAGTGWTVYPPLASNISHEGSSVDLAIFSLHMAGISSILGAMNFISTIINMYPSGMK
PDSLTLFTWSVKITAILLLLSLPVLAGAITMLLTDRNINTTFFDPSGGGDPILYQHL
>COLFA238-10|HM909149|MP00104|Agrilus_viridis
TLYFIFGAWSGMVGTALSLLIRAELGNPGALIGNDQIYNVIVTAHAFIMIFFMVMPIMMGGFGNWLVPLMLGAPDMAFPRMNNMSFWLLPPSLTLLLMSSMVESGAGTGWTVYPPLAANIAHSGGSVDLAIFSLHLAGISSILGAINFITTVINMRAV
GMTMDRVPLLVWSIAITALLLLLSLPVLAGAITMLLTDRNLNTSFFDPAGGGDPILYQHL
>COLFB756-12|KJ964444|ZMUO.001231|Schistoglossa_gemina
TLYFIFGTWAGMIGTSLSLLIRAELGNPGSLIGDDQIYNVIVTAHAFIMIFFMVMPIVIGGFGNWLIPLMLGAPDMAFPRMNNMSFWLLPPSLTLLLMSSLVESGAGTGWTVYPPLSSNIAHGGSSVDLAIFSLHLAGISSILGAVNFISTVINMRSTGISFD
RMPLFVWSVSITALLLLLSLPVLAGAITMLLTDRNLNTSFFDPAGGGDPILYQHL
>COLFC683-12|KJ963181|ZMUO.003248|Sitona_lineellus
TLYFIFGAWAGMVGTSLSVLIRTELGNPGSLIGDDQIYNVIVTAHAFIMIFFMVMPMMIGGFGNWLVPLMLGAPDMAFPRLNNMSFWLLPPSLTLLLMSSIVDKGAGTGWTVYPPLSANIAHEGASVDLAIFSLHMAGISSILGAINFISTVINMRPTG
MTLDRMTLFVWAVKITAILLLLSLPVLAGAITMLLTDRNINTSFFDPAGGGDPILYQHL
>COLFA475-12|KJ964402|ZMUO.000515|Omonadus_floralis
TLYLIFGAWAGMVGTSLSLLIRSELGNPGTLIGNDQIYNVIVTAHAFIMIFFMVMPIVIGGFGNWLVPLMLGAPDMAFPRMNNMSFWLLPPSLSLLIMSSIVESGSGTGWTVYPPLSANIAHSGSSVDLAIFSLHLAGISSILGAVNFITTVINMRPAGMS
FDRMPLFVWAVVITAVLLLLSLPVLAGAITMLLTDRNLNTSFFDPAGGGDPILYQHL
>COLFD391-12|KJ963594|ZMUO.003811|Myrmechixenus_subterraneus
TLYFIFGAWSGMVGTSLSLLIRAELGNPGSLIGDDQIYNVIVTAHAFIMIFFMVMPIMIGGFGNWLVPLMLGAPDMAFPRMNNMSFWLLPPSLTLLLMSSIVESGAGTGWTVYPPLSSNIAHGGSSVDLAIFSLHMAGISSILGAVNFITTVINMRPQG
MTFDRMPLFVWAVVITAVLLLLSLPVLAGAITMLLTDRNINTSFFDPAGGGDPILYQHL
>COLFA471-12|KJ964305|ZMUO.000511|Tachinus_basalis
TLYFIFGAWAGMVGTSLSLLIRAELGNPGTLIGDDQIYNVIVTAHAFIMIFFMVMPIVIGGFGNWLVPLMLGAPDMAFPRMNNMSFWLLPPSLTLLLMSSMVESGAGTGWTVYPPLSSNIAHGGSSVDLAIFSLHLAGISSILGAVNFITTVINMRSIG
MTFDRMPLFVWSVAITALLLLLSLPVLAGAITMLLTDRNLNTTFFDPAGGGDPILYQHL
>COLFD941-12|KJ966044|ZMUO.004551|Cionus_nigritarsis
TLYFIFGMWSGTVGTSLSMLIRTELGNPGSLIGDDQIYNTIVTAHAFIMIFFMVMPIMIGGFGNWLVPLMLGAPDMAFPRLNNMSFWLLPPSITLLLMSSIVEKGAGTGWTVYPPLSNNVTHEGASVDLAIFSLHMAGISSILGAINFISTVSNMRTSG
MDYDRTPLFVWSVNITAFLLLLSLPVLAGAITMLLTDRNINTSFFDPSGGGDPILYQHL

>COLFD824-12|KJ965301|ZMUO.004814|Quedius_cruentus
TLYFIFGAWAGMVGTSLSLLIRAELGNPGSLIGDDQIYNVIVTAHAFIMIFFMVMPIVIGGFGNWLVPLMLGAPDMAFPRMNNMSFWLLPPSLSLLLMSSMVESGAGTGWTVYPPLSSNIAHGGASVDLAIFSLHLAGISSILGAVNFITTVINMRSIG
MTFDRMPLFVWSVAITALLLLSLPVLAGAITMLLTDRNLNTSFFDPAGGGDPILYQHL
>COLFC661-12|KJ965888|ZMUO.003226|Hydnobius_spinipes
TLYFIFGSWAGMAGTSLSILIRAELGNPGSLIGDDQIYNVIVTAHAFIMIFFMVMPIVIGGFGNWLVPLMLGAPDMAFPRMNNMSFWLLPPSLSLLLMSSMVENGAGTGWTVYPPLSANIAHSGSSVDLAIFSLHLAGISSILGAVNFITTIINMRSVGM
TFDKMPLFVWSVLITAILLLSLPVLAGAITMLLTDRNLNTSFFDPAGGGDPILYQHL
>COLFF493-13|KJ962078|ZMUO.006573|Altica_opacifrons
TLYFLFGIWAGMIGTSMSLLIRTELGSPGSLIGNDQIYNVIVTAHAFIMIFFMVMPMMIGGFGNWLVPLMIGAPDMAFPRMNNMSFWLLPPSIFLLLMSSMAESGAGTGWTVYPPLSSNLAHNGPSVDLAIFSLHLTGISSILGAINFITTMINMRPQG
MSMDQMPLFVWAVFITAILLLSLPVLAGAITMLLTDRNLNTSFFEPAGGGDPILYQHL
>COLFE469-12|KJ965169|ZMUO.005029|Coelostoma_orbiculare
TLYFIFGAWAGMLGTSLSILIRTELGNPGSLIGNDQIYNVIVTAHAFIMIFFMVMPIMIGGFGNWLVPLMLGAPDMAFPRMNNMSFWLLPPSLTLLLMSSMVESGAGTGWTVYPPLSANIAHSGASVDLAIFSLHLAGISSILGAVNFITTVINMRSSN
MTYDRMPLFVWSVAITALLLLSLPVLAGAITMLLTDRNFNTSFFDPAGGGDPILYQHL
>COLFC136-12|KJ964012|ZMUO.002321|Sphindus_dubius
TLYFIFGIWSGMIGTSLSMLIRAELSTPNALIGDDQIYNVIVTAHAFIMIFFMVMPIMIGGFGNWLIPLMMGAPDMAFPRMNNMSFWLLPPSLSLLLMSSMVEKGTGTGWTVYPPLSSNVSHNGASVDLSIFSLHMAGVSSILGAINFISTIVNMRPM
NMKMDQLPLFIWAVLITAFLLVLSLPVLAGAITMLLTDRNINTSFFDPSGGGDPILYQHL
>COLFF149-13|KJ963661|ZMUO.003783|Hallomenus_axillaris
TLYFIFGSWSGMVGTSLSMLIRSELGNPGSLIGNDQIYNVIVTAHAFIMIFFMVMPIMLGGFGNWLVPLMLGAPDMAFPRMNNMSFWLLPPSLTLLIMSSMVENGAGTGWTVYPPLSSNIAHSGSSVDLAIFSLHLAGISSILGAINFITTIINMRPLGM
TLDRMPLFVWAVLITAILLLSLPVLAGAITMLLTDRNLNTSFFDPAGGGDPILYQHL
>COLFF267-13|KJ965563|ZMUO.005872|Adalia_decempunctata
TLYFLFGLWAGMVGTSLSIIIRLELGTTNSLIGNDQIYNVIVTAHAFIMIFFMVMPIMIGGFGNWLVPLMIGAPDMAFPRLNNMSFWLLPPALTLLISSSIVEMGAGTGWTVYPPLSSNLAHNGPSVDLVIFSLHLAGISSILGAVNFISTIMNMRPNGMN
LDKTPLFVWSVLITAILLLSLPVLAGAITMLLTDRNINTSFFDPTGGGDPVLYQHL
>COLFA699-12|KJ966650|ZMUO.000834|Gabrius_trossulus
TLYFIFGSWAGMVGTSLSLLIRAELGNPGSLIGDDQIYNVIVTAHAFIMIFFMVMPIVIGGFGNWLVPLMLGAPDMAFPRMNNMSFWLLPPSITLLLMSSMVESGAGTGWTVYPPLSSNIAHGGASVDLAIFSLHLAGISSILGAVNFITTVINMRSFG
MSFDRMPLFVWSVAITALLLLSLPVLAGAITMLLTDRNLNTSFFDPAGGGDPILYQHL
>COLFD372-12|KJ963501|ZMUO.004267|Cantharis_rustica
TLYFIFGAWSGSLGLALSLLIRAELGTPGTLIGNDQIYNVIVTAHAFIMIFFMVMPIMIGGFGNWLVPLMLGAPDMAFPRMNNMSFWFLPPSLMFLLMSSMVESGAGTGWTVYPPLSANIAHSGPSVDLAIFSLHMAGISSILGAVNFISTIMNMKPPS
MKFDQMPLFVWSVGITALLLLSLPVLAGAITMLLSDRNLNTSFFDPMGGGDPILYQHL
>COLFB783-12|KJ967087|ZMUO.001258|Mycetoporus_monticola
TLYFIFGSWAGMVGTSLSLLIRAELGNPGSLIGDDQIYNVIVTAHAFVMIFFMVMPIMIGGFGNWLVPLMLGAPDMAFPRMNNMSFWLLPPSLTLLLMSSMVESGAGTGWTVYPPLSSNIAHGGASVDLAIFSLHLAGISSILGAVNFITTVINMRSM
GMTFDRMPLFIWSVAITALLLLSLPVLAGAITMLLTDRNLNTSFFDPAGGGDPILYQHL
>COLFF412-13|KJ962266|ZMUO.005930|Anoplus_plantaris
TLYFIFGTWSGMIGTSLSMLIRTELGNPGSLIGDDQIYNTIVTAHAFIMIFFMVMPIMIGGFGNWLVPLMLGAPDMAFPRLNNMSFWLLPPSLILLLMSSIVDKGAGTGWTVYPPLSSNIAHEGASVDLAIFSLHMAGISSILGAVNFISTIINMRPKGMN
PDQMSLFVWAVNITAILLLSLPVLAGAITMLLTDRNINTSFFDPAGGGDPILYQHL
>COLFF564-13|KJ963808|ZMUO.006644|Sulcacis_fronticornis
TLYFLFGAWSGMVGTSMSILIRSELGAPGSLIGNDQIYNVIVTAHAFIMIFFMVMPMMIGGFGNWLVPLMLGAPDMAFPRMNNMSFWLLPPSLSLLLLSSMVENGAGTGWTVYPPLSSNIAHSGPSVDLAIFSLHLAGISSILGAVNFISTIINMRPTG
MLMEQIPLFVWAVIITAILLLSLPVLAGAITMLLTDRNFNTSFFDPAGGGDPILYQHL
>COLFG069-13|KJ963373|ZMUO.007574|Acmaeops_pratensis
VLYFIFGAWAGMVGTSLSLLIRSELGNPGSLIGNDQIYNVIVTAHAFIMIFFMVMPIMIGGFGNWLVPLMLGAPDMAFPRLNNMSFWLLPPSLTLLIMSSIVESGAGTGWTVYPPLSSNIAHGGSSVDLAIFSLHLAGISSILGAINFITTVINMRPKGMT
FDRMPLFVWAVVITAILLLSLPVLAGAITMLLTDRNLNTSFFDPAGGGDPILYQHL
>COLFE1342-13|KJ966799|ZMUO.007137|Malthodes_misellus
TLYFMFGAWSGMLGTSLSLLIRAELGSPGSLIGNDQIYNVIVTAHAFIMIFFMVMPIMIGGFGNWLVPLMLGAPDMAFPRMNNMSFWFLPPSLSLLLMSSIVENGAGTGWTVYPPLSANIAHSGSSVDLAIFSLHMAGISSILGAVNFISTVINMRSTG
MTFDRMPLFVWAVAITALLLLSLPVLAGAITMLLTDRNLNTSFFDPAGGGDPILYQHL
>COLFE1222-13|KJ966961|ZMUO.007017|Ischnosoma_splendidum
TLYFIFGAWAGMVGTSLSLLIRAELGNPGSLIGDDQIYNVIVTAHAFVMIFFMVMPIVIGGFGNWLVPLMLGAPDMAFPRMNNMSFWLLPPSLSLLLMSSLVESGAGTGWTVYPPLSSNIAHGGASVDLAIFSLHLAGISSILGAVNFITTVINMRSTG
MTFDRMPLFVWSVVITALLLLSLPVLAGAITMLLTDRNLNTSFFDPAGGGDPILYQHL
>COLFF285-13|KJ965409|ZMUO.005890|Sitona_lepidus

TLYFIFGAWAGMVGTSLSMLIRTELGNPGSLIGDDQIYNVIVTAHAFIMIFFMVMPMMIGGFGNWLVPLMLGAPDMAFPRLNNMSFWLLPPSLTLLLMSSIVDKGAGTGWTVYPPLSANIAHEGASVDLAIFSLHMAGISSILGAINFISTIINMRPKG
MTFDRTALFVWSVKITAILLLLSLPVLAGAITMLLTDRNINTSFFDPAGGGDPILYQHL
>COLFB181-12|KJ965439|ZMUO.001511|Ilybius_crassus
TLYFIFGAWAGMVGTSLSMLIRAELGNPGSLIGDDQIYNVIVTAHAFVMIFFMVMPIMIGGFGNWLVPLMLGAPDMAFPRMNNMSFWLLPPSLSLLLMSSMVESGAGTGWTVYPPLSAGIAHSGASVDLAIFSLHLAGISSILGAVNFITTIINMRSV
GMTFDRMPLFVWSVGITALLLLLSLPVLAGAITMLLTDRNLNTSFFDPAGGGDPILYQHL
>COLFD484-12|KJ962704|ZMUO.004284|Hypera_suspiciosa
TLYFIFGTWAGTVGTSLSILIRTELGNPGSLIGNDQIYNTIVTAHAFIMIFFMVMPIMIGGFGNWLVPLMLGAPDMAFPRLNNMSFWLLPPSLLLLLMSSMTDSGAGTGWTVYPPLSSNIAHEGSSVDLAIFSLHMAGVSSILGAINFISTVLNMRPSGM
SLDKMALFIWAVKITAILLLLSLPVLAGAITMLLTDRNINTSFFDPAGGGDPILYQHL
>COLFE617-13|KJ963276|ZMUO.005462|Coccinella_quinquepunctata
TLYFLFGMWAGMIGTSLSILIR--LGTTNSLIGNDKIYNVIVAAHAFIMIFFMVMPIMIGGFGNWLVPLMIGAPDMAFPRLNNMSFWLLPPALTLLIISSLVEMG--
AGWTVYPPLSSNLAHNGPSVDLVIFSLHLAGISSILGAVNFISTIMNMRPFGMNLDETPLFVWSVLITAILLLLSLPVLAGAITMLLTDRNINTSFFDPTGGGDPILYQHL
>COLFC098-12|KJ962708|ZMUO.002283|Amara_quenseli
TLYFIFGAWSGMVGTSLSMLIRAELGNPGALIGDDQIYNVIVTAHAFVMIFFMVMPIMIGGFGNWLVPLMLGAPDMAFPRMNNMSFWLLPPSLTLLLMSSMVESGAGTGWTVYPPLSSGIAHAGASVDLAIFSLHLAGISSILGAVNFITTIINMRSP
GMTFDRMPLFVWSVGITALLLLLSLPVLAGAITMLLTDRNLNTSFFDPAGGGDPILYQHL
>COLFA321-12|KJ964688|ZMUO.000456|Bembidion_bipunctatum
TLYFIFGAWAGMVGTSLSMLIRAELGNPGSLIGDDQIYNVIVTAHAFVMIFFMVMPILIGGFGNWLVPLMLGAPDMAFPRMNNMSFWLLPPSLTLLLMSSMVESGAGTGWTVYPPLSSSIAHSGASVDLAIFSLHLAGVSSILGAVNFITTIINMRSIG
MSFDRMPLFVWSVGITALLLLLSLPVLAGAITMLLTDRNLNTSFFDPAGGGDPILYQHL
>COLFB078-12|KJ963284|ZMUO.001408|Alianta_incana
TLYFIFGAWAGMVGTSLSLLIRAELGNPGSLIGDDQIYNVIVTAHAFVMIFFMVMPIMIGGFGNWLVPLMLGAPDMAFPRMNNMSFWLLPPSLSLLLMSSMVESGAGTGWTVYPPLSSNIAHGGASVDLAIFSLHLAGISSILGAVNFISTVINMRST
GITFDRMPLFVWAVAITALLLLLSLPVLAGAITMLLTDRNLNTSFFDPAGGGDPILYQHL
>COLFE796-13|KJ964573|ZMUO.005641|Ilybius_quadriguttatus
TLYFIFGAWAGMVGTSLSMLIRAELGNPGSLIGDDQIYNVIVTAHAFVMIFFMVMPIMIGGFGNWLVPLMLGAPDMAFPRMNNMSFWLLPPSLSLLLMSSMVESGAGTGWTVYPPLSAGIAHSGASVDLAIFSLHLAGISSILGAVNFITTIINMRSV
GMTFDRMPLFVWSVGITALLLLLSLPVLAGAITMLLTDRNLNTSFFDPAGGGDPILYQHL
>COLFF061-13|KJ965864|ZMUO.005761|Cercyon_marinus
TLYFIFGAWAGMVGTSLSILIRAELGNPGTLIGDDQIYNVIVTAHAFIMIFFMVMPIMIGGFGNWLVPLMLGAPDMAFPRMNNMSFWLLPPSLTLLLMSSMVESGAGTGWTVYPPLSSNIAHGGASVDLAIFSLHLAGISSILGAVNFITTVINMRSPSL
TYDRLPLFVWSVAITALLLLLSLPVLAGAITMLLTDRNLNTSFFDPAGGGDPILYQHL
>COLFC820-12|KJ962333|ZMUO.003385|Coelostoma_orbiculare
TLYFIFGAWAGMLGTSLSILIRTELGNPGSLIGNDQIYNVIVTAHAFIMIFFMVMPIMIGGFGNWLVPLMLGAPDMAFPRMNNMSFWLLPPSLTLLLMSSMVESGAGTGWTVYPPLSANIAHSGASVDLAIFSLHLAGISSILGAVNFITTVINMRSSN
MTYDRMPLFVWSVAITALLLLLSLPVLAGAITMLLTDRNFNTSFFDPAGGGDPILYQHL
>COLFF860-13|KJ966282|ZMUO.006655|Aphodius_prodromus
TLYFLFGSWAGMVGTSLSLLIRAELGNPGSLIGDDQIYNVIVTAHAFVMIFFMVMPILIGGFGNWLVPLMLGAPDMAFPRMNNMSFWLLPPSLTLLLMSSMVESGAGTGWTVYPPLSSNIAHGGASVDLAIFSLHLAGISSILGAVNFITTVINMRSPG
LTFDRMPLFVWSVAITALLLLLSLPVLAGAITMLLTDRNLNTSFFDPAGGGDPILYQHL
>COLFB017-12|KJ965909|ZMUO.001347|Enochrus_testaceus
TLYFIFGAWAGMVGTSLSILIRAELGNPGSLIGDDQIYNVIVTAHAFIMIFFMVMPIMIGGFGNWLVPLMLGAPDMAFPRMNNMSFWLLPPSLTLLLMSSMVESGAGTGWTVYPPLSSNIAHGGASVDLAIFSLHLAGISSILGAVNFITTVINMRSPS
MTYDRLPLFVWSVAITALLLLLSLPVLAGAITMLLTDRNLNTSFFDPAGGGDPILYQHL
>COLFF889-13|KJ962945|ZMUO.006684|Patrobus_atrorufus
TLYFIFGAWSGMVGTSLSMLIRAELGNPGSLIGDDQIYNVIVTAHAFVMIFFMVMPIMIGGFGNWLVPLMLGAPDMAFPRMNNMSFWLLPPSLTLLLMSSMVESGAGTGWTVYPPLSSGIAHSGASVDLAIFSLHLAGISSILGAVNFITTIINMRSVG
MTFDRMPLFVWSVGITALLLLLSLPVLAGAITMLLTDRNLNTSFFDPAGGGDPILYQHL
>COLFD568-12|KJ966946|ZMUO.004368|Bidessus_grossepunctatus
TLYFLFGAWSGMVGTSLSMLIRAELGNPGSLIGDDQIYNVIVTAHAFIMIFFMVMPIMIGGFGNWLVPLMLGAPDMAFPRMNNMSFWLLPPSLSLLMMSSMVESGAGTGWTVYPPLSSGIAHGGASVDLAIFSLHLAGISSILGAVNFITTIINMRSV
GMTFDRMPLFVWSVGITALLLLLSLPVLAGAITMLLTDRNLNTSFFDPAGGGDPILYQHL
>COLFF447-13|KJ965844|ZMUO.005965|Laccobius_striatulus
TLYFIFGAWAGMVGTSLSILIRAELGNPGTLIGDDQIYNVIVTAHAFIMIFFMVMPIMIGGFGNWLVPLMLGAPDMAFPRMNNMSFWLLPPSLTLLLMSSMVESGAGTGWTVYPPLSSNIAHGGASVDLAIFSLHLAGISSILGAVNFITTVINMRSNN
MTYDRLPLFVWSVAITALLLLLSLPVLAGAITMLLTDRNLNTSFFDPAGGGDPILYQHL
>COLFF541-13|KJ964018|ZMUO.006621|Bembidion_deletum
TLYFIFGAWSGMVGTSLSMLIRAELGNPGSLIGDDQIYNVIVTAHAFVMIFFMVMPILIGGFGNWLVPLMLGAPDMAFPRMNNMSFWLLPPSLTLLLMSSLVEKGAGTGWTVYPPLSSSIAHSGASVDLAIFSLHLAGVSSILGAVNFITTIINMRSIGM
SFDRMPLFVWSVGITALLLLLSLPVLAGAITMLLTDRNLNTSFFDPAGGGDPILYQHL

>COLFE1588-13|KJ965843|ZMUO.007478|Bembidion_pygmaeum
TLYFIFGAWSGMVGTSLSILIRAELGNPGSLIGDDQIYNVIVTAHAFIMIFFMVMPILIGGFGNWLVPLMLGAPDMAFPRMNNMSFWLLPPALSLLLMSSMVESGAGTGWTVYPPLSSSIAHSGASVDLAIFSLHLAGVSSILGAVNFITTIINMRSVGIT
FDRMPLFVWSVGITALLLLLSLPVLAGAITMLLTDRNLNTSFFDPAGGGDPILYQHL
>COLFA284-12|KJ963829|ZMUO.000419|Megarthrus_depressus
TLYFIFGAWAGMIGTSLSILIRAELGNPGSLIGDDQIYNVIVTAHAFIMIFFMVMPIVIGGFGNWLVPLMLGAPDMAFPRMNNMSFWLLPPSISLLLISSMVESGAGTGWTVYPPLSSNIAHGGASVDLAIFSLHLAGISSILGAVNFITTVINMRSMGMT
FDRMPLFVWSVAITALLLLLSLPVLAGAITMLLTDRNINTSFFDPAGGGDPILYQHL
>COLFD474-12|KJ965117|ZMUO.003894|Cantharis_lateralis
TLYFIFGAWSGSLGLALSLLIRAELGTPGTLIGNDQIYNVIVTAHAFIMIFFMVMPIMIGGFGNWLVPLMLGAPDMAFPRMNNMSFWFLPPSLMFLLMSSMVESGAGTGWTVYPPLSANIAHSGPSVDLAIFSLHMAGVSSILGAVNFISTIMNMKPP
SMKFDQMPLFVWSVGITALLLLLSLPVLAGAITMLLSDRNLNTSFFDPMGGGDPILYQHL
>COLFC595-12|KJ966222|ZMUO.003160|Malthodes_brevicollis
TLYFMFGAWSGMLGTSLSLLIRAELGSPGSLIGNDQIYNVIVTAHAFIMIFFMVMPIMIGGFGNWLVPLMLGAPDMAFPRMNNMSFWFLPPSLSLLLMSSIVENGAGTGWTVYPPLSANIAHSGSSVDLAIFSLHMAGISSILGAVNFISTVINMRSPG
MTFDRMPLFVWAVAITALLLLLSLPVLAGAITMLLTDRNLNTSFFDPAGGGDPILYQHL
>COLFF984-13|KJ965042|ZMUO.007349|Bembidion_obtusum
TLYFIFGAWSGMVGTSLSMLIRAELGNPGSLIGDDQIYNVIVTAHAFVMIFFMVMPILIGGFGNWLVPLMLGAPDMAFPRMNNMSFWLLPPSLTLLLMSSMVESGAGTGWTVYPPLSSSIAHSGASVDLAIFSLHLAGVSSILGAVNFITTIINMRSIG
MTFDRMPLFVWSVGITALLLLLSLPVLAGAITMLLTDRNLNTSFFDPAGGGDPILYQHL
>COLFA070-10|HM909088|MP00260|Carabus_clathratus
TLYFIFGAWSGMVGTSLSMLIRAELGNPGSLIGDDQIYNVIVTAHAFVMIFFMVMPIMIGGFGNWLVPLMLGAPDMAFPRMNNMSFWLLPPSLTLLLMSSVVEKGAGTGWTVYPPLSSGIAHSGASVDLAIFSLHLAGISSILGAVNFITTIINMRSVG
MTFDRMPLFVWSVGITALLLLLSLPVLAGAITMLLTDRNLNTSFFDPAGGGDPILYQHL
>COLFE1443-13|KJ962622|ZMUO.007238|Eutrichapion_viciae
TLYFIFGLWSGMVGTSLSMLIRIELGGPGSLIGDDQIYNVIVTAHAFIMIFFMVMPIMIGGFGNWLVPLMLGAPDMAFPRMNNMSFWLLPPSLTLLLMSSIVEKGAGTGWTVYPPLASNIAHGGASVDLAIFSLHLAGISSILGAVNFISTVINMYPNGL
SLDQLSLFTWAVKITAILLLLSLPVLAGAITMLLTDRNINTSFFDPAGGGDPILYQHL
>COLFC148-12|KJ963497|ZMUO.002333|Falagria_caesa
TLYFIFGAWAGMVGTSLSLLIRAELGNPGSLIGDDQIYNVIVTAHAFIMIFFMVMPIVIGGFGNWLVPLMLGAPDMAFPRMNNMSFWLLPPSLSLLLMSSMVESGAGTGWTVYPPLSSNIAHGGASVDLAIFSLHLAGISSILGAVNFISTVINMRSIGIT
FDRMPLFVWSVAITALLLLLSLPVLAGAITMLLTDRNLNTSFFDPAGGGDPILYQHL
>COLFF581-13|KJ964689|ZMUO.006471|Neocoenorrhinus_germanicus
TLYFIFGAWSGMLGTSLSLLIRAELGNPGSLIGDDQIYNVIVTAHAFIMIFFMVMPIMIGGFGNWLVPLMLGAPDMAFPRMNNMSFWLLPPSLSLLIMSSIVEKGAGTGWTVYPPLSSNIAHGGSSVDLAIFSLHLAGISSILGAVNFISTVINMRPQGM
SLDRMPLFVWAVAITALLLLSLPVLAGAITMLLTDRNINTTFFDPAGGGDPILYQHL
>COLFE917-13|KJ964422|ZMUO.006237|Lesteva_monticola
TLYFIFGAWAGMVGTSLSMLIRAELGNPGSLIGDDQIYNVIVTAHAFVMIFFMVMPIVIGGFGNWLVPLMLGAPDMAFPRMNNMSFWLLPPSLTLLLMSSMVESGAGTGWTVYPPLSSNIAHGGASVDLAIFSLHLAGISSILGAVNFITTVINMRST
GMTFDRMPLFVWSVAITALLLLLSLPVLAGAITMLLTDRNLNTSFFDPAGGGDPILYQHL
>COLFC498-12|KJ966246|ZMUO.003063|Apocatops_nigrita
TLYFIFGAWAGMVGTSLSLLIRAELGNPGSLIGDDQIYNVIVTAHAFVMIFFMVMPIVIGGFGNWLVPLMLGAPDMAFPRMNNMSFWLLPPSLSLLLMSSIVENGAGTGWTVYPPLSANIAHSGSSVDLAIFSLHLAGISSILGAVNFITTVINMRATG
MTLDKMPLFVWSVAITALLLLLSLPVLAGAITMLLTDRNLNTSFFDPAGGGDPILYQHL
>COLFD601-12|KJ964616|ZMUO.004401|Amara_brunnea
TLYFIFGTWSGMVGTSLSMLIRAELGNPGALIGDDQIYNVIVTAHAFVMIFFMVMPIMIGGFGNWLVPLMLGAPDMAFPRMNNMSFWLLPPSLSLLLMSSLVESGAGTGWTVYPPLSSNIAHAGASVDLAIFSLHLAGISSILGAVNFITTIINMRSIG
MTFDRMPLFVWSVGITALLLLLSLPVLAGAITMLLTDRNLNTSFFDPAGGGDPILYQHL
>COLFA542-12|KJ964016|ZMUO.000677|Dytiscus_circumcinctus
TLYFIFGAWAGMVGTSLSMLIRAELGNPGSLIGDDQIYNVIVTAHAFVMIFFMVMPIMIGGFGNWLVPLMLGAPDMAFPRMNNMSFWLLPPSLTLLLMSSMVESGAGTGWTVYPPLSASIAHGGASVDLAIFSLHLAGISSILGAVNFITTIINMRSV
GMTLDRMPLFVWSVGITALLLLLSLPVLAGAITMLLTDRNLNTSFFDPAGGGDPILYQHL
>COLFC665-12|KJ967501|ZMUO.003230|Sitona_lineellus
TLYFIFGAWAGMVGTSLSVLIRTELGNPGSLIGDDQIYNVIVTAHAFIMIFFMVMPMMIGGFGNWLVPLMLGAPDMAFPRLNNMSFWLLPPSLTLLLMSSIVDKGAGTGWTVYPPLSANIAHEGASVDLAIFSLHMAGISSILGAINFISTVINMRPTG
MTLDRMTLFVWAVKITAILLLLSLPVLAGAITMLLTDRNINTSFFDPAGGGDPILYQHL
>COLFF487-13|KJ964655|ZMUO.006567|Cryptolestes_ferrugineus
TLYFIFGSWAGMAGTSLSLLIRTELGTPGSLIGDDQIYNVIVTAHAFIMIFFMVMPIMIGGFGNWLVPLMLGAPDMAFPRMNNMSFWLLPPSLSLLLMSSIVEKGAGTGWTVYPPLSSNIAHGGSSVDLAIFSLHLAGISSILGAVNFISTVINMRPQGM
TLERMPLFVWAVVITAILLLLSLPVLAGAITMLLTDRNINTSFFDPAGGGDPILYQHL
>COLFE612-13|KJ964295|ZMUO.005457|Oxypselaphus_obscurus

TLYFIFGAWSGMVGTSLSMLIRAELGNPGALIGDDQIYNVIVTAHAFIMIFFMVMPIMIGGFGNWLVPLMLGAPDMAFPRMNNMSFWLLPPSLTLLLMSSMVESGAGTGWTVYPPLSSGIAHAGSSVDLAIFSLHLAGISSILGAVNFITTIINMRSVG
MTFDRMPLFVWSVGITALLLLLSLPVLAGAITMLLTDRNLNTSFFDPAGGGDPILYQHL
>COLFF1010-13|KJ963545|ZMUO.007375|Mannerheimia_arctica
TLYFVFGAWAGMVGTSLSILIRAELGNPGSLIGDDQIYNVIVTAHAFIMIFFMVMPIVIGGFGNWLVPLMLGAPDMAFPRMNNMSFWLLPPSLTLLLMSSMVESGAGTGWTVYPPLSSNIAHGGSSVDLAIFSLHLAGISSILGAVNFITTVINMRSSG
MTFDRMPLFVWSVVITALLLLLSLPVLAGAITMLLTDRNLNTSFFDPAGGGDPILYQHL
>COLFB894-12|KJ962039|ZMUO.001939|Stenostola_dubia
TLYFLFGAWAGMVGTSLSLLIRTELGTPGSLIGNDQIYNVIVTAHAFIMIFFMVMPIMIGGFGNWLVPLMLGAPDMAFPRMNNMSFWLLPPSLSLLIMSSIVDNGAGTGWTVYPPLAANIAHSGSSVDLAIFSLHLAGISSILGAVNFITTVINMRPQG
MNMDRMPLFVWAVKITAILLLLSLPVLAGAITMLLTDRNLNTSFFDPAGGGDPILYQHL
>COLFE440-12|KJ967011|ZMUO.005000|Phyllotreta_vittula
TLYFIFGIWSGMVGMSMSMLIRIELAAPGSLIGNDQIYNVIVTAHAFIMIFFMVMPIMIGGFGNWLVPLMIGAPDMAFPRMNNMSFWLLPPSLFLLIMSSIVENGAGTGWTVYPPLSANISHAGSSVDLTIFSLHLAGISSILGAINFITTIINMRPKGMS
FDRMPLFVWAVLITAILLLLSLPVLAGAITMLLTDRNLNTSFFDPMGGGDPILYQHL
>COLFC127-12|KJ966662|ZMUO.002312|Dyschirius_salinus
TLYFIFGIWSGMVGTSLSILIRAELGNPGSLIGDDQIYNVIVTAHAFIMIFFMVMPIMIGGFGNWLVPLMLGAPDMAFPRMNNMSFWLLPPSLSLLLMSSMVEKGAGTGWTVYPPLSSSIAHSGASVDLAIFSLHLAGISSILGAVNFITTIINMRSTGMT
FERMPLFIWSVGITALLLLLSLPVLAGAITMLLTDRNLNTSFFDPAGGGDPILYQHL
>COLFF019-13|KJ965998|ZMUO.005719|Cyphon_ochraceus
TLYFIFGSWSGMVGTSLSLLIRAELGTPGSLIGDDQIYNVIVTAHAFIMIFFMVMPIMIGGFGNWLVPLMLGAPDMAFPRMNNMSFWLLPPSLTLLLMSSMVENGAGTGWTVYPPLSAGIAHSGASVDLAIFSLHLAGISSILGAVNFISTVINMRSVG
MSFDRMPLFVWAVAITALLLLLSLPVLAGAITMLLTDRNLNTSFFDPAGGGDPILYQHL
>COLFF545-13|KJ961972|ZMUO.006625|Bembidion_assimile
TLYFIFGAWSGMVGTSLSMLIRAELGNPGSLIGDDQIYNVIVTAHAFVMIFFMVMPILIGGFGNWLVPLMLGAPDMAFPRMNNMSFWLLPPSLTLLLMSSMVESGAGTGWTVYPPLSSSIAHSGASVDLAIFSLHLAGVSSILGAVNFITTIINMRSTG
MTFDRMPLFVWSVGITALLLLLSLPVLAGAITMLLTDRNLNTSFFDPAGGGDPILYQHL
>COLFE1059-13|KJ963335|ZMUO.006854|Olophrum_consimile
TLYFIFGAWAGMVGTSLSILIRAELGNPGTLIGDDQIYNVIVTAHAFVMIFFMVMPILIGGFGNWLVPLMLGAPDMAFPRMNNMSFWLLPPSLTLLLMSSMVESGAGTGWTVYPPLSSNIAHGGASVDLAIFSLHLAGISSILGAVNFITTVINMRATG
MTFDRMPLFVWAVVITALLLLLSLPVLAGAITMLLTDRNLNTTFFDPAGGGDPILYQHL
>COLFF584-13|KJ964960|ZMUO.006474|Ceutorhynchus_contractus
TLYFIFGSWAGMAGTSLSMLIRTELGNPGSLIGNDQIYNSIVTAHAFIMIFFMVMPILIGGFGNWLVPLMLGAPDMAFPRLNNMSFWLLPPSLSLLLMSSIVNKGAGTGWTVYPPLSGNVAHEGMSVDLAIFSLHMAGISSILGAINFISTVMNMQPK
GMTPELMPLFVWAVEITAILLLLSLPVLAGAITMLLTDRNINTSFFDPSGGGDPILYQHL
>COLFD021-12|KJ962797|ZMUO.003916|Phratora_vulgatissima
TLYFIFGIWAGMVGTSLSMLIRSELGNPGTLIGNDQIYNVIVTAHAFIMIFFMVMPIMIGGFGNWLVPLMIGAPDMAFPRLNNMSFWLLPPSLFLLLMSSVVENGAGTGWTVYPPLSSNLAHSGSSVDLAIFSLHLAGISSILGAINFITTVINMRPEGM
GLEQTPLFVWAVLITAILLLLSLPVLAGAITMLLTDRNLNTSFFDPAGGGDPILYQHL
>COLFF272-13|KJ966279|ZMUO.005877|Tachyporus_solutus
TLYFIFGAWSGMIGTSLSLLIRAELGNPGSLIGDDQIYNVIVTAHAFIMIFFMVMPIVIGGFGNWLVPLMLGAPDMAFPRMNNMSFWLLPPSLTLLLMSSMVESGAGTGWTVYPPLSANIAHSGPSVDLAIFSLHLAGISSILGAVNFITTVINMRASGM
HFDRMPLFIWSVAITALLLLLSLPVLAGAITMLLTDRNLNTSFFDPAGGGDPILYQHL
>COLFF358-13|KJ965099|ZMUO.006058|Hydrobius_fuscipes
TLYFIFGAWAGMVGTSLSILIRAELGNPGTLIGDDQIYNVIVTAHAFIMIFFMVMPIMIGGFGNWLVPLMLGAPDMAFPRMNNMSFWLLPPSLTLLLMSSMVENGAGTGWTVYPPLSSNIAHGGASVDLAIFSLHLAGISSILGAVNFITTVINMRSPN
LTYDRLPLFVWSVAITALLLLLSLPVLAGAITMLLTDRNLNTSFFDPAGGGDPILYQHL
>COLFF559-13|KJ964373|ZMUO.006639|Tritoma_bipustulata
TLYFIFGMWAGMVGTALSMLIRSELGNPGSLIGNDQIYNVIVTAHAFIMIFFMVMPIMMGGFGNWLVPLMLGAPDMAFPRLNNMSFWLLPPSLTLLIMSSIVETGAGTGWTVYPPLSSNIAHSGASVDLAIFSLHLAGISSILGAMNFISTMMNMRP
SGMQMDQMPLFVWAVLITAILLLLSLPVLAGAITMLLTDRNINTTFFDPAGGGDPILYQHL
>COLFF716-13|KJ965090|ZMUO.006416|Cyphon_palustris
TLYFIFGSWSGMVGTSLSLLIRAELGTPGSLIGDDQIYNVIVTAHAFIMIFFMVMPIMIGGFGNWLVPLMLGAPDMAFPRMNNMSFWLLPPSLTLLLMSSMVENGAGTGWTVYPPLSAGVAHSGASVDLAIFSLHLAGISSILGAVNFISTVINMRSVG
MTFDRMPLFVWAVAITALLLLLSLPVLAGAITMLLTDRNLNTSFFDPAGGGDPILYQHL
>COLFA641-12|KJ962821|ZMUO.000776|Bisnius_puella
TLYFIFGSWAGMVGTSLSLLIRAELGNPGSLIGDDQIYNVIVTAHAFIMIFFMVMPIVIGGFGNWLVPLMLGAPDMAFPRMNNMSFWLLPPSLTLLLMSSMVESGAGTGWTVYPPLSSNIAHGGASVDLAIFSLHLAGISSILGAVNFITTVINMRSTG
MSFDRMPLFVWSVAITALLLLLSLPVLAGAITMLLTDRNLNTSFFDPAGGGDPILYQHL
>COLFB405-12|KJ965388|ZMUO.001830|Myrmecocephalus_concinnus
TLYFLFGAWAGMIGTSLSLLIRAELGNPGSLIGDDQIYNVIVTAHAFIMIFFMVMPMMIGGFGNWLVPLMLGAPDMAFPRMNNMSFWLLPPSITLLLMSSMVEKGAGTGWTVYPPLSSTIAHSGSSVDLAIFSLHLAGISSILGAVNFISTVINMRAIG
MSFDRLPLFVWSVAITALLLLLSLPVLAGAITMLLTDRNLNTSFFDPAGGGDPILYQHL

>COLFD808-12|KJ967471|ZMUO.004798|Leiodes_ruficollis
TLYFIFGAWAGMVGTSLSILIRAELGNPGSLIGDDQIYNVIVTAHAFVMIFFMVMPIVIGGFGNWLVPLMLGAPDMAFPRMNNMSFWLLPPSLTLLLMSSIVENGAGTGWTVYPPLSSNIAHSGSSVDLAIFSLHLAGISSILGAVNFITTVINMRSPGM
SFDKMPLFVWSVAITALLLLLSLPVLAGAITMLLTDRNLNTSFFDPAGGGDPILYQHL
>COLFB531-12|KJ967095|ZMUO.001006|Dicheirotrichus_placidus
TLYFIFGAWSGMVGTSLSMLIRAELGTPGALIGDDQIYNVIVTAHAFVMIFFMVMPIMIGGFGNWLVPLMLGAPDMAFPRMNNMSFWLLPPSLTLLLMSSMVEKGAGTGWTVYPPLSSGIAHGGASVDLAIFSLHLAGISSILGAVNFITTIINMRSV
GMTFDRMPLFVWSVGITALLLLLSLPVLAGAITMLLTDRNLNTSFFDPAGGGDPILYQHL
>COLFB032-12|KJ962284|ZMUO.001362|Anatis_ocellata
TLYFLFGMWAGMVGTSLSILIRLELGTTNSLIGNDQIYNVIVTAHAFIMIFFMVMPIMIGGFGNWLVPLMIGAPDMAFPRLNNMSFWLLPPALTMLIMSSIVEMGAGTGWTVYPPLSSNMAHSGSSVDLVIFSLHLAGISSILGAVNFISTIMNMRPFG
MTLEKTPLFVWSVMITAILLLLSLPVLAGAITMLLTDRNINTSFFDPMGGGDPILYQHL
>COLFG101-13|KJ964442|ZMUO.007796|Holobus_apicatus
TLYFIFGAWSGMIGTSLSLLIRAELGNPGSLIGNDQIYNVIVTAHAFIMIFFMVMPIMIGGFGNWLIPLMLGAPDMAFPRMNNMSFWLLPPALYLLLLSSIIESGSGTGWTVYPPLSSNIAHGGASVDLTIFSLHMAGISSILGAVNFISTIMNMKPQMM
TLEQMPLFVWSVMITALLLLSLPVLAGAITMLLTDRNMNTSFFDPAGGGDPILYQHL
>COLFF759-13|KJ962187|ZMUO.006459|Tribolium_confusum
TLYFMFGAWSGMVGTSLSLLIRAELGNPGSLIGDDQIYNVIVTAHAFIMIFFMVMPIMIGGFGNWLVPLMLGAPDMAFPRMNNMSFWLLPPSLSLLIMSSVVESGAGTGWTVYPPLSSNIAHGGASVDLAIFSLHLAGISSILGAVNFITTVINMRPTG
MSFERMPLFVWAVVITAILLLLSLPVLAGAITMLLTDRNINTSFFDPAGGGDPILYQHL
>COLFB070-12|KJ966113|ZMUO.001400|Bembidion_bruxellense
TLYFIFGAWSGMVGTSLSMLIRAELGNPGSLIGDDQIYNVIVTAHAFVMIFFMVMPILIGGFGNWLVPLMLGAPDMAFPRMNNMSFWLLPPSLTLLLMSSMVESGAGTGWTVYPPLSSSIAHSGASVDLAIFSLHLAGVSSILGAVNFITTIINMRSIG
MSFDRMPLFVWSVGITALLLLLSLPVLAGAITMLLTDRNLNTSFFDPAGGGDPILYQHL
>COLFF030-13|KJ963369|ZMUO.005730|Astenus_gracilis
TLYFIFGAWAGMVGTSLSLLIRAELGMPGSLIGDDQIYNVIVTAHAFVMIFFMVMPIVIGGFGNWLVPLMLGAPDMAFPRMNNMSFWLLPPSLSLLLMSSMVESGAGTGWTVYPPLSSNMAHGGASVDLAIFSLHLAGISSILGAVNFITTIINMRTK
GMSYERMPLFVWSVGITALLLLLSLPVLAGAITMLLTDRNLNTSFFDPAGGGDPILYQHL
>COLFE920-13|KJ966032|ZMUO.006240|Bisnius_subuliformis
TLYFIFGSWAGMVGTSLSLLIRAELGNPGSFIGDDQIYNVIVTAHAFIMIFFMVMPIVIGGFGNWLVPLMLGAPDMAFPRMNNMSFWLLPPSLTLLLMSSMVENGAGTGWTVYPPLSSNIAHSGASVDLAIFSLHLAGISSILGAVNFITTVINMRSTGV
TFDRMPLFVWSVAITALLLLLSLPVLAGAITMLLTDRNLNTSFFDPAGGGDPILYQHL
>COLFD081-12|KJ961979|ZMUO.003976|Cerylon_histeroides
TLYFMFGMWSGMVGTSMSMMIRLELGNPGSLIGDDQIYNVIVTAHAFVMIFFMVMPIMIGGFGNWLVPLMLGAPDMAFPRMNNMSFWLLPPSLTLLIMSSIVEKGAGTGWTVYPPLSANLTHSGSSVDLAIFSLHLAGISSILGAVNFITTVINMR
PSGMTWDRLPLFVWSVIITAVLLLLSLPVLAGAITMLLTDRNLNTSFFDPAGGGDPILYQHL
>COLFD109-12|KJ962119|ZMUO.004004|Anaspis_bohemica
TLYFIFGAWSGMVGTSLSLLIRSELGTPGSLIGDDQIYNVIVTAHAFIMIFFMVMPILIGGFGNWLVPLMLGAPDMAFPRMNNMSFWLLPPSLTLLIMSSVVENGAGTGWTVYPPLAANIAHSGSSVDLAIFSLHLAGVSSILGAVNFITTVINMRPQG
MTLDRMPLFVWAVVITAVLLLLSLPVLAGAITMLLTDRNLNTSFFDPAGGGDPILYQHL
>COLFE080-12|KJ964915|ZMUO.005305|Brachyderes_incanus
TLYFIFGAWSGMVGTSLSMLIRTELGNPGSLIGDDQIYNVIVTAHAFIMIFFMVMPMMIGGFGNWLVPLMLGAPDMAFPRMNNMSFWLLPPSLSLLLMSSIVDKGAGTGWTVYPPLSANIAHEGSSVDLAIFSLHMAGVSSILGAINFISTVINMRP
MGMSPDRMSLFIWAVKITAVLLLLSLPVLAGAITMLLTDRNVNTSFFDPAGGGDPILYQHL
>COLFA412-12|KJ962903|ZMUO.000642|Anthonomus_phyllocola
TLYFIFGAWSGTVGTSLSMLIRTELGNPGSLIGDDQIYNTIVTAHAFIMIFFMVMPIMIGGFGNWLVPLMLAAPDMAFPRLNNMSFWLLPPSLTLLLMSSIVSKGAGTGWTVYPPLSSNISHEGASVDFAIFSLHMAGISSILGAMNFISTVLNMKPKG
MKLEQMALFIWAVKITAILLLISLPVLAGAITMLLTDRNINTSFFDPAGGGDPILYQHL
>COLFG046-13|KJ966016|ZMUO.007551|Stenocorus_meridianus
TLYFIFGAWAGMVGTSLSILIRSELGNPGSLIGDDQIYNVIVTAHAFVMIFFMVMPIMIGGFGNWLVPLMLGAPDMAFPRMNNMSFWLLPPSLILLLMSSIVENGAGTGWTVYPPLSSNIAHNGSSVDLAIFSLHLAGISSILGAVNFITTVINMRPQG
MTLDRLPLFVWAVVITAILLLLSLPVLAGAITMLLTDRNLNTSFFDPAGGGDPILYQHL
>COLFF397-13|KJ963753|ZMUO.005915|Chrysomela_tremula
TLYFIFGIWAGTVGTSLSILIRAELGNPGTLIGNDQIYNVIVTAHAFIMIFFMVMPIMIGGFGNWLVPLMIGAPDMAFPRLNNMSFWLLPPSLFLLLMSSIVENGAGTGWTVYPPLSTNIAHSGSSVDLAIFSLHLAGISSILGAINFITTVINMRPEGMNFE
QTPLFVWAVLITAILLLLSLPVLAGAITMLLTDRNLNTSFFDPAGGGDPILYQHL
>COLFC434-12|KJ965700|ZMUO.002999|Dyschirius_nigricornis
TLYFIFGIWSGMVGTSLSIMIRAELGNPGSLIGDDQIYNVIVTAHAFIMIFFMVMPIMIGGFGNWLVPLMLGAPDMAFPRMNNMSFWLLPPSLSLLLMSSMVEKGAGTGWTVYPPLSSSIAHSGASVDLAIFSLHLAGVSSILGAVNFITTIINMRSTG
MTFERMPLFVWSVGITALLLLLSLPVLAGAITMLLTDRNLNTSFFDPAGGGDPILYQHL
>COLFC603-12|KJ964820|ZMUO.003168|Stenus_canaliculatus

TLYFIFGAWAGMVGTSLSLLIRAELGNPGSLIGDDQIYNVIVTAHAFVMIFFMVMPIMIGGFGNWLIPLMLGAPDMAFPRMNNMSFWLLPPSLSLLLTSSIVESGAGTGWTVYPPLSSNIAHSGSSVDLAIFSLHLAGVSSILGAINFITTIFNMRTMKLQ
LDCLPLFVWAVGITAFLLLLSLPVLAGAITMLLTDRNLNTSFFDPAGGGDPILYQHL
>COLFC805-12|KJ964466|ZMUO.003370|Thryogenes_festucae
TLYFIFGAWSGMIGTSLSILIRVELGNPGSLIGDDQIYNVIVTAHAFIMIFFMVMPIMIGGFGNWLVPLMLGAPDMAFPRLNNLSFWLLPPSLMILLMSSMIEKGAGTGWTVYPPLSSNIAHSGPSVDLAIFSLHLAGISSILGAINFISTIINMRPQGMYSD
RMTLFVWAVNITAILLLLSLPVLAGAITMLLTDRNINTSFFDPAGGGDPILYQHL
>COLFF414-13|KJ966588|ZMUO.005932|Byctiscus_populi
TLYFIFGSWAGMVGTSLSLLIRAELGNPGSLIGNDQIYNVVVTAHAFIMIFFMVMPVMIGGFGNWLVPLMLGAPDMAFPRMNNMSFWLLPPSLSLLIMSSIVENGAGTGWTVYPPLSSNIAHSGSSVDLAIFSLHLAGISSILGAVNFISTVINMRPSG
MSLDRLPLFVWAVAITALLLLLSLPVLAGAITMLLTDRNINTSFFDPAGGGDPILYQHL
>COLFE991-13|KJ963017|ZMUO.006786|Bryoporus_cernuus
TLYFIFGAWAGMVGTSLSLLIRAELGNPGSLIGDDQIYNVIVTAHAFIMIFFMVMPIVIGGFGNWLVPLMLGAPDMAFPRMNNMSFWLLPPSLTLLLMSSLVESGAGTGWTVYPPLSSNIAHGGASVDLAIFSLHLAGISSILGAVNFITTVINMRSIGM
TFDRMPLFVWSVVITALLLLLSLPVLAGAITMLLTDRNLNTSFFDPSGGGDPILYQHL
>COLFE1008-13|KJ967042|ZMUO.006803|Eucnecosum_brachypterum
TLYFIFGAWAGMVGTSLSILIRAELGNPGTLIGDDQIYNVIVTAHAFVMIFFMVMPIVIGGFGNWLVPLMLGAPDMAFPRMNNMSFWLLPPSLTLLLMSSMVESGAGTGWTVYPPLSSNIAHGGSSVDLAIFSLHLAGISSILGAVNFITTVINMRTTG
MTFDRMPLFVWSVAITALLLLLSLPVLAGAITMLLTDRNLNTSFFDPAGGGDPILYQHL
>COLFF761-13|KJ964192|ZMUO.006081|Calathus_erratus
TLYFIFGAWAGMVGTSLSMLIRAELGNPGALIGDDQVYNVIVTAHAFVMIFFMVMPIMIGGFGNWLVPLMLGAPDMAFPRMNNMSFWLLPPSLTLLLMSSMVESGAGTGWTVYPPLSSGIAHSGASVDLAIFSLHLAGISSILGAVNFITTIINMRSV
GMTFDRMPLFVWSVGITALLLLLSLPVLAGAITMLLTDRNLNTSFFDPAGGGDPILYQHL
>COLFB203-12|KJ964052|ZMUO.001533|Phratora_laticollis
TLYFIFGIWAGMVGTSLSVLIRSELGNPGTLIGNDQIYNVIVTAHAFIMIFFMVMPIMIGGFGNWLVPLMIGAPDMAFPRLNNMSFWLLPPSLFLLLMSSVVENGAGTGWTVYPPLSSNLAHSGSSVDLAIFSLHLAGISSILGAINFITTVINMRPEGMN
LEQTPLFVWAVLITAILLLLSLPVLAGAITMLLTDRNLNTSFFDPAGGGDPILYQHL
>COLFF1032-13|KJ963770|ZMUO.007397|Ceutorhynchus_gallorhenanus
TLYFIFGSWAGMAGTSLSMLIRTELGNPGSLIGNDQIYNSIVTAHAFIMIFFMVMPILIGGFGNWLVPLMLGAPDMAFPRLNNMSFWLLPPSLSLLLMSSIVNKGAGTGWTVYPPLSGNVAHEGMSVDLAIFSLHMAGISSILGAINFISTVMNMQPK
GMTPELMPLFVWAVQITAILLLISLPVLAGAITMLLTDRNINTSFFDPSGGGDPILYQHL
>COLFF678-13|KJ964560|ZMUO.006378|Altica_palustris
TLYFLFGIWAGMIGTSMSLLIRTELGSPGSLIGNDQIYNVIVTAHAFVMIFFMVMPMMIGGFGNWLVPLMIGAPDMAFPRMNNMSFWLLPPSIFLLLMSSLTESGAGTGWTVYPPLSSNLAHNGPSVDLAIFSLHLAGISSILGAINFITTMINMRPQG
MSMDQMPLFVWAVYITAILLLLSLPVLAGAITMLLTDRNLNTSFFEPAGGGDPILYQHL
>COLFA595-12|KJ962487|ZMUO.000730|Selatosomus_cruciatus
TLYFLFGAWAGMLGTSLSLLIRAELGNPGSLIGNDQIYNVIVTAHAFIMIFFMVMPIMIGGFGNWLVPLMLGAPDMAFPRMNNMSFWFLPPSLSLLLMSSIVENGAGTGWTVYPPLSSNIAHSGSSVDLAIFSLHLAGISSILGAVNFISTVINMRSTGIT
FDRMPLFVWAVAITALLLLLSLPVLAGAITMLLTDRNLNTSFFDPAGGGDPILYQHL
>COLFE851-13|KJ961986|ZMUO.005696|Phyllotreta_striolata
TLYFIFGIWSGMMGMSMSMLIRIELAAPGSLIGNDQIYNVIVTAHAFIMIFFMVMPIMIGGFGNWLVPLMIGAPDMAFPRMNNMSFWLLPPSLFLLIMSSIIENGAGTGWTVYPPLSSNVSHAGASVDLTIFSLHLAGISSILGAINFITTVINMRPKG
MTFDRMPLFVWAVLITAILLLLSLPVLAGAITMLLTDRNLNTSFFDPMGGGDPILYQHL
>COLFB251-12|KJ964145|ZMUO.001581|Hydroporus_neglectus
TLYFLFGAWSGMVGTSLSMLIRAELGNPGSLIGDDQIYNVIVTAHAFIMIFFMVMPIMIGGFGNWLVPLMLGAPDMAFPRMNNMSFWLLPPSLTLLLMSSMVENGAGTGWTVYPPLSSGIAHSGASVDLAIFSLHLAGISSILGAVNFITTIINMRSIG
MTFDRMPLFVWSVGITALLLLLSLPVLAGAITMLLTDRNLNTSFFDPAGGGDPILYQHL
>COLFD189-12|KJ966057|ZMUO.004084|Omalium_rugatum
TLYFIFGAWAWMVGTSLSLLIRAELGNPGSLIGDDQIYNVIVTAHAFIMIFFMVMPIVIGGFGNWLVPLMLGAPDMAFPRMNNMSFWLLPPSLTLLLMSSMVESGAGTGWTVYPPLSSNIAHGGSSVDLAIFSLHLAGISSILGAVNFITTVINMRAM
GMTFDRMPLFVWSVVITALLLLLSLPVLAGAITMLLTDRNLNTSFFDPAGGGDPILYQHL
>COLFE1209-13|KJ967253|ZMUO.007004|Cyphon_variabilis
TLYFIFGSWSGMVGTSLSLLIRAELGTPGSLIGDDQIYNVIVTAHAFIMIFFMVMPIMIGGFGNWLVPLMLGAPDMAFPRMNNMSFWLLPPSLTLLLMSSMVENGAGTGWTVYPPLSAGVAHSGASVDLAIFSLHLAGISSILGAVNFISTVINMRSVG
MTFDRMPLFVWAVAITALLLLLSLPVLAGAITMLLTDRNLNTSFFDPAGGGDPILYQHL
>COLFB073-12|KJ967141|ZMUO.001403|Stenus_strandi
TLYFIFGAWAGMVGTSLSLLIRAELGNPGSLIGDDQIYNVIVTAHAFVMIFFMVMPVMIGGFGNWLVPLMLGAPDMAFPRMNNMSFWLLPPSLSLLLMSSIVENGAGTGWTVYPPLSSNIAHSGASVDLAIFSLHLAGISSILGAINFITTIFNMRTMK
LHLDCLPLFVWSVGITALLLLLSLPVLAGAITMLLTDRNLNTSFFDPAGGGDPILYQHL
>COLFF277-13|KJ768192|ZMUO.005882|Agrilus_betuleti
TLYFFFGAWSGMVGTALSLLIRAELGNPGALIGNDQIYNVIVTAHAFVMIFFMVMPIMMGGFGNWLVPLMLGAPDMAFPRMNNMSFWLLPPSLTLLLMSSTVESGAGTGWTVYPPLAANIAHSGASVDLAIFSLHLAGISSILGAINFITTVINMRPP
GMTLDRIPLLVWSIAITALLLLLSLPVLAGAITMLLSDRNLNTSFFDPAGGGDPILYQHL

>COLFD830-12|KJ965296|ZMUO.004820|Antherophagus_pallens
TLYFIFGSWAGMIGTSLSLLIRSELGTPGSLIGDDQIYNVIVTAHAFVMIFFMVMPIMIGGFGNWLVPLMLGAPDMAFPRMNNMSFWLLPPSLLLLLMSSIADKGVGTGWTVYPPLSSNIAHGGSSVDLAIFSLHLAGISSILGAVNFISTIMNMNPAG
MSLDRMPLFVWAVMITAILLLLSLPVLAGAITMLLTDRNLNTSFFDPAGGGDPILYQHL
>COLFB395-12|KJ966209|ZMUO.001820|Quedius_cinctus
TLYFIFGAWAGMVGTSLSLLIRAELGNPGSMIGDDQIYNVIVTAHAFIMIFFMVMPTLIGGFGNWLVPLMLGAPDMAFPRMNNMSFWLLPPSLSLLLMSSMVESGAGTGWTVYPPLSSNIAHGGASVDLAIFSLHLAGISSILGAVNFITTVINMRSIG
MSFDRMPLFVWSVAITALLLLLSLPVLAGAITMLLTDRNLNTSFFDPAGGGDPILYQHL
>COLFE1508-13|KJ962586|ZMUO.007303|Longitarsus_exsoletus
TLYFIFGVWAGMVGTSLSILIRTELGNPGSLIGNDQIYNVIVTAHAFIMIFFMVMPIMIGGFGNWLVPLMIGAPDMAFPRMNNMSFWLLPPSLLLLVMSSMVESGAGTGWTVYPPLSSNIAHNGSSVDLAIFSLHLAGISSILGAINFITTVINMRPFGM
TLDRMPLFVWAVVITAILLLLSLPVLAGAITMLLTDRNFNTTFFDPAGGGDPILYQHL
>COLFA639-12|KJ965577|ZMUO.000774|Aleochara_lanuginosa
TLYFIFGAWAGMVGTSLSLLIRAELGNPGSLIGDDQIYNVIVTAHAFIMIFFMVMPIVIGGFGNWLVPLMLGAPDMAFPRMNNMSFWLLPPSLTLLLMSSMVESGAGTGWTVYPPLSSNIAHGGSSVDLAIFSLHLAGISSILGAVNFISTVINMRSSG
MTFDKMPLFVWSVAITALLLLLSLPVLAGAITMLLTDRNLNTSFFDPAGGGDPILYQHL
>COLFD899-12|KJ961992|ZMUO.004509|Cafius_xantholoma
TLYFIFGSWAGMVGTSLSLLIRAELGNPGTLIGDDQIYNVIVTAHAFIMIFFMVMPIVIGGFGNWLVPLMLGAPDMAFPRMNNMSFWLLPPSLTLLLMSSMVESGAGTGWTVYPPLSSNIAHGGASVDLAIFSLHLAGISSILGAVNFITTVINMRSTG
MTFDRMPLFVWSVAITALLLLLSLPVLAGAITMLLTDRNLNTSFFDPAGGGDPILYQHL
>COLFD115-12|KJ963086|ZMUO.004010|Epuraea_silacea
TLYFIFGAWSGMVGTSLSILIRTELGSPGSLIGNDQIYNVIVTAHAFIMIFFMVMPFMIGGFGNWLVPLMLGAPDMAFPRMNNMSFWLLPPSLSLLLMSSIVESGAGTGWTVYPPLSSNIAHGGSSVDLAIFSLHLAGISSILGAVNFITTIINMRPVGMT
LDRMPLFVWSVVITAILLLLSLPVLAGAITMLLTDRNLNTTFFDPSGGGDPILYQHL
>COLFF845-13|KJ963428|ZMUO.006165|Halyzia_sedecimguttata
TLYFLFGMWAGMVGTSLSIMIRLELGTTNSLIGNDQIYNVIVTAHAFIMIFFMVMPVMIGGFGNWLVPLMVGAPDMAFPRLNNMSFWLLPPALTLLIFSSMVEMGAGTGWTVYPPLSSNMAHSGSSVDLVIFSLHLAGISSILGAVNFISTIMNMRP
FGMNLDKTPLFVWSVLITAILLLLSLPVLAGAITMLLTDRNINTSFFDPMGGGDPILYQHL
>COLFE1509-13|KJ965049|ZMUO.007304|Psylliodes_napi
TLYFIFGVWSGMIGTSLSILIRAELGSPGSLIGNDQIYNVIVTAHAFIMIFFMVMPIMIGGFGNWLVPLMIGAPDMAFPRMNNMSFWLLPPSLLLLLMSSMVESGAGTGWTVYPPLSSNIAHGGSSVDLAIFSLHLAGISSILGAINFITTVINMRPEGMT
LDRMPLFVWAVVITAILLLLSLPVLAGAITMLLTDRNLNTSFFDPIGGGDPILYQHL
>COLFC069-12|KJ964913|ZMUO.002064|Helochares_obscurus
TLYFIFGAWAGMVGTSLSILIRAELGNPGSLIGDDQIYNVIVTAHAFIMIFFMVMPIMIGGFGNWLVPLMLGAPDMAFPRMNNMSFWLLPPSLTLLLMSSMVESGAGTGWTVYPPLSSNIAHGGASVDLAIFSLHLAGISSILGAVNFITTVINMRSPH
MTYDRLPLFVWSVAITALLLLLSLPVLAGAITMLLTDRNLNTSFFDPAGGGDPILYQHL
>COLFB096-12|KJ964906|ZMUO.001426|Rhynchaenus_rusci
TLYFMFGAWSGMVGTSLSMLIRTELGTPGKLIGDDQIYNTIVTAHAFIMIFFMVMPILIGGFGNWLIPLMLGAPDMAFPRLNNMSFWLLPPSLTLLLTSSIMDKGAGTGWTVYPPLSGNVSHEGASVDLAIFSLHMAGISSILGAINFISTVANMRASG
MNTDQMSLFVWAVKITAILLLLSLPVLAGAITMLLTDRNINTSFFDPAGGGDPILYQHL
>COLFE1089-13|KJ963976|ZMUO.006884|Magdalis_carbonaria
TLYLIFGAWSGMTGTSLSMLIRVELGNPGSLIGNDQIYNTIVTAHAFIMIFFMVMPIMIGSFGNWLIPLMLGAPDMAFPRLNNMSFWLLPPSLTLLLMSSIIDKGAGTGWTVYPPLSSNIAHEGSSIDLAIFSLHLAGISSILGALNFISTIINMRPTNMKLD
QMSLFIWAVKITAILLLLSLPVLAGAITMLLTDRNINTSFFDPAGGGDPILYQHL
>COLFC406-12|KJ964166|ZMUO.002971|Cryptophagus_tuberculosus
TLYFIFGAWASMIGTSLSLLIRSELGTPGSLIGDDQIYNVIVTAHAFVMIFFMVMPIMIGGFGNWLVPLMLGAPDMAFPRMNNMSFWLLPPSLTLLLMSSIADKGVGTGWTVYPPLSSNIAHGGSSVDLAIFSLHLAGISSILGAVNFISTIMNMHPTG
MTLDRMPLFVWAVMITAILLLLSLPVLAGAITMLLTDRNLNTSFFDPAGGGDPILYQHL
>COLFB106-12|KJ963327|ZMUO.001436|Rhagonycha_nigriventris
TLYFIFGAWSGSLGLALSLLIRAELGTPGTLIGNDQIYNVIVTAHAFIMIFFMVMPIMIGGFGNWLVPLMLGAPDMAFPRMNNMSFWFLPPSLMFLLMSSMVESGAGTGWTVYPPLSANIAHSGPSVDLAIFSLHMAGISSILGAVNFISTILNMKPPS
MKFDQMPLFVWSVGITALLLLLSLPVLAGAITMLLSDRNLNTSFFDPMGGGDPILYQHL
>COLFB423-12|KJ965580|ZMUO.001848|Paradromius_linearis
TLYFIFGAWAGMVGTSLSMLIRAELGNPGALIGDDQIYNVIVTAHAFIMIFFMVMPIMIGGFGNWLVPLMLGAPDMAFPRMNNMSFWLLPPSLTLLLMSSMVESGAGTGWTVYPPLSSGIAHAGASVDLAIFSLHLAGISSILGAVNFITTIINMRSIG
MTFDRMPLFVWSVGITALLLLLSLPVLAGAITMLLTDRNLNTSFFDPAGGGDPILYQHL
>COLFB351-12|KJ964407|ZMUO.001681|Dorytomus_melanophthalmus
TLYFIFGAWSGMVGTSLSMLIRTELGNPGSLIGDDQIYNTIVTAHAFIMIFFMVMPIMIGGFGNWLIPLMLGAPDMAFPRLNNMSFWLLPPSLSLLLMSSIVDKGAGTGWTVYPPLSSNIAHEGTSVDLAIFSLHMAGISSILGAMNFISTVMNMRPAG
MKTDRMSLFIWAVKITAILLLLSLPVLAGAITMLLTDRNINTSFFDPAGGGDPILYQHL
>COLFF043-13|KJ966999|ZMUO.005743|Philonthus_cognatus

TLYFIFGSWAGMVGTSLSLLIRAELGNPGSLIGDDQIYNVIVTAHAFIMIFFMVMPIMIGGFGNWLVPLMLGAPDMAFPRMNNMSFWLLPPSLTLLLMSSMVESGAGTGWTVYPPLSSNIAHDGASVDLAIFSLHLAGISSILGAVNFITTVINMRSAG
MSFDRMPLFVWSVAITALLLLLSLPVLAGAITMLLTDRNLNTTFFDPTGGGDPILYQHL
>COLFB446-12|KJ964411|ZMUO.001871|Oxymirus_cursor
TLYFVFGAWAGMVGTSLSMLIRSELGNPGSLIGDDQIYNVIVTAHAFVMIFFMVMPILIGGFGNWLVPLMLGAPDMAFPRMNNMSFWLLPPSLTLLIMSSIVENGAGTGWTVYPPLSSNIAHSGSSVDLAIFSLHLAGISSILGAVNFISTVINMRSPN
MTFDQMPLFVWAVVITAILLLLSLPVLAGAITMLLTDRNLNTSFFDPAGGGDPILYQHL
>COLFF420-13|KJ962100|ZMUO.005938|Phyllobius_maculicornis
TLYFIFGAWSGMMGTSLSILIRVELGNPGSLIGDDQIYNVIVTAHAFIMIFFMVMPMMIGGFGNWLVPLMLGAPDMAFPRLNNMSFWLLPPSLSLLLMSSIVDKGAGTGWTVYPPLSANIAHEGSSVDLAIFSLHMAGVSSILGAINFISTVINMRPSG
MSPDRMSLFIWAVKITAILLLLSLPVLAGAITMLLTDRNMNTSFFDPAGGGDPILYQHL
>COLFC302-12|KJ962383|ZMUO.002487|Bembidion_virens
TLYFIFGAWSGMVGTSLSMLIRAELGNPGSLIGDDQIYNVIVTAHAFIMIFFMVMPILIGGFGNWLVPLMLGAPDMAFPRMNNMSFWLLPPSLTLLLMSSMVESGAGTGWTVYPPLSSSIAHSGASVDLAIFSLHLAGVSSILGAVNFITTIINMRSVG
MSFDRMPLFVWSVGITALLLLLSLPVLAGAITMLLTDRNLNTTFFDPAGGGDPILYQHL
>COLFC596-12|KJ966475|ZMUO.003161|Tychius_picirostris
TLYFIFGSWSGMVGTSLSMLIRTELGNPGSLIGDDQIYNSIVTAHAFIMIFFMVMPIMIGGFGNWLIPLMLGAPDMAYPRLNNMSFWLLPPSISLLLMSSIVNKGAGTGWTVYPPLSSNIAHEGASVDLAIFSLHMAGMSSILGAINFISTMMNMRPFG
MNSEKVTLFSWAVQVTAILLLLSLPVLAGAITMLLTDRNVNTSFFDPAGGGDPILYQHL
>COLFC651-12|KJ965549|ZMUO.003216|Amara_torrida
TLYFIFGTWSGMVGTSLSMLIRAELGNPGALIGDDQIYNVIVTAHAFIMIFFMVMPIMIGGFGNWLVPLMLGAPDMAFPRMNNMSFWLLPPSLTLLLMSSMVDKGAGTGWTVYPPLSSNIAHAGASVDLAIFSLHLAGISSILGAVNFITTIINMRTV
GMTFDRMPLFVWSVGITALLLLLSLPVLAGAITMLLTDRNLNTSFFDPAGGGDPILYQHL
>COLFC370-12|KJ962251|ZMUO.002555|Cyphon_variabilis
TLYFIFGSWSGMVGTSLSLLIRAELGTPGSLIGDDQIYNVIVTAHAFIMIFFMVMPIMIGGFGNWLVPLMLGAPDMAFPRMNNMSFWLLPPSLTLLLMSSMVENGAGTGWTVYPPLSAGVAHSGASVDLAIFSLHLAGISSILGAVNFISTVINMRSVG
MTFDRMPLFVWAVAITALLLLLSLPVLAGAITMLLTDRNLNTSFFDPAGGGDPILYQHL
>COLFC137-12|KJ965578|ZMUO.002322|Ischnoglossa_obscura
TLYFIFGAWAGMVGTSLSLLIRAELGNPGSLIGDDQIYNVIVTAHAFVMIFFMVMPIMIGGFGNWLVPLMLGAPDMAFPRMNNMSFWLLPPSLTLLLMSSMVESGAGTGWTVYPPLSSNIAHGGSSVDLAIFSLHLAGISSILGAVNFISTIINMRTIG
MTFDRMPLFVWSVAITALLLLLSLPVLAGAITMLLTDRNINTTFFDPAGGGDPILYQHL
>COLFD089-12|KJ962554|ZMUO.003984|Metoecus_paradoxus
TLYFLFGAWAGMVGTSLSLLVRIELGNSGSLIGNDQIYNVIVTAHAFVMIFFMVMPIMIGGFGNWLVPLMLGAPDMAFPRMNNMSFWLLPPSLSLLMMSSMVESGAGTGWTVYPPLSSNIAHGGASVDLAIFSLHLAGISSILGAVNFITTVINMRP
EGMSFDRMPLFVWAVVITAVLLLLSLPVLAGAITMLLTDRNLNTSFFDPAGGGDPILYQHL
>COLFF133-13|KJ964214|ZMUO.003767|Cleopomiarus_distinctus
TLYFIFGTWAGMLGTSLSMIIRIELGTPGKFIGNDQIYNSIVTAHAFIMIFFMVMPIMIGGFGNWLIPLMVGAPDMSFPRLNNLSFWLLPPSICLLIMSMIIDKGVGTGWTVYPPLSTNMAHEGPAIDFAIFSLHLAGLSSILGAINFISTLMNMRPKGMK
NDRITLFSWAVKITAILLLLSLPVLAGAITMLLTDRNINTSFFDPAGGGDPILYQHL
>COLFB076-12|KJ967159|ZMUO.001406|Oodes_helopioides
TLYFIFGAWSGMVGTSLSMLIRAELGNPGALIGDDQIYNVIVTAHAFIMIFFMVMPIMIGGFGNWLVPLMLGAPDMAFPRMNNMSFWLLPPSLSLLLMSSMVESGAGTGWTVYPPLSSGIAHSGASVDLAIFSLHLAGVSSILGAVNFITTIINMRSV
GMTFDRMPLFVWSVGITALLLLLSLPVLAGAITMLLTDRNLNTSFFDPAGGGDPILYQHL
>COLFE859-13|KJ964807|ZMUO.006179|Elaphrus_uliginosus
TLYFIFGAWSGMVGTSLSMLIRAELGNPGSLIGDDQIYNVIVTAHAFIMIFFMVMPIMIGGFGNWLVPLMLGAPDMAFPRMNNMSFWLLPPSLSLLLMSSMVESGAGTGWTVYPPLSSGIAHAGASVDLAIFSLHLAGVSSILGAVNFITTIINMRSV
GMTFDRMPLFVWSVGITALLLLLSLPVLAGAITMLLTDRNLNTSFFDPAGGGDPILYQHL
>COLFB657-12|KJ965365|ZMUO.001132|Hydroporus_umbrosus
TLYFLFGAWSGMVGTSLSMLIRAELGNPGSFIGDDQIYNVIVTAHAFIMIFFMVMPIMIGGFGNWLVPLMLGAPDMAFPRMNNMSFWLLPPSLTLLLMSSMVENGAGTGWTVYPPLSSGIAHSGASVDLAIFSLHLAGISSILGAVNFITTIINMRSV
GMTFDRMPLFVWSVGITALLLLLSLPVLAGAITMLLTDRNLNTSFFDPAGGGDPILYQHL
>COLFB501-12|KJ967505|ZMUO.000976|Aphodius_fasciatus
TFYFLFGSWAGMVGTSLSLLIRAELGNPGTLIGDDQIYNVIVTAHAFVMIFFMVMPILIGGFGNWLVPLMLGAPDMAFPRMNNMSFWLLPPSLTLLLMSSMVESGAGTGWTVYPPLSSNIAHGGASVDLAIFSLHLAGISSILGAVNFITTVINMRSPG
MTFDRMPLFVWSVAITALLLLLSLPVLAGAITMLLTDRNLNTSFFDPAGGGDPILYQHL
>COLFF405-13|KJ964845|ZMUO.005923|Zeugophora_subspinosa
TLYFIFGVWAGMVGTSLSLLIRSELGTPGSLIGDDQIYNVIVTAHAFVMIFFMVMPIMIGGFGNWLVPLMLGAPDMAFPRMNNMSFWFLPPSLILLLMSSIVESGAGTGWTVYPPLSANIAHSGSSVDLAIFSLHMAGISSILGAVNFITTVINMRPMG
LNLDKMPLFVWAVVITAILLLLSLPVLAGAITMLLTDRNINTSFFDPSGGGDPILYQHL
>COLFE398-12|KJ962629|ZMUO.004958|Phratora_vitellinae
TLYFIFGIWAGMVGTSLSMLIRSELGNPGTLIGNDQIYNVIVTAHAFIMIFFMVMPIMIGGFGNWLVPLMIGAPDMAFPRLNNMSFWLLPPSLFLLLMSSVVENGAGTGWTVYPPLSSNLAHSGSSVDLAIFSLHLAGISSILGAINFITTVINMRPEGM
DLEQTPLFVWAVLITAILLLLSLPVLAGAITMLLTDRNLNTSFFDPAGGGDPILYQHL

>COLFG145-13|KJ966486|ZMUO.007840|Bibloplectus_ambiguus
TLYLIFGGWAGMIGTSLSILIRAELGNPGSLIGDDQIYNVIVTAHAFVMIFFMVMPIMIGGFGNWLVPLMLGAPDMAFPRMNNMSFWLLPPSLILLLMSSMVESGAGTGWTVYPPLSSNIAHSGASVDLTIFSLHLAGISSILGAVNFITTIINMRTMN
MKFDQLPLFVWSVGITALLLLLSLPVLAGAITMLLTDRNLNTTFFDPAGGGDPILYQHL
>COLFD499-12|KJ963924|ZMUO.004299|Carabus_granulatus
TLYFIFGAWSGMVGTSLSMLIRAELGNPGSLIGDDQIYNVIVTAHAFVMIFFMVMPIMIGGFGNWLVPLMLGAPDMAFPRMNNMSFWLLPPSLTLLLMSSMVEKGAGTGWTVYPPLSSGIAHSGASVDLAIFSLHLAGISSILGAVNFITTIINMRSV
GMTFDRMPLFVWSVGITALLLLLSLPVLAGAITMLLTDRNLNTSFFDPAGGGDPILYQHL
>COLFD144-12|KJ967381|ZMUO.004039|Harpalus_latus
TLYFIFGAWAGMVGTSLSMLIRAELGTPGALIGDDQIYNVIVTAHAFIMIFFMVMPIMIGGFGNWLVPLMLGAPDMAFPRMNNMSFWLLPPSLSLLLMSSMVESGAGTGWTVYPPLSSGIAHSGASVDLAIFSLHLAGVSSILGAVNFITTIINMRSV
GMTFDRMPLFVWSVGITALLLLLSLPVLAGAITMLLTDRNLNTSFFDPAGGGDPILYQHL
>COLFE1468-13|KJ965757|ZMUO.007263|Mordellistena
TLYFVFGAWAGMLGTSLSLLIRSELGTPGSLIGDDQIYNVIVTAHAFVMIFFMVMPVMVGGFGNWLLPLMLGAPDMAFPRLNNMSFWLLPPSLTLLILSSMVENGAGTGWTVYPPLSSNIAHGGASVDLAIFSLHLAGISSILGAINFISTMLNMHPKG
MILDQMPLFVWAIIITAILLLLSLPVLAGAITMLLTDRNLNSSFFDPAGGGDPILYQHL
>COLFE1103-13|KJ966721|ZMUO.006898|Olophrum_rotundicolle
TLYFIFGAWAGMVGTSLSILIRAELGNPGTLIGDDQIYNVIVTAHAFVMIFFMVMPILIGGFGNWLVPLMLGAPDMAFPRMNNMSFWLLPPSLTLLLTSSMVESGAGTGWTVYPPLSSNIAHGGASVDLAIFSLHLAGISSILGAVNFITTVINMRATG
MTFDRMPLFVWAVVITALLLLLSLPVLAGAITMLLTDRNLNTTFFDPAGGGDPILYQHL
>COLFE960-13|KJ966764|ZMUO.006755|Bisnius_fimetarius
TLYFIFGSWAGMVGTSLSLLIRAELGNPGTLIGDDQIYNVIVTAHAFIMIFFMVMPIVIGGFGNWLVPLMLGAPDMAFPRMNNMSFWLLPPSLTLLLMSSMVESGAGTGWTVYPPLSSNIAHGGASVDLAIFSLHLAGISSILGAVNFITTVINMRSSG
MTFDRMPLFVWSVAITALLLLLSLPVLAGAITMLLTDRNLNTSFFDPAGGGDPILYQHL
>COLFD263-12|KJ962749|ZMUO.004158|Dolichosoma_lineare
TLYFIFGAWSGMVGMSLSLLIRSELNNPGTLIGNDQIYNVIVTAHAFIMIFFMVMPILIGGFGNWLVPLMLGAPDMAFPRMNNMSFWLLPPSLTLLLMSSMVEQGAGTGWTVYPPLSANIAHGGASVDLAIFSLHLAGISSILGAVNFITTIINMRPIG
MTLDRTPLFVWAVAITALLLLLSLPVLAGAITMLLTDRNLNTSFFDPAGGGDPILYQHL
>COLFF508-13|KJ962221|ZMUO.006588|Asaphidion_pallipes
TLYFIFGAWAGMVGTSLSMLIRAELGNPGSLIGDDQIYNVIVTAHAFIMIFFMVMPILIGGFGNWLVPLMLGAPDMAFPRMNNMSFWLLPPSLTLLLMSSMVESGAGTGWTVYPPLSSSIAHSGASVDLAIFSLHLAGVSSILGAVNFITTIINMRSIG
MTFDRMPLFVWSVGITALLLLLSLPVLAGAITMLLTDRNINTSFFDPAGGGDPILYQHL
>COLFD890-12|KJ962553|ZMUO.004500|Hydroporus_angustatus
TLYFLFGAWSGMVGTSLSMLIRAELGNPGSLIGDDQIYNVIVTAHAFIMIFFMVMPIMIGGFGNWLVPLMLGAPDMAFPRMNNMSFWLLPPSLTLLLMSSMVENGAGTGWTVYPPLSSGIAHSGASVDLAIFSLHLAGISSILGAVNFITTIINMRSIG
MTFDRMPLFVWSVGITALLLLLSLPVLAGAITMLLTDRNLNTSFFDPAGGGDPILYQHL
>COLFA627-12|KJ967445|ZMUO.000762|Tachinus_marginatus
TLYFIFGAWAGMVGTSLSLLIRAELGNPGTLIGDDQIYNVIVTAHAFIMIFFMVMPIVIGGFGNWLVPLMLGAPDMAFPRMNNMSFWLLPPSLTLLLMSSMVESGAGTGWTVYPPLSSNIAHGGSSVDLAIFSLHLAGISSILGAVNFITTVINMRSIG
MTFDRMPLFVWSVAITALLLLLSLPVLAGAITMLLTDRNLNTTFFDPAGGGDPILYQHL
>COLFE558-13|KJ964468|ZMUO.005403|Chaetarthria_seminulum
TLYFIFGAWAGMVGTSLSILIRAELGNPGTLIGDDQIYNVIVTAHAFIMIFFMVMPIMIGGFGNWLVPLMLGAPDMAFPRMNNMSFWLLPPSLTLLLMSSMVESGAGTGWTVYPPLSSNIAHGGASVDLAIFSLHLAGISSILGAVNFITTVINMRSNN
MTYDRLPLFVWSVAITALLLLLSLPVLAGAITMLLTDRNLNTSFFDPAGGGDPILYQHL
>COLFB441-12|KJ966199|ZMUO.001866|Anthonomus_rubi
TLYFIFGAWSGAVGTSLSMLIRTELGNPGSLIGDDQIYNTIVTAHAFIMIFFMVMPIMIGGFGNWLIPLMLAAPDMAFPRLNNMSFWLLPPSLTLLIMSSIISKGAGTGWTVYPPLSSNLAHEGASVDFAIFSLHMAGISSILGAMNFISTILNMKPMKM
KFEQMPLFIWAVKITAILLLISLPVLAGAITMLLTDRNINTSFFDPAGGGDPILYQHL
>COLFD154-12|KJ964914|ZMUO.004049|Ochthephilum_fracticorne
TLYFIFGAWAGMVGTSLSLLIRSELANPGSLIGDDQIYNVIVTAHAFIMIFFMVMPVMIGGFGNWLVPLMLGAPDMAFPRMNNMSFWLLPPALTLLLMSSMVESGAGTGWTVYPPLSSNGFHNGSSVDLAIFSLHLAGISSILGAINFITTAINMRTTG
MNYERMPLFIWSVAITALLLLLSLPVLAGAITMLLTDRNLNTSFFDPAGGGDPILYQHL
>COLFD030-12|KJ962120|ZMUO.003925|Cryptocephalus_bipunctatus
TLYFLFGAWAGMIGTSLSLLIRIELGNPGSLIGNDQIYNTIVTAHAFIMIFFMVMPIMIGGFGNWLVPLMLGAPDMAFPRMNNMSFWLLPPSLMLLLMSSIVENGAGTGWTVYPPLSATIAHTGPSVDLAIFSLHLAGASSIMGAINFISTVINMRPQG
MMMDRTPLFVWAVLITAILLLLSLPVLAGAITMLLTDRNINTSFFDPAGGGDPILYQHL
>COLFC533-12|KJ964154|ZMUO.003098|Rhantus_grapii
TLYFIFGAWAGMVGTSLSMLIRAELGNPGSLIGDDQIYNVIVTAHAFVMIFFMVMPIMIGGFGNWLVPLMLGAPDMAFPRMNNMSFWLLPPSLTLLLMSSMVESGAGTGWTVYPPLSSGIAHGGASVDLAIFSLHLAGISSILGAVNFITTIINMRSV
GMTFDRMPLFVWSVGITALLLLLSLPVLAGAITMLLTDRNLNTSFFDPAGGGDPILYQHL
>COLFE1106-13|KJ962247|ZMUO.006901|Hydroporus_tristis

TLYFLFGAWSGMVGTSLSMLIRAELGNPGSLIGDDQIYNVIVTAHAFIMIFFMVMPIMIGGFGNWLVPLMLGAPDMAFPRMNNMSFWLLPPSLTLLLMSSMVENGAGTGWTVYPPLSSGIAHSGASVDLAIFSLHLAGVSSILGAVNFITTIINMRSI
GMTFDRMPLFVWSVGITALLLLLSLPVLAGAITMLLTDRNLNTSFFDPAGGGDPILYQHL
>COLFB470-12|KJ967305|ZMUO.001895|Hydroporus_memnonius
TLYFLFGAWSGMVGTSLSMLIRAELGNPGSLIGDDQIYNVIVTAHAFIMIFFMVMPIMIGGFGNWLVPLMLGAPDMAFPRMNNMSFWLLPPSLTLLLMSSMVENGAGTGWTVYPPLSSGIAHSGASVDLAIFSLHLAGVSSILGAVNFITTIINMRSI
GMTFDRMPLFVWSVGITALLLLLSLPVLAGAITMLLTDRNLNTSFFDPAGGGDPILYQHL
>COLFC389-12|KJ967206|ZMUO.002954|Tachinus_laticollis
TLYFIFGAWAGMVGTSLSLLIRAELGNPGTLIGDDQIYNVIVTAHAFIMIFFMVMPIVIGGFGNWLVPLMLGAPDMAFPRMNNMSFWLLPPSLSLLLMSSMVESGAGTGWTVYPPLSSNIAHGGSSVDLAIFSLHLAGISSILGAVNFITTVINMRSIGM
SFDRMPLFVWSVAITALLLLLSLPVLAGAITMLLTDRNLNTTFFDPAGGGDPILYQHL
>COLFF137-13|KJ966849|ZMUO.003771|Hypera_postica
TLYFIFGTWAGTVGTSLSILIRTELGNPGSLIGNDQIYNTIVTAHAFIMIFFMVMPIMIGGFGNWLVPLMLGAPDMAFPRLNNMSFWLLPPSLSLLLMSSMVDSGAGTGWTVYPPLSSNIAHEGSSVDLAIFSLHMAGVSSILGAINFISTVLNMRPSGM
SLDKMTLFIWAVKITAILLLLSLPVLAGAITMLLTDRNINTSFFDPAGGGDPILYQHL
>COLFC827-12|KJ963783|ZMUO.003392|Dytiscus_marginalis
TLYFIFGAWAGMVGTSLSMLIRAELGNPGSLIGDDQIYNVIVTAHAFVMIFFMVMPIMIGGFGNWLVPLMLGAPDMAFPRMNNMSFWLLPPSLTLLLMSSMVESGAGTGWTVYPPLSASIAHGGASVDLAIFSLHLAGVSSILGAVNFITTIINMRSV
GMTLDRMPLFVWSVGITALLLLLSLPVLAGAITMLLTDRNLNTSFFDPAGGGDPILYQHL
>COLFD880-12|KJ964420|ZMUO.004490|Agonum_thoreyi
TLYFIFGAWAGMVGTSLSMLIRAELGNPGALIGDDQIYNVIVTAHAFIMIFFMVMPIMIGGFGNWLVPLMLGAPDMAFPRMNNMSFWLLPPSLTLLLMSSLVESGAGTGWTVYPPLSSGIAHAGASVDLAIFSLHLAGVSSILGAVNFITTIINMRSVG
MTFDRMPLFVWSVGITALLLLLSLPVLAGAITMLLTDRNLNTSFFDPAGGGDPILYQHL
>COLFF351-13|KJ963000|ZMUO.006051|Ilybius_wasastjernae
TLYFIFGAWAGMVGTSLSMLIRAELGNPGSLIGDDQIYNVIVTAHAFVMIFFMVMPIMIGGFGNWLVPLMLGAPDMAFPRMNNMSFWLLPPSLSLLLMSSMVESGAGTGWTVYPPLSSGIAHSGASVDLAIFSLHLAGISSILGAVNFITTIINMRSV
GMTFDRMPLFVWSVGITALLLLLSLPVLAGAITMLLTDRNLNTSFFDPAGGGDPILYQHL
>COLFC145-12|KJ966399|ZMUO.002330|Gyrophaena_congrua
TLYFIFGAWSGMVGTSLSLLIRAELGNPGSLIGDDQIYNVIVTAHAFIMIFFMVMPIVIGGFGNWLVPLMLGAPDMAFPRMNNMSFWLLPPSLSLLLMSSMVESGAGTGWTVYPPLSSNIAHGGASVDLAIFSLHLAGISSILGAVNFISTIINMRAMG
MSFDRMPLFVWSVAITALLLLLSLPVLAGAITMLLTDRNLNTSFFDPAGGGDPILYQHL
>COLFE1350-13|KJ966079|ZMUO.007145|Tytthaspis_sedecimpunctata
TLYFLFGMWAGMVGTSLSILIRLELGSTGSLIGNDQMYNVIVTAHAFIMIFFMVMPIMIGGFGNWLVPLMIGAPDMAFPRLNNMSFWLLPPSLMLLLLSSMVEMGAGTGWTVYPPLSSNLAHNGSSVDLVIFSLHLAGISSILGAVNFISTIMNMRPI
GMNLDKTPLFVWSVLITAILLLLSLPVLAGAITMLLADRNLNTSFFDPTGGGDPILYQHL
>COLFC141-12|KJ967179|ZMUO.002326|Aloconota_insecta
TLYFIFGAWAGMVGTSLSLLIRAELGNPGSLIGDDQIYNVIVTAHAFIMIFFMVMPIVIGGFGNWLVPLMLGAPDMAFPRMNNMSFWLLPPSLTLLLMSSMVESGAGTGWTVYPPLSSNIAHGGSSVDLAIFSLHLAGISSILGAVNFISTVINMRSTGI
SFDRMPLFVWSVAITALLLLLSLPVLAGAITMLLTDRNLNTSFFDPAGGGDPILYQHL
>COLFB530-12|KJ964153|ZMUO.001005|Harpalus_rufipes
TLYFIFGAWAGMVGTSLSMLIRAELGTPGALIGDDQIYNVIVTAHAFIMIFFMVMPIMIGGFGNWLVPLMLGAPDMAFPRMNNMSFWLLPPSLTLLLMSSMVESGAGTGWTIYPPLSSGIAHSGASVDLAIFSLHLAGVSSILGAVNFITTIINMRSVG
MTFDRMPLFVWSVGITALLLLLSLPVLAGAITMLLTDRNLNTSFFDPAGGGDPILYQHL
>COLFF101-13|KJ965533|ZMUO.003735|Mycetochara_flavipes
TLYFMFGAWSGMVGTSLSLLIRAELGNPGSLIGDDQIYNVIVTAHAFIMIFFMVMPIMIGGFGNWLVPLMLGAPDMAFPRMNNMSFWLLPPSLTLLLMSSIVESGVGTGWTVYPPLSSNIAHGGASVDLAIFSLHLAGISSILGAVNFITTVINMKPQG
MTFDRMPLFVWAVVITAVLLLLSLPVLAGAITMLLTDRNINTSFFDPAGGGDPILYQHL
>COLFF904-13|KJ965176|ZMUO.006699|Pterostichus
TLYFIFGAWAGMVGTSLSMLIRAELGNPGSLIGDDQIYNVIVTAHAFIMIFFMVMPIMIGGFGNWLVPLMLGAPDMAFPRMNNMSFWLLPPSLTLLLMSSMVESGAGTGWTVYPPLSSGIAHAGASVDLAIFSLHLAGVSSILGAVNFITTIINMRSV
GMTFDRMPLFVWSVGITALLLLLSLPVLAGAITMLLTDRNLNTSFFDPAGGGDPILYQHL
>COLFA080-10|HM909097|MP00285|Acrotona_sylvicola
TLYFVFGTWAGMVGTSLSLLIRAELGNPGSLIGDDQIYNVIVTAHAFIMIFFMVMPIVIGGFGNWLVPLMLGAPDMAFPRMNNMSFWLLPPSLTLLLMSSMVESGAGTGWTVYPPLSSNIAHGGSSIDLAIFSLHLAGISSILGAVHFISTFINMRSVGIS
FDRMPLFVWSVVITALLLLLSLPVLAGAITMLLTDRNLNTSFFDPAGGGDPILYQHL
>COLFB537-12|KJ965906|ZMUO.001012|Atheta_vestita
TLYFIFGTWAGMIGTSLSLLIRAELGNPGSLIGDDQIYNVIVTAHAFVMIFFMVMPAMIGGFGNWLVPLMLGAPDMAFPRMNNMSFWLLPPSLTLLLMSSLVESGAGTGWTVYPPLSSNIAHGGSSVDLAIFSLHLAGISSILGAVNFISTVINMRSIGIT
FDRMPLFVWSVVITALLLLLSLPVLAGAITMLLTDRNLNTSFFDPAGGGDPILYQHL
>COLFF123-13|KJ962390|ZMUO.003757|Bledius_gallicus
TLYFIFGAWAGMVGTSLSMLIRAELGTPGSLIGNDQIYNVIVTAHAFVMIFFMVMPIVIGGFGNWLVPLMLGAPDMAFPRMNNMSFWLLPPSLTLLLMSSMVESGAGTGWTVYPPLSSNIAHSGSSVDLAIFSLHLAGISSILGAVNFISTIINMRSIG
MTFDRMPLFVWSVKITAILLLLSLPVLAGAITMLLTDRNLNTSFFDPAGGGDPILYQHL

>COLFF898-13|KJ965464|ZMUO.006693|Scymnus_nigrinus
TLYFLFGLWAGMVGTSLSILIRLELGTTSALIGNDQIYNVIVTAHAFIMIFFMVMPIMIGGFGNWLVPLMIGAPDMAFPRLNNMSFWLLPPSLTFLILSSLVESGAGTGWTVYPPLSSNIAHGGSSVDMAIFSLHLAGISSILGAVNFISTIINMRTYGMTFE
KMPLFVWSVFITAILLLLSLPVLAGAITMLLTDRNINTSFFDPAGGGDPILYQHL
>COLFC719-12|KJ966643|ZMUO.003284|Polydrusus_fulvicornis
TLYFIFGSWSGMVGTSLSMLIRTELGNPGSLIGDDQIYNVIVTAHAFIMIFFMVMPMMIGGFGNWLVPLMLGAPDMAFPRLNNMSFWLLPPSLSLLLMSSIVDKGVGTGWTVYPPLSANIAHEGSSVDLAIFSLHMAGVSSILGAINFISTVINMRPM
GMTFDRMSLFIWAVKITAILLLLSLPVLAGAITMLLTDRNINTSFFDPAGGGDPILYQHL
>COLFE1565-13|KJ966066|ZMUO.007455|Crepidodera_aurata
TLYFIFGIWSGMVGTSLSMLIRTELGNPGSLIGNDQIYNVIVTAHAFIMIFFMVMPIMIGGFGNWLVPLMIGAPDMAFPRMNNMSFWLLPPSLFLLLMSSLVESGAGTGWTVYPPLSSNIAHGGSSVDLAIFSLHLAGISSILGAINFITTIINMRPKGMS
LDRMPLFVWAVAITAILLLLSLPVLAGAITMLLTDRNLNTSFFDPIGGGDPILYQHL
>COLFF964-13|KJ962068|ZMUO.007329|Sciodrepoides_watsoni
TLYFIFGAWAGMVGTSLSLLIRAELGNPGSLIGDDQIYNVIVTAHAFVMIFFMVMPIVIGGFGNWLVPLMLGAPDMAFPRMNNMSFWLLPPSLSLLLMSSIVENGAGTGWTVYPPLSANIAHSGSSVDLAIFSLHLAGISSILGAVNFITTVINMRATG
MTLDKMPLFVWSVAITALLLLLSLPVLAGAITMLLTDRNLNTSFFDPAGGGDPILYQHL
>COLFD083-12|KJ965579|ZMUO.003978|Trypophloeus_bispinulus
TLYFIFGAWSGMVGTSLSLIIRAELGTPGNLIGNDQIYNSIVTAHAFIMIFFMVMPILIGGFGNWLVPLMLGAPDMAFPRLNNMSFWLLPPSLCFLIMSSVIDSGAGTGWTVYPPLASNIAHEGSSVDLAIFSLHMAGISSILGAMNFISTIINMSPPGMK
PEQLSLFTWAVKITAILLLLSLPVLAGAITMLLTDRNVNTSFFDPAGGGDPILYQHL
>COLFE581-13|KJ965066|ZMUO.005426|Ceutorhynchus_punctiger
TLYFIFGSWAGMTGTSLSMLIRTELGNPGMLIGDDQIYNSIVTAHAFIMIFFMVMPILIGGFGNWLVPLMLGAPDMAFPRLNNMSFWLLPPSLSLLLMSSIVNKGVGTGWTVYPPLSMNVAHEGVSVDLAIFSLHMAGISSILGAINFISTVMNMRPT
GMTAEYMPLFVWAVEITAILLLISLPVLAGAITMLLTDRNINTSFFDPTGGGDPILYQHL
>COLFE1378-13|KJ964826|ZMUO.007173|Notoxus_monoceros
TLYLIFGAWAGMVGTSLSLLIRSELGNPGSLIGDDQIYNVIVTAHAFIMIFFMVMPIVIGGFGNWLVPLMLGAPDMAFPRMNNMSFWLLPPSLTLLIMSSIVENGAGTGWTVYPPLSSNIAHSGSSVDLAIFSLHLAGVSSILGAVNFITTVINMRPSGM
SFDRMPLFVWAVVITAVLLLLSLPVLAGAITMLLTDRNLNTSFFDPAGGGDPILYQHL
>COLFB380-12|KJ965438|ZMUO.001710|Dicheirotrichus_placidus
TLYFIFGAWSGMVGTSLSMLIRAELGTPGALIGDDQIYNVIVTAHAFVMIFFMVMPIMIGGFGNWLVPLMLGAPDMAFPRMNNMSFWLLPPSLTLLLMSSMVEKGAGTGWTVYPPLSSGIAHGGASVDLAIFSLHLAGISSILGAVNFITTIINMRSV
GMTFDRMPLFVWSVGITALLLLLSLPVLAGAITMLLTDRNLNTSFFDPAGGGDPILYQHL
>COLFF045-13|KJ963431|ZMUO.005745|Notaris_scirpi
TLYFIFGAWSGMVGTSLSMLIRMELGNPGSLIGDDQIYNVIVTAHAFIMIFFMVMPIMIGGFGNWLIPLMLGAPDMAFPRLNNLSFWLLPPSLIILLSSSLIEKGTGTGWTVYPPLSSNIAHSGPSVDLAIFSLHMAGISSILGAINFISTVINMRPKGMSPD
RMTLFVWAVEITAILLLLSLPVLAGAITMLLTDRNINTSFFDPAGGGDPILYQHL
>COLFD244-12|KJ966310|ZMUO.004139|Rhagonycha_nigriventris
TLYFIFGAWSGSLGLALSLLIRAELGTPGTLIGNDQIYNVIVTAHAFIMIFFMVMPIMIGGFGNWLVPLMLGAPDMAFPRMNNMSFWFLPPSLMFLLMSSMVESGAGTGWTVYPPLSANIAHSGPSVDLAIFSLHMAGISSILGAVNFISTILNMKPPS
MKFDQMPLFVWSVGITALLLLLSLPVLAGAITMLLSDRNLNTSFFDPMGGGDPILYQHL
>COLFA437-12|KJ964034|ZMUO.000477|Aphodius_punctatosulcatus
TLYFLFGSWAGMVGTSLSLLIRAELGNPGSLIGDDQIYNVIVTAHAFVMIFFMVMPILIGGFGNWLVPLMLGAPDMAFPRMNNMSFWLLPPSLTLLLMSSMVESGAGTGWTVYPPLSSNIAHGGASVDLAIFSLHLAGISSILGAVNFITTVINMRSPG
MTFDRMPLFVWSVAITALLLLLSLPVLAGAITMLLTDRNLNTSFFDPAGGGDPILYQHL
>COLFA444-12|KJ965468|ZMUO.000484|Pterostichus_adstrictus
TLYFIFGAWSGMVGTSLSMLIRAELGNPGALIGDDQIYNVIVTAHAFVMIFFMVMPIMIGGFGNWLVPLMLGAPDMAFPRMNNMSFWLLPPSLTLLLMSSMVENGAGTGWTVYPPLSSGIAHAGASVDLAIFSLHLAGVSSILGAVNFITTIINMRS
VGMTFDRMPLFVWSVGITTLLLLLSLPVLAGAITMLLTDRNLNTSFFDPAGGGDPILYQHL
>COLFE037-12|KJ964087|ZMUO.005262|Chrysolina_marginata
TLYFIFGTWAGMVGTSLSILIRAELGNPGSLIGNDQIYNVIVTAHAFIMIFFMVMPIMIGGFGNWLVPLMLGAPDMAFPRMNNMSFWLLPPSLIFLLMSSIVENGAGTGWTVYPPLSANVAHSGPSVDLAIFSLHLAGISSILGAINFITTVINMRPTGM
KLEQMPLFSWAVLITAILLLLSLPVLAGAITMLLTDRNLNTSFFDPASGGDPILYQHL
>COLFF879-13|KJ962981|ZMUO.006674|Oxypoda_opaca
TLYFIFGAWAGMVGTSLSLLIRAELGNPGSLIGDDQIYNVIVTAHAFVMIFFMVMPIVIGGFGNWLVPLMLGAPDMAFPRMNNMSFWLLPPSLTLLLMSSMVESGAGTGWTVYPPLSSNIAHGGSSVDLAIFSLHLAGISSILGAVNFISTIINMRTSG
MTFDRMPLFVWSVAITALLLLLSLPVLAGAITMLLTDRNLNTSFFDPAGGGDPILYQHL
>COLFD902-12|KJ962319|ZMUO.004512|Aleochara_grisea
TLYFIFGAWAGMIGTSLSLLIRAELGNPGSLIGDDQIYNVIVTAHAFVMIFFMVMPIMIGGFGNWLIPLMLGAPDMAFPRMNNMSFWLLPPSLSLLLMSSMVESGAGTGWTVYPPLSTNIAHSGSSVDLAIFSLHLAGISSILGAVNFISTVINMRPKG
MSFDKMPLFIWSVIITALLLLLSLPVLAGAITMLLTDRNLNTSFFDPAGGGDPILYQHL
>COLFD099-12|KJ966886|ZMUO.003994|Triplax_russica

TLYFIFGMWAGMVGTALSMLIRSELGNPGSLIGNDQIYNVIVTAHAFIMIFFMVMPIMMGGFGNWLVPLMLGAPDMAFPRLNNMSFWLLPPSLSLLILSSIVETGAGTGWTVYPPLSSNIAHSGASVDMAIFSLHLAGISSILGAMNFITTMMNMRP
TGMTLDQMPLFVWAVLITAILLILSLPVLAGAITMLLTDRNINTTFFDPAGGGDPILYQHL
>COLFD114-12|KJ966290|ZMUO.004009|Scaphisoma_subalpinum
TLYFIFGIWSGMVGTSLSLLIRAELGMPGSLIGNDQIYNVIVTAHAFVMIFFMVMPTMIGGFGNWLVPLMLGAPDMAFPRMNNMSFWLLPPSLSLLLMSAMVESGAGTGWTVYPPLSSNIAHGGASVDLAIFSLHLAGISSILGAVNFISTILNMRAS
KMSFDQMPLFVWSVAITALLLLLSLPVLAGAITMLLTDRNLNTTFFDPAGGGDPILYQHL
>COLFE212-12|KJ963566|ZMUO.005057|Ilybius_aenescens
TLYFIFGAWAGMVGTSLSMLIRAELGNPGSLIGDDQIYNVIVTAHAFVMIFFMVMPIMIGGFGNWLVPLMLGAPDMAFPRMNNMSFWLLPPSLSLLLMSSMVESGAGTGWTVYPPLSSGIAHSGASVDLAIFSLHLAGISSILGAVNFITTIINMRSV
GMTFDRMPLFVWSVGITALLLLLSLPVLAGAITMLLTDRNLNTSFFDPAGGGDPILYQHL
>COLFC731-12|KJ966863|ZMUO.003296|Peltis_grossa
TLYFIFGSWAGMVGTSLSLLIRSELGNPGSLIGNDQIYNVIVTAHAFVMIFFMVMPILIGGFGNWLVPLMLGAPDMAFPRMNNMSFWLLPPSLTLLLLSSFVENGAGTGWTVYPPLSANISHSGSSVDLAIFSLHLAGISSILGAVNFITTVINMRPIGMT
LDRTPLFVWSVMITAILLLLSLPVLAGAITMLLTDRNINTSFFDPAGGGDPILYQHL
>COLFF288-13|KJ967145|ZMUO.005988|Tetropium_aquilonium
TLYFIFGTWAGMVGTSLSILIRSELGNPGSLIGNDQIYNVIVTAHAFIMIFFMVMPIMIGGFGNWLVPLMLGAPDMSFPRLNNLSFWFLPPSLILLIMGMIVEKGAGTGWTVYPPLSANIAHSGSSVDLTIFSLHLAGISSILSAINFITTIMNMRPKGMTL
DQMPLFVWAVMITTILLLISLPVLAGAITMLLTDRNINTSFFDPAGGGDPILYQHL
>COLFF665-13|KJ962296|ZMUO.006555|Eutrichapion_viciae
TLYFIFGLWSGMVGTSLSMLIRIELGGPGSLIGDDQIYNVIVTAHAFIMIFFMVMPIMIGGFGNWLVPLMLGAPDMAFPRMNNMSFWLLPPSLTLLLMSSIVEKGAGTGWTVYPPLASNIAHGGASVDLAIFSLHLAGISSILGAVNFISTVINMYPNGL
SLDQLSLFSWAVKITAILLLLSLPVLAGAITMLLTDRNINTSFFDPAGGGDPILYQHL
>COLFB488-12|KJ967214|ZMUO.000963|Pterostichus_minor
TLYFIFGAWAGMVGTSLSMLIRAELGNPGSLIGDDQIYNVIVTAHAFIMIFFMVMPIMIGGFGNWLVPLMLGAPDMAFPRMNNMSFWLLPPSLTLLLMSSMVESGAGTGWTVYPPLSAGIAHAGASVDLAIFSLHLAGISSILGAVNFITTIINMRSIG
MTFDRMPLFVWSVGITALLLLLSLPVLAGAITMLLTDRNLNTSFFDPAGGGDPILYQHL
>COLFD392-12|KJ967418|ZMUO.003812|Spavius_glaber
TLYFIFGAWASMVGTSLSLLIRSELGTPGSLIGDDQIYNVIVTAHAFVMIFFMVMPIMIGGFGNWLVPLMLGAPDMAFPRMNNMSFWLLPPSLSLLLMSSIAEKGVGTGWTVYPPLSSNIAHGGSSVDLAIFSLHLAGISSILGAVNFISTVMNMNPM
GMTMDRMPLFIWAVMITAILLLLSLPVLAGAITMLLTDRNLNTSFFDPAGGGDPILYQHL
>COLFA382-12|KJ961719|ZMUO.000612|Stenotrachelus_aeneus
TLYFIFGAWSGMLGTSFSLLIRSELGNPGMLIGDDQIYNVIVTSHAFVMIFFMVMPIMIGGFGNWLVPLMLGAPDMAFPRMNNMSFWLLPPSLSLLLMSSMVESGAGTGWTVYPPLSSNIAHGGASVDLAIFSLHLAGISSILGAVNFITTVINMRPQ
EMSLERMPLFVWSVVITAILLLLSLPVLAGAITMLLTDRNLNTSFFDPAGGGDPILYQHL
>COLFD614-12|KJ966436|ZMUO.004414|Pterostichus_strenuus
TLYFIFGAWAGMVGTSLSMLIRAELGNPGSLIGDDQIYNVIVTAHAFVMIFFMVMPIMIGGFGNWLVPLMLGAPDMAFPRMNNMSFWLLPPSLTLLLMSSMVESGAGTGWTVYPPLSSGIAHAGASVDLAIFSLHLAGISSILGAVNFITTIINMRSIG
MTFDRMPLFVWSVGITALLLLLSLPVLAGAITMLLTDRNLNTSFFDPAGGGDPILYQHL
>COLFE922-13|KJ966479|ZMUO.006242|Quedius_brevicornis
TLYFIFGAWAGMVGTSLSLLIRAELGNPGTLIGDDQIYNVIVTAHAFIMIFFMVMPIVIGGFGNWLVPLMLGAPDMAFPRMNNMSFWLLPPSLSLLLMSSMVESGAGTGWTVYPPLSSNIAHGGASVDLAIFSLHLAGISSILGAVNFITTVINMRSIG
MTFDRMPLFVWSVAITALLLLLSLPVLAGAITMLLTDRNLNTSFFDPAGGGDPILYQHL
>COLFF608-13|KJ966478|ZMUO.006498|Meligethes_morosus
TLYFIFGAWSSMVGTSLSMLIRTELGNPGSLIGNDQIYNVIVTAHAFVMIFFMVMPFMIGGFGNWLVPLMLGAPDMAFPRMNNMSFWLLPPSLSLLLMSSIVESGAGTGWTVYPPLSSNIAHGGASVDLAIFSLHLAGISSILGAVNFIATVINMRPSG
MTFDRMPLFVWAVAITALLLLLSLPVLAGAITMLLTDRNLNTTFFDPSGGGDPILYQHL
>COLFD655-12|KJ964772|ZMUO.004455|Trachodes_hispidus
TLYFIFGSWSGMVGTSLSMMIRTELGTPGSVIGNDQIYNTIVTAHAFIMIFFMVMPIMIGGFGNWLVPLMLGAPDMAFPRLNNMSFWLLPPSLTLLLMSSIIDKGAGTGWTVYPPLSTNIAHEGMSVDLAIFSLHLAGISSILGAMNFISTVINMHPM
GMKLDQLPLFVWSVKITAILLLLSLPVLAGAITMLLTDRNINTTFFDPAGGGDPILYQHL
>COLFB738-12|KJ965958|ZMUO.001213|Galerucella_tenella
TLYFIFGIWAGMVGTSLSILVRAELGSPGTLIGNDQIYNVIVTAHAFIMIFFMVMPIMIGGFGNWLVPLMIGAPDMAFPRMNNMSFWLLPPSLFLLIMSSIVESGAGTGWTVYPPLSSNIAHGGSSVDLAIFSLHLAGISSILGAINFITTIINMRPKGMTL
DRMPLFVWAVMITAILLLLSLPVLAGAITMLLTDRNLNTSFFDPAGGGDPILYQHL
>COLFF163-13|KJ962008|ZMUO.003797|Carabus_granulatus
TLYFIFGAWSGMVGTSLSMLIRAELGNPGSLIGDDQIYNVIVTAHAFVMIFFMVMPIMIGGFGNWLVPLMLGAPDMAFPRMNNMSFWLLPPSLTLLLMSSMVEKGAGTGWTVYPPLSSGIAHSGASVDLAIFSLHLAGISSILGAVNFITTIINMRSV
GMTFDRMPLFVWSVGITALLLLLSLPVLAGAITMLLTDRNLNTSFFDPAGGGDPILYQHL
>COLFC178-12|KJ963307|ZMUO.002363|Meotica_exilis
SLYFIFGSWAGMIGTSLSLLIRAELGNPGSLIGDDQIYNVIVTAHAFIMIFFMVMPIVIGGFGNWLVPLMLGAPDMAFPRMNNMSFWLLPPSLMLLLMSSMVESGAGTGWTVYPPLSSNIAHGGSSVDLAIFSLHLAGISSILGAVNFISTIINMRSTGM
TFDRMPLFVWSVGITALLLLLSLPVLAGAITMLLTDRNLNTSFFDPAGGGDPILYQHL

>COLFE363-12|KJ966701|ZMUO.004923|Myzia_oblongoguttata
TLYFLFGMWAGMVGTSLSILIRLELGTTNSLIGNDQIYNVIVTAHAFIMIFFMVMPVMIGGFGNWLVPLMIGAPDMAFPRLNNMSFWLLPPALTLLIFSSLVEMGAGTGWTVYPPLSANMAHSGASVDLVIFSLHLAGISSILGAVNFISTIMNMRPFG
MNLDKTPLFVWSVLITAILLLLSLPVLAGAITMLLTDRNINTSFFDPMGGGDPILYQHL
>COLFB909-12|KJ962529|ZMUO.001954|Buprestis_haemorrhoidalis
TLYFIFGAWSGMVGTALSLLIRAELGNPGALIGDDQVYNVIVTAHAFVMIFFMVMPVMMGGFGNWLVPLMLGAPDMAFPRMNNMSFWLLPPSLTLLLMSSVVENGAGTGWTVYPPLAANIAHSGASVDLAIFSLHLAGVSSILGAVNFITTVINM
RSVGMTFDRMPLFVWSVAITALLLLLSLPVLAGAITMLLTDRNFNTSFFDPAGGGDPILYQHL
>COLFE467-12|KJ963071|ZMUO.005027|Chaetocnema_mannerheimii
TLYFIFGIWSGMVGTSMSILIRAELGNPGSLIGNDQIYNVIVTAHAFVMIFFMVMPIMIGGFGNWLIPLMIGAPDMAFPRMNNMSFWLLPPSLFLLLMSSMVESGAGTGWTVYPPLSSNIAHSGSSVDLAIFSLHLAGVSSILGAINFITTIINMRPQGM
SFDQMPLFVWAVVITAILLLLSLPVLAGAITMLLTDRNLNTSFFDPMGGGDPILYQHL
>COLFE1528-13|KJ967044|ZMUO.007418|Chrysolina_fastuosa
TLYFIFGTWAGMVGTSLSILIRAELGNPGTLIGNDQIYNVIVTAHAFIMIFFMVMPIMIGGFGNWLVPLMLGAPDMAFPRMNNMSFWLLPPSLIFLLMSSIVENGVGTGWTVYPPLSANVAHSGPSVDLAIFSLHLAGISSILGAINFITTVINMRPTGM
KLEQMPLFSWAVLITAILLLLSLPVLAGAITMLLTDRNLNTSFFDPASGGDPILYQHL
>COLFC099-12|KJ962512|ZMUO.002284|Olophrum_consimile
TLYFIFGAWAGMVGTSLSILIRAELGNPGTLIGDDQIYNVIVTAHAFVMIFFMVMPILIGGFGNWLVPLMLGAPDMAFPRMNNMSFWLLPPSLTLLLMSSMVESGAGTGWTVYPPLSSNIAHGGASVDLAIFSLHLAGISSILGAVNFITTVINMRATG
MTFDRMPLFVWAVVITALLLLLSLPVLAGAITMLLTDRNLNTTFFDPAGGGDPILYQHL
>COLFF031-13|KJ965140|ZMUO.005731|Atomaria_testacea
TLYFIFGAWAGMVGTSLSMLIRTELGTPGSLIGDDQIYNVIVTAHAFIMIFFMVMPMMIGGFGNWLVPLMLGAPDMAFPRLNNMSFWLLPPSLLFLLMSSIVEKGAGTGWTVYPPLSSNVAHAGSSVDLAIFSLHLAGISSILGSVNFITTVINMRPKG
MNFDRLPLFVWAVKITTILLLLSLPVLAGAITMLLTDRNINTSFFDPAGGGDPILYQHL
>COLFB257-12|KJ966632|ZMUO.001587|Olophrum_consimile
TLYFIFGAWAGMVGTSLSILIRAELGNPGTLIGDDQIYNVIVTAHAFVMIFFMVMPILIGGFGNWLVPLMLGAPDMAFPRMNNMSFWLLPPSLTLILMSSMVESGAGTGWTVYPPLSSNIAHGGASVDLAIFSLHLAGISSILGAVNFITTVINMRATG
MTFDRMPLFVWAVVITALLLLLSLPVLAGAITMLLTDRNLNTTFFDPAGGGDPILYQHL
>COLFB323-12|KJ965538|ZMUO.001653|Halyzia_sedecimguttata
TLYFLFGMWAGMVGTSLSIMIRLELGTTNSLIGNDQIYNVIVTAHAFIMIFFMVMPVMIGGFGNWLVPLMVGAPDMAFPRLNNMSFWLLPPALTLLIFSSMVEMGAGTGWTVYPPLSSNMAHSGSSVDLVIFSLHLAGISSILGAVNFISTIMNMRP
FGMNLDKTPLFVWSVLITAILLLLSLPVLAGAITMLLTDRNINTSFFDPMGGGDPILYQHL
>COLFE1045-13|KJ963511|ZMUO.006840|Chaetarthria_seminulum
TLYFIFGAWAGMVGTSLSILIRAELGNPGTLIGDDQIYNVIVTAHAFIMIFFMVMPIMIGGFGNWLVPLMLGAPDMAFPRMNNMSFWLLPPSLTLLLMSSMVESGAGTGWTVYPPLSSNIAHSGASVDLAIFSLHLAGISSILGAVNFITTVINMRSNN
MTYDRLPLFVWSVAITALLLLLSLPVLAGAITMLLTDRNLNTSFFDPAGGGDPILYQHL
>COLFB935-12|KJ962154|ZMUO.001980|Temnocerus_tomentosus
TLYFIFGAWSGMVGTSLSILIRAELGSPGSLIGDDQIYNVIVTAHAFIMIFFMVMPIMIGGFGNWLVPLMLGAPDMAFPRMNNMSFWLLPPSLSLLIMSSIVESGAGTGWTVYPPLSSNIAHGGSSVDLAIFSLHLAGISSILGAVNFISTVINMRPMGM
NLDRMPLFVWAVAITALLLLLSLPVLAGAITMLLTDRNINTSFFDPAGGGDPILYQHL
>COLFE1253-13|KJ965860|ZMUO.007048|Atheta_brunneipennis
TLYFIFGAWAGMVGTSLSLLIRAELGNPGSLIGDDQIYNVIVTAHAFIMIFFMVMPVVIGGFGNWLVPLMLGAPDMAFPRMNNMSFWLLPPSLTLLLMSSMVESGAGTGWTVYPPLSSNIAHGGSSVDLAIFSLHLAGISSILGAVNFISTVINMRSTGI
SFDRMPLFVWSVAITALLLLLSLPVLAGAITMLLTDRNLNTSFFDPAGGGDPILYQHL
>COLFE829-13|KJ964056|ZMUO.005674|Oulema_melanopus
TLYFIFGAWSGMVGTSLSMMIRTELGNPGSLIGNDQIYNVIVTAHAFIMIFFMVMPIMIGGFGNWLIPLMLGAPDMAFPRMNNMSFWLLPPSISLLLMSSIVENGAGTGWTVYPPLSANISHNGASVDLAIFSLHLAGISSILGAVNFISTVSNMRPEG
MALDRMPLFVWAVLITAILLLLSLPVLAGAITMLLTDRNLNTSFFDPSSGGDPILYQHL
>COLFD521-12|KJ963562|ZMUO.004321|Anisodactylus_binotatus
TLYFIFGAWSGMVGTSLSMLIRAELGTPGALIGDDQIYNVIVTAHAFVMIFFMVMPIMIGGFGNWLVPLMLGAPDMAFPRMNNMSFWLLPPSLTLLLMSSMVESGAGTGWTVYPPLSSGIAHGGASVDLAIFSLHLAGVSSILGAVNFITTIINMRSV
GMTFDRMPLFVWSVGITALLLLLSLPVLAGAITMLLTDRNLNTSFFDPAGGGDPILYQHL
>COLFD417-12|KJ963765|ZMUO.003837|Adrastus_pallens
TLYFLFGAWAGMLGTSLSLLIRAELGNPGSLIGNDQIYNVIVTAHAFIMIFFMVMPIMIGGFGNWLVPLMLGAPDMAFPRMNNMSFWFLPPSLSLLLMSSIVENGAGTGWTVYPPLSSNIAHSGSSVDLAIFSLHLAGISSILGAVNFISTVINMRSVGIT
FDRMPLFVWAVAITALLLLLSLPVLAGAITMLLTDRNLNTSFFDPAGGGDPILYQHL
>COLFC634-12|KJ964216|ZMUO.003199|Hydroporus_geniculatus
TLYFLFGAWSGMVGTSLSMLIRAELGNPGSLIGDDQIYNVIVTAHAFIMIFFMVMPIMIGGFGNWLVPLMLGAPDMAFPRMNNMSFWLLPPSLTLLLMSSMVENGAGTGWTVYPPLSSGIAHSGASVDLAIFSLHLAGVSSILGAVNFITTIINMRSI
GMTFDRMPLFVWSVGITALLLLLSLPVLAGAITMLLTDRNLNTSFFDPAGGGDPILYQHL
>COLFD130-12|KJ964495|ZMUO.004025|Sericus_brunneus

TLYFIFGAWSGMLGTSLSLLIRAELGNPGALIGNDQIYNVVVTAHAFIMIFFMVMPIMIGGFGNWLVPLMLGAPDMAFPRMNNMSFWLLPPSLSLLLMSSIVENGAGTGWTVYPPLSANIAHSGSSVDLAIFSLHLAGISSILGAVNFISTVINMRSTGI
TFDRMPLFVWAVAITALLLLSLPVLAGAITMLLTDRNLNTSFFDPAGGGDPILYQHL
>COLFC119-12|KJ964837|ZMUO.002304|Cymindis_vaporariorum
TLYFIFGAWAGMVGTSLSMLIRAELGNPGALIGDDQIYNVIVTAHAFIMIFFMVMPIMIGGFGNWLVPLMLGAPDMAFPRMNNMSFWLLPPSLTLLLMSSMVESGAGTGWTVYPPLSSGIAHAGASVDLAIFSLHLAGVSSILGAVNFITTIINMRSI
GMTFDRMPLFVWSVGITALLLLSLPVLAGAITMLLTDRNLNTSFFDPAGGGDPILYQHL
>COLFB940-12|KJ962229|ZMUO.001985|Quedius
TLYFIFGAWAGMIGTSLSLLIRAELGNPGTLIGDDQIYNVIVTAHAFIMIFFMVMPVVIGGFGNWLVPLMLGAPDMAFPRMNNMSFWLLPPSLSLLLMSSMVESGAGTGWTVYPPLSSNIAHGGASVDLAIFSLHLAGISSILGAVNFITTVINMRSIG
MTFDRMPLFVWSVAITALLLLSLPVLAGAITMLLTDRNLNTSFFDPAGGGDPILYQHL
>COLFE1546-13|KJ963964|ZMUO.007436|Gymnetron_melanarium
TLYFMFGTWSGMVGTSMSMIIRTELGNPGKFIGNDQIYNSIVTAHAFIMIFFMVMPIMIGGFGNWLTPLMLGAPDMAFPRLNNMSFWLLPPSLTLLLMSSMIDKGVGTGWTVYPPLSTNVAHAGSSVDLAIFSLHMAGISSILGAMNFISTIMNMR
TNEMKPDRMPLFVWSVQITAILLLLSLPVLAGAITMLLTDRNINTSFFDPAGGGDPILYQHL
>COLFF129-13|KJ961775|ZMUO.003763|Atomaria_nitidula
TLYFIFGAWAGMVGTSLSMLIRTELGTPGSLIGDDQIYNVIVTAHAFIMIFFMVMPMMIGGFGNWLVPLMLGAPDMAFPRLNNMSFWLLPPSLMFLLMSSIVEKGAGTGWTVYPPLSANVAHAGSSVDLAIFSLHLAGISSILGSVNFITTVINMRPK
GMNFDRLPLFVWAVKITTILLLLSLPVLAGAITMLLTDRNINTSFFDPAGGGDPILYQHL
>COLFD020-12|KJ964947|ZMUO.003915|Epuraea_opalizans
TLYFIFGVWSGMVGTSLSILIRTELGSPGSLIGNDQIYNVIVTAHAFIMIFFMVMPFMIGGFGNWLVPLMLGAPDMAFPRMNNMSFWLLPPSLSLLLMSSIVESGAGTGWTVYPPLSSNIAHGGSSVDLAIFSLHLAGVSSILGAVNFITTIINMRPAGM
TLDRMPLFVWSVVITAILLLLSLPVLAGAITMLLTDRNLNTTFFDPSGGGDPILYQHL
>COLFC659-12|KJ967274|ZMUO.003224|Stenus_canaliculatus
TLYFIFGAWAGMVGTSLSLLIRAELGNPGSLIGDDQIYNVIVTAHAFVMIFFMVMPIMIGGFGNWLIPLMLGAPDMAFPRMNNMSFWLLPPSLSLLLTSSIVESGAGTGWTVYPPLSSNIAHSGSSVDLAIFSLHLAGVSSILGAINFITTIFNMRTMKLQ
LDCLPLFVWAVGITAFLLLLSLPVLAGAITMLLTDRNLNTSFFDPAGGGDPILYQHL
>COLFE246-12|KJ963221|ZMUO.005091|Rhinoncus_perpendicularis
TLYFIFGSWAGTVGTSLSMLIRTELGTPGSLIGNDQIYNSIVTAHAFIMIFFMVMPILIGGFGNWLVPLMLGAPDMAFPRLNNMSFWLLPPSILLLLMSSIVNKGVGTGWTVYPPLSSNITHEGASVDLAIFSLHMAGISSILGAINFISTIMNMRPQGMS
YEKMPLFSWAVLITAILLLLSLPVLAGAITMLLTDRNINTSFFDPAGGGDPILYQHL
>COLFE976-13|KJ961750|ZMUO.006771|Ilybius_fuliginosus
TLYFIFGAWAGMVGTSLSMLIRAELGNPGSLIGDDQIYNVIVTAHAFVMIFFMVMPIMIGGFGNWLVPLMLGAPDMAFPRMNNMSFWLLPPSLSLLLMSSMVESGAGTGWTVYPPLSSGIAHSGASVDLAIFSLHLAGISSILGAVNFITTIINMRSV
GMTFDRMPLFVWSVGITALLLLSLPVLAGAITMLLTDRNLNTSFFDPAGGGDPILYQHL
>COLFE616-13|KJ966466|ZMUO.005461|Polydrusus_mollis
TLYFIFGAWSGMVGTSLSMLIRTELGNPGSLIGDDQIYNVIVTAHAFIMIFFMVMPTMIGGFGNWLVPLMLGAPDMAFPRLNNMSFWLLPPSLSLLLMSSIVDKGAGTGWTVYPPLSANIAHEGSSVDLAIFSLHMAGVSSILGAVNFISTVINMHPK
GMTPERMPLFVWAVVITAILLLLSLPVLAGAITMLLTDRNMNTSFFDPAGGGDPILYQHL
>COLFE1580-13|KJ963313|ZMUO.007470|Melanimon_tibialis
TLYFIFGAWSGMVGTSLSLLMRAELGNPGSLIGDDQIYNVIVTAHAFIMIFFMVMPIMIGGFGNWLVPLMLGAPDMAFPRMNNMSFWLLPPSLTLLLMSSIVESGAGTGWTVYPPLSSNIAHGGSSVDLAIFSLHLAGISSILGAVNFITTVINMRPQG
MTFDQTPLFVWAVVITAVLLLLSLPVLAGAITMLLTDRNINTSFFDPAGGGDPILYQHL
>COLFB737-12|KJ967123|ZMUO.001212|Tetartopeus_terminatus
TLYFIFGAWAGMVGTSLSLLIRTELANPGSLIGDDQIYNVIVTAHAFVMIFFMVMPIVIGGFGNWLVPLMLGAPDMAFPRMNNMSFWLLPPSLSLLLASSMVESGAGTGWTVYPPLSSNIAHGGASVDLAIFSLHLAGISSILGAVNFITTIINMRSPGM
LYERMPLFVWSVGITALLLLSLPVLAGAITMLLTDRNLNTSFFDPAGGGDPILYQHL
>COLFC502-12|KJ967112|ZMUO.003067|Epuraea_boreella
TLYFIFGAWSGMVGTSLSILIRTELGSPGSLIGNDQIYNVIVTAHAFIMIFFMVMPFMIGGFGNWLVPLMLGAPDMAFPRMNNMSFWLLPPSLSLLLMSSIVESGAGTGWTVYPPLSSNIAHGGSSVDLAIFSLHLAGVSSILGAVNFITTIINMRPAGM
TLDRMPLFVWSVVITAILLLLSLPVLAGAITMLLTDRNLNTTFFDPSGGGDPILYQHL
>COLFA253-12|KJ963927|ZMUO.000388|Amara_communis
TLYFIFGAWSGMVGTSLSMLIRAELGNPGALIGDDQIYNVIVTAHAFVMIFFMVMPIMIGGFGNWLVPLMLGAPDMAFPRMNNMSFWLLPPSLTLLLMSSMVESGAGTGWTVYPPLSSGIAHAGASVDLAIFSLHLAGISSILGAVNFITTIINMRSIG
MTFDRMPLFVWSVGITALLLLSLPVLAGAITMLLTDRNLNTSFFDPAGGGDPILYQHL
>COLFC775-12|KJ966441|ZMUO.003340|Hylobius_abietis
TLYFIFGTWSGMVGTSLSMLIRTELGNPGSLIGDDQIYNTIVTAHAFIMIFFMVMPIMIGGFGNWLVPLMLGAPDMAFPRLNNMSFWLLPPSLTLLLMSSIVDKGAGTGWTVYPPLSANIAHEGASVDFAIFSLHMAGISSILGAINFISTAINMRSSGM
KSDQMSLFIWAVKITAILLLLSLPVLAGAITMLLTDRNINTSFFDPAGGGDPILYQHL
>COLFF1022-13|KJ963144|ZMUO.007387|Dermestes_szekessyi
TLYFIFGAWAGMVGTSLSMLIRTELGMPGSLIGDDQIFNVIVTAHAFIMIFFMVMPIMIGGFGNWLVPLMLGAPDMAFPRMNNMSFWLLPPSLSLLLMSSMVESGAGTGWTVYPPLSANIAHGGASVDLAIFSLHLAGISSILGAVNFITTVINMRSK
GMTPDRMPLFVWSVAITALLLLSLPVLAGAITMLLTDRNLNTSFFDPAGGGDPILYQHL

>COLFC601-12|KJ963942|ZMUO.003166|Meligethes_subaeneus
TLYFIFGAWSGMVGTSLSMLIRTELGNPGSLIGNDQIYNVIVTAHAFVMIFFMVMPFMIGGFGNWLVPLMLGAPDMAFPRMNNMSFWLLPPSLSLLLMSSIVESGAGTGWTVYPPLSSNIAHGGASVDLAIFSLHLAGISSILGAVNFITTVINMRPTG
MTFDRMPLFVWAVLITAILLLLSLPVLAGAITMLLTDRNINTTFFDPSGGGDPILYQHL
>COLFC247-12|KJ964049|ZMUO.002432|Atheta_deformis
TLYFIFGTWAGMVGTSLSLLIRAELGNPGSLIGDDQIYNVIVTAHAFIMIFFMVMPIVIGGFGNWLVPLMLGAPDMAFPRMNNMSFWLLPPSLTLLLMSSMVESGAGTGWTVYPPLASNIAHGGSSVDLAIFSLHLAGISSILGAVNFISTVINMRSTGI
SFDRMPLFVWSVAITALLLLLSLPVLAGAITMLLTDRNLNTSFFDPAGGGDPILYQHL
>COLFC023-12|KJ962944|ZMUO.002018|Anthophagus_alpinus
TLYFIFGAWAGMVGTSLSILIRSELGNPGSLIGDDQIYNVIVTAHAFVMIFFMVMPVVIGGFGNWLVPLMLGAPDMAFPRMNNMSFWLLPPSLTLLLMSSMVESGAGTGWTVYPPLSSNIAHSGASVDLAIFSLHLAGISSILGAVNFITTVINMRSTGI
TFDRMPLFVWSVAITALLLLLSLPVLAGAITMLLTDRNLNTSFFDPAGGGDPILYQHL
>COLFA492-12|KJ967438|ZMUO.000532|Hydroporus_fuscipennis
TLYFLFGAWSGMVGTSLSMLIRAELGNPGSLIGDDQIYNVIVTAHAFIMIFFMVMPIMIGGFGNWLVPLMLGAPDMAFPRMNNMSFWLLPPSLTLLLMSSMVENGAGTGWTVYPPLSSGIAHSGASVDLAIFSLHLAGVSSILGAVNFITTIINMRSI
GMTFDRMPLFVWSVGITALLLLLSLPVLAGAITMLLTDRNLNTSFFDPAGGGDPILYQHL
>COLFE470-12|KJ966213|ZMUO.005030|Cyphon_laevipennis
TLYFIFGSWSGMVGTSLSLLIRAELGTPGSLIGDDQIYNVIVTAHAFIMIFFMVMPIMIGGFGNWLVPLMLGAPDMAFPRMNNMSFWLLPPSLTLLLMSSMVENGAGTGWTVYPPLSAGVAHSGASVDLAIFSLHLAGISSILGAVNFISTVINMRSVG
MTFDRMPLFVWAVAITALLLLLSLPVLAGAITMLLTDRNLNTSFFDPAGGGDPILYQHL
>COLFA479-12|KJ962587|ZMUO.000519|Acrotona_amplicollis
TLYFIFGAWAGMIGTSLSLLIRAELGNPGSLIGDDQIYNVIVTAHAFIMIFFMVMPIMIGGFGNWLVPLMLGAPDMAFPRMNNMSFWLLPPSLTLLLMSSIVESGAGTGWTVYPPLSSNIAHGGSSVDLAIFSLHLAGISSILGAVNFISTVINMRSSGITF
DRMPLFVWAVAITALLLLLSLPVLAGAITMLLTDRNLNTSFFDPAGGGDPILYQHL
>COLFF443-13|KJ963169|ZMUO.005961|Tachyura_parvula
TLYFIFGAWAGMVGTSLSMLIRAELGNPGSLIGDDQIYNVIVTAHAFIMIFFMVMPILIGGFGNWLVPLMLGAPDMAFPRMNNMSFWLLPPSLTLLLMSSMVESGAGTGWTVYPPLSSSIAHSGASVDLAIFSLHLAGVSSILGAVNFITTIINMRSVG
MTFDRMPLFVWSVGITALLLLLSLPVLAGAITMLLTDRNLNTSFFDPAGGGDPILYQHL
>COLFF337-13|KJ966700|ZMUO.006037|Plateumaris_discolor
TLYFIFGAWSGMMGTSLSMLIRTELMNPGSLIGNDQIYNVIVTAHAFIMIFFMVMPIMIGGFGNWLIPLMLGAPDMAFPRMNNMSFWLLPPSLTFLIMSSIVENGAGTGWTVYPPLSSNIAHSGASVDLAIFSLHLAGVSSILGAVNFITTIINMRPMG
MKMDKVPLFAWAVMITAILLLLSLPVLAGAITMLLTDRNLNTSFFDPAGGGDPVLYQHL
>COLFC382-12|KJ966238|ZMUO.002947|Catops_morio
TLYFIFGAWAGMVGTSLSLLIRAELGNPGSLIGDDQIYNVIVTAHAFVMIFFMVMPIVIGGFGNWLVPLMLGAPDMAFPRMNNMSFWLLPPSLSLLLMSSIVENGAGTGWTVYPPLSANIAHSGSSVDLAIFSLHLAGISSILGAVNFITTVINMRATG
MTLDKMPLFVWSVAITALLLLLSLPVLAGAITMLLTDRNLNTSFFDPAGGGDPILYQHL
>COLFB007-12|KJ967376|ZMUO.001337|Chilocorus_renipustulatus
TLYFLFGMWSGMVGTALSMIIRLELGTPSSLIGNDQIYNVIVTAHAFIMIFFMVMPIMIGGFGNWLVPLMIGAPDMAFPRLNNMSFWLLPPSLTLLILSSMIESGAGTGWTVYPPLSSNLAHGGSSVDLAIFSLHMAGISSILGAINFISTILNMRPKGML
LEKTPLFVWSVLITAILLLLSLPVLAGAITMLLTDRNINTSFFDPTGGGDPILYQHL
>COLFB556-12|KJ965261|ZMUO.001031|Anthicus_flavipes
TLYLIFGAWAGMVGTSLSLLIRSELGNPGTLIGNDQIYNVIVTAHAFIMIFFMVMPIVIGGFGNWLVPLMLGAPDMAFPRMNNMSFWLLPPSLSLLIMSSIVESGAGTGWTVYPPLSANIAHSGSSVDLAIFSLHLAGVSSILGAVNFITTVINMRPVGM
TLDRMPLFVWAVVITAVLLLLSLPVLAGAITMLLTDRNLNTSFFDPAGGGDPILYQHL
>COLFA336-12|KJ963116|ZMUO.000471|Patrobus_assimilis
TLYFIFGAWSGMVGTSLSILIRAELGNPGSLIGDDQIYNVIVTAHAFVMIFFMVMPIMIGGFGNWLVPLMLGAPDMAFPRMNNMSFWLLPPSLTLLLMSSMVESGAGTGWTVYPPLSSGIAHSGASVDLAIFSLHLAGISSILGAVNFITTIINMRSVG
MTFDRMPLFVWSVGITALLLLLSLPVLAGAITMLLTDRNLNTSFFDPAGGGDPILYQHL
>COLFC581-12|KJ962534|ZMUO.003146|Bembidion_saxatile
TLYFIFGAWSGMVGTSLSMLIRAELGNPGSLIGDDQIYNVIVTAHAFVMIFFMVMPILIGGFGNWLVPLMLGAPDMAFPRMNNMSFWLLPPSLSLLLMSSIVESGAGTGWTVYPPLSSSIAHSGASVDLAIFSLHLAGVSSILGAVNFITTIINMRSIGM
SFDRMPLFVWSVGITALLLLLSLPVLAGAITMLLTDRNLNTSFFDPAGGGDPILYQHL
>COLFF615-13|KJ966098|ZMUO.006505|Poecilus_lepidus
TLYFIFGAWSGMVGTSLSMLIRAELGNPGSLIGDDQIYNVIVTAHAFVMIFFMVMPIMIGGFGNWLVPLMLGAPDMAFPRMNNMSFWLLPPSLTLLLMSSMVESGAGTGWTVYPPLSSGIAHAGASVDLAIFSLHLAGVSSILGAVNFITTIINMRSV
GMTFDRMPLFVWSVGITALLLLLSLPVLAGAITMLLTDRNLNTSFFDPAGGGDPILYQHL
>COLFE118-12|KJ966840|ZMUO.005153|Tomoxia_bucephala
TLYFIFGAWSGMVGTSLSLLIRAELGNPGSLIGDDQIYNTIVTAHAFIMIFFMVMPIMVGGFGNWLVPLMLGAPDMAFPRMNNMSFWLLPPSLSLLLMSSVVESGAGTGWTVYPPLSSNIAHGGSSVDLAIFSLHLAGVSSILGAINFISTMINMRPAG
MTMDRMPLFAWAILITAVLLLLSLPVLAGAITMLLTDRNLNTSFFDPAGGGDPILYQHL
>COLFC190-12|KJ964504|ZMUO.002375|Phyllodrepa_melanocephala

TLYFIFGAWAGMVGTSLSILIRAELGNPGSLIGDDQIYNVIVTAHAFVMIFFMVMPIVIGGFGNWLVPLMLGAPDMAFPRMNNMSFWLLPPSLTLLLMSSMVESGAGTGWTVYPPLSSNIAHGGSSVDLAIFSLHLAGISSILGAVNFITTVINMRSMG
MTFDRMPLFVWSVAITALLLLLSLPVLAGAITMLLTDRNLNTSFFDPAGGGDPILYQHL
>COLFA237-10|HM909148|MP00103|Agrilus_viridis
TLYFIFGAWSGMVGTALSLLIRAELGNPGALIGNDQIYNVIVTAHAFIMIFFMVMPIMMGGFGNWLVPLMLGAPDMAFPRMNNMSFWLLPPSLTLLLMSSMVESGAGTGWTVYPPLAANIAHSGGSVDLAIFSLHLAGISSILGAINFITTVINMRAV
GMTMDRVPLLVWSIAITALLLLLSLPVLAGAITMLLTDRNLNTSFFDPAGGGDPILYQHL
>COLFF979-13|KJ965989|ZMUO.007344|Bothrynoderes_affinis
TLYFIFGAWSGMVGTSLSMLIRTELGNPGSLIGDDQIYNTIVTAHAFIMIFFMVMPIMIGGFGNWLVPLMLGAPDMAFPRLNNMSFWLLPPSLTLLLMSSIVDKGAGTGWTVYPPLSTNIAHEGASVDLAIFSLHMAGISSILGAINFISTVLNMRPSG
MKPDQTTLFTWAVEITAILLLLSLPVLAGAITMLLTDRNINTSFFDPAGGGDPILYQHL
>COLFF966-13|KJ963248|ZMUO.007331|Leiodes_triepkii
TLYFIFGAWAGMTGTSLSILIRAELGNPGNLIGDDQIYNVIVTAHAFIMIFFMVMPIVIGGFGNWLVPLMLGAPDMAFPRMNNMSFWLLPPSLTLLLMSSVVENGAGTGWTVYPPLSANIAHSGPSVDLAIFSLHLAGISSILGAVNFITTVINMRPIGM
GFDKMPLFVWSVAITALLLLLSLPVLAGAITMLLTDRNLNTSFFDPAGGGDPILYQHL
>COLFC638-12|KJ964020|ZMUO.003203|Hygrotus_marklini
TLYFLFGAWSGMVGTSMSMLIRAELGNPGSLIGDDQIYNVIVTAHAFIMIFFMVMPIMIGGFGNWLVPLMLGAPDMAFPRMNNMSFWMLPPSLTLLLMSSMVESGAGTGWTVYPPLSAGIAHGGASVDLAIFSLHLAGISSILGAVNFITTIINMRS
VGMTFDRMPLFVWSVGITALLLLLSLPVLAGAITMLLTDRNLNTSFFDPAGGGDPILYQHL
>COLFA593-12|KJ965064|ZMUO.000728|Calopus_serraticornis
TLYLIFGAWAGMVGTSLSLLIRTELGNPGSLIGDDQIYNVIVTAHAFIMIFFMVMPIMIGGFGNWLIPLMLGAPDMAFPRMNNLSFWLLPPSLTLLIMSSIVENGAGTGWTVYPPLSSNIAHSGSSVDLAIFSLHLAGVSSILGAVNFITTVINMRPQGMT
LDRMPLFIWAVVITAVLLLLSLPVLAGAITMLLTDRNLNTSFFDPAGGGDPILYQHL
>COLFB941-12|KJ961874|ZMUO.001986|Zyras_humeralis
TLYFVFGAWAGMVGTSLSLLIRAELGNPGSLIGDDQIYNVIVTAHAFIMIFFMVMPIVIGGFGNWLVPLMLGAPDMAFPRMNNMSFWLLPPSLTLLLMSSMVESGAGTGWTVYPPLSSNIAHGGSSVDMAIFSLHLAGISSILGAVNFISTVINMRSV
GISFDRMPLFVWSVAITALLLLLSLPVLAGAITMLLTDRNLNTSFFDPAGGGDPILYQHL
>COLFA540-12|KJ962371|ZMUO.000675|Mylabris_sp.
TLYLIFGAWAGMVGTSLSLLIRSELGNPGTLIGDDQIYNVIVTAHAFIMIFFMVMPIVIGGFGNWLVPLMLGAPDMAFPRMNNMSFWLLPPSLTLLLMSSIVENGAGTGWTVYPPLSSNIAHGGSSVDLAIFSLHLAGVSSILGAVNFITTVINMRPAG
MSFDRMPLFVWAVAITALLLLLSLPVLAGAITMLLTDRNLNTSFFDPAGGGDPILYQHL
>COLFA647-12|KJ963906|ZMUO.000782|Atheta_atramentaria
TLYFIFGAWAGMVGTSLSLLIRAELGNPGSLIGDDQIYNVIVTAHAFIMIFFMVMPVVIGGFGNWLVPLMLGAPDMAFPRMNNMSFWLLPPSLTLLLMSSMVESGAGTGWTVYPPLSSNIAHGGSSVDLAIFSLHLAGISSILGAVNFISTVINMRSTGI
SFDRMPLFVWSVAITALLLLLSLPVLAGAITMLLTDRNLNTSFFDPAGGGDPILYQHL
>COLFD181-12|KJ966710|ZMUO.004076|Agelastica_alni
TLYFIFGVWAGMVGTSLSILIRVELGNPGSLIGNDQIYNVIVTAHAFIMIFFMVMPIMIGGFGNWLVPLMIGAPDMAFPRMNNMSFWLLPPSIFLLIMSSIVESGVGTGWTVYPPLSSNMAHNGSSVDLAIFSLHLAGISSILGAINFITTIINMRPNGMS
FDRMPLFVWAVIITAILLLLSLPVLAGAITMLLTDRNLNTSFFDPTGGGDPILYQHL
>COLFA088-10|HM909104|MP00310|Corticarina_obfuscata
SLYFLFGMWSGMVGTSLSLLIRLELGNPGSLIGDDQIYNVIVTAHAFIMIFFMVMPIMIGGFGNWLVPLMLGAPDMAFPRLNNMSFWLLPPSLSLLIMSSIVESGAGTGWTVYPPLSSNIAHGGSSVDLAIFSLHLAGISSILGAVNFITTVINMRPAGM
MLDQMPLFVWSVVITAILLLLSLPVLAGAITMLLTDRNLNTSFFDPAGGGDPILYQHL
>COLFF367-13|KJ963749|ZMUO.006067|Galerucella_sagittariae
TLYFIFGVWAGMVGTSLSILVRVELGNPGSLIGNDQIYNVIVTAHAFIMIFFMVMPIMIGGFGNWLVPLMIGAPDMAFPRMNNMSFWLLPPSLFLLIMSSIVESGAGTGWTVYPPLSSNIAHGGSSVDLAIFSLHLAGISSILGAINFITTIINMRPKGMT
LDRMPLFVWAVMITAILLLLSLPVLAGAITMLLTDRNLNTSFFDPAGGGDPILYQHL
>COLFB830-12|KJ964649|ZMUO.001305|Atheta_aeneipennis
TLYFIFGAWAGMVGTSLSLLIRAELGNPGSLIGDDQIYNVIVTAHAFIMIFFMVMPIVIGGFGNWLVPLMLGAPDMAFPRMNNMSFWLLPPSLTLLLMSSMVESGAGTGWTVYPPLSSNIAHGGSSVDLAIFSLHLAGISSILGAVNFISTVINMRSTGI
SFDRMPLFVWSVAITALLLLLSLPVLAGAITMLLTDRNLNTSFFDPAGGGDPILYQHL
>COLFA676-12|KJ963993|ZMUO.000811|Lathrobium_longulum
TLYFIFGAWAGMVGTSLSLLIRTELGNPGSLIGDDQIYNVIVTAHAFIMIFFMVMPIMIGGFGNWLVPLMLGAPDMAFPRMNNMSFWLLPPSLSLLLMSSMVESGAGTGWTVYPPLSSNIAHGGASVDLAIFSLHLAGISSILGAVNFITTIINMRSPG
MTYERMPLFVWAVAITALLLLLSLPVLAGAITMLLTDRNLNTSFFDPAGGGDPILYQHL
>COLFF830-13|KJ965367|ZMUO.006150|Philonthus_albipes
TLYFIFGAWAGMVGTSLSLLIRAELGNPGSLIGDDQIYNVIVTAHAFIMIFFMVMPIVIGGFGNWLVPLMLGAPDMAFPRMNNMSFWLLPPSLTLLLMSSMVESGAGTGWTVYPPLSSNIAHGGASVDLAIFSLHLAGISSILGAVNFITTVINMRSTGI
SFDRMPLFIWSVAITALLLLLSLPVLAGAITMLLTDRNLNTTFFDPAGGGDPILYQHL
>COLFF105-13|KJ964512|ZMUO.003739|Leiodes_picea
TLYFIFGAWSGMVGTSLSILIRAELGNPGSLIGDDQIYNVIVTAHAFIMIFFMVMPIVIGGFGNWLVPLMLGAPDMAFPRMNNMSFWLLPPSLTLLLMSSIVENGAGTGWTVYPPLSSNIAHSGSSVDLAIFSLHLAGISSILGAVNFITTVINMRPAGMS
FDKMPLFVWSVAITALLLLLSLPVLAGAITMLLTDRNLNTSFFDPAGGGDPILYQHL

>COLFB893-12|KJ966029|ZMUO.001938|Oplosia_cinerea
TLYFIFGAWAGMIGTSLSILIRSELGNSGSLIGDDQIYNTIVTAHAFIMIFFMVMPVMMGGFGNWLVPLMLGAPDMAFPRMNNMSFWLLPPSLTLLIMSSIIDNGAGTGWTVYPPLSANIAHAGSSVDLAIFSLHLAGVSSILGAVNFITTVINMRPKG
MTFDRLPLFVWAVKITAILLLLSLPVLAGAITMLLTDRNLNTSFFDPAGGGDPILYQHL
>COLFC162-12|KJ966264|ZMUO.002347|Stenus_clavicornis
TLYFIFGSWAGMVGTSLSMLIRSELGSPGSLIGDDQIYNVIVTAHAFIMIFFMVMPIMIGGFGNWLVPLMLGAPDMAFPRMNNMSFWLLPPSLSLLLMSSIVESGAGTGWTVYPPLSSNIAHSGASVDLAIFSLHLAGISSILGAINFITTIINMRTMKM
QLDCLPLFVWSVSITALLLLLSLPVLAGAITMLLTDRNLNTSFFDPAGGGDPILYQHL
>COLFD720-12|KJ962728|ZMUO.004710|Dermestes_murinus
TLYFIFGAWAGMVGTSLSMLIRTELGMPGSLIGDDQIFNVIVTAHAFIMIFFMVMPIMIGGFGNWLVPLMLGAPDMAFPRMNNMSFWLLPPSLSLLLMSSMVESGAGTGWTVYPPLSANIAHGGASVDLAIFSLHLAGISSILGAVNFITTVINMRSK
GMTPDRMPLFVWSVAITALLLLLSLPVLAGAITMLLTDRNLNTSFFDPAGGGDPILYQHL
>COLFE810-13|KJ963398|ZMUO.005655|Cymbiodyta_marginella
TLYFIFGAWAGMVGTSLSILIRAELGNPGTLIGDDQIYNVIVTAHAFIMIFFMVMPIMIGGFGNWLVPLMLGAPDMAFPRMNNMSFWLLPPSLTLLLMSSMVENGAGTGWTVYPPLSSNIAHGGASVDLAIFSLHLAGISSILGAVNFITTVINMRSNN
MTYDRLPLFVWSVAITALLLLLSLPVLAGAITMLLTDRNLNTSFFDPAGGGDPILYQHL
>COLFD062-12|KJ965214|ZMUO.003957|Mycetochara_axillaris
TLYFIFGAWSGMVGTSLSLLIRAELGNPGSLIGDDQIYNVIVTAHAFIMIFFMVMPIMIGGFGNWLVPIMLGAPDMAFPRMNNMSFWLLPPSLTLLLMSSMVESGAGTGWTVYPPLSSNIAHGGASVDLAIFSLHLAGISSILGAVNFITTVINMKPQG
MTFDRMPLFVWAVVITAVLLLLSLPVLAGAITMLLTDRNINTSFFDPAGGGDPILYQHL
>COLFD185-12|KJ967241|ZMUO.004080|Sphaerosoma_pilosum
TLYFIFGMWSGMVGTSLSLLIRLELGNPGFLIGDDQIYNVIVTAHAFIMIFFMVMPIMIGGFGNWLVPLMLGAPDMAFPRMNNMSFWLLPPSLTLLIMSSIVESGVGTGWTVYPPLSANIAHSGSSVDLAIFSLHLAGISSILGAVNFITTIINMRPINMK
WEQIPLFVWSVFITAILLLLSLPVLAGAITMLLTDRNLNTSFFDPAGGGDPILYQHL
>COLFB630-12|KJ963919|ZMUO.001105|Hydrochus_brevis
TLYFIFGAWSGMVGTSLSLLIRTELGNPGSLIGDDQIYNVIVTAHAFIMIFFMVMPIMIGGFGNWLVPLMLGAPDMAFPRMNNMSFWLLPPSLTLLLMSSMVESGAGTGWTVYPPLSSNIAHGGASVDLAIFSLHLAGISSILGAVNFITTIINMRSNN
MTYDRLPLFVWSVGITALLLLLSLPVLAGAITMLLTDRNLNTSFFDPIGGGDPILYQHL
>COLFE1013-13|KJ962841|ZMUO.006808|Rybaxis_longicornis
TLYLIFGAWAGMVGTSLSILIRAELGNPGSLIGDDQIYNVIVTAHAFIMIFFMVMPIMIGGFGNWLVPLMLGAPDMAFPRMNNMSFWLLPPSLILLLMSSMVESGAGTGWTVYPPLSSNIAHSGSSVDLTIFSLHLAGISSILGAVNFITTVINMRTMN
MKFDQLPLFVWSVIITALLLLLSLPVLAGAITMLLTDRNLNTSFFDPTGGGDPVLYQHL
>COLFF067-13|KJ963509|ZMUO.005767|Mecinus_pascuorum
TLYFIFGTWSGMMGTSMSMIIRTELGNPGKFIGNDQIYNSIVTAHAFIMIFFMVMPIMIGGFGNWLVPLMMGAPDMAFPRLNNLSFWLLPPSITMLLLSSITEKGAGTGWTVYPPLSTNIAHGGTSVDLAIFSLHMAGISSILGAMNFISTMMNMRP
LSMKLDQMPLFVWSVKITAILLLLSLPVLASAITMLLTDRNINTSFFDPAGGGDPILYQHL
>COLFB888-12|KJ963523|ZMUO.001933|Callidium_violaceum
TLYFLFGAWASMVGTSLSMLIRTELGNPGSLIGDDQIYNVIVTAHAFVMIFFMVMPIMIGGFGNWLVPLMIGAPDMAFPRMNNLSFWLLPPALILLILSSIVENGAGTGWTVYPPLSANVAHSGSSVDLAIFSLHLAGISSILGAVNFISTVINMRPMG
MTPERMPLFVWAVVITAILLLLSLPVLAGAITMLLTDRNINTSFFDPAGGGDPILYQHL
>COLFE1557-13|KJ963944|ZMUO.007447|Phratora_vitellinae
TLYFIFGIWAGMVGTSLSMLIRSELGNPGTLIGNDQIYNVIVTAHAFIMIFFMVMPIMIGGFGNWLVPLMIGAPDMAFPRLNNMSFWLLPPSLFLLLMSSVVENGAGTGWTVYPPLSSNLAHSGSSVDLAIFSLHLAGISSILGAINFITTVINMRPEGM
DLEQTPLFVWAVLITAILLLLSLPVLAGAITMLLTDRNLNTSFFDPAGGGDPILYQHL
>COLFD692-12|KJ963383|ZMUO.004682|Brachonyx_pineti
TLYFIFGMWAGSIGTSLSVLIRAELGTPGNLIGDDQIYNTIVTAHAFVMIFFMVMPIMIGGFGNWLIPLMLAAPDMAFPRLNNLSFWLLPPSLTLLIMSSVIGKGAGTGWTVYPPLSANLAHEGPSVDFAIFSLHMAGISSILGAMNFISTILNMKPMKM
KFDQMPLFIWAVKITAILLLISLPVLAGAITMLLTDRNINTSFFDPAGGGDPILYQHL
>COLFE292-12|KJ965984|ZMUO.004852|Staphylinus_caesareus
TLYFVFGSWSGMVGTSLSLLIRAELGNPGTLIGDDQIYNVIVTAHAFVMIFFMVMPIVIGGFGNWLVPLMLGAPDMAFPRMNNMSFWLLPPSLTLLLASSMAESGAGTGWTVYPPLSSNIAHSGASVDLAIFSLHLAGISSILGAVNFITTVINMRATG
MTFDRMPLFVWSVAITALLLLLSLPVLAGAITMLLTDRNLNTTFFDPAGGGDPILYQHL
>COLFE524-13|KJ967083|ZMUO.005369|Polydrusus_mollis
TLYFIFGAWSGMVGTSLSMLIRTELGNPGSLIGDDQIYNVIVTAHAFIMIFFMVMPTMIGGFGNWLVPLMLGAPDMAFPRLNNMSFWLLPPSLSLLLMSSIVDKGAGTGWTVYPPLSANIAHEGSSVDLAIFSLHMAGVSSILGAVNFISTVINMHPK
GMTPERMPLFVWAVVITAILLLLSLPVLAGAITMLLTDRNMNTSFFDPAGGGDPILYQHL
>COLFC333-12|KJ965155|ZMUO.002518|Atheta_lapponica
TLYFIFGAWAGMVGTSLSLLIRAELGNPGSLIGDDQIYNVIVTAHAFIMIFFMVMPIVIGGFGNWLVPLMLGAPDMAFPRMNNMSFWLLPPSLTLLLMSSMVESGAGTGWTVYPPLSSNIAHGGSSVDLAIFSLHLAGISSILGAVNFISTVINMRSTGI
SFDRMPLFVWSVAITALLLLLSLPVLAGAITMLLTDRNLNTSFFDPAGGGDPILYQHL
>COLFD516-12|KJ966500|ZMUO.004316|Agonum_muelleri

TLYFIFGAWAGMVGTSLSMLIRAELGNPGALIGDDQIYNVIVTAHAFIMIFFMVMPIMIGGFGNWLVPLMLGAPDMAFPRMNNMSFWLLPPSLTLLLMSSLVESGAGTGWTVYPPLSSGIAHAGASVDLAIFSLHLAGVSSILGAVNFITTIINMRSVG
MTFDRMPLFVWSVGITALLLLLSLPVLAGAITMLLTDRNLNTSFFDPAGGGDPILYQHL
>COLFE439-12|KJ962687|ZMUO.004999|Phyllotreta_vittula
TLYFIFGIWSGMVGMSMSMLIRIELAAPGSLIGNDQIYNVIVTAHAFIMIFFMVMPIMIGGFGNWLVPLMIGAPDMAFPRMNNMSFWLLPPSLFLLIMSSIVENGAGTGWTVYPPLSANISHAGSSVDLTIFSLHLAGISSILGAINFITTIINMRPKGMS
FDRMPLFVWAVLITAILLLLSLPVLAGAITMLLTDRNLNTSFFDPMGGGDPILYQHL
>COLFE937-13|KJ965014|ZMUO.006257|Leiodes_fracta
TLYFIFGAWAGMVGTSLSILIRAELGNPGSLIGDDQIYNVIVTAHAFVMIFFMVMPIVIGGFGNWLVPLMLGAPDMAFPRMNNMSFWLLPPSLSLLLMSSIVENGAGTGWTVYPPLSANIAHSGSSVDLAIFSLHLAGISSILGAVNFITTVINMRSVGM
SFDKMPLFVWSVAITALLLLLSLPVLAGAITMLLTDRNLNTSFFDPAGGGDPILYQHL
>COLFB467-12|KJ966962|ZMUO.001892|Ptinus_rufipes
TLYFVLGAWSGMIGTSLSMLIRSELGNPGALIGDDQIYNVIVTAHAFIMIFFMVMPIMIGGFGNWLIPLMLGAPDMAFPRMNNMSFWLLPPALTFLLMSSMIESGVGTGWTVYPPLSSNIAHSGASVDLAIFSLHLAGISSILGAVNFITTVINMRPMG
MTFDRMPLFIWSVAITALLLLLSLPVLAGAITMLLTDRNLNTSFFDPAGGGDPILYQHL
>COLFB345-12|KJ966558|ZMUO.001675|Eucnecosum_brachypterum
TLYFIFGAWAGMVGTSLSILIRAELGNPGTLIGDDQIYNVIVTAHAFVMIFFMVMPIVIGGFGNWLVPLMLGAPDMAFPRMNNMSFWLLPPSLTLLLMSSMVESGAGTGWTVYPPLSSNIAHGGSSVDLAIFSLHLAGISSILGAVNFITTVINMRTTG
MTFDRMPLFVWSVAITALLLLLSLPVLAGAITMLLTDRNLNTSFFDPAGGGDPILYQHL
>COLFD582-12|KJ961939|ZMUO.004382|Cantharis_obscura
TLYFIFGAWSGSLGLALSLLIRAELGTPGTLIGNDQIYNVIVTAHAFIMIFFMVMPIMIGGFGNWLVPLMLGAPDMAFPRMNNMSFWFLPPSLMFLLMSSMVESGAGTGWTVYPPLSANIAHSGPSVDLAIFSLHMAGISSILGAVNFISTIMNMKPPS
MKFDQMPLFVWSVGITALLLLLSLPVLAGAITMLLSDRNLNTSFFDPMGGGDPILYQHL
>COLFF244-13|KJ962121|ZMUO.005849|Phyllobius_argentatus
TLYFIFGAWSGMVGTSLSILIRIELGNPGSLIGDDQIYNVIVTAHAFIMIFFMVMPMMIGGFGNWLVPLMLGAPDMAFPRLNNMSFWLLPPSLSLLLMSSIVDKGAGTGWTVYPPLSANIAHEGSSVDLAIFSLHMAGVSSILGAINFISTVINMRPSG
MSPDRMSLFIWAVKITAVLLLLSLPVLAGAITMLLTDRNMNTSFFDPAGGGDPILYQHL
>COLFF901-13|KJ964144|ZMUO.006696|Philorhizus_notatus
TLYFIFGAWAGMVGTSLSMLIRAELGNPGALIGDDQIYNVIVTAHAFIMIFFMVMPIMIGGFGNWLVPLMLGAPDMAFPRMNNMSFWLLPPSLTLLLMSSMVESGTGTGWTVYPPLSSGIAHAGASVDLAIFSLHLAGISSILGAVNFITTIINMRSIG
MTFDRMPLFVWSVGITALLLLLSLPVLAGAITMLLTDRNLNTSFFDPAGGGDPILYQHL
>COLFD328-12|KJ965001|ZMUO.004223|Ischnosoma_longicorne
TLYFIFGAWAGMVGTSLSLLIRAELGNPGSLIGDDQIYNVIVTAHAFVMIFFMVMPIVIGGFGNWLVPLMLGAPDMAFPRMNNMSFWLLPPSLSLLLMSSLVESGAGTGWTVYPPLSSNIAHGGASVDLAIFSLHLAGISSILGAVNFITTIINMRSTGM
TFDRMPLFVWSVVITAFLLLLSLPVLAGAITMLLTDRNLNTSFFDPAGGGDPILYQHL
>COLFF558-13|KJ964727|ZMUO.006638|Tritoma_bipustulata
TLYFIFGMWAGMVGTALSMLIRSELGNPGSLIGNDQIYNVIVTAHAFIMIFFMVMPIMMGGFGNWLVPLMLGAPDMAFPRLNNMSFWLLPPSLTLLIMSSIVETGAGTGWTVYPPLSSNIAHSGASVDLAIFSLHLAGISSILGAMNFISTMMNMRP
SGMQMDQMPLFVWAVLITAILLLLSLPVLAGAITMLLTDRNINTTFFDPAGGGDPILYQHL
>COLFA556-12|KJ964360|ZMUO.000691|Pogonocherus_decoratus
TLYFLFGAWAGMVGTSLSLLIRSELGNPGSLIGDDQIYNVIVTAHAFVMIFFMVMPVMIGGFGNWLVPLMLGAPDMAFPRMNNMSFWLLPPSLSLLIMSSITESGAGTGWTVYPPLSSNIAHSGSSVDLAIFSLHLAGISSILGAVNFITTVINMRPTG
MIFERLPLFVWAVKFTAILLLLSLPVLAGAITMLLTDRNLNTSFFDPAGGGDPILYQHL
>COLFB101-12|KJ966936|ZMUO.001431|Silpha_carinata
TLYFIFGAWAGVVGMSLSILIRMELSTPGSLLGDDQMYNVIVTAHAFIMIFFMVMPIVIGGFGNWLVPLMLGAPDMAFPRMNNMSFWLLPPSLSLLLVSSMVESGAGTGWTVYPPLSSNIAHGGSSVDLAIFSLHLAGVSSILGAVNFITTIINMRSSG
MTFDRMPLFVWSVGVTALLLLLSLPVLAGAITMLLTDRNLNTSFFDPAGGGDPILYQHL
>COLFC768-12|KJ963764|ZMUO.003333|Dasytes_niger
TLYFIFGAWSGMVGMSLSLLIRSELNNPGTLIGNDQIYNVIVTAHAFIMIFFMVMPILIGGFGNWLVPLMLGAPDMAFPRMNNMSFWLLPPSLTLLLMSSMVEQGAGTGWTVYPPLSSNIAHGGASVDLAIFSLHLAGISSILGAVNFITTIINMRPVG
MTLDRTPLFVWAVAITALLLLLSLPVLAGAITMLLTDRNLNTSFFDPAGGGDPILYQHL
>COLFB650-12|KJ966516|ZMUO.001125|Stenus_palustris
TLYFILGAWAGLTGTSLSLLIRTELGSPGSFIGDDQIYNVIVTAHAFIMIFFMVMPIMIGGFGNWLIPLMLGAPDMAFPRMNNMSFWLLPPSLSLLLMSSIVESGAGTGWTVYPPLSSNIAHSGASVDLTIFSLHLAGISSILGAINFITTIFNMRTMKLQLD
CLPLFIWSVNVTTFLLLLSLPVLAGAITMLLTDRNVNTSFFDPGGGGDPILYQHL
>COLFC505-12|KJ966914|ZMUO.003070|Anisotoma_glabra
TLYFIFGAWSGMVGTSLSILIRAELGTPGSLIGDDQIYNVIVTAHAFVMIFFMVMPIVIGGFGNWLVPLMLGAPDMAFPRMNNMSFWLLPPSLTLLLMSSMVENGAGTGWTVYPPLSANISHSGSSVDLAIFSLHLAGISSILGAVNFITTVINMRSIGM
TFDKMPLFVWSVAITALLLLLSLPVLAGAITMLLTDRNLNTSFFDPAGGGDPILYQHL
>COLFF194-13|KJ962077|ZMUO.005799|Cryptocephalus_punctiger
TLYFLFGAWAGMIGTSLSVLIRIELGNPGSLIGNDQIYNTIVTAHAFIMIFFMVMPIMIGGFGNWLVPLMLGAPDMAFPRMNNMSFWLLPPSLTLLLMSSIVENGAGTGWTVYPPLSASLAHTGPSVDLAIFSLHLAGISSILGAINFISTVINMRPMGM
LMDRTPLFVWAVLITAVLLLLSLPVLAGAITMLLTDRNLNTSFFDPAGGGDPILYQHL

>COLFE193-12|KJ967081|ZMUO.005038|Harpalus_rubripes
TLYFIFGAWAGMVGTSLSMLIRAELGTPGALIGDDQIYNVIVTAHAFIMIFFMVMPIMIGGFGNWLVPLMLGAPDMAFPRMNNMSFWLLPPSLTLLLMSSMVESGAGTGWTVYPPLSSGIAHSGASVDLAIFSLHLAGVSSILGAVNFITTIINMRSV
GMTFDRMPLFVWSVGITALLLLLSLPVLAGAITMLLTDRNLNTSFFDPAGGGDPILYQHL
>COLFF306-13|KJ964630|ZMUO.006006|Hypera_denominanda
TLYFIFGTWAGTVGTSLSILIRTELGNPGSLIGNDQIYNTIVTAHAFIMIFFMVMPIMIGGFGNWLVPLMLGAPDMAFPRLNNMSFWLLPPSLSLLLMSSMVDSGAGTGWTVYPPLSSNIAHEGSSVDLAIFSLHMAGVSSILGAINFISTMLNMRPSG
MSLDKMALFIWAVKITAILLLLSLPVLAGAITMLLTDRNINTSFFDPAGGGDPILYQHL
>COLFE1276-13|KJ965072|ZMUO.007071|Lilioceris_merdigera
TLYFIFGAWSGMVGTSLSMLIRTELGNPGSLIGNDQIYNVIVTAHAFIMIFFMVMPIMIGGFGNWLVPLMLGAPDMAFPRMNNMSFWLLPPSLTLLLMSSIVENGAGTGWTVYPPLSANLAHNGASVDLAIFSLHLAGISSILGAVNFITTVMNMRPE
GMKMDRTPLFVWAVLITAILLLLSLPVLAGAITMLLTDRNLNTSFFDPASGGDPILYQHL
>COLFE1564-13|KJ961895|ZMUO.007454|Stenus_flavipes
TLYFIFGAWAGMVGTSLSLLIRAELGTPSSLIGDDQIYNVIVTAHAFIMIFFMVMPIMIGGFGNWLVPLMMGAPDMAFPRMNNMSFWLLPPSLSLLLMSSIVESGVGTGWTVYPPLSSNIAHSGASVDLAIFSLHLAGISSILGAINFITTIINMRTMKM
NFDCLPLFVWSVSITALLLLLSLPVLAGAITMLLTDRNLNTSFFDPAGGGDPVLYQHL
>COLFF911-13|KJ967016|ZMUO.006706|Gabrius_breviventer
TLYFIFGSWAGMVGTSLSLLIRAELGNPGTLIGDDQIYNVIVTAHAFIMIFFMVMPIVIGGFGNWLVPLMLGAPDMAFPRMNNMSFWLLPPSLTLLLMSSMVESGAGTGWTVYPPLSSNIAHGGASVDLAIFSLHLAGISSILGAVNFITTVINMRSFG
MSFDRMPLFVWSVAITALLLLLSLPVLAGAITMLLTDRNLNTSFFDPAGGGDPILYQHL
>COLFF851-13|KJ967372|ZMUO.006171|Oxystoma_subulatum
TLYFILGLWSGMVGTSLSMLIRVELGNSGSLIGDDQIYNVIVTAHAFIMIFFMVMPIMIGGFGNWLVPLMLGAPDMAFPRMNNMSFWLLPPSLTLLLMSSIVEKGAGTGWTVYPPLASNIAHSGASVDLAIFSLHLAGISSILGAVNFISTMINMHPNG
LSLDQLSLFTWAVKITAILLLLSLPVLAGAITMLLTDRNINTSFFDPAGGGDPILYQHL
>COLFB460-12|KJ967055|ZMUO.001885|Anthicus_antherinus
TLYLIFGAWAGMVGTSLSLLIRSELGNPGTFIGNDQIYNVIVTAHAFIMIFFMVMPIVIGGFGNWLVPLMLGAPDMAFPRMNNMSFWLLPPSLSLLIMSSIVESGSGTGWTVYPPLSANIAHSGSSVDLAIFSLHLAGISSILGAVNFITTVINMRPAGMT
LDRMPLFVWAVVITAVLLLLSLPVLAGAITMLLTDRNLNTSFFDPAGGGDPILYQHL
>COLFC414-12|KJ963585|ZMUO.002979|Cytilus_sericeus
TLYFIFGAWAGMVGTSLSLLIRAELGNPGSLIGDDQIYNVIVTAHAFVMIFFMVMPIMIGGFGNWLVPLMLGAPDMAFPRMNNMSFWLLPPSLSLLLMSSIVESGAGTGWTVYPPLSSNIAHSGSSVDLAIFSLHLAGISSILGAVNFISTVINMRSPG
MKFDQMSLFSWSVAITALLLLLSLPVLAGAITMLLTDRNINTSFFDPAGGGDPILYQHL
>COLFD715-12|KJ966969|ZMUO.004705|Trox_sabulosus
TLYFLFGSWAGMVGTSLSILIRAELGNPGTLIGDDQIYNVIVTAHAFIMIFFMVMPILIGGFGNWLVPLMLGAPDMAFPRMNNMSFWLLPPSLTLLLVSSLVESGAGTGWTVYPPLSSNIAHSGASVDLAIFSLHLAGISSILGAVNFITTVINMRSTGMT
FDRMPLFVWAVILTALLLLLSLPVLAGAITMLLTDRNLNTSFFDPAGGGDPVLYQHL
>COLFB308-12|KJ961794|ZMUO.001638|Bembidion_minimum
TLYFIFGAWSGMVGTSLSMLIRAELGNPGSLIGDDQIYNVIVTAHAFVMIFFMVMPILIGGFGNWLVPLMLGAPDMAFPRMNNMSFWLLPPSLTLLLMSSMVESGAGTGWTVYPPLSSSIAHSGASVDLAIFSLHLAGVSSILGAVNFITTIINMRSSG
MTFDRMPLFVWSVGITALLLLLSLPVLAGAITMLLTDRNLNTSFFDPAGGGDPILYQHL
>COLFD348-12|KJ966305|ZMUO.004243|Stenus_similis
TLYFIFGAWAGMVGTSLSLLIRAELGNPGSLIGDDQIYNVIVTAHAFIMIFFMVMPIMIGGFGNWLVPLMLGAPDMAFPRMNNMSFWLLPPSLSLLLMSSIVESGAGTGWTVYPPLSSNIAHSGASVDLAIFSLHLAGISSILGAINFITTILNMRTMKIQ
LDCLPLFVWSVGITALLLLLSLPVLAGAITMLLTDRNLNTSFFDPAGGGDPILYQHL
>COLFE1339-13|KJ967285|ZMUO.007134|Cis_punctulatus
TLYFIFGAWSGMLGTSMSMMIRSELGTPGSLIGNDQIYNVIVTAHAFIMIFFMVMPIMIGGFGNWLVPLMLGAPDMAFPRMNNMSFWLLPPSLSLLLMSSIVENGAGTGWTVYPPLSANIAHSGSSVDLAIFSLHLAGISSILGAVNFISTVINMRPIQ
MSFDRMPLFVWAVVITAVLLLLSLPVLAGAITMLLTDRNFNTTFFDPAGGGDPILYQHL
>COLFA334-12|KJ964093|ZMUO.000469|Olophrum_assimile
TLYFIFGAWAGMVGTSLSILIRAELGNPGTLIGDDQIYNVIVTAHAFVMIFFMVMPIVIGGFGNWLVPLMLGAPDMAFPRMNNMSFWLLPPSLTLLLMSSMVESGAGTGWTVYPPLSSNIAHGGSSVDLAIFSLHLAGISSILGAVNFITTVINMRSTG
MTFDRMPLFVWSVAITALLLLLSLPVLAGAITMLLTDRNLNTSFFDPAGGGDPILYQHL
>COLFE665-13|KJ962513|ZMUO.005510|Polydrusus_pilosus
TLYFIFGAWSGMVGTSLSMLIRTELGNPGSLIGDDQIYNVIVTAHAFIMIFFMVMPIMIGGFGNWLVPLMLGAPDMAFPRLNNMSFWLLPPSLTLLLMSSIVDKGAGTGWTVYPPLSMNIAHEGSSVDLAIFSLHMAGVSSILGAINFISTIINMRPTG
MSYDRMPLFVWAVKITAILLLLSLPVLAGAITMLLTDRNINTSFFDPAGGGDPILYQHL
>COLFC432-12|KJ966434|ZMUO.002997|Bledius_poppiusi
TLYFIFGAWAGMVGTSLSMLIRAELGTPGYLIGDDQIYNVVVTAHAFVMIFFMVMPIVIGGFGNWLIPLMLGAPDMAFPRMNNMSFWLLPPSLTLLLMSSMVESGAGTGWTVYPPLSSNIAHSGSSVDLAIFSLHLAGISSILGAVNFISTVINMRSIG
MTFDRMPLFVWSVKITAILLLLSLPVLAGAITMLLTDRNLNTSFFDPAGGGDPILYQHL
>COLFB457-12|KJ965607|ZMUO.001882|Anthicus_antherinus

TLYLIFGAWAGMVGTSLSLLIRSELGNPGTLIGNDQIYNVIVTAHAFIMIFFMVMPIVIGGFGNWLVPLMLGAPDMAFPRMNNMSFWLLPPSLSLLIMSSIVESGSGTGWTVYPPLSANIAHSGSSVDLAIFSLHLAGISSILGAVNFITTVINMRPAGMT
LDRMPLFVWAVVITAVLLLLSLPVLAGAITMLLTDRNLNTSFFDPAGGGDPILYQHL
>COLFE478-13|KJ964352|ZMUO.005323|Crepidodera_aurata
TLYFIFGIWSGMVGTSLSMLIRTELGNPGSLIGNDQIYNVIVTAHAFIMIFFMVMPIMIGGFGNWLVPLMIGAPDMAFPRMNNMSFWLLPPSLFLLLMSSLVESGAGTGWTVYPPLSSNIAHGGSSVDLAIFSLHLAGISSILGAINFITTIINMRPKGMS
LDRMPLFVWAVAITAILLLLSLPVLAGAITMLLTDRNLNTSFFDPIGGGDPILYQHL
>COLFB292-12|KJ967147|ZMUO.001622|Microlestes_minutulus
TLYFIFGAWAGMVGTSLSMLIRAELGNPGALIGDDQIYNVIVTAHAFIMIFFMVMPIMIGGFGNWLVPLMLGAPDMAFPRMNNMSFWLLPPSLTLLLMSSMVESGAGTGWTVYPPLSSGIAHAGASVDLAIFSLHLAGVSSILGAVNFITTIINMRSV
GMSFERMPLFVWSVGITALLLLLSLPVLAGAITMLLTDRNLNTTFFDPAGGGDPILYQHL
>COLFG103-13|KJ963293|ZMUO.007798|Phloeonomus_punctipennis
TLYFIFGAWAGMVGTSLSLLIRAELGNPGSLIGDDQIYNVIVTAHAFVMIFFMVMPIVIGGFGNWLVPLMLGAPDMAFPRMNNMSFWLLPPSLTLLLMSSMVESGAGTGWTVYPPLSSNIAHGGASVDLAIFSLHLAGISSILGAVNFITTVINMRATG
MTFDRMPLFVWSVAITALLLLLSLPVLAGAITMLLTDRNLNTSFFDPAGGGDPILYQHL
>COLFF1007-13|KJ962580|ZMUO.007372|Atrecus_pilicornis
TLYFIFGAWAGMVGTSLSLLIRAELGNPGSLIGDDQIYNVIVTAHAFVMIFFMVMPIVIGGFGNWLVPLMLGAPDMAFPRMNNMSFWLLPPSLTLLLMSSMVESGAGTGWTVYPPLSSNIAHGGASVDLAIFSLHLAGISSILGAVNFITTVINMRSIG
MTFDRMPLFIWSVAITALLLLLSLPVLAGAITMLLTDRNLNTTFFDPSGGGDPILYQHL
>COLFC338-12|KJ967017|ZMUO.002523|Tachinus_elegans
TLYFIFGAWAGMVGTSLSLLIRAELGNPGSLIGDDQIYNVIVTAHAFIMIFFMVMPVMIGGFGNWLVPLMLGAPDMAFPRMNNMSFWLLPPSLTLLLMSSMVESGAGTGWTVYPPLSSNIAHGGSSVDLAIFSLHLAGISSILGAVNFITTVINMRSIG
MTFDRMPLFVWSVAITALLLLLSLPVLAGAITMLLTDRNLNTTFFDPAGGGDPILYQHL
>COLFB664-12|KJ962877|ZMUO.001139|Brachyderes_incanus
TLYFIFGAWSGMVGTSLSMLIRTELGNPGSLIGDDQIYNVIVTAHAFIMIFFMVMPMMIGGFGNWLVPLMLGAPDMAFPRMNNMSFWLLPPSLSLLLMSSIVDKGAGTGWTVYPPLSANIAHEGSSVDLAIFSLHMAGVSSILGAINFISTVINMRP
MGMSPDRMSLFIWAVKITAVLLLLSLPVLAGAITMLLTDRNVNTSFFDPAGGGDPILYQHL
>COLFD317-12|KJ964654|ZMUO.004212|Dyschirius_thoracicus
TLYFIFGIWSGMVGTSLSILIRTELGNPGSLIGDDQIYNVIVTAHAFIMIFFMVMPIMIGGFGNWLVPLMLGAPDMAFPRMNNMSFWLLPPSLTLLLMSSMVESGAGTGWTVYPPLSSGIAHSGASVDLAIFSLHLAGISSILGAVNFITTIINMRSTGMT
FERMPLFVWSVGITALLLLLSLPVLAGAITMLLTDRNLNTSFFDPAGGGDPILYQHL
>COLFE1023-13|KJ967154|ZMUO.006818|Cyphon_variabilis
TLYFIFGSWSGMVGTSLSLLIRAELGTPGSLIGDDQIYNVIVTAHAFIMIFFMVMPIMIGGFGNWLVPLMLGAPDMAFPRMNNMSFWLLPPSLTLLLMSSMVENGAGTGWTVYPPLSAGVAHSGASVDLAIFSLHLAGISSILGAVNFISTVINMRSVG
MTFDRMPLFVWAVAITALLLLLSLPVLAGAITMLLTDRNLNTSFFDPAGGGDPILYQHL
>COLFB789-12|KJ967243|ZMUO.001264|Atheta_graminicola
TLYFIFGAWAGMVGTSLSLLIRAELGNPGSLIGDDQIYNVIVTAHAFIMIFFMVMPVVIGGFGNWLIPLMLGAPDMAFPRMNNMSFWLLPPSLTLLLMSSMVESGAGTGWTVYPPLSSNIAHSGSSVDLAIFSLHLAGISSILGAVNFISTVINMRSTGIY
FDRMPLFVWSVAITALLLLLSLPVLAGAITMLLTDRNLNTSFFDPAGGGDPILYQHL
>COLFB444-12|KJ966781|ZMUO.001869|Cis_castaneus
TLYFIFGAWSGMLGTSMSILIRSELSTPGSLIGNDQIYNVIVTAHAFIMIFFMVMPVMIGGFGNWLVPLMLGAPDMAFPRMNNMSFWLLPPSLSLLIMSSIVENGAGTGWTVYPPLSSNTAHSGSSVDLAIFSLHLAGISSILGAVNFISTIINMHPSGM
KPDQMPLFVWAVVITAVLLLLSLPVLAGAITMLLTDRNLNTSFFDPAGGGDPILYQHL
>COLFB559-12|KJ962235|ZMUO.001034|Stenus_atratulus
TLYFLFGAWAGMVGTSLSLLIRAELGNPGSFIGDDQIYNVIVTAHAFVMIFFMVMPIMIGGFGNWLVPLMLGAPDMAFPRMNNMSFWLLPPSLSLLLMSSIVENGAGTGWTVYPPLSTNIAHSGASVDLAIFSLHLAGISSILGAVNFITTIFNMRTMK
MQLDCLPLFVWSVGITALLLLLSLPVLAGAITMLLTDRNLNTSFFDPAGGGDPVLYQHL
>COLFC585-12|KJ962673|ZMUO.003150|Calvia_quatuordecimguttata
TLYFFFGMWAGMVGTSLSILIRLELGTTNSLIGNDQIYNVIVTAHAFIMIFFMVMPIMIGGFGNWLVPLMIGAPDMAFPRLNNMSFWLLPPALTLLIFSSMVEMGAGTGWTVYPPLSSNIAHSGSSVDLVIFSLHLAGISSILGAVNFISTIMNMRPFGM
NLDKTPLFVWSVLITAILLLLSLPVLAGAITMLLTDRNINTSFFDPMGGGDPILYQHL
>COLFF265-13|KJ967190|ZMUO.005870|Tetrops_praeustus
TLYFIFGAWASMVGTSLSLLIRSELGSPGSLIGNDQIYNTIVTAHAFIMIFFMVMPIMIGGFGNWLVPLMLGAPDMAFPRMNNMSFWLLPPSLTLLIISSIVENGAGTGWTVYPPLASNIAHSGSSVDLAIFSLHLAGVSSILGAVNFITTVINMRPKGMA
LDRLPLFVWAVKITAILLLLSLPVLAGAITMLLTDRNLNTSFFDPASGGDPILYQHL
>COLFE1463-13|KJ966541|ZMUO.007258|Chrysolina_varians
TLYFIFGMWAGMVGTSLSILIRAELGNPGTLIGNDQIYNVIVTAHAFIMIFFMVMPIMIGGFGNWLVPLMLGAPDMAFPRMNNMSFWLLPPSLIFLLMSSIVENGVGTGWTVYPPLSANIAHSGPSVDLAIFSLHLAGVSSILGAINFITTIINMRPAGM
QLEQMPLFSWAVLITAILLLLSLPVLAGAITMLLTDRNLNTSFFDPASGGDPILYQHL
>COLFA110-10|HQ559235|MP00371|Clytra_quadripunctata
TLYFVFGAWSGMVGTSLSLLIRVELGNPGTLIGNDQIYNTIVTAHAFIMIFFMVMPIMIGGFGNWLVPLMLGAPDMAFPRMNNMSFWLLPPSLTLLLMSSIVENGAGTGWTVYPPLSANIAHSGASVDLAIFSLHLAGISSIMGAINFISTVINMRPQG
MLLDRTPLFVWAVMITAVLLLLSLPVLAGAITMLLTDRNLNTSFFDPAGGGDPILYQHL

>COLFD059-12|KJ967269|ZMUO.003954|Anaspis_rufilabris
TLYFIFGAWSGMVGTSLSLLIRSELGTPGSLIGDDQIYNVIVTAHAFIMIFFMVMPILIGGFGNWLVPLMLGAPDMAFPRMNNMSFWLLPPSLTLLIMSSVVENGAGTGWTVYPPLAANIAHSGSSVDLAIFSLHLAGVSSILGAVNFITTVINMRPQG
MTLDRMPLFVWAVVITAVLLLLSLPVLAGAITMLLTDRNLNTSFFDPAGGGDPILYQHL
>COLFC110-12|KJ962046|ZMUO.002295|Atheta_myrmecobia
TLYFIFGAWAGMVGTSLSLLIRAELGNPGSLIGDDQIYNVIVTAHAFIMIFFMVMPVVIGGFGNWLVPLMLGAPDMAFPRMNNMSFWLLPPSLTLLLMSSMVESGAGTGWTVYPPLSSNIAHGGSSVDLAIFSLHLAGISSILGAVNFISTVINMRSTGI
SFDRMPLFVWSVVITALLLLLSLPVLAGAITMLLTDRNLNTSFFDPAGGGDPILYQHL
>COLFE141-12|KJ963812|ZMUO.005176|Cicindela_hybrida
TLYFIFGAWSGMVGTSLSMLIRAELGSPGSLIGDDQIYNVIVTAHAFVMIFFMVMPIMIGGFGNWLVPLMLGAPDMAFPRMNNMSFWLLPPSLTLLLMSSMVGDGAGTGWTVYPPLSAGIAHAGASVDLAIFSLHLAGVSSILGAVNFITTIINMRS
VGMTFDRMPLFVWSVGITALLLLLSLPVLAGAITMLLTDRNLNTSFFDPAGGGDPILYQHL
>COLFC415-12|KJ963736|ZMUO.002980|Stenus_crassus
TLYFIFGAWAGMVGTSLSLLIRAELGHPGSLIGDDQIYNVVVTAHAFVMIFFMVMPIMIGGFGNWLVPLMLGAPDMAFPRMNNMSFWLLPPSLSLLLMSSIVENGAGTGWTVYPPLSTNIAHSGASVDITIFSLHLAGISSILGAINFITTIFNMRTMKL
QLDCLPLFVWSVEITALLLLLSLPVLAGAITMLLTDRNLNTSFFDPAGGGDPILYQHL
>COLFE1382-13|KJ962911|ZMUO.007177|Galerucella_sagittariae
TLYFIFGVWAGMVGTSLSILVRVELGNPGSLIGNDQIYNVIVTAHAFIMIFFMVMPIMIGGFGNWLVPLMIGAPDMAFPRMNNMSFWLLPPSLFLLIMSSIVESGAGTGWTVYPPLSSNIAHGGSSVDLAIFSLHLAGISSILGAINFITTIINMRPKGMT
LDRMPLFVWAVMITAILLLLSLPVLAGAITMLLTDRNLNTSFFDPAGGGDPILYQHL
>COLFF366-13|KJ963786|ZMUO.006066|Galerucella_sagittariae
TLYFIFGVWAGMVGTSLSILVRVELGNPGSLIGNDQIYNVIVTAHAFIMIFFMVMPIMIGGFGNWLVPLMIGAPDMAFPRMNNMSFWLLPPSLFLLIMSSIVESGAGTGWTVYPPLSSNIAHGGSSVDLAIFSLHLAGISSILGAINFITTIINMRPKGMT
LDRMPLFVWAVMITAILLLLSLPVLAGAITMLLTDRNLNTSFFDPAGGGDPILYQHL
>COLFC761-12|KJ967194|ZMUO.003326|Elateroides_dermestoides
TLYFIFGAWAGMVGTSMSVLIRIELGTSGSLIGDDQIYNVIVTAHAFIMIFFMVMPIMIGGFGNWLVPLMLGAPDMAFPRMNNMSFWLLPPSLTLLLMSSMVESGAGTGWTVYPPLSSNIAHSGSSVDLAIFSLHLAGISSILGAANFITTIINMRPAG
MTFERMPLFVWAVLITAVLLLLSLPVLAGAITMLLTDRNLNTSFFDPAGGGDPILYQHL
>COLFA534-12|KJ963652|ZMUO.000669|Chlorophorus_herbstii
TLYFIFGAWAGMVGTSLSMLIRSELGNPGSLIGDDQIYNVIVTAHAFIMIFFMVMPIMIGGFGNWLIPLMLGAPDMAFPRMNNMSFWLLPPSLSLLIMSSIVENGAGTGWTVYPPLSANIAHSGSSVDLAIFSLHLAGISSILGAVNFISTVINMRPTGM
NLEQMPLFVWAVVITAILLLLSLPVLAGAITMLLTDRNINTSFFDPAGGGDPILYQHL
>COLFB538-12|KJ963958|ZMUO.001013|Ocalea_picata
TLYFIFGAWAGMVGTSLSLLIRAELGNPGSLIGDDQIYNVIVTAHAFVMIFFMVMPIMIGGFGNWLVPLMLGAPDMAFPRMNNMSFWLLPPSLTLLLMSSMVESGAGTGWTVYPPLSSNIAHGGASVDLAIFSLHLAGISSILGAVNFISTIINMRTSG
MTFDRMPLFVWSVAITALLLLLSLPVLAGAITMLLTDRNLNTSFFDPAGGGDPILYQHL
>COLFC223-12|KJ966395|ZMUO.002408|Drusilla_canaliculata
TLYFIFGAWAGMVGTSLSLLIRAELGNPGSLIGDDQIYNVIVTAHAFIMIFFMVMPIVIGGFGNWLIPLMLGAPDMAFPRMNNMSFWLLPPSLSLLLMSSMVESGAGTGWTVYPPLSSNIAHGGSSVDLAIFSLHLAGISSILGAVNFISTVINMRSTGIS
FDRMPLFVWSVAITALLLLLSLPVLAGAITMLLTDRNLNTSFFDPAGGGDPILYQHL
>COLFB357-12|KJ963199|ZMUO.001687|Stenus_juno
TLYFIFGSWAGMVGTSLSMLIRSELGNPGSLIGDDQIYNVIVTAHAFIMIFFMVMPIMIGGFGNWLVPLMLGAPDMAFPRMNNMSFWLLPPSLSLLLMSSIVESGAGTGWTVYPPLSSNIAHSGASVDLAIFSLHLAGISSILGAINFITTIINMRTMKLQ
LECLPLFVWSVSITALLLLLSLPVLAGAITMLLTDRNLNTSFFDPAGGGDPILYQHL
>COLFG140-13|KJ963821|ZMUO.007835|Acrotrichis_intermedia
TLYFMFGAWAGMVGTSLSILIRTELGTPGSLIGDDQIYNVIVTAHAFVMIFFMVMPILIGGFGNWLVPLMLGAPDMAFPRMNNMSFWLLPPSLILLLMSSMVESGAGTGWTVYPPLASNIAHGGASVDLAIFSLHLAGISSILGAVNFITTIINMRTPQ
MSFDQMPLFIWAVGITALLLLLSLPVLAGAITMLLTDRNLNTSFFDPAGGGDPILYQHL
>LEFIJ1812-13|KJ965407|ZMUO.004567|Ceutorhynchus_contractus
TLYFIFGSWAGMAGTSLSMLIRTELGNPGSLIGNDQIYNSIVTAHAFIMIFFMVMPILIGGFGNWLVPLMLGAPDMAFPRLNNMSFWLLPPSLSLLLMSSIVNKGAGTGWTVYPPLSGNVAHEGMSVDLAIFSLHMAGISSILGAINFISTVMNMQPK
GMTPELMPLFVWAVEITAILLLLSLPVLAGAITMLLTDRNINTSFFDPSGGGDPILYQHL
>COLFE1115-13|KJ963556|ZMUO.006910|Phratora_vitellinae
TLYFIFGIWAGMVGTSLSMLIRSELGNPGTLIGNDQIYNVIVTAHAFIMIFFMVMPIMIGGFGNWLVPLMIGAPDMAFPRLNNMSFWLLPPSLFLLLMSSVVENGAGTGWTVYPPLSSNLAHSGSSVDLAIFSLHLAGISSILGAINFITTVINMRPEGM
DLEQTPLFVWAVLITAILLLLSLPVLAGAITMLLTDRNLNTSFFDPAGGGDPILYQHL
>COLFB476-12|KJ967187|ZMUO.000951|Anisosticta_novemdecimpunctata
TLYFLFGMWAGMVGTSLSMLIRLELGSAGSLIGNDQIYNVIVTAHAFIMIFFMVMPIMIGGFGNWLVPLMIGAPDMAFPRLNNMSFWLLPPSLTLLLMSSMVEMGAGTGWTVYPPLSSNLAHNGSSVDLVIFSLHLAGISSILGAVNFISTIMNMRP
MGMSLDKTPLFVWSVLITAILLLLSLPVLAGAITMLLTDRNLNTSFFDPTGGGDPILYQHL
>COLFF856-13|KJ966191|ZMUO.006651|Carabus_glabratus

TLYFIFGTWSGMVGTSLSMLIRAELGNPGSLIGDDQIYNVIVTAHAFVMIFFMVMPIMIGGFGNWLVPLMLGAPDMAFPRMNNMSFWLLPPSLTLLLMSSMVEKGAGTGWTVYPPLSSGIAHSGASVDLAIFSLHLAGISSILGAVNFITTIINMRSVG
MTFDRMPLFVWSVGITALLLLLSLPVLAGAITMLLTDRNLNTSFFDPAGGGDPILYQHL
>COLFG042-13|KJ966760|ZMUO.007547|Necydalis_major
TLYFIFGMWAGMMGTSLSVLIRSELGNPGSLIGNNQIYNVIVTAHAFVMIFFMVMPIMIGGFGNWLIPLMLGAPDMAFPRMNNLSFWLLPPSLILLIMSSVVENGAGTGWTVYPPLSANIAHSGSSVDLAIFSLHLAGISSILGAVNFISTTMNMRPG
GMILDRLPLFVWAVMITAILLLLSLPVLAGAITMLLTDRNLNTSFFDPAGGGDPILYQHL
>COLFE393-12|KJ961973|ZMUO.004953|Phyllobius_argentatus
TLYFIFGAWSGMVGTSLSILIRIELGNPGSLIGDDQIYNVIVTAHAFIMIFFMVMPMMIGGFGNWLVPLMLGAPDMAFPRLNNMSFWLLPPSLSLLLMSSIVDKGAGTGWTVYPPLSANIAHEGSSVDLAIFSLHMAGVSSILGAINFISTVINMRPSGL
SPDRMSLFIWAVKITAVLLLLSLPVLAGAITMLLTDRNMNTSFFDPAGGGDPILYQHL
>COLFE229-12|KJ966683|ZMUO.005074|Loricera_pilicornis
TLYFIFGAWSGMVGTSLSMLIRAELGNPGSFIGDDQIYNVIVTAHAFVMIFFMVMPIMIGGFGNWLVPLMLGAPDMAFPRMNNMSFWLLPPSLTLLLLSSMVESGAGTGWTVYPPLSSVIAHSGASVDLAIFSLHLAGVSSILGAVNFISTIINMRSIG
MTFDRMPLFVWSVGITALLLLLSLPVLAGAITMLLTDRNLNTSFFDPAGGGDPILYQHL
>COLFC112-12|KJ967089|ZMUO.002297|Amischa_analis
TLYFIFGAWAGMVGTSLSLLIRAELGNPGSLIGDDQIYNVIITAHAFIMIFFMVMPIVIGGFGNWLVPLMLGAPDMAFPRMNNMSFWLLPPSLTLLLMSSMVESGAGTGWTVYPPLSSNIAHGGSSVDLAIFSLHLAGISSILGAVNFISTVINMRSTGIS
FDRMPLFVWSVAITALLLLLSLPVLAGAITMLLTDRNLNTSFFDPAGGGDPILYQHL
>COLFF504-13|KJ964907|ZMUO.006584|Chrysomela_cuprea
TLYFIFGIWAGMVGTSLSILIRAELGNPGTLIGNDQIYNVIVTAHAFIMIFFMVMPIMIGGFGNWLVPLMIGAPDMAFPRLNNMSFWLLPPSLFLLLMSSVVENGAGTGWTVYPPLSANIAHSGSSVDLAIFSLHLAGISSILGAINFITTVINMRPEGMN
FEQTPLFVWAVLITAILLLLSLPVLAGAITMLLTDRNLNTSFFDPAGGGDPILYQHL
>COLFD225-12|KJ964635|ZMUO.004120|Megarthrus_nitidulus
TLYFIFGAWAGMIGTSLSILIRAELGNPGSLIGDDQIYNVIVTAHAFIMIFFMVMPIVIGGFGNWLVPLMLGAPDMAFPRMNNMSFWLLPPSISLLLISSMVESGAGTGWTVYPPLSSNIAHGGASVDLAIFSLHLAGISSILGAVNFITTVINMRSMGMT
FDRMPLFVWSVAITALLLLLSLPVLAGAITMLLTDRNINTSFFDPAGGGDPILYQHL
>COLFE213-12|KJ967324|ZMUO.005058|Ilybius_fenestratus
TLYFIFGAWAGMVGTSLSMLIRAELGNPGSLIGDDQIYNVIVTAHAFVMIFFMVMPIMIGGFGNWLVPLMLGAPDMAFPRMNNMSFWLLPPSLSLLLMSSMVEKGAGTGWTVYPPLSSGIAHSGASVDLAIFSLHLAGISSILGAVNFITTIINMRSIG
MTFDRMPLFVWSVGITALLLLLSLPVLAGAITMLLTDRNLNTSFFDPAGGGDPILYQHL
>COLFD513-12|KJ966141|ZMUO.004313|Homalota_plana
TLYFIFGAWSGMVGTSLSLLIRAELGNPGSLIGDDQIYNVIVTAHAFIMIFFMVMPIMIGGFGNWLVPLMLGAPDMAFPRMNNMSFWLLPPSLTLLLMSSMVESGAGTGWTVYPPLSSNIAHGGASVDLAIFSLHLAGISSILGAVNFISTIINMRSMG
MSFDRMPLFVWAVAITALLLLLSLPVLAGAITMLLTDRNLNTSFFDPAGGGDPILYQHL
>COLFF425-13|KJ962461|ZMUO.005943|Tachyerges_stigma
TLYFMFGTWSGMVGTSLSMLIRTELGTPGKLIGDDQIYNTIVTAHAFIMIFFMVMPIMIGGFGNWLIPLMLGAPDMAFPRLNNMSFWLLPPSLAFLLFSSIIDKGAGTGWTVYPPLSANIAHEGTSVDMAIFSLHMAGISSILGALNFISTIMNMQPM
GMNKDQMSLFIWAVKITAILLLISLPVLAGAITMLLTDRNINTSFFDPAGGGDPILYQHL
>COLFF953-13|KJ965639|ZMUO.007318|Anisosticta_novemdecimpunctata
TLYFLFGMWAGMVGTSLSMLIRLELGSAGSLIGNDQIYNVIVTAHAFIMIFFMVMPIMIGGFGNWLVPLMIGAPDMAFPRLNNMSFWLLPPSLTLLLMSSMVEMGAGTGWTVYPPLSSNLAHNGSSVDLVIFSLHLAGISSILGAVNFISTIMNMRP
MGMSLDKTPLFVWSVLITAILLLLSLPVLAGAITMLLTDRNLNTSFFDPTGGGDPILYQHL
>COLFC526-12|KJ965793|ZMUO.003091|Saperda_scalaris
TLYFIFGAWAGMVGTSLSLLIRSELGTPGSLIGDDQIYNVIVTAHAFIMIFFMVMPIMIGGFGNWLVPLMLGAPDMAFPRMNNMSFWLLPPALSLLIMSSIVDKGAGTGWTVYPPLAANVAHNGSSVDLAIFSLHLAGISSILGAVNFITTVINMRPKG
MTLDRMPLFVWAVKITAILLLLSLPVLAGAITMLLTDRNLNTSFFDPAGGGDPILYQHL
>COLFE422-12|KJ967098|ZMUO.004982|Cercyon_analis
TLYFIFGAWAGMVGTSLSILIRAELGNPGTLIGDDQIYNVIVTAHAFIMIFFMVMPIMIGGFGNWLVPLMLGAPDMAFPRMNNMSFWLLPPSLTLLLMSSMVENGAGTGWTVYPPLSSNIAHSGSSVDLAIFSLHLAGISSILGAVNFITTVINMRSPNL
TYDRLPLFVWSVAITALLLLLSLPVLAGAITMLLTDRNLNTSFFDPAGGGDPILYQHL
>COLFC384-12|KJ967165|ZMUO.002949|Oxypoda_nigricornis
TLYFIFGAWAGMVGTSLSLLIRAELGNPGSLIGDDQIYNVIVTAHAFVMIFFMVMPIVIGGFGNWLVPLMLGAPDMAFPRMNNMSFWLLPPSLTLLLMSSMVESGAGTGWTVYPPLSSNIAHGGSSVDLAIFSLHLAGISSILGAVNFISTIINMRTSG
MSFDRMPLFVWSVAITALLLLLSLPVLAGAITMLLTDRNLNTSFFDPAGGGDPILYQHL
>COLFF551-13|KJ961848|ZMUO.006631|Dorytomus_salicinus
TLYFIFGAWSGMVGTSLSMLIRTELGNPGSLIGDDQIYNTIVTAHAFIMIFFMVMPIMIGGFGNWLIPLMLGAPDMAFPRLNNMSFWLLPPSLILLLMSSIIDKGAGTGWTVYPPLSSNIAHEGTSVDLAIFSLHMAGISSILGAMNFISTVMNMRPAG
MKPDRMSLFIWAVKITAILLLLSLPVLAGAITMLLTDRNINTSFFDPAGGGDPILYQHL
>COLFC160-12|KJ962310|ZMUO.002345|Sciaphilus_asperatus
TLYFIFGSWSGMIGTSLSMLIRTELGNPGSLIGDDQIYNVIVTAHAFIMIFFMVMPIMIGGFGNWLVPLMLGAPDMAFPRLNNMSFWLLPPSLSLLLMSSIVDKGAGTGWTVYPPLSANIAHEGSSVDLAIFSLHMAGVSSILGAINFISTVINMRPLGM
TPERMPLFVWAVKITAILLLLSLPVLAGAITMLLTDRNVNTSFFDPAGGGDPILYQHL

>LEFIJ1831-13|KJ963669|ZMUO.004586|Meligethes_aeneus
TLYFIFGAWSGMVGTSLSMLIRTELGNPGSLIGNDQIYNVIVTAHAFVMIFFMVMPFMIGGFGNWLVPLMLGAPDMAFPRMNNMSFWLLPPSLSLLLMSSIVESGAGTGWTVYPPLSSNIAHGGASVDLAIFSLHLAGISSILGAVNFITTVINMRPK
GMTFDRMPLFVWAVMITAILLLLSLPVLAGAITMLLTDRNLNTTFFDPSGGGDPILYQHL
>COLFC076-12|KJ964176|ZMUO.002071|Enochrus_coarctatus
TLYFIFGAWAGMVGTSLSILIRAELGNPGTLIGDDQIYNVIVTAHAFIMIFFMVMPIMIGGFGNWLVPLMLGAPDMAFPRMNNMSFWLLPPSLTLLLMSSMVESGAGTGWTVYPPLSSNIAHGGASVDLAIFSLHLAGISSILGAVNFITTVINMRSHS
MTYDRLPLFVWSVAITALLLLLSLPVLAGAITMLLTDRNLNTSFFDPAGGGDPILYQHL
>COLFF251-13|KJ966589|ZMUO.005856|Malthodes_pumilus
TLYFMFGAWSGMLGTSLSLLIRAELGSPGSLIGNDQIYNVIVTAHAFIMIFFMVMPIMIGGFGNWLVPLMLGAPDMAFPRMNNMSFWFLPPSLSLLLMSSLVESGAGTGWTVYPPLSSNIAHSGASVDLAIFSLHMAGISSILGAVNFISTVINMRSTG
MTFDRMPLFVWAVAITALLLLLSLPVLAGAITMLLTDRNLNTSFFDPAGGGDPILYQHL
>COLFD242-12|KJ962915|ZMUO.004137|Dyschirius_obscurus
TLYFIFGIWSGMVGTSLSILIRTELGNPGSLIGDDQIYNVIVTAHAFIMIFFMVMPIMIGGFGNWLVPLMLGAPDMAFPRMNNMSFWLLPPSLTLLLMSSMVESGAGTGWTVYPPLSSGIAHSGASVDLAIFSLHLAGISSILGAVNFITTIINMRSTGMT
FERMPLFVWSVGITALLLLLSLPVLAGAITMLLTDRNLNTSFFDPAGGGDPILYQHL
>COLFD355-12|KJ963090|ZMUO.004250|Malthodes_fibulatus
TLYFMFGAWAGMLGTSLSLLIRAELGSPGSLIGNDQIYNVIVTAHAFIMIFFMVMPIMIGGFGNWLVPLMLGAPDMAFPRMNNMSFWFLPPSLSLLLMSSIVENGAGTGWTVYPPLSANIAHSGSSVDLAIFSLHMAGISSILGAVNFISTVINMRSTG
MTFDRMPLFVWAVAITALLLLLSLPVLAGAITMLLTDRNLNTSFFDPAGGGDPILYQHL
>COLFD718-12|KJ964911|ZMUO.004708|Oxyomus_sylvestris
TLYFLFGSWAGMVGTSLSLLIRAELGNPGSLIGDDQIYNVIVTAHAFIMIFFMVMPILIGGFGNWLVPLMLGAPDMAFPRMNNMSFWLLPPSLTLLLMSSMVESGAGTGWTVYPPLSSNIAHGGASVDLAIFSLHLAGISSILGAVNFITTVINMRSPG
MTFDRMPLFVWSVAITALLLLLSLPVLAGAITMLLTDRNLNTSFFDPAGGGDPILYQHL
>COLFA010-10|HM909040|MP00140|Cyphon_padi
TLYFIFGSWSGMVGTSLSLLIRAELGTPGSLIGDDQIYNVIVTAHAFIMIFFMVMPIMIGGFGNWLVPLMLGAPDMAFPRMNNMSFWLLPPSLTLLLMSSMVENGAGTGWTVYPPLSAGVAHSGASVDLAIFSLHLAGISSILGAVNFISTVINMRSVG
MTFDRMPLFVWAVAITALLLLLSLPVLAGAITMLLTDRNLNTSFFDPAGGGDPILYQHL
>COLFE1215-13|KJ966544|ZMUO.007010|Lathrobium_brunnipes
TLYFIFGAWAGMVGTSLSLLIRTELGNPGSLIGDDQIYNVIVTAHAFVMIFFMVMPIMIGGFGNWLVPLMLGAPDMAFPRMNNMSFWLLPPSLSLLLMSSMVESGAGTGWTVYPPLSSNIAHGGASVDLAIFSLHLAGISSILGAVNFITTVINMRSPG
MTYERMPLFVWAVAITALLLLLSLPVLAGAITMLLTDRNLNTTFFDPAGGGDPILYQHL
>COLFA235-10|HM909146|MP00101|Agrilus_betuleti
TLYFFFGAWSGMVGTALSLLIRAELGNPGALIGNDQIYNVIVTAHAFVMIFFMVMPIMMGGFGNWLVPLMLGAPDMAFPRMNNMSFWLLPPSLTLLLMSSTVESGAGTGWTVYPPLAANIAHSGASVDLAIFSLHLAGISSILGAINFITTVINMRPP
GMTLDRIPLLVWSIAITALLLLLSLPVLAGAITMLLSDRNLNTSFFDPAGGGDPILYQHL
>COLFD942-12|KJ962733|ZMUO.004552|Phyllotreta_armoraciae
TLYFIFGIWSGMVGMSMSMLIRIELAAPGSLIGNDQIYNVIVTAHAFIMIFFMVMPIMIGGFGNWLIPLMIGAPDMAFPRMNNMSFWLLPPSLFLLIMSSIVENGAGTGWTVYPPLSSNVSHAGASVDLTIFSLHLAGISSILGAINFITTIINMRPKGMT
FDRMPLFVWAVFITAILLLLSLPVLAGAITMLLTDRNLNTSFFDPMGGGDPILYQHL
>COLFA319-12|KJ963154|ZMUO.000454|Ceutorhynchus_fennicus
TLYFIFGSWAGMAGTSLSMLIRTELGNPGMLIGDDQIYNSIVTAHAFIMIFFMVMPILIGGFGNWLVPLMLGAPDMAFPRLNNMSFWLLPPSLSLLLMSSIVNKGVGTGWTVYPPLSMNVAHEGMSVDLAIFSLHMAGISSILGAINFISTVLNMRPT
GMTAEYMPLFVWAVEITAILLLLSLPVLAGAITMLLTDRNINTSFFDPTGGGDPILYQHL
>COLFF482-13|KJ964155|ZMUO.006562|Oxytelus_sculptus
TLYFIFGAWSGMVGTSLSMLIRAELGAPGSLIGDDQIYNVIVTAHAFIMIFFMVMPIVIGGFGNWLVPLMLGAPDMAFPRMNNMSFWLLPPSLTLLLFSSMVESGAGTGWTVYPPLSSNIAHSGSSVDLAIFSLHLAGISSILGAVNFISTIINMRSTGM
SFDRMPLFVWSVNITAILLLLSLPVLAGAITMLLTDRNLNTSFFDPAGGGDPILYQHL
>COLFE270-12|KJ964239|ZMUO.005115|Pterostichus_melanarius
TLYFIFGAWAGMVGTSLSMLIRAELGNPGSLIGDDQIYNVIVTAHAFVMIFFMVMPIMIGGFGNWLVPLMLGAPDMAFPRMNNMSFWLLPPSLTLLLMSSMVESGAGTGWTVYPPLSSGIAHAGASVDLAIFSLHLAGVSSILGAVNFITTIINMRSI
GMTFDRMPLFVWSVGITALLLLLSLPVLAGAITMLLTDRNLNTSFFDPAGGGDPILYQHL
>COLFC482-12|KJ962450|ZMUO.003047|Pteroloma_forsstromii
TLYFIFGAWAGMVGTSLSILIRAELGNPGTLIGDDQIYNVIVTAHAFIMIFFMVMPIMIGGFGNWLVPLMLGAPDMAFPRMNNMSFWLLPPSLSLLLMSSIVENGAGTGWTVYPPLSSNIAHSGSSVDLAIFSLHLAGISSILGAVNFITTVINMRSTGM
TFDRMPLFVWAVAITALLLLLSLPVLAGAITMLLTDRNLNTSFFDPAGGGDPILYQHL
>COLFA297-12|KJ963106|ZMUO.000432|Omonadus_floralis
TLYLIFGAWAGMVGTSLSLLIRSELGNPGTLIGNDQIYNVIVTAHAFIMIFFMVMPIVIGGFGNWLVPLMLGAPDMAFPRMNNMSFWLLPPSLSLLIMSSIVESGSGTGWTVYPPLSANIAHSGSSVDLAIFSLHLAGISSILGAVNFITTVINMRPAGMS
FDRMPLFVWAVVITAVLLLLSLPVLAGAITMLLTDRNLNTSFFDPAGGGDPILYQHL
>COLFD376-12|KJ964639|ZMUO.004271|Bruchus_loti

TLYFIFGAWAGMAGTSLSMLIRAELGNPGSLIGNDQIYNVIVTAHAFIMIFFMVMPIMIGGFGNWLVPLMLGAPDMAFPRMNNMSFWLLPPSLTLLLMSSLVESGAGTGWTVYPPLASNIAHGGSSVDLAIFSLHLAGISSILGAVNFITTVINMRPSG
MSMDRMPLFSWAVVITAILLLLSLPVLAGAITMLLTDRNLNTSFFDPAGGGDPILYQHL
>COLFC320-12|KJ964114|ZMUO.002505|Patrobus_assimilis
TLYFIFGAWSGMVGTSLSILIRAELGNPGSLIGDDQIYNVIVTAHAFVMIFFMVMPIMIGGFGNWLVPLMLGAPDMAFPRMNNMSFWLLPPSLTLLLMSSMVESGAGTGWTVYPPLSSGIAHSGASVDLAIFSLHLAGISSILGAVNFITTIINMRSVG
MTFDRMPLFVWSVGITALLLLLSLPVLAGAITMLLTDRNLNTSFFDPAGGGDPILYQHL
>COLFB871-12|KJ967231|ZMUO.001916|Lepturobosca_virens
TLYFIFGAWAGMIGTSLSLLIRSELGNPGSLIGDDQIYNVIVTAHAFVMIFFMVMPIMIGGFGNWLVPLMLGAPDMAFPRMNNMSFWLLPPSLTLLIMSSIVEKGAGTGWTVYPPLAANIAHSGPSVDLAIFSLHLAGISSILGAVNFITTIINMPPVGM
KLDQMPLFVWAVMITAILLLLSLPVLAGAITMLLTDRNLNTSFFDPAGGGDPILYQHL
>COLFC181-12|KJ961860|ZMUO.002366|Placusa_depressa
TLYFIFGAWAGMVGTSLSLLIRAELGNPGSLIGDDQIYNVIVTAHAFVMIFFMVMPIVIGGFGNWLIPLMLGAPDMAFPRMNNMSFWLLPPSLTLLLMSSMVESGAGTGWTVYPPLSSNIAHSGSSVDLAIFSLHLAGISSILGAVNFISTIINMRSPGM
SFDQMSLFVWSVGLTALLLLLSLPVLAGAITMLLTDRNLNTTFFDPAGGGDPILYQHL
>COLFC002-12|KJ964103|ZMUO.001997|Ocyusa_picina
TLYFIFGAWSGMVGTSLSLLIRAELGNPGSLIGDDQIYNVIVTAHAFVMIFFMVMPIVIGGFGNWLVPLMLGAPDMAFPRMNNMSFWLLPPSLSLLLMSSMVESGAGTGWTVYPPLSSNIAHGGSSVDLAIFSLHLAGISSILGAVNFISTIMNMRTSG
MTFDRMPLFVWSVAITALLLLLSLPVLAGAITMLLTDRNLNTSFFDPAGGGDPILYQHL
>COLFF743-13|KJ966185|ZMUO.006443|Ceutorhynchus_asperifoliarum
TLYFIFGSWAGMAGTSLSMLIRAELGNPGSLIGNDQTYNSVVTAHAFIMIFFMVMPIMIGGFGNWLVPLMLGAPDMAFPRLNNMSFWLLPPSLTLLLMSSIVNKGAGTGWTVYPPLSANTAHEGMSVDLAIFSLHMAGISSILGAINFISTVMNMRP
SGMISEYMPLFVWAVEITAILLLISLPVLAGAITMLLTDRNINTSFFDPSGGGDPILYQHL
>LEFIJ1892-13|KJ965515|ZMUO.004647|Rhinoncus_pericarpius
TLYFIFGSWAGTVGTSLSMLIRTELGTPGSLIGNDQIYNSIVTAHAFIMIFFMVMPILIGGFGNWLVPLMLGAPDMAFPRLNNMSFWLLPPSIMLLLMSSIINKGAGTGWTVYPPLSSNITHEGASVDLAIFSLHMAGISSILGAINFISTIMNMRPQGMS
YDKMPLFSWAVLITATLLLLSLPVLAGAITMLLTDRNINTSFFDPAGGGDPILYQHL
>COLFA211-10|HM909130|MP00058|Agrilus_viridis
TLYFIFGAWSGMVGTALSLLIRAELGNPGALIGNDQIYNVIVTAHAFIMIFFMVMPIMMGGFGNWLVPLMLGAPDMAFPRMNNMSFWLLPPSLTLLLMSSMVESGAGTGWTVYPPLAANIAHSGGSVDLAIFSLHLAGISSILGAINFITTVINMRAV
GMTMDRVPLLVWSIAITALLLLLSLPVLAGAITMLLTDRNLNTSFFDPAGGGDPILYQHL
>COLFG181-13|KJ964614|ZMUO.007876|Atomaria_zetterstedti
TLYFIFGAWAGMVGTSLSMLIRTELGTPGSLIGDDQIYNVIVTAHAFIMIFFMVMPMMIGGFGNWLVPLMLGAPDMAFPRLNNMSFWLLPPSLMFLLMSSIVEKGAGTGWTVYPPLSSNIAHAGSSVDLAIFSLHLAGISSILGSVNFITTVINMRPKG
MNFDRLPLFVWAVKITTILLLLSLPVLAGAITMLLTDRNINTSFFDPAGGGDPILYQHL
>COLFE017-12|KJ962892|ZMUO.005242|Anoplodera_sanguinolenta
TLYFIFGAWASMVGTSLSLLIRSELGNPGSLIGNDQIYNVIVTAHAFVMIFFMVMPIMIGGFGNWLVPLMLGAPDMAFPRMNNMSFWLLPPSLTLLIMSSIVESGAGTGWTVYPPLSSNIAHSGSSVDLAIFSLHLAGISSILGAVNFITTVINMRPAGLK
PEQMPLFVWAVVITAVLLLSLPVLAGAITMLLTDRNLNTSFFDPAGGGDPILYQHL
>COLFC642-12|KJ962888|ZMUO.003207|Hygrotus_marklini
TLYFLFGAWSGMVGTSMSMLIRAELGNPGSLIGDDQIYNVIVTAHAFIMIFFMVMPIMIGGFGNWLVPLMLGAPDMAFPRMNNMSFWMLPPSLTLLLMSSMVESGAGTGWTVYPPLSAGIAHGGASVDLAIFSLHLAGISSILGAVNFITTIINMRS
VGMTFDRMPLFVWSVGITALLLLLSLPVLAGAITMLLTDRNLNTSFFDPAGGGDPILYQHL
>COLFF926-13|KJ964106|ZMUO.006721|Cortinicara_gibbosa
SLYFLFGMWAGMVGTSLSLLIRLELGNPGSLIGDDQIYNVIVTAHAFIMIFFMVMPIMIGGFGNWLVPLMLGAPDMAFPRLNNMSFWLLPPSLSLLIMSSIVESGAGTGWTVYPPLSSNIAHGGSSVDLAIFSLHLAGISSILGAVNFITTVINMRPTGM
SLDKMPLFVWSVVITAILLLLSLPVLAGAITMLLTDRNLNTSFFDPASGGDPILYQHL
>COLFF605-13|KJ965617|ZMUO.006495|Cionus_hortulanus
TLYFIFGMWSGTVGTSLSMLIRTELGNPGSLIGDDQIYNTIVTAHAFIMIFFMVMPIMIGGFGNWLVPLMLGAPDMAFPRLNNMSFWLLPPSITLLLMSSIVEKGAGTGWTVYPPLSNNVTHEGASVDLAIFSLHMAGISSILGAINFISTISNMRTKGM
DYDRTPLFVWSVNITALLLLLSLPVLAGAITMLLTDRNINTSFFDPSGGGDPILYQHL
>COLFF993-13|KJ967385|ZMUO.007358|Tachinus_marginellus
TLYFIFGAWAGMVGTSLSLLIRAELGNPGTLIGDDQIYNVIVTAHAFIMIFFMVMPIVIGGFGNWLVPLMLGAPDMAFPRMNNMSFWLLPPSLSLLLMSSMVESGAGTGWTVYPPLSSNIAHGGSSVDLAIFSLHLAGISSILGAVNFITTVINMRSIGM
TFDRMPLFVWSVAITALLLLLSLPVLAGAITMLLTDRNLNTTFFDPAGGGDPILYQHL
>COLFC599-12|KJ962843|ZMUO.003164|Tachyerges_salicis
TLYFMLGAWSGMVGTSLSMLIRTELGTPGKLIGDDQIYNTIVTAHAFVMIFFMVMPIMIGGFGNWLIPLMLGAPDMAFPRLNNMSFWLLPPSLAFLLFSSIIDKGVGTGWTVYPPLSTNISHEGASVDMAIFSLHMAGVSSILGAMNFISTAMNMFP
KGVIKDQMSLFIWAVKVTAILLLISLPVLAGAITMLLTDRNVNTSFFDPAGGGDPILYQHL
>COLFE800-13|KJ961932|ZMUO.005645|Agonum_fuliginosum
TLYFIFGAWAGMVGTSLSMLIRAELGNPGALIGDDQIYNVIVTAHAFIMIFFMVMPIMIGGFGNWLVPLMLGAPDMAFPRMNNMSFWLLPPSLTLLLMSSLVESGAGTGWTVYPPLSSGIAHAGASVDLAIFSLHLAGISSILGAVNFITTIINMRSVG
MTFDRMPLFVWSVGITALLLLLSLPVLAGAITMLLTDRNLNTSFFDPAGGGDPILYQHL

>COLFD369-12|KJ963127|ZMUO.004264|Amischa_analis
TLYFIFGAWAGMVGTSLSLLIRAELGNPGSLIGDDQIYNVIVTAHAFIMIFFMVMPIVIGGFGNWLVPLMLGAPDMAFPRMNNMSFWLLPPSLTLLLMSSMVESGAGTGWTVYPPLSSNIAHGGSSVDLAIFSLHLAGISSILGAVNFISTVINMRSTGI
SFDRMPLFVWSVAITALLLLLSLPVLAGAITMLLTDRNLNTSFFDPAGGGDPILYQHL
>COLFE429-12|KJ966603|ZMUO.004989|Plateumaris_discolor
TLYFIFGAWSGMMGTSLSMLIRTELMNPGSLIGNDQIYNVIVTAHAFIMIFFMVMPIMIGGFGNWLIPLMLGAPDMAFPRMNNMSFWLLPPSLTFLIMSSIVENGAGTGWTVYPPLSSNIAHSGASVDLAIFSLHLAGISSILGAVNFITTIINMRPMG
MKMDKVPLFAWAVMITAILLLLSLPVLAGAITMLLTDRNLNTSFFDPAGGGDPVLYQHL
>COLFC064-12|KJ966155|ZMUO.002059|Notoxus_monoceros
TLYLIFGAWAGMVGTSLSLLIRSELGNPGSLIGDDQIYNVIVTAHAFIMIFFMVMPIVIGGFGNWLVPLMLGAPDMAFPRMNNMSFWLLPPSLTLLIMSSIVENGAGTGWTVYPPLSSNIAHSGSSVDLAIFSLHLAGVSSILGAVNFITTVINMRPSGM
SFDRMPLFVWAVVITAVLLLLSLPVLAGAITMLLTDRNLNTSFFDPAGGGDPILYQHL
>COLFE1036-13|KJ961891|ZMUO.006831|Dryops_luridus
TLYFIFGSWAGLLGMSLSLLIRAELGNPGSLIGDDQIYNVIVTAHAFVMIFFMVMPIMIGGFGNWLVPLMLGAPDMAFPRMNNMSFWLLPPSLSLLLLSSMVESGAGTGWTVYPPLASNIAHSGSSVDLAIFSLHLAGISSILGAVNFITTVINMRTSGM
TFDRMPLFVWSVAITALLLLLSLPVLAGAITMLLTDRNLNTSFFDPAGGGDPILYQHL
>COLFC602-12|KJ963832|ZMUO.003167|Meligethes_subrugosus
TLYFIFGTWSGMVGTSLSILIRTELGNPGSLIGNDQIYNVIVTAHAFVMIFFMVMPFMIGGFGNWLVPLMLGAPDMAFPRMNNMSFWLLPPSLSLLLMSSIVESGAGTGWTVYPPLSSNIAHGGASVDLAIFSLHLAGISSILGAVNFITTVINMRPTG
MTFDRMPLFVWAVTITALLLLLSLPVLAGAITMLLTDRNLNTTFFDPSGGGDPILYQHL
>COLFE076-12|KJ965318|ZMUO.005301|Charopus_graminicola
TLYFIFGAWSGMIGLSLSLMIRSELSIPGTLIGNDQIYNVIVTAHAFIMIFFMVMPILIGGFGNWLVPLMLGAPDMAFPRMNNMSFWLLPPSLTLLLLSSMIENGAGTGWTVYPPLSANIAHSGASVDLAIFSLHLAGISSILGAINFITTVINMRPQGMTL
DRVPLFVWAVVITALLLLLSLPVLAGAITMLLTDRNLNTSFFDPAGGGDPILYQHL
>COLFF775-13|KJ963889|ZMUO.006095|Bembidion_tinctum
TLYFIFGAWSGMVGTSLSMLIRAELGNPGSLIGDDQIYNVIVTAHAFIMIFFMVMPILIGGFGNWLVPLMLGAPDMAFPRMNNMSFWLLPPSLSLLLMSSMVESGAGTGWTVYPPLSSSIAHSGASVDLAIFSLHLAGVSSILGAVNFITTIINMRSTG
MTFDRMPLFVWSVGITALLLLLSLPVLAGAITMLLTDRNLNTSFFDPAGGGDPILYQHL
>COLFC373-12|KJ965679|ZMUO.002558|Atomaria_vespertina
TLYFIFGAWAGMVGTSLSLLIRAELGTPGSLIGDDQIYNVIVTAHAFIMIFFMVMPMMIGGFGNWLVPLMLGAPDMAFPRLNNMSFWLLPPSLMFLLMSSLVEKGAGTGWTVYPPLSSNVAHAGASVDLAIFSLHMAGISSILGAVNFITTVINMRPK
GMNFDRLPLFVWAVKITAILLLLSLPVLAGAITMLLTDRNINTSFFDPAGGGDPILYQHL
>COLFA004-10|HM909035|MP00087|Dermestes_maculatus
TLYFIFGAWAGMVGTSLSMLIRTELGMPGSLIGDDQIFNVIVTAHAFIMIFFMVMPIMIGGFGNWLVPLMLGAPDMAFPRMNNMSFWLLPPSLSLLLMSSMVESGAGTGWTVYPPLSANIAHGGASVDLAIFSLHLAGISSILGAVNFITTVINMRSK
GMTPDRMPLFVWSVAITALLLLLSLPVLAGAITMLLTDRNLNTSFFDPAGGGDPILYQHL
>COLFC042-12|KJ966922|ZMUO.002037|Sphaeridium_lunatum
TLYFIFGAWAGMVGTSLSILIRAELGNPGTLIGDDQIYNVIVTAHAFIMIFFMVMPIMIGGFGNWLVPLMLGAPDMAFPRMNNMSFWLLPPSLTLLLMSSMVESGAGTGWTVYPPLSSNIAHGGASVDLAIFSLHLAGISSILGAVNFITTVINMRSPD
MTYDRMPLFVWSVAITALLLLLSLPVLAGAITMLLTDRNLNTSFFDPAGGGDPILYQHL
>COLFG097-13|KJ967114|ZMUO.007792|Atheta_vilis
TLYFIFGAWAGMVGTSLSLLIRAELGNPGSLIGDDQIYNVIVTAHAFVMIFFMVMPIVIGGFGNWLVPLMLGAPDMAFPRMNNMSFWLLPPSLSLLLMSSMVESGAGTGWTVYPPLSSNIAHGGSSVDLAIFSLHLAGISSILGAVNFISTVINMRSTGI
TFDRMPLFVWSVAITALLLLLSLPVLAGAITMLLTDRNLNTSFFDPAGGGDPILYQHL
>COLFD179-12|KJ966462|ZMUO.004074|Philorhizus_notatus
TLYFIFGAWAGMVGTSLSMLIRAELGNPGALIGDDQIYNVIVTAHAFIMIFFMVMPIMIGGFGNWLVPLMLGAPDMAFPRMNNMSFWLLPPSLTLLLMSSMVESGTGTGWTVYPPLSSGIAHAGASVDLAIFSLHLAGISSILGAVNFITTIINMRSIG
MTFDRMPLFVWSVGITALLLLLSLPVLAGAITMLLTDRNLNTSFFDPAGGGDPILYQHL
>COLFF842-13|KJ966049|ZMUO.006162|Limonius_minutus
TLYFLFGAWSGMLGTSLSLLIRAELGNPGSLIGNDQIYNVIVTAHAFIMIFFMVMPIMIGGFGNWLVPLMLGAPDMAFPRMNNMSFWFLPPSLSLLLMSSIVENGAGTGWTVYPPLSANIAHSGSSVDLAIFSLHLAGISSILGAVNFISTVINMRSVGIT
FDRMPLFVWAVAITALLLLLSLPVLAGAITMLLTDRNLNTSFFDPAGGGDPILYQHL
>COLFE301-12|KJ965327|ZMUO.004861|Amara_famelica
TLYFIFGAWSGMVGTSLSMLIRAELGNPGALIGDDQIYNVIVTAHAFVMIFFMVMPIMIGGFGNWLVPLMLGAPDMAFPRMNNMSFWLLPPSLTLLLMSSMVESGAGTGWTVYPPLSSGIAHAGASVDLAIFSLHLAGISSILGAVNFITTIINMRSV
GMTFDRMPLFVWSVGITALLLLLSLPVLAGAITMLLTDRNLNTSFFDPAGGGDPILYQHL
>COLFF409-13|KJ964570|ZMUO.005927|Cryptocephalus_nitidus
TLYFLFGAWAGMVGTSLSLLIRVELGNPGSLIGNDQIYNTIVTAHAFIMIFFMVMPIMIGGFGNWLVPLMLGAPDMAFPRMNNMSFWLLPPSLTLLLMSSIVENGAGTGWTVYPPLSTTIAHAGSSVDLAIFSLHLAGASSIMGAINFISTVINMRPQG
MLMDQIPLFVWAVMITAILLLLSLPVLAGAITMLLTDRNLNTSFFDPAGGGDPILYQHL
>COLFF745-13|KJ966188|ZMUO.006445|Trachys_minutus

TLYFIFGAWSGMVGTALSLLIRAELGNPGALIGNDQIYNVIVTAHAFIMIFFMVMPIMMGGFGNWLVPLMLGAPDMAFPRMNNMSFWLLPPSLSLLLMSSMVESGAGTGWTVYPPLAANIAHSGASVDLAIFSLHLAGISSILGAINFITTVINMRAY
GMTLDQMPLFVWSVAITALLLLLSLPVLAGAITMLLTDRNLNTSFFDPVGGGDPVLYQHL
>LEFIJ1845-13|KJ965939|ZMUO.004600|Rhinoncus_pericarpius
TLYFIFGSWAGTVGTSLSMLIRTELGTPGSLIGNDQIYNSIVTAHAFIMIFFMVMPILIGGFGNWLVPLMLGAPDMAFPRLNNMSFWLLPPSIMLLLMSSIINKGVGTGWTVYPPLSSNITHEGASVDLAIFSLHMAGISSILGAINFISTIMNMRPQGMS
YDKMPLFSWAVLITAILLLLSLPVLAGAITMLLTDRNINTSFFDPAGGGDPILYQHL
>COLFG010-13|KJ964877|ZMUO.007515|Dicerca_moesta
TLYFIFGAWSGMVGTGLSLLIRAELGNPGALIGDDQIYNVIVTAHAFIMIFFMVMPIMMGGFGNWLVPLMLGAPDMAFPRMNNMSFWLLPPSLTLLLMSSIVENGAGTGWTVYPPLAANVAHSGASVDLAIFSLHLAGISSILGAVNFITTVINMRST
GMSFDRMPLFVWSVAITALLLLLSLPVLAGAITMLLTDRNLNTSFFDPAGGGDPILYQHL
>COLFG136-13|KJ963143|ZMUO.007831|Atheta_dadopora
TLYFIFGAWAGMVGTSLSLLIRAELGNPGSLIGDDQIYNVIVTAHAFVMIFFMVMPIVIGGFGNWLIPLMLGAPDMAFPRMNNMSFWLLPPSLTLLLMSSMVESGAGTGWTVYPPLSSNIAHGGASVDLAIFSLHLAGISSILGAVNFISTVINMRSTGI
SFDRMPLFVWSVAITALLLLLSLPVLAGAITMLLTDRNLNTSFFDPAGGGDPILYQHL
>COLFD523-12|KJ963524|ZMUO.004323|Amara_convexiuscula
TLYFIFGAWSGMVGTSLSMLIRAELGNPGALIGDDQIYNVIVTAHAFIMIFFMVMPIMIGGFGNWLVPLMLGAPDMAFPRMNNMSFWLLPPSLTLLLMSSMVEKGAGTGWTVYPPLSSGIAHAGASVDLAIFSLHLAGISSILGAVNFITTIINMRTVG
MTFDRMPLFVWSVGITALLLLLSLPVLAGAITMLLTDRNLNTSFFDPAGGGDPILYQHL
>COLFF714-13|KJ967219|ZMUO.006414|Cerylon_histeroides
TLYFMFGMWSGMVGTSMSMMIRLELGNPGSLIGDDQIYNVIVTAHAFVMIFFMVMPIMIGGFGNWLVPLMLGAPDMAFPRMNNMSFWLLPPSLTLLIMSSIVEKGAGTGWTVYPPLSANLTHSGSSVDLAIFSLHLAGISSILGAVNFITTVINMR
PSGMTWDRLPLFVWSVIITAVLLLLSLPVLAGAITMLLTDRNLNTSFFDPAGGGDPILYQHL
>COLFC306-12|KJ963134|ZMUO.002491|Stenus_nitens
TLYFIFGAWAGMVGTSLSLLIRAELGNPGSLIGDDQIYNVIVTAHAFVMIFFMVMPIMIGGFGNWLVPLMLGAPDMAFPRMNNMSFWLLPPSLSLLLMSSIVENGAGTGWTVYPPLSSNIAHSGASVDLAIFSLHLAGISSILGAINFITTIFNMRTMKL
QLDCLPLFVWSVAITALLLLLSLPVLAGAITMLLTDRNLNTSFFDPPGGGDPILYQHL
>COLFC472-12|KJ967294|ZMUO.003037|Stenotrachelus_aeneus
TLYFIFGAWSGMLGTSFSLLIRSELGNPGMLIGDDQIYNVIVTSHAFVMIFFMVMPIMIGGFGNWLVPLMLGAPDMAFPRMNNMSFWLLPPSLSLLLMSSMVESGAGTGWTVYPPLSSNIAHGGASVDLAIFSLHLAGISSILGAVNFITTVINMRPQ
EMSLERMPLFVWSVVITAILLLLSLPVLAGAITMLLTDRNLNTSFFDPAGGGDPILYQHL
>COLFB920-12|KJ966198|ZMUO.001965|Cryptocephalus_androgyne
TLYFLFGAWSGLVGTSLSMLIRVELGNPGSLIGNDQIYNTIVTAHAFIMIFFMVMPIMIGGFGNWLVPLMLGAPDMAFPRMNNMSFWLLPPSLTLLLMSSIVENGAGTGWTVYPPLSSTIAHAGASVDLAIFSLHLAGISSIMGAINFISTVINMRPQG
MLMDRTPLFVWAVLITAILLLLSLPVLAGAITMLLTDRNLNTSFFDPAGGGDPILYQHL
>COLFD155-12|KJ966823|ZMUO.004050|Temnocerus_nanus
TLYFIFGAWSGMVGTSLSILIRAELGSPGSLIGDDQIYNVIVTAHAFIMIFFMVMPIMIGGFGNWLVPLMLGAPDMAFPRMNNMSFWLLPPSLSLLIMSSIVESGAGTGWTVYPPLSSNIAHGGSSVDLAIFSLHLAGISSILGAVNFISTVINMRPMGM
NLDRMPLFVWAVAITALLLLLSLPVLAGAITMLLTDRNINTSFFDPAGGGDPILYQHL
>COLFE827-13|KJ967422|ZMUO.005672|Rhinoncus_bruchoides
TLYFIFGSWAGTVGTSLSMLIRTELGTPGSLIGNDQIYNSIVTAHAFIMIFFMVMPILIGGFGNWLVPLMLGAPDMAFPRLNNMSFWLLPPSIMLLLMSSIVNKGAGTGWTVYPPLSSNITHEGASVDLAIFSLHMAGISSILGAINFISTIMNMRPQGM
SYDKMPLFSWAVLITAILLLLSLPVLAGAITMLLTDRNINTSFFDPAGGGDPILYQHL
>COLFD921-12|KJ965181|ZMUO.004531|Stenus_intermedius
VLYFIFGAWAGMVGTSLSLLIRTELGNPGSLIGDDQIYNVIVTAHAFVMIFFMVMPLMIGGFGNWLVPLMLGAPDMAFPRMNNMSFWLLPPSLSLLLMSSIVENGAGTGWTVYPPLSTNIAHSGASVDLAIFSLHLAGISSILGAINFITTIFNMRSMKL
QLDCLPLFIWSVEITALLLLLSLPVLAGAITMLLTDRNLNTSFFDPAGGGDPILYQHL
>COLFD919-12|KJ963994|ZMUO.004529|Stenus_incrassatus
ILYFVFGAWAGMVGTSLSLLIRAELGNPGSLIGDDQIYNVIVTAHAFVMIFFMVMPVMIGGFGNWLVPLMLGAPDMAFPRMNNMSFWLLPPSLSLLLMSSIVENGAGTGWTVYPPLSSNIAHSGASVDLAIFSLHLAGVSSILGAINFITTIFNMRTM
KLQLDCLPLFVWSVGITALLLLLSLPVLAGAITMLLTDRNLNTSFFDPAGGGDPILYQHL
>COLFB547-12|KJ963268|ZMUO.001022|Acrotona_pygmaea
TLYFVFGAWAGMVGTSLSLLIRAELGNPGSLIGDDQIYNVIVTAHAFVMIFFMVMPIVIGGFGNWLVPLMLGAPDMAFPRMNNMSFWLLPPSLTLLLMSSMVESGAGTGWTVYPPLSSNIAHGGSSVDLAIFSLHLAGISSILGAVNFISTVINMRSM
GISFDRMPLFVWSVAITALLLLLSLPVLAGAITMLLTDRNLNTSFFDPAGGGDPILYQHL
>COLFE392-12|KJ965076|ZMUO.004952|Microlestes_minutulus
TLYFIFGAWAGMVGTSLSMLIRAELGNPGALIGDDQIYNVIVTAHAFIMIFFMVMPIMIGGFGNWLVPLMLGAPDMAFPRMNNMSFWLLPPSLTLLLMSSMVESGAGTGWTVYPPLSSGIAHAGASVDLAIFSLHLAGVSSILGAVNFITTIINMRSV
GMSFERMPLFVWSVGITALLLLLSLPVLAGAITMLLTDRNLNTTFFDPAGGGDPILYQHL
>COLFG061-13|KJ967008|ZMUO.007566|Leiopus_nebulosus
TLYFIFCAWAGMVGTSLSMLIRSELGTAGSLIGDDQIYNAIVTAHAFVMIFFMVMPIMIGGFGNWLVPLMLGAPDMAFPRMNNMSFWLLPPSLTLLIMSSVVNSGAGTGWTVYPPLSSNIAHAGPSVDLAIFSLHLAGASSILGAVNFITTVINMRPK
GMSFDRLPLFVWAVKITTILLLLSLPVLAGAITMLLTDRNLNTSFFDPAGGGDPVLYQHL

>COLFF038-13|KJ967283|ZMUO.005738|Chrysobothris_chrysostigma
TLYFIFGVWSGMVGTALSLLIRAELGNPGALIGDDQIYNVIVTAHAFVMIFFMVMPIMMGGFGNWLVPLMLGAPDMAFPRMNNMSFWLLPPSLTFLLMSSIVENGAGTGWTVYPPLAANIAHSGSSVDLAIFSLHLAGISSILGAVNFITTVINMRSV
GMSFDRMPLFVWSVAITALLLLLSLPVLAGAITMLLTDRNLNTSFFDPAGGGDPILYQHL
>COLFC083-12|KJ967037|ZMUO.002078|Omalium_rivulare
TLYFIFGAWAGMVGTSLSLLIRAELGNPGSLIGDDQIYNVIVTAHAFIMIFFMVMPIVIGGFGNWLVPLMLGAPDMAFPRMNNMSFWLLPPSLTLLLMSSMVESGAGTGWTVYPPLSSNIAHGGSSVDLAIFSLHLAGISSILGAVNFITTVINMRAMG
MTFDRMPLFVWSVVITALLLLLSLPVLAGAITMLLTDRNLNTSFFDPAGGGDPILYQHL
>COLFD424-12|KJ963969|ZMUO.003844|Pseudovadonia_livida
TLYFIFGAWASMVGTSLSLLIRSELGNPGSLIGDDQIYNVIVTAHAFVMIFFMVMPIMIGGFGNWLVPLMLGAPDMAFPRMNNMSFWLLPPSLTLLIMSSVVENGAGTGWTVYPPLSANIAHSGSSVDLAIFSLHLAGISSILGAVNFITTVINMRPKG
MNLDQMPLFVWAVVITAILLLLSLPVLAGAITMLLTDRNLNTSFFDPAGGGDPILYQHL
>COLFA710-12|KJ962768|ZMUO.000845|Gabronthus_sulcifrons
TLYFIFGSWAGMVGTSLSLLIRAELGNPGSLIGDDQIYNVIVTAHAFIMIFFMVMPIVIGGFGNWLVPLMLGAPDMAFPRMNNMSFWLLPPSLTLLLMSSMVESGAGTGWTVYPPLSSNIAHGGASVDLAIFSLHLAGISSILGAVNFITTVINMRSTG
MTFDRMPLFVWSVAITALLLLLSLPVLAGAITMLLTDRNLNTTFFDPAGGGDPILYQHL
>COLFG062-13|KJ965493|ZMUO.007567|Phymatodes_testaceus
TLYFIFGAWAGMVGTSLSMLIRSELGNPGSLIGDDQIYNVIVTAHAFIMIFFMVMPVMIGGFGNWLVPLMLGAPDMAFPRMNNMSFWLLPPSLTLLIMSSIVENGAGTGWTVYPPLSANVAHSGSSVDLAIFSLHLAGVSSILGAVNFISTVINMKPA
GMTPEQMPLFVWAVLITAILLLLSLPVLAGAITMLLTDRNLNTSFFDPAGGGDPILYQHL
>COLFF648-13|KJ965350|ZMUO.006538|Phratora_polaris
TLYFIFGIWAGMVGTSLSMLIRSELGNPGTLIGNDQIYNVIVTAHAFIMIFFMVMPIMIGGFGNWLVPLMIGAPDMAFPRLNNMSFWLLPPSLFLLLMSSVVENGAGTGWTVYPPLSSNLAHSGSSVDLAIFSLHLAGISSILGAINFITTVINMRPEGM
GLEQTPLFVWAVLITAILLLLSLPVLAGAITMLLTDRNLNTSFFDPAGGGDPILYQHL
>COLFB421-12|KJ963614|ZMUO.001846|Strophosoma_capitatum
TLYFIFGAWSGMIGTSLSILIRTELGNPGSLIGDDQIYNVIVTAHAFIMIFFMVMPMMIGGFGNWLVPLMLGAPDMAFPRMNNMSFWLLPPSLSLLLMSSIVDKGAGTGWTVYPPLSANIAHEGSSVDLAIFSLHMAGVSSILGAVNFISTVINMRPM
GMSPDRMSLFIWSVKITAILLLLSLPVLAGAITMLLTDRNANTSFFDPAGGGDPILYQHL
>COLFF214-13|KJ967310|ZMUO.005819|Amara_lucida
TLYFIFGAWSGMVGTSLSMLIRAELGNPGALIGDDQIYNVIVTAHAFVMIFFMVMPIMIGGFGNWLVPLMLGAPDMAFPRMNNMSFWMLPPSLTLLLMSSMVESGAGTGWTVYPPLSSGIAHAGASVDLAIFSLHLAGISSILGAVNFITTIINMRS
VGMTFDRMPLFVWSVGITALLLLLSLPVLAGAITMLLTDRNLNTSFFDPAGGGDPILYQHL
>COLFB084-12|KJ965156|ZMUO.001414|Dyschirius_globosus
TLYFIFGIWSGMVGTSLSILIRTELGNPGSLIGDDQIYNVIVTAHAFIMIFFMVMPIMIGGFGNWLVPLMLGAPDMAFPRMNNMSFWLLPPSLTLLLMSSMVEKGAGTGWTVYPPLSSSIAHSGASVDLAIFSLHLAGISSILGAVNFITTIINMRSTGLTF
ERMPLFVWSVGITALLLLLSLPVLAGAITMLLTDRNLNTSFFDPAGGGDPILYQHL
>COLFF753-13|KJ965274|ZMUO.006453|Meligethes_tristis
TLYFIFGMWSGMIGTSLSMLIRTELGNPGSLIGNDQIYNVIVTAHAFVMIFFMVMPFMIGGFGNWLIPLMLGAPDMAFPRMNNMSFWLLPPSLCLLLMSSIVESGAGTGWTVYPPLSSNMAHGGASVDLAIFSLHLAGISSILGAVNFISTVINMRPK
GMLFDRTPLFVWAVIITALLLLLSLPVLAGAITMLLTDRNLNTTFFDPSGGGDPILYQHL
>COLFC799-12|KJ966088|ZMUO.003364|Dictyoptera_aurora
TLYFILGAWAGMMGTALSLLIRAELGTPGTLIGNDQIYNVIVTAHAFIMIFFMVMPMMIGGFGNWLIPLMLGAPDMAYPRMNNMSYWLLPPALSLLTMGSMIESGAGTGWTVYPPLASNIAHSGGSVDLTIFSLHLAGISSILGAVNFISTIINMKSKE
MTFEQMPLFVWAVGITALLLLLSLPVLAGAITMLLTDRNLNTSFFDPMGGGDPILYQHL
>COLFB694-12|KJ966801|ZMUO.001169|Poecilus_versicolor
TLYFIFGAWSGMVGTSLSMLIRAELGNPGSLIGDDQIYNVIVTAHAFVMIFFMVMPIMIGGFGNWLVPLMLGAPDMAFPRMNNMSFWLLPPSLTLLLMSSMVESGAGTGWTVYPPLSSGIAHAGASVDLAIFSLHLAGVSSILGAVNFITTIINMRSI
GMTFDRMPLFVWSVGITALLLLLSLPVLAGAITMLLTDRNLNTSFFDPAGGGDPILYQHL
>COLFD118-12|KJ963987|ZMUO.004013|Cis_comptus
ILYFIFGAWSGMVGTSMSMMIRSELGTPGSLIGNDQIYNVIVTAHAFVMIFFMVMPIMIGGFGNWLVPLMLGAPDMAFPRMNNMSFWLLPPSLSLLLMSSIVENGAGTGWTVYPPLSSNVAHSGSSVDLAIFSLHLAGISSILGAVNFISTVINMRPS
GMTPDRMPLFVWAVVITAVLLLLSLPVLAGAITMLLTDRNFNTSFFDPAGGGDPILYQHL
>COLFE975-13|KJ966645|ZMUO.006770|Ilybius_fenestratus
TLYFIFGAWAGMVGTSLSMLIRAELGNPGSLIGDDQIYNVIVTAHAFVMIFFMVMPIMIGGFGNWLVPLMLGAPDMAFPRMNNMSFWLLPPSLSLLLMSSMVESGAGTGWTVYPPLSSGIAHSGASVDLAIFSLHLAGISSILGAVNFITTIINMRSIG
MTFDRMPLFVWSVGITALLLLLSLPVLAGAITMLLTDRNLNTSFFDPAGGGDPILYQHL
>COLFF291-13|KJ964249|ZMUO.005991|Amara_alpina
TLYFIFGTWSGMVGTSLSMLIRAELGNPGALIGDDQIYNVIVTAHAFIMIFFMVMPIMIGGFGNWLVPLMLGAPDMAFPRMNNMSFWLLPPSLTLLLMSSMVDKGAGTGWTVYPPLSSNIAHAGASVDLAIFSLHLAGISSILGAVNFITTIINMRTV
GMTFDRMPLFVWSVGITALLLLLSLPVLAGAITMLLTDRNLNTSFFDPAGGGDPILYQHL
>COLFE437-12|KJ967103|ZMUO.004997|Phosphuga_atrata

TLYFIFGAWAGIVGMSLSILIRMELSTPGMFLGDDQMYNVIVTAHAFIMIFFMVMPIVIGGFGNWLVPLMLGAPDMAFPRMNNMSFWLLPPSLSLLLVSSMVESGAGTGWTVYPPLSSNIAHSGSSVDLAIFSLHLAGISSILGAVNFITTIINMRSSG
MKFDRMPLFVWSVGITALLLLLSLPVLAGAITMLLTDRNLNTSFFDPAGGGDPILYQHL
>COLFG022-13|KJ964925|ZMUO.007527|Oplosia_cinerea
TLYFIFGAWAGMIGTSLSILIRSELGNSGSLIGDDQIYNTIVTAHAFIMIFFMVMPVMMGGFGNWLVPLMLGAPDMAFPRMNNMSFWLLPPSLTLLIMSSIIDNGTGTGWTVYPPLSANIAHAGSSVDLAIFSLHLAGVSSILGAVNFITTVINMRPKG
MTFDRLPLFVWAVKITAILLLLSLPVLAGAITMLLTDRNLNTSFFDPAGGGDPILYQHL
>COLFB869-12|KJ967273|ZMUO.001914|Stictoleptura_rubra
TLYFIFGAWASMIGTSLSLLIRSELGNPGSLIGDDQIYNVIVTAHAFVMIFFMVMPIMIGGFGNWLVPLMLGAPDMAFPRMNNMSFWLLPPSLTLLIMSSIVESGAGTGWTVYPPLSANIAHGGSSVDLAIFSLHLAGISSILGAVNFITTVINMRPVGM
SPERMPLFVWAVVITAVLLLLSLPVLAGAITMLLTDRNLNTSFFDPSGGGDPILYQHL
>COLFD505-12|KJ963507|ZMUO.004305|Pissodes_pini
TLYFIFGAWSGMIGTSLSILIRTELGTPGSMIGDDQIYNTIVTAHAFIMIFFMVMPIMIGGFGNWLIPLMLGAPDMAFPRLNNMSFWLLPPSIMLLLMSSITDKGAGTGWTVYPPLSTNIAHEGPSVDLAIFSLHMAGISSILGAVNFISTVINMRPTGM
NSDQMSLFIWAVKITAVLLLLSLPVLAGAITMLLTDRNINTSFFDPAGGGDPILYQHL
>COLFC215-12|KJ963558|ZMUO.002400|Stenus_comma
TLYFIFGSWAGMVGTSLSLLIRAELGNPGSLIGDDQIYNVIVTAHAFIMIFFMVMPIMIGGFGNWLVPLMLGAPDMAFPRMNNMSFWLLPPSLSLLLMSSIVESGAGTGWTVYPPLSSNIAHSGASVDLAIFSLHLAGISSILGAINFITTIFNMRTMKLQ
LDCLPLFVWSVSITALLLLLSLPVLAGAITMLLTDRNLNTSFFDPAGGGDPILYQHL
>COLFC053-12|KJ967463|ZMUO.002048|Bembidion_hastii
TLYFIFGAWSGMVGTSLSMLIRAELGNPGSLIGDDQIYNVIVTAHAFVMIFFMVMPILIGGFGNWLVPLMLGAPDMAFPRMNNMSFWLLPPSLTLLLMSSMVESGAGTGWTVYPPLSSSIAHSGASVDLAIFSLHLAGVSSILGAVNFITTIINMRSVG
MSFDRMPLFVWSVGITALLLLLSLPVLAGAITMLLTDRNLNTTFFDPAGGGDPILYQHL
>COLFG102-13|KJ964345|ZMUO.007797|Baeocrara_japonica
TLYFIFGAWAGMVGTSLSILIRTELGTPGSLIGDDQIYNVIVTAHAFVMIFFMVMPILIGGFGNWLVPLMLGAPDMAFPRMNNMSFWLLPPSLTLLLMSSMVESGAGTGWTVYPPLASNIAHGGSSVDLAIFSLHLAGISSILGAVNFITTIINMRAPNM
KFDQMPLFVWAVGITALLLLLSLPVLAGAITMLLTDRNLNTSFFDPAGGGDPILYQHL
>COLFE898-13|KJ963702|ZMUO.006218|Synuchus_vivalis
TLYFIFGAWAGMVGTSLSMLIRAELGTPGALIGDDQIYNVIVTAHAFVMIFFMVMPIMIGGFGNWLVPLMLGAPDMAFPRMNNMSFWLLPPSLTLLLMSSMVESGAGTGWTVYPPLSAGIAHSGSSVDLAIFSLHLAGISSILGAVNFITTIINMRSIG
MTFDRMPLFVWSVGITALLLLLSLPVLAGAITMLLTDRNLNTSFFDPAGGGDPILYQHL
>COLFC050-12|KJ965025|ZMUO.002045|Bembidion_virens
TLYFIFGAWSGMVGTSLSMLIRAELGNPGSLIGDDQIYNVIVTAHAFIMIFFMVMPILIGGFGNWLVPLMLGAPDMAFPRMNNMSFWLLPPSLTLLLMSSMVESGAGTGWTVYPPLSSSIAHSGASVDLAIFSLHLAGVSSILGAVNFITTIINMRSVG
MSFDRMPLFVWSVGITALLLLLSLPVLAGAITMLLTDRNLNTTFFDPAGGGDPILYQHL
>COLFA191-10|HM909112|MP00019|Agrilus_viridis
TLYFIFGAWSGMVGTALSLLIRAELGNPGALIGNDQIYNVIVTAHAFIMIFFMVMPIMMGGFGNWLVPLMLGAPDMAFPRMNNMSFWLLPPSLTLLLMSSMVESGAGTGWTVYPPLAANIAHSGGSVDLAIFSLHLAGISSILGAINFITTVINMRAV
GMTMDRVPLLVWSIAITALLLLLSLPVLAGAITMLLTDRNLNTSFFDPAGGGDPILYQHL
>COLFD694-12|KJ967378|ZMUO.004684|Ceutorhynchus_cakilis
TLYFIFGSWAGMAGTSLSMLIRTELGNPGSLIGNDQIYNSIVTAHAFIMIFFMVMPILIGGFGNWLVPLMLGAPDMAFPRLNNMSFWLLPPSLSLLLMSSVVNKGAGTGWTVYPPLSSNVAHEGMSVDLAIFSLHMAGISSILGAINFISTVMNMQPK
GMTPELMPLFVWAVEITAILLLISLPVLAGAITMLLTDRNINTSFFDPSGGGDPILYQHL
>COLFE201-12|KJ967486|ZMUO.005046|Philonthus_sanguinolentus
TLYFIFGSWAGMVGTSLSLLIRAELGNPGSLIGDDQIYNVIVTAHAFIMIFFMVMPIVIGGFGNWLVPLMLGAPDMAFPRMNNMSFWLLPPSLTLLLMSSMVESGAGTGWTVYPPLSSNIAHGGASVDLAIFSLHLAGISSILGAVNFITTVINMRAMG
MTFDRMPLFVWSVAITALLLLLSLPVLAGAITMLLTDRNLNTTFFDPAGGGDPILYQHL
>COLFE1151-13|KJ964142|ZMUO.006946|Anthophagus_alpinus
TLYFIFGAWAGMVGTSLSILIRSELGNPGSLIGDDQIYNVIVTAHAFVMIFFMVMPVVIGGFGNWLVPLMLGAPDMAFPRMNNMSFWLLPPSLTLLLMSSMVESGAGTGWTVYPPLSSNIAHSGASVDLAIFSLHLAGISSILGAVNFITTVINMRSTGI
TFDRMPLFVWSVAITALLLLLSLPVLAGAITMLLTDRNLNTSFFDPAGGGDPILYQHL
>COLFG141-13|KJ963622|ZMUO.007836|Orthoperus_rogeri
TLYFLFGMWAGLVGTSLSLLIRLELGNPGSLIGDDQIYNVIVTAHAFIMIFFMVMPIMIGGFGNWLIPLMLGAPDMAFPRMNNMSFWLLPPSLTLLILSSIVESGVGTGWTVYPPLSANIAHNGSSVDLAIFSLHLAGISSILGAINFITTIINMRPLGMKLE
QMPLFVWSVLLTAILLLLSLPVLAGAITMLLTDRNLNTSFFDPMGGGDPILYQHL
>COLFC140-12|KJ966180|ZMUO.002325|Megarthrus_nitidulus
TLYFIFGAWAGMIGTSLSILIRAELGNPGSLIGDDQIYNVIVTAHAFIMIFFMVMPIVIGGFGNWLVPLMLGAPDMAFPRMNNMSFWLLPPSISLLLISSMVESGAGTGWTVYPPLSSNIAHGGASVDLAIFSLHLAGISSILGAVNFITTVINMRSMGMT
FDRMPLFVWSVAITALLLLLSLPVLAGAITMLLTDRNINTSFFDPAGGGDPILYQHL
>COLFA442-12|KJ962564|ZMUO.000482|Pterostichus_oblongopunctatus
TLYFIFGAWSGMVGTSLSMLIRAELGNPGALIGDDQIYNVIVTAHAFVMIFFMVMPIMIGGFGNWLVPLMLGAPDMAFPRMNNMSFWLLPPSLTLLLMSSMVENGAGTGWTVYPPLSSGIAHAGASVDLAIFSLHLAGVSSILGAVNFITTIINMRS
VGMTFDRMPLFVWSVGITALLLLLSLPVLAGAITMLLTDRNLNTSFFDPAGGGDPILYQHL

>COLFE1343-13|KJ965500|ZMUO.007138|Kateretes_pusillus
TLYFIFGAWSGMIGTSLSILIRSELGNPGSLIGDDQIYNVIVTAHAFVMIFFMVMPFMIGGFGNWLVPLMLGAPDMAFPRMNNMSFWLLPPSLSLLLMSSIVESGAGTGWTVYPPLSSNIAHGGSSVDLAIFSLHLAGISSILGAVNFITTVINMRPLGM
TFDRMPLFVWAVAITALLLLLSLPVLAGAITMLLTDRNLNTSFFDPAGGGDPVLYQHL
>COLFE1024-13|KJ966472|ZMUO.006819|Euaesthetus_laeviusculus
TLYFMFGMWSGMIGTSLSLLIRAELGNPGSLIGDDQIYNVIVTAHAFIMIFFMVMPIMIGGFGNWLVPLMLGAPDMAFPRMNNMSFWLLPPSLSLLLMSSMVEKGAGTGWTVYPPLSSNIAHGGASVDLAIFSLHLAGISSILGAINFITTIINMRSIG
MTFDRMPLFVWSVGITALLLLLSLPVLAGAITMLLTDRNLNTTFFDPAGGGDPILYQHL
>COLFE404-12|KJ965041|ZMUO.004964|Grypus_equiseti
TLYFLFGFWSGMVGTSMSMLIRTELGNPGSLIGDDQIYNSIVTAHAFVMIFFMVMPIMIGGFGNWLIPLMLGAPDMAFPRLNNLSFWLLPPSLSLLLMSSIIEKGAGTGWTVYPPLSGNISHSGVSVDLAIFSLHMAGASSILGAINFISTAINMYPKNIS
LGQLSLFIWAVCITAILLLLSLPVLAGAITMLLTDRNINTSFFDPAGGGDPILYQHL
>COLFD604-12|KJ964229|ZMUO.004404|Tropiphorus_elevatus
TLYFIFGAWSGMVGTSLSMLIRTELGNPGSLIGDDQIYNVIVTAHAFIMIFFMVMPMMIGGFGNWLIPLMLGAPDMAFPRLNNMSFWLLPPSLTLLLMSSIIDKGAGTGWTVYPPLSANIAHEGASVDLAIFSLHMAGVSSILGAINFISTVINMRSGG
MTPDRMTLFIWAVKITAILLLLSLPVLAGAITMLLTDRNVNTSFFDPAGGGDPILYQHL
>COLFD291-12|KJ965754|ZMUO.004186|Cercyon_quisquilius
TLYFIFGAWAGMVGTSLSILIRAELGNPGTLIGDDQIYNVIVTAHAFIMIFFMVMPIMIGGFGNWLVPLMLGAPDMAFPRMNNMSFWLLPPSLTLLLMSSMVESGAGTGWTVYPPLSSNIAHGGSSVDLAIFSLHLAGISSILGAVNFITTVINMRSPNL
TYDRLPLFVWSVAITALLLLLSLPVLAGAITMLLTDRNLNTSFFDPAGGGDPILYQHL
>COLFB877-12|KJ967492|ZMUO.001922|Molorchus_minor
TLYFIFGAWAGMVGTALSMLIRSELGNPGSLIGDDQIYNVIVTAHAFVMIFFMVMPIMIGGFGNWLVPLMLGAPDMAFPRMNNMSFWLLPPSLTLLLLSSIVESGAGTGWTVYPPLSSNIAHGGSSVDLAIFSLHLAGISSILGAVNFISTVINMRPEG
MNLDRMPLFIWAVMITAILLLLSLPVLAGAITMLLTDRNINTSFFDPAGGGDPILYQHL
>COLFA322-12|KJ964019|ZMUO.000457|Aphodius_niger
TLYFLFGSWAGMVGTSLSLLIRAELGNPGTLIGDDQIYNVIVTAHAFVMIFFMVMPILIGGFGNWLVPLMLGAPDMAFPRMNNMSFWLLPPSLTLLLMSSMVESGAGTGWTVYPPLSSNIAHGGASVDLAIFSLHLAGISSILGAVNFITTVINMRSPG
MTFDRMPLFVWSVAITALLLLLSLPVLAGAITMLLTDRNLNTSFFDPAGGGDPILYQHL
>COLFF124-13|KJ964175|ZMUO.003758|Bledius_gallicus
TLYFIFGAWAGMVGTSLSMLIRAELGTPGSLIGNDQIYNVIVTAHAFVMIFFMVMPIVIGGFGNWLVPLMLGAPDMAFPRMNNMSFWLLPPSLTLLLMSSMVESGAGTGWTVYPPLSSNIAHSGSSVDLAIFSLHLAGISSILGAVNFISTIINMRSIG
MTFDRMPLFVWSVKITAILLLLSLPVLAGAITMLLTDRNLNTSFFDPAGGGDPILYQHL
>COLFE1108-13|KJ966741|ZMUO.006903|Hydroporus_nigrita
TLYFMFGAWSGMVGTSLSMLIRAELGNPGSLIGDDQIYNVIVTAHAFIMIFFMVMPIMIGGFGNWLVPLMLGAPDMAFPRMNNMSFWLLPPSLTLLLMSSMVENGAGTGWTVYPPLSSGIAHSGASVDLAIFSLHLAGVSSILGAVNFITTIINMRSI
GMTFDRMPLFVWSVGITALLLLLSLPVLAGAITMLLTDRNLNTSFFDPAGGGDPILYQHL
>COLFC781-12|KJ962373|ZMUO.003346|Cassida_nobilis
TLYFIFGFWSGMVGTSLSMLIRMELGSPGSLIGNDQIYNSIVTAHAFIMIFFMVMPIMIGGFGNWLVPLMLGAPDMAFPRLNNMSFWLLPPSITFLIMSSIIESGAGTGWTVYPPLSANIAHSGASVDLAIFSLHLAGISSILGAINFISTIMNMRPSGMT
LEKMTLFVWAVIITAVLLILSLPVLAGAITMLLTDRNINTSFFDPAGGGDPILYQHL
>COLFB758-12|KJ963658|ZMUO.001233|Fagniezia_impressa
TLYLIFGAWASMIGTSLSLLIRTELGNPGSLIGDDQIYNVIVTSHAFIMIFFMVMPIMIGGFGNWLIPLMLGAPDMAFPRMNNMSFWLLPPSMILLLMSSMVESGAGTGWTVYPPLSSNIAHSGSSVDLTIFSLHLAGISSILGAVNFITTVINMRTMNM
NFDQMPLFVWSVAITALLLLLSLPVLAGAITMLLTDRNLNTSFFDPAGGGDPILYQHL
>COLFE1111-13|KJ962685|ZMUO.006906|Hygronoma_dimidiata
TLYFIFGTWAGMIGTSLSLLIRAELGNPGSLIGDDQIYNVIVTAHAFIMIFFMVMPIVIGGFGNWLIPLMLGAPDMAFPRMNNMSFWLLPPSLTLLLMSSMVESGAGTGWTVYPPLSSNIAHGGSSVDLAIFSLHLAGISSILGAVNFISTIINMRSMGISF
DRMPLFVWSVAITALLLLLSLPVLAGAITMLLTDRNLNTSFFDPAGGGDPILYQHL
>COLFF323-13|KJ967262|ZMUO.006023|Thymalus_aubei
TLYFIFGAWAGMVGTSLSLLIRSELGSPGTLIGNDQIYNVIVTAHAFIMIFFMVMPIMIGGFGNWLVPLMLGAPDMAFPRMNNMSFWLLPPSLTLLLMSSMVESGAGTGWTVYPPLSSNISHGGSSVDLAIFSLHLAGISSILGAVNFITTVINMRPNG
MTFDQMPLFVWSVSITALLLLLSLPVLAGAITMLLTDRNLNTSFFDPTGGGDPILYQHL
>COLFD657-12|KJ965838|ZMUO.004457|Phyllotreta_atra
TLYFIFGIWAGMVGMSMSMLIRIELAAPGSLIGNDQIYNVIVTAHAFIMIFFMVMPIMIGGFGNWLVPLMIGAPDMAFPRMNNMSFWLLPPSLFLLIMSSIVENGAGTGWTVYPPLSANISHAGSSVDLTIFSLHLAGISSILGAINFITTIINMRPKGMS
FDRMPLFVWAVLITAILLLLSLPVLAGAITMLLTDRNLNTSFFDPMGGGDPILYQHL
>COLFB161-12|KJ964934|ZMUO.001491|Xylotrechus_rusticus
TLYFIFGAWAGMVGTSLSILIRSELGNPGSLIGDDQIYNVIVTAHAFIMIFFMVMPIMIGGFGNWLVPLMLGAPDMAFPRMNNMSFWLLPPSLTLLIMSSIVESGAGTGWTVYPPLSANIAHSGSSVDLAIFSLHLAGVSSILGAVNFISTVINMRPTKM
SPEQMPLFVWAVAITALLLLLSLPVLAGAITMLLTDRNINTSFFDPAGGGDPILYQHL
>COLFA485-12|KJ963187|ZMUO.000525|Miarus_campanulae

TLYFIFGAWSGMLGTSLSMLIRIELGNPGKFIGDDQIYNSIVTAHAFIMIFFMVMPIMIGGFGNWLIPLMIGAPDMAFPRLNNLSFWLLPPSLCMLIFSMIINKGAGTGWTVYPPLSANITHEGASVDFAIFSLHLAGLSSILGAINFISTMMNMRPEGMK
NDQISLFTWAVQITAILLLLSLPVLAGAITMLLTDRNINTSFFDPAGGGDPILYQHL
>COLFC636-12|KJ965707|ZMUO.003201|Hydroporus_rufifrons
TLYFLFGAWSGMVGTSLSMLIRAELGNPGSLIGDDQIYNVIVTAHAFIMIFFMVMPIMIGGFGNWLVPLMLGAPDMAFPRMNNMSFWLLPPSLSLLLMSSMVENGAGTGWTVYPPLSSGIAHSGASVDLAIFSLHLAGVSSILGAVNFITTIINMRSIG
MTFDRMPLFVWSVGITALLLLLSLPVLAGAITMLLTDRNLNTSFFDPAGGGDPILYQHL
>COLFC734-12|KJ963690|ZMUO.003299|Bolitophagus_reticulatus
TLYFIFGAWAGMVGTSLSLLIRTELGNPGSLIGDDQIYNVIVTAHAFVMIFFMVMPIMIGGFGNWLVPLMLGAPDMAFPRMNNMSFWLLPPSLTLLLMSSIVENGAGTGWTVYPPLSSNIAHSGASVDLAIFSLHLAGISSILGAVNFITTVINMRPQG
MTLDRMPLFVWAVVITALLLLLSLPVLAGAITMLLTDRNINTSFFDPAGGGDPILYQHL
>COLFC839-12|KJ962494|ZMUO.003404|Agonum_thoreyi
TLYFIFGAWAGMVGTSLSMLIRAELGNPGALIGDDQIYNVIVTAHAFIMIFFMVMPIMIGGFGNWLVPLMLGAPDMAFPRMNNMSFWLLPPSLTLLLMSSLVESGAGTGWTVYPPLSSGIAHAGASVDLAIFSLHLAGVSSILGAVNFITTIINMRSVG
MTFDRMPLFVWSVGITALLLLLSLPVLAGAITMLLTDRNLNTSFFDPAGGGDPILYQHL
>COLFE1548-13|KJ963190|ZMUO.007438|Coeliastes_lamii
TLYFIFGSWAGMAGTSLSMLIRTELGNPGSLIGDDQIYNSVVTAHAFIMIFFMVMPIMIGGFGNWLVPLMLGAPDMAFPRLNNMSFWLLPPSLSLLLMSSIVNKGAGTGWTVYPPLSSNTAHEGMSVDLAIFSLHMAGISSILGAINFISTVMNMRPF
GMTSEFMPLFVWAVKITAILLLISLPVLAGAITMLLTDRNINTSFFDPSGGGDPILYQHL
>COLFC188-12|KJ966779|ZMUO.002373|Atheta_euryptera
TLYFIFGAWAGMVGTSLSLLIRAELGNPGSLIGDDQIYNVIVTAHAFIMIFFMVMPVVIGGFGNWLVPLMLGAPDMAFPRMNNMSFWLLPPSLSLLLMSSMVESGAGTGWTVYPPLSSNIAHGGSSVDLAIFSLHLAGISSILGAVNFISTVINMRSTGI
SFDRMPLFVWSVAITALLLLLSLPVLAGAITMLLTDRNLNTSFFDPAGGGDPILYQHL
>COLFD086-12|KJ961787|ZMUO.003981|Atheta_crassicornis
TLYFIFGAWAGMVGTSLSLLIRAELGNPGSLIGDDQIYNVIVTAHAFVMIFFMVMPIVIGGFGNWLVPLMLGAPDMAFPRMNNMSFWLLPPSLTLLLMSSMVESGAGTGWTVYPPLSSNIAHGGSSVDLAIFSLHLAGISSILGAVNFISTVINMRSVGI
SFDRMPLFVWSVAITALLLLLSLPVLAGAITMLLTDRNLNTSFFDPAGGGDPILYQHL
>COLFB456-12|KJ965714|ZMUO.001881|Hydroporus_angustatus
TLYFLFGAWSGMVGTSLSMLIRAELGNPGSLIGDDQIYNVIVTAHAFIMIFFMVMPIMIGGFGNWLVPLMLGAPDMAFPRMNNMSFWLLPPSLTLLLMSSMVENGAGTGWTVYPPLSSGIAHSGASVDLAIFSLHLAGISSILGAVNFITTIINMRSIG
MTFDRMPLFVWSVGITALLLLLSLPVLAGAITMLLTDRNLNTSFFDPAGGGDPILYQHL
>COLFE908-13|KJ963137|ZMUO.006228|Pissodes_harcyniae
TLYFIFGAWSGMIGTSLSIIIRTELGTPSNMIGDDQIYNTIVTAHAFIMIFFMVMPIMIGGFGNWLIPLMLGAPDMAFPRLNNMSFWLLPPSIMLLLMSSVADKGAGTGWTVYPPLSTNIAHEGPSVDLAIFSLHLAGISSILGAVNFISTIINMRPMGMN
PDQMSLFIWAVKITAILLLLSLPVLAGAITMLLTDRNINTSFFDPAGGGDPILYQHL
>COLFD045-12|KJ962236|ZMUO.003940|Dyschirius_politus
TLYFIFGIWSGMVGTSLSILIRAELGNPGSLIGDDQIYNVIVTAHAFIMIFFMVMPIMIGGFGNWLVPLMLGAPDMAFPRMNNMSFWMLPPSLSLLLMSSMVEKGAGTGWTVYPPLSSSIAHSGASVDLAIFSLHLAGISSILGAVNFITTIINMRSTGM
TFEKMPLFVWSVGITALLLLLSLPVLAGAITMLLTDRNLNTSFFDPAGGGDPILYQHL
>COLFC251-12|KJ966864|ZMUO.002436|Micrambe_abietis
TLYFIFGAWSGMVGTSLSLLIRSELGTPGSLIGDDQIYNVIVTAHAFIMIFFMVMPIMIGGFGNWLVPLMLGAPDMAFPRLNNMSFWLLPPSLTLLLMSSIAEKGVGTGWTVYPPLSSNIAHGGSSVDLAIFSLHLAGISSILGAINFISTVMNMRSVGMT
LDRMPLFVWAVLITAILLLLSLPVLAGAITMLLTDRNLNTSFFDPAGGGDPVLYQHL
>COLFA604-12|KJ965325|ZMUO.000739|Arhopalus_rusticus
TLYFIFGAWASMVGTSLSLLIRSELGSPGSLIGDDQIYNVIVTAHAFVMIFFMVMPIMIGGFGNWLVPLMLGAPDMAFPRMNNMSFWLLPPSLTLLIMSSIVESGAGTGWTVYPPLSSNIAHSGASVDLAIFSLHLAGISSILGAVNFITTVINMRPSGM
TLDRMPLFVWAVVITAILLLLSLPVLAGAITMLLTDRNLNTSFFDPSGGGDPILYQHL
>COLFC802-12|KJ966073|ZMUO.003367|Deporaus_betulae
TLYFIFGAWAGMVGTALSLLIRAELGNPGSLIGDDQIYNVIVTAHAFIMIFFMVMPIMIGGFGNWLVPLMLGAPDMAFPRMNNMSFWLLPPSLSLLIMSSIVESGAGTGWTVYPPLSANIAHGGSSVDLAIFSLHLAGISSILGAVNFISTMINMRPNG
MTLDRMPLFAWAVVITALLLLLSLPVLAGAITMLLTDRNINTTFFDPAGGGDPILYQHL
>COLFC321-12|KJ963814|ZMUO.002506|Oxypoda_soror
TLYFIFGAWAGMVGTSLSLLIRAELGNPGSLIGDDQIYNVIVTAHAFVMIFFMVMPIVIGGFGNWLIPLMLGAPDMAFPRMNNMSFWLLPPSLSLLLMSSMVESGAGTGWTVYPPLSSNIAHGGSSVDLAIFSLHLAGISSILGAVNFISTIINMRTLGM
SFDRMPLFVWSVAITALLLLLSLPVLAGAITMLLTDRNLNTSFFDPAGGGDPILYQHL
>COLFE257-12|KJ966216|ZMUO.005102|Chrysolina_sanguinolenta
TLYFIFGMWAGMVGTSLSILIRAELGNPGTLIGNDQIYNVIVTAHAFIMIFFMVMPIMIGGFGNWLVPLMLGAPDMAFPRMNNMSFWLLPPSLIFLLMSSIVENGVGTGWTVYPPLSANVAHSGPSVDLAIFSLHLAGISSILGAINFITTIINMRPSEM
KLEQMPLFSWAVLITAILLLLSLPVLAGAITMLLTDRNLNTSFFDPVSGGDPILYQHL
>COLFC120-12|KJ962854|ZMUO.002305|Gonioctena_pallida
TLYFIFGIWAGMVGTSLSILIRAELGNPGSLIGNDQIYNVIVTAHAFIMIFFMVMPIMIGGFGNWLVPLMLGAPDMAFPRMNNMSFWLLPPSLFFLIMSSVVESGAGTGWTVYPPLSANIAHSGSSVDLAIFSLHLAGISSILGAINFITTIINMRPTGMT
MDRMPLFAWAVLITTILLLLSLPVLAGAITMLLTDRNLNTSFFDPAGGGDPILYQHL

>COLFD160-12|KJ966861|ZMUO.004055|Nanophyes_marmoratus
TLYFIFGFWSGMVGTSLSLLIRLELGNPGSLIGDDQIYNVIVTAHAFIMIFFMVMPIMIGGFGNWLVPLMLGAPDMAFPRMNNMSFWLLPPSLTLLIMSSIVEKGAGTGWTVYPPLAANIAHGGASVDLAIFSLHLAGISSILGAANFISTAINMRPSKM
NLDQMPLFVWAVGITALLLLLSLPVLAGAITMLLTDRNINTSFFDPAGGGDPILYQHL
>COLFB210-12|KJ963665|ZMUO.001540|Ceutorhynchus_typhae
TLYFIFGSWAGMAGTSLSMLIRTELGNPGSLIGNDQIYNSIVTAHAFIMIFFMVMPILIGGFGNWLVPLMLGAPDMAFPRLNNMSFWLLPPSLSLLLMSSVVNKGAGTGWTVYPPLSSNVAHEGMSVDLAIFSLHMAGISSILGAINFISTVMNMQPK
GMTPELMPLFVWAVEITAILLLISLPVLAGAITMLLTDRNINTSFFDPSGGGDPILYQHL
>COLFF352-13|KJ965390|ZMUO.006052|Ilybius_wasastjernae
TLYFIFGAWAGMVGTSLSMLIRAELGNPGSLIGDDQIYNVIVTAHAFVMIFFMVMPIMIGGFGNWLVPLMLGAPDMAFPRMNNMSFWLLPPSLSLLLMSSMVESGAGTGWTVYPPLSSGIAHSGASVDLAIFSLHLAGISSILGAVNFITTIINMRSV
GMTFDRMPLFVWSVGITALLLLLSLPVLAGAITMLLTDRNLNTSFFDPAGGGDPILYQHL
>COLFA038-10|HM909063|MP00194|Pityophthorus_micrographus
TLYFIFGAWSGMVGTSLSMLIRTELGYPGSLIGDDQIYNTIVTAHAFIMIFFMVMPIMMGGFGNWLVPLMLGAPDMAFPRLNNMSFWLLPPSLTFLIMSSIIDKGAGTGWTVYPPLSSNISHEGSSVDLAIFSLHMAGVSSILGAINFISTIINMHPNGM
KPEQLPLFVWAVKITAILLLLSLPVLAGAITMLLTDRNVNTSFFDPAGGGDPILYQHL
>COLFD858-12|KJ966968|ZMUO.004468|Anotylus_rugosus
TLYFIFGAWSGMVGTSLSMLIRAELGAPGSLIGDDQIYNVVVTAHAFIMIFFMVMPIVIGGFGNWLVPLMLGAPDMAFPRMNNMSFWLLPPSLTLLLFSSIVESGAGTGWTVYPPLSSNIAHSGSSVDLAIFSLHLAGISSILGAVNFISTIINMRSTGMS
FDRMPLFVWSVNITAILLLLSLPVLAGAITMLLTDRNLNTSFFDPAGGGDPILYQHL
>COLFA257-12|KJ964143|ZMUO.000392|Ahasverus_advena
TLYFIFGSWAGMVGTSLSLLIRTELGTPNALIGDDQIYNTIVTAHAFIMIFFMVMPIMIGGFGNWLVPLMLGAPDMAFPRLNNMSFWLLPPSLTLLIMSSIVEKGAGTGWTVYPPLSSNIAHNGTSVDLAIFSLHLAGISSILGAINFISTIFNIQPNKMKM
DQIPLFCWAVLITAVLLLLSLPVLAGAITMLLTDRNLNTSFFDPSGGGDPILYQHL
>COLFD333-12|KJ966362|ZMUO.004228|Lathrobium_fulvipenne
TLYFIFGAWAGMVGTSLSLLIRTELGNPGSLIGDDQIYNVIVTAHAFVMIFFMVMPIMIGGFGNWLVPLMLGAPDMAFPRMNNMSFWLLPPSLSLLLMSSMVESGAGTGWTVYPPLSSNIAHGGASVDLAIFSLHLAGISSILGAVNFITTVINMRSPG
MTYERMPLFVWAVAITALLLLLSLPVLAGAITMLLTDRNLNTSFFDPAGGGDPILYQHL
>COLFE619-13|KJ964979|ZMUO.005464|Oxypselaphus_obscurus
TLYFIFGAWSGMVGTSLSMLIRAELGNPGALIGDDQIYNVIVTAHAFIMIFFMVMPIMIGGFGNWLVPLMLGAPDMAFPRMNNMSFWLLPPSLTLLLMSSMVESGAGTGWTVYPPLSSGIAHAGSSVDLAIFSLHLAGISSILGAVNFITTIINMRSVG
MTFDRMPLFVWSVGITALLLLLSLPVLAGAITMLLTDRNLNTSFFDPAGGGDPILYQHL
>COLFE465-12|KJ963711|ZMUO.005025|Cyphon_padi
TLYFIFGSWSGMVGTSLSLLIRAELGTPGSLIGDDQIYNVIVTAHAFIMIFFMVMPIMIGGFGNWLVPLMLGAPDMAFPRMNNMSFWLLPPSLTLLLMSSMVENGAGTGWTVYPPLSAGVAHSGASVDLAIFSLHLAGISSILGAVNFISTVINMRSVG
MTFDRMPLFVWAVAITALLLLLSLPVLAGAITMLLTDRNLNTSFFDPAGGGDPILYQHL
>COLFD046-12|KJ966126|ZMUO.003941|Dasytes_obscurus
TLYFIFGAWSGMVGMSLSLLIRSELNNPGTLIGNDQIYNVIVTAHAFIMIFFMVMPILIGGFGNWLVPLMLGAPDMAFPRMNNMSFWLLPPSLTLLLMSSMVEQGAGTGWTVYPPLSANIAHSGASVDLAIFSLHLAGISSILGAVNFITTVINMRPVG
MTLDRTPLFVWAVAITALLLLLSLPVLAGAITMLLTDRNLNTSFFDPAGGGDPILYQHL
>COLFE200-12|KJ962324|ZMUO.005045|Chaetocnema_picipes
TLYFIFGIWSGMVGTSLSVLIRTELGNPGSLIGNDQIYNVIVTAHAFIMIFFMVMPIMIGGFGNWLVPLMIGAPDMAFPRMNNMSFWLLPPSLFLLLMSSMVESGAGTGWTVYPPLSSNIAHSGSSVDLAIFSLHLAGISSILGAINFITTVINMRPQGM
TLDRLPLFVWAVVITAILLLLSLPVLAGAITMLLTDRNLNTSFFDPIGGGDPILYQHL
>COLFE019-12|KJ962562|ZMUO.005244|Magdalis_ruficornis
TLYFIFGAWSGMIGTSLSMLIRVELGNPGSLIGNDQIYNTIVTAHAFIMIFFMVMPIMIGGFGNWLIPLMLGAPDMAFPRLNNMSFWLLPPSLTLLLMSSIIDKGAGTGWTVYPPLSSNIAHEGSSVDLAIFSLHLAGISSILGAINFISTIINMRPTNMKLD
QMSLFIWAVKITAILLLLSLPVLAGAITMLLTDRNMNTSFFDPTGGGDPILYQHL
>COLFD223-12|KJ966788|ZMUO.004118|Omalium_rugatum
TLYFIFGAWAGMVGTSLSLLIRAELGNPGSLIGDDQIYNVIVTAHAFIMIFFMVMPIVIGGFGNWLVPLMLGAPDMAFPRMNNMSFWLLPPSLTLLLMSSMVESGAGTGWTVYPPLSSNIAHGGSSVDLAIFSLHLAGISSILGAVNFITTVINMRAMG
MTFDRMPLFVWSVVITALLLLLSLPVLAGAITMLLTDRNLNTSFFDPAGGGDPILYQHL
>COLFE887-13|KJ963376|ZMUO.006207|Platynus_mannerheimii
TLYFIFGAWAGMVGTSLSMLIRAELGNPGSLIGDDQIYNVIVTAHAFIMIFFMVMPIMIGGFGNWLVPLMLGAPDMAFPRMNNMSFWLLPPSLTLLLMSSMVESGAGTGWTVYPPLSSGIAHAGASVDLAIFSLHLAGVSSILGAVNFITTIINMRSV
GITFDRMPLFVWSVGITALLLLLSLPVLAGAITMLLTDRNLNTSFFDPAGGGDPILYQHL
>COLFD827-12|KJ965787|ZMUO.004817|Rhantus_exsoletus
TLYFIFGAWAGMVGTSLSMLIRAELGNPGSLIGDDQIYNVIVTAHAFVMIFFMVMPIMIGGFGNWLVPLMLGAPDMAFPRMNNMSFWLLPPSLTLLLMSSMVESGAGTGWTVYPPLSSGIAHGGASVDLAIFSLHLAGISSILGAVNFITTIINMRSV
GMTFDRMPLFVWSVGITALLLLLSLPVLAGAITMLLTDRNLNTSFFDPAGGGDPILYQHL
>COLFA282-12|KJ966107|ZMUO.000417|Atomaria_lewisi

TLYFIFGAWSGMVGTSLSMLIRTELGTPGSLISDDQIYNVIVTAHAFIMIFFMVMPMMIGGFGNWLVPLMLGAPYMAFPRLNNMSFWLLPPSLMFLLMSSIVEKGAGTGWTVYPPLSSNVAHAGSSVDLAIFSLHLAGISSILGSVNFITTVINMRPKG
MNFDRLPLFVWAVKITTILLLLSLPVLAGAITMLLTDRNINTSFFDPAGGGDPILYQHL
>COLFB236-12|KJ965991|ZMUO.001566|Laccobius_decorus
TLYFIFGAWAGMVGTSLSILIRAELGNPGTLIGDDQIYNVIVTAHAFIMIFFMVMPIMIGGFGNWLVPLMLGAPDMAFPRMNNMSFWLLPPSLTLLLMSSMVESGAGTGWTVYPPLSSNIAHGGASVDLAIFSLHLAGISSILGAVNFITTVINMRSNN
MTYDRLPLFVWSVAITALLLLLSLPVLAGAITMLLTDRNLNTSFFDPAGGGDPILYQHL
>COLFB401-12|KJ962444|ZMUO.001826|Lithocharis_nigriceps
TLYFIFGAWAGMVGTSLSLLIRAELGVPGSLIGDDQIYNVIVTAHAFVMIFFMVMPIVIGGFGNWLVPLMLGAPDMAFPRMNNMSFWLLPPSLSLLLMSSMVESGAGTGWTVYPPLSSNIAHGGASVDLAIFSLHLAGISSILGAVNFITTVINMRSSG
MTYERMPLFIWSVAITALLLLLSLPVLAGAITMLLTDRNLNTSFFDPAGGGDPILYQHL
>COLFB063-12|KJ964048|ZMUO.001393|Asaphidion_pallipes
TLYFIFGAWAGMVGTSLSMLIRAELGNPGSLIGDDQIYNVIVTAHAFVMIFFMVMPILIGGFGNWLVPLMLGAPDMAFPRMNNMSFWLLPPSLTLLLMSSMVESGAGTGWTVYPPLSSSIAHSGASVDLAIFSLHLAGVSSILGAVNFITTIINMRSIG
MTFDRMPLFVWSVGITALLLLLSLPVLAGAITMLLTDRNINTSFFDPAGGGDPILYQHL
>COLFB577-12|KJ965950|ZMUO.001052|Hydroporus_angustatus
TLYFLFGAWSGMVGTSLSMLIRAELGNPGSLIGDDQIYNVIVTAHAFIMIFFMVMPIMIGGFGNWLVPLMLGAPDMAFPRMNNMSFWLLPPSLTLLLMSSMVENGAGTGWTVYPPLSSGIAHSGASVDLAIFSLHLAGISSILGAVNFITTIINMRSIG
MTFDRMPLFVWSVGITALLLLLSLPVLAGAITMLLTDRNLNTSFFDPAGGGDPILYQHL
>COLFB169-12|KJ966193|ZMUO.001499|Hydrobius_fuscipes
TLYFIFGAWAGMVGTSLSILIRAELGNPGTLIGDDQIYNVIVTAHAFIMIFFMVMPIMIGGFGNWLVPLMLGAPDMAFPRMNNMSFWLLPPSLTLLLMSSMVENGAGTGWTVYPPLSSNIAHGGASVDLAIFSLHLAGISSILGAVNFITTVINMRSPN
LTYDRLPLFVWSVAITALLLLLSLPVLAGAITMLLTDRNLNTSFFDPAGGGDPILYQHL
>COLFB365-12|KJ965484|ZMUO.001695|Dinaraea_aequata
TLYFIFGVWAGMVGTSLSLLIRAELGTPGSLIGDDQIYNVIVTAHAFIMIFFMVMPIVIGGFGNWLIPLMLGAPDMAFPRMNNMSFWLLPPSLTLLLMSSMVESGAGTGWTVYPPLSSNIAHGGSSVDLAIFSLHLAGISSILGAVNFISTVINMRSVGIS
FDRMPLFVWSVAITALLLLLSLPVLAGAITMLLTDRNLNTSFFDPAGGGDPILYQHL
>COLFC817-12|KJ966879|ZMUO.003382|Aphthona_lutescens
TLYFIFGIWSGMVGTSLSVLIRTELGNPGSLIGNDQIYNVIVTAHAFIMIFFMVMPIMIGGFGNWLVPLMIGAPDMAFPRMNNMSFWLLPPSLIFLIMSSMVESGAGTGWTVYPPLSSNIAHGGSSVDLAIFSLHLAGISSILGAINFITTVINMRPEGMN
LDRMPLFVWAVTITAILLLLSLPVLAGAITMLLTDRNLNTSFFDPSGGGDPILYQHL
>COLFB747-12|KJ965242|ZMUO.001222|Myllaena_intermedia
TLYFIFGAWAGMIGSSLSLLIRAELGNPGSLIGDDQIYNVIVTAHAFIMIFFMVMPIVIGGFGNWLVPLMLGAPDMAFPRMNNMSFWLLPPSLSLLLMSSMVESGAGTGWTVYPPLSANIAHSGPSVDLAIFSLHLAGISSILGAVNFISTIINMRSTGMS
FDRMPLFVWSVGITALLLLLSLPVLAGAITMLLTDRNLNTSFFDPAGGGDPILYQHL
>COLFE426-12|KJ963603|ZMUO.004986|Bembidion_femoratum
TLYFIFGAWSGMVGTSLSMLIRAELGNPGSLIGDDQIYNVIVTAHAFVMIFFMVMPILIGGFGNWLVPLMLGAPDMAFPRMNNMSFWLLPPSLTLLLMSSMVESGAGTGWTVYPPLSSSIAHSGASVDLAIFSLHLAGVSSILGAVNFITTIINMRSVG
MSFDRMPLFVWSVGITALLLLLSLPVLAGAITMLLTDRNLNTSFFDPAGGGDPILYQHL
>COLFE772-13|KJ963062|ZMUO.005617|Agonum_thoreyi
TLYFIFGAWAGMVGTSLSMLIRAELGNPGALIGDDQIYNVIVTAHAFIMIFFMVMPIMIGGFGNWLVPLMLGAPDMAFPRMNNMSFWLLPPSLTLLLMSSLVESGAGTGWTVYPPLSSGIAHAGASVDLAIFSLHLAGVSSILGAVNFITTIINMRSVG
MTFDRMPLFVWSVGITALLLLLSLPVLAGAITMLLTDRNLNTSFFDPAGGGDPILYQHL
>COLFA687-12|KJ965831|ZMUO.000822|Agonum_fuliginosum
TLYFIFGAWAGMVGTSLSMLIRAELGNPGALIGDDQIYNVIVTAHAFIMIFFMVMPIMIGGFGNWLVPLMLGAPDMAFPRMNNMSFWLLPPSLTLLLMSSLVESGAGTGWTVYPPLSSGIAHAGASVDLAIFSLHLAGISSILGAVNFITTIINMRSVG
MTFDRMPLFVWSVGITALLLLLSLPVLAGAITMLLTDRNLNTSFFDPAGGGDPILYQHL
>COLFE1205-13|KJ965032|ZMUO.007000|Laccobius_minutus
TLYFLFGAWAGMVGTSLSILIRAELGNPGTLIGDDQIYNVIVTAHAFIMIFFMVMPIMIGGFGNWLVPLMLGAPDMAFPRMNNMSFWLLPPSLTLLLMSSMVESGAGTGWTVYPPLSSNIAHGGASVDLAIFSLHLAGISSILGAVNFITTVINMRSNN
MTYDRLPLFVWSVAITALLLLLSLPVLAGAITMLLTDRNLNTSFFDPAGGGDPILYQHL
>COLFE579-13|KJ962710|ZMUO.005424|Rhinoncus_pericarpius
TLYFIFGSWAGTVGTSLSMLIRTELGTPGSLIGNDQIYNSIVTAHAFIMIFFMVMPILIGGFGNWLVPLMLGAPDMAFPRLNNMSFWLLPPSIMLLLMSSIINKGAGTGWTVYPPLSSNITHEGASVDLAIFSLHMAGISSILGAINFISTIMNMRPQGMS
YDKMPLFSWAVLITAILLLLSLPVLAGAITMLLTDRNINTSFFDPAGGGDPILYQHL
>COLFE489-13|KJ963347|ZMUO.005334|Endomychus_coccineus
TLYFMFGLWAGMLGTSLSMMIRLELGNTMQLIGNDQIFNVIVTAHAFVMIFFMVMPIMIGGFGNWLVPLMLGAPDMAFPRMNNMSFWLLPPSLTLLILSSISESGVGTGWTVYPPLSSNIAHSGSSVDLAIFSLHLAGISSILGAINFITTIINMRPTG
MTLEKMPLFVWSVFITAILLLLSLPVLAGAITMLLTDRNLNTSFFDPAGGGDPILYQHL
>COLFF862-13|KJ965884|ZMUO.006657|Nebria_brevicollis
TLYFIFGAWAGMVGTSLSMLIRAELGNPGSLIGDDQIYNVIVTAHAFVMIFFMVMPIMIGGFGNWLVPLMLGAPDMAFPRMNNMSFWLLPPSLTLLLMSSMVESGAGTGWTVYPPLSSGIAHSGASVDLAIFSLHLAGISSILGAVNFITTIINMRSV
GMTFDRMPLFAWSVGITALLLLLSLPVLAGAITMLLTDRNLNTSFFDPAGGGDPILYQHL

>COLFD233-12|KJ965075|ZMUO.004128|Atomaria_peltata
TLYFIFGAWAGMVGTSLSMLIRTELGTPGSLIGDDQIYNVIVTAHAFIMIFFMVMPMMIGGFGNWLVPLMLGAPDMAFPRLNNMSFWLLPPSLMFLLMSSIVEKGAGTGWTVYPPLSSNVAHAGSSVDLAIFSLHLAGISSILGSVNFITTVINMRPK
GMNFDRLPLFVWAVKITTILLLLSLPVLAGAITMLLTDRNINTSFFDPAGGGDPILYQHL
>COLFE319-12|KJ961769|ZMUO.004879|Amara_municipalis
TLYFIFGAWSGMVGTSLSMLIRAELGNPGALIGDDQIYNVIVTAHAFVMIFFMVMPIMIGGFGNWLVPLMLGAPDMAFPRMNNMSFWLLPPSLTLLLMSSMVESGAGTGWTVYPPLSSGIAHAGASVDLAIFSLHLAGISSILGAVNFITTIINMRSV
GMTFDRMPLFVWSVGITALLLLLSLPVLAGAITMLLTDRNLNTSFFDPAGGGDPILYQHL
>COLFA689-12|KJ965067|ZMUO.000824|Chaetarthria_seminulum
TLYFIFGAWAGMVGTSLSILIRAELGNPGTLIGDDQIYNVIVTAHAFIMIFFMVMPIMIGGFGNWLVPLMLGAPDMAFPRMNNMSFWLLPPSLTLLLMSSMVESGAGTGWTVYPPLSSNIAHGGASVDLAIFSLHLAGISSILGAVNFITTVINMRSNN
MTYDRLPLFVWSVAITALLLLLSLPVLAGAITMLLTDRNLNTSFFDPAGGGDPILYQHL
>COLFF886-13|KJ962205|ZMUO.006681|Leistus_ferrugineus
TLYFIFGAWSGMVGTSLSMLIRAELGNPGSLIGDDQIYNVIVTAHAFVMIFFMVMPIMIGGFGNWLVPLMLGAPDMAFPRMNNMSFWLLPPSLTLLLMSSMVESGAGTGWTVYPPLSSGIAHSGASVDLAIFSLHLAGVSSILGAVNFITTIINMRSV
GMTFDRMPLFVWSVGITALLLLLSLPVLAGAITMLLTDRNLNTSFFDPAGGGDPILYQHL
>COLFE1176-13|KJ964181|ZMUO.006971|Stenus_tarsalis
TLYFIFGVWAGMVGTSLSLLIRTELGNPGSLIGDDQIYNVIVTAHAFIMIFFMVMPVMIGGFGNWLVPLMLGAPDMAFPRMNNMSFWLLPPSLSLLLMSSIVESGAGTGWTVYPPLSSNIAHSGASVDLAIFSLHLAGISSILGAINFITTIFNMRTMKIQ
LDCLPLFIWSVGITALLLLLSLPVLAGAITMLLTDRNINTSFFDPAGGGDPILYQHL
>COLFE1475-13|KJ966060|ZMUO.007270|Phyllobrotica_quadrimaculata
TLYFIFGIWAGLIGTSLSMLIRTELGMPGSLIGNDQIYNVIVTAHAFIMIFFMVMPIMIGGFGNWLVPLMIGAPDMAFPRMNNMSFWLLPPSLFFLIMSSTVESGAGTGWTVYPPLSSNIAHGGSSVDLAIFSLHLAGISSILGAINFITTIINMRPQGMTL
DRMPLFVWAVLITAILLLLSLPVLAGAITMLLTDRNLNTSFFDPAGGGDPILYQHL
>COLFF287-13|KJ963680|ZMUO.005987|Agonum_ericeti
TLYFIFGAWSGMVGTSLSMLIRAELGNPGALIGDDQIYNVIVTAHAFIMIFFMVMPIMIGGFGNWLVPLMLGAPDMAFPRMNNMSFWLLPPSLTLLLMSSMVESGAGTGWTVYPPLSSGIAHAGASVDLAIFSLHLAGVSSILGAVNFITTIINMRSV
GMTFDRMPLFVWSVGITALLLLLSLPVLAGAITMLLTDRNLNTSFFDPAGGGDPILYQHL
>COLFE1498-13|KJ965514|ZMUO.007293|Coccinella_undecimpunctata
TLYFLFGMWAGMIGTSLSILIRLELGTTNSLIGNDQIYNVIVTAHAFIMIFFMVMPIMIGGFGNWLVPLMIGAPDMAFPRLNNMSFWLLPPALTLLIISSLVEMGAGTGWTVYPPLSSNLAHNGPSVDLVIFSLHLAGISSILGAVNFISTIMNMRPFGM
NLDKTPLFVWSVLITAILLLLSLPVLAGAITMLLTDRNINTSFFDPTGGGDPILYQHL
>COLFD762-12|KJ961786|ZMUO.004752|Galerucella_calmariensis
TLYFIFGIWAGMVGTSLSILVRAELGSPGTLIGNDQIYNVIVTAHAFIMIFFMVMPIMIGGFGNWLVPLMIGAPDMAFPRMNNMSFWLLPPSLFLLIMSSIVESGAGTGWTVYPPLSSNIAHGGSSVDLAIFSLHLAGISSILGAINFITTIINMRPKGMTL
DRMPLFVWAVMITAILLLLSLPVLAGAITMLLTDRNLNTSFFDPAGGGDPILYQHL
>COLFB394-12|KJ964202|ZMUO.001819|Philonthus_tenuicornis
TLYFIFGSWAGMVGTSLSLLIRAELGNPGTLIGDDQIYNVIVTAHAFIMIFFMVMPIVIGGFGNWLVPLMLGAPDMAFPRMNNMSFWLLPPSLTLLLMSSMVESGAGTGWTVYPPLSSNIAHGGASVDLAIFSLHLAGISSILGAVNFITTVINMRSTG
MSFDRMPLFVWSVAITALLLLLSLPVLAGAITMLLTDRNLNTSFFDPAGGGDPILYQHL
>COLFF330-13|KJ966317|ZMUO.006030|Altica_engstromi
TLYFLFGIWAGMIGTSMSLLIRTELGSPGSLIGNDQIYNVIVTAHAFVMIFFMVMPMMIGGFGNWLVPLMIGAPDMAFPRMNNMSFWLLPPSIFLLLMSSFTESGAGTGWTVYPPLSSNLAHNGPSVDLAIFSLHLAGISSILGAINFITTMINMRPQG
MSMDQMPLFVWAVFITAILLLLSLPVLAGAITMLLTDRNLNTSFFEPAGGGDPILYQHL
>COLFB617-12|KJ965419|ZMUO.001092|Agonum_thoreyi
TLYFIFGAWAGMVGTSLSMLIRAELGNPGALIGDDQIYNVIVTAHAFIMIFFMVMPIMIGGFGNWLVPLMLGAPDMAFPRMNNMSFWLLPPSLTLLLMSSLVESGAGTGWTVYPPLSSGIAHAGASVDLAIFSLHLAGVSSILGAVNFITTIINMRSVG
MTFDRMPLFVWSVGITALLLLLSLPVLAGAITMLLTDRNLNTSFFDPAGGGDPILYQHL
>COLFF692-13|KJ963789|ZMUO.006392|Cantharis_figurata
TLYFIFGAWSGSLGLALSLLIRAELGTPGTLIGNDQIYNVIVTAHAFIMIFFMVMPIMIGGFGNWLVPLMLGAPDMAFPRMNNMSFWFLPPSLMFLLMSSMVESGAGTGWTVYPPLSANIAHSGPSVDLAIFSLHMAGISSILGAVNFISTIMNMKPPS
MKFDQMPLFVWSVGITALLLLLSLPVLAGAITMLLSDRNLNTSFFDPMGGGDPILYQHL
>COLFB821-12|KJ962002|ZMUO.001296|Liogluta_micans
TLYFIFGAWAGMVGTSLSLLIRAELGNPGSLIGDDQIYNVIVTAHAFIMIFFMVTPVVIGGFGNWLVPLMLGAPDMAFPRMNNMSFWLLPPSLTLLLMSSMVESGAGTGWTVYPPLSSNIAHSGSSVDLAIFSLHLAGISSILGAVNFISTVINMRSVG
MSFDRMPLFVWSVAITALLLLLSLPVLAGAITMLLTDRNLNTSFFDPAGGGDPILYQHL
>COLFE401-12|KJ965781|ZMUO.004961|Limnobaris_pilistriata
TLYFIFGAWAGMTGTSLSMLIRSELGNPGSLIGDDQIYNTIVTAHAFIMIFFMVMPIMIGGFGNWLVPLMLGAPDMAFPRLNNMSFWLLPPSLTLLLMSSIVDKGVGTGWTVYPPLSSNNAHEGASVDLGIFSLHMAGISSILGAMNFISTAMNMRP
TGLKSDQMSLFIWAVKITAILLLLSLPVLAGAITMLLTDRNINTSFFDPTGGGDPILYQHL
>COLFE1279-13|KJ962195|ZMUO.007074|Microcara_testacea

TLYFIFGSWAGMVGTSLSLLIRAELGTPGSLIGDDQIYNVIVTAHAFIMIFFMVMPIMIGGFGNWLVPLMLGAPDMAFPRMNNMSFWLLPPSLTLLLMSSMVENGAGTGWTVYPPLSAGMAHSGASVDLAIFSLHLAGISSILGAVNFISTVINMRSS
GMTFDRMPLFVWSVAITALLLLLSLPVLAGAITMLLTDRNLNTSFFDPAGGGDPILYQHL
>COLFD236-12|KJ963251|ZMUO.004131|Archarius_crux
TLYFIFGAWSGMVGMSLSMLIRTELGNPGSLIGDDQIYNTIVTAHAFIMIFFMVMPTLIGGFGNWLIPLMLGAPDMAFPRLNNMSFWLLPPSLFLLLLSSIADKGAGTGWTVYPPLSTNIAHEGSSVDLAIFSLHMAGISSILGAMNFISTILNMRPTSM
KPDQMSLFIWAVKITAILLLLSLPVLAGAITMLLTDRNINTSFFDPAGGGDPILYQHL
>COLFD367-12|KJ967189|ZMUO.004262|Aleochara_inconspicua
TLYFIFGAWSGMVGTSLSLLIRAELGNPGSLIGDDQIYNVIVTAHAFVMIFFMVMPIVIGGFGNWLVPLMLGAPDMAFPRMNNMSFWLLPPSLTLLLMSSMVESGAGTGWTVYPPLSSNIAHGGSSVDLAIFSLHLAGISSILGAVNFISTVINMRTMG
MSFDKMPLFVWSVAITALLLLLSLPVLAGAITMLLTDRNLNTSFFDPAGGGDPILYQHL
>COLFA559-12|KJ964578|ZMUO.000694|Monochamus_sutor
TLYFIFGAWSGMVGTSLSLLIRSELGMPGALIGDDQIYNVIVTAHAFVMIFFMVMPIMIGGFGNWLVPLMLGAPDMAFPRMNNMSFWLLPPSLTLLIMSSIVENGAGTGWTVYPPLAANVAHSGSSVDLAIFSLHLAGVSSILGAVNFITTVINMRPS
GMNLDRLPLFVWAVKITAILLLLSLPVLAGAITMLLTDRNLNTSFFDPAGGGDPILYQHL
>COLFD572-12|KJ961924|ZMUO.004372|Amara_fulva
TLYFIFGAWSGMVGTSLSMLIRTELGNPGALIGDDQIYNVIVTAHAFVMIFFMVMPIMIGGFGNWLVPLMLGAPDMAFPRMNNMSFWMLPPSLTLLLMSSMVESGAGTGWTVYPPLSSGIAHAGASVDLAIFSLHLAGISSILGAVNFITTIINMRSV
GMTFDRMPLFVWSVGITALLLLLSLPVLAGAITMLLTDRNLNTSFFDPAGGGDPILYQHL
>COLFE1004-13|KJ965236|ZMUO.006799|Pelenomus_canaliculatus
TLYFIFGSWAGTVGTSLSMIIRTELGTPGSLIGNDQIYNSIVTAHAFIMIFFMVMPILIGGFGNWLVPLMLGAPDMAFPRLNNMSFWLLPPSILLLLMSSIVNKGAGTGWTVYPPLSSNVTHEGASVDLAIFSLHMAGISSILGAINFISTIMNMRPKGMS
YDKTPLFVWAVMITAILLLLSLPVLAGAITMLLTDRNINTSFFDPAGGGDPILYQHL
>COLFC698-12|KJ965881|ZMUO.003263|Cis_jacquemartii
TLYFIFGAWSGMLGTSMSILIRSELSTPGSLIGNDQIYNVIVTAHAFIMIFFMVMPVMIGGFGNWLVPLMLGAPDMAFPRMNNMSFWLLPPSLSLLIMSSIVENGAGTGWTVYPPLSSNTAHSGSSVDLAIFSLHLAGISSILGAVNFISTIINMHPSGM
KPDQMPLFVWAVVITAVLLLLSLPVLAGAITMLLTDRNLNTSFFDPAGGGDPILYQHL
>COLFF535-13|KJ962209|ZMUO.006615|Stenus_biguttatus
TLYFLFGSWAGMVGTSLSLLIRAELGNPGSLIGDDQIYNVIVTAHAFIMIFFMVMPIMIGGFGNWLIPLMLGAPDMAFPRMNNMSFWLLPPSLSLLLMSSIVESGAGTGWTVYPPLSANIAHSGASVDLAIFSLHLAGISSILGAINFITTIFNMRTMKLQ
LDCLPLFVWSVSITALLLLLSLPVLAGAITMLLTDRNLNTSFFDPAGGGDPILYQHL
>COLFF725-13|KJ967117|ZMUO.006425|Crypticus_quisquilius
TLYFIFGAWSGMVGTSLSLLIRAELGNPGSLIGDDQIYNVIVTAHAFIMIFFMVMPIMIGGFGNWLVPLMLGAPDMAFPRMNNMSFWLLPPSLTLLLMSSIVENGAGTGWTVYPPLSSNIAHGGSSVDLAIFSLHLAGISSILGAVNFITTVINMRPQG
MTFDRMPLFVWAVVITAVLLLLSLPVLAGAITMLLTDRNINTSFFDPAGGGDPILYQHL
>COLFD909-12|KJ967105|ZMUO.004519|Carpelimus_rivularis
TLYFIFGAWSGMVGTSLSMLIRIELGTPGSLIGDDQIYNVIVTAHAFIMIFFMVMPIVIGGFGNWLVPLMLGAPDMAFPRMNNMSFWLLPPSLTLLLFSSMVESGAGTGWTVYPPLSSNIAHSGSSVDLAIFSLHLAGISSILGAVNFISTIINMRSVGMT
FDRMPLFVWSVNITAILLLLSLPVLAGAITMLLTDRNLNTSFFDPAGGGDPILYQHL
>COLFE1590-13|KJ965655|ZMUO.007480|Cytilus_auricomus
TLYFIFGAWAGMVGTSLSLLIRAELGNPGSLIGDDQIYNVIVTAHAFVMIFFMVMPIMVGGFGNWLVPLMLGAPDMAFPRMNNMSFWLLPPSLSLLLMSSIVESGAGTGWTVYPPLSSNIAHSGASVDLAIFSLHLAGISSILGAFNFISTVINMRSPG
MKFDQMSLFSWSVAITALLLLLSLPVLAGAITMLLTDRNINTSFFDPAGGGDPILYQHL
>COLFC692-12|KJ963810|ZMUO.003257|Phratora_laticollis
TLYFIFGIWAGMVGTSLSVLIRSELGNPGTLIGNDQIYNVIVTAHAFIMIFFMVMPIMIGGFGNWLVPLMIGAPDMAFPRLNNMSFWLLPPSLFLLLMSSVVENGAGTGWTVYPPLSSNLAHSGSSVDLAIFSLHLAGISSILGAINFITTVINMRPEGMN
LEQTPLFVWAVLITAILLLLSLPVLAGAITMLLTDRNLNTSFFDPAGGGDPILYQHL
>COLFD543-12|KJ965427|ZMUO.004343|Apion_frumentarium
TLYFIFGLWSGMVGTSLSMLIRVELGNPGSLIGNDQIYNMIVTAHAFIMIFFMVMPIMIGGFGNWLVPLMLGAPDMAFPRMNNMSFWLLPPSLMLLLMSSFIENGAGTGWTVYPPLASNIAHSGASVDLAIFSLHLAGISSILGAVNFISTMINMRSN
GMSLDQLSLFTWAVKITAILLLLSLPVLAGAITMLLTDRNINTSFFDPAGGGDPILYQHL
>COLFB903-12|KJ965483|ZMUO.001948|Saperda_carcharias
TLYFLFGAWASMVGTSLSLLIRSELGTPGSLIGDDQIYNVIVTAHAFIMIFFMVMPIMIGGFGNWLVPLMLGAPDMAFPRMNNMSFWLLPPSLSLLIMSSIVDNGAGTGWTVYPPLAANIAHSGSSVDLAIFSLHLAGISSILGAVNFITTVINMRPQG
MSMDRMPLFVWAVKITAILLLLSLPVLAGAITMLLTDRNLNTSFFDPAGGGDPILYQHL
>COLFD625-12|KJ963841|ZMUO.004425|Psylliodes_affinis
TLYFIFGIWSGMIGTSLSMLIRTELGAPGSLIGNDQIYNVIVTAHAFIMIFFMVMPTMIGGFGNWLIPLMIGAPDMAFPRMNNMSFWLLPPSLTLLLMSSMVENGAGTGWTVYPPLSSNIAHGGSSVDLAIFSLHLAGISSILGAINFITTVINMRPKGM
TLDRMPLFVWAVVITAILLLLSLPVLAGAITMLLTDRNLNTSFFDPIGGGDPILYQHL
>COLFF832-13|KJ964748|ZMUO.006152|Gastrophysa_polygoni
TLYFIFGVWAGMIGTSLSMLIRSELGNPGMLIGNDQIYNVIVTAHAFIMIFFMVMPIMIGGFGNWLVPLMIGAPDMAFPRMNNMSFWLLPPSLFLLLMSSVVENGAGTGWTVYPPLSANIAHSGSSVDLAIFSLHLAGISSILGAINFITTIINMRPMG
MNLEQTPLFVWAVLITAVLLLLSLPVLAGAITMLLTDRNLNTSFFDPAGGGDPILYQHL

>COLFC551-12|KJ964302|ZMUO.003116|Carabus_glabratus
TLYFIFGTWSGMVGTSLSMLIRAELGNPGSLIGDDQIYNVIVTAHAFVMIFFMVMPIMIGGFGNWLVPLMLGAPDMAFPRMNNMSFWLLPPSLTLLLMSSMVEKGAGTGWTVYPPLSSGIAHSGASVDLVIFSLHLAGISSILGAVNFITTIINMRSVG
MTFDRMPLFVWSVGITALLLLLSLPVLAGAITMLLTDRNLNTSFFDPAGGGDPILYQHL
>COLFE1246-13|KJ964288|ZMUO.007041|Anaspis_arctica
TLYFIFGAWSGMVGTSLSLLIRSELGTPGSLIGDDQIYNVIVTAHAFIMIFFMVMPILIGGFGNWLVPLMLGAPDMAFPRMNNMSFWLLPPSLTLLIMSSVVENGAGTGWTVYPPLAANIAHSGSSVDLAIFSLHLAGVSSILGAVNFITTVINMRPQG
MTLDRMPLFVWAVVITAILLLLSLPVLAGAITMLLTDRNLNTSFFDPAGGGDPILYQHL
>COLFF1005-13|KJ964279|ZMUO.007370|Pteroloma_forsstromii
TLYFIFGAWAGMVGTSLSILIRAELGNPGTLIGDDQIYNVIVTAHAFIMIFFMVMPIMIGGFGNWLVPLMLGAPDMAFPRMNNMSFWLLPPSLSLLLMSSIVENGAGTGWTVYPPLSSNIAHSGSSVDLAIFSLHLAGISSILGAVNFITTVINMRSTGM
TFDRMPLFVWAVAITALLLLLSLPVLAGAITMLLTDRNLNTSFFDPAGGGDPILYQHL
>COLFF741-13|KJ965785|ZMUO.006441|Psyllobora_vigintiduopunctata
TLYFLFGMWAGMVGTSLSILIRLELGTTNSVIGNDQIYNVIVTAHAFIMIFFMVMPIMIGGFGNWLVPLMVGAPDMAFPRLNNMSFWLLPPALTLLIFSSMVEMGAGTGWTVYPPLSSNMAHSGSSVDLVIFSLHLAGISSILGAVNFISTIMNMRPF
GMNLDKTPLFVWSVLITAILLLLSLPVLAGAITMLLTDRNINTSFFDPMGGGDPILYQHL
>COLFC411-12|KJ963695|ZMUO.002976|Gonioctena_arctica
TLYFIFGIWAGMVGTSLSILIRAELGNPGTLIGNDQIYNVIVTAHAFIMIFFMVMPIMIGGFGNWLVPLMIGAPDMAFPRMNNMSFWLLPPSLFFLIMSSVVESGAGTGWTVYPPLSANIAHSGSSVDLAIFSLHLAGISSILGAINFITTIINMRPMGMT
MDRMPLFVWAVLITAILLLLSLPVLAGAITMLLTDRNLNTSFFDPAGGGDPILYQHL
>COLFG120-13|KJ965028|ZMUO.007815|Hydrosmecta_longula
TLYFIFGAWAGMVGTSLSLLIRAELGNPGSLIGDDQIYNVIVTAHAFIMIFFMVMPIVIGGFGNWLVPLMLGAPDMAFPRMNNMSFWLLPPSLTLLLMSSMVESGAGTGWTVYPPLSSNIAHGGSSVDLAIFSLHLAGISSILGAVNFISTVINMRSTGI
SFDRMPLFVWSVAITALLLLLSLPVLAGAITMLLTDRNLNTSFFDPAGGGDPILYQHL
>COLFA473-12|KJ963649|ZMUO.000513|Hydrothassa_marginella
TLYFIFGIWSGMVGTSLSILIRSELGNPGSLIGNDQIYNVIVTAHAFIMIFFMVMPVMIGGFGNWLVPLMIGAPDMAFPRLNNMSFWLLPPSLFLLLMSSVVENGVGTGWTVYPPLSSNIAHSGSSVDLAIFSLHLAGISSILGAINFITTIINMRPKGMNL
EQTPLFVWAVLITAILLLLSLPVLAGAITMLLTDRNLNTSFFDPAGGGDPILYQHL
>COLFC667-12|KJ966321|ZMUO.003232|Cordylepherus_viridis
TLYFIFGAWSGMVGLSLSLLIRSELSIPGTLIGNDQIYNVIVTAHAFIMIFFMVMPILIGGFGNWLVPLMLGAPDMAFPRMNNMSFWLLPPSLTLLLLSSMVENGAGTGWTVYPPLSANIAHSGSSVDLAIFSLHLAGISSILGAVNFITTVINMRPQGMT
LDRTPLFVWAVVITALLLLLSLPVLAGAITMLLTDRNLNTSFFDPAGGGDPILYQHL
>COLFA619-12|KJ964425|ZMUO.000754|Mycetochara_axillaris
TLYFIFGAWSGMVGTSLSLLIRAELGNPGSLIGDDQIYNVIVTAHAFIMIFFMVMPIMIGGFGNWLVPIMLGAPDMAFPRMNNMSFWLLPPSLTLLLMSSMVESGAGTGWTVYPPLSSNIAHGGASVDLAIFSLHLAGISSILGAVNFITTVINMKPQG
MTFDRMPLFVWAVVITAVLLLLSLPVLAGAITMLLTDRNINTSFFDPAGGGDPILYQHL
>COLFB082-12|KJ963472|ZMUO.001412|Philonthus_rubripennis
TLYFIFGSWAGMVGTSLSLLIRAELGNPGTLIGDDQIYNVIVTAHAFIMIFFMVMPIVIGGFGNWLVPLMLGAPDMAFPRMNNMSFWLLPPSLTLLLMSSMVESGAGTGWTVYPPLSSNIAHGGASVDLAIFSLHLAGISSILGAVNFITTVINMRSSG
MTFDRMPLFVWSVAITALLLLLSLPVLAGAITMLLTDRNVNTTFFDPAGGGDPILYQHL
>COLFE1263-13|KJ965486|ZMUO.007058|Olophrum_boreale
TLYFIFGAWAGMVGTSLSILIRAELGNPGTLIGDDQIYNVIVTAHAFVMIFFMVMPILIGGFGNWLVPLMLGAPDMAFPRMNNMSFWLLPPSLTLLLMSSMVESGAGTGWTVYPPLSSNIAHGGASVDLAIFSLHLAGISSILGAVNFITTVINMRTTG
MTFDRMPLFVWAVAITALLLLLSLPVLAGAITMLLTDRNLNTTFFDPAGGGDPILYQHL
>COLFF985-13|KJ963203|ZMUO.007350|Bembidion_obtusum
TLYFIFGAWSGMVGTSLSMLIRAELGNPGSLIGDDQIYNVIVTAHAFVMIFFMVMPILIGGFGNWLVPLMLGAPDMAFPRMNNMSFWLLPPSLTLLLMSSMVESGAGTGWTVYPPLSSSIAHSGASVDLAIFSLHLAGVSSILGAVNFITTIINMRSIG
MTFDRMPLFVWSVGITALLLLLSLPVLAGAITMLLTDRNLNTSFFDPAGGGDPILYQHL
>COLFC653-12|KJ965374|ZMUO.003218|Deporaus_betulae
TLYFIFGAWAGMVGTALSLLIRAELGNPGSLIGDDQIYNVIVTAHAFIMIFFMVMPIMIGGFGNWLVPLMLGAPDMAFPRMNNMSFWLLPPSLSLLIMSSIVESGAGTGWTVYPPLSANIAHGGSSVDLAIFSLHLAGISSILGAVNFISTMINMRPNG
MTLDRMPLFAWAVVITALLLLLSLPVLAGAITMLLTDRNINTTFFDPAGGGDPILYQHL
>COLFE1490-13|KJ964008|ZMUO.007285|Meligethes_ochropus
TLYFIFGAWSGMVGTSLSMLIRTELGNPGSLIGNDQIYNVIVTAHAFVMIFFMVMPFMIGGFGNWLVPLMLGAPDMAFPRMNNMSFWLLPPSLSLLLMSSIVESGAGTGWTVYPPLSSNIAHGGASVDLAIFSLHLAGISSILGAVNFITTVINMRPSG
MTFDRMPLFVWAVVITAILLLLSLPVLAGAITMLLTDRNLNTTFFDPSGGGDPILYQHL
>COLFF606-13|KJ966043|ZMUO.006496|Lathrobium_longulum
TLYFIFGAWAGMVGTSLSLLIRTELGNPGSLIGDDQIYNVIVTAHAFIMIFFMVMPIMIGGFGNWLVPLMLGAPDMAFPRMNNMSFWLLPPSLSLLLMSSMVESGAGTGWTVYPPLSSNIAHGGASVDLAIFSLHLAGISSILGAVNFITTIINMHSPG
MTYERMPLFVWAVAITALLLLLSLPVLAGAITMLLTDRNLNTSFFDPAGGGDPILYQHL
>COLFD193-12|KJ964545|ZMUO.004088|Stenus_melanarius

TLYFIFGAWAGMVGTSLSLLIRAELGNPGSLIGDDQIYNVIVTAHAFVMIFFMVMPIMIGGFGNWLVPLMLGAPDMAFPRMNNMSFWLLPPSLSLLLMSSIVENGAGTGWTVYPPLSSNIAHGGASVDLAIFSLHLAGISSILGAINFITTIFNMRTMKL
QLDCLPLFVWSVGITALLLLLSLPVLAGAITMLLTDRNLNTSFFDPAGGGDPILYQHL
>COLFE1126-13|KJ967380|ZMUO.006921|Quedius_boopoides
SLYFIFGAWAGMVGTSLSLLIRAELGNPGSLIGDDQIYNVIVTAHAFIMIFFMVMPTLIGGFGNWLVPLMLGAPDMAFPRMNNMSFWLLPPSLSLLLMSSMVESGAGTGWTVYPPLSSNIAHSGASVDLAIFSLHLAGISSILGAVNFITTVINMRSTG
MTFDRMPLFVWSVAITALLLLLSLPVLAGAITMLLTDRNLNTTFFDPAGGGDPILYQHL
>COLFE340-12|KJ961771|ZMUO.004900|Bembidion_dentellum
TLYFIFGAWSGMVGTSLSMLIRAELGNPGSLIGDDQIYNVIVTAHAFIMIFFMVMPILIGGFGNWLVPLMLGAPDMAFPRMNNMSFWLLPPSLSLLLMSSMVESGAGTGWTVYPPLSSSIAHSGASVDLAIFSLHLAGVSSILGAVNFITTIINMRSTG
MTFDRMPLFVWSVGITALLLLLSLPVLAGAITMLLTDRNLNTSFFDPAGGGDPILYQHL
>COLFB688-12|KJ963323|ZMUO.001163|Atheta_elongatula
TLYFIFGAWAGMVGTSLSLLIRAELGNPGSLIGDDQIYNVIVTAHAFVMIFFMVMPIVIGGFGNWLVPLMLGAPDMAFPRMNNMSFWLLPPSLTLLLMSSMVESGAGTGWTVYPPLSSNIAHGGSSVDLAIFSLHLAGISSILGAVNFISTVINMRSTGI
SFDRMPLFVWSVAITALLLLLSLPVLAGAITMLLTDRNLNTSFFDPAGGGDPILYQHL
>COLFE1087-13|KJ962210|ZMUO.006882|Actenicerus_sjaelandicus
TLYFLFGAWAGMLGTSLSLLIRAELGNPGSLIGNDQIYNVIVTAHAFIMIFFMVMPIMIGGFGNWLVPLMLGAPDMAFPRMNNMSFWFLPPSLSLLLMSSIVENGAGTGWTVYPPLSANIAHSGSSVDLAIFSLHLAGISSILGAVNFISTVINMRSTGIT
FDRMPLFVWAVAITALLLLLSLPVLAGAITMLLTDRNLNTSFFDPAGGGDPVLYQHL
>COLFF952-13|KJ962346|ZMUO.007317|Trechus_secalis
TLYFIFGAWAGMVGTSLSMLIRAELGTPGSLIGDDQIYNVIVTAHAFIMIFFMVMPIMIGGFGNWLVPLMLGAPDMAFPRMNNMSFWLLPPSLTLLLMSSMVESGAGTGWTVYPPLSSGIAHSGASVDLAIFSLHLAGISSILGAVNFITTIINMRPMG
MTFDRMPLFVWSVGITALLLLLSLPVLAGAITMLLTDRNLNTSFFDPAGGGDPILYQHL
>COLFE1120-13|KJ966510|ZMUO.006915|Podabrus_lapponicus
TLYFIFGAWSGSLGLALSLLIRAELGTPGTLIGNDQIYNVIVTAHAFIMIFFMVMPIMIGGFGNWLVPLMLGAPDMAFPRMNNMSFWFLPPSLMFLLMSSMVESGAGTGWTVYPPLSANIAHSGPSVDLAIFSLHMAGISSILGAVNFISTIMNMKPPS
MKFDQMPLFVWSVGITALLLLLSLPVLAGAITMLLSDRNLNTSFFDPMGGGDPILYQHL
>LEFIJ1890-13|KJ964089|ZMUO.004645|Ceutorhynchus_obstrictus
TLYFIFGSWAGMAGTSLSMLIRTELGNPGSLIGNDQIYNSIVTAHAFIMIFFMVMPILIGGFGNWLVPLMLGAPDMAFPRLNNMSFWLLPPSLSLLLMSSIVNKGAGTGWTVYPPLSGNVAHEGMSVDLAIFSLHMAGISSILGAINFISTVMNMQPK
GMTPELMPLFVWAVQITAILLLISLPVLAGAITMLLTDRNINTSFFDPSGGGDPILYQHL
>COLFC265-12|KJ967500|ZMUO.002450|Trechus_rubens
TMYFIFGTWAGMVGTSLSMLIRAELGNPGSLIGDDQIYNVIVTAHAFIMIFFMVMPIMIGGFGNWLVPLMLGAPDMAFPRMNNMSFWLLPPSLMLLLMSSMVESGAGTGWTVYPPLSSGIAHSGAAVDLAIFSLHLAGISSILGAVNFITTIINMRP
MGMTFDRMPLFVWSVGITALLLLLSLPVLAGAITMLLTDRNLNTSFFDPAGGGDPILYQHL
>COLFF1019-13|KJ962363|ZMUO.007384|Lypoglossa_lateralis
TLYFIFGAWAGMIGTSLSLLIRAELGNPGSLIGDDQIYNVIVTAHAFIMIFFMVMPIVIGGFGNWLVPLMLGAPDMAFPRMNNMSFWLLPPSLTILLMSSMVESGAGTGWTVYPPLSSNIAHGGSSVDLAIFSLHLAGISSILGAVNFISTVINMRSIGITF
DRMPLFVWSVVITALLLLLSLPVLAGAITMLLTDRNLNTSFFDPAGGGDPILYQHL
>COLFD868-12|KJ964247|ZMUO.004478|Eutrichapion_ervi
TLYFIFGLWSGMVGTSLSMLIRVELGNPGSLIGDDQIYNVIVTAHAFIMIFFMVMPVMIGGFGNWLVPLMLGAPDMAFPRMNNMSFWLLPPSLTLLLMSSIVEKGAGTGWTVYPPLASNIAHGGASVDLAIFSLHLAGISSILGAVNFISTMINMPPN
GLSLDQLSLFTWAVKITAILLLLSLPVLAGAITMLLTDRNINTSFFDPAGGGDPILYQHL
>COLFB884-12|KJ965644|ZMUO.001929|Agapanthia_villosoviridescens
TLYFIFGSWAAMLGTSLSILIRSELGNPGTLIGDDQIYNVIVTAHAFVMIFFMVMPIMIGGFGNWLVPLMLGAPDMAFPRMNNMSFWLLPPSLTFLVMSSIVENGAGTGWTVYPPLAGNVAHAGCSVDLAIFSLHLAGISSILGAVNFISTVINMRPEG
MLFDRLPLFVWAVKITAILLLLSLPVLAGGITMLLTDRNINTSFFDPAGGGDPILYQHL
>COLFB541-12|KJ966598|ZMUO.001016|Atheta_vestita
TLYFIFGAWAGMIGTSLSLLIRAELGNPGSLIGDDQIYNVIVTAHAFVMIFFMVMPAMIGGFGNWLVPLMLGAPDMAFPRMNNMSFWLLPPSLTLLLMSSLVESGAGTGWTVYPPLSSNIAHGGSSVDLAIFSLHLAGISSILGAVNFISTVINMRSIGI
TFDRMPLFVWSVVITALLLLLSLPVLAGAITMLLTDRNLNTSFFDPAGGGDPILYQHL
>COLFB274-12|KJ966767|ZMUO.001604|Galerucella_tenella
TLYFIFGIWAGMVGTSLSILVRAELGSPGTLIGNDQIYNVIVTAHAFIMIFFMVMPIMIGGFGNWLVPLMIGAPDMAFPRMNNMSFWLLPPSLFLLIMSSIVESGAGTGWTVYPPLSSNIAHGGSSVDLAIFSLHLAGISSILGAINFITTIINMRPKGMTL
DRMPLFVWAVMITAILLLLSLPVLAGAITMLLTDRNLNTSFFDPAGGGDPILYQHL
>COLFG074-13|KJ965627|ZMUO.007579|Exocentrus_lusitanus
TLYFIFGAWAAMVGTSLSLLIRSELGSPGSLIGDDQIYNVIVTAHAFIMIFFMVMPIMIGGFGNWLIPLMLGAPDMAFPRMNNMSFWLLPPSLSLLIMSSIVENGAGTGWTVYPPLSANIAHSGSSVDLAIFSLHLAGISSILGAVNFITTVINMRPMGM
MFDRLSLFIWAVKITAILLLLSLPVLAGAITMLLTDRNLNTSFFDPAGGGDPILYQHL
>COLFD822-12|KJ963355|ZMUO.004812|Pyrrhalta_viburni
TLYFIFGIWAGMVGTSLSILVRAELGSPGTLIGNDQIYNVIVTAHAFIMIFFMVMPIMIGGFGNWLVPLMIGAPDMAFPRMNNMSFWLLPPSLFLLIMSSIVESGAGTGWTVYPPLSSNIAHGGSSVDLAIFSLHLAGISSILGAINFITTVINMRPKGMTL
DRMPLFVWAVVITAILLLLSLPVLAGAITMLLTDRNLNTSFFDPAGGGDPILYQHL

>COLFB061-12|KJ963553|ZMUO.001391|Nebria_rufescens
TLYFIFGAWAGMVGTSLSMLIRAELGNPGSLIGDDQIYNVIVTAHAFVMIFFMVMPIMIGGFGNWLVPLMLGAPDMAFPRMNNMSFWLLPPSLTLLLMSSMVESGAGTGWTVYPPLSSGIAHSGASVDMAIFSLHLAGVSSILGAVNFITTIINMRS
MGMTFDRMPLFVWSVGITALLLLLSLPVLAGAITMLLTDRNLNTTFFDPAGGGDPILYQHL
>COLFE081-12|KJ963766|ZMUO.005306|Cryptocephalus_quadripustulatus
TLYFLFGAWSGMVGTSLSLLIRVELGNPGSLIGNDQIYNTIVTAHAFIMIFFMVMPIMIGGFGNWLVPLMLGAPDMAFPRMNNMSFWLLPPSLTLLLMSSIVENGAGTGWTVYPPLSSTIAHAGASVDLAIFSLHLAGISSIMGAINFISTVINMRPQG
MLMDRTPLFVWAVLITAILLLLSLPVLAGAITMLLTDRNLNTSFFDPAGGGDPILYQHL
>COLFD945-12|KJ963859|ZMUO.004555|Anthonomus_pomorum
TLYFIFGAWSGTVGTSLSMLIRTELGNPGSLIGDDQIYNTIVTAHAFIMIFFMVMPIMIGGFGNWLVPLMLAAPDMAFPRLNNMSFWLLPPSLTLLIMSSIIGKGAGTGWTVYPPLSSNLAHEGASVDFAIFSLHMAGISSILGAMNFISTVLNMKPMG
MNLEQMPLFVWAVKITAILLLISLPVLAGAITMLLTDRNINTSFFDPAGGGDPILYQHL
>COLFG067-13|KJ964598|ZMUO.007572|Tetropium_castaneum
TLYFIFGAWAGMVGTSLSILIRSELGNPGSLIGNDQIYNVIVTAHAFIMIFFMVMPIMIGGFGNWLVPLMLGAPDMSFPRLNNLSFWFLPPSLILLIMGMIVEKGAGTGWTVYPPLSANIAHSGSSVDLTIFSLHLAGISSILSAINFITTIMNMRPKGMTL
DQMPLFVWAVMITTILLLISLPVLAGAITMLLTDRNINTSFFDPAGGGDPILYQHL
>COLFC809-12|KJ967023|ZMUO.003374|Bembidion_difficile
TLYFIFGAWSGMVGTSLSMLIRAELGNPGSLIGDDQIYNVIVTAHAFIMIFFMVMPILIGGFGNWLVPLMLGAPDMAFPRMNNMSFWLLPPSLSLLLMSSMVESGAGTGWTVYPPLSSSIAHSGSSVDLAIFSLHLAGVSSILGAVNFITTIINMRSTG
MSFDRMPLFVWSVGITALLLLLSLPVLAGAITMLLTDRNLNTTFFDPAGGGDPILYQHL
>COLFD349-12|KJ964245|ZMUO.004244|Stenus_similis
TLYFIFGAWAGMVGTSLSLLIRAELGNPGSLIGDDQIYNVIVTAHAFIMIFFMVMPIMIGGFGNWLVPLMLGAPDMAFPRMNNMSFWLLPPSLSLLLMSSIVESGAGTGWTVYPPLSSNIAHSGASVDLAIFSLHLAGISSILGAINFITTILNMRTMKIQ
LDCLPLFVWSVGITALLLLLSLPVLAGAITMLLTDRNLNTSFFDPAGGGDPILYQHL
>COLFD078-12|KJ962411|ZMUO.003973|Agathidium_seminulum
TLYFIFGAWSGMVGTSLSILIRAELGTPGSLIGDDQIYNVIVTAHAFVMIFFMVMPIVIGGFGNWLVPLMLGAPDMAFPRMNNMSFWLLPPSLTLLLMSSMVESGAGTGWTVYPPLSANISHSGSSVDLAIFSLHLAGISSILGAVNFITTVINMRSIGM
TFDKMPLFVWSVAITALLLLLSLPVLAGAITMLLTDRNFNTSFFDPAGGGDPILYQHL
>COLFA030-10|HM909057|MP00173|Cortinicara_gibbosa
SLYFLFGMWAGMVGTSLSLLIRLELGNPGSLIGDDQIYNVIVTAHAFIMIFFMVMPIMIGGFGNWLVPLMLGAPDMAFPRLNNMSFWLLPPSLSLLIMSSIVESGAGTGWTVYPPLSSNIAHGGSSVDLAIFSLHLAGISSILGAVNFITTVINMRPTGM
SLDKMPLFVWSVVITAILLLLSLPVLAGAITMLLTDRNLNTSFFDPAGGGDPILYQHL
>COLFE406-12|KJ963849|ZMUO.004966|Rhantus_exsoletus
TLYFIFGAWAGMVGTSLSMLIRAELGNPGSLIGDDQIYNVIVTAHAFVMIFFMVMPIMIGGFGNWLVPLMLGAPDMAFPRMNNMSFWLLPPSLTLLLMSSMVESGAGTGWTVYPPLSSGIAHGGASVDLAIFSLHLAGISSILGAVNFITTIINMRSV
GMTFDRMPLFVWSVGITALLLLLSLPVLAGAITMLLTDRNLNTSFFDPAGGGDPILYQHL
>COLFD787-12|KJ965449|ZMUO.004777|Phyllobius_pyri
TLYFIFGAWSGMVGTSLSVLIRIELGNPGSLIGDDQIYNVIVTAHAFIMIFFMVMPMMIGGFGNWLVPLMLGAPDMAFPRLNNMSFWLLPPSLSLLLMSSIVDKGAGTGWTVYPPLSANIAHEGSSVDLAIFSLHMAGVSSILGAINFISTVINMRPMG
MSPDRMSLFIWAVKITAILLLLSLPVLAGAITMLLTDRNMNTSFFDPAGGGDPILYQHL
>COLFF261-13|KJ967068|ZMUO.005866|Notiophilus_palustris
TLYFIFGAWSGMVGTSLSMLIRAELGNPGSLIGDDQIYNVIVTAHAFVMIFFMVMPIMIGGFGNWLVPLMLGAPDMAFPRMNNMSFWLLPPSLTLLLTSSMVESGAGTGWTVYPPLSSGIAHSGASVDLAIFSLHLAGVSSILGAVNFITTIINMRSVG
MTFDRMPLFVWSVGITALLLLLSLPVLAGAITMLLTDRNLNTSFFDPAGGGDPILYQHL
>COLFE1142-13|KJ961948|ZMUO.006937|Colon_appendiculatum
TLYFIFGAWAGMVGTSLSLLIRAELGNPGSLIGDDQIYNVIVTAHAFIMIFFMVMPIVIGGFGNWLVPLMLGAPDMAFPRMNNMSFWLLPPSLTLLLMSSLVESGAGTGWTVYPPLSSNIAHGGSSVDLAIFSLHLAGISSILGAVNFITTVINMRSQGM
GFDQMPLFVWSVIITAFLLLLSLPVLAGAITMLLTDRNLNTSFFDPAGGGDPILYQHL
>COLFB803-12|KJ965668|ZMUO.001278|Eucnecosum_brachypterum
TLYFIFGAWAGMVGTSLSILIRAELGNPGTLIGDDQIYNVIVTAHAFVMIFFMVMPIVIGGFGNWLVPLMLGAPDMAFPRMNNMSFWLLPPSLTLLLMSSMVESGAGTGWTVYPPLSSNIAHGGSSVDLAIFSLHLAGISSILGAVNFITTVINMRTTG
MTFDRMPLFVWSVAITALLLLLSLPVLAGAITMLLTDRNLNTSFFDPAGGGDPILYQHL
>COLFF841-13|KJ966065|ZMUO.006161|Strophosoma_melanogrammum
TLYFIFGAWSGMIGTSLSMLIRTELGNPGSLIGDDQIYNVIVTAHAFIMIFFMVMPMMIGGFGNWLVPLMLGAPDMAFPRMNNMSFWLLPPSLSLLLMSSIVDKGAGTGWTVYPPLSTNIAHEGSSVDLAIFSLHMAGVSSILGAVNFISTVINMRP
MGMSPDRVSLFIWSVKITAVLLLLSLPVLAGAITMLLTDRNVNTSFFDPAGGGDPILYQHL
>COLFD842-12|KJ965643|ZMUO.004832|Dytiscus_marginalis
TLYFIFGAWAGMVGTSLSMLIRAELGNPGSLIGDDQIYNVIVTAHAFVMIFFMVMPIMIGGFGNWLVPLMLGAPDMAFPRMNNMSFWLLPPSLTLLLMSSMVESGAGTGWTVYPPLSASIAHGGASVDLAIFSLHLAGVSSILGAVNFITTIINMRSV
GMTLDRMPLFVWSVGITALLLLLSLPVLAGAITMLLTDRNLNTSFFDPAGGGDPILYQHL
>COLFC647-12|KJ962214|ZMUO.003212|Hydroporus_geniculatus

TLYFLFGAWSGMVGTSLSMLIRAELGNPGSLIGDDQIYNVIVTAHAFIMIFFMVMPIMIGGFGNWLVPLMLGAPDMAFPRMNNMSFWLLPPSLTLLLMSSMVENGAGTGWTVYPPLSSGIAHSGASVDLAIFSLHLAGVSSILGAVNFITTIINMRSI
GMTFDRMPLFVWSVGITALLLLLSLPVLAGAITMLLTDRNLNTSFFDPAGGGDPILYQHL
>COLFE1383-13|KJ965363|ZMUO.007178|Lagria_hirta
TLYFIFGSWAGMVGTSLSLLIRVELSNPGSFIGDDQIYNVVVTAHAFIMIFFMVMPIMIGGFGNWLVPLMLGAPDMAFPRMNNMSFWLLPPSLSLLMSSLVESGAGTGWTVYPPLSSNLAHSGSSVDLAIFSLHLAGISSILGAVNFITTVINMRPLG
MKFDRLPLFVWSVMITAILLLLSLPVLAGAITMLLTDRNINTSFFDPSGGGDPILYQHL
>COLFD928-12|KJ964936|ZMUO.004538|Gyrinus_substriatus
TLYFIFGAWSGMVGTSLSMLIRAELGNPGSLIGDDQIYNVIVTAHAFIMIFFMVMPIMIGGFGNWLVPLMLGAPDMAFPRMNNMSFWLLPPSLTLLLMSSMVENGAGTGWTVYPPLSSNIAHGGASVDLAIFSLHLAGISSILGAVNFITTIINMRSIG
MTLDRMPLFVWSVGITALLLLLSLPVLAGAITMLLTDRNLNTSFFDPAGGGDPILYQHL
>COLFC166-12|KJ965021|ZMUO.002351|Dexiogyia_forticornis
TLYFIFGAWAGMVGTSLSLLIRAELGNPGSLIGDDQIYNVIVTAHAFIMIFFMVMPIVIGGFGNWLVPLMLGAPDMAFPRMNNMSFWLLPPSLTLLLMSSMVESGAGTGWTVYPPLSSNIAHGGSSVDLAIFSLHLAGISSILGAVNFISTIINMRTLGM
SFDRMPLFVWSVAITALLLLLSLPVLAGAITMLLTDRNLNTTFFDPAGGGDPILYQHL
>COLFF529-13|KJ966548|ZMUO.006609|Anisosticta_novemdecimpunctata
TLYFLFGMWAGMVGTSLSMLIRLELGSAGSLIGNDQIYNVIVTAHAFIMIFFMVMPIMIGGFGNWLVPLMIGAPDMAFPRLNNMSFWLLPPSLTLLLMSSMVEMGAGTGWTVYPPLSSNLAHNGSSVDLVIFSLHLAGISSILGAVNFISTIMNMRP
MGMSLDKTPLFVWSVLITAILLLLSLPVLAGAITMLLTDRNLNTSFFDPTGGGDPILYQHL
>COLFE639-13|KJ966941|ZMUO.005484|Cantharis_pellucida
TLYFIFGAWSGSLGLALSLLIRAELGTPGTLIGNDQIYNVIVTAHAFIMIFFMVMPIMIGGFGNWLVPLMLGAPDMAFPRMNNMSFWFLPPSLMFLLMSSMVESGAGTGWTVYPPLSANIAHSGPSVDLAIFSLHMAGISSILGAVNFISTIMNMKPPS
MKFDQMPLFVWSVGITALLLLLSLPVLAGAITMLLSDRNLNTSFFDPMGGGDPILYQHL
>COLFF254-13|KJ966785|ZMUO.005859|Malthodes_spathifer
TLYFMFGAWSGMLGTSLSLLIRAELGSPGSLIGNDQIYNVIVTAHAFIMIFFMVMPIMIGGFGNWLVPLMLGAPDMAFPRMNNMSFWFLPPSLSLLLMSSVVESGAGTGWTVYPPLSSNIAHSGASVDLAIFSLHMAGISSILGAVNFISTVINMRSTG
MTFDRMPLFVWAVAITALLLLLSLPVLAGAITMLLTDRNLNTSFFDPAGGGDPILYQHL
>COLFA398-12|KJ962726|ZMUO.000628|Melanotus_castanipes
TLYFIFGAWAGMLGTSLSLLIRAELGNPGSLIGNDQIYNVIVTAHAFIMIFFMVMPIMIGGFGNWLVPLMLGAPDMAFPRMNNMSFWLLPPSLSLLLMSSIVENGAGTGWTVYPPLSSNIAHSGSSVDLAIFSLHLAGISSILGAVNFISTVINMRSTGM
TFDRMPLFVWAVALTALLLLLSLPVLAGAITMLLTDRNLNTSFFDPAGGGDPILYQHL
>COLFB948-12|KJ961981|ZMUO.001993|Atheta_aeneipennis
TLYFIFGAWAGMVGTSLSLLIRAELGNPGSLIGDDQIYNVIVTAHAFIMIFFMVMPIVIGGFGNWLVPLMLGAPDMAFPRMNNMSFWLLPPSLTLLLMSSMVESGAGTGWTVYPPLSSNIAHGGSSVDLAIFSLHLAGISSILGAVNFISTVINMRSTGI
SFDRMPLFVWSVAITALLLLLSLPVLAGAITMLLTDRNLNTSFFDPAGGGDPILYQHL
>COLFD754-12|KJ963138|ZMUO.004744|Schizotus_pectinicornis
TLYFILGSWAGMIGTSMSLLIRTELGNSGSLIGDDQIYNVIVTAHAFIMIFFMVMPIMIGGFGNWLVPLMLGAPDMAFPRMNNMSFWLLPPSLTLLIMSSIVENGAGTGWTVYPPLSSNIAHSGSSVDLAIFSLHLAGISSILGAVNFITTVINMRPIQMT
LDKMPLFVWAVVITAVLLLSLPVLAGAITMLLTDRNLNTTFFDPIGGGDPILYQHL
>COLFB420-12|KJ962448|ZMUO.001845|Dorcatoma_robusta
TMYFIFGAWSGMIGTSMSILIRSELGNPGALIGNDQVYNVIVTAHAFVMIFFMVMPIMIGGFGNWLVPLMLGAPDMAFPRMNNMSFWLLPPSISLLLLSSAVNNGAGTGWTVYPPLSSNIAHSGASVDLAIFSLHLAGISSILGAVNFISTVINMRAN
GMSFDKMPLFVWSVAITALLLLLSLPVLAGAITMLLTDRNLNTSFFDPAGGGDPILYQHL
>COLFE441-12|KJ962174|ZMUO.005001|Batophila_rubi
TLYFIFGAWAGMVGTSLSMLIRTELGNPGSLIGNDQIYNVIVTAHAFIMIFFMVMPIMIGGFGNWLVPLMIGAPDMAFPRMNNMSFWLLPPSLSLLVSSSIVESGAGTGWTVYPPLSSNIAHSGSSVDLAIFSLHLAGISSILGAINFISTIINMRPHGML
LDRLPLFVWAVMITAILLLLSLPVLAGAITMLLTDRNLNTSFFDPAGGGDPILYQHL
>COLFC641-12|KJ966009|ZMUO.003206|Hygrotus_novemlineatus
TLYFLFGAWSGMVGTSLSMLIRAELGNPGSLIGDDQIYNVIVTAHAFIMIFFMVMPIMIGGFGNWLVPLMLGAPDMAFPRMNNMSFWMLPPSLTLLLMSSMVESGAGTGWTVYPPLSAGIAHGGASVDLAIFSLHLAGISSILGAVNFITTIINMRSV
GMTFDRMPLFVWSVGITALLLLLSLPVLAGAITMLLTDRNLNTSFFDPAGGGDPILYQHL
>COLFD728-12|KJ962570|ZMUO.004718|Atheta_subtilis
TLYFIFGAWAGMVGTSLSLLIRAELGNPGSLIGDDQIYNVIVTAHAFIMIFFMVMPVVIGGFGNWLVPLMLGAPDMAFPRMNNMSFWLLPPSLTLLLMSSMVESGAGTGWTVYPPLSSNIAHGGSSVDLAIFSLHLAGISSILGAVNFISTVINMRSTGI
SFDRMPLFVWSVAITALLLLLSLPVLAGAITMLLTDRNLNTSFFDPAGGGDPILYQHL
>COLFE044-12|KJ965876|ZMUO.005269|Acanthocinus_aedilis
TLYFIFGAWSGMVGTSLSMLIRSELGNPGTLIGNDQIYNTIVTAHAFIMIFFMVMPIMIGGFGNWLIPLMLGAPDMAFPRMNNMSFWLLPPSLTLLIMSSIVENGAGTGWTVYPPLSSNIAHSGSSVDLAIFSLHLAGISSILGAVNFITTIINMRPMGM
TFDRMPLFVWAVKITAILLLLSLPVLAGAITMLLTDRNINTSFFDPAGGGDPILYQHL
>COLFC388-12|KJ965526|ZMUO.002953|Tachinus_pallipes
TLYFIFGAWAGMVGTSLSLLIRAELGNPGTLIGDDQIYNVIVTAHAFIMIFFMVMPIVIGGFGNWLVPLMLGAPDMAFPRMNNMSFWLLPPSLTLLLMSSMVESGAGTGWTVYPPLSSNIAHGGSSVDLAIFSLHLAGISSILGAVNFITTVINMRSIG
MTFDRMPLFVWSVAITALLLLLSLPVLAGAITMLLTDRNLNTTFFDPAGGGDPILYQHL

>COLFD743-12|KJ964478|ZMUO.004733|Bembidion_grapii
TLYFIFGAWSGMVGTSLSMLIRAELGNPGSLIGDDQIYNVIVTAHAFVMIFFMVMPILIGGFGNWLVPLMLGAPDMAFPRMNNMSFWLLPPSLSLLLMSSMVESGAGTGWTVYPPLSSSIAHSGASVDLAIFSLHLAGVSSILGAVNFITTIINMRSIG
MSFDRMPLFVWSVGITALLLLLSLPVLAGAITMLLTDRNLNTSFFDPAGGGDPILYQHL
>COLFD450-12|KJ966594|ZMUO.003870|Rhinoncus_pericarpius
TLYFIFGSWAGTVGTSLSMLIRTELGTPGSLIGNDQIYNSIVTAHAFIMIFFMVMPILIGGFGNWLVPLMLGAPDMAFPRLNNMSFWLLPPSIMLLLMSSIINKGAGTGWTVYPPLSSNITHEGASVDLAIFSLHMAGISSILGAINFISTIMNMRPQGMS
YDKMPLFSWAVLITAILLLLSLPVLAGAITMLLTDRNINTSFFDPAGGGDPILYQHL
>COLFD265-12|KJ966769|ZMUO.004160|Coelostoma_orbiculare
TLYFIFGAWAGMLGTSLSILIRTELGNPGSLIGNDQIYNVIVTAHAFIMIFFMVMPIMIGGFGNWLVPLMLGAPDMAFPRMNNMSFWLLPPSLTLLLMSSMVESGVGTGWTVYPPLSANIAHSGASVDLAIFSLHLAGISSILGAVNFITTVINMRSSN
MTYDRMPLFVWSVAITALLLLLSLPVLAGAITMLLTDRNFNTSFFDPAGGGDPILYQHL
>COLFF270-13|KJ963209|ZMUO.005875|Rhagonycha_lutea
TLYFIFGAWSGSLGLALSLLIRAELGTPGTLIGNDQIYNVIVTAHAFIMIFFMVMPIMIGGFGNWLVPLMLGAPDMAFPRMNNMSFWFLPPSLMFLLMSSMVESGAGTGWTVYPPLSANIAHSGPSVDLAIFSLHMAGISSILGAVNFISTILNMKPPS
MKFDQMPLFVWSVGITALLLLLSLPVLAGAITMLLSDRNLNTSFFDPMGGGDPILYQHL
>COLFB263-12|KJ964785|ZMUO.001593|Gabrius_toxotes
TLYFIFGSWAGMVGTSLSLLIRAELGNPGTLIGDDQIYNVIVTAHAFIMIFFMVMPIVIGGFGNWLVPLMLGAPDMAFPRMNNMSFWLLPPSLTLLLMSSMVESGAGTGWTVYPPLSSNIAHGGASVDLAIFSLHLAGISSILGAVNFITTVINMRSFG
MSFDRMPLFVWSVAITALLLLLSLPVLAGAITMLLTDRNLNTSFFDPAGGGDPILYQHL
>COLFB682-12|KJ964336|ZMUO.001157|Amischa_decipiens
TLYFIFGAWAGMVGTSLSLLIRAELGNPGSLIGDDQIYNVIVTAHAFIMIFFMVMPIVIGGFGNWLVPLMLGAPDMAFPRMNNMSFWLLPPSLTLLLMSSMVESGAGTGWTVYPPLSSNIAHGGSSVDLAIFSLHLAGISSILGAVNFISTVINMRSTGI
SFDRMPLFVWSVAITALLLLLSLPVLAGAITMLLTDRNLNTSFFDPAGGGDPILYQHL
>COLFA343-12|KJ964642|ZMUO.000573|Atheta_melanocera
TLYFIFGAWAGMVGTSLSLLIRAELGNPGSLIGDDQIYNVIVTAHAFIMIFFMVMPIVIGGFGNWLVPLMLGAPDMAFPRMNNMSFWLLPPSLTLLLMSSMVESGAGTGWTVYPPLSSNIAHGGSSVDLAIFSLHLAGISSILGAVNFISTVINMRSTGI
SFDRMPLFVWSVAITALLLLLSLPVLAGAITMLLTDRNLNTSFFDPAGGGDPILYQHL
>COLFC308-12|KJ963793|ZMUO.002493|Stenus_niveus
TLYFIFGVWAGMLGTSLSLLIRAELGNPGSLIGDDQIYNVIVTAHAFIMIFFMVMPIMIGGFGNWLVPLMLGAPDMAFPRMNNMSFWLLPPSLSLLLMSSVVESGAGTGWTVYPPLSSNIAHGGASVDLAIFSLHLAGVSSILGAINFITTIFNMRTMQI
QLDCLPLFVWSVGITALLLLLSLPVLAGAITMLLTDRNLNTSFFDPAGGGDPVLYQHL
>COLFC139-12|KJ966770|ZMUO.002324|Atheta_crassicornis
TLYFIFGAWAGMVGTSLSLLIRAELGNPGSLIGDDQIYNVIVTAHAFVMIFFMVMPIVIGGFGNWLIPLMLGAPDMAFPRMNNMSFWLLPPSLTLLLMSSMVESGAGTGWTVYPPLSSNIAHGGSSVDLAIFSLHLAGISSILGAVNFISTVINMRSVGI
SFDRMPLFVWSVTITALLLLLSLPVLAGAITMLLTDRNLNTSFFDPAGGGDPILYQHL
>COLFD052-12|KJ967128|ZMUO.003947|Anthophagus_angusticollis
TLYFIFGAWAGMVGTSLSILIRSELGNPGSLIGDDQIYNVIVTAHAFVMIFFMVMPVVIGGFGNWLVPLMLGAPDMAFPRMNNMSFWLLPPSLTLLLMSSMVESGAGTGWTVYPPLSSNIAHSGSSVDLAIFSLHLAGISSILGAVNFITTVINMRSTGI
TFDRMPLFVWSVAITALLLLLSLPVLAGAITMLLTDRNLNTSFFDPAGGGDPILYQHL
>COLFB838-12|KJ961759|ZMUO.001313|Tachinus_laticollis
TLYFIFGAWAGMVGTSLSLLIRAELGNPGTLIGDDQIYNVIVTAHAFIMIFFMVMPIVIGGFGNWLVPLMLGAPDMAFPRMNNMSFWLLPPSLSLLLMSSMVESGAGTGWTVYPPLSSNIAHGGSSVDLAIFSLHLAGISSILGAVNFITTVINMRSIGM
SFDRMPLFVWSVAITALLLLLSLPVLAGAITMLLTDRNLNTTFFDPAGGGDPILYQHL
>COLFC779-12|KJ965017|ZMUO.003344|Cafius_xantholoma
TLYFIFGSWAGMVGTSLSLLIRAELGNPGTLIGDDQIYNVIVTAHAFIMIFFMVMPIVIGGFGNWLVPLMLGAPDMAFPRMNNMSFWLLPPSLTLLLMSSMVESGAGTGWTVYPPLSSNIAHGGASVDLAIFSLHLAGISSILGAVNFITTVINMRSTG
MTFDRMPLFVWSVAITALLLLLSLPVLAGAITMLLTDRNLNTSFFDPAGGGDPILYQHL
>COLFF074-13|KJ965462|ZMUO.005774|Bromius_obscurus
TLYFIFGAWSGMVGTSLSLMIRIELGSPGSLIGNDQIYNTIVTAHAFIMIFFMVMPIMIGGFGNWLVPLMLGAPDMAFPRMNNMSFWLLPPSLSLLIMSSIVESGVGTGWTVYPPLSSNIAHSGASVDLGIFSLHLAGISSILGAVNFISTVINMRPAGMI
LDRTPLFVWAVVITAILLLLSLPVLAGAITMLLTDRNLNTSFFDPSGGGDPILYQHL
>COLFA045-10|HM909069|MP00210|Attagenus_pellio
TLYFILGAWAGMVGTSLSMLIRTELGNPGSLIGDDQIFNVIVTAHAFIMIFFMVMPIMIGGFGNWLVPLMLGAPDMAFPRMNNMSFWLLPPSLSLLLMSSMVESGAGTGWTVYPPLAANIAHSGASVDLAIFSLHLAGISSILGAVNFITTAINMRSP
GMTADQMPLFVWSVAITALLLLLSLPVLAGAITMLLTDRNLNTSFFDPAGGGDPILYQHL
>COLFA445-12|KJ963263|ZMUO.000485|Ctenicera_pectinicornis
TLYFLFGAWAGMLGTSLSLLIRAELGNPGSLIGNDQIYNVIVTAHAFIMIFFMVMPIMIGGFGNWLVPLMLGAPDMAFPRMNNMSFWFLPPSLSLLLMSSIVENGAGTGWTVYPPLSANIAHSGSSVDLAIFSLHLAGISSILGAVNFISTVINMRSTGIT
FDRMPLFVWAVAITALLLLLSLPVLAGAITMLLTDRNLNTSFFDPAGGGDPILYQHL
>COLFF260-13|KJ964120|ZMUO.005865|Agonum_emarginatum

TLYFIFGAWSGMVGTSLSMLIRAELGNPGALIGDDQIYNVIVTAHAFIMIFFMVMPIMIGGFGNWLVPLMLGAPDMAFPRMNNMSFWLLPPSLTLLLMSSMVESGAGTGWTVYPPLSSGIAHAGASVDLAIFSLHLAGVSSILGAVNFITTIINMRSV
GMTFDRMPLFVWSVGITALLLLLSLPVLAGAITMLLTDRNLNTSFFDPAGGGDPILYQHL
>COLFB642-12|KJ961725|ZMUO.001117|Atheta_malleus
TLYFIFGAWAGMVGTSLSLLIRAELGNPGSLIGDDQIYNVIVTAHAFIMIFFMVMPIVIGGFGNWLVPLMLGAPDMAFPRMNNMSFWLLPPSLTLLLMSSMVESGAGTGWTVYPPLSSNIAHGGSSVDLAIFSLHLAGISSILGAVNFISTVINMRSTGI
SFDRMPLFVWSVAITALLLLLSLPVLAGAITMLLTDRNLNTSFFDPAGGGDPILYQHL
>COLFE1072-13|KJ961803|ZMUO.006867|Lagria_hirta
TLYFIFGSWAGMVGTSLSLLIRVELSNPGSFIGDDQIYNVVVTAHAFIMIFFMVMPIMIGGFGNWLVPLMLGAPDMAFPRMNNMSFWLLPPSLSLLLMSSLVESGAGTGWTVYPPLSSNLAHSGSSVDLAIFSLHLAGISSILGAVNFITTVINMRPLG
MKFDRLPLFVWSVMITAILLLLSLPVLAGAITMLLTDRNINTSFFDPSGGGDPILYQHL
>COLFD856-12|KJ963004|ZMUO.004466|Necrodes_littoralis
TLYFIFGAWAGMVGMSLSILIRAELSTPGTLLGDDQMYNVIVTAHAFIMIFFMVMPIVIGGFGNWLVPLMLGAPDMAFPRMNNMSFWLLPPSLSLLVSSMVESGAGTGWTVYPPLSSNIAHGGSSVDLAIFSLHLAGISSILGAVNFITTIINMRSSG
MTFDRMPLFVWSVAITALLLLLSLPVLAGAITMLLTDRNLNTSFFDPAGGGDPILYQHL
>COLFD237-12|KJ963412|ZMUO.004132|Bledius_subterraneus
TLYFMFGAWAGMVGTSLSMLIRAELGTPGSLIGDDQIYNVIVTAHAFVMIFFMVMPIVIGGFGNWLIPLMLGAPDMAFPRMNNMSFWLLPPSLTLLLMSSMVESGVGTGWTVYPPLSSNIAHSGSSVDLAIFSLHLAGISSILGAVNFISTIINMRSIG
MTFDRMPLFVWSVKITAILLLLSLPVLAGAITMLLTDRNLNTSFFDPAGGGDPILYQHL
>COLFF102-13|KJ965392|ZMUO.003736|Dorcatoma_robusta
TMYFIFGAWSGMIGTSMSILIRSELGNPGALIGNDQVYNVIVTAHAFVMIFFMVMPIMIGGFGNWLVPLMLGAPDMAFPRMNNMSFWLLPPSISLLLLSSAVNNGAGTGWTVYPPLSSNIAHSGASVDLAIFSLHLAGISSILGAVNFISTVINMRAN
GMSFDKMPLFVWSVAITALLLLLSLPVLAGAITMLLTDRNLNTSFFDPAGGGDPILYQHL
>COLFB337-12|KJ963167|ZMUO.001667|Bolitochara_pulchra
TLYFIFGAWAGMVGTSLSLLIRAELGNPGSLIGDDQIYNVIVTAHAFVMIFFMVMPIMIGGFGNWLVPLMLGAPDMAFPRMNNMSFWLLPPSLTLLLMSSMVESGAGTGWTVYPPLSSNIAHGGSSVDLAIFSLHLAGISSILGAVNFISTIINMRSPG
MTFDRMPLFVWSVAITALLLLLSLPVLAGAITMLLTDRNLNTSFFDPAGGGDPILYQHL
>COLFE1515-13|KJ964097|ZMUO.007310|Brachypterolus_pulicarius
TLYFIFGAWSGMVGTSLSLLIRSELGSPGSLIGDDQIYNVIVTAHAFVMIFFMVMPFMIGGFGNWLVPLMLGAPDMAFPRMNNMSFWLLPPSLFLLLMSSIIEKGAGTGWTVYPPLSGNMAHGGSSVDLAIFSLHLAGISSILGAVNFITTIINMRPSG
MMFDQMPLFVWAVVITALLLLLSLPVLAGAITMLLTDRNLNTSFFDPAGGGDPVLYQHL
>COLFE1200-13|KJ966590|ZMUO.006995|Tetartopeus_terminatus
TLYFIFGAWAGMVGTSLSLLIRTELANPGSLIGDDQIYNVIVTAHAFVMIFFMVMPIVIGGFGNWLVPLMLGAPDMAFPRMNNMSFWLLPPSLSLLLASSMVESGAGTGWTVYPPLSSNIAHGGASVDLAIFSLHLAGISSILGAVNFITTIINMRSPGM
LYERMPLFVWSVGITALLLLLSLPVLAGAITMLLTDRNLNTSFFDPAGGGDPILYQHL
>COLFA103-10|HQ559230|MP00361|Corticarina_minuta
SLYFLFGMWAGMVGTSLSLLIRLELGNPGSLIGDDQVYNVIVTAHAFIMIFFMVMPIMIGGFGNWLVPLMLGAPDMAFPRLNNMSFWLLPPSLSLLIMSSIVESGAGTGWTVYPPLSSNIAHGGSSVDLAIFSLHLAGISSILGAVNFITTVINMRPAGM
MLDQMPLFVWSVVITAILLLLSLPVLAGAITMLLTDRNLNTSFFDPAGGGDPILYQHL
>COLFF883-13|KJ961790|ZMUO.006678|Pytho_kolwensis
TLYFIFGAWSGMVGTSLSLLIRAELGNPGSLIGDDQIYNVIVTAHAFIMIFFMVMPTVIGGFGNWLVPLMLGAPDMAFPRMNNMSFWLLPPSLSLLIMSSIVENGAGTGWTVYPPLASNIAHGGSSVDLAIFSLHLAGISSILGAVNFITTVINMRPSGM
TFDRMPLFVWSVVITAVLLLLSLPVLAGAITMLLTDRNLNTSFFDPAGGGDPILYQHL
>COLFF406-13|KJ961816|ZMUO.005924|Temnocerus_tomentosus
TLYFIFGAWSGMVGTSLSILIRAELGSPGSLIGDDQIYNVIVTAHAFIMIFFMVMPIMIGGFGNWLVPLMLGAPDMAFPRMNNMSFWLLPPSLSLLIMSSIVESGAGTGWTVYPPLSSNIAHGGSSVDLAIFSLHLAGISSILGAVNFISTVINMRPMGM
NLDRMPLFVWAVAITALLLLLSLPVLAGAITMLLTDRNINTSFFDPAGGGDPILYQHL
>COLFA354-12|KJ962160|ZMUO.000584|Lythraria_salicariae
TLYFIFGGWSGMIGTSLSMLIRTELGNPGSLIGNDQIYNVIVTAHAFIMIFFMVMPIMIGGFGNWLIPLMIGAPDMAFPRMNNMSFWLLPPALFLLIMSSIVESGAGTGWTVYPPLSSNIAHGGPSVDLAIFSLHLAGISSILGAINFITTVINMRPMGMS
LDQMPLFVWSVIITAILLLLSLPVLAGAITMLLTDRNLNTSFFDPAGGGDPILYQHL
>COLFF399-13|KJ966956|ZMUO.005917|Chrysanthia_viridissima
TLYLIFGAWAGMVGTSLSLLIRTELSNPGSLIGDDQIYNVIVTAHAFIMIFFMVMPIMIGGFGNWLVPLMLGAPDMAFPRMNNMSFWLLPPSLTLLIMSSIVENGAGTGWTVYPPLSSNIAHSGSSVDLAIFSLHLAGISSILGAVNFITTIINMRPAGMT
LDRMPLFVWAVVITAVLLLLSLPVLAGAITMLLTDRNLNTSFFDPAGGGDPILYQHL
>COLFB289-12|KJ964562|ZMUO.001619|Dictyoptera_aurora
TLYFILGAWAGMMGTALSLLIRAELGTPGTLIGNDQIYNVIVTAHAFIMIFFMVMPMMIGGFGNWLIPLMLGAPDMAYPRMNNMSYWLLPPALSLLTMGSMIESGAGTGWTVYPPLASNIAHSGGSVDLTIFSLHLAGISSILGAVNFISTIINMKSKE
MTFEQMPLFVWAVGITALLLLLSLPVLAGAITMLLTDRNLNTSFFDPMGGGDPILYQHL
>COLFD262-12|KJ962089|ZMUO.004157|Propylea_quatuordecimpunctata
TLYFLFGMWAGMVGTSLSILIRLELGTTNSLIGNDQIYNVIVTAHAFIMIFFMVMPIMIGGFGNWLVPLMIGAPDMAFPRLNNMSFWLLPPALMLLIMSSMVEMGAGTGWTVYPPLSSNMAHSGSSVDLVIFSLHLAGISSILGAVNFISTIMNMRPF
GMNLDKTPLFVWSVLITAILLLLSLPVLAGAITMLLTDRNINTSFFDPMGGGDPILYQHL

>COLFB943-12|KJ964134|ZMUO.001988|Ischnosoma_longicorne
TLYFIFGAWAGMVGTSLSLLIRAELGNPGSLIGDDQIYNVIVTAHAFVMIFFMVMPIVIGGFGNWLVPLMLGAPDMAFPRMNNMSFWLLPPSLSLLLMSSLVESGAGTGWTVYPPLSSNIAHGGASVDLAIFSLHLAGISSILGAVNFITTIINMRSTGM
TFDRMPLFVWSVVITAFLLLLSLPVLAGAITMLLTDRNLNTSFFDPAGGGDPILYQHL
>COLFE1388-13|KJ963796|ZMUO.007183|Nedyus_quadrimaculatus
TLYFIFGTWAAMAGTSLSMLIRTELGNPGSLIGNDQIYNSIVTAHAFIMIFFMVMPILIGGFGNWLVPLMLGAPDMAFPRLNNMSFWLLPPALSLLLMSSIVNKGAGTGWTVYPPLSTNTAHEGMSVDLAIFSLHLAGLSSILGAINFISTVMNMRPQG
MSPEFTPLFVWAVKITAILLLLSLPVLAGAITMLLTDRNINTSFFDPSGGGDPILYQHL
>COLFE237-12|KJ967419|ZMUO.005082|Agonum_hypocrita
TLYFIFGAWSGMVGTSLSMLIRAELGNPGALIGDDQIYNVIVTAHAFIMIFFMVMPIMIGGFGNWLVPLMLGAPDMAFPRMNNMSFWLLPPSLTLLLMSSMVESGAGTGWTVYPPLSSGIAHAGASVDLAIFSLHLAGVSSILGAVNFITTIINMRSV
GMTFDRMPLFVWSVGITALLLLLSLPVLAGAITMLLTDRNLNTSFFDPAGGGDPILYQHL
>COLFC177-12|KJ962757|ZMUO.002362|Sciaphilus_asperatus
TLYFIFGSWSGMIGTSLSMLIRTELGNPGSLIGDDQIYNVIVTAHAFIMIFFMVMPIMIGGFGNWLVPLMLGAPDMAFPRLNNMSFWLLPPSLSLLLMSSIVDKGAGTGWTVYPPLSANIAHEGSSVDLAIFSLHMAGVSSILGAINFISTVINMRPLGM
TPERMPLFVWAVKITAILLLLSLPVLAGAITMLLTDRNVNTSFFDPAGGGDPILYQHL
>COLFB226-12|KJ965542|ZMUO.001556|Sphaeriestes_stockmanni
TLYFIFGSWSGMVGTSLSLLIRAELGNPGSLIGDDQTYNVIVTAHAFIMIFFMVMPIVIGGFGNWLVPLMLGAPDMAFPRMNNMSFWLLPPSLTLLIMSSIVESGAGTGWTVYPPLAANIAHSGPSVDLAIFSLHLAGISSILGAVNFITTVINMRPSGM
SFDRMPLFVWAVVITAVLLLLSLPVLAGAITMLLTDRNLNTSFFDPAGGGDPILYQHL
>COLFB685-12|KJ964329|ZMUO.001160|Myllaena_intermedia
TLYFIFGAWAGMIGSSLSLLIRAELGNPGSLIGDDQIYNVIVTAHAFIMIFFMVMPIVIGGFGNWLVPLMLGAPDMAFPRMNNMSFWLLPPSLSLLLMSSMVESGAGTGWTVYPPLSANIAHSGPSVDLAIFSLHLAGISSILGAVNFISTIINMRSTGMS
FDRMPLFVWSVGITALLLLLSLPVLAGAITMLLTDRNLNTSFFDPAGGGDPILYQHL
>COLFB741-12|KJ967012|ZMUO.001216|Lythraria_salicariae
TLYFIFGGWSGMIGTSLSMLIRTELGNPGSLIGNDQIYNVIVTAHAFIMIFFMVMPIMIGGFGNWLIPLMIGAPDMAFPRMNNMSFWLLPPALFLLIMSSIVESGAGTGWTVYPPLSSNIAHGGPSVDLAIFSLHLAGISSILGAINFITTVINMRPMGMS
LDQMPLFVWSVIITAILLLLSLPVLAGAITMLLTDRNLNTSFFDPAGGGDPILYQHL
>COLFE235-12|KJ967405|ZMUO.005080|Badister_dilatatus
TLYFIFGAWAGMVGTSLSMLIRAELGNPGSLIGDDQIYNVIVTAHAFVMIFFMVMPIMIGGFGNWLVPLMLGAPDMAFPRMNNMSFWLLPPSLTLLLMSSLVESGAGTGWTVYPPLSSGIAHSGASVDLAIFSLHLAGISSILGAVNFITTIINMRSVGI
SFDRMPLFVWSVGITALLLLLSLPVLAGAITMLLTDRNLNTSFFDPAGGGDPILYQHL
>COLFC428-12|KJ966987|ZMUO.002993|Aleochara_brundini
TLYFIFGAWAGMMGTSLSLLIRAELGNPGSLIGDDQIYNVIVTAHAFIMIFFMVMPIIIGGFGNWLVPLMLGAPDMAFPRMNNMSFWLLPPSLTLLLMSSMVESGAGTGWTVYPPLSSNMAHGGSSVDLAIFSLHLAGISSILGAVNFISTVINMRSM
GMTFDKMPLFVWSVAITALLLLLSLPVLAGAITMLLTDRNLNTSFFDPAGGGDPILYQHL
>COLFC239-12|KJ962330|ZMUO.002424|Quedius_limbatus
TLYFIFGAWAGMVGTSLSLLIRAELGNPGTLIGDDQIYNVIVTAHAFIMIFFMVMPIVIGGFGNWLVPLMLGAPDMAFPRMNNMSFWLLPPSLSLLLMSSMVESGAGTGWTVYPPLSSNIAHGGASVDLAIFSLHLAGISSILGAVNFITTVINMRSIG
MSFDRMPLFVWSVAITALLLLLSLPVLAGAITMLLTDRNLNTSFFDPAGGGDPILYQHL
>COLFF1025-13|KJ962595|ZMUO.007390|Psylliodes_dulcamarae
TLYFIFGIWSGMVGTSLSILIRTELGSPGSLIGNDQIYNVIVTAHAFIMIFFMVMPIMIGGFGNWLVPLMIGAPDMAFPRMNNMSFWLLPPSLILLLMSSMVESGAGTGWTVYPPLSSNMAHGGPSVDLAIFSLHLAGISSILGAINFITTVINMRPEGM
MLDRMPLFVWAVIITAILLLLSLPVLAGAITMLLTDRNLNTSFFDPIGGGDPILYQHL
>COLFC060-12|KJ966161|ZMUO.002055|Hypocaccus_rugifrons
TLYFIFGMWAGMVGTSLSLLIRAELGNPGSLIGDDQIYNVIVTSHAFIMIFFMVMPIMIGGFGNWLVPLMLGAPDMAFPRMNNMSFWFLPPSLTLLLMSSMVESGAGTGWTVYPPLSSNIAHSGASVDLAIFSLHLAGISSILGAVNFITTVINMRSNG
MKFDQMPLFVWSVSITALLLLLSLPVLAGAITMLLTDRNINTSFFDPAGGGDPILYQHL
>COLFF783-13|KJ963510|ZMUO.006103|Galerucella_lineola
TLYFIFGVWAGMVGTSLSILVRAELGSPGTLIGNDQIYNVIVTAHAFIMIFFMVMPIMIGGFGNWLVPLMIGAPDMAFPRMNNMSFWLLPPSLFLLIMSSIVESGAGTGWTVYPPLSSNIAHGGSSVDLAIFSLHLAGISSILGAINFITTIINMRPKGMTL
DRMPLFVWAVMITAILLLLSLPVLAGAITMLLTDRNLNTSFFDPAGGGDPILYQHL
>COLFE1242-13|KJ964601|ZMUO.007037|Tachinus_pallipes
TLYFIFGAWAGMVGTSLSLLIRAELGNPGTLIGDDQIYNVIVTAHAFIMIFFMVMPIVIGGFGNWLVPLMLGAPDMAFPRMNNMSFWLLPPSLTLLLMSSMVESGAGTGWTVYPPLSSNIAHGGSSVDLAIFSLHLAGISSILGAVNFITTVINMRSIG
MTFDRMPLFVWSVAITALLLLLSLPVLAGAITMLLTDRNLNTTFFDPAGGGDPILYQHL
>COLFB508-12|KJ962016|ZMUO.000983|Atheta_laticollis
TLYFIFGAWAGMVGTSLSLLIRAELGNPGSLIGDDQIYNVIVTAHAFIMIFFMVMPIVIGGFGNWLVPLMLGAPDMAFPRMNNMSFWLLPPSLTLLLMSSMVESGAGTGWTVYPPLSSNIAHGGSSVDLAIFSLHLAGISSILGAVNFISTVINMRSVGI
TFDRMPLFVWSVAITALLLLLSLPVLAGAITMLLTDRNLNTSFFDPAGGGDPILYQHL
>COLFD603-12|KJ966820|ZMUO.004403|Mordellochroa_abdominalis

TLYFIFGAWSGMVGTSLSLLIRTELGTPGSLIGDDQIYNVIVTAHAFVMIFFMVMPVMMGGFGNWLVPLMLGAPDMAFPRMNNMSFWLLPPSLTLLLLSSLVESGAGTGWTVYPPLSANIAHGGASVDLAIFSLHLAGISSILGAINFISTMLNMRPA
GMTLDRMPLLIWAIMITAILLLLSLPVLAGAITMLLTDRNLNTSFFDPAGGGDPILYQHL
>COLFF992-13|KJ964340|ZMUO.007357|Aphodius_fasciatus
TLYFLFGSWAGMVGTSLSLLIRAELGNPGTLIGDDQIYNVIVTAHAFVMIFFMVMPILIGGFGNWLVPLMLGAPDMAFPRMNNMSFWLLPPSLTLLLMSSMVESGAGTGWTVYPPLSSNIAHGGASVDLAIFSLHLAGISSILGAVNFITTVINMRSPG
MTFDRMPLFVWSVAITALLLLLSLPVLAGAITMLLTDRNLNTSFFDPAGGGDPILYQHL
>COLFE1456-13|KJ966862|ZMUO.007251|Aegialia_arenaria
TLYFLFGGWAGMVGTSLSLLIRAELGNPGMLIGDDQIYNVIVTAHAFVMIFFMVMPILIGGFGNWLVPLMLGAPDMAFPRMNNMSFWLLPPSLTLLLMSSMVESGAGTGWTVYPPLSSNIAHSGPSVDLAIFSLHLAGISSILGAVNFITTVINMRSIG
MSFDRMPLFVWSVALTALLLLLSLPVLAGAITMLLTDRNINTSFFDPTGGGDPILYQHL
>COLFE337-12|KJ962106|ZMUO.004897|Anisosticta_novemdecimpunctata
TLYFLFGMWAGMVGTSLSMLIRLELGSAGSLIGNDQIYNVIVTAHAFIMIFFMVMPIMIGGFGNWLVPLMIGAPDMAFPRLNNMSFWLLPPSLTLLLMSSMVEMGAGTGWTVYPPLSSNLAHNGSSVDLVIFSLHLAGISSILGAVNFISTIMNMRP
MGMSLDKTPLFVWSVLITAILLLLSLPVLAGAITMLLTDRNLNTSFFDPTGGGDPILYQHL
>COLFE1094-13|KJ963205|ZMUO.006889|Temnocerus_nanus
TLYFIFGAWSGMVGTSLSILIRAELGSPGSLIGDDQIYNVIVTAHAFIMIFFMVMPIMIGGFGNWLVPLMLGAPDMAFPRMNNMSFWLLPPSLSLLIMSSIVESGAGTGWTVYPPLSSNIAHGGSSVDLAIFSLHLAGISSILGAVNFISTVINMRPMGM
NLDRMPLFVWAVAITALLLLLSLPVLAGAITMLLTDRNINTSFFDPAGGGDPILYQHL
>COLFF224-13|KJ961812|ZMUO.005829|Mecinus_pascuorum
TLYFIFGTWSGMMGTSMSMIIRTELGNPGKFIGNDQIYNSIVTAHAFIMIFFMVMPIMIGGFGNWLVPLMMGAPDMAFPRLNNLSFWLLPPSITMLLLSSITEKGAGTGWTVYPPLSTNIAHEGTSVDLAIFSLHMAGISSILGAMNFISTVMNMRPL
SMKLDQMPLFVWSVKITAILLLLSLPVLASAITMLLTDRNINTSFFDPAGGGDPILYQHL
>COLFA008-10|HM909038|MP00136|Hydraena_gracilis
TLYFIFGGWAGMVGTSLSMLIRAELGNPGTLIGDDQIYNVIVTAHAFIMIFFMVMPILIGGFGNWLVPLMLGAPDMAFPRMNNMSFWLLPPSLSLLLMSSMVENGAGTGWTVYPPLSANIAHGGASVDLAIFSLHLAGISSILGAVNFITTIINMRSPG
MSFDQMPLFVWAVGITALLLLLSLPVLAGAITMLLTDRNLNTSFFDPAGGGDPILYQHL
>COLFE251-12|KJ965504|ZMUO.005096|Calathus_ambiguus
TLYFIFGAWAGMVGTSLSMLIRAELGNPGALIGDDQIYNVIVTAHAFVMIFFMVMPIMIGGFGNWLVPLMLGAPDMAFPRMNNMSFWLLPPSLTLLLMSSMVESGAGTGWTVYPPLSSGIAHSGASVDLAIFSLHLAGISSILGAVNFITTIINMRSV
GMTFDRMPLFVWSVGITALLLLLSLPVLAGAITMLLTDRNLNTSFFDPAGGGDPILYQHL
>COLFB097-12|KJ965292|ZMUO.001427|Brachyderes_incanus
TLYFIFGAWSGMVGTSLSMLIRTELGNPGSLIGDDQIYNVIVTAHAFIMIFFMVMPMMIGGFGNWLVPLMLGAPDMAFPRMNNMSFWLLPPSLSLLLMSSIVDKGAGTGWTVYPPLSANIAHEGSSVDLAIFSLHMAGVSSILGAINFISTVINMRP
MGMSPDRMSLFIWAVKITAVLLLLSLPVLAGAITMLLTDRNVNTSFFDPAGGGDPILYQHL
>COLFC167-12|KJ966165|ZMUO.002352|Quedius_curtipennis
TLYFIFGAWAGMVGTSLSLLIRAELGSPGSLIGDDQIYNVIVTAHAFIMIFFMVMPTVIGGFGNWLVPLMLGAPDMAFPRMNNMSFWLLPPSLSLLLMSSMVESGAGTGWTVYPPLSSNIAHSGASVDLAIFSLHLAGISSILGAVNFITTMINMRSIG
MTFDRMPLFVWSVAITALLLLLSLPVLAGAITMLLTDRNINTSFFDPAGGGDPILYQHL
>COLFE837-13|KJ963774|ZMUO.005682|Psylliodes_dulcamarae
TLYFIFGIWSGMVGTSLSILIRTELGSPGSLIGNDQIYNVIVTAHAFIMIFFMVMPIMIGGFGNWLVPLMIGAPDMAFPRMNNMSFWLLPPSLILLLMSSMVESGAGTGWTVYPPLSSNMAHGGPSVDLAIFSLHLAGISSILGAINFITTVINMRPEGM
MLDRMPLFVWAVIITAILLLLSLPVLAGAITMLLTDRNLNTSFFDPIGGGDPILYQHL
>COLFD735-12|KJ965491|ZMUO.004725|Melolontha_hippocastani
TLYFLFGSWAGMVGTSLSLLIRAELGNPGTLIGDDQIYNVIVTAHAFVMIFFMVMPIMIGGFGNWLVPLMLGAPDMAFPRMNNMSFWLLPPSLSLLLMSSLVENGAGTGWTVYPPLSSNIAHSGASVDLAIFSLHLAGISSILGAVNFITTVINMRSTG
MTFDRMPLFAWSVALTALLLLLSLPVLAGAITMLLTDRNINTSFFDPAGGGDPILYQHL
>COLFE484-13|KJ962666|ZMUO.005329|Propylea_quatuordecimpunctata
TLYFLFGMWAGMVGTSLSILIRLELGTTNSLIGNDQIYNVIVTAHAFIMIFFMVMPIMIGGFGNWLVPLMIGAPDMAFPRLNNMSFWLLPPALMLLIMSSMVEMGAGTGWTVYPPLSSNMAHSGSSVDLVIFSLHLAGISSILGAVNFISTIMNMRPF
GMNLDKTPLFVWSVLITAILLLLSLPVLAGAITMLLTDRNINTSFFDPMGGGDPILYQHL
>COLFB209-12|KJ962984|ZMUO.001539|Ceutorhynchus_typhae
TLYFIFGSWAGMAGTSLSMLIRTELGNPGSLIGNDQIYNSIVTAHAFIMIFFMVMPILIGGFGNWLVPLMLGAPDMAFPRLNNMSFWLLPPSLSLLLMSSVVNKGAGTGWTVYPPLSSNVAHEGMSVDLAIFSLHMAGISSILGAINFISTVMNMQPK
GMTPELMPLFVWAVEITAILLLISLPVLAGAITMLLTDRNINTSFFDPSGGGDPILYQHL
>COLFD311-12|KJ962763|ZMUO.004206|Stenus_fuscipes
TLYFTFGAWAGMVGTSLSLLIRAELGNPGSLIGDDQIYNVIVTAHAFVMIFFMVMPVMIGGFGNWLVPLMLGAPDMAFPRMNNMSFWLLPPSLSLLLTSSIVENGAGTGWTVYPPLSTNIAHSGASVDLAIFSLHLAGISSILGAINFITTIFNMRTMKL
QFDCLPLFVWSVGITALLLLLSLPVLAGAITMLLTDRNLNTSFFDPAGGGDPVLYQHL
>COLFE1066-13|KJ965167|ZMUO.006861|Gonioctena_flavicornis
TLYFIFGIWAGMVGTSLSILIRAELGNPGTLIGNDQIYNVIVTAHAFIMIFFMVMPIMIGGFGNWLVPLMIGAPDMAFPRMNNMSFWLLPPSLFFLIMSSIVESGAGTGWTVYPPLSANIAHSGSSVDLAIFSLHLAGISSILGAVNFITTIINMRPMGMS
MDRMPLFVWAVLITAILLLLSLPVLAGAITMLLTDRNLNTSFFDPAGGGDPILYQHL

>COLFF787-13|KJ963543|ZMUO.006107|Hydroporus_rufifrons
TLYFLFGAWSGMVGTSLSMLIRAELGNPGSLIGDDQIYNVIVTAHAFIMIFFMVMPIMIGGFGNWLVPLMLGAPDMAFPRMNNMSFWLLPPSLSLLLMSSMVENGAGTGWTVYPPLSSGIAHSGASVDLAIFSLHLAGVSSILGAVNFITTIINMRSIG
MTFDRMPLFVWSVGITALLLLLSLPVLAGAITMLLTDRNLNTSFFDPAGGGDPILYQHL
>COLFF035-13|KJ962190|ZMUO.005735|Meligethes_carinulatus
TLYFIFGAWSGMVGTSLSMLIRTELGNPGSLIGNDQIYNVIVTAHAFVMIFFMVMPFMIGGFGNWLVPLMLGAPDMAFPRMNNMSFWLLPPSLSLLLMSSIVESGAGTGWTVYPPLSSNIAHGGASVDLAIFSLHLAGISSILGAVNFITTVINMRPSG
MNFDQMPLFVWSVVITAFLLLLSLPVLAGAITMLLTDRNLNTTFFDPSGGGDPILYQHL
>COLFC252-12|KJ964757|ZMUO.002437|Anthribus_nebulosus
TLYFIFGAWAGMMGTSLSLIIRMELGNPGSLIGDDQIYNVIVTAHAFIMIFFMVMPTMIGGFGNWLVPLMLGAPDMAFPRMNNMSFWLLPPSLTLLVLSSIVESGAGTGWTVYPPLSSNIAHGGSSVDLAIFSLHLAGVSSILGAVNFITTIINMRPEG
MSFDRMPLFVWAVGITALLLLLSLPVLAGAITMLLTDRNLNTSFFDPAGGGDPILYQHL
>COLFC377-12|KJ962709|ZMUO.002562|Omalium_septentrionis
TLYFIFGAWAGMVGTSLSLLIRAELGNPGSLIGDDQIYNVIVTAHAFIMIFFMVMPIVIGGFGNWLVPLMLGAPDMAFPRMNNMSFWLLPPSLTLLLMSSMVESGAGTGWTVYPPLSSNIAHGGSSVDLAIFSLHLAGISSILGAVNFITTVINMRAMG
MTFDRMPLFVWSVVITALLLLLSLPVLAGAITMLLTDRNLNTSFFDPAGGGDPILYQHL
>COLFB060-12|KJ966822|ZMUO.001390|Philonthus_quisquiliarius
TLYFIFGSWAGMVGTSLSLLIRAELGNPGSLIGDDQIYNVIVTAHAFIMIFFMVMPIVIGGFGNWLVPLMLGAPDMAFPRMNNMSFWLLPPSLTLLLMSSMVESGAGTGWTVYPPLSSNIAHGGASVDLAIFSLHLAGISSILGAVNFITTVINMRSSG
MSFDRMPLFVWSVAITALLLLLSLPVLAGAITMLLTDRNLNTTFFDPAGGGDPILYQHL
>COLFD050-12|KJ964228|ZMUO.003945|Meligethes_matronalis
TLYFIFGAWSGMVGTSLSMLIRTELGNPGSLIGNDQIYNVIVTAHAFVMIFFMVMPFMIGGFGNWLVPLMLGAPDMAFPRMNNMSFWLLPPSLSLLLMSSIVESGAGTGWTVYPPLSSNIAHGGASVDLAIFSLHLAGVSSILGAVNFITTVINMRPT
GMTFDRMPLFVWAVLITAILLLLSLPVLAGAITMLLTDRNLNTTFFDPSGGGDPILYQHL
>COLFB080-12|KJ961735|ZMUO.001410|Agonum_viduum
TLYFIFGAWSGMVGTSLSMLIRAELGNPGALIGDDQIYNVIVTAHAFIMIFFMVMPIMIGGFGNWLVPLMLGAPDMAFPRMNNMSFWLLPPSLTLLLMSSMVESGAGTGWTVYPPLSSGIAHAGASVDLAIFSLHLAGVSSILGAVNFITTIINMRSV
GMTFDRMPLFVWSVGITALLLLLSLPVLAGAITMLLTDRNLNTSFFDPAGGGDPILYQHL
>COLFD232-12|KJ965227|ZMUO.004127|Stenichnus_collaris
TLYFILGIWSGMLGTSLSLLIRLELSSPGMMIGNDQTFNMIVTSHAFIMIFFMVMPIMIGGFGNWLVPLMLGAPDMAFPRMNNMSFWFLPPSLMLLLMSSMVESGSGTGWTVYPPLSSNIAHSGSSVDLTIFSLHLAGISSILGAVNFITTIINMRSPM
MKFDNLSLFIWAVFITAILLLLSLPVLAGAITMLLTDRNFNTSFFDPSGGGDPILYQHL
>COLFE1003-13|KJ966868|ZMUO.006798|Quedius_boopoides
SLYFIFGAWAGMVGTSLSLLIRAELGNPGSLIGDDQIYNVIVTAHAFIMIFFMVMPTLIGGFGNWLVPLMLGAPDMAFPRMNNMSFWLLPPSLSLLLMSSMVESGAGTGWTVYPPLSSNIAHSGASVDLAIFSLHLAGISSILGAVNFITTVINMRSTG
MTFDRMPLFVWSVAITALLLLLSLPVLAGAITMLLTDRNLNTTFFDPAGGGDPILYQHL
>COLFB104-12|KJ963978|ZMUO.001434|Oiceoptoma_thoracicum
TLYFIFGAWAGMVGMSLSILIRAELSTPGSLLGDDQMYNVIVTAHAFVMIFFMVMPIVIGGFGNWLVPLMLGAPDMAFPRMNNMSFWLLPPSLSLLLVSSMVESGAGTGWTVYPPLSSNIAHSGSSVDLAIFSLHLAGISSILGAVNFITTIINMRSSG
MTFDRMPLFVWSVAITALLLLLSLPVLAGAITMLLTDRNLNTSFFDPAGGGDPVLYQHL
>COLFF282-13|KJ964980|ZMUO.005887|Otiorhynchus_ligustici
TLYFIFGSWSGMVGTSLSMLIRTELGNPGSLIGDDQIYNMIVTAHAFIMIFFMVMPMMIGGFGNWLVPLMLGAPDMAFPRLNNMSFWLLPPSLSLLLMSSIIDKGAGTGWTVYPPLSSNIAHEGTSVDLAIFSLHMAGMSSILGAINFISTAINMRPG
GMSPDRMTLFIWAVKITAILLLLSLPVLASAITMLLTDRNINTSFFDPAGGGDPILYQHL
>LEFIJ1985-13|KJ965463|ZMUO.003719|Saperda_scalaris
TLYFIFGAWAGMVGTSLSLLIRSELGTPGSLIGDDQIYNVIVTAHAFIMIFFMVMPIMIGGFGNWLVPLMLGAPDMAFPRMNNMSFWLLPPALSLLIMSSIVDKGAGTGWTVYPPLAANVAHNGSSVDLAIFSLHLAGISSILGAVNFITTVINMRPKG
MTLDRMPLFVWAVKITAILLLLSLPVLAGAITMLLTDRNLNTSFFDPAGGGDPILYQHL
>COLFE064-12|KJ965764|ZMUO.005289|Chrysolina_graminis
TLYFIFGTWAGMVGTSLSVLIRTELGNPGTLIGNDQIYNVIVTAHAFIMIFFMVMPIMIGGFGNWLVPLMLGAPDMAFPRMNNMSFWLLPPSLIFLLMSSIVENGVGTGWTVYPPLSANVAHSGPSVDLAIFSLHLAGISSILGAINFITTIINMHPAKM
KLEQMPLFSWAVLITAILLLLSLPVLAGAITMLLTDRNLNTSFFDPTSGGDPILYQHL
>COLFE1360-13|KJ965628|ZMUO.007155|Dasytes_niger
TLYFIFGAWSGMVGMSLSLLIRSELNNPGTLIGNDQIYNVIVTAHAFIMIFFMVMPILIGGFGNWLVPLMLGAPDMAFPRMNNMSFWLLPPSLTLLLMSSMVEQGAGTGWTVYPPLSSNIAHGGASVDLAIFSLHLAGISSILGAVNFITTIINMRPVG
MTLDRTPLFVWAVAITALLLLLSLPVLAGAITMLLTDRNLNTSFFDPAGGGDPILYQHL
>COLFF150-13|KJ964715|ZMUO.003784|Oxytelus_laqueatus
TLYFIFGAWSGMVGTSLSMLIRAELGSPGSLIGDDQIYNVIVTAHAFVMIFFMVMPIVIGGFGNWLVPLMLGAPDMAFPRMNNMSFWLLPPSLTLLLFSSMVESGAGTGWTVYPPLSSNIAHSGSSVDLAIFSLHLAGISSILGAVNFITTIINMRATGM
TFDRMPLFVWSVNITAILLLLSLPVLAGAITMLLTDRNLNTSFFDPAGGGDPILYQHL
>COLFB927-12|KJ963438|ZMUO.001972|Cryptocephalus_labiatus

TLYFLFGAWAGMIGTSLSLLIRIELGNPGSLIGNDQIYNTIVTAHAFIMIFFMVMPIMIGGFGNWLVPLMLGAPDMAFPRMNNMSFWLLPPSLTLLLMSSIVENGAGTGWTVYPPLSTTIAHAGASVDLAIFSLHLAGISSIMGAINFISTVINMRPQGM
FMDRTPLFVWAVLITAILLLLSLPVLAGAITMLLTDRNLNTSFFDPAGGGDPILYQHL
>COLFC759-12|KJ961933|ZMUO.003324|Corticaria_lapponica
SLYFLFGMWAGMVGTSLSLLIRLELGNPGSLIGDDQIYNVIVTAHAFVMIFFMVMPIMIGGFGNWLVPLMLGAPDMAFPRLNNMSFWLLPPSLSLLIMSSIIEKGAGTGWTVYPPLSSNIAHGGSSVDLAIFSLHLAGISSILGAVNFITTVINMRPTGM
NLDQMPLFVWSVVITAILLLLSLPVLAGAITMLLTDRNLNTSFFDPAGGGDPILYQHL
>COLFE972-13|KJ964819|ZMUO.006767|Oxypoda_opaca
TLYFIFGAWAGMVGTSLSLLIRAELGNPGSLIGDDQIYNVIVTAHAFVMIFFMVMPIVIGGFGNWLVPLMLGAPDMAFPRMNNMSFWLLPPSLTLLLMSSMVESGAGTGWTVYPPLSSNIAHGGSSVDLAIFSLHLAGISSILGAVNFISTIINMRTSG
MTFDRMPLFVWSVAITALLLLLSLPVLAGAITMLLTDRNLNTSFFDPAGGGDPILYQHL
>COLFC813-12|KJ964659|ZMUO.003378|Cantharis_livida
TLYFIFGAWSGSLGLALSLLIRAELGTPGTLIGNDQIYNVIVTAHAFIMIFFMVMPIMIGGFGNWLVPLMLGAPDMAFPRMNNMSFWFLPPSLMFLLMSSMVESGAGTGWTVYPPLSANIAHSGPSVDLAIFSLHMAGVSSILGAVNFISTIMNMKPP
SMKFDQMPLFVWSVGITALLLLLSLPVLAGAITMLLSDRNLNTSFFDPMGGGDPILYQHL
>COLFB332-12|KJ964514|ZMUO.001662|Anthobium_atrocephalum
TLYFIFGAWAGMVGTSLSILIRAELGNPGTLIGDDQIYNVIVTAHAFVMIFFMVMPIVIGGFGNWLVPLMLGAPDMAFPRMNNMSFWLLPPSLTLLLMSSMVESGAGTGWTVYPPLSSNIAHGGSSVDLAIFSLHLAGISSILGAVNFITTVINMRATG
MTFDRMPLFVWSVAITALLLLLSLPVLAGAITMLLTDRNLNTSFFDPAGGGDPILYQHL
>COLFE185-12|KJ962339|ZMUO.005220|Stomis_pumicatus
TLYFIFGAWSGMVGTSLSMLIRAELGNPGALIGDDQIYNVIVTAHAFIMIFFMVMPIMMGGFGNWLVPLMLGAPDMAFPRMNNMSFWLLPPSLTLLLMSSLVENGAGTGWTVYPPLASSIAHAGASVDLAIFSLHLAGVSSILGAVNFITTIINMRSI
GMTFDRMPLFVWSVGITALLLLLSLPVLAGAITMLLTDRNLNTSFFDPAGGGDPILYQHL
>COLFC049-12|KJ965645|ZMUO.002044|Aleochara_brundini
TLYFIFGAWAGMMGTSLSLLIRAELGNPGSLIGNDQIYNVIVTAHAFIMIFFMVMPIIIGGFGNWLVPLMLGAPDMAFPRMNNMSFWLLPPSLTLLLMSSMVESGAGTGWTVYPPLSSNMAHGGSSVDLAIFSLHLAGISSILGAVNFISTVINMRSM
GMTFDKMPLFVWSVTITALLLLLSLPVLAGAITMLLTDRNLNTSFFDPAGGGDPILYQHL
>LEFIJ1828-13|KJ963561|ZMUO.004583|Meligethes_aeneus
TLYFIFGAWSGMVGTSLSMLIRTELGNPGSLIGNDQIYNVIVTAHAFVMIFFMVMPFMIGGFGNWLVPLMLGAPDMAFPRMNNMSFWLLPPSLSLLLMSSIVESGAGTGWTVYPPLSSNIAHGGASVDLAIFSLHLAGISSILGAVNFITTVINMRPK
GMTFDRMPLFVWAVMITAILLLLSLPVLAGAITMLLTDRNLNTTFFDPSGGGDPILYQHL
>COLFB868-12|KJ966417|ZMUO.001913|Aromia_moschata
TLYFIFGAWSGMVGTSLSMLIRSELGNPGSLIGDDQIYNVIVTAHAFIMIFFMVMPIMIGGFGNWLVPLMLGAPDMAFPRMNNMSFWLLPPSLTFLILSSIVESGAGTGWTVYPPLSSNIAHSGSSVDLAIFSLHLAGISSILGAVNFISTVINMRPSGMS
PDRMPLFVWAVVITAILLLLSLPVLAGAITMLLTDRNINTSFFDPAGGGDPILYQHL
>COLFF238-13|KJ961947|ZMUO.005843|Anthophagus_caraboides
TLYFIFGAWAGMVGTSLSILIRSELGNPGSLIGDDQIYNVIVTAHAFVMIFFMVMPVVIGGFGNWLVPLMLGAPDMAFPRMNNMSFWLLPPSLTLLLMSSMVESGAGTGWTVYPPLSSNIAHSGSSVDLAIFSLHLAGISSILGAVNFITTVINMRSTGI
SFDRMPLFVWSVAITALLLLLSLPVLAGAITMLLTDRNLNTSFFDPAGGGDPILYQHL
>COLFB381-12|KJ964526|ZMUO.001806|Saperda_scalaris
TLYFIFGAWAGMVGTSLSLLIRSELGTPGSLIGDDQIYNVIVTAHAFIMIFFMVMPIMIGGFGNWLVPLMLGAPDMAFPRMNNMSFWLLPPALSLLIMSSIVDKGAGTGWTVYPPLAANVAHNGSSVDLAIFSLHLAGISSILGAVNFITTVINMRPKG
MTLDRMPLFVWAVKITAILLLLSLPVLAGAITMLLTDRNLNTSFFDPAGGGDPILYQHL
>COLFF996-13|KJ965575|ZMUO.007361|Hypera_arator
TLYFIFGTWAGTVGTSLSILIRTELGNPGSLIGNDQIYNTIVTAHAFIMIFFMVMPIMIGGFGNWLVPLMLGAPDMAFPRLNNMSFWLLPPSLSLLLMSSMVDSGAGTGWTVYPPLSSNIAHEGSSVDLAIFSLHMAGVSSILGAINFISTVLNMRPSGM
SLDKMALFIWAVKITAILLLLSLPVLAGAITMLLTDRNINTSFFDPAGGGDPILYQHL
>COLFB162-12|KJ964590|ZMUO.001492|Bembidion_velox
TLYFIFGAWAGMVGTSLSMLIRAELGNPGSLIGDDQIYNVIVTAHAFVMIFFMVMPILIGGFGNWLVPLMLGAPDMAFPRMNNMSFWLLPPSLTLLLMSSMVESGAGTGWTVYPPLSSSIAHSGASVDLAIFSLHLAGVSSILGAVNFITTIINMRSVG
MTFDRMPLFVWSVGITALLLLLSLPVLAGAITMLLTDRNLNTSFFDPAGGGDPILYQHL
>COLFD222-12|KJ967237|ZMUO.004117|Omalium_rugatum
TLYFIFGAWAGMVGTSLSLLIRAELGNPGSLIGDDQIYNVIVTAHAFIMIFFMVMPIVIGGFGNWLVPLMLGAPDMAFPRMNNMSFWLLPPSLTLLLMSSMVESGAGTGWTVYPPLSSNIAHGGSSVDLAIFSLHLAGISSILGAVNFITTVINMRAMG
MTFDRMPLFVWSVVITALLLLLSLPVLAGAITMLLTDRNLNTSFFDPAGGGDPILYQHL
>COLFC267-12|KJ964693|ZMUO.002452|Dalopius_marginatus
TLYFIFGAWAGMLGTSLSLLIRAELGNPGSLIGNDQIYNVIVTAHAFIMIFFMVMPIMIGGFGNWLVPLMLGAPDMAFPRMNNMSFWFLPPSLSLLLMSSIVENGAGTGWTVYPPLSSNIAHSGSSVDLAIFSLHLAGISSILGAVNFISTVINMRSTGIT
FDRMPLFVWAVAITALLLLLSLPVLAGAITMLLTDRNLNTSFFDPAGGGDPILYQHL
>COLFF695-13|KJ964335|ZMUO.006395|Byturus_ochraceus
TLYFIFGAWAGMVGTSLSLLIRSELGNPGSLIGDDQIYNVIVTAHAFVMIFFMVMPIVIGGFGNWLVPLMLGAPDMAFPRMNNMSFWLLPPSLTLLLMSSIVESGAGTGWTVYPPLSSNIAHGGSSVDLAIFSLHLAGISSILGAVNFITTIMNMRPTG
MTLDRMPLFVWSVIITAILLLLSLPVLAGAITMLLTDRNLNTSFFDPSGGGDPILYQHL

>COLFE1340-13|KJ964546|ZMUO.007135|Limnobaris_t-album
TLYFIFGTWAGTIGTSLSMLIRSELGNPGSLIGDDQIYNTIVTAHAFIMIFFMVMPIMIGGFGNWLVPLMLGAPDMAFPRLNNMSFWLLPPSLTLLLMSSIVDKGVGTGWTVYPPLSSNNAHEGASVDLGIFSLHMAGISSILGAMNFISTAMNMRPT
GLKSDQMSLFIWAVKITAILLLLSLPVLAGAITMLLTDRNINTSFFDPIGGGDPILYQHL
>COLFC327-12|KJ962751|ZMUO.002512|Atheta_spatuloides
TLYFIFGAWAGMVGTSLSLLIRAELGNPGSLIGDDQIYNVIVTAHAFIMIFFMVMPIMIGGFGNWLIPLMLGAPDMAFPRMNNMSFWLLPPSLSLLLMSSMVESGAGTGWTVYPPLSSNIAHGGASVDLAIFSLHLAGISSILGAVNFISTVINMRSTGI
TFDRMPLFVWAVVITALLLLLSLPVLAGAITMLLTDRNLNTSFFDPAGGGDPILYQHL
>COLFB790-12|KJ965569|ZMUO.001265|Oxypoda_funebris
TLYFIFGTWAGMIGTSLSLLIRAELGNPGSLIGDDQIYNVIVTAHAFVMIFFMVMPIVIGGFGNWLIPLMLGAPDMAFPRMNNMSFWLLPPSLSLLLMSSMVESGAGTGWTVYPPLSSNIAHGGSSVDLAIFSLHLAGISSILGAVNFISTIINMRTSGMS
FDRMPLFVWSVAITALLLLLSLPVLAGAITMLLTDRNLNTSFFDPAGGGDPILYQHL
>COLFE804-13|KJ966871|ZMUO.005649|Sepedophilus_pedicularius
TLYFIFGAWSGMVGTSLSLLIRAELGNPGSLIGDDQIYNVIVTAHAFIMIFFMVMPIVIGGFGNWLVPLMLGAPDMAFPRMNNMSFWLLPPSLSLLLMSSLVESGAGTGWTVYPPLSANIAHSGSSVDLAIFSLHLAGISSILGAVNFITTVINMRTLGM
TFDRMPLFVWSVAITALLLLLSLPVLAGAITMLLTDRNLNTSFFDPAGGGDPILYQHL
>COLFB371-12|KJ966012|ZMUO.001701|Stenus_geniculatus
TLYFILGAWAGLTGTSLSLLIRTELGSPGSFIGDDQIYNVIVTAHAFIMIFFMVMPIMIGGFGNWLIPLMLGAPDMAFPRMNNMSFWLLPPSLSLLLMSSIIESGAGTGWTVYPPLSSNIAHSGASVDLTIFSLHLAGISSILGAMNFITTIFNMRTMKLQL
DCLPLFIWSVNVTTFLLLLSLPVLAGAITMLLTDRNINTSFFDPGGGGDPILYQHL
>COLFA089-10|HM909105|MP00314|Sitophilus_oryzae
TLYFIFGTWSGMVGTSLSLLIRAELGNPGSLIGNDQIYNTIVTAHAFIMIFFMVMPIMIGGFGNWLIPLMLGAPDMAFPRLNNMSFWLLPPSLTLLLMSSFIEKGAGTGWTVYPPLSSNIAHEGASVDLAIFSLHMAGISSILGAINFITTAYNMRPSGML
SERMTLFIWAVSITAILLLFSLPVLAGAITMLLTDRNINTSFFDPAGGGDPILYQHL
>COLFE259-12|KJ966320|ZMUO.005104|Ceutorhynchus_litura
TLYFIFGSWAGMMGTSLSMLIRTELGNPGTLIGNDQIYNSMVTAHAFIMIFFMVMPIMIGGFGNWLVPLMLGAPDMAFPRLNNMSFWLLPPSLTLLLMSSIVNKGAGTGWTVYPPLSASTAHEGMSVDMAIFSLHMAGISSILGAINFISTMMNM
RPSGMISEFMPLFVWAVKITAILLLISLPVLAGAITMLLTDRNINTSFFDPSGGGDPILYQHL
>COLFE1277-13|KJ964658|ZMUO.007072|Silis_ruficollis
TLYFIFGMWSGSLGLALSLLIRAELGVPGTLIGNDQIYNVIVTAHAFIMIFFMVMPIMIGGFGNWLVPLMLGAPDMAFPRLNNMSFWFLPPSLMFLLMSSMVESGAGTGWTVYPPLSANIAHSGPSVDLAIFSLHMAGISSILGAVNFISTIMNMRAP
TMKFDQMPLFVWSVGITALLLLLSLPVLAGAITMLLSDRNLNTSFFDPIGGGDPILYQHL
>COLFF275-13|KJ965269|ZMUO.005880|Atheta
TLYFIFGAWAGMVGTSLSLLIRAELGNPGSLIGDDQIYNVIVTAHAFIMIFFMVMPIMIGGFGNWLVPLMLGAPDMAFPRMNNMSFWLLPPSLTLLLMSSMVESGAGTGWTVYPPLSSNIAHGGASVDLAIFSLHLAGISSILGAVNFISTVINMRSTGI
TFDRMPLFVWAVAITALLLLLSLPVLAGAITMLLTDRNLNTSFFDPAGGGDPILYQHL
>COLFE277-12|KJ961789|ZMUO.005122|Clivina_fossor
TLYFIFGAWSGMVGTSLSMLIRAELGNPGSLIGDDQIYNVIVTAHAFVMIFFMVMPILIGGFGNWLVPLMLGAPDMAFPRMNNMSFWLLPPSLSLLLMGSMVESGAGTGWTVYPPLSSAIAHSGASVDLAIFSLHLAGVSSILGAVNFITTIINMRSTG
MTFERMPLFVWSVGITALLLLLSLPVLAGAITMLLTDRNLNTSFFDPAGGGDPILYQHL
>COLFC553-12|KJ962306|ZMUO.003118|Monochamus_urussovii
TLYFIFGAWSGMVGTSLSLLIRSELGMPGSLIGNDQIYNVIVTAHAFIMIFFMVMPIMIGGFGNWLVPLMLGAPDMAFPRMNNMSFWLLPPSLTLLIMSSIVENGAGTGWTVYPPLAANVAHSGSSVDLAIFSLHLAGVSSILGAVNFITTVINMRPSG
MNLDRLPLFVWAVKITAILLLLSLPVLAGAITMLLTDRNLNTSFFDPAGGGDPILYQHL
>COLFB233-12|KJ963895|ZMUO.001563|Leptura_quadrifasciata
TLYFIFGAWAGMVGTSLSLLIRSELGNPGSLIGDDQIYNVIVTAHAFVMIFFMVMPIMIGGFGNWLVPLMLGAPDMAFPRMNNMSFWLLPPSLTLLIMSSLVESGAGTGWTVYPPLSSNIAHGGSSVDLAIFSLHLAGISSILGAVNFITTVINMRPLG
MSPDQMPLFVWAVVITAILLLLSLPVLAGAITMLLTDRNLNTSFFDPAGGGDPILYQHL
>COLFB792-12|KJ962932|ZMUO.001267|Liogluta_micans
TLYFIFGAWAGMVGTSLSLLIRAELGNPGSLIGDDQIYNVIVTAHAFIMIFFMVMPVVIGGFGNWLVPLMLGAPDMAFPRMNNMSFWLLPPSLTLLLMSSMVESGAGTGWTVYPPLSSNIAHSGSSVDLAIFSLHLAGISSILGAVNFISTVINMRSVG
MSFDRMPLFVWSVAITALLLLLSLPVLAGAITMLLTDRNLNTSFFDPAGGGDPILYQHL
>COLFC452-12|KJ964065|ZMUO.003017|Cyphon_variabilis
TLYFIFGSWSGMVGTSLSLLIRAELGTPGSLIGDDQIYNVIVTAHAFIMIFFMVMPIMIGGFGNWLVPLMLGAPDMAFPRMNNMSFWLLPPSLTLLLMSSMVENGAGTGWTVYPPLSAGVAHSGASVDLAIFSLHLAGISSILGAVNFISTVINMRSVG
MTFDRMPLFVWAVAITALLLLLSLPVLAGAITMLLTDRNLNTSFFDPAGGGDPILYQHL
>COLFB915-12|KJ966717|ZMUO.001960|Lepturobosca_virens
TLYFIFGAWAGMIGTSLSLLIRSELGNPGSLIGDDQIYNVIVTAHAFVMIFFMVMPIMIGGFGNWLVPLMLGAPDMAFPRMNNMSFWLLPPSLTLLIMSSIVEKGAGTGWTVYPPLAANIAHSGPSVDLAIFSLHLAGISSILGAVNFITTIINMPPVGM
KLDQMPLFVWAVMITAILLLLSLPVLAGAITMLLTDRNLNTSFFDPAGGGDPILYQHL
>COLFF553-13|KJ967462|ZMUO.006633|Hololepta_plana

TLYFIFGAWAGMVGTSMSLIIRAELGNPGSLIGDDQIYNVIVTAHAFVMIFFMVMPIMIGGFGNWLVPLMLGAPDMAFPRMNNMSFWLLPPSLSLLIMSSMVESGAGTGWTVYPPLSANLAHSGASVDLAIFSLHLAGISSILGAINFITTVINMRSPG
MSFDQMPLFVWSVAITALLLLLSLPVLAGAITMLLTDRNLNTSFFDPAGGGDPILYQHL
>COLFA298-12|KJ965701|ZMUO.000433|Nephus_redtenbacheri
TLYFLFGLWAGMVGTSLSILIRLELGTPSTLIGNDQIYNVIVTAHAFIMIFFMVMPIMIGGFGNWLVPLMIGAPDMAFPRMNNMSFWLLPPSLTFLILSSIVESGAGTGWTVYPPLSSNIAHGGSSVDMAIFSLHLAGISSILGAVNFITTIINMRPSGMSF
EKMPLFVWSVLITAILLLLSLPVLAGAITMLLTDRNINTSFFDPAGGGDPILYQHL
>COLFB279-12|KJ965589|ZMUO.001609|Anacaena_lutescens
TLYFIFGAWAGMVGTSLSILIRAELGNPGTLIGDDQIYNVIVTAHAFIMIFFMVMPIMIGGFGNWLVPLMLGAPDMAFPRMNNMSFWLLPPSLTLLLMSSMVENGAGTGWTVYPPLSSNIAHGGASVDLAIFSLHLAGISSILGAVNFITTVINMRSES
MTYDRLPLFVWSVAITALLLLLSLPVLAGAITMLLTDRNLNTSFFDPAGGGDPILYQHL
>COLFE1470-13|KJ963801|ZMUO.007265|Eutrichapion_facetum
TLYFIFGLWSGMVGTSLSMLIRIELGNPSSLIGDDQIYNVIVTAHAFIMIFFMVMPVMIGGFGNWLVPLMLGAPDMAFPRMNNMSFWLLPPSLTLLLMSSIVEKGAGTGWTVYPPLASNIAHSGASVDLAIFSLHLAGISSILGAVNFISTMVNMHSNG
LSLDQLSLFTWAVKITAILLLLSLPVLAGAITMLLTDRNINTSFFDPAGGGDPILYQHL
>COLFC662-12|KJ962244|ZMUO.003227|Coccinula_quatuordecimpustulata
TLYFLFGMWAGMVGTSLSILIRLELGSTGSLIGNDQIYNVIVTAHAFIMIFFMVMPIMIGGFGNWLVPLMIGAPDMAFPRLNNMSFWLLPPSLTLLLLSSLVEMGAGTGWTVYPPLSSNLAHNGSSVDLVIFSLHLAGISSILGAVNFISTIMNMRPNG
MNLDKTPLFVWSVLITAILLLLSLPVLAGAITMLLTDRNMNTSFFDPTGGGDPILYQHL
>COLFF776-13|KJ964728|ZMUO.006096|Elaphrus_cupreus
TLYFIFGAWSGMVGTSLSMLIRAELGNPGSLIGDDQIYNVIVTAHAFIMIFFMVMPIMIGGFGNWLVPLMLGAPDMAFPRMNNMSFWLLPPSLSLLLMSSMVESGAGTGWTVYPPLSSGIAHAGASVDLAIFSLHLAGVSSILGAVNFITTIINMRSV
GMTFDRMPLFVWSVGITALLLLLSLPVLAGAITMLLTDRNLNTSFFDPAGGGDPILYQHL
>COLFC273-12|KJ963515|ZMUO.002458|Atheta_fallaciosa
TLYFIFGAWAGMIGTSLSLLIRAELGNPGSLIGDDQIYNVIVTAHAFVMIFFMVMPIVIGGFGNWLVPLMLGAPDMAFPRMNNMSFWLLPPSLTLLLMSSMVESGAGTGWTVYPPLSSNIAHSGSSVDLAIFSLHLAGISSILGAVNFISTVINMRSTGIS
FDRMPLFVWSVVITALLLLLSLPVLAGAITMLLTDRNLNTSFFDPAGGGDPILYQHL
>COLFB746-12|KJ962555|ZMUO.001221|Sepedophilus_pedicularius
TLYFIFGAWSGMVGTSLSLLIRAELGNPGSLIGDDQIYNVIVTAHAFIMIFFMVMPIVIGGFGNWLVPLMLGAPDMAFPRMNNMSFWLLPPSLSLLLMSSLVESGAGTGWTVYPPLSANIAHSGSSVDLAIFSLHLAGISSILGAVNFITTVINMRTLGM
TFDRMPLFVWSVAITALLLLLSLPVLAGAITMLLTDRNLNTSFFDPAGGGDPILYQHL
>COLFE626-13|KJ963678|ZMUO.005471|Notoxus_monoceros
TLYLIFGAWAGMVGTSLSLLIRSELGNPGSLIGDDQIYNVIVTAHAFIMIFFMVMPIVIGGFGNWLVPLMLGAPDMAFPRMNNMSFWLLPPSLTLLIMSSIVENGAGTGWTVYPPLSSNIAHSGSSVDLAIFSLHLAGVSSILGAVNFITTVINMRPSGM
SFDRMPLFVWAVVITAVLLLLSLPVLAGAITMLLTDRNLNTSFFDPAGGGDPILYQHL
>COLFC841-12|KJ964127|ZMUO.003406|Dromius_fenestratus
TLYFIFGAWAGMVGTSLSMLIRAELGNPGALIGDDQVYNVIVTAHAFIMIFFMVMPIMIGGFGNWLVPLMLGAPDMAFPRMNNMSFWLLPPSLSLLLMSSMVESGAGTGWTVYPPLSSGIAHAGASVDLAIFSLHLAGVSSILGAVNFITTIINMRSI
GMTFDRMPLFVWSVGITALLLLLSLPVLAGAITMLLTDRNLNTSFFDPAGGGDPILYQHL
>COLFF423-13|KJ965898|ZMUO.005941|Rhynchaenus_iota
TLYFMFGAWSGMVGTSLSMLIRTELGTPGKLIGNDQIYNTIVTAHAFIMIFFMVMPIMIGGFGNWLVPLMLGAPDMAFPRLNNMSFWLLPPSLTLLLLSSIMNKGAGTGWTVYPPLSSNVAHEGSSVDLAIFSLHMAGISSILGAINFISTVANMRPK
GMNTDRMSLFVWAVKITAILLLLSLPVLAGAITMLLTDRNINTSFFDPAGGGDPILYQHL
>COLFE1272-13|KJ962023|ZMUO.007067|Oedemera_croceicollis
TLYLIFGAWAGMVGTSLSLLIRTELGSPGSLIGDDQIYNVIVTAHAFIMIFFMVMPIVIGGFGNWLVPLMLGAPDMAFPRMNNMSFWLLPPSLTLLIMSSVVEKGAGTGWTVYPPLSSNIAHGGSSVDLAIFSLHLAGISSILGAVNFITTVINMRPSDM
TLDRMPLFVWAVVITAVLLLLSLPVLAGAITMLLTDRNLNTSFFDPAGGGDPILYQHL
>COLFA661-12|KJ964070|ZMUO.000796|Philonthus_albipes
TLYFIFGAWAGMVGTSLSLLIRAELGNPGSLIGDDQIYNVIVTAHAFIMIFFMVMPIVIGGFGNWLVPLMLGAPDMAFPRMNNMSFWLLPPSLTLLLMSSMVESGAGTGWTVYPPLSSNIAHGGASVDLAIFSLHLAGISSILGAVNFITTVINMRSTGI
SFDRMPLFIWSVAITALLLLLSLPVLAGAITMLLTDRNLNTTFFDPAGGGDPILYQHL
>COLFE321-12|KJ967019|ZMUO.004881|Ochthephilum_collare
TLYFIFGAWAGMVGTSLSLLIRSELANPGSLIGDDQIYNVIVTAHAFIMIFFMVMPVMIGGFGNWLVPLMLGAPDMAFPRMNNMSFWLLPPALTLLLMSSMVESGAGTGWTVYPPLSSNSFHNGSSVDLAIFSLHLAGISSILGAINFITTAINMRTTG
MNYERMPLFIWSVAITALLLLLSLPVLAGAITMLLTDRNLNTSFFDPAGGGDPILYQHL
>COLFE293-12|KJ967161|ZMUO.004853|Cryptocephalus_fulvus
TLYFLFGAWAGMVGTSLSLLIRIELGNPGSLIGNDQIYNTIVTAHAFIMIFFMVMPIMIGGFGNWLVPLMLGAPDMAFPRMNNMSFWLLPPSLTLLLMSSIVENGAGTGWTVYPPLSATIAHAGSSVDLAIFSLHLAGISSIMGAINFISTVINMRPQG
MLMDRTPLFVWAVLITAILLLLSLPVLAGAITMLLTDRNLNTSFFDPAGGGDPILYQHL
>COLFD310-12|KJ962510|ZMUO.004205|Cercyon_melanocephalus
TLYFIFGAWAGMVGTSLSILIRAELGNPGTLIGDDQIYNVIVTAHAFIMIFFMVMPIMIGGFGNWLVPLMLGAPDMAFPRMNNMSFWLLPPSLTLLLMSSMVESGAGTGWTVYPPLSSNIAHGGSSVDLAIFSLHLAGISSILGAVNFITTVINMRSPNL
TYDRLPLFVWSVIITALLLLLSLPVLAGAITMLLTDRNLNTSFFDPAGGGDPILYQHL

>COLFB050-12|KJ963223|ZMUO.001380|Enochrus_affinis
TLYFIFGAWAGMVGTSLSILIRAELGNPGTLIGDDQIYNVIVTAHAFIMIFFMVMPIMIGGFGNWLVPLMLGAPDMAFPRMNNMSFWLLPPSLTLLLMSSMVESGAGTGWTVYPPLSSNIAHGGASVDLAIFSLHLAGISSILGAVNFITTVINMRSPS
MTYDRLPLFVWSVAITALLLLLSLPVLAGAITMLLTDRNLNTSFFDPAGGGDPILYQHL
>COLFA081-10|HM909098|MP00287|Clivina_fossor
TLYFIFGAWSGMVGTSLSMLIRAELGNPGSLIGDDQIYNVIVTAHAFVMIFFMVMPILIGGFGNWLVPLMLGAPDMAFPRMNNMSFWLLPPSLSLLLMSSMVESGAGTGWTVYPPLSSAIAHSGASVDLAIFSLHLAGVSSILGAVNFITTIINMRSTG
MTFERMPLFVWSVGITALLLLLSLPVLAGAITMLLTDRNLNTSFFDPAGGGDPILYQHL
>COLFF437-13|KJ965154|ZMUO.005955|Enochrus_quadripunctatus
TLYFIFGAWAGMVGTSLSILIRAELGNPGTLIGDDQIYNVIVTAHAFIMIFFMVMPIMIGGFGNWLVPLMLGAPDMAFPRMNNMSFWLLPPSLTLLLMSSMVESGAGTGWTVYPPLSSNIAHGGASVDLAIFSLHLAGISSILGAVNFITTVINMRSPS
MTYDRLPLFVWSVAITALLLLLSLPVLAGAITMLLTDRNLNTSFFDPAGGGDPILYQHL
>COLFB033-12|KJ963418|ZMUO.001363|Thanatophilus_dispar
TLYFIFGAWAGMVGMSLSILIRAELSTPGTLLGDDQMYNVIVTAHAFIMIFFMVMPIVIGGFGNWLVPLMLGAPDMAFPRMNNMSFWLLPPSLSLLLVSSMVESGAGTGWTVYPPLSSNIAHGGSSVDLAIFSLHLAGISSILGAVNFITTIINMRASG
MTFDRMPLFVWSVAITALLLLLSLPVLAGAITMLLTDRNLNTSFFDPAGGGDPILYQHL
>COLFF527-13|KJ964792|ZMUO.006607|Anoplodera_reyi
TLYFIFGAWASMVGTSLSLLIRSELGNPGSLIGDDQIYNVIVTAHAFVMIFFMVMPIMIGGFGNWLVPLMLGAPDMAFPRMNNMSFWLLPPSLTLLIMSSIVESGAGTGWTVYPPLSSNIAHGGSSVDLAIFSLHLAGISSILGAVNFITTVINMRPVGM
SPERMPLFVWAVVITAVLLLLSLPVLAGAITMLLTDRNLNTSFFDPAGGGDPILYQHL
>COLFC111-12|KJ962931|ZMUO.002296|Amischa_bifoveolata
TLYFIFGAWAGMVGTSLSLLIRAELGNPGSLIGDDQIYNVIVTAHAFIMIFFMVMPIVIGGFGNWLVPLMLGAPDMAFPRMNNMSFWLLPPSLTLLLMSSMVESGAGTGWTVYPPLSSNIAHGGSSVDLAIFSLHLAGISSILGAVNFISTVINMRSTGI
SFDRMPLFVWSVAITALLLLLSLPVLAGAITMLLTDRNLNTSFFDPAGGGDPILYQHL
>COLFE083-12|KJ962918|ZMUO.005308|Calomicrus_pinicola
TLYFIFGIWAGMIGTSMSMIIRTELGSPGSLIGNDQIYNVIVTAHAFIMIFFMVMPIMIGGFGNWLVPLMIGAPDMAFPRMNNMSFWLLPPSLFLLLMSSMVENGAGTGWTVYPPLSSNISHSGSSIDLAIFSLHLAGISSILGAINFITTIINMRPFGMSL
DRMPLFVWSVILTAILLLLSLPVLAGAITMLLTDRNLNTSFFDPAGGGDPILYQHL
>COLFA032-10|HM909059|MP00179|Micrambe_bimaculata
SLYFIFGAWAGMIGTSLSLLIRSELGTPGSLIGDDQIYNVIVTAHAFIMIFFMVMPIMIGGFGNWLVPLMLGAPDMAFPRMNNMSFWLLPPSLCLLLMSSVAEKGVGTGWTVYPPLSSNIAHGGSSVDLAIFSLHLAGISSILGAVNFISTIMNMPPTGM
TLDRMPLFVWAVMITAVLLLLSLPVLAGAITMLLTDRNLNTSFFDPAGGGDPILYQHL
>COLFF158-13|KJ961937|ZMUO.003792|Stenopterapion_meliloti
TLYFIFGLWAGMVGTSLSMLIRIELGTPGSLIGNDQIYNVIVTAHAFIMIFFMVMPIMIGGFGNWLVPLMLGAPDMAFPRMNNMSFWLLPPSLTLLLMSSIVEKGAGTGWTVYPPLASNIAHGGASVDLAIFSLHLAGISSILGAVNFISTIINMHPNGLS
FDQMSLFTWAVKITAILLLLSLPVLAGAITMLLTDRNINTSFFDPAGGGDPILYQHL
>COLFD110-12|KJ961810|ZMUO.004005|Glischrochilus_quadripunctatus
TLYFIFGTWSGMIGTSLSLLIRSELGNPGSLIGDDQIYNVIVTAHAFIMIFFMVMPFMIGGFGNWLMPLMLGAPDMAFPRLNNMSFWLLPPSISLLLMSSIVESGAGTGWTVYPPLSSNIAHGGSSVDLAIFSLHLAGISSILGAVNFITTVINMRPSGMS
FDRMPLFAWAVVITAVLLLLSLPVLAGAITMLLTDRNLNTSFFDPAGGGDPILYQHL
>COLFE048-12|KJ965399|ZMUO.005273|Acmaeops_pratensis
VLYFIFGAWAGMVGTSLSLLIRSELGNPGSLIGNDQIYNVIVTAHAFIMIFFMVMPIMIGGFGNWLVPLMLGAPDMAFPRLNNMSFWLLPPSLTLLIMSSIVESGAGTGWTVYPPLSSNIAHGGSSVDLAIFSLHLAGISSILGAINFITTVINMRPKGMT
FDRMPLFVWAVVITAILLLLSLPVLAGAITMLLTDRNLNTSFFDPAGGGDPILYQHL
>COLFE298-12|KJ966655|ZMUO.004858|Pityogenes_chalcographus
TLYFIFGAWSGMVGTSLSMLIRTELGTPGSLIGDDQIFNTIVTAHAFIMIFFMVMPILIGGFGNWLVPLMLGAPDMAFPRLNNMSFWLLPPSLTLLIMSSITDKGAGTGWTVYPPLSSNIAHEGASVDLAIFSLHMSGISSILGAINFISTIINMHPKGVAP
EQLSLFTWAVKITAMLLLLSLPVLAGAITMLLTDRNVNTSFFDPAGGGDPILYQHL
>COLFF395-13|KJ962748|ZMUO.005913|Chrysomela_tremula
TLYFIFGIWAGTVGTSLSILIRAELGNPGTLIGNDQIYNVIVTAHAFIMIFFMVMPIMIGGFGNWLVPLMIGAPDMAFPRLNNMSFWLLPPSLFLLLMSSIVENGVGTGWTVYPPLSTNIAHSGSSVDLAIFSLHLAGISSILGAINFITTVINMRPEGMNFE
QTPLFVWAVLITAILLLLSLPVLAGAITMLLTDRNLNTSFFDPAGGGDPILYQHL
>COLFC724-12|KJ966698|ZMUO.003289|Gonioctena_linnaeana
TLYFIFGIWAGMVGTSLSILIRAELGNPGTLIGNDQIYNVIVTAHAFIMIFFMVMPIMIGGFGNWLVPLMIGAPDMAFPRMNNMSFWLLPPSLFFLIMSSIVESGAGTGWTVYPPLSANIAHSGSSVDLAIFSLHLAGISSILGAINFITTIINMRPMGMS
MDRMPLFVWAVLITAILLLLSLPVLAGAITMLLTDRNLNTSFFDPAGGGDPILYQHL
>COLFF998-13|KJ965616|ZMUO.007363|Hypera_nigrirostris
TLYFIFGTWAGTVGTSLSILIRTELGNPGSLIGNDQIYNTIVTAHAFIMIFFMVMPIMIGGFGNWLVPLMLGAPDMAFPRLNNMSFWLLPPSLSLLLMSSMVDKGAGTGWTVYPPLSSNIAHEGSSVDLAIFSLHMAGVSSILGAINFISTVLNMRPSGM
SLDKMALFIWAVKITAILLLLSLPVLAGAITMLLTDRNINTSFFDPAGGGDPILYQHL
>COLFC687-12|KJ962326|ZMUO.003252|Dyschirius_nigricornis

TLYFIFGIWSGMVGTSLSIMIRAELGNPGSLIGDDQIYNVIVTAHAFIMIFFMVMPIMIGGFGNWLVPLMLGAPDMAFPRMNNMSFWLLPPSLSLLLMSSMVEKGAGTGWTVYPPLSSSIAHSGASVDLAIFSLHLAGVSSILGAVNFITTIINIRSTGMS
FERMPLFVWSVGITALLLLLSLPVLAGAITMLLTDRNLNTSFFDPAGGGDPILYQHL
>COLFD444-12|KJ966620|ZMUO.003864|Ceutorhynchus_erysimi
TLYFIFGSWAGMAGTSLSMLIRTELGNPGSLIGNDQIYNSIVTAHAFIMIFFMVMPILIGGFGNWLVPLMLGAPDMAFPRLNNMSFWLLPPSLSLLLMSSIVNKGAGTGWTVYPPLSGNVAHEGMSVDLAIFSLHMAGISSILGAINFISTVMNMQPK
GMTPELMPLFVWAVEITAILLLLSLPVLAGAITMLLTDRNINTSFFDPSGGGDPILYQHL
>COLFC168-12|KJ962349|ZMUO.002353|Philonthus_politus
TLYFIFGSWAGMVGTSLSLLIRAELGNPGTLIGDDQIYNVIVTAHAFIMIFFMVMPIVIGGFGNWLVPLMLGAPDMAFPRMNNMSFWLLPPSLTLLLMSSMVESGAGTGWTVYPPLSSNIAHGGASVDLAIFSLHLAGISSILGAVNFITTVINMRSTG
MTFDRMPLFVWSVVITALLLLLSLPVLAGAITMLLTDRNLNTSFFDPAGGGDPILYQHL
>COLFB396-12|KJ964225|ZMUO.001821|Quedius_cinctus
TLYFIFGAWAGMVGTSLSLLIRAELGNPGSLIGDDQIYNVIVTAHAFIMIFFMVMPTLIGGFGNWLVPLMLGAPDMAFPRMNNMSFWLLPPSLSLLLMSSMVESGAGTGWTVYPPLSSNIAHGGASVDLAIFSLHLAGISSILGAVNFITTVINMRSIG
MSFDRMPLFVWSVAITALLLLLSLPVLAGAITMLLTDRNLNTSFFDPAGGGDPILYQHL
>COLFB819-12|KJ962563|ZMUO.001294|Quedius_limbatus
TLYFIFGAWAGMVGTSLSLLIRAELGNPGTLIGDDQIYNVIVTAHAFIMIFFMVMPIVIGGFGNWLVPLMLGAPDMAFPRMNNMSFWLLPPSLSLLLMSSMVESGAGTGWTVYPPLSSNIAHGGASVDLAIFSLHLAGISSILGAVNFITTVINMRSIG
MSFDRMPLFVWSVAITALLLLLSLPVLAGAITMLLTDRNLNTSFFDPAGGGDPILYQHL
>COLFF1000-13|KJ965465|ZMUO.007365|Sirocalodes_depressicollis
TLYFIFGSWAGMAGTSLSMLIRTELGNPGSLIGDDQIYNSIVTAHAFIMIFFMVMPILIGGFGNWLVPLMLGAPDMAFPRLNNMSFWLLPPSLALLLMSSIVNKGAGTGWTVYPPLSMNVAHEGMSVDLAIFSLHMAGISSILGAINFISTIMNMRPT
GMTAEYMPLFVWAVKITAILLLLSLPVLAGAITMLLTDRNINTSFFDPAGGGDPILYQHL
>COLFF697-13|KJ962234|ZMUO.006397|Abdera_affinis
TLYFIFGAWSGMLGTSLSLLIRSELGNPGSLIGDDQIYNVIVTAHAFIMIFFMVMPIMMGGFGNWMVPLMLGAPDMAFPRMNNMSFWLLPPSLTLLIMSSIVENGAGTGWTVYPPLSSNIAHSGSSVDLAIFSLHLAGVSSILGAVNFITTIINMRPAG
MTLDRMPLFVWSVAITAVLLLLSLPVLAGAITMLLTDRNLNTSFFDPAGGGDPILYQHL
>COLFF486-13|KJ961815|ZMUO.006566|Cryptolestes_ferrugineus
TLYFIFGSWAGMAGTSLSLLIRTELGTPGSLIGDDQIYNVIVTAHAFIMIFFMVMPIMIGGFGNWLVPLMLGAPDMAFPRMNNMSFWLLPPSLSLLLMSSIVEKGAGTGWTVYPPLSSNIAHGGSSVDLAIFSLHLAGISSILGAVNFISTVINMRPQGM
TLERMPLFVWAVVITAILLLLSLPVLAGAITMLLTDRNINTSFFDPAGGGDPILYQHL
>COLFA526-12|KJ962607|ZMUO.000566|Cychramus_luteus
TLYFIFGAWSGMVGTSLSILIRTELGNPGSLIGNDQIYNVIVTAHAFIMIFFMVMPFMIGGFGNWLVPLMLGAPDMAFPRMNNMSFWLLPPSLSLLLMSSIVESGAGTGWTVYPPLSSNIAHGGSSVDLAIFSLHLAGISSILGAVNFITTVINMRPNGM
SFDRMPLFVWAVVITAILLLLSLPVLAGAITMLLTDRNLNTTFFDPSGGGDPILYQHL
>COLFE1385-13|KJ965507|ZMUO.007180|Notaris_acridulus
TLYFIFGAWSGMVGTSLSMLIRMELGNPGSLIGDDQIYNVIVTAHAFIMIFFMVMPIMIGGFGNWLVPLMLGAPDMAFPRLNNLSFWLLPPSLIILLSSSLIEKGAGTGWTVYPPLSSNIAHSGPSVDLAIFSLHMAGISSILGAINFISTVINMRPMGMS
PDRMTLFVWAVEITAILLLLSLPVLAGAITMLLTDRNINTSFFDPAGGGDPILYQHL
>COLFB929-12|KJ963482|ZMUO.001974|Polydrusus_fulvicornis
TLYFIFGSWSGMVGTSLSMLIRTELGNPGSLIGDDQIYNVIVTAHAFIMIFFMVMPMMIGGFGNWLVPLMLGAPDMAFPRLNNMSFWLLPPSLSLLLMSSIVDKGAGTGWTVYPPLSANIAHEGSSVDLAIFSLHMAGVSSILGAINFISTVINMRPM
GMTFDRMSLFIWAVKITAILLLLSLPVLAGAITMLLTDRNINTSFFDPAGGGDPILYQHL
>COLFG144-13|KJ962256|ZMUO.007839|Atomaria_pusilla
TLYFIFGAWAGMVGTSLSMLIRTELGTPGSLIGDDQIYNVIVTAHAFIMIFFMVMPMMIGGFGNWLVPLMLGAPDMAFPRLNNMSFWLLPPSLMFLLMSSIVEKGAGTGWTVYPPLSSNVAHAGSSVDLAIFSLHLAGISSILGSVNFITTVINMRPN
EMNFDRLPLFVWAVKITTILLLLSLPVLAGAITMLLTDRNINTSFFDPAGGGDPILYQHL
>COLFC760-12|KJ965100|ZMUO.003325|Hylesinus_crenatus
TLYFILGAWSGMVGTSLSMIIRTELGTPGSLIGNDQIYNSIVTAHAFIMIFFMVMPIMIGGFGNWLVPLMLGAPDMAFPRLNNMSFWLLPPSLLFLLMSSIIDKGAGTGWTVYPPLANNIAHEGASVDLAIFSLHMAGVSSILGALNFISTIINMHPMG
MKLDRLTLFTWAVKITAILLLLSLPVLAGAITMLLTDRNINTSFFDPAGGGDPILYQHL
>COLFD775-12|KJ967221|ZMUO.004765|Cantharis_pellucida
TLYFIFGAWSGSLGLALSLLIRAELGTPGTLIGNDQIYNVIVTAHAFIMIFFMVMPIMIGGFGNWLVPLMLGAPDMAFPRMNNMSFWFLPPSLMFLLMSSMVESGAGTGWTVYPPLSANIAHSGPSVDLAIFSLHMAGISSILGAVNFISTIMNMKPPS
MKFDQMPLFVWSVGITALLLLLSLPVLAGAITMLLSDRNLNTSFFDPMGGGDPILYQHL
>COLFA642-12|KJ964240|ZMUO.000777|Philonthus_albipes
TLYFIFGAWAGMVGTSLSLLIRAELGNPGSLIGDDQIYNVIVTAHAFIMIFFMVMPIVIGGFGNWLVPLMLGAPDMAFPRMNNMSFWLLPPSLTLLLMSSMVESGAGTGWTVYPPLSSNIAHGGASVDLAIFSLHLAGISSILGAVNFITTVINMQSTGI
SFDRMPLFIWSVAITALLLLLSLPVLAGAITMLLTDRNLNTTFFDPAGGGDPILYQHL
>COLFC720-12|KJ964842|ZMUO.003285|Polydrusus_fulvicornis
TLYFIFGSWSGMVGTSLSMLIRTELGNPGSLIGDDQIYNVIVTAHAFIMIFFMVMPMMIGGFGNWLVPLMLGAPDMAFPRLNNMSFWLLPPSLSLLLMSSIVDKGAGTGWTVYPPLSANIAHEGSSVDLAIFSLHMAGVSSILGAINFISTVINMRPM
GMTFDRMSLFIWAVKITAILLLLSLPVLAGAITMLLTDRNINTSFFDPAGGGDPILYQHL

>COLFF524-13|KJ964981|ZMUO.006604|Platambus_maculatus
TLYFIFGAWAGMVGTSLSMLIRAELGNPGSLIGDDQIYNVIVTAHAFVMIFFMVMPIMIGGFGNWLVPLMLGAPDMAFPRMNNMSFWLLPPSLSLLLMSSLVENGAGTGWTVYPPLSSGIAHGGASVDLAIFSLHLAGISSILGAVNFITTIINMRSVG
MTFDRMPLFVWSVGITALLLLLSLPVLAGAITMLLTDRNLNTSFFDPAGGGDPILYQHL
>COLFA686-12|KJ965929|ZMUO.000821|Pterostichus_diligens
TLYFIFGAWSGMVGTSLSMLIRAELGNPGSLIGDDQIYNVIVTAHAFVMIFFMVMPIMIGGFGNWLVPLMLGAPDMAFPRMNNMSFWLLPPSLTLLLMSSMVESGAGTGWTVYPPLSSGIAHAGASVDLAIFSLHLAGISSILGAVNFITTIINMRSIG
MTFDRMPLFVWSVGITALLLLLSLPVLAGAITMLLTDRNLNTSFFDPAGGGDPILYQHL
>COLFA698-12|KJ964170|ZMUO.000833|Bembidion_guttula
TLYFIFGAWSGMVGTSLSMLIRAELGSPGSLIGDDQIYNVIVTAHAFIMIFFMVMPILIGGFGNWLVPLMLGAPDMAFPRMNNMSFWLLPPSLSLLLMSSMVENGAGTGWTVYPPLSSSIAHSGASVDLAIFSLHLAGVSSILGAVNFITTIINMRSTG
MTFDRMPLFVWSVGITALLLLLSLPVLAGAITMLLTDRNLNTSFFDPAGGGDPILYQHL
>COLFE018-12|KJ965646|ZMUO.005243|Alosterna_tabacicolor
TLYFIFGAWAGMVGTSLSLLIRSELGSPGSFIGDDQVYNVIVTAHAFIMIFFMVMPIMIGGFGNWLVPLMLGAPDMAFPRMNNMSFWLLPPSLTLLIMSSIVESGAGTGWTVYPPLSSNIAHGGSSVDLAIFSLHLAGISSILGAVNFITTVINMRPMG
MNLDRMPLFVWAVVITAILLLLSLPVLAGAITMLLTDRNLNTSFFDPAGGGDPILYQHL
>COLFE1212-13|KJ962716|ZMUO.007007|Agonum_hypocrita
TLYFIFGAWSGMVGTSLSMLIRAELGNPGALIGDDQIYNVIVTAHAFIMIFFMVMPIMIGGFGNWLVPLMLGAPDMAFPRMNNMSFWLLPPSLTLLLMSSMVESGAGTGWTVYPPLSSGIAHAGASVDLAIFSLHLAGVSSILGAVNFITTIINMRSV
GMTFDRMPLFVWSVGITALLLLLSLPVLAGAITMLLTDRNLNTSFFDPAGGGDPILYQHL
>COLFG065-13|KJ966407|ZMUO.007570|Exocentrus_lusitanus
TLYFIFGAWAAMVGTSLSLLIRSELGSPGSLIGDDQIYNVIVTAHAFIMIFFMVMPIMIGGFGNWLIPLMLGAPDMAFPRMNNMSFWLLPPSLSLLIMSSIVENGAGTGWTVYPPLSANIAHSGSSVDLAIFSLHLAGISSILGAVNFITTVINMRPMGM
MFDRLSLFIWAVKITAILLLLSLPVLAGAITMLLTDRNLNTSFFDPAGGGDPILYQHL
>COLFD560-12|KJ964640|ZMUO.004360|Harpalus_luteicornis
TLYFIFGAWAGMVGTSLSMLIRAELGTPGALIGDDQIYNVIVTAHAFIMIFFMVMPIMIGGFGNWLVPLMLGAPDMAFPRMNNMSFWLLPPSLSLLLMSSMVESGAGTGWTVYPPLSSGIAHSGASVDLAIFSLHLAGISSILGAVNFITTIINMRSVG
MTFDRMPLFVWSVGITALLLLLSLPVLAGAITMLLTDRNLNTSFFDPAGGGDPILYQHL
>COLFB500-12|KJ963068|ZMUO.000975|Aphodius_fasciatus
TLYFLFGSWAGMVGTSLSLLIRAELGNPGTLIGDDQIYNVIVTAHAFVMIFFMVMPILIGGFGNWLVPLMLGAPDMAFPRMNNMSFWLLPPSLTLLLMSSMVESGAGTGWTVYPPLSSNIAHGGASVDLAIFSLHLAGISSILGAVNFITTVINMRSPG
MTFDRMPLFVWSVAITALLLLLSLPVLAGAITMLLTDRNLNTSFFDPAGGGDPILYQHL
>COLFC419-12|KJ966658|ZMUO.002984|Oreodytes_alpinus
TLYFLFGAWSGMVGTSLSMLIRAELGNPGSLIGDDQIYNVIVTAHAFIMIFFMVMPIMIGGFGNWLVPLMLGAPDMAFPRMNNMSFWLLPPSLTLLLMSSMVENGAGTGWTVYPPLSAGIAHGGASVDLAIFSLHLAGISSILGAVNFITTIINMRSV
GMTFDRMPLFVWSVGITALLLLLSLPVLAGAITMLLTDRNLNTSFFDPAGGGDPILYQHL
>COLFF677-13|KJ967069|ZMUO.006377|Coccidula_scutellata
TLYFLFGMWAGMLGTSLSILIRLELGTPSSLIGNDQIYNVIVTAHAFIMIFFMVMPIMIGGFGNWLVPLMIGAPDMAFPRMNNMSFWLLPPALTLLILSSVVESGAGTGWTVYPPLSSNLAHSGSSVDLAIFSLHLAGISSILGAVNFITTVINMRPYGMT
FEKMPLFVWSVVITAILLLLSLPVLAGAITMLLTDRNINTSFFDPAGGGDPILYQHL
>COLFE306-12|KJ964062|ZMUO.004866|Brachonyx_pineti
TLYFIFGMWSGSIGTSLSVLIRAELGTPGNLIGDDQIYNTIVTAHAFVMIFFMVMPIMIGGFGNWLIPLMLAAPDMAFPRLNNLSFWLLPPSLTLLIMSSIIGKGAGTGWTVYPPLSANLAHEGPSVDFAIFSLHMAGISSILGAMNFISTILNMKPMKMK
FDQMPLFIWAVKITAILLLISLPVLAGAITMLLTDRNINTSFFDPAGGGDPILYQHL
>COLFF906-13|KJ967157|ZMUO.006701|Tachyporus_pusillus
TLYFIFGAWSGMVGTSLSLLIRAELGNPGSLIGDDQIYNVIVTAHAFIMIFFMVMPIVIGGFGNWLVPLMLGAPDMAFPRMNNMSFWLLPPSLTLLLMSSMVESGAGTGWTVYPPLSANIAHSGPSVDLAIFSLHLAGISSILGAVNFITTVINMRASG
MHFDRMPLFIWSVAITALLLLLSLPVLAGAITMLLTDRNLNTSFFDPAGGGDPILYQHL
>COLFD836-12|KJ967328|ZMUO.004826|Phyllodrepa_clavigera
SLYFIFGAWAGMVGTSLSILIRAELGNPGSLIGDDQIYNVIVTAHAFVMIFFMVMPIVIGGFGNWLVPLMLGAPDMAFPRMNNMSFWLLPPSLTLLLMSSMVESGAGTGWTVYPPLSSNIAHGGASVDLAIFSLHLAGISSILGAVNFITTVINMRSMG
MTFDRMPLFVWSVAITALLLLLSLPVLAGAITMLLTDRNLNTSFFDPAGGGDPILYQHL
>COLFA441-12|KJ964362|ZMUO.000481|Chrysolina_staphylaea
TLYFIFGAWAGMVGTSLSILIRAELGNPGTLIGNDQIYNVIVTAHAFIMIFFMVMPIMIGGFGNWLVPLMLGAPDMAFPRMNNMSFWFLPPSLIFLLMSSIVENGVGTGWTVYPPLSTNVAHSGSSVDLAIFSLHLAGISSILGAINFITTVINMRPTGM
KMEQMPLFSWAVLITAILLLLSLPVLAGAITMLLTDRNLNTSFFDPASGGDPILYQHL
>COLFE1563-13|KJ965356|ZMUO.007453|Meligethes_haemorrhoidalis
TLYFIFGAWSAMVGTSLSMLIRTELGNPGSLIGNDQIYNVIVTAHAFVMIFFMVMPFMIGGFGNWLVPLMLGAPDMAFPRMNNMSFWLLPPSLSLLLMSSIVESGAGTGWTVYPPLSSNIAHGGASVDLAIFSLHLAGISSILGAVNFITTVINMRPSE
MTFDRMPLFVWAVAITALLLLLSLPVLAGAITMLLTDRNLNTTFFDPSGGGDPILYQHL
>COLFF637-13|KJ961821|ZMUO.006527|Stenus_morio

TLYFIFGAWAGMVGTSLSLLIRAELGNPGSLIGDDQIYNVIVTAHAFVMIFFMVMPVMIGGFGNWLVPLMLGAPDMAFPRMNNMSFWLLPPSLSLLLMSSIVESGAGTGWTVYPPLSSNIAHGGASVDLAIFSLHLAGISSILGAINFITTIFNMRSMKI
QLDCLPLFVWSVGITALLLLLSLPVLAGAITMLLTDRNLNTSFFDPAGGGDPILYQHL
>COLFC006-12|KJ967057|ZMUO.002001|Mycetoporus_tenuis
TLYFIFGSWAGMVGTSLSLLIRAELGNPGSLIGDDQIYNVIVTAHAFVMIFFMVMPIMIGGFGNWLVPLMLGAPDMAFPRMNNMSFWLLPPSLTLLLMSSMVESGVGTGWTVYPPLSSNIAHSGASVDLAIFSLHLAGISSILGAVNFITTVINMRSM
GMTFDRMPLFIWSVAITALLLLLSLPVLAGAITMLLTDRNLNTSFFDPAGGGDPILYQHL
>COLFD535-12|KJ964331|ZMUO.004335|Chilocorus_bipustulatus
TLYFLFGMWSGMVGTALSMIIRLELGTPSSLIGNDQIYNVIVTAHAFIMIFFMVMPIMIGGFGNWLVPLMIGAPDMAFPRLNNMSFWLLPPSLTLLILSSMIESGAGTGWTVYPPLSSNLAHGGSSVDLAIFSLHMAGISSILGAINFISTILNMRPKGML
LEKTPLFVWSVLITAILLLLSLPVLAGAITMLLTDRNINTSFFDPTGGGDPILYQHL
>COLFE1591-13|KJ963860|ZMUO.007481|Hypera_venusta
TLYFIFGTWAGTVGTSLSILIRTELGNPGSLIGNDQIYNTIVTAHAFIMIFFMVMPIMIGGFGNWLVPLMLGAPDMAFPRLNNMSFWLLPPSLSLLLMSSMVDSGAGTGWTVYPPLSSNIAHEGSSVDLAIFSLHMAGVSSILGAINFISTVLNMRPSGM
SLDKMALFIWAVKITAILLLLSLPVLAGAITMLLTDRNINTSFFDPAGGGDPILYQHL
>COLFB385-12|KJ966568|ZMUO.001810|Galerucella_sagittariae
TLYFIFGVWAGMVGTSLSILVRVELGNPGSLIGNDQIYNVIVTAHAFIMIFFMVMPIMIGGFGNWLVPLMIGAPDMAFPRMNNMSFWLLPPSLFLLIMSSIVESGAGTGWTVYPPLSSNIAHGGSSVDLAIFSLHLAGISSILGAINFITTIINMRPKGMT
LDRMPLFVWAVMITAILLLLSLPVLAGAITMLLTDRNLNTSFFDPAGGGDPILYQHL
>COLFD377-12|KJ963684|ZMUO.004272|Trichosirocalus_troglodytes
TLYFIFGAWAGAAGTSLSMLIRTELGNPGSLIGNDQIYNSIVTAHAFIMIFFMVMPILIGGFGNWLVPLMLGAPDMAFPRLNNMSFWLLPPSLTLLLMSSIVNKGAGTGWTVYPPLSANVAHEGASVDLAIFSLHMAGISSILGAINFISTVMNMRPTG
MNSEFMPLFVWAVEITAVLLLLSLPVLAGAITMLLTDRNINTSFFDPAGGGDPILYQHL
>COLFA600-12|KJ964579|ZMUO.000735|Arhopalus_rusticus
TLYFIFGAWASMVGTSLSLLIRSELGSPGSLIGDDQIYNVIVTAHAFVMIFFMVMPIMIGGFGNWLVPLMLGAPDMAFPRMNNMSFWLLPPSLTLLIMSSIVESGAGTGWTVYPPLSSNIAHSGASVDLAIFSLHLAGISSILGAVNFITTVINMRPSGM
TLDRMPLFVWAVVITAILLLLSLPVLAGAITMLLTDRNLNTSFFDPSGGGDPILYQHL
>COLFB154-12|KJ966048|ZMUO.001484|Cetonia_aurata
TLYFLFGSWAGMVGTSLSLLIRAELGNPGSLIGDDQIYNVIVTAHAFIMIFFMVMPVMIGGFGNWLVPLMLGAPDMAFPRMNNMSFWLLPPSLTLLLMSSLVESGAGTGWTVYPPLSSNIAHSGASVDLAIFSLHLAGISSILGAVNFITTVINMRSTG
MTFDRMPLFVWSVVLTALLLLLSLPVLAGAITMLLTDRNINTSFFDPAGGGDPILYQHL
>COLFD789-12|KJ962641|ZMUO.004779|Cryptocephalus_pusillus
TLYFLFGAWAGMVGTSLSLLIRIELGAPGSLIGNDQIYNTIVTAHAFIMIFFMVMPIMIGGFGNWLVPLMLGAPDMAFPRMNNMSFWLLPPSLTLLLMSSIVENGAGTGWTVYPPLSTTIAHAGSSVDLAIFSLHLAGASSIMGAINFISTVINMRPQG
MLMDRTPLFVWAVLITAVLLLLSLPVLAGAITMLLTDRNLNTSFFDPAGGGDPILYQHL
>COLFE1192-13|KJ965233|ZMUO.006987|Gymnusa_brevicollis
TLYFMFGAWAGMVGTSLSLLIRAELGNPGTLIGDDQIYNVIVTAHAFIMIFFMVMPIVIGGFGNWLVPLMLGAPDMAFPRMNNMSFWLLPPSLSLLLMSSLVESGAGTGWTVYPPLSSNIAHSGSSVDLAIFSLHLAGISSILGAVNFITTVINMRSIGM
SFDQMPLFIWSVAITALLLLLSLPVLAGAITMLLTDRNLNTSFFDPSGGGDPILYQHL
>COLFA118-10|HQ559239|MP00380|Nicrophorus_vespilloides
TLYFIFGAWAGMVGMSLSMLIRVELSTPGTLLGDDQMYNVIVTAHAFIMIFFMVMPIVIGGFGNWLVPLMLGAPDMAFPRLNNMSFWLLPPSLSLLLISSMVESGAGTGWTVYPPLSANIAHSGSSVDLAIFSLHLAGISSILGAVNFITTVINMRSPG
MTFDRMPLFVWSVVITALLLLLSLPVLAGAITMLLTDRNLNTSFFDPAGGGDPILYQHL
>COLFB570-12|KJ964539|ZMUO.001045|Limnobaris_pilistriata
TLYFIFGAWAGMTGTSLSMLIRSELGNPGSLIGDDQIYNTIVTAHAFIMIFFMVMPIMIGGFGNWLVPLMLGAPDMAFPRLNNMSFWLLPPSLTLLLMSSIVDKGVGTGWTVYPPLSSNNAHEGASVDLGIFSLHMAGISSILGAMNFISTAMNMRP
TGLKSDQMSLFIWAVKITAILLLLSLPVLAGAITMLLTDRNINTSFFDPTGGGDPILYQHL
>COLFC171-12|KJ962297|ZMUO.002356|Tachinus_marginellus
TLYFIFGAWAGMVGTSLSLLIRAELGNPGTLIGDDQIYNVIVTAHAFIMIFFMVMPIVIGGFGNWLVPLMLGAPDMAFPRMNNMSFWLLPPSLSLLLMSSMVESGAGTGWTVYPPLSSNIAHGGSSVDLAIFSLHLAGISSILGAVNFITTVINMRSIGM
TFDRMPLFVWSVAITALLLLLSLPVLAGAITMLLTDRNLNTTFFDPAGGGDPILYQHL
>COLFC090-12|KJ965434|ZMUO.002085|Quedius_plagiatus
TLYFIFGAWAGMVGTSLSLLIRAELGNPGTLIGDDQIYNVIVTAHAFIMIFFMVMPIVIGGFGNWLVPLMLGAPDMAFPRMNNMSFWLLPPSLSLLLMSSMVESGAGTGWTVYPPLSSNIAHGGASVDLAIFSLHLAGISSILGAVNFITTVINMRSIG
MTFDRMPLFVWSVAITALLLLLSLPVLAGAITMLLTDRNLNTSFFDPAGGGDPILYQHL
>COLFD188-12|KJ963364|ZMUO.004083|Lathrobium_longulum
TLYFIFGVWAGMVGTSLSLLIRTELGNPGSLIGDDQIYNVIVTAHAFIMIFFMVMPIMIGGFGNWLVPLMLGAPDMAFPRMNNMSFWLLPPSLSLLLMSSMVESGAGTGWTVYPPLSSNIAHGGASVDLAIFSLHLAGISSILGAVNFITTIINMRSPG
MTYERMPLFVWAVAITALLLLLSLPVLAGAITMLLTDRNLNTSFFDPAGGGDPILYQHL
>COLFF699-13|KJ966460|ZMUO.006399|Scraptia_fuscula
TLYFIFGAWSGMVGTSLSVLIRSELGTPGSLIGDDQIYNVIVTAHAFIMIFFMVMPILIGGFGNWLVPLMLGAPDMAFPRMNNMSFWLLPPSLTLLIMSSIVENGAGTGWTVYPPLASNLAHNGSSVDLAIFSLHLAGISSILGAVNFITTVINMRPMGM
TFERMPLFVWAVVITAVLLLLSLPVLAGAITMLLTDRNLNTSFFDPAGGGDPILYQHL

>COLFD092-12|KJ967359|ZMUO.003987|Acanthoscelides_obtectus
TLYFLFGAWAGMAGTSLSILIRAELGNPGSLIGNDQIYNVIVTAHAFIMIFFMVMPIMIGGFGNWLVPLMLGAPDMAFPRMNNMSFWLLPPSLSFLMLSSLVESGAGTGWTVYPPLASNIAHSGSSVDLAIFSLHLAGISSILGAVNFITTIMNMRPNG
MSMDRMPLFSWAVMITAILLLLSLPVLAGAITMLLTDRNLNTSFFDPAGGGDPILYQHL
>COLFE1225-13|KJ962655|ZMUO.007020|Pselaphus_heisei
TLYLIFGAWAGMVGTSLSMLIRSELGNPGSLIGDDQIYNVIVTAHAFIMIFFMVMPLMIGGFGNWLVPLMLGAPDMAFPRMNNMSFWLLPPSLSLLLMSSIIENGAGTGWTVYPPLSSNIAHNGSSVDLTIFSLHLAGISSILGAVNFISTIINMRATGIIL
DRMPLFVWSVLITAILLLLSLPVLAGAITMLLTDRNLNTSFFDPAGGGDPILYQHL
>COLFE1090-13|KJ964392|ZMUO.006885|Scymnus_nigrinus
TLYFLFGLWAGMVGTSLSILIRLELGTTSALIGNDQIYNVIVTAHAFIMIFFMVMPIMIGGFGNWLVPLMIGAPDMAFPRLNNMSFWLLPPSLTFLILSSLVESGAGTGWTVYPPLSSNIAHGGSSVDMAIFSLHLAGISSILGAVNFISTIINMRTYGMTFE
KMPLFVWSVFITAILLLLSLPVLAGAITMLLTDRNINTSFFDPAGGGDPILYQHL
>COLFE1228-13|KJ962198|ZMUO.007023|Carabus_nemoralis
TLYFIFGAWSGMVGTSLSMLIRAELGNPGSLIGDDQIYNVIVTAHAFVMIFFMVMPIMIGGFGNWLVPLMLGAPDMAFPRMNNMSFWLLPPSLTLLLMSSMVEKGAGTGWTVYPPLSSGIAHSGASVDLAIFSLHLAGISSILGAVNFITTIINMRSV
GMTFDRMPLFIWSVGITALLLLSLPVLAGAITMLLTDRNLNTSFFDPAGGGDPILYQHL
>COLFB666-12|KJ963914|ZMUO.001141|Atheta_elongatula
TLYFIFGAWAGMVGTSLSLLIRAELGNPGSLIGDDQIYNVIVTAHAFVMIFFMVMPIVIGGFGNWLVPLMLGAPDMAFPRMNNMSFWLLPPSLTLLLMSSMVESGAGTGWTVYPPLSSNIAHGGSSVDLAIFSLHLAGISSILGAVNFISTVINMRSTGI
SFDRMPLFVWSVAITALLLLSLPVLAGAITMLLTDRNLNTSFFDPAGGGDPILYQHL
>COLFD451-12|KJ966552|ZMUO.003871|Oxystoma_cerdo
TLYFILGLWSGMVGTSLSMLIRIELGNSGSLIGDDQIYNVIVTAHAFIMIFFMVMPIMIGGFGNWLVPLMLGAPDMAFPRMNNMSFWMLPPSLTLLLMSSIVEKGAGTGWTVYPPLASNIAHSGASVDLAIFSLHLAGISSILGAVNFISTMINMHPNG
LSLDQLSLFTWAVKITAILLLLSLPVLAGAITMLLTDRNINTSFFDPAGGGDPILYQHL
>COLFD511-12|KJ963551|ZMUO.004311|Epuraea_pallescens
TLYFIFGAWSGMIGTSLSILIRTELGSPGSLIGNDQIYNVIVTAHAFIMIFFMVMPFMIGGFGNWLVPLMLGAPDMAFPRMNNMSFWLLPPSLSLLLMSSIVESGAGTGWTVYPPLSSNIAHGGASVDLAIFSLHLAGISSILGAVNFITTIINMRPTGMTL
DRMPLFVWSVVITAILLLLSLPVLAGAITMLLTDRNLNTTFFDPSGGGDPILYQHL
>COLFF256-13|KJ966489|ZMUO.005861|Anaspis_frontalis
TLYFIFGAWSGMVGTSLSLLIRSELGTPGSLIGDDQIYNVIVTAHAFIMIFFMVMPILIGGFGNWLVPLMLGAPDMAFPRMNNMSFWLLPPSLTLLIMSSVVENGAGTGWTVYPPLAANIAHSGSSVDLAIFSLHLAGVSSILGAVNFITTVINMRPQG
MTLDRMPLFVWAVVITAVLLLSLPVLAGAITMLLTDRNLNTSFFDPAGGGDPILYQHL
>COLFC846-12|KJ964365|ZMUO.003411|Galerucella_sp.
TLYFIFGVWAGMVGTSLSILVRVELGNPGSLIGNDQIYNVIVTAHAFIMIFFMVMPIMIGGFGNWLVPLMIGAPDMAFPRMNNMSFWLLPPSLFLLIMSSIVESGAGTGWTVYPPLSSNIAHGGSSVDLAIFSLHLAGISSILGAINFITTIINMRPKGMT
LDRMPLFVWAVMITAILLLLSLPVLAGAITMLLTDRNLNTSFFDPAGGGDPILYQHL
>COLFG113-13|KJ965886|ZMUO.007808|Cartodere_constricta
SLYFLFGMWSGMVGTSLSLLIRLELGNPGSLIGDDQIYNVIVTAHAFIMIFFMVMPLMMGGFGNWLVPLMLGAPDMAFPRLNNMSFWLLPPSLSLLIMSSVVESGVGTGWTVYPPLSSNIAHGGSSVDLAIFSLHLAGISSILGAVNFITTVMNMRPV
GMTLEQMPLFVWSVLLTAILLLLSLPVLAGAITMLLTDRNLNTSFFDPAGGGDPILYQHL
>COLFE366-12|KJ966686|ZMUO.004926|Hydrobius_fuscipes
TLYFIFGAWAGMVGTSLSILIRAELGNPGTLIGDDQIYNVIVTAHAFIMIFFMVMPIMIGGFGNWLVPLMLGAPDMAFPRMNNMSFWLLPPSLTLLLMSSMVENGAGTGWTVYPPLSSNIAHGGASVDLAIFSLHLAGISSILGAVNFITTVINMRSPN
LTYDRLPLFVWSVAITALLLLLSLPVLAGAITMLLTDRNLNTSFFDPAGGGDPILYQHL
>COLFD102-12|KJ964446|ZMUO.003997|Danosoma_conspersus
TLYFLFGAWAGMLGTSLSLLIRAELGNPGSLIGNDQIYNVVVTAHAFVMIFFMVMPVMIGGFGNWLVPLMLGAPDMAFPRMNNMSFWLLPPSLSLLLMSSIVENGAGTGWTVYPPLSANIAHSGSSVDLAIFSLHLAGISSILGAVNFISTVINMRST
GMTFDRMPLFVWAVVITALLLLLSLPVLAGAITMLLTDRNLNTTFFDPAGGGDPILYQHL
>COLFA434-12|KJ963074|ZMUO.000664|Lordithon_bimaculatus
TLYFIFGSWAGMVGTSLSLLIRAELGNPGSLIGDDQIYNVIVTAHAFIMIFFMVMPIVIGGFGNWLVPLMLGAPDMAFPRMNNMSFWLLPPSLTLLLMSSMVESGAGTGWTVYPPLSSNIAHGGASVDLAIFSLHLAGISSILGAVNFITTVINMRSTG
MTFDRMPLFIWSVAITALLLLLSLPVLAGAITMLLTDRNLNTSFFDPAGGGDPILYQHL
>COLFF092-13|KJ961846|ZMUO.005792|Stenus_boops
TLYFIFGAWAGMVGTSLSLLIRAELGNPGSLIGDDQIYNVIVTAHAFVMIFFMVMPIMIGGFGNWLVPLMLGAPDMAFPRMNNMSFWLLPPSLSLLLMSSIVESGAGTGWTVYPPLSSNIAHGGASVDLAIFSLHLAGISSILGAINFITTIFNMRTMKL
QLDCLPLFVWSVGITALLLLLSLPVLAGAITMLLTDRNLNTSFFDPAGGGDPILYQHL
>COLFF698-13|KJ966682|ZMUO.006398|Colenis_immunda
TLYFIFGAWAGMVGTSLSILIRAELGTPGSLIGDDQIYNVIVTAHAFIMIFFMVMPIVIGGFGNWLVPLMLGAPDMAFPRMNNMSFWLLPPSLTLLLMSSIVENGAGTGWTVYPPLSANIAHSGSSVDLAIFSLHLAGISSILGAVNFITTVINMRSEGMS
FDKMPLFVWSVAITALLLLLSLPVLAGAITMLLTDRNLNTSFFDPAGGGDPILYQHL
>COLFB614-12|KJ964172|ZMUO.001089|Nudobius_lentus

TLYFIFGAWAGMVGTSLSLLIRAELGNPGSLIGDDQIYNVIVTAHAFVMIFFMVMPIVIGGFGNWLVPLMLGAPDMAFPRMNNMSFWLLPPSLTLLLMSSMVENGAGTGWTVYPPLSSNIAHSGASVDLAIFSLHLAGISSILGAVNFITTIINMRSYG
MTFDRMPLFVWSVAITALLLLLSLPVLAGAITMLLTDRNLNTSFFDPTGGGDPILYQHL
>COLFC156-12|KJ963113|ZMUO.002341|Batophila_rubi
TLYFIFGAWAGMVGTSLSMLIRTELGNPGSLIGNDQIYNVIVTAHAFIMIFFMVMPIMIGGFGNWLVPLMIGAPDMAFPRMNNMSFWLLPPSLSLLVSSSIVESGAGTGWTVYPPLSSNIAHSGSSVDLAIFSLHLAGISSILGAINFISTIINMRPHGML
LDRLPLFVWAVMITAILLLLSLPVLAGAITMLLTDRNLNTSFFDPAGGGDPILYQHL
>COLFC313-12|KJ965265|ZMUO.002498|Acidota_quadrata
TLYFIFGAWAGMVGTSLSILIRAELGNPGTLIGDDQIYNVIVTAHAFVMIFFMVMPIVIGGFGNWLVPLMLGAPDMAFPRMNNMSFWLLPPSLTLLLMSSMVESGAGTGWTVYPPLSSNIAHGGSSVDLAIFSLHLAGISSILGAVNFITTVINMRATG
MTFDRMPLFVWSVAITALLLLLSLPVLAGAITMLLTDRNLNTSFFDPAGGGDPILYQHL
>COLFF264-13|KJ964629|ZMUO.005869|Cantharis_decipiens
TLYFIFGAWSGSVGLALSLLIRAELGTPGTLIGNDQIYNVIVTAHAFIMIFFMVMPIMIGGFGNWLVPLMLGAPDMAFPRMNNMSFWFLPPSLMFLLMSSMVESGAGTGWTVYPPLSANIAHSGPSVDLAIFSLHMAGISSILGAVNFISTIMNMKPP
SMKFDQMPLFVWSVGITALLLLLSLPVLAGAITMLLSDRNLNTSFFDPMGGGDPILYQHL
>COLFD412-12|KJ964189|ZMUO.003832|Notothecta_flavipes
TLYFIFGTWAGMVGTSLSLLIRAELGNPGSLIGDDQIYNVIVTAHAFIMIFFMVMPIMIGGFGNWLVPLMLGAPDMAFPRMNNMSFWLLPPSLTLLLMSSMIESGVGTGWTVYPPLSSNIAHGGSSVDLAIFSLHLAGISSILGAVNFISTVINMRSTGIS
FDRMPLFVWAVAITALLLLLSLPVLAGAITMLLTDRNLNTSFFDPAGGGDPILYQHL
>COLFC341-12|KJ961864|ZMUO.002526|Atheta_lapponica
TLYFIFGAWAGMVGTSLSLLIRAELGNPGSLIGDDQIYNVIVTAHAFIMIFFMVMPIVIGGFGNWLVPLMLGAPDMAFPRMNNMSFWLLPPSLTLLLMSSMVESGAGTGWTVYPPLSSNIAHGGSSVDLAIFSLHLAGISSILGAVNFISTVINMRSTGI
SFDRMPLFVWSVAITALLLLLSLPVLAGAITMLLTDRNLNTSFFDPAGGGDPILYQHL
>COLFB001-12|KJ962000|ZMUO.001331|Notiophilus_aquaticus
TLYFIFGAWSGMVGTSLSMLIRAELGNPGSLIGDDQIYNVIVTAHAFVMIFFMVMPIMIGGFGNWLVPLMLGAPDMAFPRMNNMSFWLLPPSLTLLLTSSMVESGAGTGWTVYPPLSSGIAHSGASVDLAIFSLHLAGVSSILGAVNFITTIINMRSVG
MTFDRMPLFVWSVGITALLLLLSLPVLAGAITMLLTDRNLNTSFFDPAGGGDPILYQHL
>COLFE197-12|KJ965555|ZMUO.005042|Ceutorhynchus_apicalis
TLYFIFGSWAGMAGTSLSMLIRTELGSPGKLIGDDQIYNSIVTAHAFIMIFFMVMPILIGGFGNWLVPLMLGAPDMAFPRLNNMSFWLLPPSLSLLLMSSVVNKGAGTGWTVYPPLSTNITHEGMSVDLAIFSLHMAGISSILGAINFISTVMNMRPTG
MTPEFTPLFVWAVEITAILLLLSLPVLAGAITMLLTDRNINTSFFDPSGGGDPILYQHL
>COLFC615-12|KJ964738|ZMUO.003180|Abdera_affinis
TLYFIFGAWSGMLGTSLSLLIRSELGNPGSLIGDDQIYNVIVTAHAFIMIFFMVMPIMMGGFGNWMVPLMLGAPDMAFPRMNNMSFWLLPPSLTLLIMSSIVENGAGTGWTVYPPLSSNIAHSGSSVDLAIFSLHLAGVSSILGAVNFITTIINMRPAG
MTLDRMPLFVWSVAITAVLLLLSLPVLAGAITMLLTDRNLNTSFFDPAGGGDPILYQHL
>COLFC290-12|KJ966034|ZMUO.002475|Malthodes_flavoguttatus
TLYFMFGAWSGMLGTSLSLLIRAELGSPGSLIGNDQIYNVIVTAHAFIMIFFMVMPIMIGGFGNWLVPLMLGAPDMAFPRMNNMSFWFLPPSLSLLLMSSIVENGAGTGWTVYPPLSANIAHSGASVDLAIFSLHMAGISSILGAVNFISTVINMRSTG
MSFDRMPLFVWAVAITALLLLLSLPVLAGAITMLLTDRNLNTSFFDPAGGGDPILYQHL
>COLFE1480-13|KJ962243|ZMUO.007275|Scopaeus_laevigatus
TLYFLFGAWSGMVGTSLSLLIRAELSAPGSMIGDDQIYNVIVTAHAFVMIFFMVMPIVIGGFGNWLVPLMLGAPDMAFPRMNNMSFWLLPPSLSLLLMSSMVESGAGTGWTVYPPLSSNIAHGGASVDLAIFSLHLAGISSILGAVNFITTVINMRSP
GMTYERMPLFVWSVAITALLLLLSLPVLAGAITMLLTDRNLNTSFFDPAGGGDPILYQHL
>COLFD940-12|KJ962415|ZMUO.004550|Longitarsus_tabidus
TLYFIFGIWAGMVGTSLSILIRTELGNPGSLIGNDQIYNVIVTAHAFIMIFFMVMPIMIGGFGNWLVPLMIGAPDMAFPRMNNMSFWLLPPSLFLLVMSSMVESGAGTGWTVYPPLSSNIAHGGSSVDLAIFSLHLAGISSILGAINFITTVINMRPIGMT
LDRMPLFVWAVVITAILLLLSLPVLAGAITMLLTDRNLNTTFFDPAGGGDPILYQHL
>COLFB700-12|KJ963966|ZMUO.001175|Trachyphloeus_rectus
TLYFIFGSWSGMIGTSLSMLIRTELGNPGSLIGDDQIYNVIVTAHAFIMIFFMIMPIMIGGFGNWLIPLMLGAPDMAFPRLNNMSFWLLPPSLSLLLMSSIVDKGAGTGWTVYPPLAANIAHEGASVDLAIFSLHMAGVSSILGAINFISTIINMRPKGMS
LDRMPLFIWSVKITTILLLLSLPVLAGAITMLLTDRNINTSFFDPAGGGDPILYQHL
>COLFF258-13|KJ966606|ZMUO.005863|Melasis_buprestoides
TLYFIFGAWSGMMGTSLSLLIRMELSSPGALIGNDQIYNVIVTAHAFVMIFFMVMPMMMGGFGNWLVPLMLGAPDMAFPRMNNMSFWLLPPSLSLLLMNSMVESGAGTGWTVYPPLSSNIAHSGASVDLAIFSLHLAGISSILGAINFITTVINMR
PKSMTFDKIPLFVWSVSLTALLLLLALPVLAGAITMLLTDRNLNTTFFDPAGGGDPILYQHL
>COLFG035-13|KJ965196|ZMUO.007540|Graphoderus_zonatus
TLYFIFGAWSGMVGTSLSMLIRAELGNPGSLIGDDQIYNVIVTAHAFIMIFFMVMPIMIGGFGNWLVPLMLGAPDMAFPRMNNMSFWLLPPSLTLLLMSSMVENGAGTGWTVYPPLSAGIAHGGASVDLAIFSLHLAGVSSILGAVNFITTIINMRSV
GMTFDRMPLFVWSVGITALLLLLSLPVLAGAITMLLTDRNLNTSFFDPAGGGDPILYQHL
>COLFE823-13|KJ965502|ZMUO.005668|Cassida_sanguinosa
TLYFIFGFWSGMVGTSLSILIRAELGNPGTLIGNDQIYNSIVTAHAFIMIFFMVMPIMIGGFGNWLVPLMLGAPDMAFPRLNNMSFWLLPPSITFLIMSSIIESGAGTGWTVYPPLSSNIAHSGASVDLAIFSLHLAGISSILGAINFISTIMNMQPAGMSL
DKMPLFVWAVIITAILLLLSLPVLAGAITMLLTDRNFNTSFFDPAGGGDPILYQHL

>COLFA498-12|KJ962171|ZMUO.000538|Psylliodes_crambicola
TLYFIFGIWSGMIGTSLSILIRAELGSPGSLIGNDQIYNVIVTAHAFIMIFFMVMPIMIGGFGNWLVPLMIGAPDMAFPRMNNMSFWLLPPSLLLLLMSSMVESGAGTGWTVYPPLSSNIAHGGSSVDLAIFSLHLAGISSILGAINFITTVINMRPEGMTL
DRMPLFVWAVVITAILLLLSLPVLAGAITMLLTDRNLNTSFFDPIGGGDPILYQHL
>COLFE1100-13|KJ965790|ZMUO.006895|Gymnusa_variegata
TLYFMFGAWAGMVGTSLSLLIRAELGNPGTLIGDDQIYNVIVTAHAFIMIFFMVMPIVIGGFGNWLVPLMLGAPDMAFPRMNNMSFWLLPPSLSLLLMSSLVESGAGTGWTVYPPLSSNIAHSGSSVDLAIFSLHLAGISSILGAVNFITTVINMRSIGM
SFDRMPLFIWSVAITALLLLLSLPVLAGAITMLLTDRNLNTSFFDPSGGGDPILYQHL
>COLFA606-12|KJ965629|ZMUO.000741|Anoplodera_reyi
TLYFIFGAWASMVGTSLSLLIRSELGNPGSLIGDDQIYNVIVTAHAFVMIFFMVMPIMIGGFGNWLVPLMLGAPDMAFPRMNNMSFWLLPPSLTLLIMSSIVESGAGTGWTVYPPLSSNIAHGGSSVDLAIFSLHLAGISSILGAVNFITTVINMRPVGM
SPERMPLFVWAVVITAVLLLLSLPVLAGAITMLLTDRNLNTSFFDPAGGGDPILYQHL
>COLFE1259-13|KJ963862|ZMUO.007054|Gnypeta_sellmani
TLYFIFGAWAGMVGTSLSLLIRAELGNPGSLIGDDQIYNVIVTAHAFVMIFFMVMPIVIGGFGNWLVPLMLGAPDMAFPRMNNMSFWLLPPSLTLLLMSSMVENGAGTGWTVYPPLSSNIAHSGSSVDLAIFSLHLAGISSILGAVNFISTVINMRSAG
MSFDRMPLFVWSVVITALLLLLSLPVLAGAITMLLTDRNLNTSFFDPAGGGDPILYQHL
>COLFA097-10|JN297960|MP00342|Cis_bidentatus
TLYFIFGAWSGMVGTSMSMLIRSELGNPGSLIGNDQIYNVIVTAHAFVMIFFMVMPIMIGGFGNWLVPLMLGAPDMAFPRMNNMSFWLLPPSLSLLLMSSIVENGAGTGWTVYPPLSSNIAHSGSSVDLTIFSLHLAGISSILGAVNFISTIINMRPM
GMNLDQMPLFVWAVLITAVLLLLSLPVLAGAITMLLTDRNFNTSFFDPAGGGDPILYQHL
>COLFB714-12|KJ965387|ZMUO.001189|Megarthrus_denticollis
TLYFIFGAWAGMIGTSLSILIRAELGNPGSLIGDDQIYNVIVTAHAFIMIFFMVMPIVIGGFGNWLVPLMLGAPDMAFPRMNNMSFWLLPPSISLLLISSMVESGAGTGWTVYPPLSSNIAHGGASVDLAIFSLHLAGISSILGAVNFITTVINMRSMGMT
FDRMPLFVWSVAITALLLLLSLPVLAGAITMLLTDRNINTSFFDPAGGGDPILYQHL
>COLFC568-12|KJ966058|ZMUO.003133|Harpalus_laevipes
TLYFIFGAWAGMVGTSLSMLIRAELGTPGALIGNDQIYNVIVTAHAFIMIFFMVMPIMIGGFGNWLVPLMLGAPDMAFPRMNNMSFWLLPPSLTLLLMSSMVESGAGTGWTVYPPLSSGIAHSGASVDLAIFSLHLAGISSILGAVNFITTIINMRSVG
MTFDRMPLFVWSVGITALLLLLSLPVLAGAITMLLTDRNLNTSFFDPAGGGDPILYQHL
>COLFE611-13|KJ966422|ZMUO.005456|Enochrus_affinis
TLYFIFGAWAGMVGTSLSILIRAELGNPGTLIGDDQIYNVIVTAHAFIMIFFMVMPIMIGGFGNWLVPLMLGAPDMAFPRMNNMSFWLLPPSLTLLLMSSMVESGAGTGWTVYPPLSSNIAHGGASVDLAIFSLHLAGISSILGAVNFITTVINMRSPS
MTYDRLPLFVWSVAITALLLLLSLPVLAGAITMLLTDRNLNTSFFDPAGGGDPILYQHL
>COLFB721-12|KJ966679|ZMUO.001196|Atheta_setigera
TLYFIFGAWSGMVGTSLSLLIRAELGNPGSLIGDDQIYNVIVTAHAFIMIFFMVMPVVIGGFGNWLVPLMLGAPDMAFPRMNNMSFWLLPPSLTLLLMSSMVESGAGTGWTVYPPLSSNIAHSGSSVDLAIFSLHLAGISSILGAVNFISTVINMRSTGI
SFDRMPLFVWSVAITALLLLLSLPVLAGAITMLLTDRNLNTSFFDPAGGGDPILYQHL
>COLFD264-12|KJ966670|ZMUO.004159|Hypera_nigrirostris
TLYFIFGTWAGTVGTSLSILIRTELGNPGSLIGNDQIYNTIVTAHAFIMIFFMVMPIMIGGFGNWLVPLMLGAPDMAFPRLNNMSFWLLPPSLSLLLMSSMVDKGAGTGWTVYPPLSSNIAHEGSSVDLAIFSLHMAGVSSILGAINFISTVLNMRPSGM
SLDKMALFIWAVKITAILLLLSLPVLAGAITMLLTDRNINTSFFDPAGGGDPILYQHL
>COLFD526-12|KJ964591|ZMUO.004326|Cantharis_paludosa
TLYFIFGAWSGSLGLALSLLIRAELGTPGTLIGNDQIYNVIVTAHAFIMIFFMVMPIMIGGFGNWLVPLMLGAPDMAFPRMNNMSFWFLPPSLMFLLMSSMVESGAGTGWTVYPPLSANIAHSGPSVDLAIFSLHMAGISSILGAVNFISTIMNMKPPS
MKFDQMPLFVWSVGITALLLLLSLPVLAGAITMLLSDRNLNTSFFDPMGGGDPILYQHL
>COLFE1606-13|KJ962770|ZMUO.007496|Longitarsus_melanocephalus
TLYFIFGIWAGMVGTSLSILIRTELGNPGSLIGNDQIYNVIVTAHAFIMIFFMVMPIMIGGFGNWLVPLMIGAPDMAFPRMNNMSFWLLPPSLFFLIMSSIVESGAGTGWTVYPPLSSNIAHGGSSVDLAIFSLHLAGISSILGAINFITTVINMRPIGMTL
DRMPLFVWAVMITAILLLLSLPVLAGAITMLLTDRNLNTTFFDPAGGGDPILYQHL
>COLFE1173-13|KJ966919|ZMUO.006968|Stenus_comma
TLYFIFGSWAGMVGTSLSLLIRAELGNPGSLIGDDQIYNVIVTAHAFIMIFFMVMPIMIGGFGNWLVPLMLGAPDMAFPRMNNMSFWLLPPSLSLLLMSSIVESGAGTGWTVYPPLSSNIAHSGASVDLAIFSLHLAGISSILGAINFITTIFNMRTMKLQ
LDCLPLFVWSVSITALLLLLSLPVLAGAITMLLTDRNLNTSFFDPAGGGDPILYQHL
>COLFB419-12|KJ963618|ZMUO.001844|Dromius_agilis
TLYFIFGAWAGMVGTSLSMLIRAELGNPGALIGDDQIYNVIVTAHAFIMIFFMVMPIMIGGFGNWLVPLMLGAPDMAFPRMNNMSFWLLPPSLSLLLMSSMVESGAGTGWTVYPPLSSGIAHAGASVDLAIFSLHLAGVSSILGAVNFITTIINMRSIG
MTFDRMPLFVWSVGITALLLLLSLPVLAGAITMLLTDRNLNTSFFDPAGGGDPILYQHL
>COLFE1473-13|KJ964696|ZMUO.007268|Sitona_lepidus
TLYFIFGAWAGMVGTSLSMLIRTELGNPGSLIGDDQIYNVIVTAHAFIMIFFMVMPMMIGGFGNWLVPLMLGAPDMAFPRLNNMSFWLLPPSLTLLLMSSIVDKGAGTGWTVYPPLSANIAHEGASVDLAIFSLHMAGISSILGAINFISTIINMRPKG
MTFDRTALFVWSVKITAILLLLSLPVLAGAITMLLTDRNINTSFFDPAGGGDPILYQHL
>COLFD171-12|KJ964242|ZMUO.004066|Odacantha_melanura

TLYFIFGAWSGMVGTSLSMLIRAELGNPGALIGDDQIYNVIVTAHAFIMIFFMVMPIMIGGFGNWLVPLMLGAPDMAFPRMNNMSFWLLPPSLTLLLMSSMVESGAGTGWTVYPPLSSGIAHAGASVDLAIFSLHLAGVSSILGAVNFITTIINMRSV
GMTFDRMPLFVWSVGITALLLLLSLPVLAGAITMLLTDRNLNTSFFDPAGGGDPILYQHL
>COLFD289-12|KJ962859|ZMUO.004184|Bembidion_schuppelii
TLYFIFGAWSGMVGTSLSMLIRAELGNPGSLIGDDQIYNVIVTAHAFVMIFFMVMPILIGGFGNWLVPLMLGAPDMAFPRMNNMSFWLLPPSLTLLLMSSTVENGAGTGWTVYPPLSSSIAHSGASVDLAIFSLHLAGVSSILGAVNFITTIINMRSTG
MTFDRMPLFVWSVGITALLLLLSLPVLAGAITMLLTDRNLNTSFFDPAGGGDPILYQHL
>COLFF495-13|KJ963952|ZMUO.006575|Hippodamia_variegata
TLYFLFGMWAGMIGTSLSLLIRLELGTTGSLIGNDQIYNVIVTAHAFIMIFFMVMPIMIGGFGNWLVPLMIGAPDMAFPRLNNMSFWLLPPALTLLLFSSIVEMGAGTGWTVYPPLSSNLAHNGPSVDLVIFSLHLAGISSILGAVNFISTIMNMRPYGM
SLDKTPLFVWSVLITAILLLLSLPVLAGAITMLLTDRNLNTSFFDPTGGGDPILYQHL
>COLFC830-12|KJ965448|ZMUO.003395|Ampedus_balteatus
TLYFIFGAWAGMLGTSLSLLIRAELGNPGSLIGNDQIYNVIVTAHAFIMIFFMVMPIMIGGFGNWLVPLMLGAPDMAFPRMNNMSFWLLPPSLSLLLMSSIVENGAGTGWTVYPPLSSNIAHSGSSVDLAIFSLHLAGISSILGAVNFISTVINMRSTGIT
FDRMPLFVWAVAITALLLLLSLPVLAGAITMLLTDRNLNTSFFDPAGGGDPILYQHL
>COLFF491-13|KJ966026|ZMUO.006571|Cordalia_obscura
TLYFIFGAWAGMVGTSLSLLIRAELGNPGSLIGDDQIYNVIVTAHAFVMIFFMVMPIVIGGFGNWLVPLMLGAPDMAFPRMNNMSFWLLPPSLTLLLMSSMVESGAGTGWTVYPPLSANIAHSGSSVDLAIFSLHLAGISSILGAVNFISTVINMRAM
GITFDRMPLFVWSVAITALLLLLSLPVLAGAITMLLTDRNLNTSFFDPAGGGDPILYQHL
>COLFD261-12|KJ962625|ZMUO.004156|Tachyporus_obtusus
TLYFIFGAWSGMIGTSLSLLIRAELGNPGSLIGDDQIYNVIVTAHAFIMIFFMVMPIVIGGFGNWLVPLMLGAPDMAFPRMNNMSFWLLPPSLTLLLMSSMVESGAGTGWTVYPPLSANIAHSGPSVDLAIFSLHLAGISSILGAVNFITTVINMRASGM
HFDRMPLFIWSVGITALLLLLSLPVLAGAITMLLTDRNLNTSFFDPAGGGDPILYQHL
>COLFB291-12|KJ962735|ZMUO.001621|Cassida_viridis
TLYFIFGFWSGMIGTSLSILIRAELGNPGNLIGNDQIYNSIVTAHAFIMIFFMVMPIMIGGFGNWLVPLMLGAPDMAFPRLNNMSFWLLPPSLMFLIMSSIIESGAGTGWTVYPPLSSNIAHSGASVDMAIFSLHLAGVSSILGAINFISTIMNMRPSEMN
LDKMPLFSWAVIITAILLLLSLPVLAGAITMLLTDRNINTSFFDPAGGGDPILYQHL
>COLFD322-12|KJ962500|ZMUO.004217|Melanophthalma_transversalis
SLYFLFGLWSGMVGTSLSLLIRLELGNPGSLIGDDQIYNVIVTAHAFIMIFFMVMPIMIGGFGNWLVPLMLGAPDMAFPRLNNMSFWLLPPSLNLLIMSSIVESGAGTGWTVYPPLSSNIAHSGSSVDLAIFSLHLAGISSILGAVNFITTVINMRPMGMT
LEKMPLFVWSVVLTAILLLLSLPVLAGAITMLLTDRNLNTSFFDPAGGGDPILYQHL
>COLFB624-12|KJ966784|ZMUO.001099|Aphthona_lutescens
TLYFIFGIWSGMVGTSLSVLIRTELGNPGSLIGNDQIYNVIVTAHAFIMIFFMVMPIMIGGFGNWLVPLMIGAPDMAFPRMNNMSFWLLPPSLIFLIMSSMVESGAGTGWTVYPPLSSNIAHGGSSVDLAIFSLHLAGISSILGAINFITTVINMRPEGMN
LDRMPLFVWAVTITAILLLLSLPVLAGAITMLLTDRNLNTSFFDPSGGGDPILYQHL
>COLFF383-13|KJ966944|ZMUO.005901|Lygistopterus_sanguineus
TLYFIFGAWAGMLGTSLSLLIRAELGTPGTLIGNDQIYNVIVTAHAFIMIFFMVMPMMIGGFGNWLVPLMLGAPDMAFPRMNNMSFWLLPPSLSLLIMSSMVESGAGTGWTVYPPLSSNIAHSGSSVDLAIFSLHLAGVSSILGAVNFISTIINMKSPG
MTYDQMPLFVWAVGITALLLLLSLPVLAGAITMLLTDRNLNTSFFDPTGGGDPILYQHL
>COLFD581-12|KJ964083|ZMUO.004381|Chrysomela_tremula
TLYFIFGIWAGTVGTSLSILIRAELGNPGTLIGNDQIYNVIVTAHAFIMIFFMVMPIMIGGFGNWLVPLMIGAPDMAFPRLNNMSFWLLPPSLFLLLMSSIVENGAGTGWTVYPPLSTNIAHSGSSVDLAIFSLHLAGISSILGAINFITTVINMRPEGMNFE
QTPLFVWAVLITAILLLLSLPVLAGAITMLLTDRNLNTSFFDPAGGGDPILYQHL
>COLFE447-12|KJ962287|ZMUO.005007|Sitona_hispidulus
TLYFIFGAWAGMVGTSLSMLIRTELGNPGSLIGDDQIYNVIVTAHAFIMIFFMVMPMMIGGFGNWLVPLMLGAPDMAFPRLNNMSFWLLPPSLTLLLMSSIVDKGAGTGWTVYPPLSANIAHEGASVDLAIFSLHMAGISSILGAINFISTIINMRPM
GMIMDRITLFVWAVKITAILLLLSLPVLAGAITMLLTDRNINTSFFDPAGGGDPILYQHL
>COLFC770-12|KJ965539|ZMUO.003335|Phratora_atrovirens
TLYFIFGIWAGMVGTSLSMLIRSELGNPGTLIGNDQIYNVIVTAHAFIMIFFMVMPIMIGGFGNWLVPLMIGAPDMAFPRLNNMSFWLLPPSLFLLLMSSVVENGAGTGWTVYPPLSSNLAHSGSSVDLAIFSLHLAGISSILGAINFITTVINMRPEGM
GLEQTPLFVWAVLITAILLLLSLPVLAGAITMLLTDRNLNTSFFDPAGGGDPILYQHL
>COLFB180-12|KJ965207|ZMUO.001510|Ilybius_crassus
TLYFIFGAWAGMVGTSLSMLIRAELGNPGSLIGDDQIYNVIVTAHAFVMIFFMVMPIMIGGFGNWLVPLMLGAPDMAFPRMNNMSFWLLPPSLSLLLMSSMVESGAGTGWTVYPPLSAGIAHSGASVDLAIFSLHLAGISSILGAVNFITTIINMRSV
GMTFDRMPLFVWSVGITALLLLLSLPVLAGAITMLLTDRNLNTSFFDPAGGGDPILYQHL
>COLFF168-13|KJ963959|ZMUO.005892|Plateumaris_discolor
TLYFIFGAWSGMMGTSLSMLIRTELMNPGSLIGNDQIYNVIVTAHAFIMIFFMVMPIMIGGFGNWLIPLMLGAPDMAFPRMNNMSFWLLPPSLTFLIMSSIVENGAGTGWTVYPPLSSNIAHSGASVDLAIFSLHLAGISSILGAVNFITTIINMRPMG
MKMDKVPLFAWAVMITAILLLLSLPVLAGAITMLLTDRNLNTSFFDPAGGGDPVLYQHL
>COLFC835-12|KJ963126|ZMUO.003400|Pelenomus_quadrituberculatus
TLYFIFGSWAGTVGTSLSMIIRTELGTPGSLIGNDQIYNSIVTAHAFIMIFFMVMPILIGGFGNWLVPLMLGAPDMAFPRLNNMSFWLLPPSILLLLMSSIVNKGAGTGWTVYPPLSSNITHEGASVDLAIFSLHMAGISSILGAINFISTIMNMRPKGMSY
DKTPLFVWAVMITAILLLISLPVLAGAITMLLTDRNINTSFFDPAGGGDPILYQHL

>COLFE539-13|KJ967086|ZMUO.005384|Chrysolina_polita
TLYFIFGIWAGMVGTSLSILIRTELGSPGTLIGNDQIYNVIVTAHAFIMIFFMVMPIMIGGFGNWLIPLMLGAPDMAFPRMNNMSFWLLPPSLLLLLMSSMMENGVGTGWTVYPPLSANIAHSGPAVDLAIFSLHLAGISSILGAINFISTTTNMRSTGM
SFEQMPLFSWAVLITVILLLLSLPVLAGAITMLLTDRNLNTSFFDPASGGDPILYQHL
>COLFF401-13|KJ768193|ZMUO.005919|Agrilus_pratensis
TLYFIFGAWSGMVGTALSLLIRAELGNPGALIGNDQIYNVIVTAHAFIMIFFMVMPIMMGGFGNWLVPLMLGAPDMAFPRMNNMSFWLLPPSLTLLLMSSMVESGAGTGWTVYPPLAANIAHSGASVDLAIFSLHLAGVSSILGAINFITTVINMRA
PGMTLDRIPLLVWSIAITALLLLLSLPVLAGAITMLLTDRNLNTSFFDPAGGGDPILYQHL
>COLFE1338-13|KJ962978|ZMUO.007133|Cytilus_auricomus
TLYFIFGAWAGMVGTSLSLLIRAELGNPGSLIGDDQIYNVIVTAHAFVMIFFMVMPIMVGGFGNWLVPLMLGAPDMAFPRMNNMSFWLLPPSLSLLLMSSIVESGAGTGWTVYPPLSSNIAHSGASVDLAIFSLHLAGISSILGAFNFISTVVNMRSPG
MKFDQMSLFSWSVAITALLLLLSLPVLAGAITMLLTDRNINTSFFDPAGGGDPILYQHL
>COLFF391-13|KJ966647|ZMUO.005909|Scaphisoma_agaricinum
TLYFIFGAWAGMIGTSLSLLIRAELGMPGSLIGDDQIYNVIVTAHAFVMIFFMVMPIVIGGFGNWLIPLMLSAPDMAFPRMNNMSFWLLPPSLSLLLLSAMVESGAGTGWTVYPPLSSNIAHGGSSVDLAIFSLHLAGISSILGAVNFISTIINMRTTGMS
FDQMSLFIWSVAITALLLLLSLPVLAGAITMLLTDRNLNTTFFDPSGGGDPILYQHL
>COLFD553-12|KJ963333|ZMUO.004353|Gyrinus_paykulli
TLYFIFGAWSGMVGTSLSMLIRAELGNPGSLIGDDQIYNVIVTAHAFIMIFFMVMPIMIGGFGNWLVPLMLGAPDMAFPRMNNMSFWLLPPSLTLLLMSSMVENGAGTGWTVYPPLSSNIAHGGASVDLAIFSLHLAGISSILGAVNFITTIINMRSIG
MTLDRMPLFVWSVGITALLLLLSLPVLAGAITMLLTDRNLNTSFFDPAGGGDPILYQHL
>COLFF014-13|KJ964486|ZMUO.005714|Amara_bifrons
TLYFIFGAWSGMVGTSLSMLIRAELGNPGALIGDDQIYNVIVTAHAFVMIFFMVMPIMIGGFGNWLVPLMLGAPDMAFPRMNNMSFWLLPPSLTLLLMSSLVESGAGTGWTVYPPLSSNIAHAGASVDLAIFSLHLAGISSILGAVNFITTIINMRSVG
MTFDRMPLFVWSVGITALLLLLSLPVLAGAITMLLTDRNLNTSFFDPAGGGDPILYQHL
>COLFE042-12|KJ966514|ZMUO.005267|Ampedus_tristis
TLYFIFGAWAGMLGTSLSLLIRAELGNPGSLIGNDQIYNVIVTAHAFIMIFFMVMPIMIGGFGNWLVPLMLGAPDMAFPRMNNMSFWLLPPSLSLLLMSSIVENGAGTGWTVYPPLSSNIAHSGSSVDLAIFSLHLAGISSILGAVNFISTVINMRSTGIT
FDRMPLFVWAVAITALLLLLSLPVLAGAITMLLTDRNLNTSFFDPAGGGDPILYQHL
>COLFE985-13|KJ966570|ZMUO.006780|Atheta_malleus
TLYFIFGAWAGMVGTSLSLLIRAELGNPGSLIGDDQIYNVIVTAHAFIMIFFMVMPIVIGGFGNWLVPLMLGAPDMAFPRMNNMSFWLLPPSLTLLLMSSMVESGAGTGWTVYPPLSSNIAHGGSSVDLAIFSLHLAGISSILGAVNFISTVINMRSTGI
SFDRMPLFVWSVAITALLLLLSLPVLAGAITMLLTDRNLNTSFFDPAGGGDPILYQHL
>COLFE282-12|KJ965474|ZMUO.005127|Amara_communis
TLYFIFGAWSGMVGTSLSMLIRAELGNPGALIGDDQIYNVIVTAHAFVMIFFMVMPIMIGGFGNWLVPLMLGAPDMAFPRMNNMSFWLLPPSLTLLLMSSMVESGAGTGWTVYPPLSSGIAHAGASVDLAIFSLHLAGISSILGAVNFITTIINMRSIG
MTFDRMPLFVWSVGITALLLLLSLPVLAGAITMLLTDRNLNTSFFDPAGGGDPILYQHL
>COLFB588-12|KJ963828|ZMUO.001063|Atheta_crassicornis
TLYFIFGAWAGMVGTSLSLLIRAELGNPGSLIGDDQIYNVIVTAHAFVMIFFMVMPIVIGGFGNWLVPLMLGAPDMAFPRMNNMSFWLLPPSLTLLLMSSMVESGAGTGWTVYPPLSSNIAHGGSSVDLAIFSLHLAGISSILGAVNFISTVINMRSVGI
SFDRMPLFVWSVAITALLLLLSLPVLAGAITMLLTDRNLNTSFFDPAGGGDPILYQHL
>COLFD679-12|KJ965002|ZMUO.004669|Brachyderes_incanus
TLYFIFGAWSGMVGTSLSMLIRTELGNPGSLIGDDQIYNVIVTAHAFIMIFFMVMPMMIGGFGNWLVPLMLGAPDMAFPRMNNMSFWLLPPSLSLLLMSSIVDKGAGTGWTVYPPLSANIAHEGSSVDLAIFSLHMAGVSSILGAINFISTVINMRP
MGMSPDRMSLFIWAVKITAVLLLLSLPVLAGAITMLLTDRNVNTSFFDPAGGGDPILYQHL
>COLFD231-12|KJ963415|ZMUO.004126|Nevraphes_angulatus
TLYFIFGIWSGMVGTSMSMLIRSELGNPGMLIGDDQIYNVIVTAHAFIMIFFMVMPIMIGGFGNWLIPLYLGAPDMAFPRMNNMSFWFLPPSLSLLLLSSMIESGAGTGWTVYPPLSSNIAHSGASVDLTIFSLHLAGISSILGAVNFISTVINMRSYGM
SFDKMPLLVWAVTITAFLLLLSLPVLAGAITMLLTDRNLNTSFFDPSGGGDPILYQHL
>COLFB795-12|KJ967030|ZMUO.001270|Eucnecosum_brunnescens
TLYFIFGAWAGMVGTSLSILIRAELGNPGTLIGDDQIYNVIVTAHAFVMIFFMVMPIVIGGFGNWLVPLMLGAPDMAFPRMNNMSFWLLPPSLTLLLMSSMVESGAGTGWTVYPPLSSNIAHGGSSVDLAIFSLHLAGISSILGAVNFITTVINMRTTG
MTFDRMPLFVWSVAITALLLLLSLPVLAGAITMLLTDRNLNTSFFDPAGGGDPILYQHL
>COLFE1479-13|KJ964073|ZMUO.007274|Rhynchites_cupreus
TLYFIFGTWAGLVGTSLSLLIRAELGNPGSLIGDDQIYNVIVTAHAFIMIFFMVMPIMIGGFGNWLVPLMLGAPDMAFPRMNNMSFWLLPPSISLLISSSIVESGAGTGWTVYPPLSSNIAHGGPSVDLAIFSLHLAGISSILGAVNFISTVINMRPKGMSF
DRMPLFVWAVAITALLLLLSLPVLAGAITMLLTDRNINTTFFDPAGGGDPILYQHL
>COLFC115-12|KJ961950|ZMUO.002300|Oxypoda_advena
TLYFIFGAWAGMVGTSLSLLIRAELGNPGSLIGDDQIYNVIVTAHAFVMIFFMVMPIVIGGFGNWLIPLMLGAPDMAFPRMNNMSFWLLPPSLTLLLMSSMVESGAGTGWTVYPPLSSNIAHGGSSVDLAIFSLHLAGISSILGAVNFISTIINMRTSGM
TFDRMPLFVWSVAITALLLLLSLPVLAGAITMLLTDRNLNTSFFDPAGGGDPILYQHL
>COLFG146-13|KJ963999|ZMUO.007841|Ptilium_exaratum

TLYFIFGAWSGMVGTSLSLLIRAELGMPGSLIGDDQIYNVIVTAHAFIMIFFMVMPILIGGFGNWLVPIMLGAPDMAFPRMNNMSFWMLPPSLTLLLMSSMVENGAGTGWTVYPPLASNLAHAGASVDLAIFSLHLAGISSILGAVNFITTIINMRAPQ
MNFEQLPLFVWSVAITALLLLLSLPVLAGAITMLLTDRNLNTSFFDPAGGGDPILYQHL
>COLFB155-12|KJ965901|ZMUO.001485|Bledius_spectabilis
TLYFIFGIWAGMVGTSLSMLIRAELGTPGSLIGDDQIYNVIVTAHAFIMIFFMVMPIMIGGFGNWLVPLMLGAPDMAFPRMNNMSFWLLPPSLTLLLMSSMVESGVGTGWTVYPPLSANIAHSGSSVDLAIFSLHLAGISSILGAVNFISTIINMRSIGM
TFDRMPLFIWSVKITAILLLLSLPVLAGAITMLLTDRNLNTSFFDPAGGGDPILYQHL
>COLFA375-12|KJ967216|ZMUO.000605|Carcinops_pumilio
TLYFIFGAWAGMVGTSLSLLIRAELGNPGTLIGDDQIYNVIVTAHAFIMIFFMVMPIMIGGFGNWLVPLMLGAPDMAFPRMNNMSFWLLPPSLSLLLLSSVVENGAGTGWTVYPPLSSNLAHNGSSVDLAIFSLHLAGISSILGAVNFITTIINMRAMG
MTFDRMPLFVWSVGITALLLLLSLPVLAGAITMLLTDRNLNTSFFDPAGGGDPILYQHL
>COLFE1086-13|KJ963121|ZMUO.006881|Chrysomela_collaris
TLYFIFGVWAGMVGTSLSILIRAELGNPGTLIGNDQIYNVIVTAHAFIMIFFMVMPIMIGGFGNWLVPLMIGAPDMAFPRLNNMSFWLLPPSLFLLLMSSIVENGAGTGWTVYPPLSSNIAHSGSSVDLAIFSLHLAGISSILGAINFITTVINMRPEGMGL
EQTPLFVWAVLITAVLLLLSLPVLAGAITMLLTDRNLNTSFFDPAGGGDPILYQHL
>COLFE1149-13|KJ966377|ZMUO.006944|Leiodes_obesa
TLYFIFGAWSGMVGTSLSILIRAELGNPGSLIGDDQVYNVIVTAHAFIMIFFMVMPIVIGGFGNWLVPLMLGAPDMAFPRMNNMSFWLLPPSLSLLLMSSIVENGAGTGWTVYPPLSSNIAHSGSSVDLAIFSLHLAGISSILGAVNFITTVINMRPAGM
SFDKMPLFVWSVAITALLLLLSLPVLAGAITMLLTDRNLNTSFFDPAGGGDPILYQHL
>COLFB641-12|KJ962904|ZMUO.001116|Alianta_incana
TLYFIFGAWAGMVGTSLSLLIRAELGNPGSLIGDDQIYNVIVTAHAFVMIFFMVMPIMIGGFGNWLVPLMLGAPDMAFPRMNNMSFWLLPPSLSLLLMSSMVESGAGTGWTVYPPLSSNIAHGGASVDLAIFSLHLAGISSILGAVNFISTVINMRST
GITFDRMPLFVWAVAITALLLLLSLPVLAGAITMLLTDRNLNTSFFDPAGGGDPILYQHL
>COLFE654-13|KJ964781|ZMUO.005499|Hylobius_abietis
TLYFIFGTWSGMVGTSLSMLIRTELGNPGSLIGDDQIYNTIVTAHAFIMIFFMVMPIMIGGFGNWLVPLMLGAPDMAFPRLNNMSFWLLPPSLTLLLMSSIVDKGAGTGWTVYPPLSANIAHEGASVDFAIFSLHMAGISSILGAINFISTAINMRSSGM
KSDQMSLFIWAVKITAILLLLSLPVLAGAITMLLTDRNINTSFFDPAGGGDPILYQHL
>COLFB281-12|KJ967250|ZMUO.001611|Gyrohypnus_angustatus
TLYFIFGAWSGMVGTSLSLLIRAELGNPGTLIGDDQIYNVIVTAHAFIMIFFMVMPIVIGGFGNWLVPLMLGAPDMAFPRMNNMSFWLLPPSLTLLLSSSLVESGAGTGWTVYPPLSSNITHSGSSVDLAIFSLHLAGVSSILGAVNFITTVINMRSSGMS
FDRMPLFVWSVAITALLLLLSLPVLAGAITMLLTDRNLNTSFFDPTGGGDPILYQHL
>COLFD893-12|KJ964902|ZMUO.004503|Bembidion_properans
TLYFIFGAWSGMVGTSLSMLIRAELGNPGSLIGDDQIYNVIVTAHAFVMIFFMVMPILIGGFGNWLVPLMLGAPDMAFPRMNNMSFWLLPPSLSLLLMSSMVESGAGTGWTVYPPLSSSIAHSGASVDLAIFSLHLAGVSSILGAVNFITTIINMRSTG
MTFDRMPLFVWSVGITALLLLLSLPVLAGAITMLLTDRNLNTSFFDPAGGGDPILYQHL
>COLFF303-13|KJ962460|ZMUO.006003|Amara_erratica
TLYFIFGAWSGMVGTSLSMLIRAELGNPGALIGDDQIYNVIVTAHAFVMIFFMVMPIMIGGFGNWLVPLMLGAPDMAFPRMNNMSFWLLPPSLTLLLMSSMVESGAGTGWTVYPPLSSGIAHAGASVDLAIFSLHLAGISSILGAVNFITTIINMRSV
GMTFDRMPLFVWSVGITALLLLLSLPVLAGAITMLLTDRNLNTSFFDPAGGGDPILYQHL
>COLFE575-13|KJ962354|ZMUO.005420|Pterostichus_niger
TLYFIFGAWSGMVGTSLSMLIRAELGNPGSLIGDDQIYNVIVTAHAFVMIFFMVMPIMIGGFGNWLVPLMLGAPDMAFPRMNNMSFWLLPPSLSLLLMSSMVENGSGTGWTVYPPLSSGIAHAGASVDLAIFSLHLAGISSILGAVNFITTIINMRST
GMTFDRMPLFVWSVGITALLLLLSLPVLAGAITMLLTDRNLNTSFFDPAGGGDPILYQHL
>COLFA451-12|KJ963637|ZMUO.000491|Ctenicera_pectinicornis
TLYFLFGAWAGMLGTSLSLLIRAELGNPGSLIGNDQIYNVIVTAHAFIMIFFMVMPIMIGGFGNWLVPLMLGAPDMAFPRMNNMSFWFLPPSLSLLLMSSIVENGAGTGWTVYPPLSANIAHSGSSVDLAIFSLHLAGISSILGAVNFISTVINMRSTGIT
FDRMPLFVWAVAITALLLLLSLPVLAGAITMLLTDRNLNTSFFDPAGGGDPILYQHL
>COLFE776-13|KJ964268|ZMUO.005621|Hydrobius_fuscipes
TLYFIFGAWAGMVGTSLSILIRAELGNPGTLIGDDQIYNVIVTAHAFIMIFFMVMPIMIGGFGNWLVPLMLGAPDMAFPRMNNMSFWLLPPSLTLLLMSSMVENGAGTGWTVYPPLSSNIAHGGASVDLAIFSLHLAGISSILGAVNFITTVINMRSPN
LTYDRLPLFVWSVAITALLLLLSLPVLAGAITMLLTDRNLNTSFFDPAGGGDPILYQHL
>COLFD671-12|KJ966477|ZMUO.004661|Oxythyrea_funesta
TLYFLFGSWAGMVGTSLSLLIRAELGNPGSLIGDDQIYNVIVTAHAFIMIFFMVMPIMIGGFGNWLVPLMLGAPDMAFPRMNNMSFWLLPPSLTLLLMSSMVESGAGTGWTVYPPLSSNIAHSGASVDLAIFSLHLAGISSILGAVNFITTVINMRSTG
MTFDRMPLFVWSVALTALLLLLSLPVLAGAITMLLTDRNINTSFFDPAGGGDPILYQHL
>COLFB918-12|KJ961917|ZMUO.001963|Oxystoma_cerdo
TLYFILGLWSGMVGTSLSMLIRIELGNSGSLIGDDQIYNVIVTAHAFIMIFFMVMPIMIGGFGNWLVPLMLGAPDMAFPRMNNMSFWMLPPSLTLLLMSSIVEKGAGTGWTVYPPLASNIAHSGASVDLAIFSLHLAGISSILGAVNFISTMINMHPNG
LSLDQLSLFTWAVKITAILLLLSLPVLAGAITMLLTDRNINTSFFDPAGGGDPILYQHL
>COLFF733-13|KJ965424|ZMUO.006433|Oedemera_lurida
TLYLIFGAWAGMVGTSLSLLIRAELGNPGSLIGDDQIYNVIVTAHAFIMIFFMVMPIVIGGFGNWLVPLMLGAPDMAFPRMNNMSFWLLPPSLTLLIMSSMVESGAGTGWTVYPPLSSNIAHSGSSVDLAIFSLHLAGVSSILGAVNFITTVINMRPVG
MTLDRMPLFVWAVVITAILLLLSLPVLAGAITMLLTDRNLNTSFFDPAGGGDPILYQHL

>LEFIJ1830-13|KJ963206|ZMUO.004585|Meligethes_aeneus
TLYFIFGAWSGMVGTSLSMLIRTELGNPGSLIGNDQIYNVIVTAHAFVMIFFMVMPFMIGGFGNWLVPLMLGAPDMAFPRMNNMSFWLLPPSLSLLLMSSIVESGAGTGWTVYPPLSSNIAHGGASVDLAIFSLHLAGISSILGAVNFITTVINMRPK
GMTFDRMPLFVWAVMITAILLLLSLPVLAGAITMLLTDRNLNTTFFDPSGGGDPILYQHL
>COLFE638-13|KJ961893|ZMUO.005483|Tenebrio_molitor
TLYFIFGAWSGMVGTSLSLLIRAELGNPGSLIGDDQIYNVIVTAHAFIMIFFMVMPIMIGGFGNWLVPLMLGAPDMAFPRMNNMSFWLLPPSLSLLLMSSIVENGAGTGWTVYPPLSSNIAHGGASVDLAIFSLHLAGISSILGAVNFITTVINMRPQG
MTFDRMPLFVWAVVITAVLLLLSLPVLAGAITMLLTDRNINTSFFDPAGGGDPILYQHL
>COLFE885-13|KJ967007|ZMUO.006205|Quedius_lundbergi
TLYFIFGAWAGMVGTSLSLLIRAELGNPGSLIGDDQIYNVIVTAHAFIMIFFMVMPIVIGGFGNWLVPLMLGAPDMAFPRMNNMSFWLLPPSLSLLLMSSMVESGAGTGWTVYPPLSSNIAHGGASVDLAIFSLHLAGISSILGAVNFITTVINMRSIG
MTFDRMPLFVWSVAITALLLLLSLPVLAGAITMLLTDRNLNTSFFDPAGGGDPILYQHL
>COLFE1399-13|KJ962503|ZMUO.007194|Trixagus_dermestoides
TLYFIFGSWAGMIGTAMSMIIRMELGTPGSLIGNDQIYNVIVTSHAFIMIFFMVMPIMIGGFGNWLVPLMIGAPDMAFPRLNNMSFWLLPPSMSLILTSNMTENGAGTGWTIYPPLSSNLAHSGPAVDFTIFSLHLAGMSSILGAINFITTSINMRSPLI
KPERLTLFAWSVSITALLLLLSLPVLAGAITMLLTDRNLNTSFFDPAGGGDPILYQHL
>COLFD885-12|KJ963253|ZMUO.004495|Stenus_nitens
TLYFIFGAWAGMVGTSLSLLIRAELGNPGSLIGDDQIYNVIVTAHAFVMIFFMVMPIMIGGFGNWLVPLMLGAPDMAFPRMNNMSFWLLPPSLSLLLMSSIVENGAGTGWTVYPPLSSNIAHSGASVDLAIFSLHLAGISSILGAINFITTIFNMRTMKL
QLDCLPLFVWSVAITALLLLLSLPVLAGAITMLLTDRNLNTSFFDPAGGGDPILYQHL
>COLFA597-12|KJ964886|ZMUO.000732|Phosphuga_atrata
TLYFIFGAWAGIVGMSLSILIRMELSTPGMFLGDDQMYNVIVTAHAFIMIFFMVMPIVIGGFGNWLVPLMLGAPDMAFPRMNNMSFWLLPPSLSLLLVSSMVESGAGTGWTVYPPLSSNIAHSGSSVDLAIFSLHLAGISSILGAVNFITTIINMRSSG
MKFDRMPLFVWSVGITALLLLLSLPVLAGAITMLLTDRNLNTSFFDPAGGGDPILYQHL
>COLFA489-12|KJ962959|ZMUO.000529|Tomicus_piniperda
TLYFIFGAWSGMVGTSLSMLIRTELGTPGSLIGDDQIYNTIVTAHAFIMIFFMVMPIMIGGFGNWLIPLMLGAPDMAFPRLNNMSFWLLPPSLTLLLLSSIIDKGAGTGWTVYPPLASNISHEGASVDFAIFSLHMAGISSILGAMNFISTILNMNPSGMK
SDRLTLFTWAVKITAILLLLSLPVLAGAITMLLTDRNINTTFFDPAGGGDPILYQHL
>COLFD527-12|KJ966519|ZMUO.004327|Bembidion_articulatum
TLYFIFGAWSGMVGTSLSMLIRAELGNPGSLIGDDQIYNVIVTAHAFVMIFFMVMPILIGGFGNWLVPLMLGAPDMAFPRMNNMSFWLLPPSLTLLLMSSMVEKGAGTGWTVYPPLSSSIAHSGASVDLAIFSLHLAGVSSILGAVNFITTIINMRSIG
MTFDRMPLFVWSVGITALLLLLSLPVLAGAITMLLTDRNLNTSFFDPAGGGDPILYQHL
>COLFC792-12|KJ961869|ZMUO.003357|Omalium_littorale
TLYFIFGAWAGMVGTSLSLLIRAELGNPGSLIGDDQIYNVIVTAHAFIMIFFMVMPIVIGGFGNWLVPLMLGAPDMAFPRMNNMSFWLLPPSLTLLLMSSMVESGAGTGWTVYPPLSSNIAHGGSSVDLAIFSLHLAGISSILGAVNFITTVINMRAMG
MTFDRMPLFVWSVVITALLLLLSLPVLAGAITMLLTDRNLNTSFFDPAGGGDPILYQHL
>COLFB428-12|KJ964804|ZMUO.001853|Oedemera_subrobusta
TLYLIFGAWAGMVGTSLSLLIRAELGNPGSLIGDDQIYNVIVTAHAFIMIFFMVMPIMIGGFGNWLVPLMLGAPDMAFPRMNNMSFWLLPPSLTLLIMSSMVESGAGTGWTVYPPLSSNIAHSGSSVDLAIFSLHLAGVSSILGAVNFITTVINMRPVG
MTLDRMPLFVWAVVITAILLLLSLPVLAGAITMLLTDRNLNTSFFDPAGGGDPILYQHL
>COLFG126-13|KJ962517|ZMUO.007821|Atheta_nesslingi
TLYFIFGAWAGMVGTSLSLLIRAELGNPGSLIGDDQIYNVIVTAHAFIMIFFMVMPVVIGGFGNWLVPLMLGAPDMAFPRMNNMSFWLLPPSLTLLLMSSMVESGAGTGWTVYPPLSSNIAHGGSSVDLAIFSLHLAGISSILGAVNFISTVINMRSTGI
SFDRMPLFVWSVAITALLLLLSLPVLAGAITMLLTDRNLNTSFFDPAGGGDPILYQHL
>COLFC163-12|KJ965878|ZMUO.002348|Acrotona_convergens
TLYFIFGTWAGMVGTSLSLLIRAELGNPGSLIGDDQIYNVIVTAHAFIMIFFMVMPIVIGGFGNWLVPLMLGAPDMAFPRMNNMSFWLLPPSLTLLLMSSMVESGAGTGWTVYPPLSSNIAHGGASVDLAIFSLHLAGISSILGAVNFISTVINMRSMGI
TFDRMPLFVWSVAITALLLLLSLPVLAGAITMLLTDRNLNTSFFDPAGGGDPILYQHL
>COLFC365-12|KJ963045|ZMUO.002550|Oxypoda_skalitzkyi
TLYFIFGAWAGMVGTSLSLLIRAELGNPGSLIGDDQIYNVIVTAHAFVMIFFMVMPIVIGGFGNWLIPLMLGAPDMAFPRMNNMSFWLLPPSLTLLLMSSMVESGAGTGWTVYPPLSSNIAHGGSSVDLAIFSLHLAGISSILGAVNFISTIINMRTSGM
SFDRMPLFIWSVAITALLLLLSLPVLAGAITMLLTDRNLNTSFFDPAGGGDPILYQHL
>COLFF080-13|KJ965151|ZMUO.005780|Bembidion_obliquum
TLYFIFGAWGGMVGTSLSMLIRAELGNPGSLIGDDQIYNVIVTAHSFEMIFFMVMPILIGGFGNWLVPLMLGAPDMAFSRMNNMSFWLLPPSLTLLLKSSMVESGAGTGWTVYPPLLSSIAHSGASVDLAIFSLHLAGVSSILGAVNFITTIINMRSTSM
TFDRMPLFVWSVGITALLLLLSLPVLAGAITMLLTDRNLNTSFFDPAGGGDPILYQHL
>COLFA308-12|KJ966812|ZMUO.000443|Protapion_apricans
TLYFIFGLWSGMIGTSLSMLIRIELGNPGSLIGNDQIYNVIVTAHAFIMIFFMVMPVMIGGFGNWLIPLMLGAPDMAFPRMNNMSFWLLPPSLTLLLMSSIIEKGAGTGWTVYPPLAANIAHSGASVDLAIFSLHLAGISSILGAVNFISTIMNMRPTGM
SLDQLSLFTWAVKITAILLLLSLPVLAGAITMLLTDRNINTSFFDPAGGGDPILYQHL
>COLFE170-12|KJ967424|ZMUO.005205|Rugilus_orbiculatus

TLYFIFGAWAGMVGTSLSLLIRAELSAPGSLIGDDQIYNVIVTAHAFVMIFFMVMPIVIGGFGNWLVPLMLGAPDMAFPRMNNMSFWLLPPSLSLLLMSSMVESGAGTGWTVYPPLSSNIAHGGASVDLAIFSLHLAGISSILGAVNFITTIINMRSKG
MTYERMPLFVWSVGITALLLLLSLPVLAGAITMLLTDRNLNTSFFDPAGGGDPILYQHL
>COLFF070-13|KJ964782|ZMUO.005770|Platambus_maculatus
TLYFIFGAWAGMVGTSLSMLIRAELGNPGSLIGDDQIYNVIVTAHAFVMIFFMVMPIMIGGFGNWLVPLMLGAPDMAFPRMNNMSFWLLPPSLSLLLMSSLVENGAGTGWTVYPPLSSGIAHGGASVDLAIFSLHLAGISSILGAVNFITTIINMRSVG
MTFDRMPLFVWSVGITALLLLLSLPVLAGAITMLLTDRNLNTSFFDPAGGGDPILYQHL
>COLFD301-12|KJ966671|ZMUO.004196|Atheta_atramentaria
TLYFIFGAWAGMVGTSLSLLIRAELGNPGSLIGDDQIYNVIVTAHAFIMIFFMVMPVVIGGFGNWLVPLMLGAPDMAFPRMNNMSFWLLPPSLTLLLMSSMVESGAGTGWTVYPPLSSNIAHGGSSVDLAIFSLHLAGISSILGAVNFISTVINMRSTGI
SFDRMPLFVWSVAITALLLLLSLPVLAGAITMLLTDRNLNTSFFDPAGGGDPILYQHL
>COLFF777-13|KJ962939|ZMUO.006097|Agonum_piceum
TLYFIFGAWAGMVGTSLSMLIRAELGNPGALIGDDQIYNVIVTAHAFIMIFFMVMPIMIGGFGNWLVPLMLGAPDMAFPRMNNMSFWLLPPSLTLLLMSSLVESGAGTGWTVYPPLSSGIAHAGASVDLAIFSLHLAGVSSILGAVNFITTIINMRSVG
MTFDRMPLFVWSVGITALLLLLSLPVLAGAITMLLTDRNLNTSFFDPAGGGDPILYQHL
>COLFF560-13|KJ966676|ZMUO.006640|Philonthus_debilis
TLYFIFGSWAGMVGTSLSLLIRAELGNPGSLIGDDQIYNVIVTAHAFIMIFFMVMPIVIGGFGNWLVPLMLGAPDMAFPRMNNMSFWLLPPSLTLLLMSSMVESGAGTGWTVYPPLSSNIAHGGASVDLAIFSLHLAGISSILGAVNFITTVINMRSTG
MTFDRMPLFVWSVAITALLLLLSLPVLAGAITMLLTDRNLNTTFFDPAGGGDPILYQHL
>COLFC255-12|KJ966485|ZMUO.002440|Placusa_incompleta
TLYFIFGAWAGMVGTSLSLLIRAELGNPGSLIGDDQIYNVIVTAHAFVMIFFMVMPVVIGGFGNWLVPLMLGAPDMAFPRMNNMSFWLLPPSLTLLLMSSMVESGAGTGWTVYPPLSSNIAHGGSSVDLAIFSLHLAGISSILGAVNFISTIINMRSPG
MTFDQMPLFVWSVGLTALLLLLSLPVLAGAITMLLTDRNINTTFFDPAGGGDPILYQHL
>COLFB793-12|KJ966720|ZMUO.001268|Oxypoda_funebris
TLYFIFGTWAGMIGTSLSLLIRAELGNPGSLIGDDQIYNVIVTAHAFVMIFFMVMPIVIGGFGNWLIPLMLGAPDMAFPRMNNMSFWLLPPSLSLLLMSSMVESGAGTGWTVYPPLSSNIAHGGSSVDLAIFSLHLAGISSILGAVNFISTIINMRTSGMS
FDRMPLFVWSVAITALLLLLSLPVLAGAITMLLTDRNLNTSFFDPAGGGDPILYQHL
>COLFE414-12|KJ963140|ZMUO.004974|Philonthus_rotundicollis
TLYFIFGSWSGMVGTSLSLLIRAELGNPGTLIGDDQIYNVIVTAHAFIMIFFMVMPIVIGGFGNWLVPLMLGAPDMAFPRMNNMSFWLLPPSLTLLLSSSMVESGAGTGWTVYPPLSSNIAHGGASVDLAIFSLHLAGISSILGAVNFITTVINMRSTGM
TFDRMPLFVWSVAITALLLLLSLPVLAGAITMLLTDRNLNTTFFDPAGGGDPILYQHL
>COLFE1433-13|KJ964764|ZMUO.007228|Carpelimus_bilineatus
TLYFIFGAWSGMVGTSLSMLIRIELGTPGSLIGDDQIYNVIVTAHAFIMIFFMVMPIVIGGFGNWLVPLMLGAPDMAFPRMNNMSFWLLPPSLTLLLFSSMVESGAGTGWTVYPPLSSNIAHSGSSVDLAIFSLHLAGISSILGAVNFISTIINMRSIGMSF
DRMPLFVWSVNITAILLLLSLPVLAGAITMLLTDRNLNTSFFDPAGGGDPILYQHL
>COLFB092-12|KJ964193|ZMUO.001422|Arpedium_quadrum
TLYFIFGAWAGMVGTSLSILIRAELGNPGTLIGDDQIYNVIVTAHAFVMIFFMVMPIVIGGFGNWLVPLMLGAPDMAFPRMNNMSFWLLPPSLTLLLMSSMVESGAGTGWTVYPPLSSNIAHGGSSVDLAIFSLHLAGISSILGAVNFITTVINMRATG
MTFDRMPLFVWSVAITALLLLLSLPVLAGAITMLLTDRNLNTSFFDPAGGGDPILYQHL
>COLFF675-13|KJ962322|ZMUO.006375|Cis_boleti
ILYFIFGTWSGMVGTSMSMLIRSELGTPGSLIGNDQIYNVIVTAHAFVMIFFMVMPIMIGGFGNWLIPLMLGAPDMAFPRMNNMSFWLLPPSLSLLLMSSIVENGAGTGWTVYPPLSSNIAHSGSSVDLAIFSLHLAGISSILGAVNFISTVINMRPQG
MNLDRMPLFVWAVVITAVLLLLSLPVLAGAITMLLTDRNFNTSFFDPAGGGDPILYQHL
>COLFB885-12|KJ962314|ZMUO.001930|Brachyta_interrogationis
TLYFIFGAWAGMVGTSLSMLIRSELGNPGSLIGDDQIYNVIVTAHAFVMIFFMVMPIMIGGFGNWLVPLMLGAPDMAFPRMNNMSFWLLPPSLILLIMSSVVESGAGTGWTVYPPLASNIAHSGSSVDLAIFSLHLTGISSILGAVNFITTVINMRPLG
MTPDRMPLFVWSVVITAILLLLSLPVLAGAITMLLTDRNLNTSFFDPAGGGDPILYQHL
>COLFF329-13|KJ966745|ZMUO.006029|Amara_erratica
TLYFIFGAWSGMVGTSLSMLIRAELGNPGALIGDDQIYNVIVTAHAFVMIFFMVMPIMIGGFGNWLVPLMLGAPDMAFPRMNNMSFWLLPPSLTLLLMSSMVESGAGTGWTVYPPLSSGIAHAGASVDLAIFSLHLAGISSILGAVNFITTIINMRSV
GMTFDRMPLFVWSVGITALLLLLSLPVLAGAITMLLTDRNLNTSFFDPAGGGDPILYQHL
>COLFC831-12|KJ966100|ZMUO.003396|Calodromius_spilotus
TLYFIFGAWAGMVGTSLSMLIRAELGNPGALIGDDQIYNVIVTAHAFIMIFFMVMPIMIGGFGNWLVPLMLGAPDMAFPRMNNMSFWLLPPSLTLLLMSSMVESGAGTGWTVYPPLSSGIAHAGASVDLAIFSLHLAGVSSILGAVNFITTIINMRSI
GMTFDRMPLFVWSVGITALLLLLSLPVLAGAITMLLTDRNLNTSFFDPAGGGDPILYQHL
>COLFA715-12|KJ962504|ZMUO.000850|Philonthus_discoideus
TLYFIFGSWAGMVGTSLSLLIRAELGNPGSLIGDDQIYNVIVTAHAFVMIFFMVMPIVIGGFGNWLVPLMLGAPDMAFPRMNNMSFWLLPPSLTLLLMSSMVESGAGTGWTVYPPLSSNIAHGGASVDLAIFSLHLAGISSILGAVNFITTVINMRSTG
MSFDRMPLFVWSVAITALLLLLSLPVLAGAITMLLTDRNLNTSFFDPAGGGDPILYQHL
>COLFE983-13|KJ967293|ZMUO.006778|Gyrinus_aeratus
TLYFIFGAWSGMVGTSLSMLIRAELGNPGSLIGDDQIYNVIVTAHAFIMIFFMVMPIMIGGFGNWLVPLMLGAPDMAFPRMNNMSFWLLPPSLTLLLMSSMVENGAGTGWTVYPPLSSNIAHGGASVDLAIFSLHLAGISSILGAVNFITTIINMRSIG
MTLDRMPLFVWSVGITALLLLLSLPVLAGAITMLLTDRNLNTSFFDPAGGGDPILYQHL

>COLFD313-12|KJ964525|ZMUO.004208|Stenus_assequens
TLYFIFGAWAGMVGTSLSLLIRSELGNPGSLIGDDQIYNVIVTAHAFIMIFFMVMPIMIGGFGNWLVPLMLGAPDMAFPRMNNMSFWLLPPSLSLLLLSSIVENGVGTGWTVYPPLSSNIGHSGASVDLAIFSLHLAGISSILGAINFITTIFNMRTMKLQ
LDCLPLFVWSVSITALLLLLSLPVLAGAITMLLTDRNLNTSFFDPAGGGDPILYQHL
>COLFF706-13|KJ963147|ZMUO.006406|Pentaphyllus_testaceus
SLYFIFGSWSGMIGTSLSLLIRTELGNPGSLIGDDQIYNVIVTAHAFIMIFFMVMPIVIGGFGNWLVPLMLGAPDMAFPRMNNMSFWLLPPSLTLLLMSSIVESGAGTGWTVYPPLSSNIAHGGSSVDLAIFSLHLAGISSILGAVNFITTVINMRPQGMT
FDRLPLFVWAVVITAMLLLLSLPVLAGAITMLLTDRNINTSFFDPAGGGDPILYQHL
>COLFE1247-13|KJ965432|ZMUO.007042|Anaspis_rufilabris
TLYFIFGAWSGMVGTSLSLLIRSELGTPGSLIGDDQIYNVIVTAHAFIMIFFMVMPILIGGFGNWLVPLMLGAPDMAFPRMNNMSFWLLPPSLTLLIMSSVVENGAGTGWTVYPPLAANIAHSGSSVDLAIFSLHLAGVSSILGAVNFITTVINMRPQG
MTLDRMPLFVWAVVITAVLLLLSLPVLAGAITMLLTDRNLNTSFFDPAGGGDPILYQHL
>COLFA018-10|HM909047|MP00150|Crepidodera_fulvicornis
TLYFIFGIWSGMVGTSLSILIRTELGSPGSLIGNDQIYNVIVTAHAFIMIFFMVMPIMIGGFGNWLIPLMIGAPDMAFPRMNNMSFWLLPPSLFLLLMSSLVESGAGTGWTVYPPLSSNIAHGGSSVDLAIFSLHLAGISSILGAINFITTIINMRPKGMNLD
RMPLFVWAVAITAILLLLSLPVLAGAITMLLTDRNMNTSFFDPIGGGDPILYQHL
>COLFE813-13|KJ962989|ZMUO.005658|Hylesinus_crenatus
TLYFILGAWSGMVGTSLSMIIRTELGTPGSLIGNDQIYNSIVTAHAFIMIFFMVMPIMIGGFGNWLVPLMLGAPDMAFPRLNNMSFWLLPPSLLFLLMSSIIDKGAGTGWTVYPPLANNIAHEGASVDLAIFSLHMAGVSSILGALNFISTIINMHPMG
MKLDRLTLFTWAVKITAILLLLSLPVLAGAITMLLTDRNINTSFFDPAGGGDPILYQHL
>COLFE1410-13|KJ965428|ZMUO.007205|Nitidula_rufipes
TLYFIFGAWSGMVGTSLSILIRTELGNPGSLIGNDQIYNVIVTAHAFIMIFFMVMPFMIGGFGNWLVPLMLGAPDMAFPRMNNMSFWLLPPSLSLLIMSSIVESGAGTGWTVYPPLSSNIAHGGSSVDLAIFSLHLAGISSILGAVNFITTVINMRPSGM
NFDRMPLFVWAVAITALLLLLSLPVLAGAITMLLTDRNLNTTFFDPSGGGDPILYQHL
>COLFA331-12|KJ962686|ZMUO.000466|Aleochara_bipustulata
TLYFIFGAWAGMVGTSLSLLIRAELGNPGSLIGDDQIYNVIVTAHAFIMIFFMVMPIVIGGFGNWLVPLMLGAPDMAFPRMNNMSFWLLPPSLTLLLMSSMVESGAGTGWTVYPPLSSNIAHGGSSVDLAIFSLHLAGISSILGAVNFISTVINMRSMG
MTFDKMPLFVWSVAITALLLLLSLPVLAGAITMLLTDRNLNTSFFDPAGGGDPILYQHL
>COLFB091-12|KJ962578|ZMUO.001421|Lathrobium_brunnipes
TLYFIFGAWAGMVGTSLSLLIRTELGNPGSLIGDDQIYNVIVTAHAFVMIFFMVMPIMIGGFGNWLVPLMLGAPDMAFPRMNNMSFWLLPPSLSLLLMSSMVESGAGTGWTVYPPLSSNIAHGGASVDLAIFSLHLAGISSILGAVNFITTVINMRSPG
MTYERMPLFVWAVAITALLLLLSLPVLAGAITMLLTDRNLNTTFFDPAGGGDPILYQHL
>COLFE1441-13|KJ961906|ZMUO.007236|Cyanapion_gyllenhalii
TLYFILGLWSGMAGTSLSMLIRIELSNSGSLIGDDQIYNVIVTAHAFIMIFFMVMPIMIGGFGNWLVPLMLGAPDMAFPRMNNMSFWLLPPSLTLLLMSSIVEKGAGTGWTVYPPLSSNIAHGGASVDLAIFSLHLAGISSILGAVNFISTMMNMYPNG
LSLDQLTLFTWAVKITALLLLLSLPVLAGAITMLLTDRNINTSFFDPAGGGDPILYQHL
>COLFA371-12|KJ962370|ZMUO.000601|Necrobia_violacea
TLYFIFGAWAGMVGTSLSLLIRSELGTPGTLIGNDQIYNVIVTAHAFIMIFFMVMPIMIGGFGNWLVPLMLGAPDMAFPRMNNMSFWLLPPSLTLLLMSSMVESGAGTGWTVYPPLSSNIAHGGSSVDLAIFSLHLAGISSILGAVNFITTVINMRPAG
MTLDRMPLFVWAVAITALLLLLSLPVLAGAITMLLTDRNLNTSFFDPAGGGDPILYQHL
>COLFF642-13|KJ965550|ZMUO.006532|Polydrusus_tereticollis
TLYFIFGSWSGMVGTSLSMLIRTELGNPGSLIGDDQIYNVIVTAHAFIMIFFMVMPMMIGGFGNWLVPLMLGAPDMAFPRLNNMSFWLLPPSLSLLLMSSIVDKGAGTGWTVYPPLSANIAHEGSSVDLAIFSLHMAGVSSILGAINFISTVINMRPSG
MSFDRMSLFIWAVKITAILLLLSLPVLAGAITMLLTDRNINTSFFDPAGGGDPILYQHL
>COLFB908-12|KJ963362|ZMUO.001953|Buprestis_haemorrhoidalis
TLYFIFGAWSGMVGTALSLLIRAELGNPGALIGDDQVYNVIVTAHAFVMIFFMVMPVMMGGFGNWLVPLMLGAPDMAFPRMNNMSFWLLPPSLTLLLMSSVVENGAGTGWTVYPPLAANIAHSGASVDLAIFSLHLAGVSSILGAVNFITTVINM
RSVGMTFDRMPLFVWSVAITALLLLLSLPVLAGAITMLLTDRNFNTSFFDPAGGGDPILYQHL
>COLFB390-12|KJ965544|ZMUO.001815|Astenus_pulchellus
TLYFIFGSWAGMVGTSLSLLIRAELGMPGSLIGDDQIYNVIVTAHAFVMIFFMVMPIVIGGFGNWLVPLMLGAPDMAFPRMNNMSFWLLPPSLSLLLMSSIVESGAGTGWTVYPPLSSNIAHSGASVDLAIFSLHLAGISSILGAVNFITTIINMRTKGM
TYERMPLFIWSVGITALLLLLSLPVLAGAITMLLTDRNLNTSFFDPAGGGDPILYQHL
>COLFD343-12|KJ964038|ZMUO.004238|Polydrusus_mollis
TLYFIFGAWSGMVGTSLSMLIRTELGNPGSLIGDDQIYNVIVTAHAFIMIFFMVMPIMIGGFGNWLVPLMLGAPDMAFPRLNNMSFWLLPPSLSLLLMSSIVDKGAGTGWTVYPPLSANIAHEGSSVDLAIFSLHMAGVSSILGTVNFISTVINMHPKG
MTPERMPLFVWAVVITAILLLLSLPVLAGAITMLLTDRNMNTSFFDPAGGGDPILYQHL
>COLFA503-12|KJ964872|ZMUO.000543|Chrysomela_collaris
TLYFIFGVWAGMVGTSLSILIRAELGNPGTLIGNDQIYNVIVTAHAFIMIFFMVMPIMIGGFGNWLVPLMIGAPDMAFPRLNNMSFWLLPPSLFLLLMSSIVENGAGTGWTVYPPLSSNIAHSGSSVDLAIFSLHLAGISSILGAINFITTVINMRPEDMGL
EQTPLFVWAVLITAVLLLLSLPVLAGAITMLLTDRNLNTSFFDPAGGGDPILYQHL
>COLFF781-13|KJ966657|ZMUO.006101|Cytilus_sericeus

TLYFIFGAWAGMVGTSLSLLIRAELGNPGSLIGDDQIYNVIVTAHAFVMIFFMVMPIMIGGFGNWLVPLMLGAPDMAFPRMNNMSFWLLPPSLSLLLMSSIVESGAGTGWTVYPPLSSNIAHSGSSVDLAIFSLHLAGISSILGAVNFISTVINMRSPG
MKFDQMSLFSWSVAITALLLLLSLPVLAGAITMLLTDRNINTSFFDPAGGGDPILYQHL
>COLFF676-13|KJ964043|ZMUO.006376|Stilbus_oblongus
TLYFIFGAWSGMVGTSLSLLIRTELGTPGSLIGDDQIYNVIVTAHAFVMIFFMVMPFMIGGFGNWLVPLMLGAPDMAFPRMNNMSFWLLPPSLSFLLMSSIVENGAGTGWTVYPPLSSNIAHGGSSVDLAIFSLHLAGISSILGAINFITTVINMRPEGM
TLDRMPLFVWSVVITAILLLLSLPVLAGAITMLLTDRNINTSFFDPAGGGDPILYQHL
>COLFC702-12|KJ964507|ZMUO.003267|Cerylon_histeroides
TLYFMFGMWSGMVGTSMSMMIRLELGNPGSLIGDDQIYNVIVTAHAFVMIFFMVMPIMIGGFGNWLVPLMLGAPDMAFPRMNNMSFWLLPPSLTLLIMSSIVEKGAGTGWTVYPPLSANLTHSGSSVDLAIFSLHLAGISSILGAVNFITTVINMR
PSGMTWDRLPLFVWSVIITAVLLLLSLPVLAGAITMLLTDRNLNTSFFDPAGGGDPILYQHL
>COLFE1037-13|KJ966599|ZMUO.006832|Anthobium_unicolor
TLYFIFGAWAGMVGTSLSILIRAELGNPGTLIGDDQIYNVIVTAHAFVMIFFMVMPIVIGGFGNWLVPLMLGAPDMAFPRMNNMSFWLLPPSLTLLLMSSMVESGAGTGWTVYPPLSSNIAHGGSSVDLAIFSLHLAGISSILGAVNFITTVINMRATG
MTFDRMPLFVWSVAITALLLLLSLPVLAGAITMLLTDRNLNTSFFDPAGGGDPILYQHL
>COLFE1280-13|KJ966967|ZMUO.007075|Microcara_testacea
TLYFIFGSWAGMVGTSLSLLIRAELGTPGSLIGDDQIYNVIVTAHAFIMIFFMVMPIMIGGFGNWLVPLMLGAPDMAFPRMNNMSFWLLPPSLTLLLMSSMVENGAGTGWTVYPPLSAGMAHSGASVDLAIFSLHLAGISSILGAVNFISTVINMRSS
GMTFDRMPLFVWSVAITALLLLLSLPVLAGAITMLLTDRNLNTSFFDPAGGGDPILYQHL
>COLFE788-13|KJ966412|ZMUO.005633|Tetartopeus_terminatus
TLYFIFGAWAGMVGTSLSLLIRTELANPGSLIGDDQIYNVIVTAHAFVMIFFMVMPIVIGGFGNWLVPLMLGAPDMAFPRMNNMSFWLLPPSLSLLLASSMVESGAGTGWTVYPPLSSNIAHGGASVDLAIFSLHLAGISSILGAVNFITTIINMRSPGM
LYERMPLFVWSVGITALLLLLSLPVLAGAITMLLTDRNLNTSFFDPAGGGDPILYQHL
>COLFC570-12|KJ963441|ZMUO.003135|Evodinus_borealis
TLYFIFGAWAGMVGTSLSMLIRSELGNPGSLIGDDQIYNVIVTAHAFVMIFFMVMPIMIGGFGNWLVPLMLGAPDMAFPRMNNMSFWLLPPSLILLIMSSIVENGAGTGWTVYPPLASNIAHSGSSVDLAIFSLHLAGISSILGAVNFITTVINMRPPG
MTPDRMPLFVWAVVITAVLLLLSLPVLAGAITMLLTDRNLNTSFFDPAGGGDPILYQHL
>COLFE338-12|KJ966806|ZMUO.004898|Dacne_bipustulata
TLYFIFGAWSGMLGTALSILIRSELGNPGSLIGNDQIYNSIVTAHAFIMIFFMVMPIMMGGFGNWLVPLMIGAPDMAFPRMNNMSFWLLPPSLNLLIMSSVVETGAGTGWTVYPPLSSNIAHAGASVDLAIFSLHLAGISSILGAANFITTMMNMRPK
QMSLDQMPLFCWAILITAILLILSLPVLAGAITMLLTDRNFNTSFFDPTGGGDPILYQHL
>COLFA280-12|KJ962909|ZMUO.000415|Silvanoprus_fagi
TLYFIFGAWAGTVGTSLSVMIRTELGTPGALIGDDQIYNVIVTAHAFIMIFFMVMPVVIGGFGNWLVPLMLGAPDMAFPRLNNMSFWLLPPSISLLLISSIVEKGAGTGWTVYPPLSANIAHNGTSVDLAIFSLHLAGISSILGAINFISTIFNMHPNKMK
MDQMPLFCWAVLLTAVLLLLSLPVLAGAITMLLTDRNLNTSFFDPSGGGDPILYQHL
>COLFE026-12|KJ967172|ZMUO.005251|Aphodius_fossor
TLYFLFGSWAGMVGTSLSLLIRAELGNPGTLIGDDQIYNVIVTAHAFVMIFFMVMPILIGGFGNWLVPLMLGAPDMAFPRMNNMSFWLLPPSLTLLLMSSMVESGAGTGWTVYPPLSSNIAHGGASVDLAIFSLHLAGISSILGAVNFITTVINMRSPG
MTFDRMPLFVWSVAITALLLLLSLPVLAGAITMLLTDRNLNTSFFDPAGGGDPILYQHL
>COLFF197-13|KJ965891|ZMUO.005802|Quedius_mesomelinus
TLYFIFGAWAGMVGTSLSLLIRAELGNPGSLIGDDQIYNVIVTAHAFIMIFFMVMPTMIGGFGNWLVPLMLGAPDMAFPRMNNMSFWLLPPSLSLLLMSSMVESGAGTGWTVYPPLSSNIAHGGASVDLAIFSLHLAGISSILGAVNFITTVINMRSIG
MTFDRMPLFVWSVAITALLLLLSLPVLAGAITMLLTDRNLNTSFFDPAGGGDPILYQHL
>COLFF634-13|KJ962334|ZMUO.006524|Donacia_thalassina
TLYFIFGAWSGMMGTSLSMLIRTELSNPGSLIGNDQLYNVIVTAHAFIMIFFMVMPIMIGGFGNWLVPLMLGAPDMAFPRMNNMSFWLLPPSLSFLIMSSITESGAGTGWTVYPPLSNNLAHSGSSVDLAIFSLHLAGISSILGAVNFISTIINMRPTG
MTLEKMPLFSWAVMITAVLLLTSLPVLAGAITMLLTDRNINTSFFDPAGGGDPILYQHL
>COLFA050-10|HM909071|MP00223|Litargus_connexus
TLYFIFGAWSGMVGTSLSMLIRSELGNPGSLIGDDQIYNVIVTAHAFIMIFFMVMPIMMGGFGNWLVPLMLGAPDMAFPRMNNMSFWLLPPSLTLLIMSSVVESGAGTGWTVYPPLSSNIAHSGSSVDLAIFSLHLAGISSILGAVNFITTVINMRPSG
MSFDRMPLFVWAVAITALLLLLSLPVLAGAITMLLTDRNLNTSFFDPAGGGDPILYQHL
>COLFA506-12|KJ965892|ZMUO.000546|Bothrynoderes_affinis
TLYFIFGAWSGMVGTSLSMLIRTELGNPGSLIGDDQIYNTIVTAHAFIMIFFMVMPIMIGGFGNWLVPLMLGAPDMAFPRLNNMSFWLLPPSLTLLLMSSIVDKGAGTGWTVYPPLSTNIAHEGASVDLAIFSLHMAGISSILGAINFISTVLNMRPSG
MKPDQTTLFTWAVEITAILLLLSLPVLAGAITMLLTDRNINTSFFDPAGGGDPILYQHL
>COLFD294-12|KJ966782|ZMUO.004189|Bembidion_prasinum
TLYFIFGAWSGMVGTSLSMLIRAELGNPGSLIGDDQIYNVIVTAHAFVMIFFMVMPILIGGFGNWLVPLMLGAPDMAFPRMNNMSFWLLPPSLSLLLMSSMVESGAGTGWTVYPPLSSSIAHSGASVDLAIFSLHLAGVSSILGAVNFITTIINMRSVG
MSFDRMPLFVWSVGITALLLLLSLPVLAGAITMLLTDRNLNTTFFDPAGGGDPILYQHL
>COLFB175-12|KJ966791|ZMUO.001505|Enochrus_melanocephalus
TLYFIFGAWAGMVGTSLSILIRAELGNPGSLIGDDQIYNVIVTAHAFIMIFFMVMPIMIGGFGNWLVPLMLGAPDMAFPRMNNMSFWLLPPSLTLLLMSSMVESGAGTGWTVYPPLSSNIAHGGASVDLAIFSLHLAGISSILGAVNFITTVINMRSPS
MTYDRLPLFVWSVAITALLLLLSLPVLAGAITMLLTDRNLNTSFFDPAGGGDPILYQHL

>COLFF047-13|KJ964701|ZMUO.005747|Enochrus_bicolor
TLYFIFGAWAGMVGTSLSILIRAELGNPGTLIGDDQIYNVIVTAHAFIMIFFMVMPIMIGGFGNWLVPLMLGAPDMAFPRMNNMSFWLLPPSLTLLLMSSMVESGAGTGWTVYPPLSSNIAHGGASVDLAIFSLHLAGISSILGAVNFITTVINMRSPS
MTYDRLPLFVWSVAITALLLLLSLPVLAGAITMLLTDRNLNTSFFDPAGGGDPILYQHL
>COLFG114-13|KJ962741|ZMUO.007809|Carpelimus_impressus
TLYFIFGAWSGMVGTSLSMLIRIELGTPGSLIGDDQIYNVIVTAHAFIMIFFMVMPIVIGGFGNWLVPLMLGAPDMAFPRMNNMSFWLLPPSLTLLLFSSMVESGAGTGWTVYPPLSSNIAHSGSSVDLAIFSLHLAGISSILGAVNFISTIINMRSIGMTF
DRMPLFVWSVNITAILLLLSLPVLAGAITMLLTDRNLNTSFFDPAGGGDPILYQHL
>COLFG153-13|KJ966153|ZMUO.007848|Ptiliolum_sahlbergi
TLYFIFGAWAGMVGTSLSLLIRAELGTPGSLIGDDQIYNVIVTAHAFVMIFFMVMPILIGGFGNWLVPLMLGAPDMAFPRMNNMSFWLLPPSLTLLLMSSMVESGAGTGWTVYPPLSSNIAHGGASVDLAIFSLHLAGISSILGAVNFITTVINMRTPS
MTFDQMPLFVWAVTITALLLLLSLPVLAGAITMLLTDRNLNTTFFDPAGGGDPILYQHL
>COLFE332-12|KJ963197|ZMUO.004892|Silvanus_bidentatus
TLYFIFGAWAGSIGTSLSVMIRTELGTPGALIGDDQIYNVIVTAHAFIMIFFMVMPVVIGGFGNWLVPLMLGAPDMAFPRLNNMSFWLLPPSISLLLMSSIVEKGAGTGWTVYPPLSANMAHNGTSVDLAIFSLHLAGISSILGAINFISTIYNMHPNKM
KMDQIPLFCWAVLVTAILLLLSLPVLAGAITMLLTDRNLNTSFFDPSGGGDPILYQHL
>COLFF867-13|KJ963021|ZMUO.006662|Aphodius_distinctus
TLYFLFGSWAGMVGTSLSLLIRAELGNPGTLIGDDQIYNVIVTAHAFVMIFFMVMPILIGGFGNWLVPLMLGAPDMAFPRMNNMSFWLLPPSLTLLLMSSMVESGAGTGWTVYPPLSSNIAHGGASVDLAIFSLHLAGISSILGAVNFITTVINMRSPG
MTFDRMPLFVWSVAITALLLLLSLPVLAGAITMLLTDRNLNTSFFDPAGGGDPILYQHL
>COLFA185-10|HQ559270|MP00447|Larinus_sturnus
TLYFIFGAWSGMVGTSLSMLIRTELGNPGSLIGDDQIYNTIVTAHAFIMIFFMVMPIMIGGFGNWLVPLMLGAPDMAFPRLNNMSFWLLPPSLTLLLMSSIVDKGAGTGWTVYPPLSTNIAHEGASVDLAIFSLHMAGISSILGAINFISTVLNMRPMG
MKPDQTTLFTWAVEITAILLLLSLPVLAGAITMLLTDRNINTSFFDPAGGGDPILYQHL
>COLFA052-10|HM909073|MP00228|Chrysolina_varians
TLYFIFGMWAGMVGTSLSILIRAELGNPGTLIGNDQIYNVIVTAHAFIMIFFMVMPIMIGGFGNWLVPLMLGAPDMAFPRMNNMSFWLLPPSLIFLLMSSIVENGVGTGWTVYPPLSANIAHSGPSVDLAIFSLHLAGVSSILGAINFITTIINMRPTGM
QLEQMPLFSWAVLITAILLLLSLPVLAGAITMLLTDRNLNTSFFDPASGGDPILYQHL
>COLFA609-12|KJ962865|ZMUO.000744|Meloe_violaceus
TLYFIFGAWAGMVGTSLSLLIRAELGNPGTLIGDDQVYNVIVTAHAFIMIFFMVMPIMIGGFGNWLVPLMLGAPDMAFPRMNNMSFWLLPPSLTLLLMSSMVENGAGTGWTVYPPLSSNIAHGGSSVDLAIFSLHLAGISSILGAVNFITTVINMRPM
GVTFDRMPLFVWAVAITALLLLLSLPVLAGAITMLLTDRNLNTSFFDPAGGGDPVLYQHL
>COLFD540-12|KJ962321|ZMUO.004340|Panagaeus_cruxmajor
TLYFIFGAWSGMVGTSLSMLIRAELGNPGSLIGDDQIYNVIVTAHAFVMIFFMVMPIMIGGFGNWLVPLMLGAPDMAFPRMNNMSFWLLPPSLSLLLMSSLVESGAGTGWTVYPPLSSGIAHSGASVDLAIFSLHLAGVSSILGAVNFITTIINMRSVG
MTFERMPLFVWSVGITALLLLLSLPVLAGAITMLLTDRNLNTSFFDPAGGGDPILYQHL
>COLFB615-12|KJ967417|ZMUO.001090|Corticaria_rubripes
SLYFLFGMWSGMVGTSLSLLIRLELGNPGSLIGDDQIYNVIVTAHAFIMIFFMVMPIMIGGFGNWLVPLMLGAPDMAFPRLNNMSFWLLPPSLSLLIMSSIVESGAGTGWTVYPPLSSNIAHGGSSVDLAIFSLHLAGISSILGAVNFITTVINMRPAGM
NLDQMPLFVWSVVITAILLLLSLPVLAGAITMLLTDRNLNTSFFDPAGGGDPILYQHL
>COLFD375-12|KJ965676|ZMUO.004270|Agrypnus_murinus
TLYFLFGAWAGMLGTSLSLLIRAELGNPGSLIGNDQIYNVVVTAHAFIMIFFMVMPIMIGGFGNWLVPLMLGAPDMAFPRMNNMSFWLLPPSLSLLLMSSIVENGAGTGWTVYPPLSANIAHSGSSVDLAIFSLHLAGISSILGAVNFISTVINMRSTGI
TFDRMPLFVWAVAITALLLLLSLPVLAGAITMLLTDRNLNTSFFDPAGGGDPILYQHL
>COLFE077-12|KJ962497|ZMUO.005302|Smaragdina_flavicollis
TLYFIFGAWSGMVGTSLSLLIRVELGNPGTLIGNDQIYNTIVTAHAFIMIFFMVMPVMIGGFGNWLVPLMLGAPDMAFPRMNNMSFWLLPPSLTLLLMSSIVENGAGTGWTVYPPLSANLAHSGASVDLAIFSLHLAGISSIMGAINFISTVINMRPQG
MLLDRTPLFVWAVVITAILLLLSLPVLAGAITMLLTDRNLNTSFFDPAGGGDPILYQHL
>COLFD136-12|KJ966901|ZMUO.004031|Triplax_russica
TLYFIFGMWAGMVGTALSMLIRSELGNPGSLIGNDQIYNVIVTAHAFIMIFFMVMPIMMGGFGNWLVPLMLGAPDMAFPRLNNMSFWLLPPSLSLLILSSIVETGAGTGWTVYPPLSSNIAHSGASVDMAIFSLHLAGISSILGAMNFITTMMNMRP
TGMTLDQMPLFVWAVLITAILLILSLPVLAGAITMLLTDRNINTTFFDPAGGGDPILYQHL
>COLFD007-12|KJ966607|ZMUO.003902|Byrrhus_fasciatus
TLYFIFGAWAGIVGTSLSMLIRTELGNPGSLIGDDQIYNVIVTAHAFIMIFFMVMPIMIGGFGNWLVPLMLGAPDMAFPRMNNMSFWLLPPSLSLLLMSSVVESGAGTGWTVYPPLSANIAHSGSSVDLAIFSLHLAGISSILGAVNFISTVINMRSPGM
SFDQMSLFSWSVVITALLLLLSLPVLAGAITMLLTDRNVNTSFFDPAGGGDPILYQHL
>COLFA033-10|HM909060|MP00182|Coccidula_rufa
TLYFLFGMWAGMVGTSLSILIRLELGTPDSLIGNDQIYNVIVTAHAFIMIFFMVMPIMIGGFGNWLVPLMIGAPDMAFPRMNNMSFWLLPPALTLLILSSLVESGAGTGWTVYPPLSSNLAHSGSSVDMAIFSLHLAGISSILGAVNFISTVINMRTPGM
TFEKMPLFVWSVIITAILLLLSLPVLAGAITMLLTDRNINTSFFDPAGGGDPILYQHL
>COLFA024-10|HM909051|MP00162|Laccobius_minutus

TLYFLFGAWAGMVGTSLSILIRAELGNPGTLIGDDQIYNVIVTAHAFIMIFFMVMPIMIGGFGNWLVPLMLGAPDMAFPRMNNMSFWLLPPSLTLLLMSSMVESGAGTGWTVYPPLSSNIAHGGASVDLAIFSLHLAGISSILGAVNFITTVINMRSNN
MTYDRLPLFVWSVAITALLLLLSLPVLAGAITMLLTDRNLNTSFFDPAGGGDPILYQHL
>COLFE506-13|KJ966202|ZMUO.005351|Coccidula_scutellata
TLYFLFGMWAGMLGTSLSILIRLELGTPSSLIGNDQIYNVIVTAHAFIMIFFMVMPIMIGGFGNWLVPLMIGAPDMAFPRMNNMSFWLLPPALTLLILSSVVESGAGTGWTVYPPLSSNLAHSGSSVDLAIFSLHLAGISSILGAVNFITTVINMRPYGMT
FEKMPLFVWSVVITAILLLLSLPVLAGAITMLLTDRNINTSFFDPAGGGDPILYQHL
>COLFB027-12|KJ965941|ZMUO.001357|Galerucella_nymphaeae
TLYFIFGVWAGMVGTSLSILVRVELGNPGSLIGNDQIYNVIVTAHAFIMIFFMVMPTMIGGFGNWLVPLMIGAPDMAFPRMNNMSFWLLPPSLFLLIMSSIVESGAGTGWTVYPPLSSNIAHGGSSVDLAIFSLHMAGISSILGAINFITTIINMRPKGM
TLDRMPLFVWAVMITAILLLLSLPVLAGAITMLLTDRNLNTSFFDPAGGGDPILYQHL
>COLFE1405-13|KJ962440|ZMUO.007200|Thinonoma_atra
TLYFIFGAWAGMVGTSLSLLIRAELGNPGSLIGDDQIYNVIVTAHAFVMIFFMVMPVVIGGFGNWLVPLMLGAPDMAFPRMNNMSFWLLPPSLTLLLMSSMVESGAGTGWTVYPPLSSNIAHGGSSVDLAIFSLHLAGISSILGAVNFISTVINMRSTG
ITFDRMPLFVWSVAITALLLLLSLPVLAGAITMLLTDRNLNTSFFDPAGGGDPILYQHL
>COLFB843-12|KJ962936|ZMUO.001318|Atheta_procera
TLYFIFGAWAGMVGTSLSLLIRAELGNPGSLIGDDQIYNVIVTAHAFIMIFFMVMPIVIGGFGNWLVPLMLGAPDMAFPRMNNMSFWLLPPSLTLLLMSSMVESGAGTGWTVYPPLSSNIAHGGSSVDLAIFSLHLAGISSILGAVNFISTVINMRSTGI
SFDRMPLFVWSVVITALLLLLSLPVLAGAITMLLTDRNLNTSFFDPAGGGDPILYQHL
>COLFF387-13|KJ966758|ZMUO.005905|Epuraea_contractula
TLYFIFGAWSGMIGTSLSILIRTELGSPGSLIGNDQIYNVIVTAHAFIMIFFMVMPFMIGGFGNWLVPLMLGAPDMAFPRMNNMSFWLLPPSLSLLLMSSIVESGAGTGWTVYPPLSSNIAHGGSSVDLAIFSLHLAGISSILGAVNFITTIINMRPVGMTL
DRMPLFVWSVMITAVLLLLSLPVLAGAITMLLTDRNLNTTFFDPSGGGDPILYQHL
>COLFC367-12|KJ966907|ZMUO.002552|Oxypoda_annularis
TLYFIFGAWAGMVGTSLSLLIRAELGNPGSLIGDDQIYNVIVTAHAFVMIFFMVMPIVIGGFGNWLVPLMLGAPDMAFPRMNNMSFWLLPPSLTLLLMSSMVESGAGTGWTVYPPLSSNIAHGGSSVDLAIFSLHLAGISSILGAVNFISTIINMRTSG
MSFDRMPLFVWSVAITALLLLLSLPVLAGAITMLLTDRNLNTSFFDPAGGGDPILYQHL
>COLFF221-13|KJ964904|ZMUO.005826|Anaspis_brunnipes
TLYFIFGAWSGMVGTSLSLLIRSELGTPGSLIGDDQIYNVIVTAHAFIMIFFMVMPILIGGFGNWLVPLMLGAPDMAFPRMNNMSFWLLPPSLTLLIMSSVVENGAGTGWTVYPPLAANIAHSGSSVDLAIFSLHLAGVSSILGAVNFITTVINMRPQG
MTLDRMPLFVWAVVITAILLLLSLPVLAGAITMLLTDRNLNTSFFDPAGGGDPILYQHL
>COLFB112-12|KJ962818|ZMUO.001442|Philonthus_carbonarius
TLYFIFGSWAGMVGTSLSLLIRAELGNPGTLIGDDQIYNVIVTAHAFIMIFFMVMPIVIGGFGNWLVPLMLGAPDMAFPRMNNMSFWLLPPSLTLLLMSSMVESGAGTGWTVYPPLSSNMAHGGASVDLAIFSLHLAGISSILGAVNFITTVINMRSTG
MNFDRMPLFVWSVAITALLLLLSLPVLAGAITMLLTDRNINTTFFDPAGGGDPILYQHL
>COLFF968-13|KJ964367|ZMUO.007333|Anchomenus_dorsalis
TLYFIFGAWAGMVGTSLSMLIRAELGNPGALIGDDQIYNVIVTAHAFIMIFFMVMPIMIGGFGNWLVPLMLGAPDMAFPRMNNMSFWLLPPSLTLLLMSSLVESGAGTGWTVYPPLSSGIAHAGASVDLAIFSLHLAGVSSILGAVNFITTIINMRSVG
MTFDRMPLFVWSVGITALLLLLSLPVLAGAITMLLTDRNLNTSFFDPAGGGDPILYQHL
>COLFE271-12|KJ962429|ZMUO.005116|Philorhizus_sigma
TLYFIFGAWAGMVGTSLSMLIRAELGNPGALIGDDQIYNVIVTAHAFIMIFFMVMPIMIGGFGNWLVPLMLGAPDMAFPRMNNMSFWLLPPSLTLLLVSSMVESGAGTGWTVYPPLSSNIAHAGASVDLAIFSLHLAGISSILGAVNFITTIINMRSIG
MTFDRMPLFVWSVGITALLLLLSLPVLAGAITMLLTDRNLNTSFFDPAGGGDPILYQHL
>COLFE1516-13|KJ962568|ZMUO.007311|Stenopterapion_tenue
TLYFIFGLWAGMVGTSLSMLIRVELGTPGSLIGNDQIYNVIVTAHAFIMIFFMVMPVMIGGFGNWLVPLMLGAPDMAFPRMNNMSFWLLPPSLTLLLMSSIVEKGAGTGWTVYPPLASNIAHGGASVDLAIFSLHLAGISSILGAVNFISTIINMHPNG
LSFDQLSLFTWAVKITAILLLLSLPVLAGAITMLLTDRNINTSFFDPAGGGDPILYQHL
>COLFB293-12|KJ962922|ZMUO.001623|Cordylepherus_viridis
TLYFIFGAWSGMVGLSLSLLIRSELSIPGTLIGNDQIYNVIVTAHAFIMIFFMVMPILIGGFGNWLVPLMLGAPDMAFPRMNNMSFWLLPPSLTLLLLSSMVENGAGTGWTVYPPLSANIAHSGSSVDLAIFSLHLAGISSILGAVNFITTVINMRPQGMT
LDRTPLFVWAVVITALLLLLSLPVLAGAITMLLTDRNLNTSFFDPAGGGDPILYQHL
>COLFG019-13|KJ962180|ZMUO.007524|Buprestis_haemorrhoidalis
TLYFIFGAWSGMVGTALSLLIRAELGNPGALIGDDQVYNVIVTAHAFVMIFFMVMPVMMGGFGNWLVPLMLGAPDMAFPRMNNMSFWLLPPSLTLLLMSSVVENGAGTGWTVYPPLAANIAHSGASVDLAIFSLHLAGVSSILGAVNFITTVINM
RSVGMTFDRMPLFVWSVAITALLLLLSLPVLAGAITMLLTDRNFNTSFFDPAGGGDPILYQHL
>COLFE1107-13|KJ964838|ZMUO.006902|Hydroporus_nigrita
TLYFMFGAWSGMVGTSLSMLIRAELGNPGSLIGDDQIYNVIVTAHAFIMIFFMVMPIMIGGFGNWLVPLMLGAPDMAFPRMNNMSFWLLPPSLTLLLMSSMVENGAGTGWTVYPPLSSGIAHSGASVDLAIFSLHLAGVSSILGAVNFITTIINMRSI
GMTFDRMPLFVWSVGITALLLLLSLPVLAGAITMLLTDRNLNTSFFDPAGGGDPILYQHL
>COLFB102-12|KJ966331|ZMUO.001432|Amara_hyperborea
TLYFIFGAWSGMVGTSLSMLIRAELGNPGALIGDDQIYNVIVTAHAFIMIFFMVMPIMIGGFGNWLVPLMLGAPDMAFPRMNNMSFWLLPPSLTLLLMSSMVESGVGTGWTVYPPLSSGIAHAGASVDLAIFSLHLAGVSSILGAVNFITTIINMRTV
GMTFDRMPLFVWSVGITALLLLLSLPVLAGAITMLLTDRNLNTSFFDPAGGGDPILYQHL

>COLFA594-12|KJ962724|ZMUO.000729|Agonum_ericeti
TLYFIFGAWSGMVGTSLSMLIRAELGNPGALIGDDQIYNVIVTAHAFIMIFFMVMPIMIGGFGNWLVPLMLGAPDMAFPRMNNMSFWLLPPSLTLLLMSSMVESGAGTGWTVYPPLSSGIAHAGASVDLAIFSLHLAGVSSILGAVNFITTIINMRSV
GMTFDRMPLFVWSVGITALLLLLSLPVLAGAITMLLTDRNLNTSFFDPAGGGDPILYQHL
>COLFD644-12|KJ962230|ZMUO.004444|Stenolophus_mixtus
TLYFIFGVWAGMVGTSLSMLIRAELGTPGALIGDDQIYNVIVTAHAFVMIFFMVMPIMIGGFGNWLVPLMLGAPDMAFPRMNNMSFWLLPPSLTLLLMSSLVENGAGTGWTVYPPLSSGIAHSGASVDLAIFSLHLAGISSILGAVNFITTIINMRSVG
MTFERMPLFVWSVGITALLLLLSLPVLAGAITMLLTDRNLNTSFFDPAGGGDPILYQHL
>COLFF146-13|KJ965010|ZMUO.003780|Deleaster_dichrous
TLYFIFGAWSGMVGTSLSMLIRTELGTPGSLIGDDQIYNVIVTAHAFIMIFFMVMPTVIGGFGNWLVPLMLGAPDMAFPRMNNMSFWLLPPSLSLLLMSSIVESGAGTGWTVYPPLSSNIAHSGSSVDLAIFSLHLAGISSILGAVNFISTIINMRSIGITF
DRMPLFVWSVGITALLLLLSLPVLAGAITMLLTDRNLNTSFFDPSGGGDPILYQHL
>COLFF253-13|KJ961748|ZMUO.005858|Anthonomus_rubi
TLYFIFGAWSGAVGTSLSMLIRTELGNPGSLIGDDQIYNTIVTAHAFIMIFFMVMPIMIGGFGNWLIPLMLAAPDMAFPRLNNMSFWLLPPSLTLLIMSSIISKGAGTGWTVYPPLSSNLAHEGASVDFAIFSLHMAGISSILGAMNFISTILNMKPMKM
KFEQMPLFIWAVKITAILLLISLPVLAGAITMLLTDRNINTSFFDPAGGGDPILYQHL
>COLFE1302-13|KJ961780|ZMUO.007097|Erichsonius_cinerascens
TLYFIFGAWAGMVGTSLSLLIRAELGNPGSLIGDDQIYNVIVTAHAFIMIFFMVMPIMIGGFGNWLVPLMLGAPDMAFPRMNNMSFWLLPPSLTLLLMSSMVESGAGTGWTVYPPLSSNIAHGGASVDLAIFSLHLAGISSILGAVNFITTVINMRSVG
MTFDRMPLFVWSVAITALLLLLSLPVLAGAITMLLTDRNLNTTFFDPAGGGDPILYQHL
>COLFF273-13|KJ962463|ZMUO.005878|Trachys_minutus
TLYFIFGAWSGMVGTALSLLIRAELGNPGALIGNDQIYNVIVTAHAFIMIFFMVMPIMMGGFGNWLVPLMLGAPDMAFPRMNNMSFWLLPPSLSLLLMSSMVESGAGTGWTVYPPLAANIAHSGASVDLAIFSLHLAGISSILGAINFITTVINMRAY
GMTLDQVPLFVWSVAITALLLLLSLPVLAGAITMLLTDRNLNTSFFDPVGGGDPVLYQHL
>COLFC012-12|KJ965205|ZMUO.002007|Paederus_fuscipes
TLYFIFGAWSGMVGTSLSLLIRAELATPGSLIGDDQIYNVIVTAHAFIMIFFMVMPIMIGGFGNWLVPLMLGAPDMAFPRMNNMSFWLLPPALTLLLMSSMVENGAGTGWTVYPPLSSNAFHNGSSVDLAIFSLHLAGISSILGAINFITTALNMRASN
MSYEQMPLFVWSVAITALLLLLSLPVLAGAITMLLTDRNLNTSFFDPSGGGDPILYQHL
>COLFE1353-13|KJ964269|ZMUO.007148|Ceutorhynchus_ignitus
TLYFIFGSWAGMAGTSLSMLIRTELGNPGSLIGNDQIYNSIVTAHAFIMIFFMVMPILIGGFGNWLVPLMLGAPDMAFPRLNNMSFWLLPPSLSLLLMSSIVNKGAGTGWTVYPPLSSNVAHEGMSVDLAIFSLHMAGISSILGAINFISTVMNMQPK
GMTPELMPLFVWAVEITAILLLLSLPVLAGAITMLLTDRNINTSFFDPSGGGDPILYQHL
>COLFA406-12|KJ966923|ZMUO.000636|Aphodius_borealis
TLYFLFGSWAWMVGTSLSLLIRAELGNPGTLIGDDQIYNVIVTAHAFVMIFFMVMPILIGGFGNWLVPLMLGAPDMAFPRMNNMSFWLLPPSLTLLLMSSMVESGAGTGWTVYPPLSSNIAHGGASVDLAIFSLHLAGISSILGAVNFITTVINMRSP
GLTFDRMPLFVWSVAITALLLLLSLPVLAGAITMLLTDRNLNTSFFDPAGGGDPILYQHL
>COLFD229-12|KJ965574|ZMUO.004124|Corticaria_polypori
SLYFMFGMWAGMVGTSLSLLIRLELGNPGSLIGDDQIYNVIVTAHAFVMIFFMVMPIMIGGFGNWLVPLMLGAPDMAFPRLNNMSFWLLPPSLSLLIMSSIVESGAGTGWTVYPPLSSNIAHSGSSVDLAIFSLHLAGISSILGAVNFITTVINMRPAG
MNLDQMPLFVWSVVITAILLLLSLPVLAGAITMLLTDRNLNTSFFDPAGGGDPILYQHL
>COLFE931-13|KJ962694|ZMUO.006251|Zeugophora_subspinosa
TLYFIFGVWAGMVGTSLSLLIRSELGTPGSLIGDDQIYNVIVTAHAFVMIFFMVMPIMIGGFGNWLVPLMLGAPDMAFPRMNNMSFWFLPPSLILLLMSSIVESGAGTGWTVYPPLSANIAHSGSSVDLAIFSLHMAGISSILGAVNFITTVINMRPMG
LNLDKMPLFVWAVVITAILLLLSLPVLAGAITMLLTDRNINTSFFDPSGGGDPILYQHL
>COLFG021-13|KJ963170|ZMUO.007526|Buprestis_dalmatina
TLYFIFGAWSGMVGTALSLLIRAELGNPGALIGDDQVYNVIVTAHAFVMIFFMVMPVMMGGFGNWLVPLMLGAPDMAFPRMNNMSFWLLPPSLTLLLMSSVVENGAGTGWTVYPPLAANIAHSGASVDLAIFSLHLAGVSSILGAVNFITTVINM
RSVGMTFDRMPLFVWSVAITALLLLLSLPVLAGAITMLLTDRNFNTSFFDPAGGGDPILYQHL
>COLFF807-13|KJ965314|ZMUO.006127|Hydraena_britteni
TLYFIFGAWAGMVGTSLSILIRAELGNPGTLIGDDQIYNVIVTAHAFIMIFFMVMPILIGGFGNWLVPLMLGAPDMAFPRMNNMSFWLLPPSLSLLLMSSMVESGAGTGWTVYPPLSSNIAHGGASVDLAIFSLHLAGISSILGAVNFITTIINMRSPGM
SFDQMPLFVWAVGITALLLLLSLPVLAGAITMLLTDRNLNTSFFDPAGGGDPILYQHL
>COLFA295-12|KJ965347|ZMUO.000430|Leptacinus_intermedius
TLYFIFGAWAGMVGTSLSLLIRAELGNPGSLIGDDQIYNVIVTAHAFIMIFFMVMPIVIGGFGNWLVPLMLGAPDMAFPRMNNMSFWLLPPSLTLLLTSSLVESGAGTGWTVYPPLSSNIAHGGASVDLAIFSLHLAGISSILGAVNFITTVLNMRSFGM
TFDRMPLFVWSVAITALLLLLSLPVLAGAITMLLTDRNLNTSFFDPMGGGDPILYQHL
>COLFA279-12|KJ967358|ZMUO.000414|Lithocharis_nigriceps
TLYFIFGAWAGMVGTSLSLLIRAELGVPGSLIGDDQIYNVIVTAHAFVMIFFMVMPIVIGGFGNWLVPLMLGAPDMAFPRMNNMSFWLLPPSLSLLLMSSMVESGAGTGWTVYPPLSSNIAHGGASVDLAIFSLHLAGISSILGAVNFITTVINMRSSG
MTYERMPLFIWSVAITALLLLLSLPVLAGAITMLLTDRNLNTSFFDPAGGGDPILYQHL
>COLFE007-12|KJ964455|ZMUO.005232|Harpalus_tardus

TLYFIFGAWAGMVGTSLSMLIRAELGTPGALIGDDQIYNVIVTAHAFIMIFFMVMPIMIGGFGNWLVPLMLGAPDMAFPRMNNMSFWLLPPSLTLLLMSSMVESGAGTGWTVYPPLSSGIAHGGASVDLAIFSLHLAGISSILGAVNFITTIINMRSTG
MTFDRMPLFVWSVGITALLLLLSLPVLAGAITMLLTDRNLNTSFFDPAGGGDPILYQHL
>COLFC560-12|KJ963338|ZMUO.003125|Agabus_fuscipennis
TLYFIFGAWAGMVGTSLSMLIRAELGNPGSLIGDDQIYNVIVTAHAFVMIFFMVMPIMIGGFGNWLVPLMLGAPDMAFPRMNNMSFWLLPPSLTLLLMSSMVENGAGTGWTVYPPLSSGIAHGGASVDLAIFSLHLAGISSILGAVNFITTIINMRSI
GMTFDRMPLFVWSVGITALLLLLSLPVLAGAITMLLTDRNLNTSFFDPAGGGDPILYQHL
>COLFE763-13|KJ962752|ZMUO.005608|Agabus_congener
TLYFIFGAWAGMVGTSLSMLIRAELGNPGSLIGDDQIYNVIVTAHAFVMIFFMVMPIMIGGFGNWLIPLMLGAPDMAFPRMNNMSFWLLPPSLTLLLMSSMVESGAGTGWTVYPPLSSGIAHGGASVDLAIFSLHLAGISSILGAVNFITTIINMRSVG
MTFDRMPLFVWSVGITALLLLLSLPVLAGAITMLLTDRNLNTSFFDPAGGGDPILYQHL
>COLFD252-12|KJ963024|ZMUO.004147|Hyphydrus_ovatus
TLYFLFGAWSGMVGTSLSMLIRAELGNPGSLIGDDQIYNVIVTAHAFIMIFFMVMPIMIGGFGNWLVPLMLGAPDMAFPRMNNMSFWLLPPSLSLLLMSSMVESGAGTGWTVYPPLSAGIAHGGSSVDLAIFSLHLAGISSILGAVNFITTIINMRSIG
MTFDRMPLFVWSVGITALLLLLSLPVLAGAITMLLTDRNLNTSFFDPAGGGDPILYQHL
>COLFE015-12|KJ963818|ZMUO.005240|Brachypterus_urticae
TLYFIFGAWSGMVGTSLSILIRSELGNPGSLIGDDQIYNVIVTAHAFVMIFFMVMPFMIGGFGNWLIPLMLGAPDMAFPRMNNMSFWLLPPSLTLLLMSSIVESGAGTGWTVYPPLSSNIAHGGSSVDLAIFSLHLAGISSILGAVNFITTVINMRPIGM
NFDRMPLFVWAVAITALLLLLSLPVLAGAITMLLTDRNLNTSFFDPAGGGDPILYQHL
>COLFD905-12|KJ964825|ZMUO.004515|Falagria_caesa
TLYFIFGAWAGMVGTSLSLLIRAELGNPGSLIGDDQIYNVIVTAHAFIMIFFMVMPIVIGGFGNWLVPLMLGAPDMAFPRMNNMSFWLLPPSLSLLLMSSMVESGAGTGWTVYPPLSSNIAHGGASVDLAIFSLHLAGISSILGAVNFISTVINMRSIGIT
FDRMPLFVWSVAITALLLLLSLPVLAGAITMLLTDRNLNTSFFDPAGGGDPILYQHL
>COLFB900-12|KJ963149|ZMUO.001945|Leiopus_nebulosus
TLYFIFGAWAGMVGTSLSMLIRSELGTAGSLIGDDQIYNAIVTAHAFVMIFFMVMPIMIGGFGNWLVPLMLGAPDMAFPRMNNMSFWLLPPSLTLLIMSSVVNSGAGTGWTVYPPLSSNIAHAGPSVDLAIFSLHLAGASSILGAVNFITTVINMRPK
GMSFDRLPLFVWAVKITTILLLLSLPVLAGAITMLLTDRNLNTSFFDPAGGGDPVLYQHL
>COLFD283-12|KJ965231|ZMUO.004178|Rhagonycha_elongata
TLYFIFGAWSGSLGLALSLLIRAELGTPGTLIGNDQIYNVIVTAHAFIMIFFMVMPIMIGGFGNWLVPLMLGAPDMAFPRMNNMSFWFLPPSLMFLLMSSMVESGAGTGWTVYPPLSANIAHSGPSVDLAIFSLHMAGISSILGAVNFISTILNMKPPS
MKFDQMPLFVWSVGITALLLLLSLPVLAGAITMLLSDRNLNTSFFDPMGGGDPILYQHL
>COLFE041-12|KJ967365|ZMUO.005266|Amara_lunicollis
TLYFIFGAWSGMVGTSLSMLIRAELGNPGALIGDDQIYNVIVTAHAFVMIFFMVMPIMIGGFGNWLVPLMLGAPDMAFPRMNNMSFWLLPPSLTLLLMSSMVENGAGTGWTVYPPLSSGIAHAGASVDLAIFSLHLAGISSILGAVNFITTIINMRSI
GMTFDRMPLFVWSVGITALLLLLSLPVLAGAITMLLTDRNLNTSFFDPAGGGDPILYQHL
>COLFD148-12|KJ966157|ZMUO.004043|Protapion_trifolii
TLYFIFGLWSGMIGTSLSMLIRIELGNPGSLIGNDQIYNVIVTAHAFIMIFFMVMPVMIGGFGNWLVPLMLGAPDMAFPRMNNMSFWLLPPSLTLLLMSSIVEKGAGTGWTVYPPLAANIAHSGASVDLAIFSLHLAGISSILGAVNFISTIMNMRPTG
MSLDQLSLFTWAVKITAILLLLSLPVLAGAITMLLTDRNINTSFFDPAGGGDPILYQHL
>COLFB198-12|KJ965394|ZMUO.001528|Cryptocephalus_parvulus
TLYFLFGAWSGLVGTSLSMLIRVELGNPGSLIGNDQIYNTIVTAHAFIMIFFMVMPIMIGGFGNWLVPLMLGAPDMAFPRMNNMSFWLLPPSLTLLLMSSIVENGAGTGWTVYPPLSSTIAHAGPSVDLAIFSLHLAGISSIMGAINFISTVINMRPQG
MMMDRTPLFVWAVLITAILLLLSLPVLAGAITMLLTDRNLNTSFFDPAGGGDPILYQHL
>COLFF636-13|KJ964636|ZMUO.006526|Ochthebius_minimus
TLYFIFGAWAGMVGTSLSILIRAELGNPGTLIGDDQIYNVIVTAHAFIMIFFMVMPIMIGGFGNWLVPLMLGAPDMAFPRMNNMSFWLLPPSLSLLLLSSMVENGAGTGWTVYPPLSSNIAHGGASVDLAIFSLHLAGISSILGAVNFITTVINMRSAG
MSFDRMPLFVWAVAITALLLLLSLPVLAGAITMLLTDRNLNTSFFDPAGGGDPILYQHL
>COLFE063-12|KJ967085|ZMUO.005288|Judolia_sexmaculata
TLYFIFGAWAGMVGTSLSLLIRSELGSPGSLIGNDQIYNVIVTAHAFVMIFFMVMPIMIGGFGNWLVPLMLGAPDMAFPRMNNMSFWLLPPSLTLLILSSMVETGAGTGWTVYPPLSSNIAHSGSSVDLAIFSLHLAGISSILGAVNFITTVINMRPVGM
NLDCMPLFVWAVVITAILLLLSLPVLAGAITMLLTDRNLNTSFFDPAGGGDPILYQHL
>COLFB794-12|KJ962479|ZMUO.001269|Oxypoda_annularis
TLYFIFGAWAGMVGTSLSLLIRAELGNPGSLIGDDQIYNVIVTAHAFVMIFFMVMPIVIGGFGNWLVPLMLGAPDMAFPRMNNMSFWLLPPSLTLLLMSSMVESGAGTGWTVYPPLSSNIAHGGSSVDLAIFSLHLAGISSILGAVNFISTIINMRTSG
MSFDRMPLFVWSVAITALLLLLSLPVLAGAITMLLTDRNLNTSFFDPAGGGYPILYQHL
>COLFA694-12|KJ963087|ZMUO.000829|Cercyon_analis
TLYFIFGAWAGMVGTSLSILIRAELGNPGTLIGDDQIYNVIVTAHAFIMIFFMVMPIMIGGFGNWLVPLMLGAPDMAFPRMNNMSFWLLPPSLTLLLMSSMVENGAGTGWTVYPPLSSNIAHSGSSVDLAIFSLHLAGISSILGAVNFITTVINMRSPNL
TYDRLPLFVWSVAITALLLLLSLPVLAGAITMLLTDRNLNTSFFDPAGGGDPILYQHL
>COLFB804-12|KJ963557|ZMUO.001279|Patrobus_septentrionis
TLYFIFGAWSGMVGTSLSMLIRAELGNPGSLIGDDQIYNVIVTAHAFVMIFFMVMPIMIGGFGNWLVPLMLGAPDMAFPRMNNMSFWLLPPSLTLLLMSSMVESGAGTGWTVYPPLSSGIAHSGASVDLAIFSLHLAGISSILGAVNFITTIINMRSVG
MSFDRMPLFVWSVGITALLLLLSLPVLAGAITMLLTDRNLNTSFFDPAGGGDPILYQHL

>COLFE084-12|KJ962491|ZMUO.005309|Magdalis_duplicata
ILYFIFGTWSGMIGTSLSMLIRVELGSPGSLIGNDQIYNTIVTAHAFIMIFFMVMPIMIGGFGNFLIPLMLGAPDMAFPRLNNLSFWLLPPSLTLLLLSSITDKGAGTGWTVYPPLSSSNFHEGTSVDLAIFSLHLAGISSILGALNFISTIINMRPKNMKLDQ
MSLFIWAVKITAILLLLSLPVLAGAITMLLTDRNINTSFFDPAGGGDPILYQHL
>COLFB349-12|KJ963255|ZMUO.001679|Acalyptus_carpini
TLYFIFGAWAGMVGTSLSMLIRTELGNPGSLIGDDQIYNTIVTAHAFIMIFFMVMPIMIGGFGNWLVPLMLGAPDMAFPRLNNMSFWLLPPSITLLLMSSIVDKGAGTGWTVYPPLSANIAHEGTSVDLAIFSLHMAGISSILGAMNFISTVINMRPM
GMKLDQMTLFVWAVKITAILLLLSLPVLAGAITMLLTDRNINTSFFDPAGGGDPILYQHL
>COLFB389-12|KJ964900|ZMUO.001814|Monochamus_sutor
TLYFIFGAWSGMVGTSLSLLIRSELGMPGALIGDDQIYNVIVTAHAFVMIFFMVMPIMIGGFGNWLVPLMLGAPDMAFPRMNNMSFWLLPPSLTLLIMSSIVENGAGTGWTVYPPLAANVAHSGSSVDLAIFSLHLAGVSSILGAVNFITTVINMRPS
GMNLDRLPLFVWAVKITAILLLLSLPVLAGAITMLLTDRNLNTSFFDPAGGGDPILYQHL
>COLFC606-12|KJ965039|ZMUO.003171|Phyllotreta_striolata
TLYFIFGIWSGMMGMSMSMLIRIELAAPGSLIGNDQIYNVIVTAHAFIMIFFMVMPIMIGGFGNWLVPLMIGAPDMAFPRMNNMSFWLLPPSLFLLIMSSIIENGAGTGWTVYPPLSSNVSHAGASVDLTIFSLHLAGISSILGAINFITTVINMRPKG
MTFDRMPLFVWAVLITAILLLLSLPVLAGAITMLLTDRNLNTSFFDPMGGGDPILYQHL
>COLFB042-12|KJ962803|ZMUO.001372|Hydrobius_fuscipes
TLYFIFGAWAGMVGTSLSILIRAELGNPGTLIGDDQIYNVIVTAHAFIMIFFMVMPIMIGGFGNWLVPLMLGAPDMAFPRMNNMSFWLLPPSLTLLLMSSMVENGAGTGWTVYPPLSSNIAHGGASVDLAIFSLHLAGISSILGAVNFITTVINMRSPN
LTYDRLPLFVWSVAITALLLLLSLPVLAGAITMLLTDRNLNTSFFDPAGGGDPILYQHL
>COLFD792-12|KJ962418|ZMUO.004782|Quedius_pseudolimbatus
TLYFIFGAWAGMVGTSLSLLIRAELGNPGTLIGDDQIYNVIVTAHAFIMIFFMVMPIVIGGFGNWLVPLMLGAPDMAFPRMNNMSYWLLPPSMSLLLMSSMVESGAGTGWTVYPPLSSNIAHSGASVDLAIFSLHLAGISSILGAVNFITTVINMRSIG
MTFDRMPLFVWSVAITALLLLLSLPVLAGAITMLLTDRNLNTSFFDPAGGGDPILYQHL
>COLFB502-12|KJ967036|ZMUO.000977|Acrotona_aterrima
TLYFIFGAWAGMVGTSLSLLIRAELGNPGSLIGDDQIYNVIVTAHAFVMIFFMVMPIVIGGFGNWLVPLMLGAPDMAFPRMNNMSFWLLPPSLSLLLMSSMVESGAGTGWTVYPPLSSNIAHGGASVDLAIFSLHLAGISSILGAVNFISTIINMRSTGI
SFDRMPLFVWSVAITALLLLLSLPVLAGAITMLLTDRNLNTSFFDPAGGGDPILYQHL
>COLFB137-12|KJ964908|ZMUO.001467|Propylea_quatuordecimpunctata
TLYFLFGMWAGMVGTSLSILIRLELGTTNSLIGNDQIYNVIVTAHAFIMIFFMVMPIMIGGFGNWLVPLMIGAPDMAFPRLNNMSFWLLPPALMLLIMSSMVEMGAGTGWTVYPPLSSNMAHSGSSVDLVIFSLHLAGISSILGAVNFISTIMNMRPF
GMNLDKTPLFVWSVLITAILLLLSLPVLAGAITMLLTDRNINTSFFDPMGGGDPILYQHL
>COLFB652-12|KJ964752|ZMUO.001127|Stenus_nitens
TLYFIFGAWAGMVGTSLSLLIRAELGNPGSLIGDDQIYNVIVTAHAFVMIFFMVMPIMIGGFGNWLVPLMLGAPDMAFPRMNNMSFWLLPPSLSLLLMSSIVENGAGTGWTVYPPLSSNIAHSGASVDLAIFSLHLAGISSILGAINFITTIFNMRTMKL
QLDCLPLFVWSVAITALLLLLSLPVLAGAITMLLTDRNLNTSFFDPAGGGDPILYQHL
>COLFD330-12|KJ965856|ZMUO.004225|Atomaria_fuscata
TLYFIFGSWAGMVGTSLSMLIRTELGTPGSLIGDDQIYNVIVTAHAFIMIFFMVMPMMIGGFGNWLVPLMLGAPDMAFPRLNNMSFWLLPPSLMFLLMSSIVEKGAGTGWTVYPPLSSNVAHAGSSVDLAIFSLHLAGISSILGSMNFITTVINMRPK
GMNFDCLPLFVWSVKITTILLLLSLPVLAGAITMLLTDRNINTSFFDPAGGGDPILYQHL
>COLFB734-12|KJ966316|ZMUO.001209|Agonum_fuliginosum
TLYFIFGAWAGMVGTSLSMLIRAELGNPGALIGDDQIYNVIVTAHAFIMIFFMVMPIMIGGFGNWLVPLMLGAPDMAFPRMNNMSFWLLPPSLTLLLMSSLVESGAGTGWTVYPPLSSGIAHAGASVDLAIFSLHLAGISSILGAVNFITTIINMRSVG
MTFDRMPLFVWSVGITALLLLLSLPVLAGAITMLLTDRNLNTSFFDPAGGGDPILYQHL
>COLFA009-10|HM909039|MP00137|Zacladus_geranii
TLYFIFGSWAGAAGTSLSMLIRTELGNPGSLIGNDQIYNSIVTAHAFIMIFFMVMPILIGGFGNWLVPLMLGAPDMAFPRLNNMSFWLLPPSLTLLLMSSIINKGAGTGWTVYPPLSANVAHEGASVDLAIFSLHMAGISSILGAINFISTIMNMRPKGM
DYDQTPLFAWAVMITAILLLLSLPVLAGAITMLLTDRNINTTFFDPSGGGDPILYQHL
>COLFF617-13|KJ964207|ZMUO.006507|Polydrusus_tereticollis
TLYFIFGSWSGMVGTSLSMLIRTELGNPGSLIGDDQIYNVIVTAHAFIMIFFMVMPMMIGGFGNWLVPLMLGAPDMAFPRLNNMSFWLLPPSLSLLLMSSIVDKGAGTGWTVYPPLSANIAHEGSSVDLAIFSLHMAGVSSILGAINFISTVINMRPSG
MSFDHMSLFIWAVKITAILLLLSLPVLAGAITMLLTDRNINTSFFDPAGGGDPILYQHL
>COLFD453-12|KJ963613|ZMUO.003873|Meligethes_difficilis
TLYFIFGAWSGMVGTSLSMLIRTELGNPGSLIGNDQIYNVIVTAHAFVMIFFMVMPFMIGGFGNWLVPLMLGAPDMAFPRMNNMSFWLLPPSLSLLLTSSIVESGAGTGWTVYPPLSSNIAHGGASVDLAIFSLHLAGISSILGAVNFITTVINMRPSG
MTFDRMPLFVWAVVITAILLLLSLPVLAGAITMLLTDRNLNTTFFDPSGGGDPILYQHL
>COLFA294-12|KJ962478|ZMUO.000429|Cordalia_obscura
TLYFIFGAWAGMVGTSLSLLIRAELGNPGSLIGDDQIYNVIVTAHAFVMIFFMVMPIVIGGFGNWLVPLMLGAPDMAFPRMNNMSFWLLPPSLTLLLMSSMVESGAGTGWTVYPPLSANIAHSGSSVDLAIFSLHLAGISSILGAVNFISTVINMRTMG
ITFDRMPLFVWSVAITALLLLLSLPVLAGAITMLLTDRNLNTSFFDPAGGGDPILYQHL
>COLFF257-13|KJ961879|ZMUO.005862|Crioceris_duodecimpunctata

TLYFIFGTWAGTVGTSLSVLIRTELGNPGSLIGNDQIYNVIVTAHAFVMIFFMVMPIMIGGFGNWLVPLMLGAPDMAFPRLNNMSFWLLPPSLSLLLMSSIVESGAGTGWTVYPPLSANLAHGGASVDLAIFSLHLAGISSILGAVNFITTVMNMRPEG
MSLDRTPLFVWAVVITAILLLLSLPVLAGAITMLLTDRNLNTSFFDPASGGDPILYQHL
>COLFB026-12|KJ965988|ZMUO.001356|Galerucella_nymphaeae
TLYFIFGVWAGMVGTSLSILVRVELGNPGSLIGNDQIYNVIVTAHAFIMIFFMVMPTMIGGFGNWLVPLMIGAPDMAFPRMNNMSFWLLPPSLFLLIMSSIVESGAGTGWTVYPPLSSNIAHGGSSVDLAIFSLHMAGISSILGAINFITTIINMRPKGM
TLDRMPLFVWAVMITAILLLLSLPVLAGAITMLLTDRNLNTSFFDPAGGGDPILYQHL
>COLFG173-13|KJ966638|ZMUO.007868|Euryptilium_saxonicum
TLYFIFGAWAGMVGTSLSLLIRAELGTPGSLIGDDQVYNVIVTAHAFVMIFFMVMPILIGGFGNWLVPLMLGAPDMAFPRLNNMSFWLLPPALSLLLMSSMVESGAGTGWTVYPPLSSNIAHGGASVDLAIFSLHLAGISSILGAVNFITTVINMRAPG
MTFDQMPLFVWAVVITALLLLLSLPVLAGAITMLLTDRNLNTSFFDPAGGGDPILYQHL
>COLFE1293-13|KJ963681|ZMUO.007088|Agabus_congener
TLYFIFGAWAGMVGTSLSMLIRAELGNPGSLIGDDQIYNVIVTAHAFVMIFFMVMPIMIGGFGNWLIPLMLGAPDMAFPRMNNMSFWLLPPSLTLLLMSSMVESGAGTGWTVYPPLSSGIAHGGASVDLAIFSLHLAGISSILGAVNFITTIINMRSVG
MTFDRMPLFVWSVGITALLLLLSLPVLAGAITMLLTDRNLNTSFFDPAGGGDPILYQHL
>COLFF638-13|KJ962953|ZMUO.006528|Cantharis_obscura
TLYFIFGAWSGSLGLALSLLIRAELGTPGTLIGNDQIYNVIVTAHAFIMIFFMVMPIMIGGFGNWLVPLMLGAPDMAFPRMNNMSFWFLPPSLMFLLMSSMVESGAGTGWTVYPPLSANIAHSGPSVDLAIFSLHMAGISSILGAVNFISTIMNMKPPS
MKFDQMPLFVWSVGITALLLLLSLPVLAGAITMLLSDRNLNTSFFDPMGGGDPILYQHL
>COLFC034-12|KJ966675|ZMUO.002029|Atheta_cribripennis
TLYFIFGAWAGMVGTSLSLLIRAELGNPGSLIGDDQIYNVIVTAHAFIMIFFMVMPIVIGGFGNWLVPLMLGAPDMAFPRMNNMSFWLLPPSLTLLLMSSMVESGAGTGWTVYPPLSSNIAHGGSSVDLAIFSLHLAGISSILGAVNFISTIINMRSTGIS
FDRMPLFVWSVAITALLLLLSLPVLAGAITMLLTDRNLNTSFFDPAGGGDPILYQHL
>COLFF689-13|KJ963807|ZMUO.006389|Corticeus_unicolor
TLYFIFGAWSGMVGTSLSLLIRAELGNPGSLIGDDQIYNVIVTAHAFIMIFFMVMPIMMGGFGNWLVPLMLGAPDMAFPRMNNMSFWLLPPSLSLLLMSSMVESGAGTGWTVYPPLSSNIAHSGSSVDLAIFSLHLAGISSILGAVNFITTVINMRPLG
MSFDRMPLFVWAVAITAVLLLLSLPVLAGAITMLLTDRNLNTSFFDPAGGGDPILYQHL
>COLFF057-13|KJ965319|ZMUO.005757|Cercyon_bifenestratus
TLYFIFGAWAGMVGTSLSILIRAELGNPGTLIGDDQIYNVIVTAHAFIMIFFMVMPIMIGGFGNWLVPLMLGAPDMAFPRMNNMSFWLLPPSLTLLLMSSMVESGAGTGWTVYPPLSSNIAHGGASVDLAIFSLHLAGISSILGAVNFITTVINMRSPN
LSYDRLPLFVWSVAITALLLLLSLPVLAGAITMLLTDRNLNTSFFDPAGGGDPILYQHL
>COLFC738-12|KJ964300|ZMUO.003303|Ampedus_pomorum
TLYFIFGAWAGMLGTSLSLLIRAELGNPGSLIGNDQIYNVIVTAHAFIMIFFMVMPIMIGGFGNWLVPLMLGAPDMAFPRMNNMSFWLLPPSLSLLLMSSIVENGAGTGWTVYPPLSSNIAHSGSSVDLAIFSLHLAGISSILGAVNFISTVINMRSTGIT
FDRMPLFVWAVAITALLLLLSLPVLAGAITMLLTDRNLNTSFFDPAGGGDPILYQHL
>LEFIJ1857-13|KJ962895|ZMUO.004612|Rhinoncus_castor
TLYFIFGSWAGTVGTSLSMLIRTELGTPGSLIGNDQIYNSIVTAHAFIMIFFMVMPILIGGFGNWLVPLMLGAPDMAFPRLNNMSFWLLPPSIMLLLMSSIVNKGAGTGWTVYPPLSSNITHEGASVDLAIFSLHMAGISSILGAINFISTIMNMRPQGM
SYDKMPLFSWAVLITAILLLLSLPVLAGAITMLLTDRNINTSFFDPAGGGDPILYQHL
>COLFD439-12|KJ967053|ZMUO.003859|Olibrus_bimaculatus
TLYFIFGAWAGMMGTSLSLLIRTELGTPGSLIGDDQIYNVIVTAHAFVMIFFMVMPFMIGGFGNWLVPLMLGAPDMAFPRMNNMSFWLLPPSLFLLLMSSLVESGAGTGWTVYPPLSSNIAHGGASVDLAIFSLHLAGISSILGAINFITTMINMRPEG
MTLDRMPLFVWAVTITAILLLLSLPVLAGAITMLLTDRNINTSFFDPAGGGDPILYQHL
>COLFC180-12|KJ965708|ZMUO.002365|Plegaderus_saucius
TLYFIFGAWAGMVGTSLSLLIRAELGNPGTLIGDDQIYNVIVTAHAFIMIFFMVMPIMMGGFGNWLVPLMLGAPDMAFPRMNNMSFWLLPPSLTLLLMSSMVESGAGTGWTVYPPLASNIAHGGASVDLAIFSLHLAGISSILGAVNFITTVINMRSP
GMSFDRMPLFVWSVAITALLLLLSLPVLAGAITMLLTDRNLNTSFFDPAGGGDPILYQHL
>COLFF687-13|KJ962766|ZMUO.006387|Dryocoetes_villosus
TLYFIFGTWSGMVGTSLSVLIRTELGTPGSLIGDDQIFNTIVTAHAFIMIFFMVMPILIGGFGNWLVPLMLGAPDMAFPRLNNMSFWLLPPSLTLLLMSSIMDKGAGTGWTVYPPLASNIAHEGASVDLAIFSLHMAGISSILGAVNFISTINNMHPAGM
KPEQLSLFTWAVKITAILLLLSLPVLAGGITMLLTDRNINTSFFDPAGGGDPILYQHL
>COLFA613-12|KJ964682|ZMUO.000748|Mycetophagus_multipunctatus
TLYFIFGSWAGMVGTSLSLLIRSELGNPGSLIGDDQIYNVIVTAHAFIMIFFMVMPIMMGGFGNWLVPLMLGAPDMAFPRMNNMSFWLLPPSLTLLIMSSIVENGAGTGWTVYPPLSANIAHSGSSVDLAIFSLHLAGISSILGAVNFITTIINMRPKG
MTFDRMPLFVWAVAITALLLLLSLPVLAGAITMLLTDRNLNTSFFDPAGGGDPILYQHL
>COLFA630-12|KJ967523|ZMUO.000765|Philonthus_addendus
TLYFIFGSWAGMVGTSLSLLIRAELGNPGTLIGDDQIYNVIVTAHAFIMIFFMVMPVVIGGFGNWLVPLMLGAPDMAFPRMNNMSFWLLPPSLTLLLMSSVVESGAGTGWTVYPPLSSNMAHGGASVDLAIFSLHLAGISSILGAVNFITTVINMRST
GMSFDRMPLFVWSVAITALLLLLSLPVLAGAITMLLTDRNLNTSFFDPAGGGDPILYQHL
>COLFC355-12|KJ962688|ZMUO.002540|Oligota_pusillima
ILYFIFGSWAGMIGTSLSLLIRAELGNPGALIGDDQIYNVIVTAHAFIMIFFMVMPIMIGGFGNWLVPLMLSAPDMAFPRMNNMSFWLLPPALMLLLFSSMVESGSGTGWTVYPPLSSNIAHSGASVDLAIFSLHMAGISSILGAINFITTILNMHPMS
MSYDQMPLFIWSVGITALLLLLSLPVLAGAITMLLTDRNMNTSFFDPAGGGDPILYQHL

>COLFD702-12|KJ965115|ZMUO.004692|Galeruca_tanaceti
TLYFIFGVWAGMVGTSLSILVRTELGSPGTLIGNDQIYNVIVTAHAFIMIFFMVMPIMIGGFGNWLVPLMIGAPDMAFPRMNNMSFWLLPPSLFLLIMSSIVESGAGTGWTVYPPLSSNIAHGGSSVDLAIFSLHLAGISSILGAINFITTVINMRPKGMT
LDRMPLFVWAVTITAILLLLSLPVLAGAITMLLTDRNLNTSFFDPAGGGDPILYQHL
>COLFC423-12|KJ961792|ZMUO.002988|Stenus_umbratilis
TLYFIFGAWAGMLGTSLSLLIRTELGNPGSFIGDDQIYNVIVTAHAFIMIFFMVMPIMIGGFGNWLVPLMLGAPDMAFPRMNNMSFWLLPPSLSLLIMSSIVESGAGTGWTVYPPLSSNIAHSGPSVDLAIFSLHLAGISSILGAINFITTILNMRTMKIQF
DCLPLFVWSVGITALLLLLSLPVLAGAITMLLTDRNLNTSFFDPAGGGDPILYQHL
>COLFE1487-13|KJ964148|ZMUO.007282|Rhamphus_oxyacanthae
TLYFLFGAWSGMVGTSLSMLIRTELGTPGKLIGDDQIYNTIVTAHAFIMIFFMVMPIMIGGFGNWLIPLMLGAPDMAFPRLNNMSFWLLPPSLTFLLLSSIMDKGAGTGWTVYPPLSANIAHEGTSVDLAIFSLHMAGVSSILGAMNFISTIINMKPKN
MSLDQMSLFVWSVKITAILLLLSLPVLAGAITMLLTDRNINTSFFDPAGGGDPILYQHL
>COLFF713-13|KJ964517|ZMUO.006413|Athous_haemorrhoidalis
TLYFLFGAWAGMLGTSLSLLIRAELGNPGSLIGNDQIYNVIVTAHAFIMIFFMVMPIMIGGFGNWLVPLMLGAPDMAFPRMNNMSFWFLPPSLSLLLMSSIVENGAGTGWTVYPPLSANIAHSGSSVDLAIFSLHLAGISSILGAVNFISTVINMRSTGIT
FDRMPLFVWAVVITALLLLLSLPVLAGAITMLLTDRNLNTSFFDPAGGGDPILYQHL
>COLFE1193-13|KJ964418|ZMUO.006988|Helophorus_strandi
TLYFIFGAWAGMVGTSLSILIRAELGNPGTLIGDDQIYNVIVTAHAFIMIFFMVMPIMIGGFGNWLVPLMLGAPDMAFPRMNNMSFWLLPPSLTLLLMSSIVESGAGTGWTVYPPLSSNIAHSGASVDLAIFSLHLAGISSILGAVNFITTVINMRSTNM
SYDRLPLFVWSVAITALLLLLSLPVLAGAITMLLTDRNLNTSFFDPAGGGDPILYQHL
>COLFC233-12|KJ965895|ZMUO.002418|Amara_lunicollis
TLYFIFGAWSGMVGTSLSMLIRAELGNPGALIGDDQIYNVIVTAHAFVMIFFMVMPIMIGGFGNWLVPLMLGAPDMAFPRMNNMSFWLLPPSLTLLLMSSMVENGAGTGWTVYPPLSSGIAHAGASVDLAIFSLHLAGISSILGAVNFITTIINMRSI
GMTFDRMPLFVWSVGITALLLLLSLPVLAGAITMLLTDRNLNTSFFDPAGGGDPILYQHL
>COLFE417-12|KJ967454|ZMUO.004977|Rhagonycha_testacea
TLYFIFGAWSGSLGLALSLLIRAELGTPGTLIGNDQIYNVIVTAHAFIMIFFMVMPIMIGGFGNWLVPLMLGAPDMAFPRMNNMSFWFLPPSLMFLLMSSMVESGAGTGWTVYPPLSANIAHSGPSVDLAIFSLHMAGISSILGAVNFISTILNMKPPS
MKFDQMPLFVWSVGITALLLLLSLPVLAGAITMLLSDRNLNTSFFDPMGGGDPILYQHL
>COLFE1344-13|KJ965330|ZMUO.007139|Phyllobius_virideaeris
TLYFIFGAWSGMIGTSLSILIRVELGNPGSLIGDDQIYNVIVTAHAFIMIFFMVMPMMIGGFGNWLVPLMLGAPDMAFPRLNNMSFWLLPPSLSLLLMSSIVDKGAGTGWTVYPPLSANIAHEGSSVDLAIFSLHMAGVSSILGAVNFISTVINMRPMG
MSPDRMSLFIWAVKITAILLLLSLPVLAGAITMLLTDRNMNTSFFDPAGGGDPILYQHL
>COLFE1071-13|KJ965954|ZMUO.006866|Notaris_aethiops
TLYFIFGAWSGMAGTSLSMLIRAELGNPGSLIGDDQIYNVIVTAHAFIMIFFMVMPIMIGGFGNWLIPLMLGAPDMAFPRLNNLSFWLLPPSLIILLSSSLIEKGAGTGWTVYPPLSSNIAHSGPSVDLAIFSLHMAGISSILGAINFISTVINMRPSGMDPD
RMTLFVWAVKITAILLLLSLPVLAGAITMLLTDRNINTSFFDPAGGGDPILYQHL
>COLFF483-13|KJ966038|ZMUO.006563|Oryzaephilus_surinamensis
TLYFIFGAWAGMVGTSLSILIRTELGTAGSLIGNDQIYNTIVTAHAFIMIFFMVMPVVIGGFGNWLIPLMIGAPDMAFPRLNNMSFWLLPPSISLLLISSIVEKGAGTGWTVYPPLSSNLAHNGTSVDLAIFSLHLAGISSILGAINFISTIFNMKPKKMNMD
QMPLFCWAVMITAVLLLLSLPVLAGAITMLLTDRNLNTSFFDPSGGGDPILYQHL
>COLFD208-12|KJ966977|ZMUO.004103|Dromius_fenestratus
TLYFIFGAWAGMVGTSLSMLIRAELGNPGALIGDDQVYNVIVTAHAFIMIFFMVMPIMIGGFGNWLVPLMLGAPDMAFPRMNNMSFWLLPPSLSLLLMSSMVESGAGTGWTVYPPLSSGIAHAGASVDLAIFSLHLAGVSSILGAVNFITTIINMRSI
GMTFDRMPLFVWSVGITALLLLLSLPVLAGAITMLLTDRNLNTSFFDPAGGGDPILYQHL
>COLFA626-12|KJ964624|ZMUO.000761|Bisnius_puella
TLYFIFGSWAGMVGTSLSLLIRAELGNPGSLIGDDQIYNVIVTAHAFIMIFFMVMPIVIGGFGNWLVPLMLGAPDMAFPRMNNMSFWLLPPSLTLLLMSSMVESGAGTGWTVYPPLSSNIAHGGASVDLAIFSLHLAGISSILGAVNFITTVINMRSTG
MSFDRMPLFVWSVAITALLLLLSLPVLAGAITMLLTDRNLNTSFFDPAGGGDPILYQHL
>COLFA139-10|HQ559250|MP00401|Arhopalus_rusticus
TLYFIFGAWASMVGTSLSLLIRSELGSPGSLIGDDQIYNVIVTAHAFVMIFFMVMPIMIGGFGNWLVPLMLGAPDMAFPRMNNMSFWLLPPSLTLLIMSSIVESGAGTGWTVYPPLSSNIAHSGASVDLAIFSLHLAGISSILGAVNFITTVINMRPSGM
TLDRMPLFVWAVVITAILLLLSLPVLAGAITMLLTDRNLNTSFFDPSGGGDPILYQHL
>COLFB723-12|KJ966085|ZMUO.001198|Atheta_laevana
TLYFIFGAWAGMVGTSLSLLIRAELGNPGSLIGDDQIYNVIVTAHAFIMIFFMVMPVVIGGFGNWLVPLMLGAPDMAFPRMNNMSFWLLPPSLSLLLMSSMVESGAGTGWTVYPPLSSNIAHGGSSVDLAIFSLHLAGISSILGAVNFISTVINMRSTGI
SFDRMPLFVWSVVITALLLLLSLPVLAGAITMLLTDRNLNTSFFDPAGGGDPILYQHL
>COLFB473-12|KJ966995|ZMUO.001898|Anaspis_rufilabris
TLYFIFGTWSGMVGTSLSLLIRSELGTPGSLIGDDQIYNVIVTAHAFIMIFFMVMPILIGGFGNWLVPLMLGAPDMAFPRMNNMSFWLLPPSLTLLIMSSVVENGAGTGWTVYPPLAANIAHSGSSVDLAIFSLHLAGVSSILGAVNFITTVINMRPQGM
TLDRMPLFVWAVVITAVLLLLSLPVLAGAITMLLTDRNLNTSFFDPAGGGDPILYQHL
>COLFF635-13|KJ965614|ZMUO.006525|Donacia_thalassina

TLYFIFGAWSGMMGTSLSMLIRTELSNPGSLIGNDQLYNVIVTAHAFIMIFFMVMPIMIGGFGNWLVPLMLGAPDMAFPRMNNMSFWLLPPSLSFLIMSSITESGAGTGWTVYPPLSNNLAHSGSSVDLAIFSLHLAGISSILGAVNFISTIINMRPTG
MTLEKMPLFSWAVMITAVLLLTSLPVLAGAITMLLTDRNINTSFFDPAGGGDPILYQHL
>COLFG147-13|KJ962499|ZMUO.007842|Ptilium_exaratum
TLYFIFGAWSGMIGTSLSLLIRAELGMSGSLIGDDQIYNVIVTAHAFIMIFFMVMPILIGGFGNWLVPIMLGAPDMAFPRMNNMSFWMLPPSLTLLLMSSMVESGAGTGWTVYPPLASNLAHMGASVDLAIFSLHLAGISSILGAVNFITTIINMRTPQ
MNFEQMPLFVWSVAITALLLLLSLPVLAGAITMLLTDRNLNTSFFDPAGGGDPILYQHL
>COLFD438-12|KJ962293|ZMUO.003858|Longitarsus_luridus
TLYFIFGIWAGMIGTSLSILIRTELGNPGSLIGNDQIYNVIVTAHAFIMIFFMVMPIMIGGFGNWLVPLMIGAPDMAFPRMNNMSFWLLPPSLFLLVMSSMVESGAGTGWTVYPPLSSNIAHGGSSVDLAIFSLHLAGISSILGAINFITTVINMRPIGMTL
DRMPLFVWAVVITAILLLLSLPVLAGAITMLLTDRNLNTTFFDPAGGGDPILYQHL
>COLFC516-12|KJ963451|ZMUO.003081|Bryophacis_maklini
TLYFIFGSWAGMVGTSLSLLIRAELGNPGSLIGDDQIYNVIVTAHAFIMIFFMVMPIVIGGFGNWLIPLMLGAPDMAFPRMNNMSFWLLPPSLTLLLMSSMVESGAGTGWTVYPPLSSNIAHSGASVDLAIFSLHLAGISSILGAVNFITTVINMRSTGM
TFDRMPLFIWSVAITALLLLLSLPVLAGAITMLLTDRNLNTSFFDPAGGGDPILYQHL
>COLFE156-12|KJ967097|ZMUO.005191|Ophonus_rufibarbis
TLYFIFGAWAGMVGTSLSMLIRAELGTPGALIGDDQIYNVIVTAHAFIMIFFMVMPIMIGGFGNWLVPLMLGAPDMAFPRMNNMSFWLLPPSLTLLLMSSMVESGAGTGWTVYPPLSSGIAHSGASVDLAIFSLHLAGISSILGAVNFITTIINMRSVG
MTFDRMPLFVWSVGITALLLLLSLPVLAGAITMLLTDRNLNTSFFDPAGGGDPILYQHL
>COLFF909-13|KJ962619|ZMUO.006704|Agonum_emarginatum
TLYFIFGAWSGMVGTSLSMLIRAELGNPGALIGDDQIYNVIVTAHAFIMIFFMVMPIMIGGFGNWLVPLMLGAPDMAFPRMNNMSFWLLPPSLTLLLMSSMVESGAGTGWTVYPPLSSGIAHAGASVDLAIFSLHLAGVSSILGAVNFITTIINMRSV
GMTFDRMPLFVWSVGITALLLLLSLPVLAGAITMLLTDRNLNTSFFDPAGGGDPILYQHL
>COLFF072-13|KJ966540|ZMUO.005772|Ilybius_crassus
TLYFIFGAWAGMVGTSLSMLIRAELGNPGSLIGDDQIYNVIVTAHAFVMIFFMVMPIMIGGFGNWLVPLMLGAPDMAFPRMNNMSFWLLPPSLSLLLMSSMVESGAGTGWTVYPPLSAGIAHSGASVDLAIFSLHLAGISSILGAVNFITTIINMRSV
GMTFDRMPLFVWSVGITALLLLLSLPVLAGAITMLLTDRNLNTSFFDPAGGGDPILYQHL
>COLFD588-12|KJ962492|ZMUO.004388|Ipidia_binotata
TLYFIFGAWSGMIGTSLSMMIRTELGNPGSLIGNDQIYNVIVTAHAFVMIFFMVMPFMIGGFGNWLVPLMLGAPDMAFPRLNNMSFWLLPPSLSLLLMSSIVESGAGTGWTVYPPLSANIAHGGSSVDLAIFSLHLAGISSILGAVNFITTVINMQPTG
MSFDKMPLFVWAVLITAILLLLSLPVLAGAITMLLTDRNLNTTFFDPSGGGDPILYQHL
>COLFA015-10|HM909044|MP00147|Cryptocephalus_distinguendus
TLYFLFGAWAGMVGTSLSLLIRIELGNPGSLIGNDQIYNTIVTAHAFIMIFFMVMPIMIGGFGNWLIPLMLGAPDMAFPRMNNLSFWLLPPSLTLLLVSSIVENGAGTGWTVYPPLSAIIAHTGASVDLAIFSLHLAGISSIMGAINFISTIINMRPQGMQ
MDCTPLFVWAVMITAVLLLLSLPVLAGAITMLLTDRNLNTSFFDPAGGGDPILYQHL
>COLFE1392-13|KJ962434|ZMUO.007187|Anidorus_nigrinus
TLYLIFGAWSGMLGTSLSLLIRSELGNPGSLIGDDQIFNVIVTAHAFIMIFFMVMPIMIGGFGNWLIPLMLGAPDMAFPRMNNMSFWLLPPSLTLLIMSSLVENGAGTGWTVYPPLSSNLTHSGSSVDLAIFSLHLAGISSILGAINFITTVINMHPKKMSF
DQMPLFVWSVMITAILLLLSLPVLAGAITMLLTDRNLNTSFFDPSGGGDPILYQHL
>COLFD034-12|KJ967412|ZMUO.003929|Monochamus_sutor
TLYFIFGAWSGMVGTSLSLLIRSELGMPGALIGDDQIYNVIVTAHAFVMIFFMVMPIMIGGFGNWLVPLMLGAPDMAFPRMNNMSFWLLPPSLTLLIMSSIVENGAGTGWTVYPPLAANVAHSGSSVDLAIFSLHLAGVSSILGAVNFITTVINMRPS
GMNLDRLPLFVWAVKITAILLLLSLPVLAGAITMLLTDRNLNTSFFDPAGGGDPILYQHL
>COLFA353-12|KJ966087|ZMUO.000583|Chaetocnema_sahlbergii
TLYFIFGIWSGMAGTSMSILIRAELGNPGSLIGNDQIYNVIVTAHAFVMIFFMVMPIMIGGFGNWLVPLMIGAPDMAFPRMNNMSFWLLPPSLFLLLMSSMVESGAGTGWTVYPPLSSNIAHGGSSVDLAIFSLHLAGISSILGAINFITTIINMRPQG
MQFDQMPLFVWAVLITAILLLLSLPVLAGAITMLLTDRNLNTSFFDPIGGGDPILYQHL
>COLFE053-12|KJ961881|ZMUO.005278|Anthribus_nebulosus
TLYFIFGAWAGMMGTSLSLIIRMELGNPGSLIGDDQIYNVIVTAHAFIMIFFMVMPTMIGGFGNWLVPLMLGAPDMAFPRMNNMSFWLLPPSLTLLVLSSIVESGAGTGWTVYPPLSSNIAHGGSSVDLAIFSLHLAGVSSILGAVNFITTIINMRPEG
MSFDRMPLFVWAVGITALLLLLSLPVLAGAITMLLTDRNLNTSFFDPAGGGDPILYQHL
>COLFE921-13|KJ966287|ZMUO.006241|Quedius_brevis
TLYFIFGAWAGMVGTSLSLLIRAELGNPGTLIGDDQIYNVIVTAHAFIMIFFMVMPIMIGGFGNWLVPLMLGAPDMAFPRMNNMSFWLLPPSLSLLLMSSMVESGVGTGWTVYPPLSSNIAHGGASVDLAIFSLHLAGISSILGAVNFITTVINMRSIG
MSFDRMPLFVWSVAITALLLLLSLPVLAGAITMLLTDRNLNTSFFDPAGGGDPILYQHL
>COLFF226-13|KJ964985|ZMUO.005831|Oxypoda_togata
TLYFIFGAWAGMIGTSLSLLIRAELGNPGSLIGDDQIYNVIVTAHAFVMIFFMVMPIMIGGFGNWLIPLMLGAPDMAFPRMNNMSFWLLPPSLTLLLMSSMVESGAGTGWTVYPPLSSNIAHSGSSVDLAIFSLHLAGISSILGAVNFISTIINMRTLGM
TFDRMPLFIWAVAITALLLLLSLPVLAGAITMLLTDRNLNTSFFDPAGGGDPILYQHL
>COLFA289-12|KJ961927|ZMUO.000424|Myrmechixenus_vaporariorum
TLYFIFGAWSGMVGTSLSLLIRAELGNPGSLIGDDQIYNVIVTAHAFIMIFFMVMPIMIGGFGNWLVPLMLGAPDMAFPRMNNMSFWLLPPSLTLLLMSSIVESGAGTGWTVYPPLSSNIAHGGSSVDLAIFSLHLAGISSILGAVNFITTVINMRPQG
MTFDRMPLFVWAVVITAVLLLLSLPVLAGAITMLLTDRNINTSFFDPAGGGDPILYQHL

>COLFF046-13|KJ963085|ZMUO.005746|Enochrus_bicolor
TLYFIFGAWAGMVGTSLSILIRAELGNPGTLIGDDQIYNVIVTAHAFIMIFFMVMPIMIGGFGNWLVPLMLGAPDMAFPRMNNMSFWLLPPSLTLLLMSSMVESGAGTGWTVYPPLSSNIAHGGASVDLAIFSLHLAGISSILGAVNFITTVINMRSPS
MTYDRLPLFVWSVAITALLLLLSLPVLAGAITMLLTDRNLNTSFFDPAGGGDPILYQHL
>COLFC468-12|KJ967342|ZMUO.003033|Ernobius_explanatus
TLYFIFGAWAGMVGTSLSMLIRSELGNPGALIGDDQIYNVIVTAHAFIMIFFMVMPIMIGGFGNWLVPLMLGAPDMAFPRMNNMSFWLLPPSLSLLLMSSAVESGAGTGWTVYPPLSANIAHSGASVDLAIFSLHLAGISSILGAVNFITTVINMRPVG
MTLDRLPLFVWSVAITALLLLLSLPVLAGAITMLLTDRNLNTSFFDPAGGGDPILYQHL
>COLFE1522-13|KJ963504|ZMUO.007412|Longitarsus_lewisii
ILYFIFGVWSGMVGTSLSILIRTELGNPGSLIGNDQIYNVIVTAHAFIMIFFMVMPIMIGGFGNWLVPLMIGAPDMAFPRMNNMSFWLLPPSLFFLIMSSVVESGAGTGWTVYPPLSSNIAHGGSSVDLAIFSLHLAGISSILGAINFITTVINMRPIGMTL
DRMPLFVWAVIITAILLLLSLPVLAGAITMLLTDRNLNTTFFDPAGGGDPILYQHL
>COLFB696-12|KJ963439|ZMUO.001171|Calathus_erratus
TLYFIFGAWAGMVGTSLSMLIRAELGNPGALIGDDQVYNVIVTAHAFVMIFFMVMPIMIGGFGNWLVPLMLGAPDMAFPRMNNMSFWLLPPSLTLLLMSSMVESGAGTGWTVYPPLSSGIAHSGASVDLAIFSLHLAGISSILGAVNFITTIINMRSV
GMTFDRMPLFVWSVGITALLLLLSLPVLAGAITMLLTDRNLNTSFFDPAGGGDPILYQHL
>COLFF079-13|KJ963609|ZMUO.005779|Nebrioporus_depressus
TLYFLFGAWSGMVGTSLSMLIRAELGNPGSLIGDDQIYNVIVTAHAFIMIFFMVMPIMIGGFGNWLVPLMLGAPDMAFPRMNNMSFWMLPPSLSLLLMSSMVENGAGTGWTVYPPLSAGLAHGGASVDLAIFSLHLAGISSILGAVNFITTIINMRS
VGMTFDRMPLFVWSVGITALLLLLSLPVLAGAITMLLTDRNLNTSFFDPAGGGDPILYQHL
>COLFC676-12|KJ963520|ZMUO.003241|Calathus_melanocephalus
TLYFIFGAWAGMVGTSLSMLIRAELGNPGALIGDDQIYNVIVTAHAFVMIFFMVMPIMIGGFGNWLVPLMLGAPDMAFPRMNNMSFWLLPPSLTLLLMSSMVESGAGTGWTVYPPLSSGIAHSGASVDLAIFSLHLAGVSSILGAVNFITTIINMRSV
GMTFDRMPLFVWSVGITALLLLLSLPVLAGAITMLLTDRNLNTSFFDPAGGGDPILYQHL
>COLFE1019-13|KJ966566|ZMUO.006814|Stenichnus_collaris
TLYFILGIWSGMLGTSLSLLIRLELSSPGMMIGNDQTFNMIVTSHAFIMIFFMVMPIMIGGFGNWLVPLMLGAPDMAFPRMNNMSFWFLPPSLMLLLMSSMVESGSGTGWTVYPPLSSNIAHSGSSVDLTIFSLHLAGISSILGAVNFITTIINMRSPM
MKFDNLSLFIWAVFITAILLLLSLPVLAGAITMLLTDRNFNTSFFDPSGGGDPILYQHL
>COLFF138-13|KJ966145|ZMUO.003772|Hypera_postica
TLYFIFGTWAGTVGTSLSILIRTELGNPGSLIGNDQIYNTIVTAHAFIMIFFMVMPIMIGGFGNWLVPLMLGAPDMAFPRLNNMSFWLLPPSLSLLLMSSMVDSGAGTGWTVYPPLSSNIAHEGSSVDLAIFSLHMAGVSSILGAINFISTVLNMRPSGM
SLDKMTLFIWAVKITAILLLLSLPVLAGAITMLLTDRNINTSFFDPAGGGDPILYQHL
>COLFD548-12|KJ963606|ZMUO.004348|Harpalus_laevipes
TLYFIFGAWAGMVGTSLSMLIRAELGTPGALIGNDQIYNVIVTAHAFIMIFFMVMPIMIGGFGNWLVPLMLGAPDMAFPRMNNMSFWLLPPSLTLLLMSSMVESGAGTGWTVYPPLSSGIAHSGASVDLAIFSLHLAGISSILGAVNFITTIINMRSVG
MTFDRMPLFVWSVGITALLLLLSLPVLAGAITMLLTDRNLNTSFFDPAGGGDPILYQHL
>COLFF916-13|KJ967448|ZMUO.006711|Thanatophilus_sinuatus
TLYFIFGAWAGMVGMSLSILIRAELSTPGTLLGDDQMYNVIVTAHAFIMIFFMVMPIVIGGFGNWLVPLMLGAPDMAFPRMNNMSFWLLPPSLSLLLVSSMVESGAGTGWTVYPPLSSNIAHGGSAVDLAIFSLHLAGISSILGAVNFITTIINMRSSG
MTFDRMPLFVWSVAITALLLLLSLPVLAGAITMLLTDRNLNTSFFDPAGGGDPILYQHL
>COLFE1002-13|KJ961758|ZMUO.006797|Quedius_boopoides
SLYFIFGAWAGMVGTSLSLLIRAELGNPGSLIGDDQIYNVIVTAHAFIMIFFMVMPTLIGGFGNWLVPLMLGAPDMAFPRMNNMSFWLLPPSLSLLLMSSMVESGAGTGWTVYPPLSSNIAHSGASVDLAIFSLHLAGISSILGAVNFITTVINMRSTG
MTFDRMPLFVWSVAITALLLLLSLPVLAGAITMLLTDRNLNTTFFDPAGGGDPILYQHL
>COLFB913-12|KJ964796|ZMUO.001958|Phymatodes_testaceus
TLYFIFGAWAGMVGTSLSMLIRSELGNPGSLIGDDQIYNVIVTAHAFIMIFFMVMPVMIGGFGNWLVPLMLGAPDMAFPRMNNMSFWLLPPSLTLLIMSSIVENGAGTGWTVYPPLSANVAHSGSSVDLAIFSLHLAGVSSILGAVNFISTVINMKPA
GMTPEQMPLFVWAVLITAILLLLSLPVLAGAITMLLTDRNLNTSFFDPAGGGDPILYQHL
>COLFB045-12|KJ966578|ZMUO.001375|Amara_majuscula
TLYFIFGAWSGMVGTSLSMLIRAELGNPGALIGDDQIYNVIVTAHAFVMIFFMVMPIMIGGFGNWLVPLMLGAPDMAFPRMNNMSFWLLPPALSLLLMSSMVESGAGTGWTVYPPLSSGIAHAGASVDLAIFSLHLAGISSILGAVNFITTIINMRSV
GMTFDRMPLFVWSVGITALLLLLSLPVLAGAITMLLTDRNMNTSFFDPAGGGDPILYQHL
>COLFA527-12|KJ965586|ZMUO.000567|Cychramus_variegatus
TLYFIFGAWSGMVGTSLSMLIRTELGNPGSLIGNDQIYNVIVTAHAFIMIFFMVMPFMIGGFGNWLVPLMLGAPDMAFPRMNNMSFWLLPPSLSLLLMSSIVESGAGTGWTVYPPLSSNIAHGGSSVDLAIFSLHLAGISSILGAVNFITTVINMRPKG
MTFDRMPLFVWAVIITAILLLLSLPVLAGAITMLLTDRNLNTTFFDPSGGGDPILYQHL
>COLFE1521-13|KJ964861|ZMUO.007411|Longitarsus_lewisii
ILYFIFGVWSGMVGTSLSILIRTELGNPGSLIGNDQIYNVIVTAHAFIMIFFMVMPIMIGGFGNWLVPLMIGAPDMAFPRMNNMSFWLLPPSLFFLIMSSVVESGAGTGWTVYPPLSSNIAHGGSSVDLAIFSLHLAGISSILGAINFITTVINMRPIGMTL
DRMPLFVWAVIITAILLLLSLPVLAGAITMLLTDRNLNTTFFDPAGGGDPILYQHL
>COLFA476-12|KJ962302|ZMUO.000516|Enochrus_affinis

TLYFIFGAWAGMVGTSLSILIRAELGNPGTLIGDDQIYNVIVTAHAFIMIFFMVMPIMIGGFGNWLVPLMLGAPDMAFPRMNNMSFWLLPPSLTLLLMSSMVESGAGTGWTVYPPLSSNIAHGGASVDLAIFSLHLAGISSILGAVNFITTVINMRSPS
MTYDRLPLFVWSVAITALLLLLSLPVLAGAITMLLTDRNLNTSFFDPAGGGDPILYQHL
>COLFE1237-13|KJ967437|ZMUO.007032|Bembidion_lapponicum
TLYFIFGAWAGMVGTSLSMLIRAELGNPGSLIGDDQIYNVIVTAHAFVMIFFMVMPILIGGFGNWLVPLMLGAPDMAFPRMNNMSFWLLPPSLTLLLMSSMVESGAGTGWTVYPPLSSSIAHSGASVDLAIFSLHLAGVSSILGAVNFITTIINMRSVG
MTFDRMPLFVWSVGITALLLLLSLPVLAGAITMLLTDRNLNTSFFDPAGGGDPILYQHL
>COLFD894-12|KJ964429|ZMUO.004504|Anacaena_lutescens
TLYFIFGAWAGMVGTSLSILIRAELGNPGTLIGDDQIYNVIVTAHAFIMIFFMVMPIMIGGFGNWLVPLMLGAPDMAFPRMNNMSFWLLPPSLTLLLMSSMVENGAGTGWTVYPPLSSNIAHGGASVDLAIFSLHLAGISSILGAVNFITTVINMRSES
MTYDRLPLFVWSVAITALLLLLSLPVLAGAITMLLTDRNLNTSFFDPAGGGDPILYQHL
>COLFF1034-13|KJ963273|ZMUO.007399|Pria_dulcamarae
TLYFIFGAWSGMVGTSLSMLIRTELGNPGSLIGNDQIYNVIVTAHAFVMIFFMVMPFMIGGFGNWLVPLMLGAPDMAFPRMNNMSFWLLPPSLSLLLMSSIVESGAGTGWTVYPPLSSNIAHGGASVDLAIFSLHLAGISSILGAVNFITTIINMRPTG
MTFDRMPLFVWAVAITALLLLLSLPVLAGAITMLLTDRNLNTTFFDPSGGGDPILYQHL
>COLFC169-12|KJ963909|ZMUO.002354|Stenus_flavipes
TLYFIFGAWAGMVGTSLSLLIRAELGTPSSLIGDDQIYNVIVTAHAFIMIFFMVMPIMIGGFGNWLVPLMMGAPDMAFPRMNNMSFWLLPPSLSLLLMSSIVESGVGTGWTVYPPLSSNIAHSGASVDLAIFSLHLAGISSILGAINFITTIINMRTMKM
NFDCLPLFVWSVSITALLLLLSLPVLAGAITMLLTDRNLNTSFFDPAGGGDPVLYQHL
>COLFF1012-13|KJ964436|ZMUO.007377|Liogluta_granigera
TLYFIFGAWAGMVGTSLSLLIRAELGNPGSLIGDDQIYNVIVTAHAFIMIFFMVMPIVIGGFGNWLVPLMLGAPDMAFPRMNNMSFWLLPPSLTLLLMSSMVESGAGTGWTVYPPLSSNIAHGGSSVDLAIFSLHLAGISSILGAVNFISTVINMRSIGIS
FDRMPLFVWSVAITALLLLLSLPVLAGAITMLLTDRNLNTSFFDPAGGGDPILYQHL
>COLFE1613-13|KJ964487|ZMUO.007503|Psammodius_asper
TLYFLFGAWSGMVGTSLSLLIRAELGNPGTLIGDDQIYNVIVTAHAFVMIFFMVMPIMIGGFGNWLIPLMLGAPDMAFPRMNNMSFWLLPPSLTFLLMSSMVESGVGTGWTVYPPLSSNIAHSGASVDLAIFSLHLAGISSILGAVNFITTVINMRSIG
MTFDRMPLFVWAVALTALLLLLSLPVLAGAITMLLTDRNLNTSFFDPSGGGDPILYQHL
>COLFE355-12|KJ962720|ZMUO.004915|Leptura_quadrifasciata
TLYFIFGAWAGMVGTSLSLLIRSELGNPGSLIGDDQIYNVIVTAHAFVMIFFMVMPIMIGGFGNWLVPLMLGAPDMAFPRMNNMSFWLLPPSLTLLIMSSLVESGAGTGWTVYPPLSSNIAHGGSSVDLAIFSLHLAGISSILGAVNFITTVINMRPLG
MSPDQMPLFVWAVVITAILLLLSLPVLAGAITMLLTDRNLNTSFFDPAGGGDPILYQHL
>COLFD782-12|KJ967453|ZMUO.004772|Telmatophilus_schoenherrii
TLYFIFGSWAGMVGTSLSLLIRSELGTPGSLIGDDQIYNVIVTAHAFIMIFFMVMPIMIGGFGNWLVPLMLGAPDMAFPRMNNMSFWLLPPSLSLLLMSSIAEKGAGTGWTVYPPLSSNIAHGGSSVDLAIFSLHLAGISSILGAVNFISTVMNMKSIGM
IMDRLPLFVWAVMITAILLLLSLPVLAGAITMLLTDRNLNTSFFDPAGGGDPVLYQHL
>COLFD887-12|KJ963666|ZMUO.004497|Myllaena_dubia
TLYFIFGAWAGMIGSSLSLLIRAELGNPGSLIGDDQIYNVIVTAHAFVMIFFMVMPIVIGGFGNWLVPLMLGAPDMAFPRMNNMSFWLLPPSLSLLLMSSMVESGAGTGWTVYPPLSSNIAHGGASVDLAIFSLHLAGISSILGAVNFISTIINMRTTGIT
FDRMPLFVWSVGITALLLLLSLPVLAGAITMLLTDRNLNTSFFDPAGGGDPILYQHL
>COLFD652-12|KJ966826|ZMUO.004452|Ceutorhynchus_litura
TLYFIFGSWAGMMGTSLSMLIRTELGNPGTLIGNDQIYNSMVTAHAFIMIFFMVMPIMIGGFGNWLVPLMLGAPDMAFPRLNNMSFWLLPPSLTLLLMSSIVNKGAGTGWTVYPPLSASTAHEGMSVDMAIFSLHMAGISSILGAINFISTMMNM
RPSGMISEFMPLFVWAVKITAILLLISLPVLAGAITMLLTDRNINTSFFDPSGGGDPILYQHL
>COLFE025-12|KJ967514|ZMUO.005250|Malthinus_flaveolus
TLYFIFGAWSGMLGTSLSLLIRAELGNPGSLIGNDQIYNVIVTAHAFIMIFFMVMPIMIGGFGNWLVPLMLGAPDMAFPRMNNMSFWFLPPSLSLLLMSSLVESGAGTGWTVYPPLSANIAHSGSSVDLAIFSLHMAGISSILGAVNFISTVINMRSVG
MSFDRMPLFVWAVAITALLLLLSLPVLAGAITMLLTDRNLNTSFFDPAGGGDPILYQHL
>COLFC143-12|KJ966108|ZMUO.002328|Gyrophaena_joyioides
TLYFIFGAWSGMVGTSLSLLIRAELGNPGSLIGDDQIYNVIVTAHAFIMIFFMVMPIVIGGFGNWLIPLMLGAPDMAFPRMNNMSFWLLPPSLSLLLMSSMVESGAGTGWTVYPPLSSNIAHGGASVDLAIFSLHLAGISSILGAVNFISTIINMRAMG
MSFDRMPLFVWSVAITALLLLLSLPVLAGAITMLLTDRNLNTSFFDPAGGGDPILYQHL
>COLFF403-13|KJ965353|ZMUO.005921|Rutidosoma_globulus
TLYFIFGSWAGSVGTSLSMLIRTELGTPGSLIGNDQIYNSIVTAHAFIMIFFMVMPILIGGFGNWLVPLMLGAPDMAFPRLNNMSFWLLPPSLMLLMMSSVINKGAGTGWTVYPPLSSNVTHEGASVDLAIFSLHMAGISSILGAINFISTIMNMRPKG
MSYDKMPLFSWAVIITAVLLLLSLPVLAGAITMLLTDRNINTSFFDPAGGGDPILYQHL
>COLFF798-13|KJ961778|ZMUO.006118|Atheta_britteni
TLYFIFGAWAGMVGTSLSLLIRAELGNPGSLIGDDQIYNVIVTAHAFIMIFFMVMPIVIGGFGNWLVPLMLGGPDMAFPRMNNMSFWLLPPSLTLLLMSSMVESGAGTGWTVYPPLSSNIAHSGSSVDLAIFSLHLAGISSILGAVNFISTIINMRSTGIS
FDRMPLFVWSVAITALLLLLSLPVLAGAITMLLTDRNLNTSFFDPAGGGDPILYQHL
>COLFC633-12|KJ964827|ZMUO.003198|Hydroporus_obscurus
TLYFLFGAWSGMVGTSLSMLIRAELGNPGSLIGDDQIYNVIVTAHAFIMIFFMVMPIMIGGFGNWLVPLMLGAPDMAFPRMNNMSFWLLPPSLSLLLMSSMVENGAGTGWTVYPPLSSGIAHSGASVDLAIFSLHLAGVSSILGAVNFITTIINMRSIG
MTFDRMPLFVWSVGITALLLLLSLPVLAGAITMLLTDRNLNTSFFDPAGGGDPILYQHL

>COLFD055-12|KJ965037|ZMUO.003950|Quedius_scitus
TLYFIFGAWAGMVGTSLSLLIRAELGNPGTLIGDDQIYNVIVTAHAFIMIFFMVMPIVIGGFGNWLVPLMLGAPDMAFPRMNNMSFWLLPPSLSLLLMSSMVESGAGTGWTVYPPLSSNIAHGGASVDLAIFSLHLAGISSILGAVNFITTVINMRSMG
MTFDRMPLFVWSVAITALLLLSLPVLAGAITMLLTDRNLNTSFFDPAGGGDPILYQHL
>COLFB303-12|KJ962010|ZMUO.001633|Corticarina_minuta
SLYFLFGMWAGMVGTSLSLLIRLELGNPGSLIGDDQVYNVIVTAHAFIMIFFMVMPIMIGGFGNWLVPLMLGAPDMAFPRLNNMSFWLLPPSLSLLIMSSIVESGAGTGWTVYPPLSSNIAHGGSSVDLAIFSLHLAGISSILGAVNFITTVINMRPAGM
MLDQMPLFVWSVVITAILLLSLPVLAGAITMLLTDRNLNTSFFDPAGGGDPILYQHL
>COLFE652-13|KJ961952|ZMUO.005497|Coccinella_septempunctata
TLYFLFGMWAGMIGTSLSILIRLELGTTNSLIGNDQIYNVIVTAHAFIMIFFMVMPIMIGGFGNWLVPLMIGAPDMAFPRLNNMSFWLLPPALTLLIISSLVEMGAGTGWTVYPPLSSNLAHNGPSVDLVIFSLHLAGISSILGAVNFISTIMNMRPFGM
NLDKTPLFVWSVLITAILLLSLPVLAGAITMLLTDRNINTSFFDPMGGGDPILYQHL
>COLFA505-12|KJ963229|ZMUO.000545|Danosoma_conspersus
TLYFLFGAWAGMLGTSLSLLIRAELGNPGSLIGNDQIYNVVVTAHAFVMIFFMVMPVMIGGFGNWLVPLMLGAPDMAFPRMNNMSFWLLPPSLSLLLMSSIVENGAGTGWTVYPPLSANIAHSGSSVDLAIFSLHLAGISSILGAVNFISTVINMRST
GMTFDRMPLFVWAVVITALLLLSLPVLAGAITMLLTDRNLNTTFFDPAGGGDPILYQHL
>COLFC530-12|KJ963846|ZMUO.003095|Saperda_perforata
TLYFLFGAWAGMVGTSLSLLIRSELGTPGTLIGNDQIYNTIVTAHAFIMIFFMVMPIMMGGFGNWLVPLMLGAPDMAFPRMNNMSFWLLPPSLTLLIMSSIVDNGAGTGWTVYPPLAANVAHSGSSVDLAIFSLHLAGISSILGAVNFITTVINMRPS
GMTLDRTSLFVWAVKITAILLLSLPVLAGAITMLLTDRNLNTSFFDPAGGGDPILYQHL
>COLFB152-12|KJ964896|ZMUO.001482|Asemum_striatum
TLYFIFGAWSGMVGTSLSMLIRSELGNPGSLIGDDQIYNVIVTAHAFIMIFFMVMPIMIGGFGNWLVPLMLGAPDMAFPRMNNMSFWLLPPSLTLLIMSSIVENGAGTGWTVYPPLSANIAHSGSSVDLAIFSLHLAGISSILGAVNFITTVINMRPKG
MMLDRMPLFVWAVVITAILLLSLPVLAGAITMLLTDRNLNTSFFDPIGGGDPILYQHL
>COLFB166-12|KJ963845|ZMUO.001496|Oodes_helopioides
TLYFIFGAWSGMVGTSLSMLIRAELGNPGALIGDDQIYNVIVTAHAFIMIFFMVMPIMIGGFGNWLVPLMLGAPDMAFPRMNNMSFWLLPPSLSLLLMSSMVESGAGTGWTVYPPLSSGIAHSGASVDLAIFSLHLAGVSSILGAVNFITTIINMRSV
GMTFDRMPLFVWSVGITALLLLSLPVLAGAITMLLTDRNLNTSFFDPAGGGDPILYQHL
>COLFA171-10|KJ963216|MP00433|Pytho_depressus
TLYFIFGAWSGMVGTSLSLLIRAELGNPGSLIGDDQIYNVIVTAHAFVMIFFMVMPTVIGGFGNWLVPLMLGAPDMAFPRMNNMSFWLLPPSLSLLIMSSIVENGAGTGWTVYPPLASNIAHGGSSVDLAIFSLHLAGISSILGAVNFITTVINMRPLG
MTFDRMPLFVWSVVITAVLLLSLPVLAGAITMLLTDRNLNTSFFDPAGGGDPILYQHL
>COLFC494-12|KJ965184|ZMUO.003059|Epuraea_rufomarginata
TLYFIFGAWSGMVGTSLSILIRTELGSPGSLIGNDQIYNVIVTAHAFVMIFFMVMPFMIGGFGNWLVPLMLGAPDMAFPRMNNMSFWLLPPSLSLLLMSSIVESGAGTGWTVYPPLSSNIAHGGSSVDLAIFSLHLAGISSILGAVNFITTIINMRPVGM
TLDRMPLFVWSVVITAILLLSLPVLAGAITMLLTDRNLNTTFFDPSGGGDPILYQHL
>COLFD609-12|KJ966404|ZMUO.004409|Opatrum_riparium
TLYFIFGAWSGMVGTSLSILIRAELGNPGSLIGDDQIYNVIVTAHAFIMIFFMVMPIMIGGFGNWLVPLMLGAPDMAFPRMNNMSFWLLPPSLTLLLMSSIVENGAGTGWTVYPPLSSNIAHGGSSVDLAIFSLHLAGISSILGAVNFITTVINMRPQG
MTFDRMPLFVWAVVITAVLLLSLPVLAGAITMLLTDRNINTSFFDPAGGGDPILYQHL
>COLFC292-12|KJ964146|ZMUO.002477|Lordithon_thoracicus
TLYFIFGSWAGMVGTSLSLLIRAELGNPGSLIGDDQIYNVIVTAHAFIMIFFMVMPIVIGGFGNWLVPLMLGAPDMAFPRMNNMSFWLLPPSLTLLLMSSMVESGAGTGWTVYPPLSSNIAHGGASVDLAIFSLHLAGISSILGAVNFITTVINMRSIGM
TFDQMPLFIWSVAITALLLLSLPVLAGAITMLLTDRNLNTTFFDPAGGGDPILYQHL
>COLFD467-12|KJ962443|ZMUO.003887|Ceutorhynchus_contractus
TLYFIFGSWAGMAGTSLSMLIRTELGNPGSLIGNDQIYNSIVTAHAFIMIFFMVMPILIGGFGNWLVPLMLGAPDMAFPRLNNMSFWLLPPSLSLLLMSSIVNKGAGTGWTVYPPLSGNVAHEGMSVDLAIFSLHMAGISSILGAINFISTVMNMQPK
GMTPELMPLFVWAVEITAILLLSLPVLAGAITMLLTDRNINTSFFDPSGGGDPILYQHL
>COLFC477-12|KJ964264|ZMUO.003042|Malthodes_guttifer
TLYFMFGAWAGMLGTSLSLLIRAELGSPGSLIGNDQIYNVIVTAHAFIMIFFMVMPIMIGGFGNWLVPLMLGAPDMAFPRMNNMSFWFLPPSLSLLLMSSIVENGAGTGWTVYPPLSANIAHSGSSVDLAIFSLHMAGISSILGAVNFISTIINMRSTG
MTFDRMPLFVWAVAITALLLLSLPVLAGAITMLLTDRNLNTSFFDPAGGGDPILYQHL
>COLFB797-12|KJ963901|ZMUO.001272|Boreostiba_sibirica
TLYFIFGTWAGMVGTSLSLLIRAELGNPGSLIGDDQIYNVIVTAHAFVMIFFMVMPVVIGGFGNWLVPLMLGAPDMAFPRMNNMSFWLLPPSLTLLLMSSMVESGAGTGWTVYPPLSSNIAHGGSSVDLAIFSLHLAGISSILGAVNFISTVINMRSTG
ISFDRMPLFVWSVVITALLLLSLPVLAGAITMLLTDRNLNTSFFDPAGGGDPILYQHL
>COLFG059-13|KJ963888|ZMUO.007564|Melanophila_acuminata
TLYFIFGAWSGMVGTALSLLIRAELGNPGALIGDDQIYNVIVTAHAFVMIFFMVMPIMMGGFGNWLVPLMLGAPDMAFPRMNNMSFWLLPPSLTLLLMSSVVENGAGTGWTVYPPLAANVAHSGASVDLAIFSLHLAGISSILGAVNFITTVINMRS
TGMTFDRMPLFVWSVAITALLLLSLPVLAGAITMLLTDRNLNTSFFDPAGGGDPILYQHL
>COLFB595-12|KJ963095|ZMUO.001070|Autalia_longicornis

TLYFIFGAWSGMVGTSLSLLIRAELGNPGSLIGDDQIYNVIVTAHAFIMIFFMVMPIVIGGFGNWLVPLMLGAPDMAFPRMNNMSFWLLPPSLSLLLMSSMVESGAGTGWTVYPPLSSNIAHGGSSVDLAIFSLHLAGISSILGAVNFISTIINMRSMGIT
FDRMPLFVWSVAITALLLLLSLPVLAGAITMLLTDRNLNTSFFDPAGGGDPILYQHL
>COLFA005-10|HM909036|MP00088|Rhynchaenus_lonicerae
TLYFMFGAWSGMVGTSLSMLIRTELGNPGKLIGDDQIYNTIVTAHAFIMIFFMVMPIMIGGFGNWLIPLMLGAPDMAFPRLNNMSFWLLPPSLTLLLMSSIMNKGAGTGWTVYPPLSGNIAHEGSSVDLAIFSLHMAGISSILGAMNFISTIMNMHP
TGMKLDQLSLFVWAVKITAILLLLSLPVLAGAITMLLTDRNINTSFFDPAGGGDPILYQHL
>COLFD493-12|KJ965224|ZMUO.004293|Ilybius_aenescens
TLYFIFGAWAGMVGTSLSMLIRAELGNPGSLIGDDQIYNVIVTAHAFVMIFFMVMPIMIGGFGNWLVPLMLGAPDMAFPRMNNMSFWLLPPSLSLLLMSSMVESGAGTGWTVYPPLSSGIAHSGASVDLAIFSLHLAGISSILGAVNFITTIINMRSV
GMTFDRMPLFVWSVGITALLLLLSLPVLAGAITMLLTDRNLNTSFFDPAGGGDPILYQHL
>COLFE832-13|KJ966795|ZMUO.005677|Dryops_ernesti
TLYFIFGSWTGPLGMSLSLLIRAELGNPGSLIGDDQIYIVIVTAHAFVMRFFMVMPIMIGGFGNW--
PLMLGAPDMAFPRMNNMSFWLLPPSPSLLLLSSMVESGAGTGWTVYPPLASNIAHSGSSVDLAIFSLHLAGISSILGAVNFITTVINMRISEMTFDRMLLFVWSVAITALLLLLSLTVLAGAITMLLTDSNMNTSFFDPA--GHPILYQHL
>COLFE1320-13|KJ965479|ZMUO.007115|Aplotarsus_incanus
TLYFLFGAWAGMLGTSLSLLIRAELGNPGSLIGNDQIYNVIVTAHAFIMIFFMVMPIMIGGFGNWLVPLMLGAPDMAFPRMNNMSFWFLPPSLSLLLMSSIVENGAGTGWTVYPPLSANIAHSGSSVDLAIFSLHLAGISSILGAVNFISTVINMRSTGIT
FDRMPLFVWAVAITALLLLLSLPVLAGAITMLLTDRNLNTSFFDPAGGGDPILYQHL
>COLFE1148-13|KJ965710|ZMUO.006943|Longitarsus_holsaticus
TLYFIFGIWAGMVGTSLSILIRTELGNPGSLIGNDQIYNVIVTAHAFIMIFFMVMPIMIGGFGNWLVPLMIGAPDMAFPRMNNMSFWLLPPSLFLLIMSSIVESGAGTGWTVYPPLSSNIAHGGSAVDLAIFSLHLAGISSILGAINFITTVINMRPTGMTL
DRMPLFVWAVIITAILLLLSLPVLAGAITMLLTDRNLNTTFFDPAGGGDPILYQHL
>COLFC540-12|KJ961722|ZMUO.003105|Nebria_nivalis
TLYFIFGAWAGMVGTSLSMLIRAELGNPGSLIGDDQIYNVIVTAHAFVMIFFMVMPIMIGGFGNWLVPLMLGAPDMAFPRMNNMSFWLLPPSLTLLLMSSMVESGAGTGWTVYPPLSSGIAHSGASVDMAIFSLHLAGVSSILGAVNFITTIINMRS
MGMTFDRMPLFVWSVGITALLLLLSLPVLAGAITMLLTDRNLNTTFFDPAGGGDPILYQHL
>COLFC206-12|KJ961882|ZMUO.002391|Atheta_allocera
TLYFIFGAWAGMVGTSLSLLIRAELGNPGSLIGDDQIYNVIVTAHAFIMIFFMVMPVVIGGFGNWLVPLMLGAPDMAFPRMNNMSFWLLPPSLTLLLMSSMVESGAGTGWTVYPPLSSNIAHSGSSVDLAIFSLHLAGISSILGAVNFISTVINMRSTGI
SFDRMPLFVWSVAITALLLLLSLPVLAGAITMLLTDRNLNTSFFDPAGGGDPILYQHL
>COLFD514-12|KJ966817|ZMUO.004314|Acupalpus_meridianus
TLYFIFGVWAGMVGTSLSMLIRAELGTPGALIGDDQIYNVIVTAHAFIMIFFMVMPIMIGGFGNWLVPLMLGAPDMAFPRMNNMSFWLLPPSLSLLLMSSLVENGAGTGWTVYPPLSSSIAHSGASVDLAIFSLHLAGVSSILGAVNFITTIINMRSVG
MTFERMPLFVWSVGITALLLLLSLPVLAGAITMLLTDRNLNTSFFDPAGGGDPILYQHL
>COLFF815-13|KJ967384|ZMUO.006135|Atomaria_rubella
TLYFIFGAWSGMVGTSLSMLIRTELGNPGSLIGDDQIYNVIVTAHAFIMIFFMVMPMMIGGFGNWLVPLMLGAPDMAFPRLNNMSFWLLPPSLMFLLMSSIVEKGAGTGWTVYPPLSSNVAHAGSSVDLAIFSLHLAGISSILGSVNFITTVINMRPK
GMNFDRLPLFVWAVKITTILLLLSLPVLAGAITMLLTDRNINTSFFDPAGGGDPILYQHL
>COLFC789-12|KJ965085|ZMUO.003354|Meligethes_aeneus
TLYFIFGAWSGMVGTSLSMLIRTELGNPGSLIGNDQIYNVIVTAHAFVMIFFMVMPFMIGGFGNWLVPLMLGAPDMAFPRMNNMSFWLLPPSLSLLLMSSIVESGAGTGWTVYPPLSSNIAHGGASVDLAIFSLHLAGISSILGAVNFITTVINMRPK
GMTFDRMPLFVWAVMITAILLLLSLPVLAGAITMLLTDRNLNTTFFDPSGGGDPILYQHL
>COLFE807-13|KJ966917|ZMUO.005652|Stenus_nitens
TLYFIFGAWAGMVGTSLSLLIRAELGNPGSLIGDDQIYNVIVTAHAFVMIFFMVMPIMIGGFGNWLVPLMLGAPDMAFPRMNNMSFWLLPPSLSLLLMSSIVENGAGTGWTVYPPLSSNIAHSGASVDLAIFSLHLAGISSILGAINFITTIFNMRTMKL
QLDCLPLFVWSVAITALLLLLSLPVLAGAITMLLTDRNLNTSFFDPAGGGDPILYQHL
>COLFD818-12|KJ966237|ZMUO.004808|Scarodytes_halensis
TLYFLFGAWSGMVGTSLSMLIRAELGNPGSLIGDDQIYNVIVTAHAFIMIFFMVMPIMIGGFGNWLVPLMLGAPDMAFPRMNNMSFWMLPPSLSLLLMSSMVESGAGTGWTVYPPLSAGIAHGGASVDLAIFSLHLAGISSILGAVNFITTIINMRSI
GMTFDRMPLFVWSVGITALLLLLSLPVLAGAITMLLTDRNLNTSFFDPAGGGDPILYQHL
>COLFD503-12|KJ965118|ZMUO.004303|Endomychus_coccineus
TLYFMFGLWAGMLGTSLSMMIRLELGNTMQLIGNDQIFNVIVTAHAFVMIFFMVMPIMIGGFGNWLVPLMLGAPDMAFPRMNNMSFWLLPPSLTLLILSSISESGVGTGWTVYPPLSSNIAHSGSSVDLAIFSLHLAGISSILGAINFITTIINMRPTG
MTLEKMPLFVWSVFITAILLLLSLPVLAGAITMLLTDRNLNTSFFDPAGGGDPILYQHL
>COLFC806-12|KJ965926|ZMUO.003371|Hydnobius_spinipes
TLYFIFGSWAGMAGTSLSILIRAELGNPGSLIGDDQIYNVIVTAHAFIMIFFMVMPIVIGGFGNWLVPLMLGAPDMAFPRMNNMSFWLLPPSLSLLLMSSMVENGAGTGWTVYPPLSANIAHSGSSVDLAIFSLHLAGISSILGAVNFITTIINMRSVGM
TFDKMPLFVWSVLITAILLLLSLPVLAGAITMLLTDRNLNTSFFDPAGGGDPILYQHL
>COLFA261-12|KJ963732|ZMUO.000396|Alphitophagus_bifasciatus
TLYFIFGAWSGMVGTSLSLLIRAELGNPGSLIGDDQIYNVIVTAHAFIMIFFMVMPIVIGGFGNWLVPLMLGAPDMAFPRMNNMSFWLLPPSLTLLLMSSIVENGAGTGWTVYPPLSSNIAHGGSSVDLAIFSLHLAGISSILGAVNFITTVINMRPQGM
TFDRMPLFVWSVVITAILLLLSLPVLAGAITMLLTDRNLNTSFFDPAGGGDPILYQHL

>COLFF736-13|KJ963723|ZMUO.006436|Syntomus_foveatus
TLYFIFGMWAGMVGTSLSILIRAELGNPGALIGDDQIYNVIVTAHAFIMIFFMVMPIMIGGFGNWLVPLMLGAPDMAFPRMNNMSFWLLPPSLTLLLMSSMVESGAGTGWTVYPPLSSGIAHAGASVDLAIFSLHLAGVSSILGAVNFITTIINMRSV
GITFDRMPLFVWSVGITALLLLLSLPVLAGAITMLLTDRNLNTSFFDPAGGGDPILYQHL
>COLFF436-13|KJ964516|ZMUO.005954|Hydroporus_planus
TLYFLFGAWSGMVGTSLSMLIRAELGNPGSLIGDDQIYNVIVTAHAFIMIFFMVMPIMIGGFGNWLVPLMLGAPDMAFPRMNNMSFWLLPPSLTLLLMSSMVENGAGTGWTVYPPLSSGIAHSGASVDLAIFSLHLAGVSSILGAVNFITTIINMRSI
GMTFDRMPLFVWSVGITALLLLLSLPVLAGAITMLLTDRNLNTSFFDPAGGGDPILYQHL
>COLFE1068-13|KJ965995|ZMUO.006863|Podabrus_alpinus
TLYFIFGAWSGSLGLALSLLIRAELGTPGTLIGNDQIYNVIVTAHAFIMIFFMVMPIMIGGFGNWLVPLMLGAPDMAFPRMNNMSFWFLPPSLMFLLMSSMVESGAGTGWTVYPPLSANIAHSGPSVDLAIFSLHMAGISSILGAVNFISTIMNMKPPS
MKFDQMPLFVWSVGITALLLLLSLPVLAGAITMLLSDRNLNTSFFDPMGGGDPILYQHL
>COLFE1208-13|KJ966352|ZMUO.007003|Hygronoma_dimidiata
TLYFIFGTWAGMIGTSLSLLIRAELGNPGSLIGDDQIYNVIVTAHAFIMIFFMVMPIVIGGFGNWLIPLMLGAPDMAFPRMNNMSFWLLPPSLTLLLMSSMVESGAGTGWTVYPPLSSNIAHGGSSVDLAIFSLHLAGISSILGAVNFISTIINMRSMGISF
DRMPLFVWSVAITALLLLLSLPVLAGAITMLLTDRNLNTSFFDPAGGGDPILYQHL
>COLFA392-12|KJ966704|ZMUO.000622|Amphicyllis_globus
TLYFIFGAWSGMVGTSLSILIRAELGTPGSLIGDDQIYNVIVTAHAFVMIFFMVMPIVIGGFGNWLVPLMLGAPDMAFPRMNNMSFWLLPPSLTLLLMSSMVENGAGTGWTVYPPLSANISHSGSSVDLAIFSLHLAGISSILGAVNFITTVINMRSIGM
TFDKMPLFVWSVAITALLLLLSLPVLAGAITMLLTDRNLNTSFFDPAGGGDPILYQHL
>COLFA362-12|KJ967347|ZMUO.000592|Bagous_longitarsis
TLYFIFGAWSSMLGTSLSMLIRLELGSPGSLIGNDQIYNTIVTAHAFIMIFFMVMPIMIGGFGNWLVPLMLGAPDMAFPRLNNMSFWLLPPSIILLILSSIVDSGAGTGWTVYPPLSSNIAHEGSSVDLAIFSLHMAGVSSILGAVNFISTVLNMRPMGML
PEQMSLFTWAVEITAILLLLSLPVLAGAITMLLTDRNINTSFFDPAGGGDPILYQHL
>COLFF866-13|KJ962597|ZMUO.006661|Aphodius_distinctus
TLYFLFGSWAGMVGTSLSLLIRAELGNPGTLIGDDQIYNVIVTAHAFVMIFFMVMPILIGGFGNWLVPLMLGAPDMAFPRMNNMSFWLLPPSLTLLLMSSMVESGAGTGWTVYPPLSSNIAHGGASVDLAIFSLHLAGISSILGAVNFITTVINMRSPG
MTFDRMPLFVWSVAITALLLLLSLPVLAGAITMLLTDRNLNTSFFDPAGGGDPILYQHL
>COLFF217-13|KJ966726|ZMUO.005822|Olisthopus_rotundatus
TLYFIFGAWSGMVGTSLSMLIRAELGNPGSLIGDDQIYNVIVTAHAFIMIFFMVMPIMIGGFGNWLVPLMLGAPDMAFPRMNNMSFWLLPPSLTLLLMSSMVESGAGTGWTVYPPLSSGIAHAGASVDLAIFSLHLAGVSSILGAVNFITTIINMRSV
GMTFDRMPLFVWSVGITALLLLLSLPVLAGAITMLLTDRNLNTSFFDPAGGGDPILYQHL
>COLFF373-13|KJ966231|ZMUO.006073|Hypera_viciae
TLYFIFGIWAGTVGTSLSILIRMELGNPGSLIGNDQIYNTIVTAHAFIMIFFMIMPIMIGGFGNWLVPLMLGAPDMAFPRLNNMSFWLLPPSLSLLLMSSMIDSGAGTGWTVYPPLSSNIAHEGSSVDLAIFSLHMAGVSSILGAINFISTVLNMRPSGMS
LDKMALFIWAVKITAILLLLSLPVLAGAITMLLTDRNINTSFFDPAGGGDPILYQHL
>COLFB197-12|KJ964201|ZMUO.001527|Cassida_flaveola
TLYFIFGFWSGMVGTSLSILIRTELGNPGSLIGNDQIYNSIVTAHAFIMIFFMVMPIMIGGFGNWLVPLMLGAPDMAFPRLNNMSFWLLPPSITFLIMSSIIESGAGTGWTVYPPLSSNIAHSGASVDMAIFSLHLAGISSILGAINFISTIMNMRPSGMTL
DKMALFVWAVIITAILLLLSLPVLAGAITMLLTDRNINTSFFDPAGGGDPILYQHL
>COLFE070-12|KJ966297|ZMUO.005295|Gastrophysa_polygoni
TLYFIFGVWAGMIGTSLSMLIRSELGNPGMLIGNDQIYNVIVTAHAFIMIFFMVMPIMIGGFGNWLVPLMIGAPDMAFPRMNNMSFWLLPPSLFLLLMSSVVENGAGTGWTVYPPLSANIAHSGSSVDLAIFSLHLAGISSILGAINFITTIINMRPMG
MNLEQTPLFVWAVLITAVLLLLSLPVLAGAITMLLTDRNLNTSFFDPAGGGDPILYQHL
>COLFB347-12|KJ965219|ZMUO.001677|Salpingus_planirostris
TLYFIFGAWSGMVGTSLSLLIRAELGNPGSLIGDDQIYNVIVTAHAFVMIFFMVMPIVIGGFGNWLVPLMLGAPDMAFPRMNNMSFWLLPPSLTLLIMSSIVESGAGTGWTVYPPLAANIAHGGSSVDLAIFSLHLAGISSILGAVNFITTVINMRPSGM
SFDRMPLFVWAVVITAVLLLLSLPVLAGAITMLLTDRNLNTTFFDPAGGGDPILYQHL
>COLFE959-13|KJ962572|ZMUO.006754|Gabrius_osseticus
TLYFIFGSWAGMVGTSLSLLIRAELGNPGTLIGDDQIYNVIVTAHAFIMIFFMVMPIVIGGFGNWLVPLMLGAPDMAFPRMNNMSFWLLPPSITLLLMSSMVESGAGTGWTVYPPLSSNIAHGGASVDLAIFSLHLAGVSSILGAVNFITTVINMRSKG
MTFDRMPLFVWSVAITALLLLLSLPVLAGAITMLLTDRNLNTSFFDPAGGGDPILYQHL
>COLFD723-12|KJ964821|ZMUO.004713|Necrobia_violacea
TLYFIFGAWAGMIGTSLSLLIRSELGTPGTLIGNDQIYNVIVTAHAFIMIFFMVMPIMIGGFGNWLVPLMLGAPDMAFPRMNNMSFWLLPPSLTLLLMSSMVESGAGTGWTVYPPLSSNIAHGGSSVDLAIFSLHLAGISSILGAVNFITTVINMRPAG
MTLDRMPLFVWAVAITALLLLLSLPVLAGAITMLLTDRNLNTSFFDPAGGGDPILYQHL
>COLFA105-10|HQ559232|MP00365|Ceutorhynchus_punctiger
TLYFIFGSWAGMTGTSLSMLIRTELGNPGMLIGDDQIYNSIVTAHAFIMIFFMVMPILIGGFGNWLVPLMLGAPDMAFPRLNNMSFWLLPPSLSLLLMSSIVNKGAGTGWTVYPPLSMNVAHEGVSVDLAIFSLHMAGISSILGAINFISTVSNMRPTG
MTAEYMPLFVWAVEITAILLLISLPVLAGAITMLLTDRNINTSFFDPTGGGDPILYQHL
>COLFF893-13|KJ965704|ZMUO.006688|Poecilus_versicolor

TLYFIFGAWSGMVGTSLSMLIRAELGNPGSLIGDDQIYNVIVTAHAFVMIFFMVMPIMIGGFGNWLVPLMLGAPDMAFPRMNNMSFWLLPPSLTLLLMSSMVESGAGTGWTVYPPLSSGIAHAGASVDLAIFSLHLAGVSSILGAVNFITTIINMRSI
GMTFDRMPLFVWSVGITALLLLLSLPVLAGAITMLLTDRNLNTSFFDPAGGGDPILYQHL
>COLFF141-13|KJ967210|ZMUO.003775|Neocrepidodera_ferruginea
TLYFIFGMWSGMIGTSLSLLIRTELGSPGSLIGNDQIYNVIVTAHAFIMIFFMVMPIMIGGFGNWLVPLMIGAPDMAFPRMNNMSFWLLPPSLFLLLMSSMVESGAGTGWTVYPPLSSNLAHSGSSVDLAIFSLHLAGISSILGAVNFITTVINMRPMG
MTLDRMPLFVWAVVITAILLLLSLPVLAGAITMLLTDRNLNTSFFDPSGGGDPILYQHL
>COLFD717-12|KJ964510|ZMUO.004707|Thanatophilus_rugosus
TLYFIFGAWAGMVGMSLSILIRAELSTPGTLLGDDQMYNVIVTAHAFVMIFFMVMPIVIGGFGNWLVPLMLGAPDMAFPRMNNMSFWLLPPSLSLLLVSSMVESGAGTGWTVYPPLSSNIAHGGSSVDLAIFSLHLAGISSILGAVNFITTIINMRSSG
MTFDRMPLFVWSVAITALLLLLSLPVLAGAITMLLTDRNLNTSFFDPAGGGDPILYQHL
>COLFE1438-13|KJ963303|ZMUO.007233|Cionus_tuberculosus
TLYFIFGMWSGTVGTSLSMLIRTELGNPGSLIGDDQIYNTIVTAHAFIMIFFMVMPIMIGGFGNWLVPLMLGAPDMAFPRLNNMSFWLLPPSITLLLMSSIVEKGAGTGWTVYPPLSNNVTHEGASVDLAIFSLHMAGISSILGAINFISTVSNMRTKG
MDYDRTPLFVWSVNITAFLLLLSLPVLAGAITMLLTDRNINTSFFDPSGGGDPILYQHL
>COLFB923-12|KJ964334|ZMUO.001968|Cryptocephalus_pini
TLYFMFGAWAGMVGTSLSLLIRIELGNPGSLIGNDQIYNTIVTAHAFIMIFFMVMPIMIGGFGNWLVPLMLGAPDMAFPRMNNMSFWLLPPSLTLLLMSSIVENGAGTGWTVYPPLSATIAHAGASVDLAIFSLHLAGVSSIMGAINFISTVINMRPQ
GMLMDRTPLFVWAVLITAVLLLLSLPVLAGAITMLLTDRNLNTSFFDPAGGGDPILYQHL
>COLFA663-12|KJ966617|ZMUO.000798|Atheta_macrocera
TLYFIFGAWSGMVGTSLSLLIRAELGNPGSLIGDDQIYNVIVTAHAFIMIFFMVMPIMIGGFGNWLVPLMLGAPDMAFPRMNNMSFWLLPPSLTLLLMSSMVESGAGTGWTVYPPLSSNIAHSGASVDLAIFSLHLAGISSILGAVNFISTVINMRSIGIT
FDRMPLFVWAVAITALLLLLSLPVLAGAITMLLTDRNLNTSFFDPAGGGDPILYQHL
>COLFE1249-13|KJ966963|ZMUO.007044|Hydroporus_obscurus
TLYFLFGAWSGMVGTSLSMLIRAELGNPGSLIGDDQIYNVIVTAHAFIMIFFMVMPIMIGGFGNWLVPLMLGAPDMAFPRMNNMSFWLLPPSLSLLLMSSMVENGAGTGWTVYPPLSSGIAHSGASVDLAIFSLHLAGVSSILGAVNFITTIINMRSIG
MTFDRMPLFVWSVGITALLLLLSLPVLAGAITMLLTDRNLNTSFFDPAGGGDPILYQHL
>COLFE564-13|KJ964428|ZMUO.005409|Scymnus_haemorrhoidalis
TLYFLFGLWAGMVGTSLSILIRLELGTTSALIGNDQIYNVIVTAHAFIMIFFMVMPIMIGGFGNWLVPLMIGAPDMAFPRLNNMSFWLLPPSLTFLILSSMVESGAGTGWTVYPPLSSNLAHGGSSVDMAIFSLHLAGISSILGAVNFISTIINMRTFGMSF
EKMPLFVWSVFITAILLLLSLPVLAGAITMLLTDRNINTSFFDPAGGGDPILYQHL
>COLFD911-12|KJ962689|ZMUO.004521|Halobrecta_puncticeps
TLYFIFGAWAGMVGTSLSLLIRAELGNPGSLIGDDQIYNVIVTAHAFIMIFFMVMPIVIGGFGNWLVPLMLGAPDMAFPRMNNMSFWLLPPSLTLLLMSSMVESGAGTGWTVYPPLSSNIAHGGSSVDLAIFSLHLAGISSILGAVNFISTVINMRSPG
MSFDRMPLFVWSVAITALLLLLSLPVLAGAITMLLTDRNLNTSFFDPAGGGDPILYQHL
>COLFA082-10|HM909099|MP00288|Cytilus_sericeus
TLYFIFGAWAGMVGTSLSLLIRAELGNPGSLIGDDQIYNVIVTAHAFVMIFFMVMPIMIGGFGNWLVPLMLGAPDMAFPRMNNMSFWLLPPSLSLLLMSSIVESGAGTGWTVYPPLSSNIAHSGSSVDLAIFSLHLAGISSILGAVNFISTVINMRSPG
MKFDQMSLFSWSVAITALLLLLSLPVLAGAITMLLTDRNINTSFFDPAGGGDPILYQHL
>COLFF727-13|KJ963712|ZMUO.006427|Otiorhynchus_porcatus
TLYFIFGSWSGMVGTSLSMLIRVELGNPGSLIGDDQIYNVIVTAHAFIMIFFMVMPMMIGGFGNWLVPLMLGAPDMAFPRLNNMSFWLLPPSLSFLLMSSIIDKGAGTGWTVYPPLSSNIAHEGSSVDLAIFSLHMAGVSSILGAINFISTAINMRPGG
MTPDRMSLFIWAVKITAILLLLSLPVLAGAITMLLTDRNINTSFFDPAGGGDPILYQHL
>COLFD807-12|KJ965866|ZMUO.004797|Oulema_obscura
TLYFIFGAWSGTVGTSLSMMIRTELGNPGSLIGNDQIYNVIVTAHAFIMIFFMVMPIMIGGFGNWLVPLMLGAPDMAFPRMNNMSFWLLPPSISLLLMSSIVENGAGTGWTVYPPLSANISHNGASVDLAIFSLHLAGISSILGAVNFISTISNMRPEG
MTFDSMPLFVWAVLITAILLLLSLPVLAGAITMLLTDRNLNTSFFDPSSGGDPILYQHL
>COLFB455-12|KJ966546|ZMUO.001880|Hydroporus_angustatus
TLYFLFGAWSGMVGTSLSMLIRAELGNPGSLIGDDQIYNVIVTAHAFIMIFFMVMPIMIGGFGNWLVPLMLGAPDMAFPRMNNMSFWLLPPSLTLLLMSSMVENGAGTGWTVYPPLSSGIAHSGASVDLAIFSLHLAGISSILGAVNFITTIINMRSIG
MTFDRMPLFVWSVGITALLLLLSLPVLAGAITMLLTDRNLNTSFFDPAGGGDPILYQHL
>COLFE1439-13|KJ967248|ZMUO.007234|Cionus_tuberculosus
TLYFIFGMWSGTVGTSLSMLIRTELGNPGSLIGDDQIYNTIVTAHAFIMIFFMVMPIMIGGFGNWLVPLMLGAPDMAFPRLNNMSFWLLPPSITLLLMSSIVEKGAGTGWTVYPPLSNNITHEGASVDLAIFSLHMAGISSILGAINFISTVSNMRTKGM
DYDRTPLFVWSVNITAFLLLLSLPVLAGAITMLLTDRNINTSFFDPSGGGDPILYQHL
>COLFB505-12|KJ962706|ZMUO.000980|Atheta_pilicornis
TLYFIFGAWAGMVGTSLSLLIRAELGNPGSLIGDDQIYNVIVTAHAFIMIFFMVMPIVIGGFGNWLVPLMLGAPDMAFPRMNNMSFWLLPPSLSLLLMSSMIESGAGTGWTVYPPLSSNIAHGGSSVDLAIFSLHLAGISSILGAVNFISTVINMRSTGIS
FDRMPLFVWSVAITALLLLLSLPVLAGAITMLLTDRNLNTSFFDPAGGGDPILYQHL
>COLFE1374-13|KJ963156|ZMUO.007169|Olibrus_norvegicus
TLYFIFGAWASMVGTSLSLLIRTELGTPGSLIGDDQIYNVIVTAHAFVMIFFMVMPFMIGGFGNWLVPLMLGAPDMAFPRMNNMSFWLLPPSLFLLLMSSLVESGAGTGWTVYPPLSSNIAHGGASVDLAIFSLHLAGISSILGAINFITTMINMRPEG
MTLDRMPLFVWAVTITAVLLLLSLPVLAGAITMLLTDRNINTSFFDPAGGGDPILYQHL

>COLFD712-12|KJ964289|ZMUO.004702|Nebria_brevicollis
TLYFIFGAWAGMVGTSLSMLIRAELGNPGSLIGDDQIYNVIVTAHAFVMIFFMVMPIMIGGFGNWLVPLMLGAPDMAFPRMNNMSFWLLPPSLTLLLMSSMVESGAGTGWTVYPPLSSGIAHSGASVDLAIFSLHLAGISSILGAVNFITTIINMRSV
GMTFDRMPLFAWSVGITALLLLLSLPVLAGAITMLLTDRNLNTSFFDPAGGGDPILYQHL
>COLFE1042-13|KJ967519|ZMUO.006837|Haliplus_immaculatus
TLYFIFGAWSGMVGTSLSMLIRAELGTPGSLIGDDQIYNVIVTAHAFIMIFFMVMPIMIGGFGNWLVPLMLGAPDMAFPRMNNMSFWLLPPSLSLLLMSSMVENGAGTGWTVYPPLSAGIAHSGASVDLAIFSLHLAGISSILGAVNFITTIINMRSM
GMTFDRMPLFVWSVGITALLLLLSLPVLAGAITMLLTDRNLNTSFFDPAGGGDPILYQHL
>COLFE1363-13|KJ964935|ZMUO.007158|Tychius_schneideri
TLYFIFGSWSGMVGTSLSMLIRTELGNPGSLIGDDQIYNSIVTAHAFIMIFFMVMPIMIGGFGNWLVPLMLGAPDMAFPRLNNMSFWLLPPSIMLLLMSSIVNKGAGTGWTVYPPLSSNMAHEGASVDLAIFSLHMAGMSSILGAINFISTVMNMRP
FGMNSERVTLFTWAVQITAILLLLSLPVLAGAITMLLTDRNVNTSFFDPAGGGDPILYQHL
>COLFF023-13|KJ964755|ZMUO.005723|Ischnopterapion_loti
TLYFIFGLWSGMAGTSLSMLIRIELGTPGSLIGNDQIYNVIVTAHAFIMIFFMVMPIMIGGFGNWLVPLMLGAPDMAFPRMNNMSFWLLPPSLTFLLMSSIVEKGAGTGWTVYPPLASNIAHGGASVDLAIFSLHLAGISSILGAVNFISTIINMHPNELS
FDQLSLFTWAVKITAILLLLSLPVLAGAITMLLTDRNINTSFFDPAGGGDPILYQHL
>COLFD742-12|KJ967303|ZMUO.004732|Harpalus_distinguendus
TLYFIFGAWAGMVGTSLSMLIRAELGTPGALIGDDQIYNVIVTAHAFIMIFFMVMPIMIGGFGNWLVPLMLGAPDMAFPRMNNMSFWLLPPSLTLLLMSSMVESGAGTGWTVYPPLSSGIAHSGASVDLAIFSLHLAGVSSILGAVNFITTIINMRSV
GMTFDRMPLFVWSVGITALLLLLSLPVLAGAITMLLTDRNLNTSFFDPAGGGDPILYQHL
>COLFB317-12|KJ966050|ZMUO.001647|Coccidula_rufa
TLYFLFGMWAGMVGTSLSILIRLELGTPDSLIGNDQIYNVIVTAHAFIMIFFMVMPIMIGGFGNWLVPLMIGAPDMAFPRMNNMSFWLLPPALTLLILSSLVESGAGTGWTVYPPLSSNLAHSGSSVDMAIFSLHLAGISSILGAVNFISTVINMRTPGM
TFEKMPLFVWSVIITAILLLLSLPVLAGAITMLLTDRNINTSFFDPAGGGDPILYQHL
>COLFE459-12|KJ965467|ZMUO.005019|Acidota_crenata
TLYFIFGAWAGMVGTSLSILIRAELGNPGTLIGDDQIYNVIVTAHAFVMIFFMVMPIVIGGFGNWLVPLMLGAPDMAFPRMNNMSFWLLPPSLSLLLMSSMVESGAGTGWTVYPPLSSNIAHGGASVDLAIFSLHLAGISSILGAVNFITTVINMRATG
MTFDRMPLFVWSVAITALLLLLSLPVLAGAITMLLTDRNLNTSFFDPAGGGDPILYQHL
>COLFF828-13|KJ966900|ZMUO.006148|Tachinus_lignorum
TLYFIFGAWAGMVGTSLSLLIRAELGNPGTLIGDDQIYNVIVTAHAFIMIFFMVMPIVIGGFGNWLVPLMLGAPDMAFPRMNNMSFWLLPPSLSLLLMSSMVESGAGTGWTVYPPLSSNIAHGGSSVDLAIFSLHLAGISSILGAVNFITTVINMRSIGM
TFDRMPLFVWSVAITALLLLLSLPVLAGAITMLLTDRNLNTTFFDPAGGGDPILYQHL
>COLFB709-12|KJ962427|ZMUO.001184|Aphodius_conspurcatus
TLYFLFGSWAGMVGTSLSLLIRAELGNPGSLIGDDQIYNVIVTAHAFVMIFFMVMPILIGGFGNWLVPLMLGAPDMAFPRMNNMSFWLLPPSLTLLLMSSMVESGAGTGWTVYPPLSSNIAHGGASVDLAIFSLHLAGISSILGAVNFITTVINMRAPG
LTFDQMPLFVWSVAITALLLLLSLPVLAGAITMLLTDRNLNTSFFDPAGGGDPILYQHL
>COLFC557-12|KJ961737|ZMUO.003122|Ocypus_ophthalmicus
TLYFIFGVWSGMVGTSLSLLIRAELGNPGTLIGDDQIYNVIVTAHAFVMIFFMVMPIMIGGFGNWLVPLMLGAPDMAFPRMNNMSFWLLPPSLTLLLMSSMAESGAGTGWTVYPPLSGNIAHSGTSVDLAIFSLHLAGISSILGAVNFITTVINMRSTG
MTFDRMPLFVWSVVITALLLLLSLPVLAGAITMLLTDRNLNTSFFDPAGGGDPILYQHL
>COLFA576-12|KJ961885|ZMUO.000711|Lamia_textor
TLYFLFGAWAGMMGTSLSILIRTELGSPGSLIGDDQIYNVIVTAHAFIMIFFMVMPIMIGGFGNWLVPLMLGAPDMAFPRMNNMSFWLLPPSLFLLIMSSIVENGAGTGWTVYPPLAANVAHNGASVDLAIFSLHLAGISSILGAVNFITTVINMRPSG
MSMDRMPLFVWSVKITAILLLLSLPVLAGAITMLLTDRNLNTSFFDPAGGGDPILYQHL
>COLFB037-12|KJ965423|ZMUO.001367|Bembidion_femoratum
TLYFIFGAWSGMVGTSLSMLIRAELGNPGSLIGDDQIYNVIVTAHAFVMIFFMVMPILIGGFGNWLVPLMLGAPDMAFPRMNNMSFWLLPPSLTLLLMSSMVESGAGTGWTVYPPLSSSIAHSGASVDLAIFSLHLAGVSSILGAVNFITTIINMRSVG
MSFDRMPLFVWSVGITALLLLLSLPVLAGAITMLLTDRNLNTSFFDPAGGGDPILYQHL
>COLFB298-12|KJ962557|ZMUO.001628|Falagria_caesa
TLYFIFGAWAGMVGTSLSLLIRAELGNPGSLIGDDQIYNVIVTAHAFIMIFFMVMPIVIGGFGNWLVPLMLGAPDMAFPRMNNMSFWLLPPSLSLLLMSSMVESGAGTGWTVYPPLSSNIAHGGASVDLAIFSLHLAGISSILGAVNFISTVINMRSIGIT
FDRMPLFVWSVAITALLLLLSLPVLAGAITMLLTDRNLNTSFFDPAGGGDPILYQHL
>COLFB660-12|KJ967209|ZMUO.001135|Helophorus_brevipalpis
TLYFIFGAWAGMVGTSLSILIRAELGNPGTLIGDDQIYNVIVTAHAFIMIFFMVMPIMIGGFGNWLVPLMLGAPDMAFPRMNNMSFWLLPPSLTLLLMSSMVESGAGTGWTVYPPLSSNIAHSGASVDLAIFSLHLAGISSILGAVNFITTVINMRSISM
TYDRLPLFVWSVAITALLLLLSLPVLAGAITMLLTDRNLNTSFFDPAGGGDPILYQHL
>COLFA670-12|KJ966439|ZMUO.000805|Atheta_atramentaria
TLYFIFGAWAGMVGTSLSLLIRAELGNPGSLIGDDQIYNVIVTAHAFIMIFFMVMPVVIGGFGNWLVPLMLGAPDMAFPRMNNMSFWLLPPSLTLLLMSSMVESGAGTGWTVYPPLSSNIAHGGSSVDLAIFSLHLAGISSILGAVNFISTVINMRSTGI
SFDRMPLFVWSVAITALLLLLSLPVLAGAITMLLTDRNLNTSFFDPAGGGDPILYQHL
>COLFA546-12|KJ964783|ZMUO.000681|Hydrophilus_aterrimus

TLYFIFGAWAGMVGTSLSILIRSELGNPGTLIGDDQIYNVIVTAHAFIMIFFMVMPIMIGGFGNWLIPLMLGAPDMAFPRMNNMSFWLLPPSLTLLLASSMVENGAGTGWTVYPPLSSNIAHSGASVDLAIFSLHLAGVSSILGAVNFISTVINMRSTNL
TYDRLPLFVWSVAITALLLLLSLPVLAGAITMLLTDRNINTSFFDPAGGGDPILYQHL
>COLFC369-12|KJ967411|ZMUO.002554|Agonum_fuliginosum
TLYFIFGAWAGMVGTSLSMLIRAELGNPGALIGDDQIYNVIVTAHAFIMIFFMVMPIMIGGFGNWLVPLMLGAPDMAFPRMNNMSFWLLPPSLTLLLMSSLVESGAGTGWTVYPPLSSGIAHAGASVDLAIFSLHLAGISSILGAVNFITTIINMRSVG
MTFDRMPLFVWSVGITALLLLLSLPVLAGAITMLLTDRNLNTSFFDPAGGGDPILYQHL
>COLFD797-12|KJ963049|ZMUO.004787|Longitarsus_reichei
TLYFIFGIWAGMVGTSLSILIRTELGNPGSLIGNDQIYNVIVTAHAFIMIFFMVMPIMIGGFGNWLVPLMIGAPDMAFPRMNNMSFWLLPPSLFFLIMSSIVESGAGTGWTVYPPLSSNIAHGGSSVDLAIFSLHLAGISSILGAINFITTVINMRPIGMTL
DRMPLFVWAVMITAILLLLSLPVLAGAITMLLTDRNLNTTFFDPAGGGDPILYQHL
>COLFC176-12|KJ963359|ZMUO.002361|Lathrobium_geminum
TLYFIFGAWAGMVGTSLSLLIRTELGNPGSLIGDDQIYNVIVTAHAFIMIFFMVMPIMIGGFGNWLVPLMLGAPDMAFPRMNNMSFWLLPPSLSLLLMSSLVESGAGTGWTVYPPLSSNIAHGGASVDLAIFSLHLAGISSILGAVNFITTVINMRSPG
MTYERMPLFVWAVAITALLLLLSLPVLAGAITMLLTDRNLNTSFFDPAGGGDPILYQHL
>COLFA337-12|KJ967484|ZMUO.000472|Donacia_thalassina
TLYFIFGAWSGMMGTSLSMLIRTELSNPGSLIGNDQLYNVIVTAHAFIMIFFMVMPIMIGGFGNWLVPLMLGAPDMAFPRMNNMSFWLLPPSLSFLIMSSITESGAGTGWTVYPPLSNNLAHSGSSVDLAIFSLHLAGISSILGAVNFISTIINMRPTG
MTLEKMPLFSWAVMITAVLLLTSLPVLAGAITMLLTDRNINTSFFDPAGGGDPILYQHL
>COLFA428-12|KJ965302|ZMUO.000658|Anisotoma_orbicularis
TLYFIFGAWSGMVGTSLSILIRAELGTPGSLIGDDQIYNVIVTAHAFIMIFFMVMPIVIGGFGNWLVPLMLGAPDMAFPRMNNMSFWLLPPSLSLLLFSSMVENGAGTGWTVYPPLSSNISHSGSSVDLAIFSLHLAGISSILGAVNFITTVINMRSMGM
TFDKMPLFVWSVAITALLLLLSLPVLAGAITMLLTDRNLNTSFFDPAGGGDPILYQHL
>COLFF912-13|KJ961738|ZMUO.006707|Stenus_atratulus
TLYFLFGAWAGMVGTSLSLLIRAELGNPGSLIGDDQIYNVIVTAHAFVMIFFMVMPIMIGGFGNWLVPLMLGAPDMAFPRMNNMSFWLLPPSLSLLLMSSIVENGAGTGWTVYPPLSTNIAHSGASVDLAIFSLHLAGISSILGAVNFITTIFNMRTMK
MQLDCLPLFVWSVGITALLLLLSLPVLAGAITMLLTDRNLNTSFFDPAGGGDPVLYQHL
>COLFE1122-13|KJ964287|ZMUO.006917|Byrrhus_arietinus
TLYYIFGAWAGMVGTSLSMLIRTELGNPGSLIGDDQIYNVIVTAHAFIMIFFMVMPIMIGGFGNWLVPLMLGAPDMAFPRMNNMSFWLLPPSLSLLLMSSVIENGAGTGWTVYPPLSANIAHSGSSVDLAIFSLHLAGISSILGAVNFISTVINMRSPG
MTFDQMSLFTWSVVITALLLLLSLPVLAGAITMLLTDRNINTSFFDPAGGGDPILYQHL
>COLFA586-12|KJ962806|ZMUO.000721|Cicindela_hybrida
TLYFIFGAWSGMVGTSLSMLIRAELGSPGSLIGDDQIYNVIVTAHAFVMIFFMVMPIMIGGFGNWLVPLMLGAPDMAFPRMNNMSFWLLPPSLTLLLMSSMVGDGAGTGWTVYPPLSAGIAHAGASVDLAIFSLHLAGVSSILGAVNFITTIINMRS
VGMTFDRMPLFVWSVGITALLLLLSLPVLAGAITMLLTDRNLNTSFFDPAGGGDPILYQHL
>COLFB212-12|KJ965337|ZMUO.001542|Danosoma_fasciata
TLYFLFGAWAGMLGTSLSLLIRAELGNPGSLIGNDQIYNVVVTAHAFVMIFFMVMPVMIGGFGNWLVPLMLGAPDMAFPRMNNMSFWLLPPSLSLLLMSSIVENGAGTGWTVYPPLSANIAHSGSSVDLAIFSLHLAGISSILGAVNFISTVINMRST
GMTFDRMPLFVWAVAITALLLLLSLPVLAGAITMLLTDRNLNTTFFDPAGGGDPILYQHL
>COLFF982-13|KJ962660|ZMUO.007347|Notaris_acridulus
TLYFIFGAWSGMVGTSLSMLIRMELGNPGSLIGDDQIYNVIVTAHAFIMIFFMVMPIMIGGFGNWLVPLMLGAPDMAFPRLNNLSFWLLPPSLIILLSSSLIEKGAGTGWTVYPPLSSNIAHSGPSVDLAIFSLHMAGISSILGAINFISTVINMRPVGMSP
DRMTLFVWAVEITAILLLLSLPVLAGAITMLLTDRNINTSFFDPAGGGDPILYQHL
>COLFA409-12|KJ966273|ZMUO.000639|Epuraea_laeviuscula
TLYFIFGAWSGMVGTSLSILIRTELGSPGSLIGNDQIYNVIVTAHAFIMIFFMVMPFMIGGFGNWLVPLMLGAPDMAFPRMNNMSFWLLPPSLSLLLMSSIVESGAGTGWTVYPPLSSNIAHGGSSVDLAIFSLHLAGISSILGAVNFITTIINMRPVGMT
LDRMPLFVWSVVITAVLLLLSLPVLAGAITMLLTDRNLNTTFFDPSGGGDPILYQHL
>COLFB594-12|KJ966229|ZMUO.001069|Atheta_crassicornis
TLYFIFGAWAGMVGTSLSLLIRAELGNPGSLIGDDQIYNVIVTAHAFVMIFFMVMPIVIGGFGNWLIPLMLGAPDMAFPRMNNMSFWLLPPSLTLLLMSSMVESGAGTGWTVYPPLSSNIAHGGSSVDLAIFSLHLAGISSILGAVNFISTVINMRSVGI
SFDRMPLFVWSVAITALLLLLSLPVLAGAITMLLTDRNLNTSFFDPAGGGDPILYQHL
>COLFA026-10|HM909053|MP00164|Stephostethus_variolosus
SLYFLFGMWSGMVGTSLSLLIRLELGNPGSLIGDDQIYNVIVTAHAFIMIFFMVMPVMMGGFGNWLVPLMLGAPDMAFPRLNNMSFWLLPPSLSLLIMSSIVESGVGTGWTVYPPLSSNIAHGGSSVDLAIFSLHLAGISSILGAVNFITTMINMRPTG
MKFELMPLFAWSVMLTAILLLLSLPVLAGAITMLLTDRNLNTSFFDPAGGGDPILYQHL
>COLFC421-12|KJ962738|ZMUO.002986|Bembidion_femoratum
TLYFIFGAWSGMVGTSLSMLIRAELGNPGSLIGDDQIYNVIVTAHAFVMIFFMVMPILIGGFGNWLVPLMLGAPDMAFPRMNNMSFWLLPPSLTLLLMSSMVESGAGTGWTVYPPLSSSIAHSGASVDLAIFSLHLAGVSSILGAVNFITTIINMRSVG
MSFDRMPLFVWSVGITALLLLLSLPVLAGAITMLLTDRNLNTSFFDPAGGGDPILYQHL
>COLFB603-12|KJ962833|ZMUO.001078|Myrmecocephalus_concinnus
TLYFLFGAWAGMIGTSLSLLIRAELGNPGSLIGDDQIYNVIVTAHAFIMIFFMVMPMMIGGFGNWLVPLMLGAPDMAFPRMNNMSFWLLPPSITLLLMSSMVEKGAGTGWTVYPPLSSTIAHSGSSVDLAIFSLHLAGISSILGAVNFISTVINMRAIG
MSFDRLPLFVWSVAITALLLLLSLPVLAGAITMLLTDRNLNTSFFDPAGGGDPILYQHL

>COLFE092-12|KJ966103|ZMUO.005317|Cryptocephalus_bipunctatus
TLYFLFGAWAGMIGTSLSLLIRIELGNPGSLIGNDQIYNTIVTAHAFIMIFFMVMPIMIGGFGNWLVPLMLGAPDMAFPRMNNMSFWLLPPSLMLLLMSSIVENGAGTGWTVYPPLSATIAHTGPSVDLAIFSLHLAGASSIMGAINFISTVINMRPQG
MMMDRTPLFVWAVLITAILLLLSLPVLAGAITMLLTDRNINTSFFDPAGGGDPILYQHL
>COLFD750-12|KJ962628|ZMUO.004740|Limonius_aeneoniger
TLYFLFGAWAGMLGTSLSLLIRAELGNPGSLIGNDQIYNVIVTAHAFIMIFFMVMPIMIGGFGNWLVPLMLGAPDMAFPRMNNMSFWFLPPSLSLLLMSSIVENGAGTGWTVYPPLSANIAHSGSSVDLAIFSLHLAGISSILGAVNFISTVINMRSVGI
TFDRMPLFVWAVAITALLLLLSLPVLAGAITMLLTDRNLNTSFFDPAGGGDPILYQHL
>COLFA128-10|HQ559247|MP00390|Peltis_grossa
TLYFIFGSWAGMVGTSLSLLIRSELGNPGSLIGNDQIYNVIVTAHAFVMIFFMVMPILIGGFGNWLVPLMLGAPDMAFPRMNNMSFWLLPPSLTLLLLSSFVENGAGTGWTVYPPLSANISHSGSSVDLAIFSLHLAGISSILGAVNFITTVINMRPIGMT
LDRTPLFVWSVMITAILLLLSLPVLAGAITMLLTDRNINTSFFDPAGGGDPILYQHL
>COLFE656-13|KJ965065|ZMUO.005501|Dromius_schneideri
TLYFIFGAWAGMVGTSLSMLIRAELGNPGALIGDDQVYNVIVTAHAFIMIFFMVMPIMIGGFGNWLVPLMLGAPDMAFPRMNNMSFWLLPPSLSLLLMSSMVESGAGTGWTVYPPLSSGIAHAGASVDLAIFSLHLAGVSSILGAVNFITTIINMRSI
GMTFDRMPLFVWSVGITALLLLLSLPVLAGAITMLLTDRNLNTSFFDPAGGGDPILYQHL
>COLFB006-12|KJ967361|ZMUO.001336|Dyschirius_globosus
TLYFIFGIWSGMVGTSLSILIRTELGNPGSLIGDDQIYNVIVTAHAFIMIFFMVMPIMIGGFGNWLVPLMLGAPDMAFPRMNNMSFWLLPPSLTLLLMSSMVEKGAGTGWTVYPPLSSSIAHSGASVDLAIFSLHLAGISSILGAVNFITTIINMRSTGLTF
ERMPLFVWSVGITALLLLLSLPVLAGAITMLLTDRNLNTSFFDPAGGGDPILYQHL
>COLFB728-12|KJ964439|ZMUO.001203|Acrolocha_pliginskii
TLYFIFGAWAGMVGTSLSILIRAELGNPGSLIGDDQIYNVIVTAHAFIMIFFMVMPIVIGGFGNWLVPLMLGAPDMAFPRMNNMSFWLLPPSLTLLLMSSMVESGAGTGWTVYPPLSSNIAHGGASVDLAIFSLHLAGISSILGAVNFITTVINMRSMG
MTFDRMPLFVWSVAITALLLLLSLPVLAGAITMLLTDRNLNTSFFDPAGGGDPILYQHL
>COLFB093-12|KJ963617|ZMUO.001423|Hydrothassa_hannoveriana
TLYFIFGIWSGMVGTSLSILIRSELGNPGSLIGNDQIYNVIVTAHAFIMIFFMVMPVMIGGFGNWLVPLMIGAPDMAFPRLNNMSFWLLPPSLFLLLMSSIVENGVGTGWTVYPPLSSNIAHSGSSVDLAIFSLHLAGISSILGAINFITTIINMRSEGMNLE
QTPLFVWAVLITAILLLLSLPVLAGAITMLLTDRNLNTSFFDPAGGGDPILYQHL
>COLFA072-10|HM909090|MP00262|Atheta_munsteri
TLYFIFGAWAGMVGTSLSLLIRAELGNPGSLIGDDQIYNVIVTAHAFIMIFFMVMPIVIGGFGNWLVPLMLGAPDMAFPRMNNMSFWLLPPSLTLLLMSSMVESGAGTGWTVYPPLSSNIAHGGSSVDLAIFSLHLAGISSILGAVNFISTVINMRSTGI
SFDRMPLFVWSVAITALLLLLSLPVLAGAITMLLTDRNLNTSFFDPAGGGDPILYQHL
>COLFA454-12|KJ966491|ZMUO.000494|Gastrophysa_viridula
TLYFIFGIWAGMVGTSLSMLIRSELGNPGTLIGNDQIYNVIVTAHAFIMIFFMVMPIMIGGFGNWLVPLMIGAPDMAFPRMNNMSFWLLPPSLFLLLMSSIVENGAGTGWTVYPPLSANIAHSGSSVDLAIFSLHLAGISSILGAINFITTIINMRPSGMN
WEQISLFVWAVMLTAILLLLSLPVLAGAITMLLTDRNLNTSFFDPAGGGDPILYQHL
>COLFB295-12|KJ964933|ZMUO.001625|Philonthus_debilis
TLYFIFGSWAGMVGTSLSLLIRAELGNPGSLIGDDQIYNVIVTAHAFIMIFFMVMPIVIGGFGNWLVPLMLGAPDMAFPRMNNMSFWLLPPSLTLLLMSSMVESGAGTGWTVYPPLSSNIAHGGASVDLAIFSLHLAGISSILGAVNFITTVINMRSTG
MTFDRMPLFVWSVAITALLLLLSLPVLAGAITMLLTDRNLNTTFFDPAGGGDPILYQHL
>COLFC488-12|KJ962845|ZMUO.003053|Orithales_serraticornis
TLYFLFGAWAGMLGTSLSLLIRAELGNPGSLIGNDQIYNVIVTAHAFIMIFFMVMPIMIGGFGNWLVPLMLGAPDMAFPRMNNMSFWFLPPSLSLLLMSSIVENGAGTGWTVYPPLSANIAHSGSSVDLAIFSLHLAGISSILGAVNFISTVINMRSTGIT
FDRMPLFVWAVAITALLLLLSLPVLAGAITMLLTDRNLNTSFFDPAGGGDPILYQHL
>COLFB126-12|KJ964262|ZMUO.001456|Triplax_aenea
TLYFIFGMWAGMVGTALSMLIRSELGNPGSLIGNDQIYNVIVTAHAFIMIFFMVMPIMMGGFGNWLVPLMLGAPDMAFPRLNNMSFWLLPPSLSLLIMSSIVETGAGTGWTVYPPLSSNIAHSGASVDMAIFSLHLAGISSILGAMNFITTMMNMR
PTGMTLDQMPLFVWAVLITAILLILSLPVLAGAITMLLTDRNVNTTFFDPAGGGDPILYQHL
>COLFF432-13|KJ964928|ZMUO.005950|Acupalpus_parvulus
TLYFIFGVWAGMVGTSLSMLIRAELGTPGALIGDDQIYNVIVTAHAFVMIFFMVMPVMIGGFGNWLVPLMLGAPDMAFPRMNNMSFWLLPPSLTLLLMSSLVESGAGTGWTVYPPLSSGIAHSGASVDLAIFSLHLAGVSSILGAVNFITTIINMRSV
GMTFERMPLFVWSVGITALLLLLSLPVLAGAITMLLTDRNLNTSFFDPAGGGDPILYQHL
>COLFF120-13|KJ963634|ZMUO.003754|Aphodius_plagiatus
TLYFLFGSWAGMVGTSLSLLIRAELGNPGTLIGDDQIYNVIVTAHAFVMIFFMVMPILIGGFGNWLVPLMLGAPDMAFPRMNNMSFWLLPPSLTLLLMSSMVEQGAGTGWTVYPPLSSNIAHGGASVDLAIFSLHLAGISSILGAVNFITTVINMRSPG
MTFDRMPLFVWSVAITALLLLLSLPVLAGAITMLLTDRNLNTSFFDPAGGGDPILYQHL
>COLFA066-10|HM909084|MP00253|Aphodius_punctatosulcatus
TLYFLFGSWAGMVGTSLSLLIRAELGNPGSLIGDDQIYNVIVTAHAFVMIFFMVMPILIGGFGNWLVPLMLGAPDMAFPRMNNMSFWLLPPSLTLLLMSSMVESGAGTGWTVYPPLSSNIAHGGASVDLAIFSLHLAGISSILGAVNFITTVINMRSPG
MTFDRMPLFVWSVAITALLLLLSLPVLAGAITMLLTDRNLNTSFFDPAGGGDPILYQHL
>COLFA104-10|HQ559231|MP00364|Dendrophilus_pygmaeus

TLYFIFGAWAGMVGTSLSLLIRAELGNPGMLIGDDQIYNVIVTSHAFIMIFFMVMPIMIGGFGNWLVPLMLGAPDMAFPRMNNMSFWLLPPSLSLLLMSSMVESGAGTGWTVYPPLSANIAHSGASVDLCIFSLHLAGISSILGAVNFITTVINMRSPG
MTFDRMPLFVWAVAITALLLLLSLPVLAGAITMLLTDRNLNTSFFDPAGGGDPILYQHL
>COLFC104-12|KJ963804|ZMUO.002289|Atheta_palustris
TLYFIFGAWAGMVGTSLSLLIRAELGNPGSLIGDDQIYNVIVTAHAFIMIFFMVMPIVIGGFGNWLVPLMLGAPDMAFPRMNNMSFWLLPPSLTLLLMSSMVESGAGTGWTVYPPLSSNIAHGGSSVDLAIFSLHLAGISSILGAVNFISTVINMRSTGI
SFDRMPLFVWSVAITALLLLLSLPVLAGAITMLLTDRNLNTSFFDPAGGGDPILYQHL
>COLFF344-13|KJ963245|ZMUO.006044|Chrysolina_marginata
TLYFIFGTWAGMVGTSLSILIRAELGNPGSLIGNDQIYNVIVTAHAFIMIFFMVMPIMIGGFGNWLVPLMLGAPDMAFPRMNNMSFWLLPPSLIFLLMSSIVENGAGTGWTVYPPLSANVAHSGPSVDLAIFSLHLAGISSILGAINFITTVINMRPTGM
KLEQMPLFSWAVLITAILLLLSLPVLAGAITMLLTDRNLNTSFFDPASGGDPILYQHL
>COLFC666-12|KJ963971|ZMUO.003231|Cordylepherus_viridis
TLYFIFGAWSGMVGLSLSLLIRSELSIPGTLIGNDQIYNVIVTAHAFIMIFFMVMPILIGGFGNWLVPLMLGAPDMAFPRMNNMSFWLLPPSLTLLLLSSMVENGAGTGWTVYPPLSANIAHSGSSVDLAIFSLHLAGISSILGAVNFITTVINMRPQGMT
LDRTPLFVWAVVITALLLLLSLPVLAGAITMLLTDRNLNTSFFDPAGGGDPILYQHL
>COLFC463-12|KJ961818|ZMUO.003028|Atomaria_sp.
TLYFIFGAWAGMVGTSLSMLIRTELGTPGSLIGDDQIYNVIVTAHAFIMIFFMVMPMMIGGFGNWLVPLMLGAPDMAFPRLNNMSFWLLPPSLMFLLMSSIVEKGAGTGWTVYPPLSSNVAHAGSSVDLAIFSLHLAGISSILGSVNFITTVINMRPK
GMNFDRLPLFVWAVKITTILLLLSLPVLAGAITMLLTDRNINTSFFDPAGGGDPILYQHL
>COLFF350-13|KJ962109|ZMUO.006050|Agabus_lapponicus
TLYFIFGAWAGMVGTSLSMLIRAELGNPGSLIGDDQIYNVIVTAHAFVMIFFMVMPIMIGGFGNWLIPLMLGAPDMAFPRMNNMSFWLLPPSLTLLLMSSMVESGAGTGWTVYPPLSSGIAHGGASVDLAIFSLHLAGISSILGAVNFITTIINMRSVG
MTFDRMPLFVWSVGITALLLLLSLPVLAGAITMLLTDRNLNTSFFDPAGGGDPILYQHL
>COLFB118-12|KJ965934|ZMUO.001448|Acanthocinus_aedilis
TLYFIFGAWSGMVGTSLSMLIRSELGNPGTLIGNDQIYNTIVTAHAFIMIFFMVMPIMIGGFGNWLIPLMLGAPDMAFPRMNNMSFWLLPPSLTLLIMSSIVENGAGTGWTVYPPLSSNIAHSGSSVDLAIFSLHLAGISSILGAVNFITTIINMRPMGM
TFDRMPLFVWAVKITAILLLLSLPVLAGAITMLLTDRNINTSFFDPAGGGDPILYQHL
>COLFF575-13|KJ965720|ZMUO.006465|Smaragdina_affinis
TLYFIFGAWSGMVGTSLSLLIRVELGNPGTLIGNDQIYNTIVTAHAFIMIFFMVMPIMIGGFGNWLVPLMLGAPDMAFPRMNNMSFWLLPPSLTLLLMSSIVENGAGTGWTVYPPLSANLAHSGASVDLAIFSLHLAGISSIMGAINFISIVINMRPQG
MLLDRTPLFVWAVVITAILLLLSLPVLAGAITMLLTDRNLNTSFFDPAGGGDPILYQHL
>COLFA101-10|HQ559228|MP00356|Corticaria_abietorum
SLYFLFGMWAGMVGTSLSLLIRLELGNPGSLIGDDQIYNVIVTAHAFVMIFFMVMPIMIGGFGNWLVPLMLGAPDMAFPRLNNMSFWLLPPSLSLLIMSSIVESGAGTGWTVYPPLSSNIAHGGSSVDLAIFSLHLAGISSILGAVNFITTVINMRPTGM
NLDQMPLFVWSVVITAILLLLSLPVLAGAITMLLTDRNLNTSFFDPAGGGDPILYQHL
>COLFE1384-13|KJ966253|ZMUO.007179|Microcara_testacea
TLYFIFGSWAGMVGTSLSLLIRAELGTPGSLIGDDQIYNVIVTAHAFIMIFFMVMPIMIGGFGNWLVPLMLGAPDMAFPRMNNMSFWLLPPSLTLLLMSSMVENGAGTGWTVYPPLSAGMAHSGASVDLAIFSLHLAGISSILGAVNFISTVINMRSS
GMTFDRMPLFVWSVAITALLLLLSLPVLAGAITMLLTDRNLNTSFFDPAGGGDPILYQHL
>COLFE394-12|KJ965812|ZMUO.004954|Harpalus_latus
TLYFIFGAWAGMVGTSLSMLIRAELGTPGALIGDDQIYNVIVTAHAFIMIFFMVMPIMIGGFGNWLVPLMLGAPDMAFPRMNNMSFWLLPPSLSLLLMSSMVESGAGTGWTVYPPLSSGIAHSGASVDLAIFSLHLAGVSSILGAVNFITTIINMRSV
GMTFDRMPLFVWSVGITALLLLLSLPVLAGAITMLLTDRNLNTSFFDPAGGGDPILYQHL
>COLFE770-13|KJ964497|ZMUO.005615|Pelenomus_quadrituberculatus
TLYFIFGSWAGTVGTSLSMIIRTELGTPGSLIGNDQIYNSIVTAHAFIMIFFMVMPILIGGFGNWLVPLMLGAPDMAFPRLNNMSFWLLPPSILLLLMSSIVNKGAGTGWTVYPPLSSNITHEGASVDLAIFSLHMAGISSILGAINFISTIMNMRPKGMSY
DKTPLFVWAVMITAILLLISLPVLAGAITMLLTDRNINTSFFDPAGGGDPILYQHL
>COLFF810-13|KJ966780|ZMUO.006130|Stephostethus_variolosus
SLYFLFGMWSGMVGTSLSLLIRLELGNPGSLIGDDQIYNVIVTAHAFIMIFFMVMPVMMGGFGNWLVPLMLGAPDMAFPRLNNMSFWLLPPSLSLLIMSSIVESGVGTGWTVYPPLSSNIAHGGSSVDLAIFSLHLAGISSILGAVNFITTMINMRPTG
MKFELMPLFAWSVMLTAILLLLSLPVLAGAITMLLTDRNLNTSFFDPAGGGDPILYQHL
>COLFD749-12|KJ967431|ZMUO.004739|Amara_tibialis
TLYFIFGAWSGMVGTSLSMLIRAELGNPGALIGDDQIYNVIVTAHAFVMIFFMVMPIMIGGFGNWLVPLMLGAPDMAFPRMNNMSFWLLPPSLTLLLMSSMVESGAGTGWTVYPPLSSGIAHAGASVDLAIFSLHLAGISSILGAVNFITTIINMRSV
GMTFDRMPLFVWSVGITALLLLLSLPVLAGAITMLLTDRNLNTSFFDPAGGGDPILYQHL
>COLFE1533-13|KJ963084|ZMUO.007423|Agriotes_lineatus
TLYFLFGAWAGMLGTSLSLLIRAELGNPGSLIGNDQIYNVIVTAHAFIMIFFMVMPIMIGGFGNWLVPLMLGAPDMAFPRMNNMSFWFLPPSLSLLLMSSIVENGAGTGWTVYPPLSSNIAHSGSSVDLAIFSLHLAGISSILGAVNFISTVINMRSTGIT
FDRMPLFVWAVAITALLLLLSLPVLAGAITMLLTDRNLNTSFFDPAGGGDPILYQHL
>COLFD629-12|KJ966848|ZMUO.004429|Notiophilus_palustris
TLYFIFGAWSGMVGTSLSMLIRAELGNPGSLIGDDQIYNVIVTAHAFVMIFFMVMPIMIGGFGNWLVPLMLGAPDMAFPRMNNMSFWLLPPSLTLLLTSSMVESGAGTGWTVYPPLSSGIAHSGASVDLAIFSLHLAGVSSILGAVNFITTIINMRSVG
MTFDRMPLFVWSVGITALLLLLSLPVLAGAITMLLTDRNLNTSFFDPAGGGDPILYQHL

>COLFF746-13|KJ967239|ZMUO.006446|Tychius_junceus
TLYFIFGSWSGMVGTSLSMLIRTELGNPGSLIGDDQIYNSIVTAHAFIMIFFMVMPIMIGGFGNWLVPLMLGAPDMAFPRLNNMSFWLLPPSISLLMMSSIVDKGAGTGWTVYPPLSSNMAHEGASVDLAIFSLHLAGASSILGAINFISTVMNMRPF
GMNSERITLFSWAVQVTAILLLLSLPVLAGAITMLLTDRNVNTTFFDPAGGGDPILYQHL
>COLFC261-12|KJ963630|ZMUO.002446|Platystethus_nodifrons
TLYFIFGAWSGMVGTSLSMIIRAELGTPGSLIGDDQIYNVVVTAHAFVMIFFMVMPIMIGGFGNWLVPLMLGAPDMAFPRMNNMSFWLLPPSLTLLLFSSVVESGAGTGWTVYPPLSSNIAHSGSSVDLAIFSLHLAGISSILGAVNFISTIINMRAVGIS
FDRMPLFIWSVNITAILLLLSLPVLAGAITMLLTDRNLNTSFFDPAGGGDPILYQHL
>COLFB222-12|KJ964646|ZMUO.001552|Stephanopachys_linearis
TLYFIFGSWAGMIGTALSILIRSELGNPGSLIGDDQIYNVIVTAHAFVMIFFMVMPMMIGGFGNWLVPLMLGAPDMAFPRMNNMSFWLLPPSLSLLLASSIVENGAGTGWTVYPPLSNNTAHSGASVDLAIFSLHLAGISSILGAVNFITTTINMRPSG
MTPERIPLFVWSVAITALLLLLSLPVLAGAITMLLTDRNLNTSFFDPAGGGDPILYQHL
>COLFB012-12|KJ964989|ZMUO.001342|Ilybius_fuliginosus
TLYFIFGAWAGMVGTSLSMLIRAELGNPGSLIGDDQIYNVIVTAHAFVMIFFMVMPIMIGGFGNWLVPLMLGAPDMAFPRMNNMSFWLLPPSLSLLLMSSMVESGAGTGWTVYPPLSSGIAHSGASVDLAIFSLHLAGISSILGAVNFITTIINMRSV
GMTFDRMPLFVWSVGITALLLLLSLPVLAGAITMLLTDRNLNTSFFDPAGGGDPILYQHL
>COLFF340-13|KJ963734|ZMUO.006040|Podabrus_flavimanus
TLYFIFGAWSGSLGLALSLLIRAELGTPGTLIGNDQIYNVIVTAHAFIMIFFMVMPIMIGGFGNWLVPLMLGAPDMAFPRMNNMSFWFLPPSLMFLLMSSMVESGAGTGWTVYPPLSANIAHSGPSVDLAIFSLHMAGISSILGAVNFISTIMNMKPPS
MKFDQMPLFVWSVGITALLLLLSLPVLAGAITMLLSDRNLNTSFFDPMGGGDPILYQHL
>COLFA591-12|KJ966132|ZMUO.000726|Dytiscus_lapponicus
TLYFIFGAWAGMVGTSLSMLIRAELGNPGSLIGDDQIYNVIVTAHAFVMIFFMVMPIMIGGFGNWLVPLMLGAPDMAFPRMNNMSFWLLPPSLTLLLMSSMVESGAGTGWTVYPPLSASIAHGGASVDLAIFSLHLAGVSSILGAVNFITTIINMRSV
GMTLDRMPLFVWSVGITALLLLLSLPVLAGAITMLLTDRNLNTSFFDPAGGGDPILYQHL
>COLFD795-12|KJ967101|ZMUO.004785|Agabus_paludosus
TLYFIFGTWAGMVGTSLSMLIRAELGNPGSLIGDDQIYNVIVTAHAFVMIFFMVMPIMIGGFGNWLVPLMLGAPDMAFPRMNNMSFWMLPPSLTLLLMSSMVEKGAGTGWTVYPPLSSSIAHGGASVDLAIFSLHLAGISSILGAVNFITTIINMRSV
GMTFDRMPLFVWSVGITALLLLLSLPVLAGAITMLLTDRNLNTSFFDPAGGGDPILYQHL
>COLFF780-13|KJ965933|ZMUO.006100|Agriotes_obscurus
TLYFLFGAWAGMLGTSLSLLIRAELGNPGSLIGNDQIYNVIVTAHAFIMIFFMVMPIMIGGFGNWLVPLMLGAPDMAFPRMNNMSFWFLPPSLSLLLMSSIVENGAGTGWTVYPPLSSNIAHSGSSVDLAIFSLHLAGISSILGAVNFISTVINMRSTGIT
FDRMPLFVWAVAITALLLLLSLPVLAGAITMLLTDRNLNTSFFDPAGGGDPILYQHL
>COLFF833-13|KJ965283|ZMUO.006153|Sphaeroderma_testaceum
TLYFIFGIWAGMVGMSLSILIRMELGNPGSLIGNDQIYNVIVTAHAFIMIFFMVMPIMIGGFGNWLVPLMIGAPDMAFPRMNNMSFWLLPPSLFFLIMSSIVESGAGTGWTVYPPLSSNIAHSGASVDLAIFSLHLAGISSILGAINFITTIINMRPNGMS
PDRMPLFVWAVLITAILLLLSLPVLAGAITMLLTDRNLNTSFFDPSGGGDPILYQHL
>COLFD559-12|KJ961915|ZMUO.004359|Cantharis_pellucida
TLYFIFGAWSGSLGLALSLLIRAELGTPGTLIGNDQIYNVIVTAHAFIMIFFMVMPIMIGGFGNWLVPLMLGAPDMAFPRMNNMSFWFLPPSLMFLLMSSMVESGAGTGWTVYPPLSANIAHSGPSVDLAIFSLHMAGISSILGAVNFISTIMNMKPPS
MKFDQMPLFVWSVGITALLLLLSLPVLAGAITMLLSDRNLNTSFFDPMGGGDPILYQHL
>COLFD199-12|KJ962158|ZMUO.004094|Falagria_caesa
TLYFIFGAWAGMVGTSLSLLIRAELGNPGSLIGDDQIYNVIVTAHAFIMIFFMVMPIVIGGFGNWLVPLMLGAPDMAFPRMNNMSFWLLPPSLSLLLMSSMVESGAGTGWTVYPPLSSNIAHGGASVDLAIFSLHLAGISSILGAVNFISTVINMRSIGIT
FDRMPLFVWSVAITALLLLLSLPVLAGAITMLLTDRNLNTSFFDPAGGGDPILYQHL
>COLFF037-13|KJ963446|ZMUO.005737|Pytho_kolwensis
TLYFIFGAWSGMVGTSLSLLIRAELGNPGSLIGDDQIYNVIVTAHAFIMIFFMVMPTVIGGFGNWLVPLMLGAPDMAFPRMNNMSFWLLPPSLSLLIMSSIVENGAGTGWTVYPPLASNIAHGGSSVDLAIFSLHLAGISSILGAVNFITTVINMRPSGM
TFDRMPLFVWSVVITAVLLLLSLPVLAGAITMLLTDRNLNTSFFDPAGGGDPILYQHL
>COLFC801-12|KJ965033|ZMUO.003366|Cicindela_campestris
TLYFIFGAWSGMVGTSLSMLIRAELGSPGSLIGDDQIYNVIVTAHAFVMIFFMVMPIMIGGFGNWLVPLMLGAPDMAFPRMNNMSFWLLPPSLTLLLMSSLVGDGAGTGWTVYPPLSAGIAHAGASVDLAIFSLHLAGVSSILGAVNFITTIINMRSV
GMTFDRMPLFVWSVGITALLLLLSLPVLAGAITMLLTDRNLNTSFFDPAGGGDPILYQHL
>COLFF549-13|KJ963178|ZMUO.006629|Anthicus_flavipes
TLYLIFGAWAGMVGTSLSLLIRSELGNPGTLIGNDQIYNVIVTAHAFIMIFFMVMPIVIGGFGNWLVPLMLGAPDMAFPRMNNMSFWLLPPSLSLLIMSSIVESGAGTGWTVYPPLSANIAHSGSSVDLAIFSLHLAGISSILGAVNFITTVINMRPVGMT
LDRMPLFVWAVVITAVLLLLSLPVLAGAITMLLTDRNLNTSFFDPAGGGDPILYQHL
>COLFF119-13|KJ965750|ZMUO.003753|Hippodamia_tredecimpunctata
TLYFLFGMWAGMVGTSLSILIRLELGTPGSLIGNDQIYNVIVTAHAFIMIFFMVMPIMIGGFGNWLVPLMIGAPDMAFPRLNNMSFWLLPPALTLLMFSSMVEMGAGTGWTVYPPLSSNLAHNGPSVDLVIFSLHLAGISSILGAVNFISTIMNMRPY
GMSMDKTPLFVWSVLITAILLLLSLPVLAGAITMLLTDRNLNTSFFDPTGGGDPILYQHL
>COLFC626-12|KJ965362|ZMUO.003191|Hydroporus_notabilis

TLYFLFGAWSGMVGTSLSMLIRAELGNPGSLIGDDQIYNVIVTAHAFIMIFFMVMPIMIGGFGNWLVPLMLGAPDMAFPRMNNMSFWLLPPSLSLLLMSSMVENGAGTGWTVYPPLSSGIAHSGASVDLAIFSLHLAGISSILGAVNFITTIINMRSIG
MTFDRMPLFVWSVGITALLLLLSLPVLAGAITMLLTDRNLNTSFFDPAGGGDPILYQHL
>COLFF097-13|KJ963442|ZMUO.003731|Cis_micans
ILYFIFGAWSGMVGTSMSMLIRSELGMPGSLIGNDQIYNVIVTAHAFVMIFFMVMPIMIGGFGNWLVPLMLGAPDMAFPRMNNMSFWLLPPSLSLLLMSSIVENGAGTGWTVYPPLSSNIAHSGSSVDLAIFSLHLAGISSILGAVNFISTVINMRPE
GMNFDRMPLFVWAVLITAILLLLSLPVLAGAITMLLTDRNFNTSFFDPAGGGDPILYQHL
>COLFA083-10|HM909100|MP00297|Heterocerus_hispidulus
TLYFILGSWSGMLGMALSMLIRAELGNPGALIGDDQIYNVIVTAHAFIMIFFMVMPIMIGGFGNWLVPLMLGAPDMAFPRMNNMSFWLLPPSLTLLLMSSMVESGAGTGWTVYPPLSANIAHSGSSVDLAIFSLHLAGISSILGAVNFITTVINMRAP
GMTFDRMPLFVWSVMITAILLLLSLPVLAGAITMLLTDRNLNTSFFDPAGGGDPILYQHL
>COLFC173-12|KJ966385|ZMUO.002358|Cercyon_sternalis
TLYFIFGAWAGMVGTSLSILIRAELGNPGTLIGDDQIYNVIVTAHAFIMIFFMVMPIMIGGFGNWLVPLMLGAPDMAFPRMNNMSFWLLPPSLSLLLMSSMVESGAGTGWTVYPPLSSNIAHGGSSVDLAIFSLHLAGISSILGAVNFITTVINMRSPSL
TYDRLPLFVWSVAITALLLLLSLPVLAGAITMLLTDRNLNTSFFDPAGGGDPILYQHL
>COLFB418-12|KJ961820|ZMUO.001843|Lampyris_noctiluca
TLYFIFGAWAGMLGTSFSLLIRAELGSAGALIGNDHIFNVIVTSHAFIMIFFMVMPIMIGGFGNWLVPLMLGAPDMAFPRMNNMSFWLLPPSLSLLIMSSLIESGAGTGWTVYPPLSANIAHSGPSVDLAIFSLHLAGVSSILGAVNFISTIINMRPNSM
MFDQMPLFVWAVLITAILLLLSLPVLAGAITMLLTDRNLNTSFFDPAGGGDPILYQHL
>COLFE1375-13|KJ964714|ZMUO.007170|Olibrus_millefolii
TLYFIFGAWAGMVGTSLSLLIRTELGTPGSLIGDDQIYNVIVTAHAFVMIFFMVMPFMIGGFGNWLVPLMLGAPDMAFPRMNNMSFWLLPPSLFLLLMSSMVESGAGTGWTVYPPLSSNIAHGGASVDLAIFSLHLAGISSILGAINFITTMINMRPE
GMTLDRMPLFVWAVTITAILLLLSLPVLAGAITMLLTDRNINTSFFDPAGGGDPILYQHL
>COLFG182-13|KJ966547|ZMUO.007877|Atomaria_turgida
TLYFIFGAWSGMVGTSLSMLIRTELGTPGSLIGDDQIYNVIVTAHAFIMIFFMVMPMMIGGFGNWLVPLMLGAPDMAFPRLNNMSFWLLPPSLMFLLMSSIVEKGAGTGWTVYPPLSANVAHAGSSVDLAIFSLHLAGISSILGSVNFITTVINMRPE
GMKFDRLPLFVWAVKITTILLLLSLPVLAGAITMLLTDRNMNTSFFDPAGGGDPILYQHL
>COLFC803-12|KJ966046|ZMUO.003368|Calathus_micropterus
TLYFIFGAWAGMVGTSLSMLIRAELGNPGALIGDDQIYNVIVTAHAFVMIFFMVMPIMIGGFGNWLVPLMLGAPDMAFPRMNNMSFWLLPPALSLLLMSSMVESGAGTGWTVYPPLSSGIAHSGASVDLAIFSLHLAGVSSILGAVNFITTIINMRSV
GMTFDRMPLFVWSVGITALLLLLSLPVLAGAITMLLTDRNLNTSFFDPAGGGDPILYQHL
>COLFA423-12|KJ964385|ZMUO.000653|Colon_bidentatum
TLYFIFGAWAGMVGTSLSLLIRAELGNPGSLIGDDQIYNVIVTAHAFIMIFFMVMPIVIGGFGNWLVPLMLGAPDMAFPRMNNMSFWLLPPSLTLLLMSSLVESGAGTGWTVYPPLSSNIAHGGSSVDLAIFSLHLAGISSILGAVNFITTVINMRSQGM
GFDQMPLFVWSVIITAFLLLLSLPVLAGAITMLLTDRNLNTSFFDPAGGGDPILYQHL
>COLFD466-12|KJ966390|ZMUO.003886|Olibrus_bimaculatus
TLYFIFGAWAGMMGTSLSLLIRTELGTPGSLIGDDQIYNVIVTAHAFVMIFFMVMPFMIGGFGNWLVPLMLGAPDMAFPRMNNMSFWLLPPSLFLLLMSSLVESGAGTGWTVYPPLSSNIAHGGASVDLAIFSLHLAGISSILGAINFITTMINMRPEG
MTLDRMPLFVWAVTITAILLLLSLPVLAGAITMLLTDRNINTSFFDPAGGGDPILYQHL
>COLFD419-12|KJ965406|ZMUO.003839|Hypera_rumicis
TLYFIFGTWAGTVGTSLSILIRTELGNPGSLIGNDQIYNTIVTAHAFIMIFFMVMPIMIGGFGNWLVPLMLGAPDMAFPRLNNMSFWLLPPSLSLLLMSSMVDSGAGTGWTVYPPLSSNIAHEGSSVDLAIFSLHMAGVSSILGAINFISTVLNMRPMG
MSLDKMALFIWAVKITAILLLLSLPVLAGAITMLLTDRNINTSFFDPAGGGDPILYQHL
>COLFE602-13|KJ964572|ZMUO.005447|Cerylon_histeroides
TLYFMFGMWSGMVGTSMSMMIRLELGNPGSLIGDDQIYNVIVTAHAFVMIFFMVMPIMIGGFGNWLVPLMLGAPDMAFPRMNNMSFWLLPPSLTLLIMSSIVEKGAGTGWTVYPPLSANLTHSGSSVDLAIFSLHLAGISSILGAVNFITTVINMR
PSGMTWDRLPLFVWSVIITAVLLLLSLPVLAGAITMLLTDRNLNTSFFDPAGGGDPILYQHL
>COLFD165-12|KJ965559|ZMUO.004060|Badister_lacertosus
TLYFIFGAWAGMVGTSLSILIRAELGNPGSLIGDDQIYNVIVTAHAFVMIFFMVMPIMIGGFGNWLIPLMLGAPDMAFPRMNNMSFWLLPPSLTLLLMSSLVESGAGTGWTVYPPLSSGIAHSGASVDLAIFSLHLAGISSILGAVNFITTIINMRSTGITF
DRMPLFVWSVGITALLLLLSLPVLAGAITMLLTDRNLNTSFFDPAGGGDPILYQHL
>COLFC134-12|KJ962062|ZMUO.002319|Atheta_incognita
TLYFIFGMWAGMVGTSLSLLIRAELGNPGSLIGDDQIYNVIVTAHAFVMIFFMVMPVMIGGFGNWLIPLMLGAPDMAFPRMNNMSFWLLPPSLTLLLMSSMVESGAGTGWTVYPPLSSNIAHGGSSVDLAIFSLHLAGISSILGAVNFISTVINMRST
GISFDRMPLFVWSVAITALLLLLSLPVLAGAITMLLTDRNLNTSFFDPAGGGDPILYQHL
>COLFF126-13|KJ962377|ZMUO.003760|Bledius_gallicus
TLYFIFGAWAGMVGTSLSMLIRAELGTPGSLIGNDQIYNVIVTAHAFVMIFFMVMPIVIGGFGNWLVPLMLGAPDMAFPRMNNMSFWLLPPSLTLLLMSSMVESGAGTGWTVYPPLSSNIAHSGSSVDLAIFSLHLAGISSILGAVNFISTIINMRSIG
MTFDRMPLFVWSVKITAILLLLSLPVLAGAITMLLTDRNLNTSFFDPAGGGDPILYQHL
>COLFC798-12|KJ963423|ZMUO.003363|Judolia_sexmaculata
TLYFIFGAWAGMVGTSLSLLIRSELGSPGSLIGNDQIYNVIVTAHAFVMIFFMVMPIMIGGFGNWLVPLMLGAPDMAFPRMNNMSFWLLPPSLTLLILSSMVETGAGTGWTVYPPLSSNIAHSGSSVDLAIFSLHLAGISSILGAVNFITTVINMRPVGM
NLDCMPLFVWAVVITAILLLLSLPVLAGAITMLLTDRNLNTSFFDPAGGGDPILYQHL

>COLFF172-13|KJ962943|ZMUO.005896|Donacia_obscura
TLYFIFGAWSGMMGTSLSMLIRSELSNPGSLIGNDQIYNVIVTAHAFIMIFFMVMPIMIGGFGNWLVPLMLGAPDMAFPRMNNMSFWLLPPSLTFLTMSSITESGAGTGWTVYPPLSNNLAHSGSSVDLAIFSLHLAGISSILGAVNFISTIINMRPAG
MTMEKMSLFSWAVMITAILLLVSLPVLAGAITMLLTDRNINTSFFDPAGGGDPILYQHL
>COLFE631-13|KJ962152|ZMUO.005476|Macroplea_appendiculata
TLYFIFGAWSSMMGTSLSILIRSELSTPGSLIENDQIYNVIVTAHAFIMIFFMVMPIMIGGFGNWLIPLMLGAPDMAFPRMNNMSFWLLPPAITFLMMSSITENGAGTGWTVYPPLSSNIAHSGTSVDLAIFSLHLAGISSILGAINFISTILNMRPIGMKM
DKMPLFVWAVMITAILLLLSLPVLAGAITMLLTDRNINTTFFDPMGGGDPILYQHL
>COLFD835-12|KJ963780|ZMUO.004825|Meligethes_denticulatus
TLYFIFGAWSGMVGTSLSILIRTELGNPGSLIGNDQIYNVIVTAHAFVMIFFMVMPFMIGGFGNWLVPLMLGAPDMAFPRMNNMSFWLLPPSLSLLLMSSIVESGAGTGWTVYPPLSSNIAHGGASVDLAIFSLHLAGISSILGAVNFITTVINMRPTG
MNFDRMPLFIWAVVITALLLLLSLPVLAGAITMLLTDRNLNTTFFDPSGGGDPILYQHL
>COLFB264-12|KJ964350|ZMUO.001594|Gabrius_toxotes
TLYFIFGSWAGMVGTSLSLLIRAELGNPGTLIGDDQIYNVIVTAHAFIMIFFMVMPIVIGGFGNWLVPLMLGAPDMAFPRMNNMSFWLLPPSMTLLLMSSMVESGAGTGWTVYPPLSSNIAHGGASVDLAIFSLHLAGISSILGAVNFITTVINMRSFG
MSFDRMPLFVWSVAITALLLLLSLPVLAGAITMLLTDRNLNTSFFDPAGGGDPILYQHL
>COLFA430-12|KJ966616|ZMUO.000660|Dryocoetes_autographus
TLYFIFGVWSGMVGTSLSVLIRTELGTPGSLIGDDQIFNTIVTAHAFIMIFFMVMPILIGGFGNWLVPLMLGAPDMAFPRLNNMSFWLLPPSLSLLLMSSIIDKGAGTGWTVYPPLASNIAHEGASVDLAIFSLHMTGVSSILGAINFISTIINMHPAGVKP
EQLSLFTWSVKITAILLLLSLPVLAGGITMLLTDRNINTSFFDPAGGGDPILYQHL
>COLFE066-12|KJ963575|ZMUO.005291|Adalia_conglomerata
TLYFLFGMWAGMVGTSLSILIRLELGTTNSLIGNDQIYNVIVTAHAFIMIFFMVMPIMIGGFGNWLVPLMIGAPDMAFPRLNNMSFWLLPPALTLLIFSSMVEMGAGTGWTVYPPLSSNLAHNGPSVDLVIFSLHLAGISSILGAVNFISTIMNMRPMG
MNLDKTPLFVWSVLITAILLLLSLPVLAGAITMLLTDRNINTSFFDPTGGGDPILYQHL
>COLFB546-12|KJ967070|ZMUO.001021|Acrotona_orbata
TLYFIFGAWAGMVGTSLSLLIRAELGNPGSLIGDDQIYNVIVTAHAFIMIFFMVMPIMIGGFGNWLVPLMLGAPDMAFPRMNNMSFWLLPPSLTLLLMSSMVESGAGTGWTVYPPLSSNIAHGGSSVDLAIFSLHLAGISSILGAVNFISTVINMRSSGI
TFDRMPLFVWAVAITALLLLLSLPVLAGAITMLLTDRNLNTSFFDPAGGGDPILYQHL
>COLFF602-13|KJ962196|ZMUO.006492|Cleopus_pulchellus
TLYFIFGMWSGTIGTSLSMLIRTELGNPGSLIGDDQIYNTIVTAHAFIMIFFMVMPIMIGGFGNWLVPLMLGAPDMAFPRLNNMSFWLLPPSITLLLMSSIVEKGAGTGWTVYPPLSNNIAHEGASVDSAIFSLHMAGISSILGAINFISTVSNMRIKGM
NYDRTPLFVWSVNITAFLLLLSLPVLAGGITMLLTDRNVNTSFFDPSGGGDPILYQHL
>COLFA108-10|HQ559234|MP00369|Coccidula_scutellata
TLYFLFGMWAGMLGTSLSILIRLELGTPSSLIGNDQIYNVIVTAHAFIMIFFMVMPIMIGGFGNWLVPLMIGAPDMAFPRMNNMSFWLLPPALTLLILSSVVESGAGTGWTVYPPLSSNLAHSGSSVDLAIFSLHLAGISSIMGAVNFITTVINMRPYGM
TFEKMPLFVWSVVITAILLLLSLPVLAGAITMLLTDRNINTSFFDPAGGGDPILYQHL
>COLFD546-12|KJ964209|ZMUO.004346|Gyrinus_marinus
TLYFIFGAWSGMVGTSLSMLIRAELGNPGSLIGDDQIYNVIVTAHAFIMIFFMVMPIMIGGFGNWLVPLMLGAPDMAFPRMNNMSFWLLPPSLTLLLMSSMVENGAGTGWTVYPPLSSNIAHGGASVDLAIFSLHLAGISSILGAVNFITTIINMRSIG
MTLDRMPLFVWSVGITALLLLLSLPVLAGAITMLLTDRNLNTSFFDPAGGGDPILYQHL
>COLFC153-12|KJ963436|ZMUO.002338|Bisnius_nigriventris
TLYFIFGSWAGMVGTSLSLLIRAELGNPGSLIGDDQIYNVIVTAHAFIMIFFMVMPIVIGGFGNWLVPLMLGAPDMAFPRMNNMSFWLLPPSLILLLMSSVVESGAGTGWTVYPPLSSNIAHGGASVDLAIFSLHLAGISSILGAVNFISTVLNMRSMGI
SFDRMPLFVWSVAITALLLLLSLPVLAGAITMLLTDRNLNTSFFDPAGGGDPILYQHL
>COLFB103-12|KJ962359|ZMUO.001433|Oiceoptoma_thoracicum
TLYFIFGAWAGMVGMSLSILIRAELSTPGSLLGDDQMYNVIVTAHAFVMIFFMVMPIVIGGFGNWLVPLMLGAPDMAFPRMNNMSFWLLPPSLSLLLVSSMVESGAGTGWTVYPPLSSNIAHSGSSVDLAIFSLHLAGISSILGAVNFITTIINMRSSG
MTFDRMPLFVWSVAITALLLLLSLPVLAGAITMLLTDRNLNTSFFDPAGGGDPVLYQHL
>COLFE1603-13|KJ967325|ZMUO.007493|Ceratomegilla_notata
TLYFLFGMWAGMIGTSLSILIRLELGTTGSLIGNDQIYNVIVTAHAFIMIFFMVMPIMIGGFGNWLVPLMIGAPDMAFPRLNNMSFWLLPPALILLLFSSLVEMGAGTGWTVYPPLSSNLAHNGPSVDLVIFSLHLAGISSILGAVNFISTIMNMRPYGM
KLDKTPLFVWSVLITAILLLLSLPVLAGAITMLLTDRNLNTSFFDPTGGGDPILYQHL
>COLFC510-12|KJ963643|ZMUO.003075|Phyllodrepa_linearis
TLYFIFGAWAGMVGTSLSILIRAELGNPGSLIGDDQIYNVIVTAHAFVMIFFMVMPIVIGGFGNWLVPLMLGAPDMAFPRMNNMSFWLLPPSLTLLLMSSMVESGAGTGWTVYPPLSSNIAHGGASVDLAIFSLHLAGISSILGAVNFITTVINMRSMG
MTFDRMPLFVWSVAITALLLLLSLPVLAGAITMLLTDRNLNTSFFDPAGGGDPILYQHL
>COLFF693-13|KJ965487|ZMUO.006393|Malthinus_frontalis
TLYFIFGAWSGMLGTSLSLLIRAELGNPGSLIGNDQIYNVIVTAHAFIMIFFMVMPIMIGGFGNWLVPLMLGAPDMAFPRMNNMSFWFLPPSLSLLLMSSLVESGAGTGWTVYPPLSANIAHSGSSVDLAIFSLHMAGISSILGAVNFISTVINMRSVG
MNFDQMPLFVWAVAITALLLLLSLPVLAGAITMLLTDRNLNTSFFDPAGGGDPILYQHL
>COLFC628-12|KJ966255|ZMUO.003193|Hygrotus_novemlineatus

TLYFLFGAWSGMVGTSLSMLIRAELGNPGSLIGDDQIYNVIVTAHAFIMIFFMVMPIMIGGFGNWLVPLMLGAPDMAFPRMNNMSFWMLPPSLTLLLMSSMVESGAGTGWTVYPPLSAGIAHGGASVDLAIFSLHLAGISSILGAVNFITTIINMRSV
GMTFDRMPLFVWSVGITALLLLLSLPVLAGAITMLLTDRNLNTSFFDPAGGGDPILYQHL
>COLFD701-12|KJ964802|ZMUO.004691|Atheta_hygrotopora
TLYFIFGAWAGMVGTSLSLLIRAELGNPGSLIGDDQIYNVIVTAHAFIMIFFMVMPIVIGGFGNWLVPLMLGAPDMAFPRMNNMSFWLLPPSLTLLLMSSMVESGAGTGWTVYPPLSSNIAHGGSSVDLAIFSLHLAGISSILGAVNFISTVINMRSAGI
SFDRMPLFVWSVVITALLLLLSLPVLAGAITMLLTDRNLNTSFFDPAGGGDPILYQHL
>COLFB549-12|KJ965080|ZMUO.001024|Omalium_riparium
TLYFIFGAWAGMVGTSLSLLIRAELGNPGSLIGDDQIYNVIVTAHAFIMIFFMVMPIVIGGFGNWLVPLMLGAPDMAFPRMNNMSFWLLPPSLTLLLMSSMVESGAGTGWTVYPPLSSNIAHGGSSVDLAIFSLHLAGISSILGAVNFITTVINMRAMG
MTFDRMPLFVWSVVITALLLLLSLPVLAGAITMLLTDRNLNTSFFDPAGGGDPILYQHL
>COLFF438-13|KJ963692|ZMUO.005956|Rhantus_frontalis
TLYFIFGAWAGMVGTSLSMLIRAELGNPGSLIGDDQIYNVIVTAHAFVMIFFMVMPIMIGGFGNWLVPLMLGAPDMAFPRMNNMSFWLLPPSLTLLLMSSMVESGAGTGWTVYPPLSSGIAHGGASVDLAIFSLHLAGISSILGAVNFITTIINMRSIG
MTFDRMPLFVWSVGITALLLLLSLPVLAGAITMLLTDRNLNTSFFDPAGGGDPILYQHL
>COLFA119-10|HQ559240|MP00381|Aromia_moschata
TLYFIFGAWSGMVGTSLSMLIRSELGNPGSLIGDDQIYNVIVTAHAFIMIFFMVMPIMIGGFGNWLVPLMLGAPDMAFPRMNNMSFWLLPPSLTFLILSSIVESGAGTGWTVYPPLSSNIAHSGSSVDLAIFSLHLAGISSILGAVNFISTVINMRPSGMS
LDRMPLFVWAVVITAILLLLSLPVLAGAITMLLTDRNINTSFFDPAGGGDPILYQHL
>COLFE632-13|KJ964851|ZMUO.005477|Macroplea_appendiculata
TLYFIFGAWSSMMGTSLSILIRSELSTPGSLIENDQIYNVIVTAHAFIMIFFMVMPIMIGGFGNWLIPLMLGAPDMAFPRMNNMSFWLLPPAITFLMMSSITENGAGTGWTVYPPLSSNIAHSGTSVDLAIFSLHLAGISSILGAINFISTILNMRPIGMKM
DKMPLFVWAVMITAILLLLSLPVLAGAITMLLTDRNINTTFFDPMGGGDPILYQHL
>COLFF986-13|KJ966440|ZMUO.007351|Oulema_melanopus
TLYFIFGTWSGMVGTSLSMMIRTELGNPGSFIGNDQIYNVIVTAHAFIMIFFMVMPIMIGGFGNWLVPLMLGAPDMAFPRMNNMSFWLLPPSISLLLMSSIVENGAGTGWTVYPPLSANISHNGASVDLAIFSLHLAGVSSILGAVNFISTVSNMRPE
GMTLDRMSLFVWAVLITAILLLLSLPVLAGAITMLLTDRNLNTSFFDPSSGGDPILYQHL
>COLFA055-10|HM909076|MP00231|Phyllobius_viridicollis
TLYFIFGAWSGMVGTSLSILIRIELGNPGSLIGDDQIYNVIVTAHAFIMIFFMVMPMMIGGFGNWLIPLMLGAPDMAFPRLNNMSFWLLPPSLSLLLMSSLVDKGAGTGWTVYPPLSANIAHEGSSVDLAIFSLHMAGVSSILGAINFISTVINMRPMG
MSPDRMSLFIWAVKITAVLLLLSLPVLAGAITMLLTDRNMNTSFFDPAGGGDPILYQHL
>COLFD065-12|KJ964099|ZMUO.003960|Colon_latum
TLYFIFGAWAGMVGTSLSLLIRAELGNPGSLIGDDQIYNVIVTAHAFVMIFFMVMPIVIGGFGNWLVPLMLGAPDMAFPRMNNMSFWLLPPSLTLLLMSSLVESGAGTGWTVYPPLSSNIAHGGSSVDLAIFSLHLAGISSILGAVNFITTVINMRSQG
MGFDQMPLFVWSVIITALLLLLSLPVLAGAITMLLTDRNLNTSFFDPAGGGDPILYQHL
>COLFE149-12|KJ962593|ZMUO.005184|Dromius_schneideri
TLYFIFGAWAGMVGTSLSMLIRAELGNPGALIGDDQVYNVIVTAHAFIMIFFMVMPIMIGGFGNWLVPLMLGAPDMAFPRMNNMSFWLLPPSLSLLLMSSMVESGAGTGWTVYPPLSSGIAHAGASVDLAIFSLHLAGVSSILGAVNFITTIINMRSI
GMTFDRMPLFVWSVGITALLLLLSLPVLAGAITMLLTDRNLNTSFFDPAGGGDPILYQHL
>COLFD240-12|KJ964550|ZMUO.004135|Phratora_vitellinae
TLYFIFGIWAGMVGTSLSMLIRSELGNPGTLIGNDQIYNVIVTAHAFIMIFFMVMPIMIGGFGNWLVPLMIGAPDMAFPRLNNMSFWLLPPSLFLLLMSSVVENGAGTGWTVYPPLSSNLAHSGSSVDLAIFSLHLAGISSILGAINFITTVINMRPEGM
DLEQTPLFVWAVLITAILLLLSLPVLAGAITMLLTDRNLNTSFFDPAGGGDPILYQHL
>COLFE1035-13|KJ962514|ZMUO.006830|Agriotes_lineatus
TLYFLFGAWAGMLGTSLSLLIRAELGNPGSLIGNDQIYNVIVTAHAFIMIFFMVMPIMIGGFGNWLVPLMLGAPDMAFPRMNNMSFWFLPPSLSLLLMSSIVENGAGTGWTVYPPLSSNIAHSGSSVDLAIFSLHLAGISSILGAVNFISTVINMRSTGIT
FDRMPLFVWAVAITALLLLLSLPVLAGAITMLLTDRNLNTSFFDPAGGGDPILYQHL
>COLFC339-12|KJ963427|ZMUO.002524|Sciodrepoides_watsoni
TLYFIFGAWAGMVGTSLSLLIRAELGNPGSLIGDDQIYNVIVTAHAFVMIFFMVMPIVIGGFGNWLVPLMLGAPDMAFPRMNNMSFWLLPPSLSLLLMSSIVENGAGTGWTVYPPLSANIAHSGSSVDLAIFSLHLAGISSILGAVNFITTVINMRATG
MTLDKMPLFVWSVAITALLLLLSLPVLAGAITMLLTDRNLNTSFFDPAGGGDPILYQHL
>COLFB939-12|KJ966036|ZMUO.001984|Zyras_humeralis
TLYFVFGAWAGMVGTSLSLLIRAELGNPGSLIGDDQIYNVIVTAHAFIMIFFMVMPIVIGGFGNWLVPLMLGAPDMAFPRMNNMSFWLLPPSLTLLLSSSMVESGAGTGWTVYPPLSSNIAHGGSSVDLAIFSLHLAGISSILGAVNFISTVINMRSVGI
SFDRMPLFVWSVAITALLLLLSLPVLAGAITMLLTDRNLNTSFFDPAGGGDPILYQHL
>COLFF022-13|KJ967268|ZMUO.005722|Malthodes_fuscus
TLYFMFGAWAGMLGTSLSLLIRAELGSPGSLIGNDQIYNVIVTAHAFIMIFFMVMPIMIGGFGNWLVPLMLGAPDMAFPRMNNMSFWFLPPSLSLLLMSSIVENGAGTGWTVYPPLSANIAHSGSSVDLAIFSLHMAGISSILGAVNFISTVINMRSTG
MTFDRMPLFVWAVAITALLLLLSLPVLAGAITMLLTDRNLNTSFFDPAGGGDPILYQHL
>COLFG077-13|KJ967232|ZMUO.007582|Xylita_laevigata
TLYFIFGAWSSMLGTSLSLLIRSELGNPGSLIGDDQIYNVIVTAHAFIMIFFMVMPILIGGFGNWLMPLMLGAPDMAFPRMNNMSFWLLPPSLILLIMSSIVEKGAGTGWTVYPPLSSNIAHNGSSVDLAIFSLHLAGISSILGAINFITTVINMRPKGMTL
DRMPLFVWSVMLTAILLLLSLPVLAGAITMLLTDRNLNTSFFDPAGGGDPVLYQHL

>COLFE307-12|KJ962276|ZMUO.004867|Charopus_graminicola
TLYFIFGAWSGMIGLSLSLMIRSELSIPGTLIGNDQIYNVIVTAHAFIMIFFMVMPILIGGFGNWLVPLMLGAPDMAFPRMNNMSFWLLPPSLTLLLLSSMIENGAGTGWTVYPPLSANIAHSGASVDLAIFSLHLAGISSILGAINFITTVINMRPQGMTL
DRVPLFVWAVVITALLLLLSLPVLAGAITMLLTDRNLNTSFFDPAGGGDPILYQHL
>COLFC092-12|KJ964829|ZMUO.002087|Phyllodrepa_sahlbergi
TLYFIFGAWAGMVGTSLSILIRAELGNPGSLIGDDQIYNVIVTAHAFVMIFFMVMPIMIGGFGNWLVPLMLGAPDMAFPRMNNMSFWLLPPSLTLLLMSSMVESGAGTGWTVYPPLSSNIAHSGSSVDLAIFSLHLAGISSILGAVNFITTVINMRSMG
MTFDRMPLFVWSVAITALLLLLSLPVLAGAITMLLTDRNLNTSFFDPAGGGDPILYQHL
>COLFA603-12|KJ966268|ZMUO.000738|Exocentrus_lusitanus
TLYFIFGAWAAMVGTSLSLLIRSELGSPGSLIGDDQIYNVIVTAHAFIMIFFMVMPIMIGGFGNWLIPLMLGAPDMAFPRMNNMSFWLLPPSLSLLIMSSIVENGAGTGWTVYPPLSANIAHSGSSVDLAIFSLHLAGISSILGAVNFITTVINMRPMGM
MFDRLSLFIWAVKITAILLLLSLPVLAGAITMLLTDRNLNTSFFDPAGGGDPILYQHL
>COLFD610-12|KJ963583|ZMUO.004410|Scaphidium_quadrimaculatum
TLYFIFGAWAGMVGTSLSLLIRAELGTQGSLIGDDQIYNVIVTAHAFVMIFFMVMPIVIGGFGNWLVPLMLGAPDMAFPRMNNMSFWLLPPSLSLLLMSSLVESGAGTGWTVYPPLSSNIAHGGASVDLAIFSLHLAGISSILGAVNFISTIINMRTIGM
SFDQMPLFIWSVGITALLLLLSLPVLAGAITMLLTDRNLNTAFFDPAGGGDPILYQHL
>COLFC123-12|KJ963676|ZMUO.002308|Eusphalerum_lapponicum
TLYFIFGAWAGMVGTSLSMLIRAELGNPGSLIGDDQIYNVIVTAHAFIMIFFMVMPIMIGGFGNWLVPLMLGAPDMAFPRMNNMSFWLLPPSLTLLLMSSMVESGAGTGWTVYPPLSSNIAHGGSSVDLAIFSLHLAGISSILGAVNFITTVINMRSM
GMTFDRMPLFVWAVAITALLLLLSLPVLAGAITMLLTDRNLNTSFFDPAGGGDPILYQHL
>COLFA342-12|KJ961733|ZMUO.000572|Pselaphus_heisei
TLYLIFGAWAGMVGTSLSMLIRSELGNPGSLIGDDQIYNVIVTAHAFIMIFFMVMPLMIGGFGNWLVPLMLGAPDMAFPRMNNMSFWLLPPSLSLLLMSSIIENGAGTGWTVYPPLSSNIAHNGSSVDLTIFSLHLAGISSILGAVNFISTIINMRATGIIL
DRMPLFVWSVLITAILLLLSLPVLAGAITMLLTDRNLNTSFFDPAGGGDPILYQHL
>COLFF877-13|KJ967287|ZMUO.006672|Megarthrus_prosseni
TLYFIFGAWAGMIGTSLSILIRAELGNPGSLIGDDQIYNVIVTAHAFIMIFFMVMPIVIGGFGNWLVPLMLGAPDMAFPRMNNMSFWLLPPSISLLLISSMVESGAGTGWTVYPPLSSNIAHGGASVDLAIFSLHLAGISSILGAVNFITTVINMRSMGMT
FDRMPLFVWSVAITALLLLLSLPVLAGAITMLLTDRNINTSFFDPAGGGDPILYQHL
>COLFB863-12|KJ965124|ZMUO.001908|Judolia_sexmaculata
TLYFIFGAWAGMVGTSLSLLIRSELGSPGSLIGNDQIYNVIVTAHAFVMIFFMVMPIMIGGFGNWLVPLMLGAPDMAFPRMNNMSFWLLPPSLTLLILSSMVETGAGTGWTVYPPLSSNIAHSGSSVDLAIFSLHLAGISSILGAVNFITTVINMRPVGM
NLDCMPLFVWAVVITAILLLLSLPVLAGAITMLLTDRNLNTSFFDPAGGGDPILYQHL
>COLFD220-12|KJ963381|ZMUO.004115|Tachyporus_chrysomelinus
TLYFIFGAWSGMVGTSLSLLIRAELGNPGSLIGDDQIYNVIVTAHAFIMIFFMVMPIVIGGFGNWLVPLMLGAPDMAFPRMNNMSFWLLPPSLTLLLMSSMVESGAGTGWTVYPPLSANIAHSGPSVDLAIFSLHLAGISSILGAVNFITTVINMRASG
MHFDRMPLFIWSVAITALLLLLSLPVLAGAITMLLTDRNLNTSFFDPAGGGDPILYQHL
>COLFD079-12|KJ962053|ZMUO.003974|Cryptophagus_confusus
TLYFIFGAWAGMVGTSLSLLIRLELSTPGSLIGDDQIYNVIVTAHAFVMIFFMVMPIMIGGFGNWLIPLMLGAPDMAFPRMNNMSFWLLPPSLSFLLMSSIAEKGVGTGWTVYPPLSSNIAHGGSSVDLAIFSLHLAGISSILGAVNFISTVMNMFPMG
MSLDRMPLFMWAVMITAFLLLLSLPVLAGAITMLLTDRNLNTSFFDPAGGGDPILYQHL
>COLFA706-12|KJ966178|ZMUO.000841|Stricticollis_tobias
TLYLIFGAWAGMVGTSLSLLIRSELGSPGTLIGNDQIYNVIVTAHAFIMIFFMVMPIVIGGFGNWLVPLMLGAPDMAFPRMNNMSFWLLPPSLSLLIMSSIVESGAGTGWTVYPPLSSNIAHSGSSVDLAIFSLHLAGISSILGAVNFITTVINMRPVGMT
LDRMPLFVWAVVITAVLLLLSLPVLAGAITMLLTDRNLNTSFFDPAGGGDPILYQHL
>COLFD661-12|KJ963114|ZMUO.004461|Tanysphyrus_lemnae
TLYFIFGAWSGMVGTSLSMLIRTELGNPGTLIGDDQIYNVIVTAHAFIMIFFMVMPIMIGGFGNWLIPLMLGAPDMAFPRMNNMSFWLLPPSLTFLLMSSIVEKGAGTGWTVYPPLSSNIAHSGSSVDLAIFSLHMAGVSSILGAINFISTIINMKTNETS
FDQLSLFTWAVGITALLLLLSLPVLAGAITMLLTDRNINTSFFDPAGGGDPILYQHL
>COLFD304-12|KJ963761|ZMUO.004199|Hippuriphila_modeeri
TLYFLFGIWSGMIGTSLSMLIRMELGNPGSLIGNDQIYNVIVTAHAFIMIFFMVMPIMIGGFGNWLIPLMIGAPDMAFPRMNNMSFWLLPPSLFLLLMSSIVESGAGTGWTVYPPLSSNIAHEGASVDLAIFSLHLAGISSILGAINFITTIINMRPKGMTL
DRMPLFVWAVLITAILLLLSLPVLAGAITMLLTDRNLNTSFFDPAGGGDPILYQHL
>COLFD124-12|KJ966610|ZMUO.004019|Anomognathus_cuspidatus
TLYFIFGAWAGMVGTSLSLLIRAELGNPGSLIGDDQIYNVIVTAHAFIMIFFMVMPIVIGGFGNWLVPLMLGAPDMAFPRMNNMSFWLLPPSLTLLLMSSMVESGAGTGWTVYPPLSSNIAHGGSSVDLAIFSLHLAGISSILGAVNFISTVINMRSPM
MTFDRMPLFVWSVAITALLLLLSLPVLAGAITMLLTDRNLNTSFFDPAGGGDPILYQHL
>COLFE1216-13|KJ963301|ZMUO.007011|Tetartopeus_sphagnetorum
TLYFIFGAWAGMVGTSLSLLIRAELSNPGSLIGNDQIYNVIVTAHAFVMIFFMVMPIVIGGFGNWLVPLMLGAPDMAFPRMNNMSFWLLPPSLSLLLMSSMVENGAGTGWTVYPPLSSNIAHGGASVDLAIFSLHLAGISSILGAVNFITTIINMRSPG
MLYERMPLFVWSVAITALLLLLSLPVLAGAITMLLTDRNLNTSFFDPAGGGDPILYQHL
>COLFB833-12|KJ962698|ZMUO.001308|Catops_alpinus

TLYFIFGAWAGMVGTSLSLLIRAELGNPGSLIGDDQIYNVIVTAHAFVMIFFMVMPIVIGGFGNWLVPLMLGAPDMAFPRMNNMSFWLLPPSLSLLLMSSIVENGAGTGWTVYPPLSANIAHSGSSVDLAIFSLHLAGISSILGAVNFITTVINMRATG
MTLDKMPLFVWSVAITALLLLLSLPVLAGAITMLLTDRNLNTSFFDPAGGGDPILYQHL
>COLFB211-12|KJ964059|ZMUO.001541|Melanophila_acuminata
TLYFIFGAWSGMVGTALSLLIRAELGNPGALIGDDQIYNVIVTAHAFVMIFFMVMPIMMGGFGNWLVPLMLGAPDMAFPRMNNMSFWLLPPSLTLLLMSSVVENGAGTGWTVYPPLAANVAHSGASVDLAIFSLHLAGISSILGAVNFITTVINMRS
TGMTFDRMPLFVWSVAITALLLLLSLPVLAGAITMLLTDRNLNTSFFDPAGGGDPILYQHL
>COLFF205-13|KJ967199|ZMUO.005810|Chrysolina_haemoptera
TLYFIFGMWAGMVGTSLSILIRAELGNPGTLIGNDQIYNVIVTAHAFIMIFFMVMPIMIGGFGNWLVPLMLGAPDMAFPRMNNMSFWLLPPSLIFLLLSSIVENGVGTGWTVYPPLSANIAHSGSSVDLAIFSLHLAGVSSILGAINFITTIINMRPKGMK
LEQMPLFSWAVLITAILLLLSLPVLAGAITMLLTDRNLNTSFFDPASGGDPILYQHL
>COLFC710-12|KJ964876|ZMUO.003275|Agonum_ericeti
TLYFIFGAWSGMVGTSLSMLIRAELGNPGALIGDDQIYNVIVTAHAFIMIFFMVMPIMIGGFGNWLVPLMLGAPDMAFPRMNNMSFWLLPPSLTLLLMSSMVESGAGTGWTIYPPLSSGIAHAGASVDLAIFSLHLAGVSSILGAVNFITTIINMRSVG
MTFDRMPLFVWSVGITALLLLLSLPVLAGAITMLLTDRNLNTSFFDPAGGGDPILYQHL
>COLFD597-12|KJ967106|ZMUO.004397|Silpha_carinata
TLYFIFGAWAGVVGMSLSILIRMELSTPGSLLGDDQMYNVIVTAHAFIMIFFMVMPIVIGGFGNWLVPLMLGAPDMAFPRMNNMSFWLLPPSLSLLLVSSMVESGAGTGWTVYPPLSSNIAHGGSSVDLAIFSLHLAGVSSILGAVNFITTIINMRSSG
MTFDRMPLFVWSVGITALLLLLSLPVLAGAITMLLTDRNLNTSFFDPAGGGDPILYQHL
>COLFB862-12|KJ965188|ZMUO.001907|Rhagium_mordax
TLYLFFGAWSGMVGTSLSLLIRSELGNPGSLIGDDQIYNVIVTAHAFIMIFFMVMPIMIGGFGNWLVPLMLGAPDMAFPRMNNMSFWLLPPSLTLLIMSSVVESGAGTGWTVYPPLSSNIAHSGSSVDLAIFSLHLAGISSILGAVNFITTVINMRPVGM
TPDRVPLFVWAVVITAILLLLSLPVLAGAITMLLTDRNLNTSFFDPAGGGDPILYQHL
>COLFE1469-13|KJ963772|ZMUO.007264|Auleutes_epilobii
TLYFIFGSWAGTVGTSMSMLIRTELGNPGSLIGDDQIYNSIVTAHAFVMIFFMVMPILIGGFGNWLVPLMLGAPDMAFPRLNNMSFWLLPPSITLLLMSSIINKGAGTGWTVYPPLSTNIAHEGASVDLAIFSLHMAGISSILGAINFISTIMNMRPKGM
EYEIMPLFSWAVMITAILLLLSLPVLAGAITMLLTDRNVNTSFFDPAGGGDPILYQHL
>COLFB749-12|KJ962420|ZMUO.001224|Philorhizus_sigma
TLYFIFGAWAGMVGTSLSMLIRAELGNPGALIGDDQIYNVIVTAHAFIMIFFMVMPIMIGGFGNWLVPLMLGAPDMAFPRMNNMSFWLLPPSLTLLLVSSMVESGAGTGWTVYPPLSSNIAHAGASVDLAIFSLHLAGISSILGAVNFITTIINMRSIG
MTFDRMPLFVWSVGITALLLLLSLPVLAGAITMLLTDRNLNTSFFDPAGGGDPILYQHL
>COLFD156-12|KJ963704|ZMUO.004051|Polydrusus_cervinus
TLYFIFGAWSGMVGTSLSMLIRTELGNPGSLIGDDQIYNVIVTAHAFIMIFFMVMPIMIGGFGNWLVPLMLGAPDMAFPRLNNMSFWLLPPSLTLLLMSSIVDKGAGTGWTVYPPLSTNIAHEGSSVDLAIFSLHMAGVSSILGAINFISTIINMRPTGM
SYDRMPLFVWAVKITAILLLLSLPVLAGAITMLLTDRNINTSFFDPAGGGDPILYQHL
>COLFF130-13|KJ963579|ZMUO.003764|Cercyon_bifenestratus
TLYFIFGAWAGMVGTSLSILIRAELGNPGTLIGDDQIYNVIVTAHAFIMIFFMVMPIMIGGFGNWLVPLMLGAPDMAFPRMNNMSFWLLPPSLTLLLMSSMVESGAGTGWTVYPPLSSNIAHGGASVDLAIFSLHLAGISSILGAVNFITTVINMRSPN
LSYDRLPLFVWSVAITALLLLLSLPVLAGAISMLLTDRNLNTSFFDPAGGGDPILYQHL
>COLFB573-12|KJ964092|ZMUO.001048|Ochthebius_minimus
TLYFIFGAWAGMVGTSLSILIRAELGNPGTLIGDDQIYNVIVTAHAFIMIFFMVMPIMIGGFGNWLVPLMLGAPDMAFPRMNNMSFWLLPPSLSLLLLSSMVENGAGTGWTVYPPLSSNIAHGGASVDLAIFSLHLAGISSILGAVNFITTVINMRSAG
MSFDRMPLFVWAVAITALLLLLSLPVLAGAITMLLTDRNLNTSFFDPAGGGDPILYQHL
>COLFD061-12|KJ962122|ZMUO.003956|Bisnius_subuliformis
TLYFIFGSWAGMVGTSLSLLIRAELGNPGSFIGDDQIYNVIVTAHAFIMIFFMVMPIVIGGFGNWLVPLMLGAPDMAFPRMNNMSFWLLPPSLTLLLMSSMVENGAGTGWTVYPPLSSNIAHSGASVDLAIFSLHLAGISSILGAVNFITTVINMRSTGV
TFDRMPLFVWSVAITALLLLLSLPVLAGAITMLLTDRNLNTSFFDPAGGGDPILYQHL
>COLFF515-13|KJ963459|ZMUO.006595|Haliplus_lineolatus
TLYFIFGAWAGMVGTSLSMLIRAELGTPGSLIGDDQIYNVIVTAHAFIMIFFMVMPIMIGGFGNWLVPLMLGAPDMAFPRMNNMSFWLLPPSLSLLLMSSMVENGAGTGWTVYPPLSAGIAHSGASVDLAIFSLHLAGISSILGAVNFITTIINMRSIG
MTFDRMPLFVWSVGITALLLLLSLPVLAGAITMLLTDRNLNTSFFDPAGGGDPILYQHL
>COLFB331-12|KJ964347|ZMUO.001661|Stenus_geniculatus
TLYFILGAWAGLTGTSLSLLIRTELGSPGSFIGDDQIYNVIVTAHAFIMIFFMVMPIMIGGFGNWLIPLMLGAPDMAFPRMNNMSFWLLPPSLSLLLMSSIVESGAGTGWTVYPPLSSNIAHSGASVDLTIFSLHLAGISSILGAMNFITTIFNMRTMKLQL
DCLPLFIWSVNVTTFLLLLSLPVLAGAITMLLTDRNINTSFFDPGGGGDPILYQHL
>COLFG082-13|KJ963023|ZMUO.007587|Platystomos_albinus
TLYFIFGAWAGMMGTSLSILIRTELGTPSSLIGDDQIYNVIVTAHAFVMIFFMVMPTMIGGFGNWLVPLMLGAPDMAFPRMNNMSFWLLPPSLILLTMSSIVEKGAGTGWTVYPPLASNIAHSGASVDLAIFSLHLAGVSSILGAVNFITTIINMRPEG
MTPDRLPLFVWAVGITALLLLLSLPVLAGAITMLLTDRNLNTTFFDPAGGGDPILYQHL
>COLFC368-12|KJ963653|ZMUO.002553|Atomaria_turgida
TLYFIFGAWSGMVGTSLSMLIRTELGTPGSLIGDDQIYNVIVTAHAFIMIFFMVMPMMIGGFGNWLVPLMLGAPDMAFPRLNNMSFWLLPPSLMFLLMSSIVEKGAGTGWTVYPPLSANVAHAGSSVDLAIFSLHLAGISSILGSVNFITTVINMRPE
GMKFDRLPLFVWAVKITTILLLLSLPVLAGAITMLLTDRNMNTSFFDPAGGGDPILYQHL

>COLFE450-12|KJ963640|ZMUO.005010|Brachygluta_fossulata
TLYLIFGAWAGMIGTSLSILIRAELGNPGSLIGDDQIYNVIVTAHAFIMIFFMVMPVMIGGFGNWLVPLMLGAPDMAFPRMNNMSFWLLPPSLILLLMSSLVESGTGTGWTVYPPLSSNIAHSGSSVDLTIFSLHLAGISSILGAVNFITTVINMRTMNM
KFDQLPLFVWSVVITALLLLLSLPVLAGAITMLLTDRNLNTSFFDPAGGGDPILYQHL
>COLFE202-12|KJ963842|ZMUO.005047|Notiophilus_aquaticus
TLYFIFGAWSGMVGTSLSMLIRAELGNPGSLIGDDQIYNVIVTAHAFVMIFFMVMPIMIGGFGNWLVPLMLGAPDMAFPRMNNMSFWLLPPSLTLLLTSSMVESGAGTGWTVYPPLSSGIAHSGASVDLAIFSLHLAGVSSILGAVNFITTIINMRSVG
MTFDRMPLFVWSVGITALLLLLSLPVLAGAITMLLTDRNLNTSFFDPAGGGDPILYQHL
>COLFA532-12|KJ964255|ZMUO.000667|Cetonischema_aeruginosa
TLYFLFGSWAGMVGTSLSLLIRAELGNPGSLIGDDQIYNVIVTAHAFIMIFFMVMPIMIGGFGNWLVPLMLGAPDMAFPRMNNMSFWLLPPSLTLLLMSSMVESGAGTGWTVYPPLSSNIAHSGASVDLAIFSLHLAGISSILGAVNFITTVINMRSTG
MTFDRMPLFVWSVALTALLLLLSLPVLAGAITMLLTDRNINTSFFDPAGGGDPILYQHL
>COLFE1535-13|KJ963406|ZMUO.007425|Oedemera_virescens
TLYLIFGAWAGMVGTSLSLLIRAELGNPGSLISDDQIYNVIVTAHAFIMIFFMVMPIVIGGFGNWLVPLMLGAPDMAFPRMNNMSFWLLPPSLTLLIMSSMVESGAGTGWTVYPPLSSNIAHSGSSVDLAIFSLHLAGVSSILGAVNFITTVINMRPVG
MTLDRMPLFVWAVVITAILLLLSLPVLAGAITMLLTDRNLNTSFFDPAGGGDPILYQHL
>COLFE1341-13|KJ964424|ZMUO.007136|Kateretes_pusillus
TLYFIFGAWSGMIGTSLSILIRSELGNPGSLIGDDQIYNVIVTAHAFVMIFFMVMPFMIGGFGNWLVPLMLGAPDMAFPRMNNMSFWLLPPSLSLLLMSSIVESGAGTGWTVYPPLSSNIAHGGSSVDLAIFSLHLAGISSILGAVNFITTVINMRPLGM
TFDRMPLFVWAVAITALLLLLSLPVLAGAITMLLTDRNLNTSFFDPAGGGDPVLYQHL
>COLFB860-12|KJ966920|ZMUO.001905|Brachyta_interrogationis
TLYFIFGAWAGMVGTSLSMLIRSELGNPGSLIGDDQIYNVIVTAHAFVMIFFMVMPIMIGGFGNWLVPLMLGAPDMAFPRMNNMSFWLLPPSLILLIMSSVVESGAGTGWTVYPPLASNIAHSGSSVDLAIFSLHLAGISSILGAVNFITTVINMRPLG
MTPDRMPLFVWSVVITAILLLLSLPVLAGAITMLLTDRNLNTSFFDPAGGGDPILYQHL
>COLFB049-12|KJ965134|ZMUO.001379|Leiodes_obesa
TLYFIFGAWSGMVGTSLSILIRAELGNPGSLIGDDQVYNVIVTAHAFIMIFFMVMPIVIGGFGNWLVPLMLGAPDMAFPRMNNMSFWLLPPSLSLLLMSSIVENGAGTGWTVYPPLSSNIAHSGSSVDLAIFSLHLAGISSILGAVNFITTVINMRPAGM
SFDKMPLFVWSVAITALLLLLSLPVLAGAITMLLTDRNLNTSFFDPAGGGDPILYQHL
>COLFF066-13|KJ967184|ZMUO.005766|Hydroglyphus_geminus
TLYFLFGAWSGMVGTSLSMLIRAELGNPGSLIGDDQIYNVIVTAHAFIMIFFMVMPIMIGGFGNWLVPLMLGAPDMAFPRMNNMSFWLLPPSLSLLMMSSMVESGAGTGWTVYPPLSSGIAHGGASVDLAIFSLHLAGISSILGAVNFITTIINMRSV
GMAFDRMPLFVWSVGITALLLLLSLPVLAGAITMLLTDRNLNTSFFDPAGGGDPILYQHL
>COLFE871-13|KJ963043|ZMUO.006191|Mordella_aculeata
TLYFIFGAWAGMLGTSLSLLIRAELGNPGSLIGDDQVYNTIVTAHAFIMIFFMVMPIMVGGFGNWLVPLMLGAPDMAFPRMNNMSFWLLPPSLSLLLMSSIVENGAGTGWTVYPPLSANIAHGGSSVDLAIFSLHLAGVSSILGAINFISTMINMRPS
GMTLDRMPLFAWAIMITAVLLLLSLPVLAGAITMLLTDRNLNTSFFDPVGGGDPILYQHL
>COLFB399-12|KJ964557|ZMUO.001824|Pterostichus_strenuus
TLYFIFGAWAGMVGTSLSMLIRAELGNPGSLIGDDQIYNVIVTAHAFVMIFFMVMPIMIGGFGNWLVPLMLGAPDMAFPRMNNMSFWLLPPSLTLLLMSSMVESGAGTGWTVYPPLSSGIAHAGASVDLAIFSLHLAGISSILGAVNFITTIINMRSIG
MTFDRMPLFVWSVGITALLLLLSLPVLAGAITMLLTDRNLNTSFFDPAGGGDPILYQHL
>COLFC763-12|KJ965050|ZMUO.003328|Ropalodontus_strandi
TLYLIFGAWSGLLGTSMSMMIRAELGTTGSLIGNDQIYNVIVTAHAFIMIFFMVMPIMIGGFGNWLVPLMLGAPDMAFPRMNNMSFWLLPPSLSLIMMSSMVESGAGTGWTVYPPLSSNTAHAGSSVDLAIFSLHLAGISSILGAVNFISTIINMRPM
GMTLDRMPLFVWAVFITAFLLLLSLPVLAGAITMLLTDRNFNTSFFDPLGGGDPILYQHL
>COLFF981-13|KJ964290|ZMUO.007346|Adalia_bipunctata
TLYFLFGLWAGMVGTSLSIIIRLELGTTNSLIGNDQIYNVIVTAHAFIMIFFMVMPIMIGGFGNWLVPLMIGAPDMAFPRLNNMSFWLLPPALTLLISSSVIEMGAGTGWTVYPPLSSNMAHNGPSVDLVIFSLHLAGISSILGAVNFISTIMNMRPNGM
NLDKTPLFVWSVLITAILLLLSLPVLAGAITMLLTDRNINTSFFDPTGGGDPVLYQHL
>COLFB235-12|KJ965144|ZMUO.001565|Rhagium_inquisitor
TLYFIFGAWSGMVGTSLSLLIRTELGNPGSLIGDDQIYNVIVTAHAFIMIFFMVMPIMIGGFGNWLVPLMLGAPDMAFPRMNNMSFWLLPPSLTLLIMSSVVENGAGTGWTVYPPLSSNIAHSGSSVDLAIFSLHLAGISSILGAVNFITTVINMRPIGM
TPDRVPLFVWAVVITAILLLLSLPVLAGAITMLLTDRNLNTSFFDPAGGGDPILYQHL
>COLFD070-12|KJ964348|ZMUO.003965|Lordithon_lunulatus
TLYFIFGSWAGMVGTSLSLLIRAELGNPGSLIGDDQIYNVIVTAHAFVMIFFMVMPIVIGGFGNWLVPLMLGAPDMAFPRMNNMSFWLLPPSLTLLLMSSMVESGAGTGWTVYPPLSSNIAHGGASVDLAIFSLHLAGISSILGAVNFITTVINMRSTG
MTFDRMPLFIWSVAITALLLLLSLPVLAGAITMLLTDRNLNTSFFDPAGGGDPILYQHL
>COLFC795-12|KJ965433|ZMUO.003360|Quedius_boops
TLYFIFGAWAGMVGTSLSLLIRAELGNPGSLIGDDQIYNVIVTAHAFIMIFFMVMPTLIGGFGNWLVPLMLGAPDMAFPRMNNMSFWLLPPSLSLLLMSSMVESGAGTGWTVYPPLSSNIAHSGASVDLAIFSLHLAGISSILGAVNFITTVINMRSIGM
TFDRMPLFVWSVAITALLLLLSLPVLAGAITMLLTDRNLNTTFFDPAGGGDPILYQHL
>COLFB016-12|KJ964058|ZMUO.001346|Laccobius_minutus

TLYFLFGAWAGMVGTSLSILIRAELGNPGTLIGDDQIYNVIVTAHAFIMIFFMVMPIMIGGFGNWLVPLMLGAPDMAFPRMNNMSFWLLPPSLTLLLMSSMVESGAGTGWTVYPPLSSNIAHGGASVDLAIFSLHLAGISSILGAVNFITTVINMRSNN
MTYDRLPLFVWSVAITALLLLLSLPVLAGAITMLLTDRNLNTSFFDPAGGGDPILYQHL
>COLFB873-12|KJ966718|ZMUO.001918|Lamia_textor
TLYFLFGAWAGMMGTSLSILIRTELGSPGSLIGDDQIYNVIVTAHAFIMIFFMVMPIMIGGFGNWLVPLMLGAPDMAFPRMNNMSFWLLPPSLFLLIMSSIVENGAGTGWTVYPPLAANVAHNGASVDLAIFSLHLAGISSILGAVNFITTVINMRPSG
MNMDRMPLFVWSVKITAILLLLSLPVLAGAITMLLTDRNLNTSFFDPAGGGDPILYQHL
>COLFD708-12|KJ963677|ZMUO.004698|Platynus_assimilis
TLYFIFGAWAGMVGTSLSMLIRAELGNPGSLIGDDQIYNVIVTAHAFIMIFFMVMPIMIGGFGNWLVPLMLGAPDMAFPRMNNMSFWLLPPSLTLLLMSSMVESGAGTGWTVYPPLSSGIAHAGASVDLAIFSLHLAGVSSILGAVNFITTIINMRSV
GMTFDRMPLFVWSVGITALLLLLSLPVLAGAITMLLTDRNLNTSFFDPAGGGDPILYQHL
>COLFC735-12|KJ967442|ZMUO.003300|Lilioceris_merdigera
TLYFIFGAWSGMVGTSLSMLIRTELGNPGSLIGNDQIYNVIVTAHAFIMIFFMVMPIMIGGFGNWLVPLMLGAPDMAFPRMNNMSFWLLPPSLTLLLMSSIVENGAGTGWTVYPPLSANLAHNGASVDLAIFSLHLAGISSILGAVNFITTVMNMRPE
GMKMDRTPLFVWAVLITAILLLLSLPVLAGAITMLLTDRNLNTSFFDPASGGDPILYQHL
>COLFC165-12|KJ964582|ZMUO.002350|Acrotona_pseudotenera
TLYFVFGAWAGMVGTSLSLLIRAELGNPGSLIGDDQIYNVIVTAHAFVMIFFMVMPIVIGGFGNWLVPLMLGAPDMAFPRMNNMSFWLLPPSLTLLLMSSMVESGAGTGWTVYPPLSSNIAHGGSSVDLAIFSLHLAGISSILGAVNFISTVINMRSTG
ISFDRMPLFVWSVAITALLLLLSLPVLAGAITMLLTDRNLNTSFFDPAGGGDPILYQHL
>COLFE548-13|KJ967142|ZMUO.005393|Hypera_pollux
TLYFIFGTWAGTVGTSLSILIRTELGNPGSLIGNDQIYNTIVTAHAFIMIFFMVMPIMIGGFGNWLVPLMLGAPDMAFPRLNNMSFWLLPPSLSLLLMSSMVDSGAGTGWTVYPPLSSNIAHEGSSVDLAIFSLHMAGVSSILGAINFISTVLNMRPSGM
SLDKMALFIWAVKITAILLLLSLPVLAGAITMLLTDRNINTSFFDPAGGGDPILYQHL
>COLFG178-13|KJ963453|ZMUO.007873|Orthoperus_corticalis
TLYFLFGMWAGLVGTSLSLLIRLELGSPGSLIGDDQIYNVIVTAHAFIMIFFMVMPIMIGGFGNWLVPLMLGAPDMAFPRMNNMSFWLLPPSLTLLILSSIVESGVGTGWTVYPPLSANIAHNGSSVDLAIFSLHLAGISSILGAINFISTIINMRPFGMKFE
QMPLFVWSVLLTAILLLLSLPVLAGAITMLLTDRNLNTSFFDPMGGGDPILYQHL
>COLFF1021-13|KJ965011|ZMUO.007386|Cassida_nobilis
TLYFIFGFWSGMVGTSLSMLIRMELGSPGSLIGNDQIYNSIVTAHAFIMIFFMVMPIMIGGFGNWLVPLMLGAPDMAFPRLNNMSFWLLPPSITFLIMSSIIESGAGTGWTVYPPLSANIAHSGASVDLAIFSLHLAGISSILGAINFISTIMNMRPSGMT
LEKMTLFVWAVIITAVLLILSLPVLAGAITMLLTDRNINTSFFDPAGGGDPILYQHL
>COLFB244-12|KJ967517|ZMUO.001574|Pterostichus_crenatus
TLYFIFGAWSGMVGTSLSMLIRAELGNPGSLIGDDQIYNVIVTAHAFVMIFFMVMPIMIGGFGNWLVPLMLGAPDMAFPRMNNMSFWLLPPSLTLLLMSSMVESGAGTGWTVYPPLSSGIAHAGASVDLAIFSLHLAGISSILGAVNFITTILNMRST
GMTFDRMPLFVWSVGITALLLLLSLPVLAGAITMLLTDRNLNTSFFDPAGGGDPILYQHL
>COLFF077-13|KJ965799|ZMUO.005777|Oreodytes_alpinus
TLYFLFGAWSGMVGTSLSMLIRAELGNPGSLIGDDQIYNVIVTAHAFIMIFFMVMPIMIGGFGNWLVPLMLGAPDMAFPRMNNMSFWLLPPSLTLLLMSSMVESGAGTGWTVYPPLSAGIAHGGASVDLAIFSLHLAGISSILGAVNFITTIINMRSV
GMTFDRMPLFVWSVGITALLLLLSLPVLAGAITMLLTDRNLNTSFFDPAGGGDPILYQHL
>COLFF112-13|KJ964431|ZMUO.003746|Colon_serripes
TLYFIFGAWAGMVGTSLSLLIRAELGNPGSLIGDDQIYNVIVTAHAFIMIFFMVMPIVIGGFGNWLVPLMLGAPDMAFPRMNNMSFWLLPPSLTLLLMSSLVESGAGTGWTVYPPLSSNIAHGGSSVDLAIFSLHLAGISSILGAVNFITTIINMRSQGM
GFDQMPLFVWSVIITAFLLLLSLPVLAGAITMLLTDRNLNTSFFDPAGGGDPILYQHL
>COLFE792-13|KJ963135|ZMUO.005637|Hydroporus_palustris
TLYFLFGAWSGMVGTSLSMLIRAELGNPGSLIGDDQIYNVIVTAHAFIMIFFMVMPIMIGGFGNWLVPLMLGAPDMAFPRMNNMSFWLLPPSLTLLLMSSMVENGAGTGWTVYPPLSSGIAHSGASVDLAIFSLHLAGVSSILGAVNFITTIINMRSI
GMTFDRMPLFVWSVGITALLLLLSLPVLAGAITMLLTDRNLNTSFFDPAGGGDPILYQHL
>COLFA256-12|KJ966309|ZMUO.000391|Ahasverus_advena
TLYFIFGSWAGMVGTSLSLLIRTELGTPNALIGDDQIYNTIVTAHAFIMIFFMVMPIMIGGFGNWLVPLMLGAPDMAFPRLNNMSFWLLPPSLTLLIMSSIVEKGAGTGWTVYPPLSSNIAHNGTSVDLAIFSLHLAGISSILGAINFISTIFNIQPNKMKM
DQIPLFCWAVLITAVLLLLSLPVLAGAITMLLTDRNLNTSFFDPSGGGDPILYQHL
>COLFC380-12|KJ965654|ZMUO.002565|Sciodrepoides_fumatus
TLYFIFGAWAGMVGTSLSLLIRAELGNPGSLIGDDQIYNVIVTAHAFVMIFFMVMPIVIGGFGNWLVPLMLGAPDMAFPRMNNMSFWLLPPSLSLLLMSSIVENGAGTGWTVYPPLSANIAHSGSSVDLAIFSLHLAGISSILGAVNFITTVINMRSTG
MSLDKMPLFVWSVAITALLLLLSLPVLAGAITMLLTDRNLNTSFFDPAGGGDPILYQHL
>COLFE1018-13|KJ962887|ZMUO.006813|Calodera_aethiops
TLYFIFGAWAGMIGTSLSLLIRAELGNPGSLIGDDQIYNVIVTAHAFVMIFFMVMPIVIGGFGNWLIPLMLGAPDMAFPRMNNMSFWLLPPSLSLLLMSSMVESGAGTGWTVYPPLSSNIAHGGSSVDLAIFSLHLAGISSILGAVNFISTIINMRTSGMS
FDRMPLFVWSVAITALLLLLSLPVLAGAITMLLTDRNLNTSFFDPAGGGDPILYQHL
>COLFB580-12|KJ964508|ZMUO.001055|Anacaena_lutescens
TLYFIFGAWAGMVGTSLSILIRAELGNPGTLIGDDQIYNVIVTAHAFIMIFFMVMPIMIGGFGNWLVPLMLGAPDMAFPRMNNMSFWLLPPSLTLLLMSSMVENGAGTGWTVYPPLSSNIAHGGASVDLAIFSLHLAGISSILGAVNFITTVINMRSES
MTYDRLPLFVWSVAITALLLLLSLPVLAGAITMLLTDRNLNTSFFDPAGGGDPILYQHL

>COLFD700-12|KJ964618|ZMUO.004690|Atheta_hygrotopora
TLYFIFGAWAGMVGTSLSLLIRAELGNPGSLIGDDQIYNVIVTAHAFIMIFFMVMPIVIGGFGNWLVPLMLGAPDMAFPRMNNMSFWLLPPSLTLLLMSSMVESGAGTGWTVYPPLSSNIAHGGSSVDLAIFSLHLAGISSILGAVNFISTVINMRSAGI
SFDRMPLFVWSVVITALLLLLSLPVLAGAITMLLTDRNLNTSFFDPAGGGDPILYQHL
>COLFF203-13|KJ963227|ZMUO.005808|Rhynchaenus_iota
TLYFMFGAWSGMVGTSLSMLIRTELGTPGKLIGNDQIYNTIVTAHAFIMIFFMVMPIMIGGFGNWLVPLMLGAPDMAFPRLNNMSFWLLPPSLTLLLSSIMNKGAGTGWTVYPPLSSNVAHEGSSVDLAIFSLHMAGISSILGAINFISTVANMRPK
GMNTDRMSLFVWAVKITAILLLLSLPVLAGAITMLLTDRNINTSFFDPAGGGDPILYQHL
>COLFA685-12|KJ967432|ZMUO.000820|Anthicus_flavipes
TLYLIFGAWAGMVGTSLSLLIRSELGNPGTLIGNDQIYNVIVTAHAFIMIFFMVMPIVIGGFGNWLVPLMLGAPDMAFPRMNNMSFWLLPPSLSLLIMSSIVESGAGTGWTVYPPLSANIAHSGSSVDLAIFSLHLAGVSSILGAVNFITTVINMRPVGM
TLDRMPLFVWAVVITAVLLLLSLPVLAGAITMLLTDRNLNTSFFDPAGGGDPILYQHL
>COLFE787-13|KJ961854|ZMUO.005632|Stenus_nitens
TLYFIFGAWAGMVGTSLSLLIRAELGNPGSLIGDDQIYNVIVTAHAFVMIFFMVMPIMIGGFGNWLVPLMLGAPDMAFPRMNNMSFWLLPPSLSLLLMSSIVENGAGTGWTVYPPLSSNIAHSGASVDLAIFSLHLAGISSILGAINFITTIFNMRTMKL
QLDCLPLFVWSVAITALLLLLSLPVLAGAITMLLTDRNLNTSFFDPAGGGDPILYQHL
>COLFE1307-13|KJ965289|ZMUO.007102|Philonthus_micans
TLYFIFGSWAGMVGTSLSLLIRAELGNPGTLIGDDQIYNVIVTAHAFIMIFFMVMPIVIGGFGNWLVPLMLGAPDMAFPRMNNMSFWLLPPSLTLLLMSSMVESGAGTGWTVYPPLSSNIAHGGASVDLAIFSLHLAGISSILGAVNFITTVINMRSSG
MTFDRMPLFVWSVAITALLLLLSLPVLAGAITMLLTDRNLNTTFFDPAGGGDPILYQHL
>COLFE1080-13|KJ964522|ZMUO.006875|Epuraea_terminalis
TLYFIFGAWSGMVGTSLSILIRTELGSPGSLIGNDQIYNVIVTAHAFIMIFFMVMPFMIGGFGNWLVPLMLGAPDMAFPRMNNMSFWLLPPSLSLLLMSSIVESGAGTGWTVYPPLSSNIAHGGSSVDLAIFSLHLAGISSILGAVNFITTIINMRPVGMT
LDRMPLFVWSVVITAVLLLLSLPVLAGAITMLLTDRNLNTTFFDPSGGGDPILYQHL
>COLFA488-12|KJ965903|ZMUO.000528|Hylastes_opacus
TLYFIFGGWSGMVGTSLSMLIRAELGTPGSLIGDDQIYNTMVTSHAFIMIFFMVMPIMIGGFGNWLIPLMLGAPDMAFPRLNNMSFWLLPPSITLLLMSSIIDKGAGTGWTVYPPLASNISHEGASVDLAIFSLHMAGISSILGAMNFISTIINMYPVGM
KPDQLSLFTWSVKITAILLLLSLPVLAGAITMLLTDRNINTTFFDPAGGGDPILYQHL
>COLFD891-12|KJ964834|ZMUO.004501|Thanasimus_formicarius
TLYFIFGAWAGMVGTSLSLLIRSELGTPGTLIGNDQIYNVIVTAHAFIMIFFMVMPIMIGGFGNWLVPLMLGAPDMAFPRMNNMSFWLLPPSLTLLLMSSMVENGAGTGWTVYPPLSSNIAHGGSSVDLAIFSLHLAGVSSILGAVNFITTVINMRPEG
MTLDRMPLFVWAVAITALLLLLSLPVLAGAITMLLTDRNLNTSFFDPAGGGDPILYQHL
>COLFD611-12|KJ964637|ZMUO.004411|Otiorhynchus_ligustici
TLYFIFGSWSGMVGTSLSMLIRTELGNPGSLIGDDQIYNMIVTAHAFIMIFFMVMPMMIGGFGNWLVPLMLGAPDMAFPRLNNMSFWLLPPSLSLLLMSSIIDKGAGTGWTVYPPLSSNIAHGGTSVDLAIFSLHMAGMSSILGAINFISTAINMRPG
GMSPDRMTLFIWAVKITAILLLLSLPVLASAITMLLTDRNINTSFFDPAGGGDPILYQHL
>COLFD722-12|KJ966129|ZMUO.004712|Creophilus_maxillosus
TLYFIFGAWAGMVGTSLSLLIRAELGNPGTLIGDDQIYNVIVTAHAFIMIFFMVMPIVIGGFGNWLVPLMLGAPDMAFPRMNNMSFWLLPPSLTLLLMSSMVESGAGTGWTVYPPLSSNIAHGGASVDLAIFSLHLAGISSILGAVNFITTVINMRSTG
MTFDRMPLFVWSVAITALLLLLSLPVLAGAITMLLTDRNLNTTFFDPAGGGDPILYQHL
>COLFE876-13|KJ964133|ZMUO.006196|Platycis_minuta
TMYFIFGAWAGMMGTSLSLLIRVELGSPNSVIGNDQIYNVIVTAHAFIMIFFMVMPMMIGGFGNWLVPLMLGAPDMAFPRMNNMSFWLLPPSITFLMMSSMVESGAGTGWTVYPPLSSNIAHSGSSVDLAIFSLHLAGISSILGAVNFISTIINMKS
PNMTSDQMPLFVWSVGITALLLLLSLPVLAGAITMLLTDRNLNTTFFDPTGGGDPILYQHL
>COLFF026-13|KJ962133|ZMUO.005726|Tachyporus
TLYFIFGAWSGMVGTSLSLLIRAELGNPGSLIGDDQIYNVIVTAHAFIMIFFMVMPIVIGGFGNWLVPLMLGAPDMAFPRMNNMSFWLLPPSLTLLLMSSMVESGAGTGWTVYPPLSANIAHSGASVDLAIFSLHLAGISSILGAVNFITTVINMRASG
MHFDRMPLFIWSVAITALLLLLSLPVLAGAITMLLTDRNLNTSFFDPAGGGDPILYQHL
>COLFF758-13|KJ963565|ZMUO.006458|Gnatocerus_cornutus
TLYFIFGAWSGMVGTSLSLLIRAELGNPGSLIGDDQIYNVIVTAHAFIMIFFMVMPIMIGGFGNWLVPLMLGAPDMAFPRMNNMSFWLLPPSLTLLLMSSIVENGAGTGWTVYPPLSSNIAHGGSSVDLAIFSLHLAGISSILGAVNFITTVINMRPQG
MSFDRMPLFVWAVVITAVLLLLSLPVLAGAITMLLTDRNINTSFFDPAGGGDPILYQHL
>COLFE328-12|KJ967457|ZMUO.004888|Notiophilus_biguttatus
TLYFIFGAWSGMVGTSLSMLIRAELGNPGSLIGDDQIYNVIVTAHAFVMIFFMVMPIMIGGFGNWLVPLMLGAPDMAFPRMNNMSFWLLPPSLTLLLTSSMVESGAGTGWTVYPPLSSGIAHSGASVDLAIFSLHLAGVSSILGAVNFITTIINMRSVG
MSFDRMPLFVWSVGITALLLLLSLPVLAGAITMLLTDRNLNTSFFDPAGGGDPILYQHL
>COLFC758-12|KJ965985|ZMUO.003323|Cis_glabratus
TLYFIFGAWSGMLGTSMSILIRSELSTPGSLIGNDQIYNVIVTAHAFIMIFFMVMPVMIGGFGNWLVPLMLGAPDMAFPRMNNMSFWLLPPSLSLLIMSSIVENGAGTGWTVYPPLSSNTAHSGSSVDLAIFSLHLAGISSILGAVNFISTIINMHPSGM
KPDQMPLFVWAVIITAVLLLLSLPVLAGAITMLLTDRNLNTSFFDPAGGGDPILYQHL
>COLFF481-13|KJ963425|ZMUO.006561|Carcinops_pumilio

TLYFIFGAWAGMVGTSLSLLIRAELGNPGTLIGDDQIYNVIVTAHAFIMIFFMVMPIMIGGFGNWLVPLMLGAPDMAFPRMNNMSFWLLPPSLSLLLLSSVVENGAGTGWTVYPPLSSNLAHNGSSVDLAIFSLHLAGISSILGAVNFITTIINMRAMG
MTFDRMPLFVWSVGITALLLLLSLPVLAGAITMLLTDRNLNTSFFDPAGGGDPILYQHL
>COLFC211-12|KJ965885|ZMUO.002396|Atheta_picipes
TLYFIFGAWAGMVGTSLSLLIRAELGNPGSLIGDDQIYNVIVTAHAFVMIFFMVMPIVIGGFGNWLVPLMLGAPDMAFPRMNNMSFWLLPPSLTLLLMSSMVESGAGTGWTVYPPLSSNIAHGGSSVDLAIFSLHLAGISSILGAVNFISTVINMRSTGI
TFDRMPLFVWSVAITALLLLLSLPVLAGAITMLLTDRNLNTSFFDPAGGGDPILYQHL
>COLFA107-10|HQ559233|MP00368|Spavius_glaber
TLYFIFGAWASMVGTSLSLLIRSELGTPGSLIGDDQIYNVIVTAHAFVMIFFMVMPIMIGGFGNWLVPLMLGAPDMAFPRMNNMSFWLLPPSLSLLLMSSIAEKGVGTGWTVYPPLSSNIAHGGSSVDLAIFSLHLAGISSILGAVNFISTVMNMNPTG
MTMDRMPLFIWAVMITAILLLLSLPVLAGAITMLLTDRNLNTSFFDPAGGGDPILYQHL
>COLFF510-13|KJ962414|ZMUO.006590|Limnius_volckmari
TLYFILGSWSGMLGMALSLLIRAELGTPGSLIGDDQIYNVIVTAHAFIMIFFMVMPIMIGGFGNWLVPLMLGAPDMAFPRMNNMSFWLLPPSLSLLLMSSLVESGVGTGWTVYPPLSANIAHSGSSVDLAIFSLHLAGISSILGAVNFITTVINMRSPGM
TFDRMPLFVWSVAITALLLLLSLPVLAGAITMLLTDRNLNTSFFDPAGGGDPILYQHL
>COLFE1346-13|KJ961942|ZMUO.007141|Phyllobius_maculicornis
TLYFIFGAWSGMIGTSLSILIRVELGNPGSLIGDDQIYNVIVTAHAFIMIFFMVMPMMIGGFGNWLVPLMLGAPDMAFPRLNNMSFWLLPPSLSLLLMSSIVDKGAGTGWTVYPPLSANIAHEGSSVDLAIFSLHMAGVSSILGAINFISTVINMRPSG
MSPDRMSLFIWAVKITAILLLLSLPVLAGAITMLLTDRNMNTSFFDPAGGGDPILYQHL
>COLFG168-13|KJ963760|ZMUO.007863|Pityophthorus_micrographus
TLYFIFGAWSGMVGTSLSMLIRTELGCPGSLIGDDQIYNTIVTAHAFIMIFFMVMPIMMGGFGNWLVPLMLGAPDMAFPRLNNMSFWLLPPSLTFLIMSSIIDKGAGTGWTVYPPLSSNISHEGSSVDLAIFSLHMAGVSSILGAINFISTIINMHPNGM
KPEQLPLFVWAVKITAILLLLSLPVLAGAITMLLTDRNVNTSFFDPAGGGDPILYQHL
>COLFE1274-13|KJ964208|ZMUO.007069|Oodes_helopioides
TLYFIFGAWSGMVGTSLSMLIRAELGNPGALIGDDQIYNVIVTAHAFIMIFFMVMPIMIGGFGNWLVPLMLGAPDMAFPRMNNMSFWLLPPSLSLLLMSSMVESGAGTGWTVYPPLSSGIAHSGASVDLAIFSLHLAGVSSILGAVNFITTIINMRSV
GMTFDRMPLFVWSVGITALLLLLSLPVLAGAITMLLTDRNLNTSFFDPAGGGDPILYQHL
>COLFE134-12|KJ966768|ZMUO.005169|Cantharis_nigra
TLYFIFGAWSGSLGLALSLLIRAELGTPGTLIGNDQIYNVIVTAHAFIMIFFMVMPIMIGGFGNWLVPLMLGAPDMAFPRMNNMSFWFLPPSLMFLLMSSMVESGAGTGWTVYPPLSANIAHSGPSVDLAIFSLHMAGISSILGAVNFISTIMNMKPPS
MKFDQMPLFVWSVGITALLLLLSLPVLAGAITMLLSDRNLNTSFFDPMGGGDPILYQHL
>COLFA460-12|KJ966154|ZMUO.000500|Lilioceris_merdigera
TLYFIFGAWSGMVGTSLSMLIRTELGNPGSLIGNDQIYNVIVTAHAFIMIFFMVMPIMIGSFGNWLVPLMLGAPDMAFPRMNNMSFWLLPPSLTLLLMSSIVENGAGTGWTVYPPLSANLAHNGASVDLAIFSLHLAGISSILGAVNFITTVMNMRPE
GMKMDRTPLFVWAVLITAILLLLSLPVLAGAITMLLTDRNLNTSFFDPASGGDPILYQHL
>COLFF673-13|KJ962893|ZMUO.006373|Cymbiodyta_marginella
TLYFIFGAWAGMVGTSLSILIRAELGNPGTLIGDDQIYNVIVTAHAFIMIFFMVMPIMIGGFGNWLVPLMLGAPDMAFPRMNNMSFWLLPPSLTLLLMSSMVENGAGTGWTVYPPLSSNIAHGGASVDLAIFSLHLAGISSILGAVNFITTVINMRSNN
MTYDRLPLFVWSVAITALLLLLSLPVLAGAITMLLTDRNLNTSFFDPAGGGDPILYQHL
>COLFE442-12|KJ963779|ZMUO.005002|Rybaxis_longicornis
TLYLIFGAWAGMVGTSLSILIRAELGNPGSLIGDDQIYNVIVTAHAFIMIFFMVMPIMIGGFGNWLVPLMLGAPDMAFPRMNNMSFWLLPPSLILLLMSSMVESGAGTGWTVYPPLSSNIAHSGSSVDLTIFSLHLAGISSILGAVNFITTVINMRTMN
MKFDQLPLFVWSVIITALLLLLSLPVLAGAITMLLTDRNLNTSFFDPTGGGDPVLYQHL
>COLFE1312-13|KJ963168|ZMUO.007107|Ochthebius_minimus
TLYFIFGAWAGMVGTSLSILIRAELGNPGTLIGDDQIYNVIVTAHAFIMIFFMVMPIMIGGFGNWLVPLMLGAPDMAFPRMNNMSFWLLPPSLSLLLLSSMVENGAGTGWTVYPPLSSNIAHGGASVDLAIFSLHLAGISSILGAVNFITTVINMRSAG
MSFDRMPLFVWAVAITALLLLLSLPVLAGAITMLLTDRNLNTSFFDPAGGGDPILYQHL
>COLFE378-12|KJ963036|ZMUO.004938|Lilioceris_merdigera
TLYFIFGAWSGMVGTSLSMLIRTELGNPGSLIGNDQIYNVIVTAHAFIMIFFMVMPIMIGGFGNWLVPLMLGAPDMAFPRMNNMSFWLLPPSLTLLLMSSIVENGAGTGWTVYPPLSANLTHNGASVDLAIFSLHLAGISSILGAVNFITTVMNMRPE
GMKMDRTPLFVWAVLITAILLLLSLPVLAGAITMLLTDRNLNTSFFDPASGGDPILYQHL
>COLFF063-13|KJ962079|ZMUO.005763|Cercyon_tristis
TLYFIFGAWAGMVGTSLSILIRAELGNPGTLIGDDQIYNVIVTAHAFIMIFFMVMPIMIGGFGNWLVPLMLGAPDMAFPRMNNMSFWLLPPSLTLLLMSSMVESGAGTGWTVYPPLSSNIAHGGSSVDLAIFSLHLAGISSILGAVNFITTVINMRSPNL
TYDRLPLFVWSVAITALLLLLSLPVLAGAITMLLTDRNLNTSFFDPAGGGDPILYQHL
>COLFD351-12|KJ964813|ZMUO.004246|Rhagonycha_nigriventris
TLYFIFGAWSGSLGLALSLLIRAELGTPGTLIGNDQIYNVIVTAHAFIMIFFMVMPIMIGGFGNWLVPLMLGAPDMAFPRMNNMSFWFLPPSLMFLLMSSMVESGAGTGWTVYPPLSANIAHSGPSVDLAIFSLHMAGISSILGAVNFISTILNMKPPS
MKFDQMPLFVWSVGITALLLLLSLPVLAGAITMLLSDRNLNTSFFDPMGGGDPILYQHL
>COLFE034-12|KJ965304|ZMUO.005259|Lebia_cruxminor
TLYFIFGAWAGMVGTSLSMLIRAELGNPGALIGDDQIYNVIVTAHAFIMIFFMVMPIMIGGFGNWLVPLMLGAPDMAFPRMNNMSFWLLPPSLTLLLMSSLVESGAGTGWTVYPPLSSGIAHAGASVDLAIFSLHLAGISSILGAVNFITTIINMRSVG
MSFDQMPLFVWSVGITALLLLLSLPVLAGAITMLLTDRNLNTSFFDPAGGGDPILYQHL

>COLFD666-12|KJ966803|ZMUO.004656|Pachyta_quadrimaculata
TLYFIFGAWAGMVGTSLSLLIRSELGNPGSLIGDDQIYNVIVTAHAFIMIFFMVMPIMIGGFGNWLVPLMLGAPDMAFPRMNNMSFWLLPPSLTLLIMSSIVESGAGTGWTVYPPLSSNIAHGGSSVDLAIFSLHLAGISSILGAVNFITTVINMRPVGM
TLDRMPLFVWAVVITAILLLLSLPVLAGAITMLLTDRNLNTSFFDPAGGGDPILYQHL
>COLFF480-13|KJ966374|ZMUO.006560|Atholus_duodecimstriatus
TLYFIFGAWAGMVGTSLSLLIRAELGAPGTLIGDDQIYNVIVTAHAFIMIFFMVMPIMIGGFGNWLVPLMLGAPDMAFPRMNNMSFWLLPPSLSLLMSSIVESGAGTGWTVYPPLSSNIAHGGASVDLAIFSLHLAGISSILGAINFITTVINMRSPGM
MFDQMPLFIWSVAITALLLLSLPVLAGAITMLLTDRNLNTSFFDPAGGGDPILYQHL
>COLFF814-13|KJ964931|ZMUO.006134|Epuraea_opalizans
TLYFIFGVWSGMVGTSLSILIRTELGSPGSLIGNDQIYNVIVTAHAFIMIFFMVMPFMIGGFGNWLVPLMLGAPDMAFPRMNNMSFWLLPPSLSLLMSSIVESGAGTGWTVYPPLSSNIAHGGSSVDLAIFSLHLAGVSSILGAVNFITTIINMRPAGM
TLDRMPLFVWSVVITAILLLLSLPVLAGAITMLLTDRNLNTTFFDPSGGGDPILYQHL
>COLFC588-12|KJ966737|ZMUO.003153|Malthinus_biguttatus
TLYFIFGAWSGMLGTSLSLLIRAELGNPGSLIGNDQIYNVIVTAHAFIMIFFMVMPIMIGGFGNWLVPLMLGAPDMAFPRMNNMSFWFLPPSLSLLMSSLVESGAGTGWTVYPPLSANIAHSGSSVDLAIFSLHMAGISSILGAVNFISTVINMRSVG
MSFDRMPLFVWAVAITALLLLSLPVLAGAITMLLTDRNLNTSFFDPAGGGDPILYQHL
>COLFB143-12|KJ963833|ZMUO.001473|Otiorhynchus_arcticus
TLYFILGAWSGMVGTSLSMLIRVELGTPGALIGDDQIYNVIVTAHAFIMIFFMVMPMMIGGFGNWLVPLMLGAPDMAFPRLNNMSFWLLPPSLSLLMSSIIDKGAGTGWTVYPPLSSNIAHEGASVDLAIFSLHMAGVSSILGAINFISTVVNMRPN
GMSPDRMTLFIWAVKITAILLLLSLPVLAGAITMLLTDRNVNTSFFDPAGGGDPILYQHL
>COLFF440-13|KJ966984|ZMUO.005958|Elaphrus_riparius
TLYFIFGAWSGMVGTSLSMLIRAELGNPGSLIGDDQIYNVIVTAHAFIMIFFMVMPIMIGGFGNWLVPLMLGAPDMAFPRMNNMSFWLLPPSLSLLMSSMVESGAGTGWTVYPPLSSGIAHAGASVDLAIFSLHLAGVSSILGAVNFITTIINMRSV
GMSFDRMPLFVWSVGITALLLLLSLPVLAGAITMLLTDRNLNTSFFDPAGGGDPILYQHL
>COLFD733-12|KJ964311|ZMUO.004723|Meloe_violaceus
TLYFIFGAWAGMVGTSLSLLIRAELGNPGTLIGDDQVYNVIVTAHAFIMIFFMVMPIMIGGFGNWLVPLMLGAPDMAFPRMNNMSFWLLPPSLTLLLMSSMVENGAGTGWTVYPPLSSNIAHGGSSVDLAIFSLHLAGISSILGAVNFITTVINMRPM
GMTFDRMPLFVWAVAITALLLLLSLPVLAGAITMLLTDRNLNTSFFDPAGGGDPVLYQHL
>COLFG068-13|KJ963072|ZMUO.007573|Anoplodera_maculicornis
TLYFIFGAWAGMIGTSLSLLIRSELGNPGSLIGDDQIYNVIVTAHAFVMIFFMVMPVMIGGFGNWLVPLMLGAPDMAFPRMNNMSFWLLPPSLTLLIMSSVVESGAGTGWTVYPPLSSNIAHSGSSVDLAIFSLHLAGISSILGAVNFITTVINMRPVG
MTPERMPLFVWAVVITAILLLLSLPVLAGAITMLLTDRNINTSFFDPAGGGDPILYQHL
>COLFC723-12|KJ963517|ZMUO.003288|Bledius_poppiusi
TLYFIFGAWAGMVGTSLSMLIRAELGTPGYLIGDDQIYNVVVTAHAFVMIFFMVMPIVIGGFGNWLIPLMLGAPDMAFPRMNNMSFWLLPPSLTLLLMSSMVESGAGTGWTVYPPLSSNIAHSGSSVDLAIFSLHLAGISSILGAVNFISTVINMRSIG
MTFDRMPLFVWSVKITAILLLLSLPVLAGAITMLLTDRNLNTSFFDPAGGGDPILYQHL
>COLFE228-12|KJ966893|ZMUO.005073|Laccophilus_hyalinus
TLYFIFGAWSGMVGTSLSLLIRAELGNPGSLIGDDQIYNVIVTAHAFIMIFFMVMPIMIGGFGNWLVPLMLGAPDMAFPRMNNMSFWLLPPSLSLLMSSMVENGAGTGWTVYPPLSSGIAHSGASVDLAIFSLHLAGISSILGAVNFITTIINMRSIGM
TFDRMPLFVWSVGITALLLLSLPVLAGAITMLLTDRNLNTSFFDPAGGGDPILYQHL
>COLFC200-12|KJ964314|ZMUO.002385|Atheta_cinnamoptera
TLYFIFGAWAGMVGTSLSLLIRAELGNPGSLIGDDQIYNVIVTAHAFIMIFFMVMPIVIGGFGNWLVPLMLGAPDMAFPRMNNMSFWLLPPSLTLLLMSSMVESGAGTGWTVYPPLSSNIAHGGSSVDLAIFSLHLAGISSILGAVNFISTVINMRSTGI
SFDRMPLFVWSVAITALLLLLSLPVLAGAITMLLTDRNLNTSFFDPAGGGDPILYQHL
>COLFE981-13|KJ962524|ZMUO.006776|Hydroporus_umbrosus
TLYFLFGAWSGMVGTSLSMLIRAELGNPGSFIGDDQIYNVIVTAHAFIMIFFMVMPIMIGGFGNWLVPLMLGAPDMAFPRMNNMSFWLLPPSLTLLLMSSMVENGAGTGWTVYPPLSSGIAHSGASVDLAIFSLHLAGISSILGAVNFITTIINMRSV
GMTFDRMPLFVWSVGITALLLLLSLPVLAGAITMLLTDRNLNTSFFDPAGGGDPILYQHL
>COLFE1287-13|KJ961873|ZMUO.007082|Anisosticta_novemdecimpunctata
TLYFLFGMWAGMVGTSLSMLIRLELGSAGSLIGNDQIYNVIVTAHAFIMIFFMVMPIMIGGFGNWLVPLMIGAPDMAFPRLNNMSFWLLPPSLTLLLMSSMVEMGAGTGWTVYPPLSSNLAHNGSSVDLVIFSLHLAGISSILGAVNFISTIMNMRP
MGMSLDKTPLFVWSVLITAILLLLSLPVLAGAITMLLTDRNLNTSFFDPTGGGDPILYQHL
>COLFC819-12|KJ963371|ZMUO.003384|Rhantus_suturellus
TLYFIFGAWAGMVGTSLSMLIRAELGNPGSLIGDDQIYNVIVTAHAFVMIFFMVMPIMIGGFGNWLVPLMLGAPDMAFPRMNNMSFWLLPPSLTLLLMSSMVESGAGTGWTVYPPLSSGIAHGGASVDLAIFSLHLAGISSILGAVNFITTIINMRSV
GMTFDRMPLFVWSVGITALLLLLSLPVLAGAITMLLTDRNLNTSFFDPAGGGDPILYQHL
>COLFB193-12|KJ965380|ZMUO.001523|Atomaria_zetterstedti
TLYFIFGAWAGMVGTSLSMLIRTELGTPGSLIGDDQIYNVIVTAHAFIMIFFMVMPMMIGGFGNWLVPLMLGAPDMAFPRLNNMSFWLLPPSLMFLLMSSIVEKGAGTGWTVYPPLSSNIAHAGSSVDLAIFSLHLAGISSILGSVNFITTVINMRPKG
MNFDRLPLFVWAVKITTILLLLSLPVLAGAITMLLTDRNINTSFFDPAGGGDPILYQHL
>COLFC614-12|KJ966615|ZMUO.003179|Abdera_affinis

TLYFIFGAWSGMLGTSLSLLIRSELGNPGSLIGDDQIYNVIVTAHAFIMIFFMVMPIMMGGFGNWMVPLMLGAPDMAFPRMNNMSFWLLPPSLTLLIMSSIVENGAGTGWTVYPPLSSNIAHSGSSVDLAIFSLHLAGVSSILGAVNFITTIINMRPAG
MTLDRMPLFVWSVAITAVLLLLSLPVLAGAITMLLTDRNLNTSFFDPAGGGDPILYQHL
>COLFC524-12|KJ965748|ZMUO.003089|Carabus_nitens
TLYFIFGAWSGMVGTSLSMLIRAELGNPGSLIGDDQIYNVIVTAHAFVMIFFMVMPIMIGGFGNWLVPLMLGAPDMAFPRMNNMSFWLLPPSLTLLLMSSMVEKGAGTGWTVYPPLSSGIAHSGASVDLAIFSLHLAGISSILGAVNFITTIINMRSV
GMTFDRMPLFVWSVGITALLLLLSLPVLAGAITMLLTDRNLNTSFFDPAGGGDPILYQHL
>COLFC825-12|KJ963250|ZMUO.003390|Ilybius_guttiger
TLYFIFGAWAGMVGTSLSMLIRAELGNPGSLIGDDQIYNVIVTAHAFVMIFFMVMPIMIGGFGNWLVPLMLGAPDMAFPRMNNMSFWLLPPSLSLLLMSSMVESGAGTGWTVYPPLSAGIAHSGASVDLAIFSLHLAGISSILGAVNFITTIINMRSV
GMTFDRMPLFVWSVGITALLLLLSLPVLAGAITMLLTDRNLNTSFFDPAGGGDPILYQHL
>COLFC033-12|KJ964369|ZMUO.002028|Cercyon_quisquilius
TLYFIFGAWAGMVGTSLSILIRAELGNPGTLIGDDQIYNVIVTAHAFIMIFFMVMPIMIGGFGNWLVPLMLGAPDMAFPRMNNMSFWLLPPSLTLLLMSSMVESGAGTGWTVYPPLSSNIAHGGSSVDLAIFSLHLAGISSILGAVNFITTVINMRSPNL
TYDRLPLFVWSVAITALLLLLSLPVLAGAITMLLTDRNLNTSFFDPAGGGDPILYQHL
>COLFE1459-13|KJ963352|ZMUO.007254|Hypocaccus_rugifrons
TLYFIFGMWAGMVGTSLSLLIRAELGNPGSLIGDDQIYNVIVTSHAFIMIFFMVMPIMIGGFGNWLVPLMLGAPDMAFPRMNNMSFWFLPPSLTLLLMSSMVESGAGTGWTVYPPLSSNIAHSGASVDLAIFSLHLAGISSILGAVNFITTVINMRSNG
MKFDQMPLFVWSVSITALLLLLSLPVLAGAITMLLTDRNINTSFFDPAGGGDPILYQHL
>COLFD471-12|KJ964194|ZMUO.003891|Lilioceris_lilii
TLYFIFGAWSGMVGTSLSMLIRTELGNPGSLIGNDQIYNVIVTAHAFIMIFFMVMPIMIGGFGNWLVPLMLGAPDMAFPRMNNMSFWLLPPSLTLLLMSSIVENGAGTGWTVYPPLSANLAHNGASVDLAIFSLHLAGISSILGAVNFITTIMNMRPE
GMKMDRTPLFVWAVLITAILLLLSLPVLAGAITMLLTDRNLNTSFFDPASGGDPILYQHL
>COLFE1168-13|KJ965103|ZMUO.006963|Nebrioporus_depressus
TLYFLFGAWSGMVGTSLSMLIRAELGNPGSLIGDDQIYNVIVTAHAFIMIFFMVMPIMIGGFGNWLVPLMLGAPDMAFPRMNNMSFWMLPPSLSLLLMSSMVENGAGTGWTVYPPLSAGLAHGGASVDLAIFSLHLAGISSILGAVNFITTIINMRS
VGMTFDRMPLFVWSVGITALLLLLSLPVLAGAITMLLTDRNLNTSFFDPAGGGDPILYQHL
>COLFA075-10|HM909093|MP00276|Hydraena_britteni
TLYFIFGAWAGMVGTSLSILIRAELGNPGTLIGDDQIYNVIVTAHAFIMIFFMVMPILIGGFGNWLVPLMLGAPDMAFPRMNNMSFWLLPPSLSLLLMSSMVESGAGTGWTVYPPLSSNIAHGGASVDLAIFSLHLAGISSILGAVNFITTIINMRSPGM
SFDQMPLFVWAVGITALLLLLSLPVLAGAITMLLTDRNLNTSFFDPAGGGDPILYQHL
>COLFF690-13|KJ966542|ZMUO.006390|Anoplodera_sexguttata
TLYFLFGAWASMVGTSLSLLIRSELGNPGSLIGDDQIYNVIVTAHAFVMIFFMVMPIMIGGFGNWLVPLMLGAPDMAFPRMNNMSFWLLPPSLTLLIMSSVVESGAGTGWTVYPPLSSNIAHSGSSVDLAIFSLHLAGISSILGAVNFITTVINMRPMG
MNLDRMPLFVWAVVVTAILLLLSLPVLAGAITMLLTDRNLNTSFFDPAGGGDPILYQHL
>COLFF131-13|KJ965920|ZMUO.003765|Cercyon_tristis
TLYFIFGAWAGMVGTSLSILIRAELGNPGTLIGDDQIYNVIVTAHAFIMIFFMVMPIMIGGFGNWLVPLMLGAPDMAFPRMNNMSFWLLPPSLTLLLMSSMVESGAGTGWTVYPPLSSNIAHGGSSVDLAIFSLHLAGISSILGAVNFITTVINMRSPNL
TYDRLPLFVWSVAITALLLLLSLPVLAGAITMLLTDRNLNTSFFDPAGGGDPILYQHL
>COLFC263-12|KJ963261|ZMUO.002448|Atomaria_nigrirostris
TLYFIFGAWAGMVGTSLSLLIRTELGTPGSLIGDDQIYNVIVTAHAFIMIFFMVMPMMIGGFGNWLVPLMLGAPDMAFPRLNNMSFWLLPPSLTFLLMSSLVEKGAGTGWTVYPPLSSNIAHAGASVDLAIFSLHLAGISSILGAVNFITTVINMRPKG
MNFDRLPLFVWAVKITAILLLLSLPVLAGAITMLLTDRNINTSFFDPAGGGDPILYQHL
>COLFB799-12|KJ962942|ZMUO.001274|Olophrum_boreale
TLYFIFGAWAGMVGTSLSILIRAELGNPGTLIGDDQIYNVIVTAHAFVMIFFMVMPILIGGFGNWLVPLMLGAPDMAFPRMNNMSFWLLPPSLTLLLMSSMVESGAGTGWTVYPPLSSNIAHGGASVDLAIFSLHLAGISSILGAVNFITTVINMRTTG
MTFDRMPLFVWAVAITALLLLLSLPVLAGAITMLLTDRNLNTTFFDPAGGGDPILYQHL
>COLFE313-12|KJ962026|ZMUO.004873|Serica_brunnea
TLYFLFGSWAGMVGTSLSMLIRAELGNPGSLIGDDQIYNVIVTAHAFVMIFFMVMPILIGGFGNWLVPLMLGAPDMAFPRMNNMSFWLLPPSLTLLLSSSLVESGAGTGWTVYPPLSANVAHSGASVDLAIFSLHLAGISSILGAVNFITTVINMRATGI
TFDRMPLFVWSVVITALLLLLSLPVLAGAITMLLTDRNLNTTFFDPSGGGDPILYQHL
>COLFD082-12|KJ964191|ZMUO.003977|Trypophloeus_bispinulus
TLYFIFGAWSGMVGTSMSLIIRAELGTPGNLIGNDQIYNSVVTAHAFIMIFFMVMPILIGGFGNWLVPLMLGAPDMAFPRLNNMSFWLLPPSLCFLIMSSVIDSGAGTGWTVYPPLASNIAHEGSSVDLAIFSLHMAGISSILGAMNFISTIINMSPPGM
KPEQLSLFTWAVKITAILLLLSLPVLAGAITMLLTDRNVNTSFFDPAGGGDPILYQHL
>COLFE1006-13|KJ965606|ZMUO.006801|Cryphalus_saltuarius
TLYFIFGAWSGMVGTSMSLIIRTELGTPSSLIGDDQIYNTIVTAHAFIMIFFMVMPILIGGFGNWLIPLMLGAPDMAFPRLNNMSFWLLPPSLVFLIMSSMINKGAGTGWTVYPPLASNIAHEGASVDFAIFSLHMAGISSILGAMNFISTIINMHPSGMK
PEQLSLFSWAVKITAILLLLSLPVLAGAITMLLTDRNINTSFFDPAGGGDPILYQHL
>COLFE1299-13|KJ963067|ZMUO.007094|Lathrobium_impressum
TLYFIFGAWAGMVGTSLSLLIRTELGNPGSLIGDDQIYNVIVTAHAFIMIFFMVMPIMIGGFGNWLVPLMLGAPDMAFPRMNNMSFWLLPPSLSLLLMSSLVESGAGTGWTVYPPLSSNIAHGGASVDLAIFSLHLAGISSILGAVNFITTVINMRSPG
MSYERMPLFVWAVAITALLLLLSLPVLAGAITMLLTDRNLNTSFFDPAGGGDPILYQHL

>COLFC304-12|KJ967449|ZMUO.002489|Atheta_cinnamoptera
TLYFIFGAWAGMVGTSLSLLIRAELGNPGSLIGDDQIYNVIVTAHAFIMIFFMVMPIVIGGFGNWLVPLMLGAPDMAFPRMNNMSFWLLPPSLTLLLMSSMVESGAGTGWTVYPPLSSNIAHGGSSVDLAIFSLHLAGISSILGAVNFISTVINMRSTGI
SFDRMPLFVWSVAITALLLLLSLPVLAGAITMLLTDRNLNTSFFDPAGGGDPILYQHL
>COLFC106-12|KJ962362|ZMUO.002291|Gabrius_appendiculatus
TLYFIFGSWAGMVGTSLSLLIRAELGNPGTLIGDDQIYNVIVTAHAFIMIFFMVMPIVIGGFGNWLVPLMLGAPDMAFPRMNNMSFWLLPPSLTLLLMSSMVESGAGTGWTVYPPLSSNIAHGGASVDLAIFSLHLAGISSILGAVNFITTVINMRSFG
MSFDRMPLFVWSVAITALLLLLSLPVLAGAITMLLTDRNLNTSFFDPAGGGDPILYQHL
>COLFE1145-13|KJ962348|ZMUO.006940|Notiophilus_germinyi
TLYFIFGAWSGMVGTSLSMLIRAELGNPGSLIGDDQIYNVIVTAHAFVMIFFMVMPIMIGGFGNWLVPLMLGAPDMAFPRMNNMSFWLLPPSLTLLLTSSMVESGAGTGWTVYPPLSSGIAHSGASVDLAIFSLHLAGVSSILGAVNFITTIINMRSVG
MTFDRMPLFVWSVGITALLLLLSLPVLAGAITMLLTDRNLNTSFFDPAGGGDPILYQHL
>COLFF739-13|KJ966796|ZMUO.006439|Trichosirocalus_troglodytes
TLYFIFGAWAGAAGTSLSMLIRTELGNPGSLIGNDQIYNSIVTAHAFIMIFFMVMPILIGGFGNWLVPLMLGAPDMAFPRLNNMSFWLLPPSLTLLLMSSIVNKGAGTGWTVYPPLSANVAHEGASVDLAIFSLHMAGISSILGAINFISTVMNMRPTG
MNSEFMPLFVWAVEITAVLLLLSLPVLAGAITMLLTDRNINTSFFDPAGGGDPILYQHL
>COLFC812-12|KJ966833|ZMUO.003377|Hypnoidus_rivularius
TLYFLFGAWSGMLGTSLSLLIRAELGNPGSLIGNDQIYNVIVTAHAFIMIFFMVMPIMIGGFGNWLVPLMLGAPDMAFPRMNNMSFWFLPPSLSLLLMSSIVENGAGTGWTVYPPLSANIAHSGSSVDLAIFSLHLAGISSILGAVNFISTVINMRSTGIT
FDRMPLFVWAVAITALLLLLSLPVLAGAITMLLTDRNLNTSFFDPAGGGDPILYQHL
>COLFE949-13|KJ965877|ZMUO.006269|Dorytomus_tremulae
ILYFIFGAWAGMVGTSLSMLIRTELGNPGSLIGDDQIYNTIVTAHAFIMIFFMVMPIMIGGFGNWLIPLMLGAPDMAFPRLNNMSFWLLPPSLILLLMSSIVDKGAGTGWTVYPPLSSNIAHEGTSVDLAIFSLHMAGISSILGAMNFISTVMNMRPAG
MKPDRMSLFIWAVKITAILLLLSLPVLAGAITMLLTDRNINTSFFDPAGGGDPILYQHL
>COLFF327-13|KJ967035|ZMUO.006027|Agabus_serricornis
TLYFIFGAWAGMVGTSLSMLIRAELGNPGSLIGDDQIYNVIVTAHAFVMIFFMVMPIMIGGFGNWLVPLMLGAPDMAFPRMNNMSFWLLPPSLTLLLMSSMVESGAGTGWTVYPPLSSGIAHGGASVDLAIFSLHLAGISSILGAVNFITTIINMRSIG
MTFDRMPLFVWSVGITALLLLLSLPVLAGAITMLLTDRNLNTSFFDPAGGGDPILYQHL
>COLFD441-12|KJ961964|ZMUO.003861|Ceutorhynchus_ignitus
TLYFIFGSWAGMAGTSLSMLIRTELGNPGSLIGNDQIYNSIVTAHAFIMIFFMVMPILIGGFGNWLVPLMLGAPDMAFPRLNNMSFWLLPPSLSLLLMSSIVNKGAGTGWTVYPPLSSNVAHEGMSVDLAIFSLHMAGISSILGAINFISTVMNMQPK
GMTPELMPLFVWAVEITAILLLLSLPVLAGAITMLLTDRNINTSFFDPSGGGDPILYQHL
>COLFC253-12|KJ962400|ZMUO.002438|Placusa_tachyporoides
TLYFIFGAWAGMVGTSLSLLIRAELGNPGSLIGDDQIYNVIVTAHAFVMIFFMVMPVVIGGFGNWLVPLMLGAPDMAFPRMNNMSFWLLPPSLTLLLMSSMVESGAGTGWTVYPPLSSNIAHGGSSVDLAIFSLHLAGISSILGAVNFISTIINMRSPG
MTFDQMPLFVWSVGLTALLLLLSLPVLAGAITMLLTDRNLNTTFFDPAGGGDPILYQHL
>COLFD096-12|KJ965168|ZMUO.003991|Acidota_crenata
TLYFIFGAWAGMVGTSLSILIRAELGNPGTLIGDDQIYNVIVTAHAFVMIFFMVMPIVIGGFGNWLVPLMLGAPDMAFPRMNNMSFWLLPPSLSLLLMSSMVESGAGTGWTVYPPLSSNIAHGGASVDLAIFSLHLAGISSILGAVNFITTVINMRATG
MTFDRMPLFVWSVAITALLLLLSLPVLAGAITMLLTDRNLNTSFFDPAGGGDPILYQHL
>COLFD772-12|KJ963743|ZMUO.004762|Phyllobius_maculicornis
TLYFIFGAWSGMIGTSLSILIRVELGNPGSLIGDDQIYNVIVTAHAFIMIFFMVMPMMIGGFGNWLVPLMLGAPDMAFPRLNNMSFWLLPPSLSLLLMSSIVDKGAGTGWTVYPPLSANIAHEGSSVDLAIFSLHMAGVSSILGAINFISTVINMRPSG
MSPDRMSLFIWAVKITAILLLLSLPVLAGAITMLLTDRNMNTSFFDPAGGGDPILYQHL
>COLFC284-12|KJ967205|ZMUO.002469|Agabus_guttatus
TLYFIFGAWAGMVGTSLSMLIRAELGNPGSLIGDDQIYNVIVTAHAFVMIFFMVMPIMIGGFGNWLVPLMLGAPDMAFPRMNNMSFWLLPPSLTLLLMSSMVEKGAGTGWTVYPPLSSGIAHSGASVDLAIFSLHLAGISSILGAVNFITTIINMRSV
GMTFDRMPLFVWSVGITALLLLLSLPVLAGAITMLLTDRNLNTSFFDPAGGGDPILYQHL
>LEFIJ1816-13|KJ965681|ZMUO.004571|Psylliodes_napi
TLYFIFGVWSGMIGTSLSILIRAELGSPGSLIGNDQIYNVIVTAHAFIMIFFMVMPIMIGGFGNWLVPLMIGAPDMAFPRMNNMSFWLLPPSLLLLLMSSMVESGAGTGWTVYPPLSSNIAHGGSSVDLAIFSLHLAGISSILGAINFITTVINMRPEGMT
LDRMPLFVWAVVITAILLLLSLPVLAGAITMLLTDRNLNTSFFDPIGGGDPILYQHL
>COLFE344-12|KJ966210|ZMUO.004904|Hylastes_opacus
TLYFIFGGWSGMVGTSLSMLIRAELGTPGSLIGDDQIYNTMVTSHAFIMIFFMVMPIMIGGFGNWLIPLMLGAPDMAFPRLNNMSFWLLPPSITLLLMSSIIDKGAGTGWTVYPPLASNISHEGASVDLAIFSLHMAGISSILGAMNFISTIINMYPVGM
KPDQLSLFTWSVKITAILLLLSLPVLAGAITMLLTDRNINTTFFDPAGGGDPILYQHL
>COLFD221-12|KJ965729|ZMUO.004116|Tachyporus_chrysomelinus
TLYFIFGAWSGMVGTSLSLLIRAELGNPGSLIGDDQIYNVIVTAHAFIMIFFMVMPIVIGGFGNWLVPLMLGAPDMAFPRMNNMSFWLLPPSLTLLLMSSMVESGAGTGWTVYPPLSANIAHSGPSVDLAIFSLHLAGISSILGAVNFITTVINMRASG
MHFDRMPLFIWSVAITALLLLLSLPVLAGAITMLLTDRNLNTSFFDPAGGGDPILYQHL
>COLFE1022-13|KJ965816|ZMUO.006817|Boreophilia_eremita

TLYFIFGAWAGMIGTSLSLLIRAELGNPGSLIGDDQIYNVIVTAHAFIMIFFMVMPIVIGGFGNWLVPLMLGAPDMAFPRMNNMSFWLLPPSLTLLLISSMVESGAGTGWTVYPPLSSNIAHGGSSVDLAIFSLHLAGISSILGAVNFISTVINMRSTGVSF
DRMPLFVWSVAITALLLLLSLPVLAGAITMLLTDRNLNTSFFDPAGGGDPILYQHL
>COLFA417-12|KJ963018|ZMUO.000647|Lordithon_lunulatus
TLYFIFGSWAGMVGTSLSLLIRAELGNPGSLIGDDQIYNVIVTAHAFVMIFFMVMPIVIGGFGNWLVPLMLGAPDMAFPRMNNMSFWLLPPSLTLLLMSSMVESGAGTGWTVYPPLSSNIAHGGASVDLAIFSLHLAGISSILGAVNFITTVINMRSTG
MTFDRMPLFIWSVAITALLLLLSLPVLAGAITMLLTDRNLNTSFFDPAGGGDPILYQHL
>COLFG138-13|KJ964412|ZMUO.007833|Baeocrara_variolosa
TLYFIFGAWAGMVGTSLSILIRTELGTPGSLIGDDQIYNVIVTAHAFVMIFFMVMPILIGGFGNWLVPLMLGAPDMAFPRMNNMSFWLLPPSLTLLLMSSMVESGAGTGWTVYPPLASNIAHGGSSVDLAIFSLHLAGISSILGAVNFITTIINMRTPNM
KFDQMPLFVWAVGITALLLLLSLPVLAGAITMLLTDRNLNTSFFDPAGGGDPILYQHL
>COLFE1171-13|KJ967122|ZMUO.006966|Oreodytes_alpinus
TLYFLFGAWSGMVGTSLSMLIRAELGNPGSLIGDDQIYNVIVTAHAFIMIFFMVMPIMIGGFGNWLVPLMLGAPDMAFPRMNNMSFWLLPPSLTLLLMSSMVESGAGTGWTVYPPLSAGIAHGGASVDLAIFSLHLAGISSILGAVNFITTIINMRSV
GMTFDRMPLFVWSVGITALLLLLSLPVLAGAITMLLTDRNLNTSFFDPAGGGDPILYQHL
>COLFF708-13|KJ965326|ZMUO.006408|Archarius_pyrrhoceras
TLYFIFGAWSGMVGTSLSMLIRTELGNPGSLIGDDQIYNTIVTAHAFIMIFFMVMPTLIGGFGNWLIPLMLGAPDMAFPRLNNMSFWLLPPSLFLLLSSIADKGAGTGWTVYPPLSTNIAHEGSSVDLAIFSLHMAGISSILGAMNFISTIMNMRPTSM
KMDQMSLFIWAVKITAILLLLSLPVLAGAITMLLTDRNINTSFFDPAGGGDPILYQHL
>COLFC573-12|KJ962788|ZMUO.003138|Polydrusus_fulvicornis
TLYFIFGSWSGMVGTSLSMLIRTELGNPGSLIGDDQIYNVIVTAHAFIMIFFMVMPMMIGGFGNWLVPLMLGAPDMAFPRLNNMSFWLLPPSLSLLLMSSIVDKGAGTGWTVYPPLSANIAHEGSSVDLAIFSLHMAGVSSILGAINFISTVINMRPM
GMTFDRMSLFIWAVKITAILLLLSLPVLAGAITMLLTDRNINTSFFDPAGGGDPILYQHL
>COLFF890-13|KJ964615|ZMUO.006685|Lathrobium_geminum
TLYFIFGAWAGMVGTSLSLLIRTELGNPGSLIGDDQIYNVIVTAHAFIMIFFMVMPIMIGGFGNWLVPLMLGAPDMAFPRMNNMSFWLLPPSLSLLMSSLVESGAGTGWTVYPPLSSNIAHGGASVDLAIFSLHLAGISSILGAVNFITTVINMRSPG
MTYERMPLFVWAVAITALLLLLSLPVLAGAITMLLTDRNLNTSFFDPAGGGDPILYQHL
>COLFE231-12|KJ963646|ZMUO.005076|Cynegetis_impunctata
TLYFLFGLWAGMVGTSLSILIRLELGTPSALIGNDQIYNVIVTAHAFIMIFFMVMPIMIGGFGNWLVPLMIGAPDMAFPRLNNMSFWLLPPALTFLILSSMVESGAGTGWTVYPPLSANIAHGGSSVDLAIFSLHLAGISSILGAVNFITTMLNMRPFGM
TLDKMPLFVWSVMITAVLLLLSLPVLAGAITMLLTDRNINTSFFDPAGGGDPILYQHL
>COLFC281-12|KJ962329|ZMUO.002466|Tetartopeus_zetterstedti
TLYFIFGAWAGMVGTSLSLLIRAELGNPGSLIGDDQIYNVIVTAHAFVMIFFMVMPIVIGGFGNWLVPLMLGAPDMAFPRMNNMSFWLLPPSLSLLLMSSMVENGAGTGWTVYPPLSSNIAHGGASVDLAIFSLHLAGISSILGAVNFITTIINMRSPG
MLYERMPLFVWSVAITALLLLLSLPVLAGAITMLLTDRNLNTSFFDPAGGGDPILYQHL
>COLFD731-12|KJ963998|ZMUO.004721|Gabrius_breviventer
TLYFIFGSWAGMVGTSLSLLIRAELGNPGTLIGDDQIYNVIVTAHAFIMIFFMVMPIVIGGFGNWLVPLMLGAPDMAFPRMNNMSFWLLPPSLTLLLMSSMVESGAGTGWTVYPPLSSNIAHGGASVDLAIFSLHLAGISSILGAVNFITTVINMRSFG
MSFDRMPLFVWSVAITALLLLLSLPVLAGAITMLLTDRNLNTSFFDPAGGGDPILYQHL
>COLFA458-12|KJ967258|ZMUO.000498|Endomychus_coccineus
TLYFMFGLWAGMLGTSLSMMIRLELGNTMQLIGNDQIFNVIVTAHAFVMIFFMVMPIMIGGFGNWLVPLMLGAPDMAFPRMNNMSFWLLPPSLTLLILSSISESGVGTGWTVYPPLSSNIAHSGSSVDLAIFSLHLAGISSILGAINFITTIINMRPTG
MTLEKMPLFVWSVFITAILLLLSLPVLAGAITMLLTDRNLNTSFFDPAGGGDPILYQHL
>COLFF109-13|KJ965832|ZMUO.003743|Bolitochara_pulchra
TLYFIFGAWAGMVGTSLSLLIRAELGNPGSLIGDDQIYNVIVTAHAFVMIFFMVMPIMIGGFGNWLVPLMLGAPDMAFPRMNNMSFWLLPPSLTLLLMSSMVESGAGTGWTVYPPLSSNIAHGGSSVDLAIFSLHLAGISSILGAVNFISTIINMRSPG
MTFDRMPLFVWSVAITALLLLLSLPVLAGAITMLLTDRNLNTSFFDPAGGGDPILYQHL
>COLFB314-12|KJ963946|ZMUO.001644|Dyschirius_globosus
TLYFIFGIWSGMVGTSLSILIRTELGNPGSLIGDDQIYNVIVTAHAFIMIFFMVMPIMIGGFGNWLVPLMLGAPDMAFPRMNNMSFWLLPPSLTLLLMSSMVEKGAGTGWTVYPPLSSSIAHSGASVDLAIFSLHLAGISSILGAVNFITTIINMRSTGLTF
ERMPLFVWSVGITALLLLLSLPVLAGAITMLLTDRNLNTSFFDPAGGGDPILYQHL
>COLFE451-12|KJ964704|ZMUO.005011|Lochmaea_suturalis
TLYFIFGIWAGMVGTSLSILVRAELGSPGTLIGNDQIYNVIVTAHAFIMIFFMVMPIMIGGFGNWLVPLMIGAPDMAFPRMNNMSFWLLPPSLFLLIMSSIVESGAGTGWTVYPPLSSNIAHGGSSVDLAIFSLHLAGISSILGAINFITTIINMRPKGMTL
DRIPLFVWAVMITAILLLLSLPVLAGAITMLLTDRNLNTSFFDPAGGGDPILYQHL
>COLFG161-13|KJ962826|ZMUO.007856|Ptenidium_nitidum
ILYFLFGSWAGMVGTSLSLLIRSELSIPNSMIGDDQIYNVIVTAHAFIMIFFMVMPILIGGFGNCLVPLMLGAPDMAFPRMNNMSFWLLPPSLSLLLMSSLVESGAGTGWTVYPPLASNIAHSGASVDLAIFSLHLAGISSILGAVNFITTIINMRTPGMA
MDQIPLFVWAVLITAILLLLSLPVLAGAITMLLTDRNLNTSFFDPAGGGDPILYQHL
>COLFG172-13|KJ962869|ZMUO.007867|Gyrophaena_fasciata
TLYFIFGAWSGMVGTSLSLLIRAELGNPGSLIGDDQIYNVIVTAHAFIMIFFMVMPIVIGGFGNWLIPLMLGAPDMAFPRMNNMSFWLLPPSLSLLLMSSMVESGAGTGWTVYPPLSSNIAHGGASVDLAIFSLHLAGISSILGAVNFISTIINMRTMGM
SFDRMPLFVWSVAITALLLLLSLPVLAGAITMLLTDRNLNTSFFDPAGGGDPILYQHL

>COLFC438-12|KJ967330|ZMUO.003003|Bembidion_difficile
TLYFIFGAWSGMVGTSLSMLIRAELGNPGSLIGDDQIYNVIVTAHAFIMIFFMVMPILIGGFGNWLVPLMLGAPDMAFPRMNNMSFWLLPPSLSLLLMSSMVESGVGTGWTVYPPLSSSIAHSGSSVDLAIFSLHLAGVSSILGAVNFITTIINMRSTGM
SFDRMPLFVWSVGITALLLLLSLPVLAGAITMLLTDRNLNTTFFDPAGGGDPILYQHL
>COLFF595-13|KJ964970|ZMUO.006485|Xantholinus_linearis
TLYFIFGAWAGMIGTSLSLLIRSELGNPGTLIGDDQIYNVIVTAHAFVMIFFMVMPIVIGGFGNWLVPLMLGAPDMAFPRMNNMSFWLLPPSLTLLLMSSMVESGAGTGWTVYPPLSSNIAHGGASVDLAIFSLHLAGISSILGAVNFITTVINMRSTG
MSFDRMPLFVWSVAITALLLLLSLPVLAGAITMLLTDRNLNTSFFDPTGGGDPILYQHL
>COLFC197-12|KJ966401|ZMUO.002382|Hippodamia_tredecimpunctata
TLYFLFGMWAGMVGTSLSILIRLELGTPGSLIGNDQIYNVIVTAHAFIMIFFMVMPIMIGGFGNWLVPLMIGAPDMAFPRLNNMSFWLLPPALTLLMFSSMVEMGAGTGWTVYPPLSSNLAHNGPSVDLVIFSLHLAGISSILGAVNFISTIMNMRPY
GMSMDKTPLFVWSVLITAILLLLSLPVLAGAITMLLTDRNLNTSFFDPTGGGDPILYQHL
>COLFE1241-13|KJ964026|ZMUO.007036|Tachinus_elongatus
TLYFIFGAWSGMVGTSLSLLIRAELGNPGTLIGDDQIYNVIVTAHAFIMIFFMVMPIVIGGFGNWLIPLMLGAPDMAFPRMNNMSFWLLPPSLSLLLMSSMVESGAGTGWTVYPPLSSNIAHGGSSVDLAIFSLHLAGISSILGAVNFITTVINMRAIGM
TFDRMPLFIWSVAITALLLLLSLPVLAGAITMLLTDRNLNTTFFDPAGGGDPILYQHL
>COLFA464-12|KJ963960|ZMUO.000504|Hypnoidus_riparius
TLYFLFGAWSGMLGTSLSLLIRAELGNPGSLIGNDQIYNVIVTAHAFIMIFFMVMPIMIGGFGNWLVPLMLGAPDMAFPRMNNMSFWFLPPSLSLLLMSSIVENGAGTGWTVYPPLSANIAHSGSSVDLAIFSLHLAGISSILGAVNFISTVINMRSTGIT
FDRMPLFVWAVAITALLLLLSLPVLAGAITMLLTDRNLNTSFFDPAGGGDPILYQHL
>COLFC241-12|KJ963002|ZMUO.002426|Hypnoidus_rivularius
TLYFLFGAWSGMLGTSLSLLIRAELGNPGSLIGNDQIYNVIVTAHAFIMIFFMVMPIMIGGFGNWLVPLMLGAPDMAFPRMNNMSFWFLPPSLSLLLMSSIVENGAGTGWTVYPPLSANIAHSGSSVDLAIFSLHLAGISSILGAVNFISTVINMRSTGIT
FDRMPLFVWAVAITALLLLLSLPVLAGAITMLLTDRNLNTSFFDPAGGGDPILYQHL
>COLFB204-12|KJ964926|ZMUO.001534|Hydroporus_pubescens
TLYFLFGAWSGMVGTSLSMLIRAELGNPGSLIGDDQIYNVIVTAHAFIMIFFMVMPIMIGGFGNWLVPLMLGAPDMAFPRMNNMSFWLLPPSLTLLLMSSMVENGAGTGWTVYPPLSSGIAHSGASVDLAIFSLHLAGVSSILGAVNFITTIINMRSI
GMTFDRMPLFVWSVGITALLLLLSLPVLAGAITMLLTDRNLNTSFFDPAGGGDPILYQHL
>COLFE1147-13|KJ965122|ZMUO.006942|Sitona_lepidus
TLYFIFGAWAGMVGTSLSMLIRTELGNPGSLIGDDQIYNVIVTAHAFIMIFFMVMPMMIGGFGNWLVPLMLGAPDMAFPRLNNMSFWLLPPSLTLLLMSSIVDKGAGTGWTVYPPLSANIAHEGASVDLAIFSLHMAGISSILGAINFISTIINMRPSG
MTFDRTALFVWSVKITAILLLLSLPVLAGAITMLLTDRNINTSFFDPAGGGDPILYQHL
>COLFF643-13|KJ967060|ZMUO.006533|Bolitophagus_reticulatus
TLYFIFGAWAGMVGTSLSLLIRTELGNPGSLIGDDQIYNVIVTAHAFVMIFFMVMPIMIGGFGNWLVPLMLGAPDMAFPRMNNMSFWLLPPSLTLLLMSSIVENGAGTGWTVYPPLSSNIAHSGASVDLAIFSLHLAGISSILGAVNFITTVINMRPQG
MTLDRMPLFVWAVVITALLLLLSLPVLAGAITMLLTDRNINTSFFDPAGGGDPILYQHL
>COLFD309-12|KJ963111|ZMUO.004204|Stenus_boops
TLYFIFGAWAGMVGTSLSLLIRAELGNPGSLIGDDQIYNVIVTAHAFVMIFFMVMPIMIGGFGNWLVPLMLGAPDMAFPRMNNMSFWLLPPSLSLLLMSSIVESGAGTGWTVYPPLSSNIAHGGASVDLAIFSLHLAGISSILGAINFITTIFNMRTMKL
QLDCLPLFVWSVGITALLLLLSLPVLAGAITMLLTDRNLNTSFFDPAGGGDPILYQHL
>COLFA697-12|KJ965585|ZMUO.000832|Bembidion_quadrimaculatum
TLYFIFGAWSGMVGTSLSMLIRAELGNPGSLIGDDQIYNVIVTAHAFVMIFFMVMPILIGGFGNWLVPLMLGAPDMAFPRMNNMSFWLLPPSLTLLLMSSMVESGAGTGWTVYPPLSSSIAHSGASVDLAIFSLHLAGVSSILGAVNFITTIINMRSTG
MTFDRMPLFVWSVGITALLLLLSLPVLAGAITMLLTDRNLNTSFFDPAGGGDPILYQHL
>COLFC329-12|KJ962189|ZMUO.002514|Atheta_macrocera
TLYFIFGAWSGMVGTSLSLLIRAELGNPGSLIGDDQIYNVIVTAHAFIMIFFMVMPIMIGGFGNWLVPLMLGAPDMAFPRMNNMSFWLLPPSLTLLLMSSMVESGAGTGWTVYPPLSSNIAHSGASVDLAIFSLHLAGISSILGAVNFISTVINMRSIGIT
FDRMPLFVWAVAITALLLLLSLPVLAGAITMLLTDRNLNTSFFDPAGGGDPILYQHL
>COLFE940-13|KJ964932|ZMUO.006260|Notiophilus_germinyi
TLYFIFGAWSGMVGTSLSMLIRAELGNPGSLIGDDQIYNVIVTAHAFVMIFFMVMPIMIGGFGNWLVPLMLGAPDMAFPRMNNMSFWLLPPSLTLLLTSSMVESGAGTGWTVYPPLSSGIAHSGASVDLAIFSLHLAGVSSILGAVNFITTIINMRSVG
MTFDRMPLFVWSVGITALLLLLSLPVLAGAITMLLTDRNLNTSFFDPAGGGDPILYQHL
>COLFD901-12|KJ967074|ZMUO.004511|Aleochara_grisea
TLYFIFGAWAGMIGTSLSLLIRAELGNPGSLIGDDQIYNVIVTAHAFVMIFFMVMPIMIGGFGNWLIPLMLGAPDMAFPRMNNMSFWLLPPSLSLLLMSSMVESGAGTGWTVYPPLSSNIAHSGSSVDLAIFSLHLAGISSILGAVNFISTVINMRPKG
MSFDKMPLFIWSVIITALLLLLSLPVLAGAITMLLTDRNLNTSFFDPAGGGDPILYQHL
>COLFB461-12|KJ966008|ZMUO.001886|Dyschirius_aeneus
TLYFIFGIWSGMVGTSLSILIRAELGNPGSLIGDDQIYNVIVTAHAFIMIFFMVMPIMIGGFGNWLVPLMLGAPDMAFPRMNNMSFWLLPPSLSLLLMSSMVEKGAGTGWTVYPPLSSSIAHSGASVDLAIFSLHLAGISSILGAVNFITTIINMRSTGMT
FERMPLFVWSVGITALLLLLSLPVLAGAITMLLTDRNLNTSFFDPAGGGDPILYQHL
>COLFB802-12|KJ961994|ZMUO.001277|Cylletron_nivale

TLYFIFGAWAGMVGTSLSILIRAELGNPGTLIGDDQIYNVIVTAHAFVMIFFMVMPIVIGGFGNWLVPLMLGAPDMAFPRMNNMSFWLLPPSLTLLLMSSMVESGAGTGWTVYPPLSSNIAHGGSSVDLAIFSLHLAGISSILGAVNFITTVINMRATG
MTFDRMPLFVWSVAITALLLLLSLPVLAGAITMLLTDRNLNTSFFDPAGGGDPILYQHL
>COLFB287-12|KJ964276|ZMUO.001617|Harpalus_tardus
TLYFIFGAWAGMVGTSLSMLIRAELGTPGALIGDDQIYNVIVTAHAFIMIFFMVMPIMIGGFGNWLVPLMLGAPDMAFPRMNNMSFWLLPPSLTLLLMSSMVESGAGTGWTVYPPLSSGIAHGGASVDLAIFSLHLAGISSILGAVNFITTIINMRSTG
MTFDRMPLFVWSVGITALLLLLSLPVLAGAITMLLTDRNLNTSFFDPAGGGDPILYQHL
>COLFB855-12|KJ967065|ZMUO.001330|Stenus_similis
TLYFIFGAWAGMVGTSLSLLIRAELGNPGSLIGDDQIYNVIVTAHAFIMIFFMVMPIMIGGFGNWLVPLMLGAPDMAFPRMNNMSFWLLPPSLSLLLMSSIVESGAGTGWTVYPPLSSNIAHSGASVDLAIFSLHLAGISSILGAINFITTILNMRTMKIQ
LDCLPLFVWSVGITALLLLLSLPVLAGAITMLLTDRNLNTSFFDPAGGGDPILYQHL
>COLFE1254-13|KJ963718|ZMUO.007049|Atheta_pinegensis
TLYFIFGAWAGMVGTSLSLLIRAELGNPGSLIGDDQIYNVIVTAHAFIMIFFMVMPIVIGGFGNWLVPLMLGAPDMAFPRMNNMSFWLLPPSLTLLLMSSMVESGAGTGWTVYPPLSSNIAHGGSSVDLAIFSLHLAGISSILGAVNFISTVINMRSAGI
SFDRMPLFVWSVVITALLLLLSLPVLAGAITMLLTDRNLNTSFFDPAGGGDPILYQHL
>COLFE989-13|KJ967136|ZMUO.006784|Hydrobius_fuscipes
TLYFIFGAWAGMVGTSLSILIRAELGNPGTLIGDDQIYNVIVTAHAFIMIFFMVMPIMIGGFGNWLVPLMLGAPDMAFPRMNNMSFWLLPPSLTLLLMSSMVENGAGTGWTVYPPLSSNIAHGGASVDLAIFSLHLAGISSILGAVNFITTVINMRSPN
LTYDRLPLFVWSVAITALLLLLSLPVLAGAITMLLTDRNLNTSFFDPAGGGDPILYQHL
>COLFD936-12|KJ962242|ZMUO.004546|Cryptocephalus_moraei
TLYFLFGVWSGMVGTSLSMLIRIELGNPGSLIGNDQIYNTIVTAHAFIMIFFLVMPIMIGGFGNWLIPLMLGAPDMAFPRMNNMSFWLLPPSLTLLLMSSIVENGVGTGWTVYPPLSTTIAHTGSSVDLAIFSLHLAGISSIMGAINFISTVINMRPQGML
MDRIPLFVWAVLITAILLLLSLPVLAGAITMLLTDRNINTSFFDPAGGGDPILYQHL
>COLFE069-12|KJ964307|ZMUO.005294|Dictyoptera_aurora
TLYFILGAWAGMMGTALSLLIRAELGTPGTLIGNDQIYNVIVTAHAFIMIFFMVMPMMIGGFGNWLIPLMLGAPDMAYPRMNNMSYWLLPPALSLLTMGSMIESGAGTGWTVYPPLASNIAHSGSSVDLTIFSLHLAGISSILGAINFISTIINMKSKE
MTFEQMPLFVWAVGITALLLLLSLPVLAGAITMLLTDRNLNTSFFDPMGGGDPILYQHL
>COLFE1326-13|KJ964333|ZMUO.007121|Gonioctena_arctica
TLYFIFGIWAGMVGTSLSILIRAELGNPGTLIGNDQIYNVIVTAHAFIMIFFMVMPIMIGGFGNWLVPLMIGAPDMAFPRMNNMSFWLLPPSLFFLIMSSVVESGAGTGWTVYPPLSANIAHSGSSVDLAIFSLHLAGISSILGAINFITTIINMRPMGMT
MDRMPLFVWAVLITAILLLLSLPVLAGAITMLLTDRNLNTSFFDPAGGGDPILYQHL
>COLFC316-12|KJ965996|ZMUO.002501|Atheta_hypnorum
TLYFIFGSWAGMVGTSLSLLIRAELGNPGSLIGDDQIYNVIVTAHAFVMIFFMVMPVVIGGFGNWLVPLMLGAPDMAFPRMNNMSFWLLPPSLTLLLMSSMVESGVGTGWTVYPPLSSNIAHGGSSVDLAIFSLHLAGISSILGAVNFISTVINMRSTG
ISFDRMPLFVWSVAITALLLLLSLPVLAGAITMLLTDRNLNTSFFDPAGGGDPILYQHL
>COLFE006-12|KJ962671|ZMUO.005231|Anomala_dubia
TLYFLFGSWAGMVGTSLSLLIRAELGNPGSLIGDDQIYNVIVTAHAFIMIFFMVMPIMIGGFGNWLVPLMLGAPDMAFPRMNNMSFWLLPPSLTLLLMSSLVENGAGTGWTVYPPLSANIAHSGASVDLAIFSLHLAGISSILGAVNFITTVINMRSTG
MTFDRMPLFVWSVVLTALLLLLSLPVLAGAITMLLTDRNINTSFFDPAGGGDPILYQHL
>COLFF218-13|KJ965675|ZMUO.005823|Olisthopus_rotundatus
TLYFIFGAWSGMVGTSLSMLIRAELGNPGSLIGDDQIYNVIVTAHAFIMIFFMVMPIMIGGFGNWLVPLMLGAPDMAFPRMNNMSFWLLPPSLTLLLMSSMVESGAGTGWTVYPPLSSGIAHAGASVDLAIFSLHLAGVSSILGAVNFITTIINMRSV
GMTFDRMPLFVWSVGITALLLLLSLPVLAGAITMLLTDRNLNTSFFDPAGGGDPILYQHL
>COLFE1038-13|KJ967018|ZMUO.006833|Haliplus_immaculatus
TLYFIFGAWSGMVGTSLSMLIRAELGTPGSLIGDDQIYNVIVTAHAFIMIFFMVMPIMIGGFGNWLVPLMLGAPDMAFPRMNNMSFWLLPPSLSLLLMSSMVENGAGTGWTVYPPLSAGIAHSGASVDLAIFSLHLAGISSILGAVNFITTIINMRSM
GMTFDRMPLFVWSVGITALLLLLSLPVLAGAITMLLTDRNLNTSFFDPAGGGDPILYQHL
>COLFE1282-13|KJ965320|ZMUO.007077|Thryogenes_festucae
TLYFIFGAWSGMIGTSLSILIRVELGNPGSLIGDDQIYNVIVTAHAFIMIFFMVMPIMIGGFGNWLVPLMLGAPDMAFPRLNNLSFWLLPPSLMILLMSSMIEKGAGTGWTVYPPLSSNIAHSGPSVDLAIFSLHLAGISSILGAINFISTIINMRPQGMYSD
RMTLFVWAVNITAILLLLSLPVLAGAITMLLTDRNINTSFFDPAGGGDPILYQHL
>COLFC550-12|KJ965817|ZMUO.003115|Byrrhus_arietinus
TLYFIFGAWAGMVGTSLSMLIRTELGNPGSLIGDDQIYNVIVTAHAFIMIFFMVMPIMIGGFGNWLVPLMLGAPDMAFPRMNNMSFWLLPPSLSLLLMSSVIENGAGTGWTVYPPLSANIAHSGSSVDLAIFSLHLAGISSILGAVNFISTVINMRSPG
MTFDQMSLFTWSVVITALLLLLSLPVLAGAITMLLTDRNINTSFFDPAGGGDPILYQHL
>COLFF662-13|KJ964506|ZMUO.006552|Crepidodera_fulvicornis
TLYFIFGIWSGMVGTSLSILIRTELGSPGSLIGNDQIYNVIVTAHAFIMIFFMVMPIMIGGFGNWLIPLMIGAPDMAFPRMNNMSFWLLPPSLFLLLMSSLVESGAGTGWTVYPPLSSNIAHGGSSVDLAIFSLHLAGISSILGAINFITTIINMRPKGMNLD
RMPLFVWAVAITAILLLLSLPVLAGAITMLLTDRNMNTSFFDPIGGGDPILYQHL
>COLFE992-13|KJ965412|ZMUO.006787|Gymnusa_brevicollis
TLYFMFGAWAGMVGTSLSLLIRAELGNPGTLIGDDQIYNVIVTAHAFIMIFFMVMPIVIGGFGNWLVPLMLGAPDMAFPRMNNMSFWLLPPSLSLLLMSSLVESGAGTGWTVYPPLSSNIAHSGSSVDLAIFSLHLAGISSILGAVNFITTVINMRSIGM
SFDRMPLFIWSVAITALLLLLSLPVLAGAITMLLTDRNLNTSFFDPSGGGDPILYQHL

>COLFE542-13|KJ964415|ZMUO.005387|Noterus_crassicornis
TLYFIFGAWSGMVGTSLSMLIRMELGNPGSLIGDDQIYNVIVTAHAFIMIFFMVMPMMIGGFGNWLVPLMLGAPDMAFPRMNNMSFWLLPPSLSLLIMSSLVENGAGTGWTVYPPLASGIAHSGASVDLAIFSLHLAGISSILGAVNFITTIINMRPM
GMSFDRMPLFVWSVGITALLLLLSLPVLAGAITMLLTDRNLNTSFFDPAGGGDPILYQHL
>COLFA617-12|KJ964556|ZMUO.000752|Spondylis_buprestoides
TLYFIFGAWAGMVGTSLSMLIRSELGSPGSLIGNDQIYNVIVTAHAFIMIFFMVMPIMIGGFGNWLVPLMLGAPDMAFPRMNNMSFWLLPPSLSLLILSSAVENGAGTGWTVYPPLSSNIAHSGASVDLAIFSLHLAGISSILGAVNFITTVINMRPYGM
SLDKMPLFVWAVVITAILLLLSLPVLAGAITMLLTDRNLNTSFFDPAGGGDPILYQHL
>COLFC464-12|KJ961857|ZMUO.003029|Rabocerus_foveolatus
TLYFIFGAWAGMVGTSLSLLIRAELGNPGALIGDDQTYNVIVTAHAFIMIFFMVMPIVIGGFGNWLVPLMLGAPDMAFPRMNNMSFWLLPPSLTLLIMSSIVETGAGTGWTVYPPLAANIAHSGPSVDLAIFSLHLAGISSILGAVNFITTVINMRPSGM
SFDRMPLFVWAVLITAILLLLSLPVLAGAITMLLTDRNLNTSFFDPAGGGDPILYQHL
>COLFE1394-13|KJ967334|ZMUO.007189|Leiodes_obesa
TLYFIFGAWSGMVGTSLSILIRAELGNPGSLIGDDQVYNVIVTAHAFIMIFFMVMPIVIGGFGNWLVPLMLGAPDMAFPRMNNMSFWLLPPSLSLLLMSSIVENGAGTGWTVYPPLSSNIAHSGSSVDLAIFSLHLAGISSILGAVNFITTVINMRPAGM
SFDKMPLFVWSVAITALLLLLSLPVLAGAITMLLTDRNLNTSFFDPAGGGDPILYQHL
>COLFD041-12|KJ965086|ZMUO.003936|Epuraea_aestiva
TLYFIFGAWSGMIGTSLSILIRTELGSPGSLIGNDQIYNVIVTAHAFIMIFFMVMPFMIGGFGNWLVPLMLGAPDMAFPRMNNMSFWLLPPSLSLLLMSSIVESGAGTGWTVYPPLSSNIAHGGSSVDLAIFSLHLAGVSSILGAVNFITTIINMRPIGMTL
DRMPLFVWSVIITAILLLLSLPVLAGAITMLLTDRNLNTTFFDPSGGGDPILYQHL
>COLFD009-12|KJ963266|ZMUO.003904|Calodera_aethiops
TLYFIFGAWAGMIGTSLSLLIRAELGNPGSLIGDDQIYNVIVTAHAFVMIFFMVMPIVIGGFGNWLVPLMLGAPDMAFPRMNNMSFWLLPPSLSLLLMSSMVESGAGTGWTVYPPLSSNIAHGGSSVDLAIFSLHLAGISSILGAVNFISTIINMRTSGM
SFDRMPLFVWSVAITALLLLLSLPVLAGAITMLLTDRNLNTSFFDPAGGGDPILYQHL
>COLFE907-13|KJ967426|ZMUO.006227|Aplocnemus_tarsalis
TLYFIFGAWSGMIGTSLSLMIRSELGNPGTLIGNDQIYNVIVTAHAFIMIFFMVMPIMIGGFGNWLVPLMLGAPDMAFPRMNNMSFWLLPPSLSLLLLSSMVESGVGTGWTVYPPLSSNIAHGGSSVDLAIFSLHLAGISSILGAVNFITTIINMRPAGM
TLDRTPLFVWSVGITALLLLLSLPVLAGAITMLLTDRNLNTSFFDPTGGGDPILYQHL
>COLFC558-12|KJ962387|ZMUO.003123|Platydracus_latebricola
TLYFIFGSWSGMVGTSLSLLIRAELGNPGTLIGDDQIYNVIVTAHAFVMIFFMVMPILIGGFGNWLVPLMLGAPDMAFPRMNNMSFWLLPPSLTLLLMSSMAESGAGTGWTVYPPLSANVAHSGASVDLAIFSLHLAGISSILGAVNFITTVINMRSTG
MTFDRMPLFVWSVAITALLLLLSLPVLAGAITMLLTDRNLNTSFFDPAGGGDPILYQHL
>COLFE1457-13|KJ965963|ZMUO.007252|Orchesia_fasciata
TLYFIFGAWSGMVGTSLSLLIRTELGNPGSLIGNDQIYNVIVTAHAFIMIFFMVMPIMMGGFGNWLVPLMLGAPDMAFPRMNNMSFWLLPPSLTLLIMSSIVENGAGTGWTVYPPLSSNIAHNGSSVDLAIFSLHLAGISSILGSVNFLTTIINMRPMG
MSFDRMPLFVWAVGITAILLLLSLPVLAGAITMLLTDRNLNTSFFDPAGGGDPILYQHL
>COLFC393-12|KJ965956|ZMUO.002958|Acidota_quadrata
TLYFIFGAWAGMVGTSLSILIRAELGNPGTLIGDDQIYNVIVTAHAFVMIFFMVMPIVIGGFGNWLVPLMLGAPDMAFPRMNNMSFWLLPPSLTLLLMSSMVESGAGTGWTVYPPLSSNIAHGGSSVDLAIFSLHLAGISSILGAVNFITTVINMRATG
MTFDRMPLFVWSVAITALLLLLSLPVLAGAITMLLTDRNLNTSFFDPAGGGDPILYQHL
>COLFB490-12|KJ965351|ZMUO.000965|Tachyporus_pulchellus
TLYFIFGAWSGMVGTSLSLLIRAELGNPGSLIGDDQIYNVIVTAHAFIMIFFMVMPIVIGGFGNWLVPLMLGAPDMAFPRMNNMSFWLLPPSLTLLLMSSMVESGAGTGWTVYPPLSANIAHSGPSVDLAIFSLHLAGISSILGAVNFITTVINMRASG
MHFDRMPLFIWSVVITALLLLLSLPVLAGAITMLLTDRNLNTSFFDPAGGGDPILYQHL
>COLFD177-12|KJ962653|ZMUO.004072|Bembidion_guttula
TLYFIFGAWSGMVGTSLSMLIRAELGSPGSLIGDDQIYNVIVTAHAFIMIFFMVMPILIGGFGNWLVPLMLGAPDMAFPRMNNMSFWLLPPSLSLLLMSSMVENGAGTGWTVYPPLSSSIAHSGASVDLAIFSLHLAGVSSILGAVNFITTIINMRSTG
MTFDRMPLFVWSVGITALLLLLSLPVLAGAITMLLTDRNLNTSFFDPAGGGDPILYQHL
>COLFF099-13|KJ965631|ZMUO.003733|Olophrum_consimile
TLYFIFGAWAGMVGTSLSILIRAELGNPGTLIGDDQIYNVIVTAHAFVMIFFMVMPILIGGFGNWLVPLMLGAPDMAFPRMNNMSFWLLPPSLTLLLMSSMVESGAGTGWTVYPPLSSNIAHGGASVDLAIFSLHLAGISSILGAVNFITTVINMRATG
MTFDRMPLFVWAVVITALLLLLSLPVLAGAITMLLTDRNLNTTFFDPAGGGDPILYQHL
>COLFD695-12|KJ964651|ZMUO.004685|Trechus_quadristriatus
TLYFIFGAWAGMVGTSLSMLIRAELGNPGSLIGDDQIYNVIVTAHAFIMIFFMVMPIMIGGFGNWLVPLMLGAPDMAFPRMNNMSFWLLPPSLTLLLMSSMVESGAGTGWTVYPPLSSGIAHSGASVDLAIFSLHLAGVSSILGAVNFITTIINMRPM
GMTFDRMPLFVWSVGITALLLLLSLPVLAGAITMLLTDRNLNTSFFDPAGGGDPILYQHL
>COLFA435-12|KJ966438|ZMUO.000665|Hydroporus_incognitus
TLYFLFGAWSGMVGTSLSMLIRAELGNPGSLIGDDQIYNVIVTAHAFIMIFFMVMPIMIGGFGNWLVPLMLGAPDMAFPRMNNMSFWLLPPSLTLLLMSSMVENGAGTGWTVYPPLSSGIAHGGASVDLAIFSLHLAGVSSILGAVNFITTIINMRSI
GMTFDRMPLFVWSVGITALLLLLSLPVLAGAITMLLTDRNLNTSFFDPAGGGDPILYQHL
>COLFB907-12|KJ963001|ZMUO.001952|Spondylis_buprestoides

TLYFIFGAWAGMVGTSLSMLIRSELGAPGSLIGNDQIYNVIVTAHAFIMIFFMVMPIMIGGFGNWLVPLMLGAPDMAFPRMNNMSFWLLPPSLSLLILSSAVENGAGTGWTVYPPLSSNIAHSGASVDLAIFSLHLAGISSILGAVNFITTVINMRPYG
MSLDKMPLFVWAVVITAILLLLSLPVLAGAITMLLTDRNLNTSFFDPAGGGDPILYQHL
>COLFF661-13|KJ965003|ZMUO.006551|Kateretes_pusillus
TLYFIFGAWSGMIGTSLSILIRSELGNPGSLIGDDQIYNVIVTAHAFVMIFFMVMPFMIGGFGNWLVPLMLGAPDMAFPRMNNMSFWLLPPSLSLLLMSSIVESGAGTGWTVYPPLSSNIAHGGSSVDLAIFSLHLAGISSILGAVNFITTVINMRPLGM
TFDRMPLFVWAVAITALLLLSLPVLAGAITMLLTDRNLNTSFFDPAGGGDPVLYQHL
>COLFE1542-13|KJ962159|ZMUO.007432|Nedyus_quadrimaculatus
TLYFIFGTWAAMAGTSLSMLIRTELGNPGSLIGNDQIYNSIVTAHAFIMIFFMVMPILIGGFGNWLVPLMLGAPDMAFPRLNNMSFWLLPPALSLLLMSSIVNKGAGTGWTVYPPLSTNTAHEGMSVDLAIFSLHLAGLSSILGAINFISTVMNMRPQG
MSPEFTPLFVWAVKITAILLLLSLPVLAGAITMLLTDRNINTSFFDPSGGGDPILYQHL
>COLFF271-13|KJ964128|ZMUO.005876|Malthodes_minimus
TLYFMFGAWAGMLGTSLSLLIRAELGSPGSLIGNDQIYNVIVTAHAFIMIFFMVMPIMIGGFGNWLVPLMLGAPDMAFPRMNNMSFWFLPPSLSLLLMSSIVENGAGTGWTVYPPLSANIAHSGSSVDLAIFSLHMAGISSILGAVNFISTVINMRSTG
MTFDRMPLFVWAVAITALLLLLSLPVLAGAITMLLTDRNLNTSFFDPAGGGDPILYQHL
>COLFC660-12|KJ967162|ZMUO.003225|Hydnobius_spinipes
TLYFIFGSWAGMAGTSLSILIRAELGNPGSLIGDDQIYNVIVTAHAFIMIFFMVMPIVIGGFGNWLVPLMLGAPDMAFPRMNNMSFWLLPPSLSLLLMSSMVENGAGTGWTVYPPLSANIAHSGSSVDLAIFSLHLAGISSILGAVNFITTIINMRSVGM
TFDKMPLFVWSVLITAILLLLSLPVLAGAITMLLTDRNLNTSFFDPAGGGDPILYQHL
>COLFA550-12|KJ965576|ZMUO.000685|Lampyris_noctiluca
TLYFIFGAWAGMLGTSFSLLIRAELGSAGTLIGNDHIFNVIVTSHAFIMIFFMVMPIMIGGFGNWLVPLMLGAPDMAFPRMNNMSFWLLPPSLSLLIMSSLIESGAGTGWTVYPPLSANIAHSGPSVDLAIFSLHLAGVSSILGAVNFISTIINMRPNSM
MFDQMPLFVWAVLITAILLLLSLPVLAGAITMLLTDRNLNTSFFDPAGGGDPILYQHL
>COLFA558-12|KJ962937|ZMUO.000693|Xylotrechus_pantherinus
TLYFIFGAWAGMVGTSLSVLIRSELGNPGSLIGDDQIYNVIVTAHAFIMIFFMVMPIMIGGFGNWLVPLMLGAPDMAFPRMNNMSFWLLPPSLALLIMSSIVESGAGTGWTVYPPLSANVAHSGSSVDLAIFSLHLAGVSSILGAVNFISTVINMRPTK
MNPEQMPLFVWAVAITALLLLLSLPVLAGAITMLLTDRNINTSFFDPAGGGDPILYQHL
>COLFF040-13|KJ964187|ZMUO.005740|Ocypus_ophthalmicus
TLYFIFGVWSGMVGTSLSLLIRAELGNPGTLIGDDQIYNVIVTAHAFVMIFFMVMPIMIGGFGNWLVPLMLGAPDMAFPRMNNMSFWLLPPSLTLLLMSSMAESGAGTGWTVYPPLSGNIAHSGTSVDLAIFSLHLAGISSILGAVNFITTVINMRSTG
MTFDRMPLFVWSVVITALLLLLSLPVLAGAITMLLTDRNLNTSFFDPAGGGDPILYQHL
>COLFA376-12|KJ964717|ZMUO.000606|Platycerus_caprea
TLYFLLGSWSGMVGTSLSMLIRAELGNPGSLIGDDQIYNVIVTAHAFVMIFFMVMPILIGGFGNWLVPLMLGAPDMAFPRMNNMSFWLLPPSLTLLLLSSMVEKGAGTGWTVYPPLSSNIAHGGASVDLAIFSLHLAGISSILGAVNFITTVINMRSIG
MTFDRMPLFVWSVVLTAILLLLSLPVLAGAITMLLTDRNINTSFFDPAGGGDPILYQHL
>COLFB522-12|KJ965417|ZMUO.000997|Gabrius_piliger
TLYFIFGSWAGMVGTSLSLLIRAELGNPGSLIGDDQIYNVIVTAHAFIMIFFMVMPIVIGGFGNWLVPLMLGAPDMAFPRMNNMSFWLLPPSLTLLLMSSMVESGAGTGWTVYPPLSSNIAHGGASVDLAIFSLHLAGISSILGAVNFITTVINMRSFG
MSFDRMPLFVWSVAITALLLLLSLPVLAGAITMLLTDRNLNTSFFDPAGGGDPILYQHL
>COLFD008-12|KJ964606|ZMUO.003903|Litargus_connexus
TLYFIFGAWSGMVGTSLSMLIRSELGNPGSLIGDDQIYNVIVTAHAFIMIFFMVMPIMMGGFGNWLVPLMLGAPDMAFPRMNNMSFWLLPPSLTLLIMSSVVESGAGTGWTVYPPLSSNIAHSGSSVDLAIFSLHLAGISSILGAVNFITTVINMRPSG
MSFDRMPLFVWAVAITALLLLLSLPVLAGAITMLLTDRNLNTSFFDPAGGGDPILYQHL
>COLFF528-13|KJ966844|ZMUO.006608|Cyphon_palustris
TLYFIFGSWSGMVGTSLSLLIRAELGTPGSLIGDDQIYNVIVTAHAFIMIFFMVMPIMIGGFGNWLVPLMLGAPDMAFPRMNNMSFWLLPPSLTLLLMSSMVENGAGTGWTVYPPLSAGVAHSGASVDLAIFSLHLAGISSILGAVNFISTVINMRSVG
MTFDRMPLFVWAVAITALLLLLSLPVLAGAITMLLTDRNLNTSFFDPAGGGDPILYQHL
>COLFG016-13|KJ967033|ZMUO.007521|Melanophila_acuminata
TLYFIFGAWSGMVGTALSLLIRAELGNPGALIGDDQIYNVIVTAHAFVMIFFMVMPIMMGGFGNWLVPLMLGAPDMAFPRMNNMSFWLLPPSLTLLLMSSVVENGAGTGWTVYPPLAANVAHSGASVDLAIFSLHLAGISSILGAVNFITTVINMRS
TGMTFDRMPLFVWSVAITALLLLLSLPVLAGAITMLLTDRNLNTSFFDPAGGGDPILYQHL
>COLFD035-12|KJ965396|ZMUO.003930|Lochmaea_caprea
TLYFIFGIWAGMVGTSLSILVRAELGSPGTLIGNDQIYNVIVTAHAFIMIFFMVMPIMIGGFGNWLVPLMIGAPDMAFPRMNNMSFWLLPPSLFLLIMSSIVESGAGTGWTVYPPLSSNIAHGGSSVDLAIFSLHLAGISSILGAINFITTIINMRPKGMTL
DRIPLFVWAVMITAILLLLSLPVLAGAITMLLTDRNLNTSFFDPAGGGDPILYQHL
>COLFB836-12|KJ965202|ZMUO.001311|Atheta_palustris
TLYFIFGAWAGMVGTSLSLLIRAELGNPGSLIGDDQIYNVIVTAHAFIMIFFMVMPIVIGGFGNWLVPLMLGAPDMAFPRMNNMSFWLLPPSLTLLLMSSMVESGAGTGWTVYPPLSSNIAHGGSSVDLAIFSLHLAGISSILGAVNFISTVINMRSTGI
SFDRMPLFVWSVAITALLLLLSLPVLAGAITMLLTDRNLNTSFFDPAGGGDPILYQHL
>COLFF585-13|KJ966839|ZMUO.006475|Ceutorhynchus_puncticollis
TLYFIFGSWAGMAGTSLSMLIRTELGNPGSLIGNDQIYNSIVTAHAFIMIFFMVMPILIGGFGNWLVPLMLGAPDMAFPRLNNMSFWLLPPSLSLLLMSSIVNKGAGTGWTVYPPLSGNVAHEGMSVDLAIFSLHMAGISSILGAINFISTVMNMQPK
GMTPELMPLFVWAVEITAILLLLSLPVLAGAITMLLTDRNINTSFFDPSGGGDPILYQHL

>COLFG183-13|KJ966217|ZMUO.007878|Atomaria_apicalis
TLYFIFGAWSGMVGTSLSMLIRTELGTPGSLIGDDQIYNVIVTAHAFIMIFFMVMPMMIGGFGNWLVPLMLGAPDMAFPRLNNMSFWLLPPSLMFLLMSSIVEKGAGTGWTVYPPLSSNVAHAGSSVDLAIFSLHLAGISSILGSVNFITTVINMRPKG
MKFDRLPLFVWAVKITTILLLLSLPVLAGAITMLLTDRNINTSFFDPAGGGDPILYQHL
>COLFD774-12|KJ964558|ZMUO.004764|Cantharis_figurata
TLYFIFGAWSGSLGLALSLLIRAELGTPGTLIGNDQIYNVIVTAHAFIMIFFMVMPIMIGGFGNWLVPLMLGAPDMAFPRMNNMSFWFLPPSLMFLLMSSMVESGAGTGWTVYPPLSANIAHSGPSVDLAIFSLHMAGISSILGAVNFISTIMNMKPPS
MKFDQMPLFVWSVGITALLLLSLPVLAGAITMLLSDRNLNTSFFDPMGGGDPILYQHL
>COLFF255-13|KJ965841|ZMUO.005860|Trixagus_dermestoides
TLYFIFGSWAGMIGTAMSMIIRMELGTPGSLIGNDQIYNVIVTSHAFIMIFFMVMPIMIGGFGNWLVPLMIGAPDMAFPRLNNMSFWLLPPSMSLILTSNMTENGAGTGWTIYPPLSSNLAHSGPAVDFTIFSLHLAGMSSILGAINFITTSINMRSPLI
KPERLTLFAWSVSITALLLLLSLPVLAGAITMLLTDRNLNTSFFDPAGGGDPILYQHL
>COLFC622-12|KJ964094|ZMUO.003187|Agabus_labiatus
TLYFIFGAWAGMVGTSLSMLIRAELGNPGSLIGDDQIYNVIVTAHAFVMIFFMVMPIMIGGFGNWLVPLMLGAPDMAFPRMNNMSFWLLPPSLTLLLMSSMVESGAGTGWTVYPPLSSGIAHGGASVDLAIFSLHLAGVSSILGAVNFITTIINMRSI
GMTFDRMPLFVWSVGITALLLLSLPVLAGAITMLLTDRNLNTSFFDPAGGGDPILYQHL
>COLFA162-10|HQ559263|MP00424|Melandrya_dubia
TLYFIFGAWSGMIGTSMSLLIRSELGNPGSLIGDDQIYNVIVTAHAFIMIFFMVMPIMMGGFGNWLVPLMLGAPDMAFPRMNNMSFWLLPPSLSLLLMSSIVENGAGTGWTVYPPLASNIAHNGSSVDLTIFSLHLAGVSSILGAVNFITTIINMRPIG
MTFDRMPLFVWAVGITAVLLLSLPVLAGAITMLLTDRNLNTSFFDPAGGGDPILYQHL
>COLFD428-12|KJ967521|ZMUO.003848|Baris_artemisiae
TLYFIFGAWSGMVGTSLSLLIRVELGSPGSLIGDDQIYNTIVTAHAFIMIFFMVMPIMIGGFGNWLVPLMLGAPDMAFPRMNNMSFWLLPPSIFLLLMSSIIDKGAGTGWTMYPPLSNNIAHEGTSMDLAIFSLHMAGISSILGAINFISTVINMHSLGM
KFDQLSLFVWAVKITAILLLSLPVLAGAITMLLTDRNINTSFFDPAGGGDPILYQHL
>COLFE1527-13|KJ967430|ZMUO.007417|Oulema_obscura
TLYFIFGAWSGTVGTSLSMMIRTELGNPGSLIGNDQIYNVIVTAHAFIMIFFMVMPIMIGGFGNWLVPLMLGAPDMAFPRMNNMSFWLLPPSISLLLMSSIVENGAGTGWTVYPPLSANISHNGASVDLAIFSLHLAGISSILGAVNFISTISNMRPEG
MTLDRMPLFVWAVLITAILLLSLPVLAGAITMLLTDRNLNTSFFDPSSGGDPILYQHL
>COLFF555-13|KJ965348|ZMUO.006635|Eblisia_minor
TLYFIFGAWSGMLGTSLSLLIRAELGNPGTLIGDDQIYNVIVTAHAFIMIFFMVMPIMIGGFGNWLVPLMLGAPDMAFPRMNNMSFWLLPPSLTLLIMSSIVENGAGTGWTVYPPLSSNISHSGASVDLAIFSLHLAGISSILGAINFITTVINMRSPGMT
FDQMPLFVWSVAITALLLLSLPVLAGAITMLLTDRNLNTSFFDPAGGGDPILYQHL
>COLFF562-13|KJ962176|ZMUO.006642|Bitoma_crenata
TLYFIFGAWSGMVGTSLSLLIRAELGNPGSLIGDDQIYNVIVTAHAFIMIFFMVMPIMIGGFGNWLVPLMLGAPDMAFPRMNNMSFWLLPPSLTLLLMSSIVENGAGTGWTVYPPLSSNIAHGGSSVDLAIFSLHLAGISSILGAVNFITTVINMRPSGM
SFDRMPLFAWAVVITAVLLLSLPVLAGAITMLLTDRNLNTSFFDPAGGGDPILYQHL
>COLFE825-13|KJ963191|ZMUO.005670|Bembidion_assimile
TLYFIFGAWSGMVGTSLSMLIRAELGNPGSLIGDDQIYNVIVTAHAFVMIFFMVMPILIGGFGNWLVPLMLGAPDMAFPRMNNMSFWLLPPSLTLLLMSSMVESGAGTGWTVYPPLSSSIAHSGASVDLAIFSLHLAGVSSILGAVNFITTIINMRSTG
MTFDRMPLFVWSVGITALLLLSLPVLAGAITMLLTDRNLNTSFFDPAGGGDPILYQHL
>COLFC778-12|KJ967217|ZMUO.003343|Protaetia_cuprea
TLYFLFGSWAGMVGTSLSLLIRAELGNPGSLIGDDQIYNVIVTAHAFIMIFFMVMPIMIGGFGNWLVPLMLGAPDMAFPRMNNMSFWLLPPSLTLLLMSSVVESGAGTGWTVYPPLSSNIAHSGASVDLAIFSLHLAGISSILGAVNFITTVINMRSTG
MTFDRMPLFVWSVALTALLLLLSLPVLAGAITMLLTDRNINTSFFDPAGGGDPILYQHL
>COLFB068-12|KJ967125|ZMUO.001398|Anthophagus_alpinus
TLYFIFGAWAGMVGTSLSILIRSELGNPGSLIGDDQIYNVIVTAHAFVMIFFMVMPVVIGGFGNWLVPLMLGAPDMAFPRMNNMSFWLLPPSLTLLLMSSMVESGAGTGWTVYPPLSSNIAHSGASVDLAIFSLHLAGISSILGAVNFITTVINMRSTGI
TFDRMPLFVWSVAITALLLLSLPVLAGAITMLLTDRNLNTSFFDPAGGGDPILYQHL
>COLFA060-10|HM909079|MP00241|Rhinoncus_pericarpius
TLYFIFGSWAGTVGTSLSMLIRTELGTPGSLIGNDQIYNSIVTAHAFIMIFFMVMPILIGGFGNWLVPLMLGAPDMAFPRLNNMSFWLLPPSIMLLLMSSIINKGAGTGWTVYPPLSSNITHEGASVDLAIFSLHMAGISSILGAINFISTIMNMRPQGMS
YDKMPLFSWAVLITAILLLSLPVLAGAITMLLTDRNINTSFFDPAGGGDPILYQHL
>COLFE216-12|KJ966494|ZMUO.005061|Porhydrus_lineatus
TLYFLFGTWAGMVGTSLSMLIRAELGNPGSLIGDDQIYNVIVTAHAFIMIFFMVMPIMIGGFGNWLVPLMLGAPDMAFPRMNNMSFWLLPPSLSLLLMSSMVESGAGTGWTVYPPLSAGIAHSGASVDLAIFSLHLAGISSILGAVNFITTIINMRSVG
MTFDRMPLFVWSVGITALLLLSLPVLAGAITMLLTDRNLNTSFFDPAGGGDPILYQHL
>COLFF002-13|KJ962731|ZMUO.005702|Carabus_glabratus
TLYFIFGTWSGMVGTSLSMLIRAELGNPGSLIGDDQIYNVIVTAHAFVMIFFMVMPIMIGGFGNWLVPLMLGAPDMAFPRMNNMSFWLLPPSLTLLLMSSMVEKGAGTGWTVYPPLSSGIAHSGASVDLAIFSLHLAGISSILGAVNFITTIINMRSVG
MTFDRMPLFVWSVGITALLLLSLPVLAGAITMLLTDRNLNTSFFDPAGGGDPILYQHL
>COLFB433-12|KJ961774|ZMUO.001858|Chaetocnema_mannerheimii

TLYFIFGIWSGMVGTSMSILIRAELGNPGSLIGNDQIYNVIVTAHAFVMIFFMVMPIMIGGFGNWLIPLMIGAPDMAFPRMNNMSFWLLPPSLFLLLMSSMVESGAGTGWTVYPPLSSNIAHSGSSVDLAIFSLHLAGVSSILGAINFITTIINMRPQGM
SFDQMPLFVWAVVITAILLLLSLPVLAGAITMLLTDRNLNTSFFDPMGGGDPILYQHL
>COLFE032-12|KJ965910|ZMUO.005257|Chrysomela_lapponica
TLYFIFGVWAGMVGTSLSILIRAELGNPGALIGNDQIYNVIVTAHAFIMIFFMVMPIMIGGFGNWLVPLMIGAPDMAFPRLNNMSFWLLPPSLFLLLMSSIVENGAGTGWTVYPPLSANTAHSGSSVDLAIFSLHLAGISSILGAINFITTVINMRPEGMN
FEQTPLFVWAVLITAILLLLSLPVLAGAITMLLTDRNLNTSFFDPAGGGDPILYQHL
>COLFA438-12|KJ964523|ZMUO.000478|Colymbetes_paykulli
TLYFIFGAWAGMVGTSLSMLIRAELGNPGSLIGDDQIYNVIVTAHAFVMIFFMVMPIMIGGFGNWLVPLMLGAPDMAFPRMNNMSFWLLPPSLTLLLMSSMVESGAGTGWTVYPPLSSGIAHGGASVDLAIFSLHLAGISSILGAVNFITTIINMRSV
GMTFDRMPLFVWSVGITALLLLLSLPVLAGAITMLLTDRNLNTSFFDPAGGGDPILYQHL
>COLFC011-12|KJ965977|ZMUO.002006|Bledius_tricornis
TLYFIFGIWAGMIGTSLSMLIRAELGTPGSLIGDDQIYNVIVTAHAFIMIFFMVMPIMIGGFGNWLVPLMLGAPDMAFPRMNNMSFWLLPPSLTLLLMSSMVESGVGTGWTVYPPLSANIAHSGSSVDLAIFSLHLAGISSILGAVNFISTIINMRSIGMT
FDRMPLFIWSVKITAILLLLSLPVLAGAITMLLTDRNLNTSFFDPAGGGDPILYQHL
>COLFC650-12|KJ961875|ZMUO.003215|Notiophilus_aquaticus
TLYFIFGAWSGMVGTSLSMLIRAELGNPGSLIGDDQIYNVIVTAHAFVMIFFMVMPIMIGGFGNWLVPLMLGAPDMAFPRMNNMSFWLLPPSLTLLLTSSMVESGAGTGWTVYPPLSSGIAHSGASVDLAIFSLHLAGVSSILGAVNFITTIINMRSVG
MTFDRMPLFVWSVGITALLLLLSLPVLAGAITMLLTDRNLNTSFFDPAGGGDPILYQHL
>COLFE339-12|KJ961920|ZMUO.004899|Harpalus_xanthopus
TLYFIFGAWAGMVGTSLSMLIRAELGTPGALIGDDQIYNVIVTAHAFIMIFFMVMPIMIGGFGNWLVPLMLGAPDMAFPRMNNMSFWLLPPSLTLLLMSSMVESGAGTGWTVYPPLSSGIAHGGASVDLAIFSLHLAGVSSILGAVNFITTIINMRSV
GITFDRMPLFVWSVGITALLLLLSLPVLAGAITMLLTDRNLNTSFFDPAGGGDPILYQHL
>COLFE1150-13|KJ967316|ZMUO.006945|Leiodes_obesa
TLYFIFGAWSGMVGTSLSILIRAELGNPGSLIGDDQVYNVIVTAHAFIMIFFMVMPIVIGGFGNWLVPLMLGAPDMAFPRMNNMSFWLLPPSLSLLLMSSIVENGAGTGWTVYPPLSSNIAHSGSSVDLAIFSLHLAGISSILGAVNFITTVINMRPAGM
SFDKMPLFVWSVAITALLLLLSLPVLAGAITMLLTDRNLNTSFFDPAGGGDPILYQHL
>COLFE269-12|KJ965789|ZMUO.005114|Rhagium_mordax
TLYFIFGAWSGMVGTSLSLLIRSELGNPGSLIGDDQIYNVIVTAHAFIMIFFMVMPIMIGGFGNWLVPLMLGAPDMAFPRMNNMSFWLLPPSLTLLIMSSVVESGAGTGWTVYPPLSSNIAHSGSSVDLAIFSLHLAGISSILGAVNFITTVINMRPVGM
TPDRVPLFVWAVVITAILLLLSLPVLAGAITMLLTDRNLNTSFFDPAGGGDPILYQHL
>COLFA254-12|KJ961995|ZMUO.000389|Silvanoprus_fagi
TLYFIFGAWAGTVGTSLSVMIRTELGTPGALIGDDQIYNVIVTAHAFIMIFFMVMPVVIGGFGNWLVPLMLGAPDMAFPRLNNMSFWLLPPSISLLLISSIVEKGAGTGWTVYPPLSANIAHNGTSVDLAIFSLHLAGISSILGAINFISTIFNMHPNKMK
MDQMPLFCWAVLLTAVLLLLSLPVLAGAITMLLTDRNLNTSFFDPSGGGDPILYQHL
>COLFB727-12|KJ966908|ZMUO.001202|Acrolocha_sulcula
TLYFIFGAWAGMVGTSLSILIRAELGNPGSLIGDDQIYNVIVTAHAFIMIFFMVMPIVIGGFGNWLVPLMLGAPDMAFPRMNNMSFWLLPPSLTLLLMSSMVESGAGTGWTVYPPLSSNIAHGGASVDLAIFSLHLAGISSILGAVNFITTVINMRSMG
MTFDRMPLFVWSVAITALLLLLSLPVLAGAITMLLTDRNLNTSFFDPAGGGDPILYQHL
>COLFE198-12|KJ962067|ZMUO.005043|Stephostethus_lardarius
SLYFMFGMWSGMIGTSLSLLIRLELGNPGSLIGDDQIYNVIVTAHAFIMIFFMVMPIMMGGFGNWLVPLMLGAPDMAFPRLNNMSFWLLPPSLTLLIMSSIVESGVGTGWTVYPPLSSNIAHGGSSVDLAIFSLHLAGISSILGAVNFITTMVNMRPSG
MNFEHMPLFAWSVNLTAILLLLSLPVLAGAITMLLTDRNLNTSFFDPAGGGDPILYQHL
>COLFF905-13|KJ964744|ZMUO.006700|Microlestes_minutulus
TLYFIFGAWAGMVGTSLSMLIRAELGNPGALIGDDQIYNVIVTAHAFIMIFFMVMPIMIGGFGNWLVPLMLGAPDMAFPRMNNMSFWLLPPSLTLLLMSSMVESGAGTGWTVYPPLSSGIAHAGASVDLAIFSLHLAGVSSILGAVNFITTIINMRSV
GMSFERMPLFVWSVGITALLLLLSLPVLAGAITMLLTDRNLNTTFFDPAGGGDPILYQHL
>COLFC107-12|KJ962135|ZMUO.002292|Xantholinus_linearis
TLYFIFGAWAGMIGTSLSLLIRSELGNPGTLIGDDQIYNVIVTAHAFVMIFFMVMPIVIGGFGNWLVPLMLGAPDMAFPRMNNMSFWLLPPSLTLLLMSSMVESGAGTGWTVYPPLSSNIAHGGASVDLAIFSLHLAGISSILGAVNFITTVINMRSTG
MSFDRMPLFVWSVAITALLLLLSLPVLAGAITMLLTDRNLNTSFFDPTGGGDPILYQHL
>COLFC289-12|KJ962139|ZMUO.002474|Rhagonycha_atra
TLYFIFGAWSGSLGLALSLLIRAELGTPGTLIGNDQIYNVIVTAHAFIMIFFMVMPIMIGGFGNWLVPLMLGAPDMAFPRMNNMSFWFLPPSLMFLLMSSMVESGAGTGWTVYPPLSANIAHSGPSVDLAIFSLHMAGISSILGAVNFISTILNMKPPS
MKFDQMPLFVWSVGITALLLLLSLPVLAGAITMLLSDRNLNTSFFDPMGGGDPILYQHL
>COLFB835-12|KJ962551|ZMUO.001310|Aleochara_moerens
TLYFIFGAWAGMVGTSLSLLIRAELGNPGSLIGDDQIYNVIVTAHAFIMIFFMVMPIVIGGFGNWLVPLMLGAPDMAFPRMNNMSFWLLPPSLTLLLMSSMVESGAGTGWTVYPPLSANIAHSGSSVDLAIFSLHLAGISSILGAVNFISTIINMRSSGM
SFDKMPLFIWSVAITALLLLLSLPVLAGAITMLLTDRNLNTSFFDPAGGGDPILYQHL
>COLFB064-12|KJ963344|ZMUO.001394|Bledius_talpa
TLYFIFGAWAGMVGTSLSMLIRTELGTPGSLIGNDQIYNVIVTAHAFVMIFFMVMPIVIGGFGNWLIPLMLGAPDMAFPRMNNMSFWLLPPSLTLLLMSSMVESGAGTGWTVYPPLSSNIAHSGSSVDLAIFSLHLAGISSILGAVNFISTIINMRSIGM
TFDRMPLFIWSVKITAILLLLSLPVLAGAITMLLTDRNLNTSFFDPAGGGDPILYQHL

>COLFD863-12|KJ963239|ZMUO.004473|Sulcacis_nitidus
TLYFLFGAWSGMVGTSMSILIRSELGTPGSLIGNDQIYNVIVTAHAFVMIFFMVMPIMIGGFGNWLVPLMLGAPDMAFPRMNNMSFWLLPPSLSLLLMSSLVENGAGTGWTVYPPLSSNIAHSGPSVDLAIFSLHLAGISSILGAVNFISTIINMRPMG
MSMDRIPLFVWAVIITAVLLLLSLPVLAGAITMLLTDRNFNTSFFDPAGGGDPILYQHL
>COLFB207-12|KJ966990|ZMUO.001537|Hydroporus_morio
TLYFLFGAWSGMVGTSLSMLIRAELGNPGSLIGDDQIYNVIVTAHAFIMIFFMVMPIMIGGFGNWLVPLMLGAPDMAFPRMNNMSFWLLPPSLSLLLMSSMVENGAGTGWTVYPPLSSGIAHSGASVDLAIFSLHLAGVSSILGAVNFITTIINMRSIG
MTFDRMPLFVWSVGITALLLLLSLPVLAGAITMLLTDRNLNTSFFDPAGGGDPILYQHL
>COLFE1295-13|KJ966380|ZMUO.007090|Ilybius_angustior
TLYFIFGAWAGMVGTSLSMLIRAELGNPGSLIGDDQIYNVIVTAHAFVMIFFMVMPIMIGGFGNWLVPLMLGAPDMAFPRMNNMSFWLLPPSLSLLLMSSMVESGAGTGWTVYPPLSAGIAHSGASVDLAIFSLHLAGISSILGAVNFITTIINMRSV
GMTFDRMPLFVWSVGITALLLLLSLPVLAGAITMLLTDRNLNTSFFDPAGGGDPILYQHL
>COLFB020-12|KJ964945|ZMUO.001350|Cercyon_sternalis
TLYFIFGAWAGMVGTSLSILIRAELGNPGTLIGDDQIYNVIVTAHAFIMIFFMVMPIMIGGFGNWLVPLMLGAPDMAFPRMNNMSFWLLPPSLSLLLMSSMVESGAGTGWTVYPPLSSNIAHGGSSVDLAIFSLHLAGISSILGAVNFITTVINMRSPSL
TYDRLPLFVWSVAITALLLLLSLPVLAGAITMLLTDRNLNTSFFDPAGGGDPILYQHL
>COLFE1446-13|KJ965948|ZMUO.007241|Malthodes_mysticus
TLYFMFGAWAGMLGTSLSLLIRAELGSPGSLIGNDQIYNVIVTAHAFIMIFFMVMPIMIGGFGNWLVPLMLGAPDMAFPRMNNMSFWFLPPSLSLLLMSSIVENGAGTGWTVYPPLSANIAHSGSSVDLAIFSLHMAGISSILGAVNFISTIINMRSTG
MTFDRMPLFVWAVAITALLLLLSLPVLAGAITMLLTDRNLNTSFFDPAGGGDPILYQHL
>COLFA094-10|HM909110|MP00330|Ephistemus_globulus
TLYFIFGAWAGMIGTSLSMLIRTELGTPGSLIGDDQIYNVIVTAHAFIMIFFMVMPMMIGGFGNWLVPLMLGAPDMAFPRLNNMSFWLLPPSLTFLLMSSIVEKGAGTGWTVYPPLSSNVAHAGSSVDLAIFSLHLAGISSILGSVNFITTVINMRPKG
MNFDRLPLFVWAVKITTILLLLSLPVLAGAITMLLTDRNINTSFFDPAGGGDPILYQHL
>COLFE594-13|KJ964243|ZMUO.005439|Hylurgops_palliatus
TLYFIFGAWSGMVGTSLSMLIRTELGTPGSLIGDDQIYNTMVTSHAFIMIFFMVMPIMIGGFGNWLIPLMLGAPDMAFPRLNNMSFWLLPPSIILLLMSSIIDKGAGTGWTVYPPLASNISHEGASVDLAIFSLHMAGISSILGAMNFISTIINMYPSGMK
PDQLSLFTWSVKITAILLLLSLPVLAGAITMLLTDRNINTTFFDPAGGGDPILYQHL
>COLFE397-12|KJ964670|ZMUO.004957|Neocoenorrhinus_germanicus
TLYFIFGAWSGMLGTSLSLLIRAELGNPGSLIGDDQIYNVIVTAHAFIMIFFMVMPIMIGGFGNWLVPLMLGAPDMAFPRMNNMSFWLLPPSLSLLIMSSIVEKGAGTGWTVYPPLSSNIAHGGSSVDLAIFSLHLAGISSILGAVNFISTVINMRPQGM
SLDRMPLFVWAVAITALLLLLSLPVLAGAITMLLTDRNINTTFFDPAGGGDPILYQHL
>COLFC678-12|KJ964454|ZMUO.003243|Bledius_talpa
TLYFIFGAWAGMVGTSLSMLIRTELGTPGSLIGNDQIYNVIVTAHAFVMIFFMVMPIVIGGFGNWLIPLMLGAPDMAFPRMNNMSFWLLPPSLTLLLMSSMVESGAGTGWTVYPPLSSNIAHSGSSVDLAIFSLHLAGISSILGAVNFISTIINMRSIGM
TFDRMPLFIWSVKITAILLLLSLPVLAGAITMLLTDRNLNTSFFDPAGGGDPILYQHL
>COLFE841-13|KJ963285|ZMUO.005686|Stilbus_atomarius
TLYFIFGAWSGMVGTSLSLLIRTELGTPGSLIGDDQIYNVIVTAHAFVMIFFMVMPFMIGGFGNWLVPLMLGAPDMAFPRMNNMSFWLLPPSLSFLLMSSIVENGAGTGWTVYPPLSSNIAHGGSSVDLAIFSLHLAGISSILGAINFITTVINMRPEGM
TLDRMPLFVWSVVITAILLLLSLPVLAGAITMLLTDRNINTSFFDPAGGGDPILYQHL
>COLFB372-12|KJ965861|ZMUO.001702|Tachyporus_pulchellus
TLYFIFGAWSGMVGTSLSLLIRAELGNPGSLIGDDQIYNVIVTAHAFIMIFFMVMPIVIGGFGNWLVPLMLGAPDMAFPRMNNMSFWLLPPSLTLLLMSSMVESGAGTGWTVYPPLSANIAHSGPSVDLAIFSLHLAGISSILGAVNFITTVINMRASG
MHFDRMPLFIWSVAITALLLLLSLPVLAGAITMLLTDRNLNTSFFDPAGGGDPILYQHL
>COLFE346-12|KJ964777|ZMUO.004906|Polydrusus_pilosus
TLYFIFGAWSGMVGTSLSMLIRTELGNPGSLIGDDQIYNVIVTAHAFIMIFFMVMPIMIGGFGNWLVPLMLGAPDMAFPRLNNMSFWLLPPSLTLLLMSSIVDKGAGTGWTVYPPLSMNIAHEGSSVDLAIFSLHMAGVSSILGAINFISTIINMRPTG
MSYDRMPLFVWAVKITAILLLLSLPVLAGAITMLLTDRNINTSFFDPAGGGDPILYQHL
>COLFF720-13|KJ963061|ZMUO.006420|Oedemera_femorata
TLYLIFGAWAGMVGTSLSLLIRAELGNPGSLIGDDQIYNVIVTAHAFIMIFFMVMPIMIGGFGNWLVPLMLGAPDMAFPRMNNMSFWLLPPSLTLLIMSSMVESGAGTGWTVYPPLSSNIAHSGSSVDLAIFSLHLAGVSSILGAVNFITTVINMRPAG
MNLDRMPLFVWAVVITAILLLLSLPVLAGAITMLLTDRNLNTSFFDPAGGGDPILYQHL
>COLFF435-13|KJ966948|ZMUO.005953|Bembidion_illigeri
TLYFIFGAWSGMVGTSLSMLIRAELGNPGSLIGDDQIYNVIVTAHAFVMIFFMVMPILIGGFGNWLVPLMLGAPDMAFPRMNNMSFWLLPPSLSLLLMSSMVESGAGTGWTVYPPLSSSIAHSGASVDLAIFSLHLAGVSSILGAVNFITTIINMRSIG
MSFDRMPLFVWSVGITALLLLLSLPVLAGAITMLLTDRNLNTSFFDPAGGGDPILYQHL
>COLFF127-13|KJ965859|ZMUO.003761|Stenus_cicindeloides
TLYFIFGAWSGMIGTSLSLLIRAELGNPGSLIGDDQIYNVIVTAHAFIMIFFMVMPAMIGGFGNWLVPLMLGAPDMAFPRMNNMSFWLLPPSLSLLLMSSIVESGAGTGWTVYPPLSSNIAHGGSSVDLAIFSLHLAGISSILGAINFITTILNMRAMKIQ
LDCMPLFVWSVGITALLLLLSLPVLAGAITMLLTDRNLNTSFFDPAGGGDPILYQHL
>COLFF963-13|KJ962029|ZMUO.007328|Coccinella_trifasciata

TLYFLFGMWAGMIGTSLSILIRLELGTTNSLIGNDQIYNVIVTAHAFIMIFFMVMPIMIGGFGNWLVPLMIGAPDMAFPRLNNMSFWFLPPALTLLIMSSLIEMGAGTGWTVYPPLSSNLAHNGPSVDLVIFSLHLAGISSILGAVNFISTIMNMRPFGM
NLDKTPLFVWSVLITAILLLLSLPVLAGAITMLLTDRNINTSFFDPTGGGDPILYQHL
>COLFB445-12|KJ963150|ZMUO.001870|Rhyncolus_ater
TLYFGFGAWSGMVGTSMSILIRTELGTPGSLIGDDQIYNTIVTAHAFIMIFFMVMPIMIGGFGNWLVPLMLGAPDMAFPRLNNMSFWLLPPSLTLLLASSAVNKGAGTGWTVYPPLSTNIAHEGPSIDLAIFSLHMAGISSILGAMNFISTIANMRPSG
MSLDRTPLFTWAVKITAVLLLISLPVLAGAITMLLTDRNINTSFFDPAGGGDPVLYQHL
>COLFC335-12|KJ962852|ZMUO.002520|Tachinus_proximus
TLYFIFGAWAGMVGTSLSLLIRAELGNPGTLIGDDQIYNVIVTAHAFIMIFFMVMPIVIGGFGNWLVPLMLGAPDMAFPRMNNMSFWLLPPSLSLLLMSSMVESGAGTGWTVYPPLSSNIAHGGSSVDLAIFSLHLAGISSILGAVNFITTVINMRSIGM
TFDRMPLFVWSVAITALLLLLSLPVLAGAITMLLTDRNLNTTFFDPAGGGDPILYQHL
>COLFG056-13|KJ966565|ZMUO.007561|Agrilus_angustulus
TLYFIFGAWSGMVGTALSLLVRAELGNPGALIGNDQIYNVIVTAHAFVMIFFMVMPIMMGGFGNWLVPLMLGAPDMAFPRMNNMSFWLLPPSLTLLLMSSMVESGAGTGWTVYPPLAANIAHSGASVDLAIFSLHLAGISSILGAINFITTVINMRA
SGMTLDRMPLLVWSIAITALLLLLSLPVLAGAITMLLTDRNLNTSFFDPAGGGDPILYQHL
>COLFD549-12|KJ963554|ZMUO.004349|Gyrinus_substriatus
TLYFIFGAWSGMVGTSLSMLIRAELGNPGSLIGDDQIYNVIVTAHAFIMIFFMVMPIMIGGFGNWLVPLMLGAPDMAFPRMNNMSFWLLPPSLTLLLMSSMVENGAGTGWTVYPPLSSNIAHGGASVDLAIFSLHLAGISSILGAVNFITTIINMRSIG
MTLDRMPLFVWSVGITALLLLLSLPVLAGAITMLLTDRNLNTSFFDPAGGGDPILYQHL
>COLFF967-13|KJ964116|ZMUO.007332|Apion_cruentatum
TLYFIFGLWSGMIGTSLSMLIRVELGNPGSLIGNDQIYNTIVTAHAFIMIFFMVMPIMIGGFGNWLVPLMLGAPDMAFPRMNNMSFWLLPPSLILLLMSSFVENGAGTGWTVYPPLASNIAHSGASVDLAIFSLHLAGISSILGAVNFISTMINMRSNG
MSLDQLSLFTWAVKITAILLLLSLPVLAGAITMLLTDRNINTSFFDPAGGGDPILYQHL
>COLFB002-12|KJ964589|ZMUO.001332|Othius_punctulatus
TLYFIFGAWAGMVGTSLSLLIRAELGNPGSLIGDDQIYNVIVTAHAFIMIFFMVMPIMIGGFGNWLVPLMLGAPDMAFPRMNNMSFWLLPPSLSLLLMSSLVESGAGTGWTVYPPLSSNIAHGGASVDLAIFSLHLAGISSILGAVNFITTVINMRSIGM
TFDRMPLFVWSVAITALLLLLSLPVLAGAITMLLTDRNLNTSFFDPAGGGDPILYQHL
>COLFF908-13|KJ963389|ZMUO.006703|Amara_famelica
TLYFIFGAWSGMVGTSLSMLIRAELGNPGALIGDDQIYNVIVTAHAFVMIFFMVMPIMIGGFGNWLVPLMLGAPDMAFPRMNNMSFWLLPPSLTLLLMSSMVESGAGTGWTVYPPLSSGIAHAGASVDLAIFSLHLAGISSILGAVNFITTIINMRSV
GMTFDRMPLFVWSVGITALLLLLSLPVLAGAITMLLTDRNLNTSFFDPAGGGDPILYQHL
>COLFF554-13|KJ965724|ZMUO.006634|Platysoma_deplanatum
TLYFIFGAWAGMLGTSLSLLIRAELGNPGTLIGDDQIYNVIVTAHAFIMIFFMVMPIMIGGFGNWLVPLMLGAPDMAFPRMNNMSFWLLPPSLTLLVMSSIVENGAGTGWTVYPPLSSNIAHGGASVDLAIFSLHLAGISSILGAINFITTVINMRSPGM
SFDQMPLFAWSVVITALLLLLSLPVLAGAITMLLTDRNLNTSFFDPAGGGDPILYQHL
>COLFF1031-13|KJ963893|ZMUO.007396|Ceutorhynchus_rapae
TLYFIFGAWSGMAGTSLSMMIRTELGNPGSLIGNDQIYNSIVTAHAFIMIFFMVMPILIGGFGNWLVPLMLGAPDMAFPRLNNMSFWLLPPSLSLLLMSSIVNKGAGTGWTVYPPLSSNVAHEGMSVDLAIFSLHMAGISSILGAINFISTVMNMQPK
GMTPELMPLFVWAVEITAILLLLSLPVLAGAITMLLTDRNINTSFFDPSGGGDPILYQHL
>COLFF514-13|KJ963535|ZMUO.006594|Haliplus_sibiricus
TLYFIFGAWAGMVGTSLSMLIRAELGTPGSLIGDDQIYNVIVTAHAFIMIFFMVMPIMIGGFGNWLVPLMLGAPDMAFPRMNNMSFWLLPPSLSLLLMSSMVENGAGTGWTVYPPLSAGIAHSGASVDLAIFSLHLAGISSILGAVNFITTIINMRSIG
MTFDRMPLFVWSVGITALLLLLSLPVLAGAITMLLTDRNLNTSFFDPAGGGDPILYQHL
>COLFB686-12|KJ961727|ZMUO.001161|Oxypoda_opaca
TLYFIFGAWAGMVGTSLSLLIRAELGNPGSLIGDDQIYNVIVTAHAFVMIFFMVMPIVIGGFGNWLVPLMLGAPDMAFPRMNNMSFWLLPPSLTLLLMSSMVESGAGTGWTVYPPLSSNIAHGGSSVDLAIFSLHLAGISSILGAVNFISTIINMRTSG
MTFDRMPLFVWSVAITALLLLLSLPVLAGAITMLLTDRNLNTSFFDPAGGGDPILYQHL
>COLFE860-13|KJ962552|ZMUO.006180|Tetropium_fuscum
TLYFIFGTWAGMVGTSLSILIRSELGNPGSLIGNDQIYNVIVTAHAFIMIFFMVMPIMIGGFGNWLVPLMLGAPDMSFPRLNNLSFWFLPPSLILLIMGMIVEKGAGTGWTVYPPLSANIAHSGSSVDLTIFSLHLAGISSILSAINFITTIMNMRPNGMTL
DQMPLFVWAVMITTILLLISLPVLAGAITMLLTDRNINTSFFDPSGGGDPILYQHL
>COLFF694-13|KJ965089|ZMUO.006394|Malthodes_minimus
TLYFMFGAWAGMLGTSLSLLIRAELGSPGSLIGNDQIYNVIVTAHAFIMIFFMVMPIMIGGFGNWLVPLMLGAPDMAFPRMNNMSFWFLPPSLSLLLMSSIVENGAGTGWTVYPPLSANIAHSGSSVDLAIFSLHMAGISSILGAVNFISTVINMRSTG
VTFDRMPLFVWAVAITALLLLLSLPVLAGAITMLLTDRNLNTSFFDPAGGGDPILYQHL
>COLFF846-13|KJ967383|ZMUO.006166|Rutidosoma_globulus
TLYFIFGSWAGSVGTSLSMLIRTELGTPGSLIGNDQIYNSIVTAHAFIMIFFMVMPILIGGFGNWLVPLMLGAPDMAFPRLNNMSFWLLPPSLMLLMMSSVINKGAGTGWTVYPPLSSNVTHEGASVDLAIFSLHMAGISSILGAINFISTIMNMRPKG
MSYDKMPLFSWAVIITAVLLLLSLPVLAGAITMLLTDRNINTSFFDPAGGGDPILYQHL
>COLFA704-12|KJ966866|ZMUO.000839|Cilea_silphoides
TLYFIFGIWAGMIGTSLSLLIRAELGNPGSLIGDDQIYNVIVTAHAFIMIFFMVMPIMIGGFGNWLVPLMLGAPDMAFPRMNNMSFWLLPPSITLLLMSSMVESGAGTGWTVYPPLSSNIAHGGSSVDLAIFSLHLAGISSILGAINFITTVINMRSPGMT
FERMPLFVWAVVITALLLLLSLPVLAGAITMLLTDRNLNTSFFDPAGGGDPILYQHL

>COLFG054-13|KJ963430|ZMUO.007559|Agrilus_angustulus
TLYFIFGAWSGMVGTALSLLVRAELGNPGALIGNDQIYNVIVTAHAFVMIFFMVMPIMMGGFGNWLVPLMLGAPDMAFPRMNNMSFWLLPPSLTLLLMSSMVESGAGTGWTVYPPLAANIAHSGASVDLAIFSLHLAGISSILGAINFITTVINMRA
SGMTLDRMPLLVWSIAITALLLLLSLPVLAGAITMLLTDRNLNTSFFDPAGGGDPILYQHL
>COLFF170-13|KJ967153|ZMUO.005894|Plateumaris_weisei
TLYFIFGAWSGMMGTSLSMLIRTELSNPGSLIGNDQTYNVIVTAHAFIMIFFMVMPIMIGGFGNWLVPLMLGAPDMAFPRMNNMSFWLLPPSLTFLIMSSIVENGVGTGWTVYPPLSSNIAHSGSSVDLAIFSLHMAGISSILGAINFITTIINMHPMG
MKMDKMPLFIWAVMITAILLLLSLPVLAGAITMLLTDRNLNTSFFDPAGGGDPILYQHL
>COLFB278-12|KJ964390|ZMUO.001608|Stenus_juno
TLYFIFGSWAGMVGTSLSMLIRSELGNPGSLIGDDQIYNVIVTAHAFIMIFFMVMPIMIGGFGNWLVPLMLGAPDMAFPRMNNMSFWLLPPSLSLLLMSSIVESGAGTGWTVYPPLSSNIAHSGASVDLAIFSLHLAGISSILGAINFITTIINMRTMKLQ
LECLPLFVWSVSITALLLLLSLPVLAGAITMLLTDRNLNTSFFDPAGGGDPILYQHL
>COLFC495-12|KJ966300|ZMUO.003060|Sepedophilus_littoreus
TLYFIFGAWAGMVGTSLSLLIRAELGNPGSLIGDDQIYNVIVTAHAFIMIFFMVMPIVIGGFGNWLVPLMLGAPDMAFPRMNNMSFWLLPPSLSLLLMSSLVESGAGTGWTVYPPLSSNIAHGGSSVDLAIFSLHLAGISSILGAVNFITTVINMRSMG
MSFDKMPLFVWSVAITALLLLLSLPVLAGAITMLLTDRNLNTSFFDPAGGGDPILYQHL
>COLFD056-12|KJ964123|ZMUO.003951|Rhyncolus_ater
TLYFGFGAWSGMVGTSMSILIRTELGTPGSVIGDDQIYNTIVTAHAFIMIFFMVMPIMIGGFGNWLVPLMLGAPDMAFPRLNNMSFWLLPPSLILLLASSAVNKGAGTGWTVYPPLSTNIAHEGPSIDLAIFSLHMAGISSILGAMNFISTIANMRPSG
MSLDRTPLFTWAVKITAVLLLISLPVLAGAITMLLTDRNINTSFFDPAGGGDPVLYQHL
>COLFD002-12|KJ963413|ZMUO.003897|Harpalus_laevipes
TLYFIFGAWAGMVGTSLSMLIRAELGTPGALIGNDQIYNVIVTAHAFIMIFFMVMPIMIGGFGNWLVPLMLGAPDMAFPRMNNMSFWLLPPSLTLLLMSSMVESGAGTGWTVYPPLSSGIAHSGASVDLAIFSLHLAGISSILGAVNFITTIINMRSVG
MTFDRMPLFVWSVGITALLLLLSLPVLAGAITMLLTDRNLNTSFFDPAGGGDPILYQHL
>COLFC705-12|KJ964458|ZMUO.003270|Platydracus_fulvipes
TLYFIFGAWSGMVGTSLSLLIRAELGNPGTLIGDDQIYNVIVTAHAFVMIFFMVMPIVIGGFGNWLVPLMLGAPDMAFPRMNNMSFWLLPPSLTLLLMSSMAESGAGTGWTVYPPLSANVAHSGTSVDLAIFSLHLAGISSILGAVNFITTVINMRSTG
MSFDRMPLFVWSVAITALLLLLSLPVLAGAITMLLTDRNLNTSFFDPAGGGDPILYQHL
>COLFE1418-13|KJ967118|ZMUO.007213|Rhizophagus_picipes
TLYFIFGAWSGMVGTSLSLLIRAELGTPGQLIGDDQIYNVIVTAHAFIMIFFMVMPILIGGFGNWLVPLMLGAPDMAFPRMNNMSFWLLPPSLTLLIMSSIVENGAGTGWTVYPPLSSNIAHSGASVDLAIFSLHLAGISSILGAVNFITTVINMRPKGMS
LDRTPLFVWAVIITAVLLLISLPVLAGAITMLLTDRNLNTSFFDPAGGGDPILYQHL
>COLFE1007-13|KJ963122|ZMUO.006802|Longitarsus_nigerrimus
TLYFIFGIWAGMVGTSLSILIRTELGNPGSLIGNDQIYNVIVTAHAFIMIFFMVMPIMIGGFGNWLVPLMIGAPDMAFPRMNNMSFWLLPPSIFLLIMSSVVENGAGTGWTVYPPLSANIAHSGSSVDLAIFSLHLAGISSILGAINFITTVINMRPIGMM
LDRMPLFVWSVLITAILLLLSLPVLAGAITMLLTDRNLNTTFFDPAGGGDPILYQHL
>COLFA452-12|KJ962332|ZMUO.000492|Colymbetes_paykulli
TLYFIFGAWAGMVGTSLSMLIRAELGNPGSLIGDDQIYNVIVTAHAFVMIFFMVMPIMIGGFGNWLVPLMLGAPDMAFPRMNNMSFWLLPPSLTLLLMSSMVESGAGTGWTVYPPLSSGIAHGGASVDLAIFSLHLAGISSILGAVNFITTIINMRSV
GMTFDRMPLFVWSVGITALLLLLSLPVLAGAITMLLTDRNLNTSFFDPAGGGDPILYQHL
>COLFC286-12|KJ965200|ZMUO.002471|Boreophilia
TLYFIFGAWAGMIGTSLSLLIRAELGNPGSLIGDDQIYNVIVTAHAFIMIFFMVMPIVIGGFGNWLVPLMLGAPDMAFPRMNNMSFWLLPPSLTLLLISSMVESGAGTGWTVYPPLSSNIAHGGSSVDLAIFSLHLAGISSILGAVNFISTVINMRSTGISF
DRMPLFVWSVAITALLLLLSLPVLAGAITMLLTDRNLNTSFFDPAGGGDPILYQHL
>COLFC620-12|KJ963513|ZMUO.003185|Hygrotus_novemlineatus
TLYFLFGAWSGMVGTSLSMLIRAELGNPGSLIGDDQIYNVIVTAHAFIMIFFMVMPIMIGGFGNWLVPLMLGAPDMAFPRMNNMSFWMLPPSLTLLLMSSMVESGAGTGWTVYPPLSAGIAHGGASVDLAIFSLHLAGISSILGAVNFITTIINMRSV
GMTFDRMPLFVWSVGITALLLLLSLPVLAGAITMLLTDRNLNTSFFDPAGGGDPILYQHL
>COLFC363-12|KJ964959|ZMUO.002548|Cryptophagus_badius
TLYFIFGAWAGMVGTSLSLLIRSELGTPGSLIGDDQIYNVIVTAHAFVMIFFMVMPIMIGGFGNWLVPLMLGAPDMAFPRMNNMSFWLLPPSLSLLLMSSIAEKGVGTGWTVYPPLSSNIAHGGSSVDLAIFSLHLAGISSILGAVNFISTVMNMHPTG
MNLDRMPLFVWAVIITAILLLLSLPVLAGAITMLLTDRNLNTSFFDPAGGGDPILYQHL
>COLFE276-12|KJ966666|ZMUO.005121|Bembidion_lampros
TLYFIFGAWSGMVGTSLSMLIRAELGNPGSLIGDDQIYNVIVTAHAFVMIFFMVMPILIGGFGNWLVPLMLGAPDMAFPRMNNMSFWLLPPSLSLLLMSSMVESGAGTGWTVYPPLSSSIAHSGASVDLAIFSLHLAGVSSILGAVNFITTIINMRSTG
MTFDRMPLFVWSVGITALLLLLSLPVLAGAITMLLTDRNLNTSFFDPAGGGDPILYQHL
>COLFD146-12|KJ965477|ZMUO.004041|Sitona_suturalis
TLYFIFGAWAGMVGTSLSMLIRTELGNPGSLIGDDQIYNVIVTAHAFIMIFFMVMPMMIGGFGNWLVPLMLGAPDMAFPRLNNMSFWLLPPSLTLLLMSSIVDKGAGTGWTVYPPLSANIAHEGASVDLAIFSLHMAGISSILGAINFISTIINMRPSG
MTFDRTTLFVWSVKITAILLLLSLPVLAGAITMLLTDRNINTTFFDPAGGGDPILYQHL
>COLFC764-12|KJ966493|ZMUO.003329|Batophila_rubi

TLYFIFGAWAGMIGTSLSMLIRTELGNPGSLIGNDQIYNVIVTAHAFIMIFFMVMPIMIGGFGNWLVPLMIGAPDMAFPRMNNMSFWLLPPSLSLLVSSSIVESGAGTGWTVYPPLSSNIAHSGSSVDLAIFSLHLAGISSILGAINFISTIINMRPHGMLL
DRLPLFVWAVMITAILLLLSLPVLAGAITMLLTDRNLNTSFFDPAGGGDPILYQHL
>COLFC322-12|KJ966119|ZMUO.002507|Atomaria_fulvipennis
TLYFIFGAWAGMVGTSLSMLIRTELGTPGSLIGDDQIYNVIVTAHAFIMIFFMVMPMMIGGFGNWLVPLMLGAPDMAFPRLNNMSFWLLPPSLMFLLMSSIVEKGAGTGWTVYPPLSANVAHAGSSVDLAIFSLHLAGISSILGSVNFITTVINMRPK
GMNFDRLPLFVWAVKITTILLLLSLPVLAGAITMLLTDRNINTSFFDPAGGGDPILYQHL
>COLFC625-12|KJ965146|ZMUO.003190|Hydroporus_notabilis
TLYFLFGAWSGMVGTSLSMLIRAELGNPGSLIGDDQIYNVIVTAHAFIMIFFMVMPIMIGGFGNWLVPLMLGAPDMAFPRMNNMSFWLLPPSLSLLLMSSMVENGAGTGWTVYPPLSSGIAHSGASVDLAIFSLHLAGISSILGAVNFITTIINMRSIG
MTFDRMPLFVWSVGITALLLLLSLPVLAGAITMLLTDRNLNTSFFDPAGGGDPILYQHL
>COLFD780-12|KJ964857|ZMUO.004770|Oedemera_femorata
TLYLIFGAWAGMVGTSLSLLIRAELGNPGSLIGDDQIYNVIVTAHAFIMIFFMVMPIMIGGFGNWLVPLMLGAPDMAFPRMNNMSFWLLPPSLTLLIMSSMVESGAGTGWTVYPPLSSNIAHSGSSVDLAIFSLHLAGVSSILGAVNFITTVINMRPAG
MNLDRMPLFVWAVVITAILLLLSLPVLAGAITMLLTDRNLNTSFFDPAGGGDPILYQHL
>COLFA273-12|KJ964699|ZMUO.000408|Cardiophorus_ruficollis
TLYFLFGAWSGMLGTSLSLLIRAELGSPGSLIGNDQIYNVIVTAHAFIMIFFMVMPIMIGGFGNWLMPLMLGAPDMAFPRMNNMSFWFLPPSLSLLLMSSIVENGAGTGWTVYPPLSSNIAHSGSSVDLAIFSLHLAGISSILGAVNFISTVINMRSPGM
TFERMPLFVWAVVITALLLLLSLPVLAGAITMLLTDRNLNTSFFDPAGGGDPILYQHL
>COLFE799-13|KJ966924|ZMUO.005644|Agonum_emarginatum
TLYFIFGAWSGMVGTSLSMLIRAELGNPGALIGDDQIYNVIVTAHAFIMIFFMVMPIMIGGFGNWLVPLMLGAPDMAFPRMNNMSFWLLPPSLTLLLMSSMVESGAGTGWTVYPPLSSGIAHAGASVDLAIFSLHLAGVSSILGAVNFITTIINMRSV
GMTFDRMPLFVWSVGITALLLLLSLPVLAGAITMLLTDRNLNTSFFDPAGGGDPILYQHL
>COLFB484-12|KJ965234|ZMUO.000959|Stenus_carbonarius
TLYFIFGSWAGMVGTSLSLLIRSELGNPGSLIGDDQIYNVIVTAHAFIMIFFMVMPVMIGGFGNWLIPLMLGAPDMAFPRMNNMSFWLLPPSLSLLLMSSIVESGAGTGWTVYPPLSSNIAHSGASVDLAIFSLHLAGISSILGAINFITTIFNMRTMNLQ
LDCLPLFVWSVGITAFLLLLSLPVLAGAITMLLTDRNLNTSFFDPAGGGDPILYQHL
>COLFA021-10|HM909049|MP00157|Dicheirotrichus_placidus
TLYFIFGAWSGMVGTSLSMLIRAELGTPGALIGDDQIYNVIVTAHAFVMIFFMVMPIMIGGFGNWLVPLMLGAPDMAFPRMNNMSFWLLPPSLTLLLMSSMVEKGAGTGWTVYPPLSSGIAHGGASVDLAIFSLHLAGISSILGAVNFITTIINMRSV
GMTFDRMPLFVWSVGITALLLLLSLPVLAGAITMLLTDRNLNTSFFDPAGGGDPILYQHL
>COLFB678-12|KJ964731|ZMUO.001153|Atheta_elongatula
TLYFIFGAWAGMVGTSLSLLIRAELGNPGSLIGDDQIYNVIVTAHAFVMIFFMVMPIVIGGFGNWLVPLMLGAPDMAFPRMNNMSFWLLPPSLTLLLMSSMVESGAGTGWTVYPPLSSNIAHGGSSVDLAIFSLHLAGISSILGAVNFISTVINMRSTGI
SFDRMPLFVWSVAITALLLLLSLPVLAGAITMLLTDRNLNTSFFDPAGGGDPILYQHL
>COLFE1044-13|KJ966573|ZMUO.006839|Proteinus_brachypterus
LLYFIFGAWAGMVGTSLSILIRAELGNPGTLIGDDQIYNVIVTAHAFIMIFFMVMPIVIGGFGNWLVPLMLGAPDMAFPRMNNMSFWLLPPSITLLLMSSMVENGAGTGWTVYPPLSSNISHSGSSVDLAIFSLHLAGISSILGAVNFITTVINMRSPGM
TFDRMPLFVWSVAITALLLLLSLPVLAGAITMLLTDRNINTTFFDPAGGGDPILYQHL
>COLFE152-12|KJ963936|ZMUO.005187|Rhagonycha_fulva
TLYFIFGAWSGSLGLALSLLIRAELGTPGTLIGNDQIYNVIVTAHAFIMIFFMVMPIMIGGFGNWLVPLMLGAPDMAFPRMNNMSFWFLPPSLMFLLMSSMVESGAGTGWTVYPPLSANIAHSGPSVDLAIFSLHMAGISSILGAVNFISTILNMKPPS
MKFDQMPLFVWSVGITALLLLLSLPVLAGAITMLLSDRNLNTSFFDPMGGGDPILYQHL
>COLFD134-12|KJ965779|ZMUO.004029|Gyrinus_opacus
TLYFIFGAWSGMVGTSLSMLIRAELGNPGSLIGDDQIYNVIVTAHAFIMIFFMVMPIMIGGFGNWLVPLMLGAPDMAFPRMNNMSFWLLPPSLTLLLMSSMVENGAGTGWTVYPPLSSNIAHGGASVDLAIFSLHLAGISSILGAVNFITTIINMRSV
GMTLDRMPLFVWSVGITALLLLLSLPVLAGAITMLLTDRNLNTSFFDPAGGGDPILYQHL
>COLFE204-12|KJ964798|ZMUO.005049|Rhantus_grapii
TLYFIFGAWAGMVGTSLSMLIRAELGNPGSLIGDDQIYNVIVTAHAFVMIFFMVMPIMIGGFGNWLVPLMLGAPDMAFPRMNNMSFWLLPPSLTLLLMSSMVESGAGTGWTVYPPLSSGIAHGGASVDLAIFSLHLAGISSILGAVNFITTIINMRSV
GMTFDRMPLFVWSVGITALLLLLSLPVLAGAITMLLTDRNLNTSFFDPAGGGDPILYQHL
>COLFE264-12|KJ962063|ZMUO.005109|Acylophorus_wagenschieberi
TLYFIFGSWAGMVGTSLSLLIRAELGNPGMLIGDDQIYNVIVTAHAFVMIFFMVMPIMIGGFGNWLVPLMLGAPDMAFPRMNNMSFWLLPPSLSLLLMSSMVESGAGTGWTVYPPLSSNIAHGGASVDLAIFSLHLAGISSILGAVNFITTVINMRSI
GMTFDRMPLFVWSVAITALLLLLSLPVLAGAITMLLTDRNLNTSFFDPTGGGDPILYQHL
>COLFD678-12|KJ962675|ZMUO.004668|Cryptocephalus_pini
TLYFIFGAWAGMVGTSLSLLIRIELGNPGSLIGNDQIYNTIVTAHAFIMIFFMVMPIMIGGFGNWLVPLMLGAPDMAFPRMNNMSFWLLPPSLTLLLMSSIVENGAGTGWTVYPPLSATIAHTGASVDLAIFSLHLAGMSSIMGAINFISTVINMRPQG
MLMDRTPLFVWAVLITAVLLLLSLPVLAGAITMLLTDRNLNTSFFDPAGGGDPILYQHL
>COLFA359-12|KJ966678|ZMUO.000589|Acrotona_fungi
TLYFIFGAWAGMVGTSLSLLIRAELGNPGSLIGDDQIYNVIVTAHAFIMIFFMVMPIMIGGFGNWLVPLMLGAPDMAFPRMNNMSFWLLPPSLTLLLMSSMVESGVGTGWTVYPPLSSNIAHGGSSVDLAIFSLHLAGISSILGAVNFISTVINMRSPGI
TFDRMPLFVWAVAITALLLLLSLPVLAGAITMLLTDRNLNTSFFDPAGGGDPILYQHL

>COLFC375-12|KJ963912|ZMUO.002560|Aleochara_curtula
TLYFIFGAWAGMVGTSLSLLIRAELGNPGSLIGDDQIYNVIVTAHAFIMIFFMVMPIVIGGFGNWLVPLMLGAPDMAFPRMNNMSFWLLPPSLTLLLMSSMVESGAGTGWTVYPPLSSNIAHGGSSVDLAIFSLHLAGISSILGAVNFISTVINMRSMG
MSFDKMPLFVWSVVITALLLLLSLPVLAGAITMLLTDRNLNTSFFDPAGGGDPILYQHL
>COLFE1271-13|KJ964417|ZMUO.007066|Hippodamia_septemmaculata
TLYFLFGMWAGMVGTSLSILIRLELGTPGSLIGNDQIYNVIVTAHAFIMIFFMVMPIMIGGFGNWLVPLMIGAPDMAFPRLNNMSFWLLPPALTLLMFSSMVEMGAGTGWTVYPPLSSNLAHNGPSVDLVIFSLHLAGISSILGAVNFISTIMNMRPY
GMSMDKTPLFVWSVLITAILLLLSLPVLAGAITMLLTDRNLNTSFFDPTGGGDPILYQHL
>COLFC259-12|KJ963792|ZMUO.002444|Gyrophaena_affinis
TLYFIFGAWSGMVGTSLSLLIRAELGNPGSLIGDDQIYNVIVTAHAFIMIFFMVMPVVIGGFGNWLVPLMLGAPDMAFPRMNNMSFWLLPPSLSLLLMSSMVESGAGTGWTVYPPLSSNIAHGGASVDLAIFSLHLAGISSILGAVNFISTIINMRALG
MSFDRMPLFVWSVAITALLLLLSLPVLAGAITMLLTDRNLNTSFFDPAGGGDPILYQHL
>COLFD316-12|KJ964248|ZMUO.004211|Dorytomus_melanophthalmus
TLYFIFGAWSGMVGTSLSMLIRTELGNPGSLIGDDQIYNTIVTAHAFIMIFFMVMPIMIGGFGNWLIPLMLGAPDMAFPRLNNMSFWLLPPSLSLLLMSSIVDKGAGTGWTVYPPLSSNIAHEGTSVDLAIFSLHMAGISSILGAMNFISTVMNMRPAG
MKTDRMSLFIWAVKITAILLLLSLPVLAGAITMLLTDRNINTSFFDPAGGGDPILYQHL
>COLFD015-12|KJ967212|ZMUO.003910|Aphodius_distinctus
TLYFLFGSWAGMVGTSLSLLIRAELGNPGTLIGDDQIYNVIVTAHAFVMIFFMVMPILIGGFGNWLVPLMLGAPDMAFPRMNNMSFWLLPPSLTLLLMSSMVESGAGTGWTVYPPLSSNIAHGGASVDLAIFSLHLAGISSILGAVNFITTVINMRSPG
MTFDRMPLFVWSVAITALLLLLSLPVLAGAITMLLTDRNLNTSFFDPAGGGDPILYQHL
>LEFIJ1893-13|KJ962311|ZMUO.004648|Meligethes_aeneus
TLYFIFGAWSGMVGTSLSMLIRTELGNPGSLIGNDQIYNVIVTAHAFVMIFFMVMPFMIGGFGNWLVPLMLGAPDMAFPRMNNMSFWLLPPSLSLLLMSSIVESGAGTGWTVYPPLSSNIAHGGASVDLAIFSLHLAGISSILGAVNFITTVINMRPK
GMTFDRMPLFVWAVMITAILLLLSLPVLAGAITMLLTDRNLNTTFFDPSGGGDPILYQHL
>COLFB560-12|KJ962807|ZMUO.001035|Chrysolina_polita
TLYFIFGIWAGMVGTSLSILIRTELGSPGTLIGNDQIYNVIVTAHAFIMIFFMVMPIMIGGFGNWLIPLMLGAPDMAFPRMNNMSFWLLPPSLLLLLMSSMMENGVGTGWTVYPPLSANIAHSGPAVDLAIFSLHLAGISSILGAINFISTTTNMRSTGM
SFEQMPLFSWAVLITVILLLLSLPVLAGAITMLLTDRNLNTSFFDPASGGDPILYQHL
>COLFA093-10|HM909109|MP00329|Sericoderus_lateralis
MLYFIFGMWAGMVGTSLSLLIRLELGNPGFLIGDDQIYNVIVTAHAFIMIFFMVMPIMMGGFGNWLVPLMLGAPDMAFPRMNNMSFWLLPPSLTLLIMSSVVESGAGTGWTVYPPLSSNIAHGGASVDLAIFSLHLAGISSILGAINFITTILNMRPA
GMNFEQMPLFVWSVLFTAILLLLSLPILAGAITMLLTDRNLNTSFFDPAGGGDPILYQHL
>COLFB163-12|KJ964565|ZMUO.001493|Magdalis_frontalis
ILYFIFGTWSGMIGTSLSMLIRVELGSPGSLIGNDQIYNTIVTAHAFIMIFFMVMPIMIGGFGNFLIPLMLGAPDMAFPRLNNLSFWLLPPSLTLLLLSSIIDKGAGTGWTVYPPLSSSIFHEGTSVDLAIFSLHLAGISSILGALNFISTIINMRPKNMKLDQMS
LFIWAVKITAILLLLSLPVLAGAITMLLTDRNINTSFFDPAGGGDPILYQHL
>COLFF592-13|KJ966410|ZMUO.006482|Protapion_assimile
TLYFIFGLWSGMIGTSLSMLIRIELGNPGSLIGNDQIYNVIVTAHAFIMIFFMVMPVMIGGFGNWLVPLMLGAPDMAFPRMNNMSFWLLPPSLTLLLMSSIVEKGAGTGWTVYPPLAANIAHSGASVDLAIFSLHLAGISSILGAVNFISTIMNMRPTG
MSLDQLSLFTWAVKITAILLLLSLPVLAGAITMLLTDRNINTSFFDPAGGGDPILYQHL
>COLFB866-12|KJ963259|ZMUO.001911|Anoplodera_sanguinolenta
TLYFIFGAWASMVGTSLSLLIRSELGNPGSLIGNDQIYNVIVTAHAFVMIFFMVMPIMIGGFGNWLVPLMLGAPDMAFPRMNNMSFWLLPPSLILLIMSSIVESGAGTGWTVYPPLSSNIAHSGSSVDLAIFSLHLAGISSILGAVNFITTVINMRPAGLK
PEQMPLFVWAVVITAVLLLLSLPVLAGAITMLLTDRNLNTSFFDPAGGGDPILYQHL
>COLFD617-12|KJ965382|ZMUO.004417|Phyllobius_oblongus
TLYFIFGTWSGMVGTSLSILIRAELGNPGSLIGDDQIYNVIVTAHAFIMIFFMVMPMMIGGFGNWLVPLMLGAPDMAFPRLNNMSFWLLPPSLSFLLMSSIVDKGAGTGWTVYPPLSANIAHEGSSVDLAIFSLHMAGVSSILGAINFISTVINMRPTG
MSFDHMSLFIWAVKITAILLLLSLPVLAGAITMLLTDRNMNTSFFDPAGGGDPILYQHL
>COLFE1104-13|KJ965375|ZMUO.006899|Olophrum_boreale
TLYFIFGAWAGMVGTSLSILIRAELGNPGTLIGDDQIYNVIVTAHAFVMIFFMVMPILIGGFGNWLVPLMLGAPDMAFPRMNNMSFWLLPPSLTLLLMSSMVESGAGTGWTVYPPLSSNIAHGGASVDLAIFSLHLAGISSILGAVNFITTVINMRTTG
MTFDRMPLFVWAVAITALLLLLSLPVLAGAITMLLTDRNLNTTFFDPAGGGDPILYQHL
>COLFE1505-13|KJ961825|ZMUO.007300|Trichosirocalus_troglodytes
TLYFIFGAWAGATGTSLSMLIRTELGNPGSLIGNDQIYNSIVTAHAFIMIFFMVMPILIGGFGNWLVPLMLGAPDMAFPRLNNMSFWLLPPSLTLLLMSSIVNKGAGTGWTVYPPLSANVAHEGASVDLAIFSLHMAGISSILGAINFISTVMNMRPTG
MNSEFMPLFVWAVEITAVLLLLSLPVLAGAITMLLTDRNINTSFFDPAGGGDPILYQHL
>COLFD738-12|KJ962624|ZMUO.004728|Diaperis_boleti
TLYFIFGAWSGMVGTSLSLLIRAELGNPGSLIGDDQIYNVIVTAHAFIMIFFMVMPIMIGGFGNWLVPLMLGAPDMAFPRMNNMSFWLLPPSLTLLLMSSIVENGAGTGWTVYPPLSSNIAHGGSSVDLAIFSLHLAGISSILGAVNFITTIINMRPQGM
TFDRMPLFVWAVIITAVLLLLSLPVLAGAITMLLTDRNINTSFFDPAGGGDPILYQHL
>COLFD685-12|KJ963933|ZMUO.004675|Anatis_ocellata

TLYFLFGMWAGMVGTSLSILIRLELGTTNSLIGNDQIYNVIVTAHAFIMIFFMVMPIMIGGFGNWLVPLMIGAPDMAFPRLNNMSFWLLPPALTMLIMSSIVEMGAGTGWTVYPPLSSNMAHSGSSVDLVIFSLHLAGISSILGAVNFISTIMNMRPFG
MTLEKTPLFVWSVMITAILLLLSLPVLAGAITMLLTDRNINTSFFDPMGGGDPILYQHL
>COLFD851-12|KJ963483|ZMUO.004841|Amphimallon_solstitiale
TLYFLFGSWAGMVGTSLSLLIRAELGNPGTLIGDDQIYNVIVTAHAFVMIFFMVMPIMIGGFGNWLVPLMLGAPDMAFPRMNNMSFWLLPPSLTLLLMSSLVESGAGTGWTVYPPLSSNIAHSGASVDLAIFSLHLAGISSILGAVNFITTVINMRSTG
MTFDRMPLFVWSVALTALLLLLSLPVLAGAITMLLTDRNINTSFFDPAGGGDPILYQHL
>COLFB435-12|KJ966183|ZMUO.001860|Perapion_curtirostre
TLYFILGVWSGLVGTSLSMLIRVELGNPGSLIGDDQIYNVIVTAHAFIMIFFMVMPIMIGGFGNWLVPLMLGAPDMAFPRMNNMSFWLLPPALTLLLMSSIVEKGAGTGWTVYPPLASNIAHEGSSVDLAIFSLHLAGISSILGAVNFISTIMNMRPSGL
SLDQLSLFTWAVKITAILLLLSLPVLAGAITMLLTDRNINTSFFDPAGGGDPILYQHL
>COLFA491-12|KJ964168|ZMUO.000531|Anotylus_rugosus
TLYFIFGAWSGMVGTSLSMLIRAELGAPGSLIGDDQIYNVIVTAHAFIMIFFMVMPIVIGGFGNWLVPLMLGAPDMAFPRMNNMSFWLLPPSLTLLLFSSIVESGAGTGWTVYPPLSSNIAHSGSSVDLAIFSLHLAGISSILGAVNFISTIINMRSTGMSF
DRMPLFVWSVNITAILLLLSLPVLAGAITMLLTDRNLNTSFFDPAGGGDPILYQHL
>COLFE323-12|KJ962024|ZMUO.004883|Diaperis_boleti
TLYFIFGAWSGMVGTSLSLLIRAELGNPGSLIGDDQIYNVIVTAHAFIMIFFMVMPIMIGGFGNWLVPLMLGAPDMAFPRMNNMSFWLLPPSLTLLLMSSIVENGAGTGWTVYPPLSSNIAHGGSSVDLAIFSLHLAGISSILGAVNFITTIINMRPQGM
TFDRMPLFVWAVIITAVLLLLSLPVLAGAITMLLTDRNINTSFFDPAGGGDPILYQHL
>COLFC038-12|KJ964080|ZMUO.002033|Aphodius_rufus
TLYFLFGSWAGMVGTSLSLLIRAELGNPGSLIGDDQIYNVIVTAHAFVMIFFMVMPILIGGFGNWLVPLMLGAPDMAFPRMNNMSFWLLPPSLTLLLMSSMVESGAGTGWTVYPPLSSNIAHGGASVDLAIFSLHLAGISSILGAVNFITTVINMRSM
GMTFDRMPLFVWSVAITALLLLLSLPVLAGAITMLLTDRNLNTSFFDPAGGGDPILYQHL
>COLFD662-12|KJ962938|ZMUO.004462|Paradromius_linearis
TLYFIFGAWAGMVGTSLSMLIRAELGNPGALIGDDQIYNVIVTAHAFIMIFFMVMPIMIGGFGNWLVPLMLGAPDMAFPRMNNMSFWLLPPSLTLLLMSSMVESGAGTGWTVYPPLSSGIAHAGASVDLAIFSLHLAGISSILGAVNFITTIINMRSIG
MTFDRMPLFVWSVGITALLLLLSLPVLAGAITMLLTDRNLNTSFFDPAGGGDPILYQHL
>COLFC565-12|KJ962175|ZMUO.003130|Amara_torrida
TLYFIFGTWSGMVGTSLSMLIRAELGNPGALIGDDQIYNVIVTAHAFIMIFFMVMPIMIGGFGNWLVPLMLGAPDMAFPRMNNMSFWLLPPSLTLLLMSSMVDKGAGTGWTVYPPLSSNIAHAGASVDLAIFSLHLAGISSILGAVNFITTIINMRTV
GMTFDRMPLFVWSVGITALLLLLSLPVLAGAITMLLTDRNLNTSFFDPAGGGDPILYQHL
>COLFD867-12|KJ964551|ZMUO.004477|Cerylon_ferrugineum
TLYFMFGMWSGMVGTSMSMMIRLELGNPGSLIGDDQIYNVIVTAHAFVMIFFMVMPIMIGGFGNWLVPLMLGAPDMAFPRMNNMSFWLLPPSLTLLIMSSIVEKGAGTGWTVYPPLSANLTHSGSSVDLAIFSLHLAGISSILGAVNFITTVINMR
PSGMTWDRLPLFVWSVIITAVLLLLSLPVLAGAITMLLTDRNLNTSFFDPAGGGDPILYQHL
>COLFD812-12|KJ965593|ZMUO.004802|Laccobius_sinuatus
TLYFIFGAWAGMVGTSLSILIRAELGNPGTLIGDDQIYNVIVTAHAFIMIFFMVMPIMIGGFGNWLVPLMLGAPDMAFPRMNNMSFWLLPPSLTLLLMSSMVESGAGTGWTVYPPLSSNIAHGGASVDLAIFSLHLAGISSILGAVNFITTVINMRSNN
MTYDRLPLFVWSVAITALLLLLSLPVLAGAITMLLTDRNLNTSFFDPAGGGDPILYQHL
>COLFE1093-13|KJ966957|ZMUO.006888|Malthodes_pumilus
TLYFMFGAWSGMLGTSLSLLIRAELGSPGSLIGNDQIYNVIVTAHAFIMIFFMVMPIMIGGFGNWLVPLMLGAPDMAFPRMNNMSFWFLPPSLSLLLMSSLVESGAGTGWTVYPPLSSNIAHSGASVDLAIFSLHMAGISSILGAVNFISTVINMRSTG
MTFDRMPLFVWAVAITALLLLLSLPVLAGAITMLLTDRNLNTSFFDPAGGGDPILYQHL
>COLFE1097-13|KJ966953|ZMUO.006892|Agabus_guttatus
TLYFIFGAWAGMVGTSLSMLIRAELGNPGSLIGDDQIYNVIVTAHAFVMIFFMVMPIMIGGFGNWLVPLMLGAPDMAFPRMNNMSFWLLPPSLTLLLMSSMVEKGAGTGWTVYPPLSSGIAHSGASVDLAIFSLHLAGISSILGAVNFITTIINMRSV
GMTFDRMPLFVWSVGITALLLLLSLPVLAGAITMLLTDRNLNTSFFDPAGGGDPILYQHL
>COLFE955-13|KJ966442|ZMUO.006750|Tachinus_schneideri
TLYFIFGAWAGMVGTSLSLLIRAELGNPGTLIGDDQIYNVIVTAHAFIMIFFMVMPIVIGGFGNWLVPLMLGAPDMAFPRMNNMSFWLLPPSLSLLLMSSMVESGAGTGWTVYPPLSSNIAHGGSSVDLAIFSLHLAGISSILGAVNFITTVINMRSIGM
TFDRMPLFVWSVAITALLLLLSLPVLAGAITMLLTDRNLNTTFFDPAGGGDPILYQHL
>COLFB156-12|KJ964115|ZMUO.001486|Amara_alpina
TLYFIFGTWSGMVGTSLSMLIRAELGNPGALIGDDQIYNVIVTAHAFIMIFFMVMPIMIGGFGNWLVPLMLGAPDMAFPRMNNMSFWLLPPSLTLLLMSSMVDKGAGTGWTIYPPLSSNIAHAGASVDLAIFSLHLAGISSILGAVNFITTIINMRTVG
MTFDRMPLFVWSVGITALLLLLSLPVLAGAITMLLTDRNLNTSFFDPAGGGDPILYQHL
>COLFF106-13|KJ963795|ZMUO.003740|Leiodes_inordinata
TLYFIFGAWAGMIGTSLSILIRAELGNPGSLIGDDQIYNVIVTAHAFIMIFFMVMPVVIGGFGNWLVPLMLGAPDMAFPRMNNMSFWLLPPSLTLLLMSSIVENGAGTGWTVYPPLSSNIAHSGSSVDLAIFSLHLAGISSILGAVNFITTVINMRSAGM
TFDKMPLFVWSVAITALLLLLSLPVLAGAITMLLTDRNLNTSFFDPAGGGDPILYQHL
>COLFB173-12|KJ964678|ZMUO.001503|Enochrus_quadripunctatus
TLYFIFGAWAGMVGTSLSILIRAELGNPGTLIGDDQIYNVIVTAHAFIMIFFMVMPIMIGGFGNWLVPLMLGAPDMAFPRMNNMSFWLLPPSLTLLLMSSMVESGAGTGWTVYPPLSSNIAHGGASVDLAIFSLHLAGISSILGAVNFITTVINMRSPS
MTYDRLPLFVWSVAITALLLLLSLPVLAGAITMLLTDRNLNTSFFDPAGGGDPILYQHL

>COLFC582-12|KJ963299|ZMUO.003147|Syneta_betulae
TLYFIFGTWSGMVGTSLSMLIRAELGNPGTLIGNDQIYNVIVTAHAFIMIFFMVMPIMIGGFGNWLVPLMLGAPDMAFPRMNNMSFWLLPPSLSLLLMSSIVENGAGTGWTVYPPLSANIAHSGSSVDLAIFSLHLAGISSILGAVNFITTVINMRPEK
MTLDRMSLFIWAVTITAILLLLSLPVLAGAITMLLTDRNLNTSFFDPAGGGDPILYQHL
>COLFC590-12|KJ966293|ZMUO.003155|Rhinusa_antirrhini
TLYFIFGAWAGMAGTSMSMIIRTELGSPGKFIGNDQIYNSIVTAHAFIMIFFMVMPVMIGGFGNWLVPLMMGAPDMAFPRLNNMSFWLLPPSLILLLSSSMVDKGAGTGWTVYPPLSANMSHEGSSVDLAIFSLHMAGISSILGAMNFISTVMNM
KPNGMKSEQSTLFIWSVKITALLLLLSLPVLAGAISMLLTDRNINTSFFDPAGGGDPVLYQHL
>COLFD004-12|KJ964978|ZMUO.003899|Orthotomicus_suturalis
TLYFIFGAWAGMVGTSLSMLIRTELGSPGSLIGDDQIFNTIVTAHAFIMIFFMVMPILIGGFGNWLVPLMLGAPDMAFPRLNNMSFWLLPPSLTFLIMSSIIDKGAGTGWTVYPPLSSNLAHEGVSVDLAIFSLHMSGVSSILGAINFISTIINMHPKGVTP
EQLTLFTWAVKITAVLLLLSLPVLAGAITMLLTDRNINTSFFDPAGGGDPILYQHL
>COLFA071-10|HM909089|MP00261|Philonthus_cognatus
TLYFIFGSWAGMVGTSLSLLIRAELGNPGSLIGDDQIYNVIVTAHAFIMIFFMVMPIMIGGFGNWLVPLMLGAPDMAFPRMNNMSFWLLPPSLTLLLMSSMVESGAGTGWTVYPPLSSNIAHDGASVDLAIFSLHLAGISSILGAVNFITTVINMRSAG
MSFDRMPLFVWSVAITALLLLLSLPVLAGAITMLLTDRNLNTTFFDPTGGGDPILYQHL
>COLFE209-12|KJ966523|ZMUO.005054|Nebria_livida
TLYFIFGAWASMVGTSLSMLIRAELGNPGSLIGDDQIYNVIVTAHAFVMIFFMVMPIMIGGFGNWLVPLMLGAPDMAFPRMNNMSFWLLPPSLTLLLMSSMVESGAGTGWTVYPPLSSSIAHSGASVDLAIFSLHLAGVSSILGAVNFITTIINMRSV
GMTFDRMPLFVWSVGITALLLLLSLPVLAGAITMLLTDRNLNTSFFDPAGGGDPILYQHL
>COLFE1034-13|KJ965208|ZMUO.006829|Philonthus_quisquiliarius
TLYFIFGSWAGMVGTSLSLLIRAELGNPGSLIGDDQIYNVIVTAHAFIMIFFMVMPIVIGGFGNWLVPLMLGAPDMAFPRMNNMSFWLLPPSLTLLLMSSMVESGAGTGWTVYPPLSSNIAHGGASVDLAIFSLHLAGISSILGAVNFITTVINMRSSG
MSFDRMPLFVWSVAITALLLLLSLPVLAGAITMLLTDRNLNTTFFDPAGGGDPILYQHL
>COLFB778-12|KJ965389|ZMUO.001253|Atheta_intermedia
TLYFIFGAWAGMIGTSLSLLIRAELGNPGSLIGDDQIYNVIVTAHAFIMIFFMVMPVVIGGFGNWLVPLMLGAPDMAFPRMNNMSFWLLPPSLTLLLMSSMVESGAGTGWTVYPPLSSNIAHGGSSVDLAIFSLHLAGISSILGAVNFISTVINMRSTGI
SFDRMPLFVWSVVITALLLLLSLPVLAGAITMLLTDRNLNTSFFDPAGGGDPILYQHL
>COLFA040-10|HM909065|MP00197|Ampedus_tristis
TLYFIFGAWAGMLGTSLSLLIRAELGNPGSLIGNDQIYNVIVTAHAFIMIFFMVMPIMIGGFGNWLVPLMLGAPDMAFPRMNNMSFWLLPPSLSLLLMSSIVENGAGTGWTVYPPLSSNIAHSGSSVDLAIFSLHLAGISSILGAVNFISTVINMRSTGIT
FDRMPLFVWAVAITALLLLLSLPVLAGAITMLLTDRNLNTSFFDPAGGGDPILYQHL
>COLFD681-12|KJ965619|ZMUO.004671|Calvia_quatuordecimguttata
TLYFLFGMWAGMVGTSLSILIRLELGTTNSLIGNDQIYNVIVTAHAFIMIFFMVMPIMIGGFGNWLVPLMIGAPDMAFPRLNNMSFWLLPPALTLLIFSSMVEMGAGTGWTVYPPLSSNIAHSGSSVDLVIFSLHLAGISSILGAVNFISTIMNMRPFGM
NLDKTPLFVWSVLITAILLLLSLPVLAGAITMLLTDRNINTSFFDPMGGGDPILYQHL
>COLFF718-13|KJ965240|ZMUO.006418|Dorcus_parallelipipedus
TLYFLLGSWSGMVGTSLSILIRAELGNPGSLIGDDQIYNVIVTAHAFIMIFFMVMPIMIGGFGNWLVPLMLGAPDMAFPRMNNMSFWLLPPSLTFLLMSSMVENGAGTGWTVYPPLSSNIAHSGASVDLAIFSLHLAGISSILGAVNFITTVINMRATGI
TFDRMPLFVWAVVLTALLLLLSLPVLAGAITMLLTDRNINTTFFDPAGGGDPILYQHL
>COLFA407-12|KJ966625|ZMUO.000637|Byturus_tomentosus
TLYFIFGAWAGMVGTSLSLLIRSELGNPGSLIGDDQIYNVIVTAHAFIMIFFMVMPIVIGGFGNWLVPLMLGAPDMAFPRMNNMSFWLLPPSLTLLLMSSIVESGAGTGWTVYPPLSSNIAHGGSSVDLAIFSLHLAGISSILGAVNFITTVINMRPAGM
TLDRMPLFVWSVVITAILLLLSLPVLAGAITMLLTDRNLNTSFFDPSGGGDPILYQHL
>COLFF122-13|KJ965160|ZMUO.003756|Aphodius_plagiatus
TLYFLFGSWAGMVGTSLSLLIRAELGNPGTLIGDDQIYNVIVTAHAFVMIFFMVMPILIGGFGNWLVPLMLGAPDMAFPRMNNMSFWLLPPSLTLLLMSSMVEQGAGTGWTVYPPLSSNIAHGGASVDLAIFSLHLAGISSILGAVNFITTVINMRSPG
MTFDRMPLFVWSVAITALLLLLSLPVLAGAITMLLTDRNLNTSFFDPAGGGDPILYQHL
>COLFF588-13|KJ964671|ZMUO.006478|Cassida_sanguinosa
TLYFIFGFWSGMVGTSLSILIRAELGNPGTLIGNDQIYNSIVTAHAFIMIFFMVMPIMIGGFGNWLVPLMLGAPDMAFPRLNNMSFWLLPPSITFLIMSSIIESGAGTGWTVYPPLSSNIAHSGASVDLAIFSLHLAGISSILGAINFISTIMNMQPAGMSL
DKMPLFVWAVIITAILLLLSLPVLAGAITMLLTDRNFNTSFFDPAGGGDPILYQHL
>COLFE1513-13|KJ963951|ZMUO.007308|Microlestes_minutulus
TLYFIFGAWAGMVGTSLSMLIRAELGNPGALIGDDQIYNVIVTAHAFIMIFFMVMPIMIGGFGNWLVPLMLGAPDMAFPRMNNMSFWLLPPSLTLLLMSSMVESGAGTGWTVYPPLSSGIAHAGASVDLAIFSLHLAGVSSILGAVNFITTIINMRSV
GMSFERMPLFVWSVGITALLLLLSLPVLAGAITMLLTDRNLNTTFFDPAGGGDPILYQHL
>COLFA154-10|KJ964620|MP00416|Ctenicera_pectinicornis
TLYFLFGAWAGMLGTSLSLLIRAELGNPGSLIGNDQIYNVIVTAHAFIMIFFMVMPIMIGGFGNWLVPLMLGAPDMAFPRMNNMSFWFLPPSLSLLLMSSIVENGAGTGWTVYPPLSANIAHSGSSVDLAIFSLHLAGISSILGAVNFISTVINMRSTGIT
FDRMPLFVWAVAITALLLLLSLPVLAGAITMLLTDRNLNTSFFDPAGGGDPILYQHL
>COLFA573-12|KJ962917|ZMUO.000708|Acmaeops_septentrionis

MLYFIFGAWAGMVGTSLSLLIRSELGNPGSLIGNDQIYNVIVTAHAFIMIFFMVMPVMIGGFGNWLVPLMLGAPDMAFPRLNNMSFWLLPPSLTLLIMSSVVESGAGTGWTVYPPLSSNIAHGGSSVDLAIFSLHLAGISSILGAINFITTVINMRPKGM
TFDRMPLFVWAVVITAILLLLSLPVLAGAITMLLTDRNLNTSFFDPAGGGDPILYQHL
>COLFB183-12|KJ965079|ZMUO.001513|Cercyon_marinus
TLYFIFGAWAGMVGTSLSILIRAELGNPGTLIGDDQIYNVIVTAHAFIMIFFMVMPIMIGGFGNWLVPLMLGAPDMAFPRMNNMSFWLLPPSLTLLLMSSMVESGAGTGWTVYPPLSSNIAHGGASVDLAIFSLHLAGISSILGAVNFITTVINMRSPSL
TYDRLPLFVWSVAITALLLLLSLPVLAGAITMLLTDRNLNTSFFDPAGGGDPILYQHL
>COLFD200-12|KJ967226|ZMUO.004095|Atomaria_lewisi
TLYFIFGAWSGMVGTSLSMLIRTELGTPGSLIGDDQIYNVIVTAHAFIMIFFMVMPMMIGGFGNWLVPLMLGAPDMAFPRLNNMSFWLLPPSLMFLLMSSIVEKGAGTGWTVYPPLSSNVAHAGSSVDLAIFSLHLAGISSILGSVNFITTVINMRPKG
MNFDRLPLFVWAVKITTILLLLSLPVLAGAITMLLTDRNINTSFFDPAGGGDPILYQHL
>COLFD098-12|KJ962087|ZMUO.003993|Orchesia_micans
TLYFIFGAWSGMVGTSLSLLIRSELGNPGSLIGNDQIYNVIVTAHAFIMIFFMVMPVMMGGFGNWLVPLMLGAPDMAFPRMNNMSFWLLPPSLTLLIMSSIVENGAGTGWTVYPPLSSNIAHNGSSVDLAIFSLHLAGISSILGSVNFLTTIINMRPTG
MSFDRMPLFVWAVGITAVLLLLSLPVLAGAITMLLTDRNLNTSFFDPAGGGDPILYQHL
>COLFE1039-13|KJ966007|ZMUO.006834|Georissus_crenulatus
TLYFIFGAWSGMVGTSLSILIRAELGNPGTLIGDDQIYNVIVTAHAFIMIFFMVMPIMIGGFGNWLVPLMLGAPDMAFPRMNNMSFWLLPPSLTLLLMSSFVESGAGTGWTVYPPLSSNIAHSGPSVDLAIFSLHLAGISSILGAINFITTVINMRSKNLT
YDRLPLFVWSVAITALLLLLSLPVLAGAITMLLTDRNLNTSFFDPAGGGDPILYQHL
>COLFD732-12|KJ962970|ZMUO.004722|Melolontha_hippocastani
TLYFLFGSWAGMVGTSLSLLIRAELGNPGTLIGDDQIYNVIVTAHAFVMIFFMVMPIMIGGFGNWLVPLMLGAPDMAFPRMNNMSFWLLPPSLSLLMSSLVENGAGTGWTVYPPLSSNIAHSGASVDLAIFSLHLAGISSILGAVNFITTVINMRSTG
MTFDRMPLFAWSVALTALLLLLSLPVLAGAITMLLTDRNINTSFFDPAGGGDPILYQHL
>COLFC325-12|KJ965548|ZMUO.002510|Atheta_spatuloides
TLYFIFGAWAGMVGTSLSLLIRAELGNPGSLIGDDQIYNVIVTAHAFIMIFFMVMPIMIGGFGNWLIPLMLGAPDMAFPRMNNMSFWLLPPSLSLLLMSSMVESGAGTGWTVYPPLSSNIAHGGASVDLAIFSLHLAGISSILGAVNFISTVINMRSTGI
TFDRMPLFVWAVVITALLLLLSLPVLAGAITMLLTDRNLNTSFFDPAGGGDPILYQHL
>COLFF509-13|KJ963182|ZMUO.006589|Haliplus_fulvus
TLYFIFGAWAGMVGTSLSMLIRAELGTPGSLIGDDQIYNVIVTAHAFIMIFFMVMPIMIGGFGNWLVPLMLGAPDMAFPRMNNMSFWLLPPSLSLLLMSSMVENGAGTGWTVYPPLSAGIAHSGSSVDLAIFSLHLAGISSILGAVNFITTIINMRSM
GMTFDRMPLFVWSVGITALLLLLSLPVLAGAITMLLTDRNLNTSFFDPSGGGDPILYQHL
>COLFC242-12|KJ967348|ZMUO.002427|Rhinoncus_bruchoides
TLYFIFGSWAGTVGTSLSMLIRTELGTPGSLIGNDQIYNSIVTAHAFIMIFFMVMPILIGGFGNWLVPLMLGAPDMAFPRLNNMSFWLLPPSIMLLLMSSIVNKGAGTGWTVYPPLSSNITHEGASVDLAIFSLHMAGISSILGAINFISTIMNMRPQGM
SYDKMPLFSWAVLITAILLLLSLPVLAGAITMLLTDRNINTSFFDPAGGGDPILYQHL
>COLFB053-12|KJ963031|ZMUO.001383|Halyzia_sedecimguttata
TLYFLFGMWAGMVGTSLSIMIRLELGTTNSLIGNDQIYNVIVTAHAFIMIFFMVMPVMIGGFGNWLVPLMVGAPDMAFPRLNNMSFWLLPPALTLLIFSSMVEMGAGTGWTVYPPLSSNMAHSGSSVDLVIFSLHLAGISSILGAVNFISTIMNMRP
FGMNLDKTPLFVWSVLITAILLLLSLPVLAGAITMLLTDRNINTSFFDPMGGGDPILYQHL
>COLFE1506-13|KJ963703|ZMUO.007301|Longitarsus_exsoletus
TLYFIFGVWAGMVGTSLSILIRTELGNPGSLIGNDQIYNVIVTAHAFIMIFFMVMPIMIGGFGNWLVPLMIGAPDMAFPRMNNMSFWLLPPSLLLLVMSSMVESGAGTGWTVYPPLSSNIAHNGSSVDLAIFSLHLAGISSILGAINFITTVINMRPFGM
TLDRMPLFVWAVVITAILLLLSLPVLAGAITMLLTDRNFNTTFFDPAGGGDPILYQHL
>COLFE1471-13|KJ967377|ZMUO.007266|Longitarsus_luridus
ILYFIFGIWAGMIGTSLSILIRTELGNPGSLIGNDQIYNVIVTAHAFIMIFFMVMPIMIGGFGNWLVPLMIGAPDMAFPRMNNMSFWLLPPSLFLLVMSSMVESGAGTGWTVYPPLSSNIAHGGSSVDLAIFSLHLAGISSILGAINFITTVINMRPIGMTL
DRMPLFVWAVVITAILLLLSLPVLAGAITMLLTDRNLNTTFFDPAGGGDPILYQHL
>COLFC643-12|KJ964942|ZMUO.003208|Agabus_lapponicus
TLYFIFGAWAGMVGTSLSMLIRAELGNPGSLIGDDQIYNVIVTAHAFVMIFFMVMPIMIGGFGNWLIPLMLGAPDMAFPRMNNMSFWLLPPSLTLLLMSSMVESGAGTGWTVYPPLSSGIAHGGASVDLAIFSLHLAGISSILGAVNFITTIINMRSVG
MTFDRMPLFVWSVGITALLLLLSLPVLAGAITMLLTDRNLNTSFFDPAGGGDPILYQHL
>COLFE312-12|KJ962141|ZMUO.004872|Anisotoma_humeralis
TLYFIFGAWSGMVGTSLSILIRAELGTPGSLIGDDQIYNVIVTAHAFVMIFFMVMPIMIGGFGNWLVPLMLGAPDMAFPRMNNMSFWLLPPSLTLLLMSSMVENGAGTGWTVYPPLSANISHSGSSVDLAIFSLHLAGISSILGAVNFITTVINMRSIG
MTFDKMPLFVWSVVITALLLLLSLPVLAGAITMLLTDRNLNTSFFDPAGGGDPILYQHL
>COLFF764-13|KJ967168|ZMUO.006084|Melanimon_tibialis
TLYFIFGAWSGMVGTSLSLLIRAELGNPGSLIGDDQIYNVIVTAHAFIMIFFMVMPIMIGGFGNWLVPLMLGAPDMAFPRMNNMSFWLLPPSLTLLLMSSIVESGAGTGWTVYPPLSSNIAHGGSSVDLAIFSLHLAGISSILGAVNFITTVINMRPQG
MTFDQMPLFVWAVVITAVLLLLSLPVLAGAITMLLTDRNINTSFFDPAGGGDPILYQHL
>COLFB025-12|KJ963213|ZMUO.001355|Galerucella_nymphaeae
TLYFIFGVWAGMVGTSLSILVRVELGNPGSLIGNDQIYNVIVTAHAFIMIFFMVMPIMIGGFGNWLVPLMIGAPDMAFPRMNNMSFWLLPPSLFLLIMSSIVESGAGTGWTVYPPLSSNIAHGGSSVDLAIFSLHLAGISSILGAINFITTIINMRPKGMT
LDRMPLFVWAVMITAILLLLSLPVLAGAITMLLTDRNLNTSFFDPAGGGDPILYQHL

>COLFD251-12|KJ964257|ZMUO.004146|Agabus_undulatus
TLYFIFGAWAGMVGTSLSMLIRAELGNPGSLIGDDQIYNVIVTAHAFVMIFFMVMPIMIGGFGNWLVPLMLGAPDMAFPRMNNMSFWLLPPSLTLLLMSSMVESGAGTGWTVYPPLSSGIAHGGASVDLAIFSLHLAGISSILGAVNFITTIINMRSV
GMTFDRMPLFVWSVGITALLLLLSLPVLAGAITMLLTDRNLNTSFFDPAGGGDPILYQHL
>COLFF078-13|KJ962576|ZMUO.005778|Nebrioporus_depressus
TLYFLFGAWSGMVGTSLSMLIRAELGNPGSLIGDDQIYNVIVTAHAFIMIFFMVMPIMIGGFGNWLVPLMLGAPDMAFPRMNNMSFWMLPPSLSLLLMSSMVENGAGTGWTVYPPLSAGLAHGGASVDLAIFSLHLAGISSILGAVNFITTIINMRS
VGMTFDRMPLFVWSVGITALLLLLSLPVLAGAITMLLTDRNLNTSFFDPAGGGDPILYQHL
>COLFE245-12|KJ964332|ZMUO.005090|Longitarsus_brunneus
TLYFIFGTWAGMIGTSLSILIRTELGSPGSLIGNDQIYNVIVTAHAFIMIFFMVMPIMIGGFGNWLVPLMIGAPDMAFPRMNNMSFWLLPPSLFLLVMSSMVESGAGTGWTVYPPLSSNIAHGGSSVDLAIFSLHLAGISSILGAINFITTVINMHPTGMS
LDQMPLFVWAVMITAILLLLSLPVLAGAITMLLTDRNLNTTFFDPAGGGDPILYQHL
>COLFA660-12|KJ963388|ZMUO.000795|Philonthus_varians
TLYFIFGSWAGMVGTSLSLLIRAELGNPGTLIGDDQIYNVIVTAHAFIMIFFMVMPVVIGGFGNWLVPLMLGAPDMAFPRMNNMSFWLLPPSLTLLLMSSMVESGAGTGWTVYPPLSSNIAHGGASVDLAIFSLHLAGISSILGAVNFITTVINMRATG
MTFDRMPLFVWSVAITALLLLLSLPVLAGAITMLLTDRNLNTTFFDPAGGGDPILYQHL
>COLFD771-12|KJ966284|ZMUO.004761|Meligethes_difficilis
TLYFIFGAWSGMVGTSLSMLIRTELGNPGSLIGNDQIYNVIVTAHAFVMIFFMVMPFMIGGFGNWLVPLMLGAPDMAFPRMNNMSFWLLPPSLSLLLTSSIVESGAGTGWTVYPPLSSNIAHGGASVDLAIFSLHLAGISSILGAVNFITTIINMRPSG
MTFDRMPLFVWAVVITAILLLLSLPVLAGAITMLLTDRNLNTTFFDPSGGGDPILYQHL
>COLFD213-12|KJ966904|ZMUO.004108|Orchesia_fasciata
TLYFIFGAWSGMVGTSLSLLIRTELGNPGSLIGNDQIYNVIVTAHAFIMIFFMVMPIMMGGFGNWLVPLMLGAPDMAFPRMNNMSFWLLPPSLTLLIMSSIVENGAGTGWTVYPPLSSNIAHNGSSVDLAIFSLHLAGISSILGSVNFLTTIINMRPMG
MSFDRMPLFVWAVGITAILLLLSLPVLAGAITMLLTDRNLNTSFFDPAGGGDPILYQHL
>COLFC493-12|KJ962993|ZMUO.003058|Sericus_brunneus
TLYFIFGAWSGMLGTSLSLLIRAELGNPGALIGNDQIYNVVVTAHAFIMIFFMVMPIMIGGFGNWLVPLMLGAPDMAFPRMNNMSFWLLPPSLSLLLMSSIVENGAGTGWTVYPPLSANIAHSGSSVDLAIFSLHLAGISSILGAVNFISTVINMRSTGI
TFDRMPLFVWAVAITALLLLLSLPVLAGAITMLLTDRNLNTSFFDPAGGGDPILYQHL
>COLFA347-12|KJ961847|ZMUO.000577|Phalacrus_caricis
TLYFMFGAWSGMIGTSLSLLIRTELGNPGSLIGDDQIYNVIVTAHAFVMIFFMVMPFMIGGFGNWLVPLMLGAPDMAFPRLNNMSFWLLPPAIILLIASSLVESGAGTGWTVYPPLSANIAHEGASVDLAIFSLHLAGISSILGAINFISTIMNMRPSGM
SMDQVPLFVWAVIITAILLLLSLPVLAGAITMLLTDRNINTSFFDPAGGGDPILYQHL
>COLFE295-12|KJ964003|ZMUO.004855|Ocalea_picata
TLYFIFGAWAGMVGTSLSLLIRAELGNPGSLIGDDQIYNVIVTAHAFVMIFFMVMPIMIGGFGNWLVPLMLGAPDMAFPRMNNMSFWLLPPSLTLLLMSSMVESGAGTGWTVYPPLSSNIAHGGASVDLAIFSLHLAGISSILGAVNFISTIINMRTSG
MTFDRMPLFVWSVAITALLLLLSLPVLAGAITMLLTDRNLNTSFFDPAGGGDPILYQHL
>COLFC773-12|KJ966881|ZMUO.003338|Dendroctonus_micans
TLYFIFGAWSGMVGTSLSLIIRTELGTPGSLIGDDQIYNTIVTAHAFIMIFFMVMPIMIGGFGNWLVPLMLGAPDMAFPRLNNMSFWLLPPSLTFLLLSSIIDKGAGTGWTVYPPLASNIAHEGPSVDCAIFSLHMAGISSILGAINFISTIMNMNPSGMKL
DRLTLFTWSVKITAILLLLSLPVLAGAITMLLTDRNINTTFFDPSGGGDPILYQHL
>COLFE1116-13|KJ963163|ZMUO.006911|Selatosomus_melancholicus
TLYFLFGAWAGMLGTSLSLLIRAELGNPGSLIGNDQIYNVIVTAHAFIMIFFMVMPIMIGGFGNWLVPLMLGAPDMAFPRMNNMSFWFLPPSLSLLLMSSIVENGAGTGWTVYPPLSANIAHSGSSVDLAIFSLHLAGISSILGAVNFISTVINMRSAGI
TFDRMPLFVWAVAITALLLLLSLPVLAGAITMLLTDRNLNTSFFDPAGGGDPILYQHL
>COLFC008-12|KJ963923|ZMUO.002003|Ocyusa_picina
TLYFIFGAWSGMVGTSLSLLIRAELGNPGSLIGDDQIYNVIVTAHAFVMIFFMVMPIVIGGFGNWLVPLMLGAPDMAFPRMNNMSFWLLPPSLSLLLMSSMVESGAGTGWTVYPPLSSNIAHGGSSVDLAIFSLHLAGISSILGAVNFISTIMNMRTSG
MTFDRMPLFVWSVAITALLLLLSLPVLAGAITMLLTDRNLNTSFFDPAGGGDPILYQHL
>COLFB360-12|KJ963938|ZMUO.001690|Heterothops_quadripunctulus
TLYFIFGAWAGMVGTSLSLLIRAELGNPGSLIGDDQIYNVIVTAHAFVMIFFMVMPIMIGGFGNWLVPLMLGAPDMAFPRMNNMSFWLLPPSLTLLLMSSMVESGAGTGWTVYPPLSSNIAHGGASVDLAIFSLHLAGISSILGAVNFITTVINMRAIG
MTFDRMPLFVWAVVITALLLLLSLPVLAGAITMLLTDRNLNTSFFDPAGGGDPILYQHL
>COLFC663-12|KJ964298|ZMUO.003228|Oxypoda_strandi
TLYFIFGAWAGMVGTSLSLLIRAELGNPGSLIGDDQIYNVIVTAHAFVMIFFMVMPIVIGGFGNWLVPLMLGAPDMAFPRMNNMSFWLLPPSLTLLLMSSMVESGAGTGWTVYPPLSSNIAHGGSSVDLAIFSLHLAGISSILGAVNFISTIINMRTSG
MSFDRMPLFVWSVAITALLLLLSLPVLAGAITMLLTDRNLNTSFFDPAGGGDPILYQHL
>COLFE046-12|KJ964511|ZMUO.005271|Malachius_bipustulatus
TLYFIFGAWSGMVGLSLSLLIRSELSIPGTLIGNDQIYNVIVTAHAFIMIFFMVMPILIGGFGNWLVPLMLGAPDMAFPRMNNMSFWLLPPSLTLLLLSSMVENGAGTGWTVYPPLSANIAHSGSSVDLAIFSLHLAGISSILGAVNFITTVINMRPQGMT
LDRTPLFVWAVVITALLLLLSLPVLAGAITMLLTDRNLNTSFFDPAGGGDPILYQHL
>COLFF198-13|KJ967415|ZMUO.005803|Deporaus_betulae

TLYFIFGAWAGMVGTALSLLIRAELGNPGSLIGDDQIYNVIVTAHAFIMIFFMVMPIMIGGFGNWLVPLMLGAPDMAFPRMNNMSFWLLPPSLSLLIMSSIVESGAGTGWTVYPPLSANIAHGGSSVDLAIFSLHLAGISSILGAVNFISTMINMRPNG
MTLDRMPLFAWAVVITALLLLLSLPVLAGAITMLLTDRNINTTFFDPAGGGDPILYQHL
>COLFB563-12|KJ966051|ZMUO.001038|Pterostichus_minor
TLYFIFGAWAGMVGTSLSMLIRAELGNPGSLIGDDQIYNVIVTAHAFIMIFFMVMPIMIGGFGNWLVPLMLGAPDMAFPRMNNMSFWLLPPSLTLLLMSSMVESGAGTGWTVYPPLSAGIAHAGASVDLAIFSLHLAGISSILGAVNFITTIINMRSIG
MTFDRMPLFVWSVGITALLLLLSLPVLAGAITMLLTDRNLNTSFFDPAGGGDPILYQHL
>COLFC319-12|KJ962884|ZMUO.002504|Hypnoidus_rivularius
TLYFLFGAWSGMLGTSLSLLIRAELGNPGSLIGNDQIYNVIVTAHAFIMIFFMVMPIMIGGFGNWLVPLMLGAPDMAFPRMNNMSFWFLPPSLSLLLMSSIVENGAGTGWTVYPPLSANIAHSGSSVDLAIFSLHLAGISSILGAVNFISTVINMRSTGIT
FDRMPLFVWAVAITALLLLLSLPVLAGAITMLLTDRNLNTSFFDPAGGGDPILYQHL
>COLFE1500-13|KJ964167|ZMUO.007295|Tetrops_starkii
TLYFIFGAWASMIGTSLSLLIRSELGNPGSLIGNDQIYNTIVTAHAFIMIFFMVMPIMIGGFGNWLIPLMLGAPDMAFPRMNNMSFWLLPPSLILLIMSSIVENGAGTGWTVYPPLASNIAHSGPSVDLAIFSLHLAGISSILGAVNFITTVINMRPKEMSL
DQLSLFIWAVKITAILLLLSLPVLAGAITMLLTDRNLNTSFFDPASGGDPILYQHL
>COLFG078-13|KJ963549|ZMUO.007583|Xylita_laevigata
TLYFIFGAWSSMLGTSLSLLIRSELGNPGSLIGDDQIYNVIVTAHAFIMIFFMVMPILIGGFGNWLMPLMLGAPDMAFPRMNNMSFWLLPPSLILLIMSSIVEKGAGTGWTVYPPLSSNIAHNGSSVDLAIFSLHLAGISSILGAINFITTVINMRPKGMTL
DRMPLFVWSVMLTAILLLLSLPVLAGAITMLLTDRNLNTSFFDPAGGGDPVLYQHL
>COLFE1098-13|KJ961776|ZMUO.006893|Agabus_guttatus
TLYFIFGAWAGMVGTSLSMLIRAELGNPGSLIGDDQIYNVIVTAHAFVMIFFMVMPIMIGGFGNWLVPLMLGAPDMAFPRMNNMSFWLLPPSLTLLLMSSMVEKGAGTGWTVYPPLSSGIAHSGASVDLAIFSLHLAGISSILGAVNFITTIINMRSV
GMTFDRMPLFVWSVGITALLLLLSLPVLAGAITMLLTDRNLNTSFFDPAGGGDPILYQHL
>COLFF445-13|KJ965378|ZMUO.005963|Mordellistena
TLYFIFGAWAGMLGTSLSLLIRSELGTPGSLIGDDQIYNVIVTAHAFVMIFFMVMPVMVGGFGNWLMPLMLGAPDMAFPRLNNMSFWLLPPSLTLLILSSMVENGAGTGWTVYPPLSSNIAHGGASVDLAIFSLHLAGISSILGAINFISTMLNMHPK
GMILDQMPLFVWAIIITAILLLLSLPVLAGAITMLLTDRNLNSSFFDPAGGGDPILYQHL
>COLFF591-13|KJ964571|ZMUO.006481|Betulapion_simile
TLYFIFGLWSGMVGTSLSMLIRIELGNPGSLIGNDQIYNVIVTAHAFIMIFFMVMPIMIGGFGNWLVPLMLGAPDMAFPRMNNMSFWLLPPSLTLLLMSSIVEKGAGTGWTVYPPLASNIAHGGASVDLAIFSLHLAGISSILGAVNFISTVINMRPTGL
SLDQLSLFTWAVKITAILLLLSLPVLAGAITMLLTDRNINTSFFDPAGGGDPILYQHL
>COLFC838-12|KJ966356|ZMUO.003403|Aphidecta_obliterata
TLYFLFGLWAGMVGTSLSILIRMELGTTGSLIGNDQIYNVIVTAHAFIMIFFMVMPIMIGGFGNWLVPLMIGAPDMAFPRLNNMSFWLLPPALTLLIFSMMVEMGAGTGWTVYPPLSSNLAHNGPSVDLVIFSLHLAGISSILGAVNFISTIMNMRPFG
MSLDKTPLFVWSVMITAILLLLSLPVLAGAITMLLTDRNLNTSFFDPTGGGDPVLYQHL
>COLFB469-12|KJ965306|ZMUO.001894|Dalopius_marginatus
TLYFIFGAWAGMLGTSLSLLIRAELGNPGSLIGNDQIYNVIVTAHAFIMIFFMVMPIMIGGFGNWLVPLMLGAPDMAFPRMNNMSFWFLPPSLSLLLMSSIVENGAGTGWTVYPPLSSNIAHSGSSVDLAIFSLHLAGISSILGAVNFISTVINMRSTGIT
FDRMPLFVWAVAITALLLLLSLPVLAGAITMLLTDRNLNTSFFDPAGGGDPILYQHL
>COLFB872-12|KJ965883|ZMUO.001917|Lamia_textor
TLYFLFGAWAGMMGTSLSILIRTELGSPGSLIGDDQIYNVIVTAHAFIMIFFMVMPIMIGGFGNWLVPLMLGAPDMAFPRMNNMSFWLLPPSFFLLIMSSIVENGAGTGWTVYPPLAANVAHNGASVDLAIFSLHLAGISSILGAVNFITTVINMRPSG
MSMDRMPLFVWSVKITAILLLLSLPVLAGAITMLLTDRNLNTSFFDPAGGGDPILYQHL
>COLFG058-13|KJ964374|ZMUO.007563|Leiopus_linnei
TLYFIFGAWAGMVGTSLSMLIRSELGTAGSLIGDDQIYNTIVTAHAFIMIFFMVMPIMIGGFGNWLVPLMLGAPDMAFPRMNNMSFWLLPPSLTLLIMSSIVNSGAGTGWTVYPPLSSNIAHAGPSVDLAIFSLHLAGASSILGAVNFITTVINMRPKG
MSFDRLPLFVWAVKITTILLLLSLPVLAGAITMLLTDRNLNTSFFDPAGGGDPVLYQHL
>COLFA325-12|KJ967198|ZMUO.000460|Oxypoda_procerula
TLYFIFGTWAGMIGTSLSLLIRAELGNPGSLIGDDQIYNVIVTAHAFVMIFFMVMPIVIGGFGNWLIPLMLGAPDMAFPRMNNMSFWLLPPSLSLLLMSSMVESGAGTGWTVYPPLSSNIAHGGSSVDLAIFSLHLAGISSILGAVNFISTIINMRTSGMS
FDRMPLFVWSVAITALLLLLSLPVLAGAITMLLTDRNLNTSFFDPAGGGDPILYQHL
>COLFB801-12|KJ966059|ZMUO.001276|Atheta_subtilis
TLYFIFGAWAGMVGTSLSLLIRAELGNPGSLIGDDQIYNVIVTAHAFIMIFFMVMPVVIGGFGNWLVPLMLGAPDMAFPRMNNMSFWLLPPSLTLLLMSSMVESGAGTGWTVYPPLSSNIAHGGSSVDLAIFSLHLAGISSILGAVNFISTVINMRSTGI
SFDRMPLFVWSVAITALLLLLSLPVLAGAITMLLTDRNLNTSFFDPAGGGDPILYQHL
>COLFF193-13|KJ961993|ZMUO.005798|Selatosomus_impressus
TLYFLFGAWAGMLGTSLSLLIRAELGNPGSLIGNDQIYNVIVTAHAFIMIFFMVMPIMIGGFGNWLVPLMLGAPDMAFPRMNNMSFWFLPPSLSLLLMSSIVENGAGTGWTVYPPLSANIAHSGSSVDLAIFSLHLAGISSILGAVNFISTVINMRSTGIT
FDRMPLFVWAVAITALLLLLSLPVLAGAITMLLTDRNLNTSFFDPAGGGDPILYQHL
>COLFD691-12|KJ967188|ZMUO.004681|Cryptophagus_parallelus
SLYFIFGAWAGMVGTSLSLLIRSELGTPGSLIGDDQIYNVIVTAHAFVMIFFMVMPIMIGGFGNWLVPLMLGAPDMAFPRMNNMSFWLLPPSLSLLLMSSIAEKGVGTGWTVYPPLSSNIAHGGSSVDLAIFSLHLAGISSILGAVNFISTIMNMCPTGL
TLDRMPLFVWAVMITAILLLLSLPVLAGAITMLLTDRNLNTSFFDPAGGGDPILYQHL

>COLFB075-12|KJ965915|ZMUO.001405|Dyschirius_globosus
TLYFIFGIWSGMVGTSLSILIRTELGNPGSLIGDDQIYNVIVTAHAFIMIFFMVMPIMIGGFGNWLVPLMLGAPDMAFPRMNNMSFWLLPPSLTLLLMSSMVEKGAGTGWTVYPPLSSSIAHSGASVDLAIFSLHLAGISSILGAVNFITTIINMRSTGLTF
ERMPLFVWSVGITALLLLLSLPVLAGAITMLLTDRNLNTSFFDPAGGGDPILYQHL
>COLFC381-12|KJ962047|ZMUO.002946|Mycetoporus_boreellus
TLYFIFGAWAGMVGTSLSLLIRAELGNPGSLIGDDQIYNVIVTAHAFVMIFFMVMPIVIGGFGNWLVPLMLGAPDMAFPRMNNMSFWLLPPSLTLLLMSSMVESGAGTGWTVYPPLSSNIAHGGASVDLAIFSLHLAGISSILGAVNFITTVINMRSTG
MTFDRMPLFIWSVAITALLLLLSLPVLAGAITMLLTDRNLNTSFFDPAGGGDPILYQHL
>COLFG090-13|KJ962798|ZMUO.007595|Ptilinus_fuscus
TLYFIFGSWSGMIGTSLSLLIRSELGNPGALIGNDQIYNVIVTAHAFIMIFFMVMPMMIGGFGNWLVPLMLGAPDMAFPRMNNMSFWLLPPSLSLLLMSMIVNSGTGTGWTVYPPLSSNIAHSGASVDLTIFSLHLAGISSILGAINFITTVINMRPMS
MTFDRMPLFVWSVVITALLLLLSLPVLAGAITMLLTDRNLNTSFFDPAGGGDPILYQHL
>COLFB375-12|KJ964632|ZMUO.001705|Anthobium_atrocephalum
TLYFIFGAWAGMVGTSLSILIRAELGNPGTLIGDDQIYNVIVTAHAFVMIFFMVMPIVIGGFGNWLVPLMLGAPDMAFPRMNNMSFWLLPPSLTLLLMSSMVESGAGTGWTVYPPLSSNIAHGGSSVDLAIFSLHLAGISSILGAVNFITTVINMRATG
MTFDRMPLFVWSVAITALLLLLSLPVLAGAITMLLTDRNLNTSFFDPAGGGDPILYQHL
>COLFC101-12|KJ966480|ZMUO.002286|Anthicus_sellatus
TLYLIFGAWAGMVGTSLSLLIRSELGNPGTLIGNDQIYNVIVTAHAFIMIFFMVMPIMIGGFGNWLVPLMLGAPDMAFPRMNNMSFWLLPPSLSLLIMSSIVESGAGTGWTVYPPLSANIAHSGSSVDLAIFSLHLAGISSILGAVNFITTVINMRPAGM
NLDRMPLFVWAVVITAILLLLSLPVLAGAITMLLTDRNLNTSFFDPAGGGDPILYQHL
>COLFB253-12|KJ967138|ZMUO.001583|Bradycellus_ruficollis
TLYFIFGTWSGMVGTSLSMLIRAELGTPGALIGDDQIYNVIVTAHAFIMIFFMVMPIMIGGFGNWLVPLMLGAPDMAFPRMNNMSFWLLPPSLSLLLMSSMVEKGAGTGWTVYPPLSSGIAHSGASVDLAIFSLHLAGVSSILGAVNFITTIINMRSVG
MTFDRMPLFVWSVGITALLLLLSLPILAGAITMLLTDRNLNTSFFDPAGGGDPILYQHL
>COLFD320-12|KJ966162|ZMUO.004215|Galerucella_lineola
TLYFIFGVWAGMVGTSLSILVRAELGSPGTLIGNDQIYNVIVTAHAFIMIFFMVMPIMIGGFGNWLVPLMIGAPDMAFPRMNNMSFWLLPPSLFLLIMSSIVESGAGTGWTVYPPLSSNIAHGGSSVDLAIFSLHLAGISSILGAINFITTIINMRPKGMTL
DRMPLFVWAVMITAILLLLSLPVLAGAITMLLTDRNLNTSFFDPAGGGDPILYQHL
>COLFF683-13|KJ963130|ZMUO.006383|Chaetocnema_sahlbergii
TLYFIFGIWSGMVGTSMSILIRAELGNPGSLIGNDQIYNVIVTAHAFVMIFFMVMPIMIGGFGNWLVPLMIGAPDMAFPRMNNMSFWLLPPSLFLLLMSSMVESGAGTGWTVYPPLSSNIAHGGSSVDLAIFSLHLAGISSILGAINFITTIINMRPQG
MQFDQMPLFVWAVLITAILLLLSLPVLAGAITMLLTDRNLNTSFFDPIGGGDPILYQHL
>COLFD019-12|KJ962057|ZMUO.003914|Hydroporus_palustris
TLYFLFGAWSGMVGTSLSMLIRAELGNPGSLIGDDQIYNVIVTAHAFIMIFFMVMPIMIGGFGNWLVPLMLGAPDMAFPRMNNMSFWLLPPSLTLLLMSSMVENGAGTGWTVYPPLSSGIAHSGASVDLAIFSLHLAGVSSILGAVNFITTIINMRSI
GMTFDRMPLFVWSVGITALLLLLSLPVLAGAITMLLTDRNLNTSFFDPAGGGDPILYQHL
>COLFC608-12|KJ965970|ZMUO.003173|Anaspis_frontalis
TLYFIFGAWSGMVGTSLSLLIRSELGTPGSLIGDDQIYNVIVTAHAFIMIFFMVMPILIGGFGNWLVPLMLGAPDMAFPRMNNMSFWLLPPSLTLLIMSSVVENGAGTGWTVYPPLAANIAHSGSSVDLAIFSLHLAGASSILGAVNFITTVINMRPQG
MTLDRMPLFVWAVVITAVLLLLSLPVLAGAITMLLTDRNLNTSFFDPAGGGDPILYQHL
>COLFC788-12|KJ962232|ZMUO.003353|Corticaria_crenulata
SLYFLFGMWSGMVGTSLSLLIRLELGNPGSLIGDDQIYNVIVTAHAFIMIFFMVMPIMIGGFGNWLVPLMLGAPDMAFPRLNNMSFWLLPPSLSLLIMSSIVESGAGTGWTVYPPLSSNIAHGGSSVDLAIFSLHLAGISSILGAVNFITTVINMRPTGM
NLDQMPLFVWSVVITAILLLLSLPVLAGAITMLLTDRNLNTSFFDPAGGGDPILYQHL
>COLFD761-12|KJ965777|ZMUO.004751|Cantharis_fusca
TLYFIFGAWSGSLGLALSLLIRAELGTPGTLIGNDQIYNVIVTAHAFIMIFFMVMPIMIGGFGNWLVPLMLGAPDMAFPRMNNMSFWFLPPSLMFLLMSSMVESGAGTGWTVYPPLSANIAHSGPSVDLAIFSLHMAGISSILGAVNFISTIMNMKPPS
MKFDQMPLFVWSVGITALLLLLSLPVLAGAITMLLSDRNLNTSFFDPMGGGDPILYQHL
>COLFA578-12|KJ962034|ZMUO.000713|Carabus_violaceus
TLYFIFGAWSGMVGTSLSMLIRAELGNPGSLIGDDQIYNVIVTAHAFVMIFFMVMPIMIGGFGNWLVPLMLGAPDMAFPRMNNMSFWLLPPSLTLLLMSSMVEKGAGTGWTVYPPLSSGIAHSGASVDLAIFSLHLAGISSILGAVNFITTIINMRSV
GMTFDRMPLFVWSVGITALLLLLSLPVLAGAITMLLTDRNLNTSFFDPAGGGDPILYQHL
>COLFF392-13|KJ963516|ZMUO.005910|Rhamphus_pulicarius
TLYFLFGGWSGMVGTSLSMLIRTELGNPGKLIGDDQIYNTIVTAHAFIMIFFMVMPIMIGGFGNWLVPLMLGAPDMAFPRLNNMSFWLLPPSLTFLLLSSVMDKGAGTGWTVYPPLSANIAHEGSSVDLAIFSLHMAGVSSILGAMNFISTIINMKPK
NMSMDQMSLFVWSVKITAILLLLSLPVLAGAITMLLTDRNINTSFFDPAGGGDPILYQHL
>COLFA557-12|KJ966163|ZMUO.000692|Xylotrechus_pantherinus
TLYFIFGAWAGMVGTSLSVLIRSELGNPGSLIGDDQIYNVIVTAHAFIMIFFMVMPIMIGGFGNWLVPLMLGAPDMAFPRMNNMSFWLLPPSLALLIMSSIVESGAGTGWTVYPPLSANVAHSGSSVDLAIFSLHLAGVSSILGAVNFISTVINMRPTK
MNPEQMPLFVWAVAITALLLLLSLPVLAGAITMLLTDRNINTSFFDPAGGGDPILYQHL
>COLFF567-13|KJ961888|ZMUO.006647|Smaragdina_salicina

TLYFIFGAWSGMVGTSLSLLIRVELGNPGTLIGNDQIYNTIVTAHAFIMIFFMVMPIMIGGFGNWLVPLMLGAPDMAFPRMNNMSFWLLPPSLTLLLMSSIVENGAGTGWTVYPPLSANLAHSGASVDLAIFSLHLAGVSSIMGAINFISTVINMRPQG
MLLDRTPLFVWAVVITAILLLLSLPVLAGAITMLLTDRNLNTSFFDPAGGGDPILYQHL
>COLFA483-12|KJ965443|ZMUO.000523|Rhinoncus_pericarpius
TLYFIFGSWAGTVGTSLSMLIRTELGTPGSLIGNDQIYNSIVTAHAFIMIFFMVMPILIGGFGNWLVPLMLGAPDMAFPRLNNMSFWLLPPSIMLLLMSSIINKGAGTGWTVYPPLSSNITHEGASVDLAIFSLHMAGISSILGAINFISTIMNMRPQGMS
YDKMPLFSWAVLITAILLLLSLPVLAGAITMLLTDRNINTSFFDPAGGGDPILYQHL
>COLFC175-12|KJ966912|ZMUO.002360|Galerucella_tenella
TLYFIFGIWAGMVGTSLSILVRAELGSPGTLIGNDQIYNVIVTAHAFIMIFFMVMPIMIGGFGNWLVPLMIGAPDMAFPRMNNMSFWLLPPSLFLLIMSSIVESGAGTGWTVYPPLSSNIAHGGSSVDLAIFSLHLAGISSILGAINFITTIINMRPKGMTL
DRMPLFVWAVMITAILLLLSLPVLAGAITMLLTDRNLNTSFFDPAGGGDPILYQHL
>COLFF1030-13|KJ962669|ZMUO.007395|Ceutorhynchus_typhae
TLYFIFGSWAGMAGTSLSMLIRTELGNPGSLIGNDQIYNSIVTAHAFIMIFFMVMPILIGGFGNWLVPLMLGAPDMAFPRLNNMSFWLLPPSLSLLLMSSVVNKGAGTGWTVYPPLSSNVAHEGMSVDLAIFSLHMAGISSILGAINFISTVMNMQPK
GMTPELMPLFVWAVEITAILLLISLPVLAGAITMLLTDRNINTSFFDPSGGGDPILYQHL
>COLFE903-13|KJ967356|ZMUO.006223|Tyrus_mucronatus
TLYLMFGAWAGMMGTSLSILIRAELGAPGSLIGDDQIYNVIVTAHAFIMIFFMVMPIMIGGFGNWLVPLMLGAPDMAFPRMNNMSFWLLPPSLILLLMSSMVENGAGTGWTVYPPLSSNIAHSGASVDLTIFSLHLAGISSILGAVNFISTIINMRTM
GMNFDQMPLFVWSVLITAILLLLSLPVLAGAITMLLTDRNLNTSFFDPAGGGDPILYQHL
>COLFE029-12|KJ965914|ZMUO.005254|Hylobius_piceus
TLYFIFGTWSGMVGTSLSMLIRTELGNPGSLIGNDQIYNSIVTAHAFIMIFFMVMPVMIGGFGNWLVPLMLGAPDMAFPRLNNMSFWLLPPSLTLLLMSNIIDKGAGTGWTVYPPLSANIAHEGASVDLAIFSLHMAGISSILGAINFISTAMNMRSSG
MNPDQMSLFTWAVKITAILLLLSLPVLAGAITMLLTDRNINTSFFDPAGGGDPILYQHL
>COLFF641-13|KJ961768|ZMUO.006531|Prasocuris_phellandrii
TLYFIFGIWAGMVGTSLSMLIRSELGNPGSLIGNDQIYNVIVTAHAFIMIFFMVMPIMIGGFGNWLVPLMIGAPDMAFPRLNNMSFWLLPPSLFLLLMSSIVENGAGTGWTVYPPLSANVAHSGSSVDLAIFSLHLAGISSILGAINFITTVINMRPEGM
NFEQTPLFVWAVLITAILLLLSLPVLAGAITMLLTDRNLNTSFFDPAGGGDPILYQHL
>COLFE658-13|KJ966031|ZMUO.005503|Alosterna_tabacicolor
TLYFIFGAWAGMVGTSLSLLIRSELGSPGSFIGDDQVYNVIVTAHAFIMIFFMVMPIMIGGFGNWLVPLMLGAPDMAFPRMNNMSFWLLPPSLTLLIMSSIVESGAGTGWTVYPPLSSNIAHGGSSVDLAIFSLHLAGISSILGAVNFITTVINMRPMG
MNLDRMPLFVWAVVITAILLLLSLPVLAGAITMLLTDRNLNTSFFDPAGGGDPILYQHL
>COLFB649-12|KJ965858|ZMUO.001124|Philonthus_micantoides
TLYFIFGSWAGMVGTSLSLLIRAELGNPGTLIGDDQIYNVIVTAHAFIMIFFMVMPIVIGGFGNWLVPLMLGAPDMAFPRMNNMSFWLLPPSLTLLLMSSMVESGAGTGWTVYPPLSSNIAHGGASVDLAIFSLHLAGISSILGAVNFITTVINMRSSG
MTFDRMPLFVWSVAITALLLLLSLPVLAGAITMLLTDRNINTTFFDPAGGGDPILYQHL
>COLFD445-12|KJ961819|ZMUO.003865|Neocrepidodera_ferruginea
TLYFIFGMWSGMIGTSLSLLIRTELGSPGSLIGNDQIYNVIVTAHAFIMIFFMVMPIMIGGFGNWLVPLMIGAPDMAFPRMNNMSFWLLPPSLFLLLMSSMVESGAGTGWTVYPPLSSNLAHSGSSVDLAIFSLHLAGISSILGAVNFITTVINMRPMG
MTLDRMPLFVWAVVITAILLLLSLPVLAGAITMLLTDRNLNTSFFDPSGGGDPILYQHL
>COLFE505-13|KJ964723|ZMUO.005350|Pterostichus_minor
TLYFIFGAWAGMVGTSLSMLIRAELGNPGSLIGDDQIYNVIVTAHAFIMIFFMVMPIMIGGFGNWLVPLMLGAPDMAFPRMNNMSFWLLPPSLTLLLMSSMVESGAGTGWTVYPPLSAGIAHAGASVDLAIFSLHLAGISSILGAVNFITTIINMRSIG
MTFDRMPLFVWSVGITALLLLLSLPVLAGAITMLLTDRNLNTSFFDPAGGGDPILYQHL
>COLFF922-13|KJ966909|ZMUO.006717|Leiodes_calcarata
TLYFIFGAWAGMVGTSLSILIRAELGNPGSLIGDDQIYNVIVTAHAFIMIFFMVMPIVIGGFGNWLVPLMLGAPDMAFPRMNNMSFWLLPPSLTLLLMSSIVENGAGTGWTVYPPLSSNIAHSGSSVDLAIFSLHLAGISSILGAVNFITTVINMRSVGMS
FDKMPLFVWSVAITALLLLLSLPVLAGAITMLLTDRNLNTSFFDPAGGGDPILYQHL
>COLFF933-13|KJ967090|ZMUO.006728|Longitarsus_succineus
TLYFIFGIWAGMIGTSLSILIRTELGNPGSLIGNDQIYNVIVTAHAFIMIFFMVMPIMIGGFGNWLVPLMIGAPDMAFPRMNNMSFWLLPPSLFLLVMSSMVESGAGTGWTVYPPLSSNIAHGGSSVDLAIFSLHLAGISSILGAINFITTVINMRPTGMT
LDRMPLFVWAVVITAILLLLSLPVLAGAITMLLTDRNLNTTFFDPAGGGDPILYQHL
>COLFF017-13|KJ964675|ZMUO.005717|Scymnus_schmidti
TLYFLFGLWAGMVGTSLSILIRLELGTTSALIGNDQIYNVIVTAHAFIMIFFMVMPIMIGGFGNWLVPLMIGAPDMAFPRLNNMSFWLLPPSLTFLILSSLVESGAGTGWTVYPPLSSNIAHGGSSVDMAIFSLHLAGISSILGAVNFISTIINMRTFGMTFE
KMPLFVWSVFITAILLLLSLPVLAGAITMLLTDRNINTSFFDPAGGGDPILYQHL
>COLFF016-13|KJ964858|ZMUO.005716|Mycetophagus_populi
TLYFIFGAWSGMVGTSLSLLIRSELGNPGSLIGDDQIYNVIVTAHAFIMIFFMVMPIMMGGFGNWLVPLMLGAPDMAFPRMNNMSFWLLPPSLMLLIMSSVTESGAGTGWTVYPPLSSNIAHSGPSVDLAIFSLHLAGISSILGAVNFITTIINMRPSG
MSFDRMPLFVWAIGITALLLLLSLPVLAGAITMLLTDRNLNTSFFDPAGGGDPILYQHL
>COLFD017-12|KJ964534|ZMUO.003912|Gyrinus_opacus
TLYFIFGAWSGMVGTSLSMLIRAELGNPGSLIGDDQIYNVIVTAHAFIMIFFMVMPIMIGGFGNWLVPLMLGAPDMAFPRMNNMSFWLLPPSLTLLLMSSMVENGAGTGWTVYPPLSSNIAHGGASVDLAIFSLHLAGISSILGAVNFITTIINMRSV
GMTLDRMPLFVWSVGITALLLLLSLPVLAGAITMLLTDRNLNTSFFDPAGGGDPILYQHL

>COLFD864-12|KJ963175|ZMUO.004474|Cerylon_histeroides
TLYFMFGMWSGMVGTSMSMMIRLELGNPGSLIGDDQIYNVIVTAHAFVMIFFMVMPIMIGGFGNWLVPLMLGAPDMAFPRMNNMSFWLLPPSLTLLIMSSIVEKGAGTGWTVYPPLSANLTHSGSSVDLAIFSLHLAGISSILGAVNFITTVINMR
PSGMTWDRLPLFVWSVIITAVLLLLSLPVLAGAITMLLTDRNLNTSFFDPAGGGDPILYQHL
>COLFD874-12|KJ962905|ZMUO.004484|Pyrrhalta_viburni
TLYFIFGIWAGMVGTSLSILVRAELGSPGTLIGNDQIYNVIVTAHAFIMIFFMVMPIMIGGFGNWLVPLMIGAPDMAFPRMNNMSFWLLPPSLFLLIMSSIVESGAGTGWTVYPPLSSNIAHGGSSVDLAIFSLHLAGISSILGAINFITTVINMRPKGMTL
DRMPLFVWAVVITAILLLLSLPVLAGAITMLLTDRNLNTSFFDPAGGGDPILYQHL
>COLFB065-12|KJ961757|ZMUO.001395|Bledius_talpa
TLYFIFGAWAGMVGTSLSMLIRTELGTPGSLIGNDQIYNVIVTAHAFVMIFFMVMPIVIGGFGNWLIPLMLGAPDMAFPRMNNMSFWLLPPSLTLLLMSSMVESGAGTGWTVYPPLSSNIAHSGSSVDLAIFSLHLAGISSILGAVNFISTIINMRSIGM
TFDRMPLFIWSVKITAILLLLSLPVLAGAITMLLTDRNLNTSFFDPAGGGDPILYQHL
>COLFE633-13|KJ965598|ZMUO.005478|Carabus_hortensis
TLYFIFGAWSGMVGTSLSMLIRAELGNPGSLIGDDQIYNVIVTAHAFVMIFFMVMPIMIGGFGNWLVPLMLGAPDMAFPRMNNMSFWLLPPSLTLLLMSSMVEKGAGTGWTVYPPLSSGIAHSGASVDLAIFSLHLAGISSILGAVNFITTIINMRSV
GMTFDRMPLFVWSVGITALLLLLSLPVLAGAITMLLTDRNLNTSFFDPAGGGDPILYQHL
>COLFB153-12|KJ963866|ZMUO.001483|Protaetia_cuprea
TLYFLFGSWAGMVGTSLSLLIRAELGNPGSLIGDDQIYNVIVTAHAFIMIFFMVMPIMIGGFGNWLVPLMLGAPDMAFPRMNNMSFWLLPPSLTLLLMSSVVESGAGTGWTVYPPLSSNIAHSGASVDLAIFSLHLAGISSILGAVNFITTVINMRSTG
MTFDRMPLFVWSVALTALLLLLSLPVLAGAITMLLTDRNINTSFFDPAGGGDPILYQHL
>COLFA480-12|KJ967402|ZMUO.000520|Amara_familiaris
TLYFIFGAWSGMVGTSLSMLIRAELGNPGALIGDDQIYNVIVTAHAFVMIFFMVMPIMIGGFGNWLVPLMLGAPDMAFPRMNNMSFWMLPPSLTLLLMSSMVESGAGTGWTVYPPLSSGIAHAGASVDLAIFSLHLAGISSILGAVNFITTIINMRS
VGMTFDRMPLFVWSVGITALLLLLSLPVLAGAITMLLTDRNLNTSFFDPAGGGDPILYQHL
>COLFE1612-13|KJ964109|ZMUO.007502|Doydirhynchus_austriacus
TLYFIFGAWAGMVGTSLSLLIRTELGNPGSLIGDDQIYNVIVTAHAFIMIFFMVMPMMIGGFGNWLVPLMLGAPDMAFPRMNNMSFWLLPPSLTLLLMSSIVESGAGTGWTVYPPLSSNIAHGGSSVDLAIFSLHLAGISSILGAVNFISTVINMRPSG
MTFDRMPLFVWAVVITALLLLLSLPVLAGAITMLLTDRNLNTSFFDPAGGGDPILYQHL
>COLFC401-12|KJ963751|ZMUO.002966|Atheta_depressicollis
TLYFIFGTWAGMIGTSLSLLIRAELGNPGSLIGDDQIYNVIVTAHAFIMIFFMVMPIVIGGFGNWLVPLMLGAPDMAFPRMNNMSFWLLPPSLSLLLMSSMVESGAGTGWTVYPPLSSNIAHGGSSVDLAIFSLHLAGISSILGAVNFISTVINMRSTGIT
FDRMPLFVWSVVITALLLLLSLPVLAGAITMLLTDRNLNTSFFDPAGGGDPILYQHL
>COLFF888-13|KJ964493|ZMUO.006683|Tasgius_melanarius
TLYFFFGTWSGMVGTSLSLLIRAELGNPGSLIGNDQIYNVIVTAHAFVMIFFMVMPIVIGGFGNWLVPLMLGAPDMAFPRMNNMSFWLLPPSLNLLLTSSMAESGAGTGWTVYPPLSANVAHSGTSVDLAIFSLHLAGISSILGAVNFITTVLNMRAT
GMTFDRMPLFVWSVTITALLLLLSLPVLAGAITMLLTDRNLNTSFFDPSGGGDPILYQHL
>COLFE262-12|KJ962253|ZMUO.005107|Phyllotreta_armoraciae
TLYFIFGIWSGMVGMSMSMLIRIELAAPGSLIGNDQIYNVIVTAHAFIMIFFMVMPIMIGGFGNWLIPLMIGAPDMAFPRMNNMSFWLLPPSLFLLIMSSIVENGAGTGWTVYPPLSSNVSHAGASVDLTIFSLHLAGISSILGAINFITTIINMRPKGMT
FDRMPLFVWAVFITAILLLLSLPVLAGAITMLLTDRNLNTSFFDPMGGGDPILYQHL
>COLFB271-12|KJ963897|ZMUO.001601|Coccidula_rufa
TLYFLFGMWAGMVGTSLSILIRLELGTPDSLIGNDQIYNVIVTAHAFIMIFFMVMPIMIGGFGNWLVPLMIGAPDMAFPRMNNMSFWLLPPALTLLILSSLVESGAGTGWTVYPPLSSNLAHSGSSVDMAIFSLHLAGISSILGAVNFISTVINMRTPGM
TFEKMPLFVWSVIITAILLLLSLPVLAGAITMLLTDRNINTSFFDPAGGGDPILYQHL
>COLFB598-12|KJ963269|ZMUO.001073|Megasternum_concinnum
TLYFIFGAWAGMVGTSLSILIRAELGNPGTLIGDDQIYNVIVTAHAFIMIFFMVMPIMIGGFGNWLVPLMLGAPDMAFPRMNNMSFWLLPPSLTLLLMSSMVESGAGTGWTVYPPLSSNIAHGGSSVDLAIFSLHLAGISSILGAVNFITTVINMRSPNL
TYDRLPLFVWSVAITALLLLLSLPVLAGAITMLLTDRNLNTSFFDPAGGGDPILYQHL
>COLFE1207-13|KJ966836|ZMUO.007002|Enochrus_affinis
TLYFIFGAWAGMVGTSLSILIRAELGNPGTLIGDDQIYNVIVTAHAFIMIFFMVMPIMIGGFGNWLVPLMLGAPDMAFPRMNNMSFWLLPPSLTLLLMSSMVESGAGTGWTVYPPLSSNIAHGGASVDLAIFSLHLAGISSILGAVNFITTVINMRSPS
MTYDRLPLFVWSVAITALLLLLSLPVLAGAITMLLTDRNLNTSFFDPAGGGDPILYQHL
>COLFA399-12|KJ966382|ZMUO.000629|Tachinus_proximus
TLYFIFGAWAGMVGTSLSLLIRAELGNPGTLIGDDQIYNVIVTAHAFIMIFFMVMPIVIGGFGNWLVPLMLGAPDMAFPRMNNMSFWLLPPSLSLLLMSSMVESGAGTGWTVYPPLSSNIAHGGSSVDLAIFSLHLAGISSILGAVNFITTVINMRSIGM
TFDRMPLFVWSVAITALLLLLSLPVLAGAITMLLTDRNLNTTFFDPAGGGDPILYQHL
>COLFD636-12|KJ963693|ZMUO.004436|Hypera_pollux
TLYFIFGTWAGTVGTSLSILIRTELGNPGSLIGNDQIYNTIVTAHAFIMIFFMVMPIMIGGFGNWLVPLMLGAPDMAFPRLNNMSFWLLPPSLSLLLMSSMVDSGAGTGWTVYPPLSSNIAHEGSSVDLAIFSLHMAGVSSILGAINFISTVLNMRPSGM
SLDKMALFIWAVKITAILLLLSLPVLAGAITMLLTDRNINTSFFDPAGGGDPILYQHL
>COLFF829-13|KJ963961|ZMUO.006149|Aleochara_intricata

TLYFIFGSWAGMVGTSLSLLIRAELGNPGSLIGNDQIYNVIVTAHAFIMIFFMVMPILIGGFGNWLVPLMLGAPDMAFPRMNNMSFWLLPPSLTLLLMSSMVESGAGTGWTVYPPLSSNIAHGGSSVDLAIFSLHLAGISSILGAVNFISTVINMRSSGM
TFDKMPLFMWAVVLTALLLLLSLPVLAGAITMLLTDRNLNTSFFDPAGGGDPILYQHL
>COLFB628-12|KJ965651|ZMUO.001103|Atheta_graminicola
TLYFIFGAWAGMVGTSLSLLIRAELGNPGSLIGDDQIYNVIVTAHAFIMIFFMVMPVVIGGFGNWLIPLMLGAPDMAFPRMNNMSFWLLPPSLTLLLMSSMVESGAGTGWTVYPPLSSNIAHSGSSVDLAIFSLHLAGISSILGAVNFISTVINMRSTGIY
FDRMPLFVWSVAITALLLLLSLPVLAGAITMLLTDRNLNTSFFDPAGGGDPILYQHL
>COLFA522-12|KJ967507|ZMUO.000562|Atheta_intermedia
TLYFIFGAWAGMIGTSLSLLIRAELGNPGSLIGDDQIYNVIVTAHAFIMIFFMVMPVVIGGFGNWLVPLMLGAPDMAFPRMNNMSFWLLPPSLTLLLMSSMVESGAGTGWTVYPPLSSNIAHGGSSVDLAIFSLHLAGISSILGAVNFISTVINMRSTGI
SFDRMPLFVWSVVITALLLLLSLPVLAGAITMLLTDRNLNTSFFDPAGGGDPILYQHL
>COLFE1496-13|KJ966715|ZMUO.007291|Anthocomus_fasciatus
TLYFIFGAWSGMVGLSLSLLIRSELSIPGTLIGNDQIYNVIVTAHAFIMIFFMVMPILIGGFGNWLVPLMLGAPDMAFPRMNNMSFWLLPPSLTLLLLSSMVENGAGTGWTVYPPLSANIAHSGSSVDLAIFSLHLAGISSILGAVNFITTVINMRPQGMT
LDRTPLFVWAVVITALLLLLSLPVLAGAITMLLTDRNLNTSFFDPAGGGDPILYQHL
>COLFB807-12|KJ963463|ZMUO.001282|Atheta_hypnorum
TLYFIFGSWAGMVGTSLSLLIRAELGNPGSLIGDDQIYNVIVTAHAFVMIFFMVMPVVIGGFGNWLVPLMLGAPDMAFPRMNNMSFWLLPPSLTLLLMSSMVESGVGTGWTVYPPLSSNIAHSGSSVDLAIFSLHLAGISSILGAVNFISTVINMRSTGI
SFDRMPLFVWSVAITALLLLLSLPVLAGAITMLLTDRNLNTSFFDPAGGGDPILYQHL
>COLFC443-12|KJ966429|ZMUO.003008|Platynus_assimilis
TLYFIFGAWAGMVGTSLSMLIRAELGNPGSLIGDDQIYNVIVTAHAFIMIFFMVMPIMIGGFGNWLVPLMLGAPDMAFPRMNNMSFWLLPPSLTLLLMSSMVESGAGTGWTVYPPLSSGIAHAGASVDLAIFSLHLAGVSSILGAVNFITTIINMRSV
GMTFDRMPLFVWSVGITALLLLLSLPVLAGAITMLLTDRNLNTSFFDPAGGGDPILYQHL
>COLFF547-13|KJ965827|ZMUO.006627|Bembidion_articulatum
TLYFIFGAWSGMVGTSLSMLIRAELGNPGSLIGDDQIYNVIVTAHAFVMIFFMVMPILIGGFGNWLVPLMLGAPDMAFPRMNNMSFWLLPPSLTLLLMSSMVEKGAGTGWTVYPPLSSSIAHSGASVDLAIFSLHLAGVSSILGAVNFITTIINMRSIG
MTFDRMPLFVWSVGITALLLLLSLPVLAGAITMLLTDRNLNTSFFDPAGGGDPILYQHL
>COLFD465-12|KJ965834|ZMUO.003885|Sitona_sulcifrons
TLYFIFGAWAGMVGTSLSVLIRTELGNPGSLIGDDQIYNVIVTAHAFIMIFFMVMPMMIGGFGNWLVPLMLGAPDMAFPRLNNMSFWLLPPSLTLLLMSSIVDKGAGTGWTVYPPLSANIAHEGASVDLAIFSLHMAGISSILGAINFISTIINMRTSG
MSFDQTALFVWSVKITAILLLLSLPVLAGAITMLLTDRNINTSFFDPAGGGDPILYQHL
>COLFB344-12|KJ964078|ZMUO.001674|Dromius_fenestratus
TLYFIFGAWAGMVGTSLSMLIRAELGNPGALIGDDQVYNVIVTAHAFIMIFFMVMPIMIGGFGNWLVPLMLGAPDMAFPRMNNMSFWLLPPSLSLLLMSSMVESGAGTGWTVYPPLSSGIAHAGASVDLAIFSLHLAGVSSILGAVNFITTIINMRSI
GMTFDRMPLFVWSVGITALLLLLSLPVLAGAITMLLTDRNLNTSFFDPAGGGDPILYQHL
>COLFD846-12|KJ964577|ZMUO.004836|Nicrophorus_investigator
TLYFIFGAWAGMVGMSLSMLIRVELSTPGTLLGDDQMYNVIVTAHAFIMIFFMVMPIVIGGFGNWLVPLMLGAPDMAFPRLNNMSFWLLPPSLSLLLISSMVESGAGTGWTVYPPLSANIAHSGSSVDLAIFSLHLAGISSILGAVNFITTVINMRSPG
MTFDRMPLFVWSVVITALLLLLSLPVLAGAITMLLTDRNLNTSFFDPAGGGDPILYQHL
>COLFB668-12|KJ965255|ZMUO.001143|Atheta_graminicola
TLYFIFGAWAGMVGTSLSLLIRAELGNPGSLIGDDQIYNVIVTAHAFIMIFFMVMPVVIGGFGNWLIPLMLGAPDMAFPRMNNMSFWLLPPSLTLLLMSSMVESGAGTGWTVYPPLSSNIAHSGSSVDLAIFSLHLAGISSILGAVNFISTVINMRSTGIY
FDRMPLFVWSVAITALLLLLSLPVLAGAITMLLTDRNLNTSFFDPAGGGDPILYQHL
>COLFD088-12|KJ962556|ZMUO.003983|Sulcacis_nitidus
TLYFLFGAWSGMVGTSMSILIRSELGTPGSLIGNDQIYNVIVTAHAFVMIFFMVMPIMIGGFGNWLVPLMLGAPDMAFPRMNNMSFWLLPPSLSLLLMSSLVENGAGTGWTVYPPLSSNIAHSGPSVDLAIFSLHLAGISSILGAVNFISTIINMRPMG
MSMDRIPLFVWAVIITAVLLLLSLPVLAGAITMLLTDRNFNTSFFDPAGGGDPILYQHL
>COLFE604-13|KJ963781|ZMUO.005449|Myzia_oblongoguttata
TLYFLFGMWAGMVGTSLSILIRLELGTTNSLIGNDQIYNVIVTAHAFIMIFFMVMPIMIGGFGNWLVPLMIGAPDMAFPRLNNMSFWLLPPALTLLIFSSLVEMGAGTGWTVYPPLSANMAHSGASVDLVIFSLHLAGISSILGAVNFISTIMNMRPFG
MNLDKTPLFVWSVLITAILLLLSLPVLAGAITMLLTDRNINTSFFDPMGGGDPILYQHL
>COLFA691-12|KJ963414|ZMUO.000826|Stilbus_oblongus
TLYFIFGAWSGMVGTSLSLLIRTELGTPGSLIGDDQIYNVIVTAHAFVMIFFMVMPFMIGGFGNWLVPLMLGAPDMAFPRMNNMSFWLLPPSLSFLLMSSIVENGAGTGWTVYPPLSSNIAHGGSSVDLAIFSLHLAGISSILGAINFITTVINMRPEGM
TLDRMPLFVWSVVITAILLLLSLPVLAGAITMLLTDRNINTSFFDPAGGGDPILYQHL
>COLFD882-12|KJ967302|ZMUO.004492|Hydrochus_megaphallus
TLYFIFGAWSGMVGTSLSLLIRAELGNPGSLIGDDQIYNVIVTAHAFIMIFFMVMPIMIGGFGNWLVPLMLGAPDMAFPRMNNMSFWLLPPSLSLLLMSSMVESGAGTGWTVYPPLSSNIAHGGASVDLAIFSLHLAGISSILGAVNFITTIINMRSKN
MTYDRLPLFVWSVGITALLLLLSLPVLAGAITMLLTDRNLNTSFFDPIGGGDPILYQHL
>COLFF446-13|KJ962592|ZMUO.005964|Laccobius_striatulus
TLYFIFGAWAGMVGTSLSILIRAELGNPGTLIGDDQIYNVIVTAHAFIMIFFMVMPIMIGGFGNWLVPLMLGAPDMAFPRMNNMSFWLLPPSLTLLLMSSMVESGAGTGWTVYPPLSSNIAHGGASVDLAIFSLHLAGISSILGAVNFITTVINMRSNN
MTYDRLPLFVWSVAITALLLLLSLPVLAGAITMLLTDRNLNTSFFDPAGGGDPILYQHL

>COLFB214-12|KJ963896|ZMUO.001544|Acanthocinus_griseus
TLYFIFGAWSGMVGTSLSMLIRSELGNPGTLIGNDQIYNTIVTAHAFIMIFFMVMPIMIGGFGNWLVPLMLGAPDMAFPRMNNMSFWLLPPSLTLLIMSSIVENGAGTGWTVYPPLSSNIAHAGSSVDLAIFSLHLAGISSILGAVNFITTIINMRPIGM
TFDRMPLFVWAVKITAILLLLSLPVLAGAITMLLTDRNINTSFFDPAGGGDPILYQHL
>COLFC654-12|KJ964021|ZMUO.003219|Hippodamia_variegata
TLYFLFGMWAGMIGTSLSLLIRLELGTTGSLIGNDQIYNVIVTAHAFIMIFFMVMPIMIGGFGNWLVPLMIGAPDMAFPRLNNMSFWLLPPALTLLLFSSIVEMGAGTGWTVYPPLSSNLAHNGPSVDLVIFSLHLAGISSILGAVNFISTIMNMRPYGM
SLDKTPLFVWSVLITAILLLLSLPVLAGAITMLLTDRNLNTSFFDPTGGGDPILYQHL
>COLFE1219-13|KJ963478|ZMUO.007014|Ochthephilum_fracticorne
TLYFIFGAWAGMVGTSLSLLIRSELANPGSLIGDDQIYNVIVTAHAFIMIFFMVMPVMIGGFGNWLVPLMLGAPDMAFPRMNNMSFWLLPPALTLLLMSSMVESGAGTGWTVYPPLSSNGFHNGSSVDLAIFSLHLAGISSILGAINFITTAINMRTTG
MNYERMPLFIWSVAITALLLLLSLPVLAGAITMLLTDRNLNTSFFDPAGGGDPILYQHL
>COLFE790-13|KJ965908|ZMUO.005635|Philonthus_micans
TLYFIFGSWAGMVGTSLSLLIRAELGNPGTLIGDDQIYNVIVTAHAFIMIFFMVMPIVIGGFGNWLVPLMLGAPDMAFPRMNNMSFWLLPPSLTLLLMSSMVESGAGTGWTVYPPLSSNIAHGGASVDLAIFSLHLAGISSILGAVNFITTVINMRSSG
MTFDRMPLFVWSVAITALLLLLSLPVLAGAITMLLTDRNLNTTFFDPAGGGDPILYQHL
>COLFD044-12|KJ965232|ZMUO.003939|Myrrha_octodecimguttata
TLYFLFGMWAGMVGTSLSMLIRLELGTTNSLIGNDQIYNVIVTAHAFIMIFFMVMPIMIGGFGNWLVPLMIGAPDMAFPRLNNMSFWLLPPALTLLIFSSMVEMGAGTGWTVYPPLSSNMAHSGSSVDLVIFSLHLAGISSILGAVNFISTIMNMRPF
GMNLDKTPLFVWSVLITAILLLLSLPVLAGAITMLLTDRNINTSFFDPMGGGDPVLYQHL
>COLFA653-12|KJ963876|ZMUO.000788|Platystethus_arenarius
TLYFIFGAWSGMVGTSLSMLIRAELGTPGSLIGDDQIYNVVVTAHAFIMIFFMVMPIVIGGFGNWLVPLMLGAPDMAFPRMNNMSFWLLPPSLTLLLFSSMVESGAGTGWTVYPPLSANIAHSGSSVDLAIFSLHLAGISSILGAVNFISTIINMRSIGM
TFDRMPLFVWSVNITAILLLLSLPVLAGAITMLLTDRNLNTSFFDPAGGGDPILYQHL
>COLFE519-13|KJ965745|ZMUO.005364|Megasternum_concinnum
TLYFIFGAWAGMVGTSLSILIRAELGNPGTLIGDDQIYNVIVTAHAFIMIFFMVMPIMIGGFGNWLVPLMLGAPDMAFPRMNNMSFWLLPPSLTLLLMSSMVESGAGTGWTVYPPLSSNIAHGGSSVDLAIFSLHLAGISSILGAVNFITTVINMRSPNL
TYDRLPLFVWSVAITALLLLLSLPVLAGAITMLLTDRNLNTSFFDPAGGGDPILYQHL
>COLFE1119-13|KJ962288|ZMUO.006914|Amara_erratica
TLYFIFGAWSGMVGTSLSMLIRAELGNPGALIGDDQIYNVIVTAHAFVMIFFMVMPIMIGGFGNWLVPLMLGAPDMAFPRMNNMSFWLLPPSLTLLLMSSMVESGAGTGWTVYPPLSSGIAHAGASVDLAIFSLHLAGISSILGAVNFITTIINMRSV
GMTFDRMPLFVWSVGITALLLLLSLPVLAGAITMLLTDRNLNTSFFDPAGGGDPILYQHL
>COLFC257-12|KJ965125|ZMUO.002442|Atheta_britteni
TLYFIFGAWAGMVGTSLSLLIRAELGNPGSLIGDDQIYNVIVTAHAFIMIFFMVMPVVIGGFGNWLVPLMLGGPDMAFPRMNNMSFWLLPPSLTLLLMSSMVESGAGTGWTVYPPLSSNIAHSGSSVDLAIFSLHLAGISSILGAVNFISTIINMRSTGIS
FDRMPLFVWSVAITALLLLLSLPVLAGAITMLLTDRNLNTSFFDPAGGGDPILYQHL
>COLFE060-12|KJ964015|ZMUO.005285|Anostirus_castaneus
TLYFLFGAWSGMLGTSLSLLIRAELGNPGSLIGNDQIYNVVVTAHAFIMIFFMVMPIMIGGFGNWLVPLMLGAPDMAFPRMNNMSFWFLPPSLSLLLMSSIVENGAGTGWTVYPPLSANIAHSGSSVDLAIFSLHLAGISSILGAVNFITTVINMRTTGI
TFDRMPLFVWAVAITALLLLLSLPVLAGAITMLLTDRNLNTSFFDPAGGGDPILYQHL
>COLFE767-13|KJ966089|ZMUO.005612|Ilybius_subtilis
TLYFIFGAWAGMVGTSLSMLIRAELGNPGSLIGDDQIYNVIVTAHAFVMIFFMVMPIMIGGFGNWLVPLMLGAPDMAFPRMNNMSFWLLPPSLSLLLMSSMVESGAGTGWTVYPPLSSGIAHSGASVDLAIFSLHLAGISSILGAVNFITTIINMRSV
GMTFDRMPLFVWSVGITALLLLLSLPVLAGAITMLLTDRNLNTSFFDPAGGGDPILYQHL
>COLFF229-13|KJ962839|ZMUO.005834|Meligethes_exilis
TLYFIFGAWSGMVGTSLSMLIRTELGNPGSLIGNDQIYNVIVTAHAFVMIFFMVMPFMIGGFGNWLVPLMLGAPDMAFPRMNNMSFWLLPPSLSLLLMSSIIESGAGTGWTVYPPLSSNIAHGGASVDLAIFSLHLAGISSILGAVNFITTVINMRPTG
MTFDRMPLFVWAVVITALLLLLSLPVLAGAITMLLTDRNLNTTFFDPSGGGDPVLYQHL
>COLFB834-12|KJ966593|ZMUO.001309|Catops_alpinus
TLYFIFGAWAGMVGTSLSLLIRAELGNPGSLIGDDQIYNVIVTAHAFVMIFFMVMPIVIGGFGNWLVPLMLGAPDMAFPRMNNMSFWLLPPSLSLLLMSSIVENGAGTGWTVYPPLSANIAHSGSSVDLAIFSLHLAGISSILGAVNFITTVINMRATG
MTLDKMPLFVWSVAITALLLLLSLPVLAGAITMLLTDRNLNTSFFDPAGGGDPILYQHL
>COLFC232-12|KJ966894|ZMUO.002417|Ampedus_sanguinolentus
TLYFIFGAWAGMLGTSLSLLIRAELGNPGSLIGNDQIYNVIVTAHAFIMIFFMVMPIMIGGFGNWLVPLMLGAPDMAFPRMNNMSFWLLPPSLSLLLMSSIVENGAGTGWTVYPPLSSNIAHSGSSVDLAIFSLHLAGISSILGAVNFISTVINMRSTGIT
FDRMPLFVWAVAITALLLLLSLPVLAGAITMLLTDRNLNTSFFDPAGGGDPILYQHL
>COLFE1396-13|KJ963673|ZMUO.007191|Anthonomus_rubi
TLYFIFGAWSGAVGTSLSMLIRTELGNPGSLIGDDQIYNTIVTAHAFIMIFFMVMPIMIGGFGNWLIPLMLAAPDMAFPRLNNMSFWLLPPSLTLLIMSSIISKGAGTGWTVYPPLSSNLAHEGASVDFAIFSLHMAGISSILGAMNFISTILNMKPMKM
KFEQMPLFIWAVKITAILLLISLPVLAGAITMLLTDRNINTSFFDPAGGGDPILYQHL
>COLFD350-12|KJ965732|ZMUO.004245|Phyllobius_argentatus

TLYFIFGAWSGMVGTSLSILIRIELGNPGSLIGDDQIYNVIVTAHAFIMIFFMVMPMMIGGFGNWLVPLMLGAPDMAFPRLNNMSFWLLPPSLSLLLMSSIVDKGAGTGWTVYPPLSANIAHEGSSVDLAIFSLHMAGVSSILGAINFISTVINMRPSG
MSPDRMSLFIWAVKITAVLLLLSLPVLAGAITMLLTDRNMNTSFFDPAGGGDPILYQHL
>COLFA571-12|KJ964790|ZMUO.000706|Pachyta_quadrimaculata
TLYFIFGAWAGMVGTSLSLLIRSELGNPGSLIGDDQIYNVIVTAHAFIMIFFMVMPIMIGGFGNWLVPLMLGAPDMAFPRMNNMSFWLLPPSLTLLIMSSIVESGAGTGWTVYPPLSSNIAHGGSSVDLAIFSLHLAGISSILGAVNFITTVINMRPVGM
TLDRMPLFVWAVVITAILLLLSLPVLAGAITMLLTDRNLNTSFFDPAGGGDPILYQHL
>COLFD859-12|KJ966809|ZMUO.004469|Pterostichus_melanarius
TLYFIFGAWAGMVGTSLSMLIRAELGNPGSLIGDDQIYNVIVTAHAFVMIFFMVMPIMIGGFGNWLVPLMLGAPDMAFPRMNNMSFWLLPPSLTLLLMSSMVESGAGTGWTIYPPLSSGIAHAGASVDLAIFSLHLAGVSSILGAVNFITTIINMRSIG
MTFDRMPLFVWSVGITALLLLLSLPVLAGAITMLLTDRNLNTSFFDPAGGGDPILYQHL
>COLFE1359-13|KJ964775|ZMUO.007154|Ceutorhynchus_pulvinatus
TLYFIFGSWAGMTGTSLSMLIRTELGNPGSLIGNDQIYNSIVTAHAFIMIFFMVMPILIGGFGNWLVPLMLGAPDMAFPRLNNMSFWLLPPSLSLLLMSSIVNKGAGTGWTVYPPLSSNVAHEGMSVDLAIFSLHMAGISSILGAINFISTVMNMQPKG
MTPESMPLFVWAVQITAILLLISLPVLAGAITMLLTDRNINTSFFDPSGGGDPILYQHL
>COLFD641-12|KJ964619|ZMUO.004441|Amara_lunicollis
TLYFIFGAWSGMVGTSLSMLIRAELGNPGALIGDDQIYNVIVTAHAFVMIFFMVMPIMIGGFGNWLVPLMLGAPDMAFPRMNNMSFWLLPPSLTLLLMSSMVENGAGTGWTVYPPLSSGIAHAGASVDLAIFSLHLAGISSILGAVNFITTIINMRSI
GMTFDRMPLFVWSVGITALLLLLSLPVLAGAITMLLTDRNLNTSFFDPAGGGDPILYQHL
>COLFA330-12|KJ964938|ZMUO.000465|Acrotona_sylvicola
TLYFVFGTWAGMVGTSLSLLIRAELGNPGSLIGDDQIYNVIVTAHAFVMIFFMVMPIVIGGFGNWLVPLMLGAPDMAFPRMNNMSFWLLPPSLTLLLMSSMVESGAGTGWTVYPPLSSNIAHGGSSVDLAIFSLHLAGISSILGAVNFISTVINMRSVG
ISFDRMPLFVWSVVITALLLLLSLPVLAGAITMLLTDRNLNTSFFDPAGGGDPILYQHL
>COLFF531-13|KJ966214|ZMUO.006611|Oulimnius_tuberculatus
TLYFILGSWSGMLGMALSLLIRAELGTPGALIGDDQIYNVIVTAHAFVMIFFMVMPIMIGGFGNWLVPLMLGAPDMAFPRMNNMSFWLLPPSLTLLLMSSMVESGAGTGWTVYPPLSANIAHSGSSVDLAIFSLHLAGISSILGAVNFITTVINMRSPG
MTFDRMPLFVWSVVITAVLLLLSLPVLAGAITMLLTDRNLNTSFFDPAGGGDPILYQHL
>COLFF386-13|KJ963456|ZMUO.005904|Mordella_holomelaena
TLYFIFGAWAGMLGTSLSLLIRAELGNPGSLIGDDQIYNTIVTAHAFIMIFFMVMPIMVGGFGNWLVPLMLGAPDMAFPRMNNMSFWLLPPSLSLLLMSSVVENGAGTGWTVYPPLSTNIAHGGSSVDLAIFSLHLAGVSSILGAINFISTMINMRPSG
MSLDRMPLFAWAIMITAVLLLLSLPVLAGAITMLLTDRNLNTSFFDPAGGGDPILYQHL
>COLFE1408-13|KJ962269|ZMUO.007203|Amara_plebeja
TLYFIFGAWSGMVGTSLSMLIRAELGNPGALIGDDQIYNVIVTAHAFVMIFFMVMPIMIGGFGNWLIPLMLGAPDMAFPRMNNMSFWLLPPSLTLLLMSSMVESGAGTGWTVYPPLSSGIAHAGASVDLAIFSLHLAGISSILGAVNFITTIINMRSM
GMTFDRMPLFVWSVGITALLLLLSLPVLAGAITMLLTDRNLNTSFFDPAGGGDPILYQHL
>COLFD561-12|KJ961764|ZMUO.004361|Chrysomela_vigintipunctata
TLYFIFGIWAGMVGTSLSILIRAELGNPGSLIGNDQIYNVIVTAHAFIMIFFMVMPIMIGGFGNWLVPLMIGAPDMAFPRLNNMSFWLLPPSLFLLLMSSIVENGAGTGWTVYPPLSTNIAHSGSSVDLAIFSLHLAGISSILGAINFITTVINMRPEGMNF
EQTPLFVWAVLITAILLLLSLPVLAGAITMLLTDRNLNTSFFDPAGGGDPILYQHL
>COLFE597-13|KJ962901|ZMUO.005442|Gyrinus_minutus
TLYFIFGAWSGMVGTSLSMLIRAELGNPGSLIGDDQVYNVIVTAHAFIMIFFMVMPIMIGGFGNWLVPLMLGAPDMAFPRMNNMSFWLLPPSLTLLLMSSMVENGAGTGWTVYPPLSSNIAHGGASVDLAIFSLHLAGISSILGAVNFITTIINMRSI
GMTLDRMPLFVWSVGITALLLLLSLPVLAGAITMLLTDRNLNTSFFDPAGGGDPILYQHL
>COLFF601-13|KJ966727|ZMUO.006491|Cionus_scrophulariae
TLYFIFGVWSGTVGTSLSMLIRTELGNPGSLIGDDQIYNTIVTAHAFIMIFFMVMPIMIGGFGNWLVPLMLGAPDMAFPRLNNMSFWLLPPSIILLLMSSIVEKGAGTGWTVYPPLSNNVTHEGASVDLAIFSLHMAGISSILGAINFISTISNMRTKGMD
YDRTPLFVWSVNITALLLLLSLPVLAGAITMLLTDRNINTSFFDPSGGGDPILYQHL
>COLFC056-12|KJ964638|ZMUO.002051|Ischnosoma_splendidum
TLYFIFGAWAGMVGTSLSLLIRAELGNPGSLIGDDQIYNVIVTAHAFVMIFFMVMPIVIGGFGNWLVPLMLGAPDMAFPRMNNMSFWLLPPSLSLLLMSSLVESGAGTGWTVYPPLSSNIAHGGASVDLAIFSLHLAGISSILGAVNFITTVINMRSTG
MTFDRMPLFVWSVVITALLLLLSLPVLAGAITMLLTDRNLNTSFFDPAGGGDPILYQHL
>COLFA470-12|KJ963816|ZMUO.000510|Lochmaea_caprea
TLYFIFGIWAGMVGTSLSILVRAELGSPGTLIGNDQIYNVIVTAHAFIMIFFMVMPIMIGGFGNWLVPLMIGAPDMAFPRMNNMSFWLLPPSLFLLIMSSIVESGAGTGWTVYPPLSSNIAHGGSSVDLAIFSLHLAGISSILGAINFITTIINMRPKGMTL
DRIPLFVWAVMITAILLLLSLPVLAGAITMLLTDRNLNTSFFDPAGGGDPILYQHL
>COLFD198-12|KJ961902|ZMUO.004093|Astenus_pulchellus
TLYFIFGSWAGMVGTSLSLLIRAELGMPGSLIGDDQIYNVIVTAHAFVMIFFMVMPIVIGGFGNWLVPLMLGAPDMAFPRMNNMSFWLLPPSLSLLLMSSIVESGAGTGWTVYPPLSSNIAHSGASVDLAIFSLHLAGISSILGAVNFITTIINMRTKGM
TYERMPLFIWSVGITALLLLLSLPVLAGAITMLLTDRNLNTSFFDPAGGGDPILYQHL
>COLFF885-13|KJ962112|ZMUO.006680|Panagaeus_cruxmajor
TLYFIFGAWSGMVGTSLSMLIRAELGNPGSLIGDDQIYNVIVTAHAFVMIFFMVMPIMIGGFGNWLVPLMLGAPDMAFPRMNNMSFWLLPPSLSLLLMSSLVESGAGTGWTVYPPLSSGIAHSGASVDLAIFSLHLAGVSSILGAVNFITTIINMRSVG
MTFERMPLFVWSVGITALLLLLSLPVLAGAITMLLTDRNLNTSFFDPAGGGDPILYQHL

>COLFF1006-13|KJ964803|ZMUO.007371|Olophrum_fuscum
TLYFIFGAWAGMVGTSLSILIRAELGNPGTLIGDDQIYNVIVTAHAFVMIFFMVMPILIGGFGNWLVPLMLGAPDMAFPRMNNMSFWLLPPSLTLLLMSSMVESGAGTGWTVYPPLSSNIAHGGASVDLAIFSLHLAGISSILGAVNFITTVINMRTTG
MTFDRMPLFVWAVAITALLLLLSLPVLAGAITMLLTDRNLNTTFFDPAGGGDPILYQHL
>COLFE362-12|KJ966261|ZMUO.004922|Clivina_fossor
TLYFIFGAWSGMVGTSLSMLIRAELGNPGSLIGDDQIYNVIVTAHAFVMIFFMVMPILIGGFGNWLVPLMLGAPDMAFPRMNNMSFWLLPPSLSLLLMSSMVESGAGTGWTVYPPLSSAIAHSGASVDLAIFSLHLAGVSSILGAVNFITTIINMRSTG
MTFERMPLFVWSVGITALLLLLSLPVLAGAITMLLTDRNLNTSFFDPAGGGDPILYQHL
>COLFC757-12|KJ964916|ZMUO.003322|Epuraea_variegata
TLYFIFGAWSSMIGTSLSILIRTELGSPGSLIGNDQIYNVIVTAHAFIMIFFMVMPFMIGGFGNWLVPLMLGAPDMAFPRMNNMSFWLLPPSLSLLLMSSIVESGAGTGWTVYPPLSSNIAHSGSSVDLAIFSLHLAGISSILGAVNFITTIINMRPIGMTLD
RMPLFVWSVMITAILLLLSLPVLAGAITMLLTDRNLNTTFFDPSGGGDPILYQHL
>COLFE761-13|KJ966501|ZMUO.005606|Badister_dilatatus
TLYFIFGAWAGMVGTSLSMLIRAELGNPGSLIGDDQIYNVIVTAHAFVMIFFMVMPIMIGGFGNWLVPLMLGAPDMAFPRMNNMSFWLLPPSLTLLLMSSLVESGAGTGWTVYPPLSSGIAHSGASVDLAIFSLHLAGISSILGAVNFITTIINMRSVGI
SFDRMPLFVWSVGITALLLLLSLPVLAGAITMLLTDRNLNTSFFDPAGGGDPILYQHL
>COLFD247-12|KJ963826|ZMUO.004142|Laccophilus_minutus
TLYFIFGAWSGMVGTSLSLLIRAELGNPGSLIGDDQIYNVIVTAHAFIMIFFMVMPIMIGGFGNWLVPLMLGAPDMAFPRMNNMSFWLLPPSLSLLLMSSMVESGAGTGWTVYPPLSSGIAHSGASVDLAIFSLHLAGISSILGAVNFITTIINMRSIGM
TFDRMPLFVWSVGITALLLLLSLPVLAGAITMLLTDRNLNTSFFDPAGGGDPILYQHL
>COLFA085-10|HM909101|MP00303|Aphidecta_obliterata
TLYFLFGLWAGMVGTSLSILIRMELSTTGSLIGNDQIYNVIVTAHAFIMIFFMVMPIMIGGFGNWLVPLMIGAPDMAFPRLNNMSFWLLPPALTLLIFSMMVEMGAGTGWTVYPPLSSNLAHNGPSVDLVIFSLHLAGISSILGAVNFISTIMNMRPFG
MSLDKTPLFVWSVMITAILLLLSLPVLAGAITMLLTDRNLNTSFFDPTGGGDPVLYQHL
>COLFF489-13|KJ961953|ZMUO.006569|Mycetaea_subterranea
TLYFIFGMWAGMVGTSLSLLIRLELGNPGSLIGDDQIYNVIVTAHAFIMIFFMVMPIMIGGFGNWLVPLMLGAPDMAFPRMNNMSFWLLPPSLSLLIMSSIIENGAGTGWTVYPPLSSNMTHSGASVDLAIFSLHLAGISSILGAVNFITTVINMRPEG
MTLEKMPLFVWSVIITAILLLLSLPVLAGAITMLLTDRNLNTSFFDPTGGGDPILYQHL
>COLFA544-12|KJ963955|ZMUO.000679|Hydrophilus_aterrimus
TLYFIFGAWAGMVGTSLSILIRSELGNPGTLIGDDQIYNVIVTAHAFIMIFFMVMPIMIGGFGNWLIPLMLGAPDMAFPRMNNMSFWLLPPSLTLLLASSMVENGAGTGWTVYPPLSSNIAHSGASVDLAIFSLHLAGVSSILGAVNFISTVINMRSTNL
TYDRLPLFVWSVAITALLLLLSLPVLAGAITMLLTDRNINTSFFDPAGGGDPILYQHL
>COLFF928-13|KJ964432|ZMUO.006723|Pityogenes_bidentatus
TLYFIFGAWSGMVGTSLSMLIRTELGTPGSLIGDDQIFNTIVTAHAFIMIFFMVMPILIGGFGNWLVPLMLGAPDMAFPRLNNMSFWLLPPSLTFLIMSSIIDKGAGTGWTVYPPLSSNIAHEGASVDLAIFSLHMSGVSSILGAINFISTIINMHPKGVAP
EQLSLFTWAVKITAILLLLSLPVLAGAITMLLTDRNVNTSFFDPAGGGDPILYQHL
>COLFB030-12|KJ966483|ZMUO.001360|Amara_apricaria
TLYFIFGAWSGMVGTSLSMLIRAELGNPGALIGDDQIYNVIVTAHAFIMIFFMVMPIMIGGFGNWLVPLMLGAPDMAFPRMNNMSFWLLPPSLSLLLMSSMVESGAGTGWTVYPPLSSGIAHAGASVDLAIFSLHLAGISSILGAVNFITTIINMRSVG
MTFDRMPLFVWSVGITALLLLLSLPVLAGAITMLLTDRNMNTSFFDPAGGGDPILYQHL
>COLFD001-12|KJ966056|ZMUO.003896|Harpalus_solitaris
TLYFIFGAWAGMVGTSLSMLIRAELGTPGALIGDDQIYNVIVTAHAFIMIFFMVMPIMIGGFGNWLVPLMLGAPDMAFPRMNNMSFWLLPPSLTLLLMSSMVESGAGTGWTVYPPLSSGIAHSGASVDLAIFSLHLAGISSILGAVNFITTIINMRSVG
MTFDRMPLFVWSVGITALLLLLSLPVLAGAITMLLTDRNLNTSFFDPAGGGDPILYQHL
>COLFA481-12|KJ965743|ZMUO.000521|Propylea_quatuordecimpunctata
TLYFLFGMWAGMVGTSLSILIRLELGTTNSLIGNDQIYNVIVTAHAFIMIFFMVMPIMIGGFGNWLVPLMIGAPDMAFPRLNNMSFWLLPPALMLLIMSSMVEMGAGTGWTVYPPLSSNMAHSGSSVDLVIFSLHLAGISSILGAVNFISTIMNMRPF
GMNLDKTPLFVWSVLITAILLLLSLPVLAGAITMLLTDRNINTSFFDPMGGGDPILYQHL
>COLFE1166-13|KJ965564|ZMUO.006961|Hippodamia_variegata
TLYFLFGMWAGMIGTSLSLLIRLELGTTGSLIGNDQIYNVIVTAHAFIMIFFMVMPIMIGGFGNWLVPLMIGAPDMAFPRLNNMSFWLLPPALTLLLFSSIVEMGAGTGWTVYPPLSSNLAHNGPSVDLVIFSLHLAGISSILGAVNFISTIMNMRPYGM
SLDKTPLFVWSVLITAILLLLSLPVLAGAITMLLTDRNLNTSFFDPTGGGDPILYQHL
>COLFG070-13|KJ965661|ZMUO.007575|Xylotrechus_rusticus
TLYFIFGAWAGMVGTSLSILIRSELGNPGSLIGDDQIYNVIVTAHAFIMIFFMVMPIMIGGFGNWLVPLMLGAPDMAFPRMNNMSFWLLPPSLTLLIMSSIVESGAGTGWTVYPPLSANIAHSGSSVDLAIFSLHLAGVSSILGAVNFISTVINMRPTKM
SPEQMPLFVWAVAITALLLLLSLPVLAGAITMLLTDRNINTSFFDPAGGGDPILYQHL
>COLFF363-13|KJ962425|ZMUO.006063|Bagous_glabrirostris
TLYFIFGAWAGMLGTSLSMLIRLELGNPGSLIGNDQIYNTIVTAHAFIMIFFMVMPIMIGGFGNWLVPLMLGAPDMAFPRLNNMSFWLLPPSILLLIMSSIVDNGAGTGWTVYPPLSSNIAHEGSSVDLAIFSLHMAGVSSILGAVNFISTVLNMRPSG
MKPEQMSLFTWAVEITAILLLLSLPVLAGAITMLLTDRNVNTSFFDPAGGGDPILYQHL
>COLFD122-12|KJ963466|ZMUO.004017|Enicmus_rugosus

SLYFIFGLWSGMVGTSLSLLIRLELGNPGSLIGDDQIYNVIVTAHAFIMIFFMVMPIMMGGFGNWLVPLMLGAPDMAFPRLNNMSFWLLPPSLSLLIMSSIVESGAGTGWTVYPPLSSNIAHNGSSVDLAIFSLHLAGISSILGAVNFITTVINMRPTGMS
LDSMPLFSWSVTLTAILLLLSLPVLAGAITMLLTDRNLNTSFFDPAGGGDPILYQHL
>COLFC624-12|KJ963057|ZMUO.003189|Hydroporus_erythrocephalus
TLYFLFGAWSGMVGTSLSMLIRAELGNPGSLIGDDQIYNVIVTAHAFIMIFFMVMPIMIGGFGNWLVPLMLGAPDMAFPRMNNMSFWLLPPSLSLLLMSSMVENGAGTGWTVYPPLSSGIAHSGASVDLAIFSLHLAGVSSILGAVNFITTIINMRSIG
MTFDRMPLFVWSVGITALLLLLSLPVLAGAITMLLTDRNLNTSFFDPAGGGDPILYQHL
>COLFD315-12|KJ961899|ZMUO.004210|Dyschirius_thoracicus
TLYFIFGIWSGMVGTSLSILIRTELGNPGSLIGDDQIYNVIVTAHAFIMIFFMVMPIMIGGFGNWLVPLMLGAPDMAFPRMNNMSFWLLPPSLTLLLMSSMVESGAGTGWTVYPPLSSGIAHSGASVDLAIFSLHLAGISSILGAVNFITTIINMRSTGMT
FERMPLFVWSVGITALLLLLSLPVLAGAITMLLTDRNLNTSFFDPAGGGDPILYQHL
>COLFD740-12|KJ965004|ZMUO.004730|Barypeithes_pellucidus
TLYFIFGAWSGMVGTSLSLLIRTELGNPGSLIGDDQIYNVIVTAHAFIMIFFMVMPIMIGGFGNWLVPLMLGAPDMAFPRLNNMSFWLLPPSLSLLLMSSIVDKGAGTGWTVYPPLSANIAHEGSSVDLAIFSLHMAGVSSILGAINFISTVINMRPTGM
SMDRMPLFVWAVKLTAILLLLSLPVLAGAITMLLTDRNVNTSFFDPAGGGDPILYQHL
>COLFB571-12|KJ962858|ZMUO.001046|Stenus_binotatus
TLYFIFGAWAGMLGTSLSLLIRAELGNPGSLIGDDQIYNVIVTAHAFIMIFFMVMPTMIGGFGNWLVPLMLGAPDMAFPRMNNMSFWLLPPSLSLLLMSSMVESGAGTGWTVYPPLSSNIAHSGASVDLAIFSLHLAGISSILGAINFITTILNMRTMK
MQLDCLPLFVWSVGITALLLLLSLPVLAGAITMLLTDRNLNTSFFDPAGGGDPILYQHL
>COLFD596-12|KJ966368|ZMUO.004396|Hyphydrus_ovatus
TLYFLFGAWSGMVGTSLSMLIRAELGNPGSLIGDDQIYNVIVTAHAFIMIFFMVMPIMIGGFGNWLVPLMLGAPDMAFPRMNNMSFWLLPPSLSLLLMSSMVESGAGTGWTVYPPLSAGIAHGGSSVDLAIFSLHLAGISSILGAVNFITTIINMRSIG
MTFDRMPLFVWSVGITALLLLLSLPVLAGAITMLLTDRNLNTSFFDPAGGGDPILYQHL
>COLFB586-12|KJ964691|ZMUO.001061|Atheta_castanoptera
TLYFIFGAWAGMVGTSLSLLIRAELGNPGSLIGDDQIYNVIVTAHAFIMIFFMVMPVVIGGFGNWLVPLMLGAPDMAFPRMNNMSFWLLPPSLTLLLMSSMVESGAGTGWTVYPPLSSNIAHGGSSVDLAIFSLHLAGISSILGAVNFISTVINMRSTGI
SFDRMPLFVWSVAITALLLLLSLPVLAGAITMLLTDRNLNTSFFDPAGGGDPILYQHL
>COLFF1009-13|KJ966875|ZMUO.007374|Mannerheimia_arctica
TLYFVFGAWAGMVGTSLSILIRTELGNPGSLIGDDQIYNVIVTAHAFIMIFFMVMPIVIGGFGNWLVPLMLGAPDMAFPRMNNMSFWLLPPSLTLLLMSSMVESGAGTGWTVYPPLSSNIAHGGSSVDLAIFSLHLAGISSILGAVNFITTVINMRSSG
MTFDRMPLFVWSVVITALLLLLSLPVLAGAITMLLTDRNLNTSFFDPAGGGDPILYQHL
>COLFE808-13|KJ967204|ZMUO.005653|Stenus_juno
TLYFIFGSWAGMVGTSLSMLIRSELGNPGSLIGDDQIYNVIVTAHAFIMIFFMVMPIMIGGFGNWLVPLMLGAPDMAFPRMNNMSFWLLPPSLSLLLMSSIVESGAGTGWTVYPPLSSNIAHSGASVDLAIFSLHLAGISSILGAINFITTIINMRTMKLQ
LECLPLFVWSVSITALLLLLSLPVLAGAITMLLTDRNLNTSFFDPAGGGDPILYQHL
>COLFE1357-13|KJ964897|ZMUO.007152|Dryophilus_pusillus
TLYFLFGAWAGMVGTSLSILIRSELGNPGTLIGNDQIYNVIVTAHAFIMIFFMVMPIMIGGFGNWLVPLMLGAPDMAFPRMNNMSFWLLPPSLSLLLMSSITENGAGTGWTVYPPLSANIAHSGSSVDLAIFSLHLAGISSILGAVNFITTIINMRPMSM
SFDQMPLFVWSVMITAILLLLSLPVLAGAITMLLTDRNLNTSFFDPAGGGDPILYQHL
>COLFB443-12|KJ964860|ZMUO.001868|Meligethes_aeneus
TLYFIFGAWSGMVGTSLSMLIRTELGNPGSLIGNDQIYNVIVTAHAFVMIFFMVMPFMIGGFGNWLVPLMLGAPDMAFPRMNNMSFWLLPPSLSLLLMSSIVESGAGTGWTVYPPLSSNIAHGGASVDLAIFSLHLAGISSILGAVNFITTVINMRPK
GMTFDRMPLFVWAVMITAILLLLSLPVLAGAITMLLTDRNLNTTFFDPSGGGDPILYQHL
>COLFF884-13|KJ962372|ZMUO.006679|Syntomus_foveatus
TLYFIFGMWAGMVGTSLSILIRAELGNPGALIGDDQIYNVIVTAHAFIMIFFMVMPIMIGGFGNWLVPLMLGAPDMAFPRMNNMSFWLLPPSLTLLLMSSMVESGAGTGWTVYPPLSSGIAHAGASVDLAIFSLHLAGVSSILGAVNFITTIINMRSV
GITFDRMPLFVWSVGITALLLLLSLPVLAGAITMLLTDRNLNTSFFDPAGGGDPILYQHL
>COLFC029-12|KJ963458|ZMUO.002024|Cercyon_castaneipennis
TLYFIFGAWAGMVGTSLSILIRAELGNPGTLIGDDQMYNVIVTAHAFIMIFFMVMPIMIGGFGNWLVPLMLGAPDMAFPRMNNMSFWLLPPSLTLLLMSSMVESGAGTGWTVYPPLSSNIAHGGSSVDLAIFSLHLAGISSILGAVNFITTVINMRSPS
LTYDRLPLFVWSVAITALLLLLSLPVLAGAITMLLTDRNLNTSFFDPAGGGDPILYQHL
>COLFA616-12|KJ962966|ZMUO.000751|Ilybius_ater
TLYFIFGAWAGMVGTSLSMLIRAELGNPGSLIGDDQIYNVIVTAHAFVMIFFMVMPIMIGGFGNWLVPLMLGAPDMAFPRMNNMSFWLLPPSLTLLLMSSMVESGAGTGWTVYPPLSAGIAHSGASVDLAIFSLHLAGISSILGAVNFITTIINMRSV
GMTFDRMPLFVWSVGITALLLLLSLPVLAGAITMLLTDRNLNTSFFDPAGGGDPILYQHL
>COLFA614-12|KJ964527|ZMUO.000749|Ampedus_balteatus
TLYFIFGAWAGMLGTSLSLLIRAELGNPGSLIGNDQIYNVIVTAHAFIMIFFMVMPIMIGGFGNWLVPLMLGAPDMAFPRMNNMSFWLLPPSLSLLLMSSIVENGAGTGWTVYPPLSSNIAHSGSSVDLAIFSLHLAGISSILGAVNFISTVINMRSTGIT
FDRMPLFVWAVAITALLLLLSLPVLAGAITMLLTDRNLNTSFFDPAGGGDPILYQHL
>COLFD381-12|KJ965692|ZMUO.003801|Oryctes_nasicornis
TLYFLFGSWSGMVGTSLSLLIRAELGTPGSLIGDDQIYNVIVTAHAFIMIFFMVMPIMIGGFGNWLVPLMLGAPDMAFPRMNNMSFWLLPPSLTLLLASSLVENGAGTGWTVYPPLSANIAHSGASVDLAIFSLHLAGISSILGAVNFITTVINMRSAGM
TFDRTPLFVWSVMLTAILLLLSLPVLAGAITMLLTDRNINTSFFDPAGGGDPILYQHL

>COLFG031-13|KJ966559|ZMUO.007536|Phaenops_cyanea
TLYFIFGAWSGMVGTALSLLIRAELGNPGALIGDDQIYNTIVTAHAFIMIFFMVMPIMMGGFGNWLVPLMLGAPDMAFPRMNNMSFWLLPPSLTLLLMSSIVENGAGTGWTVYPPLAANTAHSGASVDLAIFSLHLAGISSILGAVNFITTVINMRSV
SMTFDQMPLFVWSVAITALLLLLSLPVLAGAITMLLTDRNLNTSFFDPAGGGDPILYQHL
>COLFE1544-13|KJ964178|ZMUO.007434|Helophorus_pumilio
TLYFIFGAWAGMVGTSLSILIRAELGNPGTLIGDDQIYNVIVTAHAFIMIFFMVMPIMIGGFGNWLVPLMLGAPDMAFPRMNNMSFWLLPPSLTLLLMSSMVESGAGTGWTVYPPLSSNIAHSGASVDLAIFSLHLAGISSILGAVNFITTVINMRSVN
MTYDRLPLFVWSVAITALLLLLSLPVLAGAITMLLTDRNLNTSFFDPAGGGDPILYQHL
>COLFD275-12|KJ965890|ZMUO.004170|Malthodes_misellus
TLYFMFGAWSGMLGTSLSLLIRAELGSPGSLIGNDQIYNVIVTAHAFIMIFFMVMPIMIGGFGNWLVPLMLGAPDMAFPRMNNMSFWFLPPSLSLLLMSSIVENGAGTGWTVYPPLSANIAHSGSSVDLAIFSLHMAGISSILGAVNFISTVINMRSTG
MTFDRMPLFVWAVAITALLLLLSLPVLAGAITMLLTDRNLNTSFFDPAGGGDPILYQHL
>COLFB753-12|KJ963330|ZMUO.001228|Rybaxis_laminata
TLYLIFGAWAGMVGTSLSILIRAELGNPGSLIGDDQIYNVIVTAHAFIMIFFMVMPIMIGGFGNWLVPLMLGAPDMAFPRMNNMSFWLLPPSLILLLMSSMVESGAGTGWTVYPPLSSNIAHSGSSVDLTIFSLHLAGISSILGAVNFITTVINMRTMS
MKFDQLPLFVWSVIITALLLLLSLPVLAGAITMLLTDRNLNTSFFDPTGGGDPVLYQHL
>COLFG098-13|KJ964353|ZMUO.007793|Atheta_ischnocera
TLYFIFGAWAGMVGTSLSLLIRAELGNPGSLIGDDQIYNVIVTAHAFIMIFFMVMPIVIGGFGNWLVPLMLGAPDMAFPRMNNMSFWLLPPSLTLLLMSSMVESGAGTGWTVYPPLSSNIAHGGSSVDLAIFSLHLAGISSILGAVNFISTVINMRSTGI
SFDRMPLFVWSVAITALLLLLSLPVLAGAITMLLTDRNLNTSFFDPAGGGDPILYQHL
>COLFE971-13|KJ964291|ZMUO.006766|Philonthus_debilis
TLYFIFGSWAGMVGTSLSLLIRAELGNPGSLIGDDQIYNVIVTAHAFIMIFFMVMPIVIGGFGNWLVPLMLGAPDMAFPRMNNMSFWLLPPSLTLLLMSSMVESGAGTGWTVYPPLSSNIAHGGASVDLAIFSLHLAGISSILGAVNFITTVINMRSTG
MTFDRMPLFVWSVAITALLLLLSLPVLAGAITMLLTDRNLNTTFFDPAGGGDPILYQHL
>COLFF071-13|KJ962862|ZMUO.005771|Otiorhynchus_nodosus
TLYFIFGSWSGMVGTSLSMLIRIELGSPGSLIGDDQIYNVIVTAHAFIMIFFMVMPMMTGGFGNWLVPLMLGAPDMAFPRLNNMSFWLLPPSLSLLLMSSIIDKGSGTGWTVYPPLSSNISHEGASVDLAIFSLHMAGISSILGAINFISTAINMRPSGM
TPDRMSLFIWSVKITAILLLLSLPVLAGAITMLLTDRNINTSFFDPAGGGDPILYQHL
>COLFB548-12|KJ963730|ZMUO.001023|Acrotona_orbata
TLYFIFGAWAGMVGTSLSLLIRAELGNPGSLIGDDQIYNVIVTAHAFIMIFFMVMPIMIGGFGNWLVPLMLGAPDMAFPRMNNMSFWLLPPSLTLLLMSSMVESGAGTGWTVYPPLSSNIAHGGSSVDLAIFSLHLAGISSILGAVNFISTVINMRSSGI
TFDRMPLFVWAVAITALLLLLSLPVLAGAITMLLTDRNLNTSFFDPAGGGDPILYQHL
>COLFC330-12|KJ965247|ZMUO.002515|Atheta_vaga
TLYFIFGAWAGMVGTSLSLLIRAELGNPGSLIGDDQIYNVIVTAHAFVMIFFMVMPIVIGGFGNWLIPLMLGAPDMAFPRMNNMSFWLLPPSLTLLLMSSMVESGAGTGWTVYPPLSSNIAHGGSSVDLAIFSLHLAGISSILGAVNFISTVINMRSTGI
TFDRMPLFVWSVAITALLLLLSLPVLAGAITMLLTDRNLNTSFFDPAGGGDPILYQHL
>COLFD454-12|KJ964163|ZMUO.003874|Perapion_marchicum
TLYFIFGFWSGMVGTSLSMLIRVELGNPGSLIGDDQIYNVIVTAHAFIMIFFMVMPIMIGGFGNWLVPLMLGAPDMAFPRMNNMSFWLLPPSLTLLLMSSIIEKGAGTGWTIYPPLASNMAHGGSSVDLAIFSLHLAGISSILGAVNFISTVINMRPSGL
SLDQLSLFTWAVKITAMLLLLSLPVLAGAITMLITDRNINTSFFDPAGGGDPILYQHL
>COLFB777-12|KJ967468|ZMUO.001252|Atheta_aeneipennis
TLYFIFGAWAGMVGTSLSLLIRAELGNPGSLIGDDQIYNVIVTAHAFIMIFFMVMPIVIGGFGNWLVPLMLGAPDMAFPRMNNMSFWLLPPSLTLLLMSSMVESGAGTGWTVYPPLSSNIAHGGSSVDLAIFSLHLAGISSILGAVNFISTVINMRSTGI
SFDRMPLFVWSVAITALLLLLSLPVLAGAITMLLTDRNLNTSFFDPAGGGDPILYQHL
>COLFC248-12|KJ966654|ZMUO.002433|Epuraea_pygmaea
TLYFILGVWSGMVGTSLSILIRTELGSPGSLIGNDQIYNVIVTAHAFIMIFFMVMPFMIGGFGNWLVPLMLGAPDMAFPRMNNMSFWLLPPSLSLLLMSSIVESGAGTGWTVYPPLSSNIAHGGSSVDLAIFSLHLAGVSSILGAVNFITTIINMRPAGM
TLDRMPLFVWSVIITAILLLLSLPVLAGAITMLLTDRNLNTTFFDPSGGGDPILYQHL
>COLFA427-12|KJ966998|ZMUO.000657|Sepedophilus_littoreus
TLYFIFGAWAGMVGTSLSLLIRAELGNPGSLIGDDQIYNVIVTAHAFIMIFFMVMPIVIGGFGNWLVPLMLGAPDMAFPRMNNMSFWLLPPSLSLLLMSSLVESGAGTGWTVYPPLSSNIAHGGSSVDLAIFSLHLAGISSILGAVNFITTVINMRSMG
MSFDKMPLFVWSVAITALLLLLSLPVLAGAITMLLTDRNLNTSFFDPAGGGDPILYQHL
>COLFG137-13|KJ963409|ZMUO.007832|Cypha_tarsalis
TLYFIFGAWSGMIGTSLSLLIRAELGNPGSLIGDDQIYNVIVTSHAFIMIFFMVMPIMIGGFGNWLVPLMLSAPDMAFPRMNNMSFWLLPPALTLLLMSSMVESGSGTGWTVYPPLSSNIAHGGASVDLTIFSLHLAGISSILGAVNFISTVINMRPMG
MSFDRMPLFVWSVVITALLLLLSLPVLAGAITMLLTDRNLNTSFFDPAGGGDPILYQHL
>COLFA391-12|KJ966373|ZMUO.000621|Anisotoma_axillaris
TLYFIFGAWSGMVGTSLSILIRAELGTPGSLIGDDQIYNVIVTAHAFVMIFFMVMPIMIGGFGNWLVPLMLGAPDMAFPRMNNMSFWLLPPSLTLLLMSSMVENGAGTGWTVYPPLSANISHSGSSVDLAIFSLHLAGISSILGAVNFITTVINMRSIG
MTFDKMPLFVWSVVITALLLLLSLPVLAGAITMLLTDRNLNTSFFDPAGGGDPILYQHL
>COLFA057-10|HM909077|MP00235|Bledius_arcticus

TLYFIFGAWAGMVGTSLSMLIRAELSIPGSLIGDDQIYNVIVTAHAFVMIFFMVMPIVIGGFGNWLIPLMLGAPDMAFPRMNNMSFWLLPPSLTLLLMSSLVESGVGTGWTVYPPLSSNIAHSGSSVDLAIFSLHLAGISSILGAVNFISTVINMRSIGMT
FDRMPLFVWSVKITAILLLLSLPVLAGAITMLLTDRNLNTSFFDPAGGGDPILYQHL
>COLFC616-12|KJ963758|ZMUO.003181|Anthophagus_alpinus
TLYFIFGAWAGMVGTSLSILIRSELGNPGSLIGDDQIYNVIVTAHAFVMIFFMVMPVVIGGFGNWLVPLMLGAPDMAFPRMNNMSFWLLPPSLTLLLMSSMVESGAGTGWTVYPPLSSNIAHSGASVDLAIFSLHLAGISSILGAVNFITTVINMRSTGI
TFDRMPLFVWSVAITALLLLLSLPVLAGAITMLLTDRNLNTSFFDPAGGGDPILYQHL
>COLFG157-13|KJ962386|ZMUO.007852|Proteinus_brachypterus
TLYFIFGAWAGMVGTSLSILIRAELGNPGTLIGDDQIYNVIVTAHAFIMIFFMVMPIVIGGFGNWLVPLMLGAPDMAFPRMNNMSFWLLPPSITLLLMSSMVENGAGTGWTVYPPLSSNISHSGSSVDLAIFSLHLAGISSILGAVNFITTVINMRSPGM
TFDRMPLFVWSVAITALLLLLSLPVLAGAITMLLTDRNINTTFFDPAGGGDPILYQHL
>COLFB015-12|KJ963775|ZMUO.001345|Noterus_clavicornis
TLYFIFGAWAGMVGTSLSMLIRMELGNPGSLIGDDQIYNVIVTAHAFIMIFFMVMPIMIGGFGNWLVPLMIGAPDMAFPRMNNMSFWLLPPSLSLLIMSSLVENGAGTGWTVYPPLASGIAHSGSSVDLAIFSLHLAGISSILGAVNFITTIINMRPLG
MSFDRMPLFVWSVGITALLLLLSLPVLAGAITMLLTDRNLNTSFFDPAGGGDPILYQHL
>COLFE008-12|KJ967156|ZMUO.005233|Leptura_melanura
TLYFIFGAWAAMVGTSLSLLIRSELGNPGSLIGDDQIYNVIVTAHAFVMIFFMVMPIMIGGFGNWLVPLMLGAPDMAFPRMNNMSFWLLPPSLTLLIMSSIVETGAGTGWTVYPPLSSNIAHSGSSVDLAIFSLHLAGISSILGAVNFITTVINMHPVGM
TPDRMPLFVWAVVITAVLLLLSLPVLAGAITMLLTDRNLNTSFFDPAGGGDPILYQHL
>COLFD421-12|KJ964643|ZMUO.003841|Amara_similata
TLYFIFGAWSGMVGTSLSMLIRAELGNPGALIGDDQIYNVIVTAHAFVMIFFMVMPIMIGGFGNWLVPLMLGAPDMAFPRMNNMSFWLLPPSLSLLLMSSMVESGAGTGWTIYPPLSSGIAHAGASVDLAIFSLHLAGISSILGAVNFITTIINMRSVG
MTFDRMPLFVWSVGITALLLLLSLPVLAGAITMLLTDRNLNTSFFDPAGGGDPILYQHL
>COLFE1376-13|KJ962583|ZMUO.007171|Pityophthorus_glabratus
TLYFIFGAWSGTIGTSLSMLIRTELGCPGSLIGDDQIYNTVVTAHAFIMIFFMVMPIMMGGFGNWLVPLMLGAPDMAFPRLNNMSFWLLPPSLTFLIMSSIIDKGAGTGWTVYPPLSSNISHEGSSVDLAIFSLHMAGISSILGAINFISTIINMHPKGMK
PEQLPLFIWAVKITAVLLLLSLPVLAGAITMLLTDRNINTSFFDPTGGGDPILYQHL
>COLFD278-12|KJ967094|ZMUO.004173|Aplocnemus_tarsalis
TLYFIFGAWSGMIGTSLSLMIRSELGNPGTLIGNDQIYNVIVTAHAFIMIFFMVMPIMIGGFGNWLVPLMLGAPDMAFPRMNNMSFWLLPPSLSLLLLSSMVESGVGTGWTVYPPLSSNIAHGGSSVDLAIFSLHLAGISSILGAVNFITTIINMRPAGM
TLDRTPLFVWSVGITALLLLLSLPVLAGAITMLLTDRNLNTSFFDPTGGGDPILYQHL
>COLFB373-12|KJ967247|ZMUO.001703|Pycnoglypta_lurida
TLYFIFGAWSGMVGTSLSMLIRAELGNPGSLIGDDQIYNVIVTAHAFIMIFFMVMPIVIGGFGNWLVPLMLGAPDMAFPRMNNMSFWLLPPSLTLLLMSSMVESGAGTGWTVYPPLSSNIAHGGASVDLAIFSLHLAGISSILGAVNFITTVVNMRSM
GMTFDRMPLFVWSVAITALLLLLSLPVLAGAITMLLTDRNLNTSFFDPAGGGDPILYQHL
>COLFB806-12|KJ965456|ZMUO.001281|Boreaphilus_henningianus
TLYFIFGAWAGMVGTSLSILIRAELGNPGSLIGDDQIYNVIVTAHAFVMIFFMVMPIVIGGFGNWLVPLMLGAPDMAFPRMNNMSFWLLPPSLNLLLMSSMVESGAGTGWTVYPPLSSNIAHGGSSVDLAIFSLHLAGVSSILGAVNFITTVINMRSTG
MSFDRMPLFVWSVAITALLLLLSLPVLAGAITMLLTDRNLNTSFFDPAGGGDPILYQHL
>COLFA381-12|KJ966605|ZMUO.000611|Pterostichus_rhaeticus
TLYFIFGAWAGMVGTSLSMLIRAELGNPGSLIGDDQIYNVIVTAHAFIMIFFMVMPIMIGGFGNWLVPLMLGAPDMAFPRMNNMSFWLLPPSLTLLLMSSMVESGAGTGWTVYPPLSSGIAHAGASVDLAIFSLHLAGVSSILGAVNFITTIINMRSV
GMTFDRMPLFVWSVGITALLLLLSLPVLAGAITMLLTDRNLNTSFFDPAGGGDPILYQHL
>COLFC357-12|KJ962374|ZMUO.002542|Atheta_sodalis
TLYFIFGAWAGMVGTSLSLLIRAELGNPGSLIGDDQIYNVIVTAHAFIMIFFMVMPIMIGGFGNWLVPLMLGAPDMAFPRMNNMSFWLLPPSLTLLLMSSMVESGAGTGWTVYPPLSSNIAHGGSSVDLAIFSLHLAGISSILGAVNFISTVINMRSTGI
SFDRMPLFVWAVAITALLLLLSLPVLAGAITMLLTDRNLNTSFFDPAGGGDPILYQHL
>COLFA622-12|KJ965246|ZMUO.000757|Hippodamia_tredecimpunctata
TLYFLFGMWAGMVGTSLSILIRLELGTPGSLIGNDQIYNVIVTAHAFIMIFFMVMPIMIGGFGNWLVPLMIGAPDMAFPRLNNMSFWLLPPALTLLMFSSMVEMGAGTGWTVYPPLSSNLAHNGPSVDLVIFSLHLAGISSILGAVNFISTIMNMRPY
GMSMDKTPLFVWSVLITAILLLLSLPVLAGAITMLLTDRNLNTSFFDPTGGGDPILYQHL
>COLFE1239-13|KJ964716|ZMUO.007034|Patrobus_septentrionis
TLYFIFGAWSGMVGTSLSMLIRAELGNPGSLIGDDQIYNVIVTAHAFVMIFFMVMPIMIGGFGNWLVPLMLGAPDMAFPRMNNMSFWLLPPSLTLLLMSSMVESGAGTGWTVYPPLSSGIAHSGASVDLAIFSLHLAGISSILGAVNFITTIINMRSVG
MSFDRMPLFVWSVGITALLLLLSLPVLAGAITMLLTDRNLNTSFFDPAGGGDPILYQHL
>COLFE079-12|KJ966757|ZMUO.005304|Cryptocephalus_distinguendus
TLYFLFGAWAGMVGTSLSLLIRIELGNPGSLIGNDQIYNTIVTAHAFIMIFFMVMPIMIGGFGNWLIPLMLGAPDMAFPRMNNLSFWLLPPSLTLLLVSSIVENGAGTGWTVYPPLSAIIAHTGASVDLAIFSLHLAGISSIMGAINFISTIINMRPQGMQ
MDCTPLFVWAVMITAVLLLLSLPVLAGAITMLLTDRNLNTSFFDPAGGGDPILYQHL
>COLFD552-12|KJ966736|ZMUO.004352|Halyzia_sedecimguttata
TLYFLFGMWAGMVGTSLSIMIRLELGTTNSLIGNDQIYNVIVTAHAFIMIFFMVMPVMIGGFGNWLVPLMVGAPDMAFPRLNNMSFWLLPPALTLLIFSSMVEMGAGTGWTVYPPLSSNMAHSGSSVDLVIFSLHLAGISSILGAVNFISTIMNMRP
FGMNLDKTPLFVWSVLITAILLLLSLPVLAGAITMLLTDRNINTSFFDPMGGGDPILYQHL

>COLFB607-12|KJ962732|ZMUO.001082|Aleochara_brevipennis
TLYFIFGAWSGMVGTSLSLLIRAELGNPGSLIGDDQIYNVIVTAHAFIMIFFMVMPIMIGGFGNWLVPLMLGAPDMAFPRMNNMSFWLLPPSLTLLLMSSMVESGAGTGWTVYPPLSSNIAHGGASVDLAIFSLHLAGISSILGAVNFISTVINMRSMG
MTFDKMPLFVWAVAITALLLLLSLPVLAGAITMLLTDRNLNTSFFDPAGGGDPILYQHL
>COLFE1187-13|KJ962975|ZMUO.006982|Anisotoma_humeralis
TLYFIFGAWSGMVGTSLSILIRAELGTPGSLIGDDQIYNVIVTAHAFVMIFFMVMPIMIGGFGNWLVPLMLGAPDMAFPRMNNMSFWLLPPSLTLLLMSSMVENGAGTGWTVYPPLSANISHSGSSVDLAIFSLHLAGISSILGAVNFITTVINMRSIG
MTFDKMPLFVWSVVITALLLLLSLPVLAGAITMLLTDRNLNTSFFDPAGGGDPILYQHL
>COLFF215-13|KJ966762|ZMUO.005820|Calathus_melanocephalus
TLYFIFGAWAGMVGTSLSMLIRAELGNPGALIGDDQIYNVIVTAHAFVMIFFMVMPIMIGGFGNWLVPLMLGAPDMAFPRMNNMSFWLLPPSLTLLLMSSMVESGAGTGWTVYPPLSSGIAHSGASVDLAIFSLHLAGVSSILGAVNFITTIINMRSV
GMTFDRMPLFVWSVGITALLLLLSLPVLAGAITMLLTDRNLNTSFFDPAGGGDPILYQHL
>COLFC237-12|KJ967398|ZMUO.002422|Eanus_costalis
TLYFLFGAWAGMLGTSLSLLIRAELGNPGSLIGNDQIYNVIVTAHAFIMIFFMVMPIMIGGFGNWLVPLMLGAPDMAFPRMNNMSFWFLPPSLSLLLMSSIVENGAGTGWTVYPPLSANIAHSGSSVDLAIFSLHLAGISSILGAVNFISTVINMRSTGIT
FDRMPLFVWAVAITALLLLLSLPVLAGAITMLLTDRNLNTSFFDPAGGGDPILYQHL
>COLFE1504-13|KJ962663|ZMUO.007299|Ceutorhynchus_pallidactylus
TLYFIFGAWSGMVGTSLSMIIRTELGNPGSLIGNDQIYNSIVTAHAFIMIFFMVMPILIGGFGNWLVPLMLGAPDMAFPRLNNMSFWLLPPSLSLLLMSSIVNKGTGTGWTVYPPLSSNVAHEGMSVDLSIFSLHMAGISSILGAINFISTVMNMQPKG
MTPELMPLFVWSVEITAILLLISLPVLAGAITMLLTDRNINTSFFDPSGGGDPILYQHL
>COLFD803-12|KJ962812|ZMUO.004793|Gyrinus_natator
TLYFIFGAWSGMVGTSLSMLIRAELGNPGSLIGDDQIYNVIVTAHAFIMIFFMVMPIMIGGFGNWLVPLMLGAPDMAFPRMNNMSFWLLPPSLTLLLMSSMVENGAGTGWTVYPPLSSNIAHGGASVDLAIFSLHLAGISSILGAVNFITTIINMRSIG
MTLDRMPLFVWSVGITALLLLLSLPVLAGAITMLLTDRNLNTSFFDPAGGGDPILYQHL
>COLFC117-12|KJ966387|ZMUO.002302|Cryptopleurum_subtile
TLYFIFGAWAGMVGTSLSILIRAELGNPGTLIGDDQIYNVIVTAHAFIMIFFMVMPIMIGGFGNWLVPLMLGAPDMAFPRMNNMSFWLLPPSLTLLLMSSMVESGAGTGWTVYPPLSSNIAHGGASVDLAIFSLHLAGISSILGAVNFITTVINMRSPN
LTYDRLPLFVWSVAITALLLLLSLPVLAGAITMLLTDRNLNTSFFDPAGGGDPILYQHL
>COLFE1204-13|KJ963686|ZMUO.006999|Hydroporus_umbrosus
TLYFLFGAWSGMVGTSLSMLIRAELGNPGSFIGDDQIYNVIVTAHAFIMIFFMVMPIMIGGFGNWLVPLMLGAPDMAFPRMNNMSFWLLPPSLTLLLMSSMVENGAGTGWTVYPPLSSGIAHSGASVDLAIFSLHLAGISSILGAVNFITTIINMRSV
GMTFDRMPLFVWSVGITALLLLLSLPVLAGAITMLLTDRNLNTSFFDPAGGGDPILYQHL
>COLFD226-12|KJ964719|ZMUO.004121|Aleochara_sparsa
TLYFIFGAWAGMVGTSLSLLIRAELGNPGSLIGDDQIYNVIVTAHAFIMIFFMVMPIMIGGFGNWLIPLMLGAPDMAFPRMNNMSFWLLPPSLTLLLMSSMVESGAGTGWTVYPPLSSNIAHGGSSVDLAIFSLHLAGISSILGAVNFISTVINMRSSG
MSFDKMPLFIWSVIITALLLLLSLPVLAGAITMLLTDRNLNTSFFDPAGGGDPILYQHL
>COLFE214-12|KJ966937|ZMUO.005059|Hyphydrus_ovatus
TLYFLFGAWSGMVGTSLSMLIRAELGNPGSLIGDDQIYNVIVTAHAFIMIFFMVMPIMIGGFGNWLVPLMLGAPDMAFPRMNNMSFWLLPPSLSLLLMSSMVESGAGTGWTVYPPLSAGIAHGGSSVDLAIFSLHLAGISSILGAVNFITTIINMRSIG
MTFDRMPLFVWSVGITALLLLLSLPVLAGAITMLLTDRNLNTSFFDPAGGGDPILYQHL
>COLFG151-13|KJ963915|ZMUO.007846|Ptiliola_kunzei
TLYFFFGAWAGMVGTSLSLLIRAELGTPGSLIGDDQIYNVIVTAHAFIMIFFMVMPILIGGFGNWLVPLMLGAPDMAFPRMNNMSFWLLPPSLSLLLLSSMVESGAGTGWTVYPPLSSNIAHGGASVDLAIFSLHLAGISSILGAVNFITTIINMRAPGM
SFDQMPLFVWAVGITALLLLLSLPVLAGAITMLLTDRNINTSFFDPAGGGDPILYQHL
>COLFD211-12|KJ966424|ZMUO.004106|Rhynchaenus_lonicerae
TLYFMFGAWSGMVGTSLSMLIRTELGNPGKLIGDDQIYNTIVTAHAFIMIFFMVMPIMIGGFGNWLIPLMLGAPDMAFPRLNNMSFWLLPPSLTLLLMSSIMNKGAGTGWTVYPPLSGNIAHEGSSVDLAIFSLHMAGISSILGAMNFISTIMNMHP
TGMKLDQLSLFVWAVKITAILLLLSLPVLAGAITMLLTDRNINTSFFDPAGGGDPILYQHL
>COLFD250-12|KJ962511|ZMUO.004145|Phytoecia_nigricornis
TLYFIFGAWASMVGTSLSILIRSELGTPGSLIGDDQIYNVIVTAHAFVMIFFMVMPIMIGGFGNWLVPLMLGAPDMAFPRMNNMSFWLLPPSLSLLIMSSIVDNGAGTGWTVYPPLAANVAHNGSSVDLAIFSLHLAGISSILGAVNFITTVINMRPSG
MTLDRMPLFVWAVKITAILLLLSLPVLAGAITMLLTDRNLNTSFFDPAGGGDPILYQHL
>COLFF666-13|KJ965193|ZMUO.006366|Plateumaris_braccata
TLYFIFGAWSGMMGTSLSILIRTELSNPGSLIGNDQIYNVIVTAHAFIMIFFMVMPIMIGGFGNWLVPLMLGAPDMAFPRMNNMSFWLLPPSLTFLIMSSIVENGAGTGWTVYPPLSSNIAHSGASVDLTIFSLHLAGISSILGAVNFITTIINTRPMGMS
MDKMPLFVWAVMITAVLLLLSLPVLAGAITMLLTDRNLNTSFFDPAGGGDPILYQHL
>COLFE1404-13|KJ965955|ZMUO.007199|Atheta_elongatula
TLYFIFGAWAGMVGTSLSLLIRAELGNPGSLIGDDQIYNVIVTAHAFVMIFFMVMPIVIGGFGNWLVPLMLGAPDMAFPRMNNMSFWLLPPSLTLLLMSSMVESGAGTGWTVYPPLSSNIAHGGSSVDLAIFSLHLAGISSILGAVNFISTVINMRSTGI
SFDRMPLFVWSVAITALLLLLSLPVLAGAITMLLTDRNLNTSFFDPAGGGDPILYQHL
>COLFE005-12|KJ963192|ZMUO.005230|Cantharis_flavilabris

TLYFIFGAWSGSLGLALSLLIRAELGTPGTLIGNDQIYNVIVTAHAFIMIFFMVMPIMIGGFGNWLVPLMLGAPDMAFPRMNNMSFWFLPPSLMFLLMSSMVESGAGTGWTVYPPLSANIAHSGPSVDLAIFSLHMAGVSSILGAVNFISTIMNMKPP
SMKFDQMPLFVWSVGITALLLLLSLPVLAGAITMLLSDRNLNTSFFDPMGGGDPILYQHL
>COLFE1523-13|KJ963996|ZMUO.007413|Longitarsus
ILYFIFGVWSGMVGTSLSILIRTELGNPGSLIGNDQIYNVIVTAHAFIMIFFMVMPIMIGGFGNWLVPLMIGAPDMAFPRMNNMSFWLLPPSLFFLIMSSVVESGAGTGWTVYPPLSSNIAHGGSSVDLAIFSLHLAGISSILGAINFITTVINMRPIGMTL
DRMPLFVWAVIITAILLLLSLPVLAGAITMLLTDRNLNTTFFDPAGGGDPILYQHL
>COLFC462-12|KJ967354|ZMUO.003027|Catops_alpinus
TLYFIFGAWAGMVGTSLSLLIRAELGNPGSLIGDDQIYNVIVTAHAFVMIFFMVMPIVIGGFGNWLVPLMLGAPDMAFPRMNNMSFWLLPPSLSLLLMSSIVENGAGTGWTVYPPLSANIAHSGSSVDLAIFSLHLAGISSILGAVNFITTVINMRATG
MTLDKMPLFVWSVAITALLLLLSLPVLAGAITMLLTDRNLNTSFFDPAGGGDPILYQHL
>COLFB606-12|KJ961791|ZMUO.001081|Longitarsus_luridus
TLYFIFGIWAGMIGTSLSILIRTELGNPGSLIGNDQIYNVIVTAHAFIMIFFMVMPIMIGGFGNWLVPLMIGAPDMAFPRMNNMSFWLLPPSLFLLVMSSMVESGAGTGWTVYPPLSSNIAHGGSSVDLAIFSLHLAGISSILGAINFITTVINMRPIGMTL
DRMPLFVWAVVITAILLLLSLPVLAGAITMLLTDRNLNTTFFDPAGGGDPILYQHL
>COLFC824-12|KJ964024|ZMUO.003389|Coelostoma_orbiculare
TLYFIFGAWAGMLGTSLSILIRTELGNPGSLIGNDQIYNVIVTAHAFIMIFFMVMPIMIGGFGNWLVPLMLGAPDMAFPRMNNMSFWLLPPSLTLLLMSSMVESGAGTGWTVYPPLSANIAHSGASVDLAIFSLHLAGISSILGAVNFITTVINMRSSN
MTYDRMPLFVWSVAITALLLLLSLPVLAGAITMLLTDRNFNTSFFDPAGGGDPILYQHL
>COLFE1095-13|KJ963798|ZMUO.006890|Patrobus_septentrionis
TLYFIFGAWSGMVGTSLSMLIRAELGNPGSLIGDDQIYNVIVTAHAFVMIFFMVMPIMIGGFGNWLVPLMLGAPDMAFPRMNNMSFWLLPPSLTLLLMSSMVESGAGTGWTVYPPLSSGIAHSGASVDLAIFSLHLAGISSILGAVNFITTIINMRSVG
MSFDRMPLFVWSVGITALLLLLSLPVLAGAITMLLTDRNLNTSFFDPAGGGDPILYQHL
>COLFD579-12|KJ963275|ZMUO.004379|Ceratomegilla_notata
TLYFLFGMWAGMIGTSLSILIRLELGTTGSLIGNDQIYNVIVTAHAFIMIFFMVMPIMIGGFGNWLVPLMIGAPDMAFPRLNNMSFWLLPPALILLLFSSLVEMGAGTGWTVYPPLSSNLAHNGPSVDLVIFSLHLAGISSILGAVNFISTIMNMRPYGM
KLDKTPLFVWSVLITAILLLLSLPVLAGAITMLLTDRNLNTSFFDPTGGGDPILYQHL
>COLFD080-12|KJ964126|ZMUO.003975|Cryptophagus_quercinus
TLYFIFGAWASMVGTSLSLLIRSELGTPGSLIGDDQIYNVIVTAHAFVMIFFMVMPIMIGGFGNWLVPLMLGAPDMAFPRMNNMSFWLLPPSLSLLLMSSIAEKGVGTGWTVYPPLSSNIAHGGSSVDLAIFSLHLAGISSILGAVNFISTVMNMHPTG
MNLDRMPLFVWAVIITAILLLLSLPVLAGAITMLLTDRNLNTSFFDPAGGGDPILYQHL
>COLFA351-12|KJ966296|ZMUO.000581|Ochthebius_marinus
TLYFIFGAWAGMVGTSLSILIRAELGNPGTLIGDDQIYNVIVTAHAFIMIFFMVMPIMIGGFGNWLVPLMLGAPDMAFPRMNNMSFWLLPPSLSLLLMSSMVESGAGTGWTVYPPLSSNIAHGGASVDLAIFSLHLAGISSILGAVNFITTVINMRSAG
MTFDRMPLFVWAVVITALLLLLSLPVLAGAITMLLTDRNLNTSFFDPAGGGDPILYQHL
>COLFE001-12|KJ966915|ZMUO.005226|Acanthocinus_griseus
TLYFIFGAWSGMVGTSLSMLIRSELGNPGTLIGNDQIYNTIVTAHAFIMIFFMVMPIMIGGFGNWLVPLMLGAPDMAFPRMNNMSFWLLPPSLTLLIMSSIVENGAGTGWTVYPPLSSNIAHAGSSVDLAIFSLHLAGISSILGAVNFITTIINMRPIGM
TFDRMPLFVWAVKITAILLLLSLPVLAGAITMLLTDRNINTSFFDPAGGGDPILYQHL
>COLFD849-12|KJ967296|ZMUO.004839|Carabus_glabratus
TLYFIFGTWSGMVGTSLSMLIRAELGNPGSLIGDDQIYNVIVTAHAFVMIFFMVMPIMIGGFGNWLVPLMLGAPDMAFPRMNNMSFWLLPPSLTLLLMSSMVEKGAGTGWTVYPPLSSGIAHSGASVDLAIFSLHLAGISSILGAVNFITTIINMRSVG
MTFDRMPLFVWSVGITALLLLLSLPVLAGAITMLLTDRNLNTSFFDPAGGGDPILYQHL
>COLFC718-12|KJ963853|ZMUO.003283|Melanotus_villosus
TLYFIFGAWAGMLGTSLSLLIRAELGNPGSLIGNDQIYNVIVTAHAFIMIFFMVMPIMIGGFGNWLVPLMLGAPDMAFPRMNNMSFWLLPPSLSLLLMSSIVENGAGTGWTVYPPLSSNIAHSGSSVDLAIFSLHLAGISSILGAVNFISTVINMRSTGM
TFDRMPLFVWAVALTALLLLLSLPVLAGAITMLLTDRNLNTSFFDPAGGGDPILYQHL
>COLFF090-13|KJ962571|ZMUO.005790|Stenus_labilis
TLYFIFGAWAGMVGTSLSLLIRAELGNPGSLIGDDQIYNVIVTAHAFVMIFFMVMPIMIGGFGNWLVPLMLGAPDMAFPRMNNMSFWLLPPSLSLLLMSSIVENGAGTGWTVYPPLSSNIAHSGASVDLAIFSLHLAGVSSILGAINFITTIFNMRTMK
LQLDCLPLFVWSVGITAFLLLLSLPVLAGAITMLLTDRNLNTSFFDPAGGGDPILYQHL
>COLFD446-12|KJ962836|ZMUO.003866|Neocrepidodera
TLYFIFGMWSGMIGSSLSMLIRIELGNPGSLIGNDQIYNVIVTAHAFIMIFFMVMPIMIGGFGNWLVPLMIGAPDMAFPRMNNMSFWLLPPSLFLLLMSSMVESGAGTGWTVYPPLSSNLAHSGSSVDLAIFSLHLAGISSILGAVNFITTVINMRPKG
MTLDRMPLFVWAVVITAILLLLSLPVLAGAITMLLTDRNLNTSFFDPSGGGDPILYQHL
>COLFC027-12|KJ966379|ZMUO.002022|Atheta_nigripes
TLYFIFGAWAGMVGTSLSLLIRAELGNPGSLIGDDQIYNVIVTAHAFIMIFFMVMPIVIGGFGNWLVPLMLGAPDMAFPRMNNMSFWLLPPSLTLLLMSSMVESGAGTGWTVYPPLSSNIAHGGSSVDLAIFSLHLAGISSILGAVNFISTVINMRSTGI
SFDRMPLFVWSVAITALLLLLSLPVLAGAITMLLTDRNLNTSFFDPAGGGDPILYQHL
>COLFC194-12|KJ965364|ZMUO.002379|Olophrum_assimile
TLYFIFGAWAGMVGTSLSILIRAELGNPGTLIGDDQIYNVIVTAHAFVMIFFMVMPIVIGGFGNWLVPLMLGAPDMAFPRMNNMSFWLLPPSLTLLLMSSMVESGAGTGWTVYPPLSSNIAHGGSSVDLAIFSLHLAGISSILGAVNFITTVINMRSTG
MTFDRMPLFVWSVAITALLLLLSLPVLAGAITMLLTDRNLNTSFFDPAGGGDPILYQHL

>COLFC244-12|KJ965059|ZMUO.002429|Agonum_fuliginosum
TLYFIFGAWAGMVGTSLSMLIRAELGNPGALIGDDQIYNVIVTAHAFIMIFFMVMPIMIGGFGNWLVPLMLGAPDMAFPRMNNMSFWLLPPSLTLLLMSSLVESGAGTGWTVYPPLSSGIAHAGASVDLAIFSLHLAGISSILGAVNFITTIINMRSVG
MTFDRMPLFVWSVGITALLLLLSLPVLAGAITMLLTDRNLNTSFFDPAGGGDPILYQHL
>COLFA535-12|KJ962889|ZMUO.000670|Agapanthia_villosoviridescens
TLYFIFGSWAAMLGSSLSILIRSELGNPGTLIGDDQIYNVIVTAHAFVMIFFMVMPIMIGGFGNWLVPLMLGAPDMAFPRMNNMSFWLLPPSLTFLVMSSIVENGAGTGWTVYPPLAGNVAHAGCSVDLAIFSLHLAGISSILGAVNFISTVINMRPEG
MLFDRLPLFVWAVKITAILLLLSLPVLAGGITMLLTDRNINTSFFDPAGGGDPILYQHL
>COLFF488-13|KJ966257|ZMUO.006568|Acritus_nigricornis
TLYFIFGTWSGMVGTSLSLLIRAELGNPGSLIGDDQIYNVIVTAHAFIMIFFMVMPIVIGGFGNWLVPLMLGAPDMAFPRMNNMSFWLLPPSLTLLLMSSMVESGAGTGWTVYPPLAANIAHGGASVDLAIFSLHLAGISSILGAVNFISTVINMRTPG
MTFDRMPLFVWSVAITALLLLLSLPVLAGAITMLLTDRNINTSFFDPAGGGDPILYQHL
>COLFG091-13|KJ966890|ZMUO.007596|Ptilinus_fuscus
TLYFIFGSWSGMIGTSLSLLIRSELGNPGALIGNDQIYNVIVTAHAFIMIFFMVMPMMIGGFGNWLVPLMLGAPDMAFPRMNNMSFWLLPPSLSLLLMSMIVNSGTGTGWTVYPPLSSNIAHSGASVDLTIFSLHLAGISSILGAINFITTVINMRPMS
MTFDRMPLFVWSVVITALLLLLSLPVLAGAITMLLTDRNLNTSFFDPAGGGDPILYQHL
>COLFD339-12|KJ962598|ZMUO.004234|Melolontha_hippocastani
TLYFLFGSWAGMVGTSLSLLIRAELGNPGTLIGDDQIYNVIVTAHAFVMIFFMVMPIMIGGFGNWLVPLMLGAPDMAFPRMNNMSFWLLPPSLSLLLMSSLVENGAGTGWTVYPPLSSNIAHSGASVDLAIFSLHLAGISSILGAVNFITTVINMRSTG
MTFDRMPLFAWSVALTALLLLLSLPVLAGAITMLLTDRNINTSFFDPAGGGDPILYQHL
>COLFE647-13|KJ961919|ZMUO.005492|Lilioceris_merdigera
TLYFIFGAWSGMVGTSLSMLIRTELGNPGSLIGNDQIYNVIVTAHAFIMIFFMVMPTMIGGFGNWLVPLMLGAPDMAFPRMNNMSFWLLPPSLTLLLMSSIVENGAGTGWTVYPPLSANLAHNGASVDLAIFSLHLAGISSILGAVNFITTVMNMRP
EGMKMDRTPLFVWAVLITAILLLLSLPVLAGAITMLLTDRNLNTSFFDPASGGDPILYQHL
>COLFB584-12|KJ966140|ZMUO.001059|Proteinus_brachypterus
TLYFIFGAWAGMVGTSLSILIRAELGNPGTLIGDDQIYNVIVTAHAFIMIFFMVMPIVIGGFGNWLVPLMLGAPDMAFPRMNNMSFWLLPPSITLLLMSSMVENGAGTGWTVYPPLSSNISHSGSSVDLAIFSLHLAGISSILGAVNFITTVINMRSPGM
TFDRMPLFVWSVAITALLLLLSLPVLAGAITMLLTDRNINTTFFDPAGGGDPILYQHL
>COLFB105-12|KJ962466|ZMUO.001435|Nicrophorus_vespilloides
TLYFIFGAWAGMVGMSLSMLIRVELSTPGTLLGDDQMYNVIVTAHAFIMIFFMVMPIVIGGFGNWLVPLMLGAPDMAFPRLNNMSFWLLPPSLSLLLISSMVESGAGTGWTVYPPLSANIAHSGSSVDLAIFSLHLAGISSILGAVNFITTVINMRSPG
MTFDRMPLFVWSVVITALLLLLSLPVLAGAITMLLTDRNLNTSFFDPAGGGDPILYQHL
>COLFD190-12|KJ962038|ZMUO.004085|Longitarsus_longiseta
TLYFIFGIWSGMVGTSLSILIRVELGNPGSLIGNDQIYNVIVTAHAFIMIFFMVMPIMIGGFGNWLVPLMIGAPDMAFPRMNNLSFWMLPPSLFLLVMSSIVESGAGTGWTVYPPLSSNITHSGSSVDLAIFSLHMAGISSILGAINFITTVINMRPKGMT
LDKMPLFVWAVIITAILLLLSLPVLAGAITMLLTDRNFNTTFFDPSGGGDPILYQHL
>COLFD093-12|KJ962630|ZMUO.003988|Acanthoscelides_obtectus
TLYFLFGAWAGMAGTSLSILIRAELGNPGSLIGNDQIYNVIVTAHAFIMIFFMVMPIMIGGFGNWLVPLMLGAPDMAFPRMNNMSFWLLPPSLSFLMLSSLVESGAGTGWTVYPPLASNIAHSGSSVDLAIFSLHLAGISSILGAVNFITTIMNMRPNG
MSMDRMPLFSWAVMITAILLLLSLPVLAGAITMLLTDRNLNTSFFDPAGGGDPILYQHL
>COLFD141-12|KJ964797|ZMUO.004036|Pityogenes_bidentatus
TLYFIFGAWSGMVGTSLSMLIRTELGTPGSLIGDDQIFNTIVTAHAFIMIFFMVMPILIGGFGNWLVPLMLGAPDMAFPRLNNMSFWLLPPSLTFLIMSSIIDKGAGTGWTVYPPLSSNIAHEGASVDLAIFSLHMSGVSSILGAINFISTIINMHPKGVAP
EQLSLFTWAVKITAILLLLSLPVLAGAITMLLTDRNVNTSFFDPAGGGDPILYQHL
>COLFE798-13|KJ965228|ZMUO.005643|Pterostichus_minor
TLYFIFGAWAGMVGTSLSMLIRAELGNPGSLIGDDQIYNVIVTAHAFIMIFFMVMPIMIGGFGNWLVPLMLGAPDMAFPRMNNMSFWLLPPSLTLLLMSSMVESGAGTGWTVYPPLSAGIAHAGASVDLAIFSLHLAGISSILGAVNFITTIINMRSIG
MTFDRMPLFVWSVGITALLLLLSLPVLAGAITMLLTDRNLNTSFFDPAGGGDPILYQHL
>COLFC765-12|KJ963288|ZMUO.003330|Oxystoma_opeticum
ILYFILGLWSGMVGTSLSMLIRIELGNSGSLIGDDQIYNVIVTAHAFIMIFFMVMPVMIGGFGNWLVPLMLGAPDMAFPRMNNMSFWLLPPSLTLLLMSSIVEKGVGTGWTVYPPLASNIAHSGASVDLAIFSLHLAGISSILGAINFISTMINMYPNGLS
LDQLSLFTWAVKITAILLLLSLPVLAGAITMLLTDRNINTSFFDPAGGGDPILYQHL
>COLFA112-10|JN265888|MP00374|Colymbetes_paykulli
TLYFIFGAWAGMVGTSLSMLIRAELGNPGSLIGDDQIYNVIVTAHAFVMIFFMVMPIMIGGFGNWLVPLMLGAPDMAFPRMNNMSFWLLPPSLTLLLMSSMVESGAGTGWTVYPPLSSGIAHGGASVDLAIFSLHLAGISSILGAVNFITTIINMRSV
GMTFDRMPLFVWSVGITALLLLLSLPVLAGAITMLLTDRNLNTSFFDPAGGGDPILYQHL
>COLFA302-12|KJ961720|ZMUO.000437|Cryptopleurum_subtile
TLYFIFGAWAGMVGTSLSILIRAELGNPGTLIGDDQIYNVIVTAHAFIMIFFMVMPIMIGGFGNWLVPLMLGAPDMAFPRMNNMSFWLLPPSLTLLLMSSMVESGAGTGWTVYPPLSSNIAHGGASVDLAIFSLHLAGISSILGAVNFITTVINMRSPN
LTYDRLPLFVWSVAITALLLLLSLPVLAGAITMLLTDRNLNTSFFDPAGGGDPILYQHL
>COLFD119-12|KJ962412|ZMUO.004014|Bolitochara_pulchra

TLYFIFGAWAGMVGTSLSLLIRAELGNPGSLIGDDQIYNVIVTAHAFVMIFFMVMPIMIGGFGNWLVPLMLGAPDMAFPRMNNMSFWLLPPSLTLLLMSSMVESGAGTGWTVYPPLSSNIAHGGSSVDLAIFSLHLAGISSILGAVNFISTIINMRSPG
MTFDRMPLFVWSVAITALLLLLSLPVLAGAITMLLTDRNLNTSFFDPAGGGDPILYQHL
>COLFA153-10|KJ965267|MP00415|Pseudocistela_ceramboides
TLYFIFGAWAGMVGTSLSLLIRAELGNPGSLIGDDQIYNVIVTAHAFIMIFFMVMPIMIGGFGNWLVPLMLGAPDMAFPRMNNMSFWLLPPSLTLLLMSSVVENGAGTGWTVYPPLSSNIAHGGASVDLAIFSLHLAGISSILGAVNFITTVINMRPQG
MSFDRMPLFVWAVVITAVLLLLSLPVLAGAITMLLTDRNINTSFFDPAGGGDPILYQHL
>COLFC062-12|KJ966695|ZMUO.002057|Ischnopoda_leucopus
TLYFIFGAWAGMVGTSLSLLIRAELGNPGSLIGDDQIYNVIVTAHAFVMIFFMVMPIMIGGFGNWLVPLMLGAPDMAFPRMNNMSFWLLPPSLTLLLMSSMVESGAGTGWTVYPPLSSNIAHGGSSVDLAIFSLHLAGISSILGAVNFISTVINMRSTG
ISFDRMPLFVWSVVITALLLLLSLPVLAGAITMLLTDRNLNTSFFDPAGGGDPILYQHL
>COLFE986-13|KJ966667|ZMUO.006781|Kateretes_pusillus
TLYFIFGAWSGMVGTSLSILIRSELGSPGSLIGDDQIYNVIVTAHAFVMIFFMVMPFMIGGFGNWLVPLMLGAPDMAFPRMNNMSFWLLPPSLSLLLMSSIVESGAGTGWTVYPPLSSNIAHGGSSVDLAIFSLHLAGISSILGAVNFITTVINMRPSGM
TFDQMPLFVWAVAITALLLLLSLPVLAGAITMLLTDRNLNTSFFDPAGGGDPVLYQHL
>COLFE1337-13|KJ965979|ZMUO.007132|Phratora_polaris
TLYFIFGIWAGMVGTSLSMLIRSELGNPGTLIGNDQIYNVIVTAHAFIMIFFMVMPIMIGGFGNWLVPLMIGAPDMAFPRLNNMSFWLLPPSLFLLLMSSVVENGAGTGWTVYPPLSSNLAHSGSSVDLAIFSLHLAGISSILGAINFITTVINMRPEGM
GLEQTPLFVWAVLITAILLLLSLPVLAGAITMLLTDRNLNTSFFDPAGGGDPILYQHL
>COLFE1243-13|KJ967379|ZMUO.007038|Quedius_fulvicollis
SLYFIFGAWAGMVGTSLSLLIRAELGNPGSLIGDDQIYNVIVTAHAFIMIFFMVMPTLIGGFGNWLVPLMLGAPDMAFPRMNNMSFWLLPPSLSLLLMSSMVESGAGTGWTVYPPLSSNIAHSGASVDLAIFSLHLAGISSILGAVNFITTVINMRSTG
MTFDRMPLFVWSVAITALLLLLSLPVLAGAITMLLTDRNLNTTFFDPAGGGDPILYQHL
>COLFF840-13|KJ962811|ZMUO.006160|Ptinomorphus_imperialis
TLYFIFGAWSGMVGTSLSMLIRSELGNPGALIGDDQIYNVIVTAHAFVMIFFMVMPIMIGGFGNWLVPLMLGAPDMAFPRMNNMSFWLLPPSLSLLLASSIVENGAGTGWTVYPPLASNIAHSGASVDLAIFSLHLAGVSSILGAVNFITTVINMRPV
GMLFDSMPLFVWSVAITALLLLLSLPVLAGAITMLLTDRNLNTSFFDPAGGGDPILYQHL
>COLFE998-13|KJ967317|ZMUO.006793|Agonum_piceum
TLYFIFGAWAGMVGTSLSMLIRAELGNPGALIGDDQIYNVIVTAHAFIMIFFMVMPIMIGGFGNWLVPLMLGAPDMAFPRMNNMSFWLLPPSLTLLLMSSLVESGAGTGWTVYPPLSSGIAHAGASVDLAIFSLHLAGVSSILGAVNFITTIINMRSVG
MTFDRMPLFVWSVGITALLLLLSLPVLAGAITMLLTDRNLNTSFFDPAGGGDPILYQHL
>COLFA324-12|KJ967175|ZMUO.000459|Atheta_melanocera
TLYFIFGAWAGMVGTSLSLLIRAELGNPGSLIGDDQIYNVIVTAHAFIMIFFMVMPIVIGGFGNWLVPLMLGAPDMAFPRMNNMSFWLLPPSLTLLLMSSMVESGAGTGWTVYPPLSSNIAHGGSSVDLAIFSLHLAGISSILGAVNFISTVINMRSTGI
SFDRMPLFVWSVAITALLLLLSLPVLAGAITMLLTDRNLNTSFFDPAGGGDPILYQHL
>COLFE1206-13|KJ967469|ZMUO.007001|Enochrus_affinis
TLYFIFGAWAGMVGTSLSILIRAELGNPGTLIGDDQIYNVIVTAHAFIMIFFMVMPIMIGGFGNWLVPLMLGAPDMAFPRMNNMSFWLLPPSLTLLLMSSMVESGAGTGWTVYPPLSSNIAHGGASVDLAIFSLHLAGISSILGAVNFITTVINMRSPS
MTYDRLPLFVWSVAITALLLLLSLPVLAGAITMLLTDRNLNTSFFDPAGGGDPILYQHL
>COLFE347-12|KJ967024|ZMUO.004907|Phyllobius_pomaceus
TLYFIFGTWSGMVGTSLSILIRAELGNPGSLIGDDQIYNVIVTAHAFIMIFFMVMPMMIGGFGNWLVPLMLGAPDMAFPRLNNMSFWLLPPSLSLLLMSSIVDKGAGTGWTVYPPLSANIAHEGSSVDLAIFSLHMAGVSSILGAINFISTVINMRPSG
MSPDRMSLFIWAVKITAILLLLSLPVLAGAITMLLTDRNLNTSFFDPAGGGDPILYQHL
>COLFD314-12|KJ966972|ZMUO.004209|Thanatophilus_dispar
TLYFIFGAWAGMVGMSLSILIRAELSTPGTLLGDDQMYNVIVTAHAFIMIFFMVMPIVIGGFGNWLVPLMLGAPDMAFPRMNNMSFWLLPPSLSLLVSSMVESGAGTGWTVYPPLSSNIAHGGSSVDLAIFSLHLAGISSILGAVNFITTIINMRASG
MTFDRMPLFVWSVAITALLLLLSLPVLAGAITMLLTDRNLNTSFFDPAGGGDPILYQHL
>COLFD645-12|KJ965460|ZMUO.004445|Rhynchaenus_testaceus
TLYFMFGAWSGMVGTSLSMLIRTELGTPGKLIGDDQIYNTIVTAHAFIMIFFMVMPTMIGGFGNWLIPLMLGAPDMAFPRLNNMSFWLLPPSLALLLMSSMMNKGAGTGWTVYPPLSSNMAHEGPSVDLAIFSLHMAGISSILGAINFISTMANM
RPSGMNTDQMSLFVWAVKITAILLLLSLPVLAGAITMLLTDRNINTSFFDPAGGGDPILYQHL
>COLFE1262-13|KJ967129|ZMUO.007057|Epuraea_biguttata
TLYFIFGAWSGMVGTSLSILIRTELGSPGSLIGNDQIYNVIVTAHAFIMIFFMVMPFMIGGFGNWLIPLMLGAPDMAFPRMNNMSFWLLPPSLSLLLMSSIVESGAGTGWTVYPPLSSNIAHGGSSVDLAIFSLHLAGISSILGAVNFITTIINMRPVGMTL
DRMPLFIWSVMITAVLLLLSLPVLAGAITMLLTDRNLNTTFFDPSGGGDPILYQHL
>COLFG002-13|KJ966853|ZMUO.007507|Leptura_nigripes
TLYFIFGAWASMVGTSLSVLIRSELGNPGSLIGDDQIYNVIVTAHAFVMIFFMVMPIMIGGFGNWLVPLMLGAPDMAFPRMNNMSFWLLPPSLTLLIMSSIVESGAGTGWTIYPPLSANIAHGGSSVDLAIFSLHLAGISSILGAVNFITTVINMRPSGM
TPDQMPLFVWAVVITAILLLLSLPVLAGAITMLLTDRNLNTSFFDPAGGGDPILYQHL
>COLFF682-13|KJ962340|ZMUO.006382|Stilbus_atomarius
TLYFIFGAWSGMVGTSLSLLIRTELGTPGSLIGDDQIYNVIVTAHAFVMIFFMVMPFMIGGFGNWLVPLMLGAPDMAFPRMNNMSFWLLPPSLSFLLMSSIVENGAGTGWTVYPPLSSNIAHGGSSVDLAIFSLHLAGISSILGAINFITTVINMRPEGM
TLDRMPLFVWSVVITAILLLLSLPVLAGAITMLLTDRNINTSFFDPAGGGDPILYQHL

>COLFE623-13|KJ964766|ZMUO.005468|Galerucella_lineola
TLYFIFGVWAGMVGTSLSILVRAELGSPGTLIGNDQIYNVIVTAHAFIMIFFMVMPIMIGGFGNWLVPLMIGAPDMAFPRMNNMSFWLLPPSLFLLIMSSIVESGAGTGWTVYPPLSSNIAHGGSSVDLAIFSLHLAGISSILGAINFITTIINMRPKGMTL
DRMPLFVWAVMITAILLLLSLPVLAGAITMLLTDRNLNTSFFDPAGGGDPILYQHL
>COLFC386-12|KJ963324|ZMUO.002951|Aphodius_piceus
TLYFLFGSWAGMVGTSLSLLIRAELGNPGTLIGDDQIYNVIVTAHAFVMIFFMVMPILIGGFGNWLVPLMLGAPDMAFPRMNNMSFWLLPPSLTLLLMSSMIESGAGTGWTVYPPLSSNIAHSGASVDLAIFSLHLAGISSILGAVNFITTVINMRTPG
MTFDRMPLFVWSVAITALLLLLSLPVLAGAITMLLTDRNLNTTFFDPAGGGDPILYQHL
>COLFF997-13|KJ966267|ZMUO.007362|Cassida_sanguinosa
TLYFIFGFWSGMVGTSLSILIRAELGNPGTLIGNDQIYNSIVTAHAFIMIFFMVMPIMIGGFGNWLVPLMLGAPDMAFPRLNNMSFWLLPPSITFLIMSSIIESGAGTGWTVYPPLSSNIAHSGASVDLAIFSLHLAGISSILGAINFISTIMNMQPAGMSL
DKMPLFVWAVIITAILLLLSLPVLAGAITMLLTDRNFNTSFFDPAGGGDPILYQHL
>COLFC853-12|KJ962040|ZMUO.003418|Protapion_apricans
TLYFIFGLWSGMIGTSLSMLIRIELGNPGSLIGNDQIYNVIVTAHAFIMIFFMVMPVMIGGFGNWLIPLMLGAPDMAFPRMNNMSFWLLPPSLTLLLMSSIIEKGAGTGWTVYPPLAANIAHSGASVDLAIFSLHLAGISSILGAVNFISTIMNMRPTGM
SLDQLSLFTWAVKITAILLLLSLPVLAGAITMLLTDRNINTSFFDPAGGGDPILYQHL
>COLFC093-12|KJ965436|ZMUO.002088|Atheta_taxiceroides
TLYFIFGAWAGMVGTSLSLLIRAELGNPGSLIGDDQIYNVIVTAHAFVMIFFMVMPIVIGGFGNWLVPLMLGAPDMAFPRMNNMSFWLLPPSLTLLLMSSMVESGAGTGWTVYPPLSSNIAHGGSSVDLAIFSLHLAGISSILGAVNFISTVINMRSTGI
TFDRMPLFVWSVVITALLLLSLPVLAGAITMLLTDRNLNTSFFDPAGGGDPILYQHL
>COLFE1543-13|KJ963186|ZMUO.007433|Helophorus_granularis
TLYFIFGAWAGMVGTSLSILIRAELGNPGTLIGDDQIYNVIVTAHAFIMIFFMVMPIMIGGFGNWLVPLMLGAPDMAFPRMNNMSFWLLPPSLTLLLMSSMVESGAGTGWTVYPPLSSNIAHSGASVDLAIFSLHLAGISSILGAVNFITTVINMRSIN
MTYDRLPLFVWSVAITALLLLLSLPVLAGAITMLLTDRNLNTSFFDPAGGGDPILYQHL
>COLFB330-12|KJ965640|ZMUO.001660|Strophosoma_capitatum
TLYFIFGAWSGMIGTSLSILIRTELGNPGSLIGDDQIYNVIVTAHAFIMIFFMVMPMMIGGFGNWLVPLMLGAPDMAFPRMNNMSFWLLPPSLSLLLMSSIVDKGAGTGWTVYPPLSANIAHEGSSVDLAIFSLHMAGVSSILGAVNFISTVINMRPM
GMSPDRMSLFIWSVKITAILLLLSLPVLAGAITMLLTDRNANTSFFDPAGGGDPILYQHL
>COLFF874-13|KJ962273|ZMUO.006669|Cercyon_melanocephalus
TLYFIFGAWAGMVGTSLSILIRAELGNPGTLIGDDQIYNVIVTAHAFIMIFFMVMPIMIGGFGNWLVPLMLGAPDMAFPRMNNMSFWLLPPSLTLLLMSSMVESGAGTGWTVYPPLSSNIAHGGSSVDLAIFSLHLAGISSILGAVNFITTVINMRSPNL
TYDRLPLFVWSVIITALLLLLSLPVLAGAITMLLTDRNLNTSFFDPAGGGDPILYQHL
>COLFE967-13|KJ961877|ZMUO.006762|Lathrobium_fulvipenne
TLYFIFGAWAGMVGTSLSLLIRTELGNPGSLIGDDQIYNVIVTAHAFVMIFFMVMPIMIGGFGNWLVPLMLGAPDMAFPRMNNMSFWLLPPSLSLLLMSSMVESGAGTGWTVYPPLSSNIAHGGASVDLAIFSLHLAGISSILGAVNFITTVINMRSPG
MTYERMPLFVWAVAITALLLLLSLPVLAGAITMLLTDRNLNTSFFDPAGGGDPILYQHL
>COLFB087-12|KJ963241|ZMUO.001417|Phyllotreta_undulata
TLYFIFGIWSGMLGMSMSMLIRVELAAPGSLIGNDQIYNVIVTAHAFIMIFFMVMPIMIGGFGNWLIPLMIGAPDMAFPRMNNMSFWLLPPSLFLLIMSSIIENGAGTGWTVYPPLSSNISHAGASVDLTIFSLHLAGISSILGAINFITTVINMRPKGMT
LDRLPLFVWAVLITAILLLLSLPVLAGAITMLLTDRNLNTSFFDPMGGGDPILYQHL
>COLFB111-12|KJ962506|ZMUO.001441|Cantharis_obscura
TLYFIFGAWSGSLGLALSLLIRAELGTPGTLIGNDQIYNVIVTAHAFIMIFFMVMPIMIGGFGNWLVPLMLGAPDMAFPRMNNMSFWFLPPSLMFLLMSSMVESGAGTGWTVYPPLSANIAHSGPSVDLAIFSLHMAGISSILGAVNFISTIMNMKPPS
MKFDQMPLFVWSVGITALLLLLSLPVLAGAITMLLSDRNLNTSFFDPMGGGDPILYQHL
>COLFC716-12|KJ962620|ZMUO.003281|Rhagium_mordax
TLYFIFGAWSGMVGTSLSLLIRSELGNPGSLIGDDQIYNVIVTAHAFIMIFFMVMPIMIGGFGNWLVPLMLGAPDMAFPRMNNMSFWLLPPSLTLLIMSSVVESGAGTGWTVYPPLSSNIAHSGSSVDLAIFSLHLAGISSILGAVNFITTVINMRPMG
MTPDRVPLFVWAVVITAILLLLSLPVLAGAITMLLTDRNLNTSFFDPAGGGDPILYQHL
>COLFA499-12|KJ964380|ZMUO.000539|Arhopalus_rusticus
TLYFIFGAWASMVGTSLSLLIRSELGSPGSLIGDDQIYNVIVTAHAFVMIFFMVMPIMIGGFGNWLVPLMLGAPDMAFPRMNNMSFWLLPPSLTLLIMSSIVESGAGTGWTVYPPLSSNIAHSGASVDLAIFSLHLAGISSILGAVNFITTVINMRPSGM
TLDRMPLFVWAVVITAILLLLSLPVLAGAITMLLTDRNLNTSFFDPSGGGDPILYQHL
>COLFB592-12|KJ963882|ZMUO.001067|Lordithon_thoracicus
TLYFIFGSWAGMVGTSLSLLIRAELGNPGSLIGDDQIYNVIVTAHAFIMIFFMVMPIVIGGFGNWLVPLMLGAPDMAFPRMNNMSFWLLPPSLTLLLMSSMVESGAGTGWTVYPPLSSNIAHGGASVDLAIFSLHLAGISSILGAVNFITTVINMRSIGM
TFDQMPLFIWSVAITALLLLLSLPVLAGAITMLLTDRNLNTTFFDPAGGGDPILYQHL
>COLFD164-12|KJ962672|ZMUO.004059|Cyphon_ochraceus
TLYFIFGSWSGMVGTSLSLLIRAELGTPGSLIGDDQIYNVIVTAHAFIMIFFMVMPIMIGGFGNWLVPLMLGAPDMAFPRMNNMSFWLLPPSLTLLLMSSMVENGAGTGWTVYPPLSAGIAHSGASVDLAIFSLHLAGISSILGAVNFISTVINMRSVG
MSFDRMPLFVWAVAITALLLLLSLPVLAGAITMLLTDRNLNTSFFDPAGGGDPILYQHL
>COLFA394-12|KJ966641|ZMUO.000624|Anostirus_castaneus

TLYFLFGAWSGMLGTSLSLLIRAELGNPGSLIGNDQIYNVVVTAHAFIMIFFMVMPIMIGGFGNWLVPLMLGAPDMAFPRMNNMSFWFLPPSLSLLLMSSIVENGAGTGWTVYPPLSANIAHSGSSVDLAIFSLHLAGISSILGAVNFITTVINMRTTGI
TFDRMPLFVWAVAITALLLLSLPVLAGAITMLLTDRNLNTSFFDPAGGGDPILYQHL
>COLFF859-13|KJ965584|ZMUO.006654|Aphodius_distinctus
TLYFLFGSWAGMVGTSLSLLIRAELGNPGTLIGDDQIYNVIVTAHAFVMIFFMVMPILIGGFGNWLVPLMLGAPDMAFPRMNNMSFWLLPPSLTLLLMSSMVESGAGTGWTVYPPLSSNIAHGGASVDLAIFSLHLAGISSILGAVNFITTVINMRSPG
VTFDRMPLFVWSVAITALLLLSLPVLAGAITMLLTDRNLNTSFFDPAGGGDPILYQHL
>COLFB325-12|KJ963244|ZMUO.001655|Habrocerus_capillaricornis
TLYFIFGAWSGMVGTSLSLLIRTELGNPGSLIGDDQIYNVIVTAHAFVMIFFMVMPIVIGGFGNWLVPLMLGAPDMAFPRMNNMSFWLLPPSLTLLLMSSMVESGAGTGWTVYPPLSSNIAHGGASVDLAIFSLHLAGISSILGAVNFITTVINMRAM
GMTFDRMPLFVWSVAITALLLLSLPVLAGAITMLLTDRNLNTSFFDPSGGGDPILYQHL
>COLFB854-12|KJ966164|ZMUO.001329|Strophosoma_capitatum
TLYFIFGAWSGMIGTSLSILIRTELGNPGSLIGDDQIYNVIVTAHAFIMIFFMVMPMMIGGFGNWLVPLMLGAPDMAFPRMNNMSFWLLPPSLSLLLMSSIVDKGAGTGWTVYPPLSANIAHEGSSVDLAIFSLHMAGVSSILGAVNFISTVINMRPM
GMSPDRMSLFIWSVKITAILLLLSLPVLAGAITMLLTDRNANTSFFDPAGGGDPILYQHL
>COLFD619-12|KJ964732|ZMUO.004419|Hippodamia_tredecimpunctata
TLYFLFGMWAGMVGTSLSILIRLELGTPGSLIGNDQIYNVIVTAHAFIMIFFMVMPIMIGGFGNWLVPLMIGAPDMAFPRLNNMSFWLLPPALTLLMFSSMVEMGAGTGWTVYPPLSSNLAHNGPSVDLVIFSLHLAGISSILGAVNFISTIMNMRPY
GMSMDKTPLFVWSVLITAILLLLSLPVLAGAITMLLTDRNLNTSFFDPTGGGDPILYQHL
>COLFB447-12|KJ963030|ZMUO.001872|Judolia_sexmaculata
TLYFIFGAWAGMVGTSLSLLIRSELGSPGSLIGNDQIYNVIVTAHAFVMIFFMVMPIMIGGFGNWLVPLMLGAPDMAFPRMNNMSFWLLPPSLTLLILSSMVETGAGTGWTVYPPLSSNIAHSGSSVDLAIFSLHLAGISSILGAVNFITTVINMRPVGM
NLDCMPLFVWAVVITAILLLLSLPVLAGAITMLLTDRNLNTSFFDPAGGGDPILYQHL
>COLFD325-12|KJ961959|ZMUO.004220|Aegialia_sabuleti
TLYFLFGGWAGMTGTSLSLLIRAELGNPGMLIGDDQIYNVIVTAHAFIMIFFMVMPILIGGFGNWLVPLMLGAPDMAFPRMNNMSFWLLPPSLTLLLMSSMVESGAGTGWTVYPPLSSNIAHSGPSVDLAIFSLHLAGISSILGAVNFITTVINMRSIG
MSFDRMPLFVWSVALTALLLLSLPVLAGAITMLLTDRNINTSFFDPTGGGDPILYQHL
>COLFF573-13|KJ962847|ZMUO.006463|Luperus_flavipes
TLYFIFGIWAGMVGTSLSILIRAELGGPGSLIGNDQIYNVIVTAHAFIMIFFMVMPIMIGGFGNWLIPLMIGAPDMAFPRMNNMSFWLLPPSIFLLIMSSVVESGAGTGWTVYPPLSSNIAHGGASVDLAIFSLHLAGISSILGAINFITTVINMRPMGMSL
DRMPLFVWAVVITAILLLLSLPVLAGAITMLLTDRNLNTSFFDPAGGGDPILYQHL
>COLFD594-12|KJ965680|ZMUO.004394|Amara_famelica
TLYFIFGAWSGMVGTSLSMLIRAELGNPGALIGDDQIYNVIVTAHAFVMIFFMVMPIMIGGFGNWLVPLMLGAPDMAFPRMNNMSFWLLPPSLTLLLMSSMVESGAGTGWTVYPPLSSGIAHAGASVDLAIFSLHLAGISSILGAVNFITTIINMRSV
GMTFDRMPLFVWSVGITALLLLSLPVLAGAITMLLTDRNLNTSFFDPAGGGDPILYQHL
>COLFE607-13|KJ965147|ZMUO.005452|Chaetocnema_sahlbergii
TLYFIFGIWSGMVGTSMSILIRAELGNPGSLIGNDQIYNVIVTAHAFVMIFFMVMPIMIGGFGNWLVPLMIGAPDMAFPRMNNMSFWLLPPSLFLLLMSSMVESGAGTGWTVYPPLSSNIAHGGSSVDLAIFSLHLAGISSILGAINFITTIINMRPQG
MQFDQMPLFVWAVLITAILLLLSLPVLAGAITMLLTDRNLNTSFFDPIGGGDPILYQHL
>COLFD224-12|KJ965060|ZMUO.004119|Ischnoglossa_obscura
TLYFIFGAWAGMVGTSLSLLIRAELGNPGSLIGDDQIYNVIVTAHAFVMIFFMVMPIMIGGFGNWLVPLMLGAPDMAFPRMNNMSFWLLPPSLTLLLMSSMVESGAGTGWTVYPPLSSNIAHGGSSVDLAIFSLHLAGISSILGAVNFISTIINMRTIG
MTFDRMPLFVWSVAITALLLLSLPVLAGAITMLLTDRNINTTFFDPAGGGDPILYQHL
>COLFC282-12|KJ962018|ZMUO.002467|Geodromicus_longipes
TLYFIFGAWAGMVGTSLSILIRSELGNPGSLIGDDQIYNVIVTAHAFVMIFFMVMPVVIGGFGNWLVPLMLGAPDMAFPRMNNMSFWLLPPSLTLLLMSSMVESGAGTGWTVYPPLSSNIAHSGASVDLAIFSLHLAGISSILGAVNFITTVINMRSTGI
SFDRMPLFVWSVAITALLLLSLPVLAGAITMLLTDRNLNTSFFDPAGGGDPILYQHL
>COLFF237-13|KJ961935|ZMUO.005842|Alosterna_tabacicolor
TLYFIFGAWAGMVGTSLSLLIRSELGSPGSFIGDDQVYNVIVTAHAFIMIFFMVMPIMIGGFGNWLVPLMLGAPDMAFPRMNNMSFWLLPPSLTLLIMSSIVESGAGTGWTVYPPLSSNIAHGGSSVDLAIFSLHLAGISSILGAVNFITTVINMRPMG
MNLDRMPLFVWAVVITAILLLLSLPVLAGAITMLLTDRNLNTSFFDPAGGGDPILYQHL
>COLFA270-12|KJ964941|ZMUO.000405|Bradycellus_caucasicus
TLYFIFGAWSGMVGTSLSMLIRAELGTPGALIGDDQIYNVIVTAHAFIMIFFMVMPIMIGGFGNWLVPLMLGAPDMAFPRMNNMSFWLLPPSLSLLLMSSMVEKGAGTGWTVYPPLSSGIAHSGASVDLAIFSLHLAGVSSILGAVNFITTIINMRSVG
MTFDRMPLFVWSVGITALLLLSLPVLAGAITMLLTDRNLNTSFFDPAGGGDPILYQHL
>COLFD334-12|KJ962051|ZMUO.004229|Amara_quenseli
TLYFIFGAWSGMVGTSLSMLIRAELGNPGALIGDDQIYNVIVTAHAFVMIFFMVMPIMIGGFGNWLVPLMLGAPDMAFPRMNNMSFWLLPPSLTLLLMSSMVESGAGTGWTVYPPLSSGIAHAGASVDLAIFSLHLAGISSILGAVNFITTIINMRSP
GMTFDRMPLFVWSVGITALLLLSLPVLAGAITMLLTDRNLNTSFFDPAGGGDPILYQHL
>COLFB190-12|KJ964958|ZMUO.001520|Ampedus_pomonae
TLYFIFGAWAGMLGTSLSLLIRAELGNPGSLIGNDQIYNVIVTAHAFIMIFFMVMPIMIGGFGNWLVPLMLGAPDMAFPRMNNMSFWLLPPSLSLLLMSSIVENGAGTGWTVYPPLSSNIAHSGSSVDLAIFSLHLAGISSILGAVNFISTVINMRSTGIT
FDRMPLFVWAVAITALLLLSLPVLAGAITMLLTDRNLNTSFFDPAGGGDPILYQHL

>COLFD094-12|KJ962201|ZMUO.003989|Acanthoscelides_obtectus
TLYFLFGAWAGMAGTSLSILIRAELGNPGSLIGNDQIYNVIVTAHAFIMIFFMVMPIMIGGFGNWLVPLMLGAPDMAFPRMNNMSFWLLPPSLSFLMLSSLVESGAGTGWTVYPPLASNIAHSGSSVDLAIFSLHLAGISSILGAVNFITTIMNMRPNG
MSMDRMPLFSWAVMITAILLLLSLPVLAGAITMLLTDRNLNTSFFDPAGGGDPILYQHL
>COLFD618-12|KJ962561|ZMUO.004418|Hypera_meles
TLYFIFGTWAGTVGTSLSILIRTELGNPGSLIGNDQIYNTIVTAHAFIMIFFMVMPIMIGGFGNWLVPLMLGAPDMAFPRLNNMSFWLLPPSLSLLLMSSMVDSGAGTGWTVYPPLSSNIAHEGSSVDLAIFSLHMAGVSSILGAINFISTVLNMRPSGM
SLDKMALFIWAVKITAILLLLSLPVLAGAITMLLTDRNINTSFFDPAGGGDPILYQHL
>COLFA440-12|KJ965235|ZMUO.000480|Chrysolina_polita
TLYFIFGIWAGMVGTSLSILIRTELGNPGTLIGNDQIYNVIVTAHAFIMIFFMVMPIMIGGFGNWLIPLMLGAPDMAFPRMNNMSFWLLPPSLLLLLMSSMMENGVGTGWTVYPPLSANIAHSGPAVDLAIFSLHLAGISSILGAINFISTTTNMRSTGM
SFEQMPLFSWAVLITVILLLLSLPVLAGAITMLLTDRNLNTSFFDPASGGDPILYQHL
>COLFE1550-13|KJ961753|ZMUO.007440|Coccinula_quatuordecimpustulata
TLYFLFGMWAGMVGTSLSILIRLELGSTGSLIGNDQIYNVIVTAHAFIMIFFMVMPIMIGGFGNWLVPLMIGAPDMAFPRLNNMSFWLLPPSLTLLLLSSLVEMGAGTGWTVYPPLSSNLAHNGSSVDLVIFSLHLAGISSILGAVNFISTIMNMRPNG
MNLDKTPLFVWSVLITAILLLLSLPVLAGAITMLLTDRNMNTSFFDPTGGGDPILYQHL
>COLFA086-10|HM909102|MP00308|Oligota
ILYFILGSWSGMIGTSLSLLIRAELGNPGSLIGDDQIYNVIVTSHAFIMIFFMVMPIMIGGFGNWLIPLMLGAPDMAFPRMNNMSFWLLPPALMLLLFSSMVESGSGTGWTVYPPLSSNIAHSGASVDLAIFSLHMAGISSILGAVNFITTILNMRPISMN
YDQMPLFVWSVGITALLLLLSLPVLAGAITMLLTDRNMNTSFFDPAGGGDPILYQHL
>COLFA589-12|KJ962541|ZMUO.000724|Brachyta_interrogationis
TLYFIFGAWAGMVGTSLSMLIRSELGNPGSLIGDDQIYNVIVTAHAFVMIFFMVMPIMIGGFGNWLVPLMLGAPDMAFPRMNNMSFWLLPPSLILLIMSSVVESGAGTGWTVYPPLASNIAHSGSSVDLAIFSLHLAGISSILGAVNFITTVINMRPLG
MIPDRMPLFVWSVVITAILLLLSLPVLAGAITMLLTDRNLNTSFFDPAGGGDPILYQHL
>COLFB283-12|KJ966748|ZMUO.001613|Lathrobium_impressum
TLYFIFGAWAGMVGTSLSLLIRTELGNPGSLIGDDQIYNVIVTAHAFIMIFFMVMPIMIGGFGNWLVPLMLGAPDMAFPRMNNMSFWLLPPSLSLLLMSSLVESGAGTGWTVYPPLSSNIAHGGASVDLAIFSLHLAGISAILGAVNFITTVINMRSPG
MSYERMPLFVWAVAITALLLLLSLPVLAGAITMLLTDRNLNTSFFDPAGGGDPILYQHL
>COLFA140-10|HQ559251|MP00402|Pterostichus_niger
TLYFIFGAWSGMVGTSLSMLIRAELGNPGSLIGDDQIYNVIVTAHAFVMIFFMVMPIMIGGFGNWLVPLMLGAPDMAFPRMNNMSFWLLPPSLSLLLMSSMVENGSGTGWTVYPPLSSGIAHAGASVDLAIFSLHLAGISSILGAVNFITTIINMRST
GMTFDRMPLFVWSVGITALLLLLSLPVLAGAITMLLTDRNLNTSFFDPAGGGDPILYQHL
>COLFA536-12|KJ966756|ZMUO.000671|Purpuricenus_kaehleri
TLYFIFGAWAGMVGTSLSVLIRTELGNPGSLIGDDQIYNVIVTAHAFIMIFFMVMPIMIGGFGNWLVPLMLGAPDMAFPRMNNMSFWLLPPSLTLLIMSSIVENGAGTGWTVYPPLAANVAHEGSSVDLAIFSLHLAGISSILGAVNFISTVINMKPEG
MTPERMPLFVWAVVITAVLLLLSLPVLAGAITMLLTDRNINTSFFDPAGGGDPILYQHL
>COLFD884-12|KJ962099|ZMUO.004494|Erichsonius_cinerascens
TLYFIFGAWAGMVGTSLSLLIRAELGNPGSLIGDDQIYNVIVTAHAFIMIFFMVMPIMIGGFGNWLVPLMLGAPDMAFPRMNNMSFWLLPPSLTLLLMSSMVESGAGTGWTVYPPLSSNIAHGGASVDLAIFSLHLAGISSILGAVNFITTVINMRSVG
MTFDRMPLFVWSVAITALLLLLSLPVLAGAITMLLTDRNLNTTFFDPAGGGDPILYQHL
>COLFF632-13|KJ965097|ZMUO.006522|Aphodius_rufus
TLYFLFGSWAGMVGTSLSLLIRAELGNPGSLIGDDQIYNVIVTAHAFVMIFFMVMPILIGGFGNWLVPLMLGAPDMAFPRMNNMSFWLLPPSLTLLLMSSMVESGAGTGWTVYPPLSSNIAHGGASVDLAIFSLHLAGISSILGAVNFITTVINMRSM
GMTFDRMPLFVWSVAITALLLLLSLPVLAGAITMLLTDRNLNTSFFDPAGGGDPILYQHL
>COLFD254-12|KJ963386|ZMUO.004149|Rhantus_frontalis
TLYFIFGAWAGMVGTSLSMLIRAELGNPGSLIGDDQIYNVIVTAHAFVMIFFMVMPIMIGGFGNWLVPLMLGAPDMAFPRMNNMSFWLLPPSLTLLLMSSMVESGAGTGWTVYPPLSSGIAHGGASVDLAIFSLHLAGISSILGAVNFITTIINMRSIG
MTFDRMPLFVWSVGITALLLLLSLPVLAGAITMLLTDRNLNTSFFDPAGGGDPILYQHL
>COLFA411-12|KJ962220|ZMUO.000641|Dermestes_lardarius
TLYFIFGAWAGMVGTSLSMLIRTELGMPGSLIGNDQIFNVIVTAHAFIMIFFMVMPIMIGGFGNWLVPLMLGAPDMAFPRMNNMSFWLLPPSLSLLLMSSMVESGAGTGWTVYPPLSANIAHSGASVDLAIFSLHLAGISSILGAVNFITTVINMRSK
GMTPDRMPLFVWSVAITALLLLLSLPVLAGAITMLLTDRNLNTSFFDPAGGGDPILYQHL
>COLFF411-13|KJ962289|ZMUO.005929|Ceutorhynchus_triangulum
TLYFIFGVWAGMTGTSLSMLIRTELGNPGSLIGNDQIYNSVVTAHAFIMIFFMVMPIMIGGFGNWLVPLMLGAPDMAFPRLNNMSFWLLPPSLALLLMSSIVNKGAGTGWTVYPPLSAGTAHEGMAVDMAIFSLHMAGISSILGAINFISTIKNMLPS
GLNSELMPLFIWAVKITAILLLISLPVLASAITMLLTDRNINTSFFDPSGGGDPILYQHL
>COLFF760-13|KJ964296|ZMUO.006460|Tribolium_confusum
TLYFMFGAWSGMVGTSLSLLIRAELGNPGSLIDDDQIYNVIVTAHAFIMIFFMVMPIMIGGFGNWLVPLMLGAPDMAFPRMNNMSFWLLPPSLSLLIMSSVVESGAGTGWTVYPPLSSNIAHGGASVDLAIFSLHLAGISSILGAVNFITTVINMRPTG
MSFERMPLFVWAVVITAILLLLSLPVLAGAITMLLTDRNINTSFFDPAGGGDPILYQHL
>COLFD506-12|KJ962640|ZMUO.004306|Pissodes_pini

TLYFIFGAWSGMIGTSLSILIRTELGTPGSMIGDDQIYNTIVTAHAFIMIFFMVMPIMIGGFGNWLIPLMLGAPDMAFPRLNNMSFWLLPPSIMLLLMSSITDKGAGTGWTVYPPLSTNIAHEGPSVDLAIFSLHMAGISSILGAVNFISTVINMRPTGM
NSDQMSLFIWAVKITAVLLLLSLPVLAGAITMLLTDRNINTSFFDPAGGGDPILYQHL
>COLFC635-12|KJ961828|ZMUO.003200|Agabus_infuscatus
TLYFIFGAWAGMVGTSLSMLIRAELGNPGSLIGDDQIYNVIVTAHAFVMIFFMVMPIMIGGFGNWLVPLMLGAPDMAFPRMNNMSFWLLPPSLTLLLMSSMVESGAGTGWTVYPPLSSGIAHGGASVDLAIFSLHLAGISSILGAVNFITTIINMRSV
GMTFDRMPLFVWSVGITALLLLSLPVLAGAITMLLTDRNLNTSFFDPAGGGDPILYQHL
>COLFE797-13|KJ962824|ZMUO.005642|Pterostichus_nigrita
TLYFIFGAWAGMVGTSLSMLIRAELGNPGSLIGDDQIYNVIVTAHAFIMIFFMVMPIMIGGFGNWLVPLMLGAPDMAFPRMNNMSFWLLPPSLTLLLMSSMVESGAGTGWTVYPPLSSGIAHAGASVDLAIFSLHLAGVSSILGAVNFITTIINMRSV
GMTFDRMPLFVWSVGITALLLLSLPVLAGAITMLLTDRNLNTSFFDPAGGGDPILYQHL
>COLFC714-12|KJ967288|ZMUO.003279|Ctenicera_pectinicornis
TLYFLFGAWAGMLGTSLSLLIRAELGNPGSLIGNDQIYNVIVTAHAFIMIFFMVMPIMIGGFGNWLVPLMLGAPDMAFPRMNNMSFWFLPPSLSLLLMSSIVENGAGTGWTVYPPLSANIAHSGSSVDLAIFSLHLAGISSILGAVNFISTVINMRSTGIT
FDRMPLFVWAVAITALLLLSLPVLAGAITMLLTDRNLNTSFFDPAGGGDPILYQHL
>COLFA068-10|HM909086|MP00257|Atheta_celata
TLYFIFGAWAGMVGTSLSLLIRAELGNPGSLIGDDQIYNVIVTAHAFVMIFFMVMPIVIGGFGNWLIPLMLGAPDMAFPRMNNMSFWLLPPSLTLLLMSSMVESGAGTGWTVYPPLSSNIAHGGASVDLAIFSLHLAGISSILGAVNFISTVINMRSTGI
SFDRMPLFVWSVAITALLLLSLPVLAGAITMLLTDRNLNTSFFDPAGGGDPILYQHL
>COLFA404-12|KJ964220|ZMUO.000634|Quedius_maurus
TLYFIFGAWAGMVGTSLSLLIRAELGNPGSLIGDDQIYNVIVTAHAFIMIFFMVMPIVIGGFGNWLVPLMLGAPDMAFPRMNNMSFWLLPPSLSLLLMSSMVESGAGTGWTVYPPLSSNIAHGGASVDLAIFSLHLAGISSILGAVNFITTVINMRSIG
MTFDRMPLFVWSVAITALLLLSLPVLAGAITMLLTDRNLNTSFFDPAGGGDPILYQHL
>COLFF048-13|KJ962218|ZMUO.005748|Agriotes_obscurus
TLYFLFGAWAGMLGTSLSLLIRAELGNPGSLIGNDQIYNVIVTAHAFIMIFFMVMPIMIGGFGNWLVPLMLGAPDMAFPRMNNMSFWFLPPSLSLLLMSSIVENGAGTGWTVYPPLSSNIAHSGSSVDLAIFSLHLAGISSILGAVNFISTVINMRSTGIT
FDRMPLFVWAVAITALLLLSLPVLAGAITMLLTDRNLNTSFFDPAGGGDPILYQHL
>COLFF517-13|KJ965767|ZMUO.006597|Hygrotus_quinquelineatus
TLYFLFGGWAGMVGTSLSMLIRAELGNPGSLIGDDQIYNVIVTAHAFIMIFFMVMPIMIGGFGNWLVPLMLGAPDMAFPRMNNMSFWLLPPSLTLLLMSSMVESGAGTGWTVYPPLSAGIAHGGASVDLAIFSLHLAGISSILGAVNFITTIINMRSV
GMTFDRMPLFVWSVGITALLLLSLPVLAGAITMLLTDRNLNTSFFDPAGGGDPILYQHL
>COLFF1028-13|KJ961793|ZMUO.007393|Amalus_scortillum
TLYFIFGSWAGTVGTSLSMIIRTELGTPGSLIGNDQIYNSIVTAHAFIMIFFMVMPILIGGFGNWLVPLMIGAPDMAFPRLNNMSFWLLPPSITLLLMSSIVNKGAGTGWTVYPPLSSNVTHEGAAVDLAIFSLHMAGISSILGAINFISTIMNMRPEGMS
YDKTPLFVWAVMITAILLLLSLPVLAGAITMLLTDRNINTSFFDPAGGGDPILYQHL
>COLFE345-12|KJ965133|ZMUO.004905|Stictoleptura_rubra
TLYFIFGAWASMVGTFLSLLIRSELGNPGSLIGDDQIYNVIVTAHAFVMIFFMVMPIMIGGFGNWLVPLMLGAPDMAFPRMNNMSFWLLPPSLTLLIMSSIVESGAGTGWTVYPPLSANIAHGGSSVDLAIFSLHLAGISSILSAVNFITTVINMRPVGM
SPERMPLFVWAVVITAVLLLLSLPVLAGAITMLLTDRNLNTSFFDPSGGGDPILYQHL
>COLFB558-12|KJ963536|ZMUO.001033|Stenus_formicetorum
VLYFIFGAWAGMVGTSLSLLIRTELGNPGSLIGDDQIYNVIVTAHAFVMIFFMVMPLMIGGFGNWLVPLMLGAPDMAFPRMNNMSFWLLPPSLSLLLMSSIVENGAGTGWTVYPPLSTNIAHSGASVDLAIFSLHLAGISSILGAINFITTIFNMRSMKL
QLDCLPLFIWSVEITALLLLLSLPVLAGAITMLLTDRNLNTSFFDPAGGGDPILYQHL
>COLFE1611-13|KJ966989|ZMUO.007501|Chrysolina_geminata
TLYFIFGMWAGMVGTSLSILIRAELGNPGTLIGNDQIYNVIVTAHAFIMIFFMVMPIMIGGFGNWLVPLMLGAPDMAFPRMNNMSFWLLPPSLIFLLMSSIVENGVGTGWTVYPPLSANIAHSGPSVDLAIFSLHLAGVSSILGAINFITTIINMRPTGM
KLEQMPLFSWAVLITAILLLLSLPVLAGAITMLLTDRNLNTSFFDPASGGDPILYQHL
>COLFC685-12|KJ964963|ZMUO.003250|Lordithon_thoracicus
TLYFIFGSWAGMVGTSLSLLIRAELGNPGSLIGDDQIYNVIVTAHAFIMIFFMVMPIVIGGFGNWLVPLMLGAPDMAFPRMNNMSFWLLPPSLTLLLMSSMVESGAGTGWTVYPPLSSNIAHGGASVDLAIFSLHLAGISSILGAVNFITTVINMRSIGM
TFDQMPLFIWSVAITALLLLSLPVLAGAITMLLTDRNLNTTFFDPAGGGDPILYQHL
>COLFE890-13|KJ964157|ZMUO.006210|Danosoma_fasciata
TLYFLFGAWAGMLGTSLSLLIRAELGNPGSLIGNDQIYNVVVTAHAFVMIFFMVMPVMIGGFGNWLVPLMLGAPDMAFPRMNNMSFWLLPPSLSLLLMSSIVENGAGTGWTVYPPLSANIAHSGSSVDLAIFSLHLAGISSILGAVNFISTVINMRST
GMTFDRMPLFVWAVAITALLLLSLPVLAGAITMLLTDRNLNTTFFDPAGGGDPILYQHL
>COLFA405-12|KJ963844|ZMUO.000635|Aphodius_borealis
TLYFLFGSWAGMVGTSLSLLIRAELGNPGTLIGDDQIYNVIVTAHAFVMIFFMVMPILIGGFGNWLVPLMLGAPDMAFPRMNNMSFWLLPPSLTLLLMSSMVESGAGTGWTVYPPLSSNIAHGGASVDLAIFSLHLAGISSILGAVNFITTVINMRSPG
LTFDRMPLFVWSVAITALLLLSLPVLAGAITMLLTDRNLNTSFFDPAGGGDPILYQHL
>COLFB744-12|KJ963075|ZMUO.001219|Schistoglossa_viduata
TLYFLFGMWAGMVGTSLSMLIRLELGSAGSLIGNDQIYNVIVTAHAFIMIFFMVMPIMIGGFGNWLVPLMIGAPDMAFPRLNNMSFWLLPPSLSLLLMSSLVESGAGTGWTVYPPLSSNIAHGGASVDLAIFSLHLAGISSILGAVNFISTVINMRAVGI
SFDRMPLFIWSVVITALLLLSLPVLAGAITMLLTDRNLNTTFFDPAGGGDPILYQHL

>COLFE1025-13|KJ962979|ZMUO.006820|Loricera_pilicornis
TLYFIFGAWSGMVGTSLSMLIRAELGNPGSFIGDDQIYNVIVTAHAFVMIFFMVMPIMIGGFGNWLVPLMLGAPDMAFPRMNNMSFWLLPPSLTLLLLSSMVESGAGTGWTVYPPLSSVIAHSGASVDLAIFSLHLAGVSSILGAVNFISTIINMRSIG
MTFDRMPLFVWSVGITALLLLLSLPVLAGAITMLLTDRNLNTSFFDPAGGGDPILYQHL
>COLFD413-12|KJ964910|ZMUO.003833|Monotoma_angusticollis
TLYFIFGTWSGMVGTSLSMLIRTELGTPGSLISDDQIFNVIVTAHAFIMIFFMVMPILIGGFGNWLIPLMLGAPDMAFPRMNNMSFWLLPPSLTLLLMSSIVETGVGTGWTIYPPLSSNIAHSGAPVDLAIFSLHLAGISSILGAVNFITTVINMRPQGMTL
DRTPLFVWAVMITAILLLLSLPVLAGAITMLLTDRNLNTSFFDPAGGGDPILYQHL
>COLFF211-13|KJ962303|ZMUO.005816|Harpalus_subcylindricus
TLYFIFGAWAGMVGTSLSMLIRAELGTPGALIGDDQIYNVIVTAHAFIMIFFMVMPIMIGGFGNWLVPLMLGAPDMAFPRMNNMSFWLLPPSLTLLLMSSMVESGAGTGWTVYPPLSSGIAHSGASVDLAIFSLHLAGVSSILGAVNFITTIINMRSV
GMTFDRMPLFVWSVGITALLLLLSLPVLAGAITMLLTDRNLNTSFFDPAGGGDPILYQHL
>COLFB780-12|KJ963737|ZMUO.001255|Atheta_aeneipennis
TLYFIFGAWAGMVGTSLSLLIRAELGNPGSLIGDDQIYNVIVTAHAFIMIFFMVMPIVIGGFGNWLVPLMLGAPDMAFPRMNNMSFWLLPPSLTLLLMSSMVESGAGTGWTVYPPLSSNIAHGGSSVDLAIFSLHLAGISSILGAVNFISTVINMRSTGI
SFDRMPLFVWSVAITALLLLLSLPVLAGAITMLLTDRNLNTSFFDPAGGGDPILYQHL
>COLFA624-12|KJ966347|ZMUO.000759|Hylotrupes_bajulus
TLYFIFGAWAGMVGTSLSMLIRSELGNPGSLIGDDQIYNVIVTAHAFIMIFFMVMPIMIGGFGNWLVPLMLGAPDMAFPRMNNMSFWLLPPSLTLLVLSSIVENGAGTGWTVYPPLAANIAHSGASVDLAIFSLHLAGVSSILGAVNFISTVINMRPEG
MHPERMPLFVWAVVITAILLLLSLPVLAGAITMLLTDRNINTSFFDPAGGGDPILYQHL
>COLFA372-12|KJ964273|ZMUO.000602|Necrobia_violacea
TLYFIFGAWAGMVGTSLSLLIRSELGTPGTLIGNDQIYNVIVTAHAFIMIFFMVMPIMIGGFGNWLVPLMLGAPDMAFPRMNNMSFWLLPPSLTLLLMSSMVESGAGTGWTVYPPLSSNIAHGGSSVDLAIFSLHLAGISSILGAVNFITTVINMRPAG
MTLDRMPLFVWAVAITALLLLLSLPVLAGAITMLLTDRNLNTSFFDPAGGGDPILYQHL
>COLFD529-12|KJ963505|ZMUO.004329|Altica_carinthiaca
TLYFLFGIWAGMIGTSMSLLIRTELGSPGSLIGNDQIYNVIVTAHAFIMIFFMVMPMMIGGFGNWLVPLMIGAPDMAFPRMNNMSFWLLPPSIFLLLMSSFTESGAGTGWTVYPPLSSNLAHNGPSVDLAIFSLHLAGISSILGAINFITTMINMCPQG
MSMDQMPLFVWAVFITAILLLLSLPVLAGAITMLLTDRNFNTSFFEPAGGGDPILYQHL
>COLFF557-13|KJ962257|ZMUO.006637|Platystomos_albinus
TLYFIFGAWAGMMGTSLSILIRTELGTPSSLIGDDQIYNVIVTAHAFVMIFFMVMPTMIGGFGNWLVPLMLGAPDMAFPRMNNMSFWLLPPSLILLTMSSIVEKGAGTGWTVYPPLASNIAHSGASVDLAIFSLHLAGVSSILGAVNFITTIINMRPEG
MTPDRLPLFVWAVGITALLLLLSLPVLAGAITMLLTDRNLNTTFFDPAGGGDPILYQHL
>COLFA666-12|KJ967140|ZMUO.000801|Philonthus_marginatus
TLYFIFGSWAGMVGTSLSLLIRSELGNPGSLIGDDQIYNVIVTAHAFIMIFFMVMPTVIGGFGNWLVPLMLGAPDMAFPRMNNMSFWLLPPSLALLLMSSMVESGAGTGWTVYPPLSSNMAHGGASVDLAIFSLHLAGISSILGAVNFITTVINMRST
GMSFDRMPLFVWSVVITALLLLLSLPVLAGAITMLLTDRNLNTTFFDPAGGGDPILYQHL
>COLFD848-12|KJ965273|ZMUO.004838|Hydaticus_seminiger
TLYFIFGAWSGMVGTSLSMLIRAELGNPGSLIGDDQIYNVIVTAHAFIMIFFMVMPIMIGGFGNWLVPLMLGAPDMAFPRMNNMSFWLLPPSLTLLLMSSMVENGAGTGWTVYPPLSAGIAHGGASVDLAIFSLHLAGVSSILGAVNFITTIINMRSV
GMTFDRMPLFVWSVGITALLLLLSLPVLAGAITMLLTDRNLNTSFFDPAGGGDPILYQHL
>COLFG154-13|KJ962667|ZMUO.007849|Thinobius_ciliatus
TLYFIFGAWSGMVGTSLSMLIRAELGMPGSLIGDDQIYNVIVTAHAFIMIFFMVMPIVIGGFGNWLVPLMLGAPDMAFPRMNNMSFWLLPPSLSLLLMSSMVESGAGTGWTVYPPLSANIAHSGSSVDLAIFSLHLAGISSILGAVNFISTIINMRSPG
MTLDRMPLFVWSVNITAILLLLSLPVLAGAITMLLTDRNLNTSFFDPAGGGDPILYQHL
>COLFE769-13|KJ962222|ZMUO.005614|Hygrotus_decoratus
TLYFIFGAWSGMVGTSLSMLIRAELGNPGSLIGDDQIYNVIVTAHAFIMIFFMVMPIMIGGFGNWLVPLMLGAPDMAFPRMNNMSFWLLPPSLTLLLMSSMVESGAGTGWTVYPPLSAGIAHGGASVDLAIFSLHLAGISSILGAVNFITTIINMRSIG
MTFDRMPLFVWSVGITALLLLLSLPVLAGAITMLLTDRNLNTSFFDPAGGGDPILYQHL
>COLFB936-12|KJ965780|ZMUO.001981|Tachyerges_stigma
TLYFMFGTWSGMVGTSLSMLIRTELGTPGKLIGDDQIYNTIVTAHAFIMIFFMVMPIMIGGFGNWLIPLMLGAPDMAFPRLNNMSFWLLPPSLAFLLFSSIIDKGAGTGWTVYPPLSANIAHEGTSVDMAIFSLHMAGISSILGALNFISTIMNMQPM
GMNKDQMSLFIWAVKITAILLLISLPVLAGAITMLLTDRNINTSFFDPAGGGDPILYQHL
>COLFC021-12|KJ962701|ZMUO.002016|Acrotona_fungi
TLYFIFGAWAGMVGTSLSLLIRAELGNPGSLIGNDQIYNVIVTAHAFIMIFFMVMPIMIGGFGNWLVPLMLGAPDMAFPRMNNMSFWLLPPSLTLLLMSSMVESGVGTGWTVYPPLSSNIAHSGSSVDLAIFSLHLAGISSILGAVNFISTVINMRSPGI
TFDQMPLFVWAVSITALLLLLSLPVLAGAITMLITDRNLNTSFFDPAGGGDPILYQHL
>COLFD804-12|KJ962308|ZMUO.004794|Ellescus_bipunctatus
TLYFIFGAWSGMVGTSLSMLIRTELGNPGSLIGDDQIYNTIVTAHAFIMIFFMVMPIMIGGFGNWLIPLMLGAPDMAFPRLNNMSFWLLPPSLSLLLMSSIIDKGAGTGWTVYPPLSSNIAHEGTSVDLAIFSLHMAGISSILGAMNFISTIMNMRPAG
MKPDRMSLFIWAVKITAILLLLSLPVLAGAITMLLTDRNINTSFFDPAGGGDPILYQHL
>COLFF801-13|KJ962114|ZMUO.006121|Longitarsus_brunneus

TLYFIFGTWAGMIGTSLSILIRTELGSPGSLIGNDQIYNVIVTAHAFIMIFFMVMPIMIGGFGNWLVPLMIGAPDMAFPRMNNMSFWLLPPSLFLLVMSSMVESGAGTGWTVYPPLSSNIAHGGSSVDLAIFSLHLAGISSILGAINFITTVINMHPTGMS
LDQMPLFVWAVMITAILLLLSLPVLAGAITMLLTDRNLNTTFFDPAGGGDPILYQHL
>COLFD361-12|KJ962952|ZMUO.004256|Protapion_apricans
TLYFIFGLWSGMIGTSLSMLIRIELGNPGSLIGNDQIYNVIVTAHAFIMIFFMVMPVMIGGFGNWLIPLMLGAPDMAFPRMNNMSFWLLPPSLTLLLMSSIIEKGAGTGWTVYPPLAANIAHSGASVDLAIFSLHLAGISSILGAVNFISTIMNMRPTGM
SLDQLSLFTWAVKITAILLLLSLPVLAGAITMLLTDRNINTSFFDPAGGGDPILYQHL
>COLFB195-12|KJ962621|ZMUO.001525|Stenichnus_collaris
TLYFILGIWSGMLGTSLSLLIRLELSSPGMMIGNDQTFNMIVTSHAFIMIFFMVMPIMIGGFGNWLVPLMLGAPDMAFPRMNNMSFWFLPPSLMLLLMSSMVESGSGTGWTVYPPLSSNIAHSGSSVDLTIFSLHLAGISSILGAVNFITTIINMRSPM
MKFDNLSLFIWAVFITAILLLLSLPVLAGAITMLLTDRNFNTSFFDPSGGGDPILYQHL
>COLFE630-13|KJ967284|ZMUO.005475|Cassida_nebulosa
TLYFIFGFWSGMVGTSLSILIRMELGSPGSLIGNDQIYNSIVTAHAFIMIFFMVMPIMIGGFGNWLVPLMLGAPDMAFPRLNNMSFWLLPPSITFLIMSSIVESGAGTGWTVYPPLSSNIAHSGASVDLAIFSLHLAGISSILGAINFISTIMNMRPSGMS
MEKVALFVWAVIITAVLLLLSLPVLAGAITMLLTDRNINTSFFDPAGGGDPILYQHL
>COLFB039-12|KJ966251|ZMUO.001369|Aloconota_gregaria
TLYFIFGAWAGMVGTSLSLLIRAELGNPGSLIGDDQIYNVIVTAHAFIMIFFMVMPIVIGGFGNWLIPLMLGAPDMAFPRMNNMSFWLLPPSLSLLLMSSMVESGAGTGWTVYPPLSSNIAHGGSSVDLAIFSLHLAGISSILGAVNFISTIINMRSSGISF
DRMPLFVWSVAITALLLLLSLPVLAGAITMLLTDRNLNTSFFDPAGGGDPILYQHL
>COLFB757-12|KJ963009|ZMUO.001232|Fagniezia_impressa
TLYLIFGAWASMIGTSLSLLIRTELGNPGSLIGDDQIYNVIVTSHAFIMIFFMVMPIMIGGFGNWLIPLMLGAPDMAFPRMNNMSFWLLPPSMILLLMSSMVESGAGTGWTVYPPLSSNIAHSGSSVDLTIFSLHLAGISSILGAVNFITTVINMRTMNM
NFDQMPLFVWSVAITALLLLLSLPVLAGAITMLLTDRNLNTSFFDPAGGGDPILYQHL
>COLFF789-13|KJ964267|ZMUO.006109|Hygrotus_quinquelineatus
TLYFLFGAWAGMVGTSLSMLIRAELGNPGSLIGDDQIYNVIVTAHAFIMIFFMVMPIMIGGFGNWLVPLMLGAPDMAFPRMNNMSFWLLPPSLTLLLMSSMVESGAGTGWTVYPPLSAGIAHGGASVDLAIFSLHLAGISSILGAVNFITTIINMRSV
GMTFDRMPLFVWSVGITALLLLLSLPVLAGAITMLLTDRNLNTSFFDPAGGGDPILYQHL
>COLFD011-12|KJ964924|ZMUO.003906|Latridius_consimilis
SLYFLFGMWSGMVGTSLSLLIRLELGNPGSLIGDDQIYNVIVTAHAFIMIFFMVMPVMMGGFGNWLVPLMLGAPDMAFPRLNNMSFWLLPPSLSLLIMSSIVESGAGTGWTVYPPLSSNIAHGGSSVDLAIFSLHLAGVSSILGAVNFITTVINMRPSG
MALEQMPLFVWSVILTAILLLLSLPVLAGAITMLLTDRNLNTSFFDPAGGGDPILYQHL
>COLFD305-12|KJ964149|ZMUO.004200|Tinotus_morion
TLYFIFGAWSGMVGTSLSLLIRAELGNPGSLIGDDQIYNVIVTAHAFIMIFFMVMPIVIGGFGNWLVPLMLGAPDMAFPRMNNMSFWLLPPSLTLLLMSSMVESGAGTGWTVYPPLSSNIAHGGSSVDLAIFSLHLAGISSILGAVNFISTVINMRSMG
MSFDKMPLFVWSVAITALLLLLSLPVLAGAITMLLTDRNLNTSFFDPAGGGDPILYQHL
>COLFA300-12|KJ961980|ZMUO.000435|Monotoma_picipes
TLYFIFGAWSGMVGTSLSMLIRTELGTPGSLIGDDQIFNVIVTAHAFIMIFFMVMPILIGGFGNWLVPLMLGAPDMAFPRMNNMSFWLLPPSLSLLLMSSIVETGAGTGWTVYPPLSSNIAHGGASVDLAIFSLHLAGISSILGAVNFITTVINMRPQGM
SLDRIPLFVWAVMITAILLLLSLPVLAGAITMLLTDRNLNTSFFDPAGGGDPILYQHL
>COLFD286-12|KJ965562|ZMUO.004181|Aphodius_lapponum
TLYFLFGSWAGMVGTSLSLLIRAELGNPGTLIGDDQIYNVIVTAHAFVMIFFMVMPILIGGFGNWLVPLMLGAPDMAFPRMNNMSFWLLPPSLTLLLMSSMIESGAGTGWTVYPPLSSNIAHSGASVDLAIFSLHLAGISSILGAVNFITTVINMRTPG
MTFDRMPLFVWSVAITALLLLLSLPVLAGAITMLLTDRNLNTSFFDPAGGGDPILYQHL
>COLFC356-12|KJ964054|ZMUO.002541|Atheta_pilicornis
TLYFIFGAWAGMVGTSLSLLIRAELGNPGSLIGDDQIYNVIVTAHAFIMIFFMVMPIVIGGFGNWLVPLMLGAPDMAFPRMNNMSFWLLPPSLSLLLMSSMVESGAGTGWTVYPPLSSNIAHGGSSVDLAIFSLHLAGISSILGAVNFISTVINMRSTGI
SFDRMPLFVWSVAITALLLLLSLPVLAGAITMLLTDRNLNTSFFDPAGGGDPILYQHL
>COLFC842-12|KJ967126|ZMUO.003407|Stenus_cicindeloides
TLYFIFGAWSGMIGTSLSLLIRAELGNPGSLIGDDQIYNVIVTAHAFIMIFFMVMPAMIGGFGNWLVPLMLGAPDMAFPRMNNMSFWLLPPSLSLLLMSSIVESGAGTGWTVYPPLSSNIAHGGSSVDLAIFSLHLAGISSILGAINFITTILNMRAMKIQ
LDCMPLFVWSVGITALLLLLSLPVLAGAITMLLTDRNLNTSFFDPAGGGDPILYQHL
>COLFB639-12|KJ964032|ZMUO.001114|Pachnida_nigella
TLYFIFGAWAGMVGTSLSLLIRAELGNPGSLIGDDQIYNVIVTAHAFVMIFFMVMPIVIGGFGNWLVPLMLGAPDMAFPRMNNMSFWLLPPSLSLLLMSSMIENGAGTGWTVYPPLSSNIAHSGSSVDLAIFSLHLAGISSILGAVNFISTVINMRTPG
MSFDRMPLFVWSVAITALLLLLSLPVLAGAITMLLTDRNLNTSFFDPAGGGDPILYQHL
>COLFC337-12|KJ966888|ZMUO.002522|Tachinus_pallipes
TLYFIFGAWAGMVGTSLSLLIRAELGNPGTLIGDDQIYNVIVTAHAFIMIFFMVMPIVIGGFGNWLVPLMLGAPDMAFPRMNNMSFWLLPPSLTLLLMSSMVESGAGTGWTVYPPLSSNIAHGGSSVDLAIFSLHLAGISSILGAVNFITTVINMRSIG
MTFDRMPLFVWSVAITALLLLLSLPVLAGAITMLLTDRNLNTTFFDPAGGGDPILYQHL
>COLFA472-12|KJ962318|ZMUO.000512|Tachinus_pallipes
TLYFIFGAWAGMVGTSLSLLIRAELGNPGTLIGDDQIYNVIVTAHAFIMIFFMVMPIVIGGFGNWLVPLMLGAPDMAFPRMNNMSFWLLPPSLTLLLMSSMVESGAGTGWTVYPPLSSNIAHGGSSVDLAIFSLHLAGISSILGAVNFITTVINMRSIG
MTFDRMPLFVWSVAITALLLLLSLPVLAGAITMLLTDRNLNTTFFDPAGGGDPILYQHL

>COLFE1296-13|KJ965206|ZMUO.007091|Ilybius_quadriguttatus
TLYFIFGAWAGMVGTSLSMLIRAELGNPGSLIGDDQIYNVIVTAHAFVMIFFMVMPIMIGGFGNWLVPLMLGAPDMAFPRMNNMSFWLLPPSLSLLLMSSMVESGAGTGWTVYPPLSAGIAHSGASVDLAIFSLHLAGISSILGAVNFITTIINMRSV
GMTFDRMPLFVWSVGITALLLLLSLPVLAGAITMLLTDRNLNTSFFDPAGGGDPILYQHL
>COLFD076-12|KJ963096|ZMUO.003971|Dasytes_niger
TLYFIFGAWSGMVGMSLSLLIRSELNNPGTLIGNDQIYNVIVTAHAFIMIFFMVMPILIGGFGNWLVPLMLGAPDMAFPRMNNMSFWLLPPSLTLLLMSSMVEQGAGTGWTVYPPLSSNIAHGGASVDLAIFSLHLAGISSILGAVNFITTIINMRPVG
MTLDRTPLFVWAVAITALLLLLSLPVLAGAITMLLTDRNLNTSFFDPAGGGDPILYQHL
>COLFA683-12|KJ965329|ZMUO.000818|Pselaphus_heisei
TLYLIFGAWAGMVGTSLSMLIRSELGNPGSLIGDDQIYNVIVTAHAFIMIFFMVMPLMIGGFGNWLVPLMLGAPDMAFPRMNNMSFWLLPPSLSLLLMSSIIENGAGTGWTVYPPLSSNIAHNGSSVDLTIFSLHLAGISSILGAVNFISTIINMRATGIIL
DRMPLFVWSVLITAILLLLSLPVLAGAITMLLTDRNLNTSFFDPAGGGDPILYQHL
>COLFB256-12|KJ964397|ZMUO.001586|Paederus_riparius
TLYFIFGAWSGMVGTSLSLLIRAELATPGSLIGDDQIYNVIVTAHAFIMIFFMVMPIMIGGFGNWLVPLMLGAPDMAFPRMNNMSFWLLPPALTLLLMSSMVENGAGTGWTVYPPLSSNAFHNGSSVDLAIFSLHLAGISSILGAINFITTALNMRTSN
MSYEQMPLFVWSVAITALLLLLSLPVLAGAITMLLTDRNLNTSFFDPSGGGDPILYQHL
>COLFF959-13|KJ965111|ZMUO.007324|Stenus_sylvester
TLYFIFGSWAGMVGTSLSMLIRSELGNPGSLIGDDQIYNVIVTAHAFIMIFFMVMPIMIGGFGNWLVPLMLGAPDMAFPRMNNMSFWLLPPSLSLLLMSSIVESGAGTGWTVYPPLSSNIAHSGSSVDLAIFSLHLAGISSILGAINFITTIFNMRTMKLQ
LDCLPLFVWSVKITAFLLLLSLPVLAGAITMLLTDRNLNTSFFDPAGGGDPILYQHL
>COLFD212-12|KJ963033|ZMUO.004107|Aphidecta_obliterata
TLYFLFGLWAGMVGTSLSILIRMELGTTGSLIGNDQIYNVIVTAHAFIMIFFMVMPIMIGGFGNWLVPLMIGAPDMAFPRLNNMSFWLLPPALTLLIFSMMVEMGAGTGWTVYPPLSSNLAHNGPSVDLVIFSLHLAGISSILGAVNFISTIMNMRPFG
MSLDKTPLFVWSVMITAILLLLSLPVLAGAITMLLTDRNLNTSFFDPTGGGDPVLYQHL
>COLFF657-13|KJ967435|ZMUO.006547|Zacladus_geranii
TLYFIFGSWAGAAGTSLSMLIRTELGNPGSLIGNDQIYNSIVTAHAFIMIFFMVMPILIGGFGNWLVPLMLGAPDMAFPRLNNMSFWLLPPSLTLLLMSSIINKGAGTGWTVYPPLSANVAHEGASVDLAIFSLHMAGISSILGAINFISTIMNMRPKGM
DYDQTPLFAWAVMITAILLLLSLPVLAGAITMLLTDRNINTTFFDPSGGGDPILYQHL
>COLFB463-12|KJ963657|ZMUO.001888|Carpelimus_rivularis
TLYFIFGAWSGMVGTSLSMLIRIELGTPGSLIGDDQIYNVIVTAHAFIMIFFMVMPIVIGGFGNWLVPLMLGAPDMAFPRMNNMSFWLLPPSLTLLLFSSMVESGAGTGWTVYPPLSSNIAHSGSSVDLAIFSLHLAGISSILGAVNFISTIINMRSVGMT
FDRMPLFVWSVNITAILLLLSLPVLAGAITMLLTDRNLNTSFFDPAGGGDPILYQHL
>COLFD760-12|KJ963590|ZMUO.004750|Necrodes_littoralis
TLYFIFGAWAGMVGMSLSILIRAELSTPGTLLGDDQMYNVIVTAHAFIMIFFMVMPIVIGGFGNWLVPLMLGAPDMAFPRMNNMSFWLLPPSLSLLLVSSMVESGAGTGWTVYPPLSSNIAHGGSSVDLAIFSLHLAGISSILGAVNFITTIINMRSSG
MTFDRMPLFVWSVAITALLLLLSLPVLAGAITMLLTDRNLNTSFFDPAGGGDPILYQHL
>COLFA271-12|KJ964356|ZMUO.000406|Dicheirotrichus_rufithorax
TLYFIFGAWSGMVGTSLSMLIRAELGTPGALIGDDQIYNVIVTAHAFVMIFFMVMPIMIGGFGNWLVPLMLGAPDMAFPRMNNMSFWLLPPSLTLLLMSSMVEKGAGTGWTVYPPLSSGIAHSGSSVDLAIFSLHLAGISSILGAVNFITTIINMRSIG
MTFDRMPLFVWSVGITALLLLLSLPVLAGAITMLLTDRNLNTSFFDPAGGGDPILYQHL
>COLFE373-12|KJ962030|ZMUO.004933|Sepedophilus_marshami
TLYFIFGMWAGMVGTSLSLLIRAELGNPGSLIGDDQIYNVIVTAHAFIMIFFMVMPVIIGGFGNWLVPLMLGAPDMAFPRMNNMSFWLLPPSLSLLLMSSLVESGAGTGWTVYPPLSSNIAHGGSSVDLAIFSLHLAGISSILGAVNFITTVINMRSTG
MTFDRMPLFVWSVAITALLLLLSLPVLAGAITMLLTDRNLNTSFFDPAGGGDPILYQHL
>COLFD781-12|KJ965983|ZMUO.004771|Cassida_vibex
TLYFIFGFWSGMVGTSLSILIRAELGNPGNLIGNDQIYNSVVTAHAFIMIFFMVMPVMIGGFGNWLVPLMLGAPDMAFPRLNNMSFWLLPPSITFLIMSSIIESGAGTGWTVYPPLSSNIAHSGASVDMAIFSLHLAGISSILGAINFISTIMNMRPSGM
TLDKMPLFVWAVIITAVLLLLSLPVLAGAITMLLTDRNINTSFFDPAGGGDPILYQHL
>COLFC159-12|KJ962149|ZMUO.002344|Lathrobium_geminum
TLYFIFGAWAGMVGTSLSLLIRTELGNPGSLIGDDQIYNVIVTAHAFIMIFFMVMPIMIGGFGNWLVPLMLGAPDMAFPRMNNMSFWLLPPSLSLLLMSSLVESGAGTGWTVYPPLSSNIAHGGASVDLAIFSLHLAGISSILGAVNFITTVINMRSPG
MTYERMPLFVWAVAITALLLLLSLPVLAGAITMLLTDRNLNTSFFDPAGGGDPILYQHL
>COLFA620-12|KJ967255|ZMUO.000755|Tetrops_praeustus
TLYFIFGAWASMVGTSLSLLIRSELGSPGSLIGNDQIYNTIVTAHAFIMIFFMVMPIMIGGFGNWLVPLMLGAPDMAFPRMNNMSFWLLPPSLTLLIMSSIVENGAGTGWTVYPPLASNIAHSGSSVDLAIFSLHLAGVSSILGAVNFITTVINMRPNGM
SLDRLPLFVWAVKITAILLLLSLPVLAGAITMLLTDRNLNTSFFDPASGGDPILYQHL
>COLFC645-12|KJ963708|ZMUO.003210|Hydroporus_erythrocephalus
TLYFLFGAWSGMVGTSLSMLIRAELGNPGSLIGDDQIYNVIVTAHAFIMIFFMVMPIMIGGFGNWLVPLMLGAPDMAFPRMNNMSFWLLPPSLSLLLMSSMVENGAGTGWTVYPPLSSGIAHSGASVDLAIFSLHLAGVSSILGAVNFITTIINMRSIG
MTFDRMPLFVWSVGITALLLLLSLPVLAGAITMLLTDRNLNTSFFDPAGGGDPILYQHL
>COLFD773-12|KJ963623|ZMUO.004763|Rutidosoma_globulus

TLYFIFGSWAGSVGTSLSMLIRTELGTPGSLIGNDQIYNSIVTAHAFIMIFFMVMPILIGGFGNWLVPLMLGAPDMAFPRLNNMSFWLLPPSLMLLMMSSVINKGAGTGWTVYPPLSSNVTHEGASVDLAIFSLHMAGISSILGAINFISTIMNMRPKG
MSYDKMPLFSWAVIITAVLLLLSLPVLAGAITMLLTDRNINTSFFDPAGGGDPILYQHL
>COLFA418-12|KJ965264|ZMUO.000648|Salpingus_ruficollis
TLYFIFGAWSGMVGTSLSLLIRAELGNPGSLIGDDQIYNVIVTAHAFVMIFFMVMPVVIGGFGNWLVPLMLGAPDMAFPRMNNMSFWLLPPSLTLLIMSSIVESGAGTGWTVYPPLAANIAHGGSSVDLAIFSLHLAGVSSILGAVNFITTVINMRPSG
MSFDRMPLFVWSVVITAVLLLLSLPVLAGAITMLLTDRNLNTSFFDPAGGGDPILYQHL
>COLFB848-12|KJ963212|ZMUO.001323|Stenus_palposus
TLYFIFGAWAGMVGTSLSLLIRAELGNPGSLIGDDQIYNVIVTAHAFVMIFFMVMPVMIGGFGNWLVPLMLGAPDMAFPRMNNMSFWLLPPSLSLLLMSSIVESGAGTGWTVYPPLSSNIAHSGASVDLAIFSLHLAGISSILGAINFITTIFNMRTMKL
HLDCLSLFIWSVGITALLLLLSLPVLAGAITMLLTDRNLNTSFFDPAGGGDPILYQHL
>COLFF570-13|KJ964843|ZMUO.006650|Smaragdina_affinis
TLYFIFGAWSGMVGTSLSLLIRVELGNPGTLIGNDQIYNTIVTAHAFIMIFFMVMPIMIGGFGNWLVPLMLGAPDMAFPRMNNMSFWLLPPSLTLLLMSSIVENGAGTGWTVYPPLSANLAHSGASVDLAIFSLHLAGISSIMGAINFISTVINMRPQG
MLLDRTPLFVWAVVITAILLLLSLPVLAGAITMLLTDRNLNTSFFDPAGGGDPILYQHL
>COLFC216-12|KJ963129|ZMUO.002401|Acupalpus_parvulus
TLYFIFGVWAGMVGTSLSMLIRAELGTPGALIGDDQIYNVIVTAHAFVMIFFMVMPVMIGGFGNWLVPLMLGAPDMAFPRMNNMSFWLLPPSLTLLLMSSLVESGAGTGWTVYPPLSSGIAHSGASVDLAIFSLHLAGVSSILGAVNFITTIINMRSV
GMTFERMPLFVWSVGITALLLLLSLPVLAGAITMLLTDRNLNTSFFDPAGGGDPILYQHL
>COLFB459-12|KJ967508|ZMUO.001884|Laccobius_minutus
TLYFLFGAWAGMVGTSLSILIRAELGSPGTLIGDDQIYNVIVTAHAFIMIFFMVMPIMIGGFGNWLVPLMLGAPDMAFPRMNNMSFWLLPPSLTLLLMSSMVESGAGTGWTIYPPLSSNIAHGGASVDLAIFSLHLAGISSILGAVNFITTVINMRSNN
MTYDRLPLFVWSVAITALLLLLSLPVLAGAITMLLTDRNLNTSFFDPAGGGDPILYQHL
>COLFE1311-13|KJ965734|ZMUO.007106|Atheta_vilis
TLYFIFGAWAGMVGTSLSLLIRAELGNPGSLIGDDQIYNVIVTAHAFVMIFFMVMPIVIGGFGNWLVPLMLGAPDMAFPRMNNMSFWLLPPSLSLLLMSSMVESGAGTGWTVYPPLSSNIAHGGSSVDLAIFSLHLAGISSILGAVNFISTVINMRSTGI
TFDRMPLFVWSVAITALLLLLSLPVLAGAITMLLTDRNLNTSFFDPAGGGDPILYQHL
>COLFE1309-13|KJ965303|ZMUO.007104|Perapion_violaceum
TLYFIFGFWSGLVGTSLSMLIRVELGNPGSLIGDDQIYNVIVTAHAFIMIFFMVMPIMIGGFGNWLVPLMLGAPDMAFPRMNNMSFWLLPPSLTLLLMSSIVEKGAGTGWTVYPPLASNIAHGGSSVDLAIFSLHLAGVSSILGAVNFISTVINMRPTGV
SLDQLSLFTWAVKITAILLLLSLPVLAGAITMLLTDRNINTSFFDPAGGGDPILYQHL
>COLFE1555-13|KJ964405|ZMUO.007445|Tanysphyrus_lemnae
TLYFIFGAWSGMVGTSLSMLIRTELGNPGTLIGDDQIYNVIVTAHAFIMIFFMVMPIMIGGFGNWLIPLMLGAPDMAFPRMNNMSFWLLPPSLTFLLMSSIVEKGAGTGWTVYPPLSSNIAHSGSSVDLAIFSLHMAGVSSILGAINFISTIINMKTNETS
FDQLSLFTWAVGITALLLLLSLPVLAGAITMLLTDRNINTSFFDPAGGGDPILYQHL
>COLFE117-12|KJ963759|ZMUO.005152|Cryptocephalus_nitidulus
TLYFLFGAWSSMVGTSLSLLIRVELGNPGSLIGNDQIYNTIVTAHAFIMIFFMVMPIMIGGFGNWLVPLMLGAPDMAFPRMNNMSFWLLPPSLTLLLMSSIVENGAGTGWTVYPPLSATIAHAGASVDLAIFSLHLAGISSIMGAINFISTVINMRPKG
MLMDRTPLFVWAVLITAILLLLSLPVLAGAITMLLTDRNLNTSFFDPAGGGDPILYQHL
>COLFC769-12|KJ966587|ZMUO.003334|Galerucella_tenella
TLYFIFGIWAGMVGTSLSILVRAELGSPGTLIGNDQIYNVIVTAHAFIMIFFMVMPIMIGGFGNWLVPLMIGAPDMAFPRMNNMSFWLLPPSLFLLIMSSIVESGAGTGWTVYPPLSSNIAHGGSSVDLAIFSLHLAGISSILGAINFITTIINMRPKGMTL
DRMPLFVWAVMITAILLLLSLPVLAGAITMLLTDRNLNTSFFDPAGGGDPILYQHL
>COLFE785-13|KJ964053|ZMUO.005630|Stenus_nitens
TLYFIFGAWAGMVGTSLSLLIRAELGNPGSLIGDDQIYNVIVTAHAFVMIFFMVMPIMIGGFGNWLVPLMLGAPDMAFPRMNNMSFWLLPPSLSLLLMSSIVENGAGTGWTVYPPLSSNIAHSGASVDLAIFSLHLAGISSILGAINFITTIFNMRTMKL
QLDCLPLFVWSVAITALLLLLSLPVLAGAITMLLTDRNLNTSFFDPAGGGDPILYQHL
>COLFB583-12|KJ963626|ZMUO.001058|Oxypoda_flavicornis
TLYFIFGAWAGMVGTSLSLLIRAELGNPGSLIGDDQIYNVIVTAHAFVMIFFMVMPIVIGGFGNWLVPLMLGAPDMAFPRMNNMSFWLLPPSLTLLLMSSMVESGAGTGWTVYPPLSSNIAHGGSSVDLAIFSLHLAGISSILGAVNFISTIINMRTSG
MTFDRMPLFVWSVAITALLLLLSLPVLAGAITMLLTDRNLNTSFFDPAGGGDPILYQHL
>COLFC129-12|KJ967355|ZMUO.002314|Tychius_picirostris
TLYFIFGSWSGMVGTSLSMLIRTELGNPGSLIGDDQIYNSIVTAHAFIMIFFMVMPIMIGGFGNWLIPLMLGAPDMAYPRLNNMSFWLLPPSISLLLMSSIVNKGAGTGWTVYPPLSSNIAHEGASVDLAIFSLHMAGMSSILGAINFISTMMNMRPFG
MNSEKVTLFSWAVQVTAILLLLSLPVLAGAITMLLTDRNVNTSFFDPAGGGDPILYQHL
>COLFC073-12|KJ962853|ZMUO.002068|Chaetarthria_seminulum
TLYFIFGAWAGMVGTSLSILIRAELGNPGTLIGDDQIYNVIVTAHAFIMIFFMVMPIMIGGFGNWLVPLMLGAPDMAFPRMNNMSFWLLPPSLTLLLMSSMVESGAGTGWTVYPPLSSNIAHGGASVDLAIFSLHLAGISSILGAVNFITTVINMRSNN
MTYDRLPLFVWSVAITALLLLLSLPVLAGAITMLLTDRNLNTSFFDPAGGGDPILYQHL
>COLFB776-12|KJ965212|ZMUO.001251|Mannerheimia_arctica
TLYFVFGAWAGMVGTSLSILIRAELGNPGSLIGDDQIYNVIVTAHAFIMIFFMVMPIVIGGFGNWLVPLMLGAPDMAFPRMNNMSFWLLPPSLTLLLMSSMVESGAGTGWTVYPPLSSNIAHGGSSVDLAIFSLHLAGISSILGAVNFITTVINMRSSG
MTFDRMPLFVWSVVITALLLLLSLPVLAGAITMLLTDRNLNTSFFDPAGGGDPILYQHL

>COLFD756-12|KJ962566|ZMUO.004746|Cyphon_laevipennis
TLYFIFGSWSGMVGTSLSLLIRAELGTPGSLIGDDQIYNVIVTAHAFIMIFFMVMPIMIGGFGNWLVPLMLGAPDMAFPRMNNMSFWLLPPSLTLLLMSSMVENGAGTGWTVYPPLSAGVAHSGASVDLAIFSLHLAGISSILGAVNFISTVINMRSVG
MTFDRMPLFVWAVAITALLLLLSLPVLAGAITMLLTDRNLNTSFFDPAGGGDPILYQHL
>COLFD048-12|KJ964091|ZMUO.003943|Scymnus_suturalis
TLYFLFGLWAGMVGTSLSILIRLELGTTSALIGNDQIYNVIVTAHAFIMIFFMVMPIMIGGFGNWLVPLMIGAPDMAFPRLNNMSFWLLPPSLTFLILSSMVESGAGTGWTVYPPLSSNLAHGGSSVDMAIFSLHLAGISSILGAVNFISTIINMRTYGMT
FEKMPLFVWSVFITAILLLLSLPVLAGAITMLLTDRNINTSFFDPAGGGDPILYQHL
>COLFE416-12|KJ964535|ZMUO.004976|Adalia_bipunctata
TLYFLFGLWAGMVGTSLSIIIRLELGTTNSLIGNDQIYNVIVTAHAFIMIFFMVMPIMIGGFGNWLVPLMIGAPDMAFPRLNNMSFWLLPPALTLLISSSVIEMGAGTGWTVYPPLSSNMAHNGPSVDLVIFSLHLAGISSILGAVNFISTIMNMRPNGM
NLDKTPLFVWSVLITAILLLLSLPVLAGAITMLLTDRNINTSFFDPTGGGDPVLYQHL
>COLFA610-12|KJ966084|ZMUO.000745|Platystomos_albinus
TLYFIFGAWAGMMGTSLSILIRTELGTPSSLIGDDQIYNVIVTAHAFVMIFFMVMPTMIGGFGNWLVPLMLGAPDMAFPRMNNMSFWLLPPSLILLTMSSIVEKGAGTGWTVYPPLASNIAHSGASVDLAIFSLHLAGVSSILGAVNFITTIINMRPEG
MTPDRLPLFVWAVGITALLLLLSLPVLAGAITMLLTDRNLNTTFFDPAGGGDPILYQHL
>COLFG020-13|KJ962013|ZMUO.007525|Agrilus_mendax
TLYFIFGAWSGMVGTALSLLIRAELGNPGALIGNDQIYNVIVTAHAFIMIFFMVMPIMMGGFGNWLVPLMLGAPDMAFPRMNNMSFWLLPPSLTLLLMSSMVESGAGTGWTVYPPLASNIAHSGASVDLAIFSLHLAGISSILGAINFITTVINMRAS
GMTLDRAPLLVWSIAITALLLLLSLPVLAGAITMLLTDRNLNTSFFDPAGGGDPILYQHL
>COLFE1070-13|KJ966492|ZMUO.006865|Byctiscus_betulae
TLYFIFGSWAGMVGTSLSLLIRAELGNPGSLIGNDQIYNVIVTAHAFIMIFFMVMPVMIGGFGNWLVPLMLGAPDMAFPRMNNMSFWLLPPSLSLLIMSSIVENGAGTGWTVYPPLSSNIAHSGSSVDLAIFSLHLAGISSILGAVNFISTVINMRPSGM
SLDSMPLFVWAVSITALLLLLSLPVLAGAITMLLTDRNINTSFFDPAGGGDPILYQHL
>COLFB094-12|KJ962103|ZMUO.001424|Crepidodera_fulvicornis
TLYFIFGIWSGMVGTSLSILIRTELGSPGSLIGNDQIYNVIVTAHAFIMIFFMVMPIMIGGFGNWLIPLMIGAPDMAFPRMNNMSFWLLPPSLFLLLMSSLVESGAGTGWTVYPPLSSNIAHGGSSVDLAIFSLHLAGISSILGAINFITTIINMRPKGMNLD
RMPLFVWAVAITAILLLLSLPVLAGAITMLLTDRNMNTSFFDPIGGGDPILYQHL
>COLFC416-12|KJ965034|ZMUO.002981|Anthrenus_museorum
TLYFIFGAWAGMVGTSLSILIRTELGNPGSLIGDDQTFNVIVTAHAFIMIFFMVMPMMIGGFGNWLVPLMLGAPDMAFPRMNNMSFWLLPPSLSLLLLSSMVESGAGTGWTVYPPLSSNIAHGGASVDLAIFSLHLAGISSILGAVNFITTAINMRAA
GMTPERMPLFVWSVAITAILLLLSLPVLAGAITMLLTDRNLNTSFFDPAGGGDPILYQHL
>COLFF811-13|KJ966294|ZMUO.006131|Atomaria_basalis
TLYFIFGMWAGMVGTSLSMLIRTELGTPGSLIGDDQIYNVIVTAHAFIMIFFMVMPMMIGGFGNWLVPLMLGAPDMAFPRLNNMSFWLLPPSLMFLLMSSIVEKGAGTGWTVYPPLSSNVAHAGSSVDLAIFSLHLAGISSILGSVNFITTVINMRPK
GMNFDRLPLFVWAVKITTILLLLSLPVLAGAITMLLTDRNINTSFFDPAGGGDPILYQHL
>COLFC186-12|KJ965667|ZMUO.002371|Aleochara_moerens
TLYFIFGAWAGMVGTSLSLLIRAELGNPGSLIGDDQIYNVIVTAHAFIMIFFMVMPIVIGGFGNWLVPLMLGAPDMAFPRMNNMSFWLLPPSLTLLLMSSMVESGAGTGWTVYPPLSANIAHSGSSVDLAIFSLHLAGISSILGAVNFISTIINMRSSGM
SFDKMPLFVWSVAITALLLLLSLPVLAGAITMLLTDRNLNTSFFDPAGGGDPILYQHL
>COLFB043-12|KJ962282|ZMUO.001373|Enochrus_quadripunctatus
TLYFIFGAWAGMVGTSLSILIRAELGNPGTLIGDDQIYNVIVTAHAFIMIFFMVMPIMIGGFGNWLVPLMLGAPDMAFPRMNNMSFWLLPPSLTLLLMSSMVESGAGTGWTVYPPLSSNIAHGGASVDLAIFSLHLAGISSILGAVNFITTVINMRSPS
MTYDRLPLFVWSVAITALLLLLSLPVLAGAITMLLTDRNLNTSFFDPAGGGDPILYQHL
>COLFF765-13|KJ964045|ZMUO.006085|Philopedon_plagiatum
TLYFIFGAWSGMVGTSLSMLIRTELGNPGSLIGDDQIYNVIVTAHAFIMIFFMVMPMMIGGFGNWLVPLMLGAPDMAFPRLNNMSFWLLPPSLSLLLMSSIVDKGAGTGWTVYPPLSANIAHEGSSVDLAIFSLHMAGVSSILGAINFISTVINMRPQ
GMSPERISLFVWSVKITAVLLLLSLPVLAGAITMLLTDRNINTSFFDPAGGGDPILYQHL
>COLFE456-12|KJ965334|ZMUO.005016|Chaetocnema_hortensis
TLYFIFGIWSGMVGTSMSILIRAELGSPGSLIGNDQIYNVIVTAHAFVMIFFMVMPIMIGGFGNWLVPLMIGAPDMAFPRMNNMSFWLLPPSLFLLLMSSLVESGAGTGWTVYPPLSSNIAHGGSSVDLAIFSLHLAGVSSILGAINFITTIINMRPQGM
SFDQMPLFVWAVLITAILLLLSLPVLAGAITMLLTDRNLNTSFFDPIGGGDPILYQHL
>COLFE547-13|KJ961916|ZMUO.005392|Necrobia_violacea
TLYFIFGAWAGMVGTSLSLLIRSELGTPGTLIGNDQIYNVIVTAHAFIMIFFMVMPIMIGGFGNWLVPLMLGAPDMAFPRMNNMSFWLLPPSLTLLLMSSMVESGAGTGWTVYPPLSSNIAHGGSSVDLAIFSLHLAGISSILGAVNFITTVINMRPAG
MTLDRMPLFVWAVAITALLLLLSLPVLAGAITMLLTDRNLNTSFFDPAGGGDPILYQHL
>COLFB297-12|KJ966445|ZMUO.001627|Brachygluta_fossulata
TLYLIFGAWAGMIGTSLSILIRAELGNPGSLIGDDQIYNVIVTAHAFIMIFFMVMPVMIGGFGNWLVPLMLGAPDMAFPRMNNMSFWLLPPSLILLLMSSLVESGTGTGWTVYPPLSSNIAHSGSSVDLTIFSLHLAGISSILGAVNFITTVINMRTMNM
KFDQLPLFVWSVVITALLLLLSLPVLAGAITMLLTDRNLNTSFFDPAGGGDPILYQHL
>COLFB301-12|KJ966991|ZMUO.001631|Astenus_gracilis

TLYFIFGAWAGMVGTSLSLLIRAELGMPGSLIGDDQIYNVIVTAHAFVMIFFMVMPIVIGGFGNWLVPLMLGAPDMAFPRMNNMSFWLLPPSLSLLLMSSMVESGAGTGWTVYPPLSSNMAHGGASVDLAIFSLHLAGISSILGAVNFITTIINMRTK
GMSYERMPLFVWSVGITALLLLLSLPVLAGAITMLLTDRNLNTSFFDPAGGGDPILYQHL
>COLFE021-12|KJ963073|ZMUO.005246|Phyllopertha_horticola
TLYFLFGSWAGMVGTSLSLLIRAELGNPGSLIGDDQIYNVIVTAHAFIMIFFMVMPIMIGGFGNWLVPLMLGAPDMAFPRMNNMSFWLLPPSLSLLLMSSLVENGAGTGWTVYPPLSANIAHSGASVDLAIFSLHLAGISSILGAVNFITTVINMRSTG
MTFDRMPLFVWSVVLTALLLLLSLPVLAGAITMLLTDRNINTSFFDPAGGGDPILYQHL
>COLFF987-13|KJ964920|ZMUO.007352|Stephostethus_lardarius
SLYFMFGMWSGMIGTSLSLLIRLELGNPGSLIGDDQIYNVIVTAHAFIMIFFMVMPIMMGGFGNWLVPLMLGAPDMAFPRLNNMSFWLLPPSLTLLIMSSIVESGVGTGWTVYPPLSSNIAHGGSSVDLAIFSLHLAGISSILGAVNFITTMVNMRPSG
MNFEHMPLFAWSVNLTAILLLLSLPVLAGAITMLLTDRNLNTSFFDPAGGGDPILYQHL
>COLFB684-12|KJ962409|ZMUO.001159|Hygropora_cunctans
TLYFIFGAWAGMVGTSLSLLIRAELGNPGSLIGDDQIYNVIVTAHAFVMIFFMVMPIVIGGFGNWLIPLMLGAPDMAFPRMNNMSFWLLPPSLSLLLMSSMVESGAGTGWTVYPPLSSNIAHGGASVDLAIFSLHLAGISSILGAVNFISTIINMRTPG
MSFDRMPLFVWSVAITALLLLLSLPVLAGAITMLLTDRNLNTSFFDPAGGGDPILYQHL
>COLFB354-12|KJ962447|ZMUO.001684|Tetartopeus_zetterstedti
TLYFIFGAWAGMVGTSLSLLIRAELGNPGSLIGDDQIYNVIVTAHAFVMIFFMVMPIVIGGFGNWLVPLMLGAPDMAFPRMNNMSFWLLPPSLSLLLMSSMVENGAGTGWTVYPPLSSNIAHGGASVDLAIFSLHLAGISSILGAVNFITTIINMRSPG
MLYERMPLFVWSVAITALLLLLSLPVLAGAITMLLTDRNLNTSFFDPAGGGDPILYQHL
>COLFE033-12|KJ963236|ZMUO.005258|Coeliodes_rubicundus
TLYFIFGSWSGMVGTSLSMLIRTELGTPGSLIGDDQIYNSIVTAHAFIMIFFMVMPILIGGFGNWLVPLMLGAPDMAFPRLNNMSFWLLPPSLALLLMSSIVNKGAGTGWTVYPPLSNNIAHEGASVDLAIFSLHMAGISSILGAINFISTVMNMRPKG
METDRMPLFVWAVEITAILLLLSLPVLAGAITMLLTDRNINTSFFDPAGGGDPILYQHL
>COLFC212-12|KJ964729|ZMUO.002397|Atheta_allocera
TLYFIFGAWAGMVGTSLSLLIRAELGNPGSLIGDDQIYNVIVTAHAFIMIFFMVMPVVIGGFGNWLVPLMLGAPDMAFPRMNNMSFWLLPPSLTLLLMSSMVESGAGTGWTVYPPLSSNIAHSGSSVDLAIFSLHLAGISSILGAVNFISTVINMRSTGI
SFDRMPLFVWSVAITALLLLLSLPVLAGAITMLLTDRNLNTSFFDPAGGGDPILYQHL
>COLFB898-12|KJ964828|ZMUO.001943|Mesosa_myops
TLYFLFGAWAGMLGTSLSVLIRSELGNPGTLIGNDQIYNVIVTAHAFVMIFFMVMPIMIGGFGNWLVPLMLGAPDMAFPRMNNMSFWLLPPSLSLLIMSSIVEKGAGTGWTVYPPLAANVAHNGASVDLAIFSLHLAGISSILGAVNFITTVINMRPS
GMSFDRLPLFVWAVKITAILLLLSLPVLAGAITMLLTDRNLNTSFFDPAGGGDPILYQHL
>COLFE817-13|KJ965997|ZMUO.005662|Apion_frumentarium
TLYFIFGLWSGMVGTSLSMLIRVELGNPGSLIGNDQIYNMIVTAHAFIMIFFMVMPIMIGGFGNWLVPLMLGAPDMAFPRMNNMSFWLLPPSLMLLLMSSFVENGAGTGWTVYPPLASNIAHSGASVDLAIFSLHLAGISSILGAVNFISTMINMRS
NGMSLDQLSLFTWAVKITAILLLLSLPVLAGAITMLLTDRNINTSFFDPAGGGDPILYQHL
>COLFC539-12|KJ964718|ZMUO.003104|Nebria_nivalis
TLYFIFGAWAGMVGTSLSMLIRAELGNPGSLIGDDQIYNVIVTAHAFVMIFFMVMPIMIGGFGNWLVPLMLGAPDMAFPRMNNMSFWLLPPSLTLLLMSSMVESGAGTGWTVYPPLSSGIAHSGASVDMAIFSLHLAGVSSILGAVNFITTIINMRS
MGMTFDRMPLFVWSVGITALLLLLSLPVLAGAITMLLTDRNLNTTFFDPAGGGDPILYQHL
>COLFD676-12|KJ962604|ZMUO.004666|Pedostrangalia_pubescens
TLYFIFGAWAGMVGTSLSMLIRSELGNPGSLIGNDQIYNVIVTAHAFIMIFFMVMPIMIGGFGNWLVPLMLGAPDMAFPRMNNMSFWLLPPSLTLLIMSSVVESGAGTGWTVYPPLSSNIAHSGSSVDLAIFSLHLAGISSILGAVNFITTVINMRPVG
MTPDRMPLFVWAVVITAILLLLSLPVLAGAITMLLTDRNLNTSFFDPAGGGDPILYQHL
>COLFC607-12|KJ963834|ZMUO.003172|Betulapion_simile
TLYFIFGLWSGMVGTSLSMLIRIELGNPGSLIGNDQIYNVIVTAHAFIMIFFMVMPIMIGGFGNWLVPLMLGAPDMAFPRMNNMSFWLLPPSLTLLLMSSIVEKGAGTGWTVYPPLASNIAHGGASVDLAIFSLHLAGISSILGAVNFISTVINMRPTGL
SLDQLSLFTWAVKITAILLLLSLPVLAGAITMLLTDRNINTSFFDPAGGGDPILYQHL
>COLFB837-12|KJ962250|ZMUO.001312|Tachinus_pallipes
TLYFIFGAWAGMVGTSLSLLIRAELGNPGTLIGDDQIYNVIVTAHAFIMIFFMVMPIVIGGFGNWLVPLMLGAPDMAFPRMNNMSFWLLPPSLTLLLMSSMVESGAGTGWTVYPPLSSNIAHGGSSVDLAIFSLHLAGISSILGAVNFITTVINMRSIG
MTFDRMPLFVWSVAITALLLLLSLPVLAGAITMLLTDRNLNTTFFDPAGGGDPILYQHL
>COLFF852-13|KJ966039|ZMUO.006172|Malthodes_fuscus
TLYFMFGAWAGMLGTSLSLLIRAELGSPGSLIGNDQIYNVIVTAHAFIMIFFMVMPIMIGGFGNWLVPLMLGAPDMAFPRMNNMSFWFLPPSLSLLLMSSIVENGAGTGWTVYPPLSANIAHSGSSVDLAIFSLHMAGISSILGAVNFISTVINMRSTG
MTFDRMPLFVWAVAITALLLLLSLPVLAGAITMLLTDRNLNTSFFDPAGGGDPILYQHL
>COLFE580-13|KJ965744|ZMUO.005425|Anthonomus_rubi
TLYFIFGAWSGAVGTSLSMLIRTELGNPGSLIGDDQIYNTIVTAHAFIMIFFMVMPIMIGGFGNWLIPLMLAAPDMAFPRLNNMSFWLLPPSLTLLIMSSIISKGAGTGWTVYPPLSSNLAHEGASVDFAIFSLHMAGISSILGAMNFISTILNMKPMKM
KFEQMPLFIWAVKITAILLLISLPVLAGAITMLLTDRNINTSFFDPAGGGDPILYQHL
>COLFE402-12|KJ965558|ZMUO.004962|Ilybius_ater
TLYFIFGAWAGMVGTSLSMLIRAELGNPGSLIGDDQIYNVIVTAHAFVMIFFMVMPIMIGGFGNWLVPLMLGAPDMAFPRMNNMSFWLLPPSLTLLLMSSMVESGAGTGWTVYPPLSAGIAHSGASVDLAIFSLHLAGISSILGAVNFITTIINMRSV
GMTFDRMPLFVWSVGITALLLLLSLPVLAGAITMLLTDRNLNTSFFDPAGGGDPILYQHL

>LEFIJ1849-13|KJ964874|ZMUO.004604|Rhinoncus_pericarpius
TLYFIFGSWAGTVGTSLSMLIRTELGTPGSLIGNDQIYNSIVTAHAFIMIFFMVMPILIGGFGNWLVPLMLGAPDMAFPRLNNMSFWLLPPSIMLLLMSSIINKGVGTGWTVYPPLSSNITHEGASVDLAIFSLHMAGISSILGAINFISTIMNMRPQGMS
YDKMPLFSWAVLITAILLLLSLPVLAGAITMLLTDRNINTSFFDPAGGGDPILYQHL
>COLFF013-13|KJ966664|ZMUO.005713|Cassida_rubiginosa
TLYFIFGFWSGMVGTSLSILIRAELGNPGTLIGNDQIYNSIVTAHAFIMIFFMVMPIMIGGFGNWLVPLMLGAPDMAFPRLNNMSFWLLPPSITFLIMSSVIESGAGTGWTVYPPLSSNIAHSGASVDMAIFSLHLAGISSILGAINFISTIMNMQPSGMS
LDKMPLFVWAVIITAILLLLSLPVLAGAITMLLTDRNINTSFFDPAGGGDPILYQHL
>COLFF747-13|KJ967473|ZMUO.006447|Pseudoprotapion_astragali
TLYFIFGLWSGMVGTSLSMIIRIELGNPGSLIGNDQIYNVIVTAHAFIMIFFMVMPVMIGGFGNWLIPLMLGAPDMAFPRMNNMSFWLLPPSLSLLLMSSIVEKGAGTGWTVYPPLASNIAHAGASVDLAIFSLHLAGISSILGAVNFISTAMNMRPTG
MNLDQLTLFTWAVKITAILLLLSLPVLAGAITMLLTDRNINTSFFDPAGGGDPILYQHL
>COLFA074-10|HM909092|MP00274|Noterus_crassicornis
TLYFIFGAWSGMVGTSLSMLIRMELGNPGSLIGDDQIYNVIVTAHAFIMIFFMVMPMMIGGFGNWLVPLMLGAPDMAFPRMNNMSFWLLPPSLSLLIMSSLVENGAGTGWTVYPPLASGIAHSGASVDLAIFSLHLAGISSILGAVNFITTIINMRPM
GMSFDRMPLFVWSVGITALLLLLSLPVLAGAITMLLTDRNLNTSFFDPAGGGDPILYQHL
>COLFD271-12|KJ964317|ZMUO.004166|Boros_schneideri
TLYLIFGAWSGMIGTSLSLLIRSELSNPGSLIGDDQIYNVIVTAHAFIMIFFMVMPIMIGGFGNWLVPLMLGAPDMAFPRMNNMSFWLLPPSITLLIMSSIVENGAGTGWTVYPPLSSNIAHSGSSVDLAIFSLHLAGISSILGAVNFITTVINMRPMKMT
FDRMPLFVWAVVITAVLLLLSLPVLAGAITMLLTDRNLNTSFFDPAGGGDPILYQHL
>COLFE1255-13|KJ965291|ZMUO.007050|Atheta_diversa
TLYFIFGAWAGMVGTSLSLLIRAELGNPGSLIGDDQIYNVIVTAHAFVMIFFMVMPVVIGGFGNWLVPLMLGAPDMAFPRMNNMSFWLLPPSLTLLLMSSMVESGAGTGWTVYPPLSSNIAHGGSSVDLAIFSLHLAGISSILGAVNFISTVINMRSTG
ISFDRMPLFVWSVAITALLLLLSLPVLAGAITMLLTDRNLNTSFFDPAGGGDPILYQHL
>COLFE945-13|KJ964952|ZMUO.006265|Mycetochara_axillaris
TLYFIFGAWSGMVGTSLSLLIRAELGNPGSLIGDDQIYNVIVTAHAFIMIFFMVMPIMIGGFGNWLVPIMLGAPDMAFPRMNNMSFWLLPPSLTLLLMSSMVESGAGTGWTVYPPLSSNIAHGGASVDLAIFSLHLAGISSILGAVNFITTVINMKPQG
MTFDRMPLFVWAVVITALLLLLSLPVLAGAITMLLTDRNINTSFFDPAGGGDPILYQHL
>COLFF304-13|KJ961782|ZMUO.006004|Dissoleucas_niveirostris
TLYFIFGAWAGMMGMSLSLLIRTELGNPGSLIGDDQIYNVIVTAHAFIMIFFMVMPTMIGGFGNWLVPLMLGAPDMAFPRMNNMSFWLLPPSLTLLMMSSVVESGAGTGWTVYPPLSSNIAHGGASVDLAIFSLHLAGVSSILGAVNFITTVINMRP
VGMTLDRTPLFVWAVSITALLLLLSLPVLAGAITMLLTDRNLNTSFFDPAGGGDPILYQHL
>COLFE1462-13|KJ964383|ZMUO.007257|Oedemera_subrobusta
TLYLIFGAWAGMVGTSLSLLIRAELGNPGSLISDDQIYNVIVTAHAFIMIFFMVMPIMIGGFGNWLVPLMLGAPDMAFPRMNNMSFWLLPPSLTLLIMSSMVESGVGTGWTVYPPLSSNIAHSGSSVDLAIFSLHLAGVSSILGAVNFITTVINMRPVG
MTLDRMPLFVWAVVITAILLLLSLPVLAGAITMLLTDRNLNTSFFDPAGGGDPILYQHL
>COLFE082-12|KJ964895|ZMUO.005307|Rhagonycha_elongata
TLYFIFGAWSGSLGLALSLLIRAELGTPGTLIGNDQIYNVIVTAHAFIMIFFMVMPIMIGGFGNWLVPLMLGAPDMAFPRMNNMSFWFLPPSLMFLLMSSMVESGAGTGWTVYPPLSANIAHSGPSVDLAIFSLHMAGISSILGAVNFISTILNMKPPS
MKFDQMPLFVWSVGITALLLLLSLPVLAGAITMLLSDRNLNTSFFDPMGGGDPILYQHL
>COLFD169-12|KJ966283|ZMUO.004064|Trachodes_hispidus
TLYFIFGSWSGMVGTSLSMMIRTELGTPGSVIGNDQIYNTIVTAHAFIMIFFMVMPIMIGGFGNWLVPLMLGAPDMAFPRLNNMSFWLLPPSLTLLLMSSIIDKGAGTGWTVYPPLSTNIAHEGMSVDLAIFSLHLAGISSILGAMNFISTVINMHPTG
MKLDQLPLFVWSVKITAILLLLSLPVLAGAITMLLTDRNINTTFFDPAGGGDPILYQHL
>COLFG164-13|KJ963913|ZMUO.007859|Ptiliola_kunzei
TLYFIFGAWAGMVGTSLSLLIRAELGTPGSLIGDDQIYNVIVTAHAFIMIFFMVMPILIGGFGNWLVPLMLGAPDMAFPRMNNMSFWLLPPSLSLLLLSSMVESGAGTGWTVYPPLSSNIAHGGASVDLAIFSLHLAGISSILGAVNFITTIINMRAPGMS
FDQMPLFVWAVGITALLLLLSLPVLAGAITMLLTDRNINTSFFDPAGGGDPILYQHL
>COLFC334-12|KJ963512|ZMUO.002519|Atheta_lapponica
TLYFIFGAWAGMVGTSLSLLIRAELGNPGSLIGDDQIYNVIVTAHAFIMIFFMVMPIVIGGFGNWLVPLMLGAPDMAFPRMNNMSFWLLPPSLTLLLMSSMVESGAGTGWTVYPPLSSNIAHGGSSVDLAIFSLHLAGISSILGAVNFISTVINMRSTGI
SFDRMPLFVWSVAITALLLLLSLPVLAGAITMLLTDRNLNTSFFDPAGGGDPILYQHL
>COLFB288-12|KJ967306|ZMUO.001618|Calathus_erratus
TLYFIFGAWAGMVGTSLSMLIRAELGNPGALIGDDQVYNVIVTAHAFVMIFFMVMPIMIGGFGNWLVPLMLGAPDMAFPRMNNMSFWLLPPSLTLLLMSSMVESGAGTGWTVYPPLSSGIAHSGASVDLAIFSLHLAGISSILGAVNFITTIINMRSV
GMTFDRMPLFVWSVGITALLLLLSLPVLAGAITMLLTDRNLNTSFFDPAGGGDPILYQHL
>COLFB517-12|KJ963840|ZMUO.000992|Cercyon_quisquilius
TLYFIFGAWAGMVGTSLSILIRAELGNPGTLIGDDQIYNVIVTAHAFIMIFFMVMPIMIGGFGNWLVPLMLGAPDMAFPRMNNMSFWLLPPSLTLLLMSSMVESGAGTGWTVYPPLSSNIAHGGSSVDLAIFSLHLAGISSILGAVNFITTVINMRSPNL
TYDRLPLFVWSVAITALLLLLSLPVLAGAITMLLTDRNLNTSFFDPAGGGDPILYQHL
>COLFE1292-13|KJ966118|ZMUO.007087|Agabus_unguicularis

TLYFIFGAWAGMVGTSLSMLIRAELGNPGSLIGDDQIYNVIVTAHAFVMIFFMVMPIMIGGFGNWLVPLMLGAPDMAFPRMNNMSFWLLPPSLTLLLMSSMVEKGAGTGWTVYPPLSSGIAHSGASVDLAIFSLHLAGISSILGAVNFITTIINMRSV
GMTFDRMPLFVWSVGITALLLLLSLPVLAGAITMLLTDRNLNTSFFDPAGGGDPILYQHL
>COLFE982-13|KJ965814|ZMUO.006777|Gyrinus_aeratus
TLYFIFGAWSGMVGTSLSMLIRAELGNPGSLIGDDQIYNVIVTAHAFIMIFFMVMPIMIGGFGNWLVPLMLGAPDMAFPRMNNMSFWLLPPSLTLLLMSSMVENGAGTGWTIYPPLSSNIAHGGASVDLAIFSLHLAGISSILGAVNFITTIINMRSIG
MTLDRMPLFVWSVGITALLLLLSLPVLAGAITMLLTDRNLNTSFFDPAGGGDPILYQHL
>COLFC354-12|KJ964711|ZMUO.002539|Mycetoporus_altaicus
TLYFIFGSWAGMVGTSLSLLIRAELGNPGSFIGDDQIYNVVVTAHAFVMIFFMVMPIVIGGFGNWLVPLMLGAPDMAFPRMNNMSFWLLPPSLTLLLMSSMVESGAGTGWTVYPPLSSNIAHGGASVDLAIFSLHLAGISSILGAVNFITTVVNMRST
GMTFDRMPLFIWSVAITALLLLLSLPVLAGAITMLLTDRNLNTSFFDPAGGGDPILYQHL
>COLFC727-12|KJ962527|ZMUO.003292|Scaphidium_quadrimaculatum
TLYFIFGAWAGMVGTSLSLLIRAELGTQGSLIGDDQIYNVIVTAHAFVMIFFMVMPIVIGGFGNWLVPLMLGAPDMAFPRMNNMSFWLLPPSLSLLLMSSLVESGAGTGWTVYPPLSSNIAHGGASVDLAIFSLHLAGISSILGAVNFISTIINMRTIGM
SFDQMPLFIWSVGITALLLLLSLPVLAGAITMLLTDRNLNTAFFDPAGGGDPILYQHL
>COLFD116-12|KJ963016|ZMUO.004011|Epuraea_boreella
TLYFIFGAWSGMVGTSLSILIRTELGSPGSLIGNDQIYNVIVTAHAFIMIFFMVMPFMIGGFGNWLVPLMLGAPDMAFPRMNNMSFWLLPPSLSLLLMSSIVESGAGTGWTVYPPLSSNIAHGGSSVDLAIFSLHLAGVSSILGAVNFITTIINMRPAGM
TLDRMPLFVWSVVITAILLLLSLPVLAGAITMLLTDRNLNTTFFDPSGGGDPILYQHL
>COLFD191-12|KJ963407|ZMUO.004086|Rhynchaenus_testaceus
TLYFMFGAWSGMVGTSLSMLIRTELGTPGKLIGDDQIYNTIVTAHAFIMIFFMVMPTMIGGFGNWLIPLMLGAPDMAFPRLNNMSFWLLPPSLALLLMSSMMNKGAGTGWTVYPPLSSNMAHEGPSVDLAIFSLHMAGISSILGAINFISTMANM
RPSGMNTDQMSLFVWAVKITAILLLLSLPVLAGAITMLLTDRNINTSFFDPAGGGDPILYQHL
>COLFE1581-13|KJ966419|ZMUO.007471|Melanimon_tibialis
TLYFIFGAWSGMVGTSLSLLIRAELGNPGSLIGDDQIYNVIVTAHAFIMIFFMVMPIMIGGFGNWLVPLMLGAPDMAFPRMNNMSFWLLPPSLTLLLMSSIVESGAGTGWTVYPPLSSNIAHGGSSVDLAIFSLHLAGISSILGAVNFITTVINMRPQG
MTFDQMPLFVWAVVITAVLLLLSLPVLAGAITMLLTDRNINTSFFDPAGGGDPILYQHL
>COLFF990-13|KJ967211|ZMUO.007355|Aphodius_depressus
TLYFLFGSWAGMVGTSLSLLIRAELGNPGTLIGDDQIYNVIVTAHAFIMIFFMVMPILIGGFGNWLVPLMLGAPDMAFPRMNNMSFWLLPPSLTLLLMSSMVESGAGTGWTVYPPLSSNIAHGGASVDLAIFSLHLAGISSILGAVNFITTVINMRSPG
MTFDRMPLFVWSVAITALLLLLSLPVLAGAITMLLTDRNLNTSFFDPAGGGDPILYQHL
>COLFE828-13|KJ967313|ZMUO.005673|Rhinoncus_bruchoides
TLYFIFGSWAGTVGTSLSMLIRTELGTPGSLIGNDQIYNSIVTAHAFIMIFFMVMPILIGGFGNWLVPLMLGAPDMAFPRLNNMSFWLLPPSIMLLLMSSIVNKGAGTGWTVYPPLSSNITHEGASVDLAIFSLHMAGISSILGAINFISTIMNMRPQGM
SYDKMPLFSWAVLITAILLLLSLPVLAGAITMLLTDRNINTSFFDPAGGGDPILYQHL
>COLFD684-12|KJ966092|ZMUO.004674|Pissodes_validirostris
TLYFIFGAWSGMIGTSLSILIRTELGTPGTMIGDDQIYNTIVTAHAFIMIFFMVMPIMIGGFGNWLVPLMLGAPDMAFPRLNNMSFWLLPPSIMLLLMSSIVDKGAGTGWTVYPPLSTNIAHEGPSVDLAIFSLHMAGISSILGAVNFISTVINMRPTGM
NSDQMSLFIWAVKITAILLLLSLPVLAGAITMLLTDRNINTSFFDPAGGGDPILYQHL
>COLFE1251-13|KJ967482|ZMUO.007046|Atheta_brunneipennis
TLYFIFGAWAGMVGTSLSLLIRAELGNPGSLIGDDQIYNVIVTAHAFIMIFFMVMPVVIGGFGNWLVPLMLGAPDMAFPRMNNMSFWLLPPSLTLLLMSSMVESGAGTGWTVYPPLSSNIAHGGSSVDLAIFSLHLAGISSILGAVNFISTVINMRSTGI
SFDRMPLFVWSVAITALLLLLSLPVLAGAITMLLTDRNLNTSFFDPAGGGDPILYQHL
>COLFF873-13|KJ962341|ZMUO.006668|Cercyon_melanocephalus
TLYFIFGAWAGMVGTSLSILIRAELGNPGTLIGDDQIYNVIVTAHAFIMIFFMVMPIMIGGFGNWLVPLMLGAPDMAFPRMNNMSFWLLPPSLTLLLMSSMVESGAGTGWTVYPPLSSNIAHGGSSVDLAIFSLHLAGISSILGAVNFITTVINMRSPNL
TYDRLPLFVWSVIITALLLLLSLPVLAGAITMLLTDRNLNTSFFDPAGGGDPILYQHL
>COLFE1409-13|KJ962313|ZMUO.007204|Nitidula_rufipes
TLYFIFGAWSGMVGTSLSILIRTELGNPGSLIGNDQIYNVIVTAHAFIMIFFMVMPFMIGGFGNWLVPLMLGAPDMAFPRMNNMSFWLLPPSLSLLIMSSIVESGAGTGWTVYPPLSSNIAHGGSSVDLAIFSLHLAGISSILGAVNFITTVINMRPSGM
NFDRMPLFVWAVAITALLLLLSLPVLAGAITMLLTDRNLNTTFFDPSGGGDPILYQHL
>COLFE557-13|KJ965689|ZMUO.005402|Dyschirius_thoracicus
TLYFIFGIWSGMVGTSLSILIRTELGNPGSLIGDDQIYNVIVTAHAFIMIFFMVMPIMIGGFGNWLVPLMLGAPDMAFPRMNNMSFWLLPPSLTLLLMSSMVESGAGTGWTVYPPLSSGIAHSGASVDLAIFSLHLAGISSILGAVNFITTIINMRSTGMT
FERMPLFVWSVGITALLLLLSLPVLAGAITMLLTDRNLNTSFFDPAGGGDPILYQHL
>COLFF051-13|KJ963917|ZMUO.005751|Hydroporus_planus
TLYFLFGAWSGMVGTSLSMLIRAELGNPGSLIGDDQIYNVIVTAHAFIMIFFMVMPIMIGGFGNWLVPLMLGAPDMAFPRMNNMSFWLLPPSLTLLLMSSMVENGAGTGWTVYPPLSSGIAHSGASVDLAIFSLHLAGVSSILGAVNFITTIINMRSI
GMTFDRMPLFVWSVGITALLLLLSLPVLAGAITMLLTDRNLNTSFFDPAGGGDPILYQHL
>COLFA625-12|KJ963027|ZMUO.000760|Velleius_dilatatus
TLYFIFGAWAGMVGTSLSLLIRAELGNPGTLIGDDQIYNVIVTAHAFIMIFFMVMPIVIGGFGNWLVPLMLGAPDMAFPRMNNMSFWLLPPSLSLLLMSSMVESGAGTGWTVYPPLSSNIAHGGASVDLAIFSLHLAGISSILGAVNFITTVINMRSIG
MTFDRMPLFIWSVAITALLLLLSLPVLAGAITMLLTDRNLNTSFFDPAGGGDPILYQHL

>COLFD104-12|KJ965986|ZMUO.003999|Mycetophagus_decempunctatus
TLYFIFGAWSWMVGTSLSLLIRSELGTPGSLIGDDQIYNVIVTAHAFIMIFFMVMPIMMGGFGNWLVPLMLGAPDMAFPRMNNMSFWLLPPSLTLLIMSSIVENGAGTGWTVYPPLSSNIAHSGSSVDLAIFSLHLAGISSILGAVNFITTIINMRPQG
MTFDRMPLFVWAVGITALLLLLSLPVLAGAITMLLTDRNLNTSFFDPAGGGDPILYQHL
>COLFC538-12|KJ966663|ZMUO.003103|Harpalus_nigritarsis
TLYFIFGAWAGMVGTSLSMLIRAELGTPGALIGDDQIYNVIVTAHAFIMIFFMVMPIMIGGFGNWLVPLMLGAPDMAFPRMNNMSFWLLPPSLTLLLMSSMVESGAGTGWTVYPPLSSGIAHSGASVDLAIFSLHLAGISSILGAVNFITTIINMRSVG
MTFDRMPLFVWSVGITALLLLLSLPVLAGAITMLLTDRNLNTSFFDPAGGGDPILYQHL
>COLFC035-12|KJ966351|ZMUO.002030|Atheta_cribripennis
TLYFIFGAWAGMVGTSLSLLIRAELGNPGSLIGDDQIYNVIVTAHAFIMIFFMVMPIVIGGFGNWLVPLMLGAPDMAFPRMNNMSFWLLPPSLTLLLMSSMVESGAGTGWTVYPPLSSNIAHGGSSVDLAIFSLHLAGISSILGAVNFISTIINMRSTGIS
FDRMPLFVWSVAITALLLLLSLPVLAGAITMLLTDRNLNTSFFDPAGGGDPILYQHL
>COLFB128-12|KJ965561|ZMUO.001458|Ophonus_rufibarbis
TLYFIFGAWAGMVGTSLSMLIRAELGTPGALIGDDQIYNVIVTAHAFIMIFFMVMPIMIGGFGNWLVPLMLGAPDMAFPRMNNMSFWLLPPSLTLLLMSSMVESGAGTGWTVYPPLSSGIAHSGASVDLAIFSLHLAGISSILGAVNFITTIINMRSVG
MTFDRMPLFVWSVGITALLLLLSLPVLAGAITMLLTDRNLNTSFFDPAGGGDPILYQHL
>COLFF116-13|KJ967291|ZMUO.003750|Atheta_gagatina
TLYFIFGAWAGMIGTSLSLLIRAELGNPGSLIGDDQIYNVIVTAHAFIMIFFMVMPIVIGGFGNWLVPLMLGAPDMAFPRMNNMSFWLLPPSLSLLLMSSMVESGAGTGWTVYPPLSSNIAHGGSSVDLAIFSLHLAGISSILGAVNFISTVINMRSTGIS
FDRMPLFVWSVVITALLLLLSLPVLAGAITMLLTDRNLNTSFFDPAGGGDPILYQHL
>COLFE1478-13|KJ962398|ZMUO.007273|Cantharis_flavilabris
TLYFIFGAWSGSLGLALSLLIRAELGTPGTLIGNDQIYNVIVTAHAFIMIFFMVMPIMIGGFGNWLVPLMLGAPDMAFPRMNNMSFWFLPPSLMFLLMSSMVESGAGTGWTVYPPLSANIAHSGPSVDLAIFSLHMAGVSSILGAVNFISTIMNMKPP
SMKFDQMPLFVWSVGITALLLLLSLPVLAGAITMLLSDRNLNTSFFDPMGGGDPILYQHL
>COLFF853-13|KJ966763|ZMUO.006173|Polydrusus_mollis
TLYFIFGAWSGMVGTSLSMLIRTELGNPGSLIGDDQIYNVIVTAHAFIMIFFMVMPTMIGGFGNWLVPLMLGAPDMAFPRLNNMSFWLLPPSLSLLLMSSIVDKGAGTGWTVYPPLSANIAHEGSSVDLAIFSLHMAGVSSILGAVNFISTVINMHPK
GMTPERMPLFVWAVVITAILLLLSLPVLAGAITMLLTDRNMNTSFFDPAGGGDPILYQHL
>COLFB431-12|KJ965453|ZMUO.001856|Ceutorhynchus_typhae
TLYFIFGSWAGMAGTSLSMLIRTELGNPGSLIGNDQIYNSIVTAHAFIMIFFMVMPILIGGFGNWLVPLMLGAPDMAFPRLNNMSFWLLPPSLSLLLMSSVVNKGAGTGWTVYPPLSSNVAHEGMSVDLAIFSLHMAGISSILGAINFISTVMNMQPK
GMTPELMPLFVWAVEITAILLLISLPVLAGAITMLLTDRNINTSFFDPSGGGDPILYQHL
>COLFC639-12|KJ963271|ZMUO.003204|Hygrotus_marklini
TLYFLFGAWSGMVGTSMSMLIRAELGNPGSLIGDDQIYNVIVTAHAFIMIFFMVMPIMIGGFGNWLVPLMLGAPDMAFPRMNNMSFWMLPPSLTLLLMSSMVESGAGTGWTVYPPLSAGIAHGGASVDLAIFSLHLAGISSILGAVNFITTIINMRS
VGMTFDRMPLFVWSVGITALLLLLSLPVLAGAITMLLTDRNLNTSFFDPAGGGDPILYQHL
>COLFE1096-13|KJ965199|ZMUO.006891|Plateumaris_discolor
TLYFIFGAWSGMMGTSLSMLIRTELMNPGSLIGNDQIYNVIVTAHAFIMIFFMVMPIMIGGFGNWLIPLMLGAPDMAFPRMNNMSFWLLPPSLTFLIMSSIVENGAGTGWTVYPPLSSNIAHSGASVDLAIFSLHLAGVSSILGAVNFITTIINMRPMG
MKMDKVPLFAWAVMITAILLLLSLPVLAGAITMLLTDRNLNTSFFDPAGGGDPVLYQHL
>COLFC848-12|KJ965783|ZMUO.003413|Anthicus_ater
TLYLIFGAWAGMVGTSLSLLIRSELGNPGTLIGNDQIYNVIVTAHAFIMIFFMVMPIVIGGFGNWLVPLMLGAPDMAFPRMNNMSFWLLPPSLSLLIMSSIVESGSGTGWTVYPPLSANIAHSGSSVDLAIFSLHLAGISSILGAVNFITTVINMRPTGMS
LDRMPLFVWAVVITAVLLLLSLPVLAGAITMLLTDRNLNTSFFDPAGGGDPILYQHL
>COLFE461-12|KJ966585|ZMUO.005021|Coccinella_hieroglyphica
TLYFLFGMWAGMIGTSLSILIRLELGTTNSLIGNDQIYNVIVTAHAFIMIFFMVMPIMIGGFGNWLVPLMIGAPDMAFPRLNNMSFWLLPPALSLLIISSLVEMGAGTGWTVYPPLSSNLAHNGPSVDLVIFSLHLAGISSILGAVNFISTIMNMRPFGM
NLDKTPLFVWSVLITAILLLLSLPVLAGAITMLLTDRNINTSFFDPTGGGDPILYQHL
>COLFA657-12|KJ965244|ZMUO.000792|Philonthus_nitidus
TLYFIFGSWAGMVGTSLSLLIRAELGNPGTLIGDDQIYNVIVTAHAFIMIFFMVMPIVIGGFGNWLVPLMLGAPDMAFPRMNNMSFWLLPPSLTLLLMSSMVESGAGTGWTVYPPLSSNIAHGGASVDLAIFSLHLAGISSILGAVNFITTVINMRSTG
MSFDRMPLFVWSVAITALLLLLSLPVLAGAITMLLTDRNLNTTFFDPAGGGDPILYQHL
>COLFE194-12|KJ965201|ZMUO.005039|Ocypus_ophthalmicus
TLYFIFGVWSGMVGTSLSLLIRAELGNPGTLIGDDQIYNVIVTAHAFVMIFFMVMPIMIGGFGNWLVPLMLGAPDMAFPRMNNMSFWLLPPSLTLLLMSSMAESGAGTGWTVYPPLSGNIAHSGTSVDLAIFSLHLAGISSILGAVNFITTVINMRSTG
MTFDRMPLFVWSVVITALLLLLSLPVLAGAITMLLTDRNLNTSFFDPAGGGDPILYQHL
>COLFD173-12|KJ965218|ZMUO.004068|Pelenomus_quadrituberculatus
TLYFIFGSWAGTVGTSLSMIIRTELGTPGSLIGNDQIYNSIVTAHAFIMIFFMVMPILIGGFGNWLVPLMLGAPDMAFPRLNNMSFWLLPPSILLLLMSSIVNKGAGTGWTVYPPLSSNITHEGASVDLAIFSLHMAGISSILGAINFISTIMNMRPKGMSY
DKTPLFVWAVMITAILLLISLPVLAGAITMLLTDRNINTSFFDPAGGGDPILYQHL
>COLFE415-12|KJ965695|ZMUO.004975|Luperus_flavipes

TLYFIFGIWAGMVGTSLSILIRAELGGPGSLIGNDQIYNVIVTAHAFIMIFFMVMPIMIGGFGNWLIPLMIGAPDMAFPRMNNMSFWLLPPSIFLLIMSSVVESGAGTGWTVYPPLSSNIAHGGASVDLAIFSLHLAGISSILGAINFITTVINMRPMGMSL
DRMPLFVWAVVITAILLLLSLPVLAGAITMLLTDRNLNTSFFDPAGGGDPILYQHL
>COLFC821-12|KJ966797|ZMUO.003386|Enochrus_ochropterus
TLYFIFGAWAGMVGTSLSLLIRAELGNPGTLIGDDQIYNVIVTAHAFIMIFFMVMPIMIGGFGNWLVPLMLGAPDMAFPRMNNMSFWLLPPSLTLLLMSSMVESGAGTGWTVYPPLSSNIAHGGASVDLAIFSLHLAGISSILGAVNFITTVINMRSPS
MTYDRLPLFVWSVAITALLLLSLPVLAGAITMLLTDRNLNTSFFDPAGGGDPILYQHL
>COLFB761-12|KJ967245|ZMUO.001236|Oxypoda_islandica
TLYFIFGAWAGMVGTSLSLLIRAELGNPGSLIGDDQIYNVIVTAHAFVMIFFMVMPIVIGGFGNWLVPLMLGAPDMAFPRMNNMSFWLLPPSLTLLLMSSMVESGAGTGWTVYPPLSSNIAHGGSSVDLAIFSLHLAGISSILGAVNFISTIINMRTSG
MSFDRMPLFVWSVAITALLLLSLPVLAGAITMLLTDRNLNTSFFDPAGGGDPILYQHL
>LEFIJ1843-13|KJ964443|ZMUO.004598|Psylliodes_affinis
TLYFIFGVWSGMIGTSLSMLIRTELGAPGSLIGNDQIYNVIVTAHAFIMIFFMVMPTMIGGFGNWLIPLMIGAPDMAFPRMNNMSFWLLPPSLTLLLMSSMVENGAGTGWTVYPPLSSNIAHGGSSVDLAIFSLHLAGISSILGAINFITTVINMRPKG
MTLDRMPLFVWAVVITAILLLLSLPVLAGAITMLLTDRNLNTSFFDPIGGGDPILYQHL
>COLFE1301-13|KJ963546|ZMUO.007096|Lathrobium_elongatum
TLYFIFGAWAGMVGTSLSLLIRTELGNPGSLIGDDQIYNVIVTAHAFIMIFFMVMPIMIGGFGNWLVPLMLGAPDMAFPRMNNMSFWLLPPSLSLLLMSSLVESGAGTGWTVYPPLSSNIAHGGASVDLAIFSLHLAGISSILGAVNFITTVINMRSPG
MTYERMPLFVWAVAITALLLLSLPVLAGAITMLLTDRNLNTSFFDPAGGGDPILYQHL
>COLFD820-12|KJ962574|ZMUO.004810|Anobium_punctatum
TLYFIFGSWSGMVGTSLSILIRSELGNPGALIGDDQIYNVIVTAHAFIMIFFMVMPIMMGGFGNWLVPLMLGAPDMAFPRMNNMSFWLLPPSLSLLLMSSIVNTGAGTGWTVYPPLSSNIAHSGASVDLAIFSLHLAGASSILGAVNFITTVINMRPMS
MSFDCVPLFVWSVVITALLLLSLPVLAGAITMLLTDRNLNTSFFDPSGGGDPILYQHL
>COLFE1020-13|KJ961783|ZMUO.006815|Devia_prospera
TLYFIFGAWAGMIGTSLSLLIRAELGNPGSLIGDDQIYNVIVTAHAFVMIFFMVMPIVIGGFGNWLVPLMLGAPDMAFPRMNNMSFWLLPPSLSLLLMSSLIESGAGTGWTVYPPLSSNIAHGGSSVDLAIFSLHLAGISSILGAVNFISTIINMRTTGMY
FDRMPLFVWSVAITALLLLSLPVLAGAITMLLTDRNLNTSFFDPAGGGDPILYQHL
>COLFG005-13|KJ966109|ZMUO.007510|Nivellia_sanguinosa
TLYFIFGAWASMVGTSLSLLIRSELGNPGSLIGNDQIYNVIVTAHAFVMIFFMVMPIMIGGFGNWLVPLMLGAPDMAFPRMNNMSFWLLPPSLSLLIMSSIVEKGAGTGWTVYPPLSANIAHSGSSVDLAIFSLHLAGISSILGAVNFITTVINMRPSGM
SFDRMPLFVWAVVITAVLLLLSLPVLAGAITMLLTDRNINTSFFDPAGGGDPILYQHL
>COLFE1160-13|KJ965848|ZMUO.006955|Grypus_equiseti
TLYFLFGFWSGMVGTSMSMLIRTELGNPGSLIGDDQIYNSIVTAHAFVMIFFMVMPIMIGGFGNWLIPLMLGAPDMAFPRLNNLSFWLLPPSLSLLLMSSIIEKGAGTGWTVYPPLSGNISHSGVSVDLAIFSLHMAGASSILGAINFISTAINMYPKNIS
LDQLSLFIWAVCITAILLLLSLPVLAGAITMLLTDRNINTSFFDPAGGGDPILYQHL
>COLFE933-13|KJ962056|ZMUO.006253|Leiodes_picea
TLYFIFGAWSGMVGTSLSILIRAELGNPGSLIGDDQIYNVIVTAHAFIMIFFMVMPIVIGGFGNWLVPLMLGAPDMAFPRMNNMSFWLLPPSLTLLLMSSIVENGAGTGWTVYPPLSSNIAHSGSSVDLAIFSLHLAGISSILGAVNFITTVINMRPAGMS
FDKMPLFVWSVAITALLLLSLPVLAGAITMLLTDRNLNTSFFDPAGGGDPILYQHL
>COLFE318-12|KJ966005|ZMUO.004878|Amara_plebeja
TLYFIFGAWSGMVGTSLSMLIRAELGNPGALIGDDQIYNVIVTAHAFVMIFFMVMPIMIGGFGNWLIPLMLGAPDMAFPRMNNMSFWLLPPSLTLLLMSSMVESGAGTGWTVYPPLSSGIAHAGASVDLAIFSLHLAGISSILGAVNFITTIINMRSM
GMTFDRMPLFVWSVGITALLLLSLPVLAGAITMLLTDRNLNTSFFDPAGGGDPILYQHL
>COLFF894-13|KJ966612|ZMUO.006689|Ocypus_nitens
TLYFIFGVWSGMVGTSLSLLIRAELGNPGSLIGDDQTYNVIVTAHAFVMIFFMVMPIVIGGFGNWLVPLMLGAPDMAFPRMNNMSFWLLPPSLTLLLASSMAESGAGTGWTVYPPLSANIAHSGASVDLAIFSLHLAGISSILGAVNFITTVVNMRSTG
MTFDRMPLFVWSVAITALLLLSLPVLAGAITMLLTDRNINTSFFDPAGGGDPILYQHL
>COLFF136-13|KJ961955|ZMUO.003770|Cleopomiarus_graminis
TLYFIFGAWSGMLGTSLSMIIRIELGTPGKFIGNDQIYNSIVTAHAFIMIFFMVMPIMIGGFGNWLVPLMMGAPDMAFPRLNNLSFWLLPPSLCMLIMSMIIDKGVGTGWTVYPPLSANVAHEGSSVDFAIFSLHLAGLSSILGAINFISTMMNMRPKG
MKNDRIPLFTWAVKITAILLLLSLPVLAGAITMLLTDRNINTSFFDPAGGGDPILYQHL
>COLFC767-12|KJ963799|ZMUO.003332|Epuraea_aestiva
TLYFIFGAWSGMIGTSLSILIRTELGSPGSLIGNDQIYNVIVTAHAFIMIFFMVMPFMIGGFGNWLVPLMLGAPDMAFPRMNNMSFWLLPPSLSLLLMSSIVESGAGTGWTVYPPLSSNIAHGGSSVDLAIFSLHLAGVSSILGAVNFITTIINMRPIGMTL
DRMPLFVWSVIITAILLLLSLPVLAGAITMLLTDRNLNTTFFDPSGGGDPILYQHL
>COLFA572-12|KJ961852|ZMUO.000707|Pachyta_quadrimaculata
TLYFIFGAWAGMVGTSLSLLIRSELGNPGSLIGDDQIYNVIVTAHAFIMIFFMVMPIMIGGFGNWLVPLMLGAPDMAFPRMNNMSFWLLPPSLTLLIMSSIVESGAGTGWTVYPPLSSNIAHGGSSVDLAIFSLHLAGISSILGAVNFITTVINMRPVGM
TLDRMPLFVWAVVITAILLLLSLPVLAGAITMLLTDRNLNTSFFDPAGGGDPILYQHL
>COLFA320-12|KJ965846|ZMUO.000455|Bradycellus_caucasicus
TLYFIFGAWSGMVGTSLSMLIRAELGTPGALIGDDQIYNVIVTAHAFIMIFFMVMPIMIGGFGNWLVPLMLGAPDMAFPRMNNMSFWLLPPSLSLLLMSSMVEKGAGTGWTVYPPLSSGIAHSGASVDLAIFSLHLAGVSSILGAVNFITTIINMRSVG
MTFDRMPLFVWSVGITALLLLSLPVLAGAITMLLTDRNLNTSFFDPAGGGDPILYQHL

>COLFE1407-13|KJ965376|ZMUO.007202|Cantharis_figurata
TLYFIFGAWSGSLGLALSLLIRAELGTPGTLIGNDQIYNVIVTAHAFIMIFFMVMPIMIGGFGNWLVPLMLGAPDMAFPRMNNMSFWFLPPSLMFLLMSSMVESGAGTGWTVYPPLSANIAHSGPSVDLAIFSLHMAGISSILGAVNFISTIMNMKPPS
MKFDQMPLFVWSVGITALLLLLSLPVLAGAITMLLSDRNLNTSFFDPMGGGDPILYQHL
>COLFF231-13|KJ962097|ZMUO.005836|Byturus_ochraceus
TLYFIFGAWAGMVGTSLSLLIRSELGNPGSLIGDDQIYNVIVTAHAFVMIFFMVMPIVIGGFGNWLVPLMLGAPDMAFPRMNNMSFWLLPPSLTLLLMSSIVESGAGTGWTVYPPLSSNIAHGGSSVDLAIFSLHLAGISSILGAVNFITTIMNMRPTG
MTLDRMPLFVWSVIITAILLLLSLPVLAGAITMLLTDRNLNTSFFDPSGGGDPILYQHL
>COLFC246-12|KJ966728|ZMUO.002431|Schistoglossa_curtipennis
TLYFIFGAWAGMVGTSLSLLIRAELGNPGSLIGDDQIYNVIVTAHAFVMIFFMVMPIVIGGFGNWLIPLMLGAPDMAFPRMNNMSFWLLPPSLTLLLMSSMVESGAGTGWTVYPPLSSNIAHGGSSVDLAIFSLHLAGISSILGAVNFISTVLNMRSKGI
SFDRMPLFVWSVAITALLLLLSLPVLAGAITMLLTDRNLNTSFFDPAGGGDPILYQHL
>COLFD206-12|KJ963455|ZMUO.004101|Dromius_quadraticollis
TLYFIFGAWAGMVGTSLSMLIRAELGNPGALIGDDQIYNVIVTAHAFIMIFFMVMPIMIGGFGNWLVPLMLGAPDMAFPRMNNMSFWLLPPSLSLLLMSSMVESGAGTGWTVYPPLSSGIAHAGASVDLAIFSLHLAGVSSILGAVNFITTIINMRSIG
MTFDRMPLFVWSVGITALLLLLSLPVLAGAITMLLTDRNLNTSFFEPAGGGDPILYQHL
>COLFC822-12|KJ963041|ZMUO.003387|Plateumaris_sericea
TLYFIFGAWSGMMGTSLSMLIRTELMNPGSLIGNDQIYNVIVTAHAFIMIFFMVMPIMIGGFGNWLIPLMLGAPDMAFPRMNNMSFWLLPPSLTFLIMSSIVENGAGTGWTVYPPLSSNIAHSGASVDLAIFSLHLAGVSSILGAVNFITTIINMRPMG
MKMDKVPLFAWAVMITAILLLLSLPVLAGAITMLLTDRNLNTSFFDPAGGGDPVLYQHL
>COLFF788-13|KJ962745|ZMUO.006108|Hydroporus_submuticus
TLYFLFGAWSGMVGTSLSMLIRAELGNPGSLIGDDQIYNVIVTAHAFIMIFFMVMPIMIGGFGNWLVPLMLGAPDMAFPRMNNMSFWLLPPSLTLLLMSSMVENGAGTGWTVYPPLSSGIAHSGASVDLAIFSLHLAGVSSILGAVNFITTIINMRSI
GMTFDRMPLFVWSVGITALLLLLSLPVLAGAITMLLTDRNLNTSFFDPAGGGDPILYQHL
>COLFE222-12|KJ963022|ZMUO.005067|Rhantus_suturalis
TLYFIFGAWAGMVGTSLSMLIRAELGNPGSLIGDDQIYNVIVTAHAFVMIFFMVMPIMIGGFGNWLVPLMLGAPDMAFPRMNNMSFWLLPPSLTLLLMSSMVESGAGTGWTVYPPLSSGIAHGGASVDLAIFSLHLAGISSILGAVNFITTIINMRSV
GMTFDRMPLFVWSVGITALLLLLSLPVLAGAITMLLTDRNLNTSFFDPAGGGDPILYQHL
>COLFB715-12|KJ963863|ZMUO.001190|Atheta
TLYFIFGAWAGMVGTSLSLLIRAELGNPGSLIGDDQIYNVIVTAHAFIMIFFMVMPIVIGGFGNWLVPLMLGAPDMAFPRMNNMSFWLLPPSLTLLLMSSMVESGAGTGWTVYPPLSSNIAHGGSSVDLAIFSLHLAGISSILGAVNFISTVINMRSTGI
SFDRMPLFVWSVAITALLLLLSLPVLAGAITMLLTDRNLNTSFFDPAGGGDPILYQHL
>COLFB610-12|KJ965887|ZMUO.001085|Pachyta_lamed
TLYFIFGAWAGMVGTSLSLLIRSELGNPGSLIGDDQIYNVIVTAHAFIMIFFMVMPIMIGGFGNWLVPLMLGAPDMAFPRMNNMSFWLLPPSLTLLIMSSIVESGAGTGWTVYPPLSSNIAHGGSSVDLAIFSLHLAGISSILGAVNFITTVINMRPMG
MTLDRMPLFVWAVVITAILLLLSLPVLAGAITMLLTDRNLNTSFFDPAGGGDPILYQHL
>COLFC013-12|KJ964949|ZMUO.002008|Enochrus_bicolor
TLYFIFGAWAGMVGTSLSILIRAELGNPGTLIGDDQIYNVIVTAHAFIMIFFMVMPIMIGGFGNWLVPLMLGAPDMAFPRMNNMSFWLLPPSLTLLLMSSMVESGAGTGWTVYPPLSSNIAHGGASVDLAIFSLHLAGISSILGAVNFITTVINMRSPS
MTYDRLPLFVWSVAITALLLLLSLPVLAGAITMLLTDRNLNTSFFDPAGGGDPILYQHL
>COLFB613-12|KJ965331|ZMUO.001088|Galerucella_tenella
TLYFIFGIWAGMVGTSLSILVRAELGSPGTLIGNDQIYNVIVTAHAFIMIFFMVMPIMIGGFGNWLVPLMIGAPDMAFPRMNNMSFWLLPPSLFLLIMSSIVESGAGTGWTVYPPLSSNIAHGGSSVDLAIFSLHLAGISSILGAINFITTIINMRPKGMTL
DRMPLFVWAVMITAILLLLSLPVLAGAITMLLTDRNLNTSFFDPAGGGDPILYQHL
>COLFA028-10|HM909055|MP00169|Oxystoma_cerdo
TLYFILGLWSGMVGTSLSMLIRIELGNSGSLIGDDQIYNVIVTAHAFIMIFFMVMPIMIGGFGNWLVPLMLGAPDMAFPRMNNMSFWMLPPSLTLLLMSSIVEKGAGTGWTVYPPLASNIAHSGASVDLAIFSLHLAGISSILGAVNFISTMINMHPNG
LSLDQLSLFTWAVKITAILLLLSLPVLAGAITMLLTDRNINTSFFDPAGGGDPILYQHL
>COLFB706-12|KJ962061|ZMUO.001181|Aphodius_conspurcatus
TLYFLFGSWAGMVGTSLSLLIRAELGNPGSLIGDDQIYNVIVTAHAFVMIFFMVMPILIGGFGNWLVPLMLGAPDMAFPRMNNMSFWLLPPSLTLLLMSSMVESGAGTGWTVYPPLSSNIAHGGASVDLAIFSLHLAGISSILGAVNFITTVINMRAPG
LTFDQMPLFVWSVAITALLLLLSLPVLAGAITMLLTDRNLNTSFFDPAGGGDPILYQHL
>COLFC125-12|KJ963298|ZMUO.002310|Paederus_fuscipes
TLYFIFGAWSGMVGTSLSLLIRAELATPGSLIGDDQIYNVIVTAHAFIMIFFMVMPIMIGGFGNWLVPLMLGAPDMAFPRMNNMSFWLLPPALTLLLMSSMVENGAGTGWTVYPPLSSNAFHNGSSVDLAIFSLHLAGISSILGAINFITTALNMRASN
MSYEQMPLFVWSVAITALLLLLSLPVLAGAITMLLTDRNLNTSFFDPSGGGDPILYQHL
>COLFG027-13|KJ961841|ZMUO.007532|Clytus_arietis
TLYFIFGAWAGMVGTSLSVLIRSELGNPGSLIGDDQIYNVIVTAHAFIMIFFMVMPIMIGGFGNWLVPLMLGAPDMAFPRMNNMSFWLLPPSLALLIMSSVVESGAGTGWTVYPPLSANVAHSGSSVDLAIFSLHLAGVSSILGAVNFISTVINMRPTK
MSPEQMPLFVWAVAITALLLLLSLPVLAGAITMLLTDRNINTSFFDPAGGGDPILYQHL
>COLFD370-12|KJ964101|ZMUO.004265|Tachyporus_scitulus

TLYFIFGAWSGMVGTSLSLLIRAELGNPGSLIGDDQIYNVIVTAHAFIMIFFMVMPIVIGGFGNWLVPLMLGAPDMAFPRMNNMSFWLLPPSLTLLLMSSMVESGAGTGWTVYPPLSANIAHSGPSVDLAIFSLHLAGISSILGAVNFITTVINMRASG
MYFDRMPLFIWSVAITALLLLLSLPVLAGAITMLLTDRNLNTSFFDPAGGGDPILYQHL
>COLFB085-12|KJ965435|ZMUO.001415|Stilbus_atomarius
TLYFIFGAWSGMVGTSLSLLIRTELGTPGSLIGDDQIYNVIVTAHAFVMIFFMVMPFMIGGFGNWLVPLMLGAPDMAFPRMNNMSFWLLPPSLSFLLMSSIVENGAGTGWTVYPPLSSNIAHGGSSVDLAIFSLHLAGISSILGAINFITTVINMRPEGM
TLDRMPLFVWSVVITAILLLLSLPVLAGAITMLLTDRNINTSFFDPAGGGDPILYQHL
>COLFC430-12|KJ964471|ZMUO.002995|Fleutiauxellus_maritimus
TLYFLFGAWAGMLGTSLSLLIRAELGNPGSLIGNDQIYNVIVTAHAFIMIFFMVMPIMIGGFGNWLVPLMLGAPDMAFPRMNNMSFWFLPPSLSLLLMSSIVENGAGTGWTVYPPLSANIAHSGSSVDLAIFSLHLAGISSILGAVNFISTVINMRSTGIT
FDRMPLFVWAVAITALLLLLSLPVLAGAITMLLTDRNLNTSFFDPAGGGDPILYQHL
>COLFD805-12|KJ966859|ZMUO.004795|Rhizophagus_depressus
TLYFIFGAWAGMVGTSLSLLIRAELGTPGQLIGDDQIYNVIVTAHAFIMIFFMVMPILIGGFGNWLVPLMLGAPDMAFPRMNNMSFWLLPPSLTLLIMSSIVENGAGTGWTVYPPLSSNIAHSGSSVDLAIFSLHLAGISSILGAVNFITTVINMRPSGMT
LDRTPLFVWAVIITATLLLLSLPVLAGAITMLLTDRNLNTSFFDPAGGGDPILYQHL
>COLFC736-12|KJ962147|ZMUO.003301|Phyllobius_glaucus
TLYFIFGAWSGMVGTSLSVLIRAELGNPGSLIGDDQIYNVIVTAHAFIMIFFMVMPMMIGGFGNWLVPLMLGAPDMAFPRLNNMSFWLLPPSLSLLLMSSIVDKGAGTGWTVYPPLSANIAHEGASVDLAIFSLHMAGVSSILGAINFISTVINMRPLG
MSPDRMSLFIWAVKITAILLLLSLPVLAGAITMLLTDRNLNTSFFDPAGGGDPILYQHL
>COLFE1050-13|KJ966437|ZMUO.006845|Agabus_serricornis
TLYFIFGAWAGMVGTSLSMLIRAELGNPGSLIGDDQIYNVIVTAHAFVMIFFMVMPIMIGGFGNWLVPLMLGAPDMAFPRMNNMSFWLLPPSLTLLLMSSMVESGAGTGWTVYPPLSSGIAHGGASVDLAIFSLHLAGISSILGAVNFITTIINMRSIG
MTFDRMPLFVWSVGITALLLLLSLPVLAGAITMLLTDRNLNTSFFDPAGGGDPILYQHL
>COLFA389-12|KJ963230|ZMUO.000619|Quedius_xanthopus
TLYFVFGMWAGMVGTSLSLLIRAELGNPGTLIGDDQIYNVIVTAHAFIMIFFMVMPIVIGGFGNWLVPLMLGAPDMAFPRMNNMSFWLLPPSLSLLLVSSMVESGAGTGWTVYPPLSSNIAHGGASVDLAIFSLHLAGISSILGAVNFITTVINMRSVG
MTFDRMPLFVWSVAVTALLLLLSLPVLAGAITMLLTDRNLNTSFFDPAGGGDPILYQHL
>COLFF712-13|KJ963957|ZMUO.006412|Athous_subfuscus
TLYFLFGAWAGMLGTSLSLLIRAELGNPGSLIGNDQIYNVIVTAHAFIMIFFMVMPIMIGGFGNWLVPLMLGAPDMAFPRMNNMSFWFLPPSLSLLLMSSIVENGAGTGWTVYPPLSANIAHSGSSVDLAIFSLHLAGISSILGAVNFISTVINMRSTGIT
FDRMPLFVWAVAITALLLLLSLPVLAGAITMLLTDRNLNTSFFDPAGGGDPILYQHL
>COLFC250-12|KJ963948|ZMUO.002435|Cryptolestes_corticinus
TLYFIFGSWAGMAGTSLSLLIRTELGTPGSLIGDDQIYNVIVTAHAFIMIFFMVMPIMIGGFGNWLVPLMLGAPDMAFPRMNNMSFWLLPPSLSLLLMSSIVEKGAGTGWTVYPPLSSNIAHGGSSVDLAIFSLHLAGISSILGAVNFISTIINMRPRGMT
LERMPLFVWAVVITAILLLLSLPVLAGAITMLLTDRNINTSFFDPAGGGDPILYQHL
>COLFD323-12|KJ962003|ZMUO.004218|Rhamphus_pulicarius
TLYFLFGGWSGMVGTSLSMLIRTELGNPGKLIGDDQIYNTIVTAHAFIMIFFMVMPIMIGGFGNWLVPLMLGAPDMAFPRLNNMSFWLLPPSLTFLLLSSVMDKGAGTGWTVYPPLSANIAHEGSSVDLAIFSLHMAGVSSILGAMNFISTIINMKPK
NMSMDQMSLFVWSVKITAILLLLSLPVLAGAITMLLTDRNINTSFFDPAGGGDPILYQHL
>COLFD266-12|KJ965682|ZMUO.004161|Agabus_undulatus
TLYFIFGAWAGMVGTSLSMLIRAELGNPGSLIGDDQIYNVIVTAHAFVMIFFMVMPIMIGGFGNWLVPLMLGAPDMAFPRMNNMSFWLLPPSLTLLLMSSMVESGAGTGWTVYPPLSSGIAHGGASVDLAIFSLHLAGISSILGAVNFITTIINMRSV
GMTFDRMPLFVWSVGITALLLLLSLPVLAGAITMLLTDRNLNTSFFDPAGGGDPILYQHL
>LEFIJ1851-13|KJ965581|ZMUO.004606|Rhinoncus_castor
TLYFIFGSWAGTVGTSLSMLIRTELGTPGSLIGNDQIYNSIVTAHAFIMIFFMVMPILIGGFGNWLVPLMLGAPDMAFPRLNNMSFWLLPPSIMLLLMSSIVNKGAGTGWTVYPPLSSNITHEGASVDLAIFSLHMAGISSILGAINFISTIMNMRPQGM
SYDKMPLFSWAVLITAILLLLSLPVLAGAITMLLTDRNINTSFFDPAGGGDPILYQHL
>COLFF844-13|KJ963417|ZMUO.006164|Microcara_testacea
TLYFIFGSWAGMVGTSLSLLIRAELGTPGSLIGDDQIYNVIVTAHAFIMIFFMVMPIMIGGFGNWLVPLMLGAPDMAFPRMNNMSFWLLPPSLTLLLMSSMVENGAGTGWTVYPPLSAGMAHSGASVDLAIFSLHLAGISSILGAVNFISTVINMRSS
GMTFDRMPLFVWSVAITALLLLLSLPVLAGAITMLLTDRNLNTSFFDPAGGGDPILYQHL
>COLFD896-12|KJ965488|ZMUO.004506|Bembidion_guttula
TLYFIFGAWSGMVGTSLSMLIRAELGNPGSLIGDDQIYNVIVTAHAFIMIFFMVMPILIGGFGNWLVPLMLGAPDMAFPRMNNMSFWLLPPSLSLLLMSSMVENGAGTGWTVYPPLSSSIAHSGASVDLAIFSLHLAGVSSILGAVNFITTIINMRSTG
MTFDRMPLFVWSVGITALLLLLSLPVLAGAITMLLTDRNLNTSFFDPAGGGDPILYQHL
>COLFC227-12|KJ962191|ZMUO.002412|Stephostethus_variolosus
SLYFLFGMWSGMVGTSLSLLIRLELGNPGSLIGDDQIYNVIVTAHAFIMIFFMVMPVMMGGFGNWLVPLMLGAPDMAFPRLNNMSFWLLPPSLSLLIMSSIVESGVGTGWTVYPPLSSNIAHGGSSVDLAIFSLHLAGISSILGAVNFITTMINMRPTG
MKFELMPLFAWSVMLTAILLLLSLPVLAGAITMLLTDRNLNTSFFDPAGGGDPILYQHL
>COLFE1069-13|KJ962509|ZMUO.006864|Cantharis_rufa
TLYFIFGAWSGSLGLALSLLIRAELGTPGTLIGNDQIYNVIVTAHAFIMIFFMVMPIMIGGFGNWLVPLMLGAPDMAFPRMNNMSFWFLPPSLMFLLMSSMVESGAGTGWTVYPPLSANIAHSGPSVDLAIFSLHMAGISSILGAVNFISTIMNMKPPS
MKFDQMPLFVWSVGITALLLLLSLPVLAGAITMLLSDRNLNTSFFDPMGGGDPILYQHL

>COLFA629-12|KJ966411|ZMUO.000764|Bisnius_puella
TLYFIFGSWAGMVGTSLSLLIRAELGNPGSLIGDDQIYNVIVTAHAFIMIFFMVMPVVIGGFGNWLVPLMLGAPDMAFPRMNNMSFWLLPPSLTLLLMSSMVESGAGTGWTVYPPLSSNIAHGGASVDLAIFSLHLAGISSILGAVNFITTVINMRSTG
MSFDRMPLFVWSVAITALLLLLSLPVLAGAITMLLTDRNLNTSFFDPAGGGDPILYQHL
>COLFD012-12|KJ965159|ZMUO.003907|Latridius_consimilis
SLYFLFGMWSGMVGTSLSLLIRLELGNPGSLIGDDQIYNVIVTAHAFIMIFFMVMPVMMGGFGNWLVPLMLGAPDMAFPRLNNMSFWLLPPSLSLLIMSSIVESGAGTGWTVYPPLSSNIAHGGSSVDLAIFSLHLAGVSSILGAVNFITTVINMRPSG
MSLEQMPLFVWSVALTAILLLLSLPVLAGAITMLLTDRNLNTSFFDPAGGGDPILYQHL
>COLFC079-12|KJ962924|ZMUO.002074|Cercyon_bifenestratus
TLYFIFGAWAGMVGTSLSILIRAELGNPGTLIGDDQIYNVIVTAHAFIMIFFMVMPIMIGGFGNWLVPLMLGAPDMAFPRMNNMSFWLLPPSLTLLLMSSMVESGAGTGWTVYPPLSSNIAHGGASVDLAIFSLHLAGISSILGAVNFITTVINMRSPN
LSYDRLPLFVWSVAITALLLLLSLPVLAGAITMLLTDRNLNTSFFDPAGGGDPILYQHL
>COLFC748-12|KJ966332|ZMUO.003313|Phyllotreta_tetrastigma
TLYFIFGIWSGMIGMSMSMLIRIELAAPGSLIGNDQIYNVIVTAHAFIMIFFMVMPIMIGGFGNWLIPLMIGAPDMAFPRMNNMSFWLLPPSLFLLVMGSLIENGAGTGWTVYPPLSSNISHAGASVDLTIFSLHLAGISSILGAINFITTVINMRPKGMT
FDRMPLFVWAVLITAILLLLSLPVLAGAITMLLTDRNLNTSFFDPMGGGDPILYQHL
>COLFD663-12|KJ962455|ZMUO.004463|Harpalus_laevipes
TLYFIFGAWAGMVGTSLSMLIRAELGTPGALIGNDQIYNVIVTAHAFIMIFFMVMPIMIGGFGNWLVPLMLGAPDMAFPRMNNMSFWLLPPSLTLLLMSSMVESGAGTGWTVYPPLSSGIAHSGASVDLAIFSLHLAGISSILGAVNFITTIINMRSVG
MTFDRMPLFVWSVGITALLLLLSLPVLAGAITMLLTDRNLNTSFFDPAGGGDPILYQHL
>COLFB135-12|KJ963629|ZMUO.001465|Cordylepherus_viridis
TLYFIFGAWSGMVGLSLSLLIRSELSIPGTLIGNDQIYNVIVTAHAFIMIFFMVMPILIGGFGNWLVPLMLGAPDMAFPRMNNMSFWLLPPSLTLLLLSSMVENGAGTGWTVYPPLSANIAHSGSSVDLAIFSLHLAGISSILGAVNFITTVINMRPQGMT
LDRTPLFVWAVVITALLLLLSLPVLAGAITMLLTDRNLNTSFFDPAGGGDPILYQHL
>COLFC699-12|KJ966535|ZMUO.003264|Pityogenes_chalcographus
TLYFIFGAWSGMVGTSLSMLIRTELGTPGSLIGDDQIFNTIVTAHAFIMIFFMVMPILIGGFGNWLVPLMLGAPDMAFPRLNNMSFWLLPPSLTLLIMSSITDKGAGTGWTVYPPLSSNIAHEGASVDLAIFSLHMSGISSILGAINFISTIINMHPKGVAP
EQLSLFTWAVKITAVLLLLSLPVLAGAITMLLTDRNVNTSFFDPAGGGDPILYQHL
>COLFB031-12|KJ966929|ZMUO.001361|Cytilus_sericeus
TLYFIFGAWAGMVGTSLSLLIRAELGNPGSLIGDDQIYNVIVTAHAFVMIFFMVMPIMIGGFGNWLVPLMLGAPDMAFPRMNNMSFWLLPPSLSLLLMSSIVESGAGTGWTVYPPLSSNIAHSGSSVDLAIFSLHLAGISSILGAVNFISTVINMRSPG
MKFDQMSLFSWSVAITALLLLLSLPVLAGAITMLLTDRNINTSFFDPAGGGDPILYQHL
>COLFA067-10|HM909085|MP00255|Atheta_longicornis
TLYFIFGAWAGMVGTSLSLLIRAELGNPGSLIGDDQIYNVIVTAHAFVMIFFMVMPIVIGGFGNWLVPLMLGAPDMAFPRMNNMSFWLLPPSLTLLLMSSMVESGAGTGWTVYPPLSSNIAHGGASVDLAIFSLHLAGISSILGAVNFISTVINMRSTG
ISFDRMPLFVWSVAITALLLLLSLPVLAGAITMLLTDRNLNTSFFDPAGGGDPILYQHL
>COLFB304-12|KJ964799|ZMUO.001634|Stenus_formicetorum
VLYFIFGAWAGMVGTSLSLLIRTELGNPGSLIGDDQIYNVIVTAHAFVMIFFMVMPLMIGGFGNWLVPLMLGAPDMAFPRMNNMSFWLLPPSLSLLLMSSIVENGAGTGWTVYPPLSTNIAHSGASVDLAIFSLHLAGISSILGAINFITTIFNMRSMKL
QLDCLPLFIWSVEITALLLLLSLPVLAGAITMLLTDRNLNTSFFDPAGGGDPILYQHL
>COLFF064-13|KJ967013|ZMUO.005764|Cercyon_tristis
TLYFIFGAWAGMVGTSLSILIRAELGNPGTLIGDDQIYNVIVTAHAFIMIFFMVMPIMIGGFGNWLVPLMLGAPDMAFPRMNNMSFWLLPPSLTLLLMSSMVESGAGTGWTVYPPLSSNIAHGGSSVDLAIFSLHLAGISSILGAVNFITTVINMRSPNL
TYDRLPLFVWSVAITALLLLLSLPVLAGAITMLLTDRNLNTSFFDPAGGGDPILYQHL
>COLFC055-12|KJ964611|ZMUO.002050|Enochrus_bicolor
TLYFIFGAWAGMVGTSLSILIRAELGNPGTLIGDDQIYNVIVTAHAFIMIFFMVMPIMIGGFGNWLVPLMLGAPDMAFPRMNNMSFWLLPPSLTLLLMSSMVESGAGTGWTVYPPLSSNIAHGGASVDLAIFSLHLAGISSILGAVNFITTVINMRSPS
MTYDRLPLFVWSVAITALLLLLSLPVLAGAITMLLTDRNLNTSFFDPAGGGDPILYQHL
>COLFE1061-13|KJ963015|ZMUO.006856|Hydroporus_tristis
TLYFLFGAWSGMVGTSLSMLIRAELGNPGSLIGDDQIYNVIVTAHAFIMIFFMVMPIMIGGFGNWLVPLMLGAPDMAFPRMNNMSFWLLPPSLTLLLMSSMVENGAGTGWTVYPPLSSGIAHSGASVDLAIFSLHLAGVSSILGAVNFITTIINMRSI
GMTFDRMPLFVWSVGITALLLLLSLPVLAGAITMLLTDRNLNTSFFDPAGGGDPILYQHL
>COLFB440-12|KJ962352|ZMUO.001865|Olibrus_aeneus
TLYFIFGAWAGMVGTSLSLLIRTELGTPGSLIGDDQIYNVIVTAHAFVMIFFMVMPFMIGGFGNWLVPLMLGAPDMAFPRMNNMSFWLLPPSLFLLLMSSMVESGAGTGWTVYPPLSSNIAHGGASVDLAIFSLHLAGISSILGAINFITTMINMRPE
GMTLDRMPLFVWAVTITAILLLLSLPVLAGAITMLLTDRNINSSFFDPAGGGDPILYQHL
>COLFC771-12|KJ962424|ZMUO.003336|Rhizophagus_grandis
TLYFIFGAWAGMVGTSLSLLIRAELGTPGQLIGDDQIYNVIVTAHAFIMIFFMVMPILIGGFGNWLVPLMLGAPDMAFPRMNNMSFWLLPPSLTLLIMSSIVENGAGTGWTVYPPLSSNIAHSGSSVDLAIFSLHLAGISSILGAVNFITTVINMRPSGMT
LDRTPLFVWAVIITATLLLLSLPVLAGAITMLLTDRNFNTSFFDPAGGGDPILYQHL
>COLFC364-12|KJ964754|ZMUO.002549|Cryptophagus_badius

TLYFIFGAWAGMVGTSLSLLIRSELGTPGSLIGDDQIYNVIVTAHAFVMIFFMVMPIMIGGFGNWLVPLMLGAPDMAFPRMNNMSFWLLPPSLSLLLMSSIAEKGVGTGWTVYPPLSSNIAHGGSSVDLAIFSLHLAGISSILGAVNFISTVMNMHPTG
MNLDRMPLFVWAVIITAILLLLSLPVLAGAITMLLTDRNLNTSFFDPAGGGDPILYQHL
>COLFB056-12|KJ963363|ZMUO.001386|Cercyon_unipunctatus
TLYFIFGAWAGMVGTSLSILIRAELGNPGTLIGDDQIYNVIVTAHAFIMIFFMVMPIMIGGFGNWLVPLMLGAPDMAFPRMNNMSFWLLPPSLTLLLMSSMVESGAGTGWTVYPPLSSNIAHGGSSVDLAIFSLHLAGISSILGAVNFITTVINMRSPNL
TYDRLPLFVWSVAITALLLLLSLPVLAGAITMLLTDRNLNTSFFDPAGGGDPILYQHL
>COLFB044-12|KJ961840|ZMUO.001374|Silpha_carinata
TLYFIFGAWAGVVGMSLSILIRMELSTPGSLLGDDQMYNVIVTAHAFIMIFFMVMPIVIGGFGNWLVPLMLGAPDMAFPRMNNMSFWLLPPSLSLLLVSSMVESGAGTGWTVYPPLSSNIAHGGSSVDLAIFSLHLAGVSSILGAVNFITTIINMRSSG
MTFDRMPLFVWSVGITALLLLLSLPVLAGAITMLLTDRNLNTSFFDPAGGGDPILYQHL
>COLFD063-12|KJ967364|ZMUO.003958|Mycetochara_flavipes
TLYFMFGAWSGMVGTSLSLLIRAELGNPGSLIGDDQIYNVIVTAHAFIMIFFMVMPIMIGGFGNWLVPLMLGAPDMAFPRMNNMSFWLLPPSLTLLLMSSIVESGAGTGWTVYPPLSSNIAHGGASVDLAIFSLHLAGISSILGAVNFITTVINMKPQG
MTFDRMPLFVWAVVITAVLLLSLPVLAGAITMLLTDRNINTSFFDPAGGGDPILYQHL
>COLFG127-13|KJ966258|ZMUO.007822|Cypha_tarsalis
ILYFIFGAWSGMIGTSLSLLIRAELGNPGSLIGDDQIYNVIVTSHAFIMIFFMVMPIMIGGFGNWLVPLMLSAPDMAFPRMNNMSFWLLPPALTLLLMSSMVESGSGTGWTVYPPLSSNIAHGGASVDLTIFSLHLAGISSILGAVNFISTVINMRPMGM
SFDRMPLFVWSVVITALLLLLSLPVLAGAITMLLTDRNLNTSFFDPAGGGDPILYQHL
>COLFB353-12|KJ963104|ZMUO.001683|Mycetoporus_clavicornis
TLYFIFGAWAGMVGTSLSLLIRAELGNPGSFIGDDQIYNVIVTAHAFVMIFFMVMPIVIGGFGNWLVPLMLGAPDMAFPRMNNMSFWLLPPSLTLLLMSSMVESGAGTGWTVYPPLSANIAHSGASVDLAIFSLHLAGISSILGAVNFITTVINMRSPG
MTFDRMPLFIWSVAITALLLLLSLPVLAGAITMLLTDRNLNTSFFDPAGGGDPILYQHL
>COLFE1182-13|KJ963926|ZMUO.006977|Ceutorhynchus_querceti
TLYFIFGSWAGMAGTSLSVLIRTELGNPGKLIGNDQIYNSIVTAHAFIMIFFMVMPILIGGFGNWLIPLMLGAPDMAFPRLNNMSFWLLPPSLSLLLMSSMINKGVGTGWTVYPPLSSNVAHEGMSVDLAIFSLHMAGISSILGAINFISTIMNMQPKG
MTPELMPLFVWAVQITAILLLLSLPVLAGAITMLLTDRNINTSFFDPSGGGDPILYQHL
>COLFE530-13|KJ965216|ZMUO.005375|Psyllobora_vigintiduopunctata
TLYFLFGMWAGMVGTSLSILIRLELGTTNSVIGNDQIYNVIVTAHAFIMIFFMVMPIMIGGFGNWLVPLMVGAPDMAFPRLNNMSFWLLPPALTLLIFSSMVEMGAGTGWTVYPPLSSNMAHSGSSVDLVIFSLHLAGISSILGAVNFISTIMNMRPF
GMNLDKTPLFVWSVLITAILLLLSLPVLAGAITMLLTDRNINTSFFDPMGGGDPILYQHL
>COLFD745-12|KJ965828|ZMUO.004735|Magdalis_carbonaria
TLYLIFGAWSGMTGTSLSMLIRVELGNPGSLIGNDQIYNTIVTAHAFIMIFFMVMPIMIGSFGNWLIPLMLGAPDMAFPRLNNMSFWLLPPSLTLLLMSSIIDKGAGTGWTVYPPLSSNIAHEGSSIDLAIFSLHLAGISSILGALNFISTIINMRPTNMKLD
QMSLFIWAVKITAILLLLSLPVLAGAITMLLTDRNINTSFFDPAGGGDPILYQHL
>COLFG007-13|KJ962493|ZMUO.007512|Acmaeops_septentrionis
MLYFIFGAWAGMVGTSLSLLIRSELGNPGSLIGNDQIYNVIVTAHAFIMIFFMVMPVMIGGFGNWLVPLMLGAPDMAFPRLNNMSFWLLPPSLTLLIMSSVVESGAGTGWTVYPPLSSNIAHGGSSVDLAIFSLHLAGISSILGAINFITTVINMRPKGM
TFDRMPLFVWAVVITAILLLLSLPVLAGAITMLLTDRNLNTSFFDPAGGGDPILYQHL
>COLFD814-12|KJ967504|ZMUO.004804|Sepedophilus_marshami
TLYFIFGMWAGMVGTSLSLLIRAELGNPGSLIGDDQIYNVIVTAHAFIMIFFMVMPVIIGGFGNWLVPLMLGAPDMAFPRMNNMSFWLLPPSLSLLLMSSLVESGAGTGWTVYPPLSSNIAHGGSSVDLAIFSLHLAGISSILGAVNFITTVINMRSTG
MTFDRMPLFVWSVAITALLLLLSLPVLAGAITMLLTDRNLNTSFFDPAGGGDPILYQHL
>COLFB361-12|KJ966468|ZMUO.001691|Crepidodera_fulvicornis
TLYFIFGIWSGMVGTSLSILIRTELGSPGSLIGNDQIYNVIVTAHAFIMIFFMVMPIMIGGFGNWLIPLMIGAPDMAFPRMNNMSFWLLPPSLFLLLMSSLVESGAGTGWTVYPPLSSNIAHGGSSVDLAIFSLHLAGISSILGAINFITTIINMRPKGMNLD
RMPLFVWAVAITAILLLLSLPVLAGAITMLLTDRNMNTSFFDPIGGGDPILYQHL
>COLFD307-12|KJ965852|ZMUO.004202|Stenus_strandi
TLYFIFGAWAGMVGTSLSLLIRAELGNPGSLIGDDQIYNVIVTAHAFVMIFFMVMPVMIGGFGNWLVPLMLGAPDMAFPRMNNMSFWLLPPSLSLLLMSSIVENGAGTGWTVYPPLSSNIAHSGASVDLAIFSLHLAGISSILGAINFITTIFNMRTMK
LHLDCLPLFVWSVGITALLLLLSLPVLAGAITMLLTDRNLNTSFFDPAGGGDPILYQHL
>COLFB516-12|KJ964426|ZMUO.000991|Cercyon_terminatus
TLYFIFGAWAGMVGTSLSILIRAELGNPGTLIGDDQIYNVIVTAHAFIMIFFMVMPIMIGGFGNWLVPLMLGAPDMAFPRMNNMSFWLLPPSLTLLLMSSMVESGAGTGWTVYPPLSSNIAHGGSSVDLAIFSLHLAGISSILGAVNFITTVINMRSPSL
TYDRLPLFVWSVAITALLLLLSLPVLAGAITMLLTDRNLNTSFFDPAGGGDPILYQHL
>COLFD345-12|KJ965811|ZMUO.004240|Trachys_minutus
TLYFIFGAWSGMVGTALSLLIRAELGNPGALIGNDQIYNVIVTAHAFIMIFFMVMPIMMGGFGNWLVPLMLGAPDMAFPRMNNMSFWLLPPSLSLLLMSSMVESGAGTGWTVYPPLAANIAHSGASVDLAIFSLHLAGISSILGAINFITTVINMRAY
GMTLDQMPLFVWSVAITALLLLLSLPVLAGAITMLLTDRNLNTSFFDPVGGGDPVLYQHL
>COLFC311-12|KJ965805|ZMUO.002496|Acrulia_inflata
TLYFIFGAWSGMVGTSLSMLIRAELGNPGSLIGDDQIYNVIVTAHAFIMIFFMVMPIVIGGFGNWLVPLMLGAPDMAFPRMNNMSFWLLPPSLTLLLMSSMVESGAGTGWTVYPPLSSNIAHGGSSVDLAIFSLHLAGISSILGAVNFITTVINMRSTG
MTFDRMPLFVWSVAITALLLLLSLPVLAGAITMLLTDRNLNTSFFDPAGGGDPILYQHL

>COLFA042-10|HM909066|MP00200|Acanthocinus_aedilis
TLYFIFGAWSGMVGTSLSMLIRSELGNPGTLIGNDQIYNTIVTAHAFIMIFFMVMPIMIGGFGNWLTPLMLGAPDMAFPRMNNMSFWLLPPSLTLLIMSSIVENGAGTGWTVYPPLSSNIAHSGSSVDLAIFSLHLAGISSILGAVNFITTIINMRPMGM
TFDRMPLFVWAVKITAILLLLSLPVLAGAITMLLTDRNINTSFFDPAGGGDPILYQHL
>COLFF711-13|KJ966304|ZMUO.006411|Cryptolestes_duplicatus
TLYFIFGSWAGMAGTSLSLLIRTELGMPGSLIGDDQIYNVIVTAHAFIMIFFMVMPIMIGGFGNWLVPLMLGAPDMAFPRMNNMSFWLLPPSLSLLLMSSIVEKGAGTGWTVYPPLSSNIAHGGSSVDLAIFSLHLAGISSILGAVNFISTVINMRPQG
MTLERMPLFVWAVVITAILLLLSLPVLAGAITMLLTDRNINTSFFDPAGGGDPILYQHL
>COLFE030-12|KJ965809|ZMUO.005255|Philonthus_rectangulus
TLYFIFGSWAGMVGTSLSLLIRAELGNPGTLIGDDQIYNVIVTAHAFIMIFFMVMPIVIGGFGNWLVPLMLGAPDMAFPRMNNMSFWLLPPSLTLLLMSSMVESGAGTGWTVYPPLSSNIAHGGASVDLAIFSLHLAGISSILGAVNFITTVINMRSTG
MTFDRMPLFVWSVAITALLLLLSLPVLAGAITMLLTDRNLNTTFFDPAGGGDPILYQHL
>COLFC318-12|KJ962608|ZMUO.002503|Liogluta_alpestris
TLYFIFGAWAGMIGTSLSLLIRAELGNPGSLIGDDQIYNVIVTAHAFIMIFFMVMPVVIGGFGNWLVPLMLGAPDMAFPRMNNMSFWLLPPSLTLLLMSSMVESGAGTGWTVYPPLSSNIAHGGSSVDLAIFSLHLAGISSILGAVNFISTVINMRSVGI
SFDRMPLFVWSVAITALLLLLSLPVLAGAITMLLTDRNLNTSFFDPAGGGDPILYQHL
>COLFB201-12|KJ967176|ZMUO.001531|Ilybius_chalconatus
TLYFIFGAWAGMVGTSLSMLIRAELGNPGSLIGDDQIYNVIVTAHAFVMIFFMVMPIMIGGFGNWLVPLMLGAPDMAFPRMNNMSFWLLPPSLSLLLMSSMVESGAGTGWTVYPPLSSGIAHSGASVDLAIFSLHLAGISSILGAVNFITTIINMRSV
GMTFDRMPLFVWSVGITALLLLLSLPVLAGAITMLLTDRNLNTSFFDPAGGGDPILYQHL
>COLFC437-12|KJ964476|ZMUO.003002|Boreaphilus_henningianus
TLYFIFGAWAGMVGTSLSILIRAELGNPGSLIGDDQIYNVIVTAHAFVMIFFMVMPIVIGGFGNWLVPLMLGAPDMAFPRMNNMSFWLLPPSLNLLLMSSMVESGAGTGWTVYPPLSSNIAHGGSSVDLAIFSLHLAGVSSILGAVNFITTVINMRSTG
MSFDRMPLFVWSVAITALLLLLSLPVLAGAITMLLTDRNLNTSFFDPAGGGDPILYQHL
>COLFE391-12|KJ965547|ZMUO.004951|Cionus_hortulanus
TLYFIFGMWSGTVGTSLSMLIRTELGNPGSLIGDDQIYNTIVTAHAFIMIFFMVMPIMIGGFGNWLVPLMLGAPDMAFPRLNNMSFWLLPPSITLLLMSSIVEKGAGTGWTVYPPLSNNVTHEGASVDLAIFSLHMAGISSILGAINFISTVSNMRTKG
MDYDRTPLFVWSVNITAFLLLLSLPVLAGAITMLLTDRNINTSFFDPSGGGDPILYQHL
>COLFD270-12|KJ961788|ZMUO.004165|Haliplus_ruficollis
TLYFIFGAWAGMVGTSLSMLIRAELGTPGSLIGDDQIYNVIVTAHAFIMIFFMVMPIMIGGFGNWLVPLMLGAPDMAFPRMNNMSFWLLPPSLSLLLMSSMVENGAGTGWTVYPPLSAGIAHSGASVDLAIFSLHLAGISSILGAVNFITTIINMRSIG
MTFDRMPLFVWSVGITALLLLLSLPVLAGAITMLLTDRNLNTSFFDPAGGGDPILYQHL
>COLFD500-12|KJ964771|ZMUO.004300|Geotrupes_stercorosus
TLYFLFGSWAGMVGTSLSILIRAELGNPGSLIGDDQIFNVIVTAHAFVMIFFMVMPIMIGGFGNWLVPLMLGAPDMAFPRMNNMSFWLLPPSLTLLLMSSLVESGAGTGWTVYPPLSANIAHSGASVDLAIFSLHLAGISSILGAVNFIATVINMRSSG
MTFDRMPLFVWAVVLTALLLLLSLPVLAGAITMLLTDRNLNTSFFDPAGGGDPILYQHL
>COLFE294-12|KJ967506|ZMUO.004854|Psyllobora_vigintiduopunctata
TLYFLFGMWAGMVGTSLSILIRLELGTTNSVIGNDQIYNVIVTAHAFIMIFFMVMPIMIGGFGNWLVPLMVGAPDMAFPRLNNMSFWLLPPALTLLIFSSMVEMGAGTGWTVYPPLSSNMAHSGSSVDLVIFSLHLAGISSILGAVNFISTIMNMRPF
GMNLDKTPLFVWSVLITAILLLLSLPVLAGAITMLLTDRNINTSFFDPMGGGDPILYQHL
>COLFB876-12|KJ966600|ZMUO.001921|Callidium_aeneum
TLYFIFGAWASMVGTSLSMLIRTELGNPGSLIGDDQIYNVIVTAHAFIMIFFMVMPIMIGGFGNWLVPLMLGAPDMAFPRMNNLSFWLLPPSLTLLILSSIVENGAGTGWTVYPPLSANIAHGGSSVDLAIFSLHLAGISSILGAVNFISTIINMRPTGMTL
ERMPLFVWAVVITAILLLLSLPVLAGAITMLLTDRNINTSFFDPAGGGDPILYQHL
>COLFB023-12|KJ966753|ZMUO.001353|Necrodes_littoralis
TLYFIFGAWAGMVGMSLSILIRAELSTPGTLLGDDQMYNVIVTAHAFIMIFFMVMPIVIGGFGNWLVPLMLGAPDMAFPRMNNMSFWLLPPSLSLLLVSSMVESGAGTGWTVYPPLSSNIAHGGSSVDLAIFSLHLAGISSILGAVNFITTIINMRSSG
MTFDRMPLFVWSVAITALLLLLSLPVLAGAITMLLTDRNLNTSFFDPAGGGDPILYQHL
>COLFC656-12|KJ962652|ZMUO.003221|Elaphrus_riparius
TLYFIFGAWSGMVGTSLSMLIRAELGNPGSLIGDDQIYNVIVTAHAFIMIFFMVMPIMIGGFGNWLVPLMLGAPDMAFPRMNNMSFWLLPPSLSLLLMSSMVESGAGTGWTVYPPLSSGIAHAGASVDLAIFSLHLAGVSSILGAVNFITTIINMRSV
GMSFDRMPLFVWSVGITALLLLLSLPVLAGAITMLLTDRNLNTSFFDPAGGGDPILYQHL
>COLFB887-12|KJ964737|ZMUO.001932|Asemum_striatum
TLYFIFGAWSGMVGTSLSMLIRSELGNPGSLIGDDQIYNVIVTAHAFIMIFFMVMPIMIGGFGNWLVPLMLGAPDMAFPRMNNMSFWLLPPSLTLLIMSSIVENGAGTGWTVYPPLSANIAHSGSSVDLAIFSLHLAGISSILGAVNFITTVINMRPKG
MMLDRMPLFVWAVVITAILLLLSLPVLAGAITMLLTDRNLNTSFFDPIGGGDPILYQHL
>COLFE783-13|KJ962668|ZMUO.005628|Hydroporus_pubescens
TLYFLFGAWSGMVGTSLSMLIRAELGNPGSLIGDDQIYNVIVTAHAFIMIFFMVMPIMIGGFGNWLVPLMLGAPDMAFPRMNNMSFWLLPPSLTLLLMSSMVENGAGTGWTVYPPLSSGIAHSGASVDLAIFSLHLAGVSSILGAVNFITTIINMRSI
GMTFDRMPLFVWSVGITALLLLLSLPVLAGAITMLLTDRNLNTSFFDPAGGGDPILYQHL
>COLFE839-13|KJ966488|ZMUO.005684|Anthonomus_rubi

TLYFIFGAWSGAVGTSLSMLIRTELGNPGSLIGDDQIYNTIVTAHAFIMIFFMVMPIMIGGFGNWLIPLMLAAPDMAFPRLNNMSFWLLPPSLTLLIMSSIISKGAGTGWTVYPPLSSNLAHEGASVDFAIFSLHMAGISSILGAMNFISTILNMKPMKM
KFEQMPLFIWAVKITAILLLISLPVLAGAITMLLTDRNINTSFFDPAGGGDPILYQHL
>COLFE865-13|KJ966083|ZMUO.006185|Melandrya_dubia
TLYFIFGAWSGMIGTSMSLLIRSELGNPGSLIGDDQIYNVIVTAHAFIMIFFMVMPIMMGGFGNWLVPLMLGAPDMAFPRMNNMSFWLLPPSLSLLLMSSIVENGAGTGWTVYPPLASNIAHNGSSVDLTIFSLHLAGVSSILGAVNFITTIINMRPIG
MTFDRMPLFVWAVGITAVLLLLSLPVLAGAITMLLTDRNLNTSFFDPAGGGDPILYQHL
>COLFB661-12|KJ965788|ZMUO.001136|Helophorus_nanus
TLYFIFGAWAGMVGTSLSILIRAELGNPGTLIGDDQIYNVIVTAHAFIMIFFMVMPIMIGGFGNWLVPLMLGAPDMAFPRMNNMSFWLLPPSLTLLLMSSMVESGAGTGWTVYPPLSSNIAHSGASVDLAIFSLHLAGISSILGAVNFITTVINMRSAN
MTYDRLPLFVWSVAITALLLLLSLPVLAGAITMLLTDRNLNTSFFDPAGGGDPILYQHL
>COLFE984-13|KJ961987|ZMUO.006779|Gyrinus_aeratus
TLYFIFGAWSGMVGTSLSMLIRAELGNPGSLIGDDQIYNVIVTAHAFIMIFFMVMPIMIGGFGNWLVPLMLGAPDMAFPRMNNMSFWLLPPSLTLLLMSSMVENGAGTGWTVYPPLSSNIAHGGASVDLAIFSLHLAGISSILGAVNFITTIINMRSIG
MTLDRMPLFVWSVGITALLLLLSLPVLAGAITMLLTDRNLNTSFFDPAGGGDPILYQHL
>COLFF587-13|KJ963302|ZMUO.006477|Olibrus_millefolii
TLYFIFGAWASMVGTSLSLLIRTELGTPGSLIGDDQIYNVIVTAHAFVMIFFMVMPFMIGGFGNWLVPLMLGAPDMAFPRMNNMSFWLLPPSLFLLLMSSMVESGAGTGWTVYPPLSSNIAHGGASVDLAIFSLHLAGISSILGAINFITTMINMRPEG
MTLDRMPLFVWAVTITAILLLLSLPVLAGAITMLLTDRNINTSFFDPAGGGDPILYQHL
>COLFE385-12|KJ966854|ZMUO.004945|Oedemera_virescens
TLYLIFGAWAGMVGTSLSLLIRAELGNPGSLIGDDQIYNVIVTAHAFIMIFFMVMPIVIGGFGNWLVPLMLSAPDMAFPRMNNMSFWLLPPSLTLLIMSSMVESGAGTGWTVYPPLSSNIAHSGSSVDLAIFSLHLAGVSSILGAVNFITTVINMRPVG
MTLDRMPLFVWAVVITAILLLLSLPVLAGAITMLLTDRNLNTSFFDPAGGGDPILYQHL
>COLFE418-12|KJ967124|ZMUO.004978|Galerucella_calmariensis
TLYFIFGIWAGMVGTSLSILVRAELGSPGTLIGNDQIYNVIVTAHAFIMIFFMVMPIMIGGFGNWLVPLMIGAPDMAFPRMNNMSFWLLPPSLFLLIMSSIVESGAGTGWTVYPPLSSNIAHGGSSVDLAIFSLHLAGISSILGAINFITTIINMRPKGMTL
DRMPLFVWAVMITAILLLLSLPVLAGAITMLLTDRNLNTSFFDPAGGGDPILYQHL
>COLFA579-12|KJ962659|ZMUO.000714|Protaetia_cuprea
TLYFLFGSWAGMVGTSLSLLIRAELGNPGSLIGDDQIYNVIVTAHAFIMIFFMVMPIMIGGFGNWLVPLMLGAPDMAFPRMNNMSFWLLPPSLTLLLMSSVVESGAGTGWTVYPPLSSNIAHSGASVDLAIFSLHLAGISSILGAVNFITTVINMRSTG
MTFDRMPLFVWSVALTALLLLLSLPVLAGAITMLLTDRNINTSFFDPAGGGDPILYQHL
>COLFE1609-13|KJ962188|ZMUO.007499|Plagiosterna_aenea
TLYFIFGIWAGMVGTSLSMLIRTELGNPGSLIGNDQIYNVIVTAHAFIMIFFMVMPIMIGGFGNWLVPLMIGAPDMAFPRLNNMSFWLLPPSLFLLLMSSVVESGAGTGWTVYPPLSANITHSGSSVDLAIFSLHLAGISSILGAINFITTVINMRPEGMN
FEQTPLFVWAVLITAILLLLSLPVLAGAITMLLTDRNLNTSFFDPAGGGDPILYQHL
>COLFE055-12|KJ967071|ZMUO.005280|Bolitophagus_reticulatus
TLYFIFGAWAGMVGTSLSLLIRTELGNPGSLIGDDQIYNVIVTAHAFVMIFFMVMPIMIGGFGNWLVPLMLGAPDMAFPRMNNMSFWLLPPSLTLLLMSSIVENGAGTGWTVYPPLSSNIAHSGASVDLAIFSLHLAGISSILGAVNFITTVINMRPQG
MTLDRMPLFVWAVVITALLLLLSLPVLAGAITMLLTDRNINTSFFDPAGGGDPILYQHL
>COLFE1395-13|KJ964604|ZMUO.007190|Ceutorhynchus_punctiger
TLYFIFGSWAGMTGTSLSMLIRTELGNPGMLIGDDQIYNSIVTAHAFIMIFFMVMPILIGGFGNWLVPLMLGAPDMAFPRLNNMSFWLLPPSLSLLLMSSIVNKGAGTGWTVYPPLSMNVAHEGVSVDLAIFSLHMAGISSILGAINFISTVMNMRPT
GMTAEYMPLFVWAVEITAILLLISLPVLAGAITMLLTDRNINTSFFDPTGGGDPILYQHL
>COLFD184-12|KJ962248|ZMUO.004079|Ocalea_picata
TLYFIFGAWAGMVGTSLSLLIRAELGNPGSLIGDDQIYNVIVTAHAFVMIFFMVMPIMIGGFGNWLVPLMLGAPDMAFPRMNNMSFWLLPPSLTLLLMSSMVESGAGTGWTVYPPLSSNIAHGGASVDLAIFSLHLAGISSILGAVNFISTIINMRTSG
MTFDRMPLFVWSVAITALLLLLSLPVLAGAITMLLTDRNLNTSFFDPAGGGDPILYQHL
>COLFD033-12|KJ965119|ZMUO.003928|Actenicerus_sjaelandicus
TLYFLFGAWAGMLGTSLSLLIRAELGNPGSLIGNDQIYNVIVTAHAFIMIFFMVMPIMIGGFGNWLVPLMLGAPDMAFPRMNNMSFWFLPPSLSLLLMSSIVENGAGTGWTVYPPLSANIAHSGSSVDLAIFSLHLAGISSILGAVNFISTVINMRSTGIT
FDRMPLFVWAVAITALLLLLSLPVLAGAITMLLTDRNLNTSFFDPAGGGAPVLYQHL
>COLFD687-12|KJ962385|ZMUO.004677|Pissodes_validirostris
TLYFIFGAWSGMIGTSLSILIRTELGTPGTMIGDDQIYNTIVTAHAFIMIFFMVMPIMIGGFGNWLVPLMLGAPDMAFPRLNNMSFWLLPPSIMLLLMSSIVDKGAGTGWTVYPPLSTNIAHEGPSVDLAIFSLHMAGISSILGAVNFISTVVNMRPTG
MNSDQMSLFIWAVKITAILLLLSLPVLAGAITMLLTDRNINTSFFDPAGGGDPILYQHL
>COLFB230-12|KJ966973|ZMUO.001560|Xylita_laevigata
TLYFIFGAWSSMLGTSLSLLIRSELGNPSSLIGDDQIYNVIVTAHAFIMIFFMVMPILIGGFGNWLMPLMLGAPDMAFPRMNNMSFWLLPPSLILLIMSSIVEKGAGTGWTVYPPLSSNIAHNGSSVDLAIFSLHLAGISSILGAINFITTVINMRPKGMTLD
RMPLFVWSVMLTAILLLLSLPVLAGAITMLLTDRNLNTSFFDPAGGGDPVLYQHL
>COLFG132-13|KJ964069|ZMUO.007827|Bryaxis_puncticollis
TLYLIFGFWSGMIGTSLSILIRIQLMSPKMLIENTQIYNTIITTHAFVMIFFMIMPVLIGGFGNWLIPLMSGTPDMSFPRLNNFSFWLLPPSLILLLSSMITEHGTGTGWTIYPPLSMNMAHSSPSTDLTIFSLHMTGISSIVSSMNFITTIINMRSKGMYFNF
MPMFSWSLITTMMLLLLSLPILAGAITMLLTDRNLNTSFFDPAGGGDPVMYQHM

>COLFD796-12|KJ964875|ZMUO.004786|Haliplus_immaculatus
TLYFIFGAWSGMVGTSLSMLIRAELGTPGSLIGDDQIYNVIVTAHAFIMIFFMVMPIMIGGFGNWLVPLMLGAPDMAFPRMNNMSFWLLPPSLSLLLMSSMVENGAGTGWTVYPPLSAGIAHSGASVDLAIFSLHLAGISSILGAVNFITTIINMRSM
GMTFDRMPLFVWSVGITALLLLLSLPVLAGAITMLLTDRNLNTSFFDPAGGGDPILYQHL
>COLFF307-13|KJ963382|ZMUO.006007|Gonioctena_viminalis
TLYFIFGIWAGMVGTSLSILIRAELGNPGTLIGNDQIYNVIVTAHAFIMIFFMVMPIMIGGFGNWLVPLMIGAPDMAFPRMNNMSFWLLPPSLFFLIMSSVVESGAGTGWTVYPPLSANIAHSGSSVDLAIFSLHLAGISSILGAINFITTIINMRPTGMS
MDRMPLFVWAVLITAILLLLSLPVLAGAITMLLTDRNLNTSFFDPAGGGDPILYQHL
>COLFD149-12|KJ962320|ZMUO.004044|Protapion_gracilipes
TLYFIFGLWSGMIGTSLSMLIRIELGNPGSLIGNDQIYNVIVTAHAFIMIFFMVMPVMIGGFGNWLVPLMLGAPDMAFPRMNNMSFWLLPPSLTLLLMSSIVEKGAGTGWTVYPPLAANIAHSGASVDLAIFSLHLAGVSSILGAVNFISTIMNMRPTG
MSLDQLSLFTWAVKITAILLLLSLPVLAGAITMLLTDRNINTSFFDPAGGGDPILYQHL
>COLFB107-12|KJ965637|ZMUO.001437|Chrysolina_varians
TLYFIFGMWAGMVGTSLSILIRAELGNPGTLIGNDQIYNVIVTAHAFIMIFFMVMPIMIGGFGNWLVPLMLGAPDMAFPRMNNMSFWLLPPSLIFLLMSSIVENGVGTGWTVYPPLSANIAHSGPSVDLAIFSLHLAGVSSILGAINFITTIINMRPAGM
QLEQMPLFSWAVLITTILLLLSLPVLAGAITMLLTDRNLNTSFFDPASGGDPILYQHL
>COLFB036-12|KJ964465|ZMUO.001366|Philonthus_subvirescens
TLYFIFGSWAGMVGTSLSLLIRAELGNPGTLIGDDQIYNVIVTAHAFIMIFFMVMPIVIGGFGNWLVPLMLGAPDMAFPRMNNMSFWLLPPSLTLLLMSSLVESGAGTGWTVYPPLSSNMAHGGASVDLAIFSLHLAGISSILGAVNFITTVINMRSTG
MNFDRMPLFVWSVIITALLLLLSLPVLAGAITMLLTDRNINTTFFDPAGGGDPILYQHL
>COLFC644-12|KJ961797|ZMUO.003209|Agabus_infuscatus
TLYFIFGAWAGMVGTSLSMLIRAELGNPGSLIGDDQIYNVIVTAHAFVMIFFMVMPIMIGGFGNWLVPLMLGAPDMAFPRMNNMSFWLLPPSLTLLLMSSMVESGAGTGWTVYPPLSSGIAHGGASVDLAIFSLHLAGISSILGAVNFITTIINMRSV
GMTFDRMPLFVWSVGITALLLLLSLPVLAGAITMLLTDRNLNTSFFDPAGGGDPILYQHL
>COLFF590-13|KJ964518|ZMUO.006480|Sitona_ambiguus
TLYFIFGAWAGMVGTSLSVLIRTELGNPGSLIGDDQIYNVIVTAHAFIMIFFMVMPMMIGGFGNWLVPLMLGAPDMAFPRLNNMSFWLLPPSLTLLLMSSIVDKGAGTGWTVYPPLSSNIAHEGASVDLAIFSLHMAGISSILGAINFISTVINMRPSG
MTMERVTLFVWAVKITAILLLLSLPVLAGAITMLLTDRNINTSFFDPAGGGDPILYQHL
>COLFE1117-13|KJ965543|ZMUO.006912|Chrysolina_marginata
TLYFIFGTWAGMVGTSLSILIRAELGNPGSLIGNDQIYNVIVTAHAFIMIFFMVMPIMIGGFGNWLVPLMLGAPDMAFPRMNNMSFWLLPPSLIFLLMSSIVENGAGTGWTVYPPLSANVAHSGPSVDLAIFSLHLAGISSILGAINFITTVINMRPTGM
KLEQMPLFSWAVLITAILLLLSLPVLAGAITMLLTDRNLNTSFFDPASGGDPILYQHL
>COLFB306-12|KJ964372|ZMUO.001636|Atheta_palustris
TLYFIFGAWAGMVGTSLSLLIRAELGNPGSLIGDDQIYNVIVTAHAFIMIFFMVMPIVIGGFGNWLVPLMLGAPDMAFPRMNNMSFWLLPPSLTLLLMSSMVESGAGTGWTVYPPLSSNIAHGGSSVDLAIFSLHLAGISSILGAVNFISTVINMRSTGI
SFDRMPLFVWSVAITALLLLLSLPVLAGAITMLLTDRNLNTSFFDPAGGGDPILYQHL
>COLFE1143-13|KJ966513|ZMUO.006938|Colon_serripes
TLYFIFGAWAGMVGTSLSLLIRAELGNPGSLIGDDQIYNVIVTAHAFIMIFFMVMPIVIGGFGNWLVPLMLGAPDMAFPRMNNMSFWLLPPSLTLLLMSSLVESGAGTGWTVYPPLSSNIAHGGSSVDLAIFSLHLAGISSILGAVNFITTIINMRSQGM
GFDQMPLFVWSVIITAFLLLLSLPVLAGAITMLLTDRNLNTSFFDPAGGGDPILYQHL
>COLFG036-13|KJ967213|ZMUO.007541|Hoshihananomia_perlata
TLYFIFGAWAGMVGTSLSLLIRAELGNPGSLIGDDQIYNTIVTAHAFVMIFFMVMPIMVGGFGNWLVPLMLGAPDMAFPRMNNMSFWLLPPSLSLLLMSSIVENGAGTGWTVYPPLSSNIAHGGSSVDLAIFSLHLAGVSSILGAINFISTMINMRPS
GMTLDRMPLFVWAIVITAVLLLLSLPVLAGAITMLLTDRNLNTSFFDPAGGGDPILYQHL
>COLFE1482-13|KJ963051|ZMUO.007277|Anthonomus_conspersus
TLYFIFGAWSGTVGTSLSMLIRTELGNPGSLIGDDQIYNTIVTAHAFIMIFFMVMPIMIGGFGNWLVPLMLAAPDMAFPRLNNMSFWLLPPSLTLLIMSSIIGKGAGTGWTVYPPLSSNLAHEGASVDFAIFSLHMAGISSILGAMNFISTVLNMKPSGM
NLEQMPLFVWAVKITAVLLLISLPVLAGAITMLLTDRNINTSFFDPAGGGDPILYQHL
>COLFB179-12|KJ966099|ZMUO.001509|Anthophagus_omalinus
TLYFIFGAWAGMVGTSLSILIRSELGNPGSLIGDDQIYNVIVTAHAFVMIFFMVMPVVIGGFGNWLVPLMLGAPDMAFPRMNNMSFWLLPPSLTLLLMSSMVESGAGTGWTVYPPLSSNIAHSGASVDLAIFSLHLAGISSILGAVNFITTVINMRSTGI
SFDRMPLFVWSVAITALLLLLSLPVLAGAITMLLTDRNLNTSFFDPAGGGDPILYQHL
>COLFB942-12|KJ961726|ZMUO.001987|Helophorus_lapponicus
TLYFIFGAWAGMVGTSLSILIRAELGNPGTLIGDDQIYNVIVTAHAFIMIFFMVMPIMIGGFGNWLVPLMLGAPDMAFPRMNNMSFWLLPPSLTLLLMSSMVESGAGTGWTVYPPLSSNIAHSGASVDLAIFSLHLAGISSILGAVNFITTVINMRSISM
TYDRLPLFVWSVAITALLLLLSLPVLAGAITMLLTDRNLNTSFFDPAGGGDPILYQHL
>COLFC755-12|KJ963489|ZMUO.003320|Ceutorhynchus_cochleariae
TLYFIFGSWAGMAGTSLSMLIRTELGNPGSLIGNDQIYNSIVTAHAFIMIFFMVMPILIGGFGNWLVPLMLGAPDMAFPRLNNMSFWLLPPSLSLLLMSSVVNKGAGTGWTVYPPLSGNMAHEGMSVDLAIFSLHMAGISSILGAINFISTVMNMQP
KGMTPELLPLFVWAVQITAILLLISLPVLAGAITMLLTDRNINTSFFDPSGGGDPILYQHL
>COLFE310-12|KJ964694|ZMUO.004870|Tomicus_piniperda

TLYFIFGAWSGMVGTSLSMLIRTELGTPGSLIGDDQIYNTIVTAHAFIMIFFMVMPIMIGGFGNWLIPLMLGAPDMAFPRLNNMSFWLLPPSLTLLLLSSIIDKGAGTGWTVYPPLASNISHEGASVDFAIFSLHMAGISSILGAMNFISTILNMNPSGMK
SDRLTLFTWAVKITAILLLLSLPVLAGAITMLLTDRNINTTFFDPAGGGDPILYQHL
>COLFE453-12|KJ966571|ZMUO.005013|Polydrusus_pilosus
TLYFIFGAWSGMVGTSLSMLIRTELGNPGSLIGDDQIYNVIVTAHAFIMIFFMVMPIMIGGFGNWLVPLMLGAPDMAFPRLNNMSFWLLPPSLTLLLMSSIVDKGAGTGWTVYPPLSMNIAHEGSSVDLAIFSLHMAGVSSILGAINFISTIINMRPTG
MSYDRMPLFVWAVKITAILLLLSLPVLAGAITMLLTDRNINTSFFDPAGGGDPILYQHL
>COLFD183-12|KJ966168|ZMUO.004078|Sepedophilus_testaceus
TLYFIFGAWAGMVGTSLSLLIRAELGNPGSLIGDDQIYNVIVTAHAFIMIFFMVMPVVIGGFGNWLVPLMLGAPDMAFPRMNNMSFWLLPPSLSLLLMSSLVESGAGTGWTVYPPLSSNIAHGGSSVDLAIFSLHLAGISSILGAVNFITTVINMRSTG
MTFDRMPLFVWSVAITALLLLLSLPVLAGAITMLLTDRNLNTSFFDPAGGGDPILYQHL
>COLFF274-13|KJ962323|ZMUO.005879|Bruchus_affinis
TLYFIFGAWAGMAGTSLSMLIRAELGNPGSLIGNDQIYNVIVTAHAFIMIFFMVMPIMIGGFGNWLVPLMLGAPDMAFPRMNNMSFWLLPPSLTLLLMSSLVESGAGTGWTVYPPLASNIAHGGSSVDLAIFSLHLAGISSILGAVNFITTVINMRPSG
MSMDRMPLFSWAVVITAILLLLSLPVLAGAITMLLTDRNLNTSFFDPAGGGDPILYQHL
>COLFF805-13|KJ964505|ZMUO.006125|Enochrus_affinis
TLYFIFGAWAGMVGTSLSILIRAELGNPGTLIGDDQIYNVIVTAHAFIMIFFMVMPIMIGGFGNWLVPLMLGAPDMAFPRMNNMSFWLLPPSLTLLLMSSMVESGAGTGWTVYPPLSSNIAHGGASVDLAIFSLHLAGISSILGAVNFITTVINMRSPS
MTYDRLPLFVWSVAITALLLLLSLPVLAGAITMLLTDRNLNTSFFDPAGGGDPILYQHL
>COLFE966-13|KJ962011|ZMUO.006761|Philonthus_rectangulus
TLYFIFGSWAGMVGTSLSLLIRAELGNPGTLIGDDQIYNVIVTAHAFIMIFFMVMPIVIGGFGNWLVPLMLGAPDMAFPRMNNMSFWLLPPSLTLLLMSSMVESGAGTGWTVYPPLSSNIAHGGASVDLAIFSLHLAGISSILGAVNFITTVINMRSTG
MTFDRMPLFVWSVAITALLLLLSLPVLAGAITMLLTDRNLNTTFFDPAGGGDPILYQHL
>COLFF838-13|KJ967039|ZMUO.006158|Atheta_cauta
TLYFIFGAWAGMVGTSLSLLIRAELGNPGSLIGDDQIYNVIVTAHAFIMIFFMVMPIVIGGFGNWLVPLMLGAPDMAFPRMNNMSFWLLPPSLTLLLMSSMVESGAGTGWTVYPPLSSNIAHGGSSVDLAIFSLHLAGISSILGAVNFISTVINMRSTGI
SFDRMPLFVWSVAITALLLLLSLPVLAGAITMLLTDRNLNTSFFDPAGGGDPILYQHL
>COLFB938-12|KJ965760|ZMUO.001983|Tachinus_proximus
TLYFIFGAWAGMVGTSLSLLIRAELGNPGTLIGDDQIYNVIVTAHAFIMIFFMVMPIVIGGFGNWLVPLMLGAPDMAFPRMNNMSFWLLPPSLSLLLMSSMVESGAGTGWTVYPPLSSNIAHGGSSVDLAIFSLHLAGISSILGAVNFITTVINMRSIGM
TFDRMPLFVWSVAITALLLLLSLPVLAGAITMLLTDRNLNTTFFDPAGGGDPILYQHL
>COLFF368-13|KJ966992|ZMUO.006068|Philonthus_splendens
TLYFIFGSWAGMVGTSLSLLIRAELGNPGSLIGDDQIYNVIVTAHAFIMIFFMVMPVVIGGFGNWLVPLMLGAPDMAFPRMNNMSFWLLPPSLTLLLMSSVVESGAGTGWTVYPPLSSNIAHSGASVDLAIFSLHLAGISSILGAVNFITTVINMRSMG
MSFDQMPLFVWSVAITALLLLLSLPVLAGAITMLLTDRNLNTSFFDPAGGGDPVLYQHL
>COLFC268-12|KJ965938|ZMUO.002453|Thecturota_marchii
TLYFIFGAWAGMVGTSLSLLIRAELGNPGSLIGDDQIYNVIVTAHAFVMIFFMVMPIVIGGFGNWLVPLMLGAPDMAFPRMNNMSFWLLPPSLTLLLMSSMVESGAGTGWTVYPPLSSNIAHGGSSVDLAIFSLHLAGISSILGAVNFISTVINMRTTGI
TFDRMPLFVWSVVITALLLLLSLPVLAGAITMLLTDRNLNTSFFDPAGGGDPILYQHL
>COLFF818-13|KJ963878|ZMUO.006138|Aphodius_fossor
TLYFLFGSWAGMVGTSLSLLIRAELGNPGTLIGDDQIYNVIVTAHAFVMIFFMVMPILIGGFGNWLVPLMLGAPDMAFPRMNNMSFWLLPPSLTLLLMSSMVESGAGTGWTVYPPLSSNIAHGGASVDLAIFSLHLAGISSILGAVNFITTVINMRSPG
MTFDRMPLFVWSVAITALLLLLSLPVLAGAITMLLTDRNLNTSFFDPAGGGDPILYQHL
>COLFC234-12|KJ963588|ZMUO.002419|Atomaria_fuscata
TLYFIFGSWAGMVGTSLSMLIRTELGTPGSLIGDDQIYNVIVTAHAFIMIFFMVMPMMIGGFGNWLVPLMLGAPDMAFPRLNNMSFWLLPPSLMFLLMSSIVEKGAGTGWTVYPPLSSNVAHAGSSVDLAIFSLHLAGISSILGSMNFITTVINMRPK
GMNFDCLPLFVWSVKITTILLLLSLPVLAGAITMLLTDRNINTSFFDPAGGGDPILYQHL
>COLFG038-13|KJ962648|ZMUO.007543|Orectochilus_villosus
TLYFIFGAWSGMVGTSLSMLIRMELGNPGSLIGDDQIYNVIVTAHAFIMIFFMVMPIMIGGFGNWLVPLMLGAPDMAFPRMNNMSFWLLPPSLSLLLMSSLVESGAGTGWTVYPPLSSNIAHSGASVDLAIFSLHLAGISSILGAVNFITTIINMRSTG
MTLDRMPLFVWSVGITALLLLLSLPVLAGAITMLLTDRNLNTSFFDPAGGGDPILYQHL
>COLFF737-13|KJ964085|ZMUO.006437|Ceutorhynchus_asperifoliarum
TLYFIFGSWAGMAGTSLSMLIRAELGNPGSLIGNDQTYNSVVTAHAFIMIFFMVMPIMIGGFGNWLVPLMLGAPDMAFPRLNNMSFWLLPPSLTLLLMSSIVNKGAGTGWTVYPPLSANTAHEGMSVDLAIFSLHMAGISSILGAINFISTVMNMRP
SGMISEYMPLFVWAVEITAILLLISLPVLAGAITMLLTDRNINTSFFDPSGGGDPILYQHL
>COLFF452-13|KJ965776|ZMUO.005970|Anacaena_lutescens
TLYFIFGAWAGMVGTSLSILIRAELGNPGTLIGDDQIYNVIVTAHAFIMIFFMVMPIMIGGFGNWLVPLMLGAPDMAFPRMNNMSFWLLPPSLTLLLMSSMVENGAGTGWTVYPPLSSNIAHGGASVDLAIFSLHLAGISSILGAVNFITTVINMRSES
MTYDRLPLFVWSVAITALLLLLSLPVLAGAITMLLTDRNLNTSFFDPAGGGDPILYQHL
>COLFA531-12|KJ967299|ZMUO.000666|Plagionotus_detritus
TLYFVFGVWAGMVGTSLSVLIRSELGNPGSLIGDDQIYNVIVTAHAFIMIFFMVMPIMIGGFGNWLVPLMLGAPDMAFPRMNNMSFWLLPPSLTLLLMSSIVENGAGTGWTVYPPLSANIAHSGSSVDLAIFSLHLAGISSILGAVNFISTVINMRPTG
MSLEQMPLFVWAVVITAILLLLSLPVLAGAITMLLTDRNINTSFFDPAGGGDPILYQHL

>COLFC501-12|KJ962365|ZMUO.003066|Epuraea_marseuli
TLYFIFGAWSGMIGTSLSILIRTELGSPGSLIGNDQIYNVIVTAHAFIMIFFMVMPFMIGGFGNWLVPLMLGAPDMAFPRMNNMSFWLLPPSLSLLLMSSIVESGAGTGWTVYPPLSSNIAHGGSSVDLAIFSLHLAGISSILGAVNFITTIINMRPVGMTL
DRMPLFSWSVMITAILLLLSLPVLAGAITMLLTDRNLNTTFFDPSGGGDPILYQHL
>COLFF896-13|KJ962142|ZMUO.006691|Olophrum_assimile
TLYFIFGAWAGMVGTSLSILIRAELGNPGTLIGDDQIYNVIVTAHAFVMIFFMVMPIVIGGFGNWLVPLMLGAPDMAFPRMNNMSFWLLPPSLTLLLSSSMVESGAGTGWTVYPPLSSNIAHGGSSVDLAIFSLHLAGISSILGAVNFITTVINMRSTGM
TFDRMPLFVWSVAITALLLLLSLPVLAGAITMLLTDRNLNTSFFDPAGGGDPILYQHL
>COLFF114-13|KJ966444|ZMUO.003748|Aleochara_moerens
TLYFIFGAWAGMVGTSLSLLIRAELGNPGSLIGDDQIYNVIVTAHAFIMIFFMVMPIVIGGFGNWLVPLMLGAPDMAFPRMNNMSFWLLPPSLTLLLMSSMVESGVGTGWTVYPPLSANIAHSGSSVDLAIFSLHLAGISSILGAVNFISTIINMRSSGM
LFDKMPLFVWSVAITALLLLLSLPVLAGAITMLLTDRNLNTSFFDPAGGGDPILYQHL
>COLFD847-12|KJ961938|ZMUO.004837|Colymbetes_paykulli
TLYFIFGAWAGMVGTSLSMLIRAELGNPGSLIGDDQIYNVIVTAHAFVMIFFMVMPIMIGGFGNWLVPLMLGAPDMAFPRMNNMSFWLLPPSLTLLLMSSMVESGAGTGWTVYPPLSSGIAHGGASVDLAIFSLHLAGISSILGAVNFITTIINMRSV
GMTFDRMPLFVWSVGITALLLLLSLPVLAGAITMLLTDRNLNTSFFDPAGGGDPILYQHL
>COLFB341-12|KJ964215|ZMUO.001671|Gnathoncus_buyssoni
TLYFIFGMWAGMVGTSLSLLIRAELGSPGSLIGDDQIYNVIVTAHAFVMIFFMVMPIVIGGFGNWLVPLMLGAPDMAFPRMNNMSFWLLPPSLTLLLMSSLVESGAGTGWTVYPPLSSNIAHSGASVDLAIFSLHLAGISSILGAVNFITTVINMRSNGI
NFDQMPLFVWSVAITALLLLLSLPVLAGAITMLLTDRNINTSFFDPAGGGDPILYQHL
>COLFF007-13|KJ967493|ZMUO.005707|Otiorhynchus_singularis
TLYFILGSWSGMVGTSLSMLIRAELGTPGALIGDDQIYNVIVTAHAFIMIFFMVMPIMIGGFGNWLVPLMLGAPDMAFPRLNNMSFWLLPPSLSLLLMSSMIDKGAGTGWTVYPPLSSNIAHEGASVDLAIFSLHMAGISSILGAINFISTVANMRPSG
MSLDRMSLFVWAVKITAILLLLSLPVLAGAITMLLTDRNVNTSFFDPAGGGDPILYQHL
>COLFB881-12|KJ964768|ZMUO.001926|Saperda_perforata
TLYFLFGAWAGMVGTSLSLLIRSELGTPGTLIGNDQIYNTIVTAHAFIMIFFMVMPIMMGGFGNWLVPLMLGAPDMAFPRMNNMSFWLLPPSLTLLIMSSIVDNGAGTGWTVYPPLAANVAHSGSSVDLAIFSLHLAGISSILGAVNFITTVINMRPS
GMTLDRTSLFVWAVKITAILLLLSLPVLAGAITMLLTDRNLNTSFFDPAGGGDPILYQHL
>COLFB550-12|KJ962913|ZMUO.001025|Omalium_riparium
TLYFIFGAWAGMVGTSLSLLIRAELGNPGSLIGDDQIYNVIVTAHAFIMIFFMVMPIVIGGFGNWLVPLMLGAPDMAFPRMNNMSFWLLPPSLTLLLMSSMVESGAGTGWTVYPPLSSNIAHGGSSVDLAIFSLHLAGISSILGAVNFITTVINMRAMG
MTFDRMPLFVWSVVITALLLLLSLPVLAGAITMLLTDRNLNTSFFDPAGGGDPILYQHL
>COLFB234-12|KJ966357|ZMUO.001564|Lepturobosca_virens
TLYFIFGAWAGMIGTSLSLLIRSELGNPGSLIGDDQIYNVIVTAHAFVMIFFMVMPIMIGGFGNWLVPLMLGAPDMAFPRMNNMSFWLLPPSLTLLIMSSIVEKGAGTGWTVYPPLAANIAHSGPSVDLAIFSLHLAGISSILGAVNFITTIINMPPVGM
KLDQMPLFVWAVMITAILLLLSLPVLAGAITMLLTDRNLNTSFFDPAGGGDPILYQHL
>COLFA260-12|KJ961744|ZMUO.000395|Typhaea_stercorea
TLYFIFGAWSGMVGTSLSLLIRSELGNPGSLIGDDQIYNVIVTAHAFIMIFFMVMPIMMGGFGNWLVPLMLGAPDMAFPRMNNMSFWLLPPSLTLLIMSSVVENGAGTGWTVYPPLSSNIAHSGSSVDLAIFSLHLAGISSILGAVNFITTIINMRPVG
MSFDRMPLFVWAVAITALLLLLSLPVLAGAITMLLTDRNLNTSFFDPAGGGDPILYQHL
>COLFE443-12|KJ961897|ZMUO.005003|Sciaphilus_asperatus
TLYFIFGSWSGMIGTSLSMLIRTELGNPGSLIGDDQIYNVIVTAHAFIMIFFMVMPIMIGGFGNWLVPLMLGAPDMAFPRLNNMSFWLLPPSLSLLLMSSIVDKGAGTGWTVYPPLSANIAHEGSSVDLAIFSLHMAGVSSILGAINFISTVINMRPLGM
TPERMPLFIWAVKITAILLLLSLPVLAGAITMLLTDRNVNTSFFDPAGGGDPILYQHL
>COLFF485-13|KJ961809|ZMUO.006565|Cryptophagus_acutangulus
SLYFIFGAWAGMVGTSLSLLIRSELGTPGSLIGDDQIYNVIVTAHAFVMIFFMVMPIMIGGFGNWLVPLMLGAPDMAFPRMNNMSFWLLPPSLSLLLMSSIAEKGVGTGWTVYPPLSSNIAHGGSSVDLAIFSLHLAGISSILGAVNFISTVMNMHPTG
MSLDRMPLFVWAVMITAILLLLSLPVLAGAITMLLTDRNLNTSFFDPAGGGDPILYQHL
>COLFF117-13|KJ965024|ZMUO.003751|Atheta_gagatina
TLYFIFGAWAGMIGTSLSLLIRAELGNPGSLIGDDQIYNVIVTAHAFIMIFFMVMPIVIGGFGNWLVPLMLGAPDMAFPRMNNMSFWLLPPSLSLLLMSSMVESGAGTGWTVYPPLSSNIAHGGSSVDLAIFSLHLAGISSILGAVNFISTVINMRSTGIS
FDRMPLFVWSVVITALLLLLSLPVLAGAITMLLTDRNLNTSFFDPAGGGDPILYQHL
>COLFF778-13|KJ963694|ZMUO.006098|Agonum_gracile
TLYFIFGAWAGMVGTSLSMLIRAELGNPGALIGDDQIYNVIVTAHAFIMIFFMVMPIMIGGFGNWLVPLMLGAPDMAFPRMNNMSFWLLPPSLTLLLMSSLVESGAGTGWTVYPPLSSGIAHAGASVDLAIFSLHLAGISSILGAVNFITTIINMRSVG
MTFDRMPLFVWSVGITALLLLLSLPVLAGAITMLLTDRNLNTSFFDPAGGGDPILYQHL
>COLFC040-12|KJ962827|ZMUO.002035|Tachinus_laticollis
TLYFIFGAWAGMVGTSLSLLIRAELGNPGTLIGDDQIYNVIVTAHAFIMIFFMVMPIVIGGFGNWLVPLMLGAPDMAFPRMNNMSFWLLPPSLSLLLMSSMVESGAGTGWTVYPPLSSNIAHGGSSVDLAIFSLHLAGISSILGAVNFITTVINMRSIGM
SFDRMPLFVWSVAITALLLLLSLPVLAGAITMLLTDRNLNTTFFDPAGGGDPILYQHL
>COLFD267-12|KJ963174|ZMUO.004162|Berosus_luridus

TLYFIFGAWAGMVGTSLSLLIRAELGNPGTLIGDDQIYNVIVTAHAFIMIFFMVMPIMIGGFGNWLVPLMLGAPDMAFPRMNNMSFWLLPPSLTLLLLSSMVESGAGTGWTVYPPLSSNIAHGGASVDLAIFSLHLAGISSILGAVNFITTVINMRAPN
MTYDRMPLFVWSVAITALLLLLSLPVLAGAITMLLTDRNLNTSFFDPAGGGDPILYQHL
>COLFA533-12|KJ965630|ZMUO.000668|Xylotrechus_antilope
TLYFIFGAWAGMVGTSMSVLIRSELGTPGSLIGDDQIYNVIVTAHAFIMIFFMVMPIMIGGFGNWLVPLMLGAPDMAFPRMNNMSFWLLPPSLALLIMSSIVESGAGTGWTVYPPLSANVAHSGSSVDLAIFSLHLAGVSSILGAVNFISTVINMRPTK
MSPEQMPLFVWAVAITALLLLLSLPVLAGAITMLLTDRNINTSFFDPAGGGDPILYQHL
>COLFF426-13|KJ965567|ZMUO.005944|Phratora_laticollis
TLYFIFGIWAGMVGTSLSVLIRSELGNPGTLIGNDQIYNVIVTAHAFIMIFFMVMPIMIGGFGNWLVPLMIGAPDMAFPRLNNMSFWLLPPSLFLLLMSSVVENGAGTGWTVYPPLSSNLAHSGSSVDLAIFSLHLAGISSILGAINFITTVINMRPEGMN
LEQTPLFVWAVLITAILLLLSLPVLAGAITMLLTDRNLNTSFFDPAGGGDPILYQHL
>COLFB708-12|KJ964047|ZMUO.001183|Aphodius_prodromus
TLYFLFGSWAGMVGTSLSLLIRAELGNPGSLIGDDQIYNVIVTAHAFVMIFFMVMPILIGGFGNWLVPLMLGAPDMAFPRMNNMSFWLLPPSLTLLLMSSMVESGAGTGWTVYPPLSSNIAHGGASVDLAIFSLHLAGISSILGAVNFITTVINMRSPG
LTFDRMPLFVWSVAITALLLLLSLPVLAGAITMLLTDRNLNTSFFDPAGGGDPILYQHL
>COLFB164-12|KJ964263|ZMUO.001494|Agabus_unguicularis
TLYFIFGAWAGMVGTSLSMLIRAELGNPGSLIGDDQIYNVIVTAHAFVMIFFMVMPIMIGGFGNWLVPLMLGAPDMAFPRMNNMSFWLLPPSLTLLLMSSMVEKGAGTGWTVYPPLSSGIAHSGASVDLAIFSLHLAGISSILGAVNFITTIINMRSV
GMTFDRMPLFVWSVGITALLLLLSLPVLAGAITMLLTDRNLNTSFFDPAGGGDPILYQHL
>COLFF836-13|KJ965213|ZMUO.006156|Atheta_cauta
TLYFIFGAWAGMVGTSLSLLIRAELGNPGSLIGDDQIYNVIVTAHAFIMIFFMVMPIVIGGFGNWLVPLMLGAPDMAFPRMNNMSFWLLPPSLTLLLMSSMVESGAGTGWTVYPPLSSNIAHGGSSVDLAIFSLHLAGISSILGAVNFISTVINMRSTGI
SFDRMPLFVWSVAITALLLLLSLPVLAGAITMLLTDRNLNTSFFDPAGGGDPILYQHL
>COLFB333-12|KJ964306|ZMUO.001663|Tachyporus_nitidulus
TLYFIFGAWSGMVGTSLSLLIRAELGNPGSLIGDDQIYNVIVTAHAFIMIFFMVMPIVIGGFGNWLVPLMLGAPDMAFPRMNNMSFWLLPPSLTLLLMSSMVESGAGTGWTVYPPLSANIAHSGSSVDLAIFSLHMAGISSILGAVNFITTVINMRTSG
MHFDRMPLFIWSVVITALLLLLSLPVLAGAITMLLTDRNLNTSFFDPAGGGDPVLYQHL
>COLFC347-12|KJ963740|ZMUO.002532|Proteinus_brachypterus
TLYFIFGAWAGMVGTSLSILIRAELGNPGTLIGDDQIYNVIVTAHAFIMIFFMVMPIVIGGFGNWLVPLMLGAPDMAFPRMNNMSFWLLPPSITLLLMSSMVENGAGTGWTVYPPLSSNISHSGSSVDLAIFSLHLAGISSILGAVNFITTVINMRSPGM
TFDRMPLFVWSVAITALLLLLSLPVLAGAITMLLTDRNINTTFFDPAGGGDPILYQHL
>COLFF165-13|KJ962590|ZMUO.003799|Cantharis_paludosa
TLYFIFGAWSGSLGLALSLLIRAELGTPGTLIGNDQIYNVIVTAHAFIMIFFMVMPIMIGGFGNWLVPLMLGAPDMAFPRMNNMSFWFLPPSLMFLLMSSMVESGAGTGWTVYPPLSANIAHSGPSVDLAIFSLHMAGISSILGAVNFISTIMNMKPPS
MKFDQMPLFVWSVGITALLLLLSLPVLAGAITMLLSDRNLNTSFFDPMGGGDPILYQHL
>COLFF230-13|KJ961944|ZMUO.005835|Meligethes_exilis
TLYFIFGAWSGMVGTSLSMLIRTELGNPGSLIGNDQIYNVIVTAHAFVMIFFMVMPFMIGGFGNWLVPLMLGAPDMAFPRMNNMSFWLLPPSLSLLLMSSIIESGAGTGWTVYPPLSSNIAHGGASVDLAIFSLHLAGISSILGAVNFITTVINMRPTG
MTFDRMPLFVWAVVITALLLLLSLPVLAGAITMLLTDRNLNTTFFDPSGGGDPVLYQHL
>COLFB816-12|KJ965372|ZMUO.001291|Cephalocousya_nivicola
TLYFIFGAWAGMIGTSLSLLIRAELGNPGSLIGDDQIYNVIVTAHAFVMIFFMVMPIVIGGFGNWLVPLMLGAPDMAFPRMNNMSFWLLPPSLTLLLMSSMVESGAGTGWTVYPPLSSNIAHGGSSVDLAIFSLHLAGISSILGAVNFISTIINMRTSGM
SFDRMPLFVWSVAITALLLLLSLPVLAGAITMLLTDRNLNTSFFDPAGGGDPILYQHL
>COLFA654-12|KJ963434|ZMUO.000789|Atheta_nigripes
TLYFIFGAWAGMVGTSLSLLIRAELGNPGSLIGDDQIYNVIVTAHAFIMIFFMVMPIVIGGFGNWLVPLMLGAPDMAFPRMNNMSFWLLPPSLTLLLMSSMVESGAGTGWTVYPPLSSNIAHGGSSVDLAIFSLHLAGISSILGAVNFISTVINMRSTGI
SFDRMPLFVWSVAITALLLLLSLPVLAGAITMLLTDRNLNTSFFDPAGGGDPILYQHL
>COLFC744-12|KJ964789|ZMUO.003309|Ipidia_binotata
TLYFIFGAWSGMIGTSLSMMIRTELGNPGSLIGNDQIYNVIVTAHAFVMIFFMVMPFMIGGFGNWLVPLMLGAPDMAFPRLNNMSFWLLPPSLSLLLMSSIVESGAGTGWTVYPPLSANIAHGGSSVDLAIFSLHLAGISSILGAVNFITTVINMQPTG
MSFDKMPLFVWAVLITAILLLLSLPVLAGAITMLLTDRNLNTTFFDPSGGGDPILYQHL
>COLFC030-12|KJ964734|ZMUO.002025|Atheta_nigripes
TLYFIFGAWAGMVGTSLSLLIRAELGNPGSLIGDDQIYNVIVTAHAFIMIFFMVMPIVIGGFGNWLVPLMLGAPDMAFPRMNNMSFWLLPPSLTLLLMSSMVESGAGTGWTVYPPLSSNIAHGGSSVDLAIFSLHLAGISSILGAVNFISTVINMRSTGI
SFDRMPLFVWSVAITALLLLLSLPVLAGAITMLLTDRNLNTSFFDPAGGGDPILYQHL
>COLFF121-13|KJ966934|ZMUO.003755|Aphodius_plagiatus
TLYFLFGSWAGMVGTSLSLLIRAELGNPGTLIGDDQIYNVIVTAHAFVMIFFMVMPILIGGFGNWLVPLMLGAPDMAFPRMNNMSFWLLPPSLTLLLMSSMVEQGAGTGWTVYPPLSSNIAHGGASVDLAIFSLHLAGISSILGAVNFITTVINMRSPG
MTFDRMPLFVWSVAITALLLLLSLPVLAGTITMLLTDRNLNTSFFDPAGGGDPILYQHL
>COLFA102-10|HQ559229|MP00357|Corticaria_polypori
SLYFMFGMWAGMVGTSLSLLIRLELGNPGSLIGDDQIYNVIVTAHAFVMIFFMVMPIMIGGFGNWLVPLMLGAPDMAFPRLNNMSFWLLPPSLSLLIMSSIVESGAGTGWTVYPPLSSNIAHSGSSVDLAIFSLHLAGISSILGAVNFITTVINMRPAG
MNLDQMPLFVWSVVITAILLLLSLPVLAGAITMLLTDRNLNTSFFDPAGGGDPILYQHL

>COLFF128-13|KJ965505|ZMUO.003762|Carpelimus_obesus
TLYFIFGAWSGMVGTSLSMLIRIELGTPGSLIGDDQIYNVIVTAHAFIMIFFMVMPIVIGGFGNWLVPLMLGAPDMAFPRMNNMSFWLLPPSLTLLLFSSMVESGAGTGWTVYPPLSSNIAHSGSSVDLAIFSLHLAGISSILGAVNFISTIINMRSVGMT
FDRMPLFVWSVNITAILLLLSLPVLAGAITMLLTDRNLNTSFFDPAGGGDPILYQHL
>COLFC741-12|KJ964566|ZMUO.003306|Hylesinus_crenatus
TLYFILGAWSGMVGTSLSMIIRTELGTPGSLIGNDQIYNSIVTAHAFIMIFFMVMPIMIGGFGNWLVPLMLGAPDMAFPRLNNMSFWLLPPSLLFLLMSSIIDKGAGTGWTVYPPLANNIAHEGASVDLAIFSLHMAGVSSILGALNFISTIINMHPMG
MKLDRLTLFTWAVKITAILLLLSLPVLAGAITMLLTDRNINTSFFDPAGGGDPILYQHL
>COLFF861-13|KJ965648|ZMUO.006656|Sphaeridium_scarabaeoides
TLYFIFGAWAGMVGTSLSILIRAELGNPGTLIGDDQIYNVIVTAHAFIMIFFMVMPIMIGGFGNWLVPLMLGAPDMAFPRMNNMSFWLLPPSLTLLLMSSMVESGAGTGWTVYPPLSSNIAHGGASVDLAIFSLHLAGISSILGAVNFITTVINMRSPD
MTYDRMPLFVWSVAITALLLLLSLPVLAGAITMLLTDRNLNTSFFDPAGGGDPILYQHL
>COLFF518-13|KJ966712|ZMUO.006598|Hygrotus_quinquelineatus
TLYFLFGAWAGMVGTSLSMLIRAELGNPGSLIGDDQIYNVIVTAHAFIMIFFMVMPIMIGGFGNWLVPLMLGAPDMAFPRMNNMSFWLLPPSLTLLLMSSMVESGAGTGWTVYPPLSAGIAHGGASVDLAIFSLHLAGISSILGAVNFITTIINMRSV
GMTFDRMPLFVWSVGITALLLLLSLPVLAGAITMLLTDRNLNTSFFDPAGGGDPILYQHL
>COLFD195-12|KJ963234|ZMUO.004090|Hydroporus_angustatus
TLYFLFGAWSGMVGTSLSMLIRAELGNPGSLIGDDQIYNVIVTAHAFIMIFFMVMPIMIGGFGNWLVPLMLGAPDMAFPRMNNMSFWLLPPSLTLLLMSSMVENGAGTGWTVYPPLSSGIAHSGASVDLAIFSLHLAGISSILGAVNFITTIINMRSIG
MTFDRMPLFVWSVGITALLLLLSLPVLAGAITMLLTDRNLNTSFFDPAGGGDPILYQHL
>COLFE1011-13|KJ967155|ZMUO.006806|Schistoglossa_aubei
TLYFIFGAWAGMIGTSLSLLIRAELGNPGSLIGDDQIYNVIVTAHAFIMIFFMVMPIVIGGFGNWLVPLMLGAPDMAFPRMNNMSFWLLPPSLTLLLMSSMVESGAGTGWTVYPPLSANIAHSGSSVDLAIFSLHLAGISSILGAVNFISTVINMRSVGIS
FDRMPLFVWSVAITALLLLLSLPVLAGAITMLLTDRNLNTSFFDPAGGGDPILYQHL
>COLFG118-13|KJ965703|ZMUO.007813|Thinobius_ciliatus
TLYFIFGAWSGMVGTSLSMLIRAELGMPGSLIGDDQIYNVIVTAHAFIMIFFMVMPIVIGGFGNWLVPLMLGAPDMAFPRMNNMSFWLLPPSLSLLLMSSMVESGAGTGWTVYPPLSANIAHSGSSVDLAIFSLHLAGISSILGAVNFISTIINMRSPG
MTLDRMPLFVWSVNITAILLLLSLPVLAGAITMLLTDRNLNTSFFDPAGGGDPILYQHL
>COLFC855-12|KJ963172|ZMUO.003420|Nanophyes_marmoratus
TLYFIFGFWSGMVGTSLSLLIRLELGNPGSLIGDDQIYNVIVTAHAFIMIFFMVMPIMIGGFGNWLVPLMLGAPDMAFPRMNNMSFWLLPPSLTLLIMSSIVEKGAGTGWTVYPPLAANIAHGGASVDLAIFSLHLAGISSILGAANFISTAINMRPSKM
NLDQMPLFVWAVGITALLLLLSLPVLAGAITMLLTDRNINTSFFDPAGGGDPILYQHF
>COLFC531-12|KJ963956|ZMUO.003096|Aclypea_opaca
TLYFIFGTWAGIVGMSLSILIRLELSTPGSFLGDDQMYNVIVTAHAFIMIFFMVMPIVIGGFGNWLIPLMLGAPDMAFPRMNNMSFWLIPPSLSLLLVSSMVESGAGTGWTVYPPLSSNIAHGGSSVDMAIFSLHLAGISSILGAVNFITTIINMRSSGMT
FDRMPLFVWSVGITALLLLLSLPVLAGAITMLLTDRNLNTSFFDPAGGGDPILYQHL
>COLFD525-12|KJ966903|ZMUO.004325|Amara_aenea
TLYFIFGAWSGMVGTSLSMLIRAELGNPGALIGDDQIYNVIVTAHAFVMIFFMVMPIMIGGFGNWLVPLMLGAPDMAFPRMNNMSFWLLPPSLTLLLMSSMVESGAGTGWTVYPPLSSGIAHAGASVDLAIFSLHLAGISSILGAVNFITTIINMRSV
GMTFDRMPLFVWSVGITALLLLLSLPVLAGAITMLLTDRNLNTSFFDPAGGGDPILYQHL
>COLFE869-13|KJ964029|ZMUO.006189|Gonioctena_flavicornis
TLYFIFGIWAGMVGTSLSILIRAELGNPGTLIGNDQIYNVIVTAHAFIMIFFMVMPIMIGGFGNWLVPLMIGAPDMAFPRMNNMSFWLLPPSLFFLIMSSIVESGAGTGWTVYPPLSANIAHSGSSVDLAIFSLHLAGISSILGAVNFITTIINMRPMGMS
MDRMPLFVWAVLITAILLLLSLPVLAGAITMLLTDRNLNTSFFDPAGGGDPILYQHL
>COLFD312-12|KJ964084|ZMUO.004207|Stenus_fuscipes
TLYFTFGAWAGMVGTSLSLLIRAELGNPGSLIGDDQIYNVIVTAHAFVMIFFMVMPVMIGGFGNWLVPLMLGAPDMAFPRMNNMSFWLLPPSLSLLLTSSIVENGAGTGWTVYPPLSTNIAHSGASVDLAIFSLHLAGISSILGAINFITTIFNMRTMKL
QFDCLPLFVWSVGITALLLLLSLPVLAGAITMLLTDRNLNTSFFDPAGGGDPVLYQHL
>COLFC574-12|KJ966503|ZMUO.003139|Chrysanthia_geniculata
TLYLIFGAWAGMVGTSLSLLIRAELGNPGSLIGDDQIYNVIVTAHAFIMIFFMVMPIVIGGFGNWLVPLMLGAPDMAFPRMNNMSFWLLPPSLTLLIMSSIVENGAGTGWTVYPPLSSNIAHSGSSVDLAIFSLHLAGISSILGAVNFITTVINMRPVGM
TLDRMPLFVWAVVITAVLLLSLPILAGAITMLLTDRNLNTSFFDPAGGGDPILYQHL
>COLFC815-12|KJ966392|ZMUO.003380|Lathrobium_rufipenne
TLYFIFGAWAGMVGTSLSLLIRTELGNPGSLIGDDQIYNVIVTAHAFIMIFFMVMPIMIGGFGNWLVPLMLGAPDMAFPRMNNMSFWLLPPSLSLLMSSLVESGAGTGWTVYPPLSSNIAHGGASVDLAIFSLHLAGISSILGAVNFITTVINMRSPG
MTYERMPLFVWAVAITALLLLLSLPVLAGAITMLLTDRNLNTSFFDPAGGGDPILYQHL
>COLFC142-12|KJ967236|ZMUO.002327|Aspidiphorus_orbiculatus
TLYFIFGIWSGMIGTSLSMLIRMELSMPGALIGDDQIYNVIVTAHAFIMIFFMVMPIMIGGFGNWLIPLMMGAPDMAFPRMNNMSFWLLPPSLSLLLMSSIVEKGTGTGWTVYPPLSSNISHNGASVDLSIFSLHLAGMSSIMGAINFISTILNMRPSN
MSLDQMPLFIWAVFITAFLLALSLPVLAGAITMLLTDRNLNTSFFDPSGGGDPILYQHL
>COLFG018-13|KJ964258|ZMUO.007523|Agrilus_sulcicollis

TLYFIFGAWSGMVGTALSLLVRAELGNPGALIGNDQIYNVIVTAHAFIMIFFMVMPIMMGGFGNWLVPLMLGAPDMAFPRMNNMSFWLLPPSLTLLLMSSMVESGAGTGWTVYPPLAANIAHSGASVDLAIFSLHLAGISSILGAINFITTVINMRA
SGMTLDRMPLLVWSIAITALLLLLSLPVLAGAITMLLTDRNLNTSFFDPAGGGDPILYQHL
>COLFC199-12|KJ964041|ZMUO.002384|Xantholinus_linearis
TLYFIFGAWAGMIGTSLSLLIRSELGNPGTLIGDDQIYNVIVTAHAFVMIFFMVMPIVIGGFGNWLVPLMLGAPDMAFPRMNNMSFWLLPPSLTLLLMSSMVESGAGTGWTVYPPLSSNIAHGGASVDLAIFSLHLAGISSILGAVNFITTVINMRSTG
MSFDRMPLFVWSVAITALLLLLSLPVLAGAITMLLTDRNLNTSFFDPTGGGDPILYQHL
>COLFB518-12|KJ965015|ZMUO.000993|Atheta_ischnocera
TLYFIFGAWAGMVGTSLSLLIRAELGNPGSLIGDDQIYNVIVTAHAFIMIFFMVMPIVIGGFGNWLVPLMLGAPDMAFPRMNNMSFWLLPPSLTLLLMSSMVESGAGTGWTVYPPLSSNIAHGGSSVDLAIFSLHLAGISSILGAVNFISTVINMRSTGI
SFDRMPLFVWSVAITALLLLLSLPVLAGAITMLLTDRNLNTSFFDPAGGGDPILYQHL
>COLFA387-12|KJ963052|ZMUO.000617|Gyrinus_opacus
TLYFIFGAWSGMVGTSLSMLIRAELGNPGSLIGDDQIYNVIVTAHAFIMIFFMVMPIMIGGFGNWLVPLMLGAPDMAFPRMNNMSFWLLPPSLTLLLMSSMVENGAGTGWTVYPPLSSNIAHGGASVDLAIFSLHLAGISSILGAVNFITTIINMRSV
GMTLDRMPLFVWSVGITALLLLLSLPVLAGAITMLLTDRNLNTSFFDPAGGGDPILYQHL
>COLFF732-13|KJ962872|ZMUO.006432|Chrysanthia_viridissima
TLYLIFGAWAGMVGTSLSLLIRTELSNPGSLIGDDQIYNVIVTAHAFIMIFFMVMPIMIGGFGNWLVPLMLGAPDMAFPRMNNMSFWLLPPSLTLLIMSSIVENGAGTGWTVYPPLSSNIAHSGSSVDLAIFSLHLAGISSILGAVNFITTIINMRPAGMT
LDRMPLFVWAVVITAVLLLLSLPVLAGAITMLLTDRNLNTSFFDPAGGGDPILYQHL
>COLFF157-13|KJ965522|ZMUO.003791|Trechus_quadristriatus
TLYFIFGAWAGMVGTSLSMLIRAELGNPGSLIGDDQIYNVIVTAHAFIMIFFMVMPIMIGGFGNWLVPLMLGAPDMAFPRMNNMSFWLLPPSLTLLLMSSMVESGAGTGWTVYPPLSSGIAHSGASVDLAIFSLHLAGVSSILGAVNFITTIINMRPM
GMTFDRMPLFVWSVGITALLLLLSLPVLAGAITMLLTDRNLNTSFFDPAGGGDPILYQHL
>COLFC671-12|KJ961978|ZMUO.003236|Hydroporus_notabilis
TLYFLFGAWSGMVGTSLSMLIRAELGNPGSLIGDDQIYNVIVTAHAFIMIFFMVMPIMIGGFGNWLVPLMLGAPDMAFPRMNNMSFWLLPPSLSLLLMSSMVENGAGTGWTVYPPLSSGIAHSGASVDLAIFSLHLAGISSILGAVNFITTIINMRSIG
MTFDRMPLFVWSVGITALLLLLSLPVLAGAITMLLTDRNLNTSFFDPAGGGDPILYQHL
>COLFC039-12|KJ966376|ZMUO.002034|Tachinus_pallipes
TLYFIFGAWAGMVGTSLSLLIRAELGNPGTLIGDDQIYNVIVTAHAFIMIFFMVMPIVIGGFGNWLVPLMLGAPDMAFPRMNNMSFWLLPPSLTLLLMSSMVESGAGTGWTVYPPLSSNIAHGGSSVDLAIFSLHLAGISSILGAVNFITTVINMRSIG
MTFDRMPLFVWSVAITALLLLLSLPVLAGAITMLLTDRNLNTTFFDPAGGGDPILYQHL
>COLFB513-12|KJ964389|ZMUO.000988|Atheta_sordidula
TLYFIFGAWAGMVGTSLSLLIRAELGNPGSLIGDDQIYNVIVTAHAFVMIFFMVMPIVIGGFGNWLIPLMLGAPDMAFPRMNNMSFWLLPPSLSLLLMSSMVESGAGTGWTVYPPLSSNIAHGGASVDLAIFSLHLAGISSILGAVNFISTVINMRSVGI
SFDRMPLFVWSVAITALLLLLSLPVLAGAITMLLTDRNLNTSFFDPAGGGDPILYQHL
>COLFA183-10|HQ559268|MP00445|Oberea_oculata
TLYFIFGAWAGMVGTSLSLLIRSELGTPGSLIGDDQIYNVIVTAHAFIMIFFMVMPIMIGGFGNWLVPLMLGAPDMAFPRMNNMSFWLLPPSLSLLIMSSIIDSGAGTGWTVYPPLAANVAHNGSSVDLAIFSLHLAGISSILGAVNFITTVINMRPKGM
TLDRMPLFVWAVKITAILLLLSLPVLAGAITMLLTDRNLNTSFFDPAGGGDPILYQHL
>COLFA012-10|HM909042|MP00143|Pyrochroa_coccinea
TLYFILGAWSGMIGTSMSLIIRTELGNPGSLIGDDQIYNVIVTAHAFIMIFFMVMPIMIGGFGNWLVPLMLGAPDMAFPRMNNMSFWLLPPSLTLLIMSSIVENGAGTGWTVYPPLSSNIAHSGSSVDLAIFSLHLAGISSILGAVNFITTVINMRPSGMS
LDRMPLFVWAVVITAILLLLSLPVLAGAITMLLTDRNLNTTFFDPAGGGDPILYQHL
>COLFB764-12|KJ967318|ZMUO.001239|Trixagus_carinifrons
TLYFIFGSWAGMIGTAMSVIIRMELGTPGSMISNDQIFNVIVTSHAFIMIFFMVMPIMIGGFGNWLVPIMIGAPDMAFPRLNNMSFWLLPPSLSLIMTSNLTENGAGTGWTVYPPLSSNLAHSGPAVDFTIFSLHLAGISSILGAINFITTSINMRSPLLKP
ERLTLFTWSVSITALLLLLSLPVLAGAITMLLTDRNLNTSFFDPAGGGDPVLYQHL
>COLFE260-12|KJ966634|ZMUO.005105|Sphaeroderma_testaceum
TLYFIFGIWAGMVGMSLSILIRMELGNPGSLIGNDQIYNVIVTAHAFIMIFFMVMPIMIGGFGNWLVPLMIGAPDMAFPRMNNMSFWLLPPSLFFLIMSSIVESGAGTGWTVYPPLSSNIAHSGASVDLAIFSLHLAGISSILGAINFITTIINMRPNGMS
PDRMPLFVWAVLITAILLLLSLPVLAGAITMLLTDRNLNTSFFDPSGGGDPILYQHL
>COLFF843-13|KJ963246|ZMUO.006163|Limonius_minutus
TLYFLFGAWSGMLGTSLSLLIRAELGNPGSLIGNDQIYNVIVTAHAFIMIFFMVMPIMIGGFGNWLVPLMLGAPDMAFPRMNNMSFWFLPPSLSLLLMSSIVENGAGTGWTVYPPLSANIAHSGSSVDLAIFSLHLAGISSILGAVNFISTVINMRSVGIT
FDRMPLFVWAVAITALLLLLSLPVLAGAITMLLTDRNLNTSFFDPAGGGDPILYQHL
>COLFE866-13|KJ967338|ZMUO.006186|Diacanthous_undulatus
TLYFLFGAWAGMLGTSLSLLIRAELGNPGSLIGNDQIYNVIVTAHAFIMIFFMVMPIMIGGFGNWLVPLMLGAPDMAFPRMNNMSFWFLPPSLSLLLMSSIVENGAGTGWTVYPPLSANIAHSGSSVDLAIFSLHLAGISSILGAVNFISTVINMRSTGIT
FDRMPLFVWAVAITALLLLLSLPVLAGAITMLLTDRNLNTSFFDPAGGGDPILYQHL
>COLFE1245-13|KJ965084|ZMUO.007040|Epuraea_biguttata
TLYFIFGAWSGMVGTSLSILIRTELGSPGSLIGNDQIYNVIVTAHAFIMIFFMVMPFMIGGFGNWLVPLMLGAPDMAFPRMNNMSFWLLPPSLSLLLMSSIVESGAGTGWTVYPPLSSNIAHGGSSVDLAIFSLHLAGISSILGAVNFITTIINMRPVGMT
LDRMPLFIWSVMITAVLLLLSLPVLAGAITMLLTDRNLNTTFFDPSGGGDPILYQHL

>COLFA513-12|KJ963291|ZMUO.000553|Margarinotus_striola
TLYFIFGAWAGMVGTSLSLLIRAELGAPGTLIGDDQIYNVVVTAHAFIMIFFMVMPIMIGGFGNWLVPLMLGAPDMAFPRMNNMSFWLLPPSLSLLLMSSIVESGAGTGWTVYPPLSSNIAHGGASVDLAIFSLHLAGISSILGAINFITTVINMRSPGM
MFDQMPLFTWSVAITALLLLLSLPVLAGAITMLLTDRNLNTSFFDPAGGGDPILYQHL
>COLFE1531-13|KJ967229|ZMUO.007421|Stereonychus_fraxini
TLYFIFGLWAGAVGTSLSMLIRTELGNPGSLIGDDQIYNTIVTAHAFIMIFFMVMPIMIGGFGNWLVPLMLGAPDMAFPRLNNLSFWLLPPSITLLLMSSIVEKGAGTGWTVYPPLSNNVAHEGASVDLAIFSLHMAGISSILGAINFISTVSNMRTKGM
EYDRTPLFVWSVNITAFLLLISLPVLAGAITMLLTDRNINTSFFDPSGGGDPILYQHL
>COLFC845-12|KJ966947|ZMUO.003410|Galerucella_sp.
TLYFIFGVWAGMVGTSLSILVRVELGNPGSLIGNDQIYNVIVTAHAFIMIFFMVMPIMIGGFGNWLVPLMIGAPDMAFPRMNNMSFWLLPPSLFLLIMSSIVESGAGTGWTVYPPLSSNIAHGGSSVDLAIFSLHLAGISSILGAINFITTIINMRPKGMT
LDRMPLFVWAVMITAILLLLSLPVLAGAITMLLTDRNLNTSFFDPAGGGDPILYQHL
>COLFD157-12|KJ965592|ZMUO.004052|Polydrusus_cervinus
TLYFIFGAWSGMVGTSLSMLIRTELGNPGSLIGDDQIYNVIVTAHAFIMIFFMVMPIMIGGFGNWLVPLMLGAPDMAFPRLNNMSFWLLPPSLTLLLMSSIVDKGAGTGWTVYPPLSTNIAHEGSSVDLAIFSLHMAGVSSILGAINFISTIINMRPTGM
SYDRMPLFVWAVKITAILLLLSLPVLAGAITMLLTDRNINTSFFDPAGGGDPILYQHL
>COLFA523-12|KJ963823|ZMUO.000563|Heterocerus_fenestratus
TLYFILGSWSGMLGMALSLLIRAELGNPGALIGDDQIYNVIVTAHAFIMIFFMVMPIMIGGFGNWLVPLMLGAPDMAFPRMNNMSFWLLPPSLTLLLMSSMVESGAGTGWTVYPPLSANIAHSGSSVDLAIFSLHLAGISSILGAVNFITTVINMRAPG
MSFDRMPLFVWSVVITAVLLLLSLPVLAGAITMLLTDRNINTSFFDPAGGGDPILYQHL
>COLFC684-12|KJ964250|ZMUO.003249|Lagria_hirta
TLYFIFGSWAGMVGTSLSLLIRVELSNPGSFIGDDQVYNVVVTAHAFIMIFFMVMPIMIGGFGNWLVPLMLGAPDMAFPRMNNMSFWLLPPSLSLLLMSSLVESGAGTGWTVYPPLSSNLAHSGSSVDLAIFSLHLAGISSILGAVNFITTVINMRPLG
MKFDRLPLFVWSVMITAILLLLSLPVLAGAITMLLTDRNINTSFFDPSGGGDPILYQHL
>COLFA035-10|HM909062|MP00185|Atheta_sodalis
TLYFIFGAWAGMVGTSLSLLIRAELGNPGSLIGDDQIYNVIVTAHAFIMIFFMVMPIMIGGFGNWLVPLMLGAPDMAFPRMNNMSFWLLPPSLTLLLMSSMVESGAGTGWTVYPPLSSNIAHGGSSVDLAIFSLHLAGISSILGAVNFISTVINMRSTGI
SFDRMPLFVWAVAITALLLLLSLPVLAGAITMLLTDRNLNTSFFDPAGGGDPILYQHL
>COLFB216-12|KJ962351|ZMUO.001546|Strophosoma_capitatum
TLYFIFGAWSGMIGTSLSILIRTELGNPGSLIGDDQIYNVIVTAHAFIMIFFMVMPMMIGGFGNWLVPLMLGAPDMAFPRMNNMSFWLLPPSLSLLLMSSIVDKGAGTGWTVYPPLSANIAHEGSSVDLAIFSLHMAGVSSILGAVNFISTVINMRPM
GMSPDRMSLFIWSVKITAILLLLSLPVLAGAITMLLTDRNANTSFFDPAGGGDPILYQHL
>COLFC207-12|KJ963741|ZMUO.002392|Atheta_celata
TLYFIFGAWAGMVGTSLSLLIRAELGNPGSLIGDDQIYNVIVTAHAFVMIFFMVMPIVIGGFGNWLIPLMLGAPDMAFPRMNNMSFWLLPPSLTLLLMSSMVESGAGTGWTVYPPLSSNIAHGGASVDLAIFSLHLAGISSILGAVNFISTVINMRSTGI
SFDRMPLFVWSVAITALLLLLSLPVLAGAITMLLTDRNLNTSFFDPAGGGDPILYQHL
>COLFA310-12|KJ962495|ZMUO.000445|Cryptopleurum_minutum
TLYFIFGAWAGMVGTSLSILIRAELGNPGTLIGDDQIYNVIVTAHAFIMIFFMVMPIMIGGFGNWLVPLMLGAPDMAFPRMNNMSFWLLPPSLTLLLMSSMVESGAGTGWTVYPPLSSNIAHGGASVDLAIFSLHLAGISSILGAVNFITTVINMRSPN
LSYDRLPLFVWSVAITALLLLLSLPVLAGAITMLLTDRNLNTSFFDPAGGGDPILYQHL
>COLFF970-13|KJ961743|ZMUO.007335|Aphodius_contaminatus
TLYFLFGSWAGMVGTSLSLLIRAELGNPGSLIGDDQIYNVIVTAHAFVMIFFMVMPILIGGFGNWLVPLMLGAPDMAFPRMNNMSFWLLPPSLTLLLMSSMVESGAGTGWTVYPPLSSNIAHGGASVDLAIFSLHLAGISSILGAVNFITTVINMRSPG
MTFDRMPLFVWSVAITALLLLLSLPVLAGAITMLLTDRNLNTSFFDPAGGGDPILYQHL
>COLFD907-12|KJ961931|ZMUO.004517|Gnypeta_carbonaria
TLYFIFGAWAGMVGTSLSLLIRAELGNPGSLIGNDQIYNVIVTAHAFVMIFFMVMPIVIGGFGNWLVPLMLGAPDMAFPRMNNMSFWLLPPSLTLLLMSSMVENGAGTGWTVYPPLSSNIAHGGSSVDLAIFSLHLAGISSILGAVNFISTVINMRSTG
ISFDRMPLFVWSVAITALLLLLSLPVLAGAITMLLTDRNLNTSFFDPAGGGDPILYQHL
>COLFB557-12|KJ966778|ZMUO.001032|Stenus_incrassatus
ILYFVFGAWAGMVGTSLSLLIRAELGNPGSLIGDDQIYNVIVTAHAFVMIFFMVMPVMIGGFGNWLVPLMLGAPDMAFPRMNNMSFWLLPPSLSLLLMSSIVENGAGTGWTVYPPLSSNIAHSGASVDLAIFSLHLAGVSSILGAINFITTIFNMRTM
KLQLDCLPLFVWSVGITALLLLLSLPVLAGAITMLLTDRNLNTSFFDPAGGGDPILYQHL
>COLFA453-12|KJ962656|ZMUO.000493|Thanasimus_formicarius
TLYFIFGAWAGMVGTSLSLLIRSELGTPGTLIGNDQIYNVIVTAHAFIMIFFMVMPIMIGGFGNWLVPLMLGAPDMAFPRMNNMSFWLLPPSLTLLLMSSMVENGAGTGWTVYPPLSSNIAHGGSSVDLAIFSLHLAGVSSILGAVNFITTVINMRPEG
MTLDRMPLFVWAVAITALLLLLSLPVLAGAITMLLTDRNLNTSFFDPAGGGDPILYQHL
>COLFC637-12|KJ965290|ZMUO.003202|Agabus_labiatus
TLYFIFGAWAGMVGTSLSMLIRAELGNPGSLIGDDQIYNVIVTAHAFVMIFFMVMPIMIGGFGNWLVPLMLGAPDMAFPRMNNMSFWLLPPSLTLLLMSSMVESGAGTGWTVYPPLSSGIAHGGASVDLAIFSLHLAGVSSILGAVNFITTIINMRSI
GMTFDRMPLFVWSVGITALLLLLSLPVLAGAITMLLTDRNLNTSFFDPAGGGDPILYQHL
>COLFC567-12|KJ965656|ZMUO.003132|Cymindis_macularis

TLYFIFGAWAGMVGTSLSMLIRAELGNPGALIGDDQIYNVIVTAHAFIMIFFMVMPIMIGGFGNWLVPLMLGAPDMAFPRMNNMSFWLLPPSLTLLLMSSMVEKGAGTGWTVYPPLSSSIAHAGASVDLAIFSLHLAGVSSILGAVNFITTIINMRSIG
MTFDRMSLFVWSVGITALLLLLSLPVLAGAITMLLTDRNLNTSFFDPAGGGDPILYQHL
>COLFA003-10|HM909034|MP00084|Tropideres_dorsalis
TLYFIFGAWAGMMGTSLSILIRTELGSPGSLIGDDQIYNVIVTAHAFVMIFFMVMPTMIGGFGNWLVPLMLGAPDMAFPRMNNMSFWLLPPSLTLLTVSSIVESGAGTGWTVYPPLSSNIAHSGPSVDLAIFSLHLAGVSSILGAVNFITTILNMRPKG
MTLDRTPLFVWAVSITALLLLLSLPVLAGAITMLLTDRNLNTSFFDPAGGGDPLLYQHL
>COLFE1063-13|KJ965109|ZMUO.006858|Enochrus_affinis
TLYFIFGAWAGMVGTSLSILIRAELGNPGTLIGDDQIYNVIVTAHAFIMIFFMVMPIMIGGFGNWLVPLMLGAPDMAFPRMNNMSFWLLPPSLTLLLMSSMVESGAGTGWTVYPPLSSNIAHGGASVDLAIFSLHLAGISSILGAVNFITTVINMRSPS
MTYDRLPLFVWSVAITALLLLLSLPVLAGAITMLLTDRNLNTSFFDPAGGGDPILYQHL
>COLFD218-12|KJ963393|ZMUO.004113|Salpingus_planirostris
TLYFIFGAWSGMVGTSLSLLIRAELGNPGSLIGDDQIYNVIVTAHAFVMIFFMVMPIVIGGFGNWLVPLMLGAPDMAFPRMNNMSFWLLPPSLTLLIMSSIVESGAGTGWTVYPPLAANIAHGGSSVDLAIFSLHLAGISSILGAVNFITTVINMRPSGM
SFDRMPLFVWAVVITAVLLLLSLPVLAGAITMLLTDRNLNTTFFDPAGGGDPILYQHL
>COLFC138-12|KJ967261|ZMUO.002323|Phratora_polaris
TLYFIFGIWAGMVGTSLSMLIRSELGNPGTLIGNDQIYNVIVTAHAFIMIFFMVMPIMIGGFGNWLVPLMIGAPDMAFPRLNNMSFWLLPPSLFLLLMSSVVENGAGTGWTVYPPLSSNLAHSGSSVDLAIFSLHLAGISSILGAINFITTVINMRPEGM
GLEQTPLFVWAVLITAILLLLSLPVLAGAITMLLTDRNLNTSFFDPAGGGDPILYQHL
>COLFC584-12|KJ966023|ZMUO.003149|Lygistopterus_sanguineus
TLYFIFGAWAGMLGTSLSLLIRAELGTPGTLIGNDQIYNVIVTAHAFIMIFFMVMPMMIGGFGNWLVPLMLGAPDMAFPRMNNMSFWLLPPSLSLLIMSSMVESGAGTGWTVYPPLSSNIAHSGSSVDLAIFSLHLAGVSSILGAVNFISTIINMKSPG
MTYDQMPLFVWAVGITALLLLLSLPVLAGAITMLLTDRNLNTSFFDPTGGGDPILYQHL
>COLFE1492-13|KJ967502|ZMUO.007287|Meligethes_ochropus
TLYFIFGAWSGMVGTSLSMLIRTELGNPGSLIGNDQIYNVIVTAHAFVMIFFMVMPFMIGGFGNWLVPLMLGAPDMAFPRMNNMSFWLLPPSLSLLLMSSIVESGAGTGWTVYPPLSSNIAHGGASVDLAIFSLHLAGISSILGAVNFITTVINMRPSG
MTFDRMPLFVWAVVITAILLLLSLPVLAGAITMLLTDRNLNTTFFDPSGGGDPILYQHL
>COLFD834-12|KJ963340|ZMUO.004824|Stethorus_pusillus
TLYFMFGLWAGMVGTSLSILIRLELGTPGSLIGNDQIYNVIVTAHAFIMIFFMVMPIMIGGFGNWLVPLMIGAPDMAFPRLNNMSFWLLPPALTLLILGSMVESGAGTGWTVYPPLSSNLAHGGSAVDLSIFSLHLAGISSILGAVNFITTIINMRPTGMT
LDKTPLFVWSVFITAILLLLSLPVLAGAITMLLTDRNINTTFFDPAGGGDPLLYQHL
>COLFC307-12|KJ964921|ZMUO.002492|Coccidula_rufa
TLYFLFGMWAGMVGTSLSILIRLELGTPDSLIGNDQIYNVIVTAHAFIMIFFMVMPIMIGGFGNWLVPLMIGAPDMAFPRMNNMSFWLLPPALTLLILSSLVESGAGTGWTVYPPLSSNLAHSGSSVDMAIFSLHLAGISSILGAVNFISTVINMRTPGM
TFEKMPLFVWSVIITAILLLLSLPVLAGAITMLLTDRNINTSFFDPAGGGDPILYQHL
>COLFF479-13|KJ965108|ZMUO.006559|Atholus_duodecimstriatus
TLYFIFGAWAGMVGTSLSLLIRAELGTPGTLIGDDQIYNVIVTAHAFIMIFFMVMPIMIGGFGNWLVPLMLGAPDMAFPRMNNMSFWLLPPSLSLLLMSSIVESGAGTGWTVYPPLSSNIAHGGASVDLAIFSLHLAGISSILGAINFITTVINMRSPGM
MFDQMPLFIWSVAITALLLLLSLPVLAGAITMLLTDRNLNTSFFDPAGGGDPILYQHL
>COLFA142-10|HQ559252|MP00404|Colymbetes_dolabratus
TLYFIFGAWAGMVGTSLSMLIRAELGNPGSLIGDDQIYNVIVTAHAFVMIFFMVMPIMIGGFGNWLVPLMLGAPDMAFPRMNNMSFWLLPPSLTLLLMSSMVESGAGTGWTVYPPLSSGIAHGGASVDLAIFSLHLAGISSILGAVNFITTIINMRSV
GMTFDRMPLFVWSVGITALLLLLSLPVLAGAITMLLTDRNLNTSFFDPAGGGDPILYQHL
>COLFF847-13|KJ966464|ZMUO.006167|Malthinus_flaveolus
TLYFIFGAWSGMLGTSLSLLIRAELGNPGSLIGNDQIYNVIVTAHAFIMIFFMVMPIMIGGFGNWLVPLMLGAPDMAFPRMNNMSFWFLPPSLSLLLMSSLVESGAGTGWTVYPPLSANIAHSGSSVDLAIFSLHMAGISSILGAVNFISTVINMRSVG
MSFDRMPLFVWAVAITALLLLLSLPVLAGAITMLLTDRNLNTSFFDPAGGGDPILYQHL
>COLFB569-12|KJ962124|ZMUO.001044|Hygrotus_inaequalis
TLYFLFGAWAGMVGTSLSMLIRAELGNPGSLIGDDQIYNVIVTAHAFIMIFFMVMPIMIGGFGNWLVPLMLGAPDMAFPRMNNMSFWMLPPSLTLLLMSSMVESGAGTGWTVYPPLSAGIAHGGASVDLAIFSLHLAGISSILGAVNFITTIINMRS
VGMTFDRMALFVWSVGITALLLLLSLPVLAGAITMLLTDRNLNTSFFDPAGGGDPILYQHL
>COLFA073-10|HM909091|MP00266|Bitoma_crenata
TLYFIFGAWSGMVGTSLSLLIRAELGNPGSLIGDDQIYNVIVTAHAFIMIFFMVMPIMIGGFGNWLVPLMLGAPDMAFPRMNNMSFWLLPPSLTLLLMSSIVENGAGTGWTVYPPLSSNIAHGGSSVDLAIFSLHLAGISSILGAVNFITTVINMRPSGM
SFDRMPLFAWAVVITAVLLLLSLPVLAGAITMLLTDRNLNTSFFDPAGGGDPILYQHL
>COLFC791-12|KJ965847|ZMUO.003356|Polydrusus_pilosus
TLYFIFGAWSGMVGTSLSMLIRTELGNPGSLIGDDQIYNVIVTAHAFIMIFFMVMPIMIGGFGNWLVPLMLGAPDMAFPRLNNMSFWLLPPSLTLLLMSSIVDKGAGTGWTVYPPLSMNIAHEGSSVDLAIFSLHMAGVSSILGAINFISTIINMRPTG
MSYDRMPLFVWAVKITAILLLLSLPVLAGAITMLLTDRNINTSFFDPAGGGDPILYQHL
>COLFA274-12|KJ965975|ZMUO.000409|Anthonomus_rectirostris
TLYFIFGAWSGTIGTSLSILIRTELGTPGSLIGDDQIYNTIVTAHAFIMIFFMVMPIMIGGFGNWLIPLMLAAPDMAFPRLNNMSFWLLPPSLTLLIMSSIISKGAGTGWTVYPPLSSNLTHEGASVDFAIFSLHLAGISSILGAMNFISTVLNMKPIKMNLEQ
MPLFVWAVKITAILLLISLPVLAGAITMLLTDRNLNTSFFDPAGGGDPILYQHL

>COLFB356-12|KJ962515|ZMUO.001686|Atheta_melanocera
TLYFIFGAWAGMVGTSLSLLIRAELGNPGSLIGDDQIYNVIVTAHAFIMIFFMVMPIVIGGFGNWLVPLMLGAPDMAFPRMNNMSFWLLPPSLTLLLMSSMVESGAGTGWTVYPPLSSNIAHGGSSVDLAIFSLHLAGISSILGAVNFISTVINMRSTGI
SFDRMPLFVWSVAITALLLLLSLPVLAGAITMLLTDRNLNTSFFDPAGGGDPILYQHL
>COLFD944-12|KJ961714|ZMUO.004554|Anthonomus_pomorum
TLYFIFGAWSGAVGTSLSMLIRTELGNPGSLIGDDQIYNTIVTAHAFIMIFFMVMPIMIGGFGNWLVPLMLAAPDMAFPRLNNMSFWLLPPSLTLLIMSSIIGKGAGTGWTVYPPLSSNLAHEGASVDFAIFSLHMAGISSILGAMNFISTVLNMKPMG
MNLEQMPLFVWAVKITAILLLISLPVLAGAITMLLTDRNINTSFFDPAGGGDPILYQHL
>COLFF1013-13|KJ964031|ZMUO.007378|Calodera_riparia
TLYFIFGAWAGMIGTSLSLLIRAELGNPGSLIGDDQIYNVIVTAHAFVMIFFMVMPIVIGGFGNWLIPLMLGAPDMAFPRMNNMSFWLLPPSLSLLLMSSMVESGAGTGWTVYPPLSSNIAHGGSSVDLAIFSLHLAGISSILGAVNFISTIINMRTSGMS
FDRMPLFVWSVAITALLLLLSLPVLAGAITMLLTDRNLNTSFFDPAGGGDPILYQHL
>COLFD022-12|KJ963076|ZMUO.003917|Xyletinus_planicollis
TLYFILGAWSGMVGTSLSILIRSELGNPGALIGDDQIYNVIVTAHAFIMIFFMVMPIMIGGFGNWLVPLMLGAPDMAFPRMNNMSFWLLPPSLSLLLMSSVVNSGAGTGWTVYPPLSSNIAHSGASVDLAIFSLHLAGISSILGAVNFITTVINMRAIGM
MFDRMPLFVWSVVITAVLLLLSLPVLAGAITMLLTDRNLNTSFFDPAGGGDPILYQHL
>COLFE1258-13|KJ966841|ZMUO.007053|Atheta_pinegensis
TLYFIFGAWAGMVGTSLSLLIRAELGNPGSLIGDDQIYNVIVTAHAFIMIFFMVMPIVIGGFGNWLVPLMLGAPDMAFPRMNNMSFWLLPPSLTLLLMSSMVESGAGTGWTVYPPLSSNIAHGGSSVDLAIFSLHLAGISSILGAVNFISTVINMRSAGI
SFDRMPLFVWSVVITALLLLLSLPVLAGAITMLLTDRNLNTSFFDPAGGGDPILYQHL
>COLFB400-12|KJ967323|ZMUO.001825|Oxypoda_acuminata
TLYFIFGAWAGMVGTSLSLLIRAELGNPGSLIGDDQIYNVIVTAHAFVMIFFMVMPIVIGGFGNWLIPLMLGAPDMAFPRMNNMSFWLLPPSLTLLLMSSMVESGAGTGWTVYPPLSSNIAHGGSSVDLAIFSLHLAGISSILGAVNFISTIINMRTSGM
SFDRMPLFVWSVAITALLLLLSLPVLAGAITMLLTDRNLNTSFFDPAGGGDPILYQHL
>COLFC525-12|KJ967341|ZMUO.003090|Callidium_violaceum
TLYFLFGAWASMVGTSLSMLIRTELGNPGSLIGDDQIYNVIVTAHAFVMIFFMVMPIMIGGFGNWLVPLMIGAPDMAFPRMNNLSFWLLPPALILLILSSIVENGAGTGWTVYPPLSANVAHSGSSVDLAIFSLHLAGISSILGAVNFISTVINMRPMG
MTPERMPLFVWAVVITAILLLLSLPVLAGAITMLLTDRNINTSFFDPAGGGDPILYQHL
>COLFE1364-13|KJ966386|ZMUO.007159|Mantura_chrysanthemi
TLYFIFGIWSGMVGTSLSMLIRTELGNPGSLIGNDQIYNVIVTAHAFIMIFFMVMPIMIGGFGNWLVPLMIGAPDMAFPRMNNMSFWLLPPSLFLLLMSSVVESGAGTGWTVYPPLSSNIAHGGSSVDLAIFSLHLAGISSILGAINFITTVINMRPQGM
TLDRMPLFVWAVVITAILLLLSLPVLAGAITMLLTDRNLNTSFFDPAGGGDPILYQHL
>COLFD342-12|KJ965114|ZMUO.004237|Kateretes_pedicularius
TLYFIFGAWSGMVGTSLSLLIRSELGNPGSLIGDDQIYNVIVTAHAFVMIFFMVMPFMIGGFGNWLVPLMLGAPDMAFPRMNNMSFWLLPPSLSLLLMSSIVESGAGTGWTVYPPLSSNIAHGGSSVDLAIFSLHLAGISSILGAVNFITTVINMRPLG
MTFDRMPLFVWAVAITALLLLLSLPVLAGAITMLLTDRNLNTSFFDPAGGGDPVLYQHL
>COLFA539-12|KJ964547|ZMUO.000674|Lytta_vesicatoria
TLYLIFGAWAGMVGTSLSLLIRSELGNPGTLIGDDQIYNVIVTAHAFIMIFFMVMPIVIGGFGNWLVPLMLGAPDMAFPRMNNMSFWLLPPSLVLLIMSSIVENGAGTGWTVYPPLSSNIAHGGSSVDLAIFSLHLAGISSILGAVNFITTVINMRPMG
MSFDRMPLFVWAVVITALLLLLSLPVLAGAITMLLTDRNLNTSFFDPAGGGDPILYQHL
>COLFF891-13|KJ962599|ZMUO.006686|Quedius_fuliginosus
TLYFIFGAWAGMVGTSLSLLIRAELGNPGSLIGDDQIYNVIVTAHAFIMIFFMVMPIVIGGFGNWLVPLMLGAPDMAFPRMNNMSFWLLPPSLSLLLMSSMVESGAGTGWTVYPPLSSNIAHGGASVDLAIFSLHLAGISSILGAVNFITTVINMRSIG
MTFDRMPLFVWSVAITALLLLLSLPVLAGAITMLLTDRNLNTSFFDPAGGGDPILYQHL
>COLFB526-12|KJ965509|ZMUO.001001|Cafius_xantholoma
TLYFIFGSWAGMVGTSLSLLIRAELGNPGTLIGDDQIYNVIVTAHAFIMIFFMVMPIVIGGFGNWLVPLMLGAPDMAFPRMNNMSFWLLPPSLTLLLMSSMVESGAGTGWTVYPPLSSNIAHGGASVDLAIFSLHLAGISSILGAVNFITTVINMRSTG
MTFDRMPLFVWSVAITALLLLLSLPVLAGAITMLLTDRNLNTSFFDPAGGGDPILYQHL
>COLFA426-12|KJ965551|ZMUO.000656|Epuraea_terminalis
TLYFIFGAWSGMVGTSLSILIRTELGSPGSLIGNDQIYNVIVTAHAFIMIFFMVMPFMIGGFGNWLVPLMLGAPDMAFPRMNNMSFWLLPPSLSLLLMSSIVESGAGTGWTVYPPLSSNIAHGGSSVDLAIFSLHLAGISSILGAVNFITTIINMRPVGMT
LDRMPLFVWSVVITAVLLLLSLPVLAGAITMLLTDRNLNTTFFDPSGGGDPILYQHL
>COLFB158-12|KJ965973|ZMUO.001488|Buprestis_rustica
TLYFIFGAWSGMVGTALSLLIRAELGNPGALIGDDQVYNVIVTAHAFVMIFFMVMPVMMGGFGNWLVPLMLGAPDMAFPRMNNMSFWLLPPSLTLLLMSSVVENGAGTGWTVYPPLAANIAHSGASVDLAIFSLHLAGVSSILGAVNFITTVINM
RSVGMTFDRMPLFVWSVAITALLLLLSLPVLAGAITMLLTDRNFNTSFFDPAGGGDPILYQHL
>COLFE634-13|KJ966406|ZMUO.005479|Cortodera_femorata
TLYFIFGAWAGMVGTSLSLLIRSELGNPGSLIGDDQIYNVIVTAHAFIMIFFMVMPIMIGGFGNWLVPLMLGAPDMAFPRMNNMSFWLLPPSLTLLIMSSIVEKGAGTGWTVYPPLSSNIAHGGSSVDLAIFSLHLAGISSILGAVNFITTVINMRPSGM
TLDRMPLFVWAVVITAILLLLSLPVLAGAITMLLTDRNLNTSFFDPAGGGDPILYQHL
>COLFG029-13|KJ963776|ZMUO.007534|Lixus_iridis

TLYFIFGAWSGMVGTSLSMLIRTELGNPGSLIGDDQIYNTIVTAHAFIMIFFMVMPIMIGGFGNWLIPLMLGAPDMAFPRLNNMSFWLLPPSLTLLLMSSIVDKGAGTGWTVYPPLSANISHEGASVDLAIFSLHMAGISSILGAVNFISTVLNMRPMG
MKLDQTALFTWAVKITAILLLLSLPVLAGAITMLLTDRNINTSFFDPAGGGDPILYQHL
>COLFF103-13|KJ962997|ZMUO.003737|Cryptocephalus_aureolus
TLYFLFGAWAGMVGTSLSLLIRVELGNPSSLIGNDQIYNTIVTAHAFIMIFFMVMPIMIGGFGNWLVPLMLGAPDMAFPRMNNMSFWLLPPSLTLLLMSSIVENGAGTGWTVYPPLSSTIAHTGSSVDLAIFSLHLAGVSSILGAINFISTIINMRPQGM
FMDRTPLFVWAVLITAVLLLLSLPVLAGAITMLLTDRNLNTAFFDPAGGGDPILYQHL
>COLFC499-12|KJ963621|ZMUO.003064|Thanasimus_formicarius
TLYFIFGAWAGMVGTSLSLLIRLELGTPGTLIGNDQIYNVIVTAHAFIMIFFMVMPIMIGGFGNWLVPLMLGAPDMAFPRMNNMSFWLLPPSLTLLLMSSMVENGAGTGWTVYPPLSSNIAHGGSSVDLAIFSLHLAGVSSILGAVNFITTVINMRPEG
MTLDRMPLFVWAVAITALLLLLSLPVLAGAITMLLTDRNLNTSFFDPAGGGDPILYQHL
>COLFA512-12|KJ965615|ZMUO.000552|Alosterna_tabacicolor
TLYFIFGAWAGMVGTSLSLLIRSELGSPGSFIGDDQVYNVIVTAHAFIMIFFMVMPIMIGGFGNWLVPLMLGAPDMAFPRMNNMSFWLLPPSLTLLIMSSIVESGAGTGWTVYPPLSSNIAHGGSSVDLAIFSLHLAGISSILGAVNFITTVINMRPMG
MNLDCMPLFVWAVVITAILLLLSLPVLAGAITMLLTDRNLNTSFFDPAGGGDPILYQHL
>COLFB285-12|KJ965741|ZMUO.001615|Dasytes_niger
TLYFIFGAWSGMVGMSLSLLIRSELNNPGTLIGNDQIYNVIVTAHAFIMIFFMVMPILIGGFGNWLVPLMLGAPDMAFPRMNNMSFWLLPPSLTLLLMSSMVEQGAGTGWTVYPPLSSNIAHGGASVDLAIFSLHLAGISSILGAVNFITTIINMRPVG
MTLDRTPLFVWAVAITALLLLLSLPVLAGAITMLLTDRNLNTSFFDPAGGGDPILYQHL
>COLFE382-12|KJ962846|ZMUO.004942|Cychramus_luteus
TLYFIFGAWSGMVGTSLSILIRTELGNPGSLIGNDQIYNVIVTAHAFIMIFFMVMPFMIGGFGNWLVPLMLGAPDMAFPRMNNMSFWLLPPSLSLLLMSSIVESGAGTGWTVYPPLSSNIAHGGSSVDLAIFSLHLAGISSILGAVNFITTVINMRPNGM
SFDRMPLFVWAVVITAILLLLSLPVLAGAITMLLTDRNLNTTFFDPSGGGDPILYQHL
>COLFF321-13|KJ965671|ZMUO.006021|Lomechusoides_wellenii
TLYFIFGAWAGLVGTSLSLLIRAELGNPGSLIGNDQIYNVIVTAHAFVMIFFMVMPIVIGGFGNWLIPLMLGAPDMAFPRMNNMSFWLLPPSLTLLLMSSMVESGSGTGWTVYPPLSSNIAHGGSSVDLTIFSLHLAGISSILGAVNFISTIINMRSIGISFD
RLPLFVWSVAITALLLLLSLPVLAGAITMLLTDRNLNTSFFDPAGGGDPILYQHL
>COLFC797-12|KJ962723|ZMUO.003362|Acmaeops_pratensis
VLYFIFGAWAGMVGTSLSLLIRSELGNPGSLIGNDQIYNVIVTAHAFIMIFFMVMPIMIGGFGNWLVPLMLGAPDMAFPRLNNMSFWLLPPSLTLLIMSSIVESGAGTGWTVYPPLSSNIAHGGSSVDLAIFSLHLAGISSILGAINFITTVINMRPKGMT
FDRMPLFVWAVVITAILLLLSLPVLAGAITMLLTDRNLNTSFFDPAGGGDPILYQHL
>COLFD016-12|KJ967467|ZMUO.003911|Triplax_rufipes
TLYFIFGMWAGMVGTALSVLIRTELGNPGSLIGNDQIYNVIVTAHAFIMIFFMVMPIMMGGFGNWLVPLMLGAPDMAFPRLNNMSFWLLPPSLSLLIMSSIVETGAGTGWTVYPPLSSNIAHSGASVDLAIFSLHLAGISSILGAMNFITTMMNMRP
SGMTMDQMPLFVWAVFITSILLLLSLPVLAGGITMLLTDRNINTSFFDPAGGGDPILYQHL
>COLFA678-12|KJ963638|ZMUO.000813|Acupalpus_parvulus
TLYFIFGVWAGMVGTSLSMLIRAELGTPGALIGDDQIYNVIVTAHAFVMIFFMVMPVMIGGFGNWLVPLMLGAPDMAFPRMNNMSFWLLPPSLTLLLMSSLVESGAGTGWTVYPPLSSGIAHSGASVDLAIFSLHLAGVSSILGAVNFITTIINMRSV
GMTFERMPLFVWSVGITALLLLLSLPVLAGAITMLLTDRNLNTSFFDPAGGGDPILYQHL
>COLFE1257-13|KJ964889|ZMUO.007052|Gnypeta_caerulea
TLYFIFGAWAGMVGTSLSLLIRAELGNPGSLIGNDQIYNVIVTAHAFVMIFFMVMPIVIGGFGNWLIPLMLGAPDMAFPRMNNMSFWLLPPSLTLLLMSSMVENGAGTGWTVYPPLSSNIAHGGSSVDLAIFSLHLAGISSILGAVNFISTIINMRSTGIS
FDRMPLFVWSVAITALLLLLSLPVLAGAITMLLTDRNLNTSFFDPAGGGDPILYQHL
>COLFE1601-13|KJ966397|ZMUO.007491|Hydraena_britteni
TLYFIFGAWAGMVGTSLSILIRAELGNPGTLIGDDQIYNVIVTAHAFIMIFFMVMPILIGGFGNWLVPLMLGAPDMAFPRMNNMSFWLLPPSLSLLLMSSMVESGAGTGWTVYPPLSSNIAHGGASVDLAIFSLHLAGISSILGAVNFITTIINMRSPGM
SFDQMPLFVWAVGITALLLLLSLPVLAGAITMLLTDRNLNTSFFDPAGGGDPILYQHL
>COLFF339-13|KJ964891|ZMUO.006039|Galerucella_sagittariae
TLYFIFGVWAGMVGTSLSILVRVELGNPGSLIGNDQIYNVIVTAHAFIMIFFMVMPIMIGGFGNWLVPLMIGAPDMAFPRMNNMSFWLLPPSLFLLIMSSIVESGAGTGWTVYPPLSSNIAHGGSSVDLAIFSLHLAGISSILGAINFITTIINMRPKGMT
LDRMPLFVWAVMITAILLLLSLPVLAGAITMLLTDRNLNTSFFDPAGGGDPILYQHL
>COLFA521-12|KJ967002|ZMUO.000561|Hallomenus_axillaris
TLYFIFGSWSGMVGTSLSMLIRSELGNPGSLIGNDQIYNVIVTAHAFIMIFFMVMPIMLGGFGNWLVPLMLGAPDMAFPRMNNMSFWLLPPSLTLLIMSSMVENGAGTGWTVYPPLSSNIAHSGSSVDLAIFSLHLAGISSILGAINFITTIINMRPLGM
TLDRMPLFVWAVLITAILLLLSLPVLAGAITMLLTDRNLNTSFFDPAGGGDPILYQHL
>COLFC187-12|KJ961732|ZMUO.002372|Atheta_aeneipennis
TLYFIFGAWAGMVGTSLSLLIRAELGNPGSLIGDDQIYNVIVTAHAFIMIFFMVMPIVIGGFGNWLVPLMLGAPDMAFPRMNNMSFWLLPPSLTLLLMSSMVESGAGTGWTVYPPLSSNIAHGGSSVDLAIFSLHLAGISSILGAVNFISTVINMRSTGI
SFDRMPLFVWSVAITALLLLLSLPVLAGAITMLLTDRNLNTSFFDPAGGGDPILYQHL
>COLFB733-12|KJ966867|ZMUO.001208|Cybister_lateralimarginalis
TLYFIFGAWAGMIGTSLSLLIRAELGNPGSLIGDDQIYNVIVTAHAFIMIFFMVMPIMIGGFGNWLVPLMLGAPDMAFPRMNNMSFWLLPPSLTLLLMSSMVENGAGTGWTVYPPLSAGIAHGGASVDLAIFSLHLAGVSSILGAVNFITTIINMRSVG
MTFDRMPLFVWSVGITALLLLLSLPVLAGAITMLLTDRNLNTSFFDPAGGGDPILYQHL

>COLFE577-13|KJ963424|ZMUO.005422|Chrysolina_varians
TLYFIFGMWAGMVGTSLSILIRAELGNPGTLIGNDQIYNVIVTAHAFIMIFFMVMPIMIGGFGNWLVPLMLGAPDMAFPRMNNMSFWLLPPSLIFLLMSSIVENGVGTGWTVYPPLSANIAHSGPSVDLAIFSLHLAGVSSILGAINFITTIINMRPTGM
QLEQMPLFSWAVLITAILLLLSLPVLAGAITMLLTDRNLNTSFFDPASGGDPILYQHL
>COLFD586-12|KJ965766|ZMUO.004386|Donacia_obscura
TLYFIFGAWSGMMGTSLSMLIRSELSNPGSLIGNDQIYNVIVTAHAFIMIFFMVMPIMIGGFGNWLVPLMLGAPDMAFPRMNNMSFWLLPPSLTFLTMSSITESGAGTGWTVYPPLSNNLAHSGSSVDLAIFSLHLAGISSILGAVNFISTIINMRPAG
MTMEKMSLFSWAVMITAILLLVSLPVLAGAITMLLTDRNINTSFFDPAGGGDPILYQHL
>COLFE280-12|KJ962919|ZMUO.005125|Pterostichus_diligens
TLYFIFGAWSGMVGTSLSMLIRAELGNPGSLIGDDQIYNVIVTAHAFVMIFFMVMPIMIGGFGNWLVPLMLGAPDMAFPRMNNMSFWLLPPSLTLLLMSSMVESGAGTGWTVYPPLSSGIAHAGASVDLAIFSLHLAGISSILGAVNFITTIINMRSIG
MTFDRMPLFVWSVGITALLLLLSLPVLAGAITMLLTDRNLNTSFFDPAGGGDPILYQHL
>COLFC776-12|KJ963243|ZMUO.003341|Elateroides_dermestoides
TLYFIFGAWAGMVGTSMSVLIRIELGTSGSLIGDDQIYNVIVTAHAFIMIFFMVMPIMIGGFGNWLVPLMLGAPDMAFPRMNNMSFWLLPPSLTLLLMSSMVESGAGTGWTVYPPLSSNIAHSGSSVDLAIFSLHLAGISSILGAANFITTIINMRPAG
MTFERMPLFVWAVLITAVLLLLSLPVLAGAITMLLTDRNLNTSFFDPAGGGDPILYQHL
>COLFC728-12|KJ963858|ZMUO.003293|Ampedus_tristis
TLYFIFGAWAGMLGTSLSLLIRAELGNPGSLIGNDQIYNVIVTAHAFIMIFFMVMPIMIGGFGNWLVPLMLGAPDMAFPRMNNMSFWLLPPSLSLLLMSSIVENGAGTGWTVYPPLSSNIAHSGSSVDLAIFSLHLAGISSILGAVNFISTVINMRSTGIT
FDRMPLFVWAVAITALLLLLSLPVLAGAITMLLTDRNLNTSFFDPAGGGDPILYQHL
>COLFB772-12|KJ963611|ZMUO.001247|Mniusa_incrassata
TLYFIFGAWAGMIGTSLSLLIRAELGNPGSLIGDDQIYNVIVTAHAFVMIFFMVMPIVIGGFGNWLVPLMLGAPDMAFPRMNNMSFWLLPPSLTLLLMSSMIESGAGTGWTVYPPLSSNIAHSGASVDLAIFSLHLAGISSILGAVNFISTIINMRTSGMT
FDRMPLFVWSVAITALLLLLSLPVLAGAITMLLTDRNLNTSFFDPAGGGDPILYQHL
>COLFF579-13|KJ961734|ZMUO.006469|Ceutorhynchus_ignitus
TLYFIFGSWAGMAGTSLSMLIRTELGNPGSLIGNDQIYNSIVTAHAFIMIFFMVMPILIGGFGNWLVPLMLGAPDMAFPRLNNMSFWLLPPSLSLLLMSSIVNKGAGTGWTVYPPLSSNVAHEGMSVDLAIFSLHMTGISSILGAINFISTVMNMQPKG
MTPELMPLFVWAVEITAILLLLSLPVLAGAITMLLTDRNINTSFFDPSGGGDPILYQHL
>COLFF897-13|KJ967164|ZMUO.006692|Cassida_nobilis
TLYFIFGFWSGMVGTSLSMLIRMELGSPGSLIGNDQIYNSIVTAHAFIMIFFMVMPIMIGGFGNWLVPLMLGAPDMAFPRLNNMSFWLLPPSITFLIMSSIIESGAGTGWTVYPPLSANIAHSGASVDLAIFSLHLAGISSILGAINFISTIMNMRPSGMT
LDKMALFVWAVIITAVLLLLSLPVLAGAITMLLTDRNINTSFFDPAGGGDPILYQHL
>COLFD422-12|KJ967054|ZMUO.003842|Lagria_hirta
TLYFIFGSWAGMVGTSLSLLIRVELSNPGSFIGDDQVYNVVVTAHAFIMIFFMVMPIMIGGFGNWLVPLMLGAPDMAFPRMNNMSFWLLPPSLSLLLMSSLVESGAGTGWTVYPPLSSNLAHSGSSVDLAIFSLHLAGISSILGAVNFITTVINMRPLG
MKFDRLPLFVWSVMITAILLLLSLPVLAGAITMLLTDRNINTSFFDPSGGGDPILYQHL
>COLFE074-12|KJ967527|ZMUO.005299|Colenis_immunda
TLYFIFGAWAGMVGTSLSILIRAELGTPGSLIGDDQIYNVIVTAHAFIMIFFMVMPIVIGGFGNWLVPLMLGAPDMAFPRMNNMSFWLLPPSLTLLLMSSIVENGAGTGWTVYPPLSANIAHSGSSVDLAIFSLHLAGISSILGAVNFITTVINMRSEGMS
FDKMPLFVWSVAITALLLLLSLPVLAGAITMLLTDRNLNTSFFDPAGGGDPILYQHL
>COLFF382-13|KJ963982|ZMUO.005900|Tomoxia_bucephala
TLYFIFGAWSGMVGTSLSLLIRAELGNPGSLIGDDQIYNTIVTAHAFIMIFFMVMPIMVGGFGNWLVPLMLGAPDMAFPRMNNMSFWLLPPSLSLLLMSSVVESGAGTGWTVYPPLSSNIAHGGSSVDLAIFSLHLAGVSSILGAINFISTMINMRPAG
MTMDRMPLFAWAIMITAVLLLLSLPVLAGAITMLLTDRNLNTSFFDPAGGGDPILYQHL
>COLFE987-13|KJ962925|ZMUO.006782|Cercyon_convexiusculus
TLYFIFGAWAGMVGTSLSILIRAELGNPGTLIGDDQIYNVIVTAHAFIMIFFMVMPIMIGGFGNWLVPLMLGAPDMAFPRMNNMSFWLLPPSLSLLLMSSMVESGAGTGWTVYPPLSSNIAHGGSSVDLAIFSLHLAGISSILGAVNFITTVINMRSPNL
TYDRLPLFVWSVAITALLLLLSLPVLAGAITMLLTDRNLNTSFFDPAGGGDPILYQHL
>COLFE089-12|KJ964316|ZMUO.005314|Hypera_arator
TLYFIFGTWAGTVGTSLSILIRTELGSPGSLIGNDQIYNTIVTAHAFIMIFFMVMPIMIGGFGNWLVPLMLGAPDMAFPRLNNMSFWLLPPSLSLLLMSSMVDSGAGTGWTVYPPLSSNIAHEGSSVDLAIFSLHMAGVSSILGAINFISTVLNMRPSGM
SLDKMALFIWAVKITAILLLLSLPVLAGAITMLLTDRNINTSFFDPAGGGDPILYQHL
>COLFF169-13|KJ966846|ZMUO.005893|Plateumaris_weisei
TLYFIFGAWSGMMGTSLSMLIRTELSNPGSLIGNDQTYNVIVTAHAFIMIFFMVMPIMIGGFGNWLVPLMLGAPDMAFPRMNNMSFWLLPPSLTFLIMSSIVENGVGTGWTVYPPLSSNIAHSGSSVDLAIFSLHMAGISSILGTINFITTIINMHPMG
MKMDKMPLFIWAVMITAILLLLSLPVLAGAITMLLTDRNLNTSFFDPAGGGDPILYQHL
>COLFB697-12|KJ965447|ZMUO.001172|Licinus_depressus
TLYFIFGAWAGMVGTSLSMLIRAELGNPGSLIGDDQIYNVIVTAHAFVMIFFMVMPIMIGGFGNWLVPLMLGAPDMAFPRMNNMSFWLLPPSLTLLLMSSMVESGAGTGWTVYPPLSSGIAHSGASVDLAIFSLHLAGVSSILGAVNFITTIINMRSI
GMTLDRMPLFVWSVGITALLLLLSLPVLAGAITMLLTDRNLNTSFFDPAGGGDPILYQHL
>COLFC081-12|KJ965936|ZMUO.002076|Bolitochara_pulchra

TLYFIFGAWAGMVGTSLSLLIRAELGNPGSLIGDDQIYNVIVTAHAFVMIFFMVMPIMIGGFGNWLVPLMLGAPDMAFPRMNNMSFWLLPPSLTLLLMSSMVESGAGTGWTVYPPLSSNIAHGGSSVDLAIFSLHLAGISSILGAVNFISTIINMRSPG
MTFDRMPLFVWSVAITALLLLLSLPVLAGAITMLLTDRNLNTSFFDPAGGGDPILYQHL
>COLFE806-13|KJ966349|ZMUO.005651|Scopaeus_laevigatus
TLYFLFGAWSGMVGTSLSLLIRAELSAPGSMIGDDQIYNVIVTAHAFVMIFFMVMPIVIGGFGNWLVPLMLGAPDMAFPRMNNMSFWLLPPSLSLLLMSSMVESGAGTGWTVYPPLSSNIAHGGASVDLAIFSLHLAGISSILGAVNFITTVINMRSP
GMTYERMPLFVWSVAITALLLLLSLPVLAGAITMLLTDRNLNTSFFDPAGGGDPILYQHL
>COLFA640-12|KJ965016|ZMUO.000775|Philonthus_addendus
TLYFIFGSWAGMVGTSLSLLIRAELGNPGTLIGDDQIYNVIVTAHAFIMIFFMVMPVVIGGFGNWLVPLMLGAPDMAFPRMNNMSFWLLPPSLTLLLMSSVVESGAGTGWTVYPPLSSNMAHGGASVDLAIFSLHLAGISSILGAVNFITTVINMRST
GMSFDRMPLFVWSVAITALLLLLSLPVLAGAITMLLTDRNLNTSFFDPAGGGDPILYQHL
>COLFB327-12|KJ961855|ZMUO.001657|Othius_punctulatus
TLYFIFGAWAGMVGTSLSLLIRAELGNPGSLIGDDQIYNVIVTAHAFIMIFFMVMPIMIGGFGNWLVPLMLGAPDMAFPRMNNMSFWLLPPSLSLLLMSSLVESGAGTGWTVYPPLSSNIAHGGASVDLAIFSLHLAGISSILGAVNFITTVINMRSIGM
TFDRMPLFVWSVAITALLLLLSLPVLAGAITMLLTDRNLNTSFFDPAGGGDPILYQHL
>COLFA357-12|KJ966192|ZMUO.000587|Geostiba_circellaris
TLYFIFGAWAGMVGTSLSLLIRAELGNPGSLIGDDQIYNVIVTAHAFVMIFFMVMPIVIGGFGNWLIPLMLGAPDMAFPRMNNMSFWLLPPSLSLLLMSSMVESGAGTGWTVYPPLSSNIAHGGASVDLAIFSLHLAGISSILGAVNFISTIINMRATGI
HFDRMPLFVWSVAITALLLLLSLPVLAGAITMLLTDRNLNTSFFDPAGGGDPILYQHL
>COLFF768-13|KJ963782|ZMUO.006088|Malthodes_spathifer
TLYFMFGAWSGMLGTSLSLLIRAELGSPGSLIGNDQIYNVIVTAHAFIMIFFMVMPIMIGGFGNWLVPLMLGAPDMAFPRMNNMSFWFLPPSLSLLLMSSVVESGAGTGWTVYPPLSSNIAHSGASVDLAIFSLHMAGISSILGAVNFISTVINMRSTG
MTFDRMPLFVWAVAITALLLLLSLPVLAGAITMLLTDRNLNTSFFDPAGGGDPILYQHL
>COLFC594-12|KJ965165|ZMUO.003159|Dasytes_niger
TLYFIFGAWSGMVGMSLSLLIRSELNNPGTLIGNDQIYNVIVTAHAFIMIFFMVMPILIGGFGNWLVPLMLGAPDMAFPRMNNMSFWLLPPSLTLLLMSSMVEQGAGTGWTVYPPLSSNIAHGGASVDLAIFSLHLAGISSILGAVNFITTIINMRPVG
MTLDRTPLFVWAVAITALLLLLSLPVLAGAITMLLTDRNLNTSFFDPAGGGDPILYQHL
>COLFC623-12|KJ964625|ZMUO.003188|Berosus_luridus
TLYFIFGAWAGMVGTSLSLLIRAELGNPGTLIGDDQIYNVIVTAHAFIMIFFMVMPIMIGGFGNWLVPLMLGAPDMAFPRMNNMSFWLLPPSLTLLLLSSMVESGAGTGWTVYPPLSSNIAHGGASVDLAIFSLHLAGISSILGAVNFITTVINMRAPN
MTYDRMPLFVWSVAITALLLLLSLPVLAGAITMLLTDRNLNTSFFDPAGGGDPILYQHL
>COLFD274-12|KJ962193|ZMUO.004169|Stephanopachys_linearis
TLYFIFGSWAGMIGTALSILIRSELGNPGSLIGDDQIYNVIVTAHAFVMIFFMVMPMMIGGFGNWLVPLMLGAPDMAFPRMNNMSFWLLPPSLSLLLASSIVENGAGTGWTVYPPLSNNTAHSGASVDLAIFSLHLAGISSILGAVNFITTTINMRPSG
MTPERIPLFVWSVAITALLLLLSLPVLAGAITMLLTDRNLNTSFFDPAGGGDPILYQHL
>COLFE487-13|KJ965869|ZMUO.005332|Hippodamia_tredecimpunctata
TLYFLFGMWAGMVGTSLSILIRLELGTPGSLIGNDQIYNVIVTAHAFIMIFFMVMPIMIGGFGNWLVPLMIGAPDMAFPRLNNMSFWLLPPALILLMFSSMVEMGAGTGWTVYPPLSSNLAHNGPSVDLVIFSLHLAGISSILGAVNFISTIMNMRPYG
MSMDKTPLFVWSVLITAILLLLSLPVLAGAITMLLTDRNLNTSFFDPTGGGDPILYQHL
>COLFF670-13|KJ967049|ZMUO.006370|Odacantha_melanura
TLYFIFGAWSGMVGTSLSMLIRAELGNPGALIGDDQIYNVIVTAHAFIMIFFMVMPIMIGGFGNWLVPLMLGAPDMAFPRMNNMSFWLLPPSLTLLLMSSMVESGAGTGWTVYPPLSSGIAHAGASVDLAIFSLHLAGVSSILGAVNFITTIINMRSV
GMTFDRMPLFVWSVGITALLLLLSLPVLAGAITMLLTDRNLNTSFFDPAGGGDPILYQHL
>COLFB266-12|KJ965659|ZMUO.001596|Hygrotus_impressopunctatus
TLYFLFGAWSGMVGTSLSMLIRAELGNPGSLIGDDQIYNVIVTAHAFIMIFFMVMPIMIGGFGNWLVPLMLGAPDMAFPRMNNMSFWLLPPSLTLLLMSSMVESGAGTGWTVYPPLSAGIAHGGASVDLAIFSLHLAGISSILGAVNFITTIINMRSV
GMTFDRMPLFVWSVGITALLLLLSLPVLAGAITMLLTDRNLNTSFFDPAGGGDPILYQHL
>COLFE1286-13|KJ966469|ZMUO.007081|Pterostichus_diligens
TLYFIFGAWSGMVGTSLSMLIRAELGNPGSLIGDDQIYNVIVTAHAFVMIFFMVMPIMIGGFGNWLVPLMLGAPDMAFPRMNNMSFWLLPPSLTLLLMSSMVESGAGTGWTVYPPLSSGIAHAGASVDLAIFSLHLAGISSILGAVNFITTIINMRSIG
MTFDRMPLFVWSVGITALLLLLSLPVLAGAITMLLTDRNLNTSFFDPAGGGDPILYQHL
>COLFB199-12|KJ964355|ZMUO.001529|Zeugophora_scutellaris
TLYFIFGVWAGMVGTSLSLLIRSELGTPGSLIGDDQIYNVIVTAHAFVMIFFMVMPIMIGGFGNWLVPLMLGAPDMAFPRMNNMSFWFLPPSLTLLLMSSIVESGAGTGWTVYPPLSANIAHSGSSVDLAIFSLHMAGISSILGAVNFITTVINMRPTGL
NLDKMPLFVWAVVITAILLLLSLPVLAGAITMLLTDRNINTSFFDPSGGGDPILYQHL
>COLFF034-13|KJ966512|ZMUO.005734|Meligethes_carinulatus
TLYFIFGAWSGMVGTSLSMLIRTELGNPGSLIGNDQIYNVIVTAHAFVMIFFMVMPFMIGGFGNWLVPLMLGAPDMAFPRMNNMSFWLLPPSLSLLLMSSIVESGAGTGWTVYPPLSSNIAHGGASVDLAIFSLHLAGISSILGAVNFITTVINMRPSG
MNFDQMPLFVWSVVITAFLLLLSLPVLAGAITMLLTDRNLNTTFFDPSGGGDPILYQHL
>COLFA245-10|HM909155|MP00111|Agrilus_viridis
TLYFIFGAWSGMVGTALSLLIRAELGNPGALIGNDQIYNVIVTAHAFIMIFFMVMPIMMGGFGNWLVPLMLGAPDMAFPRMNNMSFWLLPPSLTLLLMSSMVESGAGTGWTVYPPLAANIAHSGGSVDLAIFSLHLAGISSILGAINFITTVINMRAV
GMTMDRVPLLVWSIAITALLLLLSLPVLAGAITMLLTDRNLNTSFFDPAGGGDPILYQHL

>COLFA577-12|KJ962771|ZMUO.000712|Necydalis_major
TLYFIFGMWAGMMGTSLSVLIRSELGNPGSLIGNDQIYNVIVTAHAFVMIFFMVMPIMIGGFGNWLIPLMLGAPDMAFPRMNNLSFWLLPPSLILLIMSSVVENGAGTGWTVYPPLSANIAHSGSSVDLAIFSLHLAGISSILGAVNFISTTMNMRPGG
MILDRLPLFVWAVMITAILLLLSLPVLAGAITMLLTDRNLNTSFFDPAGGGDPILYQHL
>COLFF1016-13|KJ963941|ZMUO.007381|Boreaphilus_henningianus
TLYFIFGAWAGMVGTSLSILIRAELGNPGSLIGDDQIYNVIVTAHAFVMIFFMVMPIVIGGFGNWLVPLMLGAPDMAFPRMNNMSFWLLPPSLNLLLMSSMVESGAGTGWTVYPPLSSNIAHGGSSVDLAIFSLHLAGVSSILGAVNFITTVINMRSTG
MSFDRMPLFVWSVAITALLLLLSLPVLAGAITMLLTDRNLNTSFFDPAGGGDPILYQHL
>COLFB847-12|KJ966577|ZMUO.001322|Oxyporus_rufus
TLYFIFGAWAGMVGTSLSLLIRAELGNPGSLIGDDQIYNVIVTAHAFIMIFFMVMPVVIGGFGNWLVPLMLGAPDMAFPRMNNMSFWLLPPSLTLLLMSSMVESGAGTGWTVYPPLSSNIAHGGASVDLAIFSLHLAGISSILGAVNFISTINNMRAPG
MIPERMPLFVWAVAITALLLLLSLPVLAGAITMLLTDRNLNTSFFDPAGGGDPILYQHL
>COLFC309-12|KJ962516|ZMUO.002494|Stenus_strandi
TLYFIFGAWAGMVGTSLSLLIRAELGNPGSLIGDDQIYNVIVTAHAFVMIFFMVMPVMIGGFGNWLVPLMLGAPDMAFPRMNNMSFWLLPPSLSLLLMSSIVENGAGTGWTVYPPLSSNIAHSGASVDLAIFSLHLAGISSILGAINFITTIFNMRTMK
LHLDCLPLFVWSVGITALLLLSLPVLAGAITMLLTDRNLNTSFFDPAGGGDPILYQHL
>COLFE842-13|KJ965098|ZMUO.005687|Brachypterolus_linariae
TLYFIFGAWSGMIGTSLSLLIRSELGNPGSLIGDDQIYNVIVTAHAFVMIFFMVMPFMIGGFGNWLVPLMLGAPDMAFPRMNNMSFWLLPPSLFLLLMSSIVESGAGTGWTVYPPLSSNIAHGGSSVDLAIFSLHLAGISSILGAVNFITTVINMRPSGM
TFDRMPLFIWAVVITALLLLLSLPVLAGAITMLLTDRNLNTSFFDPAGGGDPILYQHL
>COLFD276-12|KJ967121|ZMUO.004171|Abdera_triguttata
TLYFIFGAWAGLIGTSMSLIIRSELGNSSSLIGNDQIYNVIVTAHAFIMIFFMVMPIMMGGFGNWLIPLMLGAPDMAFPRMNNMSFWLLPPSLILLLMSSMVESGAGTGWTVYPPLSSNIAHSGSSVDLAIFSLHLAGVSSILGAVNFITTIINMRPIGMT
LDQMPLFVWAVGITAILLLLSLPVLAGAITMLLTDRNLNTSFFDPAGGGDPILYQHL
>COLFD555-12|KJ963094|ZMUO.004355|Cionus_tuberculosus
TLYFIFGMWSGTVGTSLSMLIRTELGNPGSLIGDDQIYNTIVTAHAFIMIFFMVMPIMIGGFGNWLVPLMLGAPDMAFPRLNNMSFWLLPPSITLLLMSSIVEKGAGTGWTVYPPLSNNITHEGASVDLAIFSLHMAGISSILGAINFISTVSNMRTKGM
DYDRTPLFVWSVNITAFLLLLSLPVLAGAITMLLTDRNINTSFFDPSGGGDPILYQHL
>COLFC149-12|KJ966971|ZMUO.002334|Oxytelus_sculptus
TLYFIFGAWSGMVGTSLSMLIRAELGAPGSLIGDDQIYNVIVTAHAFIMIFFMVMPIVIGGFGNWLVPLMLGAPDMAFPRMNNMSFWLLPPSLTLLLFSSMVESGAGTGWTVYPPLSSNIAHSGSSVDLAIFSLHLAGISSILGAVNFISTIINMRSTGM
SFDRMPLFVWSVNITAILLLLSLPVLAGAITMLLTDRNLNTSFFDPAGGGDPILYQHL
>COLFF929-13|KJ961817|ZMUO.006724|Stenus_flavipes
TLYFIFGAWAGMVGTSLSLLIRAELGAPSSLIGDDQIYNVIVTAHAFIMIFFMVMPIMIGGFGNWLVPLMMGAPDMAFPRMNNMSFWLLPPSLSLLLMSSIVESGAGTGWTVYPPLSSNIAHSGASVDLAIFSLHLAGISSILGAINFITTIINMRTMKM
NFDCLPLFVWSVSITALLLLLSLPVLAGAITMLLTDRNLNTSFFDPAGGGDPVLYQHL
>COLFD819-12|KJ966521|ZMUO.004809|Rhagonycha_fulva
TLYFIFGAWSGSLGLALSLLIRAELGTPGTLIGNDQIYNVIVTAHAFIMIFFMVMPIMIGGFGNWLVPLMLGAPDMAFPRMNNMSFWFLPPSLMFLLMSSMVESGAGTGWTVYPPLSANIAHSGPSVDLAIFSLHMAGISSILGAVNFISTILNMKPPS
MKFDQMPLFVWSVGITALLLLLSLPVLAGAITMLLSDRNLNTSFFDPMGGGDPILYQHL
>COLFG158-13|KJ966241|ZMUO.007853|Gyrophaena_strictula
TLYFIFGAWSGMVGTSLSLLIRAELGNPGSLIGDDQIYNVIVTAHAFIMIFFMVMPIVIGGFGNWLVPLMLGAPDMAFPRMNNMSFWLLPPSLSLLLMSSMVESGAGTGWTVYPPLSSNIAHGGASVDLAIFSLHLAGISSILGAVNFISTIINMRASGM
SFDRMPLFVWSVAITALLLLLSLPVLAGAITMLLTDRNLNTSFFDPAGGGDPILYQHL
>LEFIJ1841-13|KJ964954|ZMUO.004596|Psylliodes_affinis
TLYFIFGIWSGMIGTSLSMLIRTELGAPGSLIGNDQIYNVIVTAHAFIMIFFMVMPTMIGGFGNWLIPLMIGAPDMAFPRMNNMSFWLLPPSLTLLLMSSMVENGAGTGWTVYPPLSSNIAHGGSSVDLAIFSLHLAGISSILGAINFITTVINMRPKGM
TLDRMPLFVWAVVITAILLLLSLPVLAGAITMLLTDRNLNTSFFDPIGGGDPILYQHL
>COLFD455-12|KJ964344|ZMUO.003875|Meligethes_ovatus
TLYFIFGAWSGMVGTSLSMLIRTELGSPGSLIGNDQIYNVIVTAHAFVMIFFMVMPFMIGGFGNWLMPLMLGAPDMAFPRMNNMSFWLLPPSLSLLLMSSIVESGAGTGWTVYPPLSSNLAHGGASVDLAIFSLHLAGISSILGAVNFITTVINMRPS
GMTFDRMPLFIWAVVITALLLLLSLPVLAGAITMLLTDRNLNTTFFDPSGGGDPILYQHL
>COLFG142-13|KJ965912|ZMUO.007837|Cartodere_constricta
SLYFLFGMWSGMVGTSLSLLIRLELGNPGSLIGDDQIYNVIVTAHAFIMIFFMVMPLMMGGFGNWLVPLMLGAPDMAFPRLNNMSFWLLPPSLSLLIMSSVVESGVGTGWTVYPPLSSNIAHGGSSVDLAIFSLHLAGISSILGAVNFITTVMNMRPV
GMTLEQMPLFVWSVLLTAILLLLSLPVLAGAITMLLTDRNLNTSFFDPAGGGDPILYQHL
>COLFF991-13|KJ964235|ZMUO.007356|Tachinus_proximus
TLYFIFGAWAGMVGTSLSLLIRAELGNPGTLIGDDQIYNVIVTAHAFIMIFFMVMPIVIGGFGNWLVPLMLGAPDMAFPRMNNMSFWLLPPSLSLLLMSSMVESGAGTGWTVYPPLSSNIAHGGSSVDLAIFSLHLAGISSILGAVNFITTVINMRSIGM
TFDRMPLFVWSVAITALLLLLSLPVLAGAITMLLTDRNLNTTFFDPAGGGDPILYQHL
>COLFD192-12|KJ964119|ZMUO.004087|Ilybius_erichsoni

TLYFIFGAWAGMVGTSLSMLIRAELGNPGSLIGDDQIYNVIVTAHAFVMIFFMVMPIMIGGFGNWLVPLMLGAPDMAFPRMNNMSFWLLPPSLSLLLMSSMVESGAGTGWTVYPPLSSGIAHSGASVDLAIFSLHLAGISSILGAVNFITTIINMRSV
GMTFDRMPLFVWSVGITALLLLLSLPVLAGAITMLLTDRNLNTSFFDPAGGGDPILYQHL
>COLFA652-12|KJ965568|ZMUO.000787|Aleochara_lanuginosa
TLYFIFGAWAGMVGTSLSLLIRAELGNPGSLIGDDQIYNVIVTAHAFIMIFFMVMPIVIGGFGNWLVPLMLGAPDMAFPRMNNMSFWLLPPSLTLLLMSSMVESGAGTGWTVYPPLSSNIAHGGSSVDLAIFSLHLAGISSILGAVNFISTVINMRSSG
MTFDKMPLFVWSVAITALLLLLSLPVLAGAITMLLTDRNLNTSFFDPAGGGDPILYQHL
>COLFB228-12|KJ965183|ZMUO.001558|Sphaeriestes_stockmanni
TLYFIFGSWSGMVGTSLSLLIRAELGNPGSLIGDDQTYNVIVTAHAFIMIFFMVMPIVIGGFGNWLVPLMLGAPDMAFPRMNNMSFWLLPPSLTLLIMSSIVESGAGTGWTVYPPLAANIAHSGPSVDLAIFSLHLAGISSILGAVNFITTVINMRPSGM
SFDRMPLFVWAVVITAVLLLLSLPVLAGAITMLLTDRNLNTSFFDPAGGGDPILYQHL
>COLFF572-13|KJ962860|ZMUO.006462|Cardiophorus_vestigialis
TLYFLFGAWSGMLGTSLSLLIRAELGSPGSLIGNDQIYNVIVTAHAFIMIFFMVMPIMIGGFGNWLVPLMLGAPDMAFPRMNNMSFWFLPPSLSLLLMSSIVENGAGTGWTVYPPLSSNIAHSGSSVDLAIFSLHLAGISSILGAVNFISTVINMRSTGM
TFERMPLFVWAVVITALLLLLSLPVLAGAITMLLTDRNLNTSFFDPAGGGDPILYQHL
>COLFF416-13|KJ964102|ZMUO.005934|Anaspis_bohemica
TLYFIFGAWSGMVGTSLSLLIRSELGTPGSLIGDDQIYNVIVTAHAFIMIFFMVMPILIGGFGNWLVPLMLGAPDMAFPRMNNMSFWLLPPSLTLLIMSSVVENGAGTGWTVYPPLAANIAHSGSSVDLAIFSLHLAGVSSILGAVNFITTVINMRPQG
MTLDRMPLFVWAVVITAVLLLLSLPVLAGAITMLLTDRNLNTSFFDPAGGGDPILYQHL
>COLFB892-12|KJ965444|ZMUO.001937|Pytho_depressus
TLYFIFGAWSGMVGTSLSLLIRAELGNPGSLIGDDQIYNVIVTAHAFVMIFFMVMPTVIGGFGNWLVPLMLGAPDMAFPRMNNMSFWLLPPSLSLLIMSSIVENGAGTGWTVYPPLASNIAHGGSSVDLAIFSLHLAGISSILGAVNFITTVINMRPLG
MTFDRMPLFVWSVVITAVLLLLSLPVLAGAITMLLTDRNLNTSFFDPAGGGDPILYQHL
>COLFB553-12|KJ963125|ZMUO.001028|Philonthus_umbratilis
TLYFIFGSWAGMVGTSLSLLIRAELGNPGSLIGDDQIYNVIVTAHAFIMIFFMVMPIVIGGFGNWLVPLMLGAPDMAFPRMNNMSFWLLPPSLTLLLMSSMVESGAGTGWTVYPPLSSNIAHGGASVDLAIFSLHLAGISSILGAVNFITTVINMRSTGI
EFDRMPLFVWSVVITALLLLLSLPVLAGAITMLLTDRNINTTFFDPAGGGDPILYQHL
>COLFB172-12|KJ966212|ZMUO.001502|Enochrus_quadripunctatus
TLYFIFGAWAGMVGTSLSILIRAELGNPGTLIGDDQIYNVIVTAHAFIMIFFMVMPIMIGGFGNWLVPLMLGAPDMAFPRMNNMSFWLLPPSLTLLLMSSMVESGAGTGWTVYPPLSSNIAHGGASVDLAIFSLHLAGISSILGAVNFITTVINMRSPS
MTYDRLPLFVWSVAITALLLLLSLPVLAGAITMLLTDRNLNTSFFDPAGGGDPILYQHL
>COLFB596-12|KJ965728|ZMUO.001071|Atheta_crassicornis
TLYFIFGAWAGMVGTSLSLLIRAELGNPGSLIGDDQIYNVIVTAHAFVMIFFMVMPIVIGGFGNWLIPLMLGAPDMAFPRMNNMSFWLLPPSLTLLLMSSMVESGAGTGWTVYPPLSSNIAHGGSSVDLAIFSLHLAGISSILGAVNFISTVINMRSVGI
SFDRMPLFVWSVAITALLLLLSLPVLAGAITMLLTDRNLNTSFFDPAGGGDPILYQHL
>COLFC745-12|KJ967321|ZMUO.003310|Rhagonycha_testacea
TLYFIFGAWSGSLGLALSLLIRAELGTPGTLIGNDQIYNVIVTAHAFIMIFFMVMPIMIGGFGNWLVPLMLGAPDMAFPRMNNMSFWFLPPSLMFLLMSSMVESGAGTGWTVYPPLSANIAHSGPSVDLAIFSLHMAGISSILGAVNFISTILNMKPPS
MKFDQMPLFVWSVGITALLLLLSLPVLAGAITMLLSDRNLNTSFFDPMGGGDPILYQHL
>COLFC243-12|KJ964104|ZMUO.002428|Dicheirotrichus_cognatus
TLYFIFGAWSGMVGTSLSMLIRAELGTPGALIGDDQIYNVIVTAHAFVMIFFMVMPIMIGGFGNWLVPLMLGAPDMAFPRMNNMSFWLLPPSLTLLLMSSMVEKGAGTGWTVYPPLSSGIAHGGASVDLAIFSLHLAGISSILGAVNFITTIINMRSV
GMTFDRMPLFVWSVGITALLLLLSLPVLAGAITMLLTDRNLNTSFFDPAGGGDPILYQHL
>COLFE1185-13|KJ964028|ZMUO.006980|Agabus_arcticus
TLYFIFGAWAGMVGTSLSMLIRAELGNPGSLIGDDQIYNVIVTAHAFVMIFFMVMPIMIGGFGNWLVPLMLGAPDMAFPRMNNMSFWLLPPSLTLLLMSSMVESGAGTGWTVYPPLSSGIAHGGASVDLAIFSLHLAGISSILGAVNFITTIINMRSV
GMTFDRMPLFVWSVGITALLLLLSLPVLAGAITMLLTDRNLNTSFFDPAGGGDPILYQHL
>COLFE1538-13|KJ963249|ZMUO.007428|Anaspis_frontalis
TLYFIFGAWSGMVGTSLSLLIRSELGTPGSLIGDDQIYNVIVTAHAFIMIFFMVMPILIGGFGNWLVPLMLGAPDMAFPRMNNMSFWLLPPSLTLLIMSSVVENGAGTGWTVYPPLAANIAHSGSSVDLAIFSLHLAGVSSILGAVNFITTVINMRPQG
MTLDRMPLFVWAVVITAVLLLLSLPVLAGAITMLLTDRNLNTSFFDPAGGGDPILYQHL
>COLFF421-13|KJ966997|ZMUO.005939|Mordellistena
TLYFIFGAWAGMLGTALSLLIRSELGTPGSLIGDDQIYNVIVTAHAFVMIFFMVMPVMVGGFGNWLLPLMLGAPDMAFPRLNNMSFWLLPPSLTLLILSSMVENGAGTGWTVYPPLSSNIAHGGASVDLTIFSLHLAGISSILGAINFISTMLNMHPKG
MILDQMPLFVWAIIITAILLLLSLPVLAGAITMLLTDRNLNSSFFDPAGGGDPILYQHL
>COLFA265-12|KJ966631|ZMUO.000400|Migneauxia_lederi
SLYFLFGMWSGMVGTSLSLLIRLELGNPGSLIGDDQIYNVIVTAHAFIMIFFMVMPIMIGGFGNWLVPLMLGAPDMAFPRLNNMSFWLLPPSLSLLIMSSIVESGAGTGWTVYPPLSSNIAHGGSSVDLAIFSLHLAGISSILGAVNFITTVINMRPKGMS
LDRMPLFVWSVVITAVLLLLSLPVLAGAITMLLTDRNLNTSFFDPAGGGDPILYQHL
>COLFF1002-13|KJ962037|ZMUO.007367|Melanophthalma_transversalis
SLYFLFGLWSGMVGTSLSLLIRLELGNPGSLIGDDQIYNVIVTAHAFIMIFFMVMPIMIGGFGNWLVPLMLGAPDMAFPRLNNMSFWLLPPSLNLLIMSSIVESGAGTGWTVYPPLSSNIAHSGSSVDLAIFSLHLAGISSILGAVNFITTVINMRPMGMT
LEKMPLFVWSVVLTAILLLLSLPVLAGAITMLLTDRNLNTSFFDPAGGGDPILYQHL

>COLFC066-12|KJ962711|ZMUO.002061|Phyllotreta_undulata
TLYFIFGIWSGMLGMSMSMLIRVELAAPGSLIGNDQIYNVIVTAHAFIMIFFMVMPIMIGGFGNWLIPLMIGAPDMAFPRMNNMSFWLLPPSLFLLIMSSIIENGAGTGWTVYPPLSSNISHAGASVDLTIFSLHLAGISSILGAINFITTVINMRPKGMT
LDRLPLFVWAVLITAILLLLSLPVLAGAITMLLTDRNLNTSFFDPMGGGDPILYQHL
>COLFB623-12|KJ962249|ZMUO.001098|Aphthona_lutescens
TLYFIFGIWSGMVGTSLSVLIRTELGNPGSLIGNDQIYNVIVTAHAFIMIFFMVMPIMIGGFGNWLVPLMIGAPDMAFPRMNNMSFWLLPPSLIFLIMSSMVESGAGTGWTVYPPLSSNIAHGGSSVDLAIFSLHLAGISSILGAINFITTVINMRPEGMN
LDRMPLFVWAVTITAILLLLSLPVLAGAITMLLTDRNLNTSFFDPSGGGDPILYQHL
>COLFE462-12|KJ963201|ZMUO.005022|Coccinula_quatuordecimpustulata
TLYFLFGMWAGMVGTSLSILIRLELGSTGSLIGNDQIYNVIVTAHAFIMIFFMVMPIMIGGFGNWLVPLMIGAPDMAFPRLNNMSFWLLPPSLTLLLLSSLVEMGAGTGWTVYPPLSSNLAHNGSSVDLVIFSLHLAGISSILGAVNFISTIMNMRPNG
MNLDKTPLFVWSVLITAILLLLSLPVLAGAITMLLTDRNMNTSFFDPTGGGDPILYQHL
>COLFE583-13|KJ964641|ZMUO.005428|Miarus_campanulae
TLYFIFGAWSGMLGTSLSMLIRIELGNPGKFIGDDQIYNSIVTAHAFIMIFFMVMPIMIGGFGNWLIPLMIGAPDMAFPRLNNLSFWLLPPSLCMLIFSMIINKGAGTGWTVYPPLSANITHEGASVDFAIFSLHLAGLSSILGAINFISTMMNMRPEGMK
NDQISLFTWAVQITAILLLLSLPVLAGAITMLLTDRNINTSFFDPAGGGDPILYQHL
>COLFB925-12|KJ965395|ZMUO.001970|Cryptocephalus_labiatus
TLYFLFGAWAGMIGTSLSLLIRIELGNPGSLIENDQIYNTIVTAHAFIMIFFMVMPIMIGGFGNWLVPLMLGAPDMAFPRMNNMSFWLLPPSLTLLLMSSIVENGAGTGWTVYPPLSTTIAHAGASVDLAIFSLHLAGISSIMGAINFISTVINMRPQGM
FMDRTPLFVWAVLITAILLLLSLPVLAGAITMLLTDRNLNTSFFDPAGGGDPILYQHL
>COLFF430-13|KJ966042|ZMUO.005948|Meligethes
TLYFIFGAWSGMVGTSLSMLIRTELGNPGSLIGNDQIYNVIVTAHAFVMIFFMVMPFMIGGFGNWLVPLMLGAPDMAFPRMNNMSFWLLPPSLSLLLTSSIVESGAGTGWTVYPPLSSNIAHGGASVDLAIFSLHLAGISSIMGAVNFITTVINMRPS
GMNFDQMPLFVWAVTITAILLLLSLPVLAGAITMLLTDRNLNTTFFDPSGGGDPILYQHL
>COLFC483-12|KJ962799|ZMUO.003048|Deporaus_betulae
TLYFIFGAWAGMVGTALSLLIRAELGNPGSLIGDDQIYNVIVTAHAFIMIFFMVMPIMIGGFGNWLVPLMLGAPDMAFPRMNNMSFWLLPPSLSLLIMSSIVESGAGTGWTVYPPLSANIAHGGSSVDLAIFSLHLAGISSILGAVNFISTMINMRPNG
MTLDRMPLFAWAVVITALLLLLSLPVLAGAITMLLTDRNINTTFFDPAGGGDPILYQHL
>COLFB326-12|KJ963083|ZMUO.001656|Otiorhynchus_scaber
TLYFILGSWSGMVGTSLSMLIRAELGTPGALIGDDQIYNVIVTAHAFIMIFFMVMPIMIGGFGNWLVPLMLGAPDMAFPRLNNMSFWLLPPSLSLLLMSSMIDKGAGTGWTVYPPLSSNIAHEGASVDLAIFSLHMAGISSILGAINFISTVTNMRPSG
MSFDRMSLFIWAVKITAILLLLSLPVLAGAITMLLTDRNINTSFFDPAGGGDPILYQHL
>COLFC814-12|KJ964219|ZMUO.003379|Arpedium_quadrum
TLYFIFGAWAGMVGTSLSILIRAELGNPGTLIGDDQIYNVIVTAHAFVMIFFMVMPIVIGGFGNWLVPLMLGAPDMAFPRMNNMSFWLLPPSLTLLLMSSMVESGAGTGWTVYPPLSSNIAHGGSSVDLAIFSLHLAGISSILGAVNFITTVINMRATG
MTFDRMPLFVWSVAITALLLLLSLPVLAGAITMLLTDRNLNTSFFDPAGGGDPILYQHL
>COLFD778-12|KJ961739|ZMUO.004768|Ceutorhynchus_roberti
TLYFIFGSWAGMAGTSLSMLIRTELGNPGSLIGNDQIYNSIVTAHAFIMIFFMVMPILIGGFGNWLVPLMLGAPDMAFPRLNNMSFWLLPPSLSLLLMSSIVNKGAGTGWTVYPPLSGNVAHEGMSVDLAIFSLHMAGISSILGAINFISTVLNMQPKG
MTPELMPLFVWAVEITAILLLISLPVLAGAITMLLTDRNINTSFFDPSGGGDPILYQHL
>COLFB224-12|KJ964435|ZMUO.001554|Pissodes_validirostris
TLYFIFGAWSGMIGTSLSILIRTELGTPGTMIGDDQIYNTIVTAHAFIMIFFMVMPIMIGGFGNWLVPLMLGAPDMAFPRLNNMSFWLLPPSIMLLLMSSIVDKGAGTGWTVYPPLSTNIAHEGPSVDLAIFSLHMAGISSILGAVNFISTVINMRPTGM
NSDQMSLFIWAVKITAILLLLSLPVLAGAITMLLTDRNINTSFFDPAGGGDPILYQHL
>COLFE936-13|KJ963479|ZMUO.006256|Elodes_minuta
TLYFIFGAWSGMVGTSLSLLIRAELGTPGSLIGDDQIYNVIVTAHAFVMIFFMVMPIMIGGFGNWLVPLMLGAPDMAFPRMNNMSFWLLPPSLTLLLTSSMVESGAGTGWTVYPPLSSTIAHSGASVDLAIFSLHLAGISSILGAVNFISTVINMRSHG
MTFDRMPLFVWAVAITALLLLLSLPVLAGAITMLLTDRNLNTSFFDPAGGGDPILYQHL
>COLFA149-10|HQ559256|MP00411|Hypera_zoilus
TLYFIFGTWAGTIGTSLSILIRAELGNPGSLIGNDQIYNTIVTAHAFIMIFFMVMPIMIGGFGNWLVPLMLGAPDMAFPRLNNMSFWLLPPSLSLLLMSSMVNSGAGTGWTVYPPLSSNIAHEGASVDLAIFSLHMAGVSSILGAINFISTVLNMRPSGM
SLDKMALFIWAVKITAILLLLSLPVLAGAITMLLTDRNINTSFFDPAGGGDPILYQHL
>COLFG088-13|KJ961900|ZMUO.007593|Anobium_rufipes
TLYFVFGAWSGMVGTSLSMLIRSELGNPGALIGDDQIYNVIVTAHAFIMIFFMVMPIMIGGFGNWLVPLMLGAPDMAFPRMNNMSFWLLPPALSLLLMGSIVGSGAGTGWTVYPPLSSSIAHSGASVDLSIFSLHLAGISSILGAVNFITTVINMRSVG
MSFDRVSLFVWSVVITAVLLLLSLPVLAGAITMLLTDRNLNTSFFDPAGGGDPILYQHL
>COLFB265-12|KJ961724|ZMUO.001595|Chaetocnema_sahlbergii
TLYFIFGIWSGMVGTSMSILIRAELGNPGSLIGNDQIYNVIVTAHAFVMIFFMVMPIMIGGFGNWLVPLMIGAPDMAFPRMNNMSFWLLPPSLFLLLMSSMVESGAGTGWTVYPPLSSNIAHGGSSVDLAIFSLHLAGISSILGAINFITTIINMRPQG
MQFDQMPLFVWAVLITAILLLLSLPVLAGAITMLLTDRNLNTSFFDPIGGGDPILYQHL
>COLFC707-12|KJ963046|ZMUO.003272|Olophrum_rotundicolle

TLYFIFGAWAGMVGTSLSILIRAELGNPGTLIGDDQIYNVIVTAHAFVMIFFMVMPILIGGFGNWLVPLMLGAPDMAFPRMNNMSFWLLPPSLTLLLTSSMVESGAGTGWTVYPPLSSNIAHGGASVDLAIFSLHLAGISSILGAVNFITTVINMRATG
MTFDRMPLFVWAVAITALLLLLSLPVLAGAITMLLTDRNLNTTFFDPAGGGDPILYQHL
>COLFC280-12|KJ963683|ZMUO.002465|Anaspis_arctica
TLYFIFGAWSGMVGTSLSLLIRSELGTPGSLIGDDQIYNVIVTAHAFIMIFFMVMPILIGGFGNWLVPLMLGAPDMAFPRMNNMSFWLLPPSLTLLIMSSVVENGAGTGWTVYPPLAANIAHSGSSVDLAIFSLHLAGVSSILGAVNFITTVINMRPQG
MTLDRMPLFVWAVVITAILLLLSLPVLAGAITMLLTDRNLNTSFFDPAGGGDPILYQHL
>COLFF717-13|KJ962814|ZMUO.006417|Dasytes_plumbeus
TLYFIFGAWSGMVGMSLSLLIRSELNNPGTLIGNDQIYNVIVTAHAFIMIFFMVMPILIGGFGNWLVPLMLGAPDMAFPRMNNMSFWLLPPSLTLLLMSSMVEQGAGTGWTVYPPLSANIAHGGASVDLAIFSLHLAGISSILGAVNFITTVINMRPV
GMTLDRTPLFVWAVAITALLLLLSLPVLAGAITMLLTDRNLNTSFFDPAGGGDPILYQHL
>COLFE1112-13|KJ961962|ZMUO.006907|Eusphalerum_sorbicola
TLYFIFGAWAGMVGTSLSMLIRAELGNPGSLIGDDQIYNVIVTAHAFIMIFFMVMPIMIGGFGNWLVPLMLGAPDMAFPRMNNMSFWLLPPSLTLLLMSSMVESGAGTGWTVYPPLSSNIAHGGSSVDLAIFSLHLAGISSILGAVNFITTVINMRSM
GMTFDRMPLFVWAVAITALLLLLSLPVLAGAITMLLTDRNLNTSFFDPAGGGDPILYQHL
>COLFB270-12|KJ965897|ZMUO.001600|Epuraea_opalizans
TLYFIFGVWSGMVGTSLSILIRTELGSPGSLIGNDQIYNVIVTAHAFIMIFFMVMPFMIGGFGNWLVPLMLGAPDMAFPRMNNMSFWLLPPSLSLLLMSSIVESGAGTGWTVYPPLSSNIAHGGSSVDLAIFSLHLAGVSSILGAVNFITTIINMRPAGM
TLDRMPLFVWSVVITAILLLLSLPVLAGAITMLLTDRNLNTTFFDPSGGGDPILYQHL
>COLFD285-12|KJ965554|ZMUO.004180|Curimopsis_paleata
TLYFLFGAWSGMVGTSLSLLIRAELGNPGSLIGDDQIYNVIVTAHAFIMIFFMVMPIMIGGFGNWLVPLMLGAPDMAFPRMNNMSFWFLPPSLSLLLMSSIVESGAGTGWTVYPPLSSNIAHSGSSVDLAIFSLHLAGISSILGAVNFISTVLNMRSPGM
TFDQMSLFSWSVVITALLLLLSLPVLAGAITMLLTDRNLNTSFFDPAGGGDPILYQHL
>COLFE1547-13|KJ965258|ZMUO.007437|Coeliastes_lamii
TLYFIFGSWAGMAGTSLSMLIRTELGNPGSLIGDDQIYNSVVTAHAFIMIFFMVMPIMIGGFGNWLVPLMLGAPDMAFPRLNNMSFWLLPPSLSLLLMSSIVNKGAGTGWTVYPPLSSNTAHEGVSVDLAIFSLHMAGISSILGAINFISTVMNMRPF
GMTSEFMPLFVWAVEITAILLLISLPVLAGAITMLLTDRNINTSFFDPSGGGDPILYQHL
>COLFE589-13|KJ962304|ZMUO.005434|Glischrochilus_quadripunctatus
TLYFIFGTWSGMIGTSLSLLIRSELGNPGSLIGDDQIYNVIVTAHAFIMIFFMVMPFMIGGFGNWLMPLMLGAPDMAFPRLNNMSFWLLPPSISLLLMSSIVESGAGTGWTVYPPLSSNIAHGGSSVDLAIFSLHLAGISSILGAVNFITTVINMRPSGMS
FDRMPLFAWAVVITAVLLLLSLPVLAGAITMLLTDRNLNTSFFDPAGGGDPILYQHL
>COLFF849-13|KJ967367|ZMUO.006169|Epuraea_aestiva
TLYFIFGAWSGMIGTSLSILIRTELGSPGSLIGNDQIYNVIVTAHAFIMIFFMVMPFMIGGFGNWLVPLMLGAPDMAFPRMNNMSFWLLPPSLSLLLMSSIVESGAGTGWTVYPPLSSNIAHGGSSVDLAIFSLHLAGVSSILGAVNFITTIINMRPIGMTL
DRMPLFVWSVIITAILLLLSLPVLAGAITMLLTDRNLNTTFFDPSGGGDPILYQHL
>COLFB944-12|KJ964472|ZMUO.001989|Liogluta_micans
TLYFIFGAWAGMIGTSLSLLIRAELGNPGSLIGDDQIYNVIVTAHAFIMIFFMVMPVVIGGFGNWLVPLMLGAPDMAFPRMNNMSFWLLPPSLTLLLMSSMVESGAGTGWTVYPPLSSNIAHSGSSVDLAIFSLHLAGISSILGAVNFISTVINMRSVG
MSFDRMPLFVWSVAITALLLLLSLPVLAGAITMLLTDRNLNTSFFDPAGGGDPILYQHL
>COLFC172-12|KJ965341|ZMUO.002357|Tachyporus_chrysomelinus
TLYFIFGAWSGMVGTSLSLLIRAELGNPGSLIGDDQIYNVIVTAHAFIMIFFMVMPIVIGGFGNWLVPLMLGAPDMAFPRMNNMSFWLLPPSLTLLLMSSMVESGAGTGWTVYPPLSANIAHSGPSVDLAIFSLHLAGISSILGAVNFITTVINMRASG
MHFDRMPLFIWSVAITVLLLLLSLPVLAGAITMLLTDRNLNTSFFDPAGGGDPILYQHL
>COLFD534-12|KJ966706|ZMUO.004334|Trypodendron_domesticum
TLYFIFGAWAGMVGTSLSMIVRTELGTPGSLIGDDQIYNSAVTAHAFIMIFFMVMPIMIGGFGNWLVPLMLGAPDMAFPRLNNMSFWLLPPSLTFLLMSSIVDKGAGTGWTVYPPLASNIAHEGPSVDLAIFSLHMAGISSILGAMNFIATTINMHPL
GCKTEQLTLFTWAVKITAVLLLLSLPVLAGAITMLLTDRNINTSFFDPSGGGDPILYQHL
>COLFB335-12|KJ965226|ZMUO.001665|Attagenus_woodroffei
TLYFIFGAWAGMVGTSLSMLIRTELGSPGSLIGDDQIFNVIVTAHAFIMIFFLVMPIMIGGFGNWLIPLMLGAPDMAFPRMNNMSFWLLPPSLSLLLMSSMVESGAGTGWTVYPPLASNIAHGGASIDLAIFSLHLAGISSILGAVNFISTAINMRSPGM
SPEKMPLFVWSVAITALLLLLSLPVLAGAITMLLTDRNLNTSFFDPAGGGDPILYQHL
>COLFB702-12|KJ961843|ZMUO.001177|Astenus_gracilis
TLYFIFGAWAGMVGTSLSLLIRAELGMPGSLIGDDQIYNVIVTAHAFVMIFFMVMPIVIGGFGNWLVPLMLGAPDMAFPRMNNMSFWLLPPSLSLLLMSSMVESGAGTGWTVYPPLSSNMAHGGASVDLAIFSLHLAGISSILGAVNFITTIINMRTK
GMSYERMPLFVWSVGITALLLLLSLPVLAGAITMLLTDRNLNTSFFDPAGGGDPILYQHL
>COLFF439-13|KJ967228|ZMUO.005957|Philonthus_quisquiliarius
TLYFIFGSWAGMVGTSLSLLIRAELGNPGSLIGDDQIYNVIVTAHAFIMIFFMVMPIVIGGFGNWLVPLMLGAPDMAFPRMNNMSFWLLPPSLTLLLMSSMVESGAGTGWTVYPPLSSNIAHGGASVDLAIFSLHLAGISSILGAVNFITTVINMRSSG
MSFDRMPLFVWSVAITALLLLLSLPVLAGAITMLLTDRNLNTTFFDPAGGGDPILYQHL
>COLFE901-13|KJ962113|ZMUO.006221|Harpalus_laevipes
TLYFIFGAWAGMVGTSLSMLIRAELGTPGALIGNDQIYNVIVTAHAFIMIFFMVMPIMIGGFGNWLVPLMLGAPDMAFPRMNNMSFWLLPPSLTLLLMSSMVESGAGTGWTVYPPLSSGIAHSGASVDLAIFSLHLAGISSILGAVNFITTIINMRSVG
MTFDRMPLFVWSVGITALLLLLSLPVLAGAITMLLTDRNLNTSFFDPAGGGDPILYQHL

>COLFD027-12|KJ965964|ZMUO.003922|Cryptocephalus_bipunctatus
TLYFLFGAWAGMIGTSLSLLIRIELGNPGSLIGNDQIYNTIVTAHAFIMIFFMVMPIMIGGFGNWLVPLMLGAPDMAFPRMNNMSFWLLPPSLMLLLMSSIVENGAGTGWTVYPPLSATIAHTGPSVDLAIFSLHLAGASSIMGAINFISTVINMRPQG
MMMDRTPLFVWAVLITAILLLLSLPVLAGAITMLLTDRNINTSFFDPAGGGDPILYQHL
>COLFE592-13|KJ965993|ZMUO.005437|Peltis_ferruginea
TLYFIFGSWAGMVGTSLSLLIRSELGNPGSLIGNDQIYNVIVTAHAFVMIFFMVMPILIGGFGNWLVPLMLGAPDMAFPRMNNMSFWLLPPSLSLLILSSLIENGAGTGWTVYPPLSSNIAHSGSSVDLAIFSLHLAGISSILGAVNFITTVINMRPMGMT
LDRIPLFVWSVVITALLLLLSLPVLAGAITMLLTDRNINTSFFDPAGGGDPILYQHL
>COLFF1008-13|KJ966197|ZMUO.007373|Cis_dentatus
TLYFIFGAWSGMVGTSMSMLIRSELGIPGSLIGNDQIYNVIVTAHAFVMIFFMVMPIMIGGFGNWLVPLMLGAPDMAFPRMNNMSFWLLPPSLSLLLMSSIVENGAGTGWTVYPPLSANIAHSGSSVDLAIFSLHLAGISSILGAVNFISTIINMRPLG
MNLDQMPLFVWSVVITAVLLLLSLPVLAGAITMLLTDRNFNTSFFDPAGGGDPILYQHL
>COLFE1141-13|KJ965678|ZMUO.006936|Colon_pseudolatum
TLYFIFGAWAGMVGTSLSLLIRAELGNPGSLIGDDQIYNVIVTAHAFVMIFFMVMPVVIGGFGNWLVPLMLGAPDMAFPRMNNMSFWLLPPSLTLLLMSSMVESGAGTGWTVYPPLSSNIAHGGSSVDLAIFSLHLAGISSILGAVNFITTVINMRSQ
GMGFDQMPLFVWSVIITALLLLLSLPVLAGAITMLLTDRNLNTSFFDPAGGGDPILYQHL
>COLFE1191-13|KJ965384|ZMUO.006986|Philonthus_nigrita
TLYFIFGSWAGMVGTSLSLLIRAELGNPGTLIGDDQIYNVIVTAHAFIMIFFMVMPIVIGGFGNWLIPLMLGAPDMAFPRMNNMSFWLLPPSLTLLLMSSMVESGAGTGWTVYPPLSSNMAHGGASVDLAIFSLHLAGISSILGAVNFISTVINMRSTG
MTFDRMPLFIWSVAITALLLLLSLPVLAGAITMLLTDRNLNTTFFDPAGGGDPILYQHL
>COLFF921-13|KJ964349|ZMUO.006716|Leiodes_ferruginea
TLYFIFGAWAGMVGTSLSILIRTELGNPGSLIGDDQIYNVIVTAHAFIMIFFMVMPIVIGGFGNWLVPLMLGAPDMAFPRMNNMSFWLLPPSLTLLLMSSVVENGAGTGWTVYPPLSSNIAHSGSSVDLAIFSLHLAGISSILGAVNFITTVINMRPVGM
SFDKMPLFVWSVAITALLLLLSLPVLAGAITMLLTDRNLNTSFFDPAGGGDPILYQHL
>COLFB585-12|KJ962999|ZMUO.001060|Stenus_palustris
TLYFILGAWAGLTGTSLSLLIRTELGSPGSFIGDDQIYNVIVTAHAFIMIFFMVMPIMIGGFGNWLIPLMLGAPDMAFPRMNNMSFWLLPPSLSLLLMSSIVESGAGTGWTVYPPLSSNIAHSGASVDLTIFSLHLAGISSILGAINFITTIFNMRAMKLQLD
CLPLFIWSVNVTTFLLLLSLPVLAGAITMLLTDRNVNTSFFDPGGGGDPILYQHL
>COLFD879-12|KJ966120|ZMUO.004489|Agonum_fuliginosum
TLYFIFGAWAGMVGTSLSMLIRAELGNPGALIGDDQIYNVIVTAHAFIMIFFMVMPIMIGGFGNWLVPLMLGAPDMAFPRMNNMSFWLLPPSLTLLLMSSLVESGAGTGWTVYPPLSSGIAHAGASVDLAIFSLHLAGISSILGAVNFITTIINMRSVG
MTFDRMPLFVWSVGITALLLLLSLPVLAGAITMLLTDRNLNTSFFDPAGGGDPILYQHL
>COLFE1519-13|KJ965150|ZMUO.007314|Hadrobregmus_confusus
TLYFILGSWSGMIGTSLSIMIRSELSNSGTLIGNDQIYNVIVTAHAFIMIFFMVMPIMIGGFGNWLVPLMIGAPDMAFPRMNNMSFWLLPPSLSLLLLSMAVDSGVGTGWTVYPPLSSNIAHSGASVDLAIFSLHLAGVSSILGAVNFITTVINMRPIEMT
FDRMPLFVWSVSITALLLISLPVLAGAITMLVTDRNINTSFFDPSGGGDPILYQHL
>COLFB572-12|KJ961878|ZMUO.001047|Atheta_malleus
TLYFIFGAWAGMVGTSLSLLIRAELGNPGSLIGDDQIYNVIVTAHAFIMIFFMVMPIVIGGFGNWLVPLMLGAPDMAFPRMNNMSFWLLPPSLTLLLMSSMVESGAGTGWTVYPPLSSNIAHGGSSVDLAIFSLHLAGISSILGAVNFISTVINMRSTGI
SFDRMPLFVWSVAITALLLLLSLPVLAGAITMLLTDRNLNTSFFDPAGGGDPILYQHL
>COLFC504-12|KJ964884|ZMUO.003069|Quedius_tenellus
TLYFIFGAWAGMVGTSLSLLIRAELGNPGTLIGDDQIYNVIVTAHAFIMIFFMVMPIVIGGFGNWLVPLMLGAPDMAFPRMNNMSFWLLPPSLSLLLMSSMVESGAGTGWTVYPPLSSNIAHGGASVDLAIFSLHLAGISSILGAVNFITTVINMRSIG
MTFDRMPLFVWSVAITALLLLLSLPVLAGAITMLLTDRNLNTSFFDPAGGGDPILYQHL
>COLFF1020-13|KJ966025|ZMUO.007385|Agriotes_lineatus
TLYFLFGAWAGMLGTSLSLLIRAELGNPGSLIGNDQIYNVIVTAHAFIMIFFMVMPIMIGGFGNWLVPLMLGAPDMAFPRMNNMSFWFLPPSLSLLLMSSIVENGAGTGWTVYPPLSSNIAHSGSSVDLAIFSLHLAGISSILGAVNFISTVINMRSTGIT
FDRMPLFVWAVAITALLLLLSLPVLAGAITMLLTDRNLNTSFFDPAGGGDPILYQHL
>COLFB693-12|KJ965930|ZMUO.001168|Thanasimus_femoralis
TLYFIFGAWAGMVGTSLSLLIRSELGTPGTLIGNDQIYNVIVTAHAFIMIFFMVMPIMIGGFGNWLVPLMLGAPDMAFPRMNNMSFWLLPPSLTLLLMSSMVENGAGTGWTVYPPLSSNIAHGGSSVDLAIFSLHLAGVSSILGAVNFITTVINMRPEG
MTLDRMPLFVWAVAITALLLLLSLPVLAGAITMLLTDRNLNTSFFDPAGGGDPILYQHL
>COLFD577-12|KJ963010|ZMUO.004377|Neocoenorrhinus_germanicus
TLYFIFGAWSGMLGTSLSLLIRAELGNPGSLIGDDQIYNVIVTAHAFIMIFFMVMPIMIGGFGNWLVPLMLGAPDMAFPRMNNMSFWLLPPSLSLLIMSSIVEKGAGTGWTVYPPLSSNIAHGGSSVDLAIFSLHLAGISSILGAVNFISTVINMRPQGM
SLDRMPLFVWAVAITALLLLLSLPVLAGAITMLLTDRNINTTFFDPAGGGDPILYQHL
>COLFF020-13|KJ965018|ZMUO.005720|Cyphon_ochraceus
TLYFIFGSWSGMVGTSLSLLIRAELGTPGSLIGDDQIYNVIVTAHAFIMIFFMVMPIMIGGFGNWLVPLMLGAPDMAFPRMNNMSFWLLPPSLTLLLMSSMVENGAGTGWTVYPPLSAGIAHSGASVDLAIFSLHLAGISSILGAVNFISTVINMRSVG
MSFDRMPLFVWAVAITALLLLLSLPVLAGAITMLLTDRNLNTSFFDPAGGGDPILYQHL
>COLFC709-12|KJ962784|ZMUO.003274|Pterostichus_rhaeticus

TLYFIFGAWAGMVGTSLSMLIRAELGNPGSLIGDDQIYNVIVTAHAFIMIFFMVMPIMIGGFGNWLVPLMLGAPDMAFPRMNNMSFWLLPPSLTLLLMSSMVESGAGTGWTVYPPLSSGIAHAGASVDLAIFSLHLAGVSSILGAVNFITTIINMRSV
GMTFDRMPLFVWSVGITALLLLLSLPVLAGAITMLLTDRNLNTSFFDPAGGGDPILYQHL
>COLFF370-13|KJ962525|ZMUO.006070|Rhantus_exsoletus
TLYFIFGAWAGMVGTSLSMLIRAELGNPGSLIGDDQIYNVIVTAHAFVMIFFMVMPIMIGGFGNWLVPLMLGAPDMAFPRMNNMSFWLLPPSLTLLLMSSMVESGAGTGWTVYPPLSSGIAHGGASVDLAIFSLHLAGISSILGAVNFITTIINMRSV
GMTFDRMPLFVWSVGITALLLLLSLPVLAGAITMLLTDRNLNTSFFDPAGGGDPILYQHL
>COLFA402-12|KJ964627|ZMUO.000632|Leiodes_silesiaca
TLYFIFGAWAGMVGTSLSILIRAELGNPGSLIGDDQIYNVIVTAHAFVMIFFMVMPIVIGGFGNWLIPLMLGAPDMAFPRMNNMSFWLLPPSLTLLLMSSVVENGAGTGWTVYPPLSSNIAHSGSSVDLAIFSLHLAGISSILGAVNFITTVINMRSAGM
TFDKMTLFVWSVAITALLLLLSLPVLAGAITMLLTDRNLNTSFFDPAGGGDPILYQHL
>COLFD479-12|KJ964387|ZMUO.004279|Evodinus_borealis
TLYFIFGAWAGMVGTSLSMLIRSELGNPGSLIGDDQIYNVIVTAHAFVMIFFMVMPIMIGGFGNWLVPLMLGAPDMAFPRMNNMSFWLLPPSLILLIMSSIVENGAGTGWTVYPPLASNIAHSGSSVDLAIFSLHLAGISSILGAVNFITTVINMRPPG
MTPDRMPLFVWAVVITAVLLLLSLPVLAGAITMLLTDRNLNTSFFDPAGGGDPILYQHL
>COLFC044-12|KJ963921|ZMUO.002039|Cercyon_pygmaeus
TLYFIFGAWAGMVGTSLSILIRAELGNPGTLIGDDQIYNVIVTAHAFIMIFFMVMPIMIGGFGNWLVPLMLGAPDMAFPRMNNMSFWLLPPSLTLLLMSSMVESGAGTGWTVYPPLSSNIAHGGSSVDLAIFSLHLAGISSILGAVNFITTVINMRSPNL
TYDRLPLFVWSVAITALLLLLSLPVLAGAITMLLTDRNLNTSFFDPAGGGDPILYQHL
>COLFA248-12|KJ967441|ZMUO.000383|Rybaxis_longicornis
TLYLIFGAWAGMVGTSLSILIRAELGNPGSLIGDDQIYNVIVTAHAFIMIFFMVMPIMIGGFGNWLVPLMLGAPDMAFPRMNNMSFWLLPPSLILLLMSSMVESGAGTGWTVYPPLSSNIAHSGSSVDLTIFSLHLAGISSILGAVNFITTVINMRTMN
MKFDQLPLFVWSVIITALLLLLSLPVLAGAITMLLTDRNLNTSFFDPTGGGDPVLYQHL
>COLFE020-12|KJ963184|ZMUO.005245|Rhynchites_cupreus
TLYFIFGTWAGLVGTSLSLLIRAELGNPGSLIGDDQIYNVIVTAHAFIMIFFMVMPIMIGGFGNWLVPLMLGAPDMAFPRMNNMSFWLLPPSISLLISSSIVESGAGTGWTVYPPLSSNIAHGGPSVDLAIFSLHLAGISSILGAVNFISTVINMRPKGMSF
DRMPLFVWAVAITALLLLLSLPVLAGAITMLLTDRNINTTFFDPAGGGDPILYQHL
>COLFE1177-13|KJ964621|ZMUO.006972|Limnobaris_pilistriata
TLYFIFGAWAGMTGTSLSMLIRSELGNPGSLIGDDQIYNTIVTAHAFIMIFFMVMPVMIGGFGNWLVPLMLGAPDMAFPRLNNMSFWLLPPSLTLLLMSSIVDKGVGTGWTVYPPLSSNNAHEGASVDLGIFSLHMAGISSILGAMNFISTAMNMRP
TGLKSDQMSLFIWAVKITAILLLLSLPVLAGAITMLLTDRNINTSFFDPTGGGDPILYQHL
>COLFD409-12|KJ964919|ZMUO.003829|Lyprocorrhe_anceps
TLYFIFGAWAGMTGTSLSLLIRAELGNPGSLIGDDQIYNVIVTAHAFVMIFFMVMPIVIGGFGNWLIPLMLGAPDMAFPRMNNMSFWLLPPSLTLLLMSSMIESGAGTGWTVYPPLSSNLAHSGTSVDLAIFSLHLAGISSILGAVNFITTVINMRSTGIS
FDRMPLFVWSVVITALLLLLSLPVLAGAITMLLTDRNLNTSFFDPAGGGDPILYQHL
>COLFE1175-13|KJ966020|ZMUO.006970|Oreodytes_alpinus
TLYFLFGAWSGMVGTSLSMLIRAELGNPGSLIGDDQIYNVIVTAHAFIMIFFMVMPIMIGGFGNWLVPLMLGAPDMAFPRMNNMSFWLLPPSLTLLLMSSMVESGAGTGWTVYPPLSAGIAHGGASVDLAIFSLHLAGISSILGAVNFITTIINMRSV
GMTFDRMPLFVWSVGITALLLLLSLPVLAGAITMLLTDRNLNTSFFDPAGGGDPILYQHL
>COLFC046-12|KJ961835|ZMUO.002041|Atheta_nigripes
TLYFIFGAWAGMVGTSLSLLIRAELGNPGSLIGDDQIYNVIVTAHAFIMIFFMVMPIVIGGFGNWLVPLMLGAPDMAFPRMNNMSFWLLPPSLTLLLMSSMVESGAGTGWTVYPPLSSNIAHGGSSVDLAIFSLHLAGISSILGAVNFISTVINMRSTGI
SFDRMPLFVWSVAITALLLLLSLPVLAGAITMLLTDRNLNTSFFDPAGGGDPILYQHL
>COLFA078-10|HM909095|MP00282|Haliplus_nr._lineolatus
TLYFIFGAWAGMVGTSLSMLIRAELGTPGSLIGDDQIYNVIVTAHAFIMIFFMVMPIMIGGFGNWLVPLMLGAPDMAFPRMNNMSFWLLPPSLSLLLMSSMVENGAGTGWTVYPPLSAGIAHSGASVDLAIFSLHLAGISSILGAVNFITTIINMRSIG
MTFDRMPLFVWSVGITALLLLLSLPVLAGAITMLLTDRNLNTSFFDPAGGGDPILYQHL
>COLFA650-12|KJ962305|ZMUO.000785|Aleochara_lanuginosa
TLYFIFGAWAGMVGTSLSLLIRAELGNPGSLIGDDQIYNVIVTAHAFIMIFFMVMPIVIGGFGNWLVPLMLGAPDMAFPRMNNMSFWLLPPSLTLLLMSSMVESGAGTGWTVYPPLSSNIAHGGSSVDLAIFSLHLAGISSILGAVNFISTVINMRSSG
MTFDKMPLFVWSVAITALLLLLSLPVLAGAITMLLTDRNLNTSFFDPAGGGDPILYQHL
>COLFB896-12|KJ964297|ZMUO.001941|Pogonocherus_hispidus
TLYFLFGAWAGMVGTSLSLLIRSELGNPGSLIGDDQIYNVIVTAHAFVMIFFMVMPIMIGGFGNWLVPLMLGAPDMAFPRMNNMSFWLLPPSLSLLIMSSITENGAGTGWTVYPPLSSNIAHSGSSVDLAIFSLHLAGISSILGAVNFITTVINMRPAG
MIFERLPLFVWAVKITAILLLLSLPVLAGAITMLLTDRNINTSFFDPAGGGDPILYQHL
>COLFF065-13|KJ962613|ZMUO.005765|Hydroglyphus_geminus
TLYFLFGAWSGMVGTSLSMLIRAELGNPGSLIGDDQIYNVIVTAHAFIMIFFMVMPIMIGGFGNWLVPLMLGAPDMAFPRMNNMSFWLLPPSLSLLMMSSMVESGAGTGWTVYPPLSSGIAHGGASVDLAIFSLHLAGISSILGAVNFITTIINMRSV
GMTFDRMPLFVWSVGITALLLLLSLPVLAGAITMLLTDRNLNTSFFDPAGGGDPILYQHL
>COLFF415-13|KJ966372|ZMUO.005933|Crepidodera_nitidula
TLYFIFGIWSGMIGTSLSMLIRAELGNPGSLIGNDQIYNVIVTAHAFIMIFFMVMPIMIGGFGNWLIPLMIGAPDMAFPRMNNMSFWLLPPSLFLLLMSSMVESGAGTGWTVYPPLSSNIAHGGSSVDLAIFSLHLAGISSILGAINFITTVINMRPKGM
NPDQMPLFVWAVIITAILLLLSLPVLAGAITMLLTDRNLNTSFFDPIGGGDPILYQHL

>COLFD058-12|KJ966509|ZMUO.003953|Anaspis_rufilabris
TLYFIFGAWSGMVGTSLSLLIRSELGTPGSLIGDDQIYNVIVTAHAFIMIFFMVMPILIGGFGNWLVPLMLGAPDMAFPRMNNMSFWLLPPSLTLLIMSSVVENGAGTGWTVYPPLAANIAHSGSSVDLAIFSLHLAGVSSILGAVNFITTVINMRPQG
MTLDRMPLFVWAVVITAVLLLLSLPVLAGAITMLLTDRNLNTSFFDPAGGGDPILYQHL
>COLFB906-12|KJ965338|ZMUO.001951|Callidium_coriaceum
TLYFLFGAWASMVGTSLSMLIRTELGNPGSLIGDDQIYNVIVTAHAFIMIFFMVMPIMIGGFGNWLVPLMLGAPDMAFPRMNNLSFWLLPPSLTLLILSSIVENGAGTGWTVYPPLSANVAHSGSSVDLAIFSLHLAGISSILGAVNFISTIINMRPMGM
SPERMPLFVWSVVITAILLLLSLPVLAGAITMLLTDRNINTSFFDPAGGGDPILYQHL
>COLFC238-12|KJ967495|ZMUO.002423|Cryptocephalus_octopunctatus
TLYFLFGAWAGMMGTSLSLLIRIELGNPGSLIGNDQIYNTIVTAHAFIMIFFMVMPIMIGGFGNWLIPLMLGAPDMAFPRMNNMSFWFLPPSITLLFSSSIVENGAGTGWTVYPPLSTTLAHTGSSVDLAIFSLHLAGISSIMGAINFISTVINMRPQGM
LMDRTPLFVWAVLITAILLLLSLPVLAGAITMLLTDRNFNTSFFDPAGGGDPILYQHL
>COLFD624-12|KJ966891|ZMUO.004424|Trechus_quadristriatus
TLYFIFGAWAGMVGTSLSMLIRAELGNPGSLIGDDQIYNVIVTAHAFIMIFFMVMPIMIGGFGNWLVPLMLGAPDMAFPRMNNMSFWLLPPSLTLLLMSSMVESGAGTGWTVYPPLSSGIAHSGASVDLAIFSLHLAGVSSILGAVNFITTIINMRPM
GMTFDRMPLFVWSVGITALLLLLSLPVLAGAITMLLTDRNLNTSFFDPAGGGDPILYQHL
>COLFD373-12|KJ962344|ZMUO.004268|Pterostichus_melanarius
TLYFIFGAWAGMVGTSLSMLIRAELGNPGSLIGDDQIYNVIVTAHAFVMIFFMVMPIMIGGFGNWLVPLMLGAPDMAFPRMNNMSFWLLPPSLTLLLMSSMVESGAGTGWTVYPPLSSGIAHAGASVDLAIFSLHLAGVSSILGAVNFITTIINMRSI
GMTFDRMPLFVWSVGITALLLLLSLPVLAGAITMLLTDRNLNTSFFDPAGGGDPILYQHL
>COLFB891-12|KJ966110|ZMUO.001936|Asemum_striatum
TLYFIFGAWSGMVGTSLSMLIRSELGNPGSLIGDDQIYNVIVTAHAFIMIFFMVMPIMIGGFGNWLVPLMLGAPDMAFPRMNNMSFWLLPPSLTLLIMSSIVENGAGTGWTVYPPLSANIAHSGSSVDLAIFSLHLAGISSILGAVNFITTVINMRPKG
MMLDRMPLFVWAVVITAILLLLSLPVLAGAITMLLTDRNLNTSFFDPIGGGDPILYQHL
>COLFB529-12|KJ962007|ZMUO.001004|Galeruca_tanaceti
TLYFIFGVWAGMVGTSLSILVRTELGSPGTLIGNDQIYNVIVTAHAFIMIFFMVMPIMIGGFGNWLVPLMIGAPDMAFPRMNNMSFWLLPPSLFLLIMSSIVESGAGTGWTVYPPLSSNIAHGGSSVDLAIFSLHLAGISSILGAINFITTVINMRPKGMT
LDRMPLFVWAVTITAILLLLSLPVLAGAITMLLTDRNLNTSFFDPAGGGDPILYQHL
>COLFB393-12|KJ965482|ZMUO.001818|Rugilus_orbiculatus
TLYFIFGAWAGMVGTSLSLLIRAELSAPGSLIGDDQIYNVIVTAHAFVMIFFMVMPIVIGGFGNWLVPLMLGAPDMAFPRMNNMSFWLLPPSLSLLLMSSMVESGAGTGWTVYPPLSSNIAHGGASVDLAIFSLHLAGISSILGAVNFITTIINMRSKG
MTYERMSLFVWSVGITALLLLLSLPVLAGAITMLLTDRNLNTSFFDPAGGGDPILYQHL
>COLFG003-13|KJ965490|ZMUO.007508|Lamia_textor
TLYFLFGAWAGMMGTSLSILIRTELGSPGSLIGDDQIYNVIVTAHAFIMIFFMVMPIMIGGFGNWLVPLMLGAPDMAFPRMNNMSFWLLPPSFFLLIMSSIVENGAGTGWTVYPPLAANVAHNGASVDLAIFSLHLAGISSILGAVNFITTVINMRPSG
MSMDRMPLFVWSVKITAILLLLSLPVLAGAITMLLTDRNLNTSFFDPAGGGDPILYQHL
>COLFF349-13|KJ965112|ZMUO.006049|Cryptocephalus_quadripustulatus
TLYFLFGAWSGMVGTSLSLLIRVELGNPGSLIGNDQIYNTIVTAHAFIMIFFMVMPIMIGGFGNWLVPLMLGAPDMAFPRMNNMSFWLLPPSLTLLLMSSIVENGAGTGWTVYPPLSSTIAHAGASVDLAIFSLHLAGISSIMGAINFISTVINMRPQG
MLMDRTPLFVWAVLITAILLLLSLPVLAGAITMLLTDRNLNTSFFDPAGGGDPILYQHL
>COLFF858-13|KJ965969|ZMUO.006653|Aphodius_distinctus
TLYFLFGSWAGMVGTSLSLLIRAELGNPGTLIGDDQIYNVIVTAHAFVMIFFMVMPILIGGFGNWLVPLMLGAPDMAFPRMNNMSFWLLPPSLTLLLMSSMVESGAGTGWTVYPPLSSNIAHGGASVDLAIFSLHLAGISSILGAVNFITTVINMRSPG
MTFDRMPLFVWSVAITALLLLLSLPVLAGAITMLLTDRNLNTSFFDPAGGGDPILYQHL
>COLFF619-13|KJ964363|ZMUO.006509|Ampedus_balteatus
TLYFIFGAWAGMLGTSLSLLIRAELGNPGSLIGNDQIYNVIVTAHAFIMIFFMVMPIMIGGFGNWLVPLMLGAPDMAFPRMNNMSFWLLPPSLSLLLMSSIVENGAGTGWTVYPPLSSNIAHSGSSVDLAIFSLHLAGISSILGAVNFISTVINMRSTGIT
FDRMPLFVWAVAITALLLLLSLPVLAGAITMLLTDRNLNTSFFDPAGGGDPILYQHL
>COLFC296-12|KJ966942|ZMUO.002481|Hypnoidus_rivularius
TLYFLFGAWSGMLGTSLSLLIRAELGNPGSLIGNDQIYNVIVTAHAFIMIFFMVMPIMIGGFGNWLVPLMLGAPDMAFPRMNNMSFWFLPPSLSLLLMSSIVENGAGTGWTVYPPLSANIAHSGSSVDLAIFSLHLAGISSILGAVNFISTVINMRSTGIT
FDRMPLFVWAVAITALLLLLSLPVLAGAITMLLTDRNLNTSFFDPAGGGDPILYQHL
>COLFA607-12|KJ964113|ZMUO.000742|Anoplodera_sanguinolenta
TLYFIFGAWASMVGTSLSLLIRSELGNPGSLIGNDQIYNVIVTAHAFVMIFFMVMPIMIGGFGNWLVPLMLGAPDMAFPRMNNMSFWLLPPSLTLLIMSSIVESGAGTGWTVYPPLSSNIAHSGSSVDLAIFSLHLAGISSILGAVNFITTVINMRPAGLK
PEQMPLFVWAVVITAVLLLLSLPVLAGAITMLLTDRNLNTSFFDPAGGGDPILYQHL
>COLFC070-12|KJ965469|ZMUO.002065|Porhydrus_lineatus
TLYFLFGTWAGMVGTSLSMLIRAELGNPGSLIGDDQIYNVIVTAHAFIMIFFMVMPIMIGGFGNWLVPLMLGAPDMAFPRMNNMSFWLLPPSLSLLLMSSMVESGAGTGWTVYPPLSAGIAHSGASVDLAIFSLHLAGISSILGAVNFITTIINMRSVG
MTFDRMPLFVWSVGITALLLLLSLPVLAGAITMLLTDRNLNTSFFDPAGGGDPILYQHL
>COLFB305-12|KJ965940|ZMUO.001635|Stenus_incrassatus

ILYFVFGAWAGMVGTSLSLLIRAELGNPGSLIGDDQIYNVIVTAHAFVMIFFMVMPVMIGGFGNWLVPLMLGAPDMAFPRMNNMSFWLLPPSLSLLLMSSIVENGAGTGWTVYPPLSSNIAHSGASVDLAIFSLHLAGVSSILGAINFITTIFNMRTM
KLQLDCLPLFVWSVGITALLLLLSLPVLAGAITMLLTDRNLNTSFFDPAGGGDPILYQHL
>COLFE1454-13|KJ963724|ZMUO.007249|Aegialia_arenaria
TLYFLFGGWAGMVGTSLSLLIRAELGNPGMLIGDDQIYNVIVTAHAFVMIFFMVMPILIGGFGNWLVPLMLGAPDMAFPRMNNMSFWLLPPSLTLLLMSSMVESGAGTGWTVYPPLSSNIAHSGPSVDLAIFSLHLAGISSILGAVNFITTVINMRSIG
MSFDRMPLFVWSVALTALLLLLSLPVLAGAITMLLTDRNINTSFFDPTGGGDPILYQHL
>COLFF594-13|KJ964430|ZMUO.006484|Mordellistena
TLYFVFGAWAGMLGTSLSLLIRSELGTPGSLIGDDQIYNVIVTAHAFVMIFFMVMPVMVGGFGNWLLPLMLGAPDMAFPRLNNMSFWLLPPSLTLLILSSMVENGAGTGWTVYPPLSSNIAHGGASVDLAIFSLHLAGISSILGAINFISTMLNMHPKG
MILDQMPLFVWAIIITAILLLLSLPVLAGAITMLLTDRNLNSSFFDPAGGGDPILYQHL
>COLFA711-12|KJ965323|ZMUO.000846|Philonthus_rectangulus
TLYFIFGSWAGMVGTSLSLLIRAELGNPGTLIGDDQIYNVIVTAHAFIMIFFMVMPIVIGGFGNWLVPLMLGAPDMAFPRMNNMSFWLLPPSLTLLLMSSMVESGAGTGWTVYPPLSSNIAHGGASVDLAIFSLHLAGISSILGAVNFITTVINMRSTG
MTFDRMPLFVWSVAITALLLLLSLPVLAGAITMLLTDRNLNTTFFDPAGGGDPILYQHL
>COLFA027-10|HM909054|MP00168|Pycnoglypta_lurida
TLYFIFGAWSGMVGTSLSMLIRAELGNPGSLIGDDQIYNVIVTAHAFIMIFFMVMPIVIGGFGNWLVPLMLGAPDMAFPRMNNMSFWLLPPSLTLLLMSSMVESGAGTGWTVYPPLSSNIAHGGASVDLAIFSLHLAGISSILGAVNFITTVVNMRSM
GMTFDRMPLFVWSVAITALLLLLSLPVLAGAITMLLTDRNLNTSFFDPAGGGDPILYQHL
>COLFE384-12|KJ964888|ZMUO.004944|Zacladus_geranii
TLYFIFGSWAGAAGTSLSMLIRTELGNPGSLIGNDQIYNSIVTAHAFIMIFFMVMPILIGGFGNWLVPLMLGAPDMAFPRLNNMSFWLLPPSLTLLLMSSIINKGAGTGWTVYPPLSANVAHEGASVDLAIFSLHMAGISSILGAINFISTIMNMRPKGM
DYDQTPLFAWAVMITAILLLLSLPVLAGAITMLLTDRNINTTFFDPSGGGDPILYQHL
>COLFE1365-13|KJ967489|ZMUO.007160|Anaspis_frontalis
TLYFIFGAWSGMVGTSLSLLIRSELGTPGSLIGDDQIYNVIVTAHAFIMIFFMVMPILIGGFGNWLVPLMLGAPDMAFPRMNNMSFWLLPPSLTLLIMSSVVENGAGTGWTVYPPLAANIAHSGSSVDLAIFSLHLAGVSSILGAVNFITTVINMRPQG
MTLDRMPLFVWAVVITAVLLLLSLPVLAGAITMLLTDRNLNTSFFDPAGGGDPILYQHL
>COLFB454-12|KJ967382|ZMUO.001879|Pterostichus_crenatus
TLYFIFGAWSGMVGTSLSMLIRAELGNPGSLIGDDQIYNVIVTAHAFVMIFFMVMPIMIGGFGNWLVPLMLGAPDMAFPRMNNMSFWLLPPSLTLLLMSSMVESGAGTGWTVYPPLSSGIAHAGASVDLAIFSLHLAGISSILGAVNFITTILNMRST
GMTFDRMPLFVWSVGITALLLLLSLPVLAGAITMLLTDRNLNTSFFDPAGGGDPILYQHL
>COLFE608-13|KJ966470|ZMUO.005453|Chaetocnema_sahlbergii
TLYFIFGIWSGMVGTSMSILIRAELGNPGSLIGNDQIYNVIVTAHAFVMIFFMVMPIMIGGFGNWLVPLMIGAPDMAFPRMNNMSFWLLPPSLFLLLMSSMVESGAGTGWTVYPPLSSNIAHGGSSVDLAIFSLHLAGISSILGAINFITTIINMRPQG
MQFDQMPLFVWAVLITAILLLLSLPVLAGAITMLLTDRNLNTSFFDPIGGGDPILYQHL
>COLFE1485-13|KJ965951|ZMUO.007280|Rhamphus_oxyacanthae
TLYFLFGAWSGMVGTSLSMLIRTELGTPGKLIGDDQIYNTIVTAHAFIMIFFMVMPIMIGGFGNWLIPLMLGAPDMAFPRLNNMSFWLLPPSLTFLLLSSIMDKGAGTGWTVYPPLSANIAHEGTSVDLAIFSLHMAGVSSILGAMNFISTIINMKPKN
MSLDQMSLFVWSVKITAILLLLSLPVLAGAITMLLTDRNINTSFFDPAGGGDPILYQHL
>COLFD714-12|KJ966811|ZMUO.004704|Trox_sabulosus
TLYFLFGSWAGMVGTSLSILIRAELGNPGTLIGDDQIYNVIVTAHAFIMIFFMVMPILIGGFGNWLVPLMLGAPDMAFPRMNNMSFWLLPPSLTLLLVSSLVESGAGTGWTVYPPLSSNIAHSGASVDLAIFSLHLAGISSILGAVNFITTVINMRSTGMT
FDRMPLFVWAVILTALLLLLSLPVLAGAITMLLTDRNLNTSFFDPAGGGDPVLYQHL
>COLFD770-12|KJ964280|ZMUO.004760|Pachybrachis_hieroglyphicus
TLYFIFGAWSGMVGTSLSLLIRVELGNPGSLIGNDQIYNTIVTAHAFIMIFFMVMPIMIGGFGNWLVPLMLGAPDMAFPRMNNMSFWLLPPSLTLLLMSSIVENGAGTGWTVYPPLSSNLAHGGSSVDLAIFSLHLAGVSSIMGAINFISTVINMRPQG
MLLDRTPLFAWAVLITAVLLLLSLPVLAGAITMLLTDRNLNTSFFDPAGGGDPILYQHL
>COLFE330-12|KJ962767|ZMUO.004890|Cryphalus_abietis
TLYFIFGAWSGMVGTSMSLIIRTELGTPGSLIGDDQIYNTIVTAHAFIMIFFMVMPILIGGFGNWLVPLMLGAPDMAFPRLNNMSFWLLPPSLIFLVMSSIINKGAGTGWTVYPPLASNIAHEGASVDFAIFSLHMAGISSILGAMNFISTIINMHPSGMK
PEQLSLFSWAVKITAILLLLSLPVLAGAITMLLTDRNINTSFFDPAGGGDPILYQHL
>COLFC465-12|KJ964529|ZMUO.003030|Lordithon_lunulatus
TLYFIFGSWAGMVGTSLSLLIRAELGNPGSLIGDDQIYNVIVTAHAFVMIFFMVMPIVIGGFGNWLVPLMLGAPDMAFPRMNNMSFWLLPPSLTLLLMSSMVESGAGTGWTVYPPLSSNIAHGGASVDLAIFSLHLAGISSILGAVNFITTVINMRSTG
MTFDRMPLFIWSVAITALLLLLSLPVLAGAITMLLTDRNLNTSFFDPAGGGDPILYQHL
>COLFD026-12|KJ967510|ZMUO.003921|Haliplus_ruficollis
TLYFIFGAWAGMVGTSLSMLIRAELGTPGSLIGDDQIYNVIVTAHAFIMIFFMVMPIMIGGFGNWLVPLMLGAPDMAFPRMNNMSFWLLPPSLSLLLMSSMVENGAGTGWTVYPPLSAGIAHSGASVDLAIFSLHLAGISSILGAVNFITTIINMRSIG
MTFDRMPLFVWSVGITALLLLLSLPVLAGAITMLLTDRNLNTSFFDPAGGGDPILYQHL
>COLFA596-12|KJ967529|ZMUO.000731|Dasytes_cyaneus
TLYFIFGAWSGMVGMSLSLLIRSELNNPGTLIGNDQIYNVIVTAHAFIMIFFMVMPILIGGFGNWLVPLMLGAPDMAFPRMNNMSFWLLPPSLTLLLMSSMVEQGAGTGWTVYPPLSANIAHGGASVDLAIFSLHLAGISSILGAVNFITTVINMRPN
GMTLDRTPLFVWAVAITALLLLLSLPVLAGAITMLLTDRNLNTSFFDPAGGGDPILYQHL

>COLFG004-13|KJ963214|ZMUO.007509|Buprestis_novemmaculata
TLYFIFGAWSGMVGTALSLLIRAELGNPGALIGDDQVYNVIVTAHAFVMIFFMVMPVMMGGFGNWLVPLMLGAPDMAFPRMNNMSFWLLPPSLTLLLMSSVVENGAGTGWTVYPPLAANIAHSGASVDLAIFSLHLAGVSSILGAVNFITTVINM
RSVGMTFDRMPLFVWSVAITALLLLLSLPVLAGAITMLLTDRNFNTSFFDPAGGGDPILYQHL
>COLFD656-12|KJ966426|ZMUO.004456|Phyllotreta_atra
TLYFIFGIWAGMVGMSMSMLIRIELAAPGSLIGNDQIYNVIVTAHAFIMIFFMVMPIMIGGFGNWLVPLMIGAPDMAFPRMNNMSFWLLPPSLFLLIMSSIVENGAGTGWTVYPPLSANISHAGSSVDLTIFSLHLAGISSILGAINFITTIINMRPKGMS
FDRMPLFVWAVLITAILLLLSLPVLAGAITMLLTDRNLNTSFFDPMGGGDPILYQHL
>COLFE1481-13|KJ962115|ZMUO.007276|Anthonomus_conspersus
TLYFIFGAWSGTVGTSLSMLIRTELGNPGSLIGDDQIYNTIVTAHAFIMIFFMVMPIMIGGFGNWLVPLMLAAPDMAFPRLNNMSFWLLPPSLTLLIMSSIIGKGAGTGWTVYPPLSSNLAHEGASVDFAIFSLHMAGISSILGAMNFISTVLNMKPSGM
NLEQMPLFVWAVKITAVLLLISLPVLAGAITMLLTDRNINTSFFDPAGGGDPILYQHL
>COLFD628-12|KJ962994|ZMUO.004428|Pterostichus_diligens
TLYFIFGAWSGMVGTSLSMLIRAELGNPGSLIGDDQIYNVIVTAHAFVMIFFMVMPIMIGGFGNWLVPLMLGAPDMAFPRMNNMSFWLLPPSLTLLLMSSMVESGAGTGWTVYPPLSSGIAHAGASVDLAIFSLHLAGISSILGAVNFITTIINMRSIG
MTFDRMPLFVWSVGITALLLLLSLPVLAGAITMLLTDRNLNTSFFDPAGGGDPILYQHL
>COLFE256-12|KJ962644|ZMUO.005101|Lebia_chlorocephala
TLYFIFGAWAGMVGTSLSMLIRAELGNPGALIGDDQIYNVIVTAHAFIMIFFMVMPIMIGGFGNWLVPLMLGAPDMAFPRMNNMSFWLLPPSLTLLLMSSLVESGAGTGWTVYPPLSSGIAHAGASVDLAIFSLHLAGVSSILGAVNFITTIINMRSVG
MTFDRMPLFVWSVGITALLLLLSLPVLAGAITMLLTDRNLNTSFFDPAGGGDPILYQHL
>COLFD495-12|KJ963639|ZMUO.004295|Agabus_thomsoni
TLYFIFGAWAGMVGTSLSMLIRAELGNPGSLIGDDQIYNVIVTAHAFVMIFFMVMPIMIGGFGNWLIPLMLGAPDMAFPRMNNMSFWLLPPSLTLLLMSSMVESGAGTGWTVYPPLSSGIAHGGASVDLAIFSLHLAGISSILGAVNFITTIINMRSVG
MTFDRMPLFVWSVGITALLLLLSLPVLAGAITMLLTDRNLNTSFFDPAGGGDPILYQHL
>COLFA658-12|KJ962446|ZMUO.000793|Philonthus_nitidus
TLYFIFGSWAGMVGTSLSLLIRAELGNPGTLIGDDQIYNVIVTAHAFIMIFFMVMPIVIGGFGNWLVPLMLGAPDMAFPRMNNMSFWLLPPSLTLLLMSSMVESGAGTGWTVYPPLSSNIAHGGASVDLAIFSLHLAGISSILGAVNFITTVINMRSTG
MSFDRMPLFVWSVAITTLLLLLSLPVLAGAITMLLTDRNLNTTFFDPAGGGDPILYQHL
>COLFA029-10|HM909056|MP00171|Acrotrichis_nr._sitkaensis
TLYFMFGAWAGMVGTSLSILIRTELGTPGSLIGDDQIYNVIVTAHAFVMIFFMVMPILIGGFGNWLVPLMLGAPDMAFPRMNNMSFWLLPPSLTLLLMSSMVESGAGTGWTVYPPLASNIAHGGASVDLAIFSLHLAGISSILGAVNFITTIINMRAPQ
MSFDQMPLFVWAVGITALLLLLSLPVLAGAITMLLTDRNLNTSFFDPAGGGDPILYQHL
>COLFF199-13|KJ963596|ZMUO.005804|Polydrusus_cervinus
TLYFIFGAWSGMVGTSLSMLIRTELGNPGSLIGDDQIYNVIVTAHAFIMIFFMVMPIMIGGFGNWLVPLMLGAPDMAFPRLNNMSFWLLPPSLTLLLMSSIVDKGAGTGWTVYPPLSTNIAHEGSSVDLAIFSLHMAGVSSILGAINFISTIINMRPTGM
SYDRMPLFVWAVKITAILLLLSLPVLAGAITMLLTDRNINTSFFDPAGGGDPILYQHL
>COLFA175-10|HQ559267|MP00437|Judolia_sexmaculata
TLYFIFGAWAGMVGTSLSLLIRSELGSPGSLIGNDQIYNVIVTAHAFVMIFFMVMPIMIGGFGNWLVPLMLGAPDMAFPRMNNMSFWLLPPSLTLLILSSMVETGAGTGWTVYPPLSSNIAHSGSSVDLAIFSLHLAGISSILGAVNFITTVINMRPVGM
NLDCMPLFVWAVVITAILLLLSLPVLAGAITMLLTDRNLNTSFFDPAGGGDPILYQHL
>COLFE1236-13|KJ963620|ZMUO.007031|Patrobus_assimilis
TLYFIFGAWSGMVGTSLSILIRAELGNPGSLIGDDQIYNVIVTAHAFVMIFFMVMPIMIGGFGNWLVPLMLGAPDMAFPRMNNMSFWLLPPSLTLLLMSSMVESGAGTGWTVYPPLSSGIAHSGASVDLAIFSLHLAGISSILGAVNFITTIINMRSVG
MTFDRMPLFVWSVGITALLLLLSLPVLAGAITMLLTDRNLNTSFFDPAGGGDPILYQHL
>COLFE1076-13|KJ964299|ZMUO.006871|Bembidion_femoratum
TLYFIFGAWSGMVGTSLSMLIRAELGNPGSLIGDDQIYNVIVTAHAFVMIFFMVMPILIGGFGNWLVPLMLGAPDMAFPRMNNMSFWLLPPSLTLLLMSSMVESGAGTGWTVYPPLSSSIAHSGASVDLAIFSLHLAGVSSILGAVNFITTIINMRSVG
MSFDRMPLFVWSVGITALLLLLSLPVLAGAITMLLTDRNLNTSFFDPAGGGDPILYQHL
>COLFF715-13|KJ965430|ZMUO.006415|Perapion_curtirostre
TLYFILGVWSGLVGTSLSMLIRVELGNPGSLIGDDQIYNVIVTAHAFIMIFFMVMPIMIGGFGNWLVPLMLGAPDMAFPRMNNMSFWLLPPALTLLLMSSIIEKGAGTGWTVYPPLASNIAHGGSSVDLAIFSLHLAGISSILGAVNFISTIMNMRPSGL
SLDQLSLFTWAVKITAILLLLSLPVLAGAITMLLTDRNINTSFFDPAGGGDPILYQHL
>COLFB280-12|KJ966810|ZMUO.001610|Megasternum_concinnum
TLYFIFGAWAGMVGTSLSILIRAELGNPGTLIGDDQIYNVIVTAHAFIMIFFMVMPIMIGGFGNWLVPLMLGAPDMAFPRMNNMSFWLLPPSLTLLLMSSMVESGAGTGWTVYPPLSSNIAHGGSSVDLAIFSLHLAGISSILGAVNFITTVINMRSPNL
TYDRLPLFVWSVAITALLLLLSLPVLAGAITMLLTDRNLNTSFFDPAGGGDPILYQHL
>COLFB565-12|KJ963663|ZMUO.001040|Pterostichus_sp.
TLYFIFGAWAGMVGTSLSMLIRAELGNPGSLIGDDQIYNVIVTAHAFIMIFFMVMPIMIGGFGNWLVPLMLGAPDMAFPRMNNMSFWLLPPSLTLLLMSSMVESGAGTGWTVYPPLSSGIAHAGASVDLAIFSLHLAGVSSILGAVNFITTIINMRSV
GMTFDRMPLFVWSVGITALLLLLSLPVLAGAITMLLTDRNLNTSFFDPAGGGDPILYQHL
>COLFC444-12|KJ962157|ZMUO.003009|Clambus_lohsei

TLYFLFGAWSGMVGTALSLLIRIELSRPMMVIGDDQIYNTIVTAHALIMIFFMVMPILVGGFGNWLIPLMLGAPDMAFPRMNNMSFWLLPPSMTLILMSSMIESGAGTGWTIYPPLSSNIAHSGSSVDLAIFSLHLAGISSILGAVNFISTFINMRMIGM
NYTRVPLFIWSVVITAILLLLSLPVLAGAITMLLTDRNLNTSFFDPAGGGDPILFQHL
>COLFC794-12|KJ966402|ZMUO.003359|Omalium_littorale
TLYFIFGAWAGMVGTSLSLLIRAELGNPGSLIGDDQIYNVIVTAHAFIMIFFMVMPIVIGGFGNWLVPLMLGAPDMAFPRMNNMSFWLLPPSLTLLLMSSMVESGAGTGWTVYPPLSSNIAHGGSSVDLAIFSLHLAGISSILGAVNFITTVINMRAMG
MTFDRMPLFVWSVVITALLLLLSLPVLAGAITMLLTDRNLNTSFFDPAGGGDPILYQHL
>LEFIJ1977-13|KJ963809|ZMUO.003711|Saperda_perforata
TLYFLFGAWAGMVGTSLSLLIRSELGTPGTLIGNDQIYNTIVTAHAFIMIFFMVMPIMMGGFGNWLVPLMLGAPDMAFPRMNNMSFWLLPPSLTLLIMSSIVDNGAGTGWTVYPPLAANVAHSGSSVDLAIFSLHLAGISSILGAVNFITTVINMRPS
GMTLDRTSLFVWAVKITAILLLLSLPVLAGAITMLLTDRNLNTSFFDPAGGGDPILYQHL
>COLFF195-13|KJ967272|ZMUO.005800|Leptura_nigra
TLYFIFGAWASMVGTSLSLLIRSELGNPGSLIGNDQIYNVIVTAHAFVMIFFMVMPIMIGGFGNWLVPLMLGAPDMAFPRMNNMSFWLLPPSLTLLIMSSIVESGAGTGWTVYPPLSSNIAHSGSSVDLAIFSLHLAGISSILGAVNFITTVINMHPKGM
NPDQMPLFVWAVVITAILLLLSLPVLAGAITMLLTDRNLNTSFFDPAGGGDPILYQHL
>COLFB818-12|KJ963628|ZMUO.001293|Bembidion_difficile
TLYFIFGAWSGMVGTSLSMLIRAELGNPGSLIGDDQIYNVIVTAHAFIMIFFMVMPILIGGFGNWLVPLMLGAPDMAFPRMNNMSFWLLPPSLSLLLMSSMVESGVGTGWTVYPPLSSSIAHSGSSVDLAIFSLHLAGVSSILGAVNFITTIINMRSTGM
SFDRMPLFVWSVGITALLLLLSLPVLAGAITMLLTDRNLNTTFFDPAGGGDPILYQHL
>COLFA672-12|KJ964617|ZMUO.000807|Hypera_dauci
TLYFIFGIWAGTVGTSLSILIRTELGNPGSLIGNDQIYNTIVTAHAFIMIFFMVMPIMIGGFGNWLVPLMLGAPDMAFPRLNNMSFWLLPPSLSLLLMSSMVDSGAGTGWTVYPPLSSNIAHEGSSVDLAIFSLHMAGVSSILGAINFISTVLNMRPTGM
SLDKMALFVWAVKVTAILLLLSLPVLAGAITMLLTDRNINTSFFDPAGGGDPILYQHL
>COLFF278-13|KJ963240|ZMUO.005883|Neocoenorrhinus_aequatus
TLYFIFGAWSGMVGTSLSLLIRAELGNPGSLIGDDQIYNVIVTAHAFIMIFFMVMPIMIGGFGNWLVPLMLGAPDMAFPRMNNMSFWLLPPSLSLLIMSSIVESGAGTGWTVYPPLSSNIAHGGSSVDLAIFSLHLAGISSILGAVNFISTVINMRPQGM
TLDRMPLFVWAVAITALLLLLSLPVLAGAITMLLTDRNINTTFFDPAGGGDPILYQHL
>COLFF803-13|KJ966369|ZMUO.006123|Stenus_clavicornis
TLYFIFGSWAGMVGTSLSMLIRSELGSPGSLIGDDQIYNVIVTAHAFIMIFFMVMPIMIGGFGNWLVPLMLGAPDMAFPRMNNMSFWLLPPSLSLLLMSSIVESGAGTGWTVYPPLSSNIAHSGASVDLAIFSLHLAGISSILGAINFITTIINMRTMKM
QLDCLPLFVWSVSITALLLLLSLPVLAGAITMLLTDRNLNTSFFDPAGGGDPILYQHL
>COLFF700-13|KJ967464|ZMUO.006400|Tetrops_praeustus
TLYFIFGAWASMVGTSLSLLIRSELGSPGSLIGNDQIYNTIVTAHAFIMIFFMVMPIMIGGFGNWLVPLMLGAPDMAFPRMNNMSFWLLPPSLTLLIMSSIVENGAGTGWTVYPPLASNITHSGSSVDLAIFSLHLAGVSSILGAVNFITTVINMRPNGM
SLDRLPLFVWAVKITAILLLLSLPVLAGAITMLLTDRNLNTSFFDPASGGDPILYQHL
>COLFE1579-13|KJ962559|ZMUO.007469|Helophorus_nubilus
TLYFIFGAWAGMVGTSLSILIRAELGNPGTLIGDDQIYNVIVTAHAFIMIFFMVMPTMIGGFGNWLVPLMLGAPDMAFPRMNNMSFWLLPPSLTLLLMSSMVESGAGTGWTVYPPLSSNIAHSGASVDLAIFSLHLAGISSILGAVNFITTVINMRSNS
MTYDRLPLFVWSVAITALLLLLSLPVLAGAITMLLTDRNLNTSFFDPAGGGDPILYQHL
>COLFB625-12|KJ963587|ZMUO.001100|Galerucella_sagittariae
TLYFIFGVWAGMVGTSLSILVRVELGNPGSLIGNDQIYNVIVTAHAFIMIFFMVMPIMIGGFGNWLVPLMIGAPDMAFPRMNNMSFWLLPPSLFLLIMSSIVESGAGTGWTVYPPLSSNIAHGGSSVDLAIFSLHLAGISSILGAINFITTIINMRPKGMT
LDRMPLFVWAVMITAILLLLSLPVLAGAITMLLTDRNLNTSFFDPAGGGDPILYQHL
>COLFB439-12|KJ962791|ZMUO.001864|Olibrus_millefolii
TLYFIFGAWASMVGTSLSLLIRTELGTPGSLIGDDQIYNVIVTAHAFVMIFFMVMPFMIGGFGNWLVPLMLGAPDMAFPRMNNMSFWLLPPSLFLLLMSSMVESGAGTGWTVYPPLSSNIAHGGASVDLAIFSLHLAGISSILGAINFITTMINMRPEG
MTLDRMPLFVWAVTITAILLLLSLPVLAGAITMLLTDRNINTSFFDPAGGGDPILYQHL
>COLFE838-13|KJ965120|ZMUO.005683|Pria_dulcamarae
TLYFIFGAWSGMVGTSLSMLIRTELGNPGSLIGNDQIYNVIVTAHAFVMIFFMVMPFMIGGFGNWLVPLMLGAPDMAFPRMNNMSFWLLPPSLSLLLMSSIVESGAGTGWTVYPPLSSNIAHGGASVDLAIFSLHLAGISSILGAVNFITTIINMRPTG
MTFDRMPLFVWAVAITALLLLLSLPVLAGAITMLLTDRNLNTTFFDPSGGGDPILYQHL
>COLFC826-12|KJ966220|ZMUO.003391|Longitarsus_nigerrimus
TLYFIFGIWAGMVGTSLSILIRTELGNPGSLIGNDQIYNVIVTAHAFIMIFFMVMPIMIGGFGNWLVPLMIGAPDMAFPRMNNMSFWLLPPSIFLLIMSSVVENGAGTGWTVYPPLSANIAHSGSSVDLAIFSLHLAGISSILGAINFITTVINMRPIGMM
LDRMPLFVWSVLITAILLLLSLPVLAGAITMLLTDRNLNTTFFDPAGGGDPILYQHL
>COLFD018-12|KJ965223|ZMUO.003913|Hydroporus_palustris
TLYFLFGAWSGMVGTSLSMLIRAELGNPGSLIGDDQIYNVIVTAHAFIMIFFMVMPIMIGGFGNWLVPLMLGAPDMAFPRMNNMSFWLLPPSLTLLLMSSMVENGAGTGWTVYPPLSSGIAHSGASVDLAIFSLHLAGVSSILGAVNFITTIINMRSI
GMTFDRMPLFVWSVGITALLLLLSLPVLAGAITMLLTDRNLNTSFFDPAGGGDPILYQHL
>COLFC297-12|KJ961929|ZMUO.002482|Olophrum_rotundicolle
TLYFIFGAWAGMVGTSLSILIRAELGNPGTLIGDDQIYNVIVTAHAFVMIFFMVMPILIGGFGNWLVPLMLGAPDMAFPRMNNMSFWLLPPSLTLLLTSSMVESGAGTGWTVYPPLSSNIAHGGASVDLAIFSLHLAGISSILGAVNFITTVINMRATG
MTFDRMPLFVWAVAITALLLLLSLPVLAGAITMLLTDRNLNTTFFDPAGGGDPILYQHL

>COLFF958-13|KJ964676|ZMUO.007323|Stenus_juno
TLYFIFGSWAGMVGTSLSMLIRSELGNPGSLIGDDQIYNVIVTAHAFIMIFFMVMPIMIGGFGNWLVPLMLGAPDMAFPRMNNMSFWLLPPSLSLLLMSSIVESGAGTGWTVYPPLSSNIAHSGASVDLAIFSLHLAGISSILGAINFITTIINMRTMKLQ
LECLPLFVWSVSITALLLLLSLPVLAGAITMLLTDRNLNTSFFDPAGGGDPILYQHL
>COLFC476-12|KJ966323|ZMUO.003041|Epuraea_aestiva
TLYFIFGAWSGMIGTSLSILIRTELGSPGSLIGNDQIYNVIVTAHAFIMIFFMVMPFMIGGFGNWLVPLMLGAPDMAFPRMNNMSFWLLPPSLSLLLMSSIVESGAGTGWTVYPPLSSNIAHGGSSVDLAIFSLHLAGVSSILGAVNFITTIINMRPIGMTL
DRMPLFVWSVIITAILLLLSLPVLAGAITMLLTDRNLNTTFFDPSGGGDPILYQHL
>COLFB398-12|KJ963879|ZMUO.001823|Patrobus_atrorufus
TLYFIFGAWSGMVGTSLSMLIRAELGNPGSLIGDDQIYNVIVTAHAFVMIFFMVMPIMIGGFGNWLVPLMLGAPDMAFPRMNNMSFWLLPPSLTLLLMSSMVESGAGTGWTVYPPLSSGIAHSGASVDLAIFSLHLAGISSILGAVNFITTIINMRSVG
MTFDRMPLFVWSVSITALLLLLSLPVLAGAITMLLTDRNLNTSFFDPAGGGDPILYQHL
>COLFC103-12|KJ966070|ZMUO.002288|Lypoglossa_lateralis
TLYFIFGAWAGMIGTSLSLLIRAELGNPGSLIGDDQIYNVIVTAHAFIMIFFMVMPIVIGGFGNWLVPLMLGAPDMAFPRMNNMSFWLLPPSLTILLMSSMVESGAGTGWTVYPPLSSNIAHGGSSVDLAIFSLHLAGISSILGAVNFISTVINMRSIGITF
DRMPLFVWSVVITALLLLLSLPVLAGAITMLLTDRNLNTSFFDPAGGGDPILYQHL
>COLFE968-13|KJ966703|ZMUO.006763|Lathrobium_geminum
TLYFIFGAWAGMVGTSLSLLIRTELGNPGSLIGDDQIYNVIVTAHAFIMIFFMVMPIMIGGFGNWLVPLMLGAPDMAFPRMNNMSFWLLPPSLSLLLMSSLVESGAGTGWTVYPPLSSNIAHGGASVDLAIFSLHLAGISSILGAVNFITTVINMRSPG
MTYERMPLFVWAVVITALLLLLSLPVLAGAITMLLTDRNLNTSFFDPAGGGDPILYQHL
>COLFC612-12|KJ965110|ZMUO.003177|Agathidium_arcticum
TLYFIFGAWSGMVGTSLSILIRAELGTPGSLIGDDQIYNVIVTAHAFVMIFFMVMPIVIGGFGNWLVPLMLGAPDMAFPRMNNMSFWLLPPSLSLLLLSSMVENGAGTGWTVYPPLSSNISHSGSSVDLAIFSLHLAGISSILGAVNFITTVINMRSVGM
TFDKMPLFVWSVAITALLLLLSLPVLAGAITMLLTDRNLNTSFFDPAGGGDPILYQHL
>COLFC254-12|KJ964386|ZMUO.002439|Phloeopora_concolor
TLYFIFGAWAGMVGTSLSLLIRAELGNPGSLIGDDQIYNVIVTAHAFIMIFFMVMPIVIGGFGNWLVPLMLGAPDMAFPRMNNMSFWLLPPSLTLLLMSSMVESGAGTGWTVYPPLSSNIAHSGASVDLAIFSLHLAGISSILGAVNFISTIINMRTPGM
TFDRMPLFVWSVGITALLLLLSLPVLAGAITMLLTDRNLNTSFFDPAGGGDPILYQHL
>COLFF212-13|KJ965756|ZMUO.005817|Quedius_levicollis
TLYFIFGAWAGMVGTSLSLLIRAELGNPGSLIGDDQIYNVIVTAHAFIMIFFMVMPIVIGGFGNWLVPLMLGAPDMAFPRMNNMSFWLLPPSLSLLLMSSMVESGAGTGWTVYPPLSSNIAHGGASVDLAIFSLHLAGISSILGAVNFITTVINMRSIG
MTFDRMPLFVWSVAITALLLLLSLPVLAGAITMLLTDRNLNTSFFDPAGGGDPILYQHL
>COLFC518-12|KJ963098|ZMUO.003083|Phloeotribus_spinulosus
TLYFIFGAWSSMIGTSLSMLIRTELGTPNNLIGDDQIYNTIVTAHAFIMIFFMVMPILIGGFGNWLIPLMLGAPDMAFPRLNNMSFWLLPPSLILLLMSSFIDKGVGTGWTVYPPLSSNIAHEGASVDLGIFSLHMAGISSILGAMNFISTILNMFPTGMKL
DQLTLFTWAVKITAILLLLSLPVLASAITMLLTDRNINTSFFDPAGGGDPILYQHL
>COLFD095-12|KJ965796|ZMUO.003990|Mycetophagus_decempunctatus
TLYFIFGAWSGMVGTSLSLLIRSELGTPGSLIGDDQIYNVIVTAHAFIMIFFMVMPIMMGGFGNWLVPLMLGAPDMAFPRMNNMSFWLLPPSLTLLIMSSIVENGAGTGWTVYPPLSSNIAHSGSSVDLAIFSLHLAGISSILGAVNFITTIINMRPQGM
TFDRMPLFVWAVGITALLLLLSLPVLAGAITMLLTDRNLNTSFFDPAGGGDPILYQHL
>COLFE410-12|KJ965308|ZMUO.004970|Cychrus_caraboides
TLYFIFGAWSGMVGTSLSMLIRAELGNPGSLIGDDQIYNVIVTAHAFVMIFFMVMPIMIGGFGNWLVPLMLGAPDMAFPRMNNMSFWLLPPSLTLLLMSSMVESGAGTGWTVYPPLSSSIAHSGASVDLAIFSLHLAGISSILGAVNFITTIINMRSVG
MSFDRMPLFVWSVGITALLLLLSLPVLAGAITMLLTDRNLNTSFFDPAGGGDPILYQHL
>COLFF494-13|KJ962082|ZMUO.006574|Bledius_erraticus
TLYFIFGAWASMVGTSLSMLIRAELGTPGSLIGDDQIYNVIVTAHAFVMIFFMVMPVVIGGFGNWLVPLMLGAPDMAFPRMNNMSFWLLPPSLTLLLMSSMVESGAGTGWTVYPPLSSNIAHSGSSVDLAIFSLHLAGISSILGAVNFISTVINMRSIG
MTFDRMPLFVWSVKITAILLLLSLPVLAGAITMLLTDRNLNTSFFDPSGGGDPILYQHL
>COLFB336-12|KJ962055|ZMUO.001666|Atheta_vaga
TLYFIFGAWAGMVGTSLSLLIRAELGNPGSLIGDDQIYNVIVTAHAFVMIFFMVMPIVIGGFGNWLIPLMLGAPDMAFPRMNNMSFWLLPPSLTLLLMSSMVESGAGTGWTVYPPLSSNIAHGGSSVDLAIFSLHLAGISSILGAVNFISTVINMRSTGI
TFDRMPLFVWSVAITALLLLLSLPVLAGAITMLLTDRNLNTSFFDPAGGGDPILYQHL
>COLFE522-13|KJ965829|ZMUO.005367|Pocadius_ferrugineus
TLYFIFGAWSGMVGTSLSILIRTELGNPGSLIGNDQIYNVIVTAHAFIMIFFMVMPFMIGGFGNWLVPLMLGAPDMAFPRLNNMSFWLLPPALSLLLMSSIVESGAGTGWTVYPPLSSNIAHSGASVDLAIFSLHLAGISSILGAVNFITTVINMRPTGMS
FDKMPLFVWAVVITAILLLLSLPVLAGAITMLLTDRNLNTTFFDPSGGGDPVLYQHL
>COLFE1534-13|KJ962828|ZMUO.007424|Hypera_suspiciosa
TLYFIFGTWAGTVGTSLSILIRTELGNPGSLIGNDQIYNTIVTAHAFIMIFFMVMPIMIGGFGNWLVPLMLGAPDMAFPRLNNMSFWLLPPSLLLLLMSSMTDSGAGTGWTVYPPLSSNIAHEGSSVDLAIFSLHMAGVSSILGAINFISTVLNMRPSGM
SLDKMALFIWAVKITAILLLLSLPVLAGAITMLLTDRNINTSFFDPAGGGDPILYQHL
>COLFG105-13|KJ962537|ZMUO.007800|Anomognathus_cuspidatus

TLYFIFGAWAGMVGTSLSLLIRAELGNPGSLIGDDQIYNVIVTAHAFIMIFFMVMPIVIGGFGNWLVPLMLGAPDMAFPRMNNMSFWLLPPSLTLLLMSSMVESGAGTGWTVYPPLSSNIAHGGSSVDLAIFSLHLAGISSILGAVNFISTVINMRSPS
MTFDRMPLFVWSVAITALLLLLSLPVLAGAITMLLTDRNLNTSFFDPAGGGDPILYQHL
>COLFD013-12|KJ966286|ZMUO.003908|Metoecus_paradoxus
TLYFLFGAWAGMVGTSLSLLVRIELGNSGSLIGNDQIYNVIVTAHAFVMIFFMVMPIMIGGFGNWLVPLMLGAPDMAFPRMNNMSFWLLPPSLSLLMMSSMVESGAGTGWTVYPPLSSNIAHGGASVDLAIFSLHLAGISSILGAVNFITTVINMRP
EGMSFDRMPLFVWAVVITAVLLLLSLPVLAGAITMLLTDRNLNTSFFDPAGGGDPILYQHL
>COLFE1322-13|KJ963374|ZMUO.007117|Cantharis_rufa
TLYFIFGAWSGSLGLALSLLIRAELGTPGTLIGNDQIYNVIVTAHAFIMIFFMVMPIMIGGFGNWLVPLMLGAPDMAFPRMNNMSFWFLPPSLMFLLMSSMVESGAGTGWTVYPPLSANIAHSGPSVDLAIFSLHMAGISSILGAVNFISTIMNMKPPS
MKFDQMPLFVWSVGITALLLLLSLPVLAGAITMLLSDRNLNTSFFDPMGGGDPILYQHL
>COLFC673-12|KJ967425|ZMUO.003238|Eucnecosum_brunnescens
TLYFIFGAWAGMVGTSLSILIRAELGNPGTLIGDDQIYNVIVTAHAFVMIFFMVMPIVIGGFGNWLVPLMLGAPDMAFPRMNNMSFWLLPPSLTLLLMSSMVESGAGTGWTVYPPLSSNIAHGGSSVDLAIFSLHLAGISSILGAVNFITTVINMRTTG
MTFDRMPLFVWSVAITALLLLLSLPVLAGAITMLLTDRNLNTSFFDPAGGGDPILYQHL
>COLFA318-12|KJ964050|ZMUO.000453|Phytobius_leucogaster
TLYFIFGSWAGTVGTSLSMIIRTELGTPGSLIGNDQIYNSIVTAHAFIMIFFMVMPILIGGFGNWLVPLMLGAPDMAFPRLNNMSFWLLPPSILLLLMSSIVNKGAGTGWTVYPPLSSNVTHEGASVDLAIFSLHMAGISSILGAINFISTIMNMRPKGMS
YDKTPLFVWAVMITAILLLLSLPVLAGAITMLLTDRNINTSFFDPAGGGDPILYQHL
>COLFB187-12|KJ963726|ZMUO.001517|Hylobius_abietis
TLYFIFGTWSGMVGTSLSMLIRTELGNPGSLIGDDQIYNTIVTAHAFIMIFFMVMPIMIGGFGNWLVPLMLGAPDMAFPRLNNMSFWLLPPSLTLLLMSSIVDKGAGTGWTVYPPLSANIAHEGASVDFAIFSLHMAGISSILGAINFISTAINMRSSGM
KSDQMSLFIWAVKITAILLLLSLPVLAGAITMLLTDRNINTSFFDPAGGGDPILYQHL
>COLFE1536-13|KJ965624|ZMUO.007426|Hypera_meles
TLYFIFGTWAGTVGTSLSILIRTELGNPGSLIGNDQIYNTIVTAHAFIMIFFMVMPIMIGGFGNWLVPLMLGAPDMAFPRLNNMSFWLLPPSLSLLLMSSMVDSGAGTGWTVYPPLSSNIAHEGSSVDLAIFSLHMAGVSSILGAINFISTVLNMRPSGM
SLDKMALFIWAVKITAILLLLSLPVLAGAITMLLTDRNINTSFFDPAGGGDPILYQHL
>COLFB218-12|KJ962206|ZMUO.001548|Selatosomus_melancholicus
TLYFLFGAWAGMLGTSLSLLIRAELGNPGSLIGNDQIYNVIVTAHAFIMIFFMVMPIMIGGFGNWLVPLMLGAPDMAFPRMNNMSFWFLPPSLSLLLMSSIVENGAGTGWTVYPPLSANIAHSGSSVDLAIFSLHLAGISSILGAVNFISTVINMRSTGIT
FDRMPLFVWAVAITALLLLLSLPVLAGAITMLLTDRNLNTSFFDPAGGGDPILYQHL
>COLFB430-12|KJ962307|ZMUO.001855|Crepidodera_fulvicornis
TLYFIFGIWSGMVGTSLSILIRTELGSPGSLIGNDQIYNVIVTAHAFIMIFFMVMPIMIGGFGNWLIPLMIGAPDMAFPRMNNMSFWLLPPSLFLLLMSSLVESGAGTGWTVYPPLSSNIAHGGSSVDLAIFSLHLAGISSILGAINFITTIINMRPKGMNLD
RMPLFVWAVAITAILLLLSLPVLAGAITMLLTDRNMNTSFFDPIGGGDPILYQHL
>COLFG107-13|KJ963088|ZMUO.007802|Holobus_flavicornis
TLYFIFGAWSGMIGTSLSLLIRAELGNPGSLIGNDQIYNVIVTAHAFIMIFFMVMPIMIGGFGNWLVPLMLGAPDMAFPRLNNMSFWMLPPALILLLMSSIIENGSGTGWTVYPPLSSNIAHAGASVDLTIFSLHMAGISSILGAINFISTIINMHPPGMKL
DQMPLFVWSVAITALLLLLSLPVLAGAITMLLTDRNLNTSFFDPAGGGDPILYQHL
>COLFF771-13|KJ963519|ZMUO.006091|Dyschirius_thoracicus
TLYFIFGIWSGMVGTSLSILIRTELGNPGSLIGDDQIYNVIVTAHAFIMIFFMVMPIMIGGFGNWLVPLMLGAPDMAFPRMNNMSFWLLPPSLTLLLMSSMVESGAGTGWTVYPPLSSGIAHSGASVDLAIFSLHLAGISSILGAVNFITTIINMRSTGMT
FERMPLFVWSVGITALLLLLSLPVLAGAITMLLTDRNLNTSFFDPAGGGDPILYQHL
>COLFB328-12|KJ967092|ZMUO.001658|Rugilus_rufipes
TLYFIFGAWAGMVGTSLSLLIRAELGAPGSLIGDDQIYNVIVTAHAFVMIFFMVMPIVIGGFGNWLVPLMLGAPDMAFPRMNNMSFWLLPPSLSLLLMSSMVESGAGTGWTVYPPLSSNIAHGGASVDLAIFSLHLAGISSILGAVNFITTIINMRSKG
MTYERMPLFVWSVGITALLLLLSLPVLAGAITMLLTDRNLNTSFFDPAGGGDPILYQHL
>COLFA664-12|KJ962699|ZMUO.000799|Autalia_rivularis
TLYFIFGAWSGMVGTSLSLLIRAELGNPGSLIGDDQIYNVIVTAHAFIMIFFMVMPIVIGGFGNWLVPLMLGAPDMAFPRMNNMSFWLLPPSLTLLLMSSMVESGAGTGWTVYPPLSSNIAHGGSSVDLAIFSLHLAGISSILGAVNFISTIINMRSTGIS
FDRMPLFVWSVGITALLLLLSLPVLAGAITMLLTDRNLNTSFFDPAGGGDPILYQHL
>COLFE1170-13|KJ962064|ZMUO.006965|Phaedon_cochleariae
MLYFIFGIWAGMVGTSLSIMIRSELGNPGTLIGNDQIYNVIVTAHAFVMIFFMVMPLMIGGFGNWLVPLMIGAPDMAFPRMNNMSFWLLPPSLLLLMSSVVENGAGTGWTVYPPLSANIAHSGSSVDLAIFSLHLAGISSILGAINFITTVINMRPEG
MTLEQIPLFVWAVLITAILLLLSLPVLAGAITMLLTDRNLNTSFFDPAGGGDPILYQHL
>COLFB067-12|KJ962750|ZMUO.001397|Dyschirius_nigricornis
TLYFIFGIWSGMVGTSLSIMIRAELGNPGSLIGDDQIYNVIVTAHAFIMIFFMVMPIMIGGFGNWLVPLMLGAPDMAFPRMNNMSFWLLPPSLSLLLMSSMVEKGAGTGWTVYPPLSSSIAHSGASVDLAIFSLHLAGVSSILGAVNFITTIINMRSTG
MTFERMPLFVWSVGITALLLLLSLPVLAGAITMLLTDRNLNTSFFDPAGGGDPILYQHL
>COLFE629-13|KJ965530|ZMUO.005474|Cassida_nebulosa
TLYFIFGFWSGMVGTSLSILIRMELGSPGSLIGNDQIYNSIVTAHAFIMIFFMVMPIMIGGFGNWLVPLMLGAPDMAFPRLNNMSFWLLPPSITFLIMSSIVESGAGTGWTVYPPLSSNIAHSGASVDLAIFSLHLAGISSILGAINFISTIMNMRPSGMS
MEKVALFVWAVIITAVLLLLSLPVLAGAITMLLTDRNINTSFFDPAGGGDPILYQHL

>COLFB616-12|KJ962226|ZMUO.001091|Paederus_riparius
TLYFIFGAWSGMVGTSLSLLIRAELATPGSLIGDDQIYNVIVTAHAFIMIFFMVMPIMIGGFGNWLVPLMLGAPDMAFPRMNNMSFWLLPPALTLLLMSSMVENGAGTGWTVYPPLSSNAFHNGSSVDLAIFSLHLAGISSILGAINFITTALNMRTSN
MSYEQMPLFVWSVAITALLLLLSLPVLAGAITMLLTDRNLNTSFFDPSGGGDPILYQHL
>COLFE1268-13|KJ961721|ZMUO.007063|Sinodendron_cylindricum
TLYFLLGSWSGMVGTSLSILIRAELGNPGTLIGDDQIYNVIVTAHAFVMIFFMVMPILIGGFGNWLVPLMLGAPDMAFPRMNNMSFWLLPPSLTFLLMSSMVESGAGTGWTVYPPLSANIAHSGASVDLAIFSLHLAGVSSILGAVNFITTVINMRSVG
ITFDRMPLFVWSVVLTALLLLLSLPVLAGAITMLLTDRNLNTSFFDPAGGGDPILYQHL
>COLFC291-12|KJ966075|ZMUO.002476|Encephalus_complicans
TLYFIFGAWSGMIGTSLSLLIRAELGNPGSLIGDDQIYNVIVTAHAFIMIFFMVMPIVIGGFGNWLVPLMLGAPDMAFPRMNNMSFWLLPPSLSLLLMSSMVESGAGTGWTVYPPLSSNIAHGGASVDLAIFSLHLAGISSILGAVNFISTIINMRAMG
MSFDRMPLFVWSVAITALLLLLSLPVLAGAITMLLTDRNLNTSFFDPAGGGDPILYQHL
>COLFB206-12|KJ967047|ZMUO.001536|Hydroporus_morio
TLYFLFGAWSGMVGTSLSMLIRAELGNPGSLIGDDQIYNVIVTAHAFIMIFFMVMPIMIGGFGNWLVPLMLGAPDMAFPRMNNMSFWLLPPSLSLLLMSSMVENGAGTGWTVYPPLSSGIAHSGASVDLAIFSLHLAGVSSILGAVNFITTIINMRSIG
MTFDRMPLFVWSVGITALLLLLSLPVLAGAITMLLTDRNLNTSFFDPAGGGDPILYQHL
>COLFE050-12|KJ964357|ZMUO.005275|Xylotrechus_rusticus
TLYFIFGAWAGMVGTSLSILIRSELGNPGSLIGDDQIYNVIVTAHAFIMIFFMVMPIMIGGFGNWLVPLMLGAPDMAFPRMNNMSFWLLPPSLTLLIMSSIVESGAGTGWTVYPPLSANIAHSGSSVDLAIFSLHLAGVSSILGAVNFISTVINMRPTKM
SPEQMPLFVWAVAITALLLLLSLPVLAGAITMLLTDRNINTSFFDPAGGGDPILYQHL
>COLFD284-12|KJ962245|ZMUO.004179|Corticaria_orbicollis
SLYFLFGMWAGMVGTSLSLLIRLELGNPGSLIGDDQIYNVIVTAHAFVMIFFMVMPIMIGGFGNWLVPLMLGAPDMAFPRLNNMSFWLLPPSLSLLIMSSIVESGAGTGWTVYPPLSSNIAHGGSSVDLAIFSLHLAGISSILGAVNFITTVINMRPAGM
NLDQMPLFVWSVVITAILLLLSLPVLAGAITMLLTDRNLNTSFFDPAGGGDPILYQHL
>COLFC379-12|KJ962973|ZMUO.002564|Latridius_gemellatus
TLYFLFGMWSGMVGTSLSLLIRLELGNPGSLIGDDQIYNVIVTAHAFIMIFFMVMPVMMGGFGNWLVPLMLGAPDMAFPRLNNMSFWLLPPSLSLLIMSSIVESGAGTGWTVYPPLSSNIAHGGSSVDLAIFSLHLAGVSSILGAVNFITTVINMRPAG
MTLEQMPLFVWSVVLTAVLLLISLPVLAGAITMLLTDRNLNTSFFDPAGGGDPILYQHL
>COLFD869-12|KJ965818|ZMUO.004479|Sulcacis_nitidus
TLYFLFGAWSGMVGTSMSILIRSELGTPGSLIGNDQIYNVIVTAHAFVMIFFMVMPIMIGGFGNWLVPLMLGAPDMAFPRMNNMSFWLLPPSLSLLLMSSLVENGAGTGWTVYPPLSSNIAHSGPSVDLAIFSLHLAGISSILGAVNFISTIINMRPMG
MSMDRIPLFVWAVIITAVLLLLSLPVLAGAITMLLTDRNFNTSFFDPAGGGDPILYQHL
>COLFF209-13|KJ966200|ZMUO.005814|Notiophilus_aestuans
TLYFIFGAWSGMVGTSLSMLIRAELGNPGSLIGDDQIYNVIVTAHAFVMIFFMVMPIMIGGFGNWLVPLMLGAPDMAFPRMNNMSFWLLPPSLTLLLTSSMVESGAGTGWTVYPPLSSGIAHSGASVDLAIFSLHLAGVSSILGAVNFITTIINMRSVG
MTFDRMPLFVWSVGITALLLLLSLPVLAGAITMLLTDRNLNTSFFDPAGGGDPILYQHL
>COLFC135-12|KJ961766|ZMUO.002320|Atheta_paracrassicornis
TLYFIFGAWAGMVGTSLSLLIRAELGNPGSLIGDDQIYNVIVTAHAFIMIFFMVMPIVIGGFGNWLVPLMLGAPDMAFPRMNNMSFWLLPPSLTLLLMSSMVESGAGTGWTVYPPLSSNIAHGGSSVDLAIFSLHLAGISSILGAVNFISTVINMRSTGI
SFDRMPLFVWSVAITALLLLLSLPVLAGAITMLLTDRNLNTSFFDPAGGGDPILYQHL
>COLFD741-12|KJ966584|ZMUO.004731|Astenus_gracilis
TLYFIFGAWAGMVGTSLSLLIRAELGMPGSLIGDDQIYNVIVTAHAFVMIFFMVMPIVIGGFGNWLVPLMLGAPDMAFPRMNNMSFWLLPPSLSLLLMSSMVESGAGTGWTVYPPLSSNMAHGGASVDLAIFSLHLAGISSILGAVNFITTIINMRTK
GMSYERMPLFVWSVGITALLLLLSLPVLAGAITMLLTDRNLNTSFFDPAGGGDPILYQHL
>COLFE1493-13|KJ964779|ZMUO.007288|Gnypeta_caerulea
TLYFIFGAWAGMVGTSLSLLIRAELGNPGSLIGNDQIYNVIVTAHAFVMIFFMVMPIVIGGFGNWLIPLMLGAPDMAFPRMNNMSFWLLPPSLTLLLMSSMVENGAGTGWTVYPPLSSNIAHGGSSVDLAIFSLHLAGISSILGAVNFISTIINMRSTGIS
FDRMPLFVWSVAITALLLLLSLPVLAGAITMLLTDRNLNTSFFDPAGGGDPILYQHL
>COLFE1121-13|KJ964147|ZMUO.006916|Miscodera_arctica
TLYFIFGIWSGLVGTSLSLLIRSELGMPGSLIGDDQIYNVIVTAHAFVMIFFMVMPIMIGGFGNWLVPLMLGAPDMAFPRMNNMSFWLLPPALTLLLISSMVENGAGTGWTVYPPLSSNMAHGGASVDLAIFSLHLAGISSILGAVNFISTIINMRSIGIK
FDRMPLFVWSVGITALLLLLSLPVLAGAITMLLTDRNLNTSFFDPAGGGDPILYQHL
>COLFF813-13|KJ966911|ZMUO.006133|Helophorus_granularis
TLYFIFGAWAGMVGTSLSILIRAELGNPGTLIGDDQIYNVIVTAHAFIMIFFMVMPIMIGGFGNWLVPLMLGAPDMAFPRMNNMSFWLLPPSLTLLLMSSMVESGAGTGWTVYPPLSSNIAHSGASVDLAIFSLHLAGISSILGAVNFITTVINMRSIN
MTYDRLPLFVWSVAITALLLLLSLPVLAGAITMLLTDRNLNTSFFDPAGGGDPILYQHL
>COLFB117-12|KJ963700|ZMUO.001447|Anoplodera_sanguinolenta
TLYFIFGAWASMVGTSLSLLIRSELGNPGSLIGNDQIYNVIVTAHAFVMIFFMVMPIMIGGFGNWLVPLMLGAPDMAFPRMNNMSFWLLPPSLTLLIMSSIVESGAGTGWTVYPPLSSNIAHSGSSVDLAIFSLHLAGISSILGAVNFITTVINMRPAGLK
PEQMPLFVWAVVITAVLLLSLPVLAGAITMLLTDRNLNTSFFDPAGGGDPILYQHL
>COLFE929-13|KJ967178|ZMUO.006249|Dorytomus_taeniatus

ILYFIFGAWAGMVGTSLSMLIRTELGNPGSLIGDDQIYNTIVTAHAFIMIFFMVMPIMIGGFGNWLIPLMLGAPDMAFPRLNNMSFWLLPPSLILLLMSSIVDKGAGTGWTVYPPLSSNIAHEGTSVDLAIFSLHMAGISSILGAMNFISTVMNMRPAG
MKPDRMSLFIWAVKITAILLLLSLPVLAGAITMLLTDRNINTSFFDPAGGGDPILYQHL
>COLFC435-12|KJ967208|ZMUO.003000|Stenus_bilineatus
TLYFIFGSWAGLVGTSLSLLIRTELGNPGSLIGDDQIYNVIVTAHAFIMIFFMVMPIMIGGFGNWLIPLMLGAPDMAFPRMNNMSFWLLPPSLSLLLTSSIVESGAGTGWTVYPPLSSNIAHSGASVDLAIFSLHLAGISSILGAINFITTIFNMRTMKLQLD
CLPLFIWSVGITALLLLLSLPVLAGAITMLLTDRNLNTSFFDPAGGGDPILYQHL
>COLFC348-12|KJ966619|ZMUO.002533|Aphidecta_obliterata
TLYFLFGLWAGMVGTSLSILIRMELGTTGSLIGNDQIYNVIVTAHAFIMIFFMVMPIMIGGFGNWLVPLMIGAPDMAFPRLNNMSFWLLPPALTLLIFSMMVEMGAGTGWTVYPPLSSNLAHNGPSVDLVIFSLHLAGISSILGAVNFISTIMNMRPFG
MSLDKTPLFVWSVMITAILLLLSLPVLAGAITMLLTDRNLNTSFFDPTGGGDPVLYQHL
>COLFF252-13|KJ963005|ZMUO.005857|Malthodes_brevicollis
TLYFMFGAWSGMLGTSLSLLIRAELGSPGSLIGNDQIYNVIVTAHAFIMIFFMVMPIMIGGFGNWLVPLMLGAPDMAFPRMNNMSFWFLPPSLSLLLMSSIVENGAGTGWTVYPPLSANIAHSGSSVDLAIFSLHMAGISSILGAVNFISTVINMRSPG
MTFDRMPLFVWAVAITALLLLLSLPVLAGAITMLLTDRNLNTSFFDPAGGGDPILYQHL
>COLFD105-12|KJ964320|ZMUO.004000|Pediacus_fuscus
TLYFILGIWAGMIGTSMSLLIRTELGTPGSLIGDDQIYNVIVTAHAFIMIFFMVMPIMIGGFGNWLVPLMLGAPDMAFPRMNNMSFWLLPPSLSLLLMSSIVEKGVGTGWTVYPPLSANIAHSGASVDLAIFSLHLAGISSILGAINFITTIINMRPMGMT
LERMSLFIWAVNITAILLLLSLPVLAGAITMLLTDRNINTSFFDPSGGGDPILYQHL
>COLFA039-10|HM909064|MP00196|Limonius_minutus
TLYFLFGAWSGMLGTSLSLLIRAELGNPGSLIGNDQIYNVIVTAHAFIMIFFMVMPIMIGGFGNWLVPLMLGAPDMAFPRMNNMSFWFLPPSLSLLLMSSIVENGAGTGWTVYPPLSANIAHSGSSVDLAIFSLHLAGISSILGAVNFISTVINMRSVGIT
FDRMPLFVWAVAITALLLLLSLPVLAGAITMLLTDRNLNTSFFDPAGGGDPILYQHL
>COLFA192-10|HM909113|MP00020|Agrilus_viridis
TLYFIFGVWSGMVGTALSLLIRAELGNPGALIGNDQIYNVIVTAHAFIMIFFMVMPIMMGGFGNWLVPLMLGAPDMAFPRMNNMSFWLLPPSLTLLLMSSMVESGAGTGWTVYPPLAANIAHSGGSVDLAIFSLHLAGISSILGAINFITTVINMRAV
GMTMDRVPLLVWSIAITALLLLLSLPVLAGAITMLLTDRNLNTSFFDPAGGGDPILYQHL
>COLFD335-12|KJ963670|ZMUO.004230|Harpalus_solitaris
TLYFIFGAWAGMVGTSLSMLIRAELGTPGALIGDDQIYNVIVTAHAFIMIFFMVMPIMIGGFGNWLVPLMLGAPDMAFPRMNNMSFWLLPPSLTLLLMSSMVESGAGTGWTVYPPLSSGIAHSGASVDLAIFSLHLAGISSILGAVNFITTIINMRSVG
MTFDRMPLFVWSVGITALLLLLSLPVLAGAITMLLTDRNLNTSFFDPAGGGDPILYQHL
>COLFE640-13|KJ966635|ZMUO.005485|Tetropium_castaneum
TLYFIFGAWAGMVGTSLSILIRSELGNPGSLIGNDQIYNVIVTAHAFIMIFFMVMPIMIGGFGNWLVPLMLGAPDMSFPRLNNLSFWFLPPSLILLIMGMIVEKGAGTGWTVYPPLSANIAHSGSSVDLTIFSLHLAGISSILSAINFITTIMNMRPKGMTL
DQMPLFVWAVMITTILLLISLPVLAGAITMLLTDRNINTSFFDPAGGGDPILYQHL
>COLFD414-12|KJ965857|ZMUO.003834|Monotoma_angusticollis
TLYFIFGTWSGMVGTSLSMLIRTELGTPGSLISDDQIFNVIVTAHAFIMIFFMVMPILIGGFGNWLIPLMLGAPDMAFPRMNNMSFWLLPPSLTLLLMSSIVETGVGTGWTIYPPLSSNIAHSGAPVDLAIFSLHLAGISSILGAVNFITTVINMRPQGMTL
DRTPLFVWAVMITAILLLLSLPVLAGAITMLLTDRNLNTSFFDPAGGGDPILYQHL
>COLFF837-13|KJ967475|ZMUO.006157|Atheta_cauta
TLYFIFGAWAGMVGTSLSLLIRAELGNPGSLIGDDQIYNVIVTAHAFIMIFFMVMPIVIGGFGNWLVPLMLGAPDMAFPRMNNMSFWLLPPSLTLLLMSSMVESGAGTGWTVYPPLSSNIAHGGSSVDLAIFSLHLAGISSILGAVNFISTVINMRSTGI
SFDRMPLFVWSVAITALLLLLSLPVLAGAITMLLTDRNLNTSFFDPAGGGDPILYQHL
>COLFC431-12|KJ963498|ZMUO.002996|Fleutiauxellus_maritimus
TLYFLFGAWAGMLGTSLSLLIRAELGNPGSLIGNDQIYNVIVTAHAFIMIFFMVMPIMIGGFGNWLVPLMLGAPDMAFPRMNNMSFWFLPPSLSLLLMSSIVENGAGTGWTVYPPLSANIAHSGSSVDLAIFSLHLAGISSILGAVNFISTVINMRSTGIT
FDRMPLFVWAVAITALLLLLSLPVLAGAITMLLTDRNLNTSFFDPAGGGDPILYQHL
>COLFE872-13|KJ963311|ZMUO.006192|Cymindis_vaporariorum
TLYFIFGAWAGMVGTSLSMLIRAELGNPGALIGDDQIYNVIVTAHAFIMIFFMVMPIMIGGFGNWLVPLMLGAPDMAFPRMNNMSFWLLPPSLTLLLMSSMVESGAGTGWTVYPPLSSGIAHAGASVDLAIFSLHLAGVSSILGAVNFITTIINMRSI
GMTFDRMPLFVWSVGITALLLLLSLPVLAGAITMLLTDRNLNTSFFDPAGGGDPILYQHL
>COLFC315-12|KJ963280|ZMUO.002500|Boreaphilus_henningianus
TLYFIFGAWAGMVGTSLSILIRAELGNPGSLIGDDQIYNVIVTAHAFVMIFFMVMPIVIGGFGNWLVPLMLGAPDMAFPRMNNMSFWLLPPSLNLLLMSSMVESGAGTGWTVYPPLSSNIAHGGSSVDLAIFSLHLAGVSSILGAVNFITTVINMRSTG
MSFDRMPLFVWSVAITALLLLLSLPVLAGAITMLLTDRNLNTSFFDPAGGGDPILYQHL
>COLFE475-12|KJ966166|ZMUO.005035|Scymnus_frontalis
TLYFLFGLWAGMVGTSLSILIRLELGTTSALIGNDQIYNVIVTAHAFIMIFFMVMPIMIGGFGNWLVPLMIGAPDMAFPRLNNMSFWLLPPSLTFLILSSLVESGAGTGWTVYPPLSSNIAHGGSSVDMAIFSLHLAGISSILGAVNFISTIINMRTFGMTFE
KMPLFVWSVFITAILLLLSLPVLAGAITMLLTDRNINTSFFDPAGGGDPILYQHL
>COLFG177-13|KJ966327|ZMUO.007872|Crypturgus_subcribrosus
TLYFIFGAWSGMMGTSLSMIIRTELGSPGSLIGDDQIYNTIVTAHAFIMIFFMVMPIMIGGFGNWLVPLMLGAPDMAFPRLNNMSFWLLPPSLSLLIMSSIINKGAGTGWTVYPPLASNIAHEGASVDMAIFSLHMAGVSSILGAINFISTIINMHPTG
MTPERIPLFTWAVKITAILLLLSLPVLASAITMLLTDRNINTSFFDPAGGGDPILYQHL

>COLFD100-12|KJ964055|ZMUO.003995|Anisotoma_castanea
TLYFIFGAWSGMVGTSLSILIRAELGTPGSLIGDDQIYNVIVTAHAFVMIFFMVMPIVIGGFGNWLVPLMLGAPDMAFPRMNNMSFWLLPPSLTLLLMSSMVENGAGTGWTVYPPLSANISHSGSSVDLAIFSLHLAGISSILGAVNFITTVINMRSIGM
TFDKMPLFVWSVAITALLLLLSLPVLAGAITMLLTDRNLNTSFFDPAGGGDPILYQHL
>COLFA356-12|KJ962611|ZMUO.000586|Stenus_canaliculatus
TLYFIFGAWAGMVGTSLSLLIRAELGNPGSLIGDDQIYNVIVTAHAFVMIFFMVMPIMIGGFGNWLIPLMLGAPDMAFPRMNNMSFWLLPPSLSLLLTSSIVESGAGTGWTVYPPLSSNIAHSGSSVDLAIFSLHLAGVSSILGAINFITTIFNMRTMKLQ
LDCLPLFVWAVGITAFLLLLSLPVLAGAITMLLTDRNLNTSFFDPAGGGDPILYQHL
>COLFE1201-13|KJ964982|ZMUO.006996|Stenus_niveus
TLYFIFGVWAGMLGTSLSLLIRAELGNPGSLIGDDQIYNVIVTAHAFIMIFFMVMPIMIGGFGNWLVPLMLGAPDMAFPRMNNMSFWLLPPSLSLLLMSSVVESGAGTGWTVYPPLSSNIAHGGASVDLAIFSLHLAGVSSILGAINFITTIFNMRTMQI
QLDCLPLFVWSVGITALLLLLSLPVLAGAITMLLTDRNLNTSFFDPAGGGDPVLYQHL
>COLFA419-12|KJ967072|ZMUO.000649|Atheta_incognita
TLYFIFGMWAGMVGTSLSLLIRAELGNPGSLIGDDQIYNVIVTAHAFVMIFFMVMPVMIGGFGNWLIPLMLGAPDMAFPRMNNMSFWLLPPSLTLLLMSSMVESGAGTGWTVYPPLSSNIAHGGSSVDLAIFSLHLAGISSILGAVNFISTVINMRST
GISFDRMPLFVWSVAITALLLLLSLPVLAGAITMLLTDRNLNTSFFDPAGGGDPILYQHL
>COLFC130-12|KJ964801|ZMUO.002315|Tychius_picirostris
TLYFIFGSWSGMVGTSLSMLIRTELGNPGSLIGDDQIYNSIVTAHAFIMIFFMVMPIMIGGFGNWLIPLMLGAPDMAYPRLNNMSFWLLPPSISLLLMSSIVNKGAGTGWTVYPPLSSNIAHEGASVDLAIFSLHMAGMSSILGAINFISTMMNMRPFG
MNSEKVTLFSWAVQVTAILLLLSLPVLAGAITMLLTDRNVNTSFFDPAGGG?PILYQHL
>COLFD402-12|KJ961747|ZMUO.003822|Leptacinus_formicetorum
TLYFIFGAWAGMVGTSLSLLIRAELGNPGSLIGDDQIYNVIVTAHAFIMIFFMVMPIVIGGFGNWLVPLMLGAPDMAFPRMNNMSFWLLPPSLTLLLTSSLVESGAGTGWTVYPPLSSNIAHGGASVDLAIFSLHLAGISSILGAVNFITTVLNMRSFGM
TFDRMPLFVWSVA?TALLLLLSLPVLAGAITMLLTDRNLNTSFFDPMGGGDPILYQHL
>COLFD883-12|KJ965769|ZMUO.004493|Hydrochus_brevis
T?YFIFGAWSGMVGTSLSLLIRTELGNPGSLIGDDQIYNVIVTAHAFIMIFFMVMPIMIGGFGNWLVPLMLGAPDMAFPRMNNMSFWLLPPSLTLLLMSSMVESGAGTGWTVYPPLSSNIAHGGASVDLAIFSLHLAGISSILGAVNFITTIINMRSNN
MTYDRLPLFVWSVGITALLLLLSLPVLAGAITMLLTDRNLNTSFFDPIGGGDPILYQHL
>COLFE1155-13|KJ962926|ZMUO.006950|Tachyporus_pulchellus
TLYFIFGAWSGMVGTSLSLLIRAELGNPGSLIGDDQIYNVIVTAHAFIMIFFMVMPIVIGGFGNWLVPLMLGAPDMAFPRMNNMSFWLLPPSLTLLLMSSMVESGAGTGWTVYPPLSANIAHSGPSVDLAIFSLHLAGISSILGAVNFITTVINMRASG
MHFDRMPLFIWSVAITALLLLLSLPVL?GAITMLLTDRNLNTSFFDPAGGGDPILYQHL
>COLFD396-12|KJ966062|ZMUO.003816|Dienerella_vincenti
SLYFLFGMWAGMMGTSLSLIIRLELGNPGALIGDDQIYNVIVTAHAFIMIFFMVMPIMMGGFGNWLVPLMLGAPDMAFPRLNNMSFWLLPPSLSLLLSSMVESGVGTGWTVYPPLSSNI?HGGSSVDLAIFSLHMAGISSILGAVNFISTVMNMRP
NGMTLEFMPLFISSVVLTAILLLLSLPVLAGAITMLLTDRNLNTSFFDPSGGGDPILYQHL
>COLFB933-12|KJ962692|ZMUO.001978|Rhamphus_pulicarius
TLYFLFGGWSGMVGTSLSMLIRTELGNPGKLIGDDQIYNTIVTAHAFIMIFFMVMPIMIGGFGNWLVPLMLGAPDMAFPRLNNMSFWLLPPSLTFLLLSSVMDKGAGTGWTVYPPLSANIAHEGSSVDLAIFSLHMAGVSSILGAMNFISTIINMKPK
NMSMDQMSLFVWSVKI?AILLLLSLPVLAGAITMLLTDRNINTSFFDPAGGGDPILYQHL
>COLFE047-12|KJ962054|ZMUO.005272|Gonioctena_intermedia
TLYFIFGIWAGMVGTSLSILIRAELGNPGTLIGNDQIYNVIVTAHAFIMIFFMVMPIMIGGFGNWLVPLMLGAPDMAFPRMNNMSFWLLPPSLFFLIMSSIVESGAGTGWTVYPPLSANIAHSGSSVDLAIFSLHLAGISSILGAINFI?TIINMRPMGMT
MDRMPLFAWAVLITTILLLLSLPVLAGAITMLLTDRNLNTSFFDPAGGGDPILYQHL
>COLFE601-13|KJ963572|ZMUO.005446|Byturus_tomentosus
TLYFIFGAWAGMVGTSLSLLIRSELGNPGSLIGDDQIYNVIVTAHAFIMIFFMVMPIVIGGFGNWLVPLMLGAPDMAFPRMNNMSFWLLPPSLTLLLMSSIVESGAGTGWTVYPPLSSNIAHGGSSVDLAIFSLHLAGISSILGAVNFITTVINMRPAGM
TLDRMPLFVWSVVITAILLLLSLPVLAGAI?MLLTDRNLNTSFFDPSGGGDPILYQHL
>COLFB861-12|KJ967394|ZMUO.001906|Leptura_quadrifasciata
TLYFIFGAWAGMVGTS?SLLIRSELGNPGSLIGDDQIYNVIVTAHAFVMIFFMVMPIMIGGFGNWLVPLMLGAPDMAFPRMNNMSFWLLPPSLTLLIMSSLVESGAGTGWTVYPPLSSNIAHGGSSVDLAIFSLHLAGISSILGAVNFITTVINMRPLG
MSPDQMPLFVWAVVITAILLLLSLPVLAGAITMLLTDRNLNTSFFDPAGGGDPILYQHL
>COLFA338-12|KJ964371|ZMUO.000473|Donacia_thalassina
TLYFIFGAW?GMMGTSLSMLIRTELSNPGSLIGNDQLYNVIVTAHAFIMIFFMVMPIMIGGFGNWLVPLMLGAPDMAFPRMNNMSFWLLPPSLSFLIMSSITESGAGTGWTVYPPLSNNLAHSGSSVDLAIFSLHLAGISSILGAVNFISTIINMRPTG
MTLEKMPLFSWAVMITAVLLLTSLPVLAGAITMLLTDRNINTSFFDPAGGGDPILYQHL
>COLFC589-12|KJ964035|ZMUO.003154|Amphicyllis_globus
TLYFIFGAWSGMVGTSLSILIRAELGTPGSLIGDDQIYNVIVTAHAFVMIFFMVMPIVIGGFGNWLVPLMLGAPDMAFPRMNNMSFWLLPPSLTLLLMSSMVENGAGTGWTVYPPLSANISHSGSSVDLAIFSLHLAGISSIL?AVNFITTVINMRSIGM
TFDKMPLFVWSVAITALLLLLSLPVLAGAITMLLTDRNLNTSFFDPAGGGDPILYQHL
>COLFA432-12|KJ963012|ZMUO.000662|Rhizophagus_fenestralis

TLYFIFGAWAGMVGTSLSLLIRAELGTPGQLIGDDQIYNVIVTAHAFIMIFFMVMPILIGGFGNWLVPLMLGAPDMAFPRMNNMSFWLLPPSLTLLIMSSIVENGAGTGWTVYPPLSSNIAHSGSSVDLAIFSLHLAGISSILGAVNFITTVINMRPSGMT
LDRTPLFVWAVIITATLLLLSLPVLAGAITMLLTDRNLNTSFFDPAGG?DPILYQHL
>COLFD024-12|KJ961867|ZMUO.003919|Hydroporus_erythrocephalus
TLYFLFGAWS?MVGTSLSMLIRAELGNPGSLIGDDQIYNVIVTAHAFIMIFFMVMPIMIGGFGNWLVPLMLGAPDMAFPRMNNMSFWLLPPSLSLLLMSSMVENGAGTGWTVYPPLSSGIAHSGASVDLAIFSLHLAGVSSILGAVNFITTIINMRSIG
MTFDRMPLFVWSVGITALLLLLSLPVLAGAITMLLTDRNLNTSFFDPAGGGDPILYQHL
>COLFC751-12|KJ963091|ZMUO.003316|Meligethes_denticulatus
TLYFIFGAWSGMVGTSLSILIRTELGNPGSLIGNDQIYNVIVTAHAFVMLFFMVMPFMIGGFGNWLVPLMLGAPDMAFPRMNNMSFWLLPPSLSLLLMSSIVESGAGTGWTVYPPLSSNIAHG?ASVDLAIFSLHLAGISSILGAVNFITTVINMRPTG
MNFDRMPLFIWAVVITALLLLLSLPVLAGAITMLLTDRNLNTTFFDPSGGGDPILYQHL
>COLFD857-12|KJ966855|ZMUO.004467|Anotylus_insecatus
TLYFIFGAWSGMVGTSLSMLIRAELGTPGSLIGDDQIYNVVVTAHAFIMIFFMVMPIVIGGFGNWLIPLMLGAPDMAFPRMNNMSFWLLPPSLTLLLFSSIVESGAGTGWTVYPPL?SNIAHSGSSVDLAIFSLHLAGISSILGAVNFISTIINMRSVGMSF
DRMPLFVWSVNITAILLLLSLPVLAGAITMLLTDRNLNTSFFDPAGGGDPILYQHL
>COLFB258-12|KJ962073|ZMUO.001588|Tachyporus_pusillus
T?YFIFGAWSGMVGTSLSLLIRAELGNPGSLIGDDQIYNVIVTAHAFIMIFFMVMPIVIGGFGNWLVPLMLGAPDMAFPRMNNMSFWLLPPSLTLLLMSSMVESGAGTGWTVYPPLSANIAHSGPSVDLAIFSLHLAGISSILGAVNFITTVINMRASG
MHFDRMPLFIWSVAITALLLLLSLPVLAGAITMLLTDRNLNTSFFDPAGGGDPILYQHL
>COLFF402-13|KJ966457|ZMUO.005920|Halyzia_sedecimguttata
TLYFLFGMWAGMVGTSLSIMIRLELGTTNSLIGNDQIYNVI?TAHAFIMIFFMVMPVMIGGFGNWLVPLMVGAPDMAFPRLNNMSFWLLPPALTLLIFSSMVEMGAGTGWTVYPPLSSNMAHSGSSVDLVIFSLHLAGISSILGAVNFISTIMNMRP
FGMNLDKTPLFVWSVLITAILLLLSLPVLAGAITMLLTDRNINTSFFDPMGGGDPILYQHL
>COLFB525-12|KJ966421|ZMUO.001000|Omalium_rivulare
TL?FIFGAWAGMVGTSLSLLIRAELGNPGSLIGDDQIYNVIVTAHAFIMIFFMVMPIVIGGFGNWLVPLMLGAPDMAFPRMNNMSFWLLPPSLTLLLMSSMVESGAGTGWTVYPPLSSNIAHGGSSVDLAIFSLHLAGISSILGAVNFITTVINMRAMG
MTFDRMPLFVWSVVITALLLLLSLPVLAGAITMLLTDRNLNTSFFDPAGGGDPILYQHL
>COLFD897-12|KJ963346|ZMUO.004507|Stenolophus_mixtus
TLYFIFGVWAGMVGTSLSMLIRAELGTPGALIGDDQIYNVIVTAHAFVMIFFMVMPIMIGGFGNWLVPLMLGAPDMAFPRMNNMSFWLLPPSLTLLLMSSLVENGAGTGWT?YPPLSSGIAHSGASVDLAIFSLHLAGISSILGAVNFITTIINMRSVG
MTFERMPLFVWSVGITALLLLLSLPVLAGAITMLLTDRNLNTSFFDPAGGGDPILYQHL
>COLFB497-12|KJ962271|ZMUO.000972|Aploderus_caelatus
TLYFIFGAWSGMIGTSLSMLIRAELGAPGSLIGDDQIYNVVVTAHAFIMIFFMVMPIVIGGFGNWLVPLMLGAPDMAFPRMNNMSFWLLPPSLTLLLFSSMVESGAGTGWTVYPPL?SNIAHSGSSVDLAIFSLHLAGISSILGAVNFISTIINMRSVGM
SFDRMPLFIWSVNITAILLLLSLPVLAGAITMLLTDRNLNTSFFDPAGGGDPILYQHL
>COLFF582-13|KJ967182|ZMUO.006472|Otiorhynchus_ovatus
TLYFIFGSWAGMVGTSLSMLIRTELGNPGSLIGDDQIYNVIVTAHAFIMIFFMVMPMMIGGFGNWLVPLMLGAPDMAFPRMNNMSFWLLPPSLTLLLASSIVDKGAGTGWTVYPPLSANIAHEGASVDLAIFSLHMAGVSSILGAINFISTMANMRP
YGMSPD?MSLFTWAVKITAILLLLSLPVLAGAITMLLTDRNINTSFFDPAGGGDPILYQHL
>COLFB145-12|KJ964399|ZMUO.001475|Otiorhynchus_arcticus
TLYFILGAWSGMVGTSLSMLIRVELGTPGALIGDDQIYNV?VTAHAFIMIFFMVMPMMIGGFGNWLVPLMLGAPDMAFPRLNNMSFWLLPPSLSLLLMSSIIDKGAGTGWTVYPPLSSNIAHEGASVDLAIFSLHMAGVSSILGAINFISTVVNMRPN
GMSPDRMTLFIWAVKITAILLLLSLPVLAGAITMLLTDRNVNTSFFDPAGGGDPILYQHL
>COLFA592-12|KJ964685|ZMUO.000727|Thanatophilus_lapponicus
TLYFIFGAWAGMVGMSLSILIRAELS?PGTLLGDDQMYNVIVTAHAFIMIFFMVMPIVIGGFGNWLVPLMLGAPDMAFPRMNNMSFWLLPPSLSLLLVSSMVESGAGTGWTVYPPLSSNIAHGGSSVDLAIFSLHLAGISSILGAVNFITTIINMRSSG
MTFDRMPLFVWSVAITALLLLLSLPVLAGAITMLLTDRNINTSFFDPAGGGDPILYQHL
>COLFE1154-13|KJ967528|ZMUO.006949|Tachyporus_pulchellus
TLYFIFGAWSGMVGTSLSLLIRAELGNPGSLIGDDQIYNVIVTAHAFIMIFFMVMPIVIGGFGNWLVPLMLGAPDMAFPRMNNMSFWLLPPSLTLLLMSSMVESGAGTGWTVYPPLSANIAHSGPSVDLAIFSLHLAGISSILGAVNFITTVINMRASG
MHFDRMPLFIWSVAITALLLLLSLPVLAGAITMLLTDRNLNT?FFDPAGGGDPILYQHL
>COLFB527-12|KJ967335|ZMUO.001002|Cafius_xantholoma
TLYFIFGSWAGMVGTSLSLLIRAELGNPGTLIGDDQIYNVIVTAHAFIMIFFMVMPIVIGGFGNWLVPLMLGAPDMAFPRMNNLSFWLLPPSLTLLLMSSMVESGAGTGWTVYPPLSSNIAHGGASVDLAIF?LHLAGISSILGAVNFITTVINMRSTGM
TFDRMPLFVWSVAITALLLLLSLPVLAGAITMLLTDRNLNTSFFDPAGGGDPILYQHL
>COLFD923-12|KJ963208|ZMUO.004533|Bembidion_quadrimaculatum
TLYF?FGAWSGMVGTSLSMLIRAELGNPGSLIGDDQIYNVIVTAHAFVMIFFMVMPILIGGFGNWLVPLMLGAPDMAFPRMNNMSFWLLPPSLTLLLMSSMVESGAGTGWTVYPPLSSSIAHSGASVDLAIFSLHLAGVSSILGAVNFITTIINMRSTG
MTFDRMPLFVWSVGITALLLLLSLPVLAGAITMLLTDRNLNTSFFDPAGGGDPILYQHL
>COLFB114-12|KJ962695|ZMUO.001444|Lygistopterus_sanguineus
TLYFIFGAWAGMLGTSLSLLIRAELGTPGTLIGNDQIYNVIVTAHAFIMIFFMVMPMMIGGFGNWLVPLMLGAPDMAFPRMNNMSFWLLPPSLSLLIMSSMVESGAGTGWTVYPPLSSNIAHSGSSVDLAIFSLHLAGVSSILGAVNFISTIINMKSPG
MTYDQMPLFVWAVGIT?LLLLLSLPVLAGAITMLLTDRNLNTSFFDPTGGGDPILYQHL

>COLFD321-12|KJ962971|ZMUO.004216|Corticaria_impressa
SLYFLFGMWSGMVGTSLSLLIRLELGNPGSLIGDDQIYNVIVTAHAFIMIFFMVMPIMIGGFGNWLVPLMLGAPDMAFPRLNNMSFWLLPPSLSLLIMSSIVESGAGTGWTVYPPLSSNIAHGGSSVDLAIFSLHLAGISSILGAVNFITTVINMRPAGM
NLDQMPLFVWSVVITAILLLLSLPVLAGAITMLLTDRNLNTSFFDPA?GGDPILYQHL
>LEFIJ1824-13|KJ966167|ZMUO.004579|Ceutorhynchus_ignitus
TLYFIFGSWAGMAGTSLSMLIRTELGNPGSLIGNDQIYNSIVTAHAFIMIFFMVMPILIGGFGNWLVPLMLGAPDMAFPRLNNMSFWLLP?SLSLLLMSSIVNKGAGTGWTVYPPLSSNVAHEGMSVDLAIFSLHMAGISSILGAINFISTVMNMQPKG
MTPELMPLFVWAVEITAILLLLSLPVLAGAITMLLTDRNINTSFFDPSGGGDPILYQHL
>COLFE1064-13|KJ963499|ZMUO.006859|Hydroporus_erythrocephalus
TLYFLFGAWSGMVGTSLSMLIRAELGNPGSLIGDDQIYNVIVTAHAFIMIFFMVMPIMIGGFGNWLVPLMLGAPDMAFPRMNNMSFWLLPPSLSLLLMSSMVENGAGTGWTVYPPLSSGIAHSGASVDLAIFSLHLAGVSSILGAVNFITTIINMRSIG
MTFDRMPLFVWSV?ITALLLLLSLPVLAGAITMLLTDRNLNTSFFDPAGGGDPILYQHL
>COLFB034-12|KJ962533|ZMUO.001364|Dyschirius_obscurus
TLYFIFGIWSGMVGTSLSILIRTELGNPGSLIGDDQIYNVIVTAHAFIMIFFMVMPIMIGGFGNWLVPLMLGAPDMAFPRMNNMSFWLLPPSLTLLLMSSMVESGAGT?WTVYPPLSSGIAHSGASVDLAIFSLHLAGISSILGAVNFITTIINMRSTGMT
FERMPLFVWSVGITALLLLLSLPVLAGAITMLLTDRNLNTSFFDPAGGGDPILYQHL
>COLFD829-12|KJ967400|ZMUO.004819|Longitarsus_luridus
TLYFIFGIWAGMIGTSLSILIRTELGNPGSLIGNDQIYNVIVTAHAFIMIFFMVMPIMIGGFGNWLVPLMIGAPDMAFPRMNNMSFWLLPPSLFLLVMSSMVESGAGTGWTVYPPLSSNIAHGGSSVDLAIFSLHLAGISSILGAINFITTVINMRPIGMTL
DRMPLFVWAVVITAILLLLSLPVLA?AITMLLTDRNLNTTFFDPAGGGDPILYQHL
>COLFC840-12|KJ965158|ZMUO.003405|Bembidion_gilvipes
TLYFIFGAWSGMVGTSLSMLIRAELGNPGSLIGDDQIYNVIVTAHAFVMIFFMVMPILIGGFGNWLVPLMLGAPDMAFPRMNNMSFWLLPPSLTLLLMSSMVESGAGTGWTVYPPLSSSMAHSGASVDLAIFSLHLAGVSSILGAVNFITTIINMRSV
GMTFDRMPLFVWSVGITALLLLLSLPVLAGAIT?LLTDRNLNTSFFDPAGGGDPILYQHL
>COLFB069-12|KJ967028|ZMUO.001399|Bembidion_bruxellense
T?YFIFGAWSGMVGTSLSMLIRAELGNPGSLIGDDQIYNVIVTAHAFVMIFFMVMPILIGGFGNWLVPLMLGAPDMAFPRMNNMSFWLLPPSLTLLLMSSMVESGAGTGWTVYPPLSSSIAHSGASVDLAIFSLHLAGVSSILGAVNFITTIINMRSIG
MSFDRMPLFVWSVGITALLLLLSLPVLAGAITMLLTDRNLNTSFFDPAGGGDPILYQHL
>COLFE253-12|KJ964743|ZMUO.005098|Amara_municipalis
TLYFIFGAWSGMVGTSLSMLIRAELGNPGALIGDDQIYNVIVTAHAFVMIFFMVMPIMIGGFGNWLVPLMLGAPDMAFPRMNNMSFWLLPPSLTLLLMSSMVESGAGTGWTVYPPLSSGIAHAGASVDLAIFSLHLAGISSILGAVNFITTIINMRSV
GMTFDRMPLFVWSVGITALLLLLSLPVLAGAITMLLTDR?LNTSFFDPAGGGDPILYQHL
>COLFA345-12|KJ965824|ZMUO.000575|Gabrius_toxotes
TLYFIFGSWAGMVGTSLSLLIRAELGNPGTLIGDDQIYN?IVTAHAFIMIFFMVMPIVIGGFGNWLVPLMLGAPDMAFPRMNNMSFWLLPPSLTLLLMSSMVESGAGTGWTVYPPLSSNIAHGGASVDLAIFSLHLAGISSILGAVNFITTVINMRSFG
MSFDRMPLFVWSVAITALLLLLSLPVLAGAITMLLTDRNLNTSFFDPAGGGDPILYQHL
>COLFC648-12|KJ965871|ZMUO.003213|Hydroporus_geniculatus
TLYFLFGAWSGMVGTSLSMLIRAELGNPGSLIGDDQIYNVIVTAHAFIMIFFMVMPIMIGGFGNWLVPLMLGAPDMAFPRMNNMSFWLLPPSLTLLLMSSMVENGAGTGWTVYPPLSSGIAHSGASVDLAIFSLHLAGVSSILGAVNFITTIINMRSI
GMTFDRM?LFVWSVGITALLLLLSLPVLAGAITMLLTDRNLNTSFFDPAGGGDPILYQHL
>COLFA063-10|HM909081|MP00248|Upis_ceramboides
TLYFIFGAWSGMVGTSLSLMIRAELGNPGSLIGDDQIYNVIVTAHAFIMIFFMVMPIMIGGFGNWLVPLMLGAPDMAFPRMNNMSFWLLPPSLTLLLMSSIVESGAGTGWTVYPPLSSNI?HGGSSVDLAIFSLHLAGISSILGAVNFITTVINMRPQG
MTFDRMPLFVWAVVITAVLLLLSLPVLAGAITMLLTDRNINTSFFDPAGGGDPILYQHL
>COLFB370-12|KJ962338|ZMUO.001700|Liogluta_micans
TLYFIFGAWAGM?GTSLSLLIRAELGNPGSLIGDDQIYNVIVTAHAFIMIFFMVMPVVIGGFGNWLVPLMLGAPDMAFPRMNNMSFWLLPPSLTLLLMSSMVESGAGTGWTVYPPLSSNIAHSGSSVDLAIFSLHLAGISSILGAVNFISTVINMRSVG
MSFDRMPLFVWSVAITALLLLLSLPVLAGAITMLLTDRNLNTSFFDPAGGGDPILYQHL
>COLFC479-12|KJ963835|ZMUO.003044|Malthodes_fuscus
TLYFMFGAWAGMLGTSLSLLIRAELGSPGSLIGNDQIYNVIVTAHAFIMIFFMVMPIMIGGFGNWLVPLMLGAPD?AFPRMNNMSFWFLPPSLSLLLMSSIVENGAGTGWTVYPPLSANIAHSGSSVDLAIFSLHMAGISSILGAVNFISTVINMRSTG
MTFDRMPLFVWAVAITALLLLLSLPVLAGAITMLLTDRNLNTSFFDPAGGGDPILYQHL
>COLFD005-12|KJ963972|ZMUO.003900|Bryoporus_cernuus
TLYFIFGAWAGMVGTSLSLLIRAELGNPGSLIGDDQIYNVIVTAHAFIMIFFMVMPIVIGGFGNWLVPLMLGAPDMAFPRMNNMSFWLLPPSLTLLLMSSLVESGAGTGWTVYPPLSSNIAHGGASVDLAIFSLHLAGISSILGAVNFITTVINMRSIGM
TFDRMPLFVWSVVITALLLLLSLPVLAGAITMLLTDRNLNTSFFDP?GGGDPILYQHL
>COLFA465-12|KJ963699|ZMUO.000505|Bembidion_bruxellense
TLYFIFGAWSGMVGTSLSMLIRAELGN?GSLIGDDQIYNVIVTAHAFVMIFFMVMPILIGGFGNWLVPLMLGAPDMAFPRMNNMSFWLLPPSLTLLLMSSMVESGAGTGWTAYPPLSSSIAHSGASVDLAIFSLHLAGVSSILGAVNFITTIINMRSIG
MSFDRMPLFVWSVGITALLLLLSLPVLAGAITMLLTDRNLNTSFFDPAGGGDPILYQHL
>COLFE893-13|KJ964751|ZMUO.006213|Chrysolina_fastuosa

TLYFIFGTWAGMVGTSLSILIRAELGNPGTLIGNDQIYNVIVTAHAFIMIFFMVMPIMIGGFGNWLVPLMLGAPDMAFPRMNNMSFWLLPPSLIFLLMSSIVENGVGTGWTVYPPLSTNVAHSGPSVDLAIFSLHLAGISSILGAINFITTVINMRPTGM
KLEQMPLFSWAVLITAILLLLSLPVLAGAITMLLTDRNLNTSFFDPA?GGDPILYQHL
>COLFE1594-13|KJ963762|ZMUO.007484|Anaulacaspis_nigra
TLYFIFGAWAGMVGTSLSLLIRAELGNPGSLIGDDQIYNVIVTAHAFIMIFFMVMPIMIGGFGNWLVPLMLGAPDMAFPRKNNMSFWLLPPSLTLLLMSSMVESGAGTGWTVYPPLSSNIAHAGATVDLAIFSLHLAGISSILGTVNFISTVINMRSIGIS
FDRMPLFVWAVAITALLL?LSLPVLAGAITMLLTDRNLNTSFFDPAGGGDPILYQHL
>COLFB260-12|KJ962032|ZMUO.001590|Atheta_arctica
TLYFI?GVWAGMIGTSLSLLIRAELGNPGSLIGDDQIYNVIVTAHAFVMIFFMVMPIVIGGFGNWLVPLMLGAPDMAFPRMNNMSFWLLPPSLTLLLMSSMVESGAGTGWTVYPPLSSNIAHGGSSVDLAIFSLHLAGISSILGAVNFISTVINMRSTGI
SFDRMPLFVWSVAITALLLLLSLPVLAGAITMLLTDRNLNTSFFDPAGGGDPILYQHL
>COLFF317-13|KJ966814|ZMUO.006017|Gonioctena_arctica
TLYFIFGIWAGMVGTSLSLILIRAELGNPGTLIGNDQIYNVIVTAHAFIMIFFMVMPIMIGGFGNWLVPLMIGAPDMAFPRMNNMSFWLLPPSLFFLIMSSVVESGAGTGWTVYPPLSANIAHSGSSVDLAIFSLHLAGISSILGAINFITTIINMRPMGMT
MDRMPLFVWAVLITAILLLLSLPVLAGAITMLLTDRNLNTSFFDPAGG?DPILYQHL
>COLFF413-13|KJ964195|ZMUO.005931|Auleutes_epilobii
TLYFIFGSWAGTMGTSMSMLIRTELGNPGSLIGDDQIYNSIVTAHAFVMIFFMVMPILIGGFGNWLVPLMLGAPDMAFPRLNNMSFWLLPPSITLLLMSSIINKGAGTGWTVYPPLSTNIAHEGASVDLAIFSLHMAGISSILGAINFISTIMNMRPKG
MEYEIMPLFSWAVMITAILLLLSLPVLAGAITMLLTDRNVNTSFFDP?GGGDPILYQHL
>COLFF542-13|KJ967465|ZMUO.006622|Stenus_excubitor
TLYFIFGAWAGMIGTSLSLLIRAELGNPGSLIGDDQIYNVIVTAHAFVMIFFMVMPVMIGGFGNWLVPLMLGAPDMAFPRMNNMSFWLLPPSLSLLLTSSIVESGAGTGWTVYPPLSSNIAHGGASVDLAIFSLHLAGISSILGAINFITTIFNMRSMKIQ
LDCLPLFVWSVGITAFLLLLSLPVLAGAITMLLTDRNLNTSFFDPAGGGDPILY?HL
>COLFC509-12|KJ966596|ZMUO.003074|Atheta_incognita
TLYFIFGMWAGMVGTSLSLLIRAELGNPGSLIGDDQIYNVIVTAHAFVMIFFMVMPVMIGGFGNWLIPLMLGAPDMAFPRMNNMSFWLLPPSLTLLLMSSMVESGAGTGWTVYPPLSSNIAHGGSSVDLAIFSLHLAGISSILGAVNFISTVINMRST
GISFDRMPLFVWSVAITALLLLLSLPVLAGAITMLLTDRNLN?SFFDPAGGGDPILYQHL
>COLFF607-13|KJ966639|ZMUO.006497|Meligethes_difficilis
TLYFIFGAWSGMVGTSLSMLIRTELGNPGSLIGNDQIYNVIVTAHAFVMIFFMVMPFMIGGFGNWLVPLMLGAPDMAFPRMNNMSFWLLPPSLSLLLTSSIVESGAGTGWTVYPPLSSNIAHGGASVDLAIFSLHLAGISSILGAVNFITTVINMRPSG
MTFDRMPLFVWAVVITAILLLLSLPVLAGAITMLLTDR?LNTTFFDPSGGGDPILYQHL
>COLFE852-13|KJ962823|ZMUO.005697|Corticaria_impressa
SLYFLFGMWSGMVGTSLSLLIRLELGNPGSLIGDDQIYNVIVTAHAFIMIFFMVMPIMIGGFGNWLVPLMLGAPDMAFPRLNNMSFWLLPPSLSLLIMSSIVESGAGTGWTVYPPLSSNIAHGGSSVDLAIFSLHLAGISSILGAVNFITTV?NMRPAGM
NLDQMPLFVWSVVITAILLLLSLPVLAGAITMLLTDRNLNTSFFDPAGGGDPILYQHL
>COLFD175-12|KJ966143|ZMUO.004070|Quedius_umbrinus
TLYFIFGAWAGMVGTSLSLLIR?ELGNPGTLIGDDQIYNVIVTAHAFIMIFFMVMPVVIGGFGNWLVPLMLGAPDMAFPRMNNMSFWLLPPSLSLLLMSSMVESGAGM---
VYPPLSSNIAHGGASVDLAIFSLHLAGISSILGAVNFITTVINMRSIGMTFDRMPLFVWSVAITALLLLLSLPVLAGAITMLLTDRNLNTSFFDPAGGGDPILYQHL
>COLFC084-12|KJ962961|ZMUO.002079|Anotylus_clavatus
TLYFIFGAWSGMV?TSLSMLIRAELGTPGSLIGDDQIYNVIVTAHAFVMIFFMVMPIVIGGFGNWLVPLMLGAPDMAFPRMNNMSFWLLPPSLTLLLFSSMVESGAGTGWTVYPPLSSNIAHSGSSVDLAIFSLHLAGISSILGAVNFISTIINMRSIGMS
FDRMPLFVWSVNITAILLLLSLPVLAGAITMLLTDRNLNTSFFDPAGGGDPILYQHL
>COLFE1460-13|KJ961798|ZMUO.007255|Pria_dulcamarae
TLYFIFGAWSGMVGTSLSMLIRTELGNPGSLIGNDQIYNVIVTAHAFVMIFFMVMPFMIGGFGNWLVPLMLGAPDMAFPRMNNMSFWLLPPSLSLLLMSSIVESGAGTGWTVYPPLSSNIAHGGASVDLAIFSLHLAGI?SILGAVNFITTIINMRPTG
MTFDRMPLFVWAVAITALLLLLSLPVLAGAITMLLTDRNLNTTFFDPSGGGDPILYQHL
>COLFG128-13|KJ965830|ZMUO.007823|Euaesthetus_bipunctatus
TLYFIFGAWSGMIGTSLSLLIRTELGNPGSLIGNDQIYNVIVTAHAFIMIFFMVMPIMIGGFGNWLVPLMLGAPDMAFPRMNNMSFWLLPPSLSLLLMSSIVESGAGTGWTVYPPLSSNIAHSGASVDLAIFSLHLAGISSILGAVNFITTIINMRPMGMT
FDRMPLFIWSVGITALLLLLSLPVLAGAITMLLTDRNLNTTFFDPAGG?DPILYQHL
>COLFD448-12|KJ964135|ZMUO.003868|Ceutorhynchus_obstrictus
TLYFIFGSWAGMAGTSLSMLIRTELGNPGSLIGNDQIYNSIVTAHAFIMIFFMVMPILIGGFGNWLVPLMLGAPDMAFPRLNNMSFWLLPPSLSLLLMSSIVNKGAGTGWTVYPP?SGNVAHEGMSVDLAIFSLHMAGISSILGAINFISTVMNMQPK
GMTPELMPLFVWAVQITAILLLISLPVLAGAITMLLTDRNINTSFFDPSGGGDPILYQHL
>COLFB487-12|KJ962676|ZMUO.000962|Omalium_riparium
TLYFIFGAWAGMVGTSLSLLIRAELGNPGSLIGD?QIYNVIVTAHAFIMIFFMVMPIVIGGFGNWLVPLMLGAPDMAFPRMNNMSFWLLPPSLTLLLMSSMVESGAGTGWTVYPPLSSNIAHGGSSVDLAIFSLHLAGISSILGAVNFITTVINMRAMG
MTFDRMPLFVWSVVITALLLLLSLPVLAGAITMLLTDRNLNTSFFDPAGGGDPILYQHL
>COLFA719-12|KJ963443|ZMUO.000854|Astenus_pulchellus
TLYFIFGS?AGMVGTSLSLLIRAELGMPGSLIGDDQIYNVIVTAHAFVMIFFMVMPIVIGGFGNWLVPLMLGAPDMAFPRMNNMSFWLLPPSLSLLLMSSIVESGAGTGWTVYPPLSSNIAHSGASVDLAIFSLHLAGISSILGAVNFITTIINMRTKGMT
YERMPLFIWSVGITALLLLLSLPVLAGAITMLLTDRNLNTSFFDPAGGGDPILYQHL

>COLFA519-12|KJ965758|ZMUO.000559|Aphodius_borealis
TLYFLFGSWAGMVGTSLSLLIRAELGNPGTLIGDDQIYNVIVTAHAFVMIFFMVMPILIGGFGNWLVPLMLGAPDMAFPRMNNMSFWLLPPSLTLLLMSSMVESGAGTGWTVYPPLSSNIAHGGASVDLAIFSLHLAGISSILGAVNFITTVINMRSPG
LTFDRMPLFVWSVAITALLLLLSLPVLAGAITMLLTDRNLNTSFFDPAGG?DPILYQHL
>COLFB914-12|KJ962550|ZMUO.001959|Rhagium_inquisitor
TLYFIFGAWSGMVGTSLSLLIRTELGNPGSLIGDDQIYNVIVTAHAFIMIFFMVMPIMIGGFGNWLVPLMLGAPDMAFPRMNNMSFWLLPPSLTLLIMSSVVENGAGTGWTVYPPLSSNIAHSGSSVDLAIFSLHLAGISSILGAVNFITTVINMRPIGM
TPDRVPLFVWAVVITAILLLISLPVLAGAITMLLTDR?LNTSFFDPAGGGDPILYQHL
>COLFD841-12|KJ967472|ZMUO.004831|Monochamus_galloprovincialis
TLYFIFGAWSGMVGTSLSLLIRSELGMPGSLIGDDQIYNVIVTAHAFIMIFFMVMPIMIGGFGNWLVPLMLGAPDMAFPRMNNMSFWLLPPSLTLLIMSSIVENGAGTGWTVYPPLAANVAHSGSSVDLAIFSLHLAGVSSILGAVNFITTVINMRPKG
MNLDRLPLFVWAVKITAILLLLSLPVLAGAITMLLTDRN?NTSFFDPAGGGDPILYQHL
>COLFD530-12|KJ965851|ZMUO.004330|Altica_carinthiaca
TLYFLFGIWAGMIGTSMSLLIRTELGSPGSLIGNDQIYNVIVT?HAFIMIFFMVMPMMIGGFGNWLVPLMIGAPDMAFPRMNNMSFWLLPPSIFLLLMSSFTESGAGTGWTVYPPLSSNLAHNGPSVDLAIFSLHLAGISSILGAINFITTMINMCPQG
MSMDQMPLFVWAVFITAILLLLSLPVLAGAITMLLTDRNFNTSFFEPAGGGDPILYQHL
>COLFE843-13|KJ962662|ZMUO.005688|Olibrus_aeneus
TLYFIFGAWAGMVGTSLSLLIRTELGTPGSLIGDDQIYNVIVTAHAFVMIFFMVMPFMIGGFGNWLVPLMLGAPDMAFPRMNNMSFWLLPPSLFLLLMSSMVESGAGTGWTVYPPLSSNIAHGGASVDLAIFSLHLAGISSILGAINFITTMINMRPE
GMTLDRMPLFVWAVTITAILLLLSLPVLAGAITMLLTDRN?NSSFFDPAGGGDPILYQHL
>COLFE460-12|KJ966847|ZMUO.005020|Nedyus_quadrimaculatus
TLYFIFGTWAAMAGTSLSMLIRTELGNPGSLIGNDQIYNSIVTA?AFIMIFFMVMPILIGGFGNWLVPLMLGAPDMAFPRLNNMSFWLLPPALSLLLMSSIVNKGAGTGWTVYPPLSTNTAHEGMSVDLAIFSLHLAGLSSILGAINFISTVMNMRPQG
MSPEFTPLFVWAVKITAILLLLSLPVLAGAITMLLTDRNINTSFFDPSGGGDPILYQHL
>COLFD935-12|KJ966082|ZMUO.004545|Cryptocephalus_nitidus
TLYFLFG?WAGMVGTSLSLLIRVELGNPGSLIGNDQIYNTIVTAHAFIMIFFMVMPIMIGGFGNWLVPLMLGAPDMAFPRMNNMSFWLLPPSLTLLLMSSIVENGAGTGWTVYPPLSTTIAHAGSSVDLAIFSLHLAGASSIMGAINFISTVINMRPQG
MLMDQIPLFVWAVMITAILLLLSLPVLAGAITMLLTDRNLNTSFFDPAGGGDPILYQHL
>COLFB462-12|KJ966687|ZMUO.001887|Carpelimus_rivularis
TLYFIFGAWSGMVGTSLSMLIRIELGTPGSLIGDDQIYNVIVTAHAFIMIFFMVMPIVIGGFGNWLVPLMLGAPDMAFPRMNNMSFWLLPPSLTLLLFSSMVESGAGTGWTVY?PLSSNIAHSGSSVDLAIFSLHLAGISSILGAVNFISTIINMRSVGMT
FDRMPLFVWSVNITAILLLLSLPVLAGAITMLLTDRNLNTSFFDPAGGGDPILYQHL
>COLFB436-12|KJ963487|ZMUO.001861|Protapion_assimile
TLYFIFGLWSGMIGTSLSMLIRIELGNPGSLIGNDQIYNVIVTAHAFIMIFFMVMPVMIGGFGNWLVPLMLGAPDMAFPRMNNMSFWLLPPSLTLLLMSSIVEKGAGTGWTVYPPLAANIAHSGASVDLAIFSLHLAGISSILGAVNFISTIMNMRPTG
MSLDQLSLFTWAVKITAILLLLSLPVLAGAITMLLTDRNINTSFFD?AGGGDPILYQHL
>COLFC828-12|KJ965035|ZMUO.003393|Ptinus_villiger
TLYFILGAWSSMLGTSLSLLIRSELGNPGTLIGDDQIYNVIVTAHAFIMIFFMVMPIMIGGFGNWLVPLMLGAPDMAFPRMNNMSFWLLPPALTFLLVGGLVESGAGTGWTVYPPLAGNIAHSGASVDLTIFSLHLAGVSSILGAINFITTVINMRPIEM
TFDRVPLFVWSVVITAVLLL?SLPVLAGAITMLLTDRNLNTSFFDPAGGGDPILYQHL
>COLFB350-12|KJ962802|ZMUO.001680|Tachinus_corticinus
TLYFIFGAWSGMVGTSLSLLIRAELGNPGTLIGDDQIYNVIVTAHAFIMIFFMVMPIVIGGFGNWLVPLMLGAPDMAFPRMNNMSFWLLPPSLSLLLMSSMVESGAGTGWTVYPPLSSNIAHGGSSVDLAIFSLHLAGISSILGAVNFITTVINMRSIGM
TFDRMPLFVWSVAITALLLLLSLPVLAGAITMLLTDRNLNTTFFDPAGGG?PILYQHL
>COLFA490-12|KJ964892|ZMUO.000530|Atomaria_fuscata
TLYFIFGSWAGMVGTSLSMLIRTELGT?GSLIGDDQIYNVIVTAHAFIMIFFMVMPMMIGGFGNWLVPLMLGAPDMAFPRLNNMSFWLLPPSLMFLLMSSIVEKGAGTGWTVYPPLSSNVAHAGSSVDLAIFSLHLAGISSILGSMNFITTVINMRPK
GMNFDCLPLFVWSVKITTILLLLSLPVLAGAITMLLTDRNINTSFFDPAGGGDPILYQHL
>COLFB338-12|KJ962438|ZMUO.001668|Aleochara_moerens
TLYFIFGAWAGMVGTSLSLLIRAELGNPGSLIGDDQIYNVIVTAHAFIMIFFMVMPIVIGGFGNWLVPLMLGAPDMAFPRMNNMSFWLLPPSLTLLLMSSMVESGAGTGWTVYPPLSANIAHSGSSVDLAIFSLHLAGISSILGAVNFISTIINMRSSGM
SFDKMPLFVWSVAITALLLLLSLPVLAGAITMLLTDRNLNTSFFDPAGG?DPILYQHL
>COLFB932-12|KJ964001|ZMUO.001977|Malthodes_brevicollis
TLYFMFGAWSGMLGTSLSLLIRAELGSPGSLIGNDQIYNVIVTAHAFIMIFFMVMPIMIGGFGNWLVPLMLGAPDMAFPRMNNMSFWFLPPSLSLLLMSSIVENGAGTGWTVYPPLSANIAHSGSSVDLAIFSLHMAGISSILGAVNFISTVINMRSPG
MTFDRMPLFVWAVAITALLLLLSLPVLAGAITMLLTDRNLNTSFFDPAGG?DPILYQHL
>COLFB729-12|KJ963193|ZMUO.001204|Megasternum_concinnum
TLYFIFGAWAGMVGTSLSILIRAELG?PGTLIGDDQIYNVIVTAHAFIMIFFMVMPIMIGGFGNWLVPLMLGAPDMAFPRMNNMSFWLLPPSLTLLLMSSMVESGAGTGWTVYPPLSSNIAHGGSSVDLAIFSLHLAGISSILGAVNFITTVINMRSPNL
TYDRLPLFVWSVAITALLLLLSLPVLAGAITMLLTDRNLNTLFFDPAGGGDPILYQHL
>COLFB655-12|KJ966289|ZMUO.001130|Hygrotus_inaequalis

TLYFLFGAWAGMVGTSLSMLIRAELGNPGSLIGDDQIYNVIVTAHAFIMIFFMVMPIMIGGFGNWLVPLMLGAPDMAFPRMNNMSFWMLPPSLTLLLMSSMVESGAGT?WTVYPPLSAGIAHGGASVDLAIFSLHLAGISSILGAVNFITTIINMRSV
GMTFDRMALFVWSVGITALLLLLSLPVLAGAITMLLTDRNLNTSFFDPAGGGDPILYQHL
>COLFE1290-13|KJ964745|ZMUO.007085|Heterocerus_intermedius
TLYFILGSWSGMLGMALSLLIRAELGNPGALIGDDQIYNVIVTAHAFIMIFFMVMPIMIGGFGNWLVPLMLGAPDMAFPRLNNMSFWLLPPSLTLLLMSSMVESGAGTGWTVYPPLSANIAHSGSSVDLAIFSLHLAGISSILGAVNFITTVINMRAPG
MSFDR?PLFVWSVVITAVLLLLSLPVLAGAITMLLTDRNINTSFFDPAGGGDPILYQHL
>COLFE1323-13|KJ966869|ZMUO.007118|Donacia_aquatica
TLYFIFGAWSGMMGTSLSLLIRTELMNPGSLIGNDQIYNVIVTAHAFIMIFFMVMPIMIGGFGNWLVPLMLGAPDMAFPRMNNLSFWLLPPSLTFLTMSSITEKGAGTGWTVYPPLSSNIAHSGASVDLAIFSLHLAGISSILGAVNFISTVMNMRPTG
MSMEKMPLFSWAVVITAILLLLSLPVLAGAITMLLTDRNINTSFFDPAGGG?PILYQHL
>COLFB425-12|KJ965148|ZMUO.001850|Magdalis_duplicata
ILYFIFGTWSGMIGTSLSMLIRVELGSPGSLIGNDQIYNTIVTAHAFIMIFFMVMPIMIGGFGNFLIPLMLGAPDMAFPRLNNLSFWLLPPSLTLLLLSSITDKGAGTGWTVYPPLSSSIFHEGTSVDLAIFSLHLAGISSILGALNFISTIINMRPKNMKLDQM
SLFIWAVKITAILLLLSLPVLAGAITMLLTDRNINTSFFDPAGG?DPILYQHL
>COLFE428-12|KJ967031|ZMUO.004988|Aegialia_sabuleti
TLYFLFGGWAGMTGTSLSLLIRAELGNPGMLIGDDQIYNVIVTAHAFIMIFFMVMPILIGGFGNWLVPLMLGAPDMAFPRMNNMSFWL?PPSLTLLLMSSMVESGAGTGWTVYPPLSSNIAHSGPSVDLAIFSLHLAGISSILGAVNFITTVINMRSIG
MSFDRMPLFVWSVALTALLLLLSLPVLAGAITMLLTDRNINTSFFDPTGGGDPILYQHL
>COLFD713-12|KJ963651|ZMUO.004703|Trox_sabulosus
TLYFLFGSWAGMVGTSLSILIRAELGNPGTLIGDDQIYNVIVTAHAFIMIFFMVMPILIGGFGNWLVPLM?GAPDMAFPRMNNMSFWLLPPSLTLLLVSSLVESGAGTGWTVYPPLSSNIAHSGASVDLAIFSLHLAGISSILGAVNFITTVINMRSTGMT
FDRMPLFVWAVILTALLLLLSLPVLAGAITMLLTDRNLNTSFFDPAGGGDPVLYQHL
>COLFA671-12|KJ966699|ZMUO.000806|Autalia_rivularis
TLYFIFGAWSGMVGTSLSLLIRAELGNPGSLIGDDQIYNVIVTAHAFIMIFFMVMPIVIGGFGNWLVPLMLGAPDMAFPRMNNMSFWLLPPSLTLLLMSSMVESGAGTGWTVYPPLSSNIAHGGSSVDLAIFSLHLAGISSILGAVNFISTIINMRSTGIS
FDRMPLFVWSVGITALLLLLSLPVLAGAITMLLTDRNLNTSFFDPAGGG?PILYQHL
>COLFC017-12|KJ963378|ZMUO.002012|Hydroporus_geniculatus
TLYFLFGAWSGMVGTSLSMLIRAELGNPGSLIGDDQIYNVIVTAHAFIMIFFMVMP?MIGGFGNWLVPLMLGAPDMAFPRMNNMSFWLLPPSLTLLLMSSMVENGAGTGWTVYPPLSSGIAHSGASVDLAIFSLHLAGVSSILGAVNFITTIINMRSI
GMTFDRMPLFVWSVGITALLLLLSLPVLAGAITMLLTDRNLNTSFFDPAGGGDPILYQHL
>COLFA400-12|KJ963120|ZMUO.000630|Quedius_molochinus
TLYFIFGAWAGMVGTSLSLLIRAELGNPGSLIGDDQIYNVIVTAHAFIMIFFMVMPIMIGGFGNWLVPLMLGAPDMAFPRMNNMSFWLLPPSLSLLLMSSMVES?AGTGWTVYPPLSSNMAHGGASVDLAIFSLHLAGISSIFGAVNFITTVINMRSIG
MTFDRMPLFVWSVAITALLLLLSLPVLAGAITMLLSDRNLNTSFFDPAGGGDPILYQHL
>COLFB507-12|KJ966554|ZMUO.000982|Atheta_marcida
TLYFIFGAWAG?VGTSLSLLIRAELGNPGSLIGDDQIYNVIVTAHAFIMIFFMVMPIVIGGFGNWLVPLMLGAPDMAFPRMNNMSFWLLPPSLTLLLMSSMVESGAGTGWTVYPPLSSNIAHGGSSVDLAIFSLHLAGISSILGAVNFISTIINMRSTGISF
DRMPLFVWSVVITALLLLLSLPVLAGAITMLLTDRNLNTSFFDPAGGGDPILYQHL
>COLFB239-12|KJ961951|ZMUO.001569|Laccobius_minutus
TLYFLFGA?AGMVGTSLSILIRAELGNPGTLIGDDQIYNVIVTAHAFIMIFFMVMPIMIGGFGNWLVPLMLGAPDMAFPRMNNMSFWLLPPSLTLLLMSSMVESGAGTGWTVYPPLSSNIAHGGASVDLAIFSLHLAGISSILGAVNFITTVINMRSNN
MTYDRLPLFVWSVAITALLLLLSLPVLAGAITMLLTDRNLNTSFFDPAGGGDPILYQHL
>COLFE1483-13|KJ963252|ZMUO.007278|Exochomus_quadripustulatus
TLYFLFGMWAGMLGTSLSILIRLELGMSGSLIGNDQIYNVIVTAHAFIMIFFMVMPIMIGGFGNWLVPLMIGAPDMAFPRLNNMSFWLLPPALTLLILSSMMESGAGTGWTVYPPLSSNIAHSGSSVDLAIFSLHLAGISSILGAVNFITTIINMRPTGMT
LEKTPLFVWSVFITAILLLLSLPVLAGAITMLLTDRNMNTSFF?PSGGGDPILYQHL
>COLFD938-12|KJ967135|ZMUO.004548|Cassida_denticollis
TLYFIFGFWSGMVGTSLSILIRAELGNPGTLIGNDQIYNSIVTAHAFIMIFFMVMPIMIGGFGNWLVPLMLGAPDMAFPRLNNMSFWLLPPSITFLIMSSIIESGAGTGWTVYPPLSSNIAHSGASVDMAIFSLHLAGISSILGAINFISTIMNMQPSGMSL
DKMPLFVWAVIITAILLLLSLPVLAGAIT?LLTDRNINTSFFDPAGGGDPILYQHL
>COLFB732-12|KJ962059|ZMUO.001207|Stenus_nigritulus
TLYFMLGSWSGLVGTSLSLLIRAELGNPG?LIGDDQIYNVIVTAHAFIMIFFMVMPIMIGGFGNWFVPLMLGAPDMAFPRMNNMSFWLLPPSLSLLLMSSIVENGAGTGWTVYPPLSSNIAHSGASVDLAIFSLHLAGISSILGAINFITTIFNMRTMKIQ
LDCLTLFIWSVGITALLLLLSLPVLAGAITMLLTDRNLNTSFFDPAGGGDPILYQHL
>COLFB282-12|KJ966345|ZMUO.001612|Bembidion_transparens
TLYFIFGAWSGMVGTSLSMLIRAELGNPGSLIGDDQIYNVIVTAHAFVMIFFMVMPILIGGFGNWLVPLMLGAPDMVFPRMNNMSFWLLPPSLTLLLMSSMVENGAGTGWTVYPPLSSSIAHSGASVDLAIFSLHLAGVSSILGAVNFITTIINMRSM
GMTFDRMPLFVWSVGITALLLLLSLPVLAGAITMLLTDRNL?TSFFDPAGGGDPILYQHL
>COLFA608-12|KJ967494|ZMUO.000743|Leptura_quadrifasciata
TLYFIFGAWAGMVGTSLSLLIRSELGNPGSLIGDDQIYNVIVTAHAFVMIFFMVMPIMIGGFGNWLVPLMLGAPDMAFPRMNNMSFWLLPPSLTLLIMSSLVESGAGTGWTVYPPLSSNIAHGGSSVDLAIFSLHLAGISSILGAVNFITTVINMRPLG
MSPDQMPLFVWAVVITAILLLLSLPVLAGAITMLLTDRNLNTSFFDPAGGG?PILYQHL

>COLFE615-13|KJ964815|ZMUO.005460|Olophrum_assimile
TLYFIFGAWAGMVGTSLSILIRAELGNPGTLIGDDQIYNVIVTAHAFVMIFFMVMPIVIGGFGNWLVPLMLGAPDMAFPRMNNMSFWLLPPSLTLLLMSSMVESGAGTGWTVYPPLSSNIAHGGSSVDLAIFSLHLAGISSILGAVNFITTV?NMRSTG
MTFDRMPLFVWSVAITALLLLSLPVLAGAITMLLTDRNLNTSFFDPAGGGDPILYQHL
>COLFD539-12|KJ966576|ZMUO.004339|Archarius_salicivorus
TMYFIFGAWSGMVGTSLSMLIRTELGNPGSLIGDDQIYNTIVTAHAFIMIFFMVMPTLIGGFGNW?IPLMLGAPDMAFPRLNNMSFWILPPSLVLLLLSSIADKGAGTGWTVYPPLSTNIAHEGSSVDLAIFSLHMAGISSILGAMNFISSIMNMRPISM
KPDQMSLFIWAVKITAILLLLSLPVLAGAITMLLTDRNINTSFFDPAGGGDPILYQHL
>COLFA111-10|HQ559236|MP00372|Trachodes_hispidus
TLYFIFGSWSGMVGTSLSMMIRTELGTPGSVIGNDQIYNTIVTAHAFIMIFFMVMPIMIGGFGNWLVPLMLGAPDMAFPRLNNMSFWLLPPSLTLLLMSSIIDKGAGTGWTVYPPLSTNIAHEGMSVDLAIFSLHLAGISSILGAMNFISTVINMHPTG
MKLDQLPLFVWSVKITAILLLLSLPVLAGAITML?TDRNINTTFFDPAGGGDPILYQHL
>COLFB248-12|KJ963180|ZMUO.001578|Othius_punctulatus
?LYFIFGAWAGMVGTSLSL?IRAELGNPGSLIGDDQIYNVIVTAHAFIMIFFMVMPIMIGGFGNWLVPLMLGAPDMAFPRMNNMSFWLLPPSLSLLLMSSLVESGAGTGWTVYPPLSSNIAHGGASVDLAIFSLHLAGISSILGAVNFITTVINMRSIGM
TFDRMPLFVWSVAITALLLLSLPVLAGAITMLLTDRNLNTSFFDPAGGGDPILYQHL
>COLFD895-12|KJ962678|ZMUO.004505|Bembidion_guttula
TLYFIFGAWSGMVGTSLSMLIRAELG?PGSLIGDDQIYNVIVTAHAFIMIFFMVMPILIGGFGNWLVPLMLGAPDMAFPRMNNMSFWLLPPSLSLLLMSSMVENGAGTGWTVYPPLSSSIAHSGASVDLAIFSLHLAGVSSILGAVNFITTIINMRSTG
MTFDRMPLFVWSVGITALLLLSLPVLAGAITMLLTDRNLNTSFFDPAGGG?PILYQHL
>COLFB255-12|KJ962355|ZMUO.001585|Syntomus_truncatellus
?LYFIFGMWAGMVGTSLSILIRAELGNPGALIGDDQIYNVIVTAHAFIMIFFMVMPIMIGGFGNWLVPLMLGAPDMAFPRMNNMSFWLLPPSLTLLLMSSMVESGAGTGWTVYPP?SSGIAHAGASVDLAIFSLHLAGISSILGAVNFITTIINMRSVGI
TFDRMPLFVWSVGITALLLLSLPVLAGAITMLLTDRNLNTSFFDPAGGGDPILYQHL
>COLFC269-12|KJ963763|ZMUO.002454|Carpelimus_pusillus
TLYFIFGAWSGMVGTSLSMLIRIELGTPGSLIGDDQIYNVIVT?HAFIMIFFMVMPIVIGGFGNWLVPLMLGAPDMAFPRMNNMSFWLLPPSLTLLLFSSMVESGAGTGWTVYPPLSSNIAHSGSSVDLAIFSLH?AGISSILGAVNFISTIINMRSIGMTF
DRMPLFVWSVNITAVLLLLSLPVLAGAITMLLTDRNLNTSFFDPAGGGDPILYQHL
>COLFB813-12|KJ961767|ZMUO.001288|Boreostiba_sibirica
TLYF?FGTWAGM?GTSLSLLIRAELGNPGSLIGDDQIYNVIVTAHAFVMIFFMVMPVVIGGFGNWLVPLMLGAPDMAFPRMNNMSFWLLPPSLTLLLMSSMVESGAGTGWTVYPPLSSNIAHGGSSVDLAIFSLHLAGISSILGAVNFISTVINMRSTG
ISFDRMPLFVWSVVITALLLLSLPVLAGAITMLLTDRNLNTSFFDPAGGGDPILYQHL
>COLFC249-12|KJ966964|ZMUO.002434|Cerylon_histeroides
TLYFMFGMWSGMVGTSMSMMIRLELGNPGSLIGDDQIYNVIVTAHAFVMIFFMVMPIMIGGF?NWLVPLMLGAPDMAFPRMNNMSFWLLPPSLTLLIMSSIVEKGAGTGWTVYPPLSANLTHSGSSVDLAIFSLHLAGISSILGAVNFITTVINMRP
SGMTWDRLPLFVWSVIITAVLLLLSLPVLAGAITMLLTDRNLNTSFFDPAGGG?PILYQHL
>COLFD106-12|KJ965082|ZMUO.004001|Hylastes_brunneus
TLYFIF?AWAGMIGTSLSMLIRSELGTPGSLIGDDQLYNTMVTSHAFIMIFFMVMPIMIGGFGNWLIPLMLGAPDMAFPRLNNMSFWLLPPSLTLLLMSSIIDKGAGTGWTVYPPLASNISHEGSSVDLAIFSLHMAGVSSILGAMNFISTIINMYPSGM
KPDSLTLFTWSVKITAILLLLSLPVLA?AITMLLTDRNINTTFFDPSGGGDPILYQHL
>COLFB024-12|KJ963582|ZMUO.001354|Ptinus_villiger
TLYFILGAWSSMLGTSLSLLIRSELGNPGTLIGDDQIYNVIVTAHAFIMIFFMVMPIMIGGFGNWLVPLMLGAPDMAFPRMNNMSFWLLPPALTFLLVGGLV?SGAGTGWTVYPPLAGNIAHSGASVDLTIFSLHLAGVSSI?GAVNFITTVINMRPIEM
TFDRVPLFVWSVVITAVLLLLSLPVLAGAITMLLTDRNLNTSFFDPAGGGDPILYQHL
>COLFB021-12|KJ962813|ZMUO.001351|Hydroglyphus_geminus
TLYFLFGAWSGMVGTSLSMLIRAELGNPGSLIGDDQIYNVIVTAHAFIMIFFMVMPIMIGGFGNWLVPLMLGAPDMAFPRMNNMSFWLLPPSLSLLMMSSMVESGAGTGWTVYPPLSSGIAHGGASVDLAIFSLHLAGISSILGAVNFITTIINMRSV
?MTFDRMPLFVWSVGITALLLLSLPVLA?AITMLLTDRNLNTSFFDPAGGGDPILYQHL
>COLFF600-13|KJ963827|ZMUO.006490|Altica_chamaenerii
TLYFLFGIWAGMIGTSMSLLIRTELGSPGSLIGNDQIYNVIVTAHAFVMIFFMVMP?MIGGFGNWLVPLMIGAPDMAFPRMNNMSFWLL?PSIFLLLMSSFTESGAGTGWTVYPPLSSNLAHNGPSVDLAIFSLHLAGISSILGAINFITTMINMRPQG
MSMDQMPLFVWAVFITAILLLLSLPVLAGAITMLLTDRNLNTSFFEPAGGGDPILYQHL
>COLFD243-12|KJ966340|ZMUO.004138|Bembidion_bruxellense
TLYFIFGAWSGMVGTSLSMLIRAELGN??SLIGDDQIYNVIVTAHAFVMIFFMVMPILIGGFGNWLVPLMLGAPDMAFPRMNNMSFWLLPPSLTLLLMSSMVESGAGTGWTVYPPLSSSIAHSGASVDLAIFSLHLAGVSSILGAVNFITTIINMRSIG
MSFDRMPLFVWSVGITALLLLSLPVLAGAITMLLTDRNLNTSFFDPAGGGDPILYQHL
>COLFA281-12|KJ966206|ZMUO.000416|Syntomus_truncatellus
TLYFIFGMWAGMVGTSLSILIRAELGNPGALIGDDQIYNVIVTAHAFIMIFFMVMPIMIGGFGNW?VPLMLGAPDMAFPRMNNMSFWLLPPSLTLLLMSSMVESGAGTGWTVYPPLSSGIAHAGASVDLAIFSLHLAGISSILGAVNFITTIINMRSVGI
TFDRMPLF?WSVGITALLLLSLPVLAGAITMLLTDRNLNTSFFDPAGGGDPILYQHL
>COLFB189-12|KJ964346|ZMUO.001519|Ampedus_tristis

TLYFIFGAWAGMLGTSLSLLIRAELGNPGSLIGNDQIYNVIVTAHAFIMIFFMVMPIMIGGFGNWLVPLMLGAPDMAFPRMNNMSFWLLPPSLSLLLMSSIVENGAGTGWTVYPPLSSNIAHSGSSVDLAIFSLHLAGISSILGAVNFISTVINMRSTG?T
FDRMPLFVWAVAITALLLLLSLPVLAGAI?MLLTDRNLNTSFFDPAGGGDPILYQHL
>COLFE224-12|KJ963392|ZMUO.005069|Bembidion_dentellum
TLYFIFGAWSGMVGTSLSMLIRAELGNPGSLIGDDQIYNVIVTAHAFIMIFFMVMPILIGGF?NWLVPLMLGAPDMAFPRMNNMSFWLLPPSLSLLLMSSMVESGAGTGWTVYPPLSSSIAHSGASIDLAIFSLHLAGVSSILGAVNFITTIINMRSTGM
TFDRMPLFVWSVGITALLLLLSLPVLAGAITMLLTDRNLNTSFFDPAGGGDPILYQH?
>COLFE252-12|KJ964361|ZMUO.005097|Amara_eurynota
TLYFIFGAWSGMVGTSLSMLIRAELGNP?ALIGDDQIYNVIVTAHAFVMIFFMVMPIMIGGFGNWLVPLMLGAPDMAFPRMNNMSFWLLPPSLTLLLMSSMVESGAGTGWTVYPPLSSGIAHAGASVDLAIFSLHLAGISSILGAVNFITTIINMRSV
GMTFDRMPLFVWSVGITALLLLLSLPVLAGAITM?LTDRNLNTSFFDPAGGGDPILYQHL
>COLFB578-12|KJ964863|ZMUO.001053|Hydroporus_striola
TLYFLFGAWSGM?GTSLSMLIRAELGNPGSLIGDDQIYNVIVTAHAFIMIFFMVMPIMIGGFGNWLVPLMLGAPDMAFPRMNNMSFWLLPPSLTLLLMSSMVENGAGTGWTVYPPLSSGIAHGGASVDLAIF?LHLAGVSSILGAVNFITTIINMRSI
GMTFDRMPLFVWSVGITALLLLLSLPVLAGAITMLLTDRNLNTSFFDPAGGGDPILYQHL
>COLFA688-12|KJ962785|ZMUO.000823|Acupalpus_parvulus
TLYFIFGVWAGMVGTSLSMLIRAELGTPGALIGDDQIYNVIVTAHAFVMIFFMVMPVMIGGFGNWLVPLMLGAPDMAFPRMNNMSFWLLPPSLTLLLMSSLVESGAGTGWTVYPPL?SGIAHSGASVDLAIFSLHLAGVSSILGAVNFITTIINMRSV
GMTFERMPLFVWSVGITALLLLLSLPVLAGAITMLLTDRNLNTSFFD?AGGGDPILYQHL
>COLFB643-12|KJ965454|ZMUO.001118|Carpelimus_corticinus
TLYFIFGAWSGMVGTSLSMLIRIELGTPGSLIGDDQIYNVIVTAHAFIMIFFMVMPIVIGGFGNWLVPLMLGAPDMAFPRMNNMSFWLLPPSLTLLLFSSMVESGAGTGWTVYPPLSSNIAHSGSSVDLAIFSLHLAGVSSILGAVNFISTIINMRSIGMT
FDRMPLFVWSVNITAILLLLSLPVLAGAITMLLTDRNLNTSFFDPAGGG?PIL?QHL
>COLFC279-12|KJ962476|ZMUO.002464|Quedius_boopoides
SLYFIFGAWAGMVGTSLSL?IRAELGNPGSLIGDDQIYNVIVTAHAFIMIFFMV?PTLIGGFGNWLVPLMLGAPDMAFPRMNNMSFWLLPPSLSLLLMSSMVESGAGTGWTVYPPLSSNIAHSGASVDLAIFSLHLAGISSILGAVNFITTVINMRSTGM
TFDRMPLFVWSVAITALLLLLSLPVLAGAITMLLTDRNLNTTFFDPAGGGDPILYQHL
>COLFA329-12|KJ964862|ZMUO.000464|Agonum_piceum
TLYFIFGAWAGMVGTSLSMLIRAELGNPGALIGDDQI?NVIVTAHAFIMIFFMVMPIMIGGFGNWLVPLMLGAPDMAFPRMNNMSFWLLPPSLTLLLMSSLVESGAGTGWTVYPPLSSGIAHAGASVDLAIFSLHLAGVSSILGAVNFITTIINMRSVG
MTFDRMPLFVWSVGITALLLLLS?PVLAGAITMLLTDRNLNTSFFDPAGGGDPILYQHL
>COLFE010-12|KJ962458|ZMUO.005235|Amara_cursitans
TLYFIFGAWSGMVGTSLSMLIRAELGNPGALIGDDQIYNVIVTAHAFVMIFFMVMPIMIGGFGNWLVPLMLGAPDMAFPRMNNMSFWLLPPSLTLLLMSSMVESGAGTGWTIYPPLSSGIAHAGSSVDLAIFSLHLAGISSILGAVNFITTIINMRSVG
MTFDRMPLFVWSVG?TALLLLLSLPVLAGAITMLLTDRNLNTSFFDPAGGG?PILYQHL
>COLFA092-10|HM909108|MP00327|Tetratoma_fungorum
TLYFIFGAWSGMVGTSLSLLIRSELGNPGSLIGDDQIYNVIVTAHAFIMIFFMVMPVMIGGFGNWLVPLMLGAPDMAFPRLNNMSFWLLPPSLTLLIMSSIVENGAGTGWTVYPPLSSNIAHGGSSVDLAIFSLHLAGISSILGAVNFITTVINMRPVGM
TFDRMPLFVWAVVITAVLLLLSLPVLAGAITMLLTDRN?N?SFFDPAGGGDPILYQHL
>COLFA673-12|KJ963977|ZMUO.000808|Carpelimus_pusillus
TLYFIFGAWSGMVGTSLSMLIRIELGTPGSLIGDDQIYNVIVTAHAFIMIFFMVMPIVIGGFGNWLVPLMLGAPDMAFPRMNNMSFWLLPPSLTLLLFSSMVESGAGTGWTVY?PLSSNIAHSGSSVDLAIFSLHLAGISSILGAVNFISTIINMRSIGMTF
DRMPLFVWSVNITAVLLL?SLPVLAGAITMLLTDRNLNTSFFDPAGGGDPILYQHL
>COLFA355-12|KJ967202|ZMUO.000585|Melanapion_minimum
TLYFIFGVWSGLV?TSL?MLIRIELGNPGSLIGDDQIYNVIITAHAFIMIFFMVMPIMIGGFGNWLVPLMLGAPDMAFPRMNNMSFWLLPPSLTLLIMSSIVEKGAGTGWTVYPPLAANIAHSGASVDLAIFSLHLAGISSILGAVNFISTMMNMQPSGL
SLDQLSLFTWAVKITAILLLLSLPVLAGAITMLLTDRNINTSFFDPAGGGDPILYQHL
>COLFB902-12|KJ962746|ZMUO.001947|Poecilium_alni
TLYFILGAWAGMVGTSLSILIRSELGNPGSLIGDDQIYNV?VTAHAFIMIFFMVMPIMIGGFGNWLVPLMLGAPDMAFPRMNNMSFWLLPPSLSLLIMSSVVESGAGTGWTVYPPLSANIAHSGASVDLAIFSLHLAGVSSILGAVNFISTIINMKP?GM
?PEQMPLFVWAVLITAVLLLLSLPVLAGAITMLLTDRNLNTSFFDPAGGGDPILYQHL
>COLFD299-12|KJ962441|ZMUO.004194|Bembidion_bruxellense
TLYFIFGAWSGMVGTSLSMLIRAELGNPGSLIGDDQIYNVIVTAHAFVMIFFMVMPILIGGFGNWLVPLML?APDMAFPRMNNMSFWLLPPSLT?LLMSSMVESGAGTGWTVYPPLSSSIAHSGASVDLAIFSLHLAGVSSILGAVNFITTIINMRSIG
MSFDRMPLFVWSVGITALLLLLSLPVLAGAITMLLTDRNLNTSFFDPA?GGDPILYQHL
>COLFC691-12|KJ964950|ZMUO.003256|Barypeithes_pellucidus
TLYFIFGAWSGM?GTSLS??IRTELGNPGSLIGDDQIYNVIVTAHAFIMIFFMVMPIMIGGFGNWLVPLMLGAPDMAFPRLNNMSFWLLPPSLSLLLMSSIVDKGAGTGWTVYPPLSANIAHEGSSVDLAIFSLHMAGVSSILGAINFISTVINMRPTGM
SMDRMPLFVWAVKLTAILLLLSLPVLAGAITMLLTDRNVNTSFFDPAGGGDPILYQHL
>COLFB452-12|KJ965497|ZMUO.001877|Barypeithes_pellucidus
TLYFIFG?WSGMVGTSLSLLIRTELGNPGSLIGDDQIYNVIVTAHAFIMIFFMVMPIMIGGFGNWLVPLMLGAPDMA?PRLNNMSFWLLPPSLSLLLMSSIVDKGAGTGWTVYPPLSANIAHEGSSVDLAIFSLHMAGVSSI?GAINFISTVINMRPMG
MSMDRMPLFVWAVKLTAILLLLSLPVLAGAITMLLTDRNVNTSFFDPAGGGDPILYQHL

>COLFA424-12|KJ965211|ZMUO.000654|Hydroporus_incognitus
TLYFLF?AWSGMVGTSLSMLIRAELGNPGSLIGD?QIYNVIVTAHAFIMIFFMVMPIMIGGFGNWLVPLMLGAPDMAFPRMNNMSFWLLPPSLTLLLMSSMVENGAGTGWTVYPPLSSGIAHGGASVDLAIFSLHLAGVSSILGAVNFITTIINMRSIG
MTFDRMPLFVWSVGITALLLLLSLPVLAGAITMLLTDRNLNTSFFDPAGGG?PILYQHL
>COLFB186-12|KJ964318|ZMUO.001516|Ampedus_balteatus
TLYFIFGAWAGMLGTSLSLLIRAELGNPGSLIGNDQIYNVIVTAHAFIMIFFMVMPIMIGGFGNWLVPLMLGAPDMAFPRMNNMSFWLLPPSLSLLLMSSIVENGAGTGWTVYPPLSSNIAHSGSSV?LAIFSLHLAGI?SILGAVNFISTVINMRSTGITF
DRMPLFVWAVAITALLLLLSLPVLAGAITMLLTDRNLNTSFFDPAGGGD?ILYQHL
>COLFE412-12|KJ961901|ZMUO.004972|Microcara_testacea
TLYFIFGSWAGMVGTSLSLLIRAELGTPGSLIGDDQIYNVIVTAHAFIMIFFMVMPIMIGGF?NWLVP?MLGAPDMAFPRMNNMSFWLLPPSLTLLLMSSMVENGAGTGWTV?PPLSAGMAHSGASVDLAIFSLHLAGISSILGAVNFISTVINMRSS
GMTFDRMPLFVWSVAITALLLLLSLPVLAGAITMLLTDRNLNTSFFDPAGGGDPILYQHL
>COLFB644-12|KJ965705|ZMUO.001119|Carpelimus_corticinus
TLYFIFGAWSGMVGTSLSMLIRIELGTPGSLIGDDQIYNVIVTAHAFIMIFFMVMPIVIGGFGNWLVPLMLGAPDMAFPRMNNMSFWLLPPSLTLLLFSSMVES?AGTGWTVYPPLSSNIAHSGSSVDLAIFSLHLAGVSSILGAVNFISTIINMRSIGMTF
DRMPLFVWSVNITA?LLLLSLPVLAGAITMLLTDRNLNTSFFDPAGGGDPIL?QHL
>COLFF667-13|KJ963950|ZMUO.006367|Sospita_vigintiguttata
TLYFLFGMWAGMVGTSLSILI?LELGT?NSLIGNDQIYNVIVTAHAFIMIFFMVMPIMIGGFGNWLVPLMIGAPDMAFPRLNNMSFWLLPPALTLLIFSSMVEMGAGTGWTVYPPLSSNMAHSGSSVDLVIFSLHLAGISSILGAVNFISTIMNMRPFG
MNLDKTPLFVWSVLITAILLLLSLPVLAGAITMLLTDRNINTSFFDP?GGGDPILYQHL
>COLFC277-12|KJ963962|ZMUO.002462|Quedius_boopoides
SLYFIFGAWAGMVGTSLS?LIRAELGNPGSLIGDDQIYNVIVTAHAFIMIFFMV?PTLIGGFGNWLVPLMLGAPDMAFPRMNNMSFWLLPPSLSLLLMSSMVESGAGTGWTVYPPLSSNIAHSGASVDLAIFSLHLAGISSILGAVNFITTVINMRSTGM
TFDR?PLFVWSVAITALLLLLSLPVLAGAITMLLTDRNLNTTFFDPAGGGDPILYQHL
>COLFB899-12|KJ967320|ZMUO.001944|Leiopus_nebulosus
TLYFIFGAWAGMVGTSLSMLIRSELG?AGSLIGDDQIYNAIVTAHAFVMIFFMVMPIMIGGFGNWLVPLMLGAPDMAFPRMNNMSFWLLPPSLTLLIMSSVVNSGAGTGWTVYPPLSSNIAHAGPSVDLAIFSLHLAGASSILGAVNFITTVINMRPK
GMSFDRLPLFVWAVKITTILLLLSLPVLAGAITM?LTDRNLNTSFFDPAGGGDPVLYQHL
>COLFB138-12|KJ965736|ZMUO.001468|Hypocaccus_rugifrons
TLYFIFGMWAGMVGTSLSLLIRAELGNPGSLIGDDQIYNVIVTSHAFIMIFFMVMPIMIGGFGNWLVPLMLGAPDMAFPRMNNMSFWFLPPSLTLLLMSSMVESGAGTGWTVYPPLSSNIAHSGASVDLAIFSLH?AGISSILGAVNFITTVINMRSNG
MKFDQMPLFVWSVSITALLLLLSLPVLAGAITMLLTDRNINTSFF?PAGGG?PILYQHL
>COLFE073-12|KJ965461|ZMUO.005298|Dasytes_plumbeus
TLYFIFGAWSGMVGMSLSLLIRSELNNPGTLIGNDQIYNVIVTAHAFIMIFFMVMPILIGGF?NWLVPLMLGAPDMA?PRMNNMSFWLLPPSLTLLLMSSMVEQGAGTGWTVYPPLSANIAHGGASVDLAIFSLHLAGISSILGAVNFITTVINMRPIG
MTLDRTPLF?WAVAITALLLLLSLPVLAGAITMLLTDRNLNTSFFDPAGGGDPILYQHL
>COLFE1610-13|KJ966400|ZMUO.007500|Brachyderes_incanus
TLYFIFGAWSGMVGTSLSM?IRTELGNPGSLIGDDQIYNVIVTAHAFIMIFFMVMPMMIGGFGNWLV?LMLGAPDMAFPRMNNMSFWLLPPSLSLLLMSSIVDK?AGTGWTVYPPLSANIAHEGSSVDLAIFSLHMAGVSSILGAINFISTVINMRP
MGM?PDRMSLFIWAVKITAVLLLLSLPVLAGAITMLLTDRNVNTSFFDPAGGGDPILYQHL
>COLFD949-12|KJ964473|ZMUO.004559|Otiorhynchus_ovatus
TLYFIF?AWAGMVGTSLSMLIRTELGNPGSLIGDDQIYNVIVTAHAFIMIFFMVMPMMIG?FGNWLVPLMLGAPDMAFPRMNNMSFWLL?PSLTLLLTSSIVDKGAGTGWTV?PPLSANIAHEGASVDLAIFSLHMAGVSSILGAINFISTMANMRPY
GMSPDRMSLFIWAVKITAILLLLSLPVLAGAITMLLTDRNINTSFFDPAGGGDPILYQHL
>COLFF680-13|KJ965187|ZMUO.006380|Altica_chamaenerii
TLYFLFGIWAGMIGTSMSLLIRTELGSP?SLIGNDQIYNVIVTAHAFVMIFFMVMPMMIGGFGNW?VPLMIGAPDMAFPRMNNMSFWLL?PSIFLLLMSSFTESGAGTGWTVYPPLSSNLAHNGPSVDLAIFSLHLAGISSILGAINFITTMINMRPQG
?SMDQMPLFVWAVFITAILLLLSLPVLAGAITMLLTDRNLNTSFFEPAGGGDPILYQHL
>COLFF791-13|KJ964883|ZMUO.006111|Altica_engstromi
TLYFLFGIWAGMIGTSMSLLIRTELG?PGSLIGNDQIYNVIVTAHAFVMIFFMVMPMMIGGFGNWLVPLMIGAPDMAFPRMNNMSFWLL?PSIFLLLMSSFTESGAGTGWTVYPPLSSNLAHNGPSVDLAIFSLHLAGI?SILGAINFITTMI?MRPQG
MSMDQMPLFVWAVFITAILLLLSLPVLAGAITMLLTDRNLNTSFFEPAGGGDPILYQHL
>COLFB273-12|KJ966878|ZMUO.001603|Paederus_riparius
TLYFIFGAWSGMVGTSLSLLIRAELATPGSLIGDDQIYNVIVTAHAFIMIFFMVMPIMIGGFGNWLVPLMLGAPDMAFPRMNNMSFWLLPPALTLLLMSSMVENGAGTGWTVYPPLSSN?FHNGSSVDLAIFSLH?AGISSILGAINFI?TALNMRTSN
M?YEQMPLFVWSVAITALLLLLSLPVLAGAITMLLTDRNLNTSFFDPSGGGDPILYQHL
>COLFE1592-13|KJ964027|ZMUO.007482|Acrotona_pilosicollis
TLYFVFGTWAGMV?TSLSLLIRAELGNPGSLIGDDQIYNVIVTAHAFVMIFFMVMPIVIGGFGNWLIPLMLGAPDMAFPRMNNMSFWLLPPSLTLLLMSSMVESGAGTGWTVYPPLSSNIAHGGSSVDLAIFSLHLA?ISSILGAVNFISTVINMRSTGIS
FDRMPLFVWSVAITA?LLLLSLPVLAGAITMLLTDRNLNTSFFDPAGGG?PILYQHL
>COLFB901-12|KJ962401|ZMUO.001946|Poecilium_alni

TLYFI?GAWAGMVGTSLSILIRSELGNPGSLIGDDQIYNV?VTAHAFIMIFFMVMPIMIGGFGNWLVPLMLGAPDMAFPRMNNMSFWLLPPSLSLLIMSSVVESGAGTGWTVYPPLSANIAHSGASVDLAIFSLHLAGVSSILGAVNFISTIINMKP?GM
?PEQMPLFVWAVLITAVLLLLSLPVLAGAITMLLTDRNLNTSFFDPAGGGDPILYQHL
>COLFB851-12|KJ961749|ZMUO.001326|Zorochros_minimus
TLYFLFGAWAGMLGTSLSLLIRAELGNPGSLIGNDQ?YNVIVTAHAFIMIFFMVMPIMIGGFGNWLVPLMLGAPDMAFPRMNN?SFWFLPPSLSLLLMSSIVENGAGTGWTVYPPLSANIAHSGSSVDLAIFSLHLAGISSILGAVNFISTVINMRSTGIT
FDRMPLFVWAVAITALLLLLSLPVLAGAITMLLTDRN?NTSFF?PAGGGDPILYQHL
>COLFB637-12|KJ962044|ZMUO.001112|Bembidion_transparens
TLYFIFGAWSGMVGTSLSMLIRAELGNPGSLIGDDQIYNVIVTAHAFVMIFFMVMPILIGGFGNWLVPLMLGA?DM?FPRMNNMSFWLLPPSLTLLLMSSMVENGAGTGWTVY??LSSSIAHSGASVDLAIFSLHLAGVSSILGAVNFITTIINMRSMG
MTFDRMPLFVWSVGITALLLLLSLPVLAGAITMLLTDRNLNTSFFDPAGGGDPILYQHL
>COLFB832-12|KJ962485|ZMUO.001307|Megarthrus_depressus
TLYFIFGAWAGMIGTSLSILIRAELGNPGSLIGDDQIYNVIVTAHAFIMIFFMVMPIVIGGFGNWLVPLMLGAPDMAFPRMNNMSFWLLPPSISLLLISSMVESGA?TGWTVYPPLSSNIAHGGASVDLAIFSLHLAGISSILGAVNFITTVIN?RSMGMTF
DR?PLFVWSVAITALLLLLSLPVLAGAITMLLTDRNINTSFFDPAGGG?PILYQHL
>COLFF960-13|KJ967259|ZMUO.007325|Stenus_excubitor
TLYFIFGAWAGMVGTSLSLLIRAELGNPGSLIGDDQIYNVIVTAHAFVMIFFMVMPVMIGGFGNWLVPLMLGAPDMAFPRMNN?SFWLLPPSLSLLLMSSIVESGAGTGWTVYPPLSSNIAHGGASVDLAIFSLHLAGISSILGAINF?TTIFNMRSMKI
QLDCLPLFVWSVGITALLLLLSLPVLAGAITMLLTDRNLNTSFF?PAGGGD?ILYQHL
>COLFA317-12|KJ962623|ZMUO.000452|Cytilus_auricomus
TLYFIFGAWAGMVGTSLSLLIRAELGNPGSLIGDDQIYNVIVTAHAFVMIFFMVMPIMVGGFGNWLVPLMLGAPDMAFPRMNNMSFWLLPPSLSLLLMSSIVESGAGTGWTVYPPLSSNIAHSG?SVDLAIFSLHLAGISSILGA?NFISTVINMRSPG
MKFDQMSLFSWSVAITALLLLLSLPVLAG?ITMLLTDRNINTSFF?PAGG?DPILYQHL
>COLFF792-13|KJ962965|ZMUO.006112|Altica_engstromi
TLYFLFGIWAGMIGTSMSLLIRTELGSPGSLIGNDQIYNVIVTAHAFVMIFFMVMPMMIGGFGNWLVPLMIGAPDMAFPRMNNMSFWLLPPSIFLLLMSS?TESGAGT?WTVYPPLSSNLAHNGPSVDLAIFSLHLAGI?SIL?AINFITTMI?MRPQG
MSMDQMPLFVWAVFITAILLLLSLPVLAGAITMLLTDRNLNTSFFEPAGGGDPILYQHL
>COLFC556-12|KJ963905|ZMUO.003121|Rhantus_suturellus
TLYFIFGAWAGMVGTSLSMLIRAELGNPGSLIGDDQIYNVIVTAHAFVMIFFMVMPIMIGGFGNWLVPLMLGAPDMAFPRMNNMSFWLLPPSLTLLL?SSMV?SGAGTGWTVYPPLSSGIAHG?ASVDLAIFSLHLAGISSILGAVNFITTIINMRSVG
MTFDRMPLFVWSVGITALLLLLSLPVLAGAITMLLTDRNLNTSFFDPAG?GDPILYQ?L
>COLFB656-12|KJ963531|ZMUO.001131|Hydroporus_gyllenhalii
TLYFLFGAWSGMVGTSLSMLIRAELGNPGSLIGDDQIYNVIVTAHAFIMIFFMVMPIMIGGFGNWLVPLMLGAPDMAFPRMNNMSFWLLPPSLTLLLMSSMVENGAGT?WTVYPPLSSGIAHSGASVDLAIFSLHLAGVSSILGAVNFITTIINMRSIG
MTF?RMPLFVWSVGI?ALLLL?SLPVL?GAITMLLTDRNLNTSFFDPAGGGDPILYQHL
>COLFG040-13|KJ962567|ZMUO.007545|Rutpela_maculata
TLY?IFGAWASMVGT?LSLLIRSELGNPGSLIGDDQIYNVIVTAHAFVMIFFMVMPIMIGGFGNWLVPLMLG?PDMAFPRMNNMSFWLL?PSLTLLIMSSIVESGAGTGWTVYPPLSSNIAHGGSSVDLAIFSLHLAGISSIL?AVNFITTVINMRPKGMS
PDRMPLFVWAVVITAVLLLLSLPVLAGAITMLLTDRNLNTSFFDPAGGGDPILYQHL
>COLFB605-12|KJ962300|ZMUO.001080|Stenus_clavicornis
TLYFIFGSWAGMVGTSLSMLIRSELGSPGSLIGDDQIYN?IVTAHAFIMIFFMVMPIMIGGFGNWLVPLMLGAPDMAFPRMNNMSF?LLPPSLSLLLMSSIVESGAGTGWTVYPPLS?NIAHSGASVDLAIFSLHLAGISSILGAINFI?TIINMRTMKMQ
LDCLPLFVWS?SITALLLLLSLPVLAGAITMLLTDRNLNTSFFDPAGGGDPILYQHL
>COLFF790-13|KJ962940|ZMUO.006110|Altica_engstromi
TLYFLFGIWAGMIGT?MSLLIRTELGSPGSLIGNDQIYNVIVTAHAFVMIFFMVMPMMIGGFGNWLVPLMIGAPD?AFPRMNNMSFWLL?PSIFLLLMSSFTESGAGTGWTVYPPLSSNLAHNGPSVDLAIFSLHLAGI?SILGAINFITTMI?MRPQG
MSMDQMPLFVWAVFITAILLLLSLPVLAGAITMLLTDRNLNTSFFEPAGGGDPILYQHL
>COLFD950-12|KJ967513|ZMUO.004560|Otiorhynchus_ovatus
TLYFIF??WAGMVGTSLSML?RTELGNPGSLIGDDQIYNVIVTAHAFIMIFFMVMPMMIGGFGNWLVPLMLGAPDMAFPRMNNMSFWLLPPSLTLLLTSSIVDKGA?TGWTVYPPLSANIAHEGASVDLAIFSLHMAGVSSILGAINFISTMANMRP
YGMSPDRMSLFIWAVKITAILLLLSLPVLAGAITMLLTDRNINTSFFDPAGGGDPILYQHL
>COLFA346-12|KJ962181|ZMUO.000576|Bembidion_transparens
TLYFIFGAWSGMVGTSLSMLIRAELGNPGSLIGDDQI?NVIVTAHAFVMIFFMVMPILIGGFGNWLVPLMLGAPDM?FPRMNNMSFWLLPPSLTLLLMSSMVENGAGTGWTVY??LSSSIAHSGASVDLAIFSLHLAGVSSILGAVNFITTIINMRSMG
MTFDRMPLFVWSVGITALLLLLSLPVLAGAITMLLTDRNLNTSFFDPAGGG?PIL?QHL
>COLFF055-13|KJ964600|ZMUO.005755|Chaetocnema_hortensis
TLYFIFGIWSGMVGTSMSILIRAE?GSPGSLIGNDQIYNVIVTAHAFVMIF?MVMPIMI?GFGNWLVPLMIGAPDMAFPRMNNMSFWLLPPSLFLLLMSSLVESGAGTGWTVYPPLSSNIAHGGSSVD?AIFSLHLAGVSSILGAINFITTIINMRPQGM
SFDQM?LFVWAVLITAILLLLSLPVLAGAITMLLTDRNLNTSFF?PIGGGDPILYQHL
>COLFC225-12|KJ962947|ZMUO.002410|Euconnus_hirticollis
TLYFIFGIWSGLIGSSLSMLIRSELGFPGSLIGNDQIYNVIVTAHAFIMIFFMVMPIMIGGFGNWL?PLMLGAPDMAFPRMNNMSFWLLPPSLLLLLLSSLIESGSGTGWTVYP?LSSNIAHSGASVDLTIFSLHMAGISSI?GAINFITTIINMR?PGMKYD
MMPLFIWAVMITAFLLLLSLPVLAGAITMLLTDRNLNTSFFDPSGGG?PIL?QHL

Amino acid alignment of the analyzed Lepidoptera DNA barcodes (Dataset 3, FASTA format)

>LEFIC261-10|HM872105|MM03692|Abraxas_grossulariata
TLYFIFGIWAGMLGTSLSLLIRAELGNPGSLIGDDQIYNTIVTAHAFIMIFFMVMPIMIGGFGNWLIPLMLGAPDMAFPRMNNMSFWLLPPSITLLISSSIVENGAGTGWTVYPPLSSNIAHSGSSVDLAIFSLHLAGISSILGAINFITTIINMRLNNLSFDQ
MPLFIWAVGITAFLLLLSLPVLAGAITMLLTDRNLNTSFFDPAGGGDPISYQHL
>LEFIC262-10|HM872106|MM03693|Abraxas_grossulariata
TLYFIFGIWAGMLGTSLSLLIRAELGNPGSLIGDDQIYNTIVTAHAFIMIFFMVMPIMIGGFGNWLIPLMLGAPDMAFPRMNNMSFWLLPPSITLLISSSIVENGAGTGWTVYPPLSSNIAHGGSSVDLAIFSLHLAGISSILGAINFITTIINMRLNNLSFDQ
MPLFVWAVGITAFLLLLSLPVLAGAITMLLTDRNLNTSFFDPAGGGDPILYQHL
>LEFIA249-10|HM386593|MM01282|Abraxas_sylvata
TLYFIFGIWAGMLGTSLSLLIRAELGNPGSLIGDDQIYNTIVTAHAFIMIFFMVMPIMIGGFGNWLIPLMLGAPDMAFPRMNNMSFWLLPPSITLLISSSIVENGAGTGWTVYPPLSSNIAHGGSSVDLAIFSLHLAGISSILGAINFITTIINMRLNNLSFDQ
MPLFVWAVGITAFLLLLSLPVLAGAITMLLTDRNLNTSFFDPAGGGDPILYQHL
>LEFIA250-10|HM386594|MM01283|Abraxas_sylvata
TLYFIFGIWAGMLGTSLSLLIRAELGNPGSLIGDDQIYNTIVTAHAFIMIFFMVMPIMIGGFGNWLIPLMLGAPDMAFPRMNNMSFWLLPPSITLLISSSIVENGAGTGWTVYPPLSSNIAHGGSSVDLAIFSLHLAGISSILGAINFITTIINMRLNNLSFDQ
MPLFVWAVGITAFLLLLSLPVLAGAITMLLTDRNLNTSFFDPAGGGDPILYQHL
>LEFIA400-10|HM386742|MM01459|Abraxas_sylvata
TLYFIFGIWAGMLGTSLSLLIRAELGNPGSLIGDDQIYNTIVTAHAFIMIFFMVMPIMIGGFGNWLIPLMLGAPDMAFPRMNNMSFWLLPPSITLLISSSIVENGAGTGWTVYPPLSSNIAHGGSSVDLAIFSLHLAGISSILGAINFITTIINMRLNNLSFDQ
MPLFVWAVGITAFLLLLSLPVLAGAITMLLTDRNLNTSFFDPAGGGDPILYQHL
>LEFIJ165-10|KM573065|MM10848|Abraxas_sylvata
TLYFIFGIWAGMLGTSLSLLIRAELGNPGSLIGNDQIYNTIVTAHAFIMIFFMVMPIMIGGFGNWLIPLMLGAPDMAFPRMNNMSFWLLPPSITLLISSSIVENGAGTGWTVYPPLSSNIAHGGSSVDLAIFSLHLAGISSILGAINFITTIINMRLNNLSFDQ
MPLFVWAVGITAFLLLLSLPVLAGAITMLLTDRNLNTSFFDPAGGGDPILYQHL
>LEFIE433-10|HM874157|MM09179|Abrostola_asclepiadis
TLYFIFGIWAGMVGTSLSLLIRAELGTPGSLIGDDQIYNTIVTAHAFIMIFFMVMPIMIGGFGNWLIPLMLGAPDMAFPRMNNMSFWLLPPSLTLLISSSIVENGAGTGWTVYPPLSSNIAHSGSSVDLAIFSLHLAGISSILGAINFITTIINMRLNKMSFD
QMPLFIWAVSITAFLLLLSLPVLAGAITMLLTDRNLNTSFFDPAGGGDPILYQHL
>LEFIB314-10|HM871214|MM00842|Abrostola_tripartita
TLYFIFGIWAGMVGTSLSLLIRAELGTPGSLIGDDQIYNTIVTAHAFIMIFFMVMPIMIGGFGNWLVPLMLGAPDMAFPRMNNMSFWLLPPSLTLLISSSIVENGAGTGWTVYPPLSSNIAHGGSSVDLAIFSLHLAGISSILGAINFITTIINMRLNNLSFD
QMPLFIWAVGITAFLLLLSLPVLAGAITMLLTDRNLNTSFFDPAGGGDPILYQHL
>LEFIA205-10|HM396549|MM01228|Abrostola_tripartita
TLYFIFGIWAGMVGTSLSLLIRAELGTPGSLIGDDQIYNTIVTAHAFIMIFFMVMPIMIGGFGNWLVPLMLGAPDMAFPRMNNMSFWLLPPSLTLLISSSIVENGAGTGWTVYPPLSSNIAHGGSSVDLAIFSLHLAGISSILGAINFITTIINMRLNNLSFD
QMPLFIWAVGITTFLLLLSLPVLAGAITMLLTDRNLNTSFFDPAGGGDPILYQHL
>LEFIA215-10|HM396558|MM01244|Abrostola_triplasia
TLYFIFGIWAGMVGTSLSLLIRAELGTPGSLIGDDQIYNTIVTAHAFIMIFFMVMPIMIGGFGNWLIPLMLGAPDMAFPRMNNMSFWLLPPSLTLLISSSIVENGAGTGWTVYPPLSSNIAHGGSSVDLAIFSLHLAGISSILGAINFITTIINMRLNSLSFDQ
MPLFIWAVGITAFLLLLSLPVLAGAITMLLTDRNLNTSFFDPAGGGDPILYQHL
>LEFIA216-10|HM396559|MM01245|Abrostola_triplasia
TLYFIFGIWAGMVGTSLSLLIRAELGTPGSLIGDDQIYNTIVTAHAFIMIFFMVMPIMIGGFGNWLIPLMLGAPDMAFPRMNNMSFWLLPPSLTLLISSSIVENGAGTGWTVYPPLSSNIAHGGSSVDLAIFSLHLAGISSILGAINFITTIINMRLNSLSFDQ
MPLFIWAVGITAFLLLLSLPVLAGAITMLLTDRNLNTSFFDPAGGGDPILYQHL
>LEFIE152-10|HQ570358|MM08398|Acanthopsyche_atra
TLYFILGIWSGLIGTSLSFLIRAELSTPDSLIGSDQIYNTIVTAHAFIMIFFMVMPIMIGGFGNWLVPLMLGAPDMAFPRMNNMSFWLLPPSLMLLTASSFVENGAGTGWTVYPPLSSNISHSGSSVDLAIFSLHLAGISSILGAINFITTAINMRMNGMSL
DQMPLFVWSVIITAVLLLLSLPVLAGAITMLLTDRNLNTSFFDPAGGGDPILYQHL
>LEFIG123-10|HM875803|MM13930|Acanthopsyche_atra
TLYFILGIWSGLIGTSLSFLIRAELSTPDSLIGSDQIYNTIVTAHAFIMIFFMVMPIMIGGFGNWLVPLMLGAPDMAFPRMNNMSFWLLPPSLMLLTASSFVENGAGTGWTVYPPLSSNISHNGSSVDLTIFSLHLAGISSILGAINFITTAINMRMNGMSL
DQMPLFVWSVIITAVLLLLSLPVLAGAITMLLTDRNLNTSFFDPAGGGDPILYQHL
>LEFIB142-10|HM871047|MM00521|Acasis_appensata
TLYFIFGIWAGMVGTSLSLLIRAELGNPGSLIGDDQIYNTIVTAHAFIMIFFMVMPIMIGGFGNWLVPLMLGAPDMAFPRMNNMSFWLLPPSITLLISSSIVENGAGTGWTVYPPLSSNIAHGGSSVDLAIFSLHLAGISSILGAINFITTIINMRLNNMFFD
QLPLFVWAVGITAFLLLLSLPVLAGAITMLLTDRNLNTSFFDPAGGGDPILYQHL
>LEFIG970-10|HM876610|MM15834|Acasis_appensata
TLYFIFGIWAGMVGTSLSLLIRAELGNPGSLIGDDQIYNTIVTAHAFIMIFFMVMPIMIGGFGNWLVPLMLGAPDMAFPRMNNMSFWLLPPSITLLISSSVVENGAGTGWTVYPPLSSNIAHGGSSVDLAIFSLHLAGISSILGAINFITTIINMRLNNMFF
DQLPLFVWAVGITAFLLLLSLPVLAGAITMLLTDRNLNTSFFDPAGGGDPILYQHL

>LEFIC356-10|HM872199|MM03876|Acasis_viretata
TLYFIFGIWAGMVGTSLSLLIRAELGNPGSLIGDDQIYNTIVTAHAFIMIFFMVMPIMIGGFGNWLVPLMLGAPDMAFPRMNNMSFWLLPPSITLLISSSIVENGAGTGWTVYPPLSSNIAHGGSSVDLAIFSLHLAGISSILGAINFITTIINMRLNNMFFD
QLPLFVWAVGITAFLLLLSLPVLAGAITMLLTDRNLNTSFFDPAGGGDPILYQHL
>LEFIF045-10|HM874756|MM10485|Acasis_viretata
TLYFIFGIWAGMVGTSLSLLIRAELGNPGSLIGDDQIYNTIVTAHAFIMIFFMVMPIMIGGFGNWLVPLMLGAPDMAFPRMNNMSFWLLPPSITLLISSSIVENGAGTGWTVYPPLSSNIAHGGSSVDLAIFSLHLAGISSILGAINFITTIINMRLNNMFFD
QLPLFVWAVGITAFLLLLSLPVLAGAITMLLTDRNLNTSFFDPAGGGDPILYQHL
>LEFIE602-10|HM874325|MM09515|Acentria_ephemerella
TLYFIFGIWAGMVGTSLSLLIRAELGNPGSLIGDDQIYNTIVTAHAFIMIFFMVMPIMIGGFGNWLVPLMLGAPDMAFPRMNNMSFWLLPPSLTLLISSSIVENGAGTGWTVYPPLSSNIAHSGSSVDLAIFSLHLAGISSILGAINFITTIINMRINNLSFD
QMPLFVWAVGITALLLLLSLPVLAGAITMLLTDRNLNTSFFDPAGGGDPILYQHL
>LEFIA845-10|HM386985|MM09721|Acentria_ephemerella
TLYFIFGIWAGMVGTSLSLLIRAELGNPGSLIGDDQIYNTIVTAHAFIMIFFMVMPIMIGGFGNWLVPLMLGAPDMAFPRMNNMSFWLLPPSLTLLISSSIVENGAGTGWTVYPPLSSNIAHSGSSVDLAIFSLHLAGISSILGAINFITTIINMRINNLSFD
QMPLFVWAVGITALLLLLSLPVLAGAITMLLTDRNLNTSFFDPAGGGDPILYQHL
>LEFIK820-10|MM18395|Acentria_ephemerella
TLYFIFGIWAGMVGTSLSLLIRAELGNPGSLIGDDQIYNTIVTAHAFIMIFFMVMPIMIGGFGNWLVPLMLGAPDMAFPRMNNMSFWLLPPSLTLLISSSIVENGAGTGWTVYPPLSSNIAHSGSSVDLAIFSLHLAGISSILGAINFITTIINMRINNLSFD
QMPLFVWAVGITALLLLLSLPVLAGAITMLLTDRNLNTSFFDPAGGGDPILYQHL
>LEFIC311-10|HM872155|MM03813|Acerbia_alpina
TLYFIFGVWAGMVGTSLSLLIRAELGNPGSLIGDDQIYNTIVTAHAFIMIFFMVMPIMIGGFGNWLVPLMLGAPDMAFPRMNNMSFWLLPPSLTLLISSSIVENGAGTGWTVYPPLSSNIAHGGSSVDLAIFSLHLAGISSILGAINFITTIINMRLNNLSF
DQMPLFVWSVGITAFLLLLSLPVLAGAITMLLTDRNLNTSFFDPAGGGDPILYQHL
>LEFIB796-10|HM871673|MM02708|Acherontia_atropos
TLYFIFGIWAGMVGTSLSLLIRAELGNPGSLIGDDQIYNTIVTAHAFIMIFFMVMPIMIGGFGNWLVPLMLGAPDMAFPRMNNMSFWLLPPSLLLLISSSIVENGAGTGWTVYPPLSSNIAHSGSSVDLAIFSLHLAGISSILGAINFITTIINMRINNMFFD
QMPLFVWAVGITAFLLLLSLPVLAGAITMLLTDRNLNTSFFDPAGGGDPILYQHL
>LEFIA455-10|HM386795|MM01520|Achlya_flavicornis
TLYFIFGIWAGMVGTSLSLLIRAELGNPGSLIGDDQIYNTIVTAHAFIMIFFMVMPIMIGGFGNWLVPLMLGAPDMAFPRMNNMSFWMLPPSLTLLISSSIVENGAGTGWTVYPPLSSNIAHGGSSVDLAIFSLHLAGISSILGAINFITTIINMRLNNMSF
DQMPLFVWAVGITAFLLLLSLPVLAGAITMLLTDRNLNTSFFDPAGGGDPILYQHL
>LEFIF207-10|HM874901|MM10869|Achlya_flavicornis
TLYFIFGIWAGMVGTSLSLLIRAELGNPGSLIGDDQIYNTIVTAHAFIMIFFMVMPIMIGGFGNWLVPLMLGAPDMAFPRMNNMSFWMLPPSLTLLISSSIVENGAGTGWTVYPPLSSNIAHGGSSVDLAIFSLHLAGISSILGAINFITTIINMRLNNMSF
DQMPLFVWAVGITAFLLLLSLPVLAGAITMLLTDRNLNTSFFDPAGGGDPILYQHL
>LEFIK043-10|JF853921|MM17618|Achroia_grisella
TLYFIFGIWSGMVGTSLSLLIRAELGNPGSLIGDDQIYNTIVTGHAFIMIFFMVMPIMIGGFGNWLVPLMLGAPDMAFPRLNNMSFWLLPPSLTLLIFSSIVENGAGTGWTVYPPLSSNIAHSGSSVDLAIFSLHLAGISSILGAVNFITTMINMKLNGLSFD
QMPLFIWSVSITALLLLLSLPVLAGAITMLLTDRNLNTSFFDPAGGGDPILYQHL
>LEEUA206-11|JN286422|MM19614|Acleris_abietana
TLYFIFGIWAGMVGTSLSLLIRAELGNPGSLIGDDQIYNTIVTAHAFIMIFFMVMPIMIGGFGNWLVPLMLGAPDMAFPRMNNMSFWLLPPSIMLLISSSIVENGAGTGWTVYPPLSSNIAHSGSSVDLAIFSLHLAGISSILGAVNFITTIINMRPNNMSL
DQMPLFVWAVGITALLLLLSLPVLAGAITMLLTDRNLNTSFFDPAGGGDPILYQHL
>LEFIB473-10|HM871372|MM02024|Acleris_aspersana
TLYFIFGIWAGMVGTSLSLLIRAELGNPGSLIGDDQIYNTIVTAHAFIMIFFMVMPIMIGGFGNWLVPLMLGAPDMAFPRMNNMSFWLLPPSIMLLISSSIVENGAGTGWTVYPPLSSNIAHSGSSVDLTIFSLHLAGISSILGAVNFITTIINMRPNNMSL
DQMPLFIWAVGITALLLLLSLPVLAGAITMLLTDRNLNTSFFDPAGGGDPILYQHL
>LEFIF898-10|HM875580|MM13312|Acleris_aspersana
TLYFIFGIWAGMVGTSLSLLIRAELGNPGSLIGDDQIYNTIVTAHAFIMIFFMVMPIMIGGFGNWLVPLMLGAPDMAFPRMNNMSFWLLPPSIMLLISSSIVENGAGTGWTVYPPLSSNIAHSGSSVDLTIFSLHLAGISSILGAVNFITTIINMRPNNMSL
DQMPLFIWAVGITALLLLLSLPVLAGAITMLLTDRNLNTSFFDPAGGGDPILYQHL
>LEFIB463-10|HM871362|MM02005|Acleris_bergmanniana
TLYFIFGIWAGMVGTSLSLLIRAELGNPGSLIGDDQIYNTIVTAHAFIMIFFMVMPIMIGGFGNWLVPLMLGAPDMAFPRMNNMSFWLLPPSIMILISSSIVENGAGTGWTVYPPLSSNIAHSGSSVDLAIFSLHLAGISSILGAVNFITTIINMRPNNMSL
DQMPLFVWAVGITALLLLLSLPVLAGAITMLLTDRNLNTSFFDPAGGGDPILYQHL
>LEFIB474-10|HM871373|MM02026|Acleris_comariana
TLYFIFGIWAGMVGTSLSLLIRAELGNPGSLIGDDQIYNTIVTAHAFIMIFFMVMPIMIGGFGNWLVPLMLGAPDMAFPRMNNMSFWLLPPSIMLLISSSIVENGAGTGWTVYPPLSSNIAHSGSSVDLAIFSLHLAGISSILGAVNFITTIINMRPNNMSL
DQMPLFVWAVGITALLLLLSLPVLAGAITMLLTDRNLNTSFFDPAGGGDPILYQHL
>LEFIC678-10|HM872499|MM04646|Acleris_comariana
TLYFIFGIWAGMVGTSLSLLIRAELGNPGSLIGDDQIYNTIVTAHAFIMIFFMVMPIMIGGFGNWLVPLMLGAPDMAFPRMNNMSFWLLPPSIMLLISSSIVENGAGTGWTVYPPLSSNIAHSGSSVDLAIFSLHLAGISSILGAVNFITTIINMRPNNMSL

DQMPLFVWAVGITALLLLLSLPVLAGAITMLLTDRNLNTSFFDPAGGGDPILYQHL
>LEFIE062-10|HM873809|MM08189|Acleris_effractana
TLYFIFGIWAGMVGTSLSLLIRAELGNPGSLIGDDQIYNTIVTAHAFIMIFFMVMPIMIGGFGNWLVPLMLGAPDMAFPRMNNMSFWLLPPSIMLLISSSIVENGAGTGWTVYPPLSSNIAHSGSSVDLAIFSLHLAGISSILGAVNFITTIINMRPNNMSL
DQMPLFVWSVGITALLLLLSLPVLAGAITMLLTDRNLNTSFFDPAGGGDPILYQHL
>LEFIE063-10|HM873810|MM08190|Acleris_effractana
TLYFIFGIWAGMVGTSLSLLIRAELGNPGSLIGDDQIYNTIVTAHAFIMIFFMVMPIMIGGFGNWLVPLMLGAPDMAFPRMNNMSFWLLPPSIMLLISSSIVENGAGTGWTVYPPLSSNIAHSGSSVDLAIFSLHLAGISSILGAVNFITTIINMRPNNMSL
DQMPLFVWSVGITALLLLLSLPVLAGAITMLLTDRNLNTSFFDPAGGGDPILYQHL
>LEFIB265-10|HM871167|MM00743|Acleris_emargana
TLYFIFGIWAGMVGTSLSLLIRAELGNPGSLIGDDQIYNTIVTAHAFIMIFFMVMPIMIGGFGNWLVPLMLGAPDMAFPRMNNMSFWLLPPSIMLLISSSIVENGAGTGWTVYPPLSSNIAHSGSSVDLAIFSLHLAGISSILGAVNFITTIINMRPNNMSL
DQMPLFVWSVGITALLLLLSLPVLAGAITMLLTDRNLNTSFFDPAGGGDPILYQHL
>LEFIB266-10|HM871168|MM00744|Acleris_emargana
TLYFIFGIWAGMVGTSLSLLIRAELGNPGSLIGDDQIYNTIVTAHAFIMIFFMVMPIMIGGFGNWLVPLMLGAPDMAFPRMNNMSFWLLPPSIMLLISSSIVENGAGTGWTVYPPLSSNIAHSGSSVDLAIFSLHLAGISSILGAVNFITTIINMRPNNMSL
DQMPLFVWSVGITALLLLLSLPVLAGAITMLLTDRNLNTSFFDPAGGGDPILYQHL
>LEFIB267-10|HM871169|MM00745|Acleris_emargana
TLYFIFGIWAGMVGTSLSLLIRAELGNPGSLIGDDQIYNTIVTAHAFIMIFFMVMPIMIGGFGNWLVPLMLGAPDMAFPRMNNMSFWLLPPSIMLLISSSIVENGAGTGWTVYPPLSSNIAHSGSSVDLAIFSLHLAGISSILGAVNFITTIINMRPNNMSL
DQMPLFVWSVGITTLLLLLSLPVLAGAITMLLTDRNLNTSFFDPAGGGDPILYQHL
>LEFIB439-10|HM871338|MM01932|Acleris_emargana
TLYFIFGIWAGMVGTSLSLLIRAELGNPGSLIGDDQIYNTIVTAHAFIMIFFMVMPIMIGGFGNWLVPLMLGAPDMAFPRMNNMSFWLLPPSIMLLISSSIVENGAGTGWTVYPPLSSNIAHSGSSVDLAIFSLHLAGISSILGAVNFITTIINMRPNNMSL
DQMPLFVWSVGITALLLLLSLPVLAGAITMLLTDRNLNTSFFDPAGGGDPILYQHL
>LEFID043-10|HM872857|MM05505|Acleris_emargana
TLYFIFGIWAGMVGTSLSLLIRAELGNPGSLIGDDQIYNTIVTAHAFIMIFFMVMPIMIGGFGNWLVPLMLGAPDMAFPRMNNMSFWLLPPSIMLLISSSIVENGAGTGWTVYPPLSSNIAHSGSSVDLAIFSLHLAGISSILGAVNFITTIINMRPNNMSL
DQMPLFVWSVGITALLLLLSLPVLAGAITMLLTDRNLNTSFFDPAGGGDPILYQHL
>LEFIF560-10|HM875245|MM12263|Acleris_emargana
TLYFIFGIWAGMVGTSLSLLIRAELGNPGSLIGDDQIYNTIVTAHAFIMIFFMVMPIMIGGFGNWLVPLMLGAPDMAFPRMNNMSFWLLPPSIMLLISSSIVENGAGTGWTVYPPLSSNIAHSGSSVDLAIFSLHLAGISSILGAVNFITTIINMRPNNMSL
DQMPLFVWSVGITALLLLLSLPVLAGAITMLLTDRNLNTSFFDPAGGGDPILYQHL
>LEFIC498-10|HM872323|MM04204|Acleris_ferrugana
TLYFIFGIWAGMVGTSLSLLIRAELGNPGSLIGDDQIYNTIVTAHAFIMIFFMVMPIMIGGFGNWLVPLMLGAPDMAFPRMNNMSFWLLPPSIMLLISSSIVENGAGTGWTVYPPLSSNIAHSGSSVDLAIFSLHLAGISSILGAVNFITTIINMRPNNMSL
DQMPLFVWAVGITALLLLLSLPVLAGAITMLLTDRNLNTSFFDPAGGGDPILYQHL
>LEFIG803-10|HM876455|MM15667|Acleris_fimbriana
TLYFIFGIWAGMVGTSLSLLIRAELGNPGSLIGDDQIYNTIVTAHAFIMIFFMVMPIMIGGFGNWLVPLMLGAPDMAFPRMNNMSFWLLPPSIMLLISSSIVENGAGTGWTVYPPLSSNIAHSGSSVDLAIFSLHLAGISSILGAVNFITTIINMRPNNMSL
DQMPLFVWAVGITALLLLLSLPVLAGAITMLLTDRNLNTSFFDPAGGGDPILYQHL
>LEFIK012-10|JF853915|MM17587|Acleris_fimbriana
TLYFIFGIWAGMVGTSLSLLIRAELGNPGSLIGDDQIYNTIVTAHAFIMIFFMVMPIMIGGFGNWLVPLMLGAPDMAFPRMNNMSFWLLPPSIMLLISSSIVENGAGTGWTVYPPLSSNIAHSGSSVDLAIFSLHLAGISSILGAVNFITTIINMRPNNMSL
DQMPLFVWAVGITALLLLLSLPVLAGAITMLLTDRNLNTSFFDPAGGGDPILYQHL
>LEFIK692-10|MM18267|Acleris_fimbriana
TLYFIFGIWAGMVGTSLSLLIRAELGNPGSLIGDDQIYNTIVTAHAFIMIFFMVMPIMIGGFGNWLVPLMLGAPDMAFPRMNNMSFWLLPPSIMLLISSSIVENGAGTGWTVYPPLSSNIAHSGSSVDLAIFSLHLAGISSILGAVNFITTIINMRPNNMSL
DQMPLFVWAVGITALLLLLSLPVLAGAITMLLTDRNLNTSFFDPAGGGDPILYQHL
>LEFIB470-10|HM871369|MM02021|Acleris_forsskaleana
TLYFIFGIWAGMVGTSLSLLIRAELGNPGSLIGDDQIYNTIVTAHAFIMIFFMVMPIMIGGFGNWLVPLMLGAPDMAFPRMNNMSFWLLPPSIMLLISSSIVENGAGTGWTVYPPLSSNIAHSGSSVDLAIFSLHLAGISSILGAVNFITTIINMRPNNMSL
DQMPLFIWAVGITALLLLLSLPVLAGAITMLLTDRNLNTSFFDPAGGGDPILYQHL
>LEFIF875-10|HM875557|MM13267|Acleris_forsskaleana
TLYFIFGIWAGMVGTSLSLLIRAELGNPGSLIGDDQIYNTIVTAHAFIMIFFMVMPIMIGGFGNWLVPLMLGAPDMAFPRMNNMSFWLLPPSIMLLISSSIVENGAGTGWTVYPPLSSNIAHSGSSVDLAIFSLHLAGISSILGAVNFITTIINMRPNNMSL
DQMPLFIWAVGITALLLLLSLPVLAGAITMLLTDRNLNTSFFDPAGGGDPILYQHL
>LEFIB108-10|HM871015|MM00428|Acleris_hastiana
TLYFIFGIWAGMVGTSLSLLIRAELGNPGSLIGDDQIYNTIVTAHAFIMIFFMVMPIMIGGFGNWLVPLMLGAPDMAFPRMNNMSFWLLPPSIMLLISSSIVENGAGTGWTVYPPLSSNIAHSGSSVDLAIFSLHLAGISSILGAVNFITTIINMRPNNMSL
DQMPLFVWAVGITALLLLLSLPVLAGAITMLLTDRNLNTSFFDPAGGGDPILYQHL
>LEFIE071-10|HM873818|MM08206|Acleris_hastiana

TLYFIFGIWAGMVGTSLSLLIRAELGNPGSLIGDDQIYNTIVTAHAFIMIFFMVMPIMIGGFGNWLVPLMLGAPDMAFPRMNNMSFWLLPPSIMLLISSSIVENGAGTGWTVYPPLSSNIAHSGSSVDLAIFSLHLAGISSILGAVNFITTIINMRPNNMSL
DQMPLFVWAVGITALLLLLSLPVLAGAITMLLTDRNLNTSFFDPAGGGDPILYQHL
>LEFIE078-10|HM873825|MM08234|Acleris_hastiana
TLYFIFGIWAGMVGTSLSLLIRAELGNPGSLIGDDQIYNTIVTAHAFIMIFFMVMPIMIGGFGNWLVPLMLGAPDMAFPRMNNMSFWLLPPSIMLLISSSIVENGAGTGWTVYPPLSSNIAHSGSSVDLAIFSLHLAGISSILGAVNFITTIINMRPNNMSL
DQMPLFVWAVGITALLLLLSLPVLAGAITMLLTDRNLNTSFFDPAGGGDPILYQHL
>LEFIE918-10|HM874635|MM10283|Acleris_hastiana
TLYFIFGIWAGMVGTSLSLLIRAELGNPGSLIGDDQIYNTIVTAHAFIMIFFMVMPIMIGGFGNWLVPLMLGAPDMAFPRMNNMSFWLLPPSIMLLISSSIVENGAGTGWTVYPPLSSNIAHSGSSVDLAIFSLHLAGISSILGAVNFITTIINMRPNNMSL
DQMPLFVWAVGITALLLLLSLPVLAGAITMLLTDRNLNTSFFDPAGGGDPILYQHL
>LEFIF459-10|HM875144|MM11959|Acleris_hastiana
TLYFIFGMWAGMVGTSLSLLIRAELGNPGSLIGDDQIYNTIVTAHAFIMIFFMVMPIMIGGFGNWLVPLMLGAPDMAFPRMNNMSFWLLPPSIMLLISSSIVENGAGTGWTVYPPLSSNIAHSGSSVDLAIFSLHLAGISSILGAVNFITTIINMRPNNM
SLDQMPLFVWAVGITALLLLLSLPVLAGAITMLLTDRNLNTSFFDPAGGGDPILYQHL
>LEEUA448-11|KM573463|MM19856|Acleris_hastiana
TLYFIFGIWAGMVGTSLSLLIRAELGNPGSLIGDDQIYNTIVTAHAFIMIFFMVMPIMIGGFGNWLVPLMLGAPDMAFPRMNNMSFWLLPPSIMLLISSSIVENGAGTGWTVYPPLSSNIAHSGSSVDLAIFSLHLAGISSILGAVNFITTIINMRPNNMSL
DQMPLFVWAVGITALLLLLSLPVLAGAITMLLTDRNLNTSFFDPAGGGDPILYQHL
>LEEUA663-11|KM572585|MM20722|Acleris_hastiana
TLYFIFGIWAGMVGTSLSLLIRAELGNPGSLIGDDQIYNTIVTAHAFIMIFFMVMPIMIGGFGNWLVPLMLGAPDMAFPRMNNMSFWLLPPSIMLLISSSIVENGAGTGWTVYPPLSSNIAHSGSSVDLAIFSLHLAGISSILGAVNFITTIINMRPNNMSL
DQMPLFVWAVGITALLLLLSLPVLAGAITMLLTDRNLNTSFFDPAGGGDPILYQHL
>LEFIB462-10|HM871361|MM02003|Acleris_holmiana
TLYFIFGIWSGMVGTSLSLLIRAELGNPGSLIGDDQIYNTIVTAHAFIMIFFMVMPIMIGGFGNWLVPLMLGAPDMAFPRMNNMSFWLLPPSIMLLISSSIVENGAGTGWTVYPPLSSNIAHSGSSVDLAIFSLHLAGISSILGAVNFITTIINMRPNNMSL
DQMPLFVWAVGITALLLLLSLPVLAGAITMLLTDRNLNTSFFDPAGGGDPILYQHL
>LEFIC204-10|HM872048|MM03568|Acleris_holmiana
TLYFIFGIWSGMVGTSLSLLIRAELGNPGSLIGDDQIYNTIVTAHAFIMIFFMVMPIMIGGFGNWLVPLMLGAPDMAFPRMNNMSFWLLPPSIMLLISSSIVENGAGTGWTVYPPLSSNIAHSGSSVDLAIFSLHLAGISSILGAVNFITTIINMRPNNMSL
DQMPLFVWAVGITTLLLLLSLPVLAGAITMLLTDRNLNTSFFDPAGGGDPILYQHL
>LEFIG802-10|HM876454|MM15666|Acleris_hyemana
TLYFIFGIWAGMVGTSLSLLIRAELGNPGSLIGDDQIYNTIVTAHAFIMIFFMVMPIMIGGFGNWLVPLMLGAPDMAFPRMNNMSFWLLPPSIMLLISSSIVENGAGTGWTVYPPLSSNIAHSGSSVDLAIFSLHLAGISSILGAVNFITTIINMRPNNMSL
DQMPLFVWAVGITALLLLLSLPVLAGAITMLLTDRNLNTSFFDPAGGGDPILYQHL
>LEFIE109-10|HM873857|MM08297|Acleris_implexana
TLYFIFGIWAGMVGTSLSLLIRAELGNPGSLIGDDQIYNTIVTAHAFIMIFFMVMPIMIGGFGNWLVPLMLGAPDMAFPRMNNMSFWLLPPSIMLLISSSIVENGAGTGWTVYPPLSSNIAHSGSSVDLAIFSLHLAGISSILGAVNFITTIINMRPNNMSL
DQMPLFVWAVGITALLLLLSLPVLAGAITMLLTDRNLNTSFFDPAGGGDPILYQHL
>LEFIB277-10|HM871179|MM00762|Acleris_laterana
TLYFIFGIWAGMVGTSLSLLIRAELGNPGSLIGDDQIYNTIVTAHAFIMIFFMVMPIMIGGFGNWLVPLMLGAPDMAFPRMNNMSFWLLPPSIMLLISSSIVENGAGTGWTVYPPLSSNIAHSGSSVDLAIFSLHLAGISSILGAVNFITTIINMRPNNMSL
DQMPLFVWAVGITALLLLLSLPVLAGAITMLLTDRNLNTSFFDPAGGGDPILYQHL
>LEFIB475-10|HM871374|MM02029|Acleris_laterana
TLYFIFGIWAGMVGTSLSLLIRAELGNPGSLIGDDQIYNTIVTAHAFIMIFFMVMPIMIGGFGNWLVPLMLGAPDMAFPRMNNMSFWLLPPSIMLLISSSIVENGAGTGWTVYPPLSSNIAHSGSSVDLAIFSLHLAGISSILGAVNFITTIINMRPNNMSL
DQMPLFVWAVGITALLLLLSLPVLAGAITMLLTDRNLNTSFFDPAGGGDPILYQHL
>LEFIC139-10|HM871985|MM03483|Acleris_laterana
TLYFIFGIWAGMVGTSLSLLIRAELGNPGSLIGDDQIYNTIVTAHAFIMIFFMVMPIMIGGFGNWLVPLMLGAPDMAFPRMNNMSFWLLPPSIMLLISSSIVENGAGTGWTVYPPLSSNIAHSGSSVDLAIFSLHLAGISSILGAVNFITTIINMRPNNMSL
DQMPLFVWAVGITALLLLLSLPVLAGAITMLLTDRNLNTSFFDPAGGGDPILYQHL
>LEFIG095-10|HM875774|MM13787|Acleris_laterana
TLYFIFGIWAGMVGTSLSLLIRAELGNPGSLIGDDQIYNTIVTAHAFIMIFFMVMPIMIGGFGNWLVPLMLGAPDMAFPRMNNMSFWLLPPSIMLLISSSIVENGAGTGWTVYPPLSSNIAHSGSSVDLAIFSLHLAGISSILGAVNFITTIINMRPNNMSL
DQMPLFVWAVGITALLLLLSLPVLAGAITMLLTDRNLNTSFFDPAGGGDPILYQHL
>LEFIE170-10|HM873916|MM08436|Acleris_lipsiana
TLYFIFGIWAGMVGTSLSLLIRAELGNPGSLIGDDQIYNTIVTAHAFIMIFFMVMPIMIGGFGNWLVPLMLGAPDMAFPRMNNMSFWLLPPSIMLLISSSIVENGAGTGWTVYPPLSSNIAHSGSSVDLAIFSLHLAGISSILGAVNFITTIINMRPNNMSL
DQMPLFVWSVGITALLLLLSLPVLAGAITMLLTDRNLNTSFFDPAGGGDPILYQHL
>LEEUA209-11|MM19617|Acleris_literana
TLYFIFGIWAGMVGTSLSLLIRAELGNPGSLIGDDQIYNTIVTAHAFIMIFFMVMPIMIGGFGNWLVPLMLGAPDMAFPRMNNMSFWLLPPSIMLLISSSIVENGAGTGWTVYPPLSSNIAHSGSSVDLAIFSLHLAGISSILGAVNFITTIINMRPNNMSL
DQMPLFVWAVGITALLLLLSLPVLAGAITMLLTDRNLNTSFFDPAGGGDPILYQHL

>LEFIB178-10|HM871082|MM00593|Acleris_logiana
TLYFIFGIWAGMVGTSLSLLIRAELGNPGSLIGDDQIYNTIVTAHAFIMIFFMVMPIMIGGFGNWLVPLMLGAPDMAFPRMNNMSFWLLPPSIMLLISSSIVENGAGTGWTVYPPLSSNIAHSGSSVDLAIFSLHLAGISSILGAVNFITTIINMRPNNMSL
DQMPLFVWAVGITALLLLLSLPVLAGAITMLLTDRNLNTSFFDPAGGGDPILYQHL
>LEFID233-10|HM873033|MM06093|Acleris_lorquiniana
TLYFIFGIWAGMVGTSLSLLIRAELGNPGSLIGDDQIYNTIVTAHAFIMIFFMVMPIMIGGFGNWLVPLMLGAPDMAFPRMNNMSFWLLPPSIMLLISSSIVENGAGTGWTVYPPLSSNIAHSGSSVDLAIFSLHLAGISSILGAVNFITTIINMRPNNMSL
DQMPLFVWAVGITALLLLLSLPVLAGAITMLLTDRNLNTSFFDPAGGGDPILYQHL
>LEFIK181-10|MM17756|Acleris_lorquiniana
TLYFIFGIWAGMVGTSLSLLIRAELGNPGSLIGDDQIYNTIVTAHAFIMIFFMVMPIMIGGFGNWLVPLMLGAPDMAFPRMNNMSFWLLPPSIMLLISSSIVENGAGTGWTVYPPLSSNIAHSGSSVDLAIFSLHLAGISSILGAVNFITTIINMRPNNMSL
DQMPLFVWAVGITALLLLLSLPVLAGAITMLLTDRNLNTSFFDPAGGGDPILYQHL
>LEFIE066-10|HM873813|MM08197|Acleris_maccana
TLYFIFGIWAGMVGTSLSLLIRAELGNPGSLIGDDQIYNTIVTAHAFIMIFFMVMPIMIGGFGNWLVPLMLGAPDMAFPRMNNMSFWLLPPSIMLLISSSIVENGAGTGWTVYPPLSSNIAHSGSSVDLAIFSLHLAGISSILGAVNFITTIINMRPNNMSL
DQMPLFVWAVGITALLLLLSLPVLAGAITMLLTDRNLNTSFFDPAGGGDPILYQHL
>LEFIE110-10|HM873858|MM08298|Acleris_maccana
TLYFIFGIWAGMVGTSLSLLIRAELGNPGSLIGDDQIYNTIVTAHAFIMIFFMVMPIMIGGFGNWLVPLMLGAPDMAFPRMNNMSFWLLPPSIMLLISSSIVENGAGTGWTVYPPLSSNIAHSGSSVDLAIFSLHLAGISSILGAVNFITTIINMRPNNMSL
DQMPLFVWAVGITALLLLLSLPVLAGAITMLLTDRNLNTSFFDPAGGGDPILYQHL
>LEFIB326-10|HM871226|MM00864|Acleris_notana
TLYFIFGIWAGMVGTSLSLLIRAELGNPGSLIGDDQIYNTIVTAHAFIMIFFMVMPIMIGGFGNWLVPLMLGAPDMAFPRMNNMSFWLLPPSIMLLISSSIVENGAGTGWTVYPPLSSNIAHSGSSVDLAIFSLHLAGISSILGAVNFITTIINMRPNNMSL
DQMPLFVWAVGITALLLLLSLPVLAGAITMLLTDRNLNTSFFDPAGGGDPILYQHL
>LEFID004-10|HM872818|MM05424|Acleris_notana
TLYFIFGIWAGMVGTSLSLLIRAELGNPGSLIGDDQIYNTIVTAHAFIMIFFMVMPIMIGGFGNWLVPLMLGAPDMAFPRMNNMSFWLLPPSIMLLISSSIVENGAGTGWTVYPPLSSNIAHSGSSVDLAIFSLHLAGISSILGAVNFITTIINMRPNNMSL
DQMPLFVWAVGITALLLLLSLPVLAGAITMLLTDRNLNTSFFDPAGGGDPILYQHL
>LEFID471-10|HM873239|MM06413|Acleris_notana
TLYFIFGIWAGMVGTSLSLLIRAELGNPGSLIGDDQIYNTIVTAHAFIMIFFMVMPIMIGGFGNWLVPLMLGAPDMAFPRMNNMSFWLLPPSIMLLISSSIVENGAGTGWTVYPPLSSNIAHSGSSVDLAIFSLHLAGISSILGAVNFITTIINMRPNNMSL
DQMPLFVWAVGITALLLLLSLPVLAGAITMLLTDRNLNTSFFDPAGGGDPILYQHL
>LEFIB477-10|HQ570261|MM02033|Acleris_obtusana
TLYFIFGIWAGMVGTSLSLLIRAELGNPGSLIGDDQIYNTIVTAHAFIMIFFMVMPIMIGGFGNWLVPLMLGAPDMAFPRMNNMSFWLLPPSIMLLISSSIVENGAGTGWTVYPPLSSNIAHSGSSVDLAIFSLHLAGISSILGAVNFITTIINMRPNNMSL
DQMPLFVWAVGITALLLLLSLPVLAGAITMLLTDRNLNTSFFDPAGGGDPILYQHL
>LEFIC477-10|HM872309|MM04069|Acleris_obtusana
TLYFIFGIWAGMVGTSLSLLIRAELGNPGSLIGDDQIYNTIVTAHAFIMIFFMVMPIMIGGFGNWLVPLMLGAPDMAFPRMNNMSFWLLPPSIMLLISSSIVENGAGTGWTVYPPLSSNIAHSGSSVDLAIFSLHLAGISSILGAVNFITTIINMRPNNMSL
DQMPLFVWAVGITALLLLLSLPVLAGAITMLLTDRNLNTSFFDPAGSGDPILYQHL
>LEFIG096-10|HM875775|MM13821|Acleris_obtusana
TLYFIFGIWAGMVGTSLSLLIRAELGNPGSLIGDDQIYNTIVTAHAFIMIFFMVMPIMIGGFGNWLVPLMLGAPDMAFPRMNNMSFWLLPPSIMLLISSSIVENGAGTGWTVYPPLSSNIAHSGSSVDLAIFSLHLAGISSILGAVNFITTIINMRPNNMSL
DQMPLFVWAVGITALLLLLSLPVLAGAITMLLTDRNLNTSFFDPAGGGDPILYQHL
>LEEUA208-11|JN286423|MM19616|Acleris_quercinana
TLYFIFGIWAGMVGTSLSLLIRAELGNPGSLIGDDQIYNTIVTAHAFIMIFFMVMPIMIGGFGNWLVPLMLGAPDMAFPRMNNMSFWLLPPSIMLLISSSIVENGAGTGWTVYPPLSSNIAHSGSSVDLAIFSLHLAGISSILGAVNFITTIINMRPNNMSL
DQMPLFVWAVGITALLLLLSLPVLAGAITMLLTDRNLNTSFFDPAGGGDPILYQHL
>LEFIB471-10|HM871370|MM02022|Acleris_rhombana
TLYFIFGIWAGMVGTSLSLLIRAELGNPGSLIGDDQIYNTIVTAHAFIMIFFMVMPIMIGGFGNWLVPLMLGAPDMAFPRMNNMSFWLLPPSIMLLISSSIVENGAGTGWTVYPPLSSNIAHSGSSVDLAIFSLHLAGISSILGAVNFITTIINMRPNNMSL
DQMPLFVWAVGITALLLLLSLPVLAGAITMLLTDRNLNTSFFDPAGGGDPILYQHL
>LEFIF511-10|HM875196|MM12131|Acleris_roscidana
TLYFIFGIWAGMVGTSLSLLIRAELGNPGSLIGDDQIYNTIVTAHAFIMIFFMVMPIMIGGFGNWLVPLMLGAPDMAFPRMNNMSFWLLPPSIMLLISSSIVENGAGTGWTVYPPLSSNIAHSGSSVDLAIFSLHLAGISSILGAVNFITTIINMRPNNMSL
DQMPLFVWAVGITALLLLLSLPVLAGAITMLLTDRNLNTSFFDPAGGGDPILYQHL
>LEFIG804-10|HM876456|MM15668|Acleris_rufana
TLYFIFGIWAGMVGTSLSLLIRAELGNPGSLIGDDQIYNTIVTAHAFIMIFFMVMPIMIGGFGNWLVPLMLGAPDMAFPRMNNMSFWLLPPSIMLLISSSIVENGAGTGWTVYPPLSSNIAHSGSSVDLAIFSLHLAGISSILGAVNFITTIINMRPNNMSL
DQMPLFVWSVGITALLLLLSLPVLAGAITMLLTDRNLNTSFFDPAGGGDPILYQHL
>LEEUA608-11|MM20667|Acleris_scabrana
TLYFIFGIWSGLVGTSLSLLIRAELGNPGSLIGDDQIYNTIVTAHAFIMIFFMVMPIMIGGFGNWLVPLMLGAPDMAFPRMNNMSFWLLPPSIMLLISSSIVETGAGTGWTVYPPLSSNIAHSGSSVDLAIFSLHLAGISSILGAVNFITTIINMRPNNMSLD

QMPLFVWAVGITALLLLLSLPVLAGAITMLLTDRNLNTSFFDPAGGGDPILYQHL
>LEFID865-10|HM873622|MM06998|Acleris_schalleriana
TMYFIFGIWAGMVGTSLSLLIRAELGNPGSLIGDDQIYNTIVTAHAFIMIFFMVMPIMIGGFGNWLVPLMLGAPDMAFPRMNNMSFWLLPPSIMLLISSSIVENGAGTGWTVYPPLSSNIAHSGSSVDLAIFSLHLAGISSILGAVNFITTIINMRPNNMT
LDQMPLFVWAVGITALLLLLSLPVLAGAITMLLTDRNLNTSFFDPAGGGDPILYQHL
>LEFID866-10|HM873623|MM06999|Acleris_schalleriana
TMYFIFGIWAGMVGTSLSLLIRAELGNPGSLIGDDQIYNTIVTAHAFIMIFFMVMPIMIGGFGNWLVPLMLGAPDMAFPRMNNMSFWLLPPSIMLLISSSIVENGAGTGWTVYPPLSSNIAHSGSSVDLAIFSLHLAGISSILGAVNFITTIINMRPNNMT
LDQMPLFVWAVGITALLLLLSLPVLAGAITMLLTDRNLNTSFFDPAGGGDPILYQHL
>LEFIE407-10|HM874131|MM08976|Acleris_shepherdana
TLYFIFGIWAGMVGTSLSLLIRAELGNPGSLIGDDQIYNTIVTAHAFIMIFFMVMPIMIGGFGNWLVPLMLGAPDMAFPRMNNMSFWLLPPSIMLLISSSIVENGAGTGWTVYPPLSSNIAHSGSSVDLAIFSLHLAGISSILGAVNFITTIINMRPNNMLL
DQMPLFIWAVGITALLLLLSLPVLAGAITMLLTDRNLNTSFFDPAGGGDPILYQHL
>LEFIC734-10|HM872555|MM04831|Acleris_sparsana
TLYFIFGIWAGMVGTSLSLLIRAELGNPSSLIGDDQIYNTIVTAHAFIMIFFMVMPIMIGGFGNWLVPLMLGAPDMAFPRMNNMSFWLLPPSIMLLISSSIVENGAGTGWTVYPPLSSNIAHSGSSVDLAIFSLHLAGISSILGAVNFITTIINMRPNNMSL
DQMPLFVWAVGITALLLLLSLPVLAGAITMLLTDRNLNTSFFDPAGGGDPILYQHL
>LEFIB193-10|HM871097|MM00624|Acleris_umbrana
TLYFIFGIWAGMVGTSLSLLIRAELGNPGSLIGDDQIYNTIVTAHAFIMIFFMVMPIMIGGFGNWLVPLMLGAPDMAFPRMNNMSFWLLPPSILLLISSSIVENGAGTGWTVYPPLSSNIAHSGSSVDLAIFSLHLAGISSILGAVNFITTIINMRPNNMSL
DQMPLFVWAVGITALLLLLSLPVLAGAITMLLTDRNLNTSFFDPAGGGDPILYQHL
>LEFIF834-10|HM875517|MM13177|Acleris_variegana
TLYFIFGIWAGMVGTSLSLLIRAELGNPGSLIGDDQIYNTIVTAHAFIMIFFMVMPIMIGGFGNWLVPLMLGAPDMAFPRMNNMSFWLLPPSIMLLISSSIVENGAGTGWTVYPPLSSNIAHSGSSVDLAIFSLHLAGISSILGAVNFITTIINMRPNNMSL
DQMPLFVWAVGITALLLLLSLPVLAGAITMLLTDRNLNTSFFDPAGGGDPILYQHL
>LEFIF835-10|HM875518|MM13178|Acleris_variegana
TLYFIFGIWAGMVGTSLSLLIRAELGNPGSLIGDDQIYNTIVTAHAFIMIFFMVMPIMIGGFGNWLVPLMLGAPDMAFPRMNNMSFWLLPPSIMLLISSSIVENGAGTGWTVYPPLSSNIAHSGSSVDLAIFSLHLAGISSILGAVNFITTIINMRPNNMSL
DQMPLFVWAVGITALLLLLSLPVLAGAITMLLTDRNLNTSFFDPAGGGDPILYQHL
>LEFIB725-10|HM871603|MM02545|Acompsia_cinerella
TLYFIFGIWAGMLGTSLSLLIRAELGNPGSLIGDDQIYNTIVTAHAFIMIFFMVMPIMIGGFGNWLVPLMLGAPDMAFPRMNNMSFWLLPPSLTLLISSSIVENGAGTGWTVYPPLSSNIAHGGSSVDLAIFSLHLAGISSILGAINFITTIINMRVNNLSFD
QMPLFVWSVGITALLLLLSLPVLAGAITMLLTDRNLNTSFFDPAGGGDPILYQHL
>LEFIF223-10|HM874916|MM11003|Acompsia_subpunctella
TLYFIFGIWAGMLGTSLSLLIRAELGNPGSLIGDDQIYNTIVTAHAFIMIFFMVMPIMIGGFGNWLVPLMLGAPDMAFPRMNNMSFWLLPPSLTLLISSSIVENGAGTGWTVYPPLSSNIAHGGSSVDLAIFSLHLAGISSILGAINFITTIINMRVNNLSFD
QMPLFVWSVGITALLLLLSLPVLAGAITMLLTDRNLNTSFFDPAGGGDPILYQHL
>LEFIJ1215-11|MM21075|Acontia_trabealis
TLYFIFGIWAGMVGTSLSLLIRAELGNPGSLIGDDQIYNTIVTAHAFIMIFFMVMPIMIGGFGNWLVPLMLGAPDMAFPRMNNMSFWLLPPSLTLLISSSIVENGAGTGWTVYPPLSSNIAHGGSSVDLAIFSLHLAGISSILGAINFITTIINMRLNNLSFD
QMPLFVWAVGITAFLLLLSLPVLAGAITMLLTDRNLNTSFFDPAGGGDPILYQHL
>LEFIB621-10|HM871500|MM02362|Acrobasis_advenella
TLYFIFGIWAGMVGTSLSLLIRAELGTPGSLIGDDQIYNTIVTGHAFIMIFFMVMPIMIGGFGNWLVPLMLGAPDMAFPRMNNMSFWLLPPSLTLLISSSIVESGAGTGWTVYPPLSSNIAHGGSSVDLAIFSLHLAGISSILGAINFITTIINMKLNGLSFD
QMPLFVWAVGITALLLLLSLPVLAGAITMLLTDRNLNTSFFDPAGGGDPILYQHL
>LEFIC147-10|HM871993|MM03492|Acrobasis_advenella
TLYFIFGIWAGMVGTSLSLLIRAELGTPGSLIGDDQIYNTIVTGHAFIMIFFMVMPIMIGGFGNWLVPLMLGAPDMAFPRMNNMSFWLLPPSLTLLISSSIVESGAGTGWTVYPPLSSNIAHGGSSVDLAIFSLHLAGISSILGAINFITTIINMKLNGLSFD
QMPLFVWAVGITALLLLLSLPVLAGAITMLLTDRNLNTSFFDPAGGGDPILYQHL
>LEFIJ1424-12|KM573235|MM20752|Acrobasis_advenella
TLYFIFGIWAGMVGTSLSLLIRAELGTPGSLIGDDQIYNTIVTGHAFIMIFFMVMPIMIGGFGNWLVPLMLGAPDMAFPRMNNMSFWLLPPSLTLLISSSIVESGAGTGWTVYPPLSSNIAHGGSSVDLAIFSLHLAGISSILGAINFITTIINMKLNGLSFD
QMPLFVWAVGITALLLLLSLPVLAGAITMLLTDRNLNTSFFDPAGGGDPILYQHL
>LEFIC118-10|HM871965|MM03453|Acrobasis_consociella
TLYFIFGIWSGMVGTSLSLLIRAELGTPGSLIGDDQIYNTIVTGHAFIMIFFMVMPIMIGGFGNWLIPLMLGAPDMAFPRMNNMSFWLLPPSLTLLISSSIVETGAGTGWTVYPPLSSNIAHGGSSVDLAIFSLHLAGISSILGAINFITTIINMKLNGLSFDQ
MPLFIWAVGITALLLLLSLPVLAGAITMLLTDRNLNTSFFDPAGGGDPILYQHL
>LEFIC801-10|HM872620|MM04960|Acrobasis_consociella
TLYFIFGIWSGMVGTSLSLLIRAELGTPGSLIGDDQIYNTIVTGHAFIMIFFMVMPIMIGGFGNWLIPLMLGAPDMAFPRMNNMSFWLLPPSLTLLISSSIVETGAGTGWTVYPPLSSNIAHGGSSVDLAIFSLHLAGISSILGAINFITTIINMKLNGLSFDQ
MPLFIWAVGITALLLLLSLPVLAGAITMLLTDRNLNTSFFDPAGGGDPILYQHL
>LEEUA273-11|JN275974|MM19681|Acrobasis_marmorea

TLYFIFGIWSGMVGTSLSLLIRAELGTPGSLIGDDQIYNTIVTGHAFIMIFFMVMPIMIGGFGNWLVPLMLGAPDMAFPRMNNMSFWLLPPSITLLISSSIVENGAGTGWTVYPPLSSNIAHGGSSVDLAIFSLHLAGISSILGAINFITTIINMKLNGLSFDQ
MPLFVWAVGITALLLLLSLPVLAGAITMLLTDRNLNTSFFDPAGGGDPILYQHL
>LEFID701-10|HM873462|MM06754|Acrobasis_repandana
TLYFIFGIWAGMVGTSLSLLIRAELGTPGSLIGDDQIYNTIVTGHAFIMIFFMVMPIMIGGFGNWLVPLMLGAPDMAFPRMNNMSFWLLPPSLTLLISSSIVENGAGTGWTVYPPLSSNIAHGGSSVDLAIFSLHLAGISSILGAINFITTIINMKLNGLSFD
QMPLFVWAVGITALLLLLSLPVLAGAITMLLTDRNLNTSFFDPAGGGDPILYQHL
>LEFIL194-10|MM19194|Acrobasis_suavella
TLYFIFGIWSGMVGTSLSLLIRAELGTPGSLIGDDQIYNTIVTGHAFIMIFFMVMPIMIGGFGNWLVPLMLGAPDMAFPRMNNMSFWLLPPSLTLLISSSVVENGAGTGWTVYPPLSSNIAHGGSSVDLAIFSLHLAGISSILGAINFITTIINMKLNGLSFD
QMPLFVWAVGITALLLLLSLPVLAGAITMLLTDRNLNTSFFDPAGGGDPILYQHL
>LEFIL195-10|JF854524|MM19195|Acrobasis_suavella
TLYFIFGIWSGMVGTSLSLLIRAELGTPGSLIGDDQIYNTIVTGHAFIMIFFMVMPIMIGGFGNWLVPLMLGAPDMAFPRMNNMSFWLLPPSLTLLISSSVVENGAGTGWTVYPPLSSNIAHGGSSVDLAIFSLHLAGISSILGAINFITTIINMKLNGLSFD
QMPLFVWAVGITALLLLLSLPVLAGAITMLLTDRNLNTSFFDPAGGGDPILYQHL
>LEEUA271-11|MM19679|Acrobasis_tumidana
TLYFIFGIWSGMVGTSMSLLIRAELGTPGSLIGDDQIYNTIVTGHAFIMIFFMVMPIMIGGFGNWLVPLMLGAPDMAFPRMNNMSFWLLPPSLTLLISSSIVENGAGTGWTVYPPLSSNIAHGGSSVDLAIFSLHLAGISSILGAINFITTIINMKLNGLSFD
QMPLFVWAVGITALLLLLSLPVLAGAITMLLTDRNLNTSFFDPAGGGDPILYQHL
>LEFIE209-10|HM873948|MM08524|Acrocercops_brongniardella
TLYFIFGIWSGMVGTSLSLLIRAELGNPGSLIGDDQIYNTIVTAHAFIMIFFMVMPIMIGGFGNWLVPLMLGAPDMAFPRLNNMSFWLLPPSLILLIMSSIVENGAGTGWTVYPPLSSNIAHSGSSVDLAIFSLHLAGISSILGAVNFITTIINMRVNGMNF
DSMSLFSWAVGITALLLLLSLPVLAGAITMLLTDRNLNTSFFDPAGGGDPILYQHL
>LEFIE210-10|HM873949|MM08525|Acrocercops_brongniardella
TLYFIFGIWSGMVGTSLSLLIRAELGNPGSLIGDDQIYNTIVTAHAFIMIFFMVMPIMIGGFGNWLVPLMLGAPDMAFPRLNNMSFWLLPPSLILLIMSSIVENGAGTGWTVYPPLSSNIAHSGSSVDLAIFSLHLAGISSILGAVNFITTIINMRVNGMNF
DSMSLFSWAVGITALLLLLSLPVLAGAITMLLTDRNLNTSFFDPAGGGDPILYQHL
>LEFIJ577-10|MM17202|Acrolepia_autumnitella
TMYFIFGIWAGMVGTSLSLLIRAELGNPGSLIGDDQIYNTIVTAHAFIMIFFMVMPIMIGGFGNWLVPLMLGAPDMAFPRMNNMSFWLLPPSLSLLISSSIVENGAGTGWTVYPPLSSNIAHSGSSVDLAIFSLHLAGISSILGAINFITTIINMRSGSMSF
DRMPLFIWAVGITALLLLLSLPVLAGAITMLLTDRNLNTSFFDPAGGGDPILYQHL
>LEFIB616-10|HM871495|MM02352|Acrolepiopsis_assectella
TLYFIFGLWSGLIGTSLSLIIRAELGNPGSLIGDDQIYNTIVTAHAFIMIFFMVMPIMIGGFGNWLVPLMLGAPDMAFPRMNNMSFWLLPPSLTLLISSSIVENGAGTGWTVYPPLSSNIAHSGSSVDLAIFSLHLAGISSILGAINFITTIINMRSNGMSFDR
MPLFVWAVGITALLLLLSLPVLAGAITMLLTDRNLNTSFFDPAGGGDPILYQHL
>LEFIC177-10|HM872022|MM03531|Acrolepiopsis_assectella
TLYFIFGLWSGLIGTSLSLIIRAELGNPGSLIGDDQIYNTIVTAHAFIMIFFMVMPIMIGGFGNWLVPLMLGAPDMAFPRMNNMSFWLLPPSLTLLISSSIVENGAGTGWTVYPPLSSNIAHSGSSVDLAIFSLHLAGISSILGAINFITTIINMRSNGMSFDR
MPLFVWAVGITALLLLLSLPVLAGAITMLLTDRNLNTSFFDPAGGGDPILYQHL
>LEFIF962-10|HM875643|MM13539|Acrolepiopsis_assectella
TLYFIFGLWSGLIGTSLSLIIRAELGNPGSLIGDDQIYNTIVTAHAFIMIFFMVMPIMIGGFGNWLVPLMLGAPDMAFPRMNNMSFWLLPPSLTLLISSSIVENGAGTGWTVYPPLSSNIAHSGSSVDLAIFSLHLAGISSILGAINFITTIINMRSNGMSFDR
MPLFVWAVGITALLLLLSLPVLAGAITMLLTDRNLNTSFFDPAGGGDPILYQHL
>LEFIC992-10|HM872807|MM05405|Acronicta_aceris
TLYFIFGIWAGMIGTSLSLLIRAELGTPGSLIGDDQIYNTIVTAHAFIMIFFMVMPIMIGGFGNWLVPLMLGAPDMAFPRMNNMSFWLLPPSLTLLISSSIVESGAGTGWTVYPPLSSNIAHGGSSVDLAIFSLHLAGISSILGAINFITTIINMRLNNLSFDQ
MPLFIWAVGITAFLLLLSLPVLAGAITMLLTDRNLNTSFFDPAGGGDPILYQHL
>LEFIE628-10|HM874351|MM09566|Acronicta_aceris
TLYFIFGIWAGMIGTSLSLLIRAELGTPGSLIGDDQIYNTIVTAHAFIMIFFMVMPIMIGGFGNWLVPLMLGAPDMAFPRMNNMSFWLLPPSLTLLISSSIVESGAGTGWTVYPPLSSNIAHGGSSVDLAIFSLHLAGISSILGAINFITTIINMRLNNLSFDQ
MPLFIWAVGITAFLLLLSLPVLAGAITMLLTDRNLNTSFFDPAGGGDPILYQHL
>LEFIC519-10|HM872342|MM04232|Acronicta_alni
TLYFIFGIWAGMVGTSLSLLIRAELGTPGSLIGDDQIYNTIVTAHAFIMIFFMVMPIMIGGFGNWLVPLMLGAPDMAFPRMNNMSFWLLPPSLTLLISSSIVENGAGTGWTVYPPLSSNIAHGGSSVDLAIFSLHLAGISSILGAINFITTIINMRLNNMSFD
QMPLFIWAVGITAFLLLLSLPVLAGAITMLLTDRNLNTSFFDPAGGGDPILYQHL
>LEFIF350-10|HM875035|MM11171|Acronicta_alni
TLYFIFGIWAGMVGTSLSLLIRAELGTPGSLIGDDQIYNTIVTAHAFIMIFFMVMPIMIGGFGNWLVPLMLGAPDMAFPRMNNMSFWLLPPSLTLLISSSIVENGAGTGWTVYPPLSSNIAHGGSSVDLAIFSLHLAGISSILGAINFITTIINMRLNNMSFD
QMPLFIWAVGITAFLLLLSLPVLAGAITMLLTDRNLNTSFFDPAGGGDPILYQHL
>LEFII102-10|KM572450|MM18529|Acronicta_alni
TLYFIFGIWAGMVGTSLSLLIRAELGTPGSLIGDDQIYNTIVTAHAFIMIFFMVMPIMIGGFGNWLVPLMLGAPDMAFPRMNNMSFWLLPPSLTLLISSSIVENGAGTGWTVYPPLSSNIAHGGSSVDLAIFSLHLAGISSILGAINFITTIINMRLNNMSFD
QMPLFIWAVGITAFLLLLSLPVLAGAITMLLTDRNLNTSFFDPAGGGDPILYQHL

>LEFIC581-10|HM872402|MM04363|Acronicta_auricoma
TLYFIFGIWAGMVGTSLSLLIRAELGTPGSLIGDDQIYNTIVTAHAFIMIFFMVMPIMIGGFGNWLVPLMLGAPDMAFPRMNNMSFWLLPPSLTLLISSSIVENGAGTGWTVYPPLSSNIAHGGSSVDLAIFSLHLAGISSILGAINFITTIINMRLNNLSFD
QMPLFIWAVGITAFLLLLSLPVLAGAITMLLTDRNLNTSFFDPAGGGDPILYQHL
>LEFID393-10|HM873190|MM06306|Acronicta_auricoma
TLYFIFGIWAGMVGTSLSLLIRAELGTPGSLIGDDQIYNTIVTAHAFIMIFFMVMPIMIGGFGNWLVPLMLGAPDMAFPRMNNMSFWLLPPSLTLLISSSIVENGAGTGWTVYPPLSSNIAHGGSSVDLAIFSLHLAGISSILGAINFITTIINMRLNNLSFD
QMPLFIWAVGITAFLLLLSLPVLAGAITMLLTDRNLNTSFFDPAGGGDPILYQHL
>LEFIE050-10|HM873798|MM08162|Acronicta_auricoma
TLYFIFGIWAGMVGTSLSLLIRAELGTPGSLIGDDQIYNTIVTAHAFIMIFFMVMPIMIGGFGNWLVPLMLGAPDMAFPRMNNMSFWLLPPSLTLLISSSIVENGAGTGWTVYPPLSSNIAHGGSSVDLAIFSLHLAGISSILGAINFITTIINMRLNNLSFD
QMPLFIWAVGITAFLLLLSLPVLAGAITMLLTDRNLNTSFFDPAGGGDPILYQHL
>LEFIC470-10|HM872303|MM04058|Acronicta_cinerea
TLYFIFGIWAGMVGTSLSLLIRAELGTPGSLIGDDQIYNTIVTAHAFIMIFFMVMPIMIGGFGNWLVPLMLGAPDMAFPRMNNMSFWLLPPSLTLLISSSIVENGAGTGWTVYPPLSSNIAHGGSSVDLAIFSLHLAGISSILGAINFITTIINMRLNSLSFD
QMPLFIWAVGITAFLLLLSLPVLAGAITMLLTDRNLNTSFFDPAGGGDPILYQHL
>LEFID692-10|HM873453|MM06741|Acronicta_cuspis
TLYFIFGIWAGMVGTSLSLLIRAELGTPGSLIGDDQIYNTIVTAHAFIMIFFMVMPIMIGGFGNWLVPLMLGAPDMAFPRMNNMSFWLLPPSLTLLISSSIVENGAGTGWTVYPPLSSNIAHGGSSVDLAIFSLHLAGISSILGAINFITTIINMRLNNLSFD
QMPLFIWAVGITAFLLLLSLPVLAGAITMLLTDRNLNTSFFDPAGGGDPILYQHL
>LEFIB322-10|HM871222|MM00856|Acronicta_leporina
TLYFIFGIWAGMVGTSLSLLIRAELGTPGSLIGDDQIYNTIVTAHAFIMIFFMVMPIMIGGFGNWLVPLMLGAPDMAFPRMNNMSFWFLPPSLTLLISSSIVENGAGTGWTVYPPLSSNIAHGGSSVDLAIFSLHLAGISSILGAINFITTIINMRLNNLSFD
QMPLFIWAVGITAFLLLLSLPVLAGAITMLLTDRNLNTSFFDPAGGGDPILYQHL
>LEFIA460-10|HM386800|MM01525|Acronicta_leporina
TLYFIFGIWAGMVGTSLSLLIRAELGTPGSLIGDDQIYNTIVTAHAFIMIFFMVMPIMIGGFGNWLVPLMLGAPDMAFPRMNNMSFWFLPPSLTLLISSSIVENGAGTGWTVYPPLSSNIAHGGSSVDLAIFSLHLAGISSILGAINFITTIINMRLNNLSFD
QMPLFIWAVGITAFLLLLSLPVLAGAITMLLTDRNLNTSFFDPAGGGDPILYQHL
>LEFIF214-10|HQ963162|MM10910|Acronicta_leporina
TLYFIFGIWAGMVGTSLSLLIRAELGTPGSLIGDDQIYNTIVTAHAFIMIFFMVMPIMIGGFGNWLVPLMLGAPDMAFPRMNNMSFWFLPPSLTLLISSSIVENGAGTGWTVYPPLSSNIAHGGSSVDLAIFSLHLAGISSILGAINFITTIINMRLNNLSFD
QMPLFIWAVGITAFLLLLSLPVLAGAITMLLTDRNLNTSFFDPAGGGDPILYQHL
>LEFIG997-10|HM876634|MM15861|Acronicta_menyanthidis
TLYFIFGIWAGMVGTSLSLLIRAELGTPGSLIGDDQIYNTIVTAHAFIMIFFMVMPIMIGGFGNWLVPLMLGAPDMAFPRMNNMSFWLLPPSLTLLISSSIVENGAGTGWTVYPPLSSNIAHGGSSVDLAIFSLHLAGISSILGAINFITTIINMRLNNLSFD
QMPLFIWAVGITAFLLLLSLPVLAGAITMLLTDRNLNTSFFDPAGGGDPILYQHL
>LEFIA461-10|HM386801|MM01526|Acronicta_psi
TLYFIFGIWAGMVGTSLSLLIRAELGTPGSLIGDDQIYNTIVTAHAFIMIFFMVMPIMIGGFGNWLVPLMLGAPDMAFPRMNNMSFWLLPPSLTLLISSSIVENGAGTGWTVYPPLSSNIAHGGSSVDLAIFSLHLAGISSILGAINFITTIINMRLNNLSFD
QMPLFIWAVGITAFLLLLSLPVLAGAITMLLTDRNLNTSFFDPAGGGDPILYQHL
>LEFIA472-10|HM386812|MM01544|Acronicta_psi
TLYFIFGIWAGMVGTSLSLLIRAELGTPGSLIGDDQIYNTIVTAHAFIMIFFMVMPIMIGGFGNWLVPLMLGAPDMAFPRMNNMSFWLLPPSLTLLISSSIVENGAGTGWTVYPPLSSNIAHGGSSVDLAIFSLHLAGISSILGAINFITTIINMRLNNLSFD
QMPLFIWAVGITAFLLLLSLPVLAGAITMLLTDRNLNTSFFDPAGGGDPILYQHL
>LEFIC344-10|HM872187|MM03858|Acronicta_psi
TLYFIFGIWAGMVGTSLSLLIRAELGTPGSLIGDDQIYNTIVTAHAFIMIFFMVMPIMIGGFGNWLVPLMLGAPDMAFPRMNNMSFWLLPPSLTLLISSSIVENGAGTGWTVYPPLSSNIAHGGSSVDLAIFSLHLAGISSILGAINFITTIINMRLNNLSFD
QMPLFIWAVGITAFLLLLSLPVLAGAITMLLTDRNLNTSFFDPAGGGDPILYQHL
>LEFIF661-10|HM875345|MM12604|Acronicta_psi
TLYFIFGIWAGMVGTSLSLLIRAELGTPGSLIGDDQIYNTIVTAHAFIMIFFMVMPIMIGGFGNWLVPLMLGAPDMAFPRMNNMSFWLLPPSLTLLISSSIVENGAGTGWTVYPPLSSNIAHGGSSVDLAIFSLHLAGISSILGAINFITTIINMRLNNLSFD
QMPLFIWAVGITAFLLLLSLPVLAGAITMLLTDRNLNTSFFDPAGGGDPILYQHL
>LEFIA464-10|HM386804|MM01535|Acronicta_rumicis
TLYFIFGIWAGMVGTSLSLLIRAELGTPGSLIGDDQIYNTIVTAHAFIMIFFMVMPIMIGGFGNWLVPLMLGAPDMAFPRMNNMSFWLLPPSLTLLISSSIVENGAGTGWTVYPPLSSNIAHGGSSVDLAIFSLHLAGISSILGAINFITTIINMRLNNLSFD
QMPLFIWAVGITAFLLLLSLPVLAGAITMLLTDRNLNTSFFDPAGGGDPILYQHL
>LEFIA476-10|MM01553|Acronicta_strigosa
TLYFIFGIWAGMVGTSLSLLIRAELGTPGSLIGDDQIYNTIVTAHAFIMIFFMVMPIMIGGFGNWLVPLMLGAPDMAFPRMNNMSFWLLPPSLTLLISSSIVENGAGTGWTVYPPLSSNIAHGGSSVDLAIFSLHLAGISSILGAINFITTIINMRLNNLSFD
QMPLFIWAVGITAFLLLLSLPVLAGAITMLLTDRNLNTSFFDPAGGGDPILYQHL
>LEFIA477-10|MM01554|Acronicta_strigosa
TLYFIFGIWAGMVGTSLSLLIRAELGTPGSLIGDDQIYNTIVTAHAFIMIFFMVMPIMIGGFGNWLVPLMLGAPDMAFPRMNNMSFWLLPPSLTLLISSSIVENGAGTGWTVYPPLSSNIAHGGSSVDLAIFSLHLAGISSILGAINFITTIINMRLNNLSFD

QMPLFIWAVGITAFLLLLSLPVLAGAITMLLTDRNLNTSFFDPAGGGDPILYQHL
>LEFIJ335-10|MM15935|Acronicta_tridens
TLYFIFGIWAGMVGTSLSLLIRAELGTPGSLIGDDQIYNTIVTAHAFIMIFFMVMPIMIGGFGNWLVPLMLGAPDMAFPRMNNMSFWLLPPSLTLLISSSIVENGAGTGWTVYPPLSSNIAHGGSSVDLAIFSLHLAGISSILGAINFITTIINMRLNNLSFD
QMPLFIWAVGITAFLLLLSLPVLAGAITMLLTDRNLNTSFFDPAGGGDPILYQHL
>LEFIJ886-10|JF853880|MM17511|Actebia_fennica
TLYFIFGIWAGMVGTSLSLLIRAELGNPGSLIGDDQIYNTIVTAHAFIMIFFMVMPIMIGGFGNWLVPLMLGAPDMAFPRMNNMSFWLLPPSLTLLISSSIVENGAGTGWTVYPPLSSNIAHGGSSVDLAIFSLHLAGISSILGAINFITTIINMRLNSLSFD
QMPLFIWAVGITAFLLLLSLPVLAGAITMLLTDRNLNTSFFDPAGGGDPILYQHL
>LEFIH039-10|HM876670|MM15903|Actebia_praecox
TLYFIFGIWAGMVGTSLSLLIRAELGNPGSLIGDDQIYNTIVTAHAFIMIFFMVMPIMIGGFGNWLVPLMLGAPDMAFPRMNNMSFWLLPPSLTLLISSSIVENGAGTGWTVYPPLSSNIAHGGSSVDLAIFSLHLAGISSILGAINFITTIINMRLNSLSFD
QMPLFIWAVGITAFLLLLSLPVLAGAITMLLTDRNLNTSFFDPAGGGDPILYQHL
>LEFIJ367-10|MM15967|Actebia_squalida
TLYFIFGIWAGMVGTSLSLLIRAELGNPGSLIGDDQIYNTIVTAHAFIMIFFMVMPIMIGGFGNWLVPLMLGAPDMAFPRMNNMSFWLLPPSLTLLISSSIVENGAGTGWTVYPPLSSNIAHGGSSVDLAIFSLHLAGISSILGAINFITTIINMRLNSLSFD
QMPLFVWAVGITAFLLLLSLPVLAGAITMLLTDRNLNTSFFDPAGGGDPILYQHL
>LEFIC181-10|HM872025|MM03535|Actinotia_polyodon
TLYFIFGIWAGMVGTSLSLLIRAELGNPGSLIGDDQIYNTIVTAHAFIMIFFMVMPIMIGGFGNWLVPLMLGAPDMAFPRMNNMSFWLLPPSLTLLISSSIVENGAGTGWTVYPPLSSNIAHGGSSVDLAIFSLHLAGISSILGAINFITTIINMRLNSLSFD
QMPLFIWAVGITAFLLLLSLPVLAGAITMLLTDRNLNTSFFDPAGGGDPILYQHL
>LEFID073-10|HM872884|MM05682|Adaina_microdactyla
TLYFIFGIWAGMVGTSLSLLIRAELGNPGSLIGDDQIYNSIVTAHAFIMIFFMVMPIMIGGFGNWLVPLMLGAPDMAFPRMNNMSFWLLPPSIILLISSSIVENGAGTGWTVYPPLSSNIAHSGSSVDLAIFSLHLAGISSILGAINFITTIINMRLNNLMFD
QLPLFVWAVGITALLLLLSLPVLAGAITMLLTDRNINTSFFDPAGGGDPILYQHL
>LEFID314-10|HM873111|MM06199|Adela_croesella
TLYFIFGIWSGLLGTSLSLLIRTELGMPGSLIGNDQIYNTIVTAHAFIMIFFMVMPIMIGGFGNWLVPLMLGAPDMAFPRLNNMSFWLLPPSLLLTSSSLVENGAGTGWTVYPPLSSNIAHSGTSVDLAIFSLHLAGISSILGAVNFITTVINMKTMNMTF
DQMPLFVWSVAITALLLLLSLPVLAGAITMLLTDRNLNTSFFDPTGGGDPILYQHL
>LEFIC465-10|HM872298|MM04049|Adela_cuprella
TLYFIFGIWSGLLGTSLSLLIRTELGMPGSLIGNDQIYNTIVTAHAFIMIFFMVMPIMIGGFGNWLVPLMLGAPDMAFPRLNNMSFWLLPPSLLLLISSSLVESGSGTGWTVYPPLSSNVAHSGTSVDLTIFSLHLAGISSILGAVNFITTVINMRAMNMTF
DQMPLFVWSVAITALLLLLSLPVLAGAITMLLTDRNLNTSFFDPTGGGDPILYQHL
>LEFIG115-10|HM875795|MM13911|Adela_cuprella
TLYFIFGIWSGLLGTSLSLLIRTELGMPGSLIGNDQIYNTIVTAHAFIMIFFMVMPIMIGGFGNWLVPLMLGAPDMAFPRLNNMSFWLLPPSLLLLISSSLVESGSGTGWTVYPPLSSNVAHSGTSVDLTIFSLHLAGISSILGAVNFITTVINMRAMNMTF
DQMPLFVWSVAITALLLLLSLPVLAGAITMLLTDRNLNTSFFDPTGGGDPILYQHL
>LEFIB464-10|HM871363|MM02007|Adoxophyes_orana
TLYFIFGIWAGMVGTSLSLLIRAELGNPGSLLGDDQIYNTIVTAHAFIMIFFMVMPIMIGGFGNWLVPLMLGAPDMAFPRMNNMSFWLLPPSIMLLISSSIVENGAGTGWTVYPPLSSNIAHSGSSVDLAIFSLHLAGISSILGAVNFITTIINMRPNNMF
LDQMPLFVWAVGITALLLLLSLPVLAGAITMLLTDRNLNTSFFDPAGGGDPILYQHL
>LEFIB892-10|HM871769|MM02980|Adoxophyes_orana
TLYFIFGIWAGMVGTSLSLLIRAELGNPGSLLGDDQIYNTIVTAHAFIMIFFMVMPIMIGGFGNWLVPLMLGAPDMAFPRMNNMSFWLLPPSIMLLISSSIVENGAGTGWTVYPPLSSNIAHSGSSVDLAIFSLHLAGISSILGAVNFITTIINMRPNNMS
LDQMPLFVWAVGITALLLLLSLPVLAGAITMLLTDRNLNTSFFDPAGGGDPILYQHL
>LEFID566-10|HM873331|MM06560|Adscita_statices
TLYFIFGIWSGMVGTSLSLLIRTELGAPGSLIGDDQIYNTIVTAHAFIMIFFMVMPIMIGGFGNWLVPLMLGAPDMAFPRMNNMSFWLLPPSLTLLISSSIVETGAGTGWTVYPPLSSNIAHGGSSVDLTIFSLHLAGISSILGAVNFITTIINMRPNGMSF
DQMPLFVWAVGITALLLLLSLPVLAGAITMLLTDRNLNTSFFDPAGGGDPILYQHL
>LEFIJ739-10|JF853797|MM17364|Adscita_statices
TLYFIFGIWSGMVGTSLSLLIRTELGAPGSLIGDDQIYNTIVTAHAFIMIFFMVMPIMIGGFGNWLVPLMLGAPDMAFPRMNNMSFWLLPPSLTLLISSSIVETGAGTGWTVYPPLSSNIAHGGSSVDLTIFSLHLAGISSILGAVNFITTIINMRPNGMSF
DQMPLFVWAVGITALLLLLSLPVLAGAITMLLTDRNLNTSFFDPAGGGDPILY?HL
>LEFIA046-10|HM396395|MM00449|Aethalura_punctulata
TLYFIFGIWAGMVGTSLSLLIRAELGSPGSLIGDDQIYNTIVTAHAFIMIFFMVMPIMIGGFGNWLVPLMLGAPDMAFPRMNNMSFWLLPPSLTLLISSSIVENGAGTGWTVYPPLSSNIAHGGSSVDLAIFSLHLAGISSILGAINFITTIINMRLNNLSFD
QMPLFVWAVGITAFLLLLSLPVLAGAITMLLTDRNLNTSFFDPAGGGDPILYQHL
>LEFIA278-10|HM386622|MM01318|Aethalura_punctulata
TLYFIFGIWAGMVGTSLSLLIRAELGSPGSLIGDDQIYNTIVTAHAFIMIFFMVMPIMIGGFGNWLVPLMLGAPDMAFPRMNNMSFWLLPPSLTLLISSSIVENGAGTGWTVYPPLSSNIAHGGSSVDLAIFSLHLAGISSILGAINFITTIINMRLNNLSFD
QMPLFVWAVGITAFLLLLSLPVLAGAITMLLTDRNLNTSFFDPAGGGDPILYQHL
>LEFIF732-10|HM875416|MM12851|Aethalura_punctulata

TLYFIFGIWAGMVGTSLSLLIRAELGSPGSLIGDDQIYNTIVTAHAFIMIFFMVMPIMIGGFGNWLVPLMLGAPDMAFPRMNNMSFWLLPPSLTLLISSSIVENGAGTGWTVYPPLSSNIAHGGSSVDLAIFSLHLAGISSILGAINFITTIINMRLNNLSFD
QMPLFVWAVGITAFLLLLSLPVLAGAITMLLTDRNLNTSFFDPAGGGDPILYQHL
>LEFIK702-10|JF854267|MM18277|Aethes_beatricella
TLYFIFGIWAGMVGTSLSLLIRAELGNPGSLIGDDQIYNTIVTAHAFIMIFFMVMPIMIGGFGNWLVPLMLGAPDMAFPRMNNMSFWLLPPSIMLLISSSIVENGAGTGWTVYPPLSSNIAHSGSSVDLAIFSLHLAGISSILGAVNFITTIINMRPNNMKL
DQMPLFVWAVGITALLLLLSLPVLAGAITMLLTDRNLNTSFFDPAGGGDPILYQHL
>LEFIJ032-10|KM572502|MM01069|Aethes_cnicana
TLYFIFGIWAGMVGTSLSLLIRAELGNPGSLIGDDQIYNTIVTAHAFIMIFFMVMPIMIGGFGNWLVPLMLGAPDMAFPRMNNMSFWLLPPSIMLLISSSIVENGAGTGWTVYPPLSSNIAHSGSSVDLAIFSLHLAGISSILGAVNFITTIINMRPNNMSL
DQMPLFVWAVGITALLLLLSLPVLAGAITMLLTDRNLNTSFFDPAGGGDPILYQHL
>LEFIB894-10|HM871771|MM02986|Aethes_cnicana
TLYFIFGIWAGMVGTSLSLLIRAELGNPGSLIGDDQIYNTIVTAHAFIMIFFMVMPIMIGGFGNWLVPLMLGAPDMAFPRMNNMSFWLLPPSIMLLISSSIVENGAGTGWTVYPPLSSNIAHSGSSVDLAIFSLHLAGISSILGAVNFITTIINMRPNNMSL
DQMPLFVWAVGITALLLLLSLPVLAGAITMLLTDRNLNTSFFDPAGGGDPILYQHL
>LEFID671-10|HM873432|MM06710|Aethes_cnicana
TLYFIFGIWAGMVGTSLSLLIRAELGNPGSLIGDDQIYNTIVTAHAFIMIFFMVMPIMIGGFGNWLVPLMLGAPDMAFPRMNNMSFWLLPPSIMLLISSSIVENGAGTGWTVYPPLSSNIAHSGSSVDLAIFSLHLAGISSILGAVNFITTIINMRPNNMSL
DQMPLFVWAVGITALLLLLSLPVLAGAITMLLTDRNLNTSFFDPAGGGDPILYQHL
>LEFIG277-10|HM875956|MM14221|Aethes_cnicana
TLYFIFGIWAGMVGTSLSLLIRAELGNPGSLIGDDQIYNTIVTAHAFIMIFFMVMPIMIGGFGNWLVPLMLGAPDMAFPRMNNMSFWLLPPSIMLLISSSIVENGAGTGWTVYPPLSSNIAHSGSSVDLAIFSLHLAGISSILGAVNFITTIINMRPNNMSL
DQMPLFVWAVGITALLLLLSLPVLAGAITMLLTDRNLNTSFFDPAGGGDPILYQHL
>LEFIA013-10|HM396363|MM00084|Aethes_deutschiana
TLYFIFGIWAGMVGTSLSLLIRAELGNPGSLIGDDQIYNTIVTAHAFIMIFFMVMPIMIGGFGNWLVPLMLGAPDMAFPRMNNMSFWLLPPSIMLLISSSIVENGAGTGWTVYPPLSSNIAHSGSSVDLAIFSLHLAGISSILGAVNFITTIINMRPNNMSL
DQMPLFVWAVGITALLLLLSLPVLAGAITMLLTDRNLNTSFFDPAGGGDPILYQHL
>LEFID401-10|HM873198|MM06320|Aethes_deutschiana
TLYFIFGIWAGMVGTSLSLLIRAELGNPGSLIGDDQIYNTIVTAHAFIMIFFMVMPIMIGGFGNWLVPLMLGAPDMAFPRMNNMSFWLLPPSIMLLISSSIVENGAGTGWTVYPPLSSNIAHSGSSVDLAIFSLHLAGISSILGAVNFITTIINMRPNNMSL
DQMPLFVWAVGITALLLLLSLPVLAGAITMLLTDRNLNTSFFDPAGGGDPILYQHL
>LEFIF345-10|HM875030|MM11164|Aethes_fennicana
TLYFIFGIWAGMVGTSLSLLIRAELGNPGSLIGDDQIYNTIVTAHAFIMIFFMVMPIMIGGFGNWLVPLMLGAPDMAFPRMNNMSFWLLPPSIMLLISSSIVENGAGTGWTVYPPLSSNIAHSGSSVDLAIFSLHLAGISSILGAVNFITTIINMHPNNMK
LDQMPLFVWAVGITALLLLLSLPVLAGAITMLLTDRNLNTSFFDPAGGGDPILYQHL
>COLFF945-13|MM23219|Aethes_fennicana
TLYFIFGIWAGMVGTSLSLLIRAELGNPGSLIGDDQIYNTIVTAHAFIMIFFMVMPIMIGGFGNWLVPLMLGAPDMAFPRMNNMSFWLLPPSIMLLISSSIVENGAGTGWTVYPPLSSNIAHSGSSVDLAIFSLHLAGISSILGAVNFITTIINMRPNNMKL
DQMPLFVWAVGITALLLLLSLPVLAGAITMLLTDRNLNTSFFDPAGGGDPILYQHL
>COLFF946-13|MM23220|Aethes_fennicana
TLYFIFGIWAGMVGTSLSLLIRAELGNPGSLIGDDQIYNTIVTAHAFIMIFFMVMPIMIGGFGNWLVPLMLGAPDMAFPRMNNMSFWLLPPSIMLLISSSIVENGAGTGWTVYPPLSSNIAHSGSSVDLAIFSLHLAGISSILGAVNFITTIINMRPNNMKL
DQMPLFVWAVGITALLLLLSLPVLAGAITMLLTDRNLNTSFFDPAGGGDPILYQHL
>LEFIJ633-10|JF853731|MM17258|Aethes_francillana
TLYFIFGIWAGMVGTSLSLLIRAELGNPGSLIGDDQIYNTIVTAHAFIMIFFMVMPIMIGGFGNWLVPLMLGAPDMAFPRMNNMSFWLLPPSIMLLISSSIVENGAGTGWTVYPPLSSNIAHSGSSVDLAIFSLHLAGISSILGAVNFITTIINMRPNNMKL
DQMPLFVWAVGITALLLLLSLPVLAGAITMLLTDRNLNTSFFDPAGGGDPILYQHL
>LEFID185-10|HM872991|MM05974|Aethes_hartmanniana
TLYFIFGIWAGMIGTSLSLLIRAELGNPGSLIGDDQIYNTIVTAHAFIMIFFMVMPIMIGGFGNWLIPLMLGAPDMAFPRMNNMSFWLLPPSIMLLISSSIVENGAGTGWTVYPPLSSNIAHSGSSVDLAIFSLHLAGISSILGAVNFITTIINMRPNNMSLD
QMPLFVWSVGITALLLLLSLPVLAGAITMLLTDRNLNTSFFDPSGGGDPILYQHL
>LEFIG224-10|HM875904|MM14121|Aethes_hartmanniana
TLYFIFGIWAGMIGTSLSLLIRAELGNPGSLIGNDQIYNTIVTAHAFIMIFFMVMPIMIGGFGNWLIPLMLGAPDMAFPRMNNMSFWLLPPSIMLLISSSIVENGAGTGWTVYPPLSSNIAHSGSSVDLAIFSLHLAGISSILGAVNFITTIINMRPNNMSLD
QMPLFVWSVGITALLLLLSLPVLAGAITMLLTDRNLNTSFFDPSGGGDPILYQHL
>LEFIC253-10|HM872097|MM03668|Aethes_kindermanniana
TLYFIFGIWAGMVGTSLSLLIRAELGNPGSLIGDDQIYNTIVTAHAFIMIFFMVMPIMIGGFGNWLVPLMLGAPDMAFPRMNNMSFWLLPPSIMLLISSSIVENGAGTGWTVYPPLSSNIAHSGSSVDLAIFSLHLAGISSILGAVNFITTIINMRPNNMN
LDQMPLFVWAVGITALLLLLSLPVLAGAITMLLTDRNLNTSFFDPAGGGDPILYQHL
>LEFIC450-10|HM872284|MM04010|Aethes_kindermanniana
TLYFIFGIWAGMVGTSLSLLIRAELGNPGSLIGDDQIYNTIVTAHAFIMIFFMVMPIMIGGFGNWLVPLMLGAPDMAFPRMNNMSFWLLPPSIMLLISSSIVENGAGTGWTVYPPLSSNIAHSGSSVDLAIFSLHLAGISSILGAVNFITTIINMRPNNMN
LDQMPLFVWAVGITALLLLLSLPVLAGAITMLLTDRNLNTSFFDPAGGGDPILYQHL

>LEFIF880-10|HM875562|MM13281|Aethes_kindermanniana
TLYFIFGIWAGMVGTSLSLLIRAELGNPGSLIGDDQIYNTIVTAHAFIMIFFMVMPIMIGGFGNWLVPLMLGAPDMAFPRMNNMSFWLLPPSIMLLISSSIVENGAGTGWTVYPPLSSNIAHSGSSVDLAIFSLHLAGISSILGAVNFITTIINMRPNNMN
LDQMPLFVWAVGITALLLLLSLPVLAGAITMLLTDRNLNTSFFDPAGGGDPILYQHL
>LEFIF156-10|HM874850|MM10684|Aethes_kyrkii
TLYFIFGIWAGMVGTSLSLLIRAELGNPGSLIGDDQIYNTIVTAHAFIMIFFMVMPIMIGGFGNWLVPLMLGAPDMAFPRMNNMSFWLLPPSIMLLISSSIVENGAGTGWTVYPPLSSNIAHSGSSVDLAIFSLHLAGISSILGAVNFITTIINMRPNNMSL
DQMPLFVWAVGITALLLLLSLPVLAGAITMLLTDRNLNTSFFDPAGGGDPILYQHL
>LEFIB491-10|HM871386|MM02066|Aethes_margaritana
TLYFIFGIWAGMVGTSLSLLIRAELGNPGSLIGDDQIYNTIVTAHAFIMIFFMVMPIMIGGFGNWLVPLMLGAPDMAFPRMNNMSFWLLPPSIMLLISSSIVENGAGTGWTVYPPLSSNIAHSGSSVDLAIFSLHLAGISSILGAVNFITTIINMRPNNMSL
DQMPLFVWSVGITALLLLLSLPVLAGAITMLLTDRNLNTSFFDPAGGGDPILYQHL
>LEFIG222-10|HM875902|MM14117|Aethes_margaritana
TLYFIFGIWAGMVGTSLSLLIRAELGNPGSLIGDDQIYNTIVTAHAFIMIFFMVMPIMIGGFGNWLVPLMLGAPDMAFPRMNNMSFWLLPPSIMLLISSSIVENGAGTGWTVYPPLSSNIAHSGSSVDLAIFSLHLAGISSILGAVNFITTIINMRPNNMSL
DQMPLFVWSVGITALLLLLSLPVLAGAITMLLTDRNLNTSFFDPAGGGDPILYQHL
>LEFIC894-10|HM872711|MM05199|Aethes_rubigana
TLYFIFGIWAGMVGTSLSLLIRAELGNPGSLIGDDQIYNTIVTAHAFIMIFFMVMPIMIGGFGNWLVPLMLGAPDMAFPRMNNMSFWLLPPSIMLLISSSIVENGAGTGWTVYPPLSSNIAHSGSSVDLAIFSLHLAGISSILGAVNFITTIINMRPNNMSL
DQMPLFVWAVGITALLLLLSLPVLAGAITMLLTDRNLNTSFFDPAGGGDPILYQHL
>LEFID752-10|HM873509|MM06813|Aethes_rubigana
TLYFIFGVWAGMVGTSLSLLIRAELGNPGSLIGDDQIYNTIVTAHAFIMIFFMVMPIMIGGFGNWLVPLMLGAPDMAFPRMNNMSFWLLPPSIMLLISSSIVENGAGTGWTVYPPLSSNIAHSGSSVDLAIFSLHLAGISSILGAVNFITTIINMRPNNMS
LDQMPLFVWAVGITALLLLLSLPVLAGAITMLLTDRNLNTSFFDPAGGGDPILYQHL
>LEFIJ129-10|KM573080|MM08971|Aethes_rubigana
TLYFIFGIWAGMVGTSLSLLIRAELGNPGSLIGDDQIYNTIVTAHAFIMIFFMVMPIMIGGFGNWLVPLMLGAPDMAFPRMNNMSFWLLPPSIMLLISSSIVENGAGTGWTVYPPLSSNIAHSGSSVDLAIFSLHLAGISSILGAVNFITTIINMRPNNMSL
DQMPLFVWAVGITALLLLLSLPVLAGAITMLLTDRNLNTSFFDPAGGGDPILYQHL
>LEFIA004-10|HM396354|MM00075|Aethes_rutilana
TLYFIFGIWAGMVGTSLSLLIRAELGNPGSLIGDDQIYNTIVTAHAFIMIFFMVMPIMIGGFGNWLVPLMLGAPDMAFPRMNNMSFWLLPPSLMLLISSSIVENGAGTGWTVYPPLSSNIAHSGSSVDLAIFSLHLAGISSILGAVNFITTIINMRTNNMK
LDQMPLFVWSVGITALLLLLSLPVLAGAITMLLTDRNLNTSFFDPAGGGDPILYQHL
>LEFIE002-10|HM873751|MM08053|Aethes_rutilana
TLYFIFGIWAGMVGTSLSLLIRAELGNPGSLIGDDQIYNTIVTAHAFIMIFFMVMPIMIGGFGNWLVPLMLGAPDMAFPRMNNMSFWLLPPSLMLLISSSIVENGAGTGWTVYPPLSSNIAHSGSSVDLAIFSLHLAGISSILGAVNFITTIINMRTNNMK
LDQMPLFVWSVGITALLLLLSLPVLAGAITMLLTDRNLNTSFFDPAGGGDPILYQHL
>LEFIA868-10|HM387006|MM09745|Aethes_rutilana
TLYFIFGIWAGMVGTSLSLLIRTELGNPGSLIGDDQIYNTIVTAHAFIMIFFMVMPIMIGGFGNWLIPLMLGAPDMAFPRMNNMSFWLLPPSLMLLISSSIVENGAGTGWTVYPPLSSNIAHSGSSVDLAIFSLHLAGISSILGAVNFITTIINMRTNNMSL
DQMPLFVWSVGITALLLLLSLPVLAGAITMLLTDRNLNTSFFDPAGGGDPILYQHL
>LEFID400-10|HM873197|MM06319|Aethes_smeathmanniana
TLYFIFGIWAGMVGTSLSLLIRAELGNPGSLIGDDQIYNTIVTAHAFIMIFFMVMPIMIGGFGNWLVPLMLGAPDMAFPRMNNMSFWLLPPSIMLLISSSIVENGAGTGWTVYPPLSSNIAHSGSSVDLAIFSLHLAGISSILGAVNFITTIINMRPNNMSL
DQMPLFVWSVGITALLLLLSLPVLAGAITMLLTDRNLNTSFFDPAGGGDPILYQHL
>LEFIJ1423-12|KM572964|MM13141|Aethes_smeathmanniana
TLYFIFGIWAGMVGTSLSLLIRAELGNPGSLIGDDQIYNTIVTAHAFIMIFFMVMPIMIGGFGNWLVPLMLGAPDMAFPRMNNMSFWLLPPSIMLLISSSIVENGAGTGWTVYPPLSSNIAHSGSSVDLAIFSLHLAGISSILGAVNFITTIINMRPNNMSL
DQMPLFVWSVGITALLLLLSLPVLAGAITMLLTDRNLNTSFFDPAGGGDPILYQHL
>LEFID114-10|HM872925|MM05753|Aethes_tesserana
TLYFIFGIWAGMVGTSLSLLIRAELGNPGLLIGDDQIYNTIVTAHAFIMIFFMVMPIMIGGFGNWLVPLMLGAPDMAFPRMNNMSFWLLPPSIMLLISSSIVENGAGTGWTVYPPLSSNIAHSGSSVDLAIFSLHLAGISSILGAVNFITTIINMRPNNMSF
DQMPLFVWSVGITALLLLLSLPVLAGAITMLLTDRNLNTSFFDPAGGGDPILYQHL
>LEFIL220-10|MM19220|Aethes_tesserana
TLYFIFGIWAGMVGTSLSLLIRAELGNPGLLIGDDQIYNTIVTAHAFIMIFFMVMPIMIGGFGNWLVPLMLGAPDMAFPRMNNMSFWLLPPSIMLLISSSIVENGAGTGWTVYPPLSSNIAHSGSSVDLAIFSLHLAGISSILGAVNFITTIINMRPNNMSF
DQMPLFVWSVGITALLLLLSLPVLAGAITMLLTDRNLNTSFFDPAGGGDPILYQHL
>LEFIC398-10|HM872241|MM03939|Aethes_triangulana
TLYFIFGIWAGMVGTSLSLLIRAELGNPGSLIGDDQIYNTIVTAHAFIMIFFMVMPIMIGGFGNWLVPLMLGAPDMAFPRMNNMSFWLLPPSIMLLISSSIVENGAGTGWTVYPPLSSNIAHSGSSVDLAIFSLHLAGISSILGAVNFITTIINMRPNNMSL
DQMSLFIWAVGITALLLLLSLPVLAGAITMLLTDRNLNTSFFDPAGGGDPILYQHL
>LEFIF431-10|HM875116|MM11863|Aethes_triangulana
TLYFIFGIWAGMVGTSLSLLIRAELGNPGSLIGDDQIYNTIVTAHAFIMIFFMVMPIMIGGFGNWLIPLMLGAPDMAFPRMNNMSFWLLPPSIMLLISSSIVENGAGTGWTVYPPLSSNIAHSGSSVDLAIFSLHLAGISSILGAVNFITTIINMRPNNMSL

DQMSLFIWAVGITALLLLLSLPVLAGAITMLLTDRNLNTSFFDPAGGGDPILYQHL
>LEFIG812-10|HM876464|MM15676|Aethes_triangulana
TLYFIFGIWAGMVGTSLSLLIRAELGNPGSLIGDDQIYNTIVTAHAFIMIFFMVMPIMIGGFGNWLVPLMLGAPDMVFPRMNNMSFWLLPPSIMLLISSSIVENGAGTGWTVYPPLSSNIAHSGSSVDLAIFSLHLAGISSILGAVNFITTIINMRPNNMSL
DQMSLFIWAVGITALLLLLSLPVLAGAITMLLTDRNLNTSFFDPAGGGDPILYQHL
>LEFID757-10|HM873514|MM06820|Agapeta_hamana
TLYFIFGIWSGMLGTSLSMLIRAELGNPGSLIGDDQIYNTIVTAHAFIMIFFMVMPIMIGGFGNWLVPLMLGAPDMAFPRMNNMSFWLLPPSIMILISSSIVENGAGTGWTVYPPLSSNIAHSGSSVDLAIFSLHLAGISSILGAVNFITTIINMRPNNMKL
DQLPLFIWAVGITALLLLLSLPVLAGAITMLLTDRNLNTSFFDPAGGGDPILYQHL
>LEFIF409-10|HM875094|MM11780|Agapeta_hamana
TLYFIFGIWSGMLGTSLSMLIRAELGNPGSLIGDDQIYNTIVTAHAFIMIFFMVMPIMIGGFGNWLVPLMLGAPDMAFPRMNNMSFWLLPPSIMILISSSIVENGAGTGWTVYPPLSSNIAHSGSSVDLAIFSLHLAGISSILGAVNFITTIINMRPNNMKL
DQLPLFIWAVGITALLLLLSLPVLAGAITMLLTDRNLNTSFFDPAGGGDPILYQHL
>LEFID813-10|HM873570|MM06900|Agapeta_zoegana
TLYFIFGIWAGMIGTSLSLLIRAELGNPGSLIGDDQIYNTIVTAHAFIMIFFMVMPIMIGGFGNWLVPLMLGAPDMAFPRMNNMSFWLLPPSIMLLISSSIVENGAGTGWTVYPPLSSNIAHSGSSVDLAIFSLHLAGISSILGAVNFITTIINMRPNNMKL
DQMPLFVWAVGITALLLLLSLPVLAGAITMLLTDRNLNTSFFDPAGGGDPILYQHL
>LEFIF830-10|HM875513|MM13171|Agapeta_zoegana
TLYFIFGIWAGMIGTSLSLLIRAELGNPGSLIGDDQIYNTIVTAHAFIMIFFMVMPIMIGGFGNWLVPLMLGAPDMAFPRMNNMSFWLLPPSIMLLISSSIVENGAGTGWTVYPPLSSNIAHSGSSVDLAIFSLHLAGISSILGAVNFITTIINMRPNNMKL
DQMPLFVWAVGITALLLLLSLPVLAGAITMLLTDRNLNTSFFDPAGGGDPILYQHL
>LEEUA259-11|JN261744|MM19667|Agdistis_bennetii
TLYFIFGIWAGMLGTSLSMLIRAELGNPGSLIGDDQIYNTIVTAHAFIMIFFMVMPIMIGGFGNWLIPLMMGAPDMAFPRMNNMSFWLLPPSIFLLISSSIVENGAGTGWTVYPPLSSNLTHSGSSVDLAIFSLHLAGISSILGAINFITTIINMRLNKMMF
DQMPLFIWAVGITAILLLLSLPVLAGAITMLLTDRNLNTSFFDPAGGGDPILYQHL
>LEFIB081-10|HM870990|MM00383|Aglais_io
TLYFIFGIWAGMVGTSLSLLIRTELGNPGSLIGDDQIYNTIVTAHAFIMIFFMVMPIMIGGFGNWLIPLMLGAPDMAFPRMNNMSFWLLPPSLMLLISSSIVENGAGTGWTVYPPLSSNIAHSGSSVDLAIFSLHLAGISSILGAINFITTIINMRVNSMSFD
QMPLFVWAVGITALLLLLSLPVLAGAITMLLTDRNINTSFFDPAGGGDPILYQHL
>LEFIB080-10|HM870989|MM00382|Aglais_urticae
TLYFIFGIWAGMVGTSLSLLIRTELGNPGSLIGDDQIYNTIVTAHAFIMIFFMVMPIMIGGFGNWLVPLMLGAPDMAFPRMNNMSFWLLPPSLMLLISSSIVENGAGTGWTVYPPLSSNIAHSGSSVDLAIFSLHLAGISSILGAINFITTIINMRVNSMSF
DQMPLFVWAVGITALLLLLSLPVLAGAITMLLTDRNINTSFFDPAGGGDPILYQHL
>LEFIJ529-10|JF853648|MM17154|Aglais_urticae
TLYFIFGIWAGMVGTSLSLLIRTELGNPGSLIGDDQIYNTIVTAHAFIMIFFMVMPIMIGGFGNWLVPLMLGAPDMAFPRMNNMSFWLLPPSLMLLISSSIVENGAGTGWTVYPPLSSNIAHSGSSVDLAIFSLHLAGISSILGAINFITTIINMRVNSMSF
DQMPLFVWAVGITALLLLLSLPVLAGAITMLLTDRNINTSFFDPAGGGDPILYQHL
>LEFIB006-10|HM870919|MM00017|Aglia_tau
TLYFIFGIWAGMVGTSLSLLIRAELGTPGSLIGDDQIYNTIVTAHAFIMIFFMVMPIMIGGFGNWLVPLMLGAPDMAFPRMNNMSFWLLPPSLTLLISSSIVENGAGTGWTVYPPLSSNIAHSGTSVDLAIFSLHLAGISSILGAINFITTIINMRLNNMSFD
QMPLFVWAVGITAFLLLLSLPVLAGAITMLLTDRNLNTSFFDPAGGGDPILYQHL
>LEFIE959-10|HM874676|MM10365|Aglossa_pinguinalis
TLYFIFGIWSGMVGTSLSLLIRAELGNPGSLIGDDQIYNTIVTAHAFIMIFFMVMPIMIGGFGNWLVPLMLGAPDMAFPRMNNMSFWLLPPSLTLLISSSIVENGAGTGWTVYPPLSSNIAHGGSSVDLAIFSLHLAGISSILGAVNFITTIINMKLNNLSFD
QMPLFVWSVGITALLLLLSLPVLAGAITMLLTDRNLNTSFFDPAGGGDPILYQHL
>LEFIG659-10|HM876320|MM15523|Agnathosia_mendicella
TLYFIFGIWAGMLGTSLSVLIRMELGNPGSLIGNDQIYNTVVTAHAFIMIFFMVMPIMMGGFGNWLVPLMLGAPDMAFPRMNNMSFWLLPPSLMLLITSSIVENGSGTGWTVYPPLSSNIAHSGSSVDLTIFSLHLAGISSILGAVNFITTMFNMKPIN
MKLNQMPLFVWSVGITALLLLLSLPVLAGAITMLLTDRNLNTSFFDPAGGGDPILYQHL
>LEFIC144-10|HM871990|MM03489|Agonopterix_alstromeriana
TLYFIFGIWAGMVGTSLSLLIRAELGNPGSLIGDDQIYNTIVTAHAFIMIFFMVMPIMIGGFGNWLVPLMLGAPDMAFPRMNNMSFWLLPPSLTLLISSSIVENGAGTGWTVYPPLSSNIAHGGSSVDLAIFSLHLAGISSILGAINFITTIINMRLNNMSF
DRLPLFVWAVGITALLLLLSLPVLAGAITMLLTDRNLNTSFFDPAGGGDPILYQHL
>LEFIB557-10|HM871439|MM02196|Agonopterix_angelicella
TLYFIFGIWAGMVGTSLSLLIRAELGNPGSLIGDDQIYNTIVTAHAFIMIFFMVMPIMIGGFGNWLVPLMLGAPDMAFPRMNNMSFWLLPPSLTLLISSSIVENGAGTGWTVYPPLSSNIAHGGSSVDLAIFSLHLAGISSILGAINFITTIINMRLNNMSF
DRLPLFVWAVGITALLLLLSLPVLAGAITMLLTDRNLNTSFFDPAGGGDPILYQHL
>LEFIE072-10|HM873819|MM08211|Agonopterix_angelicella
TLYFIFGIWAGMVGTSLSLLIRAELGNPGSLIGDDQIYNTIVTAHAFIMIFFMVMPIMIGGFGNWLVPLMLGAPDMAFPRMNNMSFWLLPPSLTLLISSSIVENGAGTGWTVYPPLSSNIAHGGSSVDLAIFSLHLAGISSILGAINFITTIINMRLNNMSF
DRLPLFVWAVGITALLLLLSLPVLAGAITMLLTDRNLNTSFFDPAGGGDPILYQHL
>LEFIJ920-10|JF853899|MM17545|Agonopterix_arctica

TLYFIFGIWAGMVGTSLSLLIRAELGNPGSLIGDDQIYNTIVTAHAFIMIFFMVMPIMIGGFGNWLVPLMLGAPDMAFPRMNNMSFWLLPPSLTLLISSSIVENGAGTGWTVYPPLSSNIAHGGSSVDLAIFSLHLAGISSILGAINFITTIINMRLNNMSF
DQLPLFVWAVGITALLLLLSLPVLAGAITMLLTDRNLNTSFFDPAGGGDPILYQHL
>LEFIF919-10|HM875601|MM13348|Agonopterix_arenella
TLYFIFGIWAGMVGTSLSLLIRAELGNPGSLIGDDQIYNTIVTAHAFIMIFFMVMPIMIGGFGNWLVPLMLGAPDMAFPRMNNMSFWLLPPSLTLLISSSIVENGAGTGWTVYPPLSSNIAHGGSSVDLAIFSLHLAGISSILGAINFITTIINMRLNNMSF
DRLPLFVWAVGITALLLLLSLPVLAGAITMLLTDRNLNTSFFDPAGGGDPILYQHL
>LEEUA096-11|MM19504|Agonopterix_assimilella
TLYFIFGIWAGMVGTSLSLLIRAELGNPGSLIGDDQIYNTIVTAHAFIMIFFMVMPIMIGGFGNWLVPLMLGAPDMAFPRMNNMSFWLLPPSLTLLISSSIVENGAGTGWTVYPPLSSNIAHGGSSVDLAIFSLHLAGISSILGAINFITTIINMRLNNMSF
DRLPLFVWSVGITALLLLLSLPVLAGAITMLLTDRNLNTSFFDPAGGGDPILYQHL
>LEFIC284-10|HM872128|MM03749|Agonopterix_astrantiae
TLYFIFGIWAGMVGTSLSLLIRAELGNPGSLIGDDQIYNTIVTAHAFIMIFFMVMPIMIGGFGNWLVPLMLGAPDMAFPRMNNMSFWLLPPSLTLLISSSIVENGAGTGWTVYPPLSSNIAHSGSSVDLAIFSLHLAGISSILGAINFITTIINMRLNNMSFD
RLPLFVWAVGITALLLLLSLPVLAGAITMLLTDRNLNTSFFDPAGGGDPILYQHL
>LEEUA095-11|JN266642|MM19503|Agonopterix_atomella
TLYFIFGIWAGMVGTSLSLLIRAELGNPGSLIGDDQIYNTIVTAHAFIMIFFMVMPIMIGGFGNWLVPLMLGAPDMAFPRMNNMSFWLLPPSLTLLISSSIVENGAGTGWTVYPPLSSNIAHGGSSVDLAIFSLHLAGISSILGAINFITTIINMRLNNMSF
DRLPLFVWAVGITALLLLLSLPVLAGAITMLLTDRNLNTSFFDPAGGGDPILYQHL
>LEFIE111-10|HM873859|MM08300|Agonopterix_broennoeensis
TLYFIFGIWAGMVGTSLSLLIRAELGNPGSLIGDDQIYNTIVTAHAFIMIFFMVMPIMIGGFGNWLVPLMLGAPDMAFPRMNNMSFWLLPPSLTLLISSSIVENGAGTGWTVYPPLSSNIAHGGSSVDLAIFSLHLAGISSILGAINFITTIINMRLNNMSF
DRLPLFVWAVGITALLLLLSLPVLAGAITMLLTDRNLNTSFFDPAGGGDPILYQHL
>LEFIE232-10|HM873966|MM08550|Agonopterix_broennoeensis
TLYFIFGIWAGMVGTSLSLLIRAELGNPGSLIGDDQIYNTIVTAHAFIMIFFMVMPIMIGGFGNWLVPLMLGAPDMAFPRMNNMSFWLLPPSLTLLISSSIVENGAGTGWTVYPPLSSNIAHGGSSVDLAIFSLHLAGISSILGAINFITTIINMRLNNMSF
DRLPLFVWAVGITALLLLLSLPVLAGAITMLLTDRNLNTSFFDPAGGGDPILYQHL
>LEFIE236-10|HM873970|MM08556|Agonopterix_capreolella
TLYFIFGIWAGMVGTSLSLLIRAELGNPGSLIGDDQIYNTIVTAHAFIMIFFMVMPIMIGGFGNWLVPLMLGAPDMAFPRMNNMSFWLLPPSLTLLISSSIVENGAGTGWTVYPPLSSNIAHGGSSVDLAIFSLHLAGISSILGAINFITTIINMRLNSMSFD
RLPLFVWAVGITALLLLLSLPVLAGAITMLLTDRNLNTSFFDPAGGGDPILYQHL
>LEFIE237-10|HM873971|MM08557|Agonopterix_capreolella
TLYFIFGIWAGMVGTSLSLLIRAELGNPGSLIGDDQIYNTIVTAHAFIMIFFMVMPIMIGGFGNWLVPLMLGAPDMAFPRMNNMSFWLLPPSLTLLISSSIVENGAGTGWTVYPPLSSNIAHGGSSVDLAIFSLHLAGISSILGAINFITTIINMRLNSMSFD
RLPLFVWAVGITALLLLLSLPVLAGAITMLLTDRNLNTSFFDPAGGGDPILYQHL
>LEFIE238-10|HM873972|MM08558|Agonopterix_capreolella
TLYFIFGIWAGMVGTSLSLLIRAELGNPGSLIGDDQIYNTIVTAHAFIMIFFMVMPIMIGGFGNWLVPLMLGAPDMAFPRMNNMSFWLLPPSLTLLISSSIVENGAGTGWTVYPPLSSNIAHGGSSVDLAIFSLHLAGISSILGAINFITTIINMRLNNMSF
DRLPLFVWAVGITALLLLLSLPVLAGAITMLLTDRNLNTSFFDPAGGGDPILYQHL
>LEFIG459-10|HM876135|MM14500|Agonopterix_capreolella
TLYFIFGIWAGMVGTSLSLLIRAELGNPGSLIGDDQIYNTIVTAHAFIMIFFMVMPIMIGGFGNWLVPLMLGAPDMAFPRMNNMSFWLLPPSLTLLISSSIVENGAGTGWTVYPPLSSNIAHGGSSVDLAIFSLHLAGISSILGAINFITTIINMRLNNMSF
DRLPLFVWAVGITALLLLLSLPVLAGAITMLLTDRNLNTSFFDPAGGGDPILYQHL
>LEFIC507-10|HM872332|MM04215|Agonopterix_ciliella
TLYFIFGIWAGMVGTSLSLLIRAELGNPGSLIGDDQIYNTIVTAHAFIMIFFMVMPIMIGGFGNWLVPLMLGAPDMAFPRMNNMSFWLLPPSLTLLISSSIVENGAGTGWTVYPPLSSNIAHGGSSVDLAIFSLHLAGISSILGAINFITTIINMRLNNMSF
DRLPLFVWAVGITALLLLLSLPVLAGAITMLLTDRNLNTSFFDPAGGGDPILYQHL
>LEFIE230-10|HM873964|MM08548|Agonopterix_ciliella
TLYFIFGIWAGMVGTSLSLLIRAELGNPGSLIGDDQIYNTIVTAHAFIMIFFMVMPIMIGGFGNWLVPLMLGAPDMAFPRMNNMSFWLLPPSLTLLISSSIVENGAGTGWTVYPPLSSNIAHGGSSVDLAIFSLHLAGISSILGAINFITTIINMRLNNMSF
DRLPLFVWAVGITALLLLLSLPVLAGAITMLLTDRNLNTSFFDPAGGGDPILYQHL
>LEFIK569-10|JF854160|MM18144|Agonopterix_ciliella
TLYFIFGIWAGMVGTSLSLLIRAELGNPGSLIGDDQIYNTIVTAHAFIMIFFMVMPIMIGGFGNWLVPLMLGAPDMAFPRMNNMSFWLLPPSLTLLISSSIVENGAGTGWTVYPPLSSNIAHGGSSVDLAIFSLHLAGISSILGAINFITTIINMRLNNMSF
DRLPLFVWAVGITALLLLLSLPVLAGAITMLLTDRNLNTSFFDPAGGGDPILYQHL
>LEEUA098-11|MM19506|Agonopterix_cnicella
TLYFIFGIWAGMVGTSLSLLIRAELGNPGSLIGDDQIYNTIVTAHAFIMIFFMVMPIMIGGFGNWLVPLMLGAPDMAFPRMNNMSFWLLPPSLTLLISSSIVENGAGTGWTVYPPLSSNIAHGGSSVDLAIFSLHLAGISSILGAINFITTIINMRLNNMSF
DRLPLFVWAVGITALLLLLSLPVLAGAITMLLTDRNLNTSFFDPAGGGDPILYQHL
>LEFIB280-10|HM871182|MM00768|Agonopterix_conterminella
TLYFIFGIWAGMVGTSLSLLIRAELGTPGSLIGDDQIYNTIVTAHAFIMIFFMVMPIMIGGFGNWLVPLMLGAPDMAFPRMNNMSFWLLPPSITLLISSSIVENGAGTGWTVYPPLSSNIAHGGSSVDLAIFSLHLAGISSILGAINFITTIINMRLNNMFFD
QLPLFVWAVGITALLLLLSLPVLAGAITMLLTDRNLNTSFFDPAGGGDPILYQHL

>LEFIE913-10|HM874630|MM10277|Agonopterix_conterminella
TLYFIFGIWAGMVGTSLSLLIRAELGTPGSLIGDDQIYNTIVTAHAFIMIFFMVMPIMIGGFGNWLVPLMLGAPDMAFPRMNNMSFWLLPPSITLLISSSIVENGAGTGWTVYPPLSSNIAHGGSSVDLAIFSLHLAGISSILGAINFITTIINMRLNNMFFD
QLPLFVWAVGITALLLLLSLPVLAGAITMLLTDRNLNTSFFDPAGGGDPILYQHL
>LEFIL591-10|JF854676|MM18889|Agonopterix_conterminella
TLYFIFGIWAGMVGTSLSLLIRAELGTPGSLIGDDQIYNTIVTAHAFIMIFFMVMPIMIGGFGNWLVPLMLGAPDMAFPRMNNMSFWLLPPSITLLISSSIVENGAGTGWTVYPPLSSNIAHGGSSVDLAIFSLHLAGISSILGAINFITTIINMRLNNMFFD
QLPLFVWAVGITALLLLLSLPVLAGAITMLLTDRNLNTSFFDPAGGGDPILYQHL
>LEEUA097-11|MM19505|Agonopterix_curvipunctosa
TLYFIFGIWAGMVGTSLSLLIRAELGNPGSLIGDDQIYNTIVTAHAFIMIFFMVMPIMIGGFGNWLVPLMLGAPDMAFPRMNNMSFWLLPPSLTLLISSSIVENGAGTGWTVYPPLSSNIAHGGSSVDLAIFSLHLAGISSILGAINFITTIINMRLNNMSF
DRLPLFVWAVGITALLLLLSLPVLAGAITMLLTDRNLNTSFFDPAGGGDPILYQHL
>LEFIB359-10|HM871259|MM00911|Agonopterix_heracliana
TLYFIFGIWAGMVGTSLSLLIRAELGNPGSLIGDDQIYNTIVTAHAFIMIFFMVMPIMIGGFGNWLVPLMLGAPDMAFPRMNNMSFWLLPPSLTLLISSSIVENGAGTGWTVYPPLSSNIAHGGSSVDLAIFSLHLAGISSILGAINFITTIINMRLNNMSF
DRLPLFVWAVGITALLLLLSLPVLAGAITMLLTDRNLNTSFFDPAGGGDPILYQHL
>LEFIA138-10|HM396484|MM01086|Agonopterix_heracliana
TLYFIFGIWAGMVGTSLSLLIRAELGNPGSLIGDDQIYNTIVTAHAFIMIFFMVMPIMIGGFGNWLVPLMLGAPDMAFPRMNNMSFWLLPPSLTLLISSSIVENGAGTGWTVYPPLSSNIAHGGSSVDLAIFSLHLAGISSILGAINFITTIINMRLNNMSF
DRLPLFVWAVGITALLLLLSLPVLAGAITMLLTDRNLNTSFFDPAGGGDPILYQHL
>LEFIA139-10|HM396485|MM01087|Agonopterix_heracliana
TLYFIFGIWAGMVGTSLSLLIRAELGNPGSLIGDDQIYNTIVTAHAFIMIFFMVMPIMIGGFGNWLVPLMLGAPDMAFPRMNNMSFWLLPPSLTLLISSSIVENGAGTGWTVYPPLSSNIAHGGSSVDLAIFSLHLAGISSILGAINFITTIINMRLNNMSF
DRLPLFVWAVGITALLLLLSLPVLAGAITMLLTDRNLNTSFFDPAGGGDPILYQHL
>LEFIE235-10|HM873969|MM08554|Agonopterix_hypericella
TLYFIFGIWAGMVGTSLSLLIRAELGNPGSLIGDDQIYNTIVTAHAFIMIFFMVMPIMIGGFGNWLVPLMLGAPDMAFPRMNNMSFWLLPPSLTLLISSSIVENGAGTGWTVYPPLSSNIAHGGSSVDLAIFSLHLAGISSILGAINFITTIINMRLNNMSF
DQLPLFVWAVGITALLLLLSLPVLAGAITMLLTDRNLNTSFFDPAGGGDPILYQHL
>LEFIB783-10|HM871660|MM02681|Agonopterix_kaekeritziana
TLYFIFGIWAGMVGTSLSLLIRAELGNPGSLIGDDQIYNTIVTAHAFIMIFFMVMPIMIGGFGNWLVPLMLGAPDMAFPRMNNMSFWLLPPSLTLLISSSIVENGAGTGWTVYPPLSSNIAHGGSSVDLAIFSLHLAGISSILGAINFITTIINMRLNNMSF
DRLPLFVWAVGITALLLLLSLPVLAGAITMLLTDRNLNTSFFDPAGGGDPILYQHL
>LEFIG579-10|HM876250|MM14764|Agonopterix_kaekeritziana
TLYFIFGIWAGMVGTSLSLLIRAELGNPGSLIGDDQIYNTIVTAHAFIMIFFMVMPIMIGGFGNWLVPLMLGAPDMAFPRMNNMSFWLLPPSLTLLISSSIVENGAGTGWTVYPPLSSNIAHGGSSVDLAIFSLHLAGISSILGAINFITTIINMRLNNMSF
DRLPLFVWAVGITALLLLLSLPVLAGAITMLLTDRNLNTSFFDPAGGGDPILYQHL
>LEFIL699-10|KM573635|MM18997|Agonopterix_kaekeritziana
TLYFIFGIWAGMVGTSLSLLIRAELGNPGSLIGDDQIYNTIVTAHAFIMIFFMVMPIMIGGFGNWLVPLMLGAPDMAFPRMNNMSFWLLPPSLTLLISSSIVENGAGTGWTVYPPLSSNIAHSGSSVDLAIFSLHLAGISSILGAINFITTIINMRLNNMSFD
RLPLFVWAVGITALLLLLSLPVLAGAITMLLTDRNLNTSFFDPAGGGDPILYQHL
>LEEUA093-11|JN266640|MM19501|Agonopterix_laterella
TLYFIFGIWAGMVGTSLSLLIRAELGNPGSLIGDDQIYNTIVTAHAFIMIFFMVMPIMIGGFGNWLVPLMLGAPDMAFPRMNNMSFWLLPPSLTLLISSSIVENGAGTGWTVYPPLSSNIAHGGSSVDLAIFSLHLAGISSILGAINFITTIINMRLNNMSF
DRLPLFVWAVGITALLLLLSLPVLAGAITMLLTDRNLNTSFFDPAGGGDPILYQHL
>LEFID754-10|HM873511|MM06815|Agonopterix_liturosa
TLYFIFGIWAGMVGTSLSLLIRAELGNPGSLIGDDQIYNTIVTAHAFIMIFFMVMPIMIGGFGNWLVPLMLGAPDMAFPRMNNMSFWLLPPSLTLLISSSIVENGAGTGWTVYPPLSSNIAHGGSSVDLAIFSLHLAGISSILGAINFITTIINMRLNNMSF
DQLPLFVWAVGITALLLLLSLPVLAGAITMLLTDRNLNTSFFDPAGGGDPILYQHL
>LEFIF858-10|HM875540|MM13226|Agonopterix_liturosa
TLYFIFGIWAGMVGTSLSLLIRAELGNPGSLIGDDQIYNTIVTAHAFIMIFFMVMPIMIGGFGNWLVPLMLGAPDMAFPRMNNMSFWLLPPSLTLLISSSIVENGAGTGWTVYPPLSSNIAHGGSSVDLAIFSLHLAGISSILGAINFITTIINMRLNNMSF
DQLPLFVWAVGITALLLLLSLPVLAGAITMLLTDRNLNTSFFDPAGGGDPILYQHL
>LEFIC456-10|HM872290|MM04039|Agonopterix_multiplicella
TLYFIFGIWAGMVGTSLSLLIRAELGNPGSLIGDDQIYNTIVTAHAFIMIFFMVMPIMIGGFGNWLVPLMLGAPDMAFPRMNNMSFWLLPPSLTLLISSSIVENGAGTGWTVYPPLSSNIAHGGSSVDLAIFSLHLAGISSILGAINFITTIINMRLNNMSF
DRLPLFVWAVGITALLLLLSLPVLAGAITMLLTDRNLNTSFFDPAGGGDPILYQHL
>LEFID256-10|HM873054|MM06121|Agonopterix_nervosa
TLYFIFGIWSGMVGTSLSLLIRAELGNPGSLIGDDQIYNTIVTAHAFIMIFFMVMPIMIGGFGNWLVPLMLGAPDMAFPRMNNMSFWLLPPSLTLLISSSIVENGAGTGWTVYPPLSSNIAHGGSSVDLAIFSLHLAGISSILGAINFITTIINMRLNNMSF
DRLPLFVWAVGITTLLLLLSLPVLAGAITMLLTDRNLNTSFFDPAGGGDPILYQHL
>LEFIF527-10|HM875212|MM12167|Agonopterix_nervosa
TLYFIFGIWSGMVGTSLSLLIRAELGNPGSLIGDDQIYNTIVTAHAFIMIFFMVMPIMIGGFGNWLVPLMLGAPDMAFPRMNNMSFWLLPPSLTLLISSSIVENGAGTGWTVYPPLSSNIAHGGSSVDLAIFSLHLAGISSILGAINFITTIINMRLNNMSF

DRLPLFVWAVGITALLLLLSLPVLAGAITMLLTDRNLNTSFFDPAGGGDPILYQHL
>LEFIK572-10|MM18147|Agonopterix_nervosa
TLYFIFGIWSGMVGTSLSLLIRAELGNPGSLIGDDQIYNTIVTAHAFIMIFFMVMPIMIGGFGNWLVPLMLGAPDMAFPRMNNMSFWLLPPSLTLLISSSIVENGAGTGWTVYPPLSSNIAHGGSSVDLAIFSLHLAGISSILGAINFITTIINMRLNNMSF
DRLPLFVWAVGITALLLLLSLPVLVGAITMLLTDRNLNTSFFDPAGGGDPILYQHL
>LEFIB113-10|HM871020|MM00461|Agonopterix_ocellana
TLYFIFGIWAGMVGTSLSLLIRAELGNPGSLIGDDQIYNTIVTAHAFIMIFFMVMPIMIGGFGNWLVPLMLGAPDMAFPRMNNMSFWLLPPSLTLLISSSIVENGAGTGWTVYPPLSSNIAHGGSSVDLAIFSLHLAGISSILGAINFITTIINMRLNNMSF
DRLPLFVWAVGITALLLLLSLPVLAGAITMLLTDRNLNTSFFDPAGGGDPILYQHL
>LEEUA568-11|MM20627|Agonopterix_parilella
TLYFIFGI?AGMVGTSLSLLIRAELGNPGSLIGDDQIYNTIVTAHAFIMIFFMVMPIMIGGFGNWLVPLMLGAPDMAFPRMNNMSFWLLPPSLTLLISSSIVENGAGTGWTVYPPLSSNIAHGGSSVDLAIFSLHLAGISSILGAINFITTIINMRLYNMSFD
RLPLFVWAVGITALLLLLSLPVLAGAITMLLTDRNLNTSFFDPAGGGDPILYQHL
>LEFID188-10|HM872994|MM05980|Agonopterix_propinquella
TLYFIFGIWAGMVGTSLSLLIRAELGNPGSLIGDDQIYNTIVTAHAFIMIFFMVMPIMIGGFGNWLVPLMLGAPDMAFPRMNNMSFWLLPPSLTLLISSSIVENGAGTGWTVYPPLSSNIAHSGSSVDLAIFSLHLAGISSILGAINFITTIINMRLNNMSFD
RLPLFVWAVGITALLLLLSLPVLAGAITMLLTDRNLNTSFFDPAGGGDPILYQHL
>LEEUA100-11|MM19508|Agonopterix_purpurea
TLYFIFGIWAGMVGTSLSLLIRAELGNPGSLIGDDQIYNTIVTAHAFIMIFFMVMPIMIGGFGNWLVPLMLGAPDMAFPRMNNMSFWLLPPSLTLLISSSIVENGAGTGWTVYPPLSSNIAHGGSSVDLAIFSLHLAGISSILGAINFITTIINMRLNNMSF
DRLPLFVWAVGITALLLLLSLPVLAGAITMLLTDRNLNTSFFDPAGGGDPILYQHL
>LEFIB037-10|HM870946|MM00302|Agonopterix_quadripunctata
TLYFIFGIWAGMVGTSLSLLIRAELGNPGSLIGDDQIYNTIVTAHAFIMIFFMVMPIMIGGFGNWLVPLMLGAPDMAFPRMNNMSFWLLPPSLTLLISSSIVENGAGTGWTVYPPLSSNIAHSGTSVDLAIFSLHLAGISSILGAINFITTIINMRLNNMSF
DRLPLFVWAVGITALLLLLSLPVLAGAITMLLTDRNLNTSFFDPAGGGDPILYQHL
>LEFIG689-10|HM876346|MM15553|Agonopterix_quadripunctata
TLYFIFGIWAGMVGTSLSLLIRAELGNPGSLIGDDQIYNTIVTAHAFIMIFFMVMPIMIGGFGNWLVPLMLGAPDMAFPRMNNMSFWLLPPSLTLLISSSIVENGAGTGWTVYPPLSSNIAHSGTSVDLAIFSLHLAGISSILGAINFITTIINMRLNNMSF
DRLPLFVWAVGITALLLLLSLPVLAGAITMLLTDRNLNTSFFDPAGGGDPILYQHL
>LEFIG690-10|HM876347|MM15554|Agonopterix_quadripunctata
?LYFIFGIWAGMVGTSLSLLIRAELGNPGSLIGDDQIYNTIVTAHAFIMIFFMVMPIMIGGFGNWLVPLMLGAPDMAFPRMNNMSFWLLPPSLTLLISSSIVENGAGTGWTVYPPLSSNIAHSGTSVDLAIFSLHLAGISSILGAINFITTIINMRLNNMSFD
RLPLFVWAVGITALLLLLSLPVLAGAITMLLTDRNLNTSFFDPAGGGDPILYQHL
>LEEUA094-11|JN266641|MM19502|Agonopterix_scopariella
TLYFIFGIWAGMVGTSLSLLIRAELGNPGSLIGDDQIYNTIVTAHAFIMIFFMVMPIMIGGFGNWLVPLMLGAPDMAFPRMNNMSFWLLPPSLTLLISSSIVENGAGTGWTVYPPLSSNIAHGGSSVDLAIFSLHLAGISSILGAINFITTIINMRLNNMSF
DRLPLFVWAVGITALLLLLSLPVLAGAITMLLTDRNLNTSFFDPAGGGDPILYQHL
>LEFIA654-10|HM870902|MM01771|Agonopterix_selini
TLYFIFGIWAGMVGTSLSLLIRTELGNPGSLIGDDQIYNTIVTAHAFIMIFFMVMPIMIGGFGNWLVPLMLGAPDMAFPRMNNMSFWLLPPSLTLLISSSIVETGAGTGWTVYPPLSSNIAHSGSSVDLAIFSLHLAGISSILGAINFITTIINMRLNNMSFD
RLPLFVWAVGITALLLLLSLPVLAGAITMLLTDRNLNTSFFDPAGGGDPILYQHL
>LEFIB405-10|HM871304|MM01773|Agonopterix_selini
TLYFIFGIWAGMVGTSLSLLIRTELGNPGSLIGDDQIYNTIVTAHAFIMIFFMVMPIMIGGFGNWLVPLMLGAPDMAFPRMNNMSFWLLPPSLTLLISSSIVENGAGTGWTVYPPLSSNIAHSGSSVDLAIFSLHLAGISSILGAINFITTIINMRLNNMSFD
RLPLFVWAVGITALLLLLSLPVLAGAITMLLTDRNLNTSFFDPAGGGDPILYQHL
>LEEUA101-11|MM19509|Agonopterix_subpropinquella
TLYFIFGIWAGMIGTSLSLLIRAELGTPGSLIGDDQIYNTIVTAHAFIMIFFMVMPIMIGGFGNWLVPLMLGAPDMAFPRMNNMSFWLLPPSLILLISSSIVENGAGTGWTVYPPLSSNIAHSGSSVDLAIFSLHLAGISSILGAINFITTIINMRLNNMSFDR
LPLFVWAVGITALLLLLSLPVLAGAITMLLTDRNLNTSFFDPAGGGDPILYQHL
>LEFIL614-10|MM18912|Agonopterix_yeatiana
TLYFIFGIWAGMVGTSLSLLIRAELGNPGSLIGDDQIYNTIVTAHAFIMIFFMVMPIMIGGFGNWLVPLMLGAPDMAFPRMNNMSFWLLPPSLTLLISSSIVENGAGTGWTVYPPLSSNIAHGGSSVDLAIFSLHLAGISSILGAINFITTIINMRLNNMSF
DRLPLFVWAVGITALLLLLSLPVLAGAITMLLTDRNLNTSFFDPAGGGDPILYQHL
>LEFIL374-10|MM18684|Agriades_aquilo
TLYFIFGIWAGMVGTSLSILIRMELSTPGSLIGDDQIYNTIVTAHAFIMIFFMVMPIMIGGFGNWLVPLMLGAPDMAFPRMNNMSFWLLPPSLMLLISSSIVENGAGTGWTVYPPLSSNIAHSGSSVDLAIFSLHLAGISSILGAINFITTIINMRVNNLSFD
QMSLFIWAVGITALLLLLSLPVLAGAITMLLTDRNLNTSFFDPAGGGDPILYQHL
>LEFIE088-10|HM873835|MM08266|Agriopis_aurantiaria
TLYFIFGIWAGMLGTSLSLLIRAELGNPGSLIGDDQIYNTIVTAHAFIMIFFMVMPIMIGGFGNWLVPLMLGAPDMAFPRMNNMSFWLLPPSITLLISSSIVENGAGTGWTVYPPLSSNIAHGGSSVDLAIFSLHLAGISSILGAINFITTIINMRLNNLSFD
QMPLFVWAVGITAFLLLLSLPVLAGAITMLLTDRNLNTSFFDPAGGGDPILYQHL
>LEFIG975-10|HM876615|MM15839|Agriopis_aurantiaria

TLYFIFGIWAGMLGTSLSLLIRAELGNPGSLIGDDQIYNTIVTAHAFIMIFFMVMPIMIGGFGNWLVPLMLGAPDMAFPRMNNMSFWLLPPSITLLISSSIVENGAGTGWTVYPPLSSNIAHGGSSVDLAIFSLHLAGISSILGAINFITTIINMRLNNLSFD
QMPLFVWAVGITAFLLLLSLPVLAGAITMLLTDRNLNTSFFDPAGGGDPILYQHL
>LEFID898-10|HM873648|MM07217|Agriopis_marginaria
TLYFIFGIWAGMLGTSLSLLIRAELGNPGSLIGDDQIYNTIVTAHAFIMIFFMVMPIMIGGFGNWLVPLMLGAPDMAFPRMNNMSFWLLPPSITLLISSSIVENGAGTGWTVYPPLSSNIAHGGSSVDLAIFSLHLAGISSILGAINFITTIINMRLNNLSFD
QMPLFVWAVGITAFLLLLSLPVLAGAITMLLTDRNLNTSFFDPAGGGDPILYQHL
>LEFIG582-10|HM876253|MM14771|Agriphila_biarmicus
TLYFIFGIWAGMVGTSLSLLIRAELGNPGSLIGDDQIYNTIVTAHAFIMIFFMVMPIMIGGFGNWLIPLMLGSPDMAFPRMNNMSFWLLPPSLTLLISSSMVETGAGTGWTVYPPLSSNIAHGGSSVDLAIFSLHLAGISSILGAINFITTIINMRINGLSFN
QMPLFVWSVGITAILLLLSLPVLAGAITMLLTDRNLNTSFFDPAGGGDPILYQHL
>LEFIL551-10|JF854654|MM18849|Agriphila_deliella
TLYFIFGIWAGMVGTSLSLLIRAELGNPGCLIGDDQIYNTIVTAHAFIMIFFMVMPIMIGGFGNWLVPLMLGAPDMAFPRMNNMSFWLLPPSLTLLISSSIVETGAGTGWTVYPPLSSNIAHSGSSVDLAIFSLHLAGISSILGAINFITTIINMRINGLSFDQ
MPLFVWSVGITALLLLLSLPVLAGAITMLLTDRNLNTSFFDPAGGGDPILYQHL
>LEEUA280-11|JN266124|MM19688|Agriphila_geniculea
TLYFIFGIWAGMVGTSLSLLIRAELGNPGSLIGDDQIYNTIVTAHAFIMIFFMVMPIMIGGFGNWLVPLMLGAPDMAFPRMNNMSFWLLPPSLTLLISSSIVENGAGTGWTVYPPLSSNIAHGGSSVDLAIFSLHLAGISSILGAINFITTIINMRINGLSFD
QMPLFVWSVGITALLLLLSLPVLAGAITMLLTDRNLNTSFFDPAGGGDPILYQHL
>LEFIB414-10|HM871313|MM01886|Agriphila_inquinatella
TLYFIFGIWAGMVGTSLSLLIRAELGNPGSLIGDDQIYNTIVTAHAFIMIFFMVMPIMIGGFGNWLVPLMLGAPDMAFPRMNNMSFWLLPPSLTLLISSSIVENGAGTGWTVYPPLSSNIAHSGSSVDLAIFSLHLAGISSILGAINFITTIINMRINGLSFD
QMPLFVWSVGITALLLLLSLPVLAGAITMLLTDRNLNTSFFDPAGGGDPILYQHL
>LEEUA279-11|JN266123|MM19687|Agriphila_latistria
TLYFIFGIWAGMVGTSLSLLIRAELGNPGSLIGDDQIYNTIVTAHAFIMIFFMVMPIMIGGFGNWLVPLMLGAPDMAFPRMNNMSFWLLPPSLTLLISSSIVENGAGTGWTVYPPLSSNIAHGGSSVDLAIFSLHLAGISSILGAINFITTIINMRINGLSFD
QMPLFVWSVGITALLLLLSLPVLAGAITMLLTDRNLNTSFFDPAGGGDPILYQHL
>LEFIB415-10|HM871314|MM01888|Agriphila_selasella
TLYFIFGIWAGMVGTSLSLLIRAELGNPGCLIGDDQIYNTIVTAHAFIMIFFMVMPIMIGGFGNWLVPLMLGAPDMAFPRMNNMSFWLLPPSLTLLISSSIVETGAGTGWTVYPPLSSNIAHSGSSVDLAIFSLHLAGISSILGAINFITTIINMRINGLSFDQ
MPLFVWSVGITALLLLLSLPVLAGAITMLLTDRNLNTSFFDPAGGGDPILYQHL
>LEFID840-10|HM873597|MM06960|Agriphila_selasella
TLYFIFGIWAGMVGTSLSLLIRAELGNPGCLIGDDQIYNTIVTAHAFIMIFFMVMPIMIGGFGNWLVPLMLGAPDMAFPRMNNMSFWLLPPSLTLLISSSIVETGAGTGWTVYPPLSSNIAHSGSSVDLAIFSLHLAGISSILGAINFITTIINMRINGLSFDQ
MPLFVWSVGITALLLLLSLPVLAGAITMLLTDRNLNTSFFDPAGGGDPILYQHL
>LEFIB428-10|HM871327|MM01913|Agriphila_straminella
TLYFIFGIWAGMVGTSLSLLIRAELGTPGSLINDDQIYNTIVTAHAFIMIFFMVMPIMIGGFGNWLVPLMLGAPDMAFPRMNNMSFWLLPPSLTLLISSSIVENGAGTGWTVYPPLSSNIAHSGSSVDLAIFSLHLAGISSILGAINFITTIINMRINSLSFDQ
MPLFVWSVGITALLLLLSLPVLAGAITMLLTDRNLNTSFFDPAGGGDPILYQHL
>LEFIJ123-10|KM572310|MM08487|Agriphila_straminella
TLYFIFGIWAGMVGTSLSLLIRAELGTPGSLINDDQIYNTIVTAHAFIMIFFMVMPIMIGGFGNWLVPLMLGAPDMAFPRMNNMSFWLLPPSLTLLISSSIVENGAGTGWTVYPPLSSNIAHSGSSVDLAIFSLHLAGISSILGAINFITTIINMRINSLSFDQ
MPLFVWSVGITALLLLLSLPVLAGAITMLLTDRNLNTSFFDPAGGGDPILYQHL
>LEFIF789-10|HM875473|MM13031|Agriphila_straminella
TLYFIFGIWAGMVGTSLSLLIRAELGIPGSLINDDQIYNTIITAHAFIMIFFMVMPIMIGGFGNWLVPLMLGAPDMAFPRMNNMSFWLLPPSLTLLISSSIVENGAGTGWTVYPPLSSNIAHSGSSVDLAIFSLHLAGISSILGAINFITTIINMRINGLSFDQ
MPLFVWSVGITALLLLLSLPVLAGAITMLLTDRNLNTSFFDPAGGGDPILYQHL
>LEFIB427-10|HM871326|MM01912|Agriphila_tristella
TLYFIFGIWAGMVGTSLSLLIRAELGNPGSLIGDDQIYNTIVTAHAFIMIFFMVMPIMIGGFGNWLIPLMLGAPDMAFPRMNNMSFWLLPPSLTLLISSSIVENGAGTGWTVYPPLSSNIAHGGSSVDLAIFSLHLAGISSILGAINFITTIINMRINSLSFDQ
MPLFVWSVGITALLLLLSLPVLAGAITMLLTDRNLNTSFFDPAGGGDPILYQHL
>LEFIB204-10|HM871108|MM00652|Agrius_convolvuli
TLYFIFGIWAGMVGTSLSLLIRAELGNPGSLIGDDQIYNTIVTAHAFIMIFFMVMPIMIGGFGNWLVPLMLGAPDMAFPRMNNMSFWLLPPSLMLLISSSIVENGAGTGWTVYPPLSSNIAHSGSSVDLAIFSLHLAGISSILGAINFITTIINMRVNNMSF
DQMPLFVWAVGITAFLLLLSLPVLAGAITMLLTDRNLNTSFFDPAGGGDPILYQHL
>LEFIC739-10|HM872560|MM04842|Agrochola_circellaris
TLYFIFGIWAGMVGTSLSLLIRAELGNPGSLIGDDQIYNTIVTAHAFIMIFFMVMPIMIGGFGNWLVPLMLGAPDMAFPRLNNMSFWLLPPSLTLLISSSIVENGAGTGWTVYPPLSSNIAHGGSSVDLAIFSLHLAGISSILGAINFITTIINMRLNSLSFDQ
MPLFIWAVGITAFLLLLSLPVLAGAITMLLTDRNLNTSFFDPAGGGDPILYQHL
>LEFID862-10|HM873619|MM06994|Agrochola_circellaris
TLYFIFGIWAGMVGTSLSLLIRAELGNPGSLIGDDQIYNTIVTAHAFIMIFFMVMPIMIGGFGNWLVPLMLGAPDMAFPRLNNMSFWLLPPSLTLLISSSIVENGAGTGWTVYPPLSSNIAHGGSSVDLAIFSLHLAGISSILGAINFITTIINMRLNSLSFDQ
MPLFIWAVGITAFLLLLSLPVLAGAITMLLTDRNLNTSFFDPAGGGDPILYQHL

>LEFID942-10|HM873692|MM07738|Agrochola_helvola
TLYFIFGIWAGMVGTSLSLLIRAELGNPGSLIGDDQIYNTIVTAHAFIMIFFMVMPIMIGGFGNWLVPLMLGAPDMAFPRLNNMSFWLLPPSLTLLISSSIVENGAGTGWTVYPPLSSNIAHGGSSVDLAIFSLHLAGISSILGAINFITTIINMRLNNLSFDQ
MPLFIWAVGITAFLLLLSLPVLAGAITMLLTDRNLNTSFFDPAGGGDPILYQHL
>LEFIE070-10|HM873817|MM08205|Agrochola_helvola
TLYFIFGIWAGMVGTSLSLLIRAELGNPGSLIGDDQIYNTIVTAHAFIMIFFMVMPIMIGGFGNWLVPLMLGAPDMAFPRLNNMSFWLLPPSLTLLISSSIVENGAGTGWTVYPPLSSNIAHGGSSVDLAIFSLHLAGISSILGAINFITTIINMRLNNLSFDQ
MPLFIWAVGITAFLLLLSLPVLAGAITMLLTDRNLNTSFFDPAGGGDPILYQHL
>LEFIC740-10|HM872561|MM04843|Agrochola_litura
TLYFIFGIWAGMVGTSLSLLIRAELGNPGSLIGDDQIYNTIVTAHAFIMIFFMVMPIMIGGFGNWLVPLMLGAPDMAFPRLNNMSFWLLPPSLTLLISSSIVENGAGTGWTVYPPLSSNIAHGGSSVDLAIFSLHLAGISSILGAINFITTIINMRLNSLSFDQ
MPLFIWAVGITAFLLLLSLPVLAGAITMLLTDRNLNTSFFDPAGGGDPILYQHL
>LEFIF671-10|HM875355|MM12647|Agrochola_litura
TLYFIFGIWAGMVGTSLSLLIRAELGNPGSLIGDDQIYNTIVTAHAFIMIFFMVMPIMIGGFGNWLVPLMLGAPDMAFPRLNNMSFWLLPPSLTLLISSSIVENGAGTGWTVYPPLSSNIAHGGSSVDLAIFSLHLAGISSILGAINFITTIINMRLNSLSFDQ
MPLFIWAVGITAFLLLLSLPVLAGAITMLLTDRNLNTSFFDPAGGGDPILYQHL
>LEFIB788-10|HM871665|MM02694|Agrochola_lota
TLYFIFGIWAGMVGTSLSLLIRAELGNPGSLIGDDQIYNTIVTAHAFIMIFFMVMPIMIGGFGNWLVPLMLGAPDMAFPRLNNMSFWLLPPSLTLLISSSIVENGAGTGWTVYPPLSSNIAHGGSSVDLAIFSLHLAGISSILGAINFITTIINMRLNSLSFDQ
MPLFIWAVGITAFLLLLSLPVLAGAITMLLTDRNLNTSFFDPAGGGDPILYQHL
>LEFIB987-10|HM871859|MM03181|Agrochola_lota
TLYFIFGIWAGMVGTSLSLLIRAELGNPGSLIGDDQIYNTIVTAHAFIMIFFMVMPIMIGGFGNWLVPLMLGAPDMAFPRLNNMSFWLLPPSLTLLISSSIVENGAGTGWTVYPPLSSNIAHGGSSVDLAIFSLHLAGISSILGAINFITTIINMRLNSLSFDQ
MPLFIWAVGITAFLLLLSLPVLAGAITMLLTDRNLNTSFFDPAGGGDPILYQHL
>LEFIK466-10|JF854079|MM18041|Agrochola_lota
TLYFIFGIWAGMVGTSLSLLIRAELGNPGSLIGDDQIYNTIVTAHAFIMIFFMVMPIMIGGFGNWLVPLMLGAPDMAFPRLNNMSFWLLPPSLTLLISSSIVENGAGTGWTVYPPLSSNIAHGGSSVDLAIFSLHLAGISSILGAINFITTIINMRLNSLSFDQ
MPLFIWAVGITAFLLLLSLPVLAGAITMLLTDRNLNTSFFDPAGGGDPILYQHL
>LEFIB988-10|HM871860|MM03182|Agrochola_macilenta
TLYFIFGIWAGMVGTSLSLLIRAELGNPGSLIGDDQIYNTIVTAHAFIMIFFMVMPIMIGGFGNWLVPLMLGAPDMAFPRLNNMSFWLLPPSLTLLISSSIVENGAGTGWTVYPPLSSNIAHGGSSVDLAIFSLHLAGISSILGAINFITTIINMRLNNLSFDQ
MPLFIWAVGITAFLLLLSLPVLAGAITMLLTDRNLNTSFFDPAGGGDPILYQHL
>LEFIB989-10|HM871861|MM03183|Agrochola_macilenta
TLYFIFGIWAGMVGTSLSLLIRAELGNPGSLIGDDQIYNTIVTAHAFIMIFFMVMPIMIGGFGNWLVPLMLGAPDMAFPRLNNMSFWLLPPSLTLLISSSIVENGAGTGWTVYPPLSSNIAHGGSSVDLAIFSLHLAGISSILGAINFITTIINMRLNNLSFDQ
MPLFIWAVGITAFLLLLSLPVLAGAITMLLTDRNLNTSFFDPAGGGDPILYQHL
>LEFIC675-10|HM872496|MM04642|Agrochola_macilenta
TLYFIFGIWAGMVGTSLSLLIRAELGNPGSLIGDDQIYNTIVTAHAFIMIFFMVMPIMIGGFGNWLVPLMLGAPDMAFPRLNNMSFWLLPPSLTLLISSSIVENGAGTGWTVYPPLSSNIAHGGSSVDLAIFSLHLAGISSILGAINFITTIINMRLNNLSFDQ
MPLFIWAVGITAFLLLLSLPVLAGAITMLLTDRNLNTSFFDPAGGGDPILYQHL
>LEFIJ350-10|JF853553|MM15950|Agrochola_nitida
TLYFIFGIWAGMVGTSLSLLIRAELGNPGSLIGDDQIYNTIVTAHAFIMIFFMVMPIMIGGFGNWLVPLMLGAPDMAFPRLNNMSFWLLPPSLTLLISSSIVENGAGTGWTVYPPLSSNIAHGGSSVDLAIFSLHLAGISSILGAINFITTIINMRLNNLSFDQ
MPLFIWAVGITAFLLLLSLPVLAGAITMLLTDRNLNTSFFDPAGGGDPILYQHL
>LEFIL607-10|KM572474|MM18905|Agrochola_nitida
TLYFIFGIWAGMVGTSLSLLIRAELGNPGSLIGDDQIYNTIVTAHAFIMIFFMVMPIMIGGFGNWLVPLMLGAPDMAFPRLNNMSFWLLPPSLTLLISSSIVENGAGTGWTVYPPLSSNIAHGGSSVDLAIFSLHLAGISSILGAINFITTIINMRLNNLSFDQ
MPLFIWAVGITAFLLLLSLPVLAGAITMLLTDRNLNTSFFDPAGGGDPILYQHL
>LEFII260-11|MM19910|Agrotera_nemoralis
TLYFIFGIWAGMVGTSLSLLIRAELGNPGSLIGDDQIYNTIVTAHAFIMIFFMVMPIMIGGFGNWLIPLMLGAPDMAFPRMNNMSFWLLPPSLILLISSSIVENGAGTGWTVYPPLSSNIAHGGSSVDLAIFSLHLAGISSILGAINFITTIINMRINGLSFDQ
MPLFVWAVGITALLLLLSLPVLAGAITMLLTDRNLNTSFFDPAGGGDPILYQHL
>LEFIA562-10|KM573319|MM01656|Agrotis_clavis
TLYFIFGIWAGMVGTSLSLLIRAELGNPGSLIGDDQIYNTIVTAHAFIMIFFMVMPIMIGGFGNWLVPLMLGAPDMAFPRMNNMSFWLLPPSLTLLISSSIVENGAGTGWTVYPPLSSNIAHGGSSVDLAIFSLHLAGISSILGAINFITTIINMRLNSLSFD
QMPLFIWAVGITAFLLLLSLPVLAGAITMLLTDRNLNTSFFDPAGGGDPILYQHL
>LEFIE587-10|HM874310|MM09495|Agrotis_clavis
TLYFIFGIWAGMVGTSLSLLIRAELGNPGSLIGDDQIYNTIVTAHAFIMIFFMVMPIMIGGFGNWLVPLMLGAPDMAFPRMNNMSFWLLPPSLTLLISSSIVENGAGTGWTVYPPLSSNIAHGGSSVDLAIFSLHLAGISSILGAINFITTIINMRLNSLSFD
QMPLFIWAVGITAFLLLLSLPVLAGAITMLLTDRNLNTSFFDPAGGGDPILYQHL
>LEFIC626-10|HM872447|MM04568|Agrotis_exclamationis
TLYFIFGIWAGMVGTSLSLLIRAELGNPGSLIGDDQIYNTIVTAHAFIMIFFMVMPIMIGGFGNWLVPLMLGAPDMAFPRMNNMSFWLLPPSLTLLISSSIVENGAGTGWTVYPPLSSNIAHGGSSVDLAIFSLHLAGISSILGAINFITTIINMRLNSLSFD

QMPLFIWAVGITAFLLLLSLPVLAGAITMLLTDRNLNTSFFDPAGGGDPILYQHL
>LEFIJ587-10|KM573001|MM17212|Agrotis_ipsilon
TLYFIFGIWAGMVGTSLSLLIRAELGNPGSLIGDDQIYNTIVTAHAFIMIFFMVMPIMIGGFGNWLVPLMLGAPDMAFPRMNNMSFWLLPPSLTLLISSSIVENGAGTGWTVYPPLSSNIAHGGSSVDLAIFSLHLAGISSILGAINFITTIINMRLNNLSFD
QMPLFIWAVGITAFLLLLSLPVLAGAITMLLTDRNLNTSFFDPAGGGDPILYQHL
>LEFIK425-10|JF854054|MM18000|Agrotis_ipsilon
TLYFIFGIWAGMVGTSLSLLIRAELGNPGSLIGDDQIYNTIVTAHAFIMIFFMVMPIMIGGFGNWLVPLMLGAPDMAFPRMNNMSFWLLPPSLTLLISSSIVENGAGTGWTVYPPLSSNIAHGGSSVDLAIFSLHLAGISSILGAINFITTIINMRLNNLSFD
QMPLFIWAVGITAFLLLLSLPVLAGAITMLLTDRNLNTSFFDPAGGGDPILYQHL
>LEEUA334-11|MM19742|Agrotis_puta
TLYFIFGIWAGMVGTSLSLLIRAELGNPGSLIGDDQIYNTIVTAHAFIMIFFMVMPIMIGGFGNWLVPLMLGAPDMAFPRLNNMSFWLLPPSLTLLISSSIVENGAGTGWTVYPPLSSNIAHGGSSVDLAIFSLHLAGISSILGAINFITTIINMRLNSLSFDQ
MPLFIWAVGITAFLLLLSLPVLAGAITMLLTDRNLNTSFFDPAGGGDPILYQHL
>LEEUA521-11|MM20580|Agrotis_ripae
TLYFIFGIWAGMVGTSLSLLIRAELGNPGSLIGDDQIYNTIVTAHAFIMIFFMVMPIMIGGFGNWLVPLMLGAPDMAFPRMNNMSFWLLPPSLTLLISSSIVENGAGTGWTVYPPLSSNIAHGGSSVDLAIFSLHLAGISSILGAINFITTIINMRLNSLSFD
QMPLFIWAVGITAFLLLLSLPVLAGAITMLLTDRNLNTSFFDPAGGGDPILYQHL
>LEFIF321-10|HM875006|MM11111|Agrotis_segetum
TLYFIFGIWAGMVGTSLSLLIRAELGNPGSLIGDDQIYNTIVTAHAFIMIFFMVMPIMIGGFGNWLVPLMLGAPDMAFPRMNNMSFWLLPPSLTLLISSSIVENGAGTGWTVYPPLSSNIAHGGSSVDLAIFSLHLAGISSILGAINFITTIINMRLNSLSFD
QMPLFIWAVGITAFLLLLSLPVLAGAITMLLTDRNLNTSFFDPAGGGDPILYQHL
>LEFIH040-10|HM876671|MM15904|Agrotis_segetum
TLYFIFGIWAGMVGTSLSLLIRAELGNPGSLIGDDQIYNTIVTAHAFIMIFFMVMPIMIGGFGNWLVPLMLGAPDMAFPRMNNMSFWLLPPSLTLLISSSIVENGAGTGWTVYPPLSSNIAHGGSSVDLAIFSLHLAGISSILGAINFITTIINMRLNSLSFD
QMPLFIWAVGITAFLLLLSLPVLAGAITMLLTDRNLNTSFFDPAGGGDPILYQHL
>LEFIF681-10|HM875365|MM12684|Agrotis_vestigialis
TLYFIFGIWAGMVGTSLSLLIRAELGNPGSLIGDDQIYNTIVTAHAFIMIFFMVMPIMIGGFGNWLVPLMLGAPDMAFPRMNNMSFWLLPPSLTLLISSSIVENGAGTGWTVYPPLSSNIAHGGSSVDLAIFSLHLAGISSILGAINFITTIINMRLNSLSFD
QMPLFVWAVGITAFLLLLSLPVLAGAITMLLTDRNLNTSFFDPAGGGDPILYQHL
>LEFIH042-10|HM876673|MM15906|Agrotis_vestigialis
TLYFIFGIWAGMVGTSLSLLIRAELGNPGSLIGDDQIYNTIVTAHAFIMIFFMVMPIMIGGFGNWLVPLMLGAPDMAFPRMNNMSFWLLPPSLTLLISSSIVENGAGTGWTVYPPLSSNIAHGGSSVDLAIFSLHLAGISSILGAINFITTIINMRLNSLSFD
QMPLFVWAVGITAFLLLLSLPVLAGAITMLLTDRNLNTSFFDPAGGGDPILYQHL
>LEFIH043-10|HM876674|MM15907|Agrotis_vestigialis
TLYFIFGIWAGMVGTSLSLLIRAELGNPGSLIGDDQIYNTIVTAHAFIMIFFMVMPIMIGGFGNWLVPLMLGAPDMAFPRMNNMSFWLLPPSLTLLISSSIVENGAGTGWTVYPPLSSNIAHGGSSVDLAIFSLHLAGISSILGAINFITTIINMRLNSLSFD
QMPLFVWAVGITAFLLLLSLPVLAGAITMLLTDRNLNTSFFDPAGGG?PILYQHL
>LEFID222-10|HM873023|MM06079|Alcis_bastelbergeri
TLYFIFGIWAGMVGTSLSLLIRAELGNPGSLIGDDQIYNTIVTAHAFIMIFFMVMPIMIGGFGNWLVPLMLGAPDMAFPRMNNMSFWLLPPSITLLISSSIVESGAGTGWTVYPPLSSNIAHGGSSVDLAIFSLHLAGISSILGAINFITTIINMRLNNLSFD
QMPLFIWSVGITAFLLLLSLPVLAGAITMLLTDRNLNTSFFDPAGGGDPILYQHL
>LEFIK288-10|MM17863|Alcis_bastelbergeri
TLYFIFGIWAGMVGTSLSLLIRAELGNPGSLIGDDQIYNTIVTAHAFIMIFFMVMPIMIGGFGNWLVPLMLGAPDMAFPRMNNMSFWLLPPSITLLISSSIVENGAGTGWTVYPPLSSNIAHGGSSVDLAIFSLHLAGISSILGAINFITTIINMRLNNLSFD
QMPLFIWSVGITAFLLLLSLPVLAGAITMLLTDRNLNTSFFDPAGGGDPILYQHL
>LEFIF614-10|HM875298|MM12493|Alcis_jubata
TLYFIFGIWAGMVGTSLSLLIRAELGNPGSLIGDDQIYNTIVTAHAFIMIFFMVMPIMIGGFGNWLVPLMLGAPDMAFPRMNNMSFWLLPPSLTLLISSSIVENGAGTGWTVYPPLSSNIAHGGSSVDLAIFSLHLAGISSILGAINFITTIINMRLNNLLFD
QMPLFVWSVGITAFLLLLSLPVLAGAITMLLTDRNLNTSFFDPAGGGDPILYQHL
>LEFIA452-10|HM386793|MM01516|Alcis_repandata
TLYFIFGIWAGMVGTSLSLLIRAELGNPGSLIGDDQIYNTIVTAHAFIMIFFMVMPIMIGGFGNWLVPLMLGAPDMAFPRMNNMSFWLLPPSITLLISSSIVENGAGTGWTVYPPLSSNIAHGGSSVDLAIFSLHLAGISSILGAINFITTIINMRLNNLSFD
QMPLFIWSVGITAFLLLLSLPVLAGAITMLLTDRNLNTSFFDPAGGGDPILYQHL
>LEFIF876-10|HM875558|MM13268|Aleimma_loeflingiana
TLYFIFGIWAGMIGTSLSLLIRAELGNPGSLIGDDQIYNTIVTAHAFIMIFFMVMPIMIGGFGNWLVPLMLGAPDMAFPRMNNMSFWLLPPSIMLLISSSIVENGAGTGWTVYPPLSSNIAHSGSSVDLAIFSLHLAGISSILGAVNFITTIINMRPNNMSL
DQMPLFVWAVGITALLLLLSLPVLAGAITMLLTDRNLNTSFFDPAGGGDPILYQHL
>LEFIF146-10|HM874840|MM10662|Alloclemensia_mesospilella
TLYFIFGIWSGMVGTSLSLLIRTELGMPGSLIGNDQIYNTIVTAHAFIMIFFMVMPIMIGGFGNWLVPLMLGAPDMAFPRLNNMSFWLLPPSLTLLISSSLVENGAGTGWTVYPPLSSNISHAGSSVDLAIFSLHLAGISSILGAVNFITTVINMRSNGMSF
DQMPLFVWAVAITALLLLLSLPVLAGAITMLLTDRNLNTSFFDPAGGGDPILYQHL
>LEFIF150-10|HM874844|MM10668|Alloclemensia_mesospilella

TLYFIFGIWSGMVGTSLSLLIRTELGMPGSLIGNDQIYNTIVTAHAFIMIFFMVMPIMIGGFGNWLVPLMLGAPDMAFPRLNNMSFWLLPPSLTLLISSSLVENGAGTGWTVYPPLSSNISHAGSSVDLAIFSLHLAGISSILGAVNFITTVINMRSNGMSF
DQMPLFVWAVAITALLLLLSLPVLAGAITMLLTDRNLNTSFFDPAGGGDPILYQHL
>LEFIK357-10|JF853993|MM17932|Alloclemensia_mesospilella
TLYFIFGIWSGMVGTSLSLLIRTELGMPGSLIGNDQIYNTIVTAHAFIMIFFMVMPIMIGGFGNWLVPLMLGAPDMAFPRLNNMSFWLLPPSLTLLISSSLVENGAGTGWTVYPPLSSNISHAGSSVDLAIFSLHLAGISSILGAVNFITTVINMRSNGMSF
DQMPLFVWAVAITALLLLLSLPVLAGAITMLLTDRNLNTSFFDPAGGGDPILYQHL
>LEEUA034-11|JN303401|MM19347|Alloclemensia_mesospilella
TLYFIFGIWSGMVGTSLSLLIRTELGMPGSLIGNDQIYNTIVTAHAFIMIFFMVMPIMIGGFGNWLVPLMLGAPDMAFPRLNNMSFWLLPPSLTLLISSSLVENGAGTGWTVYPPLSSNISHAGSSVDLAIFSLHLAGISSILGAVNFITTVINMRSNGMSF
DQMPLFVWAVAITALLLLLSLPVLAGAITMLLTDRNLNTSFFDPAGGGDPILYQHL
>LEFIA629-10|HM870878|MM01744|Allophyes_oxyacanthae
TLYFIFGIWAGMVGTSLSLLIRAELGTPGSLIGDDQIYNTIVTAHAFIMIFFMVMPIMIGGFGNWLVPLMLGAPDMAFPRMNNMSFWLLPPSLTLLISSSIVENGAGTGWTVYPPLSSNIAHGGSSVDLAIFSLHLAGISSILGAINFITTIINMRLNSLSFD
QMPLFIWAVGITAFLLLLSLPVLAGAITMLLTDRNLNTSFFDPAGGGDPILYQHL
>LEEUA506-11|MM20565|Alsophila_aescularia
TLYFIFGIWAGMVGTSLSLLIRAELGNPGSLIGDDQIYNTIVTAHAFIMIFFMVMPIMIGGFGNWLVPLMLGAPDMAFPRMNNMSFWLLPPSLTLLISSSIVENGAGTGWTVYPPLSSNIAHGGSAVDLAIFSLHLAGISSILGAINFITTIINMRLNNLSFD
QMPLFIWSVGITAFLLLLSLPVLAGAITMLLTDRNLNTSFFDPAGGGDPILYQHL
>LEFIC367-10|HM872210|MM03894|Altenia_perspersella
TLYFIFGIWAGMVGTSLSLLIRAELGNPGSLIGDDQIYNTIVTAHAFIMIFFMVMPIMIGGFGNWLVPLMLGAPDMAFPRMNNMSFWLLPPSLTLLISSSIVENGAGTGWTVYPPLSSNIAHSGSSVDLTIFSLHLAGISSILGAINFITTIINMRIYNLSFDQ
MPLFVWAVGITALLLLLSLPVLAGAITMLLTDRNLNTSFFDPAGGGDPILYQHL
>LEFID457-10|HM873228|MM06396|Altenia_perspersella
TLYFIFGIWAGMVGTSLSLLIRAELGNPGSLIGDDQIYNTIVTAHAFIMIFFMVMPIMIGGFGNWLVPLMLGAPDMAFPRMNNMSFWLLPPSLTLLISSSIVENGAGTGWTVYPPLSSNIAHSGSSVDLAIFSLHLAGISSILGAINFITTIINMRIYNLSFDQ
MPLFVWAVGITALLLLLSLPVLAGAITMLLTDRNLNTSFFDPAGGGDPILYQHL
>LEFIG176-10|HM875856|MM14043|Altenia_perspersella
TLYFIFGIWAGMVGTSLSLLIRAELGNPGSLIGDDQIYNTIVTAHAFIMIFFMVMPIMIGGFGNWLVPLMLGAPDMAFPRMNNMSFWLLPPSLTLLISSSIVENGAGTGWTVYPPLSSNIAHSGSSVDLAIFSLHLAGISSILGAINFITTIINMRIYNLSFDQ
MPLFVWAVGITTLLLLLSLPVLAGAITMLLTDRNLNTSFFDPAGGGDPILYQHL
>LEFIG898-10|HM876542|MM15762|Alucita_hexadactyla
TLYFIFGIWAGLLGTSLSLLIRAELGNPGSLIGDDQIYNTIVTAHAFIMIFFMVMPIMIGGFGNWLVPLMLGAPDMAFPRMNNMSFWLLPPSITLLISSSIVENGAGTGWTVYPPLSSNIAHSGSSVDLAIFSLHLAGISSILGAINFITTIINMKINGLMFDQ
MPLFVWAVGITALLLLLSLPVLAGAITMLLTDRNLNTSFFDPAGGGDPILYQHL
>LEFIK036-10|JF853918|MM17611|Alucita_hexadactyla
TLYFIFGIWAGLLGTSLSLLIRAELGNPGSLIGDDQIYNTIVTAHAFIMIFFMVMPIMIGGFGNWLVPLMLGAPDMAFPRMNNMSFWLLPPSITLLISSSIVENGAGTGWTVYPPLSSNIAHSGSSVDLAIFSLHLAGISSILGAINFITTIINMKINGLMFDQ
MPLFVWAVGITALLLLLSLPVLAGAITMLLTDRNLNTSFFDPAGGGDPILYQHL
>LEFIE629-10|HM874352|MM09567|Amblyptilia_acanthadactyla
TLYFIFGIWAGMVGTSLSLLIRAELGTPGSLIGDDQIYNSIVTAHAFIMIFFMVMPIMIGGFGNWLVPLMLGAPDMAFPRMNNMSFWLLPPSLSLLISSSIVENGAGTGWTVYPPLSSNIAHGGPSVDLAIFSLHLAGISSILGAINFISTIINMRLNKMMF
DQLPLFVWAVGITALLLLLSLPVLAGAITMLLTDRNLNTSFFDPAGGGDPILYQHL
>LEFIE630-10|HM874353|MM09568|Amblyptilia_acanthadactyla
TLYFIFGIWAGMVGTSLSLLIRAELGTPGSLIGDDQIYNSIVTAHAFIMIFFMVMPIMIGGFGNWLVPLMLGAPDMAFPRMNNMSFWLLPPSLSLLISSSIVENGAGTGWTVYPPLSSNIAHGGPSVDLAIFSLHLAGISSILGAINFISTIINMRLNKMMF
DQLPLFVWAVGITALLLLLSLPVLAGAITMLLTDRNLNTSFFDPAGGGDPILYQHL
>LEFIE631-10|HM874354|MM09569|Amblyptilia_acanthadactyla
TLYFIFGIWAGMVGTSLSLLIRAELGTPGSLIGDDQIYNSIVTAHAFIMIFFMVMPIMIGGFGNWLVPLMLGAPDMAFPRMNNMSFWLLPPSLSLLISSSIVENGAGTGWTVYPPLSSNIAHGGPSVDLAIFSLHLAGISSILGAINFISTIINMRLNKMMF
DQLPLFVWAVGITALLLLLSLPVLAGAITMLLTDRNLNTSFFDPAGGGDPILYQHL
>LEFIB125-10|HM871031|MM00489|Amblyptilia_punctidactyla
TLYFIFGIWAGMVGTSLSLLIRAELGTPGSLIGDDQIYNSIVTAHAFIMIFFMVMPIMIGGFGNWLVPLMLGAPDMAFPRMNNMSFWLLPPSLSLLISSSIVENGAGTGWTVYPPLSSNIAHGGPSVDLAIFSLHLAGISSILGAINFISTIINMRLNKMMF
DQLPLFVWAVGITALLLLLSLPVLAGAITMLLTDRNLNTSFFDPAGGGDPILYQHL
>LEFIB576-10|HM871457|MM02248|Amblyptilia_punctidactyla
TLYFIFGIWAGMVGTSLSLLIRAELGTPGSLIGDDQIYNSIVTAHAFIMIFFMVMPIMIGGFGNWLVPLMLGAPDMAFPRMNNMSFWLLPPSLSLLISSSIVENGAGTGWTVYPPLSSNIAHGGPSVDLAIFSLHLAGISSILGAINFISTIINMRLNKMMF
DQLPLFVWAVGITALLLLLSLPVLAGAITMLLTDRNLNTSFFDPAGGGDPILYQHL
>LEFIA888-10|HM387025|MM09765|Amblyptilia_punctidactyla
TLYFIFGIWAGMVGTSLSLLIRAELGTPGSLIGDDQIYNSIVTAHAFIMIFFMVMPIMIGGFGNWLVPLMLGAPDMAFPRMNNMSFWLLPPSLSLLISSSIVENGAGTGWTVYPPLSSNIAHGGPSVDLAIFSLHLAGISSILGAINFISTIINMRLNKMMF
DQLPLFVWAVGITALLLLLSLPVLAGAITMLLTDRNLNTSFFDPAGGGDPILYQHL

>LEFIG204-10|HM875884|MM14088|Amblyptilia_punctidactyla
TLYFIFGIWAGMVGTSLSLLIRAELGTPGSLIGDDQIYNSIVTAHAFIMIFFMVMPIMIGGFGNWLVPLMLGAPDMAFPRMNNMSFWLLPPSLSLLISSSIVENGAGTGWTVYPPLSSNIAHGGPSVDLAIFSLHLAGISSILGAINFISTIINMRLNKMMF
DQLPLFVWAVGITALLLLLSLPVLAGAITMLLTDRNLNTSFFDPAGGGDPILYQHL
>LEFIH007-10|HM876643|MM15871|Ammoconia_caecimacula
TMYFIFGIWAGMVGTSLSLLIRAELGNPGSLIGDDQIYNTIVTAHAFIMIFFMVMPIMIGGFGNWLVPLMLGAPDMAFPRMNNMSFWLLPPSLTLLISSSIVENGAGTGWTVYPPLSSNIAHGGSSVDLAIFSLHLAGISSILGAINFITTIINMRLNNLSF
DQMPLFIWAVGITAFLLLLSLPVLAGAITMLLTDRNLNTSFFDPAGGGDPILYQHL
>LEFIK461-10|JF854077|MM18036|Ammoconia_caecimacula
TMYFIFGIWAGMVGTSLSLLIRAELGNPGSLIGDDQIYNTIVTAHAFIMIFFMVMPIMIGGFGNWLVPLMLGAPDMAFPRMNNMSFWLLPPSLTLLISSSIVENGAGTGWTVYPPLSSNIAHGGSSVDLAIFSLHLAGISSILGAINFITTIINMRLNNLSF
DQMPLFIWAVGITAFLLLLSLPVLAGAITMLLTDRNLNTSFFDPAGGGDPILYQHL
>LEFIA051-10|HM396399|MM00957|Amphipoea_crinanensis
TLYFIFGIWAGMVGTSLSLLIRAELGNPGSLIGDDQIYNTIVTAHAFIMIFFMVMPIMIGGFGNWLVPLMLGAPDMAFPRMNNMSFWLLPPSLTLLISSSIVENGAGTGWTVYPPLSSNIAHGGSSVDLAIFSLHLAGISSILGAINFITTIINMRLNNLSFD
QMPLFIWAVGITAFLLLLSLPVLAGAITMLLTDRNLNTSFFDPAGGGDPILYQHL
>LEFIB635-10|HM871514|MM02391|Amphipoea_crinanensis
TLYFIFGIWAGMVGTSLSLLIRAELGNPGSLIGDDQIYNTIVTAHAFIMIFFMVMPIMIGGFGNWLVPLMLGAPDMAFPRMNNMSFWLLPPSLTLLISSSIVENGAGTGWTVYPPLSSNIAHGGSSVDLAIFSLHLAGISSILGAINFITTIINMRLNNLSFD
QMPLFIWAVGITAFLLLLSLPVLAGAITMLLTDRNLNTSFFDPAGGGDPILYQHL
>LEFIB402-10|HM871301|MM01674|Amphipoea_fucosa
TLYFIFGIWAGMVGTSLSLLIRAELGNPGSLIGDDQIYNTIVTAHAFIMIFFMVMPIMIGGFGNWLVPLMLGAPDMAFPRMNNMSFWLLPPSLTLLISSSIVENGAGTGWTVYPPLSSNIAHGGSSVDLAIFSLHLAGISSILGAINFITTIINMRLNNLSFD
QMPLFIWAVGITAFLLLLSLPVLAGAITMLLTDRNLNTSFFDPAGGGDPILYQHL
>LEFIB403-10|HM871302|MM01675|Amphipoea_fucosa
TLYFIFGIWASMVGTSLSLLIRAELGNPGSLIGDDQIYNTIVTAHAFIMIFFMVMPIMIGGFGNWLVPLMLGAPDMAFPRMNNMSFWLLPPSLTLLISSSIVENGAGTGWTVYPPLSSNIAHGGSSVDLAIFSLHLAGISSILGAINFITTIINMRLNNLSFD
QMPLFIWAVGITAFLLLLSLPVLAGAITMLLTDRNLNTSFFDPAGGGDPILYQHL
>LEFIB404-10|HM871303|MM01676|Amphipoea_fucosa
TLYFIFGIWAGMVGTSLSLLIRAELGNPGSLIGDDQIYNTIVTAHAFIMIFFMVMPIMIGGFGNWLVPLMLGAPDMAFPRMNNMSFWLLPPSLTLLISSSIVENGAGTGWTVYPPLSSNIAHGGSSVDLAIFSLHLAGISSILGAINFITTIINMRLNNLSFD
QMPLFIWAVGITAFLLLLSLPVLAGAITMLLTDRNLNTSFFDPAGGGDPILYQHL
>LEFIF686-10|HM875370|MM12701|Amphipoea_fucosa
TLYFIFGIWAGMVGTSLSLLIRAELGNPGSLIGDDQIYNTIVTAHAFIMIFFMVMPIMIGGFGNWLVPLMLGAPDMAFPRMNNMSFWLLPPSLTLLISSSIVENGAGTGWTVYPPLSSNIAHGGSSVDLAIFSLHLAGISSILGAINFITTIINMRLNNLSFD
QMPLFIWAVGITAFLLLLSLPVLAGAITMLLTDRNLNTSFFDPAGGGDPILYQHL
>LEFIF687-10|HM875371|MM12702|Amphipoea_fucosa
TLYFIFGIWAGMVGTSLSLLIRAELGNPGSLIGDDQIYNTIVTAHAFIMIFFMVMPIMIGGFGNWLVPLMLGAPDMAFPRMNNMSFWLLPPSLTLLISSSIVENGAGTGWTVYPPLSSNIAHGGSSVDLAIFSLHLAGISSILGAINFITTIINMRLNNLSFD
QMPLFIWAVGITAFLLLLSLPVLAGAITMLLTDRNLNTSFFDPAGGGDPILYQHL
>LEFIB304-10|HM871205|MM00823|Amphipoea_lucens
TLYFIFGIWAGMVGTSLSLLIRAELGNPGSLIGDDQIYNTIVTAHAFIMIFFMVMPIMIGGFGNWLVPLMLGAPDMAFPRMNNMSFWLLPPSLTLLISSSIVENGAGTGWTVYPPLSSNIAHGGSSVDLAIFSLHLAGISSILGAINFITTIINMRLNNLSFD
QMPLFIWAVGITAFLLLLSLPVLAGAITMLLTDRNLNTSFFDPAGGGDPILYQHL
>LEFID929-10|HM873679|MM07395|Amphipoea_lucens
TLYFIFGIWAGMVGTSLSLLIRAELGNPGSLIGDDQIYNTIVTAHAFIMIFFMVMPIMIGGFGNWLVPLMLGAPDMAFPRMNNMSFWLLPPSLTLLISSSIVENGAGTGWTVYPPLSSNIAHGGSSVDLAIFSLHLAGISSILGAINFITTIINMRLNNLSFD
QMPLFIWAVGITAFLLLLSLPVLAGAITMLLTDRNLNTSFFDPAGGGDPILYQHL
>LEFIB301-10|HM871202|MM00819|Amphipoea_oculea
TLYFIFGIWAGMVGTSLSLLIRAELGNPGSLIGDDQIYNTIVTAHAFIMIFFMVMPIMIGGFGNWLVPLMLGAPDMAFPRMNNMSFWLLPPSLTLLISSSIVENGAGTGWTVYPPLSSNIAHGGSSVDLAIFSLHLAGISSILGAINFITTIINMRLNNLSFD
QMPLFIWAVGITAFLLLLSLPVLAGAITMLLTDRNLNTSFFDPAGGGDPILYQHL
>LEFIC745-10|HM872566|MM04849|Amphipyra_berbera
TLYFIFGIWAGMVGTSLSLLIRAELGNPGSLIGDDQIYNTIVTAHAFIMIFFMVMPIMIGGFGNWLVPLMLGAPDMAFPRMNNMSFWLLPPSLTLLISSSIVENGAGTGWTVYPPLSSNIAHGGSSVDLAIFSLHLAGISSILGAINFITTIINMRLNSLSFD
QMPLFIWAVGITAFLLLLSLPVLAGAITMLLTDRNLNTSFFDPAGGGDPILYQHL
>LEFIC574-10|HM872395|MM04353|Amphipyra_perflua
TLYFIFGIWAGMVGTSLSLLIRAELGNPGSLIGDDQIYNTIVTAHAFIMIFFMVMPIMIGGFGNWLVPLMLGAPDMAFPRMNNMSFWLLPPSLTLLISSSIVETGAGTGWTVYPPLSSNIAHSGSSVDLAIFSLHLAGISSILGAINFITTIINMRLNSLSFDQ
MPLFIWAVGITAFLLLLSLPVLAGAITMLLTDRNLNTSFFDPAGGGDPILYQHL
>LEFIC744-10|HM872565|MM04847|Amphipyra_pyramidea
TLYFIFGIWAGMVGTSLSLLIRAELGNPGSLIGDDQIYNTIVTAHAFIMIFFMVMPIMIGGFGNWLVPLMLGAPDMAFPRMNNMSFWLLPPSLTLLISSSIVENGAGTGWTVYPPLSSNIAHGGSSVDLAIFSLHLAGISSILGAINFITTIINMRLNSLSFD

QMPLFIWAVGITAFLLLLSLPVLAGAITMLLTDRNLNTSFFDPAGGGDPILYQHL
>LEFIG397-10|HM876074|MM14419|Amphipyra_pyramidea
TLYFIFGIWAGMVGTSLSLLIRAELGNPGSLIGDDQIYNTIVTAHAFIMIFFMVMPIMIGGFGNWLVPLMLGAPDMAFPRMNNMSFWLLPPSLTLLISSSIVENGAGTGWTVYPPLSSNIAHGGSSVDLAIFSLHLAGISSILGAINFITTIINMRLNSLSFD
QMPLFIWAVGITAFLLLLSLPVLAGAITMLLTDRNLNTSFFDPAGGGDPILYQHL
>LEFII105-10|KM572494|MM18532|Amphipyra_pyramidea
TLYFIFGIWAGMVGTSLSLLIRAELGNPGSLIGDDQIYNTIVTAHAFIMIFFMVMPIMIGGFGNWLVPLMLGAPDMAFPRMNNMSFWLLPPSLTLLISSSIVENGAGTGWTVYPPLSSNIAHGGSSVDLAIFSLHLAGISSILGAINFITTIINMRLNSLSFD
QMPLFIWAVGITAFLLLLSLPVLAGAITMLLTDRNLNTSFFDPAGGGDPILYQHL
>LEFIA616-10|HM870865|MM01730|Amphipyra_tragopoginis
TLYFIFGIWAGMVGTSLSLLIRAELGNPGSLIGDDQIYNTIVTAHAFIMIFFMVMPIMIGGFGNWLVPLMLGAPDMAFPRMNNMSFWLLPPSLTLLISSSIVENGAGTGWTVYPPLSSNIAHGGSSVDLAIFSLHLAGISSILGAINFITTIINMRLNNLSFD
QMPLFIWAVGITAFLLLLSLPVLAGAITMLLTDRNLNTSFFDPAGGGDPILYQHL
>LEFIA617-10|HM870866|MM01731|Amphipyra_tragopoginis
TLYFIFGIWAGMVGTSLSLLIRAELGNPGSLIGDDQIYNTIVTAHAFIMIFFMVMPIMIGGFGNWLVPLMLGAPDMAFPRMNNMSFWLLPPSLTLLISSSIVENGAGTGWTVYPPLSSNIAHGGSSVDLAIFSLHLAGISSILGAINFITTIINMRLNNLSFD
QMPLFIWAVGITAFLLLLSLPVLAGAITMLLTDRNLNTSFFDPAGGGDPILYQHL
>LEFIC716-10|HM872537|MM04766|Amphipyra_tragopoginis
TLYFIFGIWAGMVGTSLSLLIRAELGNPGSLIGDDQIYNTIVTAHAFIMIFFMVMPIMIGGFGNWLVPLMLGAPDMAFPRMNNMSFWLLPPSLTLLISSSIVENGAGTGWTVYPPLSSNIAHGGSSVDLAIFSLHLAGISSILGAINFITTIINMRLNNLSFD
QMPLFIWAVGITAFLLLLSLPVLAGAITMLLTDRNLNTSFFDPAGGGDPILYQHL
>LEFIB607-10|HM871486|MM02324|Anacampsis_blattariella
TLYFIFGIWAGMVGTSLSLLIRAELGNPGQLIGDDQIYNTIVTAHAFIMIFFMVMPIMIGGFGNWLVPLMLGAPDMAFPRMNNMSFWLLPPSLTLLISSSIVENGAGTGWTVYPPLSSNIAHGGSSVDLAIFSLHLAGISSILGAINFITTIINMRINGMSF
DQMPLFVWAVGITALLLLLSLPVLAGAITMLLTDRNLNTSFFDPAGGGDPILYQHL
>LEFIF221-10|HM874914|MM11001|Anacampsis_fuscella
TLYFIFGIWAGMVGTSLSLLIRAELGNPGQLIGDDQIYNTIVTAHAFIMIFFMVMPIMIGGFGNWLVPLMLGAPDMAFPRMNNMSFWLLPPSLTLLISSSIVENGAGTGWTVYPPLSSNIAHGGSSVDLAIFSLHLAGISSILGAINFITTIINMRISGLSFD
QMPLFVWAVGITALLLLLSLPVLAGAITMLLTDRNLNTSFFDPAGGGDPILYQHL
>LEFIC239-10|HM872083|MM03644|Anacampsis_populella
TLYFIFGIWAGMVGTSLSLLIRAELGNPGQLIGDDQIYNTIVTAHAFIMIFFMVMPIMIGGFGNWLVPLMLGAPDMAFPRMNNMSFWLLPPSLTLLISSSIVENGAGTGWTVYPPLSSNIAHGGSSVDLAIFSLHLAGISSILGAINFITTIINMRINGMSF
DQMPLFVWAVGITALLLLLSLPVLAGAITMLLTDRNLNTSFFDPAGGGDPILYQHL
>LEFID552-10|HM873317|MM06541|Anacampsis_temerella
TLYFIFGIWAGMVGTSLSLLIRAELGNPGQLIGDDQIYNTIVTAHAFIMIFFMVMPIMIGGFGNWLVPLMLGAPDMAFPRMNNMSFWLLPPSLTLLISSSIVENGAGTGWTVYPPLSSNIAHGGSSVDLAIFSLHLAGISSILGAINFITTIINMRINGLSFD
QMPLFVWAVGITALLLLLSLPVLAGAITMLLTDRNLNTSFFDPAGGGDPILYQHL
>LEFIA713-10|HM386857|MM01868|Anania_coronata
TLYFIFGIWSGMVGTSLSLLIRAELGNPGSLIGDDQIYNTIVTAHAFIMIFFMVMPIMIGGFGNWLVPLMLGAPDMAFPRMNNMSFWLLPPSLTLLISSSIVENGAGTGWTVYPPLSSNIAHGGSSVDLAIFSLHLAGISSILGAINFITTIINMRINSLSFDQ
MPLFVWAVGITALLLLLSLPVLAGAITMLLTDRNLNTSFFDPAGGGDPILYQHL
>LEFIE637-10|HM874360|MM09585|Anania_crocealis
TLYFIFGIWSGMVGTSLSLLIRAELGNPGSLIGDDQIYNTIVTAHAFIMIFFMVMPIMIGGFGNWLIPLMLGAPDMAFPRMNNMSFWLLPPSLTLLISSSIVENGAGTGWTVYPPLSSNIAHGGSSVDLAIFSLHLAGISSILGAINFITTIINMRINGMSFD
QMPLFVWAVGITALLLLLSLPVLAGAITMLLTDRNLNTSFFDPAGGGDPILYQHL
>LEFIE638-10|HM874361|MM09586|Anania_crocealis
TLYFIFGIWSGMVGTSLSLLIRAELGNPGSLIGDDQIYNTIVTAHAFIMIFFMVMPIMIGGFGNWLIPLMLGAPDMAFPRMNNMSFWLLPPSLTLLISSSIVENGAGTGWTVYPPLSSNIAHGGSSVDLAIFSLHLAGISSILGAINFITTIINMRINGMSFD
QMPLFVWAVGITALLLLLSLPVLAGAITMLLTDRNLNTSFFDPAGGGDPILYQHL
>LEFID435-10|HQ570331|MM06366|Anania_funebris
TLYFIFGIWSSMVGTSLSLLIRAELGNPGSLIGDDQIYNTIVTAHAFIMIFFMVMPIMIGGFGNWLVPLMLGAPDMAFPRMNNMSFWLLPPSLTLLISSSIVENGAGTGWTVYPPLSSNIAHGGGSVDLAIFSLHLAGISSILGAINFITTIINMRINGMSFD
QMPLFVWAVGITALLLLLSLPVLAGAITMLLTDRNLNTSFFDPAGGGDPILYQHL
>LEFIK825-10|JN277455|MM18400|Anania_funebris
TLYFIFGIWSGMVGTSLSLLIRAELGNPGSLIGDDQIYNTIVTAHAFIMIFFMVMPIMIGGFGNWLVPLMLGAPDMAFPRMNNMSFWLLPPSLTLLISSSIVENGAGTGWTVYPPLSSNIAHGGGSVDLAIFSLHLAGISSILGAINFITTIINMRINGMSF
DQMPLFVWAVGITALLLLLSLPVLAGAITMLLTDRNLNTSFFDPAGGGDPILYQHL
>LEFIA711-10|HM386856|MM01866|Anania_fuscalis
TLYFIFGIWSGMVGTSLSLLIRAELGNPGSLIGDDQIYNTIVTAHAFIMIFFMVMPIMIGGFGNWLIPLMLGAPDMAFPRMNNMSFWLLPPSLTLLISSSIVENGAGTGWTVYPPLSSNIAHGGSSVDLAIFSLHLAGISSILGAINFITTIINMRINGMSFD
QMPLFVWAVGITALLLLLSLPVLAGAITMLLTDRNLNTSFFDPAGGGDPILYQHL
>LEFIA712-10|HQ963157|MM01867|Anania_fuscalis

TLYFIFGIWSGMVGTSLSLLIRAELGNPGSLIGDDQIYNTIVTAHAFIMIFFMVMPIMIGGFGNWLIPLMLGAPDMAFPRMNNMSFWLLPPSLTLLISSSIVENGAGTGWTVYPPLSSNIAHGGSSVDLAIFSLHLAGISSILGAINFITTIINMRINGMSFD
QMPLFVWAVGITALLLLLSLPVLAGAITMLLTDRNLNTSFFDPAGGGDPILYQHL
>LEFIA173-10|HM396518|MM01154|Anania_hortulata
TLYFIFGIWSGMVGTSLSLLIRAELGNPGSLIGDDQIYNTIVTAHAFIMIFFMVMPIMIGGFGNWLVPLMLGAPDMAFPRMNNMSFWLLPPSLTLLISSSIVENGAGTGWTVYPPLSSNIAHSGSSVDLAIFSLHLAGISSILGAINFITTIINMRINGMSFD
QMPLFIWAVGITALLLLLSLPVLAGAITMLLTDRNLNTSFFDPAGGGDPILYQHL
>LEFIK269-10|KM572783|MM17844|Anania_lancealis
TLYFIFGIWSGMVGTSLSLLIRAELGNPGSLIGDDQIYNTIVTAHAFIMIFFMVMPIMIGGFGNWLVPLMLGAPDMAFPRMNNMSFWLLPPSLTLLISSSIVENGAGTGWTVYPPLSSNIAHSGSSVDLAIFSLHLAGISSILGAINFITTIINMRIKGMSFD
QMPLFVWAVGITALLLLLSLPVLAGAITMLLTDRNLNTSFFDPAGGGDPILYQHL
>LEFIK312-10|KM572481|MM17887|Anania_lancealis
TLYFIFGIWSGMVGTSLSLLIRAELGNPGSLIGDDQIYNTIVTAHAFIMIFFMVMPIMIGGFGNWLVPLMLGAPDMAFPRMNNMSFWLLPPSLTLLISSSIVENGAGTGWTVYPPLSSNIAHSGSSVDLAIFSLHLAGISSILGAINFITTIINMRIKGMSFD
QMPLFVWAVGITALLLLLSLPVLAGAITMLLTDRNLNTSFFDPAGGGDPILYQHL
>LEFIA709-10|HM386854|MM01864|Anania_perlucidalis
TLYFIFGIWSGMVGTSLSLLIRAELGNPGSLIGDDQIYNTIVTAHAFIMIFFMVMPIMIGGFGNWLVPLMLGAPDMAFPRMNNMSFWLLPPSLTLLISSSIVENGAGTGWTVYPPLSSNIAHGGSSVDLAIFSLHLAGISSILGAINFITTIINMRINGLSFD
QMPLFVWAVGITALLLLLSLPVLAGAITMLLTDRNLNTSFFDPAGGGDPILYQHL
>LEFID662-10|HM873424|MM06695|Anania_stachydalis
TLYFIFGIWSGMVGTSLSLLIRAELGNPGSLIGDDQIYNTIVTAHAFIMIFFMVMPIMIGGFGNWLIPLMLGAPDMAFPRMNNMSFWLLPPSLTLLISSSIVENGAGTGWTVYPPLSSNIAHGGSSVDLAIFSLHLAGISSILGAINFITTIINMRINGMSFD
QMPLFVWAVGITALLLLLSLPVLAGAITMLLTDRNLNTSFFDPAGGGDPILYQHL
>LEFIB666-10|HM871544|MM02447|Anania_terrealis
TLYFIFGIWSGMVGTSLSLLIRAELGNPGSLIGDDQIYNTIVTAHAFIMIFFMVMPIMIGGFGNWLIPLMLGAPDMAFPRMNNMSFWLLPPSLTLLISSSIVENGAGTGWTVYPPLSSNIAHGGSSVDLAIFSLHLAGISSILGAINFITTIINMRINGMSFD
QMPLFVWAVGITALLLLLSLPVLAGAITMLLTDRNLNTSFFDPAGGGDPILYQHL
>LEFIE137-10|HM873885|MM08366|Anania_terrealis
TLYFIFGIWSGMVGTSLSLLIRAELGNPGSLIGDDQIYNTIVTAHAFIMIFFMVMPIMIGGFGNWLIPLMLGAPDMAFPRMNNMSFWLLPPSLTLLISSSIVENGAGTGWTVYPPLSSNIAHGGSSVDLAIFSLHLAGISSILGAINFITTIINMRINGMSFD
QMPLFVWAVGITALLLLLSLPVLAGAITMLLTDRNLNTSFFDPAGGGDPILYQHL
>LEFIJ737-10|KM573089|MM17362|Anania_verbascalis
TLYFIFGIWSGMVGTSLSLLIRAELGNPGSLIGDDQIYNTIVTAHAFIMIFFMVMPIMIGGFGNWLVPLMLGAPDMAFPRMNNMSFWLLPPSLTLLISSSIVENGAGTGWTVYPPLSSNIAHGGSSVDLAIFSLHLAGISSILGAINFITTIINMRINGMSFD
QMPLFVWAVGITALLLLLSLPVLAGAITMLLTDRNLNTSFFDPAGGGDPILYQHL
>LEFIK315-10|KM572425|MM17890|Anania_verbascalis
TLYFIFGIWSGMVGTSLSLLIRAELGNPGSLIGDDQIYNTIVTAHAFIMIFFMVMPIMIGGFGNWLVPLMLGAPDMAFPRMNNMSFWLLPPSLTLLISSSIVENGAGTGWTVYPPLSSNIAHGGSSVDLAIFSLHLAGISSILGAINFITTIINMRINGMSFD
QMPLFVWAVGITALLLLLSLPVLAGAITMLLTDRNLNTSFFDPAGGGDPILYQHL
>LEEUA285-11|MM19693|Anania_verbascalis
TLYFIFGIWSGMVGTSLSLLIRAELGNPGSLIGDDQIYNTIVTAHAFIMIFFMVMPIMIGGFGNWLVPLMLGAPDMAFPRMNNMSFWLLPPSLTLLISSSIVENGAGTGWTVYPPLSSNIAHGGSSVDLAIFSLHLAGISSILGAINFITTIINMRINGMSFD
QMPLFVWAVGITALLLLLSLPVLAGAITMLLTDRNLNTSFFDPAGGGDPILYQHL
>LEFIA549-10|KM572060|MM01638|Anaplectoides_prasina
TLYFIFGIWAGMVGTSLSLLIRAELGNPGSLIGDDQIYNTIVTAHAFIMIFFMVMPIMIGGFGNWLVPLMLGAPDMAFPRMNNMSFWLLPPSLTLLISSSIVENGAGTGWTVYPPLSSNIAHGGSSVDLAIFSLHLAGISSILGAINFITTIINMRLNNLSFD
QMPLFIWAVGITAFLLLLSLPVLAGAITMLLTDRNLNTSFFDPAGGGDPILYQHL
>LEFIE192-10|HM873937|MM08507|Anaproutia_norvegica
TLYFILGIWSGLIGTSLSVMIRAELGTPGSLIGSDQIYNTLVTAHAFIMIFFMVMPIMIGGFGNWLIPLMLGAPDMAFPRMNNMSFWLLPPSLMLLTASSIVENGAGTGWTVYPPLSSNIAHDGRAVDLAIFSLHLAGISSILGAVNFITTTINMRPYEMTL
DMMPLFVWSVAITALLLMLSLPVLAGAITMLLTDRNLNTSFFDPAGGGDPILYQHL
>LEFIC837-10|HM872655|MM05037|Anarsia_lineatella
TLYFIFGIWSGMVGTSLSLLIRAELGNPGSLIGDDQIYNTIVTAHAFIMIFFMVMPIMIGGFGNWLVPLMLGAPDMAFPRMNNMSFWLLPPSITLLISSSIVETGAGTGWTVYPPLSSNIAHSGGSVDLAIFSLHLAGISSILGAINFITTIINMRINGLSFDQ
MPLFVWAVGITALLLLLSLPVLAGAITMLLTDRNLNTSFFDPAGGGDPILYQHL
>LEEUA393-11|JN264932|MM19801|Anarsia_lineatella
TLYFIFGIWAGMVGTSLSLLIRAELGNPGSLIGDDQIYNTIVTAHAFIMIFFMVMPIMIGGFGNWLVPLMLGAPDMAFPRMNNMSFWLLPPSITLLISSSIVETGAGTGWTVYPPLSSNIAHSGGSVDLAIFSLHLAGISSILGAINFITTIINMRINGLSFDQ
MPLFVWAVGITALLLLLSLPVLAGAITMLLTDRNLNTSFFDPAGGGDPILYQHL
>LEFIA749-10|HM386893|MM04103|Anarta_melanopa
TLYFIFGIWAGMVGTSLSLLIRAELGNPGSLIGDDQIYNTIVTAHAFIMIFFMVMPIMIGGFGNWLVPLMLGAPDMAFPRMNNMSFWLLPPSLTLLISSSVVENGAGTGWTVYPPLSSNIAHGGSSVDLAIFSLHLAGISSILGAINFITTIINMRLNNLSF
DQMPLFIWAVGITAFLLLLSLPVLAGAITMLLTDRNLNTSFFDPAGGGDPILYQHL

>LEFIK422-10|JF854051|MM17997|Anarta_melanopa
TLYFIFGIWAGMVGTSLSLLIRAELGNPGSLIGDDQIYNTIVTAHAFIMIFFMVMPIMIGSFGNWLVPLMLGAPDMAFPRMNNMSFWLLPPSLTLLISSSVVENGAGTGWTVYPPLSSNIAHGGSSVDLAIFSLHLAGISSILGAINFITTIINMRLNNLSFD
QMPLFIWAVGITAFLLLLSLPVLAGAITMLLTDRNLNTSFFDPAGGGDPILYQHL
>LEFIK447-10|JF854069|MM18022|Anarta_myrtilli
TLYFIFGIWAGMVGTSLSLLIRAELGNPGSLIGDDQIYNTIVTAHAFIMIFFMVMPIMIGGFGNWLVPLMLGAPDMAFPRMNNMSFWLLPPSLTLLISSSIVENGAGTGWTVYPPLSSNIAHGGSSVDLAIFSLHLAGISSILGAINFITTIINMRLNNLSFD
QMPLFIWAVGITAFLLLLSLPVLAGAITMLLTDRNLNTSFFDPAGGGDPILYQHL
>LEFID051-10|HM872865|MM05525|Anarta_trifolii
TLYFIFGIWAGMVGTSLSLLIRAELGNPGSLIGDDQIYNTIVTAHAFIMIFFMVMPIMIGGFGNWLVPLMLGAPDMAFPRMNNMSFWLLPPSLTLLISSSIVENGAGTGWTVYPPLSSNIAHGGSSVDLAIFSLHLAGISSILGAINFITTIINMRLNSLSFD
QMPLFIWAVGITAFLLLLSLPVLAGAITMLLTDRNLNTSFFDPAGGGDPILYQHL
>LEEUA132-11|MM19540|Anchinia_cristalis
TLYFIFGIWSGMVGTSLSLLIRAELGNPGSLIGDDQIYNTIVTAHAFIMIFFMVMPIMIGGFGNWLVPLMLGAPDMAFPRMNNMSFWLLPPSLTLLISSSIVETGAGTGWTVYPPLSSNIAHSGSSVDLAIFSLHLAGISSILGAINFITTIINMKINNMNFD
QMPLFVWSVGITALLLLLSLPVLAGAITMLLTDRNLNTSFFDPAGGGDPILYQHL
>LEFIE372-10|HM874096|MM08880|Anchinia_daphnella
TLYFIFGIWAGMVGTSLSLLIRTELGNPGSLIGDDQIYNTIVTAHAFIMIFFMVMPIMIGGFGNWLVPLMLGAPDMAFPRMNNMSFWILPPSLTLIISSSIVENGAGTGWTVYPPLSSNIAHSGSSVDLAIFSLHLAGISSILGAINFITTIINMKINNLNFDQ
MPLFVWSVGITALLLLLSLPVLAGAITMLLTDRNLNTSFFDPAGGGDPILYQHL
>LEFIE373-10|HM874097|MM08881|Anchinia_daphnella
TLYFIFGIWAGMVGTSLSLLIRTELGNPGSLIGDDQIYNTIVTAHAFIMIFFMVMPIMIGGFGNWLVPLMLGAPDMAFPRMNNMSFWILPPSLTLIISSSIVENGAGTGWTVYPPLSSNIAHSGSSVDLAIFSLHLAGISSILGAINFITTIINMKINNLNFDQ
MPLFVWSVGITALLLLLSLPVLAGAITMLLTDRNLNTSFFDPAGGGDPILYQHL
>LEFIK205-10|JF853941|MM17780|Ancylis_achatana
TLYFIFGIWAGMVGTSLSLLIRAELGNTGSLIGDDQIYNTIVTAHAFIMIFFMVMPIMIGGFGNWLVPLMLGAPDMAFPRMNNMSFWLLPPSIMLLISSSIVENGA?TGWTVYPPLSSNIAHSGSSVDLAIFSLHLAGISSILGAVNFITTIINMRPNNMSL
DQMPLFVWAVGITALLLLLSLPVLAGAITMLLTDRNLNTSFFDPAGGGDPILYQHL
>LEFIC091-10|HM871959|MM03392|Ancylis_apicella
TLYFIFGIWAGMVGTSLSLLIRAELGNPGSLIGDDQIYNTIVTAHAFIMIFFMVMPIMIGGFGNWLVPLMLGAPDMAFPRMNNMSFWLLPPSIMLLISSSIVENGAGTGWTVYPPLSSNIAHSGSSVDLAIFSLHLAGISSILGAVNFITTIINMRPNNMSL
DQMPLFVWAVGITALLLLLSLPVLAGAITMLLTDRNLNTSFFDPAGGGDPILYQHL
>LEFIB501-10|HM871396|MM02087|Ancylis_badiana
TLYFIFGIWAGMVGTSLSLLIRAELGNPGSLIGDDQIYNTIVTAHAFIMIFFMVMPIMIGGFGNWLVPLMLGAPDMAFPRMNNMSFWLLPPSIMLLISSSIVENGAGTGWTVYPPLSSNIAHSGSSVDLAIFSLHLAGISSILGAVNFITTIINMRPNNMSL
DQMPLFVWAVGITALLLLLSLPVLAGAITMLLTDRNLNTSFFDPAGGGDPILYQHL
>LEFIB533-10|HM871419|MM02144|Ancylis_badiana
TLYFIFGIWAGMVGTSLSLLIRAELGNPGSLIGDDQIYNTIVTAHAFIMIFFMVMPIMIGGFGNWLVPLMLGAPDMAFPRMNNMSFWLLPPSIMLLISSSIVENGAGTGWTVYPPLSSNIAHSGSSVDLAIFSLHLAGISSILGAVNFITTIINMRPNNMSL
DQMPLFVWAVGITALLLLLSLPVLAGAITMLLTDRNLNTSFFDPAGGGDPILYQHL
>LEFIC557-10|HM872378|MM04334|Ancylis_badiana
TLYFIFGIWAGMVGTSLSLLIRAELGNPGSLIGDDQIYNTIVTAHAFIMIFFMVMPIMIGGFGNWLVPLMLGAPDMAFPRMNNMSFWLLPPSIMLLISSSIVENGAGTGWTVYPPLSSNIAHSGSSVDLAIFSLHLAGISSILGAVNFITTIINMRPNNMSL
DQMPLFVWAVGITALLLLLSLPVLAGAITMLLTDRNLNTSFFDPAGGGDPILYQHL
>LEFIK745-10|JF854305|MM18320|Ancylis_badiana
TLYFIFGIWAWMVGTSLSLLIRAELGNPGSLIGDDQIYNTIVTAHAFIMIFFMVMPIMIGGFGNWLVPLMLGAPDMAFPRMNNMSFWLLPPSIMLLISSSIVENGAGTGWTVYPPLSSNIAHSGSSVDLAIFSLHLAGISSILGAVNFITTIINMRPNNMS
LDQMPLFVWAVGITALLLLLSLPVLAGAITMLLTDRNLNTSFFDPAGGGDPILYQHL
>LEFIE306-10|HM874032|MM08663|Ancylis_comptana
TLYFIFGIWAGMVGTSLSLMIRAELGNPGSLIGDDQIYNTIVTAHAFIMIFFMVMPIMIGGFGNWLVPLMLGAPDMAFPRMNNMSFWLLPPSIMLLISSSIVENGAGTGWTVYPPLSSNIAHSGSSVDLAIFSLHLAGISSILGAVNFITTIINMRPNNMS
LDQMPLFVWAVGITAILLLLSLPVLAGAITMLLTDRNLNTSFFDPAGGGDPILYQHL
>LEFIE464-10|HM874188|MM09236|Ancylis_comptana
TLYFIFGIWAGMVGTSLSLLIRAELGNPGSLIGDDQIYNTIVTAHAFIMIFFMVMPIMIGGFGNWLVPLMLGAPDMAFPRMNNMSFWLLPPSIMLLISSSIVENGAGTGWTVYPPLSSNIAHSGSSVDLAIFSLHLAGISSILGAVNFITTIINMRPNNMSL
DQMPLFVWAVGITAILLLLSLPVLAGAITMLLTDRNLNTSFFDPAGGGDPILYQHL
>LEFIE465-10|HM874189|MM09237|Ancylis_comptana
TLYFIFGIWAGMVGTSLSLLIRAELGNPGSLIGDDQIYNTIVTAHAFIMIFFMVMPIMIGGFGNWLVPLMLGAPDMAFPRMNNMSFWLLPPSIMLLISSSIVENGAGTGWTVYPPLSSNIAHSGSSVDLAIFSLHLAGISSILGAVNFITTIINMRPNNMSL
DQMPLFVWAVGITAILLLLSLPVLAGAITMLLTDRNLNTSFFDPAGGGDPILYQHL
>LEFIB511-10|HQ570265|MM02105|Ancylis_diminutana
TLYFIFGVWAGMVGTSLSLLIRAELGNPGSLIGNDQIYNTIVTAHAFIMIFFMVMPIMIGGFGNWLVPLMLGAPDMAFPRMNNMSFWLLPPSMMLLISSSIVENGAGTGWTVYPPLSSNIAHSGSSVDLTIFSLHLAGISSILGAVNFITTIINMRPNSM

SLDQMPLFVWAVAITALLLLLSLPVLAGAITMLLTDRNLNTSFFDPAGGGDPILYQHL
>LEFIB530-10|HQ570271|MM02141|Ancylis_geminana
TLYFIFGIWAGMVGTSLSLLIRAELGNPGSLIGNDQIYNTIVTAHAFIMIFFMVMPIMIGGFGNWLVPLMLGAPDMAFPRMNNMSFWLLPPSIMLLISSSIVENGAGTGWTVYPPLSSNIAHSGSSVDLAIFSLHLAGISSILGAVNFITTIINMRPNNMSL
DQMPLFVWAVAITALLLLLSLPVLAGAITMLLTDRNLNTSFFDPAGGGDPILYQHL
>LEFIG098-10|HM875777|MM13831|Ancylis_habeleri
TLYFIFGIWAGMVGTSLSLLIRAELGNPGSLIGDDQIYNTIVTAHAFIMIFFMVMPIMIGGFGNWLVPLMLGAPDMAFPRMNNMSFWLLPPSIMLLISSSIVENGAGTGWTVYPPLSSNIAHSGSSVDLAIFSLHLAGISSILGAVNFITTIINMRPNNMSL
DQMPLFVWAVGITALLLLLSLPVLAGAITMLLTDRNLNTSFFDPAGGGDPILYQHL
>LEFIJ669-10|JX034607|MM17294|Ancylis_habeleri
TLYFIFGIWAGMVGTSLSLLIRAELGNPGSLIGDDQIYNTIVTAHAFIMIFFMVMPIMIGGFGNWLVPLMLGAPDMAFPRMNNMSFWLLPPSIMLLISSSIVENGAGTGWTVYPPLSSNIAHSGSSVDLAIFSLHLAGISSILGAVNFITTIINMRPNNMSL
DQMPLFVWAVGITALLLLLSLPVLAGAITMLLTDRNLNTSFFDPAGGGDPILYQHL
>LEFIB624-10|HM871503|MM02369|Ancylis_kenneli
TLYFIFGVWAGMVGTSLSLLIRAELGNPGSLIGDDQIYNTIVTAHAFIMIFFMVMPIMIGGFGNWLVPLMLGAPDMAFPRMNNMSFWLLPPSIMLLISSSIVENGAGTGWTVYPPLSSNIAHSGSSVDLAIFSLHLAGISSILGAVNFITTIINMRPNNMS
LDQMPLFVWAVGITALLLLLSLPVLAGAITMLLTDRNLNTSFFDPAGGGDPILYQHL
>LEFIB147-10|HM871052|MM00532|Ancylis_laetana
TLYFIFGVWAGMVGTSLSLLIRAELGNPGSLIGDDQIYNTIVTAHAFIMIFFMVMPIMIGGFGNWLVPLMLGAPDMAFPRMNNMSFWLLPPSIMLLISSSIVENGAGTGWTVYPPLSSNIAHSGSSVDLAIFSLHLAGISSILGAVNFITTIINMRPNNMS
LDQMPLFVWAVGITALLLLLSLPVLAGAITMLLTDRNLNTSFFDPAGGGDPILYQHL
>LEFIC337-10|HM872181|MM03844|Ancylis_mitterbacheriana
TLYFIFGIWAGMIGTSLSLLIRAELGNPGSLIGDDQIYNTIVTAHAFIMIFFMVMPIMIGGFGNWLVPLMLGAPDMAFPRMNNMSFWLLPPSIMLLISSSIVENGAGTGWTVYPPLSSNIAHSGSSVDLAIFSLHLAGISSILGAVNFITTIINMRPNSMSL
DQMPLFVWAVGITALLLLLSLPVLAGAITMLLTDRNLNTSFFDPAGGGDPILYQHL
>LEFIB500-10|HM871395|MM02085|Ancylis_myrtillana
TLYFIFGIWAGMVGTSLSLLIRAELGNPGSLIGDDQIYNTIVTAHAFIMIFFMVMPIMIGGFGNWLVPLMLGAPDMAFPRMNNMSFWLLPPSIMLLISSSIVENGAGTGWTVYPPLSSNIAHSGSSVDLAIFSLHLAGISSILGAVNFITTIINMRPNNMSL
DQMPLFVWAVGITALLLLLSLPVLAGAITMLLTDRNLNTSFFDPAGGGDPILYQHL
>LEFIC369-10|HM872212|MM03897|Ancylis_obtusana
TLYFIFGIWAGMVGTSLSLMIRAELGNPGSLIGDDQIYNTIVTAHAFIMIFFMVMPIMIGGFGNWLIPLMLGAPDMAFPRMNNMSFWLLPPSIMLLISSSIVENGAGTGWTVYPPLSSNIAHSGSSVDLAIFSLHLAGISSILGAVNFITTIINMRPNNMS
LDQMPLFVWAVGITALLLLLSLPVLAGAITMLLTDRNLNTSFFDPAGGGDPILYQHL
>LEFID205-10|HM873007|MM06033|Ancylis_obtusana
TLYFIFGIWAGMVGTSLSLMIRAELGNPGSLIGDDQIYNTIVTAHAFIMIFFMVMPIMIGGFGNWLIPLMLGAPDMAFPRMNNMSFWLLPPSIMLLISSSIVENGAGTGWTVYPPLSSNIAHSGSSVDLAIFSLHLAGISSILGAVNFISTIINMRPNNMS
LDQMPLFVWAVGITALLLLLSLPVLAGAITMLLTDRNLNTSFFDPAGGGDPILYQHL
>LEFIG239-10|HM875918|MM14155|Ancylis_paludana
TLYFIFGIWAGMVGTSLSLLIRAELGNPGSLIGDDQIYNTIVTAHAFIMIFFMVMPIMIGGFGNWLVPLMLGAPDMAFPRMNNMSFWLLPPSIMLLISSSIVENGAGTGWTVYPPLSSNIAHSGSSVDLAIFSLHLAGISSILGAVNFITTIINMRPNNMSL
DQMPLFVWAVGITALLLLLSLPVLAGAITMLLTDRNLNTSFFDPAGGGDPILYQHL
>LEFIG254-10|HM875933|MM14178|Ancylis_paludana
TLYFIFGIWAGMVGTSLSLLIRAELGNPGSLIGDDQIYNTIVTAHAFIMIFFMVMPIMIGGFGNWLVPLMLGAPDMAFPRMNNMSFWLLPPSIMLLISSSIVENGAGTGWTVYPPLSSNIAHSGSSVDLAIFSLHLAGISSILGAVNFITTIINMRPNNMSL
DQMPLFVWAVGITALLLLLSLPVLAGAITMLLTDRNLNTSFFDPAGGGDPILYQHL
>LEFIF234-10|HM874927|MM11014|Ancylis_selenana
TLYFIFGIWAGMVGTSLSLLIRAELGNPGSLIGDDQIYNTIVTAHAFIMIFFMVMPIMIGGFGNWLVPLMLGAPDMAFPRMNNMSFWLLPPSIMLLISSSIVENGAGTGWTVYPPLSSNIAHSGSSVDLAIFSLHLAGISSILGAVNFITTIINMRPNNMSL
DQMPLFVWAVAITAILLLLSLPVLAGAITMLLTDRNLNTSFFDPAGGGDPILYQHL
>LEFIK739-10|JF854299|MM18314|Ancylis_selenana
TLYFIFGIWAGMVGTSLSLLIRAELGNPGSLIGDDQIYNTIVTAHAFIMIFFMVMPIMIGGFGNWLVPLMLGAPDMAFPRMNNMSFWLLPPSIMLLISSSIVENGAGTGWTVYPPLSSNIAHSGSSVDLAIFSLHLAGISSILGAVNFITTIINMRPNNMSL
DQMPLFVWAVAITAILLLLSLPVLAGAITMLLTDRNLNTSFFDPAGGGDPILYQHL
>LEFID377-10|HM873174|MM06288|Ancylis_subarcuana
TLYFIFGVWAGMVGTSLSLLIRAELGNPGSLIGNDQIYNTIVTAHAFIMIFFMVMPIMIGGFGNWLVPLMLGAPDMAFPRMNNMSFWLLPPSIMLLISSSIVENGAGTGWTVYPPLSSNIAHSGSSVDLAIFSLHLAGISSILGAVNFITTIINMRPNNMS
LDQMPLFVWAVAITALLLLLSLPVLAGAITMLLTDRNLNTSFFDPAGGGDPILYQHL
>LEFID479-10|HM873245|MM06421|Ancylis_subarcuana
TLYFIFGVWAGMVGTSLSLLIRAELGNPGSLIGNDQIYNTIVTAHAFIMIFFMVMPIMIGGFGNWLVPLMLGAPDMAFPRMNNMSFWLLPPSIMLLISSSIVENGAGTGWTVYPPLSSNIAHSGSSVDLAIFSLHLAGISSILGAVNFITTIINMRPNNMS
LDQMPLFVWAVAITALLLLLSLPVLAGAITMLLTDRNLNTSFFDPAGGGDPILYQHL
>LEFIE172-10|HM873918|MM08441|Ancylis_subarcuana

TLYFIFGVWAGMMGTSLSLLIRAELGNPGSLIGNDQIYNTIVTAHAFIMIFFMVMPIMIGGFGNWLVPLMLGAPDMAFPRMNNMSFWLLPPSIMLLTSSSIVENGAGTGWTVYPPLSSNIAHSGSSVDLAIFSLHLAGISSILGAVNFITTIINMRPNNM
SLDQMPLFVWAVAITALLLLLSLPVLAGAITMLLTDRNLNTSFFDPAGGGDPILYQHL
>LEFIB222-10|HM871126|MM00676|Ancylis_tineana
TMYFIFGIWAGMIGTSLSLLIRAELGNPGSLIGDDQIYNTIVTAHAFIMIFFMVMPIMIGGFGNWLVPLMLGAPDMAFPRMNNMSFWLLPPSIMLLISSSIVENGAGTGWTVYPPLSSNIAHSGSSVDLAIFSLHLAGISSILGAVNFITTIINMRPNSMSL
DQMPLFVWAVGITALLLLLSLPVLAGAITMLLTDRNLNTSFFDPAGGGDPILYQHL
>LEFIK740-10|JF854300|MM18315|Ancylis_tineana
TMYFIFGIWAGMIGTSLSLLIRAELGNPGSLIGDDQIYNTIVTAHAFIMIFFMVMPIMIGGFGNWLVPLMLGAPDMAFPRMNNMSFWLLPPSIMLLISSSIVENGAGTGWTVYPPLSSNIAHSGSSVDLAIFSLHLAGISSILGAVNFITTIINMRPNSMSL
DQMPLFVWAVGITALLLLLSLPVLAGAITMLLTDRNLNTSFFDPAGGGDPILYQHL
>LEFIB180-10|HM871084|MM00596|Ancylis_uncella
TLYFIFGIWAGMVGTSLSLLIRAELGNPGSLIGDDQIYNTIVTAHAFIMIFFMVMPIMIGGFGNWLVPLMLGAPDMAFPRMNNMSFWLLPPSLMLLISSSIVENGAGTGWTVYPPLSSNIAHSGSSVDLAIFSLHLAGISSILGAVNFITTIINMRPNNMS
LDQMPLFVWAVGITALLLLLSLPVLAGAITMLLTDRNLNTSFFDPAGGGDPILYQHL
>LEFIG163-10|HM875843|MM14022|Ancylis_uncella
TLYFIFGIWAGMVGTSLSLLIRAELGNPGSLIGDDQIYNTIVTAHAFIMIFFMVMPIMIGGFGNWLVPLMLGAPDMAFPRMNNMSFWLLPPSLMLLISSSIVENGAGTGWTVYPPLSSNIAHSGSSVDLAIFSLHLAGISSILGAVNFITTIINMRPNNMS
LDQMPLFVWAVGITALLLLLSLPVLAGAITMLLTDRNLNTSFFDPAGGGDPILYQHL
>LEFIB756-10|HM871633|MM02610|Ancylis_unculana
TLYFIFGVWAGMVGTSLSLLIRAELGNPGSLIGDDQIYNTIVTAHAFIMIFFMVMPIMIGGFGNWLVPLMLGAPDMAFPRMNNMSFWLLPPSIMLLISSSIVENGAGTGWTVYPPLSSNIAHSGSSVDLAIFSLHLAGISSILGAVNFITTIINMRPNNMS
LDQMPLFIWAVGITALLLLLSLPVLAGAITMLLTDRNLNTSFFDPAGGGDPILYQHL
>LEFIB179-10|HM871083|MM00595|Ancylis_unguicella
TLYFIFGIWAGMVGTSLSLLIRAELGNPGSLIGDDQIYNTIVTAHAFIMIFFMVMPIMIGGFGNWLVPLMLGAPDMAFPRMNNMSFWLLPPSLMLLISSSIVENGAGTGWTVYPPLSSNIAHSGSSVDLAIFSLHLAGISSILGAVNFITTIINMRPNNMS
LDQMPLFVWSVGITALLLLLSLPVLAGAITMLLTDRNLNTSFFDPAGGGDPILYQHL
>LEFIC110-10|HQ570301|MM03438|Ancylis_unguicella
TLYFIFGIWAGMVGTSLSLLIRAELGNPGSLIGDDQIYNTIVTAHAFIMIFFMVMPIMIGGFGNWLVPLMLGAPDMAFPRMNNMSFWLLPPSLMLLISSSIVENGAGTGWTVYPPLSSNIAHSGSSVDLAIFSLHLAGISSILGAVNFITTIINMRPNNMS
LDQMPLFVWSVGITALLLLLSLPVLAGAITMLLTDRNLNTSFFDPAGGGDPILYQHL
>LEFIC349-10|HM872192|MM03865|Ancylis_upupana
TLYFIFGIWSGMVGTSLSLLIRAELGNPGSLIGDDQIYNTIVTAHAFIMIFFMVMPIMIGGFGNWLVPLMLGAPDMAFPRMNNMSFWLLPPSIMLLISSSIVENGAGTGWTVYPPLSSNIAHSGSSVDLAIFSLHLAGISSILGAVNFITTIINMRPNNMSL
DQMPLFVWAVGITALLLLLSLPVLAGAITMLLTDRNLNTSFFDPAGGGDPILYQHL
>LEFIE141-10|HM873889|MM08376|Ancylis_upupana
TLYFIFGIWSGMVGTSLSLLIRAELGNPGSLIGDDQIYNTIVTAHAFIMIFFMVMPIMIGGFGNWLVPLMLGAPDMAFPRMNNMSFWLLPPSIMLLISSSIVENGAGTGWTVYPPLSSNIAHSGSSVDLAIFSLHLAGISSILGAVNFITTIINMRPNNMSL
DQMPLFVWAVGITALLLLLSLPVLAGAITMLLTDRNLNTSFFDPAGGGDPILYQHL
>LEFIB053-10|HM870962|MM00336|Anerastia_lotella
TLYFIFGIWSGMVGTSLSLLIRAELGTPGSLIGDDQIYNTIVTGHAFIMIFFMVMPIMIGGFGNWLVPLMLGAPDMAFPRMNNMSFWLLPPSLTLLISSSIVENGAGTGWTVYPPLSSNIAHSGSSVDLAIFSLHLAGISSILGAINFITTIINMKLNGLLFDQ
MPLFVWSVGITALLLLLSLPVLAGAITMLLTDRNLNTSFFDPAGGGDPILYQHL
>LEFID572-10|HM873337|MM06567|Anerastia_lotella
TLYFIFGIWSGMVGTSLSLLIRAELGIPGSLIGDDQIYNTIVTGHAFIMIFFMVMPIMIGGFGNWLVPLMLGAPDMAFPRMNNMSFWLLPPSLTLLISSSIVENGAGTGWTVYPPLSSNIAHSGSSVDLAIFSLHLAGISSILGAINFITTIINMKLNGLSFDQ
MPLFVWSVGITALLLLLSLPVLAGAITMLLTDRNLNTSFFDPAGGGDPILYQHL
>LEFIF783-10|HM875467|MM13019|Anerastia_lotella
TLYFIFGIWSGMVGTSLSLLIRAELGTPGSLIGDDQIYNTIVTGHAFIMIFFMVMPIMIGGFGNWLVPLMLGAPDMAFPRMNNMSFWLLPPSLTLLISSSIVENGAGTGWTVYPPLSSNIAHSGSSVDLAIFSLHLAGISSILGAINFITTIINMKLNGLSFDQ
MPLFVWSVGITALLLLLSLPVLAGAITMLLTDRNLNTSFFDPAGGGDPILYQHL
>LEFIA237-10|HM386581|MM01270|Angerona_prunaria
TLYFIFGIWAGMVGTSLSLLIRAELGNPGSLIGNDQIYNTIVTAHAFIMIFFMVMPIMIGGFGNWLVPLMLGAPDMAFPRMNNMSFWLLPPSITLLISSSIVENGAGTGWTVYPPLSSNIAHGGSSVDLAIFSLHLAGISSILGAINFITTIINMRLNNLSFD
QMPLFVWSVGITAFLLLLSLPVLAGAITMLLTDRNLNTSFFDPAGGGDPILYQHL
>LEFIA238-10|HM386582|MM01271|Angerona_prunaria
TLYFIFGIWAGMVGTSLSLLIRAELGNPGSLIGNDQIYNTIVTAHAFIMIFFMVMPIMIGGFGNWLIPLMLGAPDMAFPRMNNMSFWLLPPSITLLISSSIVENGAGTGWTVYPPLSSNIAHGGSSVDLAIFSLHLAGISSILGAINFITTIINMRLNNLSFDQ
MPLFVWSVGITAFLLLLSLPVLAGAITMLLTDRNLNTSFFDPAGGGDPILYQHL
>LEFIC562-10|HM872383|MM04339|Anorthoa_munda
TLYFIFGIWAGMIGTSLSLLIRAELGNPGSLIGDDQIYNTIVTAHAFIMIFFMVMPIMIGGFGNWLVPLMLGAPDMAFPRMNNMSFWLLPPSLTLLISSSIVENGAGTGWTVYPPLSSNIAHGGSSVDLAIFSLHLAGISSILGAINFITTIINMRLNNLSFD
QMPLFIWAVGITAFLLLLSLPVLAGAITMLLTDRNLNTSFFDPAGGGDPILYQHL

>LEFIB129-10|HM871035|MM00495|Anthocharis_cardamines
TLYFIFGIWSGMVGTSLSLLIRTELGNPGSLIGDDQIYNTIVTAHAFIMIFFMVMPIMIGGFGNWLIPLMLGAPDMAFPRMNNMSFWLLPPSLTLLISSSIVENGAGTGWTVYPPLSSNIAHSGSSVDLAIFSLHLAGISSILGAINFITTIINMRINNMSFDQ
MPLFVWAVGITALLLLLSLPVLAGAITMLLTDRNLNTSFFDPAGGGDPILYQHL
>LEFIB131-10|HM871037|MM00500|Anthocharis_cardamines
TLYFIFGIWSGMVGTSLSLLIRTELGNPGSLIGDDQIYNTIVTAHAFIMIFFMVMPIMIGGFGNWLIPLMLGAPDMAFPRMNNMSFWLLPPSLTLLISSSIVENGAGTGWTVYPPLSSNIAHSGSSVDLAIFSLHLAGISSILGAINFITTIINMRINNMSFDQ
MPLFVWAVGITALLLLLSLPVLAGAITMLLTDRNLNTSFFDPAGGGDPILYQHL
>LEFIB612-10|HM871491|MM02342|Anthophila_fabriciana
TLYFIFGIWSGMVGTSLSLLIRAELGNPGSLIGDDQIYNTIVTAHAFIMIFFMVMPIMIGGFGNWLVPLMLGAPDMAFPRMNNMSFWLLPPSLTLLISSSIVETGAGTGWTVYPPLSSNIAHGGSSVDLAIFSLHLAGISSILGAINFITTIINMKPNNMTLD
QMPLFVWSVQITAILLLLSLPVLAGAITMLLTDRNLNTSFFDPAGGGDPILYQHL
>LEFIG102-10|HM875782|MM13886|Anticlea_derivata
TLYFIFGIWAGMIGTSLSLLIRAELGNPGSLIGDDQIYNTIVTAHAFIMIFFMVMPIMIGGFGNWLVPLMLGAPDMAFPRMNNMSFWLLPPSITLLISSSIVENGAGTGWTVYPPLSSNIAHGGSSVDLAIFSLHLAGISSILGAINFITTIINMRLNNMFFD
QLPLFVWAVGITAFLLLLSLPVLAGAITMLLTDRNLNTSFFDPAGGGDPILYQHL
>LEFIJ810-10|JF853835|MM17435|Anticlea_derivata
TLYFIFGIWAGMIGTSLSLLIRAELGNPGSLIGDDQIYNTIVTAHAFIMIFFMVMPIMIGGFGNWLVPLMLGAPDMAFPRMNNMSFWLLPPSITLLISSSIVENGAGTGWTVYPPLSSNIAHGGSSVDLAIFSLHLAGISSILGAINFITTIINMRLNNMFFD
QLPLFVWAVGITAFLLLLSLPVLAGAITMLLTDRNLNTSFFDPAGGGDPILYQHL
>LEFIC794-10|HM872613|MM04942|Anticollix_sparsata
TLYFIFGIWAGMIGTSLSLLIRAELGNPGSLIGDDQIYNTIVTAHAFIMIFFMVMPIMIGGFGNWLVPLMLGAPDMAFPRMNNMSFWLLPPSITLLISSSVVENGAGTGWTVYPPLSSNIAHGGSSVDLAIFSLHLAGISSILGAINFITTIINMRLNNMFFD
QLPLFVWAVGITAFLLLLSLPVLAGAITMLLTDRNLNTSFFDPAGGGDPILYQHL
>LEFIG266-10|HM875945|MM14199|Anticollix_sparsata
TLYFIFGIWAGMIGTSLSLLIRAELGNPGSLIGDDQIYNTIVTAHAFIMIFFMVMPIMIGGFGNWLVPLMLGAPDMAFPRMNNMSFWLLPPSITLLISSSVVENGAGTGWTVYPPLSSNIAHGGSSVDLAIFSLHLAGISSILGAINFITTIINMRLNNMFFD
QLPLFVWAVGITAFLLLLSLPVLAGAITMLLTDRNLNTSFFDPAGGGDPILYQHL
>LEFIB791-10|HM871668|MM02699|Antitype_chi
TLYFIFGIWAGMVGTSLSLLIRAELGNPGSLIGDDQIYNTIVTAHAFIMIFFMVMPIMIGGFGNWLVPLMLGAPDMAFPRMNNMSFWLLPPSLTLLISSSIVESGAGTGWTVYPPLSSNIAHGGSSVDLAIFSLHLAGISSILGAINFITTIINMRLNNLSFD
QMPLFIWAVGITAFLLLLSLPVLAGAITMLLTDRNLNTSFFDPAGGGDPILYQHL
>LEFIF294-10|HM874984|MM11076|Apamea_anceps
TLYFIFGIWAGMVGTSLSLLIRAELGNPGSLIGDDQIYNTIVTAHAFIMIFFMVMPIMIGGFGNWLVPLMLGAPDMAFPRMNNMSFWLLPPSLTLLISSSIVENGAGTGWTVYPPLSSNIAHGGSSVDLAIFSLHLAGISSILGAINFITTIINMRLNNLSFD
QMPLFIWAVGITAFLLLLSLPVLAGAITMLLTDRNLNTSFFDPAGGGDPILYQHL
>LEFIA183-10|HM396528|MM01172|Apamea_crenata
TLYFIFGIWAGMVGTSLSLLIRAELGNPGSLIGDDQIYNTIVTAHAFIMIFFMVMPIMIGGFGNWLVPLMLGAPDMAFPRMNNMSFWLLPPSLTLLISSSIVENGAGTGWTVYPPLSSNIAHGGSSVDLAIFSLHLAGISSILGAINFITTIINMRLNSLSFD
QMPLFIWAVGITAFLLLLSLPVLAGAITMLLTDRNLNTSFFDPAGGGDPILYQHL
>LEFIA184-10|HM396529|MM01173|Apamea_crenata
TLYFIFGIWAGMVGTSLSLLIRAELGNPGSLIGDDQIYNTIVTAHAFIMIFFMVMPIMIGGFGNWLVPLMLGAPDMAFPRMNNMSFWLLPPSLTLLISSSIVENGAGTGWTVYPPLSSNIAHGGSSVDLAIFSLHLAGISSILGAINFITTIINMRLNNLSFD
QMPLFIWAVGITAFLLLLSLPVLAGAITMLLTDRNLNTSFFDPAGGGDPILYQHL
>LEFIC702-10|HM872523|MM04727|Apamea_crenata
TLYFIFGIWAGMVGTSLSLLIRAELGNPGSLIGDDQIYNTIVTAHAFIMIFFMVMPIMIGGFGNWLVPLMLGAPDMAFPRMNNMSFWLLPPSLTLLISSSIVENGAGTGWTVYPPLSSNIAHGGSSVDLAIFSLHLAGISSILGAINFITTIINMRLNNLSFD
QMPLFIWAVGITAFLLLLSLPVLAGAITMLLTDRNLNTSFFDPAGGGDPILYQHL
>LEFIL275-10|JF854565|MM19275|Apamea_epomidion
TLYFIFGIWAGMVGTSLSLLIRAELGNPGSLIGDDQIYNTIVTAHAFIMIFFMVMPIMIGGFGNWLVPLMLGAPDMAFPRMNNMSFWLLPPSLTLLISSSIVESGAGTGWTVYPPLSSNIAHGGSSVDLAIFSLHLAGISSILGAINFITTIINMRLNNLSFD
QMPLFIWAVGITAFLLLLSLPVLAGAITMLLTDRNLNTSFFDPAGGGDPILYQHL
>LEFIC777-10|HM872596|MM04908|Apamea_furva
TLYFIFGIWAGMVGTSLSLMIRAELGNPGSLIGDDQIYNTIVTAHAFIMIFFMVMPIMIGGFGNWLVPLMLGAPDMAFPRMNNMSFWLLPPSLTLLISSSIVENGAGTGWTVYPPLSSNIAHSGSSVDLAIFSLHLAGISSILGAINFITTIINMRLNNLSFD
QMPLFIWAVGITAFLLLLSLPVLAGAITMLLTDRNLNTSFFDPAGGGDPILYQHL
>LEFIF191-10|HM874885|MM10783|Apamea_furva
TLYFIFGIWAGMVGTSLSLMIRAELGNPGSLIGDDQIYNTIVTAHAFIMIFFMVMPIMIGGFGNWLVPLMLGAPDMAFPRMNNMSFWLLPPSLTLLISSSIVENGAGTGWTVYPPLSSNIAHSGSSVDLAIFSLHLAGISSILGAINFITTIINMRLNNLSFD
QMPLFIWAVGITAFLLLLSLPVLAGAITMLLTDRNLNTSFFDPAGGGDPILYQHL
>LEFIK458-10|JF854075|MM18033|Apamea_furva
TLYFIFGIWAGMVGTSLSLMIRAELGNPGSLIGDDQIYNTIVTAHAFIMIFFMVMPIMIGGFGNWLVPLMLGAPDMAFPRMNNMSFWLLPPSLTLLISSSIVENGAGTGWTVYPPLSSNIAHSGSSVDLAIFSLHLAGISSILGAINFITTIINMRLNNLSFD

QMPLFIWAVGITAFLLLLSLPVLAGAITMLLTDRNLNTSFFDPAGGGDPILYQHL
>LEFIF663-10|HM875347|MM12610|Apamea_illyria
TLYFIFGIWAGMVGTSLSLLIRAELGNPGSLIGDDQIYNTIVTAHAFIMIFFMVMPIMIGGFGNWLVPLMLGAPDMAFPRMNNMSFWLLPPSLTLLISSSIVENGAGTGWTVYPPLSSNIAHGGSSVDLAIFSLHLAGISSILGAINFITTIINMRLNNLSFD
QMPLFIWAVGITAFLLLLSLPVLAGAITMLLTDRNLNTSFFDPAGGGDPILYQHL
>LEFIA620-10|HM870869|MM01734|Apamea_lateritia
TLYFIFGIWAGMVGTSLSLLIRAELGTPGSLIGDDQIYNTIVTAHAFIMIFFMVMPIMIGGFGNWLVPLMLGAPDMAFPRMNNMSFWLLPPSLTLLISSSIVENGAGTGWTVYPPLSSNIAHGGSSVDLAIFSLHLAGISSILGAINFITTIINMRLNSLSFD
QMPLFIWAVGITAFLLLLSLPVLAGAITMLLTDRNLNTSFFDPAGGGDPILYQHL
>LEFIA621-10|HM870870|MM01735|Apamea_lateritia
TLYFIFGIWAGMVGTSLSLLIRAELGTPGSLIGDDQIYNTIVTAHAFIMIFFMVMPIMIGGFGNWLVPLMLGAPDMAFPRMNNMSFWLLPPSLTLLISSSIVENGAGTGWTVYPPLSSNIAHGGSSVDLAIFSLHLAGISSILGAINFITTIINMRLNSLSFD
QMPLFIWAVGITAFLLLLSLPVLAGAITMLLTDRNLNTSFFDPAGGGDPILYQHL
>LEFIE775-10|HM874494|MM09935|Apamea_lateritia
TLYFIFGIWAGMVGTSLSLLIRAELGTPGSLIGDDQIYNTIVTAHAFIMIFFMVMPIMIGGFGNWLVPLMLGAPDMAFPRMNNMSFWLLPPSLTLLISSSIVENGAGTGWTVYPPLSSNIAHGGSSVDLAIFSLHLAGISSILGAINFITTIINMRLNSLSFD
QMPLFIWAVGITAFLLLLSLPVLAGAITMLLTDRNLNTSFFDPAGGGDPILYQHL
>LEFIH014-10|HM876650|MM15878|Apamea_lithoxylaea
TLYFIFGIWAGMVGTSLSLLIRAELGNPGSLIGDDQIYNTIVTAHAFIMIFFMVMPIMIGGFGNWLVPLMLGAPDMAFPRMNNMSFWLLPPSLTLLISSSIVENGAGTGWTVYPPLSSNIAHGGSSVDLAIFSLHLAGISSILGAINFITTIINMRLNNLSFD
QMPLFIWAVGITAFLLLLSLPVLAGAITMLLTDRNLNTSFFDPAGGGDPILYQHL
>LEFIC575-10|HM872396|MM04354|Apamea_monoglypha
TLYFIFGIWAGMVGTSLSLLIRAELGNPGSLIGDDQIYNTIVTAHAFIMIFFMVMPIMIGGFGNWLVPLMLGAPDMAFPRMNNMSFWLLPPSLTLLISSSIVENGAGTGWTVYPPLSSNIAHGGSSVDLAIFSLHLAGISSILGAINFITTIINMRLNSLSFD
QMPLFIWAVGITAFLLLLSLPVLAGAITMLLTDRNLNTSFFDPAGGGDPILYQHL
>LEFIF673-10|HM875357|MM12653|Apamea_monoglypha
TLYFIFGIWAGMVGTSLSLLIRAELGNPGSLIGDDQIYNTIVTAHAFIMIFFMVMPIMIGGFGNWLVPLMLGAPDMAFPRMNNMSFWLLPPSLTLLISSSIVENGAGTGWTVYPPLSSNIAHGGSSVDLAIFSLHLAGISSILGAINFITTIINMRLNSLSFD
QMPLFIWAVGITAFLLLLSLPVLAGAITMLLTDRNLNTSFFDPAGGGDPILYQHL
>LEFID048-10|HM872862|MM05521|Apamea_oblonga
TLYFIFGIWAGMVGTSLSLLIRAELGNPGSLIGDDQIYNTIVTAHAFIMIFFMVMPIMIGGFGNWLVPLMLGAPDMAFPRMNNMSFWLLPPSLTLLISSSIVENGAGTGWTVYPPLSSNIAHGGSSVDLAIFSLHLAGISSILGAINFITTIINMRLNNLSFD
QMPLFIWAVGITAFLLLLSLPVLAGAITMLLTDRNLNTSFFDPAGGGDPILYQHL
>LEFIG456-10|HM876132|MM14497|Apamea_oblonga
TLYFIFGIWAGMVGTSLSLLIRAELGNPGSLIGDDQIYNTIVTAHAFIMIFFMVMPIMIGGFGNWLVPLMLGAPDMAFPRMNNMSFWLLPPSLTLLISSSIVENGAGTGWTVYPPLSSNIAHGGSSVDLAIFSLHLAGISSILGAINFITTIINMRLNNLSFD
QMPLFIWAVGITAFLLLLSLPVLAGAITMLLTDRNLNTSFFDPAGGGDPILYQHL
>LEFIG457-10|HM876133|MM14498|Apamea_oblonga
TLYFIFGIWAGMVGTSLSLLIRAELGNPGSLIGDDQIYNTIVTAHAFIMIFFMVMPIMIGGFGNWLVPLMLGAPDMAFPRMNNMSFWLLPPSLTLLISSSIVENGAGTGWTVYPPLSSNIAHGGSSVDLAIFSLHLAGISSILGAINFITTIINMRLNNLSFD
QMPLFIWAVGITAFLLLLSLPVLAGAITMLLTDRNLNTSFFDPAGGGDPILYQHL
>LEFIA632-10|HM870881|MM01747|Apamea_remissa
TLYFIFGIWAGMVGTSLSLLIRAELGNPGSLIGDDQIYNTIVTAHAFIMIFFMVMPIMIGGFGNWLVPLMLGAPDMAFPRMNNMSFWLLPPSLTLLISSSIVENGAGTGWTVYPPLSSNIAHGGSSVDLAIFSLHLAGISSILGAINFITTIINMRLNNLSFD
QMPLFIWAVGITAFLLLLSLPVLAGAITMLLTDRNLNTSFFDPAGGGDPILYQHL
>LEFID212-10|HM873014|MM06042|Apamea_rubrirena
TLYFIFGIWAGMVGTSLSLLIRAELGNPGSLIGDDQIYNTIVTAHAFIMIFFMVMPIMIGGFGNWLVPLMLGAPDMAFPRMNNMSFWLLPPSLTLLISSSIVENGAGTGWTVYPPLSSNIAHGGSSVDLAIFSLHLAGISSILGAINFITTIINMRLNSLSFD
QMPLFIWAVGITAFLLLLSLPVLAGAITMLLTDRNLNTSFFDPAGGGDPILYQHL
>LEFIK457-10|KM573685|MM18032|Apamea_rubrirena
TLYFIFGIWAGMVGTSLSLLIRAELGNPGSLIGDDQIYNTIVTAHAFIMIFFMVMPIMIGGFGNWLVPLMLGAPDMAFPRMNNMSFWLLPPSLTLLISSSIVENGAGTGWTVYPPLSSNIAHGGSSVDLAIFSLHLAGISSILGAINFITTIINMRLNSLSFD
QMPLFIWAVGITAFLLLLSLPVLAGAITMLLTDRNLNTSFFDPAGGGDPILYQHL
>LEFIE442-10|HM874166|MM09205|Apamea_schildei
TLYFIFGIWAGMVGTSLSLLIRAELGNPGSLIGDDQIYNTIVTAHAFIMIFFMVMPIMIGGFGNWLVPLMLGAPDMAFPRMNNMSFWLLPPSLTLLISSSIVENGAGTGWTVYPPLSSNIAHGGSSVDLAIFSLHLAGISSILGAINFITTIINMRLNSLSFD
QMPLFIWAVGITAFLLLLSLPVLAGAITMLLTDRNLNTSFFDPAGGGDPILYQHL
>LEFIF292-10|HM874982|MM11074|Apamea_schildei
TLYFIFGIWAGMVGTSLSLLIRAELGTPGSLIGDDQIYNTIVTAHAFIMIFFMVMPIMIGGFGNWLVPLMLGAPDMAFPRMNNMSFWLLPPSLTLLISSSIVENGAGTGWTVYPPLSSNIAHGGSSVDLAIFSLHLAGISSILGAINFITTIINMRLNSLSFD
QMPLFIWAVGITAFLLLLSLPVLAGAITMLLTDRNLNTSFFDPAGGGDPILYQHL
>LEFIJ849-10|JF853860|MM17474|Apamea_schildei

TLYFIFGIWAGMVGTSLSLLIRAELGNPGSLIGDDQIYNTIVTAHAFIMIFFMVMPIMIGGFGNWLVPLMLGAPDMAFPRMNNMSFWLLPPSLTLLISSSIVENGAGTGWTVYPPLSSNIAHGGSSVDLAIFSLHLAGISSILGAINFITTIINMRLNSLSFD
QMPLFIWAVGITAFLLLLSLPVLAGAITMLLTDRNLNTSFFDPAGGGDPILYQHL
>LEFIA574-10|HM870823|MM01668|Apamea_scolopacina
TLYFIFGIWAGMVGTSLSLLIRAELGNPGSLIGDDQIYNTIVTAHAFIMIFFMVMPIMIGGFGNWLVPLMLGAPDMAFPRMNNMSFWLLPPSLTLLISSSIVENGAGTGWTVYPPLSSNIAHGGSSVDMAIFSLHLAGISSILGAINFITTIINMRLNNLSF
DQMPLFIWAVGITAFLLLLSLPVLAGAITMLLTDRNLNTSFFDPAGGGDPILYQHL
>LEFIC775-10|HM872594|MM04905|Apamea_scolopacina
TLYFIFGIWAGMVGTSLSLLIRAELGNPGSLIGDDQIYNTIVTAHAFIMIFFMVMPIMIGGFGNWLVPLMLGAPDMAFPRMNNMSFWLLPPSLTLLISSSIVENGAGTGWTVYPPLSSNIAHGGSSVDLAIFSLHLAGISSILGAINFITTIINMRLNNLSFD
QMPLFIWAVGITAFLLLLSLPVLAGAITMLLTDRNLNTSFFDPAGGGDPILYQHL
>LEFIA531-10|MM01615|Apamea_sordens
TLYFIFGIWAGMVGTSLSLMIRAELGNPGSLIGDDQIYNTIVTAHAFIMIFFMVMPIMIGGFGNWLVPLMLGAPDMAFPRMNNMSFWLLPPSLTLLISSSIVENGAGTGWTVYPPLSSNIAHGGSSVDLAIFSLHLAGISSILGAINFITTIINMRLNNLSF
DQMPLFIWAVGITAFLLLLSLPVLAGAITMLLTDRNLNTSFFDPAGGGDPILYQHL
>LEFIG366-10|HM876043|MM14369|Apamea_sublustris
TLYFIFGIWAGMVGTSLSLLIRAELGNPGSLIGDDQIYNTIVTAHAFIMIFFMVMPIMIGGFGNWLVPLMLGAPDMAFPRMNNMSFWLLPPSLTLLISSSIVENGAGTGWTVYPPLSSNIAHGGSSVDLAIFSLHLAGISSILGAINFITTIINMRLNNLSFD
QMPLFIWAVGITAFLLLLSLPVLAGAITMLLTDRNLNTSFFDPAGGG?PILYQHL
>LEFIH013-10|HM876649|MM15877|Apamea_sublustris
TLYFIFGIWAGMVGTSLSLLIRAELGNPGSLIGDDQIYNTIVTAHAFIMIFFMVMPIMIGGFGNWLVPLMLGAPDMAFPRMNNMSFWLLPPSLTLLISSSIVENGAGTGWTVYPPLSSNIAHGGSSVDLAIFSLHLAGISSILGAINFITTIINMRLNNLSFD
QMPLFIWAVGITAFLLLLSLPVLAGAITMLLTDRNLNTSFFDPAGGGDPILYQHL
>LEFIJ846-10|KM572608|MM17471|Apamea_sublustris
TLYFIFGIWAGMVGTSLSLLIRAELGNPGSLIGDDQIYNTIVTAHAFIMIFFMVMPIMIGGFGNWLVPLMLGAPDMAFPRMNNMSFWLLPPSLTLLISSSIVENGAGTGWTVYPPLSSNIAHGGSSVDLAIFSLHLAGISSILGAINFITTIINMRLNNLSFD
QMPLFIWAVGITAFLLLLSLPVLAGAITMLLTDRNLNTSFFDPAGGGDPILYQHL
>LEFIB638-10|HM871517|MM02396|Apamea_unanimis
TLYFIFGIWAGMVGTSLSLLIRAELGNPGSLIGDDQIYNTIVTAHAFIMIFFMVMPIMIGGFGNWLVPLMLGAPDMAFPRMNNMSFWLLPPSLTLLISSSIVENGAGTGWTVYPPLSSNIAHGGSSVDLAIFSLHLAGISSILGAINFITTIINMRLNNLSFD
QMPLFIWAVGITAFLLLLSLPVLAGAITMLLTDRNLNTSFFDPAGGGDPILYQHL
>LEFIK456-10|KM572893|MM18031|Apamea_unanimis
TLYFIFGIWAGMVGTSLSLLIRAELGNPGSLIGDDQIYNTIVTAHAFIMIFFMVMPIMIGGFGNWLVPLMLGAPDMAFPRMNNMSFWLLPPSLTLLISSSIVENGAGTGWTVYPPLSSNIAHGGSSVDLAIFSLHLAGISSILGAINFITTIINMRLNNLSFD
QMPLFIWAVGITAFLLLLSLPVLAGAITMLLTDRNLNTSFFDPAGGGDPILYQHL
>LEFID153-10|HM872960|MM05916|Apatura_ilia
TLYFIFGIWAGMVGTSLSMLIRTELGNPGSLIGDDQIYNTIVTAHAFIMIFFMVMPIMIGGFGNWLVPLMLGAPDMAFPRMNNMSFWLLPPSLVLLISSSIVENGAGTGWTVYPPLSSNIAHGGSSVDLAIFSLHLAGISSILGAINFITTIINMRVNNLSF
DQMPLFVWAVGITALLLLLSLPVLAGAITMLLTDRNINTSFFDPAGGGDPILYQHL
>LEFIB065-10|HM870974|MM00353|Apatura_iris
TLYFIFGIWAGMVGTSLSMLIRTELGNPGSLIGDDQIYNTIVTAHAFIMIFFMVMPIMIGGFGNWLVPLMLGAPDMAFPRMNNMSFWLLPPSLILLISSSIVENGAGTGWTVYPPLSSNIAHSGSSVDLAIFSLHLAGISSILGAINFITTIINMRVNNLSFD
QMPLFVWAVGITALLLLLSLPVLAGAITMLLTDRNINTSFFDPAGGGDPILYQHL
>LEFIA439-10|HM386780|MM01503|Apeira_syringaria
TLYFIFGIWAGMVGTSLSLLIRAELGNPGSLIGDDQIYNTIVTAHAFIMIFFMVMPIMIGGFGNWLVPLMLGAPDMAFPRMNNMSFWLLPPSITLLISSSIVENGAGTGWTVYPPLSSNIAHNGSSVDLAIFSLHLAGISSILGAINFITTIINMRLNNLSFD
QMPLFVWAVGITAFLLLLSLPVLAGAITMLLTDRNLNTSFFDPAGGGDPILYQHL
>LEFIC025-10|HM871895|MM03238|Aphantopus_hyperantus
TLYFIFGIWAGMVGTSLSLIIRTELGNPGFLIGDDQIYNTIVTAHAFIMIFFMVMPIMIGGFGNWLVPLMLGAPDMAFPRMNNMSFWLLPPSLILLISSSIVENGAGTGWTVYPPLSSNIAHGGSSVDLAIFSLHLAGISSILGAINFITTIINMRVNNMSYD
QMPLFVWAVGITALLLLLSLPVLAGAITMLLTDRNLNTSFFDPAGGGDPILYQHL
>LEFIJ559-10|JF853674|MM17184|Aphantopus_hyperantus
TLYFIFGIWAGMVGTSLSLIIRTELGNPGFLIGDDQIYNTIVTAHAFIMIFFMVMPIMIGGFGNWLVPLMLGAPDMAFPRMNNMSFWLLPPSLILLISSSIVENGAGTGWTIYPPLSSNITHSGSSVDLAIFSLHLAGISSILGAINFITTIINMRVNNMLYDQ
MPLFVWAVGITALLLLLSLPVLAGAITMLLTDRNLNTSFFDPAGGGDPILYQHL
>LEFIB060-10|HM870969|MM00345|Aphelia_paleana
TLYFIFGIWAGMVGTSLSLLIRAELGNPGSLIGDDQIYNTIVTAHAFIMIFFMVMPIMIGGFGNWLVPLMLGAPDMAFPRMNNMSFWLLPPSIMLLISSSIVENGAGTGWTVYPPLSSNIAHSGSSVDLTIFSLHLAGISSILGAVNFITTIINMRPNNMSL
DQMPLFVWSVGITALLLLLSLPVLAGAITMLLTDRNLNTSFFDPAGGGDPILYQHL
>LEFIC816-10|HM872635|MM04997|Aphelia_paleana
TLYFIFGIWAGMVGTSLSLLIRAELGNPGSLIGDDQIYNTIVTAHAFIMIFFMVMPIMIGGFGNWLVPLMLGAPDMAFPRMNNMSFWLLPPSIMLLISSSIVENGAGTGWTVYPPLSSNIAHSGSSVDLAIFSLHLAGISSILGAVNFITTIINMRPNNMSL
DQMPLFVWSVGITALLLLLSLPVLAGAITMLLTDRNLNTSFFDPAGGGDPILYQHL

>LEFIE371-10|HM874095|MM08875|Aphelia_paleana
TLYFIFGIWAGMVGTSLSLLIRAELGNPGSLIGDDQIYNTIVTAHAFIMIFFMVMPIMIGGFGNWLVPLMLGAPDMAFPRMNNMSFWLLPPSIMLLISSSIVENGAGTGWTVYPPLSSNIAHSGSSVDLAIFSLHLAGISSILGAVNFITTIINMRPNNMSL
DQMPLFVWSVGITALLLLLSLPVLAGAITMLLTDRNLNTSFFDPAGGGDPILYQHL
>LEFIK685-10|KM573402|MM18260|Aphelia_paleana
TLYFIFGIWAGMVGTSLSLLIRAELGNPGSLIGDDQIYNTIVTAHAFIMIFFMVMPIMIGGFGNWLVPLMLGAPDMAFPRMNNMSFWLLPPSIMLLISSSIVENGAGTGWTVYPPLSSNIAHSGSSVDLAIFSLHLAGISSILGAVNFITTIINMRPNNMSL
DQMPLFVWSVGITALLLLLSLPVLAGAITMLLTDRNLNTSFFDPAGGGDPILYQHL
>LEFID264-10|HM873062|MM06129|Aphelia_unitana
TLYFIFGIWAGMVGTSLSLLIRAELGNPGSLIGDDQIYNTIVTAHAFIMIFFMVMPIMIGGFGNWLVPLMLGAPDMAFPRMNNMSFWLLPPSIMLLISSSIVENGAGTGWTVYPPLSSNIAHSGSSVDLAIFSLHLAGISSILGAVNFITTIINMRPNNMSL
DQMPLFVWSVGITALLLLLSLPVLAGAITMLLTDRNLNTSFFDPAGGGDPILYQHL
>LEFIG791-10|HM876443|MM15655|Aphelia_unitana
TLYFIFGIWAGMVGTSLSLLIRAELGNPGSLIGDDQIYNTIVTAHAFIMIFFMVMPIMIGGFGNWLVPLMLGAPDMAFPRMNNMSFWLLPPSIMLLISSSIVENGAGTGWTVYPPLSSNIAHSGSSVDLAIFSLHLAGISSILGAVNFITTIINMRPNNMSL
DQMPLFVWSVGITALLLLLSLPVLAGAITMLLTDRNLNTSFFDPAGGGDPILYQHL
>LEFIG793-10|HM876445|MM15657|Aphelia_unitana
TLYFIFGIWAGMVGTSLSLLIRAELGNPGSLIGDDQIYNTIVTAHAFIMIFFMVMPIMIGGFGNWLVPLMLGAPDMAFPRMNNMSFWLLPPSIMLLISSSIVENGAGTGWTVYPPLSSNIAHSGSSVDLAIFSLHLAGISSILGAVNFITTIINMRPNNMSL
DQMPLFVWSVGITALLLLLSLPVLAGAITMLLTDRNLNTSFFDPAGGGDPILYQHL
>LEEUA221-11|JN286433|MM19629|Aphelia_unitana
TLYFIFGIWAGMVGTSLSLLIRAELGSPGSLIGDDQIYNTIVTAHAFIMIFFMVMPIMIGGFGNWLVPLMLGAPDMAFPRMNNMSFWLLPPSIMLLISSSIVENGAGTGWTVYPPLSSNIAHSGSSVDLAIFSLHLAGISSILGAVNFITTIINMRPNNMSL
DQMPLFVWSVGITALLLLLSLPVLAGAITMLLTDRNLNTSFFDPAGGGDPILYQHL
>LEFIC301-10|HM872145|MM03785|Aphelia_viburniana
TLYFIFGIWAGMVGTSLSLMIRAELGNPGSLIGDDQIYNTIVTAHAFIMIFFMVMPIMIGGFGNWLVPLMLGAPDMAFPRMNNMSFWLLPPSIMLLISSSIVENGAGTGWTVYPPLSSNIAHSGSSVDLAIFSLHLAGISSILGAVNFITTIINMRPNNMS
LDQMPLFVWSVGITALLLLLSLPVLAGAITMLLTDRNLNTSFFDPAGGGDPILYQHL
>LEFIJ385-10|JF853572|MM15990|Aphelia_viburniana
TLYFIFGIWAGMVGTSLSLMIRAELGNPGSLIGDDQIYNTIVTAHAFIMIFFMVMPIMIGGFGNWLVPLMLGAPDMAFPRMNNMSFWLLPPSIMLLISSSIVENGAGTGWTVYPPLSSNIAHSGSSVDLAIFSLHLAGISSILGAVNFITTIINMRPNNMS
LDQMPLFVWSVGITALLLLLSLPVLAGAITMLLTDRNLNTSFFDPAGGGDPILYQHL
>LEFIC457-10|HM872291|MM04040|Aphomia_sociella
TLYFIFGIWSGMVGTSLSLLIRAELGNPGSLIGDDQIYNTIVTGHAFIMIFFMVMPIMIGGFGNWLIPLMLGAPDMAFPRMNNMSFWLLPPSLTLLIFSSIVENGAGTGWTVYPPLSANIAHSGSSVDLAIFSLHLAGISSILGAINFITTVINMKLNGLSFD
QMPLFVWAVIITALLLLLSLPVLAGAITMLLTDRNLNTSFFDPAGGGDPILYQHL
>LEFIE699-10|HM874420|MM09681|Aphomia_sociella
TLYFIFGIWSGMVGTSLSLLIRAELGNPGSLIGDDQIYNTIVTGHAFIMIFFMVMPIMIGGFGNWLIPLMLGAPDMAFPRMNNMSFWLLPPSLTLLIFSSIVENGAGTGWTVYPPLSANIAHSGSSVDLAIFSLHLAGISSILGAINFITTVINMKLNGLSFD
QMPLFVWAVIITALLLLLSLPVLAGAITMLLTDRNLNTSFFDPAGGGDPILYQHL
>LEFIJ1293-11|MM21153|Aphomia_zelleri
TLYFIFGIWSGMVGTSLSLLIRAELGNPGSLIGDDQIYNTIVTGHAFIMIFFMVMPIMIGGFGNWLVPLMLGAPDMAFPRMNNMSFWLLPPSLTLLIFSSIVENGAGTGWTVYPPLSSNIAHSGSSVDLAIFSLHLAGISSILGAINFITTVINMKLNGLSFD
QMPLFVWAVSITALLLLLSLPVLAGAITMLLTDRNLNTSFFDPAGGGDPILYQHL
>LEFIJ1294-11|MM21154|Aphomia_zelleri
TLYFIFGIWSGMVGTSLSLLIRAELGNPGSLIGDDQIYNTIVTGHAFIMIFFMVMPIMIGGFGNWLVPLMLGAPDMAFPRMNNMSFWLLPPSLTLLIFSSIVENGAGTGWTVYPPLSSNIAHSGSSVDLAIFSLHLAGISSILGAINFITTVINMKLNGLSFD
QMPLFVWAVSITALLLLLSLPVLAGAITMLLTDRNLNTSFFDPAGGGDPILYQHL
>LEEUA314-11|JN279401|MM19722|Aplocera_efformata
TLYFIFGIWAGMVGTSLSLLIRAELGTPGSLIGDDQIYNTIVTAHAFIMIFFMVMPIMIGGFGNWLVPLMLGAPDMAFPRMNNMSFWLLPPSITLLISSSIVETGAGTGWTVYPPLSSNIAHGGSSVDLAIFSLHLAGISSILGAINFITTIINMRLNNMFFD
QLPLFVWAVGITAFLLLLSLPVLAGAITMLLTDRNLNTSFFDPAGGGDPILYQHL
>LEFIF654-10|HM875338|MM12581|Aplocera_plagiata
TLYFIFGIWAGMVGTSLSLLIRAELGTPGSLIGDDQIYNTIVTAHAFIMIFFMVMPIMIGGFGNWLVPLMLGAPDMAFPRMNNMSFWLLPPSITLLISSSIVETGAGTGWTVYPPLSSNIAHGGSSVDLAIFSLHLAGISSILGAINFITTIINMRLNNMFFD
QLPLFVWAVGITAFLLLLSLPVLAGAITMLLTDRNLNTSFFDPAGGGDPILYQHL
>LEFIF655-10|HM875339|MM12582|Aplocera_plagiata
TLYFIFGIWAGMVGTSLSLLIRAELGTPGSLIGDDQIYNTIVTAHAFIMIFFMVMPIMIGGFGNWLVPLMLGAPDMAFPRMNNMSFWLLPPSITLLISSSIVETGAGTGWTVYPPLSSNIAHGGSSVDLAIFSLHLAGISSILGAINFITTIINMRLNNMFFD
QLPLFVWAVGITAFLLLLSLPVLAGAITMLLTDRNLNTSFFDPAGGGDPILYQHL
>LEFIG451-10|HM876127|MM14489|Aplocera_plagiata
TLYFIFGIWAGMVGTSLSLLIRAELGTPGSLIGDDQIYNTIVTAHAFIMIFFMVMPIMIGGFGNWLVPLMLGAPDMAFPRMNNMSFWLLPPSITLLISSSIVETGAGTGWTVYPPLSSNIAHGGSSVDLAIFSLHLAGISSILGAINFITTIINMRLNNMFFD

QLPLFVWAVGITAFLLLLSLPVLAGAITMLLTDRNLNTSFFDPAGGGDPILYQHL
>LEFIA407-10|HM386749|MM01468|Aplocera_praeformata
TLYFIFGIWAGMVGTSLSLLIRAELGTPGSLIGDDQIYNTIVTAHAFIMIFFMVMPIMIGGFGNWLVPLMLGTPDMAFPRMNNMSFWLLPPSITLLISSSIVETGAGTGWTVYPPLSSNIAHGGSSVDLAIFSLHLAGISSILGAINFITTIINMRLNNMFFD
QLPLFVWAVGITAFLLLLSLPVLAGAITMLLTDRNLNTSFFDPAGGGDPILYQHL
>LEFIK073-10|JX034675|MM17648|Aplota_nigricans
TLYFIFGIWAGMLGTSLSLLIRTELGNPGSLIGDDQIYNTLVTAHAFIMIFFMVMPIMIGGFGNWLVPLMLGAPDMAFPRMNNMSFWLLPPSLTLLTSSSMVENGAGTGWTVYPPLSSNIAHSGSSVDLAIFSLHLAGISSILGAINFITTVINMRTNNM
SFDQMPLFIWSVSITALLLLLSLPVLAGAITMLLTDRNLNTSFFDPAGGGDPILYQHL
>LEEUA131-11|MM19539|Aplota_palpella
TLYFIFGIWAGMVGTSLSLLIRAELGNPGSLIGDDQIYNTLVTAHAFIMIFFMVMPIMIGGFGNWLVPLMLGAPDMAFPRMNNMSFWLLPPSLTLLISSSMVENGAGTGWTVYPPLSSNIAHGGSSVDLAIFSLHLAGISSILGAINFITTIINMRINNMS
LDQMPLFVWSVGITALLLLLSLPVLAGAITMLLTDRNLNTSFFDPAGGGDPILYQHL
>LEFIE641-10|HM874364|MM09591|Apodia_bifractella
TLYFIFGIWAGMVGTSLSLLIRAELGNPGSLIGDDQIYNTIVTAHAFIMIFFMVMPIMIGGFGNWLVPLMLGAPDMAFPRMNNMSFWLLPPSLILLISSSIVENGAGTGWTVYPPLSSNIAHGGSSVDLAIFSLHLAGISSILGAINFITTIINMKINGLSFDQ
MPLFVWAVGITALLLLLSLPVLAGAITMLLTDRNLNTSFFDPAGGGDPILYQHL
>LEFIB664-10|HM871542|MM02444|Apomyelois_bistriatella
TLYFIFGIWSGLIGTSLSLLIRTELGTPSSLIGDDQIYNTIVTGHAFIMIFFMVMPIMIGGFGNWLVPLMLGAPDMAFPRMNNMSFWLLPPSLTLLISSSIVENGAGTGWTVYPPLSSNIAHSGSSVDLAIFSLHLAGISSILGAINFITTIINMKLNGMFFDQ
MPLFVWAVGITALLLLLSLPVLAGAITMLLTDRNLNTSFFDPAGGGDPILYQHL
>LEFIA024-10|HM396374|MM00095|Aporia_crataegi
TLYFIFGIWSGMVGTSLSLLIRTELGNPGSLIGNDQIYNTIVTAHAFIMIFFMVMPIMIGGFGNWLIPLMLGAPDMAFPRMNNMSFWLLPPSLTLLISSSIVENGAGTGWTVYPPLSSNIAHSGSSVDLTIFSLHLAGISSILGAINFITTIINMRINNMSFDQ
MPLFVWAVGITALLLLLSLPVLAGAITMLLTDRNLNTSFFDPAGGGDPILYQHL
>LEFIJ505-10|JF853628|MM17130|Aporia_crataegi
TLYFIFGIWSGMVGTSLSLLIRTELGNPGSLIGNDQIYNTIVTAHAFIMIFFMVMPIMIGGFGNWLIPLMLGAPDMAFPRMNNMSFWLLPPSLTLLISSSIVENGAGTGWTVYPPLSSNIAHGGSSVDLTIFSLHLAGISSILGAINFITTIINMRINNMSFDR
MPLFVWAVGITALLLLLSLPVLAGAITMLLTDRNLNTSFFDPAGGGDPILYQHL
>LEEUA322-11|JN266515|MM19730|Aporophyla_lueneburgensis
TLYFIFGIWAGMVGTSLSLLIRAELGNPGSLIGDDQIYNTIVTAHAFIMIFFMVMPIMIGGFGNWLVPLMLGAPDMAFPRMNNMSFWLLPPSLTLLISSSIVENGAGTGWTVYPPLSSNIAHGGSSVDLAIFSLHLAGISSILGAINFITTIINMRLNNLSFD
QMPLFIWAVGITAFLLLLSLPVLAGAITMLLTDRNLNTSFFDPAGGGDPILYQHL
>LEEUA512-11|MM20571|Aporophyla_lueneburgensis
TLYFIFGIWAGMVGTSLSLLIRAELGNPGSLIGDDQIYNTIVTAHAFIMIFFMVMPIMIGGFGNWLVPLMLGAPDMAFPRMNNMSFWLLPPSLTLLISSSIVENGAGTGWTVYPPLSSNIAHGGSSVDLAIFSLHLAGISSILGAINFITTIINMRLNNLSFD
QMPLFIWAVGITAFLLLLSLPVLAGAITMLLTDRNLNTSFFDPAGGGDPILYQHL
>LEFID070-10|HM872882|MM05679|Apotomis_algidana
TLYFIFGIWAGMVGTSLSLLIRAELGNPGSLIGDDQIYNTIVTAHAFIMIFFMVMPIMIGGFGNWLVPLMLGAPDMAFPRMNNMSFWLLPPSIMLLISSSIVENGAGTGWTVYPPLSSNIAHSGSSVDLAIFSLHLAGISSILGAVNFITTIINMRPNSMSL
DQMPLFVWAVGITALLLLLSLPVLAGAITMLLTDRNLNTSFFDPAGGGDPILYQHL
>LEFIG829-10|HM876479|MM15693|Apotomis_algidana
TLYFIFGIWAGMVGTSLSLLIRAELGNPGSLIGDDQIYNTIVTAHAFIMIFFMVMPIMIGGFGNWLVPLMLGAPDMAFPRMNNMSFWLLPPSIMLLISSSIVENGAGTGWTVYPPLSSNIAHSGSSVDLAIFSLHLAGISSILGAVNFITTIINMRPNSMSL
DQMPLFVWAVGITALLLLLSLPVLAGAITMLLTDRNLNTSFFDPAGGGDPILYQHL
>LEFIB278-10|HM871180|MM00765|Apotomis_betuletana
TLYFIFGIWAGMVGTSLSLLIRAELGNPGSLIGDDQIYNTIVTAHAFIMIFFMVMPIMIGGFGNWLVPLMLGAPDMAFPRMNNMSFWLLPPSIMLLISSSIVENGAGTGWTVYPPLSSNIAHSGSSVDLAIFSLHLAGISSILGAVNFITTIINMRPNSMSL
DQMPLFVWAVGITALLLLLSLPVLAGAITMLLTDRNLNTSFFDPAGGGDPILYQHL
>LEFIB887-10|HM871764|MM02972|Apotomis_betuletana
TLYFIFGIWAGMVGTSLSLLIRAELGNPGSLIGDDQIYNTIVTAHAFIMIFFMVMPIMIGGFGNWLVPLMLGAPDMAFPRMNNMSFWLLPPSIMLLISSSIVENGAGTGWTVYPPLSSNIAHSGSSVDLAIFSLHLAGISSILGAVNFITTIINMRPNSMSL
DQMPLFVWAVGITALLLLLSLPVLAGAITMLLTDRNLNTSFFDPAGGGDPILYQHL
>LEFIF412-10|HM875097|MM11786|Apotomis_betuletana
TLYFIFGIWAGMVGTSLSLLIRAELGNPGSLIGDDQIYNTIVTAHAFIMIFFMVMPIMIGGFGNWLVPLMLGAPDMAFPRMNNMSFWLLPPSIMLLISSSIVENGAGTGWTVYPPLSSNIAHSGSSVDLAIFSLHLAGISSILGAVNFITTIINMRPNSMSL
DQMPLFVWAVGITALLLLLSLPVLAGAITMLLTDRNLNTSFFDPAGGGDPILYQHL
>LEFIG512-10|HM876185|MM14612|Apotomis_betuletana
TLYFIFGIWAGMVGTSLSLLIRAELGNPGSLIGNDQIYNTIVTAHAFIMIFFMVMPIMIGGFGNWLVPLMLGAPDMAFPRMNNMSFWLLPPSIMLLISSSIVENGAGTGWTVYPPLSSNIAHSGSSVDLAIFSLHLAGISSILGAVNFITTIINMRPNSMSL
DQMPLFVWAVGITALLLLLSLPVLAGAITMLLTDRNLNTSFFDPAGGGDPILYQHL
>LEFIB457-10|HM871356|MM01991|Apotomis_capreana

TLYFIFGIWAGMVGTSLSLLIRAELGNPGSLIGDDQIYNTIVTAHAFIMIFFMVMPIMIGGFGNWLVPLMLGAPDMAFPRMNNMSFWLLPPSIMLLISSSIVENGAGTGWTVYPPLSSNIAHSGSSVDLAIFSLHLAGISSILGAVNFITTIINMRPNSMSL
DQMPLFVWAVGITALLLLLSLPVLAGAITMLLTDRNLNTSFFDPAGGGDPILYQHL
>LEFIC818-10|HM872637|MM04999|Apotomis_capreana
TLYFIFGIWAGMVGTSLSLLIRAELGNPGSLIGDDQIYNTIVTAHAFIMIFFMVMPIMIGGFGNWLVPLMLGAPDMAFPRMNNMSFWLLPPSIMLLISSSIVENGAGTGWTVYPPLSSNIAHSGSSVDLAIFSLHLAGISSILGAVNFITTIINMRPNSMSL
DQMPLFVWAVGITALLLLLSLPVLAGAITMLLTDRNLNTSFFDPAGGGDPILYQHL
>LEFIK198-10|MM17773|Apotomis_demissana
TLYFIFGIWAGMVGTSLSLLIRAELGNPGSLIGDDQIYNTIVTAHAFIMIFFMVMPIMIGGFGNWLVPLMLGAPDMAFPRMNNMSFWLLPPSIMLLISSSIVENGAGTGWTVYPPLSSNIAHSGSSVDLAIFSLHLAGISSILGAVNFITTIINMRPNSMSL
DQMPLFVWAVGITALLLLLSLPVLAGAITMLLTDRNLNTSFFDPAGGGDPILYQHL
>LEFII255-11|MM19905|Apotomis_demissana
TLYFIFGIWAGMVGTSLSLLIRAELGNPGSLIGDDQIYNTIVTAHAFIMIFFMVMPIMIGGFGNWLVPLMLGAPDMAFPRMNNMSFWLLPPSIMLLISSSIVENGAGTGWTVYPPLSSNIAHSGSSVDLAIFSLHLAGISSILGAVNFITTIINMRPNSMSL
DQMPLFVWAVGITALLLLLSLPVLAGAITMLLTDRNLNTSFFDPAGGGDPILYQHL
>LEFII280-11|MM19930|Apotomis_fraterculana
TLYFIFGIWAGMVGTSLSLLIRAELGNPGSLIGDDQIYNTIVTAHAFIMIFFMVMPIMIGGFGNWLVPLMLGAPDMAFPRMNNMSFWLLPPSIMLLISSSIVENGAGTGWTVYPPLSSNIAHSGSSVDLAIFSLHLAGISSILGAVNFITTIINMRPNSMSL
DQMPLFVWAVGITALLLLLSLPVLAGAITMLLTDRNLNTSFFDPAGGGDPILYQHL
>LEFII281-11|MM19931|Apotomis_fraterculana
TLYFIFGIWAGMVGTSLSLLIRAELGNPGSLIGDDQIYNTIVTAHAFIMIFFMVMPIMIGGFGNWLVPLMLGAPDMAFPRMNNMSFWLLPPSIMLLISSSIVENGAGTGWTVYPPLSSNIAHSGSSVDLAIFSLHLAGISSILGAVNFITTIINMRPNSMSL
DQMPLFVWAVGITALLLLLSLPVLAGAITMLLTDRNLNTSFFDPAGGGDPILYQHL
>LEFIB453-10|HM871352|MM01981|Apotomis_infida
TLYFIFGIWAGMVGTSLSLLIRAELGNPGSLIGDDQIYNTIVTAHAFIMIFFMVMPIMIGGFGNWLVPLMLGAPDMAFPRMNNMSFWLLPPSIMLLISSSIVENGAGTGWTVYPPLSSNIAHSGSSVDLAIFSLHLAGISSILGAVNFITTIINMRPNSMSL
DQMPLFVWAVGITALLLLLSLPVLAGAITMLLTDRNLNTSFFDPAGGGDPILYQHL
>LEFIB454-10|HM871353|MM01982|Apotomis_infida
TLYFIFGIWAGMVGTSLSLLIRAELGNPGSLIGDDQIYNTIVTAHAFIMIFFMVMPIMIGGFGNWLVPLMLGAPDMAFPRMNNMSFWLLPPSIMLLISSSIVENGAGTGWTVYPPLSSNIAHSGSSVDLAIFSLHLAGISSILGAVNFITTIINMRPNSMSL
DQMPLFVWAVGITALLLLLSLPVLAGAITMLLTDRNLNTSFFDPAGGGDPILYQHL
>LEFIB455-10|HM871354|MM01983|Apotomis_infida
TLYFIFGIWAGMVGTSLSLLIRAELGNPGSLIGDDQIYNTIVTAHAFIMIFFMVMPIMIGGFGNWLVPLMLGAPDMAFPRMNNMSFWLLPPSIMLLISSSIVENGAGTGWTVYPPLSSNIAHSGSSVDLAIFSLHLAGISSILGAVNFITTIINMRPNSMSL
DQMPLFVWAVGITALLLLLSLPVLAGAITMLLTDRNLNTSFFDPAGGGDPILYQHL
>LEFIB883-10|HM871760|MM02968|Apotomis_infida
TLYFIFGIWAGMVGTSLSLLIRAELGNPGSLIGDDQIYNTIVTAHAFIMIFFMVMPIMIGGFGNWLVPLMLGAPDMAFPRMNNMSFWLLPPSIMLLISSSIVENGAGTGWTVYPPLSSNIAHSGSSVDLAIFSLHLAGISSILGAVNFITTIINMRPNSMSL
DQMPLFVWAVGITALLLLLSLPVLAGAITMLLTDRNLNTSFFDPAGGGDPILYQHL
>LEFIJ038-10|MM01990|Apotomis_inundana
TLYFIFGIWAGMVGTSLSLLIRAELGNPGSLIGDDQIYNTIVTAHAFIMIFFMVMPIMIGGFGNWLVPLMLGAPDMAFPRMNNMSFWLLPPSIMLLISSSIVENGAGTGWTVYPPLSSNIAHSGSSVDLAIFSLHLAGISSILGAVNFITTIINMRPNSMSL
DQMPLFVWAVGITALLLLLSLPVLAGAITMLLTDRNLNTSFFDPAGGGDPILYQHL
>LEFIA822-10|HM386963|MM04176|Apotomis_lemniscatana
TLYFIFGIWAGMIGTSLSLLIRAELGNPGSLIGDDQIYNTIVTAHAFIMIFFMVMPIMIGGFGNWLVPLMLGAPDMAFPRMNNMSFWLLPPSIMLLISSSVVENGAGTGWTVYPPLSSNIAHSGSSVDLAIFSLHLAGISSILGAVNFITTIINMRPNSMSL
DQMPLFVWAVGITALLLLLSLPVLAGAITMLLTDRNLNTSFFDPAGGGDPILYQHL
>LEFIA823-10|HM386964|MM04177|Apotomis_lemniscatana
TLYFIFGIWAGMIGTSLSLLIRAELGNPGSLIGDDQIYNTIVTAHAFIMIFFMVMPIMIGGFGNWLVPLMLGAPDMAFPRMNNMSFWLLPPSIMLLISSSVVENGAGTGWTVYPPLSSNIAHSGSSVDLAIFSLHLAGISSILGAVNFITTIINMRPNSMSL
DQMPLFVWAVGITALLLLLSLPVLAGAITMLLTDRNLNTSFFDPAGGGDPILYQHL
>LEFID140-10|HM872950|MM05900|Apotomis_lemniscatana
TLYFIFGIWAGMIGTSLSLLIRAELGNPGSLIGDDQIYNTIVTAHAFIMIFFMVMPIMIGGFGNWLVPLMLGAPDMAFPRMNNMSFWLLPPSIMLLISSSIVENGAGTGWTVYPPLSSNIAHSGSSVDLAIFSLHLAGISSILGAVNFITTIINMRPNSMSL
DQMPLFVWAVGITALLLLLSLPVLAGAITMLLTDRNLNTSFFDPAGGGDPILYQHL
>LEFIJ733-10|JF853792|MM17358|Apotomis_lineana
TLYFIFGIWAGMVGTSLSLLIRAELGNPGSLIGDDQIYNTIVTAHAFIMIFFMVMPIMIGGFGNWLVPLMLGAPDMAFPRMNNMSFWLLPPSIMLLISSSIVENGAGTGWTVYPPLSSNIAHSGSSVDLAIFSLHLAGISSILGAVNFITTIINMRPNSMSL
DQMPLFVWAVGITALLLLLSLPVLAGAITMLLTDRNLNTSFFDPAGGGDPILYQHL
>LEFIJ735-10|JF853794|MM17360|Apotomis_lineana
TLYFIFGIWAGMVGTSLSLLIRAELGNPGSLIGDDQIYNTIVTAHAFIMIFFMVMPIMIGGFGNWLVPLMLGAPDMAFPRMNNMSFWLLPPSIMLLISSSIVENGAGTGWTVYPPLSSNIAHSGSSVDLAIFSLHLAGISSILGAVNFITTIINMRPNSMSL
DQMPLFVWAVGITALLLLLSLPVLAGAITMLLTDRNLNTSFFDPAGGGDPILYQHL

>LEFIA828-10|HM386968|MM04182|Apotomis_sauciana
TLYFIFGIWAGMVGTSLSLLIRAELGNPGSLIGDDQIYNTIVTAHAFIMIFFMVMPIMIGGFGNWLVPLMLGAPDMAFPRMNNMSFWLLPPSIMLLISSSIVENGAGTGWTVYPPLSSNIAHSGSSVDLAIFSLHLAGISSILGAVNFITTIINMRPNSMSL
DQMPLFVWAVGITALLLLLSLPVLAGAITMLLTDRNLNTSFFDPAGGGDPILYQHL
>LEFIE131-10|HM873879|MM08347|Apotomis_sauciana
TLYFIFGIWAGMVGTSLSLLIRAELGNPGSLIGDDQIYNTIVTAHAFIMIFFMVMPIMIGGFGNWLVPLMLGAPDMAFPRMNNMSFWLLPPSIMLLISSSIVENGAGTGWTVYPPLSSNIAHSGSSVDLAIFSLHLAGISSILGAVNFITTIINMRPNSMSL
DQMPLFVWAVGITALLLLLSLPVLAGAITMLLTDRNLNTSFFDPAGGGDPILYQHL
>LEFIG832-10|HM876481|MM15696|Apotomis_sauciana
TLYFIFGIWAGMVGTSLSLLIRAELGNPGSLIGDDQIYNTIVTAHAFIMIFFMVMPIMIGGFGNWLVPLMLGAPDMAFPRMNNMSFWLLPPSIMLLISSSIVENGAGTGWTVYPPLSSNIAHSGSSVDLAIFSLHLAGISSILGAVNFITTIINMRPNNMSL
DQMPLFVWAVGITALLLLLSLPVLAGAITMLLTDRNLNTSFFDPAGGGDPILYQHL
>LEFIJ1205-11|KM573092|MM21065|Apotomis_sauciana
TLYFIFGIWAGMVGTSLSLLIRAELGNPGSLIGDDQIYNTIVTAHAFIMIFFMVMPIMIGGFGNWLVPLMLGAPDMAFPRMNNMSFWLLPPSIMLLISSSIVENGAGTGWTVYPPLSSNIAHSGSSVDLAIFSLHLAGISSILGAVNFITTIINMRPNSMSL
DQMPLFVWAVGITALLLLLSLPVLAGAITMLLTDRNLNTSFFDPAGGGDPILYQHL
>LEFIJ2221-14|MM23401|Apotomis_sauciana
TLYFIFGIWAGMVGTSLSLLIRAELGNPGSLIGDDQIYNTIVTAHAFIMIFFMVMPIMIGGFGNWLVPLMLGAPDMAFPRMNNMSFWLLPPSIMLLISSSIVENGAGTGWTVYPPLSSNIAHSGSSVDLAIFSLHLAGISSILGAVNFITTIINMRPNSMSL
DQMPLFVWAVGITALLLLLSLPVLAGAITMLLTDRNLNTSFFDPAGGGDPILYQHL
>LEFIJ2222-14|MM23402|Apotomis_sauciana
TLYFIFGIWAGMVGTSLSLLIRAELGNPGSLIGDDQIYNTIVTAHAFIMIFFMVMPIMIGGFGNWLVPLMLGAPDMAFPRMNNMSFWLLPPSIMLLISSSIVENGAGTGWTVYPPLSSNIAHSGSSVDLAIFSLHLAGISSILGAVNFITTIINMRPNSMSL
DQMPLFVWAVGITALLLLLSLPVLAGAITMLLTDRNLNTSFFDPAGGGDPILYQHL
>LEFIB459-10|HM871358|MM01996|Apotomis_sororculana
TLYFIFGIWAGMVGTSLSLLIRAELGNPGSLIGDDQIYNTIVTAHAFIMIFFMVMPIMIGGFGNWLVPLMLGAPDMAFPRMNNMSFWLLPPSIMLLISSSIVENGAGTGWTVYPPLSSNIAHSGSSVDLAIFSLHLAGISSILGAVNFITTIINMRPNSMSL
DQMPLFVWAVGITALLLLLSLPVLAGAITMLLTDRNLNTSFFDPAGGGDPILYQHL
>LEFIB458-10|HM871357|MM01993|Apotomis_turbidana
TLYFIFGIWAGMIGTSLSLLIRAELGNPGSLIGDDQIYNTIVTAHAFIMIFFMVMPIMIGGFGNWLVPLMLGAPDMAFPRMNNMSFWLLPPSIMLLISSSIVENGAGTGWTVYPPLSSNIAHSGSSVDLAIFSLHLAGISSILGAVNFITTIINMRPNSMSL
DQMPLFVWAVGITALLLLLSLPVLAGAITMLLTDRNLNTSFFDPAGGGDPILYQHL
>LEFIB889-10|HM871766|MM02977|Apotomis_turbidana
TLYFIFGIWAGMIGTSLSLLIRAELGNPGSLIGDDQIYNTIVTAHAFIMIFFMVMPIMIGGFGNWLVPLMLGAPDMAFPRMNNMSFWLLPPSIMLLISSSIVENGAGTGWTVYPPLSSNIAHSGSSVDLAIFSLHLAGISSILGAVNFITTIINMRPNSMSL
DQMPLFVWAVGITALLLLLSLPVLAGAITMLLTDRNLNTSFFDPAGGGDPILYQHL
>LEFIF410-10|HM875095|MM11783|Apotomis_turbidana
TLYFIFGIWAGMIGTSLSLLIRAELGNPGSLIGDDQIYNTVVTAHAFIMIFFMVMPIMIGGFGNWLVPLMLGAPDMAFPRMNNMSFWLLPPSIMLLISSSIVENGAGTGWTVYPPLSSNIAHSGSSVDLAIFSLHLAGISSILGAVNFITTIINMRPNSMSL
DQMPLFVWAVGITALLLLLSLPVLAGAITMLLTDRNLNTSFFDPAGGGDPILYQHL
>LEFIC492-10|HQ570315|MM04196|Aproaerema_anthyllidella
TLYFIFGIWAGMVGTSLSLLIRAELGNPGQLIGDDQIYNTIVTAHAFIMIFFMVMPIMIGGFGNWLVPLMLGAPDMAFPRMNNMSFWLLPPSLTLLISSSIVENGAGTGWTVYPPLSSNIAHSGSSVDLAIFSLHLAGISSILGAINFITTIINMRINGMMF
DQMPLFVWAVGITALLLLLSLPVLAGAITMLLTDRNLNTSFFDPAGGGDPILYQHL
>LEFID445-10|HQ570341|MM06379|Aproaerema_anthyllidella
TLYFIFGIWAGMVGTSLSLLIRAELGNPGQLIGDDQIYNTIVTAHAFIMIFFMVMPIMIGGFGNWLVPLMLGAPDMAFPRMNNMSFWLLPPSLTLLISSSIVENGAGTGWTVYPPLSSNIAHSGSSVDLAIFSLHLAGISSILGAINFITTIINMRINGMMF
DQMPLFVWAVGITALLLLLSLPVLAGAITMLLTDRNLNTSFFDPAGGGDPILYQHL
>LEFIE588-10|HM874311|MM09496|Aproaerema_anthyllidella
TLYFIFGIWAGMVGTSLSLLIRAELGNPGQLIGDDQIYNTIVTAHAFIMIFFMVMPIMIGGFGNWLVPLMLGAPDMAFPRMNNMSFWLLPPSLTLLISSSIVENGAGTGWTVYPPLSSNIAHSGSSVDLAIFSLHLAGISSILGAINFITTIINMRINGMMF
DQMPLFVWAVGITALLLLLSLPVLAGAITMLLTDRNLNTSFFDPAGGGDPILYQHL
>LEFIE589-10|HM874312|MM09497|Aproaerema_anthyllidella
TLYFIFGIWAGMVGTSLSLLIRAELGNPGQLIGDDQIYNTIVTAHAFIMIFFMVMPIMIGGFGNWLVPLMLGAPDMAFPRMNNMSFWLLPPSLTLLISSSIVENGAGTGWTVYPPLSSNIAHSGSSVDLAIFSLHLAGISSILGAINFITTIINMRINGMMF
DQMPLFVWAVGITALLLLLSLPVLAGAITMLLTDRNLNTSFFDPAGGGDPILYQHL
>LEFIJ630-10|JF853728|MM17255|Aproaerema_anthyllidella
TLYFIFGIWAGMVGTSLSLLIRAELGNPGQLIGDDQIYNTIVTAHAFIMIFFMVMPIMIGGFGNWLVPLMLGAPDMAFPRMNNMSFWLLPPSLTLLISSSIVENGAGTGWTVYPPLSSNIAHSGSSVDLAIFSLHLAGISSILGAINFITTIINMRINGMMF
DQMPLFVWAVGITALLLLLSLPVLAGAITMLLTDRNLNTSFFDPAGGGDPILYQHL
>LEEUA190-11|MM19598|Aproaerema_anthyllidella
TLYFIFGIWAGMVGTSLSLLIRAELGNPGQLIGDDQIYNTIVTAHAFIMIFFMVMPIMIGGFGNWLVPLMLGAPDMAFPRMNNMSFWLLPPSLTLLISSSIVENGAGTGWTVYPPLSSNIAHSGSSVDLAIFSLHLAGISSILGAINFITTIINMRINGMMF

DQMPLFVWAVGITALLLLLSLPVLAGAITMLLTDRNLNTSFFDPAGGGDPILYQHL
>LEFIC246-10|HM872090|MM03656|Apterogenum_ypsillon
TLYFIFGIWAGMVGTSLSLLIRAELGNPGSLIGDDQIYNTIVTAHAFIMIFFMVMPIMIGGFGNWLVPLMLGAPDMAFPRLNNMSFWLLPPSLTLLISSSIVENGAGTGWTVYPPLSSNIAHGGSSVDLAIFSLHLAGISSILGAINFITTIINMRLNNLSFDQ
MPLFIWAVGITAFLLLLSLPVLAGAITMLLTDRNLNTSFFDPAGGGDPILYQHL
>LEFID200-10|HM873003|MM06026|Araschnia_levana
TLYFIFGIWAGMVGTSLSLLIRTELGNPGSLIGDDQIYNTIVTAHAFIMIFFMVMPIMIGGFGNWLIPLMLGAPDMAFPRMNNMSFWLLPPSLTLLIFSSIVENGAGTGWTVYPPLSSNIAHSGSSVDLAIFSLHLAGISSILGAINFITTIINMRINNMSFD
QMSLFIWAVGITAILLLLSLPVLAGAITMLLTDRNINTSFFDPAGGGDPILYQHL
>LEFIH021-10|HM876657|MM15885|Archanara_dissoluta
TLYFIFGIWAGMVGTSLSLLIRAELGHPGSLIGDDQIYNTIVTAHAFIMIFFMVMPIMIGGFGNWLVPLMLGAPDMAFPRMNNMSFWLLPPSLTLLISSSIVENGAGTGWTVYPPLSSNIAHGGSSVDLAIFSLHLAGISSILGAINFITTIINMRLNNLSFD
QMPLFIWAVGITAFLLLLSLPVLAGAITMLLTDRNLNTSFFDPAGGGDPILYQHL
>LEEUA327-11|MM19735|Archanara_neurica
TLYFIFGIWAGMVGTSLSLLIRAELGNPGSLIGDDQIYNTIVTAHAFIMIFFMVMPIMIGGFGNWLVPLMLGAPDMAFPRMNNMSFWLLPPSLTLLISSSIVENGAGTGWTVYPPLSSNIAHGGSSVDLAIFSLHLAGISSILGAINFITTIINMRLNNLSFD
QMPLFIWAVGITAFLLLLSLPVLAGAITMLLTDRNLNTSFFDPAGGGDPILYQHL
>LEFIB077-10|HM870986|MM00378|Archiearis_parthenias
TLYFIFGIWAGMMGTSLSLLIRAELGNPGSLIGDDQIYNTIVTAHAFIMIFFMVMPIMIGGFGNWLVPLMLGAPDMAFPRMNNMSFWLLPPSITLLISSSIVENGAGTGWTVYPPLSSNIAHGGSSVDLAIFSLHLAGISSILGAINFITTIINMRLNNMM
FDQMPLFVWAVGITAFLLLLSLPVLAGAITMLLTDRNLNTSFFDPAGGGDPILYQHL
>LEFIA042-10|HM396391|MM00444|Archiearis_parthenias
TLYFIFGIWAGMMGTSLSLLIRAELGNPGSLIGDDQIYNTIVTAHAFIMIFFMVMPIMIGGFGNWLVPLMLGAPDMAFPRMNNMSFWLLPPSITLLISSSIVENGAGTGWTVYPPLSSNIAHGGSSVDLAIFSLHLAGISSILGAINFITTIINMRLNNMM
FDQMPLFVWAVGITAFLLLLSLPVLAGAITMLLTDRNLNTSFFDPAGGGDPILYQHL
>LEFIB593-10|HM871472|MM02301|Archinemapogon_yildizae
TLYFIFGIWASMIGTSLSILIRTELSNPGSFINNDQIYNSIVTAHAFIMIFFMIMPIMIGGFGNWLVPLMLGAPDMAFPRMNNMSFWFLPPSLMLLISSMMMENGAGTGWTVYPPLSSNIAHSGSSVDLTIFSLHLAGISSILGAINFITTMINMRPINMS
LDMMPLLVWAIFITVVLLLLSLPVLAGAITMLLTDRNLNTSFFDPSGGGDPILYQHL
>LEFIK168-10|JF853937|MM17743|Archips_betulana
TLYFIFGIWAGMVGTSLSLLIRAELGNPGSLIGDDQIYNTIVTAHAFIMIFFMVMPIMIGGFGNWLVPLMLGAPDMAFPRMNNMSFWLLPPSIMLLISSSIVENGAGTGWTVYPPLSSNIAHSGSSVDLAIFSLHLAGISSILGAVNFITTIINMRPNNMTL
DQMPLFVWAVGITALLLLLSLPVLAGAITMLLTDRNLNTSFFDPAGGGDPILYQ?L
>LEFIL242-10|MM19242|Archips_betulana
TLYFIFGIWAGMVGTSLSLLIRAELGNPGSLIGDDQIYNTIVTAHAFIMIFFMVMPIMIGGFGNWLVPLMLGAPDMAFPRMNNMSFWLLPPSIMLLISSSIVENGAGTGWTVYPPLSSNIAHSGSSVDLAIFSLHLAGISSILGAVNFITTIINMRPNNMTL
DQMPLFVWAVGITALLLLLSLPVLAGAITMLLTDRNLNTSFFDPAGGGDPILYQHL
>LEFIL262-10|MM19262|Archips_crataegana
TLYFIFGIWAGMVGTSLSLLIRAELGNPGSLIGDDQIYNTIVTAHAFIMIFFMVMPIMIGGFGNWLVPLMLGAPDMAFPRMNNMSFWLLPPSIMLLISSSIVENGAGTGWTVYPPLSSNIAHSGSSVDLAIFSLHLAGISSILGAVNFITTIINMRPNNMTL
DQMPLFVWAVGITALLLLLSLPVLAGAITMLLTDRNLNTSFFDPAGGGDPILYQHL
>LEFII254-11|MM19904|Archips_crataegana
TLYFIFGIWAGMVGTSLSLLIRAELGNPGSLIGDDQIYNTIVTAHAFIMIFFMVMPIMIGGFGNWLVPLMLGAPDMAFPRMNNMSFWLLPPSIMLLISSSIVENGAGTGWTVYPPLSSNIAHSGSSVDLAIFSLHLAGISSILGAVNFITTIINMRPNNMTL
DQMPLFVWAVGITALLLLLSLPVLAGAITMLLTDRNLNTSFFDPAGGGDPILYQHL
>LEFIB469-10|HM871368|MM02019|Archips_oporana
TLYFIFGIWAGMIGTSLSMMIRAELGNPGSLIGDDQIYNTIVTAHAFIMIFFMVMPIMIGGFGNWLVPLMLGAPDMAFPRMNNMSFWLLPPSIMLLISSSIVENGAGTGWTVYPPLSSNIAHSGSSVDLAIFSLHLAGISSILGAVNFITTIINMRPNNM
MLDQMPLFVWAVGITALLLLLSLPVLAGAITMLLTDRNLNTSFFDPAGGGDPILYQHL
>LEFIC906-10|HM872723|MM05228|Archips_podana
TLYFIFGIWAGMVGTSLSLLIRAELGNPGSLIGDDQIYNTIVTAHAFIMIFFMVMPIMIGGFGNWLVPLMLGAPDMAFPRMNNMSFWLLPPSIMLLISSSIVENGAGTGWTVYPPLSSNIAHSGSSVDLAIFSLHLAGISSILGAVNFITTIINMRPNNMTL
DQMPLFVWAVGITALLLLLSLPVLAGAITMLLTDRNLNTSFFDPAGGGDPILYQHL
>LEFIG789-10|HM876441|MM15653|Archips_podana
TLYFIFGIWAGMVGTSLSLLIRAELGNPGSLIGDDQIYNTIVTAHAFIMIFFMVMPIMIGGFGNWLVPLMLGAPDMAFPRMNNMSFWLLPPSIMLLISSSIVENGAGTGWTVYPPLSSNIAHSGSSVDLAIFSLHLAGISSILGAVNFITTIINMRPNNMTL
DQMPLFVWAVGITALLLLLSLPVLAGAITMLLTDRNLNTSFFDPAGGGDPILYQHL
>LEFIB468-10|HM871367|MM02016|Archips_rosana
TLYFIFGIWAGMVGTSLSLLIRAELGNPGSLIGDDQIYNTIVTAHAFIMIFFMVMPIMIGGFGNWLVPLMLGAPDMAFPRMNNMSFWLLPPSIMLLISSSIVENGAGTGWTVYPPLSSNIAHSGSSVDLAIFSLHLAGISSILGAVNFITTIINMRPNNMA
LDQMPLFVWAVGITALLLLLSLPVLAGAITMLLTDRNLNTSFFDPAGGGDPILYQHL
>LEFIC811-10|HM872630|MM04987|Archips_xylosteana

TLYFIFGIWAGMVGTSLSLLIRAELGNPGSLIGDDQIYNTIVTAHAFIMIFFMVMPIMIGGFGNWLVPLMLGAPDMAFPRMNNMSFWLLPPSIMLLISSSIVENGAGTGWTVYPPLSSNIAHSGSSVDLAIFSLHLAGISSILGAVNFITTIINMRPNNMTL
DQMPLFVWAVGITALLLLLSLPVLAGAITMLLTDRNLNTSFFDPAGGGDPILYQHL
>LEFIE585-10|HM874308|MM09490|Archips_xylosteana
TLYFIFGIWAGMVGTSLSLLIRAELGNPGSLIGDDQIYNTIVTAHAFIMIFFMVMPIMIGGFGNWLVPLMLGAPDMAFPRMNNMSFWLLPPSIMLLISSSIVENGAGTGWTVYPPLSSNIAHSGSSVDLAIFSLHLAGISSILGAVNFITTIINMRPNNMA
LDQMPLFVWAVGITALLLLLSLPVLAGAITMLLTDRNLNTSFFDPAGGGDPILYQHL
>LEFIC785-10|HM872604|MM04921|Arctia_caja
TLYFIFGIWAGMVGTSLSLLIRAELGNPGSLIGDDQIYNTIVTAHAFIMIFFMVMPIMIGGFGNWLVPLMLGAPDMAFPRMNNMSFWLLPPSLTLLISSSIVENGAGTGWTVYPPLSSNIAHGGSSVDLAIFSLHLAGISSILGAINFITTIINMRLNNLSFD
QMPLFVWAVGITAFLLLLSLPVLAGAITMLLTDRNLNTSFFDPAGGGDPILYQHL
>LEFID959-10|HM873709|MM07925|Arctia_caja
TLYFIFGIWAGMVGTSLSLLIRAELGNPGSLIGDDQIYNTIVTAHAFIMIFFMVMPIMIGGFGNWLVPLMLGAPDMAFPRMNNMSFWLLPPSLTLLISSSIVENGAGTGWTVYPPLSSNIAHGGSSVDLAIFSLHLAGISSILGAINFITTIINMRLNNLSFD
QMPLFVWAVGITAFLLLLSLPVLAGAITMLLTDRNLNTSFFDPAGGGDPILYQHL
>LEFIA864-10|HM387002|MM09741|Arctia_caja
TLYFIFGIWAGMVGTSLSLLIRAELGNPGSLIGDDQIYNTIVTAHAFIMIFFMVMPIMIGGFGNWLVPLMLGAPDMAFPRMNNMSFWLLPPSLTLLISSSIVENGAGTGWTVYPPLSSNIAHGGSSVDLAIFSLHLAGISSILGAINFITTIINMRLNNLSFD
QMPLFVWAVGITAFLLLLSLPVLAGAITMLLTDRNLNTSFFDPAGGGDLILYQHL
>LEEUA664-11|KM573193|MM20723|Arctia_caja
TLYFIFGIWAGMVGTSLSLLIRAELGNPGSLIGDDQIYNTIVTAHAFIMIFFMVMPIMIGGFGNWLVPLMLGAPDMAFPRMNNMSFWLLPPSLTLLISSSIVENGAGTGWTVYPPLSSNIAHGGSSVDLAIFSLHLAGISSILGAINFITTIINMRLNNLSFD
QMPLFVWAVGITAFLLLLSLPVLAGAITMLLTDRNLNTSFFDPAGGG?PILYQHL
>LEFIJ323-10|KM572905|MM15923|Arctornis_l-nigrum
TLYFIFGIWAGMVGTSLSLLIRAELGNPGSLIGNDQIYNTIVTAHAFIMIFFMVMPIMIGGFGNWLVPLMLGAPDMAFPRMNNMSFWLLPPSLTLLISSSIVENGAGTGWTVYPPLSSNIAHSGSSVDLAIFSLHLAGISSILGAINFITTIINMRLNNLSFD
QMPLFVWAVGITAFLLLLSLPVLAGAITMLLTDRNLNTSFFDPAGGGDPILYQHL
>LEFIL170-10|MM19170|Arctornis_l-nigrum
TLYFIFGIWAGMVGTSLSLLIRAELGNPGSLIGNDQIYNTIVTAHAFIMIFFMVMPIMIGGFGNWLVPLMLGAPDMAFPRMNNMSFWLLPPSLTLLISSSIVENGAGTGWTVYPPLSSNIAHSGSSVDLAIFSLHLAGISSILGAINFITTIINMRLNNLSFD
QMPLFVWAVGITAFLLLLSLPVLAGAITMLLTDRNLNTSFFDPAGGGDPILYQHL
>LEFIB064-10|HM870973|MM00352|Arenostola_phragmitidis
TLYFIFGIWAGMVGTSLSLLIRAELGNPGSLIGDDQIYNTIVTAHAFIMIFFMVMPIMIGGFGNWLVPLMLGAPDMAFPRMNNMSFWLLPPSLTLLISSSIVENGAGTGWTVYPPLSSNIAHGGSSVDLAIFSLHLAGISSILGAINFITTIINMRLNNLSFD
QMPLFIWAVGITAFLLLLSLPVLAGAITMLLTDRNLNTSFFDPAGGGDPILYQHL
>LEFIB601-10|HM871480|MM02315|Argolamprotes_micella
TLYFIFGIWAGMVGTSLSLLIRAELGNPGSLIGDDQIYNTIVTAHAFIMIFFMVMPIMIGGFGNWLVPLMLGAPDMAFPRMNNMSFWLLPPSLTLLISSSLVENGAGTGWTVYPPLSSNIAHGGSSVDLAIFSLHLAGISSILGAINFITTIINMKINGLSFD
QMPLFIWAVGITALLLLLSLPVLAGAITMLLTDRNLNTSFFDPAGGGDPILYQHL
>LEFIA915-10|HM387051|MM09793|Argolamprotes_micella
TLYFIFGIWAGMVGTSLSLLIRAELGNPGSLISDDQIYNTIVTAHAFIMIFFMVMPIMIGGFGNWLVPLMLGAPDMAFPRMNNMSFWLLPPSLTLLISSSLVENGAGTGWTVYPPLSSNIAHGGSSVDLAIFSLHLAGISSILGAINFITTIINMKINGLSFD
QMPLFIWAVGITALLLLLSLPVLAGAITMLLTDRNLNTSFFDPAGGGDPILYQHL
>LEFIG384-10|HM876061|MM14397|Argynnis_adippe
TLYFIFGIWAGMVGTSLSLLIRTELGNPGSLIGDDQIYNTIVTAHAFIMIFFMVMPIMIGGFGNWLIPLMLGAPDMAFPRMNNMSFWLLPPSLILLISSSIVENGAGTGWTVYPPLSSNIAHEGSSVDLAIFSLHLAGISSILGAINFITTIINMRINGMSFDQ
MPLFVWAVGITALLLLLSLPVLAGAITMLLTDRNLNTSFFDPAGGGDPILYQHL
>LEFIJ539-10|JF853656|MM17164|Argynnis_adippe
TLYFIFGIWAGMVGTSLSLLIRTELGNPGSLIGDDQIYNTIVTAHAFIMIFFMVMPIMIGGFGNWLIPLMLGAPDMAFPRMNNMSFWLLPPSLILLISSSIVENGAGTGWTVYPPLSSNIAHEGSSVDLAIFSLHLAGISSILGAINFITTIINMRINGMSFDQ
MPLFVWAVGITALLLLLSLPVLAGAITMLLTDRNLNTSFFDPAGGGDPILYQHL
>LEFIC070-10|HM871939|MM03355|Argynnis_aglaja
TLYFIFGIWAGMVGTSLSLLIRTELGNPGSLIGDDQIYNTIVTAHAFIMIFFMVMPIMIGGFGNWLVPLMLGAPDMAFPRMNNMSFWLLPPSLILLISSSIVENGAGTGWTVYPPLSSNIAHSGSSVDLAIFSLHLAGISSILGAINFITTIINMRINSMSFDQ
MPLFVWAVGITALLLLLSLPVLAGAITMLLTDRNLNTSFFDPAGGGDPILYQHL
>LEFIJ311-10|JF853546|MM15911|Argynnis_laodice
TLYFIFGIWAGMVGTSLSLLIRTELGNPGSLIGDDQIYNTIVTAHAFIMIFFMVMPIMIGGFGNWLVPLMLGAPDMAFPRMNNMSFWLLPPSLILLISSSIVENGAGTGWTVYPPLSSNIAHGGSSVDLAIFSLHLAGISSILGAINFITTIINMRINNMTFD
QMPLFVWAVGITALLLLLSLPVLAGAITMLLTDRNLNTSFFDPAGGGDPILYQHL
>LEFIJ279-10|JF853516|MM14895|Argynnis_niobe
TLYFIFGIWAGMVGTSLSLLIRTELGNPGSLIGDDQIYNTIVTAHAFIMIFFMVMPIMIGGFGNWLIPLMLGAPDMAFPRMNNMSFWLLPPSLILLISSSIVENGAGTGWTVYPPLSSNIAHEGSSVDLAIFSLHLAGISSILGAINFITTIINMRINGMSFDQ
MPLFVWAVGITALLLLLSLPVLAGAITMLLTDRNLNTSFFDPAGGGDPILYQHL

>LEFIC085-10|HM871953|MM03384|Argynnis_paphia
TLYFIFGIWAGMVGTSLSLLIRTELGNPGSLIGDDQIYNTIVTAHAFIMIFFMVMPIMIGGFGNWLIPLMLGAPDMAFPRMNNMSFWLLPPSLMLLISSSIVENGAGTGWTVYPPLSSNISHGGSSVDLAIFSLHLAGISSILGAINFITTIINMRINSMSFD
QMPLFVWAVGITALLLLLSLPVLAGAITMLLTDRNLNTSFFDPAGGGDPILYQHL
>LEFIB964-10|HM871839|MM03140|Argyresthia_abdominalis
TLYFIFGIWSGMVGTSMSLLIRAELGNPGSLIGDDQIYNTIVTAHAFIMIFFMVMPIMIGGFGNWLIPLMLGAPDMAFPRMNNMSFWLLPPSLTLLISSSIVESGAGTGWTVYPPLSSNIAHGGSSVDLAIFSLHLAGISSILGAINFITTIINMKSNGLTFD
RMPLFVWSVSITAILLLLSLPVLAGAITMLLTDRNLNTSFFDPSGGGDPILYQHL
>LEFID719-10|HM873476|MM06773|Argyresthia_albistria
TLYFIFGIWSGMVGTSLSLMIRAELGNPGSLIGDDQIYNTIVTAHAFIMIFFMVMPIMIGGFGNWLVPLMLGAPDMAFPRMNNMSFWLLPPSLTLLISSSIVENGAGTGWTVYPPLSSNIAHSGSSVDLAIFSLHLAGISSILGAINFITTIINMKSNGLNF
DQMPLFIWSVKITAILLLLSLPVLAGAITMLLTDRNLNTSFFDPSGGGDPILYQHL
>LEFIC429-10|HM872265|MM03977|Argyresthia_arceuthina
TLYFIFGIWSGMVGTSLSLLIRAELGNPGSLIGDDQIYNTIVTAHAFIMIFFMVMPIMIGGFGNWLVPLMLGAPDMAFPRMNNMSFWLLPPSLTLLISSSIVESGAGTGWTVYPPLSSNIAHSGSSVDLAIFSLHLAGISSILGAINFITTIINMKSNGLTFDR
MPLFVWSVMITAILLLLSLPVLAGAITMLLTDRNLNTSFFDPSGGGDPILYQHL
>LEFIB764-10|HM871641|MM02627|Argyresthia_aurulentella
TLYFIFGIWSGLVGTSLSLLIRAELGNPGSLIGDDQIYNTIVTAHAFIMIFFMVMPIMIGGFGNWLVPLMLGAPDMAFPRMNNMSFWLLPPSLTLLISSSIVESGAGTGWTVYPPLSSNIAHGGSSVDLAIFSLHLAGISSILGAINFITTIINMKSNGLTFDR
MPLFVWSVSITAVLLLLSLPVLAGAITMLLTDRNLNTSFFDPSGGGDPILYQHL
>LEFIG525-10|HM876198|MM14643|Argyresthia_aurulentella
TLYFIFGIWSGLVGTSLSLLIRAELGNPGSLIGDDQIYNTIVTAHAFIMIFFMVMPIMIGGFGNWLVPLMLGAPDMAFPRMNNMSFWLLPPSLTLLISSSIVESGAGTGWTVYPPLSSNIAHGGSSVDLAIFSLHLAGISSILGAINFITTIINMKSNGLTFDR
MPLFVWSVSITAILLLLSLPVLAGAITMLLTDRNLNTSFFDPSGGGDPILYQHL
>LEFIE466-10|HM874190|MM09238|Argyresthia_bergiella
TLYFIFGIWSGMVGTSLSLLIRAELGNPGSLIGDDQIYNTIVTAHAFIMIFFMVMPIMIGGFGNWLVPLMLGAPDMAFPRMNNMSFWLLPPSLTLLISSSIVESGAGTGWTVYPPLSSNIAHSGSSVDLAIFSLHLAGISSILGAINFITTIINMKSNGLTFDR
MPLFIWAVMITAILLLLSLPVLAGAITMLLTDRNLNTSFFDPSGGGDPILYQHL
>LEFIE733-10|HM874453|MM09832|Argyresthia_bergiella
TLYFIFGIWSGMVGTSLSLLIRAELGSPGSLIGDDQIYNTIVTAHAFIMIFFMVMPIMIGGFGNWLVPLMLGAPDMAFPRMNNMSFWLLPPSLTLLISSSIVESGAGTGWTVYPPLSSNIAHSGSSVDLAIFSLHLAGISSILGAINFITTIINMKSNGLTFDR
MPLFIWAVMITAILLLLSLPVLAGAITMLLTDRNLNTSFFDPSGGGDPILYQHL
>LEFIK539-10|JF854136|MM18114|Argyresthia_bergiella
TLYFIFGIWSGMVGTSLSLLIRAELGNPGSLIGDDQIYNTIVTAHAFIMIFFMVMPIMIGGFGNWLVPLMLGAPDMAFPRMNNMSFWLLPPSLTLLISSSIVESGAGTGWTVYPPLSSNIAHSGSSVDLAIFSLHLAGISSILGAINFITTIINMKSNGLTFDR
MPLFIWAVMITAILLLLSLPVLAGAITMLLTDRNLNTSFFDPSGGGDPILYQHL
>LEFIK541-10|MM18116|Argyresthia_bergiella
TLYFIFGIWSGMVGTSLSLLIRAELGNPGSLIGDDQIYNTIVTAHAFIMIFFMVMPIMIGGFGNWLVPLMLGAPDMAFPRMNNMSFWLLPPSLTLLISSSIVESGAGTGWTVYPPLSSNIAHSGSSVDLAIFSLHLAGISSILGAINFITTIINMKSNGLTFDR
MPLFIWAVMITAILLLLSLPVLAGAITMLL?DRNLNTSFFDPSGGGDPILYQHL
>LEFID687-10|HM873448|MM06733|Argyresthia_bonnetella
TLYFIFGIWSGMVGTSLSLLIRAELGNPGSLIGDDQIYNTIVTAHAFIMIFFMVMPIMIGGFGNWLIPLMLGAPDMAFPRMNNMSFWMLPPSLMLLISSSIVENGAGTGWTVYPPLSSNIAHSGSSVDLAIFSLHLAGISSILGAINFITTIINMKSNGMNF
DQMPLFIWAVKITAILLLLSLPVLAGAITMLLTDRNLNTSFFDPSGGGDPILYQHL
>LEFID688-10|HM873449|MM06734|Argyresthia_bonnetella
TLYFIFGIWSGMVGTSLSLLIRAELGNPGSLIGDDQIYNTIVTAHAFIMIFFMVMPIMIGGFGNWLIPLMLGAPDMAFPRMNNMSFWMLPPSLMLLISSSIVENGAGTGWTVYPPLSSNIAHSGSSVDLAIFSLHLAGISSILGAINFITTIINMKSNGMNF
DQMPLFIWAVKITAILLLLSLPVLAGAITMLLTDRNLNTSFFDPSGGGDPILYQHL
>LEFIB959-10|HM871835|MM03133|Argyresthia_brockeella
TLYFIFGIWSGMLGTSLSLLIRAELGNPGSLIGDDQIYNTIVTAHAFIMIFFMVMPIMIGGFGNWLIPLMLGAPDMAFPRMNNMSFWLLPPSLMLLISSSIVENGAGTGWTVYPPLSSNIAHSGGSVDLAIFSLHLAGISSILGAINFITTIINMKSNGLTFD
RLPLFVWAVKITAILLLLSLPVLAGAITMLLTDRNLNTSFFDPSGGGDPILYQHL
>LEFIB590-10|HM871469|MM02292|Argyresthia_conjugella
TLYFIFGIWSGMVGTSLSLLIRAELGNPGSLIGDDQIYNTIVTAHAFIMIFFMVMPIMIGGFGNWLVPLMLGAPDMAFPRMNNMSFWLLPPSLTLLISSSIVENGAGTGWTVYPPLSSNIAHGGSSVDLAIFSLHLAGISSILGAINFITTIINMKSNGLSFD
QMPLFIWAVKITAVLLLLSLPVLAGAITMLLTDRNLNTSFFDPSGGGDPILYQHL
>LEFIB962-10|HM871838|MM03137|Argyresthia_conjugella
TLYFIFGIWSGMVGTSLSLLIRAELGNPGSLIGDDQIYNTIVTAHAFIMIFFMVMPIMIGGFGNWLVPLMLGAPDMAFPRMNNMSFWLLPPSLTLLISSSIVENGAGTGWTVYPPLSSNIAHGGSSVDLAIFSLHLAGISSILGAINFITTIINMKSNGLSFD
QMPLFIWAVKITAVLLLLSLPVLAGAITMLLTDRNLNTSFFDPSGGGDPILYQHL
>LEFID772-10|HM873529|MM06838|Argyresthia_conjugella
TLYFIFGIWSGMVGTSLSLLIRAELGNPGSLIGDDQIYNTIVTAHAFIMIFFMVMPIMIGGFGNWLVPLMLGAPDMAFPRMNNMSFWLLPPSLTLLISSSIVENGAGTGWTVYPPLSSNIAHGGSSVDLAIFSLHLAGISSILGAINFITTIINMKSNGLSFD

QMPLFIWAVKITAVLLLLSLPVLAGAITMLLTDRNLNTSFFDPSGGGDPILYQHL
>LEFIE144-10|HM873892|MM08380|Argyresthia_conjugella
TLYFIFGIWSGMVGTSLSLLIRAELGNPGSLIGDDQIYNTIVTAHAFIMIFFMVMPIMIGGFGNWLVPLMLGAPDMAFPRMNNMSFWLLPPSLTLLISSSIVENGAGTGWTVYPPLSSNIAHGGSSVDLAIFSLHLAGISSILGAINFITTIINMKSNGLSFD
QMPLFIWAVKITAVLLLLSLPVLAGAITMLLTDRNLNTSFFDPSGGGDPILYQHL
>LEFID779-10|HM873536|MM06847|Argyresthia_curvella
TLYFMFGIWSGMVGTSLSLLIRAELGNPGSLIGDDQIYNTIVTAHAFIMIFFMVMPIMIGGFGNWLVPLMLGAPDMAFPRMNNMSFWLLPPSLMLLISSSIIENGAGTGWTVYPPLSSNIAHSGSSVDLAIFSLHLAGISSILGAINFITTIINMKSNGMN
FDQMPLFIWAVMITAILLLLSLPVLAGAITMLLTDRNLNTSFFDPSGGGDPILYQHL
>LEFIG086-10|HM875765|MM13745|Argyresthia_curvella
TLYFMFGIWSGMVGTSLSLLIRAELGNPGSLIGDDQIYNTIVTAHAFIMIFFMVMPIMIGGFGNWLVPLMLGAPDMAFPRMNNMSFWLLPPSLMLLISSSIIENGAGTGWTVYPPLSSNIAHSGSSVDLAIFSLHLAGISSILGAINFITTIINMKSNGMN
FDQMPLFIWAVMITAILLLLSLPVLAGAITMLLTDRNLNTSFFDPSGGGDPILYQHL
>LEFID737-10|HM873494|MM06796|Argyresthia_dilectella
TLYFIFGIWSGMMGTSLSLLIRAELGNPGSLIGNDQIYNTIVTAHAFIMIFFMVMPIMIGGFGNWLVPLMLGAPDMAFPRMNNMSFWLLPPSLTLLISSSIVESGAGTGWTVYPPLSSNIAHSGSSVDLAIFSLHLAGISSILGAINFITTIINMKSNGLTFD
RMPLFVWAVSITAVLLLLSLPVLAGAITMLLTDRNLNTSFFDPSGGGDPILYQHL
>LEFIF492-10|HM875177|MM12058|Argyresthia_dilectella
TLYFIFGIWSGMMGTSLSLLIRAELGNPGSLIGNDQIYNTIVTAHAFIMIFFMVMPIMIGGFGNWLVPLMLGAPDMAFPRMNNMSFWLLPPSLTLLISSSIVESGAGTGWTVYPPLSSNIAHSGSSVDLAIFSLHLAGISSILGAINFITTIINMKSNGLTFD
RMPLFVWAVSITAVLLLLSLPVLAGAITMLLTDRNLNTSFFDPSGGGDPILYQHL
>LEFIG360-10|HM876037|MM14358|Argyresthia_dilectella
TLYFIFGIWSGMMGTSLSLLIRAELGNPGSLIGNDQIYNTIVTAHAFIMIFFMVMPIMIGGFGNWLVPLMLGAPDMAFPRMNNMSFWLLPPSLTLLISSSIVESGAGTGWTVYPPLSSNIAHSGSSVDLAIFSLHLAGISSILGAINFITTIINMKSNGLTFD
RMPLFVWAVSITAVLLLLSLPVLAGAITMLLTDRNLNTSFFDPSGGGDPILYQHL
>LEFIC035-10|HM871905|MM03262|Argyresthia_glabratella
TLYFIFGIWSWMVGTSLSLLIRAELGNPGSLIGDDQIYNTIVTAHAFIMIFFMVMPIMIGGFGNWLVPLMLGAPDMAFPRMNNMSFWLLPPSLTLLISSSIVESGAGTGWTVYPPLSSNIAHSGSSVDLAIFSLHLAGISSILGAINFITTIINMKSNGLTFD
RMPLFIWAVSITAVLLLLSLPVLAGAITMLLTDRNLNTSFFDPSGGGDPILYQHL
>LEFIG258-10|HM875937|MM14184|Argyresthia_glabratella
TLYFIFGIWSGMVGTSLSLLIRAELGNPGSLIGDDQIYNTIVTAHAFIMIFFMVMPIMIGGFGNWLVPLMLGAPDMAFPRMNNMSFWLLPPSLTLLISSSIVESGAGTGWTVYPPLSSNIAHSGSSVDLAIFSLHLAGISSILGAINFITTIINMKSNGLTFDR
MPLFIWAVSITAVLLLLSLPVLAGAITMLLTDRNLNTSFFDPSGGGDPILYQHL
>LEFIK534-10|JF854131|MM18109|Argyresthia_glabratella
TLYFIFGIWSGMVGTSLSLLIRAELGNPGSLIGDDQIYNTIVTAHAFIMIFFMVMPIMIGGFGNWLVPLMLGAPDMAFPRMNNMSFWLLPPSLTLLISSSIVESGAGTGWTVYPPLSSNIAHSGSSVDLAIFSLHLAGISSILGAINFITTIINMKSNGLTFDR
MPLFIWAVSITAVLLLLSLPVLAGAITMLLTDRNLNTSFFDPSGGGDPILYQHL
>LEFIK536-10|JF854133|MM18111|Argyresthia_glabratella
TLYFIFGIWSGMVGTSLSLLIRAELGNPGSLIGDDQIYNTIVTAHAFIMIFFMVMPIMIGGFGNWLVPLMLGAPDMAFPRMNNMSFWLLPPSLTLLISSSIVESGAGTGWTVYPPLSSNIAHSGSSVDLAIFSLHLAGISSILGAINFITTIINMKSNGLTFDR
MPLFIWAVSITAVLLLLSLPVLAGAITMLLTDRNLNTSFFDPSGGGDPILYQHL
>LEFIA857-10|HM386995|MM09734|Argyresthia_glaucinella
TLYFIFGIWSGMLGTSLSLLIRAELGNPGSLIGDDQIYNTIVTAHAFIMIFFMVMPIMIGGFGNWLIPLMLGAPDMAFPRMNNMSFWLLPPSLTLLISSSIVESGAGTGWTVYPPLSSNIAHSGSSVDLAIFSLHLAGISSILGAINFITTIINMKSNGLTFDQ
MSLFVWAVMITAILLLLSLPVLAGAITMLLTDRNMNTSFFDPSGGGDPILYQHL
>LEFIB611-10|HM871490|MM02340|Argyresthia_goedartella
TLYFIFGIWSGMVGTSLSLLIRAELGNPGSLIGDDQIYNTIVTAHAFIMIFFMVMPIMIGGFGNWLVPLMLGAPDMAFPRMNNMSFWLLPPSLTLLISSSIVENGAGTGWTVYPPLSSNIAHGGSSVDLAIFSLHLAGISSILGAINFITTIINMKSNGMTF
DRMPLFIWAVKITAVLLLLSLPVLAGAITMLLTDRNLNTSFFDPSGGGDPILYQHL
>LEFIB961-10|HM871837|MM03136|Argyresthia_goedartella
TLYFIFGIWSGMVGTSLSLLIRAELGNPGSLIGDDQIYNTIVTAHAFIMIFFMVMPIMIGGFGNWLVPLMLGAPDMAFPRMNNMSFWLLPPSLTLLISSSIVENGAGTGWTVYPPLSSNIAHGGSSVDLAIFSLHLAGISSILGAINFITTIINMKSNGMTF
DRMPLFIWAVKITAVLLLLSLPVLAGAITMLLTDRNLNTSFFDPSGGGDPILYQHL
>LEFIF474-10|HM875159|MM12019|Argyresthia_goedartella
TLYFIFGIWSGMVGTSLSLLIRAELGNPGSLIGDDQIYNTIVTAHAFIMIFFMVMPIMIGGFGNWLVPLMLGAPDMAFPRMNNMSFWLLPPSLTLLISSSIVENGAGTGWTVYPPLSSNIAHGGSSVDLAIFSLHLAGISSILGAINFITTIINMKSNGMTF
DRMPLFIWAVKITAVLLLLSLPVLAGAITMLLTDRNLNTSFFDPSGGGDPILYQHL
>LEFIG680-10|HM876340|MM15544|Argyresthia_laevigatella
TLYFIFGIWSGMVGTSLSLLIRAELGNPGSLIGDDQIYNTIVTAHAFIMIFFMVMPIMIGGFGNWLVPLMLGAPDMAFPRMNNMSFWLLPPSLTLLISSSIVEFGAGTGWTVYPPLSSNIAHSGSSVDLAIFSLHLAGISSILGAINFITTIINMKSNGLTFDR
MPLFVWAVLITAILLLLSLPVLAGAITMLLTDRNLNTSFFDPSGGGDPILYQHL
>LEFIL688-10|MM18986|Argyresthia_praecocella

TLYFIFGIWSGMVGTSLSLLIRAELGNPGSLIGDDQIYNTIVTAHAFIMIFFMVMPIMIGGFGNWLVPLMLGAPDMAFPRMNNMSFWLLPPSLTLLISSSVVESGAGTGWTVYPPLSSNIAHSGSSVDLAIFSLHLAGISSILGAINFITTIINMKSNGLTYD
QMPLFVWAVSITAVLLLLSLPVLAGAITMLLTDRNLNTSFFDPSGGGDPILYQHL
>LEFIE615-10|HM874338|MM09531|Argyresthia_pruniella
TLYFIFGIWSGMVGTSLSLLIRAELGNPGSLIGDDQIYNTIVTAHAFIMIFFMVMPIMIGGFGNWLVPLMLGAPDMAFPRMNNMSFWLLPPSLMLLISSSIVENGAGTGWTVYPPLSSNIAHGGSSVDLAIFSLHLAGISSILGAINFITTIINMKSNGMTF
DQMPLFIWAVKITAILLLLSLPVLAGAITMLLTDRNLNTSFFDPSGGGDPILYQHL
>LEFIK375-10|JF854008|MM17950|Argyresthia_pruniella
TLYFIFGIWSGMVGTSLSLLIRAELGNPGSLIGDDQIYNTIVTAHAFIMIFFMVMPIMIGGFGNWLVPLMLGAPDMAFPRMNNMSFWLLPPSLMLLISSSIVENGAGTGWTVYPPLSSNIAHGGSSVDLAIFSLHLAGISSILGAINFITTIINMKSNGMTF
DQMPLFIWAVKITAILLLLSLPVLAGAITMLLTDRNLNTSFFDPSGGGDPILYQHL
>LEFIK530-10|JF854127|MM18105|Argyresthia_pruniella
TLYFIFGIWSGMVGTSLSLLIRAELGNPGSLIGDDQIYNTIVTAHAFIMIFFMVMPIMIGGFGNWLVPLMLGAPDMAFPRMNNMSFWLLPPSLMLLISSSIVENGAGTGWTVYPPLSSNIAHGGSSVDLAIFSLHLAGISSILGAINFITTIINMKSNGMTF
DQMPLFIWAVKITAILLLLSLPVLAGAITMLLTDRNLNTSFFDPSGGGDPILYQHL
>LEFIB592-10|HM871471|MM02299|Argyresthia_pulchella
TLYFIFGIWSGMVGTSLSLLIRAELGNPGSLIGDDQIYNTIVTAHAFIMIFFMVMPIMIGGFGNWLVPLMLGAPDMAFPRMNNMSFWLLPPSLTLLISSSIVENGAGTGWTVYPPLSSNIAHGGSSVDLAIFSLHLAGISSILGAINFITTIINMKSNGMTF
DQMPLFIWAVKITAILLLLSLPVLAGAITMLLTDRNLNTSFFDPSGGGDPILYQHL
>LEFIB743-10|HM871621|MM02585|Argyresthia_pygmaeella
TLYFMFGIWSGMVGTSLSLLIRAELGNPGSLIGDDQIYNTIVTAHAFIMIFFMVMPIMIGGFGNWLVPLMLGAPDMAFPRMNNMSFWLLPPSLTLLISSSIVENGAGTGWTVYPPLSSNIAHSGSSVDLAIFSLHLAGISSILGAINFITTIINMKSNGMTF
DQMPLFIWAVKITAILLLLSLPVLAGAITMLLTDRNLNTSFFDPSGGGDPILYQHL
>LEFIB960-10|HM871836|MM03135|Argyresthia_pygmaeella
TLYFMFGIWSGMVGTSLSLLIRAELGNPGSLIGDDQIYNTIVTAHAFIMIFFMVMPIMIGGFGNWLVPLMLGAPDMAFPRMNNMSFWLLPPSLTLLISSSIVENGAGTGWTVYPPLSSNIAHSGSSVDLAIFSLHLAGISSILGAINFITTIINMKSNGMTF
DQMPLFIWAVKITAILLLLSLPVLAGAITMLLTDRNLNTSFFDPSGGGDPILYQHL
>LEFIB963-10|HQ570278|MM03138|Argyresthia_retinella
TLYFIFGIWSGMVGTSLSLLIRAELGNPGSLIGDDQIYNTIVTAHAFIMIFFMVMPIMIGGFGNWLVPLMLGAPDMAFPRMNNMSFWLL?PSLTLLISSSIVENGAGTGWTVYPPLSSNIAHGGSSVDLAIFSLHLAGISSILGAINFITTIINMKSNGLTFD
RMPLFIWAVKITAVLLLLSLPVLAGAITMLLTDRNLNTSFFDPSGGGDPILYQHL
>LEFIF990-10|HM875671|MM13594|Argyresthia_retinella
TLYFIFGIWSGMVGTSLSLLIRAELGNPGSLIGDDQIYNTIVTAHAFIMIFFMVMPIMIGGFGNWLVPLMLGAPDMAFPRMNNMSFWLLPPSLTLLISSSIVENGAGTGWTVYPPLSSNIAHGGSSVDLAIFSLHLAGISSILGAINFITTIINMKSNGLTFD
RMPLFIWAVKITAVLLLLSLPVLAGAITMLLTDRNLNTSFFDPSGGGDPILYQHL
>LEFIK531-10|JF854128|MM18106|Argyresthia_semifusca
TLYFMFGIWSGMVGTSLSLLIRAELGNPGSLIGDDQIYNTIVTAHAFIMIFFMVMPIMIGGFGNWLVPLMLGAPDMAFPRMNNMSFWLLPPSLTLLISSSIVENGAGTGWTVYPPLSSNIAHSGSSVDLAIFSLHLAGISSILGAINFITTIINMKSNGMTF
DQMPLFIWAVKITAILLLLSLPVLAGAITMLLTDRNLNTSFFDPSGGGDPILYQHL
>LEFIB766-10|HM871643|MM02629|Argyresthia_sorbiella
TLYFIFGIWSGMVGTSLSLLIRAELGNPGSLIGDDQIYNTIVTAHAFIMIFFMVMPIMIGGFGNWLIPLMLGAPDMAFPRMNNMSFWLLPPSLTLLISSSIVENGTGTGWTVYPPLSSNIAHSGSSVDLAIFSLHLAGISSILGAINFITTIINMKSNGMTFD
QMPLFIWAVKITAILLLLSLPVLAGAITMLLTDRNLNTSFFDPSGGGDPILY?HL
>LEFID713-10|HM873474|MM06767|Argyresthia_sorbiella
TLYFIFGIWSGMVGTSLSLLIRAELGNPGSLIGDDQIYNTIVTAHAFIMIFFMVMPIMIGGFGNWLIPLMLGAPDMAFPRMNNMSFWLLPPSLTLLISSSIVENGTGTGWTVYPPLSSNIAHSGSSVDLAIFSLHLAGISSILGAINFITTIINMKSNGMTFD
QMPLFIWAVKITAILLLLSLPVLAGAITMLLTDRNLNTSFFDPSGGGDPILYQHL
>LEFID720-10|HM873477|MM06775|Argyresthia_spinosella
TLYFIFGIWSGMVGTSLSLLIRAELGNPGSLIGDDQIYNTIVTAHAFIMIFFMVMPIMIGGFGNWLVPLMLGAPDMAFPRMNNMSFWLLPPSLMLLISSSIVENGAGTGWTVYPPLSSNIAHSGSSVDMAIFSLHLAGISSILGAINFITTIINMKNNGMN
FDQMPLFIWAVKITAILLLLSLPVLAGAITMLLTDRNLNTSFFDPSGGGDPILYQHL
>GRAFW2433-13|MM23510|Argyresthia_spinosella
TLYFIFGIWSGMVGTSLSLLIRAELGNPGSLIGDDQIYNTIVTAHAFIMIFFMVMPIMIGGFGNWLVPLMLGAPDMAFPRMNNMSFWLLPPSLMLLISSSIVENGAGTGWTVYPPLSSNIAHSGSSVDMAIFSLHLAGISSILGAINFITTIINMKNNGMN
FDQMPLFIWAVKITAILLLLSLPVLAGAITMLLTDRNLNTSFFDPSGGGDPILYQHL
>LEFID467-10|HM873235|MM06408|Argyresthia_svenssoni
TLYFIFGIWSGMVGTSLSLLIRAELGSPGSLIGDDQIYNTIVTAHAFIMIFFMVMPIMIGGFGNWLVPLMLGAPDMAFPRMNNMSFWLLPPSLTLLISSGIVESGAGTGWTVYPPLSSNIAHSGSSVDLAIFSLHLAGISSILGAINFITTIINMKSNGLTFDQ
MPLFVWAVSITAVLLLLSLPVLAGAITMLLTDRNLNTSFFDPSGGGDPILYQHL
>LEFIK533-10|JF854130|MM18108|Argyresthia_svenssoni
TLYFIFGIWSGMVGTSLSLLIRAELGSPGSLIGDDQIYNTIVTAHAFIMIFFMVMPIMIGGFGNWLVPLMLGAPDMAFPRMNNMSFWLLPPSLTLLISSGIVESGAGTGWTVYPPLSSNIAHSGSSVDLAIFSLHLAGISSILGAINFITTIINMKSNGLTFDQ
MPLFVWAVSITAVLLLLSLPVLAGAITMLLTDRNLNTSFFDPSGGGDPILYQHL

>LEFIK535-10|JF854132|MM18110|Argyresthia_svenssoni
TLYFIFGIWSGMVGTSLSLLIRAELGSPGSLIGDDQIYNTIVTAHAFIMIFFMVMPIMIGGFGNWLVPLMLGAPDMAFPRMNNMSFWLLPPSLTLLISSGIVESGAGTGWTVYPPLSSNIAHSGSSVDLAIFSLHLAGISSILGAINFITTIINMKSNGLTFDQ
MPLFVWAVSITAVLLLLSLPVLAGAITMLLTDRNLNTSFFDPPGGGDPILYQHL
>LEFIK540-10|KM572856|MM18115|Argyresthia_svenssoni
TLYFIFGIWSGMVGTSLSLLIRAELGSPGSLIGDDQIYNTIVTAHAFIMIFFMVMPIMIGGFGNWLVPLMLGAPDMAFPRMNNMSFWLLPPSLTLLISSGIVESGTGTGWTVYPPLSSNIAHSGSSVDLAIFSLHLAGISSILGAINFITTIINMKSNGLTFDQ
MPLFVWAVSITAVLLLLSLPVLAGAITMLLTDRNLNTSFFDPSGGGDPILYQHL
>LEEUA077-11|JN263009|MM19390|Argyresthia_trifasciata
TLYFIFGIWSGMVGTSLSLLIRAELGNPGSLIGDDQIYNTIVTAHAFIMIFFMVMPIMIGGFGNWLVPLMLGAPDMAFPRMNNMSFWLLPPSLTLLISSSIVESGAGTGWTVYPPLSSNIAHSGSSVDLAIFSLHLAGISSILGAINFITTIINMKSNGLTFDR
MPLFVWSVMITAILLLLSLPVLAGAITMLLTDRNLNTSFFDPSGGGDPILYQHL
>LEFIA011-10|HM396361|MM00082|Argyroploce_aquilonana
TLYFIFGIWAGMIGTSLSLLIRAELGNPGSLIGDDQIYNTIVTAHAFIMIFFMVMPIMIGGFGNWLVPLMLGAPDMAFPRMNNMSFWLLPPSIMLLISSSIVENGAGTGWTVYPPLSSNIAHSGSSVDLAIFSLHLAGISSILGAVNFITTIINMRPNNMSL
DQMPLFVWAVGITALLLLLSLPVLAGAITMLLTDRNLNTSFFDPAGGGDPILYQHL
>LEFIC112-10|HQ570303|MM03442|Argyroploce_arbutella
TLYFIFGIWAGMIGTSLSLLIRAELGNPGSLIGDDQIYNTIVTAHAFIMIFFMVMPIMIGGFGNWLVPLMLGAPDMAFPRMNNMSFWLLPPSIMLLISSSIVENGAGTGWTVYPPLSSNIAHSGSSVDLAIFSLHLAGISSILGAVNFITTIINMRPNNMSL
DQMPLFVWAVGITALLLLLSLPVLAGAITMLLTDRNLNTSFFDPAGGGDPILYQHL
>LEFIF582-10|HM875266|MM12391|Argyroploce_arbutella
TLYFIFGIWAGMIGTSLSLLIRAELGNPGSLIGDDQIYNTIVTAHAFIMIFFMVMPIMIGGFGNWLVPLMLGAPDMAFPRMNNMSFWLLPPSIMLLISSSIVENGAGTGWTVYPPLSSNIAHSGSSVDLAIFSLHLAGISSILGAVNFITTIINMHPNNMSL
DQMPLFVWAVGITALLLLLSLPVLAGAITMLLTDRNLNTSFFDPAGGGDPILYQHL
>LEFIG473-10|HM876149|MM14517|Argyroploce_arbutella
TLYFIFGIWAGMIGTSLSLLIRAELGNPGSLIGDDQIYNTIVTAHAFIMIFFMVMPIMIGGFGNWLVPLMLGAPDMAFPRMNNMSFWLLPPSIMLLISSSIVENGAGTGWTVYPPLSSNIAHSGSSVDLAIFSLHLAGISSILGAVNFITTIINMRPNNMSL
DQMPLFVWAVGITALLLLLSLPVLAGAITMLLTDRNLNTSFFDPAGGGDPILYQHL
>LEFIA810-10|HM386951|MM04164|Argyroploce_concretana
TLYFIFGIWSGMIGTSLSLLIRAELGNPGSLIGDDQIYNTIVTAHAFIMIFFMVMPIMIGGFGNWLVPLMLGAPDMAFPRMNNMSFWLLPPSIMLLISSSIVENGAGTGWTVYPPLSSNIAHSGSSVDLAIFSLHLAGISSILGAVNFITTIINMRPNNMSL
DQMPLFVWAVGITALLLLLSLPVLAGAITMLLTDRNLNTSFFDPAGGGDPILYQHL
>LEFIG819-10|HM876471|MM15683|Argyroploce_externa
TLYFIFGIWSGMIGTSLSLLIRAELGNPGSLIGDDQIYNTIVTAHAFIMIFFMVMPIMIGGFGNWLVPLMLGAPDMAFPRMNNMSFWLLPPSIMLLISSSVVENGAGTGWTVYPPLSSNIAHSGSSVDLAIFSLHLAGISSILGAVNFITTIINMRPNNMLL
DQMPLFVWAVGITALLLLLSLPVLAGAITMLLTDRNLNTSFFDPAGGGDPILYQHL
>LEFIC444-10|HM872278|MM04002|Argyroploce_lediana
TLYFIFGVWAGMIGTSLSLLIRAELGNPGSLIGDDQIYNTIVTAHAFIMIFFMVMPIMIGGFGNWLVPLMLGAPDMAFPRMNNMSFWLLPPSIMLLISSSVVESGAGTGWTVYPPLSSNIAHSGSSVDLAIFSLHLAGISSILGAVNFITTIINMRPNNMS
LDQMPLFVWAVGITALLLLLSLPVLAGAITMLLTDRNINTSFFDPAGGGDPILYQHL
>LEFIA012-10|HM396362|MM00083|Argyroploce_noricana
TLYFIFGIWAGMIGTSLSLLIRAELGNPGSLIGDDQIYNTIVTAHAFIMIFFMVMPIMIGGFGNWLVPLMLGAPDMAFPRMNNMSFWLLPPSIMLLISSSIVENGAGTGWTVYPPLSSNIAHSGSSVDLAIFSLHLAGISSILGAVNFITTIINMRPNNMSL
DQMPLFVWAVGITALLLLLSLPVLAGAITMLLTDRNLNTSFFDPAGGGDPILYQHL
>LEFIB234-10|HM871137|MM00696|Argyroploce_roseomaculana
TLYFIFGIWAGMIGTSLSLLIRAELGNPGSLIGDDQIYNTIVTAHAFIMIFFMVMPIMIGGFGNWLVPLMLGAPDMAFPRMNNMSFWLLPPSIMLLISSSVVENGAGTGWTVYPPLSSNIAHSGSSVDLAIFSLHLAGISSILGAINFITTIINMRPNNMSL
DQMPLFVWSVGITALLLLLSLPVLAGAITMLLTDRNLNTSFFDPAGGGDPILYQHL
>LEFID027-10|HM872841|MM05462|Argyrotaenia_ljungiana
TLYFIFGIWAGMVGTSLSLLIRAELGNPGSLIGDDQIYNTIVTAHAFIMIFFMVMPIMIGGFGNWLVPLMLGAPDMAFPRMNNMSFWLLPPSIMLLISSSIVENGAGTGWTVYPPLSSNIAHSGSSVDLAIFSLHLAGISSILGAVNFITTIINMRPNNMSL
DQMPLFVWSVGITALLLLLSLPVLAGAITMLLTDRNLNTSFFDPAGGGDPILYQHL
>LEFIA305-10|HM386648|MM01351|Arichanna_melanaria
TLYFIFGIWAGMVGTSLSLLIRAELGNPGSLIGDDQIYNTIVTAHAFIMIFFMVMPIMIGGFGNWLVPLMLGAPDMAFPRMNNMSFWLLPPSITLLISSSIVENGAGTGWTVYPPLSSNIAHGGSSVDLAIFSLHLAGISSILGAINFITTIINMRLNSLSFD
QMPLFIWAVGITAFLLLLSLPVLAGAITMLLTDRNLNTSFFDPAGGGDPILYQHL
>LEFIA306-10|HM386649|MM01352|Arichanna_melanaria
TLYFIFGIWAGMVGTSLSLLIRAELGNPGSLIGDDQIYNTIVTAHAFIMIFFMVMPIMIGGFGNWLVPLMLGAPDMAFPRMNNMSFWLLPPSITLLISSSIVENGAGTGWTVYPPLSSNIAHGGSSVDLAIFSLHLAGISSILGAINFITTIINMRLNNLSFD
QMPLFIWAVGITAFLLLLSLPVLAGAITMLLTDRNLNTSFFDPAGGGDPILYQHL
>LEFIE035-10|HM873784|MM08115|Arichanna_melanaria
TLYFIFGIWAGMVGTSLSLLIRAELGNPGSLIGDDQIYNTIVTAHAFIMIFFMVMPIMIGGFGNWLVPLMLGAPDMAFPRMNNMSFWLLPPSITLLISSSIVENGAGTGWTVYPPLSSNIAHGGSSVDLAIFSLHLAGISSILGAINFITTIINMRLNNLSFD

QMPLFIWAVGITAFLLLLSLPVLAGAITMLLTDRNLNTSFFDPAGGGDPILYQHL
>LEFIC063-10|HM871932|MM03347|Aricia_artaxerxes
TLYFIFGIWAGMVGTSLSILIRMELSIPGSLIGDDQIYNTIVTAHAFIMIFFMVMPIMIGGFGNWLVPLMLGAPDMAFPRMNNMSFWLLPPSLMLLISSSIVENGAGTGWTVYPPLSSNIAHGGSSVDLAIFSLHLAGISSILGAINFITTIINMRVNNLSFD
QMSLFIWAVGITALLLLLSLPVLAGAITMLLTDRNINTSFFDPAGGGDPILYQHL
>LEFIC059-10|HM871928|MM03341|Aricia_nicias
TLYFIFGIWAGMVGTSLSILIRMELSIPGSLIGDDQIYNTIVTAHAFIMIFFMVMPIMIGGFGNWLVPLMLGAPDMAFPRMNNMSFWLLPPSLMLLISSSIVENGAGTGWTVYPPLSSNIAHSGSSVDLAIFSLHLAGISSILGAINFITTIINMRVNNLSFD
QMSLFIWAVGITALLLLLSLPVLAGAITMLLTDRNINTSFFDPAGGGDPILYQHL
>LEFID248-10|HM873047|MM06112|Aricia_nicias
TLYFIFGIWAGMVGTSLSILIRMELSIPGSLIGDDQIYNTIVTAHAFIMIFFMVMPIMIGGFGNWLVPLMLGAPDMAFPRMNNMSFWLLPPSLMLLISSSIVENGAGTGWTVYPPLSSNIAHSGSSVDLAIFSLHLAGISSILGAINFITTIINMRVNNLSFD
QMSLFIWAVGITALLLLLSLPVLAGAITMLLTDRNINTSFFDPAGGGDPILYQHL
>LEFIG534-10|HM876207|MM14661|Aricia_nicias
TLYFIFGIWAGMVGTSLSILIRMELSIPGSLIGDDQIYNTIVTAHAFIMIFFMVMPIMIGGFGNWLVPLMLGAPDMAFPRMNNMSFWLLPPSLMLLISSSIVENGAGTGWTVYPPLSSNIAHSGSSVDLAIFSLHLAGISSILGAINFITTIINMRVNNLSFD
QMSLFIWAVGITALLLLLSLPVLAGAITMLLTDRNINTSFFDPAGGGDPILYQHL
>LEEUA594-11|MM20653|Aristotelia_baltica
TLYFIFGIWAGMVGTSLSLLIRAELGNPGSLIGDDQIYNTIVTAHAFIMIFFMVMPIMIGGFGNWLVPLMLGAPDMAFPRMNNMSFWLLPPSLTLLISSSIVENGAGTGWTVYPPLSSNIAHSGSSVDLAIFSLHLAGISSILGAINFITTIINMKINGLSFD
QMPLFVWAVGITALLLLLSLPVLAGAITMLLTDRNLNTSFFDPAGGGDPILYQHL
>LEFIK636-10|JF854208|MM18211|Aristotelia_brizella
TLYFIFGIWAGMLGTSLSLLIRAELGNPGSLIGDDQIYNTIVTAHAFIMIFFMVMPIMIGGFGNWLVPLMLGAPDMAFPRMNNMSFWLLPPSLTLLISSSIVENGAGTGWTVYPPLSSNIAHGGSSVDLAIFSLHLAGISSILGAINFITTIINMKINGLSFD
QMPLFVWAVGITALLLLLSLPVLAGAITMLLTDRNLNTSFFDPAGGGDPILYQHL
>LEFIB243-10|HM871146|MM00707|Aristotelia_ericinella
TLYFIFGIWAGMVGTSLSLLIRAELGNPGSLIGDDQIYNTIVTAHAFIMIFFMVMPIMIGGFGNWLVPLMLGAPDMAFPRMNNMSFWLLPPSLTLLISSSIVENGAGTGWTVYPPLSSNIAHGGSSVDLAIFSLHLAGISSILGAINFITTIINMKINGLSFD
QMPLFVWSVGITALLLLLSLPVLAGAITMLLTDRNLNTSFFDPAGGGDPILYQHL
>LEFIG756-10|HQ570408|MM15620|Aristotelia_heliacella
TLYFIFGIWAGMVGTSLSLLIRAELGNPGSLIGDDQIYNTIVTAHAFIMIFFMVMPIMIGGFGNWLVPLMLGAPDMAFPRMNNMSFWLLPPSLTLLISSSIVENGAGTGWTVYPPLSSNIAHSGSSVDLAIFSLHLAGISSILGAINFITTIINMKINGLNFD
QMPLFVWSVGITALLLLLSLPVLAGAITMLLTDRNLNTSFFDPAGGGDPILYQHL
>LEFIJ637-10|JF853735|MM17262|Aristotelia_subdecurtella
TLYFIFGIWAGMVGTSLSLLIRAELGSPGSLIGDDQIYNTIVTAHAFIMIFFMVMPIMIGGFGNWLVPLMLGAPDMAFPRMNNMSFWLLPPSLTLLISSSIVENGAGTGWTVYPPLSSNIAHGGSSVDLAIFSLHLAGISSILGAINFITTIINMKVNGLLFD
RMPLFVWSVGITAILLLLSLPVLAGAITMLLTDRNLNTSFFDPAGGGDPILYQHL
>LEFIK634-10|JF854207|MM18209|Aristotelia_subdecurtella
TLYFIFGIWAGMVGTSLSLLIRAELGNPGSLIGDDQIYNTIVTAHAFIMIFFMVMPIMIGGFGNWLVPLMLGAPDMAFPRMNNMSFWLLPPSLTLLISSSIVENGAGTGWTVYPPLSSNIAHGGSSVDLAIFSLHLAGISSILGAINFITTIINMKINGLSFD
QMPLFVWSVGITALLLLLSLPVLAGAITMLLTDRNLNTSFFDPAGGGDPILYQHL
>LEFIB751-10|HM871629|MM02598|Aroga_velocella
TLYFIFGIWAGMVGTSLSLLIRAELGNPGSLIGDDQIYNTIVTAHAFIMIFFMVMPIMIGGFGNWLVPLMLGAPDMAFPRMNNMSFWLLPPSLTLLISSSIVENGAGTGWTVYPPLSSNIAHGGSSVDLAIFSLHLAGISSILGAINFITTIINMRINGLSFD
QMPLFVWAVGITALLLLLSLPVLAGAITMLLTDRNLNTSFFDPAGGGDPILYQHL
>LEFIJ093-10|JF853440|MM06571|Aroga_velocella
TLYFIFGIWAGMVGTSLSLLIRAELGNPGSLIGDDQIYNTIVTAHAFIMIFFMVMPIMIGGFGNWLVPLMLGAPDMAFPRMNNMSFWLLPPSLTLLISSSIVENGAGTGWTVYPPLSSNIAHGGSSVDLAIFSLHLAGISSILGAINFITTIINMRINGLSFD
QMPLFVWAVGITALLLLLSLPVLAGAITMLLTDRNLNTSFFDPAGGGDPILYQHL
>LEFIE425-10|HM874149|MM09036|Aroga_velocella
TLYFIFGIWAGMVGTSLSLLIRAELGNPGSLIGDDQIYNTIVTTHAFIMIFFMVMPIMIGGFGNWLVPLMLGAPDMAFPRMNNMSFWLLPPSLTLLISSSIVENGAGTGWTVYPPLSSNIAHGGSSVDLAIFSLHLAGISSILGAINFITTIINMRINGLSFD
QMPLFVWAVGITALLLLLSLPVLAGAITMLLTDRNLNTSFFDPAGGGDPILYQHL
>LEFIF456-10|HM875141|MM11947|Aroga_velocella
TLYFIFGIWAGMVGTSLSLLIRAELGNPGSLIGDDQIYNTIVTAHAFIMIFFMVMPIMIGGFGNWLVPLMLGAPDMAFPRMNNMSFWLLPPSLTLLISSSIVENGAGTGWTVYPPLSSNIAHGGSSVDLAIFSLHLAGISSILGAINFITTIINMRINGLSFD
QMPLFVWAVGITALLLLLSLPVLAGAITMLLTDRNLNTSFFDPAGGGDPILYQHL
>LEFIG236-10|HM875915|MM14147|Aroga_velocella
TLYFIFGIWAGMVGTSLSLLIRAELGNPGSLIGDDQIYNTIVTAHAFIMIFFMVMPIMIGGFGNWLVPLMLGAPDMAFPRMNNMSFWLLPPSLTLLISSSIVENGAGTGWTVYPPLSSNIAHGGSSVDLAIFSLHLAGISSILGAINFITTIINMRINGLSFD
QMPLFVWAVGITALLLLLSLPVLAGAITMLLTDRNLNTSFFDPAGGGDPILYQHL
>LEFIE454-10|HM874178|MM09218|Aspilapteryx_tringipennella

TLYFIFGIWSGMIGTSLSMLIRAELGNPGHLIGDDQIYNTIVTAHAFIMIFFMVMPIMIGGFGNWLVPLMLGAPDMAFPRLNNMSFWLLPPSLILLISSSIVENGAGTGWTVYPPLSSNIAHSGSSVDLAIFSLHLAGISSILGAINFITTVINMRTNGMMF
DSMSLFIWAVSITALLLLLSLPVLAGAITMLLTDRNLNTSFFDPAGGGDPILYQHL
>LEFIG365-10|HM876042|MM14366|Aspilapteryx_tringipennella
TLYFIFGIWSGMIGTSLSMLIRAELGNPGHLIGDDQIYNTIVTAHAFIMIFFMVMPIMIGGFGNWLVPLMLGAPDMAFPRLNNMSFWLLPPSLILLISSSIVENGAGTGWTVYPPLSSNIAHSGSSVDLAIFSLHLAGISSILGAINFITTVINMRTSGMMF
DSMSLFIWAVSITALLLLLSLPVLAGAITMLLTDRNLNTSFFDPAGGGDPILYQHL
>LEFIJ818-10|JF853840|MM17443|Aspitates_gilvaria
TLYFIFGIWAGMVGTSLSLLIRAELGNPGSLIGDDQIYNTIVTAHAFIMIFFMVMPIMIGGFGNWLVPLMLGAPDMAFPRMNNMSFWLLPPSITLLISSSIVENGAGTGWTVYPPLSSNIAHGGSSVDLAIFSLHLAGISSILGAINFITTIINMRLNNMSFD
QMPLFVWAVGITAFLLLLSLPVLAGAITMLLTDRNLNTSFFDPAGGGDPILYQHL
>LEFIB659-10|HM871537|MM02432|Assara_terebrella
TLYFIFGIWSGMVGTSLSLLIRAELGTPGSLIGDDQIYNTIVTSHAFIMIFFMVMPIMIGGFGNWLVPLMLGAPDMAFPRMNNMSFWLLPPSLTLLISSSMVENGAGTGWTVYPPLSSNIAHGGSSVDLAIFSLHLAGISSILGAINFITTIINMKLNGLSFD
QMPLFVWSVGITALLLLLSLPVLAGAITMLLTDRNLNTSFFDPAGGGDPILYQHL
>LEFID243-10|HM873042|MM06107|Asthena_albulata
TLYFIFGIWAGMVGTSLSLLIRAELGNPGSLIGDDQIYNTIVTAHAFIMIFFMVMPIMIGGFGNWLVPLMLGAPDMAFPRMNNMSFWLLPPSITLLISSSIVENGAGTGWTVYPPLSSNIAHGGSSVDLAIFSLHLAGISSILGAINFITTIINMRLNNMFFD
QLPLFVWAVGITAFLLLLSLPVLAGAITMLLTDRNLNTSFFDPAGGGDPILYQHL
>LEFIF378-10|HM875063|MM11624|Asthena_albulata
TLYFIFGIWAGMVGTSLSLLIRAELGNPGSLIGDDQIYNTIVTAHAFIMIFFMVMPIMIGGFGNWLVPLMLGAPDMAFPRMNNMSFWLLPPSITLLISSSIVENGAGTGWTVYPPLSSNIAHGGSSVDLAIFSLHLAGISSILGAINFITTIINMRLNNMFFD
QLPLFVWAVGITAFLLLLSLPVLAGAITMLLTDRNLNTSFFDPAGGGDPILYQHL
>LEFIF379-10|HM875064|MM11625|Asthena_albulata
TLYFIFGIWAGMVGTSLSLLIRAELGNPGSLIGDDQIYNTIVTAHAFIMIFFMVMPIMIGGFGNWLVPLMLGAPDMAFPRMNNMSFWLLPPSITLLISSSIVENGAGTGWTVYPPLSSNIAHGGSSVDLAIFSLHLAGISSILGAINFITTIINMRLNNMFFD
QLPLFVWAVGITAFLLLLSLPVLAGAITMLLTDRNLNTSFFDPAGGGDPILYQHL
>LEFIL554-10|JF854657|MM18852|Asthena_anseraria
TLYFIFGIWAGMVGTSLSLLIRAELGNPGSLIGDDQIYNTIVTAHAFIMIFFMVMPIMIGGFGNWLIPLMLGAPDMAFPRMNNMSFWLLPPSITLLISSSIVENGAGTGWTVYPPLSSNIAHGGSSVDLAIFSLHLAGISSILGAINFITTIINMRLNNMFFD
QLPLFVWAVGITAFLLLLSLPVLAGAITMLLTDRNLNTSFFDPAGGGDPILYQHL
>LEFID089-10|HM872900|MM05701|Atemelia_torquatella
TLYFIFGIWAGMVGTSLSLLIRAELGNPGSLIGDDQIYNTIVTAHAFIMIFFMVMPIMIGGFGNWLVPLMLGAPDMAFPRMNNMSFWLLPPSLTLLISSSIVENGAGTGWTVYPPLSSNIAHSGSSVDLAIFSLHLAGISSILGAINFITTIINMKSNGMSFD
QMPLFVWAVGITALLLLLSLPVLAGAITMLLTDRNLNSSFFDPAGGGDPILYQHL
>LEFID895-10|HM873645|MM07214|Aterpia_chalybeia
TLYFIFGIWAGMVGTSLSLLIRAELGNPGSLIGDDQIYNTIVTAHAFIMIFFMVMPIMIGGFGNWLVPLMLGAPDMAFPRMNNMSFWLLPPSIMLLISSSIVENGAGTGWTVYPPLSSNIAHSGSSVDLAIFSLHLAGISSILGAVNFITTIINMRPNNMSL
DQMPLFVWSVGITALLLLLSLPVLAGAITMLLTDRNLNTSFFDPAGGGDPILYQHL
>LEFID094-10|HM872905|MM05706|Athetis_gluteosa
TLYFIFGIWAGMVGTSLSLLIRAELGNPGSLIGDDQIYNTIVTAHAFIMIFFMVMPIMIGGFGNWLVPLMLGAPDMAFPRMNNMSFWLLPPSLTLLISSSIVENGAGTGWTVYPPLSSNIAHGGSSVDLAIFSLHLAGISSILGAINFITTIINMRLNSLSFD
QMPLFVWAVGITAFLLLLSLPVLAGAITMLLTDRNLNTSFFDPAGGGDPILYQHL
>LEFID459-10|HM873230|MM06399|Athetis_lepigone
TLYFIFGIWAGMVGTSLSLLIRAELGNPGSLIGDDQIYNTIVTAHAFIMIFFMVMPIMIGGFGNWLVPLMLGAPDMAFPRMNNMSFWLLPPSLTLLISSSIVENGAGTGWTVYPPLSSNIAHGGSSVDLAIFSLHLAGISSILGAINFITTIINMRLNSLSFD
QMPLFIWAVGITAFLLLLSLPVLAGAITMLLTDRNLNTSFFDPAGGGDPILYQHL
>LEFIA456-10|HM386796|MM01521|Athetis_pallustris
TLYFIFGIWAGMVGTSLSLLIRAELGNPGSLIGDDQIYNTIVTAHAFIMIFFMVMPIMIGGFGNWLVPLMLGAPDMAFPRMNNMSFWLLPPSLTLLISSSIVENGAGTGWTVYPPLSSNIAHGGSSVDLAIFSLHLAGISSILGAINFITTIINMRLNNLSFD
QMPLFVWAVGITAFLLLLSLPVLAGAITMLLTDRNLNTSFFDPAGGGDPILYQHL
>LEFIB829-10|HM871706|MM02778|Athetis_pallustris
TLYFIFGIWAGMVGTSLSLLIRAELGNPGSLIGDDQIYNTIVTAHAFIMIFFMVMPIMIGGFGNWLVPLMLGAPDMAFPRMNNMSFWLLPPSLTLLISSSIVENGAGTGWTVYPPLSSNIAHGGSSVDLAIFSLHLAGISSILGAINFITTIINMRLNNLSFD
QMPLFVWAVGITAFLLLLSLPVLAGAITMLLTDRNLNTSFFDPAGGGDPILYQHL
>LEFIK142-10|JN270950|MM17717|Athrips_amoenella
TLYFIFGIWAGMVGTSLSLLIRAELGNPGSLIGDDQIYNTIVTAHAFIMIFFMVMPIMIGGFGNWLVPLMLGAPDMAFPRMNNMSFWLLPPSLTLLISSSIVENGAGTGWTVYPPLSSNIAHGGSSVDLAIFSLHLAGISSILGAINFITTIINMRINGLSFD
QMPLFVWAVGITALLLLLSLPVLAGAITMLLTDRNLNTSFFDPAGGGDPILYQHL
>LEFIC849-10|HM872667|MM05061|Athrips_mouffetella
TLYFIFGVWSGMVGTSLSLLIRAELGNPGSLIGDDQIYNTIVTAHAFIMIFFMVMPIMIGGFGNWLVPLMLGAPDMAFPRMNNMSFWLLPPSLTLLISSSIVENGAGTGWTVYPPLSSNIAHSGSSVDLAIFSLHLAGISSILGAINFITTIINMRINALSFD
QMPLFVWSVGITALLLLLSLPVLAGAITMLLTDRNLNTSFFDPAGGGDPILYQHL

>LEFIC321-10|HM872165|MM03825|Athrips_pruinosella
TLYFIFGIWSGMVGTSLSLLIRAELGNPGSLIGDDQIYNTIVTAHAFIMIFFMVMPIMIGGFGNWLVPLMLGAPDMAFPRMNNMSFWLLPPSLTLLISSSIVENGAGTGWTVYPPLSSNIAHGGSSVDLAIFSLHLAGISSILGAINFITTIINMRINGLSFD
QMPLFVWSVGITALLLLLSLPVLAGAITMLLTDRNLNTSFFDPAGGGDPILYQHL
>LEFIA797-10|HM386938|MM04151|Athrips_pruinosella
TLYFIFGIWSGMVGTSLSLLIRAELGNPGSLIGDDQIYNTIVTAHAFIMIFFMVMPIMIGGFGNWLVPLMLGAPDMAFPRMNNMSFWLLPPSLTLLISSSIVENGAGTGWTVYPPLSSNIAHGGSSVDLAIFSLHLAGISSILGAINFITTIINMRINGLSFD
QMPLFVWSVGITALLLLLSLPVLAGAITMLLTDRNLNTSFFDPAGGG?PILYQHL
>LEFIB598-10|HM871477|MM02312|Athrips_tetrapunctella
TLYFIFGVWAGMVGTSLSLLIRAELGNPGSLIGDDQIYNTIVTAHAFIMIFFMVMPIMIGGFGNWLVPLMLGAPDMAFPRMNNMSFWLLPPSLTLLISSSVVENGAGTGWTVYPPLSSNIAHGGSSVDLAIFSLHLAGISSILGAINFITTIINMRINGLSF
DQMPLFVWAVGITALLLLLSLPVLAGAITMLLTDRNLNTSFFDPAGGGDPILYQHL
>LEFIC390-10|HM872233|MM03928|Athrips_tetrapunctella
TLYFIFGVWAGMVGTSLSLLIRAELGNPGSLIGDDQIYNTIVTAHAFIMIFFMVMPIMIGGFGNWLVPLMLGAPDMAFPRMNNMSFWLLPPSLTLLISSSVVENGAGTGWTVYPPLSSNIAHGGSSVDLAIFSLHLAGISSILGAINFITTIINMRINGLSF
DQMPLFVWAVGITALLLLLSLPVLAGAITMLLTDRNLNTSFFDPAGGGDPILYQHL
>LEFIA095-10|HM396442|MM01017|Atolmis_rubricollis
TLYFIFGIWAGMLGTSLSLLIRAELGNPGSLIGDDQIYNTIVTAHAFIMIFFMVMPIMIGGFGNWLIPLMLGAPDMAFPRMNNMSFWLLPPSLTLLISSSIVENGAGTGWTVYPPLSSNIAHSGSSVDLAIFSLHLAGISSILGAINFITTIINMRLNKLMFD
QMPLFVWAVGITAFLLLLSLPVLAGAITMLLTDRNLNTSFFDPAGGGDPILYQHL
>LEFIJ341-10|MM15941|Atypha_pulmonaris
TLYFIFGIWAGMVGTSLSLLIRAELGNPGSLIGDDQIYNTIVTAHAFIMIFFMVMPIMIGGFGNWLVPLMLGAPDMAFPRLNNMSFWLLPPSLTLLISSSIVENGAGTGWTVYPPLSSNIAHGGSSVDLAIFSLHLAGISSILGAINFITTIINMRLNNLSFDQ
MPLFIWAVGITAFLLLLSLPVLAGAITMLLTDRNLNTSFFDPAGGGDPILYQHL
>LEFIA209-10|HM396553|MM01232|Autographa_bractea
TLYFIFGIWAGMVGTSLSLLIRAELGTPGSLIGDDQIYNTIVTAHAFIMIFFMVMPIMIGGFGNWLVPLMLGAPDMAFPRMNNMSFWLLPPSLTLLISSSIVENGAGTGWTVYPPLSSNIAHGGSSVDLAIFSLHLAGISSILGAINFITTIINMRLNSLSFD
QMPLFIWAVGITAFLLLLSLPVLAGAITMLLTDRNLNTSFFDPAGGGDPILYQHL
>LEFIA191-10|HM396536|MM01184|Autographa_buraetica
TLYFIFGIWAGMVGTSLSLLIRAELGTPGSLIGDDQIYNTIVTAHAFIMIFFMVMPIMIGGFGNWLVPLMLGAPDMAFPRMNNMSFWLLPPSLTLLISSSIVENGAGTGWTVYPPLSSNIAHGGSSVDLAIFSLHLAGISSILGAINFITTIINMRLNSLSFD
QMPLFIWAVGITAFLLLLSLPVLAGAITMLLTDRNLNTSFFDPAGGGDPILYQHL
>LEFIA233-10|HM396576|MM01265|Autographa_buraetica
TLYFIFGIWAGMVGTSLSLLIRAELGTPGSLIGDDQIYNTIVTAHAFIMIFFMVMPIMIGGFGNWLVPLMLGAPDMAFPRMNNMSFWLLPPSLTLLISSSIVENGAGTGWTVYPPLSSNIAHGGSSVDLAIFSLHLAGISSILGAINFITTIINMRLNSLSFD
QMPLFIWAVGITAFLLLLSLPVLAGAITMLLTDRNLNTSFFDPAGGGDPILYQHL
>LEFIC594-10|HM872415|MM04514|Autographa_buraetica
TLYFIFGIWAWMVGTSLSLLIRAELGTPGSLIGDDQIYNTIVTAHAFIMIFFMVMPIMIGGFGNWLVPLMLGAPDMAFPRMNNMSFWLLPPSLTLLISSSIVENGAGTGWTVYPPLSSNIAHGGSSVDLAIFSLHLAGISSILGAINFITTIINMRLNSLSFD
QMPLFIWAVGITAFLLLLSLPVLAGAITMLLTDRNLNTSFFDPAGGGDPILYQHL
>LEFIC600-10|HM872421|MM04520|Autographa_buraetica
TLYFIFGIWAGMVGTSLSLLIRAELGTPGSLIGDDQIYNTIVTAHAFIMIFFMVMPIMIGGFGNWLVPLMLGAPDMAFPRMNNMSFWLLPPSLTLLISSSIVENGAGTGWTVYPPLSSNIAHGGSSVDLAIFSLHLAGISSILGAINFITTIINMRLNSLSFD
QMPLFIWAVGITAFLLLLSLPVLAGAITMLLTDRNLNTSFFDPAGGGDPILYQHL
>LEFIF169-10|HM874863|MM10718|Autographa_buraetica
TLYFIFGIWAGMVGTSLSLLIRAELGTPGSLIGDDQIYNTIVTAHAFIMIFFMVMPIMIGGFGNWLVPLMLGAPDMAFPRMNNMSFWLLPPSLTLLISSSIVENGAGTGWTVYPPLSSNIAHGGSSVDLAIFSLHLAGISSILGAINFITTIINMRLNSLSFD
QMPLFIWAVGITAFLLLLSLPVLAGAITMLLTDRNLNTSFFDPAGGGDPILYQHL
>LEFIH172-10|HM876792|MM15140|Autographa_buraetica
TLYFIFGIWAGMVGTSLSLLIRAELGTPGSLIGDDQIYNTIVTAHAFIMIFFMVMPIMIGGFGNWLVPLMLGAPDMAFPRMNNMSFWLLPPSLTLLISSSIVENGAGTGWTVYPPLSSNIAHGGSSVDLAIFSLHLAGISSILGAINFITTIINMRLNSLSFD
QMPLFIWAVGITAFLLLLSLPVLAGAITMLLTDRNLNTSFFDPAGGGDPILYQHL
>LEFIA056-10|HM396404|MM00962|Autographa_excelsa
TLYFIFGIWAGMVGTSLSLLIRAELGTPGSLIGDDQIYNTIVTAHAFIMIFFMVMPIMIGGFGNWLVPLMLGAPDMAFPRMNNMSFWLLPPSLTLLISSSIVENGAGTGWTVYPPLSSNIAHGGSSVDLAIFSLHLAGISSILGAINFITTIINMRLNSLSFD
QMPLFIWAVGITAFLLLLSLPVLAGAITMLLTDRNLNTSFFDPAGGGDPILYQHL
>LEFIC580-10|HM872401|MM04361|Autographa_gamma
TLYFIFGIWAGMVGTSLSLLIRAELGTPGSLIGDDQIYNTIVTAHAFIMIFFMVMPIMIGGFGNWLVPLMLGAPDMAFPRMNNMSFWLLPPSLTLLISSSIVENGAGTGWTVYPPLSSNIAHGGSSVDLAIFSLHLAGISSILGAINFITTIINMRLNSLSFD
QMPLFIWAVGITAFLLLLSLPVLAGAITMLLTDRNLNTSFFDPAGGGDPILYQHL
>LEFIA190-10|HM396535|MM01183|Autographa_jota
TLYFIFGIWAGMVGTSLSLLIRAELGTPGSLIGDDQIYNTIVTAHAFIMIFFMVMPIMIGGFGNWLVPLMLGAPDMAFPRMNNMSFWLLPPSLTLLISSSIVENGAGTGWTVYPPLSSNIAHGGSSVDLAIFSLHLAGISSILGAINFITTIINMRLNSLSFD

QMPLFIWAVGITAFLLLLSLPVLAGAITMLLTDRNLNTSFFDPAGGGDPILYQHL
>LEFIG994-10|HM876631|MM15858|Autographa_macrogamma
TLYFIFGIWAGMVGTSLSLLIRAELGTPGSLIGDDQIYNTIVTAHAFIMIFFMVMPIMIGGFGNWLVPLMLGAPDMAFPRMNNMSFWLLPPSLTLLISSSIVENGAGTGWTVYPPLSSNIAHGGSSVDLAIFSLHLAGISSILGAINFITTIINMRLNSLSFD
QMPLFIWAVGITAFLLLLSLPVLAGAITMLLTDRNLNTSFFDPAGGGDPILYQHL
>LEFIG995-10|HM876632|MM15859|Autographa_macrogamma
TLYFIFGIWAGMVGTSLSLLIRAELGTPGSLIGDDQIYNTIVTAHAFIMIFFMVMPIMIGGFGNWLVPLMLGAPDMAFPRMNNMSFWLLPPSLTLLISSSIVENGAGTGWTVYPPLSSNIAHGGSSVDLAIFSLHLAGISSILGAINFITTIINMRLNSLSFD
QMPLFIWAVGITAFLLLLSLPVLAGAITMLLTDRNLNTSFFDPAGGGDPILYQHL
>LEFIA630-10|HM870879|MM01745|Autographa_mandarina
TLYFIFGIWAGMVGTSLSLLIRAELGTPGSLIGDDQIYNTIVTAHAFIMIFFMVMPIMIGGFGNWLVPLMLGAPDMAFPRMNNMSFWLLPPSLTLLISSSIVENGAGTGWTVYPPLSSNIAHGGSSVDLAIFSLHLAGISSILGAINFITTIINMRLNSLSFD
QMPLFIWAVGITAFLLLLSLPVLAGAITMLLTDRNLNTSFFDPAGGGDPILYQHL
>LEFIA193-10|HM396538|MM01186|Autographa_pulchrina
TLYFIFGIWAGMVGTSLSLLIRAELGTPGSLIGDDQIYNTIVTAHAFIMIFFMVMPIMIGGFGNWLVPLMLGAPDMAFPRMNNMSFWLLPPSLTLLISSSIVENGAGTGWTVYPPLSSNIAHGGSSVDLAIFSLHLAGISSILGAINFITTIINMRLNSLSFD
QMPLFIWAVGITAFLLLLSLPVLAGAITMLLTDRNLNTSFFDPAGGGDPILYQHL
>LEFIA195-10|HM396540|MM01188|Autographa_pulchrina
TLYFIFGIWAGMVGTSLSLLIRAELGTPGSLIGDDQIYNTIVTAHAFIMIFFMVMPIMIGGFGNWLVPLMLGAPDMAFPRMNNMSFWLLPPSLTLLISSSIVENGAGTGWTVYPPLSSNIAHGGSSVDLAIFSLHLAGISSILGAINFITTIINMRLNSLSFD
QMPLFIWAVGITAFLLLLSLPVLAGAITMLLTDRNLNTSFFDPAGGGDPILYQHL
>LEFIA196-10|HM396541|MM01189|Autographa_pulchrina
TLYFIFGIWAWMVGTSLSLLIRAELGTPGSLIGDDQIYNTIVTAHAFIMIFFMVMPIMIGGFGNWLVPLMLGAPDMAFPRMNNMSFWLLPPSLTLLISSSIVENGAGTGWTVYPPLSSNIAHGGSSVDLAIFSLHLAGISSILGAINFITTIINMRLNSLSFD
QMPLFIWAVGITAFLLLLSLPVLAGAITMLLTDRNLNTSFFDPAGGGDPILYQHL
>LEFIB393-10|HM871292|MM01197|Autographa_pulchrina
TLYFIFGIWAGMVGTSLSLLIRAELGTPGSLIGDDQIYNTIVTAHAFIMIFFMVMPIMIGGFGNWLVPLMLGAPDMAFPRMNNMSFWLLPPSLTLLISSSIVENGAGTGWTVYPPLSSNIAHGGSSVDLAIFSLHLAGISSILGAINFITTIINMRLNSLSFD
QMPLFIWAVGITAFLLLLSLPVLAGAITMLLTDRNLNTSFFDPAGGGDPILYQHL
>LEFIB398-10|HM871297|MM01202|Autographa_pulchrina
TLYFIFGIWAGMVGTSLSLLIRAELGTPGSLIGDDQIYNTIVTAHAFIMIFFMVMPIMIGGFGNWLVPLMLGAPDMAFPRMNNMSFWLLPPSLTLLISSSIVENGAGTGWTVYPPLSSNIAHGGSSVDLAIFSLHLAGISSILGAINFITTIINMRLNSLSFD
QMPLFIWAVGITAFLLLLSLPVLAGAITMLLTDRNLNTSFFDPAGGGDPILYQHL
>LEFIC605-10|HM872426|MM04525|Autographa_pulchrina
TLYFIFGIWAGMVGTSLSLLIRAELGTPGSLIGDDQIYNTIVTAHAFIMIFFMVMPIMIGGFGNWLVPLMLGAPDMAFPRMNNMSFWLLPPSLTLLISSSIVENGAGTGWTVYPPLSSNIAHGGSSVDLAIFSLHLAGISSILGAINFITTIINMRLNSLSFD
QMPLFIWAVGITAFLLLLSLPVLAGAITMLLTDRNLNTSFFDPAGGGDPILYQHL
>LEFIE121-10|HM873869|MM08326|Autographa_pulchrina
TLYFIFGIWAGMVGTSLSLLIRAELGTPGSLIGDDQIYNTIVTAHAFIMIFFMVMPIMIGGFGNWLVPLMLGAPDMAFPRMNNMSFWLLPPSLTLLISSSIVENGAGTGWTVYPPLSSNIAHGGSSVDLAIFSLHLAGISSILGAINFITTIINMRLNSLSFD
QMPLFIWAVGITAFLLLLSLPVLAGAITMLLTDRNLNTSFFDPAGGGDPILYQHL
>LEFIE175-10|HM873921|MM08448|Autographa_pulchrina
TLYFIFGIWDGMVGTSLSLLIRAELGTPGSLIGDDQIYNTIVTAHAFIMIFFMVMPIMIGGFGNWLVPLMLGAPDMAFPRMNNMSFWLLPPSLTLLISSSIVENGAGTGWTVYPPLSSNIAHGGSSVDLAIFSLHLAGISSILGAINFITTIINMRLNSLSFD
QMPLFIWAVGITAFLLLLSLPVLAGAITMLLTDRNLNTSFFDPAGGGDPILYQHL
>LEFIF658-10|HM875342|MM12595|Autographa_pulchrina
TLYFIFGIWAGMVGTSLSLLIRAELGTPGSLIGDDQIYNTIVTAHAFIMIFFMVMPIMIGGFGNWLVPLMLGAPDMAFPRMNNMSFWLLPPSLTLLISSSIVENGAGTGWTVYPPLSSNIAHGGSSVDLAIFSLHLAGISSILGAINFITTIINMRLNSLSFD
QMPLFIWAVGITAFLLLLSLPVLAGAITMLLTDRNLNTSFFDPAGGGDPILYQHL
>LEFIG201-10|HM875881|MM14084|Autographa_pulchrina
TLYFIFGIWAWMVGTSLSLLIRAELGTPGSLIGDDQIYNTIVTAHAFIMIFFMVMPIMIGGFGNWLVPLMLGAPDMAFPRMNNMSFWLLPPSLTLLISSSIVENGAGTGWTVYPPLSSNIAHGGSSVDLAIFSLHLAGISSILGAINFITTIINMRLNSLSFD
QMPLFIWAVGITAFLLLLSLPVLAGAITMLLTDRNLNTSFFDPAGGGDPILYQHL
>LEFIH156-10|HM876776|MM15093|Autographa_pulchrina
TLYFIFGIWAGMVGTSLSLLIRAELGTPGSLIGDDQIYNTIVTAHAFIMIFFMVMPIMIGGFGNWLVPLMLGAPDMAFPRMNNMSFWLLPPSLTLLISSSIVENGAGTGWTVYPPLSSNIAHGGSSVDLAIFSLHLAGISSILGAINFITTIINMRLNSLSFD
QMPLFIWAVGITAFLLLLSLPVLAGAITMLLTDRNLNTSFFDPAGGGDPILYQHL
>LEFIC869-10|HM872687|MM05142|Axylia_putris
TLYFIFGIWAGMVGTSLSLLIRAELGNPGSLIGDDQIYNTIVTAHAFIMIFFMVMPIMIGGFGNWLVPLMLGAPDMAFPRMNNMSFWLLPPSLTLLISSSIVENGAGTGWTVYPPLSSNIAHGGSSVDLAIFSLHLAGISSILGAINFITTIINMRLNSLSFD
QMPLFIWAVGITAFLLLLSLPVLAGAITMLLTDRNLNTSFFDPAGGGDPILYQHL
>LEFIB654-10|HM871533|MM02423|Bactra_furfurana

TLYFIFGIWAGMVGTSLSLLIRAELGNPGSLIGDDQIYNTIVTAHAFIMIFFMVMPIMIGGFGNWLVPLMLGAPDMAFPRMNNMSFWLLPPSIMLLISSSIVENGAGTGWTVYPPLSSNIAHSGSSVDLTIFSLHLAGISSILGAVNFITTIINMRPNKMSL
DQMPLFVWAVGITALLLLLSLPVLAGAITMLLTDRNLNTSFFDPAGGGDPILYQHL
>LEFID546-10|HM873311|MM06534|Bactra_furfurana
TLYFIFGIWAGMVGTSLSLLIRAELGNPGSLIGDDQIYNTIVTAHAFIMIFFMVMPIMIGGFGNWLVPLMLGAPDMAFPRMNNMSFWLLPPSIMLLISSSIVENGAGTGWTVYPPLSSNIAHSGSSVDLTIFSLHLAGISSILGAVNFITTIINMRPNKMSL
DQMPLFVWAVGITALLLLLSLPVLAGAITMLLTDRNLNTSFFDPAGGGDPILYQHL
>LEFID769-10|HM873526|MM06835|Bactra_lacteana
TLYFIFGIWAGMVGTSLSLLIRAELGNPGSLIGDDQIYNTIVTAHAFIMIFFMVMPIMIGGFGNWLVPLMLGAPDMAFPRMNNMSFWLLPPSIMLLISSSIVENGAGTGWTVYPPLSSNIAHSGSSVDLTIFSLHLAGISSILGAINFITTIINMRPNKMSL
DQMPLFVWAVGITALLLLLSLPVLAGAITMLLTDRNLNTSFFDPAGGGDPILYQHL
>LEFIG300-10|HM875979|MM14258|Bactra_lacteana
TLYFIFVIWAGMVGTSLSLLIRAELGNPGSLIGDDQIYNTIVTAHAFIMIFFMVMPIMIGGFGNWLVPLMLGAPDMAFPRMNNMSFWLLPPSIMLLISSSIVENGAGTGWTVYPPLSSNIAHSGSSVDLTIFSLHLAGISSILGAINFITTIINMRPNKMSLD
QMPLFVWAVGITALLLLLSLPVLAGAITMLLTDRNLNTSFFDPAGGGDPILYQHL
>LEFIB670-10|HM871548|MM02453|Bactra_lancealana
TLYFIFGIWAGMVGTSLSLLIRAELGNPGSLIGDDQIYNTIVTAHAFIMIFFMVMPIMIGGFGNWLVPLMLGAPDMAFPRMNNMSFWLLPPSIMLLISSSIVENGAGTGWTVYPPLSSNIAHSGSSVDLAIFSLHLAGISSILGAVNFITTIINMRPNNMSL
DQMPLFVWSVGITALLLLLSLPVLAGAITMLLTDRNLNTSFFDPAGGGDPILYQHL
>LEFIB671-10|HM871549|MM02454|Bactra_lancealana
TLYFIFGIWAGMVGTSLSLLIRAELGNPGSLIGDDQIYNTIVTAHAFIMIFFMVMPIMIGGFGNWLVPLMLGAPDMAFPRMNNMSFWLLPPSIMLLISSSIVENGAGTGWTVYPPLSSNIAHSGSSVDLAIFSLHLAGISSILGAVNFITTIINMRPNNMSL
DQMPLFVWSVGITALLLLLSLPVLAGAITMLLTDRNLNTSFFDPAGGGDPILYQHL
>LEFIB917-10|HM871794|MM03043|Bactra_lancealana
TLYFIFGIWAGMVGTSLSLLIRAELGNPGSLIGDDQIYNTIVTAHAFIMIFFMVMPIMIGGFGNWLVPLMLGAPDMAFPRMNNMSFWLLPPSIMLLISSSIVENGAGTGWTVYPPLSSNIAHSGSSVDLAIFSLHLAGISSILGAVNFITTIINMRPNNMSL
DQMPLFVWSVGITALLLLLSLPVLAGAITMLLTDRNLNTSFFDPAGGGDPILYQHL
>LEFIC377-10|HM872220|MM03907|Bactra_lancealana
TLYFIFGIWAGMVGTSLSLLIRAELGNPGSLIGDDQIYNTIVTAHAFIMIFFMVMPIMIGGFGNWLVPLMLGAPDMAFPRMNNMSFWLLPPSIMLLISSSIVENGAGTGWTVYPPLSSNIAHSGSSVDLAIFSLHLAGISSILGAVNFITTIINMRPNNMSL
DQMPLFVWSVGITALLLLLSLPVLAGAITMLLTDRNLNTSFFDPAGGGDPILYQHL
>LEFIE724-10|HM874444|MM09817|Bactra_lancealana
TLYFIFGIWAGMVGTSLSLLIRAELGNPGSLIGDDQIYNTIVTAHAFIMIFFMVMPIMIGGFGNWLVPLMLGAPDMAFPRMNNMSFWLLPPSIMLLISSSIVENGAGTGWTVYPPLSSNIAHSGSSVDLAIFSLHLAGISSILGAVNFITTIINMRPNNMSL
DQMPLFVWSVGITALLLLLSLPVLAGAITMLLTDRNLNTSFFDPAGGGDPILYQHL
>LEFIG249-10|HM875928|MM14172|Bactra_lancealana
TLYFIFGIWAGMVGTSLSLLIRAELGNPGSLIGDDQIYNTIVTAHAFIMIFFMVMPIMIGGFGNWLVPLMLGAPDMAFPRMNNMSFWLLPPSIMLLISSSIVENGAGTGWTVYPPLSSNIAHSGSSVDLAIFSLHLAGISSILGAVNFITTIINMRPNNMSL
DQMPLFVWSVGITALLLLLSLPVLAGAITMLLTDRNLNTSFFDPAGGGDPILYQHL
>LEFIK203-10|KM573430|MM17778|Bactra_lancealana
TLYFIFGIWAGMVGTSLSLLIRAELGNPGSLIGDDQIYNTIVTAHAFIMIFFMVMPIMIGGFGNWLVPLMLGAPDMAFPRMNNMSFWLLPPSIMLLISSSIVENGAGTGWTVYPPLSSNIAHSGSSVDLAIFSLHLAGISSILGAVNFITTIINMRPNNMSL
DQMPLFVWSVGITALLLLLSLPVLAGAITMLLTDRNLNTSFFDPAGGGDPILYQHL
>LEFIF235-10|HM874928|MM11015|Bactra_robustana
TLYFIFGIWAGMVGTSLSLLIRAELGNPGSLIGDDQIYNTIVTAHAFIMIFFMVMPIMIGGFGNWLVPLMLGAPDMAFPRMNNMSFWLLPPSIMLLISSSIVENGAGTGWTVYPPLSSNIAHSGSSVDLAIFSLHLAGISSILGAVNFITTIINMRPNNMSL
DQMPLFVWSVGITALLLLLSLPVLAGAITMLLTDRNLNTSFFDPAGGGDPILYQHL
>LEFIK022-10|MM17597|Bactra_robustana
TLYFIFGIWAGMVGTSLSLLIRAELGNPGSLIGDDQIYNTIVTAHAFIMIFFMVMPIMIGGFGNWLVPLMLGAPDMAFPRMNNMSFWLLPPSIMLLISSSIVENGAGTGWTVYPPLSSNIAHSGSSVDLAIFSLHLAGISSILGAVNFITTIINMRPNNMSL
DQMPLFVWSVGITALLLLLSLPVLAGAITMLLTDRNLNTSFFDPAGGGDPILYQHL
>LEFIJ635-10|JF853733|MM17260|Bactra_suedana
TLYFIFGIWAGMVGTSLSLLIRAELGNPGSLIGNDQIYNTIVTAHAFIMIFFMVMPIMIGGFGNWLVPLMLGAPDMAFPRMNNMSFWLLPPSIMLLISSSIVENGAGTGWTVYPPLSSNIAHSGSSVDLTIFSLHLAGISSILGAINFITTIINMRPNKMSL
DQMPLFVWAVGITALLLLLSLPVLAGAITMLLTDRNLNTSFFDPAGGGDPILYQHL
>LEEUA224-11|JN274922|MM19632|Bactra_suedana
TLYFIFGIWAGMVGTSLSLLIRAELGNPGSLIGNDQIYNTIVTAHAFIMIFFMVMPIMIGGFGNWLVPLMLGAPDMAFPRMNNMSFWLLPPSIMLLISSSIVENGAGTGWTVYPPLSSNIAHSGSSVDLTIFSLHLAGISSILGAINFITTIINMRPNKMSL
DQMPLFVWAVGITALLLLLSLPVLAGAITMLLTDRNLNTSFFDPAGGGDPILYQHL
>LEEUA045-11|JN285985|MM19358|Bankesia_conspurcatella
TLYFILGIWSGMMGASLSMLIRVELGSPNSLLDSDQIYNTIVTAHALIMIFFMVMPIMIGGFGNWLLPLMLGAPDMAFPRMNNMSFWLLPPSLMLLITSSIVEDGVGTGWTLYPPLSSNLSHSGSSVDLAIFSLHLAGISSILGAVNFITTVINMRPINMS
LDQMPLFVWSVAITALLLLLSLPVLAGAITMLLTDRNLNTSFFDPAGGGDPILFQHL

>LEFIF031-10|HM874742|MM10471|Baptria_tibiale
TLYFIFGIWAGMIGTSLSLLIRAELGNPGSLIGDDQIYNTIVTAHAFIMIFFMVMPIMIGGFGNWLVPLMLGAPDMAFPRMNNMSFWLLPPSIVLLISSSIVENGAGTGWTVYPPLSSNIAHGGSSVDLAIFSLHLAGISSILGAINFITTIINMRLNNMFFD
QLPLFVWAVGITAFLLLLSLPVLAGAITMLLTDRNLNTSFFDPAGGGDPILYQHL
>LEFIJ811-10|JF853836|MM17436|Baptria_tibiale
TLYFIFGIWAGMIGTSLSLLIRAELGNPGSLIGDDQIYNTIVTAHAFIMIFFMVMPIMIGGFGNWLVPLMLGAPDMAFPRMNNMSFWLLPPSIVLLISSSIVENGAGTGWTVYPPLSSNIAHGGSSVDLAIFSLHLAGISSILGAINFITTIINMRLNNMFFD
QLPLFVWAVGITAFLLLLSLPVLAGAITMLLTDRNLNTSFFDPAGGGDPILYQHL
>LEFID647-10|HM873409|MM06674|Batrachedra_pinicolella
TLYFIFGIWAGMVGTSLSLLIRAELGNPGSLIGDDQIYNTIVTAHAFIMIFFMVMPIMIGGFGNWLVPLMLGAPDMAFPRMNNMSFWLLPPSLTLLISSSIVENGAGTGWTVYPPLSSNIAHSGSSVDLAIFSLHLAGISSILGAINFITTIINMRLTNLSFD
QLPLFVWSVGITALLLLLSLPVLAGAITMLLTDRNLNTSFFDPAGGGDPILYQHL
>LEFID667-10|HM873429|MM06705|Batrachedra_pinicolella
TLYFIFGIWAGMVGTSLSLLIRAELGNPGSLIGDDQIYNTIVTAHAFIMIFFMVMPIMIGGFGNWLIPLMLGAPDMAFPRMNNMSFWLLPPSLMLLISSSIVENGAGTGWTVYPPLSSNIAHSGSSVDLTIFSLHLAGISSILGAINFITTIINMRLTNLSFD
QLPLFVWSVGITALLLLLSLPVLAGAITMLLTDRNLNTSFFDPAGGGDPILYQHL
>LEFIC842-10|HM872660|MM05053|Batrachedra_praeangusta
TLYFIFGIWAGMVGTSLSLLIRAELGNPGSLIGDDQIYNTIVTAHAFIMIFFMVMPIMIGGFGNWLVPLMLGAPDMAFPRMNNMSFWLLPPSLTLLISSSIVENGAGTGWTVYPPLSSNIAHSGSSVDLAIFSLHLAGISSILGAINFITTIINMRLSNLSFD
QLPLFVWSVGITALLLLLSLPVLAGAITMLLTDRNLNTSFFDPAGGGDPILYQHL
>LEFIL296-10|MM19296|Bembecia_ichneumoniformis
TLYFIFGIWAGLVGTTLSLLIRAELGTPGSLIGDDQIYNTIVTAHAFIMIFFMVMPIMIGGFGNWLVPLMLGAPDMAFPRMNNMSFWLLPPSLLLLISSSIVENGAGTGWTVYPPLSSNIAHGGGSVDLAIFSLHLAGISSILGAVNFITTIINMRPINMSLD
QMPLFVWSVGITALLLLLSLPVLAGAITMLLTDRNLNTSFFDPAGGGDPILYQHL
>LEFIL297-10|MM19297|Bembecia_ichneumoniformis
TLYFIFGIWAGLVGTTLSLLIRAELGTPGSLIGDDQIYNTIVTAHAFIMIFFMVMPIMIGGFGNWLVPLMLGAPDMAFPRMNNMSFWLLPPSLLLLISSSIVENGAGTGWTVYPPLSSNIAHGGGSVDLAIFSLHLAGISSILGAVNFITTIINMRPINMSLD
QMPLFVWSVGITALLLLLSLPVLAGAITMLLTDRNLNTSFFDPAGGGDPILYQHL
>LEEUA197-11|JN279253|MM19605|Bembecia_ichneumoniformis
TLYFIFGIWAGLVGTTLSLLIRAELGTPGSLIGDDQIYNTIVTAHAFIMIFFMVMPIMIGGFGNWLVPLMLGAPDMAFPRMNNMSFWLLPPSLLLLISSSIVENGAGTGWTVYPPLSSNIAHGGGSVDLAIFSLHLAGISSILGAVNFITTIINMRPINMSLD
QMPLFVWSVGITALLLLLSLPVLAGAITMLLTDRNLNTSFFDPAGGGDPILYQHL
>LEFIB256-10|HM871159|MM00728|Bena_bicolorana
TLYFIFGIWAGMVGTSLSLLIRAELGNPGSLIGDDQIYNTIVTAHAFIMIFFMVMPIMIGGFGNWLVPLMLGAPDMAFPRMNNMSFWLLPPSLTLLISSSIVENGAGTGWTVYPPLSSNIAHGGSSVDLAIFSLHLAGISSILGAINFITTIINMRLNNLSFD
QMPLFVWAVGITAFLLLLSLPVLAGAITMLLTDRNLNTSFFDPAGGGDPILYQHL
>LEFIA890-10|HM387026|MM09767|Bena_bicolorana
TLYFIFGIWAGMVGTSLSLLIRAELGNPGSLIGDDQIYNTIVTAHAFIMIFFMVMPIMIGGFGNWLVPLMLGAPDMAFPRMNNMSFWLLPPSLTLLISSSIVENGAGTGWTVYPPLSSNIAHGGSSVDLAIFSLHLAGISSILGAINFITTIINMRLNNLSFD
QMPLFVWAVGITAFLLLLSLPVLAGAITMLLTDRNLNTSFFDPAGGGDPILYQHL
>LEFIE479-10|HM874203|MM09254|Bisigna_procerella
TLYFIFGIWAGMVGTSLSLLIRAELGNPGSLIGDDQIYNTIVTAHAFIMIFFMVMPIMIGGFGNWLIPLMLGAPDMAFPRMNNMSFWLLPPSLTLLISSSIVENGAGTGWTVYPPLSSNIAHGGSSVDLAIFSLHLAGISSILGAINFITTIINMRMNNMSF
DQMPLFVWAVGITALLLLLSLPVLAGAITMLLTDRNLNTSFFDPAGGGDPILYQHL
>LEFIF984-10|HM875665|MM13578|Bisigna_procerella
TLYFIFGIWAGMVGTSLSLLIRAELGNPGSLIGDDQIYNTIVTAHAFIMIFFMVMPIMIGGFGNWLIPLMLGAPDMAFPRMNNMSFWLLPPSLTLLISSSIVENGAGTGWTVYPPLSSNIAHGGSSVDLAIFSLHLAGISSILGAINFITTIINMRMNNMSF
DQMPLFVWAVGITALLLLLSLPVLAGAITMLLTDRNLNTSFFDPAGGGDPILYQHL
>LEFIB836-10|HM871713|MM02799|Biston_betularia
TLYFIFGIWAGMIGTSLSLLIRAELGNPGSLIGDDQIYNTIVTAHAFIMIFFMVMPIMIGGFGNWLVPLMLGAPDMAFPRMNNMSFWLLPPSITLLISSSIVESGAGTGWTVYPPLSSNIAHGGSSVDLAIFSLHLAGISSILGAINFITTIINMRLNKLSFDQ
MPLFVWAVGITAFLLLLSLPVLAGAITMLLTDRNLNTSFFDPAGGGDPILYQHL
>LEFIB622-10|HM871501|MM02367|Biston_strataria
TLYFIFGIWAGMVGTSLSLLIRAELGNPGSLIGDDQIYNTIVTAHAFIMIFFMVMPIMIGGFGNWLVPLMLGAPDMAFPRMNNMSFWLLPPSITLLISSSIVESGAGTGWTVYPPLSSNIAHGGSSVDLAIFSLHLAGISSILGAINFITTIINMRLNKLSFDQ
MPLFVWAVGITAFLLLLSLPVLAGAITMLLTDRNLNTSFFDPAGGGDPILYQHL
>LEFIC654-10|HM872475|MM04607|Biston_strataria
TLYFIFGIWAGMVGTSLSLLIRAELGNPGSLIGDDQIYNTIVTAHAFIMIFFMVMPIMIGGFGNWLVPLMLGAPDMAFPRMNNMSFWLLPPSITLLISSSIVESGAGTGWTVYPPLSSNIAHGGSSVDLAIFSLHLAGISSILGAINFITTIINMRLNKLSFDQ
MPLFVWAVGITAFLLLLSLPVLAGAITMLLTDRNLNTSFFDPAGGGDPILYQHL
>LEFII244-11|MM19894|Blastodacna_atra
TLYFIFGIWAGMVGTSLSLLIRAELGNPGMLIGDDQIYNTIVTAHAFIMIFFMVMPIMIGGFGNWLVPLMLSAPDMAFPRMNNMSFWLLPPSIILLISSSLVETGAGTGWTVYPPLSSNIAHSGSSVDLAIFSLHLAGISSILGAINFITTIINMRLNNMSFD

QMPLFVWAVGITALLLLLSLPVLAGAITMLLTDRNLNTSFFDPAGGGDPILYQHL
>LEEUA115-11|MM19523|Blastodacna_hellerella
TLYFIFGIWAGMVGTSLSLLIRAELGNPGMLIGDDQIYNTIVTAHAFIMIFFMVMPIMIGGFGNWLVPLMLGAPDMAFPRMNNMSFWLLPPSIILLISSSLVETGAGTGWTVYPPLSSNIAHSGSSVDLAIFSLHLAGISSILGAINFITTIINMRLNNMSFD
QMPLFVWAVGITALLLLLSLPVLAGAITMLLTDRNLNTSFFDPAGGGDPILYQHL
>LEFID989-10|HM873739|MM08022|Blepharita_amica
TLYFIFGIWAGMVGTSLSLLIRAELGNPGSLIGDDQIYNTIVTAHAFIMIFFMVMPIMIGGFGNWLVPLMLGAPDMAFPRMNNMSFWLLPPSLTLLISSSIVENGAGTGWTVYPPLSSNIAHGGSSVDLAIFSLHLAGISSILGAINFITTIINMRLNNLSFD
QMPLFIWAVGITAFLLLLSLPVLAGAITMLLTDRNLNTSFFDPAGGGDPILYQHL
>LEFID783-10|HM873540|MM06852|Bohemannia_pulverosella
TMYFIFGIWSGMVGTSLSLLIRAELGNPGSLIGDDQIYNTIVTAHAFIMIFFMVMPIMIGGFGNWLVPLMLGAPDMAFPRLNNMSFWLLPPSLMLLVSSSMVENGAGTGWTVYPPLSTNIAHSGSSVDLTIFSLHLAGISSILGAINFITTVINMRPNKM
SFDQMPLFVWAVVITALLLLLSLPVLAGAITMLLTDRNLNTSFFDPAGGGDPILYQHL
>LEFIE310-10|HM874036|MM08668|Bohemannia_pulverosella
TMYFIFGIWSGMVGTSLSLLIRAELGNPGSLIGDDQIYNTIVTAHAFIMIFFMVMPIMIGGFGNWLVPLMLGAPDMAFPRLNNMSFWLLPPSLMLLVSSSMVENGAGTGWTVYPPLSTNIAHSGSSVDLTIFSLHLAGISSILGAINFITTVINMRPNKM
SFDQMPLFVWAVVITALLLLLSLPVLAGAITMLLTDRNLNTSFFDPAGGGDPILYQHL
>LEFIC066-10|HM871935|MM03351|Boloria_aquilonaris
TLYFIFGIWAGMVGTSLSLLIRTELGNPGSLIGDDQIYNTIVTAHAFIMIFFMVMPIMIGGFGNWLVPLMLGAPDMAFPRMNNMSFWLLPPSLILLISSSIVENGAGTGWTVYPPLSSNIAHSGSSVDLAIFSLHLAGISSILGAINFITTIINMRINNMSFD
QMPLFVWAVGITALLLLLSLPVLAGAITMLLTDRNLNTSFFDPAGGGDPILYQHL
>LEFIG493-10|HQ570403|MM14566|Boloria_aquilonaris
TLYFIFGIWAGMVGTSLSLLIRTELGNPGSLIGDDQIYNTIVTAHAFIMIFFMVMPIMIGGFGNWLVPLMLGAPDMAFPRMNNMSFWLLPPSLILLISSSIVENGAGTGWTVYPPLSSNIAHSGSSVDLAIFSLHLAGISSILGAINFITTIINMRINNMSFD
QMPLFVWAVGITALLLLLSLPVLAGAITMLLTDRNLNTSFFDPAGGGDPILYQHL
>LEFID417-10|HM873213|MM06341|Boloria_chariclea
TLYFIFGIWAGMVGTSLSLLIRTELGNPGSLIGDDQIYNTIVTAHAFIMIFFMVMPIMIGGFGNWLVPLMLGAPDMAFPRMNNMSFWLLPPSLILLISSSIVENGAGTGWTVYPPLSSNIAHSGASVDLAIFSLHLAGISSILGAINFITTIINMRINNMSFD
QMPLFVWAVGITALLLLLSLPVLAGAITMLLTDRNLNTSFFDPAGGGDPILYQHL
>LEFID124-10|HM872934|MM05860|Boloria_dia
TLYFIFGIWAGMLGTSLSLLIRTELGNPGSLIGDDQIYNTIVTAHAFIMIFFMVMPIMIGGFGNWLIPLMLGAPDMAFPRMNNMSFWLLPPSLILLISSSIVENGAGTGWTVYPPLSSNIAHSGASVDLAIFSLHLAGISSILGAINFITTIINMRINNMSFDQ
MPLFVWAVGITALLLLLSLPVLAGAITMLLTDRNLNTSFFDPAGGGDPILYQHL
>LEFIG143-10|HM875823|MM13982|Boloria_eunomia
TLYFIFGIWAGMVGTSLSLLIRTELGNPGSLIGDDQIYNTIVTAHAFIMIFFMVMPIMIGGFGNWLVPLMLGAPDMAFPRMNNMSFWLLPPSLILLISSSIVENGAGTGWTVYPPLSSNIAHSGSSVDLAIFSLHLAGISSILGAINFITTIMNMRINNMSF
DQMPLFVWAVGITALLLLLSLPVLAGAITMLLTDRNLNTSFFDPAGGGDPILYQHL
>LEFIG144-10|HM875824|MM13983|Boloria_eunomia
TLYFIFGIWAGMVGTSLSLLIRTELGNPGSLIGDDQIYNTIVTAHAFIMIFFMVMPIMIGGFGNWLVPLMLGAPDMAFPRMNNMSFWLLPPSLILLISSSIVENGAGTGWTVYPPLSSNIAHSGSSVDLAIFSLHLAGISSILGAINFITTIMNMRINNMSF
DQMPLFVWAVGITALLLLLSLPVLAGAITMLLTDRNLNTSFFDPAGGGDPILYQHL
>LEFIJ544-10|JF853660|MM17169|Boloria_eunomia
PLYFIFGIWAGMVGTSLSLLIRTELGNPGSLIGDDQIYNTIVTAHAFIMIFFMVMPIMIGGFGNWLVPLMLGAPDMAFPRMNNMSFWLLPPSLILLISSSIVENGAGTGWTVYPPLSSNIAHSGSSVDLAIFSLHLAGISSILGAINFITTIMNMRINNMSF
DQMPLFVWAVGITALLLLLSLPVLAGAITMLLTDRNLNTSFFDPAGGGDPILYQHL
>LEFID495-10|HM873260|MM06452|Boloria_euphrosyne
TLYFIFGIWAGMLGTSLSLLIRTELGNPGSLIGDDQIYNTIVTAHAFIMIFFMVMPIMIGGFGNWLVPLMLGAPDMAFPRMNNMSFWLLPPSLILLISSSIVENGAGTGWTVYPPLSSNIAHSGASVDLAIFSLHLAGISSILGAINFITTIINMRINNMSFD
QMPLFVWAVGITALLLLLSLPVLAGAITMLLTDRNLNTSFFDPAGGGDPILYQHL
>LEFIJ548-10|JF853663|MM17173|Boloria_euphrosyne
TLYFIFGIWAGMLGTSFSLLIRTELGNPGSLIEDDQIYNTIVTAHAFIMIFFMVMPIMIGGFGNWLVPLMLGAPDMAFPRMNNMSFWLLPPSLILLISSSIVENGTGTGWTIYPPLSSNIAHSGASVDLAIFSLHLAGISSILGAINFITTIINMRINNMSFDQ
MPLFVWAVGITALLLLLSLPVLAGAITMLLTDRNLNTSFFDPAGGGDPILYQHL
>LEFIB217-10|HM871121|MM00671|Boloria_freija
TLYFIFGIWAGMVGTSLSLLIRTELGNPGSLIGDDQIYNTIVTAHAFIMIFFMVMPIMIGGFGNWLVPLMLGAPDMAFPRMNNMSFWLLPPSLILLISSSIVENGAGTGWTVYPPLSSNIAHSGASVDLAIFSLHLAGISSILGAINFITTIINMRINNMSFD
QMPLFVWAVGITALLLLLSLPVLAGAITMLLTDRNLNTSFFDPAGGGDPILYQHL
>LEFIA741-10|HM386885|MM04095|Boloria_freija
TLYFIFGIWAGMVGTSLSLLIRTELGNPGSLIGDDQIYNTIVTAHAFIMIFFMVMPIMIGGFGNWLVPLMLGAPDMAFPRMNNMSFWLLPPSLILLISSSIVENGAGTGWTVYPPLSSNIAHSGASVDLAIFSLHLAGISSILGAINFITTIINMRINNMSFD
QMPLFVWAVGITALLLLLSLPVLAGAITMLLTDRNLNTSFFDPAGGGDPILYQHL
>LEFIG145-10|HM875825|MM13984|Boloria_frigga

TLYFIFGIWAGMVGTSLSLLIRTELGNPGSLIGDDQIYNTIVTAHAFIMIFFMVMPIMIGGFGNWLVPLMLGAPDMAFPRMNNMSFWLLPPSLILLMSSSIVETGAGTGWTVYPPLSSNIAHSGASVDLAIFSLHLAGISSILGAINFITTIINMRVNNMSF
DQMPLFVWAVGITALLLLLSLPVLAGAITMLLTDRNLNTSFFDPAGGGDPILYQHL
>LEFIJ546-10|JF853662|MM17171|Boloria_frigga
TLYFIFGIWAGMVGTSLSLLIRTELGNPGSLIGDDQIYNTIVTAHAFIMIFFMVMPIMIGGFGNWLVPLMLGAPDMAFPRMNNMSFWLLPPSLILLISSSIVENGAGTGWTVYPPLSSNIAHSGASVDLAIFSLHLAGISSILGAINFITTIINMRVNNMSFD
QMPLFVWAVGITALLLLLSLPVLAGAITMLLTDRNLNTSFFDPAGGGDPILYQHL
>LEFIG488-10|HM876164|MM14553|Boloria_improba
TLYFIFGIWAGMVGTSLSLLIRTELGNPGSLIGDDQIYNTIVTAHAFIMIFFMVMPIMIGGFGNWLVPLMLGAPDMAFPRMNNMSFWLLPPSLILLISSSIVENGAGTGWTVYPPLSSNIAHSGASVDLAIFSLHLAGISSILGAINFITTIINMRINNMSFD
QMPLFVWAVGITALLLLLSLPVLAGAITMLLTDRNLNTSFFDPAGGGDPILYQHL
>LEFIG489-10|HM876165|MM14554|Boloria_improba
TLYFIFGIWAGMVGTSLSLLIRTELGNPGSLIGDDQIYNTIVTAHAFIMIFFMVMPIMIGGFGNWLVPLMLGAPDMAFPRMNNMSFWLLPPSLILLISSSIVENGAGTGWTVYPPLSSNIAHSGASVDLAIFSLHLAGISSILGAINFITTIINMRINNMSFD
QMPLFVWAVGITALLLLLSLPVLAGAITMLLTDRNLNTSFFDPAGGGDPILYQHL
>LEFIG491-10|HM876167|MM14556|Boloria_improba
TLYFIFGIWAGMVGTSLSLLIRTELGNPGSLIGDDQIYNTIVTAHAFIMIFFMVMPIMIGGFGNWLVPLMLGAPDMAFPRMNNMSFWLLPPSLILLISSSIVENGAGTGWTVYPPLSSNIAHSGASVDLAIFSLHLAGISSILGAINFITTIINMRINNMSFD
QMPLFVWAVGITALLLLLSLPILAGAITMLLTDRNLNTSFFDPAGGGDPILYQHL
>LEFIC094-10|HQ570285|MM03407|Boloria_napaea
TLYFIFGIWAGMVGTSLSLLIRTELGNPGSLIGDDQIYNTIVTAHAFIMIFFMVMPIMIGGFGNWLVPLMLGAPDMAFPRMNNMSFWLLPPSLILLISSSIVENGAGTGWTVYPPLSSNIAHSGSSVDLAIFSLHLAGISSILGAINFITTIINMRINNMSFD
QMPLFVWAVGITALLLLLSLPVLAGAITMLLTDRNLNTSFFDPAGGGDPILYQHL
>LEFID420-10|HM873216|MM06346|Boloria_polaris
TLYFIFGIWAGMVGTSLSLLIRTELGNPGSLIGDDQIYNTIVTAHAFIMIFFMVMPIMIGGFGNWLVPLMLGAPDMAFPRMNNMSFWLLPPSLILLISSSIVENGAGTGWTVYPPLSSNIAHSGASVDLAIFSLHLAGISSILGAINFITTIINMRINNMSFD
QMPLFVWAVGITALLLLLSLPVLAGAITMLLTDRNLNTSFFDPAGGGDPILYQHL
>LEFIB997-10|HM871868|MM03195|Boloria_selene
TLYFIFGIWAGMVGTSLSLLIRTELGNPGSLIGDDQIYNTIVTAHAFIMIFFMVMPIMIGGFGNWLIPLMLGAPDMAFPRMNNMSFWLLPPSLILLISSSIVENGAGTGWTVYPPLSSNLAHSGTSVDLAIFSLHLAGISSILGAINFITTIINMRINNMSFD
QMPLFVWAVGITALLLLLSLPVLAGAITMLLTDRNLNTSFFDPAGGGDPILYQHL
>LEFIC043-10|HM871913|MM03274|Boloria_selene
TLYFIFGIWAGMVGTSLSLLIRTELGNPGSLIGDDQIYNTIVTAHAFIMIFFMVMPIMIGGFGNWLIPLMLGAPDMAFPRMNNMSFWLLPPSLILLISSSIVENGAGTGWTVYPPLSSNLAHSGTSVDMAIFSLHLAGISSILGAINFITTIINMRINNMSFD
QMPLFVWAVGITALLLLLSLPVLAGAITMLLTDRNLNTSFFDPAGGGDPILYQHL
>LEFIJ545-10|JF853661|MM17170|Boloria_selene
TLYFIFGIWAGMVGTSLSLLIRTELGNPGSLIGDDQIYNTIVTAHAFIMIFFMVMPIMIGGFGNWLIPLMLGAPDMAFPRMNNMSFWLLPPSLILLISSSIVENGAGTGWTVYPPLSSNLAHSGTSVDLAIFSLHLAGISSILGAINFITTIINMRINNMSFD
QMPLFVWAVGITALLLLLSLPVLAGAITMLLTDRNLNTSFFDPAGGGDPILYQHL
>LEFIC095-10|HQ570286|MM03408|Boloria_thore
TLYFIFGIWAGMVGTSLSLLIRTELGNPGSLIGDDQIYNTIVTAHAFIMIFFMVMPIMIGGFGNWLVPLMLGAPDMAFPRMNNMSFWLLPPSLILLISSSIVENGAGTGWTVYPPLSSNLAHSGASVDLAIFSLHLAGISSILGAINFITTIINMRINNMSFD
QMPLFVWAVGITALLLLLSLPVLAGAITMLLTDRNLNTSFFDPAGGGDPILYQHL
>LEFIA740-10|HM386884|MM04094|Boloria_thore
TLYFIFGIWAGMVGTSLSLLIRTELGNPGSLIGDDQIYNTIVTAHAFIMIFFMVMPIMIGGFGNWLVPLMLGAPDMAFPRMNNMSFWLLPPSLILLISSSIVENGAGTGWTVYPPLSSNLAHSGASVDLAIFSLHLAGISSILGAINFITTIINMRINNMSFD
QMPLFVWAVGITALLLLLSLPVLAGAITMLLTDRNLNTSFFDP?GGGDPILYQHL
>LEFIB655-10|HM871534|MM02424|Borkhausenia_fuscescens
TLYFIFGIWAGMLGTSLSLLIRAELGNPGSLIGDDQIYNTIVTAHAFIMIFFMVMPIMIGGFGNWLIPLMLGAPDMAFPRMNNMSFWLLPPSLILLISSSIVENGAGTGWTVYPPLSSNIAHSGSSVDLAIFSLHLAGISSILGAINFITTIINMRLNHMMFD
QMPLFVWSVSITALLLLLSLPVLAGAITMLLTDRNLNTSFFDPAGGGDPILYQHL
>LEFIJ1518-12|KM572092|MM22786|Borkhausenia_fuscescens
TLYFIFGIWAGMLGTSLSLLIRAELGNPGSLIGDDQIYNTIVTAHAFIMIFFMVMPIMIGGFGNWLIPLMLGAPDMAFPRMNNMSFWLLPPSLILLISSGIVENGAGTGWTVYPPLSSNIAHSGSSVDLAIFSLHLAGISSILGAINFITTIINMRLNHMMFD
QMPLFVWSVSITALLLLLSLPVLAGAITMLLTDRNLNTSFFDPAGGGDPILYQHL
>LEFIB722-10|HM871600|MM02542|Borkhausenia_luridicomella
TLYFIFGIWAGMVGTSLSLLIRAELGNPGSLIGDDQIYNTIVTAHAFIMIFFMVMPIMIGGFGNWLVPLMLGAPDMAFPRMNNMSFWLLPPSLTLLISSSMVENGAGTGWTVYPPLSSNIAHGGSSVDLAIFSLHLAGISSILGAINFITTIINMRINNMM
FDQMPLFVWAVGITALLLLLSLPVLAGAITMLLTDRNLNTSFFDPAGGGDPILYQHL
>LEFID649-10|HM873411|MM06676|Borkhausenia_luridicomella
TLYFIFGIWAGMVGTSLSLLIRAELGNPGSLIGDDQIYNTIVTAHAFIMIFFMVMPIMIGGFGNWLVPLMLGAPDMAFPRMNNMSFWLLPPSLTLLISSSMVENGAGTGWTVYPPLSSNIAHGGSSVDLAIFSLHLAGISSILGAINFITTIINMRINNMM
FDQMPLFVWAVGITALLLLLSLPVLAGAITMLLTDRNLNTSFFDPAGGGDPILYQHL

>LEFIF999-10|HM875680|MM13607|Borkhausenia_luridicomella
TLYFIFGIWAGMVGTSLSLLIRAELGNPGSLIGDDQIYNTIVTAHAFIMIFFMVMPIMIGGFGNWLVPLMLGAPDMAFPRMNNMSFWLLPPSLTLLISSSMVENGAGTGWTVYPPLSSNIAHGGSSVDLAIFSLHLAGISSILGAINFITTIINMRINNMM
FDQMPLFVWAVGITALLLLLSLPVLAGAITMLLTDRNLNTSFFDPAGGGDPILYQHL
>LEEUA126-11|MM19534|Borkhausenia_minutella
TLYFIFGIWAGMMGTSLSLLIRAELGNPGSFIGDDQIYNTIVTAHAFIMIFFMVMPIMIGGFGNWLIPLMLGAPDMAFPRMNNMSFWLLPPSLTLLISSSIVENGAGTGWTVYPPLSSNIAHSGSSVDLAIFSLHLAGISSILGAINFITTIINMRLNNMMF
DQMPLFVWAVGITALLLLLSLPVLAGAITMLLTDRNLNTSFFDPAGGGDPILYQHL
>LEFIF017-10|HM874731|MM10456|Boudinotiana_notha
TLYFIFGIWAGMVGTSLSLLIRAELGNPGSLIGDDQIYNTIVTAHAFIMIFFMVMPIMIGGFGNWLVPLMLGAPDMAFPRMNNMSFWLLPPSITLLISSSIVENGAGTGWTVYPPLSSNIAHSGSSVDLAIFSLHLAGISSILGAINFITTIINMRINNMMF
DQMPLFVWAVGITAFLLLLSLPVLAGAITMLLTDRNLNTSFFDPAGGGDPILYQHL
>LEFIA471-10|HM386811|MM01543|Brachionycha_nubeculosa
TLYFIFGIWAGMVGTSLSLLIRAELGNPGSLIGDDQIYNTIVTAHAFIMIFFMVMPIMIGGFGNWLVPLMLGAPDMAFPRMNNMSFWLLPPSLTLLISSSIVENGAGTGWTVYPPLSSNIAHGGSSVDLAIFSLHLAGISSILGAINFITTIINMRLNSLSFD
QMPLFIWAVGITAFLLLLSLPVLAGAITMLLTDRNLNTSFFDPAGGGDPILYQHL
>LEFIA530-10|KM572649|MM01614|Brachionycha_nubeculosa
TLYFIFGIWAGMVGTSLSLLIRAELGNPGSLIGDDQIYNTIVTAHAFIMIFFMVMPIMIGGFGNWLVPLMLGAPDMAFPRMNNMSFWLLPPSLTLLISSSIVENGAGTGWTVYPPLSSNIAHGGSSVDLAIFSLHLAGISSILGAINFITTIINMRLNSLSFD
QMPLFIWAVGITAFLLLLSLPVLAGAITMLLTDRNLNTSFFDPAGGGDPILYQHL
>LEFIB825-10|HM871702|MM02767|Brachionycha_nubeculosa
TLYFIFGIWAGMVGTSLSLLIRAELGNPGSLIGDDQIYNTIVTAHAFIMIFFMVMPIMIGGFGNWLVPLMLGAPDMAFPRMNNMSFWLLPPSLTLLISSSIVENGAGTGWTVYPPLSSNIAHGGSSVDLAIFSLHLAGISSILGAINFITTIINMRLNSLSFD
QMPLFIWAVGITAFLLLLSLPVLAGAITMLLTDRNLNTSFFDPAGGGDPILYQHL
>LEFIE302-10|HM874028|MM08656|Brachmia_blandella
TLYFIFGIWAGMLGTSLSLLIRAELGNPGSLIGDDQIYNTIVTAHAFIMIFFMVMPIMIGGFGNWLVPLMLGAPDMAFPRMNNMSFWLLPPSLSLLISSSIVENGAGTGWTVYPPLSSNIAHGGSSVDLAIFSLHLAGISSILGAINFITTIINMRVNNMSF
DQMPLFVWAVGITALLLLLSLPVLAGAITMLLTDRNLNTSFFDPAGGGDPILYQHL
>LEFIK007-10|KM573094|MM17582|Brachmia_blandella
TLYFIFGIWAGMLGTSLSLLIRAELGNPGSLIGDDQIYNTIVTAHAFIMIFFMVMPIMIGGFGNWLVPLMLGAPDMAFPRMNNMSFWLLPPSLSLLISSSIVENGAGTGWTVYPPLSSNIAHGGSSVDLAIFSLHLAGISSILGAINFITTIINMRVNNMSF
DQMPLFVWAVGITALLLLLSLPVLAGAITMLLTDRNLNTSFFDPAGGGDPILYQHL
>LEFIK678-10|JF854246|MM18253|Brachmia_blandella
TLYFIFGIWAGMLGTSLSLLIRAELGNPGSLIGDDQIYNTIVTAHAFIMIFFMVMPIMIGGFGNWLVPLMLGAPDMAFPRMNNMSFWLLPPSLSLLISSSIVENGAGTGWTVYPPLSSNIAHGGSSVDLAIFSLHLAGISSILGAINFITTIINMRVNNMSF
DQMPLFVWAVGITALLLLLSLPVLAGAITMLLTDRNLNTSFFDPAGGGDPILYQHL
>LEFID829-10|HM873586|MM06945|Brachmia_dimidiella
TLYFIFGIWSGMLGTSLSLLIRAELGNPGSLIGDDQIYNTIVTAHAFIMIFFMVMPIMIGGFGNWLVPLMLGAPDMAFPRMNNMSFWLLPPSLSLLISSSIVENGAGTGWTVYPPLSSNIAHGGSSVDLAIFSLHLAGISSILGAINFITTIINMRVNNMSF
DQMPLFVWAVGITALLLLLSLPVLAGAITMLLTDRNLNTSFFDPAGGGDPILYQHL
>LEFIG785-10|HM876437|MM15649|Brachmia_inornatella
TLYFIFGIWAGMLGTSLSLLIRAELGNPGSLIGDDQIYNTIVTAHAFIMIFFMVMPIMIGGFGNWLVPLMLGAPDMAFPRMNNMSFWLLPPSLSLLISSSIVENGAGTGWTVYPPLSSNIAHGGSSVDLAIFSLHLAGISSILGAINFITTIINMRINHMSFD
QMPLFVWSVGITALLLLLSLPVLAGAITMLLTDRNLNTSFFDPAGGGDPILYQHL
>LEFIB311-10|HM871211|MM00835|Brachylomia_viminalis
TLYFIFGIWAGMVGTSLSLLIRAELGNPGSLIGDDQIYNTIVTAHAFIMIFFMVMPIMIGGFGNWLVPLMLGAPDMAFPRMNNMSFWLLPPSLTLLISSSIVENGAGTGWTVYPPLSSNIAHGGSSVDLAIFSLHLAGISSILGAINFITTIINMRLNSLSFD
QMPLFIWAVGITAFLLLLSLPVLAGAITMLLTDRNLNTSFFDPAGGGDPILYQHL
>LEFIA627-10|HM870876|MM01742|Brachylomia_viminalis
TLYFIFGIWAGMVGTSLSLLIRAELGNPGSLIGDDQIYNTIVTAHAFIMIFFMVMPIMIGGFGNWLVPLMLGAPDMAFPRMNNMSFWLLPPSLTLLISSSIVENGAGTGWTVYPPLSSNIAHGGSSVDLAIFSLHLAGISSILGAINFITTIINMRLNSLSFD
QMPLFIWAVGITAFLLLLSLPVLAGAITMLLTDRNLNTSFFDPAGGGDPILYQ?L
>LEFIC068-10|HM871937|MM03353|Brenthis_ino
TLYFIFGIWAGMVGTSLSLLIRTELGNPGSLIGDDQIYNTIVTAHAFIMIFFMVMPIMIGGFGNWLIPLMLGAPDMAFPRMNNMSFWLLPPSLILLISSSIVENGAGTGWTVYPPLSSNIAHSGSSVDLAIFSLHLAGISSILGAINFITTIINMRINNMSFDQ
MPLFVWAVGITALLLLLSLPVLAGAITMLLTDRNLNTSFFDPAGGGDPILYQHL
>LEEUA311-11|JN263383|MM19719|Bryophila_domestica
TLYFIFGIWAGMVGTSLSLLIRAELGTPGSLIGDDQIYNTIVTAHAFIMIFFMVMPIMIGGFGNWLVPLMLGAPDMAFPRMNNMSFWLLPPSLTLLISSSIVENGAGTGWTVYPPLSSNIAHGGSSVDLAIFSLHLAGISSILGAINFITTIINMRLNSLSFD
QMPLFIWAVGITAFLLLLSLPVLAGAITMLLTDRNLNTSFFDPAGGGDPILYQHL
>LEFIF220-10|HM874913|MM10971|Bryophila_raptricula
TLYFIFGIWAGMVGTSLSLLIRAELGTPGSLIGDDQIYNTIVTAHAFIMIFFMVMPIMIGGFGNWLIPLMLGAPDMAFPRMNNMSFWLLPPSLTLLISSSIVENGAGTGWTVYPPLSSNIAHGGSSVDLAIFSLHLAGISSILGAINFITTIINMRLNSLSFDQ

MPLFIWAVGITAFLLLLSLPVLAGAITMLLTDRNLNTSFFDPAGGGDPILYQHL
>LEFIF694-10|HM875378|MM12732|Bryophila_raptricula
TLYFIFGIWAGMVGTSLSLLIRAELGTPGSLIGDDQIYNTIVTAHAFIMIFFMVMPIMIGGFGNWLIPLMLGAPDMAFPRMNNMSFWLLPPSLTLLISSSIVENGAGTGWTVYPPLSSNIAHGGSSVDLAIFSLHLAGISSILGAINFITTIINMRLNSLSFDQ
MPLFIWAVGITAFLLLLSLPVLAGAITMLLTDRNLNTSFFDPAGGGDPILYQHL
>LEFID087-10|HM872898|MM05699|Bryotropha_affinis
TLYFIFGIWAGMIGTSLSLLIRAELGNPGSLIGDDQIYNTIVTAHAFIMIFFMVMPIMIGGFGNWLVPLMLGAPDMAFPRMNNMSFWLLPPSLTLLISSSIVENGAGTGWTVYPPLSSNIAHGGSSVDLAIFSLHLAGISSILGAINFITTIINMKINGLSFDQ
MPLFVWAVGITALLLLLSLPVLAGAITMLLTDRNLNTSFFDPAGGGDPILYQHL
>LEEUA174-11|MM19582|Bryotropha_affinis
TLYFIFGIWAGMIGTSLSLLIRAELGNPGSLIGDDQIYNTIVTAHAFIMIFFMVMPIMIGGFGNWLVPLMLGAPDMAFPRMNNMSFWLLPPSLTLLISSSIVENGAGTGWTVYPPLSSNIAHGGSSVDLAIFSLHLAGISSILGAINFITTIINMKINGLSFDQ
MPLFVWAVGITALLLLLSLPVLAGAITMLLTDRNLNTSFFDPAGGGDPILYQHL
>LEEUA175-11|MM19583|Bryotropha_affinis
TLYFIFGIWAGMIGTSLSLLIRAELGNPGSLIGDDQIYNTIVTAHAFIMIFFMVMPIMIGGFGNWLVPLMLGAPDMAFPRMNNMSFWLLPPSLTLLISSSIVENGAGTGWTVYPPLSSNIAHGGSSVDLAIFSLHLAGISSILGAINFITTIINMKINGLSFDQ
MPLFVWAVGITALLLLLSLPVLAGAITMLLTDRNLNTSFFDPAGGGDPILYQHL
>LEFIE001-10|HM873750|MM08052|Bryotropha_boreella
TLYFIFGIWASMIGTSLSLLIRTELGNPGSFIGDDQIYNTIVTAHAFIMIFFMVMPIMIGGFGNWLVPLMLGAPDMAFPRMNNMSFWLLPPSLILLISSSIVENGAGTGWTVYPPLSSNIAHSGSSVDLTIFSLHLAGISSILGAINFITTIINMKINGLSFDQ
MPLFVWAVGITALLLLLSLPVLAGAITMLLTDRNLNTSFFDPAGGGDPILYQHL
>LEFIC317-10|HM872161|MM03820|Bryotropha_desertella
TLYFIFGIWAGMVGTSLSLLIRAELGNPGSLIGDDQIYNTIVTAHAFIMIFFMVMPIMIGGFGNWLVPLMLGAPDMAFPRMNNMSFWLLPPSLTLLISSSIVENGAGTGWTVYPPLSSNIAHGGSSVDLAIFSLHLAGISSILGAINFITTIINMKINGLSFD
QMPLFVWSVGITALLLLLSLPVLAGAITMLLTDRNLNTSFFDPAGGGDPILYQHL
>LEFIC047-10|HQ570283|MM03294|Bryotropha_galbanella
TLYFIFGIWAGMIGTSLSLLIRSELGNPGSFIGDDQIYNTIVTAHAFIMIFFMVMPIMIGGFGNWLVPLMLGAPDMAFPRMNNMSFWLLPPSLILLISSSIVENGAGTGWTVYPPLSSNIAHSGSSVDLAIFSLHLAGISSILGAINFITTIINMKINGLSFDQ
MPLFVWAVSITALLLLLSLPVLAGAITMLLTDRNLNTSFFDPAGGGDPILYQHL
>LEFIC037-10|HM871907|MM03265|Bryotropha_plantariella
TLYFIFGIWAGMIGTSLSLLIRAELGNPGSLIGDDQIYNTIVTAHAFIMIFFMVMPIMIGGFGNWLVPLMLGAPDMAFPRMNNMSFWLLPPSLTLLISSSIVENGAGTGWTVYPPLSSNIAHGGSSVDLAIFSLHLAGISSILGAINFITTIINMKINGLSFDQ
MPLFVWSVGITALLLLLSLPVLAGAITMLLTDRNLNTSFFDPAGGGDPILYQHL
>LEFIG252-10|HM875931|MM14176|Bryotropha_plantariella
TLYFIFGIWAGMIGTSLSLLIRAELGNPGSLIGDDQIYNTIVTAHAFIMIFFMVMPIMIGGFGNWLVPLMLGAPDMAFPRMNNMSFWLLPPSLTLLISSSIVENGTGTGWTVYPPLSSNIAHGGSSVDLAIFSLHLAGISSILGAINFITTIINMKINGLSFDQ
MPLFVWSVGITALLLLLSLPVLAGAITMLLTDRNLNTSFFDPAGGGDPILYQHL
>LEFIC361-10|HM872204|MM03886|Bryotropha_purpurella
TLYFIFGIWAGMVGTSLSLLIRAELGNPGSLIGDDQIYNTIVTAHAFIMIFFMVMPIMIGGFGNWLVPLMLGAPDMAFPRMNNMSFWLLPPSLTLLISSSIVENGAGTGWTVYPPLSSNIAHGGSSVDLAIFSLHLAGISSILGAINFITTIINMKINGMSFD
QMPLFVWSVGITALLLLLSLPVLAGAITMLLTDRNLNTSFFDPAGGGDPILYQHL
>LEFID645-10|HM873407|MM06672|Bryotropha_senectella
TLYFIFGIWAGMIGTSLSLLIRAELGNPGSLIGDDQIYNTIVTAHAFIMIFFMVMPIMIGGFGNWLIPLMLGAPDMAFPRMNNMSFWLLPPSLTLLISSSIVENGAGTGWTVYPPLSSNIAHSGSSVDLAIFSLHLAGISSILGAINFITTIINMKINGLSFDQ
MPLFVWSVGITALLLLLSLPVLAGAITMLLTDRNLNTSFFDPAGGGDPILYQHL
>LEFIE613-10|HM874336|MM09528|Bryotropha_senectella
TLYFIFGIWAGMIGTSLSLLIRAELGNPGSLIGDDQIYNTIVTAHAFIMIFFMVMPIMIGGFGNWLIPLMLGAPDMAFPRMNNMSFWLLPPSLTLLISSSIVENGAGTGWTVYPPLSSNIAHSGSSVDLAIFSLHLAGISSILGAINFITTIINMKINGLSFDQ
MPLFVWSVGITALLLLLSLPVLAGAITMLLTDRNLNTSFFDPAGGGDPILYQHL
>LEFIE948-10|HM874665|MM10345|Bryotropha_senectella
TLYFIFGIWAGMIGTSLSLLIRAELGNPGSLIGDDQIYNTIVTAHAFIMIFFMVMPIMIGGFGNWLIPLMLGAPDMAFPRMNNMSFWLLPPSLTLLISSSIVENGAGTGWTVYPPLSSNIAHSGSSVDLAIFSLHLAGISSILGAINFITTIINMKINGLSFDQ
MPLFVWSVGITALLLLLSLPVLAGAITMLLTDRNLNTSFFDPAGGGDPILYQHL
>LEFIB750-10|HM871628|MM02596|Bryotropha_similis
TLYFIFGIWAGMIGTSLSLLIRAELGNPGSLIGDDQIYNTIVTAHAFIMIFFMVMPIMIGGFGNWLVPLMLGAPDMAFPRMNNMSFWLLPPSLTLLISSSIVENGAGTGWTVYPPLSSNIAHGGSSVDLAIFSLHLAGISSILGAINFITTIINMKINGLSFDQ
MPLFVWAVGITALLLLLSLPVLAGAITMLLTDRNLNTSFFDPAGGGDPILYQHL
>LEFIJ399-10|JF853585|MM06816|Bryotropha_similis
TLYFIFGIWAGMIGTSLSLLIRAELGNPGSLIGDDQIYNTIVTAHAFIMIFFMVMPIMIGGFGNWLVPLMLGAPDMAFPRMNNMSFWLLPPSLTLLISSSIVENGAGTGWTVYPPLSSNIAHGGSSVDLAIFSLHLAGISSILGAINFITTIINMKINGLSFDQ
MPLFVWAVGITALLLLLSLPVLAGAITMLLTDRNLNTSFFDPAGGGDPILYQHL
>LEFIE845-10|HM874564|MM10066|Bryotropha_similis

TLYFIFGIWAGMIGTSLSLLIRAELGNPGSLIGDDQIYNTIVTAHAFIMIFFMVMPIMIGGFGNWLVPLMLGAPDMAFPRMNNMSFWLLPPSLTLLISSSIVENGAGTGWTVYPPLSSNIAHGGSSVDLAIFSLHLAGISSILGAINFITTIINMKINGLSFDQ
MPLFVWAVGITALLLLLSLPVLAGAITMLLTDRNLNTSFFDPAGGGDPILYQHL
>LEFIG010-10|HM875690|MM13627|Bryotropha_similis
TLYFIFGIWAGMIGTSLSLLIRAELGNPGSLIGNDQIYNTIVTAHAFIMIFFMVMPIMIGGFGNWLVPLMLGAPDMAFPRMNNMSFWLLPPSLTLLISSSIVENGAGTGWTVYPPLSSNIAHGGSSVDLAIFSLHLAGISSILGAINFITTIINMKINGLSFDQ
MPLFVWAVGITALLLLLSLPVLAGAITMLLTDRNLNTSFFDPAGGGDPILYQHL
>LEFIG486-10|HM876162|MM14548|Bryotropha_similis
TLYFIFGIWAGMIGTSLSLLIRAELGNPGSLIGDDQIYNTIVTAHAFIMIFFMVMPIMIGGFGNWLVPLMLGAPDMAFPRMNNMSFWLLPPSLTLLISSSIVENGAGTGWTVYPPLSSNIAHGGSSVDLTIFSLHLAGISSILGAINFITTIINMKINGLSFDQ
MPLFVWAVGITALLLLLSLPVLAGAITMLLTDRNLNTSFFDPAGGGDPILYQHL
>LEFIJ2203-14|MM23392|Bryotropha_similis
TLYFIFGIWAGMIGTSLSLLIRAELGNPGSLIGDDQIYNTIVTAHAFIMIFFMVMPIMIGGFGNWLVPLMLGAPDMAFPRMNNMSFWLLPPSLTLLISSSIVENGAGTGWTVYPPLSSNIAHGGSSVDLAIFSLHLAGISSILGAINFITTIINMKINGLSFDQ
MPLFVWAVGITALLLLLSLPVLAGAITMLLTDRNLNTSFFDPAGGGDPILYQHL
>LEFIB749-10|HM871627|MM02595|Bryotropha_terrella
TLYFIFGIWAGMVGTSLSLLIRAELGNPGSLIGDDQIYNTIVTAHAFIMIFFMVMPIMIGGFGNWLVPLMLGAPDMAFPRMNNMSFWLLPPSLTLLISSSIVENGAGTGWTVYPPLSSNIAHGGSSVDLAIFSLHLAGISSILGAINFITTIINMKINGLSFD
QMPLFVWSVGITALLLLLSLPVLAGAITMLLTDRNLNTSFFDPAGGGDPILYQHL
>LEFIF518-10|HM875203|MM12146|Bryotropha_terrella
TLYFIFGIWAGMVGTSLSLLIRAELGNPGSLIGDDQIYNTIVTAHAFIMIFFMVMPIMIGGFGNWLVPLMLGAPDMAFPRMNNMSFWLLPPSLTLLISSSIVENGAGTGWTVYPPLSSNIAHGGSSVDLAIFSLHLAGISSILGAINFITTIINMKINGLSFD
QMPLFVWSVGITALLLLLSLPVLAGAITMLLTDRNLNTSFFDPAGGGDPILYQHL
>LEEUA470-11|JN270944|MM19878|Bryotropha_terrella
TLYFIFGIWAGMMGTSLSLLIRAELGNPGSLIGDDQIYNTIVTAHAFIMIFFMVMPIMIGGFGNWLVPLMLGAPDMAFPRMNNMSFWLLPPSLTLLISSSIVENGAGTGWTVYPPLSSNIAHGGSSVDLAIFSLHLAGISSILGAINFITTIINMKINGLSFD
QMPLFVWSVGITALLLLLSLPVLAGAITMLLTDRNLNTSFFDPAGGGDPILYQHL
>LEFID369-10|HM873166|MM06278|Bryotropha_umbrosella
TLYFIFGIWAGMIGTSLSLLIRAELGNPGSLIGDDQIYNTIVTAHAFIMIFFMVMPIMIGGFGNWLVPLMLGAPDMAFPRMNNMSFWLLPPSLTLLISSSIVENGAGTGWTVYPPLSSNIAHGGSSVDLAIFSLHLAGISSILGAINFITTIINMKINGLSFDQ
MPLFVWAVGITALLLLLSLPVLAGAITMLLTDRNLNTSFFDPAGGGDPILYQHL
>LEFIJ094-10|JF853441|MM06574|Bryotropha_umbrosella
TLYFIFGIWAGMIGTSLSLLIRAELGNPGSLIGDDQIYNTIVTAHAFIMIFFMVMPIMIGGFGNWLVPLMLGAPDMAFPRMNNMSFWLLPPSLTLLISSSIVENGAGTGWTVYPPLSSNIAHGGSSVDLAIFSLHLAGISSILGAINFITTIINMKINGLSFDQ
MPLFVWAVGITALLLLLSLPVLAGAITMLLTDRNLNTSFFDPAGGGDPILYQHL
>LEFIJ945-10|JF853908|MM17570|Bryotropha_umbrosella
TLYFIFGIWAGMIGTSLSLLIRAELGNPGSLIGDDQIYNTIVTAHAFIMIFFMVMPIMIGGFGNWLVPLMLGAPDMAFPRMNNMSFWLLPPSLTLLISSSIVENGAGTGWTVYPPLSSNIAHGGSSVDLAIFSLHLAGISSILGAINFITTIINMKINGLSFDQ
MPLFVWAVGITALLLLLSLPVLAGAITMLLTDRNLNTSFFDPAGGGDPILYQHL
>LEFIJ947-10|JF853910|MM17572|Bryotropha_umbrosella
TLYFIFGIWAGMIGTSLSLLIRAELGNPGSLIGDDQIYNTIVTAHAFIMIFFMVMPIMIGGFGNWLVPLMLGAPDMAFPRMNNMSFWLLPPSLTLLISSSIVENGAGTGWTVYPPLSSNIAHGGSSVDLAIFSLHLAGISSILGAINFITTIINMKINGLSFDQ
MPLFVWAVGITALLLLLSLPVLAGAITMLLTDRNLNTSFFDPAGGGDPILYQHL
>LEFIL366-10|JN270958|MM18676|Bryotropha_umbrosella
TLYFIFGIWAGMIGTSLSLLIRAELGNPGSLIGDDQIYNTIVTAHAFIMIFFMVMPIMIGGFG?WLVPLMLGAPDMAFPRMNNMSFWLLPPSLTLLISSSIVENGAGTGWTVYPPLSSNIAHGGSSVDLAIFSLHLAGISSILGAINFITTIINMKINGLSFDQ
MPLFVWAVGITALLLLLSLPVLAGAITMLLTDRNLNTSFFDPAGGGDPILYQHL
>LEFIK052-10|MM17627|Bucculatrix_albedinella
TLYFIFGIWAGMVGTSLSLLIRAELGNPGSLIGNDQIYNTIVTAHAFIMIFFMVMPIMIGGFGNWLVPLMLGAPDMAFPRMNNMSFWLLPPSLLLLISSSIVEMGAGTGWTVYPPLSSNIAHGGSSVDLAIFSLHLAGISSILGAINFITTIINMRTNNMSL
DQMPLFVWAVGITALLLLLSLPVLAGAITMLLTDRNLNTSFFDPAGGGDPILYQHL
>LEFIC407-10|HM872250|MM03951|Bucculatrix_argentisignella
TLYFIFGIWAGMIGTSLSLLIRAELGSPGSLIGNDQIYNTIVTAHAFIMIFFMVMPIMIGGFGNWLVPLMLGAPDMAFPRMNNMSFWLLPPSLLLLISSSIVEMGAGTGWTVYPPLSSNIAHGGSSVDLAIFSLHLAGISSILGAINFITTIINMRTNKMSFD
QMPLFVWAVGITALLLLLSLPVLAGAITMLLTDRNLNTSFFDPAGGGDPILYQHL
>LEFIC408-10|HM872251|MM03952|Bucculatrix_argentisignella
TLYFIFGIWAGMIGTSLSLLIRAELGSPGSLIGNDQIYNTIVTAHAFIMIFFMVMPIMIGGFGNWLVPLMLGAPDMAFPRMNNMSFWLLPPSLLLLISSSIVEMGAGTGWTVYPPLSSNIAHGGSSVDLAIFSLHLAGISSILGAINFITTIINMRTNKMSFD
QMPLFVWAVGITALLLLLSLPVLAGAITMLLTDRNLNTSFFDPAGGGDPILYQHL
>LEFIC401-10|HM872244|MM03944|Bucculatrix_artemisiella
TLYFIFGIWAGMVGTSLSLLIRAELGNPGSLIGNDQIYNTIVTAHAFIMIFFMVMPIMIGGFGNWLVPLMLGAPDMAFPRMNNMSFWLLPPSLMLLISSSIVEMGAGTGWTVYPPLSSNIAHSGSSVDLAIFSLHLAGISSILGAINFITTIINMRTNKMSF
DQMPLFVWAVGITALLLLLSLPVLAGAITMLLTDRNLNTSFFDPAGGGDPILYQHL

>LEFIB771-10|HM871648|MM02642|Bucculatrix_bechsteinella
TLYFIFGIWAGMVGTSLSLLIRAELGNPGSLIGNDQIYNTIVTAHAFIMIFFMVMPIMIGGFGNWLVPLMLGAPDMAFPRMNNMSFWLLPPSILLLISSSIVEMGAGTGWTVYPPLSSNIAHGGSSVDLAIFSLHLAGISSILGAINFITTIINMRTNKMSF
DQMPLFVWAVGITALLLLLSLPVLAGAITMLLTDRNLNTSFFDPAGGGDPILYQHL
>LEFID303-10|HM873100|MM06181|Bucculatrix_bechsteinella
TLYFIFGIWAGMVGTSLSLLIRAELGNPGSLIGNDQIYNTIVTAHAFIMIFFMVMPIMIGGFGNWLVPLMLGAPDMAFPRMNNMSFWLLPPSILLLISSSIVEMGAGTGWTVYPPLSSNIAHGGSSVDLAIFSLHLAGISSILGAINFITTIINMRTNKMSF
DQMPLFVWAVGITALLLLLSLPVLAGAITMLLTDRNLNTSFFDPAGGGDPILYQHL
>LEFIG089-10|HM875768|MM13757|Bucculatrix_bechsteinella
TLYFIFGIWAGMVGTSLSLLIRAELGNPGSLIGNDQIYNTIVTAHAFIMIFFMVMPIMIGGFGNWLVPLMLGAPDMAFPRMNNMSFWLLPPSILLLISSSIVEMGAGTGWTVYPPLSSNIAHGGSSVDLAIFSLHLAGISSILGAINFITTIINMRTNKMSF
DQMPLFVWAVGITALLLLLSLPVLAGAITMLLTDRNLNTSFFDPAGGGDPILYQHL
>LEFIB780-10|HM871657|MM02655|Bucculatrix_cidarella
TLYFIFGIWAGMVGTSLSLLIRAELGNPGSLIGNDQIYNTIVTAHAFIMIFFMVMPIMIGGFGNWLVPLMLGAPDMAFPRMNNMSFWLLPPSILLLISSSIVEMGAGTGWTVYPPLSSNIAHGGGSVDLAIFSLHLAGISSILGAINFITTIINMRTNKMSF
DQMPLFVWAVGITALLLLLSLPVLAGAITMLLTDRNLNTSFFDPAGGGDPILYQHL
>LEFID302-10|HM873099|MM06180|Bucculatrix_cidarella
TLYFIFGIWAGMVGTSLSLLIRAELGNPGSLIGNDQIYNTIVTAHAFIMIFFMVMPIMIGGFGNWLVPLMLGAPDMAFPRMNNMSFWLLPPSILLLISSSIVEMGAGTGWTVYPPLSSNIAHGGGSVDLAIFSLHLAGISSILGAINFITTIINMRTNKMSF
DQMPLFVWAVGITALLLLLSLPVLAGAITMLLTDRNLNTSFFDPAGGGDPILYQHL
>LEFIJ1031-11|KM573380|MM16607|Bucculatrix_cidarella
TLYFIFGIWAGMVGTSLSLLIRAELGNPGSLIGNDQIYNTIVTAHAFIMIFFMVMPIMIGGFGNWLVPLMLGAPDMAFPRMNNMSFWLLPPSILLLISSSIVEMGAGTGWTVYPPLSSNIAHGGGSVDLAIFSLHLAGISSILGAINFITTIINMRTNKMSF
DQMPLFVWAVGITALLLLLSLPVLAGAITMLLTDRNLNTSFFDPAGGGDPILYQHL
>LEFIC409-10|HM872252|MM03954|Bucculatrix_cristatella
TLYFIFGIWAGMVGTSLSLLIRAELGNPGSLIGNDQIYNTIVTAHAFIMIFFMVMPIMIGGFGNWLVPLMLGAPDMAFPRMNNMSFWLLPPSLMLLISSSIVEMGAGTGWTVYPPLSSNIAHGGSSVDLAIFSLHLAGISSILGAINFITTIINMRTNKMS
FDQMPLFVWAVGITALLLLLSLPVLAGAITMLLTDRNLNTSFFDPAGGGDPILYQHL
>LEFID534-10|HM873299|MM06517|Bucculatrix_cristatella
TLYFIFGIWAGMVGTSLSLLIRAELGNPGSLIGNDQIYNTIVTAHAFIMIFFMVMPIMIGGFGNWLVPLMLGAPDMAFPRMNNMSFWLLPPSLMLLISSSIVEMGAGTGWTVYPPLSSNIAHGGSSVDLAIFSLHLAGISSILGAINFITTIINMRTNKMS
FDQMPLFVWAVGITALLLLLSLPVLAGAITMLLTDRNLNTSFFDPAGGGDPILYQHL
>LEFID535-10|HM873300|MM06518|Bucculatrix_cristatella
TLYFIFGIWAGMVGTSLSLLIRAELGNPGSLIGNDQIYNTIVTAHAFIMIFFMVMPIMIGGFGNWLVPLMLGAPDMAFPRMNNMSFWLLPPSLMLLISSSIVEMGAGTGWTVYPPLSSNIAHGGSSVDLAIFSLHLAGISSILGAINFITTIINMRTNKMS
FDQMPLFVWAVGITALLLLLSLPVLAGAITMLLTDRNLNTSFFDPAGGG?PILYQHL
>LEFIE946-10|HM874663|MM10335|Bucculatrix_cristatella
TLYFIFGIWAGMVGTSLSLLIRAELGNPGSLIGNDQIYNTIVTAHAFIMIFFMVMPIMIGGFGNWLVPLMLGAPDMAFPRMNNMSFWLLPPSLMLLISSSIVEMGAGTGWTVYPPLSSNIAHGGSSVDLAIFSLHLAGISSILGAINFITTIINMRTNKMS
FDQMPLFVWAVGITALLLLLSLPVLAGAITMLLTDRNLNTSFFDPAGGGDPILYQHL
>LEFIG433-10|HM876109|MM14469|Bucculatrix_cristatella
TLYFIFGIWAGMVGTSLSLLIRAELGNPGSLIGNDQIYNTIVTAHAFIMIFFMVMPIMIGGFGNWLVPLMLGAPDMAFPRMNNMSFWLLPPSLMLLISSSIVEMGAGTGWTVYPPLSSNIAHGGSSVDLAIFSLHLAGISSILGAINFITTIINMRTNKMS
FDQMPLFVWAVGITALLLLLSLPVLAGAITMLLTDRNLNTSFFDPAGGGDPILYQHL
>LEFIK377-10|JF854010|MM17952|Bucculatrix_cristatella
TLYFIFGIWAGMVGTSLSLLIRAELGNPGSLIGNDQIYNTIVTAHAFIMIFFMVMPIMIGGFGNWLVPLMLGAPDMAFPRMNNMSFWLLPPSLMLLISSSIVEMGAGTGWTVYPPLSSNIAHGGSSVDLAIFSLHLAGISSILGAINFITTIINMRTNKMS
FDQMPLFVWAVGITALLLLLSLPVLAGAITMLLTDRNLNTSFFDPAGGGDPILYQHL
>LEFID304-10|HM873101|MM06182|Bucculatrix_demaryella
TLYFIFGIWAGMVGTSLSLLIRAELGNPGSLIGNDQIYNTIVTAHAFIMIFFMVMPIMIGGFGNWLIPLMLGAPDMAFPRMNNMSFWLLPPSILLLISSSIVEMGAGTGWTVYPPLSSNIAHGGSSVDLAIFSLHLAGISSILGAINFITTIINMRTNKMSFD
QMPLFVWAVGITALLLLLSLPVLAGAITMLLTDRNLNTSFFDPAGGGDPILYQHL
>LEFID301-10|HM873098|MM06179|Bucculatrix_frangutella
TLYFIFGIWASMVGTSLSLLIRAELGNPGSLIGNDQIYNTIVTAHAFIMIFFMVMPIMIGGFGNWLVPLMLGAPDMAFPRMNNMSFWLLPPSLILLISSSIVEMGAGTGWTVYPPLSSNIAHGGGSVDLAIFSLHLAGISSILGAINFITTIINMRVNNMSF
DQMPLFVWAVGITALLLLLSLPVLAGAITMLLTDRNLNTSFFDPAGGGDPILYQHL
>LEFIK506-10|KM572943|MM18081|Bucculatrix_frangutella
TLYFIFGIWAGMVGTSLSLLIRAELGNPGSLIGNDQIYNTIVTAHAFIMIFFMVMPIMIGGFGNWLVPLMLGAPDMAFPRMNNMSFWLLPPSLLLLISSSIVEMGAGTGWTVYPPLSSNIAHGGGSVDLAIFSLHLAGISSILGAINFITTIINMRVNNMS
FDQMPLFVWAVGITALLLLLSLPVLAGAITMLLTDRNLNTSFFDPAGGGDPILYQHL
>LEFIC462-10|HM872295|MM04046|Bucculatrix_humiliella
TLYFIFGIWAGMVGTSLSLLIRAELGNPGSLIGNDQIYNTIVTAHAFIMIFFMVMPIMIGGFGNWLVPLMLGAPDMAFPRMNNMSFWLLPPSLLLLISSSVVEMGAGTGWTVYPPLSSNIAHSGSSVDLAIFSLHLAGISSILGAINFITTIINMRTNKMSF

DQMPLFVWAVGITALLLLLSLPVLAGAITMLLTDRNLNTSFFDPAGGGDPILYQHL
>LEFIC399-10|HM872242|MM03942|Bucculatrix_latviaella
TLYFIFGIWAGMVGTSLSLLIRAELGNPGSLIGNDQIYNTIVTAHAFIMIFFMVMPIMIGGFGNWLVPLMLGAPDMAFPRMNNMSFWLLPPSLILLISSSIVEMGVGTGWTVYPPLSSNIAHNGSSVDLAIFSLHLAGISSILGAINFITTIINMRTNQMTF
DQMPLFVWAVGITALLLLLSLPVLAGAITMLLTDRNLNTSFFDPAGGGDPILYQHL
>LEFIC400-10|HM872243|MM03943|Bucculatrix_latviaella
TLYFIFGIWAGMVGTSLSLLIRAELGNPGSLIGNDQIYNTIVTAHAFIMIFFMVMPIMIGGFGNWLVPLMLGAPDMAFPRMNNMSFWLLPPSLILLISSSIVEMGVGTGWTVYPPLSSNIAHNGSSVDLAIFSLHLAGISSILGAINFITTIINMRTNQMTF
DQMPLFVWAVGITALLLLLSLPVLAGAITMLLTDRNLNTSFFDPAGGGDPILYQHL
>LEFIE213-10|HM873951|MM08528|Bucculatrix_maritima
TLYFIFGIWAGMVGTSLSLLIRAELGNPGSLIGNDQIYNTIVTAHAFIMIFFMVMPIMIGGFGNWLVPLMLGAPDMAFPRMNNMSFWLLPPSLILLISSSIVEMGAGTGWTVYPPLSSNIAHSGSSVDLAIFSLHLAGISSILGAINFITTIINMRTNQMTF
DQMPLFVWAVGITALLLLLSLPVLAGAITMLLTDRNLNTSFFDPAGGGDPILYQHL
>LEFIB763-10|HM871640|MM02626|Bucculatrix_nigricomella
TLYFIFGIWASMIGTSLSLLIRAELGSPGSLIGNDQIYNTIVTAHAFIMIFFMVMPIMIGGFGNWLVPLMLGAPDMAFPRMNNMSFWLLPPSLILLISSSVVEMGAGTGWTVYPPLSSNIAHNGSSVDLAIFSLHLAGISSILGAINFITTIINMRMNKMTF
DQMPLFIWAVGITALLLLLSLPVLAGAITMLLTDRNLNTSFFDPAGGGDPILYQHL
>LEFIE777-10|HM874496|MM09948|Bucculatrix_nigricomella
TLYFIFGIWASMIGTSLSLLIRAELGSPGSLIGNDQIYNTIVTAHAFIMIFFMVMPIMIGGFGNWLVPLMLGAPDMAFPRMNNMSFWLLPPSLILLISSSVVEMGAGTGWTVYPPLSSNIAHNGSSVDLAIFSLHLAGISSILGAINFITTIINMRMNKMTF
DQMPLFIWAVGITALLLLLSLPVLAGAITMLLTDRNLNTSFFDPAGGGDPILYQHL
>LEFIC405-10|HM872248|MM03949|Bucculatrix_ratisbonensis
TLYFIFGIWAGMVGTSLSLLIRAELGNPGSLIGNDQIYNTIVTAHAFIMIFFMVMPIMIGGFGNWLVPLMLGAPDMAFPRMNNMSFWLLPPSLMLLISSSVVEMGAGTGWTVYPPLSSNIAHGGSSVDLAIFSLHLAGISSILGAINFITTIINMRTNKMS
FDQMPLFVWAVGITALLLLLSLPVLAGAITMLLTDRNLNTSFFDPAGGGDPILYQHL
>LEFIG674-10|HM876334|MM15538|Bucculatrix_thoracella
TLYFIFGIWAGMVGTSLSLLIRAELGNPGSLIGNDQIYNTIVTAHAFIMIFFMVMPIMIGGFGNWLVPLMLGAPDMAFPRMNNMSFWLLPPSILLLISSSVVEMGAGTGWTVYPPLSSNIAHGGSSVDLAIFSLHLAGISSILGAINFITTIINMRANKMSF
DQMPLFVWAVGITALLLLLSLPVLAGAITMLLTDRNLNTSFFDPAGGGDPILYQHL
>LEFIK509-10|JF854111|MM18084|Bucculatrix_thoracella
TLYFIFGIWAGMIGTSLSLLIRAELGNPGSLIGNDQIYNTIVTAHAFIMIFFMVMPIMIGGFGNWLVPLMLGAPDMAFPRMNNMSFWLLPPSILLLISSSVVEMGAGTGWTVYPPLSSNIAHGGSSVDLAIFSLHLAGISSILGAINFITTIINMRANKMSF
DQMPLFVWAVGITALLLLLSLPVLAGAITMLLTDRNLNTSFFDPAGGGDPILYQHL
>LEFID077-10|HM872888|MM05686|Bucculatrix_ulmella
TLYFIFGIWAGMVGTSLSLLIRAELGNPGSLIGNDQIYNTIVTAHAFIMIFFMVMPIMIGGFGNWLVPLMLGAPDMAFPRMNNMSFWLLPPSILLLISSSIVEMGAGTGWTVYPPLSSNIAHGGSSVDLAIFSLHLAGISSILGAINFITTIINMRTNKMSF
DQMPLFVWAVGITALLLLLSLPVLAGAITMLLTDRNLNTSFFDPAGGGDPILYQHL
>LEFIJ1015-11|KM572086|MM09290|Buckleria_paludum
TLYFIFGIWAGMIGTSLSLLIRAELSTPSSLIGDDQIYNSIVTAHAFIMIFFMVMPIMIGGFGNWLVPLMLGAPDMAFPRMNNMSFWLLPPSILLLISSSIVENGVGTGWTVYPPLSSNIAHGGPSVDLAIFSLHLAGISSILGAINFISTIINMRLNKMMFD
QMPLFVWAVGITALLLLLSLPVLAGAITMLLTDRNLNTSFFDPAGGGDPILYQHL
>LEFIK039-10|KM572572|MM17614|Buckleria_paludum
TLYFIFGIWAGMIGTSLSLLIRAELSTPSSLIGDDQIYNSIVTAHAFIMIFFMVMPIMIGGFGNWLVPLMLGAPDMAFPRMNNMSFWLLPPSILLLISSSIVENGVGTGWTVYPPLSSNIAHGGPSVDLAIFSLHLAGISSILGAINFISTIINMRLNKMMFD
QMPLFVWAVGITALLLLLSLPVLAGAITMLLTDRNLNTSFFDPAGGGDPILYQHL
>LEFIJ1250-11|KM572117|MM21110|Buckleria_paludum
TLYFIFGIWAGMIGTSLSLLIRAELSTPSSLIGDDQIYNSIVTAHAFIMIFFMVMPIMIGGFGNWLVPLMLGAPDMAFPRMNNMSFWLLPPSILLLISSSIVENGVGTGWTVYPPLSSNIAHGGPSVDLAIFSLHLAGISSILGAINFISTIINMRLNKMMFD
QMPLFVWAVGITALLLLLSLPVLAGAITMLLTDRNLNTSFFDPAGGG?PILYQHL
>LEFID006-10|HM872820|MM05427|Bupalus_piniaria
TLYFIFGIWAGMVGTSLSLLIRAELGNPGSLISNDQIYNTIVTAHAFIMIFFMVMPIMIGGFGNWLVPLMLGAPDMAFPRMNNMSFWLLPPSITLLISSSIVENGAGTGWTVYPPLSSNIAHGGSSVDLAIFSLHLAGISSILGAINFITTIINMRLNNLSFD
QMPLFIWAVGITAFLLLLSLPVLAGAITMLLTDRNLNTSFFDPAGGGDPILYQHL
>LEEUA264-11|JN277216|MM19672|Buszkoiana_capnodactylus
TMYFMFGIWAGMIGTSLSLLIRAELGTPGSLIGNDQIYNSIVTAHAFIMIFFMVMPIMIGGFGNWLVPLMLGAPDMAFPRMNNMSFWLLPPSICLLISSSIVENGVGTGWTVYPPLSSNIAHSGPSVDLAIFSLHLAGISSILGAINFISTIINMRLNSMM
FDQLPLFVWSVGITALLLLLSLPVLAGAITMLLTDRNLNTSFFDPAGGGDPILYQHL
>LEFIB199-10|HM871103|MM00640|Cabera_exanthemata
TLYFIFGIWAGMVGTSLSLLIRAELGNPGSLIGDDQIYNTIVTAHAFIMIFFMVMPIMIGGFGNWLVPLMLGAPDMAFPRMNNMSFWLLPPSITLLISSSIVENGAGTGWTVYPPLSSNIAHGGSSVDLAIFSLHLAGISSILGAINFITTIINMRLNNLSFD
QMPLFVWAVGITAFLLLLSLPVLAGAITMLLTDRNLNTSFFDPAGGGDPILYQHL
>LEFIA267-10|HM386611|MM01306|Cabera_exanthemata

TLYFIFGIWAGMVGTSLSLLIRAELGNPGSLIGDDQIYNTIVTAHAFIMIFFMVMPIMIGGFGNWLVPLMLGAPDMAFPRMNNMSFWLLPPSITLLISSSIVENGAGTGWTVYPPLSSNIAHGGSSVDLAIFSLHLAGISSILGAINFITTIINMRLNNLSFD
QMPLFVWAVGITAFLLLLSLPVLAGAITMLLTDRNLNTSFFDPAGGGDPILYQHL
>LEFIB176-10|HM871080|MM00589|Cabera_pusaria
TLYFIFGIWAGMVGTSLSLLIRAELGTPGSLIGDDQIYNTIVTAHAFIMIFFMVMPIMIGGFGNWLVPLMLGAPDMAFPRMNNMSFWLLPPSITLLISSSIVENGAGTGWTVYPPLSSNIAHGGSSVDLAIFSLHLAGISSILGAINFITTIINMRLNNLSFD
QMPLFVWAVGITAFLLLLSLPVLAGAITMLLTDRNLNTSFFDPAGGGDPILYQHL
>LEFIA265-10|HM386609|MM01304|Cabera_pusaria
TLYFIFGIWAGMVGTSLSLLIRAELGTPGSLIGDDQIYNTIVTAHAFIMIFFMVMPIMIGGFGNWLVPLMLGAPDMAFPRMNNMSFWLLPPSITLLISSSIVENGAGTGWTVYPPLSSNIAHGGSSVDLAIFSLHLAGISSILGAINFITTIINMRLNNLSFD
QMPLFVWAVGITAFLLLLSLPVLAGAITMLLTDRNLNTSFFDPAGGGDPILYQHL
>LEFIA266-10|HM386610|MM01305|Cabera_pusaria
TLYFIFGIWAGMVGTSLSLLIRAELGTPGSLIGDDQIYNTIVTAHAFIMIFFMVMPIMIGGFGNWLVPLMLGAPDMAFPRMNNMSFWLLPPSITLLISSSIVENGAGTGWTVYPPLSSNIAHGGSSVDLAIFSLHLAGISSILGAINFITTIINMRLNNLSFD
QMPLFVWAVGITAFLLLLSLPVLAGAITMLLTDRNLNTSFFDPAGGGDPILYQHL
>LEFIL708-10|JF854696|MM13136|Cadra_cautella
TLYFIFGIWSGMVGTSLSLLIRAELGTPGSLIGDDQIYNTIVTGHAFIMIFFMVMPIMIGGFGNWLVPLMLGAPDMAFPRLNNMSFWLLPPSLSLLISSSIVENGAGTGWTVYPPLSSNIAHGGSSVDLAIFSLHLAGISSILGAINFITTIINMKLNGMMFD
QMPLFVWAVGITALLLLLSLPVLAGAITMLLTDRNLNTSFFDPAGGGDPILYQHL
>LEEUA277-11|JN275977|MM19685|Cadra_cautella
TLYFIFGIWSGMVGTSLSLLIRAELGTPGSLIGDDQIYNTIVTGHAFIMIFFMVMPIMIGGFGNWLVPLMLGAPDMAFPRLNNMSFWLLPPSLSLLISSSIVENGAGTGWTVYPPLSSNIAHGGSSVDLAIFSLHLAGISSILGAINFITTIINMKLNGMMFD
QMPLFVWAVGITALLLLLSLPVLAGAITMLLTDRNLNTSFFDPAGGGDPILYQHL
>LEFIH017-10|HM876653|MM15881|Calamia_tridens
TLYFIFGIWAGMVGTSLSLLIRAELGTPGSLIGDDQIYNTIVTAHAFIMIFFMVMPIMIGGFGNWLVPLMLGAPDMAFPRMNNMSFWLLPPSLTLLISSSIVENGAGTGWTVYPPLSSNIAHGGSSVDLAIFSLHLAGISSILGAINFITTIINMRLNSLSFD
QMPLFIWAVGITAFLLLLSLPVLAGAITMLLTDRNLNTSFFDPAGGGDPILYQHL
>LEFIC156-10|HM872002|MM03502|Calamotropha_paludella
TLYFIFGIWAGMVGTSLSLLIRAELGNPGSLIGDDQIYNTIVTAHAFIMIFFMVMPIMIGGFGNWLIPLMLGAPDMAFPRMNNMSFWLLPPSLTLLISSSIVENGAGTGWTAYPPLSSNIAHGGSSVDLAIFSLHLAGISSILGAINFITTIINMRINGLSFDQ
MPLFVWSVGITALLLLLSLPVLAGAITMLLTDRNLNTSFFDPAGGGDPILYQHL
>LEFIF780-10|HM875464|MM13014|Calamotropha_paludella
TLYFIFGIWAGMVGTSLSLLIRAELGNPGSLIGDDQIYNTIVTAHAFIMIFFMVMPIMIGGFGNWLIPLMLGAPDMAFPRMNNMSFWLLPPSLTLLISSSIVENGAGTGWTAYPPLSSNIAHGGSSVDLAIFSLHLAGISSILGAINFITTIINMRINGLSFDQ
MPLFVWSVGITALLLLLSLPVLAGAITMLLTDRNLNTSFFDPAGGGDPILYQHL
>LEFIJ828-10|JF853848|MM17453|Callimorpha_dominula
TLYFIFGIWAGMVGTSLSLLIRAELGNPGSLIGDDQIYNTIVTAHAFIMIFFMVMPIMIGGFGNWLIPLMLGAPDMAFPRMNNMSFWLLPPSLTLLISSSIVENGAGTGWTVYPPLSSNIAHGGSSVDLAIFSLHLAGISSILGAINFITTIINMRLNSLSFDQ
MPLFIWAVGITAFLLLLSLPVLAGAITMLLTDRNLNTSFFDPAGGGDPILYQHL
>LEFIE207-10|HQ570365|MM08522|Callisto_coffeella
TLYFIFGIWSGMVGTSLSLLIRAELGNPGSLIGDDQIYNTIVTAHAFIMIFFMVMPIMIGGFGNWLVPLMLGAPDMAFPRLNNMSFWLLPPSLILLISSSVVENGAGTGWTVYPPLSSNIAHSGSSVDLAIFSLHLAGISSILGAINFITTIINMRPAGMKLD
KMPLFVWAVLITAILLLLSLPVLAGAITMLLTDRNLNTSFFDPAGGGDPILYQHL
>LEFIE208-10|HQ570366|MM08523|Callisto_coffeella
TLYFIFGIWSGMVGTSLSLLIRAELGNPGSLIGDDQIYNTIVTAHAFIMIFFMVMPIMIGGFGNWLVPLMLGAPDMAFPRLNNMSFWLLPPSLILLISSSVVENGAGTGWTVYPPLSSNIAHGGSSVDLAIFSLHLAGISSILGAINFITTIINMRPAGMKLD
KMPLFVWAVLITAILLLLSLPVLAGAITMLLTDRNLNTSFFDPAGGGDPILYQHL
>LEFIE246-10|HM873980|MM08573|Callisto_coffeella
TLYFIFGIWSGMVGTSLSLLIRAELGNPGSLIGDDQIYNTIVTAHAFIMIFFMVMPIMIGGFGNWLVPLMLGAPDMAFPRLNNMSFWLLPPSLILLISSSVVENGAGTGWTVYPPLSSNIAHGGSSVDLAIFSLHLAGISSILGAINFITTIINMRPAGMKLD
KMPLFVWAVLITAILLLLSLPVLAGAITMLLTDRNLNTSFFDPAGGGDPILYQHL
>LEFIK359-10|JF853995|MM17934|Callisto_denticulella
TLYFIFGIWSGMVGTSLSLLIRAELGNPGSLIGDDQIYNTIVTAHAFIMIFFMVMPIMIGGFGNWLVPLMLGAPDMAFPRLNNMSFWLLPPSLILLISSSVVENGAGTGWTVYPPLSSNIAHGGSSVDLAIFSLHLAGISSILGAINFITTIINMRPDSMSLD
KMPLFVWAVMITAVLLLLSLPVLAGAITMLLTDRNLNTSFFDPAGGGDPILYQHL
>LEFIJ1327-11|MM21187|Callisto_denticulella
TLYFIFGIWSGMVGTSLSLLIRAELGNPGSLIGDDQIYNTIVTAHAFIMIFFMVMPIMIGGFGNWLVPLMLGAPDMAFPRLNNMSFWLLPPSLILLISSSVVENGAGTGWTVYPPLSSNIAHGGSSVDLAIFSLHLAGISSILGAINFITTIINMRPDSMSLD
KMPLFVWAVMITTVLLLLSLPVLAGAITMLLTDRNLNTSFFDPAGGGDPILYQHL
>LEFIK050-10|JF853924|MM17625|Callisto_insperatella
TLYFIFGIWSGMLGTSLSLLIRAELGNPGSLIGDDQIYNTIVTAHAFIMIFFMVMPIMIGGFSNWLVPLMLGAPDMAFPRLNNMSFWLLPPSLILLISSSVVENGAGTGWTVYPPLSSNIAHGGSSVDLTIFSLHLAGISSILGAINFITTIINMRPNGMKLDK
MPLFVWSVLITAILLLLSLPVLAGAITMLLTDRNLNTSFFDPAGGGDPILYQHL

>LEFIL690-10|MM18988|Callisto_insperatella
TLYFIFGIWSGMLGTSLSLLIRAELGNPGSLIGDDQIYNTIVTAHAFIMIFFMVMPIMIGGFSNWLVPLMLGAPDMAFPRLNNMSFWLLPPSLILLISSSVVENGTGTGWTVYPPLSSNIAHGGSSVDLTIFSLHLAGISSILGAINFITTIINMRPNGMKLDK
MPLFVWSVLITAILLLLSLPVLAGAITMLLTDRNLNTSFFDPAGGGDPILYQHL
>LEFIC163-10|HM872009|MM03512|Calliteara_abietis
TLYFIFGIWAGMVGTSLSLLIRAELGNPGSLIGNDQIYNTIVTAHAFIMIFFMVMPIMIGGFGNWLVPLMLGAPDMAFPRMNNMSFWLLPPSLTLLISSSIVENGAGTGWTVYPPLSSNIAHGGSSVDLAIFSLHLAGISSILGAINFITTIINMRLNNLSFD
QMPLFVWAVGITAFLLLLSLPVLAGAITMLLTDRNLNTSFFDPAGGGDPILYQHL
>LEFIB323-10|HM871223|MM00859|Calliteara_pudibunda
TLYFIFGIWAGMIGTSLSLLIRAELGNPGSLIGNDQIYNTIVTAHAFIMIFFMVMPIMIGGFGNWLVPLMLGAPDMAFPRMNNMSFWLLPPSLTLLISSSIVENGAGTGWTVYPPLSSNIAHSGSSVDLAIFSLHLAGISSILGAINFITTIINMRLNNLSFDQ
MPLFVWAVGITAFLLLLSLPVLAGAITMLLTDRNLNTSFFDPAGGGDPILYQHL
>LEFIB084-10|HM870993|MM00386|Callophrys_rubi
TLYFIFGIWAGMLGTSLSILIRMELGTPGSLIGDDQIYNTIVTAHAFIMIFFMVMPIMIGGFGNWLVPLMLGAPDMAFPRMNNMSFWLLPPSLMLLISSSIVENGAGTGWTVYPPLSSNIAHGGSSVDLAIFSLHLAGISSILGAINFITTIINMRINNLSFD
QMSLFIWSVGITALLLLLSLPVLAGAITMLLTDRNLNTSFFDPAGGGDPILYQHL
>LEFIJ1505-12|MM22773|Callopistria_juventina
TLYFIFGIWAGMVGTSLSLLIRAELGTPGSLIGDDQIYNTIVTAHAFIMIFFMVMPIMIGGFGNWLVPLMLGAPDMAFPRMNNMSFWLLPPSLTLLISSSIVENGAGTGWTVYPPLSSNIAHGGSSVDLAIFSLHLAGISSILGAINFITTIINMRLNNLSFD
QMPLFIWAVGITAFLLLLSLPVLAGAITMLLTDRNLNTSFFDPAGGGDPILYQHL
>LEFIF665-10|HM875349|MM12623|Calophasia_lunula
TLYFIFGIWAGMVGTSLSLLIRAELGNPGSLIGDDQIYNTIVTAHAFIMIFFMVMPIMIGGFGNWLVPLMLGAPDMAFPRMNNMSFWLLPPSLTLLISSSIVENGAGTGWTVYPPLSSNIAHGGSSVDLAIFSLHLAGISSILGAINFITTIINMRLNNLSFD
QMPLFIWAVGITAFLLLLSLPVLAGAITMLLTDRNLNTSFFDPAGGGDPILYQHL
>LEFIF666-10|HM875350|MM12624|Calophasia_lunula
TLYFIFGIWAGMVGTSLSLLIRAELGNPGSLIGDDQIYNTIVTAHAFIMIFFMVMPIMIGGFGNWLVPLMLGAPDMAFPRMNNMSFWLLPPSLTLLISSSIVENGAGTGWTVYPPLSSNIAHGGSSVDLAIFSLHLAGISSILGAINFITTIINMRLNNLSFD
QMPLFIWAVGITAFLLLLSLPVLAGAITMLLTDRNLNTSFFDPAGGGDPILYQHL
>LEFIF337-10|HM875022|MM11151|Caloptilia_alchimiella
TLYFIFGIWSGMLGTSLSMLIRAELGNPGSLIGDDQIYNTIVTAHAFIMIFFMVMPIMIGGFGNWLVPLMLGAPDMAFPRLNNMSFWLLPPSLILLISSSIVETGVGTGWTVYPPLSSNIAHGGSSVDLAIFSLHLAGISSILGAINFITTIINMRANGMMF
DSMSLFAWAVSITALLLLLSLPVLAGAITMLLTDRNLNTSFFDPAGGGDPILYQHL
>LEFIB098-10|HM871005|MM00409|Caloptilia_betulicola
TLYFIFGIWAGMLGTSLSMLIRAELGNPGSLIGDDQIYNTIVTAHAFIMIFFMVMPIMIGGFGNWLVPLMLGAPDMAFPRLNNMSFWLLPPSLILLISSSIVETGVGTGWTVYPPLSSNIAHGGSSVDLAIFSLHLAGISSILGAINFITTIINMRVNGMMF
DSMSLFTWAVSITALLLLLSLPVLAGAITMLLTDRNLNTSFFDPAGGGDPILYQHL
>LEFIB672-10|HM871550|MM02456|Caloptilia_betulicola
TLYFIFGIWAGMLGTSLSMLIRAELGNPGSLIGDDQIYNTIVTAHAFIMIFFMVMPIMIGGFGNWLVPLMLGAPDMAFPRLNNMSFWLLPPSLILLISSSIVETGVGTGWTVYPPLSSNIAHGGSSVDLAIFSLHLAGISSILGAINFITTIINMRVNGMMF
DSMSLFTWAVSITALLLLLSLPVLAGAITMLLTDRNLNTSFFDPAGGGDPILYQHL
>LEFIE202-10|HQ570362|MM08517|Caloptilia_cuculipennella
TLYFIFGIWSGMVGTSLSMLIRAELGTPGSLIGDDQIYNTIVTAHAFIMIFFMVMPIMIGGFGNWLVPLMLGAPDMAFPRLNNMSFWLLPPSLILLISSSIVENGAGTGWTVYPPLSSNIAHGGSSVDLAIFSLHLAGISSILGAINFITTIINMRANGMMF
DSMSLFTWAVSITALLLLLSLPVLAGAITMLLTDRNLNTSFFDPAGGGDPILYQHL
>LEFIE203-10|HQ570363|MM08518|Caloptilia_cuculipennella
TLYFIFGIWSGMVGTSLSMLIRAELGTPGSLIGDDQIYNTIVTAHAFIMIFFMVMPIMIGGFGNWLVPLMLGAPDMAFPRLNNMSFWLLPPSLILLISSSIVENGAGTGWTVYPPLSSNIAHGGSSVDLAIFSLHLAGISSILGAINFITTIINMRANGMMF
DSMSLFTWAVSITALLLLLSLPVLAGAITMLLTDRNLNTSFFDPAGGGDPILYQHL
>LEFIB109-10|HM871016|MM00447|Caloptilia_elongella
TLYFIFGIWSGMLGTSLSMLIRAELGNPGSLIGDDQIYNTIVTAHAFIMIFFMVMPIMIGGFGNWLVPLMLGAPDMAFPRLNNMSFWLLPPSLILLISSSIVETGVGTGWTVYPPLSSNIAHGGSSVDLAIFSLHLAGISSILGAINFITTIINMRVNGMMF
DSMSLFTWAVSITALLLLLSLPVLAGAITMLLTDRNLNTSFFDPAGGGDPILYQHL
>LEFID279-10|HM873077|MM06153|Caloptilia_elongella
TLYFIFGIWSGMLGTSLSMLIRAELGNPGSLIGDDQIYNTIVTAHAFIMIFFMVMPIMIGGFGNWLVPLMLGAPDMAFPRLNNMSFWLLPPSLILLISSSIVETGVGTGWTVYPPLSSNIAHGGSSVDLAIFSLHLAGISSILGAINFITTIINMRVNGMMF
DSMSLFTWAVSITALLLLLSLPVLAGAITMLLTDRNLNTSFFDPAGGGDPILYQHL
>LEFIE420-10|HM874144|MM09017|Caloptilia_elongella
TLYFIFGIWSGMLGTSLSMLIRAELGNPGSLIGDDQIYNTIVTAHAFIMIFFMVMPIMIGGFGNWLVPLMLGAPDMAFPRLNNMSFWLLPPSLILLISSSIVETGVGTGWTVYPPLSSNIAHGGSSVDLAIFSLHLAGISSILGAINFITTIINMRVNGMMF
DSMSLFTWAVSITALLLLLSLPVLAGAITMLLTDRNLNTSFFDPAGGGDPILYQHL
>LEFID082-10|HM872893|MM05693|Caloptilia_falconipennella
TLYFIFGIWSGMLGTSLSMLIRAELGNPGSLIGDDQIYNTIVTAHAFIMIFFMVMPIMIGGFGNWLVPLMLGAPDMAFPRLNNMSFWLLPPSLILLISSSIVETGVGTGWTVYPPLSSNIAHGGSSVDLAIFSLHLAGISSILGAINFITTIINMRANGMMF

DSMSLFTWAVSITALLLLLSLPVLAGAITMLLTDRNLNTSFFDPAGGGDPILYQHL
>LEFIJ904-10|MM17529|Caloptilia_falconipennella
TLYFIFGIWSGMLGTSLSMLIRAELGNPGSLIGDDQIYNTIVTAHAFIMIFFMVMPIMIGGFGNWLVPLMLGAPDMAFPRLNNMSFWLLPPSLILLISSSIVETGVGTGWTVYPPLSSNIAHGGSSVDLAIFSLHLAGISSILGAINFITTIINMRANGMMF
DSMSLFTWAVSITALLLLLSLPVLAGAITMLLTDRNLNTSFFDPAGGGDPILYQHL
>LEFIJ905-10|MM17530|Caloptilia_falconipennella
TLYFIFGIWSGMLGTSLSMLIRAELGNPGSLIGDDQIYNTIVTAHAFIMIFFMVMPIMIGGFGNWLVPLMLGAPDMAFPRLNNMSFWLLPPSLILLVSSSIVETGVGTGWTVYPPLSSNIAHGGSSVDLAIFSLHLAGISSILGAINFITTIINMRANGMMF
DSMSLFTWAVSITALLLLLSLPVLAGAITMLLTDRNLNTSFFDPAGGGDPILYQHL
>LEFIJ717-10|MM17342|Caloptilia_hemidactylella
TLYFIFGIWSGMLGTSLSMLIRAELGNPGSLIGDDQIYNTIVTAHAFIMIFFMVMPIMIGGFGNWLVPLMLGAPDMAFPRLNNMSFWLLPPSLMLLISSSIVETGVGTGWTVYPPLSSNIAHGGSSVDLAIFSLHLAGISSILGAINFITTIINMRVNGMMF
DSMSLFTWAVSITALLLLLSLPVLAGAITMLLTDRNLNTSFFDPAGGGDPILYQHL
>LEFIK358-10|JF853994|MM17933|Caloptilia_hemidactylella
TLYFIFGIWSGMLGTSLSMLIRAELGNPGSLIGDDQIYNTIVTAHAFIMIFFMVMPIMIGGFGNWLVPLMLGAPDMAFPRLNNMSFWLLPPSLMLLISSSIVETGVGTGWTVYPPLSSNIAHGGSSVDLAIFSLHLAGISSILGAINFITTIINMRVNGMMF
DSMSLFTWAVSITALLLLLSLPVLAGAITMLLTDRNLNTSFFDPAGGGDPILYQHL
>LEFIE476-10|HM874200|MM09250|Caloptilia_jurateae
TLYFIFGIWSGMLGTSLSMLIRAELGTPGSLIGDDQIYNTIVTAHAFIMIFFMVMPIMIGGFGNWLVPLMLGAPDMAFPRLNNMSFWLLPPSLMLLISSSVVETGVGTGWTVYPPLSSNIAHGGSSVDLAIFSLHLAGISSILGAINFITTIINMRVNGMM
FDSMSLFTWAVSITALLLLLSLPVLAGAITMLLTDRNLNTSFFDPAGGGDPILYQHL
>LEFIJ722-10|JN271967|MM17347|Caloptilia_jurateae
TLYFIFGIWSGMLGTSLSMLIRAELGTPGSLIGDDQIYNTIVTAHAFIMIFFMVMPIMIGGFGNWLVPLMLGAPDMAFPRLNNMSFWLLPPSLMLLISSSVVETGVGTGWTVYPPLSSNIAHGGSSVDLAIFSLHLAGISSILGAINFITTIINMRVNGMM
FDSMSLFTWAVSITALLLLLSLPVLAGAITMLLTDRNLNTSFFDPPGGGDPILYQHL
>LEFIC348-10|HM872191|MM03864|Caloptilia_populetorum
TLYFIFGIWSGMLGTSLSMLIRAELGNPGSLIGDDQIYNTIVTAHAFIMIFFMVMPIMIGGFGNWLVPLMLGAPDMAFPRLNNMSFWLLPPSLILLISSSIVENGAGTGWTVYPPLSSNIAHGGSSVDLAIFSLHLAGISSILGAINFITTIINMRANGMMF
DSMSLFTWAVSITALLLLLSLPVLAGAITMLLTDRNLNTSFFDPAGGGDPILYQHL
>LEFIC124-10|HM871971|MM03461|Caloptilia_robustella
TLYFIFGIWSGMLGTSLSMLIRAELGNPGSLIGDDQIYNTIVTAHAFIMIFFMVMPIMIGGFGNWLVPLMLGAPDMAFPRLNNMSFWLLPPSLILLISSSIVETGVGTGWTVYPPLSSNIAHGGSSVDLAIFSLHLAGISSILGAINFITTIINMRANGMMF
DSMSLFAWAVSITALLLLLSLPVLAGAITMLLTDRNLNTSFFDPAGGGDPILYQHL
>LEFIE204-10|HM873946|MM08519|Caloptilia_robustella
TLYFIFGIWSGMLGTSLSMLIRAELGNPGSLIGDDQIYNTIVTAHAFIMIFFMVMPIMIGGFGNWLVPLMLGAPDMAFPRLNNMSFWLLPPSLILLISSSIVETGVGTGWTVYPPLSSNIAHGGSSVDLAIFSLHLAGISSILGAINFITTIINMRANGMMF
DSMSLFAWAVSITALLLLLSLPVLAGAITMLLTDRNLNTSFFDPAGGGDPILYQHL
>LEFIB114-10|HM871021|MM00462|Caloptilia_stigmatella
TLYFIFGIWSGMLGTSLSMLIRAELGNPGSLIGDDQIYNTIVTAHAFIMIFFMVMPIMIGGFGNWLVPLMLGAPDMAFPRLNNMSFWLLPPSLTLLISSSIVETGVGTGWTVYPPLSSNIAHGGSSVDLAIFSLHLAGISSILGAINFITTIINMRANGMMF
DSMSLFTWAVSITALLLLLSLPVLAGAITMLLTDRNLNTSFFDPAGGGDPILYQHL
>LEFIB190-10|HM871094|MM00619|Caloptilia_stigmatella
TLYFIFGIWSGMLGTSLSMLIRAELGNPGSLIGDDQIYNTIVTAHAFIMIFFMVMPIMIGGFGNWLVPLMLGAPDMAFPRLNNMSFWLLPPSLTLLISSSIVETGVGTGWTVYPPLSSNIAHGGSSVDLAIFSLHLAGISSILGAINFITTIINMRANGMMF
DSMSLFTWAVSITALLLLLSLPVLAGAITMLLTDRNLNTSFFDPAGGGDPILYQHL
>LEFIA040-10|HM396389|MM00442|Caloptilia_suberinella
TMYFIFGIWSGMLGTSLSMLIRAELGNPGSLIGDDQIYNTIVTAHAFIMIFFMVMPIMIGGFGNWLVPLMLGAPDMAFPRLNNMSFWLLPPSLILLISSSIVENGAGTGWTVYPPLSSNIAHGGSSVDLAIFSLHLAGISSILGAINFITTIINMRANGMM
FDSMSLFTWAVSITALLLLLSLPVLAGAITMLLTDRNLNTSFFDPAGGGDPILYQHL
>LEFIA043-10|HM396392|MM00445|Caloptilia_suberinella
TMYFIFGIWSGMLGTSLSMLIRAELGNPGSLIGDDQIYNTIVTAHAFIMIFFMVMPIMIGGFGNWLVPLMLGAPDMAFPRLNNMSFWLLPPSLILLISSSIVENGAGTGWTVYPPLSSNIAHGGSSVDLAIFSLHLAGISSILGAINFITTIINMRANGMM
FDSMSLFTWAVSITALLLLLSLPVLAGAITMLLTDRNLNTSFFDPAGGGDPILYQHL
>LEFIB127-10|HM871033|MM00491|Caloptilia_suberinella
TMYFIFGIWSGMLGTSLSMLIRAELGNPGSLIGDDQIYNTIVTAHAFIMIFFMVMPIMIGGFGNWLVPLMLGAPDMAFPRLNNMSFWLLPPSLILLISSSIVENGAGTGWTVYPPLSSNIAHGGSSVDLAIFSLHLAGISSILGAINFITTIINMRANGMM
FDSMSLFTWAVSITALLLLLSLPVLAGAITMLLTDRNLNTSFFDPAGGGDPILYQHL
>LEFIA038-10|HM396387|MM00437|Calybites_phasianipennella
TLYFIFGIWSGMVGTSLSILIRAELGTPGSLIGDDQIYNTIVTAHAFIMIFFMVMPIMIGGFGNWLVPLMLGAPDMAFPRLNNMSFWLLPPSIVLLISSSIVENGAGTGWTVYPPLSSNIAHSGSSVDLAIFSLHLAGISSILGAINFITTIINMRVSGMMFDS
MSLFTWAVGITALLLLLSLPVLAGAITMLLTDRNLNTSFFDPAGGGDPILYQHL
>LEFID148-10|HM872955|MM05910|Calyciphora_albodactylus

TLYFIFGIWSGMIGTSLSLLIRAELGNPGSLIGDDQIYNTIVTAHAFIMIFFMVMPIMIGGFGNWLVPLMLGAPDMAFPRMNNMSFWLLPPSIILLISSSVVENGAGTGWTVYPPLSANIAHSGTSVDLAIFSLHLAGISSILGAINFITTIINMRLNKMMFD
QLPLFVWAVGITALLLLLSLPVLAGAITMLLTDRNLNTSFFDPAGGGDPILYQHL
>LEFIB798-10|HM871675|MM02711|Calyptra_thalictri
TLYFIFGIWAGMVGTSLSLLIRAELGNPGSLIGDDQIYNTIVTAHAFIMIFFMVMPIMIGGFGNWLVPLMLGAPDMAFPRMNNMSFWLLPPSLTLLISSSIVENGAGTGWTVYPPLSSNIAHSGSSVDLAIFSLHLAGISSILGAINFITTIINMRLNNLSFD
QMPLFIWAVGITAFLLLLSLPVLAGAITMLLTDRNLNTSFFDPAGGGDPILYQHL
>LEFID981-10|HM873731|MM08013|Calyptra_thalictri
TLYFIFGIWAGMVGTSLSLLIRAELGNPGSLIGDDQIYNTIVTAHAFIMIFFMVMPIMIGGFGNWLVPLMLGAPDMAFPRMNNMSFWLLPPSLTLLISSSIVENGAGTGWTVYPPLSSNIAHSGSSVDLAIFSLHLAGISSILGAINFITTIINMRLNNLSFD
QMPLFIWAVGITAFLLLLSLPVLAGAITMLLTDRNLNTSFFDPAGGGDPILYQHL
>LEFID254-10|HM873052|MM06119|Cameraria_ohridella
TLYFIFGIWAGMVGSSLSIMIRAELGNPGSLIGDDQIYNTIVTAHAFIMIFFMVMPIMIGGFGNWLVPLMLGAPDMAFPRLNNMSFWLLPPSMLLLISSSVVENGVGTGWTVYPPLSSNIAHSGSSVDLAIFSLHLAGISSILGAINFITTIINMRTNGMSF
DNMPLFVWAVGITALLLLLSLPVLAGAITMLLTDRNLNTSFFDPAGGGDPILYQHL
>LEFID696-10|HM873457|MM06747|Campaea_margaritaria
TLYFIFGIWAGMVGTSLSLLIRAELGNPGSLIGDDQIYNTIVTAHAFIMIFFMVMPIMIGGFGNWLIPLMLGAPDMAFPRMNNMSFWLLPPSITLLISSSIVENGAGTGWTVYPPLSSNIAHGGSSVDLAIFSLHLAGISSILGAINFITTIINMRLNNLSFDQ
MPLFVWAVGITAFLLLLSLPVLAGAITMLLTDRNLNTSFFDPAGGGDPILYQHL
>LEFIC184-10|HM872028|MM03540|Camptogramma_bilineata
TLYFIFGIWAGMVGTSLSLLIRAELGNPGSLIGDDQIYNTIVTAHAFIMIFFMVMPIMIGGFGNWLVPLMLGAPDMAFPRMNNMSFWLLPPSITLLISSSIVENGAGTGWTVYPPLSSNIAHGGSSVDLAIFSLHLAGISSILGAINFITTIINMRLNNMFFD
QLPLFVWAVGITAFLLLLSLPVLAGAITMLLTDRNLNTSFFDPAGGGDPILYQHL
>LEFIC270-10|HM872114|MM03702|Camptogramma_bilineata
TLYFIFGIWAGMIGTSLSLLIRAELGNPGSLIGDDQIYNTIVTAHAFIMIFFMVMPIMIGGFGNWLVPLMLGAPDMAFPRMNNMSFWLLPPSITLLISSSIVENGAGTGWTVYPPLSSNIAHGGSSVDLAIFSLHLAGISSILGAINFITTIINMRLNNMFFD
QLPLFVWAVGITAFLLLLSLPVLAGAITMLLTDRNLNTSFFDPAGGGDPILYQHL
>LEFIF571-10|HM875256|MM12329|Camptogramma_bilineata
TLYFIFGIWAGMVGTSLSLLIRAELGNPGSLIGDDQIYNTIVTAHAFIMIFFMVMPIMIGGFGNWLVPLMLGAPDMAFPRMNNMSFWLLPPSITLLISSSIVENGAGTGWTVYPPLSSNIAHGGSSVDLAIFSLHLAGISSILGAINFITTIINMRLNNMFFD
QLPLFVWAVGITAFLLLLSLPVLAGAITMLLTDRNLNTSFFDPAGGGDPILYQHL
>LEFIJ738-10|JF853796|MM17363|Canephora_hirsuta
TLYFILGVWSGLVGTSLSLLIRAELGTPGTLIGSDQIYNTIVTAHAFIMIFFMVMPIMIGGFGNWLVPLMLGAPDMAFPRMNNMSFWLLPPSLMLLTASSLVESGAGTGWTVYPPLSSNISHNGSSVDLAIFSLHLAGISSILGAINFITTAINMRMNGMS
LDQMPLFVWAVIITAVLLLLSLPVLAGAITMLLTDRNLNTSFFDPAGGGDPILYQHL
>LEFIL214-10|MM19214|Capperia_trichodactyla
TLYFIFGIWAGMIGTSLSLLIRAELSTPSSLIGDDQIYNSIVTAHAFIMIFFMVMPIMIGGFGNWLVPLMLGAPDMAFPRMNNMSFWLLPPSILLLISSSIVENGAGTGWTVYPPLSSNIAHGGPSVDLAIFSLHLAGISSILGAINFISTIINMRLNKMMFD
QMPLFVWAVGITALLLLLSLPVLAGAITMLLTDRNLNTSFFDPAGGGDPILYQHL
>LEFIE926-10|HM874643|MM10302|Capricornia_boisduvaliana
TLYFIFGIWAGMIGTSLSLLIRAELGNPGLLIGDDQIYNTIVTAHAFIMIFFMVMPIMIGGFGNWLVPLMLGAPDMAFPRMNNMSFWLLPPSIMLLISSSIVENGAGTGWTVYPPLSSNIAHSGSSVDLAIFSLHLAGISSILGAVNFITTIINMRPNNMSL
DQMPLFVWSVGITALLLLLSLPVLAGAITMLLTDRNLNTSFFDPAGGGDPILYQHL
>LEFIC309-10|HM872153|MM03806|Capua_vulgana
TLYFIFGIWAGMVGTSLSLLIRAELGNPGSLIGDDQIYNTIVTAHAFIMIFFMVMPIMIGGFGNWLVPLMLGAPDMAFPRMNNMSFWLLPPSIMLLISSSIVENGAGTGWTVYPPLSSNIAHSGSSVDLAIFSLHLAGISSILGAVNFITTIINMRPNNMSL
DQMPLFVWSVGITALLLLLSLPVLAGAITMLLTDRNLNTSFFDPAGGGDPILYQHL
>LEFIC388-10|HM872231|MM03925|Capua_vulgana
TLYFIFGIWAGMVGTSLSLLIRAELGNPGSLIGDDQIYNTIVTAHAFIMIFFMVMPIMIGGFGNWLVPLMLGAPDMAFPRMNNMSFWLLPPSIMLLISSSIVENGAGTGWTVYPPLSSNIAHSGSSVDLAIFSLHLAGISSILGAVNFITTIINMRPNNMSL
DQMPLFVWSVGITALLLLLSLPVLAGAITMLLTDRNLNTSFFDPAGGGDPILYQHL
>LEFIF288-10|HM874978|MM11070|Caradrina_albina
TLYFIFGIWAGMVGTSLSLLIRAELGNPGSLIGDDQIYNTIVTAHAFIMIFFMVMPIMIGGFGNWLVPLMLGAPDMAFPRMNNMSFWLLPPSLTLLISSSIVENGAGTGWTVYPPLSSNIAHGGSSVDLAIFSLHLAGISSILGAINFITTIINMRLNSLSFD
QMPLFIWAVGITAFLLLLSLPVLAGAITMLLTDRNLNTSFFDPAGGGDPILYQHL
>LEFIC454-10|HM872288|MM04037|Caradrina_clavipalpis
TLYFIFGIWAGMVGTSLSLLIRAELGNPGSLIGDDQIYNTIVTAHAFIMIFFMVMPIMIGGFGNWLVPLMLGAPDMAFPRMNNMSFWLLPPSLTLLISSSIVENGAGTGWTVYPPLSSNIAHGGSSVDLAIFSLHLAGISSILGAINFITTIINMRLNSLSFD
QMPLFIWAVGITAFLLLLSLPVLAGAITMLLTDRNLNTSFFDPAGGGDPILYQHL
>LEFIC682-10|HM872503|MM04652|Caradrina_clavipalpis
TLYFIFGIWAGMVGTSLSLLIRAELGNPGSLIGDDQIYNTIVTAHAFIMIFFMVMPIMIGGFGNWLVPLMLGAPDMAFPRMNNMSFWLLPPSLTLLISSSIVENGAGTGWTVYPPLSSNIAHGGSSVDLAIFSLHLAGISSILGAINFITTIINMRLNSLSFD
QMPLFIWAVGITAFLLLLSLPVLAGAITMLLTDRNLNTSFFDPAGGGDPILYQHL

>LEFID260-10|HM873058|MM06125|Caradrina_clavipalpis
TLYFIFGIWAGMVGTSLSLLIRAELGNPGSLIGDDQIYNTIVTAHAFIMIFFMVMPIMIGGFGNWLVPLMLGAPDMAFPRMNNMSFWLLPPSLTLLISSSIVENGAGTGWTVYPPLSSNIAHGGSSVDLAIFSLHLAGISSILGAINFITTIINMRLNSLSFD
QMPLFIWAVGITAFLLLLSLPVLAGAITMLLTDRNLNTSFFDPAGGGDPILYQHL
>LEFID808-10|HM873565|MM06894|Caradrina_clavipalpis
TLYFIFGIWAGMVGTSLSLLIRAELGNPGSLIGDDQIYNTIVTAHAFIMIFFMVMPIMIGGFGNWLVPLMLGAPDMAFPRMNNMSFWLLPPSLTLLISSSIVENGAGTGWTVYPPLSSNIAHGGSSVDLAIFSLHLAGISSILGAINFITTIINMRLNSLSFD
QMPLFIWAVGITAFLLLLSLPVLAGAITMLLTDRNLNTSFFDPAGGGDPILYQHL
>LEFIE912-10|HM874629|MM10276|Caradrina_montana
TLYFIFGIWAGMVGTSLSLLIRAELGNPGSLIGDDQIYNTIVTAHAFIMIFFMVMPIMIGGFGNWLVPLMLGAPDMAFPRMNNMSFWLLPPSLTLLISSSIVENGAGTGWTVYPPLSSNIAHGGSSVDLAIFSLHLAGISSILGAINFITTIINMRLNSLSFD
QMPLFIWAVGITAFLLLLSLPVLAGAITMLLTDRNLNTSFFDPAGGGDPILYQHL
>LEFIA556-10|MM01649|Caradrina_morpheus
TLYFIFGIWAGMVGTSLSLLIRAELGNPGSLIGDDQIYNTIVTAHAFIMIFFMVMPIMIGGFGNWLVPLMLGAPDMAFPRMNNMSFWLLPPSLTLLISSSIVENGAGTGWTVYPPLSSNIAHGGSSVDLAIFSLHLAGISSILGAINFITTIINMRLNNLSFD
QMPLFIWAVGITAFLLLLSLPVLAGAITMLLTDRNLNTSFFDPAGGGDPILYQHL
>LEFIB209-10|HM871113|MM00661|Caradrina_selini
TLYFIFGIWAGMVGTSLSLLIRAELGNPGSLIGDDQIYNTIVTAHAFIMIFFMVMPIMIGGFGNWLVPLMLGAPDMAFPRMNNMSFWLLPPSLTLLISSSIVENGAGTGWTVYPPLSSNIAHGGSSVDLAIFSLHLAGISSILGAINFITTIINMRLNSLSFD
QMPLFIWAVGITAFLLLLSLPVLAGAITMLLTDRNLNTSFFDPAGGGDPILYQHL
>LEFIC195-10|HM872039|MM03556|Carpatolechia_alburnella
TLYFIFGIWAGMVGTSLSLLIRAELGNPGSLIGDDQIYNTIVTAHAFIMIFFMVMPIMIGGFGNWLVPLMLGAPDMAFPRMNNMSFWLLPPSLTLLISSSIVENGAGTGWTVYPPLSSNIAHGGSSVDLAIFSLHLAGISSILGAINFITTIINMKINGLSFD
QMPLFVWAVGITALLLLLSLPVLAGAITMLLTDRNLNTSFFDPAGGGDPILYQHL
>LEFIE716-10|HM874436|MM09805|Carpatolechia_alburnella
TLYFIFGIWAGMVGTSLSLLIRAELGNPGSLIGDDQIYNTIVTAHAFIMIFFMVMPIMIGGFGNWLVPLMLGAPDMAFPRMNNMSFWLLPPSLTLLISSSIVENGAGTGWTVYPPLSSNIAHGGSSVDLAIFSLHLAGISSILGAINFITTIINMKINGLSFD
QMPLFVWAVGITALLLLLSLPVLAGAITMLLTDRNLNTSFFDPAGGGDPILYQHL
>LEFIF591-10|HM875275|MM12437|Carpatolechia_alburnella
TLYFIFGIWAGMVGTSLSLLIRAELGNPGSLIGDDQIYNTIVTAHAFIMIFFMVMPIMIGGFGNWLVPLMLGAPDMAFPRMNNMSFWLLPPSLTLLISSSIVENGAGTGWTVYPPLSSNIAHGGSSVDLAIFSLHLAGISSILGAINFITTIINMKINGLSFD
QMPLFVWAVGITALLLLLSLPVLAGAITMLLTDRNLNTSFFDPAGGGDPILYQHL
>LEFIE087-10|HM873834|MM08264|Carpatolechia_epomidella
TLYFIFGIWAGMVGTSLSLLIRAELGNPGSLIGDDQIYNTIVTAHAFIMIFFMVMPIMIGGFGNWLVPLMLGAPDMAFPRMNNMSFWLLPPSLTLLISSSIVENGAGTGWTVYPPLSSNIAHGGSSVDLAIFSLHLAGISSILGAINFITTIINMKINGLSFD
QMPLFVWSVGITALLLLLSLPVLAGAITMLLTDRNLNTSFFDPAGGGDPILYQHL
>LEFIF475-10|HM875160|MM12021|Carpatolechia_fugitivella
TLYFIFGIWAGMVGTSLSLLIRAELGNPGSLIGDDQIYNTIVTAHAFIMIFFMVMPIMIGGFGNWLVPLMLGAPDMAFPRMNNMSFWLLPPSLTLLISSSIVENGAGTGWTVYPPLSSNIAHGGSSVDLAIFSLHLAGISSILGAINFITTIINMKINGMSFD
QMPLFVWAVGITALLLLLSLPVLAGAITMLLTDRNLNTSFFDPAGGGDPILYQHL
>LEFIG761-10|HM876414|MM15625|Carpatolechia_fugitivella
TLYFIFGIWAGMVGTSLSLLIRAELGNPGSLIGDDQIYNTIVTAHAFIMIFFMVMPIMIGGFGNWLVPLMLGAPDMAFPRMNNMSFWLLPPSLTLLISSSIVENGAGTGWTVYPPLSSNIAHGGSSVDLAIFSLHLAGISSILGAINFITTIINMKINGMSFD
QMPLFVWAVGITALLLLLSLPVLAGAITMLLTDRNLNTSFFDPAGGGDPILYQHL
>LEFIG279-10|HM875958|MM14223|Carpatolechia_notatella
TLYFIFGIWAGMVGTSLSLLIRAELGNPGSLIGDDQIYNTIVTAHAFIMIFFMVMPIMIGGFGNWLVPLMLGAPDMAFPRMNNMSFWLLPPSLTLLISSSIVENGAGTGWTVYPPLSSNIAHGGSSVDLAIFSLHLAGISSILGAINFITTIINMKINGLPFD
QMPLFVWAVGITALLLLLSLPVLAGAITMLLTDRNLNTSFFDPAGGGDPILYQHL
>LEFIJ373-10|JF853560|MM15974|Carpatolechia_notatella
TLYFIFGIWAGMVGTSLSLLIRAELGNPGSLIGDDQIYNTIVTAHAFIMIFFMVMPIMIGGFGNWLVPLMLGAPDMAFPRMNNMSFWLLPPSLTLLISSSIVENGAGTGWTVYPPLSSNIAHGGSSVDLAIFSLHLAGISSILGAINFITTIINMKINGLPFD
QMPLFVWAVGITALLLLLSLPVLAGAITMLLTDRNLNTSFFDPAGGGDPILYQHL
>LEFIB145-10|HM871050|MM00527|Carpatolechia_proximella
TLYFIFGIWAGMVGTSLSLLIRAELGNPGSLIGDDQIYNTIVTAHAFIMIFFMVMPIMIGGFGNWLVPLMLGAPDMAFPRMNNMSFWLLPPSLTLLISSSIVENGAGTGWTVYPPLSSNIAHGGSSVDLAIFSLHLAGISSILGAINFITTIINMKINGLSFD
QMPLFVWAVGITALLLLLSLPVLAGAITMLLTDRNLNTSFFDPAGGGDPILYQHL
>LEFIA381-10|HM386723|MM01438|Carsia_sororiata
TLYFIFGIWAGMVGTSLSLLIRAELGTPGSLIGDDQIYNTIVTAHAFIMIFFMVMPIMIGGFGNWLVPLMLGAPDMAFPRMNNMSFWLLPPSITLLISSSIVETGAGTGWTVYPPLSSNIAHGGSSVDLAIFSLHLAGISSILGAINFITTIINMRLNNMFFD
QLPLFVWAVGITAFLLLLSLPVLAGAITMLLTDRNLNTSFFDPAGGGDPILYQHL
>LEFIB866-10|HM871743|MM02919|Carsia_sororiata
TLYFIFGIWAGMVGTSLSLLIRAELGTPGSLIGDDQIYNTIVTAHAFIMIFFMVMPIMIGGFGNWLVPLMLGAPDMAFPRMNNMSFWLLPPSITLLISSSIVETGAGTGWTVYPPLSSNIAHGGSSVDLAIFSLHLAGISSILGAINFITTIINMRLNNMFFD

QLPLFVWAVGITAFLLLLSLPVLAGAITMLLTDRNLNTSFFDPAGGGDPILYQHL
>LEFIE036-10|HM873785|MM08117|Carsia_sororiata
TLYFIFGIWAGMVGTSLSLLIRAELGTPGSLIGDDQIYNTIVTAHAFIMIFFMVMPIMIGGFGNWLVPLMLGAPDMAFPRMNNMSFWLLPPSITLLISSSIVETGAGTGWTVYPPLSSNIAHGGSSVDLAIFSLHLAGISSILGAINFITTIINMRLNNMFFD
QLPLFVWAVGITAFLLLLSLPVLAGAITMLLTDRNLNTSFFDPAGGGDPILYQHL
>LEFIB139-10|HM871044|MM00511|Carterocephalus_palaemon
TLYFIFGIWAGMVGTSLSLLIRTELGNPGSLIGDDQIYNTIVTAHAFIMIFFMVMPIMIGGFGNWLVPLMLGAPDMAFPRMNNMSFWMLPPSLTLLISSSIVENGAGTGWTVYPPLSSNIAHQGSSVDLAIFSLHLAGISSILGAINFITTIINMRIKNLSFD
QMSLFIWAVGITALLLLLSLPVLAGAITMLLTDRNLNTSFFDPAGGGDPILYQHL
>LEFIE935-10|HM874652|MM10322|Carterocephalus_palaemon
TLYFIFGIWAGMVGTSLSLLIRTELGNPGSLIGDDQIYNTIVTAHAFIMIFFMVMPIMIGGFGNWLVPLMLGAPDMAFPRMNNMSFWMLPPSLTLLISSSIVENGAGTGWTVYPPLSSNIAHQGSSVDLAIFSLHLAGISSILGAINFITTIINMRIKNLSFD
QMSLFIWAVGITALLLLLSLPVLAGAITMLLTDRNLNTSFFDPAGGGDPILYQHL
>LEFIG184-10|HM875864|MM14052|Carterocephalus_silvicola
TLYFIFGIWAGMVGTSLSLLIRTELGNPGSLIGDDQIYNTIVTAHAFIMIFFMVMPIMIGGFGNWLVPLMLGAPDMAFPRMNNMSFWMLPPSLTLLISSSIVENGAGTGWTVYPPLSSNIAHQGSSVDLAIFSLHLAGISSILGAINFITTIINMRIKNLSFD
QMSLFIWAVGITALLLLLSLPVLAGAITMLLTDRNLNTSFFDPAGGGDPILYQHL
>LEFIF522-10|HM875207|MM12154|Caryocolum_amaurella
TLYFIFGIWAGMVGTSLSLLIRAELGNPGSLIGDDQIYNTIVTAHAFIMIFFMVMPIMIGGFGNWLVPLMLGAPDMAFPRMNNMSFWLLPPSLTLLISSSIVENGAGTGWTVYPPLSSNIAHGGSSVDLAIFSLHLAGISSILGAINFITTIINMRINNLSFD
QMPLFVWAVGITALLLLLSLPVLAGAITMLLTDRNLNTSFFDPAGGGDPILYQHL
>LEFIF976-10|HM875657|MM13561|Caryocolum_amaurella
TLYFIFGIWAGMVGTSLSLLIRAELGNPGSLIGDDQIYNTIVTAHAFIMIFFMVMPIMIGGFGNWLVPLMLGTPDMAFPRMNNMSFWLLPPSLTLLISSSIVENGAGTGWTIYPPLSSNIAHGGSSVDLAIFSLHLAGISSILGAINFITTIINMRINNLSFDQ
MPLFVWAVGITALLLLLSLPVLAGAITMLLTDRNLNTSFFDPAGGGDPILYQHL
>LEFIB755-10|HM871632|MM02608|Caryocolum_blandella
TLYFIFGIWAGMVGTSLSLLIRAELGNPGSLIGDDQIYNTIVTAHAFIMIFFMVMPIMIGGFGNWLVPLMLGAPDMAFPRMNNMSFWMLPPSLTLLISSSIVENGAGTGWTVYPPLSSNIAHGGSSVDLAIFSLHLAGISSILGAINFITTIINMRINNLSFD
QMPLFVWAVGITALLLLLSLPVLAGAITMLLTDRNLNTSFFDPAGGGDPILYQHL
>LEFIC179-10|HM872024|MM03533|Caryocolum_blandella
TLYFIFGIWAGMVGTSLSLLIRAELGNPGSLIGDDQIYNTIVTAHAFIMIFFMVMPIMIGGFGNWLVPLMLGAPDMAFPRMNNMSFWMLPPSLTLLISSSIVENGAGTGWTVYPPLSSNIAHGGSSVDLAIFSLHLAGISSILGAINFITTIINMRINNFSF
DQMPLFVWAVGITALLLLLSLPVLAGAITMLLTDRNLNTSFFDPAGGGDPILYQHL
>LEFIJ1299-11|KJ427043|MM21159|Caryocolum_blandella
TLYFIFGIWAGMVGTSLSLLIRAELGNPGSLIGDDQIYNTIVTAHAFIMIFFMVMPIMIGGFGNWLVPLMLGAPDMAFPRMNNMSFWMLPPSLTLLISSSIVENGAGTGWTVYPPLSSNIAHGGSSVDLAIFSLHLAGISSILGAINFITTIINMRINNLSFD
QMPLFVWAVGITALLLLLSLPVLAGAITMLLTDRNLNTSFFDPAGGGDPILYQHL
>LEFIF059-10|HM874770|MM10499|Caryocolum_blandelloides
TLYFIFGIWAGMVGTSLSLLIRAELGNPGSLIGDDQIYNTIVTAHAFIMIFFMVMPIMIGGFGNWLVPLMLGAPDMAFPRMNNMSFWMLPPSLTLLISSSIVENGAGTGWTVYPPLSSNIAHGGSSVDLAIFSLHLAGISSILGAINFITTIINMRINNLSFD
QMPLFVWAVGITALLLLLSLPVLAGAITMLLTDRNLNTSFFDPAGGGDPILYQHL
>LEFIL286-10|JN270954|MM19286|Caryocolum_blandulella
TLYFIFGIWAGMVGTSLSLLIRAELGNPGSLIGDDQIYNTIVTAHAFIMIFFMVMPIMIGGFGNWLVPLMLGAPDMAFPRMNNMSFWLLPPSLTLLISSSIVENGAGTGWTVYPPLSSNIAHGGSSVDLAIFSLHLAGISSILGAINFITTIINMRINNLSFD
QMPLFVWAVGITALLLLLSLPVLAGAITMLLTDRNLNTSFFDPAGGGDPILYQHL
>LEFIL287-10|KJ427098|MM19287|Caryocolum_blandulella
TLYFIFGIWAGMVGTSLSLLIRAELGNPGSLIGDDQIYNTIVTAHAFIMIFFMVMPIMIGGFGNWLVPLMLGAPDMAFPRMNNMSFWLLPPSLTLLISSSIVENGAGTGWTVYPPLSSNIAHGGSSVDLAIFSLHLAGISSILGAINFITTIINMRINNLSFD
QMPLFVWAVGITALLLLLSLPVLAGAITMLLTDRNLNTSFFDPAGGGDPILYQHL
>LEEUA184-11|KJ427095|MM19592|Caryocolum_blandulella
TLYFIFGIWAGMVGTSLSLLIRAELGNPGSLIGDDQIYNTIVTAHAFIMIFFMVMPIMIGGFGNWLVPLMLGAPDMAFPRMNNMSFWLLPPSLTLLISSSIVENGAGTGWTVYPPLSSNIAHGGSSVDLAIFSLHLAGISSILGAINFITTIINMRINNLSFD
QMPLFVWAVGITALLLLLSLPVLAGAITMLLTDRNLNTSFFDPAGGGDPILYQHL
>LEFIE046-10|HM873794|MM08149|Caryocolum_cassella
TLYFIFGIWAGMVGTSLSLLIRAELGNPGSLIGDDQIYNTIVTAHAFIMIFFMVMPIMIGGFGNWLVPLMLGAPDMAFPRMNNMSFWLLPPSLTLLISSSIVENGAGTGWTVYPPLSSNIAHGGSSVDLAIFSLHLAGISSILGAINFITTIINMRVNNLSFD
QMPLFVWAVGITALLLLLSLPVLAGAITMLLTDRNLNTSFFDPAGGGDPILYQHL
>LEFIE633-10|HM874356|MM09575|Caryocolum_cassella
TLYFIFGIWAGMVGTSLSLLIRAELGNPGSLIGDDQIYNTIVTAHAFIMIFFMVMPIMIGGFGNWLVPLMLGAPDMAFPRMNNMSFWLLPPSLTLLISSSIVENGAGTGWTVYPPLSSNIAHGGSSVDLAIFSLHLAGISSILGAINFITTIINMRVNNLSFD
QMPLFIWAVGITALLLLLSLPVLAGAITMLLTDRNLNTSFFDPAGGGDPILYQHL
>LEFIF586-10|HM875270|MM12412|Caryocolum_cassella

TLYFIFGIWAGMVGTSLSLLIRAELGNPGSLIGDDQIYNTIVTAHAFIMIFFMVMPIMIGGFGNWLVPLMLGAPDMAFPRMNNMSFWLLPPSLTLLISSSIVENGAGTGWTVYPPLSSNIAHGGSSVDLAIFSLHLAGISSILGAINFITTIINMRVNNLSFD
QMPLFVWAVGITALLLLLSLPVLAGAITMLLTDRNLNTSFFDPAGGGDPILYQHL
>LEFIJ2446-14|MM23792|Caryocolum_cassella
TLYFIFGIWAGMVGTSLSLLIRAELGNPGSLIGDDQIYNTIVTAHAFIMIFFMVMPIMIGGFGNWLVPLMLGAPDMAFPRMNNMSFWLLPPSLTLLISSSIVENGAGTGWTVYPPLSSNIAHGGSSVDLAIFSLHLAGISSILGAINFITTIINMRVNNLSFD
QMPLFVWAVGITALLLLLSLPVLAGAITMLLTDRNLNTSFFDPAGGGDPILYQHL
>LEFIJ776-10|JF853817|MM17401|Caryocolum_cauligenella
TLYFIFGIWAGMVGTSLSLLIRTELGNPGSLIGDDQIYNTIVTAHAFIMIFFMVMPIMIGGFGNWLVPLMLGAPDMAFPRMNNMSFWLLPPSLTLLISSSIVENGAGTGWTVYPPLSSNIAHGGSSVDLAIFSLHLAGISSILGAINFITTIINMRINNMSFD
QMPLFVWAVGITALLLLLSLPVLAGAITMLLTDRNLNTSFFDPAGGGDPILYQHL
>LEFIF971-10|HM875652|MM13555|Caryocolum_fischerella
TLYFIFGIWAGMVGTSLSLLIRAELGNPGSLIGDDQIYNTIVTAHAFIMIFFMVMPIMIGGFGNWLVPLMLGAPDMAFPRMNNMSFWLLPPSLTLLISSSVVENGAGTGWTVYPPLSSNIAHSGSSVDLTIFSLHLAGISSILGAINFITTIINMRINNLSFD
QMPLFVWAVGITALLLLLSLPVLAGAITMLLTDRNLNTSFFDPAGGGDPILYQHL
>LEFIF480-10|HM875165|MM12032|Caryocolum_fraternella
TLYFIFGIWAGMVGTSLSLLIRAELGNPGSLIGDDQIYNTIVTAHAFIMIFFMVMPIMIGGFGNWLVPLMLGAPDMAFPRMNNMSFWLLPPSLTLLISSSIVENGTGTGWTVYPPLSSNIAHGGSSVDLTIFSLHLAGISSILGAINFITTIINMRINNLSFD
QMPLFVWAVGITALLLLLSLPVLAGAITMLLTDRNLNTSFFDPAGGGDPILYQHL
>LEFIL705-10|JF854695|MM05724|Caryocolum_junctella
TLYFIFGIWAGMVGTSLSLLIRAELGNPGSLIGDDQIYNTIVTAHAFIMIFFMVMPIMIGGFGNWLVPLMLGAPDMAFPRMNNMSFWLLPPSLTLLISSSIVENGAGTGWTVYPPLSSNIAHGGSSVDLAIFSLHLAGISSILGAINFITTIINMRINNLSFD
QMPLFVWAVGITALLLLLSLPVLAGAITMLLTDRNLNTSFFDPAGGGDPILYQHL
>LEFIE482-10|HM874206|MM09258|Caryocolum_kroesmanniella
TLYFIFGIWAGMVGTSLSLLIRAELGNPGSLIGDDQIYNTIVTAHAFIMIFFMVMPIMIGGFGNWLVPLMLGAPDMAFPRMNNMSFWLLPPSLTLLISSSIVENGAGTGWTVYPPLSSNIAHGGSSVDLAIFSLHLAGISSILGAINFITTIINMRVNNLSFD
QMPLFVWAVGITALLLLLSLPVLAGAITMLLTDRNLNTSFFDPAGGGDPILYQHL
>LEFIF521-10|HM875206|MM12153|Caryocolum_kroesmanniella
TLYFIFGIWAGMVGTSLSLLIRAELGNPGSLIGDDQIYNTIVTAHAFIMIFFMVMPIMIGGFGNWLVPLMLGAPDMAFPRMNNMSFWLLPPSLTLLISSSIVENGAGTGWTVYPPLSSNIAHGGSSVDLAIFSLHLAGISSILGAINFITTIINMRVNNLSFD
QMPLFVWAVGITALLLLLSLPVLAGAITMLLTDRNLNTSFFDPAGGGDPILYQHL
>LEEUA182-11|KJ427041|MM19590|Caryocolum_marmorea
TLYFIFGIWAGMVGTSLSLLIRAELGNPGSLIGDDQIYNTIVTAHAFIMIFFMVMPIMIGGFGNWLVPLMLGAPDMAFPRMNNMSFWLLPPSLTLLISSSIVENGAGTGWTVYPPLSSNIAHGGSSVDLAIFSLHLAGISSILGAINFITTIINMRINNLSFD
QMPLFVWAVGITALLLLLSLPVLAGAITMLLTDRNLNTSFFDPAGGGDPILYQHL
>LEFIJ1014-11|KJ427089|MM09288|Caryocolum_petrophila
TLYFIFGIWAGMVGTSLSLLIRAELGNPGSLIGDDQIYNTIVTAHAFIMIFFMVMPIMIGGFGNWLVPLMLGAPDMAFPRMNNMSFWLLPPSLTLLISSSIVENGAGTGWTVYPPLSSNIAHGGSSVDLAIFSLHLAGISSILGAINFITTIINMRINNLSFD
QMPLFVWAVGITALLLLLSLPVLAGAITMLLTDRNLNTSFFDPAGGGDPILYQHL
>LEFIG784-10|HM876436|MM15648|Caryocolum_petryi
TLYFIFGIWAGMVGTSLSLLIRAELGNPGSLIEDDQIYNTIVTAHAFIMIFFMVMPIMIGGFGNWLVPLMLGAPDMAFPRMNNMSFWLLPPSLTLLISSSIVENGAGTGWTVYPPLSSNIAHGGSSVDLAIFSLHLAGISSILGAINFITTIINMRINNLSFD
QMPLFVWAVGITALLLLLSLPVLAGAITMLLTDRNLNTSFFDPAGGGDPILYQHL
>LEFIB286-10|HM871187|MM00778|Caryocolum_pullatella
TLYFIFGIWAGMVGTSLSLLIRAELGNPGSLIGDDQIYNTIVTAHAFIMIFFMVMPIMIGGFGNWLVPLMLGAPDMAFPRMNNMSFWLLPPSLTLLISSSIVENGAGTGWTVYPPLSSNIAHGGSSVDLAIFSLHLAGISSILGAINFITTIINMRVDNLSFD
QMPLFVWAVGITALLLLLSLPVLAGAITMLLTDRNLNTSFFDPAGGGDPILYQHL
>LEFIB936-10|HM871813|MM03085|Caryocolum_pullatella
TLYFIFGIWAGMVGTSLSLLIRAELGNPGSLIGDDQIYNTIVTAHAFIMIFFMVMPIMIGGFGNWLVPLMLGAPDMAFPRMNNMSFWLLPPSLTLLISSSIVENGAGTGWTVYPPLSSNIAHGGSSVDLAIFSLHLAGISSILGAINFITTIINMRVNNLSFD
QMPLFVWAVGITALLLLLSLPVLAGAITMLLTDRNLNTSFFDPAGGGDPILYQ?L
>LEFIE429-10|HM874153|MM09053|Caryocolum_pullatella
TLYFIFGIWAGMVGTSLSLLIRAELGNPGSLIGDDQIYNTIVTAHAFIMIFFMVMPIMIGGFGNWLVPLMLGAPDMAFPRMNNMSFWLLPPSLTLLISSSIVENGAGTGWTVYPPLSSNIAHGGSSVDLAIFSLHLAGISSILGAINFITTIINMRVDNLSFD
QMPLFVWAVGITALLLLLSLPVLAGAITMLLTDRNLNTSFFDPAGGGDPILYQHL
>LEFIJ779-10|JF853820|MM17404|Caryocolum_schleichi
TLYFIFGIWAGMMGTSLSLLIRAELGNPGSLIGDDQIYNTIVTAHAFIMIFFMVMPIMIGGFGNWLIPLMLGAPDMAFPRMNNMSFWLLPPSLTLLISSSIVENGAGTGWTVYPPLSSNIAHGGSSVDLAIFSLHLAGISSILGAINFITTIINMRINNLSFD
QMPLFVWAVGITALLLLLSLPVLAGAITMLLTDRNLNTSFFDPAGGGDPILYQHL
>LEFIL552-10|JF854655|MM18850|Caryocolum_schleichi
TLYFIFGIWAGMMGTSLSLLIRAELGSPGSLIGDDQIYNTIVTAHAFIMIFFMVMPIMIGGFGNWLIPLMLGAPDMAFPRMNNMSFWLLPPSLTLLISSSIVENGAGTGWTVYPPLSSNIAHGGSSVDLAIFSLHLAGISSILGAINFITTIINMRISNLSFDQ
MPLFVWAVGITALLLLLSLPVLAGAITMLLTDRNLNTSFFDPAGGGDPILYQHL

>LEFIC280-10|HM872124|MM03743|Caryocolum_tischeriella
TLYFIFGIWAGMVGTSLSLLIRAELGNPGSLIGNDQIYNTIVTAHAFIMIFFMVMPIMIGGFGNWLVPLMLGAPDMAFPRMNNMSFWLLPPSLTLLISSSIVENGAGTGWTVYPPLSSNIAHSGSSVDLAIFSLHLAGISSILGAINFITTIINMRINNLSFD
QMPLFVWAVGITALLLLLSLPVLAGAITMLLTDRNLNTSFFDPAGGGDPILYQHL
>LEFIF467-10|HM875152|MM11992|Caryocolum_tricolorella
TLYFIFGIWAGMVGTSLSLLIRAELGNPGSLIGDDQIYNTIVTAHAFIMIFFMVMPIMIGGFGNWLVPLMLGAPDMAFPRMNNMSFWLLPPSLTLLISSSIVENGAGTGWTVYPPLSSNIAHGGSSVDLAIFSLHLAGISSILGAINFITTIINMRINNLSFD
QMPLFVWSVGITALLLLLSLPVLAGAITMLLTDRNLNTSFFDPAGGGDPILYQHL
>LEFIF974-10|HM875655|MM13558|Caryocolum_vicinella
TLYFIFGIWAGMVGTSLSLLIRAELGNPGSLIGDDQIYNTIVTAHAFIMIFFMVMPIMIGGFGNWLVPLMLGAPDMAFPRMNNMSFWLLPPSLTLLISSSIVENGAGTGWTVYPPLSSNIAHGGSSVDLAIFSLHLAGISSILGAINFITTIINMRINNLSFD
QMPLFVWAVGITALLLLLSLPVLAGAITMLLTDRNLNTSFFDPAGGGDPILYQHL
>LEFIB285-10|KJ427085|MM00777|Caryocolum_viscariella
TLYFIFGIWAGMVGTSLSLLIRAELGNPGSLIGDDQIYNTIVTAHAFIMIFFMVMPIMIGGFGNWLVPLMLGAPDMAFPRMNNMSFWLLPPSLTLLISSSIVENGAGTGWTVYPPLSSNIAHGGSSVDLAIFSLHLAGISSILGAINFITTIINMRINNLSFD
QMPLFVWAVGITALLLLLSLPVLAGAITMLLTDRNLNTSFFDPAGGGDPILYQHL
>LEFIF469-10|HM875154|MM11997|Caryocolum_viscariella
TLYFIFGIWAGMVGTSLSLLIRAELGNPGSLIGDDQIYNTIVTAHAFIMIFFMVMPIMIGGFGNWLVPLMLGAPDMAFPRMNNMSFWLLPPSLTLLISSSIVENGAGTGWTVYPPLSSNIAHGGSSVDLAIFSLHLAGISSILGAINFITTIINMRINNLSFD
QMPLFVWAVGITALLLLLSLPVLAGAITMLLTDRNLNTSFFDPAGGGDPILYQHL
>LEFIF470-10|HM875155|MM11998|Caryocolum_viscariella
TLYFIFGIWAGMVGTSLSLLIRAELGNPGSLIGDDQIYNTIVTAHAFIMIFFMVMPIMIGGFGNWLVPLMLGAPDMAFPRMNNMSFWLLPPSLTLLISSSIVENGAGTGWTVYPPLSSNIAHGGSSVDLAIFSLHLAGISSILGAINFITTIINMRINNLSFD
QMPLFVWAVGITALLLLLSLPVLAGAITMLLTDRNLNTSFFDPAGGGDPILYQHL
>LEFID270-10|HM873068|MM06141|Cataclysta_lemnata
TLYFLFGIWAGMTGTSLSLLIRAELGNPGSLIGDDQIYNTIVTAHAFIMIFFMVMPIMIGGFGNWLVPLMLGAPDMAFPRMNNMSFWLLPPSLMLLISSSIVENGAGTGWTVYPPLSSNIAHGGSSVDLAIFSLHLAGISSILGAINFITTIINMRINNLTF
DQMPLFVWAVGITALLLLLSLPVLAGAITMLLTDRNLNTSFFDPAGGGDPILYQHL
>LEFID677-10|HM873438|MM06718|Catarhoe_cuculata
TLYFIFGIWAGMIGTSLSLLIRAELGNPGSLIGDDQIYNTIVTAHAFIMIFFMVMPIMIGGFGNWLVPLMLGAPDMAFPRMNNMSFWLLPPSITLLISSSIVENGAGTGWTVYPPLSSNIAHGGSSVDLAIFSLHLAGISSILGAINFITTIINMRLNNMYFD
QLPLFVWAVGITAFLLLLSLPVLAGAITMLLTDRNLNTSFFDPAGGGDPILYQHL
>LEFIB062-10|HM870971|MM00349|Catarhoe_rubidata
TLYFIFGIWAGMIGTSLSLLIRAELGNPGSLIGDDQIYNTIVTAHAFIMIFFMVMPIMIGGFGNWLVPLMLGAPDMAFPRMNNMSFWLLPPSITLLISSSVVENGAGTGWTVYPPLSSNIAHGGSSVDLAIFSLHLAGISSILGAINFITTIINMRLNNMFFD
QLPLFVWAVGITAFLLLLSLPVLAGAITMLLTDRNLNTSFFDPAGGGDPILYQHL
>LEFIA348-10|HM386691|MM01395|Catarhoe_rubidata
TLYFIFGIWAGMIGTSLSLLIRAELGNPGSLIGDDQIYNTIVTAHAFIMIFFMVMPIMIGGFGNWLVPLMLGAPDMAFPRMNNMSFWLLPPSITLLISSSIVENGAGTGWTVYPPLSSNIAHGGSSVDLAIFSLHLAGISSILGAINFITTIINMRLNNMFFD
QLPLFVWAVGITAFLLLLSLPVLAGAITMLLTDRNLNTSFFDPAGGGDPILYQHL
>LEFIF085-10|HQ570381|MM10534|Catastia_marginea
TLYFIFGIWSGMVGTSLSLLIRAELGTPGSLIGDDQIYNTIVTGHAFIMIFFMVMPIMIGGFGNWLVPLMLGAPDMAFPRMNNMSFWLLPPSLTLLISSSIVENGAGTGWTVYPPLSSNIAHSGSSVDLAIFSLHLAGISSILGAINFITTIINMKLNGLSFDQ
MPLFVWAVGITALLLLLSLPVLAGAITMLLTDRNLNTSFFDPAGGGDPILYQHL
>LEFIB316-10|HM871216|MM00845|Catocala_adultera
TLYFIFGIWAGMVGTSLSLLIRAELGNPGSLIGDDQIYNTIVTAHAFIMIFFMVMPIMIGGFGNWLVPLMLGAPDMAFPRMNNMSFWLLPPSLTLLISSSIVENGTGTGWTIYPPLSSNIAHSGSSVDLAIFSLHLAGISSILGAINFITTIINMRLNNLMFD
QMPLFIWAVGITAFLLLLSLPVLAGAITMLLTDRNLNTSFFDPAGGGDPILYQHL
>LEFIB317-10|HM871217|MM00846|Catocala_adultera
TLYFIFGIWAGMVGTSLSLLIRAELGNPGSLIGDDQIYNTIVTAHAFIMIFFMVMPIMIGGFGNWLVPLMLGAPDMAFPRMNNMSFWLLPPSLTLLISSSIVENGTGTGWTIYPPLSSNIAHSGSSVDLAIFSLHLAGISSILGAINFITTIINMRLNNLMFD
QMPLFIWAVGITAFLLLLSLPVLAGAITMLLTDRNLNTSFFDPAGGGDPILYQHL
>COLFE1614-13|MM23226|Catocala_elocata
TLYFIFGIWAGMVGTSLSLLIRAELGNPGSLIGDDQIYNTIVTAHAFIMIFFMVMPIMIGGFGNWLVPLMLGAPDMAFPRMNNMSFWLLPPSLTLLISSSIVENGAGTGWTVYPPLSSNIAHSGSSVDLAIFSLHLAGISSILGAINFITTIINMRLNNLMFD
QMPLFIWAVGITAFLLLLSLPVLAGAITMLLTDRNLNTSFFDPAGGGDPILYQHL
>LEFIA695-10|HM386840|MM01840|Catocala_fraxini
TLYFIFGIWAGMVGTSLSLLIRAELGNPGSLIGDDQIYNTIVTAHAFIMIFFMVMPIMIGGFGNWLVPLMLGAPDMAFPRMNNMSFWLLPPSLTLLISSSIVENGAGTGWTVYPPLSSNIAHSGSSVDLAIFSLHLAGISSILGAINFITTIINMRLNNLMFD
QMPLFIWAVGITAFLLLLSLPVLAGAITMLLTDRNLNTSFFDPAGGGDPILYQHL
>LEFIB832-10|HM871709|MM02788|Catocala_fraxini
TLYFIFGIWAGMVGTSLSLLIRAELGNPGSLIGDDQIYNTIVTAHAFIMIFFMVMPIMIGGFGNWLVPLMLGAPDMAFPRMNNMSFWLLPPSLTLLISSSIVENGAGTGWTVYPPLSSNIAHSGSSVDLAIFSLHLAGISSILGAINFITTIINMRLNSLMFD

QMPLFIWAVGITAFLLLLSLPVLAGAITMLLTDRNLNTSFFDPAGGGDPILYQHL
>LEFID902-10|HM873652|MM07250|Catocala_fulminea
TLYFIFGIWAGMVGTSLSLLIRAELGNPGSLIGDDQIYNTIVTAHAFIMIFFMVMPIMIGGFGNWLVPLMLGAPDMAFPRMNNMSFWLLPPSLTLLISSSIVENGAGTGWTVYPPLSSNIAHSGSSVDLAIFSLHLAGISSILGAINFITTIINMRLNNLMFD
QMPLFIWAVGITAFLLLLSLPVLAGAITMLLTDRNLNTSFFDPAGGGDPILYQHL
>LEFID983-10|HM873733|MM08015|Catocala_fulminea
TLYFIFGIWAGMVGTSLSLLIRAELGNPGSLIGDDQIYNTIVTAHAFIMIFFMVMPIMIGGFGNWLVPLMLGAPDMAFPRMNNMSFWLLPPSLTLLISSSIVENGAGTGWTVYPPLSSNIAHSGSSVDLAIFSLHLAGISSILGAINFITTIINMRLNNLMFD
QMPLFIWAVGITAFLLLLSLPVLAGAITMLLTDRNLNTSFFDPAGGGDPILYQHL
>LEFIC508-10|HM872333|MM04216|Catocala_nupta
TLYFIFGIWAGMVGTSLSLLIRAELGNPGSLIGDDQIYNTIVTAHAFIMIFFMVMPIMIGGFGNWLVPLMLGAPDMAFPRMNNMSFWLLPPSLTLLISSSIVENGAGTGWTVYPPLSSNIAHSGSSVDLAIFSLHLAGISSILGAINFITTIINMRLNNLMFD
QMPLFIWAVGITAFLLLLSLPVLAGAITMLLTDRNLNTSFFDPAGGGDPILYQHL
>LEFIG987-10|HM876626|MM15851|Catocala_nupta
TLYFIFGIWAGMVGTSLSLLIRAELGNPGSLIGDDQIYNTIVTAHAFIMIFFMVMPIMIGGFGNWLVPLMLGAPDMAFPRMNNMSFWLLPPSLTLLISSSIVENGAGTGWTVYPPLSSNIAHSGSSVDLAIFSLHLAGISSILGAINFITTIINMRLNNLMFD
QMPLFIWAVGITAFLLLLSLPVLAGAITMLLTDRNLNTSFFDPAGGGDPILYQHL
>LEFIK946-10|MM18521|Catocala_pacta
TLYFIFGIWAGMVGTSLSLLIRAELGNPGSLIGDDQIYNTIVTAHAFIMIFFMVMPIMIGGFGNWLVPLMLGAPDMAFPRMNNMSFWLLPPSLTLLISSSIVENGAGTGWTVYPPLSSNIAHSGSSVDLAIFSLHLAGISSILGAINFITTIINMRLNNLMFD
QMPLFIWAVGITAFLLLLSLPVLAGAITMLLTDRNLNTSFFDPAGGGDPILYQHL
>LEFIC577-10|HM872398|MM04357|Catocala_promissa
TLYFIFGIWAGMVGTSLSLLIRAELGNPGSLIGDDQIYNTIVTAHAFIMIFFMVMPIMIGGFGNWLVPLMLGAPDMAFPRMNNMSFWLLPPSLTLLISSSIVENGAGTGWTVYPPLSSNIAHSGSSVDLAIFSLHLAGISSILGAINFITTIINMRLNNLMFD
QMPLFIWAVGITAFLLLLSLPVLAGAITMLLTDRNLNTSFFDPAGGGDPILYQHL
>LEFIB986-10|HM871858|MM03180|Catocala_sponsa
TLYFIFGIWAGMVGTSLSLLIRAELGNPGSLIGDDQIYNTIVTAHAFIMIFFMVMPIMIGGFGNWLVPLMLGAPDMAFPRMNNMSFWLLPPSLTLLISSSIVENGAGTGWTVYPPLSSNIAHSGSSVDLAIFSLHLAGISSILGAINFITTIINMRLNNLMFD
QMPLFIWAVGITAFLLLLSLPVLAGAITMLLTDRNLNTSFFDPAGGGDPILYQHL
>LEFIB432-10|HM871331|MM01920|Catoptria_falsella
TLYFIFGIWAGMVGTSLSLLIRAELGNPGSLIGDDQIYNTIVTAHAFIMIFFMVMPIMIGGFGNWLIPLMLGAPDMAFPRMNNMSFWLLPPSLTLLISSSIVENGAGTGWTVYPPLSSNIAHGGSSVDLAIFSLHLAGISSILGAINFITTIINMRINGLSFDQ
MPLFIWSVGITALLLLLSLPVLAGAITMLLTDRNLNTSFFDPAGGGDPILYQHL
>LEFIC800-10|HM872619|MM04958|Catoptria_falsella
TLYFIFGIWAGMVGTSLSLLIRAELGNPGSLIGDDQIYNTIVTAHAFIMIFFMVMPIMIGGFGNWLIPLMLGAPDMAFPRMNNMSFWLLPPSLTLLISSSIVENGAGTGWTVYPPLSSNIAHGGSSVDLAIFSLHLAGISSILGAINFITTIINMRINGLSFDQ
MPLFIWSVGITALLLLLSLPVLAGAITMLLTDRNLNTSFFDPAGGGDPILYQHL
>LEFIF796-10|HM875480|MM13050|Catoptria_falsella
TLYFIFGIWAGMVGTSLSLLIRAELGNPGSLIGDDQIYNTIVTAHAFIMIFFMVMPIMIGGFGNWLIPLMLGAPDMAFPRMNNMSFWLLPPSLTLLISSSIVENGAGTGWTVYPPLSSNIAHGGSSVDLAIFSLHLAGISSILGAINFITTIINMRINGLSFDQ
MPLFIWSVGITALLLLLSLPVLAGAITMLLTDRNLNTSFFDPAGGGDPILYQHL
>LEFIG386-10|HM876063|MM14403|Catoptria_fulgidella
TLYFIFGIWAGMVGTSLSLLIRAELGNPGSLIGDDQIYNTIVTAHAFIMIFFMVMPIMIGGFGNWLVPLMLGAPDMAFPRMNNMSFWLLPPSLTLLISSSIVENGAGTGWTVYPPLSSNIAHGGSSVDLAIFSLHLAGISSILGAINFITTIINMRVNGLSFD
QMPLFVWSVGITALLLLLSLPVLAGAITMLLTDRNLNTSFFDPAGGGDPILYQHL
>LEFIA006-10|HM396356|MM00077|Catoptria_furcatellus
TLYFIFGIWAGMLGTSLSLLIRAELGNPGSLIGDDQIYNTIVTAHAFIMIFFMVMPIMIGGFGNWLIPLMLGTPDMAFPRMNNMSFWLLPPSLTLLISSSIVENGAGTGWTVYPPLSSNFAHSGSSVDLAIFSLHLAGISSILGAINFITTIINMRINGLSFDQ
MPLFVWSVGITALLLLLSLPVLAGAITMLLTDRNLNTSFFDPAGGGDPILYQHL
>LEFIL206-10|JF854528|MM19206|Catoptria_lythargyrella
TLYFIFGIWAGMVGTSLSLLIRAELGNPGSLIGDDQIYNTIVTAHAFIMIFFMVMPIMIGGFGNWLVPLMLGAPDMAFPRMNNMSFWLLPPSLTLLISSSIVENGAGTGWTVYPPLSSNIAHGGSSVDLAIFSLHLAGISSILGAINFITTIINMRINGLSFD
QMPLFVWSVGITALLLLLSLPVLAGAITMLLTDRNLNTSFFDPAGGGDPILYQHL
>LEFIA789-10|HM386930|MM04143|Catoptria_maculalis
TLYFIFGIWAGMVGTSLSLLIRAELGNPGTLIGDDQIYNTIVTAHAFIMIFFMVMPIMIGGFGNWLIPLMLGAPDMAFPRMNNMSFWLLPPSLTLLISSSIVETGAGTGWTVYPPLSSNIAHSGSSVDLAIFSLHLAGISSILGAINFITTIINMRINGLSFDQ
MPLFVWSVGITALLLLLSLPVLAGAITMLLTDRNLNTSFFDPAGGGDPILYQHL
>LEFIE474-10|HM874198|MM09248|Catoptria_maculalis
TLYFIFGIWAGMVGTSLSLLIRAELGNPGTLIGDDQIYNTIVTAHAFIMIFFMVMPIMIGGFGNWLIPLMLGAPDMAFPRMNNMSFWLLPPSLTLLISSSIVETGAGTGWTVYPPLSSNIAHSGSSVDLAIFSLHLAGISSILGAINFITTIINMRINGLSFDQ
MPLFVWSVGITALLLLLSLPVLAGAITMLLTDRNLNTSFFDPAGGGDPILYQHL
>LEFIB878-10|HM871755|MM02951|Catoptria_margaritella

TLYFIFGIWAGMVGTSLSLLIRAELGTPGSLIGDDQIYNTIVTAHAFIMIFFMVMPIMIGGFGNWLVPLMLGAPDMAFPRMNNMSFWLLPPSLTLLISSSIVENGAGTGWTVYPPLSSNIAHSGSSVDLAIFSLHLAGISSILGAINFITTIINMRINMMSFD
QMPLFVWSVGITALLLLLSLPVLAGAITMLLTDRNLNTSFFDPAGGGDPILYQHL
>LEFIC807-10|HM872626|MM04977|Catoptria_margaritella
TLYFIFGIWAGMVGTSLSLLIRAELGTPGSLIGDDQIYNTIVTAHAFIMIFFMVMPIMIGGFGNWLVPLMLGAPDMAFPRMNNMSFWLLPPSLTLLISSSIVENGAGTGWTVYPPLSSNIAHSGSSVDLAIFSLHLAGISSILGAINFITTIINMRINMMSFD
QMPLFVWSVGITALLLLLSLPVLAGAITMLLTDRNLNTSFFDPAGGGDPILYQHL
>LEFIF823-10|HM875507|MM13152|Catoptria_margaritella
TLYFIFGIWAGMVGTSLSLLIRAELGTPGSLIGDDQIYNTIVTAHAFIMIFFMVMPIMIGGFGNWLVPLMLGAPDMAFPRMNNMSFWLLPPSLTLLISSSIVENGAGTGWTVYPPLSSNIAHSGSSVDLAIFSLHLAGISSILGAINFITTIINMRINMMSFD
QMPLFVWSVGITALLLLLSLPVLAGAITMLLTDRNLNTSFFDPAGGGDPILYQHL
>LEEUA281-11|MM19689|Catoptria_osthelderi
TLYFIFGIWAGTVGTSLSLLIRAELGTPGSLIGDDQIYNTIVTAHAFIMIFFMVMPIMIGGFGNWLIPLMLGAPDMAFPRMNNMSFWLLPPSLTLLISSSIVENGAGTGWTVYPPLSSNIAHSGSSVDLAIFSLHLAGISSILGAINFITTIINMRINGLSFDQ
MPLFVWSVGITALLLLLSLPVLAGAITMLLTDRNLNTSFFDPAGGGDPILYQHL
>LEFIG920-10|HM876564|MM15784|Catoptria_permiacus
TLYFIFGIWAGMVGTSLSLLIRAELGNPGSLIGDDQIYNTIVTAHAFIMIFFMVMPIMIGGFGNWLVPLMLGAPDMAFPRMNNMSFWLLPPSLTLLISSSIVETGAGTGWTVYPPLSSNIAHSGSSVDLAIFSLHLAGISSILGAINFITTIINMKINGLSFDQ
MPLFVWSVGITALLLLLSLPVLAGAITMLLTDRNLNTSFFDPAGGGDPILYQHL
>LEFIG921-10|HM876565|MM15785|Catoptria_permiacus
TLYFIFGIWAGMVGTSLSLLIRAELGNPGSLIGDDQIYNTIVTAHAFIMIFFMVMPIMIGGFGNWLVPLMLGAPDMAFPRMNNMSFWLLPPSLTLLISSSIVETGAGTGWTVYPPLSSNIAHSGSSVDLAIFSLHLAGISSILGAINFITTIINMKINGLSFDQ
MPLFIWSVGITALLLLLSLPVLAGAITMLLTDRNLNTSFFDPAGGGDPILYQHL
>LEFIB420-10|HM871319|MM01899|Catoptria_permutatellus
TLYFIFGIWAGMVGTSLSLLIRAELGTPSSLIGDDQIYNTIVTAHAFIMIFFMVMPIMIGGFGNWLIPLMLGAPDMAFPRMNNMSFWLLPPSLTLLISSSIVENGAGTGWTVYPPLSSNIAHSGSSVDLAIFSLHLAGISSILGAINFITTIINMRINGLSFDQ
MPLFVWSVGITALLLLLSLPVLAGAITMLLTDRNLNTSFFDPAGGGDPILYQHL
>LEFIB619-10|HM871498|MM02357|Catoptria_pinella
TLYFIFGIWAGMVGTSLSLLIRAELGNPGSLIGDDQIYNTIVTAHAFIMIFFMVMPIMIGGFGNWLVPLMLGAPDMAFPRMNNMSFWLLPPSLTLLISSSIVENGAGTGWTVYPPLSSNIAHGGSSVDLAIFSLHLAGISSILGAINFITTIINMRINGLSFD
QMPLFVWSVGITALLLLLSLPVLAGAITMLLTDRNLNTSFFDPAGGGDPILYQHL
>LEFIC212-10|HM872056|MM03598|Catoptria_pinella
TLYFIFGIWAGMVGTSLSLLIRAELGNPGSLIGDDQIYNTIVTAHAFIMIFFMVMPIMIGGFGNWLVPLMLGAPDMAFPRMNNMSFWLLPPSLTLLISSSIVENGAGTGWTVYPPLSSNIAHGGSSVDLAIFSLHLAGISSILGAINFITTIINMRINGLSFD
QMPLFVWSVGITALLLLLSLPVLAGAITMLLTDRNLNTSFFDPAGGGDPILYQHL
>LEFIF390-10|HM875075|MM11690|Catoptria_pinella
TLYFIFGIWAGMVGTSLSLLIRAELGNPGSLIGDDQIYNTIVTAHAFIMIFFMVMPIMIGGFGNWLVPLMLGAPDMAFPRMNNMSFWLLPPSLTLLISSSIVENGAGTGWTVYPPLSSNIAHGGSSVDLAIFSLHLAGISSILGAINFITTIINMRINGLSFD
QMPLFVWSVGITALLLLLSLPVLAGAITMLLTDRNLNTSFFDPAGGGDPILYQHL
>LEFIG514-10|HM876187|MM14614|Catoptria_pinella
TLYFIFGIWAGMVGTSLSLLIRAELGNPGSLIGDDQIYNTIVTAHAFIMIFFMVMPIMIGGFGNWLVPLMLGAPDMAFPRMNNMSFWLLPPSLTLLISSSIVENGAGTGWTVYPPLSSNIAHGGSSVDLAIFSLHLAGISSILGAINFITTIINMRINGLSFD
QMPLFVWSVGITALLLLLSLPVLAGAITMLLTDRNLNTSFFDPAGGGDPILYQHL
>LEFIB433-10|HM871332|MM01922|Catoptria_verellus
TLYFIFGIWAGMVGTSLSLLIRAELGNPGSLIGDDQIYNTIVTAHAFIMIFFMVMPIMIGGFGNWLVPLMLGAPDMAFPRMNNMSFWLLPPSLTLLISSSIVENGAGTGWTVYPPLSSNIAHGGSSVDLAIFSLHLAGISSILGAINFITTIINMRINNLSFD
QMPLFVWSVGITALLLLLSLPVLAGAITMLLTDRNLNTSFFDPAGGGDPILYQHL
>LEFIC193-10|HM872037|MM03554|Catoptria_verellus
TLYFIFGIWAGMVGTSLSLLIRAELGNPGSLIGDDQIYNTIVTAHAFIMIFFMVMPIMIGGFGNWLVPLMLGAPDMAFPRMNNMSFWLLPPSLTLLISSSIVENGAGTGWTVYPPLSSNIAHGGSSVDLAIFSLHLAGISSILGAINFITTIINMRINNLSFD
QMPLFVWSVGITALLLLLSLPVLAGAITMLLTDRNLNTSFFDPAGGGDPILYQHL
>LEFIC417-10|HQ570306|MM03963|Cauchas_breviantennella
TLYFIFGIWSGLLGTSLSLIIRTELGMPGSLIGNDQIYNTIVTAHAFIMIFFMVMPIMIGGFGNWLVPLMLGAPDMAFPRLNNMSFWLLPPSLTLLISGSLVENGAGTGWTVYPPLSSNMFHSGTSVDLTIFSLHLAGISSILGAINFITTVINMRTMNMMF
DQMPLFVWSVAITALLLLLSLPVLAGAITMLLTDRNLNTSFFDPMGGGDPILYQHL
>LEFID262-10|HM873060|MM06127|Cauchas_fibulella
TLYFFFGIWSGLLGTSLSLLIRTELGMPGSLIGNDQIYNTIVTAHAFIMIFFMVMPIMIGGFGNWLVPLMLGAPDMAFPRLNNMSFWLLPPSLMLLISSSLVENGSGTGWTVYPPLSSNISHSGTSVDLTIFSLHLAGISSILGAINFITTVINMRTMNMTF
DQMSLFTWSVAITALLLLLSLPVLAGAITMLLTDRNLNTSFFDPMGGGDPILYQHL
>LEFIB019-10|HM870931|MM00047|Cauchas_rufimitrella
TLYFIFGIWSGLLGTSLSLLIRTELGMPGSLIGNDQIYNTIVTAHAFIMIFFMVMPIMIGGFGNWLVPLMLGAPDMAFPRLNNMSFWLLPPSLLLLTSSSLVESGSGTGWTVYPPLSSNVAHSGTSVDLTIFSLHLAGVSSILGAVNFITTVINMRAMNMT
FDQMPLFVWSVAITALLLLLSLPVLAGAITMLLTDRNLNTSFFDPTGGGDPILYQHL

>LEFIE282-10|HM874014|MM08628|Cauchas_rufimitrella
TLYFIFGIWSGLLGTSLSLLIRTELGMPGSLIGNDQIYNTIVTAHAFIMIFFMVMPIMIGGFGNWLVPLMLGAPDMAFPRLNNMSFWLLPPSLLLLTSSSLVESGSGTGWTVYPPLSSNVAHSGTSVDLTIFSLHLAGVSSILGAVNFITTVINMRAMNMT
FDQMPLFVWSVAITALLLLLSLPVLAGAITMLLTDRNLNTSFFDPTGGGDPILYQHL
>LEFIB221-10|HM871125|MM00675|Cedestis_gysseleniella
TLYFIFGIWSGMVGTSLSLLIRAELGNPGSLIGNDQIYNTIVTAHAFIMIFFMVMPIMIGGFGNWLVPLMLGAPDMAFPRMNNMSFWLLPPSLTLLISSSIVETGAGTGWTVYPPLSSNIAHSGSSVDMAIFSLHLAGISSILGAINFITTIINMKSNGMTF
DQMPLFVWAVGITALLLLLSLPVLAGAITMLLTDRNLNTSFFDPAGGGDPILYQHL
>LEFIB721-10|HM871599|MM02540|Cedestis_gysseleniella
TLYFIFGIWSGMVGTSLSLLIRAELGNPGSLIGNDQIYNTIVTAHAFIMIFFMVMPIMIGGFGNWLVPLMLGAPDMAFPRMNNMSFWLLPPSLTLLISSSIVETGAGTGWTVYPPLSSNIAHSGSSVDMAIFSLHLAGISSILGAINFITTIINMKSNGMTF
DQMPLFVWAVGITALLLLLSLPVLAGAITMLLTDRNLNTSFFDPAGGGDPILYQHL
>LEFIG082-10|HM875761|MM13735|Cedestis_gysseleniella
TLYFIFGIWSGMVGTSLSLLIRAELGNPGSLIGNDQIYNTIVTAHAFIMIFFMVMPIMIGGFGNWLVPLMLGAPDMAFPRMNNMSFWLLPPSLTLLISSSIVETGAGTGWTVYPPLSSNIAHSGSSVDMAIFSLHLAGISSILGAINFITTIINMKSNGMTF
DQMPLFVWAVGITALLLLLSLPVLAGAITMLLTDRNLNTSFFDPAGGGDPILYQHL
>LEFID022-10|HM872836|MM05452|Cedestis_subfasciella
TLYFIFGIWSGMLGTSLSLLIRAELGNPGSLIGDDQIYNTIVTAHAFIMIFFMVMPIMIGGFGNWLVPLMLGAPDMAFPRMNNMSFWLLPPSLTLLISSSIVETGAGTGWTVYPPLSSNIAHSGSSVDMAIFSLHLAGISSILGAINFITTIINMKNNGMSF
DQMPLFVWAVGITALLLLLSLPVLAGAITMLLTDRNLNTSFFDPAGGGDPILYQHL
>LEFIG670-10|HM876331|MM15534|Cedestis_subfasciella
TLYFIFGIWSGMLGTSLSLLIRAELGNPGSLIGDDQIYNTIVTAHAFIMIFFMVMPIMIGGFGNWLVPLMLGAPDMAFPRMNNMSFWLLPPSLTLLISSSIVETGAGTGWTVYPPLSSNIAHSGSSVDMAIFSLHLAGISSILGAINFITTIINMKNNGMSF
DQMPLFVWAVGITALLLLLSLPVLAGAITMLLTDRNLNTSFFDPAGGGDPILYQHL
>LEFIB298-10|HM871199|MM00810|Celaena_haworthii
TLYFIFGIWAGMVGTSLSLLIRAELGNPGSLIGDDQIYNTIVTAHAFIMIFFMVMPIMIGGFGNWLVPLMLGAPDMAFPRMNNMSFWLLPPSLTLLISSSIVENGAGTGWTVYPPLSSNIAHGGSSVDLAIFSLHLAGISSILGAINFITTIINMRLNNLSFD
QMPLFIWAVGITAFLLLLSLPVLAGAITMLLTDRNLNTSFFDPAGGGDPILYQHL
>LEFIB124-10|HM871030|MM00488|Celastrina_argiolus
TLYFIFGIWAGMLGTSLSILIRMELGTPGSLIGDDQIYNTIVTAHAFIMIFFMVMPIMIGGFGNWLIPLMLGAPDMAFPRMNNMSFWLLPPSLMLLISSSIVENGAGTGWTVYPPLSSNIAHSGSSVDLAIFSLHLAGISSILGAINFITTIINMRVNNLSFD
QMSLFIWAVGITALLLLLSLPVLAGAITMLLTDRNLNTSFFDPAGGGDPILYQHL
>LEEUA616-11|MM20675|Celypha_aurofasciana
TLYFIFGIWAGMIGTSLSLLIRAELGNPGSLIGDDQIYNTIVTAHAFIMIFFMVMPIMIGGFGNWLVPLMLGAPDMAFPRMNNMSFWLLPPSIMLLISSSIVENGAGTGWTVYPPLSSNIAHSGSSVDLAIFSLHLAGISSILGAVNFITTIINMRPNNMSL
DQMPLFVWAVGITALLLLLSLPVLAGAITMLLTDRNLNTSFFDPAGGGDPILYQHL
>LEEUA615-11|MM20674|Celypha_capreolana
TLYFIFGIWAGMIGTSLSLLIRAELGNPSSLIGDDQIYNTIVTAHAFIMIFFMVMPIMIGGFGNWLVPLMLGAPDMAFPRMNNMSFWLLPPSIMLLISSSVVENGAGTGWTVYPPLSSNIAHSGSSVDLAIFSLHLAGISSILGAVNFITTIINMRPNNMSL
DQMPLFVWAVGITALLLLLSLPVLAGAITMLLTDRNLNTSFFDPAGGGDPILYQHL
>LEFID556-10|HM873321|MM06545|Celypha_cespitana
TLYFIFGVWAGMIGTSLSLLIRAELGNPGSLIGDDQIYNTIVTAHAFIMIFFMVMPIMIGGFGNWLVPLMLGAPDMAFPRMNNMSFWLLPPSLMLLISSSIVENGAGTGWTVYPPLSSNIAHSGSSVDLAIFSLHLAGISSILGAVNFITTIINMRPNNMS
LDQMPLFVWAVGITALLLLLSLPVLAGAITMLLTDRNLNTSFFDPAGGGDPILYQHL
>LEFID710-10|HM873471|MM06764|Celypha_cespitana
TLYFIFGVWAGMIGTSLSLLIRAELGNPGSLIGDDQIYNTIVTAHAFIMIFFMVMPIMIGGFGNWLVPLMLGAPDMAFPRMNNMSFWLLPPSLMLLISSSIVENGAGTGWTVYPPLSSNIAHSGSSVDLAIFSLHLAGISSILGAVNFITTIINMRPNNMS
LDQMPLFVWAVGITALLLLLSLPVLAGAITMLLTDRNLNTSFFDPAGGGDPILYQHL
>LEFIF420-10|HM875105|MM11829|Celypha_cespitana
TLYFIFGVWAGMIGTSLSLLIRAELGNPGSLIGDDQIYNTIVTAHAFIMIFFMVMPIMIGGFGNWLVPLMLGAPDMAFPRMNNMSFWLLPPSLMLLISSSIVENGAGTGWTVYPPLSSNIAHSGSSVDLAIFSLHLAGISSILGAVNFITTIINMRPNNMS
LDQMPLFVWAVGITALLLLLSLPVLAGAITMLLTDRNLNTSFFDPAGGGDPILYQHL
>COLFF463-13|KM573059|MM23196|Celypha_cespitana
TLYFIFGVWAGMIGTSLSLLIRAELGNPGSLIGDDQIYNTIVTAHAFIMIFFMVMPIMIGGFGNWLVPLMLGAPDMAFPRMNNMSFWLLPPSLMLLISSSIVENGAGTGWTVYPPLSSNIAHSGSSVDLAIFSLHLAGISSILGAVNFITTIINMRPNNMS
LDQMPLFVWAVGITALLLLLSLPVLAGAITMLLTDRNLNTSFFDPAGGGDPILYQHL
>LEFIB493-10|HM871388|MM02070|Celypha_lacunana
TLYFIFGVWAGMIGTSLSLLIRAELGNPGSLIGDDQIYNTIVTAHAFIMIFFMVMPIMIGGFGNWLVPLMLGAPDMAFPRMNNMSFWLLPPSIMLLISSSIVENGAGTGWTVYPPLSSNIAHSGSSVDLAIFSLHLAGISSILGAVNFITTIINMRPNNMSL
DQMPLFVWAVGITALLLLLSLPVLAGAITMLLTDRNLNTSFFDPAGGGDPILYQHL
>LEEUA231-11|JN274929|MM19639|Celypha_lacunana
TLYFIFGVWAGMIGTSLSLLIRAELGNPGSLIGDDQIYNTIVTAHAFIMIFFMVMPIMIGGFGNWLVPLMLGAPDMAFPRMNNMSFWLLPPSIMLLISSSIVENGAGTGWTVYPPLSSNIAHSGSSVDLAIFSLHLAGISSILGAVNFITTIINMRPNNMSL

DQMPLFVWAVGITALLLLLSLPVLAGAITMLLTDRNLNTSFFDPAGGGDPILYQHL
>LEFIB494-10|HM871389|MM02072|Celypha_rivulana
TLYFIFGIWAGMIGTSLSLLIRAELGNPGSLIGDDQIYNTIVTAHAFIMIFFMVMPIMIGGFGNWLVPLMLGAPDMAFPRMNNMSFWLLPPSIMLLISSSIVENGAGTGWTVYPPLSSNIAHSGSSVDLAIFSLHLAGISSILGAVNFITTIINMRPNNMSL
DQMPLFVWAVGITALLLLLSLPVLAGAITMLLTDRNLNTSFFDPAGGGDPILYQHL
>LEFIB900-10|HM871777|MM03001|Celypha_rivulana
TLYFIFGIWAGMIGTSLSLLIRAELGNPGSLIGDDQIYNTIVTAHAFIMIFFMVMPIMIGGFGNWLVPLMLGAPDMAFPRMNNMSFWLLPPSIMLLISSSIVENGAGTGWTVYPPLSSNIAHSGSSVDLAIFSLHLAGISSILGAVNFITTIINMRPNNMSL
DQMPLFVWAVGITALLLLLSLPVLAGAITMLLTDRNLNTSFFDPAGGGDPILYQHL
>LEFIF886-10|HM875568|MM13292|Celypha_rivulana
TLYFIFGIWAGMIGTSLSLLIRAELGNPGSLIGDDQIYNTIVTAHAFIMIFFMVMPIMIGGFGNWLVPLMLGAPDMAFPRMNNMSFWLLPPSIMLLISSSIVENGAGTGWTVYPPLSSNIAHSGSSVDLAIFSLHLAGISSILGAVNFITTIINMRPNNMSL
DQMPLFVWAVGITALLLLLSLPVLAGAITMLLTDRNLNTSFFDPAGGGDPILYQHL
>LEFIB544-10|HM871429|MM02163|Celypha_rosaceana
TLYFIFGIWAGMIGTSLSLLIRAELGNPGSLIGDDQIYNTIVTAHAFIMIFFMVMPIMIGGFGNWLVPLMLGAPDMAFPRMNNMSFWLLPPSIMLLISSSIVENGAGTGWTVYPPLSSNIAHSGSSVDLAIFSLHLAGISSILGAVNFITTIINMRPNNMYL
DQMPLFIWAVGITALLLLLSLPVLAGAITMLLTDRNLNTSFFDPAGGGDPILYQHL
>LEFIC924-10|HM872741|MM05268|Celypha_rosaceana
TLYFIFGIWAGMIGTSLSLLIRAELGNPGSLIGDDQIYNTIVTAHAFIMIFFMVMPIMIGGFGNWLVPLMLGAPDMAFPRMNNMSFWLLPPSIMLLISSSIVENGAGTGWTVYPPLSSNIAHSGSSVDLAIFSLHLAGISSILGAVNFITTIINMRPNNMYL
DQMPLFIWAVGITALLLLLSLPVLAGAITMLLTDRNLNTSFFDPAGGGDPILYQHL
>LEFIF440-10|HM875125|MM11903|Celypha_rosaceana
TLYFIFGIWAGMIGTSLSLLIRAELGNPGSLIGDDQIYNTIVTAHAFIMIFFMVMPIMIGGFGNWLVPLMLGAPDMAFPRMNNMSFWLLPPSIMLLISSSIVENGAGTGWTVYPPLSSNIAHSGSSVDLAIFSLHLAGISSILGAVNFITTIINMRPNNMYL
DQMPLFIWAVGITALLLLLSLPVLAGAITMLLTDRNLNTSFFDPAGGGDPILYQHL
>LEFIC050-10|HM871919|MM03309|Celypha_rufana
TLYFIFGVWAGMIGTSLSLLIRAELGNPGSLIGDDQIYNTIVTAHAFIMIFFMVMPIMIGGFGNWLVPLMLGAPDMAFPRMNNMSFWLLPPSLMLLISSSIVETGAGTGWTVYPPLSSNIAHSGSSVDLAIFSLHLAGISSILGAVNFITTIINMRPNNMSL
DQMPLFVWAVGITALLLLLSLPVLAGAITMLLTDRNLNTSFFDPAGGGDPILYQHL
>LEFIG479-10|HM876155|MM14531|Celypha_rufana
TLYFIFGVWAGMIGTSLSLLIRAELGNPGSLIGDDQIYNTIVTAHAFIMIFFMVMPIMIGGFGNWLVPLMLGAPDMAFPRMNNMSFWLLPPSLMLLISSSIVETGAGTGWTVYPPLSSNIAHSGSSVDLAIFSLHLAGISSILGAVNFITTIINMRPNNMSL
DQMPLFVWAVGITALLLLLSLPVLAGAITMLLTDRNLNTSFFDPAGGGDPILYQHL
>LEFIB907-10|HM871784|MM03011|Celypha_rurestrana
TLYFIFGIWAGMIGTSLSLLIRAELGNPSSLIGDDQIYNTIVTAHAFIMIFFMVMPIMIGGFGNWLVPLMLGAPDMAFPRMNNMSFWLLPPSIMLLTSSSIVENGAGTGWTVYPPLSSNIAHSGSSVDLAIFSLHLAGISSILGAVNFITTIINMRPNNMSL
DQMPLFVWAVGITALLLLLSLPVLAGAITMLLTDRNLNTSFFDPAGGGDPILYQHL
>LEFIB508-10|HM871402|MM02101|Celypha_siderana
TLYFIFGIWAGMIGTSLSLLIRAELGNPGSLIGDDQIYNTIVTAHAFIMIFFMVMPIMIGGFGNWLVPLMLGAPDMAFPRMNNMSFWLLPPSILLLISSSIVENGAGTGWTVYPPLSSNIAHSGSSVDLAIFSLHLAGISSILGAVNFITTIINMRPNNMML
DQMPLFIWAVGITALLLLLSLPVLAGAITMLLTDRNLNTSFFDPAGGGDPILYQHL
>LEFIB506-10|HM871400|MM02098|Celypha_striana
TLYFIFGIWAGMIGTSLSLLIRAELGNPGSLIGDDQIYNTIVTAHAFIMIFFMVMPIMIGGFGNWLVPLMLGTPDMAFPRMNNMSFWLLPPSIMLLISSSLVENGAGTGWTVYPPLSSNIAHSGSSVDLAIFSLHLAGISSILGAVNFITTIINMRPNNMNL
DQMPLFIWAVGITALLLLLSLPVLAGAITMLLTDRNLNTSFFDPAGGGDPILYQHL
>LEFIB507-10|HM871401|MM02100|Celypha_tiedemanniana
TLYFIFGVWAGMIGTSLSLLIRAELGNPGSLIGDDQIYNTIVTAHAFIMIFFMVMPIMIGGFGNWLVPLMLGAPDMAFPRMNNMSFWLLPPSIMLLISSSIVENGAGTGWTVYPPLSSNIAHSGSSVDLAIFSLHLAGISSILGAVNFITTIINMRPNNMSL
DQMPLFVWAVGITALLLLLSLPVLAGAITMLLTDRNLNTSFFDPAGGGDPILYQHL
>LEFIA283-10|HM386627|MM01323|Cepphis_advenaria
TLYFIFGIWAGMVGTSLSLLIRAELGNPGSLIGDDQIYNTIVTAHAFIMIFFMVMPIMIGGFGNWLVPLMLGAPDMAFPRMNNMSFWLLPPSITLLISSSIVENGAGTGWTVYPPLSSNIAHGGSSVDLAIFSLHLAGISSILGAINFITTIINMRLNNLMFD
QMPLFVWSVGITAFLLLLSLPVLAGAITMLLTDRNLNTSFFDPAGGGDPILYQHL
>LEFIF372-10|HM875057|MM11594|Cepphis_advenaria
TLYFIFGIWAGMVGTSLSLLIRAELGNPGSLIGDDQIYNTIVTAHAFIMIFFMVMPIMIGGFGNWLVPLMLGAPDMAFPRMNNMSFWLLPPSITLLISSSIVENGAGTGWTVYPPLSSNIAHGGSSVDLAIFSLHLAGISSILGAINFITTIINMRLNNLMFD
QMPLFVWSVGITAFLLLLSLPVLAGAITMLLTDRNLNTSFFDPAGGGDPILYQHL
>LEFIB306-10|HM871207|MM00826|Ceramica_pisi
TLYFIFGIWAGMVGTSLSLLIRAELGNPGSLIGDDQIYNTIVTAHAFIMIFFMVMPIMIGGFGNWLVPLMLGAPDMAFPRMNNMSFWLLPPSLTLLISSSIVENGAGTGWTVYPPLSSNIAHGGSSVDLAIFSLHLAGISSILGAINFITTIINMRLNNLSFD
QMPLFIWAVGITAFLLLLSLPVLAGAITMLLTDRNLNTSFFDPAGGGDPILYQHL
>LEFIA528-10|KM572110|MM01612|Cerapteryx_graminis

TLYFIFGIWAGMVGTSLSLLIRAELGNPGSLIGDDQIYNTIVTAHAFIMIFFMVMPIMIGGFGNWLVPLMLGAPDMAFPRMNNMSFWLLPPSLTLLISSSIVENGAGTGWTVYPPLSSNIAHGGSSVDLAIFSLHLAGISSILGAINFITTIINMRLNSLSFD
QMPLFIWAVGITAFLLLLSLPVLAGAITMLLTDRNLNTSFFDPAGGGDPILYQHL
>LEFIC455-10|HM872289|MM04038|Cerastis_leucographa
TLYFIFGIWAGMVGTSLSLLIRAELGNPGSLIGDDQIYNTIVTAHAFIMIFFMVMPIMIGGFGNWLVPLMLGAPDMAFPRMNNMSFWLLPPSLTLLISSSIVENGAGTGWTVYPPLSSNIAHGGSSVDLAIFSLHLAGISSILGAINFITTIINMRLNSLSFD
QMPLFIWAVGITAFLLLLSLPVLAGAITMLLTDRNLNTSFFDPAGGGDPILYQHL
>LEFIB099-10|HM871006|MM00410|Cerastis_rubricosa
TLYFIFGIWAGMVGTSLSLLIRAELGNPGSLIGDDQIYNTIVTAHAFIMIFFMVMPIMIGGFGNWLVPLMLGAPDMAFPRMNNMSFWLLPPSLTLLISSSIVENGAGTGWTVYPPLSSNIAHGGSSVDLAIFSLHLAGISSILGAINFITTIINMRLNSLSFD
QMPLFIWAVGITAFLLLLSLPVLAGAITMLLTDRNLNTSFFDPAGGGDPILYQHL
>LEFID221-10|HM873022|MM06078|Cerura_erminea
TLYFIFGIWAGMVGTSLSLLIRAELGNPGSLIGDDQIYNTIVTAHAFIMIFFMVMPIMIGGFGNWLVPLMLGAPDMAFPRMNNMSFWMLPPSLTLLISSSIVENGAGTGWTVYPPLSSNIAHGGSSVDLAIFSLHLAGISSILGAINFITTIINMRLNNMSF
DQMPLFIWAVGITAFLLLLSLPVLAGAITMLLTDRNLNTSFFDPAGGGDPILYQHL
>LEFID519-10|HM873284|MM06492|Cerura_erminea
TLYFIFGIWAGMVGTSLSLLIRAELGNPGSLIGDDQIYNTIVTAHAFIMIFFMVMPIMIGGFGNWLVPLMLGAPDMAFPRMNNMSFWMLPPSLTLLISSSIVENGAGTGWTVYPPLSSNIAHGGSSVDLAIFSLHLAGISSILGAINFITTIINMRLNNMSF
DQMPLFIWAVGITAFLLLLSLPVLAGAITMLLTDRNLNTSFFDPAGGGDPILYQHL
>LEFIA067-10|HM396414|MM00980|Cerura_vinula
TLYFIFGIWAGMVGTSLSLLIRAELGNPGSLIGDDQIYNTIVTAHAFIMIFFMVMPIMIGGFGNWLVPLMLGAPDMAFPRMNNMSFWMLPPSLTLLISSSIVENGAGTGWTVYPPLSSNIAHGGSSVDLAIFSLHLAGISSILGAINFITTIINMRLNNMSF
DQMPLFVWAVGITAFLLLLSLPVLAGAITMLLTDRNLNTSFFDPAGGGDPILYQHL
>LEFIF271-10|HM874964|MM11053|Cerura_vinula
TLYFIFGIWAGMVGTSLSLLIRAELGNPGSLIGDDQIYNTIVTAHAFIMIFFMVMPIMIGGFGNWLVPLMLGAPDMAFPRMNNMSFWMLPPSLTLLISSSIVENGAGTGWTVYPPLSSNIAHGGSSVDLAIFSLHLAGISSILGAINFITTIINMRLNNMSF
DQMPLFVWAVGITAFLLLLSLPVLAGAITMLLTDRNLNTSFFDPAGGGDPILYQHL
>LEFIA467-10|HM386807|MM01538|Charanyca_ferruginea
TLYFIFGIWAGMVGTSLSLLIRAELGNPGSLIGDDQIYNTIVTAHAFIMIFFMVMPIMIGGFGNWLVPLMLGAPDMAFPRMNNMSFWLLPPSLTLLISSSIVENGAGTGWTVYPPLSSNIAHGGSSVDLAIFSLHLAGISSILGAINFITTIINMRLNSLSFD
QMPLFIWAVGITAFLLLLSLPVLAGAITMLLTDRNLNTSFFDPAGGGDPILYQHL
>LEFIF649-10|HM875333|MM12562|Charanyca_trigrammica
TLYFIFGIWAGMVGTSLSLLIRAELGNPGSLIGDDQIYNTIVTAHAFIMIFFMVMPIMIGGFGNWLIPLMLGAPDMAFPRMNNMSFWLLPPSLTLLISSSIVENGAGTGWTVYPPLSSNIAHGGSSVDLAIFSLHLAGISSILGAINFITTIINMRLNSLSFDQ
MPLFIWAVGITAFLLLLSLPVLAGAITMLLTDRNLNTSFFDPAGGGDPILYQHL
>LEFIC208-10|HM872052|MM03579|Charissa_obscurata
TLYFIFGIWAGMIGTSLSLLIRAELGNPGSLIGDDQIYNTIVTAHAFIMIFFMVMPIMIGGFGNWLVPLMLGAPDMAFPRMNNMSFWLLPPSITLLISSSIVENGAGTGWTVYPPLSSNIAHGGGSVDLAIFSLHLAGISSILGAINFITTIINMRLNNLSFD
QMPLFVWSVGITAFLLLLSLPVLAGAITMLLTDRNLNTSFFDPAGGGDPILYQHL
>LEFID904-10|HM873654|MM07258|Charissa_obscurata
TLYFIFGIWAGMIGTSLSLLIRAELGNPGSLIGDDQIYNTIVTAHAFIMIFFMVMPIMIGGFGNWLVPLMLGAPDMAFPRMNNMSFWLLPPSITLLISSSIVENGAGTGWTVYPPLSSNIAHGGSSVDLAIFSLHLAGISSILGAINFITTIINMRLNNLSFDQ
MPLFVWSVGITAFLLLLSLPVLAGAITMLLTDRNLNTSFFDPAGGGDPILYQHL
>LEFIL313-10|MM19313|Chersotis_andereggii
TLYFIFGIWAGMVGTSLSLLIRAELGNPGSLIGDDQIYNTIVTAHAFIMIFFMVMPIMIGGFGNWLVPLMLGAPDMAFPRMNNMSFWLLPPSLTLLISSSIVENGAGTGWTVYPPLSSNIAHGGSSVDLAIFSLHLAGISSILGAINFITTIINMRLNNLSFD
QMPLFIWAVGITAFLLLLSLPVLAGAITMLLTDRNLNTSFFDPAGGGDPILYQHL
>LEFIA608-10|HM870857|MM01720|Chersotis_cuprea
TLYFIFGIWAGMVGTSLSLLIRAELGNPGSLIGDDQIYNTIVTAHAFIMIFFMVMPIMIGGFGNWLVPLMLGAPDMAFPRMNNMSFWLLPPSLTLLISSSIVENGAGTGWTVYPPLSSNIAHGGSSVDLAIFSLHLAGISSILGAINFITTIINMRLNNLSFD
QMPLFIWAVGITAFLLLLSLPVLAGAITMLLTDRNLNTSFFDPAGGGDPILYQHL
>LEEUA313-11|JN279400|MM19721|Chesias_legatella
TLYFIFGIWAGMIGTSLSLMIRAELGSPGSLIGDDQIYNTIVTAHAFIMIFFMVMPIMIGGFGNWLVPLMLGAPDMAFPRMNNMSFWLLPPSITLLISSSIVENGAGTGWTVYPPLSSNIAHGGSSVDLAIFSLHLAGISSILGAINFITTIINMRLNNMFFD
QLPLFVWSVGITAFLLLLSLPVLAGAITMLLTDRNLNTSFFDPAGGGDPILYQHL
>LEFIA245-10|HM386589|MM01278|Chiasmia_clathrata
TLYFIFGIWAGMVGTSLSLLIRAELGNPGSLIGDDQIYNTIVTAHAFIMIFFMVMPIMIGGFGNWLVPLMLGAPDMAFPRMNNMSFWLLPPSITLLISSSIVENGAGTGWTVYPPLSSNIAHGGSSVDLAIFSLHLAGISSILGAINFITTIINMRLNNLSFD
QMPLFVWAVGITAFLLLLSLPVLAGAITMLLTDRNLNTSFFDPAGGGDPILYQHL
>LEFIA246-10|HM386590|MM01279|Chiasmia_clathrata
TLYFIFGIWAGMVGTSLSLLIRAELGNPGSLIGDDQIYNTIVTAHAFIMIFFMVMPIMIGGFGNWLVPLMLGAPDMAFPRMNNMSFWLLPPSITLLISSSIVENGAGTGWTVYPPLSSNIAHGGSSVDLAIFSLHLAGISSILGAINFITTIINMRLNNLSFD
QMPLFVWAVGITAFLLLLSLPVLAGAITMLLTDRNLNTSFFDPAGGGDPILYQHL

>LEFIG130-10|HM875810|MM13945|Chiasmia_clathrata
TLYFIFGIWAGMVGTSLSLLIRAELGNPGSLIGDDQIYNTIVTAHAFIMIFFMVMPIMIGGFGNWLVPLMLGAPDMAFPRMNNMSFWLLPPSITLLISSSIVENGAGTGWTVYPPLSSNIAHGGSSVDLAIFSLHLAGISSILGAINFITTIINMRLNNLSFD
QMPLFVWAVGITAFLLLLSLPVLAGAITMLLTDRNLNTSFFDPAGGGDPILYQHL
>LEFIB667-10|HM871545|MM02448|Chilo_phragmitella
TLYFIFGIWAGMIGTSLSLLIRAELGTPGSLIGDDQIYNTIVTAHAFIMIFFMVMPIMIGGFGNWLVPLMLGAPDMAFPRMNNMSFWLLPPSLTLLISSSIVENGAGTGWTVYPPLSSNIAHAGSSVDLAIFSLHLAGISSILGAINFITTIINMRINGLSFDQ
MPLLIWSIGITALLLLLSLPVLAGAITMLLTDRNLNTSFFDPAGGGDPILYQHL
>LEFIA873-10|HM387011|MM09750|Chilo_phragmitella
TLYFIFGIWAGMIGTSLSLLIRAELGTPGSLIGDDQIYNTIVTAHAFIMIFFMVMPIMIGGFGNWLVPLMLGAPDMAFPRMNNMSFWLLPPSLTLLISSSIVENGAGTGWTVYPPLSSNIAHAGSSVDLAIFSLHLAGISSILGAINFITTIINMRINGLSFDQ
MPLLIWSIGITALLLLLSLPVLAGAITMLLTDRNLNTSFFDPAGGGDPILYQHL
>LEFIF770-10|HM875454|MM12991|Chilo_phragmitella
TLYFIFGIWAGMIGTSLSLLIRAELGTPGSLIGDDQIYNTIVTAHAFIMIFFMVMPIMIGGFGNWLVPLMLGAPDMAFPRMNNMSFWLLPPSLTLLISSSIVENGAGTGWTVYPPLSSNIAHAGSSVDLAIFSLHLAGISSILGAINFITTIINMRINGLSFDQ
MPLLIWSIGITALLLLLSLPVLAGAITMLLTDRNLNTSFFDPAGGGDPILYQHL
>LEFIG259-10|HM875938|MM14190|Chilo_phragmitella
TLYFIFGIWAGMIGTSLSLLIRAELGTPGSLIGDDQIYNTIVTAHAFIMIFFMVMPIMIGGFGNWLVPLMLGAPDMAFPRMNNMSFWLLPPSLTLLISSSIVENGAGTGWTVYPPLSSNIAHAGSSVDLAIFSLHLAGISSILGAINFITTIINMRINGLSFDQ
MPLLIWSIGITALLLLLSLPVLAGAITMLLTDRNLNTSFFDPAGGGDPILYQHL
>LEFIC621-10|HM872442|MM04555|Chilodes_maritima
TLYFIFGIWAGMVGTSLSLLIRAELGNPGSLIGDDQIYNTIVTAHAFIMIFFMVMPIMIGGFGNWLVPLMLGAPDMAFPRMNNMSFWLLPPSLTLLISSSIVENGAGTGWTVYPPLSSNIAHGGSSVDLAIFSLHLAGISSILGAINFITTIINMRLNSLSFD
QMPLFIWAVGITAFLLLLSLPVLAGAITMLLTDRNLNTSFFDPAGGGDPILYQHL
>LEFIF380-10|HM875065|MM11626|Chilodes_maritima
TLYFIFGIWAGMVGTSLSLLIRAELGNPGSLIGDDQIYNTIVTAHAFIMIFFMVMPIMIGGFGNWLVPLMLGAPDMAFPRMNNMSFWLLPPSLTLLISSSIVENGAGTGWTVYPPLSSNIAHGGSSVDLAIFSLHLAGISSILGAINFITTIINMRLNSLSFD
QMPLFIWAVGITAFLLLLSLPVLAGAITMLLTDRNLNTSFFDPAGGGDPILYQHL
>LEFIG478-10|HM876154|MM14529|Chilodes_maritima
TLYFIFGIWAGMVGTSLSLLIRAELGNPGSLIGDDQIYNTIVTAHAFIMIFFMVMPIMIGGFGNWLVPLMLGAPDMAFPRMNNMSFWLLPPSLTLLISSSIVENGAGTGWTVYPPLSSNIAHGGSSVDLAIFSLHLAGISSILGAINFITTIINMRLNSLSFD
QMPLFIWAVGITAFLLLLSLPVLAGAITMLLTDRNLNTSFFDPAGGGDPILYQHL
>LEFIC378-10|HM872221|MM03908|Chionodes_continuella
TLYFIFGIWAGMVGTSLSLLIRAELGNPGSLIGDDQIYNTIVTAHAFIMIFFMVMPIMIGGFGNWLIPLMLGAPDMAFPRMNNMSFWLLPPSLTLLISSSIVENGAGTGWTVYPPLSSNIAHDGSSVDLAIFSLHLAGISSILGAINFITTIINMRISGMSFD
QMPLFVWSVGITALLLLLSLPVLAGAITMLLTDRNLNTSFFDPAGGGDPILYQHL
>LEFIF453-10|HM875138|MM11942|Chionodes_continuella
TLYFIFGIWAGMVGTSLSLLIRAELGNPGSLIGDDQIYNTIVTAHAFIMIFFMVMPIMIGGFGNWLVPLMLGAPDMAFPRMNNMSFWLLPPSLTLLISSSIVENGAGTGWTVYPPLSSNIAHDGSSVDLAIFSLHLAGISSILGAINFITTIINMRISGMSFD
QMPLFVWSVGITALLLLLSLPVLAGAITMLLTDRNLNTSFFDPAGGGDPILYQHL
>LEFIF941-10|HM875622|MM13396|Chionodes_continuella
TLYFIFGIWAGMVGTSLSLLIRAELGNPGSLIGDDQIYNTIVTAHAFIMIFFMVMPIMIGGFGNWLVPLMLGAPDMAFPRMNNMSFWLLPPSLTLLISSSIVENGAGTGWTVYPPLSSNIAHGGSSVDLAIFSLHLAGISSILGAINFITTIINMRISGMSFD
QMPLFVWSVGITALLLLLSLPVLAGAITMLLTDRNLNTSFFDPAGGGDPILYQHL
>LEFID565-10|HM873330|MM06558|Chionodes_distinctella
TLYFIFGIWAGMVGTSLSLLIRAELGNPGSLIGDDQIYNTIVTAHAFIMIFFMVMPIMIGGFGNWLVPLMLGAPDMAFPRMNNMSFWLLPPSLTLLISSSIVENGAGTGWTVYPPLSSNIAHGGSSVDLAIFSLHLAGISSILGAINFITTIINMRINGMSFD
QMPLFVWAVGITALLLLLSLPVLAGAITMLLTDRNLNTSFFDPAGGGDPILYQHL
>LEFIE796-10|HM874515|MM09981|Chionodes_distinctella
TLYFIFGIWAGMVGTSLSLLIRAELGNPGSLIGDDQIYNTIVTAHAFIMIFFMVMPIMIGGFGNWLVPLMLGAPDMAFPRMNNMSFWLLPPSLTLLISSSIVENGAGTGWTVYPPLSSNIAHGGSSVDLAIFSLHLAGISSILGAINFITTIINMRINGMSFD
QMPLFVWAVGITALLLLLSLPVLAGAITMLLTDRNLNTSFFDPAGGGDPILYQHL
>LEFIJ142-10|KM373601|MM09982|Chionodes_distinctella
TLYFIFGIWAGMVGTSLSLLIRAELGNPGSLIGDDQIYNTIVTAHAFIMIFFMVMPIMIGGFGNWLVPLMLGAPDMAFPRMNNMSFWLLPPSLTLLISSSIVENGAGTGWTVYPPLSSNIAHGGSSVDLAIFSLHLAGISSILGAINFITTIINMRINGMSFD
QMPLFVWAVGITALLLLLSLPVLAGAITMLLTDRNLNTSFFDPAGGGDPILYQHL
>LEFIE939-10|HM874656|MM10327|Chionodes_distinctella
TLYFIFGIWAGMVGTSLSLLIRAELGNPGSLIGDDQIYNTIVTAHAFIMIFFMVMPIMIGGFGNWLVPLMLGAPDMAFPRMNNMSFWLLPPSLTLLISSSIVENGAGTGWTVYPPLSSNIAHGGSSVDLAIFSLHLAGISSILGAINFITTIINMRINGMSFD
QMPLFVWAVGITALLLLLSLPVLAGAITMLLTDRNLNTSFFDPAGGGDPILYQHL
>LEFIJ1289-11|MM21149|Chionodes_distinctella
TLYFIFGIWAGMVGTSLSLLIRAELGNPGSLIGDDQIYNTIVTAHAFIMIFFMVMPIMIGGFGNWLVPLMLGAPDMAFPRMNNMSFWLLPPSLTLLISSSIVENGAGTGWTVYPPLSSNIAHGGSSVDLAIFSLHLAGISSILGAINFITTIINMRINGMSFD

QMPLFVWAVGITALLLLLSLPVLAGAITMLLTDRNLNTSFFDPAGGGDPILYQHL
>LEFIB231-10|HM871134|MM00691|Chionodes_electella
TLYFIFGIWAGMMGTSLSLLIRAELGNPGSLIGDDQIYNTIVTAHAFIMIFFMVMPIMIGGFGNWLVPLMLGAPDMAFPRMNNMSFWLLPPSIILLISSSIVENGAGTGWTVYPPLSSNIAHSGSSVDLAIFSLHLAGISSILGAINFITTIINMRINGLSFDQ
MPLFVWAVGITALLLLLSLPVLAGAITMLLTDRNLNTSFFDPAGGGDPILYQHL
>LEFIA913-10|HM387049|MM09791|Chionodes_electella
TLYFIFGIWAGMMGTSLSLLIRAELGNPGSLIGDDQIYNTIVTAHAFIMIFFMVMPIMIGGFGNWLVPLMLGAPDMAFPRMNNMSFWLLPPSIILLISSSIVENGAGTGWTVYPPLSSNIAHSGSSVDLAIFSLHLAGISSILGAINFITTIINMRINGLSFDQ
MPLFVWAVGITALLLLLSLPVLAGAITMLLTDRNLNTSFFDPAGGGDPILYQHL
>LEFIJ057-10|JF853419|MM03589|Chionodes_fumatella
TLYFIFGIWAGMVGTSLSLLIRAELGNPGSLIGDDQIYNTIVTAHAFIMIFFMVMPIMIGGFGNWLVPLMLGAPDMAFPRMNNMSFWLLPPSLTLLISSSIVENGAGTGWTVYPPLSSNIAHGGSSVDLAIFSLHLAGISSILGAINFITTIINMRINGMSFD
QMPLFVWAVGITALLLLLSLPVLAGAITMLLTDRNLNTSFFDPAGGGDPILYQHL
>LEFIJ155-10|JF853468|MM10282|Chionodes_fumatella
TLYFIFGIWAGMMGTSLSLLIRAELGNPGSLIGDDQIYNTIVTAHAFIMIFFMVMPIMIGGFGNWLVPLMLGAPDMAFPRMNNMSFWLLPPSLTLLISSSIVENGAGTGWTVYPPLSSNIAHGGSSVDLAIFSLHLAGISSILGAINFITTIINMRINGMSF
DQMPLFVWAVGITALLLLLSLPVLAGAITMLLTDRNLNTSFFDPAGGGDPILYQHL
>LEFIE938-10|HM874655|MM10326|Chionodes_fumatella
TLYFIFGIWAGMVGTSLSLLIRAELGNPGSLIGDDQIYNTIVTAHAFIMIFFMVMPIMIGGFGNWLVPLMLGAPDMAFPRMNNMSFWLLPPSLTLLISSSIVENGAGTGWTVYPPLSSNIAHGGSSVDLAIFSLHLAGISSILGAINFITTIINMRINGMSFD
QMPLFVWAVGITALLLLLSLPVLAGAITMLLTDRNLNTSFFDPAGGGDPILYQHL
>LEFIJ235-10|KM373599|MM13633|Chionodes_fumatella
TLYFIFGIWAGMVGTSLSLLIRAELGNPGSLIGDDQIYNTIVTAHAFIMIFFMVMPIMIGGFGNWLVPLMLGAPDMAFPRMNNMSFWLLPPSLTLLISSSIVENGAGTGWTVYPPLSSNIAHGGSSVDLAIFSLHLAGISSILGAINFITTIINMRINGMSFD
QMPLFVWAVGITALLLLLSLPVLAGAITMLLTDRNLNTSFFDPAGGGDPILYQHL
>LEFIJ627-10|JF853725|MM17252|Chionodes_fumatella
TLYFIFGIWAGMVGTSLSLLIRAELGNPGSLIGDDQIYNTIVTAHAFIMIFFMVMPIMIGGFGNWLVPLMLGAPDMAFPRMNNMSFWLLPPSLTLLISSSIVENGAGTGWTVYPPLSSNIAHGGSSVDLAIFSLHLAGISSILGAINFITTIINMRINGMSFD
QMPLFVWAVGITALLLLLSLPVLAGAITMLLTDRNLNTSFFDPAGGGDPILYQHL
>LEFII173-10|JF853382|MM18600|Chionodes_fumatella
TLYFIFGIWAGMVGTSLSLLIRAELGNPGSLIGDDQIYNTIVTAHAFIMIFFMVMPIMIGGFGNWLVPLMLGAPDMAFPRMNNMSFWLLPPSLTLLISSSIVENGAGTGWTVYPPLSSNIAHGGSSVDLAIFSLHLAGISSILGAINFITTIINMRINGMSFD
QMPLFVWAVGITALLLLLSLPVLAGAITMLLTDRNLNTSFFDPAGGGDPILYQHL
>LEFII174-10|JF853383|MM18601|Chionodes_fumatella
TLYFIFGIWAGMVGTSLSLLIRAELGNPGSLIGDDQIYNTIVTAHAFIMIFFMVMPIMIGGFGNWLVPLMLGAPDMAFPRMNNMSFWLLPPSLTLLISSSIVENGAGTGWTVYPPLSSNIAHGGSSVDLAIFSLHLAGISSILGAINFITTIINMRINGMSFD
QMPLFVWAVGITALLLLLSLPVLAGAITMLLTDRNLNTSFFDPAGGGDPILYQHL
>LEFII175-10|JF853384|MM18602|Chionodes_fumatella
TLYFIFGIWAGMVGTSLSLLIRAELGNPGSLIGDDQIYNTIVTAHAFIMIFFMVMPIMIGGFGNWLVPLMLGAPDMAFPRMNNMSFWLLPPSLTLLISSSIVENGAGTGWTVYPPLSSNIAHGGSSVDLAIFSLHLAGISSILGAINFITTIINMRINGMSFD
QMPLFVWAVGITALLLLLSLPVLAGAITMLLTDRNLNTSFFDPAGGGDPILYQHL
>LEFII178-10|JF853387|MM18605|Chionodes_fumatella
TLYFIFGIWAGMVGTSLSLLIRAELGNPGSLIGDDQIYNTIVTAHAFIMIFFMVMPIMIGGFGNWLVPLMLGAPDMAFPRMNNMSFWLLPPSLTLLISSSIVENGAGTGWTVYPPLSSNIAHGGSSVDLAIFSLHLAGISSILGAINFITTIINMRINGMSFD
QMPLFVWAVGITALLLLLSLPVLAGAITMLLTDRNLNTSFFDPAGGGDPILYQHL
>LEFIL343-10|JN270957|MM18653|Chionodes_fumatella
TLYFIFGIWAGMVGTSLSLLIRAELGNPGSLIGDDQIYNTIVTAHAFIMIFFMVMPIMIGGFGNWLVPLMLGAPDMAFPRMNNMSFWLLPPSLTLLISSSIVENGAGTGWTVYPPLSSNIAHGGSSVDLAIFSLHLAGISSILGAINFITTIINMRINGMSFD
QMPLFVWAVGITALLLLLSLPVLAGAITMLLTDRNLNTSFFDPAGGGDPILYQHL
>LEFIB739-10|HM871617|MM02573|Chionodes_holosericella
TLYFIFGIWAGMVGTSLSLLIRAELGNPGSLIGDDQIYNTIVTAHAFIMIFFMVMPIMIGGFGNWLVPLMLGAPDMAFPRMNNMSFWLLPPSLTLLISSSIVENGAGTGWTVYPPLSSNIAHSGSSVDLAIFSLHLAGISSILGAINFITTIINMRINGLTFD
QMPLFVWAVGITALLLLLSLPVLAGAITMLLTDRNLNTSFFDPAGGGDPILYQHL
>LEFIE025-10|HM873774|MM08090|Chionodes_holosericella
TLYFIFGIWAGMVGTSLSLLIRAELGNPGSLIGDDQIYNTIVTAHAFIMIFFMVMPIMIGGFGNWLVPLMLGAPDMAFPRMNNMSFWLLPPSLTLLISSSIVENGAGTGWTVYPPLSSNIAHSGSSVDLAIFSLHLAGISSILGAINFITTIINMRINGLTFD
QMPLFVWAVGITALLLLLSLPVLAGAITMLLTDRNLNTSFFDPAGGGDPILYQHL
>LEFIJ706-10|JF853782|MM17331|Chionodes_ignorantella
TLYFIFGIWAGMVGTSLSLLIRAELGNPGSLIGDDQIYNTIVTAHAFIMIFFMVMPIMIGGFGNWLVPLMLGAPDMAFPRMNNMSFWLLPPSLTLLISSSIVENGAGTGWTVYPPLSSNIAHGGSSVDLTIFSLHLAGISSILGAINFITTIINMRINGMSFD
QMPLFVWAVGITALLLLLSLPVLAGAITMLLTDRNLNTSFFDPAGGGDPILYQHL
>LEFIJ773-10|JF853814|MM17398|Chionodes_ignorantella

TLYFIFGIWAGMVGTSLSLLIRAELGNPGSLIGDDQIYNTIVTAHAFIMIFFMVMPIMIGGFGNWLVPLMLGAPDMAFPRMNNMSFWLLPPSLTLLISSSIVENGAGTGWTVYPPLSSNIAHGGSSVDLTIFSLHLAGISSILGAINFITTIINMRINSMSFD
QMPLFVWAVGITALLLLLSLPVLAGAITMLLTDRNLNTSFFDPAGGGDPILYQHL
>LEFIB232-10|HM871135|MM00693|Chionodes_luctuella
TLYFIFGIWAGMVGTSLSLLIRAELGNPGSLIGDDQIYNTIVTAHAFIMIFFMVMPIMIGGFGNWLVPLMLGAPDMAFPRMNNMSFWLLPPSLTLLISSSIVENGAGTGWTVYPPLSSNIAHGGSSVDLAIFSLHLAGISSILGAINFITTIINMRINGLSFD
QMPLFVWAVGITALLLLLSLPVLAGAITMLLTDRNLNTSFFDPAGGGDPILYQHL
>LEFIA900-10|HM387036|MM09778|Chionodes_luctuella
TLYFIFGIWAGMVGTSLSLLIRAELGNPGSLIGDDQIYNTIVTAHAFIMIFFMVMPIMIGGFGNWLVPLMLGAPDMAFPRMNNMSFWLLPPSLTLLISSSIVENGAGTGWTVYPPLSSNIAHGGSSVDLAIFSLHLAGISSILGAINFITTIINMRINGLSFD
QMPLFVWAVGITALLLLLSLPVLAGAITMLLTDRNLNTSFFDPAGGGDPILYQHL
>LEFIC032-10|HM871902|MM03257|Chionodes_lugubrella
TLYFIFGIWAGMVGTSLSLLIRAELGAPGSLIGDDQIYNTIVTAHAFIMIFFMVMPIMIGGFGNWLVPLMLGAPDMAFPRMNNMSFWLLPPSLTLLISSSIVENGAGTGWTVYPPLSSNIAHGGSSVDLAIFSLHLAGISSILGAINFITTIINMRMNGLSF
DQMPLFVWSVGITALLLLLSLPVLAGAITMLLTDRNLNTSFFDPAGGGDPILYQHL
>LEFIF947-10|HM875628|MM13505|Chionodes_lugubrella
TLYFIFGIWAGMVGTSLSLLIRAELGAPGSLIGDDQIYNTIVTAHAFIMIFFMVMPIMIGGFGNWLVPLMLGAPDMAFPRMNNMSFWLLPPSLTLLISSSIVENGAGTGWTVYPPLSSNIAHGGSSVDLAIFSLHLAGISSILGAINFITTIINMRMNGLSF
DQMPLFVWSVGITALLLLLSLPVLAGAITMLLTDRNLNTSFFDPAGGGDPILYQHL
>LEFIB938-10|HM871815|MM03088|Chionodes_nubilella
TLYFIFGIWAGMVGTSLSLLIRAELGNPGSLIGDDQIYNTIVTAHAFIMIFFMVMPIMIGGFGNWLVPLMLGAPDMAFPRMNNMSFWLLPPSLTLLISSSIVENGAGTGWTVYPPLSSNIAHGGSSVDLAIFSLHLAGISSILGAINFITTIINMRINGLSFD
QMPLFVWSVGITALLLLLSLPVLAGAITMLLTDRNLNTSFFDPAGGGDPILYQHL
>LEFIA775-10|HM386917|MM04129|Chionodes_nubilella
TLYFIFGIWAGMVGTSLSLLIRAELGNPGSLIGDDQIYNTIVTAHAFIMIFFMVMPIMIGGFGNWLVPLMLGAPDMAFPRMNNMSFWLLPPSLTLLISSSIVENGAGTGWTVYPPLSSNIAHGGSSVDLAIFSLHLAGISSILGAINFITTIINMRINGLSFD
QMPLFVWSVGITALLLLLSLPVLAGAITMLLTDRNLNTSFFDPAGGGDPILYQHL
>LEFIA796-10|HM386937|MM04150|Chionodes_nubilella
TLYFIFGIWAGMVGTSLSLLIRAELGNPGSLIGDDQIYNTIVTAHAFIMIFFMVMPIMIGGFGNWLVPLMLGAPDMAFPRMNNMSFWLLPPSLTLLISSSIVENGAGTGWTVYPPLSSNIAHGGSSVDLAIFSLHLAGISSILGAINFITTIINMRINGLSFD
QMPLFVWSVGITALLLLLSLPVLAGAITMLLTDRNLNTSFFDPAGGGDPILYQHL
>LEFIF052-10|HM874763|MM10492|Chionodes_tragicella
TLYFIFGIWAGMVGTSLSLLIRAELGNPGSLIGDDQIYNTIVTAHAFIMIFFMVMPIMIGGFGNWLVPLMLGAPDMAFPRMNNMSFWLLPPSITLLISSSIVETGAGTGWTVYPPLSSNIAHGGSSVDLAIFSLHLAGISSILGAINFITTIINMKINGLSFDQ
MPLFVWAVGITALLLLLSLPVLAGAITMLLTDRNLNTSFFDPAGGGDPILYQHL
>LEFIJ772-10|JF853813|MM17397|Chionodes_tragicella
TLYFIFGIWAGMVGTSLSLLIRAELGNPGSLIGDDQIYNTIVTAHAFIMIFFMVMPIMIGGFGNWLVPLMLGAPDMAFPRMNNMSFWLLPPSITLLISSSIVETGAGTGWTVYPPLSSNIAHGGSSVDLAIFSLHLAGISSILGAINFITTIINMKINGLSFDQ
MPLFVWAVGITALLLLLSLPVLAGAITMLLTDRNLNTSFFDPAGGGDPILYQHL
>LEFIA015-10|HM396365|MM00086|Chionodes_viduella
TLYFIFGIWAGMVGTSLSLLIRAELGNPGSLIGDDQIYNTIVTAHAFIMIFFMVMPIMIGGFGNWLVPLMLGAPDMAFPRMNNMSFWLLPPSLTLLITSSIVENGAGTGWTVYPPLSSNIAHGGSSVDLAIFSLHLAGISSILGAINFITTIINMRINGMSFD
QMPLFVWAVGITALLLLLSLPVLAGAITMLLTDRNLNTSFFDPAGGGDPILYQHL
>LEFIC366-10|HM872209|MM03893|Chionodes_viduella
TLYFIFGIWAGMVGTSLSLLIRAELGNPGSLIGDDQIYNTIVTAHAFIMIFFMVMPIMIGGFGNWLVPLMLGAPDMAFPRMNNMSFWLLPPSLTLLITSSIVENGAGTGWTVYPPLSSNIAHGGSSVDLAIFSLHLAGISSILGAINFITTIINMRINGMSFD
QMPLFVWAVGITALLLLLSLPVLAGAITMLLTDRNLNTSFFDPAGGGDPILYQHL
>LEFID374-10|HM873171|MM06284|Chionodes_viduella
TLYFIFGIWAGMVGTSLSLLIRAELGNPGSLIGDDQIYNTIVTAHAFIMIFFMVMPIMIGGFGNWLVPLMLGAPDMAFPRMNNMSFWLLPPSLTLLITSSIVENGAGTGWTVYPPLSSNIAHGGSSVDLAIFSLHLAGISSILGAINFITTIINMRINGMSFD
QMPLFVWAVGITALLLLLSLPVLAGAITMLLTDRNLNTSFFDPAGGGDPILYQHL
>LEFIE471-10|HM874195|MM09244|Chionodes_violacea
TLYFIFGIWAGMVGTSLSLLIRAELGNPGSLIGDDQIYNTIVTAHAFIMIFFMVMPIMIGGFGNWLVPLMLGAPDMAFPRMNNMSFWLLPPSLTLLISSSIVENGAGTGWTVYPPLSSNIAHGGSSVDLAIFSLHLAGISSILGAINFITTIINMRISGMSFD
QMPLFIWAVGITALLLLLSLPVLAGAITMLLTDRNLNTSFFDPAGGGDPILYQHL
>LEFIF053-10|HM874764|MM10493|Chionodes_violacea
TLYFIFGIWAGMVGTSLSLLIRAELGNPGSLIGDDQIYNTIVTAHAFIMIFFMVMPIMIGGFGNWLVPLMLGAPDMAFPRMNNMSFWLLPPSLTLLISSSIVENGAGTGWTVYPPLSSNIAHGGSSVDLAIFSLHLAGISSILGAINFITTIINMRISGMSFD
QMPLFIWAVGITALLLLLSLPVLAGAITMLLTDRNLNTSFFDPAGGGDPILYQHL
>LEFIF625-10|HM875309|MM12514|Chloantha_hyperici
TLYFIFGIWAGMVGTSLSLLIRAELGNPGSLIGDDQIYNTIVTAHAFIMIFFMVMPIMIGGFGNWLIPLMLGAPDMAFPRMNNMSFWLLPPSLTLLISSSIVENGAGTGWTVYPPLSSNIAHGGSSVDLAIFSLHLAGISSILGAINFITTIINMRLNNLSFD
QMPLFIWAVGITAFLLLLSLPVLAGAITMLLTDRNLNTSFFDPAGGGDPILYQHL

>LEFIC353-10|HM872196|MM03871|Chlorissa_viridata
TLYFIFGIWAGMIGTSLSLLIRAELGNPGSLIGDDQIYNTIVTAHAFIMIFFMVMPIMIGGFGNWLVPLMLGAPDMAFPRMNNMSFWLLPPSLTLLISSSVVENGAGTGWTVYPPLSSNIAHGGGSVDLAIFSLHLAGISSILGAINFITTIINMRLNNLSFD
QMPLFVWAVGITAFLLLLSLPVLAGAITMLLTDRNLNTSFFDPAGGGDPILYQHL
>LEFIE832-10|HM874551|MM10048|Chlorissa_viridata
TLYFIFGIWAGMIGTSLSLLIRAELGNPGSLIGDDQIYNTIVTAHAFIMIFFMVMPIMIGGFGNWLVPLMLGAPDMAFPRMNNMSFWLLPPSLTLLISSSVVENGAGTGWTVYPPLSSNIAHGGGSVDLAIFSLHLAGISSILGAINFITTIINMRLNNLSFD
QMPLFVWAVGITAFLLLLSLPVLAGAITMLLTDRNLNTSFFDPAGGGDPILYQHL
>LEFIG160-10|HM875840|MM14018|Chlorissa_viridata
TLYFIFGIWAGMIGTSLSLLIRAELGNPGSLIGDDQIYNTIVTAHAFIMIFFMVMPIMIGGFGNWLVPLMLGAPDMAFPRMNNMSFWLLPPSLTLLISSSVVENGAGTGWTVYPPLSSNIAHGGGSVDLAIFSLHLAGISSILGAINFITTIINMRLNNLSFD
QMPLFVWAVGITAFLLLLSLPVLAGAITMLLTDRNLNTSFFDPAGGGDPILYQHL
>LEFIA258-10|HM386602|MM01295|Chloroclysta_miata
TLYFIFGIWAGMVGTSLSLLIRAELGNPGSLIGDDQIYNTIVTAHAFIMIFFMVMPIMIGGFGNWLVPLMLGAPDMAFPRMNNMSFWLLPPSITLLISSSIVENGAGTGWTVYPPLSSNIAHGGSSVDLAIFSLHLAGISSILGAINFITTIINMRLNNMFFD
QLPLFVWAVGITAFLLLLSLPVLAGAITMLLTDRNLNTSFFDPAGGGDPILYQHL
>LEFIC669-10|HM872490|MM04634|Chloroclysta_miata
TLYFIFGIWAGMVGTSLSLLIRAELGNPGSLIGDDQIYNTIVTAHAFIMIFFMVMPIMIGGFGNWLVPLMLGAPDMAFPRMNNMSFWLLPPSITLLISSSIVENGAGTGWTVYPPLSSNIAHGGSSVDLAIFSLHLAGISSILGAINFITTIINMRLNNMFFD
QLPLFVWAVGITAFLLLLSLPVLAGAITMLLTDRNLNTSFFDPAGGGDPILYQHL
>LEFIB333-10|HM871233|MM00874|Chloroclysta_siterata
TLYFIFGIWAGMVGTSLSLLIRAELGNPGSLIGDDQIYNTIVTAHAFIMIFFMVMPIMIGGFGNWLVPLMLGAPDMAFPRMNNMSFWLLPPSITLLISSSIVENGAGTGWTVYPPLSSNIAHGGSSVDLAIFSLHLAGISSILGAINFITTIINMRLNNMFFD
QLPLFVWAVGITAFLLLLSLPVLAGAITMLLTDRNLNTSFFDPAGGGDPILYQHL
>LEFIC670-10|HM872491|MM04635|Chloroclysta_siterata
TLYFIFGIWAGMVGTSLSLLIRAELGNPGSLIGDDQIYNTIVTAHAFIMIFFMVMPIMIGGFGNWLVPLMLGAPDMAFPRMNNMSFWLLPPSITLLISSSIVENGAGTGWTVYPPLSSNIAHGGSSVDLAIFSLHLAGISSILGAINFITTIINMRLNNMFFD
QLPLFVWAVGITAFLLLLSLPVLAGAITMLLTDRNLNTSFFDPAGGG?PILYQHL
>LEFIF728-10|HM875412|MM12822|Chloroclysta_siterata
TLYFIFGIWAGMVGTSLSLLIRAELGNPGSLIGDDQIYNTIVTAHAFIMIFFMVMPIMIGGFGNWLVPLMLGAPDMAFPRMNNMSFWLLPPSITLLISSSIVENGAGTGWTVYPPLSSNIAHGGSSVDLAIFSLHLAGISSILGAINFITTIINMRLNNMFFD
QLPLFVWAVGITAFLLLLSLPVLAGAITMLLTDRNLNTSFFDPAGGGDPILYQHL
>LEFIF038-10|HM874749|MM10478|Chloroclystis_v-ata
TLYFIFGIWAGMIGTSLSLLIRAELGTPGSLIGDDQIYNTIVTAHAFIMIFFMVMPIMIGGFGNWLVPLMLGAPDMAFPRMNNMSFWLLPPSITLLISSSIVENGAGTGWTVYPPLSSNIAHGGSSVDLAIFSLHLAGISSILGAINFITTIINMRLNNMFFD
QLPLFVWAVGITAFLLLLSLPVLAGAITMLLTDRNLNTSFFDPAGGGDPILYQHL
>LEFIF040-10|HM874751|MM10480|Chloroclystis_v-ata
TLYFIFGIWAGMIGTSLSLLIRAELGTPGSLIGDDQIYNTIVTAHAFIMIFFMVMPIMIGGFGNWLVPLMLGAPDMAFPRMNNMSFWLLPPSITLLISSSIVENGAGTGWTVYPPLSSNIAHGGSSVDLAIFSLHLAGISSILGAINFITTIINMRLNNMFFD
QLPLFVWAVGITAFLLLLSLPVLAGAITMLLTDRNLNTSFFDPAGGGDPILYQHL
>LEFIF042-10|HM874753|MM10482|Chloroclystis_v-ata
TLYFIFGIWAGMIGTSLSLLIRAELGTPGSLIGDDQIYNTIVTAHAFIMIFFMVMPIMIGGFGNWLVPLMLGAPDMAFPRMNNMSFWLLPPSITLLISSSIVENGAGTGWTVYPPLSSNIAHGGSSVDLAIFSLHLAGISSILGAINFITTIINMRLNNMFFD
QLPLFVWAVGITAFLLLLSLPVLAGAITMLLTDRNLNTSFFDPAGGGDPILYQHL
>LEFIB076-10|HM870985|MM00377|Choreutis_diana
TLYFIFGIWSGMVGTSLSLLIRAELGNPGSLIGDDQIYNTIVTAHAFIMIFFMVMPIMIGGFGNWLVPLMLGAPDMAFPRMNNMSFWLLPPSLTLLISSSIVETGAGTGWTVYPPLSSNMAHGGSSVDLAIFSLHLAGISSILGAINFITTIINMKPNNMSL
DQMPLFVWSVQITAILLLLSLPVLAGAITMLLTDRNLNTSFFDPAGGGDPILYQHL
>LEFIE909-10|HM874626|MM10271|Choreutis_diana
TLYFIFGIWSGMVGTSLSLLIRAELGNPGSLIGDDQIYNTIVTAHAFIMIFFMVMPIMIGGFGNWLVPLMLGAPDMAFPRMNNMSFWLLPPSLTLLISSSIVETGAGTGWTVYPPLSSNMAHGGSSVDLAIFSLHLAGISSILGAINFITTIINMKPNNMSL
DQMPLFVWSVQITAILLLLSLPVLAGAITMLLTDRNLNTSFFDPAGGGDPILYQHL
>LEFIB010-10|HM870923|MM00024|Choreutis_pariana
TLYFIFGIWSGMVGTSLSLLIRAELGNPGSLIGDDQIYNTIVTAHAFIMIFFMVMPIMIGGFGNWLVPLMLGAPDMAFPRMNNMSFWLLPPSLTLLISSSIVETGAGTGWTVYPPLSSNMAHGGSSVDLAIFSLHLAGISSILGAINFITTIINMKPNNMSL
DQMPLFVWSVQITAILLLLSLPVLAGAITMLLTDRNLNTSFFDPAGGGDPILYQHL
>LEFIB328-10|HM871228|MM00866|Choreutis_pariana
TLYFIFGIWSGMVGTSLSLLIRAELGNPGSLIGDDQIYNTIVTAHAFIMIFFMVMPIMIGGFGNWLVPLMLGAPDMAFPRMNNMSFWLLPPSLTLLISSSIVETGAGTGWTVYPPLSSNMAHGGSSVDLAIFSLHLAGISSILGAINFITTIINMKPNNMSL
DQMPLFVWSVQITAILLLLSLPVLAGAITMLLTDRNLNTSFFDPAGGGDPILYQHL
>LEFIA014-10|HM396364|MM00085|Choristoneura_albaniana
TLYFIFGIWSGMVGTSLSLLIRAELGNPGSLIGDDQIYNTIVTAHAFIMIFFMVMPIMIGGFGNWLVPLMLGAPDMAFPRMNNMSFWLLPPSIMLLISSSIVENGAGTGWTVYPPLSSNIAHSGSSVDLTIFSLHLAGISSILGAVNFITTIINMRPNNMSL

DQMPLFVWAVGITALLLLLSLPVLAGAITMLLTDRNLNTSFFDPAGGGDPILYQHL
>LEFIF528-10|HM875213|MM12172|Choristoneura_diversana
TLYFIFGIWAGMVGTSLSLLIRAELGNPGSLIGDDQIYNTIVTAHAFIMIFFMVMPIMIGGFGNWLVPLMLGAPDMAFPRMNNMSFWLLPPSIMLLISSSIVENGAGTGWTVYPPLSSNIAHSGSSVDLAIFSLHLAGISSILGAVNFITTIINMRPNNMSL
DQMPLFVWSVGITALLLLLSLPVLAGAITMLLTDRNLNTSFFDPAGGGDPILYQHL
>LEFIL715-10|JF854699|MM16005|Chrysoclista_lathamella
TLYFIFGIWAGMLGTSLSLLIRAELGNPGSLIGDDQIYNTIVTAHAFIMIFFMVMPIMIGGFGNWLVPLMLGAPDMAFPRLNNMSFWLLPPSITLLISSSLVENGAGTGWTVYPPLSSNIAHSGSSVDLAIFSLHLAGISSILGAINFITTIINMRLTNMFFDQ
MPLFVWSVGITALLLLLSLPVLAGAITMLLTDRNLNTSFFDPAGGGDPILYQHL
>LEFIJ629-10|JF853727|MM17254|Chrysoclista_lathamella
TLYFIFGIWSGMLGTSLSLLIRSELGNPGSLIGDDQIYNTIVTAHAFIMIFFMVMPIMIGGFGNWLVPLMLGAPDMAFPRMNNMSFWLLPPSITLLISSSLVENGAGTGWTVYPPLSSNIAHSGSSVDLAIFSLHLAGISSILGAINFITTIINMRLTNMFFD
QMPLFVWSVGITALLLLLSLPVLAGAITMLLTDRNLNTSFFDPAGGGDPILYQHL
>LEFIJ1198-11|MM21058|Chrysoclista_linneella
TLYFIFGIWAVMMGTSLSLLIRTELGNPGSLIGDDQIYNTIVTAHAFIMIFFMVMPIMIGGFGNWLVPLMLGAPDMAFPRMNNMSFWLLPPSITLLISSSVVETGAGTGWTVYPPLSSNIAHSGSSVDLAIFSLHLAGISSILGAINFITTIINMRLTNMSFD
QMPLFVWSVGITALLLLLSLPVLAGAITMLLTDRNLNTSFFDPMGGGDPILYQHL
>LEFID092-10|HM872903|MM05704|Chrysoesthia_drurella
TLYFIFGIWAGMLGTSLSLLIRAELGNPGSLIGDDQIYNTIVTAHAFIMIFFMVMPIMIGGFGNWLVPLMLGAPDMAFPRMNNMSFWLLPPSILLLISSSIVETGAGTGWTVYPPLSSNIAHSGSSVDLAIFSLHLAGISSILGAINFITTIINMKINGLSFDQ
MPLFVWAVGITALLLLLSLPVLAGAITMLLTDRNLNTSFFDPAGGGDPILYQHL
>LEFIC439-10|HM872273|MM03994|Chrysoesthia_sexguttella
TLYFIFGIWAGMVGTSLSLLIRAELGNPGSLIGDDQIYNTIVTAHAFIMIFFMVMPIMIGGFGNWLVPLMLGAPDMAFPRMNNMSFWLLPPSLTLLISSSVVENGAGTGWTVYPPLSSNIAHGGSSVDLAIFSLHLAGISSILGAINFITTIINMKINGLSFD
QMPLFVWAVGITALLLLLSLPVLAGAITMLLTDRNLNTSFFDPAGGGDPILYQHL
>LEFIG755-10|HM876410|MM15619|Chrysoesthia_sexguttella
TLYFIFGIWAGMVGTSLSLLIRAELGNPGSLIGDDQIYNTIVTAHAFIMIFFMVMPIMIGGFGNWLVPLMLGAPDMAFPRMNNMSFWLLPPSLTLLISSSVVENGAGTGWTVYPPLSSNIAHGGSSVDLAIFSLHLAGISSILGAINFITTIINMKINGLSFD
QMPLFVWAVGITALLLLLSLPVLAGAITMLLTDRNLNTSFFDPAGGGDPILYQHL
>LEFIB194-10|HM871098|MM00626|Chrysoteuchia_culmella
TLYFIFGIWAGMVGTSLSLLIRAELGNPGSLIGDDQIYNTIVTAHAFIMIFFMVMPIMIGGFGNWLIPLMLGAPDMAFPRMNNMSFWLLPPSLTLLISSSIVENGAGTGWTVYPPLSSNIAHGGSSVDLAIFSLHLAGISSILGAINFITTIINMRINGLSFDQ
MPLFVWSVGITALLLLLSLPVLAGAITMLLTDRNLNTSFFDPAGGGDPILYQHL
>LEFIA302-10|HM386645|MM01347|Cidaria_fulvata
TLYFIFGIWAGMVGTSLSLLIRAELGTPGSLIGDDQIYNTIVTAHAFIMIFFMVMPIMIGGFGNWLVPLMLGAPDMAFPRMNNMSFWLLPPSITLLISSSIVENGAGTGWTVYPPLSSNIAHGGSSVDLAIFSLHLAGISSILGAINFITTIINMRLNNMFFD
QLPLFVWAVGITAFLLLLSLPVLAGAITMLLTDRNLNTSFFDPAGGGDPILYQHL
>LEEUA245-11|JN274941|MM19653|Clavigesta_purdeyi
TLYFIFGIWAGMVGTSLSLLIRAELGNPGSLIGDDQIYNTIVTAHAFIMIFFMVMPIMIGGFGNWLVPLMLGAPDMAFPRMNNMSFWLLPPSIMLLISSSIVENGAGTGWTVYPPLSSNIAHSGSSVDLAIFSLHLAGISSILGAVNFITTIINMRPNNMSL
DQMPLFVWAVGITALLLLLSLPVLAGAITMLLTDRNLNTSFFDPAGGGDPILYQHL
>LEFIA047-10|HM396396|MM00450|Cleora_cinctaria
TLYFIFGIWAGMIGTSLSLLIRAELGNPGSLIGDDQIYNTIVTAHAFIMIFFMVMPIMIGGFGNWLVPLMLGAPDMAFPRMNNMSFWLLPPSITLLISSSIVENGAGTGWTVYPPLSSNIAHGGSSVDLAIFSLHLAGISSILGAINFITTIINMRLNSLSFDQ
MPLFVWAVGITAFLLLLSLPVLAGAITMLLTDRNLNTSFFDPAGGGDPILYQHL
>LEFIA261-10|HM386605|MM01299|Cleora_cinctaria
TLYFIFGIWAGMIGTSLSLLIRAELGNPGSLIGDDQIYNTIVTAHAFIMIFFMVMPIMIGGFGNWLVPLMLGAPDMAFPRMNNMSFWLLPPSITLLISSSIVENGAGTGWTVYPPLSSNIAHGGSSVDLAIFSLHLAGISSILGAINFITTIINMRLNSLSFDQ
MPLFVWAVGITAFLLLLSLPVLAGAITMLLTDRNLNTSFFDPAGGGDPILYQHL
>LEFIC668-10|HM872489|MM04632|Cleora_cinctaria
TLYFIFGIWAGMIGTSLSLLIRAELGNPGSLIGDDQIYNTIVTAHAFIMIFFMVMPIMIGGFGNWLVPLMLGAPDMAFPRMNNMSFWLLPPSITLLISSSIVENGAGTGWTVYPPLSSNIAHGGSSVDLAIFSLHLAGISSILGAINFITTIINMRLNSLSFDQ
MPLFVWAVGITAFLLLLSLPVLAGAITMLLTDRNLNTSFFDPAGGGDPILYQHL
>LEFIF047-10|HM874758|MM10487|Cleorodes_lichenaria
TLYFIFGIWAGMVGTSLSLLIRAELGNPGSLIGDDQIYNTIVTAHAFIMIFFMVMPIMIGGFGNWLVPLMLGAPDMAFPRMNNMSFWLLPPSITLLISSSIVENGAGTGWTVYPPLSSNIAHGGSSVDLAIFSLHLAGISSILGAINFITTIINMRLNNLSFD
QMPLFVWSVGITAFLLLLSLPVLAGAITMLLTDRNLNTSFFDPAGGGDPILYQHL
>LEEUA222-11|JN286434|MM19630|Clepsis_consimilana
TLYFIFGIWAGMVGTSLSLLIRAELGSPGSLIGDDQIYNTIVTAHAFIMIFFMVMPIMIGGFGNWLVPLMLGAPDMAFPRMNNMSFWLLPPSIMLLISSSIVENGAGTGWTVYPPLSSNIAHSGSSVDLAIFSLHLAGISSILGAVNFITTIINMRPNNMSL
DQMSLFIWAVGITALLLLLSLPVLAGAITMLLTDRNLNTSFFDPAGGGDPILYQHL
>LEFID466-10|HM873234|MM06407|Clepsis_illustrana

TLYFIFGIWAGMVGTSLSLLIRAELGNPGSLIGDDQIYNTIVTAHAFIMIFFMVMPIMIGGFGNWLVPLMLGAPDMAFPRMNNMSFWLLPPSIMLLISSSIVENGAGTGWTVYPPLSSNIAHSGSSVDLAIFSLHLAGISSILGAVNFITTIINMRPKNMSL
DQMPLFVWAVGITALLLLLSLPVLAGAITMLLTDRNLNTSFFDPAGGGDPILYQHL
>LEFIG786-10|HM876438|MM15650|Clepsis_illustrana
TLYFIFGIWAGMVGTSLSLLIRAELGNPGSLIGDDQIYNTIVTAHAFIMIFFMVMPIMIGGFGNWLVPLMLGAPDMAFPRMNNMSFWLLPPSIMLLISSSIVENGAGTGWTVYPPLSSNIAHSGSSVDLAIFSLHLAGISSILGAVNFITTIINMRPKNMSL
DQMPLFVWAVGITALLLLLSLPVLAGAITMLLTDRNLNTSFFDPAGGGDPILYQHL
>LEFID208-10|HM873010|MM06037|Clepsis_lindebergi
TLYFIFGIWAGMVGTSLSLMIRAELGNPGSLIGDDQIYNTIVTAHAFIMIFFMVMPIMIGGFGNWLVPLMLGAPDMAFPRMNNMSFWLLPPSIMLLISSSIVENGAGTGWTVYPPLSSNIAHSGGSVDLAIFSLHLAGISSILGAVNFITTIINMRPNNM
FLDQMSLFVWSVGITALLLLLSLPVLAGAITMLLTDRNLNTSFFDPAGGGDPILYQHL
>LEFIJ2108-14|MM23294|Clepsis_mehli
TLYFIFGIWAGMVGTSLSLMIRAELGNPGSLIGDDQIYNTIVTAHAFIMIFFMVMPIMIGGFGNWLVPLMLGAPDMAFPRMNNMSFWLLPPSIMLLISSSIVENGAGTGWTVYPPLSSNIAHSGSSVDLAIFSLHLAGISSILGAVNFITTIINMRPNNMS
LDQMSLFIWSVGITALLLLLSLPVLAGAITMLLTDRNLNTSFFDPAGGGDPILYQHL
>LEFIG795-10|HM876447|MM15659|Clepsis_neglectana
TLYFIFGIWAGMVGTSLSLLIRAELGNPGLLIGDDQIYNTIVTAHAFIMIFFMVMPIMIGGFGNWLVPLMLGAPDMAFPRMNNMSFWLLPPSIMLLISSSIVENGAGTGWTVYPPLSSNIAHSGSSVDLAIFSLHLAGISSILGAVNFITTIINMRPNNMSL
DQMSLFIWAVGITALLLLLSLPVLAGAITMLLTDRNLNTSFFDPAGGGDPILYQHL
>LEFIG794-10|HM876446|MM15658|Clepsis_nybomi
TLYF?FGIWAGMVGTSLSLLIRAELGSPGSLIGDDQIYNTIVTAHAFIMIFFMVMPIMIGGFGNWLIPLMLGAPDMAFPRMNNMSFWLLPPSIMLLISSSIVENGAGTGWTVYPPLSSNIAHSGSSVDLAIFSLHLAGISSILGAVNFITTIINMRPNNMSL
DQMPLFVWSVGITALLLLLSLPVLAGAITMLLTDRNLNTSFFDPAGGGDPILYQHL
>LEFIK008-10|JF853914|MM17583|Clepsis_pallidana
TLYFIFGIWSGMMGTSLSLLIRAELGNPGSLIGNDQIYNTIVTAHAFIMIFFMVMPIMIGGFGNWLVPLMLGAPDMAFPRMNNMSFWLLPPSIMLLISSSIVENGAGTGWTVYPPLSSNIAHSGSSVDLTIFSLHLAGISSILGAVNFITTIINMRPNNMS
LDQMPLFVWSVGITALLLLLSLPVLAGAITMLLTDRNLNTSFFDPAGGGDPILYQHL
>LEFIK009-10|MM17584|Clepsis_pallidana
TLYFIFGIWSGMMGTSLSLLIRAELGNPGSLIGNDQIYNTIVTAHAFIMIFFMVMPIMIGGFGNWLVPLMLGAPDMAFPRMNNMSFWLLPPSIMLLISSSIVENGAGTGWTVYPPLSSNIAHSGSSVDLTIFSLHLAGISSILGAVNFITTIINMRPNNMS
LDQMPLFVWSVGITALLLLLSLPVLAGAITMLLTDRNLNTSFFDPAGGGDPILYQHL
>LEFIJ1422-12|MM22501|Clepsis_rogana
TLYFIFGIWAGMVGTSLSLLIRAELGSPGSLIGDDQIYNTIVTAHAFIMIFFMVMPIMIGGFGNWLVPLMLGAPDMAFPRMNNMSFWLLPPSIMLLISSSIVENGAGTGWTVYPPLSSNIAHSGSSVDLAIFSLHLAGISSILGAVNFITTIINMRPNNMSL
DQMPLFVWSVGITALLLLLSLPVLAGAITMLLTDRNLNTSFFDPAGGGDPILYQHL
>LEFIB465-10|HM871364|MM02009|Clepsis_rurinana
TLYFIFGIWAGMVGTSLSLLIRAELGNPGSLIGDDQIYNTIVTAHAFIMIFFMVMPIMIGGFGNWLVPLMLGAPDMAFPRMNNMSFWLLPPSIMLLISSSIVENGAGTGWTVYPPLSSNIAHSGSSVDLAIFSLHLAGISSILGAVNFITTIINMRPNSMSL
DQMPLFVWAVGITALLLLLSLPVLAGAITMLLTDRNLNTSFFDPAGGGDPILYQHL
>LEFIB148-10|HM871053|MM00533|Clepsis_senecionana
TLYFIFGIWAGMVGTSLSLMIRAELGNPGSLIGNDQIYNTIVTAHAFIMIFFMVMPIMIGGFGNWLVPLMLGAPDMAFPRMNNMSFWLLPPSFMLLISSSIVENGAGTGWTVYPPLSSNIAHSGSSVDLAIFSLHLAGISSILGAVNFITTIINMRPNNM
SLDQMPLFVWSVGITALLLLLSLPVLAGAITMLLTDRNLNTSFFDPAGGGDPILYQHL
>LEFIE852-10|HM874570|MM10084|Clepsis_spectrana
TMYFIFGIWASMVGTSLSLLIRTELGNPGSLIGDDQIYNTIVTAHAFIMIFFMVMPIMIGGFGNWLVPLMLGAPDMAFPRMNNMSFWLLPPSIMLLISSSIVENGAGTGWTVYPPLSSNIAHSGSSVDLAIFSLHLAGISSILGAVNFITTIINMRPNNMS
LDQMPLFVWSVGITALLLLLSLPVLAGAITMLLTDRNLNTSFFDPAGGGDPILYQHL
>LEFIF396-10|HM875081|MM11756|Clepsis_spectrana
TMYFIFGIWASMVGTSLSLLIRTELGNPGSLIGDDQIYNTIVTAHAFIMIFFMVMPIMIGGFGNWLVPLMLGAPDMAFPRMNNMSFWLLPPSIMLLISSSIVENGAGTGWTVYPPLSSNIAHSGSSVDLAIFSLHLAGISSILGAVNFITTIINMRPNNMS
LDQMPLFVWSVGITALLLLLSLPVLAGAITMLLTDRNLNTSFFDPAGGGDPILYQHL
>LEFID935-10|HM873685|MM07683|Clostera_anachoreta
TLYFIFGIWAGMVGTSLSLLIRAELGNPGSLIGDDQIYNTIVTAHAFIMIFFMVMPIMIGGFGNWLIPLMLGAPDMAFPRMNNMSFWLLPPSLTLLISSSIVENGAGTGWTVYPPLSSNIAHGGSSVDLAIFSLHLAGISSILGAINFITTIINMRLNNMSFD
QMPLFVWAVGITAFLLLLSLPVLAGAITMLLTDRNLNTSFFDPAGGGDPILYQHL
>LEFIG211-10|HM875891|MM14099|Clostera_anachoreta
TLYFIFGIWAGMVGTSLSLLIRAELGNPGSLIGDDQIYNTIVTAHAFIMIFFMVMPIMIGGFGNWLIPLMLGAPDMAFPRMNNMSFWLLPPSLTLLISSSIVENGAGTGWTVYPPLSSNIAHGGSSVDLAIFSLHLAGISSILGAINFITTIINMRLNNMSFD
QMPLFVWAVGITAFLLLLSLPVLAGAITMLLTDRNLNTSFFDPAGGGDPILYQHL
>LEFID655-10|HM873417|MM06682|Clostera_anastomosis
TLYFIFGIWAGMVGTSLSLLIRAELGNPGSLIGDDQIYNTIVTAHAFIMIFFMVMPIMIGGFGNWLVPLMLGAPDMAFPRLNNMSFWLLPPSLTLLISSSIVENGAGTGWTVYPPLSSNIAHSGSSVDLAIFSLHLAGISSILGAINFITTIINMRLNNMSFD
QMPLFVWAVGITAFLLLLSLPVLAGAITMLLTDRNLNTSFFDPAGGGDPILYQHL

>LEFID656-10|HM873418|MM06683|Clostera_anastomosis
TLYFIFGIWAGMVGTSLSLLIRAELGNPGSLIGDDQIYNTIVTAHAFIMIFFMVMPIMIGGFGNWLVPLMLGAPDMAFPRLNNMSFWLLPPSLTLLISSSIVENGAGTGWTVYPPLSSNIAHSGSSVDLAIFSLHLAGISSILGAINFITTIINMRLNNMSFD
QMPLFVWAVGITAFLLLLSLPVLAGAITMLLTDRNLNTSFFDPAGGGDPILYQHL
>LEFIA084-10|HM396431|MM01003|Clostera_curtula
TLYFIFGIWAGMVGTSLSLLIRAELGNPGSLIGDDQIYNTIVTAHAFIMIFFMVMPIMIGGFGNWLIPLMLGAPDMAFPRMNNMSFWLLPPSLTLLISSSVVENGAGTGWTVYPPLSSNIAHGGSSVDLAIFSLHLAGISSILGAINFITTIINMRLNNMSF
DQMPLFVWAVGITAFLLLLSLPVLAGAITMLLTDRNLNTSFFDPAGGGDPILYQHL
>LEFIC631-10|HM872452|MM04573|Clostera_curtula
TLYFIFGIWAGMVGTSLSLLIRAELGNPGSLIGDDQIYNTIVTAHAFIMIFFMVMPIMIGGFGNWLIPLMLGAPDMAFPRMNNMSFWLLPPSLTLLISSSVVENGAGTGWTVYPPLSSNIAHGGSSVDLAIFSLHLAGISSILGAINFITTIINMRLNNMSF
DQMPLFVWAVGITAFLLLLSLPVLAGAITMLLTDRNLNTSFFDPAGGGDPILYQHL
>LEFIB133-10|HM871039|MM00502|Clostera_pigra
TLYFIFGIWAGMVGTSLSLLIRAELGNPGSMIGDDQIYNTIVTAHAFIMIFFMVMPIMIGGFGNWLIPLMLGAPDMAFPRMNNMSFWLLPPSLTLLISSSVVENGAGTGWTVYPPLSSNIAHGGSSVDLAIFSLHLAGISSILGAINFITTIINMRINNMSF
DQLPLFVWAVGITAFLLLLSLPVLAGAITMLLTDRNLNTSFFDPAGGGDPILYQHL
>LEFID776-10|HM873533|MM06842|Cnaemidophorus_rhododactyla
TLYFIFGIWAGMIGTSLSLLIRAELGTSGSLIGDDQIYNSIVTAHAFIMIFFMVMPIMIGGFGNWLVPLMLGAPDMAFPRMNNMSFWLLPPSILILISSSIVENGVGTGWTVYPPLSSNIAHGGSSVDLAIFSLHLAGISSILGAINFISTIINMRLNKMMFD
QLPLFVWAVGITALLLLLSLPVLAGAITMLLTDRNLNTSFFDPAGGGDPILYQHL
>LEFIE278-10|HM874010|MM08619|Cnephasia_alticolana
TLYFIFGIWAGMVGTSLSLLIRAELGNPGSLIGDDQIYNTIVTAHAFIMIFFMVMPIMIGGFGNWLVPLMLGAPDMAFPRMNNMSFWLLPPSIMLLISSSIVENGAGTGWTVYPPLSSNIAHSGSSVDLAIFSLHLAGISSILGAVNFITTIINMRPNNMSL
DQMPLFVWAVGITALLLLLSLPVLAGAITMLLTDRNLNTSFFDPAGGGDPILYQHL
>LEFIB529-10|HM871417|MM02140|Cnephasia_asseclana
TLYFIFGIWAGMVGTSLSLLIRAELGNPGSLIGDDQIYNTIVTAHAFIMIFFMVMPIMIGGFGNWLVPLMLGAPDMAFPRMNNMSFWLLPPSIMLLISSSIVENGAGTGWTVYPPLSSNIAHSGSSVDLAIFSLHLAGISSILGAVNFITTIINMRPNNMSL
DQMPLFVWAVGITALLLLLSLPVLAGAITMLLTDRNLNTSFFDPAGGGDPILYQHL
>LEFIC915-10|HM872732|MM05256|Cnephasia_asseclana
TLYFIFGIWAGMVGTSLSLLIRAELGNPGSLIGDDQIYNTIVTAHAFIMIFFMVMPIMIGGFGNWLVPLMLGAPDMAFPRMNNMSFWLLPPSIMLLISSSIVENGAGTGWTVYPPLSSNIAHSGSSVDLAIFSLHLAGISSILGAVNFITTIINMRPNNMSL
DQMPLFVWAVGITALLLLLSLPVLAGAITMLLTDRNLNTSFFDPAGGGDPILYQHL
>LEFID557-10|HM873322|MM06548|Cnephasia_asseclana
TLYFIFGIWAGMVGTSLSLLIRAELGNPGSLIGDDQIYNTIVTAHAFIMIFFMVMPIMIGGFGNWLVPLMLGAPDMAFPRMNNMSFWLLPPSIMLLISSSIVENGAGTGWTVYPPLSSNIAHSGSSVDLAIFSLHLAGISSILGAVNFITTIINMRPNNMSL
DQMPLFVWAVGITALLLLLSLPVLAGAITMLLTDRNLNTSFFDPAGGGDPILYQHL
>LEFIJ2195-14|MM23384|Cnephasia_communana
TLYFIFGIWAGMVGTSLSLLIRAELGNPGSLIGDDQIYNTIVTAHAFIMIFFMVMPIMIGGFGNWLVPLMLGAPDMAFPRMNNMSFWLLPPSIMLLISSSIVENGAGTGWTVYPPLSSNIAHSGSSVDLAIFSLHLAGISSILGAVNFITTIINMRPNNMSL
DQMPLFVWAVGITALLLLLSLPVLAGAITMLLTDRNLNTSFFDPAGGGDPILYQHL
>LEEUA211-11|JN286425|MM19619|Cnephasia_genitalana
TLYFIFGIWAGMVGTSLSLLIRAELGNPGSLIGDDQIYNTIVTAHAFIMIFFMVMPIMIGGFGNWLVPLMLGAPDMAFPRMNNMSFWLLPPSIMLLISSSIVENGAGTGWTVYPPLSSNIAHSGSSVDLAIFSLHLAGISSILGAVNFITTIINMRPNNMSL
DQMPLFVWAVGITALLLLLSLPVLAGAITMLLTDRNLNTSFFDPAGGGDPILYQHL
>LEEUA212-11|JN286426|MM19620|Cnephasia_genitalana
TLYFIFGIWAGMVGTSLSLLIRAELGNPGSLIGDDQIYNTIVTAHAFIMIFFMVMPIMIGGFGNWLVPLMLGAPDMAFPRMNNMSFWLLPPSIMLLISSSIVENGAGTGWTVYPPLSSNIAHSGSSVDLAIFSLHLAGISSILGAVNFITTIINMRPNNMSL
DQMPLFVWAVGITALLLLLSLPVLAGAITMLLTDRNLNTSFFDPAGGGDPILYQHL
>LEFIC296-10|HM872140|MM03769|Cnephasia_incertana
TLYFIFGIWAGMVGTSLSLLIRAELGNPGSLIGDDQIYNTIVTAHAFIMIFFMVMPIMIGGFGNWLVPLMLGAPDMAFPRMNNMSFWLLPPSIMLLISSSIVENGAGTGWTVYPPLSSNIAHSGSSVDLAIFSLHLAGISSILGAVNFITTIINMRPNNMSL
DQMPLFVWAVGITALLLLLSLPVLAGAITMLLTDRNLNTSFFDPAGGGDPILYQHL
>LEFIK173-10|JN286438|MM17748|Cnephasia_incertana
TLYFIFGIWAGMVGTSLSLLIRAELGNPGSLIGDDQIYNTIVTAHAFIMIFFMVMPIMIGGFGNWLVPLMLGAPDMAFPRMNNMSFWLLPPSIMLLISSSIVENGAGTGWTVYPPLSSNIAHSGSSVDLAIFSLHLAGISSILGAVNFITTIINMRPNNMSL
DQMPLFVWAVGITALLLLLSLPVLAGAITMLLTDRNLNTSFFDPAGGGDPILYQHL
>LEEUA215-11|JN286428|MM19623|Cnephasia_longana
TLYFIFGIWAGMVGTSLSLLIRAELGNPGSLIGDDQIYNTIVTAHAFIMIFFMVMPIMIGGFGNWLVPLMLGAPDMAFPRMNNMSFWLLPPSIMLLISSSIVENGAGTGWTVYPPLSSNIAHSGSSVDLAIFSLHLAGISSILGAVNFITTIINMRPNNMSL
DQMPLFVWAVGITALLLLLSLPVLAGAITMLLTDRNLNTSFFDPAGGGDPILYQHL
>LEFII253-11|MM19903|Cnephasia_pasiuana
TLYFIFGIWAGMVGTSLSLLIRAELGNPGSLIGDDQIYNTIVTAHAFIMIFFMVMPIMIGGFGNWLVPLMLGAPDMAFPRMNNMSFWLLPPSIMLLISSSIVENGAGTGWTVYPPLSSNIAHSGSSVDLAIFSLHLAGISSILGAVNFITTIINMRPNSMSL

DQMPLFVWAVGITALLLLLSLPVLAGAITMLLTDRNLNTSFFDPAGGGDPILYQHL
>LEFIB525-10|HM871413|MM02129|Cnephasia_stephensiana
TLYFIFGIWAGMVGTSLSLLIRAELGNPGSLIGDDQIYNTIVTAHAFIMIFFMVMPIMIGGFGNWLVPLMLGAPDMAFPRMNNMSFWLLPPSIMLLISSSIVENGAGTGWTVYPPLSSNIAHSGSSVDLAIFSLHLAGISSILGAVNFITTIINMRPNNMTL
DQMPLFVWAVGITALLLLLSLPVLAGAITMLLTDRNLNTSFFDPAGGGDPILYQHL
>LEFIC914-10|HM872731|MM05255|Cnephasia_stephensiana
TLYFIFGIWAGMVGTSLSLLIRTELGNPGSLIGDDQIYNTIVTAHAFIMIFFMVMPIMIGGFGNWLVPLMLGAPDMAFPRMNNMSFWLLPPSIMLLISSSIVENGAGTGWTVYPPLSSNIAHSGSSVDLAIFSLHLAGISSILGAVNFITTIINMRPNNMTL
DQMPLFVWAVGITALLLLLSLPVLAGAITMLLTDRNLNTSFFDPAGGGDPILYQHL
>LEFIF891-10|HM875573|MM13298|Cochylidia_heydeniana
TLYFIFGIWAGMVGTSLSLLIRAELGNPGSLIGDDQIYNTIVTAHAFIMIFFMVMPIMIGGFGNWLVPLMLGAPDMAFPRMNNMSFWLLPPSIMLLISSSIVENGAGTGWTVYPPLSSNIAHGGSSVDLAIFSLHLAGISSILGAVNFITTIINMRPNNMN
LDQMPLFVWSVGITALLLLLSLPVLAGAITMLLTDRNLNTSFFDPAGGGDPILYQHL
>LEFIJ725-10|MM17350|Cochylidia_heydeniana
TLYFIFGIWAGMVGTSLSLLIRAELGNPGSLIGDDQIYNTIVTAHAFIMIFFMVMPIMIGGFGNWLVPLMLGAPDMAFPRMNNMSFWLLPPSIMLLISSSIVENGAGTGWTVYPPLSSNIAHGGSSVDLAIFSLHLAGISSILGAVNFITTIINMRPNNMN
LDQMPLFVWSVGITALLLLLSLPVLAGAITMLLTDRNLNTSFFDPAGGGDPILYQHL
>LEFIC225-10|HM872069|MM03623|Cochylidia_implicitana
TLYFIFGIWAGMVGTSLSLLIRAELGNPGSLIGDDQIYNTIVTAHAFIMIFFMVMPIMIGGFGNWLVPLMLGAPDMAFPRMNNMSFWLLPPSIMLLISSSIVENGAGTGWTVYPPLSSNIAHGGSSVDLAIFSLHLAGISSILGAVNFITTIINMRPNNMN
LDQMPLFVWSVGITALLLLLSLPVLAGAITMLLTDRNLNTSFFDPAGGGDPILYQHL
>LEFIC241-10|HM872085|MM03650|Cochylidia_implicitana
TLYFIFGIWAGMVGTSLSLLIRAELGNPGSLIGDDQIYNTIVTAHAFIMIFFMVMPIMIGGFGNWLVPLMLGAPDMAFPRMNNMSFWLLPPSIMLLISSSIVENGAGTGWTVYPPLSSNIAHGGSSVDLAIFSLHLAGISSILGAVNFITTIINMRPNNMN
LDQMPLFVWSVGITALLLLLSLPVLAGAITMLLTDRNLNTSFFDPAGGGDPILYQHL
>LEFIF231-10|HM874924|MM11011|Cochylidia_implicitana
TLYFIFGIWAGMVGTSLSLLIRAELGNPGSLIGDDQIYNTIVTAHAFIMIFFMVMPIMIGGFGNWLVPLMLGAPDMAFPRMNNMSFWLLPPSIMLLISSSIVENGAGTGWTVYPPLSSNIAHGGSSVDLAIFSLHLAGISSILGAVNFSTTIINMRPNNM
NLDQMPLFVWSVGITALLLLLSLPVLAGAITMLLTDRNLNTSFFDPAGGGDPILYQHL
>LEFIJ638-10|JF853736|MM17263|Cochylidia_moguntiana
TLYFIFGIWAGMVGTSLSLLIRAELGSPGSLIGDDQIYNTIVTAHAFIMIFFMVMPIMIGGFGNWLVPLMLGAPDMAFPRMNNMSFWLLPPSIMLLISSSIVENGAGTGWTVYPPLSSNIAHSGSSVDLAIFSLHLAGISSILGAVNFITTIINMRPNNMNL
DQMPLFVWAVGITALLLLLSLPVLAGAITMLLTDRNLNTSFFDPAGGGDPILYQHL
>LEFIJ890-10|JF853882|MM17515|Cochylidia_moguntiana
TLYFIFGIWAGMVGTSLSLLIRAELGNPGSLIGDDQIYNTIVTAHAFIMIFFMVMPIMIGGFGNWLVPLMLGAPDMAFPRMNNMSFWLLPPSIMLLISSSIVENGAGTGWTVYPPLSSNIAHSGSSVDLAIFSLHLAGISSILGAVNFITTIINMRPNNMN
LDQMPLFVWAVGITALLLLLSLPVLAGAITMLLTDRNLNTSFFDPAGGGDPILYQHL
>LEFID079-10|HM872890|MM05688|Cochylidia_richteriana
TLYFIFGIWAGMVGTSLSLLIRAELGNPGSLIGDDQIYNTIVTAHAFIMIFFMVMPIMIGGFGNWLVPLMLGAPDMAFPRMNNMSFWLLPPSIMILISSSIVENGAGTGWTVYPPLSSNIAHGGSSVDLAIFSLHLAGISSILGAINFITTIINMRPNNMNL
DQMPLFVWAVGITALLLLLSLPVLAGAITMLLTDRNLNTSFFDPAGGGDPILYQHL
>LEFIF232-10|HM874925|MM11012|Cochylidia_richteriana
TLYFIFGIWAGMVGTSLSLLIRAELGNPGSLIGDDQIYNTIVTAHAFIMIFFMVMPIMIGGFGNWLVPLMLGAPDMAFPRMNNMSFWLLPPSIMILISSSIVENGAGTGWTVYPPLSSNIAHGGSSVDLAIFSLHLAGISSILGAVNFITTIINMRPNNMN
LDQMPLFVWAVGITALLLLLSLPVLAGAITMLLTDRNLNTSFFDPAGGGDPILYQHL
>LEFIK187-10|JF853940|MM17762|Cochylidia_rupicola
TLYFIFGIWAGMIGTSLSLLIRAELGNPGSLISDDQIYNTIVTAHAFIMIFFMVMPIMIGGFGNWLVPLMLGAPDMAFPRMNNMSFWLLPPSIMLLISSSIVENGAGTGWTVYPPLSSNIAHSGSSVDLAIFSLHLAGISSILGAVNFITTIINMRPNNMNL
DQMPLFVWAVGITALLLLLSLPVLAGAITMLLTDRNLNTSFFDPAGGGDPILYQHL
>LEFIB731-10|HM871609|MM02558|Cochylidia_subroseana
TLYFIFGIWAGMVGTSLSLLIRAELGNPGSLIGDDQIYNTIVTAHAFIMIFFMVMPIMIGGFGNWLVPLMLGAPDMAFPRMNNMSFWLLPPSIMLLISSSMVENGAGTGWTVYPPLSSNIAHGGSSVDLAIFSLHLAGISSILGAVNFITTIINMRPNNM
NLDQMPLFVWAVGITALLLLLSLPVLAGAITMLLTDRNLNTSFFDPAGGGDPILYQHL
>LEFIB896-10|HM871773|MM02991|Cochylidia_subroseana
TLYFIFGIWAGMVGTSLSLLIRAELGNPGSLIGDDQIYNTIVTAHAFIMIFFMVMPIMIGGFGNWLVPLMLGAPDMAFPRMNNMSFWLLPPSIMLLISSSMVENGAGTGWTVYPPLSSNIAHGGSSVDLAIFSLHLAGISSILGAVNFITTIINMRPNNM
NLDQMPLFVWAVGITALLLLLSLPVLAGAITMLLTDRNLNTSFFDPAGGGDPILYQHL
>LEFIC277-10|HM872121|MM03736|Cochylimorpha_alternana
TLYFIFGIWAGMVGTSLSLLIRAELGNPGSLIGDDQIYNTIVTAHAFIMIFFMVMPIMIGGFGNWLVPLMLGAPDMAFPRMNNMSFWLLPPSIMLLISSSIVENGAGTGWTVYPPLSSNIAHSGSSVDLAIFSLHLAGISSILGAVNFITTIINMRPNNMSL
DQMPLFVWSVGITALLLLLSLPVLAGAITMLLTDRNLNTSFFDPAGGGDPILYQHL
>LEEUA204-11|JN286420|MM19612|Cochylis_atricapitana

TLYFIFGIWAGMLGTSLSLLIRTELGNPGSFIGDDQIYNTIVTAHAFIMIFFMVMPIMIGGFGNWLVPLMLGAPDMAFPRMNNMSFWLLPPSILLLISSSIVENGAGTGWTVYPPLSSNIAHSGSSVDLAIFSLHLAGISSILGAVNFITTIINMRPNNMNL
DQMPLFVWAVGITALLLLLSLPVLAGAITMLLTDRNLNTSFFDPAGGGDPIL?QHL
>LEFIB727-10|HM871605|MM02551|Cochylis_dubitana
TLYFIFGIWSGMVGTSLSLLIRAELGNPGSLIGDDQIYNTIVTAHAFIMIFFMVMPIMIGGFGNWLVPLMLGAPDMAFPRMNNMSFWLLPPSIMLLISSSIVENGAGTGWTVYPPLSSNIAHGGSSVDLAIFSLHLAGISSILGAVNFITTIINMRPNNMSL
DQMPLFVWAVGITALLLLLSLPVLAGAITMLLTDRNLNTSFFDPAGGGDPILYQHL
>LEFIA817-10|HM386958|MM04171|Cochylis_dubitana
TLYFIFGIWSGMVGTSLSLLIRAELGNPGSLIGDDQIYNTIVTAHAFIMIFFMVMPIMIGGFGNWLVPLMLGAPDMAFPRMNNMSFWLLPPSIMLLISSSIVENGAGTGWTVYPPLSSNIAHSGSSVDLAIFSLHLAGISSILGAVNFITTIINMRPNNMSL
DQMPLFVWAVGITALLLLLSLPVLAGAITMLLTDRNLNTSFFDPAGGGDPILYQHL
>LEFIA818-10|HM386959|MM04172|Cochylis_dubitana
TLYFIFGIWSGMVGTSLSLLIRAELGNPGSLIGDDQIYNTIVTAHAFIMIFFMVMPIMIGGFGNWLVPLMLGAPDMAFPRMNNMSFWLLPPSIMLLISSSIVENGAGTGWTVYPPLSSNIAHGGSSVDLAIFSLHLAGISSILGAVNFITTIINMRPNNMSL
DQMPLFVWAVGITALLLLLSLPVLAGAITMLLTDRNLNTSFFDPA?GGDPILYQHL
>LEFIE949-10|HM874666|MM10346|Cochylis_dubitana
TLYFIFGIWSGMVGTSLSLLIRAELGNPGSLIGDDQIYNTIVTAHAFIMIFFMVMPIMIGGFGNWLVPLMLGAPDMAFPRMNNMSFWLLPPSIMLLISSSIVENGAGTGWTVYPPLSSNIAHGGSSVDLAIFSLHLAGISSILGAVNFITTIINMRPNNMSL
DQMPLFVWAVGITALLLLLSLPVLAGAITMLLTDRNLNTSFFDPAGGGDPILYQHL
>LEFIF883-10|HM875565|MM13286|Cochylis_dubitana
TLYFIFGIWSGMVGTSLSLLIRAELGNPGSLIGDDQIYNTIVTAHAFIMIFFMVMPIMIGGFGNWLVPLMLGAPDMAFPRMNNMSFWLLPPSIMLLISSSIVENGAGTGWTVYPPLSSNIAHGGSSVDLAIFSLHLAGISSILGAVNFITTIINMRPNNMSL
DQMPLFVWAVGITALLLLLSLPVLAGAITMLLTDRNLNTSFFDPAGGGDPILYQHL
>LEFID597-10|HM873362|MM06608|Cochylis_flaviciliana
TLYFIFGIWAGMVGTSLSLLIRAELGNPGSLIGDDQIYNTIVTAHAFIMIFFMVMPIMIGGFGNWLVPLMLGAPDMAFPRMNNMSFWLLPPSIMLLISSSIVENGAGTGWTVYPPLSSNIAHSGSSVDLAIFSLHLAGISSILGAVNFITTIINMRPNNMSL
DQMPLFVWAVGITALLLLLSLPVLAGAITMLLTDRNLNTSFFDPAGGGDPILYQHL
>LEFIL709-10|JF854697|MM13137|Cochylis_hybridella
TLYFIFGIWSGMVGTSLSLLIRAELGNPGSLIGDDQIYNTIVTAHAFIMNFFMVMPIMIGGFGNWLVPLMLGAPDMAFPRMNNMSFWLLPPSIMLLISSSIVENGAGTGWTVYPPLSSNIAHGGSSVDLAIFSLHLAGISSILGAVNFITTIINMRPNNMS
LDQMPLFVWAVGITALLLLLSLPVLAGAITMLLTDRNLNTSFFDPAGGGDPILYQHL
>LEFIL531-10|JF854643|MM18829|Cochylis_hybridella
TLYFIFGIWSGMVGTSLSLLIRAELGNPGSLIGDDQIYNTIVTAHAFIMIFFMVMPIMIGGFGNWLVPLMLGAPDMAFPRMNNMSFWLLPPSIMLLISSSIVENGAGTGWTVYPPLSSNIAHGGSSVDLAIFSLHLAGISSILGAVNFITTIINMRPNNMSL
DQMPLFVWAVGITALLLLLSLPVLAGAITMLLTDRNLNTSFFDPAGGGDPILYQHL
>LEFIC001-10|HM871871|MM03201|Cochylis_nana
TLYFIFGIWAGMVGTSLSLLIRAELGNPGSLIGDDQIYNTIVTAHAFIMIFFMVMPIMIGGFGNWLVPLMLGAPDMAFPRMNNMSFWLLPPSIMLLISSSIVENGAGTGWTVYPPLSSNIAHSGSSVDLAIFSLHLAGISSILGAVNFITTIINMRPNNMSL
DQMPLFVWAVGITALLLLLSLPVLAGAITMLLTDRNLNTSFFDPAGGGDPILYQHL
>LEFIB728-10|HM871606|MM02553|Cochylis_pallidana
TLYFIFGIWAGMVGTSLSLLIRAELGNPGSLIGDDQIYNTIVTAHAFIMIFFMVMPIMIGGFGNWLVPLMLGAPDMAFPRMNNMSFWLLPPSIMLLISSSIVENGAGTGWTVYPPLSSNIAHSGSSVDLAIFSLHLAGISSILGAVNFITTIINMRPNNMSL
DQMPLFVWAVGITALLLLLSLPVLAGAITMLLTDRNLNTSFFDPAGGGDPILYQHL
>LEFID350-10|HM873147|MM06248|Cochylis_pallidana
TLYFIFGIWAGMVGTSLSLLIRAELGNPGSLIGDDQIYNTIVTAHAFIMIFFMVMPIMIGGFGNWLVPLMLGAPDMAFPRMNNMSFWLLPPSIMLLISSSIVENGAGTGWTVYPPLSSNIAHGGSSVDLAIFSLHLAGISSILGAVNFITTIINMRPNNMS
LDQMPLFVWAVGITALLLLLSLPVLAGAITMLLTDRNLNTSFFDPAGGGDPILYQHL
>LEFID351-10|HM873148|MM06249|Cochylis_pallidana
TLYFIFGIWAGMVGTSLSLLIRAELGNPGSLIGDDQIYNTIVTAHAFIMIFFMVMPIMIGGFGNWLVPLMLGAPDMAFPRMNNMSFWLLPPSIMLLISSSIVENGAGTGWTVYPPLSSNIAHGGSSVDLAIFSLHLAGISSILGAVNFITTIINMRPNNMN
LDQMPLLVWAVGITALLLLLSLPVLAGAITMLLTDRNLNTSFFDPAGGGDPILYQHL
>LEFID708-10|HM873469|MM06762|Cochylis_pallidana
TLYFIFGIWAGMVGTSLSLLIRAELGNPGSLIGDDQIYNTIVTAHAFIMIFFMVMPIMIGGFGNWLVPLMLGAPDMAFPRMNNMSFWLLPPSIMLLISSSIVENGAGTGWTVYPPLSSNIAHSGSSVDLAIFSLHLAGISSILGAVNFITTIINMRPNNMSL
DQMPLFVWAVGITALLLLLSLPVLAGAITMLLTDRNLNTSFFDPAGGGDPILYQHL
>LEFIK189-10|MM17764|Cochylis_posterana
TLYFIFGIWAGMVGTSLSLLIRAELGNPGSLIGDDQIYNTIVTAHAFIMIFFMVMPIMIGGFGNWLVPLMLGAPDMAFPRMNNMSFWLLPPSIMLLISSSIVENGAGTGWTVYPPLSSNIAHGGSSVDLAIFSLHLAGISSILGAVNFITTIINMRPNNMK
LDQMPLFVWAVGITALLLLLSLPVLAGAITMLLTDRNLNTSFFDPAGGGDPILYQHL
>LEFIJ1309-11|MM21169|Cochylis_posterana
TLYFIFGIWAGMVGTSLSLLIRAELGNPGSLIGDDQIYNTIVTAHAFIMIFFMVMPIMIGGFGNWLVPLMLGAPDMAFPRMNNMSFWLLPPSIMLLISSSIVENGAGTGWTVYPPLSSNIAHGGSSVDLAIFSLHLAGISSILGAVNFITTIINMRPNNMK
LDQMPLFVWAVGITALLLLLSLPVLAGAITMLLTDRNLNTSFFDPAGGGDPILYQHL

>LEEUA203-11|JN286419|MM19611|Cochylis_roseana
TLYFIFGIWAGMVGTSLSLLIRAELGNPGSLIGDDQIYNTIVTAHAFIMIFFMVMPIMIGGFGNWLVPLMLGAPDMAFPRMNNMSFWLLPPSIMLLISSSIVENGAGTGWTVYPPLSSNIAHGGSSVDLAIFSLHLAGISSILGAVNFITTI?NMRPNNM
NLDQMPLFVWAVGITALLLLLSLPVLAGAITMLLTDRNLNTSFFDPAGGGDPILYQHL
>LEEUA328-11|JN261875|MM19736|Coenobia_rufa
TLYFIFGIWAGMVGTSLSLLIRAELGNPGSLIGDDQIYNTIVTAHAFIMIFFMVMPIMIGGFGNWLVPLMLGAPDMAFPRMNNMSFWLLPPSLTLLISSSIVENGAGTGWTVYPPLSSNIAHGGSSVDLAIFSLHLAGISSILGAINFITTIINMRLNNLSFD
QMPLFIWAVGITAFLLLLSLPVLAGAITMLLTDRNLNTSFFDPAGGGDPILYQHL
>LEFIE038-10|HM873787|MM08121|Coenocalpe_lapidata
TLYFIFGIWAGMIGTSLSLLIRAELGTPGSLIGDDQIYNTIVTAHAFIMIFFMVMPIMIGGFGNWLVPLMLGAPDMAFPRMNNMSFWLLPPSITLLISSSIVETGAGTGWTVYPPLSSNIAHSGSSVDLAIFSLHLAGISSILGAINFITTIINMRLNNMFFDQ
LPLFVWAVGITAFLLLLSLPVLAGAITMLLTDRNLNTSFFDPAGGGDPILYQHL
>LEEUA526-11|MM20585|Coenonympha_arcania
TLYFIFGIWAGMVGTSLSLIIRTELGNPGSLIGDDQIYNTIVTAHAFIMIFFMVMPIMIGGFGNWLVPLMLGAPDMAFPRMNNMSFWLLPPSLILLISSSIVENGAGTGWTVYPPLSSNIAHGGSSVDLAIFSLHLAGISSILGAINFITTIINMRANGMSYD
QMPLFIWAVGITALLLLLSLPVLAGAITMLLTDRNLNTSFFDPAGGGDPILYQHL
>LEFID313-10|HM873110|MM06198|Coenonympha_glycerion
TLYFIFGIWAGMVGTSLSLIIRTELGTPGFLIGDDQIYNTIVTAHAFIMIFFMVMPIMIGGFGNWLIPLMLGAPDMAFPRMNNMSFWLLPPSLILLISSSIVENGAGTGWTVYPPLSSNIAHGGSSVDLAIFSLHLAGISSILGAINFITTIINMRINGMSYDQ
MPLFVWAVGITALLLLLSLPVLAGAITMLLTDRNLNTSFFDPAGGGDPILYQHL
>LEFIE850-10|HM874569|MM10081|Coenonympha_glycerion
TLYFIFGIWAGMVGTSLSLIIRTELGTPGFLIGDDQIYNTIVTAHAFIMIFFMVMPIMIGGFGNWLIPLMLGAPDMAFPRMNNMSFWLLPPSLILLISSSIVENGAGTGWTVYPPLSSNIAHGGSSVDLAIFSLHLAGISSILGAINFITTIINMRINGMSYDQ
MPLFVWAVGITALLLLLSLPVLAGAITMLLTDRNLNTSFFDPAGGGDPILYQHL
>LEFIG385-10|HM876062|MM14398|Coenonympha_glycerion
TLYFIFGIWAGMVGTSLSLIIRTELGTPGFLIGDDQIYNTIVTAHAFIMIFFMVMPIMIGGFGNWLIPLMLGAPDMAFPRMNNMSFWLLPPSLILLISSSIVENGAGTGWTVYPPLSSNIAHGGSSVDLAIFSLHLAGISSILGAINFITTIINMRINGMSYDQ
MPLFIWAVGITALLLLLSLPVLAGAITMLLTDRNLNTSFFDPAGGGDPILYQHL
>LEFIL045-10|MM19045|Coenonympha_hero
TLYFIFGVWAGMVGTSLSLIIRTELGNPGSLIGDDQIYNTIVTAHAFIMIFFMVMPIMIGGFGNWLVPLMLGAPDMAFPRMNNMSFWLLPPSLILLISSSIVENGAGTGWTVYPPLSSNIAHGGSSVDLAIFSLHLAGISSILGAINFITTIINMRVNGMSY
DQMPLFIWAVGITALLLLLSLPVLAGAITMLLTDRNLNTSFFDPAGGGDPILYQHL
>LEFIJ558-10|JF853673|MM17183|Coenonympha_pamphilus
TLYFIFGIWAGMVGTSLSLIIRTELGNPGSLIGDDQIYNTIVTAHAFIMIFFMVMPIMIGGFGNWLVPLMLGAPDMAFPRMNNMSFWLLPPSLVLLISSSIVENGAGTGWTVYPPLSSNIAHGGSSVDLAIFSLHLAGISSILGAINFITTIINMRVNGMSY
DQMPLFVWAVGITALLLLLSLPVLAGAITMLLTDRNLNTSFFDPAGGGDPILYQHL
>LEFIC021-10|HM871891|MM03232|Coenonympha_tullia
TLYFIFGIWAGMVGTSLSLIIRTELGNPGFLIGDDQIYNTIVTAHAFIMIFFMVMPIMIGGFGNWLIPLMLGAPDMAFPRMNNMSFWLLPPSLILLISSSIVENGAGTGWTVYPPLSSNIAHGGSSVDLAIFSLHLAGISSILGAINFITTIINMRINGMSYDQ
MPLFVWAVGITALLLLISLPVLAGAITMLLTDRNLNTSFFDPAGGGDPILYQHL
>LEFIG583-10|HM876254|MM14772|Coenonympha_tullia
TLYFIFGIWAGMVGTSLSLIIRTELGNPGFLIGDDQIYNTIVTAHAFIMIFFMVMPIMIGGFGNWLIPLMLGAPDMAFPRMNNMSFWLLPPSLILLISSSIVENGAGTGWTVYPPLSSNIAHGGSSVDLAIFSLHLAGISSILGAINFITTIINMRINGMSYDQ
MPLFVWAVGITALLLLISLPVLAGAITMLLTDRNLNTSFFDPAGGGDPILYQHL
>LEFIA619-10|HM870868|MM01733|Coenophila_subrosea
TLYFIFGIWAGMVGTSLSLLIRAELGNPGSLIGDDQIYNTIVTAHAFIMIFFMVMPIMIGGFGNWLVPLMLGAPDMAFPRMNNMSFWLLPPSLTLLISSSIVENGAGTGWTVYPPLSSNIAHGGSSVDLAIFSLHLAGISSILGAINFITTIINMRLNNLSFD
QMPLFIWSVGITAFLLLLSLPVLAGAITMLLTDRNLNTSFFDPAGGGDPILYQ?L
>LEFIB375-10|HM871274|MM00947|Coleophora_absinthii
TLYFIFGIWAGMVGTSLSLLIRAELGNPGSLIGDDQIYNTIVTAHAFIMIFFMVMPIMIGGFGNWLVPLMLGAPDMAFPRMNNMSFWLLPPSLTLLISSSIVENGTGTGWTVYPPLSSNIAHSGSSVDLSIFSLHLAGISSILGAINFITTIINMRLNNMSFD
QLPLFVWAVGITALLLLLSLPVLAGAITMLLTDRNLNTSFFDPAGGGDPILYQHL
>LEFIJ277-10|JF853514|MM14893|Coleophora_adelogrammella
TLYFIFGIWAGMVGTSLSLLIRAELGNPGSLIGDDQIYNTIVTAHAFIMIFFMVMPIMIGGFGNWLVPLMLGAPDMAFPRMNNMSFWLLPPSLTLLISGSIVENGTGTGWTVYPPLSSNIAHSGSSVDLSIFSLHLAGISSILGAINFITTIINMRLNNMSFD
QLPLFVWAVGITALLLLLSLPVLAGAITMLLTDRNLNTSFFDPAGGGDPILYQHL
>LEFIK610-10|JF854187|MM18185|Coleophora_adelogrammella
TLYFIFGIWAGMVGTSLSLLIRAELGNPGSLIGDDQIYNTIVTAHAFIMIFFMVMPIMIGGFGNWLVPLMLGAPDMAFPRMNNMSFWLLPPSLTLLISGSIVENGTGTGWTVYPPLSSNIAHSGSSVDLSIFSLHLAGISSILGAINFITTIINMRLNNMSFD
QLPLFVWAVGITALLLLLSLPVLAGAITMLLTDRNLNTSFFDPAGGGDPILYQHL
>LEFID560-10|HM873325|MM06553|Coleophora_adjunctella
TLYFIFGIWAGMVGTSLSLLIRAELGNPGSLIGDDQIYNTIVTAHAFIMIFFMVMPIMIGGFGNWLVPLMLGAPDMAFPRMNNMSFWLLPPSIMLLISSSIVENGTGTGWTVYPPLSSNIAHSGSSVDLSIFSLHLAGISSILGAINFITTIINMRLNNMSFD

QLPLFVWAVGITALLLLLSLPVLAGAITMLLTDRNLNTSFFDPAGGGDPILYQHL
>LEFIF006-10|HM874720|MM10444|Coleophora_adspersella
TLYFIFGIWAGMVGTSLSLLIRAELGNPGSLIGDDQIYNTIVTAHAFIMIFFMVMPIMIGGFGNWLVPLMLGAPDMAFPRMNNMSFWLLPPSLTLLISSSIVENGTGTGWTVYPPLSSNIAHGGSSVDLSIFSLHLAGISSILGAINFITTIINMRLNNMSFD
QLPLFVWAVGITALLLLLSLPVLAGAITMLLTDRNLNTSFFDPAGGGDPILYQHL
>LEFIG035-10|HM875714|MM13656|Coleophora_adspersella
TLYFIFGIWAGMVGTSLSLLIRAELGNPGSLIGDDQIYNTIVTAHAFIMIFFMVMPIMIGGFGNWLVPLMLGAPDMAFPRMNNMSFWLLPPSLTLLISSSIVENGTGTGWTVYPPLSSNIAHGGSSVDLSIFSLHLAGISSILGAINFITTIINMRLNNMSFD
QLPLFVWAVGITALLLLLSLPVLAGAITMLLTDRNLNTSFFDPAGGGDPILYQHL
>LEFIG628-10|HM876294|MM14849|Coleophora_ahenella
TLYFIFGIWAGMMGTSLSLLIRAELGNPGSLIGDDQIYNVIVTAHAFIMIFFMVMPIMIGGFGNWLVPLMLGAPDMAFPRMNNMSFWFLPPSLMLLISSSIVENGTGTGWTVYPPLSSNIAHGGSSVDLSIFSLHLAGISSILGAINFITTIINMRLNNLSF
DQLSLFIWSVGITALLLLLSLPVLAGAITMLLTDRNLNTSFFDPAGGGDPILYQHL
>LEFIE995-10|HM874710|MM10433|Coleophora_albella
TLYFIFGIWAGMVGTSLSLLIRAELGNPGSLIGDDQIYNTIVTAHAFIMIFFMVMPIMIGGFGNWLVPLMLGAPDMAFPRLNNMSFWLLPPSLTLLISSSIVENGTGTGWTVYPPLSSNIAHGGSSVDLSIFSLHLAGISSILGAINFITTIINMRLNNMSFD
QLPLFVWSVGITALLLLLSLPVLAGAITMLLTDRNLNTSFFDPAGGGDPILYQHL
>LEFIG731-10|HM876387|MM15595|Coleophora_albicans
TLYFIFGIWAGMVGTSLSLLIRAELGNPGSLIGDDQIYNTIVTAHAFIMIFFMVMPIMIGGFGNWLVPLMLGAPDMAFPRMNNMSFWLLPPSLTLLISSSIVENGTGTGWTVYPPLSSNIAHSGSSVDLSIFSLHLAGISSILGAINFITTIINMRLNNMSFD
QLPLFVWAVGITALLLLLSLPVLAGAITMLLTDRNLNTSFFDPAGGGDPILYQHL
>LEFIB696-10|HM871574|MM02496|Coleophora_albidella
TLYFIFGIWAGMVGTSLSLLIRAELGNPGSLIGDDQIYNTIVTAHAFIMIFFMVMPIMIGGFGNWLVPLMLGAPDMAFPRMNNMSFWLLPPSMTLLISSSMVETGTGTGWTVYPPLSSNIAHSGSSVDLSIFSLHLAGISSILGAINFITTIINMRLNNLSF
DQLPLFVWAVGITAVLLLLSLPVLAGAITMLLTDRNLNTSFFDPAGGGDPILYQHL
>LEFID542-10|HM873307|MM06528|Coleophora_albidella
TLYFIFGIWAGMVGTSLSLLIRAELGNPGSLIGDDQIYNTIVTAHAFIMIFFMVMPIMIGGFGNWLVPLMLGAPDMAFPRMNNMSFWLLPPSMTLLISSSMVETGTGTGWTVYPPLSSNIAHSGSSVDLSIFSLHLAGISSILGAINFITTIINMRLNNLSF
DQLPLFVWAVGITAILLLLSLPVLAGAITMLLTDRNLNTSFFDPAGGGDPILYQHL
>LEFIJ930-10|KM572151|MM17555|Coleophora_albidella
TLYFIFGIWAGMVGTSLSLLIRAELGNPGSLIGDDQIYNTIVTAHAFIMIFFMVMPIMIGGFGNWLVPLMLGAPDMAFPRMNNMSFWLLPPSMTLLISSSMVETGTGTGWTVYPPLSSNIAHSGSSVDLSIFSLHLAGISSILGAINFITTIINMRLNNLSF
DQLPLFVWAVGITAVLLLLSLPVLAGAITMLLTDRNLNTSFFDPAGGGDPILYQHL
>LEFIG566-10|HM876237|MM14714|Coleophora_albitarsella
TLYFIFGIWAGMVGSSLSLLIRTELGNPGSLISDDQIYNTIVTAHAFIMIFFMVMPIMIGGFGNWLVPLMLGSPDMAFPRMNNMSFWLLPPSLTLLISSSIVENGTGTGWTVYPPLSSNIAHSGSSVDLSIFSLHLAGISSILGAINFITTIINMRLNNLSFDQ
LPLFVWAVGITALLLLLSLPVLAGAITMLLTDRNLNTSFFDPAGGGDPILYQHL
>LEFIG567-10|HM876238|MM14715|Coleophora_albitarsella
TLYFIFGIWAGMVGSSLSLLIRTELGNPGSLISDDQIYNTIVTAHAFIMIFFMVMPIMIGGFGNWLVPLMLGSPDMAFPRMNNMSFWLLPPSLTLLISSSIVENGTGTGWTVYPPLSSNIAHSGSSVDLSIFSLHLAGISSILGAINFITTIINMRLNNLSFDQ
LPLFVWAVGITALLLLLSLPVLAGAITMLLTDRNLNTSFFDPAGGGDPILYQHL
>LEFIB718-10|HM871596|MM02535|Coleophora_alcyonipennella
TLYFIFGIWAGMVGTSLSLLIRAELGNPGSLIGDDQIYNTIVTAHAFIMIFFMVMPIMIGGFGNWLVPLMLGAPDMAFPRMNNMSFWLLPPSLTLLISSSIVENGTGTGWTVYPPLSSNIAHGGSSVDLSIFSLHLAGISSILGAINFITTIINMRLNNMSFD
QLPLFVWAVGITALLLLLSLPVLAGAITMLLTDRNLNTSFFDPAGGGDPILYQHL
>LEFIG358-10|HM876035|MM14356|Coleophora_alcyonipennella
TLYFIFGIWAGMVGTSLSLLIRAELGNPGSLIGDDQIYNTIVTAHAFIMIFFMVMPIMIGGFGNWLVPLMLGAPDMAFPRMNNMSFWLLPPSLTLLISSSIVENGTGTGWTVYPPLSSNIAHGGSSVDLSIFSLHLAGISSILGAINFITTIINMRLNNMSFD
QLPLFVWAVGITALLLLLSLPVLAGAITMLLTDRNLNTSFFDPAGGGDPILYQHL
>LEFIG710-10|HM876366|MM15574|Coleophora_alnifoliae
TLYFIFGIWAGMVGTSLSLLIRTELGNPGSLIGDDQIYNTIVTAHAFIMIFFMVMPIMIGGFGNWLVPLMLGAPDMAFPRMNNMSFWLLPPSLTLLISSSIVENGTGTGWTVYPPLSSNIAHSGSSVDLSIFSLHLAGISSILGAINFITTIINMRLNSMLFD
QLSLFIWAVGITAILLLLSLPVLAGAITMLLTDRNLNTSFFDPAGGGDPILYQHL
>LEFIL349-10|JN265342|MM18659|Coleophora_alnifoliae
TLYFIFGIWAGMVGTSLSLLIRTELGNPGSLIGDDQIYNTIVTAHAFIMIFFMVMPIMIGGFGNWLVPLMLGAPDMAFPRMNNMSFWLLPPSLTLLISSSIVENGTGTGWTVYPPLSSNIAHSGSSVDLSIFSLHLAGISSILGAINFITTIINMRLNNMLFD
QLSLFIWAVGITAILLLLSLPVLAGAITMLLTDRNLNTSFFDPAGGGDPILYQHL
>LEFIL696-10|MM18994|Coleophora_alnifoliae
TLYFIFGIWAGMVGTSLSLLIRTELGNPGSLIGDDQIYNTIVTAHAFIMIFFMVMPIMIGGFGNWLVPLMLGAPDMAFPRMNNMSFWLLPPSLTLLISSSIVENGTGTGWTVYPPLSSNIAHSGSSVDLSIFSLHLAGISSILGAINFITTIINMRLNNMLFD
QLSLFIWAVGITAILLLLSLPVLAGAITMLLTDRNLNTSFFDPAGGGDPILYQHL
>LEFIB674-10|HM871552|MM02463|Coleophora_alticolella

TLYFIFGIWAGMVGTSLSLLIRAELGNPGSLIGDDQIYNTIVTAHAFIMIFFMVMPIMIGGFGNWLVPLMLGAPDMAFPRMNNMSFWLLPPSLMLLISSSIVENGTGTGWTVYPPLSSNIAHSGSSVDLSIFSLHLAGISSILGAINFITTIINMRLNNMSF
DQLPLFVWAVGITALLLLLSLPVLAGAITMLLTDRNLNTSFFDPAGGGDPILYQHL
>LEFID448-10|HQ570344|MM06383|Coleophora_alticolella
TLYFIFGIWAGMVGTSLSLLIRAELGNPGSLIGDDQIYNTIVTAHAFIMIFFMVMPIMIGGFGNWLVPLMLGAPDMAFPRMNNMSFWLLPPSLMLLISSSIVENGTGTGWTVYPPLSSNIAHSGSSVDLSIFSLHLAGISSILGAINFITTIINMRLNNMSF
DQLPLFVWAVGITALLLLLSLPVLAGAITMLLTDRNLNTSFFDPAGGGDPILYQHL
>LEFID561-10|HM873326|MM06554|Coleophora_alticolella
TLYFIFGIWAGMVGTSLSLLIRAELGNPGSLIGDDQIYNTIVTAHAFIMIFFMVMPIMIGGFGNWLVPLMLGAPDMAFPRMNNMSFWLLPPSLMLLISSSIVENGTGTGWTVYPPLSSNIAHSGSSVDLSIFSLHLAGISSILGAINFITTIINMRLNNMSF
DQLPLFVWAVGITALLLLLSLPVLAGAITMLLTDRNLNTSFFDPAGGGDPILYQHL
>LEFIC360-10|HM872203|MM03884|Coleophora_amellivora
TLYFIFGIWAGMVGTSLSLLIRAELGNPGSLIGDDQIYNTIVTAHAFIMIFFMVMPIMIGGFGNWLVPLMLGAPDMAFPRMNNMSFWLLPPSLTLLISSSIVENGTGTGWTVYPPLSSNIAHGGSSVDLSIFSLHLAGISSILGAINFITTIINMRLNNMSFD
QLPLFVWSVGITALLLLLSLPVLAGAITMLLTDRNLNTSFFDPAGGGDPILYQHL
>LEFID845-10|HM873602|MM06969|Coleophora_amellivora
TLYFIFGIWAGMVGTSLSLLIRAELGNPGSLIGDDQIYNTIVTAHAFIMIFFMVMPIMIGGFGNWLVPLMLGAPDMAFPRMNNMSFWLLPPSLTLLISSSIVENGTGTGWTVYPPLSSNIAHGGSSVDLSIFSLHLAGISSILGAINFITTIINMRLNNMSFD
QLPLFVWSVGITALLLLLSLPVLAGAITMLLTDRNLNTSFFDPAGGGDPILYQHL
>LEFIB150-10|HM871055|MM00538|Coleophora_antennariella
TLYFIFGIWAGMVGTSLSLLIRTELGNPGSLISDDQIYNTIVTAHAFIMIFFMVMPIMIGGFGNWLVPLMLGAPDMAFPRMNNMSFWLLPPSLTLLISSSIVENGTGTGWTVYPPLSSNIAHSGNSVDLSIFSLHLAGISSILGAINFITTIINMRLNNMSFD
QLPLFVWAVGITALLLLLSLPVLAGAITMLLTDRNLNTSFFDPAGGGDPILYQHL
>LEFIB151-10|HM871056|MM00539|Coleophora_antennariella
TLYFIFGIWAGMVGTSLSLLIRTELGNPGSLISDDQIYNTIVTAHAFIMIFFMVMPIMIGGFGNWLVPLMLGAPDMAFPRMNNMSFWLLPPSLTLLISSSIVENGTGTGWTVYPPLSSNIAHSGNSVDLSIFSLHLAGISSILGAINFITTIINMRLNNMSFD
QLPLFVWAVGITTLLLLLSLPVLAGAITMLLTDRNLNTSFFDPAGGGDPILYQHL
>LEFIC425-10|HQ570309|MM03971|Coleophora_arctostaphyli
TLYFIFGIWAGMMGTSLSLLIRAELGNPGSLIGNDQIYNVIVTAHAFIMIFFMVMPIMIGGFGNWLVPLMLGAPDMAFPRMNNMSFWLLPPSLMILISSSIVENGTGTGWTVYPPLSSNIAHSGSSVDLSIFSLHLAGISSILGAINFITTIINMRLNNLSFD
QLPLFVWSVGITALLLLLSLPVLAGAITMLLTDRNLNTSFFDPAGGGDPILYQHL
>LEFIE604-10|HM874327|MM09517|Coleophora_argentula
TLYFIFGIWAGMVGTSLSLLIRAELGNPGSLIGDDQIYNTIVTAHAFIMIFFMVMPIMIGGFGNWLVPLMLGAPDMAFPRMNNMSFWLLPPSLTLLISSSIVENGTGTGWTVYPPLSSNIAHGGSSVDLSIFSLHLAGISSILGAINFITTIINMRLNNMSFD
QLPLFVWAVGITALLLLLSLPVLAGAITMLLTDRNLNTSFFDPAGGGDPILYQHL
>LEFIE941-10|HM874658|MM10330|Coleophora_argentula
TLYFIFGIWAGMVGTSLSLLIRAELGNPGSLIGDDQIYNTIVTAHAFIMIFFMVMPIMIGGFGNWLVPLMLGAPDMAFPRMNNMSFWLLPPSLTLLISSSIVENGTGTGWTVYPPLSSNIAHGGSSVDLSIFSLHLAGISSILGAINFITTIINMRLNNMSFD
QLPLFVWAVGITALLLLLSLPVLAGAITMLLTDRNLNTSFFDPAGGGDPILYQHL
>LEFIE943-10|HM874660|MM10332|Coleophora_argentula
TLYFIFGIWAGMVGTSLSLLIRAELGNPGSLIGDDQIYNTIVTAHAFIMIFFMVMPIMIGGFGNWLVPLMLGAPDMAFPRMNNMSFWLLPPSLTLLISSSIVENGTGTGWTVYPPLSSNIAHGGSSVDLSIFSLHLAGISSILGAINFITTIINMRLNNMSFD
QLPLFVWAVGITALLLLLSLPVLAGAITMLLTDRNLNTSFFDPAGGGDPILYQHL
>LEFIJ1321-11|KF808470|MM21181|Coleophora_argentula
TLYFIFGIWAGMVGTSLSLLIRAELGNPGSLIGDDQIYNTIVTAHAFIMIFFMVMPIMIGGFGNWLVPLMLGAPDMAFPRMNNMSFWLLPPSLTLLISSSIVENGTGTGWTVYPPLSSNIAHGGSSVDLSIFSLHLAGISSILGAINFITTIINMRLNNMSFD
QLPLFVWAVGITALLLLLSLPVLAGAITMLLTDRNLNTSFFDPAGGGDPILYQHL
>LEFIB695-10|HM871573|MM02495|Coleophora_artemisicolella
TLYFIFGIWAGMVGTSLSLLIRAELGNPGSLIGDDQIYNTIVTAHAFIMIFFMVMPIMIGGFGNWLVPLMLGAPDMAFPRMNNMSFWLLPPSLTLLISSSIVENGTGTGWTVYPPLSSNIAHGGSSVDLSIFSLHLAGISSILGAINFITTIINMRLNNMSFD
QLPLFVWAVGITALLLLLSLPVLAGAITMLLTDRNLNTSFFDPAGGGDPILYQHL
>LEEUA153-11|MM19561|Coleophora_asteris
TLYFIFGIWAGMVGTSLSLLIRAELGNPGSLIGDDQIYNTIVTAHAFIMIFFMVMPIMIGGFGNWLIPLMLGAPDMAFPRMNNMSFWLLPPSLTLLISSSIVENGTGTGWTVYPPLSSNIAHSGASVDLSIFSLHLAGISSILGAINFITTIINMRLNNMSFD
QLPLFVWAVGITALLLLLSLPVLAGAITMLLTDRNLNTSFFDPAGGGDPILYQHL
>LEFIJ398-10|JF853584|MM06579|Coleophora_atriplicis
TLYFIFGIWSGMVGTSLSLLIRAELGNPGSLIGDDQIYNTIVTAHAFIMIFFMVMPIMIGGFGNWLVPLMLGAPDMAFPRMNNMSFWLLPPSLTLLISSSIVENGTGTGWTVYPPLSSNIAHSGSSVDLSIFSLHLAGISSILGAINFITTIINMRLNNLSFDQ
LPLFVWSVGITALLLLLSLPVLAGAITMLLTDRNLNTSFFDPAGGGDPILYQHL
>LEFIE339-10|HM874064|MM08733|Coleophora_badiipennella
TLYFIFGIWAGMVGTSLSLLIRTELGNPGSLIGDDQIYNTIVTAHAFIMIFFMVMPIMIGGFGNWLVPLMLGAPDMAFPRMNNMSFWLLPPSLTLLISSSIVENGTGTGWTVYPPLSSNIAHSGSSVDLSIFSLHLAGISSILGAINFITTIINMRLNNMLFD
QLSLFIWAVGITAILLLLSLPVLAGAITMLLTDRNLNTSFFDPAGGGDPILYQHL

>LEFIE432-10|HM874156|MM09178|Coleophora_bernoulliella
TLYFIFGIWAGMIGTSLSLLIRAELGNPGSLIGDDQIYNTIVTAHAFIMIFFMVMPIMIGGFGNWLVPLMLGAPDMAFPRMNNMSFWLLPPSMTLLISSSMVETGTGTGWTVYPPLSSNIAHSGSSVDLSIFSLHLAGISSILGAINFITTIINMRLNNLSFD
QLPLFVWAVGITAILLLLSLPVLAGAITMLLTDRNLNTSFFDPAGGGDPILYQHL
>LEFID604-10|HM873369|MM06617|Coleophora_betulella
TLYFIFGIWAGMIGTSLSLLIRTELGNPGSLIGNDQIYNTIVTAHAFIMIFFMVMPIMIGGFGNWLVPLMLGAPDMAFPRMNNMSFWLLPPSLTLLISSSMVETGTGTGWTVYPPLSSNISHSGSSVDLSIFSLHLAGISSILGAINFITTIINMRLNNLSFDQ
LPLFIWAVGITAILLLLSLPVLAGAITMLLTDRNLNTSFFDPAGGGDPILYQHL
>LEFIA919-10|HM387055|MM09797|Coleophora_betulella
TLYFIFGIWAGMIGTSLSLLIRTELGNPGSLIGNDQIYNTIVTAHAFIMIFFMVMPIMIGGFGNWLVPLMLGAPDMAFPRMNNMSFWLLPPSLTLLISSSMVETGTGTGWTVYPPLSSNISHSGSSVDLSIFSLHLAGISSILGAINFITTIINMRLNNLSFDQ
LPLFIWAVGITAILLLLSLPVLAGAITMLLTDRNLNTSFFDPAGGGDPILYQHL
>LEFIK596-10|JF854178|MM18171|Coleophora_betulella
TLYFIFGIWAGMIGTSLSLLIRTELGNPGSLIGNDQIYNTIVTAHAFIMIFFMVMPIMIGGFGNWLVPLMLGAPDMAFPRMNNMSFWLLPPSLTLLISSSMVETGTGTGWTVYPPLSSNISHSGSSVDLSIFSLHLAGISSILGAINFITTIINMRLNNLSFDQ
LPLFIWAVGITAILLLLSLPVLAGAITMLLTDRNLNTSFFDPAGGGDPILYQHL
>LEFIE519-10|HM874242|MM09351|Coleophora_binderella
TLYFIFGIWAGMMGTSLSLLIRAELGNPGSLIGDDQIYNVIVTAHAFIMIFFMVMPIMIGGFGNWLVPLMLGAPDMAFPRMNNMSFWFLPPSLMLLISSSIVENGTGTGWTVYPPLSSNIAHSGSSVDLSIFSLHLAGISSILGAINFITTIINMRLNNLSF
DQLPLFVWSVGITALLLLLSLPVLAGAITMLLTDRNLNTSFFDPAGGGDPILYQHL
>LEFIE549-10|HM874272|MM09434|Coleophora_binderella
TLYFIFGIWAGMMGTSLSLLIRTELGNPGSLIGDDQIYNVIVTAHAFIMIFFMVMPIMIGGFGNWLVPLMLGAPDMAFPRMNNMSFWFLPPSLMLLISSSIVENGTGTGWTVYPPLSSNIAHSSSSVDLSIFSLHLAGISSILGAINFITTIINMRLNNLSFD
QLPLFVWSVGITALLLLLSLPVLAGAITMLLTDRNLNTSFFDPAGGGDPILYQHL
>LEFID195-10|HM872998|MM05994|Coleophora_boreella
TLYFIFGIWAGMMGTSLSLLIRAELGNPGSLIGDDQIYNTIVTAHAFIMIFFMVMPIMIGGFGNWLVPLMLGAPDMAFPRMNNMSFWLLPPSLTLLISSSIVENGTGTGWTVYPPLSSNIAHGGSSVDLSIFSLHLAGISSILGAINFITTIINMRLNNMSF
DQLPLFVWSVGITALLLLLSLPVLAGAITMLLTDRNLNTSFFDPAGGGDPILYQHL
>ELACA488-10|JF847558|MM16714|Coleophora_boreella
TLYFIFGIWAGMMGTSLSLLIRAELGNPGSLIGDDQIYNTIVTAHAFIMIFFMVMPIMIGGFGNWLVPLMLGAPDMAFPRMNNMSFWLLPPSLTLLISSSIVENGTGTGWTVYPPLSSNIAHGGSSVDLSIFSLHLAGISSILGAINFITTIINMRLNNMSF
DQLPLFVWSVGITALLLLLSLPVLAGAITMLLTDRNLNTSFFDPAGGGDPILYQHL
>LEFIC288-10|HM872132|MM03758|Coleophora_brevipalpella
TLYFIFGIWAGMVGTSLSLLIRTELGNPGSFIGDDQIYNTIVTAHAFIMIFFMVMPIMIGGFGNWLVPLMLGAPDMAFPRMNNMSFWLLPPSLMLLISSSIVENGTGTGWTVYPPLSSNIAHSGSSVDLSIFSLHLAGISSILGAINFITTIINMRLNNLSFD
QLPLFVWAVGITALLLLLSLPVLAGAITMLLTDRNLNTSFFDPAGGGDPILYQHL
>ELACA2052-12|MM21606|Coleophora_burmanni
TLYFIFGIWAGMVGTSLSLLIRAELGNPGSLIGDDQIYNTIVTAHAFIMIFFMVMPIMIGGFGNWLVPLMLGAPDMAFPRMNNMSFWLLPPSLTLLISSSIVENGTGTGWTVYPPLSSNIAHSGSSVDLSIFSLHLAGISSILGAINFITTIINMRLNNMSFD
QLPLFVWAVGITALLLLLSLPVLAGAITMLLTDRNLNTSFFDPAGGGDPILYQHL
>LEFIC285-10|HM872129|MM03752|Coleophora_caelebipennella
TLYFIFGIWAGMIGTSLSLLIRAELGNPSSLIGDDQIYNTIVTAHAFIMIFFMVMPIMIGGFGNWLVPLMLGAPDMAFPRMNNMSFWLLPPSLTLLISSSIVETGTGTGWTVYPPLSSNIAHSGSSVDLSIFSLHLAGISSILGAINFITTIINMRLNNLSFDQL
PLFVWSVGITALLLLLSLPVLAGAITMLLTDRNLNTSFFDPAGGGDPILYQHL
>LEFID327-10|HM873124|MM06220|Coleophora_caespititiella
TLYFIFGIWAGMVGTSLSLLIRAELGNPGSLIGDDQIYNTIVTAHAFIMIFFMVMPIMIGGFGNWLVPLMLGAPDMAFPRMNNMSFWLLPPSLTLLISSSVVENGTGTGWTVYPPLSSNIAHGGSSVDLSIFSLHLAGISSILGAINFITTIINMRLNNMSF
DQLPLFVWAVGITALLLLLSLPVLAGAITMLLTDRNLNTSFFDPAGGGDPILYQHL
>LEFIE759-10|HM874478|MM09883|Coleophora_carelica
TLYFIFGIWAGMVGTSLSLLIRAELGNPGSLIGDDQIYNTIVTAHAFIMIFFMVMPIMIGGFGNWLVPLMLGAPDMAFPRMNNMSFWLLPPSLTLLISSSIVENGTGTGWTVYPPLSSNIAHGGSSVDLSIFSLHLAGISSILGAINFITTIINMRLNNMSFD
QLPLFVWAVGITALLLLLSLPVLAGAITMLLTDRNLNTSFFDPAGGGDPILYQHL
>LEFID085-10|HM872896|MM05697|Coleophora_chrysanthemi
TLYFIFGIWAGMLGTSLSLLIRAELGNPGSLIGDDQIYNTIVTAHAFIMIFFMVMPIMIGGFGNWLVPLMLGAPDMAFPRMNNMSFWLLPPSLTLLISSSIVENGTGTGWTVYPPLSSNIAHGGSSVDLSIFSLHLAGISSILGAINFITTIINMRLNNMSFD
QLPLFVWSVGITALLLLLSLPVLAGAITMLLTDRNLNTSFFDPAGGGDPILYQHL
>LEFID355-10|HM873152|MM06258|Coleophora_chrysanthemi
TLYFIFGIWAGMLGTSLSLLIRAELGNPGSLIGDDQIYNTIVTAHAFIMIFFMVMPIMIGGFGNWLVPLMLGAPDMAFPRMNNMSFWLLPPSLTLLISSSIVENGTGTGWTVYPPLSSNIAHGGSSVDLSIFSLHLAGISSILGAINFITTIINMRLNNMSFD
QLPLFVWSVGITALLLLLSLPVLAGAITMLLTDRNLNTSFFDPAGGGDPILYQHL
>LEFIJ1237-11|MM21097|Coleophora_clypeiferella
TLYFIFGIWAGMVGTSLSLLIRAELGNPGSLIGDDQIYNTIVTAHAFIMIFFMVMPIMIGGFGNWLVPLMLGAPDMAFPRMNNMSFWLLPPSLTLLISSSIVENGTGTGWTVYPPLSSNIAHGGSSVDLSIFSLHLAGISSILGAINFITTIINMRLNNMSFD

QLPLFVWAVGITALLLLLSLPVLAGAITMLLTDRNLNTSFFDPAGGGDPILYQHL
>LEFIJ371-10|JF853558|MM15971|Coleophora_colutella
TLYFIFGIWAGMVGTSLSLLIRAELGTPGSLIGDDQIYNTIVTAHAFIMIFFMVMPIMIGGFGNWLVPLMLSAPDMAFPRMNNMSFWLLPPSLTLLISSSMVENGTGTGWTVYPPLSSNIAHSGSSVDLSIFSLHLAGISSILGAINFITTIINMRLNNLSFD
QLPLFVWSVGITALLLLLSLPVLAGAITMLLTDRNLNTSFFDPAGGGDPILYQHL
>ELACA870-11|MM20108|Coleophora_colutella
TLYFIFGIWAGMVGTSLSLLIRAELGTPGSLIGDDQIYNTIVTAHAFIMIFFMVMPIMIGGFGNWLVPLMLSAPDMAFPRMNNMSFWLLPPSLTLLISSSMVENGTGTGWTVYPPLSSNIAHSGSSVDLSIFSLHLAGISSILGAINFITTIINMRLNNLSFD
QLPLFVWSVGITALLLLLSLPVLAGAITMLLTDRNLNTSFFDPAGGG?PILYQHL
>LEFIB075-10|HM870984|MM00371|Coleophora_conspicuella
TLYFIFGIWAGMIGTSLSLLIRAELGNPGSLIGDDQIYNTIVTAHAFIMIFFMVMPIMIGGFGNWLIPLMLGAPDMAFPRMNNMSFWLLPPSLTLLISSSIVETGTGTGWTVYPPLSSNIAHSGSSVDMSIFSLHLAGISSILGAINFITSIINMRLNNLSFDQ
LPLFIWAVGITALLLLLSLPVLAGAITMLLTDRNLNTSFFDPAGGGDPILYQHL
>LEFIJ933-10|MM17558|Coleophora_conspicuella
TLYFIFGIWAGMIGTSLSLLIRAELGNPGSLIGDDQIYNTIVTAHAFIMIFFMVMPIMIGGFGNWLIPLMLGAPDMAFPRMNNMSFWLLPPSLTLLISSSIVETGTGTGWTVYPPLSSNIAHSGSSVDMSIFSLHLAGISSILGAINFITSIINMRLNNLLFDQ
LPLFIWAVGITALLLLLSLPVLAGAITMLLTDRNLNTSFFDPAGGGDPILYQHL
>ELACA236-10|MM16531|Coleophora_conyzae
TLYFIFGIWAGMMGTSLSLLIRAELGNPGFLIGDDQIYNTIVTAHAFIMIFFMVMPIMIGGFGNWLVPLMLGAPDMAFPRMNNMSFWLLPPSLMLLISSSVVENGTGTGWTVYPPLSSNIAHSGTSVDLSIFSLHLAGISSILGAINFITTIINMRLNNFSF
DQLPLFIWAIGITTLLLLLSLPVLAGAITMLLTDRNLNTSFFDPAGGGDPILYQHL
>LEFIL304-10|JN265341|MM19304|Coleophora_conyzae
TLYFIFGIWAGMMGTSLSLLIRAELGNPGFLIGDDQIYNTIVTAHAFIMIFFMVMPIMIGGFGNWLVPLMLGAPDMAFPRMNNMSFWLLPPSLMLLISSSVVENGTGTGWTVYPPLSSNIAHSGTSVDLSIFSLHLAGISSILGAINFITTIINMRLNNFSF
DQLPLFIWAIGITTLLLLLSLPVLAGAITMLLTDRNLNTSFFDPAGGGDPILYQHL
>LEFIJ301-10|JF853538|MM15188|Coleophora_cornutella
TLYFIFGIWAGMMGTSLSLLIRAELGNPGSLIGDDQIYNVIVTAHAFIMIFFMVMPIMIGGFGNWLVPLMLGAPDMAFPRMNNMSFWFLPPSLMILISSSIVENGTGTGWTVYPPLSSNIAHSGSSVDLSIFSLHLAGISSILGAINFITTIINMRLNNLSFD
QLPLFVWSVGITALLLLLSLPVLAGAITMLLTDRNLNTSFFDPAGGGDPILYQHL
>LOBU080-12|MM22022|Coleophora_cornutella
TLYFIFGIWAGMMGTSLSLLIRAELGNPGSLIGDDQIYNVIVTAHAFIMIFFMVMPIMIGGFGNWLVPLMLGAPDMAFPRMNNMSFWFLPPSLMILISSSIVENGTGTGWTVYPPLSSNIAHSGSSVDLSIFSLHLAGISSILGAINFITTIINMRLNNLSFD
QLPLFVWSVGITALLLLLSLPVLAGAITMLLTDRNLNTSFFDPAGGGDPILYQHL
>LEFIB716-10|HM871594|MM02529|Coleophora_deauratella
TLYFIFGIWAGMVGTSLSLLIRAELGNPGSLIGDDQIYNTIVTAHAFIMIFFMVMPIMIGGFGNWLVPLMLGAPDMAFPRMNNMSFWLLPPSLTLLISSSIVENGTGTGWTVYPPLSSNIAHGGSSVDLSIFSLHLAGISSILGAINFITTIINMRLNNMSFD
QLPLFVWAVGITALLLLLSLPVLAGAITMLLTDRNLNTSFFDPAGGGDPILYQHL
>LEFIB975-10|HM871848|MM03167|Coleophora_deauratella
TLYFIFGIWAGMVGTSLSLLIRAELGNPGSLIGDDQIYNTIVTAHAFIMIFFMVMPIMIGGFGNWLVPLMLGAPDMAFPRMNNMSFWLLPPSLTLLISSSIVENGTGTGWTVYPPLSSNIAHGGSSVDLSIFSLHLAGISSILGAINFITTIINMRLNNMSFD
QLPLFVWAVGITALLLLLSLPVLAGAITMLLTDRNLNTSFFDPAGGGDPILYQHL
>LEFID266-10|HM873064|MM06134|Coleophora_deauratella
TLYFIFGIWAGMVGTSLSLLIRAELGNPGSLIGDDQIYNTIVTAHAFIMIFFMVMPIMIGGFGNWLVPLMLGAPDMAFPRMNNMSFWLLPPSLTLLISSSIVENGTGTGWTVYPPLSSNIAHGGSSVDLSIFSLHLAGISSILGAINFITTIINMRLNNMSFD
QLPLFVWAVGITALLLLLSLPVLAGAITMLLTDRNLNTSFFDPAGGGDPILYQHL
>LEFID601-10|HM873366|MM06614|Coleophora_deauratella
TLYFIFGIWAGMVGTSLSLLIRAELGNPGSLIGDDQIYNTIVTAHAFIMIFFMVMPIMIGGFGNWLVPLMLGAPDMAFPRMNNMSFWLLPPSLTLLISSSIVENGTGTGWTVYPPLSSNIAHGGSSVDLSIFSLHLAGISSILGAINFITTIINMRLNNMSFD
QLPLFVWAVGITALLLLLSLPVLAGAITMLLTDRNLNTSFFDPAGGGDPILYQHL
>LEFIG014-10|HM875694|MM13635|Coleophora_deauratella
TLYFIFGIWAGMVGTSLSLLIRAELGNPGSLIGDDQIYNTIVTAHAFIMIFFMVMPIMIGGFGNWLVPLMLGAPDMAFPRMNNMSFWLLPPSLTLLISSSIVENGTGTGWTVYPPLSSNIAHGGSSVDLSIFSLHLAGISSILGAINFITTIINMRLNNMSFD
QLPLFVWAVGITALLLLLSLPVLAGAITMLLTDRNLNTSFFDPAGGGDPILYQHL
>LEFIG015-10|HM875695|MM13636|Coleophora_deauratella
TLYFIFGIWAGMVGTSLSLLIRAELGNPGSLIGDDQIYNTIVTAHAFIMIFFMVMPIMIGGFGNWLVPLMLGAPDMAFPRMNNMSFWLLPPSLTLLISSSIVENGTGTGWTVYPPLSSNIAHGGSSVDLSIFSLHLAGISSILGAINFITTIINMRLNNMSFD
QLPLFVWAVGITALLLLLSLPVLAGAITMLLTDRNLNTSFFDPAGGGDPILYQHL
>LEFIB697-10|HM871575|MM02498|Coleophora_dianthi
TLYFIFGIWAGMVGTSLSLLIRAELGNPGSLIGDDQIYNTIVTAHAFIMIFFMVMPIMIGGFGNWLIPLMMGAPDMAFPRMNNMSFWLLPPSLTLLISSSIVENGTGTGWTVYPPLSSNIAHSGSSVDLSIFSLHLAGISSILGAINFITTIINMRLNNMSFN
QLPLFVWAVGITALLLLLSLPVLAGAITMLLTDRNLNTSFFDPAGGGDPILYQHL
>LEFID086-10|HM872897|MM05698|Coleophora_dianthi

TLYFIFGIWAGMMGTSLSLLIRAELGNPGSLIGDDQIYNTIVTAHAFIMIFFMVMPIMIGGFGNWLVPLMLGAPDMAFPRMNNMSFWLLPPSLTLLISSSIVENGTGTGWTVYPPLSSNIAHSGSSVDLSIFSLHLAGISSILGAINFITTIINMRLNNMSF
DQLPLFVWAVGITALLLLLSLPVLAGAITMLLTDRNLNTSFFDPAGGGDPILYQHL
>LEFIE269-10|HM874002|MM08607|Coleophora_dianthi
TLYFIFGIWAGMVGTSLSLLIRAELGSPGSLIGDDQIYNTIVTAHAFIMIFFMVMPIMIGGFGNWLVPLMLGAPDMAFPRMNNMSFWLLPPSLTLLISSSIVENGTGTGWTVYPPLSSNIAHSGSSVDLSIFSLHLAGISSILGAINFITTIINMRLNNMLFD
QLPLFVWAVGITALLLLLSLPVLAGAITMLLTDRNLNTSFFDPAGGGDPILYQHL
>LEFIJ137-10|MM09881|Coleophora_dianthi
TLYFIFGIWAGMMGTSLSLLIRAELGNPGSLIGDDQIYNTIVTAHAFIMIFFMVMPIMIGGFGNWLIPLMLGAPDMAFPRMNNMSFWLLPPSLTLLISSSIVENGTGTGWTVYPPLSSNIAHSGSSVDLSIFSLHLAGISSILGAINFITTIINMRLNNMSFD
RLPLFVWAVGITALLLLLSLPVLAGAITMLLTDRNLNTSFFDPAGGGDPILYQHL
>LEFIJ138-10|MM09882|Coleophora_dianthi
TLYFIFGIWAGMVGTSLSLLIRAELGNPGSLIGDNQIYNTIVTAHAFIMIFFMVMPIMIGGFGNWLVPLMLGAPDMAFPRMNNMSFWLLPPSLTLLISSSIVENGTGTGWTVYPPLSSNIAHSGSSVDLSIFSLHLAGISSILGAINFITTIINMRLNNMSFD
QLPLFVWAVGITALLLLLSLPVLAGAITMLLTDRNLNTSFFDPAGGGDPILYQHL
>LEFIE829-10|HM874548|MM10041|Coleophora_dianthi
TLYFIFGIWAGMVGTSLSLLIRAELGNPGSLIGDDQIYNTIVTAHAFIMIFFMVMPIMIGGFGNWLIPLMLGAPDMAFPRMNNMSFWLLPPSLTLLISSSIVENGTGTGWTVYPPLSSNIAHSGSSVDLSIFSLHLAGISSILGAINFITTIINMRLNNMSFD
QLPLFVWAVGITALLLLLSLPVLAGAITMLLTDRNLNTSFFDPAGGGDPILYQHL
>LEFIG730-10|HM876386|MM15594|Coleophora_dianthi
TLYFIFGIWAGMMGTSLSLLIRAELGNPGSLINDDQIYNTIVTAHAFIMIFFMVMPIMIGGFGNWLVPLMMGAPDMAFPRMNNMSFWLLPPSLTLLISSSIVENGTGTGWTVYPPLSSNIAHSGSSVDLSIFSLHLAGISSILGAINFITTIINMRLNNMSF
DQLPLFVWAVGITALLLLLSLPVLAGAITMLLTDRNLNTSFFDPAGGGDPILYQHL
>LEFIK616-10|JF854192|MM18191|Coleophora_dianthi
TLYFIFGIWAGMMGTSLSLLIRAELGNPGSLIGDDQIYNTIVTAHAFIMIFFMVMPIMIGGFGNWLIPLMLGAPDMAFPRMNNMSFWLLPPSLTLLISSSIVENGTGTGWTVYPPLSSNIAHSGSSVDLSIFSLHLAGISSILGAINFITTIINMRLNNMSFD
QLPLFVWAVGITALLLLLSLPVLAGAITMLLTDRNLNTSFFDPAGGGDPILYQHL
>LEFIF491-10|HM875176|MM12048|Coleophora_directella
TLYFIFGIWAGMVGTSLSLLIRAELGNPGSLIGDDQIYNTIVTAHAFIMIFFMVMPIMIGGFGNWLVPLMLGAPDMAFPRMNNMSFWLLPPSLTLLISSSIVENGTGTGWTVYPPLSSNIAHGGSSVDLSIFSLHLAGISSILGAINFITTIINMRLNNMSFD
QLPLFVWAVGITALLLLLSLPVLAGAITMLLTDRNLNTSFFDPAGGGDPILYQHL
>LEFID788-10|HM873545|MM06858|Coleophora_discordella
TLYFIFGIWAGMVGTSLSLLIRAELGNPGSLIGDDQIYNTIVTAHAFIMIFFMVMPIMIGGFGNWLVPLMLGAPDMAFPRMNNMSFWLLPPSLTLLISSSVVENGTGTGWTVYPPLSSNIAHSGSSVDLSIFSLHLAGISSILGAINFITTIINMRLNNLSFD
QLPLFVWSVGITALLLLLSLPVLAGAITMLLTDRNLNTSFFDPAGGGDPILYQHL
>LEFIK595-10|JF854177|MM18170|Coleophora_discordella
TLYFIFGIWAGMVGTSLSLLIRAELGNPGSLIGDDQIYNTIVTAHAFIMIFFMVMPIMIGGFGNWLVPLMLGAPDMAFPRMNNMSFWLLPPSLTLLISSSVVENGTGTGWTVYPPLSSNIAHSGSSVDLSIFSLHLAGISSILGAINFITTIINMRLNNLSFD
QLPLFVWSVGITALLLLLSLPVLAGAITMLLTDRNLNTSFFDPAGGGDPILYQHL
>LEFIK612-10|JF854188|MM18187|Coleophora_expressella
TLYFIFGIWAGMVGTSLSLLIRAELGNPGSLIGDDQIYNTIVTAHAFIMIFFMVMPIMIGGFGNWLVPLMLGAPDMAFPRMNNMSFWLLPPSLTLLISSSIVENGTGTGWTVYPPLSSNIAHGGSSVDLSIFSLHLAGISSILGAINFITTIINMRLNNMSFD
QLPLFVWAVGITALLLLLSLPVLAGAITMLLTDRNLNTSFFDPAGGGDPILYQHL
>LEFIE487-10|HM874211|MM09268|Coleophora_filaginella
TLYFIFGIWAGMVGTSLSLLIRAELGNPGSLIGDDQIYNTIVTAHAFIMIFFMVMPIMIGGFGNWLVPLMLGAPDMAFPRMNNMSFWLLPPSLTLLISSSIVENGTGTGWTVYPPLSSNIAHSGSSVDLSIFSLHLAGISSILGAINFITTIINMRLNNMSFD
QLPLFVWSVGITALLLLLSLPVLAGAITMLLTDRNLNTSFFDPAGGGDPILYQHL
>LEFID666-10|HM873428|MM06704|Coleophora_flavipennella
TLYFIFGIWAGMVGTSLSLLIRAELGNPGSLIGDDQIYNTIVTAHAFIMIFFMVMPVMIGGFGNWLVPLMLGAPDMAFPRMNNMSFWLLPPSLTLLISSSIVENGTGTGWTVYPPLSSNIAHSGSSVDLSIFSLHLAGISSILGAINFITTIINMRLNNLSFD
QLPLFVWAVGITALLLLLSLPVLAGAITMLLTDRNLNTSFFDPAGGGDPILYQHL
>LEFIE711-10|HM874431|MM09776|Coleophora_flavipennella
TLYFIFGIWAGMVGTSLSLLIRAELGNPGSLIGDDQIYNTIVTAHAFIMIFFMVMPVMIGGFGNWLVPLMLGAPDMAFPRMNNMSFWLLPPSLTLLISSSIVENGTGTGWTVYPPLSSNIAHSGSSVDLSIFSLHLAGISSILGAINFITTIINMRLNNLSFD
QLPLFVWAVGITALLLLLSLPVLAGAITMLLTDRNLNTSFFD?AEGGDPILYQHL
>LEFIL298-10|MM19298|Coleophora_follicularis
TLYFIFGIWASMVGTSLSLLIRAELGNPGSLIGDDQIYNTIVTAHAFIMIFFMVMPIMIGGFGNWLVPLMLGAPDMAFPRMNNMSFWLLPPSLTLLISSSIVENGTGTGWTVYPPLSSNIAHGGSSVDLSIFSLHLAGISSILGAINFITTIINMRLNNMLFD
QLPLFVWAVGITALLLLLSLPVLAGAITMLLTDRNLNTSFFDPAGGGDPILYQHL
>LEFIG419-10|HM876095|MM14446|Coleophora_frischella
TLYFIFGIWAGMVGTSLSLLIRAELGNPGSFIGDDQIYNTIVTAHAFIMIFFMVMPIMIGGFGNWLVPLMLGAPDMAFPRMNNMSFWLLPPSLTLLISSSIVENGTGTGWTVYPPLSSNIAHSGSSVDLSIFSLHLAGISSILGAINFITTIINMRLNNMSFD
QLPLFVWAVGITALLLLLSLPVLAGAITMLLTDRNLNTSFFDPAGGGDPILYQHL

>LEFIG425-10|HM876101|MM14458|Coleophora_frischella
TLYFIFGIWAGMVGTSLSLLIRAELGNPGSFIGDDQIYNTIVTAHAFIMIFFMVMPIMIGGFGNWLVPLMLGAPDMAFPRMNNMSFWLLPPSLTLLISSSIVENGTGTGWTVYPPLSSNIAHSGSSVDLSIFSLHLAGISSILGAINFITTIINMRLNNMSFD
QLPLFVWAVGITALLLLLSLPVLAGAITMLLTDRNLNTSFFDPAGGGDPILYQHL
>LEFIG711-10|HM876367|MM15575|Coleophora_fuscocuprella
TLYFIFGIWAGMMGTSLSLLIRAELGNPGSLIGDDQIYNVIVTAHAFIMIFFMVMPIMIGGFGNWLVPLMLGAPDMAFPRMNNMSFWFLPPSLMLLISSSIVENGTGTGWTVYPPLSSNIAHSGSSVDLSIFSLHLAGISSILGAINFITTIINMRLNNLSF
DQLPLFVWSVGITALLLLLSLPVLAGAITMLLTDRNLNTSFFDPAGGGDPILYQHL
>LEFIL301-10|JN265340|MM19301|Coleophora_gallipennella
TLYFIFGIWAGMVGTSLSLLIRAELGNPGSLIGDDQIYNTIVTAHAFIMIFFMVMPIMIGGFGNWLVPLMLGAPDMAFPRMNNMSFWLLPPSLMLLISSSIVENGTGTGWTVYPPLSSNIAHSGSSVDLSIFSLHLAGISSILGAINFITTIINMRLNNMSF
DQLPLFVWAVGITALLLLLSLPVLAGAITMLLTDRNLNTSFFDPAGGGDPILYQHL
>LEFIH367-10|HM876983|MM13704|Coleophora_gardesanella
TLYFIFGIWASMVGTSLSLLIRAELGNPGSLISDDQIYNTIVTAHAFIMIFFMVMPIMIGGFGNWLVPLMLGAPDMAFPRMNNMSFWLLPPSLMLLISSSIVENGTGTGWTVYPPLSSNIAHSGSSVDLSIFSLHLAGISSILGAINFITTIINMRLNNMSFD
QLSLFVWAVGITALLLLLSLPVLAGAITMLLTDRNLNTSFFDPAGGGDPILYQHL
>LEFIB675-10|HM871553|MM02467|Coleophora_glaucicolella
TLYFIFGIWSGMVGTSLSLLIRAELGNPGSLIGDDQIYNTIVTAHAFIMIFFMVMPIMIGGFGNWLVPLMLGAPDMAFPRMNNMSFWLLPPSLMLLISSSVVENGTGTGWTVYPPLSSNIAHSGSSVDLSIFSLHLAGISSILGAINFISTIINMRLNNMSF
DQLPLFVWAVGITALLLLLSLPVLAGAITMLLTDRNLNTSFFDPAGGGDPILYQHL
>LEFIC040-10|HM871910|MM03271|Coleophora_glaucicolella
TLYFIFGIWSGMVGTSLSLLIRAELGNPGSLIGDDQIYNTIVTAHAFIMIFFMVMPIMIGGFGNWLVPLMLGAPDMAFPRMNNMSFWLLPPSLMLLISSSVVENGTGTGWTVYPPLSSNIAHSGSSVDLSIFSLHLAGISSILGAINFISTIINMRLNNMSF
DQLPLFVWAVGITALLLLLSLPVLAGAITMLLTDRNLNTSFFDPAGGGDPILYQHL
>LEFID512-10|HM873277|MM06479|Coleophora_glaucicolella
TLYFIFGIWSGMVGTSLSLLIRAELGNPGSLIGDDQIYNTIVTAHAFIMIFFMVMPIMIGGFGNWLVPLMLGAPDMAFPRMNNMSFWLLPPSLMLLISSSVVENGTGTGWTVYPPLSSNIAHSGSSVDLSIFSLHLAGISSILGAINFISTIINMRLNNMSF
DQLPLFVWAVGITALLLLLSLPVLAGAITMLLTDRNLNTSFFDPAGGGDPILYQHL
>LEFIB978-10|HM871851|MM03171|Coleophora_glitzella
TLYFIFGIWAGMMGTSLSLLIRAELGTPGSLIGDDQIYNVIVTAHAFIMIFFMVMPIMIGGFGNWLVPLMLGAPDMAFPRMNNMSFWFLPPSLMLLTSSSIVENGTGTGWTVYPPLSSNIAHSGSSVDLSIFSLHLAGISSILGAINFITTIINMRLNNLSF
DQLPLFVWSVGITALLLLLSLPVLAGAITMLLTDRNLNTSFFDPAGGGDPILYQHL
>LEFIA812-10|HM386953|MM04166|Coleophora_glitzella
TLYFIFGIWAGMMGTSLSLLIRAELGTPGSLIGDDQIYNVIVTAHAFIMIFFMVMPIMIGGFGNWLVPLMLGAPDMAFPRMNNMSFWFLPPSLMLLTSSSIVENGTGTGWTVYPPLSSNIAHSGSSVDLSIFSLHLAGISSILGAINFITTIINMRLNNLSF
DQLPLFVWSVGITALLLLLSLPVLAGAITMLLTDRNLNTSFFDPAGGGDPILYQHL
>LEFIE005-10|HM873754|MM08056|Coleophora_glitzella
TLYFIFGIWAGMMGTSLSLLIRAELGTPGSLIGDDQIYNVIVTAHAFIMIFFMVMPIMIGGFGNWLVPLMLGAPDMAFPRMNNMSFWFLPPSLMLLTSSSIVENGTGTGWTVYPPLSSNIAHSGSSVDLSIFSLHLAGISSILGAINFITTIINMRLNNLSF
DQLPLFVWSVGITALLLLLSLPVLAGAITMLLTDRNLNTSFFDPAGGGDPILYQHL
>LEFID513-10|HM873278|MM06480|Coleophora_graminicolella
TLYFIFGIWAGMVGTSLSLLIRAELGNPGSLIGDDQIYNTIVTAHAFIMIFFMVMPIMIGGFGNWLVPLMLGAPDMAFPRMNNMSFWLLPPSLTLLISSSIVENGTGTGWTVYPPLSSNIAHGGSSVDLSIFSLHLAGISSILGAINFITTIINMRLNNMSFD
QLPLFVWAVGITALLLLLSLPVLAGAITMLLTDRNLNTSFFDPAGGGDPILYQHL
>LEFIG022-10|HM875702|MM13643|Coleophora_graminicolella
TLYFIFGIWAGMVGTSLSLLIRAELGNPGSLIGDDQIYNTIVTAHAFIMIFFMVMPIMIGGFGNWLVPLMLGAPDMAFPRMNNMSFWLLPPSLTLLISSSIVENGTGTGWTVYPPLSSNIAHGGSSVDLSIFSLHLAGISSILGAINFITTIINMRLNNMSFD
QLPLFVWAVGITALLLLLSLPVLAGAITMLLTDRNLNTSFFDPAGGGDPILYQHL
>LEFIG023-10|HM875703|MM13644|Coleophora_graminicolella
TLYFIFGIWAGMVGTSLSLLIRAELGNPGSLIGDDQIYNTIVTAHAFIMIFFMVMPIMIGGFGNWLVPLMLGAPDMAFPRMNNMSFWLLPPSLTLLISSSIVENGTGTGWTVYPPLSSNIAHGGSSVDLSIFSLHLAGISSILGAINFITTIINMRLNNMSFD
QLPLFVWAVGITALLLLLSLPVLAGAITMLLTDRNLNTSFFDPAGGGDPILYQHL
>LEFIG052-10|HM875731|MM13673|Coleophora_graminicolella
TLYFIFGIWAGMVGTSLSLLIRAELGNPGSLIGDDQIYNTIVTAHAFIMIFFMVMPIMIGGFGNWLVPLMLGAPDMAFPRMNNMSFWLLPPSLTLLISSSIVENGTGTGWTVYPPLSSNIAHGGSSVDLSIFSLHLAGISSILGAINFITTIINMRLNNMSFD
QLPLFVWAVGITALLLLLSLPVLAGAITMLLTDRNLNTSFFDPAGGGDPILYQHL
>LEFIL716-10|JF854700|MM16006|Coleophora_graminicolella
TLYFIFGIWAGMVGTSLSLLIRAELGNPGSLIGDDQIYNTIVTAHAFIMIFFMVMPIMIGGFGNWLVPLMLGAPDMAFPRMNNMSFWLLPPSLTLLISSSIVENGTGTGWTVYPPLSSNIAHGGSSVDLSIFSLHLAGISSILGAINFITTIINMRLNNMSFD
QLPLFVWAVGITALLLLLSLPVLAGAITMLLTDRNLNTSFFDPAGGGDPILYQHL
>LEFIC392-10|HM872235|MM03932|Coleophora_gryphipennella
TLYFIFGIWAGMVGTSLSLLIRAELGNPGSLIGDDQIYNTIVTAHAFIMIFFMVMPIMIGGFGNWLVPLMLGAPDMAFPRMNNMSFWLLPPSLTLLISSSIVENGTGTGWTVYPPLSSNIAHSGSSVDLSIFSLHLAGISSILGAINFITTIINMRLNNMFFD

QLPLFVWAVGITALLLLLSLPVLAGAITMLLTDRNLNTSFFDPAGGGDPILYQHL
>LEFIC393-10|HM872236|MM03933|Coleophora_hackmani
TLYFIFGIWAGMVGTSLSLLIRAELGNPGSLIGDDQIYNTIVTAHAFIMIFFMVMPIMIGGFGNWLVPLMLGAPDMAFPRMNNMSFWLLPPSLTLLISSSIVENGTGTGWTVYPPLSSNIAHGGSSVDLSIFSLHLAGISSILGAINFITTIINMRLNNMSFD
QLPLFVWAVGITALLLLLSLPVLAGAITMLLTDRNLNTSFFDPAGGGDPILYQHL
>LEFIL665-10|MM18963|Coleophora_hemerobiella
TLYFIFGIWAGMVGTSLSLLIRTELGNPGSLIGDDQIYNTIVTAHAFIMIFFMVMPIMIGGFGNWLVPLMLGAPDMAFPRMNNMSFWLLPPSLTLLISSSIVENGTGTGWTVYPPLSSNIAHSGSSVDLSIFSLHLAGISSILGAINFITTIINMRLNNMSFD
QLPLFVWAVGITALLLLLSLPVLAGAITMLLTDRNLNTSFFDPAGGGDPILYQHL
>LEFIG134-10|HM875814|MM13955|Coleophora_idaeella
TLYFIFGIWAGMMGTSLSLLIRAELGNPGSLIGDDQIYNVIVTAHAFIMIFFMVMPIMIGGFGNWLVPLMLGAPDMAFPRMNNMSFWFLPPSLMLLISSSIVENGTGTGWTVYPPLSSNIAHSGSSVDLSIFSLHLAGISSILGAINFITTIINMRLNNLSF
DQLPLFVWSVGITALLLLLSLPVLAGAITMLLTDRNLNTSFFDPAGGGDPILYQHL
>LEFIG135-10|HM875815|MM13956|Coleophora_idaeella
TLYFIFGIWAGMMGTSLSLLIRAELGNPGSLIGDDQIYNVIVTAHAFIMIFFMVMPIMIGGFGNWLVPLMLGAPDMAFPRMNNMSFWFLPPSLMLLISSSIVENGTGTGWTVYPPLSSNIAHSGSSVDLSIFSLHLAGISSILGAINFITTIINMRLNNLSF
DQLPLFVWSVGITALLLLLSLPVLAGAITMLLTDRNLNTSFFDPAGGGDPILYQHL
>LEFIK591-10|JF854173|MM18166|Coleophora_idaeella
TLYFIFGIWAGMMGTSLSLLIRAELGNPGSLIGDDQIYNVIVTAHAFIMIFFMVMPIMIGGFGNWLVPLMLGAPDMAFPRMNNMSFWFLPPSLMILISSSIVENGTGTGWTVYPPLSSNIAHSGSSVDLSIFSLHLAGISSILGAINFITTIINMRLNNLSFD
QLPLFVWSVGITALLLLLSLPVLAGAITMLLTDRNLNTSFFDPAGGGDPILYQHL
>LEFIG723-10|HM876379|MM15587|Coleophora_inulae
TLYFIFGTWAGMVGTSLSLLIRAELGNPSSLIGDDQIYNTIITAHAFIMIFFMVMPIMIGGFGNWLVPLMLGAPDMAFPRMNNMSFWLLPPSLTLLISSSIVENGTGTGWTVYPPLSSNITHSGSSVDLSIFSLHLAGISSILGAINFITTIINMRLNNMSFD
QLPLFVWAVGITALLLLLSLPVLAGAITMLLTDRNLNTSFFDPAGGGDPILYQHL
>LEFIJ2233-14|MM23413|Coleophora_jaernaensis
TLYFIFGIWAGMMGTSLSLLIRAELGNPGSLIGDDQIYNTIVTAHAFIMIFFMVMPIMIGGFGNWLVPLMLGAPDMAFPRMNNMSFWLLPPSLTLLIFSSIVENGTGTGWTVYPPLSSNIAHSGSSVDLSIFSLHLAGISSILGAINFITTIINMRLNNMSF
DQLPLFVWAVGITALLLLLSLPVLAGAITMLLTDRNLNTSFFDPAGGGDPILYQHL
>LEFIE349-10|HM874073|MM08785|Coleophora_juncicolella
TLYFIFGIWAGMMGMSLSLLIRAELGSPGSLIKNDQIYNVIVTAHAFIMIFFMVMPIMIGGFGNWLVPLMLGSPDMAFPRMNNMSFWFLPPSLMLLISSSIVENGTGTGWTVYPPLSSNIAHSGSSVDLSIFSLHLAGISSILGAINFITTIINMRLNNLSF
DQLPLFVWSVGITALLLLLSLPVLAGAITMLLTDRNLNTSFFDPAGGGDPILYQHL
>LEFIL669-10|KM573538|MM18967|Coleophora_kuehnella
TLYFIFGIWASMVGTSLSLLIRAELGNPGSLIGDDQIYNTIVTAHAFIMIFFMVMPIMIGGFGNWLVPLMLGAPDMAFPRMNNMSFWLLPPSLTLLISSSIVETGTGTGWTVYPPLSSNIAHSGSSVDLSIFSLHLAGISSILGAINFITTIINMRLNNLPFDQ
LPLFIWAVGITAILLLLSLPVLAGAITMLLTDRNLNTSFFDPAGGGDPILYQHL
>LEFIG715-10|HM876371|MM15579|Coleophora_laricella
TLYFIFGIWAGMMGTSLSLLIRAELGNPGSLIGDDQIYNVIVTAHAFIMIFFMVMPIMIGGFGNWLVPLMLGAPDMAFPRMNNMSFWFLPPSLMILISSSIVENGTGTGWTVYPPLSSNIAHSGSSVDLSIFSLHLAGISSILGAINFITTIINMRLNNLSFD
QLPLFVWSVSITALLLLLSLPVLAGAITMLLTDRNLNTSFFDPAGGGDPILYQHL
>LEFID320-10|HM873117|MM06208|Coleophora_ledi
TLYFIFGIWAGMMGTSLSLLIRAELGNPGSLIGDDQIYNVIVTAHAFIMIFFMVMPIMIGGFGNWLVPLMLGAPDMAFPRMNNMSFWFLPPSLMILISSSVVENGTGTGWTVYPPLSSNIAHSGSSVDLSIFSLHLAGISSILGAINFITTIINMRLNNLSF
DQLPLFVWSVGITALLLLLSLPVLAGAITMLLTDRNLNTSFFDPAGGGDPILYQHL
>LEFIJ259-10|JF853500|MM14873|Coleophora_ledi
TLYFIFGIWAGMMGTSLSLLIRAELGNPGSLIGDDQIYNVIVTAHAFIMIFFMVMPIMIGGFGNWLVPLMLGAPDMAFPRMNNMSFWFLPPSLMILISSSVVENGTGTGWTVYPPLSSNIAHSGSSVDLSIFSLHLAGISSILGAINFITTIINMRLNNLSF
DQLPLFVWSVGITALLLLLSLPVLAGAITMLLTDRNLNTSFFDPAGGGDPILYQHL
>LEFIJ260-10|JF853501|MM14874|Coleophora_ledi
TLYFIFGIWAGMMGTSLSLLIRAELGNPGSLIGDDQIYNVIVTAHAFIMIFFMVMPIMIGGFGNWLVPLMLGAPDMAFPRMNNMSFWFLPPSLMILISSSVVENGTGTGWTVYPPLSSNIAHSGSSVDLSIFSLHLAGISSILGAINFITTIINMRLNNLLF
DQLPLFVWSVGITALLLLLSLPVLAGAITMLLTDRNLNTSFFDPAGGGDPILYQHL
>LEFIJ299-10|JF853536|MM15186|Coleophora_ledi
TLYFIFGIWAGMMGTSLSLLIRAELGNPGSLIGNDQIYNVIVTAHAFIMIFFMVMPIMIGGFGNWLVPLMLGAPDMAFPRMNNMSFWFLPPSLMILISSSIVENGTGTGWTVYPPLSSNIAHSGSSVDLSIFSLHLAGISSILGAINFITTIINMRLNNLSFD
QLPLFVWSVGITALLLLLSLPVLAGAITMLLTDRNLNTSFFDPAGGGDPILYQHL
>LEFIK585-10|MM18160|Coleophora_limosipennella
TLYFIFGIWAGMVGTSLSLMIRTELGNPGSLIGDDQIYNTIVTAHAFIMIFFMVMPIMIGGFGNWLVPLMLGAPDMAFPRMNNMSFWLLPPSLTLLISSSIVENGTGTGWTIYPPLSSNIAHSGSSVDLSIFSLHLAGISSILGAINFITTIINMRLNNMLFD
QLSLFIWAVGITAILLLLSLPVLAGSITMLLTDRNLNTSFFDPAGGGDPILYQHL
>LEFIE467-10|HM874191|MM09239|Coleophora_lithargyrinella

TLYFIFGIWAGMVGTSLSLLIRSELGNPGSLIGDDQIYNTIVTAHAFIMIFFMVMPIMIGGFGNWLVPLMLGAPDMAFPRMNNMSFWLLPPSLTLLISSSAVENGTGTGWTVYPPLSSNIAHSGSSVDLSIFSLHLAGISSILGAINFITTIINMRLNNMFFD
QLPLFVWSVGITALLLLLSLPVLAGAITMLLTDRNLNTSFFDPAGGGDPILYQHL
>LEFIF002-10|HM874716|MM10440|Coleophora_lixella
TLYFIFGVWAGMVGTSLSLLIRAELGNPGSLIGDDQIYNTIVTAHAFIMIFFMVMPIMIGGFGNWLVPLMLGAPDMAFPRMNNMSFWLLPPSLTLLISSSIVENGTGTGWTVYPPLSSNIAHSGGSVDLSIFSLHLAGISSILGAINFITTIINMRLNNLMF
DQLPLFVWAVGITALLLLLSLPVLAGAITMLLTDRNLNTSFFDPAGGGDPILYQHL
>LEFIF003-10|HM874717|MM10441|Coleophora_lixella
TLYFIFGIWAGMMGTSLSLLIRAELGNPGSLIGDDQIYNTIITAHAFIMIFFMVMPIMIGGFGNWLVPLMLGAPDMAFPRMNNMSFWLLPPSLTLLISSSIVENGTGTGWTVYPPLSSNIAHSGGSVDLSIFSLHLAGISSILGAINFITTIINMRLNSLMFD
QLPLFVWAVGITALLLLLSLPVLAGAITMLLTDRNLNTSFFDPAGGGDPILYQHL
>LEFIK597-10|JF854179|MM18172|Coleophora_lixella
TLYFIFGVWAGMVGTSLSLLIRAELGNPGSLIGDDQIYNTIVTAHAFIMIFFMVMPIMIGGFGNWLVPLMLGAPDMAFPRMNNMSFWLLPPSLTLLISSSIVENGTGTGWTVYPPLSSNIAHSGGSVDLSIFSLHLAGISSILGAINFITTIINMRLNNLMF
DQLPLFVWAVGITALLLLLSLPVLAGAITMLLTDRNLNTSFFDPAGGGDPILYQHL
>LEFIA055-10|HM396403|MM00961|Coleophora_lusciniaepennella
TLYFIFGIWAGMMGTSLSLLIRAELGNPGSLIGDDQIYNVIVTAHAFIMIFFMVMPIMIGGFGNWLVPIMLGAPDMAFPRMNNMSFWFLPPSLMLLISSSIVENGTGTGWTVYPPLSSNIAHSGSSVDLSIFSLHLAGISSILGAINFITTIINMRLSNLSFD
QLPLFVWSVGITALLLLLSLPVLAGAITMLLTDRNLNTSFFDPAGGGDPILYQHL
>LEFID059-10|HM872873|MM05659|Coleophora_lutipennella
TLYFIFGIWAGMVGTSLSLLIRAELGNPGSLIGDDQIYNTIVTAHAFIMIFFMVMPIMIGGFGNWLVPLMLGAPDMAFPRMNNMSFWLLPPSLTLLISSSIVENGTGTGWTVYPPLSSNIAHSGSSVDLSIFSLHLAGISSILGAINFITTIINMRLNNLSFD
QLSLFVWAVGITALLLLLSLPVLAGAITMLLTDRNLNTSFFDPAGGGDPILYQHL
>LEFIB717-10|HM871595|MM02532|Coleophora_mayrella
TLYFIFGIWAGMVGTSLSLLIRAELGNPGSLIGDDQIYNTIVTAHAFIMIFFMVMPIMIGGFGNWLVPLMLGAPDMAFPRMNNMSFWLLPPSLTLLISSSIVENGTGTGWTVYPPLSSNIAHSGSSVDLSIFSLHLAGISSILGAINFITTIINMRLNNMSFD
QLPLFVWAVGITALLLLLSLPVLAGAITMLLTDRNLNTSFFDPAGGGDPILYQHL
>LEFIG484-10|HM876160|MM14543|Coleophora_millefolii
TLYFIFGIWAGMVGTSLSLLIRAELGNPGSLLGDDQIYNTIVTAHAFIMIFFMVMPIMIGGFGNWLVPLMLGAPDMAFPRMNNMSFWLLPPSLTLLISSSIVENGTGTGWTVYPPLSSNIAHGGSSVDLSIFSLHLAGISSILGAINFITTIINMRLNNMSF
DQLPLFVWSVGITALLLLLSLPVLAGAITMLLTDRNLNTSFFDPAGGGDPILYQHL
>LEFIB679-10|HM871557|MM02475|Coleophora_milvipennis
TLYFIFGIWAGMVGTSLSLLIRTELGNPGSLIGDDQIYNTIVTAHAFIMIFFMVMPIMIGGFGNWLVPLMLGAPDMAFPRMNNMSFWLLPPSLTLLISSSIVENGTGTGWTVYPPLSSNIAHSGSSVDLSIFSLHLAGISSILGAINFITTIINMRLNNMLFD
QLSLFIWAVGITAILLLLSLPVLAGAITMLLTDRNLNTSFFDPAGGGDPILYQHL
>LEFID508-10|HM873273|MM06475|Coleophora_milvipennis
TLYFIFGIWAGMVGTSLSLLIRTELGNPGSLIGDDQIYNTIVTAHAFIMIFFMVMPIMIGGFGNWLVPLMLGAPDMAFPRMNNMSFWLLPPSLTLLISSSIVENGTGTGWTVYPPLSSNIAHSGSSVDLSIFSLHLAGISSILGAINFITTIINMRLNNMLFD
QLSLFIWAVGITAILLLLSLPVLAGAITMLLTDRNLNTSFFDPAGGGDPILYQHL
>LEFID610-10|HM873375|MM06624|Coleophora_milvipennis
TLYFIFGIWAGMVGTSLSLLIRTELGNPGSLIGDDQIYNTIVTAHAFIMIFFMVMPIMIGGFGNWLVPLMLGAPDMAFPRMNNMSFWLLPPSLTLLISSSIVENGTGTGWTVYPPLSSNIAHSGSSVDLSIFSLHLAGISSILGAINFITTIINMRLNNMLFD
QLSLFVWAVGITAILLLLSLPVLAGAITMLLTDRNLNTSFFDPAGGGDPILYQHL
>LEFIB690-10|HM871568|MM02490|Coleophora_murinella
TLYFIFGIWAGMMGTSLSLLIRAELGTPGSLIGDDQIYNVIVTAHAFIMIFFMVMPIMIGGFGNWLVPLMLGAPDMAFPRMNNMSFWFLPPSLMLLTSSSIVENGTGTGWTVYPPLSSNIAHSGSSVDLSIFSLHLAGISSILGAINFITTIINMRLNNLSF
DQLPLFVWSVGITALLLLLSLPVLAGAITMLLTDRNLNTSFFDPAGGGDPILYQHL
>LEFIB691-10|HM871569|MM02491|Coleophora_murinella
TLYFIFGIWAGMMGTSLSLLIRAELGTPGSLIGDDQIYNVIVTAHAFIMIFFMVMPIMIGGFGNWLVPLMLGAPDMAFPRMNNMSFWFLPPSLMLLTSSSIVENGTGTGWTVYPPLSSNIAHSGSSVDLSIFSLHLAGISSILGAINFITTIINMRLNNLSF
DQLPLFVWSVGITALLLLLSLPVLAGAITMLLTDRNLNTSFFDPAGGGDPILYQHL
>LEFIL726-10|JF854702|MM16015|Coleophora_nr._virgaureae
TLYFIFGIWAGMVGTSLSLLIRAELGNPGSLIGDDQIYNTIVTAHAFIMIFFMVMPIMIGGFGNWLVPLMLGAPDMAFPRMNNMSFWLLPPSLTLLISSSIVENGTGTGWTVYPPLSSNIAHSGASVDLSIFSLHLAGISSILGAINFITTIINMRLNSMLFD
QLPLFVWAVGITALLLLLSLPVLAGAITMLLTDRNLNTSFFDPAGGGDPILYQHL
>LEFIK620-10|JF854195|MM18195|Coleophora_nr._virgaureae
TLYFIFGIWAGMVGTSLSLLIRAELGNPGSLIGDDQIYNTIVTAHAFIMIFFMVMPIMIGGFGNWLVPLMLGAPDMAFPRMNNMSFWLLPPSLTLLISSSIVENGTGTGWTVYPPLSSNIAHSGASVDLSIFSLHLAGISSILGAINFITTIINMRLNSMLFD
QLPLFVWAVGITALLLLLSLPVLAGAITMLLTDRNLNTSFFDPAGGGDPILYQHL
>LEFIC396-10|HM872239|MM03937|Coleophora_nutantella
TLYFIFGIWAGMVGTSLSLLIRAELGNPGSLIGDDQIYNTIVTAHAFIMIFFMVMPIMIGGFGNWLVPLMLGAPDMAFPRMNNMSFWLLPPSLTLLISSSIVENGTGTGWTVYPPLSSNIAHGGSSVDLSIFSLHLAGISSILGAINFITTIINMRLNNMSFD
QLPLFVWAVGITALLLLLSLPVLAGAITMLLTDRNLNTSFFDPAGGGDPILYQHL

>LEFID138-10|HM872948|MM05898|Coleophora_obscuripalpella
TLYFIFGIWAGMMGTSLSLLIRAELGNPGSLIGDDQIYNVIVTAHAFIMIFFMVMPIMIGGFGNWLVPLMLGAPDMAFPRMNNMSFWFLPPSLMLLISSSIVENGTGTGWTVYPPLSSNIAHSGSSVDLSIFSLHLAGISSILGAINFITTIINMRLNNLSF
DQLPLFVWSVGITALLLLLSLPVLAGAITMLLTDRNLNTSFFDPAGGGDPILYQHL
>LEFIE264-10|HM873997|MM08601|Coleophora_obscuripalpella
TLYFIFGIWAGMMGTSLSLLIRAELGNPGSLIGDDQIYNVIVTAHAFIMIFFMVMPIMIGGFGNWLVPLMLGAPDMAFPRMNNMSFWFLPPSLMLLISSSIVENGTGTGWTVYPPLSSNIAHSGSSVDLSIFSLHLAGISSILGAINFITTIINMRLNNLSF
DQLPLFVWSVGITALLLLLSLPVLAGAITMLLTDRNLNTSFFDPAGGGDPILYQHL
>COEUA050-11|MM20220|Coleophora_onobrychiella
TLYFIFGIWAGMVGTSLSLLIRAELGNPGSFIGDDQIYNTIVTAHAFIMIFFMVMPIMIGGFGNWLVPLMLGAPDMAFPRMNNMSFWLLPPSLTLLISSSIVENGTGTGWTVYPPLSSNIAHGGSSVDLSIFSLHLAGISSILGAINFITTIINMRLNNLSFD
QLPLFVWSVGITALLLLLSLPVLAGAITMLLTDRNLNTSFFDPAGGGDPILYQHL
>LEFIE500-10|HM874224|MM09315|Coleophora_orbitella
TLYFIFGIWAGMMGTSLSLLIRAELGNPGSLIGDDQIYNVIVTAHAFIMIFFMVMPIMIGGFGNWLVPLMLGAPDMAFPRMNNMSFWFLPPSLMILISSSIVENGTGTGWTVYPPLSSNIAHSGSSVDLSIFSLHLAGISSILGAINFITTIINMRLNNLSFD
QLPLFVWSVGITALLLLLSLPVLAGAITMLLTDRNLNTSFFDPAGGGDPILYQHL
>LEFIE548-10|HM874271|MM09433|Coleophora_orbitella
TLYFIFGIWAGMMGTSLSLLIRAELGNPGSLIGDDQIYNVIVTAHAFIMIFFMVMPIMIGGFGNWLVPLMLGAPDMAFPRMNNMSFWFLPPSLMLLISSSIVENGTGTGWTVYPPLSSNIAHSGSSVDLSIFSLHLAGISSILGAINFITTIINMRLNNLSF
DQLPLFVWSVGITALLLLLSLPVLAGAITMLLTDRNLNTSFFDPAGGGDPILYQHL
>LEFIB681-10|HM871559|MM02478|Coleophora_otidipennella
TLYFIFGIWAGMVGTSLSLLIRAELGNPGSLIGDDQIYNTIVTAHAFIMIFFMVMPIMIGGFGNWLVPLMLGAPDMAFPRMNNMSFWLLPPSLMLLISSSIVENGTGTGWTVYPPLSSNIAHSGSSVDLSIFSLHLAGISSILGAINFITTIINMRLNNMLF
DQLPLFVWAVGITALLLLLSLPVLAGAITMLLTDRNLNTSFFDPAGGGDPILYQHL
>LEFIB682-10|HM871560|MM02479|Coleophora_otidipennella
TLYFIFGIWAGMVGTSLSLLIRAELGNPGSLIGDDQIYNTIVTAHAFIMIFFMVMPIMIGGFGNWLVPLMLGAPDMAFPRMNNMSFWLLPPSLMLLISSSIVENGTGTGWTVYPPLSSNIAHSGSSVDLSIFSLHLAGISSILGAINFITTIINMRLNNMLF
DQLPLFVWAVGITALLLLLSLPVLAGAITMLLTDRNLNTSFFDPAGGGDPILYQHL
>LEFIC389-10|HM872232|MM03927|Coleophora_pappiferella
TLYFIFGIWASMVGTSLSLLIRAELGNPGSLIGDDQIYNTIVTAHAFIMIFFMVMPIMIGGFGNWLVPLMLGAPDMAFPRMNNMSFWLLPPSLTLLISSSIVENGTGTGWTVYPPLSSNIAHSGSSVDLSIFSLHLAGISSILGAINFITTIINMRLNNMSFD
QLPLFVWAVGITALLLLLSLPVLAGAITMLLTDRNLNTSFFDPAGGGDPILYQHL
>LEFID449-10|HQ570345|MM06384|Coleophora_pappiferella
TLYFIFGIWASMVGTSLSLLIRAELGNPGSLIGDDQIYNTIVTAHAFIMIFFMVMPIMIGGFGNWLVPLMLGAPDMAFPRMNNMSFWLLPPSLTLLISSSIVENGTGTGWTVYPPLSSNIAHSGSSVDLSIFSLHLAGISSILGAINFITTIINMRLNNMSFD
QLPLFVWAVGITALLLLLSLPVLAGAITMLLTDRNLNTSFFDPAGGGDPILYQHL
>LEFIB366-10|HM871265|MM00929|Coleophora_paripennella
TLYFIFGIWASMVGTSLSLLIRAELGNPGSLIGDDQIYNTIVTAHAFIMIFFMVMPIMIGGFGNWLVPLMLGAPDMAFPRMNNMSFWLLPPSLTLLISSSIVENGTGTGWTVYPPLSSNIAHSGSSVDLSIFSLHLAGISSILGAINFITTIINMRLNNMSFD
QLPLFVWAVGITALLLLLSLPVLAGAITMLLTDRNLNTSFFDPAGGGDPILYQHL
>LEFID356-10|HM873153|MM06259|Coleophora_paripennella
TLYFIFGIWASMVGTSLSLLIRAELGNPGSLIGDDQIYNTIVTAHAFIMIFFMVMPIMIGGFGNWLVPLMLGAPDMAFPRMNNMSFWLLPPSLTLLISSSIVENGTGTGWTVYPPLSSNIAHGGSSVDLSIFSLHLAGISSILGAINFITTIINMRLNNMSFD
QLPLFVWAVGITALLLLLSLPVLAGAITMLLTDRNLNTSFFDPAGGGDPILYQHL
>COEUA055-11|MM20225|Coleophora_parthenogenella
TLYFIFGIWAGMVGTSLSLLIRAELGMPGSLIGDDQIYNVIVTAHAFIMIFFMVMPIMIGGFGNWLVPLMLGAPDMAFPRMNNMSFWLLPPSLTLLISSSIVENGTGTGWTVYPPLSSNIAHGGSSVDLSIFSLHLAGISSILGAINFITTIINMRLNNLSFD
QLPLFVWSVGITALLLLLSLPVLAGAITMLLTDRNLNTSFFDPAGGGDPILYQHL
>LEFID486-10|HM873252|MM06436|Coleophora_partitella
TLYFIFGIWAGMIGTSLSLMIRTELGIPGSFIGDDQIYNTIVTAHAFIMIFFMVMPIMIGGFGNWLVPLMLGAPDMAFPRMNNMSFWLLPPSLILLISSSIVETGTGTGWTVYPPLSSNIAHSGSSVDLSIFSLHLAGISSILGAINFITTIINMRLNNMSFDQ
LPLFVWAVGITALLLLLSLPVLAGAITMLLTDRNLNTSFFDPAGGGDPILYQHL
>LEEUA149-11|MM19557|Coleophora_pennella
TLYFIFGIWAGMVGTSLSLLIRAELGNPGSLIGDDQIYNTIVTAHAFIMIFFMVMPIMIGGFGNWLVPLMLGAPDMAFPRMNNMSFWLLPPSLTLLISSSIVENGTGTGWTVYPPLSSNIAHSGSSVDLSIFSLHLAGISSILGAINFITTIINMRLNNLSFD
QLPLFVWAVGITALLLLLSLPVLAGAITMLLTDRNLNTSFFDPAGGGDPILYQHL
>ELACA937-11|MM20175|Coleophora_peri
TLYFIFGIWAGMMGTSLSLLIRAELGNPGSLIGDDQIYNVIVTAHAFIMIFFMVMPIMIGGFGNWLVPLMLGAPDMAFPRMNNMSFWFLPPSLMLLISSSIVENGTGTGWTVYPPLSSNIAHSGSSVDLSIFSLHLAGISSILGAINFITTIINMRLNNLSF
DQLPLFVWSVGITALLLLLSLPVLAGAITMLLTDRNLNTSFFDPAGGGDPILYQHL
>LEFIB346-10|HM871246|MM00894|Coleophora_peribenanderi
TLYFIFGIWAGMVGTSLSLLIRAELGNPGSLIGDDQIYNTIVTAHAFIMIFFMVMPIMIGGFGNWLVPLMLGTPDMAFPRMNNMSFWLLPPSLTLLISSSIVENGTGTGWTVYPPLSSNIAHSGSSVDLSIFSLHLAGISSILGAINFITTIINMRLNNMLFD

QLPLFVWAVGITALLLLLSLPVLAGAITMLLTDRNLNTSFFDPAGGGDPILYQHL
>ELACA858-11|MM20096|Coleophora_pilosae
TLYFIFGIWAGMVGTSLSLLIRAELGTPGSLIGDDQIYNTIVTAHAFIMIFFMVMPIMIGGFGNWLVPLMLGAPDMAFPRMNNMSFWLLPPSLTLLISGSIVENGTGTGWTVYPPLSSNIAHSGSSVDLSIFSLHLAGISSILGAINFITTIINMRLNNMSFD
QLPLFVWSVGITALLLLLSLPVLAGAITMLLTDRNLNTSFFDPAGGGDPILYQHL
>LEFIE263-10|HM873996|MM08599|Coleophora_plumbella
TLYFIFGIWAGMMGTSLSLLIRAELGNPGSLIGDDQIYNVIVTAHAFIMIFFMVMPIMIGGFGNWLVPLMLGAPDMAFPRMNNMSFWFLPPSLMILISSSIVENGTGTGWTVYPPLSSNIAHSGSSVDLSIFSLHLAGISSILGAINFITTIINMRLNNLSFD
QLPLFVWSVGITALLLLLSLPVLAGAITMLLTDRNLNTSFFDPAGGGDPILYQHL
>LEFIG558-10|HM876230|MM14704|Coleophora_plumbella
TLYFIFGIWAGMMGTSLSLLIRAELGNPGSLIGDDQIYNVIVTAHAFIMIFFMVMPIMIGGFGNWLVPLMLGAPDMAFPRMNNMSFWFLPPSLMILISSSIVENGTGTGWTVYPPLSSNIAHSGSSVDLSIFSLHLAGISSILGAINFITTIINMRLNNLSFD
QLPLFVWSVGITALLLLLSLPVLAGAITMLLTDRNLNTSFFDPAGGGDPILYQHL
>ELACA1482-12|MM20321|Coleophora_polonicella
TLYFIFGIWSGMVGTSLSLLIRAELGNPGSLIGDDQIYNTIVTAHAFIM?FFMVMPIMIGGFGNWLVPLMLGAPDMAFPRMNNMSFWLLPPSLMLLISSSIVETGTGTGWTVYPPLSSNIAHSGSSVDLSIFSLHLAGISSILGAINFITTIINMRLNNLSFD
QLPLFVWAVGITALLLLLSLPVLAGAITMLLTDRNLNTSFFDPAGGGDPILYQHL
>ELACA1483-12|MM20322|Coleophora_polonicella
TLYFIFGIWSGMVGTSLSLLIRAELGNPGSLIGDDQIYNTIVTAHAFIMIFFMVMPIMIGGFGNWLVPLMLGAPDMAFPRMNNMSFWLLPPSLMLLISSSIVETGTGTGWTVYPPLSSNIAHSGSSVDLSIFSLHLAGISSILGAINFITTIINMRLNNLSFD
QLPLFVWAVGITALLLLLSLPVLAGAITMLLTDRNLNTSFFDPAGGGDPILYQHL
>LEFIC414-10|HM872257|MM03960|Coleophora_potentillae
TLYFIFGIWAGMMGTSLSLLIRAELGNPGSLIGDDQIYNVIVTAHAFIMIFFMVMPIMIGGFGNWLVPLMLGAPDMAFPRMNNMSFWFLPPSLMLLISSSIVENGTGTGWTVYPPLSSNIAHGGSSVDLSIFSLHLAGISSILGAINFITTIINMRLNNLSF
DQLSLFIWSVGITALLLLLSLPVLAGAITMLLTDRNLNTSFFDPAGGGDPILYQHL
>LEFIG152-10|HM875832|MM13999|Coleophora_pyrrhulipennella
TLYFIFGIWASMVGTSLSLLIRAELGNPGSLIGDDQIYNTIVTAHAFIMIFFMVMPIMIGGFGNWLVPLMLGAPDMAFPRMNNMSFWFLPPSLTLLISSSIVETGTGTGWTVYPPLSSNIAHSGSSVDLSIFSLHLAGISSILGAINFITTIINMRLNNLSFDQ
LPLFVWAVGITAILLLLSLPVLAGAITMLLTDRNLNTSFFDPAGGGDPILYQHL
>LEFIG213-10|HM875893|MM14103|Coleophora_pyrrhulipennella
TLYFIFGIWASMVGTSLSLLIRAELGNPGSLIGDDQIYNTIVTAHAFIMIFFMVMPIMIGGFGNWLVPLMLGAPDMAFPRMNNMSFWFLPPSLTLLISSSIVETGTGTGWTVYPPLSSNIAHSGSSVDLSIFSLHLAGISSILGAINFITTIINMRLNNLSFDQ
LPLFVWAVGITAILLLLSLPVLAGAITMLLTDRNLNTSFFDPAGGGDPILYQHL
>LEFIF005-10|HM874719|MM10443|Coleophora_ramosella
TLYFIFGIWAGMVGTSLSLLIRAELGNPGSLIGDDQIYNTIVTAHAFIMIFFMVMPIMIGGFGNWLVPLMLGAPDMAFPRMNNMSFWLLPPSLTLLISSSIVENGTGTGWTVYPPLSSNIAHGGSSVDLSIFSLHLAGISSILGAINFISTIINMRLNNMLFD
QLPLFVWAVGITALLLLLSLPVLAGAITMLLTDRNLNTSFFDPAGGGDPILYQHL
>LEFIG722-10|HM876378|MM15586|Coleophora_ramosella
TLYFIFGIWAGMVGTSLSLLIRAELGNPGSLIGDDQIYNTIVTAHAFIMIFFMVMPIMIGGFGNWLVPLMLGAPDMAFPRMNNMSFWLLPPSLTLLISSSIVENGTGTGWTVYPPLSSNIAHGGSSVDLSIFSLHLAGISSILGAINFISTIINMRLNNMLFD
QLPLFVWAVGITALLLLLSLPVLAGAITMLLTDRNLNTSFFDPAGGGDPILYQHL
>LEFIE295-10|HQ570370|MM08647|Coleophora_salicorniae
TLYFIFGIWAGMVGTSLSLLIRAELGNPGSLIGDDQIYNTIVTAHAFIMIFFMVMPIMIGGFGNWLVPLMLGAPDMAFPRMNNMSFWLLPPSLTLLISSSIVENGTGTGWTVYPPLSSNIAHGGSSVDLSIFSLHLAGISSILGAINFITTIINMRLNNMSFD
QLPLFVWAVGITALLLLLSLPVLAGAITMLLTDRNLNTSFFDPAGGGDPILYQHL
>LEFIB702-10|HM871580|MM02505|Coleophora_saxicolella
TLYFIFGIWAGMVGTSLSLLIRAELGNPGSLIGDDQIYNTIVTAHAFIMIFFMVMPIMIGGFGNWLVPLMLGAPDMAFPRMNNMSFWLLPPSLTLLISSSIVENGTGTGWTVYPPLSSNIAHGGSSVDLSIFSLHLAGISSILGAINFITTIINMRLNNMSFD
QLPLFVWSVGITALLLLLSLPVLAGAITMLLTDRNLNTSFFDPAGGGDPILYQHL
>LEFIG043-10|HM875722|MM13664|Coleophora_saxicolella
TLYFIFGIWAGMVGTSLSLLIRAELGNPGSLIGDDQIYNTIVTAHAFIMIFFMVMPIMIGGFGNWLVPLMLGAPDMAFPRMNNMSFWLLPPSLTLLISSSIVENGTGTGWTVYPPLSSNIAHGGSSVDLSIFSLHLAGISSILGAINFITTIINMRLNNMSFD
QLPLFVWSVGITALLLLLSLPVLAGAITMLLTDRNLNTSFFDPAGGGDPILYQHL
>ELACA493-10|KF808863|MM16719|Coleophora_saxicolella
TLYFIFGIWAGMVGTSLSLLIRAELGNPGSLIGDDQIYNTIVTAHAFIMIFFMVMPIMIGGFGNWLVPLMLGAPDMAFPRMNNMSFWLLPPSLTLLISSSIVENGTGTGWTVYPPLSSNIAHGGSSVDLSIFSLHLAGISSILGAINFITTIINMRLNNMSFD
QLPLFVWSVGITALLLLLSLPVLAGAITMLLTERNLNTSFFDPAGGGDPILYQHL
>LEFIB238-10|HM871141|MM00700|Coleophora_serratella
TLYFIFGIWAGMVGTSLSLLIRAELGNPGSLIGDDQIYNTIVTAHAFIMIFFMVMPIMIGGFGNWLVPLMLGAPDMAFPRMNNMSFWLLPPSLTLLISSSIVENGTGTGWTVYPPLSSNIAHSGSSVDLSIFSLHLAGISSILGAINFITTIINMRLNNMSFD
QLPLFVWAVGITALLLLLSLPVLAGAITMLLTDRNLNTSFFDPAGGGDPILYQHL
>LEFIB699-10|HM871577|MM02502|Coleophora_serratella

TLYFIFGIWAGMVGTSLSLLIRAELGNPGSLIGDDQIYNTIVTAHAFIMIFFMVMPIMIGGFGNWLVPLMLGAPDMAFPRMNNMSFWLLPPSLTLLISSSIVENGTGTGWTVYPPLSSNIAHSGSSVDLSIFSLHLAGISSILGAINFITTIINMRLNNMSFD
QLPLFVWAVGITALLLLLSLPVLAGAITMLLTDRNLNTSFFDPAGGGDPILYQHL
>LEFIE632-10|HM874355|MM09574|Coleophora_serratella
TLYFIFGIWAGMVGTSLSLLIRAELGNPGSLIGDDQIYNTIVTAHAFIMIFFMVMPIMIGGFGNWLVPLMLGAPDMAFPRMNNMSFWLLPPSLTLLISSSIVENGTGTGWTVYPPLSSNIAHSGSSVDLSIFSLHLAGISSILGAINFITTIINMRLNNMSFD
QLPLFVWAVGITALLLLLSLPVLAGAITMLLTDRNLNTSFFDPAGGGDPILYQHL
>LEFIE877-10|HM874595|MM10149|Coleophora_sibiricella
TLYFIFGIWAGMMGTSLSLLIRAELGNPGSLIGDDQIYNVIVTAHAFIMIFFMVMPIMIGGFGNWLVPLMLGAPDMAFPRMNNMSFWFLPPSLMILISSSIVENGTGTGWTVYPPLSSNIAHSGSSVDLSIFSLHLAGISSILGAINFITTIINMRLNNLSFD
QLPLFVWSVSITALLLLLSLPVLAGAITMLLTDRNLNTSFFDPAGGGDPILYQHL
>LEFIL737-10|MM16026|Coleophora_sibiricella
TLYFIFGIWAGMMGTSLSLLIRAELGNPGSLIGDDQIYNVIVTAHAFIMIFFMVMPIMIGGFGNWLVPLMLGAPDMAFPRMNNMSFWFLPPSLMILISSSIVENGTGTGWTVYPPLSSNIAHSGSSVDLSIFSLHLAGISSILGAINFITTIINMRLNNLSFD
QLPLFVWSVSITALLLLLSLPVLAGAITMLLTDRNLNTSFFDPAGGGDPILYQHL
>LEFIL739-10|MM16028|Coleophora_sibiricella
TLYFIFGIWAGMMGTSLSLLIRAELGNPGSLIGDDQIYNVIVTAHAFIMIFFMVMPIMIGGFGNWLVPLMLGAPDMAFPRMNNMSFWFLPPSLMILISSSIVENGTGTGWTVYPPLSSNIAHSGSSVDLSIFSLHLAGISSILGAINFITTIINMRLNNLSFD
QLPLFVWSVSITALLLLLSLPVLAGAITMLLTDRNLNTSFFDPAGGGDPILY?HL
>LEFIB707-10|HM871585|MM02511|Coleophora_siccifolia
TLYFIFGIWSSMVGTSLSLLIRAELGNPGFLIGNDQIYNTIVTAHAFIMIFFMVMPIMIGGFGNWLLPLMLGAPDMAFPRMNNMSFWLLPPSLTLLISSSMVENGTGTGWTVYPPLSSNIAHSGSSVDLSIFSLHLAGISSILGAINFITTIINMRLNNMSFD
QLSLFIWSVGITALLLLLSLPVLAGAITMLLTDRNLNTSFFDPIGGGDPILYQHL
>ELACA249-10|MM16544|Coleophora_silenella
TLYFIFGIWAGMVGTSLSLLIRAELGNPGSLIGDDQIYNTIVTAHAFIMIFFMVMPIMIGGFGNWLVPLMLGAPDMAFPRMNNMSFWLLPPSLTLLISSSIVENGTGTGWTVYPPLSSNIAHGGSSVDLSIFSLHLAGISSILGAINFITTIINMRLNNMSFD
QLPLFVWAVGITALLLLLSLPVLAGAITMLLTDRNLNTSFFDPAGGGDPILYQHL
>COEUA001-11|MM16581|Coleophora_sirella
TLYFIFGIWAGMMGTSLSLLIRAELGNPGSLIGDDQIYNVIVTAHAFIMIFFMVMPIMIGGFGNWLVPLMLGAPDMAFPRMNNMSFWFLPPSLMLLISSSIVENGTGTGWTVYPPLSSNIAHSGSSVDLSIFSLHLAGISSILGAINFITTIINMRMNNLSF
DQLPLFVWSVGITALLLLLSLPVLAGAITMLLTDRNLNTSFFDPAGGGDPILYQHL
>COEUA007-11|MM16587|Coleophora_sirella
TLYFIFGIWAGMMGTSLSLLIRAELGNPGSLIGDDQIYNVIVTAHAFIMIFFMVMPIMIGGFGNWLVPLMLGAPDMAFPRMNNMSFWFLPPSLMLLISSSIVENGTGTGWTVYPPLSSNIAHSGSSVDLSIFSLHLAGISSILGAINFITTIINMRMNNLSF
DQLPLFVWSVGITALLLLLSLPVLAGAITMLLTDRNLNTSFFDPAGGGDPILYQ?L
>LEFIK617-10|MM18192|Coleophora_solitariella
TLYFIFGIWASMVGTSLSLLIRAELGNPGSLIGDDQIYNTIVTAHAFIMIFFMVMPIMIGGFGNWLVPLMLGAPDMAFPRMNNMSFWLLPPSLTLLISSSIVENGTGTGWTVYPPLSSNIAHSGSSVDLSIFSLHLAGISSILGAINFITTIINMRLNNMLFD
QLPLFVWAVGITALLLLLSLPVLAGAITMLLTDRNLNTSFFDPAGGGDPILYQHL
>LEFIJ2269-14|MM23453|Coleophora_spinella
TLYFIFGIWAGMVGTSLSLLIRAELGNPGSLIGDDQIYNTIVTAHAFIMIFFMVMPIMIGGFGNWLVPLMLGAPDMAFPRMNNMSFWLLPPSLTLLISSSIVENGTGTGWTVYPPLSSNIAHSGSSVDLSIFSLHLAGISSILGAINFITTIINMRLNNMSFD
QLPLFVWAVGITALLLLLSLPVLAGAITMLLTDRNLNTSFFDPAGGGDPILYQHL
>LEFID081-10|HM872892|MM05692|Coleophora_squalorella
TLYFIFGIWAGMVGTSLSLLIRAELGNPGSLIGDDQIYNTIVTAHAFIMIFFMVMPIMIGGFGNWLVPLMLGAPDMAFPRMNNMSFWLLPPSLTLLISSSIVENGTGTGWTVYPPLSSNIAHGGSSVDLSIFSLHLAGISSILGAINFITTIINMRLNNMSFD
QLPLFVWAVGITALLLLLSLPVLAGAITMLLTDRNLNTSFFDPAGGGDPILYQHL
>LEFIK117-10|MM17692|Coleophora_squalorella
TLYFIFGIWAGMVGTSLSLLIRAELGNPGSLIGDDQIYNTIVTAHAFIMIFFMVMPIMIGGFGNWLVPLMLGAPDMAFPRMNNMSFWLLPPSLTLLISSSIVENGTGTGWTVYPPLSSNIAHGGSSVDLSIFSLHLAGISSILGAINFITTIINMRLNNMSFD
QLPLFVWAVGITALLLLLSLPVLAGAITMLLTDRNLNTSFFDPAGGGDPILYQHL
>LEFIK627-10|JF854201|MM18202|Coleophora_squalorella
TLYFIFGIWAGMVGTSLSLLIRAELGNPGSLIGDDQIYNTIVTAHAFIMIFFMVMPIMIGGFGNWLVPLMLGAPDMAFPRMNNMSFWLLPPSLTLLISSSIVENGTGTGWTVYPPLSSNIAHSGSSVDLSIFSLHLAGISSILGAINFITTIINMRLNNMSFD
QLPLFVWAVGITALLLLLSLPVLAGAITMLLTDRNLNTSFFDPAGGGDPILYQHL
>LEFID337-10|HM873134|MM06231|Coleophora_squamosella
TLYFIFGIWAGMVGTSLSLLIRAELGNPGSLIGDDQIYNTIVTAHAFIMIFFMVMPIMIGGFGNWLVPLMLGAPDMAFPRMNNMSFWLLPPSLTLLISSSIVENGTGTGWTVYPPLSSNIAHSGASVDLSIFSLHLAGISSILGAINFITTIINMRLNNMSFD
QLPLFVWAVGITALLLLLSLPVLAGAITMLLTDRNLNTSFFDPAGGGDPILYQHL
>LEFIB701-10|HM871579|MM02504|Coleophora_sternipennella
TLYFIFGIWAGMVGTSLSLLIRAELGNPGSLIGDDQIYNTIVTAHAFIMIFFMVMPIMIGGFGNWLVPLMLGAPDMAFPRMNNMSFWFLPPSLTILISSSVVENGTGTGWTVYPPLSSNIAHSGSSVDLSIFSLHLAGISSILGAINFITTIINMRLNNMSFD
QLPLFVWSVGITALLLLLSLPVLAGAITMLLTDRNLNTSFFDPAGGGDPILYQHL

>LEFIE606-10|HM874329|MM09520|Coleophora_sternipennella
TLYFIFGIWAGMVGTSLSLLIRAELGNPGSLIGDDQIYNTIVTAHAFIMIFFMVMPIMIGGFGNWLVPLMLGAPDMAFPRMNNMSFWFLPPSLTILISSSVVENGTGTGWTVYPPLSSNIAHSGSSVDLSIFSLHLAGISSILGAINFITTIINMRLNNMSFD
QLPLFVWSVGITALLLLLSLPVLAGAITMLLTDRNLNTSFFDPAGGGDPILYQHL
>LEFIG059-10|HM875738|MM13680|Coleophora_sternipennella
TLYFIFGIWAGMVGTSLSLLIRAELGNPGSLIGDDQIYNTIVTAHAFIMIFFMVMPIMIGGFGNWLVPLMLGAPDMAFPRMNNMSFWFLPPSLTILISSSVVENGTGTGWTVYPPLSSNIAHSGSSVDLSIFSLHLAGISSILGAINFITTIINMRLNNMSFD
QLPLFVWSVGITALLLLLSLPVLAGAITMLLTDRNLNTSFFDPAGGGDPILYQHL
>LEFIB683-10|HM871561|MM02481|Coleophora_striatipennella
TLYFIFGIWAGMVGTSLSLLIRAELGNPGSLIGDDQIYNTIVTAHAFIMIFFMVMPIMIGGFGNWLVPLMLGTPDMAFPRMNNMSFWLLPPSLTLLISSSIVENGTGTGWTVYPPLSSNIAHGGSSVDLSIFSLHLAGISSILGAINFITTIINMRLNNMSFD
QLPLFVWAVGITALLLLLSLPVLAGAITMLLTDRNLNTSFFDPAGGGDPILYQHL
>LEFIB684-10|HM871562|MM02482|Coleophora_striatipennella
TLYFIFGIWAGMVGTSLSLLIRAELGNPGSLIGDDQIYNTIVTAHAFIMIFFMVMPIMIGGFGNWLVPLMLGTPDMAFPRMNNMSFWLLPPSLTLLISSSIVENGTGTGWTVYPPLSSNIAHGGSSVDLSIFSLHLAGISSILGAINFITTIINMRLNNMSFD
QLPLFVWAVGITALLLLLSLPVLAGAITMLLTDRNLNTSFFDPAGGGDPILYQHL
>LEFID763-10|HM873520|MM06828|Coleophora_striatipennella
TLYFIFGIWAGMVGTSLSLLIRAELGNPGSLIGDDQIYNTIVTAHAFIMIFFMVMPIMIGGFGNWLVPLMLGTPDMAFPRMNNMSFWLLPPSLTLLISSSIVENGTGTGWTVYPPLSSNIAHGGSSVDLSIFSLHLAGISSILGAINFITTIINMRLNNMSFD
QLPLFVWAVGITALLLLLSLPVLAGAITMLLTDRNLNTSFFDPAGGGDPILYQHL
>LEFIL660-10|MM18958|Coleophora_succursella
TLYFIFGIWAGMLGTSLSLLIRAELGNPGSLIGDDQIYNTIVTAHAFIMIFFMVMPIMIGGFGNWLVPLMLGAPDMAFPRMNNMSFWLLPPSLTLLISSSVVENGTGTGWTVYPPLSSNIAHSGSSVDLSIFSLHLAGISSILGAINFITTIINMRLNNMTFD
QLPLFVWSVGITALLLLLSLPVLAGAITMLLTDRNLNTSFFDPAGGGDPILYQHL
>LEFIL662-10|MM18960|Coleophora_succursella
TLYFIFGIWAGMLGTSLSLLIRAELGNPGSLIGDDQIYNTIVTAHAFIMIFFMVMPIMIGGFGNWLVPLMLGAPDMAFPRMNNMSFWLLPPSLTLLISSSVVENGTGTGWTVYPPLSSNIAHSGSSVDLSIFSLHLAGISSILGAINFITTIINMRLNNMTFD
QLPLFVWSVGITALLLLLSLPVLAGAITMLLTDRNLNTSFFDPAGGGDPILYQHL
>LEFIA027-10|HM396377|MM00098|Coleophora_svenssoni
TLYFIFGIWAGMVGTSLSLLIRAELGNPGSLIGDDQIYNTIVTAHAFIMIFFMVMPIMIGGFGNWLVPLMLGAPDMAFPRMNNMSFWLLPPSLTLLISSSIVENGTGTGWTVYPPLSSNIAHSGSSVDLSIFSLHLAGISSILGAINFITTIINMRLNNLSFD
QLPLFVWSVGITALLLLLSLPVLAGAITMLLTDRNLNTSFFDPAGGGDPILYQHL
>LEFIB680-10|HM871558|MM02476|Coleophora_taeniipennella
TLYFIFGIWAGMVGTSLSLLIRAELGNPGSLIGDDQIYNTIVTAHAFIMIFFMVMPIMIGGFGNWLVPLMLGAPDMAFPRMNNMSFWLLPPSLMLLISSSIVENGTGTGWTVYPPLSSNIAHSGSSVDLSIFSLHLAGISSILGAINFITTIINMRLNNMSF
DQLPLFVWAVGITALLLLLSLPVLAGAITMLLTDRNLNTSFFDPAGGGDPILYQHL
>LEFIB698-10|HM871576|MM02500|Coleophora_taeniipennella
TLYFIFGIWAGMVGTSLSLLIRAELGNPGSLIGDDQIYNTIVTAHAFIMIFFMVMPIMIGGFGNWLVPLMLGAPDMAFPRMNNMSFWLLPPSLMLLISSSVVENGTGTGWTVYPPLSSNIAHSGSSVDLSIFSLHLAGISSILGAINFITTIINMRLNNMSF
DQLPLFVWAVGITALLLLLSLPVLAGAITMLLTDRNLNTSFFDPAGGGDPILYQHL
>LEFIJ934-10|JF853904|MM17559|Coleophora_tamesis
TLYFIFGIWAGMVGTSLSLLIRAELGNPGSLIGDDQIYNTIVTAHAFIMIFFMVMPIMIGGFGNWLVPLMLGAPDMAFPRMNNMSFWLLPPSLMILISSSIVENGTGTGWTVYPPLSSNIAHSGSSVDLSIFSLHLAGISSILGAINFITTIINMRLNNMSFD
QLPLFVWAVGITALLLLLSLPVLAGAITMLLTDRNLNTSFFDPAGGGDPILYQHL
>LEFID827-10|HM873584|MM06939|Coleophora_tanaceti
TLYFIFGIWAGMVGTSLSLLIRAELGNPGSLIGDDQIYNTIVTAHAFIMIFFMVMPIMIGGFGNWLVPLMLGAPDMAFPRMNNMSFWLLPPSLTLLISSSIVENGTGTGWTVYPPLSSNIAHSGSSVDLSIFSLHLAGISSILGAINFITTIINMRLNNMLFD
QLPLFVWAVGITALLLLLSLPVLAGAITMLLTDRNLNTSFFDPAGGGDPILYQHL
>LEFIF482-10|HM875167|MM12034|Coleophora_therinella
TLYFIFGIWAGMVGTSLSLLIRAELGNPGSLIGDDQIYNTIVTAHAFIMIFFMVMPIMIGGFGNWLVPLMLGAPDMAFPRMNNMSFWLLPPSLTLLISSSIVENGTGTGWTVYPPLSSNIAHGGGSVDLSIFSLHLAGISSILGAINFITTIINMRLNNMSF
DQLPLFVWAVGLTALLLLLSLPVLAGAITMLLTDRNLNTSFFDPAGGGDPILYQHL
>LEFIF483-10|HM875168|MM12035|Coleophora_therinella
TLYFIFGIWAGMVGTSLSLLIRAELGNPGSLIGDDQIYNTIVTAHAFIMIFFMVMPIMIGGFGNWLVPLMLGAPDMAFPRMNNMSFWLLPPSLTLLISSSIVENGTGTGWTVYPPLSSNIAHGGGSVDLSIFSLHLAGISSILGAINFITTIINMRLNNLSFD
QLPLFVWAVGLTALLLLLSLPVLAGAITMLLTDRNLNTSFFDPAGGGDPILYQHL
>LEFIG030-10|HM875709|MM13651|Coleophora_therinella
TLYFIFGIWAGMVGTSLSLLIRAELGNPGSLIGDDQIYNTIVTAHAFIMIFFMVMPIMIGGFGNWLVPLMLGAPDMAFPRMNNMSFWLLPPSLTLLISSSIVENGTGTGWTVYPPLSSNIAHGGGSVDLSIFSLHLAGISSILGAINFITTIINMRLNNMSF
DQLPLFVWAVGLTALLLLLSLPVLAGAITMLLTDRNLNTSFFDPAGGGDPILYQHL
>LEFIG034-10|HM875713|MM13655|Coleophora_therinella
TLYFIFGIWAGMVGTSLSLLIRAELGNPGSLIGDDQIYNTIVTAHAFIMIFFMVMPIMIGGFGNWLVPLMLGAPDMAFPRMNNMSFWLLPPSLTLLISSSIVENGTGTGWTVYPPLSSNIAHGGGSVDLSIFSLHLAGISSILGAINFITTIINMRLNNMSF

DQLPLFVWAVGLTALLLLLSLPVLAGAITMLLTDRNLNTSFFDPAGGGDPILYQHL
>LEFIG056-10|HM875735|MM13677|Coleophora_therinella
TLYFIFGIWAGMVGTSLSLLIRAELGNPGSLIGDDQIYNTIVTAHAFIMIFFMVMPIMIGGFGNWLVPLMLGAPDMAFPRMNNMSFWLLPPSLTLLISSSIVENGTGTGWTVYPPLSSNIAHGGGSVDLSIFSLHLAGISSILGAINFITTIINMRLNNMSF
DQLPLFVWAVGLTALLLLLSLPVLAGAITMLLTDRNLNTSFFDPAGGGDPILYQHL
>LEFIF489-10|HM875174|MM12044|Coleophora_trifolii
TLYFIFGIWAGMVGTSLSLLIRAELGNPGSLIGDDQIYNTIVTAHAFIMIFFMVMPIMIGGFGNWLVPLMLGAPDMAFPRMNNMSFWLLPPSLTLLISSSIVENGTGTGWTVYPPLSSNIAHSGSSVDLSIFSLHLAGISSILGAINFITTIINMRLNNMSFD
QLPLFVWAVGITALLLLLSLPVLAGAITMLLTDRNLNTSFFDPAGGGDPILYQHL
>LEFIE998-10|HM874713|MM10436|Coleophora_trigeminella
TLYFIFGIWAGMTGTSLSLLIRAELGNPGSLIGDDQIYNTIVTAHAFIMIFFMVMPIMIGGFGNWLVPLMLGAPDMAFPRMNNMSFWLLPPSLTLLISSSIVENGTGTGWTVYPPLSSNIAHSGSSVDLSIFSLHLAGISSILGAINFITTIINMRLNNMSFD
QLPLFIWAVGITALLLLLSLPVLAGAITMLLTDRNLNTSFFDPAGGGDPILYQHL
>LEFIK107-10|JN265335|MM17682|Coleophora_trigeminella
TLYFIFGIWAGMTGTSLSLLIRAELGNPGSLIGDDQIYNTIVTAHAFIMIFFMVMPIMIGGFGNWLVPLMLGAPDMAFPRMNNMSFWLLPPSLTLLISSSIVENGTGTGWTVYPPLSSNIAHSGSSVDLSIFSLHLAGISSILGAINFITTIINMRLNNMSFD
QLPLFIWAVGITALLLLLSLPVLAGAITMLLTDRNLNTSFFDPAGGGDPILYQHL
>LEFIB687-10|HM871565|MM02487|Coleophora_trochilella
TLYFIFGIWAGMVGTSLSLLVRAELGNPGSLIGDDQIYNTIVTAHAFIMIFFMVMPIMIGGFGNWLVPLMLGAPDMAFPRMNNMSFWLLPPSLTLLISSSIVENGTGTGWTVYPPLSSNIAHGGSSVDLSIFSLHLAGISSILGAINFITTIINMRLNNMSF
DQLPLFVWAVGITALLLLLSLPILAGAITMLLTDRNLNTSFFDPAGGGDPILYQHL
>LEFIJ377-10|JF853564|MM15981|Coleophora_uliginosella
TLYFIFGIWSSMVGTSLSLLIRAELGNPGFLIGNDQIYNTIVTAHAFIMIFFMVMPIMIGGFGNWLLPLMLGAPDMAFPRMNNMSFWLLPPSLTLLISSSMVENGTGTGWTVYPPLSSNIAHSGSSVDLSIFSLHLAGISSILGAINFITTIINMRLNNMSFD
QLSLFIWSVGITALLLLLSLPVLAGAITMLLTDRNLNTSFFDPIGGGDPILYQHL
>LEEUA481-11|KM572046|MM20540|Coleophora_uliginosella
TLYFILGIWSSMVGTSLSLLIRAELGNPGFLIGNDQIYNTIVTAHAFIMIFFMVMPIMIGGFGNWLLPLMLGAPDMAFPRMNNMSFWLLPPSLTLLISSSMVENGTGTGWTVYPPLSSNIAHSGSSVDLSIFSLHLAGISSILGAINFITTIINMRLNNMSFD
QLSLFIWSVGITALLLLLSLPVLAGAITMLLTDRNLNTSFFDPIGGGDPILYQHL
>LEFIA028-10|HM396378|MM00099|Coleophora_unigenella
TLYFIFGIWSGMMGTSLSLLIRAELGNPGSLIGDDQIYNVIVTAHAFIMIFFMVMPIMIGGFGNWLVPLMLGAPDMAFPRMNNMSFWFLPPSLMLLISSSIVETGTGTGWTVYPPLSSNIAHSGSSVDLSIFSLHLAGISSILGAINFITTIINMRLNNLSFD
QLPLFVWSVGITALLLLLSLPVLAGAITMLLTDRNLNTSFFDPAGGGDPILYQHL
>LEFIA003-10|HM396353|MM00074|Coleophora_vacciniella
TLYFIFGIWAGMMGTSLSLLIRAELGNPGSLIGDDQIYNVIVTAHAFIMIFFMVMPIMIGGFGNWLVPLMLGAPDMAFPRMNNMSFWFLPPSLMLLISSSIVENGTGTGWTVYPPLSSNIAHSGSSVDLSIFSLHLAGISSILGAINFITTIINMRLNNLSF
DQLPLFVWSVGITALLLLLSLPVLAGAITMLLTDRNLNTSFFDPAGGGDPILYQHL
>LEFIB376-10|HM871275|MM00948|Coleophora_vacciniella
TLYFIFGIWAGMMGTSLSLLIRAELGNPGSLIGDDQIYNVIVTAHAFIMIFFMVMPIMIGGFGNWLVPLMLGAPDMAFPRMNNMSFWFLPPSLMLLISSSIVENGTGTGWTVYPPLSSNIAHSGSSVDLSIFSLHLAGISSILGAINFITTIINMRLNNLSF
DQLPLFVWSVGITALLLLLSLPVLAGAITMLLTDRNLNTSFFDPAGGGDPILYQHL
>LEFIC364-10|HM872207|MM03891|Coleophora_vacciniella
TLYFIFGIWAGMMGTSLSLLIRAELGNPGSLIGDDQIYNVIVTAHAFIMIFFMVMPIMIGGFGNWLVPLMLGAPDMAFPRMNNMSFWFLPPSLMLLISSSIVENGTGTGWTVYPPLSSNIAHSGSSVDLSIFSLHLAGISSILGAINFITTIINMRLNNLSF
DQLPLFVWSVGITALLLLLSLPVLAGAITMLLTDRNLNTSFFDPAGGGDPILYQHL
>LEFID378-10|HM873175|MM06289|Coleophora_vacciniella
TLYFIFGIWAGMMGTSLSLLIRAELGNPGSLIGDDQIYNVIVTAHAFIMIFFMVMPIMIGGFGNWLVPLMLGAPDMAFPRMNNMSFWFLPPSLMLLISSSIVENGTGTGWTVYPPLSSNIAHSGSSVDLSIFSLHLAGISSILGAINFITTIINMRLNNLSF
DQLPLFVWSVGITALLLLLSLPVLAGAITMLLTDRNLNTSFFDPAGGGDPILYQHL
>LEFIJ464-10|JF853592|MM10168|Coleophora_vacciniella
TLYFIFGIWAGMMGTSLSLLIRAELGNPGSLIGDDQIYNVIVTAHAFIMIFFMVMPIMIGGFGNWLVPLMLGAPDMAFPRMNNMSFWFLPPSLMLLISSSIVENGTGTGWTVYPPLSSNIAHSGSSVDLSIFSLHLAGISSILGAINFITTIINMRLNNLSF
DQLPLFVWSVGITALLLLLSLPVLAGAITMLLTDRNLNTSFFDPAGGGDPILY?HL
>LEFIB700-10|HM871578|MM02503|Coleophora_versurella
TLYFIFGIWAGMMGTSLSLLIRAELGNPGSLIGDDQIYNTIVTAHAFIMIFFMVMPIMIGGFGNWLVPLMLGAPDMAFPRMNNMSFWLLPPSLTLLISSSIVENGTGTGWTVYPPLSSNIAHSGSSVDLSIFSLHLAGISSILGAINFITTIINMRLNNMSF
DQLPLFVWAVGITALLLLLSLPVLAGAITMLLTDRNLNTSFFDPAGGGDPILYQHL
>LEFIF486-10|HM875171|MM12038|Coleophora_versurella
TLYFIFGIWAGMMGTSLSLLIRAELGNPGSLIGDDQIYNTIVTAHAFIMIFFMVMPIMIGGFGNWLVPLMLGAPDMAFPRMNNMSFWLLPPSLTLLISSSIVENGTGTGWTVYPPLSSNIAHSGSSVDLSIFSLHLAGISSILGAINFITTIINMRLNNMSF
DQLPLFVWAVGITALLLLLSLPVLAGAITMLLTDRNLNTSFFDPAGGGDPILYQHL
>LEFIK605-10|MM18180|Coleophora_versurella

TLYFIFGIWAGMMGTSLSLLIRAELGNPGSLIGDDQIYNTIVTAHAFIMIFFMVMPIMIGGFGNWLVPLMLGAPDMAFPRMNNMSFWLLPPSLTLLISSSIVENGTGTGWTVYPPLSSNIAHSGSSVDLSIFSLHLAGISSILGAINFITTIINMRLNNMSF
DQLPLFVW?VGITALLLLLSLPVLAGAITMLLTDRNLNTSFFDPAGGGDPILYQHL
>LEFIE942-10|HM874659|MM10331|Coleophora_vestianella
TLYFIFGIWAGMVGTSLSLLIRAELGNPGSLIGDDQIYNTIVTAHAFIMIFFMVMPIMIGGFGNWLVPLMLGAPDMAFPRMNNMSFWLLPPSLTLLISSSIVENGTGTGWTVYPPLSSNIAHGGSSVDLSIFSLHLAGISSILGAINFITTIINMRLNNMSFD
QLPLFVWAVGITALLLLLSLPVLAGAITMLLTDRNLNTSFFDPAGGGDPILYQHL
>LEFIG061-10|HM875740|MM13706|Coleophora_vestianella
TLYFIFGIWAGMVGTSLSLLIRAELGNPGSLIGDDQIYNTIVTAHAFIMIFFMVMPIMIGGFGNWLVPLMLGAPDMAFPRMNNMSFWLLPPSLTLLISSSIVENGTGTGWTVYPPLSSNIAHGGSSVDLSIFSLHLAGISSILGAINFITTIINMRLNNMSFD
QLPLFVWAVGITALLLLLSLPVLAGAITMLLTDRNLNTSFFDPAGGGDPILYQHL
>ELACA911-11|MM20149|Coleophora_vibicigerella
TLYFIFGIWAGMVGTSLSLLIRAELGNPGSLIGDDQIYNTIVTAHAFIMIFFMVMPIMIGGFGNWLVPLMLGAPDMAFPRMNNMSFWLLPPSLMLLISSSTVETGTGTGWTVYPPLSSNIAHSGSSVDLSIFSLHLAGISSILGAINFITTIINMRLNNLSFD
QLPLFVWAVGITALLLLLSLPVLAGAITMLLTDRNLNTSFFDPAGGGDPILYQHL
>LEFIC413-10|HM872256|MM03959|Coleophora_violacea
TLYFIFGIWSGMMGTSLSLLIRAELGNPGSLIGDDQIYNVIVTAHAFIMIFFMVMPIMIGGFGNWLVPLMLGAPDMAFPRMNNMSFWFLPPSLMILISSSIVENGTGTGWTVYPPLSSNIAHSGSSVDLSIFSLHLAGISSILGAINFITTIINMRLNNLSFD
QLSLFVWSVGITALLLLLSLPVLAGAITMLLTDRNLNTSFFDPAGGGDPILYQHL
>LEFIB692-10|HM871570|MM02492|Coleophora_virgaureae
TLYFIFGIWAGMVGTSLSLLIRAELGNPGSLIGDDQIYNTIVTAHAFIMIFFMVMPIMIGGFGNWLVPLMLGAPDMAFPRMNNMSFWLLPPSLTLLIFSSIVENGTGTGWTVYPPLSSNIAHSGASVDLSIFSLHLAGISSILGAINFITTIINMRLNNMSFD
QLPLFVWAVGITALLLLLSLPVLAGAITMLLTDRNLNTSFFDPAGGGDPILYQHL
>LEFIB979-10|HM871852|MM03172|Coleophora_virgaureae
TLYFIFGIWAGMVGTSLSLLIRAELGNPGSLIGDDQIYNTIVTAHAFIMIFFMVMPIMIGGFGNWLVPLMLGAPDMAFPRMNNMSFWLLPPSLTLLIFSSIVENGTGTGWTVYPPLSSNIAHSGASVDLSIFSLHLAGISSILGAINFITTIINMRLNNMSFD
QLPLFVWAVGITALLLLLSLPVLAGAITMLLTDRNLNTSFFDPAGGGDPILYQHL
>LEFIB980-10|HM871853|MM03173|Coleophora_virgaureae
TLYFIFGIWAGMVGTSLSLLIRAELGNPGSLIGDDQIYNTIVTAHAFIMIFFMVMPIMIGGFGNWLVPLMLGAPDMAFPRMNNMSFWLLPPSLTLLIFSSIVENGTGTGWTVYPPLSSNIAHSGASVDLSIFSLHLAGISSILGAINFITTIINMRLNNMSFD
QLPLFVWAVGITALLLLLSLPVLAGAITMLLTDRNLNTSFFDPAGGGDPILYQHL
>ELACA281-10|MM16576|Coleophora_virgaureae
TLYFIFGIWAGMVGTSLSLLIRAELGNPGSLIGDDQIYNTIVTAHAFIMIFFMVMPIMIGGFGNWLVPLMLGAPDMAFPRMNNMSFWLLPPSLTLLIFSSIVENGTGTGWTVYPPLSSNIAHSGASVDLSIFSLHLAGISSILGAINFITTIINMRLNNMSFD
QLPLFVWAVGITALLLLLSLPVLAGAITMLLTDRNLNTSFFVLAGGGDPILYQHL
>LEFIG112-10|HM875792|MM13905|Coleophora_vitisella
TLYFIFGIWAGMMGTSLSLLIRAELGNPGSLIGDDQIYNVIVTAHAFIMIFFMVMPIMIGGFGNWLVPLMLGAPDMAFPRMNNMSFWFLPPSLMILISSSIVENGTGTGWTVYPPLSSNIAHSGSSVDLSIFSLHLAGISSILGAINFITTIINMRLNNLSFD
QLPLFIWSVGITALLLLLSLPVLAGAITMLLTDRNLNTSFFDPAGGGDPILYQHL
>LEEUA582-11|MM20641|Coleophora_vulnerariae
TLYFIFGIWAGMVGTSLSLLIRAELGNPGSLIGDDQIYNTIVTAHAFIMIFFMVMPIMIGGFGNWLVPLMLGAPDMAFPRMNNMSFWLLPPSLILLISSSIVENGTGTGWTVYPPLSSNIAHGGSSVDLSIFSLHLAGISSILGAINFITTIINMRLNNMSFD
QLPLFVWAVGITALLLLLSLPVLAGAITMLLTDRNLNTSFFDPAGGGDPILYQHL
>LEFIC096-10|HQ570287|MM03410|Colias_hecla
TLYFIFGVWAGMIGTSLSLLIRTELGNPGSLIGDDQIYNTIVTAHAFIMIFFMVMPIMIGGFGNWLIPLMLGAPDMAFPRMNNMSFWLLPPSLTLLISSSIVENGAGTGWTVYPPLSSNIAHSGSSVDLAIFSLHLAGISSILGAINFITTIINMRINNMSFD
QMPLFVWAVGITALLLLLSLPVLAGAITMLLTDRNLNTSFFDPAGGGDPILYQHL
>LEFID428-10|HQ570324|MM06358|Colias_hecla
TLYFIFGVWAGMIGTSLSLLIRTELGNPGSLIGDDQIYNTIVTAHAFIMIFFMVMPIMIGGFGNWLIPLMLGAPDMAFPRMNNMSFWLLPPSLTLLISSSIVENGAGTGWTVYPPLSSNIAHSGSSVDLAIFSLHLAGISSILGAINFITTIINMRINNMSFD
QMPLFVWAVGITALLLLLSLPVLAGAITMLLTDRNLNTSFFDPAGGGDPILYQHL
>LEFID236-10|HM873036|MM06097|Colias_hyale
TLYFIFGVWAGMIGTSLSLLIRTELGNPGSLIGDDQIYNTIVTAHAFIMIFFMVMPIMIGGFGNWLIPLMLGAPDMAFPRMNNMSFWLLPPSLVLLISSSIVENGAGTGWTVYPPLSSNIAHSGSSVDLAIFSLHLAGISSILGAINFITTIINMRINNMSFD
QMPLFVWAVGITALLLLLSLPVLAGAITMLLTDRNLNTSFFDPAGGGDPILYQHL
>LEFIJ508-10|JF853631|MM17133|Colias_hyale
TLYFIFGVWAGMIGTSLSLLIRTELGNPGSLIGDDQIYNTIVTAHAFIMIFFMVMPIMIGGFGNWLIPLMLGAPDMAFPRMNNMSFWLLPPSLILLISSSIVENGAGTGWTVYPPLSSNIAHSGSSVDLAIFSLHLAGISSILGAINFITTIINMRINNMSFDQ
MPLFVWAVGITALLLLLSLPVLAGAITMLLTDRNLNTSFFDPAGGGDPILYQHL
>LEFIC097-10|HQ570288|MM03411|Colias_palaeno
TLYFIFGVWAGMIGTSLSLLIRTELGNPGSLIGDDQIYNTIVTAHAFIMIFFMVMPIMIGGFGNWLIPLMLGAPDMAFPRMNNMSFWLLPPSLTLLISSSIVENGAGTGWTVYPPLSSNIAHSGSSVDLAIFSLHLAGISSILGAINFITTIINMRINNMSFD
QMPLFVWAVGITALLLLLSLPVLAGAITMLLTDRNLNTSFFDPAGGGDPILYQHL

>LEFIA022-10|HM396372|MM00093|Colias_tyche
TLYFIFGVWAGMIGTSLSLLIRTELGNPGSLIGDDQIYNTIVTAHAFIMIFFMVMPIMIGGFGNWLIPLMLGAPDMAFPRMNNMSFWLLPPSLTLLISSSIVENGAGTGWTVYPPLSSNIAHSGSSVDLAIFSLHLAGISSILGAINFITTIINMRINNMSFD
QMPLFVWAVGITALLLLLSLPVLAGAITMLLTDRNLNTSFFDPAGGGDPILYQHL
>LEFIA738-10|HM386882|MM04092|Colias_tyche
TLYFIFGVWAGMIGTSLSLLIRTELGNPGSLIGDDQIYNTIVTAHAFIMIFFMVMPIMIGGFGNWLIPLMLGAPDMAFPRMNNMSFWLLPPSLTLLISSSIVENGAGTGWTVYPPLSSNIAHSGSSVDLAIFSLHLAGISSILGAINFITTIINMRINNMSFD
QMPLFVWAVGITALLLLLSLPVLAGAITMLLTDRNLNTSFFDPAGGGDPILYQHL
>LEFIA368-10|HM386710|MM01425|Colobochyla_salicalis
TLYFIFGIWAGMVGTSLSLLIRAELGNPGSLIGDDQIYNTIVTAHAFIMIFFMVMPIMIGGFGNWLIPLMLGAPDMAFPRMNNMSFWLLPPSITLLISSSIVENGAGTGWTVYPPLSSNIAHSGSSVDLAIFSLHLAGISSILGAINFITTIINMRLNNLSFDQ
MPLFIWAVGITAFLLLLSLPVLAGAITMLLTDRNLNTSFFDPAGGGDPILYQHL
>LEFIA458-10|HM386798|MM01523|Colocasia_coryli
TLYFIFGIWAGMVGTSLSLLIRAELGTPGSLIGDDQIYNTIVTAHAFIMIFFMVMPIMIGGFGNWLVPLMLGAPDMAFPRMNNMSFWLLPPSLTLLISSSIVENGAGTGWTVYPPLSSNIAHGGSSVDLAIFSLHLAGISSILGAINFITTIINMRLNNLSFD
QMPLFIWAVGITAFLLLLSLPVLAGAITMLLTDRNLNTSFFDPAGGGDPILYQHL
>LEFIE163-10|HM873909|MM08417|Colocasia_coryli
TLYFIFGIWAGMVGTSLSLLIRAELGTPGSLIGDDQIYNTIVTAHAFIMIFFMVMPIMIGGFGNWLVPLMLGAPDMAFPRMNNMSFWLLPPSLTLLISSSIVENGAGTGWTVYPPLSSNIAHGGSSVDLAIFSLHLAGISSILGAINFITTIINMRLNNLSFD
QMPLFIWAVGITAFLLLLSLPVLAGAITMLLTDRNLNTSFFDPAGGGDPILYQHL
>LEFIA324-10|HM386667|MM01371|Colostygia_aptata
TLYFIFGIWAGMIGTSLSLLIRAELGNPGSLIGDDQIYNTIVTAHAFIMIFFMVMPIMIGGFGNWLVPLMLGAPDMAFPRMNNMSFWLLPPSITLLISSSIVENGAGTGWTVYPPLSSNIAHGGSSVDLAIFSLHLAGISSILGAINFITTIINMRLNNMFFD
QLPLFVWSVGITAFLLLLSLPVLAGAITMLLTDRNLNTSFFDPAGGGDPILYQHL
>LEFIC791-10|HM872610|MM04930|Colostygia_aptata
TLYFIFGIWAGMIGTSLSLLIRAELGNPGSLIGDDQIYNTIVTAHAFIMIFFMVMPIMIGGFGNWLVPLMLGAPDMAFPRMNNMSFWLLPPSITLLISSSIVENGAGTGWTVYPPLSSNIAHGGSSVDLAIFSLHLAGISSILGAINFITTIINMRLNNMFFD
QLPLFVWSVGITAFLLLLSLPVLAGAITMLLTDRNLNTSFFDPAGGGDPILYQHL
>LEFIE623-10|HM874346|MM09552|Colostygia_olivata
TLYFIFGIWAGMIGTSLSLLIRAELGNPGSLIGDDQIYNTIVTAHAFIMIFFMVMPIMIGGFGNWLVPLMLGAPDMAFPRMNNMSFWLLPPSITLLISSSIVENGAGTGWTVYPPLSSNIAHGGSSVDLAIFSLHLAGISSILGAINFITTIINMRLNNMFFD
QLPLFVWSVGITAFLLLLSLPVLAGAITMLLTDRNLNTSFFDPAGGGDPILYQHL
>LEFIB867-10|HM871744|MM02922|Colostygia_pectinataria
TLYFIFGIWAGMIGTSLSLLIRAELGNPGSLIGDDQIYNTIVTAHAFIMIFFMVMPIMIGGFGNWLVPLMLGAPDMAFPRMNNMSFWLLPPSITLLISSSIVENGAGTGWTVYPPLSSNIAHGGSSVDLAIFSLHLAGISSILGAINFITTIINMRLNNMFFD
QLPLFVWAVGITAFLLLLSLPVLAGAITMLLTDRNLNTSFFDPAGGGDPILYQHL
>LEFIF030-10|HM874741|MM10469|Colostygia_turbata
TLYFIFGIWAGMIGTSLSLLIRAELGNPGSLIGDDQIYNTIVTAHAFIMIFFMVMPIMIGGFGNWLVPLMLGAPDMAFPRMNNMSFWLLPPSITLLISSSIVENGAGTGWTVYPPLSSNIAHGGSSVDLAIFSLHLAGISSILGAINFITTIINMRLNNMFFD
QLPLFVWSVGITAFLLLLSLPVLAGAITMLLTDRNLNTSFFDPAGGGDPILYQHL
>LEFIB787-10|HM871664|MM02693|Colotois_pennaria
TLYFIFGIWAGMVGTSLSLLIRAELGNPGSLIGDDQIYNTIVTAHAFIMIFFMVMPIMIGGFGNWLVPLMLGAPDMAFPRMNNMSFWLLPPSLTLLISSSVVENGAGTGWTVYPPLSSNIAHGGSSVDLAIFSLHLAGISSILGAINFITTIINMRLNNMSF
DQMPLFVWAVGITAFLLLLSLPVLAGAITMLLTDRNLNTSFFDPAGGGDPILYQHL
>LEFIG932-10|HM876574|MM15796|Comibaena_bajularia
TLYFIFGIWAGMIGTSLSLLIRAELGNPGSLIGNDQIYNTIVTAHAFIMIFFMVMPIMIGGFGNWLIPLMLGAPDMAFPRMNNMSFWLLPPSITLLISSSIVENGAGTGWTVYPPLSSNIAHGGSSVDLAIFSLHLAGISSILGAINFITTIINMRLNNMSFD
QMPLFVWAVGITAFLLLLSLPVLAGAITMLLTDRNLNTSFFDPAGGGDPILYQHL
>LEFIK836-10|JN271714|MM18411|Comibaena_bajularia
TLYFIFGIWAGMIGTSLSLLIRAELGNPGSLIGNDQIYNTIVTAHAFIMIFFMVMPIMIGGFGNWLIPLMLGAPDMAFPRMNNMSFWLLPPSITLLISSSIVENGAGTGWTVYPPLSSNIAHGGSSVDLAIFSLHLAGISSILGAINFITTIINMRLNNMSFD
QMPLFVWAVGITAFLLLLSLPVLAGAITMLLTDRNLNTSFFDPAGGGDPILYQHL
>LEEUA518-11|MM20577|Conisania_leineri
TLYFIFGIWAGMVGTSLSLLIRAELGNPGSLIGDDQIYNTIVTAHAFIMIFFMVMPIMIGGFGNWLVPLMLGAPDMAFPRMNNMSFWLLPPSLTLLISSSIVENGAGTGWTVYPPLSSNIAHGGSSVDLAIFSLHLAGISSILGAINFITTIINMRLNSLSFD
QMPLFIWAVGITAFLLLLSLPVLAGAITMLLTDRNLNTSFFDPAGGGDPILYQHL
>LEFIJ354-10|MM15954|Conisania_luteago
TLYFIFGIWAGMVGTSLSLLIRAELGNPGSLIGDDQIYNTIVTAHAFIMIFFMVMPIMIGGFGNWLVPLMLGAPDMAFPRMNNMSFWLLPPSLTLLISSSIVENGAGTGWTVYPPLSSNIAHGGSSVDLAIFSLHLAGISSILGAINFITTIINMRLNNLSFD
QMPLFIWAVGITAFLLLLSLPVLAGAITMLLTDRNLNTSFFDPAGGGDPILYQHL
>LEFIB990-10|HM871862|MM03185|Conistra_erythrocephala
TLYFIFGIWAGMVGTSLSLLIRAELGNPGSLIGDDQIYNTIVTAHAFIMIFFMVMPIMIGGFGNWLVPLMLGAPDMAFPRLNNMSFWLLPPSLTLLISSSIVENGAGTGWTVYPPLSSNIAHGGSSVDLAIFSLHLAGISSILGAINFITTIINMRLNNLSFDQ

MPLFIWAVGITAFLLLLSLPVLAGAITMLLTDRNLNTSFFDPAGGGDPILYQHL
>LEFIC665-10|HM872486|MM04625|Conistra_rubiginea
TLYFIFGIWAGMVGTSLSLLIRAELGNPGSLIGDDQIYNTIVTAHAFIMIFFMVMPIMIGGFGNWLVPLMLGAPDMAFPRLNNMSFWLLPPSLTLLISSSIVENGAGTGWTVYPPLSSNIAHGGSSVDLAIFSLHLAGISSILGAINFITTIINMRLNNLSFDQ
MPLFIWAVGITAFLLLLSLPVLAGAITMLLTDRNLNTSFFDPAGGGDPILYQHL
>LEFIF534-10|HM875219|MM12191|Conistra_rubiginea
TLYFIFGIWAGMVGTSLSLLIRAELGNPGSLIGDDQIYNTIVTAHAFIMIFFMVMPIMIGGFGNWLVPLMLGAPDMAFPRLNNMSFWLLPPSLTLLISSSIVENGAGTGWTVYPPLSSNIAHGGSSVDLAIFSLHLAGISSILGAINFITTIINMRLNNLSFDQ
MPLFIWAVGITAFLLLLSLPVLAGAITMLLTDRNLNTSFFDPAGGGDPILYQHL
>LEFIG413-10|HM876089|MM14439|Conistra_rubiginosa
TLYFIFGIWAGMVGTSLSLLIRAELGNPGSLIGDDQIYNTIVTAHAFIMIFFMVMPIMIGGFGNWLVPLMLGAPDMAFPRLNNMSFWLLPPSLTLLISSSIVENGAGTGWTVYPPLSSNIAHGGSSVDLAIFSLHLAGISSILGAINFITTIINMRLNSLSFDQ
MPLFIWAVGITAFLLLLSLPVLAGAITMLLTDRNLNTSFFDPAGGGDPILYQHL
>LEFIB104-10|HM871011|MM00416|Conistra_vaccinii
TLYFIFGIWAGMVGTSLSLLIRAELGNPGSLIGDDQIYNTIVTAHAFIMIFFMVMPIMIGGFGNWLVPLMLGAPDMAFPRLNNMSFWLLPPSLTLLISSSIVENGAGTGWTVYPPLSSNIAHGGSSVDLAIFSLHLAGISSILGAINFITTIINMRLNSLSFDQ
MPLFIWAVGITAFLLLLSLPVLAGAITMLLTDRNLNTSFFDPAGGGDPILYQHL
>LEFIB342-10|HM871242|MM00889|Coptotriche_angusticollella
TLYFIFGIWAGMLGTSLSLIIRAELGMPGSLIGNDQIYNTIVTAHAFIMIFFMVMPIMIGGFGNWLVPLMLGAPDMAFPRLNNMSFWLLPPSLLLLISSSVVENGAGTGWTVYPPLSSNIAHNGSSVDLTIFSLHLAGISSILGAINFITTMVNMRTQGMT
FDQMPLFVWAVMITTILLLLSLPVLAGAITMLLTDRNLNTSFFDPAGGGDPILYQHL
>LEFIB334-10|HM871234|MM00876|Coptotriche_heinemanni
TLYFIFGIWAGMVGTSLSLLIRAELGTPGSLIGDDQIYNTIVTAHAFIMIFFMVMPIMIGGFGNWLVPLMLGAPDMAFPRLNNMSFWLLPPSLLLLISSSVVENGAGTGWTVYPPLSSNIAHTGSSVDLTIFSLHLAGISSILGAINFITTMLNMRAQGMSF
DQMPLFVWAVAITTVLLLLSLPVLAGAITMLLTDRNLNTSFFDPAGGGDPILYQHL
>LEFIE331-10|HM874057|MM08720|Coptotriche_marginea
TLYFIFGIWAGMVGTSLSLLIRAELGTPGSLIGDDQIYNTIVTAHAFIMIFFMVMPIMIGGFGNWLVPLMLGAPDMAFPRLNNMSFWLLPPSLLLLISSSVVENGAGTGWTVYPPLSSNIAHTGSSVDLTIFSLHLAGISSILGAINFITTMLNMRAQGMSF
DQMPLFVWAVAITTVLLLLSLPVLAGAITMLLTDRNLNTSFFDPAGGGDPILYQHL
>LEFIC354-10|HM872197|MM03873|Coranarta_cordigera
TLYFIFGIWAGMVGTSLSLLIRAELGNPGSLIGDDQIYNTIVTAHAFIMIFFMVMPIMIGGFGNWLVPLMLGAPDMAFPRMNNMSFWLLPPSLTLLISSSIVENGAGTGWTVYPPLSSNIAHGGSSVDLAIFSLHLAGISSILGAINFITTIINMRLNSLSFD
QMPLFIWAVGITAFLLLLSLPVLAGAITMLLTDRNLNTSFFDPAGGGDPILYQHL
>LEFIG141-10|HM875821|MM13976|Coranarta_cordigera
TLYFIFGIWAGMVGTSLSLLIRAELGNPGSLIGDDQIYNTIVTAHAFIMIFFMVMPIMIGGFGNWLVPLMLGAPDMAFPRMNNMSFWLLPPSLTLLISSSIVENGAGTGWTVYPPLSSNIAHGGSSVDLAIFSLHLAGISSILGAINFITTIINMRLNSLSFD
QMPLFIWAVGITAFLLLLSLPVLAGAITMLLTDRNLNTSFFDPAGGGDPILYQHL
>LEFIG171-10|HM875851|MM14034|Coranarta_cordigera
TLYFIFGIWAGMVGTSLSLLIRAELGNPGSLIGDDQIYNTIVTAHAFIMIFFMVMPIMIGGFGNWLVPLMLGAPDMAFPRMNNMSFWLLPPSLTLLISSSIVENGAGTGWTVYPPLSSNIAHGGSSVDLAIFSLHLAGISSILGAINFITTIINMRLNSLSFD
QMPLFIWAVGITAFLLLLSLPVLAGAITMLLTDRNLNTSFFDPAGGGDPILYQHL
>LEFIK319-10|MM17894|Corticivora_piniana
TLYFIFGIWSGMIGTSLSLLIRAELGNPGSLIGDDQIYNTIVTAHAFIMIFFMVMPIMIGGFGNWLVPLMLGAPDMAFPRMNNMSFWLLPPSIFLLISSSIVETGAGTGWTVYPPLSSNIAHSGSSVDLAIFSLHLAGISSILGAVNFITTIINMRPNNMSLD
QMPLFVWAVGITALLLLLSLPVLAGAITMLLTDRNLNTSFFDPAGGGDPILYQHL
>LEFID630-10|HM873393|MM06649|Coscinia_cribraria
TLYFIFGIWAGMVGTSLSLLIRAELGNPGSLIGDDQIYNTIVTAHAFIMIFFMVMPIMIGGFGNWLVPLMLGAPDMAFPRMNNMSFWLLPPSLTLLISSSIVENGAGTGWTVYPPLSSNIAHSGSSVDLAIFSLHLAGISSILGAINFITTIINMRLNNLSFD
QMPLFVWSVGITAFLLLLSLPVLAGAITMLLTDRNLNTSFFDPAGGGDPILYQHL
>LEFID778-10|HM873535|MM06846|Coscinia_cribraria
TLYFIFGIWAGMVGTSLSLLIRAELGNPGSLIGDDQIYNTIVTAHAFIMIFFMVMPIMIGGFGNWLVPLMLGAPDMAFPRMNNMSFWLLPPSLTLLISSSIVENGAGTGWTVYPPLSSNIAHSGSSVDLAIFSLHLAGISSILGAINFITTIINMRLNNLSFD
QMPLFVWSVGITAFLLLLSLPVLAGAITMLLTDRNLNTSFFDPAGGGDPILYQHL
>LEEUA602-11|KM373611|MM20661|Cosmardia_moritzella
TLYFIFGIWAGMVGTSLSLLIRAELGNPGSLIGDDQIYNTIVTAHAFIMIFFMVMPIMIGGFGNWLVPLMLGAPDMAFPRMNNMSFWLLPPSLTLLISSSIVENGAGTGWTVYPPLSSNIAHGGSSVDLAIFSLHLAGISSILGAINFITTIINMRINNLSFD
QMPLFVWAVGITALLLLLSLPVLAGAITMLLTDRNLNTSFFDP?GGGDPILYQHL
>LEFIJ1013-11|KM572331|MM09284|Cosmia_affinis
TLYFIFGIWAGMVGTSLSLLIRAELGNPGSLIGDDQIYNTIVTAHAFIMIFFMVMPIMIGGFGNWLVPLMLGAPDMAFPRLNNMSFWLLPPSLTLLISSSIVENGAGTGWTVYPPLSSNIAHGGSSVDLAIFSLHLAGISSILGAINFITTIINMRLNNLSFDQ
MPLFVWAVGITAFLLLLSLPVLAGAITMLLTDRNLNTSFFDPAGGGDPILYQHL
>LEFIJ346-10|KM573099|MM15946|Cosmia_pyralina

TLYFIFGIWAGMVGTSLSLLIRAELGNPGSLIGDDQIYNTIVTAHAFIMIFFMVMPIMIGGFGNWLVPLMLGAPDMAFPRLNNMSFWLLPPSLTLLISSSIVENGAGTGWTVYPPLSSNIAHGGSSVDLAIFSLHLAGISSILGAINFITTIINMRLNSLSFDQ
MPLFIWAVGITAFLLLLSLPVLAGAITMLLTDRNLNTSFFDPAGGGDPILYQHL
>LEFIA586-10|HM870835|MM01696|Cosmia_trapezina
TLYFIFGIWAGMVGTSLSLLIRAELGNPGSLIGDDQIYNTIVTAHAFIMIFFMVMPIMIGGFGNWLVPLMLGAPDMAFPRLNNMSFWLLPPSLTLLISSSIVENGAGTGWTVYPPLSSNIAHGGSSVDLAIFSLHLAGISSILGAINFITTIINMRLNNLSFDQ
MPLFVWAVGITAFLLLLSLPVLAGAITMLLTDRNLNTSFFDPAGGGDPILYQHL
>LEFIA587-10|HM870836|MM01697|Cosmia_trapezina
TLYFIFGIWAGMVGTSLSLLIRAELGNPGSLIGDDQIYNTIVTAHAFIMIFFMVMPIMIGGFGNWLVPLMLGAPDMAFPRLNNMSFWLLPPSLTLLISSSIVENGAGTGWTVYPPLSSNIAHGGSSVDLAIFSLHLAGISSILGAINFITTIINMRLNNLSFDQ
MPLFVWAVGITAFLLLLSLPVLAGAITMLLTDRNLN?SFFDPAGGGDPILYQHL
>LEFIG741-10|HM876397|MM15605|Cosmopterix_lienigiella
TLYFIFGIWAGMVGTSLSLLIRAELGNPGSLIGDDQIYNTIVTAHAFIMIFFMVMPIMIGGFGNWLVPLMLGAPDMAFPRMNNMSFWLLPPSLTLLISSSIVETGAGTGWTVYPPLSSNIAHSGSSVDLAIFSLHLAGISSILGAINFITTIINMRVNNLSFD
QMPLFVWAVGITALLLLLSLPVLAGAITMLLTDRNLNTSFFDPAGGGDPILYQHL
>LEFID299-10|HM873096|MM06176|Cosmopterix_orichalcea
TLYFIFGIWAGMVGTSLSLLIRAELGNPGSLIGDDQIYNTIVTAHAFIMIFFMVMPIMIGGFGNWLVPLMLGAPDMAFPRMNNMSFWLLPPSLTLLISSSIVETGAGTGWTVYPPLSSNIAHSGSSVDLAIFSLHLAGISSILGAINFITTIINMRINNLSFDQ
MPLFVWAVGITALLLLLSLPVLAGAITMLLTDRNLNTSFFDPAGGGDPILYQHL
>LEFIK632-10|KM572113|MM18207|Cosmopterix_orichalcea
TLYFIFGIWAGMVGTSLSLLIRAELGNPGSLIGDDQIYNTIVTAHAFIMIFFMVMPIMIGGFGNWLVPLMLGAPDMAFPRMNNMSFWLLPPSLTLLISSSIVETGAGTGWTVYPPLSSNIAHSGSSVDLAIFSLHLAGISSILGAINFITTIINMRINNLSFDQ
MPLFVWAVGITALLLLLSLPVLAGAITMLLTDRNLNTSFFDPAGGGDPILYQHL
>LEFIJ2453-14|MM23890|Cosmopterix_orichalcea
TLYFIFGIWAGMVGTSLSLLIRAELGNPGSLIGDDQIYNTIVTAHAFIMIFFMVMPIMIGGFGNWLVPLMLGAPDMAFPRMNNMSFWLLPPSLTLLISSSIVETGAGTGWTVYPPLSSNIAHSGSSVDLAIFSLHLAGISSILGAINFITTIINMRINNLSFDQ
MPLFVWAVGITALLLLLSLPVLAGAITMLLTDRNLNTSFFDPAGGGDPILYQHL
>LEFIK120-10|MM17695|Cosmopterix_scribaiella
TLYFIFGIWAGMVGTSLSLLIRAELGNPGSLIGDDQIYNTIVTAHAFIMIFFMVMPIMIGGFGNWLVPLMLGAPDMAFPRMNNMSFWLLPPSLTLLISSSIVETGAGTGWTVYPPLSSNIAHSGSSVDLAIFSLHLAGISSILGAINFITTIINMRVNNLSFD
QMPLFVWAVGITALLLLLSLPVLAGAITMLLTDRNLNTSFFDPAGGGDPILYQHL
>LEEUA161-11|MM19569|Cosmopterix_zieglerella
TLYFIFGIWAGMVGTSLSLLIRAELGNPGSLIGNDQIYNTIVTAHAFIMIFFMVMPIMIGGFGNWLVPLMLGAPDMAFPRMNNMSFWLLPPSLTLLISSSIVETGAGTGWTVYPPLSSNIAHSGSSVDLAIFSLHLAGISSILGAINFITTIINMRINNLSFDQ
MPLFVWAVGITALLLLLSLPVLAGAITMLLTDRNLNTSFFDPAGGGDPILYQHL
>LEFIA219-10|HM396562|MM01250|Cosmorhoe_ocellata
TLYFIFGIWAGMIGTSLSLLIRAELGNPGSLIGDDQIYNTIVTAHAFIMIFFMVMPIMIGGFGNWLVPLMLGAPDMAFPRMNNMSFWLLPPSITLLISSSIVENGAGTGWTVYPPLSSNIAHGGSSVDLAIFSLHLAGISSILGAINFITTIINMRLNNMFFD
QLPLFVWAVGITAFLLLLSLPVLAGAITMLLTDRNLNTSFFDPAGGGDPILYQHL
>LEFID927-10|HM873677|MM07392|Cosmotriche_lobulina
TLYFIFGIWAGMVGTSLSLLIRAELGTPGSLIGDDQIYNTIVTAHAFIMIFFMVMPIMIGGFGNWLVPLMLGAPDMAFPRMNNMSFWLLPPSLTLLISSSIVENGAGTGWTVYPPLSSNIAHGGSSVDLAIFSLHLAGISSILGAINFITTIINMRLNNMSFD
QMPLFVWAVGITAFLLLLSLPVLAGAITMLLTDRNLNTSFFDPAGGGDPILYQHL
>LEFIB066-10|HM870975|MM00354|Cossus_cossus
TLYFIFGIWSGMVGTSLSLLIRAELGNPGSLIGNDQIYNTIVTAHAFIMIFFMVMPIMIGGFGNWLVPLMLGAPDMAFPRMNNMSFWLLPPSLALLISSSIVENGAGTGWTVYPPLSSNIAHGGSSVDLAIFSLHLAGISSILGAINFITTIINMRPNNMSF
DQMPLFVWAVGITALLLLLSLPVLAGAITMLLTDRNLNTSFFDPAGGGDPILYQHL
>LEFID742-10|HM873499|MM06801|Cossus_cossus
TLYFIFGIWSGMVGTSLSLLIRAELGNPGSLIGNDQIYNTIVTAHAFIMIFFMVMPIMIGGFGNWLVPLMLGAPDMAFPRMNNMSFWLLPPSLALLISSSIVENGAGTGWTVYPPLSSNIAHGGSSVDLAIFSLHLAGISSILGAINFITTIINMRPNNMSF
DQMPLFVWAVGITALLLLLSLPVLAGAITMLLTDRNLNTSFFDPAGGGDPILYQHL
>LEFIC017-10|HM871887|MM03227|Crambus_alienellus
TLYFIFGIWAGMVGTSLSLLIRAELGNPGFLIGDDQIYNTIVTAHAFIMIFFMVMPIMIGGFGNWLVPLMLGAPDMAFPRMNNMSFWLLPPSLTLLISSSIVETGAGTGWTVYPPLSSNIAHSGSSVDLAIFSLHLAGISSILGAINFITTIINMRINGLSFDQ
MPLFVWSVGITALLLLLSLPVLAGAITMLLTDRNLNTSFFDPAGGGDPILYQHL
>LEFIC121-10|HM871968|MM03457|Crambus_ericella
TLYFIFGIWAGMVGTSLSLLIRAELGNPGFLIGDDQIYNTIVTAHAFIMIFFMVMPIMIGGFGNWLVPLMLGAPDMAFPRMNNMSFWLLPPSLTLLISSSIVENGAGTGWTVYPPLSSNIAHGGSSVDLAIFSLHLAGISSILGAINFITTIINMRINGLSFD
QMPLFVWSVGITALLLLLSLPVLAGAITMLLTDRNLNTSFFDPAGGGDPILYQHL
>LEFID437-10|HQ570333|MM06369|Crambus_ericella
TLYFIFGIWAGMVGTSLSLLIRAELGNPGFLIGDDQIYNTIVTAHAFIMIFFMVMPIMIGGFGNWLVPLMLGAPDMAFPRMNNMSFWLLPPSLTLLISSSIVENGAGTGWTVYPPLSSNIAHGGSSVDLAIFSLHLAGISSILGAINFITTIINMRINGLSFD
QMPLFVWSVGITALLLLLSLPVLAGAITMLLTDRNLNTSFFDPAGGGDPILYQHL

>LEFIE368-10|HM874092|MM08839|Crambus_hamella
TLYFIFGIWAGMVGTSLSLLIRAELGNPGFLIGDDQIYNTIVTAHAFIMIFFMVMPIMIGGFGNWLVPLMLGAPDMAFPRMNNMSFWLLPPSLTLLISSSIVENGAGTGWTVYPPLSSNIAHGGSSVDLTIFSLHLAGISSILGAINFITTIINMRINGLSFD
QMPLFVWSVGITALLLLLSLPVLAGAITMLLTDRNLNTSFFDPAGGGDPILYQHL
>LEFIF581-10|HM875265|MM12383|Crambus_hamella
TLYFIFGIWAGMVGTSLSLLIRAELGNPGFLIGDDQIYNTIVTAHAFIMIFFMVMPIMIGGFGNWLVPLMLGAPDMAFPRMNNMSFWLLPPSLTLLISSSIVENGAGTGWTVYPPLSSNIAHGGSSVDLTIFSLHLAGISSILGAINFITTIINMRINGLSFD
QMPLFVWSVGITALLLLLSLPVLAGAITMLLTDRNLNTSFFDPAGGGDPILYQHL
>LEFIF564-10|HM875249|MM12314|Crambus_heringiellus
TLYFIFGIWAGMVGTSLSLLIRAELGNPGFLIGDDQIYNTIVTAHAFIMIFFMVMPIMIGGFGNWLVPLMLGAPDMAFPRMNNMSFWLLPPSLTLLISSSIVENGAGTGWTVYPPLSSNIAHSGSSVDLAIFSLHLAGISSILGAINFITTIINMRINGLSFD
QMPLFVWSVGITALLLLLSLPVLAGAITMLLTDRNLNTSFFDPAGGGDPILYQHL
>LEFIB431-10|HM871330|MM01918|Crambus_lathoniellus
TLYFIFGIWAGMVGTSLSLLIRAELGNPGFLIGDDQIYNTIVTAHAFIMIFFMVMPIMIGGFGNWLVPLMLGAPDMAFPRMNNMSFWLLPPSLTLLISSSIVENGAGTGWTVYPPLSSNIAHSGSSVDLAIFSLHLAGISSILGAINFITTIINMRINSLSFDQ
MPLFVWSVGITALLLLLSLPVLAGAITMLLTDRNLNTSFFDPAGGGDPILYQHL
>LEFIF415-10|HM875100|MM11810|Crambus_lathoniellus
TLYFIFGIWAGMVGTSLSLLIRAELGNPGFLIGDDQIYNTIVTAHAFIMIFFMVMPIMIGGFGNWLVPLMLGAPDMAFPRMNNMSFWLLPPSLTLLISSSIVENGAGTGWTVYPPLSSNIAHSGSSVDLAIFSLHLAGISSILGAINFITTIINMRINSLSFDQ
MPLFVWSVGITALLLLLSLPVLAGAITMLLTDRNLNTSFFDPAGGGDPILYQHL
>LEFIC805-10|HM872624|MM04975|Crambus_pascuella
TLYFIFGIWAGMVGTSLSLLIRAELGNPGFLIGDDQIYNTIVTAHAFIMIFFMVMPIMIGGFGNWLVPLMLGAPDMAFPRMNNMSFWLLPPSLTLLISSSIVENGAGTGWTVYPPLSSNIAHSGSSVDLAIFSLHLAGISSILGAINFITTIINMRINGLSFD
QMPLFVWSVGITALLLLLSLPVLAGAITMLLTDRNLNTSFFDPAGGGDPILYQHL
>LEFIE903-10|HM874620|MM10229|Crambus_pascuella
TLYFIFGIWAGMVGTSLSLLIRAELGNPGFLIGDDQIYNTIVTAHAFIMIFFMVMPIMIGGFGNWLVPLMLGAPDMAFPRMNNMSFWLLPPSLTLLISSSIVENGAGTGWTVYPPLSSNIAHSGSSVDLAIFSLHLAGISSILGAINFITTIINMRINGLSFD
QMPLFVWSVGITALLLLLSLPVLAGAITMLLTDRNLNTSFFDPAGGGDPILYQHL
>LEFIC806-10|HM872625|MM04976|Crambus_perlella
TLYFIFGIWAGMIGTSLSLLIRAELGNPGFLIGDDQIYNTIVTAHAFIMIFFMVMPIMIGGFGNWLVPLMLGAPDMAFPRMNNMSFWLLPPSLTLLISSSIVENGAGTGWTVYPPLSSNIAHSGSSVDLAIFSLHLAGISSILGAINFITTIINMRINGLSFDQ
MPLFVWSVGITALLLLLSLPVLAGAITMLLTDRNLNTSFFDPAGGGDPILYQHL
>LEFID559-10|HM873324|MM06551|Crambus_perlella
TLYFIFGIWAGMIGTSLSLLIRAELGNPGFLIGDDQIYNTIVTAHAFIMIFFMVMPIMIGGFGNWLVPLMLGAPDMAFPRMNNMSFWLLPPSLTLLISSSIVENGAGTGWTVYPPLSSNIAHSGSSVDLAIFSLHLAGISSILGAINFITTIINMRINGLSFDQ
MPLFVWSVGITALLLLLSLPVLAGAITMLLTDRNLNTSFFDPAGGGDPILYQHL
>LEFIE100-10|HM873848|MM08284|Crambus_perlella
TLYFIFGIWAGMIGTSLSLLIRAELGNPGFLIGDDQIYNTIVTAHAFIMIFFMVMPIMIGGFGNWLVPLMLGAPDMAFPRMNNMSFWLLPPSLTLLISSSIVENGAGTGWTVYPPLSSNIAHSGSSVDLAIFSLHLAGISSILGAINFITTIINMRINGLSFDQ
MPLFVWSVGITALLLLLSLPVLAGAITMLLTDRNLNTSFFDPAGGGDPILYQHL
>LEFIC033-10|HM871903|MM03258|Crambus_pratella
TLYFIFGIWAGMVGTSLSLLIRAELGNPGFLIGDDQIYNTIVTAHAFIMIFFMVMPIMIGGFGNWLVPLMLGAPDMAFPRMNNMSFWLLPPSLNLLISSSIVENGAGTGWTVYPPLSSNIAHSGSSIDLAIFSLHLAGISSILGAINFITTIINMRINGLSFDQ
MPLFVWSVGITALLLLLSLPVLAGAITMLLTDRNLNTSFFDPAGGGDPILYQHL
>LEFIE028-10|HM873777|MM08095|Crambus_pratella
TLYFIFGIWAGMVGTSLSLLIRAELGNPGFLIGDDQIYNTIVTAHAFIMIFFMVMPIMIGGFGNWLVPLMLGAPDMAFPRMNNMSFWLLPPSLNLLISSSIVENGAGTGWTVYPPLSSNIAHSGSSIDLAIFSLHLAGISSILGAINFITTIINMRINGLSFDQ
MPLFVWSVGITALLLLLSLPVLAGAITMLLTDRNLNTSFFDPAGGGDPILYQHL
>LEFIF794-10|HM875478|MM13048|Crambus_silvella
TLYFIFGIWAGMIGTSLSLLIRAELGNPGFLIGDDQIYNTIVTAHAFIMIFFMVMPIMIGGFGNWLVPLMLGAPDMAFPRMNNMSFWLLPPSLTLLISSSIVENGVGTGWTVYPPLSSNIAHSGSSVDLAIFSLHLAGISSILGAINFITTIINMRINGLSFDQ
MPLFIWSVGITALLLLLSLPVLAGAITMLLTDRNLNTSFFDPAGGGDPILYQHL
>LEFIJ1228-11|KM572796|MM21088|Crambus_silvella
TLYFIFGIWAGMIGTSLSLLIRAELGNPGFLIGDDQIYNTIVTAHAFIMIFFMVMPIMIGGFGNWLVPLMLGAPDMAFPRMNNMSFWLLPPSLTLLISSSIVENGVGTGWTVYPPLSSNIAHSGSSVDLAIFSLHLAGISSILGAINFITTIINMRINGLSFDQ
MPLFIWSVGITALLLLLSLPVLAGAITMLLTDRNLNTSFFDPAGGGDPILYQHL
>LEFIC074-10|HM871943|MM03363|Crambus_uliginosellus
TLYFIFGIWAGMVGTSLSLLIRAELGNPGFLIGDDQIYNTIVTAHAFIMIFFMVMPIMIGGFGNWLVPLMLGAPDMAFPRMNNMSFWLLPPSLTLLISSSIVETGAGTGWTVYPPLSSNIAHSGSSVDLAIFSLHLAGISSILGAINFITTIINMRINGLSFDQ
MPLFIWSVGITAILLLLSLPVLAGAITMLLTDRNLNTSFFDPAGGGDPILYQHL
>LEFIB034-10|HM870943|MM00137|Craniophora_ligustri
TLYFIFGIWAGMMGTSLSLLIRAELGTPGSLIGDDQIYNTIVTAHAFIMIFFMVMPIMIGGFGNWLVPLMLGAPDMAFPRMNNMSFWLLPPSLTLLISSSIVENGAGTGWTVYPPLSSNIAHGGSSVDLAIFSLHLAGISSILGAINFITTIINMRLNNLSFD

QMPLFIWGVGITAFLLLLSLPVLAGAITMLLTDRNLNTSFFDPAGGGDPILYQHL
>LEFIC332-10|HM872176|MM03838|Crassa_tinctella
TLYFIFGIWAGMVGTSLSLLIRAELGNPGSLIGDDQIYNTIVTAHAFIMIFFMVMPIMIGGFGNWLVPLMLGAPDMAFPRMNNMSFWLLPPSLTLLISSSIVENGAGTGWTVYPPLSSNIAHGGSSVDLAIFSLHLAGISSILGAINFITTIINMRMNNMSF
DQMPLFVWAVGITALLLLLSLPVLAGAITMLLTDRNLNTSFFDPAGGGDPILYQHL
>LEFIF960-10|HM875641|MM13534|Crassa_tinctella
TLYFIFGIWAGMVGTSLSLLIRAELGNPGSLIGDDQIYNTIVTAHAFIMIFFMVMPIMIGGFGNWLVPLMLGAPDMAFPRMNNMSFWLLPPSLTLLISSSIVENGAGTGWTVYPPLSSNIAHGGSSVDLAIFSLHLAGISSILGAINFITTIINMRMNNMSF
DQMPLFVWAVGITALLLLLSLPVLAGAITMLLTDRNLNTSFFDPAGGGDPILYQHL
>LEFIA320-10|HM386663|MM01367|Crocallis_elinguaria
TLYFIFGIWAGMVGTSLSLLIRAELGNPGSLIGDDQIYNTIVTAHAFIMIFFMVMPIMIGGFGNWLVPLMLGAPDMAFPRMNNMSFWLLPPSITLLISSSVVENGAGTGWTVYPPLSSNIAHGGSSVDLAIFSLHLAGISSILGAINFITTIINMRLNNLSFD
QMPLFIWAVGITAFLLLLSLPVLAGAITMLLTDRNLNTSFFDPAGGGDPILYQHL
>LEFIJ1085-11|MM14801|Crombrugghia_distans
TMYFIFGIWAGMIGTSLSLLIRAELGNPGSLIGDDQIYNSIVTAHAFIMIFFMVMPIMIGGFGNWLVPLMLGAPDMAFPRMNNMSFWLLPPSILLLISSSIVENGVGTGWTVYPPLSSNIAHSGPSVDLAIFSLHLAGISSILGAINFISTIINMRLNKMMF
DQLPLFVWAVWITAVLLLLSLPVLAGAITMLLTDRNLNTSFFDPAGGGDPILYQHL
>LEFIJ1087-11|MM14803|Crombrugghia_distans
TMYFIFGIWAGMIGTSLSLLIRAELGNPGSLIGDDQIYNSIVTAHAFIMIFFMVMPIMIGGFGNWLVPLMLGAPDMAFPRMNNMSFWLLPPSILLLISSSIVENGVGTGWTVYPPLSSNIAHSGPSVDLAIFSLHLAGISSILGAINFISTIINMRLNKMMF
DQLPLFVWAVWITAVLLLLSLPVLAGAITMLLTDRNLNTSFFDPAGGGDPILYQHL
>LEFIB309-10|HM871210|MM00832|Crypsedra_gemmea
TLYFIFGIWAGMVGTSLSLLIRAELGTPGSLIGDDQIYNTIVTAHAFIMIFFMVMPIMIGGFGNWLVPLMLGAPDMAFPRMNNMSFWLLPPSLTLLISSSIVENGAGTGWTVYPPLSSNIAHGGSSVDLAIFSLHLAGISSILGAINFITTIINMRLNNMSFD
QMPLFIWAVGITAFLLLLSLPVLAGAITMLLTDRNLNTSFFDPAGGGDPILYQHL
>LEFIA625-10|HM870874|MM01740|Crypsedra_gemmea
TLYFIFGIWAGMVGTSLSLLIRAELGTPGSLIGDDQIYNTIVTAHAFIMIFFMVMPIMIGGFGNWLVPLMLGAPDMAFPRMNNMSFWLLPPSLTLLISSSIVENGAGTGWTVYPPLSSNIAHGGSSVDLAIFSLHLAGISSILGAINFITTIINMRLNNMSFD
QMPLFIWAVGITAFLLLLSLPVLAGAITMLLTDRNLN?SFFDPAGGGDPILYQHL
>LEFIF080-10|HM874787|MM10529|Cryptoblabes_bistriga
TLYFIFGIWAGMVGTSLSLLIRAELGTPGSLIGDDQIYNTIVTGHAFIMIFFMVMPIMIGGFGNWLVPLMLGAPDMAFPRMNNMSFWLLPPSLTLLISSSIVENGAGTGWTVYPPLSSNIAHGGSSVDLAIFSLHLAGISSILGAINFITTIINMKLNGLFFD
QMPLFIWAVGITALLLLLSLPVLAGAITMLLTDRNLNTSFFDPAGGGDPILYQHL
>LEFIG905-10|HM876549|MM15769|Cryptoblabes_bistriga
TLYFIFGIWAGMVGTSLSLLIRAELGTPGSLIGDDQIYNTIVTGHAFIMIFFMVMPIMIGGFGNWLVPLMLGAPDMAFPRMNNMSFWLLPPSLTLLISSSIVENGAGTGWTVYPPLSSNIAHGGSSVDLAIFSLHLAGISSILGAINFITTIINMKLNGLFFD
QMPLFIWAVGITALLLLLSLPVLAGAITMLLTDRNLNTSFFDPAGGGDPILYQHL
>LEFIC065-10|HM871934|MM03350|Cryptocala_chardinyi
TLYFIFGIWAGMVGTSLSLLIRAELGNPGSLIGDDQIYNTIVTAHAFIMIFFMVMPIMIGGFGNWLVPLMLGAPDMAFPRMNNMSFWLLPPSLTLLISSSIVENGAGTGWTVYPPLSSNIAHGGSSVDLAIFSLHLAGISSILGAINFITTIINMRLNSLSFD
QMPLFIWAVGITAFLLLLSLPVLAGAITMLLTDRNLNTSFFDPAGGGDPILYQHL
>LEFID009-10|HM872823|MM05431|Cucullia_absinthii
TLYFIFGIWAGMVGTSLSLLIRAELGTPGSLIGDDQIYNTIVTAHAFIMIFFMVMPIMIGGFGNWLIPLMLGAPDMAFPRMNNMSFWLLPPSLTLLISSSIVENGAGTGWTVYPPLSSNIAHGGSSVDLAIFSLHLAGISSILGAINFITTIINMRLNNLSFDQ
MPLFIWAVGITAFLLLLSLPVLAGAITMLLTDRNLNTSFFDPAGGGDPILYQHL
>LEFIF289-10|HM874979|MM11071|Cucullia_argentea
TLYFIFGIWAGMVGTSLSLLIRAELGTPGSLIGDDQIYNTIVTAHAFIMIFFMVMPIMIGGFGNWLVPLMLGAPDMAFPRMNNMSFWLLPPSLTLLISSSIVENGAGTGWTVYPPLSSNIAHGGSSVDLAIFSLHLAGISSILGAINFITTIINMRLNNLSFD
QMPLFIWAVGITAFLLLLSLPVLAGAITMLLTDRNLNTSFFDPAGGGDPILYQHL
>LEFIE456-10|HM874180|MM09220|Cucullia_asteris
TLYFIFGIWAGMVGTSLSLLIRAELGTPGSLIGDDQIYNTIVTAHAFIMIFFMVMPIMIGGFGNWLVPLMLGAPDMAFPRMNNMSFWLLPPSLTLLISSSIVENGAGTGWTVYPPLSSNIAHGGSSVDLAIFSLHLAGISSILGAINFITTIINMRLNNLSFD
QMPLFIWAVGITAFLLLLSLPVLAGAITMLLTDRNLNTSFFDPAGGGDPILYQHL
>LEFIJ750-10|JF853801|MM17375|Cucullia_chamomillae
TLYFIFGIWAGMVGTSLSLLIRAELGTPGSLIGDDQIYNTIVTAHAFIMIFFMVMPIMIGGFGNWLVPLMLGAPDMAFPRMNNMSFWLLPPSLTLLISSSIVENGAGTGWTVYPPLSSNIAHSGSSVDLAIFSLHLAGISSILGAINFITTIINMRLNSLSFDQ
MPLFIWAVGITAFLLLLSLPVLAGAITMLLTDRNLNTSFFDPAGGGDPILYQHL
>LEFID227-10|HM873028|MM06085|Cucullia_fraudatrix
TLYFIFGIWAGMVGTSLSLLIRAELGTPGSLIGDDQIYNTIVTAHAFIMIFFMVMPIMIGGFGNWLVPLMLGAPDMAFPRMNNMSFWLLPPSLTLLISSSIVENGAGTGWTVYPPLSSNIAHGGSSVDLAIFSLHLAGISSILGAINFITTIINMRLNNLSFD
QMPLFIWAVGITAFLLLLSLPVLAGAITMLLTDRNLNTSFFDPAGGGDPILYQHL
>LEFIJ343-10|MM15943|Cucullia_fraudatrix

TLYFIFGIWAGMVGTSLSLLIRAELGTPGSLIGDDQIYNTIVTAHAFIMIFFMVMPIMIGGFGNWLIPLMLGAPDMAFPRMNNMSFWLLPPSLTLLISSSIVENGAGTGWTVYPPLSSNIAHGGSSVDLAIFSLHLAGISSILGAINFITTIINMRLNNLSFDQ
MPLFIWAVGITAFLLLLSLPVLAGAITMLLTDRNLNTSFFDPAGGGDPILYQHL
>LEFIJ751-10|JF853802|MM17376|Cucullia_gnaphalii
TLYFIFGIWAGMVGTSLSLLIRAELGTPGSLIGDDQIYNTIVTAHAFIMIFFMVMPIMIGGFGNWLVPLMLGAPDMAFPRMNNMSFWLLPPSLTLLISSSIVENGAGTGWTVYPPLSSNIAHGGSSVDLAIFSLHLAGISSILGAINFITTIINMRLNNLSFD
QMPLFIWAVGITAFLLLLSLPVLAGAITMLLTDRNLNTSFFDPAGGGDPILYQHL
>LEFIJ863-10|JF853866|MM17488|Cucullia_gnaphalii
TLYFIFGIWAGMVGTSLSLLIRAELGTPGSLIGDDQIYNTIVTAHAFIMIFFMVMPIMIGGFGNWLVPLMLGAPDMAFPRMNNMSFWLLPPSLTLLISSSIVENGAGTGWTVYPPLSSNIAHGGSSVDLAIFSLHLAGISSILGAINFITTIINMRLNNLSFD
QMPLFIWAVGITAFLLLLSLPVLAGAITMLLTDRNLNTSFFDPAGGGDPILYQHL
>LEFIC880-10|HM872698|MM05158|Cucullia_lactucae
TLYFIFGIWAGMVGTSLSLLIRAELGTPGSLIGDDQIYNTIVTAHAFIMIFFMVMPIMIGGFGNWLIPLMLGAPDMAFPRMNNMSFWLLPPSLTLLISSSIVENGAGTGWTVYPPLSSNIAHSGSSVDLAIFSLHLAGISSILGAINFITTIINMRLNNLSFDQ
MPLFIWAVGITAFLLLLSLPVLAGAITMLLTDRNLNTSFFDPAGGGDPILYQHL
>LEFIJ861-10|JF853864|MM17486|Cucullia_lucifuga
TLYFIFGIWAGMVGTSLSLLIRAELGTPGSLIGDDQIYNTIVTAHAFIMIFFMVMPIMIGGFGNWLIPLMLGAPDMAFPRMNNMSFWLLPPSITLLISSSIVENGAGTGWTVYPPLSSNIAHSGSSVDLAIFSLHLAGISSILGAINFITTIINMRLNNLSFDQ
MPLFIWAVGITAFLLLLSLPVLAGAITMLLTDRNLNTSFFDPAGGGDPILYQHL
>LEEUA317-11|JN266513|MM19725|Cucullia_praecana
TLYFIFGIWAGMVGTSLSLLIRAELGTPGSLIGDDQIYNTIVTAHAFIMIFFMVMPIMIGGFGNWLVPLMLGAPDMAFPRMNNMSFWLLPPSLTLLISSSIVENGAGTGWTVYPPLSSNIAHGGSSVDLAIFSLHLAGISSILGAINFITTIINMRLNNLSFD
QMPLFIWAVGITAFLLLLSLPVLAGAITMLLTDRNLNTSFFDPAGGGDPILYQHL
>LEFIC782-10|HM872601|MM04914|Cucullia_umbratica
TLYFIFGIWAGMVGTSLSLLIRAELGTPGSLIGDDQIYNTIVTAHAFIMIFFMVMPIMIGGFGNWLVPLMLGAPDMAFPRMNNMSFWLLPPSLTLLISSSIVENGAGTGWTVYPPLSSNIAHGGSSVDLAIFSLHLAGISSILGAINFITTIINMRLNNLSFD
QMPLFIWAVGITAFLLLISLPVLAGAITMLLTDRNLNTSFFDPAGGGDPILYQHL
>LEFID693-10|HM873454|MM06742|Cucullia_umbratica
TLYFIFGIWAGMVGTSLSLLIRAELGTPGSLIGDDQIYNTIVTAHAFIMIFFMVMPIMIGGFGNWLVPLMLGAPDMAFPRMNNMSFWLLPPSLTLLISSSIVENGAGTGWTVYPPLSSNIAHGGSSVDLAIFSLHLAGISSILGAINFITTIINMRLNNLSFD
QMPLFIWAVGITAFLLLISLPVLAGAITMLLTDRNLNTSFFDPAGGGDPILYQHL
>LEFIF125-10|HM874826|MM10581|Cupido_argiades
TLYFIFGIWAGMLGTSLSILIRMELGTPGSLIGDDQIYNTIVTAHAFIMIFFMVMPIMIGGFGNWLVPLMLGAPDMAFPRMNNMSFWLLPPSLMLLISSSIVENGAGTGWTVYPPLSSNIAHSGSSVDLAIFSLHLAGISSILGAINFITTIINMRVNNLSFD
QMSLFIWAVGITALLLLLSLPVLAGAITMLLTDRNLNTSFFDPAGGGDPILYQHL
>LEFIF123-10|HM874824|MM10579|Cupido_minimus
TLYFIFGIWAGMLGTSLSILIRMELGTPGSLIGDDQIYNTIVTAHAFIMIFFMVMPIMIGGFGNWLVPLMLGAPDMAFPRMNNMSFWLLPPSLMLLISSSIVENGAGTGWTVYPPLSSNIAHGGSSVDLAIFSLHLAGISSILGAINFITTIINMRVNNLSF
DQMSLFIWAVGITALLLLLSLPVLAGAITMLLTDRNLNTSFFDPAGGGDPILYQHL
>LEFIL356-10|JN276922|MM18666|Cupido_minimus
TLYFIFGIWAGMLGTSLSILIRMELGTPGSLIGDDQIYNTIVTAHAFIMIFFMVMPIMIGGFGNWLVPLMLGAPDMAFPRMNNMSFWLLPPSLMLLISSSIVENGAGTGWTVYPPLSSNIAHGGSSVDLAIFSLHLAGISSILGAINFITTIINMRVNNLSF
DQMSLFIWAVGITALLLLLSLPVLAGAITMLLTDRNLNTSFFDPAGGGDPILYQHL
>LEFIJ518-10|JF853640|MM17143|Cyaniris_semiargus
TLYFIFGIWAGMVGTSLSILIRMELSTPGSLIGDDQIYNTIVTAHAFIMIFFMVMPIMIGGFGNWLVPLMLGAPDMAFPRMNNMSFWLLPPSLMLLISSSIVENGAGTGWTVYPPLSSNIAHGGSSVDLAIFSLHLAGISSILGAINFITTIINMRVNNLSF
DQMSLFIWAVGITALLLLLSLPVLAGAITMLLTDRNLNTSFFDPAGGGDPILYQHL
>LEFIA106-10|HM396453|MM01029|Cybosia_mesomella
TLYFIFGVWAGMVGTSLSLLIRAELGNPGSLIGDDQIYNTIVTAHAFIMIFFMVMPIMIGGFGNWLVPLMLGAPDMAFPRMNNMSFWLLPPSLTLLISSSIVENGAGTGWTVYPPLSSNIAHSGSSVDLAIFSLHLAGISSILGAINFITTIINMRLNKMMF
DQMPLFVWAVGITAFLLLLSLPVLAGAITMLLTDRNLNTSFFDPAGGGDPILYQHL
>LEFIA107-10|HM396454|MM01030|Cybosia_mesomella
TLYFIFGVWAGMVGTSLSLLIRAELGNPGSLIGDDQIYNTIVTAHAFIMIFFMVMPIMIGGFGNWLVPLMLGAPDMAFPRMNNMSFWLLPPSLTLLISSSIVENGAGTGWTVYPPLSSNIAHSGSSVDLAIFSLHLAGISSILGAINFITTIINMRLNKMMF
DQMPLFVWAVGITAFLLLLSLPVLAGAITMLLTDRNLNTSFFDPAGGGDPILYQHL
>LEFIA274-10|HM386618|MM01314|Cyclophora_albipunctata
TLYFIFGIWAGMIGTSLSLLIRAELGNPGSLIGDDQIYNTIVTAHAFIMIFFMVMPIMIGGFGNWLVPLMLGAPDMAFPRMNNMSFWLLPPSITLLISSSIVESGAGTGWTVYPPLSSNIAHSGSSVDLAIFSLHLAGISSILGAINFITTIINMRLNNMSFDQ
LPLFVWAVGITAFLLLLSLPVLAGAITMLLTDRNLNTSFFDPSGGGDPILYQHL
>LEFIE149-10|HM873897|MM08392|Cyclophora_albipunctata
TLYFIFGIWAGMIGTSLSLLIRAELGNPGSLIGDDQIYNTIVTAHAFIMIFFMVMPIMIGGFGNWLVPLMLGAPDMAFPRMNNMSFWLLPPSITLLISSSIVESGAGTGWTVYPPLSSNIAHSGSSVDLAIFSLHLAGISSILGAINFITTIINMRLNNMSFDQ
LPLFVWAVGITAFLLLLSLPVLAGAITMLLTDRNLNTSFFDPSGGGDPILYQHL

>LEFID055-10|HM872869|MM05654|Cyclophora_annularia
TLYFIFGIWAGMIGTSLSLLIRAELGNPGSLIGDDQIYNTIVTAHAFIMIFFMVMPIMIGGFGNWLVPLMLGAPDMAFPRMNNMSFWLLPPSITLLISSSIVESGAGTGWTVYPPLSSNIAHSGSSVDLAIFSLHLAGISSILGAINFITTIINMRLNNMSFDQ
LPLFVWAVGITAFLLLLSLPVLAGAITMLLTDRNLNTSFFDPSGGGDPILYQHL
>LEFIJ318-10|KF807317|MM15918|Cyclophora_linearia
TLYFIFGIWAGMIGTSLSLLIRAELGNPGSLIGDDQIYNTIVTAHAFIMIFFMVMPIMIGGFGNWLVPLMLGAPDMAFPRMNNMSFWLLPPSITLLISSSIVESGAGTGWTVYPPLSSNIAHSGSSVDLAIFSLHLAGISSILGAINFITTIINMRLNNMSFDQ
LPLFIWAVGITAFLLLLSLPVLAGAITMLLTDRNLNTSFFDPSGGGDPILYQHL
>LEFIA354-10|HM386697|MM01402|Cyclophora_pendularia
TLYFIFGIWAGMIGTSLSLLIRAELGNPGSLIGDDQIYNTIVTAHAFIMIFFMVMPIMIGGFGNWLVPLMLGAPDMAFPRMNNMSFWLLPPSITLLISSSIVESGAGTGWTVYPPLSSNIAHSGSSVDLAIFSLHLAGISSILGAINFITTIINMRLNNMSFDQ
LPLFVWAVGITAFLLLLSLPVLAGAITMLLTDRNLNTSFFDPSGGGDPILYQHL
>LEFIC217-10|HM872061|MM03609|Cyclophora_punctaria
TLYFIFGIWAGMIGTSLSLLIRAELGNPGFLIGDDQIYNTIVTAHAFIMIFFMVMPIMIGGFGNWLVPLMLGAPDMAFPRMNNMSFWLLPPSITLLISSSIVESGAGTGWTVYPPLSSNIAHSGSSVDLAIFSLHLAGISSILGAINFITTIINMRLNNMSFDQ
LPLFVWAVGITAFLLLLSLPVLAGAITMLLTDRNLNTSFFDPSGGGDPILYQHL
>LEFIC694-10|HM872515|MM04677|Cyclophora_punctaria
TLYFIFGIWAGMIGTSLSLLIRAELGNPGFLIGDDQIYNTIVTAHAFIMIFFMVMPIMIGGFGNWLVPLMLGAPDMAFPRMNNMSFWLLPPSITLLISSSIVESGAGTGWTVYPPLSSNIAHSGSSVDLAIFSLHLAGISSILGAINFITTIINMRLNNMSFDQ
LPLFVWAVGITAFLLLLSLPVLAGAITMLLTDRNLNTSFFDPSGGGDPILYQHL
>LEFIK287-10|KF807301|MM17862|Cyclophora_quercimontaria
TLYFIFGIWAGMIGTSLSLLIRAELGNPGFLIGDDQIYNTIVTAHAFIMIFFMVMPIMIGGFGNWLVPLMLGAPDMAFPRMNNMSFWLLPPSITLLISSSIVESGAGTGWTVYPPLSSNIAHSGSSVDLAIFSLHLAGISSILGAINFITTIINMRLNNMSFDQ
LPLFVWAVGITAFLLLLSLPVLAGAI?MLLTDRNLNTSFFDPSGGGDPILYQHL
>LEFIE102-10|HM873850|MM08286|Cydia_cognatana
TLYFIFGIWSGMVGTSLSLLIRAELGNPSSLIGDDQIYNTIVTAHAFIMIFFMVMPIMIGGFGNWLVPLMLGAPDMAFPRMNNMSFWLLPPSLTLLISSSIVENGAGTGWTVYPPLSSNIAHSGSSVDLAIFSLHLAGISSILGAVNFITTIINMRPNNMSL
DQMPLFVWAVGITALLLLLSLPVLAGAITMLLTDRNLNTSFFDPAGGGDPILYQHL
>LEFIG871-10|HM876516|MM15735|Cydia_cognatana
TLYFIFGIWSGMVGTSLSLLIRAELGNPSSLIGDDQIYNTIVTAHAFIMIFFMVMPIMIGGFGNWLVPLMLGAPDMAFPRMNNMSFWLLPPSLTLLISSSIVENGAGTGWTVYPPLSSNIAHSGSSVDLAIFSLHLAGISSILGAVNFITTIINMRPNNMSL
DQMPLFVWAVGITALLLLLSLPVLAGAITMLLTDRNLNTSFFDPAGGGDPILYQHL
>LEFID184-10|HM872990|MM05973|Cydia_coniferana
TLYFIFGVWAGMVGTSLSLLIRAELGNPGSLIGDDQIYNTIVTAHAFIMIFFMVMPIMIGGFGNWLVPLMLGAPDMAFPRMNNMSFWLLPPSIMLLISSSIVENGAGTGWTVYPPLSSNIAHSGSSVDLAIFSLHLAGISSILGAVNFITTIINMRPNNMS
LDQMPLFVWAVGITALLLLLSLPVLAGAITMLLTDRNLNTSFFDPAGGGDPILYQHL
>LEFID443-10|HQ570339|MM06376|Cydia_coniferana
TLYFIFGVWAGMVGTSLSLLIRAELGNPGSLIGDDQIYNTIVTAHAFIMIFFMVMPIMIGGFGNWLVPLMLGAPDMAFPRMNNMSFWLLPPSIMLLISSSIVENGAGTGWTVYPPLSSNIAHSGSSVDLAIFSLHLAGISSILGAVNFITTIINMRPNNMS
LDQMPLFVWAVGITALLLLLSLPVLAGAITMLLTDRNLNTSFFDPAGGGDPILYQHL
>LEFIF524-10|HM875209|MM12162|Cydia_coniferana
TLYFIFGVWAGMVGTSLSLLIRAELGNPGSLIGDDQIYNTIVTAHAFIMIFFMVMPIMIGGFGNWLVPLMLGAPDMAFPRMNNMSFWLLPPSIMLLISSSIVENGAGTGWTVYPPLSSNIAHSGSSVDLAIFSLHLAGISSILGAVNFITTIINMRPNNMS
LDQMPLFVWAVGITALLLLLSLPVLAGAITMLLTDRNLNTSFFDPAGGGDPILYQHL
>LEFIA922-10|HM387058|MM13838|Cydia_coniferana
TLYFIFGVWAGMVGTSLSLLIRAELGNPGSLIGDDQIYNTIVTAHAFIMIFFMVMPIMIGGFGNWLVPLMLGAPDMAFPRMNNMSFWLLPPSIMLLISSSIVENGAGTGWTVYPPLSSNIAHSGSSVDLAIFSLHLAGISSILGAVNFITTIINMRPNNMS
LDQMPLFVWAVGITALLLLLSLPVLAGAITMLLTDRNLNTSFFDPAGGGDPILYQHL
>LEFIC474-10|HM872307|MM04066|Cydia_cornucopiae
TLYFIFGIWAGMVGTSLSLLIRAELGNPGSLIGDDQIYNTIVTAHAFIMIFFMVMPIMIGGFGNWLVPLMLGAPDMAFPRMNNMSFWLLPPSIMLLISSSIVETGAGTGWTVYPPLSSNIAHSGSSVDLAIFSLHLAGISSILGAVNFITTIINMRPNNMSL
DQMPLFVWAVGITALLLLLSLPVLAGAITMLLTDRNLNTSFFDPAGGGDPILYQHL
>LEFIF257-10|HM874950|MM11039|Cydia_corollana
TLYFIFGIWAGMVGTSLSMLIRAELGNPGSLIGDDQIYNTIVTAHAFIMIFFMVMPIMIGGFGNWLVPLMLGAPDMAFPRMNNMSFWLLPPSIMLLISSSIVENGAGTGWTVYPPLSSNIAHSGSSVDLAIFSLHLAGISSILGAVNFITTIINMRPNNMS
LDQMPLFVWSVKITALLLLLSLPVLAGAITMLLTDRNLNTSFFDPAGGGDPILYQHL
>LEFIG876-10|HM876521|MM15740|Cydia_corollana
TLYFIFGIWAGMVGTSLSMLIRAELGNPGSLIGDDQIYNTIVTAHAFIMIFFMVMPIMIGGFGNWLVPLMLGAPDMAFPRMNNMSFWLLPPSIMLLISSSIVENGAGTGWTVYPPLSSNIAHSGSSVDLAIFSLHLAGISSILGAVNFITTIINMRPNNMS
LDQMPLFVWSVKITALLLLLSLPVLAGAITMLLTDRNLNTSFFDPAGGGDPILYQHL
>LEFIE259-10|HM873993|MM08594|Cydia_cosmophorana
TLYFIFGIWAGMIGTSLSLIIRAELGNPGSLIGDDQIYNTIVTAHAFIMIFFMVMPIMIGGFGNWLVPLMLGAPDMAFPRMNNMSFWLLPPSILLLISSSIVENGAGTGWTVYPPLSSNIAHSGSSVDLAIFSLHLAGISSILGAVNFITTIINMRPNNMSLD

QMPLFVWAVGITALLLLLSLPVLAGAITMLLTDRNLNTSFFDPAGGGDPILYQHL
>LEFIA921-10|HM387057|MM13837|Cydia_cosmophorana
TLYFIFGIWAGMIGTSLSLIIRAELGNPGSLIGDDQIYNTIVTAHAFIMIFFMVMPIMIGGFGNWLVPLMLGAPDMAFPRMNNMSFWLLPPSILLLISSSIVENGAGTGWTVYPPLSSNIAHSGSSVDLAIFSLHLAGISSILGAVNFITTIINMRPNNMSLD
QMPLFVWAVGITALLLLLSLPVLAGAITMLLTDRNLNTSFFDPAGGGDPILYQHL
>LEFIB916-10|HM871793|MM03040|Cydia_duplicana
TLYFIFGIWAGMMGTSLSLLIRAELGNPGSLINDDQIYNTIVTAHAFIMIFFMVMPIMIGGFGNWLVPLMLGAPDMAFPRMNNMSFWLLPPSIMLLISSSIVENGAGTGWTVYPPLSSNIAHSGSSVDLAIFSLHLAGISSILGAVNFITTIINMRPHNMS
LDQMPLFIWAVGITALLLLLSLPVLAGAITMLLTDRNLNTSFFDPAGGGDPILYQHL
>LEEUA250-11|JN274944|MM19658|Cydia_fagiglandana
TLYFIFGIWAGMVGTSLSLLIRAELGNPGSLIGDDQIYNTIVTAHAFIMIFFMVMPIMIGGFGNWLVPLMLGAPDMAFPRMNNMSFWLLPPSIMLLISSSIVENGAGTGWTVYPPLSSNIAHSGSSVDLAIFSLHLAGISSILGAVNFITTIINMRPNNMSL
DQMPLFVWAVGITALLLLLSLPVLAGAITMLLTDRNLNTSFFDPAGGGDPILYQHL
>LEEUA249-11|JN274943|MM19657|Cydia_grunertiana
TLYFIFGIWAGMVGTSLSLLIRAELGNPGSLIGDDQIYNTIVTAHAFIMIFFMVMPIMIGGFGNWLVPLMLGAPDMAFPRMNNMSFWLLPPSIMLLISSSVVENGAGTGWTVYPPLSSNIAHSGSSVDLAIFSLHLAGISSILGAINFITTIINMRPNNMSL
DQMPLFVWAVGITALLLLLSLPVLAGAITMLLTDRNLNTSFFDPAGGGDPILYQHL
>LEFIB380-10|HM871279|MM00964|Cydia_illutana
TLYFIFGIWAGMVGTSLSLLIRAELGNPGSLIGDDQIYNTIVTAHAFIMIFFMVMPIMIGGFGNWLVPLMLGAPDMAFPRMNNMSFWLLPPSIMLLISSSIVENGAGTGWTVYPPLSSNIAHSGSSVDLAIFSLHLAGISSILGAVNFITTIINMRPNNMSL
DQMPLFVWAVGITALLLLLSLPVLAGAITMLLTDRNLNTSFFDPAGGGDPILYQHL
>LEFIG874-10|HM876519|MM15738|Cydia_indivisa
TLYFIFGIWAGMLGTSLSLLIRAELGTPGSLIGDDQIYNTIVTAHAFIMIFFMVMPIMIGGFGNWLVPLMLGAPDMAFPRMNNMSFWLLPPSIMLLISSSVVENGAGTGWTVYPPLSSNIAHSGSSVDLAIFSLHLAGISSILGAVNFITTIINMRPNNMSL
DQMPLFVWAVGITALLLLLSLPVLAGAITMLLTDRNLNTSFFDPAGGGDPILYQHL
>LEFIC336-10|HM872180|MM03843|Cydia_inquinatana
TLYFIFGIWAGMVGTSLSLLIRAELGNPGSLIGDDQIYNTIVTAHAFIMIFFMVMPIMIGGFGNWLVPLMLGAPDMAFPRMNNMSFWLLPPSIMLLISSSIVENGAGTGWTVYPPLSSNIAHSGSSVDLAIFSLHLAGISSILGAVNFITTIINMRPNNMSL
DQMPLFVWSVGITALLLLLSLPVLAGAITMLLTDRNLNTSFFDPAGGGDPILYQHL
>LEFIC826-10|HM872645|MM05013|Cydia_inquinatana
TLYFIFGIWAGMVGTSLSLLIRAELGNPGSLIGDDQIYNTIVTAHAFIMIFFMVMPIMIGGFGNWLVPLMLGAPDMAFPRMNNMSFWLLPPSIMLLISSSIVENGAGTGWTVYPPLSSNIAHSGSSVDLAIFSLHLAGISSILGAVNFITTIINMRPNNMSL
DQMPLFVWSVGITALLLLLSLPVL?GAITMLLTDRNLNTSFFDPAGGGDPILYQHL
>LEFIJ1010-11|MM04309|Cydia_leguminana
TLYFIFGIWAGMVGTSLSLLIRAELGNPGSLIGDDQIYNTIVTAHAFIMIFFMVMPIMIGGFGNWLVPLMLGAPDMAFPRMNNMSFWLLPPSIMLLISSSIVENGAGTGWTVYPPLSSNIAHSGSSVDLAIFSLHLAGISSILGAVNFITTIINMRPNNMSL
DQMPLFVWAVGITALLLLLSLPVLAGAITMLLTDRNLNTSFFDPAGGGDPILYQHL
>LEFIE301-10|HM874027|MM08655|Cydia_medicaginis
TLYFIFGIWAGMIGTSLSLLIRAELGNPGSLIGDDQIYNTIVTAHAFIMIFFMVMPIMIGGFGNWLVPLMLGAPDMAFPRMNNMSFWLLPPSIMLLISSSIVENGAGTGWTVYPPLSSNIAHSGSSVDLAIFSLHLAGISSILGAVNFITTIINMRPNNMSL
DQMPLFVWAVGITALLLLLSLPVLAGAITMLLTDRNLNTSFFDPAGGGDPILYQHL
>LEFIG870-10|HM876515|MM15734|Cydia_medicaginis
TLYFIFGIWAGMIGTSLSLLIRAELGNPGSLIGDDQIYNTIVTAHAFIMIFFMVMPIMIGGFGNWLVPLMLGAPDMAFPRMNNMSFWLLPPSIMLLISSSIVENGAGTGWTVYPPLSSNIAHSGSSVDLAIFSLHLAGISSILGAVNFITTIINMRPNNMSL
DQMPLFVWAVGITALLLLLSLPVLAGAITMLLTDRNLNTSFFDPAGGGDPILYQHL
>LEFIB384-10|HM871283|MM00968|Cydia_nigricana
TLYFIFGIWAGMIGTSLSLLIRAELGNPGSLIGDDQIYNTIVTAHAFIMIFFMVMPIMIGGFGNWLVPLMLGAPDMAFPRMNNMSFWLLPPSIMLLISSSIVENGAGTGWTVYPPLSSNIAHSGSSVDLAIFSLHLAGISSILGAVNFITTIINMRPNNMSL
DQMPLFVWAVGITALLLLLSLPVLAGAITMLLTDRNLNTSFFDPAGGGDPILYQHL
>LEFIE552-10|HM874275|MM09437|Cydia_nigricana
TLYFIFGIWAGMIGTSLSLLIRAELGNPGSLIGDDQIYNTIVTAHAFIMIFFMVMPIMIGGFGNWLVPLMLGAPDMAFPRMNNMSFWLLPPSIMLLISSSIVENGAGTGWTVYPPLSSNIAHSGSSVDLAIFSLHLAGISSILGAVNFITTIINMRPNNMSL
DQMPLFVWAVGITALLLLLSLPVLAGAITMLLTDRNLNTSFFDPAGGGDPILYQHL
>LEFIK327-10|JF853973|MM17902|Cydia_nigricana
TLYFIFGIWAGMIGTSLSLLIRAELGNPGSLIGDDQIYNTIVTAHAFIMIFFMVMPIMIGGFGNWLVPLMLGAPDMAFPRMNNMSFWLLPPSIMLLISSSIVENGAGTGWTVYPPLSSNIAHSGSSVDLAIFSLHLAGISSILGAVNFITTIINMRPNNMSL
DQMPLFVWAVGITALLLLLSLPVLAGAITMLLTDRNLNTSFFDPAGGGDPILYQHL
>LEFIK328-10|JF853974|MM17903|Cydia_nigricana
TLYFIFGIWAGMIGTSLSLLIRAELGNPGSLIGDDQIYNTIVTAHAFIMIFFMVMPIMIGGFGNWLVPLMLGAPDMAFPRMNNMSFWLLPPSIMLLISSSIVENGAGTGWTVYPPLSSNIAHSGSSVDLAIFSLHLAGISSILGAVNFITTIINMRPNNMSL
DQMPLFVWAVGITALLLLLSLPVLAGAITMLLTDRNLNTSFFDPAGG?DPILYQHL
>LEFIE797-10|HM874516|MM09983|Cydia_pactolana

TLYFIFGIWAGMVGTSLSLLIRAELGNPGSLIGDDQIYNTIVTAHAFIMIFFMVMPIMIGGFGNWLVPLMLGAPDMAFPRMNNMSFWLLPPSIMLLISSSVVENGAGTGWTVYPPLSSNIAHSGSSVDLAIFSLHLAGISSILGAINFITTIINMRPNNMSL
DQMPLFVWAVGITALLLLLSLPVLAGAITMLLTDRNLNTSFFDPAGGGDPILYQHL
>LEFIC136-10|HM871982|MM03480|Cydia_pomonella
TLYFIFGIWAGMVGTSLSLLIRAELGNPGSLIGDDQIYNTIVTAHAFIMIFFMVMPIMIGGFGNWLVPLMLGAPDMAFPRMNNMSFWLLPPSIMLLISSSIVENGAGTGWTVYPPLSSNIAHSGSSVDLAIFSLHLAGISSILGAVNFITTIINMRPNNMSL
DQMPLFVWAVGITALLLLLSLPVLAGAITMLLTDRNLNTSFFDPAGGGDPILYQHL
>LEFIE564-10|HM874287|MM09453|Cydia_pomonella
TLYFIFGIWAGMVGTSLSLLIRAELGNPGSLIGDDQIYNTIVTAHAFIMIFFMVMPIMIGGFGNWLVPLMLGAPDMAFPRMNNMSFWLLPPSIMLLISSSIVENGAGTGWTVYPPLSSNIAHSGSSVDLAIFSLHLAGISSILGAVNFITTIINMRPNNMSL
DQMPLFVWAVGITALLLLLSLPVLAGAITMLLTDRNLNTSFFDPAGGGDPILYQHL
>LEFIL636-10|MM18934|Cydia_servillana
TLYFIFGIWAGMVGTSLSLLIRAELGNPGSLIGDDQIYNTIVTAHAFIMIFFMVMPIMIGGFGNWLIPLMLGAPDMAFPRMNNMSFWLLPPSIMLLISSSIVENGAGTGWTVYPPLSSNIAHSGSSVDLAIFSLHLAGISSILGAINFITTIINMRPNNMSLD
QMPLFVWAVGITALLLLLSLPVLAGAITMLLTDRNLNTSFFDPAGGGDPILYQHL
>LEFIL637-10|MM18935|Cydia_servillana
TLYFIFGIWAGMVGTSLSLLIRAELGNPGSLIGDDQIYNTIVTAHAFIMIFFMVMPIMIGGFGNWLIPLMLGAPDMAFPRMNNMSFWLLPPSIMLLISSSIVENGAGTGWTVYPPLSSNIAHSGSSVDLAIFSLHLAGISSILGAINFITTIINMRPNNMSLD
QMPLFVWAVGITALLLLLSLPVLAGAITMLLTDRNLNTSFFDPAGGGDPILYQHL
>LEFIC122-10|HM871969|MM03458|Cydia_splendana
TLYFIFGIWAGMVGTSLSLLIRAELGNPGSLIGDDQIYNTIVTAHAFIMIFFMVMPIMIGGFGNWLVPLMLGAPDMAFPRMNNMSFWLLPPSIMLLISSSIVENGAGTGWTVYPPLSSNIAHSGSSVDLAIFSLHLAGISSILGAVNFITTIINMRPNNMSL
DQMPLFVWAVGITALLLLLSLPVLAGAITMLLTDRNLNTSFFDPAGGGDPILYQHL
>LEFIC203-10|HM872047|MM03565|Cydia_splendana
TLYFIFGIWAGMVGTSLSLLIRAELGNPGSLIGDDQIYNTIVTAHAFIMIFFMVMPIMIGGFGNWLVPLMLGAPDMAFPRMNNMSFWLLPPSIMLLISSSIVENGAGTGWTVYPPLSSNIAHSGSSVDLAIFSLHLAGISSILGAVNFITTIINMRPNNMSL
DQMPLFVWAVGITALLLLLSLPVLAGAITMLLTDRNLNTSFFDPAGGGDPILYQHL
>LEFIE286-10|HM874018|MM08633|Cydia_splendana
TLYFIFGIWAGMVGTSLSLLIRAELGNPGSLIGDDQIYNTIVTAHAFIMIFFMVMPIMIGGFGNWLVPLMLGAPDMAFPRMNNMSFWLLPPSIMLLISSSIVENGAGTGWTVYPPLSSNIAHSGSSVDLAIFSLHLAGISSILGAVNFITTIINMRPNNMSL
DQMPLFVWAVGITALLLLLSLPVLAGAITMLLTDRNLNTSFFDPAGGGDPILYQHL
>LEFIF833-10|HM875516|MM13175|Cydia_splendana
TLYFIFGIWAGMVGTSLSLLIRAELGNPGSLIGDDQIYNTIVTAHAFIMIFFMVMPIMIGGFGNWLVPLMLGAPDMAFPRMNNMSFWLLPPSIMLLISSSIVENGAGTGWTVYPPLSSNIAHSGSSVDLAIFSLHLAGISSILGAVNFITTIINMRPNNMSL
DQMPLFVWAVGITALLLLLSLPVLAGAITMLLTDRNLNTSFFDPAGGGDPILYQHL
>LEFID380-10|HM873177|MM06291|Cydia_strobilella
TLYFIFGIWAGMVGTSLSLLIRAELGNPGSLIGDDQIYNTIVTAHAFIMIFFMVMPIMIGGFGNWLVPLMLGAPDMAFPRMNNMSFWLLPPSIMLLISSSIVENGAGTGWTVYPPLSSNIAHSGSSVDLAIFSLHLAGISSILGAVNFITTIINMRPNNMSL
DQMPLFVWAVGITALLLLLSLPVLAGAITMLLTDRNLNTSFFDPAGGGDPILYQHL
>LEFID470-10|HM873238|MM06412|Cydia_strobilella
TLYFIFGIWAGMVGTSLSLLIRAELGNPGSLIGDDQIYNTIVTAHAFIMIFFMVMPIMIGGFGNWLVPLMLGAPDMAFPRMNNMSFWLLPPSIMLLISSSIVENGAGTGWTVYPPLSSNIAHSGSSVDLAIFSLHLAGISSILGAVNFITTIINMRPNNMSL
DQMPLFVWAVGITALLLLLSLPVLAGAITMLLTDRNLNTSFFDPAGGGDPILYQHL
>LEFIF255-10|HM874948|MM11037|Cydia_succedana
TLYFIFGIWAGMIGTSLSLLIRAELGNPGSLIGDDQIYNTIVTAHAFIMIFFMVMPIMIGGFGNWLVPLMLGAPDMAFPRMNNMSFWLLPPSIMLLISSSIVENGAGTGWTVYPPLSSNIAHSGSSVDLAIFSLHLAGISSILGAVNFITTIINMRPNNMSL
DQMPLFVWAVGITALLLLLSLPVLAGAITMLLTDRNLNTSFFDPAGGGDPILYQHL
>LEFIF256-10|HM874949|MM11038|Cydia_succedana
TLYFIFGIWAGMIGTSLSLLIRAELGNPGSLIGDDQIYNTIVTAHAFIMIFFMVMPIMIGGFGNWLVPLMLGAPDMAFPRMNNMSFWLLPPSIMLLISSSVVENGAGTGWTVYPPLSSNIAHSGSSVDLAIFSLHLAGISSILGAVNFITTIINMRPNNMSL
DQMPLFVWAVGITALLLLLSLPVLAGAITMLLTDRNLNTSFFDPAGGGDPILYQHL
>LEFIK776-10|JN274962|MM18351|Cydia_succedana
TLYFIFGIWAGMIGTSLSLLIRAELGNPGSLIGDDQIYNTIVTAHAFIMIFFMVMPIMIGGFGNWLVPLMLGAPDMAFPRMNNMSFWLLPPSIMLLISSSIVENGAGTGWTVYPPLSSNIAHSGSSVDLAIFSLHLAGISSILGAVNFITTIINMRPNNMSL
DQMPLFVWAVGITALLLLLSLPVLAGAITMLLTDRNLNTSFFDPAGGGDPILYQHL
>LEFIC160-10|HM872006|MM03506|Cymolomia_hartigiana
TMYFIFGVWAGMIGTSLSLLIRAELGNPGSLIGDDQIYNTIVTAHAFIMIFFMVMPIMIGGFGNWLVPLMLGAPDMAFPRMNNMSFWLLPPSIMLLISSSIVESGAGTGWTVYPPLSSNIAHGGSSVDLAIFSLHLAGISSILGAVNFITTIINMRPNNMP
LDQMPLFVWAVGITALLLLLSLPVLAGAITMLLTDRNLNTSFFDPAGGGDPILYQHL
>LEFIE956-10|HM874673|MM10356|Cymolomia_hartigiana
TMYFIFGVWAGMIGTSLSLLIRAELGNPGSLIGDDQIYNTIVTAHAFIMIFFMVMPIMIGGFGNWLVPLMLGAPDMAFPRMNNMSFWLLPPSIMLLISSSIVESGAGTGWTVYPPLSSNIAHGGSSVDLAIFSLHLAGISSILGAVNFITTIINMRPNNMS
LDQMPLFVWAVGITALLLLLSLPVLAGAITMLLTDRNLNTSFFDPAGGGDPILYQHL

>LEFIE248-10|HM873982|MM08576|Cynaeda_dentalis
TLYFIFGIWSGMVGTSLSLMIRAELGNPGSLIGDDQIYNTIVTAHAFIMIFFMVMPIMIGGFGNWLIPLMLGAPDMAFPRMNNMSFWLLPPSLTLLISSSIVENGAGTGWTVYPPLSSNIAHGGSSVDLAIFSLHLAGISSILGAINFITTIINMRISGLMFD
QMPLFVWAVGITALLLLLSLPVLAGAITMLLTDRNLNTSFFDPAGGGDPILYQHL
>LEEUA466-11|JN283836|MM19874|Dahlica_lichenella
TLYFILGVWSGVLGTSLSMLIRTELGIPQSLIGDDQIYNTIVTAHAFIMIFFMVMPIMIGGFGNWLVPLMLGAPDMAFPRMNNMSFWLLPPSLMLLIMSSITEDGAGTGWTVYPPLSSNIAHSGSSIDLAIFSLHLAGISSILGAVNFITTTINMRPNYMTL
DRMPLFVWSIAITALLLLLSLPVLAGAITMLLTDRNLNTSFFDPAGGGDPILFQHL
>LEFIE696-10|HM874417|MM09674|Dahlica_triquetrella
TLYFILGVWSGVLGTSLSMLIRTELGIPQSLIGDDQIYNTIVTAHAFIMIFFMVMPIMIGGFGNWLVPLMLGAPDMAFPRMNNMSFWLLPPSLMLLVMASITEDGAGTGWTVYPPLSSNIAHSGSSIDLAIFSLHLAGISSILGAVNFITTTINMRPNYM
ALDQMPLFVWSIAITALLLLLSLPVLAGAITMLLTDRNLNTSFFDPAGGGDPILFQHL
>LEFIG568-10|HM876239|MM14716|Dahlica_triquetrella
TLYFILGVWSGVLGTSLSMLIRTELGIPQSLIGDDQIYNTIVTAHAFIMIFFMVMPIMIGGFGNWLVPLMLGAPDMAFPRMNNMSFWLLPPSLMLLIMASITEDGAGTGWTVYPPLSSNIAHSGSSIDLAIFSLHLAGISSILGAVNFITTTINMRPNYMT
LDRMPLFVWSIAITALLLLLSLPVLAGAITMLLTDRNLNTSFFDPAGGGDPILFQHL
>LEFIB793-10|HM871670|MM02702|Dasypolia_templi
TLYFIFGIWAGMVGTSLSLLIRAELGNPGSLIGDDQIYNTIVTAHAFIMIFFMVMPIMIGGFGNWLVPLMLGAPDMAFPRMNNMSFWLLPPSLTLLISSSIVENGAGTGWTVYPPLSSNIAHGGSSVDLAIFSLHLAGISSILGAINFITTIINMRLNNLSFD
QMPLFIWAVGITAFLLLLSLPVLAGAITMLLTDRNLNTSFFDPAGGGDPILYQHL
>LEFIG692-10|HM876349|MM15556|Dasystoma_salicella
TLYFIFGIWAGMVGTSLSLLIRAELGNPGSFIGDDQIYNTIVTAHAFIMIFFMVMPIMIGGFGNWLVPLMLGAPDMAFPRMNNMSFWLLPPSLTLLISSSIVENGAGTGWTVYPPLSSNIAHGGSSVDLAIFSLHLAGISSILGAINFITTIINMRLNNMYF
DQMPLFVWSVGITALLLLLSLPVLAGAITMLLTDRNLNTSFFDPAGGGDPILYQHL
>LEFIK072-10|JF853932|MM17647|Decantha_borkhausenii
TLYFIFGIWAGMVGTSLSLLIRAELGNPGSLIGDDQIYNTIVTAHAFIMIFFMVMPIMIGGFGNWLVPLMLGAPDMAFPRMNNMSFWLLPPSLTLLISSSIVENGAGTGWTVYPPLSSNIAHGGSSVDLAIFSLHLAGISSILGAINFITTIINMRMHNMSF
DQMPLFVWAVGITALLLLLSLPVLAGAITMLLTDRNLNTSFFDPAGGGDPILYQHL
>LEFIA059-10|HM396406|MM00971|Deilephila_elpenor
TLYFIFGIWAGMVGTSLSLLIRAELGTPGSLIGDDQIYNTIVTAHAFIMIFFMVMPIMIGGFGNWLIPLMLGAPDMAFPRMNNMSFWLLPPSLTLLISSSIVENGAGTGWTVYPPLSSNIAHSGSSVDLAIFSLHLAGISSIMGAVNFITTIINMRINNLSFD
QMPLFVWAVGITAFLLLLSLPVLAGAITMLLTDRNLNTSFFDPAGGGDPILYQHL
>LEFIB212-10|HM871116|MM00664|Deilephila_porcellus
TLYFIFGIWAGMVGTSLSLLIRAELGTPGSLIGDDQIYNTIVTAHAFIMIFFMVMPIMIGGFGNWLVPLMLGAPDMAFPRMNNMSFWLLPPSLTLLISSSIVENGAGTGWTVYPPLSSNIAHSGSSVDLAIFSLHLAGISSIMGAVNFITTIINMRINNLSFD
QMPLFVWAVGITAFLLLLSLPVLAG?ITMLLTDRNLNTSFFDPAGGGDPILYQHL
>LEFIC642-10|HM872463|MM04588|Deileptenia_ribeata
TLYFIFGIWAGMVGTSLSLLIRAELGNPGSLIGDDQIYNTIVTAHAFIMIFFMVMPIMIGGFGNWLVPLMLGAPDMAFPRMNNMSFWLLPPSITLLISSSIVENGAGTGWTVYPPLSSNIAHGGSSVDLAIFSLHLAGISSILGAINFITTIINMRLNNLSFD
QMPLFVWAVGITAFLLLLSLPVLAGAITMLLTDRNLNTSFFDPAGGGDPILYQHL
>LEFID621-10|HM873386|MM06638|Deileptenia_ribeata
TLYFIFGIWAGMVGTSLSLLIRAELGNPGSLIGDDQIYNTIVTAHAFIMIFFMVMPIMIGGFGNWLVPLMLGAPDMAFPRMNNMSFWLLPPSITLLISSSIVENGAGTGWTVYPPLSSNIAHGGSSVDLAIFSLHLAGISSILGAINFITTIINMRLNNLSFD
QMPLFVWAVGITAFLLLLSLPVLAGAITMLLTDRNLNTSFFDPAGGGDPILYQHL
>LEFID660-10|HM873422|MM06692|Deileptenia_ribeata
TLYFIFGIWAGMVGTSLSLLIRAELGNPGSLIGDDQIYNTIVTAHAFIMIFFMVMPIMIGGFGNWLVPLMLGAPDMAFPRMNNMSFWLLPPSITLLISSSIVENGAGTGWTVYPPLSSNIAHGGSSVDLAIFSLHLAGISSILGAINFITTIINMRLNNLSFD
QMPLFVWAVGITAFLLLLSLPVLAGAITMLLTDRNLNTSFFDPAGGGDPILYQHL
>LEFIB055-10|HM870964|MM00338|Delplanqueia_dilutella
TLYFIFGIWSGMVGTSLSLLIRAELGTPGSLIGDDQIYNTIVTGHAFIMIFFMVMPIMIGGFGNWLVPLMLGAPDMAFPRMNNMSFWLLPPSLTLLISSSIVENGAGTGWTVYPPLSSNIAHSGSSVDLAIFSLHLAGISSILGAINFITTIINMKLNNLSFDQ
MPLFVWAVGITALLLLLSLPVLAGAITMLLTDRNLNTSFFDPAGGGDPILYQHL
>LEFIA636-10|HM870885|MM01751|Deltote_bankiana
TLYFIFGIWAGMVGTSLSLLIRAELGTPGSLIGDDQIYNTIVTAHAFIMIFFMVMPIMIGGFGNWLIPLMLGAPDMAFPRMNNMSFWLLPPSLTLLISSSIVENGAGTGWTVYPPLSSNIAHGGSSVDLAIFSLHLAGISSILGAINFITTIINMRLNSLNFDQ
MPLFIWAVGITAFLLLLSLPVLAGAITMLLTDRNLNTSFFDPAGGGDPILYQHL
>LEFIA448-10|HM386789|MM01512|Deltote_pygarga
TLYFIFGIWAGMVGTSLSLLIRAELGTPGSLIGDDQIYNTIVTAHAFIMIFFMVMPIMIGGFGNWLIPLMLGAPDMAFPRMNNMSFWLLPPSITLLISSSIVENGAGTGWTVYPPLSSNIAHGGSSVDLAIFSLHLAGISSILGAINFITTIINMRLNNLHFDQ
MPLFIWAVGITAFLLLLSLPVLAGAITMLLTDRNLNTSFFDPAGGGDPILYQHL
>LEFIB868-10|HM871745|MM02925|Deltote_uncula
TLYFIFGIWAGMVGTSLSLLIRAELGTPGSLIGDDQIYNTIVTAHAFIMIFFMVMPIMIGGFGNWLVPLMLGAPDMAFPRMNNMSFWLLPPSLTLLISSSIVENGAGTGWTVYPPLSSNIAHGGSSVDLAIFSLHLAGISSILGAINFITTIINMRLNNLYFD

QMPLFIWAVGITAFLLLLSLPVLAGAITMLLTDRNLNTSFFDPAGGGDPILYQHL
>LEFIK948-10|JF854386|MM18523|Deltote_uncula
TLYFIFG?WAGMVGTSLSLLIRAELGTPGSLIGDDQIYNTIVTAHAFIMIFFMVMPIMIGGFGNWLVPLMLGAPDMAFPRMNNMSFWLLPPSLTLLISSSIVENGAGTGWTVYPPLSSNIAHGGSSVDLAIFSLHLAGISSILGAINFITTIINMRLNNLYFD
QMPLFIWAVGITAFLLLLSLPVLAGAITMLLTDRNLNTSFFDPAGGGDPILYQHL
>LEFIA125-10|HM396472|MM01054|Dendrolimus_pini
TLYFIFGIWAGMVGTSLSLLIRAELGTPGSLIGDDQIYNTIVTAHAFIMIFFMVMPIMIGGFGNWLVPLMLGAPDMAFPRMNNMSFWLLPPSLTLLISSSIVESGAGTGWTVYPPLSSNIAHSGSSVDLAIFSLHLAGISSILGAINFITTIINMKLNNMSFD
QMPLFVWAVGITAFLLLLSLPVLAGAITMLLTDRNLNTSFFDPAGGGDPILYQHL
>LEFIE113-10|HM873861|MM08302|Dendrolimus_pini
TLYFIFGIWAGMVGTSLSLLIRAELGTPGSLIGDDQIYNTIVTAHAFIMIFFMVMPIMIGGFGNWLVPLMLGAPDMAFPRMNNMSFWLLPPSLTLLISSSIVESGAGTGWTVYPPLSSNIAHSGSSVDLAIFSLHLAGISSILGAINFITTIINMKLNNMSFD
QMPLFVWAVGITAFLLLLSLPVLAGAITMLLTDRNLNTSFFDPAGGGDPILYQHL
>LEFIL575-10|JF854670|MM18873|Denisia_luticiliella
TLYFIFGIWAGMIGTSLSLLIRAELGNPGSLIGDDQIYNTIVTAHAFIMIFFMVMPIMIGGFGNWLIPLMLGAPDMAFPRMNNMSFWLLPPSLTLLISSSIVENGAGTGWTVYPPLSSNIAHGGSSVDLAIFSLHLAGISSILGAINFITTIINMRMNNMSFD
QMPLFVWAVGITALLLLLSLPVLAGAITMLLTDRNLNTSFFDPAGGGDPILYQHL
>LEFIE323-10|HM874049|MM08702|Denisia_obscurella
TLYFIFGIWAGMVGTSLSLLIRAELGNPGSLIGDDQIYNTIVTAHAFIMIFFMVMPIMIGGFGNWLIPLMLGAPDMAFPRMNNMSFWLLPPSLTLLISSSIVESGAGTGWTVYPPLSSNIAHGGSSVDLAIFSLHLAGISSILGAINFITTIINMRMNNLSFD
QMPLFVWSVGITALLLLLSLPVLAGAITMLLTDRNLNTSFFDPAGGGDPILYQHL
>LEFIK573-10|JF854163|MM18148|Denisia_obscurella
TLYFIFGIWAGMVGTSLSLLIRAELGNPGSLIGDDQIYNTIVTAHAFIMIFFMVMPIMIGGFGNWLIPLMLGAPDMAFPRMNNMSFWLLPPSLTLLISSSIVESGAGTGWTVYPPLSSNIAHGGSSVDLAIFSLHLAGISSILGAINFITTIINMRMNNLSFD
QMPLFVWSVGITALLLLLSLPVLAGAITMLLTDRNLNTSFFDPAGGGDPILYQHL
>LEFIC326-10|HM872170|MM03830|Denisia_similella
TLYFIFGIWAGMVGTSLSLLIRAELGNPGSLIGDDQIYNTIVTAHAFIMIFFMVMPIMIGGFGNWLIPLMLGAPDMAFPRMNNMSFWLLPPSLTLLISSSIVENGAGTGWTVYPPLSSNIAHGGSSVDLAIFSLHLAGISSILGAINFITTIINMRMNNMSF
DQMPLFVWAVGITALLLLLSLPVLAGAITMLLTDRNLNTSFFDPAGGGDPILYQHL
>LEFID997-10|HM873747|MM08046|Denisia_stipella
TLYFIFGIWAGMIGTSLSLLIRAELGNPGSLIGDDQIYNTIVTAHAFIMIFFMVMPIMIGGFGNWLIPLMLGAPDMAFPRMNNMSFWLLPPSLTLLISSSIVENGAGTGWTVYPPLSSNIAHGGSSVDLAIFSLHLAGISSILGAINFITTIINMRMNNMSFD
QMPLFVWAVGITALLLLLSLPVLAGAITMLLTDRNLNTSFFDPAGGGDPILYQHL
>LEFIB308-10|HM871209|MM00830|Denticucullus_pygmina
TLYFIFGIWAGMMGTSLSLLIRAELGMPGSLIGDDQIYNTIVTAHAFIMIFFMVMPIMIGGFGNWLIPLMLGAPDMAFPRMNNMSFWLLPPSLTLLISSSIVENGAGTGWTVYPPLSSNIAHGGSSVDLAIFSLHLAGISSILGAINFITTIINMRLNNLSFD
QMPLFIWAVGITAFLLLLSLPVLAGAITMLLTDRNLNTSFFDPAGGGDPILYQHL
>LEFIJ2205-14|MM23393|Depressaria_artemisiae
TLYFIFGIWAGMVGTSLSLLIRAELGNPGSLIGDDQIYNTIVTAHAFIMIFFMVMPIMIGGFGNWLVPLMLGAPDMAFPRMNNMSFWLLPPSLTLLISSSIVENGAGTGWTVYPPLSSNIAHGGSSVDLAIFSLHLAGISSILGAINFITTIINMRLNNMSF
DQLPLFVWAVGITALLLLLSLPVLAGAITMLLTDRNLNTSFFDPAGGGDPILYQHL
>LEFIB561-10|HM871443|MM02203|Depressaria_badiella
TLYFIFGIWAGMVGTSLSLLIRAELGNPGSLIGDDQIYNTIVTAHAFIMIFFMVMPIMIGGFGNWLVPLMLGAPDMAFPRMNNMSFWLLPPSLTLLISSSIVENGAGTGWTVYPPLSSNIAHGGSSVDLAIFSLHLAGISSILGAINFITTIINMRLNNMSF
DQLPLFVWAVGITALLLLLSLPVLAGAITMLLTDRNLNTSFFDPAGGGDPILYQHL
>TYPFN060-11|MM16892|Depressaria_douglasella
TLYFIFGIWAGMVGTSLSLLIRAELGNPGSLIGDDQIYNTIVTAHAFIMIFFMVMPIMIGGFGNWLVPLMLGAPDMAFPRMNNMSFWLLPPSLTLLISSSIVENGAGTGWTVYPPLSSNIAHGGSSVDLAIFSLHLAGISSILGAINFITTIINMRLNNMSF
DQLPLFVWAVGITALLLLLSLPVLAGAITMLLTDRNLNTSFFDPAGGGDPILYQHL
>LEFIE228-10|HM873962|MM08545|Depressaria_chaerophylli
TLYFIFGIWAGMVGTSLSLLIRAELGNPGSLIGDDQIYNTIVTAHAFIMIFFMVMPIMIGGFGNWLVPLMLGAPDMAFPRMNNMSFWLLPPSITLLISSSIVENGAGTGWTVYPPLSSNIAHGGSSVDLAIFSLHLAGISSILGAINFITTIINMRLNNMSFD
QLPLFVWAVGITALLLLLSLPVLAGAITMLLTDRNLNTSFFDPAGGGDPILYQHL
>LEFIB560-10|HM871442|MM02202|Depressaria_daucella
TLYFIFGIWAGMVGTSLSLLIRAELGNPGSLIGDDQIYNTIVTAHAFIMIFFMVMPIMIGGFGNWLVPLMLGAPDMAFPRMNNMSFWLLPPSLTLLISSSIVENGAGTGWTVYPPLSSNIAHGGSSVDLAIFSLHLAGISSILGAINFITTIINMRLNNMSF
DQLPLFVWAVGITALLLLLSLPVLAGAITMLLTDRNLNTSFFDPAGGGDPILYQHL
>LEFIC939-10|HM872754|MM05298|Depressaria_daucella
TLYFIFGIWAGMVGTSLSLLIRAELGNPGSLIGDDQIYNTIVTAHAFIMIFFMVMPIMIGGFGNWLVPLMLGAPDMAFPRMNNMSFWLLPPSLTLLISSSIVENGAGTGWTVYPPLSSNIAHGGSSVDLAIFSLHLAGISSILGAINFITTIINMRLNNMSF
DQLPLFVWAVGITALLLLLSLPVLAGAITMLLTDRNLNTSFFDPAGGGDPILYQHL
>LEFIE384-10|HM874108|MM08920|Depressaria_daucella

TLYFIFGIWAGMVGTSLSLLIRAELGNPGSLIGDDQIYNTIVTAHAFIMIFFMVMPIMIGGFGNWLVPLMLGAPDMAFPRMNNMSFWLLPPSLTLLISSSIVENGAGTGWTVYPPLSSNIAHGGSSVDLAIFSLHLAGISSILGAINFITTIINMRLNNMSF
DQLPLFVWAVGITALLLLLSLPVLAGAITMLLTDRNLNTSFFDPAGGGDPILYQHL
>LEFIB563-10|HM871445|MM02210|Depressaria_depressana
TLYFIFGIWAGMVGTSLSLLIRAELGNPGSLIGDDQIYNTIVTAHAFIMIFFMVMPIMIGGFGNWLVPLMLGAPDMAFPRMNNMSFWLLPPSLTLLISSSIVENGAGTGWTVYPPLSSNIAHGGSSVDLAIFSLHLAGISSILGAINFITTIINMRLNNMSF
DQLPLFVWAVGITALLLLLSLPVLAGAITMLLTDRNLNTSFFDPAGGGDPILYQHL
>LEEUA103-11|MM19511|Depressaria_douglasella
TLYFIFGIWAGMVGTSLSLLIRAELGNPGSLIGDDQIYNTIVTAHAFIMIFFMVMPIMIGGFGNWLVPLMLGAPDMAFPRMNNMSFWLLPPSLTLLISSSIVENGAGTGWTVYPPLSSNIAHGGSSVDLAIFSLHLAGISSILGAINFITTIINMRLNNMSF
DQLPLFVWAVGITALLLLLSLPVLAGAITMLLTDRNLNTSFFDPAGGGDPILYQHL
>LEFIC941-10|HM872756|MM05301|Depressaria_emeritella
TLYFIFGIWAGMVGTSLSLLIRAELGNPGSLIGDDQIYNTIVTAHAFIMIFFMVMPIMIGGFGNWLVPLMLGAPDMAFPRMNNMSFWLLPPSLTLLISSSIVENGAGTGWTVYPPLSSNIAHGGSSVDLAIFSLHLAGISSILGAINFITTIINMRLNNMSF
DQLPLFVWAVGITALLLLLSLPVLAGAITMLLTDRNLNTSFFDPAGGGDPILYQHL
>LEFII313-11|MM19963|Depressaria_emeritella
TLYFIFGIWAGMVGTSLSLLIRAELGNPGSLIGDDQIYNTIVTAHAFIMIFFMVMPIMIGGFGNWLVPLMLGAPDMAFPRMNNMSFWLLPPSLTLLISSSIVENGAGTGWTVYPPLSSNIAHGGSSVDLAIFSLHLAGISSILGAINFITTIINMRLNNMSF
DQLPLFVWAVGITALLLLLSLPVLAGAITMLLTDRNLNTSFFDPAGSWDPILYQHL
>LEFID258-10|HM873056|MM06123|Depressaria_leucocephala
TLYFIFGIWAGMVGTSLSLLIRAELGNPGSLIGDDQIYNTIVTAHAFIMIFFMVMPIMIGGFGNWLVPLMLGAPDMAFPRMNNMSFWLLPPSLTLLISSSIVENGAGTGWTVYPPLSSNIAHGGSSVDLAIFSLHLAGISSILGAINFITTIINMRLNNMSF
DQLPLFVWAVGITALLLLLSLPVLAGAITMLLTDRNLNTSFFDPAGGGDPILYQHL
>LEFIB072-10|HM870981|MM00362|Depressaria_libanotidella
TLYFIFGIWAGMVGTSLSLLIRAELGNPGSLIGDDQIYNTIVTAHAFIMIFFMVMPIMIGGFGNWLVPLMLGAPDMAFPRMNNMSFWLLPPSLTLLISSSIVENGAGTGWTVYPPLSSNIAHGGSSVDLAIFSLHLAGISSILGAINFITTIINMRLNNMSF
DQLPLFVWAVGITALLLLLSLPVLAGAITMLLTDRNLNTSFFDPAGGGDPILYQHL
>LEFIC089-10|HM871957|MM03390|Depressaria_libanotidella
TLYFIFGIWAGMVGTSLSLLIRAELGNPGSLIGDDQIYNTIVTAHAFIMIFFMVMPIMIGGFGNWLVPLMLGAPDMAFPRMNNMSFWLLPPSLTLLISSSIVENGAGTGWTVYPPLSSNIAHGGSSVDLAIFSLHLAGISSILGAINFITTIINMRLNNMSF
DQLPLFVWAVGITALLLLLSLPVLAGAITMLLTDRNLNTSFFDPAGGGDPILYQHL
>LEFIJ1374-12|MM22045|Depressaria_libanotidella
TLYFIFGIWAGMVGTSLSLLIRAELGNPGSLIGDDQIYNTIVTAHAFIMIFFMVMPIMIGGFGNWLVPLMLGAPDMAFPRMNNMSFWLLPPSLTLLISSSIVENGAGTGWTVYPPLSSNIAHGGSSVDLAIFSLHLAGISSILGAINFITTIINMRLNNMSF
DQLPLFV?AVGITALLLLLSLPVLAGAITMLLTDRNLNTSFFDPAGGGDPILYQHL
>LEFIJ1376-12|MM22047|Depressaria_libanotidella
TLYFIFGIWAGMVGTSLSLLIRAELGNPGSLIGDDQIYNTIVTAHAFIMIFFMVMPIMIGGFGNWLVPLMLGAPDMAFPRMNNMSFWLLPPSLTLLISSSIVENGAGTGWTVYPPLSSNIAHGGSSVDLAIFSLHLAGISSILGAINFITTIINMRLNNMSF
DQLPLFVWAVGITALLLLLSLPVLAGAITMLLTDRNLNTSFFDPAGGGDPILYQHL
>LEFIK064-10|JF853930|MM17639|Depressaria_nemolella
TLYFIFGIWAGMVGTSLSLLIRAELGNPGSLIGDDQIYNTIVTAHAFIMIFFMVMPIMIGGFGNWLVPLMLGAPDMAFPRMNNMSFWLLPPSLTLLISSSIVENGAGTGWTVYPPLSSNIAHGGSSVDLAIFSLHLAGISSILGAINFITTIINMRLNNMSF
DQLPLFVWAVGITALLLLLSLPVLAGAITMLLTDRNLNTSFFDPAGGGDPILYQHL
>LEFIB566-10|HM871448|MM02216|Depressaria_olerella
TLYFIFGIWAGMVGTSLSLLIRAELGNPGSLIGDDQIYNTIVTAHAFIMIFFMVMPIMIGGFGNWLVPLMLGAPDMAFPRMNNMSFWLLPPSLTLLISSSIVENGAGTGWTVYPPLSSNIAHGGSSVDLAIFSLHLAGISSILGAINFITTIINMRLNNMSF
DQLPLFVWAVGITALLLLLSLPVLAGAITMLLTDRNLNTSFFDPAGGGDPILYQHL
>LEFIF920-10|HM875602|MM13352|Depressaria_olerella
TLYFIFGIWAGMVGTSLSLLIRAELGNPGSLIGDDQIYNTIVTAHAFIMIFFMVMPIMIGGFGNWLVPLMLGAPDMAFPRMNNMSFWLLPPSLTLLISSSIVENGAGTGWTVYPPLSSNIAHGGSSVDLAIFSLHLAGISSILGAINFITTIINMRLNNMSF
DQLPLFVWAVGITALLLLLSLPVLAGAITMLLTDRNLNTSFFDPAGGGDPILYQHL
>LEFIK564-10|JF854155|MM18139|Depressaria_olerella
TLYFIFGIWAGMVGTSLSLLIRAELGNPGSLIGDDQIYNTIVTAHAFIMIFFMVMPIMIGGFGNWLVPLMLGAPDMAFPRMNNMSFWLLPPSLTLLISSSIVENGAGTGWTVYPPLSSNIAHGGSSVDLAIFSLHLAGISSILGAINFITTIINMRLNNMSF
DQLPLFVWAVGITALLLLLSLPVLAGAITMLLTDRNLNTSFFDPAGGGDPILYQHL
>LEFIA039-10|HM396388|MM00438|Depressaria_pimpinellae
TLYFIFGIWAGMVGTSLSLLIRAELGNPGSLIGDDQIYNTIVTAHAFIMIFFMVMPIMIGGFGNWLVPLMLGAPDMAFPRMNNMSFWLLPPSLTLLISSSIVENGAGTGWTVYPPLSSNIAHGGSSVDLAIFSLHLAGISSILGAINFITTIINMRLNNMSF
DQLPLFVWAVGITALLLLLSLPVLAGAITMLLTDRNLNTSFFDPAGGGDPILYQHL
>LEFIE226-10|HM873960|MM08543|Depressaria_pulcherrimella
TLYFIFGIWAGMVGTSLSLLIRAELGNPGSLIGDDQIYNTIVTAHAFIMIFFMVMPIMIGGFGNWLVPLMLGAPDMAFPRMNNMSFWLLPPSLTLLISSSIVENGAGTGWTVYPPLSSNIAHGGSSVDLAIFSLHLAGISSILGAINFITTIINMRLNNMSF
DQLPLFVWAVGITALLLLLSLPVLAGAITMLLTDRNLNTSFFDPAGGGDPILYQHL

>LEFID175-10|HM872982|MM05956|Depressaria_radiella
TLYFIFGIWAGMVGTSLSLLIRAELGNPGSLIGDDQIYNTIVTAHAFIMIFFMVMPIMIGGFGNWLVPLMLGAPDMAFPRMNNMSFWLLPPSLTLLISSSVVENGAGTGWTVYPPLSSNIAHGGSSVDLAIFSLHLAGISSILGAINFITTIINMRLNNMSF
DQLPLFVWAVGITALLLLLSLPVLAGAITMLLTDRNLNTSFFDPAGGGDPILYQHL
>LEFIA049-10|HM396397|MM00955|Depressaria_silesiaca
TLYFIFGIWAGMVGTSLSLLIRAELGNPGSLIGDDQIYNTIVTAHAFIMIFFMVMPIMIGGFGNWLVPLMLGAPDMAFPRMNNMSFWLLPPSLTLLISSSIVENGAGTGWTVYPPLSSNIAHGGSSVDLAIFSLHLAGISSILGAINFITTIINMRLNNMSF
DQLPLFVWAVGITALLLLLSLPVLAGAITMLLTDRNLNTSFFDPAGGGDPILYQHL
>LEFIC560-10|HM872381|MM04337|Depressaria_silesiaca
TLYFIFGIWAGMVGTSLSLLIRAELGNPGSLIGDDQIYNTIVTAHAFIMIFFMVMPIMIGGFGNWLVPLMLGAPDMAFPRMNNMSFWLLPPSLTLLISSSIVENGAGTGWTVYPPLSSNIAHGGSSVDLAIFSLHLAGISSILGAINFITTIINMRLNNMSF
DQLPLFVWAVGITALLLLLSLPVLAGAITMLLTDRNLNTSFFDPAGGGDPILYQHL
>LEFIB565-10|HM871447|MM02213|Depressaria_sordidatella
TLYFIFGIWAGMVGTSLSLLIRAELGNPGSLIGDDQIYNTIVTAHAFIMIFFMVMPIMIGGFGNWLVPLMLGAPDMAFPRMNNMSFWLLPPSLTLLISSSIVENGAGTGWTVYPPLSSNIAHGGSSVDLAIFSLHLAGISSILGAINFITTIINMRLNNMSF
DQLTLFVWAVGITALLLLLSLPVLAGAITMLLTDRNLNTSFFDPAGGGDPILYQHL
>LEFIE047-10|HM873795|MM08151|Depressaria_sordidatella
TLYFIFGIWAGMVGTSLSLLIRAELGNPGSLIGDDQIYNTIVTAHAFIMIFFMVMPIMIGGFGNWLVPLMLGAPDMAFPRMNNMSFWLLPPSLTLLISSSIVENGAGTGWTVYPPLSSNIAHGGSSVDLAIFSLHLAGISSILGAINFITTIINMRLNNMSF
DQLTLFVWAVGITALLLLLSLPVLAGAITMLLTDRNLNTSFFDPAGGGDPILYQHL
>LEFIL707-10|KF809178|MM13135|Depressaria_sordidatella
TLYFIFGIWAGMVGTSLSLLIRAELGNPGSLIGDDQIYNTIVTAHAFIMIFFMVMPIMIGGFGNWLVPLMLGAPDMAFPRMNNMSFWLLPPSLTLLISSSIVENGAGTGWTVYPPLSSNIAHGGSSVDLAIFSLHLAGISSILGAINFITTIINMRLNNMSF
DQLTLFVWAVGITALLLLLSLPVLAGAITMLLTDRNLNTSFFDPAGGGDPILYQHL
>TYPFN061-11|MM16893|Depressaria_sordidatella
TLYFIFGIWAGMVGTSLSLLIRAELGNPGSLIEDDQIYNTIVTAHAFIMIFFMVMPIMIGGFGNWLVPLMLGAPDMAFPRMNNMSFWLLPPSLTLLISSSIVENGAGTGWTVYPPLSSNVAHGGSSVDLAIFSLHLAGISSILGAINFITTIINMRLNNMSF
DQLTLFIWAVGITALLLLLSLPVLAGAITMLLTDRNLNTSFFDPAGGGDPILYQHL
>LEEUA102-11|MM19510|Depressaria_ultimella
TLYFIFGIWAGMVGTSLSLLIRAELGNPGSLIGDDQIYNTIVTAHAFIMIFFMVMPIMIGGFGNWLVPLMLGAPDMAFPRMNNMSFWLLPPSLTLLISSSIVENGAGTGWTVYPPLSSNIAHGGSSVDLAIFSLHLAGISSILGAINFITTIINMRLNNMSF
DQLPLFVWAVGITALLLLLSLPVLAGAITMLLTDRNLNTSFFDPAGGGDPILYQHL
>LEFIA140-10|HM396486|MM01088|Diachrysia_chrysitis
TLYFIFGIWAGMVGTSLSLLIRAELGTPGSLIGDDQIYNTIVTAHAFIMIFFMVMPIMIGGFGNWLIPLMLGAPDMAFPRMNNMSFWLLPPSLTLLISSSIVENGAGTGWTVYPPLSSNIAHGGSSVDLAIFSLHLAGISSILGAINFITTIINMRLNNLSFDQ
MPLFIWAVGITAFLLLLSLPVLAGAITMLLTDRNLNTSFFDPAGGGDPILYQHL
>LEFIA141-10|HM396487|MM01089|Diachrysia_chrysitis
TLYFIFGIWAGMVGTSLSLLIRAELGTPGSLIGDDQIYNTIVTAHAFIMIFFMVMPIMIGGFGNWLIPLMLGAPDMAFPRMNNMSFWLLPPSLTLLISSSIVENGAGTGWTVYPPLSSNIAHGGSSVDLAIFSLHLAGISSILGAINFITTIINMRLNNLSFDQ
MPLFIWAVGITAFLLLLSLPVLAGAITMLLTDRNLNTSFFDPAGGGDPILYQHL
>LEFIA144-10|HM396490|MM01092|Diachrysia_chrysitis
TLYFIFGIWAGMVGTSLSLLIRAELGTPGSLIGDDQIYNTIVTAHAFIMIFFMVMPIMIGGFGNWLIPLMLGAPDMAFPRMNNMSFWLLPPSLTLLISSSIVENGAGTGWTVYPPLSSNIAHGGSSVDLAIFSLHLAGISSILGAINFITTIINMRLNNLSFDQ
MPLFIWAVGITAFLLLLSLPVLAGAITMLLTDRNLNTSFFDPAGGGDPILYQHL
>LEFID851-10|HM873608|MM06977|Diachrysia_chrysitis
TLYFIFGIWAGMVGTSLSLLIRAELGTPGSLIGDDQIYNTIVTAHAFIMIFFMVMPIMIGGFGNWLIPLMLGAPDMAFPRMNNMSFWLLPPSLTLLISSSIVENGAGTGWTVYPPLSSNIAHGGSSVDLAIFSLHLAGISSILGAINFITTIINMRLNNLSFDQ
MPLFIWAVGITAFLLLLSLPVLAGAITMLLTDRNLNTSFFDPAGGGDPILYQHL
>LEFID911-10|HM873661|MM07312|Diachrysia_chrysitis
TLYFIFGIWAGMVGTSLSLLIRAELGTPGSLIGDDQIYNTIVTAHAFIMIFFMVMPIMIGGFGNWLIPLMLGAPDMAFPRMNNMSFWLLPPSLTLLISSSIVENGAGTGWTVYPPLSSNIAHGGSSVDLAIFSLHLAGISSILGAINFITTIINMRLNNLSFDQ
MPLFIWAVGITAFLLLLSLPVLAGAITMLLTDRNLNTSFFDPAGGGDPILYQHL
>LEFIH134-10|HM876754|MM15036|Diachrysia_chrysitis
TLYFIFGIWAGMVGTSLSLLIRAELGTPGSLIGDDQIYNTIVTAHAFIMIFFMVMPIMIGGFGNWLIPLMLGAPDMAFPRMNNMSFWLLPPSLTLLISSSIVENGAGTGWTVYPPLSSNIAHGGSSVDLAIFSLHLAGISSILGAINFITTIINMRLNNLSFDQ
MPLFIWAVGITAFLLLLSLPVLAGAITMLLTDRNLNTSFFDPAGGGDPILYQHL
>LEFIH139-10|HM876759|MM15041|Diachrysia_chrysitis
TLYFIFGIWAGMVGTSLSLLIRAELGTPGSLIGDDQIYNTIVTAHAFIMIFFMVMPIMIGGFGNWLIPLMLGAPDMAFPRMNNMSFWLLPPSLTLLISSSIVENGAGTGWTVYPPLSSNIAHGGSSVDLAIFSLHLAGISSILGAINFITTIINMRLNNLSFDQ
MPLFIWAVGITAFLLLLSLPVLAGAITMLLTDRNLNTSFFDPAGGGDPILYQHL
>LEFIA118-10|HM396465|MM01043|Diacrisia_sannio
TLYFIFGIWSGMVGTSLSLLIRAELGAPGSLIGDDQIYNTIVTAHAFIMIFFMVMPIMIGGFGNWLVPLMLGAPDMAFPRMNNMSFWLLPPSLTLLISSSIVENGAGTGWTVYPPLSSNIAHGGSSVDLAIFSLHLAGISSILGAINFITTIINMRLNNLSFD

QMPLFVWAVGITAFLLLLSLPVLAGAITMLLTDRNLNTSFFDPAGGGDPILYQHL
>LEFIA110-10|HM396457|MM01033|Diaphora_mendica
TLYFIFGVWAGMVGTSLSLLIRAELGNPGSLIGDDQIYNTIVTAHAFIMIFFMVMPIMIGGFGNWLIPLMLGAPDMAFPRMNNMSFWLLPPSLTLLISSSIVENGAGTGWTVYPPLSSNIAHGGSSVDLAIFSLHLAGISSILGAINFITTIINMRLNNLSFD
QMPLFVWAVGITAFLLLLSLPVLAGAITMLLTDRNLNTSFFDPAGGGDPILYQHL
>LEFIA111-10|HM396458|MM01034|Diaphora_mendica
TLYFIFGVWAGMVGTSLSLLIRAELGNPGSLIGDDQIYNTIVTAHAFIMIFFMVMPIMIGGFGNWLIPLMLGAPDMAFPRMNNMSFWLLPPSLTLLISSSIVENGAGTGWTVYPPLSSNIAHGGSSVDLAIFSLHLAGISSILGAINFITTIINMRLNNLSFD
QMPLFVWAVGITAFLLLLSLPVLAGAITMLLTDRNLNTSFFDPAGGGDPILYQHL
>LEFIA559-10|HQ963151|MM01653|Diarsia_brunnea
TLYFIFGIWAGMVGTSLSLLIRAELGNPGSLIGDDQIYNTIVTAHAFIMIFFMVMPIMIGGFGNWLVPLMLGAPDMAFPRMNNMSFWLLPPSLTLLISSSIVENGAGTGWTVYPPLSSNIAHGGSSVDLAIFSLHLAGISSILGAINFITTIINMRLNNLSFD
QMPLFIWAVGITAFLLLLSLPVLAGAITMLLTDRNLNTSFFDPAGGGDPILYQHL
>LEFIK435-10|KM573123|MM18010|Diarsia_brunnea
TLYFIFGIWAGMVGTSLSLLIRAELGNPGSLIGDDQIYNTIVTAHAFIMIFFMVMPIMIGGFGNWLVPLMLGAPDMAFPRMNNMSFWLLPPSLTLLISSSIVENGAGTGWTVYPPLSSNIAHGGSSVDLAIFSLHLAGISSILGAINFITTIINMRLNNLSFD
QMPLFIWAVGITAFLLLLSLPVLAGAITMLLTDRNLNTSFFDPAGGGDPILYQHL
>LEFIA598-10|HM870847|MM01710|Diarsia_dahlii
TLYFIFGIWAGMVGTSLSLLIRAELGNPGSLIGDDQIYNTIVTAHAFIMIFFMVMPIMIGGFGNWLVPLMLGAPDMAFPRMNNMSFWLLPPSLTLLISSSIVENGAGTGWTVYPPLSSNIAHGGSSVDLAIFSLHLAGISSILGAINFITTIINMRLNNLSFD
QMPLFIWAVGITAFLLLLSLPVLAGAITMLLTDRNLNTSFFDPAGGGDPILYQHL
>LEFIA599-10|HM870848|MM01711|Diarsia_dahlii
TLYFIFGIWAGMVGTSLSLLIRAELGNPGSLIGDDQIYNTIVTAHAFIMIFFMVMPIMIGGFGNWLVPLMLGAPDMAFPRMNNMSFWLLPPSLTLLISSSIVENGAGTGWTVYPPLSSNIAHGGSSVDLAIFSLHLAGISSILGAINFITTIINMRLNNLSFD
QMPLFIWAVGITAFLLLLSLPVLAGAITMLLTDRNLNTSFFDPAGGGDPILYQHL
>LEFIA553-10|KM573557|MM01646|Diarsia_mendica
TLYFIFGIWAGMVGTSLSLLIRAELGNPGSLIGDDQIYNTIVTAHAFIMIFFMVMPIMIGGFGNWLVPLMLGAPDMAFPRMNNMSFWLLPPSLTLLISSSIVENGAGTGWTVYPPLSSNIAHSGSSVDLAIFSLHLAGISSILGAINFITTIINMRLNNLSFD
QMPLFIWSVGITAFLLLLSLPVLAGAITMLLTDRNLNTSFFDPAGGGDPILYQHL
>LEFIJ047-10|KM572872|MM02777|Diarsia_mendica
TLYFIFGIWAGMVGTSLSLLIRAELGNPGSLIGDDQIYNTIVTAHAFIMIFFMVMPIMIGGFGNWLVPLMLGAPDMAFPRMNNMSFWLLPPSLTLLISSSIVENGAGTGWTVYPPLSSNIAHGGSSVDLAIFSLHLAGISSILGAINFITTIINMRLNNLSFD
QMPLFIWAVGITAFLLLLSLPVLAGAITMLLTDRNLNTSFFDPAGGGDPILYQHL
>LEFIJ068-10|KM573116|MM05112|Diarsia_mendica
TLYFIFGIWAGMVGTSLSLLIRAELGNPGSLIGDDQIYNTIVTAHAFIMIFFMVMPIMIGGFGNWLVPLMLGAPDMAFPRMNNMSFWLLPPSLTLLISSSIVENGAGTGWTVYPPLSSNIAHGGSSVDLAIFSLHLAGISSILGAINFITTIINMRLNNLSFD
QMPLFIWAVGITAFLLLLSLPVLAGAITMLLTDRNLNTSFFDPAGGGDPILYQHL
>LEFIK340-10|KM573297|MM17915|Diarsia_mendica
TLYFIFGIWAGMVGTSLSLLIRAELGNPGSLIGDDQIYNTIVTAHAFIMIFFMVMPIMIGGFGNWLVPLMLGAPDMAFPRMNNMSFWLLPPSLTLLISSSIVENGAGTGWTVYPPLSSNIAHSGSSVDLAIFSLHLAGISSILGAINFITTIINMRLNNLSFD
QMPLFIWSVGITAFLLLLSLPVLAGAITMLLTDRNLNTSFFDPAGGGDPILYQHL
>LEFIB400-10|HM871299|MM01642|Diarsia_rubi
TLYFIFGIWAGMVGTSLSLLIRAELGNPGSLIGDDQIYNTIVTAHAFIMIFFMVMPIMIGGFGNWLMPLMLGAPDMAFPRMNNMSFWLLPPSLTLLISSSIVENGAGTGWTVYPPLSSNIAHGGSSVDLAIFSLHLAGISSILGAINFITTIINMRLNNLSF
DQMPLFIWSVGITAFLLLLSLPVLAGAITMLLTDRNLNTSFFDPAGGGDPILYQHL
>LEFIB401-10|HM871300|MM01643|Diarsia_rubi
TLYFIFGIWAGMVGTSLSLLIRAELGNPGSLIGDDQIYNTIVTAHAFIMIFFMVMPIMIGGFGNWLVPLMLGAPDMAFPRMNNMSFWLLPPSLTLLISSSIVENGAGTGWTVYPPLSSNIAHGGSSVDLAIFSLHLAGISSILGAINFITTIINMRLNNLSFD
QMPLFIWSVGITAFLLLLSLPVLAGAITMLLTDRNLNTSFFDPAGGGDPILYQHL
>LEFIA551-10|KM571953|MM01644|Diarsia_rubi
TLYFIFGIWAGMVGTSLSLLIRAELGNPGSLIGDDQIYNTIVTAHAFIMIFFMVMPIMIGGFGNWLVPLMLGAPDMAFPRMNNMSFWLLPPSLTLLISSSIVENGAGTGWTVYPPLSSNIAHGGSSVDLAIFSLHLAGISSILGAINFITTIINMRLNNLSFD
QMPLFIWSVGITAFLLLLSLPVLAGAITMLLTDRNLNTSFFDPAGGGAPILYQHL
>LEFIC858-10|HM872676|MM05102|Diarsia_rubi
TLYFIFGIWAGMVGTSLSLLIRAELGNPGSLIGDDQIYNTIVTAHAFIMIFFMVMPIMIGGFGNWLVPLMLGAPDMAFPRMNNMSFWLLPPSLTLLISSSIVENGAGTGWTVYPPLSSNIAHGGSSVDLAIFSLHLAGISSILGAINFITTIINMRLNNLSFD
QMPLFIWSVGITAFLLLLSLPVLAGAITMLLTDRNLNTSFFDPAGGGDPILYQHL
>LEFIF113-10|HM874816|MM10568|Diasemia_reticularis
TLYFIFGVWAGMVGTSLSLLIRAELGNPGSLIGDDQIYNTIVTAHAFIMIFFMVMPIMIGGFGNWLVPLMLGAPDMAFPRMNNMSFWLLPPSLLLLISSSIVENGAGTGWTVYPPLSSNIAHSGSSVDLAIFSLHLAGISSILGAINFITTIINMRINGLSFD
QMPLFVWAVGITALLLLLSLPVLAGAITMLLTDRNLNTSFFDPAGGGDPILYQHL
>LEFID695-10|HM873456|MM06746|Dicallomera_fascelina

TLYFIFGIWAGMIGTSLSLLIRAELGNPGSLIGNDQIYNTIVTAHAFIMIFFMVMPIMIGGFGNWLVPLMLGAPDMAFPRMNNMSFWLLPPSLTLLISSSIVENGAGTGWTVYPPLSSNIAHGGSSVDLAIFSLHLAGISSILGAINFITTIINMRLNNLSFD
QMPLFVWAVGITAFLLLLSLPVLAGAITMLLTDRNLNTSFFDPAGGGDPILYQHL
>LEFID702-10|HM873463|MM06755|Dichelia_histrionana
TLYFIFGIWAGMVGTSLSLLIRAELGNPGSLIGDDQIYNTIVTAHAFIMIFFMVMPIMIGGFGNWLVPLMLGAPDMAFPRMNNMSFWLLPPSIMLLISSSVVENGAGTGWTVYPPLSSNIAHSGSSVDLAIFSLHLAGISSILGAVNFITTIINMRPNNMS
LDQMPLFVWSVGITALLLLLSLPVLAGAITMLLTDRNLNTSFFDPAGGGDPILYQHL
>LEFIC559-10|HM872380|MM04336|Dichomeris_alacella
TLYFIFGIWAGLMGTSLSLLIRAELGNPGYLIGDDQIYNTIVTAHAFIMIFFMVMPIMIGGFGNWLIPLMLGAPDMAFPRMNNMSFWLLPPSLTLLISSSIVENGVGTGWTVYPPLSSNIAHSGSSVDLAIFSLHLAGISSILGAINFITTIINMRINNLSFDQ
MPLFIWAVGITALLLLLSLPVLAGAITMLLTDRNLNTSFFDPAGGGDPILYQHL
>LEFID801-10|HM873558|MM06878|Dichomeris_alacella
TLYFIFGIWAGLMGTSLSLLIRAELGNPGYLIGDDQIYNTIVTTHAFIMIFFMVMPIMIGGFGNWLIPLMLGAPDMAFPRMNNMSFWLLPPSLTLLISSSIVENGVGTGWTVYPPLSSNIAHSGSSVDLAIFSLHLAGISSILGAINFITTIINMRINNLSFDQ
MPLFIWAVGITALLLLLSLPVLAGAITMLLTDRNLNTSFFDPAGGGDPILYQHL
>LEEUA605-11|MM20664|Dichomeris_derasella
TLYFIFGIWAGMLGTSLSLLIRAELGNPGSLIGDDQIYNTIVTAHAFIMIFFMVMPIMIGGFGNWLVPLMLGAPDMAFPRMNNMSFWLLPPSLTLLISSSIVENGVGTGWTVYPPLSSNIAHGGSSVDLAIFSLHLAGISSILGAINFITTIINMRINNLSFD
QMPLFVWAVGITALLLLLSLPVLAGAITMLLTDRNLNTSFFDPAGGGDPILYQHL
>LEFIE095-10|HM873842|MM08274|Dichomeris_juniperella
TLYFIFGIWAGMLGTSLSLLIRAELGNPGSLIGDDQIYNTIVTAHAFIMIFFMVMPIMIGGFGNWLVPLMLGAPDMAFPRMNNMSFWLLPPSLTLLIFSSIVENGAGTGWTVYPPLSSNIAHSGSSVDLAIFSLHLAGISSILGAINFITTIINMKINNLSFDQ
MPLFVWAVGITALLLLLSLPVLAGAITMLLTDRNLNTSFFDPAGGGDPILYQHL
>LEFIF451-10|HM875136|MM11939|Dichomeris_juniperella
TLYFIFGIWAGMLGTSLSLLIRAELGNPGSLIGDDQIYNTIVTAHAFIMIFFMVMPIMIGGFGNWLVPLMLGAPDMAFPRMNNMSFWLLPPSLTLLIFSSIVENGAGTGWTVYPPLSSNIAHSGSSVDLAIFSLHLAGISSILGAINFITTIINMKINNLSFDQ
MPLFVWAVGITALLLLLSLPVLAGAITMLLTDRNLNTSFFDPAGGGDPILYQHL
>LEFIE738-10|HM874458|MM09839|Dichomeris_latipennella
TLYFIFGIWAGLVGTSLSLLIRAELGNPGSLIGDDQIYNTIVTAHAFIMIFFMVMPIMIGGFGNWLIPLMLGAPDMAFPRMNNMSFWLLPPSLTLLISSSIVENGVGTGWTVYPPLSSNIAHGGSSVDLAIFSLHLAGISSILGAINFITTIINMRINNLSFDQ
MPLFVWAVGITALLLLLSLPVLAGAITMLLTDRNLNTSFFDPAGGGDPILYQHL
>LEFIK154-10|KM573163|MM17729|Dichomeris_limosellus
TLYFIFGIWAGMLGTSLSLLIRAELGNPGSLIGDDQIYNTIVTAHAFIMIFFMVMPIMIGGFGNWLVPLMLGAPDMAFPRMNNMSFWLLPPSLTLLISSSIVENGVGTGWTVYPPLSSNIAHGGSSVDLAIFSLHLAGISSILGAINFITTIINMRINNLSFD
QMPLFVWSVGITALLLLLSLPVLAGAITMLLTDRNLNTSFFDPAGGGDPILYQHL
>LEEUA193-11|JN266692|MM19601|Dichomeris_marginella
TLYFIFGIWAGMLGTSLSLLIRAELGNPGSLIGDDQIYNTIVTAHAFIMIFFMVMPIMIGGFGNWLVPLMLGAPDMAFPRMNNMSFWLLPPSLTLLISSSIVENGAGTGWTVYPPLSSNIAHGGSSVDLAIFSLHLAGISSILGAINFITTIINMKINNLSFD
QMPLFVWAVGITALLLLLSLPVLAGAITMLLTDRNLNTSFFDPAGGGDPILYQHL
>LEFIJ2089-13|MM23174|Dichomeris_rasilella
TLYFIFGIWAGMLGTSLSLLIRAELGNPGSLIGDDQIYNTIVTAHAFIMIFFMVMPIMIGGFGNWLVPLMLGAPDMAFPRMNNMSFWLLPPSISLLISSSIVENGAGTGWTVYPPLSSNIAHSGSSVDLTIFSLHLAGISSILGAINFITTIINMRINNLSFDQ
MPLFVWAVGITALLLLLSLPVLAGAITMLLTDRNLNTSFFDPAGGGDPILYQHL
>LEEUA194-11|JN266693|MM19602|Dichomeris_ustalella
TLYFIFGIWAGMLGTSLSLLIRAELGNPGSLIGDDQIYNTIVTAHAFIMIFFMVMPIMIGGFGNWLVPLMLGAPDMAFPRMNNMSFWLLPPSLTLLISSSIVENGVGTGWTVYPPLSSNIAHGGSSVDLAIFSLHLAGISSILGAINFITTIINMRINNLSFD
QMPLFVWAVGITALLLLLSLPVLAGAITMLLTDRNLNTSFFDPAGGGDPILYQHL
>LEFIB490-10|HM871385|MM02064|Dichrorampha_acuminatana
TLYFIFGIWAGMIGTSLSLLIRAELGNPGSLIGDDQIYNTIVTAHAFIMIFFMVMPIMIGGFGNWLVPLMLGAPDMAFPRMNNMSFWLLPPSIMLLISSSIVENGAGTGWTVYPPLSSNIAHSGSSVDLAIFSLHLAGISSILGAVNFITTIINMRPNNMSL
DQMPLFVWAVGITALLLLISLPVLAGAITMLLTDRNLNTSFFDPAGGGDPILYQHL
>LEFIC174-10|HM872019-WITHDRAWN|MM03526|Dichrorampha_acuminatana
TLYFIFGIWAGMIGTSLSLLIRAELGNPGSLIGDDQIYNTIVTAHAFIMIFFMVMPIMIGGFGNWLVPLMLGAPDMAFPRMNNMSFWLLPPSIMLLISSSIVENGAGTGWTVYPPLSSNIAHSGSSVDLAIFSLHLAGISSILGAVNFITTIINMRPNNMSL
DQMPLFVWAVGITALLLLISLPVLAGAITMLLTDRNLNTSFFDPAGGGDPILYQHL
>LEFIE726-10|HM874446|MM09820|Dichrorampha_acuminatana
TLYFIFGIWAGMIGTSLSLLIRAELGNPGSLIGDDQIYNTIVTAHAFIMIFFMVMPIMIGGFGNWLVPLMLGAPDMAFPRMNNMSFWLLPPSIMLLISSSIVENGAGTGWTVYPPLSSNIAHSGSSVDLAIFSLHLAGISSILGAVNFITTIINMRPNNMSL
DQMPLFVWAVGITALLLLISLPVLAGAITMLLTDRNLNTSFFDPAGGGDPILYQHL
>LEFIF904-10|HM875586|MM13318|Dichrorampha_acuminatana
TLYFIFGIWAGMIGTSLSLLIRAELGNPGSLIGDDQIYNTIVTAHAFIMIFFMVMPIMIGGFGNWLVPLMLGAPDMAFPRMNNMSFWLLPPSIMLLISSSIVENGAGTGWTVYPPLSSNIAHSGSSVDLAIFSLHLAGISSILGAVNFITTIINMRPNNMSL
DQMPLFVWAVGITALLLLISLPVLAGAITMLLTDRNLNTSFFDPAGGGDPILYQHL

>LEFID348-10|HM873145|MM06244|Dichrorampha_aeratana
TLYFIFGVWAGMIGTSLSLLIRAELGNPGSLIGDDQIYNTIVTAHAFIMIFFMVMPIMIGGFGNWLVPLMLGAPDMAFPRMNNMSFWLLPPSIMLLISSSIVENGAGTGWTVYPPLSSNIAHSGSSVDLAIFSLHLAGISSILGAVNFITTIINMRPNNMSL
DQMPLFVWAVGITALLLLLSLPVLAGAITMLLTDRNLNTSFFDPAGGGDPILYQHL
>LEFIJ1304-11|MM21164|Dichrorampha_aeratana
TLYFIFGVWAGMIGTSLSLLIRAELGNPGSLIGDDQIYNTIVTAHAFIMIFFMVMPIMIGGFGNWLVPLMLGAPDMAFPRMNNMSFWLLPPSIMLLISSSIVENGAGTGWTVYPPLSSNIAHSGSSVDLAIFSLHLAGISSILGAVNFITTIINMRPNNMSL
DQMPLFVWAVGITALLLLLSLPVLAGAITMLLTDRNLNTSFFDPAGGGDPILYQHL
>LEFIC006-10|HM871876|MM03206|Dichrorampha_agilana
TLYFIFGIWAGMIGTSLSLLIRAELGNPGSLIGDDQIYNTIVTAHAFIMIFFMVMPIMIGGFGNWLVPLMLGAPDMAFPRMNNMSFWLLPPSIMLLISSSIVENGAGTGWTVYPPLSSNIAHSGSSVDLTIFSLHLAGISSILGAVNFITTIINMRPNNMSL
DQMPLFVWAVGITALLLLLSLPVLAGAITMLLTDRNLNTSFFDPAGGGDPILYQHL
>LEFII186-10|JF853395|MM18613|Dichrorampha_agilana
TLYFIFGIWAGMIGTSLSLLIRAELGNPGSLIGDDQIYNTIVTAHAFIMIFFMVMPIMIGGFGNWLVPLMLGAPDMAFPRMNNMSFWLLPPSIMLLISSSIVENGAGTGWTVYPPLSSNIAHSGSSVDLTI?SLHLAGISSILGAVNFITTIINMRPNNMSL
DQMPLFVWAVGITALLLLLSLPVLAGAITMLLTDRNLNTSFFDPAGGGDPILYQHL
>LEFIF263-10|HM874956|MM11045|Dichrorampha_alpinana
TLYFIFGIWAGMIGTSLSLLIRAELGNPGSLIGDDQIYNTIVTAHAFIMIFFMVMPIMIGGFGNWLVPLMLGAPDMAFPRMNNMSFWLLPPSIMLLISSSIVENGAGTGWTVYPPLSSNIAHSGSSVDLAIFSLHLAGISSILGAVNFITTIINMRPNNMSL
DQMPLFVWAVGITALLLLLSLPVLAGAITMLLTDRNLNTSFFDPAGGGDPILYQHL
>LEFIF264-10|HM874957|MM11046|Dichrorampha_alpinana
TLYFIFGIWAGMIGTSLSLLIRAELGNPGSLIGDDQIYNTIVTAHAFIMIFFMVMPIMIGGFGNWLVPLMLGAPDMAFPRMNNMSFWLLPPSIMLLISSSIVENGAGTGWTVYPPLSSNIAHSGSSVDLAIFSLHLAGISSILGAVNFITTIINMRPNNMSL
DQMPLFVWAVGITALLLLLSLPVLAGAITMLLTDRNLNTSFFDPAGGGDPILYQHL
>LEFIE582-10|HM874305|MM09481|Dichrorampha_cinerascens
TLYFIFGIWAGMIGTSLSLLIRTELGNPGSLIGNDQIYNTIVTAHAFIMIFFMVMPIMIGGFGNWLVPLMLGAPDMAFPRMNNMSFWLLPPSIMLLISGSVVENGAGTGWTVYPPLSSNIAHSGSSVDLAIFSLHLAGISSILGAVNFITTIINMRPNNMS
LDQMPLFVWAVGITALLLLLSLPVLAGAITMLLTDRNLNTSFFDPAGGGDPILYQHL
>LEFIF265-10|HM874958|MM11047|Dichrorampha_cinerascens
TLYFIFGIWAGMIGTSLSLLIRAELGNPGSLIGNDQIYNTIVTAHAFIMIFFMVMPIMIGGFGNWLVPLMLGAPDMAFPRMNNMSFWLLPPSIMLLISSSVVENGAGTGWTVYPPLSSNIAHSGSSVDLAIFSLHLAGISSILGAVNFITTIINMRPNNMSL
DQMPLFVWAVGITALLLLLSLPVLAGAITMLLTDRNLNTSFFDPAGGGDPILYQHL
>LEFIE610-10|HM874333|MM09524|Dichrorampha_consortana
TLYFIFGIWAGMTGTSLSLLIRAELGNPGSLIGDDQIYNTIVTAHAFIMIFFMVMPIMIGGFGNWLVPLMLGAPDMAFPRMNNMSFWLLPPSIMLLISGSIVENGAGTGWTVYPPLSSNIAHSGSSVDLTIFSLHLAGISSILGAVNFITTIINMRPNNMSL
DQMPLFVWAVGITALLLLLSLPVLAGAITMLLTDRNLNTSFFDPAGGGDPILYQHL
>LEFIG417-10|HM876093|MM14443|Dichrorampha_consortana
TLYFIFGIWAGMTGTSLSLLIRAELGNPGSLIGDDQIYNTIVTAHAFIMIFFMVMPIMIGGFGNWLVPLMLGAPDMAFPRMNNMSFWLLPPSIMLLISGSIVENGAGTGWTVYPPLSSNIAHSGSSVDLTIFSLHLAGISSILGAVNFITTIINMRPNNMSL
DQMPLFVWAVGITALLLLLSLPVLAGAITMLLTDRNLNTSFFDPAGGGDPILYQHL
>LEFIG543-10|HM876216|MM14682|Dichrorampha_flavidorsana
TLYFIFGIWAGMIGTSLSLLIRAELGNPGSLIGDDQIYNTIVTAHAFIMIFFMVMPIMIGGFGNWLVPLMLGAPDMAFPRMNNMSFWLLPPSIMLLISSSIVENGAGTGWTVYPPLSSNIAHSGSSVDLAIFSLHLAGISSILGAVNFITTIINMRPNNMSL
DQMPLFVWAVGITALLLLLSLPVLAGAITMLLTDRNLNTSFFDPAGGGDPILYQHL
>LEFIC255-10|HM872099|MM03670|Dichrorampha_heegerana
TLYFIFGIWSGMIGTSLSLLIRAELGNPGSLIGDDQIYNTIVTAHAFIMIFFMVMPIMIGGFGNWLVPLMLGAPDMAFPRMNNMSFWLLPPSIMLLISSSIVENGAGTGWTVYPPLSSNIAHGGSSVDLAIFSLHLAGISSILGAVNFITTIINMRPNNMSL
DQMPLFVWAVGITALLLLLSLPVLAGAITMLLTDRNLNTSFFDPAGGGDPILYQHL
>LEFIE311-10|HM874037|MM08670|Dichrorampha_obscuratana
TLYFIFGIWSGMIGTSLSLLIRAELGNPGSLIGDDQIYNTIVTAHAFIMIFFMVMPIMIGGFGNWLVPLMLGAPDMAFPRMNNMSFWLLPPSIMLLISSSIVENGAGTGWTVYPPLSSNIAHSGSSVDLAIFSLHLAGISSILGAVNFITTIINMRPNNMSL
DQMPLFVWSVGITALLLLLSLPVLAGAITMLLTDRNLNTSFFDPAGGGDPILYQHL
>LEFIE843-10|HM874562|MM10062|Dichrorampha_obscuratana
TLYFIFGIWSGMIGTSLSLLIRAELGNPGSLIGDDQIYNTIVTAHAFIMIFFMVMPIMIGGFGNWLVPLMLGAPDMAFPRMNNMSFWLLPPSIMLLISSSIVENGAGTGWTVYPPLSSNIAHSGSSVDLAIFSLHLAGISSILGAVNFITTIINMRPNNMSL
DQMPLFVWSVGITALLLLLSLPVLAGAITMLLTDRNLNTSFFDPAGGGDPILYQHL
>LEFII184-10|JF853393|MM18611|Dichrorampha_obscuratana
TLYFIFGIWSGMIGTSLSLLIRAELGNPGSLIGDDQIYNTIVTAHAFIMIFFMVMPIMIGGFGNWLVPLMLGAPDMAFPRMNNMSFWLLPPSIMLLISSSIVENGAGTGWTVYPPLSSNIAHSGSSVDLAIFSLHLAGISSILGAVNFITTIINMRPNNMSL
DQMPLFVWSVGITALLLLLSLPVLAGAITMLLTDRNLNTSFFDPAGGGDPILYQHL
>LEFIJ1441-12|MM22709|Dichrorampha_obscuratana
TLYFIFGIWSGMIGTSLSLLIRAELGNPGSLIGDDQIYNTIVTAHAFIMIFFMVMPIMIGGFGNWLVPLMLGAPDMAFPRMNNMSFWLLPPSIMLLISSSIVENGAGTGWTVYPPLSSNIAHSGSSVDLAIFSLHLAGISSILGAVNFITTIINMRPNNMSL

DQMPLFVWSVGITALLLLLSLPVLAGAITMLLTDRNLNTSFFDPAGGGDPILYQHL
>LEFIJ1442-12|MM22710|Dichrorampha_obscuratana
TLYFIFGIWSGMIGTSLSLLIRAELGNPGSLIGDDQIYNTIVTAHAFIMIFFMVMPIMIGGFGNWLVPLMLGAPDMAFPRMNNMSFWLLPPSIMLLISSSIVENGAGTGWTVYPPLSSNIAHSGSSVDLAIFSLHLAGISSILGAVNFITTIINMRPNNMSL
DQMPLFVWSVGITALLLLLSLPVLAGAITMLLTDRNLNTSFFDPAGGG?PILYQHL
>LEFID729-10|HM873486|MM06785|Dichrorampha_petiverella
TLYFIFGIWAGMIGTSLSLLIRAELGNPGSLIGDDQIYNTIVTAHAFIMIFFMVMPIMIGGFGNWLVPLMLGAPDMAFPRMNNMSFWLLPPSIMLLISSSIVENGAGTGWTVYPPLSSNIAHSGSSVDLAIFSLHLAGISSILGAVNFITTIINMRPNNMSL
DQMPLFVWAVGITALLLLLSLPVLAGAITMLLTDRNLNTSFFDPAGGGDPILYQHL
>LEFIE793-10|HM874512|MM09975|Dichrorampha_petiverella
TLYFIFGIWAGMIGTSLSLLIRAELGNPGSLIGDDQIYNTIVTAHAFIMIFFMVMPIMIGGFGNWLVPLMLGAPDMAFPRMNNMSFWLLPPSIMLLISSSIVENGAGTGWTVYPPLSSNIAHSGSSVDLAIFSLHLAGISSILGAVNFITTIINMRPNNMSL
DQMPLFVWAVGITALLLLLSLPVLAGAITMLLTDRNLNTSFFDPAGGGDPILYQHL
>LEFIF417-10|HM875102|MM11822|Dichrorampha_petiverella
TLYFIFGIWAGMIGTSLSLLIRAELGNPGSLIGNDQIYNTIVTAHAFIMIFFMVMPIMIGGFGNWLVPLMLGAPDMAFPRMNNMSFWLLPPSIMLLISSSIVENGAGTGWTVYPPLSSNIAHSGSSVDLAIFSLHLAGISSILGAVNFITTIINMHPNNMSL
DQMPLFVWAVGITALLLLLSLPVLAGAITMLLTDRNLNTSFFDPAGGGDPILYQHL
>LEFIG428-10|HM876104|MM14462|Dichrorampha_petiverella
TLYFIFGIWAGMIGTSLSLLIRAELGNPGSLIGDDQIYNTIVTAHAFIMIFFMVMPIMIGGFGNWLVPLMLGAPDMAFPRMNNMSFWLLPPSIMLLISSSIVENGAGTGWTVYPPLSSNIAHSGSSVDLAIFSLHLAGISSILGAVNFITTIINMRPNNMSL
DQMPLFVWAVGITALLLLLSLPVLAGAITMLLTDRNLNTSFFDPAGGGDPILYQHL
>LEFID274-10|HM873072|MM06148|Dichrorampha_plumbagana
TLYFIFGIWSGMIGTSLSLLIRAELGNPGSLIGDDQIYNTIVTAHAFIMIFFMVMPIMIGGFGNWLVPLMLGAPDMAFPRMNNMSFWLLPPSIMLLISSSIVENGAGTGWTVYPPLSSNIAHSGSSVDLAIFSLHLAGISSILGAVNFITTIINMRPNNMSL
DQMPLFVWAVGITALLLLLSLPVLAGAITMLLTDRNLNTSFFDPAGGGDPILYQHL
>LEFIC036-10|HM871906|MM03263|Dichrorampha_plumbana
TLYFIFGVWAGMIGTSLSLLIRAELGNPGSLIGDDQIYNTIVTAHAFIMIFFMVMPIMIGGFGNWLVPLMLGAPDMAFPRMNNMSFWLLPPSIMLLISSSIVENGAGTGWTVYPPLSSNIAHSGSSVDLAIFSLHLAGISSILGAVNFITTIINMHPNNMS
LDQMPLFVWAVGITALLLLLSLPVLAGAITMLLTDRNLNTSFFDPAGGGDPILYQHL
>LEFID554-10|HM873319|MM06543|Dichrorampha_plumbana
TLYFIFGVWAGMIGTSLSLLIRAELGNPGSLIGDDQIYNTIVTAHAFIMIFFMVMPIMIGGFGNWLVPLMLGAPDMAFPRMNNMSFWLLPPSIMLLISSSIVENGAGTGWTVYPPLSSNIAHSGSSVDLAIFSLHLAGISSILGAVNFITTIINMRPNNMSL
DQMPLFVWAVGITALLLLLSLPVLAGAITMLLTDRNLNTSFFDPAGGGDPILYQHL
>LEFIJ2236-14|MM23416|Dichrorampha_sedatana
TLYFIFGVWAGMIGTSLSLLIRAELGNPGSLIGDDQIYNTIVTAHAFIMIFFMVMPIMIGGFGNWLVPLMLGAPDMAFPRMNNMSFWLLPPSIMLLISSSIVENGAGTGWTVYPPLSSNIAHSGSSVDLAIFSLHLAGISSILGAVNFITTIINMRPNNMSL
DQMPLFVWAVGITALLLLLSLPVLAGAITMLLTDRNLNTSFFDPAGGGDPILYQHL
>LEEUA258-11|JN274949|MM19666|Dichrorampha_sequana
TLYFIFGVWAGMIGTSLSLLIRAELGNPGSLIGDDQIYNTIVTAHAFIMIFFMVMPIMIGGFGNWLVPLMLGAPDMAFPRMNNMSFWFLPPSIMLLISSSIVENGAGTGWTVYPPLSSNIAHSGSSVDLAIFSLHLAGISSILGAVNFITTVINMRPNNMS
LDQMPLFVWAVSITALLLLLSLPVLAGAITMLLTDRNLNTSFFDPAGGGDPILYQHL
>LEFIB545-10|HM871430|MM02165|Dichrorampha_simpliciana
TLYFIFGIWAGMIGTSLSLLIRAELGNPGSLIGDDQIYNTIVTAHAFIMIFFMVMPIMIGGFGNWLVPLMLGAPDMAFPRMNNMSFWLLPPSIMLLISSSIVENGAGTGWTVYPPLSSNIAHGGSSVDLAIFSLHLAGISSILGAVNFITTIINMRPNNMSL
DQMPLFVWAVGITALLLLLSLPVLAGAITMLLTDRNLNTSFFDPAGGGDPILYQHL
>LEFIF905-10|HM875587|MM13320|Dichrorampha_simpliciana
TLYFIFGIWAGMIGTSLSLLIRAELGNPGSLIGDDQIYNTIVTAHAFIMIFFMVMPIMIGGFGNWLVPLMLGAPDMAFPRMNNMSFWLLPPSIMLLISSSIVENGAGTGWTVYPPLSSNIAHGGSSVDLAIFSLHLAGISSILGAVNFITTIINMRPNNMSL
DQMPLFVWAVGITALLLLLSLPVLAGAITMLLTDRNLNTSFFDPAGGGDPILYQHL
>LEFIE556-10|HM874279|MM09441|Dichrorampha_sylvicolana
TLYFIFGIWAGMIGTSLSLLIRAELGNPGSLIGDDQIYNTIVTAHAFIMIFFMVMPIMIGGFGNWLVPLMLGAPDMAFPRMNNMSFWLLPPSIMLLISSSIVENGAGTGWTVYPPLSSNIAHGGSSVDLAIFSLHLAGISSILGAVNFITTIINMRPNNMSL
DQMPLFVWAVGITALLLLLSLPVLAGAITMLLTDRNLNTSFFDPAGGGDPILYQHL
>LEFIC013-10|HM871883|MM03219|Dichrorampha_vancouverana
TLYFIFGIWAGMIGTSLSLLIRAELGNPGSLIGDDQIYNTIVTAHAFIMIFFMVMPIMIGGFGNWLVPLMLGAPDMAFPRMNNMSFWLLPPSIMLLISSSVVENGAGTGWTVYPPLSSNIAHSGSSVDLAIFSLHLAGISSILGAVNFITTIINMRPNNMSL
DQMPLFVWAVGITALLLLLSLPVLAGAITMLLTDRNLNTSFFDPAGGGDPILYQHL
>LEFID570-10|HM873335|MM06564|Dichrorampha_vancouverana
TLYFIFGIWAGMIGTSLSLLIRAELGNPGSLIGDDQIYNTIVTAHAFIMIFFMVMPIMIGGFGNWLVPLMLGAPDMAFPRMNNMSFWLLPPSIMLLISSSVVENGAGTGWTVYPPLSSNIAHSGSSVDLAIFSLHLAGISSILGAVNFITTIINMRPNNMSL
DQMPLFVWAVGITALLLLLSLPVLAGAITMLLTDRNLNTSFFDPAGGGDPILYQHL
>LEFIL098-10|MM19098|Dicycla_oo

TLYFIFGIWAGMVGTSLSLLIRAELGNPGSLIGDDQIYNTIVTAHAFIMIFFMVMPIMIGGFGNWLVPLMLGAPDMAFPRLNNMSFWLLPPSLTLLISSSIVESGAGTGWTVYPPLSSNIAHGGGSVDLAIFSLHLAGISSILGAINFITTIINMRLNNMSFD
QMPLFVWAVGITAFLLLLSLPVLAGAITMLLTDRNLNTSFFDPAGGGDPILYQHL
>LEFID207-10|HM873009|MM06036|Digitivalva_reticulella
TLYFIFGIWSGMIGTSLSLLIR?ELGNPGSLIGDDQIYNTIVTAHAFIMIFFMVMPIMIGGFGNWLVPLMLGAPDMAFPRMNNMSFWLLPPSLTLLISSSIVENGAGTGWTVYPPLSSNIAHSGSSVDLAIFSLHLAGISSILGAINFITTIINMRSNGMSFD
RMPLFVWAVGITALLLLLSLPVLAGAITMLLTDRNLNTSFFDPAGGGDPILYQHL
>LEFID596-10|HM873361|MM06606|Digitivalva_reticulella
TLYFIFGIWSGMIGTSLSLLIRAELGNPGSLIGDDQIYNTIVTAHAFIMIFFMVMPIMIGGFGNWLVPLMLGAPDMAFPRMNNMSFWLLPPSLTLLISSSIVENGAGTGWTVYPPLSSNIAHSGSSVDLAIFSLHLAGISSILGAINFITTIINMRSNGMSFD
RMPLFVWAVGITALLLLLSLPVLAGAITMLLTDRNLNTSFFDPAGGGDPILYQHL
>LEFIJ326-10|KM571949|MM15926|Diloba_caeruleocephala
TLYFIFGIWAGMVGTSLSLLIRAELGNPGSLIGDDQIYNTIVTAHAFIMIFFMVMPIMIGGFGNWLVPLMLGAPDMAFPRMNNMSFWLLPPSLTLLISSSIVENGAGTGWTVYPPLSSNIAHGGSSVDLAIFSLHLAGISSILGAINFITTIINMRLNNMSF
DQMPLFIWAVGITAFLLLLSLPVLAGAITMLLTDRNLNTSFFDPAGGGDPILYQHL
>LEFIJ1189-11|KM573425|MM21049|Diloba_caeruleocephala
TLYFIFGIWAGMVGTSLSLLIRAELGNPGSLIGDDQIYNTIVTAHAFIMIFFMVMPIMIGGFGNWLVPLMLGAPDMAFPRMNNMSFWLLPPSLTLLISSSIVENGAGTGWTVYPPLSSNIAHGGSSVDLAIFSLHLAGISSILGAINFITTIINMRLNNMSF
DQMPLFIWAVGITAFLLLLSLPVLAGAITMLLTDRNLNTSFFDPAGGGDPILYQHL
>LEFIB785-10|HM871662|MM02690|Dioryctria_abietella
TLYFIFGIWSGMVGTSLSLLIRAELGTPGSLIGDDQIYNTIVTGHAFIMIFFMVMPIMIGGFGNWLVPLMLGAPDMAFPRMNNMSFWLLPPSLTLLISSSIVENGAGTGWTVYPPLSSNIAHSGSSVDLAIFSLHLAGISSILGAINFITTIINMKLNGLSFDQ
MPLFVWAVGITALLLLLSLPVLAGAITMLLTDRNLNTSFFDPAGGGDPILYQHL
>LEFIB668-10|HM871546|MM02451|Dioryctria_schuetzeella
TLYFIFGIWSGMVGTSLSLLIRAELGTPGSLIGDDQIYNTIVTGHAFIMIFFMVMPIMIGGFGNWLVPLMLGAPDMAFPRMNNMSFWLLPPSLTLLISSSIVENGAGTGWTVYPPLSSNIAHSGSSVDLAIFSLHLAGISSILGAINFITTIINMKLNGLAFD
QMPLFVWAVGITALLLLLSLPVLAGAITMLLTDRNLNTSFFDPAGGGDPILYQHL
>LEFIB669-10|HM871547|MM02452|Dioryctria_schuetzeella
TLYFIFGIWSGMVGTSLSLLIRAELGTPGSLIGDDQIYNTIVTGHAFIMIFFMVMPIMIGGFGNWLVPLMLGAPDMAFPRMNNMSFWLLPPSLTLLISSSIVENGAGTGWTVYPPLSSNIAHSGSSVDLAIFSLHLAGISSILGAINFITTIINMKLNGLAFD
QMPLFVWAVGITALLLLLSLPVLAGAITMLLTDRNLNTSFFDPAGGGDPILYQHL
>LEFIE776-10|HM874495|MM09947|Dioryctria_schuetzeella
TLYFIFGIWSGMVGTSLSLLIRAELGTPGSLIGDDQIYNTIVTGHAFIMIFFMVMPIMIGGFGNWLVPLMLGAPDMAFPRMNNMSFWLLPPSLTLLISSSIVENGAGTGWTVYPPLSSNIAHSGSSVDLAIFSLHLAGISSILGAINFITTIINMKLNGLAFD
QMPLFVWAVGITALLLLLSLPVLAGAITMLLTDRNLNTSFFDPAGGGDPILYQHL
>LEFIF438-10|HM875123|MM11900|Dioryctria_simplicella
TLYFIFGIWSGMVGTSLSLLIRAELGTPGSLIGDDQIYNTIVTGHAFIMIFFMVMPIMIGGFGNWLVPLMLGAPDMAFPRMNNMSFWLLPPSLTLLISSSIVENGAGTGWTVYPPLSSNIAHSGSSVDLAIFSLHLAGISSILGAINFITTIINMKLNGLSFDQ
MPLFVWAVGITALLLLLSLPVLAGAITMLLTDRNLNTSFFDPAGGGDPILYQHL
>LEFIC183-10|HM872027|MM03538|Dioryctria_sylvestrella
TLYFIFGIWSGMVGTSLSLLIRAELGTPGSLIGDDQIYNTIVTGHAFIMIFFMVMPIMIGGFGNWLVPLMLGAPDMAFPRMNNMSFWLLPPSLNLLISSSIVENGAGTGWTVYPPLSSNIAHSGSSVDLAIFSLHLAGISSILGAINFITTIINMKLNGMSFD
QMPLFVWAVGITALLLLLSLPVLAGAITMLLTDRNLNTSFFDPAGGGD?ILYQHL
>LEFIF509-10|HM875194|MM12120|Dioryctria_sylvestrella
TLYFIFGIWSGMVGTSLSLLIRAELGTPGSLIGDDQIYNTIVTGHAFIMIFFMVMPIMIGGFGNWLVPLMLGAPDMAFPRMNNMSFWLLPPSLNLLISSSIVENGAGTGWTVYPPLSSNIAHSGSSVDLAIFSLHLAGISSILGAINFITTIINMKLNGMSFD
QMPLFVWAVGITALLLLLSLPVLAGAITMLLTDRNLNTSFFDPAGGGDPILYQHL
>LEFID095-10|HM872906|MM05707|Diplodoma_laichartingella
TLYFILGIWAGMIGTSLSFLIRVELGIPNSFLGSDQIYNTIVTAHALIMIFFMVMPIMIGGFGNWLVPLMLGAPDMAFPRMNNMSFWLLPPSLMLLIMSSIVENGAGTGWTIYPPLSSNLTHSGSSVDLAIFSLHLAGISSILGAVNFITTIINMRPSNMSL
DQMPLFVWSVAITAVLLLLSLPVLAGAITMLLTDRNLNTSFFDPAGGGDPILFQHL
>LEFIE867-10|HM874585|MM10121|Diplodoma_laichartingella
TLYFILGIWAGMIGTSLSFLIRVELGIPNSFLGSDQIYNTIVTAHALIMIFFMVMPIMIGGFGNWLVPLMLGAPDMAFPRMNNMSFWLLPPSLMLLIMSSIVENGAGTGWTIYPPLSSNLTHSGSSVDLAIFSLHLAGISSILGAVNFITTIINMRPSNMSL
DQMPLFVWSVAITAVLLLLSLPVLAGAITMLLTDRNLNTSFFDPAGGGDPILFQHL
>LEEUA216-11|JN286429|MM19624|Ditula_angustiorana
TLYFIFGIWSGLVGTSLSLLIRAELGNPGSLIGDDQIYNTIVTAHAFIMIFFMVMPIMIGGFGNWLVPLMLGAPDMAFPRMNNMSFWLLPPSIMLLISSSIVENGAGTGWTVYPPLSSNIAHSGSSVDLAIFSLHLAGISSILGAVNFITTIINMRPHNMSLD
QMPLFVWAVGITALLLLLSLPVLAGAITMLLTDRNLNTSFFDPAGGGDPILYQHL
>LEFIB984-10|HM871856|MM03178|Diurnea_lipsiella
TLYFIFGIWAGMIGSSLSLLIRAELGNPGSLIGDDQIYNTIVTAHAFIMIFFMVMPIMIGGFGNWLVPLMLGAPDMAFPRMNNMSFWLLPPSLTLLISSSVVENGAGTGWTVYPPLSSNIAHSGSSVDLAIFSLHLAGISSILGAINFITTIINMRLNNMYFD
QMPLFVWAVGITALLLLLSLPVLAGAITMLLTDRNLNTSFFDPAGGGDPILYQHL

>LEFIK828-10|MM18403|Dolicharthria_punctalis
TLYFIFGIWAGMIGTSLSLLIRAELGNPGSLIGDDQIYNTIVTAHAFIMIFFMVMPIMIGGFGNWLVPLMLGAPDMAFPRMNNMSFWLLPPSLTLLISSSIVENGAGTGWTVYPPLSSNIAHSGTSVDLAIFSLHLAGISSILGAINFITTIINMRINGLSFDQ
MPLFVWAVGITALLLLLSLPVLAGAITMLLTDRNLNTSFFDPAGGGDPILYQHL
>LEFIK010-10|MM17585|Dolopioca_punctulana
TLYFIFGIWAGMVGTSLSLLIRAELGNPGSLIGDDQIYNTIVTAHAFIMIFFMVMPIMIGGFGNWLVPLMLGAPDMAFPRMNNMSFWLLPPSIMLLISSSIVENGAGTGWTVYPPLSSNIAHSGSSVDLAIFSLHLAGISSILGAVNFITTIINMRPNNMSL
DQMPLFVWAVGITALLLLLSLPVLAGAITMLLTDRNLNTSFFDPAGGGDPILYQHL
>LEFIL656-10|MM18954|Donacaula_forficella
TLYFIFGIWAGMLGTSLSLLIRAELGNPGSLIGDDQIYNTIVTAHAFIMIFFMVMPIMIGGFGNWLVPLMLGAPDMAFPRMNNMSFWLLPPSLTLLISSSIVENGAGTGWTVYPPLSSNIAHSGTSVDLAIFSLHLAGISSILGAINFITTIINMRINGLSFDQ
MPLFVWSVGITALLLLLSLPVLAGAITMLLTDRNLNTSFFDPAGGGDPILYQHL
>LEFIL657-10|JF854684|MM18955|Donacaula_forficella
TLYFIFGIWAGMLGTSLSLLIRAELGNPGSLIGDDQIYNTIVTAHAFIMIFFMVMPIMIGGFGNWLVPLMLGAPDMAFPRMNNMSFWLLPPSLTLLISSSIVENGAGTGWTVYPPLSSNIAHSGTSVDLAIFSLHLAGISSILGAINFITTIINMRINGLSFDQ
MPLFVWSVGITALLLLLSLPVLAGAITMLLTDRNLNTSFFDPA?GGDPILYQHL
>LEFIJ2198-14|MM23387|Donacaula_forficella
TLYFIFGIWAGMLGTSLSLLIRAELGNPGSLIGDDQIYNTIVTAHAFIMIFFMVMPIMIGGFGNWLVPLMLGAPDMAFPRMNNMSFWLLPPSLTLLISSSIVENGAGTGWTVYPPLSSNIAHSGTSVDLAIFSLHLAGISSILGAINFITTIINMRINGLSFDQ
MPLFVWSVGITALLLLLSLPVLAGAITMLLTDRNLNTSFFDPAGGGDPILYQHL
>LEFIE979-10|HM874696|MM10413|Donacaula_mucronella
TLYFIFGIWSGMVGTSLSLLIRAELGSPGSLIGDDQIYNSIVTAHAFIMIFFMVMPIMIGGFGNWLVPLMLGAPDMAFPRMNNMSFWMLPPSLTLLISSSIVENGVGTGWTVYPPLSSNIAHSGTSVDLAIFSLHLAGISSILGAINFISTIINMRINGLSFD
QMPLFVWAVGITALLLLLSLPVLAGAITMLLTDRNLNTSFFDPCGGGDPILYQHL
>LEFIF782-10|HM875466|MM13018|Donacaula_mucronella
TLYFIFGIWSGMVGTSLSLLIRAELGNPGSLIGDDQIYNSIVTAHAFIMIFFMVMPIMIGGFGNWLVPLMLGAPDMAFPRMNNMSFWMLPPSLTLLISSSIVENGVGTGWTVYPPLSSNIAHSGTSVDLAIFSLHLAGISSILGAINFISTIINMRINGLSFD
QMPLFVWAVGITALLLLLSLPVLAGAITMLLTDRNLNTSFFDPCGGGDPILYQHL
>LEFIA227-10|HM396570|MM01259|Drepana_curvatula
TLYFIFGIWAGMIGTSLSLLIRAELGNPGSLIGNDQIYNTIVTAHAFIMIFFMVMPIMIGGFGNWLVPLMLGAPDMAFPRMNNMSFWMLPPSLTLLISSSMVENGAGTGWTVYPPLSSNIAHSGSSVDLAIFSLHLAGISSILGAINFITTIINMRLNNM
MFDQMPLFVWAVGITAFLLLLSLPVLAGAITMLLTDRNLNTSFFDPAGGGDPILYQHL
>LEFIA223-10|HM396566|MM01254|Drepana_falcataria
TLYFIFGIWSGMIGTSLSLLIRAELGNPGSLIGNDQIYNTIVTAHAFIMIFFMVMPIMIGGFGNWLVPLMLGAPDMAFPRMNNMSFWMLPPSLTLLISSSIVENGAGTGWTVYPPLSSNIAHGGSSVDLAIFSLHLAGISSILGAINFITTIINMRLNNMMF
DQMPLFVWAVGITAFLLLLSLPVLAGAITMLLTDRNLNTSFFDPAGGGDPILYQHL
>LEFIA224-10|HM396567|MM01255|Drepana_falcataria
TLYFIFGIWSGMIGTSLSLLIRAELGNPGSLIGNDQIYNTIVTAHAFIMIFFMVMPIMIGGFGNWLVPLMLGAPDMAFPRMNNMSFWMLPPSLTLLISSSIVENGAGTGWTVYPPLSSNIAHGGSSVDLAIFSLHLAGISSILGAINFITTIINMRLNNMMF
DQMPLFVWAVGITAFLLLLSLPVLAGAITMLLTDRNLNTSFFDPAGGGDPILYQHL
>LEFID127-10|HM872937|MM05865|Drymonia_ruficornis
TLYFIFGIWAGMVGTSLSLLIRAELGNPGSLIGDDQIYNTIVTAHAFIMIFFMVMPIMIGGFGNWLVPLMLGAPDMAFPRMNNMSFWLLPPSLTLLISSSIVENGAGTGWTVYPPLSSNIAHGGSSVDLAIFSLHLAGISSILGAINFITTIINMRLNGMSF
DQMPLFVWAVGITAFLLLLSLPVLAGAITMLLTDRNLNTSFFDPAGGGDPILYQHL
>LEFIH005-10|HM876641|MM15869|Dryobotodes_eremita
TLYFIFGIWAGMVGTSLSLLIRAELGNPGSLIGDDQIYNTIVTAHAFIMIFFMVMPIMIGGFGNWLVPLMLGAPDMAFPRMNNMSFWLLPPSLTLLISSSIVENGAGTGWTVYPPLSSNIAHGGSSVDLAIFSLHLAGISSILGAINFITTIINMRLNNLSFD
QMPLFIWAVGITAFLLLLSLPVLAGAITMLLTDRNLNTSFFDPAGGGDPILYQHL
>LEFIJ586-10|MM17211|Dryobotodes_eremita
TLYFIFGIWAGMVGTSLSLLIRAELGNPGSLIGDDQIYNTIVTAHAFIMIFFMVMPIMIGGFGNWLVPLMLGAPDMAFPRMNNMSFWLLPPSLTLLISSSIVENGAGTGWTVYPPLSSNIAHGGSSVDLAIFSLHLAGISSILGAINFITTIINMRLNNLSFD
QMPLFIWAVGITAFLLLLSLPVLAGAITMLLTDRNLNTSFFDPAGGGDPILYQHL
>LEFIJ792-10|JF853826|MM17417|Duponchelia_fovealis
TLYFIFGIWSGMIGTSLSLLIRAELGNPGSLIGDDQIYNTIVTAHAFIMIFFMVMPIMIGGFGNWLVPLMLGAPDMAFPRMNNMSFWLLPPSLTLLISSSIVENGAGTGWTVYPPLSSNIAHGGSSVDLAIFSLHLAGISSILGAINFITTIINMRINGLSFDQ
MPLFVWAVGITALLLLLSLPVLAGAITMLLTDRNLNTSFFDPAGGGDPILYQHL
>LEFIC868-10|HM872686|MM05138|Dypterygia_scabriuscula
TLYFIFGIWAGMVGTSLSLLIRAELGTPGSLIGDDQIYNTIVTAHAFIMIFFMVMPIMIGGFGNWLVPLMLGAPDMAFPRMNNMSFWLLPPSLTLLISSSIVENGAGTGWTVYPPLSSNIAHGGSSVDLAIFSLHLAGISSILGAINFITTIINMRLNNLSFD
QMPLFIWAVGITAFLLLLSLPVLAGAITMLLTDRNLNTSFFDPAGGGDPILYQHL
>LEFIF211-10|HM874905|MM10887|Dypterygia_scabriuscula
TLYFIFGIWAGMVGTSLSLLIRAELGTPGSLIGDDQIYNTIVTAHAFIMIFFMVMPIMIGGFGNWLVPLMLGAPDMAFPRMNNMSFWLLPPSLTLLISSSIVENGAGTGWTVYPPLSSNIAHGGSSVDLAIFSLHLAGISSILGAINFITTIINMRLNNLSFD

QMPLFIWAVGITAFLLLLSLPVLAGAITMLLTDRNLNTSFFDPAGGGDPILYQHL
>LEFIF212-10|HM874906|MM10888|Dypterygia_scabriuscula
TLYFIFGIWAGMVGTSLSLLIRAELGTPGSLIGDDQIYNTIVTAHAFIMIFFMVMPIMIGGFGNWLVPLMLGAPDMAFPRMNNMSFWLLPPSLTLLISSSIVENGAGTGWTVYPPLSSNIAHGGSSVDLAIFSLHLAGISSILGAINFITTIINMRLNNLSFD
QMPLFIWAVGITAFLLLLSLPVLAGAITMLLTDRNLNTSFFDPAGGGDPILYQHL
>LEFIJ674-10|JF853762|MM17299|Dyseriocrania_subpurpurella
TLYFIFGIWSGMVGTSLSILIRSELGNPGSLIGDDQIYNVIVTAHAFIMIFFMVMPIMIGGFGNWLVPLMLGAPDMAFPRLNNMSFWLLPPSLTLLISSSFVENGAGTGWTVYPPLSSNIAHAGSSVDLAIFSLHLAGISSILGAVNFITTVINMRPYGMSL
DRMPLFVWAVVITALLLLSLPVLAGAITMLLTDRNLNTSFFDPAGGGDPILYQHL
>LEFIJ675-10|JF853763|MM17300|Dyseriocrania_subpurpurella
TLYFIFGIWSGMVGTSLSILIRSELGNPGSLIGDDQIYNVIVTAHAFIMIFFMVMPIMIGGFGNWLVPLMLGAPDMAFPRLNNMSFWLLPPSLTLLISSSFVENGAGTGWTVYPPLSSNIAHAGSSVDLAIFSLHLAGISSILGAVNFITTVINMRPYGMSL
DRMPLFVWAVVITALLLLSLPVLAGAITMLLTDRNLNTSFFDPAGGGDPILYQHL
>LEFIB295-10|HM871196|MM00799|Dysstroma_citrata
TLYFIFGIWAGMIGTSLSLLIRAELGNPGSLIGDDQIYNTIVTAHAFIMIFFMVMPIMIGGFGNWLVPLMLGAPDMAFPRMNNMSFWLLPPSITLLISSSIVENGAGTGWTVYPPLSSNIAHGGSSVDLAIFSLHLAGISSILGAINFITTIINMRLNNMFFD
QLPLFVWAVGITAFLLLLSLPVLAGAITMLLTDRNLNTSFFDPAGGGDPILYQHL
>LEFIA375-10|HM386717|MM01432|Dysstroma_citrata
TLYFIFGIWAGMIGTSLSLLIRAELGNPGSLIGDDQIYNTIVTAHAFIMIFFMVMPIMIGGFGNWLVPLMLGAPDMAFPRMNNMSFWLLPPSITLLISSSIVENGAGTGWTVYPPLSSNIAHGGSSVDLAIFSLHLAGISSILGAINFITTIINMRLNNMFFD
QLPLFVWAVGITAFLLLLSLPVLAGAITMLLTDRNLNTSFFDPAGGGDPILYQHL
>LEFIC072-10|HM871941|MM03359|Dysstroma_infuscata
TLYFIFGIWAGMIGTSLSLLIRAELGNPGSLIGDDQIYNTIVTAHAFIMIFFMVMPIMIGGFGNWLVPLMLGAPDMAFPRMNNMSFWLLPPSITLLISSSIVENGAGTGWTVYPPLSSNIAHSGSSVDLAIFSLHLAGISSILGAINFITTIINMRLNNMFFD
QLPLFVWAVGITAFLLLLSLPVLAGAITMLLTDRNLNTSFFDPAGGGDPILYQHL
>LEFIC046-10|HM871916|MM03289|Dysstroma_latefasciata
TLYFIFGIWAGMIGTSLSLLIRAELGNPGSLIGDDQIYNTIVTAHAFIMIFFMVMPIMIGGFGNWLVPLMLGAPDMAFPRMNNMSFWLLPPSITLLISSSIVENGAGTGWTVYPPLSSNIAHSGSSVDLAIFSLHLAGISSILGAINFITTIINMRLNNMFFD
QLPLFVWAVGITAFLLLLSLPVLAGAITMLLTDRNLNTSFFDPAGGGDPILYQHL
>LEFIA416-10|HM386758|MM01478|Dysstroma_truncata
TLYFIFGIWAGMIGTSLSLLIRAELGNPGSLIGDDQIYNTIVTAHAFIMIFFMVMPIMIGGFGNWLVPLMLGAPDMAFPRMNNMSFWLLPPSITLLISSSIVESGAGTGWTVYPPLSSNIAHGGSSVDLAIFSLHLAGISSILGAINFITTIINMRLNNMFFD
QLPLFVWAVGITAFLLLLSLPVLAGAITMLLTDRNLNTSFFDPAGGGDPILYQHL
>LEFIJ069-10|KM572719|MM05175|Dysstroma_truncata
TLYFIFGIWAGMIGTSLSLLIRAELGNPGSLIGDDQIYNTIVTAHAFIMIFFMVMPIMIGGFGNWLVPLMLGAPDMAFPRMNNMSFWLLPPSITLLISSSIVENGAGTGWTVYPPLSSNIAHSGSSVDLAIFSLHLAGISSILGAINFITTIINMRLNNMFFD
QLPLFVWAVGITAFLLLLSLPVLAGAITMLLTDRNLNTSFFDPAGGGDPILYQHL
>LEFIJ174-10|KM572667|MM11589|Dysstroma_truncata
TLYFIFGIWAGMIGTSLSLLIRAELGNPGSLIGDDQIYNTIVTAHAFIMIFFMVMPIMIGGFGNWLVPLMLGAPDMAFPRMNNMSFWLLPPSITLLISSSIVENGAGTGWTVYPPLSSNIAHSGSSVDLAIFSLHLAGISSILGAINFITTIINMRLNNMFFD
QLPLFVWAVGITAFLLLLSLPVLAGAITMLLTDRNLNTSFFDPAGGGDPILYQHL
>LEFIK856-10|JF854318|MM18431|Dysstroma_truncata
TLYFIFGIWAGMIGTSLSLLIRAELGNPGSLIGDDQIYNTIVTAHAFIMIFFMVMPIMIGGFGNWLVPLMLGAPDMAFPRMNNMSFWLLPPSITLLISSSIVENGAGTGWTVYPPLSSNIAHSGSSVDLAIFSLHLAGISSILGAINFITTIINMRLNNMFFD
QLPLFVWAVGITAFLLLLSLPVLAGAITMLLTDRNLNTSFFDPAGGGDPILYQHL
>LEFIC913-10|HM872730|MM05253|Eana_argentana
TLYFIFGIWAGMVGTSLSLLIRAELGNPGSLIGDDQIYNTIVTAHAFIMIFFMVMPIMIGGFGNWLVPLMLGAPDMAFPRMNNMSFWLLPPSIMLLISSSIVENGAGTGWTVYPPLSSNIAHGGSSVDLAIFSLHLAGISSILGAVNFITTIINMRPNNMS
LDQMPLFVWSVGITALLLLLSLPVLAGAITMLLTDRNLNTSFFDPAGGGDPILYQHL
>LEFIB897-10|HM871774|MM02993|Eana_osseana
TLYFIFGIWAGMVGTSLSLLIRAELGNPGSLIGDDQIYNTIVTAHAFIMIFFMVMPIMIGGFGNWLVPLMLGAPDMAFPRMNNMSFWLLPPSIMLLISSSIVENGAGTGWTVYPPLSSNIAHSGSSVDLAIFSLHLAGISSILGAVNFITTIINMRPNNMSL
DQMPLFVWSVGITALLLLLSLPVLAGAITMLLTDRNLNTSFFDPAGGGDPILYQHL
>LEFIA786-10|HM386927|MM04140|Eana_osseana
TLYFIFGIWAGMVGTSLSLLIRAELGNPGSLIGDDQIYNTIVTAHAFIMIFFMVMPIMIGGFGNWLVPLMLGAPDMAFPRMNNMSFWLLPPSIMLLISSSIVENGAGTGWTVYPPLSSNIAHSGSSVDLAIFSLHLAGISSILGAVNFITTIINMRPNNMSL
DQMPLFVWSVGITALLLLLSLPVLAGAITMLLTDRNLNTSFFDPAGGGDPILYQHL
>LEFIC119-10|HM871966|MM03455|Eana_penziana
TLYFIFGIWAGMVGTSLSLLIRTELGNPGSLIGDDQIYNTIVTAHAFIMIFFMVMPIMIGGFGNWLVPLMLGAPDMAFPRMNNMSFWLLPPSIMLLISSSIVENGAGTGWTVYPPLSSNIAHSGSSVDLAIFSLHLAGISSILGAVNFITTIINMRPNNMSL
DQMPLFVWSVGITALLLLLSLPVLAGAITMLLTDRNLNTSFFDPAGGGDPILYQHL
>LEFIC154-10|HM872000|MM03500|Eana_penziana

TLYFIFGIWAGMVGTSLSLLIRTELGNPGSLIGDDQIYNTIVTAHAFIMIFFMVMPIMIGGFGNWLVPLMLGAPDMAFPRMNNMSFWLLPPSIMLLISSSIVENGAGTGWTVYPPLSSNIAHSGSSVDLAIFSLHLAGISSILGAVNFITTIINMRPNNMSL
DQMPLFVWSVGITALLLLLSLPVLAGAITMLLTDRNLNTSFFDPAGGGDPILYQHL
>LEFIE117-10|HM873865|MM08314|Eana_penziana
TLYFIFGIWAGMVGTSLSLLIRTELGNPGSLIGDDQIYNTIVTAHAFIMIFFMVMPIMIGGFGNWLVPLMLGAPDMAFPRMNNMSFWLLPPSIMLLISSSIVENGAGTGWTVYPPLSSNIAHSGSSVDLAIFSLHLAGISSILGAVNFITTIINMRPNNMSL
DQMPLFVWSVGITALLLLLSLPVLAGAITMLLTDRNLNTSFFDPAGGGDPILYQHL
>LEFIB028-10|HM870939|MM00114|Earias_clorana
TLYFIFGIWAGMVGTSLSLLIRAELGTPGSLIGDDQIYNTIVTAHAFIMIFFMVMPIMIGGFGNWLVPLMLGAPDMAFPRMNNMSFWLLPPSITLLISSSIVENGAGTGWTVYPPLSSNIAHGGSSVDLAIFSLHLAGISSILGAINFITTIINMRLNNLSFD
QMPLFVWAVGITAFLLLLSLPVLAGAITMLLTDRNLNTSFFDPAGGGDPILYQHL
>LEFID176-10|HM872983|MM05959|Earophila_badiata
TLYFIFGIWAGMIGTSLSLLIRAELGNPGSLIGDDQIYNTIVTAHAFIMIFFMVMPIMIGGFGNWLVPLMLGAPDMAFPRMNNMSFWLLPPSITLLISSSIVESGAGTGWTVYPPLSSNIAHGGSSVDLAIFSLHLAGISSILGAINFITTIINMRLNNMYFD
QLPLFVWAVGITAFLLLLSLPVLAGAITMLLTDRNLNTSFFDPAGGGDPILYQHL
>LEFID199-10|HM873002|MM06000|Earophila_badiata
TLYFIFGIWAGMIGTSLSLLIRAELGNPGSLIGDDQIYNTIVTAHAFIMIFFMVMPIMIGGFGNWLVPLMLGAPDMAFPRMNNMSFWLLPPSITLLISSSIVESGAGTGWTVYPPLSSNIAHGGSSVDLAIFSLHLAGISSILGAINFITTIINMRLNNMYFD
QLPLFVWAVGITAFLLLLSLPVLAGAITMLLTDRNLNTSFFDPAGGGDPILYQHL
>LEEUA631-11|MM20690|Eccopisa_effractella
TLYFIFGIWAGMVGTSLSLLIRAELGTPGSLIGDDQIYNTIVTGHAFIMIFFMVMPIMIGGFGNWLVPLMLGAPDMAFPRMNNMSFWLLPPSLTLLISSSIVENGAGTGWTVYPPLSSNIAHSGSSVDLAIFSLHLAGISSILGAINFITTIINMKLNGLSFD
QMPLFVWAVGITALLLLLSLPVLAGAITMLLTDRNLNTSFFDPAGGGDPILYQHL
>LEFIF029-10|HM874740|MM10468|Ecliptopera_capitata
TLYFIFGIWAGMIGTSLSLLIRAELGNPESLIGDDQIYNTIVTAHAFIMIFFMVMPIMIGGFGNWLVPLMLGAPDMAFPRMNNMSFWLLPPSIALLISSSIVENGVGTGWTVYPPLSSNIAHGGSSVDLAIFSLHLAGISSILGAINFITTIINMRLNSMFFD
QLPLFVWAVGITAFLLLLSLPVLAGAITMLLTDRNLNTSFFDPAGGGDPILYQHL
>LEFIK851-10|JN279411|MM18426|Ecliptopera_capitata
TLYFIFGIWAGMIGTSLSLLIRAELGNPGSLIGDDQIYNTIVTAHAFIMIFFMVMPIMIGGFGNWLVPLMLGAPDMAFPRMNNMSFWLLPPSITLLISSSIVENGVGTGWTVYPPLSSNIAHGGSSVDLAIFSLHLAGISSILGAINFITTIINMRLNSMFFD
QLPLFVWAVGITAFLLLLSLPVLAGAITMLLTDRNLNTSFFDPAGGGDPILY?HL
>LEFIA281-10|HM386625|MM01321|Ecliptopera_silaceata
TLYFIFGIWAGMIGTSLSLLIRAELGNPGSLIGDDQIYNTIVTAHAFIMIFFMVMPIMIGGFGNWLVPLMLGAPDMAFPRMNNMSFWLLPPSITLLISSSIVENGAGTGWTVYPPLSSNIAHGGSSVDLAIFSLHLAGISSILGAINFITTIINMRLNNMFFD
QLPLFVWAVGITAFLLLLSLPVLAGAITMLLTDRNLNTSFFDPAGGGDPILYQHL
>LEFIE688-10|HM874409|MM09664|Ectoedemia_agrimoniae
TLYFMFGIWSGLVGTSLSLLIRAELGNPGSLIGDDQIYNTIVTAHAFIMIFFMVMPIMIGGFGNWLVPLMLGAPDMAFPRLNNMSFWLLPPSLTLLISSSIVENGVGTGWTVYPPLSANIAHSGSSVDLAIFSLHLAGISSILGAINFITTVINMRTNMMSF
DQMPLFVWAVAITALLLLLSLPVLAGAITMLLTDRNLNTSFFDPMGGGDPILYQHL
>LEFIG468-10|HM876144|MM14509|Ectoedemia_albibimaculella
TLYFMFGIWSGMIGTSLSLLIRAELGNPGSLIGDDQIYNTIVTAHAFIMIFFMVMPIMIGGFGNWLVPIMLGAPDMAFPRLNNMSFWLLPPSLTLLISSSIVETGVGTGWTVYPPLSSNIAHSGSSVDLAIFSLHLAGISSILGAINFITTVINMRTYGMSFD
QMPLFIWAVTITALLLLLSLPVLAGAITMLLTDRNLNTSFFDPMGGGDPILYQHL
>LEFIG469-10|HM876145|MM14510|Ectoedemia_albibimaculella
TLYFMFGIWSGMIGTSLSLLIRAELGNPGSLIGDDQIYNTIVTAHAFIMIFFMVMPIMIGGFGNWLVPIMLGAPDMAFPRLNNMSFWLLPPSLTLLISSSIVETGVGTGWTVYPPLSSNIAHSGSSVDLAIFSLHLAGISSILGAINFITTVINMRTYGMSFD
QMPLFIWAVTITALLLLLSLPVLAGAITMLLTDRNLNTSFFDPMGGGDPILYQHL
>LEFIB360-10|HM871260|MM00917|Ectoedemia_albifasciella
TLYFMFGIWSGMIGTSLSLLIRAELGNPGSLIGDDQIYNTIVTAHAFIMIFFMVMPIMIGGFGNWLVPLMLGAPDMAFPRLNNMSFWLLPPSLTLLVSSSIVENGVGTGWTVYPPLSANIAHSGASVDLAIFSLHLAGISSILGAINFITTVINMRAYGMSF
DQMPLFVWAVAITALLLLLSLPVLAGAITMLLTDRNLNTSFFDPMGGGDPILYQHL
>LEFIE693-10|HM874414|MM09671|Ectoedemia_albifasciella
TLYFMFGIWSGMIGTSLSLLIRAELGNPGSLIGDDQIYNTIVTAHAFIMIFFMVMPIMIGGFGNWLVPLMLGAPDMAFPRLNNMSFWLLPPSLTLLVSSSIVENGVGTGWTVYPPLSANIAHSGASVDLAIFSLHLAGISSILGAINFITTVINMRAYGMSF
DQMPLFVWAVAITALLLLLSLPVLAGAITMLLTDRNLNTSFFDPMGGGDPILYQHL
>LEEUA028-11|MM19341|Ectoedemia_amani
TLYFIFG?WSGMIGTSLSLLIRAELGNPGSLIGDDQIYNSIVTAHAFIMIFFMVMPIMIGGFGNWLVPLMLGAPDMAFPRLNNMSFWLLPPSLMLLISSSIVENGVGTGWTVYPPLSSNIAHSGSSVDLAIFSLHLAGISSILGAINFITTVINMRTNKMSFD
QMPLFVWAVAITALLLLLSLPVLAGAITMLLTDRNLNTSFFDPMGGGDPILYQHL
>LEFIB339-10|HM871239|MM00885|Ectoedemia_angulifasciella
TLYFMFGIWSGLLGTSLSLLIRAELGNPGSLIGDDQIYNTIVTAHAFIMIFFMVMPIMIGGFGNWLVPLMLGAPDMAFPRLNNMSFWLLPPSLTLLISSSIVENGAGTGWTVYPPLSANIAHSGSSVDLAIFSLHLAGISSILGAINFITTVINMRAYGMSFD
QMPLFVWAVAITALLLLLSLPVLAGAITMLLTDRNLNTSFFDPMGGGDPILYQHL

>LEFIE655-10|HM874378|MM09617|Ectoedemia_angulifasciella
TLYFMFGIWSGLLGTSLSLLIRAELGNPGSLIGDDQIYNTIVTAHAFIMIFFMVMPIMIGGFGNWLVPLMLGAPDMAFPRLNNMSFWLLPPSLTLLISSSIVENGAGTGWTVYPPLSANIAHSGSSVDLAIFSLHLAGISSILGAINFITTVINMRAYGMSFD
QMPLFVWAVAITALLLLLSLPVLAGAITMLLTDRNLNTSFFDPMGGGDPILYQHL
>LEFIE666-10|HM874388|MM09631|Ectoedemia_arcuatella
TLYFMFGIWSGLLGTSLSLLIRAELGNPGSLIGDDQIYNTIVTAHAFIMIFFMVMPIMIGGFGNWLVPLMLGAPDMAFPRLNNMSFWLLPPSLTLLISSSIVENGVGTGWTVYPPLSANIAHSGSSVDLAIFSLHLAGISSILGAINFITTVINMRAYAMSFD
QMPLFVWAVAITALLLLLSLPVLAGAITMLLTDRNLNTSFFDPMGGGDPILYQHL
>LEFIJ1041-11|MM20732|Ectoedemia_arcuatella
TLYFMFGIWSGLLGTSLSLLIRAELGNPGSLIGDDQIYNTIVTAHAFIMIFFMVMPIMIGGFGNWLVPLMLGAPDMAFPRLNNMSFWLLPPSLTLLISSSIVENGVGTGWTVYPPLSANIAHSGSSVDLAIFSLHLAGISSILGAINFITTVINMRAYGMSFD
QMPLFVWAVAITALLLLLSLPVLAGAITMLLTDRNLNTSFFDPMGGGDPILYQHL
>LEFIJ1042-11|MM20736|Ectoedemia_arcuatella
TLYFMFGIWSGLLGTSLSLLIRAELGNPGSLIGDDQIYNTIVTAHAFIMIFFMVMPIMIGGFGNWLVPLMLGAPDMAFPRLNNMSFWLLPPSLTLLISSSIVENGVGTGWTVYPPLSANIAHSGSSVDLAIFSLHLAGISSILGAINFITTVINMRAYGMSFD
QMPLFVWAVAITALLLLLSLPVLAGAITMLLTDRNLNTSFFDPMGGGDPILYQHL
>LEFIG090-10|HM875769|MM13758|Ectoedemia_argyropeza
TLYFMFGIWSGMVGTSLSLLIRAELGNPGSLIGDDQIYNTIVTAHAFIMIFFMVMPIMIGGFGNWLVPLMLGAPDMAFPRLNNMSFWLLPPSLTLLISSSVVENGVGTGWTVYPPLSANIAHSGSSVDLAIFSLHLAGISSILGAINFITTVINMRTNGMS
FDQMPLFVWAVAITALLLLLSLPVLAGAITMLLTDRNLNTSFFDPMGGGDPILYQHL
>LEFIJ305-10|JF853540|MM15194|Ectoedemia_argyropeza
TLYFMFGIWSGMVGTSLSLLIRAELGNPGSLIGDDQIYNTIVTAHAFIMIFFMVMPIMIGGFGNWLVPLMLGAPDMAFPRLNNMSFWLLPPSLTLLISSSVVENGVGTGWTVYPPLSANIAHSGSSVDLAIFSLHLAGISSILGAINFITTVINMRTNGMS
FDQMPLFVWAVAITALLLLLSLPVLAGAITMLLTDRNLNTSFFDPMGGGDPILYQHL
>LEEUA026-11|MM19339|Ectoedemia_decentella
TLYFMFGIWSGMIGTSLSLLIRMELGNPGSLIGDDQIYNTIVTAHAFIMIFFMVMPIMIGGFGNWLVPIMLGAPDMAFPRLNNMSFWLLPPSLTLLISSSIVETGVGTGWTVYPPLSSNIAHSGSSVDLAIFSLHLAGISSILGAINFITTVINMRAYGMSFD
QMSLFIWAVAITALLLLLSLPVLAGAITMLLTDRNLNTSFFDPMGGGDPILYQHL
>LEEUA029-11|JN284003|MM19342|Ectoedemia_hannoverella
TLYFMFGIWSGMVGTSLSLLIRAELGNPGSLIGDDQIYNTIVTAHAFIMIFFMVMPIMIGGFGNWLIPLMLGSPDMAFPRLNNMSFWLLPPSLTLLISSSVVENGAGTGWTVYPPLSANIAHSGSSVDLAIFSLHLAGISSILGAINFITTVINMRTNGMSF
DQMPLFVWAVAITALLLLLSLPVLAGAITMLLTDRNLNTSFFDPMGGGDPILYQHL
>LEFIK486-10|JF854096|MM18061|Ectoedemia_intimella
TLYFMFGIWSGMVGTSLSLLIRAELGNPGSLIGDDQIYNTIVTAHAFIMIFFMVMPIMIGGFGNWLVPLMLGAPDMAFPRLNNMSFWLLPPSLTLLISSSIVENGAGTGWTVYPPLSANIAHSGSSVDLAIFSLHLAGISSILGAINFITTVINMRTNGMSF
DQMPLFVWAVAITALLLLLSLPVLAGAITMLLTDRNLNTSFFDPMGGGDPILYQHL
>LEEUA025-11|MM19338|Ectoedemia_louisella
TLYFMFGIWSGMVGTSLSLLIRMELGNPGSLIGDDQIYNTIVTAHAFIMIFFMVMPIMIGGFGNWLVPIMLGAPDMAFPRLNNMSFWLLPPSLTLLISSSIVETGVGTGWTVYPPLSSNIAHSGSSVDLAIFSLHLAGISSILGAINFITTVINMRAYGMSF
DQMSLFIWAVAITALLLLLSLPVLAGAITMLLTDRNLNTSFFDPMGGGDPILYQHL
>LEFIG540-10|HM876213|MM14678|Ectoedemia_minimella
TLYFIFGIWSGLVGTSLSLLIRAELGNPGSLIGDDQIYNTIVTAHAFIMIFFMVMPIMIGGFGNWLVPLMLGAPDMAFPRLNNMSFWLLPPSLTLLISSSIIENGVGTGWTVYPPLSANIAHSGSSVDLAIFSLHLAGISSILGAINFITTVINMRTNGMSFDQ
MPLFVWAVAITALLLLLSLPVLAGAITMLLTDRNLNTSFFDPMGGGDPILYQHL
>LEFIK378-10|JF854011|MM17953|Ectoedemia_minimella
TLYFIFGIWSGLVGTSLSLLIRAELGNPGSLIGDDQIYNTIVTAHAFIMIFFMVMPIMIGGFGNWLVPLMLGAPDMAFPRLNNMSFWLLPPSLTLLISSSIIENGVGTGWTVYPPLSANIAHSGSSVDLAIFSLHLAGISSILGAINFITTVINMRTNGMSFDQ
MPLFVWAVAITALLLLLSLPVLAGAITMLLTDRNLNTSFFDPMGGGDPILYQHL
>LEFIB329-10|HM871229|MM00868|Ectoedemia_occultella
TLYFIFGIWSGLVGTSLSLLIRAELGNPGSFIGDDQIYNTIVTAHAFIMIFFMVMPIMIGGFGNWLVPLMLGAPDMAFPRLNNMSFWLLPPSLTLLISSSIVENGAGTGWTVYPPLSSNIAHSGSSVDLAIFSLHLAGISSILGAINFITTVINMRTNGMSFD
QMPLFVWAVAITALLLLLSLPVLAGAITMLLTDRNLNTSFFDPMGGGDPILYQHL
>LEFIG194-10|HM875874|MM14074|Ectoedemia_occultella
TLYFIFGIWSGLVGTSLSLLIRAELGNPGSFIGDDQIYNTIVTAHAFIMIFFMVMPIMIGGFGNWLVPLMLGAPDMAFPRLNNMSFWLLPPSLTLLISSSIVENGAGTGWTVYPPLSSNIAHSGSSVDLAIFSLHLAGISSILGAINFITTVINMRTNGMSFD
QMPLFVWAVAITALLLLLSLPVLAGAITMLLTDRNLNTSFFDPMGGGDPILYQHL
>LEFIB354-10|HM871254|MM00905|Ectoedemia_rubivora
TLYFMFGIWSGLLGTSLSLLIRAELGNPGSLIGDDQIYNTIVTAHAFIMIFFMVMPIMIGGFGNWLVPLMLGAPDMAFPRLNNMSFWLLPPSLTLLISSSIVENGVGTGWTVYPPLSANIAHSGSSVDLAIFSLHLAGISSILGAINFITTVINMRAYGMSFD
QMPLFVWAVAITALLLLLSLPVLAGAITMLLTDRNLNTSFFDPMGGGDPILYQHL
>LEFIE317-10|HM874043|MM08694|Ectoedemia_rubivora
TLYFMFGIWSGLLGTSLSLLIRAELGNPGSLIGDDQIYNTIVTAHAFIMIFFMVMPIMIGGFGNWLVPLMLGAPDMAFPRLNNMSFWLLPPSLTLLISSSIVENGVGTGWTVYPPLSANIAHSGSSVDLAIFSLHLAGISSILGAINFITTVINMRAYGMSFD

QMPLFVWAVAITALLLLLSLPVLAGAITMLLTDRNLNTSFFDPMGGGDPILYQHL
>LEFIG619-10|HM876285|MM14835|Ectoedemia_septembrella
TLYFIFGIWSGMLGTSLSLLIRAELGNPGSLIGNDQIYNTIVTAHAFIMIFFMVMPIMIGGFGNWLVPLMLGAPDMAFPRLNNMSFWLLPPSLTLLISSSIVESGAGTGWTVYPPLSSNIAHSGSSVDLAIFSLHLAGISSILGAINFITTVINMRTNGMSFD
QMPLFVWAVAITALLLLLSLPVLAGAITMLLTDRNLNTSFFDPMGGGDPILYQHL
>LEFIJ264-10|JF853504|MM14880|Ectoedemia_sericopeza
TLYFMFGIWSGMVGTSLSLLIRMELGNPGSLIGDDQIYNTIVTAHAFIMIFFMVMPIMIGGFGNWLVPIMLGAPDMAFPRLNNMSFWLLPPSLTLLISSSIVETGVGTGWTVYPPLSSNIAHSGSSVDLAIFSLHLAGISSILGAINFITTVINMRAYGMSF
DQMSLFIWAVAITALLLLLSLPVLAGAITMLLTDRNLNTSFFDPMGGGDPILYQHL
>LEFIJ1177-11|KM572908|MM21037|Ectoedemia_sericopeza
TLYFMFGIWSGMVGTSLSLLIRMELGNPGSLIGDDQIYNTIVTAHAFIMIFFMVMPIMIGGFGNWLVPIMLGAPDMAFPRLNNMSFWLLPPSLTLLISSSIVETGVGTGWTVYPPLSSNIAHSGSSVDLAIFSLHLAGISSILGAINFITTVINMRAYGMSF
DQMSLFIWAVAITALLLLLSLPVLAGAITMLLTDRNLNTSFFDPMGGGDPILYQHL
>LEFIE672-10|HM874394|MM09639|Ectoedemia_subbimaculella
TLYFMFGIWSGMIGTSLSLLIRAELGNPGSLIGDDQIYNTIVTAHAFIMIFFMVMPIMIGGFGNWLVPLMLGAPDMAFPRLNNMSFWLLPPSLTLLISSSVVENGAGTGWTVYPPLSANIAHSGASVDLAIFSLHLAGISSILGAINFITTVINMRAYGMSF
DQMPLFVWAVAITALLLLLSLPVLAGAITMLLTDRNLNTSFFDPMGGGDPILYQHL
>LEFIE761-10|HM874480|MM09899|Ectoedemia_turbidella
TLYFMFGIWSGMVGTSLSLLIRAELGNPGSLIGDDQIYNTIVTAHAFIMIFFMVMPIMIGGFGNWLVPLMLGAPDMAFPRLNNMSFWLLPPSLTLLISSSVVENGVGTGWTVYPPLSANIAHSGSSVDLAIFSLHLAGISSILGAINFITTVINMRTNGMS
FDQMPLFVWAVAITALLLLLSLPVLAGAITMLLTDRNLNTSFFDPMGGGDPILYQHL
>LEFIE763-10|HM874482|MM09901|Ectoedemia_turbidella
TLYFMFGIWSGMVGTSLSLLIRAELGNPGSLIGDDQIYNTIVTAHAFIMIFFMVMPIMIGGFGNWLVPLMLGAPDMAFPRLNNMSFWLLPPSLTLLISSSVVENGVGTGWTVYPPLSANIAHSGSSVDLAIFSLHLAGISSILGAINFITTVINMRTNGMS
FDQMPLFVWAVAITALLLLLSLPVLAGAITMLLTDRNLNTSFFDPMGGGDPILYQHL
>LEFIB769-10|HM871646|MM02640|Ectoedemia_weaveri
TLYFIFGTWSGMVGTSLSLLIRAELGNPGSLIGNDQIYNTIVTAHAFIMIFFMVMPIMIGGFGNWLIPLMLGAPDMAFPRLNNMSFWLLPPSLTLLISSSIVESGAGTGWTVYPPLSSNIAHSGSSVDLAIFSLHLAGISSILGAINFITTIINMRTNGMSFDQ
MSLFIWAVAITALLLLLSLPVLAGAITMLLTDRNLNTSFFDPMGGGDPILYQHL
>LEFIB118-10|HM871024|MM00472|Ectropis_crepuscularia
TLYFIFGIWAGMVGTSLSLLIRAELGNPGSLIGDDQIYNTIVTAHAFIMIFFMVMPIMIGGFGNWLVPLMLGAPDMAFPRMNNMSFWLLPPSLTLLISSSIVENGAGTGWTVYPPLSSNIAHGGSSVDLAIFSLHLAGISSILGAINFITTIINMRLNNLSFD
QMPLFVWAVGITAFLLLLSLPVLAGAITMLLTDRNLNTSFFDPAGGGDPILYQHL
>LEFIB200-10|HM871104|MM00643|Ectropis_crepuscularia
TLYFIFGIWAGMVGTSLSLLIRAELGNPGSLIGDDQIYNTIVTAHAFIMIFFMVMPIMIGGFGNWLVPLMLGAPDMAFPRMNNMSFWLLPPSLTLLISSSIVENGAGTGWTVYPPLSSNIAHGGSSVDLAIFSLHLAGISSILGAINFITTIINMRLNNLSFD
QMPLFVWAVGITAFLLLLSLPVLAGAITMLLTDRNLNTSFFDPAGGGDPILYQHL
>LEFIA256-10|HM386600|MM01293|Ectropis_crepuscularia
TLYFIFGIWAGMVGTSLSLLIRAELGNPGSLIGDDQIYNTIVTAHAFIMIFFMVMPIMIGGFGNWLVPLMLGAPDMAFPRMNNMSFWLLPPSLTLLISSSIVENGAGTGWTVYPPLSSNIAHGGSSVDLAIFSLHLAGISSILGAINFITTIINMRLNNLSFD
QMPLFVWAVGITAFLLLLSLPVLAGAITMLLTDRNLNTSFFDPAGGGDPILYQHL
>LEFIA371-10|HM386713|MM01428|Ectropis_crepuscularia
TLYFIFGIWAGMVGTSLSLLIRAELGNPGSLIGDDQIYNTIVTAHAFIMIFFMVMPIMIGGFGNWLVPLMLGAPDMAFPRMNNMSFWLLPPSLTLLISSSIVENGAGTGWTVYPPLSSNIAHGGSSVDLAIFSLHLAGISSILGAINFITTIINMRLNNLSFD
QMPLFVWAVGITAFLLLLSLPVLAGAITMLLTDRNLNTSFFDPAGGGDPILYQHL
>LEFID322-10|HM873119|MM06210|Ectropis_crepuscularia
TLYFIFGIWAGMVGTSLSLLIRAELGNPGSLIGDDQIYNTIVTAHAFIMIFFMVMPIMIGGFGNWLVPLMLGAPDMAFPRMNNMSFWLLPPSLTLLISSSIVENGAGTGWTVYPPLSSNIAHGGSSVDLAIFSLHLAGISSILGAINFITTIINMRLNNLSFD
QMPLFVWAVGITAFLLLLSLPVLAGAITMLLTDRNLNTSFFDPAGGGDPILYQHL
>LEFID949-10|HM873699|MM07791|Ectropis_crepuscularia
TLYFIFGIWAGMVGTSLSLLIRAELGNPGSLIGDDQIYNTIVTAHAFIMIFFMVMPIMIGGFGNWLVPLMLGAPDMAFPRMNNMSFWLLPPSLTLLISSSIVENGAGTGWTVYPPLSSNIAHGGSSVDLAIFSLHLAGISSILGAINFITTIINMRLNNLSFD
QMPLFVWAVGITAFLLLLSLPVLAGAITMLLTDRNLNTSFFDPAGGGDPILYQHL
>LEFIJ104-10|KM573109|MM07792|Ectropis_crepuscularia
TLYFIFGIWAGMVGTSLSLLIRAELGNPGSLIGDDQIYNTIVTAHAFIMIFFMVMPIMIGGFGNWLVPLMLGAPDMAFPRMNNMSFWLLPPSLTLLISSSIVENGAGTGWTVYPPLSSNIAHGGSSVDLAIFSLHLAGISSILGAINFITTIINMRLNNLSFD
QMPLFVWAVGITAFLLLLSLPVLAGAITMLLTDRNLNTSFFDPAGGGDPILYQHL
>LEFIJ175-10|KM573644|MM11593|Ectropis_crepuscularia
TLYFIFGIWAGMVGTSLSLLIRAELGNPGSLIGDDQIYNTIVTAHAFIMIFFMVMPIMIGGFGNWLVPLMLGAPDMAFPRMNNMSFWLLPPSLTLLISSSIVENGAGTGWTVYPPLSSNIAHGGSSVDLAIFSLHLAGISSILGAINFITTIINMRLNNLSFD
QMPLFVWAVGITAFLLLLSLPVLAGAITMLLTDRNLNTSFFDPAGGGDPILYQHL
>LEFIF734-10|HM875418|MM12856|Ectropis_crepuscularia

TLYFIFGIWAGMVGTSLSLLIRAELGNPGSLIGDDQIYNTIVTAHAFIMIFFMVMPIMIGGFGNWLVPLMLGAPDMAFPRMNNMSFWLLPPSLTLLISSSIVENGAGTGWTVYPPLSSNIAHGGSSVDLAIFSLHLAGISSILGAINFITTIINMRLNNLSFD
QMPLFVWAVGITAFLLLLSLPVLAGAITMLLTDRNLNTSFFDPAGGGDPILYQHL
>LEFIJ745-10|JF853800|MM17370|Ectropis_crepuscularia
TLYFIFGIWAGMVGTSLSLLIRAELGNPGSLIGDDQIYNTIVTAHAFIMIFFMVMPIMIGGFGNWLVPLMLGAPDMAFPRMNNMSFWLLPPSLTLLISSSIVENGAGTGWTVYPPLSSNIAHGGSSVDLAIFSLHLAGISSILGAINFITTIINMRLNNLSFD
QMPLFVWAVGITAFLLLLSLPVLAGAITMLLTDRNLNTSFFDPAGGGDPILYQHL
>LEFIK417-10|JF854046|MM17992|Ectropis_crepuscularia
TLYFIFGIWAGMVGTSLSLLIRAELGNPGSLIGDDQIYNTIVTAHAFIMIFFMVMPIMIGGFGNWLVPLMLGAPDMAFPRMNNMSFWLLPPSLTLLISSSIVENGAGTGWTVYPPLSSNIAHGGSSVDLAIFSLHLAGISSILGAINFITTIINMRLNNLSFD
QMPLFVWAVGITAFLLLLSLPVLAGAITMLLTDRNLNTSFFDPAGGGDPILYQHL
>LEFIE702-10|HM874423|MM09687|Eidophasia_messingiella
TLYFLFGIWSGMLGTSLSLLIRAELGNPGSLIGDDQIYNTIVTAHAFIMIFFMVMPIVIGGFGNWLIPLMLGAPDMAFPRMNNMSFWLLPPSLTLLISSSIVENGAGTGWTVYPPLSSNIAHSGSSVDLAIFSLHLAGISSILGAINFITTIINMKSNGMSFDR
MPLFVWAVGITAILLLLSLPVLAGAITMLLTDRNLNTSFFDPAGGGDPILYQHL
>LEFIA101-10|HM396448|MM01024|Eilema_complana
TLYFIFGIWAGMVGTSLSLLIRAELGNPGSLIGDDQIYNTIVTAHAFIMIFFMVMPIMIGGFGNWLVPLMLGAPDMAFPRMNNMSFWLLPPSLMLLISSSIVENGAGTGWTVYPPLSSNIAHSGSSVDLAIFSLHLAGISSILGAINFITTIINMRLNKLMF
DQMPLFVWAVGITAFLLLLSLPVLAGAITMLLTDRNLNTSFFDPAGGGDPILYQHL
>LEFIA108-10|HM396455|MM01031|Eilema_depressa
TLYFIFGIWAGMVGTSLSLLIRAELGNPGSLIGDDQIYNTIVTAHAFIMIFFMVMPIMIGGFGNWLIPLMLGAPDMAFPRMNNMSFWLLPPSLTLLISSSIVENGAGTGWTVYPPLSSNIAHSGSSVDLAIFSLHLAGISSILGAINFITTIINMRLNKLMFD
QMPLFVWAVGITAFLLLLSLPVLAGAITMLLTDRNLNTSFFDPAGGGDPILYQHL
>LEFIA103-10|HM396450|MM01026|Eilema_griseola
TLYFIFGVWAGMVGTSLSLLIRAELGNPGSLIGDDQIYNTIVTAHAFIMIFFMVMPIMIGGFGNWLVPLMLGAPDMAFPRMNNMSFWLLPPSLTLLISSSIVENGAGTGWTVYPPLSSNIAHSGSSVDLAIFSLHLAGISSILGAINFITTIINMRLNKLMF
DQMPLFVWAVGITAFLLLLSLPVLAGAITMLLTDRNLNTSFFDPAGGGDPILYQHL
>LEFIF381-10|HM875066|MM11628|Eilema_griseola
TLYFIFGVWAGMVGTSLSLLIRAELGNPGSLIGDDQIYNTIVTAHAFIMIFFMVMPIMIGGFGNWLVPLMLGAPDMAFPRMNNMSFWLLPPSLTLLISSSIVENGAGTGWTVYPPLSSNIAHSGSSVDLAIFSLHLAGISSILGAINFITTIINMRLNKLMF
DQMPLFVWAVGITAFLLLLSLPVLAGAITMLLTDRNLNTSFFDPAGGGDPILYQHL
>LEFIA099-10|HM396446|MM01022|Eilema_lurideola
TLYFIFGIWAGMVGTSLSLLIRAELGNPGSLIGDDQIYNTIVTAHAFIMIFFMVMPIMIGGFGNWLIPLMLGAPDMAFPRMNNMSFWLLPPSLTLLISSSIVENGAGTGWTVYPPLSSNIAHSGSSVDLAIFSLHLAGISSILGAINFITTIINMRLNKLMFD
QMPLFVWAVGITAFLLLLSLPVLAGAITMLLTDRNLNTSFFDPAGGGDPILYQHL
>LEFIJ574-10|JF853687|MM17199|Eilema_lurideola
TLYFIFGIWAGMVGTSLSLLIRAELGNPGSLIGDDQIYNTIVTAHAFIMIFFMVMPIMIGGFGNWLIPLMLGAPDMAFPRMNNMSFWLLPPSLTLLISSSIVENGAGTGWTVYPPLSSNIAHSGSSVDLAIFSLHLAGISSILGAINFITTIINMRLNKLMFD
QMPLFVWAVGITAFLLLLSLPVLAGAITMLLTDRNLNTSFFDPAGGGDPILYQHL
>LEFIA097-10|HM396444|MM01020|Eilema_lutarella
TLYFIFGIWAGMVGTSLSLLIRAELGNPGSLIGDDQIYNTIVTAHAFIMIFFMVMPIMIGGFGNWLVPLMLGAPDMAFPRMNNMSFWLLPPSLTLLISSSIVENGAGTGWTVYPPLSSNIAHSGSSVDLAIFSLHLAGISSILGAINFITTIINMRLNKLMFD
QMPLFVWAVGITAFLLLLSLPVLAGAITMLLTDRNLNTSFFDPAGGGDPILYQHL
>LEFIA098-10|HM396445|MM01021|Eilema_lutarella
TLYFIFGIWAGMVGTSLSLLIRAELGNPGSLIGDDQIYNTIVTAHAFIMIFFMVMPIMIGGFGNWLVPLMLGAPDMAFPRMNNMSFWLLPPSLTLLISSSIVENGAGTGWTVYPPLSSNIAHSGSSVDLAIFSLHLAGISSILGAINFITTIINMRLNKLMFD
QMPLFVWAVGITAFLLLLSLPVLAGAITMLLTDRNLNTSFFDPAGGGDPILYQHL
>LEFIJ825-10|MM17450|Eilema_lutarella
TLYFIFGIWAGMVGTSLSLLIRAELGNPGSLIGDDQIYNTIVTAHAFIMIFFMVMPIMIGGFGNWLVPLMLGAPDMAFPRMNNMSFWLLPPSLTLLISSSIVENGAGTGWTVYPPLSSNIAHSGSSVDLAIFSLHLAGISSILGAINFITTIINMRLNKLMFD
QMPLFVWAVGITAFLLLLSLPVLAGAITMLLTDRNLNTSFFDPAGGGDPILYQHL
>LEFIF134-10|HM874832|MM10591|Eilema_pygmaeola
TLYFIFGIWAGMVGTSLSLLIRAELGNPGSLIGDDQIYNTIVTAHAFIMIFFMVMPIMIGGFGNWLVPLMLGAPDMAFPRMNNMSFWLLPPSLTLLISSSIVENGAGTGWTVYPPLSSNIAHSGSSVDLAIFSLHLAGISSILGAINFITTIINMRLNKLMFD
QMPLFVWSVGITAFLLLLSLPVLAGAITMLLTDRNLNTSFFDPAGGGDPILYQHL
>LEFIA690-10|HM386835|MM01831|Eilema_sororcula
TLYFIFGIWAGMVGTSLSLLIRAELGNPGSLIGDDQIYNTIVTAHAFIMIFFMVMPIMIGGFGNWLIPLMLGAPDMAFPRMNNMSFWLLPPSLTLLISSSIVENGAGTGWTVYPPLSSNIAHSGSSVDLAIFSLHLAGISSILGAINFITTIINMRLNSLMFD
QMPLFVWAVGITAFLLLLSLPVLAGAITMLLTDRNLNTSFFDPAGGGDPILYQHL
>LEFIK103-10|JN267143|MM17678|Elachista_abiskoella
TLYFIFGIWAGLMGTSLSLLIRTELGNPGSLIGNDQIYNTIVTAHAFIMIFFMVMPIMIGGFGNWLVPLMLGAPDMAFPRMNNMSFWLLPPSLTLLISSSIVENGAGTGWTVYPPLSSNIAHSGSSVDLAIFSLHLAGISSILGAINFITTVINMRLNNMMF
DQMPLFVWAVSITALLLLLSLPVLAGAITMLLTDRNLNTSFFDP?GGGDPILYQHL

>LEFII167-10|KF809277|MM18594|Elachista_abiskoella
TLYFIFGIWAGLMGTSLSLLIRTELGNPGSLIGNDQIYNTIVTAHAFIMIFFMVMPIMIGGFGNWLVPLMLGAPDMAFPRMNNMSFWLLPPSLTLLISSSIVENGAGTGWTVYPPLSSNIAHSGSSVDLAIFSLHLAGISSILGAINFITTVINMRLNNMMF
DQMPLFVWAVSITALLLLLSLPVLAGAITMLLTDRNLNTSFFDPAGGGDPILYQHL
>LEFIA722-10|HM386866|MM02658|Elachista_adscitella
TLYFIFGIWAGMVGTSLSLLIRAELGNPGSLIGNDQIYNTIVTAHAFIMIFFMVMPIMIGGFGNWLVPLMLGAPDMAFPRMNNMSFWLLPPSLTLLIFSSVVENGSGTGWTVYPPLSSNIAHSGSSVDMAIFSLHLAGISSILGAINFITTIINMRLNNMM
FDQMPLFVWAVGITALLLLLSLPVLAGAITMLLTDRNLNTSFFDPAGGGDPILYQHL
>LEFID683-10|HM873444|MM06727|Elachista_adscitella
TLYFIFGIWAGMVGTSLSLLIRAELGNPGSLIGNDQIYNTIVTAHAFIMIFFMVMPIMIGGFGNWLVPLMLGAPDMAFPRMNNMSFWLLPPSLTLLIFSSVVENGSGTGWTVYPPLSSNIAHSGSSVDMAIFSLHLAGISSILGAINFITTIINMRLNNMM
FDQMPLFVWAVGITALLLLLSLPVLAGAITMLLTDRNLNTSFFDPAGGGDPILYQHL
>LEFIE484-10|HM874208|MM09261|Elachista_adscitella
TLYFIFGIWAGMVGTSLSLLIRAELGNPGSLIGNDQIYNTIVTAHAFIMIFFMVMPIMIGGFGNWLVPLMLGAPDMAFPRMNNMSFWLLPPSLTLLIFSSVVENGSGTGWTVYPPLSSNIAHSGSSVDMAIFSLHLAGISSILGAINFITTIINMRLNNMM
FDQMPLFVWAVGITALLLLLSLPVLAGAITMLLTDRNLNTSFFDPAGGGDPILYQHL
>LEFIC381-10|HM872224|MM03911|Elachista_albidella
TLYFIFGIWAGMVGTSLSLLIRAELGNPGSLIGNDQIYNTIVTAHAFIMIFFMVMPIMIGGFGNWLVPLMLGAPDMAFPRMNNMSFWLLPPSLTLLISSSIVENGAGTGWTVYPPLSSNIAHSGSSVDLAIFSLHLAGISSILGAINFITTIINMRLNNLMFD
QMPLFVWAVGITALLLLLSLPVLAGAITMLLTDRNLNTSFFDPAGGGDPILYQHL
>LEFIC382-10|HM872225|MM03912|Elachista_albidella
TLYFIFGIWAGMMGTSLSLLIRAELGNPGSLIGNDQIYNTIVTAHAFIMIFFMVMPIMIGGFGNWLVPLMLGAPDMAFPRMNNMSFWLLPPSLTLLISSSIVENGAGTGWTVYPPLSSNIAHSGSSVDLAIFSLHLAGISSILGAINFITTIINMRLNNLMF
DQMPLFVWAVGITALLLLLSLPVLAGAITMLLTDRNLNTSFFDPAGGGDPILYQHL
>LEFIE994-10|HQ570377|MM10432|Elachista_albidella
TLYFIFGIWAGMVGTSLSLLIRAELGNPGSLIGNDQIYNTIVTAHAFIMIFFMVMPIMIGGFGNWLVPLMLGAPDMAFPRMNNMSFWLLPPSLTLLISSSIVENGAGTGWTVYPPLSSNIAHSGSSVDLAIFSLHLAGISSILGAINFITTIINMRLNNLMFD
QMPLFVWAVGITALLLLLSLPVLAGAITMLLTDRNLNTSFFDPAGGGDPILYQHL
>LEFIG708-10|HM876364|MM15572|Elachista_albidella
TLYFIFGIWAGMVGTSLSLLIRAELGNPGSLIGNDQIYNTIVTAHAFIMIFFMVMPIMIGGFGNWLVPLMLGAPDMAFPRMNNMSFWLLPPSLTLLISSSIVENGAGTGWTVYPPLSSNIAHSGSSVDLAIFSLHLAGISSILGAINFITTIINM?LNNLMFD
QMPLFVWAVGITALLLLLSLPVLAGAITMLLTDRNLNTSFFDPAGGGDPILYQHL
>ELACA298-10|JF847438|MM16213|Elachista_albidella
TLYFIFGIWAGMVGTSLSLLIRAELGNPGSLIGNDQIYNTIVTAHAFIMIFFMVMPIMIGGFGNWLVPLMLGAPDMAFPRMNNMSFWLLPPSLTLLISSSIVENGVGTGWTVYPPLSSNIAHSGSSVDLAIFSLHLAGISSILGAINFITTIINMRLNNLMFD
QMPLFVWAVGITALLLLLSLPVLAGAITMLLTDRNLNTSFFDPAGGGDPILYQHL
>LEFIA725-10|HM386869|MM02662|Elachista_albifrontella
TLYFIFGIWAGMVGTSLSLLIRAELGNPGSLIGNDQIYNTIVTAHAFIMIFFMVMPIMIGGFGNWLVPLMLGAPDMAFPRMNNMSFWLLPPSLTLLISSSIVENGAGTGWTVYPPLSSNIAHNGSSVDLAIFSLHLAGISSILGAINFITTIINMRLNDLMF
DQMPLFVWAVGITALLLLLSLPVLAGAITMLLTDRNLNTSFFDPA?GGDPILYQHL
>LEFID286-10|HM873084|MM06161|Elachista_albifrontella
TLYFIFGIWAGMVGTSLSLLIRAELGNPGSLIGNDQIYNTIVTAHAFIMIFFMVMPIMIGGFGNWLVPLMLGAPDMAFPRMNNMSFWLLPPSLTLLISSSIVENGAGTGWTVYPPLSSNIAHNGSSVDLAIFSLHLAGISSILGAINFITTIINMRLNDLMF
DQMPLFVWAVGITALLLLLSLPVLAGAITMLLTDRNLNTSFFDPAGGGDPILYQHL
>LEFID531-10|HM873296|MM06514|Elachista_albifrontella
TLYFIFGIWAGMVGTSLSLLIRAELGNPGSLIGNDQIYNTIVTAHAFIMIFFMVMPIMIGGFGNWLVPLMLGAPDMAFPRMNNMSFWLLPPSLTLLISSSIVENGAGTGWTVYPPLSSNIAHNGSSVDLAIFSLHLAGISSILGAINFITTIINMRLNDLMF
DQMPLFVWAVGITALLLLLSLPVLAGAITMLLTDRNLNTSFFDPAGGGDPILYQHL
>LEFIF479-10|HM875164|MM12029|Elachista_albifrontella
TLYFIFGIWAGMVGTSLSLLIRAELGNPGSLIGNDQIYNTIVTAHAFIMIFFMVMPIMIGGFGNWLVPLMLGAPDMAFPRMNNMSFWLLPPSLTLLISSSIVENGAGTGWTVYPPLSSNIAHNGSSVDLAIFSLHLAGISSILGAINFITTIINMRLNDLMF
DQMPLFVWAVGITALLLLLSLPVLAGAITMLLTDRNLNTSFFDPAGGGDPILYQHL
>LEFIG287-10|HM875966|MM14235|Elachista_albifrontella
TLYFIFGIWAGMVGTSLSLLIRAELGNPGSLIGNDQIYNTIVTAHAFIMIFFMVMPIMIGGFGNWLVPLMLGAPDMAFPRMNNMSFWLLPPSLTLLISSSIVENGAGTGWTVYPPLSSNIAHNGSSVDLAIFSLHLAGISSILGAINFITTIINMRLNDLMF
DQMPLFVWAVGITALLLLLSLPVLAGAITMLLTDRNLNTSFFDPAGGGDPILYQHL
>LEFIG333-10|HM876012|MM14309|Elachista_albifrontella
TLYFIFGIWAGTVGTSLSLLIRAELGNPGSLIGNDQIYNTIVTAHAFIMIFFMVMPIMIGGFGNWLVPLMLGAPDMAFPRMNNMSFWLLPPSLTLLISSSIVENGAGTGWTVYPPLSSNIAHNGSSVDLAIFSLHLAGISSILGAINFITTIINMRLNDLMFD
QMPLFVWAVGITALLLLLSLPVLAGAITMLLTDRNLNTSFFDPAGGGDPILYQHL
>LEFID823-10|HM873580|MM06929|Elachista_alpinella
TLYFIFGIWAGMVGTSLSLLIRAELGNPGSLIGNDQIYNTIVTAHAFIMIFFMVMPIMIGGFGNWLVPLMLGAPDMAFPRMNNMSFWLLPPSLTLLISGSIVENGAGTGWTVYPPLSSNIAHSGSSVDLTIFSLHLAGISSILGAINFITTIINMRLNNLMF

DQMPLFVWAVGITALLLLLSLPVLAGAITMLLTDRNLNTSFFDPAGGGDPILYQHL
>LEFII129-10|JF853358|MM18556|Elachista_anserinella
TLYFIFGIWAGMMGTSLSLLIRAELGNPGSLIGNDQIYNTIVTAHAFIMIFFMVMPIMIGGFGNWLVPLMLGAPDMAFPRMNNMSFWLLPPSLTLLISSSIVENGAGTGWTVYPPLSSNIAHSGSSVDLAIFSLHLAGISSILGAINFITTIINMRLNNLMF
DQMSLFVWAVGITALLLLLSLPVLAGAITMLLTDRNLNTSFFDPAGGGDPILYQHL
>LEFIA723-10|HM386867|MM02659|Elachista_apicipunctella
TLYFIFGIWAGMVGTSLSLLIRAELGNPGSLIGNDQIYNTIVTAHAFIMIFFMVMPIMIGGFGNWLVPLMLGAPDMAFPRMNNMSFWLLPPSLTLLISSSLVENGAGTGWTVYPPLSANIAHNGSSVDLAIFSLHLAGISSILGAINFITTIINMRLNNLMF
DQMPLFVWAVGITALLLLLSLPVLAGAITMLLTDRNLNTSFFDPAGGGDPILYQHL
>LEFIG231-10|HM875910|MM14139|Elachista_apicipunctella
TLYFIFGIWAGMVGTSLSLLIRAELGNPGSLIGNDQIYNTIVTAHAFIMIFFMVMPIMIGGFGNWLVPLMLGAPDMAFPRMNNMSFWLLPPSLTLLISSSLVENGAGTGWTVYPPLSANIAHNGSSVDLAIFSLHLAGISSILGAINFITTIINMRLNNLMF
DQMPLFVWAVGITALLLLLSLPVLAGAITMLLTDRNLNTSFFDPAGGGDPILYQHL
>LEFIG063-10|HM875742|MM13708|Elachista_argentella
TLYFIFGIWAGLVGTSLSLLIRAELGNPGSLIGNDQIYNTIVTAHAFIMIFFMVMPIMIGGFGNWLVPLMLGAPDMAFPRMNNMSFWLLPPSLTLLIFSSVVENGAGTGWTVYPPLSSNIAHGGSSVDMAIFSLHLAGISSILGAINFITTIINMRLNNMM
FDQMPLFVWAVGITALLLLLSLPVLAGAITMLLTDRNLNTSFFDPAGGGDPILYQHL
>ELACA308-10|KF809278|MM16223|Elachista_atricomella
TLYFIFGIWAGMVGTSLSLLIRAELGNPGSLIGNDQIYNTIVTAHAFIMIFFMVMPIMIGGFGNWLVPLMLGAPDMAFPRMNNMSFWLLPPSLTLLISSSIVENGAGTGWTVYPPLSSNIAHSGSSVDLAIFSLHLAGISSILGAINFITTIINMRLNNLIFD
QMPLFVWAVGITALLLLLSLPVLAGAITMLLTDRNLNTSFFDPAGGGDPILYQHL
>LEFIJ632-10|JF853730|MM17257|Elachista_atricomella
TLYFIFGIWAGMVGTSLSLLIRAELGNPGSLIGNDQIYNTIVTAHAFIMIFFMVMPIMIGGFGNWLVPLMLGAPDMAFPRMNNMSFWLLPPSLTLLISSSIVENGAGTGWTVYPPLSSNIAHSGSSVDLAIFSLHLAGISSILGAINFITTIINMRLNNLIFD
QMPLFVWAVGITALLLLLSLPVLAGAITMLLTDRNLNTSFFDPAGGGDPILYQHL
>LEFID450-10|HQ570346|MM06385|Elachista_baltica
TLYFIFGIWAGMVGTSLSLLIRAELGNPGSLIGNDQIYNTIVTAHAFIMIFFMVMPIMIGGFGNWLVPLMLGAPDMAFPRMNNMSFWLLPPSLTLLISSSVVENGAGTGWTVYPPLSSNIAHSGSSVDLAIFSLHLAGISSILGAINFITTIINMRLNNLMF
DQMPLFVWAVGITALLLLLSLPVLAGAITMLLTDRNLNTSFFDPAGGGDPILYQHL
>LEFII143-10|JN267142|MM18570|Elachista_bedellella
TLYFIFGIWAGMVGTSLSLLIRAELGNPGSLIGNDQIYNTIVTAHAFIMIFFMVMPIMIGGFGNWLVPLMLGAPDMAFPRMNNMSFWLLPPSLTLLISSSVVENGAGTGWTVYPPLSSNIAHSGSSVDMAIFSLHLAGISSILGAINFITTIINMRLNNM
MFDQMPLFVWAVGITAILLLLSLPVLAGAITMLLTDRNLNTSFFDPAGGGDPILYQHL
>LEFII144-10|MM18571|Elachista_bedellella
TLYFIFGIWAGMVGTSLSLLIRAELGNPGSLIGNDQIYNTIVTAHAFIMIFFMVMPIMIGGFGNWLVPLMLGAPDMAFPRMNNMSFWLLPPSLTLLISSSVVENGAGTGWTVYPPLSSNIAHSGSSVDMAIFSLHLAGISSILGAINFITTIINMRLNNM
MFDQMPLFVWAVGITAILLLLSLPVLAGAITMLLTDRNLNTSFFDPAGGGDPILYQHL
>LEFII161-10|JF853373|MM18588|Elachista_bedellella
TLYFIFGIWAGMVGTSLSLLIRAELGNPGSLIGNDQIYNTIVTAHAFIMIFFMVMPIMIGGFGNWLVPLMLGAPDMAFPRMNNMSFWLLPPSLTLLISSSVVENGAGTGWTVYPPLSSNIAHSGSSVDMAIFSLHLAGISSILGAINFITTIINMRLNNM
MFDQMPLFVWAVGITAILLLLSLPVLAGAITMLLTDRNLNTSFFDPAGGGDPILYQHL
>LEEUA465-11|JN267141|MM19873|Elachista_bedellella
TLYFIFGIWAGMVGTSLSLLIRAELGNPGSLIGNDQIYNTIVTAHAFIMIFFMVMPIMIGGFGNWLVPLMLGAPDMAFPRMNNMSFWLLPPSLTLLISSSVVENGAGTGWTVYPPLSSNIAHSGSSVDMAIFSLHLAGISSILGAINFITTIINMRLNNM
MFDQMPLFVWAVGITAILLLLSLPVLAGAITMLLTDRNLNTSFFDPAGGGDPILYQHL
>LEFII115-10|JF853349|MM18542|Elachista_biatomella
TLYFIFGIWAGMLGTSLSLLIRAELGNPGSLIGNDQIYNTIVTAHAFIMIFFMVMPIMIGGFGNWLVPLMLGAPDMAFPRMNNMSFWLLPPSLSLLISSSIVENGAGTGWTVYPPLSSNIAHSGSSVDLAIFSLHLAGISSILGAINFITTIINMRLNNLMFD
QMPLFVWAVGITALLLLLSLPVLAGAITMLLTDRNLNTSFFDPAGGGDPILYQHL
>ELACA429-10|JF847517|MM16162|Elachista_bifasciella
TLYFIFGIWAGMVGTSLSLLIRAELGNPGSLIGNDQIYNTIVTAHAFIMIFFMVMPIMIGGFGNWLVPLMLGAPDMAFPRMNNMSFWLLPPSLTLLISSSVVENGAGTGWTVYPPLSSNIAHSGSSVDLAIFSLHLAGISSILGAINFITTIINMRLNNLMF
DQMPLFVWAVGITALLLLLSLPVLAGAITMLLTDRNLNTSFFDPAGGGDPILYQHL
>LEFIA720-10|HM386864|MM02656|Elachista_bisulcella
TLYFIFGIWAGMVGTSLSLLIRAELGNPGSLIGNDQIYNTIVTAHAFIMIFFMVMPIMIGGFGNWLVPLMLGAPDMAFPRMNNMSFWLLPPSLTLLIFSSVVENGSGTGWTVYPPLSSNIAHSGSSVDMAIFSLHLAGISSILGAINFITTIINMRLNNMM
FDQMPLFVWAVGITALLLLLSLPVLAGAITMLLTDRNLNTSFFDPAGGGDPILYQHL
>LEFIC422-10|HM872262|MM03968|Elachista_bisulcella
TLYFIFGIWAGMVGTSLSLLIRAELGNPGSLIGNDQIYNTIVTAHAFIMIFFMVMPIMIGGFGNWLVPLMLGAPDMAFPRMNNMSFWLLPPSLTLLIFSSVVENGSGTGWTVYPPLSSNIAHSGSSVDMAIFSLHLAGISSILGAINFITTIINMRLNNMM
FDQMPLFVWAVGITALLLLLSLPVLAGAITMLLTDRNLNTSFFDPAGGGDPILYQHL
>LEFIE590-10|HM874313|MM09499|Elachista_bisulcella

TLYFIFGIWAGMVGTSLSLLIRAELGNPGSLIGNDQIYNTIVTAHAFIMIFFMVMPIMIGGFGNWLVPLMLGAPDMAFPRMNNMSFWLLPPSLTLLIFSSVVENGSGTGWTVYPPLSSNIAHSGSSVDMAIFSLHLAGISSILGAINFITTIINMRLNNMM
FDQMPLFVWAVGITALLLLLSLPVLAGAITMLLTDRNLNTSFFDPAGGGDPILYQHL
>LEFIG068-10|HM875747|MM13717|Elachista_bisulcella
TLYFIFGIWAGMVGTSLSLLIRAELGNPGSLIGNDQIYNTIVTAHAFIMIFFMVMPIMIGGFGNWLVPLMLGAPDMAFPRMNNMSFWLLPPSLTLLIFSSVVENGSGTGWTVYPPLSSNIAHSGSSVDMAIFSLHLAGISSILGAINFITTIINMRLNNMM
FDQMPLFVWAVGITALLLLLSLPVLAGAITMLLTDRNLNTSFFDPAGGGDPILYQHL
>LEFID247-10|HM873046|MM06111|Elachista_bruuni
TMYFIFGIWSGLVGTSLSLLIRAELGNPGSLIGNDQIYNTIVTAHAFIMIFFMVMPIMIGGFGNWLVPLMLGAPDMAFPRMNNMSFWLLPPSLTLLIFSSLVENGAGTGWTVYPPLSSNIAHSGSSVDLTIFSLHLAGISSILGAINFITTIINMRLNNMMF
DQMPLFVWAVGITAILLLLSLPVLAGAITMLLTDRNLNTSFFDPAGGGDPILYQHL
>LEFIA729-10|HM386873|MM02669|Elachista_canapennella
TLYFIFGIWAGMVGTSLSLLIRAELGNPGSLIGNDQIYNTIVTAHAFIMIFFMVMPIMIGGFGNWLVPLMLGAPDMAFPRMNNMSFWLLPPSLTLLISSSIVENGAGTGWTVYPPLSSNIAHSGSSVDLAIFSLHLAGISSILGAINFITTIINMRLNNLMFD
QMPLFVWAVGITALLLLLSLPVLAGAITMLLTDRNLNTSFFDPAGGGDPILYQHL
>LEFII137-10|JF853362|MM18564|Elachista_chrysodesmella
TLYFIFGIWAGMLGTSLSLLIRAELGNPGSLIGNDQIYNTIVTAHAFIMIFFMVMPIMIGGFGNWLVPLMLGAPDMAFPRMNNMSFWLLPPSITLLIFSSVVENGAGTGWTVYPPLSSNIAHGGSSVDMAIFSLHLAGISSILGAINFITTIINMRLNNMM
FDQMPLFVWAVGITALLLLLSLPVLAGAITMLLTDRNLNTSFFDPAGGGDPILYQHL
>ELACA305-10|MM16220|Elachista_cinereopunctella
TLYFIFGIWAGMMGTSLSLLIRAELGNPGSLIGNDQIYNTIVTAHAFIMIFFMVMPIMIGGFGNWLVPLMLGAPDMAFPRMNNMSFWLLPPSLTLLISSSIVENGAGTGWTVYPPLSSNIAHGGSSVDLAIFSLHLAGISSILGAINFITTIINMRLNNLMF
DQMPLFVWAVGITALLLLLSLPVLAGAITMLLTDRNLNTSFFDPAGGGDPILYQHL
>LEFID332-10|HM873129|MM06226|Elachista_cingillella
TLYFIFGIWAGMVGTSLSLLIRAELGNPGSLIGNDQIYNTIVTAHAFIMIFFMVMPIMIGGFGNWLVPLMLGAPDMAFPRMNNMSFWLLPPSLTLLIFSSVVENGSGTGWTVYPPLSSNIAHSGSSVDMAIFSLHLAGISSILGAINFITTIINMRLNNMM
FDQMPLFVWAVGITALLLLLSLPVLAGAITMLLTDRNLNTSFFDPAGGGDPILYQHL
>LEFID333-10|HM873130|MM06227|Elachista_cingillella
TLYFIFGIWAGMVGTSLSLLIRAELGNPGSLIGNDQIYNTIVTAHAFIMIFFMVMPIMIGGFGNWLVPLMLGAPDMAFPRMNNMSFWLLPPSLTLLIFSSVVENGSGTGWTVYPPLSSNIAHSGSSVDMAIFSLHLAGISSILGAINFITTIINMRLNNMM
FDQMPLFVWAVGITALLLLLSLPVLAGAITMLLTDRNLNTSFFDPAGGGDPILYQHL
>ELACA792-11|JN267116|MM20030|Elachista_cingillella
TLYFIFGIWAGMVGTSLSLLIRAELGNPGSLIGNDQIYNTIVTAHAFIMIFFMVMPIMIGGFGNWLVPLMLGAPDMAFPRMNNMSFWLLPPSLTLLIFSSVVENGSGTGWTVYPPLSSNIAHSGSSVDMAIFSLHLAGISSILGAINFITTIINMRLNNMM
FDQMPLFVWAVGITALLLLLSLPVLAGAITMLLTDRNLNTSFFDPAGGGDPILYQHL
>LEFIE933-10|HM874650|MM10317|Elachista_compsa
TLYFIFGIWAGMMGTSLSLLIRAELGNPGSLIGNDQIYNTIVTAHAFIMIFFMVMPIMIGGFGNWLVPLMLGAPDMAFPRMNNMSFWLLPPSLTLLVSSSIVENGAGTGWTVYPPLSSNIAHNGSSVDLAIFSLHLAGISSILGAINFITTIINMHLNKLM
FDQMPLFVWAVGITALLLLLSLPVLAGAITMLLTDRNLNTSFFDPAGGGDPILYQHL
>LEFII148-10|JF853364|MM18575|Elachista_consortella
TLYFIFGIWAGMVGTSLSLLIRAELGNPGSLIGNDQIYNTIVTAHAFIMIFFMVMPIMIGGFGNWLVPLMLGAPDMAFPRMNNMSFWLLPPSLTLLIASGVVENGAGTGWTVYPPLSSNIAHSGSSVDLAIFSLHLAGISSILGAINFITTIINMRLNNLMF
DQMPLFVWAVGITALLLLLSLPVLAGAITMLLTDRNLNTSFFDPAGGGDPILYQHL
>LEFII149-10|JF853365|MM18576|Elachista_consortella
TLYFIFGIWAGMVGTSLSLLIRAELGNPGSLIGNDQIYNTIVTAHAFIMIFFMVMPIMIGGFGNWLVPLMLGAPDMAFPRMNNMSFWLLPPSLTLLIASGVVENGAGTGWTVYPPLSSNIAHSGSSVDLAIFSLHLAGISSILGAINFITTIINMRLNNLMF
DQMPLFVWAVGITALLLLLSLPVLAGAITMLLTDRNLNTSFFDPAGGGDPILYQHL
>LEEUA105-11|MM19513|Elachista_consortella
TLYFIFGIWAGMVGTSLSLLIRAELGNPGSLIGNDQIYNTIVTAHAFIMIFFMVMPIMIGGFGNWLVPLMLGAPDMAFPRMNNMSFWLLPPSLTLLIASGVVENGAGTGWTVYPPLSSNIAHSGSSVDLAIFSLHLAGISSILGAINFITTIINMRLNNLMF
DQMPLFVWAVGITALLLLLSLPVLAGAITMLLTDRNLNTSFFDPAGGGDPILYQHL
>ELACA999-11|MM20941|Elachista_consortella
TLYFIFGIWAGMVGTSLSLLIRAELGNPGSLIGNDQIYNTIVTAHAFIMIFFMVMPIMIGGFGNWLVPLMLG?PDMAFPRMNNMSFWLLPPSLTLLIASGVVENGAGTGWTVYPPLSSNIAHSGSSVDLAIFSLHLAGISSILGAINFITTIINMRLNNLMF
DQMPLFVWAVGITALLLLLSLPVLAGAITMLLTDRNLNTSFFDPAGGGDPILYQHL
>LEFIG154-10|HM875834|MM14008|Elachista_deriventa
TLYFIFGIWAGMVGTSLSLLIRAELGNPGSLIGNDQIYNTIVTAHAFIMIFFMVMPIMIGGFGNWLVPLMLGAPDMAFPRMNNMSFWLLPPSLTLLISSSIVENGAGTGWTVYPPLSSNIAHSGSSVDLAIFSLHLAGISSILGAINFITTIINMRLNNLMFD
QMTLFIWAVGITALLLLLSLPVLAGAITMLLTDRNLNTSFFDPAGGGDPILYQHL
>LEFIG157-10|HM875837|MM14011|Elachista_deriventa
TLYFIFGIWAGMMGTSLSLLIRAELGNPGSLIGNDQIYNTIVTAHAFIMIFFMVMPIMIGGFGNWLVPLMLGAPDMAFPRMNNMSFWLLPPSLTLLISSSIVENGAGTGWTVYPPLSSNIAHSGSSVDLAIFSLHLAGISSILGAINFITTIINMRLNNLMF
DQMTLFIWAVGITALLLLLSLPVLAGAITMLLTDRNLNTSFFDPAGGGDPILYQHL

>LEFIG158-10|HM875838|MM14012|Elachista_deriventa
TLYFIFGIWAGMVGTSLSLLIRAELGNPGSLIGNDQIYNTIVTAHAFIMIFFMVMPIMIGGFGNWLVPLMLGAPDMAFPRMNNMSFWLLPPSLTLLISSSIVENGAGTGWTVYPPLSSNIAHSGSSVDLAIFSLHLAGISSILGAINFITTIINMRLNNLMFD
QMTLFIWAVGITALLLLLSLPVLAGAITMLLTDRNLNTSFFDPAGGGDPILYQHL
>LEFID068-10|HM872880|MM05677|Elachista_diederichsiella
TLYFIFGIWAGMVGTSLSLLIRAELGNPGSLIGNDQIYNTIVTAHAFIMIFFMVMPIMIGGFGNWLVPLMLGAPDMAFPRMNNMSFWLLPPSLTLLISSSIVENGAGTGWTVYPPLSSNIAHSGSSVDLAIFSLHLAGISSILGAINFITTIINMRLNNLMFD
QMPLFVWAVGITALLLLLSLPVLAGAITMLLTDRNLNTSFFDPAGGGDPILYQHL
>LEFID330-10|HM873127|MM06224|Elachista_diederichsiella
TLYFIFGIWAGMVGTSLSLLIRAELGNPGSLIGNDQIYNTIVTAHAFIMIFFMVMPIMIGGFGNWLVPLMLGAPDMAFPRMNNMSFWLLPPSLTLLISSSIVENGAGTGWTVYPPLSSNIAHSGSSVDLAIFSLHLAGISSILGAINFITTIINMRLNNLMFD
QMPLFVWAVGITALLLLLSLPVLAGAITMLLTDRNLNTSFFDPAGGGDPILYQHL
>LEFIE123-10|HM873871|MM08330|Elachista_diederichsiella
TLYFIFGIWAGMVGTSLSLLIRAELGNPGSLIGNDQIYNTIVTAHAFIMIFFMVMPIMIGGFGNWLVPLMLGAPDMAFPRMNNMSFWLLPPSLTLLISSSIVENGAGTGWTVYPPLSSNIAHSGSSVDLAIFSLHLAGISSILGAINFITTIINMRLNNLMFD
QMPLFVWAVGITALLLLLSLPVLAGAITMLLTDRNLNTSFFDPAGGGDPILYQHL
>ELACA459-10|JF847535|MM16192|Elachista_diederichsiella
TLYFIFGIWAGMVGTSLSLLIRAELGNPGSLIGNDQIYNTIVTAHAFIMIFFMVMPIMIGGFGNWLVPLMLGAPDMAFPRMNNMSFWLLPPSLTLLISSSIVENGAGTGWTVYPPLSSNIAHSGSSVDLAIFSLHLAGISSILGAINFITTIINMRLNNLMFD
QMPLFVWAVGITALLLLLSLPVLAGAITMLLTDRNLNTSFFDPAGGGDPILYQHL
>ELACA460-10|JF847536|MM16193|Elachista_diederichsiella
TLYFIFGIWAGMVGTSLSLLIRAELGNPGSLIGNDQIYNTIVTAHAFIMIFFMVMPIMIGGFGNWLVPLMLGAPDMAFPRMNNMSFWLLPPSLTLLISSSIVENGAGTGWTVYPPLSSNIAHSGSSVDLAIFSLHLAGISSILGAINFITTIINMRLNNLMFD
QMPLFVWAVGITALLLLLSLPVLAGAITMLLTDRNLNTSFFDPAGGGDPILYQHL
>ELACA115-10|KF809321|MM16315|Elachista_diederichsiella
TLYFIFGIWAGMVGTSLSLLIRAELGNPGSLIGNDQIYNTIVTAHAFIMIFFMVMPIMIGGFGNWLVPLMLGAPDMAFPRMNNMSFWLLPPSLTLLISSSIVENGAGTGWTVYPPLSSNIAHSGSSVDLAIFSLHLAGISSILGAINFITTIINMRLNNLMFD
QMPLFVWAVGITALLLLLSLPVLAGAITMLLTDRNLNTSFFDPAGGGDPILYQHL
>ELACA116-10|KF809290|MM16316|Elachista_diederichsiella
TLYFIFGIWAGMVGTSLSLLIRAELGNPGSLIGNDQIYNTIVTAHAFIMIFFMVMPIMIGGFGNWLVPLMLGAPDMAFPRMNNMSFWLLPPSLTLLISSSIVENGAGTGWTVYPPLSSNIAHSGSSVDLAIFSLHLAGISSILGAINFITTIINMRLNNLMFD
QMPLFVWAVGITALLLLLSLPVLAGAITMLLTDRNLNTSFFDPAGGGDPILYQHL
>COEUA053-11|KJ130922|MM20223|Elachista_dispunctella
TLYFIFGIWAGLVGTSLSLLIRAELGNPGSLIGNDQIYNTIVTAHAFIMIFFMVMPIMIGGFGNWLVPLMLGAPDMAFPRMNNMSFWLLPPSLTLLIFSSLVENGAGTGWTVYPPLSSNIAHGGSSVDLAIFSLHLAGISSILGAINFITTIINMRLNNMMF
DQMPLFVWAVGITAVLLLLSLPVLAGAITMLLTDRNLNTSFFDPAGGGDPILYQHL
>LEFID739-10|HM873496|MM06798|Elachista_distigmatella
TLYFIFGIWAGMVGTSLSLLIRAELGNPGSLIGNDQIYNTIVTAHAFIMIFFMVMPIMIGGFGNWLVPLMLGAPDMAFPRMNNMSFWLLPPSLTLLIFSSLVENGAGTGWTVYPPLSSNIAHSGSSVDLAIFSLHLAGISSILGAINFITTIINMRLNNMMF
DQMPLFVWAVGITAVLLLLSLPVLAGAITMLLTDRNLNTSFFDPAGGGDPILYQHL
>LEFIG305-10|HM875984|MM14268|Elachista_distigmatella
TLYFIFGIWAGMVGTSLSLLIRAELGNPGSLIGNDQIYNTIVTAHAFIMIFFMVMPIMIGGFGNWLVPLMLGAPDMAFPRMNNMSFWLLPPSLTLLIFSSLVENGAGTGWTVYPPLSSNIAHSGSSVDLAIFSLHLAGISSILGAINFITTIINMRLNNMMF
DQMPLFVWAVGITAVLLLLSLPVLAGAITMLLTDRNLNTSFFDPAGGGDPILYQHL
>LEFIF069-10|HM874779|MM10509|Elachista_elegans
TLYFIFGIWAGMVGTSLSLLIRAELGNPGSLIGNDQIYNTIVTAHAFIMIFFMVMPIMIGGFGNWLVPLMLGAPDMAFPRMNNMSFWLLPPSLTLLISSSIVENGAGTGWTVYPPLSSNIAHSGSSVDLAIFSLHLAGISSILGAINFITTIINMRLNNLMFD
QMPLFVWAVGITALLLLLSLPVLAGAITMLLTDRNLNTSFFDPAGGGDPILYQHL
>LEFIJ1298-11|KF809233|MM21158|Elachista_elegans
TLYFIFGIWAGMVGTSLSLLIRAELGNPGSLIGNDQIYNTIVTAHAFIMIFFMVMPIMIGGFGNWLVPLMLGAPDMAFPRMNNMSFWLLPPSLTLLISSSIVENGAGTGWTVYPPLSSNIAHSGSSVDLAIFSLHLAGISSILGAINFITTIINMRLNNLMFD
QMPLFVWAVGITALLLLLSLPVLAGAITMLLTDRNLNTSFFDPAGGGDPILYQHL
>LEFIC030-10|HM871900|MM03254|Elachista_eleochariella
TLYFIFGIWAGMVGTSLSLLIRAELGNPGSLIGNDQIYNTIVTAHAFIMIFFMVMPIMIGGFGNWLVPLMLGAPDMAFPRMNNMSFWLLPPSLTLLISSSIVENGAGTGWTVYPPLSSNIAHGGSSVDLAIFSLHLAGISSILGAINFITTIINMRLNNLMF
DQMPLFVWAVGITALLLLLSLPVLAGAITMLLTDRNLNTSFFDPAGGGDPILYQHL
>LEFIK361-10|JF853996|MM17936|Elachista_eleochariella
TLYFIFGIWAGMVGTSLSLLIRAELGNPGSLIGSDQIYNTIVTAHAFIMIFFMVMPIMIGGFGNWLVPLMLGAPDMAFPRMNNMSFWLLPPSLTLLISSSIVENGAGTGWTVYPPLSSNIAHGGSSVDLAIFSLHLAGISSILGAINFITTIINMRLNNLMFD
QMPLFVWAVGITALLLLLSLPVLAGAITMLLTDRNLNTSFFDPAGGGDPILYQHL
>LEFIE159-10|HM873905|MM08409|Elachista_eskoi
TLYFIFGIWSGMVGTSLSLLIRAELGNPGSLIGNDQIYNTIVTAHAFIMIFFMVMPIMIGGFGNWLVPLMLGAPDMAFPRMNNMSFWLLPPSLTLLISSSVVENGAGTGWTVYPPLSSNIAHSGSSVDLTIFSLHLAGISSILGAINFITTIINMRLNNLMF

DQMPLFVWAVGITALLLLLSLPVLAGAITMLLTDRNLNTSFFDPAGGGDPILYQHL
>LEFIE990-10|HM874706|MM10428|Elachista_eskoi
TLYFIFGIWSGMVGTSLSLLIRAELGNPGSLIGNDQIYNTIVTAHAFIMIFFMVMPIMIGGFGNWLVPLMLGAPDMAFPRMNNMSFWLLPPSLTLLISSSVVENGAGTGWTVYPPLSSNIAHSGSSVDLTIFSLHLAGISSILGAINFITTIINMRLNNLMF
DQMPLFVWAVGITALLLLLSLPVLAGAITMLLTDRNLNTSFFDPAGGGDPILYQHL
>ELACA787-11|JN267111|MM20025|Elachista_eskoi
TLYFIFGIWSGMVGTSLSLLIRAELGSPGSLIGNDQIYNTIVTAHAFIMIFFMVMPIMIGGFGNWLVPLMLGAPDMAFPRMNNMSFWLLPPSLTLLISSSVVENGAGTGWTVYPPLSSNIAHSGSSVDLTIFSLHLAGISSILGAINFITTIINMRLNNLMFD
QMPLFVWAVGITALLLLLSLPVLAGAITMLLTDRNLNTSFFDPAGGGDPILYQHL
>LEFIB158-10|HM871063|MM00548|Elachista_exactella
TLYFIFGIWAGMVGTSLSLLIRAELGNPGSLIGNDQIYNTIVTAHAFIMIFFMVMPIMIGGFGNWLVPLMLGAPDMAFPRMNNMSFWLLPPSLTLLISSSVVENGAGTGWTVYPPLSSNIAHSGSSVDLAIFSLHLAGISSILGAINFITTIINMRLNNLMF
DQMPLFVWAVGITALLLLLSLPVLAGAITMLLTDRNLNTSFFDPAGGGDPILYQHL
>LEFIC125-10|HM871972|MM03462|Elachista_exactella
TLYFIFGIWAGMVGTSLSLLIRAELGNPGSLIGNDQIYNTIVTAHAFIMIFFMVMPIMIGGFGNWLVPLMLGAPDMAFPRMNNMSFWLLPPSLTLLISSSVVENGAGTGWTVYPPLSSNIAHSGSSVDLAIFSLHLAGISSILGAINFITTIINMRLNNLMF
DQMPLFVWAVGITALLLLLSLPVLAGAITMLLTDRNLNTSFFDPAGGGDPILYQHL
>ELACA332-10|JF847449|MM16247|Elachista_exactella
TLYFIFGIWAGMVGTSLSLLIRAELGNPGSLIGNDQIYNTIVTAHAFIMIFFMVMPIMIGGFGNWLVPLMLGAPDMAFPRMNNMSFWLLPPSLTLLISSSVVENGAGTGWTVYPPLSSNIAHSGSSVDLAIFSLHLAGISSILGAINFITTIINMRLNNLMF
DQMPLFVWAVGITALLLLLSLPVLAGAITMLLTDRNLNTSFFDPAGGGDPILYQHL
>LEFIC111-10|HQ570302|MM03441|Elachista_excelsicola
TLYFIFGIWAGMVGTSLSLLIRAELGNPGSLIGNDQIYNTIVTAHAFIMIFFMVMPIMIGGFGNWLVPLMLGAPDMAFPRMNNMSFWLLPPSLTLLISSSIVENGAGTGWTVYPPLSSNIAHNGSSVDLTIFSLHLAGISSILGAINFITTIINMRLNNLMF
DQMPLFVWAVGITALLLLLSLPVLAGAITMLLTDRNLNTSFFDPAGGGDPILYQHL
>LEFIE255-10|HM873989|MM08590|Elachista_excelsicola
TLYFIFGIWAGMVGTSLSLLIRAELGSPGSLIGNDQIYNTIVTAHAFIMIFFMVMPIMIGGFGNWLVPLMLGAPDMAFPRMNNMSFWLLPPSLTLLISSSIVENGAGTGWTVYPPLSSNIAHNGSSVDLTIFSLHLAGISSILGAINFITTIINMRLNNLMFD
QMPLFVWAVGITALLLLLSLPVLAGAITMLLTDRNLNTSFFDPAGGGDPILYQHL
>LEFIG698-10|HM876355|MM15562|Elachista_excelsicola
TLYFIFGIWAGMVGTSLSLLIRAELGNPGSLIGNDQIYNTIVTAHAFIMIFFMVMPIMIGGFGNWLVPLMLGAPDMAFPRMNNMSFWLLPPSLTLLISSSIVENGAGTGWTVYPPLSSNIAHNGSSVDLTIFSLHLAGISSILGAINFITTIINMRLNNLMF
DQMPLFVWAVGITALLLLLSLPVLAGAITMLLTDRNLNTSFFDPAGGGDPILYQHL
>LEFIJ1323-11|KF809352|MM21183|Elachista_excelsicola
TLYFIFGIWAGMVGTSLSLLIRAELGSPGSLIGNDQIYNTIVTAHAFIMIFFMVMPIMIGGFGNWLVPLMLGAPDMAFPRMNNMSFWLLPPSLTLLISSSIVENGAGTGWTVYPPLSSNIAHNGSSVDLTIFSLHLAGISSILGAINFITTIINMRLNNLMFD
QMPLFVWAVGITALLLLLSLPVLAGAITMLLTDRNLNTSFFDPAGGGDPILYQHL
>LEFIJ962-11|KF809273|MM21202|Elachista_excelsicola
TLYFIFGIWAGMVGTSLSLLIRAELGNPGSLIGNDQIYNTIVTAHAFIMIFFMVMPIMIGGFGNWLVPLMLGAPDMAFPRMNNMSFWLLPPSLTLLISSSIVENGAGTGWTVYPPLSSNIAHNGSSVDLTIFSLHLAGISSILGAINFITTIINMRLNNLMF
DQMPLFVWAVGITALLLLLSLPVLAGAITMLLTDRNLNTSFFDPAGGGDPILYQHL
>LEFII133-10|MM18560|Elachista_festucicolella
TLYFIFGIWAGMVGTSLSLLIRAELGNPGSLIGNDQIYNTIVTAHAFIMIFFMVMPIMIGGFGNWLVPLMLGAPDMAFPRMNNMSFWLLPPSLMLLIFSSLVENGAGTGWTVYPPLSSNIAHSGSSVDLAIFSLHLAGISSILGAINFITTIINMRINNMM
FDQMPLFVWAVGITAVLLLLSLPVLAGAITMLLTDRNLNTSFFDPAGGGDPILYQHL
>LEFIG072-10|HM875751|MM13722|Elachista_freyerella
TLYFIFGIWAGMVGTSLSLLIRAELGNPGSLIGNDQIYNTIVTAHAFIMIFFMVMPIMIGGFGNWLVPLMLGAPDMAFPRMNNMSFWLLPPSLTLLISSSVVENGAGTGWTVYPPLSSNIAHSGSSVDLAIFSLHLAGISSILGAINFITTIINMRLNNLMF
DQMPLFVWAVGITALLLLLSLPVLAGAITMLLTDRNLNTSFFDPAGGGDPILYQHL
>LEFIG073-10|HM875752|MM13723|Elachista_freyerella
TLYFIFGIWAGMVGTSLSLLIRAELGNPGSLIGNDQIYNTIVTAHAFIMIFFMVMPIMIGGFGNWLVPLMLGAPDMAFPRMNNMSFWLLPPSLTLLISSSVVENGAGTGWTVYPPLSSNIAHSGSSVDLAIFSLHLAGISSILGAINFITTIINMRLNNLMF
DQMPLFVWAVGITALLLLLSLPVLAGAITMLLTDRNLNTSFFDPAGGGDPILYQHL
>ELACA442-10|JF847523|MM16175|Elachista_fuscofrontella
TLYFIFGIWAGMMGTSLSLLIRAELGNPGSLIGNDQIYNTIVTAHAFIMIFFMVMPIMIGGFGNWLVPLMLGAPDMAFPRMNNMSFWLLPPSLTLLISSSIVENGAGTGWTVYPPLSSNIAHSGSSVDLAIFSLHLAGISSILGAINFITTIINMRLNNLMF
DQMPLFVWAVGITALLLLLSLPVLAGAITMLLTDRNLNTSFFDPAGGGDPILYQHL
>LEFII157-10|JF853370|MM18584|Elachista_gangabella
TLYFIFGIWAGMMGTSLSLLIRAELGSPGSLIGNDQIYNTIVTAHAFIMIFFMVMPIMIGGFGNWLVPLMLGAPDMAFPRMNNMSFWLLPPSLMLLVFSSVVENGSGTGWTVYPPLSSNIAHSGSSVDMAIFSLHLAGISSILGAINFITTIINMRLNNM
MFDQMPLFVWAVGITALLLLLSLPVLAGAITMLLTDRNLNTSFFDPAGGGDPILYQHL
>LEFIC441-10|HM872275|MM03999|Elachista_gleichenella

TLYFIFGIWAGMVGTSLSLLIRAELGNPGSLIGNDQIYNTIVTAHAFIMIFFMVMPIMIGGFGNWLVPLMLGAPDMAFPRMNNMSFWLLPPSLTLLISSSIVENGAGTGWTVYPPLSSNIAHSGSSVDLAIFSLHLAGISSILGAINFITTIINMRLNSLMFD
QMPLFIWAVGITALLLLLSLPVLAGAITMLLTDRNLNTSFFDPAGGGDPILYQHL
>LEFIB325-10|HM871225|MM00863|Elachista_humilis
TLYFIFGIWAGMVGTSLSLLIRAELGNPGSLIGNDQIYNTIVTAHAFIMIFFMVMPIMIGGFGNWLVPLMLGAPDMAFPRMNNMSFWLLPPSLTLLISSGVVENGAGTGWTVYPPLSSNIAHSGSSVDLAIFSLHLAGISSILGAINFITTIINMRLNNLMF
DQMPLFVWAVGITALLLLLSLPVLAGAITMLLTDRNLNTSFFDPAGGGDPILYQHL
>LEFIA726-10|HM386870|MM02663|Elachista_humilis
TLYFIFGIWAGMVGTSLSLLIRAELGNPGSLIGNDQIYNTIVTAHAFIMIFFMVMPIMIGGFGNWLVPLMLGAPDMAFPRMNNMSFWLLPPSLTLLISSGVVENGAGTGWTVYPPLSSNIAHSGSSVDLAIFSLHLAGISSILGAINFITTIINMRLNNLMF
DQMPLFVWAVGITALLLLLSLPVLAGAITMLLTDRNLNTSFFDPAGGGDPILYQHL
>LEFIE012-10|HM873761|MM08064|Elachista_humilis
TLYFIFGIWAGMVGTSLSLLIRAELGNPGSLIGNDQIYNTIVTAHAFIMIFFMVMPIMIGGFGNWLVPLMLGAPDMAFPRMNNMSFWLLPPSLTLLISSGVVENGAGTGWTVYPPLSSNIAHSGSSVDLAIFSLHLAGISSILGAINFITTIINMRLNNLMF
DQMPLFVWAVGITALLLLLSLPVLAGAITMLLTDRNLNTSFFDPAGGGDPILYQHL
>LEFIK367-10|JF854001|MM17942|Elachista_humilis
TLYFIFGIWAGMVGTSLSLLIRAELGNPGSLIGNDQIYNTIVTAHAFIMIFFMVMPIMIGGFGNWLVPLMLGAPDMAFPRMNNMSFWLLPPSLTLLISSGVVENGAGTGWTVYPPLSSNIAHNGSSVDLAIFSLHLAGISSILGAINFITTIINMRLNNLM
FDQMPLFVWAVGITALLLLLSLPVLAGAITMLLTDRNLNTSFFDPAGGGDPILYQHL
>LEFIG701-10|HM876358|MM15565|Elachista_imatrella
TLYFIFGIWAGM?GTSLSLLIRAELGNPGSLIGNDQIYNTIVTAHAFIMIFFMVMPIMIGGFGNWLVPLMLGAPDMAFPRMNNMSFWLLPPSITLLISSSIVENGAGTGWTVYPPLSSNIAHGGSSVDLAIFSLHLAGISSILGAINFITTIINMRLNNLMFD
QMPLFVWAVGITALLLLLSLPVLAGAITMLLTDRNLNTSFFDPAGGGDPILYQHL
>LEFIG702-10|HQ570407|MM15566|Elachista_kebneella
TLYFIFGIWAGMVGTSLSLLIRAELGNPGSLIGNDQIYNTIVTAHAFIMIFFMVMPIMIGGFGNWLVPLMLGAPDMAFPRMNNMSFWLLPPSLTLLISSSIVENGAGTGWTVYPPLSSNIAHGGSSVDLAIFSLHLAGISSILGAINFITTIINMRLNNLMF
DQMPLFVWAVGITALLLLLSLPVLAGAITMLLTDRNLNTSFFDPAGGGDPILYQHL
>LEFIK580-10|JF854168|MM18155|Elachista_kebneella
TLYFIFGIWAGMVGTSLSLLIRAELGNPGSLIGNDQIYNTIVTAHAFIMIFFMVMPIMIGGFGNWLVPLMLGAPDMAFPRMNNMSFWLLPPSLTLLISSSIVENGAGTGWTVYPPLSSNIAHGGSSVDLAIFSLHLAGISSILGAINFITTIINMRLNNLMF
DQMPLFVWAVGITALLLLLSLPVLAGAITMLLTDRNLNTSFFDPAGGGDPILYQHL
>LEFIB228-10|HM871131|MM00687|Elachista_kilmunella
TLYFIFGIWAGMVGTSLSLLIRAELGNPGSLIGNDQIYNTIVTAHAFIMIFFMVMPIMIGGFGNWLVPLMLGAPDMAFPRMNNMSFWLLPPSLTLLISSSIVENGAGTGWTVYPPLSSNIAHNGSSVDLTIFSLHLAGISSILGAINFITTIINMRLNNLMF
DQMPLFVWAVGITALLLLLSLPVLAGAITMLLTDRNLNTSFFDPAGGGDPILYQHL
>LEFIC445-10|HM872279|MM04003|Elachista_kilmunella
TLYFIFGIWAGMVGTSLSLLIRAELGNPGSLIGNDQIYNTIVTAHAFIMIFFMVMPIMIGGFGNWLVPLMLGAPDMAFPRMNNMSFWLLPPSLTLLISSSIVENGAGTGWTVYPPLSSNIAHNGSSVDLTIFSLHLAGISSILGAINFITTIINMRLNNLMF
DQMPLFVWAVGITALLLLLSLPVLAGAITMLLTDRNLNTSFFDPAGGGDPILYQHL
>ELACA292-10|JF847436|MM16207|Elachista_kilmunella
TLYFIFGIWAGMVGTSLSLLIRAELGNPGSLIGNDQIYNTIVTAHAFIMIFFMVMPIMIGGFGNWLVPLMLGAPDMAFPRMNNMSFWLLPPSLTLLISSSIVENGAGTGWTVYPPLSSNIAHNGSSVDLTIFSLHLAGISSILGAINFITTIINMRLNNLMF
DQMPLFVWAVGITALLLLLSLPVLAGAITMLLTDRNLNTSFFDPAGGGDPILYQHL
>LEFII152-10|JF853366|MM18579|Elachista_kilmunella
TLYFIFGIWAGMVGTSLSLLIRAELGNPGSLIGNDQIYNTIVTAHAFIMIFFMVMPIMIGGFGNWLVPLMLGAPDMAFPRMNNMSFWLLPPSLTLLISSSIVENGAGTGWTVYPPLSSNIAHNGSSVDLTIFSLHLAGISSILGAINFITTIINMRLNNLMF
DQMPLFVWAVGITALLLLLSLPVLAGAITMLLTDRNLNTSFFDPAGGGDPILYQHL
>ELACA766-11|JN267094|MM20004|Elachista_kilmunella
TLYFIFGIWAGMVGTSLSLLIRAELGNPGSLIGNDQIYNTIVTAHAFIMIFFMVMPIMIGGFGNWLVPLMLGAPDMAFPRMNNMSFWLLPPSLTLLISSSIVENGAGTGWTVYPPLSSNIAHNGSSVDLTIFSLHLAGISSILGAINFITTIINMRLNNLMF
DQMPLFVWAVGITALLLLLSLPVLAGAITMLLTDRNLNTSFFDPAGGGDPILYQHL
>LEFIJ961-11|KF809249|MM21201|Elachista_kilmunella
TLYFIFGIWAGMVGTSLS?LIRAELGNPGSLIGNDQIYNTIVTAHAFIMIFFMVMPIMIGGFGNWLVPLMLGAPDMAFPRMNNMSFWLLPPSLTLLISSSIVENGAGTGWTVYPPLSSNIAHNGSSVDLTIFSLHLAGISSILGAINFITTIINMRLNNLMF
DQMPLFVWAVGITALLLLLSLPVLAGAITMLLTDRNLNTSFFDPAGGGDPILYQHL
>LEFIG262-10|HM875941|MM14194|Elachista_krogeri
TLYFIFGIWAGMIGTSLSLLIRAELGNPGSLIGNDQIYNTIVTAHAFIMIFFMVMPIMIGGFGNWLVPLMLGAPDMAFPRMNNMSFWLLPPSLTLLISSSIVENGAGTGWTVYPPLSSNIAHSGSSVDLAIFSLHLAGISSILGAINFITTIINMRLNNLMFD
QMPLFVWAVGITALLLLLSLPVLAGAITMLLTDRNLNTSFFDPAGGGDPILYQHL
>LEEUA113-11|MM19521|Elachista_littoricola
TLYFIFGIWAGMVGTSLSLLIRAELGNPGSLIGNDQIYNTIVTAHAFIMIFFMVMPIMIGGFGNWLVPLMLGAPDMAFPRMNNMSFWLLPPSLTLLISSSVVENGAGTGWTVYPPLSSNIAHSGSSVDMAIFSLHLAGISSILGAINFITTIINMRLNNM
MFDQMPLFVWAVGITALLLLLSLPVLAGAITMLLTDRNLNTSFFDPAGGGDPILYQHL

>ELACA799-11|JN267123|MM20037|Elachista_littoricola
TLYFIFGIWAGMVGTSLSLLIRAELGNPGSLIGNDQIYNTIVTAHAFIMIFFMVMPIMIGGFGNWLVPLMLGAPDMAFPRMNNMSFWLLPPSLTLLISSSVVENGAGTGWTVYPPLSSNIAHSGSSVDMAIFSLHLAGISSILGAINFITTIINMRLNNM
MFDQMPLFVWAVGITALLLLLSLPVLAGAITMLLTDRNLNTSFFDPAGGGDPILYQHL
>LEFID625-10|HM873390|MM06643|Elachista_luticomella
TLYFIFGIWAGMVGTSLSLLIRAELGNPGSLIGNDQIYNTIVTAHAFIMIFFMVMPIMIGGFGNWLVPLMLGAPDMAFPRMNNMSFWLLPPSLTLLISSSIVENGAGTGWTVYPPLSSNIAHSGSSVDLAIFSLHLAGISSILGAINFITTIINMRLNNLMFD
QMPLFVWAVGITALLLLLSLPVLAGAITMLLTDRNLNTSFFDPAGGGDPILYQHL
>LEFIB324-10|HM871224|MM00861|Elachista_maculicerusella
TLYFILGIWAGMVGTSLSLLIRAELGNPGSLINNDQIYNTIVTSHAFIMIFFMVMPIMIGGFGNWLVPLMLGAPDMAFPRMNNMSFWLLPPSLTLLISSSIVENGAGTGWTVYPPLSSNIAHSGSSVDLAIFSLHLAGISSILGAINFITTIINMRINNLMFD
QMSLFVWAVGITALLLLLSLPVLAGAITMLLTDRNLNTSFFDPAGGGDPILYQHL
>LEFIB773-10|HM871650|MM02647|Elachista_maculicerusella
TLYFILGIWAGMVGTSLSLLIRAELGNPGSLIGNDQIYNTIVTSHAFIMIFFMVMPIMIGGFGNWLVPLMLGAPDMAFPRMNNMSFWLLPPSLTLLISSSIVENGAGTGWTVYPPLSSNIAHSGSSVDLAIFSLHLAGISSILGAINFITTIINMRINNLMFD
QMSLFVWAVGITALLLLLSLPVLAGAITMLLTDRNLNTSFFDPAGGGDPILYQHL
>LEFIC433-10|HM872268|MM03985|Elachista_maculicerusella
TLYFILGIWAGMVGTSLSLLIRAELGNPGSLIGNDQIYNTIVTSHAFIMIFFMVMPIMIGGFGNWLVPLMLGAPDMAFPRMNNMSFWLLPPSLTLLISSSIVENGAGTGWTVYPPLSSNIAHSGSSVDLAIFSLHLAGISSILGAINFITTIINMRINNLMFD
QMSLFVWAVGITALLLLLSLPVLAGAITMLLTDRNLNTSFFDPAGGGDPILYQHL
>LEFIA856-10|HQ570259|MM09733|Elachista_maculicerusella
TLYFILGIWAGMVGTSLSLLIRAELGNPGSLIGNDQIYNTIVTSHAFIMIFFMVMPIMIGGFGNWLIPLMLGAPDMAFPRMNNMSFWLLPPSLTLLISSSIVENGAGTGWTVYPPLSSNIAHSGSSVDLAIFSLHLAGISSILGAINFITTIINMRINNLMFD
QMSLFVWAVGITALLLLLSLPVLAGAITMLLTDRNLNTSFFDPAGGGDPILYQHL
>LEFIF476-10|HM875161|MM12025|Elachista_maculicerusella
TLYFILGIWAGMVGTSLSLLIRAELGNPGSLIGNDQIYNTIVTSHAFIMIFFMVMPIMIGGFGNWLVPLMLGAPDMAFPRMNNMSFWLLPPSLTLLISSSIVENGAGTGWTVYPPLSSNIAHSGSSVDLAIFSLHLAGISSILGAINFITTIINMRINNLMFD
QMSLFVWAVGITALLLLLSLPVLAGAITMLLTDRNLNTSFFDPAGGGDPILYQHL
>ELACA309-10|JF847440|MM16224|Elachista_nielswolffi
TLYFIFGIWAGMIGTSLSLLIRAELGNPGSLIGNDQIYNTIVTAHAFIMIFFMVMPIMIGGFGNWLVPLMLGAPDMAFPRMNNMSFWLLPPSLALLISSSVVENGAGTGWTVYPPLSSNIAHSGSSVDLAIFSLHLAGISSILGAINFITTIINMRLNNLMF
DQMPLFVWAVGITALLLLLSLPVLAGAITMLLTDRNLNTSFFDPAGGGDPILYQHL
>ELACA311-10|JF847441|MM16226|Elachista_nielswolffi
TLYFIFGIWAGMIGTSLSLLIRAELGNPGSLIGNDQIYNTIVTAHAFIMIFFMVMPIMIGGFGNWLVPLMLGAPDMAFPRMNNMSFWLLPPSLALLISSSVVENGAGTGWTVYPPLSSNIAHSGSSVDLAIFSLHLAGISSILGAINFITTIINMRLNNLMF
DQMPLFVWAVGITALLLLLSLPVLAGAITMLLTDRNLNTSFFDPAGGGDPILYQHL
>LEFID284-10|HM873082|MM06159|Elachista_nobilella
TLYFIFGIWAGMVGTSLSLLIRAELGNPGSLIGNDQIYNTIVTAHAFIMIFFMVMPIMIGGFGNWLVPLMLGAPDMAFPRMNNMSFWLLPPSLTLLISSSLVENGAGTGWTVYPPLSSNIAHNGSSVDLAIFSLHLAGISSILGAINFITTIINMRLNNLMF
DQMPLFVWAVGITALLLLLSLPVLAGAITMLLTDRNLNTSFFDPAGGGDPILYQHL
>LEFIE755-10|HM874475|MM09877|Elachista_obliquella
TLYFIFGIWAGMVGTSLSLLIRAELGNPGSLIGNDQIYNTIVTAHAFIMIFFMVMPIMIGGFGNWLVPLMLGAPDMAFPRMNNMSFWLLPPSLTLLIFSSVVENGSGTGWTVYPPLSSNIAHSGSSVDMAIFSLHLAGISSILGAINFITTIINMRLNNMM
FDQMPLFVWAVGITALLLLLSLPVLAGAITMLLTDRNLNTSFFDPAGGGDPILYQHL
>LEFIC415-10|HM872258|MM03961|Elachista_occidentalis
TLYFIFGIWAGMVGTSLSLLIRAELGNPGSLIGNDQIYNTIVTAHAFIMIFFMVMPIMIGGFGNWLVPLMLGAPDMAFPRMNNMSFWLLPPSLTLLISSSIVENGAGTGWTVYPPLSSNIAHGGSSVDLAIFSLHLAGISSILGAINFITTIINMRLNNLMF
DQMPLFVWAVGITALLLLLSLPVLAGAITMLLTDRNLNTSFFDPAGGGDPILYQHL
>LEFIC430-10|HM872266|MM03979|Elachista_occidentalis
TLYFIFGIWAGMVGTSLSLLIRAELGNPGSLIGNDQIYNTIVTAHAFIMIFFMVMPIMIGGFGNWLVPLMLGAPDMAFPRMNNMSFWLLPPSLTLLISSSIVENGAGTGWTVYPPLSSNIAHGGSSVDLAIFSLHLAGISSILGAINFITTIINMRLNNLMF
DQMPLFVWAVGITALLLLLSLPVLAGAITMLLTDRNLNTSFFDPAGGGDPILYQHL
>ELACA1276-12|KM572884|MM21339|Elachista_occidentalis
TLYFIFGIWAGMVGTSLSLLIRAELGNPGSLIGNDQIYNTIVTAHAFIMIFFMVMPIMIGGFGNWLVPLMLGAPDMAFPRMNNMSFWLLPLSLTLLISSSIVENG?GTGWTVYPPLSSNIAHGGSSVDLAIFSLHLAGISSILGAINFITTIINMRLNNLMFD
QMPLFVWAVGITALLLLLSLPVLAGAITMLLTDRNLNTSFFDPAGGGDPILYQHL
>LEFIE270-10|HM874003|MM08608|Elachista_ornithopodella
TLYFIFGIWAGMVGTSLSLLIRAELGNPGSLIGNDQIYNTIVTAHAFIMIFFMVMPIMIGGFGNWLVPLMLGAPDMAFPRMNNMSFWLLPPSLTLLISSSVVESGAGTGWTVYPPLSSNIAHGGSSVDLAIFSLHLAGISSILGAINFITTIINMRLNNLMF
DQMPLFVWSVGITALLLLLSLPVLAGAITMLLTDRNLNTSFFDPAGGGDPILYQHL
>LEFII125-10|JF853356|MM18552|Elachista_orstadii
TLYFIFGIWAGMVGTSLSLLIRAELGNPGSLIGNDQIYNTIVTAHAFIMIFFMVMPIMIGGFGNWLVPLMLGAPDMAFPRMNNMSFWLLPPSLTLLISSSIVENGAGTGWTVYPPLSSNIAHSGSSVDLAIFSLHLAGISSILGAINFITTIINMRLNNLMFD

QMPLFVWAVGITALLLLLSLPVLAGAITMLLTDRNLNTSFFDPAGGGDPILYQHL
>LEFII126-10|KF809353|MM18553|Elachista_orstadii
TLYFIFGIWAGMVGTSLSLLIRAELGNPGSLIGNDQIYNTIVTAHAFIMIFFMVMPIMIGGFGNWLVPLMLGAPDMAFPRMNNMSFWLLPPSLTLLISSSIVENGAGTGWTVYPPLSSNIAHSGSSVDLAIFSLHLAGISSILGAINFITTIINMRLNNLMFD
QMPLFVWAVGITALLLLLSLPVLAGAITMLLTDRNLNTSFFDPAGGGDPILYQHL
>LEFIG694-10|HM876351|MM15558|Elachista_poae
TLYFIFGIWAGMVGTSLSLLIRAELGNPGSLIGNDQIYNTIVTAHAFIMIFFMVMPIMIGGFGNWLVPLMLGAPDMAFPRMNNMSFWLLPPSLTLLISSSIVENGAGTGWTVYPPLSSNIAHSGSSVDLAIFSLHLAGISSILGAINFITTIINMRLNNLMFD
QMPLFVWAVGITALLLLLSLPVLAGAITMLLTDRNLNTSFFDPAGGGDPILYQHL
>LEFID291-10|HM873089|MM06166|Elachista_pollinariella
TLYFIFGIWAGMLGTSLSLLIRAELGTPGSLIGNDQIYNTIVTAHAFIMIFFMVMPIMIGGFGNWLIPLMLGAPDMAFPRMNNMSFWLLPPSLILLTFSSVVENGAGTGWTVYPPLSSNIAHSGSSVDMTIFSLHLAGISSILGAINFITTIINMRLNNMMF
DQMPLFVWAVGITALLLLLSLPVLAGAITMLLTDRNLNTSFFDPAGGGDPILYQHL
>LEFIA727-10|HM386871|MM02664|Elachista_pomerana
TLYFILGIWAGMVGTSLSLLIRAELGNPGSLIGNDQIYNTIVTAHAFIMIFFMVMPIMIGGFGNWLVPLMLGAPDMAFPRMNNMSFWLLPPSLTLLISSSIVENGAGTGWTVYPPLSSNIAHNGSSVDLAIFSLHLAGISSILGAINFITTIINMRLNNLMF
DQMPLFVWAVGITALLLLLSLPVLAGAITMLLTDRNLNTSFFDPAGGGDPILYQHL
>LEFIE287-10|HM874019|MM08634|Elachista_pomerana
TLYFILGIWAGMVGTSLSLLIRAELGNPGSLIGNDQIYNTIVTAHAFIMIFFMVMPIMIGGFGNWLVPLMLGAPDMAFPRMNNMSFWLLPPSLTLLISSSIVENGAGTGWTVYPPLSSNIAHNGSSVDLAIFSLHLAGISSILGAINFITTIINMRLNNLMF
DQMPLFVWAVGITALLLLLSLPVLAGAITMLLTDRNLNTSFFDPAGGGDPILYQHL
>ELACA776-11|JN267103|MM20014|Elachista_pomerana
TLYFILGIWAGMVGTSLSLLIRAELGNPGSLIGNDQIYNTIVTAHAFIMIFFMVMPIMIGGFGNWLVPLMLGAPDMAFPRMNNMSFWLLPPSLTLLISSSIVENGAGTGWTVYPPLSSNIAHNGSSVDLAIFSLHLAGISSILGAINFITTIINMRLNNLMF
DQMPLFVWAVGITALLLLL?LPVLAGAITMLLTDRNLNTSFFDPAGGGDPILYQHL
>LEFID287-10|HM873085|MM06162|Elachista_pullicomella
TLYFIFGIWAGMVGTSLSLLIRAELGNPGSLIGNDQIYNTIVTAHAFIMIFFMVMPIMIGGFGNWLVPLMLGAPDMAFPRMNNMSFWLLPPSLTLLISSSVVEVGAGTGWTVYPPLSSNIAHSGSSVDLAIFSLHLAGISSILGAINFITTIINMRLNNMM
FDQMPLFVWAVGITAVLLLLSLPVLAGAITMLLTDRNLNTSFFDPAGGGDPILYQHL
>LEFIG069-10|HM875748|MM13718|Elachista_pullicomella
TLYFIFGIWAGMVGTSLSLLIRAELGNPGSLIGNDQIYNTIVTAHAFIMIFFMVMPIMIGGFGNWLVPLMLGAPDMAFPRMNNMSFWLLPPSLTLLISSSVVEVGAGTGWTVYPPLSSNIAHSGSSVDLAIFSLHLAGISSILGAINFITTIINMRLNNMM
FDQMPLFVWAVGITAVLLLLSLPVLAGAITMLLTDRNLNTSFFDPAGGGDPILYQHL
>ELACA771-11|JN267098|MM20009|Elachista_pullicomella
TLYFIFGIWAGMVGTSLSLLIRAELGNPGSLIGNDQIYNTIVTAHAFIMIFFMVMPIMIGGFGNWLVPLMLGAPDMAFPRMNNMSFWLLPPSLTLLISSSVVEVGAGTGWTVYPPLSSNIAHSGSSVDLAIFSLHLAGISSILGAINFITTIINMRLNNMM
FDQMPLFVWAVGITAVLLLLSLPVLAGAITMLLTDRNLNTSFFDPAGGGDPILYQHL
>LEFII117-10|JF853350|MM18544|Elachista_scirpi
TLYFIFGIWAGMMGTSLSLLIRAELGNPGSLIGNDQIYNTIVTAHAFIMIFFMVMPIMIGGFGNWLVPLMLGAPDMAFPRMNNMSFWLLPPSITLLISSSIVENGAGTGWTVYPPLSSNIAHSGSSVDLAIFSLHLAGISSILGAINFITTIINMRLNNLMF
DQMPLFVWAVGITALLLLLSLPVLAGAITMLLTDRNLNTSFFDPAGGGDPILYQHL
>LEFID822-10|HM873579|MM06928|Elachista_serricornis
TLYFIFGIWAGMVGTSLSLLIRAELGTPGSLIGNDQIYNTIVTAHAFIMIFFMVMPIMIGGFGNWLVPLMLGAPDMAFPRMNNMSFWLLPPSLTLLISSSIVENGAGTGWTVYPPLSSNIAHGGSSVDLAIFSLHLAGISSILGAINFITTIINMRLNNLMFD
QMPLFVWAVGITALLLLLSLPVLAGAITMLLTDRNLNTSFFDPAGGGDPILYQHL
>LEFIE485-10|HM874209|MM09264|Elachista_serricornis
TLYFIFGIWAGMVGTSLSLLIRAELGTPGSLIGNDQIYNTIVTAHAFIMIFFMVMPIMIGGFGNWLVPLMLGAPDMAFPRMNNMSFWLLPPSLTLLISSSIVENGAGTGWTVYPPLSSNIAHGGSSVDLAIFSLHLAGISSILGAINFITTIINMRLNNLMFD
QMPLFVWAVGITALLLLLSLPVLAGAITMLLTDRNLNTSFFDPAGGGDPILYQHL
>LEFII146-10|MM18573|Elachista_stabilella
TLYFIFGIWAGMVGTSLSLLIRAELGNPGSLIGNDQIYNTIVTAHAFIMIFFMVMPIMIGGFGNWLVPLMLGAPDMAFPRMNNMSFWLLPPSLTLLISSSVVENGAGTGWTVYPPLSSNIAHSGSSVDLAIFSLHLAGISSILGAINFITTIINMRLNNLMF
DQMPLFVWAVGITALLLLLSLPVLAGAITMLLTDRNLNTSFFDPAGGGDPILYQHL
>LEFII147-10|MM18574|Elachista_stabilella
TLYFIFGIWAGMVGTSLSLLIRAELGNPGSLIGNDQIYNTIVTAHAFIMIFFMVMPIMIGGFGNWLVPLMLGAPDMAFPRMNNMSFWLLPPSLTLLISSSVVENGAGTGWTVYPPLSSNIAHSGSSVDLAIFSLHLAGISSILGAINFITTIINMRLNNLMF
DQMPLFVWAVGITALLLLLSLPVLAGAITMLLTDRNLNTSFFDPAGGGDPILYQHL
>LEFIB775-10|HM871652|MM02650|Elachista_subalbidella
TLYFIFGIWAGMVGTSLSLLIRAELGNPGSLIGNDQIYNTIVTAHAFIMIFFMVMPIMIGGFGNWLVPLMLGAPDMAFPRMNNMSFWLLPPSLTLLIFSSVVENGSGTGWTVYPPLSSNIAHSGSSVDMAIFSLHLAGISSILGAINFITTIINMRLNNMM
FDQMPLFVWAVGITALLLLLSLPVLAGAITMLLTDRNLNTSFFDPAGGGDPILYQHL
>ELACA382-10|JF847489|MM15299|Elachista_subnigrella

TLYFIFGIWAGMVGTSLSLLIRAELGNPGSLIGNDQIYNTIVTAHAFIMIFFMVMPIMIGGFGNWLVPLMLGAPDMAFPRMNNMSFWLLPPSLTLLISSSIVENGAGTGWTVYPPLSSNIAHSGSSVDLAIFSLHLAGISSILGAINFITTIINMRLNNLMFD
QMPLFVWAVGITALLLLLSLPVLAGAITMLLTDRNLNTSFFDPAGGGDPILYQHL
>ELACA399-10|JF847497|MM16132|Elachista_subnigrella
TLYFIFGIWAGMVGTSLSLLIRAELGNPGSLIGNDQIYNTIVTAHAFIMIFFMVMPIMIGGFGNWLVPLMLGAPDMAFPRMNNMSFWLLPPSLTLLISSSIVENGAGTGWTVYPPLSSNIAHSGSCVDLAIFSLHLAGISSILGAINFITTIINMRLNNLMF
DQMPLFVWAVGITALLLLLSLPVLAGAITMLLTDRNLNTSFFDPAGGGDPILYQHL
>LEFII123-10|JF853354|MM18550|Elachista_subnigrella
TLYFIFGIWAGMVGTSLSLLIRAELGNPGSLIGNDQIYNTIVTAHAFIMIFFMVMPIMIGGFGNWLVPLMLGAPDMAFPRMNNMSFWLLPPSLTLLISSSIVENGAGTGWTVYPPLSSNIAHSGSSVDLAIFSLHLAGISSILGAINFITTIINMRLNNLMFD
QMPLFVWAVGITALLLLLSLPVLAGAITMLLTDRNLNTSFFDPAGGGDPILYQHL
>LEFII124-10|JF853355|MM18551|Elachista_subnigrella
TLYFIFGIWAGMVGTSLSLLIRAELGNPGSLIGNDQIYNTIVTAHAFIMIFFMVMPIMIGGFGNWLVPLMLGAPDMAFPRMNNMSFWLLPPSLTLLISSSIVENGTGTGWTVYPPLSSNIAHSGSSVDLAIFSLHLAGISSILGAINFITTIINMRLNNLMFD
QMPLFVWAVGITALLLLLSLPVLAGAITMLLTDRNLNTSFFDPAGGGDPILYQHL
>LEFIF061-10|HM874771|MM10501|Elachista_subocellea
TLYFIFGIWAGMVGTSLSLMIRAELGNPGSLIGNDQIYNTIVTAHAFIMIFFMVMPIMIGGFGNWLVPLMLGAPDMAFPRMNNMSFWLLPPSLTLLIFSSVVENGAGTGWTVYPPLSSNIAHSGSSVDMAIFSLHLAGISSILGAINFITTIINMRLNNM
MFDQMPLFVWAVGITALLLLLSLPVLAGAITMLLTDRNLNTSFFDPAGGGDPILYQHL
>LEFIF065-10|HM874775|MM10505|Elachista_subocellea
TLYFIFGIWAGMVGTSLSLMIRAELGNPGSLIGNDQIYNTIVTAHAFIMIFFMVMPIMIGGFGNWLVPLMLGAPDMAFPRMNNMSFWLLPPSLTLLIFSSVVENGAGTGWTVYPPLSSNIAHSGSSVDMAIFSLHLAGISSILGAINFITTIINMRLNNM
MFDQMPLFVWAVGITALLLLLSLPVLAGAITMLLTDRNLNTSFFDPAGGGDPILYQHL
>LEFIF068-10|HM874778|MM10508|Elachista_subocellea
TLYFIFGIWAG?VGTSLSLMIRAELGNPGSLIGNDQIYNTIVTAHAFIMIFFMVMPIMIGGFGNWLVPLMLGAPDMAFPRMNNMSFWLLPPSLTLLIFSSVVENGAGTGWTVYPPLSSNIAHSGSSVDMAIFSLHLAGISSILGAINFITTIINMRLNNM
MFDQMPLFVWAVGITALLLLLSLPVLAGAITMLLTDRNLNTSFFDPAGGGDPILYQHL
>ELACA421-10|JF847513|MM16154|Elachista_tanaella
TLYFIFGIWAGMIGTSLSLLIRAELGNPGSLIGNDQIYNTIVTAHAFIMIFFMVMPIMIGGFGNWLVPLMLGAPDMAFPRMNNMSFWLLPPSLTLLISSTIVENGTGTGWTVYPPLSSNISHNGSSVDLTIFSLHLAGISSILGAINFITTIINMRLNKLMFD
QMPLFVWAVGITALLLLLSLPVLAGAITMLLTDRNLNTSFFDPAGGGDPILYQHL
>LEFIC997-10|HM872812|MM05413|Elachista_tengstromi
TLYFIFGIWAGLVGTSLSLLIRAELGNPGSLIGNDQIYNTIVTAHAFIMIFFMVMPIMIGGFGNWLVPLMLGAPDMAFPRMNNMSFWLLPPSLTLLISSSIVENGAGTGWTVYPPLSSNIAHGGSSVDLAIFSLHLAGISSILGAINFITTIINMRLNNMMF
DQMPLFVWAVGITALLLLLSLPVLAGAITMLLTDRNLNTSFFDPAGGGDPILYQHL
>LEFIG426-10|HM876102|MM14459|Elachista_tengstromi
TLYFIFGIWAGLVGTSLSLLIRAELGNPGSLIGNDQIYNTIVTAHAFIMIFFMVMPIMIGGFGNWLVPLMLGAPDMAFPRMNNMSFWLLPPSLTLLISSSIVENGAGTGWTVYPPLSSNIAHGGSSVDLAIFSLHLAGISSILGAINFITTIINMRLNNMMF
DQMPLFVWAVGITALLLLLSLPVLAGAITMLLTDRNLNTSFFDPAGGGDPILYQHL
>ELACA129-10|JF847352|MM16329|Elachista_tetragonella
TLYFIFGIWAGMVGTSLSLLIRAELGNPGSLIGNDQIYNTIVTAHAFIMIFFMVMPIMIGGFGNWLVPLMLGAPDMAFPRMNNMSFWLLPPSLTLLISSSIVENGAGTGWTVYPPLSSNIAHGGSSVDLAIFSLHLAGISSILGAINFITTIINMRLNNLMF
DQMPLFVWAVGITALLLLLSLPVLAGAITMLLTDRNLNTSFFDPAGGGDPILYQHL
>LEFIE993-10|HM874709|MM10431|Elachista_trapeziella
TLYFIFGIWAGMIGTSLSLLIRAELGNPGSLIGNDQIYNTIVTAHAFIMIFFMVMPIMIGGFGNWLVPLMLGAPDMAFPRMNNMSFWLLPPSLTLLISSSIVENGAGTGWTVYPPLSSNIAHSGSSVDLAVFSLHLAGISSILGAINFITTIINMRLNNLMFD
QMPLFVWAVGITALLLLLSLPVLAGAITMLLTDRNLNTSFFDPAGGGDPILYQHL
>ELACA119-10|JF847350|MM16319|Elachista_trapeziella
TLYFIFGIWAGMIGTSLSLLIRAELGNPGSLIGNDQIYNTIVTAHAFIMIFFMVMPIMIGGFGNWLVPLMLGAPDMAFPRMNNMSFWLLPPSLTLLISSSIVENGAGTGWTVYPPLSSNIAHSGSSVDLAVFSLHLAGISSILGAINFITTIINMRLNNLMFD
QMPLFVWAVGITALLLLLSLPVLAGAITMLLTDRNLNTSFFDPAGGGDPILYQHL
>ELACA764-11|JN267092|MM20002|Elachista_trapeziella
TLYFIFGIWAGMIGTSLSLLIRAELGNPGSLIGNDQIYNTIVTAHAFIMIFFMVMPIMIGGFGNWLVPLMLG?PDMAFPRMNNMSFWLLPPSLTLLISSSIVENGAGTGWTVYPPLSSNIAHSGSSVDLAVFSLHLAGISSILGAINFITTIINMRLNNLMFD
QMPLFVWAVGITALLLLLSLPVLAGAITMLLTDRNLNTSFFDPAGGGDPILYQHL
>COLFF464-13|MM23197|Elachista_trapeziella
TLYFIFGIWAGMIGTSLSLLIRAELGNPGSLIGNDQIYNTIVTAHAFIMIFFMVMPIMIGGFGNWLVPLMLGAPDMAFPRMNNMSFWLLPPSLTLLISSSIVENGAGTGWTVYPPLSSNIAHSGSSVDLAVFSLHLAGISSILGAINFITTIINMRLNNLMFD
QMPLFVWAVGITALLLLLSLPVLAGAITMLLTDRNLNTSFFDPAGGGDPILYQHL
>LEFID816-10|HM873573|MM06903|Elachista_triatomea
TLYFIFGIWAGMVGTSLSLLIRAELGNPGSLIGNDQIYNMIVTAHAFIMIFFMVMPIMIGGFGNWLVPLMLGAPDMAFPRMNNMSFWLLPPSLTLLISSSVVENGAGTGWTVYPPLSSNIAHSGSSVDLAIFSLHLAGISSILGAINFITTIVNMKTNNLM
FDQMPLFIWAVGITAVLLLLSLPVLAGAITMLLTDRNLNTSFFDPAGGGDPILYQHL

>LEFII140-10|MM18567|Elachista_unifasciella
TLYFIFGIWAGMVGTSLSLLIRAELGNPGSLIGNDQIYNTIVTAHAFIMIFFMVMPIMIGGFGNWLVPLMLGAPDMAFPRMNNMSFWLLPPSLTLLIFSSMVENGAGTGWTVYPPLSSNIAHSGSSVDMAIFSLHLAGISSILGAINFITTIINMRFNNM
MFDQMPLFIWAVGITALLLLLSLPVLAGAITMLLTDRNLNTSFFDPAGGGDPILYQHL
>LEFID741-10|HM873498|MM06800|Elachista_utonella
TLYFIFGIWAGMVGTSLSLLIRAELGNPGSLIGNDQIYNTIVTAHAFIMIFFMVMPIMIGGFGNWLVPLMLGAPDMAFPRMNNMSFWLLPPSLTLLISSSIVENGAGTGWTVYPPLSSNIAHGGSSVDLAIFSLHLAGISSILGAINFITTIINMRLNNLMF
DQMPLFVWAVGITALLLLLSLPVLAGAITMLLTDRNLNTSFFDPAGGGDPILYQHL
>LEFIE290-10|HM874022|MM08637|Elachista_utonella
TLYFIFGIWAGMVGTSLSLLIRAELGNPGSLIGNDQIYNTIVTAHAFIMIFFMVMPIMIGGFGNWLVPLMLGAPDMAFPRMNNMSFWLLPPSLTLLISSSIVENGAGTGWTVYPPLSSNIAHGGSSVDLAIFSLHLAGISSILGAINFITTIINMRLNNLMF
DQMPLFVWAVGITALLLLLSLPVLAGAITMLLTDRNLNTSFFDPAGGGDPILYQHL
>LEFIE620-10|HM874343|MM09545|Elachista_utonella
TLYFIFGIWAGMVGTSLSLLIRAELGNPGSLIGNDQIYNTIVTAHAFIMIFFMVMPIMIGGFGNWLVPLMLGAPDMAFPRMNNMSFWLLPPSLTLLISSSIVENGAGTGWTVYPPLSSNIAHGGSSVDLAIFSLHLAGISSILGAINFITTIINMRLNNLMF
DQMPLFVWAVGITALLLLLSLPVLAGAITMLLTDRNLNTSFFDPAGGGDPILYQHL
>LEFIE621-10|HM874344|MM09546|Elachista_utonella
TLYFIFGIWAGMVGTSLSLLIRAELGNPGSLIGNDQIYNTIVTAHAFIMIFFMVMPIMIGGFGNWLVPLMLGAPDMAFPRMNNMSFWLLPPSLTLLISSSIVENGAGTGWTVYPPLSSNIAHGGSSVDLAIFSLHLAGISSILGAINFITTIINMRLNNLMF
DQMPLFVWAVGITALLLLLSLPVLAGAITMLLTDRNLNTSFFDPAGGGDPILYQHL
>LEFIE975-10|HM874692|MM10403|Elachista_utonella
TLYFIFGIWAGMVGTSLSLLIRAELGNPGSLIGNDQIYNTIVTAHAFIMIFFMVMPIMIGGFGNWLVPLMLGAPDMAFPRMNNMSFWLLPPSLTLLISSSIVENGAGTGWTVYPPLSSNIAHGGSSVDLAIFSLHLAGISSILGAINFITTIINMRLNNLMF
DQMPLFVWAVGITALLLLLSLPVLAGAITMLLTDRNLNTSFFDPAGGGDPILYQHL
>ELACA286-10|JF847434|MM16201|Elachista_utonella
TLYFIFGIWAGMVGTSLSLLIRAELGNPGSLIGNDQIYNTIVTAHAFIMIFFMVMPIMIGGFGNWLVPLMLGAPDMAFPRMNNMSFWLLPPSLTLLISSSIVENGAGTGWTVYPPLSSNIAHGGSSVDLAIFSLHLAGISSILGAINFITTIINMRLNNLMF
DQMPLFVWAVGITALLLLLSLPVLAGAITMLLTDRNLNTSFFDPAGGGDPILYQHL
>ELACA287-10|JF847435|MM16202|Elachista_utonella
TLYFIFGIWAGMVGTSLSLLIRAELGNPGSLIGNDQIYNTIVTAHAFIMIFFMVMPIMIGGFGNWLVPLMLGAPDMAFPRMNNMSFWLLPPSLTLLISSSIVENGAGTGWTVYPPLSSNIAHGGSSVDLAIFSLHLAGISSILGAINFITTIINMRLNNLMF
DQMPLFVWAVGITALLLLLSLPVLAGAITMLLTDRNLNTSFFDPAGGGDPILYQHL
>LEFID533-10|HM873298|MM06516|Elachista_vonschantzi
TLYFIFGIWAGMVGTSLSLLIRAELGNPGSLIGNDQIYNTIVTAHAFIMIFFMVMPIMIGGFGNWLVPLMLGAPDMAFPRMNNMSFWLLPPSLTLLISSSIVENGAGTGWTVYPPLSSNIAHSGSSVDLAIFSLHLAGISSILGAINFITTIINMRLNNLMFD
QMPLFVWAVGITALLLLLSLPVLAGAITMLLTDRNLNTSFFDPAGGGDPILYQHL
>LEFIK079-10|KF809257|MM17654|Elachista_zernyi
TLYFIFGIWAGMVGTSLSLLIRAELGNPGSLIGNDQIYNTIVTAHAFIMIFFMVMPIMIGGFGNWLVPLMLGAPDMAFPRMNNMSFWLLPPSLTLLISSSIVENGAGTGWTVYPPLSSNIAHSGSSVDLAIFSLHLAGISSILGAINFITTIINMRLNNLMFD
QMPLFVWAVGITALLLLLSLPVLAGAITMLLTDRNLNTSFFDPAGGGDPILYQHL
>LEFII127-10|KF809311|MM18554|Elachista_zernyi
TLYFIFGIWAGMVGTSLSLLIRAELGNPGSLIGNDQIYNTIVTAHAFIMIFFMVMPIMIGGFGNWLVPLMLGAPDMAFPRMNNMSFWLLPPSLTLLISSSIVENGAGTGWTVYPPLSSNIAHSGSSVDLAIFSLHLAGISSILGAINFITTIINMRLNNLMFD
QMPLFVWAVGITALLLLLSLPVLAGAITMLLTDRNLNTSFFDPAGGGDPILYQHL
>LEFID605-10|HM873370|MM06618|Elaphria_venustula
TLYFIFGIWAGMVGTSLSLLIRAELGTPGSLIGDDQIYNTIVTAHAFIMIFFMVMPIMIGGFGNWLIPLMLGAPDMAFPRMNNMSFWLLPPSLTLLISSSIVENGAGTGWTVYPPLSSNIAHGGSSVDLAIFSLHLAGISSILGAINFITTIINMRLNSLSFDQ
MPLFIWAVGITAFLLLLSLPVLAGAITMLLTDRNLNTSFFDPAGGGDPILYQHL
>LEFIE201-10|HM873945|MM08516|Elatobia_fuliginosella
TLYFIFGIWSGMLGTSLSLLIRTELSSPGSLIGNDQIYNTIVTAHAFIMIFFMVMPIMIGGFGNWLVPLMLGAPDMAFPRMNNMSFWLLPPSLLLLICSSIIENGAGTGWTVYPPLSSNISHLGSSVDLTIFSLHLAGISSILGAINFITTIINMQSKGMSFDQ
MPLFVWAVGITALLLLLSLPVLAGAITMLLTDRNLNTSFFDPAGGGDPILYQHL
>LEFIA244-10|HM386588|MM01277|Electrophaes_corylata
TLYFIFGIWAGMIGTSLSLLIRAELGNPGSLIGDDQIYNTIVTAHAFIMIFFMVMPIMIGGFGNWLVPLMLGAPDMAFPRMNNMSFWLLPPSITLLISSSIVENGAGTGWTVYPPLSSNIAHGGSSVDLAIFSLHLAGISSILGAINFITTIINMRLNNMFFD
QLPLFVWAVGITAFLLLLSLPVLAGAITMLLTDRNLNTSFFDPAGGGDPILYQHL
>LEFIF376-10|HM875061|MM11619|Electrophaes_corylata
TLYFIFGIWAGMIGTSLSLLIRAELGNPGSLIGDDQIYNTIVTAHAFIMIFFMVMPIMIGGFGNWLVPLMLGAPDMAFPRMNNMSFWLLPPSITLLISSSIVENGAGTGWTVYPPLSSNIAHGGSSVDLAIFSLHLAGISSILGAINFITTIINMRLNNMFFD
QLPLFVWAVGITAFLLLLSLPVLAGAITMLLTDRNLNTSFFDPAGGGDPILYQHL
>LEEUA269-11|JN275971|MM19677|Elegia_similella
TLYFIFGIWAGMVGTSLSLLIRAELGTPGSLIGDDQIYNTIVTGHAFIMIFFMVMPIMIGGFGNWLVPLMLGAPDMAFPRMNNMSFWLLPPSLTLLISSSIVESGAGTGWTVYPPLSSNIAHSGSSVDLAIFSLHLAGISSILGAINFITTIINMKLNGLSFDQ

MPLFVWAVGITALLLLLSLPVLAGAITMLLTDRNLNTSFFDPAGGGDPILYQHL
>LEFIC732-10|HM872553|MM04808|Elophila_nymphaeata
TLYFIFGIWAGMVGTSLSLLIRAELGNPGSLIGDDQIYNTIVTAHAFIMIFFMVMPIMIGGFGNWLVPLMLGAPDMAFPRMNNMSFWLLPPSLTLLISSSIVENGAGTGWTVYPPLSSNIAHGGSSVDLAIFSLHLAGISSILGAINFITTIINMRINNLFFD
QMPLFVWAVGITAFLLLLSLPVLAGAITMLLTDRNLNTSFFDPAGGGDPILYQHL
>LEFIB844-10|HM871721|MM02824|Elophos_vittaria
TLYFIFGIWAGMVGTSLSLLIRAELGNPGSLIGDDQIYNTIVTAHAFIMIFFMVMPIMIGGFGNWLVPLMLGAPDMAFPRMNNMSFWLLPPSITLLISSSIVENGAGTGWTVYPPLSSNIAHGGSSVDLAIFSLHLAGISSILGAINFITTIINMRLNNMSFD
QMPLFVWSVGITAFLLLLSLPVLAGAITMLLTDRNLNTSFFDPAGGGDPILYQHL
>LEFIA782-10|HM386923|MM04136|Elophos_vittaria
TLYFIFGIWAGMVGTSLSLLIRAELGNPGSLIGDDQIYNTIVTAHAFIMIFFMVMPIMIGGFGNWLVPLMLGAPDMAFPRMNNMSFWLLPPSITLLISSSIVENGAGTGWTVYPPLSSNIAHGGSSVDLAIFSLHLAGISSILGAINFITTIINMRLNNMSFD
QMPLFVWSVGITAFLLLLSLPVLAGAITMLLTDRNLNTSFFDPAGGGDPILYQHL
>LEFIB132-10|HM871038|MM00501|Ematurga_atomaria
TLYFIFGIWAWMVGTSLSLLIRAELGNPGSLIGDDQIYNTIVTAHAFIMIFFMVMPIMIGGFGNWLVPLMLGAPDMAFPRMNNMSFWLLPPSITLLISSSIVENGAGTGWTVYPPLSSNIAHGGSSVDLAIFSLHLAGISSILGAINFITTIINMRLNNLSFD
QMPLFVWAVGITAFLLLLSLPVLAGAITMLLTDRNLNTSFFDPAGGGDPILYQHL
>LEFIB165-10|HM871069|MM00562|Ematurga_atomaria
TLYFIFGIWAGMVGTSLSLLIRAELGNPGSLIGDDQIYNTIVTAHAFIMIFFMVMPIMIGGFGNWLVPLMLGAPDMAFPRMNNMSFWLLPPSITLLISSSIVENGAGTGWTVYPPLSSNIAHGGSSVDLAIFSLHLAGISSILGAINFITTIINMRLNNLSFD
QMPLFVWAVGITAFLLLLSLPVLAGAITMLLTDRNLNTSFFDPAGGGDPILYQHL
>LEFIK935-10|JF854377|MM18510|Ematurga_atomaria
TLYFIFGIWAGMVGTSLSLLIRAELGNPGSLIGDDQIYNTIVTAHAFIMIFFMVMPIMIGGFGNWLVPLMLGAPDMAFPRMNNMSFWLLPPSITLLISSSIVENGAGTGWTVYPPLSSNIAHGGSSVDLAIFSLHLAGISSILGAINFITTIINMRLNNLSFD
QMPLFVWAVGITAFLLLLSLPVLAGAITMLLTDRNLNTSFFDPAGGGDPILYQHL
>LEFIC808-10|HM872627|MM04982|Emmelina_monodactyla
TLYFIFGIWAGMVGTSLSLLIRAELGNPGSLIGDDQIYNTIVTAHAFIMIFFMVMPIMIGGFGNWLVPLMLGAPDMAFPRMNNMSFWLLPPSITLLISSSIVENGAGTGWTVYPPLSSNIAHSGASVDLAIFSLHLAGISSILGAINFITTIINMKLSNLMFD
QLPLFVWAVGITALLLLLSLPVLAGAITMLLTDRNLNTSFFDPAGGGDPILYQHL
>LEFIA602-10|HM870851|MM01714|Enargia_paleacea
TLYFIFGIWAGMVGTSLSLLIRAELGNPGSLIGDDQIYNTIVTAHAFIMIFFMVMPIMIGGFGNWLVPLMLGAPDMAFPRLNNMSFWLLPPSLTLLISSSIVENGAGTGWTVYPPLSSNIAHGGSSVDLAIFSLHLAGISSILGAINFITTIINMRLNNLSFDQ
MPLFIWAVGITAFLLLLSLPVLAGAITMLLTDRNLNTSFFDPAGGGDPILYQHL
>LEFIA603-10|HM870852|MM01715|Enargia_paleacea
TLYFIFGIWAGMVGTSLSLLIRAELGNPGSLIGDDQIYNTIVTAHAFIMIFFMVMPIMIGGFGNWLVPLMLGAPDMAFPRLNNMSFWLLPPSLTLLISSTIVENGAGTGWTVYPPLSSNIAHGGSSVDLAIFSLHLAGISSILGAINFITTIINMRLNNLSFDQ
MPLFIWAVGITAFLLLLSLPVLAGAITMLLTDRNLN?SFFDPAGGGDPILYQHL
>LEFIB811-10|HM871688|MM02745|Enargia_paleacea
TLYFIFGIWAGMVGTSLSLLIRAELGNPGSLIGDDQIYNTIVTAHAFIMIFFMVMPIMIGGFGNWLVPLMLGAPDMAFPRLNNMSFWLLPPSLTLLISSSIVENGAGTGWTVYPPLSSNIAHGGSSVDLAIFSLHLAGISSILGAINFITTIINMRLNNLSFDQ
MPLFIWAVGITAFLLLLSLPVLAGAITMLLTDRNLNTSFFDPAGGGDPILYQHL
>LEFIF645-10|HM875329|MM12553|Enargia_paleacea
TLYFIFGIWAGMVGTSLSLLIRAELGNPGSLIGDDQIYNTIVTAHAFIMIFFMVMPIMIGGFGNWLVPLMLGAPDMAFPRLNNMSFWLLPPSLTLLISSTIVENGAGTGWTVYPPLSSNIAHGGSSVDLAIFSLHLAGISSILGAINFITTIINMRLNNLSFDQ
MPLFIWAVGITAFLLLLSLPVLAGAITMLLTDRNLNTSFFDPAGGGDPILYQHL
>LEFIB495-10|HM871390|MM02074|Enarmonia_formosana
TLYFIFGIWAGMIGTSLSLLIRAELGNPGSLIGDDQIYNTIVTAHAFIMIFFMVMPIMIGGFGNWLVPLMLGAPDMAFPRMNNMSFWLLPPSIMLLISSSIVENGAGTGWTVYPPLSSNIAHSGSSVDLAIFSLHLAGISSILGAVNFITTIINMRPNNMSL
DQMPLFVWAVGITALLLLLSLPVLAGAITMLLTDRNLNTSFFDPAGGGDPILYQHL
>LEFIF865-10|HM875547|MM13238|Endothenia_ericetana
TLYFIFGVWAGMVGTSLSLLIRAELGNPGSLIGNDQIYNTIVTAHAFIMIFFMVMPIMIGGFGNWLIPLMLGAPDMAFPRMNNMSFWLLPPSIMLLISSSIVENGAGTGWTVYPPLSSNIAHSGSSVDLAIFSLHLAGISSILGAVNFITTIINMRPNNMSL
DQMPLFVWAVGITALLLLLSLPVLAGAITMLLTDRNLNTSFFDPAGGGDPILYQHL
>LEFIG845-10|HM876493|MM15709|Endothenia_ericetana
TLYFIFGVWAGMVGTSLSLLIRAELGNPGSLIGNDQIYNTIVTAHAFIMIFFMVMPIMIGGFGNWLIPLMLGAPDMAFPRMNNMSFWLLPPSIMLLISSSIVENGAGTGWTVYPPLSSNIAHSGSSVDLAIFSLHLAGISSILGAVNFITTIINMRPNNMSL
DQMPLFVWAVGITALLLLLSLPVLAGAITMLLTDRNLNTSFFDPAGGGDPILYQHL
>LEFID206-10|HM873008|MM06034|Endothenia_gentianaeana
TLYFIFGIWAGMVGTSLSLLIRAELGNPGSLIGNDQIYNTIVTAHAFIMIFFMVMPIMIGGFGNWLIPLMLGAPDMAFPRMNNMSFWLLPPSIMLLISSSIVENGAGTGWTVYPPLSSNIAHSGSSVDLAIFSLHLAGISSILGAVNFITTIINMRPNNMTL
DQMPLFVWAVGITALLLLLSLPVLAGAITMLLTDRNLNSSFFDPAGGGDPILYQHL
>LEFIB538-10|HM871424|MM02151|Endothenia_marginana

TLYFIFGVWAGMMGTSLSLLIRAELGNPGSLIGDDQIYNTIVTAHAFIMIFFMVMPIMIGGFGNWLVPLMLGAPDMAFPRMNNMSFWLLPPSIMLLISSSVVENGAGTGWTVYPPLSSNIAHSGSSVDLAIFSLHLAGISSILGAVNFITTIINMRPNNM
TLDQMPLFIWAVGITALLLLLSLPILAGAITMLLTDRNLNSSFFDPAGGGDPILYQHL
>LEFIB539-10|HM871425|MM02152|Endothenia_marginana
TLYFIFGVWAGMMGTSLSLLIRAELGNPGSLIGDDQIYNTIVTAHAFIMIFFMVMPIMIGGFGNWLVPLMLGAPDMAFPRMNNMSFWLLPPSIMLLISSSVVENGAGTGWTVYPPLSSNIAHSGSSVDLAIFSLHLAGISSILGAVNFITTIINMRPNNM
TLDQMPLFIWAVGITALLLLLSLPVLAGAITMLLTDRNLNSSFFDPAGGGDPILYQHL
>LEFIF419-10|HM875104|MM11827|Endothenia_marginana
TLYFIFGVWAGMMGTSLSLLIRAELGNPGSLIGDDQIYNTIVTAHAFIMIFFMVMPIMIGGFGNWLVPLMLGAPDMAFPRMNNMSFWLLPPSIMLLISSSVVENGAGTGWTVYPPLSSNIAHSGSSVDLAIFSLHLAGISSILGAVNFITTIINMRPNNM
TLDQMPLFIWAVGITALLLLLSLPVLAGAITMLLTDRNLNSSFFDPAGGGDPILYQHL
>LEFID159-10|HM872966|MM05923|Endothenia_nigricostana
TLYFIFGIWAGMVGTSLSLLIRAELGNPGSLIGDDQIYNTIVTAHAFIMIFFMVMPIMIGGFGNWLVPLMLGAPDMAFPRMNNMSFWLLPPSIMLLISSSVVENGAGTGWTVYPPLSSNIAHSGSSVDLAIFSLHLAGISSILGAVNFITTIINMRPNNMS
LDQLPLFIWAVGITALLLLLSLPVLAGAITMLLTDRNLNTSFFDPAGGGDPILYQHL
>LEEUA229-11|JN274927|MM19637|Endothenia_nigricostana
TLYFIFGIWAGMVGTSLSLLIRAELGNPGSLIGDDQIYNTIVTAHAFIMIFFMVMPIMIGGFGNWLVPLMLGAPDMAFPRMNNMSFWLLPPSIMLLISSSVVENGAGTGWTVYPPLSSNIAHSGSSVDLAIFSLHLAGISSILGAVNFITTIINMRPNNMS
LDQLPLFIWAVGITALLLLLSLPVLAGAITMLLTDRNLNTSFFDPAGGGDPILYQHL
>LEEUA226-11|JN274924|MM19634|Endothenia_oblongana
TLYFIFGIWAGMVGTSLSLLIRAELGNPGSLIGDDQIYNTIVTAHAFIMIFFMVMPIMIGGFGNWLVPLMLGAPDMAFPRMNNMSFWLLPPSIMLLISSSIVENGAGTGWTVYPPLSSNIAHSGSSVDLAIFSLHLAGISSILGAVNFITTIINMRPNNMTL
DQMPLFVWAVGITALLLLLSLPVLAGAITMLLTDRNLNSSFFDPAGGGDPILYQHL
>LEEUA228-11|JN274926|MM19636|Endothenia_pullana
T?YFIFGIWAGMVGTSLSLLIRAELGNPGSLIGDDQIYNTIVTAHAFIMIFFMVMPIMIGGFGNWLVPLMLGAPDMAFPRMNNMSFWLLPPSIMLLISSSVVENGAGTGWTVYPPLSSNIAHSGSSVDLAIFSLHLAGISSILGAVNFITTIINMRPNNMS
LDQLPLFIWAVGITALLLLLSLPVLAGAITMLLTDRNLNTSFFDPAGGGDPILYQHL
>LEFIB514-10|HM871406|MM02110|Endothenia_quadrimaculana
TLYFIFGIWAGMIGTSLSLLIRAELGNPGSLIGDDQIYNTIVTAHAFIMIFFMVMPIMIGGFGNWLIPLMLGAPDMAFPRMNNMSFWLLPPSIMLLISSSIVENGAGTGWTVYPPLSSNIAHSGSSVDLAIFSLHLAGISSILGAVNFITTIINMRPNNMSLD
QMPLFVWAVGITALLLLLSLPVLAGAITMLLTDRNLNTSFFDPAGGGDPILYQHL
>LEFIC810-10|HM872629|MM04986|Endothenia_quadrimaculana
TLYFIFGIWAGMIGTSLSLLIRAELGNPGSLIGDDQIYNTIVTAHAFIMIFFMVMPIMIGGFGNWLIPLMLGAPDMAFPRMNNMSFWLLPPSIMLLISSSIVENGAGTGWTVYPPLSSNIAHSGSSVDLAIFSLHLAGISSILGAVNFITTIINMRPNNMSLD
QMPLFVWAVGITALLLLLSLPVLAGAITMLLTDRNLNTSFFDPAGGGDPILYQHL
>LEEUA227-11|JN274925|MM19635|Endothenia_ustulana
TLYFIFGIWAGMVGTSLSLLIRAELGNPGSLIGDDQIYNTIVTAHAFIMIFFMVMPIMIGGFGNWLIPLMLGAPDMAFPRMNNMSFWLLPPSIMLLISSSIVENGAGTGWTVYPPLSSNIAHSGSSVDLAIFSLHLAGISSILGAVNFITTIINMRPNNMTL
DQMPLFVWAVGITALLLLLSLPVLAGAITMLLTDRNLNSSFFDPAGGGDPILYQHL
>LEFIF530-10|HM875215|MM12175|Endromis_versicolora
TLYFIFGIWAGMVGTSLSLLIRAELGNPGSLIGDDQIYNTIVTAHAFIMIFFMVMPIMIGGFGNWLIPLMLGAPDMAFPRMNNMSFWLLPPSLTLLISSSIVENGAGTGWTVYPPLSSNIAHGGSSVDLAIFSLHLAGISSIMGAINFITTIINMRLNNLSLD
QMPLFVWAVGITAFLLLLSLPVLAGAITMLLTDRNLNTSFFDPAGGGDPILYQHL
>LEFIB042-10|HM870951|MM00316|Endrosis_sarcitrella
TLYFIFGIWAGMVGTSLSLLIRAELGNPGSLIGDDQIYNTIVTAHAFIMIFFMVMPIMIGGFGNWLVPLMLGAPDMAFPRMNNMSFWLLPPSLTLLISSSIVENGAGTGWTVYPPLSSNIAHGGSSVDLAIFSLHLAGISSILGAINFITTIINMRLNNMMF
DQMPLFVWAVGITALLLLLSLPVLAGAITMLLTDRNLNTSFFDPAGGGDPILYQHL
>LEFIC591-10|HM872412|MM04510|Ennomos_alniaria
TLYFIFGIWAGMVGTSLSLLIRAELGNPGSLIGDDQIYNTIVTAHAFIMIFFMVMPIMIGGFGNWLVPLMLGAPDMAFPRMNNMSFWLLPPSITLLISSSIVENGAGTGWTVYPPLSSNIAHGGSSVDLAIFSLHLAGISSILGAINFITTIINMRLNNMSFD
QMPLFVWAVGITAFLLLLSLPVLAGAITMLLTDRNLNTSFFDPAGGGDPILYQHL
>LEFIF717-10|HM875401|MM12770|Ennomos_alniaria
TLYFIFGIWAGMVGTSLSLLIRAELGNPGSLIGDDQIYNTIVTAHAFIMIFFMVMPIMIGGFGNWLVPLMLGAPDMAFPRMNNMSFWLLPPSITLLISSSIVENGAGTGWTVYPPLSSNIAHGGSSVDLAIFSLHLAGISSILGAINFITTIINMRLNNMSFD
QMPLFVWAVGITAFLLLLSLPVLAGAITMLLTDRNLNTSFFDPAGGGDPILYQHL
>LEFIA317-10|HM386660|MM01364|Ennomos_autumnaria
TLYFIFGIWAGMVGTSLSLLIRAELGNPGSLIGDDQIYNTIVTAHAFIMIFFMVMPIMIGGFGNWLVPLMLGAPDMAFPRMNNMSFWLLPPSITLLISSSIVENGAGTGWTVYPPLSSNIAHGGSSVDLAIFSLHLAGISSILGAINFITTIINMRLNNMSFD
QMPLFVWAVGITAFLLLLSLPVLAGAITMLLTDRNLNTSFFDPAGGGDPILYQHL
>LEFIB842-10|HM871719|MM02811|Ennomos_autumnaria
TLYFIFGIWAGMVGTSLSLLIRAELGNPGSLIGDDQIYNTIVTAHAFIMIFFMVMPIMIGGFGNWLVPLMLGAPDMAFPRMNNMSFWLLPPSITLLISSSIVENGAGTGWTVYPPLSSNIAHGGSSVDLAIFSLHLAGISSILGAINFITTIINMRLNNMSFD
QMPLFVWAVGITAFLLLLSLPVLAGAITMLLTDRNLNTSFFDPAGGGDPILYQHL

>LEFIC748-10|HM872568|MM04853|Ennomos_erosaria
TLYFIFGIWAGMVGTSLSLLIRAELGNPGSLIGDDQIYNTIVTAHAFIMIFFMVMPIMIGGFGNWLVPLMLGAPDMAFPRMNNMSFWLLPPSITLLISSSIVENGAGTGWTVYPPLSSNIAHGGSSVDLAIFSLHLAGISSILGAINFITTIINMRLNNMSFD
QMPLFIWAVGITAFLLLLSLPVLAGAITMLLTDRNLNTSFFDPAGGGDPILYQHL
>LEFIF216-10|HM874909|MM10929|Ennomos_fuscantaria
TLYFIFGIWAGMVGTSLSLLIRAELGNPGSLIGDDQIYNTIVTAHAFIMIFFMVMPIMIGGFGNWLVPLMLGAPDMAFPRMNNMSFWLLPPSITLLISSSIVENGAGTGWTVYPPLSSNIAHGGSSVDLAIFSLHLAGISSILGAINFITTIINMRLNNMPF
DQMPLFVWAVGITAFLLLLSLPVLAGAITMLLTDRNLNTSFFDPAGGGDPILYQHL
>LEFIB857-10|HM871734|MM02878|Entephria_caesiata
TLYFIFGIWSGMIGTSLSLLIRAELGNPGSLIGDDQIYNTIVTAHAFIMIFFMVMPIMIGGFGNWLIPLMLGAPDMAFPRMNNMSFWLLPPSITLLISSSIVESGAGTGWTVYPPLSSNIAHSGSSVDLAIFSLHLAGISSILGAINFITTIINMRLNNMFFDQL
PLFVWAVGITAFLLLLSLPVLAGAITMLLTDRNLNTSFFDPAGGGDPILYQHL
>LEFIF026-10|HQ570378|MM10465|Entephria_flavicinctata
TLYFIFGIWSGMIGTSLSLLIRAELGNPGSLIGDDQIYNTIVTAHAFIMIFFMVMPIMIGGFGNWLVPLMLGAPDMAFPRMNNMSFWLLPPSITLLISSSIVESGAGTGWTVYPPLSSNIAHSGSSVDLAIFSLHLAGISSILGAINFITTIINMRLNNMFFDQ
LPLFVWAVGITAFLLLLSLPVLAGAITMLLTDRNLNTSFFDPAGGGDPILYQHL
>LEFIL402-10|JX034689|MM18700|Entephria_nobiliaria
TLYFIFGIWSGMIGTSLSLLIRAELGNPGSLIGDDQIYNTIVTAHAFIMIFFMVMPIMIGGFGNWLIPLMLGAPDMAFPRMNNMSFWLLPPSITLLISSSIVESGAGTGWTVYPPLSSNIAHSGSSVDLAIFSLHLAGISSILGAINFITTIINMRLNNMFFDQL
PLFVWAVGITAFLLLLSLPVLAGAITMLLTDRNLNTSFFDPAGGGDPILYQHL
>LEFIG941-10|HQ570419|MM15805|Entephria_polata
TLYFIFGIWSGMIGTSLSLLIRAELGNPGSLIGDDQIYNTIVTAHAFIMIFFMVMPIMIGGFGNWLIPLMLGAPDMAFPRMNNMSFWLLPPSITLLISSSIVESGAGTGWTVYPPLSSNIAHSGSSVDLAIFSLHLAGISSILGAINFITTIINMRLNNMFFDQL
PLFVWAVGITAFLLLLSLPVLAGAITMLLTDRNLNTSFFDPAGGGDPILYQHL
>LEFIC102-10|HQ570293|MM03418|Entephria_punctipes
TLYFIFGIWSGMIGTSLSLLIRAELGNPGSLIGDDQIYNTIVTAHAFIMIFFMVMPIMIGGFGNWLVPLMLGAPDMAFPRMNNMSFWLLPPSITLLISSSIVESGAGTGWTVYPPLSSNIAHSGSSVDLAIFSLHLAGISSILGAINFITTIINMRLNNMFFDQ
LPLFVWAVGITAFLLLLSLPVLAGAITMLLTDRNLNTSFFDPAGGGDPILYQHL
>LEFIC103-10|HQ570294|MM03419|Entephria_punctipes
TLYFIFGIWSGMIGTSLSLLIRAELGNPGSLIGDDQIYNTIVTAHAFIMIFFMVMPIMIGGFGNWLVPLMLGAPDMAFPRMNNMSFWLLPPSITLLISSSIVESGAGTGWTVYPPLSSNIAHSGSSVDLAIFSLHLAGISSILGAINFITTIINMRLNNMFFDQ
LPLFVWAVGITAFLLLLSLPVLAGAITMLLTDRNLNTSFFDPAGGGDPILYQHL
>LEEUA004-11|JN283989|MM19317|Enteucha_acetosae
TLYFMFGIWSGMLGTSLSLLIRIELGNPGSLIGDDQIYNSIVTAHAFIMIFFMVMPIMIGGFGNWLVPLMLGAPDMAFPRMNNMSFWFLPPSLTLLVSSGIVENGVGTGWTVYPPLSSNIAHSGASVDLGIFSLHLAGISSILGAVNFITTVINMRPKGM
SFDQMPLFVWAVVITALLLLLSLPVLAGAITMLLTDRNLNTSFFDPAGGGDPILYQHL
>LEEUA539-11|MM20598|Enteucha_acetosae
TLYFMFGIWSGMLGTSLSLLIRIELGNPGSLIGDDQIYNSIVTAHAFIMIFFMVMPIMIGGFGNWLVPLMLGAPDMAFPRMNNMSFWFLPPSLTLLVSSGIVENGVGTGWTVYPPLSSNIAHSGASVDLGIFSLHLAGISSILGAVNFITTVINMRPKGM
SFDQMPLFVWAVVITALLLLLSLPVLAGAITMLLTDRNLNTSFFDPAGGG?PILYQHL
>LEFIB893-10|HM871770|MM02982|Epagoge_grotiana
TLYFIFGIWAGMVGTSLSLLIRAELGNPGSLIGDDQIYNTIVTAHAFIMIFFMVMPIMIGGFGNWLVPLMLGAPDMAFPRMNNMSFWLLPPSLMLLISSSVVENGAGTGWTVYPPLSSNIAHSGSSVDLAIFSLHLAGISSILGAVNFITTIINMRPNNM
SLDQMPLFVWAVGITALLLLLSLPVLAGAITMLLTDRNLNTSFFDPAGGGDPILYQHL
>LEFIF877-10|HM875559|MM13269|Epagoge_grotiana
TLYFIFGIWAGMVGTSLSLLIRAELGNPGSLIGDDQIYNTIVTAHAFIMIFFMVMPIMIGGFGNWLVPLMLGAPDMAFPRMNNMSFWLLPPSLMLLISSSVVENGAGTGWTVYPPLSSNIAHSGSSVDLAIFSLHLAGISSILGAVNFITTIINMRPNNM
SLDQMPLFVWAVGITALLLLLSLPVLAGAITMLLTDRNLNTSFFDPAGGGDPILYQHL
>LEFIA041-10|HM396390|MM00443|Epermenia_chaerophyllella
TLYFIFGIWAGMVGTSLSLLIRAELGTPSSLIGDDQIYNTIVTAHAFIMIFFMVMPIMIGGFGNWLIPLMLGAPDMAFPRMNNMSFWLLPPSLFLLISSSIVENGAGTGWTVYPPLSSNIAHSGSSVDLAIFSLHLAGISSILGAINFITTIINMRLNGMMFD
QMPLFVWAVGITALLLLLSLPVLAGAITMLLTDRNLNTSFFDPAGGGDPILYQHL
>LEFIA875-10|HM387013|MM09752|Epermenia_chaerophyllella
TLYFIFGIWAGMVGTSLSLLIRAELGTPSSLIGDDQIYNTIVTAHAFIMIFFMVMPIMIGGFGNWLVPLMLGAPDMAFPRMNNMSFWLLPPSLFLLISSSIVENGAGTGWTVYPPLSSNIAHSGSSVDLAIFSLHLAGISSILGAINFITTIINMRLNGMMF
DQMPLFVWAVGITALLLLLSLPVLAGAITMLLTDRNLNTSFFDPAGGGDPILYQHL
>LEFIG153-10|HM875833|MM14007|Epermenia_chaerophyllella
TLYFIFGIWAGMVGTSLSLLIRAELGTPSSLIGDDQIYNTIVTAHAFIMIFFMVMPIMIGGFGNWLVPLMLGAPDMAFPRMNNMSFWLLPPSLFLLISSSIVENGAGTGWTVYPPLSSNIAHSGSSVDLAIFSLHLAGISSILGAINFITTIINMRLNGMMF
DQMPLFVWAVGITALLLLLSLPVLAGAITMLLTDRNLNTSFFDPAGGGDPILYQHL
>LEFIB956-10|HM871832|MM03122|Epermenia_falciformis
TLYFIFGVWAGMVGTSLSLLIRAELGTPNSLIGDDQIYNTIVTAHAFIMIFFMVMPIMIGGFGNWLVPLMLGAPDMAFPRMNNMSFWLLPPSLLLLISSSVVENGAGTGWTVYPPLSSNIAHGGSSVDLAIFSLHLAGISSILGAINFITTIINMRINGMM

FDQMPLFIWAVGITALLLLLSLPVLAGAITMLLTDRNLNTSFFDPAGGGDPILYQHL
>LEFIB715-10|HM871593|MM02527|Epermenia_illigerella
TLYFIFGIWAGMVGTSLSILIRAELGTPNSLIGDDQIYNTIVTAHAFIMIFFMVMPIMIGGFGNWLVPLMLGAPDMAFPRMNNMSFWLLPPSLLLLISSSIVENGAGTGWTVYPPLSSNIAHGGSSVDLAIFSLHLAGISSILGAINFITTIINMRLNGLMFD
QMPLFVWAVGITALLLLLSLPVLAGAITMLLTDRNLNTSFFDPAGGGDPILYQHL
>LEFIC142-10|HM871988|MM03487|Epermenia_illigerella
TLYFIFGIWAGMVGTSLSILIRAELGTPNSLIGDDQIYNTIVTAHAFIMIFFMVMPIMIGGFGNWLVPLMLGAPDMAFPRMNNMSFWLLPPSLLLLISSSIVENGAGTGWTVYPPLSSNIAHGGSSVDLAIFSLHLAGISSILGAINFITTIINMRLNGLMFD
QMPLFVWAVGITALLLLLSLPVLAGAITMLLTDRNLNTSFFDPAGGGDPILYQHL
>LEFIC900-10|HM872717|MM05213|Epermenia_illigerella
TLYFIFGIWAGMMGTSLSILIRAELGTPNSLIGDDQIYNTIVTAHAFIMIFFMVMPIMIGGFGNWLVPLMLGAPDMAFPRMNNMSFWLLPPSLLLLISSSIVENGAGTGWTVYPPLSSNIAHGGSSVDLAIFSLHLAGISSILGAINFITTIINMRLNGLMF
DQMPLFVWAVGITALLLLLSLPVLAGAITMLLTDRNLNTSFFDPAGGGDPILYQHL
>LEFID215-10|HM873017|MM06046|Epermenia_profugella
TLYFIFGIWAGMVGTSLSLLIRAELGTPSSLIGDDQIYNTIVTAHAFIMIFFMVMPIMIGGFGNWLVPLMLGAPDMAFPRMNNMSFWLLPPSLLLLISSSIVENGAGTGWTVYPPLSSNIAHSGSSVDLAIFSLHLAGISSILGAINFITTIINMRLNGLMFD
QMPLFVWAVGITALLLLLSLPVLAGAITMLLTDRNLNTSFFDPAGGGDPILYQHL
>LEFIK791-10|JN269619|MM18366|Epermenia_profugella
TLYFIFGIWAGMVGTSLSLLIRAELGTPSSLIGDDQIYNTIVTAHAFIMIFFMVMPIMIGGFGNWLVPLMLGAPDMAFPRMNNMSFWLLPPSLLLLISSSIVENGAGTGWTVYPPLSSNIAHSGSSVDLAIFSLHLAGISSILGAINFITTIINMRLNGLMFD
QMPLFVWAVGITALLLLLSLPVLAGAITMLLTDRNLNTSFFDPAGGGDPILYQHL
>LEFID790-10|HM873547|MM06861|Ephestia_elutella
TLYFIFGIWSGMVGTSLSLLIRAELGTPGSLIGDDQIYNTIVTGHAFIMIFFMVMPIMIGGFGNWLVPLMLGAPDMAFPRMNNMSFWLLPPSLSLLISSSIVENGAGTGWTVYPPLSSNIAHSGSSVDLAIFSLHLAGISSILGAINFITTIINMKLNGMMF
DQMPLFVWAVGITALLLLLSLPVLAGAITMLLTDRNLNTSFFDPAGGGDPILYQHL
>LEFIE962-10|HM874679|MM10368|Ephestia_elutella
TLYFIFGIWSGMVGTSLSLLIRAELGTPGSLIGDDQIYNTIVTGHAFIMIFFMVMPIMIGGFGNWLVPLMLGAPDMAFPRMNNMSFWLLPPSLSLLISSSIVENGAGTGWTVYPPLSSNIAHSGSSVDLAIFSLHLAGISSILGAINFITTIINMKLNGMMF
DQMPLFVWAVGITALLLLLSLPVLAGAITMLLTDRNLNTSFFDPAGGGDPILYQHL
>LEFIC988-10|HM872803|MM05396|Ephestia_kuehniella
TLYFIFGIWSGMVGTSLSLLIRAELGTPESLIGDDQIYNTIVTGHAFIMIFFMVMPIMIGGFGNWLVPLMLGAPDMAFPRLNNMSFWLLPPSLTLLISSSIVENGTGTGWTVYPPLSSNIAHGGSSVDLAIFSLHLAGISSILGAINFITTIINMKLNGMMFD
QMPLFVWAVGITALLLLLSLPVLAGAITMLLTDRNLNTSFFDPAGGGDPILYQHL
>LEFIL203-10|MM19203|Ephestia_mistralella
TLYFIFGIWSGMVGTSLSLLIRAELGTPGSLIGDDQIYNTIVTGHAFIMIFFMVMPIMIGGFGNWLVPLMLGAPDMAFPRMNNMSFWLLPPSLSLLISSSIVENGAGTGWTVYPPLSSNIAHSGSSVDLAIFSLHLAGISSILGAINFITTIINMKLNGMMF
DQMPLFVWAVGITALLLLLSLPVLAGAITMLLTDRNLNTSFFDPAGGGDPILYQHL
>LEFIB451-10|HM871350|MM01976|Epiblema_cirsiana
TLYFIFGIWSGMIGTSLSLLIRAELGNPGSLIGDDQIYNTIVTAHAFIMIFFMVMPIMIGGFGNWLVPLMLGAPDMAFPRMNNMSFWLLPPSILLLISSSIVENGAGTGWTVYPPLSSNIAHSGSSVDLAIFSLHLAGISSILGAVNFITTIINMRPNNMSLD
QMPLFVWAVGITALLLLLSLPVLAGAITMLLTDRNLNTSFFDPAGGGDPILYQHL
>LEFIB452-10|HM871351|MM01977|Epiblema_cirsiana
TLYFIFGIWSGMIGTSLSLLIRAELGNPGSLIGDDQIYNTIVTAHAFIMIFFMVMPIMIGGFGNWLVPLMLGAPDMAFPRMNNMSFWLLPPSILLLISSSIVENGAGTGWTVYPPLSSNIAHSGSSVDLAIFSLHLAGISSILGAVNFITTIINMRPNNMSLD
QMPLFVWAVGITALLLLLSLPVLAGAITMLLTDRNLNTSFFDPAGGGDPILYQHL
>LEFIB911-10|HM871788|MM03020|Epiblema_cirsiana
TLYFIFGIWSGMIGTSLSLLIRAELGNPGSLIGDDQIYNTIVTAHAFIMIFFMVMPIMIGGFGNWLVPLMLGAPDMAFPRMNNMSFWLLPPSILLLISSSIVENGAGTGWTVYPPLSSNIAHSGSSVDLAIFSLHLAGISSILGAVNFITTIINMRPNNMSLD
QMPLFVWAVGITALLLLLSLPVLAGAITMLLTDRNLNTSFFDPAGGGDPILYQHL
>LEFIB450-10|HM871349|MM01974|Epiblema_foenella
TLYFIFGIWAGMIGTSLSLLIRAELGNPGSLIGDDQIYNTIVTAHAFIMIFFMVMPIMIGGFGNWLVPLMLGAPDMAFPRMNNMSFWLLPPSIMLLISSSIVENGAGTGWTVYPPLSSNIAHSGSSVDLAIFSLHLAGISSILGAVNFITTIINMRPNNMSL
DQMPLFVWAVGITALLLLLSLPVLAGAITMLLTDRNLNTSFFDPAGGGDPILYQHL
>LEFIF831-10|HM875514|MM13172|Epiblema_foenella
TLYFIFGIWAGMIGTSLSLLIRAELGNPGSLIGDDQIYNTIVTAHAFIMIFFMVMPIMIGGFGNWLVPLMLGAPDMAFPRMNNMSFWLLPPSIMLLISSSIVENGAGTGWTVYPPLSSNIAHSGSSVDLAIFSLHLAGISSILGAVNFITTIINMRPNNMSL
DQMPLFVWAVGITALLLLLSLPVLAGAITMLLTDRNLNTSFFDPAGGGDPILYQHL
>LEFIE325-10|HM874051|MM08704|Epiblema_grandaevana
TLYFIFGMWAWMIGTSLSLLIRAELGNPGSLIGDDQIYNTIVTAHAFIMIFFMVMPIMIGGFGNWLVPLMLGAPDMAFPRMNNMSFWLLPPSILLLISSSVVENGAGTGWTVYPPLSSNIAHSGSSVDLAIFSLHLAGISSILGAVNFITTIINMRPNNMS
LDQMPLFVWAVGITALLLLLSLPVLAGAITMLLTDRNLNTSFFDPAGGGDPILYQHL
>LEFIF587-10|HM875271|MM12415|Epiblema_grandaevana

TLYFIFGMWAGMIGTSLSLLIRAELGNPGSLIGDDQIYNTIVTAHAFIMIFFMVMPIMIGGFGNWLVPLMLGAPDMAFPRMNNMSFWLLPPSILLLISSSVVENGAGTGWTVYPPLSSNIAHSGSSVDLAIFSLHLAGISSILGAVNFITTIINMRPNNMS
LDQMPLFVWAVGITALLLLLSLPVLAGAITMLLTDRNLNTSFFDPAGGGDPILYQHL
>LEFIG275-10|HM875954|MM14216|Epiblema_grandaevana
TLYFIFGMWAGMIGTSLSLLIRAELGNPGSLIGDDQIYNTIVTAHAFIMIFFMVMPIMIGGFGNWLVPLMLGAPDMAFPRMNNMSFWLLPPSILLLISSSVVENGAGTGWTVYPPLSSNIAHSGSSVDLAIFSLHLAGISSILGAVNFITTIINMRPNNMS
LDQMPLFVWAVGITALLLLLSLPVLAGAITMLLTDRNLNTSFFDPAGGGDPILYQHL
>LEFIJ1398-12|KM573221|MM22090|Epiblema_grandaevana
TLYFIFGMWAGMIGTSLSLLIRAELGNPGSLIGDDQIYNTIVTAHAFIMIFFMVMPIMIGGFGNWLVPLMLGAPDMAFPRMNNMSFWLLPPSILLLISSSVVENGAGTGWTVYPPLSSNIAHSGSSVDLAIFSLHLAGISSILGAVNFITTIINMRPNNMS
LDQMPLFVWAVGITALLLLLSLPVLAGAITMLLTDRNLNTSFFDPAGGGDPILYQHL
>LEFID609-10|HM873374|MM06623|Epiblema_graphana
TLYFIFGIWAGMIGTSLSLLIRAELGNPGSFIGDDQIYNTIITAHAFIMIFFMVMPIMIGGFGNWLVPLMLGAPDMAFPRMNNMSFWLLPPSIMLLISSSIVENGAGTGWTVYPPLSSNIAHGGSSVDLAIFSLHLAGISSILGAINFITTIINMRPNNMSLD
QMPLFVWSVSITALLLLLSLPVLAGAITMLLTDRNLNTSFFDPAGGGDPILYQHL
>LEFIE836-10|HM874555|MM10053|Epiblema_graphana
TLYFIFGIWAGMIGTSLSLLIRAELGNPGSFIGDDQIYNTIVTAHAFIMIFFMVMPIMIGGFGNWLVPLMLGAPDMAFPRMNNMSFWLLPPSIMLLISSSIVENGAGTGWTVYPPLSSNIAHGGSSVDLAIFSLHLAGISSILGAINFITTIINMRPNNMSL
DQMPLFVWSVSITALLLLLSLPVLAGAITMLLTDRNLNTSFFDPAGGGDPILYQHL
>LEFIE837-10|HM874556|MM10054|Epiblema_graphana
TLYFIFGIWAGMIGTSLSLLIRAELGNPGSFIGDDQIYNTIITAHAFIMIFFMVMPIMIGGFGNWLVPLMLGAPDMAFPRMNNMSFWLLPPSIMLLISSSIVENGAGTGWTVYPPLSSNIAHGGSSVDLAIFSLHLAGISSILGAINFITTIINMRPNNMSLD
QMPLFVWSVSITALLLLLSLPVLAGAITMLLTDRNLNTSFFDPAGGGDPILYQHL
>LEFIJ387-10|JF853574|MM15992|Epiblema_graphana
TLYFIFGIWAGMIGTSLSLLIRAELGNPGSFIGDDQIYNTIVTAHAFIMIFFMVMPIMIGGFGNWLVPLMLGAPDMAFPRMNNMSFWLLPPSIMLLISSSIVENGAGTGWTVYPPLSSNIAHGGSSVDLAIFSLHLAGISSILGAINFITTIINMRPNNMSL
DQMPLFVWSVSITALLLLLSLPVLAGAITMLLTDRNLNTSFFDPAGGGDPILYQHL
>LEFIJ1519-12|MM22787|Epiblema_graphana
TLYFIFWIWAGMIGTSLSLLIRAELGNPGSFIGDDQIYNTIVTAHAFIMIFFMVMPIMIGGFGNWLVPLMLGAPDMAFPRMNNMSFWLLPPSIMLLISSSIVENGAGTGWTVYPPLSSNIAHGGSSVDLAIFSLHLAGISSILGAINFITTIINMRPNNMSL
DQMPLFVWSVSITALLLLLSLPVLAGAITMLLTDRNLNTSFFDPAGGGDPILYQHL
>LEFID078-10|HM872889|MM05687|Epiblema_inulivora
TLYFIFGIWAGMIGTSLSLLIRAELGNPGSLIGDDQIYNTIVTAHAFIMIFFMVMPIMIGGFGNWLVPLMLGAPDMAFPRMNNMSFWLLPPSIMLLISSSIVENGAGTGWTVYPPLSSNIAHSGSSVDLAIFSLHLAGISSILGAVNFITTIINMRPNNMSL
DQMPLFVWSVGITALLLLLSLPVLAGAITMLLTDRNLNTSFFDPAGGGDPILYQHL
>LEFIG328-10|HM876007|MM14301|Epiblema_inulivora
TLYFIFGIWAGMIGTSLSLLIRAELGNPGSLIGDDQIYNTIVTAHAFIMIFFMVMPIMIGGFGNWLVPLMLGAPDMAFPRMNNMSFWLLPPSIMLLISSSIVENGAGTGWTVYPPLSSNIAHSGSSVDLAIFSLHLAGISSILGAVNFITTIINMRPNNMSL
DQMPLFVWSVGITALLLLLSLPVLAGAITMLLTDRNLNTSFFDPAGGGDPILYQHL
>LEFIJ641-10|JF853738|MM17266|Epiblema_scutulana
TLYFIFGIWAGMIGTSLSLLIRAELGNPGSLIGDDQIYNTIVTAHAFIMIFFMVMPIMIGGFGNWLVPLMLGAPDMAFPRMNNMSFWLLPPSILLLISSSIVENGAGTGWTVYPPLSSNIAHSGSSVDLAIFSLHLAGISSILGAVNFITTIINMRPNNMSLD
QMPLFVWAVGITALLLLLSLPVLAGAITMLLTDRNLNTSFFDPAGGGDPILYQHL
>LEFIA002-10|HM396352|MM00073|Epiblema_simploniana
TMYFIFGIWAGMIGTSLSLLIRAELGNPGSLIGDDQIYNTIVTAHAFIMIFFMVMPIMIGGFGNWLVPLMLGAPDMAFPRMNNMSFWLLPPSIMLLISSSIVENGAGTGWTVYPPLSSNIAHSGSSVDLAIFSLHLAGISSILGAVNFITTIINMRPNNMS
LDQMPLFIWAVGITALLLLLSLPVLAGAITMLLTDRNLNTSFFDPAGGGDPILYQHL
>LEFIA820-10|HM386961|MM04174|Epiblema_simploniana
TMYFIFGIWAGMIGTSLSLLIRAELGNPGSLIGDDQIYNTIVTAHAFIMIFFMVMPIMIGGFGNWLVPLMLGAPDMAFPRMNNMSFWLLPPSIMLLISSSIVENGAGTGWTVYPPLSSNIAHSGSSVDLAIFSLHLAGISSILGAVNFITTIINMRPNNMS
LDQMPLFIWAVGITALLLLLSLPVLAGAITMLLTDRNLNTSFFDPAGGGDPILYQHL
>LEFIA821-10|HM386962|MM04175|Epiblema_simploniana
TMYFIFGIWAGMIGTSLSLLIRAELGNPGSLIGDDQIYNTIVTAHAFIMIFFMVMPIMIGGFGNWLVPLMLGAPDMAFPRMNNMSFWLLPPSIMLLISSSVVENGAGTGWTVYPPLSSNIAHSGSSVDLAIFSLHLAGISSILGAVNFITTIINMRPNNMS
LDQMPLFVWAVGITALLLLLSLPVLAGAITMLLTDRNLNTSFFDPAGGGDPILYQHL
>LEFIG505-10|HM876179|MM14592|Epiblema_simploniana
TMYFIFGIWAGMIGTSLSLLIRAELGNPGSLIGDDQIYNTIVTAHAFIMIFFMVMPIMIGGFGNWLVPLMLGAPDMAFPRMNNMSFWLLPPSIMLLISSSVVENGAGTGWTVYPPLSSNIAHSGSSVDLAIFSLHLAGISSILGAVNFITTIINMRPNNMS
LDQMPLFVWAVGITALLLLLSLPVLAGAITMLLTDRNLNTSFFDPAGGGDPILYQHL
>LEFIE800-10|HM874519|MM09989|Epiblema_sticticana
TLYFIFGMWAGMIGTSFSLLIRAELGNPGSLIGNDQIYNTIVTAHAFIMIFFMVMPIMIGGFGNWLVPLMLGAPDMAFPRMNNMSFWLLPPSILILISSSVVENGVGTGWTVYPPLSSNIAHSGSSVDLAIFSLHLAGISSILGAVNFITTIINMRPNNMT
LDQMPLFVWAVGITALLLLLSLPVLAGAITMLLTDRNLNTSFFDPAGGGDPILYQHL

>LEEUA563-11|MM20622|Epicallima_formosella
TLYFIFGIWAGMVGTSLSLLIRAELGNPGSLIGDDQIYNTIVTAHAFIMIFFMVMPIMIGGFGNWLVPLMLGAPDMAFPRMNNMSFWLLPPSLTLLISSSIVENGAGTGWTVYPPLSSNIAHGGASVDLAIFSLHLAGISSILGAINFITTIINMRMNNLSF
DQMPLFVWAVGITALLLLLSLPVLAGAITMLLTDRNLNTSFFDPAGGGDPILYQHL
>LEEUA548-11|MM20607|Epichnopterix_plumella
TLYFILGIWSGLVGTSLSVLIRAELGTPGSLIGNDQIYNTLVTAHAFIMIFFMVMPIMIGGFGNWLVPLMLGAPDMAFPRMNNMSFWLLPPSLMLLTASSIVENGAGTGWTVYPPLSSNIAHDGGAVDLAIFSLHLAGISSILGAVNFITTTINMRPYEM
TLDRMPLFVWSVAITALLLLLSLPVLAGAITMLLTDRNLNTSFFDPAGGGDPILYQHL
>LEFIA871-10|HM387009|MM09748|Epinotia_abbreviana
TLYFIFGIWSGMVGTSLSLLIRAELGNPGSLIGDDQIYNTIVTAHAFIMIFFMVMPIMIGGFGNWLVPLMLGAPDMAFPRMNNMSFWLLPPSIMLLISSSIVENGAGTGWTVYPPLSSNIAHSGSSVDLAIFSLHLAGISSILGAVNFITTIINMRPNNMSL
DQMPLFVWAVGITALLLLLSLPVLAGAITMLLTDRNLNTSFFDPAGGGDPILYQHL
>LEFIA872-10|HM387010|MM09749|Epinotia_abbreviana
TLYFIFGIWSGMVGTSLSLLIRAELGNPGSLIGDDQIYNTIVTAHAFIMIFFMVMPIMIGGFGNWLVPLMLGAPDMAFPRMNNMSFWLLPPSIMLLISSSIVENGAGTGWTVYPPLSSNIAHSGSSVDLAIFSLHLAGISSILGAVNFITTIINMRPNNMSL
DQMPLFVWAVGITALLLLLSLPVLAGAITMLLTDRNLNTSFFDPAGGGDPILYQHL
>LEFIG852-10|HM876500|MM15716|Epinotia_bilunana
TLYFIFGIWAGMIGTSLSLLIRAELGNPGSLIGNDQIYNTIVTAHAFIMIFFMVMPIMIGGFGNWLVPLMLGAPDMAFPRMNNMSFWLLPPSIMLLISSSMVENGAGTGWTVYPPLSSNIAHSGSSVDLAIFSLHLAGISSILGAVNFITTIINMRPNNMS
LDQMPLFVWAVGITALLLLLSLPVLAGAITMLLTDRNLNTSFFDPAGGGDPILYQHL
>LEFIB271-10|HM871173|MM00751|Epinotia_brunnichana
TLYFIFGIWAGMVGTSLSLLIRAELGNPGSLIGDDQIYNTIVTAHAFIMIFFMVMPIMIGGFGNWLVPLMLGAPDMAFPRMNNMSFWLLPPSIMLLISSSIVENGAGTGWTVYPPLSSNIAHSGSSVDLAIFSLHLAGISSILGAVNFITTIINMRPNNMSL
DQMPLFVWAVGITALLLLLSLPVLAGAITMLLTDRNLNTSFFDPAGGGDPILYQHL
>LEFIK747-10|JF854307|MM18322|Epinotia_caprana
TLYFIFGIWAGMIGTSLSLLIRAELGNPGSLIGDDQIYNTIVTAHAFIMIFFMVMPIMIGGFGNWLVPLMLGAPDMAFPRMNNMSFWLLPPSIMLLISSSIVENGAGTGWTVYPPLSSNIAHSGSSVDLAIFSLHLAGISSILGAVNFITTIINMRPNNMSL
DQMPLFVWAVGITALLLLLSLPVLAGAITMLLTDRNLNTSFFDPAGGGDPILYQHL
>LEFIK748-10|JF854308|MM18323|Epinotia_caprana
TLYFIFGIWAGMIGTSLSLLIRAELGNPGSLIGDDQIYNTIVTAHAFIMIFFMVMPIMIGGFGNWLVPLMLGAPDMAFPRMNNMSFWLLPPSIMLLISSSIVENGAGTGWTVYPPLSSNIAHSGSSVDLAIFSLHLAGISSILGAVNFITTIINMRPNNMSL
DQMPLFVWAVGITALLLLLSLPVLAGAITMLLTDRNLNTSFFDPAGGGDPILYQHL
>LEFIB484-10|HM871379|MM02050|Epinotia_cinereana
TLYFIFGIWAGMIGTSLSLLIRAELGNPGSLIGDDQIYNTIVTAHAFIMIFFMVMPIMIGGFGNWLVPLMLGAPDMAFPRMNNMSFWLLPPSIMLLISSSIVENGAGTGWTVYPPLSSNIAHSGSSVDLAIFSLHLAGISSILGAVNFITTIINMRPNNMSL
DQMPLFVWAVGITALLLLLSLPVLAGAITMLLTDRNLNTSFFDPAGGGDPILYQHL
>LEFIJ039-10|JQ775175|MM02051|Epinotia_cinereana
TLYFIFGIWAGMIGTSLSLLIRAELGNPGSLIGDDQIYNTIVTAHAFIMIFFMVMPIMIGGFGNWLVPLMLGAPDMAFPRMNNMSFWLLPPSIMLLISSSIVENGAGTGWTVYPPLSSNIAHSGSSVDLAIFSLHLAGISSILGAVNFITTIINMRPNNMSL
DQMPLFVWAVGITALLLLLSLPVLAGAITMLLTDRNLNTSFFDPAGGGDPILYQHL
>LEFIJ051-10|JQ775176|MM03022|Epinotia_cinereana
TLYFIFGIWAGMIGTSLSLLIRAELGNPGSLIGDDQIYNTIVTAHAFIMIFFMVMPIMIGGFGNWLVPLMLGAPDMAFPRMNNMSFWLLPPSIMLLISSSIVENGAGTGWTVYPPLSSNIAHSGSSVDLAIFSLHLAGISSILGAVNFITTIINMRPNNMSL
DQMPLFVWAVGITALLLLLSLPVLAGAITMLLTDRNLNTSFFDPAGGGDPILYQHL
>LEFIJ100-10|JF853446|MM06933|Epinotia_cinereana
TLYFIFGIWAGMIGTSLSLLIRAELGNPGSLIGDDQIYNTIVTAHAFIMIFFMVMPIMIGGFGNWLVPLMLGAPDMAFPRMNNMSFWLLPPSIMLLISSSIVENGAGTGWTVYPPLSSNIAHSGSSVDLAIFSLHLAGISSILGAVNFITTIINMRPNNMSL
DQMPLFVWAVGITALLLLLSLPVLAGAITMLLTDRNLNTSFFDPAGGGDPILYQHL
>LEFIJ114-10|JQ775173|MM08218|Epinotia_cinereana
TLYFIFGIWAGMIGTSLSLLIRAELGNPGSLIGDDQIYNTIVTAHAFIMIFFMVMPIMIGGFGNWLVPLMLGAPDMAFPRMNNMSFWLLPPSIMLLISSSIVENGAGTGWTVYPPLSSNIAHSGSSVDLAIFSLHLAGISSILGAVNFITTIINMRPNNMSL
DQMPLFVWAVGITALLLLLSLPVLAGAITMLLTDRNLNTSFFDPAGGGDPILYQHL
>LEFIB115-10|HM871022|MM00466|Epinotia_crenana
TLYFIFGIWAGMVGTSLSLLIRAELGNPGSLIGDDQIYNTIVTAHAFIMIFFMVMPIMIGGFGNWLVPLMLGAPDMAFPRMNNMSFWLLPPSIMLLISSSIVENGAGTGWTVYPPLSSNIAHSGSSVDLAIFSLHLAGISSILGAVNFITTIINMRPNNMSL
DQMPLFVWAVGITALLLLLSLPVLAGAITMLLTDRNLNTSFFDPAGGGDPILYQHL
>LEFIK753-10|JF854311|MM18328|Epinotia_crenana
TLYFIFGIWAGMVGTSLSLLIRAELGNPGSLIGDDQIYNTIVTAHAFIMIFFMVMPIMIGGFGNWLVPLMLGAPDMAFPRMNNMSFWLLPPSIMLLISSSIVENGAGTGWTVYPPLSSNIAHSGSSVDLAIFSLHLAGISSILGAVNFITTIINMRPNNMSL
DQMPLFVWAVGITALLLLLSLPVLAGAITMLLTDRNLNTSFFDPAGGGDPILYQHL
>LEFIC053-10|HM871922|MM03323|Epinotia_cruciana
TLYFIFGIWAGMIGTSLSLLIRAELGNPGSLIGDDQIYNTIVTAHAFIMIFFMVMPIMIGGFGNWLVPLMLGAPDMAFPRMNNMSFWLLPPSILLLISSSVVENGAGTGWTVYPPLSSNIAHSGSSVDLAIFSLHLAGISSILGAVNFITTIINMRPNNMSL

DQMPLFVWAVGITALLLLLSLPVLAGAITMLLTDRNLNTSFFDPAGGGDPILYQHL
>LEFID555-10|HM873320|MM06544|Epinotia_cruciana
TLYFIFGIWAGMIGTSLSLLIRAELGNPGSLIGDDQIYNTIVTAHAFIMIFFMVMPIMIGGFGNWLVPLMLGAPDMAFPRMNNMSFWLLPPSILLLISSSVVENGAGTGWTVYPPLSSNIAHSGSSVDLAIFSLHLAGISSILGAVNFITTIINMRPNNMSL
DQMPLFVWAVGITALLLLLSLPVLAGAITMLLTDRNLNTSFFDPAGGGDPILYQHL
>LEFID821-10|HM873578|MM06921|Epinotia_cruciana
TLYFIFGIWAGMIGTSLSLLIRAELGNPGSLIGDDQIYNTIVTAHAFIMIFFMVMPIMIGGFGNWLVPLMLGAPDMAFPRMNNMSFWLLPPSILLLISSSVVENGAGTGWTVYPPLSSNIAHSGSSVDLAIFSLHLAGISSILGAVNFITTIINMRPNNMSL
DQMPLFVWAVGITALLLLLSLPVLAGAITMLLTDRNLNTSFFDPAGGGDPILYQHL
>LEFIE133-10|HM873881|MM08349|Epinotia_cruciana
TLYFIFGIWAGMIGTSLSLLIRAELGNPGSLIGDDQIYNTIVTAHAFIMIFFMVMPIMIGGFGNWLVPLMLGAPDMAFPRMNNMSFWLLPPSILLLISSSVVENGAGTGWTVYPPLSSNIAHSGSSVDLAIFSLHLAGISSILGAVNFITTIINMRPNNMSL
DQMPLFVWAVGITALLLLLSLPVLAGAITMLLTDRNLNTSFFDPAGGGDPILYQHL
>LEFIB275-10|HM871177|MM00759|Epinotia_demarniana
TLYFIFGIWAGMIGTSLSLLIRAELGNPGSLIGDDQIYNTIVTAHAFIMIFFMVMPIMIGGFGNWLVPLMLGAPDMAFPRMNNMSFWLLPPSIMLLISSSIVENGAGTGWTVYPPLSSNIAHSGSSVDLAIFSLHLAGISSILGAVNFITTIINMRPNNMSL
DQMPLFVWAVGITALLLLLSLPVLAGAITMLLTDRNLNTSFFDPAGGGDPILYQHL
>LEEUA236-11|JN274933|MM19644|Epinotia_fraternana
TLYFIFGIWAGMIGTSLSLLIRAELGNPGSLIGDDQIYNTIVTAHAFIMIFFMVMPIMIGGFGNWLVPLMLGAPDMAFPRMNNMSFWLLPPSIMLLISSSIVENGAGTGWTVYPPLSSNIAHSGSSVDLAIFSLHLAGISSILGAVNFITTIINMRPNNMSL
DQMPLFVWAVGITALLLLLSLPVLAGAITMLLTDRNLNTSFFDPAGGGDPILYQHL
>LEEUA237-11|JN274934|MM19645|Epinotia_fraternana
TLYFIFGIWAGMIGTSLSLLIRAELGNPGSLIGDDQIYNTIVTAHAFIMIFFMVMPIMIGGFGNWLVPLMLGAPDMAFPRMNNMSFWLLPPSIMLLISSSIVENGAGTGWTVYPPLSSNIAHSGSSVDLAIFSLHLAGISSILGAVNFITTIINMRPNNMSL
DQMPLFVWAVGITALLLLLSLPVLAGAITMLLTDRNLNTSFFDPAGGGDPILYQHL
>LEFIB244-10|HM871147|MM00710|Epinotia_gimmerthaliana
TLYFIFGIWAGMIGTSLSLLIRAELGNPGSLIGDDQIYNTIVTAHAFIMIFFMVMPIMIGGFGNWLVPLMLGAPDMAFPRMNNMSFWLLPPSIMLLISSSIVENGAGTGWTVYPPLSSNIAHSGSSVDLAIFSLHLAGISSILGAVNFITTIINMRPNNMSL
DQMPLFVWAVGITALLLLLSLPVLAGAITMLLTDRNLNTSFFDPAGGGDPILYQHL
>LEFIG855-10|HM876503|MM15719|Epinotia_granitana
TLYFIFGIWAGMVGTSLSLLIRAELGNPGSLIGDDQIYNTIVTAHAFIMIFFMVMPIMIGGFGNWLVPLMLGAPDMAFPRMNNMSFWLLPPSIMLLISSSIVENGAGTGWTVYPPLSSNIAHSGSSVDLAIFSLHLAGISSILGAVNFITTIINMRPNNMSL
DQMPLFVWAVGITALLLLLSLPVLAGAITMLLTDRNLNTSFFDPAGGGDPILYQHL
>LEFIB540-10|HQ570273|MM02153|Epinotia_immundana
TLYFIFGIWAGMIGTSLSLLIRAELGNPGSLISDDQIYNTIVTAHAFIMIFFMVMPIMIGGFGNWLIPLMLGAPDMAFPRMNNMSFWLLPPSIMLLISSSIVENGAGTGWTVYPPLSSNIAHSGSSVDLAIFSLHLAGISSILGAVNFITTIINMRPNNMSLD
QMPLFVWAVGITALLLLLSLPVLAGAITMLLTDRNLNTSFFDPAGGGDPILYQHL
>LEFID028-10|HM872842|MM05463|Epinotia_immundana
TLYFIFGIWAGMIGTSLSLLIRAELGNPGSLIGDDQIYNTIVTAHAFIMIFFMVMPIMIGGFGNWLVPLMLGAPDMAFPRMNNMSFWLLPPSIMLLISSSIVENGAGTGWTVYPPLSSNIAHSGSSVDLAIFSLHLAGISSILGAVNFITTIINMRPNNMSL
DQMPLFVWAVGITALLLLLSLPVLAGAITMLLTDRNLNTSFFDPAGGGDPILYQHL
>LEFIF432-10|HM875117|MM11865|Epinotia_immundana
TLYFIFGIWAGMIGTSLSLLIRAELGNPGSLISDDQIYNTIVTAHAFIMIFFMVMPIMIGGFGNWLIPLMLGAPDMAFPRMNNMSFWLLPPSIMLLISSSIVENGAGTGWTVYPPLSSNIAHSGSSVDLAIFSLHLAGISSILGAVNFITTIINMRPNNMSLD
QMPLFVWAVGITALLLLLSLPVLAGAITMLLTDRNLNTSFFDPAGGGDPILYQHL
>LEFIB926-10|HM871803|MM03067|Epinotia_indecorana
TLYFIFGVWAGMVGTSLSLLIRAELGNPGSLIGDDQIYNTIVTAHAFIMIFFMVMPIMIGGFGNWLVPLMLGAPDMAFPRMNNMSFWLLPPSIMLLISSSIVENGAGTGWTVYPPLSSNIAHSGSSVDLAIFSLHLAGISSILGAVNFITTIINMRPNNMS
LDQMPLFVWAVGITALLLLLSLPVLAGAITMLLTDRNLNTSFFDPAGGGDPILYQHL
>LEFID856-10|HM873613|MM06982|Epinotia_indecorana
TLYFIFGIWAGMVGTSLSLLIRAELGNPGSLIGDDQIYNTIVTAHAFIMIFFMVMPIMIGGFGNWLVPLMLGAPDMAFPRMNNMSFWLLPPSIMLLISSSIVENGAGTGWTVYPPLSSNIAHSGSSVDLAIFSLHLAGISSILGAVNFITTIINMRPNNMSL
DQMPLFVWAVGITALLLLLSLPVLAGAITMLLTDRNLNTSFFDPAGGGDPILYQHL
>LEFID858-10|HM873615|MM06987|Epinotia_indecorana
TLYFIFGVWAGMVGTSLSLLIRAELGNPGSLIGDDQIYNTIVTAHAFIMIFFMVMPIMIGGFGNWLVPLMLGAPDMAFPRMNNMSFWLLPPSIMLLISSSIVENGAGTGWTVYPPLSSNIAHSGSSVDLAIFSLHLAGISSILGAVNFITTIINMRPNNMP
LDQMPLFVWAVGITALLLLLSLPVLAGAITMLLTDRNLNTSFFDPAGGGDPILYQHL
>LEFIB273-10|HM871175|MM00756|Epinotia_maculana
TLYFIFGIWAGMVGTSLSLLIRAELGNPGSLIGDDQIYNTIVTAHAFIMIFFMVMPIMIGGFGNWLVPLMLGAPDMAFPRMNNMSFWLLPPSIMLLISSSIVENGAGTGWTVYPPLSSNIAHSGSSVDLAIFSLHLAGISSILGAVNFITTIINMRPNNMSL
DQMPLFVWAVGITALLLLLSLPVLAGAITMLLTDRNLNTSFFDPAGGGDPILYQHL
>LEFIB442-10|HM871341|MM01956|Epinotia_maculana

TLYFIFGIWAGMVGTSLSLLIRAELGNPGSLIGDDQIYNTIVTAHAFIMIFFMVMPIMIGGFGNWLVPLMLGAPDMAFPRMNNMSFWLLPPSIMLLISSSIVENGAGTGWTVYPPLSSNIAHSGSSVDLAIFSLHLAGISSILGAVNFITTIINMRPNNMSL
DQMPLFVWAVGITALLLLLSLPVLAGAITMLLTDRNLNTSFFDPAGGGDPILYQHL
>LEFID217-10|HM873018|MM06048|Epinotia_mercuriana
TLYFIFGIWAGMIGTSLSLLIRAELGNPGSLIGDDQIYNTIVTAHAFIMIFFMVMPIMIGGFGNWLVPLMLGAPDMAFPRMNNMSFWLLPPSILLLISSSVVENGAGTGWTVYPPLSSNIAHSGSSVDLAIFSLHLAGISSILGAVNFITTIINMRPNNMSL
DQMPLFVWAVGITALLLLLSLPVLAGAITMLLTDRNLNTSFFDPAGGGDPILYQHL
>LEFIG522-10|HM876195|MM14635|Epinotia_mercuriana
TLYFIFGIWAGMIGTSLSLLIRAELGNPGSLIGDDQIYNTIVTAHAFIMIFFMVMPIMIGGFGNWLVPLMLGAPDMAFPRMNNMSFWLLPPSILLLTSSSVVENGAGTGWTVYPPLSSNIAHSGSSVDLAIFSLHLAGISSILGAVNFITTIINMRPNNMSL
DQMPLFVWAVGITALLLLLSLPVLAGAITMLLTDRNLNTSFFDPAGGGDPILYQHL
>LEFIB235-10|HM871138|MM00697|Epinotia_nanana
TLYFILGIWAGMIGTSLSLLIRAELGNPGSLIGDDQIYNTIVTAHAFIMIFFMVMPIMIGGFGNWLVPLMLGAPDMAFPRMNNMSFWLLPPSIMLLISSSVVENGAGTGWTVYPPLSSNIAHSGSSVDLAIFSLHLAGISSILGAVNFITTIINMRPNNMSL
DQMPLFVWAVGITALLLLLSLPVLAGAITMLLTDRNLNTSFFDPAGGGDPILYQHL
>LEFIE972-10|HM874689|MM10398|Epinotia_nanana
TLYFIFGIWAGMIGTSLSLLIRAELGNPGSLIGDDQIYNTIVTAHAFIMIFFMVMPIMIGGFGNWLVPLMLGAPDMAFPRMNNMSFWLLPPSIMLLISSSVVENGAGTGWTVYPPLSSNIAHSGSSVDLAIFSLHLAGISSILGAVNFITTIINMRPNNMSL
DQMPLFVWAVGITALLLLLSLPVLAGAITMLLTDRNLNTSFFDPAGGGDPILYQHL
>LEFIC375-10|HM872218|MM03905|Epinotia_nemorivaga
TLYFIFGIWAGMVGTSLSLLIRAELGNPGSLIGDDQIYNTIVTAHAFIMIFFMVMPIMIGGFGNWLVPLMLGAPDMAFPRMNNMSFWLLPPSIMLLISSSIVENGAGTGWTVYPPLSSNIAHSGSSVDLAIFSLHLAGISSILGAVNFITTIINMRPNNMSL
DQMPLFVWAVGITALLLLLSLPVLAGAITMLLTDRNLNTSFFDPAGGGDPILYQHL
>LEFIA819-10|HM386960|MM04173|Epinotia_nemorivaga
TLYFIFGIWAGMVGTSLSLLIRAELGNPGSLIGDDQIYNTIVTAHAFIMIFFMVMPIMIGGFGNWLVPLMLGAPDMAFPRMNNMSFWLLPPSIMLLISSSIVENGAGTGWTVYPPLSSNIAHSGSSVDLAIFSLHLAGISSILGAVNFITTIINMRPKNMSL
DQMPLFVWAVGITALLLLLSLPMLAGAITMLLTDRNLNTSFFDPAGGGDPILYQHL
>LEFIE791-10|HM874510|MM09973|Epinotia_nemorivaga
TLYFIFGIWAGMIGTSLSLLIRAELGNPGSLIGDDQIYNTIVTAHAFIMIFFMVMPIMIGGFGNWLVPLMLGAPDMAFPRMNNMSFWLLPPSIMLLISSSIVENGAGTGWTVYPPLSSNIAHSGSSVDLAIFSLHLAGISSILGAVNFITTIINMRPNNMSL
DQMPLFVWAVGITALLLLLSLPVLAGAITMLLTDRNLNTSFFDPAGGGDPILYQHL
>LEFIB274-10|HM871176|MM00758|Epinotia_nisella
TLYFIFGIWAGMIGTSLSLLIRAELGNPGSLIGDDQIYNTIVTAHAFIMIFFMVMPIMIGGFGNWLVPLMLGAPDMAFPRMNNMSFWLLPPSIMLLISSSVVENGAGTGWTVYPPLSSNIAHSGSSVDLAIFSLHLAGISSILGAVNFITTIINMRPNNMSL
DQMPLFVWAVGITALLLLLSLPVLAGAITMLLTDRNLNTSFFDPAGGGDPILYQHL
>LEFIJ126-10|JQ775241|MM08948|Epinotia_nisella
TLYFIFGIWAGMIGTSLSLLIRAELGNPGSLIGDDQIYNTIVTAHAFIMIFFMVMPIMIGGFGNWLVPLMLGAPDMAFPRMNNMSFWLLPPSIMLLISSSVVENGAGTGWTVYPPLSSNIAHSGSSVDLAIFSLHLAGISSILGAVNFITTIINMRPNNMSL
DQMPLFVWAVGITALLLLLSLPVLAGAITMLLTDRNLNTSFFDPAGGGDPILYQHL
>LEFIF435-10|HM875120|MM11880|Epinotia_nisella
TLYFIFGIWAGMIGTSLSLLIRAELGNPGSLIGDDQIYNTIVTAHAFIMIFFMVMPIMIGGFGNWLVPLMLGAPDMAFPRMNNMSFWLLPPSIMLLISSSVVENGAGTGWTVYPPLSSNIAHSGSSVDLAIFSLHLAGISSILGAVNFITTIINMRPNNMSL
DQMPLFVWAVGITALLLLLSLPVLAGAITMLLTDRNLNTSFFDPAGGGDPILYQHL
>LEFIJ189-10|JQ775243|MM12338|Epinotia_nisella
TLYFIFGIWAGMIGTSLSLLIRAELGNPGSLIGDDQIYNTIVTAHAFIMIFFMVMPIMIGGFGNWLVPLMLGAPDMAFPRMNNMSFWLLPPSIMLLISSSVVENGAGTGWTVYPPLSSNIAHSGSSVDLAIFSLHLAGISSILGAVNFITTIINMRPNNMSL
DQMPLFVWAVGITALLLLLSLPVLAGAITMLLTDRNLNTSFFDPAGGGDPILYQHL
>LEFIJ190-10|JQ775244|MM12339|Epinotia_nisella
TLYFIFGIWAGMIGTSLSLLIRAELGNPGSLIGDDQIYNTIVTAHAFIMIFFMVMPIMIGGFGNWLVPLMLGAPDMAFPRMNNMSFWLLPPSIMLLISSSVVENGAGTGWTVYPPLSSNIAHSGSSVDLAIFSLHLAGISSILGAVNFITTIINMRPNNMSL
DQMPLFVWAVGITALLLLLSLPVLAGAITMLLTDRNLNTSFFDPAGGGDPILYQHL
>LEFIJ191-10|JQ775247|MM12340|Epinotia_nisella
TLYFIFGIWAGMIGTSLSLLIRAELGNPGSLIGDDQIYNTIVTAHAFIMIFFMVMPIMIGGFGNWLVPLMLGAPDMAFPRMNNMSFWLLPPSIMLLISSSVVENGAGTGWTVYPPLSSNIAHSGSSVDLAIFSLHLAGISSILGAVNFITTIINMRPNNMSL
DQMPLFVWAVGITALLLLLSLPVLAGAITMLLTDRNLNTSFFDPAGGGDPILYQHL
>LEFIJ192-10|JQ775248|MM12341|Epinotia_nisella
TLYFIFGIWAGMIGTSLSLLIRAELGNPGSLIGDDQIYNTIVTAHAFIMIFFMVMPIMIGGFGNWLVPLMLGAPDMAFPRMNNMSFWLLPPSIMLLISSSVVENGAGTGWTVYPPLSSNIAHSGSSVDLAIFSLHLAGISSILGAVNFITTIINMRPNNMSL
DQMPLFVWAVGITALLLLLSLPVLAGAITMLLTDRNLNTSFFDPAGGGDPILYQHL
>LEFIK756-10|JQ775240|MM18331|Epinotia_nisella
TLYFIFGIWAGMIGTSLSLLIRAELGNPGSLIGDDQIYNTIVTAHAFIMIFFMVMPIMIGGFGNWLVPLMLGAPDMAFPRMNNMSFWLLPPSIMLLISSSVVENGAGTGWTVYPPLSSNIAHSGSSVDLAIFSLHLAGISSILGAVNFITTIINMRPNNMSL
DQMPLFVWAVGITALLLLLSLPVLAGAITMLLTDRNLNTSFFDPAGGGDPILYQHL

>LEFIC463-10|HM872296|MM04047|Epinotia_pygmaeana
TLYFIFGIWAGMVGTSLSLLIRAELGNPGSLIGDDQIYNTIVTAHAFIMIFFMVMPIMIGGFGNWLVPLMLGAPDMAFPRMNNMSFWLLPPSIMLLISSSIVENGAGTGWTVYPPLSSNIAHSGSSVDLAIFSLHLAGISSILGAVNFITTIINMRPNNMSL
DQMPLFVWAVGITALLLLLSLPVLAGAITMLLTDRNLNTSFFDPAGGGDPILYQHL
>LEFIB226-10|HM871130|MM00681|Epinotia_ramella
TLYFIFGIWAGMIGTSLSLLIRAELGNPGSLIGDDQIYNTIVTAHAFIMIFFMVMPIMIGGFGNWLVPLMLGAPDMAFPRMNNMSFWLLPPSIMLLISSSIVENGMGTGWTVYPPLSSNIAHSGSSVDLAIFSLHLAGISSILGAINFITTIINMRPNNMSL
DQMPLFIWAVGITALLLLMSLPVLAGAITMLLTDRNLNTSFFDPAGGGDPILYQHL
>LEFIJ037-10|KM572107|MM01966|Epinotia_ramella
TLYFIFGIWAGMIGTSLSLLIRAELGNPGSLIGDDQIYNTIVTAHAFIMIFFMVMPIMIGGFGNWLVPLMLGAPDMAFPRMNNMSFWLLPPSIMLLISSSIVENGAGTGWTVYPPLSSNIAHSGSSVDLAIFSLHLAGISSILGAVNFITTIINMRPNNMSL
DQMPLFVWAVGITALLLLLSLPVLAGAITMLLTDRNLNTSFFDPAGGGDPILYQHL
>LEFIE391-10|HM874115|MM08933|Epinotia_ramella
TLYFIFGIWAGMVGTSLSLLIRAELGNPGSLIGDDQIYNTIVTAHAFIMIFFMVMPIMIGGFGNWLVPLMLGAPDMAFPRMNNMSFWLLPPSIMLLISSSIVENGAGTGWTVYPPLSSNIAHSGSSVDLAIFSLHLAGISSILGAVNFITTIINMRPNNMSL
DQMPLFVWAVGITALLLLLSLPVLAGAITMLLTDRNLNTSFFDPAGGGDPILYQHL
>LEFID768-10|HM873525|MM06834|Epinotia_rubiginosana
TLYFIFGIWAGMIGTSLSLLIRAELGNPSSLIGDDQIYNTIVTAHAFIMIFFMVMPIMIGGFGNWLVPLMLGAPDMAFPRMNNMSFWLLPPSLMLLISSSIVENGAGTGWTVYPPLSSNIAHSGSSVDLAIFSLHLAGISSILGAVNFITTIINMRPNNMSL
DQMPLFVWAVGITALLLLLSLPVLAGAITMLLTDRNLNTSFFDPAGGGDPILYQHL
>LEFIC134-10|HM871980|MM03477|Epinotia_signatana
TLYFIFGIWAGMVGTSLSLLIRAELGNPGSLIGDDQIYNTIVTAHAFIMIFFMVMPIMIGGFGNWLVPLMLGAPDMAFPRMNNMSFWLLPPSIMLLISSSIVENGAGTGWTVYPPLSSNIAHSGSSVDLAIFSLHLAGISSILGAVNFITTIINMRPNNMSL
DQMPLFVWAVGITALLLLLSLPVLAGAITMLLTDRNLNTSFFDPAGGGDPILYQHL
>LEFIB272-10|HM871174|MM00754|Epinotia_solandriana
TLYFIFGIWAGMIGTSLSLLIRAELGNPGSLIGDDQIYNTIVTAHAFIMIFFMVMPIMIGGFGNWLVPLMLGAPDMAFPRMNNMSFWLLPPSIMLLISSSIVENGAGTGWTVYPPLSSNIAHSGSSVDLAIFSLHLAGISSILGAVNFITTIINMKPNNMSL
DQMPLFVWAVGITALLLLLSLPVLAGAITMLLTDRNLNTSFFDPAGGGDPILYQHL
>LEFIE085-10|HM873832|MM08258|Epinotia_solandriana
TLYFIFGIWAGMVGTSLSLLIRAELGNPGSLIGDDQIYNTIVTAHAFIMIFFMVMPIMIGGFGNWLVPLMLGAPDMAFPRMNNMSFWLLPPSIMLLISSSIVENGAGTGWTVYPPLSSNIAHSGSSVDLAIFSLHLAGISSILGAVNFITTIINMRPNNMSL
DQMPLFVWAVGITALLLLLSLPVLAGAITMLLTDRNLNTSFFDPAGGGDPILYQHL
>LEFIF842-10|HM875525|MM13187|Epinotia_solandriana
TLYFIFGIWAGMVGTSLSLLIRAELGNPGSLIGDDQIYNTIVTAHAFIMIFFMVMPIMIGGFGNWLVPLMLGAPDMAFPRMNNMSFWLLPPSIMLLISSSIVENGAGTGWTVYPPLSSNIAHSGSSVDLAIFSLHLAGISSILGAVNFITTIINMRPNNMSL
DQMPLFVWAVGITALLLLLSLPVLAGAITMLLTDRNLNTSFFDPAGGGDPILYQHL
>LEEUA234-11|MM19642|Epinotia_sordidana
TLYFIFGIWAGMVGTSLSLLIRAELGNPGSLIGDDQIYNTIVTAHAFIMIFFMVMPIMIGGFGNWLVPLMLGAPDMAFPRMNNMSFWLLPPSILLLISSSMVENGAGTGWTVYPPLSSNIAHSGSSVDLAIFSLHLAGISSILGAVNFITTIINMRPNNMS
LDQMPLFVWAVGITALLLLLSLPVLAGAITMLLTDRNLNTSFFDPAGGGDPILYQHL
>LEFIE252-10|HM873986|MM08585|Epinotia_subocellana
TLYFIFGIWAGMVGTSLSLLIRAELGNPGSLIGDDQIYNTIVTAHAFIMIFFMVMPIMIGGFGNWLVPLMLGAPDMAFPRMNNMSFWLLPPSIMLLISSSIVENGAGTGWTVYPPLSSNIAHSGSSVDLAIFSLHLAGISSILGAVNFITTIINMRPNNMSL
DQMPLFVWAVGITALLLLLSLPVLAGAITMLLTDRNLNTSFFDPAGGGDPILYQHL
>LEFIF913-10|HM875595|MM13338|Epinotia_tedella
TLYFIFGIWAGMIGTSLSLLIRAELGNPGSLIGDDQIYNTIVTAHAFIMIFFMVMPIMIGGFGNWLVPLMLGAPDMAFPRMNNMSFWLLPPSIMLLISSSIVENGAGTGWTVYPPLSSNIAHSGSSVDLAIFSLHLAGISSILGAVNFITTIINMRPNNMSL
DQMPLFVWAVGITALLLLLSLPVLAGAITMLLTDRNLNTSFFDPAGGGDPILYQHL
>LEFIB914-10|HM871791|MM03024|Epinotia_tenerana
TLYFIFGIWAGMIGTSLSLLIRAELGNPGSLIGDDQIYNTIVTAHAFIMIFFMVMPIMIGGFGNWLVPLMLGAPDMAFPRMNNMSFWLLPPSIMLLISSSIVENGAGTGWTVYPPLSSNIAHSGSSVDLAIFSLHLAGISSILGAVNFITTIINMRPNNMSL
DQMPLFVWAVGITALLLLLSLPVLAGAITMLLTDRNLNTSFFDPAGGGDPILYQHL
>LEFIF906-10|HM875588|MM13323|Epinotia_tenerana
TLYFIFGIWAGMIGTSLSLLIRAELGNPGSLIGDDQIYNTIVTAHAFIMIFFMVMPIMIGGFGNWLVPLMLGAPDMAFPRMNNMSFWLLPPSIMLLISSSIVENGAGTGWTVYPPLSSNIAHSGSSVDLAIFSLHLAGISSILGAVNFITTIINMRPNNMSL
DQMPLFVWAVGITALLLLLSLPVLAGAITMLLTDRNLNTSFFDPAGGGDPILYQHL
>LEFIB153-10|HM871058|MM00543|Epinotia_tetraquetrana
TLYFIFGVWAGMVGTSLSLLIRAELGNPGSLIGDDQIYNTIVTAHAFIMIFFMVMPIMIGGFGNWLVPLMLGAPDMAFPRMNNMSFWLLPPSIMLLISSSIVENGAGTGWTVYPPLSSNIAHSGSSVDLAIFSLHLAGISSILGAVNFITTIINMRPNNMS
LDQMPLFVWAVGITALLLLLSLPVLAGAITMLLTDRNLNTSFFDPAGGGDPILYQHL
>LEFIB154-10|HM871059|MM00544|Epinotia_tetraquetrana
TLYFIFGVWAGMVGTSLSLLIRAELGNPGSLIGDDQIYNTIVTAHAFIMIFFMVMPIMIGGFGNWLVPLMLGAPDMAFPRMNNMSFWLLPPSIMLLISSSIVENGAGTGWTVYPPLSSNIAHSGSSVDLAIFSLHLAGISSILGAVNFITTIINMRPNNMS

LDQMPLFVWAVGITALLLLLSLPVLAGAITMLLTDRNLNTSFFDPAGGGDPILYQHL
>LEFIJ388-10|JF853575|MM15993|Epinotia_tetraquetrana
TLYFIFGVWAGMVGTSLSLLIRAELGNPGSLIGDDQIYNTIVTAHAFIMIFFMVMPIMIGGFGNWLVPLMLGAPDMAFPRMNNMSFWLLPPSIMLLISSSIVENGAGTGWTVYPPLSSNIAHSGSSVDLAIFSLHLAGISSILGAVNFITTIINMRPNNMS
LDQMPLFVWAVGITALLLLLSLPVLAGAITMLLTDRNLNTSFFDPAGGGDPILYQHL
>LEFIB481-10|HQ570263|MM02047|Epinotia_trigonella
TLYFIFGIWAGMVGTSLSLLIRAELGNPGSLIGDDQIYNTIVTAHAFIMIFFMVMPIMIGGFGNWLVPLMLGAPDMAFPRMNNMSFWLLPPSIMLLISSSIVENGAGTGWTVYPPLSSNIAHSGSSVDLAIFSLHLAGISSILGAVNFITTIINMRPNNMSL
DQMPLFVWAVGITALLLLLSLPVLAGAITMLLTDRNLNTSFFDPAGGGDPILYQHL
>LEFIC679-10|HM872500|MM04647|Epinotia_trigonella
TLYFIFGIWAGMVGTSLSLLIRAELGNPGSLIGDDQIYNTIVTAHAFIMIFFMVMPIMIGGFGNWLVPLMLGAPDMAFPRMNNMSFWLLPPSIMLLISSSIVENGAGTGWTVYPPLSSNIAHSGSSVDLAIFSLHLAGISSILGAVNFITTIINMRPNNMSL
DQMPLFVWAVGITALLLLLSLPVLAGAITMLLTDRNLNTSFFDPAGGGDPILYQHL
>LEFIE022-10|HM873771|MM08081|Epione_repandaria
TLYFIFGIWAGMIGTSLSLLIRAELGNPGSLIGDDQIYNTIVTAHAFIMIFFMVMPIMIGGFGNWLVPLMLGAPDMAFPRMNNMSFWLLPPSITLLISSSIVENGAGTGWTVYPPLSSNIAHGGSSVDLAIFSLHLAGISSILGAINFITTIINMRLNNLSFDQ
MPLFVWAVGITAFLLLLSLPVLAGAITMLLTDRNLNTSFFDPAGGGDPILYQHL
>LEFIA435-10|HM386776|MM01499|Epione_vespertaria
TLYFIFGVWAGMIGTSLSLLIRAELGNPGSLIGDDQIYNTIVTAHAFIMIFFMVMPIMIGGFGNWLVPLMLGAPDMAFPRMNNMSFWLLPPSITLLISSSIVENGAGTGWTVYPPLSSNIAHGGSSVDLAIFSLHLAGISSILGAINFITTIINMRLNNLSFD
QMPLFVWAVGITAFLLLLSLPVLAGAITMLLTDRNLNTSFFDPAGGGDPILYQHL
>LEFIA436-10|HM386777|MM01500|Epione_vespertaria
TLYFIFGVWAGMIGTSLSLLIRAELGNPGSLIGDDQIYNTIVTAHAFIMIFFMVMPIMIGGFGNWLVPLMLGAPDMAFPRMNNMSFWLLPPSITLLISSSIVENGAGTGWTVYPPLSSNIAHGGSSVDLAIFSLHLAGISSILGAINFITTIINMRLNNLSFD
QMPLFVWAVGITAFLLLLSLPVLAGAITMLLTDRNLNTSFFDPAGGGDPILYQHF
>LEFIC247-10|HM872091|MM03657|Epipsilia_grisescens
TLYFIFGIWAGMVGTSLSLLIRAELGNPGSLIGDDQIYNTIVTAHAFIMIFFMVMPIMIGGFGNWLVPLMLGAPDMAFPRMNNMSFWLLPPSLTLLISSSIVENGAGTGWTVYPPLSSNIAHGGSSVDLAIFSLHLAGISSILGAINFITTIINMRLNNLSFD
QMPLFIWAVGITAFLLLLSLPVLAGAITMLLTDRNLNTSFFDPAGGGDPILYQHL
>LEFIA231-10|HM396574|MM01263|Epirranthis_diversata
TLYFIFGIWAGMVGTSLSLLIRAELGNPGSLIGDDQIYNTIVTAHAFIMIFFMVMPIMIGGFGNWLVPLMLGAPDMAFPRMNNMSFWLLPPSITLLISSSVVENGAGTGWTVYPPLSSNIAHGGSSVDLAIFSLHLAGISSILGAINFITTIINMRLNNLSFD
QMPLFVWAVGITAFLLLLSLPVLAGAITMLLTDRNLNTSFFDPAGGGDPILYQHL
>LEFIB188-10|HM871092|MM00615|Epirrhoe_alternata
TLYFIFGIWAGMIGTSLSLLIRAELGNPGSLIGDDQIYNTIVTAHAFIMIFFMVMPIMIGGFGNWLIPLMLGAPDMAFPRMNNMSFWLLPPSITLLISSSIVENGAGTGWTVYPPLSSNIAHGGSSVDLAIFSLHLAGISSILGAINFITTIINMRLNNMFFD
QLPLFVWAVGITAFLLLLSLPVLAGAITMLLTDRNLNTSFFDPAGGGDPILYQHL
>LEFID953-10|HM873703|MM07844|Epirrhoe_alternata
TLYFIFGIWAGMIGTSLSLLIRAELGNPGSLIGDDQIYNTIVTAHAFIMIFFMVMPIMIGGFGNWLIPLMLGAPDMAFPRMNNMSFWLLPPSITLLISSSIVENGAGTGWTIYPPLSSNIAHGGSSVDLAIFSLHLAGISSILGAINFITTIINMRLNNMFFDQ
LPLFVWAVGITAFLLLLSLPVLAGAITMLLTDRNLNTSFFDPAGGGDPILYQHL
>LEFIF377-10|HM875062|MM11620|Epirrhoe_alternata
TLYFIFGIWAGMIGTSLSLLIRAELGNPGSLIGDDQIYNTIVTAHAFIMIFFMVMPIMIGGFGNWLIPLMLGAPDMAFPRMNNMSFWLLPPSITLLISSSIVENGAGTGWTVYPPLSSNIAHGGSSVDLAIFSLHLAGISSILGAINFITTIINMRLNNMFFD
QLPLFVWAVGITAFLLLLSLPVLAGAITMLLTDRNLNTSFFDPAGGGDPILYQHL
>COLFF459-13|KM573339|MM23192|Epirrhoe_alternata
TLYFIFGIWAGMIGTSLSLLIRAELGNPGSLIGDDQIYNTIVTAHAFIMIFFMVMPIMIGGFGNWLIPLMLGAPDMAFPRMNNMSFWLLPPSITLLISSSIVENGAGTGWTVYPPLSSNIAHGGSSVDLAIFSLHLAGISSILGAINFITTIINMRLNNMFFD
QLPLFVWAVGITAFLLLLSLPVLAGAITMLLTDRNLNTSFFDPAGGGDPILYQHL
>LEFIB056-10|HM870965|MM00339|Epirrhoe_galiata
TLYFIFGIWAGMIGTSLSLLIRAELGNPGSLIGDDQIYNTIVTAHAFIMIFFMVMPIMIGGFGNWLIPLMLGAPDMAFPRMNNMSFWLLPPSITLLISSSIVENGAGTGWTVYPPLSSNIAHGGSSVDLAIFSLHLAGISSILGAINFITTIINMRLNNMFFD
QLPLFVWAVGITAFLLLLSLPVLAGAITMLLTDRNLNTSFFDPAGGGDPILYQHL
>LEFIF025-10|HM874739|MM10464|Epirrhoe_hastulata
TLYFIFGMWAGMIGTSLSLLIRAELGNPGSLIGDDQIYNTIVTAHAFIMIFFMVMPIMIGGFGNWLVPLMLGAPDMAFPRMNNMSFWLLPPSITLLISSSIVENGAGTGWTVYPPLSSNIAHGGSSVDLAIFSLHLAGISSILGAINFITTIINMRLNNMFF
DQLPLFVWSVGITAFLLLLSLPVLAGAITMLLTDRNLNTSFFDPAGGGDPILYQHL
>LEFIG938-10|HM876580|MM15802|Epirrhoe_hastulata
TLYFIFGMWAGMIGTSLSLLIRAELGNPGSLIGDDQIYNTIVTAHAFIMIFFMVMPIMIGGFGNWLVPLMLGAPDMAFPRMNNMSFWLLPPSITLLISSSIVENGAGTGWTVYPPLSSNIAHGGSSVDLAIFSLHLAGISSILGAINFITTIINMRLNNMFF
DQLPLFVWSVGITAFLLLLSLPVLAGAITMLLTDRNLNTSFFDPAGGGDPILYQHL
>LEFIJ808-10|JF853834|MM17433|Epirrhoe_hastulata

TLYFIFGMWAGMIGTSLSLLIRAELGNPGSLIGDDQIYNTIVTAHAFIMIFFMVMPIMIGGFGNWLVPLMLGAPDMAFPRMNNMSFWLLPPSITLLISSSIVENGAGTGWTVYPPLSSNIAHGGSSVDLAIFSLHLAGISSILGAINFITTIINMRLNNMFF
DQLPLFVWSVGITAFLLLLSLPVLAGAITMLLTDRNLNTSFFDPAGGGDPILYQHL
>LEFIE441-10|HM874165|MM09203|Epirrhoe_pupillata
TLYFIFGIWAGMIGTSLSLLIRAELGNPGSLIGDDQIYNTIVTAHAFIMIFFMVMPIMIGGFGNWLIPLMLGAPDMAFPRMNNMSFWLLPPSIMLLISSSIVENGAGTGWTVYPPLSSNIAHSGSSVDLAIFSLHLAGISSILGAINFITTIINMRLNNMFFD
QLPLFVWAVGITAFLLLLSLPVLAGAITMLLTDRNLNTSFFDPAGGGDPILYQHL
>LEFIJ806-10|MM17431|Epirrhoe_pupillata
TLYFIFGIWAGMIGTSLSLLIRAELGNPGSLIGDDQIYNTIVTAHAFIMIFFMVMPIMIGGFGNWLIPLMLGAPDMAFPRMNNMSFWLLPPSIMLLISSSIVENGAGTGWTVYPPLSSNIAHSGSSVDLAIFSLHLAGISSILGAINFITTIINMRLNNMFFD
QLPLFVWAVGITAFLLLLSLPVLAGAITMLLTDRNLNTSFFDPAGGGDPILYQHL
>LEFIJ807-10|MM17432|Epirrhoe_pupillata
TLYFIFGIWAGMIGTSLSLLIRAELGNPGSLIGDDQIYNTIVTAHAFIMIFFMVMPIMIGGFGNWLIPLMLGAPDMAFPRMNNMSFWLLPPSIMLLISSSIVENGAGTGWTVYPPLSSNIAHSGSSVDLAIFSLHLAGISSILGAINFITTIINMRLNNMFFD
QLPLFVWAVGITAFLLLLSLPVLAGAITMLLTDRNLNTSFFDPAGGGDPILYQHL
>LEFID156-10|HM872963|MM05919|Epirrhoe_rivata
TLYFIFGIWAGMIGTSLSLLIRAELGNPGSLIGDDQIYNTIVTAHAFIMIFFMVMPIMIGGFGNWLIPLMLGAPDMAFPRMNNMSFWLLPPSITLLISSSIVENGAGTGWTVYPPLSSNIAHGGSSVDLAIFSLHLAGISSILGAINFITTIINMRLNNMFFD
QLPLFVWAVGITAFLLLLSLPVLAGAITMLLTDRNLNTSFFDPAGGGDPILYQHL
>LEFIH045-10|HM876676|MM15909|Epirrhoe_rivata
TLYFIFGIWAGMIGTSLSLLIRAELGNPGSLIGDDQIYNTIVTAHAFIMIFFMVMPIMIGGFGNWLIPLMLGAPDMAFPRMNNMSFWLLPPSITLLISSSIVENGAGTGWTVYPPLSSNIAHGGSSVDLAIFSLHLAGISSILGAINFITTIINMRLNNMFFD
QLPLFVWAVGITAFLLLLSLPVLAGAITMLLTDRNLNTSFFDPAGGGDPILYQHL
>LEFID316-10|HM873113|MM06201|Epirrhoe_tristata
TLYFIFGIWAGMIGTSLSLLIRAELGNPGSLIGDDQIYNTIVTAHAFIMIFFMVMPIMIGGFGNWLVPLMLGAPDMAFPRMNNMSFWLLPPSITLLISSSIVENGAGTGWTVYPPLSSNIAHGGSSVDLAIFSLHLAGISSILGAINFITTIINMRLNNMFFD
QLPLFVWSVGITAFLLLLSLPVLAGAITMLLTDRNLNTSFFDPAGGGDPILYQHL
>LEFID749-10|HM873506|MM06810|Epirrhoe_tristata
TLYFIFGIWAGMIGTSLSLLIRAELGNPGSLIGDDQIYNTIVTAHAFIMIFFMVMPIMIGGFGNWLVPLMLGAPDMAFPRMNNMSFWLLPPSITLLISSSIVENGAGTGWTVYPPLSSNIAHGGSSVDLAIFSLHLAGISSILGAINFITTIINMRLNNMFFD
QLPLFVWSVGITAFLLLLSLPVLAGAITMLLTDRNLNTSFFDPAGGGDPILYQHL
>LEFIE578-10|HM874301|MM09475|Epirrhoe_tristata
TLYFIFGIWAGMIGTSLSLLIRAELGNPGSLIGDDQIYNTIVTAHAFIMIFFMVMPIMIGGFGNWLVPLMLGAPDMAFPRMNNMSFWLLPPSITLLISSSIVENGAGTGWTVYPPLSSNIAHGGSSVDLAIFSLHLAGISSILGAINFITTIINMRLNNMFFD
QLPLFVWSVGITAFLLLLSLPVLAGAITMLLTDRNLNTSFFDPAGGGDPILYQHL
>LEFIB297-10|HM871198|MM00807|Epirrita_autumnata
TLYFIFGIWAGMIGTSLSLLIRAELGNPGSLIGDDQIYNTIVTAHAFIMIFFMVMPIMIGGFGNWLVPLMLGAPDMAFPRMNNMSFWLLPPSITLLISSSIVENGAGTGWTVYPPLSSNIAHGGSSVDLAIFSLHLAGISSILGAINFITTIINMRLNNMFFD
QLPLFVWAVGITAFLLLLSLPVLAGAITMLLTDRNLNTSFFDPAGGGDPILYQHL
>LEFIJ031-10|KM573210|MM00808|Epirrita_autumnata
TLYFIFGIWAGMIGTSLSLLIRAELGNPGSLIGDDQIYNTIVTAHAFIMIFFMVMPIMIGGFGNWLVPLMLGAPDMAFPRMNNMSFWLLPPSITLLISSSIVENGAGTGWTVYPPLSSNIAHGGSSVDLAIFSLHLAGISSILGAINFITTIINMRLNNMFFD
QLPLFVWAVGITAFLLLLSLPVLAGAITMLLTDRNLNTSFFDPAGGGDPILYQHL
>LEFIB784-10|HM871661|MM02686|Epirrita_autumnata
TLYFIFGIWAGMIGTSLSLLIRAELGNPGSLIGDDQIYNTIVTAHAFIMIFFMVMPIMIGGFGNWLVPLMLGAPDMAFPRMNNMSFWLLPPSITLLISSSIVENGAGTGWTVYPPLSSNIAHGGSSVDLAIFSLHLAGISSILGAINFITTIINMRLNNMFFD
QLPLFVWAVGITAFLLLLSLPVLAGAITMLLTDRNLNTSFFDPAGGGDPILYQHL
>LEFIJ045-10|KM573511|MM02687|Epirrita_autumnata
TLYFIFGIWAGMIGTSLSLLIRAELGNPGSLIGDDQIYNTIVTAHAFIMIFFMVMPIMIGGFGNWLVPLMLGAPDMAFPRMNNMSFWLLPPSITLLISSSIVENGAGTGWTVYPPLSSNIAHGGSSVDLAIFSLHLAGISSILGAINFITTIINMRLNNMFFD
QLPLFVWAVGITAFLLLLSLPVLAGAITMLLTDRNLNTSFFDPAGGGDPILYQHL
>LEFIC590-10|HM872411|MM04509|Epirrita_autumnata
TLYFIFGIWAGMIGTSLSLLIRAELGNPGSLIGDDQIYNTIVTAHAFIMIFFMVMPIMIGGFGNWLVPLMLGAPDMAFPRMNNMSFWLLPPSITLLISSSIVENGAGTGWTVYPPLSSNIAHGGSSVDLAIFSLHLAGISSILGAINFITTIINMRLNNMFFD
QLPLFVWAVGITAFLLLLSLPVLAGAITMLLTDRNLNTSFFDPAGGGDPILYQHL
>LEFIJ108-10|KM572524|MM07901|Epirrita_autumnata
TLYFIFGIWAGMIGTSLSLLIRAELGNPGSLIGDDQIYNTIVTAHAFIMIFFMVMPIMIGGFGNWLVPLMLGAPDMAFPRMNNMSFWLLPPSITLLISSSIVENGAGTGWTVYPPLSSNIAHGGSSVDLAIFSLHLAGISSILGAINFITTIINMRLNNMFFD
QLPLFVWAVGITAFLLLLSLPVLAGAITMLLTDRNLNTSFFDPAGGGDPILYQHL
>LEFIJ198-10|KM572806|MM12813|Epirrita_autumnata
TLYFIFGIWAGMIGTSLSLLIRAELGNPGSLIGDDQIYNTIVTAHAFIMIFFMVMPIMIGGFGNWLVPLMLGAPDMAFPRMNNMSFWLLPPSITLLISSSIVENGAGTGWTVYPPLSSNIAHGGSSVDLAIFSLHLAGISSILGAINFITTIINMRLNNMFFD
QLPLFVWAVGITAFLLLLSLPVLAGAITMLLTDRNLNTSFFDPAGGGDPILYQHL

>LEFIG570-10|HM876241|MM14724|Epirrita_christyi
TLYFIFGIWAGMIGTSLSLLIRAELGSPGSLIGDDQIYNTIVTAHAFIMIFFMVMPIMIGGFGNWLVPLMLGAPDMAFPRMNNMSFWLLPPSITLLISSSIVENGAGTGWTVYPPLSSNIAHGGSSVDLAIFSLHLAGISSILGAINFITTIINMRLNNMFFD
QLPLFVWAVGITAFLLLLSLPVLAGAITMLLTDRNLNTSFFDPAGGGDPILYQHL
>LEFIG574-10|HM876245|MM14730|Epirrita_christyi
TLYFIFGIWAGMIGTSLSLLIRAELGSPGSLIGDDQIYNTIVTAHAFIMIFFMVMPIMIGGFGNWLVPLMLGAPDMAFPRMNNMSFWLLPPSITLLISSSIVENGAGTGWTVYPPLSSNIAHGGSSVDLAIFSLHLAGISSILGAINFITTIINMRLNNMFFD
QLPLFVWAVGITAFLLLLSLPVLAGAITMLLTDRNLNTSFFDPAGGGDPILYQHL
>LEFIB994-10|HQ570282|MM03191|Epirrita_dilutata
TLYFIFGIWAGMIGTSLSLLIRAELGNPGSLIGDDQIYNTIVTAHAFIMIFFMVMPIMIGGFGNWLVPLMLGAPDMAFPRMNNMSFWLLPPSITLLISSSIVENGAGTGWTVYPPLSSNIAHGGSSVDLAIFSLHLAGISSILGAINFITTIINMRLNNMFFD
QLPLFVWAVGITAFLLLLSLPVLAGAITMLLTDRNLNTSFFDPAGGGDPILYQHL
>LEFIF512-10|HM875197|MM12133|Episcythrastis_tetricella
TLYFIFGIWSGMVGTSLSLLIRAELGTSGSLIGDDQIYNTIVTGHAFIMIFFMVMPIMIGGFGNWLVPLMLGAPDMAFPRMNNMSFWLLPPSLTLLISSSIVENGAGTGWTVYPPLSSNIAHSGSSVDLAIFSLHLAGISSILGAINFITTIINMKINGMSFD
QMPLFVWAVGITALLLLLSLPVLAGAITMLLTDRNLNTSFFDPAGGGDPILYQHL
>LEFIG908-10|HM876552|MM15772|Episcythrastis_tetricella
TLYFIFGIWSGMVGTSLSLLIRAELGTSGSLIEDDQIYNTIVTGHAFIMIFFMVMPIMIGGFGNWLVPLMLGAPDMAFPRMNNMSFWLLPPSLTLLISSSIVENGAGTGWTVYPPLSSNIAHSGSSVDLAIFSLHLAGISSILGAINFITTIINMKINGMSFD
QMPLFVWAVGITALLLLLSLPVLAGAITMLLTDRNLNTSFFDPAGGGDPILYQHL
>LEFIF548-10|HM875233|MM12237|Erannis_defoliaria
TLYFIFGIWAGMVGTSLSLLIRAELGNPGSLIGDDQIYNTIVTAHAFIMIFFMVMPIMIGGFGNWLVPLMLGAPDMAFPRMNNMSFWLLPPSITLLISSSIVENGAGTGWTVYPPLSSNIAHGGSSVDLAIFSLHLAGISSILGAINFITTIINMRLNNLSFD
QMPLFVWSVGITAFLLLLSLPVLAGAITMLLTDRNLNTSFFDPAGGGDPILYQHL
>LEFIK940-10|JF854380|MM18515|Erannis_defoliaria
TLYFIFGIWAGMVGTSLSLLIRAELGNPGSLIGDDQIYNTIVTAHAFIMIFFMVMPIMIGGFGNWLVPLMLGAPDMAFPRMNNMSFWLLPPSITLLISSSIVENGAGTGWTVYPPLSSNIAHGGSSVDLAIFSLHLAGISSILGAINFITTIINMRLNNLSFD
QMPLFVWSVGITAFLLLLSLPVLAGAITMLLTDRNLNTSFFDPAGGGDPILYQHL
>LEEUA523-11|MM20582|Erebia_aethiops
TLYFIFGIWAGMVGTSLSLIIRTELGNPGSLIGNDQIYNTIVTAHAFIMIFFMVMPIMIGGFGNWLVPLMLGAPDMAFPRMNNMSFWLLPPSLTLLISSSIVENGAGTGWTVYPPLSSNIAHSGSSVDLAIFSLHLAGISSILGAINFITTIINMRINNMSYD
QMPLFVWAVGITALLLLLSLPVLAGAITMLLTDRNLNTSFFDPAGGGDPILYQHL
>LEFIJ554-10|JF853669|MM17179|Erebia_disa
TLYFIFGIWAGMVGTSLSLIIRTELGNPGSLIGDDQIYNTIVTAHAFIMIFFMVMPIMIGGFGNWLIPLMLGAPDMAFPRMNNMSFWLLPPSLILLISSSIVENGAGTGWTVYPPLSSNIAHSGASVDLAIFSLHLAGISSILGAINFITTIINMRINNMSYDQ
MPLFVWAVGITALLLLLSLPVLAGAITMLLTDRNLNTSFFDPAGGGDPILYQHL
>LEFID492-10|HM873257|MM06449|Erebia_embla
TLYFIFGIWAGMVGTSLSLIIRTELGNPGSLIGDDQIYNTIVTAHAFIMIFFMVMPIMIGGFGNWLIPLMLGAPDMAFPRMNNMSFWLLPPSLILLISSSIVENGAGTGWTVYPPLSSNIAHSGASVDLAIFSLHLAGISSILGAINFITTIINMRINNMSYDQ
MPLFVWAVGITALLLLLSLPVLAGAITMLLTDRNLNTSFFDPAGGGDPILYQHL
>LEFIC041-10|HM871911|MM03272|Erebia_ligea
TLYFIFGIWAGMVGTSLSLIIRTELGNPGSLIGDDQIYNTIVTAHAFIMIFFMVMPIMIGGFGNWLVPLMLGAPDMAFPRMNNMSFWLLPPSLVLLISSSIVENGAGTGWTVYPPLSSNIAHGGSSVDLAIFSLHLAGISSILGAINFITTIINMRINNMAYD
QMPLFVWAVGITALLLLLSLPVLAGAITMLLTDRNLNTSFFDPAGGGDPILYQHL
>LEFID112-10|HM872923|MM05735|Erebia_ligea
TLYFIFGIWAGMVGTSLSLIIRTELGNPGSLIGDDQIYNTIVTAHAFIMIFFMVMPIMIGGFGNWLVPLMLGAPDMAFPRMNNMSFWLLPPSLVLLISSSIVENGAGTGWTVYPPLSSNIAHGGSSVDLAIFSLHLAGISSILGAINFITTIINMRINNMAYD
QMPLFVWAVGITALLLLLSLPVLAGAITMLLTDRNLNTSFFDPAGGGDPILYQHL
>LEFIA020-10|HM396370|MM00091|Erebia_pandrose
TLYFIFGIWAGMMGTSLSLIIRTELGNPGSLIGNDQIYNTIVTAHAFIMIFFMVMPIMIGGFGNWLIPLMLGAPDMAFPRMNNMSFWLLPPSLILLISSSIVENGTGTGWTVYPPLSSNIAHSGASVDLAIFSLHLAGISSILGAINFITTIINMRINNMSYD
QMPLFVWAVGITALLLLLSLPVLAGAITMLLTDRNLNTSFFDPAGGGDPILYQHL
>LEFIF127-10|HQ570388|MM10583|Erebia_polaris
TLYFIFGIWAGMVGTSLSLIIRTELNTPGSLIGDDQIYNTVVTAHAFIMIFFMVMPIMIGGFGNWLIPLMLGAPDMAFPRMNNMSFWLLPPSLMLLISSSIVENGAGTGWTVYPPLSSNIAHSGSSVDLAIFSLHLAGISSILGAINFITTIINMRINNMSYD
QMPLFIWAVGITALLLLLSLPVLAGAITMLLTDRNLNTSFFDPAGGGDPILYQHL
>LEFIA037-10|HM396386|MM00436|Eriocrania_cicatricella
TLYFIFGIWSGMVGTSLSILIRSELGNPGSLIGDDQIYNVIVTAHAFIMIFFMVMPIMIGGFGNWLVPLMLGAPDMAFPRLNNMSFWLLPPSLTLLISSSFVENGAGTGWTVYPPLSSNIAHAGSSVDLAIFSLHLAGISSILGAVNFITTVINMRPYGMSL
DRMPLFVWAVVITALLLLLSLPVLAGAITMLLTDRNLNTSFFDPAGGGDPILYQHL
>LEFIE353-10|HM874077|MM08792|Eriocrania_cicatricella
TLYFIFGIWSGMVGTSLSILIRSELGNPGSLIGDDQIYNVIVTAHAFIMIFFMVMPIMIGGFGNWLVPLMLGAPDMAFPRLNNMSFWLLPPSLTLLISSSFVENGAGTGWTVYPPLSSNIAHAGSSVDLAIFSLHLAGISSILGAVNFITTVINMRPYGMSL

DRMPLFVWAVVITALLLLLSLPVLAGAITMLLTDRNLNTSFFDPAGGGDPILYQHL
>LEFIB087-10|HM870995|MM00391|Eriocrania_sangii
TLYFIFGIWSGMVGTSLSILIRSELGNPGSLIGDDQIYNVIVTAHAFIMIFFMVMPIMIGGFGNWLVPLMLGAPDMAFPRLNNMSFWLLPPSLTLLISSSFVENGAGTGWTVYPPLSSNIAHAGSSVDLAIFSLHLAGISSILGAVNFITTVINMRPYGMSL
DRMPLFVWAVVITALLLLLSLPVLAGAITMLLTDRNLNTSFFDPAGGGDPILYQHL
>LEFIJ084-10|JF853435|MM05747|Eriocrania_sangii
TLYFIFGIWSGMVGTSLSILIRSELGNPGSLIGDDQIYNVIVTAHAFIMIFFMVMPIMIGGFGNWLVPLMLGAPDMAFPRLNNMSFWLLPPSLTLLISSSFVENGAGTGWTVYPPLSSNIAHAGSSVDLAIFSLHLAGISSILGAVNFITTVINMRPYGMSL
DRMPLFVWAVVITALLLLLSLPVLAGAITMLLTDRNLNTSFFDPAGGGDPILYQHL
>LEFIJ087-10|KM572683|MM05750|Eriocrania_sangii
TLYFIFGIWSGMVGTSLSILIRSELGNPGSLIGDDQIYNVIVTAHAFIMIFFMVMPIMIGGFGNWLVPLMLGAPDMAFPRLNNMSFWLLPPSLTLLISSSFVENGAGTGWTVYPPLSSNIAHAGSSVDLAIFSLHLAGISSILGAVNFITTVINMRPYGMSL
DRMPLFVWAVVITALLLLLSLPVLAGAITMLLTDRNLNTSFFDPAGGGDPILYQHL
>LEFIF495-10|HM875180|MM12061|Eriocrania_sangii
TLYFIFGIWSGMVGTSLSILIRSELGNPGSLIGDDQIYNVIVTAHAFIMIFFMVMPIMIGGFGNWLVPLMLGAPDMAFPRLNNMSFWLLPPSLTLLISSSFVENGAGTGWTVYPPLSSNIAHAGSSVDLAIFSLHLAGISSILGAVNFITTVINMRPYGMSL
DRMPLFVWAVVITALLLLLSLPVLAGAITMLLTDRNLNTSFFDPAGGGDPILYQHL
>LEFIK349-10|JF853985|MM17924|Eriocrania_sangii
TLYFIFGIWSGMVGTSLSILIRSELGNPGSLIGDDQIYNVIVTAHAFIMIFFMVMPIMIGGFGNWLVPLMLGAPDMAFPRLNNMSFWLLPPSLTLLISSSFVENGAGTGWTVYPPLSSNIAHAGSSVDLAIFSLHLAGISSILGAVNFITTVINMRPYGMSL
DRMPLFVWAVVITALLLLLSLPVLAGAITMLLTDRNLNTSFFDPAGGGDPILYQHL
>LEFIK352-10|JF853988|MM17927|Eriocrania_sangii
TLYFIFGIWSGMVGTSLSILIRSELGNPGSLIGDDQIYNVIVTAHAFIMIFFMVMPIMIGGFGNWLVPLMLGAPDMAFPRLNNMSFWLLPPSLTLLISSSFVENGAGTGWTVYPPLSSNIAHAGSSVDLAIFSLHLAGISSILGAVNFITTVINMRPYGMSL
DRMPLFVWAVVITALLLLLSLPVLAGAITMLLTDRNLNTSFFDPAGGGDPILYQHL
>LEFIK354-10|JF853990|MM17929|Eriocrania_sangii
TLYFIFGIWSGMVGTSLSILIRSELGNPGSLIGDDQIYNVIVTAHAFIMIFFMVMPIMIGGFGNWLVPLMLGAPDMAFPRLNNMSFWLLPPSLTLLISSSFVENGAGTGWTVYPPLSSNIAHAGSSVDLAIFSLHLAGISSILGAVNFITTVINMRPYGMKL
DRMPLFVWAVVITALLLLLSLPVLAGAITMLLTDRNLNTSFFDPAGGGDPILYQHL
>LEEUA351-11|JN303414|MM19759|Eriocrania_sangii
TLYFIFGIWSGMVGTSLSILIRSELGNPGSLIGDDQIYNVIVTAHAFIMIFFMVMPIMIGGFGNWLVPLMLGAPDMAFPRLNNMSFWLLPPSLTLLISSSFVENGAGTGWTVYPPLSSNIAHAGSSVDLAIFSLHLAGISSILGAVNFITTVINMRPYGMSL
DRMPLFVWAVVITALLLLLSLPVLAGAITMLLTDRNLNTSFFDPAGGGDPILYQHL
>LEEUA358-11|JN303419|MM19766|Eriocrania_sangii
TLYFIFGIWSGMVGTSLSILIRSELGNPGSLIGDDQIYNVIVTAHAFIMIFFMVMPIMIGGFGNWLVPLMLGAPDMAFPRLNNMSFWLLPPSLTLLISSSFVENGAGTGWTVYPPLSSNIAHAGSSVDLAIFSLHLAGISSILGAVNFITTVINMRPYGMSL
DRMPLFVWAVVITALLLLLSLPVLAGAITMLLTDRNLNTSFFDPAGGGDPILYQHL
>LEEUA359-11|JN303420|MM19767|Eriocrania_sangii
TLYFIFGIWSGMVGTSLSILIRSELGNPGSLIGDDQIYNVIVTAHAFIMIFFMVMPIMIGGFGNWLVPLMLGAPDMAFPRLNNMSFWLLPPSLTLLISSSFVENGAGTGWTVYPPLSSNIAHAGSSVDLAIFSLHLAGISSILGAVNFITTVINMRPYGMSL
DRMPLFVWAVVITALLLLLSLPVLAGAITMLLTDRNLNTSFFDPAGGGDPILYQHL
>LEFIJ983-11|KM573159|MM21223|Eriocrania_sangii
TLYFIFGIWSGMVGTSLSILIRSELGNPGSLIGDDQIYNVIVTAHAFIMIFFMVMPIMIGGFGNWLVPLMLGAPDMAFPRLNNMSFWLLPPSLTLLISSSFVENGAGTGWTVYPPLSSNIAHAGSSVDLAIFSLHLAGISSILGAVNFITTVINMRPYGMSL
DRMPLFVWAVVITALLLLLSLPVLAGAITMLLTDRNLNTSFFDPAGGGDPILYQHL
>LEFIB095-10|HM871002|MM00404|Eriocrania_semipurpurella
TLYFIFGIWSGMVGTSLSILIRSELGNPGSLIGDDQIYNVIVTAHAFIMIFFMVMPIMIGGFGNWLVPLMLGAPDMAFPRLNNMSFWLLPPSLTLLISSSFVENGAGTGWTVYPPLSSNIAHAGSSVDLAIFSLHLAGISSILGAVNFITTVINMRPYGMSL
DRMPLFVWAVVITALLLLLSLPVLAGAITMLLTDRNLNTSFFDPAGGGDPILYQHL
>LEFIB096-10|HM871003|MM00405|Eriocrania_semipurpurella
TLYFIFGIWSGMVGTSLSILIRSELGNPGSLIGDDQIYNVIVTAHAFIMIFFMVMPIMIGGFGNWLVPLMLGAPDMAFPRLNNMSFWLLPPSLTLLISSSFVENGAGTGWTVYPPLSSNIAHAGSSVDLAIFSLHLAGISSILGAVNFITTVINMRPYGMSL
DRMPLFVWAVVITALLLLLSLPVLAGAITMLLTDRNLNTSFFDPAGGGDPILYQHL
>LEFIA031-10|HM396381|MM00430|Eriocrania_semipurpurella
TLYFIFGIWSGMVGTSLSILIRSELGNPGSLIGDDQIYNVIVTAHAFIMIFFMVMPIMIGGFGNWLVPLMLGAPDMAFPRLNNMSFWLLPPSLTLLISSSFVENGAGTGWTVYPPLSSNIAHAGSSVDLAIFSLHLAGISSILGAVNFITTVINMRPYGMSL
DRMPLFVWAVVITALLLLLSLPVLAGAITMLLTDRNLNTSFFDPAGGGDPILYQHL
>LEFIA035-10|HM396385|MM00434|Eriocrania_semipurpurella
TLYFIFGIWSGMVGTSLSILIRSELGNPGSLIGDDQIYNVIVTAHAFIMIFFMVMPIMIGGFGNWLVPLMLGAPDMAFPRLNNMSFWLLPPSLTLLISSSFVENGAGTGWTVYPPLSSNIAHAGSSVDLAIFSLHLAGISSILGAVNFITTVINMRPYGMSL
DRMPLFVWAVVITALLLLLSLPVLAGAITMLLTDRNLNTSFFDPAGGGDPILYQHL
>LEFIE421-10|HM874145|MM09028|Eriocrania_semipurpurella

TLYFIFGIWSGMVGTSLSILIRSELGNPGSLIGDDQIYNVIVTAHAFIMIFFMVMPIMIGGFGNWLVPLMLGAPDMAFPRLNNMSFWLLPPSLTLLISSSFVENGAGTGWTVYPPLSSNIAHAGSSVDLAIFSLHLAGISSILGAVNFITTVINMRPYGMSL
DRMPLFVWAVVITALLLLLSLPVLAGAITMLLTDRNLNTSFFDPAGGGDPILYQHL
>LEFIJ209-10|JF853477|MM13110|Eriocrania_semipurpurella
TLYFIFGIWSGMVGTSLSILIRSELGNPGSLIGDDQIYNVIVTAHAFIMIFFMVMPIMIGGFGNWLVPLMLGAPDMAFPRLNNMSFWLLPPSLTLLISSSFVENGAGTGWTVYPPLSSNIAHAGSSVDLAIFSLHLAGISSILGAVNFITTVINMRPYGMSL
DRMPLFVWAVVITALLLLLSLPVLAGAITMLLTDRNLNTSFFDPAGGGDPILYQHL
>LEFIJ210-10|KM572778|MM13111|Eriocrania_semipurpurella
TLYFIFGIWSGMVGTSLSILIRSELGNPGSLIGDDQIYNVIVTAHAFIMIFFMVMPIMIGGFGNWLVPLMLGAPDMAFPRLNNMSFWLLPPSLTLLISSSFVENGAGTGWTVYPPLSSNIAHAGSSVDLAIFSLHLAGISSILGAVNFITTVINMRPYGMSL
DRMPLFVWAVVITALLLLLSLPVLAGAITMLLTDRNLNTSFFDPAGGGDPILYQHL
>LEFIJ216-10|KM572084|MM13117|Eriocrania_semipurpurella
TLYFIFGIWSGMVGTSLSILIRSELGNPGSLIGDDQIYNVIVTAHAFIMIFFMVMPIMIGGFGNWLVPLMLGAPDMAFPRLNNMSFWLLPPSLTLLISSSFVENGAGTGWTVYPPLSSNIAHAGSSVDLAIFSLHLAGISSILGAVNFITTVINMRPYGMSL
DRMPLFVWAVVITALLLLLSLPVLAGAITMLLTDRNLNTSFFDPAGGGDPILYQHL
>LEFIJ225-10|JF853481|MM13127|Eriocrania_semipurpurella
TLYFIFGIWSGMVGTSLSILIRSELGNPGSLIGDDQIYNVIVTAHAFIMIFFMVMPIMIGGFGNWLVPLMLGAPDMAFPRLNNMSFWLLPPSLTLLISSSFVENGAGTGWTVYPPLSSNIAHAGSSVDLAIFSLHLAGISSILGAVNFITTVINMRPYGMSL
DRMPLFVWAVVITALLLLLSLPVLAGAITMLLTDRNLNTSFFDPAGGGDPILYQHF
>LEFIJ228-10|KM573481|MM13130|Eriocrania_semipurpurella
TLYFIFGIWSGMVGTSLSILIRSELGNPGSLIGDDQIYNVIVTAHAFIMIFFMVMPIMIGGFGNWLVPLMLGAPDMAFPRLNNMSFWLLPPSLTLLISSSFVENGAGTGWTVYPPLSSNIAHAGSSVDLAIFSLHLAGISSILGAVNFITTVINMRPYGMKL
DRMPLFVWAVVITALLLLLSLPVLAGAITMLLTDRNLNTSFFDPAGGGDPILYQHL
>LEFIG590-10|HM876261|MM14779|Eriocrania_semipurpurella
TLYFIFGIWSGMVGTSLSILIRSELGNPGSLIGDDQIYNVIVTAHAFIMIFFMVMPIMIGGFGNWLVPLMLGAPDMAFPRLNNMSFWLLPPSLTLLISSSFVENGAGTGWTVYPPLSSNIAHAGSSVDLAIFSLHLAGISSILGAVNFITTVINMRPYGMSL
DRMPLFVWAVVITALLLLLSLPVLAGAITMLLTDRNLNTSFFDPAGGGDPILYQHL
>LEFIG592-10|HM876263|MM14781|Eriocrania_semipurpurella
TLYFIFGIWSGMVGTSLSILIRSELGNPGSLIGDDQIYNVIVTAHAFIMIFFMVMPIMIGGFGNWLVPLMLGAPDMAFPRLNNMSFWLLPPSLTLLISSSFVENGAGTGWTVYPPLSSNIAHAGSSVDLAIFSLHLAGISSILGAVNFITTVINMRPYGMSL
DRMPLFVWAVVITALLLLLSLPVLAGAITMLLTDRNLNTSFFDPAGGGDPILYQHL
>LEFIJ1090-11|KM572152|MM16676|Eriocrania_semipurpurella
TLYFIFGIWSGMVGTSLSILIRSELGNPGSLIGDDQIYNVIVTAHAFIMIFFMVMPIMIGGFGNWLVPLMLGAPDMAFPRLNNMSFWLLPPSLTLLISSSFVENGAGTGWTVYPPLSSNIAHAGSSVDLAIFSLHLAGISSILGAVNFITTVINMRPYGMSL
DRMPLFVWAVVITALLLLLSLPVLAGAITMLLTDRNLNTSFFDPAGGGDPILYQHL
>LEFIK380-10|JF854013|MM17955|Eriocrania_semipurpurella
TLYFIFGIWSGMVGTSLSILIRSELGNPGSLIGDDQIYNVIVTAHAFIMIFFMVMPIMIGGFGNWLVPLMLGAPDMAFPRLNNMSFWLLPPSLTLLISSSFVENGAGTGWTVYPPLSSNIAHAGSSVDLAIFSLHLAGISSILGAVNFITTVINMRPYGMSL
DRMPLFVWAVIITALLLLLSLPVLAGAITMLLTDRNLNTSFFDPAGGGDPILYQHL
>LEEUA365-11|JN303425|MM19773|Eriocrania_semipurpurella
TLYFIFGIWSGMVGTSLSILIRSELGNPGSLIGDDQIYNVIVTAHAFIMIFFMVMPIMIGGFGNWLVPLMLGAPDMAFPRLNNMSFWLLPPSLTLLISSSFVENGAGTGWTVYPPLSSNIAHAGSSVDLAIFSLHLAGISSILGAMNFITTVINMRPYGMSL
DRMPLFVWAVVITALLLLLSLPVLAGAITMLLTDRNLNTSFFDPAGGGDPILYQHL
>LEEUA425-11|JN303432|MM19833|Eriocrania_semipurpurella
TLYFIFGIWSGMVGTSLSILIRSELGNPGSLIGDDQIYNVIVTAHAFIMIFFMVMPIMIGGFGNWLVPLMLGAPDMAFPRLNNMSFWLLPPSLTLLISSSFVENGAGTGWTVYPPLSSNIAHAGSSVDLAIFSLHLAGISSILGAVNFITTVINMRPYGMSL
DRMPLFVWAVVITALLLLLSLPVLAGAITMLLTDRNLNTSFFDPAGGGDPILYQHL
>LEFIG117-10|HM875797|MM13914|Eriocrania_sparrmannella
TLYFIFGIWSGMVGTSLSILIRSELGNPGSLIGDDQIYNVIVTAHAFIMIFFMVMPIMIGGFGNWLVPLMLGAPDMAFPRLNNMSFWLLPPSLTLLISSSFVENGAGTGWTVYPPLSSNIAHAGSSVDLAIFSLHLAGISSILGAVNFITTVINMRPYGMSL
DRMPLFVWSVVITALLLLLSLPVLAGAITMLLTDRNLNTSFFDPAGGGDPILYQHL
>LEFIG519-10|HM876192|MM14627|Eriocrania_sparrmannella
TLYFIFGIWSGMVGTSLSILIRSELGNPGSLIGDDQIYNVIVTAHAFIMIFFMVMPIMIGGFGNWLVPLMLGAPDMAFPRLNNMSFWLLPPSLTLLISSSFVENGAGTGWTVYPPLSSNIAHAGSSVDLAIFSLHLAGISSILGAVNFITTVINMRPYGMSL
DRMPLFVWSVVITALLLLLSLPVLAGAITMLLTDRNLNTSFFDPAGGGDPILYQHL
>LEFIC168-10|HM872014|MM03520|Eriogaster_lanestris
TLYFIFGIWAGMVGTSLSLLIRAELGTPGSLIGDDQIYNTIVTAHAFIMIFFMVMPIMIGGFGNWLVPLMLGAPDMAFPRMNNMSFWLLPPSLSLLISSSIVENGAGTGWTVYPPLSSNIAHGGSSVDLAIFSLHLAGISSILGAINFITTIINMRLNNMSFD
QMPLFVWAVGITAFLLLLSLPVLAGAITMLLTDRNLNTSFFDPAGGGDPILYQHL
>LEFIA928-10|HM387062|MM13856|Eriogaster_lanestris
TLYFIFGIWAGMVGTSLSLLIRAELGTPGSLIGDDQIYNTIVTAHAFIMIFFMVMPIMIGGFGNWLVPLMLGAPDMAFPRMNNMSFWLLPPSLSLLISSSIVENGAGTGWTVYPPLSSNIAHGGSSVDLAIFSLHLAGISSILGAINFITTIINMRLNNMSFD
QMPLFVWAVGITAFLLLLSLPVLAGAITMLLTDRNLNTSFFDPAGGGDPILYQHL

>LEFIJ568-10|JF853682|MM17193|Eriogaster_lanestris
TLYFIFGIWAGMVGTSLSLLIRAELGTPGSLIGDDQIYNTIVTAHAFIMIFFMVMPIMIGGFGNWLVPLMLGAPDMAFPRMNNMSFWLLPPSLSLLISSSIVENGAGTGWTVYPPLSSNIAHGGSSVDLAIFSLHLAGISSILGAINFITTIINMRLNNMSFD
QMPLFVWAVGITAFLLLLSLPVLAGAITMLLTDRNLNTSFFDPAGGGDPILYQHL
>LEFIA768-10|HM386911|MM04122|Eriopsela_quadrana
TLYFIFGVWAGMVGTSLSLLIRAELGNPGHLIGDDQIYNTIVTAHAFIMIFFMVMPIMIGGFGNWLIPLMLGAPDMAFPRMNNMSFWLLPPSIMLLISSSIVENGAGTGWTVYPPLSSNIAHSGSSVDLAIFSLHLAGISSILGAVNFITTIINMRPNNMS
LDQMPLLVWAVGITALLLLLSLPVLAGAITMLLTDRNLNTSFFDPAGGGDPILYQHL
>LEFIA769-10|HM386912|MM04123|Eriopsela_quadrana
TLYFIFGVWAGMVGTSLSLLIRAELGNPGHLIGDDQIYNTIVTAHAFIMIFFMVMPIMIGGFGNWLIPLMLGAPDMAFPRMNNMSFWLLPPSIMLLISSSIVENGAGTGWTVYPPLSSNIAHSGSSVDLAIFSLHLAGISSILGAVNFITTIINMRPNNMS
LDQMPLLVWAVGITALLLLLSLPVLAGAITMLLTDRNLNTSFFDPAGGGDPILYQHL
>LEFID340-10|HM873137|MM06234|Eteobalea_anonymella
TLYFIFGIWSGMVGTSLSMLIRTELGNPGFLISDDQIYNTIVTAHAFIMIFFMVMPIMIGGFGNWLVPLMLGAPDMAFPRMNNMSFWLLPPSLILLISSSIVENGAGTGWTVYPPLSSNITHSGSSVDLAIFSLHLAGISSILGAINFITTIINMRINNLVFDQ
MPLFVWSVGITALLLLLSLPVLAGAITMLLTDRNLNTSFFDPAGGGDPILYQHL
>LEFID155-10|HM872962|MM05918|Ethmia_bipunctella
TLYFIFGIWAGMVGTSLSLLIRAELGNPGSLIGDDQIYNTIVTAHAFIMIFFMVMPIMIGGFGNWLVPLMLGAPDMAFPRMNNMSFWLLPPSLTLLISSSIVENGAGTGWTVYPPLSSNIAHGGSSVDLAIFSLHLAGISSILGAINFITTIINMRLNNMSF
DQMPLFVWAVGITALLLLLSLPVLAGAITMLLTDRNLNTSFFDPAGGGDPILYQHL
>LEFIF628-10|HM875312|MM12518|Ethmia_bipunctella
TLYFIFGIWAGMVGTSLSLLIRAELGNPGSLIGDDQIYNTIVTAHAFIMIFFMVMPIMIGGFGNWLVPLMLGAPDMAFPRMNNMSFWLLPPSLTLLISSSIVENGAGTGWTVYPPLSSNIAHGGSSVDLAIFSLHLAGISSILGAINFITTIINMRLNNMSF
DQMPLFVWAVGITALLLLLSLPVLAGAITMLLTDRNLNTSFFDPAGGGDPILYQHL
>LEFIK070-10|MM17645|Ethmia_dodecea
TLYFIFGIWAGMVGTSLSLLIRAELGNPGSLIGDDQIYNTIVTAHAFIMIFFMVMPIMIGGFGNWLVPLMLGAPDMAFPRMNNMSFWLLPPSLTLLISSSIVENGAGTGWTVYPPLSSNIAHGGSSVDLAIFSLHLAGISSILGAINFITTIINMRLTNMSFD
QMPLFVWAVGITALLLLLSLPVLAGAITMLLTDRNLNTSFFDPAGGGDPILYQHL
>LEFIA200-10|HM396545|MM01223|Ethmia_pusiella
TLYFIFGIWAGMVGTSLSLLIRAELGNPGSLIGDDQIYNTIVTAHAFIMIFFMVMPIMIGGFGNWLVPLMLGAPDMAFPRMNNMSFWLLPPSLTLLISSSIVENGAGTGWTVYPPLSSNIAHSGSSVDLAIFSLHLAGISSILGAINFITTIINMRLNNMSFD
QMPLFVWAVGITALLLLLSLPVLAGAITMLLTDRNLNTSFFDPAGGGDPILYQHL
>LEFIC554-10|HM872375|MM04315|Ethmia_pyrausta
TLYFIFGIWAGMIGTSLSLLIRAELGNPGSLIGNDQIYNTIVTAHAFIMIFFMVMPIMIGGFGNWLVPLMLGAPDMAFPRMNNMSFWLLPPSLTLLITSSVVENGAGTGWTVYPPLSSNITHSGSSVDLTIFSLHLAGISSILGAINFITTIINMRLNNMSFD
QMPLFVWAVGITALLLLLSLPVLAGAITMLLTDRNLNTSFFDPAGGGDPILYQHL
>LEFIE984-10|HM874700|MM10421|Ethmia_pyrausta
TLYFIFWNWAGMIGTSLSLLIRAELGNPGSLIGNDQIYNTIVTAHAFIMIFFMVMPIMIGGFGNWLVPLMLGAPDMAFPRMNNMSFWLLPPSLTLLITSSVVENGAGTGWTVYPPLSSNITHSGSSVDLTIFSLHLAGISSILGAINFITTIINMRLNNMSF
DQMPLFVWAVGITALLLLLSLPVLAGAITMLLTDRNLNTSFFDPAGGGDPILYQHL
>LEFIB625-10|HM871504|MM02370|Ethmia_quadrillella
TLYFIFGIWAGMVGTSLSLLIRAELGNPGSLIGDDQIYNTIVTAHAFIMIFFMVMPIMIGGFGNWLVPLMLGAPDMAFPRMNNMSFWLLPPSLTLLISSSIVENGAGTGWTVYPPLSSNIAHGGSSVDLAIFSLHLAGISSILGAINFITTIINMRLTNMSFD
QMPLFVWAVGITALLLLLSLPVLAGAITMLLTDRNLNTSFFDPAGGGDPILYQHL
>LEFIB045-10|HM870954|MM00319|Ethmia_terminella
TLYFIFGIWAGMVGTSLSLLIRAELGNPGSLIGDDQIYNTIVTAHAFIMIFFMVMPIMIGGFGNWLVPLMLGAPDMAFPRMNNMSFWLLPPSLTLLISSSIVENGAGTGWTVYPPLSSNIAHGGSSVDLAIFSLHLAGISSILGAINFITTIINMRLNNMSF
DQMPLFVWAVGITALLLLLSLPVLAGAITMLLTDRNLNTSFFDPAGGGDPILYQHL
>LEFIJ327-10|MM15927|Eublemma_minutata
TLYFIFGIWAGMVGTSLSLLIRAELGNPGSLIGDDQIYNTIVTAHAFIMIFFMVMPIMIGGFGNWLVPLMLGAPDMAFPRMNNMSFWLLPPSLTLLISSSVVENGAGTGWTVYPPLSSNIAHGGSSVDLAIFSLHLAGISSILGAINFISTIINMRLNNLSF
DQMPLFIWAVGITAFLLLLSLPVLAGAITMLLTDRNLNTSFFDPAGGGDPILYQHL
>LEFIJ328-10|MM15928|Eublemma_parva
TLYFIFGIWAGMVGTSLSLLIRAELGNPGSLIGDDQIYNTIVTAHAFIMIFFMVMPIMIGGFGNWLVPLMLGAPDMAFPRMNNMSFWLLPPSLTLLISSSIVENGAGTGWTVYPPLSSNI?HGGSSVDLAIFSLHLAGISSILGAINFISTIINMRLNNLSFD
QMPLFIWAVGITAFLLLLSLPVLAGAITMLLTDRNLNTSFFDPAGGGDPILYQHL
>LEFID064-10|HM872878|MM05673|Eucarta_virgo
TLYFIFGIWAGMVGTSLSLLIRAELGTPGSLIGDDQIYNTIVTAHAFIMIFFMVMPIMIGGFGNWLVPLMLGAPDMAFPRMNNMSFWLLPPSLTLLISSSIVENGAGTGWTVYPPLSSNIAHGGSSVDLAIFSLHLAGISSILGAINFITTIINMRLNSLSFD
QMPLFIWAVGITAFLLLLSLPVLAGAITMLLTDRNLNTSFFDPAGGGDPILYQHL
>LEFID235-10|HM873035|MM06095|Eucarta_virgo
TLYFIFGIWAGMVGTSLSLLIRAELGTPGSLIGDDQIYNTIVTAHAFIMIFFMVMPIMIGGFGNWLVPLMLGAPDMAFPRMNNMSFWLLPPSLTLLISSSIVENGAGTGWTVYPPLSSNIAHGGSSVDLAIFSLHLAGISSILGAINFITTIINMRLNSLSFD

QMPLFIWAVGITAFLLLLSLPVLAGAITMLLTDRNLNTSFFDPAGGGDPILYQHL
>LEFIJ748-10|MM17373|Eucarta_virgo
TLYFIFGIWAGMVGTSLSLLIRAELGTPGSLIGDDQIYNTIVTAHAFIMIFFMVMPIMIGGFGNWLVPLMLGAPDMAFPRMNNMSFWLLPPSLTLLISSSIVENGAGTGWTVYPPLSSNIAHGGSSVDLAIFSLHLAGISSILGAINFITTIINMRLNSLSFD
QMPLFIWAVGITAFLLLLSLPVLAGAITMLLTDRNLNTSFFDPAGGGDPILYQHL
>LEFID011-10|HM872825|MM05433|Euchalcia_modestoides
TLYFIFGIWAGMVGTSLSLLIRAELGTPGSLIGDDQIYNTIVTAHAFIMIFFMVMPIMIGGFGNWLVPLMLGAPDMAFPRMNNMSFWLLPPSLTLLISSSIVENGAGTGWTVYPPLSSNIAHGGSSVDLAIFSLHLAGISSILGAINFITTIINMRLNNLSFD
QMPLFIWAVGITAFLLLLSLPVLAGAITMLLTDRNLNTSFFDPAGGGDPILYQHL
>LEFIK949-10|JF854387|MM18524|Euchalcia_modestoides
TLYFIFGIWAGMVGTSLSLLIRAELGTPGSLIGDDQIYNTIVTAHAFIMIFFMVMPIMIGGFGNWLVPLMLGAPDMAFPRMNNMSFWLLPPSLTLLISSSIVENGAGTGWTVYPPLSSNIAHGGSSVDLAIFSLHLAGISSILGAINFITTIINMRLNNLSFD
QMPLFIWAVGITAFLLLLSLPVLAGAITMLLTDRNLNTSFFDPAGGGDPILYQHL
>LEFIJ334-10|JF853551|MM15934|Euchalcia_variabilis
TLYFIFGIWAGMVGTSLSLLIRAELGTPGSLIGDDQIYNTIVTAHAFIMIFFMVMPIMIGGFGNWLVPLMLGAPDMAFPRMNNMSFWLLPPSLTLLISSSIVENGAGTGWTVYPPLSSNIAHSGSSVDLAIFSLHLAGISSILGAINFITTIINMRLNNLSFD
QMPLFIWAVGITAFLLLLSLPVLAGAITMLLTDRNLNTSFFDPAGGGDPILYQHL
>LEFIA276-10|HM386620|MM01316|Euchoeca_nebulata
TLYFIFGIWAGMIGTSLSLLIRAELGNPGSLIGDDQIYNTIVTAHAFIMIFFMVMPIMIGGFGNWLVPLMLGAPDMAFPRMNNMSFWLLPPSITLLISSSIVENGAGTGWTVYPPLSSNIAHGGSSVDLAIFSLHLAGISSILGAINFITTIINMRLNKMFFD
QLPLFVWAVGITAFLLLLSLPVLAGAITMLLTDRNLNTSFFDPAGGGDPILYQHL
>LEFIA277-10|HM386621|MM01317|Euchoeca_nebulata
TLYFIFGIWAGMIGTSLSLLIRAELGNPGSLIGDDQIYNTIVTAHAFIMIFFMVMPIMIGGFGNWLVPLMLGAPDMAFPRMNNMSFWLLPPSITLLISSSIVENGAGTGWTVYPPLSSNIAHGGSSVDLAIFSLHLAGISSILGAINFITTIINMRLNKMFFD
QLPLFVWAVGITAFLLLLSLPVLAGAITMLLTDRNLNTSFFDPAGGGDPILYQHL
>LEFIF095-10|HM874798|MM10544|Euchromius_ocellea
TLYFIFGIWAGMVGTSLSLLIRAELGNPGSLIGDDQIYNTIVTAHAFIMIFFMVMPIMIGGFGNWLVPLMLGAPDMAFPRMNNMSFWLLPPSLTLLISSSIVENGAGTGWTVYPPLSSNIAHGGSSVDLAIFSLHLAGISSILGAINFITTIINMRINGLSFD
QMPLFVWSVGITALLLLLSLPVLAGAITMLLTDRNLNTSFFDPAGGGDPILYQHL
>LEFIF097-10|HM874800|MM10546|Euchromius_ocellea
TLYFIFGIWAGMVGTSLSLLIRAELGNPGSLIGDDQIYNTIVTAHAFIMIFFMVMPIMIGGFGNWLVPLMLGAPDMAFPRMNNMSFWLLPPSLTLLISSSIVENGAGTGWTVYPPLSSNIAHGGSSVDLAIFSLHLAGISSILGAINFITTIINMRINGLSFD
QMPLFVWSVGITALLLLLSLPVLAGAITMLLTDRNLNTSFFDPAGGGDPILYQHL
>LEFIB830-10|HM871707|MM02785|Euclidia_glyphica
TLYFIFGIWAGMVGTSLSLLIRAELGNPGSLIGDDQIYNTIVTAHAFIMIFFMVMPIMIGGFGNWLVPLMLGAPDMAFPRMNNMSFWLLPPSLTLLISSSIVENGAGTGWTVYPPLSSNIAHSGSSVDLAIFSLHLAGISSILGAINFITTIINMRLNNLMFD
QMPLFVWAVGITAFLLLLSLPVLAGAITMLLTDRNLNTSFFDPAGGGDPILYQHL
>LEFIB012-10|HM870925|MM00026|Euclidia_mi
TLYFIFGIWAGMVGTSLSLLIRAELGNPGSLIGDDQIYNTIVTAHAFIMIFFMVMPIMIGGFGNWLVPLMLGAPDMAFPRMNNMSFWLLPPSLTLLISSSIVENGAGTGWTVYPPLSSNIAHSGSSVDLAIFSLHLAGISSILGAINFITTIINMRLNNLMFD
QMPLFVWAVGITAFLLLLSLPVLAGAITMLLTDRNLNTSFFDPAGGGDPILYQHL
>LEFIB488-10|HM871383|MM02062|Eucosma_aemulana
TLYFIFGIWAGMIGTSLSLLIRAELGNPGSLIGDDQIYNTIVTAHAFIMIFFMVMPIMIGGFGNWLVPLMLGAPDMAFPRMNNMSFWLLPPSIMLLVSSSIVENGAGTGWTVYPPLSSNIAHSGSSVDLAIFSLHLAGISSILGAVNFITTIINMRPNNMSL
DQMPLFIWAVGITALLLLLSLPVLAGAITMLLTDRNLNTSFFDPAGGGDPILYQHL
>LEFID020-10|HM872834|MM05449|Eucosma_aspidiscana
TLYFIFGIWAGMIGTSLSLLIRAELGTPGSLIGDDQIYNTIVTAHAFIMIFFMVMPIMIGGFGNWLVPLMLGAPDMAFPRMNNMSFWLLPPSIMLLISSSIVENGAGTGWTVYPPLSSNIAHSGSSVDLAIFSLHLAGISSILGAVNFITTIINMRPNNMSL
DQMPLFVWAVGITALLLLLSLPVLAGAITMLLTDRNLNTSFFDPAGGGDPILYQHL
>LEFIJ140-10|JF853454|MM09962|Eucosma_aspidiscana
TLYFIFGIWAGMIGTSLSLLIRAELGTPGSLIGDDQIYNTIVTAHAFIMIFFMVMPIMIGGFGNWLVPLMLGAPDMAFPRMNNMSFWLLPPSIMLLISSSIVENGAGTGWTVYPPLSSNIAHSGSSVDLAIFSLHLAGISSILGAVNFITTIINMRPNNMSL
DQMPLFIWAVGITALLLLLSLPVLAGAITMLLTDRNLNTSFFDPAGGGDPILYQHL
>LEFIF245-10|HM874938|MM11026|Eucosma_aspidiscana
TLYFIFGIWAGMIGTSLSLLIRAELGAPGSLIGDDQIYNTIVTAHAFIMIFFMVMPIMIGGFGNWLVPLMLGAPDMAFPRMNNMSFWLLPPSIMLLISSSIVENGAGTGWTVYPPLSSNIAHSGSSVDLAIFSLHLAGISSILGAVNFITTIINMRPNNMSL
DQMPLFVWAVGITALLLLLSLPVLAGAITMLLTDRNLNTSFFDPAGGGDPILYQHL
>LEFIF248-10|HM874941|MM11029|Eucosma_aspidiscana
TLYFIFGIWAGMIGTSLSLLIRAELGTPGSLIGDDQIYNTIVTAHAFIMIFFMVMPIMIGGFGNWLVPLMLGAPDMAFPRMNNMSFWLLPPSIMLLISSSIVENGAGTGWTVYPPLSSNIAHSGSSVDLAIFSLHLAGISSILGAVNFITTIINMRPNNMSL
DQMPLFIWAVGITALLLLLSLPVLAGAITMLLTDRNLNTSFFDPAGGGDPILYQHL
>LEFIK762-10|JN274952|MM18337|Eucosma_aspidiscana

TLYFIFGIWTGMIGTSLSLLIRAELGTPGSLIGDDQIYNTIVTAHAFIMIFFMVMPIMIGGFGNWLVPLMLGAPDMAFPRMNNMSFWLLPPSIMLLISSSIVENGAGTGWTVYPPLSSNIAHSGSSVDLAIFSLHLAGISSILGAINFITTIINMRPNNMSLD
QMPLFIWAVGITALLLLLSLPVLAGAITMLLTDRNLNTSFFDPAGGGDPILYQHL
>LEFIF523-10|HM875208|MM12160|Eucosma_campoliliana
TLYFIFGVWAGMIGTSLSLLIRAELGMPGSLIGDDQIYNTIVTAHAFIMIFFMVMPIMIGGFGNWLVPLMLGAPDMAFPRMNNMSFWLLPPSIMLLISSSIVENGAGTGWTVYPPLSSNIAHSGSSVDLAIFSLHLAGISSILGAVNFITTIINMRPNNMS
LDQMPLFVWAVGITALLLLLSLPVLAGAITMLLTDRNLNTSFFDPAGGGDPILYQHL
>LEFIJ651-10|JF853746|MM17276|Eucosma_campoliliana
TLYFIFGVWAGMIGTSLSLLIRAELGMPGSLIGDDQIYNTIVTAHAFIMIFFMVMPIMIGGFGNWLVPLMLGAPDMAFPRMNNMSFWLLPPSIMLLISSSIVENGAGTGWTVYPPLSSNIAHSGSSVDLAIFSLHLAGISSILGAVNFITTIINMRPNNMS
LDQMPLFVWAVGITALLLLLSLPVLAGAITMLLTDRNLNTSFFDPAGGGDPILYQHL
>LEFIB513-10|HM871405|MM02107|Eucosma_cana
TLYFIFGIWAGMIGTSLSLMIRAELGNPGSLIGDDQIYNTIVTAHAFIMIFFMVMPIMIGGFGNWLVPLMLGAPDMAFPRMNNMSFWLLPPSIMLLISSSIVENGAGTGWTVYPPLSSNIAHSGSSVDLAIFSLHLAGISSILGAINFITTIINMRPNNMSL
DQMPLFIWAVGITALLLLLSLPVLAGAITMLLTDRNLNTSFFDPAGGGDPILYQHL
>LEFIE400-10|HM874124|MM08961|Eucosma_cana
TLYFIFGIWAGMIGTSLSLMIRAELGNPGSLIGDDQIYNTIVTAHAFIMIFFMVMPIMIGGFGNWLVPLMLGAPDMAFPRMNNMSFWLLPPSIMLLISSSIVENGAGTGWTVYPPLSSNIAHSGSSVDLAIFSLHLAGISSILGAINFITTIINMRPNNMSL
DQMPLFIWAVGITALLLLLSLPVLAGAITMLLTDRNLNTSFFDPAGGGDPILYQHL
>LEFIE401-10|HM874125|MM08963|Eucosma_cana
TLYFIFGIWAGMIGTSLSLMIRAELGNPGSLIGDDQIYNTIVTAHAFIMIFFMVMPIMIGGFGNWLVPLMLGAPDMAFPRMNNMSFWLLPPSIMLLISSSIVENGAGTGWTVYPPLSSNIAHSGSSVDLAIFSLHLAGISSILGAINFITTIINMRPNNMSL
DQMPLFIWAVGITALLLLLSLPVLAGAITMLLTDRNLNTSFFDPAGGGDPILYQHL
>LEFIF841-10|HM875524|MM13186|Eucosma_cana
TLYFIFGIWAGMIGTSLSLMIRAELGNPGSLIGDDQIYNTIVTAHAFIMIFFMVMPIMIGGFGNWLVPLMLGAPDMAFPRMNNMSFWLLPPSIMLLISSSIVENGAGTGWTVYPPLSSNIAHSGSSVDLAIFSLHLAGISSILGAINFITTIINMRPNNMSL
DQMPLFIWAVGITALLLLLSLPVLAGAITMLLTDRNLNTSFFDPAGGGDPILYQHL
>LEFIC568-10|HM872389|MM04346|Eucosma_conterminana
TLYFIFGIWAGMIGTSLSLLIRAELGNPGSLIGDDQIYNTIVTAHAFIMIFFMVMPIMIGGFGNWLVPLMLGAPDMAFPRMNNMSFWLLPPSIMLLISSSIVENGAGTGWTVYPPLSSNIAHSGSSVDLAIFSLHLAGISSILGAVNFITTIINMRPNNMSL
DQMPLFVWAVGITALLLLLSLPVLAGAITMLLTDRNLNTSFFDPAGGGDPILYQHL
>LEFID803-10|HM873560|MM06887|Eucosma_conterminana
TLYFIFGIWAGMIGTSLSLLIRAELGNPGSLIGDDQIYNTIVTAHAFIMIFFMVMPIMIGGFGNWLVPLMLGAPDMAFPRMNNMSFWLLPPSIMLLISSSIVENGAGTGWTVYPPLSSNIAHSGSSVDLAIFSLHLAGISSILGAVNFITTIINMRPNNMSL
DQMPLFVWAVGITALLLLLSLPVLAGAITMLLTDRNLNTSFFDPAGGGDPILYQHL
>LEFIE617-10|HM874340|MM09536|Eucosma_conterminana
TLYFIFGIWAGMIGTSLSLLIRAELGNPGSLIGDDQIYNTIVTAHAFIMIFFMVMPIMIGGFGNWLVPLMLGAPDMAFPRMNNMSFWLLPPSIMLLISSSIVENGAGTGWTVYPPLSSNIAHSGSSVDLAIFSLHLAGISSILGAVNFITTIINMRPNNMSL
DQMPLFVWAVGITALLLLLSLPVLAGAITMLLTDRNLNTSFFDPAGGGDPILYQHL
>LEFIJ1608-12|KM572413|MM22624|Eucosma_conterminana
TLYFIFGIWAGMIGTSLSLLIRAELGNPGSLIGDDQIYNTIVTAHAFIMIFFMVMPIMIGGFGNWLVPLMLGAPDMAFPRMNNMSFWLLPPSIMLLISSSIVENGAGTGWTVYPPLSSNIAHSGSSVDLAIFSLHLAGISSILGAVNFITTIINMRPNNMSL
DQMPLFVWAVGITALLLLLSLPVLAGAITMLLTDRNLNTSFFDPAGGGDPILYQHL
>LEFID214-10|HM873016|MM06045|Eucosma_flavispecula
TLYFIFGIWAGMVGTSLSLLIRAELGNPGSLIGDDQIYNTIVTAHAFIMIFFMVMPIMIGGFGNWLVPLMLGAPDMAFPRMNNMSFWLLPPSIMLLISSSIVENGAGTGWTVYPPLSSNIAHSGSSVDLAIFSLHLAGISSILGAVNFITTIINMRPNNMSL
DQMPLFVWAVGITALLLLLSLPVLAGAITMLLTDRNLNTSFFDPAGGGDPILYQHL
>LEFIL713-10|MM16003|Eucosma_flavispecula
TLYFIFGIWAGMVGTSLSLLIRAELGNPGSLIGDDQIYNTIVTAHAFIMIFFMVMPIMIGGFGNWLVPLMLGAPDMAFPRMNNMSFWLLPPSIMLLISSSIVENGAGTGWTVYPPLSSNIAHSGSSVDLAIFSLHLAGISSILGAVNFITTIINMRPNNMSL
DQMPLFVWAVGITALLLLLSLPVLAGAITMLLTDRNLNTSFFDPAGGGDPILYQHL
>LEFID735-10|HM873492|MM06793|Eucosma_fulvana
TLYFIFGIWSGMVGTSLSLLIRAELGNPGYLIGDDQIYNTIVTAHAFIMIFFMVMPIMIGGFGNWLVPLMLGAPDMAFPRMNNMSFWLLPPSIMLLISSSMVENGAGTGWTVYPPLSSNIAHSGSSVDLAIFSLHLAGISSILGAVNFITTIINMRPNNM
SLDQMPLFIWAVGITALLLLLSLPVLAGAITMLLTDRNLNTSFFDPAGGGDPILYQHL
>LEFID736-10|HM873493|MM06794|Eucosma_fulvana
TLYFIFGIWSGMVGTSLSLLIRAELGNPGYLIGDDQIYNTIVTAHAFIMIFFMVMPIMIGGFGNWLVPLMLGAPDMAFPRMNNMSFWLLPPSIMLLISSSMVENGAGTGWTVYPPLSSNIAHSGSSVDLAIFSLHLAGISSILGAVNFITTIINMRPNNM
SLDQMPLFIWAVGITALLLLLSLPVLAGAITMLLTDRNLNTSFFDPAGGGDPILYQHL
>LEFIF240-10|HM874933|MM11021|Eucosma_fulvana
TLYFIFGIWSGMVGTSLSLLIRAELGNPGYLIGDDQIYNTIVTAHAFIMIFFMVMPIMIGGFGNWLVPLMLGTPDMAFPRMNNMSFWLLPPSIMLLISSSMVENGAGTGWTVYPPLSSNIAHSGSSVDLAIFSLHLAGISSILGAVNFITTIINMRPNNMS
LDQMPLFIWAVGITALLLLLSLPVLAGAITMLLTDRNLNTSFFDPAGGGDPILYQHL

>LEFIF243-10|HM874936|MM11024|Eucosma_fulvana
TLYFIFGIWSGMVGTSLSLLIRAELGNPGYLIGDDQIYNTIVTAHAFIMIFFMVMPIMIGGFGNWLVPLMLGAPDMAFPRMNNMSFWLLPPSIMLLISSSMVENGAGTGWTVYPPLSSNIAHSGSSVDLAIFSLHLAGISSILGAVNFITTIINMRPNNM
SLDQMPLFIWAVGITALLLLLSLPVLAGAITMLLTDRNLNTSFFDPAGGGDPILYQHL
>LEFIG465-10|HM876141|MM14506|Eucosma_fulvana
TLYFIFGIWSGMVGTSLSLLIRAELGNPGYLIGDDQIYNTIVTAHAFIMIFFMVMPIMIGGFGNWLVPLMLGAPDMAFPRMNNMSFWLLPPSIMLLISSSMVENGAGTGWTVYPPLSSNIAHSGSSVDLAIFSLHLAGISSILGAVNFITTIINMRPNNM
SLDQMPLFIWAVGITALLLLLSLPVLAGAITMLLTDRNLNTSFFDPAGGGDPILYQHL
>LEFIG467-10|HM876143|MM14508|Eucosma_fulvana
TLYFIFGIWSGMVGTSLSLLIRAELGNPGYLIGDDQIYNTIVTAHAFIMIFFMVMPIMIGGFGNWLVPLMLGAPDMAFPRMNNMSFWLLPPSIMLLISSSMVENGAGTGWTVYPPLSSNIAHSGSSVDLAIFSLHLAGISSILGAVNFITTIINMRPNNM
SLDQMPLFIWAVGITALLLLLSLPVLAGAITMLLTDRNLNTSFFDPAGGGDPILYQHL
>LEEUA238-11|JN274935|MM19646|Eucosma_fulvana
TLYFIFGIWSGMVGTSLSLLIRAELGNPGYLIGDDQIYNTIVTAHAFIMIFFMVMPIMIGGFGNWLVPLMLGAPDMAFPRMNNMSFWLLPPSIMLLISSSMVENGAGTGWTVYPPLSSNIAHSGSSVDLAIFSLHLAGISSILGAVNFITTIINMRPNNM
SLDQMPLFIWAVGITALLLLLSLPVLAGAITMLLTDRNLNTSFFDPAGGGDPILYQHL
>LEEUA239-11|JN274936|MM19647|Eucosma_fulvana
TLYFIFGIWSGMVGTSLSLLIRAELGNPGYLIGDDQIYNTIVTAHAFIMIFFMVMPIMIGGFGNWLVPLMLGAPDMAFPRMNNMSFWLLPPSIMLLISSSMVENGAGTGWTVYPPLSSNIAHSGSSVDLAIFSLHLAGISSILGAVNFITTIINMRPNNM
SLDQMPLFIWAVGITALLLLLSLPVLAGAITMLLTDRNLNTSFFDPAGGGDPILYQHL
>LEFID434-10|HQ570330|MM06365|Eucosma_guentheri
TLYFIFGIWAGMIGTSLSLLIRAELGNPGSLIGDDQIYNTIVTAHAFIMIFFMVMPIMIGGFGNWLIPLMLGAPDMAFPRMNNMSFWLLPPSIMLLISSSIVENGAGTGWTVYPPLSSNIAHSGSSVDLAIFSLHLAGISSILGAVNFITTIINMRPNNMLLD
QMPLFVWAVGITALLLLLSLPVLAGAITMLLTDRNLNTSFFDPAGGGDPILYQHL
>LEFID615-10|HM873380|MM06630|Eucosma_hohenwartiana
TLYFIFGIWSGMVGTSLSLLIRAELGNPGYLIGDDQIYNTIVTAHAFIMIFFMVMPIMIGGFGNWLVPLMLGAPDMAFPRMNNMSFWLLPPSIMLLISSSMVENGAGTGWTVYPPLSSNIAHSGSSVDLAIFSLHLAGISSILGAVNFITTIINMRPNNM
SLDQMPLFIWAVGITALLLLLSLPVLAGAITMLLTDRNLNTSFFDPAGGGDPILYQHL
>LEFID812-10|HM873569|MM06899|Eucosma_hohenwartiana
TLYFIFGIWSGMVGTSLSLLIRAELGNPGYLIGDDQIYNTIVTAHAFIMIFFMVMPIMIGGFGNWLVPLMLGAPDMAFPRMNNMSFWLLPPSIMLLISSSMVENGAGTGWTVYPPLSSNIAHSGSSVDLAIFSLHLAGISSILGAVNFITTIINMRPNNM
SLDQMPLFIWAVGITALLLLLSLPVLAGAITMLLTDRNLNTSFFDPAGGGDPILYQHL
>LEFIE554-10|HM874277|MM09439|Eucosma_hohenwartiana
TLYFIFGIWSGMVGTSLSLLIRAELGNPGYLIGDDQIYNTIVTAHAFIMIFFMVMPIMIGGFGNWLVPLMLGAPDMAFPRMNNMSFWLLPPSIMLLISSSMVENGAGTGWTVYPPLSSNIAHSGSSVDLAIFSLHLAGISSILGAVNFITTIINMRPNNM
SLDQMPLFIWAVGITALLLLLSLPVLAGAITMLLTDRNLNTSFFDPAGGGDPILYQHL
>LEFIG461-10|HM876137|MM14502|Eucosma_hohenwartiana
TLYFIFGIWSGMVGTSLSLLIRAELGNPGYLIGDDQIYNTIVTAHAFIMIFFMVMPIMIGGFGNWLVPLMLGAPDMAFPRMNNMSFWLLPPSIMLLISSSMVENGAGTGWTVYPPLSSNIAHSGSSVDLAIFSLHLAGISSILGAVNFITTIINMRPNNM
SLDQMPLFIWAVGITALLLLLSLPVLAGAITMLLTDRNLNTSFFDPAGGGDPILYQHL
>LEFIJ1605-12|KM572998|MM22621|Eucosma_hohenwartiana
TLYFIFGIWAGMVGTSLSLLIRAELGNPGYLIGDDQIYNTIVTAHAFIMIFFMVMPIMIGGFGNWLVPLMLGAPDMAFPRMNNMSFWLLPPSIMLLISSSMVENGAGTGWTVYPPLSSNIAHSGSSVDLAIFSLHLAGISSILGAVNFITTIINMRPNNM
SLDQMPLFIWAVGITALLLLLSLPVLAGAITMLLTDRNLNTSFFDPAGGGDPILYQHL
>LEFIJ1606-12|KM572351|MM22622|Eucosma_hohenwartiana
TLYFIFGIWSGMVGTSLSLLIRAELGNPGYLIGDDQIYNTIVTAHAFIMIFFMVMPIMIGGFGNWLVPLMLGAPDMAFPRMNNMSFWLLPPSIMLLISSSMVENGAGTGWTVYPPLSSNIAHSGSSVDLAIFSLHLAGISSILGAVNFITTIINMRPNNM
SLDQMPLFIWA?GITALLLLLSLPVLAGAITMLLTDRNLNTSFFDPAGGGDPILYQHL
>LEEUA240-11|JN274937|MM19648|Eucosma_krygeri
TLYFIFGIWAGMIGTSLSLLIRAELGTPGSLIGDDQIYNTIVTAHAFIMIFFMVMPIMIGGFGNWLVPLMLGAPDMAFPRMNNMSFWLLPPSIMLLISSSIVENGAGTGWTVYPPLSSNIAHSGSSVDLAIFSLHLAGISSILGAVNFITTIINMRPNNMSL
DQMPLFVWAVGITALLLLLSLPVLAGAITMLLTDRNLNTSFFDPAGGGDPILYQHL
>LEFIE616-10|HM874339|MM09535|Eucosma_lacteana
TLYFIFGIWAGMIGTSLSLLIRAELGNPGSLIGDDQIYNTIVTAHAFIMIFFMVMPIMIGGFGNWLVPLMLGAPDMAFPRMNNMSFWLLPPSIMLLISSSIVENGAGTGWTVYPPLSSNIAHSGSSVDLAIFSLHLAGISSILGAVNFITTIINMRPNNMSL
DQMPLFVWAVGITALLLLLSLPVLAGAITMLLTDRNLNTSFFDPAGGGDPILYQHL
>LEFIF857-10|HM875539|MM13224|Eucosma_lacteana
TLYFIFGIWAGMIGTSLSLLIRAELGNPGSLIGDDQIYNTIVTAHAFIMIFFMVMPIMIGGFGNWLVPLMLGAPDMAFPRMNNMSFWLLPPSIMLLISSSIVENGAGTGWTVYPPLSSNIAHSGSSVDLAIFSLHLAGISSILGAVNFITTIINMRPNNMSL
DQMPLFVWAVGITALLLLLSLPVLAGAITMLLTDRNLNTSFFDPAGGGDPILYQHL
>LEFIL712-10|JF854698|MM13150|Eucosma_messingiana
TLYFIFGIWAGMIGTSLSLLIRAELGNPGSLIGDDQIYNTIVTAHAFIMIFFMVMPIMIGGFGNWLVPLMLGAPDMAFPRMNNMSFWLLPPSIMLLISSSIVENGAGTGWTVYPPLSSNIAHSGSSVDLAIFSLHLAGISSILGAINFITTIINMRPNNMSLD

QMPLFVWAVGITALLLLLSLPVLAGAITMLLTDRNLNTSFFDPAGGGDPILYQHL
>LEFIA641-10|HM870890|MM01756|Eucosma_metzneriana
TLYFIFGIWAGMIGTSLSLLIRAELGTPGSLIGDDQIYNTIVTAHAFIMIFFMVMPIMIGGFGNWLVPLMLGAPDMAFPRMNNMSFWLLPPSIMLLISSSIVENGAGTGWTVYPPLSSNIAHSGSSVDLAIFSLHLAGISSILGAVNFITTIINMRPNNMSL
DQMPLFVWAVGITALLLLLSLPVLAGAITMLLTDRNLNTSFFDPAGGGDPILYQHL
>LEFIJ040-10|MM02168|Eucosma_obumbratana
TLYFIFGIWAGMVGTSLSLLIRAELGNPGSLIGDDQIYNTIVTAHAFIMIFFMVMPIMIGGFGNWLVPLMLGAPDMAFPRMNNMSFWLLPPSIMLLISSSIVENGAGTGWTVYPPLSSNIAHSGSSVDLAIFSLHLAGISSILGAVNFITTIINMRPNNMSL
DQMPLFVWAVGITALLLLLSLPVLAGAITMLLTDRNLNTSFFDPAGGGDPILYQHL
>LEFID804-10|HM873561|MM06888|Eucosma_obumbratana
TLYFIFGIWAGMIGTSLSLLIRAELGNPGSLIGDDQIYNTIVTAHAFIMIFFMVMPIMIGGFGNWLVPLMLGAPDMAFPRMNNMSFWLLPPSIMLLISSSIVENGAGTGWTVYPPLSSNIAHSGSSVDLAIFSLHLAGISSILGAVNFITTIINMRPNNMSL
DQMPLFVWAVGITALLLLLSLPVLAGAITMLLTDRNLNTSFFDPAGGGDPILYQHL
>LEFIJ156-10|JF853469|MM10329|Eucosma_obumbratana
TLYFIFGIWAGMVGTSLSLLIRAELGNPGSLIGDDQIYNTIVTAHAFIMIFFMVMPIMIGGFGNWLVPLMLGAPDMAFPRMNNMSFWLLPPSIMLLISSSIVENGAGTGWTVYPPLSSNIAHSGSSVDLAIFSLHLAGISSILGAVNFITTIINMRPNNMSL
DQMPLFVWAVGITALLLLLSLPVLAGAITMLLTDRNLNTSFFDPAGGGDPILYQHL
>LEFIF563-10|HM875248|MM12303|Eucosma_obumbratana
TLYFIFGIWAGMVGTSLSLLIRAELGNPGSLIGDDQIYNTIVTAHAFIMIFFMVMPIMIGGFGNWLVPLMLGAPDMAFPRMNNMSFWLLPPSIMLLISSSIVENGAGTGWTVYPPLSSNIAHSGSSVDLAIFSLHLAGISSILGAVNFITTIINMRPNNMSL
DQMPLLVWAVGITALLLLLSLPVLAGAITMLLTDRNLNTSFFDPAGGGDPILYQHL
>LEFIJ186-10|MM12306|Eucosma_obumbratana
TLYFIFGIWAGMVGTSLSLLIRAELGNPGSLIGDDQIYNTIVTAHAFIMIFFMVMPIMIGGFGNWLVPLMLGAPDMAFPRMNNMSFWLLPPSIMLLISSSIVENGAGTGWTVYPPLSSNIAHSGSSVDLAIFSLHLAGISSILGAVNFITTIINMRPNNMSL
DQMPLFVWAVGITALLLLLSLPVLAGAITMLLTDRNLNTSFFDPAGGGDPILYQHL
>LEFIK768-10|JN274956|MM18343|Eucosma_pupillana
TLYFIFGIWAGMIGTSLSLLIRAELGNPGSLIGDDQIYNTIVTAHAFIMIFFMVMPIMIGGFGNWLVPLMLGAPDMAFPRMNNMSFWLLPPSIMLLISSSIVENGAGTGWTVYPPLSSNIAHSGSSVDLAIFSLHLAGISSILGAVNFITTIINMRPNNMSL
DQMPLFVWAVGITALLLLLSLPVLAGAITMLLTDRNLNTSFFDPAGGGDPILYQHL
>LEFIE929-10|HM874646|MM10308|Eucosma_saussureana
TLYFIFGIWAGMIGTSLSLLIRAELGNPGSLIGDDQIYNTIVTAHAFIMIFFMVMPIMIGGFGNWLVPLMLGAPDMAFPRMNNMSFWLLPPSIMLLISSSIVENGAGTGWTVYPPLSSNIAHGGSSVDLAIFSLHLAGISSILGAINFITTIINMRPNNMSL
DQMPLFVWAVGITALLLLLSLPVLAGAITMLLTDRNLNTSFFDPAGGGDPILYQHL
>LEFIG864-10|HM876512|MM15728|Eucosma_saussureana
TLYFIFGIWAGMIGTSLSLLIRAELGNPGSLIGDDQIYNTIVTAHAFIMIFFMVMPIMIGGFGNWLVPLMLGAPDMAFPRMNNMSFWLLPPSIMLLISSSIVENGAGTGWTVYPPLSSNIAHSGSSVDLAIFSLHLAGISSILGAINFITTIINMRPNNMSLD
QMPLFVWAVGITALLLLLSLPVLAGAITMLLTDRNLNTSFFDPAGGGDPILYQHL
>TYPFN051-11|MM16883|Eucosma_scorzonerana
TLYFIFGIWAGMVGTSLSLLIRAELGNPGSLIGDDQIYNTIVTAHAFIMIFFMVMPIMIGGFGNWLVPLMLGAPDMAFPRMNNMSFWLLPPSIMLLISSSIVENGAGTGWTVYPPLSSNIAHSGSSVDLAIFSLHLAGISSILGAVNFITTIINMRPNNMSL
DQMPLFVWAVGITALLLLLSLPVLAGAITMLLTDRNLNTSFFDPAGGGDPILYQHL
>LEFID084-10|HM872895|MM05696|Eucosma_suomiana
TLYFIFGIWAGMIGTSLSLMIRAELGNPGSLIGDDQIYNTIVTAHAFIMIFFMVMPIMIGGFGNWLVPLMLGAPDMAFPRMNNMSFWLLPPSIMLLISSSIVENGAGTGWTVYPPLSSNIAHSGSSVDLAIFSLHLAGISSILGAVNFITTIINMRPNNMS
LDQMPLFVWAVGITALLLLLSLPVLAGAITMLLTDRNLNTSFFDPAGGGDPILYQHL
>LEFIF249-10|HM874942|MM11030|Eucosma_suomiana
TLYFIFGIWAGMIGTSLSLMIRAELGNPGSLIGDDQIYNTIVTAHAFIMIFFMVMPIMIGGFGNWLVPLMLGAPDMAFPRMNNMSFWLLPPSIMLLISSSIVENGAGTGWTVYPPLSSNIAHSGSSVDLAIFSLHLAGISSILGAVNFITTIINMRPNNMS
LDQMPLFVWAVGITALLLLLSLPVLAGAITMLLTDRNLNTSFFDPAGGGDPILYQHL
>LEFIG299-10|HM875978|MM14257|Eucosmomorpha_albersana
TLYFIFGVWAGMVGTSLSLLIRAELGNPGSLIGDDQIYNTIVTAHAFIMIFFMVMPIMIGGFGNWLVPLMLGAPDMAFPRMNNMSYWLLPPSIMLLISSSVVENGAGTGWTVYPPLSSNIAHSGSSVDLAIFSLHLAGISSILGAVNFITTIINMRPNNM
SLDQMPLFVWAVGITALLLLLSLPVLAGAITMLLTDRNLNTSFFDPAGGGDPILYQHL
>LEFIC893-10|HM872710|MM05198|Eudemis_porphyrana
TMYFIFGIWAGMVGTSLSLLIRAELGNPGSLIGDDQIYNTIVTAHAFIMIFFMVMPIMIGGFGNWLVPLMLGAPDMAFPRMNNMSFWLLPPSIMLLISSSIVENGAGTGWTVYPPLSSNIAHSGSSVDLAIFSLHLAGISSILGAVNFITTIINMRPNNMS
LDQMPLFVWAVGITALLLLLSLPVLAGAITMLLTDRNLNTSFFDPAGGGDPILYQHL
>LEFIC891-10|HM872708|MM05194|Eudemis_profundana
TMYFIFGIWAGMMGTSLSLLIRAELGNPGSLIGDDQIYNTIVTAHAFIMIFFMVMPIMIGGFGNWLVPLMLGAPDMAFPRMNNMSFWLLPPSIMLLISSSIVENGAGTGWTVYPPLSSNIAHGGSSVDLAIFSLHLAGISSILGAINFITTIINMRPNNM
SLDQMPLFIWAVGITALLLLLSLPVLAGAITMLLTDRNLNTSFFDPAGGGDPILYQHL
>LEFIE705-10|HM874426|MM09697|Eudemis_profundana

TMYFIFGIWAGMVGTSLSLLIRAELGNPGSLIGDDQIYNTIVTAHAFIMIFFMVMPIMIGGFGNWLVPLMLGAPDMAFPRMNNMSFWLLPPSIMLLISSSIVENGAGTGWTVYPPLSSNIAHSGSSVDLAIFSLHLAGISSILGAVNFITTIINMRPNNMS
LDQMPLFVWAVGITALLLLLSLPVLAGAITMLLTDRNLNTSFFDPAGGGDPILYQHL
>LEFIE706-10|HM874427|MM09698|Eudemis_profundana
TMYFIFGIWAGMVGTSLSLLIRAELGNPGSLIGDDQIYNTIVTAHAFIMIFFMVMPIMIGGFGNWLVPLMLGAPDMAFPRMNNMSFWLLPPSIMLLISSSIVENGAGTGWTVYPPLSSNIAHSGSSVDLAIFSLHLAGISSILGAVNFITTIINMRPNNMS
LDQMPLFVWAVGITALLLLLSLPVLAGAITMLLTDRNLNTSFFDPAGGGDPILYQHL
>LEFIG919-10|HM876563|MM15783|Eudonia_aequalis
TLYFIFGIWAGMIGTSLSLLIRAELGNPGSLIGDDQIYNTIVTAHAFIMIFFMVMPIMIGGFGNWLIPLMLGAPDMAFPRMNNMSFWLLPPSLTLLISSSIVENGAGTGWTVYPPLSSNIAHSGSSVDLAIFSLHLAGISSILGAINFITTIINMRINGLSFDQ
MPLFVWAVGITALLLLLSLPVLAGAITMLLTDRNLNTSFFDPAGGGDPILYQHL
>LEFIB191-10|HM871095|MM00622|Eudonia_alpina
TLYFIFGIWAGMVGTSLSLLIRAELGNPGSLIGNDQIYNTIVTAHAFIMIFFMVMPIMIGGFGNWLVPLMLGAPDMAFPRMNNMSFWLLPPSLTLLISSSIVENGAGTGWTVYPPLSSNIAHSGSSVDLAIFSLHLAGISSILGAINFITTIINMRINGLSFD
QMPLFVWAVGITALLLLLSLPILAGAITMLLTDRNLNTSFFDPAGGGDPILYQHL
>LEFID438-10|HQ570334|MM06370|Eudonia_alpina
TLYFIFGIWAGMVGTSLSLLIRAELGNPGSLIGNDQIYNTIVTAHAFIMIFFMVMPIMIGGFGNWLVPLMLGAPDMAFPRMNNMSFWLLPPSLTLLISSSIVENGAGTGWTVYPPLSSNIAHSGSSVDLAIFSLHLAGISSILGAINFITTIINMRINGLSFD
QMPLFVWAVGITALLLLLSLPILAGAITMLLTDRNLNTSFFDPAGGGDPILYQHL
>LEFIC804-10|HM872623|MM04969|Eudonia_lacustrata
TLYFIFGIWAGMIGTSLSLLIRAELGNPGSLIGDDQIYNTIVTAHAFIMIFFMVMPIMIGGFGNWLIPLMLGAPDMAFPRMNNMSFWLLPPSLTLLISSSIVENGAGTGWTVYPPLSSNIAHSGSSVDLAIFSLHLAGISSILGAINFITTIINMRINGLSFDQ
MPLFVWAVGITALLLLLSLPVLAGAITMLLTDRNLNTSFFDPAGGGDPILYQHL
>LEFIF924-10|HM875606|MM13363|Eudonia_laetella
TLYFIFGIWAGMVGTSLSLLIRAELGNPGSLIGDDQIYNTIVTAHAFIMIFFMVMPIMIGGFGNWLIPLMLGAPDMAFPRMNNMSFWLLPPSLTLLISSSIVENGAGTGWTVYPPLSSNIAHSGSSVDLAIFSLHLAGISSILGAINFITTIINMRINSLSFDQ
MPLFVWAVGITALLLLLSLPVLAGAITMLLTDRNLNTSFFDPAGGGDPILYQHL
>LEFIJ599-10|JF853699|MM17224|Eudonia_laetella
TLYFIFGIWAGMVGTSLSLLIRAELGNPGSLIGDDQIYNTIVTAHAFIMIFFMVMPIMIGGFGNWLIPLMLGAPDMAFPRMNNMSFWLLPPSLTLLISSSIVENGAGTGWTVYPPLSSNIAHSGSSVDLAIFSLHLAGISSILGAINFITTIINMRINSLSFDQ
MPLFVWAVGITALLLLLSLPVLAGAITMLLTDRNLNTSFFDPAGGGDPILYQHL
>LEFIJ1249-11|KM573604|MM21109|Eudonia_laetella
TLYFIFGIWAGMVGTSLSLLIRAELGNPGSLIGDDQIYNTIVTAHAFIMIFFMVMPIMIGGFGNWLIPLMLGAPDMAFPRMNNMSFWLLPPSLTLLISSSIVENGAGTGWTIYPPLSSNIAHSGSSVDLAIFSLHLAGISSILGAINFITTIINMRINSLSFDQ
MPLFVWAVGITALLLLLSLPVLAGAITMLLTDRNLNTSFFDPAGGGDPILYQHL
>LEFID636-10|HM873399|MM06660|Eudonia_mercurella
TLYFIFGIWAGMVGTSLSLLIRAELGNPGSLIGDDQIYNTIVTAHAFIMIFFMVMPIMIGGFGNWLVPLMLGAPDMAFPRMNNMSFWLLPPSLTLLISSSIVENGAGTGWTVYPPLSSNIAHGGSSVDLAIFSLHLAGISSILGAINFITTIINMRINGLSFD
QMPLFVWAVGITALLLLLSLPVLAGAITMLLTDRNLNTSFFDPAGGGDPILYQHL
>LEFIK814-10|JN279061|MM18389|Eudonia_mercurella
TLYFIFGIWAGMVGTSLSLLIRAELGNPGSLIGDDQIYNTIVTAHAFIMIFFMVMPIMIGGFGNWLVPLMLGAPDMAFPRMNNMSFWLLPPSLTLLISSSIVENGAGTGWTVYPPLSSNIAHGGSSVDLAIFSLHLAGISSILGAINFITTIINMRINGLSFD
QMPLFVWAVGITALLLLLSLPVLAGAITMLLTDRNLNTSFFDPAGGGDPILYQHL
>LEFIB881-10|HM871758|MM02954|Eudonia_murana
TLYFIFGIWAGMVGTSLSLLIRAELGNPGSLIGDDQIYNTIVTAHAFIMIFFMVMPIMIGGFGNWLIPLMLGAPDMAFPRMNNMSFWLLPPSLTLLISSSIVENGAGTGWTVYPPLSSNIAHSGSSVDLAIFSLHLAGISSILGAINFITTIINMRINNLSFDQ
MPLFVWAVGITALLLLLSLPVLAGAITMLLTDRNLNTSFFDPAGGGDPILYQHL
>LEFIC196-10|HM872040|MM03557|Eudonia_pallida
TLYFIFGIWAGMLGTSLSLLIRAELGNPGSLIGDDQIYNTIVTAHAFIMIFFMVMPIMIGGFGNWLIPLMLGAPDMAFPRMNNMSFWLLPPSLTLLISSSIVENGAGTGWTVYPPLSSNIAHSGSSVDLAIFSLHLAGISSILGAINFITTIINMRINGLFFDQ
MPLFIWAVGITALLLLLSLPVLAGAITMLLTDRNLNTSFFDPAGGGDPILYQHL
>LEFIB248-10|HM871151|MM00717|Eudonia_sudetica
TLYFIFGVWAGTVGTSLSLLIRAELGNPGSLIGDDQIYNTIVTAHAFIMIFFMVMPIMIGGFGNWLIPLMLGAPDMAFPRMNNMSFWLLPPSLTLLISSSIVENGAGTGWTVYPPLSSNIAHSGSSVDLAIFSLHLAGISSILGAINFITTIINMRINNLSFDQ
MPLFVWAVGITALLLLLSLPILAGAITMLLTDRNLNTSFFDPAGGGDPILYQHL
>LEFIB282-10|HM871184|MM00771|Eudonia_truncicolella
TLYFIFGIWAGMIGTSLSLLIRAELGNPGSLIGDDQIYNTIVTAHAFIMIFFMVMPIMIGGFGNWLIPLMLGAPDMAFPRMNNMSFWLLPPSLTLLISSSIVENGAGTGWTVYPPLSSNIAHSGSSVDLAIFSLHLAGISSILGAINFITTIINMRINGLSFDQ
MPLFIWAVGITALLLLLSLPVLAGAITMLLTDRNLNTSFFDPAGGGDPILYQHL
>LEFIC582-10|HM872403|MM04364|Eugnorisma_depuncta
TLYFIFGIWAGMVGTSLSLLIRAELGNPGSLIGDDQIYNTIVTAHAFIMIFFMVMPIMIGGFGNWLVPLMLGAPDMAFPRMNNMSFWLLPPSLTLLISSSIVENGAGTGWTVYPPLSSNIAHGGSSVDLAIFSLHLAGISSILGAINFITTIINMRLNNLSFD
QMPLFIWAVGITAFLLLLSLPVLAGAITMLLTDRNLNTSFFDPAGGGDPILYQHL

>LEFIF678-10|HM875362|MM12668|Eugnorisma_depuncta
TLYFIFGIWAGMVGTSLSLLIRAELGNPGSLIGDDQIYNTIVTAHAFIMIFFMVMPIMIGGFGNWLVPLMLGAPDMAFPRMNNMSFWLLPPSLTLLISSSIVENGAGTGWTVYPPLSSNIAHGGSSVDLAIFSLHLAGISSILGAINFITTIINMRLNNLSFD
QMPLFIWAVGITAFLLLLSLPVLAGAITMLLTDRNLNTSFFDPAGGGDPILYQHL
>LEFIL080-10|JF854441|MM19080|Eugnorisma_glareosa
TLYFIFGIWAGMVGTSLSLLIRAELGNPGSLIGDDQIYNTIVTAHAFIMIFFMVMPIMIGGFGNWLVPLMLGAPDMAFPRMNNMSFWLLPPSLTLLISSSIVENGAGTGWTVYPPLSSNIAHGGSSVDLAIFSLHLAGISSILGAINFITTIINMRLNSLSFD
QMPLFIWAVGITAFLLLLSLPVLAGAITMLLTDRNLNTSFFDPAGGGDPILYQHL
>LEFIJ363-10|KM572336|MM15963|Eugraphe_sigma
T?YFIFGIWAGMVGTSLSLLIRAELGNPGSLIGDDQIYNTIVTAHAFIMIFFMVMPIMIGGFGNWLVPLMLGAPDMAFPRMNNMSFWLLPPSLTLLISSSIVENGAGTGWTVYPPLSSNIAHGGSSVDLAIFSLHLAGISSILGAINFITTIINMRLNNLFFD
QMPLFIWAVGITAFLLLLSLPVLAGAITMLLTDRNLNTSFFDPAGGGDPILYQHL
>LEFIL174-10|JF854511|MM19174|Eugraphe_sigma
TLYFIFGIWAGMVGTSLSLLIRAELGNPGSLIGDDQIYNTIVTAHAFIMIFFMVMPIMIGGFGNWLVPLMLGAPDMAFPRMNNMSFWLLPPSLTLLISSSIVENGAGTGWTVYPPLSSNIAHGGSSVDLAIFSLHLAGISSILGAINFITTIINMRLNSLFFD
QMPLFIWAVGITAFLLLLSLPVLAGAITMLLTDRNLNTSFFDPAGGGDPILYQHL
>LEFII271-11|KM572714|MM19921|Eugraphe_sigma
TLYFIFGIWAGMVGTSLSLLIRAELGNPGSLIGDDQIYNTIVTAHAFIMIFFMVMPIMIGGFGNWLVPLMLGAPDMAFPRMNNMSFWLLPPSLTLLISSSIVENGAGTGWTVYPPLSSNIAHGGSSVDLAIFSLHLAGISSILGAINFITTIINMRLNSLFFD
QMPLFIWAVGITAFLLLLSLPVLAGAITMLLTDRNLNTSFFDPAGGGDPILYQHL
>LEFID639-10|HM873402|MM06664|Euhyponomeuta_stannella
TLYFIFGIWSGMVGTSLSLLIRAELGNPGSLIGDDQIYNTIVTAHAFIMIFFMVMPIMIGGFGNWLVPLMLGAPDMAFPRMNNMSFWLLPPSLILLISSSMVETGAGTGWTVYPPLSSNIAHSGSSVDMAIFSLHLAGISSILGAINFITTIINMKSNGMSF
DQMPLFVWSVGITALLLLLSLPVLAGAITMLLTDRNLNTSFFDPAGGGDPILYQHL
>LEFIB965-10|HM871840|MM03145|Euhyponomeutoides_albithoracellus
TLYFIFGIWSGMVGTSLSLLIRAELGNPGSLIGDDQIYNTIVTAHAFIMIFFMVMPIMIGGFGNWLIPLMLGAPDMAFPRMNNMSFWLLPPSLTLLISSSIVENGAGTGWTVYPPLSSNIAHSGSSVDLAIFSLHLAGISSILGAINFITTIINMKSNGMMFD
QMPLFVWAVGITALLLLLSLPVLAGAITMLLTDRNLNTSFFDPAGGGDPILYQHL
>LEFIF987-10|HM875668|MM13587|Euhyponomeutoides_albithoracellus
TLYFIFGIWSGMVGTSLSLLIRAELGNPGSLIGDDQIYNTIVTAHAFIMIFFMVMPIMIGGFGNWLIPLMLGAPDMAFPRMNNMSFWLLPPSLTLLISSSIVENGAGTGWTVYPPLSSNIAHSGSSVDLAIFSLHLAGISSILGAINFITTIINMKSNGMMFD
QMPLFVWAVGITALLLLLSLPVLAGAITMLLTDRNLNTSFFDPAGGGDPILYQHL
>LEFID019-10|HM872833|MM05446|Euhyponomeutoides_ribesiella
TLYFIFGIWSGMVGTSLSLLIRAELGNPGSLIGDDQIYNTIVTAHAFIMIFFMVMPIMIGGFGNWLVPLMLGAPDMAFPRMNNMSFWLLPPSLTLLICSSIVENGAGTGWTVYPPLSSNIAHSGSSVDLAIFSLHLAGISSILGAINFITTIINMKSNGMTFD
QMPLFVWAVGITALLLLLSLPVLAGAITMLLTDRNLNTSFFDPAGGGDPILYQHL
>LEFIB724-10|HM871602|MM02544|Eulamprotes_atrella
TLYFIFGIWAGMVGTSLSLLIRAELGNPGSLIGDDQIYNTIVTAHAFIMIFFMVMPIMIGGFGNWLVPLMLGAPDMAFPRMNNMSFWLLPPSLTLLISSSIVENGAGTGWTVYPPLSSNIAHSGSSVDLAIFSLHLAGISSILGAINFITTIINMKIKGLSFDQ
MPLFVWSVGITALLLLLSLPVLAGAITMLLTDRNLNTSFFDPAGGGDPILYQHL
>LEFIG752-10|HM876407|MM15616|Eulamprotes_superbella
TLYFIFGIWAGLVGTSLSLLIRAELGTPGSLIGDDQIYNTIVTAHAFIMIFFMVMPIMIGGFGNWLVPLMLGAPDMAFPRMNNMSFWLLPPSLTLLISSSIVENGAGTGWTVYPPLSSNIAHSGSSVDLAIFSLHLAGISSILGAINFITTIINMKINGLSFDQ
MPLFVWSVGITALLLLLSLPVLAGAITMLLTDRNLNTSFFDPAGGGDPILYQHL
>LEFIG753-10|HM876408|MM15617|Eulamprotes_superbella
TLYFIFGIWAGLVGTSLSLLIRAELGTPGSLIGDDQIYNTIVTAHAFIMIFFMVMPIMIGGFGNWLVPLMLGAPDMAFPRMNNMSFWLLPPSLTLLISSSIVENGAGTGWTVYPPLSSNIAHSGSSVDLAIFSLHLAGISSILGAINFITTIINMKINGLSFDQ
MPLFVWSVGITALLLLLSLPVLAGAITMLLTDRNLNTSFFDPAGGGDPILYQHL
>LEFIB734-10|HM871612|MM02563|Eulamprotes_unicolorella
TLYFIFGIWAGMVGTSLSLLIRAELGNPGSLIGDDQIYNTIVTTHAFIMIFFMVMPIMIGGFGNWLVPLMLGAPDMAFPRMNNMSFWLLPPSLTLLISSSIVENGAGTGWTVYPPLSSNIAHGGSSVDLAIFSLHLAGISSILGAINFITTIINMKINGLSFD
QMPLFVWAVGITALLLLLSLPVLAGAITMLLTDRNLNTSFFDPAGGGDPILYQHL
>LEFIF499-10|HM875184|MM12068|Eulamprotes_wilkella
TLYFIFGIWAGLIGTSLSLLIRAELGTPGSLIEDDQIYNTIVTAHAFIMIFFMVMPIMIGGFGNWLVPLMLGAPDMAFPRMNNMSFWLLPPSLTLLISSSIVENGAGTGWTVYPPLSSNIAHGGSSVDLAIFSLHLAGISSILGAINFITTIINMKISGLSFDQM
PLFVWSVGITALLLLLSLPVLAGAITMLLTDRNLNTSFFDPAGGGDPILYQHL
>LEFIG081-10|HM875760|MM13733|Eulamprotes_wilkella
TLYFIFGIWAGLIGTSLSLLIRAELGTPGSLIGDDQIYNTIVTAHAFIMIFFMVMPIMIGGFGNWLVPLMLGAPDMAFPRMNNMSFWLLPPSLTLLISSSIVENGAGTGWTVYPPLSSNIAHGGSSVDLAIFSLHLAGISSILGAINFITTIINMKISGLSFDQM
PLFIWSVGITTLLLLLSLPVLAGAITMLLTDRNLNTSFFDPAGGGDPILYQHL
>LEFIG474-10|HM876150|MM14521|Eulamprotes_wilkella
TLYFIFGIWAGLIGTSLSLLIRAELGTPGSLIGDDQIYNTIVTAHAFIMIFFMVMPIMIGGFGNWLVPLMLGAPDMAFPRMNNMSFWLLPPSLTLLISSSIVENGAGTGWTVYPPLSSNIAHGGSSVDLAIFSLHLAGISSILGAINFITTIINMKISGLSFDQM

PLFVWSVGITALLLLLSLPVLAGAITMLLTDRNLNTSFFDPAGGGDPILYQHL
>LEFIB024-10|HM870935|MM00064|Eulia_ministrana
TLYFIFGIWAGMIGTSLSMLIRAELGNPGYLIGDDQIYNTIVTAHAFIMIFFMVMPIMIGGFGNWLVPLMLGAPDMAFPRMNNMSFWLLPPSILLLISSSIVENGAGTGWTVYPPLSSNIAHSGSSVDLAIFSLHLAGISSILGAVNFITTIINMRPQNMSL
DQMPLFVWAVMITAILLLLSLPVLAGAITMLLTDRNLNTSFFDPAGGGDPILYQHL
>LEFIB026-10|HM870937|MM00109|Eulia_ministrana
TLYFIFGIWAGMIGTSLSMLIRAELGNPGYLIGDDQIYNTIVTAHAFIMIFFMVMPIMIGGFGNWLVPLMLGAPDMAFPRMNNMSFWLLPPSILLLISSSIVENGAGTGWTVYPPLSSNIAHSGSSVDLAIFSLHLAGISSILGAVNFITTIINMRPQNMSL
DQMPLFVWAVMITAVLLLLSLPVLAGAITMLLTDRNLNTSFFDPAGGGDPILYQHL
>LEFIA132-10|HM396479|MM01066|Eulia_ministrana
TLYFIFGIWAGMIGTSLSMLIRAELGNPGYLIGDDQIYNTIVTAHAFIMIFFMVMPIMIGGFGNWLVPLMLGAPDMAFPRMNNMSFWLLPPSILLLISSSIVENGAGTGWTVYPPLSSNIAHSGSSVDLAIFSLHLAGISSILGAVNFITTIINMRPQNMSL
DQMPLFVWAVIITAILLLLSLPVLAGAITMLLTDRNLNTSFFDPAGGGDPILYQHL
>LEFIA425-10|HM386767|MM01488|Eulithis_mellinata
TLYFIFGIWAGMIGTSLSLLIRAELGTPGSLIGDDQIYNTIVTAHAFIMIFFMVMPIMIGGFGNWLVPLMLGAPDMAFPRMNNMSFWLLPPSITLLISSSIVENGAGTGWTVYPPLSSNIAHGGSSVDLAIFSLHLAGISSILGAINFITTIINMRLNNMFFD
QLPLFVWAVGITAFLLLLSLPVLAGAITMLLTDRNLNTSFFDPAGGGDPILYQHL
>LEFIB846-10|HM871723|MM02830|Eulithis_mellinata
TLYFIFGIWAGMIGTSLSLLIRAELGTPGSLIGDDQIYNTIVTAHAFIMIFFMVMPIMIGGFGNWLVPLMLGAPDMAFPRMNNMSFWLLPPSITLLISSSIVENGAGTGWTVYPPLSSNIAHGGSSVDLAIFSLHLAGISSILGAINFITTIINMRLNNMFFD
QLPLFVWAVGITAFLLLLSLPVLAGAITMLLTDRNLNTSFFDPAGGGDPILYQHL
>LEFIB293-10|HM871194|MM00797|Eulithis_populata
TLYFIFGIWAGMIGTSLSLLIRAELGTPGSLIGDDQIYNTIVTAHAFIMIFFMVMPIMIGGFGNWLVPLMLGAPDMAFPRMNNMSFWLLPPSITLLISSSIVENGAGTGWTVYPPLSSNIAHSGSSVDLAIFSLHLAGISSILGAINFITTIINMRLNNMFFD
QLPLFVWAVGITAFLLLLSLPVLAGAITMLLTDRNLNTSFFDPAGGGDPILYQHL
>LEFIE024-10|HM873773|MM08084|Eulithis_populata
TLYFIFGIWAGMIGTSLSLLIRAELGTPGSLIGDDQIYNTIVTAHAFIMIFFMVMPIMIGGFGNWLVPLMLGAPDMAFPRMNNMSFWLLPPSITLLISSSIVENGAGTGWTVYPPLSSNIAHSGSSVDLAIFSLHLAGISSILGAINFITTIINMRLNNMFFD
QLPLFVWAVGITAFLLLLSLPVLAGAITMLLTDRNLNTSFFDPAGGGDPILYQHL
>LEFIB294-10|HM871195|MM00798|Eulithis_prunata
TLYFIFGIWAGMIGTSLSLLIRAELGTPGSLIGDDQIYNTIVTAHAFIMIFFMVMPIMIGGFGNWLVPLMLGAPDMAFPRMNNMSFWLLPPSITLLISSSIVENGAGTGWTVYPPLSSNIAHSGSSVDLAIFSLHLAGISSILGAINFITTIINMRLNNMFFD
QLPLFVWAVGITAFLLLLSLPVLAGAITMLLTDRNLNTSFFDPAGGGDPILYQHL
>LEFIA429-10|HM386770|MM01492|Eulithis_prunata
TLYFIFGIWAGMIGTSLSLLIRAELGTPGSLIGDDQIYNTIVTAHAFIMIFFMVMPIMIGGFGNWLVPLMLGAPDMAFPRMNNMSFWLLPPSITLLISSSIVENGAGTGWTVYPPLSSNIAHSGSSVDLAIFSLHLAGISSILGAINFITTIINMRLNNMFFD
QLPLFVWAVGITAFLLLLSLPVLAGAITMLLTDRNLNTSFFDPAGGGDPILYQHL
>LEFID211-10|HM873013|MM06041|Eulithis_pyropata
TLYFIFGIWAGMIGTSLSLLIRAELGTPGSLIGDDQIYNTIVTAHAFIMIFFMVMPIMIGGFGNWLVPLMLGAPDMAFPRMNNMSFWLLPPSITLLISSSIVENGAGTGWTVYPPLSSNIAHSGSSVDLAIFSLHLAGISSILGAINFITTIINMRLNNMFFD
QLPLFVWAVGITAFLLLLSLPVLAGAITMLLTDRNLNTSFFDPAGGGDPILYQHL
>LEFIB291-10|HM871192|MM00794|Eulithis_testata
TLYFIFGIWAGMIGTSLSLLIRAELGTPGSLIGDDQIYNTIVTAHAFIMIFFMVMPIMIGGFGNWLVPLMLGAPDMAFPRMNNMSFWLLPPSITLLISSSIVENGAGTGWTVYPPLSSNIAHSGSSVDLAIFSLHLAGISSILGAINFITTIINMRLNNMFFD
QLPLFVWAVGITAFLLLLSLPVLAGAITMLLTDRNLNTSFFDPAGGGDPILYQHL
>LEFIC009-10|HM871879|MM03212|Eumedonia_eumedon
TLYFIFGIWAGMVGTSLSILIRMELSTPGSLIGDDQIYNTIVTAHAFIMIFFMVMPIMIGGFGNWLVPLMLGAPDMAFPRMNNMSFWLLPPSLMLLISSSIVETGAGTGWTVYPPLSSNIAHSGSSVDLAIFSLHLAGISSILGAINFITTIINMRVNNLSFD
QLSLFIWAVGITALLLLLSLPVLAGAITMLLTDRNLNTSFFDPAGGGDPILYQHL
>LEFIG228-10|HM875908|MM14128|Eumedonia_eumedon
TLYFIFGIWAGMVGTSLSILIRMELSTPGSLIGDDQIYNTIVTAHAFIMIFFMVMPIMIGGFGNWLVPLMLGAPDMAFPRMNNMSFWLLPPSLMLLISSSIVETGAGTGWTVYPPLSSNIAHSGSSVDLAIFSLHLAGISSILGAINFITTIINMRVNNLSFD
QLSLFIWAVGITALLLLLSLPVLAGAITMLLTDRNLNTSFFDPAGGGDPILYQHL
>LEFID126-10|HM872936|MM05864|Euphydryas_aurinia
TLYFIFGIWAGMMGTSLSLLIRTELGNPGSLIGDDQIYNTIVTTHAFIMIFFMVMPIMIGGFGNWLIPLMLGAPDMAFPRMNNMSFWLLPPSLMLLISSSIVENGAGTGWTIYPPLSSNIAHSGSSVDLAIFSLHLAGISSILGAINFITTIINMRINNMSFD
QMPLFVWAVGITALLLLLSLPVLAGAITMLLTDRNINTSFFDPAGGGDPILYQHL
>LEFIA735-10|HM386879|MM04089|Euphydryas_iduna
TLYFIFGIWAGMVGTSLSLLIRTELGNPGSLIGDDQIYNTIVTAHAFIMIFFMVMPIMIGGFGNWLVPLMLGAPDMAFPRMNNMSFWLLPPSLMLLISSSIVENGAGTGWTVYPPLSSNIAHSGSSVDLAIFSLHLAGISSILGAINFITTIINMRINNMSF
DQMPLFVWAVGITALLLLLSLPVLAGAITMLLTDRNINTSFFDPAGGGDPILYQHL
>LEFID416-10|HM873212|MM06340|Euphydryas_iduna

TLYFIFGIWAGMVGTSLSLLIRTELGNPGSLIGDDQIYNTIVTAHAFIMIFFMVMPIMIGGFGNWLVPLMLGAPDMAFPRMNNMSFWLLPPSLMLLISSSIVENGAGTGWTVYPPLSSNIAHSGSSVDLAIFSLHLAGISSILGAINFITTIINMRINNMSF
DQMPLFVWAVGITALLLLLSLPVLAGAITMLLTDRNINTSFFDPAGGGDPILYQHL
>LEFIJ550-10|JF853665|MM17175|Euphydryas_maturna
TLYFIFGIWAGMVGTSLSLLIRTELGNPGSLIGDDQIYNTIVTAHAFIMIFFMVMPIMIGGFGNWLVPLMLGAPDMAFPRMNNMSFWLLPPSLMLLISSSIVENGAGTGWTVYPPLSSNIAHSGSSVDLAIFSLHLAGISSILGAINFITTIINMRVNNMSF
DQMPLFVWAVGITALLLLLSLPVLAGAITMLLTDRNINTSFFDPAGGGDPILYQHL
>LEFIL394-10|JF854581|MM18692|Euphydryas_maturna
TLYFIFGIWAGMVGTSLSLLIRTELGNPGSLIGDDQIYNTIVTAHAFIMIFFMVMPIMIGGFGNWLVPLMLGAPDMAFPRMNNMSFWLLPPSLMLLISSSIVENGAGTGWTVYPPLSSNIAHSGSSVDLAIFSLHLAGISSILGAINFITTIINMRVNNMSF
DQMPLFVWAVGITALLLLLSLPVLAGAITMLLTDRNINTSFFDPAGGGDPILYQHL
>LEFIA406-10|HM386748|MM01466|Euphyia_unangulata
TLYFIFGIWAGMVGTSLSLLIRAELGNPGSLIGNDQIYNTIVTAHAFIMIFFMVMPIMIGGFGNWLVPLMLGAPDMAFPRMNNMSFWLLPPSITLLISSSIVENGAGTGWTVYPPLSSNIAHGGSSVDLAIFSLHLAGISSILGAINFITTIINMRLNNMFF
DQLPLFVWAVGITAFLLLLSLPVLAGAITMLLTDRNLNTSFFDPAGGGDPILYQHL
>LEFIB858-10|HM871735|MM02880|Euphyia_unangulata
TLYFIFGIWAGMVGTSLSLLIRAELGNPGSLIGDDQIYNTIVTAHAFIMIFFMVMPIMIGGFGNWLVPLMLGAPDMAFPRMNNMSFWLLPPSITLLISSSIVENGAGTGWTVYPPLSSNIAHGGSSVDLAIFSLHLAGISSILGAINFITTIINMRLNNMFFD
QLPLFVWAVGITAFLLLLSLPVLAGAITMLLTDRNLNTSFFDPAGGGDPILYQHL
>LEFIE700-10|HM874421|MM09682|Euphyia_unangulata
TLYFIFGIWAGMVGTSLSLLIRAELGNPGSLIGDDQIYNTIVTAHAFIMIFFMVMPIMIGGFGNWLVPLMLGAPDMAFPRMNNMSFWLLPPSITLLISSSIVENGAGTGWTVYPPLSSNIAHGGSSVDLAIFSLHLAGISSILGAINFITTIINMRLNNMFFD
QLPLFVWAVGITAFLLLLSLPVLAGAITMLLTDRNLNTSFFDPAGGGDPILYQHL
>LEFID777-10|HM873534|MM06844|Eupithecia_abietaria
TLYFIFGIWAGMVGTSLSLLIRAELGTPGSLIGDDQIYNTIVTAHAFIMIFFMVMPIMIGGFGNWLIPLMLGAPDMAFPRMNNMSFWLLPPSITLLISSSIVENGAGTGWTVYPPLSSNIAHGGSSVDLAIFSLHLAGISSILGAINFITTIINMRLNNMFFD
QLPLFVWAVGITAFLLLLSLPVLAGAITMLLTDRNLNTSFFDPAGGGDPILYQHL
>LEFIA902-10|HM387038|MM09780|Eupithecia_abietaria
TLYFIFGIWAGMVGTSLSLLIRAELGTPGSLIGDDQIYNTIVTAHAFIMIFFMVMPIMIGGFGNWLIPLMLGAPDMAFPRMNNMSFWLLPPSITLLISSSIVENGAGTGWTVYPPLSSNIAHGGSSVDLAIFSLHLAGISSILGAINFITTIINMRLNNMFFD
QLPLFVWAVGITAFLLLLSLPVLAGAITMLLTDRNLNTSFFDPAGGGDPILYQHL
>LEFIA681-10|HM386827|MM01820|Eupithecia_absinthiata
TLYFIFGIWAGMIGTSLSLLIRAELGTPGSLIGDDQIYNTIVTAHAFIMIFFMVMPIMIGGFGNWLVPLMLGAPDMAFPRMNNMSFWLLPPSITLLISSSIVENGAGTGWTVYPPLSSNIAHGGSSVDLAIFSLHLAGISSILGAINFITTIINMRLNNMFFD
QLPLFVWAVGITAFLLLLSLPVLAGAITMLLTDRNLNTSFFDPAGGGDPILYQHL
>LEFIF762-10|HM875446|MM12972|Eupithecia_absinthiata
TLYFIFGIWAGMIGTSLSLLIRAELGTPGSLIGDDQIYNTIVTAHAFIMIFFMVMPIMIGGFGNWLVPLMLGAPDMAFPRMNNMSFWLLPPSITLLISSSIVENGAGTGWTVYPPLSSNIAHGGSSVDLAIFSLHLAGISSILGAINFITTIINMRLNNMFFD
QLPLFVWAVGITAFLLLLSLPVLAGAITMLLTDRNLNTSFFDPAGGGDPILYQHL
>LEFIJ392-10|JF853578|MM15997|Eupithecia_absinthiata
TLYFIFGIWAGMIGTSLSLLIRAELGTPGSLIGDDQIYNTIVTAHAFIMIFFMVMPIMIGGFGNWLVPLMLGAPDMAFPRMNNMSFWLLPPSITLLISSSIVENGAGTGWTVYPPLSSNIAHGGSSVDLAIFSLHLAGISSILGAINFITTIINMRLNNMFFD
QLPLFVWAVGITAFLLLLSLPVLAGAITMLLTDRNLNTSFFDPAGGGDPILYQHL
>LEFIJ393-10|JF853579|MM15998|Eupithecia_absinthiata
TLYFIFGIWAGMIGTSLSLLIRAELGTPGSLIGDDQIYNTIVTAHAFIMIFFMVMPIMIGGFGNWLVPLMLGAPDMAFPRMNNMSFWLLPPSITLLISSSIVENGAGTGWTVYPPLSSNIAHGGSSVDLAIFSLHLAGISSILGAINFITTIINMRLNNMFFD
QLPLFVWAVGITAFLLLLSLPVLAGAITMLLTDRNLNTSFFDPAGGGDPILYQHL
>LEFIJ619-10|JF853719|MM17244|Eupithecia_absinthiata
TLYFIFGIWAGMIGTSLSLLIRAELGTPGSLIGDDQIYNTIVTAHAFIMIFFMVMPIMIGGFGNWLVPLMLGAPDMAFPRMNNMSFWLLPPSITLLISSSIVENGAGTGWTVYPPLSSNIAHSGSSVDLAIFSLHLAGISSILGAINFITTIINMRLNNMFFD
QLPLFVWAVGITAFLLLLSLPVLAGAITMLLTDRNLNTSFFDPAGGGDPILYQHL
>LEFIA663-10|HM870911|MM01793|Eupithecia_actaeata
TLYFIFGIWAGMIGTSLSLLIRAELGTPGSLIGDDQIYNTIVTAHAFIMIFFMVMPIMIGGFGNWLVPLMLGAPDMAFPRMNNMSFWLLPPSITLLISSSIVENGAGTGWTVYPPLSSNIAHGGSSVDLAIFSLHLAGISSILGAINFITTIINMRLNNMFFD
QLPLFVWAVGITAFLLLLSLPVLAGAITMLLTDRNLNTSFFDPAGGGDPILYQHL
>LEFIC697-10|HM872518|MM04685|Eupithecia_actaeata
TLYFIFGIWAGMIGTSLSLLIRAELGTPGSLIGDDQIYNTIVTAHAFIMIFFMVMPIMIGGFGNWLVPLMLGAPDMAFPRMNNMSFWLLPPSITLLISSSIVENGAGTGWTVYPPLSSNIAHGGSSVDLAIFSLHLAGISSILGAINFITTIINMRLNNMFFD
QLPLFVWAVGITAFLLLLSLPVLAGAITMLLTDRNLNTSFFDPAGGGDPILYQHL
>LEFIF328-10|HM875013|MM11133|Eupithecia_actaeata
TLYFIFGIWAGMIGTSLSLLIRAELGTPGSLIGDDQIYNTIVTAHAFIMIFFMVMPIMIGGFGNWLVPLMLGAPDMAFPRMNNMSFWLLPPSITLLISSSIVENGAGTGWTVYPPLSSNIAHGGSSVDLAIFSLHLAGISSILGAINFITTIINMRLNNMFFD
QLPLFVWAVGITAFLLLLSLPVLAGAITMLLTDRNLNTSFFDPAGGGDPILYQHL

>LEFID034-10|HM872848|MM05486|Eupithecia_analoga
TLYFIFGIWAGMVGTSLSLLIRAELGTPGSLIGDDQIYNTIVTAHAFIMIFFMVMPIMIGGFGNWLIPLMLGAPDMAFPRMNNMSFWLLPPSITLLISSSIVENGAGTGWTVYPPLSSNIAHGGSSVDLAIFSLHLAGISSILGAINFITTIINMRLNNMFFD
QLPLFVWAVGITAFLLLLSLPVLAGAITMLLTDRNLNTSFFDPAGGGDPILYQHL
>LEFID518-10|HM873283|MM06490|Eupithecia_assimilata
TLYFIFGIWAGMIGTSLSLLIRAELGTPGSLIGDDQIYNTIVTAHAFIMIFFMVMPIMIGGFGNWLVPLMLGAPDMAFPRMNNMSFWLLPPSITLLISSSIVENGAGTGWTVYPPLSSNIAHGGSSVDLAIFSLHLAGISSILGAINFITTIINMRLNNMFFD
QLPLFVWAVGITAFLLLLSLPVLAGAITMLLTDRNLNTSFFDPAGGGDPILYQHL
>LEFID674-10|HM873435|MM06714|Eupithecia_assimilata
TLYFIFGIWAGMIGTSLSLLIRAELGTPGSLIGDDQIYNTIVTAHAFIMIFFMVMPIMIGGFGNWLVPLMLGAPDMAFPRMNNMSFWLLPPSITLLISSSIVENGAGTGWTVYPPLSSNIAHGGSSVDLAIFSLHLAGISSILGAINFITTIINMRLNNMFFD
QLPLFVWAVGITAFLLLLSLPVLAGAITMLLTDRNLNTSFFDPAGGGDPILYQHL
>LEFIG226-10|HM875906|MM14124|Eupithecia_assimilata
TLYFIFGIWAGMIGTSLSLLIRAELGTPGSLIGDDQIYNTIVTAHAFIMIFFMVMPIMIGGFGNWLVPLMLGAPDMAFPRMNNMSFWLLPPSITLLISSSIVENGAGTGWTVYPPLSSNIAHGGSSVDLAIFSLHLAGISSILGAINFITTIINMRLNNMFFD
QLPLFVWAVGITAFLLLLSLPVLAGAITMLLTDRNLNTSFFDPAGGGDPILYQHL
>LEFIG954-10|HM876594|MM15818|Eupithecia_cauchiata
TLYFIFGIWAGMIGTSLSLLIRAELGTPGSLIGDDQIYNTIVTAHAFIMIFFMVMPIMIGGFGNWLVPLMLGAPDMAFPRMNNMSFWLLPPSITLLISSSIVENGAGTGWTVYPPLSSNIAHGGSSVDLAIFSLHLAGISSILGAINFITTIINMRLNNMFFD
QLPLFVWAVGITAFLLLLSLPVLAGAITMLLTDRNLNTSFFDPAGGGDPILYQHL
>LEFIG955-10|HM876595|MM15819|Eupithecia_cauchiata
TLYFIFGIWAGMIGTSLSLLIRAELGTPGSLIGDDQIYNTIVTAHAFIMIFFMVMPIMIGGFGNWLVPLMLGAPDMAFPRMNNMSFWLLPPSITLLISSSIVENGAGTGWTVYPPLSSNIAHGGSSVDLAIFSLHLAGISSILGAINFITTIINMRLNNMFFD
QLPLFVWAVGITAFLLLLSLPVLAGAITMLLTDRNLNTSFFDPAGGGDPILYQHL
>LEFIG956-10|HM876596|MM15820|Eupithecia_cauchiata
TLYFIFGIWAGMIGTSLSLLIRAELGTPGSLIGDDQIYNTIVTAHAFIMIFFMVMPIMIGGFGNWLVPLMLGAPDMAFPRMNNMSFWLLPPSITLLISSSIVENGAGTGWTVYPPLSSNIAHGGSSVDLAIFSLHLAGISSILGAINFITTIINMRLNNMFFD
QLPLFVWAVGITAFLLLLSLPVLAGAITMLLTDRNLNTSFFDPAGGGDPILYQHL
>LEFIC683-10|HM872504|MM04654|Eupithecia_centaureata
TLYFIFGIWAGMIGTSLSLLIRAELGTPGSLIGDDQIYNTIVTAHAFIMIFFMVMPIMIGGFGNWLVPLMLGAPDMAFPRMNNMSFWLLPPSITLLISSSIVESGAGTGWTVYPPLSSNIAHGGSSVDLAIFSLHLAGISSILGAINFITTIINMRLNNMFFDQ
LPLFVWAVGITAFLLLLSLPVLAGAITMLLTDRNLNTSFFDPAGGGDPILYQHL
>LEFID574-10|HM873339|MM06569|Eupithecia_centaureata
TLYFIFGIWAGMIGTSLSLLIRAELGTPGSLIGDDQIYNTIVTAHAFIMIFFMVMPIMIGGFGNWLVPLMLGAPDMAFPRMNNMSFWLLPPSITLLISSSIVESGAGTGWTVYPPLSSNIAHGGSSVDLAIFSLHLAGISSILGAINFITTIINMRLNNMFFDQ
LPLFVWAVGITAFLLLLSLPVLAGAITMLLTDRNLNTSFFDPAGGGDPILYQHL
>LEFIG177-10|HM875857|MM14044|Eupithecia_centaureata
TLYFIFGIWAGMIGTSLSLLIRAELGTPGSLIGDDQIYNTIVTAHAFIMIFFMVMPIMIGGFGNWLVPLMLGAPDMAFPRMNNMSFWLLPPSITLLISSSIVESGAGTGWTVYPPLSSNIAHGGSSVDLAIFSLHLAGISSILGAINFITTIINMRLNNMFFDQ
LPLFVWAVGITAFLLLLSLPVLAGAITMLLTDRNLNTSFFDPAGGGDPILYQHL
>LEFIB168-10|HM871072|MM00569|Eupithecia_conterminata
TLYFIFGIWAGMIGTSLSLLIRAELGTPGSLIGDDQIYNTIVTAHAFIMIFFMVMPIMIGGFGNWLVPLMLGAPDMAFPRMNNMSFWLLPPSITLLISSSIVENGAGTGWTVYPPLSSNIAHGGSSVDLAIFSLHLAGISSILGAINFITTIINMRLNNMFFD
QLPLFVWAVGITAFLLLLSLPVLAGAITMLLTDRNLNTSFFDPAGGGDPILYQHL
>LEFID698-10|HM873459|MM06751|Eupithecia_denotata
TLYFIFGIWAGMIGTSLSLLIRAELGTPGSLIGDDQIYNTIVTAHAFIMIFFMVMPIMIGGFGNWLVPLMLGAPDMAFPRMNNMSFWLLPPSITLLISSSIVENGAGTGWTVYPPLSSNIAHGGSSVDLAIFSLHLAGISSILGAINFITTIINMRLNNMFFD
QLPLFVWAVGITAFLLLLSLPVLAGAITMLLTDRNLNTSFFDPAGGGDPILYQHL
>LEFIE905-10|HM874622|MM10236|Eupithecia_denotata
TLYFIFGIWAGMIGTSLSLLIRAELGTPGSLIGDDQIYNTIVTAHAFIMIFFMVMPIMIGGFGNWLVPLMLGAPDMAFPRMNNMSFWLLPPSITLLISSSIVENGAGTGWTVYPPLSSNIAHGGSSVDLAIFSLHLAGISSILGAINFITTIINMRLNNMFFD
QLPLFVWAVGITAFLLLLSLPVLAGAITMLLTDRNLNTSFFDPAGGGDPILYQHL
>LEFIG317-10|HM875996|MM14284|Eupithecia_denotata
TLYFIFGIWAGMIGTSLSLLIRAELGTPGSLIGDDQIYNTIVTAHAFIMIFFMVMPIMIGGFGNWLVPLMLGAPDMAFPRMNNMSFWLLPPSITLLISSSIVENGAGTGWTVYPPLSSNIAHGGSSVDLAIFSLHLAGISSILGAINFITTIINMRLNNMFFD
QLPLFVWAVGITAFLLLLSLPVLAGAITMLLTDRNLNTSFFDPAGGGDPILYQHL
>LEFIJ2187-14|MM23378|Eupithecia_distinctaria
TLYFIFGIWAGMIGTSLSLLIRAELGTPGSLIGDDQIYNTIVTAHAFIMIFFMVMPIMIGGFGNWLVPLMLGAPDMAFPRMNNMSFWLLPPSITLLISSSIVESGAGTGWTVYPPLSSNIAHGGSSVDLAIFSLHLAGISSILGAINFITTIINMRLNNMFFDQ
LPLFVWAVGITAFLLLLSLPVLAGAITMLLTDRNLNTSFFDPAGGGDPILYQHL
>LEFID180-10|HM872986|MM05966|Eupithecia_dodoneata
TLYFIFGIWAGMIGTSLSLLIRAELGTPGSLIGDDQIYNTIVTAHAFIMIFFMVMPIMIGGFGNWLVPLMLGAPDMAFPRMNNMSFWLLPPSITLLISSSIVENGAGTGWTVYPPLSSNIAHGGSSVDLAIFSLHLAGISSILGAINFITTIINMRLNNMFFD

QLPLFVWAVGITAFLLLLSLPVLAGAITMLLTDRNLNTSFFDPAGGGDPILYQHL
>LEFIL622-10|MM18920|Eupithecia_dodoneata
TLYFIFGIWAGMIGTSLSLLIRAELGTPGSLIGDDQIYNTIVTAHAFIMIFFMVMPIMIGGFGNWLVPLMLGAPDMAFPRMNNMSFWLLPPSITLLISSSIVENGAGTGWTVYPPLSSNIAHGGSSVDLAIFSLHLAGISSILGAINFITTIINMRLNNMFFD
QLPLFVWAVGITAFLLLLSLPVLAGAITMLLTDRNLNTSFFDPAGGGDPILYQHL
>LEFID057-10|HM872871|MM05657|Eupithecia_egenaria
TLYFIFGIWAGMIGTSLSLLIRAELGTPGSLIGDDQIYNTIVTAHAFIMIFFMVMPIMIGGFGNWLVPLMLGAPDMAFPRMNNMSFWLLPPSITLLISSSIVENGAGTGWTVYPPLSSNIAHGGSSVDLAIFSLHLAGISSILGAINFITTIINMRLNNMFFD
QLPLFVWAVGITAFLLLLSLPVLAGAITMLLTDRNLNTSFFDPAGGGDPILYQHL
>LEFIL406-10|JF854585|MM18704|Eupithecia_egenaria
TLYFIFGIWAGMIGTSLSLLIRAELGTPGSLIGDDQIYNTIVTAHAFIMIFFMVMPIMIGGFGNWLVPLMLGAPDMAFPRMNNMSFWLLPPSITLLISSSIVENGAGTGWTVYPPLSSNIAHGGSSVDLAIFSLHLAGISSILGAINFITTIINMRLNNMFFD
QLPLFVWAVGITAFLLLLSLPVLAGAITMLLTDRNLNTSFFDPAGGGDPILYQHL
>LEFIA652-10|HM870901|MM01769|Eupithecia_exiguata
TLYFIFGIWAGMIGTSLSLLIRAELGTPGSLIGDDQIYNTIVTAHAFIMIFFMVMPIMIGGFGNWLVPLMLGAPDMAFPRMNNMSFWLLPPSITLLISSSIVENGAGTGWTVYPPLSSNIAHGGSSVDLAIFSLHLAGISSILGAINFITTIINMRLNNMFFD
QLPLFVWAVGITAFLLLLSLPVLAGAITMLLTDRNLNTSFFDPAGGGDPILYQHL
>LEFIC339-10|HM872183|MM03846|Eupithecia_exiguata
TLYFIFGIWAGMIGTSLSLLIRAELGTPGSLIGDDQIYNTIVTAHAFIMIFFMVMPIMIGGFGNWLVPLMLGAPDMAFPRMNNMSFWLLPPSITLLISSSIVENGAGTGWTVYPPLSSNIAHGGSSVDLAIFSLHLAGISSILGAINFITTIINMRLNNMFFD
QLPLFVWAVGITAFLLLLSLPVLAGAITMLLTDRNLNTSFFDPAGGGDPILYQHL
>LEFIA026-10|HM396376|MM00097|Eupithecia_fennoscandica
TLYFIFGIWAGMIGTSLSLLIRAELGTPGSLIGDDQIYNTIVTAHAFIMIFFMVMPIMIGGFGNWLVPLMLGAPDMAFPRMNNMSFWLLPPSITLLISSSIVENGAGTGWTVYPPLSSNIAHGGSSVDLAIFSLHLAGISSILGAINFITTIINMRLNNMFFD
QLPLFVWAVGITAFLLLLSLPVLAGAITMLLTDRNLNTSFFDPAGGGDPILYQHL
>LEFIG962-10|HM876602|MM15826|Eupithecia_gelidata
TLYFIFGIWAGMIGTSLSLLIRAELGTPGSLIGDDQIYNTIVTAHAFIMIFFMVMPIMIGGFGNWLVPLMLGAPDMAFPRMNNMSFWLLPPSIILLISSSTVENGAGTGWTVYPPLSSNIAHGGSSVDLAIFSLHLAGISSILGAINFITTIINMRLNNMFFD
QLPLFVWAVGITAFLLLLSLPVLAGAITMLLTDRNLNTSFFDPAGGGDPILYQHL
>LEFIG963-10|HM876603|MM15827|Eupithecia_gelidata
TLYFIFGIWAGMIGTSLSLLIRAELGTPGSLIGDDQIYNTIVTAHAFIMIFFMVMPTMIGGFGNWLVPLMLGAPDMAFPRMNNMSFWLLPPSITLLISSSIVENGAGTGWTVYPPLSSNIAHGGSSVDLAIFSLHLAGISSILGAINFITTIINMRLNNMFFD
QLPLFVWAVGITAFLLLLSLPVLAGAITMLLTDRNLNTSFFDPAGGGDPILYQHL
>LEFIK408-10|JF854037|MM17983|Eupithecia_gelidata
TLYFIFGIWAGMVGTSLSLLIRAELGTPGSLIGDDQIYNTIVTAHAFIMIFFMVMPIMIGGFGNWLVPLMLGAPDMAFPRMNNMSFWLLPPSIILLISSSIVENGAGTGWTVYPPLSSNIAHGGSSVDLAIFSLHLAGISSILGAINFITTIINMRLNNMFFD
QLPLFVWAVGITAFLLLLSLPVLAGAITMLLTDRNLNTSFFDPAGGGDPILYQHL
>LEFIL344-10|JN279418|MM18654|Eupithecia_gelidata
TLYFIFGIWAGMIGTSLSLLIRAELGTPGSLIGDDQIYNTIVTAHAFIMIFFMVMPIMIGGFGNWLVPLMLGAPDMAFPRMNNMSFWLLPPSITLLISSSIVENGAGTGWTVYPPLSSNIAHGGSSVDLAIFSLHLAGISSILGAINFITTIINMRLNNMFFD
QLPLFVWAVGITAFLLLLSLPVLAGAITMLLTDRNLNTSFFDPAGGGDPILYQHL
>LEFIJ816-10|MM17441|Eupithecia_goossensiata
TLYFIFGIWAGMIGTSLSLLIRAELGTPGSLIGDDQIYNTIVTAHAFIMIFFMVMPIMIGGFGNWLVPLMLGAPDMAFPRMNNMSFWLLPPSITLLISSSIVENGAGTGWTVYPPLSSNIAHGGSSVDLAIFSLHLAGISSILGAINFITTIINMRLNNMFFD
QLPLFVWAVGITAFLLLLSLPVLAGAITMLLTDRNLNTSFFDPAGGGDPILYQHL
>LEFIL407-10|JF854586|MM18705|Eupithecia_goossensiata
TLYFIFGIWAGMIGTSLSLLIRAELGTPGSLIGDDQIYNTIVTAHAFIMIFFMVMPIMIGGFGNWLVPLMLGAPDMAFPRMNNMSFWLLPPSITLLISSSIVENGAGTGWTVYPPLSSNIAHGGSSVDLAIFSLHLAGISSILGAINFITTIINMRLNNMFFD
QLPLFVWAVGITAFLLLLSLPVLAGAITMLLTDRNLNTSFFDPAGGGDPILYQHL
>LEFIL408-10|JF854587|MM18706|Eupithecia_goossensiata
TLYFIFGIWAGMIGTSLSLLIRAELGTPGSLIGDDQIYNTIVTAHAFIMIFFMVMPIMIGGFGNWLVPLMLGAPDMAFPRMNNMSFWLLPPSITLLISSSIVENGAGTGWTVYPPLSSNIAHGGSSVDLAIFSLHLAGISSILGAINFITTIINMRLNNMFFD
QLPLFVWAVGITAFLLLLSLPVLAGAITMLLTDRNLNTSFFDPAGGGDPILYQHL
>LEFIG586-10|HM876257|MM14775|Eupithecia_groenblomi
TLYFIFGIWAGMIGTSLSLLIRAELGTPGSLIGDDQIYNTIVTAHAFIMIFFMVMPIMIGGFGNWLVPLMLGAPDMAFPRMNNMSFWLLPPSITLLISSSIVENGAGTGWTVYPPLSSNIAHGGSSVDLAIFSLHLAGISSILGAINFITTIINMRLNNMFFD
QLPLFVWAVGITAFLLLLSLPVLAGAITMLLTDRNLNTSFFDPAGGGDPILYQHL
>LEFIA666-10|HQ963153|MM01798|Eupithecia_icterata
TLYFIFGIWAGMIGTSLSLLIRAELGTPGSLIGDDQIYNTIVTAHAFIMIFFMVMPIMIGGFGNWLVPLMLGAPDMAFPRMNNMSFWLLPPSITLLISSSIVENGAGTGWTVYPPLSSNIAHGGSSVDLAIFSLHLAGISSILGAINFITTIINMRLNNMFFD
QLPLFVWAVGITAFLLLLSLPVLAGAITMLLTDRNLNTSFFDPAGGGDPILYQHL
>LEFIC793-10|HM872612|MM04941|Eupithecia_icterata

TLYFIFGIWAGMIGTSLSLLIRAELGTPGSLIGDDQIYNTIVTAHAFIMIFFMVMPIMIGGFGNWLVPLMLGAPDMAFPRMNNMSFWLLPPSITLLISSSIVENGAGTGWTVYPPLSSNIAHGGSSVDLAIFSLHLAGISSILGAINFITTIINMRLNNMFFD
QLPLFVWAVGITAFLLLLSLPVLAGAITMLLTDRNLNTSFFDPAGGGDPILYQHL
>LEFIA638-10|HM870887|MM01753|Eupithecia_immundata
TLYFIFGIWAGMIGTSLSLLIRAELGTPGSLIGDDQIYNTIVTAHAFIMIFFMVMPIMIGGFGNWLVPLMLGAPDMAFPRMNNMSFWLLPPSITLLISSSIVENGAGTGWTVYPPLSSNIAHGGSSVDLAIFSLHLAGISSILGAINFITTIINMRLNNMFFD
QLPLFVWAVGITAFLLLLSLPVLAGAITMLLTDRNLNTSFFDPAGGGDPILYQHL
>LEFIA639-10|HM870888|MM01754|Eupithecia_immundata
TLYFIFGIWAGMIGTSLSLLIRAELGTPGSLIGDDQIYNTIVTAHAFIMIFFMVMPIMIGGFGNWLVPLMLGAPDMAFPRMNNMSFWLLPPSITLLISSSIVENGAGTGWTVYPPLSSNIAHGGSSVDLAIFSLHLAGISSILGAINFITTIINMRLNNMFFD
QLPLFVWAVGITAFLLLLSLPVLAGAITMLLTDRNLNTSFFDPAGGGDPI?YQHL
>LEFIG380-10|HM876057|MM14387|Eupithecia_immundata
TLYFIFGIWAGMIGTSLSLLIRAELGTPGSLIGDDQIYNTIVTAHAFIMIFFMVMPIMIGGFGNWLVPLMLGAPDMAFPRMNNMSFWLLPPSITLLISSSIVENGAGTGWTVYPPLSSNIAHGGSSVDLAIFSLHLAGISSILGAINFITTIINMRLNNMFFD
QLPLFVWAVGITAFLLLLSLPVLAGAITMLLTDRNLNTSFFDPAGGGDPILYQHL
>LEFIB167-10|HM871071|MM00568|Eupithecia_indigata
TLYFIFGIWAGMIGTSLSLLIRAELGTPGSLIGDDQIYNTIVTAHAFIMIFFMVMPIMIGGFGNWLVPLMLGAPDMAFPRMNNMSFWLLPPSITLLISSSIVENGAGTGWTVYPPLSSNIAHGGSSVDLAIFSLHLAGISSILGAINFITTIINMRLNNMFFD
QLPLFVWAVGITAFLLLLSLPVLAGAITMLLTDRNLNTSFFDPAGGGDPILYQHL
>LEFIF745-10|HM875429|MM12909|Eupithecia_indigata
TLYFIFGIWAGMIGTSLSLLIRAELGTPGSLIGDDQIYNTIVTAHAFIMIFFMVMPIMIGGFGNWLVPLMLGAPDMAFPRMNNMSFWLLPPSITLLISSSIVENGAGTGWTVYPPLSSNIAHGGSSVDLAIFSLHLAGISSILGAINFITTIINMRLNNMFFD
QLPLFVWAVGITAFLLLLSLPVLAGAITMLLTDRNLNTSFFDPAGGGDPILYQHL
>LEFIE940-10|HM874657|MM10328|Eupithecia_innotata
TLYFIFGIWAGMIGTSLSLLIRAELGTPGSLIGDDQIYNTIVTAHAFIMIFFMVMPIMIGGFGNWLVPLMLGAPDMAFPRMNNMSFWLLPPSITLLISSSIVENGAGTGWTVYPPLSSNIAHGGSSVDLAIFSLHLAGISSILGAINFITTIINMRLNNMFFD
QLPLFVWAVGITAFLLLLSLPVLAGAITMLLTDRNLNTSFFDPAGGGDPILYQHL
>LEFIF764-10|HM875448|MM12974|Eupithecia_innotata
TLYFIFGIWAGMIGTSLSLLIRAELGTPGSLIGDDQIYNTIVTAHAFIMIFFMVMPIMIGGFGNWLVPLMLGAPDMAFPRMNNMSFWLLPPSITLLISSSIVENGAGTGWTVYPPLSSNIAHGGSSVDLAIFSLHLAGISSILGAINFITTIINMRLNNVFFDQ
LPLFVWAVGITAFLLLLSLPVLAGAITMLLTDRNLNTSFFDPAGGGDPILYQHL
>LEFIG964-10|HM876604|MM15828|Eupithecia_innotata
TLYFIFGIWAGMIGTSLSLLIRAELGTPGSLIGDDQIYNTIVTAHAFIMIFFMVMPIMIGGFGNWLVPLMLGAPDMAFPRMNNMSFWLLPPSITLLISSSIVENGAGTGWTVYPPLSSNIAHGGSSVDLAIFSLHLAGISSILGAINFITTIINMRLNNMFFD
QLPLFVWAVGITAFLLLLSLPVLAGAITMLLTDRNLNTSFFDPAGGGDPILYQHL
>LEFIG966-10|HM876606|MM15830|Eupithecia_innotata
TLYFIFGIWAGMIGTSLSLLIRAELGTPGSLIGDDQIYNTIVTAHAFIMIFFMVMPIMIGGFGNWLVPLMLGAPDMAFPRMNNMSFWLLPPSITLLISSSIVENGAGTGWTVYPPLSSNIAHGGSSVDLAIFSLHLAGISSILGAINFITTIINMRLNNMFFD
QLPLFVWAVGITAFLLLLSLPVLAGAITMLLTDRNLNTSFFDPAGGGDPILYQHL
>LEFIJ382-10|JF853569|MM15987|Eupithecia_innotata
TLYFIFGIWAGMIGTSLS?LIRAELGTPGSLIGDDQIYNTIVTAHAFIMIFFMVMPIMIGGFGNWLVPLMLGAPDMAFPRMNNMSFWLLPPSITLLISSSIVENGAGTGWTVYPPLSSNIAHGGSSVDLAIFSLHLAGISSILGAINFITTIINMRLNNMFFD
QLPLFVWAVGITAFLLLLSLPVLAGAITMLLTDRNLNTSFFDPAGGGDPILYQHL
>LEFIA672-10|HM386820|MM01806|Eupithecia_intricata
TLYFIFGIWAGMIGTSLSLMIRAELGTPGSLIGDDQIYNTIVTAHAFIMIFFMVMPIMIGGFGNWLVPLMLGAPDMAFPRMNNMSFWLLPPSITLLISSSTVENGAGTGWTVYPPLSSNIAHGGSSVDLAIFSLHLAGISSILGAINFITTIINMRLNNMFF
DQLPLFVWAVGITAFLLLLSLPVLAGAITMLLTDRNLNTSFFDPAGGGDPILYQHL
>LEFIC853-10|HM872671|MM05083|Eupithecia_inturbata
TLYFIFGIWAGMIGTSLSLLIRAELGTPGSLIGDDQIYNTIVTAHAFIMIFFMVMPIMIGGFGNWLVPLMLGAPDMAFPRMNNMSFWLLPPSITLLISSSIVENGAGTGWTVYPPLSSNIAHGGSSVDLAIFSLHLAGISSILGAINFITTIINMRLNNMFFD
QLPLFVWAVGITAFLLLLSLPVLAGAITMLLTDRNLNTSFFDPAGGGDPILYQHL
>LEFID972-10|HM873722|MM07979|Eupithecia_inturbata
TLYFIFGIWAGMIGTSLSLLIRAELGTPGSLIGDDQIYNTIVTAHAFIMIFFMVMPIMIGGFGNWLVPLMLGAPDMAFPRMNNMSFWLLPPSITLLISSSIVENGAGTGWTVYPPLSSNIAHGGSSVDLAIFSLHLAGISSILGAINFITTIINMRLNNMFFD
QLPLFVWAVGITAFLLLLSLPVLAGAITMLLTDRNLNTSFFDPAGGGDPILYQHL
>LEFIL627-10|MM18925|Eupithecia_irriguata
TLYFIFGIWAGMIGTSLSLLIRAELGTPGSLIGDDQIYNTIVTAHAFIMIFFMVMPIMIGGFGNWLVPLMLGAPDMAFPRMNNMSFWLLPPSITLLISSSIVENGAGTGWTVYPPLSSNIAHGGSSVDLAIFSLHLAGISSILGAINFITTIINMRLNNMFFD
QLPLFVWAVGITAFLLLLSLPVLAGAITMLLTDRNLNTSFFDPAGGGDPILYQHL
>LEFIL628-10|JF854681|MM18926|Eupithecia_irriguata
TLYFIFGIWAGMIGTSLSLLIRAELGTPGSLIGDDQIYNTIVTAHAFIMIFFMVMPIMIGGFGNWLVPLMLGAPDMAFPRMNNMSFWLLPPSITLLISSSIVENGAGTGWTVYPPLSSNIAHGGSSVDLAIFSLHLAGISSILGAINFITTIINMRLNNMFFD
QLPLFVWAVGITAFLLLLSLPVLAGAITMLLTDRNLNTSFFDPAGGGDPILYQHL

>LEFID018-10|HM872832|MM05445|Eupithecia_lanceata
TLYFIFGIWAGMIGTSLSLLIRAELGTPGSLIGDDQIYNTIVTAHAFIMIFFMVMPIMIGGFGNWLVPLMLGAPDMAFPRMNNMSFWLLPPSITLLISSSIVENGAGTGWTVYPPLSSNIAHGGSSVDLAIFSLHLAGISSILGAINFITTIINMRLNNMFFD
QLPLFIWAVGITAFLLLLSLPVLAGAITMLLTDRNLNTSFFDPAGGGDPILYQHL
>LEFIF383-10|HM875068|MM11647|Eupithecia_lanceata
TLYFIFGIWAGMIGTSLSLLIRAELGTPGSLIGDDQIYNTIVTAHAFIMIFFMVMPIMIGGFGNWLVPLMLGAPDMAFPRMNNMSFWLLPPSITLLISSSIVENGAGTGWTVYPPLSSNIAHGGSSVDLAIFSLHLAGISSILGAINFITTIINMRLNNMFFD
QLPLFVWAVGITAFLLLLSLPVLAGAITMLLTDRNLNTSFFDPAGGGDPILYQHL
>LEFID970-10|HM873720|MM07976|Eupithecia_lariciata
TLYFIFGIWAGMIGTSLSLLIRAELGTPGSLIGDDQIYNTIVTAHAFIMIFFMVMPIMIGGFGNWLVPLMLGAPDMAFPRMNNMSFWLLPPSITLLISSSIVENGAGTGWTVYPPLSSNIAHGGSSVDLAIFSLHLAGISSILGAINFITTIINMRLNNMFFD
QLPLFVWAVGITAFLLLLSLPVLAGAITMLLTDRNLNTSFFDPAGGGDPILYQHL
>LEFIK929-10|JF854372|MM18504|Eupithecia_lariciata
TLYFIFGIWAGMIGTSLSLLIRAELGTPGSLIGDDQIYNTIVTAHAFIMIFFMVMPIMIGGFGNWLVPLMLGAPDMAFPRMNNMSFWLLPPSITLLISSSIVENGAGTGWTVYPPLSSNIAHGGSSVDLAIFSLHLAGISSILGAINFITTIINMRLNNMFFD
QLPLFVWAVGITAFLLLLSLPVLAGAITMLLTDRNLNTSFFDPAGGGDPILYQHL
>LEFIA657-10|HM870905|MM01784|Eupithecia_linariata
TLYFIFGIWAGMIGTSLSLLIRAELGTPGSLIGDDQIYNTIVTAHAFIMIFFMVMPIMIGGFGNWLVPLMLGAPDMAFPRMNNMSFWLLPPSITLLISSSIVENGAGTGWTVYPPLSSNIAHGGSSVDLAIFSLHLAGISSILGAINFITTIINMRLNNMFFD
QLPLFVWAVGITAFLLLLSLPVLAGAITMLLTDRNLNTSFFDPAGGGDPILYQHL
>LEFIF384-10|HM875069|MM11648|Eupithecia_linariata
TLYFIFGIWAGMIGTSLSLLIRAELGTPGSLIGDDQIYNTIVTAHAFIMIFFMVMPIMIGGFGNWLVPLMLGAPDMAFPRMNNMSFWLLPPSITLLISSSIVENGAGTGWTVYPPLSSNIAHGGSSVDLAIFSLHLAGISSILGAINFITTIINMRLNNMFFD
QLPLFVWAVGITAFLLLLSLPVLAGAITMLLTDRNLNTSFFDPAGGGDPILYQHL
>LEFIE566-10|HM874289|MM09455|Eupithecia_millefoliata
TLYFIFGIWAGMIGTSLSLLIRAELGTPGSLIGDDQIYNTIVTAHAFIMIFFMVMPIMIGGFGNWLVPLMLGAPDMAFPRMNNMSFWLLPPSIMLLISSSIVENGAGTGWTVYPPLSSNIAHGGSSVDLAIFSLHLAGISSILGAINFITTIINMRLNNMFFD
QLPLFVWAVGITAFLLLLSLPVLAGAITMLLTDRNLNTSFFDPAGGGDPILYQHL
>LEFIG961-10|HM876601|MM15825|Eupithecia_millefoliata
TLYFIFGIWAGMIGTSLSLLIRAELGTPGSLIGDDQIYNTIVTAHAFIMIFFMVMPIMIGGFGNWLVPLMLGAPDMAFPRMNNMSFWLLPPSIMLLISSSIVENGAGTGWTVYPPLSSNIAHGGSSVDLAIFSLHLAGISSILGAINFITTIINMRLNNMFFD
QLPLFVWAVGITAFLLLLSLPVLAGAITMLLTDRNLNTSFFDPAGGGDPILYQHL
>LEFID676-10|HM873437|MM06717|Eupithecia_nanata
TLYFIFGIWAGMVGTSLSLLIRAELGTPGSLIGDDQIYNTIVTAHAFIMIFFMVMPIMIGGFGNWLVPLMLGAPDMAFPRMNNMSFWLLPPSIILLISSSIVENGAGTGWTVYPPLSSNIAHGGSSVDLAIFSLHLAGISSILGAINFITTIINMRLNNMFFD
QLPLFVWAVGITAFLLLLSLPVLAGAITMLLTDRNLNTSFFDPAGGGDPILYQHL
>LEFIF757-10|HM875441|MM12966|Eupithecia_nanata
TLYFIFGIWAGMIGTSLSLLIRAELGTPGSLIGDDQIYNTIVTAHAFIMIFFMVMPIMIGGFGNWLVPLMLGAPDMAFPRMNNMSFWLLPPSITLLISSSIVENGAGTGWTVYPPLSSNIAHGGSSVDLAIFSLHLAGISSILGAINFITTIINMRLNNMFFD
QLPLFVWAVGITAFLLLLSLPVLAGAITMLLTDRNLNTSFFDPAGGGDPILYQHL
>LEFIK927-10|JF854370|MM18502|Eupithecia_nanata
TLYFIFGIWAGMIGTSLSLLIRAELGTPGSLIGDDQIYNTIVTAHAFIMIFFMVMPIMIGGFGNWLVPLMLGAPDMAFPRMNNMSFWLLPPSITLLISSSIVENGAGTGWTVYPPLSSNIAHGGSSVDLAIFSLHLAGISSILGAINFITTIINMRLNNMFFD
QLPLFVWAVGITAFLLLLSLPVLAGAITMLLTDRNLNTSFFDPAGGGDPILYQHL
>LEFIJ612-10|JF853712|MM17237|Eupithecia_ochridata
TLYFIFGIWAGMVGTSLSLLIRAELGTPGSLIGDDQIYNTIVTAHAFIMIFFMVMPIMIGGFGNWLVPLMLGAPDMAFPRMNNMSFWLLPPSIILLISSSIVENGAGTGWTVYPPLSSNIAHGGSSVDLAIFSLHLAGISSILGAINFITTIINMRLNNMFFD
QLPLFVWAVGITAFLLLLSLPVLAGAITMLLTDRNLNTSFFDPAGGGDPILYQHL
>LEFII262-11|MM19912|Eupithecia_ochridata
TLYFIFGIWAGMVGTSLSLLIRAELGTPGSLIGDDQIYNTIVTAHAFIMIFFMVMPIMIGGFGNWLVPLMLGAPDMAFPRMNNMSFWLLPPSIILLISSSIVENGAGTGWTVYPPLSSNIAHGGSSVDLAIFSLHLAGISSILGAINFITTIINMRLNNMFFD
QLPLFVWAVGITAFLLLLSLPVLAGAITMLLTDRNLNTSFFDPAGGGDPILYQHL
>LEFIG952-10|HM876592|MM15816|Eupithecia_pernotata
TLYFIFGIWAGMIGTSLSLLIRAELGTPGSLIGDDQIYNTIVTAHAFIMIFFMVMPIMIGGFGNWLVPLMLGAPDMAFPRMNNMSFWLLPPSITLLISSSIVENGAGTGWTVYPPLSSNIAHGGSSVDLAIFSLHLAGISSILGAINFITTIINMRLNNMFFD
QLPLFVWAVGITAFLLLLSLPVLAGAITMLLTDRNLNTSFFDPAGGGDPILYQHL
>LEFIA662-10|HM870910|MM01792|Eupithecia_pimpinellata
TLYFIFGIWAGMIGTSLSLLIRAELGTPGSLIGDDQIYNTIVTAHAFIMIFFMVMPIMIGGFGNWLVPLMLGAPDMAFPRMNNMSFWLLPPSITLLISSSIVENGAGTGWTVYPPLSSNIAHGGSSVDLAIFSLHLAGISSILGAINFITTIINMRLNNMFFD
QLPLFVWAVGITAFLLLLSLPVLAGAITMLLTDRNLNTSFFDPAGGGDPILYQHL
>LEFIC120-10|HM871967|MM03456|Eupithecia_pimpinellata
TLYFIFGIWAGMIGTSLSLLIRAELGTPGSLIGDDQIYNTIVTAHAFIMIFFMVMPIMIGGFGNWLVPLMLGAPDMAFPRMNNMSFWLLPPSITLLISSSIVESGAGTGWTVYPPLSSNIAHGGSSVDLAIFSLHLAGISSILGAINFITTIINMRLNNMFFDQ

LPLFVWAVGITAFLLLLSLPVLAGAITMLLTDRNLNTSFFDPAGGGDPILYQHL
>LEFIA675-10|HM386823|MM01811|Eupithecia_plumbeolata
TLYFIFGIWAGMIGTSLSLLIRAELGTPGSLIGDDQIYNTIVTAHAFIMIFFMVMPIMIGGFGNWLVPLMLGAPDMAFPRMNNMSFWLLPPSITLLISSSIVENGAGTGWTVYPPLSSNIAHGGSSVDLAIFSLHLAGISSILGAINFITTIINMRLNNMFFD
QLPLFVWAVGITAFLLLLSLPVLAGAITMLLTDRNLNTSFFDPAGGGDPILYQHL
>LEFIA676-10|HM386824|MM01812|Eupithecia_plumbeolata
TLYFIFGIWAGMIGTSLSLLIRAELGTPGSLIGDDQIYNTIVTAHAFIMIFFMVMPIMIGGFGNWLVPLMLGAPDMAFPRMNNMSFWLLPPSITLLISSSIVENGAGTGWTVYPPLSSNIAHGGSSVDLAIFSLHLAGISSILGAINFITTIINMRLNNMFFD
QLPLFVWAVGITAFLLLLSLPVLAGAITMLLTDRNLNTSFFDPAGGGDPILYQHL
>LEFIJ036-10|KM573024|MM01814|Eupithecia_plumbeolata
TLYFIFGIWAGMIGTSLSLLIRAELGTPGSLIGDDQIYNTIVTAHAFIMIFFMVMPIMIGGFGNWLVPLMLGAPDMAFPRMNNMSFWLLPPSITLLISSSIVENGAGTGWTVYPPLSSNIAHGGSSVDLAIFSLHLAGISSILGAINFITTIINMRLNNMFFD
QLPLFVWAVGITAFLLLLSLPVLAGAITMLLTDRNLNTSFFDPAGGGDPILYQHL
>LEFID530-10|HM873295|MM06512|Eupithecia_plumbeolata
TLYFIFGIWAGMIGTSLSLLIRAELGTPGSLIGDDQIYNTIVTAHAFIMIFFMVMPIMIGGFGNWLVPLMLGAPDMAFPRMNNMSFWLLPPSITLLISSSIVENGAGTGWTVYPPLSSNIAHGGSSVDLAIFSLHLAGISSILGAINFITTIINMRLNNMFFD
QLPLFVWAVGITAFLLLLSLPVLAGAITMLLTDRNLNTSFFDPAGGGDPILYQHL
>LEFID629-10|HM873392|MM06648|Eupithecia_plumbeolata
TLYFIFGIWAGMIGTSLSLLIRAELGTPGSLIGDDQIYNTIVTAHAFIMIFFMVMPIMIGGFGNWLVPLMLGAPDMAFPRMNNMSFWLLPPSITLLISSSIVENGAGTGWTVYPPLSSNIAHGGSSVDLAIFSLHLAGISSILGAINFITTIINMRLNNMFFD
QLPLFVWAVGITAFLLLLSLPVLAGAITMLLTDRNLNTSFFDPAGGGDPILYQHL
>LEFIG283-10|HM875962|MM14229|Eupithecia_plumbeolata
TLYFIFGIWAGMIGTSLSLLIRAELGTPGSLIGDDQIYNTIVTAHAFIMIFFMVMPIMIGGFGNWLVPLMLGAPDMAFPRMNNMSFWLLPPSITLLISSSIVENGAGTGWTVYPPLSSNIAHGGSSVDLAIFSLHLAGISSILGAINFITTIINMRLNNMFFD
QLPLFVWAVGITAFLLLLSLPVLAGAITMLLTDRNLNTSFFDPAGGGDPILYQHL
>LEFIK899-10|KM573070|MM18474|Eupithecia_plumbeolata
TLYFIFGIWAGMIGTSLSLLIRAELGTPGSLIGDDQIYNTIVTAHAFIMIFFMVMPIMIGGFGNWLVPLMLGAPDMAFPRMNNMSFWLLPPSITLLISSSIVENGAGTGWTVYPPLSSNIAHGGSSVDLAIFSLHLAGISSILGAINFITTIINMRLNNMFFD
QLPLFVWAVGITAFLLLLSLPVLAGAITMLLTDRNLNTSFFDPAGGGDPILYQHL
>LEEUA508-11|MM20567|Eupithecia_pulchellata
TLYFIFGIWAGMIGTSLSLLIRAELGTPGSLIGDDQIYNTIVTAHAFIMIFFMVMPIMIGGFGNWLVPLMLGAPDMAFPRMNNMSFWLLPPSITLLISSSIVENGAGTGWTVYPPLSSNIAHGGSSVDLAIFSLHLAGISSILGAINFITTIINMRLNNMFFD
QLPLFVWAVGITAFLLLLSLPVLAGAITMLLTDRNLNTSFFDPAGGGDPILYQHL
>LEFIA658-10|HM870906|MM01785|Eupithecia_pusillata
TLYFIFGIWAGMIGTSLSLLIRAELGTPGSLIGDDQIYNTIVTAHAFIMIFFMVMPIMIGGFGNWLVPLMLGAPDMAFPRMNNMSFWLLPPSITLLISSSIVENGAGTGWTVYPPLSSNIAHGGSSVDLAIFSLHLAGISSILGAINFITTIINMRLNNMFFD
QLPLFVWAVGITAFLLLLSLPVLAGAITMLLTDRNLNTSFFDPAGGGDPILYQHL
>LEFIE045-10|HM873793|MM08143|Eupithecia_pusillata
TLYFIFGIWAGMIGTSLSLLIRAELGTPGSLIGDDQIYNTIVTAHAFIMIFFMVMPIMIGGFGNWLVPLMLGAPDMAFPRMNNMSFWLLPPSITLLISSSIVENGAGTGWTVYPPLSSNIAHGGSSVDLAIFSLHLAGISSILGAINFITTIINMRLNNMFFD
QLPLFVWAVGITAFLLLLSLPVLAGAITMLLTDRNLNTSFFDPAGGGDPILYQHL
>LEFIJ588-10|KM573453|MM17213|Eupithecia_pusillata
TLYFIFGIWAGMIGTSLSLLIRAELGTPGSLIGDDQIYNTIVTAHAFIMIFFMVMPIMIGGFGNWLVPLMLGAPDMAFPRMNNMSFWLLPPSITLLISSSIVENGAGTGWTVYPPLSSNIAHGGSSVDLAIFSLHLAGISSILGAINFITTIINMRLNNMFFD
QLPLFVWAVGITAFLLLLSLPVLAGAITMLLTDRNLNTSFFDPAGGGDPILYQHL
>LEFIC004-10|HM871874|MM03204|Eupithecia_pygmaeata
TLYFIFGIWAGMIGTSLSLLIRAELGTPGSLIGDDQIYNTIVTAHAFIMIFFMVMPIMIGGFGNWLVPLMLGAPDMAFPRMNNMSFWLLPPSITLLISSSIVENGAGTGWTVYPPLSSNIAHGGSSVDLAIFSLHLAGISSILGAINFITTIINMRLNNMFFD
QLPLFVWAVGITAFLLLLSLPVLAGAITMLLTDRNLNTSFFDPAGGGDPILYQHL
>LEFIJ026-10|JF853414|MM00567|Eupithecia_satyrata
TLYFIFGIWAGMIGTSLSLLIRAELGTPGSLIGDDQIYNTIVTAHAFIMIFFMVMPIMIGGFGNWLVPLMLGAPDMAFPRMNNMSFWLLPPSITLLISSSIVENGAGTGWTVYPPLSSNIAHGGSSVDLAIFSLHLAGISSILGAINFITTIINMRLNNMFFD
QLPLFVWAVGITAFLLLLSLPVLAGAITMLLTDRNLNTSFFDPAGGGDPILYQHL
>LEFIJ028-10|JF853416|MM00633|Eupithecia_satyrata
TLYFIFGIWAGMIGTSLSLLIRAELGTPGSLIGDDQIYNTIVTAHAFIMIFFMVMPIMIGGFGNWLVPLMLGAPDMAFPRMNNMSFWLLPPSITLLISSSIVENGAGTGWTVYPPLSSNIAHGGSSVDLAIFSLHLAGISSILGAINFITTIINMRLNNMFFD
QLPLFVWAVGITAFLLLLSLPVLAGAITMLLTDRNLNTSFFDPAGGGDPILYQHL
>LEFIJ034-10|KM572182|MM01789|Eupithecia_satyrata
TLYFIFGIWAGMIGTSLSLLIRAELGTPGSLIGDDQIYNTIVTAHAFIMIFFMVMPIMIGGFGNWLVPLMLGAPDMAFPRMNNMSFWLLPPSITLLISSSIVENGAGTGWTVYPPLSSNIAHGGSSVDLAIFSLHLAGISSILGAINFITTIINMRLNNMFFD
QLPLFVWAVGITAFLLLLSLPVLAGAITMLLTDRNLNTSFFDPAGGGDPILYQHL
>LEFIJ035-10|KM572620|MM01790|Eupithecia_satyrata

TLYFIFGIWAGMIGTSLSLLIRAELGTPGSLIGDDQIYNTIVTAHAFIMIFFMVMPIMIGGFGNWLVPLMLGAPDMAFPRMNNMSFWLLPPSITLLISSSIVENGAGTGWTVYPPLSSNIAHGGSSVDLAIFSLHLAGISSILGAINFITTIINMRLNNMFFD
QLPLFVWAVGITAFLLLLSLPVLAGAITMLLTDRNLNTSFFDPAGGGDPILYQHL
>LEFIA671-10|HM386819|MM01805|Eupithecia_satyrata
TLYFIFGIWAGMIGTSLSLLIRAELGTPGSLIGDDQIYNTIVTAHAFIMIFFMVMPIMIGGFGNWLVPLMLGAPDMAFPRMNNMSFWLLPPSITLLISSSIVENGAGTGWTVYPPLSSNIAHGGSSVDLAIFSLHLAGISSILGAINFITTIINMRLNNMFFD
QLPLFVWAVGITAFLLLLSLPVLAGAITMLLTDRNLNTSFFDPAGGGDPILYQHL
>LEFIC699-10|HM872520|MM04689|Eupithecia_satyrata
TLYFIFGIWAGMIGTSLSLLIRAELGTPGSLIGDDQIYNTIVTAHAFIMIFFMVMPIMIGGFGNWLVPLMLGAPDMAFPRMNNMSFWLLPPSITLLISSSIVENGAGTGWTVYPPLSSNIAHGGSSVDLAIFSLHLAGISSILGAINFITTIINMRLNNMFFD
QLPLFVWAVGITAFLLLLSLPVLAGAITMLLTDRNLNTSFFDPAGGGDPILYQHL
>LEFIJ111-10|KM572141|MM07962|Eupithecia_satyrata
TLYFIFGIWAGMIGTSLSLLIRAELGTPGSLIGDDQIYNTIVTAHAFIMIFFMVMPIMIGGFGNWLVPLMLGAPDMAFPRMNNMSFWLLPPSITLLISSSIVENGAGTGWTVYPPLSSNIAHGGSSVDLAIFSLHLAGISSILGAINFITTIINMRLNNMFFD
QLPLFVWAVGITAFLLLLSLPVLAGAITMLLTDRNLNTSFFDPAGGGDPILYQHL
>LEFID056-10|HM872870|MM05655|Eupithecia_selinata
TLYFIFGIWAGMIGTSLSLLIRAELGTPGSLIGDDQIYNTIVTAHAFIMIFFMVMPIMIGGFGNWLVPLMLGAPDMAFPRMNNMSFWLLPPSITLLISSSIVENGAGTGWTVYPPLSSNIAHGGSSVDLAIFSLHLAGISSILGAINFITTIINMRLNNMFFD
QLPLFVWAVGITAFLLLLSLPVLAGAITMLLTDRNLNTSFFDPAGGGDPILYQHL
>LEFIB051-10|HM870960|MM00334|Eupithecia_simpliciata
TLYFIFGIWAGMIGTSLSLLIRAELGTPGSLIGDDQIYNTIVTAHAFIMIFFMVMPIMIGGFGNWLVPLMLGAPDMAFPRMNNMSFWLLPPSITLLISSSIVENGAGTGWTVYPPLSSNIAHGGSSVDLAIFSLHLAGISSILGAINFITTIINMRLNNMFFD
QLPLFVWAVGITAFLLLLSLPVLAGAITMLLTDRNLNTSFFDPAGGGDPILYQHL
>LEFIB050-10|HM870959|MM00332|Eupithecia_sinuosaria
TLYFIFGIWAGMIGTSLSLLIRAELGTPGSLIGDDQIYNTIVTAHAFIMIFFMVMPIMIGGFGNWLVPLMLGAPDMAFPRMNNMSFWLLPPSITLLISSSIVENGAGTGWTVYPPLSSNIAHGGSSVDLAIFSLHLAGISSILGAINFITTIINMRLNNMFFD
QLPLFVWAVGITAFLLLLSLPVLAGAITMLLTDRNLNTSFFDPAGGGDPILYQHL
>LEFIB186-10|HM871090|MM00613|Eupithecia_subfuscata
TLYFIFGIWAGMIGTSLSLLIRAELGTPGSLIGDDQIYNTIVTAHAFIMIFFMVMPIMIGGFGNWLVPLMLGAPDMAFPRMNNMSFWLLPPSITLLISSSIVENGAGTGWTVYPPLSSNIAHGGSSVDLAIFSLHLAGISSILGAINFITTIINMRLNNMFFD
QLPLFVWAVGITAFLLLLSLPVLAGAITMLLTDRNLNTSFFDPAGGGDPILYQHL
>LEFIA677-10|HQ963154|MM01816|Eupithecia_subfuscata
TLYFIFGIWAGMIGTSLSLLIRAELGTPGSLIGDDQIYNTIVTAHAFIMIFFMVMPIMIGGFGNWLVPLMLGAPDMAFPRMNNMSFWLLPPSITLLISSSIVENGAGTGWTVYPPLSSNIAHGGSSVDLAIFSLHLAGISSILGAINFITTIINMRLNNMFFD
QLPLFVWAVGITAFLLLLSLPVLAGAITMLLTDRNLNTSFFDPAGGGDPILYQHL
>LEFID628-10|HM873391|MM06647|Eupithecia_subfuscata
TLYFIFGIWAGMIGTSLSLLIRAELGTPGSLIGDDQIYNTIVTAHAFIMIFFMVMPIMIGGFGNWLVPLMLGAPDMAFPRMNNMSFWLLPPSITLLISSSIVENGAGTGWTVYPPLSSNIAHGGSSVDLAIFSLHLAGISSILGAINFITTIINMRLNNMFFD
QLPLFVWAVGITAFLLLLSLPVLAGAITMLLTDRNLNTSFFDPAGGGDPILYQHL
>LEFIC308-10|HM872152|MM03805|Eupithecia_subumbrata
TLYFIFGIWAGMIGTSLSLLIRAELGTPGSLIGDDQIYNTIVTAHAFIMIFFMVMPIMIGGFGNWLVPLMLGAPDMAFPRMNNMSFWLLPPSIMLLISSSIVENGAGTGWTVYPPLSSNIAHGGSSVDLAIFSLHLAGISSILGAINFITTIINMRLNNMFFD
QLPLFVWAVGITAFLLLLSLPVLAGAITMLLTDRNLNTSFFDPAGGGDPILYQHL
>LEFIF756-10|HM875440|MM12965|Eupithecia_subumbrata
TLYFIFGIWAGMIGTSLSLLIRAELGTPGSLIGDDQIYNTIVTAHAFIMIFFMVMPIMIGGFGNWLVPLMLGAPDMAFPRMNNMSFWLLPPSIMLLISSSIVENGAGTGWTVYPPLSSNIAHGGSSVDLAIFSLHLAGISSILGAINFITTIINMRLNNMFFD
QLPLFVWAVGITAFLLLLSLPVLAGAITMLLTDRNLNTSFFDPAGGGDPILYQHL
>LEFIG355-10|HM876032|MM14352|Eupithecia_subumbrata
TLYFIFGIWAGMIGTSLSLLIRAELGTPGSLIGDDQIYNTIVTAHAFIMIFFMVMPIMIGGFGNWLVPLMLGAPDMAFPRMNNMSFWLLPPSIMLLISSSIVENGAGTGWTVYPPLSSNIAHGGSSVDLAIFSLHLAGISSILGAINFITTIINMRLNNMFFD
QLPLFVWAVGITAFLLLLSLPVLAGAITMLLTDRNLNTSFFDPAGGGDPILYQHL
>LEFIK919-10|JF854364|MM18494|Eupithecia_subumbrata
TLYFIFGIWAGMIGTSLSLLIRAELGTPGSLIGDDQIYNTIVTAHAFIMIFFMVMPIMIGGFGNWLVPLMLGAPDMAFPRMNNMSFWLLPPSIMLLISSSIVENGAGTGWTVYPPLSSNIAHGGSSVDLAIFSLHLAGISSILGAINFITTIINMRLNNMFFD
QLPLFVWAVGITAFLLLLSLPVLAGAITMLLTDRNLNTSFFDPAGGGDPILYQHL
>LEFIA650-10|HM870899|MM01767|Eupithecia_succenturiata
TLYFIFGIWAGMIGTSLS?LIRAELGTPGSLIGDDQIYNTIVTAHAFIMIFFMVMPIMIGGFGNWLVPLMLGAPDMAFPRMNNMSFWLLPPSITLLISSSIVENGAGTGWTVYPPLSSNIAHGGSSVDLAIFSLHLAGISSILGAINFITTIINMRLNNMFFD
QLPLFVWAVGITAFLLLLSLPVLAGAITMLLTDRNLNTSFFDPAGGGDPILYQHL
>LEFIA651-10|HM870900|MM01768|Eupithecia_succenturiata
TLYFIFGIWAGMIGTSLSLLIRAELGTPGSLIGDDQIYNTIVTAHAFIMIFFMVMPIMIGGFGNWLVPLMLGAPDMAFPRMNNMSFWLLPPSITLLISSSIVENGAGTGWTVYPPLSSNIAHGGSSVDLAIFSLHLAGISSILGAINFITTIINMRLNNMFFD
QLPLFVWAVGITAFLLLLSLPVLAGAITMLLTDRNLNTSFFDPAGGGDPILYQHL

>LEFIC051-10|HM871920|MM03320|Eupithecia_succenturiata
TLYFIFGIWAGMIGTSLSLLIRAELGTPGSLIGDDQIYNTIVTAHAFIMIFFMVMPIMIGGFGNWLVPLMLGAPDMAFPRMNNMSFWLLPPSITLLISSSIVENGAGTGWTVYPPLSSNIAHGGSSVDLAIFSLHLAGISSILGAINFITTIINMRLNNMFFD
QLPLFVWAVGITAFLLLLSLPVLAGAITMLLTDRNLNTSFFDPAGGGDPILYQHL
>LEFIC341-10|HM872185|MM03848|Eupithecia_tantillaria
TLYFIFGIWAGMIGTSLSLLIRAELGTPGSLIGDDQIYNTIVTAHAFIMIFFMVMPIMIGGFGNWLVPLMLGAPDMAFPRMNNMSFWLLPPSITLLISSSIVENGAGTGWTVYPPLSSNIAHGGSSVDLAIFSLHLAGISSILGAINFITTIINMRLNNMFFD
QLPLFVWAVGITAFLLLLSLPVLAGAITMLLTDRNLNTSFFDPAGGGDPILYQHL
>LEFID966-10|HM873716|MM07961|Eupithecia_tantillaria
TLYFIFGIWAGMIGTSLSLLIRAELGTPGSLIGDDQIYNTIVTAHAFIMIFFMVMPIMIGGFGNWLVPLMLGAPDMAFPRMNNMSFWLLPPSITLLISSSIVENGAGTGWTVYPPLSSNIAHGGSSVDLAIFSLHLAGISSILGAINFITTIINMRLNNMFFD
QLPLFVWAVGITAFLLLLSLPVLAGAITMLLTDRNLNTSFFDPAGGGDPILYQHL
>LEFIE783-10|HM874502|MM09961|Eupithecia_tantillaria
TLYFIFGIWAGMIGTSLSLLIRAELGTPGSLIGDDQIYNTIVTAHAFIMIFFMVMPIMIGGFGNWLVPLMLGAPDMAFPRMNNMSFWLLPPSITLLISSSIVENGAGTGWTVYPPLSSNIAHGGSSVDLAIFSLHLAGISSILGAINFITTIINMRLNNMFFD
QLPLFVWAVGITAFLLLLSLPVLAGAITMLLTDRNLNTSFFDPAGGGDPILYQHL
>LEFIA679-10|HM386825|MM01818|Eupithecia_tenuiata
TLYFIFGIWAGMIGTSLSLLIRAELGTPGSLIGDDQIYNTIVTAHAFIMIFFMVMPIMIGGFGNWLVPLMLGAPDMAFPRMNNMSFWLLPPSITLLISSSIVENGAGTGWTVYPPLSSNIAHGGSSVDLAIFSLHLAGISSILGAINFITTIINMRLNNMFFD
QLPLFVWAVGITAFLLLLSLPVLAGAITMLLTDRNLNTSFFDPAGGGDPILYQHL
>LEFIF759-10|HM875443|MM12968|Eupithecia_tenuiata
TLYFIFGIWAGMIGTSLSLLIRAELGTPGSLIGDDQIYNTIVTAHAFIMIFFMVMPIMIGGFGNWLVPLMLGAPDMAFPRMNNMSFWLLPPSITLLISSSIVENGAGTGWTVYPPLSSNIAHGGSSVDLAIFSLHLAGISSILGAINFITTIINMRLNNMFFD
QLPLFVWAVGITAFLLLLSLPVLAGAITMLLTDRNLNTSFFDPAGGGDPILYQHL
>LEFID323-10|HM873120|MM06211|Eupithecia_tripunctaria
TLYFIFGIWAGMIGTSLSLLIRAELGTPGSLIGDDQIYNTIVTAHAFIMIFFMVMPIMIGGFGNWLVPLMLGAPDMAFPRMNNMSFWLLPPSITLLISSSIVENGAGTGWTVYPPLSSNIAHGGSSVDLAIFSLHLAGISSILGAINFITTIINMRLNTMFFD
QLPLFIWAVGITAFLLLLSLPVLAGAITMLLTDRNLNTSFFDPAGGGDPILYQHL
>LEFID675-10|HM873436|MM06716|Eupithecia_trisignaria
TLYFIFGIWAGMIGTSLSLLIRAELGTPGSLIGDDQIYNTIVTAHAFIMIFFMVMPIMIGGFGNWLVPLMLGAPDMAFPRMNNMSFWLLPPSITLLISSSIVESGAGTGWTVYPPLSSNIAHGGSSVDLAIFSLHLAGISSILGAINFITTIINMRLNNMFFDQ
LPLFVWAVGITAFLLLLSLPVLAGAITMLLTDRNLNTSFFDPAGGGDPILYQHL
>LEFIE698-10|HM874419|MM09680|Eupithecia_trisignaria
TLYFIFGIWAGMIGTSLSLLIRAELGTPGSLIGDDQIYNTIVTAHAFIMIFFMVMPIMIGGFGNWLVPLMLGAPDMAFPRMNNMSFWLLPPSITLLISSSIVESGAGTGWTVYPPLSSNIAHGGSSVDLAIFSLHLAGISSILGAINFITTIINMRLNNMFFDQ
LPLFVWAVGITAFLLLLSLPVLAGAITMLLTDRNLNTSFFDPAGGGDPILYQHL
>LEFIK917-10|JF854363|MM18492|Eupithecia_trisignaria
TLYFIFGIWAGMIGTSLSLLIRAELGTPGSLIGDDQIYNTIVTAHAFIMIFFMVMPIMIGGFGNWLVPLMLGAPDMAFPRMNNMSFWLLPPSITLLISSSIVESGAGTGWTVYPPLSSNIAHGGSSVDLAIFSLHLAGISSILGAINFITTIINMRLNNMFFDQ
LPLFVWAVGITAFLLLLSLPVLAGAITMLLTDRNLNTSFFDPAGGGDPILYQHL
>LEFIA905-10|HM387041|MM09783|Eupithecia_valerianata
TLYFIFGIWAGMIGTSLSLLIRAELGTPGSLIGDDQIYNTIVTAHAFIMIFFMVMPIMIGGFGNWLVPLMLGAPDMAFPRMNNMSFWLLPPSITLLISSSIVENGAGTGWTVYPPLSSNIAHGGSSVDLAIFSLHLAGISSILGAINFITTIINMRLNNMFFD
QLPLFVWAVGITAFLLLLSLPVLAGAITMLLTDRNLNTSFFDPAGGGDPILYQHL
>LEFIK902-10|JF854351|MM18477|Eupithecia_valerianata
TLYFIFGIWAGMIGTSLSLLIRAELGTPGSLIGDDQIYNTIVTAHAFIMIFFMVMPIMIGGFGNWLVPLMLGAPDMAFPRMNNMSFWLLPPSITLLISSSIVENGAGTGWTVYPPLSSNIAHGGSSVDLAIFSLHLAGISSILGAINFITTIINMRLNNMFFD
QLPLFVWAVGITAFLLLLSLPVLAGAITMLLTDRNLNTSFFDPAGGGDPILYQHL
>LEFIG957-10|HM876597|MM15821|Eupithecia_venosata
TLYFIFGIWAGMIGTSLSLLIRAELGTPGSLIGDDQIYNTIVTAHAFIMIFFMVMPIMIGGFGNWLVPLMLGAPDMAFPRMNNMSFWLLPPSIMLLISSSIVENGAGTGWTVYPPLSSNIAHGGSSVDLAIFSLHLAGISSILGAINFITTIINMRLNNMFFD
QLPLFIWAVGITAFLLLLSLPVLAGAITMLLTDRNLNTSFFDPAGGGDPILYQHL
>LEFIG958-10|HM876598|MM15822|Eupithecia_venosata
TLYFIFGIWAGMIGTSLSLLIRAELGTPGSLIGDDQIYNTIVTAHAFIMIFFMVMPIMIGGFGNWLVPLMLGAPDMAFPRMNNMSFWLLPPSIMLLISSSIVENGAGTGWTVYPPLSSNIAHGGSSVDLAIFSLHLAGISSILGAINFITTIINMRLNNMFFD
QLPLFIWAVGITAFLLLLSLPVLAGAITMLLTDRNLNTSFFDPAGGGDPILYQHL
>LEFIK903-10|KM572014|MM18478|Eupithecia_venosata
TLYFIFGIWAGMIGTSLSLLIRAELGTPGSLIGDDQIYNTIVTAHAFIMIFFMVMPIMIGGFGNWLVPLMLGAPDMAFPRMNNMSFWLLPPSIMLLISSSIVENGAGTGWTVYPPLSSNIAHGGSSVDLAIFSLHLAGISSILGAINFITTIINMRLNNMFFD
QLPLFIWAVGITAFLLLLSLPVLAGAITMLLTDRNLNTSFFDPAGGGDPILYQHL
>LEFIA665-10|HM870913|MM01797|Eupithecia_virgaureata
TLYFIFGIWAGMIGTSLSLLIRAELGTPGSLIGDDQIYNTIVTAHAFIMIFFMVMPIMIGGFGNWLVPLMLGAPDMAFPRMNNMSFWLLPPSITLLISSSIVENGAGTGWTVYPPLSSNIAHGGSSVDLAIFSLHLAGISSILGAINFITTIINMRLNNMFFD

QLPLFVWAVGITAFLLLLSLPVLAGAITMLLTDRNLNTSFFDPAGGGDPILYQHL
>LEFID382-10|HM873179|MM06293|Eupithecia_virgaureata
TLYFIFGIWAGMIGTSLSLLIRAELGTPGSLIGDDQIYNTIVTAHAFIMIFFMVMPIMIGGFGNWLVPLMLGAPDMAFPRMNNMSFWLLPPSITLLISSSIVENGAGTGWTVYPPLSSNIAHGGSSVDLAIFSLHLAGISSILGAINFITTIINMRLNNMFFD
QLPLFVWAVGITAFLLLLSLPVLAGAITMLLTDRNLNTSFFDPAGGGDPILYQHL
>LEFID472-10|HM873240|MM06414|Eupithecia_virgaureata
TLYFIFGIWAGMIGTSLSLLIRAELGTPGSLIGDDQIYNTIVTAHAFIMIFFMVMPIMIGGFGNWLVPLMLGAPDMAFPRMNNMSFWLLPPSITLLISSSIVENGAGTGWTVYPPLSSNIAHGGSSVDLAIFSLHLAGISSILGAINFITTIINMRLNNMFFD
QLPLFVWAVGITAFLLLLSLPVLAGAITMLLTDRNLNTSFFDPAGGGDPILYQHL
>LEFIE862-10|HM874580|MM10109|Eupithecia_virgaureata
TLYFIFGIWAGMIGTSLSLLIRAELGTPGSLIGDDQIYNTIVTAHAFIMIFFMVMPIMIGGFGNWLVPLMLGAPDMAFPRMNNMSFWLLPPSITLLISSSIVENGAGTGWTVYPPLSSNIAHGGSSVDLAIFSLHLAGISSILGAINFITTIINMRLNNMFFD
QLPLFVWAVGITAFLLLLSLPVLAGAITMLLTDRNLNTSFFDPAGGGDPILYQHL
>LEFIB196-10|HM871100|MM00632|Eupithecia_vulgata
TLYFIFGIWAGMIGTSLSLLIRAELGTPGSLIGDDQIYNTIVTAHAFIMIFFMVMPIMIGGFGNWLVPLMLGAPDMAFPRMNNMSFWLLPPSITLLISSSIVENGAGTGWTVYPPLSSNIAHGGSSVDLAIFSLHLAGISSILGAINFITTIINMRLNNMFFD
QLPLFVWAVGITAFLLLLSLPVLAGAITMLLTDRNLNTSFFDPAGGGDPILYQHL
>LEFIA667-10|HM386816|MM01799|Eupithecia_vulgata
TLYFIFGIWAGMIGTSLSLLIRAELGTPGSLIGDDQIYNTIVTAHAFIMIFFMVMPIMIGGFGNWLVPLMLGAPDMAFPRMNNMSFWLLPPSITLLISSSIVENGAGTGWTVYPPLSSNIAHGGSSVDLAIFSLHLAGISSILGAINFITTIINMRLNNMFFD
QLPLFVWAVGITAFLLLLSLPVLAGAITMLLTDRNLNTSFFDPAGGGDPILYQHL
>LEFIE055-10|HM873802|MM08171|Eupithecia_vulgata
TLYFIFGIWAGMIGTSLSLLIRAELGTPGSLIGDDQIYNTIVTAHAFIMIFFMVMPIMIGGFGNWLVPLMLGAPDMAFPRMNNMSFWLLPPSITLLISSSIVENGAGTGWTVYPPLSSNIAHGGSSVDLAIFSLHLAGISSILGAINFITTIINMRLNNMFFD
QLPLFVWAVGITAFLLLLSLPVLAGAITMLLTDRNLNTSFFDPAGGGDPILYQHL
>LEFIA469-10|HM386809|MM01540|Euplexia_lucipara
TLYFIFGIWAGMVGTSLSLLIRAELGNPGSLIGDDQIYNTIVTAHAFIMIFFMVMPIMIGGFGNWLVPLMLGAPDMAFPRMNNMSFWLLPPSLTLLISSSIVESGAGTGWTVYPPLSSNIAHGGSSVDLAIFSLHLAGISSILGAINFITTIINMRLNNLSFD
QMPLFIWAVGITAFLLLLSLPVLAGAITMLLTDRNLNTSFFDPAGGGDPILYQHL
>LEFIA470-10|HM386810|MM01541|Euplexia_lucipara
TLYFIFGIWAGMVGTSLSLLIRAELGNPGSLIGDDQIYNTIVTAHAFIMIFFMVMPIMIGGFGNWLVPLMLGAPDMAFPRMNNMSFWLLPPSLTLLISSSIVESGAGTGWTVYPPLSSNIAHGGSSVDLAIFSLHLAGISSILGAINFITTIINMRLNNLSFD
QMPLFIWAVGITAFLLLLSLPVLAGAITMLLTDRNLNTSFFDPAGGGDPILYQHL
>LEFID505-10|HM873270|MM06468|Eupoecilia_ambiguella
TLYFIFGIWAGMIGTSLSLLIRAELGSPGSLIGDDQIYNTIVTAHAFIMIFFMVMPIMIGGFGNWLVPLMLGAPDMAFPRMNNMSFWLLPPSIMLLISSSIVENGAGTGWTVYPPLSSNIAHSGSSVDLAIFSLHLAGISSILGAVNFITTIINMRPNNMKL
DQMPLFVWAVGVTALLLLLSLPVLAGAITMLLTDRNLNTSFFDPAGGGDPILYQHL
>LEFIG808-10|HM876460|MM15672|Eupoecilia_ambiguella
TLYFIFGIWAGMIGTSLSLLIRAELGSPGSLIGDDQIYNTIVTAHAFIMIFFMVMPIMIGGFGNWLVPLMLGAPDMAFPRMNNMSFWLLPPSIMLLISSSIVENGAGTGWTVYPPLSSNIAHSGSSVDLAIFSLHLAGISSILGAVNFITTIINMRPNNMKL
DQMPLFVWAVGITALLLLLSLPVLAGAITMLLTDRNLNTSFFDPAGGGDPILYQHL
>LEFIG809-10|HM876461|MM15673|Eupoecilia_ambiguella
TLYFIFGIWAGMIGTSLSLLIRAELGSPGSLIGDDQIYNTIVTAHAFIMIFFMVMPIMIGGFGNWLVPLMLGAPDMAFPRMNNMSFWLLPPSIMLLISSSIVENGAGTGWTVYPPLSSNIAHSGSSVDLAIFSLHLAGISSILGAVNFITTIINMRPNNMKL
DQMPLFVWAVGITALLLLLSLPVLAGAITMLLTDRNLNTSFFDPAGGGDPILYQHL
>LEFIF425-10|HM875110|MM11846|Eupoecilia_angustana
TLYFMFGIWAGMIGTSLSLLIRAELGNPGSLIGDDQIYNTIVTAHAFIMIFFMVMPIMIGGFGNWLIPLMLGAPDMAFPRMNNMSFWLLPPSIMLLISSSIVENGAGTGWTVYPPLSSNIAHSGSSVDLAIFSLHLAGISSILGAVNFITTIINMRPNNMKL
DQMPLFVWAVGITALLLLLSLPVLAGAITMLLTDRNLNTSFFDPAGGGDPILYQHL
>LEFIC263-10|HM872107|MM03694|Euproctis_similis
TLYFIFGIWAGMIGSSLSLMIRAELGTPGSLINNDQIFNTIITAHAFIMIFFMVMPIMIGGFGNWLVPLMLGAPDMAFPRMNNMSFWLLPPSLILLISSSIIENGAGTGWTVYPPLSGNIAHSGSSVDLTIFSLHLAGISSILGAINFITTIINMRLNNLSFDQ
MPLFVWAVGITAFLLLLSLPVLAGAITMLLTDRNLNTSFFDPAGGGDPILYQHL
>LEFIC264-10|HM872108|MM03695|Euproctis_similis
TLYFIFGIWAGMIGSSLSLMIRAELGTPGSLINNDQIFNTIITAHAFIMIFFMVMPIMIGGFGNWLVPLMLGAPDMAFPRMNNMSFWLLPPSLILLISSSIIENGAGTGWTVYPPLSGNIAHSGSSVDLTIFSLHLAGISSILGAINFITTIINMRLNNLSFDQ
MPLFVWAVGITAFLLLLSLPVLAGAITMLLTDRNLNTSFFDPAGGGDPILYQHL
>LEFIK943-10|JF854383|MM18518|Euproctis_similis
TLYFIFGIWAGMIGSSLSLMIRAELGTPGSLINNDQIFNTIITAHAFIMIFFMVMPIMIGGFGNWLVPLMLGAPDMAFPRMNNMSFWLLPPSLILLISSSIIENGAGTGWTVYPPLSGNIAHSGSSVDLTIFSLHLAGISSILGAINFITTIINMRLNNLSFDQ
MPLFVWAVGITAFLLLLSLPVLAGAITMLLTDRNLNTSFFDPAGGGDPILYQHL
>LEFIC662-10|HM872483|MM04621|Eupsilia_transversa

TLYFIFGIWAGMVGTSLSLLIRAELGNPGSLIGDDQIYNTIVTAHAFIMIFFMVMPIMIGGFGNWLVPLMLGAPDMAFPRLNNMSFWLLPPSLTLLISSSIVENGAGTGWTVYPPLSSNIAHGGSSVDLAIFSLHLAGISSILGAINFITTIINMRLNNLSFDQ
MPLFIWAVGITAFLLLLSLPVLAGAITMLLTDRNLNTSFFDPAGGGDPILYQHL
>LEFIH003-10|HM876639|MM15867|Eupsilia_transversa
TLYFIFGIWAGMVGTSLSLLIRAELGNPGSLIGDDQIYNTIVTAHAFIMIFFMVMPIMIGGFGNWLVPLMLGAPDMAFPRLNNMSFWLLPPSLTLLISSSIVENGAGTGWTVYPPLSSNIAHGGSSVDLAIFSLHLAGISSILGAINFITTIINMRLNNLSFDQ
MPLFIWAVGITAFLLLLSLPVLAGAITMLLTDRNLNTSFFDPAGGGDPILYQHL
>LEFIL248-10|JF854552|MM19248|Eurhodope_cirrigerella
TLYFIFGIWSGMVGTSLSLLIRAELGTPGSLIGDDQIYNTIVTGHAFIMIFFMVMPIMIGGFGNWLVPLMLGAPDMAFPRMNNMSFWLLPPSLTLLISSSIVENGAGTGWTVYPPLSSNIAHSGSSVDLAIFSLHLAGISSILGAINFITTIINMKLNGMMF
DQMPLFVWAVGITALLLLLSLPVLAGAITMLLTDRNLNTSFFDPAGGGDPILYQHL
>LEFIB831-10|HM871708|MM02786|Eurois_occulta
TLYFIFGIWAGMVGTSLSLLIRAELGNPGSLIGDDQIYNTIVTAHAFIMIFFMVMPIMIGGFGNWLVPLMLGAPDMAFPRMNNMSFWLLPPSLTLLISSSIVENGAGTGWTVYPPLSSNIAHGGSSVDLAIFSLHLAGISSILGAINFITTIINMRLNSLSFD
QMPLFIWAVGITAFLLLLSLPVLAGAITMLLTDRNLNTSFFDPAGGGDPILYQHL
>LEEUA283-11|JN274703|MM19691|Eurrhypis_pollinalis
TLYFIFGIWAGMVGTSLSLLIRAELGNPGSLIGDDQIYNTIVTAHAFIMIFFMVMPIMIGGFGNWLVPLMLGAPDMAFPRMNNMSFWLLPPSLLLLISSSIVENGAGTGWTVYPPLSSNIAHGGSSVDLAIFSLHLAGISSILGAINFITTIINMRINGLSFD
QMPLFVWAVGITALLLLLSLPVLAGAITMLLTDRNLNTSFFDPAGGGDPILYQHL
>LEFIG107-10|HM875787|MM13894|Euspilapteryx_auroguttella
TLYFIFGIWAGMLGTSLSMLIRAELGNPGSLIGDDQIYNTIVTAHAFIMIFFMVMPIMIGGFGNWLVPLMLGAPDMAFPRLNNMSFWLLPPSLILLISSSIVENGAGTGWTVYPPLSSNIAHGGSSVDLAIFSLHLAGISSILGAINFITTIINMRANGMMF
DSMSLFTWAVSITALLLLLSLPVLAGAITMLLTDRNLNTSFFDPAGGGDPILYQHL
>LEFIG190-10|HM875870|MM14063|Euspilapteryx_auroguttella
TLYFIFGIWAGMLGTSLSMLIRAELGNPGSLIGDDQIYNTIVTAHAFIMIFFMVMPIMIGGFGNWLVPLMLGAPDMAFPRLNNMSFWLLPPSLILLISSSIVENGAGTGWTVYPPLSSNIAHGGSSVDLAIFSLHLAGISSILGAINFITTIINMRANGMMF
DSMSLFTWAVSITALLLLLSLPVLAGAITMLLTDRNLNTSFFDPAGGGDPILYQHL
>LEFIB627-10|HM871506|MM02373|Eustroma_reticulata
TLYFIFGIWAGMIGTSLSLLIRAELGNPGSLIGDDQIYNTIVTAHAFIMIFFMVMPIMIGGFGNWLVPLMLGAPDMAFPRMNNMSFWLLPPSITLLISSSIVENGAGTGWTVYPPLSSNIAHGGSSVDLAIFSLHLAGISSILGAINFITTIINMRLNNMFFD
QLPLFVWAVGITAFLLLLSLPVLAGAITMLLTDRNLNTSFFDPAGGGDPILYQHL
>LEFIC182-10|HM872026|MM03537|Eustroma_reticulata
TLYFIFGIWAGMIGTSLSLLIRAELGNPGSLIGDDQIYNTIVTAHAFIMIFFMVMPIMIGGFGNWLVPLMLGAPDMAFPRMNNMSFWLLPPSITLLISSSIVENGAGTGWTVYPPLSSNIAHGGSSVDLAIFSLHLAGISSILGAINFITTIINMRLNNMFFD
QLPLFVWAVGITAFLLLLSLPVLAGAITMLLTDRNLNTSFFDPAGGGDPILYQHL
>LEFIA126-10|HM396473|MM01056|Euthrix_potatoria
TLYFIFGIWAGMVGTSLSLLIRAELGTSGSLIGDDQIYNTIVTAHAFIMIFFMVMPIMIGGFGNWLVPLMLGAPDMAFPRMNNMSFWLLPPSLTLLISSSIVENGAGTGWTVYPPLSSNIAHSGSSVDLAIFSLHLAGISSILGAINFITTIINMRLNNMSFD
QMPLFVWSVGITAFLLLLSLPVLAGAITMLLTDRNLNTSFFDPAGGGDPILYQHL
>LEFIJ1016-11|MM09291|Euxoa_adumbrata
TLYFIFGIWAGMVGTSLSLLIRAELGNPGSLIGDDQIYNTIVTAHAFIMIFFMVMPIMIGGFGNWLVPLMLGAPDMAFPRMNNMSFWLLPPSLTLLISSSIVENGAGTGWTVYPPLSSNIAHGGSSVDLAIFSLHLAGISSILGAINFITTIINMRLNSLSFD
QMPLFIWAVGITAFLLLLSLPVLAGAITMLLTDRNLNTSFFDPAGGGDPILYQHL
>LEFID830-10|HM873587|MM06947|Euxoa_cursoria
TLYFIFGIWAGMVGTSLSLLIRAELGNPGSLIGDDQIYNTIVTAHAFIMIFFMVMPIMIGGFGNWLVPLMLGAPDMAFPRMNNMSFWLLPPSLTLLISSSIVENGAGTGWTVYPPLSSNIAHGGSSVDLAIFSLHLAGISSILGAINFITTIINMRLNSLSFD
QMPLFIWAVGITAFLLLLSLPVLAGAITMLLTDRNLNTSFFDPAGGGDPILYQHL
>LEFIC221-10|HM872065|MM03617|Euxoa_nigricans
TLYFIFGIWAGMVGTSLSLLIRAELGNPGSLIGDDQIYNTIVTAHAFIMIFFMVMPIMIGGFGNWLVPLMLGAPDMAFPRMNNMSFWLLPPSLTLLISSSIVENGAGTGWTVYPPLSSNIAHGGSSVDLAIFSLHLAGISSILGAINFITTIINMRLNSLSFD
QMPLFIWAVGITAFLLLLSLPVLAGAITMLLTDRNLNTSFFDPAGGGDPILYQHL
>LEFIC774-10|HM872593|MM04904|Euxoa_nigricans
TLYFIFGIWAGMVGTSLSLLIRAELGNPGSLIGDDQIYNTIVTAHAFIMIFFMVMPIMIGGFGNWLVPLMLGAPDMAFPRMNNMSFWLLPPSLTLLISSSIVENGAGTGWTVYPPLSSNIAHGGSSVDLAIFSLHLAGISSILGAINFITTIINMRLNSLSFD
QMPLFIWAVGITAFLLLLSLPVLAGAITMLLTDRNLNTSFFDPAGGGDPILYQHL
>LEFID838-10|HM873595|MM06955|Euxoa_nigricans
TLYFIFGIWAGMVGTSLSLLIRAELGNPGSLIGDDQIYNTIVTAHAFIMIFFMVMPIMIGGFGNWLVPLMLGAPDMAFPRMNNMSFWLLPPSLTLLISSSIVENGAGTGWTVYPPLSSNIAHGGSSVDLAIFSLHLAGISSILGAINFITTIINMRLNSLSFD
QMPLFIWAVGITAFLLLLSLPVLAGAITMLLTDRNLNTSFFDPAGGGDPILYQHL
>LEFIG551-10|HM876224|MM14694|Euxoa_nigricans
TLYFIFGIWAGMVGTSLSLLIRAELGNPGSLIGDDQIYNTIVTAHAFIMIFFMVMPIMIGGFGNWLVPLMLGAPDMAFPRMNNMSFWLLPPSLTLLISSSIVENGAGTGWTVYPPLSSNIAHGGSSVDLAIFSLHLAGISSILGAINFITTIINMRLNSLSFD
QMPLFIWAVGITAFLLLLSLPVLAGAITMLLTDRNLNTSFFDPAGGGDPILYQHL

>LEFIF710-10|HM875394|MM12748|Euxoa_obelisca
TLYFIFGIWAGMVGTSLSLLIRAELGNPGSLIGDDQIYNTIVTAHAFIMIFFMVMPIMIGGFGNWLVPLMLGAPDMAFPRMNNMSFWLLPPSLTLLISSSIVENGAGTGWTVYPPLSSNIAHGGSSVDLAIFSLHLAGISSILGAINFITTIINMRLNNLSFD
QMPLFIWAVGITAFLLLLSLPVLAGAITMLLTDRNLNTSFFDPAGGGDPILYQHL
>LEFIJ1459-12|MM22727|Euxoa_obelisca
TLYFIFGIWAGMVGTSLSLLIRAELGNPGSLIGDDQIYNTIVTAHAFIMIFFMVMPIMIGGFGNWLIPLMLGAPDMAFPRMNNMSFWLLPPSLTLLISSSIVENGAGTGWTVYPPLSSNIAHGGSSVDLAIFSLHLAGISSILGAINFITTIINMRLNNLSFD
QMPLFIWAVGITAFLLLLSLPVLAGAITMLLTDRNLNTSFFDPAGGGDPILYQHL
>LEFIB260-10|HM871163|MM00732|Euxoa_ochrogaster
TLYFIFGIWAGMVGTSLSLLIRAELGNPGSLIGDDQIYNTIVTAHAFIMIFFMVMPIMIGGFGNWLVPLMLGAPDMAFPRMNNMSFWLLPPSLTLLISSSIVENGAGTGWTVYPPLSSNIAHGGSSVDLAIFSLHLAGISSILGAINFITTIINMRLNNLSFD
QMPLFIWAVGITAFLLLLSLPVLAGAITMLLTDRNLNTSFFDPAGGGDPILYQHL
>LEFIB258-10|HM871161|MM00730|Euxoa_recussa
TLYFIFGIWAGMVGTSLSLLIRAELGNPGSLIGDDQIYNTIVTAHAFIMIFFMVMPIMIGGFGNWLIPLMLGAPDMAFPRMNNMSFWLLPPSLTLLISSSIVENGAGTGWTVYPPLSSNIAHGGSSVDLAIFSLHLAGISSILGAINFITTIINMRLNNLSFD
QMPLFIWAVGITAFLLLLSLPVLAGAITMLLTDRNLNTSFFDPAGGGDPILYQHL
>LEFIC741-10|HM872562|MM04844|Euxoa_tritici
TLYFIFGIWAGMVGTSLSLLIRAELGNPGSLIGDDQIYNTIVTAHAFIMIFFMVMPIMIGGFGNWLVPLMLGAPDMAFPRMNNMSFWLLPPSLTLLISSSIVENGAGTGWTVYPPLSSNIAHGGSSVDLAIFSLHLAGISSILGAINFITTIINMRLNNLSFD
QMPLFIWAVGITAFLLLLSLPVLAGAITMLLTDRNLNTSFFDPAGGGDPILYQHL
>LEFIF540-10|HM875225|MM12219|Euxoa_tritici
TLYFIFGIWAGMVGTSLSLLIRAELGNPGSLIGDDQIYNTIVTAHAFIMIFFMVMPIMIGGFGNWLVPLMLGAPDMAFPRMNNMSFWLLPPSLTLLISSSIVENGAGTGWTVYPPLSSNIAHGGSSVDLAIFSLHLAGISSILGAINFITTIINMRLNNLSFD
QMPLFIWAVGITAFLLLLSLPVLAGAITMLLTDRNLNTSFFDPAGGGDPILYQHL
>LEFIH124-10|HM876744|MM15018|Euxoa_tritici
TLYFIFGIWAGMIGTSLSLLIRAELGNPGSLIGDDQIYNTIVTAHAFIMIFFMVMPIMIGGFGNWLVPLMLGAPDMAFPRMNNMSFWLLPPSLTLLISSSIVENGAGTGWTVYPPLSSNIAHGGSSVDLAIFSLHLAGISSILGAINFITTIINMRLNNLSFD
QMPLFIWAVGITAFLLLLSLPVLAGAITMLLTDRNLNTSFFDPAGGGDPILYQHL
>LEFIJ432-10|MM17033|Euxoa_tritici
TLYFIFGIWAGMVGTSLSLLIRAELGNPGSLIGDDQIYNTIVTAHAFIMIFFMVMPIMIGGFGNWLVPLMLGAPDMAFPRMNNMSFWLLPPSLTLLISSSIVENGAGTGWTVYPPLSSNIAHGGSSVDLAIFSLHLAGISSILGAINFITTIINMRLNNLSFD
QMPLFIWAVGITAFLLLLSLPVLAGAITMLLTDRNLNTSFFDPAGGGDPILYQHL
>LEFIF786-10|HM875470|MM13024|Euzophera_cinerosella
TLYFIFGIWSGLVGTSLSLLIRTELGTPNSLIGDDQIYNTIVTGHAFIMIFFMVMPIMIGGFGNWLVPLMLGAPDMAFPRMNNMSFWLLPPSLTLLISSSIVENGAGTGWTVYPPLSSNIAHSGSSVDLAIFSLHLAGISSILGAINFITTIINMKLNGMSFDQ
MPLFVWAVGITALLLLLSLPVLAGAITMLLTDRNLNTSFFDPAGGGDPILYQHL
>LEFIF787-10|HM875471|MM13025|Euzophera_cinerosella
TLYFIFGIWSGLVGTSLSLLIRTELGTPNSLIGDDQIYNTIVTGHAFIMIFFMVMPIMIGGFGNWLVPLMLGAPDMAFPRMNNMSFWLLPPSLTLLISSSIVENGAGTGWTVYPPLSSNIAHSGSSVDLAIFSLHLAGISSILGAINFITTIINMKLNGMSFDQ
MPLFVWAVGITALLLLLSLPVLAGAITMLLTDRNLNTSFFDPAGGGDPILYQHL
>LEFIC200-10|HM872044|MM03562|Euzophera_fuliginosella
TLYFIFGIWSGLVGTSLSLLIRAELGTPGSLIGDDQIYNTIVTGHAFIMIFFMVMPIMIGGFGNWLVPLMLGAPDMAFPRLNNMSFWLLPPSILLLISSSIVENGAGTGWTVYPPLSSNIAHGGSSVDLAIFSLHLAGISSILGAINFITTIINMKLNGLSFDQM
PLFIWAVGITALLLLLSLPVLAGAITMLLTDRNLNTSFFDPAGGGDPILYQHL
>LEFIK811-10|JN275980|MM18386|Euzophera_fuliginosella
TLYFIFGIWSGLVGTSLSLLIRAELGTPGSLIGDDQIYNTIVTGHAFIMIFFMVMPIMIGGFGNWLVPLMLGAPDMAFPRLNNMSFWLLPPSILLLISSSIVENGAGTGWTVYPPLSSNIAHGGSSVDLAIFSLHLAGISSILGAINFITTIINMKLNGLSFDQM
PLFVWAVGITALLLLLSLPVLAGAITMLLTDRNLNTSFFDPAGGGDPILYQHL
>LEFID775-10|HM873532|MM06841|Euzophera_pinguis
TLYFIFGIWSGLIGTSLSLLIRTELGTPNSLIGDDQIYNTIVTGHAFIMIFFMVMPIMIGGFGNWLVPLMLGAPDMAFPRMNNMSFWLLPPSLTLLISSSIVENGVGTGWTVYPPLSSNIAHSGSSVDLAIFSLHLAGISSILGAINFITTIINMKLNGMSFDQ
MPLFVWAVGITALLLLLSLPVLAGAITMLLTDRNLNTSFFDPAGGGDPILYQHL
>LEFIC911-10|HM872728|MM05249|Evergestis_aenealis
TLYFIFGIWAGMVGTSLSLLIRAELGNPGSLIGDDQIYNTIVTAHAFIMIFFMVMPIMIGGFGNWLVPLMLGAPDMAFPRMNNMSFWLLPPSLTLLISSSIVENGAGTGWTVYPPLSSNIAHGGSSVDLAIFSLHLAGISSILGAINFITTIINMRINGLSFD
QMPLFVWAVGITALLLLLSLPVLAGAITMLLTDRNLNTSFFDPAGGGDPILYQHL
>LEFIA703-10|HM386848|MM01858|Evergestis_extimalis
TLYFIFGIWAGMVGTSLSLLIRAELGNPGSLIGDDQIYNTIVTAHAFIMIFFMVMPIMIGGFGNWLVPLMLGAPDMAFPRMNNMSFWLLPPSLTLLISSSIVENGAGTGWTVYPPLSSNIAHGGSSVDLAIFSLHLAGISSILGAINFITTIINMRINGLSFD
QMPLFVWAVGITALLLLLSLPVLAGAITMLLTDRNLNTSFFDPAGGGDPILYQHL
>LEFIA704-10|HM386849|MM01859|Evergestis_extimalis
TLYFIFGIWAGMVGTSLSLLIRAELGNPGSLIGDDQIYNTIVTAHAFIMIFFMVMPIMIGGFGNWLVPLMLGAPDMAFPRMNNMSFWLLPPSLTLLISSSIVENGAGTGWTVYPPLSSNIAHGGSSVDLAIFSLHLAGISSILGAINFITTIINMRINGLSFD

QMPLFVWAVGITALLLLLSLPVLAGAITMLLTDRNLNTSFFDPAGGGDPILYQHL
>LEFIF748-10|HM875432|MM12936|Evergestis_extimalis
TLYFIFGIWAGMVGTSLSLLIRAELGNPGSLIGDDQIYNTIVTAHAFIMIFFMVMPIMIGGFGNWLVPLMLGAPDMAFPRMNNMSFWLLPPSLTLLISSSIVENGAGTGWTVYPPLSSNIAHGGSSVDLAIFSLHLAGISSILGAINFITTIINMRINGLSFD
QMPLFVWAVGITALLLLLSLPVLAGAITMLLTDRNLNTSFFDPAGGGDPILYQHL
>LEFIA719-10|HM386863|MM01874|Evergestis_forficalis
TLYFIFGIWAGMVGTSLSLLIRAELGNPGSLIGDDQIYNTIVTAHAFIMIFFMVMPIMIGGFGNWLVPLMLGAPDMAFPRMNNMSFWLLPPSLTLLISSSIVENGAGTGWTVYPPLSSNIAHGGSSVDLAIFSLHLAGISSILGAINFITTIINMRINGLSFD
QMPLFVWAVGITALLLLLSLPVLAGAITMLLTDRNLNTSFFDPAGGGDPILYQHL
>LEFIC569-10|HM872390|MM04347|Evergestis_limbata
TLYFIFGVWAGMVGTSLSLLIRAELGNPGSLIGDDQIYNTIVTAHAFIMIFFMVMPIMIGGFGNWLVPLMLGAPDMAFPRMNNMSFWLLPPSLTLLISSSIVENGAGTGWTVYPPLSSNIAHGGSSVDLAIFSLHLAGISSILGAINFITTIINMRINGLSFD
QMPLFVWAVGITALLLLLSLPVLAGAITMLLTDRNLNTSFFDPAGGGDPILYQHL
>LEFIC570-10|HM872391|MM04348|Evergestis_limbata
TLYFIFGVWAGMVGTSLSLLIRAELGNPGSLIGDDQIYNTIVTAHAFIMIFFMVMPIMIGGFGNWLVPLMLGAPDMAFPRMNNMSFWLLPPSLTLLISSSIVENGAGTGWTVYPPLSSNIAHGGSSVDLAIFSLHLAGISSILGAINFITTIINMRINGLSFD
QMPLFVWAVGITALLLLLSLPVLAGAITMLLTDRNLNTSFFDPAGGGDPILYQHL
>LEFIA701-10|HM386846|MM01856|Evergestis_pallidata
TLYFIFGIWAGMVGTSLSLLIRAELGNPGSLIGDDQIYNTIVTAHAFIMIFFMVMPIMIGGFGNWLVPLMLGAPDMAFPRMNNMSFWLLPPSLTLLISSSIVENGAGTGWTVYPPLSSNIAHGGSSVDLAIFSLHLAGISSILGAINFITTIINMRINGLSFD
QMPLFVWAVGITALLLLLSLPVLAGAITMLLTDRNLNTSFFDPAGGGDPILYQHL
>LEFIB569-10|HM871451|MM02223|Exaeretia_allisella
TLYFIFGIWAGMVGTSLSLMIRAELGNPGSLIGDDQIYNTIVTAHAFIMIFFMVMPIMIGGFGNWLVPLMLGAPDMAFPRMNNMSFWLLPPSLTLLISSSIVENGAGTGWTVYPPLSSNIAHGGSSVDLAIFSLHLAGISSILGAINFITTIINMKLNNMSF
DQLPLFVWAVGITALLLLLSLPVLAGAITMLLTDRNLNTSFFDPAGGGDPILYQHL
>LEFIB107-10|HM871014|MM00427|Exaeretia_ciniflonella
TLYFIFGIWAGMIGTSLSLLIRAELGNPGSLIGDDQIYNTIVTAHAFIMIFFMVMPIMIGGFGNWLVPLMLGAPDMAFPRMNNMSFWLLPPSLTLLISSSIVENGAGTGWTVYPPLSSNIAHSGSSVDLAIFSLHLAGISSILGAINFITTIINMRLNSMSFD
RLPLFVWAVGITALLLLLSLPVLAGAITMLLTDRNLNTSFFDPAGGGDPILYQHL
>LEFIC938-10|HM872753|MM05297|Exaeretia_ciniflonella
TLYFIFGIWAGMIGTSLSLLIRAELGNPGSLIGDDQIYNTIVTAHAFIMIFFMVMPIMIGGFGNWLVPLMLGAPDMAFPRMNNMSFWLLPPSLTLLISSSIVENGAGTGWTVYPPLSSNIAHSGSSVDLAIFSLHLAGISSILGAINFITTIINMRLNSMSFD
RLPLFVWAVGITALLLLLSLPVLAGAITMLLTDRNLNTSFFDPAGGGDPILYQHL
>LEFIB800-10|HM871677|MM02714|Exapate_congelatella
T?YFIFGIWAGLMGTSLSLLIRMELGNPGSLIGDDQIYNTIVTAHAFIMIFFMVMPIMIGGFGNWLVPLMLGAPDMAFPRMNNMSFWLLPPSIILLISSSIVENGVGTGWTVYPPLSSNIAHSGSSVDLTIFSLHLAGISSILGAINFITTIINMRPNNMNLD
QMPLFVWSVGITALLLLLSLPILAGAITMLLTDRNLNTSFFDPAGGGDPILYQHL
>LEFIE898-10|HM874616|MM10195|Exapate_congelatella
TLYFIFGIWAGLMGTSLSLLIRMELGNPGSLIGDDQIYNTIVTAHAFIMIFFMVMPIMIGGFGNWLVPLMLGAPDMAFPRMNNMSFWLLPPSIILLISSSIVENGAGTGWTVYPPLSSNIAHSGSSVDLTIFSLHLAGISSILGAINFITTIINMRPNNMNLD
QMPLFVWSVGITALLLLLSLPILAGAITMLLTDRNLNTSFFDPAGGGDPILYQHL
>LEFIE899-10|HM874617|MM10196|Exapate_congelatella
TLYFIFGIWAGLMGTSLSLLIRMELGNPGSLIGDDQIYNTIVTAHAFIMIFFMVMPIMIGGFGNWLVPLMLGAPDMAFPRMNNMSFWLLPPSIILLISSSIVENGAGTGWTVYPPLSSNIAHSGSSVDLTIFSLHLTGISSILGAINFITTIINMRPNNMNLD
QMPLFVWSVGITALLLLLSLPILAGAITMLLTDRNLNTSFFDPAGGGDPILYQHL
>LEFIC386-10|HM872229|MM03918|Exoteleia_dodecella
TLYFIFGIWAGMVGTSLSLLIRAELGNPGSLIGDDQIYNTIVTAHAFIMIFFMVMPIMIGGFGNWLIPLMLGAPDMAFPRMNNMSFWLLPPSLTLLISSSIVENGAGTGWTVYPPLSSNIAHGGSSVDLAIFSLHLAGISSILGAINFITTIINMRINGLSFDQ
MPLFVWAVGITALLLLLSLPVLAGAITMLLTDRNLNTSFFDPAGGGDPILYQHL
>LEFIF291-10|HM874981|MM11073|Fabula_zollikoferi
TLYFIFGIWAGMVGTSLSLLIRAELGNPGSLIGDDQIYNTIVTAHAFIMIFFMVMPIMIGGFGNWLVPLMLGAPDMAFPRMNNMSFWLLPPSLTLLISSSIVENGAGTGWTVYPPLSSNIAHGGSSVDLAIFSLHLAGISSILGAINFITTIINMRLNSLSFD
QMPLFIWAVGITAFLLLLSLPVLAGAITMLLTDRNLNTSFFDPAGGGDPILYQHL
>LEFIL436-10|JF854596|MM18734|Fabula_zollikoferi
TLYFIFGIWAGMVGTSLSLLIRAELGNPGSLIGDDQIYNTIVTAHAFIMIFFMVMPIMIGGFGNWLVPLMLGAPDMAFPRMNNMSFWLLPPSLTLLISSSIVENGAGTGWTVYPPLSSNIAHGGSSVDLAIFSLHLAGISSILGAINFITTIINMRLNSLSFD
QMPLFIWAVGITAFLLLLSLPVLAGAITMLLTDRNLNTSFFDPAGGGDPILYQHL
>LEFIA225-10|HM396568|MM01257|Falcaria_lacertinaria
TLYFIFGIWAGMLGTSLSLLIRAELGNPGSLIGDDQIYNTIVTAHAFIMIFFMVMPIMIGGFGNWLVPLMLGAPDMAFPRMNNMSFWMLPPSLTLLISSSIVENGAGTGWTVYPPLSSNIAHSGSSVDLAIFSLHLAGISSILGAINFITTIINMRLNNMM
FDQMPLFVWAVGITAFLLLLSLPVLAGAITMLLTDRNLNTSFFDPAGGGDPILYQHL
>LEEUA205-11|JN286421|MM19613|Falseuncaria_degreyana

TLYFIFGIWAGMVGTSLSLLIRAELGNPGSLIGDDQIYNTIVTAHAFIMIFFMVMPIMIGGFGNWLVPLMLGAPDMAFPRMNNMSFWLLPPSIMLLISSSIVENGAGTGWTVYPPLSSNIAHSGSSVDLAIFSLHLAGISSILGAVNFITTIINMRPNNMSL
DQMPLFVWAVGITALLLLLSLPVLAGAITMLLTDRNLNTSFFDPAGGGDPILYQHL
>LEFIE607-10|HM874330|MM09521|Falseuncaria_ruficiliana
TLYFIFGIWAGMVGTSLSLLIRAELGNPGSLIGDDQIYNTIVTAHAFIMIFFMVMPIMIGGFGNWLVPLMLGAPDMAFPRMNNMSFWLLPPSIMLLISSSIVENGAGTGWTVYPPLSSNIAHSGSSVDLAIFSLHLAGISSILGAVNFITTIINMRPNNMSL
DQMPLFVWSVGITALLLLLSLPVLAGAITMLLTDRNLNTSFFDPAGGGDPILYQHL
>LEFIF893-10|HM875575|MM13300|Falseuncaria_ruficiliana
TLYFIFGIWAGMVGTSLSLLIRAELGNPGSLIGDDQIYNTIVTAHAFIMIFFMVMPIMIGGFGNWLVPLMLGAPDMAFPRMNNMSFWLLPPSIMLLISSSIVENGAGTGWTVYPPLSSNIAHSGSSVDLAIFSLHLAGISSILGAVNFITTIINMRPNNMSL
DQMPLFVWSVGITALLLLLSLPVLAGAITMLLTDRNLNTSFFDPAGGGDPILYQHL
>LEFIK703-10|JF854268|MM18278|Falseuncaria_ruficiliana
TLYFIFGIWAGMVGTSLSLLIRAELGNPGSLIGDDQIYNTIVTAHAFIMIFFMVMPIMIGGFGNWLVPLMLGAPDMAFPRMNNMSFWLLPPSIMLLISSSIVENGAGTGWTVYPPLSSNIAHSGSSVDLAIFSLHLAGISSILGAVNFITTIINMRPNNMSL
DQMPLFVWSVGITALLLLLSLPVLAGAITMLLTDRNLNTSFFDPAGGGDPILYQHL
>LEFIB001-10|HM870914|MM00001|Favonius_quercus
TLYFIFGIWAGMLGTSLSILIRMELGTPGSLIGNDQIYNTIVTAHAFIMIFFMVMPIMIGGFGNWLVPLMLGAPDMAFPRMNNMSFWLLPPSLMLLISSSIVENGAGTGWTVYPPLSSNIAHSGASVDLAIFSLHLAGISSILGAINFITTIINMRISNLSFD
QMSLFIWAVGITALLLLLSLPVLAGAITMLLTDRNLNTSFFDPAGGGDPILYQHL
>LEFID029-10|HM872843|MM05466|Filatima_incomptella
TLYFIFGIWAGMLGTSLSLLIRAELGNPGSLIGDDQIYNTIVTAHAFIMIFFMVMPIMIGGFGNWLIPLMLGAPDMAFPRMNNMSFWLLPPSLTLLISSSIVENGAGTGWTVYPPLSSNIAHGGSSVDLAIFSLHLAGISSILGAINFITTIINMRINSLSFDK
MPLFVWAVGITALLLLLSLPVLAGAITMLLTDRNLNTSFFDPAGGGDPILYQHL
>LEFIG618-10|HM876284|MM14834|Filatima_incomptella
TLYFIFGIWAGMLGTSLSLLIRAELGNPGSLIGDDQIYNTIVTAHAFIMIFFMVMPIMIGGFGNWLIPLMLGAPDMAFPRMNNMSFWLLPPSLTLLISSSIVENGAGTGWTVYPPLSSNIAHGGSSVDLAIFSLHLAGISSILGAINFITTIINMRINSLSFDK
MPLFVWAVSITALLLLLSLPVLAGAITMLLTDRNLNTSFFDPAGGGDPILYQHL
>LEFIB002-10|HM870915|MM00004|Furcula_bicuspis
TLYFIFGIWAGMVGTSLSLLIRAELGNPGSLIGDDQIYNTIVTAHAFIMIFFMVMPIMIGGFGNWLVPLMLGAPDMAFPRMNNMSFWMLPPSLTLLISSSIVENGAGTGWTVYPPLSSNIAHGGSSVDLAIFSLHLAGISSILGAINFITTIINMRLNNMM
FDQMPLFIWAVGITAFLLLLSLPVLAGAITMLLTDRNLNTSFFDPAGGGDPILYQHL
>LEFIC293-10|HM872137|MM03765|Furcula_bicuspis
TLYFIFGIWAGMVGTSLSLLIRAELGNPGSLIGDDQIYNTIVTAHAFIMIFFMVMPIMIGGFGNWLVPLMLGAPDMAFPRMNNMSFWMLPPSLTLLISSSIVENGAGTGWTVYPPLSSNIAHGGSSVDLAIFSLHLAGISSILGAINFITTIINMRLNNMM
FDQMPLFIWAVGITAFLLLLSLPVLAGAITMLLTDRNLNTSFFDPAGGGDPILYQHL
>LEFIB630-10|HM871509|MM02379|Furcula_bifida
TLYFIFGIWAGMVGTSLSLLIRAELGNPGSLIGDDQIYNTIVTAHAFIMIFFMVMPIMIGGFGNWLVPLMLGAPDMAFPRMNNMSFWMLPPSLALLISSSIVENGAGTGWTVYPPLSSNIAHGGSSVDLAIFSLHLAGISSILGAINFITTIINMRLNNM
MFDQMPLFIWAVGITAFLLLLSLPVLAGAITMLLTDRNLNTSFFDPAGGGDPILYQHL
>LEFID934-10|HM873684|MM07681|Furcula_bifida
TLYFIFGIWAGMVGTSLSLLIRAELGNPGSLIGDDQIYNTIVTAHAFIMIFFMVMPIMIGGFGNWLVPLMLGAPDMAFPRMNNMSFWMLPPSLALLISSSIVENGAGTGWTVYPPLSSNIAHGGSSVDLAIFSLHLAGISSILGAINFITTIINMRLNNM
MFDQMPLFIWAVGITAFLLLLSLPVLAGAITMLLTDRNLNTSFFDPAGGGDPILYQHL
>LEFID933-10|HM873683|MM07680|Furcula_furcula
TLYFIFGIWAGMVGTSLSLLIRAELGNPGSLIGDDQIYNTIVTAHAFIMIFFMVMPIMIGGFGNWLVPLMLGAPDMAFPRMNNMSFWMLPPSLTLLISSSIVENGAGTGWTVYPPLSSNIAHGGSSVDLAIFSLHLAGISSILGAINFITTIINMRLNNMM
FDQMPLFIWAVGITAFLLLLSLPVLAGAITMLLTDRNLNTSFFDPAGGGDPILYQHL
>LEFIG980-10|HM876619|MM15844|Furcula_furcula
TLYFIFGIWAGMVGTSLSLLIRAELGNPGSLIGDDQIYNTIVTAHAFIMIFFMVMPIMIGGFGNWLVPLMLGAPDMAFPRMNNMSFWMLPPSLTLLISSSIVENGAGTGWTVYPPLSSNIAHGGSSVDLAIFSLHLAGISSILGAINFITTIINMRLNNMM
FDQMPLFIWAVGITAFLLLLSLPVLAGAITMLLTDRNLNTSFFDPAGGGDPILYQHL
>LEFID697-10|HM873458|MM06750|Gagitodes_sagittata
TLYFIFGIWAGMIGTSLSLLIRAELGSPGSLIGDDQIYNTIVTAHAFIMIFFMVMPIMIGGFGNWLVPLMLGAPDMAFPRMNNMSFWLLPPSITLLISSSIVENGAGTGWTVYPPLSSNIAHGGSSVDLAIFSLHLAGISSILGAINFITTIINMRLNNMFFD
QLPLFVWAVGITAFLLLLSLPVLAGAITMLLTDRNLNTSFFDPAGGGDPILYQHL
>LEFIF373-10|HM875058|MM11603|Gagitodes_sagittata
TLYFIFGIWAGMIGTSLSLLIRAELGSPGSLIGDDQIYNTIVTAHAFIMIFFMVMPIMIGGFGNWLVPLMLGAPDMAFPRMNNMSFWLLPPSVTLLISSSIVENGAGTGWTVYPPLSSNIAHGGSSVDLAIFSLHLAGISSILGAINFITTIINMRLNNMFFD
QLPLFVWAVGITAFLLLLSLPVLAGAITMLLTDRNLNTSFFDPAGGGDPILYQHL
>LEFIK041-10|MM17616|Galleria_mellonella
TLYFIFGIWSGMVGTSLSLLIRAELGNPGSLIGDDQIYNTIVTGHAFIMIFFMVMPIMIGGFGNWLVPLMLGAPDMAFPRLNNMSFWLLPPSLTLLIFSSIVENGAGTGWTVYPPLSSNIAHSGSSVDLAIFSLHLAGISSILGAVNFITTVINMKLNGLSFD
QMPLFIWSVSITALLLLLSLPVLAGAITMLLTDRNLNTSFFDPAGGGDPILYQHL

>LEFIC209-10|HM872053|MM03583|Gandaritis_pyraliata
TLYFIFGIWAGMIGTSLSLLIRAELGNPGSLIGDDQIYNTIVTAHAFIMIFFMVMPIMIGGFGNWLVPLMLGAPDMAFPRMNNMSFWLLPPSITLLISSSIVENGAGTGWTIYPPLSSNIAHSGSSVDLAIFSLHLAGISSILGAINFITTIINMRLNNMFFDQ
LPLFVWAVGITAFLLLLSLPVLAGAITMLLTDRNLNTSFFDPAGGGDPILYQHL
>LEFIF726-10|HM875410|MM12812|Gandaritis_pyraliata
TLYFIFGIWAGMIGTSLSLLIRAELGNPGSLIGDDQIYNTIVTAHAFIMIFFMVMPIMIGGFGNWLVPLMLGAPDMAFPRMNNMSFWLLPPSITLLISSSIVENGAGTGWTVYPPLSSNIAHGGSSVDLAIFSLHLAGISSILGAINFITTIINMRLNNMFFD
QLPLFVWAVGITAFLLLLSLPVLAGAITMLLTDRNLNTSFFDPAGGGDPILYQHL
>LEFID239-10|HM873039|MM06102|Gastropacha_populifolia
TLYFIFGIWAGMVGTSLSLLIRAELGTPGSLIGDDQIYNTIVTAHAFIMIFFMVMPIMIGGFGNWLVPLMLGAPDMAFPRMNNMSFWLLPPSLTLLISSSIVENGAGTGWTVYPPLSSNIAHGGSSVDLAIFSLHLAGISSILGAINFITTIINMKLNNMSFD
QMPLFVWAVGITAFLLLLSLPVLAGAITMLLTDRNLNTSFFDPAGGGDPILYQHL
>LEFIF131-10|HM874829|MM10587|Gastropacha_quercifolia
TLYFIFGIWAGMIGTSLSLLIRAELGISGSLIGDDQIYNTIVTAHAFIMIFFMVMPIMIGGFGNWLVPLMLGAPDMAFPRMNNMSFWLLPPSLTLLISSSIVESGAGTGWTVYPPLSSNIAHSGSSVDLAIFSLHLAGISSILGAINFITTIINMRLNNMSLDQ
MPLFVWAVGITAFLLLLSLPVLAGAITMLLTDRNLNTSFFDPAGGGDPILYQHL
>LEFID218-10|HM873019|MM06049|Gazoryctra_fuscoargenteus
TLYFIFGIWAGMVGTSLSLMIRTELGTPSSLIGDDQIYNVIVTAHAFIMIFFMVMPLMIGGFGNWLIPLMLGAPDMAFPRLNNMSFWLLPPSLMLLISSSIVENGAGTGWTVYPPLSSNIAHAGASVDLAIFSLHLAGISSILGAINFITTVINMRSKGMSF
DRMPLFVWSVAITALLLLLSLPVLAGAITMLLTDRNLNTSFFDPAGGGDPILYQHL
>LEFIF617-10|HM875301|MM12505|Gazoryctra_ganna
TLYFIFGLWAGMVGTSLSLMIRTELGTPGSLIGDDQIYNVIVTAHAFIMIFFMVMPLMIGGFGNWLIPLMLGAPDMAFPRLNNMSFWLLPPSLMLLISSSIVENGAGTGWTVYPPLSSNIAHAGGSVDLTIFSLHLAGVSSILGAINFITTVINMRSKGMS
FDRMPLFVWSVVITALLLLLSLPVLAGAITMLLTDRNLNTSFFDPAGGGDPILYQHL
>LEFIF618-10|HM875302|MM12506|Gazoryctra_ganna
TLYFIFGLWAGMVGTSLSLMIRTELGTPGSLIGDDQIYNVIVTAHAFIMIFFMVMPLMIGGFGNWLIPLMLGAPDMAFPRLNNMSFWLLPPSLMLLISSSIVENGAGTGWTVYPPLSSNIAHAGGSVDLTIFSLHLAGISSILGAINFITTVINMRSKGMS
FDRMPLFVWSVVITALLLLLSLPVLAGAITMLLTDRNLNTSFFDPAGGGDPILYQHL
>LEFIB949-10|HM871826|MM03107|Geina_didactyla
TLYFIFGIWAGMIGTSLSLLIRTELSTPSSLIGDDQIYNSIVTAHAFIMIFFMVMPIMIGGFGNWLVPLMLGAPDMAFPRMNNMSFWLLPPSILLLISSSIVENGVGTGWTVYPPLSSNIAHSGPSVDLAIFSLHLAGISSILGAINFISTIINMRLNKMMFDQ
MPLFVWAVGITAILLLLSLPVLAGAITMLLTDRNLNTSFFDPAGGGDPILYQHL
>LEFIA050-10|HM396398|MM00956|Gelechia_cuneatella
TLYFIFGIWAGMVGTSLSLLIRAELGNPGSLIGDDQIYNTIVTAHAFIMIFFMVMPIMIGGFGNWLVPLMLGAPDMAFPRMNNMSFWLLPPSLTLLISSSIVENGAGTGWTVYPPLSSNIAHGGSSVDLAIFSLHLAGISSILGAINFITTIINMKINGLTFD
QMPLFVWAVGITALLLLLSLPVLAGAITMLLTDRNLNTSFFDPAGGGDPILYQHL
>LEFIG762-10|HM876415|MM15626|Gelechia_hippophaella
TLYFIFGIWAGMVGTSLSLLIRAELGNPGSLIGDDQIYNTIVTAHAFIMIFFMVMPIMIGGFGNWLIPLMLGAPDMAFPRMNNMSFWLLPPSLTLLISSSIVENGAGTGWTVYPPLSSNIAHGGSSVDLAIFSLHLAGISSILGAINFITTIINMKINGLTFDQ
MPLFVWAVGITALLLLLSLPVLAGAITMLLTDRNLNTSFFDPAGGGDPILYQHL
>LEFIJ771-10|JF853812|MM17396|Gelechia_hippophaella
TLYFIFGIWAGMVGTSLSLLIRAELGNPGSLIGDDQIYNTIVTAHAFIMIFFMVMPIMIGGFGNWLIPLMLGAPDMAFPRMNNMSFWLLPPSLTLLISSSIVENGAGTGWTVYPPLSSNIAHGGSSVDLAIFSLHLAGISSILGAINFITTIINMKINGLTFDQ
MPLFVWAVGITALLLLLSLPVLAGAITMLLTDRNLNTSFFDPAGGGDPILYQHL
>LEFIB603-10|HM871482|MM02319|Gelechia_jakovlevi
TLYFIFGVWAGMVGTSLSLLIRAELGNPGSLIGDDQIYNTIVTAHAFIMIFFMVMPIMIGGFGNWLVPLMLGAPDMAFPRMNNMSFWLLPPSLTLLISSSIVENGAGTGWTVYPPLSSNIAHGGSSVDLAIFSLHLAGISSILGAINFITTIINMKINGLTFD
QMPLFVWAVGITALLLLLSLPVLAGAITMLLTDRNLNTSFFDPAGGGDPILYQHL
>LEFIC135-10|HM871981|MM03478|Gelechia_muscosella
TLYFIFGIWAGMVGTSLSLLIRAELGNPGSLIGDDQIYNTIVTAHAFIMIFFMVMPIMIGGFGNWLVPLMLGAPDMAFPRMNNMSFWLLPPSLTLLISSSIVENGAGTGWTVYPPLSSNIAHGGSSVDLAIFSLHLAGISSILGAINFITTIINMKINGLTFD
QMPLFVWAVGITALLLLLSLPVLAGAITMLLTDRNMNTSFFDPAGGGDPILYQHL
>LEFIE717-10|HM874437|MM09807|Gelechia_muscosella
TLYFIFGIWAGMVGTSLSLLIRAELGNPGSLIGDDQIYNTIVTAHAFIMIFFMVMPIMIGGFGNWLVPLMLGAPDMAFPRMNNMSFWLLPPSLTLLISSSIVENGAGTGWTVYPPLSSNIAHGGSSVDLAIFSLHLAGISSILGAINFITTIINMKINGLTFD
QMPLFVWAVGITALLLLLSLPVLAGAITMLLTDRNMNTSFFDPAGGGDPILYQHL
>LEFIC835-10|HM872653|MM05033|Gelechia_nigra
TLYFIFGIWAGMVGTSLSLLIRAELGNPGSLIGNDQIYNTIVTAHAFIMIFFMVMPIMIGGFGNWLVPLMLGAPDMAFPRMNNMSFWLLPPSLTLLISSSIVENGAGTGWTVYPPLSSNIAHGGSSVDLAIFSLHLAGISSILGAINFITTIINMKINGLTFD
QMPLFVWAVGITALLLLLSLPVLAGAITMLLTDRNLNTSFFDPAGGGDPILYQHL
>LEFIF526-10|HM875211|MM12166|Gelechia_nigra
TLYFIFGIWAGMVGTSLSLLIRAELGNPGSLIGNDQIYNTIVTAHAFIMIFFMVMPIMIGGFGNWLVPLMLGAPDMAFPRMNNMSFWLLPPSLTLLISSSIVENGAGTGWTVYPPLSSNIAHGGSSVDLAIFSLHLAGISSILGAINFITTIINMKINGLTFD

QMPLFVWAVGITALLLLLSLPVLAGAITMLLTDRNLNTSFFDPAGGGDPILYQHL
>LEFIB736-10|HM871614|MM02568|Gelechia_rhombella
TLYFIFGIWAGMVGTSLSLLIRAELGNPGSLIGDDQIYNTIVTAHAFIMIFFMVMPIMIGGFGNWLVPLMLGAPDMAFPRMNNMSFWLLPPSLTLLISSSIVENGAGTGWTVYPPLSSNIAHSGSSVDLAIFSLHLAGISSILGAINFITTIINMKINGMTFD
QMPLFVWAVGITALLLLLSLPVLAGAITMLLTDRNLNTSFFDPAGGGDPILYQHL
>LEEUA599-11|MM20658|Gelechia_rhombelliformis
TLYFIFGIWAGMVGTSLSLLIRAELGNPGSLIGDDQIYNTIVTAHAFIMIFFMVMPIMIGGFGNWLVPLMLGAPDMAFPRMNNMSFWLLPPSLTLLISSSIVENGAGTGWTVYPPLSSNIAHGGSSVDLAIFSLHLAGISSILGAINFITTIINMKINGLNFD
QMPLFVWAVGITALLLLLSLPVLAGAITMLLTDRNLNTSFFDPAGGGDPILYQHL
>LEFID257-10|HM873055|MM06122|Gelechia_sabinellus
TLYFIFGIWAGMVGTSLSLLIRAELGNPGSLIGDDQIYNTIVTAHAFIMIFFMVMPIMIGGFGNWLVPLMLGAPDMAFPRMNNMSFWLLPPSLTLLISSSIVENGAGTGWTVYPPLSSNIAHGGSSVDLAIFSLHLAGISSILGAINFITTIINMKINGLSFD
QMPLFVWAVGITALLLLLSLPVLAGAITMLLTDRNLNTSFFDPAGGGDPILYQHL
>LEFIF949-10|HM875630|MM13511|Gelechia_sabinellus
TLYFIFGIWAGMVGTSLSLLIRAELGNPGSLIGDDQIYNTIVTAHAFIMIFFMVMPIMIGGFGNWLVPLMLGAPDMAFPRMNNMSFWLLPPSLTLLISSSIVENGAGTGWTVYPPLSSNIAHSGSSVDLAIFSLHLAGISSILGAINFITTIINMKINGLSFD
QMPLFVWAVGITALLLLLSLPVLAGAITMLLTDRNLNTSFFDPAGGGDPILYQHL
>LEFIK001-10|KM373633|MM17576|Gelechia_sabinellus
TLYFIFGIWAGMVGTSLSLLIRAELGNPGSLIGDDQIYNTIVTAHAFIMIFFMVMPIMIGGFGNWLVPLMLGAPDMAFPRMNNMSFWLLPPSLTLLISSSIVENGAGTGWTVYPPLSSNIAHGGSSVDLAIFSLHLAGISSILGAINFITTIINMKINGLSFD
QMPLFVWAVGITALLLLLSLPVLAGAITMLLTDRNLNTSFFDPAGGGDPILYQHL
>LEFIG398-10|HM876075|MM14421|Gelechia_sestertiella
TLYFIFGIWAGMVGTSLSLLIRAELGNPGSLIGDDQIYNTIVTAHAFIMIFFMVMPIMIGGFGNWLVPLMLGAPDMAFPRMNNMSFWLLPPSLTLLISSSIVENGAGTGWTVYPPLSSNIAHGGSSVDLAIFSLHLAGISSILGAINFITTIINMKINGLMFD
QMPLFVWAVGITALLLLLSLPVLAGAITMLLTDRNLNTSFFDPAGGGDPILYQHL
>LEFIB214-10|HM871118|MM00668|Gelechia_sororculella
TLYFIFGIWAGMVGTSLSLLIRAELGNPSFLIGDDQIYNTIVTAHAFIMIFFMVMPIMIGGFGNWLVPLMLGAPDMAFPRMNNMSFWLLPPSLTLLISSSVVENGAGTGWTVYPPLSSNIAHGGSSVDLAIFSLHLAGISSILGAINFITTIINMKINGLTFD
QMPLFVWAVGITALLLLLSLPVLAGAITMLLTDRNLNTSFFDPAGGGDPILYQHL
>LEFIB215-10|HM871119|MM00669|Gelechia_sororculella
TLYFIFGIWAGMVGTSLSLLIRAELGNPSFLIGDDQIYNTIVTAHAFIMIFFMVMPIMIGGFGNWLVPLMLGAPDMAFPRMNNMSFWLLPPSLTLLISSSVVENGAGTGWTVYPPLSSNIAHGGSSVDLAIFSLHLAGISSILGAINFITTIINMKINGLTFD
QMPLFVWAVGITALLLLLSLPVLAGAITMLLTDRNLNTSFFDPAGGGDPILYQHL
>LEFIG599-10|HM876265|MM14796|Gelechia_turpella
TLYFIFGIWAGMVGTSLSLLIRAELGNPGSLIGDDQIYNTIVTAHAFIMIFFMVMPIMIGGFGNWLVPLMLGAPDMAFPRMNNMSFWLLPPSLTLLISSSIVENGAGTGWTVYPPLSSNIAHGGSSVDLAIFSLHLAGISSILGAINFITTIINMKINGMTFD
QMPLFVWAVGITALLLLLSLPVLAGAITMLLTDRNLNTSFFDPAGGGDPILYQHL
>LEFIA170-10|HM396515|MM01147|Geometra_papilionaria
TLYFIFGIWAGMIGTSLSLLIRAELGNPGSLIGDDQIYNTIVTAHAFIMIFFMVMPIMIGGFGNWLVPLMLGAPDMAFPRMNNMSFWLLPPSITLLISSSIVENGAGTGWTVYPPLSSNIAHGGSSVDLAIFSLHLAGISSILGAINFITTIINMRLNNMSFD
QMPLFVWAVGITAFLLLLSLPVLAGAITMLLTDRNLNTSFFDPAGGGDPILYQHL
>LEFIG911-10|HM876555|MM15775|Gesneria_centuriella
TLYFIFGIWAGMVGTSLSLLIRAELGNPGSLIGDDQIYNTIVTAHAFIMIFFMVMPIMIGGFGNWLIPLMLGAPDMAFPRMNNMSFWLLPPSLTLLISSSIVENGAGTGWTVYPPLSSNIAHSGSSVDLAIFSLHLAGISSILGAINFITTIINMRINKLTFDQ
MPLFVWAVGITALLLLLSLPVLAGAITMLLTDRNLNTSFFDPAGGGDPILYQHL
>LEFIB516-10|HQ570267|MM02115|Gibberifera_simplana
TLYFIFGIWAGMVGTSLSLLIRAELGNPGSLIGDDQIYNTIVTAHAFIMIFFMVMPIMIGGFGNWLVPLMLGAPDMAFPRMNNMSFWLLPPSIMLLISSSIVENGAGTGWTVYPPLSSNIAHSGSSVDLAIFSLHLAGISSILGAVNFITTIINMRPNNMSL
DQMPLFVWAVGITALLLLLSLPVLAGAITMLLTDRNLNTSFFDPAGGGDPILYQHL
>LEFID839-10|HM873596|MM06957|Gillmeria_ochrodactyla
TLYFIFGIWAGMVGTSLSLLIRAELGTPGSLIGDDQIYNSIVTAHAFIMIFFMVMPIMIGGFGNWLVPLMLGAPDMAFPRMNNMSFWLLPPSICLLISSSIVENGVGTGWTVYPPLSSNIAHGGPAVDLAIFSLHLAGISSILGAINFISTIINMRLNKMMF
DQLPLFVWAVGITALLLLLSLPVLAGAITMLLTDRNLNTSFFDPAGGGDPILYQHL
>LEFIF936-10|HM875618|MM13380|Gillmeria_ochrodactyla
TLYFIFGIWAGMVGTSLSLLIRAELGTPGSLIGDDQIYNSIVTAHAFIMIFFMVMPIMIGGFGNWLVPLMLGAPDMAFPRMNNMSFWLLPPSICLLISSSIVENGVGTGWTVYPPLSSNIAHGGPAVDLAIFSLHLAGISSILGAINFISTIINMRLNKMMF
DQLPLFVWAVGITALLLLLSLPVLAGAITMLLTDRNLNTSFFDPAGGGDPILYQHL
>LEFIB945-10|HM871822|MM03101|Gillmeria_pallidactyla
TLYFIFGIWAGMIGTSLSLLIRAELGTPGSLIGDDQIYNSIVTAHAFIMIFFMVMPIMIGGFGNWLVPLMLGAPDMAFPRMNNMSFWLLPPSICLLISSSIVENGVGTGWTVYPPLSSNIAHSGPAVDLAIFSLHLAGISSILGAINFISTIINMRLNKMMFD
QLPLFVWAVGITALLLLLSLPVLAGAITMLLTDRNLNTSFFDPAGGGDPILYQHL
>LEFIC888-10|HM872706|MM05188|Gillmeria_pallidactyla

TLYFIFGIWAGMIGTSLSLLIRAELGTPGSLIGDDQIYNSIVTAHAFIMIFFMVMPIMIGGFGNWLVPLMLGAPDMAFPRMNNMSFWLLPPSICLLISSSIVENGVGTGWTVYPPLSSNIAHSGPAVDLAIFSLHLAGISSILGAINFISTIINMRLNKMMFD
QLPLFVWAVGITALLLLLSLPVLAGAITMLLTDRNLNTSFFDPAGGGDPILYQHL
>LEFIA018-10|HM396368|MM00089|Glacies_coracina
TLYFIFGVWAGMVGTSLSLLIRAELGNPGSLIGDDQIYNTIVTAHAFIMIFFMVMPIMIGGFGNWLVPLMLGAPDMAFPRMNNMSFWLLPPSLTLLISSSIVENGAGTGWTVYPPLSSNIAHGGSSVDLAIFSLHLAGISSILGAINFITTIINMRLNNLSF
DQMPLFIWSVGITAFLLLLSLPVLAGAITMLLTDRNLNTSFFDPAGGGDPILYQHL
>LEFID395-10|HM873192|MM06309|Glacies_coracina
TLYFIFGVWAGMVGTSLSLLIRAELGNPGSLIGDDQIYNTIVTAHAFIMIFFMVMPIMIGGFGNWLVPLMLGAPDMAFPRMNNMSFWLLPPSLTLLISSSIVENGAGTGWTVYPPLSSNIAHGGSSVDLAIFSLHLAGISSILGAINFITTIINMRLNNLSF
DQMPLFIWSVGITAFLLLLSLPVLAGAITMLLTDRNLNTSFFDPAGGGDPILYQHL
>LEFIC345-10|HM872188|MM03860|Glaucopsyche_alexis
TLYFIFGIWAGMLGTSLSILIRMELGTPGSLIGDDQIYNTIVTAHAFIMIFFMVMPIMIGGFGNWLVPLMLGAPDMAFPRMNNMSFWLLPPSLMLLISSSIVENGAGTGWTVYPPLSSNIAHSGSSVDLAIFSLHLAGISSILGAINFITTIINMRVNNLSFD
QMSLFIWAVGITALLLLLSLPVLAGAITMLLTDRNINTSFFDPAGGGDPILYQHL
>LEFIK307-10|MM17882|Globia_algae
TLYFIFGIWAGMVGTSLSLLIRAELGSPGSLIGDDQIYNTIVTAHAFIMIFFMVMPIMIGGFGNWLVPLMLGAPDMAFPRMNNMSFWLLPPSLTLLISSSIVENGAGTGWTVYPPLSSNIAHGGSSVDLAIFSLHLAGISSILGAINFITTIINMRLNSLSFD
QMPLFIWAVGITAFLLLLSLPVLAGAITMLLTDRNLNTSFFDPAGGGDPILYQHL
>LEFIB786-10|HM871663|MM02691|Globia_sparganii
TLYFIFGIWAGMVGTSLSLLIRAELGNPGSLIGDDQIYNTIVTAHAFIMIFFMVMPIMIGGFGNWLVPLMLGAPDMAFPRMNNMSFWLLPPSLTLLISSSIVENGAGTGWTVYPPLSSNIAHGGSSVDLAIFSLHLAGISSILGAINFITTIINMRLNSLSFD
QMPLFIWAVGITAFLLLLSLPVLAGAITMLLTDRNLNTSFFDPAGGGDPILYQHL
>LEFIA091-10|HM396438|MM01013|Gluphisia_crenata
TLYFIFGIWAGMVGTSLSLLIRAELGNPGSLIGDDQIYNTIVTAHAFIMIFFMVMPIMIGGFGNWLIPLMLGAPDMAFPRMNNMSFWLLPPSLTLLISSSIVENGAGTGWTVYPPLSSNIAHGGSSVDLAIFSLHLAGISSILGAINFITTIINMRLNNMSFD
QMTLFVWAVGITAFLLLLSLPVLAGAITMLLTDRNLNTSFFDPAGGGDPILYQHL
>LEFIE223-10|HM873959|MM08540|Glyphipterix_bergstraesserella
TLYFIFGIWSGMIGTSLSLLIRAELGTPGSFIGDDQIYNTIVTAHAFIMIFFMVMPIMIGGFGNWLVPLMLGAPDMAFPRMNNMSFWLLPPSLTLLISSSLTETGAGTGWTVYPPLSSNIAHSGSSVDLTIFSLHLAGISSILGAINFITTIINMRPNKMNFDR
MPLFVWAVGITALLLLLSLPVLAGAITMLLTDRNLNTSFFDPAGGGDPILYQHL
>LEFIG085-10|HM875764|MM13740|Glyphipterix_equitella
TLYFIFGIWSGMVGTSLSLLIRAELGNPGSLIGDDQIYNTIVTAHAFIMIFFMVMPIMIGGFGNWLVPLMLGAPDMAFPRMNNMSFWLLPPSLSLLIFSSLTETGAGTGWTVYPPLSSNIAHSGSSVDLAIFSLHLAGISSILGAINFITTIINMRPNNMSFD
RMPLFVWAVGITALLLLLSLPVLAGAITMLLTDRNLNTSFFDPAGGGDPILYQHL
>LEFIG363-10|HM876040|MM14362|Glyphipterix_equitella
TLYFIFGIWSGMVGTSLSLLIRAELGNPGSLIGNDQIYNTIVTAHAFIMIFFMVMPIMIGGFGNWLVPLMLGAPDMAFPRMNNMSFWLLPPSLSLLIFSSLTETGAGTGWTVYPPLSSNIAHSGSSVDLAIFSLHLAGISSILGAINFITTIINMRPNNMSFD
RMPLFVWAVGITALLLLLSLPVLAGAITMLLTDRNLNTSFFDPAGGGDPILYQHL
>LEFIG364-10|HM876041|MM14363|Glyphipterix_equitella
TLYFIFGIWSGMVGTSLSLLIRAELGNPGSLIGDDQIYNTIVTAHAFIMIFFMVMPIMIGGFGNWLVPLMLGAPDMAFPRMNNMSFWLLPPSLSLLIFSSLTETGAGTGWTVYPPLSSNIAHSGSSVDLAIFSLHLAGISSILGAINFITTIINMRPNNMSFD
RMPLFVWAVGITALLLLLSLPVLAGAITMLLTDRNLNTSFFDPAGGGDPILYQHL
>LEFIJ914-10|MM17539|Glyphipterix_equitella
TLYFIFGIWSGMVGTSLSLLIRAELGNPGSLIGDDQIYNTIVTAHAFIMIFFMVMPIMIGGFGNWLIPLMLGAPDMAFPRMNNMSFWLLPPSLSLLIFSSLTETGAGTGWTVYPPLSSNIAHSGSSVDLAIFSLHLAGISSILGAINFITTIINMRPNNMSFD
RMPLFVWAVGITALLLLLSLPVLAGAITMLLTDRNLNTSFFDPAGGGDPILYQHL
>LEFIK547-10|MM18122|Glyphipterix_equitella
TLYFIFGIWSGMVGTSLSLLIRAELGNPGSLIGDDQIYNTIVTAHAFIMIFFMVMPIMIGGFGNWLVPLMLGAPDMAFPRMNNMSFWLLPPSLSLLIFSSLTETGAGTGWTVYPPLSSNIAHSGSSVDLAIFSLHLAGISSILGAINFITTIINMRPNNMSFD
RMPLFVWAVGITALLLLLSLPVLAGAITMLLTDRNLNTSFFDPAGGGDPILYQHL
>LEFIK548-10|JF854142|MM18123|Glyphipterix_equitella
TLYFIFGIWSGMVGTSLSLLIRAELGNPGSLIGDDQIYNTIVTAHAFIMIFFMVMPIMIGGFGNWLVPLMLGAPDMAFPRMNNMSFWLLPPSLSLLIFSSLTETGAGTGWTVYPPLSSNIAHSGSSVDLAIFSLHLAGISSILGAINFITTIINMRPNNMSFD
RMPLFVWAVGITALLLLLSLPVLAGAITMLLTDRNLNTSFFDPAGGGDPILYQHL
>LEFIK551-10|JF854145|MM18126|Glyphipterix_equitella
TLYFIFGIWSGMVGTSLSLLIRAELGNPGSLIGDDQIYNTIVTAHAFIMIFFMVMPIMIGGFGNWLVPLMLGAPDMAFPRMNNMSFWLLPPSLSLLIFSSLTETGAGTGWTVYPPLSSNIAHSGSSVDLAIFSLHLAGISSILGAINFITTIINMRPNNMSFD
RMPLFVWAVGITALLLLLSLPVLAGAITMLLTDRNLNTSFFDPAGGGDPILYQHL
>LEEUA085-11|JN271884|MM19398|Glyphipterix_equitella
TLYFIFGIWSGMVGTSLSLLIRAELGNPGSLIGDDQIYNTIVTAHAFIMIFFMVMPIMIGGFGNWLVPLMLGAPDMAFPRMNNMSFWLLPPSLSLLIFSSLTETGAGTGWTVYPPLSSNIAHSGSSVDLAIFSLHLAGISSILGAINFITTIINMRPNNMSFD
RMPLFVWAVGITALLLLLSLPVLAGAITMLLTDRNLNTSFFDPAGGGDPILYQHL

>LEFIC442-10|HM872276|MM04000|Glyphipterix_forsterella
TLYFIFGIWAGMVGTSLSLLIRAELGNPGSLIGDDQIYNTIVTAHAFIMIFFMVMPIMIGGFGNWLVPLMLGAPDMAFPRMNNMSFWLLPPSLSLLIFSGMAETGAGTGWTVYPPLSSNIAHSGSSVDLAIFSLHLAGISSILGAINFITTIINMRPNNMS
FDRMPLFIWAVGITALLLLLSLPVLAGAITMLLTDRNLNTSFFDPAGGGDPILYQHL
>LEFIG681-10|HM876341|MM15545|Glyphipterix_forsterella
TLYFIFGIWAGMVGTSLSLLIRAELGNPGSLIGDDQIYNTIVTAHAFIMIFFMVMPIMIGGFGNWLVPLMLGAPDMAFPRMNNMSFWLLPPSLSLLIFSGMAETGAGTGWTVYPPLSSNIAHSGSSVDLAIFSLHLAGISSILGAINFITTIINMRPNNMS
FDRMPLFIWAVGITALLLLLSLPVLAGAITMLLTDRNLNTSFFDPAGGGDPILYQHL
>LEFIG682-10|HM876342|MM15546|Glyphipterix_forsterella
TLYFIFGIWAGMVGTSLSLLIRAELGNPGSLIGDDQIYNTIVTAHAFIMIFFMVMPIMIGGFGNWLVPLMLGAPDMAFPRMNNMSFWLLPPSLSLLIFSGMAETGAGTGWTVYPPLSSNIAHSGSSVDLAIFSLHLAGISSILGAINFITTIINMRPNNMS
FDRMPLFIWAVGITALLLLLSLPVLAGAITMLLTDRNLNTSFFDPAGGGDPILYQHL
>LEFIJ1306-11|KM572035|MM21166|Glyphipterix_forsterella
TLYFIFGIWAGMVGTSLSLLIRAELGNPGSLIGDDQIYNTIVTAHAFIMIFFMVMPIMIGGFGNWLVPLMLGAPDMAFPRMNNMSFWLLPPSLSLLIFSGLAETGAGTGWTVYPPLSSNIAHSGSSVDLAIFSLHLAGISSILGAINFITTIINMRPNNMSF
DRMPLFIWAVGITALLLLLSLPVLAGAITMLLTDRNLNTSFFDPAGGGDPILYQHL
>LEFIG118-10|HM875798|MM13922|Glyphipterix_haworthana
TLYFIFGIWAGMVGTSLSLIIRAELGNPGSLIGDDQIYNTIVTAHAFIMIFFMVMPIMIGGFGNWLVPLMLGAPDMAFPRMNNMSFWLLPPSLSLLIFSGLAEFGAGTGWTVYPPLSSNIAHSGSSVDLAIFSLHLAGISSILGAINFITTIINMRPNNMSF
DRMPLFIWAVGITALLLLLSLPVLAGAITMLLTDRNLNTSFFDPAGGGDPILYQHL
>LEFIG253-10|HM875932|MM14177|Glyphipterix_haworthana
TLYFIFGIWAGMVGTSLSLIIRAELGNPGSLIGDDQIYNTIVTAHAFIMIFFMVMPIMIGGFGNWLVPLMLGAPDMAFPRMNNMSFWLLPPSLSLLIFSGLAEFGAGTGWTVYPPLSSNIAHSGSSVDLAIFSLHLAGISSILGAINFITTIINMRPNNMSF
DRMPLFIWAVGITALLLLLSLPVLAGAITMLLTDRNLNTSFFDPAGGGDPILYQHL
>LEFIB760-10|HM871637|MM02619|Glyphipterix_simpliciella
TLYFIFGIWSGMVGTSLSLLIRAELGNPGSLIGDDQIYNTIVTAHAFIMIFFMVMPIMIGGFGNWLVPLMLGAPDMAFPRMNNMSFWLLPPSLSLLIFSSLAETGAGTGWTVYPPLSSNIAHSGSSVDLAIFSLHLAGISSILGAINFITTIINMRPNNMKF
DQMPLFVWAVGITALLLLLSLPVLAGAITMLLTDRNLNTSFFDPAGGGDPILYQHL
>LEFIK546-10|JF854141|MM18121|Glyphipterix_simpliciella
TLYFIFGIWSGMVGTSLSLLIRAELGNPGSLIGDDQIYNTIVTAHAFIMIFFMVMPIMIGGFGNWLVPLMLGAPDMAFPRMNNMSFWLLPPSLSLLIFSSLAETGAGTGWTVYPPLSSNIAHSGSSVDLAIFSLHLAGISSILGAINFITTIINMRPNNMKF
DQMPLFVWAVGITALLLLLSLPVLAGAITMLLTDRNLNTSFFDPAGGGDPILYQHL
>LEFIK549-10|JF854143|MM18124|Glyphipterix_simpliciella
TLYFIFGIWSGMVGTSLSLLIRAELGNPGSLIGDDQIYNTIVTAHAFIMIFFMVMPIMIGGFGNWLIPLMLGAPDMAFPRMNNMSFWLLPPSLSLLIFSSLAETGAGTGWTVYPPLSSNIAHSGSSVDLAIFSLHLAGISSILGAINFITTIINMRPNNMKFD
QMPLFVWAVGITALLLLLSLPVLAGAITMLLTDRNLNTSFFDPAGGGDPILYQHL
>LEFIE643-10|HM874366|MM09594|Glyphipterix_thrasonella
TLYFIFGIWSGLVGTSLSLLIRAELGNPGSLIGDDQIYNTIVTAHAFIMIFFMVMPIMIGGFGNWLVPLMLGAPDMAFPRMNNMSFWLLPPSLTLLISSSLAESGAGTGWTVYPPLSSNIAHSGSSVDLTIFSLHLAGISSILGAINFITTIINMRPHNMSFDR
MPLFVWAVGITALLLLLSLPVLAGAITMLLTDRNLNTSFFDPAGGGDPILYQHL
>LEFIF740-10|HM875424|MM12883|Gnophos_obfuscata
TLYFIFGIWAGMVGTSLSLLIRAELGNPGSLIGDDQIYNTIVTAHAFIMIFFMVMPIMIGGFGNWLVPLMLGAPDMAFPRMNNMSFWLLPPSITLLISSSIVENGAGTGWTVYPPLSSNIAHGGSSVDLAIFSLHLAGISSILGAINFITTIINMRLNNLSFD
QMPLFVWSVGITAFLLLLSLPVLAGAITMLLTDRNLNTSFFDPAGGGDPILYQHL
>LEFIG978-10|HM876617|MM15842|Gnophos_obfuscata
TLYFIFGIWAGMVGTSLSLLIRAELGNPGSLIGDDQIYNTIVTAHAFIMIFFMVMPIMIGGFGNWLVPLMLGAPDMAFPRMNNMSFWLLPPSITLLISSSIVENGAGTGWTVYPPLSSNIAHGGSSVDLAIFSLHLAGISSILGAINFITTIINMRLNNLSFD
QMPLFVWSVGITAFLLLLSLPVLAGAITMLLTDRNLNTSFFDPAGGGDPILYQHL
>LEFID213-10|HM873015|MM06043|Gnorimoschema_epithymella
TLYFIFGIWAGMVGTSLSLLIRAELGNPGSLIGDDQIYNTIVTAHAFIMIFFMVMPIMIGGFGNWLVPLMLGAPDMAFPRMNNMSFWLLPPSLTLLISSSIVENGAGTGWTVYPPLSSNIAHGGSSVDLAIFSLHLAGISSILGAINFITTIINMRINGLSFD
QMPLFVWAVGITALLLLLSLPVLAGAITMLLTDRNLNTSFFDPAGGGDPILYQHL
>LEFIE937-10|HM874654|MM10325|Gnorimoschema_epithymella
TLYFIFGIWAGMVGTSLSLLIRAELGNPGSLIGDDQIYNTIVTAHAFIMIFFMVMPIMIGGFGNWLVPLMLGAPDMAFPRMNNMSFWLLPPSLTLLISSSIVENGAGTGWTVYPPLSSNIAHGGSSVDLAIFSLHLAGISSILGAINFITTIINMRIYGLSFD
QMPLFVWAVGITALLLLLSLPVLAGAITMLLTDRNLNTSFFDPAGGGDPILYQHL
>LEFIF054-10|HM874765|MM10494|Gnorimoschema_herbichii
TLYFIFGIWAGMVGTSLSLLIRAELGNPGSLIGDDQIYNTIVTAHAFIMIFFMVMPIMIGGFGNWLVPLMLGAPDMAFPRMNNMSFWLLPPSLTLLISSSIVENGAGTGWTVYPPLSSNIAHGGSSVDLAIFSLHLAGISSILGAINFITTIINMRINGLSFD
QMPLFVWAVGITALLLLLSLPVLAGAITMLLTDRNLNTSFFDPAGGGDPILYQHL
>LEFIG764-10|HM876417|MM15628|Gnorimoschema_herbichii
TLYFIFGIWAGMVGTSLSLLIRAELGNPGSLIGDDQIYNTIVTAHAFIMIFFMVMPIMIGGFGNWLVPLMLGAPDMAFPRMNNMSFWLLPPSLTLLISSSIVENGAGTGWTVYPPLSSNIAHGGSSVDLAIFSLHLAGISSILGAINFITTIINM?INGLSFD

QMPLFVWAVGITALLLLLSLPVLAGAITMLLTDRNLNTSFFDPAGGGDPILYQHL
>LEFIK659-10|JF854229|MM18234|Gnorimoschema_herbichii
TLYFIFGIWAGMVGTSLSLLIRAELGNPGSLIGDDQIYNTIVTAHAFIMIFFMVMPIMIGGFGNWLVPLMLGAPDMAFPRMNNMSFWLLPPSLTLLISSSIVENGAGTGWTVYPPLSSNIAHGGSSVDLAIFSLHLAGISSILGAINFITTIINMRINGLSFD
QMPLFVWAVGITALLLLLSLPVLAGAITMLLTDRNLNTSFFDPAGGGDPILYQHL
>LEFIJ1359-12|MM22041|Gnorimoschema_herbichii
TLYFIFGIWAGMVGTSLSLLIRAELGNPGSLIGDDQIYNTIVTAHAFIMIFFMVMPIMIGGFGNWLVPLMLGAPDMAFPRMNNMSFWLLPPSLTLLISSSIVENGAGTGWTVYPPLSSNIAHGGSSVDLAIFSLHLAGISSILGAINFITTIINMRINGLSFD
QMPLFVWAVGITALLLLLSLPVLAGAITMLLTDRNLNTSFFDPAGGGDPILYQHL
>LEFIJ1360-12|MM22043|Gnorimoschema_herbichii
TLYFIFGIWAGMVGTSLSLLIRAELGNPGSLIGDDQIYNTIVTAHAFIMIFFMVMPIMIGGFGNWLVPLMLGAPDMAFPRMNNMSFWLLPPSLTLLISSSIVENGAGTGWTVYPPLSSNIAHGGSSVDLAIFSLHLAGISSILGAINFITTIINMRINGLSFD
QMPLFVWAVGITALLLLLSLPVLAGAITMLLTDRNLNTSFFDPAGGGDPILYQHL
>LEFID344-10|HM873141|MM06240|Gnorimoschema_nordlandicolella
TLYFIFGIWAGMVGTSLSLLIRAELGNPGSLIGDDQIYNTIVTAHAFIMIFFMVMPIMIGGFGNWLVPLMLGAPDMAFPRMNNMSFWLLPPSLTLLISSSVVENGAGTGWTVYPPLSSNIAHSGSSVDLAIFSLHLAGISSILGAINFITTIINMRINGLSFD
QMPLFVWAVGITALLLLLSLPVLAGAITMLLTDRNLNTSFFDPAGGGDPILYQHL
>LEFIE983-10|HM874699|MM10420|Gnorimoschema_nordlandicolella
TLYFIFGIWAGMVGTSLSLLIRAELGNPGSLIGDDQIYNTIVTAHAFIMIFFMVMPIMIGGFGNWLVPLMLGAPDMAFPRMNNMSFWLLPPSLTLLISSSIVENGAGTGWTVYPPLSSNIAHSGSSVDLAIFSLHLAGISSILGAINFITTIINMRINGLSFD
QMPLFVWAVGITALLLLLSLPVLAGAITMLLTDRNLNTSFFDPAGGGDPILYQHL
>LEFIG767-10|HM876420|MM15631|Gnorimoschema_streliciella
TLYFIFGIWAGMIGTSLSLLIRAELGNPGSLIGDDQIYNTIVTAHAFIMIFFMVMPIMIGGFGNWLVPLMLGAPDMAFPRMNNMSFWLLPPSLTLLISSSIVENGAGTGWTVYPPLSSNIAHGGSSVDLAIFSLHLAGISSILGAINFITTIINMRINGLSFDQ
MPLFVWAVGITALLLLLSLPVLAGAITMLLTDRNLNTSFFDPAGGGDPILYQHL
>LEFIG768-10|HQ570410|MM15632|Gnorimoschema_streliciella
TLYFIFGIWAGMIGTSLSLLIRAELGNPGSLIGDDQIYNTIVTAHAFIMIFFMVMPIMIGGFGNWLVPLMLGAPDMAFPRMNNMSFWLLPPSLTLLISSSIVENGAGTGWTVYPPLSSNIAHGGSSVDLAIFSLHLAGISSILGAINFITTIINMRINGLSFDQ
MPLFVWAVGITALLLLLSLPVLAGAITMLLTDRNLNTSFFDPAGGGDPILYQHL
>LEFID444-10|HQ570340|MM06377|Gnorimoschema_valesiella
TLYFIFGIWAGMVGTSLSLLIRAELGNPGSLIGNDQIYNTIVTAHAFIMIFFMVMPIMIGGFGNWLVPLMLGAPDMAFPRMNNMSFWLLPPSLTLLISSSIVENGAGTGWTVYPPLSSNIAHSGSSVDLAIFSLHLAGISSILGAINFITTIINMRINGLSFD
QMPLFVWAVGITALLLLLSLPVLAGAITMLLTDRNLNTSFFDPAGGGDPILYQHL
>LEFIB372-10|HM871271|MM00941|Gonepteryx_rhamni
TLYFIFGIWSGMVGTSLSLLIRTELGNPGSLIGDDQIYNTIVTAHAFIMIFFMVMPIMIGGFGNWLVPLMLGAPDMAFPRMNNMSFWLLPPSLTLLISSSIVENGAGTGWTVYPPLSSNIAHSGASVDLAIFSLHLAGISSILGAINFITTIINMRINNMSFD
QMPLFVWAVGITALLLLLSLPVLAGAITMLLTDRNLNTSFFDPAGGGDPILYQHL
>LEFIJ499-10|JF853623|MM17124|Gonepteryx_rhamni
TLYFIFGIWSGMVGTSLSLLIRTELGNPGSLIGDDQIYNTIVTAHAFIMIFFMVMPIMIGGFGNWLVPLMLGAPDMAFPRMNNMSFWLLPPSLTLLISSSIVENGAGTGWTVYPPLSSNIAHSGASVDLAIFSLHLAGISSILGAINFITTIINMRINNMSFD
QMPLFVWAVGITALLLLLSLPVLAGAITMLLTDRNLNTSFFDPAGGGDPILYQHL
>LEFIA592-10|HM870841|MM01704|Gortyna_flavago
TLYFIFGIWAGMVGTSLSLLIRAELGNPGSLIGDDQIYNTIVTAHAFIMIFFMVMPIMIGGFGNWLVPLMLGAPDMAFPRMNNMSFWLLPPSLTLLISSSIVENGAGTGWTVYPPLSSNIAHGGSSVDLAIFSLHLAGISSILGAINFITTIINMRLNSLSFD
QMPLFIWAVGITAFLLLLSLPVLAGAITMLLTDRNLNTSFFDPAGGGDPILYQHL
>LEFIB331-10|HM871231|MM00872|Gracillaria_syringella
TLYFIFGIWSGMVGTSLSMLIRAELGNPGSLIGDDQIYNTIVTAHAFIMIFFMVMPIMIGGFGNWLVPLMLGAPDMAFPRLNNMSFWLLPPSLILLISSSIVETGAGTGWTVYPPLSSNIAHSGSSVDLAIFSLHLAGISSILGAINFITTIINMRTNGMMFD
SMSLFTWAVSITALLLLLSLPVLAGAITMLLTDRNLNTSFFDPAGGGDPILYQHL
>LEFIF473-10|HM875158|MM12012|Gracillaria_syringella
TLYFIFGIWSGMVGTSLSMLIRAELGNPGSLIGDDQIYNTIVTAHAFIMIFFMVMPIMIGGFGNWLVPLMLGAPDMAFPRLNNMSFWLLPPSLILLISSSIVETGAGTGWTVYPPLSSNIAHSGSSVDLAIFSLHLAGISSILGAINFITTIINMRTNGMMFD
SMSLFTWAVSITALLLLLSLPVLAGAITMLLTDRNLNTSFFDPAGGGDPILYQHL
>LEFIF135-10|HQ570391|MM10592|Grammia_quenseli
TLYFIFGIWAGMVGTSLSLLIRAELGNPGSLIGDDQIYNTIVTAHAFIMIFFMVMPIMIGGFGNWLVPLMLGAPDMAFPRMNNMSFWLLPPSLTLLISSSIVENGAGTGWTVYPPLSSNIAHGGSSVDLAIFSLHLAGISSILGAINFITTIINMRLNNLSFD
QMPLFIWAVGITAFLLLLSLPVLAGAITMLLTDRNLNTSFFDPAGGGDPILYQHL
>LEFIB820-10|HM871697|MM02757|Graphiphora_augur
TLYFIFGIWAGMVGTSLSLLIRAELGNPGSLIGDDQIYNTIVTAHAFIMIFFMVMPIMIGGFGNWLVPLMLGAPDMAFPRMNNMSFWLLPPSLTLLISSSIVENGAGTGWTVYPPLSSNIAHGGSSVDLAIFSLHLAGISSILGAINFITTIINMRLNSLSFD
QMPLFIWAVGITAFLLLLSLPVLAGAITMLLTDRNLNTSFFDPAGGGDPILYQHL
>LEFIE019-10|HM873768|MM08074|Graphiphora_augur

TLYFIFGIWAGMVGTSLSLLIRAELGNPGSLIGDDQIYNTIVTAHAFIMIFFMVMPIMIGGFGNWLVPLMLGAPDMAFPRMNNMSFWLLPPSLTLLISSSIVENGAGTGWTVYPPLSSNIAHGGSSVDLAIFSLHLAGISSILGAINFITTIINMRLNSLSFD
QMPLFIWAVGITAFLLLLSLPVLAGAITMLLTDRNLNTSFFDPAGGGDPILYQHL
>LEFIA010-10|HM396360|MM00081|Grapholita_aureolana
TLYFIF?IWAGMIGTSLSLLIRAELGNPGSLIGDDQIYNTIVTAHAFIMIFFMVMPIMIGGFGNWLVPLMLGAPDMAFPRMNNMSFWLLPPSIMLLISSSIVENGAGTGWTVYPPLSSNIAHSGSSVDLAIFSLHLAGISSILGAVNFITTIINMRPNNMSL
DQMPLFVWAVGITALLLLLSLPVLAGAITMLLTDRNLNTSFFDPAGGGDPILYQHL
>LEFIA774-10|HM386916|MM04128|Grapholita_aureolana
TLYFIFGIWAGMIGTSLSLLIRAELGNPGSLIGDDQIYNTIVTAHAFIMIFFMVMPIMIGGFGNWLVPLMLGAPDMAFPRMNNMSFWLLPPSIMLLISSSIVENGAGTGWTVYPPLSSNIAHSGSSVDLAIFSLHLAGISSILGAVNFITTIINMRPNNMSL
DQMPLFVWAVGITALLLLLSLPVLAGAITMLLTDRNLNTSFFDPAGGGDPILYQHL
>LEFIJ681-10|JF853768|MM17306|Grapholita_caecana
TLYFIFGIWAGMVGTSLSLLIRAELGNPGSLIGDDQIYNTIVTAHAFIMIFFMVMPIMIGGFGNWLVPLMLGAPDMAFPRMNNMSFWLLPPSIMLLISSSIVENGAGTGWTVYPPLSSNIAHSGSSVDLAIFSLHLAGISSILGAVNFITTIINMRPNNMSL
DQMPLFVWAVGITALLLLLSLPVLAGAITMLLTDRNLNTSFFDPAGGGDPILYQHL
>LEFIC007-10|HM871877|MM03207|Grapholita_compositella
TLYFIFGIWAGMIGTSLSLLIRAELGNPGSLIGDDQIYNTIVTAHAFIMIFFMVMPIMIGGFGNWLVPLMLGAPDMAFPRMNNMSFWLLPPSIMLLISSSIVENGAGTGWTVYPPLSSNISHSGSSVDLAIFSLHLAGISSILGAVNFITTIINMRPNSMSLD
QMPLFVWAVGITALLLLLSLPVLAGAITMLLTDRNLNTSFFDPAGGGDPILYQHL
>LEFIG110-10|HM875790|MM13898|Grapholita_compositella
TLYFIFGIWAGMIGTSLSLLIRAELGNPGSLIGDDQIYNTIVTAHAFIMIFFMVMPIMIGGFGNWLVPLMLGAPDMAFPRMNNMSFWLLPPSIMLLISSSIVENGAGTGWTVYPPLSSNISHSGSSVDLAIFSLHLAGISSILGAVNFITTIINMRPNSMSLD
QMPLFVWAVGITALLLLLSLPVLAGAITMLLTDRNLNTSFFDPAGGGDPILYQHL
>LEFIK782-10|JN274966|MM18357|Grapholita_compositella
TLYFIFGIWAGMIGTSLSLLIRAELGNPGSLIGDDQIYNTIVTAHAFIMIFFMVMPIMIGGFGNWLVPLMLGAPDMAFPRMNNMSFWLLPPSIMLLISSSIVENGAGTGWTVYPPLSSNISHSGSSVDLAIFSLHLAGISSILGAVNFITTIINMRPNSMSLD
QMPLFVWAVGITALLLLLSLPVLAGAITMLLTDRNLNTSFFDPAGGGDPILYQHL
>LEFIL646-10|JF854683|MM18944|Grapholita_discretana
TLYFIFGIWSGMIGTSLSLLIRAELGNPGSLIGDDQIYNTIVTAHAFIMIFFMVMPIMIGGFGNWLVPLMLGAPDMAFPRMNNMSFWLLPPSIMLLISSSIVENGAGTGWTVYPPLSSNIAHSGSSVDLAIFSLHLAGISSILGAINFITTIINMRPNNMSLD
QMPLFIWAVGITALLLLLSLPVLAGAITMLLTDRNLNTSFFDPAGGGDPILYQHL
>LEFID767-10|HM873524|MM06833|Grapholita_funebrana
TLYFIFGIWAGMVGTSLSLLIRAELGNPGSLIGDDQIYNTIVTAHAFIMIFFMVMPIMIGGFGNWLIPLMLGAPDMAFPRMNNMSFWLLPPSIMLLISSSIVENGAGTGWTVYPPLSSNIAHSGSSVDLAIFSLHLAGISSILGAVNFITTIINMRPNNMSL
DQMPLFVWAVGITALLLLLSLPVLAGAITMLLTDRNLNTSFFDPAGGGDPILYQHL
>LEFID727-10|HM873484|MM06783|Grapholita_janthinana
TLYFIFGIWAGMVGTSLSLLIRAELGNPGSLIGDDQIYNTIVTAHAFIMIFFMVMPIMIGGFGNWLVPLMLGAPDMAFPRMNNMSFWLLPPSIMLLISSSIVENGAGTGWTVYPPLSSNIAHSGSSVDLAIFSLHLAGISSILGAINFITTIINMRPNNMSL
DQMPLFVWAVGITAILLLLSLPVLAGAITMLLTDRNLNTSFFDPAGGGDPILYQHL
>LEFID173-10|HM872980|MM05953|Grapholita_jungiella
TLYFIFGIWAGMVGTSLSLLIRAELGNPGSLIGDDQIYNTIVTAHAFIMIFFMVMPIMIGGFGNWLVPLMLGAPDMAFPRMNNMSFWLLPPSIMLLISSSIVENGAGTGWTVYPPLSSNIAHSGSSVDLAIFSLHLAGISSILGAVNFITTIINMRPNNMSL
DQMPLFVWAVGITALLLLLSLPVLAGAITMLLTDRNLNTSFFDPAGGGDPILYQHL
>LEFID186-10|HM872992|MM05975|Grapholita_jungiella
TLYFIFGIWAGMVGTSLSLLIRAELGNPGSLIGDDQIYNTIVTAHAFIMIFFMVMPIMIGGFGNWLVPLMLGAPDMAFPRMNNMSFWLLPPSIMLLISSSIVENGAGTGWTVYPPLSSNIAHSGSSVDLAIFSLHLAGISSILGAVNFITTIINMRPNNMSL
DQMPLFVWAVGITALLLLLSLPVLAGAITMLLTDRNLNTSFFDPAGGGDPILYQHL
>LEFIG111-10|HM875791|MM13899|Grapholita_jungiella
TLYFIFGIWAGMVGTSLSLLIRAELGNPGSLIGDDQIYNTIVTAHAFIMIFFMVMPIMIGGFGNWLVPLMLGAPDMAFPRMNNMSFWLLPPSIMLLISSSIVENGAGTGWTVYPPLSSNIAHSGSSVDLAIFSLHLAGISSILGAVNFITTIINMRPNNMSL
DQMPLFVWAVGITALLLLLSLPVLAGAITMLLTDRNLNTSFFDPAGGGDPILYQHL
>LEFIJ488-10|JF853613|MM17113|Grapholita_lobarzewskii
TLYFIFGIWAGMVGTSLSLLIRAELGNPGSLIGDDQIYNTIVTAHAFIMIFFMVMPIMIGGFGNWLVPLMLGAPDMAFPRMNNMSFWLLPPSIMLLISSSIVENGAGTGWTVYPPLSSNIAHSGSSVDLAIFSLHLAGISSILGAVNFITTIINMRPNNMSL
DQMPLFVWAVGITALLLLLSLPVLAGAITMLLTDRNLNTSFFDPAGGGDPILYQHL
>LEEUA247-11|JN274942|MM19655|Grapholita_lunulana
TLYFIFGIWAGMVGTSLSLLIRAELGNPGSLIGDDQIYNTIVTAHAFIMIFFMVMPIMIGGFGNWLVPLMLGAPDMAFPRMNNMSFWLLPPSIMLLISSSIVENGAGTGWTVYPPLSSNIAHSGSSVDLAIFSLHLAGISSILGAVNFITTIINMRPNNMSL
DQMPLFVWAVGITALLLLLSLPVLAGAITMLLTDRNLNTSFFDPAGGGDPILYQHL
>LEFID244-10|HM873043|MM06108|Grapholita_orobana
TLYFIFGVWAGMIGTSLSLLIRAELGNPGSLIGDDQIYNTIVTAHAFIMIFFMVMPIMIGGFGNWLVPLMLGAPDMAFPRMNNMSFWLLPPSIMLLISSSIVENGAGTGWTVYPPLSSNIAHSGSSVDLAIFSLHLAGISSILGAVNFITTIINMRPNNMSL
DQMPLFVWAVGITALLLLLSLPVLAGAITMLLTDRNLNTSFFDPAGGGDPILYQHL

>LEFIB658-10|HM871536|MM02430|Grapholita_tenebrosana
TLYFIFGIWAGMVGTSLSLLIRAELGNPGSLIGDDQIYNTIVTAHAFIMIFFMVMPIMIGGFGNWLIPLMLGAPDMAFPRMNNMSFWLLPPSIMLLISSSIVENGAGTGWTVYPPLSSNIAHSGSSVDLAIFSLHLAGISSILGAVNFITTIINMRPNNMSL
DQMPLFVWAVGITAILLLLSLPVLAGAITMLLTDRNLNTSFFDPAGGGDPILYQHL
>LEFID506-10|HM873271|MM06472|Grapholita_tenebrosana
TLYFIFGIWAGMVGTSLSLLIRAELGNPGSLIGDDQIYNTIVTAHAFIMIFFMVMPIMIGGFGNWLVPLMLGAPDMAFPRMNNMSFWLLPPSIMLLISSSIVENGAGTGWTVYPPLSSNIAHSGSSVDLAIFSLHLAGISSILGAINFITTIINMRPNNMSL
DQMPLFVWAVGITAILLLLSLPVLAGAITMLLTDRNLNTSFFDPAGGGDPILYQHL
>LEFIJ643-10|JF853740|MM17268|Grapholita_tenebrosana
TLYFIFGIWAGMVGTSLSLLIRAELGNPGSLIGDDQIYNTIVTAHAFIMIFFMVMPIMIGGFGNWLVPLMLGAPDMAFPRMNNMSFWLLPPSIMLLISSSIVENGAGTGWTVYPPLSSNIAHSGSSVDLAIFSLHLAGISSILGAVNFITTIINMRPNNMSL
DQMPLFVWAVGITAILLLLSLPVLAGAITMLLTDRNLNTSFFDPAGGGDPILYQHL
>LEFIJ644-10|JF853741|MM17269|Grapholita_tenebrosana
TLYFIFGIWAGMVGTSLSLLIRAELGNPGSLIGDDQIYNTIVTAHAFIMIFFMVMPIMIGGFGNWLIPLMLGAPDMAFPRMNNMSFWLLPPSIMLLISSSIVENGAGTGWTVYPPLSSNIAHSGSSVDLAIFSLHLAGISSILGAVNFITTIINMRPNNMSL
DQMPLFVWAVGITAILLLLSLPVLAGAITMLLTDRNLNTSFFDPAGGGDPILYQHL
>LEFIC713-10|HM872534|MM04757|Griposia_aprilina
TLYFIFGIWAGMVGTSLSLLIRAELGNPGSLIGDDQIYNTIVTAHAFIMIFFMVMPIMIGGFGNWLVPLMLGAPDMAFPRMNNMSFWLLPPSLTLLISSSIVENGAGTGWTVYPPLSSNIAHGGSSVDLAIFSLHLAGISSILGAINFITTIINMRLNNLSFD
QMPLFIWAVGITAFLLLLSLPVLAGAITMLLTDRNLNTSFFDPAGGGDPILYQHL
>LEFIA683-10|HM386829|MM01823|Gymnoscelis_rufifasciata
TLYFIFGIWAGMIGTSLSLLIRAELGTPGSLIGDDQIYNTIVTAHAFIMIFFMVMPIMIGGFGNWLVPLMLGAPDMAFPRMNNMSFWLLPPSITLLISSSIVENGAGTGWTVYPPLSSNIAHGGSSVDLAIFSLHLAGISSILGAINFITTIFNMRLNNMFFD
QLPLFIWAVGITAFLLLLSLPVLAGAITMLLTDRNLNTSFFDPAGGGDPILYQHL
>LEFIA684-10|HM386830|MM01824|Gymnoscelis_rufifasciata
TLYFIFGIWAGMIGTSLSLLIRAELGTPGSLIGDDQIYNTIVTAHAFIMIFFMVMPIMIGGFGNWLVPLMLGAPDMAFPRMNNMSFWLLPPSITLLISSSIVENGAGTGWTVYPPLSSNIAHGGSSVDLAIFSLHLAGISSILGAINFITTIFNMRLNNMFFD
QLPLFIWAVGITAFLLLLSLPVLAGAITMLLTDRNLNTSFFDPAGGGDPILYQHL
>LEFIF751-10|HM875435|MM12960|Gymnoscelis_rufifasciata
TLYFIFGIWAGMIGTSLSLLIRAELGTPGSLIGDDQIYNTIVTAHAFIMIFFMVMPIMIGGFGNWLVPLMLGAPDMAFPRMNNMSFWLLPPSITLLISSSIVENGAGTGWTVYPPLSSNIAHGGSSVDLAIFSLHLAGISSILGAINFITTIFNMRLNNMFFD
QLPLFIWAVGITAFLLLLSLPVLAGAITMLLTDRNLNTSFFDPAGGGDPILYQHL
>LEFIF272-10|HM874965|MM11054|Gynaephora_selenitica
TLYFIFGIWAGMIGTSLSLLIRAELGNPGTLIGNDQIYNTIVTAHAFIMIFFMVMPIMIGGFGNWLVPLMLGAPDMAFPRMNNMSFWLLPPSLTLLISSSIVENGAGTGWTVYPPLSSNIAHSGSSVDLAIFSLHLAGISSILGAINFITTIINMRLNNLSFDQ
MPLFVWAVGITAFLLLLSLPVLAGAITMLLTDRNLNTSFFDPAGGGDPILYQHL
>LEFIJ711-10|JF853786|MM17336|Gynnidomorpha_alismana
TLYFIFGIWAGMVGTSLSLLIRAELGNPGSLIGDDQIYNTIVTAHAFIMIFFMVMPIMIGGFGNWLVPLMLGAPDMAFPRMNNMSFWLLPPSIMLLISSSIVENGAGTGWTVYPPLSSNIAHSGSSVDLAIFSLHLAGISSILGAVNFITTIINMRPNNMSL
DQMPLFVWAVGITALLLLLSLPVLAGAITMLLTDRNLNTSFFDPAGGGDPILYQHL
>LEFIG807-10|HM876459|MM15671|Gynnidomorpha_luridana
TLYFIFGIWAGMVGTSLSLLIRAELGNPGSLIGDDQIYNTIVTAHAFIMIFFMVMPIMIGGFGNWLVPLMLGAPDMAFPRMNNMSFWLLPPSIMLLISSSIVENGAGTGWTVYPPLSSNIAHSGSSVDLAIFSLHLAGISSILGAVNFITTIINMRPNNMN
LDQMPLFVWAVGITALLLLLSLPVLAGAITMLLTDRNLNTSFFDPAGGGDPILYQHL
>LEFIC380-10|HM872223|MM03910|Gynnidomorpha_minimana
TLYFIFGIWAGMVGTSLSLLIRAELGNPGSLIGDDQIYNTIVTAHAFIMIFFMVMPIMIGGFGNWLVPLMLGAPDMAFPRMNNMSFWLLPPSIMLLISSSIVENGAGTGWTVYPPLSSNIAHSGSSVDLAIFSLHLAGISSILGAVNFITTIINMRPNNMN
LDQMPLFVWAVGITALLLLLSLPVLAGAITMLLTDRNLNTSFFDPAGGGDPILYQHL
>LEFIB729-10|HM871607|MM02555|Gynnidomorpha_permixtana
TLYFIFGIWAGMVGTSLSLLIRAELGNPGSLIGDDQIYNTIVTAHAFIMIFFMVMPIMIGGFGNWLVPLMLGAPDMAFPRMNNMSFWLLPPSIMLLISSSIVENGAGTGWTVYPPLSSNIAHSGSSVDLAIFSLHLAGISSILGAVNFITTIINMRPNNMN
LDQMPLFVWAVGITALLLLLSLPVLAGAITMLLTDRNLNTSFFDPAGGGDPILYQHL
>LEFIK184-10|JQ775260|MM17759|Gynnidomorpha_permixtana
TLYFIFGIWAGMVGTSLSLLIRAELGNPGSLIGDDQIYNTIVTAHAFIMIFFMVMPIMIGGFGNWLVPLMLGAPDMAFPRMNNMSFWLLPPSIMLLISSSIVENGAGTGWTVYPPLSSNIAHSGSSVDLAIFSLHLAGISSILGAVNFITTIINMRPNNMN
LDQMPLFVWAVGITALLLLLSLPVLAGAITMLLTDRNLNTSFFDPAGGGDPILYQHL
>LEFIJ1182-11|MM21042|Gynnidomorpha_permixtana
TLYFIFGIWAGMVGTSLSLLIRAELGNPGSLIGDDQIYNTIVTAHAFIMIFFMVMPIMIGGFGNWLVPLMLGAPDMAFPRMNNMSFWLLPPSIMLLISSSIVENGAGTGWTVYPPLSSNIAHSGSSVDLAIFSLHLAGISSILGAVNFITTIINMRPNNMN
LDQMPLFVWAVGITALLLLLSLPVLAGAITMLLTDRNLNTSFFDPAGGGDPILYQHL
>LEFIC176-10|HM872021|MM03530|Gynnidomorpha_vectisana
TLYFIFGIWAGMVGTSLSLLIRAELGNPGSLIGDDQIYNTIVTAHAFIMIFFMVMPIMIGGFGNWLVPLMLGAPDMAFPRMNNMSFWLLPPSIMLLISSSIVENGAGTGWTVYPPLSSNIAHSGSSVDLAIFSLHLAGISSILGAVNFITTIINMRPNNMSL

DQMPLFVWAVGITALLLLLSLPVLAGAITMLLTDRNLNTSFFDPAGGGDPILYQHL
>LEEUA241-11|JN274938|MM19649|Gypsonoma_aceriana
TLYFIFGIWAGMIGTSLSLLIRAELGNPGSLIGDDQIYNTIVTAHAFIMIFFMVMPIMIGGFGNWLVPLMLGAPDMAFPRMNNMSFWLLPPSIMLLISSSIVENGAGTGWTVYPPLSSNIAHSGSSVDLAIFSLHLAGISSILGAVNFITTIINMRPNNMTL
DQMPLFVWAVGITAILLLLSLPVLAGAITMLLTDRNLNTSFFDPAGGGDPILYQHL
>LEFIC138-10|HM871984|MM03482|Gypsonoma_dealbana
TLYFIFGIWAGMVGTSLSLLIRAELGNPGSLIGDDQIYNTIVTAHAFIMIFFMVMPIMIGGFGNWLVPLMLGAPDMAFPRMNNMSFWLLPPSIMLLISSSIVENGAGTGWTVYPPLSSNIAHSGSSVDLAIFSLHLAGISSILGAVNFITTIINMRPNNMSL
DQMPLFVWAVGITALLLLLSLPVLAGAITMLLTDRNLNTSFFDPAGGGDPILYQHL
>LEFIE725-10|HM874445|MM09819|Gypsonoma_dealbana
TLYFIFGIWAGMVGTSLSLLIRAELGNPGSLIGDDQIYNTIVTAHAFIMIFFMVMPIMIGGFGNWLVPLMLGAPDMAFPRMNNMSFWLLPPSIMLLISSSIVENGAGTGWTVYPPLSSNIAHSGSSVDLAIFSLHLAGISSILGAVNFITTIINMRPNNMSL
DQMPLFVWAVGITALLLLLSLPVLAGAITMLLTDRNLNTSFFDPAGGGDPILYQHL
>LEFID642-10|HM873404|MM06668|Gypsonoma_minutana
TLYFIFGIWAGMIGTSLSLLIRAELGNPGSLIGDDQIYNTIVTAHAFIMIFFMVMPIMIGGFGNWLVPLMLGAPDMAFPRMNNMSFWLLPPSIMLLISSSVVENGAGTGWTVYPPLSSNIAHSGSSVDLAIFSLHLAGISSILGAVNFITTIINMRPNNMSL
DQMPLFVWAVGITALLLLLSLPVLAGAITMLLTDRNLNTSFFDPAGGGDPILYQHL
>LEFIB497-10|HM871392|MM02078|Gypsonoma_nitidulana
TLYFIFGIWAGMVGTSLSLLIRAELGTPGSLIGDDQIYNTIVTAHAFIMIFFMVMPIMIGGFGNWLVPLMLGAPDMAFPRMNNMSFWLLPPSIMLLISSSIVENGAGTGWTVYPPLSSNIAHSGSSVDLAIFSLHLAGISSILGAVNFITTIINMRPNNMSL
DQMPLFVWSVGITALLLLLSLPVLAGAITMLLTDRNLNTSFFDPAGGGDPILYQHL
>LEFIC513-10|HM872337|MM04221|Gypsonoma_nitidulana
TLYFIFGIWAGMVGTSLSLLIRAELGTPGSLIGDDQIYNTIVTAHAFIMIFFMVMPIMIGGFGNWLVPLMLGAPDMAFPRMNNMSFWLLPPSIMLLISSSIVENGAGTGWTVYPPLSSNIAHSGSSVDLAIFSLHLAGISSILGAVNFITTIINMRPNNMSL
DQMPLFVWSVGITALLLLLSLPVLAGAITMLLTDRNLNTSFFDPAGGGDPILYQHL
>LEFID439-10|HQ570335|MM06371|Gypsonoma_nitidulana
TLYFIFGIWAGMVGTSLSLLIRAELGNPGSLIGDDQIYNTIVTAHAFIMIFFMVMPIMIGGFGNWLVPLMLGAPDMAFPRMNNMSFWLLPPSIMLLISSSIVENGAGTGWTVYPPLSSNIAHSGSSVDLAIFSLHLAGISSILGAVNFITTIINMRPNNMSL
DQMPLFVWSVGITALLLLLSLPVLAGAITMLLTDRNLNTSFFDPAGGGDPILYQHL
>LEFIF862-10|HM875544|MM13234|Gypsonoma_nitidulana
TLYFIFGIWAGMVGTSLSLLIRAELGTPGSLIGDDQIYNTIVTAHAFIMIFFMVMPIMIGGFGNWLVPLMLGAPDMAFPRMNNMSFWLLPPSIMLLISSSIVENGAGTGWTVYPPLSSNIAHSGSSVDLAIFSLHLAGISSILGAVNFITTIINMRPNNMSL
DQMPLFVWSVGITALLLLLSLPVLAGAITMLLTDRNLNTSFFDPAGGGDPILYQHL
>LEFIG178-10|HM875858|MM14045|Gypsonoma_nitidulana
TLYFIFGIWAGMVGTSLSLLIRAELGNPGSLIGDDQIYNTIVTAHAFIMIFFMVMPIMIGGFGNWLVPLMLGAPDMAFPRMNNMSFWLLPPSIMLLISSSIVENGAGTGWTVYPPLSSNIAHSGSSVDLAIFSLHLAGISSILGAVNFITTIINMRPNNMSL
DQMPLFVWSVGITALLLLLSLPVLAGAITMLLTDRNLNTSFFDPAGGGDPILYQHL
>LEFIK758-10|JF854315|MM18333|Gypsonoma_nitidulana
TLYFIFGIWAGMVGTSLSLLIRAELGNPGSLIGDDQIYNTIVTAHAFIMIFFMVMPIMIGGFGNWLVPLMLGAPDMAFPRMNNMSFWLLPPSIMLLISSSIVENGAGTGWTVYPPLSSNIAHSGSSVDLAIFSLHLAGISSILGAVNFITTIINMRPNNMSL
DQMPLFVWSVGITALLLLLSLPVLAGAITMLLTDRNLNTSFFDPAGGGDPILYQHL
>LEFIE447-10|HM874171|MM09211|Gypsonoma_oppressana
?LYFIFGIWAGMVGTSLSLLIRAELGSPGSLIGDDQIYNTIVTAHAFIMIFFMVMPIMIGGFGNWLVPLMLGAPDMAFPRMNNMSFWLLPPSIMLLISSSVVENGAGTGWTVYPPLSSNIAHSGSSVDLAIFSLHLAGISSILGAVNFITTIINMRPNNMS
LDQMPLFVWAVGITALLLLLSLPVLAGAITMLLTDRNLNTSFFDPAGGGDPILYQHL
>LEFIG860-10|HM876508|MM15724|Gypsonoma_oppressana
TLYFIFGIWAGMVGTSLSLLIRAELGSPGSLIGDDQIYNTIVTAHAFIMIFFMVMPIMIGGFGNWLVPLMLGAPDMAFPRMNNMSFWLLPPSIMLLISSSVVENGAGTGWTVYPPLSSNIAHSGSSVDLAIFSLHLAGISSILGAVNFITTIINMRPNNMS
LDQMPLFVWAVGITALLLLLSLPVLAGAITMLLTDRNLNTSFFDPAGGGDPILYQHL
>LEFIB541-10|HM871426|MM02154|Gypsonoma_sociana
TLYFIFGIWAGMVGTSLSLLIRAELGSPGSLIGDDQIYNTIVTAHAFIMIFFMVMPIMIGGFGNWLVPLMLGAPDMAFPRMNNMSFWLLPPSVMLLISSSIVENGAGTGWTVYPPLSSNIAHSGSSVDLAIFSLHLAGISSILGAVNFITTIINMRPNNMS
LDQMPLFVWSVGITALLLLLSLPVLAGAITMLLTDRNLNTSFFDPAGGGDPILYQHL
>LEFIB999-10|HM871870|MM03199|Gypsonoma_sociana
TLYFIFGIWAGMVGTSLSLLIRAELGNPGSLIGDDQIYNTIVTAHAFIMIFFMVMPIMIGGFGNWLVPLMLGAPDMAFPRMNNMSFWLLPPSVMLLISSSIVENGAGTGWTVYPPLSSNIAHSGSSVDLAIFSLHLAGISSILGAVNFITTIINMRPNNMS
LDQMPLFVWSVGITALLLLLSLPVLAGAITMLLTDRNLNTSFFDPAGGGDPILYQHL
>LEFIA846-10|HM386986|MM09722|Gypsonoma_sociana
TLYFIFGIWAGMVGTSLSLLIRAELGSPGSLIGDDQIYNTIVTAHAFIMIFFMVMPIMIGGFGNWLVPLMLGAPDMAFPRMNNMSFWLLPPSVMLLISSSIVENGAGTGWTVYPPLSSNIAHSGSSVDLAIFSLHLAGISSILGAVNFITTIINMRPNNMS
LDQMPLFVWSVGITALLLLLSLPVLAGAITMLLTDRNLNTSFFDPAGGGDPILYQHL
>LEFIJ801-10|KM572311|MM17426|Habrosyne_pyritoides

TLYFIFGIWAGMVGTSLSLLIRAELGNPGSLIGDDQIYNTIVTAHAFIMIFFMVMPIMIGGFGNWLVPLMLGAPDMAFPRMNNMSFWMLPPSLTLLISSSIVENGAGTGWTVYPPLSSNIAHSGSSVDLAIFSLHLAGISSILGAINFITTIINMRLNNMSF
DQMPLFVWAVGITAFLLLLSLPVLAGAITMLLTDRNLNTSFFDPAGGGDPILYQHL
>LEFIL361-10|KM572498|MM18671|Habrosyne_pyritoides
TLYFIFGIWAGMVGTSLSLLIRAELGNPGSLIGDDQIYNTIVTAHAFIMIFFMVMPIMIGGFGNWLVPLMLGAPDMAFPRMNNMSFWMLPPSLTLLISSSIVENGAGTGWTVYPPLSSNIAHSGSSVDLAIFSLHLAGISSILGAINFITTIINMRLNNMSF
DQMPLFVWAVGITAFLLLLSLPVLAGAITMLLTDRNLNTSFFDPAGGGDPILYQHL
>LEFIA504-10|KM572551|MM01586|Hada_plebeja
TLYFIFGIWAGMVGTSLSLLIRAELGTPGSLIGDDQIYNTIVTAHAFIMIFFMVMPIMIGGFGNWLVPLMLGAPDMAFPRMNNMSFWLLPPSLTLLISSSIVENGAGTGWTVYPPLSSNIAHGGSSVDLAIFSLHLAGISSILGAINFITTIINMRLNNLSFD
QMPLFIWAVGITAFLLLLSLPVLAGAITMLLTDRNLNTSFFDPAGGGDPILYQHL
>LEFIA744-10|HM386888|MM04098|Hada_plebeja
TLYFIFGIWAGMVGTSLSLLIRAELGTPGSLIGDDQIYNTIVTAHAFIMIFFMVMPIMIGGFGNWLVPLMLGAPDMAFPRMNNMSFWLLPPSLTLLISSSIVENGAGTGWTVYPPLSSNIAHGGSSVDLAIFSLHLAGISSILGAINFITTIINMRLNNLSFD
QMPLFIWAVGITAFLLLLSLPVLAGAITMLLTDRNLNTSFFDPAGGGDPILYQHL
>LEFIB206-10|HM871110|MM00656|Hadena_albimacula
TLYFIFGIWAGMVGTSLSLLIRAELGNPGSLIGDDQIYNTIVTAHAFIMIFFMVMPIMIGGFGNWLVPLMLGAPDMAFPRMNNMSFWLLPPSLILLISSSIVENGAGTGWTVYPPLSSNIAHGGSSVDLAIFSLHLAGISSILGAINFITTIINMRLNNLSFD
QMPLFIWAVGITAFLLLLSLPVLAGAITMLLTDRNLNTSFFDPAGGGDPILYQHL
>LEFIH025-10|HM876661|MM15889|Hadena_albimacula
TLYFIFGIWAGMVGTSLSLLIRAELGNPGSLIGDDQIYNTIVTAHAFIMIFFMVMPIMIGGFGNWLVPLMLGAPDMAFPRMNNMSFWLLPPSLILLISSSIVENGAGTGWTVYPPLSSNIAHGGSSVDLAIFSLHLAGISSILGAINFITTIINMRLNNLSFD
QMPLFIWAVGITAFLLLLSLPVLAGAITMLLTDRNLNTSFFDPAGGGDPILYQHL
>LEFIL719-10|MM16009|Hadena_bicruris
TLYFIFGIWAGMVGTSLSLLIRAELGNPGSLIGDDQIYNTIVTAHAFIMIFFMVMPIMIGGFGNWLVPLMLGAPDMAFPRMNNMSFWFLPPSLTLLISSSIVENGAGTGWTVYPPLSSNIAHGGSSVDLAIFSLHLAGISSILGAINFITTIINMRLNSLSFD
QMPLFIWAVGITAFLLLLSLPVLAGAITMLLTDRNLNTSFFDPAGGGDPILYQHL
>LEFIJ1504-12|MM22772|Hadena_bicruris
TLYFIFGIWAGMVGTSLSLLIRAELGNPGSLIGDDQIYNTIVTAHAFIMIFFMVMPIMIGGFGNWLVPLMLGAPDMAFPRMNNMSFWFLPPSLTLLISSSIVENGAGTGWTVYPPLSSNIAHGGSSVDLAIFSLHLAGISSILGAINFITTIINMRLNSLSFD
QMPLFIWAVGITAFLLLLSLPVLAGAITMLLTDRNLNTSFFDPAGGGDPILYQHL
>LEFIJ1506-12|MM22774|Hadena_caesia
TLYFIFGIWAGMVGTSLSLLIRAELGNPGSLIGDDQIYNTIVTAHAFIMIFFMVMPIMIGGFGNWLIPLMLGAPDMAFPRMNNMSFWLLPPSLTLLISSSIVENGAGTGWTVYPPLSSNIAHGGSSVDLAIFSLHLAGISSILGAINFITTIINMRLNNLSFD
QMPLFIWAVGITAFLLLLSLPVLAGAITMLLTDRNLNTSFFDPAGGGDPILYQHL
>LEFIA536-10|MM01620|Hadena_capsincola
TLYFIFGIWAGMVGTSLSLLIRAELGNPGSLIGDDQIYNTIVTAHAFIMIFFMVMPIMIGGFGNWLVPLMLGAPDMAFPRMNNMSFWFLPPSLTLLISSSIVENGAGTGWTVYPPLSSNIAHGGSSVDLAIFSLHLAGISSILGAINFITTIINMRLNSLSFD
QMPLFIWAVGITAFLLLLSLPVLAGAITMLLTDRNLNTSFFDPAGGGDPILYQHL
>LEFIC628-10|HM872449|MM04570|Hadena_compta
TLYFIFGIWAGMVGTSLSLLIRAELGNPGSLIGDDQIYNTIVTAHAFIMIFFMVMPIMIGGFGNWLIPLMLGAPDMAFPRMNNMSFWLLPPSLTLLISSSIVENGAGTGWTVYPPLSSNIAHGGSSVDLAIFSLHLAGISSILGAINFITTIINMRLNSLSFDQ
MPLFIWAVGITAFLLLLSLPVLAGAITMLLTDRNLNTSFFDPAGGGDPILYQHL
>LEFIC771-10|HM872590|MM04898|Hadena_compta
TLYFIFGIWAGMVGTSLSLLIRAELGNPGSLIGDDQIYNTIVTAHAFIMIFFMVMPIMIGGFGNWLIPLMLGAPDMAFPRMNNMSFWLLPPSLTLLISSSIVENGAGTGWTVYPPLSSNIAHGGSSVDLAIFSLHLAGISSILGAINFITTIINMRLNSLSFDQ
MPLFIWAVGITAFLLLLSLPVLAGAITMLLTDRNLNTSFFDPAGGGDPILYQHL
>LEFIB207-10|HM871111|MM00657|Hadena_confusa
TLYFIFGIWAGMVGTSLSLLIRAELGNPGSLIGDDQIYNTIVTAHAFIMIFFMVMPIMIGGFGNWLVPLMLGAPDMAFPRMNNMSFWLLPPSLTLLISSSIVENGAGTGWTVYPPLSSNIAHGGSSVDLAIFSLHLAGISSILGAINFITTIINMRLNNLSFD
QMPLFIWAVGITAFLLLLSLPVLAGAITMLLTDRNLNTSFFDPAGGGDPILYQHL
>LEFIL095-10|JF854454|MM19095|Hadena_irregularis
TLYFIFGIWAGMVGTSLSLLIRAELGNPGSLIGDDQIYNTIVTAHAFIMIFFMVMPIMIGGFGNWLVPLMLGAPDMAFPRMNNMSFWLLPPSLTLLISSSIVENGAGTGWTVYPPLSSNIAHGGSSVDLAIFSLHLAGISSILGAINFITTIINMRLNNLSFD
QMPLFIWAVGITAFLLLLSLPVLAGAITMLLTDRNLNTSFFDPAGGGDPILYQHL
>LEFIB208-10|HM871112|MM00658|Hadena_perplexa
TLYFIFGIWAGMVGTSLSLLIRAELGNPGSLIGDDQIYNTIVTAHAFIMIFFMVMPIMIGGFGNWLVPLMLGAPDMAFPRLNNMSFWLLPPSLTLLISSSIVENGAGTGWTVYPPLSSNIAHGGSSVDLAIFSLHLAGISSILGAINFITTIINMRLNNLSFDQ
MPLFIWAVGITAFLLLLSLPVLAGAITMLLTDRNLNTSFFDPAGGGDPILYQHL
>LEFIF670-10|HM875354|MM12635|Hadena_perplexa
TLYFIFGIWAGMVGTSLSLLIRAELGNPGSLIGDDQIYNTIVTAHAFIMIFFMVMPIMIGGFGNWLVPLMLGAPDMAFPRLNNMSFWLLPPSLTLLISSSIVENGAGTGWTVYPPLSSNIAHGGSSVDLAIFSLHLAGISSILGAINFITTIINMRLNNLSFDQ
MPLFIWAVGITAFLLLLSLPVLAGAITMLLTDRNLNTSFFDPAGGGDPILYQHL

>LEFIJ894-10|JF853885|MM17519|Haplotinea_ditella
TLYFIFGIWSGMLGTSLSLLIRAELGNPGSLIGDDQIYNTIVTAHAFIMIFFMVMPIMIGGFGNWLVPLMLGAPDMAFPRMNNMSFWLLPPSLTLLISSSIVENGAGTGWTVYPPLSSNIAHGGSSVDLTIFSLHLAGISSILGAVNFITTVINMRSYGMSF
DQMPLFVWAVAITALLLLLSLPVLAGAITMLLTDRNLNTSFFDPAGGGDPILYQHL
>LEFIB253-10|HM871156|MM00723|Haplotinea_insectella
TLYFIFGIWSGMLGTSLSLLIRAELGNPGSLIGDDQIYNTIVTAHAFIMIFFMVMPIMIGGFGNWLVPLMLGAPDMAFPRMNNMSFWLLPPSLTLLISSSIVENGAGTGWTVYPPLSSNIAHGGSSVDLTIFSLHLAGISSILGAVNFITTVINMRSYGMSF
DQMPLFVWAVAITALLLLLSLPVLAGAITMLLTDRNLNTSFFDPAGGGDPILYQHL
>LEFIB254-10|HM871157|MM00724|Haplotinea_insectella
TLYFIFGIWSGMLGTSLSLLIRAELGNPGSLIGDDQIYNTIVTAHAFIMIFFMVMPIMIGGFGNWLVPLMLGAPDMAFPRMNNMSFWLLPPSLTLLISSSIVENGAGTGWTVYPPLSSNIAHGGSSVDLTIFSLHLAGISSILGAVNFITTVINMRSYGMSF
DQMPLFVWAVAITALLLLLSLPVLAGAITMLLTDRNLNTSFFDPAGGGDPILYQHL
>LEFID591-10|HM873356|MM06600|Haplotinea_insectella
TLYFIFGIWSGMLGTSLSLLIRAELGNPGSLIGDDQIYNTIVTAHAFIMIFFMVMPIMIGGFGNWLVPLMLGAPDMAFPRMNNMSFWLLPPSLTLLISSSIVENGAGTGWTVYPPLSSNIAHGGSSVDLTIFSLHLAGISSILGAVNFITTVINMRSYGMSF
DQMPLFVWAVAITALLLLLSLPVLAGAITMLLTDRNLNTSFFDPAGGGDPILYQHL
>LEFIE583-10|HM874306|MM09485|Harpella_forficella
TLYFIFGIWAGMVGTSLSLLIRAELGNPGSLIGDDQIYNTIVTAHAFIMIFFMVMPIMIGGFGNWLVPLMLGAPDMAFPRMNNMSFWLLPPSLTLLISSSIVENGAGTGWTVYPPLSSNIAHGGSSVDLAIFSLHLAGISSILGAINFITTIINMRMNNMSF
DQMPLFIWSVGITALLLLLSLPVLAGAITMLLTDRNLNTSFFDPAGGGDPILYQHL
>LEFIB631-10|HM871510|MM02382|Hecatera_bicolorata
TLYFIFGIWAGMVGTSLSLLIRAELGNPGSLIGDDQIYNTIVTAHAFIMIFFMVMPIMIGGFGNWLVPLMLGAPDMAFPRMNNMSFWLLPPSLTLLISSSIVENGAGTGWTVYPPLSSNIAHGGSSVDLAIFSLHLAGISSILGAINFITTIINMRLNNLSFD
QMPLFIWAVGITAFLLLLSLPVLAGAITMLLTDRNLNTSFFDPAGGGDPILYQHL
>LEFIB445-10|HM871344|MM01962|Hedya_dimidiana
TLYFIFGIWAGMIGTSLSLLIRAELGNPGSLIGDDQIYNTIVTAHAFIMIFFMVMPIMIGGFGNWLVPLMLGAPDMAFPRMNNMSFWLLPPSIMLLISSSIVENGAGTGWTVYPPLSSNIAHSGSSVDLAIFSLHLAGISSILGAVNFITTIINMRPNNMSL
DQMPLFVWAVGITALLLLLSLPVLAGAITMLLTDRNLNTSFFDPAGGGDPILYQHL
>LEFID315-10|HM873112|MM06200|Hedya_dimidiana
TLYFIFGIWAGMIGTSLSLLIRAELGNPGSLIGDDQIYNTIVTAHAFIMIFFMVMPIMIGGFGNWLVPLMLGAPDMAFPRMNNMSFWLLPPSIMLLISSSIVENGAGTGWTVYPPLSSNIAHSGSSVDLAIFSLHLAGISSILGAVNFITTIINMRPNNMSL
DQMPLFVWAVGITALLLLLSLPVLAGAITMLLTDRNLNTSFFDPAGGGDPILYQHL
>LEFIB446-10|HM871345|MM01964|Hedya_nubiferana
TLYFIFGIWAGMIGTSLSLLIRAELGNPGSLIGDDQIYNTIVTAHAFIMIFFMVMPIMIGGFGNWLVPLMLGAPDMAFPRMNNMSFWLLPPSIMLLISSSIVENGAGTGWTVYPPLSSNIAHSGSSVDLAIFSLHLAGISSILGAVNFITTIINMRPNNMSL
DQMPLFVWAVGITALLLLLSLPVLAGAITMLLTDRNLNTSFFDPAGGGDPILYQHL
>LEFIC961-10|HM872776|MM05336|Hedya_nubiferana
TLYFIFGIWAGMIGTSLSLLIRAELGNPGSLIGDDQIYNTIVTAHAFIMIFFMVMPIMIGGFGNWLVPLMLGAPDMAFPRMNNMSFWLLPPSIMLLISSSIVENGAGTGWTVYPPLSSNIAHSGSSVDLAIFSLHLAGISSILGAVNFITTIINMRPNNMSL
DQMPLFVWAVGITALLLLLSLPVLAGAITMLLTDRNLNTSFFDPAGGGDPILYQHL
>LEFIF867-10|HM875549|MM13244|Hedya_nubiferana
TLYFIFGIWAGMIGTSLSLLIRAELGNPGSLIGDDQIYNTIVTAHAFIMIFFMVMPIMIGGFGNWLVPLMLGAPDMAFPRMNNMSFWLLPPSIMLLISSSIVENGAGTGWTVYPPLSSNIAHSGSSVDLAIFSLHLAGISSILGAVNFITTIINMRPNNMSL
DQMPLFVWAVGITALLLLLSLPVLAGAITMLLTDRNLNTSFFDPAGGGDPILYQHL
>LEFIA876-10|HM387014|MM09753|Hedya_pruniana
TLYFIFGIWAGMIGTSLSLLIRAELGNPGSLIGDDQIYNTIVTAHAFIMIFFMVMPIMIGGFGNWLVPLMLGAPDMAFPRMNNMSFWLLPPSIMLLISSSIVENGAGTGWTVYPPLSSNIAHSGSSVDLAIFSLHLAGISSILGAVNFITTIINMRPNNMSL
DQMPLFVWAVGITALLLLLSLPVLAGAITMLLTDRNLNTSFFDPAGGGDPILYQHL
>LEFIC148-10|HM871994|MM03493|Hedya_salicella
TLYFIFGIWSGMIGTSLSLLIRAELGNPGSLIGDDQIYNTIVTAHAFIMIFFMVMPIMIGGFGNWLVPLMLGAPDMAFPRMNNMSFWLLPPSIMLLISSSIVENGAGTGWTVYPPLSSNIAHSGSSVDLAIFSLHLAGISSILGAVNFITTIINMRPNNMSL
DQMPLFVWAVGITALLLLLSLPVLAGAITMLLTDRNLNTSFFDPAGGGDPILYQHL
>LEFIE444-10|HM874168|MM09207|Heinemannia_laspeyrella
TLYFIFGIWAGLIGTSLSLLIRTELGNPGSLIGDDQIYNTIVTAHAFIMIFFMVMPIMIGGFGNWLVPLMLGAPDMAFPRMNNMSFWLLPPSITLLISSSVVENGAGTGWTVYPPLSSNIAHSGSSVDLAIFSLHLAGISSILGAINFITTIINMRLNNLSFDQ
MPLFVWAVGITALLLLLSLPVLAGAITMLLTDRNLNTSFFDPAGGGDPILYQHL
>LEFIK356-10|JF853992|MM17931|Heinemannia_laspeyrella
TLYFIFGIWAGLIGTSLSLLIRTELGNPGSLIGDDQIYNTIVTAHAFIMIFFMVMPIMIGGFGNWLVPLMLGAPDMAFPRMNNMSFWLLPPSITLLISSSVVENGAGTGWTVYPPLSSNIAHSGSSVDLAIFSLHLAGISSILGAINFITTIINMRLNNLSFDQ
MPLFVWAVGITALLLLLSLPVLAGAITMLLTDRNLNTSFFDPAGGGDPILYQHL
>LEFIC515-10|HM872339|MM04224|Helcystogramma_lineolella
TLYFIFGIWAGMLGTSLSLLIRAELGNPGSLIGNDQIYNTIVTAHAFIMIFFMVMPIMIGGFGNWLIPLMLGAPDMAFPRMNNMSFWLLPPSLMLLISSSIVENGAGTGWTVYPPLSSNIAHSGSSVDLTIFSLHLAGISSILGAINFITTIINMRMNNLLFD

QMPLFVWSVGITALLLLLSLPVLAGAITMLLTDRNLNTSFFDPAGGGDPILYQHL
>LEFIF959-10|HM875640|MM13533|Helcystogramma_lineolella
TLYFIFGIWAGMLGTSLSLLIRAELGNPGSLIGNDQIYNTIVTAHAFIMIFFMVMPIMIGGFGNWLIPLMLGAPDMAFPRMNNMSFWLLPPSLMLLISSSIVENGAGTGWTVYPPLSSNIAHSGSSVDLTIFSLHLAGISSILGAINFITTIINMRMNNLLFD
QMPLFVWSVGITALLLLLSLPVLAGAITMLLTDRNLNTSFFDPAGGGDPILYQHL
>LEFIF513-10|HM875198|MM12134|Helcystogramma_lutatella
TLYFIFGIWAGMLGTSLSLLIRAELGNPGSLIGDDQIYNTIVTAHAFIMIFFMVMPIMIGGFGNWLVPLMLGAPDMAFPRMNNMSFWLLPPSLTLLISSSIVENGAGTGWTVYPPLSSNIAHGGSSVDLAIFSLHLAGISSILGAINFITTIINMRMNNLSF
DQMPLFVWSVGITALLLLLSLPVLAGAITMLLTDRNLNTSFFDPAGGGDPILYQHL
>LEFIF950-10|HM875631|MM13512|Helcystogramma_lutatella
TLYFIFGIWAGMLGTSLSLLIRAELGNPGSLIGDDQIYNTIVTAHAFIMIFFMVMPIMIGGFGNWLVPLMLGAPDMAFPRMNNMSFWLLPPSLTLLISSSIVENGAGTGWTVYPPLSSNIAHGGSSVDLAIFSLHLAGISSILGAINFITTIINMRMNNLSF
DQMPLFVWSVGITALLLLLSLPVLAGAITMLLTDRNLNTSFFDPAGGGDPILYQHL
>LEFIB934-10|HM871811|MM03080|Helcystogramma_rufescens
TLYFIFGIWSGMLGTSLSLLIRMELGNPGSLIGDDQIYNTIVTAHAFIMIFFMVMPIMIGGFGNWLVPLMLGAPDMAFPRMNNMSFWLLPPSIMLLISSSIVENGAGTGWTVYPPLSSNIAHSGSSVDLAIFSLHLAGISSILGAINFITTIINMRVNNLLFD
QMPLFVWAVGITALLLLLSLPVLAGAITMLLTDRNLNTSFFDPAGGGDPILYQHL
>LEFIE370-10|HM874094|MM08862|Helcystogramma_rufescens
TLYFIFGIWSGMLGTSLSLLIRMELGNPGSLIGDDQIYNTIVTAHAFIMIFFMVMPIMIGGFGNWLVPLMLGAPDMAFPRMNNMSFWLLPPSIMLLISSSIVENGTGTGWTVYPPLSSNIAHSGSSVDLAIFSLHLAGISSILGAINFITTIINMRVNNLLFD
QMPLFVWAVGITALLLLLSLPVLAGAITMLLTDRNLNTSFFDPAGGGDPILYQHL
>LEFIF322-10|HM875007|MM11112|Helicoverpa_armigera
TLYFIFGIWAGMVGTSLSLLIRAELGNPGSLIGDDQIYNTIVTAHAFIMIFFMVMPIMIGGFGNWLVPLMLGAPDMAFPRMNNMSFWLLPPSLTLLISSSIVENGAGTGWTVYPPLSSNIAHGGSSVDLAIFSLHLAGISSILGAINFITTIINMKLNSLSFD
QMPLFIWAVGITAFLLLLSLPVLAGAITMLLTDRNLNTSFFDPAGGGDPILYQHL
>LEFIJ865-10|JF853867|MM17490|Helicoverpa_armigera
TLYFIFGIWAGMVGTSLSLLIRAELGNPGSLIGDDQIYNTIVTAHAFIMIFFMVMPIMIGGFGNWLVPLMLGAPDMAFPRMNNMSFWLLPPSLTLLISSSIVENGAGTGWTVYPPLSSNIAHGGSSVDLAIFSLHLAGISSILGAINFITTIINMKLNSLSFD
QMPLFIWAVGITAFLLLLSLPVLAGAITMLLTDRNLNTSFFDPAGGGDPILYQHL
>LEFIJ866-10|JF853868|MM17491|Helicoverpa_armigera
TLYFIFGIWAGMVGTSLSLLIRAELGNPGSLIGDDQIYNTIVTAHAFIMIFFMVMPIMIGGFGNWLVPLMLGAPDMAFPRMNNMSFWLLPPSLTLLISSSIVENGAGTGWTVYPPLSSNIAHGGSSVDLAIFSLHLAGISSILGAINFITTIINMKLNSLSFD
QMPLFIWAVGITAFLLLLSLPVLAGAITMLLTDRNLNTSFFDPAGGGDPILYQHL
>LOBU079-12|MM22021|Heliothela_wulfeniana
TLYFMFGIWAGMVGTSLSLLIRAELGNPGSLIGDDQIYNTIVTAHAFIMIFFMVMPIMIGGFGNWLVPLMLGAPDMAFPRMNNMSFWLLPPSLTLLISSSIVENGAGTGWTVYPPLSSNISHGGSSVDLAIFSLHLAGISSILGAINFITTIINMRINNLSF
DQMPLFVWSVGITALLLLLSLPVLAGAITMLLTDRNLNTSFFDPAGGGDPILYQHL
>LEEUA320-11|JN272570|MM19728|Heliothis_maritima
TLYFIFGIWAGMVGTSLSLLIRAELGNPGSLIGDDQIYNTIVTAHAFIMIFFMVMPIMIGGFGNWLVPLMLGAPDMAFPRMNNMSFWLLPPSLTLLISSSIVENGAGTGWTVYPPLSSNIAHGGSSVDLAIFSLHLAGISSILGAINFITTIINMKLNNLSFD
QMPLFVWAVGITAFLLLLSLPVLAGAITMLLTDRNLNTSFFDPAGGGDPILYQHL
>LEFIF323-10|HM875008|MM11113|Heliothis_nubigera
TLYFIFGIWAGMVGTSLSLLIRAELGNPGSLIGDDQIYNTIVTAHAFIMIFFMVMPIMIGGFGNWLVPLMLGAPDMAFPRMNNMSFWLLPPSLTLLISSSIVENGAGTGWTVYPPLSSNIAHGGSSVDLAIFSLHLAGISSILGAINFITTIINMKLNNLSFD
QMPLFVWAVGITAFLLLLSLPVLAGAITMLLTDRNLNTSFFDPAGGGDPILYQHL
>LEFID152-10|HM872959|MM05915|Heliothis_viriplaca
TLYFIFGIWAGMVGTSLSLLIRAELGNPGSLIGDDQIYNTIVTAHAFIMIFFMVMPIMIGGFGNWLVPLMLGAPDMAFPRMNNMSFWLLPPSLTLLISSSIVENGAGTGWTVYPPLSSNIAHGGSSVDLAIFSLHLAGISSILGAINFITTIINMKLNNLSFD
QMPLFVWAVGITAFLLLLSLPVLAGAITMLLTDRNLNTSFFDPAGGGDPILYQHL
>LEFIK475-10|JF854085|MM18050|Heliothis_viriplaca
TLYFIFGIWAGMVGTSLSLLIRAELGNPGSLIGDDQIYNTIVTAHAFIMIFFMVMPIMIGGFGNWLVPLMLGAPDMAFPRMNNMSFWLLPPSLTLLISSSIVENGAGTGWTVYPPLSSNIAHGGSSVDLAIFSLHLAGISSILGAINFITTIINMKLNNLSFD
QMPLFVWAVGITAFLLLLSLPVLAGAITMLLTDRNLNTSFFDPAGGGDPILYQHL
>LEFIB575-10|HM871456|MM02246|Hellinsia_didactylites
TLYFIFGIWAGMVGTSLSLLIRAELGNPGSLIGDDQIYNSIVTAHAFIMIFFMVMPIMIGGFGNWLVPLMLGAPDMAFPRMNNMSFWLLPPSIMLLISSSIVENGAGTGWTVYPPLSSNIAHSGSSVDLAIFSLHLAGISSILGAINFITTIINMRLNNMMF
DQLPLFVWAVGITALLLLLSLPVLAGAITMLLTDRNLNTSFFDPAGGGDPILYQHL
>LEFIK037-10|JF853919|MM17612|Hellinsia_distinctus
TLYFIFGIWAGMVGTSLSLLIRAELGNPGSLIGDDQIYNSIVTAHAFIMIFFMVMPIMIGGFGNWLVPLMLGAPDMAFPRMNNMSFWLLPPSIMLLISSSIVENGAGTGWTVYPPLSSNIAHSGSSVDLAIFSLHLAGISSILGAINFITTIINMRLNNMMF
DQLPLFVWAVGITALLLLLSLPVLAGAITMLLTDRNLNTSFFDPAGGGDPILYQHL
>LEFIB573-10|HM871455|MM02243|Hellinsia_lienigianus

TLYFIFGIWAGMVGTSLSLLIRAELGNPGSLIGDDQIYNTIVTAHAFIMIFFMVMPIMIGGFGNWLVPLMLGAPDMAFPRMNNMSFWLLPPSIILLISSSIVENGAGTGWTVYPPLSSNIAHSGTSVDLAIFSLHLAGISSILGAINFITTIINMRLNNMMF
DQLPLFVWAVGITALLLLLSLPVLAGAITMLLTDRNLNTSFFDPAGGGDPILYQHL
>LEFIB948-10|HM871825|MM03105|Hellinsia_osteodactylus
TLYFIFGIWAGMIGTSLSLLIRAELGNPGSLICDDQIYNSIVTAHAFIMIFFMVMPIMIGGFGNWLVPLMLGAPDMAFPRMNNMSFWLLPPSIILLISSSIVENGAGTGWTVYPPLSSNIAHSGSSVDLAIFSLHLAGISSILGAINFITTIINMRLNNMMFD
QLPLFVWAVGITALLLLLSLPVLAGAITMLLTDRNLNTSFFDPAGGGDPILYQHL
>LEFID427-10|HQ570323|MM06357|Hellinsia_osteodactylus
TLYFIFGIWAGMIGTSLSLLIRAELGNPGSLICDDQIYNSIVTAHAFIMIFFMVMPIMIGGFGNWLVPLMLGAPDMAFPRMNNMSFWLLPPSIILLISSSIVENGAGTGWTVYPPLSSNIAHSGSSVDLAIFSLHLAGISSILGAINFITTIINMRLNNMMFD
QLPLFVWAVGITALLLLLSLPVLAGAITMLLTDRNLNTSFFDPAGGGDPFLYQHL
>LEFIB946-10|HM871823|MM03103|Hellinsia_tephradactyla
TLYFIFGIWAGMVGTSLSLLIRAELGNPGSLISDDQIYNTIVTAHAFIMIFFMVMPIMIGGFGNWLVPLMLGAPDMAFPRMNNMSFWLLPPSIILLISSSLVENGAGTGWTVYPPLSSNIAHSGTSVDLAIFSLHLAGISSILGAINFITTIINMRLNNLMFD
QLPLFVWAVGITALLLLLSLPVLAGAITMLLTDRNINTSFFDPAGGGDPILYQHL
>LEFIB947-10|HM871824|MM03104|Hellinsia_tephradactyla
TLYFIFGIWAGMVGTSLSLLIRAELGNPGSLISDDQIYNTIVTAHAFIMIFFMVMPIMIGGFGNWLVPLMLGAPDMAFPRMNNMSFWLLPPSIILLISSSLVENGAGTGWTVYPPLSSNIAHSGTSVDLAIFSLHLAGISSILGAINFITTIINMRLNNLMFD
QLPLFVWAVGITALLLLLSLPVLAGAITMLLTDRNINTSFFDPAGGGDPILYQHL
>LEFIA628-10|HM870877|MM01743|Helotropha_leucostigma
TLYFIFGIWAGMVGTSLSLLIRAELGNPGSLIGDDQIYNTIVTAHAFIMIFFMVMPIMIGGFGNWLVPLMLGAPDMAFPRMNNMSFWLLPPSLTLLISSSIVENGAGTGWTVYPPLSSNIAHGGSSVDLAIFSLHLAGISSILGAINFITTIINMRLNNLSFD
QMPLFIWAVGITAFLLLLSLPVLAGAITMLLTDRNLNTSFFDPAGGGDPILYQHL
>LEFID032-10|HM872846|MM05477|Hemaris_fuciformis
TLYFIFGIWAGMVGTSLSLLIRAELGNPGSLIGDDQIYNTIVTAHAFIMIFFMVMPIMIGGFGNWLVPLMLGAPDMAFPRMNNMSFWLLPPSLTLLISSSIVENGAGTGWTVYPPLSANIAHSGSSVDLAIFSLHLAGISSILGAINFITTIINMRINNLSFD
QMPLFVWAVGITAFLLLLSLPVLAGAITMLLTDRNLNTSFFDPAGGGDPILYQHL
>LEFIE337-10|HM874062|MM08731|Hemaris_tityus
TLYFIFGIWAGMVGTSLSLLIRAELGNPGSLIGDDQIYNTIVTAHAFIMIFFMVMPIMIGGFGNWLVPLMLGAPDMAFPRMNNMSFWLLPPSLTLLISSSIVENGAGTGWTVYPPLSANIAHSGSSVDLAIFSLHLAGISSILGAINFITTIINMRINNLSFD
QMPLFVWAVGITAFLLLLSLPVLAGAITMLLTDRNLNTSFFDPAGGGDPILYQHL
>LEFIJ565-10|JF853680|MM17190|Hemaris_tityus
TLYFIFGIWAGMVGTSLSLLIRAELGNPGSLIGDDQIYNTIVTAHAFIMIFFMVMPIMIGGFGNWLVPLMLGAPDMAFPRMNNMSFWLLPPSLTLLISSSIVENGAGTGWTVYPPLSANIAHSGSSVDLAIFSLHLAGISSILGAINFITTIINMRINNLSFD
QMPLFVWAVGITAFLLLLSLPVLAGAITMLLTDRNLNTSFFDPAGGGDPILYQHL
>LEFID237-10|HM873037|MM06098|Hemithea_aestivaria
TLYFIFGIWAGMIGTSLSLLIRAELGNPGSLIGDDQIYNTIVTAHAFIMIFFMVMPIMIGGFGNWLVPLMLGAPDMAFPRMNNMSFWLLPPSLTLLISSSIVENGAGTGWTVYPPLSSNIAHGGSSVDLAIFSLHLAGISSILGAINFITTIINMRLNNLSFD
QMPLFIWAVGITAFLLLLSLPVLAGAITMLLTDRNLNTSFFDPAGGGDPILYQHL
>LEFIF208-10|HM874902|MM10874|Hemithea_aestivaria
TLYFIFGIWAGMIGTSLSLLIRAELGNPGSLIGDDQIYNTIVTAHAFIMIFFMVMPIMIGGFGNWLVPLMLGAPDMAFPRMNNMSFWLLPPSLTLLISSSIVENGAGTGWTVYPPLSSNIAHGGSSVDLAIFSLHLAGISSILGAINFITTIINMRLNNLSFD
QMPLFVWAVGITAFLLLLSLPVLAGAITMLLTDRNLNTSFFDPAGGGDPILYQHL
>LEFIA130-10|HM396477|MM01061|Hepialus_humuli
TLYFIFGIWSGMVGTSLSLLIRTELGNPGSLIGDDQIYNVIVTAHAFIMIFFMVMPIMIGGFGNWLIPLMLGAPDMAFPRLNNMSFWLLPPSLMLLISSSIVENGAGTGWTVYPPLSSNIAHNGSSVDLAIFSLHLAGISSILGAINFITTVINMRSNGMSFD
RMPLFIWSVVITALLLLLSLPVLAGAITMLLTDRNLNTSFFDPAGGGDPILYQHL
>LEFIG319-10|HM875998|MM14287|Hepialus_humuli
TLYFIFGIWSGMVGTSLSLLIRTELGNPGSLIGDDQIYNVIVTAHAFIMIFFMVMPIMIGGFGNWLIPLMLGAPDMAFPRLNNMSFWLLPPSLMLLISSSIVENGAGTGWTVYPPLSSNIAHNGSSVDLAIFSLHLAGISSILGAINFITTVINMRSNGMSFD
RMPLFVWSVVITALLLLLSLPVLAGAITMLLTDRNLNTSFFDPAGGGDPILYQHL
>LEFIA034-10|HM396384|MM00433|Heringocrania_unimaculella
TLYFIFGIWSGMVGTSLSILIRSELGNPGSLIGDDQIYNVIVTAHAFIMIFFMVMPIMIGGFGNWLVPLMLGAPDMAFPRLNNMSFWLLPPSLTLLISSSFVENGAGTGWTVYPPLSSNIAHAGSSVDLAIFSLHLAGISSILGAVNFITTVINMRPYGMSL
DRMPLFVWAVAITALLLLLSLPVLAGAITMLLTDRNLNTSFFDPAGGGDPILYQHL
>LEFIF274-10|HM874967|MM11056|Herminia_tarsicrinalis
TLYFIFGIWAGMVGTSLSLLIRAELGNPGSLIGDDQIYNTIVTAHAFIMIFFMVMPIMIGGFGNWLVPLMLGAPDMAFPRMNNMSFWLLPPSLTLLISSSIVENGAGTGWTVYPPLSSNIAHSGSSVDLAIFSLHLAGISSILGAINFITTIINMRLNNLSFD
QMPLFIWAVGITAFLLLLSLPVLAGAITMLLTDRNLNTSFFDPAGGGDPILYQHL
>LEFIJ595-10|JF853695|MM17220|Herminia_tarsicrinalis
TLYFIFGMWAGMVGTSLSLLIRAELGNPGSLIGDDQIYNTIVTAHAFIMIFFMVMPIMIGGFGNWLVPLMLGAPDMAFPRMNNMSFWLLPPSLTLLISSSIVENGAGTGWTVYPPLSSNIAHSGSSVDLAIFSLHLAGISSILGAINFITTIINMRLNNLSF
DQMPLFIWAVGITAFLLLLSLPVLAGAITMLLTDRNLNTSFFDPAGGGDPILYQHL

>LEFIC641-10|HM872462|MM04587|Herminia_tarsipennalis
TLYFIFGIWAGMVGTSLSLLIRAELGNPGSLIGDDQIYNTIVTAHAFIMIFFMVMPIMIGGFGNWLIPLMLGAPDMAFPRMNNMSFWLLPPSLTLLISSSIVENGAGTGWTVYPPLSSNIAHGGSSVDLAIFSLHLAGISSILGAINFITTIINMRLNNLSFD
QMPLFIWAVGITAFLLLLSLPVLAGAITMLLTDRNLNTSFFDPAGGGDPILYQHL
>LEFID810-10|HM873567|MM06896|Hesperia_comma
TLYFIFGIWAGMLGTSLSLLIRTELGNPGSLIGDDQIYNTIVTAHAFIMIFFMVMPIMIGGFGNWLVPLMLGAPDMAFPRMNNMSFWMLPPSLTLLISSSIVENGTGTGWTVYPPLSSNIAHQGSSVDLTIFSLHLAGISSILGAINFITTIINMRIKNLSFD
QMPLFVWSVGITALLLLLSLPVLAGAITMLLTDRNLNTSFFDPAGGGDPILYQHL
>LEFIC552-10|HM872373|MM04311|Heterogenea_asella
TLYFIFGIWAGMVGTSLSLMIRAELGNPGSLIGDDQIYNTIVTAHAFIMIFFMVMPIMIGGFGNWLVPLMLGAPDMAFPRMNNMSFWLLPPSLMLLVSSSIVENGAGTGWTVYPPLSSNIAHSGSSVDLAIFSLHLAGISSILGAVNFITTIINMRPNGM
AFDQMPLFVWAVGITALLLLLSLPVLAGAITMLLTDRNLNTSFFDPAGGGDPILYQHL
>LEFID062-10|HM872876|MM05667|Heterogenea_asella
TLYFIFGIWAGMVGTSLSLMIRAELGNPGSLIGDDQIYNTIVTAHAFIMIFFMVMPIMIGGFGNWLVPLMLGAPDMAFPRMNNMSFWLLPPSLMLLISSSIVENGAGTGWTVYPPLSSNIAHSGSSVDLAIFSLHLAGISSILGAVNFITTIINMRPNGM
AFDQMPLFVWAVGITALLLLLSLPVLAGAITMLLTDRNLNTSFFDPAGGGDPILYQHL
>LEFIG947-10|HM876587|MM15811|Heterothera_serraria
TMYFIFGIWAGMVGTSLSLLIRAELGNPGSLIGDDQIYNTIVTAHAFIMIFFMVMPIMIGGFGNWLVPLMLGAPDMAFPRMNNMSFWLLPPSITLLISSSIVENGAGTGWTVYPPLSSNIAHGGSSVDLAIFSLHLAGISSILGAINFITTIINMRLNNMFF
DQLSLFVWAVGITAFLLLLSLPVLAGAITMLLTDRNLNTSFFDPAGGGDPILYQHL
>LEFIG948-10|HM876588|MM15812|Heterothera_serraria
TMYFIFGIWAGMVGTSLSLLIRAELGSPGSLIGDDQIYNTIVTAHAFIMIFFMVMPIMIGGFGNWLVPLMLGAPDMAFPRMNNMSFWLLPPSITLLISSSIVENGAGTGWTVYPPLSSNIAHGGSSVDLAIFSLHLAGISSILGAINFITTIINMRLNNMFF
DQLPLFVWAVGITAFLLLLSLPVLAGAITMLLTDRNLNTSFFDPAGGGDPILYQHL
>LEFIG949-10|HM876589|MM15813|Heterothera_serraria
TMYFIFGIWAGMVGTSLSLLIRAELGNPGSLIGDDQIYNTIVTAHAFIMIFFMVMPIMIGGFGNWLVPLMLGAPDMAFPRMNNMSFWLLPPSITLLISSSIVENGAGTGWTVYPPLSSNIAHGGSSVDLAIFSLHLAGISSILGAINFITTIINMRLNNMFF
DQLPLFVWAVGITAFLLLLSLPVLAGAITMLLTDRNLNTSFFDPAGGGDPILYQHL
>LEFIE030-10|HM873779|MM08102|Hillia_iris
TLYFIFGIWAGMVGTSLSLLIRAELGNPGSLIGDDQIYNTIVTAHAFIMIFFMVMPIMIGGFGNWLVPLMLGAPDMAFPRLNNMSFWLLPPSLTLLISSSIVENGAGTGWTVYPPLSSNIAHGGSSVDLAIFSLHLAGISSILGAINFITTIINMRLNSLSFDQ
MPLFIWAVGITAFLLLLSLPVLAGAITMLLTDRNLNTSFFDPAGGGDPILYQHL
>LEEUA524-11|MM20583|Hipparchia_hermione
TLYFIFGIWAGMVGTSLSLIIRTELGNPGFLIGDDQIYNTIVTAHAFIMIFFMVMPIMIGGFGNWLVPLMLGAPDMAFPRMNNMSFWLLPPSLMLLISSSIVENGAGTGWTVYPPLSSNIAHGGSSVDLAIFSLHLAGISSILGAINFITTIINMRVNSMTY
DQMPLFVWAVGITALLLLLSLPVLAGAITMLLTDRNLNTSFFDPAGGGDPILYQHL
>LEFID811-10|HM873568|MM06897|Hipparchia_semele
TLYFIFGIWAGMVGTSLSLIIRTELGNPGFLIGDDQIYNTIVTAHAFIMIFFMVMPIMIGGFGNWLVPLMLGAPDMAFPRMNNMSFWLLPPSLMLLISSSIVENGAGTGWTVYPPLSSNIAHGGSSVDLAIFSLHLAGISSILGAINFITTIINMRINSMTYD
QMPLFVWAVGITALLLLLSLPVLAGAITMLLTDRNLNTSFFDPAGGGDPILYQHL
>LEFIJ551-10|JF853666|MM17176|Hipparchia_semele
TLYFIFGIWAGMVGTSLSLIIRTELGNPGFLIGDDQIYNTIVTAHAFIMIFFMVMPIMIGGFGNWLVPLMLGAPDMAFPRMNNMSFWLLPPSLMLLISSSIVENGAGTGWTVYPPLSSNIAHGGSSVDLAIFSLHLAGISSILGAINFITTIINMRINSMTYD
QMPLFVWAVGITALLLLLSLPVLAGAITMLLTDRNLNTSFFDPAGGGDPILYQHL
>LEFIJ552-10|JF853667|MM17177|Hipparchia_semele
TLYFIFGIWAGMVGTSLSLIIRTELGNPGFLIGDDQIYNTIVTAHAFIMIFFMVMPIMIGGFGNWLVPLMLGAPDMAFPRMNNMSFWLLPPSLMLLISSSIVENGAGTGWTVYPPLSSNIAHGGSSVDLAIFSLHLAGISSILGAINFITTIINMRINSMTYD
QMPLFVWAVGITALLLLLSLPVLAGAITMLLTDRNLNTSFFDPAGGGDPILYQHL
>LEFIE964-10|HM874681|MM10372|Hofmannophila_pseudospretella
TLYFIFGIWAGMVGTSLSLLIRAELGNPGSLIGDDQIYNTIVTAHAFIMIFFMVMPIMIGGFGNWLVPLMLGAPDMAFPRMNNMSFWLLPPSLTLLISSSIVENGAGTGWTVYPPLSSNIAHGGSSVDLAIFSLHLAGISSILGAINFITTIINMRLSNMSFD
QMPLFVWAVGITALLLLLSLPVLAGAITMLLTDRNLNTSFFDPAGGGDPILYQHL
>LEFIE965-10|HM874682|MM10373|Hofmannophila_pseudospretella
TLYFIFGIWAGMVGTSLSLLIRAELGNPGSLIGDDQIYNTIVTAHAFIMIFFMVMPIMIGGFGNWLVPLMLGAPDMAFPRMNNMSFWLLPPSLTLLISSSIVENGAGTGWTVYPPLSSNIAHGGSSVDLAIFSLHLAGISSILGAINFITTIINMRLSNMSFD
QMPLFVWAVGITALLLLLSLPVLAGAITMLLTDRNLNTSFFDPAGGGDPILYQHL
>LEFIL246-10|JF854550|MM19246|Holoarctia_puengeleri
TLYFIFGIWAGMVGTSLSLLIRAELGNPGSLIGDDQIYNTIVTAHAFIMIFFMVMPIMIGGFGNWLVPLMLGAPDMAFPRMNNMSFWLLPPSLTLLISSSIVENGAGTGWTVYPPLSSNIAHGGSSVDLAIFSLHLAGISSILGAINFITTIINMRLNNLSFD
QMPLFVWAVGITAFLLLLSLPVLAGAITMLLTDRNLNTSFFDPAGGGDPILYQHL
>LEFIF091-10|HM874795|MM10540|Homoeosoma_nebulella
TLYFIFGIWSGMVGTSLSLLIRAELGTPGSLIGDDQIYNTIVTGHAFIMIFFMVMPIMIGGFGNWLVPLMLGAPDMAFPRMNNMSFWLLPPSLTLLISSSIVENGAGTGWTVYPPLSSNIAHSGSSVDLAIFSLHLAGISSILGAINFITTIINMKLNGMSFD

QMPLFVWAVGITALLLLLSLPVLAGAITMLLTDRNLNTSFFDPAGGGDPILYQHL
>LEEUA275-11|JN275975|MM19683|Homoeosoma_nimbella
TLYFIFGIWSGMVGTSLSLLIRAELGTPGSLIGDDQIYNTIVTGHAFIMIFFMVMPIMIGGFGNWLVPLMLGAPDMAFPRMNNMSFWLLPPSLTLLISSSIVENGAGTGWTVYPPLSSNIAHSGSSVDLAIFSLHLAGISSILGAINFITTIINMKLNGMSFD
QMPLFVWAVGITALLLLLSLPVLAGAITMLLTDRNLNTSFFDPAGGGDPILYQHL
>LEFIC090-10|HM871958|MM03391|Homoeosoma_sinuella
TLYFIFGIWSGMVGTSLSLLIRAELGTPGSLIGDDQIYNTIVTGHAFIMIFFMVMPIMIGGFGNWLVPLMLGAPDMAFPRMNNMSFWLLPPSLTLLISSSIVENGAGTGWTVYPPLSSNIAHSGSSVDLAIFSLHLAGISSILGAINFITTIINMKLNGMSFD
QMPLFVWAVGITALLLLLSLPVLAGAITMLLTDRNLNTSFFDPAGGGDPILYQHL
>LEFIC866-10|HM872684|MM05130|Hoplodrina_blanda
TLYFIFGIWAGMVGTSLSLLIRAELGNPGSLIGDDQIYNTIVTAHAFIMIFFMVMPIMIGGFGNWLVPLMLGAPDMAFPRMNNMSFWLLPPSLTLLISSSIVENGAGTGWTVYPPLSSNIAHGGSSVDLAIFSLHLAGISSILGAINFITTIINMRLNSLSFD
QMPLFIWAVGITAFLLLLSLPVLAGAITMLLTDRNLNTSFFDPAGGGDPILYQHL
>LEFIJ740-10|KM572665|MM17365|Hoplodrina_blanda
TLYFIFGIWAGMVGTSLSLLIRAELGNPGSLIGDDQIYNTIVTAHAFIMIFFMVMPIMIGGFGNWLVPLMLGAPDMAFPRMNNMSFWLLPPSLTLLISSSIVENGAGTGWTVYPPLSSNIAHGGSSVDLAIFSLHLAGISSILGAINFITTIINMRLNSLSFD
QMPLFIWAVGITAFLLLLSLPVLAGAITMLLTDRNLNTSFFDPAGGGDPILYQHL
>LEFID932-10|HM873682|MM07463|Hoplodrina_octogenaria
TLYFIFGIWAGMVGTSLSLLIRAELGNPGSLIGDDQIYNTIVTAHAFIMIFFMVMPIMIGGFGNWLVPLMLGAPDMAFPRMNNMSFWLLPPSLTLLISSSIVENGAGTGWTVYPPLSSNIAHGGSSVDLAIFSLHLAGISSILGAINFITTIINMRLNSLSFD
QMPLFIWAVGITAFLLLLSLPVLAGAITMLLTDRNLNTSFFDPAGGGDPILYQHL
>LEFIC347-10|HM872190|MM03863|Horisme_tersata
TLYFIFGIWAGMVGTSLSLLIRAELGTPGSLIGDDQIYNTIVTAHAFIMIFFMVMPIMIGGFGNWLVPLMLGAPDMAFPRMNNMSFWLLPPSITLLISSSIVENGAGTGWTVYPPLSSNIAHSGSSVDLAIFSLHLAGISSILGAINFITTIINMRLNNMFFD
QLPLFVWAVGITAFLLLLSLPVLAGAITMLLTDRNLNTSFFDPAGGGDPILYQHL
>LEFIF719-10|HM875403|MM12781|Horisme_tersata
TLYFIFGIWAGMVGTSLSLLIRAELGTPGSLIGDDQIYNTIVTAHAFIMIFFMVMPIMIGGFGNWLVPLMLGAPDMAFPRMNNMSFWLLPPSITLLISSSIVENGAGTGWTVYPPLSSNIAHSGSSVDLAIFSLHLAGISSILGAINFITTIINMRLNNMFFD
QLPLFVWAVGITAFLLLLSLPVLAGAITMLLTDRNLNTSFFDPAGGGDPILYQHL
>LEFIG295-10|HM875974|MM14251|Horisme_tersata
TLYFIFGIWAGMVGTSLSLLIRAELGTPGSLIGDDQIYNTIVTAHAFIMIFFMVMPIMIGGFGNWLVPLMLGAPDMAFPRMNNMSFWLLPPSITLLISSSIVENGAGTGWTVYPPLSSNIAHSGSSVDLAIFSLHLAGISSILGAINFITTIINMRLNNMFFD
QLPLFVWAVGITAFLLLLSLPVLAGAITMLLTDRNLNTSFFDPAGGGDPILYQHL
>LEFIB305-10|HM871206|MM00824|Hydraecia_micacea
TLYFIFGIWAGMVGTSLSLLIRAELGNPGSLIGDDQIYNTIVTAHAFIMIFFMVMPIMIGGFGNWLVPLMLGAPDMAFPRMNNMSFWLLPPSLTLLISSSIVENGAGTGWTVYPPLSSNIAHGGSSVDLAIFSLHLAGISSILGAINFITTIINMRLNNLSFD
QMPLFIWAVGITAFLLLLSLPVLAGAITMLLTDRNLNTSFFDPAGGGDPILYQHL
>LEFIA610-10|HM870859|MM01722|Hydraecia_micacea
TLYFIFGIWAGMVGTSLSLLIRAELGNPGSLIGDDQIYNTIVTAHAFIMIFFMVMPIMIGGFGNWLVPLMLGAPDMAFPRMNNMSFWLLPPSLTLLISSSIVENGAGTGWTVYPPLSSNIAHGGSSVDLAIFSLHLAGISSILGAINFITTIINMRLNNLSFD
QMPLFIWAVGITAFLLLLSLPVLAGAITMLLTDRNLNTSFFDPAGGGDPILYQHL
>LEFIA611-10|HM870860|MM01723|Hydraecia_micacea
TLYFIFGIWAGMVGTSLSLLIRAELGNPGSLIGDDQIYNTIVTAHAFIMIFFMVMPIMIGGFGNWLVPLMLGAPDMAFPRMNNMSFWLLPPSLTLLISSSIVENGAGTGWTVYPPLSSNIAHGGSSVDLAIFSLHLAGISSILGAINFITTIINMRLNNLSFD
QMPLFIWAVGITAFLLLLSLPVLAGAITMLLTDRNLNTSFFDPAGGGDPILYQHL
>LEFID980-10|HM873730|MM08012|Hydraecia_micacea
TLYFIFGIWAGMVGTSLSLLIRAELGSPGSLIGDDQIYNTIVTAHAFIMIFFMVMPIMIGGFGNWLVPLMLGAPDMAFPRMNNMSFWLLPPSLTLLISSSIVENGAGTGWTVYPPLSSNIAHGGSSVDLAIFSLHLAGISSILGAINFITTIINMRLNNLSFD
QMPLFIWAVGITAFLLLLSLPVLAGAITMLLTDRNLNTSFFDPAGGGDPILYQHL
>LEFIC503-10|HM872328|MM04211|Hydraecia_nordstroemi
TLYFIFGIWAGMVGTSLSLLIRAELGNPGSLIGDDQIYNTIVTAHAFIMIFFMVMPIMIGGFGNWLVPLMLGAPDMAFPRMNNMSFWLLPPSLTLLISSSIVENGAGTGWTVYPPLSSNIAHGGSSVDLAIFSLHLAGISSILGAINFITTIINMRLNSLSFD
QMPLFVWAVGITAFLLLLSLPVLAGAITMLLTDRNLNTSFFDPAGGGDPILYQHL
>LEFIF202-10|HM874896|MM10826|Hydraecia_nordstroemi
TLYFIFGIWAGMVGTSLSLLIRAELGNPGSLIGDDQIYNTIVTAHAFIMIFFMVMPIMIGGFGNWLVPLMLGAPDMAFPRMNNMSFWLLPPSLTLLISSSIVENGAGTGWTVYPPLSSNIAHGGSSVDLAIFSLHLAGISSILGAINFITTIINMRLNSLSFD
QMPLFVWAVGITAFLLLLSLPVLAGAITMLLTDRNLNTSFFDPAGGGDPILYQHL
>LEFIF204-10|HM874898|MM10828|Hydraecia_nordstroemi
TLYFIFGIWAGMVGTSLSLLIRAELGNPGSLIGDDQIYNTIVTAHAFIMIFFMVMPIMIGGFGNWLVPLMLGAPDMAFPRMNNMSFWLLPPSLTLLISSSIVENGAGTGWTVYPPLSSNIAHGGSSVDLAIFSLHLAGISSILGAINFITTIINMRLNSLSFD
QMPLFIWAVGITAFLLLLSLPVLAGAITMLLTDRNLNTSFFDPAGGGDPILYQHL
>LEFIG406-10|HM876082|MM14432|Hydraecia_petasitis

TLYFIFGIWAGMVGTSLSLLIRAELGNPGSLIGDDQIYNTIVTAHAFIMIFFMVMPIMIGGFGNWLVPLMLGAPDMAFPRMNNMSFWLLPPSLTLLISSSIVENGAGTGWTVYPPLSSNIAHGGSSVDLAIFSLHLAGISSILGAINFITTIINMRLNSLSFD
QMPLFVWAVGITAFLLLLSLPVLAGAITMLLTDRNLNTSFFDPAGGGDPILYQHL
>LEFIA053-10|HM396401|MM00959|Hydraecia_ultima
TLYFIFGIWAGMVGTSLSLLIRAELGNPGSLIGDDQIYNTIVTAHAFIMIFFMVMPIMIGGFGNWLVPLMLGAPDMAFPRMNNMSFWLLPPSLTLLISSSIVENGAGTGWTVYPPLSSNIAHGGSSVDLAIFSLHLAGISSILGAINFITTIINMRLNSLSFD
QMPLFIWAVGITAFLLLLSLPVLAGAITMLLTDRNLNTSFFDPAGGGDPILYQHL
>LEFIA373-10|HM386715|MM01430|Hydrelia_flammeolaria
TLYFIFGIWAGMIGTSLSLLIRAELGNPGSLIGDDQIYNTIVTAHAFIMIFFMVMPIMIGGFGNWLVPLMLGAPDMAFPRMNNMSFWLLPPSITLLISSSIVENGAGTGWTVYPPLSSNIAHGGSSVDLAIFSLHLAGISSILGAINFITTIINMRLNNMFFD
QLPLFVWAVSITAFLLLLSLPVLAGAITMLLTDRNLNTSFFDPAGGGDPILYQHL
>LEFIA384-10|HM386726|MM01441|Hydrelia_sylvata
TLYFIFGIWAGMIGTSLSLLIRAELGNPGSLIGDDQIYNTIVTAHAFIMIFFMVMPIMIGGFGNWLVPLMLGAPDMAFPRMNNMSFWLLPPSITLLISSSIVENGAGTGWTVYPPLSSNIAHGGSSVDLAIFSLHLAGISSILGAINFITTIINMRLNNMFFD
QLPLFVWAVGITAFLLLLSLPVLAGAITMLLTDRNLNTSFFDPAGGGDPILYQHL
>LEFIA385-10|HM386727|MM01442|Hydrelia_sylvata
TLYFIFGIWAGMIGTSLSLLIRAELGNPGSLIGDDQIYNTIVTAHAFIMIFFMVMPIMIGGFGNWLVPLMLGAPDMAFPRMNNMSFWLLPPSITLLISSSIVENGAGTGWTVYPPLSSNIAHGGSSVDLAIFSLHLAGISSILGAINFITTIINMRLNNMFFD
QLPLFVWAVGITAFLLLLSLPVLAGAITMLLTDRNLNTSFFDPAGGGDPILYQHF
>LEFIC452-10|HM872286|MM04034|Hydria_cervinalis
TLYFIFGIWAGMIGTSLSLLIRAELGNPGSLIGDDQIYNTIVTAHAFIMIFFMVMPIMIGGFGNWLVPLMLGAPDMAFPRMNNMSFWLLPPSITLLISSSIVENGAGTGWTVYPPLSSNIAHGGSSVDLAIFSLHLAGISSILGAINFITTIINMRLNNMFFD
QLPLFVWAVGITAFLLLLSLPVLAGAITMLLTDRNLNTSFFDPAGGGDPILYQHL
>LEFIA403-10|HM386745|MM01462|Hydria_undulata
TLYFIFGIWAGMVGTSLSLLIRAELGNPGSLIGDDQIYNTIVTAHAFIMIFFMVMPIMIGGFGNWLVPLMLGAPDMAFPRMNNMSFWLLPPSITLLISSSIVENGAGTGWTVYPPLSSNIAHGGSSVDLAIFSLHLAGISSILGAINFITTIINMRLNNMFFD
QLPLFVWAVGITAFLLLLSLPVLAGAITMLLTDRNLNTSFFDPAGGGDPILYQHL
>LEFIA389-10|HM386731|MM01446|Hydriomena_furcata
TLYFIFGIWAGMIGTSLSLLIRAELGNPGSLIGDDQIYNTIVTAHAFIMIFFMVMPIMIGGFGNWLVPLMLGAPDMAFPRMNNMSFWLLPPSITLLISSSVVENGAGTGWTVYPPLSSNIAHGGSSVDLAIFSLHLAGISSILGAINFITTIINMRLNNMFFD
QLPLFVWAVGITAFLLLLSLPVLAGAITMLLTDRNLNTSFFDPAGGGDPILYQHL
>LEFIA390-10|HM386732|MM01447|Hydriomena_furcata
TLYFIFGIWAGMIGTSLSLLIRAELGNPGSLIGDDQIYNTIVTAHAFIMIFFMVMPIMIGGFGNWLVPLMLGAPDMAFPRMNNMSFWLLPPSITLLISSSVVENGAGTGWTVYPPLSSNIAHGGSSVDLAIFSLHLAGISSILGAINFITTIINMRLNNMFFD
QLPLFVWAVGITAFLLLLSLPVLAGAITMLLTDRNLNTSFFDPAGGGDPILYQHL
>LEFIA259-10|HM386603|MM01296|Hydriomena_impluviata
TLYFIFGIWAGMIGTSLSLLIRAELGNPGSLIGDDQIYNTIVTAHAFIMIFFMVMPIMIGGFGNWLVPLMLGAPDMAFPRMNNMSFWLLPPSITLLISSSVVENGAGTGWTVYPPLSSNIAHGGSSVDLAIFSLHLAGISSILGAINFITTIINMRLNNMFFD
QLPLFVWAVGITAFLLLLSLPVLAGAITMLLTDRNLNTSFFDPAGGGDPILYQHL
>LEFIB849-10|HM871726|MM02844|Hydriomena_impluviata
TLYFIFGIWAGMIGTSLSLLIRAELGNPGSLIGDDQIYNTIVTAHAFIMIFFMVMPIMIGGFGNWLVPLMLGAPDMAFPRMNNMSFWLLPPSITLLISSSVVENGAGTGWTVYPPLSSNIAHGGSSVDLAIFSLHLAGISSILGAINFITTIINMRLNNMFFD
QLPLFVWAVGITAFLLLLSLPVLAGAITMLLTDRNLNTSFFDPAGGGDPILYQHL
>LEFIC632-10|HM872453|MM04574|Hydriomena_impluviata
TLYFIFGIWAGMIGTSLSLLIRAELGNPGSLIGDDQIYNTIVTAHAFIMIFFMVMPIMIGGFGNWLVPLMLGAPDMAFPRMNNMSFWLLPPSITLLISSSVVENGAGTGWTVYPPLSSNIAHGGSSVDLAIFSLHLAGISSILGAINFITTIINMRLNNMFFD
QLPLFVWAVGITAFLLLLSLPVLAGAITMLLTDRNLNTSFFDPAGGGDPILYQHL
>LEFIB123-10|HM871029|MM00485|Hydriomena_ruberata
TLYFIFGIWAGMIGTSLSLLIRAELGNPGSLIGDDQIYNTIVTAHAFIMIFFMVMPIMIGGFGNWLVPLMLGAPDMAFPRMNNMSFWLLPPSITLLISSSVVENGAGTGWTIYPPLSSNIAHGGSSVDLAIFSLHLAGISSILGAINFITTIINMRLNNMFFD
QLPLFVWAVGITAFLLLLSLPVLAGAITMLLTDRNLNTSFFDPAGGGDPILYQHL
>LEFIA252-10|HM386596|MM01289|Hydriomena_ruberata
TLYFIFGIWAGMIGTSLSLLIRAELGNPGSLIGDDQIYNTIVTAHAFIMIFFMVMPIMIGGFGNWLVPLMLGAPDMAFPRMNNMSFWLLPPSITLLISSSVVENGAGTGWTVYPPLSSNIAHGGSSVDLAIFSLHLAGISSILGAINFITTIINMRLNNMFFD
QLPLFVWAVGITAFLLLLSLPVLAGAITMLLTDRNLNTSFFDPAGGGDPILYQHL
>LEFIE169-10|HM873915|MM08431|Hydriomena_ruberata
TLYFIFGIWAGMIGTSLSLLIRAELGNPGSLIGDDQIYNTIVTAHAFIMIFFMVMPIMIGGFGNWLVPLMLGAPDMAFPRMNNMSFWLLPPSITLLISSSVVENGAGTGWTVYPPLSSNIAHGGSSVDLAIFSLHLAGISSILGAINFITTIINMRLNNMFFD
QLPLFVWAVGITAFLLLLSLPVLAGAITMLLTDRNLNTSFFDPAGGGDPILYQHL
>LEFID526-10|HM873291|MM06506|Hylaea_fasciaria
TLYFIFGIWAGMVGTSLSLLIRAELGNPGSLIGDDQIYNTIVTAHAFIMIFFMVMPIMIGGFGNWLIPLMLGAPDMAFPRMNNMSFWLLPPSITLLISSSIVENGAGTGWTVYPPLSSNIAHGGSSVDLAIFSLHLAGISSILGAINFITTIINMRLNNLSFDQ
MPLFVWAVGITAFLLLLSLPVLAGAITMLLTDRNLNTSFFDPAGGGDPILYQHL

>LEFIF165-10|HM874859|MM10703|Hyles_gallii
TLYFIFGIWAGMVGTSLSLLIRAELGTPGSLIGDDQIYNTIVTAHAFIMIFFMVMPIMIGGFGNWLVPLMLGAPDMAFPRMNNMSFWLLPPSLTLLISSSIVENGAGTGWTVYPPLSSNIAHSGSSVDLAIFSLHLAGISSIMGAVNFITTIINMRINNLSFD
QMPLFVWAVGITAFLLLLSLPVLAGAITMLLTDRNLNTSFFDPAGGGDPILYQHL
>LEFIF633-10|HM875317|MM12530|Hyles_gallii
TLYFIFGIWAGMVGTSLSLLIRAELGTPGSLIGDDQIYNTIVTAHAFIMIFFMVMPIMIGGFGNWLVPLMLGAPDMAFPRMNNMSFWLLPPSLTLLISSSIVENGAGTGWTVYPPLSSNIAHSGSSVDLAIFSLHLAGISSIMGAVNFITTIINMRINNLSFD
QMPLFVWAVGITAFLLLLSLPVLAGAITMLLTDRNLNTSFFDPAGGGDPILYQHL
>LEFIF142-10|HM874836|MM10650|Hyles_livornica
TLYFIFGIWAGMVGTSLSLLIRAELGTPGSLIGDDQIYNTIVTAHAFIMIFFMVMPIMIGGFGNWLVPLMLGAPDMAFPRMNNMSFWLLPPSLTLLISSSIVENGAGTGWTVYPPLSSNIAHSGSSVDLAIFSLHLAGISSIMGAVNFITTIINMRINNLSFD
QMPLFVWAVGITAFLLLLSLPVLAGAITMLLTDRNLNTSFFDPAGGGDPILYQHL
>LEFIB551-10|HM871434|MM02182|Hypatima_rhomboidella
TLYFIFGIWAGMVGTSLSLLIRAELGNPGSLIGDDQIYNTIVTAHAFIMIFFMVMPIMIGGFGNWLVPLMLGAPDMAFPRMNNMSFWLLPPSLTLLISSSIVETGAGTGWTVYPPLSSNIAHGGSSVDLAIFSLHLAGISSILGAINFITTIINMRINGISFDQ
MPLFIWAVGITALLLLLSLPVLAGAITMLLTDRNLNTSFFDPAGGGDPILYQHL
>LEFIE042-10|HM873791|MM08129|Hypatima_rhomboidella
TLYFIFGIWAGMVGTSLSLLIRAELGNPGSLIGDDQIYNTIVTAHAFIMIFFMVMPIMIGGFGNWLVPLMLGAPDMAFPRMNNMSFWLLPPSLTLLISSSIVETGAGTGWTVYPPLSSNIAHGGSSVDLAIFSLHLAGISSILGAINFITTIINMRINGISFDQ
MPLFIWAVGITALLLLLSLPVLAGAITMLLTDRNLNTSFFDPAGGGDPILYQHL
>LEFIF994-10|HM875675|MM13601|Hypatima_rhomboidella
TLYFIFGIWAGMVGTSLSLLIRAELGNPGSLIGDDQIYNTIVTAHAFIMIFFMVMPIMIGGFGNWLIPLMLGAPDMAFPRMNNMSFWLLPPSLTLLISSSIVETGAGTGWTVYPPLSSNIAHGGSSVDLAIFSLHLAGISSILGAINFITTIINMRINGISFDQ
MPLFIWAVGITALLLLLSLPVLAGAITMLLTDRNLNTSFFDPAGGGDPILYQHL
>LEFIE084-10|HM873831|MM08251|Hypatopa_binotella
TLYFIFGIWAGMVGTSLSLLIRAELGNPDSLIGDDQIYNTIVTAHAFIMIFFMVMPIMIGGFGNWLVPLMLGAPDMAFPRMNNMSFWLLPPSLTLLISSSIVENGAGTGWTVYPPLSSNIAHGGSSVDLAIFSLHLAGISSILGAINFITTIINMRPNNMSF
DQMPLFVWSVGITALLLLLSLPVLAGAITMLLTDRNLNTSFFDPAGGGDPILYQHL
>LEFIF961-10|HM875642|MM13538|Hypatopa_binotella
TLYFIFGIWAGMVGTSLSLLIRAELGNPDSLIGDDQIYNTIVTAHAFIMIFFMVMPIMIGGFGNWLVPLMLGAPDMAFPRMNNMSFWLLPPSLTLLISSSIVENGAGTGWTVYPPLSSNIAHGGSSVDLAIFSLHLAGISSILGAINFITTIINMRPNNMSF
DQMPLFVWSVGITALLLLLSLPVLAGAITMLLTDRNLNTSFFDPAGGGDPILYQHL
>LEFIG747-10|HM876403|MM15611|Hypatopa_binotella
TLYFIFGIWAGMVGTSLSLLIRAELGNPDSLIGDDQIYNTIVTAHAFIMIFFMVMPIMIGGFGNWLVPLMLGAPDMAFPRMNNMSFWLLPPSLTLLISSSIVENGAGTGWTVYPPLSSNIAHGGSSVDLAIFSLHLAGISSILGAINFITTIINMRPNNMSF
DQMPLFVWSVGITALLLLLSLPVLAGAITMLLTDRNLNTSFFDPAGGGDPILYQHL
>LEFID549-10|HM873314|MM06537|Hypatopa_inunctella
TLYFIFGIWAGMVGTSLSLLIRAELGNPGSLIGDDQIYNTIVTAHAFIMIFFMVMPIMIGGFGNWLVPLMLGAPDMAFPRMNNMSFWLLPPSLTLLISSSIVENGAGTGWTVYPPLSSNIAHSGSSVDLAIFSLHLAGISSILGAINFITTIINMRPNNMSF
DQMPLFIWSVGITALLLLLSLPVLAGAITMLLTDRNLNTSFFDPAGGGDPILYQHL
>LEFIK122-10|JX034589|MM17697|Hypatopa_segnella
TLYFIFGIWAGMVGTSLSLLIRAELGNPGSLIGDDQIYNTIVTAHAFIMIFFMVMPIMIGGFGNWLVPLMLGAPDMAFPRMNNMSFWLLPPSLTLLISSSIVENGAGTGWTVYPPLSSNIAHGGSSVDLAIFSLHLAGISSILGAINFITTIINMRPNNMSF
DQMPLFVWSVGITALLLLLSLPVLAGAITMLLTDRNLNTSFFDPAGGGDPILYQHL
>LEFIA597-10|HM870846|MM01709|Hypena_crassalis
TLYFIFGIWAGMVGTSLSLLIRAELGTPGSLIGDDQIYNTIVTAHAFIMIFFMVMPIMIGGFGNWLVPLMLGAPDMAFPRMNNMSFWLLPPSLILLISSSVVENGAGTGWTVYPPLSSNIAHSGSSVDLAIFSLHLAGISSILGAINFITTIINMRLTNLSFDQ
MPLFVWAVGITAFLLLLSLPVLAGAITMLLTDRNLNTSFF?PAGGGDPILYQHL
>LEFIJ833-10|KM572972|MM17458|Hypena_crassalis
TLYFIFGIWAGMVGTSLSLLIRAELGTPGSLIGDDQIYNTIVTAHAFIMIFFMVMPIMIGGFGNWLVPLMLGAPDMAFPRMNNMSFWLLPPSLILLISSSVVENGAGTGWTVYPPLSSNIAHSGSSVDLAIFSLHLAGISSILGAINFITTIINMRLTNLSFDQ
MPLFVWAVGITAFLLLLSLPVLAGAITMLLTDRNLNTSFFDPAGGGDPILYQHL
>LEFIA473-10|HM386813|MM01546|Hypena_proboscidalis
TLYFIFGIWAGMVGTSLSLLIRAELGTPGSLIGDDQIYNTIVTAHAFIMIFFMVMPIMIGGFGNWLVPLMLGAPDMAFPRMNNMSFWLLPPSLTLLISSSIVENGAGTGWTVYPPLSSNIAHSGSSVDLAIFSLHLAGISSILGAINFITTIINMRLTNLSFDQ
MPLFVWAVGITAFLLLLSLPVLAGAITMLLTDRNLNTSFFDPAGGGDPILYQHL
>LEFIG990-10|HM876629|MM15854|Hypena_rostralis
TLYFIFGIWAGMVGTSLSLLIRAELGTPGSLIGDDQIYNTIVTAHAFIMIFFMVMPIMIGGFGNWLVPLMLGAPDMAFPRMNNMSFWLLPPSLTLLISSSIVENGAGTGWTVYPPLSSNIAHSGSSVDLAIFSLHLAGISSILGAINFITTIINMRLTNLSFDQ
MPLFVWAVGITAFLLLLSLPVLAGAITMLLTDRNLNTSFFDPAGGGDPILYQHL
>LEFIB930-10|HM871807|MM03071|Hypenodes_humidalis
TLYFIFGIWAGMVGTSLSLLIRAELGAPGSLIGDDQIYNTIVTAHAFIMIFFMVMPIMIGGFGNWLVPLMLGAPDMAFPRMNNMSFWLLPPSLTLLISSSIVENGAGTGWTVYPPLSSNIAHGGSSVDLAIFSLHLAGISSILGAINFITTIINMRLSNLSFD

QMPLFIWAVGITAFLLLLSLPVLAGAITMLLTDRNLNTSFFDPAGGGDPILYQHL
>LEFIJ055-10|JF853418|MM03315|Hypenodes_humidalis
TLYFIFGIWAGMVGTSLSLLIRAELGTPGSLIGDDQIYNTIVTAHAFIMIFFMVMPIMIGGFGNWLVPLMLGAPDMAFPRMNNMSFWLLPPSLTLLISSSIVENGAGTGWTVYPPLSSNIAHGGSSVDLAIFSLHLAGISSILGAINFITTIINMRLSNLSFD
QMPLFIWAVGITAFLLLLSLPVLAGAITMLLTDRNLNTSFFDPAGGGDPILYQHL
>LEFIC789-10|HM872608|MM04927|Hypenodes_humidalis
TLYFIFGIWAGMVGTSLSLLIRAELGTPGSLIGDDQIYNTIVTAHAFIMIFFMVMPIMIGGFGNWLVPLMLGAPDMAFPRMNNMSFWLLPPSLTLLISSSIVENGAGTGWTVYPPLSSNIAHGGSSVDLAIFSLHLAGISSILGAINFITTIINMRLSNLSFD
QMPLFIWAVGITAFLLLLSLPVLAGAITMLLTDRNLNTSFFDPAGGGDPILYQHL
>LEFIE184-10|HM873929|MM08491|Hypenodes_humidalis
TLYFIFGIWAGMVGTSLSLLIRAELGTPGSLIGDDQIYNTIVTAHAFIMIFFMVMPIMIGGFGNWLVPLMLGAPDMAFPRMNNMSFWLLPPSLTLLISSSIVENGAGTGWTVYPPLSSNIAHGGSSVDLAIFSLHLAGISSILGAINFITTIINMRLSNLSFD
QMPLFIWAVGITAFLLLLSLPVLAGAITMLLTDRNLNTSFFDPAGGGDPILYQHL
>LEFIJ710-10|MM17335|Hypenodes_humidalis
TLYFIFGIWAGMVGTSLSLLIRAELGTPGSLIGDDQIYNTIVTAHAFIMIFFMVMPIMIGGFGNWLVPLMLGAPDMAFPRMNNMSFWLLPPSLTLLISSSIVENGAGTGWTVYPPLSSNIAHGGSSVDLAIFSLHLAGISSILGAINFITTIINMRLSNLSFD
QMPLFIWAVGITAFLLLLSLPVLAGAITMLLTDRNLNTSFFDPAGGGDPILYQHL
>LEFID730-10|HM873487|MM06787|Hypercallia_citrinalis
TLYFIFGIWSGMVGTSLSLLIRTELGNPGSLIGNDQIYNTIVTAHAFIMIFFMVMPIMIGGFGNWLIPLMLGAPDMAFPRMNNMSFWLLPPSLTLLISSSIVENGAGTGWTVYPPLSSNIAHNGSSVDLTIFSLHLAGISSILGAINFITTIINMKINYMSFDQ
MPLFVWAVGITALLLLLSLPVLAGAITMLLTDRNLNTSFFDPAGGGDPILYQHL
>LEFIE293-10|HM874025|MM08641|Hypercallia_citrinalis
TLYFIFGIWSGMVGTSLSLLIRTELGNPGSLIGNDQIYNTIVTAHAFIMIFFMVMPIMIGGFGNWLIPLMLGAPDMAFPRMNNMSFWLLPPSLTLLISSSIVENGAGTGWTVYPPLSSNIAHNGSSVDLTIFSLHLAGISSILGAINFITTIINMKLNMMSFD
QMPLFVWAVGITALLLLLSLPVLAGAITMLLTDRNLNTSFFDPAGGGDPILYQHL
>LEFIA865-10|HM387003|MM09742|Hypercallia_citrinalis
TLYFIFGIWSGMVGTSLSLLIRTELGNPGSLIGNDQIYNTIVTAHAFIMIFFMVMPIMIGGFGNWLIPLMLGAPDMAFPRMNNMSFWLLPPSLTLLISSSIVENGAGTGWTVYPPLSSNIAHNGSSVDLTIFSLHLAGISSILGAINFITTIINMKLNMMSFD
QMPLFVWAVGITALLLLLSLPVLAGAITMLLTDRNLNTSFFDPAGGGDPILHQHL
>LEFIE345-10|HM874069|MM08771|Hyphoraia_aulica
TLYFIFGVWAGMVGTSLSLLIRAELGNPGSLIGDDQIYNTIVTAHAFIMIFFMVMPIMIGGFGNWLVPLMLGAPDMAFPRMNNMSFWLLPPSLTLLISSSIVENGAGTGWTVYPPLSSNIAHGGSSVDLAIFSLHLAGISSILGAINFITTIINMRLNNMSF
DQMPLFVWAVGITAFLLLLSLPVLAGAITMLLTDRNLNTSFFDPAGGGDPILYQHL
>LEFIB411-10|HM871310|MM01880|Hypochalcia_ahenella
TLYFIFGIWSGMVGTSLSLLIRTELGTPSSLIGDDQIYNTIVTGHAFIMIFFMVMPIMIGGFGNWLVPLMLGAPDMAFPRMNNMSFWLLPPSLTLLISSSVVENGAGTGWTVYPPLSSNIAHSGSSVDLAIFSLHLAGISSILGAINFITTIINMKLNSLFFDQ
MPLFVWAVGITALLLLLSLPVLAGAITMLLTDRNLNTSFFDPAGGGDPILYQHL
>LEFIG357-10|HM876034|MM14355|Hypochalcia_ahenella
TLYFIFGIWSGMVGTSLSLLIRTELGTPSSLIGDDQIYNTIVTGHAFIMIFFMVMPIMIGGFGNWLVPLMLGAPDMAFPRMNNMSFWLLPPSLTLLISSSVVENGAGTGWTVYPPLSSNIAHSGSSVDLAIFSLHLAGISSILGAINFITTIINMKLNSLFFDQ
MPLFVWAVGITALLLLLSLPVLAGAITMLLTDRNLNTSFFDPAGGGDPILYQHL
>LEFIA247-10|HM386591|MM01280|Hypomecis_punctinalis
TLYFIFGIWAGMVGTSLSLLIRAELGNPGSLIGDDQIYNTIVTAHAFIMIFFMVMPIMIGGFGNWLVPLMLGAPDMAFPRMNNMSFWLLPPSITLLISSSIVENGAGTGWTVYPPLSSNIAHGGSSVDLAIFSLHLAGISSILGAINFITTIINMRLNNLSFD
QMPLFVWAVGITAFLLLLSLPVLAGAITMLLTDRNLNTSFFDPAGGGDPILYQHL
>LEFIA248-10|HM386592|MM01281|Hypomecis_punctinalis
TLYFIFGIWAGMVGTSLSLLIRAELGNPGSLIGDDQIYNTIVTAHAFIMIFFMVMPIMIGGFGNWLVPLMLGAPDMAFPRMNNMSFWLLPPSITLLISSSIVENGAGTGWTVYPPLSSNIAHGGSSVDLAIFSLHLAGISSILGAINFITTIINMRLNNLSFD
QMPLFVWAVGITAFLLLLSLPVLAGAITMLLTDRNLNTSFFDPAGGGDPILYQHL
>LEFIA408-10|HM386750|MM01470|Hypomecis_roboraria
TLYFIFWIWAGMVGTSLSLLIRAELGNPGSLIGDDQIYNTIVTAHAFIMIFFMVMPIMIGGFGNWLVPLMLGAPDMAFPRMNNMSFWLLPPSITLLISSSIVENGAGTGWTVYPPLSSNIAHGGSSVDLAIFSLHLAGISSILGAINFITTIINMRLNNLSFD
QMPLFVWAVGITAFLLLLSLPVLAGAITMLLTDRNLNTSFFDPAGGGDPILYQHL
>LEFIC867-10|HM872685|MM05135|Hypomecis_roboraria
TLYFIFGIWAGMVGTSLSLLIRAELGNPGSLIGDDQIYNTIVTAHAFIMIFFMVMPIMIGGFGNWLVPLMLGAPDMAFPRMNNMSFWLLPPSITLLISSSIVENGAGTGWTVYPPLSSNIAHGGSSVDLAIFSLHLAGISSILGAINFITTIINMRLNNLSFD
QMPLFVWAVGITAFLLLLSLPVLAGAITMLLTDRNLNTSFFDPAGGGDPILYQHL
>LEFIK298-10|JF853952|MM17873|Hyponephele_lycaon
TLYFIFGIWAGMVGTSLSLIIRTELGNPGFLIGDDQIYNTIVTAHAFIMIFFMVMPIMIGGFGNWLVPLMLGAPDMAFPRMNNMSFWLLPPSLILLISSSIVENGAGTGWTVYPPLSSNIAHGGASVDLAIFSLHLAGISSILGAINFITTIINMRVNSMSYD
QMPLFVWAVGITALLLLLSLPVLAGAITMLLTDRNLNTSFFDPAGGGDPILYQHL
>LEFIF533-10|HM875218|MM12189|Hypoxystis_pluviaria

TLYFIFGIWAGMVGTSLSLLIRAELGNPGSLIGDDQIYNTIVTAHAFIMIFFMVMPIMIGGFGNWLVPLMLGAPDMAFPRMNNMSFWLLPPSLTLLISSSIVENGAGTGWTVYPPLSSNIAHGGSSVDLAIFSLHLAGISSILGAINFITTIINMRLNNMSF
DQMPLFVWAVGITAFLLLLSLPVLAGAITMLLTDRNLNTSFFDPAGGGDPILYQHL
>LEFIK416-10|JF854045|MM17991|Hypoxystis_pluviaria
TLYFIFGIWAGMVGTSLSLLIRAELGNPGSLIGDDQIYNTIVTAHAFIMIFFMVMPIMIGGFGNWLVPLMLGAPDMAFPRMNNMSFWLLPPSLTLLISSSIVENGAGTGWTVYPPLSSNIAHGGSSVDLAIFSLHLAGISSILGAINFITTIINMRLNNMSF
DQMPLFVWAVGITAFLLLLSLPVLAGAITMLLTDRNLNTSFFDPAGGGDPILYQHL
>LEFIB201-10|HM871105|MM00646|Hyppa_rectilinea
TLYFIFGIWAGMVGTSLSLLIRAELGNPGSLIGDDQIYNTIVTAHAFIMIFFMVMPIMIGGFGNWLVPLMLGAPDMAFPRMNNMSFWLLPPSLTLLISSSVVENGAGTGWTVYPPLSSNIAHGGSSVDLAIFSLHLAGISSILGAINFITTIINMRLNGLSF
DQMPLFIWAVGITAFLLLLSLPVLAGAITMLLTDRNLNTSFFDPAGGGDPILYQHL
>LEFIG378-10|HM876055|MM14384|Hyppa_rectilinea
TLYFIFGIWAGMVGTSLSLLIRAELGNPGSLIGDDQIYNTIVTAHAFIMIFFMVMPIMIGGFGNWLVPLMLGAPDMAFPRMNNMSFWLLPPSLTLLISSSVVENGAGTGWTVYPPLSSNIAHGGSSVDLAIFSLHLAGISSILGAINFITTIINMRLNGLSF
DQMPLFIWAVGITAFLLLLSLPVLAGAITMLLTDRNLNTSFFDPAGGGDPILYQHL
>LEFID700-10|HM873461|MM06753|Hypsopygia_costalis
TLYFIFGIWSGMVGTSLSLLIRAELGNPGSLIGDDQIYNTIVTAHAFIMIFFMVMPIMIGGFGNWLVPLMLGAPDMAFPRMNNMSFWLLPPSLTLLISSSIVENGAGTGWTVYPPLSSNIAHGGSSVDLAIFSLHLAGISSILGAVNFITTIINMKLNNLFFD
QMPLFVWSVGITALLLLLSLPVLAGAITMLLTDRNLNTSFFDPAGGGDPILYQHL
>LEFIF408-10|HM875093|MM11778|Hypsopygia_costalis
TLYFIFGIWSGMVGTSLSLLIRAELGNPGSLIGDDQIYNTIVTAHAFIMIFFMVMPIMIGGFGNWLVPLMLGAPDMAFPRMNNMSFWLLPPSLTLLISSSIVENGAGTGWTVYPPLSSNIAHGGSSVDLAIFSLHLAGISSILGAVNFITTIINMKLNNLFFD
QMPLFVWSVGITALLLLLSLPVLAGAITMLLTDRNLNTSFFDPAGGGDPILYQHL
>LEFIA717-10|HM386861|MM01872|Hypsopygia_glaucinalis
TLYFIFGIWSGMVGTSLSLLIRAELGNPGSLIGDDQIYNTIVTAHAFIMIFFMVMPIMIGGFGNWLVPLMLGAPDMAFPRMNNMSFWLLPPSLTLLISSSIVENGAGTGWTVYPPLSSNIAHGGSSVDLAIFSLHLAGISSILGAVNFITTIINMKLNNMSF
DQMPLFVWSVGITALLLLLSLPVLAGAITMLLTDRNLNTSFFDPAGGGDPILYQHL
>LEFIA430-10|HM386771|MM01493|Idaea_aversata
TLYFIFGIWAGMVGTSLSLMIRAELGNPGSLIGDDQIYNTIVTAHAFIMIFFMVMPIMIGGFGNWLVPLMLGAPDMAFPRMNNMSFWLLPPSITLLISSSIVENGAGTGWTVYPPLSSNIAHGGSSVDLTIFSLHLAGISSILGAINFITTIINMRLNNMSF
DQLPLFVWAVGITAFLLLLSLPVLAGAITMLLTDRNLNTSFFDPAGGGDPILYQHL
>LEFIA431-10|HM386772|MM01494|Idaea_aversata
TLYFIFGIWAGMVGTSLSLMIRAELGNPGSLIGDDQIYNTIVTAHAFIMIFFMVMPIMIGGFGNWLVPLMLGAPDMAFPRMNNMSFWLLPPSITLLISSSIVENGAGTGWTVYPPLSSNIAHGGSSVDLTIFSLHLAGISSILGAINFITTIINMRLNNMSF
DQLPLFVWAVGITAFLLLLSLPVLAGAITMLLTDRNLNTSFFDPAGGGDPILYQHL
>LEFIA444-10|HM386785|MM01508|Idaea_biselata
TLYFIFGIWAGMVGTSLSLMIRAELGNPGSLIGDDQIYNTIVTAHAFIMIFFMVMPIMIGGFGNWLVPLMLGAPDMAFPRMNNMSFWLLPPSITLLISSSIVENGAGTGWTVYPPLSSNIAHSGSSVDLAIFSLHLAGISSILGAINFITTIINMRLNNLSFD
QLSLFIWSVGITAFLLLLSLPVLAGAITMLLTDRNLNTSFFDPAGGGDPILYQHL
>LEFID699-10|HM873460|MM06752|Idaea_deversaria
TLYFIFGIWAGMVGTSLSLMIRAELGNPGSLIGDDQIYNTIVTAHAFIMIFFMVMPIMIGGFGNWLVPLMLGAPDMAFPRMNNMSFWLLPPSIILLISSSIVENGAGTGWTVYPPLSSNIAHGGSSVDLTIFSLHLAGISSILGAINFITTIINMRLNNMSF
DQLPLFVWAVGITAFLLLLSLPVLAGAITMLLTDRNLNTSFFDPAGGGDPILYQHL
>LEFIG480-10|HM876156|MM14535|Idaea_dimidiata
TLYFISEFWAGMVGTSLSLMIRAELGNPGSLIGDDQIYNTIVTAHAFIMIFFMVMPIMIGGFGNWLVPLMLGAPDMAFPRMNNMSFWLLPPSITLLISSSIVENGAGTGWTVYPPLSSNIAHGGSSVDLAIFSLHLAGISSILGAINFITTIINMRLNNMSF
DQLSLFIWSVGITAFLLLLSLPVLAGAITMLLTDRNLNTSFFDPAGGGDPILYQHL
>LEFIA355-10|HM386698|MM01403|Idaea_emarginata
TLYFIFGIWAGMVGTSLSLMIRAELGNPGSLIGDDQIYNTIVTAHAFIMIFFMVMPIMIGGFGNWLVPLMLGAPDMAFPRMNNMSFWLLPPSITLLISSSIVENGAGTGWTVYPPLSSNIAHGGSSVDLAIFSLHLAGISSILGAINFITTIINMRLNNMSF
DQLPLFVWAVGITAFLLLLSLPVLAGAITMLLTDRNLNTSFFDPAGGGDPILYQHL
>LEFID731-10|HM873488|MM06789|Idaea_humiliata
TLYFIFGIWAGMVGTSLSLMIRAELGNPGSLIGDDQIYNTIVTAHAFIMIFFMVMPIMIGGFGNWLVPLMLGAPDMAFPRMNNMSFWLLPPSITLLISSSIVENGAGTGWTVYPPLSSNIAHGGSSVDLAIFSLHLAGISSILGAINFITTIINMRLNNMSF
DQLPLFVWAVGITAFLLLLSLPVLAGAITMLLTDRNLNTSFFDPAGGGDPILYQHL
>LEFID789-10|HM873546|MM06859|Idaea_humiliata
TLYFIFGIWAGMVGTSLSLMIRAELGNPGSLIGDDQIYNTIVTAHAFIMIFFMVMPIMIGGFGNWLVPLMLGAPDMAFPRMNNMSFWLLPPSITLLISSSIVENGAGTGWTVYPPLSSNIAHGGSSVDLAIFSLHLAGISSILGAINFITTIINMRLNNMSF
DQLPLFVWAVGITAFLLLLSLPVLAGAITMLLTDRNLNTSFFDPAGGGDPILYQHL
>LEFID761-10|HM873518|MM06826|Idaea_muricata
TLYFIFGIWAGMIGTSLSLMIRAELGNPGSLIGNDQIYNTIVTAHAFIMIFFMVMPIMIGGFGNWLVPLMLGAPDMAFPRMNNMSFWLLPPSITLLISSSIVENGAGTGWTVYPPLSSNIAHGGSSVDLAIFSLHLAGISSILGAINFITTIINMRLNNMSF
DQLPLFVWAVGITAFLLLLSLPVLAGAITMLLTDRNLNTSFFDPAGGGDPILYQHL

>LEFIA280-10|HM386624|MM01320|Idaea_pallidata
TLYFIFGIWAGMMGTSLSLMIRTELGNPGSLIGDDQIYNTIVTAHAFIMIFFMVMPIMIGGFGNWLVPLMLGAPDMAFPRMNNMSFWLLPPSIILLISSSIVESGTGTGWTVYPPLSSNIAHSGSSVDLTIFSLHLAGISSILGAINFITTIINMRLNNMSLD
QLPLFVWSVGITAFLLLLSLPVLAGAITMLLTDRNLNTSFFDPAGGGDPILYQHL
>LEFIF735-10|HM875419|MM12861|Idaea_pallidata
TLYFIFGIWAGMMGTSLSLMIRTELGNPGSLIGDDQIYNTIVTAHAFIMIFFMVMPIMIGGFGNWLVPLMLGAPDMAFPRMNNMSFWLLPPSIILLISSSIVESGAGTGWTVYPPLSSNIAHSGSSVDLTIFSLHLAGISSILGAINFITTIINMRLNNMSLD
QLPLFVWSVGITAFLLLLSLPVLAGAITMLLTDRNLNTSFFDPAGGGDPILYQHL
>LEFIK840-10|JN285638|MM18415|Idaea_pallidata
TLYFIFGIWAGMMGTSLSLMIRTELGNPGSLIGDDQIYNTIVTAHAFIMIFFMVMPIMIGGFGNWLVPLMLGAPDMAFPRMNNMSFWLLPPSIILLISSSIVESGAGTGWTVYPPLSSNIAHSGSSVDLTIFSLHLAGISSILGAINFITTIINMRLNNMSLD
QLPLFVWSVGITAFLLLLSLPVLAGAITMLLTDRNLNTSFFDPAGGGDPILYQHL
>LEFID806-10|HM873563|MM06892|Idaea_seriata
TLYFIFGIWSGMVGTSLSLMIRAELGNPGSLIGDDQIYNTIVTAHAFIMIFFMVMPIMIGGFGNWLVPLMLGAPDMAFPRMNNMSFWLLPPSITLLISSSIVENGAGTGWTVYPPLSSNIAHGGSSVDLAIFSLHLAGISSILGAINFITTIINMRLNSMSF
DQLPLFVWAVGITAFLLLLSLPVLAGAITMLLTDRNLNTSFFDPAGGGDPILYQHL
>LEFIC003-10|HM871873|MM03203|Idaea_serpentata
TLYFIFGIWAGMMGTSLSLMIRAELGNPGSLIGDDQIYNTIVTAHAFIMIFFMVMPIMIGGFGNWLVPLMLGAPDMAFPRMNNMSFWLLPPSLTLLISSSIVENGAGTGWTVYPPLSSNIAHSGSSVDLAIFSLHLAGISSILGAINFITTIINMRLNNMS
FDQLPLFVWSVGITAFLLLLSLPVLAGAITMLLTDRNLNTSFFDPAGGGDPILYQHL
>LEFIE955-10|HM874672|MM10355|Idaea_serpentata
TLYFIFGIWAGMMGTSLSLMIRAELGNPGSLIGDNQIYNTIVTAHAFIMIFFMVMPIMIGGFGNWLVPLMLGAPDMAFPRMNNMSFWLLPPSLTLLISSSIVENGAGTGWTVYPPLSSNIAHSGSSVDLAIFSLHLAGISSILGAINFITTIINMRLNNMS
FDQLPLFVWSVGITAFLLLLSLPVLAGAITMLLTDRNLNTSFFDPAGGGDPILYQHL
>LEFIE576-10|HM874299|MM09472|Idaea_straminata
TLYFIFGIWAGMVGTSLSLMIRAELGNPGSLIGDDQIYNTIVTAHAFIMIFFMVMPIMIGGFGNWLVPLMLGAPDMAFPRMNNMSFWLLPPSITLLISSSIVENGAGTGWTVYPPLSSNIAHGGSSVDLAIFSLHLAGISSILGAINFITTIINMRLNNMYF
DQLPLFVWAVGITAFLLLLSLPVLAGAITMLLTDRNLNTSFFDPAGGGDPILYQHL
>LEFIF749-10|HM875433|MM12944|Idaea_straminata
TLYFIFGIWAGMVGTSLSLMIRAELGNPGSLIGDDQIYNTIVTAHAFIMIFFMVMPIMIGGFGNWLVPLMLGAPDMAFPRMNNMSFWLLPPSITLLISSSIVENGAGTGWTVYPPLSSNIAHGGSSVDLAIFSLHLAGISSILGAINFITTIINMRLNNMSF
DQLPLFVWAVSITAFLLLLSLPVLAGAITMLLTDRNLNTSFFDPAGGGDPILYQHL
>LEFIB054-10|HM870963|MM00337|Idaea_sylvestraria
TLYFIFGIWAGMIGTSLSLMIRAELGNPGSLIGDDQIYNTIVTAHAFIMIFFMVMPIMIGGFGNWLVPLMLGAPDMAFPRMNNMSFWLLPPSITLLISSSIVENGAGTGWTVYPPLSSNIAHGGSSVDLAIFSLHLAGISSILGAINFITTIINMRLNNMSF
DQLPLFVWAVGITAFLLLLSLPVLAGAITMLLTDRNLNTSFFDPAGGGDPILYQHL
>LEFIC719-10|HM872540|MM04774|Idaea_sylvestraria
TLYFIFGIWAGMIGTSLSLMIRAELGNPGSLIGNDQIYNTIVTAHAFIMIFFMVMPIMIGGFGNWLVPLMLGAPDMAFPRMNNMSFWLLPPSITLLISSSIVENGAGTGWTVYPPLSSNIAHGGSSVDLAIFSLHLAGISSILGAINFITTIINMRLNNMSF
DQLPLFVWAVGITAFLLLLSLPVLAGAITMLLTDRNLNTSFFDPAGGGDPILYQHL
>LEFID573-10|HM873338|MM06568|Idaea_sylvestraria
TLYFIFGIWAGMIGTSLSLMIRAELGNPGSLIGDDQIYNTIVTAHAFIMIFFMVMPIMIGGFGNWLVPLMLGAPDMAFPRMNNMSFWLLPPSITLLISSSIVENGAGTGWTVYPPLSSNIAHGGSSVDLAIFSLHLAGISSILGAINFITTIINMRLNNMSF
DQLPLFVWAVGITAFLLLLSLPVLAGAITMLLTDRNLNTSFFDPAGGGDPILYQHL
>LEFIE082-10|HM873829|MM08248|Incurvaria_circulella
TLYFIFGIWAGMVGTSLSLLIRSELGMPGSFIGDDQIYNTIVTAHAFIMIFFMVMPIMIGGFGNWLVPLMLGAPDMAFPRLNNMSFWLLPPSLTLLISSSLVENGAGTGWTVYPPLASNIAHAGSSVDLAIFSLHLAGISSILGAVNFITTVINMRTNGMS
FDQMPLFVWAVAITALLLLLSLPVLAGAITMLLTDRNLNTSFFDPAGGGDPILYQHL
>LEFIA924-10|HM387060|MM13849|Incurvaria_circulella
TLYFIFGIWAGMVGTSLSLLIRSELGMPGSFIGDDQIYNTIVTAHAFIMIFFMVMPIMIGGFGNWLVPLMLGAPDMAFPRLNNMSFWLLPPSLTLLISSSLVENGAGTGWTVYPPLASNIAHAGSSVDLAIFSLHLAGISSILGAVNFITTVINMRTNGMS
FDQMPLFVWAVAITALLLLLSLPVLAGAITMLLTDRNLNTSFFDPAGGGAPILYQHL
>LEFIG101-10|HM875781|MM13883|Incurvaria_circulella
TLYFIFGIWAGMVGTSLSLLIRSELGMPGSFIDDDQIYNTIVTAHAFIMIFFMVMPIMIGGFGNWLVPLMLGAPDMAFPRLNNMSFWLLPPSLTLLISSSLVENGAGTGWTVYPPLASNIAHAGSSVDLAIFSLHLAGISSILGAVNFITTVINMRTNGMS
FDQMPLFVWAVAITALLLLLSLPVLAGAITMLLTDRNLNTSFFDPAGGGDPILYQHL
>LEFIK373-10|MM17948|Incurvaria_circulella
TLYFIFGIWAGMVGTSLSLLIRSELGMPGSFIGDDQIYNTIVTAHAFIMIFFMVMPIMIGGFGNWLVPLMLGAPDMAFPRLNNMSFWLLPPSLTLLISSSLVENGAGTGWTVYPPLAANIAHAGSSVDLAIFSLHLAGISSILGAVNFITTVINMRTNGMS
FDQMPLFVWAVAITALLLLLSLPVLAGAITMLLTDRNLNTSFFDPAGGGDPILYQHL
>LEFIL314-10|KM573248|MM18624|Incurvaria_masculella
TLYFIFGIWAGMVGTSLSMLIRTELGMPGSFIGNDQIYNTIVTAHAFIMIFFMVMPIMIGGFGNWLVPLMLGAPDMAFPRLNNMSFWLLPPSLTLLISSSLVENGAGTGWTVYPPLSSNIAHAGSSVDLAIFSLHLAGISSILGAVNFITTVINMRTNGM

SFDQMPLFVWAVAITALLLLLSLPVLAGAITMLLTDRNLNTSFFDPAGGGDPILYQHL
>LEFIC365-10|HM872208|MM03892|Incurvaria_oehlmanniella
TLYFIFGIWAGMIGTSLSMLIRTELGMPGSFIGNDQIYNTIVTAHAFIMIFFMVMPIMIGGFGNWLIPLMLGAPDMAFPRLNNMSFWLLPPSLTLLISSSLVENGAGTGWTVYPPLSSNIAHAGSSVDLAIFSLHLAGISSILGAVNFITTVINMRTNGMSF
DQMPLFVWAVAITALLLLLSLPVLAGAITMLLTDRNLNTSFFDPAGGGDPILYQHL
>LEFIA802-10|HM386943|MM04156|Incurvaria_oehlmanniella
TLYFIFGIWAGMIGTSLSMLIRTELGMPGSFIGNDQIYNTIVTAHAFIMIFFMVMPIMIGGFGNWLIPLMLGAPDMAFPRLNNMSFWLLPPSLTLLISSSLVENGAGTGWTVYPPLSSNIAHAGSSVDLAIFSLHLAGISSILGAVNFITTVINMRTNGMSF
DQMPLFVWAVAITALLLLLSLPVLAGAITMLLTDRNLNTSFFDPAGGGDPILYQHL
>LEFID362-10|HM873159|MM06270|Incurvaria_oehlmanniella
TLYFIFGIWAGMIGTSLSMLIRTELGMPGSFIGNDQIYNTIVTAHAFIMIFFMVMPIMIGGFGNWLIPLMLGAPDMAFPRLNNMSFWLLPPSLTLLISSSLVENGAGTGWTVYPPLSSNIAHAGSSVDLAIFSLHLAGISSILGAVNFITTVINMRTNGMSF
DQMPLFVWAVAITALLLLLSLPVLAGAITMLLTDRNLNTSFFDPAGGGDPILYQHL
>LEFID052-10|HM872866|MM05651|Incurvaria_pectinea
TLYFIFGIWAGMVGTSLSLLIRTELGMPGSLIGNDQIYNTIVTAHAFIMIFFMVMPIMIGGFGNWLVPLMLGAPDMAFPRLNNMSFWLLPPSLTLLISSSLVENGAGTGWTVYPPLSSNIAHAGSSVDLAIFSLHLAGISSILGAVNFITTVINMRANGMS
FDQMPLFVWAVAITALLLLLSLPVLAGAITMLLTDRNLNTSFFDPAGGGDPILYQHL
>LEFID172-10|HM872979|MM05952|Incurvaria_pectinea
TLYFIFGIWAGMVGTSLSLLIRTELGMPGSLIGNDQIYNTIVTAHAFIMIFFMVMPIMIGGFGNWLVPLMLGAPDMAFPRLNNMSFWLLPPSLTLLISSSLVENGAGTGWTVYPPLSSNIAHAGSSVDLAIFSLHLAGISSILGAVNFITTVINMRANGMS
FDQMPLFVWAVAITALLLLLSLPVLAGAITMLLTDRNLNTSFFDPAGGGDPILYQHL
>LEFIB583-10|HM871463|MM02272|Incurvaria_praelatella
TLYFIFGIWAGMVGTSLSLLIRTELGMTGSLIGNDQIYNTIVTAHAFIMIFFMVMPIMIGGFGNWLVPLMLGAPDMAFPRLNNMSFWLLPPSLTLLISSSLIENGAGTGWTVYPPLSSNISHAGSSVDLTIFSLHLAGISSILGAVNFITTVINMRANGMSF
DQMPLFVWAVAITALLLLLSLPVLAGAITMLLTDRNINTSFFDPAGGGDPILYQHL
>LEFIC329-10|HM872173|MM03834|Incurvaria_praelatella
TLYFIFGIWAGMVGTSLSLLIRTELGMTGSLIGNDQIYNTIVTAHAFIMIFFMVMPIMIGGFGNWLVPLMLGAPDMAFPRLNNMSFWLLPPSLTLLISSSLIENGTGTGWTVYPPLSSNISHAGSSVDLTIFSLHLAGISSILGAVNFITTVINMRANGMSF
DQMPLFVWAVAITALLLLLSLPVLAGAITMLLTDRNINTSFFDPAGGGDPILYQHL
>LEFID996-10|HM873746|MM08043|Incurvaria_praelatella
TLYFIFGIWAGMVGTSLSLLIRTELGMTGSLIGNDQIYNTIVTAHAFIMIFFMVMPIMIGGFGNWLVPLMLGAPDMAFPRLNNMSFWLLPPSLTLLISSSLIENGAGTGWTVYPPLSSNISHAGSSVDLTIFSLHLAGISSILGAVNFITTVINMRANGMSF
DQMPLFVWAVAITALLLLLSLPVLAGAITMLLTDRNINTSFFDPAGGGDPILYQHL
>LEFIA008-10|HM396358|MM00079|Incurvaria_vetulella
TLYFIFGIWAGMVGTSLSMLIRTELGMPGSFIGNDQIYNTIVTAHAFIMIFFMVMPIMIGGFGNWLIPLMLGAPDMAFPRLNNMSFWLLPPSLTLLISSSLVENGAGTGWTVYPPLSSNIAHAGSSVDLAIFSLHLAGISSILGAVNFITTVINMRTNGMS
FDQMPLFVWAVAITALLLLLSLPVLAGAITMLLTDRNLNTSFFDPAGGGDPILYQHL
>LEFIA806-10|HM386947|MM04160|Incurvaria_vetulella
TLYFIFGIWAGMAGTSLSMLIRTELGMPGSFIGNDQIYNTIVTAHAFIMIFFMVMPIMIGGFGNWLIPLMLGAPDMAFPRLNNMSFWLLPPSLTLLISSSLVENGAGTGWTVYPPLSSNIAHAGSSVDLAIFSLHLAGISSILGAVNFITTVINMRTNGMS
FDQMPLFVWAVAITALLLLLSLPVLAGAITMLLTDRNLNTSFFDPAGGGDPILYQHL
>LEFIA925-10|HM387061|MM13850|Incurvaria_vetulella
TLYFIFGIWAGMVGTSLSMLIRTELGMPGSFIGNDQIYNTIVTAHAFIMIFFMVMPIMIGGFGNWLIPLMLGAPDMAFPRLNNMSFWLLPPSLTLLISSSLVENGAGTGWTVYPPLSSNIAHAGSSVDLAIFSLHLAGISSILGAVNFITTVINMRTNGMS
FDQMPLFVWAVAITALLLLLSLPVLAGAITMLLTDRNLNTSFFDPAGGGDPILYQHL
>LEFID780-10|HM873537|MM06848|Infurcitinea_ignicomella
TLYFIFGIWSGMVGTSLSLLIRAELGNPGSLIGDDQIYNTIVTAHAFIMIFFMVMPIMIGGFGNWLVPLMLGAPDMAFPRMNNMSFWLLPPSLTLLISSSIVENGAGTGWTVYPPLSSNIAHGGGSVDLAIFSLHLAGISSILGAVNFITTVINMRSQGMS
FDRMPLFVWAVAITALLLLLSLPVLAGAITMLLTDRNLNTSFFDPAGGGDPILYQHL
>LEFIF985-10|HM875666|MM13579|Infurcitinea_ignicomella
TLYFIFGIWSGMVGTSLSLLIRAELGNPGSLIGDDQIYNTIVTAHAFIMIFFMVMPIMIGGFGNWLVPLMLGAPDMAFPRMNNMSFWLLPPSLTLLISSSIVENGAGTGWTVYPPLSSNIAHGGGSVDLAIFSLHLAGISSILGAVNFITTVINMRSQGMS
FDRMPLFVWAVAITALLLLLSLPVLAGAITMLLTDRNLNTSFFDPAGGGDPILYQHL
>LEFIG083-10|HM875762|MM13737|Infurcitinea_ignicomella
TLYFIFGIWSGMVGTSLSLLIRAELGNPGSLIGDDQIYNTIVTAHAFIMIFFMVMPIMIGGFGNWLVPLMLGAPDMAFPRMNNMSFWLLPPSLTLLISSSIVENGAGTGWTVYPPLSSNIAHGGGSVDLAIFSLHLAGISSILGAVNFITTVINMRSQGMS
FDRMPLFVWAVAITALLLLLSLPVLAGAITMLLTDRNLNTSFFDPAGGGDPILYQHL
>LEFIJ349-10|MM15949|Ipimorpha_contusa
TLYFIFGIWAGMVGTSLSLLIRAELGNPGSLIGDDQIYNTIVTAHAFIMIFFMVMPIMIGGFGNWLVPLMLGAPDMAFPRLNNMSFWLLPPSLTLLISSSIVENGAGTGWTVYPPLSSNIAHGGSSVDLAIFSLHLAGISSILGAINFITTIINMRLNNLSFDQ
MPLFVWAVGITAFLLLLSLPVLAGAITMLLTDRNLNTSFFDPAGGGDPILYQHL
>LEFII266-11|MM19916|Ipimorpha_contusa

TLYFIFGIWAGMVGTSLSLLIRAELGNPGSLIGDDQIYNTIVTAHAFIMIFFMVMPIMIGGFGNWLVPLMLGAPDMAFPRLNNMSFWLLPPSLTLLISSSIVENGAGTGWTVYPPLSSNIAHGGSSVDLAIFSLHLAGISSILGAINFITTIINMRLNNLSFDQ
MPLFVWAVGITAFLLLLSLPVLAGAITMLLTDRNLNTSFFDPAGGGDPILYQHL
>LEFIA590-10|HM870839|MM01701|Ipimorpha_retusa
TLYFIFGIWAGMVGTSLSLLIRAELGNPGSLIGDDQIYNTIVTAHAFIMIFFMVMPIMIGGFGNWLVPLMLGAPDMAFPRLNNMSFWLLPPSLTLLISSSIVENGAGTGWTVYPPLSSNIAHGGSSVDLAIFSLHLAGISSILGAINFITTIINMRLNSLSFDQ
MPLFIWAVGITAFLLLLSLPVLAGAITMLLTDRNLN?SFFDPAGGGDPILYQHL
>LEFIA591-10|HM870840|MM01702|Ipimorpha_retusa
TLYFIFGIWAGMVGTSLSLLIRAELGNPGSLIGDDQIYNTIVTAHAFIMIFFMVMPIMIGGFGNWLVPLMLGAPDMAFPRLNNMSFWLLPPSLTLLISSSIVENGAGTGWTVYPPLSSNIAHGGSSVDLAIFSLHLAGISSILGAINFITTIINMRLNSLSFDQ
MPLFIWAVGITAFLLLLSLPVLAGAITMLLTDRNLNTSFFDPAGGGDPILYQHL
>LEFIK469-10|JF854081|MM18044|Ipimorpha_subtusa
TLYFIFGIWAGMVGTSLSLLIRAELGNPGSLIGDDQIYNTIVTAHAFIMIFFMVMPIMIGGFGNWLVPLMLGAPDMAFPRLNNMSFWLLPPSLTLLISSSIVENGAGTGWTVYPPLSSNIAHGGSSVDLAIFSLHLAGISSILGAINFITTIINMRLNSLSFDQ
MPLFIWAVGITAFLLLLSLPVLAGAITMLLTDRNLNTSFFDPAGGGDPILYQHL
>LEFIB745-10|HM871623|MM02589|Isophrictis_striatella
TLYFIFGIWAGMVGTSLSLLIRAELGTPGSLIGDDQIYNTIVTAHAFIMIFFMVMPIMIGGFGNWLVPLMLGAPDMAFPRMNNMSFWLLPPSLTLLISSSIVENGAGTGWTVYPPLSSNIAHGGSSVDLAIFSLHLAGISSILGAINFITTIINMKINGLSFD
QMPLFVWAVGITALLLLLSLPVLAGAITMLLTDRNLNTSFFDPAGGGDPILYQHL
>LEFID644-10|HM873406|MM06670|Isophrictis_striatella
TLYFIFGIWAGMVGTSLSLLIRAELGTPGSLIGDDQIYNTIVTAHAFIMIFFMVMPIMIGGFGNWLVPLMLGAPDMAFPRMNNMSFWLLPPSLTLLISSSIVENGAGTGWTVYPPLSSNIAHGGSSVDLAIFSLHLAGISSILGAINFITTIINMKINGLSFD
QMPLFVWAVGITALLLLLSLPVLAGAITMLLTDRNLNTSFFDPAGGGDPILYQHL
>LEFIF995-10|HM875676|MM13602|Isophrictis_striatella
TLYFIFGVWAGMVGTSLSLLIRAELGTPGSLIGDDQIYNTIVTAHAFIMIFFMVMPIMIGGFGNWLVPLMLGAPDMAFPRMNNMSFWLLPPSLTLLISSSIVENGAGTGWTVYPPLSSNIAHGGSSVDLAIFSLHLAGISSILGAINFITTIINMKINGLSFD
QMPLFVWAVGITALLLLLSLPVLAGAITMLLTDRNLNTSFFDPAGGGDPILYQHL
>LEFIF996-10|HM875677|MM13603|Isophrictis_striatella
TLYFNFGIWAGMVGTSLSLLIRAELGTPGSLIGDDQIYNTIVTAHAFIMIFFMVMPIMIGGFGNWLVPLMLGAPDMAFPRMNNMSFWLLPPSLTLLISSSIVENGAGTGWTVYPPLSSNIAHGGSSVDLAIFSLHLAGISSILGAINFITTIINMKINGLSFD
QMPLFVWAVGITALLLLLSLPVLAGAITMLLTDRNLNTSFFDPAGGGDPILYQHL
>LEFIJ1288-11|MM21148|Isophrictis_striatella
TLYFIFGIWAGMVGTSLSLLIRAELGTPGSLIGDDQIYNTIVTAHAFIMIFFMVMPIMIGGFGNWLVPLMLGAPDMAFPRMNNMSFWLLPPSLTLLISSSIVENGAGTGWTVYPPLSSNIAHGGSSVDLAIFSLHLAGISSILGAINFITTIINMKINGLSFD
QMPLFVWAVGITALLLLLSLPVLAGAITMLLTDRNLNTSFFDPAGGGDPILYQHL
>LEFIG105-10|HM875785|MM13889|Issoria_lathonia
TLYFIFGIWAGMVGTSLSLLIRTELGNPGSLIGDDQIYNTIVTAHAFIMIFFMVMPIMIGGFGNWLVPLMLGAPDMAFPRMNNMSFWLLPPSLILLISSSIVENGAGTGWTVYPPLSSNIAHSGSSVDLAIFSLHLAGISSILGAINFITTIINMRINNMSFD
QMPLFVWAVGITALLLLLSLPVLAGAITMLLTDRNLNTSFFDPAG?GDPILYQHL
>LEFIG353-10|HM876030|MM14350|Issoria_lathonia
TLYFIFGIWAGMVGTSLSLLIRTELGNPGSLIGDDQIYNTIVTAHAFIMIFFMVMPIMIGGFGNWLVPLMLGAPDMAFPRMNNMSFWLLPPSLILLISSSIVENGAGTGWTVYPPLSSNIAHSGSSVDLAIFSLHLAGISSILGAINFITTIINMRINNMSFD
QMPLFVWAVGITALLLLLSLPVLAGAITMLLTDRNLNTSFFDPAGGGDPILYQHL
>LEFIA440-10|HM386781|MM01504|Jodis_lactearia
TLYFIFGIWAGMIGTSLSLLIRAELGNPGSLIGDDQIYNTIVTAHAFIMIFFMVMPIMIGGFGNWLVPLMLGAPDMAFPRMNNMSFWLLPPSLTLLISSSIVENGAGTGWTVYPPLSSNIAHGGSSVDLAIFSLHLAGISSILGAINFITTIINMRLNNMSFD
QMPLFVWAVGITAFLLLLSLPVLAGAITMLLTDRNLNTSFFDPAGGGDPILYQHL
>LEFIG322-10|HM876001|MM14292|Jodis_lactearia
TLYFIFGIWAGMIGTSLSLLIRAELGNPGSLIGDDQIYNTIVTAHAFIMIFFMVMPIMIGGFGNWLVPLMLGAPDMAFPRMNNMSFWLLPPSLTLLISSSIVENGAGTGWTVYPPLSSNIAHGGSSVDLAIFSLHLAGISSILGAINFITTIINMRLNNMSFD
QMPLFVWAVGITAFLLLLSLPVLAGAITMLLTDRNLNTSFFDPAGGGDPILYQHL
>LEFIA442-10|HM386783|MM01506|Jodis_putata
TLYFIFGIWAGMIGTSLSLLIRAELGNPGSLIGDDQIYNTIVTAHAFIMIFFMVMPIMIGGFGNWLVPLMLGAPDMAFPRMNNMSFWLLPPSLTLLISSSIVENGAGTGWTVYPPLSSNIAHGGSSVDLAIFSLHLAGISSILGAINFITTIINMRLNNMSFD
QMPLFVWAVGITAFLLLLSLPVLAGAITMLLTDRNLNTSFFDPAGGGDPILYQHL
>LEFIB126-10|HM871032|MM00490|Kessleria_fasciapennella
TLYFIFGIWSGMLGTSLSLLIRAELGNPGSLIGDDQIYNTIVTAHAFIMIFFMVMPIMIGGFGNWLVPLMLGAPDMAFPRMNNMSFWLLPPSLTLLISSSIVENGAGTGWTVYPPLSSNIAHSGSSVDLTIFSLHLAGISSILGAINFITTIINMKSNSMKFD
QMPLFVWAVGITALLLLLSLPVLAGAITMLLTDRNLNTSFFDPAGGGDPILYQHL
>LEFIB327-10|HM871227|MM00865|Kessleria_fasciapennella
TLYFIFGIWSGMLGTSLSLLIRAELGNPGSLIGDDQIYNTIVTAHAFIMIFFMVMPIMIGGFGNWLVPLMLGAPDMAFPRMNNMSFWLLPPSLTLLISSSIVENGAGTGWTVYPPLSSNIAHSGSSVDLAIFSLHLAGISSILGAINFITTIINMKSNSMKFD
QMPLFVWAVGITALLLLLSLPVLAGAITMLLTDRNLNTSFFDPAGGGDPILYQHL

>LEFIE216-10|HM873953|MM08531|Klimeschia_transversella
TLYFIFGIWSGLLGTSLSLLIRAELGNPGSLLGDDQIYNTIVTAHAFIMIFFMVMPIMIGGFGNWLVPLMIGAPDMAFPRLNNLSFWLLPPSISLLLTSSMVETGAGTGWTVYPPLSSNIAHSGSSVDLAIFSLHLAGISSILGAMNFITTIINMRPNNLLYDQ
MSLFTWAVGITALLLLLSLPVLAGAITMLLTDRNLNTSFFDPAGGGDPILYQHL
>LEFIF980-10|HM875661|MM13567|Klimeschiopsis_kiningerella
TLYFIFGIWAGMMGTSLSLLIRTELGTPGSLIGDDQIYNTIVTAHAFIMIFFMVMPIMIGGFGNWLVPLMLGAPDMAFPRMNNMSFWLLPPSFMLLISSSIVENGAGTGWTVYPPLSSNIAHSGSSVDLTIFSLHLAGISSILGAINFITTIINMKMNNLLF
DQMPLFVWSVGITALLLLLSLPVLAGAITMLLTDRNLNTSFFDPAGGGDPILYQHL
>LEFIC639-10|HM872460|MM04584|Lacanobia_contigua
TLYFIFGIWAGMVGTSLSLLIRAELGNPGSLIGDDQIYNTIVTAHAFIMIFFMVMPIMIGGFGNWLVPLMLGAPDMAFPRMNNMSFWLLPPSLTLLISSSIVENGAGTGWTVYPPLSSNIAHGGSSVDLAIFSLHLAGISSILGAINFITTIINMRLNNLSFD
QMPLFIWAVGITAFLLLLSLPVLAGAITMLLTDRNLNTSFFDPAGGGDPILYQHL
>LEFIA533-10|KM572889|MM01617|Lacanobia_oleracea
TLYFIFGIWAGMVGTSLSLLIRAELGNPGSLIGDDQIYNTIVTAHAFIMIFFMVMPIMIGGFGNWLVPLMLGAPDMAFPRMNNMSFWLLPPSLTLLISSSIVENGAGTGWTVYPPLSSNIAHGGSSVDLAIFSLHLAGISSILGAINFITTIINMRLNNLSFD
QMPLFIWAVGITAFLLLLSLPVLAGAITMLLTDRNLNTSFFDPAGGGDPILYQHL
>LEFIA534-10|KM573599|MM01618|Lacanobia_oleracea
TLYFIFGIWAGMVGTSLSLLIRAELGNPGSLIGDDQIYNTIVTAHAFIMIFFMVMPIMIGGFGNWLVPLMLGAPDMAFPRMNNMSFWLLPPSLTLLISSSIVENGAGTGWTVYPPLSSNIAHGGSSVDLAIFSLHLAGISSILGAINFITTIINMRLNSLSFD
QMPLFIWAVGITAFLLLLSLPVLAGAITMLLTDRNLNTSFFDPAGGGDPILYQHL
>LEFIJ1226-11|KM572069|MM21086|Lacanobia_oleracea
TLYFIFGIWAGMVGTSLSLLIRAELGNPGSLIGDDQIYNTIVTAHAFIMIFFMVMPIMIGGFGNWLVPLMLGAPDMAFPRMNNMSFWLLPPSLTLLISSSIVENGAGTGWTVYPPLSSNIAHGGSSVDLAIF?LHLAGISSILGAINFITTIINMRLNSLSFD
QMPLFIWAVGITAFLLLLSLPVLAGAITMLLTDRNLNTSFFDPAGGGDPILYQHL
>LEFIF674-10|HM875358|MM12654|Lacanobia_suasa
TLYFIFGIWAGMVGTSLSLLIRAELGNPGSLIGDDQIYNTIVTAHAFIMIFFMVMPIMIGGFGNWLVPLMLGAPDMAFPRMNNMSFWLLPPSLTLLISSSIVENGAGTGWTVYPPLSSNIAHGGSSVDLAIFSLHLAGISSILGAINFITTIINMRLNNLSFD
QMPLFIWAVGITAFLLLLSLPVLAGAITMLLTDRNLNTSFFDPAGGGDPILYQHL
>LEFIJ660-10|JF853754|MM17285|Lacanobia_suasa
TLYFIFGIWAGMVGTSLSLLIRAELGNPGSLIGDDQIYNTIVTAHAFIMIFFMVMPIMIGGFGNWLVPLMLGAPDMAFPRMNNMSFWLLPPSLTLLISSSIVENGAGTGWTVYPPLSSNIAHGGSSVDLAIFSLHLAGISSILGAINFITTIINMRLNNLSFD
QMPLFIWAVGITAFLLLLSLPVLAGAITMLLTDRNLNTSFFDPAGGGDPILYQHL
>LEFIB183-10|HM871087|MM00608|Lacanobia_thalassina
TLYFIFGIWAGMVGTSLSLLIRAELGNPGSLIGDDQIYNTIVTAHAFIMIFFMVMPIMIGGFGNWLVPLMLGAPDMAFPRMNNMSFWLLPPSLTLLISSSIVENGAGTGWTVYPPLSSNIAHGGSSVDLAIFSLHLAGISSILGAINFITTIINMRLNNLSFD
QMPLFIWAVGITAFLLLLSLPVLAGAITMLLTDRNLNTSFFDPAGGGDPILYQHL
>LEFIJ353-10|KM573034|MM15953|Lacanobia_w-latinum
TLYFIFGIWAGMVGTSLSLLIRAELGNPGSLIGDDQIYNTIVTAHAFIMIFFMVMPIMIGGFGNWLIPLMLGAPDMAFPRMNNMSFWLLPPSLTLLISSSIVENGAGTGWTVYPPLSSNIAHGGSSVDLAIFSLHLAGISSILGAINFITTIINMRLNNLSFD
QMPLFIWAVGITAFLLLLSLPVLAGAITMLLTDRNLNTSFFDPAGGGDPILYQHL
>LEFID521-10|HM873286|MM06496|Lampronia_capitella
TLYFIFGIWSGMVGTSLSLLIRAELGTPGSLIGNDQIYNTIVTAHAFIMIFFMVMPIMIGGFGNWLVPLMLGAPDMAFPRLNNMSFWLLPPSLTLLISSSLVENGAGTGWTVYPPLSSNIAHSGGSVDLAIFSLHLAGISSILGAVNFITTVINMKPNNMM
YDNMPLFVWAVAITALLLLLSLPVLAGAITMLLTDRNLNTSFFDPAGGGDPILYQHL
>LEFIG648-10|HM876311|MM15512|Lampronia_capitella
TLYFIFGIWSGMVGTSLSLLIRAELGTPGSLIGNDQIYNTIVTAHAFIMIFFMVMPIMIGGFGNWLVPLMLGAPDMAFPRLNNMSFWLLPPSLTLLISSSLVENGAGTGWTVYPPLSSNIAHSGGSVDLAIFSLHLAGISSILGAVNFITTVINMKPNNMM
YDNMPLFVWAVAITALLLLLSLPVLAGAITMLLTDRNLNTSFFDPAGGGDPILYQHL
>LEFIG649-10|HM876312|MM15513|Lampronia_capitella
TLYFIFGIWSGMVGTSLSILIRAELGTPGSLIGNDQIYNTIVTAHAFIMIFFMVMPIMIGGFGNWLVPLMLSAPDMAFPRLNNMSFWLLPPSLTLLISSSLVENGAGTGWTVYPPLSSNIAHSGGSVDLAIFSLHLAGISSILGAVNFITTVINMKPNNMMY
DNMPLFVWAVAITALLLLLSLPVLAGAITMLLTDRNLNTSFFDPAGGGDPILYQHL
>LEFIG655-10|HM876316|MM15519|Lampronia_corticella
TLYFIFGIWSGMVGTSLSLLIRAELSTPGALIGDDQIYNTIVTAHAFIMIFFMVMPIMIGGFGNWLVPLMLGAPDMAFPRLNNMSFWLLPPSLTLLISSSLVENGAGTGWTVYPPLSSNLTHTGSSVDLAIFSLHLAGISSILGAVNFITTVINMRPMNMSF
DSMPLFVWAVAITALLLLLSLPVLAGAITMLLTDRNLNTSFFDPAGGGDPILYQHL
>LEFIC324-10|HM872168|MM03828|Lampronia_flavimitrella
TLYFIFGIWSGMVGTSLSLLIRAELGTSGALIGDDQIYNTIVTAHAFIMIFFMVMPIMIGGFGNWLVPLMLGAPDMAFPRLNNMSFWLLPPSLTLLISSSLIENGAGTGWTVYPPLSSNITHTGSSVDLAIFSLHLAGISSILGAVNFITTVINMRPMNMMF
DNMPLFVWAVAITALLLLLSLPVLAGAITMLLTDRNLNTSFFDPAGGGDPILYQHL
>LEFID879-10|HM873636|MM07018|Lampronia_fuscatella
TLYFIFGIWSGMVGTSLSLLIRAELGIPGSFIESDQIYNTIVTAHAFIMIFFMVMPIMIGGFGNWLVPLMLGAPDMAFPRLNNMSFWLLPPSLMLLISSSLVENGTGTGWTIYPPLSSNIAHSGSSVDLTIFSLHLAGISSILGAINFITTVINMKINNMSYDM

MPLFVWSVTITALLLLLSLPVLAGAITMLLTDRNLNTSFFDPAGGGDPILYQHL
>LEFIE254-10|HM873988|MM08587|Lampronia_fuscatella
TLYFIFGIWSGMVGTSLSLLIRAELGIPGSFIESDQIYNTIVTAHAFIMIFFVVMPIMIGGFGNWLVPLMLGAPDMAFPRLNNMSFWLLPPSLMLLISSSLVENGTGTGWTIYPPLSSNIAHSGSSVDLTIFSLHLAGISSILGAINFITTVINMKINNMSYDM
MPLFVWSVTITALLLLLSLPVLAGAITMLLTDRNLNTSFFDPAGGGDPILYQHL
>LEFIG652-10|HM876313|MM15516|Lampronia_luzella
TLYFIFGIWSGMIGTSLSLLIRAELGTSGALIGDDQIYNTIVTAHAFIMIFFMVMPIMIGGFGNWLVPLMLGAPDMAFPRLNNMSFWLLPPSLTLLISSSLVESGAGTGWTVYPPLSSNITHTGSSVDLAIFSLHLAGISSILGAVNFITTVINMRPMNMSFD
NMPLFVWAVAITALLLLLSLPVLAGAITMLLTDRNLNTSFFDPAGGGDPILYQHL
>LEFIG653-10|HM876314|MM15517|Lampronia_luzella
TLYFIFGIWSGMIGTSLSLLIRAELGTSGALIGDDQIYNTIVTAHAFIMIFFMVMPIMIGGFGNWLVPLMLGAPDMAFPRLNNMSFWLLPPSLTLLISSSLVESGAGTGWTIYPPLSSNITHTGSSVDLAIFSLHLAGISSILGAVNFITTVINMRPMNMSFD
NMPLFVWAVAITALLLLLSLPVLAGAITMLLTDRNLNTSFFDPAGGGDPILYQHL
>LEFIK491-10|JF854098|MM18066|Lampronia_luzella
TLYFIFGIWSGMIGTSLSLLIRAELGTSGALIGDDQIYNTIVTAHAFIMIFFMVMPIMIGGFGNWLVPLMLGAPDMAFPRLNNMSFWLLPPSLTLLISSSLVESGAGTGWTVYPPLSSNITHTGSSVDLAIFSLHLAGISSILGAVNFITTVINMRPMNMSFD
NMPLFVWAVAITALLLLLSLPVLAGAITMLLTDRNLNTSFFDPAGGGDPILYQHL
>LEFIC294-10|HM872138|MM03767|Lampronia_morosa
TLYFIFGIWSGMVGTSLSLLIRAELGTSGALIGDDQIYNTIVTAHAFIMIFFMVMPIMIGGFGNWLVPLMLGAPDMAFPRLNNMSFWLLPPSLTLLISSSLVENGAGTGWTVYPPLSSNITHTGSSVDLAIFSLHLAGISSILGAVNFITTVINMRPTNMMF
DNMPLFVWAVAITALLLLLSLPVLAGAITMLLTDRNLNTSFFDPAGGGDPILYQHL
>LEFID520-10|HM873285|MM06494|Lampronia_rupella
TLYFIFGIWSGMIGTSLSLLIRAELGTPSSLIGNDQIYNTIVTAHAFIMIFFMVMPIMIGGFGNWLIPLMLGAPDMAFPRLNNMSFWLLPPSLTLLISSSLVENGAGTGWTVYPPLSSNISHSGGSVDLAIFSLHLAGISSILGAVNFITTVINMKPNNMSYD
NMPLFVWSVVITALLLLLSLPVLAGAITMLLTDRNLNTSFFDPAGGGDPILYQHL
>LEFID880-10|HM873637|MM07022|Lampronia_standfussiella
TLYFIFGIWSGMIGTSLSLLIRAELGTSGALIGDDQIYNTIVTAHAFIMIFFMVMPIMIGGFGNWLVPLMLGAPDMAFPRLNNMSFWLLPPSLTLLISSSLVENGAGTGWTVYPPLSSNITHTGSSVDLAIFSLHLAGISSILGAVNFITTVINMRPTNMMF
DNMPLFVWAVAITALLLLLSLPVLAGAITMLLTDRNLNTSFFDPAGGGDPILYQHL
>LEFIK493-10|JF854100|MM18068|Lampronia_standfussiella
TLYFIFGIWSGMIGTSLSLLIRAELGTSGALIGDDQIYNTIVTAHAFIMIFFMVMPIMIGGFGNWLVPLMLGAPDMAFPRLNNMSFWLLPPSLTLLISSSLVENGAGTGWTVYPPLSSNITHTGSSVDLAIFSLHLAGISSILGAVNFITTVINMRPTNMMF
DNMPLFVWAVAITALLLLLSLPVLAGAITMLLTDRNLNTSFFDPAGGGDPILYQHL
>LEFIA392-10|HM386734|MM01449|Lampropteryx_otregiata
TLYFIFGIWAGMIGTSLSLLIRAELGNPGSLIGDDQIYNTIVTAHAFIMIFFMVMPIMIGGFGNWLVPLMLGAPDMAFPRMNNMSFWLLPPSITLLISSSIVENGAGTGWTVYPPLSSNIAHGGSSVDLAIFSLHLAGISSILGAINFITTIINMRLNNMFFD
QLPLFVWAVGITAFLLLLSLPVLAGAITMLLTDRNLNTSFFDPAGGGDPILYQHL
>LEFIA414-10|HM386756|MM01476|Lampropteryx_otregiata
TLYFIFGIWAGMIGTSLSLLIRAELGNPGSLIGDDQIYNTIVTAHAFIMIFFMVMPIMIGGFGNWLVPLMLGAPDMAFPRMNNMSFWLLPPSITLLISSSIVENGAGTGWTVYPPLSSNIAHGGSSVDLAIFSLHLAGISSILGAINFITTIINMRLNNMFFD
QLPLFVWAVGITAFLLLLSLPVLAGAITMLLTDRNLNTSFFDPAGGGDPILYQHL
>LEFIA264-10|HM386608|MM01302|Lampropteryx_suffumata
TLYFIFGIWAGMIGTSLSLLIRAELGNPGSLIGDDQIYNTIVTAHAFIMIFFMVMPIMIGGFGNWLVPLMLGAPDMAFPRMNNMSFWLLPPSITLLISSSIVENGAGTGWTVYPPLSSNIAHGGSSVDLAIFSLHLAGISSILGAINFITTIINMRLNNMFFD
QLPLFVWAVGITAFLLLLSLPVLAGAITMLLTDRNLNTSFFDPAGGGDPILYQHL
>LEFIF366-10|HM875051|MM11570|Lampropteryx_suffumata
TLYFIFGIWAGMIGTSLSLLIRAELGNPGSLIGDDQIYNTIVTAHAFIMIFFMVMPIMIGGFGNWLVPLMLGAPDMAFPRMNNMSFWLLPPSITLLISSSIVENGAGTGWTVYPPLSSNIAHGGSSVDLAIFSLHLAGISSILGAINFITTIINMRLNNMFFD
QLPLFVWAVGITAFLLLLSLPVLAGAITMLLTDRNLNTSFFDPAGGGDPILYQHL
>LEFIA929-10|HM387063|MM13857|Lampropteryx_suffumata
TLYFIFGIWAGMIGTSLSLLIRAELGNPGSLIGDDQIYNTIVTAHAFIMIFFMVMPIMIGGFGNWLVPLMLGAPDMAFPRMNNMSFWLLPPSITLLISSSIVENGAGTGWTVYPPLSSNIAHGGSSVDLAIFSLHLAGISSILGAINFITTIINMRLNNMFFD
QLPLFVWAVGITAFLLLLSLPVLAGAITMLLTDRNLNTSFFDPAGGGDPILYQHL
>LEFIB007-10|HM870920|MM00018|Lamprotes_c-aureum
TLYFIFGIWAGMVGTSLSLLIRAELGTPGSLIGDDQIYNTIVTAHAFIMIFFMVMPIMIGGFGNWLVPLMLGAPDMAFPRMNNMSFWLLPPSLTLLISSSIVENGAGTGWTVYPPLSSNIAHGGSSVDLAIFSLHLAGISSILGAINFITTIINMRLNNLSFD
QMPLFIWAVGITAFLLLLSLPVLAGAITMLLTDRNLNTSFFDPAGGGDPILYQHL
>LEFIF792-10|HM875476|MM13040|Laodamia_faecella
TLYFIFGIWSGMVGTSLSLLIRAELGTPGSLIGDDQIYNTIVTGHAFIMIFFMVMPIMIGGFGNWLVPLMLGAPDMAFPRMNNMSFWLLPPSLTLLISSSIVENGAGTGWTVYPPLSSNIAHGGSSVDLAIFSLHLAGISSILGAINFITTIINMKLNGLSFD
QMPLFVWAVGITALLLLLSLPVLAGAITMLLTDRNLNTSFFDPAGGGDPILYQHL
>LEFIA062-10|HM396409|MM00974|Laothoe_amurensis

TLYFIFGIWAGMVGTSLSLLIRAELGNPGSLIGDDQIYNTIVTAHAFIMIFFMVMPIMIGGFGNWLVPLMLGAPDMAFPRMNNMSFWLLPPSLTLLISSSIVENGAGTGWTVYPPLSSNIAHSGSSVDLAIFSLHLAGISSILGAINFITTIINMRINNMSFD
QMPLFVWAVGITAFLLLLSLPVLAGAITMLLTDRNLNTSFFDPAGGGDPILYQHL
>LEFIA064-10|HM396411|MM00977|Laothoe_populi
TLYFIFGIWAGMVGTSLSLLIRAELGNPGSLIGDDQIYNTIVTAHAFIMIFFMVMPIMIGGFGNWLVPLMLGAPDMAFPRMNNMSFWLLPPSLTLLISSSIVENGAGTGWTVYPPLSSNIAHSGSSVDLAIFSLHLAGISSILGAINFITTIINMRINNMSFD
QMPLFVWAVGITAFLLLLSLPVLAGAITMLLTDRNLNTSFFDPAGGGDPILYQHL
>LEFIF535-10|HM875220|MM12202|Larentia_clavaria
TLYFIFGIWAGMIGTSLSLLIRAELGNPGSLIGDDQIYNTIVTAHAFIMIFFMVMPIMIGGFGNWLVPLMLGAPDMAFPRMNNMSFWLLPPSITLLISSSIVENGAGTGWTVYPPLSSNIAHGGSSVDLAIFSLHLAGISSILGAINFITTIINMRLNNMFFD
QLPLFVWSVGITAFLLLLSLPVLAGAITMLLTDRNLNTSFFDPAGGGDPILYQHL
>LEFIB078-10|HM870987|MM00380|Lasiocampa_quercus
SLYFIFGIWAGMVGTSLSLLIRAELGTPGSLIGDDQIYNTIVTAHAFIMIFFMVMPIMIGGFGNWLVPLMLGAPDMAFPRMNNMSFWLLPPSLTLLISSSIVENGTGTGWTVYPPLSSNIAHGGSSVDLAIFSLHLAGISSILGAINFITTIINMRLNNMSFD
QMPLLVWAVGITAFLLLLSLPVLAGAITMLLTDRNLNTSFFDPAGGGDPILYQHL
>LEFID618-10|HM873383|MM06634|Lasiocampa_quercus
TLYFIFGIWAGMVGTSLSLLIRAELGTPGSLIGDDQIYNTIVTAHAFIMIFFMVMPIMIGGFGNWLVPLMLGAPDMAFPRMNNMSFWLLPPSLTLLISSSIVENGAGTGWTVYPPLSSNIAHGGSSVDLAIFSLHLAGISSILGAINFITTIINMRLNNMSFD
QMPLFIWAVGITAFLLLLSLPVLAGAITMLLTDRNLNTSFFDPAGGGDPILYQHL
>LEFID691-10|HM873452|MM06739|Lasiocampa_quercus
TLYFIFGIWAGMVGTSLSLLIRAELGTPGSLIGDDQIYNTIVTAHAFIMIFFMVMPIMIGGFGNWLVPLMLGAPDMAFPRMNNMSFWLLPPSLTLLISSSIVENGAGTGWTVYPPLSSNIAHGGSSVDLAIFSLHLAGISSILGAINFITTIINMRLNNMSFD
QMPLFIWAVGITAFLLLLSLPVLAGAITMLLTDRNLNTSFFDPAGGGDPILYQHL
>LEFIF356-10|HM875041|MM11191|Lasiocampa_quercus
TLYFIFGIWAGMVGTSLSLLIRAELGTPGSLIGDDQIYNTIVTAHAFIMIFFMVMPIMIGGFGNWLVPLMLGAPDMAFPRMNNMSFWLLPPSLTLLISSSIVENGAGTGWTVYPPLSSNIAHGGSSVDLAIFSLHLAGISSILGAINFITTIINMRLNNMSFD
QMPLFIWAIGITAFLLLLSLPVLAGAITMLLTDRNLNTSFFDPAGGGDPILYQHL
>LEFIJ196-10|KM573509|MM12528|Lasiocampa_quercus
TLYFIFGIWAGMVGTSLSLLIRAELGTPGSLIGDDQIYNTIVTAHAFIMIFFMVMPIMIGGFGNWLVPLMLGAPDMAFPRMNNMSFWLLPPSLTLLISSSIVENGAGTGWTVYPPLSSNIAHGGSSVDLAIFSLHLAGISSILGAINFITTIINMRLNNMSFD
QMPLFIWAVGITAFLLLLSLPVLAGAITMLLTDRNLNTSFFDPAGGGDPILYQHL
>LEFIG273-10|HM875952|MM14213|Lasiocampa_quercus
TLYFIFGIWAGMVGTSLSLLIRAELGTPGSLIGDDQIYNTIVTAHAFIMIFFMVMPIMIGGFGNWLVPLMLGAPDMAFPRMNNMSFWLLPPSLMLLISSSIVENGAGTGWTVYPPLSSNIAHGGSSVDLAIFSLHLAGISSILGAINFITTIINMRLNNMSF
DQMPLFIWAVGITAFLLLLSLPVLAGAITMLLTDRNLNTSFFDPAGGGDPILYQHL
>LEFIK423-10|JF854052|MM17998|Lasiocampa_quercus
TLYFIFGIWAGMVGTSLSLLIRAELGTPGSLIGDDQIYNTIVTAHAFIMIFFMVMPIMIGGFGNWLVPLMLGAPDMAFPRMNNMSFWLLPPSLTLLISSSIVENGAGTGWTVYPPLSSNIAHGGSSVDLAIFSLHLAGISSILGAINFITTIINMRLNNMSFD
QMPLFIWAVGITAFLLLLSLPVLAGAITMLLTDRNLNTSFFDPAGGGDPILYQHL
>LEFIF635-10|HM875319|MM12534|Lasiocampa_trifolii
TLYFIFGIWAGMVGTSLSLLIRAELGTPGSLIGDDQIYNTIVTAHAFIMIFFMVMPIMIGGFGNWLVPLMLGAPDMAFPRMNNMSFWLLPPSLTLLISSSIVENGAGTGWTVYPPLSSNIAHGGSSVDLAIFSLHLAGISSILGAINFITTIINMRLNNMSFD
QMPLFVWAVGITAFLLLLSLPVLAGAITMLLTDRNLNTSFFDPAGGGDPILYQHL
>LEFID593-10|HM873358|MM06603|Lasiommata_maera
TLYFIFGIWAGMVGTSLSLIIRTELGNPGFLIGDDQIYNTIVTAHAFIMIFFMVMPIMIGGFGNWLIPLMLGAPDMAFPRMNNMSFWLLPPSLILLISSSIVENGAGTGWTVYPPLSSNIAHSGSSVDLAIFSLHLAGISSILGAINFITTIINMRINNLSYDQ
MPLFVWAVGITALLLLLSLPVLAGAITMLLTDRNLNTSFFDPAGGGDPILYQHL
>LEFIB177-10|HM871081|MM00591|Lasiommata_petropolitana
TLYFIFGIWAGMVGTSLSLIIRTELGNPGFLIGDDQIYNTIVTAHAFIMIFFMVMPIMIGGFGNWLIPLMLGAPDMAFPRMNNMSFWLLPPSLILLISSSIVENGAGTGWTVYPPLSSNIAHSGSSVDLAIFSLHLAGISSILGAINFITTIINMRINNMSYDQ
MPLFVWAVGITALLLLLSLPVLAGAITMLLTDRNLNTSFFDPAGGGDPILYQHL
>LEFIA526-10|KM573003|MM01609|Lasionycta_imbecilla
TLYFIFGIWAGMVGTSLSLLIRAELGNPGSLIGDDQIYNTIVTAHAFIMIFFMVMPIMIGGFGNWLVPLMLGAPDMAFPRMNNMSFWLLPPSLTLLISSSIVENGAGTGWTVYPPLSSNIAHGGSSVDLAIFSLHLAGISSILGAINFITTIINMRLNSLSFD
QMPLFIWAVGITAFLLLLSLPVLAGAITMLLTDRNLNTSFFDPAGGGDPILYQHL
>LEFIA733-10|HM386877|MM04087|Lasionycta_leucocycla
TLYFIFGIWAGMVGTSLSLLIRAELGNPGSLIGDDQIYNTIVTAHAFIMIFFMVMPIMIGGFGNWLVPLMLGAPDMAFPRMNNMSFWLLPPSLTLLISSSIVENGAGTGWTVYPPLSSNIAHSGSSVDLAIFSLHLAGISSILGAINFITTIINMRLNNLSFD
QMPLFIWAVGITAFLLLLSLPVLAGAITMLLTDRNLNTSFFDPAGGGDPILYQHL
>LEFIA734-10|HM386878|MM04088|Lasionycta_leucocycla
TLYFIFGIWAGMVGTSLSLLIRAELGNPGSLIGDDQIYNTIVTAHAFIMIFFMVMPIMIGGFGNWLVPLMLGAPDMAFPRMNNMSFWLLPPSLTLLISSSIVENGAGTGWTVYPPLSSNIAHSGSSVDLAIFSLHLAGISSILGAINFITTIINMRLNNLSFD
QMPLFIWAVGITAFLLLLSLPVLAGAITMLLTDRNLNTSFFDPAGGGDPILYQHL

>LEFIH026-10|HM876662|MM15890|Lasionycta_proxima
TLYFIFGIWAGMVGTSLSLLIRAELGNPGSLIGDDQIYNTIVTAHAFIMIFFMVMPIMIGGFGNWLVPLMLGAPDMAFPRMNNMSFWLLPPSLTLLISSSIVENGAGTGWTVYPPLSSNIAHGGSSVDLAIFSLHLAGISSILGAINFITTIINMRLNNLSFD
QMPLFIWAVGITAFLLLLSLPVLAGAITMLLTDRNLNTSFFDPAGGGDPILYQHL
>LEFIH027-10|HM876663|MM15891|Lasionycta_proxima
TLYFIFGIWAGMVGTSLSLLIRAELGNPGSLIGDDQIYNTIVTAHAFIMIFFMVMPIMIGGFGNWLVPLMLGAPDMAFPRMNNMSFWLLPPSLTLLISSSIVENGAGTGWTVYPPLSSNIAHGGSSVDLAIFSLHLAGISSILGAINFITTIINMRLNNLSFD
QMPLFIWAVGITAFLLLLSLPVLAGAITMLLTDRNLNTSFFDPAGGGDPILYQHL
>LEFIK436-10|JF854059|MM18011|Lasionycta_secedens
TLYFIFGIWAGMVGTSLSLLIRAELGSPGSLIGDDQIYNTIVTAHAFIMIFFMVMPIMIGGFGNWLVPLMLGAPDMAFPRMNNMSFWLLPPSLTLLISSSIVENGAGTGWTVYPPLSSNIAHGGSSVDLAIFSLHLAGISSILGAINFITTIINMRLNNLSFD
QMPLFIWAVGITAFLLLLSLPVLAGAITMLLTDRNLNTSFFDPAGGGDPILYQHL
>LEFIL470-10|MM18768|Lasionycta_skraelingia
TLYFIFGIWAGMVGTSLSLLIRAELGNPGSLIGDDQIYNTIVTAHAFIMIFFMVMPIMIGGFGNWLVPLMLGAPDMAFPRMNNMSFWLLPPSLTLLISSSIVENGAGTGWTVYPPLSSNIAHGGSSVDLAIFSLHLAGISSILGAINFITTIINMRLNSLSFD
QMPLFIWAVGITAFLLLLSLPVLAGAITMLLTDRNLNTSFFDPAGGGDPILYQHL
>COLFF176-13|MM23203|Lasionycta_skraelingia
TLYFIFGIWAGMVGTSLSLLIRAELGNPGSLIGDDQIYNTIVTAHAFIMIFFMVMPIMIGGFGNWLVPLMLGAPDMAFPRMNNMSFWLLPPSLTLLISSSIVENGAGTGWTVYPPLSSNIAHGGSSVDLAIFSLHLAGISSILGAINFITTIINMRLNSLSFD
QMPLFIWAVGITAFLLLLSLPVLAGAITMLLTDRNLNTSFFDPAGGGDPILYQHL
>LEFIK438-10|JF854061|MM18013|Lasionycta_staudingeri
TLYFIFGIWAGMVGTSLSLLIRAELGNPGSLIGDDQIYNTIVTAHAFIMIFFMVMPIMIGGFGNWLVPLMLGAPDMAFPRMNNMSFWLLPPSLTLLISSSIVENGAGTGWTVYPPLSSNIAHSGSSVDLAIFSLHLAGISSILGAINFITTIINMRLNNLSFD
QMPLFIWAVGITAFLLLLSLPVLAGAITMLLTDRNLNTSFFDPAGGGDPILYQHL
>LEFIA359-10|HM386702|MM01415|Laspeyria_flexula
TLYFIFGIWAGMVGTSLSMLIRAELGNPGSLIGDDQIYNTIVTAHAFIMIFFMVMPIMIGGFGNWLVPLMLGAPDMAFPRMNNMSFWLLPPSLTLLISSSIVENGAGTGWTVYPPLSSNIAHGGSSVDLAIFSLHLAGISSILGAINFISTIINMKLNNLSF
DQMPLFIWAVGITAFLLLLSLPVLAGAITMLLTDRNLNTSFFDPAGGGDPILYQHL
>LEFIC854-10|HM872672|MM05088|Laspeyria_flexula
TLYFIFGIWAGMVGTSLSMLIRAELGNPGSLIGDDQIYNTIVTAHAFIMIFFMVMPIMIGGFGNWLVPLMLGAPDMAFPRMNNMSFWLLPPSLTLLISSSIVENGAGTGWTVYPPLSSNIAHGGSSVDLAIFSLHLAGISSILGAINFISTIINMKLNNLSF
DQMPLFIWAVGITAFLLLLSLPVLAGAITMLLTDRNLNTSFFDPAGGGDPILYQHL
>LEFIC756-10|HM872575|MM04871|Lateroligia_ophiogramma
TLYFIFGIWAGMVGTSLSLLIRAELGNPGSLIGDDQIYNTIVTAHAFIMIFFMVMPIMIGGFGNWLVPLMLGAPDMAFPRMNNMSFWLLPPSLTLLISSSIVENGAGTGWTVYPPLSSNIAHGGSSVDLAIFSLHLAGISSILGAINFITTIINMRLNNLSFD
QMPLFIWAVGITAFLLLLSLPVLAGAITMLLTDRNLNTSFFDPAGGGDPILYQHL
>LEFIB499-10|HM871394|MM02083|Lathronympha_strigana
TLYFIFGIWAGMVGTSLSLLIRAELGNPGSLIGDDQIYNTIVTAHAFIMIFFMVMPIMIGGFGNWLVPLMLGAPDMAFPRMNNMSFWLLPPSLMLLISSSVVENGAGTGWTVYPPLSSNIAHSGSSVDLAIFSLHLAGISSILGAVNFITTIINMRPNNM
SLDQMPLFVWSVGITALLLLLSLPVLAGAITMLLTDRNLNTSFFDPAGGGDPILYQHL
>LEFIF132-10|HM874830|MM10588|Lemonia_dumi
TLYFIFGIWAGMVGTSLSLLIRAELGNPGSLIGDDQIYNTIVTAHAFIMIFFMVMPIMIGGFGNWLVPLMLGAPDMAFPRMNNMSFWLLPPSLTLLISSSIVENGAGTGWTVYPPLSSNIAHGGSSVDLAIFSLHLAGASSIMGAINFITTIINMRLNNMS
FDQMPLFVWAVGITAFLLLLSLPVLAGAITMLLTDRNLNTSFFDPAGGGDPILFQHL
>LEFIL358-10|JN303439|MM18668|Lemonia_dumi
TLYFIFGIWAGMVGTSLSLLIRTELGNPGSLIGDDQIYNTIVTAHAFIMIFFMVMPIMIGGFGNWLVPLMLGAPDMAFPRMNNMSFWLLPPSLTLLISSSIVENGAGTGWTVYPPLSSNIAHGGSSVDLAIFSLHLAGASSIMGAINFITTIINMRLNNMS
FDQMPLFVWAVGITAFLLLLSLPVLAGAITMLLTDRNLNTSFFDPAGGGDPILYQHL
>LEFIB067-10|HM870976|MM00355|Lenisa_geminipuncta
TLYFIFGIWAGMVGTSLSLLIRAELGTPGSLIGDDQIYNTIVTAHAFIMIFFMVMPIMIGGFGNWLVPLMLGAPDMAFPRMNNMSFWLLPPSLTLLISSSIVENGAGTGWTVYPPLSSNIAHGGSSVDLAIFSLHLAGISSILGAINFITTIINMRLNNLSFD
QMPLFIWAVGITAFLLLLSLPVLAGAITMLLTDRNLNTSFFDPAGGGDPILYQHL
>LEFIJ727-10|MM17352|Lenisa_geminipuncta
TLYFIFGIWAGMVGTSLSLLIRAELGTPGSLIGDDQIYNTIVTAHAFIMIFFMVMPIMIGGFGNWLVPLMLGAPDMAFPRMNNMSFWLLPPSLTLLISSSIVENGAGTGWTVYPPLSSNIAHGGSSVDLAIFSLHLAGISSILGAINFITTIINMRLNNLSFD
QMPLFIWAVGITAFLLLLSLPVLAGAITMLLTDRNLNTSFFDPAGGGDPILYQHL
>LEFIJ2040-13|KM572124|MM22892|Leptidea_juvernica
TLYFIFGIWAGMVGTSLSLLIRTELGNPGSLIGDDQIYNTIVTAHAFIMIFFMVMPIMIGGFGNWLIPLMLGAPDMAFPRMNNMSFWMLPPSLLILITSSVVENGAGTGWTVYPPLSSNIAHSGSSVDLAIFSLHLAGISSILGAINFITTIINMRINNLSFD
QMPLFVWAVGITALLLLLSLPVLAGAITMLLTDRNLNTSFFDPAGGGDPILYQHL
>LEFIJ2181-14|MM23256|Leptidea_juvernica
TLYFIFGIWAGMVGTSLSLLIRTELGNPGSLIGDDQIYNTIVTAHAFIMIFFMVMPIMIGGFGNWLIPLMLGAPDMAFPRMNNMSFWMLPPSLLILITSSVVENGAGTGWTVYPPLSSNIAHSGSSVDLAIFSLHLAGISSILGAINFITTIINMRINNLSFD

QMPLFVWAVGITALLLLLSLPVLAGAITMLLTDRNLNTSFFDPAGGGDPILYQHL
>LEFIC521-10|HM872344|MM04238|Leptidea_sinapis
TLYFIFGIWAGMVGTSLSLLIRTELGNPGSLIGDDQVYNTIVTAHAFIMIFFMVMPIMIGGFGNWLIPLMLGAPDMAFPRMNNMSFWMLPPSLLILITSSVVENGAGTGWTVYPPLSSNIAHSGSSVDLAIFSLHLAGISSILGAINFITTIINMRINNLSFD
QMPLFVWAVGITALLLLLSLPVLAGAITMLLTDRNLNTSFFDPAGGGDPILYQHL
>LEFID110-10|HM872921|MM05733|Leptidea_sinapis
TLYFIFGIWAGMVGTSLSLLIRTELGNPGSLIGDDQVYNTIVTAHAFIMIFFMVMPIMIGGFGNWLIPLMLGAPDMAFPRMNNMSFWMLPPSLLILITSSVVENGAGTGWTVYPPLSSNIAHSGSSVDLAIFSLHLAGISSILGAINFITTIINMRINNLSFD
QMPLFVWAVGITALLLLLSLPVLAGAITMLLTDRNLNTSFFDPAGGGDPILYQHL
>LEFIE586-10|HM874309|MM09493|Leucania_comma
TLYFIFGIWAGMVGTSLSLLIRAELGTPGSLIGDDQIYNTIVTAHAFIMIFFMVMPIMIGGFGNWLVPLMLGAPDMAFPRMNNMSFWLLPPSLTLLISSSIVENGAGTGWTVYPPLSSNIAHGGSSVDLAIFSLHLAGISSILGAINFITTIINMRLNSLSFD
QMPLFIWAVGITAFLLLLSLPVLAGAITMLLTDRNLNTSFFDPAGGGDPILYQHL
>LEFIB210-10|HM871114|MM00662|Leucania_obsoleta
TLYFIFGIWAGMVGTSLSLLIRAELGTPGSLIGDDQIYNTIVTAHAFIMIFFMVMPIMIGGFGNWLVPLMLGAPDMAFPRMNNMSFWLLPPSLTLLISSSIVENGAGTGWTVYPPLSSNIAHGGSSVDLAIFSLHLAGISSILGAINFITTIINMRLNNLSFD
QMPLFIWAVGITAFLLLLSLPVLAGAITMLLTDRNLNTSFFDPAGGGDPILYQHL
>LEFIA089-10|HM396436|MM01011|Leucodonta_bicoloria
TLYFIFGIWAGMVGTSLSLLIRAELGNPGSLIGDDQIYNTIVTAHAFIMIFFMVMPIMIGGFGNWLVPLMLGAPDMAFPRMNNMSFWMLPPSLTLLISSSIVESGAGTGWTVYPPLSSNIAHSGSSVDLAIFSLHLAGISSILGAINFITTIINMRLNNMTF
DQMPLFVWAVGITAFLLLLSLPVLAGAITMLLTDRNLNTSFFDPAGGGDPILYQHL
>LEFIA839-10|HM386979|MM09714|Leucoma_salicis
TLYFIFGIWAGMIGTSLSLLIRAELGNPGSLIDNDQIYNTIVTAHAFIMIFFMVMPIMMGGFGNWLIPLMLGAPDMAFPRMNNMSFWLLPPSLTLLISSSIVENGAGTGWTVYPPLSSNIAHSGSSVDLAIFSLHLAGISSILGAINFITTIINMRLNNLSFD
QMPLFVWAVGITAFLLLLSLPVLAGAITMLLTDRNLNTSFFDPAGGGDPILYQHL
>LEEUA091-11|MM19404|Leucoptera_lathyrifoliella
TLYFIFGIWAGLIGTSLSILIRAELGNPGSLIGDDQIYNTIVTAHAFIMIFFMVMPIMIGGFGNWLVPLMLGAPDMAFPRMNNMSFWLLPPSIILLISSSIVESGAGTGWTVYPPLSSNIAHSGSSVDLAIFSLHLAGISSILGAINFITTIINMRSKNMNFDQ
MPLFVWAVGITALLLLLSLPVLAGAITMLLTDRNLNTSFFDPAGGGDPILYQHL
>LEFIJ915-10|JF853896|MM17540|Leucoptera_lustratella
TLYFIFGIWSGMIGTSMSILIRAELGNPGSLIGDDQIYNTIVTAHAFIMIFFMVMPIMIGGFGNWLVPLMLGAPDMAFPRMNNMSFWLLPPSMILLISSSIVENGAGTGWTVYPPLSSNIAHSGSSVDLTIFSLHLAGISSILGAINFITTIINMRSDNMNLE
QMPLFVWAVGITAILLLLSLPVLAGAITMLLTDRNLNTSFFDPAGGGDAILYQHL
>LEFIG185-10|HM875865|MM14053|Leucoptera_malifoliella
TLYFIFGIWAGMIGTSLSIMIRAELGNSGSLIGDDQIYNTIVTAHAFIMIFFMVMPIMIGGFGNWLVPLMLGAPDMAFPRLNNMSFWLLPPSIFLLISSSIIEMGVGTGWTVYPPLSANIAHSGSSVDLAIFSLHLAGISSILGAINFITTIINMRSKNMNFD
QMPLFVWAVGITALLLLLSLPVLAGAITMLLTDRNLNTSFFDPAGGGDPILYQHL
>LEFIJ267-10|JF853507|MM14883|Leucoptera_orobi
TLYFIFGIWAGLIGTSLSILIRAELGNPGSLIGDDQIYNTIVTAHAFIMIFFMVMPIMIGGFGNWLVPLMLGAPDMAFPRMNNMSFWLLPPSIVLLISSSVVESGAGTGWTVYPPLSSNIAHSGSSVDLAIFSLHLAGISSILGAINFITTIINMRSKNMNFDQ
MPLFVWAVGITALLLLLSLPVLAGAITMLLTDRNLNTSFFDPAGGGDPILYQHL
>LEFID311-10|HM873108|MM06192|Leucoptera_sinuella
TMYFIFGIWSGLIGTMLSMLIRLELGNPGSFLSNDQIYNTVVTAHAFIMIFFMVMPIMIGGFGNWLVPLMLGAPDMAFPRMNNMSFWLLPPSLMLLISSSIVENGSGTGWTVYPPLSSNIAHSGSSVDLTIFSLHLAGISSILGAINFITTVINMRSNNLS
LDQMPLFVWAVMITAVLLLLSLPVLAGAITMLLTDRNLNTSFFDPAGGGDPILYQHL
>LEEUA088-11|MM19401|Leucoptera_spartifoliella
TLYFIFGIWAGMIGTSLSILIRAELGNPGSLIGDDQIYNTIVTAHAFIMIFFMVMPIMIGGFGNWLVPLMLGAPDMAFPRMNNMSFWLLPPSIILLISSSIVESGAGTGWTVYPPLSSNIAHSGSSVDLAIFSLHLAGISSILGAINFITTIINMRSKNMNFDQ
MPLFVWAVGITALLLLLSLPVLAGAITMLLTDRNLNTSFFDPAGGGDPILYQHL
>LEFID878-10|HM873635|MM07017|Leucospilapteryx_omissella
TLYFIFGIWSGMVGTSLSMLIRVELGNPGSLIGNDQIYNTIVTAHAFIMIFFMVMPIMIGGFGNWLVPLMLGAPDMAFPRLNNMSFWLLPPSLILLISSSIVETGAGTGWTVYPPLSSNIAHGGSSVDLAIFSLHLAGISSILGAINFITTIINMRPNSMNFD
KMSLFSWAVGITAMLLLLSLPVLAGAITMLLTDRNLNTSFFDPAGGGDPILYQHL
>LEFIG580-10|HM876251|MM14769|Levipalpus_hepatariella
TLYFIFGVWAGMLGTSLSLLIRAELGNPGSLIGDDQIYNTIVTAHAFIMIFFMVMPIMIGGFGNWLVPLMLGAPDMAFPRMNNMSFWLLPPSLTLLISSSIVENGAGTGWTVYPPLSSNIAHSGSSVDLAIFSLHLAGISSILGAINFITTIINMRLNNMSF
DRLPLFVWSVGITALLLLLSLPVLAGAITMLLTDRNLNTSFFDPAGGGDPILYQHL
>LEFIJ1222-11|MM21082|Levipalpus_hepatariella
TLYFIFGVWAGMLGTSLSLLIRAELGNPGSLIGDDQIYNTIVTAHAFIMIFFMVMPIMIGGFGNWLVPLMLGAPDMAFPRMNNMSFWLLPPSLTLLISSSIVENGAGTGWTVYPPLSSNIAHSGSSVDLAIFSLHLAGISSILGAINFITTIINMRLNNMSF
DRLPLFIWSVGITALLLLLSLPVLAGAITMLLTDRNLNTSFFDPAGGGDPILYQHL
>LEFIL037-10|JF854411|MM19037|Ligdia_adustata

TLYFIFGIWAGMVGTSLSLLIRAELGNPGSLIGDDQIYNTIVTAHAFIMIFFMVMPIMIGGFGNWLIPLMLGAPDMAFPRMNNMSFWLLPPSIMLLISSSIVENGAGTGWTVYPPLSSNIAHGGSSVDLAIFSLHLAGISSILGAINFITTIINMRLNNLSFD
QMPLFVWAVGITAFLLLLSLPVLAGAITMLLTDRNLNTSFFDPAGGGDPILYQHL
>LEFIJ1008-11|MM21248|Limenitis_camilla
TLYFIFGIWAGMVGTSLSLLIRTELGNPGSLIGDDQIYNTIVTAHAFIMIFFMVMPIMIGGFGNWLVPLMLGAPDMAFPRMNNMSFWLLPPSLILLISSSIVENGAGTGWTVYPPLSSNIAHGGSSVDLAIFSLHLAGISSILGAINFITTIINMRINNMSFD
QMPLFVWSVGITALLLLLSLPVLAGAITMLLTDRNLNTSFFDPAGGGDPILYQHL
>LEFIC084-10|HM871952|MM03383|Limenitis_populi
TLYFIFGIWAGMVGTSLSLLIRTELGNPGSLIGDDQIYNTIVTAHAFIMIFFMVMPIMIGGFGNWLVPLMLGAPDMAFPRMNNMSFWLLPPSLILLISSSVVENGAGTGWTVYPPLSSNIAHSGSSVDLAIFSLHLAGISSILGAINFITTIINMRINHMSFD
QMPLFIWSVGITALLLLLSLPVLAGAITMLLTDRNLNTSFFDPAGGGDPILYQHL
>LEFID365-10|HM873162|MM06273|Limenitis_populi
TLYFIFGIWAGMVGTSLSLLIRTELGNPGSLIGDDQIYNTIVTAHAFIMIFFMVMPIMIGGFGNWLVPLMLGAPDMAFPRMNNMSFWLLPPSLILLISSSVVENGAGTGWTVYPPLSSNIAHSGSSVDLAIFSLHLAGISSILGAINFITTIINMRINHMSFD
QMPLFIWSVGITALLLLLSLPVLAGAITMLLTDRNLNTSFFDPAGGGDPILYQHL
>LEFID765-10|HM873522|MM06831|Limnaecia_phragmitella
TLYFIFGIWSGMVGTSLSLLIRAELGNPGSLIGDDQIYNTIVTAHAFIMIFFMVMPIMIGGFGNWLIPLMLGAPDMAFPRMNNMSFWLLPPSLTLLISSSIVETGAGTGWTVYPPLSSNIAHGGSSVDLAIFSLHLAGISSILGAINFITTIINMRVNNLSFDQ
MPLFVWAVGITALLLLLSLPVLAGAITMLLTDRNLNTSFFDPAGGGDPILYQHL
>LEFIA478-10|KM573328|MM01555|Lithophane_consocia
TLYFIFGIWAGMVGTSLSLLIRAELGNPGSLIGDDQIYNTIVTAHAFIMIFFMVMPIMIGGFGNWLVPLMLGAPDMAFPRLNNMSFWLLPPSLTLLISSSIVENGAGTGWTVYPPLSSNIAHGGSSVDLAIFSLHLAGISSILGAINFITTIINMRLNNLSFDQ
MPLFIWAVGITAFLLLLSLPVLAGAITMLLTDRNLNTSFFDPAGGGDPILYQHL
>LEFIF198-10|HM874892|MM10812|Lithophane_furcifera
TLYFIFGIWAGMVGTSLSLLIRAELGNPGSLIGDDQIYNTIVTAHAFIMIFFMVMPIMIGGFGNWLVPLMLGAPDMAFPRLNNMSFWLLPPSLTLLISSSIVENGAGTGWTVYPPLSSNIAHGGSSVDLAIFSLHLAGISSILGAINFITTIINMRLNNLSFDQ
MPLFIWAVGITAFLLLLSLPVLAGAITMLLTDRNLNTSFFDPAGGGDPILYQHL
>LEFIC666-10|HM872487|MM04627|Lithophane_lamda
TLYFIFGIWAGMVGTSLSLLIRAELGNPGSLIGDDQIYNTIVTAHAFIMIFFMVMPIMIGGFGNWLVPLMLGAPDMAFPRMNNMSFWLLPPSLTLLISSSIVENGAGTGWTVYPPLSSNIAHGGSSVDLAIFSLHLAGISSILGAINFITTIINMRLNNLSFD
QMPLFIWAVGITAFLLLLSLPVLAGAITMLLTDRNLNTSFFDPAGGGDPILYQHL
>LEFIE099-10|HM873846|MM08281|Lithophane_lamda
TLYFIFGIWAGMVGTSLSLLIRAELGNPGSLIGDDQIYNTIVTAHAFIMIFFMVMPIMIGGFGNWLVPLMLGAPDMAFPRMNNMSFWLLPPSLTLLISSSIVENGAGTGWTVYPPLSSNIAHGGSSVDLAIFSLHLAGISSILGAINFITTIINMRLNNLSFD
QMPLFIWAVGITAFLLLLSLPVLAGAITMLLTDRNLNTSFFDPAGGGDPILYQHL
>LEFIJ661-10|MM17286|Lithophane_ornitopus
TLYFIFGIWAGMVGTSLSLLIRAELGNPGSLIGDDQIYNTIVTAHAFIMIFFMVMPIMIGGFGNWLVPLMLGAPDMAFPRLNNMSFWLLPPSLTLLISSSIVENGAGTGWTVYPPLSSNIAHGGSSVDLAIFSLHLAGISSILGAINFITTIINMRLNNLSFDQ
MPLFIWAVGITAFLLLLSLPVLAGAITMLLTDRNLNTSFFDPAGGGDPILYQHL
>LEEUA324-11|JN286982|MM19732|Lithophane_semibrunnea
TLYFIFGIWAGMVGTSLSLLIRAELGNPGSLIGDDQIYNTIVTAHAFIMIFFMVMPIMIGGFGNWLVPLMLGAPDMAFPRLNNMSFWLLPPSLTLLISSSIVENGAGTGWTVYPPLSSNIAHGGSSVDLAIFSLHLAGISSILGAINFITTIINMRLNNLSFDQ
MPLFIWAVGITAFLLLLSLPVLAGAITMLLTDRNLNTSFFDPAGGGDPILYQHL
>LEFIA480-10|MM01557|Lithophane_socia
TLYFIFGIWAGMVGTSLSLLIRAELGNPGSLIGDDQIYNTIVTAHAFIMIFFMVMPIMIGGFGNWLVPLMLGAPDMAFPRLNNMSFWLLPPSLTLLISSSIVENGAGTGWTVYPPLSSNIAHGGSSVDLAIFSLHLAGISSILGAINFITTIINMRLNNLSFDQ
MPLFIWAVGITAFLLLLSLPVLAGAITMLLTDRNLNTSFFDPAGGGDPILYQHL
>LEFIG399-10|HM876076|MM14422|Lithosia_quadra
TLYFIFGIWAGMVGTSLSLLIRAELGNPGSLIGDDQIYNTIVTAHAFIMIFFMVMPIMIGGFGNWLVPLMLGAPDMAFPRMNNMSFWLLPPSLTLLISSSIVENGAGTGWTVYPPLSSNIAHSGSSVDLAIFSLHLAGISSILGAINFITTIINMRLNKLMFD
QMPLFVWAVGITAFLLLLSLPVLAGAITMLLTDRNLNTSFFDPAGGGDPILYQHL
>LEFIF363-10|HM875048|MM11537|Litoligia_literosa
TLYFIFGIWAGMVGTSLSLLIRAELGNPGSLIGDDQIYNTIVTAHAFIMIFFMVMPIMIGGFGNWLVPLMLGAPDMAFPRMNNMSFWLLPPSLTLLISSSIVENGAGTGWTVYPPLSSNIAHGGSSVDLAIFSLHLAGISSILGAINFITTIINMRLNNLSFD
QMPLFIWAVGITAFLLLLSLPVLAGAITMLLTDRNLNTSFFDPAGGGDPILYQHL
>LEFIF418-10|HM875103|MM11824|Lobesia_bicinctana
TLYFIFGIWAGMIGTSLSLLIRAELGNPGSLIGDDQIYNTIVTAHAFIMIFFMVMPIMIGGFGNWLVPLMLGAPDMAFPRMNNMSFWLLPPSIMLLISSSIVENGAGTGWTVYPPLSSNIAHSGSSVDLAIFSLHLAGISSILGAVNFITTIINMRPNNMSL
DQMPLFVWAVGITAILLLLSLPVLAGAITMLLTDRNLNTSFFDPAGGGDPILYQHL
>LEEUA233-11|JN274931|MM19641|Lobesia_littoralis
TLYFIFGIWAGMVGTSLSLLIRAELGNPGSLIGDDQIYNTIVTAHAFIMIFFMVMPIMIGGFGNWLVPLMLGAPDMAFPRMNNMSFWLLPPSIMLLISSSIVENGAGTGWTVYPPLSSNIAHSGSSVDLAIFSLHLAGISSILGAVNFITTIINMRPNNMSL
DQMPLFVWAVGITALLLLLSLPVLAGAITMLLTDRNLNTSFFDPAGGGDPILYQHL

>LEFID273-10|HM873071|MM06146|Lobesia_reliquana
TLYFIFGIWAGMIGTSLSLLIRAELGNPGSLIGDDQIYNTIVTAHAFIMIFFMVMPIMIGGFGNWLVPLMLGAPDMAFPRMNNMSFWLLPPSIMLLISSSIVENGAGTGWTVYPPLSSNIAHSGSSVDLAIFSLHLAGISSILGAVNFITTIINMRPNNMSL
DQMPLFVWAVGITALLLLLSLPVLAGAITMLLTDRNLNTSFFDPAGGGDPILYQHL
>LEFIF434-10|HM875119|MM11878|Lobesia_reliquana
TLYFIFGIWAGMIGTSLSLLIRAELGNPGSLIGDDQIYNTIVTAHAFIMIFFMVMPIMIGGFGNWLVPLMLGAPDMAFPRMNNMSFWLLPPSIMLLISSSIVENGAGTGWTVYPPLSSNIAHSGSSVDLAIFSLHLAGISSILGAVNFITTIINMRPNNMSL
DQMPLFVWAVGITALLLLLSLPVLAGAITMLLTDRNLNTSFFDPAGGGDPILYQHL
>LEFIG847-10|HM876495|MM15711|Lobesia_reliquana
TLYFIFGIWAGMIGTSLSLLIRAELGNPGSLIGDDQIYNTIVTAHAFIMIFFMVMPIMIGGFGNWLVPLMLGAPDMAFPRMNNMSFWLLPPSIMLLISSSIVENGAGTGWTVYPPLSSNIAHSGSSVDLAIFSLHLAGISSILGAVNFITTIINMRPNNMSL
DQMPLFVWAVGITALLLLLSLPVLAGAITMLLTDRNLNTSFFDPAGGGDPILYQHL
>LEFIC368-10|HM872211|MM03895|Lobesia_virulenta
TLYFIFGVWAGMVGTSLSLLIRAELGNPGSLIGDDQIYNTIVTAHAFIMIFFMVMPIMIGGFGNWLVPLMLGAPDMAFPRMNNMSFWLLPPSIMLLISSSIVENGAGTGWTVYPPLSSNIAHSGSSVDLAIFSLHLAGISSILGAVNFITTIINMRPNNMS
LDQMPLFVWAVGITALLLLLSLPVLAGAITMLLTDRNLNTSFFDPAGGGDPILYQHL
>LEFID360-10|HM873157|MM06268|Lobesia_virulenta
TLYFIFGVWAGMVGTSLSLLIRAELGNPGSLIGDDQIYNTIVTAHAFIMIFFMVMPIMIGGFGNWLVPLMLGAPDMAFPRMNNMSFWLLPPSIMLLISSSIVENGAGTGWTVYPPLSSNIAHSGSSVDLAIFSLHLAGISSILGAVNFITTIINMRPNNMS
LDQMPLFVWAVGITALLLLLSLPVLAGAITMLLTDRNLNTSFFDPAGGGDPILYQHL
>LEFIA241-10|HM386585|MM01274|Lobophora_halterata
TLYFIFGIWAGMIGTSLSLLIRAELGNPGSLIGDDQIYNTIVTAHAFIMIFFMVMPIMIGGFGNWLVPLMLGAPDMAFPRMNNMSFWLLPPSITLLISSSIVENGAGTGWTVYPPLSSNIAHGGSSVDLAIFSLHLAGISSILGAINFITTIINMRLNNMFFD
QLPLFVWAVGITAFLLLLSLPVLAGAITMLLTDRNLNTSFFDPAGGGDPILYQHL
>LEFIA242-10|HM386586|MM01275|Lobophora_halterata
TLYFIFGIWAGMIGTSLSLLIRAELGNPGSLIGDDQIYNTIVTAHAFIMIFFMVMPIMIGGFGNWLVPLMLGAPDMAFPRMNNMSFWLLPPSITLLISSSIVENGAGTGWTVYPPLSSNIAHGGSSVDLAIFSLHLAGISSILGAINFITTIINMRLNNMFFD
QLPLFVWAVGITAFLLLLSLPVLAGAITMLLTDRNLNTSFFDPAGGGDPILYQHL
>LEFIB169-10|HM871073|MM00572|Lomaspilis_marginata
TLYFIFGVWAGMVGTSLSLLIRAELGTPGSLIGDDQIYNTIVTAHAFIMIFFMVMPIMIGGFGNWLVPLMLGAPDMAFPRMNNMSFWLLPPSITLLISSSIVENGAGTGWTVYPPLSSNIAHGGSSVDLAIFSLHLAGISSILGAINFITTIINMRLNNLSFD
QMPLFVWAVGITAFLLLLSLPVLAGAITMLLTDRNLNTSFFDPAGGGDPILYQHL
>LEFIC306-10|HM872150|MM03802|Lomaspilis_opis
TLYFIFGVWAGMVGTSLSLLIRAELGTPGSLIGDDQIYNTIVTAHAFIMIFFMVMPIMIGGFGNWLVPLMLGAPDMAFPRMNNMSFWLLPPSITLLISSSIVENGAGTGWTVYPPLSSNIAHGGSSVDLAIFSLHLAGISSILGAINFITTIINMRLNNLSFD
QMPLFVWAVGITAFLLLLSLPVLAGAITMLLTDRNLNTSFFDPAGGGDPILYQHL
>LEFIA270-10|HM386614|MM01310|Lomographa_bimaculata
TLYFIFGIWAGMVGTSLSLLIRAELGNPGSLIGDDQIYNTIVTAHAFIMIFFMVMPIMIGGFGNWLVPLMLGAPDMAFPRMNNMSFWLLPPSITLLISSSIVESGAGTGWTVYPPLSSNIAHGGSSVDLAIFSLHLAGISSILGAINFITTIINMRLNNMSFD
QMPLFVWAVGITAFLLLLSLPVLAGAITMLLTDRNLNTSFFDPAGGGDPILYQHL
>LEFIA269-10|HM386613|MM01309|Lomographa_temerata
TLYFIFGIWAGMVGTSLSLLIRAELGNPGSLIGDDQIYNTIVTAHAFIMIFFMVMPIMIGGFGNWLVPLMLGAPDMAFPRMNNMSFWLLPPSITLLISSSIVESGAGTGWTVYPPLSSNIAHGGSSVDLAIFSLHLAGISSILGAINFITTIINMRLNNMSFD
QMPLFVWAVGITAFLLLLSLPVLAGAITMLLTDRNLNTSFFDPAGGGDPILYQHL
>LEFIB049-10|HM870958|MM00331|Longalatedes_elymi
TLYFIFGIWAGMVGTSLSLLIRAELGNPGSLIGDDQIYNTIVTAHAFIMIFFMVMPIMIGGFGNWLVPLMLGAPDMAFPRMNNMSFWLLPPSLTLLISSSIVENGAGTGWTVYPPLSSNIAHGGSSVDLAIFSLHLAGISSILGAINFITTIINMRLNNLSFD
QMPLFIWAVGITAFLLLLSLPVLAGAITMLLTDRNLNTSFFDPAGGGDPILYQHL
>LEFIJ317-10|MM15917|Lopinga_achine
TLYFIFGIWAGMVGTSLSLIIRTELGNPGFLIGDDQIYNTIVTAHAFIMIFFMVMPIMIGGFGNWLIPLMLGAPDMAFPRMNNMSFWLLPPSLILLISSSIVENGAGTGWTVYPPLSSNIAHGGSSVDLAIFSLHLAGISSILGAINFITTIINMRINNMSYDQ
MPLFVWAVGITALLLLLSLPVLAGAITMLLTDRNLNTSFFDPAGGGDPILYQHL
>LEFIC352-10|HM872195|MM03870|Loxostege_commixtalis
TLYFIFGIWSGMVGTSLSLLIRAELGNPGSLIGDDQIYNTIVTAHAFIMIFFMVMPIMIGGFGNWLVPLMLGAPDMAFPRMNNMSFWLLPPSLTLLISSSIVENGAGTGWTVYPPLSSNIAHGGSSVDLAIFSLHLAGISSILGAINFITTIINMRVNGMSF
DQMPLFVWAVGITALLLLLSLPVLAGAITMLLTDRNLNTSFFDPAGGGDPILYQHL
>LEFID494-10|HM873259|MM06451|Loxostege_commixtalis
TLYFIFGIWSGMVGTSLSLLIRAELGNPGSLIGDDQIYNTIVTAHAFIMIFFMVMPIMIGGFGNWLVPLMLGAPDMAFPRMNNMSFWLLPPSLTLLISSSIVENGAGTGWTVYPPLSSNIAHGGSSVDLAIFSLHLAGISSILGAINFITTIINMRVNGMSF
DQMPLFVWAVGITALLLLLSLPVLAGAITMLLTDRNLNTSFFDPAGGGDPILYQHL
>LEFIF112-10|HQ570385|MM10565|Loxostege_ephippialis
TLYFIFGIWSGMVGTSLSLLIRAELGNPGSLIGDDQIYNTIVTAHAFIMIFFMVMPIMIGGFGNWLVPLMLGAPDMAFPRMNNMSFWLLPPSLTLLISSSIVENGAGTGWTVYPPLSSNIAHGGSSVDLAIFSLHLAGISSILGAINFITTIINMRINGMSFD

QMPLFVWAVGITALLLLLSLPVLAGAITMLLTDRNLNTSFFDPAGGGDPILYQHL
>LEFIF109-10|HM874813|MM10562|Loxostege_sticticalis
TLYFIFGIWSGMVGTSLSLLIRAELGNPGSLIGDDQIYNTIVTAHAFIMIFFMVMPIMIGGFGNWLVPLMLGAPDMAFPRMNNMSFWLLPPSLTLLISSSIVENGAGTGWTVYPPLSSNIAHGGSSVDLAIFSLHLAGISSILGAINFITTIINMRINGMSFD
QMPLFVWAVGITALLLLLSLPVLAGAITMLLTDRNLNTSFFDPAGGGDPILYQHL
>LEFIF110-10|HM874814|MM10563|Loxostege_sticticalis
TLYFIFGIWSGMVGTSLSLLIRAELGNPGSLIGDDQIYNTIVTAHAFIMIFFMVMPIMIGGFGNWLVPLMLGAPDMAFPRMNNMSFWLLPPSLTLLISSSIVENGAGTGWTVYPPLSSNIAHGGSSVDLAIFSLHLAGISSILGAINFITTIINMRINGMSFD
QMPLFVWAVGITALLLLLSLPVLAGAITMLLTDRNLNTSFFDPAGGGDPILYQHL
>LEFIF111-10|HM874815|MM10564|Loxostege_sticticalis
TLYFIFGIWSGMVGTSLSLLIRAELGNPGSLIGDDQIYNTIVTAHAFIMIFFMVMPIMIGGFGNWLVPLMLGAPDMAFPRMNNMSFWLLPPSLTLLISSSIVENGAGTGWTVYPPLSSNIAHGGSSVDLAIFSLHLAGISSILGAINFITTIINMRINGMSFD
QMPLFVWAVGITALLLLLSLPVLAGAITMLLTDRNLNTSFFDPAGGGDPILYQHL
>LEFIB367-10|HM871266|MM00933|Loxostege_turbidalis
TLYFIFGIWSGMVGTSLSLLIRAELGNPGSLIGDDQIYNTIVTAHAFIMIFFMVMPIMIGGFGNWLVPLMLGAPDMAFPRMNNMSFWLLPPSLTLLISSSIVENGAGTGWTVYPPLSSNIAHGGSSVDLAIFSLHLAGISSILGAINFITTIINMRINGMSFD
QMPLFVWAVGITALLLLLSLPVLAGAITMLLTDRNLNTSFFDPAGGGDPILYQHL
>LEFIB423-10|HM871322|MM01906|Lozotaenia_forsterana
TLYFIFGIWAGMMGTSLSLLIRAELGNPGSLIGDDQIYNTIVTAHAFIMIFFMVMPIMIGGFGNWLVPLMLGAPDMAFPRMNNMSFWLLPPSIMLLISSSIVENGVGTGWTVYPPLSSNIAHSGSSVDLAIFSLHLAGISSILGAVNFITTIINMRPNNMS
LDQMPLFVWSVGITALLLLLSLPVLAGAITMLLTDRNLNTSFFDPAGGGDPILYQHL
>LEFIB890-10|HM871767|MM02978|Lozotaenia_forsterana
TLYFIFGIWAGMVGTSLSLLIRAELGNPGSLIGDDQIYNTIVTAHAFIMIFFMVMPIMIGGFGNWLVPLMLGAPDMAFPRMNNMSFWLLPPSIMLLISSSIVENGAGTGWTVYPPLSSNIAHSGSSVDLAIFSLHLAGISSILGAVNFITTIINMRPNNMSL
DQMPLFVWSVGITALLLLLSLPVLAGAITMLLTDRNLNTSFFDPAGGGDPILYQHL
>LEFIA794-10|HM386935|MM04148|Lozotaenia_forsterana
TLYFIFGIWAGMVGTSLSLLIRAELGNPGSLIGDDQIYNTIVTAHAFIMIFFMVMPIMIGGFGNWLVPLMLGAPDMAFPRMNNMSFWLLPPSIMLLISSSIVENGAGTGWTVYPPLSSNIAHSGSSVDLAIFSLHLAGISSILGAVNFITTIINMRPNNMSL
DQMPLFVWSVGITALLLLLSLPVLAGAITMLLTDRNLNTSFFDPAGGGDPILYQHL
>LEFIC912-10|HM872729|MM05252|Lozotaenia_forsterana
TLYFIFGIWAGMVGTSLSLLIRAELGNPGSLIGDDQIYNTIVTAHAFIMIFFMVMPIMIGGFGNWLVPLMLGAPDMAFPRMNNMSFWLLPPSIMLLISSSIVENGAGTGWTVYPPLSSNIAHSGSSVDLAIFSLHLAGISSILGAVNFITTIINMRPNNMSL
DQMPLFVWSVGITALLLLLSLPVLAGAITMLLTDRNLNTSFFDPAGGGDPILYQHL
>LEFID678-10|HM873439|MM06721|Lozotaenia_forsterana
TLYFIFGIWAGMVGTSLSLLIRAELGNPGSLIGDDQIYNTIVTAHAFIMIFFMVMPIMIGGFGNWLVPLMLGAPDMAFPRMNNMSFWLLPPSIMLLISSSIVENGAGTGWTVYPPLSSNIAHSGSSVDLAIFSLHLAGISSILGAVNFITTIINMRPNNMSL
DQMPLFVWSVGITALLLLLSLPVLAGAITMLLTDRNLNTSFFDPAGGGDPILYQHL
>LEFIA885-10|HM387022|MM09762|Lozotaenia_forsterana
TLYFIFGIWAGMVGTSLSLLIRAELGNPGSLIGDDQIYNTIVTAHAFIMIFFMVMPIMIGGFGNWLVPLMLGAPDMAFPRMNNMSFWLLPPSIMLLISSSIVENGAGTGWTVYPPLSSNIAHSGSSVDLAIFSLHLAGISSILGAVNFITTIINMRPNNMSL
DQMPLFVWSVGITALLLLLSLPVLAGAITMLLTDRNLNTSFFDPAGGGDPILYQHL
>LEFIF394-10|HM875079|MM11746|Lozotaenia_forsterana
TLYFIFGIWAGMMGTSLSLLIRAELGNPGSLIGDDQIYNTIVTAHAFIMIFFMVMPIMIGGFGNWLVPLMLGAPDMAFPRMNNMSFWLLPPSIMLLISSSIVENGVGTGWTVYPPLSSNIAHSGSSVDLAIFSLHLAGISSILGAVNFITTIINMRPNNMS
LDQMPLFVWSVGITALLLLLSLPVLAGAITMLLTDRNLNTSFFDPAGGGDPILYQHL
>LEFIF570-10|HM875255|MM12326|Lozotaenia_forsterana
TLYFIFGIWAGMMGTSLSLLIRAELGNPGSLIGDDQIYNTIVTAHAFIMIFFMVMPIMIGGFGNWLVPLMLGAPDMAFPRMNNMSFWLLPPSIMLLISSSIVENGVGTGWTVYPPLSSNIAHSGSSVDLAIFSLHLAGISSILGAVNFITTIINMRPNNMS
LDQMPLFVWSVGITALLLLLSLPVLAGAITMLLTDRNLNTSFFDPAGGGDPILYQHL
>LEFIJ851-10|MM17476|Luperina_testacea
TLYFIFGIWAGMVGTSLSLLIRAELGNPGSLIGDDQIYNTIVTAHAFIMIFFMVMPIMIGGFGNWLVPLMLGAPDMAFPRMNNMSFWLLPPSLTLLISSSIVENGAGTGWTVYPPLSSNIAHGGSSVDLAIFSLHLAGISSILGAINFITTIINMRLNSLSFD
QMSLFIWAVGITAFLLLLSLPVLAGAITMLLTDRNLNTSFFDPAGGGDPILYQHL
>LEFIJ853-10|MM17478|Luperina_testacea
TLYFIFGIWAGMVGTSLSLLIRAELG?PGSLIGDDQIYNTIVTAHAFIMIFFMVMPIMIGGFGNWLVPLMLGAPDMAFPRMNNMSFWLLPPSLTLLISSSIVENGAGTGWTVYPPLSSNIAHGGSSVDLAIFSLHLAGISSILGAINFITTIINMRLNSLSFD
QMSLFIWAVGITAFLLLLSLPVLAGAITMLLTDRNLNTSFFDPAGGGDPILYQHL
>LEEUA092-11|JN266639|MM19405|Luquetia_lobella
TLYFIFGIWAGMVGLSLSLLIRAELGNPGSLIGDDQIYNTIVTAHAFIMIFFMVMPIMIGGFGNWLVPLMLGAPDMAFPRMNNMSFWLLPPSLILLISSSIVENGAGTGWTVYPPLSSNIAHSGSSVDLAIFSLHLAGISSILGAINFITTIINMRLNNMFFD
QLPLFVWAVGITALLLLLSLPVLAGAITMLLTDRNINTSFFDPAGGGDPILYQHL
>LEEUA566-11|MM20625|Luquetia_lobella

TLYFIFGIWAGMVGLSLSLLIRAELGNPGSLIGDDQIYNTIVTAHAFIMIFFMVMPIMIGGFGNWLVPLMLGAPDMAFPRMNNMSFWLLPPSLILLISSSIVENGAGTGWTVYPPLSSNIAHSGSSVDLAIFSLHLAGISSILGAINFITTIINMRLNNMFFD
QLPLFVWAVGITALLLLLSLPVLAGAITMLLTDRNINTSFFDPAGGGDPILYQHL
>LEFIJ310-10|JF853545|MM15910|Lycaena_dispar
TLYFIFGIWAGMVGTSLSILIRLELGNPGSLIGDDQIYNTIVTAHAFIMIFFMVMPIMIGGFGNWLVPLMLGAPDMAFPRMNNMSFWLLPPSLLLLISSSIVENGAGTGWTVYPPLSSNIAHSGSSVDLAIFSLHLAGISSILGAINFITTIINMRINNLSFDQ
MSLFIWSVGITALLLLLSLPVLAGAITMLLTDRNLNTSFFDPAGGGDPILYQHL
>LEFIF121-10|HM874822|MM10577|Lycaena_helle
TLYFIFGIWAGMVGTSLSILIRLELGTPGSLIGDDQIYNTIVTAHAFIMIFFMVMPIMIGGFGNWLVPLMLGAPDMAFPRMNNMSFWLLPPSLLLLISSSIVENGAGTGWTVYPPLSSNIAHSGSSVDLAIFSLHLAGISSILGAINFITTIINMRINNLSFDQ
MSLFIWAVGITALLLLLSLPVLAGAITMLLTDRNLNTSFFDPAGGGDPILYQHL
>LEFID354-10|HM873151|MM06257|Lycaena_hippothoe
TLYFIFGIWAGMIGTSLSILIRLELGTSGSLIGDDQIYNTIVTAHAFIMIFFMVMPIMIGGFGNWLVPLMLGAPDMAFPRMNNMSFWLLPPSLLLLISSSIVENGAGTGWTVYPPLSSNIAHSGSSVDLAIFSLHLAGISSILGAINFITTIINMRINNLSFDQM
SLFIWAVGITALLLLLSLPVLAGAITMLLTDRNLNTSFFDPAGGGDPILYQHL
>LEFIC267-10|HM872111|MM03698|Lycaena_phlaeas
TLYFIFGIWAGMVGTSLSILIRLELGTPGSLIGDDQIYNTIVTAHAFIMIFFMVMPIMIGGFGNWLVPLMLGAPDMAFPRMNNMSFWLLPPSLLLLISSSIVENGAGTGWTVYPPLSSNIAHSGSSVDLAIFSLHLAGISSILGAINFITTIINMRINNLSFDQ
MSLFIWAVGITALLLLLSLPVLAGAITMLLTDRNLNTSFFDPAGGGDPILYQHL
>LEFID122-10|HM872932|MM05858|Lycaena_tityrus
TLYFIFGIWAGMIGTSLSILIRLELGTPGSLIGDDQIYNTIVTAHAFIMIFFMVMPIMIGGFGNWLIPLMLGAPDMAFPRMNNMSFWLLPPSLLLLISSSIVENGAGTGWTVYPPLSSNIAHSGSSVDLAIFSLHLAGISSILGAINFITTIINMRINNLSFDQM
SLFIWAVGITALLLLLSLPVLAGAITMLLTDRNLNTSFFDPAGGGDPILYQHL
>LEFIG533-10|HM876206|MM14659|Lycaena_virgaureae
TLYFIFGIWAGMIGTSLSILIRLELGTPGSLIGDDQIYNTIVTAHAFIMIFFMVMPIMIGGFGNWLVPLMLGAPDMAFPRMNNMSFWLLPPSLLLLISSSIVENGAGTGWTVYPPLSSNMAHSGPSVDLAIFSLHLAGISSILGAINFITTIINMRINNLSFDQ
MSLFIWAVGITALLLLLSLPVLAGAITMLLTDRNLNTSFFDPAGGGDPILYQHL
>LEFIJ496-10|JF853620|MM17121|Lycaena_virgaureae
TLYFIFGIWAGMIGTSLSILIRLELGTPGSLIGDDQIYNTIVTAHAFIMIFFMVMPIMIGGFGNWLVPLMLGAPDMAFPRMNNMSFWLLPPSLLLLISSSIVENGAGTGWTVYPPLSSNMAHSGPSVDLAIFSLHLAGISSILGAINFITTIINMRINNLSFDQ
MSLFIWAVGITALLLLLSLPVLAGAITMLLTDRNLNTSFFDPAGGGDPILYQHL
>LEFIA421-10|HM386763|MM01483|Lycia_hirtaria
TLYFIFGFWAGMVGTSLSLLIRAELGNPGSLIGDDQIYNTIVTAHAFIMIFFMVMPIMIGGFGNWLVPLMLGAPDMAFPRMNNMSFWLLPPSITLLISSSIVENGAGTGWTVYPPLSSNIAHGGSSVDLAIFSLHLAGISSILGAINFITTIINMRLNNLSFD
QMPLFVWAVGITAFLLLLSLPVLAGAITMLLTDRNLNTSFFDPAGGGDPILYQHL
>LEFIA422-10|HM386764|MM01484|Lycia_hirtaria
TLYFIFGIWAGMVGTSLSLLIRAELGNPGSLIGDDQIYNTIVTAHAFIMIFFMVMPIMIGGFGNWLVPLMLGAPDMAFPRMNNMSFWLLPPSITLLISSSIVENGAGTGWTVYPPLSSNIAHGGSSVDLAIFSLHLAGISSILGAINFITTIINMRLNNLSFD
QMPLFVWAVGITAFLLLLSLPVLAGAITMLLTDRNLNTSFFDPAGGGDPILYQHL
>LEFIB840-10|HM871717|MM02807|Lycia_hirtaria
TLYFIFGIWAGMVGTSLSLLIRAELGNPGSLIGDDQIYNTIVTAHAFIMIFFMVMPIMIGGFGNWLVPLMLGAPDMAFPRMNNMSFWLLPPSITLLISSSIVENGAGTGWTVYPPLSSNIAHGGSSVDLAIFSLHLAGISSILGAINFITTIINMRLNNLSFD
QMPLFVWAVGITAFLLLLSLPVLAGAITMLLTDRNLNTSFFDPAGGGDPILYQHL
>LEFIB086-10|HM870994|MM00388|Lycia_lapponaria
TLYFIFGIWAGMVGTSLSLLIRAELGNPGSLIGDDQIYNTIVTAHAFIMIFFMVMPIMIGGFGNWLVPLMLGAPDMAFPRMNNMSFWLLPPSITLLISSSIVENGAGTGWTVYPPLSSNIAHGGSSVDLAIFSLHLAGISSILGAINFITTIINMRLNNMSFD
QMPLFVWAVGITAFLLLLSLPVLAGAITMLLTDRNLNTSFFDPAGGGDPILYQHL
>LEFIG974-10|HM876614|MM15838|Lycia_lapponaria
TLYFIFGIWAGMVGTSLSLLIRAELGNPGSLIGDDQIYNTIVTAHAFIMIFFMVMPIMIGGFGNWLVPLMLGAPDMAFPRMNNMSFWLLPPSITLLISSSIVENGAGTGWTVYPPLSSNIAHGGSSVDLAIFSLHLAGISSILGAINFITTIINMRLNNMSFD
QMPLFVWAVGITAFLLLLSLPVLAGAITMLLTDRNLNTSFFDPAGGGDPILYQHL
>LEFIK939-10|JF854379|MM18514|Lycia_lapponaria
TLYFIFGIWAGMVGTSLSLLIRAELGNPGSLIGDDQIYNTIVTAHAFIMIFFMVMPIMIGGFGNWLVPLMLGAPDMAFPRMNNMSFWLLPPSITLLISSSIVENGAGTGWTVYPPLSSNIAHGGSSVDLAIFSLHLAGISSILGAINFITTIINMRLNNMSFD
QMPLFVWAVGITAFLLLLSLPVLAGAITMLLTDRNLNTSFFDPAGGGDPILYQHL
>LEFIJ025-10|MM00463|Lycia_pomonaria
TLYFIFGIWAGMVGTSLSLLIRAELGNPGSLIGDDQIYNTIVTAHAFIMIFFMVMPIMIGGFGNWLVPLMLGAPDMAFPRMNNMSFWLLPPSITLLISSSIVENGAGTGWTVYPPLSSNIAHGGSSVDLAIFSLHLAGISSILGAINFITTIINMRLNNMSFD
QMPLFVWAVGITAFLLLLSLPVLAGAITMLLTDRNLNTSFFDPAGGGDPILYQHL
>LEFIA232-10|HM396575|MM01264|Lycia_pomonaria
TLYFIFGIWAGMVGTSLSLLIRAELGNPGSLIGDDQIYNTIVTAHAFIMIFFMVMPIMIGGFGNWLVPLMLGAPDMAFPRMNNMSFWLLPPSITLLISSSIVENGAGTGWTVYPPLSSNIAHGGSSVDLAIFSLHLAGISSILGAINFITTIINMRLNNMSFD
QMPLFVWAVGITAFLLLLSLPVLAGAITMLLTDRNLNTSFFDPAGGGDPILYQHL

>LEFIE112-10|HM873860|MM08301|Lycia_pomonaria
TLYFIFGIWAGMVGTSLSLLIRAELGNPGSLIGDDQIYNTIVTAHAFIMIFFMVMPIMIGGFGNWLVPLMLGAPDMAFPRMNNMSFWLLPPSITLLISSSIVENGAGTGWTVYPPLSSNIAHGGSSVDLTIFSLHLAGISSILGAINFITTIINMRLNNMSFD
QMPLFVWAVGITAFLLLLSLPVLAGAITMLLTDRNLNTSFFDPAGGGDPILYQHL
>LEFIB822-10|HM871699|MM02760|Lycophotia_porphyrea
TLYFIFGIWAGMVGTSLSLLIRAELGNPGSLIGDDQIYNTIVTAHAFIMIFFMVMPIMIGGFGNWLVPLMLGAPDMAFPRMNNMSFWLLPPSLTLLISSSIVENGAGTGWTVYPPLSSNIAHGGSSVDLAIFSLHLAGISSILGAINFITTIINMRLNSLSFD
QMPLFIWAVGITAFLLLLSLPVLAGAITMLLTDRNLNTSFFDPAGGGDPILYQHL
>LEFIC877-10|HM872695|MM05151|Lycophotia_porphyrea
TLYFIFGIWAGMVGTSLSLLIRAELGNPGSLIGDDQIYNTIVTAHAFIMIFFMVMPIMIGGFGNWLVPLMLGAPDMAFPRMNNMSFWLLPPSLTLLISSSIVENGAGTGWTVYPPLSSNIAHGGSSVDLAIFSLHLAGISSILGAINFITTIINMRLNSLSFD
QMPLFIWAVGITAFLLLLSLPVLAGAITMLLTDRNLNTSFFDPAGGGDPILYQHL
>LEFIF623-10|HM875307|MM12512|Lygephila_craccae
TLYFIFGIWAGMVGTSLSLLIRAELGNPGSLIGDDQIYNTIVTAHAFIMIFFMVMPIMIGGFGNWLVPLMLGAPDMAFPRMNNMSFWLLPPSLTLLISSSIVENGAGTGWTVYPPLSSNIAHGGSSVDLAIFSLHLAGISSILGAINFITTIINMRLNSLSFD
QMPLFVWAVGITAFLLLLSLPVLAGAITMLLTDRNLNTSFFDPAGGGDPILYQHL
>LEFIA152-10|HM396498|MM01121|Lygephila_pastinum
TLYFIFGIWAGMVGTSLSLLIRAELGTPGSLIGDDQIYNTIVTAHAFIMIFFMVMPIMIGGFGNWLVPLMLGAPDMAFPRMNNMSFWLLPPSLTLLISSSIVENGAGTGWTVYPPLSSNIAHSGSSVDLAIFSLHLAGISSILGAINFITTIINMRLNNLSFD
QMPLFVWAVGITAFLLLLSLPVLAGAITMLLTDRNLNTSFFDPAGGGDPILYQHL
>LEFIJ331-10|KM573214|MM15931|Lygephila_viciae
TLYFIFGIWAGMVGTSLSLLIRAELGNPGSLIGDDQIYNTIVTAHAFIMIFFMVMPIMIGGFGNWLVPLMLGAPDMAFPRMNNMSFWLLPPSLTLLISSSIVESGAGTGWTVYPPLSSNIAHGGSSVDLAIFSLHLAGISSILGAINFITTIINMRLNNLSFD
QMPLFIWAVGITAFLLLLSLPVLAGAITMLLTDRNLNTSFFDPAGGGDPILYQHL
>LEFIA122-10|HM396469|MM01050|Lymantria_monacha
TLYFIFGIWSGMVGTSLSLLIRAELGNPGSLIGNDQIYNTIVTAHAFIMIFFMVMPIMIGGFGNWLVPLMLGAPDMAFPRMNNMSFWLLPPSLTLLISSSIVENGAGTGWTVYPPLSSNIAHGGSSVDLAIFSLHLAGISSILGAINFITTIINMRLTNLSFD
QMPLFIWAVGITAFLLLLSLPVLAGAITMLLTDRNLNTSFFDPAGGGDPILYQHL
>LEFIC242-10|HM872086|MM03651|Lymantria_monacha
TLYFIFGIWSGMVGTSLSLLIRAELGNPGSLIGNDQIYNTIVTAHAFIMIFFMVMPIMIGGFGNWLVPLMLGAPDMAFPRMNNMSFWLLPPSLTLLISSSIVENGAGTGWTVYPPLSSNIAHGGSSVDLAIFSLHLAGISSILGAINFITTIINMRLTNLSFD
QMPLFIWAVGITAFLLLLSLPVLAGAITMLLTDRNLNTSFFDPAGGGDPILYQHL
>LEFIB374-10|HM871273|MM00946|Lyonetia_clerkella
ILYFIFGIWAGLIGTSLSLLIRAELGNPGSLIGDDQIYNTIVTAHAFIMIFFMVMPILIGGFGNWLIPLMLGAPDMAFPRMNNMSFWLLPPSLTLLISSSMVENGAGTGWTVYPPLSSNIAHGGSSVDLAIFSLHLAGISSILGAINFITTIINMRSNGMTFDR
MPLFVWAVGITALLLLLSLPVLAGAITMLLTDRNLNTSFFDPAGGGDPILYQHL
>LEFIB761-10|HM871638|MM02621|Lyonetia_clerkella
ILYFIFGIWAGLIGTSLSLLIRAELGNPGSLIGDDQIYNTIVTAHAFIMIFFMVMPILIGGFGNWLVPLMLGAPDMAFPRMNNMSFWLLPPSLTLLISSSMVENGAGTGWTVYPPLSSNIAHGGSSVDLAIFSLHLAGISSILGAINFITTIINMRSNGMTFDR
MPLFVWAVGITALLLLLSLPVLAGAITMLLTDRNLNTSFFDPAGGGDPILYQHL
>LEFIG084-10|HM875763|MM13739|Lyonetia_clerkella
ILYFIFGIWAGLIGTSLSLLIRAELGNPGSLIGDDQIYNTIVTAHAFIMIFFMVMPILIGGFGNWLIPLMLGAPDMAFPRMNNMSFWLLPPSLTLLISSSMVENGAGTGWTVYPPLSSNIAHGGSSVDLAIFSLHLAGISSILGAINFITTIINMRSNGMTFDR
MPLFVWAVGITALLLLLSLPVLAGAITMLLTDRNLNTSFFDPAGGGDPILYQHL
>LEFIJ1392-12|KM573505|MM22063|Lyonetia_clerkella
ILYFIFGIWAGLIGTSLSLLIRAELGNPGSLIGDDQIYNTIVTAHAFIMIFFMVMPILIGGFGNWLVPLMLGAPDMAFPRMNNMSFWLLPPSLTLLISSSMVENGAGTGWTVYPPLSSNIAHGGSSVDLAIFSLHLAGISSILGAINFITTIINMRSNGMTFDR
MPLFVWAVGITALLLLLSLPVLAGAITMLLTDRNLNTSFFDPAGGGDPILYQHL
>LEFID465-10|HM873233|MM06406|Lyonetia_ledi
ILYFIFGIWSGMVGTSLSLLIRAELGNPGSLIGDDQIYNTIVTAHAFIMIFFMVMPILIGGFGNWLVPLMLGAPDMAFPRMNNMSFWLLPPSLTLLISSSIVENGAGTGWTVYPPLSSNIAHGGSSVDLAIFSLHLAGISSILGAINFITTIINMRSDNMTFDR
MPLFVWAVGITALLLLLSLPVLAGAITMLLTDRNLNTSFFDPAGGGDPILYQHL
>LEFID740-10|HM873497|MM06799|Lyonetia_prunifoliella
ILYFIFGIWAGMVGTSLSLLIRAELGNPGSLIGDDQIYNTIVTAHAFIMIFFMVMPILIGGFGNWLVPLMLGAPDMAFPRMNNMSFWLLPPSLTLLISSSIVENGAGTGWTVYPPLSSNIAHGGSSVDLAIFSLHLAGISSILGAINFITTIINMRSDSMSFDR
MPLFVWAVGITALLLLLSLPVLAGAITMLLTDRNLNTSFFDPAGGGDPILYQHL
>LEFIA851-10|HM386991|MM09728|Lyonetia_prunifoliella
ILYFIFGIWAGMVGTSLSLLIRAELGNPGSLIGDDQIYNTIVTAHAFIMIFFMVMPILIGGFGNWLVPLMLGAPDMAFPRMNNMSFWLLPPSLTLLISSSIVENGAGTGWTVYPPLSSNIAHGGSSVDLAIFSLHLAGISSILGAINFITTIINMRSDSMSFDR
MPLFVWAVGITALLLLLSLPVLAGAITMLLTDRNLNTSFFDPAGGGDPILYQHL
>LEFIL391-10|MM16831|Lyonetia_prunifoliella
ILYFIFGIWSGMVGTSLSLLIRAELGNPGSLIGDDQIYNTIVTAHAFIMIFFMVMPILIGGFGNWLVPLMLGAPDMAFPRMNNMSFWLLPPSLTLLISSSIVETGAGTGWTVYPPLSSNIAHSGSSVDLAIFSLHLAGISSILGAINFITTIINMRSNGMSFDR

MPLFVWAVGITALLLLLSLPVLAGAITMLLTDRNLNTSFFDPAGGGDPILYQHL
>LEFIL392-10|MM16832|Lyonetia_prunifoliella
ILYFIFGIWSGMVGTSLSLLIRAELGNPGSLIGDDQIYNTIVTAHAFIMIFFMVMPILIGGFGNWLVPLMLGAPDMAFPRMNNMSFWLLPPSLTLLISSSIVETGAGTGWTVYPPLSSNIAHSGSSVDLAIFSLHLAGISSILGAINFITTIINMRSNGMSFDR
MPLFVWAVGITALLLLLSLPVLAGAITMLLTDRNLNTSFFDPAGGGDPILYQHL
>LEFIJ967-11|MM21207|Lyonetia_prunifoliella
ILYFIFGIWSGMVGTSLSLLIRAELGNPGSLIGDDQIYNTIVTAHAFIMIFFMVMPILIGGFGNWLVPLMLGAPDMAFPRMNNMSFWLLPPSLTLLISSSIVETGAGTGWTVYPPLSSNIAHSGSSVDLAIFSLHLAGISSILGAINFITTIINMRSNGMSFDR
MPLFVWAVGITALLLLLSLPVLAGAITMLLTDRNLNTSFFDPAGGGDPILYQHL
>LEFIG687-10|HM876344|MM15551|Lyonetia_pulverulentella
ILYFIFGIWSGMVGTSLSLLIRAELGNPGSLIGDDQIYNTIVTAHAFIMIFFMVMPILIGGFGNWLVPLMLGAPDMAFPRMNNMSFWLLPPSLTLLISSSIVENGAGTGWTVYPPLSSNIAHSGSSVDLAIFSLHLAGISSILGAINFITTIINMRSNNMSFDR
MPLFVWAVGITALLLLLSLPVLAGAITMLLTDRNLNTSFFDPAGGGDPILYQHL
>LEFIK553-10|KF808523|MM18128|Lyonetia_pulverulentella
ILYFIFGIWSGMVGTSLSLLIRAELGNPGSLIGDDQIYNTIVTAHAFIMIFFMVMPILIGGFGNWLVPLMLGAPDMAFPRMNNMSFWLLPPSLTLLISSSIVENGAGTGWTVYPPLSSNIAHSGSSVDLAIFSLHLAGISSILGAINFITTIINMRSNNMSFDR
MPLFVWAVGITALLLLLSLPVLAGAITMLLTDRNLNTSFFDPAGGGDPILYQHL
>LEFIC491-10|HQ570314|MM04195|Lypusa_maurella
TLYFIFGIWAGMVGTSLSLLIRAELGTPGSLIGDDQIYNTIVTAHAFIMIFFMVMPIMIGGFGNWLVPLMLGAPDMAFPRMNNMSFWLLPPSLTLLISSSIVENGAGTGWTVYPPLSSNIAHSGSSVDLAIFSLHLAGISSILGAINFITTIINMRMNNMFF
DQMPLFVWSVGITALLLLLSLPVLAGAITMLLTDRNLNTSFFDPAGGGDPILYQHL
>LEFIG179-10|HM875859|MM14046|Lypusa_maurella
TLYFIFGIWAGMVGTSLSLLIRAELGTPGSLIGDDQIYNTIVTAHAFIMIFFMVMPIMIGGFGNWLIPLMLGAPDMAFPRMNNMSFWLLPPSLTLLISSSIVENGAGTGWTVYPPLSSNIAHSGSSVDLAIFSLHLAGISSILGAINFITTIINMRMNNMFFD
QMPLFVWSVGITALLLLLSLPVLAGAITMLLTDRNLNTSFFDPAGGGDPILYQHL
>LEFIK842-10|JN279402|MM18417|Lythria_cruentaria
TLYFIFGIWAGMIGTSLSLLIRAELGNPGHLIGDDQIYNTIVTAHAFIMIFFMVMPIMIGGFGNWLVPLMLGAPDMAFPRMNNMSFWMLPPSITLLISSSIVENGAGTGWTVYPPLSSNIAHSGSSVDLAIFSLHLAGISSILGAINFITTIINMRLNNMSF
DQLPLFVWAVGITAFLLLLSLPVLAGAITMLLTDRNLNTSFFDPAGGGDPILYQHL
>LEFIA290-10|HM386633|MM01334|Macaria_alternata
TLYFIFGIWAGMVGTSLSLLIRAELGNPGSLIGDDQIYNTIVTAHAFIMIFFMVMPIMIGGFGNWLVPLMLGAPDMAFPRMNNMSFWLLPPSITLLISSSIVENGAGTGWTVYPPLSSNIAHGGSSVDLAIFSLHLAGISSILGAINFITTIINMRLNNLSFD
QMPLFVWAVGITAFLLLLSLPVLAGAITMLLTDRNLNTSFFDPAGGGDPILYQHL
>LEFID246-10|HM873045|MM06110|Macaria_artesiaria
TLYFIFGIWAGMVGTSLSLLIRAELGNPGSLIGDDQIYNTIVTAHAFIMIFFMVMPIMIGGFGNWLVPLMLGAPDMAFPRMNNMSFWLLPPSITLLISSSIVENGAGTGWTVYPPLSSNIAHGGSSVDLAIFSLHLAGISSILGAINFITTIINMRLNNLSFD
QMPLFVWAVGITAFLLLLSLPVLAGAITMLLTDRNLNTSFFDPAGGGDPILYQHL
>LEFIF046-10|HM874757|MM10486|Macaria_artesiaria
TLYFIFGIWAGMVGTSLSLLIRAELGNPGSLIGDDQIYNTIVTAHAFIMIFFMVMPIMIGGFGNWLVPLMLGAPDMAFPRMNNMSFWLLPPSITLLISSSIVENGAGTGWTVYPPLSSNIAHGGSSVDLAIFSLHLAGISSILGAINFITTIINMRLNNLSFD
QMPLFVWAVGITAFLLLLSLPVLAGAITMLLTDRNLNTSFFDPAGGGD?ILYQHL
>LEFIA396-10|HM386738|MM01455|Macaria_brunneata
TLYFIFGIWAGMLGTSLSLLIRAELGNPGSLIGDDQIYNTIVTAHAFIMIFFMVMPIMIGGFGNWLVPLMLGAPDMAFPRMNNMSFWLLPPSITLLISSSIVENGSGTGWTVYPPLSSNIAHGGSSVDLAIFSLHLAGISSILGAINFITTIINMRLNNLSFDQ
MPLFVWAVGITAFLLLLSLPVLAGAITMLLTDRNLNTSFFDPAGGGDPILYQHL
>LEFID480-10|HM873246|MM06422|Macaria_carbonaria
TLYFIFGIWAGMVGTSLSLLIRAELGNPGSLIGDDQIYNTIVTAHAFIMIFFMVMPIMIGGFGNWLVPLMLGAPDMAFPRMNNMSFWLLPPSITLLISSSIVENGAGTGWTVYPPLSSNIAHGGSSVDLAIFSLHLAGISSILGAINFITTIINMRLNNLSFD
QMPLFVWAVGITAFLLLLSLPVLAGAITMLLTDRNLNTSFFDPAGGGDPILYQHL
>LEFIE241-10|HM873975|MM08562|Macaria_carbonaria
TLYFIFGIWAGMVGTSLSLLIRAELGNPGSLIGDDQIYNTIVTAHAFIMIFFMVMPIMIGGFGNWLVPLMLGAPDMAFPRMNNMSFWLLPPSITLLISSSIVENGAGTGWTVYPPLSSNIAHGGSSVDLAIFSLHLAGISSILGAINFITTIINMRLNNLSFD
QMPLFVWAVGITAFLLLLSLPVLAGAITMLLTDRNLNTSFFDPAGGGDPILYQHL
>LEFIG116-10|HM875796|MM13912|Macaria_carbonaria
TLYFIFGIWAGMVGTSLSLLIRAELGNPGSLIGDDQIYNTIVTAHAFIMIFFMVMPIMIGGFGNWLVPLMLGAPDMAFPRMNNMSFWLLPPSITLLISSSIVENGAGTGWTVYPPLSSNIAHGGSSVDLAIFSLHLAGISSILGAINFITTIINMRLNNLSFD
QMPLFVWAVGITAFLLLLSLPVLAGAITMLLTDRNLNTSFFDPAEEGDPILYQHL
>LEFIC104-10|HQ570295|MM03420|Macaria_fusca
TLYFIFGIWAGMMGTSLSLLIRAELGNPGSLIGDDQIYNTIVTAHAFIMIFFMVMPIMIGGFGNWLVPLMLGAPDMAFPRMNNMSFWLLPPSITLLISSSIVENGAGTGWTVYPPLSSNIAHGGSSVDLAIFSLHLAGISSILGAINFITTIINMRLNNLSFD
QMPLFVWAVGITAFLLLLSLPVLAGAITMLLTDRNLNTSFFDPAGGGDPILYQHL
>LEFIG503-10|HM876177|MM14587|Macaria_fusca

TLYFIFGIWAGMMGTSLSLLIRAELGNPGSLIGDDQIYNTIVTAHAFIMIFFMVMPIMIGGFGNWLVPLMLGAPDMAFPRMNNMSFWLLPPSITLLISSSIVENGAGTGWTVYPPLSSNIAHGGSSVDLAIFSLHLAGISSILGAINFITTIINMRLNNLSFD
QMPLFVWAVGITAFLLLLSLPVLAGAITMLLTDRNLNTSFFDPAGGGDPILYQHL
>LEFIA419-10|HM386761|MM01481|Macaria_liturata
TLYFIFGIWAGMVGTSLSLLIRAELGNPGSLIGDDQIYNTIVTAHAFIMIFFMVMPIMIGGFGNWLVPLMLGAPDMAFPRMNNMSFWLLPPSITLLISSSIVENGAGTGWTVYPPLSSNIAHGGSSVDLAIFSLHLAGISSILGAINFITTIINMRLNNLSFD
QMPLFVWAVGITAFLLLLSLPVLAGAITMLLTDRNLNTSFFDPAGGGDPILYQHL
>LEFIC751-10|HM872570|MM04865|Macaria_liturata
TLYFIFGIWAGMVGTSLSLLIRAELGNPGSLIGDDQIYNTIVTAHAFIMIFFMVMPIMIGGFGNWLVPLMLGAPDMAFPRMNNMSFWLLPPSITLLISSSIVENGAGTGWTVYPPLSSNIAHGGSSVDLAIFSLHLAGISSILGAINFITTIINMRLNNLSFD
QMPLFVWAVGITAFLLLLSLPVLAGAITMLLTDRNLNTSFFDPAGGGDPILYQHL
>LEFIE020-10|HM873769|MM08077|Macaria_loricaria
TLYFIFGIWAGMVGTSLSLLIRAELGNPGSLIGDDQIYNTIVTAHAFIMIFFMVMPIMIGGFGNWLVPLMLGAPDMAFPRMNNMSFWLLPPSITLLISSSIVENGAGTGWTVYPPLSSNIAHGGSSVDLAIFSLHLAGISSILGAINFITTIINMRLNNLSFD
QMPLFVWAVGITAFLLLLSLPVLAGAITMLLTDRNLNTSFFDPAGGGDPILYQHL
>LEFIB175-10|HM871079|MM00582|Macaria_notata
TLYFIFGIWAGMVGTSLSLLIRAELGNPGSLIGDDQIYNTIVTAHAFIMIFFMVMPIMIGGFGNWLVPLMLGAPDMAFPRMNNMSFWLLPPSITLLISSSIVESGAGTGWTVYPPLSSNIAHGGSSVDLAIFSLHLAGISSILGAINFITTIINMRLNNLSFD
QMPLFIWAVGITAFLLLLSLPVLAGAITMLLTDRNLNTSFFDPAGGGDPILYQHL
>LEFID202-10|HM873005|MM06029|Macaria_signaria
TLYFIFGIWAGMVGTSLSLLIRAELGNPGSLIGDDQIYNTIVTAHAFIMIFFMVMPIMIGGFGNWLVPLMLGAPDMAFPRMNNMSFWLLPPSITLLISSSIVENGAGTGWTVYPPLSSNIAHGGSSVDLAIFSLHLAGISSILGAINFITTIINMRLNNLSFD
QMPLFVWAVGITAFLLLLSLPVLAGAITMLLTDRNLNTSFFDPAGGGDPILYQHL
>LEFIB856-10|HM871733|MM02870|Macaria_wauaria
TLYFIFGIWAGMVGTSLSLLIRAELGNPGSLIGDDQIYNTIVTAHAFIMIFFMVMPIMIGGFGNWLVPLMLGAPDMAFPRMNNMSFWLLPPSITLLISSSIVENGAGTGWTVYPPLSSNIAHGGSSVDLAIFSLHLAGISSILGAINFITTIINMRLNNLSFD
QMPLFVWAVGITAFLLLLSLPVLAGAITMLLTDRNLNTSFFDPAGGGDPILYQHL
>LEFIF537-10|HM875222|MM12215|Macdunnoughia_confusa
TLYFIFGIWAGMVGTSLSLLIRAELGTPGSLIGDDQIYNTIVTAHAFIMIFFMVMPIMIGGFGNWLVPLMLGAPDMAFPRMNNMSFWLLPPSLTLLISSSIVENGAGTGWTVYPPLSSNIAHGGSSVDLAIFSLHLAGISSILGAINFITTIINMRLNSLSFD
QMPLFIWAVGITAFLLLLSLPVLAGAITMLLTDRNLNTSFFDPAGGGDPILYQHL
>LEFIF675-10|HM875359|MM12657|Macdunnoughia_confusa
TLYFIFGIWAGMVGTSLSLLIRAELGTPGSLIGDDQIYNTIVTAHAFIMIFFMVMPIMIGGFGNWLVPLMLGAPDMAFPRMNNMSFWLLPPSLTLLISSSIVENGAGTGWTVYPPLSSNIAHGGSSVDLAIFSLHLAGISSILGAINFITTIINMRLNSLSFD
QMPLFIWAVGITAFLLLLSLPVLAGAITMLLTDRNLNTSFFDPAGGGDPILYQHL
>LEFIC765-10|HM872584|MM04888|Macrochilo_cribrumalis
TLYFIFGIWAGMVGTSLSLLIRAELGNPGSLIGDDQIYNTIVTAHAFIMIFFMVMPIMIGGFGNWLVPLMLGAPDMAFPRMNNMSFWLLPPSLTLLISSSIVENGAGTGWTVYPPLSSNIAHSGSSVDLAIFSLHLAGISSILGAINFITTIINMRLNNLSFD
QMPLFVWAVGITAFLLLLSLPVLAGAITMLLTDRNLNTSFFDPAGGGDPILYQHL
>LEFIB172-10|HM871076|MM00578|Macroglossum_stellatarum
TLYFIFGIWAGMVGTSLSLLIRAELGTPGSLIGDDQIYNTIVTAHAFIMIFFMVMPIMIGGFGNWLVPLMLGAPDMAFPRMNNMSFWLLPPSLTLLISSSIVENGAGTGWTVYPPLSSNIAHSGSSVDLAIFSLHLAGISSIMGAVNFITTIINMRINNLSFD
QMPLFVWAVGITAFLLLLSLPVLAGAITMLLTDRNLNTSFFDPAGGGDPILYQHL
>LEFIA128-10|HM396475|MM01059|Macrothylacia_rubi
TLYFIFGIWAGMVGTSLSLLIRAELGTPGSLIGDDQIYNTIVTAHAFIMIFFMVMPIMIGGFGNWLVPLMLGAPDMAFPRMNNMSFWLLPPSLTLLISSSIVENGAGTGWTVYPPLSSNIAHSGSSVDLAIFSLHLAGISSILGAINFITTIINMRLNNMSFD
QMPLFVWAVGITAFLLLLSLPVLAGAITMLLTDRNLNTSFFDPAGGGDPILYQHL
>LEFIC990-10|HM872805|MM05401|Malacodea_regelaria
TLYFIFGIWAGMIGTSLSLLIRAELGNPGSLIGDDQIYNTIVTAHAFIMIFFMVMPIMIGGFGNWLVPLMLGAPDMAFPRMNNMSFWMLPPSIMLLISSSIVENGAGTGWTVYPPLSSNIAHSGSSVDLAIFSLHLAGISSILGAINFITTIINMRLNNMYF
DQLPLFVWAVGITAFLLLLSLPVLAGAITMLLTDRNLNTSFFDPAGGGDPILYQHL
>LEFIC245-10|HM872089|MM03655|Malacosoma_castrensis
TLYFIFGIWAGMVGTSLSLLIRAELGTPGSLIGDDQIYNTIVTAHAFIMIFFMVMPIMIGGFGNWLVPLMLGTPDMAFPRMNNMSFWLLPPSLTLLISSSMVENGAGTGWTVYPPLSSNIAHGGSSVDLAIFSLHLAGISSILGAINFITTIINMRLNKMSF
DQMPLFVWAVGITAFLLLLSLPVLAGAITMLLTDRNLNTSFFDPAGGGDPILYQHL
>LEFIJ592-10|JF853692|MM17217|Malacosoma_neustria
TLYFIFGIWAGMVGTSLSLLIRAELGTPGSLIGDDQIYNTIVTAHAFIMIFFMVMPIMIGGFGNWLVPLMLGAPDMAFPRMNNMSFWLLPPSLTLLISSSMVENGAGTGWTVYPPLSSNIAHGGSSVDLAIFSLHLAGISSILGAINFITTIINMRLNKMSF
DQMPLFVWSVGITAFLLLLSLPVLAGAITMLLTDRNLNTSFFDPAGGGDPILYQHL
>LEFIC778-10|HM872597|MM04910|Mamestra_brassicae
TLYFIFGIWAGMVGTSLSLLIRAELGNPGSLIGDDQIYNTIVTAHAFIMIFFMVMPIMIGGFGNWLVPLMLGAPDMAFPRMNNMSFWLLPPSLTLLISSSIVENGAGTGWTVYPPLSSNIAHGGSSVDLAIFSLHLAGISSILGAINFITTIINMRLNSLSFD
QMPLFIWAVGITAFLLLLSLPVLAGAITMLLTDRNLNTSFFDPAGGGDPILYQHL

>LEFID787-10|HM873544|MM06856|Maniola_jurtina
TLYFIFGIWAGMVGTSLSLIIRMELGNPGSLIGNDQIYNTIVTAHAFIMIFFMVMPIMIGGFGNWLVPLMLGAPDMAFPRMNNMSFWLLPPSLMLLISSSIVENGAGTGWTVYPPLSSNIAHSGSSVDLAIFSLHLAGISSILGAINFITTIINMRINNMSY
DQMPLFVWAVGITALLLLLSLPVLAGAITMLLTDRNLNTSFFDPAGGGDPILYQHL
>LEFID814-10|HM873571|MM06901|Maniola_jurtina
TLYFIFGIWAGMVGTSLSLIIRTELGNPGSLIGDDQIYNTIVTAHAFIMIFFMVMPIMIGGFGNWLVPLMLGAPDMAFPRMNNMSFWLLPPSLVLLISSSIVENGAGTGWTVYPPLSSNIAHGGSSVDLAIFSLHLAGISSILGAINFITTIINMRINNMSYD
QMPLFVWAVGITALLLLLSLPVLAGAITMLLTDRNLNTSFFDPAGGGDPILYQHL
>LEFID815-10|HM873572|MM06902|Maniola_jurtina
TLYFIFGIWAGMVGTSLSLIIRTELGNPGSLIGDDQIYNTIVTAHAFIMIFFMVMPIMIGGFGNWLVPLMLGAPDMAFPRMNNMSFWLLPPSLILLISSSIVENGAGTGWTVYPPLSSNIAHGGSSVDLAIFSLHLAGISSILGAINFITTIINMRINNMSYD
QMPLFVWAVGITALLLLLSLPVLAGAITMLLTDRNLNTSFFDPAGGGDPILYQHL
>LEFIF269-10|HM874962|MM11051|Marasmarcha_lunaedactyla
TLYFIFGIWAGMIGTSLSLLIRAELGMPGMLIGNDQIYNSIVTAHAFIMIFFMVMPIMIGGFGNWLVPLMLGAPDMAFPRMNNMSFWLLPPSIILLISSSMVENGAGTGWTVYPPLSANIAHAGPSVDLAIFSLHLAGISSILGAINFISTIINMRLNKMM
FDQLPLFVWAVGITALLLLLSLPVLAGAITMLLTDRNLNTSFFDPAGGGDPILYQHL
>LEFIA391-10|HM386733|MM01448|Martania_taeniata
TLYFIFGIWASMIGTSLSLLIRAELGTPGSLIGDDQIYNTIVTAHAFIMIFFMVMPIMIGGFGNWLVPLMLGAPDMAFPRMNNMSFWLLPPSITLLISSSIVESGAGTGWTVYPPLSSNIAHGGSSVDLAIFSLHLAGISSILGAINFITTIINMRLNNMFFDQ
LPLFVWAVGITAFLLLLSLPVLAGAITMLLTDRNLNTSFFDPAGGGDPILYQHL
>LEFIE180-10|HM873926|MM08468|Martania_taeniata
TLYFIFGIWAGMIGTSLSLLIRAELGTPGSLIGDDQIYNTIVTAHAFIMIFFMVMPIMIGGFGNWLVPLMLGAPDMAFPRMNNMSFWLLPPSITLLISSSIVESGAGTGWTVYPPLSSNIAHGGSSVDLAIFSLHLAGISSILGAINFITTIINMRLNNMFFDQ
LPLFVWAVGITAFLLLLSLPVLAGAITMLLTDRNLNTSFFDPAGGGDPILYQHL
>LEFID631-10|HM873394|MM06652|Matilella_fusca
TLYFIFGIWSGMVGTSLSLLIRAELGTPGSLIGDDQIYNTIVTGHAFIMIFFMVMPIMIGGFGNWLVPLMLGAPDMAFPRMNNMSFWLLPPSLTLLISSSVVENGAGTGWTVYPPLSSNIAHGGSSVDLAIFSLHLAGISSILGAINFITTIINMKLNGLSFD
QMPLFVWAVGITALLLLLSLPVLAGAITMLLTDRNLNTSFFDPAGGGDPILYQHL
>LEFIE574-10|HM874297|MM09468|Matilella_fusca
TLYFIFGIWSGMVGTSLSLLIRAELGTPGSLIGDDQIYNTIVTGHAFIMIFFMVMPIMIGGFGNWLVPLMLGAPDMAFPRMNNMSFWLLPPSLTLLISSSVVENGAGTGWTVYPPLSSNIAHGGSSVDLAIFSLHLAGISSILGAINFITTIINMKLNGLSFD
QMPLFVWAVGITALLLLLSLPVLAGAITMLLTDRNLNTSFFDPAGGGDPILYQHL
>LEFIE567-10|HM874290|MM09456|Mecyna_flavalis
TLYFIFGIWAGMIGTSLSLLIRAELGNPGSLIGDDQIYNTIVTAHAFIMIFFMVMPIMIGGFGNWLVPLMLGAPDMAFPRMNNMSFWLLPPSLTLLISSSIVENGAGTGWTVYPPLSSNIAHGGSSVDLAIFSLHLAGISSILGAINFITTIINMRINGMSFD
QMPLFVWAVGITALLLLLSLPVLAGAITMLLTDRNLNTSFFDPAGGGDPILYQHL
>LEFIG927-10|HM876571|MM15791|Mecyna_flavalis
TLYFIFGIWAGMIGTSLSLLIRAELGNPGSLIGDDQIYNTIVTAHAFIMIFFMVMPIMIGGFGNWLVPLMLGAPDMAFPRMNNMSFWLLPPSLTLLISSSIVENGAGTGWTVYPPLSSNIAHGGSSVDLAIFSLHLAGISSILGAINFITTIINMRINGMSFD
QMPLFVWAVGITALLLLLSLPVLAGAITMLLTDRNLNTSFFDPAGGGDPILYQHL
>LEFIC692-10|HM872513|MM04673|Meganola_strigula
TLYFIFGIWAGMVGTSLSLLIRAELGNPGSLIGDDQIYNTIVTAHAFIMIFFMVMPIMIGGFGNWLVPLMLGAPDMAFPRMNNMSFWLLPPSLTLLISSSIVENGAGTGWTVYPPLSSNIAHSGSSVDLAIFSLHLAGISSILGAINFITTIINMRLNNLSFD
QMSLFIWAVGITAFLLLLSLPVLAGAITMLLTDRNLNTSFFDPAGGGDPILYQHL
>LEFIA515-10|KM573690|MM01597|Melanchra_persicariae
TLYFIFGIWAGMVGTSLSLLIRAELGNPGSLIGDDQIYNTIVTAHAFIMIFFMVMPIMIGGFGNWLIPLMLGAPDMAFPRMNNMSFWLLPPSLTLLISSSIVENGAGTGWTVYPPLSSNIAHGGSSVDLAIFSLHLAGISSILGAINFITTIINMRLNNMSFD
QMPLFIWAVGITAFLLLLSLPVLAGAITMLLTDRNLNTSFFDPAGGGDPILYQHL
>LEFID245-10|HM873044|MM06109|Melitaea_athalia
TLYFIFGIWAGMLGTSLSLLIRTELGNPGSLIGDDQIYNTIVTAHAFIMIFFMVMPIMIGGFGNWLVPLMLGAPDMAFPRMNNMSFWLLPPSLILLISSSIVENGAGTGWTVYPPLSSNIAHSGSSVDLAIFSLHLAGISSILGAINFITTIINMRVNNMSFD
QMPLFVWAVGITALLLLLSLPVLAGAITMLLTDRNINTSFFDPAGGGDPILYQHL
>LEFIJ549-10|JF853664|MM17174|Melitaea_athalia
TLYFIFGIWAGMLGTSLSLLIRTELGNPGSLIGDDQIYNTIVTAHAFIMIFFMVMPIMIGGFGNWLVPLMLGAPDMAFPRMNNMSFWLLPPSLILLISSSIVENGAGTGWTVYPPLSSNIAHSGSSVDLAIFSLHLAGISSILGAINFITTIINMRVNNMSFD
QMPLFVWAVGITALLLLLSLPVLAGAITMLLTDRNINTSFFDPAGGGDPILYQHL
>LEEUA530-11|MM20589|Melitaea_aurelia
TLYFIFGIWAGMLGTSLSLLIRTELGNPGSLIGDDQIYNTIVTAHAFIMIFFMVMPIMIGGFGNWLVPLMLGAPDMAFPRMNNMSFWLLPPSLILLISSSIVENGAGTGWTVYPPLSSNIAHSGSSVDLAIFSLHLAGISSILGAINFITTIINMRVNNMSFD
QMPLFVWAVGITALLLLLSLPVLAGAITMLLTDRNINTSFFDPAGGGDPILYQHL
>LEFIB318-10|HM871218|MM00849|Melitaea_cinxia
TLYFIFGIWAGMLGTSLSLLIRTELGNPGSLIGDDQIYNTIVTAHAFIMIFFMVMPIMIGGFGNWLIPLMLGAPDMAFPRMNNMSFWLLPPSLMLLISSSIVENGAGTGWTVYPPLSSNIAHSGSSVDLAIFSLHLAGISSILGAINFITTIINMRINNMSFD

QMPLFVWAVGITAVLLLLSLPVLAGAITMLLTDRNINTSFFDPAGGGDPILYQHL
>LEFIL379-10|JN274637|MM18689|Melitaea_diamina
TLYFIFGIWAGMLGTSLSLLIRTELGNPGSLIGDDQIYNTIVTAHAFVMIFFMVMPIMIGGFGNWLVPLMLGAPDMAFPRMNNMSFWLLPPSLILLISSSIVENGAGTGWTVYPPLSSNIAHSGSSVDLAIFSLHLAGISSILGAINFITTIINMRINNMSFD
QMPLFVWAVGITALLLLSLPVLAGAITMLLTDRNINTSFFDPAGGGDPILYQHL
>LEFIK806-10|JN277229|MM18381|Merrifieldia_baliodactylus
TLYFIFGIWAGMVGTSLSLLIRAELGNPGSLIGDDQIYNTIVTAHAFIMIFFMVMPIMIGGFGNWLIPLMLGSPDMAFPRMNNMSFWLLPPSITLLISSSIVENGAGTGWTVYPPLSSNIAHSGTSVDLAIFSLHLAGISSILGAINFITTIINMRLNNLMFD
QLPLFVWSVGITALLLLLSLPVLAGAITMLLTDRNLNTSFFDPAGGGDPILYQHL
>LEFID353-10|HM873150|MM06254|Merrifieldia_leucodactyla
TLYFIFGIWAGMIGTSLSLLIRAELGNPGSLIGDDQIYNTIVTAHAFIMIFFMVMPIMIGGFGNWLVPLMLGAPDMAFPRMNNMSFWLLPPSITLLISSSIVENGAGTGWTVYPPLSSNIAHSGTSVDLAIFSLHLAGISSILGAINFITTIINMRLNNLMFD
QLPLFVWSVGITALLLLLSLPVLAGAITMLLTDRNLNTSFFDPAGGGDPILYQHL
>LEFIC081-10|HM871949|MM03377|Merrifieldia_tridactyla
TLYFIFGIWAGMIGTSLSLLIRAELGNPGSLIGDDQIYNTIVTAHAFIMIFFMVMPIMIGGFGNWLVPLMLGAPDMAFPRMNNMSFWLLPPSLTLLISSSIVENGAGTGWTVYPPLSSNIAHGGTSVDLAIFSLHLAGISSILGAINFITTIINMRLNNLMFD
QLPLFVWSVGITALLLLLSLPVLAGAITMLLTDRNLNTSFFDPAGGGDPILYQHL
>LEFIC769-10|HM872588|MM04895|Mesapamea_secalis
TLYFIFGIWAGMVGTSLSLLIRAELGNPGSLIGDDQIYNTIVTAHAFIMIFFMVMPIMIGGFGNWLVPLMLGAPDMAFPRMNNMSFWLLPPSLTLLISSSIVENGAGTGWTVYPPLSSNIAHGGSSVDLAIFSLHLAGISSILGAINFITTIINMRLNNLSFD
QMPLFIWAVGITAFLLLLSLPVLAGAITMLLTDRNLNTSFFDPAGGGDPILYQHL
>LEFIF689-10|HM875373|MM12711|Mesapamea_secalis
TLYFIFGIWAGMVGTSLSLLIRAELGNPGSLIGDDQIYNTIVTAHAFIMIFFMVMPIMIGGFGNWLVPLMLGAPDMAFPRMNNMSFWLLPPSLTLLISSSIVENGAGTGWTVYPPLSSNIAHGGSSVDLAIFSLHLAGISSILGAINFITTIINMRLNNLSFD
QMPLFIWAVGITAFLLLLSLPVLAGAITMLLTDRNLNTSFFDPAGGGDPILYQHL
>LEFIJ2234-14|MM23414|Mesapamea_secalis
TLYFIFGIWAGMVGTSLSLLIRAELGNPGSLIGDDQIYNTIVTAHAFIMIFFMVMPIMIGGFGNWLVPLMLGAPDMAFPRMNNMSFWLLPPSLTLLISSSIVENGAGTGWTVYPPLSSNIAHGGSSVDLAIFSLHLAGISSILGAINFITTIINMRLNNLSFD
QMPLFIWAVGITAFLLLLSLPVLAGAITMLLTDRNLNTSFFDPAGGGDPILYQHL
>LEFIL428-10|KM572154|MM18726|Mesogona_oxalina
TLYFIFGIWAGMVGTSLSLLIRAELGTPGSLIGDDQIYNTIVTAHAFIMIFFMVMPIMIGGFGNWLVPLMLGAPDMAFPRLNNMSFWLLPPSLTLLISSSIVENGAGTGWTVYPPLSSNIAHGGSSVDLAIFSLHLAGISSILGAINFITTIINMRLNNLFFDQ
MPLFVWAVGITAFLLLLSLPVLAGAITMLLTDRNLNTSFFDPAGGGDPILYQHL
>LEFIA330-10|HM386673|MM01377|Mesoleuca_albicillata
TLYFIFGIWSGMIGTSLSLLIRAELGNPGSLIGDDQIYNTIVTAHAFIMIFFMVMPIMIGGFGNWLVPLMLGAPDMAFPRMNNMSFWLLPPSIMMLISSSIVESGAGTGWTVYPPLSSNIAHSGSSVDLAIFSLHLAGISSILGAINFITTIINMRLNNMFFD
QLPLFVWAVGITAFLLLLSLPVLAGAITMLLTDRNLNTSFFDPAGGGDPILYQHL
>LEFID403-10|HM873200|MM06322|Mesoleuca_albicillata
TLYFIFGIWSGMVGTSLSLLIRAELGNPGSLIGDDQIYNTIVTAHAFIMIFFMVMPIMIGGFGNWLVPLMLGAPDMAFPRMNNMSFWLLPPSIMMLISSSIVESGAGTGWTVYPPLSSNIAHGGSSVDLAIFSLHLAGISSILGAINFITTIINMRLNNMFF
DQLPLFVWAVGITAFLLLLSLPVLAGAITMLLTDRNLNTSFFDPAGGSDPILYQHL
>LEFIJ125-10|JF853448|MM08673|Mesoleuca_albicillata
TLYFIFGIWSGMVGTSLSLLIRAELGNPGSLIGDDQIYNTIVTAHAFIMIFFMVMPIMIGGFGNWLVPLMLGAPDMAFPRMNNMSFWLLPPSIMMLISSSIVESGAGTGWTVYPPLSSNIAHGGSSVDLAIFSLHLAGISSILGAINFITTIINMRLNNMFF
DQLPLFVWAVGITAFLLLLSLPVLAGAITMLLTDRNLNTSFFDPAGGGDPILYQHL
>LEFIG297-10|HM875976|MM14254|Mesoleuca_albicillata
TLYFIFGIWSGMVGTSLSLLIRAELGNPGSLIGDDQIYNTIVTAHAFIMIFFMVMPIMIGGFGNWLVPLMLGAPDMAFPRMNNMSFWLLPPSIMMLISSSIVESGAGTGWTVYPPLSSNIAHGGSSVDLAIFSLHLAGISSILGAINFITTIINMRLNNMFF
DQLPLFVWAVGITAFLLLLSLPVLAGAITMLLTDRNLNTSFFDPAGGGDPILYQHL
>LEFIA613-10|HM870862|MM01727|Mesoligia_furuncula
TLYFIFGIWAGMVGTSLSLLIRAELGNPGSLIGDDQIYNTIVTAHAFIMIFFMVMPIMIGGFGNWLVPLMLGAPDMAFPRMNNMSFWLLPPSLTLLISSSIVENGAGTGWTVYPPLSSNIAHGGSSVDLAIFSLHLAGISSILGAINFITTIINMRLNSLSFD
QMPLFIWAVGITAFLLLLSLPVLAGAITMLLTDRNLNTSFFDPAGGGDPILYQHL
>LEFID075-10|HM872886|MM05684|Mesophleps_silacella
TLYFLFGIWAGMVGTSLSLLIRAELGNPGQLIGDDQIYNTIVTAHAFIMIFFMVMPIMMGGFGNWLVPLMLGAPDMAFPRMNNMSFWLLPPSLTLLISSSIVENGAGTGWTVYPPLSSNIAHGGSSVDLAIFSLHLAGISSILGAINFITTIINMKINGLSF
DQMPLFVWAVGITALLLLLSLPVLAGAITMLLTDRNLNTSFFDPAGGGDPILYQHL
>LEFIF148-10|HM874842|MM10664|Mesophleps_silacella
TLYFLFGIWAGMVGTSLSLLIRAELGNPGQLIGDDQIYNTIVTAHAFIMIFFMVMPIMIGGFGNWLVPLMLGAPDMAFPRMNNMSFWLLPPSLTLLISSSIVENGAGTGWTVYPPLSSNIAHGGSSVDLAIFSLHLAGISSILGAINFITTIINMKINGLSFD
QMPLFVWAVGITALLLLLSLPVLAGAITMLLTDRNLNTSFFDPAGGGDPILYQHL
>LEFIA308-10|HM386651|MM01354|Mesotype_didymata

TLYFIFGIWAGMIGTSLSLLIRAELGNPGSLIGDDQIYNTIVTAHAFIMIFFMVMPIMIGGFGNWLVPLMLGAPDMAFPRMNNMSFWLLPPSITLLISSSIVENGAGTGWTVYPPLSSNIAHGGSSVDLAIFSLHLAGISSILGAINFITTIINMRLNNMFFD
QLPLFVWAVGITAFLLLLSLPVLAGAITMLLTDRNLNTSFFDPAGGGDPILYQHL
>LEFIB048-10|HM870957|MM00329|Metalampra_cinnamomea
TLYFIFGIWSGMVGTSLSLLIRAELGNPGSLIGDDQIYNTIVTAHAFIMIFFMVMPIMIGGFGNWLIPLMLGAPDMAFPRMNNMSFWLLPPSLTLLISSSIVENGAGTGWTVYPPLSSNIAHGGSSVDLAIFSLHLAGISSILGAINFITTIINMRMNNMSF
DQMPLFVWAVGITALLLLLSLPVLAGAITMLLTDRNLNTSFFDPAGGGDPILYQHL
>LEFIF100-10|HM874804|MM10552|Metaxmeste_schrankiana
TLYFIFGIWAGMVGTSLSLLIRAELGNPGSLIGDDQIYNTIVTAHAFIMIFFMVMPIMIGGFGNWLVPLMLGAPDMAFPRMNNMSFWLLPPSLTLLISSSIVENGAGTGWTVYPPLSSNIAHGGSSVDLAIFSLHLAGISSILGAINFITTIINMRINSLSFD
QMPLFVWSVGITALLLLLSLPVLAGAITMLLTDRNLNTSFFDPAGGGDPILYQHL
>LEFIG119-10|HM875799|MM13925|Metaxmeste_schrankiana
TLYFIFGIWAGMVGTSLSLLIRAELGNPGSLIGDDQIYNTIVTAHAFIMIFFMVMPIMIGGFGNWLVPLMLGAPDMAFPRMNNMSFWLLPPSLTLLISSSIVENGAGTGWTVYPPLSSNIAHGGSSVDLAIFSLHLAGISSILGAINFITTIINMRINSLSFD
QMPLFVWSVGITALLLLLSLPVLAGAITM?LTDRNLNTSFFDPAGGGDPILYQHL
>LEFIB444-10|HM871343|MM01960|Metendothenia_atropunctana
TLYFIFGIWAGMIGTSLSLLIRAELGNPGSLIGDDQIYNTIVTAHAFIMIFFMVMPIMIGGFGNWLVPLMLGAPDMAFPRMNNMSFWLLPPSIMLLISSSIVEYGAGTGWTVYPPLSSNIAHSGSSVDLAIFSLHLAGISSILGAVNFITTIINMRPNNMSL
DQMPLFVWAVGITALLLLLSLPVLAGAITMLLTDRNLNTSFFDPAGGGDPILYQHL
>LEEUA134-11|MM19542|Metriotes_lutarea
TLYFIFGIWAGMVGTSLSLLIRAELGNPGSLI?DDQIYNTIVTAHAFIMIFFMVMPIMIGGFGNWLVPLMLGAPDMAFPRMNNMSFWLLPPSLTLLISSSIVENGTGTGWTVYPPLSSNIAHGGSSVDLSIFSLHLAGISSILGAINFITTIINMRLNNMSFD
QLPLFVWAVGITALLLLLSLPVLAGAITMLLTDRNLNTSFFDPAGGGDPILYQHL
>LEEUA482-11|MM20541|Metriotes_lutarea
TLYFIFGIWAGMVGTSLSLLIRAELGNPGSLIGDDQIYNTIVTAHAFIMIFFMVMPIMIGGFGNWLVPLMLGAPDMAFPRMNNMSFWLLPPSLTLLISSSIVENGTGTGWTVYPPLSSNIAHGGSSVDLSIFSLHLAGISSILGAINFITTIINMRLNNMSFD
QLPLFVWAVGITALLLLLSLPVLAGAITMLLTDRNLNTSFFDPAGGGDPILYQHL
>LEFIE858-10|HM874576|MM10100|Metzneria_aestivella
TLYFIFGIWAGMVGTSLSLLIRAELGNPGSLIGDDQIYNTIVTAHAFIMIFFMVMPIMIGGFGNWLVPLMLGAPDMAFPRMNNMSFWLLPPSLTLLISSSIVENGAGTGWTVYPPLSSNIAHGGSSVDLAIFSLHLAGISSILGAINFITTIINMKINGLSFD
QMPLFVWAVGITALLLLLSLPVLAGAITMLLTDRNLNTSFFDPAGGGDPILYQHL
>LEFIF147-10|HM874841|MM10663|Metzneria_aprilella
TLYFIFGIWAGMVGTSLSLLIRAELGNPGSLIGDDQIYNTIVTAHAFIMIFFMVMPIMIGGFGNWLVPLMLGAPDMAFPRMNNMSFWLLPPSLTLLISSSIVENGAGTGWTVYPPLSSNIAHGGSSVDLAIFSLHLAGISSILGAINFITTIINMKINGLSFD
QMPLFVWAVGITALLLLLSLPVLAGAITMLLTDRNLNTSFFDPAGGGDPILYQHL
>LEEUA170-11|MM19578|Metzneria_ehikeella
TLYFIFGIWAGMVGTSLSLLIRAELGNPGSLIGDDQIYNTIVTAHAFIMIFFMVMPIMIGGFGNWLVPLMLGAPDMAFPRMNNMSFWLLPPSLTLLISSSIVENGAGTGWTVYPPLSSNIAHGGSSVDLAIFSLHLAGISSILGAINFITTIINMKINGLSFD
QMPLFVWAVGITALLLLLSLPVLAGAITMLLTDRNLNTSFFDPAGGGDPILYQHL
>LEFIB713-10|HM871591|MM02522|Metzneria_lappella
TLYFIFGIWAGMVGTSLSLLIRAELGNPGSLIGDDQIYNTIVTAHAFIMIFFMVMPIMIGGFGNWLVPLMLGAPDMAFPRMNNMSFWLLPPSLTFLISSSIVENGAGTGWTVYPPLSSNIAHGGSSVDLAIFSLHLAGISSILGAINFITTIINMKINGLSFD
QMPLFVWAVGITALLLLLSLPVLAGAITMLLTDRNLNTSFFDPAGGGDPILYQHL
>LEFIB712-10|HM871590|MM02520|Metzneria_metzneriella
TLYFIFGIWAGMVGTSLSLLIRAELGNPGSLIGDDQIYNTIVTAHAFIMIFFMVMPIMIGGFGNWLVPLMLGAPDMAFPRMNNMSFWLLPPSLTLLISSSIVENGAGTGWTVYPPLSSNIAHGGSSVDLAIFSLHLAGISSILGAINFITTIINMKINGLSFD
QMPLFVWAVGITALLLLLSLPVLAGAITMLLTDRNLNTSFFDPAGGGDPILYQHL
>LEFIB714-10|HM871592|MM02523|Metzneria_neuropterella
TLYFIFGIWAGMVGTSLSLLIRAELGNPGSLIGDDQIYNTIVTAHAFIMIFFMVMPIMIGGFGNWLVPLMLGAPDMAFPRMNNMSFWLLPPSLTLLISSSIVENGAGTGWTVYPPLSSNIAHGGSSVDLAIFSLHLAGISSILGAINFITTIINMKINGLSFD
QMPLFVWAVGITALLLLLSLPVLAGAITMLLTDRNLNTSFFDPAGGGDPILYQHL
>LEFIG748-10|HM876404|MM15612|Metzneria_neuropterella
TLYFIFGIWAGMVGTSLSLLIRAELGNPGSLIGDDQIYNTIVTAHAFIMIFFMVMPIMIGGFGNWLIPLMLGAPDMAFPRMNNMSFWLLPPSLTLLISSSIVENGTGTGWTVYPPLSSNIAHGGSSVDLTIFSLHLAGISSILGAINFITTIINMKINSLSFDQ
MPLFVWAVGITALLLLLSLPVLAGAITMLLTDRNLNTSFFDPAGGGDPILYQHL
>LEFIJ626-10|JF853724|MM17251|Metzneria_neuropterella
TLYFIFGIWAGMVGTSLSLLIRAELGNPGSLIGDDQIYNTIVTAHAFIMIFFMVMPIMIGGFGNWLVPLMLGAPDMAFPRMNNMSFWLLPPSLTLLISSSIVENGAGTGWTVYPPLSSNIAHGGSSVDLAIFSLHLAGISSILGAINFITTIINMKINGLSFD
QMPLFVWAVGITALLLLLSLPVLAGAITMLLTDRNLNTSFFDPAGGGDPILYQHL
>LEFIE818-10|HM874537|MM10020|Metzneria_santolinella
TLYFIFGIWAGMVGTSLSLLIRAELGNPGSLIGDDQIYNTIVTAHAFIMIFFMVMPIMIGGFGNWLVPLMLGAPDMAFPRMNNMSFWLLPPSLTLLISSSIVENGAGTGWTVYPPLSSNIAHGGSSVDLAIFSLHLAGISSILGAINFITTIINMKINGLSFD
QMPLFVWSVGITALLLLLSLPVLAGAITMLLTDRNLNTSFFDPAGGGDPILYQHL

>LEFIK641-10|JF854212|MM18216|Metzneria_santolinella
TLYFIFGIWAGMVGTSLSLLIRAELGNPGSLIGDDQIYNTIVTAHAFIMIFFMVMPIMIGGFGNWLVPLMLGAPDMAFPRMNNMSFWLLPPSLTLLISSSIVENGAGTGWTVYPPLSSNIAHGGSSVDLAIFSL?LAGISSILGAINFITTIINMKINGLSFD
QMPLFVWSVGITALLLLLSLPVLAGAITMLLTDRNLNTSFFDPAGGGDPILYQHL
>LEFIG341-10|HM876018|MM14326|Micropterix_aruncella
ILYFLFGMWNGLIGMMFSMLIRIELSIPNYFLNNDQIFNLIITSHAFIMIFFMVMPILIGGFGNWLVPLMLGSPDMAFPRMNNLSFWLLPPSINLLLLSSFMELGTGTGWTMYPPLSSSIYHSGISVDLTIFSLHLAGISSILGAINFISTIMNMKIYNLNFNQI
PLFVWSVKITALLLLLSLPVLAGAITMLLTDRNLNTSFFDPAGGGDPILFQHL
>LEFIJ262-10|JF853503|MM14876|Micropterix_aureatella
MLYFLFGMWNGLIGMMFSMLIRIELSIPNYFLNNDQIFNLIITSHAFIMIFFMVMPILIGGFGNWLVPLMLGSPDMAFPRMNNLSFWLLPPSINLLLLSSFMELGTGTGWTMYPPLSSSIYHSGISVDLTIFSLHLAGISSILGAINFISTIMNMKIYNLNFN
QIPLFVWSVKITALLLLLSLPVLAGAITMLLTDRNLNTSFFDPAGGGDPILFQHL
>LEFIJ270-10|JF853510|MM14886|Micropterix_calthella
ILYFLFGMWNGLIGMMFSMLIRIELSIPNYFLNNDQIFNLIITSHAFIMIFFMVMPILIGGFGNWLVPLMLGSPDMAFPRMNNLSFWLLPPSINLLLLSSFMELGTGTGWTLYPPLSSSIYHSGISVDLTIFSLHLAGISSILGAINFISTIMNMKIYNLNFNQIP
LFVWSVKITALLLLLSLPVLAGAITMLLTDRNLNTSFFDPAGGGDPILFQHL
>LEEUA538-11|MM20597|Micropterix_tunbergella
ILYFLFGMWNGLIGMMFSMLIRIELSIPNYFLNNDQIFNLIITSHAFIMIFFMVMPILIGGFGNWLVPLMLGSPDMAFPRMNNLSFWLLPPSINLLLLSSFMELGTGTGWTLYPPLSSSIYHSGISVDLTIFSLHLAGISSILGAINFISTIMNMKIYNLNFNQIP
LFVWSVKITALLLLLSLPVLAGAITMLLTDRNLNTSFFDPAGGGDPILFQHL
>LEFIE211-10|HM873950|MM08526|Micrurapteryx_gradatella
TLYFLFGIWSGMVGTSLSLLIRAELGNPGSLIGDDQIYNTIVTAHAFIMIFFMVMPIMIGGFGNWIVPLMLGAPDMAFPRLNNMSFWLLPPSLLLLISSSMVENGAGTGWTVYPPLSSNIAHSGSSVDLAIFSLHLAGISSILGAINFISTIINMRPNGMNF
DSMPLFAWAVGITALLLLLSLPVLAGAITMLLTDRNLNTSFFDPAGGGDPILYQHL
>LEFIA104-10|HM396451|MM01027|Miltochrista_miniata
TLYFIFGIWAGMVGTSLSLLIRAELGNPGSLIGDDQIYNTIVTAHAFIMIFFMVMPIMIGGFGNWLVPLMLGAPDMAFPRMNNMSFWLLPPSLTLLISSSIVENGAGTGWTVYPPLSSNIAHSGSSVDLAIFSLHLAGISSILGAINFITTIINMRLNSLTFD
QMPLFVWAVGITAFLLLLSLPVLAGAITMLLTDRNLNTSFFDPAGGGDPILYQHL
>LEFIA057-10|HM396405|MM00969|Mimas_tiliae
TLYFIFGIWAGMVGTSLSLLIRAELGNPGSLIGDDQIYNTIVTAHAFIMIFFMVMPIMIGGFGNWLVPLMLGAPDMAFPRMNNMSFWLLPPSLTLLISSSIVENGAGTGWTVYPPLSSNIAHSGSSVDLAIFSLHLAGISSILGAINFITTIINMRINNMSFD
QMPLFVWAVGITAFLLLLSLPVLAGAITMLLTDRNLNTSFFDPAGGGDPILYQHL
>COLFG192-13|MM23258|Minucia_lunaris
TLYFIFGIWAGMVGTSLSLLIRAELGNPGSLIGDDQIYNTIVTAHAFIMIFFMVMPIMIGGFGNWLVPLMLGAPDMAFPRMNNMSFWLLPPSLTLLISSSIVENGAGTGWTVYPPLSSNIAHSGSSVDLAIFSLHLAGISSILGAINFITTIINMRLNNLMFD
QMPLFVWSVGITAFLLLLSLPVLAGAITMLLTDRNLNTSFFDPAGGGDPILYQHL
>LEFIL557-10|JF854660|MM18855|Mirificarma_lentiginosella
TLYFIFGIWAGMVGTSLSLLIRAELGNPGSLIGDDQIYNTIVTAHAFIMIFFMVMPIMIGGFGNWLVPLMLGAPDMAFPRMNNMSFWLLPPSLTLLISSSIVENGAGTGWTVYPPLSSNIAHSGSSVDLAIFSLHLAGISSILGAINFITTIINMRINGMFFD
QMPLFVWAVGITALLLLLSLPVLAGAITMLLTDRNLNTSFFDPAGGGDPILYQHL
>LEFIB170-10|HM871074|MM00575|Mniotype_adusta
TLYFIFGIWAGMVGTSLSLLIRAELGNPGSLIGDDQIYNTIVTAHAFIMIFFMVMPIMIGGFGNWLVPLMLGAPDMAFPRMNNMSFWLLPPSLTLLISSSIVENGAGTGWTVYPPLSSNIAHGGSSVDLAIFSLHLAGISSILGAINFITTIINMRLNSLSFD
QMPLFIWAVGITAFLLLLSLPVLAGAITMLLTDRNLNTSFFDPAGGGDPILYQHL
>LEFIH011-10|HM876647|MM15875|Mniotype_adusta
TLYFIFGIWAGMVGTSLSLLIRAELGNPGSLIGDDQIYNTIVTAHAFIMIFFMVMPIMIGGFGNWLVPLMLGAPDMAFPRMNNMSFWLLPPSLTLLISSSIVENGAGTGWTVYPPLSSNIAHGGSSVDLAIFSLHLAGISSILGAINFITTIINMRLNSLSFD
QMPLFIWAVGITAFLLLLSLPVLAGAITMLLTDRNLNTSFFDPAGGGDPILYQHL
>LEFIJ844-10|JF853858|MM17469|Mniotype_adusta
TLYFIFGIWAGMVGTSLSLLIRAELGNPGSLIGDDQIYNTIVTAHAFIMIFFMVMPIMIGGFGNWLVPLMLGAPDMAFPRMNNMSFWLLPPSLTLLISSSIVENGAGTGWTVYPPLSSNIAHGGSSVDLAIFSLHLAGISSILGAINFITTIINMRLNSLSFD
QMPLFIWAVGITAFLLLLSLPVLAGAITMLLTDRNLNTSFFDPAGGGDPILYQHL
>LEFID093-10|HM872904|MM05705|Mniotype_bathensis
TLYFIFGIWAGMVGTSLSLLIRAELGNPGSLIGDDQIYNTIVTAHAFIMIFFMVMPIMIGGFGNWLVPLMLGAPDMAFPRMNNMSFWLLPPSLTLLISSSIVENGAGTGWTVYPPLSSNIAHGGSSVDLAIFSLHLAGISSILGAINFITTIINMRLNSLSFD
QMPLFIWAVGITAFLLLLSLPVLAGAITMLLTDRNLNTSFFDPAGGGDPILYQHL
>LEFIJ1020-11|MM09298|Mniotype_bathensis
TLYFIFGIWAGMVGTSLSLLIRAELGNPGSLIGDDQIYNTIVTAHAFIMIFFMVMPIMIGGFGNWLVPLMLGAPDMAFPRMNNMSFWLLPPSLTLLISSSIVENGAGTGWTVYPPLSSNIAHGGSSVDLAIFSLHLAGISSILGAINFITTIINMRLNSLSFD
QMPLFIWAVGITAFLLLLSLPVLAGAITMLLTDRNLNTSFFDPAGGGDPILYQHL
>LEFID918-10|HM873668|MM07372|Mniotype_satura
TLYFIFGIWAGMVGTSLSLLIRAELGHPGSLIGDDQIYNTIVTAHAFIMIFFMVMPIMIGGFGNWLVPLMLGAPDMAFPRMNNMSFWLLPPSLTLLISSSIVENGAGTGWTVYPPLSSNIAHGGSSVDLAIFSLHLAGISSILGAINFITTIINMRLNNLSFD

QMPLFIWAVGITAFLLLLSLPVLAGAITMLLTDRNLNTSFFDPAGGGDPILYQHL
>LEFIC291-10|HM872135|MM03763|Moma_alpium
TLYFIFGIWAGMVGTSLSLLIRAELGNPGSLIGDDQIYNTIVTAHAFIMIFFMVMPIMIGGFGNWLVPLMLGAPDMAFPRMNNMSFWLLPPSLTLLISSSIVENGAGTGWTVYPPLSSNIAHSGSSVDLAIFSLHLAGISSILGAINFITTIINMRLNNLSFD
QMPLFVWAVGITAFLLLLSLPVLAGAITMLLTDRNLNTSFFDPAGGGDPILYQHL
>LEFID991-10|HM873741|MM08028|Mompha_conturbatella
TLYFIFGIWAGMVGTSLSLLIRAELGNPGSLIGDDQIYNTIVTAHAFIMIFFMVMPIMIGGFGNWLVPLMLGAPDMAFPRMNNMSFWLLPPSLTLLISSSIVENGAGTGWTVYPPLSSNIAHGGSSVDLAIFSLHLAGISSILGAINFITTIINMRLNNLEFD
QMPLFVWAVGITALLLLLSLPVLAGAITMLLTDRNLNTSFFDPAGGGDPILYQHL
>LEFIE415-10|HM874139|MM09001|Mompha_conturbatella
TLYFIFGIWAGMVGTSLSLLIRAELGNPGSLIGDDQIYNTIVTAHAFIMIFFMVMPIMIGGFGNWLVPLMLGAPDMAFPRMNNMSFWLLPPSLTLLISSSIVENGAGTGWTVYPPLSSNIAHGGSSVDLAIFSLHLAGISSILGAINFITTIINMRLNNLEFD
QMPLFVWAVGITALLLLLSLPVLAGAITMLLTDRNLNTSFFDPAGGGDPILYQHL
>LEFID067-10|HM872879|MM05676|Mompha_divisella
TLYFIFGIWAGMVGTSLSLLIRAELGNPGSLIGDDQIYNTIVTAHAFIMIFFMVMPIMIGGFGNWLVPLMLGAPDMAFPRMNNMSFWLLPPSLTLLISSSIVENGAGTGWTVYPPLSSNIAHGGSSVDLAIFSLHLAGISSILGAINFITTIINMRLNNLMF
DQMPLFVWAVGITALLLLLSLPVLAGAITMLLTDRNLNTSFFDPAGGGDPILYQHL
>LEFIE438-10|HM874162|MM09195|Mompha_epilobiella
TLYFIFGIWAGMVGTSLSLLIRAELGNPGSLIGDDQIYNTIVTAHAFIMIFFMVMPIMIGGFGNWLVPLMLGAPDMAFPRMNNMSFWLLPPSLTLLISSSIVENGAGTGWTVYPPLSSNIAHSGSSVDLAIFSLHLAGISSILGAINFITTIINMRLNNLKFD
QMPLFVWAVGITALLLLLSLPVLAGAITMLLTDRNLNTSFFDPAGGGDPILYQHL
>LEFIB586-10|HM871466|MM02278|Mompha_idaei
TLYFIFGIWAGMMGTSLSLLIRAELGNPGSLIGDDQIYNTIVTAHAFIMIFFMVMPIMIGGFGNWLIPLMLGAPDMAFPRMNNMSFWLLPPSLTLLISSSMVENGAGTGWTVYPPLSSNIAHSGSSVDLTIFSLHLAGISSILGAINFITTIINMRLNNLMF
DQMPLFVWAVGITALLLLLSLPVLAGAITMLLTDRNLNTSFFDPAGGGDPILYQHL
>LEFIB958-10|HM871834|MM03131|Mompha_idaei
TLYFIFGIWAGMMGTSLSLLIRAELGNPGSLIGDDQIYNTIVTAHAFIMIFFMVMPIMIGGFGNWLIPLMLGAPDMAFPRMNNMSFWLLPPSLTLLISSSMVENGAGTGWTVYPPLSSNIAHSGSSVDLTIFSLHLAGISSILGAINFITTIINMRLNNLMF
DQMPLFVWAVGITALLLLLSLPVLAGAITMLLTDRNLNTSFFDPAGGGDPILYQHL
>LEFID847-10|HM873604|MM06971|Mompha_lacteella
TLYFIFGIWAGMVGTSLSLLIRAELGNPGSLIGDDQIYNTIVTAHAFIMIFFMVMPIMIGGFGNWLVPLMLGAPDMAFPRMNNMSFWLLPPSLTLLISSSIVENGAGTGWTVYPPLSSNIAHSGSSVDLAIFSLHLAGISSILGAINFITTIINMRLNNLAFD
QMPLFVWAVGITALLLLLSLPVLAGAITMLLTDRNLNTSFFDPAGGGDPILYQHL
>LEFIC566-10|HM872387|MM04343|Mompha_langiella
TLYFIFGIWAGMVGTSLSLLIRAELGNPGSLIGDDQIYNTIVTAHAFIMIFFMVMPIMIGGFGNWLVPLMLGAPDMAFPRMNNMSFWLLPPSLTLLISSSIVENGAGTGWTVYPPLSSNIAHGGSSVDLAIFSLHLAGISSILGAINFITTIINMRLSNLSFD
QMPLFVWAVGITALLLLLSLPVLAGAITMLLTDRNLNTSFFDPAGGGDPILYQHL
>LEFIG415-10|HM876091|MM14441|Mompha_locupletella
TLYFIFGIWAGMIGTSLSLLIRAELGNPGSLIGDDQIYNTIVTAHAFIMIFFMVMPLMIGGFGNWLVPLMLGAPDMAFPRMNNMSFWLLPPSLTLLLSSSLVENGAGTGWTVYPPLSSNIAHSGSSVDLAIFSLHLAGISSILGAINFITTIINMRLNNLMF
DQMPLFVWAVGITALLLLLSLPVLAGAITMLLTDRNLNTSFFDPAGGGDPILYQHL
>LEFIK381-10|KM573421|MM17956|Mompha_locupletella
TLYFIFGIWAGMIGTSLSLLIRAELGNPGSLIGDDQIYNTIVTAHAFIMIFFMVMPLMIGGFGNWLVPLMLGAPDMAFPRMNNMSFWLLPPSLTLLLSSSLVENGAGTGWTVYPPLSSNIAHSGSSVDLAIFSLHLAGISSILGAINFITTIINMRLNNLMF
DQMPLFVWAVGITALLLLLSLPVLAGAITMLLTDRNLNTSFFDPAGGGDPILYQHL
>LEFIJ769-10|MM17394|Mompha_miscella
TLYFIFGIWAGMIGTSLSLMIRAELGNPGSLIGDDQIYNTIVTAHAFIMIFFMVMPIMIGGFGNWLVPLMLGAPDMAFPRMNNMSFWLLPPSLTLLISSSVVENGAGTGWTVYPPLSSNIAHSGSSVDLAIFSLHLAGISSILGAINFITTIINMRLNNLMF
DQMPLFVWAVGITALLLLLSLPVLAGAITMLLTDRNLNTSFFDPAGGGDPILYQHL
>LEEUA166-11|MM19574|Mompha_ochraceella
TLYFIFGIWAGMVGTSLSLLIRAELGNPGSLIGDDQIYNTIVTAHAFIMIFFMVMPIMIGGFGNWLVPLMLGAPDMAFPRMNNMSFWLLPPSLTLLISSSIVENGAGTGWTVYPPLSSNIAHSGSSVDLAIFSLHLAGISSILGAINFITTIINMRLNNLAFD
QMPLFVWAVGITALLLLLSLPVLAGAITMLLTDRNLNTSFFDPAGGGDPILYQHL
>LEFIB777-10|HM871654|MM02652|Mompha_propinquella
TLYFIFGIWAGMVGTSLSLLIRAELGNPGSLIGDDQIYNTIVTAHAFIMIFFMVMPIMIGGFGNWLVPLMLGAPDMAFPRMNNMSFWLLPPSLTLLISSSIVENGAGTGWTVYPPLSSNIAHSGSSVDLAIFSLHLAGISSILGAINFITTIINMRLNNLAFD
QMPLFVWAVGITALLLLLSLPVLAGAITMLLTDRNLNTSFFDPAGGGDPILYQHL
>LEFIB149-10|HM871054|MM00537|Mompha_raschkiella
TLYFIFGIWAGMVGTSLSLMIRMELGNPGSLIGDDQIYNTIVTAHAFIMIFFMVMPIMIGGFGNWLVPLMLGAPDMAFPRMNNMSFWLLPPSLTLLMSSSMVENGVGTGWTVYPPLSSNIAHSGSSVDLAIFSLHLAGISSILGAINFITTIINMRLNK
MMFDQMSLFIWAVGITALLLLLSLPVLAGAITMLLTDRNLNTSFFDPAGGGDPILYQHL
>LEFIG734-10|HM876390|MM15598|Mompha_sexstrigella

TLYFIFGIWAGMVGTSLSLLIRMELGNPGSLIGDDQIYNTIVTAHAFIMIFFMVMPIMIGGFGNWLVPLMVGAPDMAFPRMNNMSFWLLPPSLTLLLSSSVVENGAGTGWTVYPPLSSNIAHSGSSVDLAIFSLHLAGISSILGAINFITTIINMRLNHM
MFDQMPLFVWAVGITALLLLLSLPVLAGAITMLLTDRNLNTSFFDPAGGGDPILYQHL
>LEFIA044-10|HM396393|MM00446|Mompha_sturnipennella
TLYFIFGIWAGMVGTSLSLLIRAELGNPGSLIGDDQIYNTIVTAHAFIMIFFMVMPIMIGGFGNWLVPLMLGAPDMAFPRMNNMSFWLLPPSLTLLISSSIVENGAGTGWTVYPPLSSNIAHGGSSVDLAIFSLHLAGISSILGAINFITTIINMRLNNLAFD
QMPLFVWAVGITALLLLLSLPVLAGAITMLLTDRNLNTSFFDPAGGGDPILYQHL
>LEFIG736-10|HM876392|MM15600|Mompha_subbistrigella
T?YFIFGIWAGMVGTSLSLLIRAELGNPGSLIGDDQIYNTIVTAHAFIMIFFMVMPIMIGGFGNWLVPLMLGAPDMAFPRMNNMSFWLLPPSLTLLISSSIVENGAGTGWTVYPPLSSNMAHSGSSVDLAIFSLHLAGISSILGAINFITTIINMRLNNLKF
DQMPLFVWAVGITALLLLLSLPVLAGAITMLLTDRNLNTSFFDPAGGGDPILYQHL
>LEFIG737-10|HM876393|MM15601|Mompha_subbistrigella
T?YFIFGIWAGMVGTSLSLLIRAELGNPGSLIGDDQIYNTIVTAHAFIMIFFMVMPIMIGGFGNWLVPLMLGAPDMAFPRMNNMSFWLLPPSLTLLISSSIVENGAGTGWTVYPPLSSNMAHSGSSVDLAIFSLHLAGISSILGAINFITTIINMRLNNLKF
DQMPLFVWAVGITALLLLLSLPVLAGAITMLLTDRNLNTSFFDPAGGGDPILYQHL
>LEFIJ938-10|JF853905|MM17563|Mompha_subbistrigella
TLYFIFGIWAGMVGTSLSLLIRAELGNPGSLIGDDQIYNTIVTAHAFIMIFFMVMPIMIGGFGNWLVPLMLGAPDMAFPRMNNMSFWLLPPSLTLLISSSIVENGAGTGWTVYPPLSSNMAHSGSSVDLAIFSLHLAGISSILGAINFITTIINMRLNNLKF
DQMPLFVWAVGITALLLLLSLPVLAGAITMLLTDRNLNTSFFDPAGGGDPILYQHL
>LEFIJ1241-11|KM573553|MM21101|Mompha_subbistrigella
TLYFIFGIWAGMVGTSLSLLIRAELGNPGSLIGDDQIYNTIVTAHAFIMIFFMVMPIMIGGFGNWLVPLMLGAPDMAFPRMNNMSFWLLPPSLTLLISSSIVENGAGTGWTVYPPLSSNMAHSGSSVDLAIFSLHLAGISSILGAINFITTIINMRLNNLKF
DQMPLFVWAVGITALLLLLSLPVLAGAITMLLTDRNLNTSFFDPAGGGDPILYQHL
>COLFF465-13|KM572225|MM23198|Mompha_subbistrigella
TLYFIFGIWAGMVGTSLSLLIRAELGNPGSLIGDDQIYNTIVTAHAFIMIFFMVMPIMIGGFGNWLVPLMLGAPDMAFPRMNNMSFWLLPPSLTLLISSSIVENGAGTGWTVYPPLSSNMAHSGSSVDLAIFSLHLAGISSILGAINFITTIINMRLNNLKF
DQMPLFVWAVGITALLLLLSLPVLAGAITMLLTDRNLNTSFFDPAGGGDPILYQHL
>LEFIL706-10|KM573663|MM05725|Mompha_terminella
TLYFIFGIWAGMVGTSLSLLIRAELGTPGSLIGDDQIYNTIVTAHAFIMIFFMVMPIMIGGFGNWLVPLMLGAPDMAFPRMNNMSFWLLPPSLTLLISSSVVENGAGTGWTVYPPLSSNIAHSGSSVDLAIFSLHLAGISSILGAINFITTIINMRLNNLMF
DQMPLFVWAVGITALLLLLSLPVLAGAITMLLTDRNLNTSFFDPAGGGDPILYQHL
>LEEUA164-11|MM19572|Mompha_terminella
TLYFIFGIWAGMVGTSLSLLIRAELGTPGSLIGDDQIYNTIVTAHAFIMIFFMVMPIMIGGFGNWLVPLMLGAPDMAFPRMNNMSFWLLPPSLTLLISSSVVENGAGTGWTVYPPLSSNIAHSGSSVDLAIFSLHLAGISSILGAINFITTIINMRLNNLMF
DQMPLFVWAVGITALLLLLSLPVLAGAITMLLTDRNLNTSFFDPAGGGDPILYQHL
>LEFIB757-10|HM871634|MM02612|Monochroa_arundinetella
TLYFIFGIWAGMVGTSLSMLIRAELGNPGSLISDDQIYNTIVTAHAFIMIFFMVMPIMIGGFGNWLVPLMLGAPDMAFPRMNNMSFWLLPPSLTLLISSSIVESGAGTGWTVYPPLSSNIAHSGSSVDLAIFSLHLAGISSILGAINFITTIINMKINGLSFD
QMPLFVWAVGITALLLLLSLPVLAGAITMLLTDRNLNTSFFDPAGGGDPILYQHL
>LEFIK131-10|JN262184|MM17706|Monochroa_arundinetella
TLYFIFGIWAGMVGTSLSMLIRAELGNPGSLIGDDQIYNTIVTAHAFIMIFFMVMPIMIGGFGNWLVPLMLGAPDMAFPRMNNMSFWLLPPSLTLLISSSIVESGAGTGWTVYPPLSSNIAHSGSSVDLAIFSLHLAGISSILGAINFITTIINMKINGLSFD
QMPLFVWAVGITALLLLLSLPVLAGAITMLLTDRNLNTSFFDPAGGGDPILYQHL
>LEFIB733-10|HM871611|MM02562|Monochroa_conspersella
TLYFIFGIWAGMMGTSLSLLIRAELGNPGSLIGDDQIYNTIVTAHAFIMIFFMVMPIMIGGFGNWLVPLMLGAPDMAFPRMNNMSFWLLPPSLTLLISSSIVENGAGTGWTVYPPLSSNIAHSGSSVDLAIFSLHLAGISSILGAINFITTIINMKINGLSFD
QMPLFVWAVGITALLLLLSLPVLAGAITMLLTDRNLNTSFFDPAGGGDPILYQHL
>LEFIB744-10|HM871622|MM02587|Monochroa_cytisella
TLYFIFGIWAGMVGTSLSLLIRAELGNPGSLIGDDQIYNTIVTAHAFIMIFFMVMPIMIGGFGNWLVPLMLGAPDMAFPRMNNMSFWLLPPSLTLLISSSIVETGAGTGWTVYPPLSSNIAHGGSSVDLAIFSLHLAGISSILGAINFITTIINMKINGLSFD
QMPLFVWAVGITALLLLLSLPVLAGAITMLLTDRNLNTSFFDPAGGGDPILYQHL
>LEFIE712-10|HM874432|MM09798|Monochroa_cytisella
TLYFIFGIWAGMMGTSLSLLIRAELGNPGSLIGDDQIYNTIVTAHAFIMIFFMVMPIMIGGFGNWLVPLMLGAPDMAFPRMNNMSFWLLPPSLTLLISSSIVETGAGTGWTVYPPLSSNIAHGGSSVDLAIFSLHLAGISSILGAINFITTIINMKINGLSFD
QMPLFVWAVGITALLLLLSLPVLAGAITMLLTDRNLNTSFFDPAGGGDPILYQHL
>LEEUA596-11|MM20655|Monochroa_divisella
TLYFIFGIWAGMVGTSLSLLIRAELGNPGSLIGDDQIYNTIVTAHAFIMIFFMVMPIMIGGFGNWLVPLMLGAPDMAFPRMNNMSFWLLPPSLTLLISSSIVEMGAGTGWTVYPPLSSNIAHSGSSVDLAIFSLHLAGISSILGAINFITTIINMKINNMSF
DQMPLFVWAVGITALLLLLSLPVLAGAITMLLTDRNLNTSFFDPAGGGDPILYQHL
>LEFIJ705-10|JF853781|MM17330|Monochroa_elongella
TLYFIFGIWSGMVGTSLSLLIRAELGNPGSLIGDDQIYNTIVTAHAFIMIFFMVMPIMIGGFGNWLVPLMLGAPDMAFPRMNNMSFWLLPPSLTLLISSSIVESGAGTGWTVYPPLSSNIAHSGSSVDLAIFSLHLAGISSILGAINFITTIINMKINGLSFDQ
MPLFVWAVGITALLLLLSLPVLAGAITMLLTDRNLNTSFFDPAGGGDPILYQHL

>LEFIC383-10|HM872226|MM03913|Monochroa_ferrea
TLYFIFGIWAGMVGTSLSLLIRAELGNPGSLIGDDQIYNTIVTAHAFIMIFFMVMPIMIGGFGNWLVPLMLGAPDMAFPRMNNMSFWLLPPSLTLLISSSIVENGAGTGWTVYPPLSSNIAHGGSSVDLAIFSLHLAGISSILGAINFITTIINMKINGLSFD
QMPLFVWAVGITALLLLLSLPVLAGAITMLLTDRNLNTSFFDPAGGGDPILYQHL
>LEFIC384-10|HM872227|MM03914|Monochroa_ferrea
TLYFIFGIWAGMVGTSLSLLIRAELGNPGSLIGDDQIYNTIVTAHAFIMIFFMVMPIMIGGFGNWLVPLMLGAPDMAFPRMNNMSFWLLPPSLTLLISSSIVENGAGTGWTVYPPLSSNIAHGGSSVDLAIFSLHLAGISSILGAINFITTIINMKINGLSFD
QMPLFVWAVGITALLLLLSLPVLAGAITMLLTDRNLNTSFFDPAGGGDPILYQHL
>LEFIG481-10|HM876157|MM14536|Monochroa_ferrea
TLYFIFGIWAGMVGTSLSLLIRAELGNPGSLIGDDQIYNTIVTAHAFIMIFFMVMPIMIGGFGNWLVPLMLGAPDMAFPRMNNMSFWLLPPSLTLLISNSIVENGAGTGWTVYPPLSSNIAHSGSSVDLAIFSLHLAGISSILGAINFITTIINMKINGLSFD
QMPLFVWAVGITALLLLLSLPVLAGAITMLLTDRNLNTSFFDPAGGGDPILYQHL
>LEFIB752-10|HM871630|MM02599|Monochroa_hornigi
TLYFIFGIWAGMVGTSLSLLIRAELGNPGSLIGDDQIYNTIVTAHAFIMIFFMVMPIMIGGFGNWLVPLMLGAPDMAFPRMNNMSFWLLPPSLTLLISSSIVETGAGTGWTVYPPLSSNIAHGGSSVDLAIFSLHLAGISSILGAINFITTIINMKINGLSFD
QMPLFVWAVGITALLLLLSLPVLAGAITMLLTDRNLNTSFFDPAGGGDPILYQHL
>LEFIE129-10|HM873877|MM08344|Monochroa_lucidella
TLYFIFGIWAGMLGTSLSLLIRAELGNPGSLIGDDQIYNTIVTAHAFIMIFFMVMPIMIGGFGNWLVPLMLGAPDMAFPRMNNMSFWLLPPSLTLLISSSIVETGAGTGWTVYPPLSSNIAHSGSSVDLAIFSLHLAGISSILGAINFITTIINMKINGLSFDQ
MPLFVWAVGITALLLLLSLPVLAGAITMLLTDRNLNTSFFDPAGGGDPILYQHL
>LEFIG302-10|HM875981|MM14260|Monochroa_lucidella
TLYFIFGIWAGMLGTSLSLLIRAELGNPGSLIGDDQIYNTIVTAHAFIMIFFMVMPIMIGGFGNWLVPLMLGAPDMAFPRMNNMSFWLLPPSLTLLISSSIVETGAGTGWTVYPPLSSNIAHSGSSVDLAIFSLHLAGISSILGAINFITTIINMKINGLSFDQ
MPLFVWAVGITALLLLLSLPVLAGAITMLLTDRNLNTSFFDPAGGGDPILYQHL
>LEFIB737-10|HM871615|MM02570|Monochroa_lutulentella
TLYFIFGIWAGMVGTSLSLLIRAELGNPGSLIGDDQIYNTIVTAHAFIMIFFMVMPIMIGGFGNWLVPLMLGAPDMAFPRMNNMSFWLLPPSLTLLISSSIVETGAGTGWTVYPPLSSNIAHSGSSVDLAIFSLHLAGISSILGAINFITTIINMKINGLSFDQ
MPLFVWAVGITALLLLLSLPVLAGAITMLLTDRNLNTSFFDPAGGGDPILYQHL
>LEFIC127-10|HM871974|MM03466|Monochroa_lutulentella
TLYFIFGIWAGMMGTSLSLLIRAELGNPGSLIGDDQIYNTIVTAHAFIMIFFMVMPIMIGGFGNWLVPLMLGAPDMAFPRMNNMSFWLLPPSLTLLISSSIVETGAGTGWTVYPPLSSNIAHSGSSVDLAIFSLHLAGISSILGAINFITTIINMKINGLSFD
QMPLFVWAVGITALLLLLSLPVLAGAITMLLTDRNLNTSFFDPAGGGDPILYQHL
>LEFIF502-10|HM875187|MM12077|Monochroa_lutulentella
TLYFIFGIWAGMVGTSLSLLIRAELGNPGSLIGDDQIYNTIVTAHAFIMIFFMVMPIMIGGFGNWLVPLMLGAPDMAFPRMNNMSFWLLPPSLTLLISSSIVETGAGTGWTVYPPLSSNIAHSGSSVDLAIFSLHLAGISSILGAINFITTIINMKINGLSFDQ
MPLFVWAVGITALLLLLSLPVLAGAITMLLTDRNLNTSFFDPAGGGDPILYQHL
>LEEUA171-11|MM19579|Monochroa_niphognatha
TLYFIFGIWAGMVGTSLSLLIRAELGNPGSLIGDDQIYNTIVTAHAFIMIFFMVMPIMIGGFGNWLVPLMLGAPDMAFPRMNNMSFWLLPPSLTLLISSSIVETGAGTGWTVYPPLSSNIAHSGSSVDLAIFSLHLAGISSILGAINFITTIINMKINGLSFDQ
MPLFIWAVGITALLLLLSLPVLAGAITMLLTDRNLNTSFFDPAGGGDPILYQHL
>LEFIF015-10|HM874729|MM10453|Monochroa_palustrellus
TLYFIFGIWAGMVGTSLSLLIRAELGNPGSLIGDDQIYNTIVTAHAFIMIFFMVMPIMIGGFGNWLVPLMLGAPDMAFPRMNNMSFWLLPPSLILLISSSIVENGAGTGWTVYPPLSSNIAHSGSSVDLAIFSLHLAGISSILGAINFITTIINMKINNLSFDQ
MPLFVWAVGITALLLLLSLPVLAGAITMLLTDRNLNTSFFDPAGGGDPILYQHL
>LEFIL700-10|MM18998|Monochroa_rumicetella
TLYFIFGIWAGMIGTSLSLLIRAELGNPGSLIGDDQIYNTIVTAHAFIMIFFMVMPIMIGGFGNWLVPLMLGAPDMAFPRMNNMSFWLLPPSLTLLISSSIVENGAGTGWTVYPPLSSNIAHGGSSVDLTIFSLHLAGISSILGAINFITTIINMKINGLSFDQ
MPLFVWAVGITALLLLLSLPVLAGAITMLLTDRNLNTSFFDPAGGGDPILYQHL
>LEFIL238-10|MM19238|Monochroa_rumicetella
TLYFIFGIWAGMIGTSLSLLIRAELGNPGSLIGNDQIYNTIVTAHAFIMIFFMVMPIMIGGFGNWLVPLMLGAPDMAFPRMNNMSFWLLPPSLTLLISSSIVENGAGTGWTVYPPLSSNIAHGGSSVDLAIFSLHLAGISSILGAINFITTIINMKINGLSFDQ
MPLFVWAVGITALLLLLSLPVLAGAITMLLTDRNLNTSFFDPAGGGDPILYQHL
>LEFID339-10|HM873136|MM06233|Monochroa_sepicolella
TLYFIFGIWAGMVGTSLSLLIRAELGNPGSLIGDDQIYNTIVTAHAFIMIFFMVMPIMIGGFGNWLVPLMLGAPDMAFPRMNNMSFWLLPPSLTLLISSSMVENGAGTGWTVYPPLSSNIAHGGSSVDLAIFSLHLAGISSILGAINFITTIINMKINGMSF
DQMPLFVWAVGITALLLLLSLPVLAGAITMLLTDRNLNTSFFDPAGGGDPILYQHL
>LEFIF014-10|HM874728|MM10452|Monochroa_servella
TLYFIFGIWAGMMGTSLSLLIRAELGNPGFLIGDDQIYNTIVTAHAFIMIFFMVMPIMIGGFGNWLVPLMLGAPDMAFPRMNNMSFWLLPPSLTLLISSSIVENGAGTGWTVYPPLSSNIAHSGSSVDLTIFSLHLAGISSILGAINFITTIINMKINGLFFD
QMPLFVWAVGITALLLLLSLPVLAGAITMLLTDRNLNTSFFDPAGGGDPILYQHL
>LEFIG751-10|HM876406|MM15615|Monochroa_suffusella
TLYFIFGIWAGMVGTSLSMLIRAELGNPGSLIGDDQIYNTIVTAHAFIMIFFMVMPIMIGGFGNWLVPLMLGAPDMAFPRMNNMSFWLLPPSLTLLISSSIVESGAGTGWTVYPPLSSNIAHSGSSVDLAIFSLHLAGISSILGAINFITTIINMKINGLSFD

QMPLFVWAVGITALLLLLSLPVLAGAITMLLTDRNLNTSFFDPAGGGDPILYQHL
>LEFID352-10|HM873149|MM06251|Monochroa_tenebrella
TLYFIFGIWAGMVGTSLSLLIRTELGNPGSFIGDDQIYNTIVTAHAFIMIFFMVMPIMIGGFGNWLVPLMLGAPDMAFPRMNNMSFWLLPPSLTLLISSSIVENGAGTGWTVYPPLSSNIAHGGSSVDLAIFSLHLAGISSILGAINFITTIINMKINGLSFD
QMPLFIWSVGITALLLLLSLPVLAGAITMLLTDRNLNTSFFDPAGGGDPILYQHL
>LEFIE300-10|HQ570375|MM08654|Monochroa_tetragonella
TLYFIFGIWAGMMGTSLSLLIRAELGNPGSLIGDDQIYNTIVTAHAFIMIFFMVMPIMIGGFGNWLVPLMLGAPDMAFPRMNNMSFWLLPPSLTLLISSSVVENGAGTGWTVYPPLSSNIAHSGSSVDLAIFSLHLAGISSILGAINFITTIINMKINGLSFD
QMPLFVWSVGITALLLLLSLPVLAGAITMLLTDRNLNTSFFDPAGGGDPILYQHL
>LEFIE196-10|HM873941|MM08511|Monopis_fenestratella
TLYFIFGIWSGMVGTSLSLLIRAELGNPGLLIGDDQIYNTIVTAHAFVMIFFMVMPIMIGGFGNWLVPLMLGAPDMAFPRLNNMSFWLLPPSLILLTSSSIVENGAGTGWTVYPPLSSNVAHGGSSVDLAIFSLHLAGISSILGAVNFITTVINMRPKSMSL
DQMPLFVWAVVITALLLLLSLPVLAGAITMLLTDRNLNTSFFDPAGGGDPILYQHL
>LEFII216-11|MM16083|Monopis_imella
TLYFLFGIWSGMVGTSLSLLIRMELGTPGLLIGDDQIYNTIVTAHAFIMIFFMVMPIMIGGFGNWLVPLMLSAPDMAFPRMNNMSFWLLPPSLLLLTSSSIVETGAGTGWTVYPPLSSNISHGGASVDLAIFSLHLAGISSILGAINFITTMINMKPSKMTF
DQLPLFVWAVTITALLLLLSLPVLAGAITMLLTDRNLNTSFFDPAGGGDPILYQHL
>LEFIL600-10|MM18898|Monopis_imella
TLYFLFGIWSGMVGTSLSLLIRMELGTPGLLIGDDQIYNTIVTAHAFIMIFFMVMPIMIGGFGNWLVPLMLSAPDMAFPRMNNMSFWLLPPSLLLLTSSSIVETGAGTGWTVYPPLSSNISHGGASVDLAIFSLHLAGISSILGAINFITTMINMKPPKMSF
DQLPLFVWAVTITALLLLLSLPVLAGAITMLLTDRNLNTSFFDPAGGGDPILYQHL
>LEFIL601-10|MM18899|Monopis_imella
TLYFLFGI?SGMVGTSLSLLIRMELGTPGLLIGDDQIYNTIVTAHAFIMIFFMVMPIMIGGFGNWLVPLMLSAPDMAFPRMNNMSFWLLPPSLLLLTSSSIVETGAGTGWTVYPPLSSNISHGGASVDLAIFSLHLAGISSILGAINFITTMINMKPPKMSF
DQLPLFVWAVTITALLLLLSLPVLAGAITMLLTDRNLNTSFFDPAGGGDPILYQHL
>LEFIE866-10|HM874584|MM10119|Monopis_laevigella
TLYFLFGIWAGMVGTSLSILIRMELGTPGLLIGDDQIYNTIITAHAFIMIFFMVMPIMIGGFGNWLVPLMLNAPDMAFPRMNNMSFWLLPPSLLLLTSSSIVETGAGTGWTVYPPLSSNIAHGGASVDLAIFSLHLAGISSILGAINFITTMINMKPQNLSF
DQLPLFVWAVSITALLLLLSLPVLAGAITMLLTDRNLNTSFFDPAGGGDPILYQHL
>LEFIG662-10|HM876323|MM15526|Monopis_laevigella
TLYFLFGIWAGMVGTSLSILIRMELGTPGLLIGDDQIYNTIITAHAFIMIFFMVMPIMIGGFGNWLVPLMLNAPDMAFPRMNNMSFWLLPPSLLLLTSSSIVETGAGTGWTVYPPLSSNIAHGGASVDLAIFSLHLAGISSILGAINFITTMINMKPQNLNF
DQLPLFVWAVSITALLLLLSLPVLAGAITMLLTDRNLNTSFFDPAGGGDPILYQHL
>LEFIJ678-10|JF853766|MM17303|Monopis_laevigella
TLYFLFGIWAGMVGTSLSILIRMELGTPGLLIGDDQIYNTIITAHAFIMIFFMVMPIMIGGFGNWLVPLMLNAPDMAFPRMNNMSFWLLPPSLLLLTSSSIVETGAGTGWTVYPPLSSNIAHGGASVDLAIFSLHLAGISSILGAINFITTMINMKPQNLSF
DQLPLFVWAVSITALLLLLSLPVLAGAITMLLTDRNLNTSFFDPAGGGDPILYQHL
>LEFIJ897-10|JF853886|MM17522|Monopis_laevigella
TLYFLFGIWAGMVGTSLSILIRMELGTPGLLIGDDQIYNTIITAHAFIMIFFMVMPIMIGGFGNWLVPLMLNAPDMAFPRMNNMSFWLLPPSLLLLTSSSIVETGAGTGWTVYPPLSSNIAHGGASVDLAIFSLHLAGISSILGAINFITTMINMKPQNLSF
DQLPLFVWAVSITALLLLLSLPVLAGAITMLLTDRNLNTSFFDPAGGGDPILYQHL
>LEFIJ901-10|JF853889|MM17526|Monopis_laevigella
TLYFLFGIWAGMVGTSLSILIRMELGTPGLLIGDDQIYNTIITAHAFIMIFFMVMPIMIGGFGNWLVPLMLNAPDMAFPRMNNMSFWLLPPSLLLLTSSSIVETGAGTGWTVYPPLSSNIAHGGASVDLAIFSLHLAGISSILGAINFITTMINMKPQNLSF
DQLPLFVWAVSITALLLLLSLPVLAGAITMLLTDRNLNTSFFDPAGGGDPILYQHL
>LEFIL315-10|JN286245|MM18625|Monopis_laevigella
TLYFLFGIWAGMVGTSLSILIRMELGTPGLLIGDDQIYNTIITAHAFIMIFFMVMPIMIGGFGNWLVPLMLNAPDMAFPRMNNMSFWLLPPSLLLLTSSSIVETGAGTGWTVYPPLSSNIAHGGASVDLAIFSLHLAGISSILGAINFITTMINMKPQNLSF
DQLPLFVWAVSITALLLLLSLPVLAGAITMLLTDRNLNTSFFDPAGGGDPILYQHL
>LEEUA042-11|JN286244|MM19355|Monopis_laevigella
TLYFLFGIWAGMVGTSLSILIRMELGTPGLLIGDDQIYNTIITAHAFIMIFFMVMPVMIGGFGNWLVPLMLNAPDMAFPRMNNMSFWLLPPSLLLLTSSSIVETGAGTGWTVYPPLSSNIAHGGASVDLAIFSLHLAGISSILGAINFITTMINMKPQNLS
FDQLPLFVWAVSITALLLLLSLPVLAGAITMLLTDRNLNTSFFDPAGGGDPILYQHL
>LEFIF450-10|HM875135|MM11934|Monopis_monachella
TLYFLFGIWTGLIGTSLSLLIRIELGTPGFLIGDDQIYNTIVTAHAFIMIFFMVMPIMIGGFGNWLVPLMINAPDMAFPRMNNMSFWLLPPSLLLLTSSSIVENGAGTGWTVYPPLSSTIAHSGASVDLAIFSLHLAGISSILGAINFITTMINMKPQEMKFD
QLPLFVWAVSITALLLLLSLPVLAGAITMLLTDRNLNTSFFDPAGGGDPILYQHL
>LEFID732-10|HM873489|MM06790|Monopis_obviella
TLYFLFGIWAGMVGTSLSLLIRMELGTPGLLIGDDQIYNTIVTAHAFIMIFFMVMPIMIGGFGNWLVPLMLSAPDMAFPRMNNMSFWLLPPSLLLLTSSSIVETGAGTGWTVYPPLSSNIAHGGASVDLAIFSLHLAGISSILGAINFITTMINMKPPKMN
FDQLPLFVWAVSITALLLLLSLPVLAGAITMLLTDRNLNTSFFDPAGGGDPILYQHL
>LEFIB595-10|HM871474|MM02304|Monopis_spilotella

TLYFLFGIWAGMVGTSLSLLIRMELGTPGLLIGDDQIYNTIVTAHAFIMIFFMVMPIMIGGFGNWLVPLMLNAPDMAFPRMNNMSFWLLPPSLLLLTSSSIVETGAGTGWTVYPPLSSTIAHGGASVDLAIFSLHLAGISSILGAINFITTMINMKPQTMK
FDQLPLFVWAVSITALLLLLSLPVLAGAITMLLTDRNLNTSFFDPAGGGDPILYQHL
>LEFIB970-10|HM871844|MM03158|Monopis_spilotella
TLYFLFGIWAGMVGTSLSLLIRMELGTPGLLIGDDQIYNTIVTTHAFIMIFFMVMPIMIGGFGNWLVPLMLNAPDMAFPRMNNMSFWLLPPSLLLLTSSSIVETGAGTGWTVYPPLSSTIAHGGASVDLAIFSLHLAGISSILGAINFITTMINMKPQTMK
FDQLPLFVWAVSITALLLLLSLPVLAGAITMLLTDRNLNTSFFDPAGGGDPILYQHL
>LEFIA804-10|HM386945|MM04158|Monopis_weaverella
TLYFLFGIWAGMVGTSLSILIRMELGTPGLLIGDDQIYNTIITAHAFIMIFFMVMPIMIGGFGNWLVPLMLTAPDMAFPRMNNMSFWLLPPSLLLLTSSSIVETGAGTGWTVYPPLSSNIAHGGTSVDLAIFSLHLAGISSILGAINFITTMINMKPQNLSF
DQLPLFVWAVSITALLLLLSLPVLAGAITMLLTDRNLNTSFFDPAGGGDPILYQHL
>LEFIB972-10|HQ570280|MM03162|Montescardia_tessulatellus
TLYFIFGIWSGMVGMSLSLIIRMELGTPGSLIGNDQIYNTIVTAHAFIMIFFMVMPIMIGGFGNWLVPLMLGAPDMAFPRMNNMSFWFLPPSLTLLLSSSLLENGVGTGWTVYPPLSANIAHSGSSVDLSIFSLHLAGISSILGAVNFITTVINMRSN-
ISFDQLPLFVWAVSITALLLLLSLPVLAGAITMLLTDRNLNTSFFDPAGGGDPILYQHL
>LEFIE414-10|HM874138|MM08999|Montescardia_tessulatellus
TLYFIFGIWSGMVGMSLSLIIRMELGTPGSLIGNDQIYNTIVTAHAFIMIFFMVMPIMIGGFGNWLVPLMLGAPDMAFPRMNNMSFWFLPPSLTLLLSSSLLENGVGTGWTVYPPLSANIAHSGSSVDLSIFSLHLAGISSILGAVNFITTVINMRSN-
ISFDQLPLFVWAVSITALLLLLSLPVLAGAITMLLTDRNLNTSFFDPAGGGDPILYQHL
>LEFIK495-10|JF854102|MM18070|Montescardia_tessulatellus
TLYFIFGIWSGMVGMSLSLIIRMELGTPGSLIGNDQIYNTIVTAHAFIMIFFMVMPIMIGGFGNWLVPLMLGAPDMAFPRMNNMSFWFLPPSLTLLLSSSLLENGVGTGWTVYPPLSANIAHSGSSVDLSIFSLHLAGISSILGAVNFITTVINMRSN-
ISFDQLPLFVWAVSITALLLLLSLPVLAGAITMLLTDRNLNTSFFDPAGGGDPILYQHL
>LEFIJ342-10|KM572960|MM15942|Mormo_maura
TLYFIFGIWAGMVGTSLSLLIRAELGNPGSLIGDDQIYNTIVTAHAFIMIFFMVMPIMIGGFGNWLVPLMLGAPDMAFPRMNNMSFWLLPPSLTLLISSSIVENGAGTGWTVYPPLSSNIAHGGSSVDLAIFSLHLAGISSILGAINFITTIINMRLNNLSFD
QMPLFVWAVGITAFLLLLSLPVLAGAITMLLTDRNLNTSFFDPAGGGDPILYQHL
>LEFIB579-10|HM871460|MM02260|Morophaga_choragella
TLYFIFGIWSGLVGTSLSLIIRTELGNPGSLIGNDQIYNTIVTAHAFIMIFFMVMPIMIGGFGNWLVPLMLGAPDMAFPRMNNMSFWFLPPSLTLLLSGSMVENGAGTGWTVYPPLSSNITHSGTSVDLSIFSLHLAGISSILGAINFITTMINMRSN-
MQFDQLPLFVWAVSITALLLLLSLPVLAGAITMLLTDRNLNTSFFDPAGGGDPILYQHL
>LEFID734-10|HM873491|MM06792|Myelois_circumvoluta
TLYFIFGVWSGMIGTSLSLLIRAELGTPGSLIGDDQIYNTIVTGHAFIMIFFMVMPIMIGGFGNWLVPLMLGAPDMAFPRMNNMSFWLLPPSLTLLISSSIVENGAGTGWTVYPPLSSNIAHGGSSVDLAIFSLHLAGISSILGAINFITTIINMKLNGMAF
DQMPLFVWAVGITALLLLLSLPVLAGAITMLLTDRNLNTSFFDPAGGGDPILYQHL
>LEFIE096-10|HM873843|MM08275|Myrmecozela_ochraceella
TLYFIFGIWSGMVGTSLSLLIRAELGNPGSLIGDDQIYNTIVTAHAFIMIFFMVMPIMIGGFGNWLVPLMLGAPDMAFPRMNNMSFWLLPPSLTLLISSSIVENGAGTGWTVYPPLSSNIAHGGGSVDLAIFSLHLAGISSILGAVNFITTVINMRTNGMS
FDRMPLFVWSVAITALLLLLSLPVLAGAITMLLTDRNLNTSFFDPAGGGDPILYQHL
>LEFIG658-10|HM876319|MM15522|Myrmecozela_ochraceella
TLYFIFGIWSGMVGTSLSLLIRAELGNPGSLIGDDQIYNTIVTAHAFIMIFFMVMPIMIGGFGNWLVPLMLGAPDMAFPRMNNMSFWLLPPSLTLLISSSIVENGAGTGWTVYPPLSSNIAHGGGSVDLAIFSLHLAGISSILGAVNFITTVINMRTNGMS
FDRMPLFVWSVAITALLLLLSLPVLAGAITMLLTDRNLNTSFFDPAGGGDPILYQHL
>LEFIA524-10|KM572250|MM01607|Mythimna_conigera
TLYFIFGIWAGMVGTSLSLLIRAELGTPGSLIGDDQIYNTIVTAHAFIMIFFMVMPIMIGGFGNWLVPLMLGAPDMAFPRMNNMSFWLLPPSLTLLISSSIVENGAGTGWTVYPPLSSNIAHGGSSVDLAIFSLHLAGISSILGAINFITTIINMRLNNLSFD
QMPLFIWAVGITAFLLLLSLPVLAGAITMLLTDRNLNTSFFDPAGGGDPILYQHL
>LEFIC757-10|HM872576|MM04874|Mythimna_conigera
TLYFIFGIWAGMVGTSLSLLIRAELGTPGSLIGDDQIYNTIVTAHAFIMIFFMVMPIMIGGFGNWLVPLMLGAPDMAFPRMNNMSFWLLPPSLTLLISSSIVENGAGTGWTVYPPLSSNIAHGGSSVDLAIFSLHLAGISSILGAINFITTIINMRLNNLSFD
QMPLFIWAVGITAFLLLLSLPVLAGAITMLLTDRNLNTSFFDPAGGGDPILYQHL
>LEEUA336-11|JN272154|MM19744|Mythimna_favicolor
TLYFIFGIWAGMVGTSLSLLIRAELGTPGSLIGDDQIYNTIVTAHAFIMIFFMVMPIMIGGFGNWLVPLMLGAPDMAFPRMNNMSFWLLPPSLTLLISSSIVENGAGTGWTVYPPLSSNIAHGGSSVDLAIFSLHLAGISSILGAINFITTIINMRLNSLSFD
QMPLFIWAVGITAFLLLLSLPVLAGAITMLLTDRNLNTSFFDPAGGGDPILYQHL
>LEFIA544-10|KM572440|MM01631|Mythimna_ferrago
TLYFIFGIWAGMVGTSLSLLIRAELGTPGSLIGDDQIYNTIVTAHAFIMIFFMVMPIMIGGFGNWLVPLMLGAPDMAFPRMNNMSFWLLPPSLTLLISSSIVENGAGTGWTVYPPLSSNIAHGGSSVDLAIFSLHLAGISSILGAINFITTIINMRLNSLSFD
QMPLFIWAVGITAFLLLLSLPVLAGAITMLLTDRNLNTSFFDPAGGGDPILYQHL
>LEFIF219-10|HM874912|MM10961|Mythimna_ferrago
TLYFIFGIWAGMVGTSLSLLIRAELGTPGSLIGDDQIYNTIVTAHAFIMIFFMVMPIMIGGFGNWLVPLMLGAPDMAFPRMNNMSFWLLPPSLTLLISSSIVENGAGTGWTVYPPLSSNIAHGGSSVDLAIFSLHLAGISSILGAINFITTIINMRLNSLSFD
QMPLFIWAVGITAFLLLLSLPVLAGAITMLLTDRNLNTSFFDPAGGGDPILYQHL

>LEFIJ1185-11|KM573622|MM21045|Mythimna_ferrago
TLYFIFGIWAGMVGTSLSLLIRAELGTPGSLIGDDQIYNTIVTAHAFIMIFFMVMPIMIGGFGNWLVPLMLGAPDMAFPRMNNMSFWLLPPSLTLLISSSIVENGAGTGWTVYPPLSSNIAHGGSSVDLAIFSLHLAGISSILGAINFITTIINMRLNSLSFD
QMPLFIWAVGITAFLLLLSLPVLAGAITMLLTDRNLNTSFFDPAGGGDPILYQHL
>LEFIA522-10|KM572706|MM01605|Mythimna_impura
TLYFIFGIWAGMVGTSLSLLIRAELGTPGSLIGDDQIYNTIVTAHAFIMIFFMVMPIMIGGFGNWLVPLMLGAPDMAFPRMNNMSFWLLPPSLTLLISSSIVENGAGTGWTVYPPLSSNIAHGGSSVDLAIFSLHLAGISSILGAINFITTIINMRLNNLSFD
QMPLFIWAVGITAFLLLLSLPVLAGAITMLLTDRNLNTSFFDPAGGGDPILYQHL
>LEFIC865-10|HM872683|MM05124|Mythimna_impura
TLYFIFGIWAGMVGTSLSLLIRAELGTPGSLIGDDQIYNTIVTAHAFIMIFFMVMPIMIGGFGNWLVPLMLGAPDMAFPRMNNMSFWLLPPSLTLLISSSIVENGAGTGWTVYPPLSSNIAHGGSSVDLAIFSLHLAGISSILGAINFITTIINMRLNNLSFD
QMPLFIWAVGITAFLLLLSLPVLAGAITMLLTDRNLNTSFFDPAGGGDPILYQHL
>LEFIJ355-10|KM572217|MM15955|Mythimna_l-album
TLYFIFGIWAGMVGTSLSLMIRAELGTPGSLIGDDQIYNTIVTAHAFIMIFFMVMPIMIGGFGNWLVPLMLGAPDMAFPRMNNMSFWLLPPSLTLLISSSIVESGAGTGWTVYPPLSSNIAHGGSSVDLAIFSLHLAGISSILGAINFITTIINMRLNNLSFD
QMPLFIWAVGITAFLLLLSLPVLAGAITMLLTDRNLNTSFFDPAGGGDPILYQHL
>LEFIF217-10|HM874910|MM10930|Mythimna_pudorina
TLYFIFGIWAGMVGTSLSLLIRAELGTPGSLIGDDQIYNTIVTAHAFIMIFFMVMPIMIGGFGNWLVPLMLGAPDMAFPRMNNMSFWLLPPSLTLLISSSIVENGAGTGWTVYPPLSSNIAHGGSSVDLAIFSLHLAGISSILGAINFITTIINMRLNSLSFD
QMPLFVWAVGITAFLLLLSLPVLAGAITMLLTDRNLNTSFFDPAGGGDPILYQHL
>LEFIK441-10|JF854064|MM18016|Mythimna_pudorina
TLYFIFGIWAGMVGTSLSLLIRAELGTPGSLIGDDQIYNTIVTAHAFIMIFFMVMPIMIGGFGNWLVPLMLGAPDMAFPRMNNMSFWLLPPSLTLLISSSIVENGAGTGWTVYPPLSSNIAHGGSSVDLAIFSLHLAGISSILGAINFITTIINMRLNSLSFD
QMPLFVWAVGITAFLLLLSLPVLAGAITMLLTDRNLNTSFFDPAGGGDPILYQHL
>LEFIL442-10|JF854597|MM18740|Mythimna_pudorina
TLYFIFGIWAGMVGTSLSLLIRAELGTPGSLIGDDQIYNTIVTAHAFIMIFFMVMPIMIGGFGNWLVPLMLGAPDMAFPRMNNMSFWLLPPSLTLLISSSIVENGAGTGWTVYPPLSSNIAHGGSSVDLAIFSLHLAGISSILGAINFITTIINMRLNSLSFD
QMPLFVWAVGITAFLLLLSLPVLAGAITMLLTDRNLNTSFFDPAGGGDPILYQHL
>LEFIC879-10|HM872697|MM05155|Mythimna_straminea
TLYFIFGIWAGMVGTSLSLLIRAELGTPGSLIGDDQIYNTIVTAHAFIMIFFMVMPIMIGGFGNWLVPLMLGAPDMAFPRMNNMSFWLLPPSLTLLISSSIVENGAGTGWTVYPPLSSNIAHGGSSVDLAIFSLHLAGISSILGAINFITTIINMRLNNLSFD
QMPLFIWAVGITAFLLLLSLPVLAGAITMLLTDRNLNTSFFDPAGGGDPILYQHL
>LEFIF215-10|HM874908|MM10923|Mythimna_straminea
TLYFIFGIWAGMVGTSLSLLIRAELGTPGSLIGDDQIYNTIVTAHAFIMIFFMVMPIMIGGFGNWLVPLMLGAPDMAFPRMNNMSFWLLPPSLTLLISSSIVENGAGTGWTVYPPLSSNIAHGGSSVDLAIFSLHLAGISSILGAINFITTIINMRLNNLSFD
QMPLFIWAVGITAFLLLLSLPVLAGAITMLLTDRNLNTSFFDPAGGGDPILYQHL
>LEFIJ889-10|JF853881|MM17514|Mythimna_turca
TLYFIFGIWAGMVGTSLSLLIRAELGTPGSLIGDDQIYNTIVTAHAFIMIFFMVMPIMIGGFGNWLVPLMLGAPDMAFPRMNNMSFWLLPPSLTLLISSSIVENGAGTGWTVYPPLSSNIAHGGSSVDLAIFSLHLAGISSILGAINFITTIINMRLNNLSFD
QMPLFVWAVGITAFLLLLSLPVLAGAITMLLTDRNLNTSFFDPAGGGDPILYQHL
>LEFIL609-10|KM573625|MM18907|Mythimna_turca
TLYFIFGIWAGMVGTSLSLLIRAELGTPGSLIGDDQIYNTIVTAHAFIMIFFMVMPIMIGGFGNWLVPLMLGAPDMAFPRMNNMSFWLLPPSLTLLISSSIVENGAGTGWTVYPPLSSNIAHGGSSVDLAIFSLHLAGISSILGAINFITTIINMRLNNLSFD
QMPLFVWAVGITAFLLLLSLPVLAGAITMLLTDRNLNTSFFDPAGGGDPILYQHL
>LEFIC623-10|HM872444|MM04557|Naenia_typica
TLYFIFGIWAGMVGTSLSLLIRAELGNPGSLIGDDQIYNTIVTAHAFIMIFFMVMPIMIGGFGNWLVPLMLGAPDMAFPRMNNMSFWLLPPSLTLLISSSIVENGAGTGWTVYPPLSSNIAHGGSSVDLAIFSLHLAGISSILGAINFITTIINMRLNSLSFD
QMPLFIWAVGITAFLLLLSLPVLAGAITMLLTDRNLNTSFFDPAGGGDPILYQHL
>LEFIA863-10|HM387001|MM09740|Naenia_typica
TLYFIFGIWAGMVGTSLSLLIRAELGNPGSLIGDDQIYNTIVTAHAFIMIFFMVMPIMIGGFGNWLVPLMLGAPDMAFPRMNNMSFWLLPPSLTLLISSSIVENGAGTGWTVYPPLSSNIAHGGSSVDLAIFSLHLAGISSILGAINFITTIINMRLNSLSFD
QMPLFIWAVGITAFLLLLSLPVLAGAITMLLTDRNLNTSFFDPAGGGDPILYQHL
>LEEUA043-11|JN283834|MM19356|Narycia_duplicella
TLYFILGVWSGILGTSLSLLIRTELGTPQSLIGNDQIYNTIVTAHAFIMIFFMVMPIMIGGFGNWLVPLMLGAPDMAFPRMNNMSFWLLPPSLLLLIMSSIVENGAGTGWTVYPPLSSNIAHSGSSVDLAIFSLHLAGISSILGAVNFITTTINMRPNNMSF
DRMPLFVWSVAITALLLLLSLPVLAGAITMLLTDRNLNTSFFDPAGGGDPILFQHL
>LEFIA644-10|HM870893|MM01760|Nascia_cilialis
TLYFIFGIWSGMVGTSLSLLIRAELGNPGSLIGDDQIYNTIVTAHAFIMIFFMVMPIMIGGFGNWLVPLMLGAPDMAFPRMNNMSFWLLPPSLTLLISSSIVENGAGTGWTVYPPLSSNIAHGGSSVDLAIFSLHLAGISSILGAINFITTIINMRVNGMSF
DQMPLFVWSVGITALLLLLSLPVLAGAITMLLTDRNLN?SFFDPAGGGDPILYQHL
>LEFIK826-10|JN277456|MM18401|Nascia_cilialis
TLYFIFGIWSGMVGTSLSLLIRAELGNPGSLIGDDQIYNTIVTAHAFIMIFFMVMPIMIGGFGNWLVPLMLGAPDMAFPRMNNMSFWLLPPSLTLLISSSIVENGAGTGWTVYPPLSSNIAHGGSSVDLAIFSLHLAGISSILGAINFITTIINMRVNGMSF

DQMPLFVWSVGITALLLLLSLPVLAGAITMLLTDRNLNTSFFDPAGGGDPILYQHL
>LEFIL338-10|JN277459|MM18648|Nascia_cilialis
TLYFIFGIWSGMVGTSLSLLIRAELGNPGSLIGDDQIYNTIVTAHAFIMIFFMVMPIMIGGFGNWLVPLMLGAPDMAFPRMNNMSFWLLPPSLTLLISSSIVENGAGTGWTVYPPLSSNIAHSGSSVDLAIFSLHLAGISSILGAINFITTIINMRVNGMSFD
QMPLFVWSVGITALLLLLSLPVLAGAITMLLTDRNLNTSFFDPAGGGDPILYQHL
>LEFIJ696-10|JF853777|MM17321|Nemapogon_clematella
TLYFIFGIWSSMVGTSLSILIRTELSIPGSLINNDQIYNSIVTAHAFIMIFFMIMPIMIGGFGNWLVPLMLGAPDMAFPRMNNMSFWFLPPSLMLLISSMVMENGAGTGWTVYPPLSSNISHSGSSVDLTIFSLHLAGISSILGAINFITTMINMRPINMSL
DMMPLLVWAIFITVVLLLLSLPVLAGAITMLLTDRNLNTSFFDPSGGGDPILYQHL
>LEFIL598-10|MM18896|Nemapogon_clematella
TLYFIFGIWSSMVGTSLSILIRTELSIPGSLINNDQIYNSIVTAHAFIMIFFMIMPIMIGGFGNWLVPLMLGAPDMAFPRMNNMSFWFLPPSLMLLISSMVMENGAGTGWTVYPPLSSNISHSGSSVDLTIFSLHLAGISSILGAINFITTMINMRPINMSL
DMMPLLVWAIFITVVLLLLSLPVLAGAITMLLTDRNLNTSFFDPSGGGDPILYQHL
>LEFIB591-10|HM871470|MM02294|Nemapogon_cloacella
TLYFIFGIWASMIGTSLSILIRTELSNPGSFINNDQIYNSIVTAHAFIMIFFMIMPIMIGGFGNWLIPLMLGAPDMAFPRMNNMSFWFLPPSLMLLISSMMMENGAGTGWTVYPPLSSNIAHSGTSVDLTIFSLHLAGISSILGAINFITTMINMRPLNMSL
DMMPLLVWAIFITVVLLLLSLPVLAGAITMLLTDRNLNTSFFDPSGGGDPILYQHL
>LEFIB971-10|HM871845|MM03160|Nemapogon_cloacella
TLYFIFGIWASMIGTSLSILIRTELSNPGSFINNDQIYNSIVTAHAFIMIFFMIMPIMIGGFGNWLIPLMLGAPDMAFPRMNNMSFWFLPPSLMLLISSMMMENGAGTGWTVYPPLSSNIAHSGTSVDLTIFSLHLAGISSILGAINFITTMINMRPLNMSL
DMMPLLVWAIFITVVLLLLSLPVLAGAITMLLTDRNLNTSFFDPSGGGDPILYQHL
>LEFIC331-10|HM872175|MM03836|Nemapogon_cloacella
TLYFIFGIWASMIGTSLSILIRTELSNPGSFINNDQIYNSIVTAHAFIMIFFMIMPIMIGGFGNWLIPLMLGAPDMAFPRMNNMSFWFLPPSLMLLISSMMMENGAGTGWTVYPPLSSNIAHSGTSVDLTIFSLHLAGISSILGAINFITTMINMRPLNMSL
DMMPLLVWAIFITVVLLLLSLPVLAGAITMLLTDRNLNTSFFDPSGGGDPILYQHL
>LEFIC484-10|HM872316|MM04081|Nemapogon_fungivorella
TLYFIFGIWASMIGTSLSILIRTELSSPGSFINNDQIYNSIVTAHAFIMIFFMIMPIMIGGFGNWLVPLMLGAPDMAFPRMNNMSFWFLPPSLMLLISSMMMENGAGTGWTVYPPLSSNIAHSGSSVDLTIFSLHLAGISSILGAINFITTMINMRPMNM
SLDMMPLLVWAIFITVVLLLLSLPVLAGAITMLLTDRNLNTSFFDPSGGGDPILYQHL
>LEFIE188-10|HM873933|MM08503|Nemapogon_granella
TLYFIFGIWASMVGTSLSILIRTELSNPGSFINNDQIYNSIVTAHAFIMIFFMIMPIMIGGFGNWLVPLMLGAPDMAFPRMNNMSFWFLPPSLMLLISSMMMENGAGTGWTVYPPLSSNIAHSGSSVDLTIFSLHLAGISSILGAINFITTMINMRPLNM
SLDMMPLLVWAILITVVLLLLSLPVLAGAITMLLTDRNLNTSFFDPSGGGDPILYQHL
>LEFID753-10|HM873510|MM06814|Nemapogon_nigralbella
TLYFIFGIWASMVGTSLSILIRTELSSPGSFINNDQIYNSIVTAHAFIMIFFMIMPIMIGGFGNWLVPLMLGAPDMAFPRMNNMSFWFLPPSLMLLISSMMMENGAGTGWTVYPPLSSNIAHSGSSVDLTIFSLHLAGISSILGAINFITTMINMRPSNMS
LDMMPLLVWAILITVVLLLLSLPVLAGAITMLLTDRNLNTSFFDPSGGGDPILYQHL
>LEFIB594-10|HM871473|MM02302|Nemapogon_picarella
TLYFIFGIWASMVGTSLSILIRTELSSPGSFINNDQIYNSIVTAHAFIMIFFMIMPIMIGGFGNWLVPLMLGAPDMAFPRMNNMSFWFLPPSLMLLISSMMMENGAGTGWTVYPPLSSNIAHSGSSVDLTIFSLHLAGISSILGAINFITTMINMRPSNMS
LDMMPLLVWAILITVVLLLLSLPVLAGAITMLLTDRNLNTSFFDPSGGGDPILYQHL
>LEFIC322-10|HM872166|MM03826|Nemapogon_picarella
TLYFIFGIWASMVGTSLSILIRTELSSPGSFINNDQIYNSIVTAHAFIMIFFMIMPIMIGGFGNWLVPLMLGAPDMAFPRMNNMSFWFLPPSLMLLISSMMMENGAGTGWTVYPPLSSNIAHSGSSVDLTIFSLHLAGISSILGAINFITTMINMRPSNMS
LDMMPLLVWAILITVVLLLLSLPVLAGAITMLLTDRNLNTSFFDPSGGGDPILYQHL
>LEFIG308-10|HM875987|MM14272|Nemapogon_picarella
TLYFIFGIWASMVGTSLSILIRTELSSPGSFINNDQIYNSIVTAHAFIMIFFMIMPIMIGGFGNWLVPLMLGAPDMAFPRMNNMSFWFLPPSLMLLISSMMMENGAGTGWTVYPPLSSNIAHSGSSVDLTIFSLHLAGISSILGAINFITTMINMRPSNMS
LDMMPLLVWAILITVVLLLLSLPVLAGAITMLLTDRNLNTSFFDPSGGGDPILYQHL
>LEFIK498-10|JF854105|MM18073|Nemapogon_variatella
TLYFIFGIWASMVGTSLSILIRTELSNPGSLINNDQIYNSIVTAHAFIMIFFMIMPIMIGGFGNWLVPLMLGAPDMAFPRMNNMSFWFLPPSLMLLISSMMMENGAGTGWTVYPPLSSNIAHSGSSVDLTIFSLHLAGISSILGAINFITTMINMRPLNM
SLDMMPLLVWAILITVVLLLLSLPVLAGAITMLLTDRNLNTSFFDPSGGGDPILYQHL
>LEFIJ1242-11|KF808859|MM21102|Nemapogon_variatella
TLYFIFGIWASMVGTSLSILIRTELSNPGSLINNDQIYNSIVTAHAFIMIFFMIMPIMIGGFGNWLVPLMLGAPDMAFPRMNNMSFWFLPPSLMLLISSMMMENGAGTGWTVYPPLSSNIAHSGSSVDLTIFSLHLAGISSILGAINFITTMINMRPLNM
SLDMMPLLVWAILITVVLLLLSLPVLAGAITMLLTDRNLNTSFFDPSGGGDPILYQHL
>LEFID998-10|HM873748|MM08048|Nemapogon_wolffiella
TLYFIFGIWASMIGTSLSILIRTELSSPGSFINNDQIYNSIVTAHAFIMIFFMIMPIMIGGFGNWLVPLMLGAPDMAFPRMNNMSFWFLPPSLMLLISSMMMENGAGTGWTVYPPLSSNIAHSGSSVDLTIFSLHLAGISSILGAINFITTMINMRPLNMS
LDMMPLLVWAIFITVVLLLLSLPVLAGAITMLLTDRNLNTSFFDPSGGGDPILYQHL
>LEFIB581-10|HM871461|MM02269|Nematopogon_magna

TLYFIFGIWSGLLGTSMSILIRTELSMPGTLIGNDQIYNTIVTAHAFIMIFFMVMPIMIGGFGNWLVPLMLGAPDMAFPRLNNMSFWMLPPSLTLLISSSLAENGAGTGWTVYPPLSSNISHSGASVDLTIFSLHLAGISSILGAVNFITTVINMRTKNMAF
DQMPLFVWSVAITALLLLLSLPVLAGAITMLLTDRNLNTSFFDPAGGGDPILYQHL
>LEFIB969-10|HM871843|MM03156|Nematopogon_magna
TLYFIFGIWSGLLGTSMSILIRTELSMPGTLIGNDQIYNTIVTAHAFIMIFFMVMPIMIGGFGNWLVPLMLGAPDMAFPRLNNMSFWMLPPSFTLLISSSLAENGAGTGWTVYPPLSSNISHSGASVDLTIFSLHLAGISSILGAVNFITTVINMRTKNMAF
DQMPLFVWSVAITALLLLLSLPVLAGAITMLLTDRNLNTSFFDPAGGGDPILYQHL
>LEFIC300-10|HM872144|MM03782|Nematopogon_metaxella
TLYFIFGIWSGLIGTSLSILIRTELSMPGTLIGNDQIYNTIVTAHAFIMIFFMVMPIMIGGFGNWLVPLMLGAPDMAFPRLNNMSFWLLPPSLTLLLSSSLTENGAGTGWTVYPPLSSNIAHAGTSVDLTIFSLHLAGISSILGAVNFITTVINMRTKNMSFD
QMPLFVWSVAITALLLLLSLPVLAGAITMLLTDRNLNTSFFDPAGGGDPILYQHL
>LEFIJ061-10|KM573238|MM03896|Nematopogon_pilella
TLYFIFGIWSGLLGTSMSILIRTELSMPGTLIGNDQIYNTIVTAHAFIMIFFMVMPIMIGGFGNWLVPLMLGAPDMAFPRLNNMSFWLLPPSLTLLLSSSLTENGAGTGWTVYPPLSSNIAHAGASVDLTIFSLHLAGISSILGAVNFITTVINMRTKNMAF
DQMPLFVWSVAITALLLLLSLPVLAGAITMLLTDRNLNTSFFDPAGGGDPILYQHL
>LEFIE247-10|HM873981|MM08574|Nematopogon_pilella
TLYFIFGIWSGLLGTSMSILIRTELSMPGTLIGNDQIYNTIVTAHAFIMIFFMVMPIMIGGFGNWLVPLMLGAPDMAFPRLNNMSFWLLPPSLTLLLSSSLTENGAGTGWTVYPPLSSNIAHAGASVDLTIFSLHLAGISSILGAVNFITTVINMRTKNMAF
DQMPLFVWSVAITALLLLLSLPVLAGAITMLLTDRNLNTSFFDPAGGGDPILYQHL
>LEFIC079-10|HM871947|MM03375|Nematopogon_robertella
TMYFIFGIWSGLLGTSMSILIRTELSMPGTLIGNDQIYNTIVTAHAFIMIFFMVMPIMIGGFGNWLVPLMLGAPDMAFPRLNNMSFWLLPPSLTLLISSSLAENGAGTGWTVYPPLSSNIAHAGASVDLTIFSLHLAGISSILGAVNFITTVINMRTKNMAF
DQMPLFVWSVAITALLLLLSLPVLAGAITMLLTDRNLNTSFFDPAGGGDPILYQHL
>LEFIE814-10|HM874533|MM10013|Nematopogon_schwarziellus
TLYFIFGIWSGLIGTSLSILIRTELSMPGTLIGNDQIYNTIVTAHAFIMIFFMVMPIMIGGFGNWLVPLMLGAPDMAFPRLNNMSFWLLPPSLTLLLSSSLAENGAGTGWTVYPPLSSNIAHAGASVDLTIFSLHLAGISSILGAVNFITTVINMRTKNMSFD
QMPLFVWSVAITALLLLLSLPVLAGAITMLLTDRNLNTSFFDPAGGGDPILYQHL
>LEFIG169-10|HM875849|MM14030|Nematopogon_schwarziellus
TLYFIFGIWSGLIGTSLSILIRTELSMPGTLIGNDQIYNTIVTAHAFIMIFFMVMPIMIGGFGNWLVPLMLGAPDMAFPRLNNMSFWLLPPSLTLLLSSSLAENGAGTGWTVYPPLSSNIAHAGASVDLTIFSLHLAGISSILGAVNFITTVINMRTKNMSFD
QMSLFVWSVAITALLLLLSLPVLAGAITMLLTDRNLNTSFFDPAGGGDPILYQHL
>LEFIF929-10|HM875611|MM13371|Nematopogon_swammerdamella
TLYFIFGIWSGLVGTSLSILIRTELSMPGTLIGNDQIYNTIVTAHAFIMIFFMVMPIMIGGFGNWLVPLMLGAPDMAFPRLNNMSFWLLPPSLTLLLSSSLTENGAGTGWTVYPPLSSNIAHAGASVDLTIFSLHLAGISSILGAVNFITTVINMRTKNMTFD
QMPLFVWSVAITALLLLLSLPVLAGAITMLLTDRNLNTSFFDPAGGGDPILYQHL
>LEFIG140-10|HM875820|MM13975|Nematopogon_swammerdamella
TLYFIFGIWSGLVGTSLSILIRTELSMPGTLIGNDQIYNTIVTAHAFIMIFFMVMPIMIGGFGNWLVPLMLGAPDMAFPRLNNMSFWLLPPSLTLLLSSSLAENGAGTGWTVYPPLSSNIAHAGASVDLTIFSLHLAGISSILGAVNFITTVINMRTKNMTFD
QMPLFVWSVAITALLLLLSLPVLAGAITMLLTDRNLNTSFFDPAGGGDPILYQHL
>LEFIB015-10|HM870928|MM00036|Nemaxera_betulinella
TLYFIFGIWASMVGTSLSILIRTELSNPGSFINNDQIYNSIVTAHAFIMIFFMIMPIMIGGFGNWLVPLMLGAPDMAFPRMNNMSFWFLPPSLMLLISSMMMENGAGTGWTVYPPLSSNIAHSGSSVDLTIFSLHLAGISSILGAINFITTMINMRPLNM
SLDMMPLLVWAIFITVVLLLLSLPVLAGAITMLLTDRNLNTSFFDPSGGGDPILYQHL
>LEFIB229-10|HM871132|MM00688|Nemophora_amatella
TLYFFFGIWSGLLGTSLSLLIRTELGMPGSLIGNDQIYNTIVTAHAFIMIFFMVMPIMIGGFGNWLVPLMLGAPDMAFPRLNNMSFWLLPPSLMLLISSSLVENGAGTGWTVYPPLSSNISHSGTSVDLTIFSLHLAGISSILGAINFITTVINMRMINMTFD
QMSLFTWSVAITALLLLLSLPVLAGAITMLLTDRNLNTSFFDPMGGGDPILYQHL
>LEFIA788-10|HM386929|MM04142|Nemophora_bellela
TLYFFFGIWSGLLGTSLSLLIRTELGMPGSLIGNDQIYNTIVTAHAFIMIFFMVMPIMIGGFGNWLVPLMLGAPDMAFPRLNNMSFWLLPPSLMILISSSLVENGAGTGWTVYPPLSSNISHSGTSVDLTIFSLHLAGISSILGAINFITTVINMRMINMTFD
QMSLFTWSVAITALLLLLSLPVLAGAITMLLTDRNLNTSFFDPMGGGDPILYQHL
>LEFIK488-10|JF854097|MM18063|Nemophora_bellela
TLYFFFGIWSGLLGTSLSLLIRTELGMPGSLIGNDQIYNTIVTAHAFIMIFFMVMPIMIGGFGNWLVPLMLGAPDMAFPRLNNMSFWLLPPSLMLLISSSLVENGAGTGWTVYPPLSSNISHSGTSVDLTIFSLHLAGISSILGAINFITTVINMRMINMTFD
QMSLFTWSVVITALLLLLSLPVLAGAITMLLTDRNLNTSFFDPMGGGDPILYQHL
>LEFIL243-10|JF854548|MM19243|Nemophora_cupriacella
TLYFFFGIWSGLLGTSLSLLIRTELGMPGSLIGNDQIYNTIVTAHAFIMIFFMVMPIMIGGFGNWLVPLMLGAPDMAFPRLNNMSFWLLPPSLMLLISSSLVENGSGTGWTVYPPLSSNISHSGTSVDLTIFSLHLAGISSILGAINFITTVINMRTMNMTF
DQMSLFTWSVAITALLLLLSLPVLAGAITMLLTDRNLNTSFFDPMGGGDPIL?QHL
>LEFID582-10|HM873347|MM06587|Nemophora_degeerella
TLYFFFGIWSGLLGTSLSLLIRTELGMPGSLIGNDQIYNTIVTAHAFIMIFFMVMPIMIGGFGNWLVPLMLGAPDMAFPRLNNMSFWLLPPSLMLLISSSLVENGAGTGWTVYPPLSSNISHSGTSVDLTIFSLHLAGISSILGAINFITTVINMRMINMTFD
QMSLFTWSVAITALLLLLSLPVLAGAITMLLTDRNLNTSFFDPMGGGDPILYQHL

>LEFIE624-10|HM874347|MM09554|Nemophora_degeerella
TLYFFFGIWSGLLGTSLSLLIRTELGMPGSLIGNDQIYNTIVTAHAFIMIFFMVMPIMIGGFGNWLVPLMLGAPDMAFPRLNNMSFWLLPPSLMLLISSSLVENGAGTGWTVYPPLSSNISHSGTSVDLTIFSLHLAGISSILGAINFITTVINMRMINMTFD
QMSLFTWSVAITALLLLLSLPVLAGAITMLLTDRNLNTSFFDPMGGGDPILYQHL
>LEFID606-10|HM873371|MM06620|Nemophora_metallica
TLYFFFGIWSGLLGTSLSLLIRTELGMPGSLIGNDQIYNTIVTAHAFIMIFFMVMPIMIGGFGNWLVPLMLGAPDMAFPRLNNMSFWLLPPSLMLLISSSLVESGSGTGWTVYPPLSSNISHSGTSVDLTIFSLHLAGISSILGAINFITTVINMRTMNMTFD
QMSLFTWSVAITALLLLLSLPVLAGAITMLLTDRNLNTSFFDPMGGGDPILYQHL
>LEEUA033-11|JN261650|MM19346|Nemophora_ochsenheimerella
TLYFFFGIWSGLLGTSLSLLIRTELGMPGSLIGNDQIYNTIVTAHAFIMIFFMVMPIMIGGFGNWLVPLMLGAPDMAFPRMNNMSFWLLPPSLMLLISGSLVENGAGTGWTVYPPLSSNISHSGTSVDLTIFSLHLAGISSILGAINFITTVINMRMMNM
TFDQMSLFTWSVAITALLLLLSLPVLAGAITMLLTDRNLNTSFFDPMGGGDPILYQHL
>LEFIC379-10|HM872222|MM03909|Neofaculta_ericetella
TLYFIFGIWAGMVGTSLSLLIRAELGNPGSLIGDDQIYNTIVTAHAFIMIFFMVMPIMIGGFGNWLVPLMLGAPDMAFPRMNNMSFWLLPPSLTLLISSSIVENGAGTGWTVYPPLSSNIAHGGSSVDLAIFSLHLAGISSILGAINFITTIINMRVNGMSF
DQMPLFVWAVGITALLLLLSLPVLAGAITMLLTDRNLNTSFFDPAGGGDPILYQHL
>LEFIG138-10|HM875818|MM13970|Neofaculta_ericetella
TLYFIFGIWAGMVGTSLSLLIRAELGNPGSLIGDDQIYNTIVTAHAFIMIFFMVMPIMIGGFGNWLVPLMLGAPDMAFPRMNNMSFWLLPPSLTLLISSSIVENGAGTGWTVYPPLSSNIAHGGSSVDLAIFSLHLAGISSILGAINFITTIINMRVNGMSF
DQMPLFVWAVGITALLLLLSLPVLAGAITMLLTDRNLNTSFFDPAGGGDPILYQHL
>LEFIG139-10|HM875819|MM13971|Neofaculta_ericetella
TLYFIFGIWAGMVGTSLSLLIRAELGNPGSLIGDDQIYNTIVTAHAFIMIFFMVMPIMIGGFGNWLVPLMLGAPDMAFPRMNNMSFWLLPPSLTLLISSSIVENGAGTGWTVYPPLSSNIAHGGSSVDLAIFSLHLAGISSILGAINFITTIINMRVNGMSF
DQMPLFVWAVGITALLLLLSLPVLAGAITMLLTDRNLNTSFFDPAGGGDPILYQHL
>LEFIC109-10|HQ570300|MM03437|Neofaculta_infernella
TLYFIFGIWAGMVGTSLSLLIRAELGNPGSLIGDDQIYNTIVTAHAFIMIFFMVMPIMIGGFGNWLVPLMLGAPDMAFPRMNNMSFWLLPPSLTLLISSSIVENGAGTGWTVYPPLSSNIAHGGSSVDLAIFSLHLAGISSILGAINFITTIINMRANGMSF
DQMPLFVWAVGITALLLLLSLPVLAGAITMLLTDRNLNTSFFDPAGGGDPILYQHL
>LEFIC514-10|HM872338|MM04223|Neofaculta_infernella
TLYFIFGIWAGMVGTSLSLLIRAELGNPGSLIGDDQIYNTIVTAHAFIMIFFMVMPIMIGGFGNWLVPLMLGAPDMAFPRMNNMSFWLLPPSLTLLISSSIVENGAGTGWTVYPPLSSNIAHSGSSVDLAIFSLHLAGISSILGAINFITTIINMRINGMSFD
QMPLFVWAVGITALLLLLSLPVLAGAITMLLTDRNLNTSFFDPAGGGDPILYQHL
>LEFID030-10|HM872844|MM05472|Neofaculta_infernella
TLYFIFGIWAGMVGTSLSLLIRAELGNPGSLIGDDQIYNTIVTAHAFIMIFFMVMPIMIGGFGNWLVPLMLGAPDMAFPRMNNMSFWLLPPSLTLLISSSIVENGAGTGWTVYPPLSSNIAHGGSSVDLAIFSLHLAGISSILGAINFITTIINMRANGMSF
DQMPLFVWAVGITALLLLLSLPVLAGAITMLLTDRNLNTSFFDPAGGGDPILYQHL
>LEFIK675-10|JF854243|MM18250|Neofaculta_infernella
TLYFIFGIWAGMVGTSLSLLIRAELGNPGSLIGDDQIYNTIVTAHAFIMIFFMVMPIMIGGFGNWLVPLMLGAPDMAFPRMNNMSFWLLPPSLTLLISSSIVENGAGTGWTVYPPLSSNIAHGGSSVDLAIFSLHLAGISSILGAINFITTIINMRANGMSF
DQMPLFVWAVGITALLLLLSLPVLAGAITMLLTDRNLNTSFFDPAGGGDPILYQHL
>LEFIC843-10|HM872661|MM05055|Neofriseria_peliella
TLYFIFGIWAGMVGTSLSLLIRAELGNPGSLIGDDQIYNTIVTAHAFIMIFFMVMPIMIGGFGNWLVPLMLGAPDMAFPRMNNMSFWLLPPSLTLLISSSIVENGAGTGWTVYPPLSSNIAHGGSSVDLAIFSLHLAGISSILGAINFITTIINMRVNGLSFD
QMPLFVWAVGITALLLLLSLPVLAGAITMLLTDRNLNTSFFDPAGGGDPILYQHL
>LEFIF468-10|HM875153|MM11996|Neofriseria_peliella
TLYFIFGIWAGMVGTSLSLLIRAELGNPGSLIGDDQIYNTIVTAHAFIMIFFMVMPIMIGGFGNWLVPLMLGAPDMAFPRMNNMSFWLLPPSLTLLISSSIVENGAGTGWTVYPPLSSNIAHGGSSVDLAIFSLHLAGISSILGAINFITTIINMRVNGLSFD
QMPLFVWAVGITALLLLLSLPVLAGAITMLLTDRNLNTSFFDPAGGGDPILYQHL
>LEFIF966-10|HM875647|MM13547|Neofriseria_peliella
TLYFIFGIWAGMVGTSLSLLIRAELGNPGSLIGDDQIYNTIVTAHAFIMIFFMVMPIMIGGFGNWLVPLMLGAPDMAFPRMNNMSFWLLPPSLTLLISSSIVENGAGTGWTVYPPLSSNIAHGGSSVDLAIFSLHLAGISSILGAINFITTIINMRVNGLSFD
QMPLFVWAVGITALLLLLSLPVLAGAITMLLTDRNLNTSFFDPAGGGDPILYQHL
>LEFIL530-10|JF854642|MM18828|Neofriseria_peliella
TLYFIFGIWAGMVGTSLSLLIRAELGNPGSLIGDDQIYNTIVTAHAFIMIFFMVMPIMIGGFGNWLVPLMLGAPDMAFPRMNNMSFWLLPPSLTLLISSSIVENGAGTGWTVYPPLSSNIAHGGSSVDLAIFSLHLAGISSILGAINFITTIINMRVNGLSFD
QMPLFVWAVGITALLLLLSLPVLAGAITMLLTDRNLNTSFFDPAGGGDPILYQHL
>LEFIK144-10|JN270951|MM17719|Neofriseria_singula
TLYFIFGIWAGMVGTSLSLLIRAELGNPGSLIGDDQIYNTIVTAHAFIMIFFMVMPIMIGGFGNWLVPLMLGAPDMAFPRMNNMSFWLLPPSLTLLISSSIVENGAGTGWTVYPPLSSNIAHGGSSVDLAIFSLHLAGISSILGAINFITTIINMRVNGLSFD
QMPLFVWAVGITALLLLLSLPVLAGAITMLLTDRNLNTSFFDPAGGGDPILYQHL
>LEFIK145-10|JN270952|MM17720|Neofriseria_singula
TLYFIFGIWAGMVGTSLSLLIRAELGNPGSLIGDDQIYNTIVTAHAFIMIFFMVMPIMIGGFGNWLVPLMLGAPDMAFPRMNNMSFWLLPPSLTLLISSSIVENGAGTGWTVYPPLSSNIAHGGSSVDLAIFSLHLAGISSILGAINFITTIINMRVNGLSFD

QMPLFVWAVGITALLLLLSLPVLAGAITMLLTDRNLNTSFFDPAGGGDPILYQHL
>LEFID038-10|HM872852|MM05492|Neosphaleroptera_nubilana
TLYFIFGIWAGMIGTSLSLLIRAELGNPGSLIGDDQIYNTIVTAHAFIMIFFMVMPIMIGGFGNWLVPLMLGAPDMAFPRMNNMSFWLLPPSILLLISSSIVENGAGTGWTVYPPLSSNIAHSGSSVDLTIFSLHLAGISSILGAVNFITTIINMRPNNMML
DQMPLFVWAVGITALLLLLSLPVLAGAITMLLTDRNLNTSFFDPAGGGDPILYQHL
>LEFIK137-10|JN270947|MM17712|Neotelphusa_sequax
TLYFIFGIWAGMVGTSLSLLIRAELGNPGSLIGDDQIYNTIVTAHAFIMIFFMVMPIMIGGFGNWLVPLMLGAPDMAFPRMNNMSFWLLPPSLTLLISSSIVENGAGTGWTVYPPLSSNIAHGGSSVDLAIFSLHLAGISSILGAINFITTIINMKINGLSFD
QMPLFVWAVGITALLLLLSLPVLAGAITMLLTDRNLNTSFFDPAGGGDPILYQHL
>LEFIJ609-10|JF853709|MM17234|Nephopterix_angustella
TLYFIFGIWSGMVGTSLSLLIRAELGTPGSLIGDDQIYNTIVTGHAFIMIFFMVMPIMIGGFGNWLVPLMLGAPDMAFPRMNNMSFWLLPPSLTLLISSSIVENGAGTGWTVYPPLSSNIAHSGSSVDLAIFSLHLAGISSILGAINFITTIINMKLNGLSFDQ
MPLFVWAVGITALLLLLSLPVLAGAITMLLTDRNLNTSFFDPAGGGDPILYQHL
>LEEUA270-11|JN275972|MM19678|Nephopterix_angustella
TLYFIFGIWSGMVGTSLSLLIRAELGTPGSLIGDDQIYNTIVTGHAFIMIFFMVMPIMIGGFGNWLVPLMLGAPDMAFPRMNNMSFWLLPPSLTLLISSSIVENGAGTGWTVYPPLSSNIAHSGSSVDLAIFSLHLAGISSILGAINFITTIINMKLNGLSFDQ
MPLFVWAVGITALLLLLSLPVLAGAITMLLTDRNLNTSFFDPAGGGDPILYQHL
>LEFIC010-10|HM871880|MM03213|Niditinea_fuscella
TLYFIFGIWAGMLGTSLSLLIRAELGNPGLLIGDDQIYNTIVTAHAFIMIFFMVMPIMIGGFGNWLIPLMLGAPDMAFPRLNNMSFWLLPPSLLLLTSSSLVENGAGTGWTVYPPLSSNIAHGGSSVDLAIFSLHLAGISSILGAINFITTMINMRPMKMSL
DQMPLFAWAVLITAVLLLLSLPVLAGAITMLLTDRNLNTSFFDPAGGGDPILYQHL
>LEFIB797-10|HM871674|MM02709|Niditinea_striolella
TLYFIFGIWAGMLGTSLSLLIRTELGNPGLLIGDDQIYNTIVTAHAFIMIFFMVMPIMIGGFGNWLVPLMLGAPDMAFPRLNNMSFWLLPPSLLLLTSSSIVENGAGTGWTVYPPLSSNIAHGGSSVDLAIFSLHLAGISSILGAINFITTMINMRPMKMSL
DQMPLFAWAVLITAVLLLLSLPVLAGAITMLLTDRNLNTSFFDPAGGGDPILYQHL
>LEFIG372-10|HM876049|MM14377|Niditinea_striolella
TLYFIFGIWAGMLGTSLSLLIRTELGNPGLLIGDDQIYNTIVTAHAFIMIFFMVMPIMIGGFGNWLVPLMLGAPDMAFPRLNNMSFWLLPPSLLLLTSSSIVENGAGTGWTVYPPLSSNIAHGGSSVDLAIFSLHLAGISSILGAINFITTMINMRPMKMSL
DQMPLFAWAVLITAVLLLLSLPVLAGAITMLLTDRNLNTSFFDPAGGGDPILYQHL
>LEFIB216-10|HM871120|MM00670|Niditinea_truncicolella
TLYFIFGIWAGMVGTSLSLLIRAELGNPGLLIGDDQIYNTIVTAHAFVMIFFMVMPIMIGGFGNWLVPLMLGAPDMAFPRLNNMSFWLLPPSLLLLTSSSLVENGAGTGWTVYPPLSSNIAHGGSSVDLAIFSLHLAGISSILGAINFITTMINMRPMKM
SMDQMPLFVWAVLITAVLLLLSLPVLAGAITMLLTDRNLNTSFFDPAGGGDPILYQHL
>LEFIL629-10|JF854682|MM18927|Niditinea_truncicolella
TLYFIFGIWAGMVGTSLSLLIRAELGNPGLLIGDDQIYNTIVTAHAFVMIFFMVMPIMIGGFGNWLVPLMLGAPDMAFPRLNNMSFWLLPPSLLLLTSSSLVENGAGTGWTVYPPLSSNIAHGGSSVDLAIFSLHLAGISSILGAINFITTMINMRPMKM
SMDQMPLFVWAVLITAVLLLLSLPVLAGAITMLLTDRNLNTSFFDPAGGGDPILYQHL
>LEFIF304-10|HM874991|MM11092|Noctua_comes
TLYFIFGIWAGMVGTSLSLLIRAELGNPGSLIGDDQIYNTIVTAHAFIMIFFMVMPIMIGGFGNWLVPLMLGAPDMAFPRMNNMSFWLLPPSLTLLISSSIVENGAGTGWTVYPPLSSNIAHGGSSVDLAIFSLHLAGISSILGAINFITTIINMRLNSLSFD
QMPLFIWAVGITAFLLLLSLPVLAGAITMLLTDRNLNTSFFDPAGGGDPILYQHL
>LEFIC711-10|HM872532|MM04753|Noctua_fimbriata
TLYFIFGIWAGMVGTSLSLLIRAELGNPGSLIGDDQIYNTIVTAHAFIMIFFMVMPIMIGGFGNWLVPLMLGAPDMAFPRMNNMSFWLLPPSLTLLISSSIVENGAGTGWTVYPPLSSNIAHGGSSVDLAIFSLHLAGISSILGAINFITTIINMRLNSLSFD
QMPLFIWAVGITAFLLLLSLPVLAGAITMLLTDRNLNTSFFDPAGGGDPILYQHL
>LEFIJ876-10|JF853875|MM17501|Noctua_interjecta
TLYFIFGIWAGMVGTSLSLLIRAELGNPGSLIGDDQIYNTIVTAHAFIMIFFMVMPIMIGGFGNWLVPLMLGAPDMAFPRMNNMSFWLLPPSLTLLISSSIVENGAGTGWTVYPPLSSNIAHGGSSVDLAIFSLHLAGISSILGAINFITTIINMRLNSLSFD
QMPLFIWAVGITAFLLLLSLPVLAGAITMLLTDRNLNTSFFDPAGGGDPILYQHL
>LEFID158-10|HM872965|MM05922|Noctua_interposita
TLYFIFGIWAGMVGTSLSLLIRAELGNPGSLIGDDQIYNTIVTAHAFIMIFFMVMPIMIGGFGNWLVPLMLGAPDMAFPRMNNMSFWLLPPSLTLLISSSIVENGAGTGWTVYPPLSSNIAHGGSSVDLAIFSLHLAGISSILGAINFITTIINMRLNSLSFD
QMPLFIWAVGITAFLLLLSLPVLAGAITMLLTDRNLNTSFFDPAGGGDPILYQHL
>LEFIF303-10|HM874990|MM11090|Noctua_interposita
TLYFIFGIWAGMVGTSLSLLIRAELGNPGSLIGDDQIYNTIVTAHAFIMIFFMVMPIMIGGFGNWLVPLMLGAPDMAFPRMNNMSFWLLPPSLTLLISSSIVENGAGTGWTVYPPLSSNIAHGGSSVDLAIFSLHLAGISSILGAINFITTIINMRLNSLSFD
QMPLFIWAVGITAFLLLLSLPVLAGAITMLLTDRNLNTSFFDPAGGGDPILYQHL
>LEFII270-11|KM573337|MM19920|Noctua_janthe
TLYFIFGIWAGMVGTSLSLLIRAELGNPGSLIGDDQIYNTIVTAHAFIMIFFMVMPIMIGGFGNWLVPLMLGAPDMAFPRMNNMSFWLLPPSLTLLISSSIVENGAGTGWTVYPPLSSNIAHGGSSVDLAIFSLHLAGISSILGAINFITTIINMRLNSLSFD
QMPLFIWAVGITAFLLLLSLPVLAGAITMLLTDRNLNTSFFDPAGGGDPILYQHL
>LEFIJ361-10|KM572780|MM15961|Noctua_janthina

TLYFIFGIWAGMVGTSLSLLIRAELGNPGSLIGDDQIYNTIVTAHAFIMIFFMVMPIMIGGFGNWLVPLMLGAPDMAFPRMNNMSFWLLPPSLTLLISSSIVENGVGTGWTVYPPLSSNIAHGGSSVDLAIFSLHLAGISSILGAINFITTIINMRLNSLSFD
QMPLFIWAVGITAFLLLLSLPVLAGAITMLLTDRNLNTSFFDPAGGGDPILYQHL
>LEFIJ867-10|JF853869|MM17492|Noctua_janthina
TLYFIFGIWAGMVGTSLSLLIRAELGNPGSLIGDDQIYNTIVTAHAFIMIFFMVMPIMIGGFGNWLVPLMLGAPDMAFPRMNNMSFWLLPPSLTLLISSSIVENGAGTGWTVYPPLSSNIAHGGSSVDLAIFSLHLAGISSILGAINFITTIINMRLNSLSFD
QMPLFIWAVGITAFLLLLSLPVLAGAITMLLTDRNLNTSFFDPAGGGDPILYQHL
>LEFIF302-10|HM874989|MM11089|Noctua_orbona
TLYFIFGIWAGMVGTSLSLLIRAELGNPGSLIGDDQIYNTIVTAHAFIMIFFMVMPIMIGGFGNWLVPLMLGAPDMAFPRMNNMSFWLLPPSLTLLISSSIVENGAGTGWTVYPPLSSNIAHGGSSVDLAIFSLHLAGISSILGAINFITTIINMRLNSLSFD
QMPLFIWAVGITAFLLLLSLPVLAGAITMLLTDRNLNTSFFDPAGGGDPILYQHL
>LEFIJ868-10|MM17493|Noctua_orbona
TLYFIFGIWAGMVGTSLSLLIRAELGNPGSLIGDDQIYNTIVTAHAFIMIFFMVMPIMIGGFGNWLVPLMLGAPDMAFPRMNNMSFWLLPPSLTLLISSSIVENGAGTGWTVYPPLSSNIAHGGSSVDLAIFSLHLAGISSILGAINFITTIINMRLNSLSFD
QMPLFIWAVGITAFLLLLSLPVLAGAITMLLTDRNLNTSFFDPAGGGDPILYQHL
>LEFIA580-10|HM870829|MM01685|Noctua_pronuba
TLYFIFGIWAGMVGTSLSLLIRAELGNPGSLIGDDQIYNTIVTAHAFIMIFFMVMPIMIGGFGNWLVPLMLGAPDMAFPRMNNMSFWLLPPSLTLLISSSIVENGAGTGWTVYPPLSSNIAHGGSSVDLAIFSLHLAGISSILGAINFITTIINMRLNSLSFD
QMPLFIWAVGITAFLLLLSLPVLAGAITMLLTDRNLNTSFFDPAGGGDPILYQHL
>LEFIA581-10|HM870830|MM01686|Noctua_pronuba
TLYFIFGIWAGMVGTSLSLLIRAELGNPGSLIGDDQIYNTIVTAHAFIMIFFMVMPIMIGGFGNWLVPLMLGAPDMAFPRMNNMSFWLLPPSLTLLISSSIVENGAGTGWTVYPPLSSNIAHGGSSVDLAIFSLHLAGISSILGAINFITTIINMRLNSLSFD
QMPLFIWAVGITAFLLLLSLPVLAGAITMLLTDRNLNTSFFDPAGGGDPILYQHL
>LEFIC780-10|HM872599|MM04912|Noctua_pronuba
TLYFIFGIWAGMVGTSLSLLIRAELGNPGSLIGDDQIYNTIVTAHAFIMIFFMVMPIMIGGFGNWLVPLMLGAPDMAFPRMNNMSFWLLPPSLTLLISSSIVENGAGTGWTVYPPLSSNIAHGGSSVDLAIFSLHLAGISSILGAINFITTIINMRLNSLSFD
QMPLFIWAVGITAFLLLLSLPVLAGAITMLLTDRNLNTSFFDPAGGGDPILYQHL
>LEFID584-10|HM873349|MM06590|Nola_aerugula
TLYFIFGIWAGMVGTSLSLLIRAELGNPGSLIGDDQIYNTIVTAHAFIMIFFMVMPIMIGGFGNWLVPLMLGAPDMAFPRMNNMSFWLLPPSITLLISSSIVETGAGTGWTVYPPLSSNIAHGGSSVDLAIFSLHLAGISSILGAINFITTIINMRLNNMSFD
QMSLFIWAVGITAFLLLLSLPVLAGAITMLLTDRNLNTSFFDPAGGGDPILYQHL
>LEFIC298-10|HM872142|MM03773|Nola_confusalis
TLYFIFGIWAGMVGTSLSLLIRAELGNPGSLIGDDQIYNTIVTAHAFIMIFFMVMPIMIGGFGNWLVPLMLGAPDMAFPRMNNMSFWLLPPSLTLLISSSIVETGAGTGWTVYPPLSSNIAHGGSSVDLAIFSLHLAGISSILGAINFITTIINMRLNNMSFD
QMSLFIWAVGITAFLLLLSLPVLAGAITMLLTDRNLNTSFFDPAGGGDPILYQHL
>LEFIC237-10|HM872081|MM03640|Nola_cucullatella
TLYFIFGVWAGMVGTSLSLLIRAELGTPGSLIGDDQIYNTIVTAHAFIMIFFMVMPIMIGGFGNWLVPLMLGAPDMAFPRMNNMSFWLLPPSLTLLISSSIVENGAGTGWTVYPPLSSNIAHSGSSVDLAIFSLHLAGISSILGAINFITTIINMRLNNLSFD
QMSLFIWAVGITAFLLLLSLPVLAGAITMLLTDRNLNTSFFDPAGGGDPILYQHL
>LEFIC788-10|HM872607|MM04925|Nola_cucullatella
TLYFIFGIWAGMVGTSLSLLIRAELGTPGSLIGDDQIYNTIVTAHAFIMIFFMVMPIMIGGFGNWLVPLMLGAPDMAFPRMNNMSFWLLPPSLTLLISSSIVENGAGTGWTVYPPLSSNIAHSGSSVDLAIFSLHLAGISSILGAINFITTIINMRLNNLSFD
QMSLFIWAVGITAFLLLLSLPVLAGAITMLLTDRNLNTSFFDPAGGGDPILYQHL
>LEFIF275-10|HM874968|MM11057|Nola_karelica
TLYFIFGIWAGMVGTSLSLLIRAELGNPGSLIGDDQIYNTIVTAHAFIMIFFMVMPIMIGGFGNWLVPLMLGAPDMAFPRMNNMSFWLLPPSLTLLISSSIVETGAGTGWTVYPPLSSNIAHSGSSVDLAIFSLHLAGISSILGAINFITTIINMRLNNLSFD
QMSLFIWAVGITAFLLLLSLPVLAGAITMLLTDRNLNTSFFDPAGGGDPILYQHL
>LEFIF277-10|HM874970|MM11059|Nola_karelica
TLYFIFGIWAGMVGTSLSLLIRAELGNPGSLIGDDQIYNTIVTAHAFIMIFFMVMPIMIGGFGNWLVPLMLGAPDMAFPRMNNMSFWLLPPSLTLLISSSIVETGAGTGWTVYPPLSSNIAHSGSSVDLAIFSLHLAGISSILGAINFITTIINMRLNNLSFD
QMSLFIWAVGITAFLLLLSLPVLAGAITMLLTDRNLNTSFFDPAGGGDPILYQHL
>LEFIC499-10|HM872324|MM04206|Nomophila_noctuella
TLYFIFGIWAGMVGTSLSLLIRAELGNPGSLIGDDQIYNTIVTAHAFIMIFFMVMPIMIGGFGNWLVPLMLGAPDMAFPRMNNMSFWLLPPSLTLLISSSIVENGAGTGWTVYPPLSSNIAHGGSSVDLAIFSLHLAGISSILGAINFITTIINMRINGLSFD
QMPLFVWAVGITALLLLLSLPVLAGAITMLLTDRNLNTSFFDPAGGGDPILYQHL
>LEFIJ716-10|JF853789|MM17341|Nomophila_noctuella
TLYFIFGIWAGMVGTSLSLLIRAELGNPGSLIGDDQIYNTIVTAHAFIMIFFMVMPIMIGGFGNWLVPLMLGAPDMAFPRMNNMSFWLLPPSLTLLISSSIVENGAGTGWTVYPPLSSNIAHGGSSVDLAIFSLHLAGISSILGAINFITTIINMRINGLSFD
QMPLFVWAVGITALLLLLSLPVLAGAITMLLTDRNLNTSFFDPAGGGDPILY?HL
>LEFIE902-10|HM874619|MM10213|Nonagria_typhae
TLYFIFGIWAGMVGTSLSLLIRAELGNPGSLIGDDQIYNTIVTAHAFIMIFFMVMPIMIGGFGNWLVPLMLGAPDMAFPRMNNMSFWLLPPSLTLLISSSIVENGAGTGWTVYPPLSSNIAHGGSSVDLAIFSLHLAGISSILGAINFITTIINMRLNNLSFD
QMPLFIWAVGITAFLLLLSLPVLAGAITMLLTDRNLNTSFFDPAGGGDPILYQHL

>LEFIB063-10|HM870972|MM00351|Nothris_lemniscellus
TLYFIFGIWAGMVGTSLSLLIRAELGNPGSLIGDDQIYNTIVTAHAFIMIFFMVMPIMIGGFGNWLVPLMLGAPDMAFPRMNNMSFWLLPPSLTLLISSSIVENGAGTGWTVYPPLSSNIAHSGSSVDLAIFSLHLAGISSILGAINFITTIINMKINGLSFD
QMPLFVWSVGITALLLLLSLPVLAGAITMLLTDRNLNTSFFDPAGGGDPILYQHL
>LEFIA029-10|HM396379|MM00100|Nothris_verbascella
TLYFIFGIWAGMIGTSLSLLIRAELGNPGSLIGDDQIYNTIVTAHAFIMIFFMVMPIMIGGFGNWLVPLMLGAPDMAFPRMNNMSFWLLPPSLTLLISSSIVENGAGTGWTVYPPLSSNIAHSGSSVDLAIFSLHLAGISSILGAINFITTIINMKINGLSFDQ
MPLFVWAVGITALLLLLSLPVLAGAITMLLTDRNLNTSFFDPAGGGDPILYQHL
>LEFIB910-10|HM871787|MM03018|Notocelia_cynosbatella
TLYFIFGVWAGMVGTSLSLLVRAELGNPGSLIGDDQIYNTIVTAHAFIMIFFMVMPIMIGGFGNWLVPLMLGAPDMAFPRMNNMSFWLLPPSIMLLISSSIVENGAGTGWTVYPPLSSNIAHGGSSVDLAIFSLHLAGISSILGAVNFITTIINMRPNNM
SLDQMPLFVWAVGITALLLLLSLPVLAGAITMLLTDRNLNTSFFDPAGGGDPILYQHL
>LEFIF840-10|HM875523|MM13185|Notocelia_cynosbatella
TLYFIFGVWAGMVGTSLSLLIRAELGNPGSLIGDDQIYNTIVTAHAFIMIFFMVMPIMIGGFGNWLVPLMLGAPDMAFPRMNNMSFWLLPPSIMLLISSSIVENGAGTGWTVYPPLSSNIAHGGSSVDLAIFSLHLAGISSILGAVNFITTIINMRPNNM
SLDQMPLFVWAVGITALLLLLSLPVLAGAITMLLTDRNLNTSFFDPAGGGDPILYQHL
>LEFIJ2047-13|KM572843|MM22899|Notocelia_cynosbatella
TLYFIFGVWAGMVGTSLSLLIRAELGNPGSLIGDDQIYNTIVTAHAFIMIFFMVMPIMIGGFGNWLVPLMLGAPDMAFPRMNNMSFWLLPPSIMLLISSSIVENGAGTGWTVYPPLSSNIAHGGSSVDLAIFSLHLAGISSILGAVNFITTIINMRPNNM
SLDQMPLFVWAVGITALLLLLSLPVLAGAITMLLTDRNLNTSFFDPAGGGDPILYQHL
>LEFID986-10|HM873736|MM08018|Notocelia_incarnatana
TLYFIFGIWAGMVGTSLSLLIRAELGNPGSLIGDDQIYNTIVTAHAFIMIFFMVMPIMIGGFGNWLVPLMLGAPDMAFPRMNNMSFWLLPPSMMLLISSSIVENGAGTGWTVYPPLSSNIAHSGSSVDLAIFSLHLAGISSILGAVNFITTIINMRPNNM
SLDQMPLFIWAVGITALLLLLSLPVLAGAITMLLTDRNLNTSFFDPAGGGDPILYQHL
>LEFIF238-10|HM874931|MM11019|Notocelia_incarnatana
TLYFIFGIWAGMVGTSLSLLIRAELGNPGSLIGDDQIYNTIVTAHAFIMIFFMVMPIMIGGFGNWLVPLMLGAPDMAFPRMNNMSFWLLPPSMMLLISSSIVENGAGTGWTVYPPLSSNIAHSGSSVDLAIFSLHLAGISSILGAVNFITTIINMRPNNM
SLDQMPLFIWAVGITALLLLLSLPVLAGAITMLLTDRNLNTSFFDPAGGGDPILYQHL
>LEFIB447-10|HM871346|MM01968|Notocelia_roborana
TLYFIFGIWAGMIGTSLSLLIRAELGNPGSLIGDDQIYNTIVTAHAFIMIFFMVMPIMIGGFGNWLVPLMLGAPDMAFPRMNNMSFWLLPPSILLLISSSIVENGAGTGWTVYPPLSSNIAHSGSSVDLAIFSLHLAGISSILGAVNFITTIINMRPNKMSLD
QMPLFVWAVGITALLLLLSLPVLAGAITMLLTDRNLNTSFFDPAGGGDPILYQHL
>LEFIF885-10|HM875567|MM13291|Notocelia_roborana
TLYFIFGIWAGMIGTSLSLLIRAELGNPGSLIGDDQIYNTIVTAHAFIMIFFMVMPIMIGGFGNWLVPLMLGAPDMAFPRMNNMSFWLLPPSILLLISSSIVENGAGTGWTVYPPLSSNIAHSGSSVDLAIFSLHLAGISSILGAVNFITTIINMRPNKMSLD
QMPLFVWAVGITALLLLLSLPVLAGAITMLLTDRNLNTSFFDPAGGGDPILYQHL
>LEFID987-10|HM873737|MM08020|Notocelia_rosaecolana
TLYFIFGVWSGMVGTSLSLLIRAELGNPGSLIGDDQIYNTIVTAHAFIMIFFMVMPIMIGGFGNWLVPLMLGAPDMAFPRMNNMSFWLLPPSIMLLISSSVVENGAGTGWTVYPPLSSNIAHSGSSVDLAIFSLHLAGISSILGAVNFITTIINMRPNNM
SLDQMPLFVWAVGITALLLLLSLPVLAGAITMLLTDRNLNTSFFDPAGGGDPILYQHL
>LEFIL240-10|KM573601|MM19240|Notocelia_tetragonana
TLYFIFGIWAGMIGTSLSLLIRAELGNPGSLIGDDQIYNTIVTAHAFIMIFFMVMPIMIGGFGNWLVPLMLGAPDMAFPRMNNMSFWLLPPSLMLLISSSVVENGAGTGWTVYPPLSSNIAHSGSSVDLAIFSLHLAGISSILGAVNFITTIINMRPNNMS
LDQMPLFIWAVGITALLLLLSLPVLAGAITMLLTDRNLNTSFFDPAGGGDPILYQHL
>LEEUA244-11|JN274940|MM19652|Notocelia_trimaculana
TLYFIFGIWAGMIGTSLSLLIRAELGNPGSLIGDDQIYNTIVTAHAFIMIFFMVMPIMIGGFGNWLVPLMLGAPDMAFPRMNNMSFWLLPPSLMLLISSSIVENGAGTGWTVYPPLSSNIAHSGSSVDLAIFSLHLAGISSILGAVNFITTIINMRPNNMNL
DQMPLFIWAVGITALLLLLSLPVLAGAITMLLTDRNLNTSFFDPAGGGDPILYQHL
>LEFIB030-10|HM870941|MM00121|Notodonta_dromedarius
TLYFIFGIWAGMVGTSLSLLIRAELGNPGSLIGDDQIYNTIVTAHAFIMIFFMVMPIMIGGFGNWLVPLMLGAPDMAFPRMNNMSFWLLPPSLTLLISSSIVENGAGTGWTVYPPLSSNIAHGGSSVDLAIFSLHLAGISSILGAINFITTIINMRLNGMSF
DQMPLFVWAVGITAFLLLLSLPVLAGAITMLLTDRNLNTSFFDPAGGGDPILYQHL
>LEFIA080-10|HM396427|MM00999|Notodonta_torva
TLYFIFGIWAGMVGTSLSLLIRAELGNPGSLIGDDQIYNTIVTAHAFIMIFFMVMPIMIGGFGNWLVPLMLGAPDMAFPRMNNMSFWLLPPSLTLLISSSIVENGAGTGWTVYPPLSSNIAHGGSSVDLAIFSLHLAGISSILGAINFITTIINMRLNGMSF
DQMPLFVWAVGITAFLLLLSLPVLAGAITMLLTDRNLNTSFFDPAGGGDPILYQHL
>LEFIC612-10|HM872433|MM04542|Notodonta_torva
TLYFIFGIWAGMVGTSLSLLIRAELGNPGSLIGDDQIYNTIVTAHAFIMIFFMVMPIMIGGFGNWLVPLMLGAPDMAFPRMNNMSFWLLPPSLTLLISSSIVENGAGTGWTVYPPLSSNIAHGGSSVDLAIFSLHLAGISSILGAINFITTIINMRLNGMSF
DQMPLFVWAVGITAFLLLLSLPVLAGAITMLLTDRNLNTSFFDPAGGGDPILYQHL
>LEFIA054-10|HM396402|MM00960|Notodonta_tritophus
TLYFIFGIWAGMVGTSLSLLIRAELGNPGSLIGDDQIYNTIVTAHAFIMIFFMVMPIMIGGFGNWLVPLMLGAPDMAFPRMNNMSFWLLPPSLTLLISSSIVENGAGTGWTVYPPLSSNIAHGGSSVDLAIFSLHLAGISSILGAINFITTIINMRLNGMSF

DQMPLFVWAVGITAFLLLLSLPVLAGAITMLLTDRNLNTSFFDPAGGGDPILYQHL
>LEFIF647-10|HM875331|MM12560|Notodonta_tritophus
TLYFIFGIWAGMVGTSLSLLIRAELGNPGSLIGDDQIYNTIVTAHAFIMIFFMVMPIMIGGFGNWLVPLMLGAPDMAFPRMNNMSFWLLPPSLTLLISSSIVENGAGTGWTVYPPLSSNIAHGGSSVDLAIFSLHLAGISSILGAINFITTIINMRLNGMSF
DQMPLFVWAVGITAFLLLLSLPVLAGAITMLLTDRNLNTSFFDPAGGGDPILYQHL
>LEFIA082-10|HM396429|MM01001|Notodonta_ziczac
TLYFIFGIWAGMVGTSLSLLIRAELGNPGSLIGDDQIYNTIVTAHAFIMIFFMVMPIMIGGFGNWLVPLMLGAPDMAFPRMNNMSFWLLPPSLTLLISSSIVENGAGTGWTVYPPLSSNIAHGGSSVDLAIFSLHLAGISSILGAINFITTIINMRLNGMSF
DQMPLFVWAVGITAFLLLLSLPVLAGAITMLLTDRNLNTSFFDPAGGGDPILYQHL
>LEFIA083-10|HM396430|MM01002|Notodonta_ziczac
TLYFIFGIWAGMVGTSLSLLIRAELGNPGSLIGDDQIYNTIVTAHAFIMIFFMVMPIMIGGFGNWLVPLMLGAPDMAFPRMNNMSFWLLPPSLTLLISSSIVENGAGTGWTVYPPLSSNIAHGGSSVDLAIFSLHLAGISSILGAINFITTIINMRLNGMSF
DQMPLFVWAVGITAFLLLLSLPVLAGAITMLLTDRNLNTSFFDPAGGGDPILYQHL
>LEFIJ824-10|JF853845|MM17449|Nudaria_mundana
TLYFIFGIWAGMVGTSLSLLIRAELGNPGSLIGDDQIYNTIVTAHAFIMIFFMVMPIMIGGFGNWLVPLMLGAPDMAFPRMNNMSFWLLPPSLTLLISSSIVENGAGTGWTVYPPLSSNIAHSGSSVDLAIFSLHLAGISSILGAINFITTIINMRLNNLSFD
QMPLFIWAVGITAFLLLLSLPVLAGAITMLLTDRNLNTSFFDPAGGGDPILYQHL
>LEFIL414-10|JF854588|MM18712|Nudaria_mundana
TLYFIFGIWAGMVGTSLSLLIRAELGNPGSLIGDDQIYNTIVTAHAFIMIFFMVMPIMIGGFGNWLVPLMLGAPDMAFPRMNNMSFWLLPPSLTLLISSSIVENGAGTGWTVYPPLSSNIAHSGSSVDLAIFSLHLAGISSILGAINFITTIINMRLNNLSFD
QMPLFIWAVGITAFLLLLSLPVLAGAITMLLTDRNLNTSFFDPAGGGDPILYQHL
>LEFID758-10|HM873515|MM06821|Nyctegretis_lineana
TLYFIFGIWSGMVGTSLSLLIRAELGTPGSLIGDDQIYNTIVTGHAFIMIFFMVMPIMIGGFGNWLVPLMLGAPDMAFPRMNNMSFWLLPPSLTLLISSSVVENGAGTGWTVYPPLSSNIAHSGSSVDLAIFSLHLAGISSILGAINFITTIINMKLNGLSFD
QMPLFVWAVGITALLLLLSLPVLAGAITMLLTDRNLNTSFFDPAGGGDPILYQHL
>LEFIF278-10|HM874971|MM11060|Nycteola_asiatica
TLYFIFGIWAGMVGTSLSLLIRAELGNPGSLIGDDQIYNTIVTAHAFIMIFFMVMPIMIGGFGNWLVPLMLGAPDMAFPRMNNMSFWLLPPSLTLLISSSIVENGAGTGWTVYPPLSSNIAHGGSSVDLAIFSLHLAGISSILGAINFITTIINMRLNNLSFD
QMPLFIWAVGITAFLLLLSLPVLAGAITMLLTDRNLNTSFFDPAGGGDPILYQHL
>LEFIA691-10|HM386836|MM01834|Nycteola_degenerana
TLYFIFGIWAGMVGTSLSLLIRAELGNPGSLIGDDQIYNTIVTAHAFIMIFFMVMPIMIGGFGNWLVPLMLGAPDMAFPRMNNMSFWLLPPSLTLLISSSIVENGAGTGWTVYPPLSSNIAHGGSSVDLAIFSLHLAGISSILGAINFITTIINMRLNSLSFD
QMPLFIWAVGITAFLLLLSLPVLAGAITMLLTDRNLNTSFFDPAGGGDPILYQHL
>LEFIA692-10|HM386837|MM01835|Nycteola_degenerana
TLYFIFGIWAGMVGTSLSLLIRAELGNPGSLIGDDQIYNTIVTAHAFIMIFFMVMPIMIGGFGNWLVPLMLGAPDMAFPRMNNMSFWLLPPSLTLLISSSIVENGAGTGWTVYPPLSSNIAHGGSSVDLAIFSLHLAGISSILGAINFITTIINMRLNSLSFD
QMPLFIWAVGITAFLLLLSLPVLAGAITMLLTDRNLNTSFFDPAGGGDPILYQHL
>LEFIC663-10|HM872484|MM04623|Nycteola_degenerana
TLYFIFGIWAGMVGTSLSLLIRAELGNPGSLIGDDQIYNTIVTAHAFIMIFFMVMPIMIGGFGNWLVPLMLGAPDMAFPRMNNMSFWLLPPSLTLLISSSIVENGAGTGWTVYPPLSSNIAHGGSSVDLAIFSLHLAGISSILGAINFITTIINMRLNSLSFD
QMPLFIWAVGITAFLLLLSLPVLAGAITMLLTDRNLNTSFFDPAGGGDPILYQHL
>LEFID198-10|HM873001|MM05998|Nycteola_revayana
TLYFIFGIWAGMVGTSLSLLIRAELGNPGSLIGDDQIYNTIVTAHAFIMIFFMVMPIMIGGFGNWLVPLMLGAPDMAFPRMNNMSFWLLPPSLTLLISSSIVENGAGTGWTVYPPLSSNIAHGGSSVDLAIFSLHLAGISSILGAINFITTIINMRLNSLSFD
QMPLFIWAVGITAFLLLLSLPVLAGAITMLLTDRNLNTSFFDPAGGGDPILYQHL
>LEFIG452-10|HM876128|MM14490|Nycteola_revayana
TLYFIFGIWAGMVGTSLSLLIRAELGNPGSLIGDDQIYNTIVTAHAFIMIFFMVMPIMIGGFGNWLVPLMLGAPDMAFPRMNNMSFWLLPPSLTLLISSSIVENGAGTGWTVYPPLSSNIAHGGSSVDLAIFSLHLAGISSILGAINFITTIINMRLNSLSFD
QMPLFIWAVGITAFLLLLSLPVLAGAITMLLTDRNLNTSFFDPAGGGDPILYQHL
>LEFIJ530-10|MM17155|Nymphalis_antiopa
TLYFIFGIWAGMVGTSLSLLIRTELGNPGSLIGDDQIYNTIVTAHAFIMIFFMVMPIMIGGFGNWLVPLMLGAPDMAFPRMNNMSFWLLPPSLILLISSSIVENGAGTGWTVYPPLSSNIAHSGASVDLAIFSLHLAGISSILGAINFITTIINMRINNMSFD
QMPLFVWAVGITALLLLLSLPVLAGAITMLLTDRNINTSFFDPAGGGDPILYQHL
>LEFID220-10|HM873021|MM06077|Nymphalis_polychloros
TLYFIFGIWAGMVGTSLSLLIRTELGNPGSLIGDDQIYNTIVTAHAFIMIFFMVMPIMIGGFGNWLVPLMLGAPDMAFPRMNNMSFWLLPPSLMLLISSSIVENGAGTGWTVYPPLSSNIAHSGASVDLAIFSLHLAGISSILGAINFITTIINMRINSMSF
DQMPLFVWAVGITALLLLLSLPVLAGAITMLLTDRNINTSFFDPAGGGDPILYQHL
>LEFIJ1026-11|MM14865|Nymphalis_xanthomelas
TLYFIFGIWAGMVGTSLSLLIRTELGNPGSLIGDDQIYNTIVTAHAFIMIFFMVMPIMIGGFGNWLVPLMLGAPDMAFPRMNNMSFWLLPPSLILLISSSIVENGAGTGWTVYPPLSSNIAHSGASVDLAIFSLHLAGISSILGAINFITTIINMRINNMSFD
QMPLFVWAVGITALLLLLSLPVLAGAITMLLTDRNINTSFFDPAGGGDPILYQHL
>LEFIJ313-10|MM15913|Nymphalis_xanthomelas

TLYFIFGIWAGMVGTSLSLLIRTELGNPGSLIGDDQIYNTIVTAHAFIMIFFMVMPIMIGGFGNWLVPLMLGAPDMAFPRMNNMSFWLLPPSLILLISSSIVENGAGTGWTVYPPLSSNIAHSGASVDLAIFSLHLAGISSILGAINFITTIINMRINNMSFD
QMPLFVWAVGITALLLLLSLPVLAGAITMLLTDRNINTSFFDPAGGGDPILYQHL
>LEFIF775-10|HM875459|MM13005|Nymphula_nitidulata
TLYFIFGIWSGMVGTSLSLLIRAELGSPGSLIGDDQIYNTIVTAHAFIMIFFMVMPIMIGGFGNWLVPLMLGAPDMAFPRMNNMSFWLLPPSLTLLISSSIVENGAGTGWTVYPPLSSNIAHGGSSVDLAIFSLHLAGISSILGAINFITTIINMRINNLSFDQ
MPLFVWAVGITALLLLLSLPVLAGAITMLLTDRNLNTSFFDPAGGGDPILYQHL
>LEFIJ487-10|JF853612|MM17112|Ochlodes_sylvanus
TLYFIFGIWAGMLGTSLSLLIRTELGNPGSLIGDDQIYNTIVTAHAFIMIFFMVMPIMIGGFGNWLVPLMLGAPDMAFPRMNNMSFWMLPPSLTLLISSSIVENGAGTGWTVYPPLSSNIAHQGSSVDLAIFSLHLAGISSILGAINFITTIINMRIKNLSFD
QMPLFVWSVGITALLLLLSLPVLAGAITMLLTDRNLNTSFFDPAGGGDPILYQHL
>LEFIL196-10|MM19196|Ochromolopis_ictella
TLYFIFGIWAGMVGTSLSLLIRAELGTPNSLIGDDQIYNTIVTAHAFIMIFFMVMPIMIGGFGNWLVPLMLGAPDMAFPRMNNMSFWLLPPSLTLLISSSIVENGAGTGWTVYPPLSSNIAHSGSSVDLAIFSLHLAGISSILGAINFITTIINMKLNNLMFD
QMPLFVWAVGITALLLLLSLPVLAGAITMLLTDRNLNTSFFDPAGGGDPILYQHL
>LEFIA207-10|HM396551|MM01230|Ochropacha_duplaris
TLYFIFGIWAGMVGTSLSLLIRAELGNPGSLIGDDQIYNTIVTAHAFIMIFFMVMPIMIGGFGNWLVPLMLGAPDMAFPRMNNMSFWMLPPSLTLLISSSIVENGAGTGWTVYPPLSSNIAHGGSSVDLAIFSLHLAGISSILGAINFITTIINMRLNNMSF
DQMPLFVWAVGITAFLLLLSLPVLAGAITMLLTDRNLNTSFFDPAGGGDPILYQHL
>LEFIA208-10|HM396552|MM01231|Ochropacha_duplaris
TLYFIFGIWAGMVGTSLSLLIRAELGNPGSLIGDDQIYNTIVTAHAFIMIFFMVMPIMIGGFGNWLVPLMLGAPDMAFPRMNNMSFWMLPPSLTLLISSSIVENGAGTGWTVYPPLSSNIAHGGSSVDLAIFSLHLAGISSILGAINFITTIINMRLNNMSF
DQMPLFVWAVGITAFLLLLSLPVLAGAITMLLTDRNLNTSFFDPAGGGDPILYQHL
>LEFIA503-10|KM573020|MM01585|Ochropleura_plecta
TLYFIFGIWAGMVGTSLSLLIRAELGNPGSLIGDDQIYNTIVTAHAFIMIFFMVMPIMIGGFGNWLVPLMLGAPDMAFPRMNNMSFWLLPPSLTLLISSSIVENGAGTGWTVYPPLSSNIAHGGSSVDLAIFSLHLAGISSILGAINFITTIINMRLNNLSFD
QMPLFIWAVGITAFLLLLSLPVLAGAITMLLTDRNLNTSFFDPAGGGDPILYQHL
>LEFIJ2042-13|KM572277|MM22894|Ochropleura_plecta
TLYFIFGIWAGMVGTSLSLLIRAELGNPGSLIGDDQIYNTIVTAHAFIMIFFMVMPIMIGGFGNWLVPLMLGAPDMAFPRMNNMSFWLLPPSLTLLISSSIVENGAGTGWTVYPPLSSNIAHGGSSVDLAIFSLHLAGISSILGAINFITTIINMRLNNLSFD
QMPLFIWAVGITAFLLLLSLPVLAGAITMLLTDRNLNTSFFDPAGGGDPILYQHL
>LEFIE952-10|HM874669|MM10349|Ochsenheimeria_urella
TLYFIFGIWSGMVGTSLSLLIRAELGNPGSLIGDDQIYNTIVTAHAFIMIFFMVMPIMIGGFGNWLVPLMLGAPDMAFPRMNNMSFWLLPPSLTLLISSSIVETGAGTGWTVYPPLSSNIAHGGSSVDLAIFSLHLAGISSILGAINFITTIINMRSNGMSFD
RMPLFVWAVGITALLLLLSLPVLAGAITMLLTDRNLNTSFFDPAGGGDPILYQHL
>LEFIG537-10|HM876210|MM14672|Ochsenheimeria_urella
TLYFIFGIWSGMVGTSLSLLIRAELGNPGSLIGDDQIYNTIVTAHAFIMIFFMVMPIMIGGFGNWLVPLMLGAPDMAFPRMNNMSFWLLPPSLTLLISSSIVETGAGTGWTVYPPLSSNIAHGGSSVDLAIFSLHLAGISSILGAINFITTIINMRSNGMSFD
RMPLFVWAVGITALLLLLSLPVLAGAITMLLTDRNLNTSFFDPAGGGDPILYQHL
>LEFIJ2391-14|MM23774|Ochsenheimeria_urella
TLYFIFGIWSGMVGTSLSL?IRAELGNPGSLIGDDQIYNTIVTAHAFIMIFFMVMPIMIGGFGNWLVPLMLGAPDMAFPRMNNMSFWLLPPSLTLLISSSIVETGAGTGWTVYPPLSSNIAHGGSSVDLAIFSLHLAGISSILGAINFITTIINMRSNGMSFD
RMPLFVWAVGITALLLLLSLPVLAGAITMLLTDRNLNTSFFDPAGGGDPILYQHL
>LEFIJ1467-12|MM22735|Ochsenheimeria_vacculella
TLYFIFGIWSGMVGTSLSLLIRAELGNPGSLIGDDQIYNTIVTAHAFIMIFFMVMPIMIGGFGNWLVPLMLGAPDMAFPRMNNMSFWLLPPSLTLLISSSIVETGAGTGWTVYPPLSSNIAHSGSSVDLAIFSLHLAGISSILGAINFITTIINMRSNGMSFD
RMPLFVWAVGITALLLLLSLPVLAGAITMLLTDRNLNTSFFDPAGGGDPILYQHL
>LEFIG671-10|HM876332|MM15535|Ocnerostoma_friesei
TLYFIFGIWSGMVGTSLSLLIRAELGNPSSLIGDDQIYNTIVTAHAFIMIFFMVMPIMIGGFGNWLVPLMLGAPDMAFPRMNNMSFWLLPPSLMLLISSSIVETGAGTGWTVYPPLSSNIAHSGSSVDLAIFSLHLAGISSILGAINFITTIINMKNNSMMF
DQMPLFVWAVGITALLLLLSLPVLAGAITMLLTDRNLNTSFFDPAGGGDPILYQHL
>LEFIG672-10|HM876333|MM15536|Ocnerostoma_friesei
TLYFIFGIWSGMVGTSLSLLIRAELGNPSSLIGDDQIYNTIVTAHAFIMIFFMVMPIMIGGFGNWLVPLMLGAPDMAFPRMNNMSFWLLPPSLMLLISSSIVETGAGTGWTVYPPLSSNIAHSGSSVDLAIFSLHLAGISSILGAINFITTIINMKNNSMMF
DQMPLFVWAVGITALLLLLSLPVLAGAITMLLTDRNLNTSFFDPAGGGDPILYQHL
>LEFIL727-10|JF854703|MM16016|Ocnerostoma_friesei
TLYFIFGIWSGMVGTSLSLLIRAELGNPSSLIGDDQIYNTIVTAHAFIMIFFMVMPIMIGGFGNWLVPLMLGAPDMAFPRMNNMSFWLLPPSLMLLISSSIVETGAGTGWTVYPPLSSNIAHSGSSVDLAIFSLHLAGISSILGAINFITTIINMKNNSMMF
DQMPLFVWAVGITALLLLLSLPVLAGAITMLLTDRNLNTSFFDPAGGGDPILYQHL
>LEFID283-10|HM873081|MM06158|Ocnerostoma_piniariella
TLYFMFGIWSGMVGTSLSLLIRAELGNPGSLIGDDQIYNTIVTAHAFIMIFFMVMPIMIGGFGNWLVPLMLGAPDMAFPRMNNMSFWLLPPSLMLLISSSIVETGAGTGWTVYPPLSSNIAHSGSSVDLAIFSLHLAGISSILGAINFITTIINMKNNSML
FDQMPLFVWAVGITALLLLLSLPVLAGAITMLLTDRNLNTSFFDPAGGGDPILYQHL

>LEFIC026-10|HM871896|MM03239|Odezia_atrata
TLYFIFGIWAGMIGTSLSLLIRAELGSPGSLIGDDQIYNTIVTAHAFIMIFFMVMPIMIGGFGNWLVPLMLGAPDMAFPRMNNMSFWLLPPSITLLISSSIVENGAGTGWTVYPPLSSNIAHGGSSVDLAIFSLHLAGISSILGAINFITTIINMRLNNMFFD
QLPLFVWAVGITAFLLLLSLPVLAGAITMLLTDRNLNTSFFDPAGGGDPILYQHL
>LEFIL139-10|JF854488|MM19139|Odonestis_pruni
TLYFIFGIWAGMVGTSLSLLIRAELGTPGSLIGDDQIYNTIVTAHAFIMIFFMVMPIMIGGFGNWLVPLMLGAPDMAFPRMNNMSFWLLPPSLTLLISSSIVENGAGTGWTVYPPLSSNIAHGGSSVDLAIFSLHLAGISSILGAINFITTIINMRLNNMSFD
QMPLFVWAVGITAFLLLLSLPVLAGAITMLLTDRNLNTSFFDPAGGGDPILYQHL
>LEFIB203-10|HM871107|MM00648|Odontopera_bidentata
TLYFIFGIWAGMIGTSLSLLIRAELGNPGSLIGDDQIYNTIVTAHAFIMIFFMVMPIMIGGFGNWLVPLMLGAPDMAFPRMNNMSFWLLPPSITLLISSSIVENGAGTGWTVYPPLSSNIAHGGSSVDLAIFSLHLAGISSILGAINFITTIINMRLNNLSFDQ
MPLFIWAVGITAFLLLLSLPVLAGAITMLLTDRNLNTSFFDPAGGGDPILYQHL
>LEFIA069-10|HM396416|MM00987|Odontosia_carmelita
TLYFIFGIWAGMVGTSLSLLIRAELGNPGSLIGDDQIYNTIVTAHAFIMIFFMVMPIMIGGFGNWLVPLMLGAPDMAFPRMNNMSFWLLPPSLTLLISSSIVENGAGTGWTVYPPLSSNIAHGGSSVDLAIFSLHLAGISSILGAINFITTIINMRLNNMSF
DQMPLFVWAVGITAFLLLLSLPVLAGAITMLLTDRNLNTSFFDPAGGGDPILYQHL
>LEFIB090-10|HM870997|MM00396|Odontosia_sieversii
TLYFIFGIWAGMVGTSLSLLIRAELGNPGSLIGDDQIYNTIVTAHAFIMIFFMVMPIMIGGFGNWLVPLMLGAPDMAFPRMNNMSFWLLPPSLTLLISSSIVENGAGTGWTVYPPLSSNIAHGGSSVDLAIFSLHLAGISSILGAINFITTIINMRLNNMSF
DQMPLFVWAVGITAFLLLLSLPVLAGAITMLLTDRNLNTSFFDPAGGGDPILYQHL
>LEFIC335-10|HM872179|MM03842|Oecophora_bractella
TLYFIFGVWAGMVGTSLSLLIRAELGNPGSLIGDDQIYNTIVTAHAFIMIFFMVMPIMIGGFGNWLVPLMLGAPDMAFPRMNNMSFWLLPPSLTLLISSSIVENGAGTGWTVYPPLSSNIAHGGSSVDLAIFSLHLAGISSILGAINFITTIINMRMNNMS
FDQMPLFVWAVGITALLLLLSLPVLAGAITMLLTDRNLNTSFFDPAGGGDPILYQHL
>LEFIF472-10|HM875157|MM12011|Oegoconia_deauratella
TLYFIFGIWAGMVGTSLSLLIRAELGNPGSLIGDDQIYNTIVTAHAFIMIFFMVMPIMIGGFGNWLVPLMLGAPDMAFPRMNNMSFWLLPPSLTLLISSSIVETGAGTGWTVYPPLSSNIAHSGSSVDLAIFSLHLAGISSILGAINFITTIINMRLNNMSFD
QMPLFVWAVGITALLLLLSLPVLAGAITMLLTDRNLNTSFFDPAGGGDPILYQHL
>LEEUA169-11|KF808712|MM19577|Oegoconia_novimundi
TLYFIFGIWAGMVGTSLSLLIRAELGNPGSLIGDDQIYNTIVTAHAFIMIFFMVMPIMIGGFGNWLVPLMLGAPDMAFPRMNNMSFWLLPPSLTLLISSSIVETGAGTGWTVYPPLSSNIAHSGSSVDLAIFSLHLAGISSILGAINFITTIINMRLYNMSFD
QMPLFVWAVGITALLLLLSLPVLAGAITMLLTDRNLNTSFFDPAGGGDPILYQHL
>LEFIF129-10|HM874828|MM10585|Oeneis_bore
TLYFILGIWAGMVGTSLSLIIRTELGNPGSLIGNDQIYNTIVTAHAFIMIFFMVMPIMIGGFGNWLIPLMLGAPDMAFPRMNNMSFWLLPPSLMLLISSSIVENGAGTGWTIYPPLSSNIAHSGSSVDLAIFSLHLAGISSILGAINFITTIINMRINNMTYD
QMPLFVWAVGITALLLLLSLPVLAGAITMLLTDRNLNTSFFDPAGGGDPILYQHL
>LEFIJ557-10|JF853672|MM17182|Oeneis_bore
TLYFILGIWAGMVGTSLSLIIRTELGNPGSLIGNDQIYNTIVTAHAFIMIFFMVMPIMIGGFGNWLIPLMLGAPDMAFPRMNNMSFWLLPPSLMLLISSSIVENGAGTGWTIYPPLSSNIAHSGSSVDLAIFSLHLAGISSILGAINFITTIINMRINNMTYD
QMPLFVWAVGITALLLLLSLPVLAGAITMLLTDRNLNTSFFDPAGGGDPILYQHL
>LEFIC071-10|HM871940|MM03356|Oeneis_jutta
TLYFIFGIWAGMVGTSLSLIIRTELGNPGSLIGDDQIYNTIVTAHAFIMIFFMVMPIMIGGFGNWLIPLMLGAPDMAFPRMNNMSFWLLPPSLMLLISSSIVENGAGTGWTVYPPLSSNIAHSGSSVDLAIFSLHLAGISSILGAINFITTIINMRINNMNYD
QMPLFVWAVGITALLLLLSLPVLAGAITMLLTDRNLNTSFFDPAGGGDPILYQHL
>LEFIJ556-10|JF853671|MM17181|Oeneis_jutta
TLYFIFGIWAGMVGTSLSLIIRTELGNPGSLIGDDQIYNTIVTAHAFIMIFFMVMPIMIGGFGNWLIPLMLGAPDMAFPRMNNMSFWLLPPSLMLLISSSIVENGAGTGWTVYPPLSSNIAHSGSSVDLAIFSLHLAGISSILGAINFITTIINMRINNMNYD
QMPLFVWAVGITALLLLLSLPVLAGAITMLLTDRNLNTSFFDPAGGGDPILYQHL
>LEFIA021-10|HM396371|MM00092|Oeneis_norna
TLYFIFGIWAGMVGTSLSLIIRTELGNPGSLIGDDQIYNTIVTAHAFIMIFFMVMPIMIGGFGNWLIPLMLGAPDMAFPRMNNMSFWLLPPSLMLLISSSIVENGAGTGWTVYPPLSSNIAHSGSSVDLAIFSLHLAGISSILGAINFITTIINMRINNMNYD
QMPLFVWAVGITALLLLLSLPVLAGAITMLLTDRNLNTSFFDPAGGGDPILYQHL
>LEFIJ2156-14|MM23181A|Oeneis_norna
TLYFIFGIWAGMVGTSLSLIIRTELGNPGSLIGDDQIYNTIVTAHAFIMIFFMVMPIMIGGFGNWLIPLMLGAPDMAFPRMNNMSFWLLPPSLMLLISSSIVENGAGTGWTVYPPLSSNIAHSGSSVDLAIFSLHLAGISSILGAINFITTIINMRINNMNYD
QMPLFVWAVGITALLLLLSLPVLAGAITMLLTDRNLNTSFFDPAGGGDPILYQHL
>COLFF474-13|MM23184|Oeneis_norna
TLYFIFGIWAGMVGTSLSLIIRTELGNPGSLIGDDQIYNTIVTAHAFIMIFFMVMPIMIGGFGNWLIPLMLGAPDMAFPRMNNMSFWLLPPSLMLLISSSIVENGAGTGWTVYPPLSSNIAHSGSSVDLAIFSLHLAGISSILGAINFITTIINMRINNMNYD
QMPLFVWAVGITALLLLLSLPVLAGAITMLLTDRNLNTSFFDPAGGGDPILYQHL
>COLFF475-13|MM23185|Oeneis_norna
TLYFIFGIWAGMVGTSLSLIIRTELGNPGSLIGDDQIYNTIVTAHAFIMIFFMVMPIMIGGFGNWLIPLMLGAPDMAFPRMNNMSFWLLPPSLMLLISSSIVENGAGTGWTVYPPLSSNIAHSGSSVDLAIFSLHLAGISSILGAINFITTIINMRINNMNYD

QMPLFVWAVGI?ALLLLLSLPVLAGAITMLLTDRNLNTSFFDPAGGGDPILYQHL
>LEFIJ2154-14|MM23340|Oeneis_norna
TLYFIFGIWAGMVGTSLSLIIRTELGNPGSLIGDDQIYNTIVTVHAFIMIFFMVMPIMIGGFGNWLIPLMLGAPDMAFPRMNNMSFWLLPPSLMLLISSSIVENGVGTGWTIYPPLSSNIAHSGSSVDLAIFSLHLAGISSILGAINFITTIINMRINNMTYDQ
MPLFIWAVGITALLLLLSLPVLAGAITMLLTDRNLNTSFFDPAGGGDPILYQHL
>LEFIE639-10|HM874362|MM09587|Oidaematophorus_lithodactyla
TLYFIFGIWAGMVGTSLSLLIRAELGNPGSLIGDDQIYNTIVTAHAFIMIFFMVMPIMIGGFGNWLVPLMLGAPDMAFPRMNNMSFWLLPPSLILLISSSIVENGAGTGWTVYPPLSSNIAHSGTSVDLAIFSLHLAGISSILGAINFITTIINMRLNNLMFD
QLPLFVWSVGITALLLLLSLPVLAGAITMLLTDRNLNTSFFDPAGGGDPILYQHL
>LEFIE971-10|HM874688|MM10393|Oidaematophorus_lithodactyla
TLYFIFGIWAGMVGTSLSLLIRAELGNPGSLIGDDQIYNTIVTAHAFIMIFFMVMPIMIGGFGNWLVPLMLGAPDMAFPRMNNMSFWLLPPSLILLISSSIVENGAGTGWTVYPPLSSNIAHSGTSVDLAIFSLHLAGISSILGAINFITTIINMRLNNLMFD
QLPLFVWSVGITALLLLLSLPVLAGAITMLLTDRNLNTSFFDPAGGGDPILYQHL
>LEFID454-10|HM873225|MM06393|Oidaematophorus_rogenhoferi
TLYFIFGIWAGLVGTSLSLLIRAELGNPGSLIGDDQIYNTIVTAHAFIMIFFMVMPIMIGGFGNWLVPLMLGAPDMAFPRMNNMSFWLLPPSIILLISSSIVENGAGTGWTVYPPLSSNIAHSGASVDLAIFSLHLAGISSILGAINFITTIINMRLNNMMFD
QLPLFVWATGITALLLLLSLPVLAGAITMLLTDRNLNTSFFDPAGGGDPILYQHL
>LEFID579-10|HM873344|MM06582|Oidaematophorus_rogenhoferi
TLYFIFGIWAGLVGTSLSLLIRAELGNPGSLIGDDQIYNTIVTAHAFVMIFFMVMPIMIGGFGNWLVPLMLGAPDMAFPRMNNMSFWLLPPSIILLISSSIVENGAGTGWTVYPPLSSNIAHSGTSVDLAIFSLHLAGISSILGAINFITTIINMRLNNMMFD
QLPLFVWATGITALLLLLSLPVLAGAITMLLTDRNLNTSFFDPAGGGDPILYQHL
>LEFIL230-10|MM19230|Oidaematophorus_vafradactylus
TLYFIFGIWAGMVGTSLSLLIRAELGNPGSLIGDDQIYNTIVTAHAFIMIFFMVMPIMIGGFGNWLVPLMLGAPDMAFPRMNNMSFWLLPPSFILLISSSIVENGAGTGWTVYPPLSSNIAHSGASVDLAIFSLHLAGISSILGAINFITTIINMRLNNLMFD
QLPLFVWSVGITALLLLLSLPVLAGAITMLLTDRNLNTSFFDPAGGGDPILYQHL
>LEFIE626-10|HM874349|MM09557|Olethreutes_arcuella
TLYFIFGIWAGMIGTSLSLLIRAELGNPGSLIGDDQIYNTIVTAHAFIMIFFMVMPIMIGGFGNWLVPLMLGAPDMAFPRMNNMSFWLLPPSIMLLISSSIVENGAGTGWTVYPPLSSNIAHSGSSVDLAIFSLHLAGISSILGAVNFITTIINMRPNNMSL
DQMPLFIWAVGITALLLLLSLPVLAGAITMLLTDRNLNTSFFDPAGGGDPILYQHL
>LEFIF888-10|HM875570|MM13294|Olethreutes_arcuella
TLYFIFGIWAGMIGTSLSLLIRAELGNPGLLIGDDQIYNTIVTAHAFIMIFFMVMPIMIGGFGNWLVPLMLGAPDMAFPRMNNMSFWLLPPSIMLLISSSIVENGAGTGWTVYPPLSSNIAHSGSSVDLAIFSLHLAGISSILGAVNFITTIINMRPNNMSL
DQMPLFIWAVGITALLLLLSLPVLAGAITMLLTDRNLNTSFFDPAGGGDPILYQHL
>LEFID229-10|HM873029|MM06088|Oligia_fasciuncula
TLYFIFGIWAGMVGTSLSLLIRAELGAPGSLIGDDQIYNTIVTAHAFIMIFFMVMPIMIGGFGNWLVPLMLGAPDMAFPRMNNMSFWLLPPSLTLLISSSIVENGAGTGWTVYPPLSSNIAHGGSSVDLAIFSLHLAGISSILGAINFITTIINMRLTNLSFD
QMPLFIWAVGITAFLLLLSLPVLAGAITMLLTDRNLNTSFFDPAGGGDPILYQHL
>LEFIA570-10|MM01664|Oligia_latruncula
TLYFIFGIWAGMVGTSLSLLIRAELGTPGSLIGDDQIYNTIVTAHAFIMIFFMVMPIMIGGFGNWLVPLMLGAPDMAFPRMNNMSFWLLPPSLTLLISSSIVENGAGTGWTVYPPLSSNIAHGGSSVDLAIFSLHLAGISSILGAINFITTIINMRLNNLSFD
QMPLFIWAVGITAFLLLLSLPVLAGAITMLLTDRNLNTSFFDPAGGGDPILYQHL
>LEFID619-10|HM873384|MM06635|Oligia_latruncula
TLYFIFGIWAGMVGTSLSLLIRAELGTPGSLIGDDQIYNTIVTAHAFIMIFFMVMPIMIGGFGNWLVPLMLGAPDMAFPRMNNMSFWLLPPSLTLLISSSIVENGAGTGWTVYPPLSSNIAHGGSSVDLAIFSLHLAGISSILGAINFITTIINMRLNNLSFD
QMPLFIWAVGITAFLLLLSLPVLAGAITMLLTDRNLNTSFFDPAGGGDPILYQHL
>LEFIA572-10|HM870821|MM01666|Oligia_strigilis
TLYFIFGIWAGMVGTSLSLLIRAELGTPGSLIGDDQIYNTIVTAHAFIMIFFMVMPIMIGGFGNWLVPLMLGAPDMAFPRMNNMSFWLLPPSLTLLISSSIVENGAGTGWTVYPPLSSNIAHGGSSVDLAIFSLHLAGISSILGAINFITTIINMRLTNLSFD
QMPLFIWAVGITAFLLLLSLPVLAGAITMLLTDRNLNTSFFDPAGGGDPILYQHL
>LEFID115-10|HM872926|MM05755|Oligia_versicolor
TLYFIFGIWAGMVGTSLSLLIRAELGTPGSLIGDDQIYNTIVTAHAFIMIFFMVMPIMIGGFGNWLVPLMLGAPDMAFPRMNNMSFWLLPPSLTLLISSSIVENGAGTGWTVYPPLSSNIAHGGSSVDLAIFSLHLAGISSILGAINFITTIINMRLNNLSFD
QMPLFIWAVGITAFLLLLSLPVLAGAITMLLTDRNLNTSFFDPAGGGDPILYQHL
>LEFID063-10|HM872877|MM05669|Olindia_schumacherana
TLYFIFGIWSGMIGTSLSLLIRAELGNPGSLIGDDQIYNTIVTAHAFIMIFFMVMPIMIGGFGNWLVPLMLGAPDMAFPRMNNMSFWLLPPSIMLLISSSIVENGAGTGWTVYPPLSSNIAHSGGSVDLAIFSLHLAGISSILGAVNFITTIINMRPNNMSL
DQMPLFVWAVGITALLLLLSLPVLAGAITMLLTDRNLNTSFFDPAGGGDPILYQHL
>LEFIC207-10|HM872051|MM03576|Oncocera_semirubella
TLYFIFGIWSGMVGTSLSLLIRAELGTPGSLIGDDQIYNTIVTGHAFIMIFFMVMPIMIGGFGNWLVPLMLGAPDMAFPRMNNMSFWLLPPSLTLLISSSIVENGAGTGWTVYPPLSSNIAHGGSSVDLAIFSLHLAGISSILGAINFITTIINMKLNGLSFD
QMPLFVWAVGITALLLLLSLPVLAGAITMLLTDRNLNTSFFDPAGGGDPILYQHL
>LEFIE091-10|HM873838|MM08270|Operophtera_brumata

TLYFIFGIWAGMIGTSLSLLIRAELGNPGSLIGDDQIYNTIVTAHAFIMIFFMVMPIMIGGFGNWLVPLMLGAPDMAFPRMNNMSFWLLPPSITLLISSSIVENGAGTGWTVYPPLSSNIAHGGSSVDLAIFSLHLAGISSILGAINFITTIINMRLNNMFFD
QLPLFVWAVGITAFLLLLSLPVLAGAITMLLTDRNLNTSFFDPAGGGDPILYQHL
>LEFIG572-10|HM876243|MM14726|Operophtera_brumata
TLYFIFGIWAGMIGTSLSLLIRAELGNPGSLIGDDQIYNTIVTAHAFIMIFFMVMPIMIGGFGNWLVPLMLGAPDMAFPRMNNMSFWLLPPSITLLISSSIVENGAGTGWTVYPPLSSNIAHGGSSVDLAIFSLHLAGISSILGAINFITTIINMRLNNMFFD
QLPLFVWAVGITAFLLLLSLPVLAGAITMLLTDRNLNTSFFDPAGGGDPILYQHL
>LEFIB790-10|HM871667|MM02698|Operophtera_fagata
TLYFIFGIWAGMIGTSLSLLIRAELGNPGSLIGDDQIYNTIVTAHAFIMIFFMVMPIMIGGFGNWLVPLMLGAPDMAFPRMNNMSFWLLPPSITLLISSSIVENGAGTGWTVYPPLSSNIAHSGSSVDLAIFSLHLAGISSILGAINFITTIINMRLNNMFFD
QLPLFVWAVGITAFLLLLSLPVLAGAITMLLTDRNLNTSFFDPAGGGDPILYQHL
>LEFIE090-10|HM873837|MM08269|Operophtera_fagata
TLYFIFGIWAGMIGTSLSLLIRAELGNPGSLIGDDQIYNTIVTAHAFIMIFFMVMPIMIGGFGNWLVPLMLGAPDMAFPRMNNMSFWLLPPSITLLISSSIVENGAGTGWTVYPPLSSNIAHSGSSVDLAIFSLHLAGISSILGAINFITTIINMRLNNMFFD
QLPLFVWAVGITAFLLLLSLPVLAGAITMLLTDRNLNTSFFDPAGGGDPILYQHL
>LEFIH030-10|HM876666|MM15894|Opigena_polygona
TLYFIFGIWAGMVGTSLSLLIRAELGNPGSLIGDDQIYNTIVTAHAFIMIFFMVMPIMIGGFGNWLVPLMLGAPDMAFPRMNNMSFWLLPPSLTLLISSSIVENGAGTGWTVYPPLSSNIAHGGSSVDLAIFSLHLAGISSILGAINFITTIINMRLNNLSFD
QMPLFIWAVGITAFLLLLSLPVLAGAITMLLTDRNLNTSFFDPAGGGDPILYQHL
>LEFIJ364-10|MM15964|Opigena_polygona
TLYFIFGIWAGMVGTSLSLLIRAELGNPGSLIGDDQIYNTIVTAHAFIMIFFMVMPIMIGGFGNWLVPLMLGAPDMAFPRMNNMSFWLLPPSLTLLISSSIVENGAGTGWTVYPPLSSNIAHGGSSVDLAIFSLHLAGISSILGAINFITTIINMRLNNLSFD
QMPLFIWAVGITAFLLLLSLPVLAGAITMLLTDRNLNTSFFDPAGGGDPILYQHL
>LEFIC691-10|HM872512|MM04672|Opisthograptis_luteolata
TLYFIFGIWAGMVGTSLSLLIRAELGNPGSLIGDDQIYNTIVTAHAFIMIFFMVMPIMIGGFGNWLVPLMLGAPDMAFPRMNNMSFWLLPPSITLLISSSIVENGAGTGWTVYPPLSSNIAHGGSSVDLAIFSLHLAGISSILGAINFITTIINMRLNNLSFD
QMPLFVWAVGITAFLLLLSLPVLAGAITMLLTDRNLNTSFFDPAGGGDPILYQHL
>LEFIJ1380-12|MM22051|Opogona_sacchari
TLYFILGIWAGLVGTSLSILIRMELGNPGSMIGNDQIYNTIVTAHAFIMIFFMVMPIMIGGFGNWLVPLMLGSPDMAFPRMNNMSFWLLPPSLTLLISSSMVENGAGTGWTVYPPLSSNIAHGGGSVDLAIFSLHLAGISSILGAANFITTTINMRPDN
MSFDKMPLFVWSVAITALLLLLSLPVLAGAITMLLTDRNLNTSFFDPAGGGDPILYQHL
>LEFIC435-10|HM872270|MM03987|Opostega_salaciella
TLYFLFGIWSGMVGTSLSILIRAELGNPGFLIGDDQIYNSIVTAHAFIMIFFMVMPIMIGGFGNWLVPLMLGAPDMAFPRLNNMSFWMLPPSLLLLISSSIVENGVGTGWTVYPPLSSNIAHGGSSVDLAIFSLHLAGISSILGAINFITTVINMKSSGMSF
DRMPLFVWSVAITALLLMLSLPVLAGAITMLLTDRNLNTSFFDPAGGGDPILYQHL
>LEFIE745-10|HM874465|MM09850|Opostega_salaciella
TLYFLFGIWSGMVGTSLSILIRAELGNPGFLIGDDQIYNSIVTAHAFIMIFFMVMPIMIGGFGNWLVPLMLGAPDMAFPRLNNMSFWMLPPSLLLLISSSIVENGVGTGWTVYPPLSSNIAHGGSSVDLAIFSLHLAGISSILGAINFITTVINMKSSGMSF
DRMPLFVWSVAITALLLMLSLPVLAGAITMLLTDRNLNTSFFDPAGGGDPILYQHL
>LEFIC718-10|HM872539|MM04770|Orgyia_antiqua
TLYFIFGIWAGMIGTSLSLLIRAELGNPGSLIGNDQIYNTIVTAHAFIMIFFMVMPIMIGGFGNWLVPLMLGAPDMAFPRMNNMSFWLLPPSLTLLISSSIVENGAGTGWTVYPPLSSNIAHSGSSVDLAIFSLHLAGISSILGAINFITTIINMRLNNLSFDQ
MPLFIWAVGITAFLLLLSLPVLAGAITMLLTDRNLNTSFFDPAGGGDPILYQHL
>LEFIE512-10|HM874236|MM09339|Orgyia_antiqua
TLYFIFGIWAGMIGTSLSLLIRAELGNPGSLIGNDQIYNTIVTAHAFIMIFFMVMPIMIGGFGNWLVPLMLGAPDMAFPRMNNMSFWLLPPSLTLLISSSIVENGAGTGWTVYPPLSSNIAHSGSSVDLAIFSLHLAGISSILGAINFITTIINMRLNNLSFDQ
MPLFIWAVGITAFLLLLSLPVLAGAITMLLTDRNLNTSFFDPAGGGDPILYQHL
>LEFIF538-10|HM875223|MM12217|Orgyia_antiqua
TLYFIFGIWAGMIGTSLSLLIRAELGNPGSLIGNDQIYNTIVTAHAFIMIFFMVMPIMIGGFGNWLVPLMLGAPDMAFPRMNNMSFWLLPPSLTLLISSSIVENGAGTGWTVYPPLSSNIAHSGSSVDLAIFSLHLAGISSILGAINFITTIINMRLNNLSFDQ
MPLFIWAVGITAFLLLLSLPVLAGAITMLLTDRNLNTSFFDPAGGGDPILYQHL
>LEFIG982-10|HM876621|MM15846|Orgyia_antiquoides
TLYFIFGVWAGMIGTSLSLLIRTELGNPGSFIGNDQIYNTIVTAHAFIMIFFMVMPIMIGGFGNWLVPLMLGAPDMAFPRMNNMSFWLLPPSLTLLISSSIVENGAGTGWTVYPPLSSNIAHSGSSVDLAIFSLHLAGISSILGAINFITTIINMRLNNLSFD
QMPLFVWAVGITAFLLLLSLPVLAGAITMLLTDRNLNTSFFDPAGGGDPILYQHL
>LEFIE245-10|HM873979|MM08572|Orgyia_recens
TLYFIFGIWAGMIGTSLSLLIRAELGAPGSLIGNDQIYNTIVTAHAFIMIFFMVMPIMIGGFGNWLVPLMLGAPDMAFPRMNNMSFWLLPPSLTLLISSSIVENGAGTGWTVYPPLSSNIAHSGSSVDLAIFSLHLAGISSILGAINFITTIINMRLNNLTFDQ
MPLFVWAVGITAFLLLLSLPVLAGAITMLLTDRNLNTSFFDPAGGGDPILYQHL
>LEFIJ821-10|JF853843|MM17446|Orgyia_recens
TLYFIFGIWAGMIGTSLSLLIRAELGTPGSLIGNDQIYNTIVTAHAFIMIFFMVMPIMIGGFGNWLVPLMLGAPDMAFPRMNNMSFWLLPPSLTLLISSSIVENGAGTGWTVYPPLSSNIAHSGSSVDLAIFSLHLAGISSILGAINFITTIINMRLNNLTFDQ
MPLFVWAVGITAFLLLLSLPVLAGAITMLLTDRNLNTSFFDPAGGGDPILYQHL

>LEFIK306-10|JF853960|MM17881|Orgyia_recens
TLYFIFGIWAGMIGTSLSLLIRAELGTPGSLIGNDQIYNTIVTAHAFIMIFFMVMPIMIGGFGNWLVPLMLGAPDMAFPRMNNMSFWLLPPSLTLLISSSIVENGAGTGWTVYPPLSSNIAHSGSSVDLAIFSLHLAGISSILGAINFITTIINMRLNNLTFDQ
MPLFVWAVGITAFLLLLSLPVLAGAITMLLTDRNLNTSFFDPAGGGDPILY?HL
>LEFIB726-10|HM871604|MM02547|Orophia_ferrugella
TLYFIFGIWAGMLGTSLSLLIRAELGNPGSLIGDDQIYNTIVTAHAFIMIFFMVMPIMIGGFGNWLVPLMLGAPDMAFPRMNNMSFWLLPPSITLLISSSIVESGAGTGWTVYPPLSSNIAHSGSSVDLAIFSLHLAGISSILGAINFITTVINMRVNNMSFD
QMPLFVWAVSITALLLLLSLPVLAGAITMLLTDRNLNTSFFDPAGGGDPILYQHL
>LEFID724-10|HM873481|MM06780|Orophia_ferrugella
TLYFIFGIWAGMLGTSLSLLIRAELGNPGSLIGDDQIYNTIVTAHAFIMIFFMVMPIMIGGFGNWLVPLMLGAPDMAFPRMNNMSFWLLPPSITLLISSSIVESGAGTGWTVYPPLSSNIAHSGSSVDLAIFSLHLAGISSILGAINFITTVINMRVNNMSFD
QMPLFVWAVSITALLLLLSLPVLAGAITMLLTDRNLNTSFFDPAGGGDPILYQHL
>LEFIE873-10|HM874591|MM10143|Ortholepis_betulae
TLYFIFGIWSGMVGTSLSLLIRAELGTPGSLIGDDQIYNTIVTGHAFIMIFFMVMPIMIGGFGNWLVPLMLGAPDMAFPRMNNMSFWLLPPSLTLLISSSIVENGAGTGWTVYPPLSSNIAHSGSSVDLAIFSLHLAGISSILGAINFITTIINMKLNGLSFDQ
MPLFVWAVGITALLLLLSLPVLAGAITMLLTDRNLNTSFFDPAGGGDPILYQHL
>LEFIF081-10|HM874788|MM10530|Ortholepis_vacciniella
TLYFIFGIWSGMVGTSLSLLIRAELGTPGSLIGDDQIYNTIVTGHAFIMIFFMVMPIMIGGFGNWLVPLMLGAPDMAFPRMNNMSFWLLPPSLTLLISSSIVENGAGTGWTVYPPLSSNIAHSGSSVDLAIFSLHLAGISSILGAINFITTIINMKLNGLSFDQ
MPLFVWAVGITALLLLLSLPVLAGAITMLLTDRNLNTSFFDPAGGGDPILYQHL
>LEFIL400-10|KM573680|MM18698|Orthonama_obstipata
TLYFIFGIWAGMIGTSLSLLIRAELGNPGSLIGDDQIYNTIVTAHAFIMIFFMVMPIMIGGFGNWLVPLMLGAPDMAFPRMNNMSFWLLPPSITLLISSSIVENGAGTGWTVYPPLSSNIAHGGSSVDLAIFSLHLAGISSILGAINFITTIINMRLNNMFFD
QLPLFVWAVGITAFLLLLSLPVLAGAITMLLTDRNLNTSFFDPAGGGDPILYQHL
>LEFID527-10|HM873292|MM06507|Orthonama_vittata
TLYFIFGIWAGMIGTSLSLLIRAELGNPGSLIGDDQIYNTIVTAHAFIMIFFMVMPIMIGGFGNWLVPLMLGAPDMAFPRMNNMSFWLLPPSITLLISSSIVENGAGTGWTVYPPLSSNIAHSGSSVDLAIFSLHLAGISSILGAINFITTIINMRLNNMFFD
QLPLFVWAVGITAFLLLLSLPVLAGAITMLLTDRNLNTSFFDPAGGGDPILYQHL
>LEFID528-10|HM873293|MM06508|Orthonama_vittata
TLYFIFGIWAGMIGTSLSLLIRAELGNPGSLIGDDQIYNTIVTAHAFIMIFFMVMPIMIGGFGNWLVPLMLGAPDMAFPRMNNMSFWLLPPSITLLISSSIVENGAGTGWTVYPPLSSNIAHSGSSVDLAIFSLHLAGISSILGAINFITTIINMRLNNMFFD
QLPLFVWAVGITAFLLLLSLPVLAGAITMLLTDRNLNTSFFDPAGGGDPILYQHL
>LEFID661-10|HM873423|MM06694|Orthonama_vittata
TLYFIFGIWAGMIGTSLSLLIRAELGNPGSLIGDDQIYNTIVTAHAFIMIFFMVMPIMIGGFGNWLVPLMLGAPDMAFPRMNNMSFWLLPPSITLLISSSIVENGAGTGWTVYPPLSSNIAHSGSSVDLTIFSLHLAGISSILGAINFITTIINMRLNNMFFD
QLPLFVWAVGITAFLLLLSLPVLAGAITMLLTDRNLNTSFFDPAGGGDPILYQHL
>LEFIC656-10|HM872477|MM04612|Orthosia_cerasi
TLYFIFGIWAGMVGTSLSLLIRAELGTPGSLIGDDQIYNTIVTAHAFIMIFFMVMPIMIGGFGNWLIPLMLGAPDMAFPRMNNMSFWLLPPSLTLLISSSIVENGAGTGWTVYPPLSSNIAHGGSSVDLAIFSLHLAGISSILGAINFITTIINMRLNNLSFDQ
MPLFIWAVGITAFLLLLSLPVLAGAITMLLTDRNLNTSFFDPAGGGDPILYQHL
>LEFIC659-10|HM872480|MM04616|Orthosia_cruda
TLYFIFGIWAGMVGTSLSLLIRAELGNPGSLIGDDQIYNTIVTAHAFIMIFFMVMPIMIGGFGNWLVPLMLGAPDMAFPRMNNMSFWLLPPSLTLLISSSIVENGAGTGWTVYPPLSSNIAHGGSSVDLAIFSLHLAGISSILGAINFITTIINMRLNNLSFD
QMPLFIWAVGITAFLLLLSLPVLAGAITMLLTDRNLNTSFFDPAGGGDPILYQHL
>LEFIC661-10|HM872482|MM04618|Orthosia_cruda
TLYFIFGIWAGMVGTSLSLLIRAELGNPGSLIGDDQIYNTIVTAHAFIMIFFMVMPIMIGGFGNWLVPLMLGAPDMAFPRMNNMSFWLLPPSLTLLISSSIVENGAGTGWTVYPPLSSNIAHGGSSVDLAIFSLHLAGISSILGAINFITTIINMRLNNLSFD
QMPLFIWAVGITAFLLLLSLPVLAGAITMLLTDRNLNTSFFDPAGGGDPILYQHL
>LEFIA492-10|KM572558|MM01573|Orthosia_gothica
TLYFIFGIWAGMVGTSLSLLIRAELGNPGSLIGDDQIYNTIVTAHAFIMIFFMVMPIMIGGFGNWLIPLMLGAPDMAFPRMNNMSFWLLPPSLTLLISSSIVENGAGTGWTVYPPLSSNIAHGGSSVDLAIFSLHLAGISSILGAINFITTIINMRLNSLSFDQ
MPLFIWAVGITAFLLLLSLPVLAGAITMLLTDRNLNTSFFDPAGGGDPILYQHL
>LEFIA489-10|KM573640|MM01567|Orthosia_gracilis
TLYFIFGIWAGMVGTSLSLLIRAELGNPGSLIGDDQIYNTIVTAHAFIMIFFMVMPIMIGGFGNWLVPLMLGAPDMAFPRMNNMSFWLLPPSLTLLISSSIVENGAGTGWTVYPPLSSNIAHGGSSVDLAIFSLHLAGISSILGAINFITTIINMRLNNLSFD
QMPLFIWAVGITAFLLLLSLPVLAGAITMLLTDRNLNTSFFDPAGGGDPILYQHL
>LEFIA486-10|KM571964|MM01563|Orthosia_incerta
TLYFIFGIWAGMVGTSLSLLIRAELGNPGSLIGDDQIYNTIVTAHAFIMIFFMVMPIMIGGFGNWLIPLMLGAPDMAFPRMNNMSFWLLPPSLTLLISSSIVENGAGTGWTVYPPLSSNIAHGGSSVDLAIFSLHLAGISSILGAINFITTIINMRLNNLSFD
QMPLFIWAVGITAFLLLLSLPVLAGAITMLLTDRNLNTSFFDPAGGGDPILYQHL
>LEFIB103-10|HM871010|MM00415|Orthosia_opima
TLYFIFGIWAGMVGTSLSLLIRAELGNPGSLIGDDQIYNTIVTAHAFIMIFFMVMPIMIGGFGNWLVPLMLGAPDMAFPRMNNMSFWLLPPSLTLLISSSIVENGAGTGWTVYPPLSSNIAHGGSSVDLAIFSLHLAGISSILGAINFITTIINMRLNNLSFD

QMPLFIWAVGITAFLLLLSLPVLAGAITMLLTDRNLNTSFFDPAGGGDPILYQHL
>LEFIB111-10|HM871018|MM00453|Orthosia_populeti
TLYFIFGIWAGMVGTSLSLLIRAELGNPGSLIGDDQIYNTIVTAHAFIMIFFMVMPIMIGGFGNWLVPLMLGAPDMAFPRMNNMSFWLLPPSLTLLISSSIVENGAGTGWTVYPPLSSNIAHGGSSVDLAIFSLHLAGISSILGAINFITTIINMRLNNLSFD
QMPLFIWAVGITAFLLLLSLPVLAGAITMLLTDRNLNTSFFDPAGGGDPILYQHL
>LEFIB908-10|HM871785|MM03014|Orthotaenia_undulana
TLYFIFGIWAGMVGTSLSLLIRAELGNPGSLIGDDQIYNTIVTAHAFIMIFFMVMPIMIGGFGNWLVPLMLGAPDMAFPRMNNMSFWLLPPSIMLLISSSIVENGAGTGWTVYPPLSSNIAHSGSSVDLAIFSLHLAGISSILGAVNFITTIINMRPNNMSL
DQMPLFVWAVGITALLLLLSLPVLAGAITMLLTDRNLNTSFFDPAGGGDPILYQHL
>LEFIE182-10|HM873927|MM08483|Orthotaenia_undulana
TLYFIFGIWAGMVGTSLSLLIRAELGNPGSLIGDDQIYNTIVTAHAFIMIFFMVMPIMIGGFGNWLVPLMLGAPDMAFPRMNNMSFWLLPPSIMLLISSSIVENGAGTGWTVYPPLSSNIAHSGSSVDLAIFSLHLAGISSILGAVNFITTIINMRPNNMSL
DQMPLFVWAVGITALLLLLSLPVLAGAITMLLTDRNLNTSFFDPAGGGDPILYQHL
>LEFIF887-10|HM875569|MM13293|Orthotaenia_undulana
TLYFIFGIWAGMVGTSLSLLIRAELGNPGSLIGDDQIYNTIVTAHAFIMIFFMVMPIMIGGFGNWLVPLMLGAPDMAFPRMNNMSFWLLPPSIMLLISSSIVENGAGTGWTVYPPLSSNIAHSGSSVDLAIFSLHLAGISSILGAVNFITTIINMRPNNMSL
DQMPLFVWAVGITALLLLLSLPVLAGAITMLLTDRNLNTSFFDPAGGGDPILYQHL
>LEFIE437-10|HM874161|MM09192|Orthotelia_sparganella
TLYFIFGIWSGMIGTSLSLLIRAELGTPGSLIGDDQIYNTIVTAHAFIMIFFMVMPIMIGGFGNWLVPLMLGAPDMAFPRMNNMSFWLLPPSLTLLISSSIVENGAGTGWTVYPPLSSNIAHSGSSVDLAIFSLHLAGISSILGAINFITTIINMKNYSMKFDQ
MPLFVWSVGITALLLLLSLPVLAGAITMLLTDRNLNTSFFDPAGGGDPILYQHL
>LEFIA643-10|HM870892|MM01759|Ostrinia_nubilalis
TLYFIFGIWSGMVGTSLSLLIRAELGNPGSLIGDDQIYNTIVTAHAFIMIFFMVMPIMIGGFGNWLVPLMLGAPDMAFPRMNNMSFWLLPPSLTLLISSSIVENGAGTGWTVYPPLSSNIAHGGSSVDLAIFSLHLAGISSILGAINFITTIINMRINGMSFD
QMPLFVWSVGITALLLLLSLPVLAGAITMLLTDRNLNTSFFDPAGGGDPILYQHL
>LEFIF330-10|HM875015|MM11135|Ostrinia_nubilalis
TLYFIFGIWSGMVGTSLSLLIRAELGNPGSLIGDDQIYNTIVTAHAFIMIFFMVMPIMIGGFGNWLVPLMLGAPDMAFPRMNNMSFWLLPPSLTLLISSSIVENGAGTGWTVYPPLSSNIAHGGSSVDLAIFSLHLAGISSILGAINFITTIINMRINGMSFD
QMPLFVWSVGITALLLLLSLPVLAGAITMLLTDRNLNTSFFDPAGGGDPILYQHL
>LEFIL720-10|MM16010|Ostrinia_palustralis
TLYFIFGIWSGMVGTSLSLLIRAELGNPGSLIGDDQIYNTIVTAHAFIMIFFMVMPIMIGGFGNWLVPLMLGAPDMAFPRMNNMSFWLLPPSLTLLISSSIVENGAGTGWTVYPPLSSNIAHGGSSVDLAIFSLHLAGISSILGAINFITTIINMRVNGMSF
DQMPLFVWAVGITALLLLLSLPVLAGAITMLLTDRNLNTSFFDPAGGGDPILYQHL
>LEFIA171-10|HM396516|MM01149|Ourapteryx_sambucaria
TLYFIFGIWAGMVGTSLSLLIRAELGSPGSLIGDDQIYNTIVTAHAFIMIFFMVMPIMIGGFGNWLVPLMLGAPDMAFPRMNNMSFWLLPPSITLLISSSIVENGAGTGWTVYPPLSSNIAHGGSSVDLAIFSLHLAGISSILGAINFITTIINMRLNNLSFD
QMPLFVWAVGITAFLLLLSLPVLAGAITMLLTDRNLNTSFFDPAGGGDPILYQHL
>LEFIA172-10|HM396517|MM01150|Ourapteryx_sambucaria
TLYFIFGIWAGMVGTSLSLLIRAELGNPGSLIGDDQIYNTIVTAHAFIMIFFMVMPIMIGGFGNWLVPLMLGAPDMAFPRMNNMSFWLLPPSITLLISSSIVENGAGTGWTVYPPLSSNIAHGGSSVDLAIFSLHLAGISSILGAINFITTIINMRLNNLSFD
QMPLFVWAVGITAFLLLLSLPVLAGAITMLLTDRNLNTSFFDPAGGGDPILYQHL
>LEFIA840-10|HM386980|MM09715|Ourapteryx_sambucaria
TLYFIFGIWAGMVGTSLSLLIRAELGNPGSLIGDDQIYNTIVTAHAFIMIFFMVMPIMIGGFGNWLVPLMLGAPDMAFPRMNNMSFWLLPPSITLLISSSIVENGAGTGWTVYPPLSSNIAHGGSSVDLAIFSLHLAGISSILGAINFITTIINMRLNNLSFD
QMPLFIWAVGITAFLLLLSLPVLAGAITMLLTDRNLNTSFFDPAGGGDPILYQHL
>LEFIA895-10|HM387031|MM09772|Ourapteryx_sambucaria
TLYFIFGIWAGMVGTSLSLLIRAELGNPGSLIGDDQIYNTIVTAHAFIMIFFMVMPIMIGGFGNWLVPLMLGAPDMAFPRMNNMSFWLLPPSITLLISSSIVENGAGTGWTVYPPLSSNIAHGGSSVDLAIFSLHLAGISSILGAINFITTIINMRLNNLSFD
QMPLFVWAVGITAFLLLLSLPVLAGAITMLLTDRNLNTSFFDPAGGGDPILYQHL
>LEFIF588-10|HM875272|MM12427|Oxyptilus_chrysodactyla
TMYFIFGIWAGMVGTSLSLLIRAELGNPGSLIGDDQIYNSIVTAHAFIMIFFMVMPIMIGGFGNWLVPLMLGAPDMAFPRMNNMSFWLLPPSILLLISSSIVENGVGTGWTVYPPLSSNIAHSGPSVDLAIFSLHLAGISSILGAINFISTIINMRLNKMMF
DQLPLFVWAVWITAVLLLLSLPVLAGAITMLLTDRNLNTSFFDPAGGGDPILYQHL
>LEFIJ230-10|MM13386|Oxyptilus_chrysodactyla
TMYFIFGIWAGMVGTSLSLLIRAELGNPGSLIGDDQIYNSIVTAHAFIMIFFMVMPIMIGGFGNWLVPLMLGAPDMAFPRMNNMSFWLLPPSILLLISSSIVENGVGTGWTVYPPLSSNIAHSGPSVDLAIFSLHLAGISSILGAINFISTIINMRLNKMMF
DQLPLFVWAVWITAVLLLLSLPVLAGAITMLLTDRNLNTSFFDPAGGGDPILYQHL
>LEFIJ1264-11|MM21124|Oxyptilus_chrysodactyla
TMYFIFGIWAGMVGTSLSLLIRAELGNPGSLIGDDQIYNSIVTAHAFIMIFFMVMPIMIGGFGNWLVPLMLGAPDMAFPRMNNMSFWLLPPSILLLISSSIVENGVGTGWTVYPPLSSNIAHSGPSVDLAIFSLHLAGISSILGAINFISTIINMRLNKMMF
DQLPLFVWAVWITAVLLLLSLPVLAGAITMLLTDRNLNTSFFDPAGGGDPILYQHL
>LEFID828-10|HM873585|MM06944|Oxyptilus_ericetorum

TMYFIFGIWAGMIGTSLSLLIRAELGNPGSLIGDDQIYNSIVTAHAFIMIFFMVMPIMIGGFGNWLVPLMLGAPDMAFPRMNNMSFWLLPPSILLLISSSIVENGVGTGWTVYPPLSSNIAHSGPSVDLAIFSLHLAGISSILGAINFISTIINMRLNKMMF
DQLPLFVWAVWITAVLLLLSLPVLAGAITMLLTDRNLNTSFFDPAGGGDPILYQHL
>LEFIE795-10|HM874514|MM09979|Oxyptilus_parvidactyla
TLYFIFGIWAGMIGTSLSLLIRAELGNPGFLIGDDQIYNSIVTAHAFIMIFFMVMPIMIGGFGNWLVPLMLGAPDMAFPRMNNMSFWLLPPSILLLISSSIVENGVGTGWTVYPPLSSNIAHSGPSVDLAIFSLHLAGISSILGAINFISTIINMRLNKMMFD
QLPLFVWAVWITAVLLLLSLPVLAGAITMLLTDRNLNTSFFDPAGGGDPILYQHL
>LEFIJ058-10|JF853420|MM03674|Oxyptilus_pilosellae
TLYFIFGIWAGMIGTSLSLLIRAELGNPGSLIGDDQIYNSIVTAHAFIMIFFMVMPIMIGGFGNWLVPLMLGAPDMAFPRMNNMSFWLLPPSILLLISSSIVENGVGTGWTVYPPLSSNIAHSGPSVDLAIFSLHLAGISSILGAINFISTIINMRLNKMMFD
QLPLFVWAVWITAVLLLLSLPVLAGAITMLLTDRNLNTSFFDPAGGGDPILYQHL
>LEFIJ098-10|JF853444|MM06818|Oxyptilus_pilosellae
TLYFIFGIWAGMIGTSLSLLIRAELGNPGSLIGDDQIYNSIVTAHAFIMIFFMVMPIMIGGFGNWLVPLMLGAPDMAFPRMNNMSFWLLPPSILLLISSSIVENGVGTGWTVYPPLSSNIAHSGPSVDLAIFSLHLAGISSILGAINFISTIINMRLNKMMFD
QLPLFVWAVWITAVLLLLSLPVLAGAITMLLTDRNLNTSFFDPAGGGDPILYQHL
>LEFIE619-10|HM874342|MM09543|Oxyptilus_pilosellae
TLYFIFGIWAGMIGTSLSLLIRAELGNPGSLIGDDQIYNSIVTAHAFIMIFFMVMPIMIGGFGNWLVPLMLGAPDMAFPRMNNMSFWLLPPSILLLISSSIVENGVGTGWTVYPPLSSNIAHSGPSVDLAIFSLHLAGISSILGAINFISTIINMRLNKMMFD
QLPLFVWAVWITAVLLLLSLPVLAGAITMLLTDRNLNTSFFDPAGGGDPILYQHL
>LEFIF583-10|HM875267|MM12395|Oxyptilus_pilosellae
TLYFIFGIWAGMIGTSLSLLIRAELGNPGSLIGDDQIYNSIVTAHAFIMIFFMVMPIMIGGFGNWLVPLMLGAPDMAFPRMNNMSFWLLPPSILLLISSSIVENGVGTGWTVYPPLSSNIAHSGPSVDLAIFSLHLAGISSILGAINFISTIINMRLNKMMFD
QLPLFVWAVWITAVLLLLSLPVLAGAITMLLTDRNLNTSFFDPAGGGDPILYQHL
>LEFIK805-10|JN277228|MM18380|Oxyptilus_pilosellae
TLYFIFGIWAGMIGTSLSLLIRAELGNPGSLIGDDQIYNSIVTAHAFIMIFFMVMPIMIGGFGNWLVPLMLGAPDMAFPRMNNMSFWLLPPSILLLISSSIVENGVGTGWTVYPPLSSNIAHSGPSVDLAIFSLHLAGISSILGAINFISTIINMRLNKMMFD
QLPLFVWAVWITAVLLLLSLPVLAGAITMLLTDRNLNTSFFDPAGGGDPILYQHL
>LEFID047-10|HM872861|MM05519|Pabulatrix_pabulatricula
TLYFIFGIWAGMVGTSLSLLIRAELGNPGSLIGDDQIYNTIVTAHAFIMIFFMVMPIMIGGFGNWLVPLMLGAPDMAFPRMNNMSFWLLPPSLTLLISSSIVENGAGTGWTVYPPLSSNIAHGGSSVDLAIFSLHLAGISSILGAINFITTIINMRLNSLSFD
QMPLFIWAVGITAFLLLLSLPVLAGAITMLLTDRNLNTSFFDPAGGGDPILYQHL
>LEFIJ357-10|KM572927|MM15957|Pachetra_sagittigera
TLYFIFG?WAGMVGTSLSLLIRAELGTPGSLIGDDQIYNTIVTAHAFIMIFFMVMPIMIGGFGNWLVPLMLGAPDMAFPRMNNMSFWLLPPSLTLLISSSIVENGAGTGWTVYPPLSSNIAHGGSSVDLAIFSLHLAGISSILGAINFITTIINMRLNSLSFD
QMPLFIWAVGITAFLLLLSLPVLAGAITMLLTDRNLNTSFFDPAGGGDPILYQHL
>LEFIL611-10|KM573018|MM18909|Pachetra_sagittigera
TLYFIFGIWAGMVGTSLSLLIRAELGTPGSLIGDDQIYNTIVTAHAFIMIFFMVMPIMIGGFGNWLVPLMLGAPDMAFPRMNNMSFWLLPPSLTLLISSSIVENGAGTGWTVYPPLSSNIAHGGSSVDLAIFSLHLAGISSILGAINFITTIINMRLNSLSFD
QMPLFIWAVGITAFLLLLSLPVLAGAITMLLTDRNLNTSFFDPAGGGDPILYQHL
>LEEUA316-11|JN268643|MM19724|Pachycnemia_hippocastanaria
TLYFIFGIWAGMIGTSLSLLIRAELGNPGSLIGDDQIYNTIVTAHAFIMIFFMVMPIMIGGFGNWLVPLMLGAPDMAFPRMNNMSFWLLPPSITLLISSSIVENGAGTGWTVYPPLSSNIAHGGSSVDLAIFSLHLAGISSILGAINFITTIINMRLNNLSFDQ
MPLFIWAVGITAFLLLLSLPVLAGAITMLLTDRNLNTSFFDPAGGGDPILYQHL
>LEEUA514-11|MM20573|Pachycnemia_hippocastanaria
TLYFIFGIWAGMIGTSLSLLIRAELGNPGSLIGDDQIYNTIVTAHAFIMIFFMVMPIMIGGFGNWLVPLMLGAPDMAFPRMNNMSFWLLPPSITLLISSSIVENGAGTGWTVYPPLSSNIAHGGSSVDLAIFSLHLAGISSILGAINFITTIINMRLNNLSFDQ
MPLFIWAVGITAFLLLLSLPVLAGAITMLLTDRNLNTSFFDPAG?GDPILYQHL
>LEFIG520-10|HM876193|MM14629|Pachythelia_villosella
TLYFILGIWSGLIGTSLSFLIRAELGTPNSLIGNDQIYNTIVTAHAFIMIFFMVMPIMIGGFGNWLVPLMLGAPDMAFPRMNNMSFWLLPPSLMLLTASSLVENGAGTGWTVYPPLSSNISHNGGSVDLAIFSLHLAGISSILGAINFITTAINMRMNNMS
LDQMPLFVWSVIITAVLLLLSLPVLAGAITMLLTDRNLNTSFFDPAGGGDPILYQHL
>LEFIL340-10|MM18650|Palpita_vitrealis
TLYFIFGIWSGMVGTSLSLLIRAELGNPGSLIGDDQIYNTIVTAHAFIMIFFMVMPIMIGGFGNWLVPLMLGAPDMAFPRMNNMSFWLLPPSLTLLISSSIVENGAGTGWTVYPPLSSNIAHGGSSVDLAIFSLHLAGISSILGAINFITTIINMRVNGLSFD
QMPLFVWAVGITALLLLLSLPVLAGAITMLLTDRNLNTSFFDPAGGGDPILYQHL
>LEFIC391-10|HM872234|MM03931|Pammene_albuginana
TLYFIFGIWAGMVGTSLSLLIRAELGNPGSLIGDDQIYNTIVTAHAFIMIFFMVMPIMIGGFGNWLVPLMLGAPDMAFPRMNNMSFWLLPPSIMLLISSSIVENGAGTGWTVYPPLSSNIAHSGSSVDLAIFSLHLAGISSILGAVNFITTIINMRPNNMSL
DQMPLFVWAVGITALLLLLSLPVLAGAITMLLTDRNLNTSFFDPAGGGDPILYQHL
>LEFIG584-10|HM876255|MM14773|Pammene_argyrana
TLYFIFGIWAGMVGTSLSLLIRAELGNPGSLIGDDQIYNTIVTAHAFIMIFFMVMPIMIGGFGNWLVPLMLGAPDMAFPRMNNMSFWLLPPSIMLLISSSIVENGAGTGWTVYPPLSSNIAHSGSSVDLAIFSLHLAGISSILGAVNFITTIINMRPNNMSL
DQMPLFVWAVGITALLLLLSLPVLAGAITMLLTDRNLNTSFFDPAGGGDPILYQHL

>LEFIK032-10|MM17607|Pammene_aurana
TLYFIFGIWAGMVGTSLSLLIRAELGNPGSLIGDDQIYNTIVTAHAFIMIFFMVMPIMIGGFGNWLVPLMLGAPDMAFPRMNNMSFWLLPPSILLLISSSVVENGAGTGWTVYPPLSSNIAHSGSSVDLAIFSLHLAGISSILGAVNFITTIINMRPNNMSL
DQMPLFVWAVGITALLLLLSLPVLAGAITMLLTDRNLNTSFFDPAGGGDPILYQHL
>LEEUA251-11|MM19659|Pammene_aurana
TLYFIFGIWAGMVGTSLSLLIRAELGNPGSLIGDDQIYNTIVTAHAFIMIFFMVMPIMIGGFGNWLVPLMLGAPDMAFPRMNNMSFWLLPPSILLLISSSVVENGAGTGWTVYPPLSSNIAHSGSSVDLAIFSLHLAGISSILGAVNFITTIINMRPNNMSL
DQMPLFVWAVGITALLLLLSLPVLAGAITMLLTDRNLNTSFFDPAGGGDPILYQHL
>LEEUA256-11|JN274948|MM19664|Pammene_aurita
TLYFIFGVWAGMVGTSLSLLIRAELGNPGSLIGDDQIYNTIVTAHAFIMIFFMVMPIMIGGFGNWLVPLMLGAPDMAFPRMNNMSFWLLPPSILLLISSSIVENGAGTGWTVYPPLSSNIAHSGSSVDLAIFSLHLAGISSILGAVNFITTIINMRPNNMSL
DQMPLFVWAVGITALLLLLSLPVLAGAITMLLTDRNLNTSFFDPAGGGDPILYQHL
>LEFID406-10|HM873202|MM06328|Pammene_clanculana
TLYFIFGIWAGMVGTSLSLLIRAELGNPGSFIGDDQIYNTIVTAHAFIMIFFMVMPIMIGGFGNWLVPLMLGAPDMAFPRMNNMSFWLLPPSIMLLISSSIVENGAGTGWTVYPPLSSNIAHSGSSVDLAIFSLHLAGISSILGAVNFITTIINMRPNNMSL
DQMPLFVWAVGITALLLLLSLPVLAGAITMLLTDRNLNTSFFDPAGGGDPILYQHL
>LEFID484-10|HM873250|MM06427|Pammene_clanculana
TLYFIFGIWAGMVGTSLSLLIRAELGNPGSFIGDDQIYNTIVTAHAFIMIFFMVMPIMIGGFGNWLVPLMLGAPDMAFPRMNNMSFWLLPPSIMLLISSSIVENGAGTGWTVYPPLSSNIAHSGSSVDLAIFSLHLAGISSILGAVNFITTIINMRPNNMSL
DQMPLFVWAVGITALLLLLSLPVLAGAITMLLTDRNLNTSFFDPAGGGDPILYQHL
>LEFIC323-10|HM872167|MM03827|Pammene_fasciana
TLYFIFGIWAGMVGTSLSLLIRAELGNPGSLIGDDQIYNTIVTAHAFIMIFFMVMPIMIGGFGNWLVPLMLGAPDMAFPRMNNMSFWLLPPSIMLLISSSIVENGAGTGWTVYPPLSSNIAHSGSSVDLAIFSLHLAGISSILGAVNFITTIINMRPNNMSL
DQMPLFVWAVGITALLLLLSLPVLAGAITMLLTDRNLNTSFFDPAGGGDPILYQHL
>LEFIE481-10|HM874205|MM09256|Pammene_gallicana
TLYFIFGIWAGMVGTSLSLLIRAELGNPGSLIGDDQIYNTIVTAHAFIMIFFMVMPIMIGGFGNWLVPLMLGAPDMAFPRMNNMSFWLLPPSILLLISSSIVENGAGTGWTVYPPLSSNIAHSGSSVDLAIFSLHLAGISSILGAVNFITTIINMRPNNMSL
DQMPLFVWAVGITALLLLLSLPVLAGAITMLLTDRNLNTSFFDPAGGGDPILYQHL
>LEFIK033-10|MM17608|Pammene_gallicana
TLYFIFGIWAGMVGTSLSLLIRAELGNPGSLIGDDQIYNTIVTAHAFIMIFFMVMPIMIGGFGNWLVPLMLGAPDMAFPRMNNMSFWLLPPSILLLISSSIVENGAGTGWTVYPPLSSNIAHSGSSVDLAIFSLHLAGISSILGAVNFITTIINMRPNNMSL
DQMPLFVWAVGITALLLLLSLPVLAGAITMLLTDRNLNTSFFDPAGGGDPILYQHL
>LEFIK785-10|JN274969|MM18360|Pammene_gallicana
TLYFIFGIWAGMVGTSLSLLIRAELGNPGSLIGDDQIYNTIVTAHAFIMIFFMVMPIMIGGFGNWLVPLMLGAPDMAFPRMNNMSFWLLPPSILLLISSSIVENGAGTGWTVYPPLSSNIAHSGSSVDLAIFSLHLAGISSILGAVNFITTIINMRPNNMSL
DQMPLFVWAVGITALLLLLSLPVLAGAITMLLTDRNLNTSFFDPAGGGDPILYQHL
>LEFID080-10|HM872891|MM05689|Pammene_germmana
TLYFIFGIWAGMVGTSLSLLIRAELGNPGSLIGDDQIYNTIVTAHAFIMIFFMVMPIMIGGFGNWLVPLMLGAPDMAFPRMNNMSFWLLPPSIMLLISSSIVENGAGTGWTVYPPLSSNIAHSGSSVDLAIFSLHLAGISSILGAVNFITTIINMRPNNMSL
DQMPLFVWAVGITALLLLLSLPVLAGAITMLLTDRNLNTSFFDPAGGGDPILYQHL
>LEEUA252-11|JN274945|MM19660|Pammene_giganteana
TLYFIFGIWAGMVGTSLSLLIRAELGNPGSLIGDDQIYNTIVTAHAFIMIFFMVMPIMIGGFGNWLVPLMLGAPDMAFPRMNNMSFWLLPPSIMLLISSSIVENGAGTGWTVYPPLSSNIAHSGSSVDLAIFSLHLAGISSILGAVNFITTIINMRPNNMSL
DQMPLFVWAVGITALLLLLSLPVLAGAITMLLTDRNLNTSFFDPAGGGDPILYQHL
>LEFIC551-10|HM872372|MM04310|Pammene_ignorata
TLYFIFGVWAGMVGTSLSLLIRAELGNPGSLIGDDQIYNTIVTAHAFIMIFFMVMPIMIGGFGNWLVPLMLGAPDMAFPRMNNMSFWLLPPSIMLLISSSIVENGAGTGWTVYPPLSSNIAHSGSSVDLAIFSLHLAGISSILGAVNFITTIINMRPNNMS
LDQMPLFVWAVGITALLLLLSLPVLAGAITMLLTDRNLNTSFFDPAGGGDPILYQHL
>LEFIE478-10|HM874202|MM09253|Pammene_ignorata
TLYFIFGIWAGMVGTSLSLLIRAELGNPGSLIGDDQIYNTIVTAHAFIMIFFMVMPIMIGGFGNWLVPLMLGAPDMAFPRMNNMSFWLLPPSIMLLISSSIVENGAGTGWTVYPPLSSNIAHSGSSVDLAIFSLHLAGISSILGAINFITTIINMRPNNMSL
DQMPLFVWAVGITALLLLLSLPVLAGAITMLLTDRNLNTSFFDPAGGGDPILYQHL
>LEFIF262-10|HM874955|MM11044|Pammene_ignorata
TLYFIFGVWAGMVGTSLSLLIRAELGNPGSLIGDDQIYNTIVTAHAFIMIFFMVMPIMIGGFGNWLVPLMLGAPDMAFPRMNNMSFWLLPPSIMLLISSSIVENGAGTGWTVYPPLSSNIAHSGSSVDLAIFSLHLAGISSILGAVNFITTIINMRPNNMS
LDQMPLFVWAVGITALLLLLSLPVLAGAITMLLTDRNLNTSFFDPAGGGDPILYQHL
>LEFIE806-10|HM874525|MM09998|Pammene_insulana
TLYFIFGIWAGMVGTSLSLLIRAELGNPGSLIGDDQIYNTIVTAHAFIMIFFMVMPIMIGGFGNWLIPLMLGAPDMAFPRMNNMSFWLLPPSIMLLISSSIVENGAGTGWTVYPPLSSNIAHSGSSVDLAIFSLHLAGISSILGAVNFITTIINMRPNNMSL
DQMPLFVWAVGITALLLLLSLPVLAGAITMLLTDRNLNTSFFDPAGGGDPILYQHL
>LEFIG880-10|HM876525|MM15744|Pammene_insulana
TLYFIFGIWAGMVGTSLSLLIRAELGNPGSLIGDDQIYNTIVTAHAFIMIFFMVMPIMIGGFGNWLVPLMLGAPDMAFPRMNNMSFWLLPPSIMLLISSSIVENGAGTGWTVYPPLSSNIAHSGSSVDLAIFSLHLAGISSILGAVNFITTIINMRPNNMSL

DQMPLFVWAVGITALLLLLSLPVLAGAITMLLTDRNLNTSFFDPAGGGDPILYQHL
>LEFIL633-10|MM18931|Pammene_luculentana
TLYFIFGIWAGMVGTSLSLLIRAELGNPGSLIGDDQIYNTIVTAHAFIMIFFMVMPIMIGGFGNWLIPLMLGAPDMAFPRMNNMSFWLLPPSIMLLISSSIVENGAGTGWTVYPPLSSNIAHSGSSVDLAIFSLHLAGISSILGAVNFITTIINMRPNNMSL
DQMPLFVWAVGITALLLLLSLPVLAGAITMLLTDRNLNTSFFDPAGGGDPILYQHL
>LEFIL634-10|MM18932|Pammene_luculentana
TLYFIFGIWAGMVGTSLSLLIRAELGNPGSLIGDDQIYNTIVTAHAFIMIFFMVMPIMIGGFGNWLVPLMLGAPDMAFPRMNNMSFWLLPPSIMLLISSSIVENGAGTGWTVYPPLSSNIAHSGSSVDLAIFSLHLAGISSILGAVNFITTIINMRPNNMSL
DQMPLFVWAVGITALLLLLSLPVLAGAITMLLTDRNLNTSFFDPAGGGDPILYQHL
>LEFIE242-10|HM873976|MM08563|Pammene_luedersiana
TLYFIFGIWAGMVGTSLSLLIRAELGNPGSLIGDDQIYNTIVTAHAFIMIFFMVMPIMIGGFGNWLIPLMLGAPDMAFPRMNNMSFWLLPPSIMLLISSSIVENGAGTGWTVYPPLSSNIAHSGSSVDLAIFSLHLAGISSILGAVNFITTIINMRPNNMSL
DQMPLFVWAVGITALLLLLSLPVLAGAITMLLTDRNLNTSFFDPAGGGDPILYQHL
>LEFID502-10|HM873267|MM06462|Pammene_obscurana
TLYFIFGIWAGMVGTSLSLLIRAELGNPGSFIGDDQIYNTIVTAHAFIMIFFMVMPIMIGGFGNWLVPLMLGAPDMAFPRMNNMSFWLLPPSIMLLISSSIVENGAGTGWTVYPPLSSNIAHSGSSVDLAIFSLHLAGISSILGAVNFITTIINMRPNNMSL
DQMPLFVWAVGITALLLLLSLPVLAGAITMLLTDRNLNTSFFDPAGGGDPILYQHL
>LEFIE155-10|HM873902|MM08401|Pammene_obscurana
TLYFIFGIWAGMVGTSLSLLIRAELGNPGSFIGDDQIYNTIVTAHAFIMIFFMVMPIMIGGFGNWLVPLMLGAPDMAFPRMNNMSFWLLPPSIMLLISSSIVENGAGTGWTVYPPLSSNIAHSGSSVDLAIFSLHLAGISSILGAVNFITTIINMRPNNMSL
DQMPLFVWAVGITALLLLLSLPVLAGAITMLLTDRNLNTSFFDPAGGGDPILYQHL
>LEFIE740-10|HM874460|MM09843|Pammene_ochsenheimeriana
TLYFIFGIWAGMVGTSLSLLIRAELGNPGSLIGDDQIYNTIVTAHAFIMIFFMVMPIMIGGFGNWLVPLMLGAPDMAFPRMNNMSFWLLPPSLMLLISSSIVENGAGTGWTVYPPLSSNIAHSGSSVDLAIFSLHLAGISSILGAVNFITTIINMRPNNMS
LDQMPLFVWAVGITALLLLLSLPVLAGAITMLLTDRNLNTSFFDPAGGGDPILYQ?L
>LEFIE741-10|HM874461|MM09844|Pammene_ochsenheimeriana
TLYFIFGIWAGMVGTSLSLLIRAELGNPGSLIGDDQIYNTIVTAHAFIMIFFMVMPIMIGGFGNWLVPLMLGAPDMAFPRMNNMSFWLLPPSLMLLISSSIVENGAGTGWTVYPPLSSNIAHSGSSVDLAIFSLHLAGISSILGAVNFITTIINMRPNNMS
LDQMPLFVWAVGITALLLLLSLPVLAGAITMLLTDRNLNTSFFDPAGGGDPILYQHL
>LEFIL335-10|JN274983|MM18645|Pammene_populana
TLYFIFGIWAGMVGTSLSLLIRAELGNPGSLIGDDQIYNTIVTAHAFIMIFFMVMPIMIGGFGNWLVPLMLGAPDMAFPRMNNMSFWLLPPSIMLLISSSIVENGAGTGWTVYPPLSSNIAHSGSSVDLAIFSLHLAGISSILGAVNFITTIINMRPNNMSL
DQMPLFVWAVGITALLLLLSLPVLAGAITMLLTDRNLNTSFFDPAGGGDPILYQHL
>LEFIL511-10|MM18809|Pammene_populana
TLYFIFGIWAGMVGTSLSLLIRAELGNPGSLIGDDQIYNTIVTAHAFIMIFFMVMPIMIGGFGNWLVPLMLGAPDMAFPRMNNMSFWLLPPSIMLLISSSIVENGAGTGWTVYPPLSSNIAHSGSSVDLAIFSLHLAGISSILGAVNFITTIINMRPNNMSL
DQMPLFVWAVGITALLLLLSLPVLAGAITMLLTDRNLNTSFFDPAGGGDPILYQHL
>LEFIL215-10|MM19215|Pammene_regiana
TLYFIFGIWAGMVGTSLSLLIRAELGNPGSLIGDDQIYNTIVTAHAFIMIFFMVMPIMIGGFGNWLVPLMLGAPDMAFPRMNNMSFWLLPPSILLLISSSIVENGAGTGWTVYPPLSSNIAHSGSSVDLAIFSLHLAGISSILGAVNFITTIINMRPNNMSL
DQMPLFVWAVGITALLLLLSLPVLAGAITMLLTDRNLNTSFFDPAGGGDPILY?HL
>LEEUA255-11|JN274947|MM19663|Pammene_regiana
TLYFIFGIWAGMVGTSLSLLIRAELGNPGSLIGDDQIYNTIVTAHAFIMIFFMVMPIMIGGFGNWLVPLMLGAPDMAFPRMNNMSFWLLPPSILLLISSSIVENGAGTGWTVYPPLSSNIAHSGSSVDLAIFSLHLAGISSILGAVNFITTIINMRPNNMSL
DQMPLFVWAVGITALLLLLSLPVLAGAITMLLTDRNLNTSFFDPAGGGDPILYQHL
>LEEUA625-11|MM20684|Pammene_regiana
TLYFIFGIWAGMVGTSLSLLIRAELGNPGSLIGDDQIYNTIVTAHAFIMIFFMVMPIMIGGFGNWLIPLMLGAPDMAFPRMNNMSFWLLPPSILLLISSSIVENGAGTGWTVYPPLSSNIAHSGSSVDLAIFSLHLAGISSILGAVNFITTIINMRPNNMSLD
QMPLFVWAVGITALLLLLSLPVLAGAITMLLTDRNLNTSFFDPAGGGDPILYQHL
>LEFIC363-10|HM872206|MM03890|Pammene_rhediella
TLYFIFGIWAGMVGTSLSLLIRAELGNPGSLIGDDQIYNTIVTAHAFIMIFFMVMPIMIGGFGNWLVPLMLGAPDMAFPRMNNMSFWLLPPSIMLLISSSIVENGAGTGWTVYPPLSSNIAHSGSSVDLAIFSLHLAGISSILGAVNFITTIINMRPNNMSL
DQMPLFVWAVGITALLLLLSLPVLAGAITMLLTDRNLNTSFFDPAGGGDPILYQHL
>LEFIL574-10|JF854669|MM18872|Pammene_spiniana
TLYFIFGIWAGMVGTSLSLLIRAELGNPGSLIGDDQIYNTIVTAHAFIMIFFMVMPIMIGGFGNWLVPLMLGAPDMAFPRMNNMSFWLLPPSIMLLISSSIVENGAGTGWTVYPPLSSNIAHSGSSVDLAIFSLHLAGISSILGAVNFITTIINMRPNNMSL
DQMPLFVWAVGITALLLLLSLPVLAGAITMLLTDRNLNTSFFDPAGGGDPILYQHL
>LEFIL505-10|MM18803|Pammene_splendidulana
TLYFIFGIWAGMVGTSLSLLIRAELGNPGSLIGDDQIYNTIVTAHAFIMIFFMVMPIMIGGFGNWLVPLMLGAPDMAFPRMNNMSFWLLPPSIMLLISSSIVENGAGTGWTVYPPLSSNIAHSGSSVDLAIFSLHLAGISSILGAVNFITTIINMRPNNMSL
DQMPLFVWAVGITALLLLLSLPVLAGAITMLLTDRNLNTSFFDPAGGGDPILYQHL
>LEFIL621-10|MM18919|Pammene_splendidulana

TLYFIFGIWAGMVGTSLSLLIRAELGNPGSLIGDDQIYNTIVTAHAFIMIFFMVMPIMIGGFGNWLVPLMLGAPDMAFPRMNNMSFWLLPPSIMLLISSSIVENGAGTGWTVYPPLSSNIAHSGSSVDLAIFSLHLAGISSILGAVNFITTIINMRPNNMSL
DQMPLFVWAVGITALLLLLSLPVLAGAITMLLTDRNLNTSFFDPAGGGDPILYQHL
>LEFIL503-10|MM18801|Pammene_suspectana
TLYFIFGIWAGMVGTSLSLLIRAELGNPGSLIGDDQIYNTIVTAHAFIMIFFMVMPIMIGGFGNWLVPLMLGAPDMAFPRMNNMSFWLLPPSIMLLISSSIVENGAGTGWTVYPPLSSNIAHSGSSVDLAIFSLHLAGISSILGAVNFITTIINMRPNNMSL
DQMPLFVWAVGITALLLLLSLPVLAGAITMLLTDRNLNTSFFDPAGGGDPILYQHL
>LEFIL504-10|MM18802|Pammene_suspectana
TLYFIFGIWAGMVGTSLSLLIRAELGNPGSLIGDDQIYNTIVTAHAFIMIFFMVMPIMIGGFGNWLVPLMLGAPDMAFPRMNNMSFWLLPPSIMLLISSSIVENGAGTGWTVYPPLSSNIAHSGSSVDLAIFSLHLAGISSILGAVNFITTIINMRPNNMSL
DQMPLFVWAVGITALLLLLSLPVLAGAITMLLTDRNLNTSFFDPPGGGDPILYQHL
>LEFIE781-10|HM874500|MM09954|Pancalia_schwarzella
TLYFIFGIWAGMVGTSLSLLIRAELGNPGSLIGDDQIYNTIVTAHAFIMIFFMVMPIMIGGFGNWLVPLMLGAPDMAFPRMNNMSFWLLPPSLTLLISSSIVESGAGTGWTVYPPLSSNIAHGGSSVDLAIFSLHLAGISSILGAINFITTIINMRVNNLSFD
QMPLFVWAVGITALLLLLSLPVLAGAITMLLTDRNLNTSFFDPAGGGDPILYQHL
>LEFIB466-10|HM871365|MM02012|Pandemis_cerasana
TLYFIFGIWAGMVGTSLSLLIRAELGNPGSLIGDDQIYNTIVTAHAFIMIFFMVMPIMIGGFGNWLVPLMLGAPDMAFPRMNNMSFWLLPPSLMLLISSSIVENGAGTGWTVYPPLSSNIAHSGSSVDLAIFSLHLAGISSILGAVNFITTIINMRPNNMS
LDQMPLFVWAVGITALLLLLSLPVLAGAITMLLTDRNLNTSFFDPAGGGDPILYQHL
>LEFIA135-10|HM396482|MM01076|Pandemis_cinnamomeana
TLYFIFGIWAGMVGTSLSLLIRAELGNPGSLIGDDQIYNTIVTAHAFIMIFFMVMPIMIGGFGNWLVPLMLGAPDMAFPRMNNMSFWLLPPSIMLLISSSIVENGAGTGWTVYPPLSSNIAHSGSSVDLAIFSLHLAGISSILGAVNFITTIINMRPNNMSL
DQMPLFVWAVGITALLLLLSLPVLAGAITMLLTDRNLNTSFFDPAGGGDPILYQHL
>LEFIC075-10|HM871944|MM03365|Pandemis_cinnamomeana
TLYFIFGIWAGMVGTSLSLLIRAELGNPGSLIGDDQIYNTIVTAHAFIMIFFMVMPIMIGGFGNWLVPLMLGAPDMAFPRMNNMSFWLLPPSIMLLISSSIVENGAGTGWTVYPPLSSNIAHSGSSVDLAIFSLHLAGISSILGAVNFITTIINMRPNNMSL
DQMPLFVWAVGITALLLLLSLPVLAGAITMLLTDRNLNTSFFDPAGGGDPILYQHL
>LEFIF847-10|HM875530|MM13198|Pandemis_cinnamomeana
TLYFIFGIWAGMVGTSLSLLIRAELGNPGSLIGDDQIYNTIVTAHAFIMIFFMVMPIMIGGFGNWLVPLMLGAPDMAFPRMNNMSFWLLPPSIMLLISSSIVENGAGTGWTVYPPLSSNIAHSGSSVDLAIFSLHLAGISSILGAVNFITTIINMRPNNMSL
DQMPLFVWAVGITALLLLLSLPVLAGAITMLLTDRNLNTSFFDPAGGGDPILYQHL
>LEFIF228-10|HM874921|MM11008|Pandemis_corylana
TLYFIFGIWAGMVGTSLSLLIRAELGAPGSLIGDDQIYNTIVTAHAFIMIFFMVMPIMIGGFGNWLVPLMLGAPDMAFPRMNNMSFWLLPPSIMLLISSSIVENGAGTGWTVYPPLSSNIAHSGSSVDLAIFSLHLAGISSILGAVNFITTIINMRPNNMSL
DQMPLFVWAVGITALLLLLSLPVLAGAITMLLTDRNLNTSFFDPAGGGDPILYQHL
>LEFIJ646-10|JF853742|MM17271|Pandemis_corylana
TLYFIFGIWAGMVGTSLSLLIRAELGAPGSLIGDDQIYNTIVTAHAFIMIFFMVMPIMIGGFGNWLVPLMLGAPDMAFPRMNNMSFWLLPPSIMLLISSSIVENGAGTGWTVYPPLSSNIAHSGSSVDLAIFSLHLAGISSILGAVNFITTIINMRPNNMSL
DQMPLFVWAVGITALLLLLSLPVLAGAITMLLTDRNLNTSFFDPAGGGDPILYQHL
>LEFIC161-10|HM872007|MM03507|Pandemis_dumetana
TLYFIFGIWSGMVGTSLSLLIRAELGNPGSLIGDDQIYNTIVTAHAFIMIFFMVMPIMIGGFGNWLVPLMLGAPDMAFPRMNNMSFWLLPPSIMLLISSSIVENGAGTGWTVYPPLSSNIAHSGSSVDLAIFSLHLAGISSILGAVNFITTIINMRPNNMSL
DQMPLFVWAVAITAILLLLSLPVLAGAITMLLTDRNLNTSFFDPAGGGDPILYQHL
>LEFIB467-10|HM871366|MM02014|Pandemis_heparana
TLYFIFGIWAGMVGTSLSLLIRAELGNPGSLIGDDQIYNTIVTAHAFIMIFFMVMPIMIGGFGNWLVPLMLGAPDMAFPRMNNMSFWLLPPSIMLLISSSIVENGAGTGWTVYPPLSSNIAHSGSSVDLAIFSLHLAGISSILGAVNFITTIINMRPNNMSL
DQMPLFVWAVGITALLLLLSLPVLAGAITMLLTDRNLNTSFFDPAGGGDPILYQHL
>LEFIB003-10|HM870916|MM00006|Panemeria_tenebrata
TLYFIFGIWAGMVGTSLSLLIRAELGTPGSLIGDDQIYNTIVTAHAFIMIFFMVMPIMIGGFGNWLIPLMLGAPDMAFPRMNNMSFWLLPPSLTLLISSSIVENGAGTGWTVYPPLSSNIAHGGSSVDLAIFSLHLAGISSILGAINFITTIINMRLNNLSFDQ
MPLFIWAVGITAFLLLLSLPVLAGAITMLLTDRNLNTSFFDPAGGGDPILYQHL
>LEFIE794-10|HM874513|MM09978|Panemeria_tenebrata
TLYFIFGIWAGMVGTSLSLLIRAELGTPGSLIGDDQIYNTIVTAHAFIMIFFMVMPIMIGGFGNWLIPLMLGAPDMAFPRMNNMSFWLLPPSLTLLISSSIVENGAGTGWTVYPPLSSNIAHGGSSVDLAIFSLHLAGISSILGAINFITTIINMRLNNLSFDQ
MPLFIWAVGITAFLLLLSLPVLAGAITMLLTDRNLNTSFFDPAGGGDPILYQHL
>LEFIB091-10|HM870998|MM00397|Panolis_flammea
TLYFIFGIWAGMVGTSLSLLIRAELGNPGSLIGDDQIYNTIVTAHAFIMIFFMVMPIMIGGFGNWLVPLMLGAPDMAFPRMNNMSFWLLPPSLTLLISSSIVENGAGTGWTVYPPLSSNIAHGGSSVDLAIFSLHLAGISSILGAINFITTIINMRLNNLSFD
QMPLFIWAVGITAFLLLLSLPVLAGAITMLLTDRNLNTSFFDPAGGGDPILYQHL
>LEFID150-10|HM872957|MM05913|Panthea_coenobita
TLYFIFGIWAGMVGTSLSLLIRAELGTPGSLIGDDQIYNTIVTAHAFIMIFFMVMPIMIGGFGNWLVPLMLGAPDMAFPRMNNMSFWLLPPSLTLLISSSIVENGAGTGWTVYPPLSSNIAHAGSSVDLAIFSLHLAGISSILGAINFITTIINMRLNNLSFD
QMPLFIWAVGITAFLLLLSLPVLAGAITMLLTDRNLNTSFFDPAGGGDPILYQHL

>LEFIF629-10|HM875313|MM12519|Papestra_biren
TLYFIFGIWAGMVGTSLSLLIRAELGNPGSLIGDDQIYNTIVTAHAFIMIFFMVMPIMIGGFGNWLVPLMLGAPDMAFPRMNNMSFWLLPPSLTLLISSSIVENGAGTGWTVYPPLSSNIAHGGSSVDLAIFSLHLAGISSILGAINFITTIINMRLNNLSFD
QMPLFIWAVGITAFLLLLSLPVLAGAITMLLTDRNLNTSFFDPAGGGDPILYQHL
>LEFIB255-10|HM871158|MM00727|Papilio_machaon
TLYFIFGIWASMLGTSLSLLIRTELGTPGSLIGDDQIYNTIVTAHAFIMIFFMVMPIMIGGFGNWLIPLMLGAPDMAFPRMNNMSFWLLPPSLTLLISSMIVENGAGTGWTVYPPLSSNIAHGSSSVDLVIFSLHLAGISSILGAINFITTIINMRINNMSFDQ
MPLFVWAVGITALLLLLSLPVLAGAITMLLTDRNLNTSFFDPAGGGDPILYQHL
>LEFID021-10|HM872835|MM05450|Papilio_machaon
TLYFIFGIWASMLGTSLSLLIRTELGTPGSLIGDDQIYNTIVTAHAFIMIFFMVMPIMIGGFGNWLIPLMLGAPDMAFPRMNNMSFWLLPPSLTLLISSMIVENGAGTGWTVYPPLSSNIAHGSSSVDLVIFSLHLAGISSILGAINFITTIINMRINNMSFDQ
MPLFVWAVGITALLLLLSLPVLAGAITMLLTDRNLNTSFFDPAGGGDPILYQHL
>LEFID090-10|HM872901|MM05702|Papilio_machaon
TLYFIFGIWASMLGTSLSLLIRTELGTPGSLIGDDQIYNTIVTAHAFIMIFFMVMPIMIGGFGNWLIPLMLGAPDMAFPRMNNMSFWLLPPSLTLLISSMIVENGAGTGWTVYPPLSSNIAHGSSSVDLVIFSLHLAGISSILGAINFITTIINMRINNMSFDQ
MPLFVWAVGITALLLLLSLPVLAGAITMLLTDRNLNTSFFDPAGGGDPILYQHL
>LEFID209-10|HM873011|MM06038|Parachronistis_albiceps
TLYFIFGIWAGMVGTSLSLLIRAELGNPGSLIGDDQIYNTIVTAHAFIMIFFMVMPIMIGGFGNWLVPLMLGAPDMAFPRMNNMSFWLLPPSLTLLISSSIVENGAGTGWTVYPPLSSNIAHGGSSVDLAIFSLHLAGISSILGAINFITTIINMRMNSLSFD
QMPLFVWAVGITALLLLLSLPVLAGAITMLLTDRNLNTSFFDPAGGGDPILYQHL
>LEFID690-10|HM873451|MM06737|Parachronistis_albiceps
TLYFIFGIWAGMVGTSLSLLIRAELGNPGSLIGDDQIYNTIVTAHAFIMIFFMVMPIMIGGFGNWLVPLMLGAPDMAFPRMNNMSFWLLPPSLTLLISSSIVENGAGTGWTVYPPLSSNIAHGGSSVDLAIFSLHLAGISSILGAINFITTIINMRMNSLSFD
QMPLFVWAVGITALLLLLSLPVLAGAITMLLTDRNLNTSFFDPAGGGDPILYQHL
>LEFIC787-10|HM872606|MM04924|Paracolax_tristalis
TLYFLFGIWAGMVGTSLSLLIRAELGTPGSLIGDDQIYNTIVTAHAFIMIFFMVMPIMIGGFGNWLVPLMLGAPDMAFPRMNNMSFWLLPPSLTLLISSSIVENGAGTGWTVYPPLSSNIAHGGSSVDLAIFSLHLAGISSILGAINFITTIINMRLNNLSFD
QMPLFVWAVGITAFLLLLSLPVLAGAITMLLTDRNLNTSFFDPAGGGDPILYQHL
>LEFID802-10|HM873559|MM06882|Paracolax_tristalis
TLYFLFGIWAGMVGTSLSLLIRAELGTPGSLIGDDQIYNTIVTAHAFIMIFFMVMPIMIGGFGNWLVPLMLGAPDMAFPRMNNMSFWLLPPSLTLLISSSIVENGAGTGWTVYPPLSSNIAHGGSSVDLAIFSLHLAGISSILGAINFITTIINMRLNNLSFD
QMPLFVWAVGITAFLLLLSLPVLAGAITMLLTDRNLNTSFFDPAGGGDPILYQHL
>LEFIA255-10|HM386599|MM01292|Paradarisa_consonaria
TLYFIFGIWAGMVGTSLSLLIRAELGNPGSLIGDDQIYNTIVTAHAFIMIFFMVMPIMIGGFGNWLVPLMLGAPDMAFPRMNNMSFWLLPPSITLLISSSIVENGAGTGWTVYPPLSSNIAHGGSSVDLAIFSLHLAGISSILGAINFITTIINMRLNNLSFD
QMPLFVWAVGITAFLLLLSLPVLAGAITMLLTDRNLNTSFFDPAGGGDPILYQHL
>LEFIA451-10|HM386792|MM01515|Paradarisa_consonaria
TLYFIFGIWAGMVGTSLSLLIRAELGNPGSLIGDDQIYNTIVTAHAFIMIFFMVMPIMIGGFGNWLVPLMLGAPDMAFPRMNNMSFWLLPPSITLLISSSIVENGAGTGWTVYPPLSSNIAHGGSSVDLAIFSLHLAGISSILGAINFITTIINMRLNNLSFD
QMPLFVWAVGITAFLLLLSLPVLAGAITMLLTDRNLNTSFFDPAGGGDPILYQHL
>LEFIJ1022-11|MM09300|Paradiarsia_punicea
TLYFIFGIWAGMVGTSLSLLIRAELGNPGSLIGDDQIYNTIVTAHAFIMIFFMVMPIMIGGFGNWLVPLMLGAPDMAFPRMNNMSFWLLPPSLTLLISSSIVENGAGTGWTVYPPLSSNIAHGGSSVDLAIFSLHLAGISSILGAINFITTIINMRLNSLSFD
QMPLFIWAVGITAFLLLLSLPVLAGAITMLLTDRNLNTSFFDPAGGGDPILYQHL
>LEFIJ360-10|MM15960|Paradiarsia_punicea
TLYFIFGIWAGMVGTSLSLLIRAELGNPGSLIGDDQIYNTIVTAHAFIMIFFMVMPIMIGGFGNWLVPLMLGAPDMAFPRMNNMSFWLLPPSLTLLISSSIVENGAGTGWTVYPPLSSNIAHGGSSVDLAIFSLHLAGISSILGAINFITTIINMRLNSLSFD
QMPLFIWAVGITAFLLLLSLPVLAGAITMLLTDRNLNTSFFDPAGGGDPILYQHL
>LEFIL624-10|MM18922|Paradiarsia_punicea
TLYFIFGIWAGMVGTSLSLLIRAELGNPGSLIGDDQIYNTIVTAHAFIMIFFMVMPIMIGGFGNWLVPLMLGAPDMAFPRMNNMSFWLLPPSLTLLISSSIVENGAGTGWTVYPPLSSNIAHGGSSVDLAIFSLHLAGISSILGAINFITTIINMRLNSLSFD
QMPLFIWAVGITAFLLLLSLPVLAGAITMLLTDRNLNTSFFDPAGGGDPILYQHL
>LEFIL015-10|JF854396|MM19015|Paradiarsia_punicea
TLYFIFGIWAGMVGTSLSLLIR?ELGNPGSLIGDDQIYNTIVTAHAFIMIFFMVMPIMIGGFGNWLVPLMLGAPDMAFPRMNNMSFWLLPPSLTLLISSSIVENGAGTGWTVYPPLSSNIAHGGSSVDLAIFSLHLAGISSILGAINFITTIINMRLNSLSFD
QMPLFIWAVGITAFLLLLSLPVLAGAITMLLTDRNLNTSFFDPAGGGDPILYQHL
>LEFIC821-10|HM872640|MM05005|Paramesia_gnomana
TLYFIFGIWAGMIGTSLSLLIRAELGTPGSLIGDDQIYNTIVTAHAFIMIFFMVMPIMIGGFGNWLVPLMLGAPDMAFPRMNNMSFWLLPPSIMLLISSSLVENGAGTGWTVYPPLSSNIAHSGSSVDLAIFSLHLAGISSILGAVNFITTIINMRPNNMAL
DQMPLFVWAVGITALLLLLSLPVLAGAITMLLTDRNLNTSFFDPAGGGDPILYQHL
>LEFIE150-10|HM873898|MM08396|Paranthrene_tabaniformis
TLYFIFGIWSGLIGTSLSLLIRAELGMSGSLIGDDQIYNTIVTAHAFIMIFFMVMPIMIGGFGNWLVPLMLGAPDMAFPRMNNMSFWLLPPSLTLLISSSVVESGAGTGWTVYPPLSSNIAHGSSSVDLTIFSLHLAGISSILGAINFITTIINMRSLNMSFDQ

MPLFVWAVGITALLLLLSLPVLAGAITMLLTDRNLNTSFFDPAGGGDPILYQHL
>LEFIG892-10|HM876537|MM15756|Paranthrene_tabaniformis
TLYFIFGIWSGLIGTSLSLLIRAELGMSGSLIGDDQIYNTIVTAHAFIMIFFMVMPIMIGGFGNWLIPLMLGAPDMAFPRMNNMSFWLLPPSLTLLISSSVVESGAGTGWTVYPPLSSNIAHGSSSVDLTIFSLHLAGISSILGAINFITTIINMRSLNMSFDQ
MPLFVWAVGITALLLLLSLPVLAGAITMLLTDRNLNTSFFDPAGGGDPILYQHL
>LEFIC186-10|HM872030|MM03544|Parapoynx_stratiotata
TLYFIFGIWSGMIGTSLSLLIRAELGNPGSLIGDDQIYNTIVTAHAFIMIFFMVMPIMIGGFGNWLVPLMLGAPDMAFPRMNNMSFWLLPPSLTLLISSSIVENGAGTGWTVYPPLSSNIAHSGSSVDLAIFSLHLAGISSILGAINFITTIINMKMNGLSFD
QMSLFVWAVGITALLLLLSLPVLAGAITMLLTDRNLNTSFFDPAGGGDPILYQHL
>LEFIE364-10|HM874088|MM08813|Parapoynx_stratiotata
TLYFIFGIWSGMIGTSLSLLIRAELGNPGSLIGDDQIYNTIVTAHAFIMIFFMVMPIMIGGFGNWLVPLMLGAPDMAFPRMNNMSFWLLPPSLTLLISSSIVENGAGTGWTVYPPLSSNIAHSGSSVDLAIFSLHLAGISSILGAINFITTIINMKMNGLSFD
QMSLFVWAVGITALLLLLSLPVLAGAITMLLTDRNLNTSFFDPAGGGDPILYQHL
>LEFIJ477-10|JF853605|MM17102|Pararctia_lapponica
TLYFIFGVWAGMVGSSLSLLIRAELGNPGSFIEDDQIYNTIVTAHAFIMIFFMVMPIMIGGFGNWLVPLMLGAPDMAFPRMNNMSFWLLPPSLTLLISSSIVENGAGTGWTVYPPLSSNIAHGGSSVDLAIFSLHLAGISSILGAINFITTIINMRLNNMSF
DQMPLFVWSVGITAFLLLLSLPVLAGAITMLLTDRNLNTSFFDPAGGGDPILYQHL
>LEFIJ560-10|JF853675|MM17185|Pararge_aegeria
TLYFIFGIWAGMVGTSLSLIIRTELGNPGFLIGDDQIYNTIVTAHAFIMIFFMVMPIMIGGFGNWLIPLMLGAPDMAFPRMNNMSFWLLPPSLMLLISSSIVENGAGTGWTVYPPLSSNIAHSGSSVDLAIFSLHLAGISSILGAINFITTIINMRINSMSYD
QMPLFVWAVGITALLLLLSLPVLAGAITMLLTDRNLNTSFFDPAGGGDPILYQHL
>LEFIA357-10|HM386700|MM01412|Parascotia_fuliginaria
TLYFIFGIWAGMVGTSLSLLIRAELGNPGSLIGDDQIYNTIVTAHAFIMIFFMVMPIMIGGFGNWLVPLMLGAPDMAFPRMNNMSFWLLPPSLTLLISSSIVENGAGTGWTVYPPLSSNIAHGGSSVDLAIFSLHLAGISSILGAINFISTIINMRLSNLSFD
QMPLFIWAVGITAFLLLLSLPVLAGAITMLLTDRNLNTSFFDPAGGGDPILYQHL
>LEEUA576-11|MM20635|Parascythris_muelleri
TLYFIFGIWAGMIGTSLSLLIRAELGNPGSLIGDDQIYNTIVTAHAFIMIFFMVMPIMIGGFGNWLIPLMLGAPDMAFPRMNNMSFWLLPPSLILLISSSIVENGAGTGWTVYPPLSSNIAHSGSSVDLAIFSLHLAGISSILGAINFITTIINMRISNLSFDQM
PLFVWAVGITALLLLLSLPVLAGAITMLLTDRNLNTSFFDPAGGGDPILYQHL
>LEFIB998-10|HM871869|MM03197|Parasemia_plantaginis
TLYFIFGIWAGMVGTSLSLLIRAELGNPGSLIGDDQIYNTIVTAHAFIMIFFMVMPIMIGGFGNWLVPLMLGAPDMAFPRMNNMSFWLLPPSLTLLISSSIVENGAGTGWTVYPPLSSNIAHGGSSVDLAIFSLHLAGISSILGAINFITTIINMRLNNLSFD
QMPLFVWAVGITAFLLLLSLPVLAGAITMLLTDRNLNTSFFDPAGGGDPILYQHL
>LEFIB824-10|HM871701|MM02764|Parastichtis_suspecta
TLYFIFGIWAGMVGTSLSLLIRAELGNPGSLIGDDQIYNTIVTAHAFIMIFFMVMPIMIGGFGNWLVPLMLGAPDMAFPRLNNMSFWLLPPSLTLLISSSIVENGAGTGWTVYPPLSSNIAHGGSSVDLAIFSLHLAGISSILGAINFITTIINMRLNNLSFDQ
MPLFIWAVGITAFLLLLSLPVLAGAITMLLTDRNLNTSFFDPAGGGDPILYQHL
>LEFIK056-10|KF808762|MM17631|Paraswammerdamia_albicapitella
TLYFIFGIWSGMVGTSLSLLIRAELGNPGSLIGDDQIYNTIVTAHAFIMIFFMVMPIMIGGFGNWLVPLMLGAPDMAFPRMNNMSFWLLPPSLTLLISSSIVENGAGTGWTVYPPLSSNIAHGGSSVDLAIFSLHLAGISSILGAINFITTIINMKSNGMSF
DQMPLFVWAVGITALLLLLSLPVLAGAITMLLTDRNLNTSFFDPAGGGDPILYQHL
>LEFIB242-10|HM871145|MM00705|Paraswammerdamia_conspersella
TLYFIFGIWSGMVGTSLSLLIRAELGNPGSLIGDDQIYNTIVTAHAFIMIFFMVMPIMIGGFGNWLVPLMLGAPDMAFPRMNNMSFWLLPPSLTLLISSSIVENGAGTGWTVYPPLSSNIAHSGSSVDLAIFSLHLAGISSILGAINFITTIINMKSNGMSFD
QMPLFVWAVGITALLLLLSLPVLAGAITMLLTDRNLNTSFFDPAGGGDPILYQHL
>LEFID993-10|HM873743|MM08039|Paraswammerdamia_conspersella
TLYFIFGIWSGMVGTSLSLLIRAELGNPGSLIGDDQIYNTIVTAHAFIMIFFMVMPIMIGGFGNWLVPLMLGAPDMAFPRMNNMSFWLLPPSLTLLISSSIVENGAGTGWTVYPPLSSNIAHSGSSVDLAIFSLHLAGISSILGAINFITTIINMKSNGMSFD
QMPLFVWAVGITALLLLLSLPVLAGAITMLLTDRNLNTSFFDPAGGGDPILYQHL
>LEFIB240-10|HM871143|MM00702|Paraswammerdamia_lapponica
TLYFIFGIWSGMVGTSLSLLIRAELGNPGSLIGDDQIYNTIVTAHAFIMIFFMVMPIMIGGFGNWLVPLMLGAPDMAFPRMNNMSFWLLPPSLTLLISSSIVENGAGTGWTVYPPLSSNIAHSGSSVDLAIFSLHLAGISSILGAINFITTIINMKSNGMSFD
QMPLFVWAVGITALLLLLSLPVLAGAITMLLTDRNLNTSFFDPAGGGDPILYQHL
>LEFIB758-10|HM871635|MM02613|Paraswammerdamia_nebulella
TLYFIFGIWSGMVGTSLSLLIRAELGNPGSLIGDDQIYNTIVTAHAFIMIFFMVMPIMIGGFGNWLVPLMLGAPDMAFPRMNNMSFWLLPPSLTLLISSSIVENGAGTGWTVYPPLSSNIAHGGSSVDLAIFSLHLAGISSILGAINFITTIINMKSNGMNF
DQMPLFVWAVGITALLLLLSLPVLAGAITMLLTDRNLNTSFFDPAGGGDPILYQHL
>LEFID686-10|HM873447|MM06731|Paraswammerdamia_nebulella
TLYFIFGIWSGMVGTSLSLLIRAELGNPGSLIGDDQIYNTIVTAHAFIMIFFMVMPIMIGGFGNWLVPLMLGAPDMAFPRMNNMSFWLLPPSLTLLISSSIVENGAGTGWTVYPPLSSNIAHGGSSVDLAIFSLHLAGISSILGAINFITTIINMKSNGMNF
DQMPLFVWAVGITALLLLLSLPVLAGAITMLLTDRNLNTSFFDPAGGGDPILYQHL
>LEFIB629-10|HM871508|MM02377|Paratalanta_hyalinalis

TLYFIFGIWSGLLGTSLSLLIRAELGNPGSFIGDDQIYNTIVTAHAFIMIFFMVMPIMIGGFGNWLIPLMLGAPDMAFPRMNNMSFWLLPPSLTLLLSSSIVENGAGTGWTVYPPLSSNIAHGGSSVDLAIFSLHLAGISSILGAINFITTIINMRVNGMSFD
QMPLFVWSVGITALLLLLSLPVLAGAITMLLTDRNLNTSFFDPAGGGDPILYQHL
>LEFIA698-10|HM386843|MM01852|Paratalanta_pandalis
TLYFIFGIWSGMLGTSLSLLIRAELGNPGSFIGDDQIYNTIVTAHAFIMIFFMVMPIMIGGFGNWLIPLMLGAPDMAFPRMNNMSFWLLPPSITLLISSSIVETGAGTGWTVYPPLSSNIAHSGSSVDLAIFSLHLAGISSILGAINFITTIINMRVNGMSFDQ
MPLFVWAVGITALLLLLSLPVLAGAITMLLTDRNLNTSFFDPAGGGDPILYQHL
>LEFIC008-10|HM871878|MM03209|Parectopa_ononidis
TLYFLFGIWSGMVGTSLSFLIRAELGNPGSLIGDDQIYNTIVTAHAFIMIFFMVMPIMIGGFGNWLVPLMLGAPDMAFPRLNNMSFWLLPPSILLLISSSVVENGAGTGWTVYPPLSSNIAHGGSSVDLAIFSLHLAGISSILGAINFISTIINMRPYEMSLD
KMPLFAWAVGITALLLLLSLPVLAGAITMLLTDRNLNTSFFDPAGGGDPILYQHL
>LEFIG362-10|HM876039|MM14361|Parectopa_ononidis
TLYFLFGIWSGMVGTSLSFLIRAELGNPGSLIGDDQIYNTIVTAHAFIMIFFMVMPIMIGGFGNWLVPLMLGAPDMAFPRLNNMSFWLLPPSILLLISSSVVENGAGTGWTVYPPLSSNIAHGGSSVDLAIFSLHLAGISSILGAINFISTIINMRPYEMSLD
KMPLFAWAVGITALLLLLSLPVLAGAITMLLTDRNLNTSFFDPAGGGDPILYQHL
>LEFIG434-10|HM876110|MM14470|Parectopa_ononidis
TLYFLFGIWSGMVGTSLSFLIRAELGNPGSLIGDDQIYNTIVTAHAFIMIFFMVMPIMIGGFGNWLVPLMLGAPDMAFPRLNNMSFWLLPPSILLLISSSVVENGAGTGWTVYPPLSSNIAHGGSSVDLAIFSLHLAGISSILGAINFISTIINMRPYEMSLD
KMPLFAWAVGITALLLLLSLPVLAGAITMLLTDRNLNTSFFDPAGGGDPILYQHL
>LEFIL267-10|JF854559|MM19267|Parectropis_similaria
TLYFIFGIWAGMVGTSLSLLIRAELGNPGSLIGDDQIYNTIVTAHAFIMIFFMVMPIMIGGFGNWLVPLMLGAPDMAFPRMNNMSFWLLPPSITLLISSSIVENGAGTGWTVYPPLSSNIAHGGSSVDLAIFSLHLAGISSILGAINFITTIINMRLNNMSFD
QMPLFVWAVGITAFLLLLSLPVLAGAITMLLTDRNLNTSFFDPAGGGDPILYQHL
>LEEUA505-11|MM20564|Parectropis_similaria
TLYFIFGVWAGMVGTSLSLLIRAELGSPGSLIGDDQIYNTIVTAHAFIMIFFMVMPIMIGGFGNWLVPLMLGAPDMAFPRMNNMSFWLLPPSITLLISSSIVENGAGTGWTVYPPLSSNIAHGGSSVDLAIFSLHLAGISSILGAINFITTIINMRLNNMSF
DQMPLFIWAVGITAFLLLLSLPVLAGAITMLLTDRNLNTSFFDPAGGGDPILYQHL
>LEFIL578-10|MM18876|Pareulype_berberata
TLYFIFGIWAGMIGTSLSLLIRAELGNPGSLIGDDQIYNTIVTAHAFIMIFFMVMPIMIGGFGNWLIPLMLGAPDMAFPRMNNMSFWLLPPSITLLISSSIVENGAGTGWTVYPPLSSNIAHGGSSVDLAIFSLHLAGISSILGAINFITTIINMRLNNMFFD
QLPLFVWAVGITAFLLLLSLPVLAGAITMLLTDRNLNTSFFDPAGGGDPILYQHL
>LEFID119-10|HM872929|MM05853|Parnassius_mnemosyne
TLYFIFGIWAGMVGTSLSLLIRTELGNPGSLIGDDQIYNTIVTAHAFIMIFFMVMPIMIGGFGNWLIPLMLGAPDMAFPRMNNMSFWLLPPSLTLLISSSIVENGAGTGWTVYPPLSSNIAHSGSSVDLAIFSLHLAGISSILGAINFITTIINMRINHMSFD
QMPLFVWAVGITALLLLLSLPVLAGAITMLLTDRNLNTSFFDPAGGGDPILYQHL
>LEFIB230-10|HM871133|MM00689|Parornix_anglicella
TLYFIFGIWSGMVGTSLSLLIRAELGNPGSLIGDDQIYNTIVTAHAFIMIFFMVMPIMIGGFGNWLVPLMLGAPDMAFPRLNNMSFWLLPPSLILLISSSVVENGAGTGWTVYPPLSSNIAHGGSSVDLAIFSLHLAGISSILGAINFITTIINMRPNGMKFD
SMPLFVWSVLITAILLLLSLPVLAGAITMLLTDRNLNTSFFDPAGGGDPILYQHL
>LEFID305-10|HM873102|MM06184|Parornix_anglicella
TLYFIFGIWSGMVGTSLSLLIRAELGNPGSLIGDDQIYNTIVTAHAFIMIFFMVMPIMIGGFGNWLVPLMLGAPDMAFPRLNNMSFWLLPPSLILLISSSVVENGAGTGWTVYPPLSSNIAHGGSSVDLAIFSLHLAGISSILGAINFITTIINMRPNGMKFD
SMPLFVWSVLITAILLLLSLPVLAGAITMLLTDRNLNTSFFDPAGGGDPILYQHL
>LEFID306-10|HM873103|MM06186|Parornix_betulae
TLYFIFGIWSGMVGTSLSLLIRAELGNPSSLIGDDQIYNTIVTAHAFIMIFFMVMPIMIGGFGNWLVPLMLGAPDMAFPRLNNMSFWLLPPSLILLISSSVVESGAGTGWTVYPPLSSNIAHGGSSVDLAIFSLHLAGISSILGAINFITTIINMRPNEMKFDS
MPLFVWSVLITTILLLLSLPVLAGAITMLLTDRNLNTSFFDPAGGGDPILYQHL
>LEFID387-10|HM873184|MM06299|Parornix_betulae
TLYFIFGIWSGMVGTSLSLLIRAELGNPGSLIGDDQIYNTIVTAHAFIMIFFMVMPIMIGGFGNWLVPLMLGAPDMAFPRLNNMSFWLLPPSLILLISSSVVESGAGTGWTVYPPLSSNIAHGGSSVDLAIFSLHLAGISSILGAINFITTIINMRPNGMKFD
SMPLFVWSVLITAILLLLSLPVLAGAITMLLTDRNLNTSFFDPAGGGDPILYQHL
>LEFIK399-10|JF854031|MM17974|Parornix_betulae
TLYFIFGIWSGMVGTSLSLLIRAELGNPGSLIGDDQIYNTIVTAHAFIMIFFMVMPIMIGGFGNWLVPLMLGAPDMAFPRLNNMSFWLLPPSLILLISSSVVESGAGTGWTVYPPLSSNIAHGGSSVDLAIFSLHLAGISSILGAINFITTIINMRPNGMKFD
SMPLFVWSVLITAILLLLSLPVLAGAITMLLTDRNLNTSFFDPAGGGDPILYQHL
>LEEUA053-11|JN271965|MM19366|Parornix_carpinella
TLYFIFGIWSGMVGTSLSLLIRAELGNPGSLIGDDQIYNTIVTAHAFIMIFFMVMPIMIGGFGNWLVPLMLGAPDMAFPRLNNMSFWLLPPSLILLISSSVVENGAGTGWTVYPPLSSNIAHGGSSVDLAIFSLHLAGISSILGAINFITTIINMRPNGMKFD
SMPLFVWSVLITAILLLLSLPVLAGAITMLLTDRNLNTSFFDPAGGGDPILYQHL
>LEFID874-10|HM873631|MM07010|Parornix_devoniella
TLYFIFGIWSGMVGTSLSLLIRAELGNPGSLIGDDQIYNTIVTAHAFIMIFFMVMPIMIGGFGNWLVPLMLGAPDMAFPRLNNMSFWLLPPSLMLLISSSVVENGAGTGWTVYPPLSSNIAHGGSSVDLAIFSLHLAGISSILGAINFITTIINMRPNEMKF
DSMPLFVWSVLITAILLLLSLPVLAGAITMLLTDRNLNTSFFDPAGGGDPILYQHL

>LEFIE650-10|HM874373|MM09610|Parornix_devoniella
TLYFIFGIWSGMVGTSLSLLIRAELGNPGSLIGDDQIYNTIVTAHAFIMIFFMVMPIMIGGFGNWLVPLMLGAPDMAFPRLNNMSFWLLPPSLMLLISSSVVENGAGTGWTVYPPLSSNIAHGGSSVDLAIFSLHLAGISSILGAINFITTIINMRPNEMKF
DSMPLFVWSVLITAILLLLSLPVLAGAITMLLTDRNLNTSFFDPAGGGDPILYQHL
>LEFIK514-10|JF854114|MM18089|Parornix_finitimella
TLYFIFGIWSGMVGTSLSLLIRAELGNPGSLIGDDQIYNTIVTAHAFIMIFFMVMPIMIGGFGNWLVPLMLGAPDMAFPRLNNMSFWLLPPSLLLLISSSVVESGSGTGWTVYPPLSSNIAHGGSSVDLAIFSLHLAGISSILGAINFITTIINMRPNGMKFD
NMPLFVWSVLITAILLLLSLPVLAGAITMLLTDRNLNTSFFDPAGGGDPILYQHL
>LEFIC426-10|HQ570310|MM03972|Parornix_loganella
TLYFIFGIWSGMVGTSLSLLIRAELGNPGSLIGDDQIYNTIVTAHAFIMIFFMVMPIMIGGFGNWLVPLMLGAPDMAFPRLNNMSFWLLPPSLILLISSSVVESGAGTGWTVYPPLSSNIAHGGSSVDLAIFSLHLAGISSILGAINFITTIINMRPNGMKFD
NMPLFVWSVLITAILLLLSLPVLAGAITMLLTDRNLNTSFFDPAGGGDPILYQHL
>LEFID491-10|HM873256|MM06448|Parornix_loganella
TLYFIFGIWSGMVGTSLSLLIRAELGNPGSLIGDDQIYNTIVTAHAFIMIFFMVMPIMIGGFGNWLVPLMLGAPDMAFPRLNNMSFWLLPPSLILLISSSVVESGAGTGWTVYPPLSSNIAHGGSSVDLAIFSLHLAGISSILGAINFITTIINMRPNGMKFD
NMPLFVWSVLITAILLLLSLPVLAGAITMLLTDRNLNTSFFDPAGGGDPILYQHL
>LEFIE173-10|HM873919|MM08444|Parornix_loganella
TLYFIFGIWSGMVGTSLSLLIRAELGNPGSLIGDDQIYNTIVTAHAFIMIFFMVMPIMIGGFGNWLVPLMLGAPDMAFPRLNNMSFWLLPPSLILLISSSVVESGAGTGWTVYPPLSSNIAHGGSSVDLAIFSLHLAGISSILGAINFITTIINMRPNGMKFD
NMPLFVWSVLITAILLLLSLPVLAGAITMLLTDRNLNTSFFDPAGGGDPILYQHL
>LEFID388-10|HM873185|MM06300|Parornix_polygrammella
TLYFIFGIWSGMVGTSLSLLIRAELGNPGSLIGDDQIYNTIVTAHAFIMIFFMVMPIMIGGFGNWLVPLMLGAPDMAFPRLNNMSFWLLPPSLIMLISSSVVENGAGTGWTVYPPLSSNIAHGGSSVDLAIFSLHLAGISSILGAINFITTIINMRPNGMKF
DSMPLFVWSVLITAILLLLSLPVLAGAITMLLTDRNLNTSFFDPAGGGDPILYQHL
>LEFID409-10|HM873205|MM06333|Parornix_polygrammella
TLYFIFGIWSGMVGTSLSLLIRAELGNPGSLIGDDQIYNTIVTAHAFIMIFFMVMPIMIGGFGNWLVPLMLGAPDMAFPRLNNMSFWLLPPSLILLISSSVVENGAGTGWTVYPPLSSNIAHGGSSVDLAIFSLHLAGISSILGAINFITTIINMRPNGMKFD
SMPLFVWSVLITAILLLLSLPVLAGAITMLLTDRNLNTSFFDPAGGGDPILYQHL
>LEFIK515-10|JF854115|MM18090|Parornix_polygrammella
TLYFIFGIWSGMVGTSLSLLIRAELGNPGSLIGDDQIYNTIVTAHAFIMIFFMVMPIMIGGFGNWLIPLMLGAPDMAFPRLNNMSFWLLPPSLILLISSSVVENGAGTGWTVYPPLSSNIAHGGSSVDLAIFSLHLAGISSILGAINFITTIINMRPNGMKFDS
MPLFVWSVLITAILLLLSLPILAGAITMLLTDRNLNTSFFDPAGGGDPILYQHL
>LEEUA055-11|JN271966|MM19368|Parornix_torquillella
TLYFIFGIWSGMVGTSLSLLIRAELGNPGSLIGDDQIYNTIVTAHAFIMIFFMVMPIMIGGFGNWLVPLMLGAPDMAFPRLNNMSFWLLPPSLILLISSSVVENGVGTGWTVYPPLSSNIAHGGSSVDLAIFSLHLAGISSILGAINFITTIINMKPNGMKFD
SMPLFVWAVLITAILLLLSLPVLAGAITMLLTDRNLNTSFFDPAGGGDPILYQHL
>LEEUA054-11|MM19367|Parornix_traugotti
TLYFIFGIWSGMVGTSLSLLIRAKLGNPGSLIGDDQIFNTIVTAYAFIMFFFMVMPIMIGGFGNWLVPLMLGAPDMAFPRLNNMSFCLLPPSLILLISSSVVENGAGTGWTVYLPLSSNIAHGGSSVDLAIFSLHLAGISSILGAINFITTIINMRPNGMKFDS
MPLFVWSVLITAILLLLSLPVLAGAITMLLTDRNLNTSFFDPAGGGDPILY?HL
>LEFIB246-10|HM871149|MM00713|Pasiphila_chloerata
TLYFIFGIWAGMIGTSLSLLIRAELGTPGSLIGDDQIYNTIVTAHAFIMIFFMVMPIMIGGFGNWLVPLMLGAPDMAFPRMNNMSFWLLPPSITLLISSSIVENGAGTGWTVYPPLSSNIAHGGSSVDLAIFSLHLAGISSILGAINFITTIINMRLNNMFFD
QLPLFVWSVGITAFLLLLSLPVLAGAITMLLTDRNLNTSFFDPAGGGDPILYQHL
>LEFID058-10|HM872872|MM05658|Pasiphila_chloerata
TLYFIFGIWAGMIGTSLSLLIRAELGTPGSLIGDDQIYNTIVTAHAFIMIFFMVMPIMIGGFGNWLVPLMLGAPDMAFPRMNNMSFWLLPPSITLLISSSIVENGAGTGWTVYPPLSSNIAHGGSSVDLAIFSLHLAGISSILGAINFITTIINMRLNNMFFD
QLPLFVWSVGITAFLLLLSLPVLAGAITMLLTDRNLNTSFFDPAGGGDPILYQHL
>LEFIB247-10|HM871150|MM00715|Pasiphila_debiliata
TLYFIFGIWAGMVGTSLSLLIRAELGTPGSLIGDDQIYNTIVTAHAFIMIFFMVMPIMIGGFGNWLVPLMLGAPDMAFPRMNNMSFWLLPPSITLLISSSIVENGAGTGWTVYPPLSSNIAHGGSSVDLAIFSLHLAGISSILGAINFITTIINMRLNNMFFD
QLPLFVWSVGITAFLLLLSLPVLAGAITMLLTDRNLNTSFFDPAGGGDPILYQHL
>LEFIA687-10|HM386833|MM01828|Pasiphila_debiliata
TLYFIFGIWAGMVGTSLSLLIRAELGTPGSLIGDDQIYNTIVTAHAFIMIFFMVMPIMIGGFGNWLVPLMLGAPDMAFPRMNNMSFWLLPPSITLLISSSIVENGAGTGWTVYPPLSSNIAHGGSSVDLAIFSLHLAGISSILGAINFITTIINMRLNNMFFD
QLPLFVWSVGITAFLLLLSLPVLAGAITMLLTDRNLNTSFFDPAGGGDPILYQHL
>LEFIB245-10|HM871148|MM00712|Pasiphila_rectangulata
TLYFIFGIWAGMIGTSLSLLIRAELGTPGSLIGDDQIYNTIVTAHAFIMIFFMVMPIMIGGFGNWLVPLMLGAPDMAFPRMNNMSFWLLPPSITLLISSSIVENGAGTGWTVYPPLSSNIAHGGSSVDLAIFSLHLAGISSILGAINFITTIINMRLNNMFFD
QLPLFVWSVGITAFLLLLSLPVLAGAITMLLTDRNLNTSFFDPAGGGDPILYQHL
>LEFIA685-10|HM386831|MM01825|Pasiphila_rectangulata
TLYFIFGIWAGMIGTSLSLLIRAELGTPGSLIGDDQIYNTIVTAHAFIMIFFMVMPIMIGGFGNWLVPLMLGAPDMAFPRMNNMSFWLLPPSITLLISSSIVENGAGTGWTVYPPLSSNIAHGGSSVDLAIFSLHLAGISSILGAINFITTIINMRLNNMFFD

QLPLFVWSVGITAFLLLLSLPVLAGAITMLLTDRNLNTSFFDPAGGGDPILYQHL
>LEFIA686-10|HM386832|MM01826|Pasiphila_rectangulata
TLYFIFGIWAGMIGTSLSLLIRAELGTPGSLIGDDQIYNTIVTAHAFIMIFFMVMPIMIGGFGNWLVPLMLGAPDMAFPRMNNMSFWLLPPSITLLISSSIVENGAGTGWTVYPPLSSNIAHGGSSVDLAIFSLHLAGISSILGAINFITTIINMRLNNMFFD
QLPLFVWSVGITAFLLLLSLPVLAGAITMLLTDRNLNTSFFDPAGGGDPILYQHL
>LEFID973-10|HM873723|MM07981|Pasiphila_rectangulata
TLYFIFGIWAGMIGTSLSLLIRAELGTPGSLIGDDQIYNTIVTAHAFIMIFFMVMPIMIGGFGNWLVPLMLGAPDMAFPRMNNMSFWLLPPSITLLISSSIVENGAGTGWTVYPPLSSNIAHGGSSVDLAIFSLHLAGISSILGAINFITTIINMRLNNMFFD
QLPLFVWSVGITAFLLLLSLPVLAGAITMLLTDRNLNTSFFDPAGGGDPILYQHL
>LEFIA251-10|HM386595|MM01288|Pechipogo_strigilata
TLYFIFGIWAGMVGTSLSLLIRAELGNPGSLIGDDQIYNTIVTAHAFIMIFFMVMPIMIGGFGNWLIPLMLGAPDMAFPRMNNMSFWLLPPSLTLLISSSIVENGAGTGWTVYPPLSSNIAHGGSSVDLAIFSLHLAGISSILGAINFITTIINMRLNNLSFD
QMPLFIWAVGITAFLLLLSLPVLAGAITMLLTDRNLNTSFFDPAGGGDPILYQHL
>LEFID562-10|HM873327|MM06555|Pediasia_aridella
TLYFIFGIWAGMVGTSLSLLIRAELGNPGSLIGDDQIYNTIVTAHAFIMIFFMVMPIMIGGFGNWLVPLMLGAPDMAFPRMNNMSFWLLPPSLTLLISSSIVENGAGTGWTVYPPLSSNIAHGGSSVDLAIFSLHLAGISSILGAINFITTIINMRINGLSFD
QMPLFVWSVGITALLLLLSLPVLAGAITMLLTDRNLNTSFFDPAGGGDPILYQHL
>LEFIF327-10|HM875012|MM11132|Pediasia_contaminella
TLYFIFGIWAGMVGTSLSLLIRAELGNPGSLIGDDQIYNTIVTAHAFIMIFFMVMPIMIGGFGNWLVPLMLGAPDMAFPRMNNMSFWLLPPSLTLLISSSIVENGAGTGWTVYPPLSSNIAHGGSSVDLAIFSLHLAGISSILGAINFISTIINMRINGLSFD
QMPLFVWSVGITALLLLLSLPVLAGAITMLLTDRNLNTSFFDPAGGGDPILYQHL
>LEFIB058-10|HM870967|MM00342|Pediasia_fascelinella
TLYFIFGIWAGMIGTSLSLLIRAELGNPGFLIGDDQIYNTIVTAHAFIMIFFMVMPIMIGGFGNWLVPLMLGAPDMAFPRMNNMSFWLLPPSLSLLVSSSIVENGAGTGWTVYPPLSSNIAHSGSSVDLAIFSLHLAGISSILGAINFITTIINMRINGLSFD
QMPLFVWSVGITALLLLLSLPVLAGAITMLLTDRNLNTSFFDPAGGGDPILYQHL
>LEFIL261-10|MM19261|Pediasia_luteella
TLYFIFGIWAGMIGTSLSLLIRAELGNPGFLIGDDQIYNTIVTAHAFIMIFFMVMPIMIGGFGNWLVPLMLGAPDMAFPRMNNMSFWLLPPSLSLLISSSIVENGAGTGWTVYPPLSSNIAHGGSSVDLAIFSLHLAGISSILGAINFITTIINMRINGLSFDQ
MPLFVWSVGITALLLLLSLPVLAGAITMLLTDRNLNTSFFDPAGGGDPILYQHL
>LEEUA634-11|MM20693|Pediasia_luteella
TLYFIFGIWAGMIGTSLSLLIRAELGNPGFLIGDDQIYNTIVTAHAFIMIFFMVMPIMIGGFGNWLVPLMLGAPDMAFPRMNNMSFWLLPPSLSLLISSSIVENGAGTGWTVYPPLSSNIAHGGSSVDLAIFSLHLAGISSILGAINFITTIINMRINGLSFDQ
MPLFVWSVGITALLLLLSLPVLAGAITMLLTDRNLNTSFFDPAGGGDPILYQHL
>LEFIK818-10|JN266126|MM18393|Pediasia_truncatellus
TLYFIFGIWAGMVGTSLSLLIRAELGNPGSLIGDDQIYNTIVTAHAFIMIFFMVMPIMIGGFGNWLVPLMLGAPDMAFPRMNNMSFWLLPPSLTLLIFSSIVENGAGTGWTVYPPLSSNIAHGGSSVDLAIFSLHLAGISSILGAINFITTIINMRINGMSFD
QMPLFVWSVGITALLLLLSLPVLAGAITMLLTDRNLNTSFFDPAGGGDPILYQHL
>LEFIK819-10|JN266127|MM18394|Pediasia_truncatellus
TLYFIFGIWAGMVGTSLSLLIRAELGNPGSLIGDDQIYNTIVTAHAFIMIFFMVMPIMIGGFGNWLVPLMLGAPDMAFPRMNNMSFWLLPPSLTLLISSSIVENGAGTGWTVYPPLSSNIAHSGSSVDLAIFSLHLAGISSILGAINFITTIINMRINGMSFD
QMPLFVWSVGITALLLLLSLPVLAGAITMLLTDRNLNTSFFDPAGGGDPILYQHL
>LEFID154-10|HM872961|MM05917|Pelochrista_caecimaculana
TLYFIFGIWSGMIGTSLSLLIRAELGNPGSLIGDDQIYNTIVTAHAFIMIFFMVMPIMIGGFGNWLVPLMLGAPDMAFPRMNNMSFWLLPPSIMLLISSSIVENGAGTGWTVYPPLSSNIAHSGSSVDLAIFSLHLAGISSILGAVNFITTIINMRPNNMSL
DQMPLFVWAVAITALLLLLSLPVLAGAITMLLTDRNLNTSFFDPAGGGDPILYQHL
>LEFIL332-10|KM573580|MM18642|Pelochrista_caecimaculana
TLYFIFGIWSGMIGTSLSLLIRAELGNPGSLIGDDQIYNTIVTAHAFIMIFFMVMPIMIGGFGNWLVPLMLGAPDMAFPRMNNMSFWLLPPSIMLLISSSIVENGAGTGWTVYPPLSSNIAHSGSSVDLAIFSLHLAGISSILGAVNFITTIINMRPNNMSL
DQMPLFVWAVAITALLLLLSLPVLAGAITMLLTDRNLNTSSFDPAGGGDPILYQHL
>LEFIE827-10|HM874546|MM10037|Pelochrista_huebneriana
TLYFIFGIWAGMIGTSLSLLIRAELGNPGSLIGDDQIYNTIVTAHAFIMIFFMVMPIMIGGFGNWLIPLMLGAPDMAFPRMNNMSFWLLPPSIMLLISSSIVENGAGTGWTVYPPLSSNIAHSGSSVDLAIFSLHLAGISSILGAVNFITTIINMRPNNMSLD
QMPLFVWAVSITALLLLLSLPVLAGAITMLLTDRNLNTSFFDPAGGGDPILYQHL
>LEFIF825-10|HM875508|MM13158|Pelochrista_huebneriana
TLYFIFGIWAGMIGTSLSLLIRAELGNPGSLIGDDQIYNTIVTAHAFIMIFFMVMPIMIGGFGNWLIPLMLGAPDMAFPRMNNMSFWLLPPSIMLLISSSIVENGAGTGWTVYPPLSSNIAHSGSSVDLAIFSLHLAGISSILGAVNFITTIINMRPNNMSLD
QMPLFVWAVGITALLLLLSLPVLAGAITMLLTDRNLNTSFFDPAGGGDPILYQHL
>LEFIG390-10|HM876067|MM14407|Pelochrista_infidana
TLYFIFGIWAGMIGTSLSLLIRAELGNPGSLFGNDQIYNTIVTAHAFIMIFFMVMPIMIGGFGNWLIPLMLGAPDMAFPRMNNMSFWLLPPSIMLLISSSIVENGAGTGWTVYPPLSSNIAHSGSSVDLAIFSLHLAGISSILGAVNFITTIINMRPNNMSL
DQMPLFIWAVGITAILLLLSLPVLAGAITMLLTDRNLNTSFFDPAGGGDPILYQHL
>LEFIL268-10|MM19268|Pelochrista_mollitana

TLYFIFGIWSGMIGTSLSLLIRAELGNPGSLIGDDQIYNTIVTAHAFIMIFFMVMPIMIGGFGNWLVPLMLGAPDMAFPRMNNMSFWLLPPSIMLLISSSIVENGAGTGWTVYPPLSSNIAHSGSSVDLAIFSLHLAGISSILGAVNFITTIINMRPNNMSL
DQMPLFVWAVGITALLLLLSLPVLAGAITMLLTDRNLNTSFFDPAGGGDPILYQHL
>LEFIA094-10|HM396441|MM01016|Pelosia_muscerda
TLYFIFGIWAGMVGTSLSLLIRAELGNPGSLIGDDQIYNTIVTAHAFIMIFFMVMPIMIGGFGNWLVPLMLGAPDMAFPRMNNMSFWLLPPSLTLLISSSIVENGAGTGWTVYPPLSSNIAHSGSSVDLAIFSLHLAGISSILGAINFITTIINMRLNNLMFD
QMPLFVWAVGITAFLLLLSLPVLAGAITMLLTDRNLNTSFFDPAGGGDPILYQHL
>LEFIF742-10|HM875426|MM12903|Pelosia_muscerda
TLYFIFGIWAGMVGTSLSLLIRAELGNPGSLIGDDQIYNTIVTAHAFIMIFFMVMPIMIGGFGNWLVPLMLGAPDMAFPRMNNMSFWLLPPSLTLLISSSIVENGAGTGWTVYPPLSSNIAHSGSSVDLAIFSLHLAGISSILGAINFITTIINMRLNNLMFD
QMPLFVWAVGITAFLLLLSLPVLAGAITMLLTDRNLNTSFFDPAGGGDPILYQHL
>LEFIE572-10|HM874295|MM09465|Pelurga_comitata
TLYFIFGIWAGMVGTSLSLLIRAELGNPGSLIGDDQIYNTIVTAHAFIMIFFMVMPIMIGGFGNWLVPLMLGAPDMAFPRMNNMSFWLLPPSITLLISSSIVENGAGTGWTVYPPLSSNIAHGGSSVDLAIFSLHLAGISSILGAINFITTIINMRLNNMFFD
QLPLFVWAVGITAFLLLLSLPVLAGAITMLLTDRNLNTSFFDPAGGGDPILYQHL
>LEFIC131-10|HM871977|MM03472|Pempelia_palumbella
TLYFIFGIWSGMVGTSLSLLIRAELGTPGSLIGDDQIYNTIVTGHAFIMIFFMVMPIMIGGFGNWLVPLMLGAPDMAFPRMNNMSFWLLPPSLTLLISSSIVENGAGTGWTVYPPLSSNIAHGGSSVDLAIFSLHLAGISSILGAINFITTIINMKLNGLSFD
QMPLFVWAVGITALLLLLSLPVLAGAITMLLTDRNLNTSFFDPAGGGDPILYQHL
>LEFIF083-10|HM874790|MM10532|Pempeliella_ornatella
TLYFIFGIWSGMVGTSLSLLIRAELGTPGSLIGDDQIYNTIVTGHAFIMIFFMVMPIMIGGFGNWLVPLMLGAPDMAFPRMNNMSFWLLPPSLTLLISSSIVENGAGTGWTVYPPLSSNIAHGGSSVDLAIFSLHLAGISSILGAINFITTIINMKLNGLSFD
QMPLFVWAVGITALLLLLSLPVLAGAITMLLTDRNLNTSFFDPAGGGDPILYQHL
>LEFIF084-10|HM874791|MM10533|Pempeliella_ornatella
TLYFIFGIWSGMVGTSLSLLIRAELGTPGSLIGDDQIYNTIVTGHAFIMIFFMVMPIMIGGFGNWLVPLMLGAPDMAFPRMNNMSFWLLPPSLTLLISSSIVENGAGTGWTVYPPLSSNIAHGGSSVDLAIFSLHLAGISSILGAINFITTIINMKLNGLSFD
QMPLFVWAVGITALLLLLSLPVLAGAITMLLTDRNLNTSFFDPAGGGDPILYQHL
>LEFIF510-10|HM875195|MM12127|Pempeliella_ornatella
TLYFIFGIWSGMVGTSLSLLIRAELGTPGSLIGDDQIYNTIVTGHAFIMIFFMVMPIMIGGFGNWLVPLMLGAPDMAFPRMNNMSFWLLPPSLTLLISSSIVENGAGTGWTVYPPLSSNIAHGGSSVDLAIFSLHLAGISSILGAINFITTIINMKLNGLSFD
QMPLFVWAVGITALLLLLSLPVLAGAITMLLTDRNLNTSFFDPAGGGDPILYQHL
>LEFID842-10|HM873599|MM06964|Pennisetia_hylaeiformis
TLYFIFGIWAGLLGTSLSLLIRLELGTPGLFIGNDQIYNSIVTAHAFIMIFFMVMPIMIGGFGNWLVPLMLGAPDMAFPRMNNMSFWLLPPSLLLLISSSVIEMGVGTGWTVYPPLSSNIAHGGSSVDLAIFSLHLAGISSILGAINFITTILNMKPINMNFD
QMPLFVWSVGITALLLLLSLPVLAGAITMLLTDRNLNTSFFDPAGGGDPILYQHL
>LEFIC729-10|HM872550|MM04805|Pennithera_firmata
TLYFIFGIWAGMIGTSLSLLIRAELGSPGSLIGDDQIYNTIVTAHAFIMIFFMVMPIMIGGFGNWLVPLMLGAPDMAFPRMNNMSFWLLPPSITLLISSSIVENGAGTGWTVYPPLSSNIAHGGSSVDLAIFSLHLAGISSILGAINFITTIINMRLNNMFFD
QLPLFVWAVGITAFLLLLSLPVLAGAITMLLTDRNLNTSFFDPAGGGDPILYQHL
>LEFIF736-10|HM875420|MM12868|Perconia_strigillaria
TLYFIFGVWAGMVGTSLSLLIRAELGNPGSLIGDDQIYNTIVTAHAFIMIFFMVMPIMIGGFGNWLVPLMLGAPDMAFPRMNNMSFWLLPPSLTLLISSSIVENGAGTGWTVYPPLSSNIAHGGSSVDLAIFSLHLAGISSILGAINFITTIINMRLNNLSF
DQMPLFVWSVGITAFLLLLSLPVLAGAITMLLTDRNLNTSFFDPAGGGDPILYQHL
>LEFIG977-10|HM876616|MM15841|Perconia_strigillaria
TLYFIFGVWAGMVGTSLSLLIRAELGNPGSLIGDDQIYNTIVTAHAFIMIFFMVMPIMIGGFGNWLVPLMLGAPDMAFPRMNNMSFWLLPPSLTLLISSSIVENGAGTGWTVYPPLSSNIAHGGSSVDLAIFSLHLAGISSILGAINFITTIINMRLNNLSF
DQMPLFVWSVGITAFLLLLSLPVLAGAITMLLTDRNLNTSFFDPAGGGDPILYQHL
>LEFIK934-10|JF854376|MM18509|Perconia_strigillaria
TLYFIFGVWAGMVGTSLSLLIRAELGNPGSLIGDDQIYNTIVTTHAFIMIFFMVMPIMIGGFGNWLVPLMLGAPDMAFPRMNNMSFWLLPPSLTLLISSSIVENGAGTGWTVYPPLSSNIAHGGSSVDLAIFSLHLAGISSILGAINFITTIINMRLNNLSFD
QMPLFVWSVGITAFLLLLSLPVLAGAITMLLTDRNLNTSFFDPAGGGDPILYQHL
>LEFID745-10|HM873502|MM06806|Peribatodes_secundaria
TLYFIFGIWAGMVGTSLSLLIRAELGNPGSLIGDDQIYNTIVTAHAFIMIFFMVMPIMIGGFGNWLVPLMLGAPDMAFPRMNNMSFWLLPPSITLLISSSIVENGAGTGWTVYPPLSSNIAHGGSSVDLAIFSLHLAGISSILGAINFITTIINMRLNNLSFD
QMPLFVWSVGITAFLLLLSLPVLAGAITMLLTDRNLNTSFFDPAGGGDPILYQHL
>LEEUA217-11|JN286430|MM19625|Periclepsis_cinctana
TLYFIFGIWAGMVGTSLSLLIRTELGMPGSLIGDDQIYNTIVTAHAFIMIFFMVMPIMIGGFGNWLVPLMLGAPDMAFPRMNNMSFWLLPPSLMLLISSSIVENGAGTGWTVYPPLSSNIAHSGSSVDLAIFSLHLAGISSILGAVNFITTIINMRPDNMT
LDQMPLFVWSVGITALLLLLSLPVLAGAITMLLTDRNLNTSFFDPAGGGDPILYQHL
>LEFID121-10|HM872931|MM05856|Peridea_anceps
TLYFIFGVWAGMVGTSLSLLIRAELGNPGSLIGDDQIYNTIVTAHAFIMIFFMVMPIMIGGFGNWLVPLMLGAPDMAFPRMNNMSFWLLPPSLTLLISSSIVETGAGTGWTVYPPLSSNIAHGGSSVDLAIFSLHLAGISSILGAINFITTIINMRLNGMSF
DQMPLFVWAVGITAFLLLLSLPVLAGAITMLLTDRNLNTSFFDPAGGGDPILYQHL

>LEFID224-10|HM873025|MM06082|Peridroma_saucia
TLYFIFGIWAGMVGTSLSLLIRAELGNPGSLIGDDQIYNTIVTAHAFIMIFFMVMPIMIGGFGNWLVPLMLGAPDMAFPRMNNMSFWLLPPSLTLLISSSIVENGAGTGWTVYPPLSSNIAHGGSSVDLAIFSLHLAGISSILGAINFITTIINMRLNSLSFD
QMPLFIWAVGITAFLLLLSLPVLAGAITMLLTDRNLNTSFFDPAGGGDPILYQHL
>LEFID335-10|HM873132|MM06229|Perittia_farinella
TLYFIFGIWAGMVGTSLSLLIRAELGNPGSLIGNDQIYNTIVTTHAFIMIFFMVMPIMIGGFGNWLIPLMLGAPDMAFPRMNNMSFWLLPPSLILLISSSIVENGAGTGWTVYPPLSSNIAHSGSSVDLTIFSLHLAGISSILGAINFITTIINMRLYNMMFD
QMPLFVWAVGITALLLLLSLPVLAGAITMLLTDRNLNTSFFDPAGGGDPILYQHL
>LEFID334-10|HM873131|MM06228|Perittia_herrichiella
TLYFIFGIWAGMVGTSLSLLIRAELGNPGSLIGNDQIYNTIVTAHAFIMIFFMVMPIMIGGFGNWLVPLMLGAPDMAFPRMNNMSFWLLPPSLTLLISSSIVETGAGTGWTVYPPLSSNIAHSGSSVDLAIFSLHLAGISSILGAINFITTIINMRLNNMMF
DQMPLFVWAVGITALLLLLSLPVLAGAITMLLTDRNLNTSFFDPAGGGDPILYQHL
>LEFIE332-10|HM874058|MM08724|Perittia_herrichiella
TLYFIFGIWAGMVGTSLSLLIRAELGNPGSLIGNDQIYNTIVTAHAFIMIFFMVMPIMIGGFGNWLVPLMLGAPDMAFPRMNNMSFWLLPPSLTLLISSSIVETGAGTGWTVYPPLSSNIAHSGSSVDLAIFSLHLAGISSILGAINFITTIINMRLNNMMF
DQMPLFVWAVGITALLLLLSLPVLAGAITMLLTDRNLNTSFFDPAGGGDPILYQHL
>LEFIK574-10|MM18149|Perittia_obscurepunctella
TLYFIFGIWAGMVGTSLSLLIRAELGNPGSLIGNDQIYNTIVTAHAFIMIFFMVMPIMIGGFGNWLVPLMLGAPDMAFPRMNNMSFWLLPPSLTLLISSSIVETGAGTGWTVYPPLSSNIAHSGSSVDLAIFSLHLAGISSILGAINFITTIINMRLNNMMF
DQMPLFVWAVGITALLLLLSLPVLAGAITMLLTDRNLNTSFFDPAGGGDPILYQHL
>LEFIA023-10|HM396373|MM00094|Perizoma_affinitata
TLYFIFGIWAGMIGTSLSLLIRAELGNPGSLIGDDQIYNTIVTAHAFIMIFFMVMPIMIGGFGNWLVPLMLGAPDMAFPRMNNMSFWLLPPSITLLISSSIVENGAGTGWTVYPPLSSNIAHGGSSVDLAIFSLHLAGISSILGAINFITTIINMRLNNMFFD
QLPLFVWAVGITAFLLLLSLPVLAGAITMLLTDRNLNTSFFDPAGGGDPILYQHL
>LEFIA298-10|HM386641|MM01343|Perizoma_affinitata
TLYFIFGIWAGMIGTSLSLLIRAELGNPGSLIGDDQIYNTIVTAHAFIMIFFMVMPIMIGGFGNWLVPLMLGAPDMAFPRMNNMSFWLLPPSITLLISSSIVENGAGTGWTVYPPLSSNIAHGGSSVDLAIFSLHLAGISSILGAINFITTIVNMRLNNMFFD
QLPLFVWAVGITAFLLLLSLPVLAGAITMLLTDRNLNTSFFDPAGGGDPILYQHL
>LEFIK894-10|JF854345|MM18469|Perizoma_affinitata
TLYFIFGIWAGMIGTSLSLLIRAELGNPGSLIGDDQIYNTIVTAHAFIMIFFMVMPIMIGGFGNWLVPLMLGAPDMAFPRMNNMSFWLLPPSITLLISSSIVENGAGTGWTVYPPLSSNIAHGGSSVDLAIFSLHLAGISSILGAINFITTIINMRLNNMFFD
QLPLFVWAVGITAFLLLLSLPVLAGAITMLLTDRNLNTSFFDPAGGGDPILYQHL
>LEFID399-10|HM873196|MM06317|Perizoma_albulata
TLYFIFGIWAGMIGTSLSLLIRAELGNPGSLIGDDQIYNTIVTAHAFIMIFFMVMPIMIGGFGNWLVPLMLGAPDMAFPRMNNMSFWLLPPSITLLISSSIVENGAGTGWTVYPPLSSNIAHGGSSVDLAIFSLHLAGISSILGAINFITTIINMRLNNMFFD
QLPLFVWAVGITAFLLLLSLPVLAGAITMLLTDRNLNTSFFDPAGGGDPILYQHL
>LEFIB250-10|HM871153|MM00719|Perizoma_alchemillata
TLYFIFGIWAGMIGTSLSLLIRAELGNPGSLIGDDQIYNTIVTAHAFIMIFFMVMPIMIGGFGNWLVPLMLGAPDMAFPRMNNMSFWLLPPSITLLISSSIVENGAGTGWTVYPPLSSNIAHGGSSVDLAIFSLHLAGISSILGAINFITTIINMRLNNMFFD
QLPLFVWAVGITAFLLLLSLPVLAGAITMLLTDRNLNTSFFDPAGGGDPILYQHL
>LEFIA395-10|HM386737|MM01454|Perizoma_alchemillata
TLYFIFGIWAGMIGTSLSLLIRAELGNPGSLIGDDQIYNTIVTAHAFIMIFFMVMPIMIGGFGNWLVPLMLGAPDMAFPRMNNMSFWLLPPSITLLISSSIVENGAGTGWTVYPPLSSNIAHGGSSVDLAIFSLHLAGISSILGAINFITTIINMRLNNMFFD
QLPLFVWAVGITAFLLLLSLPVLAGAITMLLTDRNLNTSFFDPAGGGDPILYQHL
>LEFIA372-10|HM386714|MM01429|Perizoma_bifaciata
TLYFIFGIWAGMIGTSLSLLIRAELGNPGSLIGDDQIYNTIVTAHAFIMIFFMVMPIMIGGFGNWLVPLMLGAPDMAFPRMNNMSFWLLPPSITLLISSSIVENGAGTGWTVYPPLSSNIAHGGSSVDLAIFSLHLAGISSILGAINFITTIINMRLNNMFFD
QLPLFVWAVGITAFLLLLSLPVLAGAITMLLTDRNLNTSFFDPAGGGDPILYQHL
>LEFIA646-10|HM870895|MM01763|Perizoma_bifaciata
TLYFIFGIWAGMIGTSLSLLIRAELGNPGSLIGDDQIYNTIVTAHAFIMIFFMVMPIMIGGFGNWLVPLMLGAPDMAFPRMNNMSFWLLPPSITLLISSSIVENGAGTGWTVYPPLSSNIAHSGSSVDLAIFSLHLAGISSILGAINFITTIINMRLNNMFFD
QLPLFVWAVGITAFLLLLSLPVLAGAITMLLTDRNLNTSFFDPAGGGDPILYQHL
>LEFIB052-10|HM870961|MM00335|Perizoma_blandiata
TLYFIFGIWAGMIGTSLSLLIRAELGNPGSLIGDDQIYNTIVTAHAFIMIFFMVMPIMIGGFGNWLVPLMLGAPDMAFPRMNNMSFWLLPPSITLLISSSIVENGAGTGWTVYPPLSSNIAHGGSSVDLAIFSLHLAGISSILGAINFITTIINMRLNNMFFD
QLPLFVWAVGITAFLLLLSLPVLAGAITMLLTDRNLNTSFFDPAGGGDPILYQHL
>LEFIA382-10|HM386724|MM01439|Perizoma_flavofasciata
TLYFIFGIWAGMIGTSLSLLIRAELGNPGSLIGDDQIYNTIVTAHAFIMIFFMVMPIMIGGFGNWLVPLMLGAPDMAFPRMNNMSFWLLPPSITLLISSSIVENGAGTGWTVYPPLSSNIAHGGSSVDLAIFSLHLAGISSILGAINFITTIINMRLNNMFFD
QLPLFVWAVGITAFLLLLSLPVLAGAITMLLTDRNLNTSFFDPAGGGDPILYQHL
>LEFIC698-10|HM872519|MM04686|Perizoma_hydrata
TLYFIFGIWAGMIGTSLSLLIRAELGNPGSLIGDDQIYNTIVTAHAFIMIFFMVMPIMIGGFGNWLVPLMLGAPDMAFPRMNNMSFWLLPPSITLLISSSIVENGAGTGWTVYPPLSSNIAHGGSSVDLAIFSLHLAGISSILGAINFITTIINMRLNNMFFD

QLPLFVWAVGITAFLLLLSLPVLAGAITMLLTDRNLNTSFFDPAGGGDPILYQHL
>LEFIL577-10|MM18875|Perizoma_lugdunaria
TLYFIFGIWAGMIGTSLSLLIRAELGNPGSLIGDDQIYNTIVTAHAFIMIFFMVMPIMIGGFGNWLVPLMLGAPDMAFPRMNNMSFWLLPPSITLLISSSIVENGAGTGWTVYPPLSSNIAHGGSSVDLAIFSLHLAGISSILGAINFITTIINMRLNNMFFD
QLPLFVWAVGITAFLLLLSLPVLAGAITMLLTDRNLNTSFFDPAGGGDPILYQHL
>LEFIA019-10|HM396369|MM00090|Perizoma_minorata
TLYFIFGIWAGMIGTSLSLLIRAELGNPGSLIGDDQIYNTIVTAHAFIMIFFMVMPIMIGGFGNWLVPLMLGAPDMAFPRMNNMSFWLLPPSITLLISSSIVENGAGTGWTVYPPLSSNIAHGGSSVDLAIFSLHLAGISSILGAINFITTIINMRLNNMFFD
QLPLFVWAVGITAFLLLLSLPVLAGAITMLLTDRNLNTSFFDPAGGGDPILYQHL
>LEFIC588-10|HM872409|MM04506|Petrophora_chlorosata
TLYFIFGIWAGMIGTSLSLLIRAELGNPGSLIGDDQIYNTIVTAHAFIMIFFMVMPIMIGGFGNWLVPLMLGAPDMAFPRMNNMSFWLLPPSITLLISSSIVENGAGTGWTVYPPLSSNIAHGGSSVDLAIFSLHLAGISSILGAINFITTIINMRLNNMSFD
QMPLFIWAVGITAFLLLLSLPVLAGAITMLLTDRNLNTSFFDPAGGGDPILYQHL
>LEFIC999-10|HM872814|MM05420|Petrophora_chlorosata
TLYFIFGIWAGMIGTSLSLLIRAELGNPGSLIGDDQIYNTIVTAHAFIMIFFMVMPIMIGGFGNWLVPLMLGAPDMAFPRMNNMSFWLLPPSITLLISSSIVENGAGTGWTVYPPLSSNIAHGGSSVDLAIFSLHLAGISSILGAINFITTIINMRLNNMSFD
QMPLFIWAVGITAFLLLLSLPVLAGAITMLLTDRNLNTSFFDPAGGGDPILYQHL
>LEFID781-10|HM873538|MM06850|Pexicopia_malvella
TLYFIFGIWAGMMGTSLSLLIRAELGNPGSLIGDDQIYNTIVTAHAFIMIFFMVMPIMIGGFGNWLVPLMLGAPDMAFPRMNNMSFWLLPPSLTLLISSSIVENGAGTGWTVYPPLSSNIAHGGSSVDLAIFSLHLAGISSILGAINFITTIINMRINGLSFD
QMPLFVWAVGITALLLLLSLPVLAGAITMLLTDRNLNTSFFDPAGGGDPILYQHL
>LEFIF589-10|HM875273|MM12429|Pexicopia_malvella
TLYFIFGIWAGMVGTSLSLLIRAELGNPGSLIGDDQIYNTIVTAHAFIMIFFMVMPIMIGGFGNWLVPLMLGAPDMAFPRMNNMSFWLLPPSLTLLISSSIVENGAGTGWTVYPPLSSNIAHGGSSVDLAIFSLHLAGISSILGAINFITTIINMRINGLSFD
QMPLFVWAVGITALLLLLSLPVLAGAITMLLTDRNLNTSFFDPAGGGDPILYQHL
>LEFID037-10|HM872851|MM05491|Phalacropterix_graslinella
TLYFILGIWSGLIGTSLSLLIRAELGTPGSLIGNDQIYNTIVTAHAFIMIFFMVMPIMIGGFGNWLIPLMLGAPDMAFPRMNNMSFWLLPPSLMLLTASSLVENGAGTGWTVYPPLSSNIAHNGGSVDLAIFSLHLAGISSILGAVNFISTTINMRMDSMSL
DQMPLFVWSVAITALLLLLSLPVLAGAITMLLTDRNLNTSFFDPAGGGDPILYQHL
>LEFIA068-10|HM396415|MM00983|Phalera_bucephala
TLYFIFGIWAGMVGTSLSLLIRAELGNPGSLIGDDQIYNTIVTAHAFIMIFFMVMPIMIGGFGNWLIPLMLGAPDMAFPRMNNMSFWLLPPSLTLLISSSIVENGAGTGWTVYPPLSSNIAHGGSSVDLAIFSLHLAGISSILGAINFITTIINMRLNNMSFD
QMPLFIWAVGITAFLLLLSLPVLAGAITMLLTDRNLNTSFFDPAGGGDPILYQHL
>LEFIB648-10|HM871527|MM02414|Phalonidia_curvistrigana
TLYFIFGIWAGMVGTSLSLLIRAELGNPGSLIGDDQIYNTIVTAHAFIMIFFMVMPIMIGGFGNWLVPLMLGAPDMAFPRMNNMSFWLLPPSIMLLISSSIVENGAGTGWTVYPPLSSNIAHSGSSVDLAIFSLHLAGISSILGAVNFITTIINMRPNNMN
LDQMPLFVWAVGITALLLLLSLPVLAGAITMLLTDRNLNTSFFDPAGGGDPILYQHL
>LEFIG285-10|HM875964|MM14232|Phalonidia_manniana
TLYFIFGIWAGMVGTSLSMLIRAELGNPGSLIGDDQIYNTIVTAHAFIMIFFMVMPIMIGGFGNWLIPLMLGAPDMAFPRMNNMSFWLLPPSIMLLISSSIVENGAGTGWTVYPPLSSNIAHSGSSVDLAIFSLHLAGISSILGAVNFITTIINMRPNNMN
LDQMPLFVWAVGITALLLLLSLPVLAGAITMLLTDRNLNTSFFDPAGGGDPILYQHL
>LEFIJ1024-11|MM14233|Phalonidia_manniana
TLYFIFGIWAGMVGTSLSMLIRAELGNPGSLIGDDQIYNTIVTAHAFIMIFFMVMPIMIGGFGNWLVPLMLGAPDMAFPRMNNMSFWLLPPSIMLLISSSIVENGAGTGWTVYPPLSSNIAHSGSSVDLAIFSLHLAGISSILGAVNFITTIINMRPNNM
NLDQMPLFVWAVGITALLLLLSLPVLAGAITMLLTDRNLNTSFFDPAGGGDPILYQHL
>LEFIJ1037-11|MM20726|Phalonidia_manniana
TLYFIFGIWAGMVGTSLSMLIRAELGNPGSLIGDDQIYNTIVTAHAFIMIFFMVMPIMIGGFGNWLVPLMLGAPDMAFPRMNNMSFWLLPPSIMLLISSSIVENGAGTGWTVYPPLSSNIAHSGSSVDLAIFSLHLAGISSILGAVNFITTIINMRPNNM
NLDQMPLFVWAVGITALLLLLSLPVLAGAITMLLTDRNLNTSFFDPAGGGDPILYQHL
>LEFID265-10|HM873063|MM06131|Phalonidia_udana
TLYFIFGIWAGMVGTSLSMLIRAELGNPGSLIGDDQIYNTIVTAHAFIMIFFMVMPIMIGGFGNWLVPLMLGAPDMAFPRMNNMSFWLLPPSIMLLISSSIVENGAGTGWTVYPPLSSNIAHSGSSVDLAIFSLHLAGISSILGAVNFITTIINMRPNNM
NLDQMPLFVWAVGITALLLLLSLPVLAGAITMLLTDRNLNTSFFDPAGGGDPILYQHL
>LEFIE856-10|HM874574|MM10088|Phalonidia_udana
TLYFIFGIWAGMVGTSLSMLIRAELGNPGSLIGDDQIYNTIVTAHAFIMIFFMVMPIMIGGFGNWLVPLMLGAPDMAFPRMNNMSFWLLPPSIMLLISSSIVENGAGTGWTVYPPLSSNIAHSGSSVDLAIFSLHLAGISSILGAVNFITTIINMRPNNM
NLDQMPLFVWAVGITALLLLLSLPVLAGAITMLLTDRNLNTSFFDPAGGGDPILYQHL
>LEFIB973-10|HM871846|MM03163|Pharmacis_fusconebulosa
TLYFIFGIWAGMVGTSLSLLIRTELGNPGSLIGDDQIYNVIVTAHAFIMIFFMVMPIMIGGFGNWLVPLMLGAPDMAFPRLNNMSFWLLPPSLMLLISSSIVENGAGTGWTVYPPLSSNIAHAGSSVDLAIFSLHLAGISSILGAINFITTMINMRSKGMSF
DQMPLFIWSIMITTLLLLLSLPVLAGAITMLLTDRNLNTSFFDPAGGGDPILYQHL
>LEFIL179-10|KM572697|MM19179|Pharmacis_lupulina

TLYFIFGIWAGMVGTSLSLLIRTELGNPGSLIGDDQIYNVIVTAHAFIMIFFMVMPIMIGGFGNWLVPLMLGAPDMAFPRLNNMSFWLLPPSLMLLISSSIVENGAGTGWTVYPPLSSNIAHAGSSVDLAIFSLHLAGISSILGAINFITTVINMRSKGMSF
DQMPLFVWSIVITTLLLLLSLPVLAGAITMLLTDRNLNTSFFDPAGGGDPILYQHL
>LEFIG886-10|HM876531|MM15750|Phaulernis_dentella
TLYFIFGIWAGMVGTSLSLLIRAELGTPSSLIGDDQIYNTIVTAHAFIMIFFMVMPIMIGGFGNWLIPLMLGAPDMAFPRMNNMSFWLLPPSLLLLISSSLVENGAGTGWTVYPPLSSNIAHGGSSVDLAIFSLHLAGISSILGAINFITTIINMRLNGMMFD
QMPLFIWAVGITALLLLLSLPVLAGAITMLLTDRNLNTSFFDPAGGGDPILYQHL
>LEFIG526-10|HM876199|MM14645|Phaulernis_fulviguttella
TLYFIFGIWAGMVGTSLSLLIRAELGTPSSLIEDDQIYNTIVTAHAFIMIFFMVMPIMIGGFGNWLIPLMLGAPDMAFPRMNNMSFWLLPPALLLLISSSIVENGAGTGWTVYPPLSSNIAHSGSSVDLAIFSLHLAGISSILGAINFITTIINMRINGMMFD
QMPLFIWAVGITALLLLLSLPVLAGAITMLLTDRNLNTSFFDPAGGGDPILYQHL
>LEFIG887-10|HM876532|MM15751|Phaulernis_fulviguttella
TLYFIFGIWAGMVGTSLSLLIRAELGTPSSLIGDDQIYNTIVTAHAFIMIFFMVMPIMIGGFGNWLIPLMLGAPDMAFPRMNNMSFWLLPPALLLLISSSIVENGAGTGWTVYPPLSSNIAHSGSSVDLAIFSLHLAGISSILGAINFITTIINMRINGMMFD
QMPLFIWAVGITALLLLLSLPVLAGAITMLLTDRNLNTSFFDPAGGGDPILYQHL
>LEFIL606-10|KM573007|MM18904|Phaulernis_fulviguttella
TLYFIFGIWAGMVGTSLSLLIRAELGTPSSLIGDDQIYNTIVTAHAFIMIFFMVMPIMIGGFGNWLIPLMLGAPDMAFPRMNNMSFWLLPPALLLLISSSIVENGAGTGWTVYPPLSSNIAHSGSSVDLAIFSLHLAGISSILGAINFITTIINMRINGMMFD
QMPLFIWAVGITALLLLLSLPVLAGAITMLLTDRNLNTSFFDPAGGGDPILYQHL
>LEEUA502-11|MM20561|Phengaris_arion
TLYFIFGIWAGMLGMSLSILIRMELGTPGSLIGNDQIYNTIVTAHAFIMIFFMVMPIMIGGFGNWLVPLMLGAPDMAFPRMNNMSFWLLPPSLMLLISSSIVENGAGTGWTVYPPLSSNIAHSGSSVDLAIFSLHLAGISSILGAINFITTIINMKVNNLFF
DQMSLFIWAVGITALLLLLSLPVLAGAITMLLTDRNLNTSFFDPAGGGDPILYQHL
>LEFIA078-10|HM396425|MM00996|Pheosia_gnoma
TLYFIFGIWAGMVGTSLSLLIRAELGIPGSLIGDDQIYNTIVTAHAFIMIFFMVMPIMIGGFGNWLVPLMLGAPDMAFPRMNNMSFWLLPPSLTLLISSSIVENGAGTGWTVYPPLSSNIAHGGSSVDLAIFSLHLAGISSILGAINFITTIINMRLNNMSFD
QMPLFVWAVGITAFLLLLSLPVLAGAITMLLTDRNLNTSFFDPAGGGDPILYQHL
>LEFIA076-10|HM396423|MM00994|Pheosia_tremula
TLYFIFGIWAGMVGTSLSLLIRAELGTPGSLIGDDQIYNTIVTAHAFIMIFFMVMPIMIGGFGNWLVPLMLGAPDMAFPRLNNMSFWLLPPSLTLLISSSIVENGAGTGWTVYPPLSSNIAHGGSSVDLAIFSLHLAGISSILGAINFITTIINMRLNNMSFD
QMPLFVWAVGITAFLLLLSLPVLAGAITMLLTDRNLNTSFFDPAGGGDPILYQHL
>LEFIA077-10|HM396424|MM00995|Pheosia_tremula
TLYFIFGIWAGMVGTSLSLLIRAELGTPGSLIGDDQIYNTIVTAHAFIMIFFMVMPIMIGGFGNWLVPLMLGAPDMAFPRLNNMSFWLLPPSLTLLISSSIVENGAGTGWTVYPPLSSNIAHGGSSVDLAIFSLHLAGISSILGAINFITTIINMRLNNMSFD
QMPLFMWAVGITAFLLLLSLPVLAGAITMLLTDRNLNTSFFDPAGGGDPILYQHL
>LEFIE125-10|HM873873|MM08333|Pheosia_tremula
TLYFIFGIWAGMVGTSLSLLIRAELGTPGSLIGDDQIYNTIVTAHAFIMIFFMVMPIMIGGFGNWLVPLMLGAPDMAFPRLNNMSFWLLPPSLTLLISSSIVENGAGTGWTVYPPLSSNIAHGGSSVDLAIFSLHLAGISSILGAINFITTIINMRLNNMSFD
QMPLFVWAVGITAFLLLLSLPVLAGAITMLLTDRNLNTSFFDPAGGGDPILYQHL
>LEFIB902-10|HM871779|MM03005|Phiaris_bipunctana
TLYFIFGVWAGMVGTSLSLLIRAELGNPGSLIGDDQIYNTIVTAHAFIMIFFMVMPIMIGGFGNWLVPLMLGAPDMAFPRMNNMSFWLLPPSIMLLISSSIVENGAGTGWTVYPPLSSNIAHSGSSVDLAIFSLHLAGISSILGAVNFITTIINMRPNNMS
PDQMPLFVWSVGITALLLLLSLPVLAGAITMLLTDRNLNTSFFDPAGGGDPILYQHL
>LEFIK711-10|JF854274|MM18286|Phiaris_bipunctana
TLYFIFGVWAGMVGTSLSLLIRAELGNPGSLIGDDQIYNTIVTAHAFIMIFFMVMPIMIGGFGNWLVPLMLGAPDMAFPRMNNMSFWLLPPSIMLLISSSIVENGAGTGWTVYPPLSSNIAHSGSSVDLAIFSLHLAGISSILGAVNFITTIINMRPNNMS
PDQMPLFVWSVGITALLLLLSLPVLAGAITMLLTDRNLNTSFFDPAGGGDPILYQHL
>LEFIB903-10|HM871780|MM03007|Phiaris_dissolutana
TLYFIFGVWAGMIGTSLSLLIRAELGNPGSLIGDDQIYNTIVTAHAFIMIFFMVMPIMIGGFGNWLVPLMLGAPDMAFPRMNNMSFWLLPPSLMLLISSSIVENGAGTGWTVYPPLSSNIAHSGSSVDLAIFSLHLAGISSILGAVNFITTIINMRPNKMSL
DQMPLFIWAVGITALLLLLSLPVLAGAITMLLTDRNLNTSFFDPAGGGDPILYQHL
>LEFIB904-10|HM871781|MM03008|Phiaris_dissolutana
TLYFIFGVWAGMIGTSLSLLIRAELGNPGSLIGDDQIYNTIVTAHAFIMIFFMVMPIMIGGFGNWLVPLMLGAPDMAFPRMNNMSFWLLPPSLMLLISSSIVENGAGTGWTVYPPLSSNIAHSGSSVDLAIFSLHLAGISSILGAVNFITTIINMRPNKMSL
DQMPLFIWAVGITALLLLLSLPVLAGAITMLLTDRNLNTSFFDPAGGGDPILYQHL
>LEFIG821-10|HM876472|MM15685|Phiaris_heinrichana
TLYFIFGVWAGMIGTSLSLLIRAELGNPGSLIGDDQIYNTIVTAHAFIMIFFMVMPIMIGGFGNWLVPLMLGAPDMAFPRMNNMSFWLLPPSIMLLISSSIVENGAGTGWTVYPPLSSNIAHSGSSVDLAIFSLHLAGISSILGAVNFITTIINMRPNNMSL
DQMPLFVWSVGITALLLLFSLPVLAGAITMLLTDRNINTSFFDPAGGGDPILYQHL
>LEFIG822-10|HM876473|MM15686|Phiaris_heinrichana
TLYFIFGVWAGMIGTSLSLLIRAELGNPGSLIGDDQIYNTIVTAHAFIMIFFMVMPIMIGGFGNWLVPLMLGAPDMAFPRMNNMSFWLLPPSIMLLISSSIVENGAGTGWTVYPPLSSNIAHSGSSVDLAIFSLHLAGISSILGAVNFITTIINMRPNNMSL
DQMPLFVWSVGITALLLLLSLPVLAGAITMLLTDRNINTSFFDPAGGGDPILYQHL

>LEFIB905-10|HM871782|MM03009|Phiaris_metallicana
TLYFIFGVWSGMIGTSLSLLIRAELGNPGSLIGDDQIYNTIVTAHAFIMIFFMVMPIMIGGFGNWLVPLMLGAPDMAFPRMNNMSFWLLPPSIVLLISSSIVENGAGTGWTVYPPLSSNIAHSGSSVDLAIFSLHLAGISSILGAVNFITTIINMRPNNMSL
DQMPLFVWSVGITALLLLLSLPVLAGAITMLLTDRNLNTSFFDPAGGGDPILYQHL
>LEFIJ684-10|JF853771|MM17309|Phiaris_metallicana
TLYFIFGVWSGMIGTSLSLLIRAELGNPGSLIGDDQIYNTIVTAHAFIMIFFMVMPIMIGGFGNWLVPLMLGAPDMAFPRMNNMSFWLLPPSIVLLISSSIVENGAGTGWTVYPPLSSNIAHSGSSVDLAIFSLHLAGISSILGAVNFITTIINMRPNNMSL
DQMPLFVWSVGITALLLLLSLPVLAGAITMLLTDRNLNTSFFDPAGGGDPILYQHL
>LEFIC054-10|HM871923|MM03330|Phiaris_micana
TLYFIFGIWSGMIGTSLSLLIRAELGNPGSLIGNDQIYNTIVTAHAFIMIFFMVMPIMIGGFGNWLVPLMLGAPDMAFPRMNNMSFWLLPPSITLLISSSIVENGAGTGWTVYPPLSSNIAHSGSSVDLAIFSLHLAGISSILGAVNFITTIINMRPINMSLD
QMPLFIWSVGITALLLLLSLPVLAGAITMLLTDRNLNTSFFDPAGGGDPILYQHL
>LEFIA762-10|HM386905|MM04116|Phiaris_obsoletana
TLYFIFGVWSGMVGTSLSLLIRAELGNPGSLIGDDQIYNTIVTAHAFIMIFFMVMPIMIGGFGNWLVPLMLGAPDMAFPRMNNMSFWLLPPSIMLLISSSIVENGAGTGWTVYPPLSSNIAHSGSSVDLAIFSLHLAGISSILGAVNFITTIINMRPNNMS
LDQMPLFVWAVGITALLLLLSLPVLAGAITMLLTDRNLNTSFFDPAGGGDPILYQHL
>LEFIG820-10|HQ570411|MM15684|Phiaris_obsoletana
TLYFIFGVWSGMVGTSLSLLIRAELGNPGSLIGDDQIYNTIVTAHAFIMIFFMVMPIMIGGFGNWLVPLMLGAPDMAFPRMNNMSFWLLPPSIMLLISSSIVENGAGTGWTVYPPLSSNIAHSGSSVDLAIFSLHLAGISSILGAVNFITTIINMRPNNMS
LDQMSLFVWAVGITALLLLLSLPVLAGAITMLLTDRNLNTSFFDPAGGGDPILYQHL
>LEFIB485-10|HM871380|MM02052|Phiaris_palustrana
TLYFIFGIWAGMIGTSLSLLIRAELGNPGSLIGDDQIYNTIVTAHAFIMIFFMVMPIMIGGFGNWLVPLMLGAPDMAFPRMNNMSFWLLPPSIMLLISSSIVENGAGTGWTVYPPLSSNIAHSGSSVDLAIFSLHLAGISSILGAVNFITTIINMRPINMSLD
QMPLFIWSVGITALLLLLSLPVLAGAITMLLTDRNLNTSFFDPAGGGDPILYQHL
>LEFIC303-10|HM872147|MM03787|Phiaris_schulziana
TLYFIFGVWAGMIGTSLSLLIRAELGNPGSLIGDDQIYNTIVTAHAFIMIFFMVMPIMIGGFGNWLVPLMLGAPDMAFPRMNNMSFWLLPPSIMLLISSSIVENGAGTGWTVYPPLSSNIAHSGSSVDLAIFSLHLAGISSILGAVNFITTIINMRPNNMSL
DQMPLFVWSVGITALLLLLSLPVLAGAITMLLTDRNLNTSFFDPAGGGDPILYQHL
>LEFIA764-10|HM386907|MM04118|Phiaris_schulziana
TLYFIFGVWAGMIGTSLSLLIRAELGNPGSLIGDDQIYNTIVTAHAFIMIFFMVMPIMIGGFGNWLVPLMLGAPDMAFPRMNNMSFWLLPPSIMLLISSSIVENGAGTGWTVYPPLSSNIAHSGSSVDLAIFSLHLAGISSILGAVNFITTIINMRPNNMSL
DQMPLFVWSVGITALLLLLSLPVLAGAITMLLTDRNLNTSFFDPAGGGDPILYQHL
>LEFID318-10|HM873115|MM06203|Phiaris_schulziana
TLYFIFGVWAGMIGTSLSLLIRAELGNPGSLIGDDQIYNTIVTAHAFIMIFFMVMPIMIGGFGNWLVPLMLGAPDMAFPRMNNMSFWLLPPSIMLLISSSIVENGAGTGWTVYPPLSSNIAHSGSSVDLAIFSLHLAGISSILGAVNFITTIINMRPNNMSL
DQMPLFVWSVGITALLLLLSLPVLAGAITMLLTDRNLNTSFFDPAGGGDPILYQHL
>LEFID423-10|HM873219|MM06351|Phiaris_schulziana
TLYFIFGVWAGMIGTSLSLLIRAELGNPGSLIGDDQIYNTIVTAHAFIMIFFMVMPIMIGGFGNWLVPLMLGAPDMAFPRMNNMSFWLLPPSIMLLISSSIVENGAGTGWTVYPPLSSNIAHSGSSVDLAIFSLHLAGISSILGAVNFITTIINMRPNNMSL
DQMPLFVWSVGITALLLLLSLPVLAGAITMLLTDRNLNTSFFDPAGGGDPILYQHL
>LEFIJ122-10|JX034661|MM08475|Phiaris_schulziana
TLYFIFGVWAGMIGTSLSLLIRAELGNPGSLIGDDQIYNTIVTAHAFIMIFFMVMPIMIGGFGNWLVPLMLGAPDMAFPRMNNMSFWLLPPSIMLLISSSIVENGAGTGWTVYPPLSSNIAHSGSSVDLAIFSLHLAGISSILGAVNFITTIINMRPNNMSL
DQMPLFVWSVGITALLLLLSLPVLAGAITMLLTDRNLNTSFFDPAGGGDPILYQHL
>LEEUA232-11|JN274930|MM19640|Phiaris_schulziana
TLYFIFGVWAGMIGTSLSLLIRAELGNPGSLIGDDQIYNTIVTAHAFIMIFFMVMPIMIGGFGNWLVPLMLGAPDMAFPRMNNMSFWLLPPSIMLLISSSIVENGAGTGWTVYPPLSSNIAHSGSSVDLAIFSLHLAGISSILGAVNFITTIINMRPNNMSL
DQMPLFVWSVGITALLLLLSLPVLAGAITMLLTDRNLNTSFFDPAGGGDPILYQHL
>LEFIJ1183-11|KM573009|MM21043|Phiaris_schulziana
TLYFIFGVWAGMIGTSLSLLIRAELGNPGSLIGDDQIYNTIVTAHAFIMIFFMVMPIMIGGFGNWLVPLMLGAPDMAFPRMNNMSFWLLPPSIMLLISSSIVENGAGTGWTVYPPLSSNIAHSGSSVDLAIFSLHLAGISSILGAVNFITTIINMRPNNMSL
DQMPLFAWSVGITALLLLLSLPVLAGAITMLLTDRNLNTSFFDPAGGGDPILYQHL
>LEFID456-10|HM873227|MM06395|Phiaris_septentrionana
TLYFIFGVWAGMIGTSLSLLIRAELGNPGSLIGDDQIYNTIVTAHAFIMIFFMVMPIMIGGFGNWLVPLMLGAPDMAFPRMNNMSFWLLPPSIMLLISSSIVENGAGTGWTVYPPLSSNIAHSGSSVDLAIFSLHLAGISSILGAVNFITTIINMRPNNMSL
DQMPLFIWSVGITALLLLLSLPVLAGAITMLLTDRNLNTSFFDPAGGGDPILYQHL
>LEFIG823-10|HQ570412|MM15687|Phiaris_septentrionana
TLYFIFGVWAGMIGTSLSLLIRAELGNPGSLIGDDQIYNTIVTAHAFIMIFFMVMPIMIGGFGNWLVPLMLGAPDMAFPRMNNMSFWLLPPSIMLLISSSIVENGAGTGWTVYPPLSSNIAHSGSSVDLAIFSLHLAGISSILGAVNFITTIINMRPNNMSL
DQMPLFIWSVGITALLLLLSLPVLAGAITMLLTDRNLNTSFFDPAGGGDPILYQHL
>LEFIC018-10|HM871888|MM03228|Phiaris_turfosana
TLYFIFGVWAGMIGTSLSLLIRAELGNPGSLIGDDQIYNTIVTAHAFIMIFFMVMPIMIGGFGNWLVPLMLGAPDMAFPRMNNMSFWLLPPSIMLLISSSIVENGAGTGWTVYPPLSSNIAHSGSSVDLAIFSLHLAGISSILGAVNFITTIINMRPNNMSL

DQMPLFVWSVGITALLLLLSLPVLAGAITMLLTDRNLNTSFFDPAGGGDPILYQHL
>LEFIC028-10|HM871898|MM03249|Phiaris_umbrosana
TLYFIFGVWAGMVGTSLSLLIRAELGNPGSLIGDDQIYNTIVTAHAFIMIFFMVMPIMIGGFGNWLVPLMLGAPDMAFPRMNNMSFWLLPPSIMLLISSSIVENGAGTGWTVYPPLSSNIAHSGSSVDLAIFSLHLAGISSILGAVNFITTIINMRPNNMS
LDQMPLFVWAVGITALLLLLSLPVLAGAITMLLTDRNLNTSFFDPAGGGDPILYQHL
>LEFID157-10|HM872964|MM05920|Phibalapteryx_virgata
TLYFIFGIWAGMIGTSLSLLIRAELGTPGSLIGDDQIYNTIVTAHAFIMIFFMVMPIMIGGFGNWLVPLMLGAPDMAFPRMNNMSFWLLPPSIMLLISSSIVENGAGTGWTVYPPLSSNIAHGGSSVDLAIFSLHLAGISSILGAINFITTIINMRLNNMFFD
QLPLFVWSVGITAFLLLLSLPVLAGAITMLLTDRNLNTSFFDPAGGGDPILYQHL
>LEFIA420-10|HM386762|MM01482|Phigalia_pilosaria
TLYFIFGIWAGMVGTSLSLLIRAELGNPGSFIGDDQIYNTIVTAHAFIMIFFMVMPIMIGGFGNWLVPLMLGAPDMAFPRMNNMSFWLLPPSITLLISSSIVENGAGTGWTVYPPLSSNIAHGGSSVDLAIFSLHLAGISSILGAINFITTIINMRLNNMSF
DQMPLFVWSVGITAFLLLLSLPVLAGAITMLLTDRNLNTSFFDPAGGGDPILYQHL
>LEFID941-10|HM873691|MM07736|Phigalia_pilosaria
TLYFIFGIWAGMVGTSLSLLIRAELGNPGSFIGDDQIYNTIVTAHAFIMIFFMVMPIMIGGFGNWLVPLMLGAPDMAFPRMNNMSFWLLPPSITLLISSSIVENGAGTGWTVYPPLSSNIAHGGSSVDLAIFSLHLAGISSILGAINFITTIINMRLNNMSF
DQMPLFVWSVGITAFLLLLSLPVLAGAITMLLTDRNLNTSFFDPAGGGDPILYQHL
>LEFIG796-10|HM876448|MM15660|Philedone_gerningana
TLYFLFGIWAGLIGTSLSLLIRMELGNPGSLIGDDQIYNTIVTAHAFIMIFFMVMPIMIGGFGNWLVPLMLGAPDMAFPRMNNMSFWLLPPSILLLISSSIVENGAGTGWTVYPPLSSNIAHSGSSVDLAIFSLHLAGISSILGAINFITTIINMRPNNMSLD
QMPLFVWAVGITALLLLLSLPVLAGAITMLLTDRNLNTSFFDPAGGGDPILYQHL
>LEFIG797-10|HM876449|MM15661|Philedone_gerningana
TLYFLFGIWAGLIGTSLSLLIRMELGNPGSLIGDDQIYNTIVTAHAFIMIFFMVMPIMIGGFGNWLVPLMLGAPDMAFPRMNNMSFWLLPPSILLLISSSIVENGAGTGWTVYPPLSSNIAHSGSSVDLAIFSLHLAGISSILGAINFITTIINMRPNNMSLD
QMPLFVWAVGITALLLLLSLPVLAGAITMLLTDRNLNTSFFDPAGGGDPILYQHL
>LEFIC468-10|HM872301|MM04056|Philedonides_lunana
TLYFIFGIWAGMVGTSLSMLIRAELGNPGSLIGDDQIYNTIVTAHAFIMIFFMVMPIMIGGFGNWLVPLMLGAPDMAFPRMNNMSFWLLPPSIMLLISSSIVENGAGTGWTVYPPLSSNIAHSGSSVDLAIFSLHLAGISSILGAVNFITTIINMRPNNMT
LDQMPLFVWSVGITALLLLLSLPVLAGAITMLLTDRNLNTSFFDPAGGGDPILYQHL
>LEFIC986-10|HM872801|MM05393|Philedonides_lunana
TLYFIFGIWAGMVGTSLSMLIRAELGNPGSLIEDDQIYNTIVTAHAFIMIFFMVMPIMIGGFGNWLVPLMLGAPDMAFPRMNNMSFWLLPPSIMLLISSSIVENGAGTGWTVYPPLSSNIAHSGSSVDLAIFSLHLAGISSILGAVNFITTIINMRPNNMT
LDQMPLFVWSVGITALLLLLSLPVLAGAITMLLTDRNLNTSFFDPAGGGDPILYQHL
>LEFIE342-10|HM874066|MM08757|Philedonides_lunana
TLYFIFGIWAGMVGTSLSLLIRAELGNPGSLIGDDQIYNTIVTAHAFIMIFFMVMPIMIGGFGNWLVPLMLGAPDMAFPRMNNMSFWLLPPSITLLISSSIVENGAGTGWTVYPPLSSNIAHSGSSVDLAIFSLHLAGISSILGAVNFITTIINMRPNNMKL
DQMPLFVWSVGITALLLLLSLPVLAGAITMLLTDRNLNTSFFDPAGGGDPILYQHL
>LEFIK688-10|JF854254|MM18263|Philedonides_lunana
TLYFIFGIWAGMVGTSLSLLIRAELGNPGSLIGDDQIYNTIVTAHAFIMIFFMVMPIMIGGFGNWLVPLMLGAPDMAFPRMNNMSFWLLPPSIMLLISSSIVENGAGTGWTVYPPLSSNIAHSGSSVDLAIFSLHLAGISSILGAVNFITTIINMRPNNMTL
DQMPLFVWSVGITALLLLLSLPVLAGAITMLLTDRNLNTSFFDPAGGGDPILYQHL
>LEFIK689-10|JF854255|MM18264|Philedonides_lunana
TLYFIFGIWAGMVGTSLSLLIRAELGNPGSLIGNDQIYNTIVTAHAFIMIFFMVMPIMIGGFGNWLVPLMLGAPDMAFPRMNNMSFWLLPPSIMLLISSSIVENGVGTGWTVYPPLSSNIAHSGSSVDLAIFSLHLAGISSILGAVNFITTIINMRPNSMTL
DQMPLFVWSVGITALLLLLSLPVLAGAITMLLTDRNLNTSFFDPAGGGDPILYQHL
>LEFIJ972-11|MM21212|Philedonides_lunana
TLYFIFGIWAGMVGTSLSLLIRAELGNPGSLIGNDQIYNTIVTAHAFIMIFFMVMPIMIGGFGNWLVPLMLGAPDMAFPRMNNMSFWLLPPSIMLLISSSIVENGVGTGWTVYPPLSSNIAHSGSSVDLAIFSLHLAGISSILGAVNFITTIINMRPNNMTL
DQMPLFVWSVGITALLLLLSLPVLAGAITMLLTDRNLNTSFFDPAGGGDPILYQHL
>LEFIJ973-11|MM21213|Philedonides_lunana
TLYFIFGIWAGMVGTSLSMLIRAELGNPGSLIGDDQIYNTIVTAHAFIMIFFMVMPIMIGGFGNWLVPLMLGAPDMAFPRMNNMSFWLLPPSIMLLISSSIVENGAGTGWTVYPPLSSNIAHSGSSVDLAIFSLHLAGISSILGAVNFITTIINMRPNNM
ALDQMPLFVWSVGITALLLLLSLPVLAGAITMLLTDRNLNTSFFDPAGGGDPILYQHL
>LEFIE978-10|HM874695|MM10411|Philereme_transversata
TMYFIFGIWAGMIGTSLSLLIRAELGNPGSLIGDDQIYNTIVTAHAFIMIFFMVMPIMIGGFGNWLVPLMLGAPDMAFPRMNNMSFWLLPPSITLLISSSIVENGAGTGWTVYPPLSSNIAHGGSSVDLAIFSLHLAGISSILGAINFITTIINMRLNNMFF
DQLPLFVWAVGITAFLLLLSLPVLAGAITMLLTDRNLNTSFFDPAGGGDPILYQHL
>LEFIK874-10|KM572753|MM18449|Philereme_transversata
TMYFIFGIWAGMVGTSLSLLIRAELGNPGSLIGDDQIYNTIVTAHAFIMIFFMVMPIMIGGFGNWLVPLMLGAPDMAFPRMNNMSFWLLPPSITLLISSSIVENGAGTGWTVYPPLSSNIAHGGSSVDLAIFSLHLAGISSILGAINFITTIINMRLNNMFF
DQLPLFVWAVGITAFLLLLSLPVLAGAITMLLTDRNLNTSFFDPAGGGDPILYQHL
>LEFIA903-10|HM387039|MM09781|Philereme_vetulata

TMYFIFGIWAGMIGTSLSLLIRAELGNPGSLIGDDQIYNTIVTAHAFIMIFFMVMPIMIGGFGNWLVPLMLGAPDMAFPRMNNMSFWLLPPSITLLISSSIVENGAGTGWTVYPPLSSNIAHGGSSVDLAIFSLHLAGISSILGAINFITTIINMRLNNMFF
DQLPLFVWAVGITAFLLLLSLPVLAGAITMLLTDRNLNTSFFDPAGGGDPILYQHL
>LEFIA904-10|HM387040|MM09782|Philereme_vetulata
TMYFIFGIWAGMIGTSLSLLIRAELGNPGSLIGDDQIYNTIVTAHAFIMIFFMVMPIMIGGFGNWLVPLMLGAPDMAFPRMNNMSFWLLPPSITLLISSSIVENGAGTGWTVYPPLSSNIAHGGSSVDLAIFSLHLAGISSILGAINFITTIINMRLNNMFF
DQLPLFVWAVGITAFLLLLSLPVLAGAITMLLTDRNLNTSFFDPAGGGDPILYQHL
>LEFIK873-10|KM572560|MM18448|Philereme_vetulata
TMYFIFGIWAGMIGTSLSLLIRAELGNPGSLIGDDQIYNTIVTAHAFIMIFFMVMPIMIGGFGNWLVPLMLGAPDMAFPRMNNMSFWLLPPSITLLISSSIVENGAGTGWTVYPPLSSNIAHGGSSVDLAIFSLHLAGISSILGAINFITTIINMRLNNMFF
DQLPLFVWAVGITAFLLLLSLPVLAGAITMLLTDRNLNTSFFDPAGGGDPILYQHL
>LEFIG410-10|HM876086|MM14436|Phlogophora_meticulosa
TLYFIFGIWAGMVGTSLSLLIRAELGNPGSLIGDDQIYNTIVTAHAFIMIFFMVMPIMIGGFGNWLVPLMLGAPDMAFPRMNNMSFWLLPPSLTLLISSSIVESGAGTGWTVYPPLSSNIAHGGSSVDLAIFSLHLAGISSILGAINFITTIINMRLNNLSFD
QMPLFIWAVGITAFLLLLSLPVLAGAITMLLTDRNLNTSFFDPAGGGDPILYQHL
>LEFIB633-10|HM871512|MM02389|Photedes_captiuncula
TLYFIFGIWAGMVGTSLSLLIRAELGNPGSLIGDDQIYNTIVTAHAFIMIFFMVMPIMIGGFGNWLVPLMLGAPDMAFPRMNNMSFWLLPPSLTLLISSSIVENGAGTGWTVYPPLSSNIAHGGSSVDLAIFSLHLAGISSILGAINFITTIINMRLNNLSFD
QMPLFIWAVGITAFLLLLSLPVLAGAITMLLTDRNLNTSFFDPAGGGDPILYQHL
>LEFIK452-10|JF854073|MM18027|Photedes_captiuncula
TLYFIFGIWAGMVGTSLSLLIRAELGNPGSLIGDDQIYNTIVTAHAFIMIFFMVMPIMIGGFGNWLVPLMLGAPDMAFPRMNNMSFWLLPPSLTLLISSSIVENGAGTGWTVYPPLSSNIAHGGSSVDLAIFSLHLAGISSILGAINFITTIINMRLNNLSFD
QMPLFIWAVGITAFLLLLSLPVLAGAITMLLTDRNLNTSFFDPAGGGDPILYQHL
>LEFIK453-10|MM18028|Photedes_captiuncula
TLYFIFGIWAGMVGTSLSLLIRAELGNPGSLIGDDQIYNTIVTAHAFIMIFFMVMPIMIGGFGNWLVPLMLGAPDMAFPRMNNMSFWLLPPSLTLLISSSIVENGAGTGWTVYPPLSSNIAHGGSSVDLAIFSLHLTGISSILGAINFITTIINMRLNNLSFD
QMPLFIWAVGITAFLLLLSLPVLAGAITMLLTDRNLNTSFFDPAGGGDPILYQHL
>LEFIC340-10|HM872184|MM03847|Photedes_extrema
TLYFIFGIWAGMVGTSLSLLIRAELGTPGSLIGDDQIYNTIVTAHAFIMIFFMVMPIMIGGFGNWLVPLMLGAPDMAFPRMNNMSFWLLPPSMTLLISSSIVENGAGTGWTVYPPLSSNIAHGGSSVDLAIFSLHLAGISSILGAINFITTIINMRLNSLSFD
QMPLFIWAVGITAFLLLLSLPVLAGAITMLLTDRNLNTSFFDPAGGGDPILYQHL
>LEFIE568-10|HM874291|MM09457|Photedes_extrema
TLYFIFGIWAGMVGTSLSLLIRAELGTPGSLIGDDQIYNTIVTAHAFIMIFFMVMPIMIGGFGNWLVPLMLGAPDMAFPRMNNMSFWLLPPSLTLLISSSIVENGAGTGWTVYPPLSSNIAHGGSSVDLAIFSLHLAGISSILGAINFITTIINMRLNSLSFD
QMPLFIWAVGITAFLLLLSLPVLAGAITMLLTDRNLNTSFFDPAGGGDPILYQHL
>LEFIA539-10|KM572669|MM01626|Photedes_fluxa
TLYFIFGIWAGMVGTSLSLLIRAELGNPGSLIGDDQIYNTIVTAHAFIMIFFMVMPIMIGGFGNWLVPLMLGAPDMAFPRMNNMSFWLLPPSLTLLISSSIVENGAGTGWTVYPPLSSNIAHGGSSVDLAIFSLHLAGISSILGAINFITTIINMRLNNLSFD
QMPLFIWAVGITAFLLLLSLPVLAGAITMLLTDRNLNTSFFDPAGGGDPILYQHL
>LEFIA540-10|KM573434|MM01627|Photedes_fluxa
TLYFIFGIWAGMVGTSLSLLIRAELGNPGSLIGDDQIYNTIVTAHAFIMIFFMVMPIMIGGFGNWLVPLMLGAPDMAFPRMNNMSFWLLPPSLTLLISSSIVENGAGTGWTVYPPLSSNIAHGGSSVDLAIFSLHLAGISSILGAINFITTIINMRLNNLSFD
QMPLFIWAVGITAFLLLLSLPVLAGAITMLLTDRNLNTSFFDPAGGGDPILYQHL
>LEFIK454-10|MM18029|Photedes_minima
TLYFIFGIWAGMMGTSLSLLIRAELGNPGSLIGDDQIYNTIVTAHAFIMIFFMVMPIMIGGFGNWLVPLMLGAPDMAFPRMNNMSFWLLPPSLTLLISSSIVENGAGTGWTVYPPLSSNIAHGGSSVDLAIFSLHLAGISSILGAINFITTIINMRLNNLSF
DQMPLFIWAVGITAFLLLLSLPVLAGAITMLLTDRNLNTSFFDPAGGGDPILYQHL
>LEFIF226-10|HM874919|MM11006|Phragmataecia_castaneae
TLYFILGIWSGMVGTSLSLLIRAELGTPGFLIGDDQIYNTIVTAHAFIMIFFMVMPIMIGGFGNWLVPLMLGAPDMAFPRMNNMSFWLLPPSLMLLISSSIVENGAGTGWTIYPPLSANIAHGGSSVDLAIFSLHLAGISSILGAINFITTIINMRPNKMSFD
QMPLFVWAVGITALLLLLSLPVLAGAITMLLTDRNLNTSFFDPAGGGDPILYQHL
>LEFIJ1023-11|MM13489|Phragmatiphila_nexa
TLYFIFGIWAGMVGTSLSLLIRAELGTPGSLIGDDQIYNTIVTAHAFIMIFFMVMPIMIGGFGNWLIPLMLGAPDMAFPRMNNMSFWLLPPSLTLLISSSIVENGAGTGWTVYPPLSSNIAHGGSSVDLAIFSLHLAGISSILGAINFITTIINMRLNNLSFDQ
MPLFIWAVGITAFLLLLSLPVLAGAITMLLTDRNLNTSFFDPAGGGDPILYQHL
>LEFIB218-10|HM871122|MM00672|Phragmatobia_fuliginosa
TLYFIFGIWAGMVGTSLSLLIRAELGTPGSLIGDDQIYNTIVTAHAFIMIFFMVMPIMIGGFGNWLIPLMLGAPDMAFPRMNNMSFWLLPPSLTLLISSSIVENGAGTGWTVYPPLSSNIAHGGSSVDLAIFSLHLAGISSILGAINFITTIINMRLNNMSFD
QMPLFVWAVGITAFLLLLSLPVLAGAITMLLTDRNLNTSFFDPAGGGDPILYQHL
>LEFIC784-10|HM872603|MM04920|Phragmatobia_fuliginosa
TLYFIFGIWAGMVGTSLSLLIRAELGTPGSLIGDDQIYNTIVTAHAFIMIFFMVMPIMIGGFGNWLIPLMLGAPDMAFPRMNNMSFWLLPPSLTLLISSSIVENGAGTGWTVYPPLSSNIAHGGSSVDLAIFSLHLAGISSILGAINFITTIINMRLNNMSFD
QMPLFVWAVGITAFLLLLSLPVLAGAITMLLTDRNLNTSFFDPAGGGDPILYQHL

>LEFID113-10|HM872924|MM05751|Phragmatobia_luctifera
TLYFIFGIWAGMVGTSLSLLIRAELGNPGSLIGDDQIYNTIVTAHAFIMIFFMVMPIMIGGFGNWLVPLMLGAPDMAFPRMNNMSFWLLPPSLTLLISSSIVENGAGTGWTVYPPLSSNIAHGGSSVDLAIFSLHLAGISSILGAINFITTIINMRLSNLSFD
QMPLFVWSVGITAFLLLLSLPVLAGAITMLLTDRNLNTSFFDPAGGGDPILYQHL
>LEFIB517-10|HQ570268|MM02117|Phtheochroa_inopiana
TLYFIFGIWAGMVGTSLSLLIRAELGNPGSLIGDDQIYNTIVTAHAFIMIFFMVMPIMIGGFGNWLVPLMLGAPDMAFPRMNNMSFWLLPPSIMLLISSSIVENGAGTGWTVYPPLSSNIAHSGSSVDLAIFSLHLAGISSILGAVNFITTIINMRPNNMKL
DQMPLFVWAVGITALLLLLSLPVLAGAITMLLTDRNLNTSFFDPAGGGDPILYQHL
>LEFIK176-10|MM17751|Phtheochroa_sodaliana
TLYFIFG?WAGMIGTSLSLLIRAELGSPGSLIGDDQIYNTIVTAHAFIMIFFMVMPIMIGGFGNWLVPLMLGAPDMAFPRMNNMSFWLLPPSIMLLISSSIVENGAGTGWTVYPPLSSNIAHSGSSVDLAIFSLHLAGISSILGAVNFITTIINMRPNNMSL
DQMPLFVWAVGITALLLLLSLPVLAGAITMLLTDRNLNTSFFDPAGGGDPILYQHL
>LEFIK177-10|JQ775270|MM17752|Phtheochroa_sodaliana
TLYFIFGIWAGMIGTSLSLLIRAELGSPGSLIGDDQIYNTIVTAHAFIMIFFMVMPIMIGGFGNWLVPLMLGAPDMAFPRMNNMSFWLLPPSIMLLISSSIVENGAGTGWTVYPPLSSNIAHSGSSVDLAIFSLHLAGISSILGAVNFITTIINMRPNNMSL
DQMPLFVWAVGITALLLLLSLPVLAGAITMLLTDRNLNTSFFDPAGGGDPILYQHL
>LEFIA808-10|HM386949|MM04162|Phtheochroa_vulneratana
TLYFIFGIWSGMIGTSLSLLIRAELGNPGSLIGDDQIYNTIVTAHAFIMIFFMVMPIMIGGFGNWLIPLMLGAPDMAFPRMNNMSFWLLPPSIMLLISSSIVENGAGTGWTVYPPLSSNIAHSGSSVDLAIFSLHLAGISSILGAVNFITTIINMRPNNMSLD
QMPLFVWAVGITALLLLLSLPVLAGAITMLLTDRNLNTSFFDPAGGGDPILYQHL
>LEFID232-10|HM873032|MM06092|Phtheochroa_vulneratana
TLYFIFGIWSGMIGTSLSLLIRAELGNPGSLIGDDQIYNTIVTAHAFIMIFFMVMPIMIGGFGNWLVPLMLGAPDMAFPRMNNMSFWLLPPSIMLLISSSIVENGAGTGWTVYPPLSSNIAHSGSSVDLAIFSLHLAGISSILGAVNFITTIINMRPNNMSL
DQMPLFVWAVGITALLLLLSLPVLAGAITMLLTDRNLNTSFFDPAGGGDPILYQHL
>LEFIC797-10|HM872616|MM04952|Phycita_roborella
TLYFIFGIWSGMVGTSLSLLIRAELGTPGSLIGDDQIYNTIVTGHAFIMIFFMVMPIMIGGFGNWLVPLMLGAPDMAFPRMNNMSFWLLPPSLTLLISSSIVENGAGTGWTVYPPLSSNIAHGGSSVDLAIFSLHLAGISSILGAINFITTIINMKLNGLSFD
QMPLFVWAVGITALLLLLSLPVLAGAITMLLTDRNLNTSFFDPAGGGDPILYQHL
>LEFIC892-10|HM872709|MM05197|Phycita_roborella
TLYFIFGIWSGMVGTSLSLLIRAELGTPGSLIGDDQIYNTIVTGHAFIMIFFMVMPIMIGGFGNWLVPLMLGAPDMAFPRMNNMSFWLLPPSLTLLISSSIVENGAGTGWTVYPPLSSNIAHGGSSVDLAIFSLHLAGISSILGAINFITTIINMKLNGLSFD
QMPLFVWAVGITALLLLLSLPVLAGAITMLLTDRNLNTSFFDPAGGGDPILYQHL
>LEFIC188-10|HM872032|MM03547|Phycitodes_albatella
TLYFIFGIWSGMVGTSLSLLIRAELGTPGSLIGDDQIYNTIVTGHAFIMIFFMVMPIMIGGFGNWLIPLMLGAPDMAFPRMNNMSFWLLPPSLTLLISSSIVENGAGTGWTVYPPLSSNIAHGGSSVDLAIFSLHLAGISSILGAINFITTIINMKLNGMSFD
QMPLFVWAVGITALLLLLSLPVLAGAITMLLTDRNLNTSFFDPAGGGDPILYQHL
>LEFIC374-10|HM872217|MM03904|Phycitodes_albatella
TLYFIFGIWSGMVGTSLSLLIRAELGTPGSLIGDDQIYNTIVTGHAFIMIFFMVMPIMIGGFGNWLIPLMLGAPDMAFPRMNNMSFWLLPPSLTLLISSSIVENGAGTGWTVYPPLSSNIAHGGSSVDLAIFSLHLAGISSILGAINFITTIINMKLNGMSFD
QMPLFVWAVGITALLLLLSLPVLAGAITMLLTDRNLNTSFFDPAGGGDPILYQHL
>LEFIF455-10|HM875140|MM11945|Phycitodes_albatella
TLYFIFGIWSGMVGTSLSLLIRAELGTPGSLIGDDQIYNTIVTSHAFIMIFFMVMPIMIGGFGNWLIPLMLGAPDMAFPRMNNMSFWLLPPSLTLLISSSIVENGAGTGWTVYPPLSSNIAHGGSSVDLAIFSLHLAGISSILGAINFITTIINMKLNGMSFD
QMPLFVWAVGITALLLLLSLPVLAGAITMLLTDRNLNTSFFDPAGGGDPILYQHL
>LEFIJ791-10|MM17416|Phycitodes_albatella
TLYFIFGIWSGMVGTSLSLLIRAELGTPGSLIGDDQIYNTIVTGHAFIMIFFMVMPIMIGGFGNWLIPLMLGAPDMAFPRMNNMSFWLLPPSLTLLISSSIVENGAGTGWTVYPPLSSNIAHGGSSVDLAIFSLHLAGISSILGAINFITTIINMKLNGMSFD
QMPLFVWAVGITALLLLLSLPVLAGAITMLLTDRNLNTSFFDPAGGGDPILYQHL
>LEFIB660-10|HM871538|MM02437|Phycitodes_binaevella
TLYFIFGIWSGMVGTSLSLLIRAELGTPGSLIGDDQIYNTIVTGHAFIMIFFMVMPIMIGGFGNWLVPLMLGAPDMAFPRMNNMSFWLLPPSLTLLISSSIVENGAGTGWTVYPPLSSNIAHSGSSVDLAIFSLHLAGISSILGAINFITTIINMKLNGMSFD
QMPLFVWAVGITALLLLLSLPVLAGAITMLLTDRNLNTSFFDPAGGGDPILYQHL
>LEFIK812-10|JN275981|MM18387|Phycitodes_binaevella
TLYFIFGIWSGMVGTSLSLLIRAELGTPGSLIGDDQIYNTIVTGHAFIMIFFMVMPIMIGGFGNWLVPLMLGAPDMAFPRMNNMSFWLLPPSLTLLISSSIVENGAGTGWTVYPPLSSNIAHSGSSVDLAIFSLHLAGISSILGAINFITTIINMKLNGMSFD
QMPLFVWAVGITALLLLLSLPVLAGAITMLLTDRNLNTSFFDPAGGGDPILYQHL
>LEFIB262-10|HM871165|MM00736|Phycitodes_lacteella
TLYFIFGIWSGMMGTSLSLLIRAELGTPGSLIGDDQIYNTIVTGHAFIMIFFMVMPIMIGGFGNWLVPLMLGAPDMAFPRMNNMSFWLLPPSLTLLISSSIVENGAGTGWTVYPPLSSNIAHGGSSVDLAIFSLHLAGISSILGAINFITTIINMKLNGMSF
DQMPLFVWAVGITALLLLLSLPVLAGAITMLLTDRNLNTSFFDPAGGGDPILYQHL
>LEFIG219-10|HM875899|MM14111|Phycitodes_maritima
TLYFIFGIWSGMVGTSLSLLIRAELGTPGSLIGDDQIYNTIVTGHAFIMIFFMVMPIMIGGFGNWLVPLMLGAPDMAFPRMNNMSFWLLPPSLTLLISSSIVENGAGTGWTVYPPLSSNIAHSGSSVDLAIFSLHLAGISSILGAINFITTIINMKLNGMSFD

QMPLFVWAVGITALLLLLSLPVLAGAITMLLTDRNLNTSFFDPAGGGDPILYQHL
>LEFIJ718-10|JF853790|MM17343|Phycitodes_maritima
TLYFIFGIWSGMVGTSLSLLIRAELGTPGSLIGDDQIYNTIVTGHAFIMIFFMVMPIMIGGFGNWLVPLMLGAPDMAFPRMNNMSFWLLPPSLTLLISSSIVENGAGTGWTVYPPLSSNIAHGGSSVDLAIFSLHLAGISSILGAINFITTIINMKLNGMSFD
QMPLFVWAVGITALLLLLSLPVLAGAITMLLTDRNLNTSFFDPAGGGDPILYQHL
>LEFIJ719-10|MM17344|Phycitodes_maritima
TLYFIFGIWSGMVGTSLSLLIRAELGTPGSLIGDDQIYNTIVTGHAFIMIFFMVMPIMIGGFGNWLVPLMLGAPDMAFPRMNNMSFWLLPPSLTLLISSSIVENGAGTGWTVYPPLSSNIAHSGSSVDLAIFSLHLAGISSILGAINFITTIINMKLNGMSFD
QMPLFVWAVGITALLLLLSLPVLAGAITMLLTDRNLNTSFFDPAGGGDPILYQHL
>LEFIC235-10|HM872079|MM03635|Phycitodes_saxicola
TLYFIFGIWSGMVGTSLSLLIRAELGTPGSLIGDDQIYNTIVTGHAFIMIFFMVMPIMIGGFGNWLIPLMLGAPDMAFPRMNNMSFWLLPPSLTLLISSSIVENGAGTGWTVYPPLSSNIAHGGSSVDLAIFSLHLAGISSILGAINFITTIINMKLNGMSFD
QMPLFVWAVGITALLLLLSLPVLAGAITMLLTDRNLNTSFFDPAGGGDPILYQHL
>LEFID005-10|HM872819|MM05425|Phyllocnistis_labyrinthella
TLYFLFGIWSGMVGTSLSLLIRMELGNPGSLIGDDQIYNTIVTAHAFIMIFFMVMPMMIGGFGNWLVPIMLGAPDMAFPRLNNMSFWLLPPSLLLLISSSLVENGAGTGWTVYPPLSSNIAHGGSSVDLAIFSLHLAGISSILGAINFITTIINMRINKMTL
DSMSLFVWSVGITALLLLLSLPVLAGAITMLLTDRNLNTSFFDPAGGGDPILYQHL
>LEFID017-10|HM872831|MM05443|Phyllocnistis_labyrinthella
TLYFLFGIWSGMVGTSLSLLIRMELGNPGSLIGDDQIYNTIVTAHAFIMIFFMVMPMMIGGFGNWLVPIMLGAPDMAFPRLNNMSFWLLPPSLLLLISSSLVENGAGTGWTVYPPLSSNIAHGGSSVDLAIFSLHLAGISSILGAINFITTIINMRINKMTL
DSMSLFVWSVGITALLLLLSLPVLAGAITMLLTDRNLNTSFFDPAGGGDPILYQHL
>LEFIG124-10|HM875804|MM13931|Phyllocnistis_labyrinthella
TLYFLFGIWSGMVGTSLSLLIRMELGNPGSLIGDDQIYNTIVTAHAFIMIFFMVMPMMIGGFGNWLVPIMLGAPDMAFPRLNNMSFWLLPPSLLLLISSSLVENGAGTGWTVYPPLSSNIAHGGSSVDLAIFSLHLAGISSILGAINFITTIINMRINKMTL
DSMSLFVWSVGITALLLLLSLPVLAGAITMLLTDRNLNTSFFDPAGGGDPILYQHL
>LEFIG125-10|HM875805|MM13932|Phyllocnistis_labyrinthella
TLYFLFGIWSGMVGTSLSLLIRMELGNPGSLIGDDQIYNTIVTAHAFIMIFFMVMPMMIGGFGNWLVPIMLGAPDMAFPRLNNMSFWLLPPSLLLLISSSLVENGAGTGWTVYPPLSSNIAHGGSSVDLAIFSLHLAGISSILGAINFITTIINMRINKMTL
DSMSLFVWSVGITALLLLLSLPVLAGAITMLLTDRNLNTSFFDPAGGGDPILYQHL
>LEEUA071-11|JN276187|MM19384|Phyllocnistis_labyrinthella
TLYFLFGIWSGMVGTSLSLLIRMELGNPGSLIGDDQIYNTIVTAHAFIMIFFMVMPMMIGGFGNWLVPIMLGAPDMAFPRLNNMSFWLLPPSLLLLISSSLVENGAGTGWTVYPPLSSNIAHGGSSVDLAIFSLHLAGISSILGAINFITTIINMRINKMTL
DSMSLFVWSVGITALLLLLSLPVLAGAITMLLTDRNLNTSFFDPAGGGDPILYQHL
>LEFII240-11|MM19890|Phyllocnistis_labyrinthella
TLYFLFGIWSGMVGTSLSLLIRMELGNPGSLIGDDQIYNTIVTAHAFIMIFFMVMPMMIGGFGNWLVPIMLGAPDMAFPRLNNMSFWLLPPSLLLLISSSLVENGAGTGWTVYPPLSSNIAHGGSSVDLAIFSLHLAGISSILGAINFITTIINMRINKMTL
DSMSLFVWSVGITALLLLLSLSVLAGAITMLLTDRNLNTSFFDPAGGGDPILYQHL
>LEFIJ1468-12|MM22736|Phyllocnistis_saligna
TLYFLFGIWSGMVGTSLSLLIRMELGNPGSLIGDDQIYNTIVTAHAFIMIFFMVMPMMIGGFGNWLVPIMLGAPDMAFPRLNNMSFWLLPPSLLLLISSSLVENGAGTGWTVYPPLSSNIAHGGSSVDLAIFSLHLAGISSILGAINFITTIINMRINKMTL
DSMSLFVWSVGITALLLLLSLPVLAGAITMLLTDRNLNTSFFDPAGGGDPILYQHL
>LEFIK524-10|JF854121|MM18099|Phyllocnistis_unipunctella
TLYFLFGIWSGMVGTSLSLLIRMELGNPGSLIGDDQIYNTIVTAHAFIMIFFMVMPMMIGGFGNWLVPIMLGAPDMAFPRLNNMSFWLLPPSLLLLISSSLVENGAGTGWTVYPPLSSNIAHGGSSVDLAIFSLHLAGISSILGAINFITTIINMRINKMTL
DSMSLFVWSVGITALLLLLSLPVLAGAITMLLTDRNLNTSFFDPAGGGDPILYQHL
>LEFIK525-10|JF854122|MM18100|Phyllocnistis_unipunctella
TLYFLFGIWSGMVGTSLSLLIRMELGNPGSLIGDDQIYNTIVTAHAFIMIFFMVMPMMIGGFGNWLVPIMLGAPDMAFPRLNNMSFWLLPPSLLLLISSSLVENGAGTGWTVYPPLSSNIAHGGSSVDLAIFSLHLAGISSILGAINFITTIINMRINKMTL
DSMSLFVWSVGITALLLLLSLPVLAGAITMLLTDRNLNTSFFDPAGGGDPILYQHL
>LEFIC466-10|HM872299|MM04052|Phyllodesma_ilicifolia
TLYFIFGIWAGMVGTSLSLLIRAELGTPGSLIGDDQIYNTIVTAHAFIMIFFMVMPIMIGGFGNWLVPLMLGAPDMAFPRMNNMSFWLLPPSLTLLISSSIVENGAGTGWTVYPPLSSNIAHSGSSVDLAIFSLHLAGISSILGAINFITTIINMRLNNMSFD
QMPLFVWAVGITAFLLLLSLPVLAGAITMLLTDRNLNTSFFDPAGGGDPILYQHL
>LEFID940-10|HM873690|MM07733|Phyllodesma_ilicifolia
TLYFIFGIWAGMVGTSLSLLIRAELGTPGSLIGDDQIYNTIVTAHAFIMIFFMVMPIMIGGFGNWLVPLMLGAPDMAFPRMNNMSFWLLPPSLTLLISSSIVENGAGTGWTVYPPLSSNIAHSGSSVDLAIFSLHLAGISSILGAINFITTIINMRLNNMSFD
QMPLFVWAVGITAFLLLLSLPVLAGAITMLLTDRNLNTSFFDPAGGGDPILYQHL
>LEFIJ116-10|MM08299|Phyllodesma_ilicifolia
TLYFIFGIWAGMVGTSLSLLIRAELGTPGSLIGDDQIYNTIVTAHAFIMIFFMVMPIMIGGFGNWLVPLMLGAPDMAFPRMNNMSFWLLPPSLTLLISSSIVENGAGTGWTVYPPLSSNIAHSGSSVDLAIFSLHLAGISSILGAINFITTIINMRLNNMSFD
QMPLFVWAVGITAFLLLLSLPVLAGAITMLLTDRNLNTSFFDPAGGGDPILYQHL
>LEFIE165-10|HM873911|MM08420|Phyllodesma_ilicifolia

TLYFIFGIWAGMVGTSLSLLIRAELGTPGSLIGDDQIYNTIVTAHAFIMIFFMVMPIMIGGFGNWLVPLMLGAPDMAFPRMNNMSFWLLPPSLTLLISSSIVENGAGTGWTVYPPLSSNIAHSGSSVDLAIFSLHLAGISSILGAINFITTIINMRLNNMSFD
QMPLFVWAVGITAFLLLLSLPVLAGAITMLLTDRNLNTSFFDPAGGGDPILYQHL
>LEFID120-10|HM872930|MM05854|Phyllodesma_japonica
TLYFIFGIWAGMVGTSLSLLIRAELGTPGSLIGDDQIYNTIVTAHAFIMIFFMVMPIMIGGFGNWLVPLMLGAPDMAFPRMNNMSFWLLPPSLTLLISSSIVENGAGTGWTVYPPLSSNIAHSGSSVDLAIFSLHLAGISSILGAINFITTIINMRLNNMSFD
QMPLFVWAVGITAFLLLLSLPVLAGAITMLLTDRNLNTSFFDPAGGGDPILYQHL
>LEFIG186-10|HM875866|MM14054|Phyllonorycter_anderidae
TLYFIFGIWAGMIGSSLSIMIRAELGTPGSLIGDDQIYNTIVTAHAFIMIFFMVMPIMIGGFGNWLVPLMLGAPDMAFPRLNNMSFWLLPPSILLLISSSVVENGVGTGWTVYPPLSSNIAHGGGSVDLAIFSLHLAGISSILGAINFITTIINMRANGMKFD
NMPLFVWAVGITALLLLLSLPVLAGAITMLLTDRNLNTSFFDPAGGGDPILYQHL
>LEFIG675-10|HM876335|MM15539|Phyllonorycter_anderidae
TLYFIFGIWAGMIGSSLSIMIRAELGTPGSLIGDDQIYNTIVTAHAFIMIFFMVMPIMIGGFGNWLVPLMLGAPDMAFPRLNNMSFWLLPPSILLLISSSVIENGVGTGWTVYPPLSSNIAHGGGSVDLAIFSLHLAGISSILGAINFITTIINMRANGMKFD
NMPLFVWAVGITALLLLLSLPVLAGAITMLLTDRNLNTSFFDPAGGGDPILYQHL
>LEFIK521-10|JF854118|MM18096|Phyllonorycter_anderidae
TLYFIFGIWAGMIGSSLSIMIRAELGTPGSLIGDDQIYNTIVTAHAFIMIFFMVMPIMIGGFGNWLVPLMLGAPDMAFPRLNNMSFWLLPPSILLLISSSVVENGVGTGWTVYPPLSSNIAHGGGSVDLAIFSLHLAGISSILGAINFITTIINMRANGMKFD
NMPLFVWAVGITALLLLLSLPVLAGAITMLLTDRNLNTSFFDPAGGGDPILYQHL
>LEFIL348-10|JN280378|MM18658|Phyllonorycter_anderidae
TLYFIFGIWAGMIGSSLSIMIRAELGTPGSLIGDDQIYNTIVTAHAFIMIFFMVMPIMIGGFGNWLVPLMLGAPDMAFPRLNNMSFWLLPPSILLLISSSVVENGVGTGWTVYPPLSSNIAHGGGSVDLAIFSLHLAGISSILGAINFITTIINMRANGMKFD
NMPLFVWAVGITALLLLLSLPVLAGAITMLLTDRNLNTSFFDPAGGGDPILYQHL
>LEFIE919-10|HM874636|MM10285|Phyllonorycter_apparella
TLYFIFGIWAGMVGSSLSIMIRAELGNPGSLIGDDQIYNTIVTAHAFIMIFFMVMPIMIGGFGNWLVPLMLGAPDMAFPRLNNMSFWLLPPSILLLISSSIVENGAGTGWTVYPPLSSNVAHGGSSVDLAIFSLHLAGISSILGAINFITTIINMRANGMMF
DNMPLFVWAVGITALLLLLSLPVLAGAITMLLTDRNLNTSFFDPAGGGDPILYQHL
>LEFIF332-10|HM875017|MM11138|Phyllonorycter_blancardella
TLYFIFGIWAGMIGSSLSIMIRAELGNPGSLIGDDQIYNTIVTAHAFIMIFFMVMPIMIGGFGNWLVPLMLGAPDMAFPRLNNMSFWLLPPSILLLISSSVVENGAGTGWTVYPPLSSNIAHGGSSVDLAIFSLHLAGISSILGAINFITTIINMRTNGMKFD
NMPLFVWAVGITALLLLLSLPVLAGAITMLLTDRNLNTSFFDPAGGGDPILYQHL
>LEFII155-10|JF853368|MM18582|Phyllonorycter_blancardella
TLYFIFGIWAGMIGSSLSIMIRAELGNPGSLIGDDQIYNTIVTAHAFIMIFFMVMPIMIGGFGNWLVPLMLGAPDMAFPRLNNMSFWLLPPSILLLISSSVVENGAGTGWTVYPPLSSNIAHGGSSVDLAIFSLHLAGISSILGAINFITTIINMRTNGMKFD
NMPLFVWAVGITALLLLLSLPVLAGAITMLLTDRNLNTSFFDPAGGGDPILYQHL
>LEFIB164-10|HM871068|MM00555|Phyllonorycter_cavella
TLYFIFGIWAGMIGSSLSIMIRAELGNPGSLIGDDQIYNTIVTAHAFIMIFFMVMPIMIGGFGNWLVPLMLGAPDMAFPRLNNMSFWLLPPSILLLISSSIVENGAGTGWTVYPPLSSNIAHGGSSVDLAIFSLHLAGISSILGAINFITTIINMRTNGMKFD
NMPLFVWAVGITALLLLLSLPVLAGAITMLLTDRNLNTSFFDPAGGGDPILYQHL
>LEFIF334-10|HM875019|MM11141|Phyllonorycter_cerasicolella
TLYFIFGIWAGMIGSSLSIMIRAELGNPGSLIGDDQIYNTIVTAHAFIMIFFMVMPIMIGGFGNWLVPLMLGAPDMAFPRLNNMSFWLLPPSILLLISSSVVENGVGTGWTVYPPLSSNIAHGGSSVDLAIFSLHLAGISSILGAINFITTIINMRTNGMKFD
NMPLFVWAVGITALLLLLSLPVLAGAITMLLTDRNLNTSFFDPAGGGDPILYQHL
>LEFIC170-10|HM872016|MM03522|Phyllonorycter_connexella
TLYFIFGIWAGMIGSSLSIMIRAELGNPGSLIGDDQIYNTIVTAHAFIMIFFMVMPIMIGGFGNWLVPLMLGAPDMAFPRLNNMSFWLLPPSMLLLISSSIVENGAGTGWTVYPPLSSNIAHAGSSVDLTIFSLHLAGISSILGAINFITTIINMRTNGMMF
DNMPLFVWAVGITALLLLLSLPVLAGAITMLLTDRNLNTSFFDPAGGGDPILYQHL
>LEFID875-10|HM873632|MM07011|Phyllonorycter_coryli
TLYFIFGIWAGMIGSSLSLMIRAELGNPGSLIGDDQIYNTIVTAHAFIMIFFMVMPIMIGGFGNWLVPLMLGAPDMAFPRLNNMSFWLLPPSILLLISSSVVENGVGTGWTVYPPLSSNIAHGGSSVDLAIFSLHLAGISSILGAINFITTIINMRTNGMKFD
NMPLFVWAVGITALLLLLSLPVLAGAITMLLTDRNLNTSFFDPAGGGVPILYQHL
>LEFID876-10|HM873633|MM07012|Phyllonorycter_coryli
TLYFIFGIWAGMIGSSLSIMIRAELGNPGSLIGDDQIYNTIVTAHAFIMIFFMVMPIMIGGFGNWLVPLMLGAPDMAFPRLNNMSFWLLPPSILLLISSSVVENGVGTGWTVYPPLSSNIAHGGSSVDLAIFSLHLAGISSILGAINFITTIINMRTNGMKFD
NMPLFVWAVGITALLLLLSLPVLAGAITMLLTDRNLNTSFFDPAGGGDPILYQHL
>LEFIB156-10|HM871061|MM00546|Phyllonorycter_corylifoliella
TLYFIFGIWAGMIGSSLSIMIRAELGNPGSLIGDDQIYNTIVTAHAFIMIFFMVMPIMIGGFGNWLVPLMLGAPDMAFPRLNNMSFWLLPPSILLLISSSVVENGAGTGWTVYPPLSSNIAHGGSSVDLAIFSLHLAGISSILGAINFITTIINMRANGMKFD
NMPLFVWAVGITALLLLLSLPVLAGAITMLLTDRNLNTSFFDPAGGGDPILYQHL
>LEFIB184-10|HM871088|MM00610|Phyllonorycter_corylifoliella
TLYFIFGIWAGMIGSSLSIMIRAELGNPGSLIGDDQIYNTIVTAHAFIMIFFMVMPIMIGGFGNWLVPLMLGAPDMAFPRLNNMSFWLLPPSILLLISSSVVENGAGTGWTVYPPLSSNIAHGGSSVDLAIFSLHLAGISSILGAINFITTIINMRANGMKFD
NMPLFVWAVGITALLLLLSLPVLAGAITMLLTDRNLNTSFFDPAGGGDPILYQHL

>LEFIB220-10|HM871124|MM00674|Phyllonorycter_corylifoliella
TLYFIFGIWAGMIGSSLSIMIRAELGNPGSLIGDDQIYNTIVTAHAFIMIFFMVMPIMIGGFGNWLVPLMLGAPDMAFPRLNNMSFWLLPPSILLLISSSVVENGAGTGWTVYPPLSSNIAHGGSSVDLAIFSLHLAGISSILGAINFITTIINMRANGMKFD
NMPLFVWAVGITALLLLLSLPVLAGAITMLLTDRNLNTSFFDPAGGGDPILYQHL
>LEFIC546-10|HM872367|MM04297|Phyllonorycter_dubitella
TLYFIFGIWAGMIGSSLSIMIRAELGNPGSLIGDDQIYNTIVTAHAFIMIFFMVMPIMIGGFGNWLVPLMLGAPDMAFPRLNNMSFWLLPPSILLLISSSVVENGAGTGWTVYPPLSSNIAHGGSSVDLAIFSLHLAGISSILGAINFITTIINMRSNGMKFD
NMPLFVWAVGITALLLLLSLPVLAGAITMLLTDRNLNTSFFDPAGGGDPILYQHL
>LEFIC547-10|HM872368|MM04300|Phyllonorycter_dubitella
TLYFIFGIWAGMIGSSLSIMIRAELGNPGSLIGDDQIYNTIVTAHAFIMIFFMVMPIMIGGFGNWLVPLMLGAPDMAFPRLNNMSFWLLPPSILLLISSSVVENGAGTGWTVYPPLSSNIAHGGSSVDLAIFSLHLAGISSILGAINFITTIINMRSNGMKFD
NMPLFVWAVGITALLLLLSLPVLAGAITMLLTDRNLNTSFFDPAGGGDPILYQHL
>LEFIK522-10|JF854119|MM18097|Phyllonorycter_emberizaepenella
TLYFIFGIWAGMIGSSLSIMIRAELGNPGSLIGDDQIYNTIVTAHAFIMIFFMVMPIMIGGFGNWLVPLMLGAPDMAFPRLNNMSFWLLPPSILLLISSSIVENGVGTGWTVYPPLSSNIAHGGSSVDLAIFSLHLAGISSILGAINFITTIINMRTNGMMFD
NMPLFVWAVGITALLLLLSLPVLAGAITMLLTDRNLNTSFFDPAGGGDPILYQHL
>LEFIE316-10|HM874042|MM08688|Phyllonorycter_froelichiella
TLYFIFGIWAGMIGSSLSIMIRAELGNPGSLIGDDQIYNTIVTAHAFIMIFFMVMPIMIGGFGNWLIPLMLGAPDMAFPRLNNMSFWLLPPSILLLISSSIVENGVGTGWTVYPPLSSNIAHGGSSVDLAIFSLHLAGISSILGAINFITTIINMRANGMKFDN
MPLFVWAVGITALLLLLSLPVLAGAITMLLTDRNLNTSFFDPAGGGDPILYQHL
>LEFIF336-10|HM875021|MM11144|Phyllonorycter_froelichiella
TLYFIFGIWAGMIGSSLSIMIRAELGNPGSLIGDDQIYNTIVTAHAFIMIFFMVMPIMIGGFGNWLIPLMLGAPDMAFPRLNNMSFWLLPPSILLLISSSIVENGVGTGWTVYPPLSSNIAHGGSSVDLAIFSLHLAGISSILGAINFITTIINMRANGMKFDN
MPLFVWAVGITALLLLLSLPVLAGAITMLLTDRNLNTSFFDPAGGGDPILYQHL
>LEFIJ238-10|MM14072|Phyllonorycter_froelichiella
TLYFIFGIWAGMIGSSLSIMIRAELGNPGSLIGDDQIYNTIVTAHAFIMIFFMVMPIMIGGFGNWLIPLMLGAPDMAFPRLNNMSFWLLPPSILLLISSSIVENGVGTGWTVYPPLSSNIAHGGSSVDLAIFSLHLAGISSILGAINFITTIINMRANEMKFDN
MPLFVWAVGITALLLLLSLPVLAGAITMLLTDRNLNTSFFDPAGGGDPILYQHL
>LEFIF331-10|HM875016|MM11137|Phyllonorycter_harrisella
TLYFIFGIWAGMIGSSLSIMIRAELGNPGSLIGDDQIYNTIVTAHAFIMIFFMVMPIMIGGFGNWLVPLMLGAPDMAFPRLNNMSFWLLPPSILLLISSSIVENGVGTGWTVYPPLSSNIAHGGSSVDLAIFSLHLAGISSILGAINFITTIINMRTNGMMFD
NMPLFVWAVGITALLLLLSLPVLAGAITMLLTDRNLNTSFFDPAGGGDPILYQHL
>LEFIF341-10|HM875026|MM11157|Phyllonorycter_harrisella
TLYFIFGIWAGMIGSSLSIMIRAELGNPGSLIGDDQIYNTIVTAHAFIMIFFMVMPIMIGGFGNWLVPLMLGAPDMAFPRLNNMSFWLLPPSILLLISSSIVENGVGTGWTVYPPLSSNIAHGGSSVDLAIFSLHLAGISSILGAINFITTIINMRTNGMMFD
NMPLFVWAVGITALLLLLSLPVLAGAITMLLTDRNLNTSFFDPAGGGDPILYQHL
>LEFIF342-10|HM875027|MM11158|Phyllonorycter_harrisella
TLYFIFGIWAGMIGSSLSIMIRAELGNPGSLIGDDQIYNTIVTAHAFIMIFFMVMPIMIGGFGNWLVPLMLGAPDMAFPRLNNMSFWLLPPSILLLISSSIVENGVGTGWTVYPPLSSNIAHGGSSVDLAIFSLHLAGISSILGAINFITTIINMRTNGMMFD
NMPLFVWAVGITALLLLLSLPVLAGAITMLLTDRNLNTSFFDPAGGGDPILYQHL
>LEFIF343-10|HM875028|MM11159|Phyllonorycter_heegeriella
TLYFIFGIWAGMIGSSLSIMIRAELGNPGSLIGDDQIYNTIVTAHAFIMIFFMVMPIMIGGFGNWLVPLMLGAPDMAFPRLNNMSFWLLPPSILLLISSSIVENGVGTGWTVYPPLSSNIAHGGSSVDLAIFSLHLAGISSILGAINFITTIINMRTNGMKFD
NMPLFVWAVGITALLLLLSLPVLAGAITMLLTDRNLNTSFFDPAGGGDPILYQHL
>LEFIC541-10|HM872362|MM04288|Phyllonorycter_hilarella
TLYFIFGIWAGMIGSSLSIMIRAELGNPGSLIGDDQIYNTIVTAHAFIMIFFMVMPIMIGGFGNWLVPLMLGAPDMAFPRLNNMSFWLLPPSILLLISSSVVENGAGTGWTVYPPLSSNIAHGGSSVDLAIFSLHLAGISSILGAINFITTIINMRANGMKFD
NMPLFVWAVGITALLLLLSLPVLAGAITMLLTDRNLNTSFFDPAGGGDPILYQHL
>LEFIC542-10|HM872363|MM04290|Phyllonorycter_hilarella
TLYFIFGIWAGMIGSSLSIMIRAELGNPGSLIGDDQIYNTIVTAHAFIMIFFMVMPIMIGGFGNWLVPLMLGAPDMAFPRLNNMSFWLLPPSILLLISSSVVENGAGTGWTVYPPLSSNIAHGGSSVDLAIFSLHLAGISSILGAINFITTIINMRANGMKFD
NMPLFVWAVGITALLLLLSLPVLAGAITMLLTDRNLNTSFFDPAGGGDPILYQHL
>LEFID385-10|HM873182|MM06296|Phyllonorycter_hilarella
TLYFIFGIWAGMIGSSLSIMIRAELGNPGSLIGDDQIYNTIVTAHAFIMIFFMVMPIMIGGFGNWLVPLMLGAPDMAFPRLNNMSFWLLPPSILLLISSSVVENGAGTGWTVYPPLSSNIAHGGSSVDLAIFSLHLAGISSILGAINFITTIINMRANGMKFD
NMPLFVWAVGITALLLLLSLPVLAGAITMLLTDRNLNTSFFDPAGGGDPILYQHL
>LEFIE669-10|HM874391|MM09634|Phyllonorycter_insignitella
TLYFIFGIWAGMIGSSLSIMIRAELGNPGSLIGDDQIYNTIVTAHAFIMIFFMVMPIMIGGFGNWLVPLMLGAPDMAFPRLNNMSFWLLPPSILLLISSSVVESGAGTGWTVYPPLSSNIAHNGSSVDLAIFSLHLAGISSILGAINFITTIINMRVNGMKFD
NMPLFVWAVGITALLLLLSLPVLAGAITMLLTDRNLNTSFFDPAGGGDPILYQHL
>LEFIE670-10|HM874392|MM09635|Phyllonorycter_insignitella
TLYFIFGIWAGMIGSSLSIMIRAELGNPGSLIGDDQIYNTIVTAHAFIMIFFMVMPIMIGGFGNWLVPLMLGAPDMAFPRLNNMSFWLLPPSILLLISSSVVESGAGTGWTVYPPLSSNIAHNGSSVDLAIFSLHLAGISSILGAINFITTIINMRVNGMKFD

NMPLFVWAVGITALLLLLSLPVLAGAITMLLTDRNLNTSFFDPAGGGDPILYQHL
>LEFIB340-10|HM871240|MM00887|Phyllonorycter_issikii
TLYFIFGIWAGMIGSSLSIMIRAELGNPGSLIGDDQIYNTIVTAHAFIMIFFMVMPIMIGGFGNWLVPLMLGAPDMAFPRLNNMSFWLLPPSILLLISSSVVENGVGTGWTVYPPLSSNIAHSGSSVDLAIFSLHLAGISSILGAINFITTIINMRTNGMKFD
NMPLFVWAVGITALLLLLSLPVLAGAITMLLTDRNLNTSFFDPAGGGDPILYQHL
>LEFIB341-10|HM871241|MM00888|Phyllonorycter_issikii
TLYFIFGIWAGMIGSSLSIMIRAELGNPGSLIGDDQIYNTIVTAHAFIMIFFMVMPIMIGGFGNWLVPLMLGAPDMAFPRLNNMSFWLLPPSILLLISSSVVENGVGTGWTVYPPLSSNIAHSGSSVDLAIFSLHLAGISSILGAINFITTIINMRTNGMKFD
NMPLFVWAVGITALLLLLSLPVLAGAITMLLTDRNLNTSFFDPAGGGDPILYQHL
>LEFIJ2189-14|MM23380|Phyllonorycter_issikii
TLYFIFGIWAGMIGSSLSIMIRAELGNPGSLIGDDQIYNTIVTAHAFIMIFFMVMPIMIGGFGNWLVPLMLGAPDMAFPRLNNMSFWLLPPSILLLISSSVVENGVGTGWTVYPPLSSNIAHSGSSVDLAIFSLHLAGISSILGAINFITTIINMRTNGMKFD
NMPLFVWAVGITALLLLLSLPVLAGAITMLLTDRNLNTSFFDPAGGGDPILYQHL
>LEFID807-10|HM873564|MM06893|Phyllonorycter_joannisi
TLYFIFGIWAGMIGSSLSIMIRAELGNPGSLIGDDQIYNTIVTAHAFIMIFFMVMPIMIGGFGNWLVPLMLGAPDMAFPRLNNMSFWLLPPSILLLISSSVVENGAGTGWTVYPPLSSNIAHSGSSVDLAIFSLHLAGISSILGAINFITTIINMRTNGMKFD
NMPLFVWAVGITALLLLLSLPVLAGAITMLLTDRNLNTSFFDPAGGGDPILYQHL
>LEFIC048-10|HM871917|MM03296|Phyllonorycter_junoniella
TLYFIFGIWAGMIGSSLSIMIRAELGNPGSLIGDDQIYNTIVTAHAFIMIFFMVMPIMIGGFGNWLVPLMLGAPDMAFPRLNNMSFWLLPPSILLLISSSVVENGAGTGWTVYPPLSSNIAHSGSSVDLAIFSLHLAGISSILGAINFITTIINMRTNGMKFD
NMPLFVWAVGITALLLLLSLPVLAGAITMLLTDRNLNTSFFDPAGGGDPILYQHL
>LEFIE010-10|HM873759|MM08061|Phyllonorycter_junoniella
TLYFIFGIWAGMIGSSLSIMIRAELGNPGSLIGDDQIYNTIVTAHAFIMIFFMVMPIMIGGFGNWLVPLMLGAPDMAFPRLNNMSFWLLPPSILLLISSSVVENGAGTGWTVYPPLSSNIAHSGSSVDLAIFSLHLAGISSILGAINFITTIINMRTNGMKFD
NMPLFVWAVGITALLLLLSLPVLAGAITMLLTDRNLNTSFFDPAGGGDPILYQHL
>LEFIF344-10|HM875029|MM11161|Phyllonorycter_klemannella
TLYFIFGIWAGMIGSSLSIMIRAELGNPSSLIGDDQIYNTIVTAHAFIMIFFMVMPIMIGGFGNWLVPLMLGAPDMAFPRLNNMSFWLLPPSILLLISSSVVESGVGTGWTVYPPLSSNIAHGGSSVDLAIFSLHLAGISSILGAINFITTIINMRTNGMKFD
NMPLFVWAVGITALLLLLSLPVLAGAITMLLTDRNLNTSFFDPAGGGDPILYQHL
>LEFIJ1371-12|MM20749|Phyllonorycter_lantanella
?LYFIFGIWAGMIGSSLSIMIRAELGNPGSLIGDDQIYNTIVTAHAFIMIFFMVMPIMIGGFGNWLVPLMLGAPDMAFPRLNNMSFWLLPPSILLLISSGVVETGAGTGWTVYPPLSSNIAHSGSSVDLAIFSLHLAGISSILGAINFITTIINMRTNGMKFD
NMPLFVWSVGITALLLLLSLPVLAGAITMLLTDRNLNTSFFDPAGGGDPILYQHL
>LEFIJ1372-12|MM20750|Phyllonorycter_lantanella
TLYFIFGIWAGMIGSSLSIMIRAELGNPGSLIGDDQIYNTIVTAHAFIMIFFMVMPIMIGGFGNWLVPLMLGAPDMAFPRLNNMSFWLLPPSILLLISSGVVETGAGTGWTVYPPLSSNIAHSGSSVDLAIFSLHLAGISSILGAINFITTIINMRTNGMKFD
NMPLFVWSVGITALLLLLSLPVLAGAITMLLTDRNLNTSFFDPAGGGDPILYQHL
>LEFIE692-10|HM874413|MM09670|Phyllonorycter_lautella
TLYFIFGIWAGMIGSSLSIMIRAELGNPGSLIGDDQIYNTIVTAHAFIMIFFMVMPIMIGGFGNWLVPLMLGAPDMAFPRLNNMSFWLLPPSILLLISSSVVENGVGTGWTVYPPLSSNIAHGGSSVDLAIFSLHLAGISSILGAINFITTIINMRTNGMKFD
NMPLFIWAVGITALLLLLSLPVLAGAITMLLTDRNLNTSFFDPAGGGDPILYQHL
>LEFIF340-10|HM875025|MM11156|Phyllonorycter_lautella
TLYFIFGIWAGMIGSSLSIMIRAELGNPGSLIGDDQIYNTIVTAHAFIMIFFMVMPIMIGGFGNWLVPLMLGAPDMAFPRLNNMSFWLLPPSILLLISSSVVENGVGTGWTVYPPLSSNIAHGGSSVDLAIFSLHLAGISSILGAINFITTIINMRTNGMKFD
NMPLFIWAVGITALLLLLSLPVLAGAITMLLTDRNLNTSFFDPAGGGDPILYQHL
>LEFIL563-10|MM18861|Phyllonorycter_leucographella
TLYFIFGIWAGMIGSSLSIMIRAELGNPGSLIGDDQIYNTIVTAHAFIMIFFMVMPIMIGGFGNWLVPLMLGAPDMAFPRLNNMSFWLLPPSILLLISSSVVENGAGTGWTVYPPLSSNIAHSGSSVDLAIFSLHLAGISSILGAINFITTIINMRTNGMKFD
NMPLFVWAVGITALLLLLSLPVLAGAITMLLTDRNLNTSFFDPAGGGDPILYQHL
>LEFIK051-10|KF808554|MM17626|Phyllonorycter_maestingella
TLYFIFGIWAGMIGSSLSIMIRAELGNPGSLIGDDQIYNTIVTAHAFIMIFFMVMPIMIGGFGNWLVPLMLGAPDMAFPRLNNMSFWLLPPSILLLISSSVVENGAGTGWTVYPPLSSNIAHGGSSVDLAIFSLHLAGISSILGAINFITTIINMRTNGMKFD
NMPLFVWAVGITALLLLLSLPVLAGAITMLLTDRNLNTSFFDPAGGGDPILYQHL
>LEFIJ1369-12|KM573359|MM20747|Phyllonorycter_maestingella
TLYFIFGIWAGMIGSSLSIMIRAELGNPGSLIGDDQIYNTIVTAHAFIMIFFMVMPIMIGGFGNWLVPLMLGAPDMAFPRLNNMSFWLLPPSILLLISSSVVENGAGTGWTVYPPLSSNIAHGGSSVDLAIFSLHLAGISSILGAINFITTIINMRANGMKFD
NMPLFVWAVGITALLLLLSLPVLAGAITMLLTDRNLNTSFFDPAGGGDPILYQHL
>LEEUA057-11|MM19370|Phyllonorycter_messaniella
TLYFIFGIWAGMIGSSLSIMIRAELGNPGSLIGDDQIYNTIVTAHAFIMIFFMVMPIMIGGFGNWLVPLMLGAPDMAFPRLNNMSFWLLPPSILLLISSSVVENGAGTGWTVYPPLSSNIAHGGSSVDLAIFSLHLAGISSILGAINFITTIINMRTNGMKFD
NMPLFVWAVGITALLLLLSLPVLAGAITMLLTDRNLNTSFFDPAGGGDPILYQHL
>LEFIE652-10|HM874375|MM09612|Phyllonorycter_nicellii

TLYFIFGIWAGMIGSSLSIMIRAELGNPGSLIGDDQIYNTIVTAHAFIMIFFMVMPIMIGGFGNWLVPLMLGAPDMAFPRLNNMSFWLLPPSILLLISSSIVENGVGTGWTVYPPLSSNIAHGGSSVDLAIFSLHLAGISSILGAINFITTIINMRANGMKFD
NMPLFVWAVGITALLLLLSLPVLAGAITMLLTDRNLNTSFFDPAGGGDPILYQHL
>LEFIE653-10|HM874376|MM09614|Phyllonorycter_nicellii
TLYFIFGIWAGMIGSSLSIMIRAELGNPGSLIGDDQIYNTIVTAHAFIMIFFMVMPIMIGGFGNWLVPLMLGAPDMAFPRLNNMSFWLLPPSILLLISSSIVENGVGTGWTVYPPLSSNIAHGGSSVDLAIFSLHLAGISSILGAINFITTIINMRANGMKFD
NMPLFVWAVGITALLLLLSLPVLAGAITMLLTDRNLNTSFFDPAGGGDPILYQHL
>LEFIB768-10|HM871645|MM02638|Phyllonorycter_nigrescentella
TLYFIFGIWAGMIGSSLSIMIRAELGNPGSLIGDDQIYNTIVTAHAFIMIFFMVMPIMIGGFGNWLVPLMLGAPDMAFPRLNNMSFWLLPPSILLLISSSVVENGAGTGWTVYPPLSSNIAHGGSSVDLAIFSLHLAGISSILGAINFITTIINMRANGMKFD
NMPLFVWAVGITALLLLLSLPVLAGAITMLLTDRNLNTSFFDPAGGGDPILYQHL
>LEFIF325-10|HM875010|MM11126|Phyllonorycter_nigrescentella
TLYFIFGIWAGMIGSSLSIMIRAELGNPGSLIGDDQIYNTIVTAHAFIMIFFMVMPIMIGGFGNWLVPLMLGAPDMAFPRLNNMSFWLLPPSILLLISSSVVENGAGTGWTVYPPLSSNIAHGGSSVDLAIFSLHLAGISSILGAINFITTIINMRANGMKFD
NMPLFVWAVGITALLLLLSLPVLAGAITMLLTDRNLNTSFFDPAGGGDPILYQHL
>LEFIL693-10|JF854692|MM18991|Phyllonorycter_nigrescentella
TLYFIFGIWAGMIGSSLSIMIRAELGNPGSLIGDDQIYNTIVTAHAFIMIFFMVMPIMIGGFGNWLVPLMLGAPDMAFPRLNNMSFWLLPPSILLLISSSVVENGAGTGWTVYPPLSSNIAHGGSSVDLAIFSLHLAGISSILGAINFITTIINMRANGMKFD
NMPLFVWAVGITALLLLLSLPVLAGAITMLLTDRNLNTSFFDPAGGGDPILYQHL
>LEFIJ1387-12|MM22058|Phyllonorycter_nigrescentella
TLYFIFGIWAGMIGSSLSIMIRAELGNPGSLIGDDQIYNTIVTAHAFIMIFFMVMPIMIGGFGNWLVPLMLGAPDMAFPRLNNMSFWLLPPSILLLISSSVVENGAGTGWTVYPPLSSNIAHGGSSVDLAIFSLHLAGISSILGAINFITTIINMRANGMKFD
NMPLFVWAVGITALLLLLSLPVLAGAITMLLTDRNLNTSFF?PAGGGDPILYQHL
>LEFIE920-10|HM874637|MM10288|Phyllonorycter_pastorella
TLYFIFGIWAGMVGSSLSIMIRAELGNPGSLIGDDQIYNTIVTAHAFIMIFFMVMPIMIGGFGNWLVPLMLGAPDMAFPRLNNMSFWLLPPSILLLISSSIVENGAGTGWTVYPPLSANVAHSGSSVDLAIFSLHLAGISSILGAINFITTIINMRANGMMF
DNMPLFVWAVGITALLLLLSLPVLAGAITMLLTDRNLNTSFFDPAGGGDPILYQHL
>LEFIL568-10|MM18866|Phyllonorycter_platani
TLYFIFGIWAGMIGSSLSIMIRAELGNPGSLIGDDQIYNTIVTAHAFIMIFFMVMPIMIGGFGNWLVPLMLGAPDMAFPRLNNMSFWLLPPSILLLISSSIVENGVGTGWTVYPPLSSNIAHGGSSVDLAIFSLHLAGISSILGAINFITTIINMRTNGMKFD
NMPLFVWAVGITALLLLLSLPVLAGAITMLLTDRNLNTSFFDPAGGGDPILYQHL
>LEFIG401-10|HM876078|MM14424|Phyllonorycter_populifoliella
TLYFIFGIWAGMVGSSLSIMIRAELGNPGSLIGDDQIYNTIVTAHAFIMIFFMVMPIMIGGFGNWLVPLMLGAPDMAFPRLNNMSFWLLPPSILLLISSSIVENGAGTGWTVYPPLSSNIAHSGSSVDLAIFSLHLAGISSILGAINFITTIINMRANGMMF
DNMPLFVWAVGITALLLLLSLPVLAGAITMLLTDRNLNTSFFDPAGGGDPILYQHL
>LEFIG402-10|HM876079|MM14425|Phyllonorycter_populifoliella
TLYFIFGIWAGMVGSSLSIMIRAELGNPGSLIGDDQIYNTIVTAHAFIMIFFMVMPIMIGGFGNWLVPLMLGAPDMAFPRLNNMSFWLLPPSILLLISSSIVENGAGTGWTVYPPLSSNIAHSGSSVDLAIFSLHLAGISSILGAINFITTIINMRANGMMF
DNMPLFVWAVGITALLLLLSLPVLAGAITMLLTDRNLNTSFFDPAGGGDPILYQHL
>LEFIE334-10|HM874059|MM08726|Phyllonorycter_pyrifoliella
TLYFIFGIWAGMIGSSLSIMIRAELGNPGSLIGDDQIYNTIVTAHAFIMIFFMVMPIMIGGFGNWLVPLMLGAPDMAFPRLNNMSFWLLPPSILLLISSSVVENGAGTGWTVYPPLSSNIAHGGSSVDLAIFSLHLAGISSILGAINFITTIINMRTNGMKFD
NMPLFVWAVGITALLLLLSLPVLAGAITMLLTDRNLNTSFFDPAGGGDPILYQHL
>LEFID183-10|HM872989|MM05971|Phyllonorycter_quercifoliella
TLYFIFGIWAGMIGSSLSIMIRAELGNPGSLIGDDQIYNTIVTAHAFIMIFFMVMPIMIGGFGNWLVPLMLGAPDMAFPRLNNMSFWLLPPSILLLISSSVVENGVGTGWTVYPPLSSNIAHGGSSVDLAIFSLHLAGISSILGAINFITTIINMRTNGMKFD
NMPLFVWAVGITALLLLLSLPVLAGAITMLLTDRNLNTSFFDPAGGGDPILYQHL
>LEFIF326-10|HM875011|MM11130|Phyllonorycter_quercifoliella
TLYFIFGIWAGMIGSSLSIMIRAELGNPGSLIGDDQIYNTIVTAHAFIMIFFMVMPIMIGGFGNWLVPLMLGAPDMAFPRLNNMSFWLLPPSILLLISSSVVENGVGTGWTVYPPLSSNIAHGGSSVDLAIFSLHLAGISSILGAINFITTIINMRTNGMKFD
NMPLFVWAVGITALLLLLSLPVLAGAITMLLTDRNLNTSFFDPAGGGDPILYQHL
>LEFIF335-10|HM875020|MM11142|Phyllonorycter_quercifoliella
TLYFIFGIWAGMIGSSLSIMIRAELGNPGSLIGDDQIYNTIVTAHAFIMIFFMVMPIMIGGFGNWLVPLMLGAPDMAFPRLNNMSFWLLPPSILLLISSSVVENGVGTGWTVYPPLSSNIAHGGSSVDLAIFSLHLAGISSILGAINFITTIINMRTNGMKFD
NMPLFVWAVGITALLLLLSLPVLAGAITMLLTDRNLNTSFFDPAGGGDPILYQHL
>LEFIC980-10|HM872795|MM05376|Phyllonorycter_quinqueguttella
TLYFIFGIWAGMIGSSLSIMIRAELGNPGSLIGDDQIYNTIVTAHAFIMIFFMVMPIMIGGFGNWLVPLMLGAPDMAFPRLNNMSFWLLPPSMLLLISSSIVENGAGTGWTVYPPLSSNIAHAGSSVDLTIFSLHLAGISSILGAINFITTIINMRANGMMF
DNMPLFVWAVGITALLLLLSLPVLAGAITMLLTDRNLNTSFFDPAGGGDPILYQHL
>LEFID371-10|HM873168|MM06280|Phyllonorycter_quinqueguttella
TLYFIFGIWAGMIGSSLSIMIRAELGNPGSLIGDDQIYNTIVTAHAFIMIFFMVMPIMIGGFGNWLVPLMLGAPDMAFPRLNNMSFWLLPPSMLLLISSSIVENGAGTGWTVYPPLSSNIAHAGSSVDLTIFSLHLAGISSILGAINFITTIINMRANGMMF
DNMPLFVWAVGITALLLLLSLPVLAGAITMLLTDRNLNTSFFDPAGGGDPILYQHL

>LEFIF144-10|HM874838|MM10658|Phyllonorycter_quinqueguttella
TLYFIFGIWAGMIGSSLSIMIRAELGNPGSLIGDDQIYNTIVTAHAFIMIFFMVMPIMIGGFGNWLVPLMLGAPDMAFPRLNNMSFWLLPPSMLLLISSSIVENGAGTGWTVYPPLSSNIAHAGSSVDLTIFSLHLAGISSILGAINFITTIINMRANGMMF
DNMPLFVWAVGITALLLLLSLPVLAGAITMLLTDRNLNTSFFDPAGGGDPILYQHL
>LEFIF353-10|HM875038|MM11180|Phyllonorycter_rajella
TLYFIFGIWAGMIGSSLSIMIRAELGNPGSLIGDDQIYNTIVTAHAFIMIFFMVMPIMIGGFGNWLVPLMLGAPDMAFPRLNNMSFWLLPPSILLLISSSIVENGVGTGWTVYPPLSSNIAHSGSSVDLAIFSLHLAGISSILGAINFITTIINMRTNGMMFD
NMPLFVWAVGITALLLLLSLPVLAGAITMLLTDRNLNTSFFDPAGGGDPILYQHL
>LEFIE459-10|HM874183|MM09226|Phyllonorycter_roboris
TLYFIFGIWAGMIGSSLSIMIRAELGNPGSLIGDDQIYNTIVTAHAFIMIFFMVMPIMIGGFGNWLVPLMLGAPDMAFPRLNNMSFWLLPPSILLLISSSIVENGVGTGWTVYPPLSSNIAHSGSSVDLAIFSLHLAGISSILGAINFITTIINMRTNGMKFDN
MPLFVWAVGITALLLLLSLPVLAGAITMLLTDRNLNTSFFDPAGGGDPILYQHL
>LEFIG678-10|HM876338|MM15542|Phyllonorycter_rolandi
TLYFIFGIWAGMIGSSLSIMIRAELGNPGSLIGDDQIYNTIVTAHAFIMIFFMVMPIMIGGFGNWLVPLMLGAPDMAFPRLNNMSFWLLPPSILLLISSSVVENGAGTGWTVYPPLSSNIAHGGSSVDLAIFSLHLAGISSILGAINFITTIINMRANGMKFD
NMPLFVWAVGITALLLLLSLPVLAGAITMLLTDRNLNTSFFDPAGGGDPILYQHL
>LEFIG679-10|HM876339|MM15543|Phyllonorycter_rolandi
TLYFIFGIWAGMIGSSLSIMIRAELGNPGSLIGDDQIYNTIVTAHAFIMIFFMVMPIMIGGFGNWLVPLMLGAPDMAFPRLNNMSFWLLPPSILLLISSSVVENGAGTGWTVYPPLSSNIAHGGSSVDLAIFSLHLAGISSILGAINFITTIINMRANGMKFD
NMPLFVWAVGITALLLLLSLPVLAGAITMLLTDRNLNTSFFDPAGGGDPILYQHL
>LEFID859-10|HM873616|MM06988|Phyllonorycter_sagitella
TLYFIFGNWAGKVGSSLSIMIRAELGNPGSLIGDDQIYNTIVTAHAFIMIFFMVMPIMIGGFGNWLVPLMLGAPDMAFPRLNNMSFWLLPPSILLLISSSVVENGAGTGWTVYPPLSANMAHSGSSVDLAIFSLHLAGISSILGAINFITTIINMRTNGM
MFDNMPLFVWAVGITALLLLLSLPVLAGAITMLLTDRNLNTSFFDPAGGGDPILYQHL
>LEFIG087-10|HM875766|MM13754|Phyllonorycter_sagitella
TLYFIFGIWAGMVGSSLSIMIRAELGNPGSLIGDDQIYNTIVTAHAFIMIFFMVMPIMIGGFGNWLVPLMLGAPDMAFPRLNNMSFWLLPPSILLLISSSVVENGAGTGWTVYPPLSANMAHSGSSVDLAIFSLHLAGISSILGAINFITTIINMRTNGMM
FDNMPLFVWAVGITALLLLLSLPVLAGAITMLLTDRNLNTSFFDPAGGGDPILYQHL
>LEFIC981-10|HM872796|MM05377|Phyllonorycter_salicicolella
TLYFIFGIWAGMIGSSLSIMIRAELGNPGSLIGDDQIYNTIVTAHAFIMIFFMVMPIMIGGFGNWLIPLMLGAPDMAFPRLNNMSFWLLPPSILLLISSSVVENGAGTGWTVYPPLSSNIAHSGSSVDLAIFSLHLAGISSILGAINFITTIINMRANGMKFD
NMPLFVWAVGITALLLLLSLPVLAGAITMLLTDRNLNTSFFDPAGGGDPILYQHL
>LEFID386-10|HM873183|MM06297|Phyllonorycter_salicicolella
TLYFIFGIWAGMIGSSLSIMIRAELGNPGSLIGDDQIYNTIVTAHAFIMIFFMVMPIMIGGFGNWLVPLMLGAPDMAFPRLNNMSFWLLPPSILLLISSSVVENGAGTGWTVYPPLSSNIAHGGSSVDLAIFSLHLAGISSILGAINFITTIINMRANGMKFD
NMPLFVWAVGITALLLLLSLPVLAGAITMLLTDRNLNTSFFDPAGGGDPILYQHL
>LEFIF159-10|HM874853|MM10689|Phyllonorycter_salicicolella
TLYFIFGIWAGMIGSSLSIMIRAELGNPGSLIGDDQIYNTIVTAHAFIMIFFMVMPIMIGGFGNWLVPLMLGAPDMAFPRLNNMSFWLLPPSILLLISSSVVENGAGTGWTVYPPLSSNIAHGGSSVDLAIFSLHLAGISSILGAINFITTIINMRANGMKFD
NMPLFVWAVGITALLLLLSLPVLAGAITMLLTDRNLNTSFFDPAGGGDPILYQHL
>LEFIF160-10|HM874854|MM10690|Phyllonorycter_salicicolella
TLYFIFGIWAGMIGSSLSIMIRAELGNPGSLIGDDQIYNTIVTAHAFIMIFFMVMPIMIGGFGNWLVPLMLGAPDMAFPRLNNMSFWLLPPSILLLISSSVVENGAGTGWTVYPPLSSNIAHGGSSVDLAIFSLHLAGISSILGAINFITTIINMRANGMKFD
NMPLFVWAVGITALLLLLSLPVLAGAITMLLTDRNLNTSFFDPAGGGDPILYQHL
>LEFIF161-10|HM874855|MM10691|Phyllonorycter_salicicolella
TLYFIFGIWAGMIGSSLSIMIRAELGNPGSLIGDDQIYNTIVTAHAFIMIFFMVMPIMIGGFGNWLVPLMLGAPDMAFPRLNNMSFWLLPPSILLLISSSVVENGAGTGWTVYPPLSSNIAHGGSSVDLAIFSLHLAGISSILGAINFITTIINMRANGMKFD
NMPLFVWAVGITALLLLLSLPVLAGAITMLLTDRNLNTSFFDPAGGGDPILYQHL
>LEFIF162-10|HM874856|MM10693|Phyllonorycter_salicicolella
TLYFIFGIWAGMIGSSLSIMIRAELGNPGSLIGDDQIYNTIVTAHAFIMIFFMVMPIMIGGFGNWLVPLMLGAPDMAFPRLNNMSFWLLPPSILLLISSSVVENGAGTGWTVYPPLSSNIAHGGSSVDLAIFSLHLAGISSILGAINFITTIINMRANGMKFD
NMPLFVWAVGITALLLLLSLPVLAGAITMLLTDRNLNTSFFDPAGGGDPILYQHL
>LEFIC523-10|HM872346|MM04243|Phyllonorycter_salictella
TLYFIFGIWAGMIGSSLSIMIRAELGNPGSLIGDDQIYNTIVTAHAFIMIFFMVMPIMIGGFGNWLVPLMLGAPDMAFPRLNNMSFWLLPPSILLLISSSVVENGAGTGWTVYPPLSSNIAHSGSSVDLTIFSLHLAGISSILGAINFITTIINMRTNGMKFD
NMPLFVWAVGITALLLLLSLPVLAGAITMLLTDRNLNTSFFDPAGGGDPILYQHL
>LEFIC530-10|HM872351|MM04261|Phyllonorycter_salictella
TLYFIFGIWAGMIGSSLSIMIRAELGNPGSLIGDDQIYNTIVTAHAFIMIFFMVMPIMIGGFGNWLVPLMLGAPDMAFPRLNNMSFWLLPPSILLLISSSVVENGAGTGWTVYPPLSSNIAHGGSSVDLAIFSLHLAGISSILGAINFITTIINMRANGMKFD
NMPLFVWAVGITALLLLLSLPVLAGAITMLLTDRNLNTSFFDPAGGGDPILYQHL
>LEFIC535-10|HM872356|MM04274|Phyllonorycter_salictella
TLYFIFGIWAGMIGSSLSIMIRAELGNPGSLIGDDQIYNTIVTAHAFIMIFFMVMPIMIGGFGNWLVPLMLGAPDMAFPRLNNMSFWLLPPSILLLISSSVVENGAGTGWTVYPPLSSNIAHSGSSVDLTIFSLHLAGISSILGAINFITTIINMRTNGMKFD

NMPLFVWAVGITALLLLLSLPVLAGAITMLLTDRNLNTSFFDPAGGGDPILYQHL
>LEFIC537-10|HM872358|MM04278|Phyllonorycter_salictella
TLYFILGIWAGMIGSSLSIMIRAELGNPGSLIGDDQIYNTIVTAHAFIMIFFMVMPIMIGGFGNWLVPLMLGAPDMAFPRLNNMSFWLLPPSILLLISSSVVENGAGTGWTVYPPLSSNIAHGGSSVDLAIFSLHLAGISSILGAINFITTIINMRANGMKFD
NMPLFVWAVGITALLLLLSLPVLAGAITMLLTDRNLNTSFFDPAGGGDPILYQHL
>LEFIE493-10|HM874217|MM09280|Phyllonorycter_salictella
TLYFIFGIWAGMIGSSLSIMIRAELGNPGSLIGDDQIYNTIVTAHAFIMIFFMVMPIMIGGFGNWLVPLMLGAPDMAFPRLNNMSFWLLPPSILLLISSSVVENGAGTGWTVYPPLSSNIAHGGSSVDLAIFSLHLAGISSILGAINFITTIINMRANGMKFD
NMPLFVWAVGITALLLLLSLPVLAGAITMLLTDRNLNTSFFDPAGGGDPILYQHL
>LEFIA852-10|HM386992|MM09729|Phyllonorycter_salictella
TLYFIFGIWAGMIGSSLSIMIRAELGNPGSLIGDDQIYNTIVTAHAFIMIFFMVMPIMIGGFGNWLVPLMLGAPDMAFPRLNNMSFWLLPPSILLLISSSVVENGAGTGWTVYPPLSSNIAHGGSSVDLAIFSLHLAGISSILGAINFITTIINMRANGMKFD
NMPLFVWAVGITALLLLLSLPVLAGAITMLLTDRNLNTSFFDPAGGGDPILYQHL
>LEFIF357-10|HM875042|MM11192|Phyllonorycter_salictella
TLYFIFGIWAGMIGSSLSIMIRAELGNPGSLIGDDQIYNTIVTAHAFIMIFFMVMPIMIGGFGNWLVPLMLGAPDMAFPRLNNMSFWLLPPSILLLISSSVVENGAGTGWTVYPPLSSNIAHGGSSVDLAIFSLHLAGISSILGAINFITTIINMRANGMKFD
NMPLFVWAVGITALLLLLSLPVLAGAITMLLTDRNLNTSFFDPAGGGDPILYQHL
>LEEUA061-11|JN280372|MM19374|Phyllonorycter_salictella
TLYFIFGIWAGMIGSSLSIMIRAELGNPGSLIGDDQIYNTIVTAHAFIMIFFMVMPIMIGGFGNWLVPLMLGAPDMAFPRLNNMSFWLLPPSILLLISSSVVENGAGTGWTVYPPLSSNIAHGGSSVDLAIFSLHLAGISSILGAINFITTIINMRANGMKFD
NMPLFVWAVGITALLLLLSLPVLAGAITMLLTDRNLNTSFFDPAGGGDPILYQHL
>LEEUA062-11|JN280373|MM19375|Phyllonorycter_salictella
TLYFIFGIWAGMIGSSLSIMIRAELGNPGSLIGDDQIYNTIVTAHAFIMIFFMVMPIMIGGFGNWLVPLMLGAPDMAFPRLNNMSFWLLPPSILLLISSSVVENGAGTGWTVYPPLSSNIAHGGSSVDLAIFSLHLAGISSILGAINFITTIINMRANGMKFD
SMPLFVWAVGITALLLLLSLPVLAGAITMLLTDRNLNTSFFDPAGGGDPILYQHL
>LEEUA063-11|JN280374|MM19376|Phyllonorycter_salictella
TLYFIFGIWAGMIGSSLSIMIRAELGNPGSLIGDDQIYNTIVTAHAFIMIFFMVMPIMIGGFGNWLVPLMLGAPDMAFPRLNNMSFWLLPPSILLLISSSVVENGAGTGWTVYPPLSSNIAHGGSSVDLAIFSLHLAGISSILGAINFITTIINMRANGMKFD
NMPLFVWAVGITALLLLLSLPVLAGAITMLLTDRNLNTSFFDPAGGGDPILYQHL
>LEEUA066-11|JN280375|MM19379|Phyllonorycter_scopariella
TLYFIFGIWAGMIGSSLSIMIRAELGNPGSLIGDDQIYNTIVTAHAFIMIFFMVMPIMIGGFGNWLVPLMLGAPDMAFPRLNNMSFWLLPPSILLLISSSVVENGAGTGWTVYPPLSSNIAHGGSSVDLAIFSLHLAGISSILGAINFITTIINMRANGMKFD
NMPLFVWAVGITALLLLLSLPVLAGAITMLLTDRNLNTSFFDPAGGGDPILYQHL
>LEFIB161-10|HM871065|MM00552|Phyllonorycter_sorbi
[truncated: 248,566 more chars]
